# Concordance of Gene Expression and Functional Correlation Patterns across the NCI-60 Cell Lines and the Cancer Genome Atlas Glioblastoma Samples

**DOI:** 10.1371/journal.pone.0040062

**Published:** 2012-07-26

**Authors:** Barry R. Zeeberg, Kurt W. Kohn, Ari Kahn, Vladimir Larionov, John N. Weinstein, William Reinhold, Yves Pommier

**Affiliations:** 1 Laboratory of Molecular Pharmacology, Center for Cancer Research, National Cancer Institute, National Institutes of Health, Bethesda, Maryland, United States of America; 2 SRA International, Inc., Fairfax, Virginia, United States of America; 3 Departments of Bioinformatics and Computational Biology and Systems Biology, M.D. Anderson Cancer Center, Houston, Texas, United States of America; University of Navarra, Spain

## Abstract

**Background:**

The NCI-60 is a panel of 60 diverse human cancer cell lines used by the U.S. National Cancer Institute to screen compounds for anticancer activity. We recently clustered genes based on correlation of expression profiles across the NCI-60. Many of the resulting clusters were characterized by cancer-associated biological functions. The set of curated glioblastoma (GBM) gene expression data from the Cancer Genome Atlas (TCGA) initiative has recently become available. Thus, we are now able to determine which of the processes are robustly shared by both the immortalized cell lines and clinical cancers.

**Results:**

Our central observation is that some sets of highly correlated genes in the NCI-60 expression data are also highly correlated in the GBM expression data. Furthermore, a “double fishing” strategy identified many sets of genes that show Pearson correlation ≥0.60 in both the NCI-60 and the GBM data sets relative to a given “bait” gene. The number of such gene sets far exceeds the number expected by chance.

**Conclusion:**

Many of the gene-gene correlations found in the NCI-60 do not reflect just the conditions of cell lines in culture; rather, they reflect processes and gene networks that also function *in vivo*. A number of gene network correlations co-occur in the NCI-60 and GBM data sets, but there are others that occur only in NCI-60 or only in GBM. In sum, this analysis provides an additional perspective on both the utility and the limitations of the NCI-60 in furthering our understanding of cancers *in vivo.*

## Introduction

The NCI-60 [1] is a panel of 60 human cancer cell lines used by the Developmental Therapeutics Program (DTP) of the U.S. National Cancer Institute to screen >100,000 compounds plus natural products since 1990. The NCI-60 panel includes cancers of colorectal (CO), renal (RE), ovarian (OV), prostate (PR), lung (LC), breast (BR), and central nervous system (CNS) origin, as well as leukemias (LE) and melanomas (ME). We and our many colleagues around the world have profiled the NCI-60 more comprehensively at the DNA, RNA, protein, mutation, functional, and pharmacological levels than any other panel of diverse cell types in existence. The NCI-60 data have been widely used in cancer research and bioinformatics, but the multiple datasets may be most informative for the recognition of complex ‘biosignatures’ (a ‘biosignature’ involves an ensemble of genes whose features are predictive). Analysis of such biosignatures has led to increased understanding of cell phenotypes and pathway relationships.

When we recently clustered genes based on correlation of expression profiles across the NCI-60 [2], many of the clusters were associated with cancer-related biological functions. The number of such clusters far exceeded what would be expected by chance. One of the clusters, designated as “cluster 52 of the 160-cut,” was comprised of significant categories that generally reflected neuron development, immune response, and epithelial to mesenchymal transition (EMT) in addition to cell migration. In contrast, cluster 68 of the 160-cut was focused strongly on a single biological process, namely immune function.

**Figure 1:**
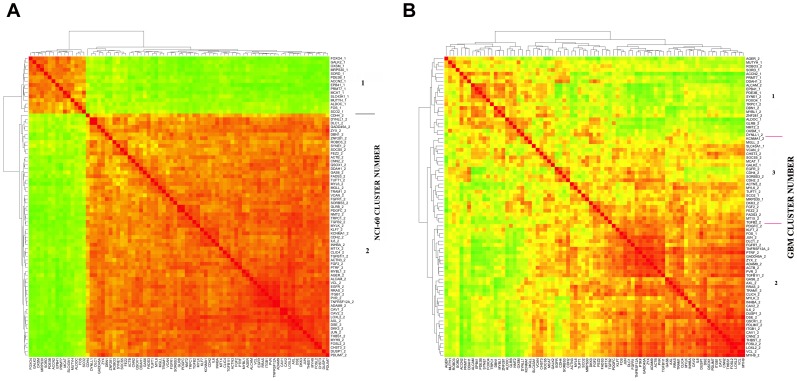
Thumbnails of gene correlation clustering for Cluster 52 genes across (A) NCI-60 cell lines and (B) TCGA GBM samples. The full size figures are available as Figures S1 and S2. The numbers appended after the gene name refer to the NCI-60 cluster in which that gene appeared.

A previous study [3] compared the gene expression profiles between cell lines and breast tumor tissue samples. The authors noted that: “cell lines and tumors share many aspects of their gene expression patterns that can be related to the normal and pathological physiology that distinguishes breast cell types *in vivo*. These gene sets include 1) the basal epithelial cluster, 2) the luminal epithelial/ER+ cluster, 3) the ERBB2+ amplicon cluster, 4) the proliferation cluster, and 5) the interferon cluster.”

**Table 1:** Contingency table for cluster 52 re-clustered across NCI-60 and across GBM.

		GBM CLUSTER			MARGINALS
		1	2	3	
**NCI-60 CLUSTER**	**1**	9	1	5	15
	**2**	12	34	18	64
**MARGINALS**		21	35	23	79

The Fisher Exact p-value corresponding to this contingency table was 0.00039.

Cancer cells in culture are subject to very different conditions than tumor cells in the host. They have been removed from their physiological milieu of other cell types, tissue architecture, hormonal influences, and autocrine/paracrine signals. So the question remained: “What does such a pattern of association in cell culture tell us about cancer cells in vivo?”.

To explore that question, we analyzed the highly curated glioblastoma (GBM) transcript expression data set generated by the The Cancer Genome Atlas (TCGA) initiative [4]. TCGA was established to build a comprehensive catalogue of genomic and phenotypic abnormalities that drive carcinogenesis and potentially affect therapy in >20 different tumor types. In particular, TCGA has now provided a detailed view of genomic aberrations in a GBM cohort consisting of 206 patient samples. Verhaak and Hoadley *et al.* [5] recently described a gene expression-based molecular classification of GBM into Proneural, Neural, Classical and Mesenchymal subtypes and integrated multiple types of genomic data to establish patterns of somatic mutation, DNA copy number change, and gene expression.

**Table 2:** Fisher Exact p-values for concordance of re-clustering cluster 52 across NCI-60 and across GBM.

NUMBER OF CLUSTERS (parameter “k” of cutree())						
2	3	4	5	6	7	8
0.00254	**0.00039**	0.00103	0.00093	0.00167	0.00157	0.00189

The Fisher’s Exact p-value for 100 randomizations corresponding to k = 3 was 0.464±0.279. The bold value indicates the lowest p-value model for the reals. This was the model used for the remainder of the cluster 52 analysis.

**Table 3:** Concordance of cluster 52 genes in NCI-60 and GBM clusters.

		GBM CLUSTER		
		1	2	3
**NCI-60 CLUSTER**	**1**	ACCN2	FOS	GALK2
		ALDOC		MCAT
		EPB41		MRPS30
		FOXO4		SCO2
		MUTYH		SLC43A1
		OXSM		
		PDE3B		
		PRMT7		
		SORD		
	**2**	AGER	ACTB	ACTN3
		ALCAM	ADAM9	CDH2
		DBN1	AXL	CDH4
		DDAH1	CAV1	CHST3
		DYNLL1	CAV2	DKK3
		GLRB	CLIC4	EGFR
		MYBL1	CNN2	FADS3
		NMT2	DLC1	FEZ2
		ROBO3	DSE	FGF2
		SYNE1	DUSP1	KCNMA1
		TRPC1	FGFR1	MGLL
		ZNF281	FOSL2	MT1X
			GADD45A	MYL6
			GAS6	SOCS5
			IL6	SORBS3
			INHBA	TGFB2
			ITGB1	TUFT1
			JUN	VCAN
			KLF7	
			LOXL2	
			MYH9	
			MYLK	
			PDGFC	
			PDLIM7	
			PTRF	
			PVR	
			QSOX1	
			RRAS	
			TGFB1I1	
			THBS1	
			TNFRSF12A	
			TRAM1	
			VCL	
			ZYX	

In the present analysis, we tested whether sets of genes that we previously found to be (1) highly co-expressed across the NCI-60, and (2) functionally coherent were also highly co-expressed across the GBM samples. We then extended that basic analysis by a “double fishing” strategy. That is, we identified sets of genes that showed correlation ≥0.60 in both the NCI-60 and GBM data sets relative to a given “bait” gene. We found that the number of such gene sets far exceeded the number expected by chance. That analysis does not mean that cancer cells in culture share all, or even most, of their characteristics with cells in vivo, but it does indicate similarities.

**Table 4:** Contingency table for cluster 68 re-clustered across NCI-60 and across GBM.

		GBM CLUSTER				MARGINALS
		1	2	3	4	
**NCI-60 CLUSTER**	**1**	8	11	0	5	24
	**2**	11	12	38	14	75
**MARGINALS**		19	23	38	19	99

The Fisher Exact p-value corresponding to this contingency table was 0.00001.

**Table 5:** Fisher Exact p-values for concordance of re-clustering cluster 68 across NCI-60 and across GBM.

NUMBER OF CLUSTERS (parameter “k” of *cutree()*)						
2	3	4	5	6	7	8
0.09162	0.10917	**0.00001**	0.00001	0.00001	0.00001	0.00001

The Fisher’s Exact p-value for 100 randomizations corresponding to k = 3 was 0.529±0.283. The bold value indicates the lowest p-value model for the reals. This was the model used for the remainder of the cluster 68 analysis.

## Methods

### Datasets

For GBM expression data, the files *unifiedScaled.txt* (which contains a complete set of expression data, referred to as *TCGA.GBM.complete*) *TCGA_unified_CORE_ClaNC840.txt* (which includes the subtype tags of each sample) were downloaded from the TCGA web site http://tcga-data.nci.nih.gov/docs/publications/gbm_exp/.

**Table 6:** Concordance of cluster 68 genes in NCI-60 and GBM clusters.

		GBM CLUSTER			
		1	2	3	4
**NCI-60 CLUSTER**	**1**	ACVR2A	ADAM15		APP
		CSNK1G3	AGPAT3		DOCK1
		CTBP2	AHNAK		MLF1
		MAP3K13	DUSP3		NOL3
		SMAD5	EMP2		OAT
		YES1	GRN		
		ZNF205	LMNA		
		ZNF35	MGAT4B		
			PLP2		
			SPR		
			ZFHX3		
	**2**	CHRNA3	ADA	AIF1	C9
		CYFIP2	ALDH1A2	ARHGDIB	CD1A
		ELOVL4	CD1E	CCR4	GDF10
		GNB1L	CD79A	CCR7	GRAP
		GRIK5	GP5	CD1D	LY6H
		MYB	IGLL1	CD27	NKX2-5
		NFKBIL1	KRT1	CD3D	PRKCQ
		SLIT1	LAX1	CD3E	RAG2
		SMPD3	LTA	CD3G	RASGRP2
		TSPAN7	PTGDR	CD4	RORB
		USP20	RAG1	CD5	SLC15A2
			SEPT6	CD52	SLC18A2
				CD7	TAL1
				CD84	VPREB1
				CD96	
				CECR1	
				CTSW	
				FLI1	
				FYB	
				GFI1	
				GMFG	
				GNA15	
				GRAP2	
				IL12RB1	
				IL2RG	
				ITGAL	
				ITGB2	
				ITK	
				LST1	
				MAP4K1	
				RHOH	
				SELL	
				SH2D1A	
				SIT1	
				SPN	
				TRAT1	
				TREML2	
				ZAP70	

We used all 202 GBM samples that are available, representing roughly comparable numbers of samples of each subtype. Since the calculated correlation values will be more accurate if they come from a more diverse sampling population, we wanted to retain as much diversity as possible by looking at all subtypes together, so we did not report co-expression within or between subtypes.

**Figure 2:**
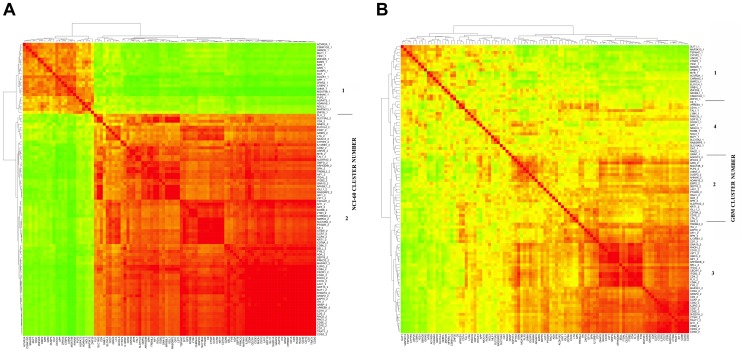
Thumbnails of gene correlation clustering for Cluster 68 genes across (A) the NCI-60 cell lines and GBM samples (B). The full size figures are available as Figures S3 and S4. The numbers appended after the gene name refer to the NCI-60 cluster in which that gene appeared.

**Figure 3:**
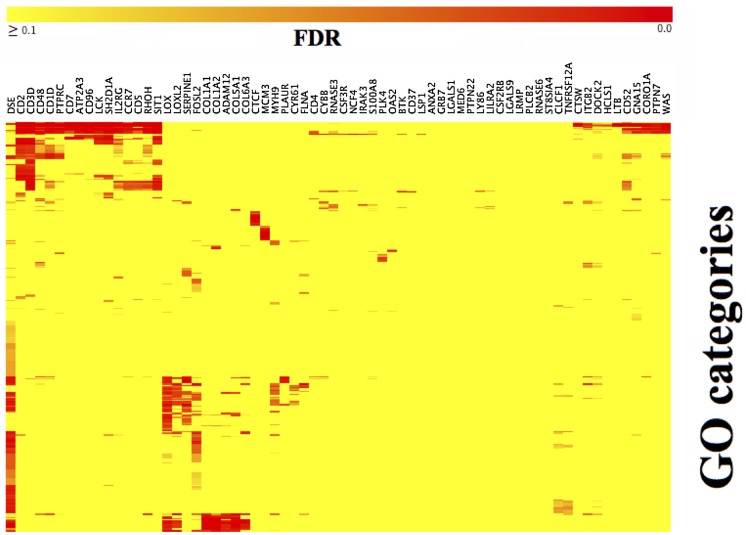
Thumbnail of GO category *versus* gene list CIM for sets of genes with correlation ≥0.60 across both the NCI-60 and GBM samples. The full size CIM is available as Figure S5. The gene name given as the column header is the representative of a list of genes. The full list of genes is available in the HTGM Download S1.

NCI-60 expression data were obtained from CellMiner [6]. Determination of composite expression levels for each gene was performed as described previously [7]–[9]. A special request was made to the system administrator for the complete set of gene expression profiles (referred to as *NCI-60.complete*). That download would have been too large to perform through the standard web interface. Further details are provided in [2]). Briefly, *NCI-60.complete* was pre-processed by selecting only those genes that have both an HGNC symbol and annotation in the GO Biological Process ontology. Each gene profile vector was scaled to zero mean and unit variance. That reduced dataset is referred to here as *NCI-60.BP.*

As mentioned above for the GBM samples, we are trying to achieve as high a degree of diversity as possible in the cell lines, so that the highly heterogeneous mixture of cell lines represented by the NCI-60 is ideal. For illustration, consider two genes. We are looking to see if the expression levels of those two genes go up and down together as we traverse the 60 cell lines. If all of the cells lines were essentially identical to one another, there would be no variation and we could not see how the two genes relate in different conditions.

For most of the studies reported here, the expression data for GBM and for NCI-60 were restricted to those genes that were present in both *TCGA.GBM.complete* and *NCI-60.BP*.

**Table 7:** Summary of high ranking genes with correlation ≥0.60 across both NCI-60 and GBM.

rank	Designated gene (G)	number of genes with correlation ≥0.60	generalized functional correlation	number of genes incommon with cluster 52	Number of genes in common with cluster 68
1	WAS	50	immune	0	21
2	IL2RG	37	immune	0	23
3	CD4	37	immune	0	9
4	CD48	36	immune	0	13
5	PTPRC	35	immune	0	15
6	PTPN7	35	immune	0	12
7	HCLS1	35	immune	0	12
8	CORO1A	35	immune	0	11
9	CD37	34	immune	0	8
10	PLCB2	33	immune	0	12
11	RHOH	32	immune	0	16
12	LILRA2	31	immune	0	4
13	LRMP	29	immune	0	9
14	RNASE6	28	immune	0	3
15	NCF4	28	immune	0	0
16	CD3D	28	immune	0	20
17	CSF3R	27	immune	0	4
18	CYBB	26	immune	0	0
19	SIT1	23	immune	0	19
20	DOCK2	23	immune	0	9
21	CD1D	23	immune	0	14
22	MYH9	21	angiogenesis	7	0
23	CD2	21	immune	0	15
24	SERPINE1	20	angiogenesis	4	0
25	LOXL2	20	angiogenesis	4	0
26	CYR61	20	angiogenesis	6	0
27	SH2D1A	19	immune	0	14
28	GNA15	19	immune	0	10
29	COL5A1	19	extracellular matrix	2	0
30	BTK	19	immune	0	2
31	LY86	18	immune	0	0
32	LOX	18	angiogenesis	2	0
33	FLNA	18	cell-cell junciton	6	0
34	CD52	18	immune	0	13
35	S100A8	17	immune	0	2
36	RNASE3	17	immune	0	2
37	LGALS9	17	immune	0	8
38	CSF2RB	17	immune	0	0
39	CD5	17	immune	0	13
40	TNFRSF12A	16	chemotaxis	5	0
41	ST8SIA4	16	tyrosine phosphorylation	0	5
42	PLK4	16	mitosis	0	0
43	OAS2	16	immune	0	0
44	MCM3	16	DNA repair	0	0
45	LSP1	16	immune	0	2
46	ITGB2	16	immune	0	10
47	CD96	16	immune	0	14
48	CD7	16	immune	0	11
49	ATP2A3	16	immune	0	12
50	COL1A2	15	extracellular matrix	0	0
51	CCR7	15	immune	0	13
52	ADAM12	15	extracellular matrix	1	0
53	MED6	14	NA	0	0
54	LGALS1	14	NA	0	0
55	LCK	14	immune	0	10
56	FOSL2	14	angiogenesis	4	0
57	DSE	14	immune	4	0
58	COL6A3	14	extracellular matrix	0	0
59	COL1A1	14	extracellular matrix	1	0
60	CLCF1	14	tyrosine phosphorylation	7	0
61	ANXA2	14	NA	3	0
62	PTPN22	13	NA	0	4
63	PLAUR	13	immune	1	0
64	LTB	13	immune	0	11
65	CTSW	13	immune	0	13
66	IRAK3	12	immune	0	0
67	GRB7	12	NA	0	0
68	CTCF	12	chromatin assembly	0	0

The designated gene (G) appearing in the gene column is the representative of a group of genes that correlate strongly with G.

“NA” indicates that the gene set did not map to any statistically significant GO categories.

The complete High-Throughput GoMiner (HTGM) download is provided in file Download S1. The files in the subdirectory work2026406846/inputFileDir are named according to each gene G. Each such file contains the complete list of genes correlating with G.

### R Language

R language code [10] was developed to read and integrate the data in the two downloaded files, as well as to provide support for both basic and more complex queries [*e.g.*, automatically find sets of genes meeting certain conditions with respect to both NCI-60 and GBM and then generate a relevant series of expression or correlation clustered image maps (CIMs)]. Historically, CIMs were first introduced in [11], [12].

### Studies Predicated on Pre-existing Correlations Across the NCI-60

The key question we addressed here was whether genes that co-clustered with respect to their expression profiles across the NCI-60 cells also co-clustered with respect to their expression profiles across the GBM samples. To facilitate that analysis, we took advantage of the R language function *cutree()*. A key parameter in *cutree()* is “k” the number of clusters into which the cluster tree is to be divided. In the cluster 52 and cluster 68 studies (*i.e.,* sets of genes reported in [2]), preliminary studies showed that k = 2 was optimal for NCI-60 expression clusters. Each such gene set had been derived from a clustering study using an absolute correlation metric, and therefore had two major partitionings (*e.g.*, Figures 1A, S1). The two partitionings are designated as “cluster 1” and “cluster 2,” and are delineated by the number appended to each gene name on the right of the CIM. The genes within a single partitioning are mutually positively-correlated, and all genes in cluster 1 are negatively-correlated with all genes in cluster 2. We colloquially refer to the larger cluster (in the case of Figures 1A, S1, this would be cluster 2) as the “positively-correlated” genes and the smaller cluster as the “negatively-correlated” genes. In contrast to k = 2 for NCI-60, there was no *a priori* basis for selecting a particular value of k for the clustering across GBM, so we allowed k for GBM to range from 2 through 8.

To determine the optimal value of k, we constructed a 2×k contingency table (*e.g.,* Table 1), each cell_i,j_ of which contains the number of genes that are both in the i^th^ cluster of the NCI-60 clustering and the j^th^ cluster of the GBM clustering. We computed a Fisher’s exact p-value for the null hypothesis that a distribution as extreme as the observed distribution could have occurred by chance. In addition, we randomized the gene names between performing the NCI-60 and GBM clusterings, to determine if the observed Fisher’s exact p-value could be achieved for a random gene set.

### De novo Identification of Sets of Genes with Correlation ≥0.60 Across both NCI-60 and GBM

Without reference to any prior clustering analysis, the program constructed *de novo* a list of all pairs of genes having correlation ≥0.60 with respect to both NCI-60 and GBM expression profiles. The threshold of 0.60 was chosen for the calculations because it had been used in an earlier study of gene-gene correlations to minimize the number of false positives. Genes were ranked with respect to frequency of appearance in that list. Each gene “G” with frequency ≥5 was then used to “represent” the set of genes that showed correlation ≥0.60 with G. The top-ranking G gene was WAS (49 genes had correlation ≥0.60 with WAS). Many of the gene lists constructed by that method were highly redundant with respect to one another (*i.e.*, pairs of lists may have many gene in common). To alleviate the redundancy problem, we computed the Jaccard similarity metric (the Jaccard coefficient measures similarity between sample sets, and is defined as the size of the intersection divided by the size of the union of the sample sets [13]) we eliminated highly redundant (Jaccard value ≥0.90; 0.90 was determined to be optimal in preliminary studies not shown here) gene sets from further analysis. Thus, we used a less-redundant set of 68 gene sets (from an initial selection of the top (possibly redundant) 100 gene sets) for the analysis.

We wished to determine if the number of pairs of genes having correlation ≥0.60 with respect to both NCI-60 and GBM expression profiles exceeded the number expected by chance. We therefore performed a set of 10 studies in which we randomized the gene names in the GBM expression profiles. The number of such pairs obtained in the real study was 2708. In contrast, the number in the randomization studies was small in comparison (193±14).

### Functional Categorization

Functional categorization of gene lists was performed using the High-Throughput GoMiner (HTGM) program [14]. The parameters used in running HTGM are tabulated in Table S1.

### Clustered Image Maps

We used either the Genesis clustering program [15] or our own in-house R language code to construct CIMs presented here.

## Results and Discussion

### Studies Predicated on Existing Correlations Across the NCI-60

We recently clustered genes based on correlation of expression profiles across the NCI-60 [2]. Many of those clusters were characterized by cancer-associated biological functions.

Using the expression profiles for the cluster 52 genes across the NCI-60 cell lines and also across the GBM samples, we were able to generate expression correlation CIMs across both of those sets of expression profiles (Figures 1A, S1, 1B, S2). The distinct patterns of red and green in the NCI-60 correlation CIM (Figures 1A, S1) results from the fact that cluster 52 had been derived by clustering the expression profiles in the NCI-60 cell lines using an absolute correlation metric. Thus, cluster 52 is composed of “negatively” and “positively” correlated subgroups. Not surprisingly, the patterns of red and green are less distinct in the GBM correlation CIM (Figures 1B, S2), since cluster 52 had been defined relative to NCI-60, not GBM, expression patterns. Although less distinct than for NCI-60, the GBM pattern is highly correlated with the pattern for NCI-60. That relationship is obvious by visual inspection. The quantitative analysis below confirms the visual impression.

In the correlation CIMs, we appended a number (1 or 2) to the gene names, corresponding to membership in the two major clusters in the NCI-60 CIM. Those same numbers were retained in the gene names for the GBM CIM to allow identification of the cluster to which that gene belonged in the NCI-60 CIM. The pattern of clustering in the GBM correlation CIM (Figures 1B, S2) is markedly similar to that in the NCI-60 CIM. That observation shows that some gene co-expression patterns in the NCI-60 human tumor cell line panel are preserved in clinical glioblastoma, and supports our conjecture that NCI-60 gene expression correlations can indicate widely applicable gene-gene relationships.

More precisely, Table 1 shows that there are 15 genes in cluster 1 and 64 genes in cluster 2, relative to the NCI-60 expression profile. Thirty-four of the 64 cluster 2 genes are the predominant members of GBM cluster 2. The remaining NCI-60 cluster 2 genes are distributed across GBM clusters 1 and 3. The concordance between the clustering patterns in NCI-60 and GBM is highly significant (Table 2). The Fisher’s exact p-value for k = 3 (0.00039) is strikingly lower than for the randomized controls (0.46±0.28). Furthermore, the large majority of the genes that were mutually correlated or anti-correlated in the NCI-60 preserved that relationship in the GBM tissue samples. The identities of the relevant genes are shown in Table 3.

A notable finding is that nearly half of the genes in GBM cluster 2 (Figures 1B, S2) are genes that were previously found to be involved in cell adhesion/migration and to form a mutually-high correlation subset of the cluster 52 genes [16]. Moreover, those genes were found to function coherently in a particular aspect of the cell migration process. With the exception of ALCAM and EGFR, the cell adhesion/migration tight cluster genes fall within GBM cluster 2. Sixteen of twenty-four genes of that tight cluster fall into GBM cluster 2. Thus, a set of genes previously found to be closely related in both gene expression and function in the NCI-60 cell lines [2], [16] are now found to be co-expressed also in clinical glioblastoma samples.

To investigate other potential examples of coherence between gene expression clusters in NCI-60 cell lines and GBM samples, we repeated that analysis with the immune system-related cluster 68 genes [2] (Tables 4–6; Figures 2A, S3, 2B, S4). Again, the Fisher’s exact p-value (0.00001) (Table 5) validates the visual impression that there is a significant concordance between the NCI-60 and the GBM clustering.

### De novo Identification of Sets of Genes with Correlation ≥0.60 Across both NCI-60 and TCGA GBM

There were 34,865 gene pairs with correlation ≥0.60 in the NCI-60 data set but not in GBM, 87,556 in GBM but not in the NCI-60, and 2,708 in both the NCI-60 and GBM. The highest-ranking gene of the 2,708 was WAS; 49 genes showed correlation ≥0.60 with WAS. Of the top 100 genes (*i.e.*, genes with the highest number of correlations ≥0.60), 68 were non-redundant (*i.e.*, the lists of correlating genes had Jaccard value ≤0.90). Functional categorization of those 68 gene lists by High-Throughput GoMiner (HTGM) revealed a complex set of significant categories (Figures 3, S5). The number of genes and the generalized functional correlations for the top 68 non-redundant gene sets are listed in Table 7. As is evident from Table 7, immune categories dominated, but Table 7 and Figure S5 reveal that there were also categories representing *e.g.* apoptosis, chemotaxis, DNA repair, chromatin assembly, angiogenesis, and adhesion.

The genes in cluster 52 or cluster 68 had been obtained by prior clustering of the gene expression profiles across NCI-60 cell lines, but not across TCGA GBM samples. We expect to find that some of the *do novo* gene lists derived from simultaneous consideration of both NCI-60 cell lines and TCGA GBM samples might overlap with genes in the cluster 52 or cluster 68 gene lists. In fact, Table 7 shows that the genes in certain of the *de novo* gene lists overlapped with the genes in NCI-60 clusters 52 (cell migration) and 68 (immune). Such overlap is particularly strong for cluster 68.

This analysis shows ways in which strong gene-gene correlations and functional categorization (*ie.,* GO assignments) obtained for the NCI-60 cell lines across tumor types can reflect *in vivo* relationships. It also shows the limitations of such similarity. The two types of sample sets represent major initiatives of the National Cancer Institute (NCI), in terms of both expense and research investment. Hence, a delineation of the similarities and differences remains a subject of considerable practical importance.

## Supporting Information

Figure S1 Full version of Figure 1A.
(PDF)

Figure S2 Full version of Figure 1B.
(TIF)

Figure S3 Full version of Figure 2A.
(PDF)

Figure S4 Full version of Figure 2B.
(PDF)

Figure S5 HTGM GO categories *versus* gene set CIM for sets of genes with correlation ≥ 0.60 simultaneously in both NCI-60 and TCGA GBM.
(PNG)

Table S1 The parameters used in running HTGM.
(JPG)

Download S1 Zip archive of HTGM results.
(ZIP)
