# Supplementary figures and images for: Concordance of Gene Expression and Functional Correlation Patterns across the NCI-60 Cell Lines and the Cancer Genome Atlas Glioblastoma Samples
Source: PLoS One. 2012 Jul 26;7(7):e40062. doi: 10.1371/journal.pone.0040062 (PMC3406063; doi:10.1371/journal.pone.0040062)

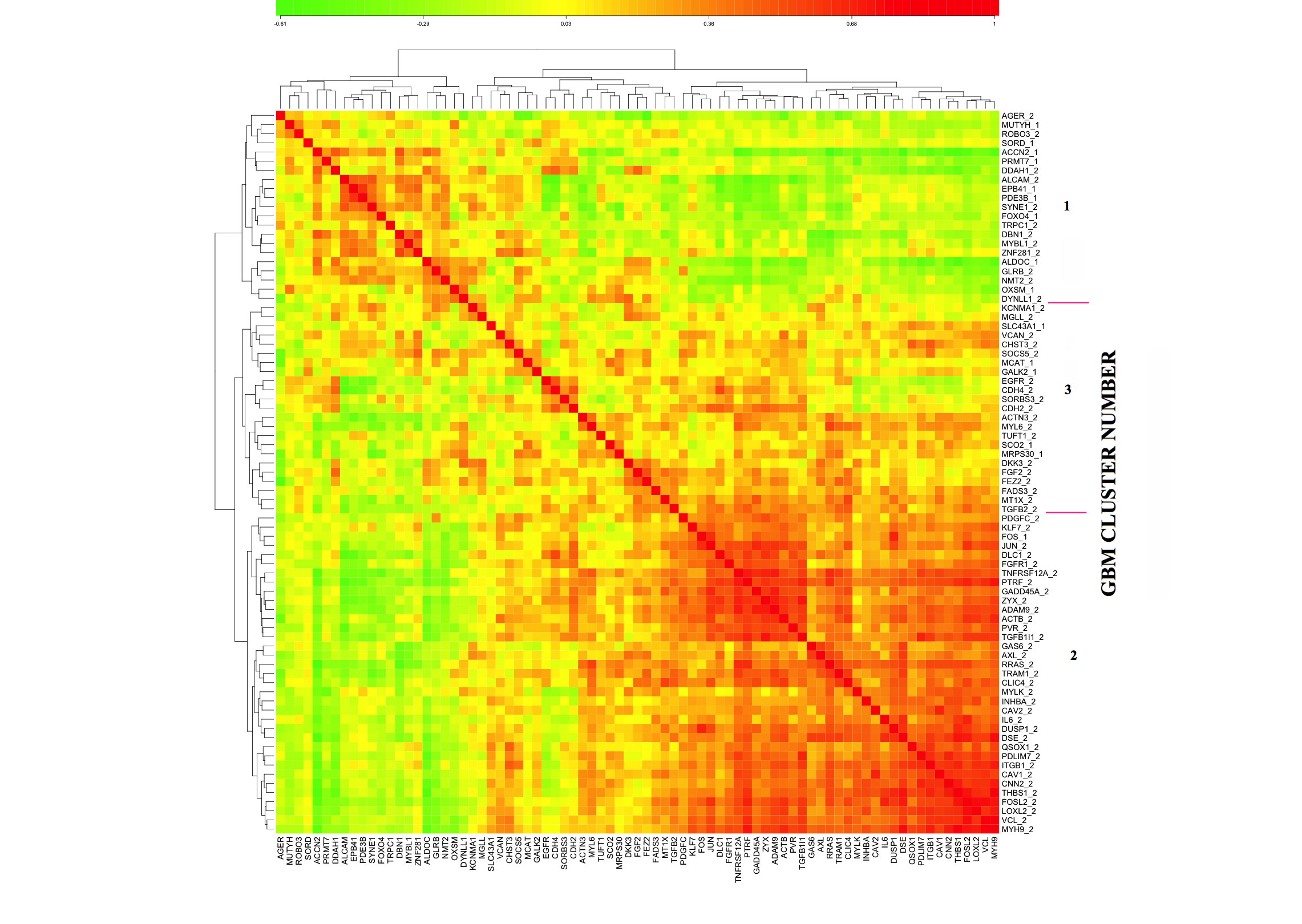

Supplement: Figure S2 — Full version of Figure 1B. (TIF) [file pone.0040062.s002.tiff]

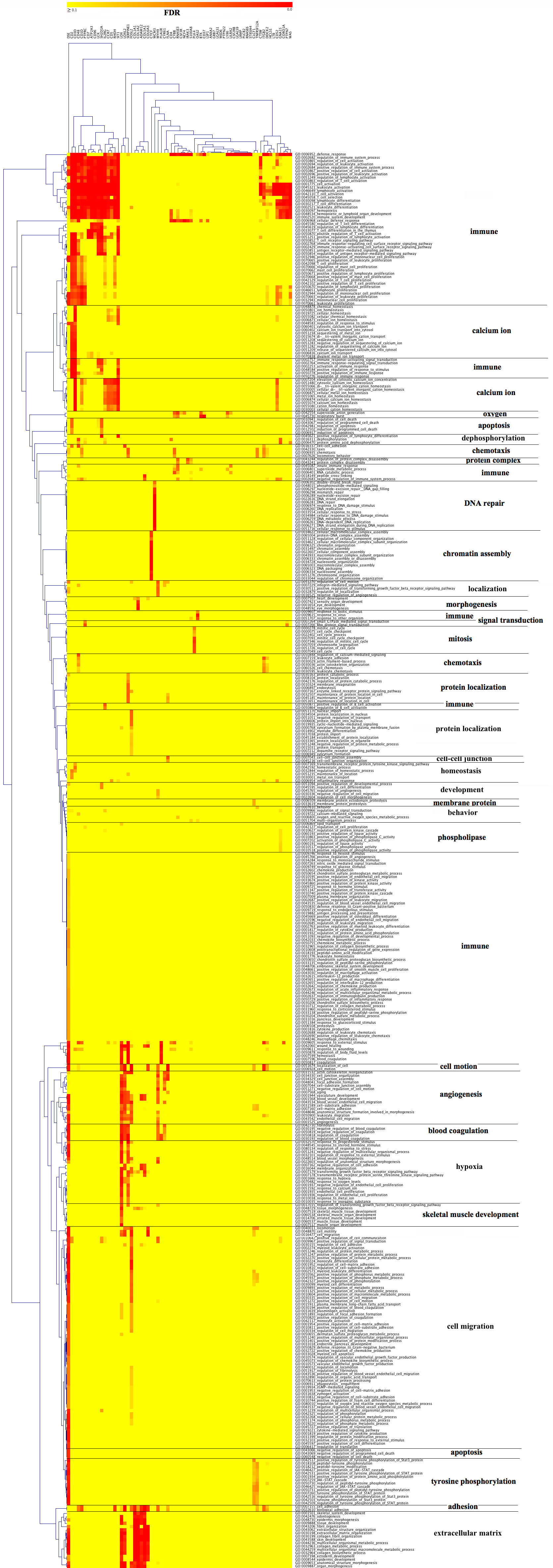

Supplement: Figure S5 — HTGM GO categories versus gene set CIM for sets of genes with correlation ≥ 0.60 simultaneously in both NCI-60 and TCGA GBM. (PNG) [file pone.0040062.s005.png]

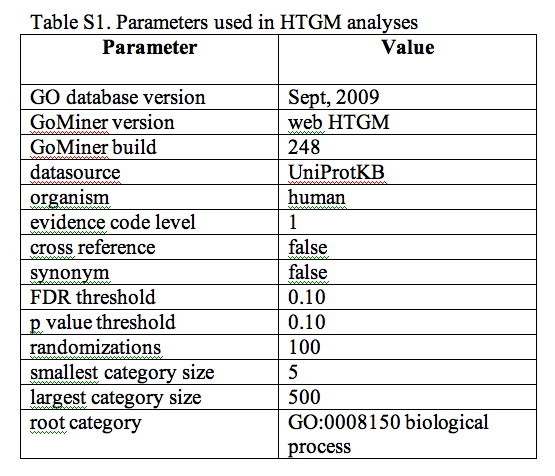

Supplement: Table S1 — The parameters used in running HTGM. (JPG) [file pone.0040062.s006.jpg]

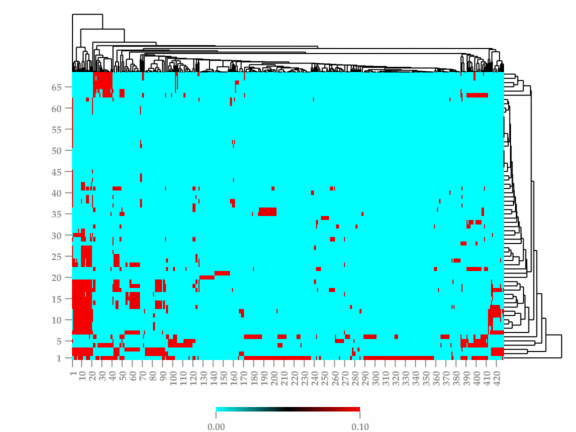

Supplement: Download S1 — Zip archive of HTGM results. (ZIP) [file pone.0040062.s007.zip › work2026406846/Generated_Total2026406846.dir/Generated_Total.change.series.CIM.1.dir/cgi_user_matrix.gif]

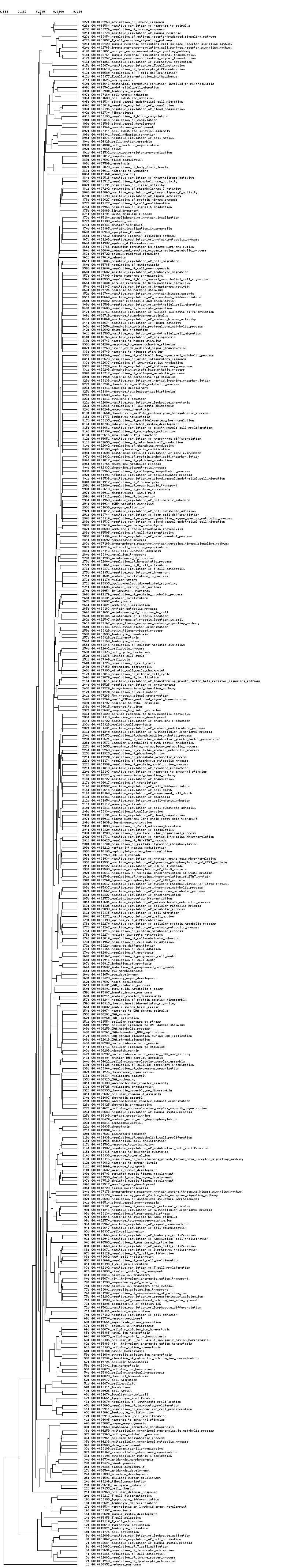

Supplement: Download S1 — Zip archive of HTGM results. (ZIP) [file pone.0040062.s007.zip › work2026406846/Generated_Total2026406846.dir/Generated_Total.change.series.CIM.1.dir/xplclust.gif]

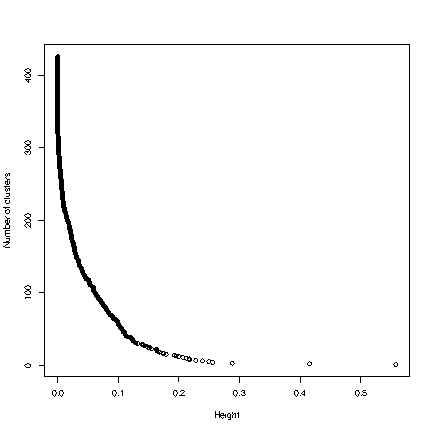

Supplement: Download S1 — Zip archive of HTGM results. (ZIP) [file pone.0040062.s007.zip › work2026406846/Generated_Total2026406846.dir/Generated_Total.change.series.CIM.1.dir/xplot.png]

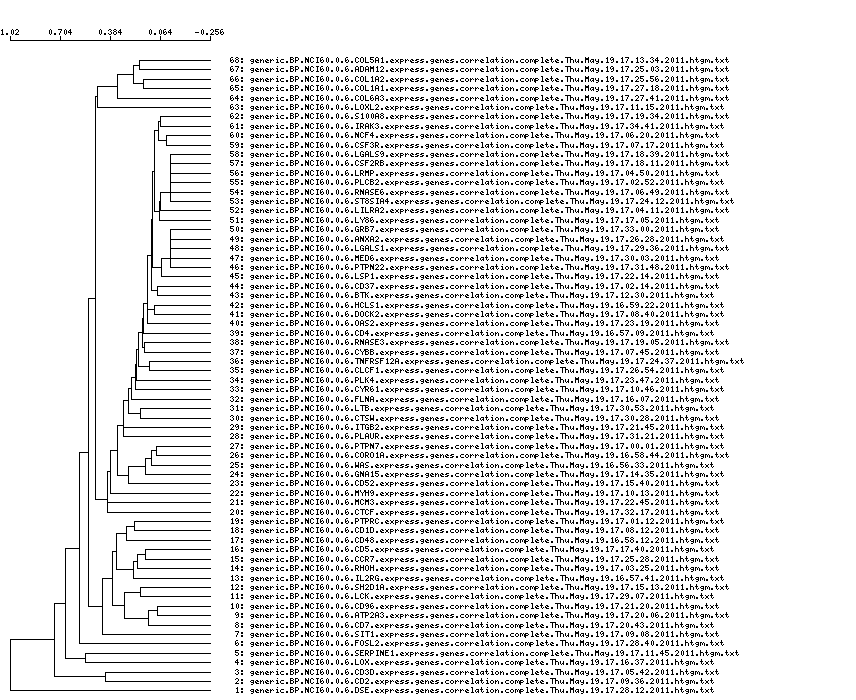

Supplement: Download S1 — Zip archive of HTGM results. (ZIP) [file pone.0040062.s007.zip › work2026406846/Generated_Total2026406846.dir/Generated_Total.change.series.CIM.1.dir/yplclust.gif]

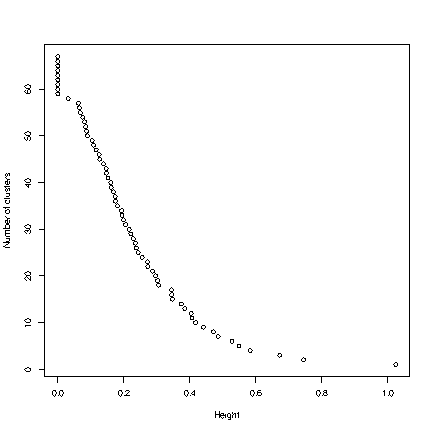

Supplement: Download S1 — Zip archive of HTGM results. (ZIP) [file pone.0040062.s007.zip › work2026406846/Generated_Total2026406846.dir/Generated_Total.change.series.CIM.1.dir/yplot.png]

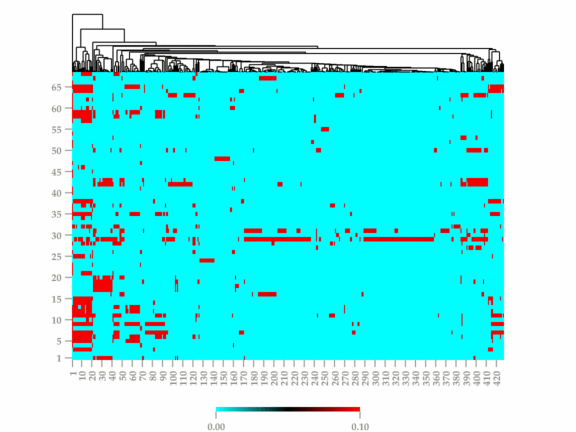

Supplement: Download S1 — Zip archive of HTGM results. (ZIP) [file pone.0040062.s007.zip › work2026406846/Generated_Total2026406846.dir/Generated_Total.change.series.CIM.2.dir/cgi_user_matrix.gif]

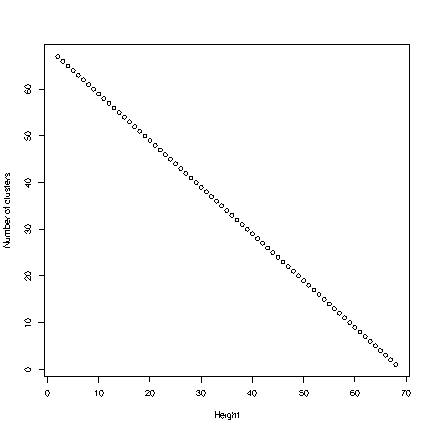

Supplement: Download S1 — Zip archive of HTGM results. (ZIP) [file pone.0040062.s007.zip › work2026406846/Generated_Total2026406846.dir/Generated_Total.change.series.CIM.2.dir/yplot.png]

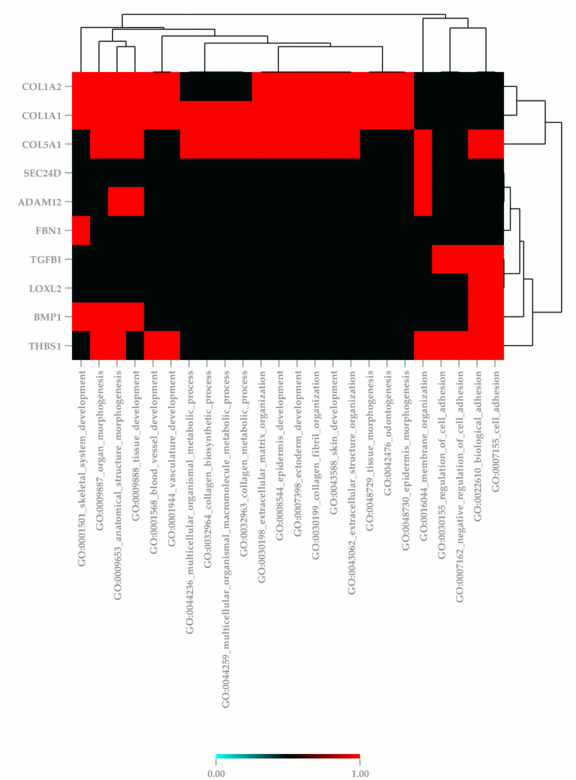

Supplement: Download S1 — Zip archive of HTGM results. (ZIP) [file pone.0040062.s007.zip › work2026406846/Generated_Total2026406846.dir/generic.BP.NCI60.0.6.ADAM12.express.genes.correlation.complete.Thu.May.19.17.25.03.2011.htgm.txt.dir/generic.BP.NCI60.0.6.ADAM12.express.genes.correlation.complete.Thu.May.19.17.25.03.2011.htgm.txt.change.gce.CIM.dir/cgi_user_matrix.gif]

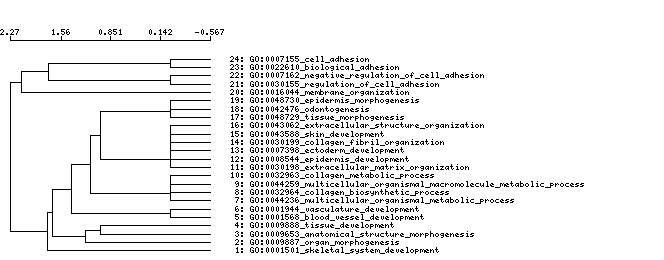

Supplement: Download S1 — Zip archive of HTGM results. (ZIP) [file pone.0040062.s007.zip › work2026406846/Generated_Total2026406846.dir/generic.BP.NCI60.0.6.ADAM12.express.genes.correlation.complete.Thu.May.19.17.25.03.2011.htgm.txt.dir/generic.BP.NCI60.0.6.ADAM12.express.genes.correlation.complete.Thu.May.19.17.25.03.2011.htgm.txt.change.gce.CIM.dir/xplclust.gif]

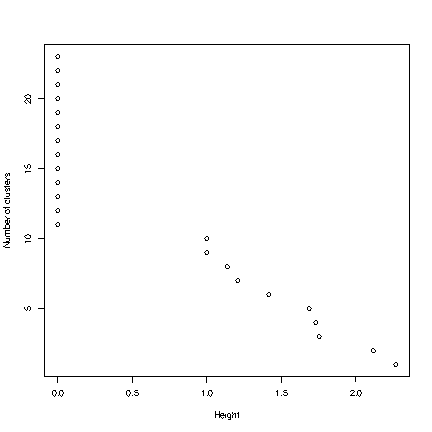

Supplement: Download S1 — Zip archive of HTGM results. (ZIP) [file pone.0040062.s007.zip › work2026406846/Generated_Total2026406846.dir/generic.BP.NCI60.0.6.ADAM12.express.genes.correlation.complete.Thu.May.19.17.25.03.2011.htgm.txt.dir/generic.BP.NCI60.0.6.ADAM12.express.genes.correlation.complete.Thu.May.19.17.25.03.2011.htgm.txt.change.gce.CIM.dir/xplot.png]

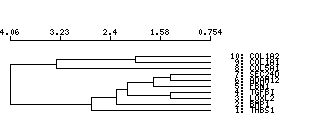

Supplement: Download S1 — Zip archive of HTGM results. (ZIP) [file pone.0040062.s007.zip › work2026406846/Generated_Total2026406846.dir/generic.BP.NCI60.0.6.ADAM12.express.genes.correlation.complete.Thu.May.19.17.25.03.2011.htgm.txt.dir/generic.BP.NCI60.0.6.ADAM12.express.genes.correlation.complete.Thu.May.19.17.25.03.2011.htgm.txt.change.gce.CIM.dir/yplclust.gif]

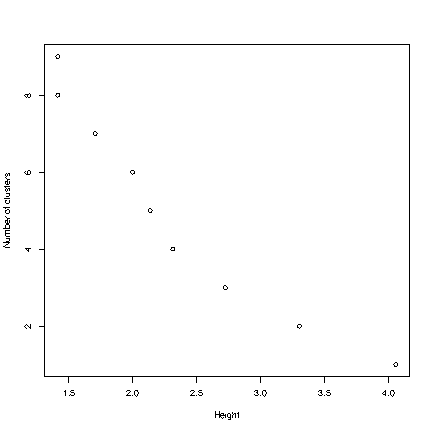

Supplement: Download S1 — Zip archive of HTGM results. (ZIP) [file pone.0040062.s007.zip › work2026406846/Generated_Total2026406846.dir/generic.BP.NCI60.0.6.ADAM12.express.genes.correlation.complete.Thu.May.19.17.25.03.2011.htgm.txt.dir/generic.BP.NCI60.0.6.ADAM12.express.genes.correlation.complete.Thu.May.19.17.25.03.2011.htgm.txt.change.gce.CIM.dir/yplot.png]

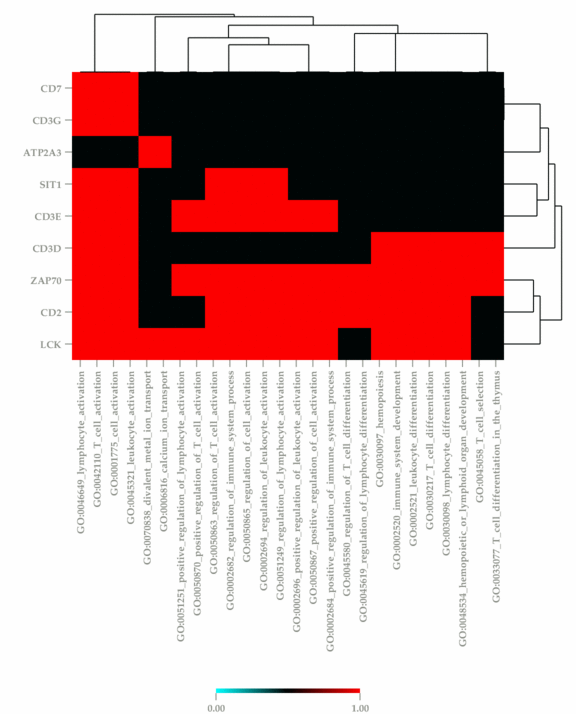

Supplement: Download S1 — Zip archive of HTGM results. (ZIP) [file pone.0040062.s007.zip › work2026406846/Generated_Total2026406846.dir/generic.BP.NCI60.0.6.ATP2A3.express.genes.correlation.complete.Thu.May.19.17.20.06.2011.htgm.txt.dir/generic.BP.NCI60.0.6.ATP2A3.express.genes.correlation.complete.Thu.May.19.17.20.06.2011.htgm.txt.change.gce.CIM.dir/cgi_user_matrix.gif]

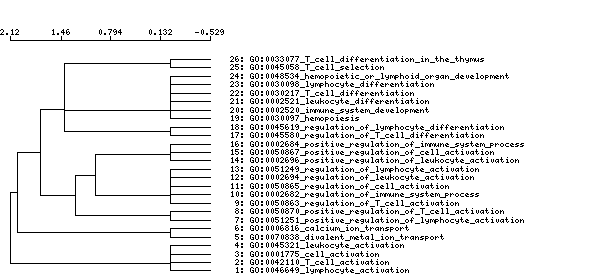

Supplement: Download S1 — Zip archive of HTGM results. (ZIP) [file pone.0040062.s007.zip › work2026406846/Generated_Total2026406846.dir/generic.BP.NCI60.0.6.ATP2A3.express.genes.correlation.complete.Thu.May.19.17.20.06.2011.htgm.txt.dir/generic.BP.NCI60.0.6.ATP2A3.express.genes.correlation.complete.Thu.May.19.17.20.06.2011.htgm.txt.change.gce.CIM.dir/xplclust.gif]

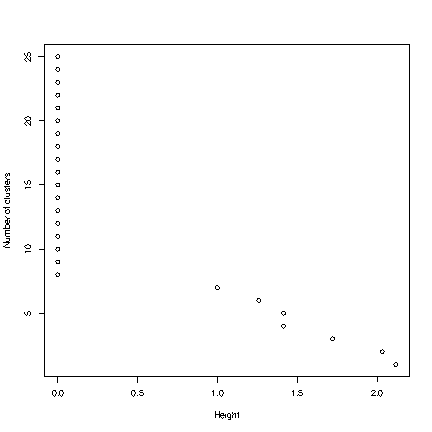

Supplement: Download S1 — Zip archive of HTGM results. (ZIP) [file pone.0040062.s007.zip › work2026406846/Generated_Total2026406846.dir/generic.BP.NCI60.0.6.ATP2A3.express.genes.correlation.complete.Thu.May.19.17.20.06.2011.htgm.txt.dir/generic.BP.NCI60.0.6.ATP2A3.express.genes.correlation.complete.Thu.May.19.17.20.06.2011.htgm.txt.change.gce.CIM.dir/xplot.png]

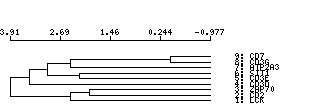

Supplement: Download S1 — Zip archive of HTGM results. (ZIP) [file pone.0040062.s007.zip › work2026406846/Generated_Total2026406846.dir/generic.BP.NCI60.0.6.ATP2A3.express.genes.correlation.complete.Thu.May.19.17.20.06.2011.htgm.txt.dir/generic.BP.NCI60.0.6.ATP2A3.express.genes.correlation.complete.Thu.May.19.17.20.06.2011.htgm.txt.change.gce.CIM.dir/yplclust.gif]

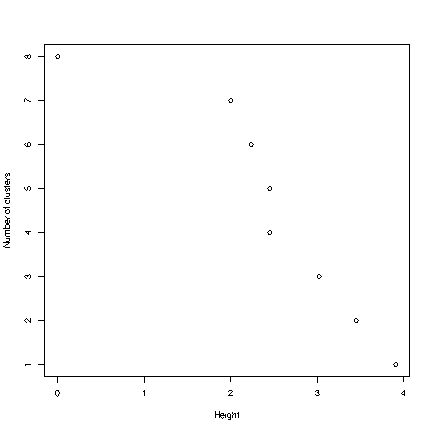

Supplement: Download S1 — Zip archive of HTGM results. (ZIP) [file pone.0040062.s007.zip › work2026406846/Generated_Total2026406846.dir/generic.BP.NCI60.0.6.ATP2A3.express.genes.correlation.complete.Thu.May.19.17.20.06.2011.htgm.txt.dir/generic.BP.NCI60.0.6.ATP2A3.express.genes.correlation.complete.Thu.May.19.17.20.06.2011.htgm.txt.change.gce.CIM.dir/yplot.png]

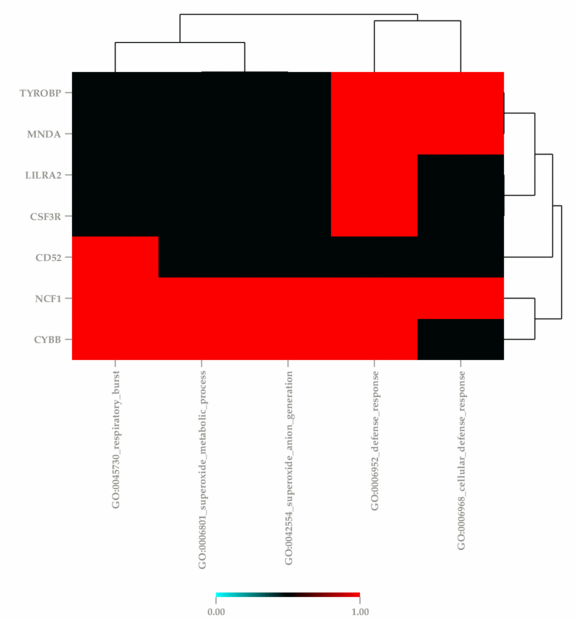

Supplement: Download S1 — Zip archive of HTGM results. (ZIP) [file pone.0040062.s007.zip › work2026406846/Generated_Total2026406846.dir/generic.BP.NCI60.0.6.BTK.express.genes.correlation.complete.Thu.May.19.17.12.30.2011.htgm.txt.dir/generic.BP.NCI60.0.6.BTK.express.genes.correlation.complete.Thu.May.19.17.12.30.2011.htgm.txt.change.gce.CIM.dir/cgi_user_matrix.gif]

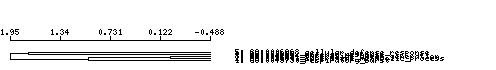

Supplement: Download S1 — Zip archive of HTGM results. (ZIP) [file pone.0040062.s007.zip › work2026406846/Generated_Total2026406846.dir/generic.BP.NCI60.0.6.BTK.express.genes.correlation.complete.Thu.May.19.17.12.30.2011.htgm.txt.dir/generic.BP.NCI60.0.6.BTK.express.genes.correlation.complete.Thu.May.19.17.12.30.2011.htgm.txt.change.gce.CIM.dir/xplclust.gif]

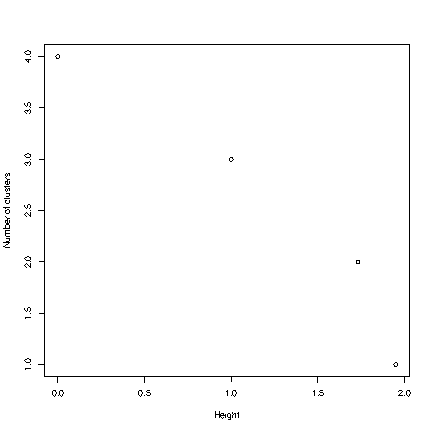

Supplement: Download S1 — Zip archive of HTGM results. (ZIP) [file pone.0040062.s007.zip › work2026406846/Generated_Total2026406846.dir/generic.BP.NCI60.0.6.BTK.express.genes.correlation.complete.Thu.May.19.17.12.30.2011.htgm.txt.dir/generic.BP.NCI60.0.6.BTK.express.genes.correlation.complete.Thu.May.19.17.12.30.2011.htgm.txt.change.gce.CIM.dir/xplot.png]

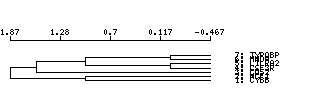

Supplement: Download S1 — Zip archive of HTGM results. (ZIP) [file pone.0040062.s007.zip › work2026406846/Generated_Total2026406846.dir/generic.BP.NCI60.0.6.BTK.express.genes.correlation.complete.Thu.May.19.17.12.30.2011.htgm.txt.dir/generic.BP.NCI60.0.6.BTK.express.genes.correlation.complete.Thu.May.19.17.12.30.2011.htgm.txt.change.gce.CIM.dir/yplclust.gif]

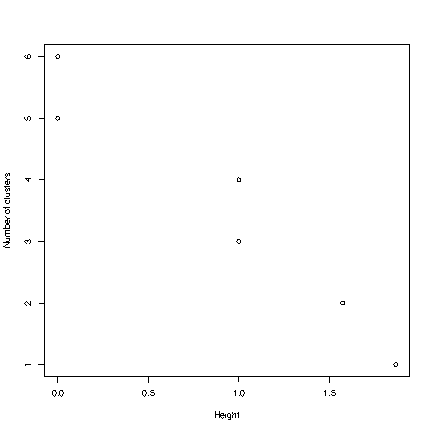

Supplement: Download S1 — Zip archive of HTGM results. (ZIP) [file pone.0040062.s007.zip › work2026406846/Generated_Total2026406846.dir/generic.BP.NCI60.0.6.BTK.express.genes.correlation.complete.Thu.May.19.17.12.30.2011.htgm.txt.dir/generic.BP.NCI60.0.6.BTK.express.genes.correlation.complete.Thu.May.19.17.12.30.2011.htgm.txt.change.gce.CIM.dir/yplot.png]

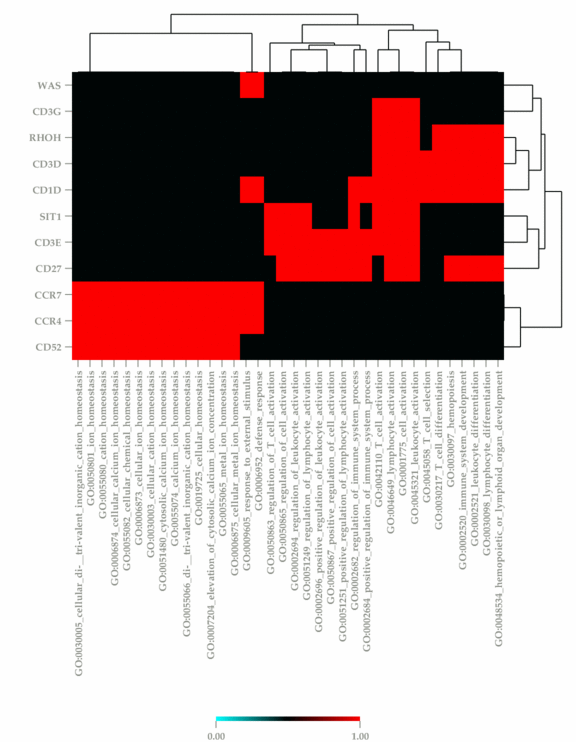

Supplement: Download S1 — Zip archive of HTGM results. (ZIP) [file pone.0040062.s007.zip › work2026406846/Generated_Total2026406846.dir/generic.BP.NCI60.0.6.CCR7.express.genes.correlation.complete.Thu.May.19.17.25.28.2011.htgm.txt.dir/generic.BP.NCI60.0.6.CCR7.express.genes.correlation.complete.Thu.May.19.17.25.28.2011.htgm.txt.change.gce.CIM.dir/cgi_user_matrix.gif]

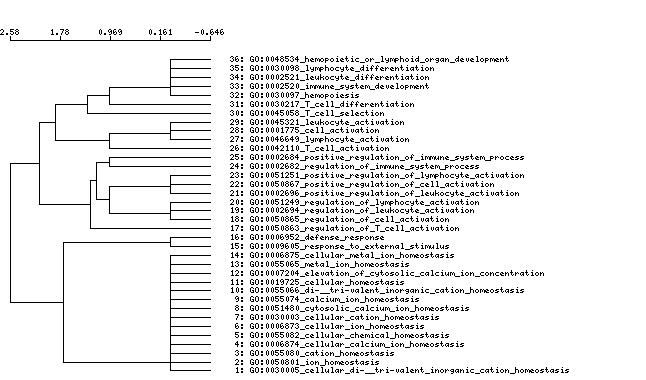

Supplement: Download S1 — Zip archive of HTGM results. (ZIP) [file pone.0040062.s007.zip › work2026406846/Generated_Total2026406846.dir/generic.BP.NCI60.0.6.CCR7.express.genes.correlation.complete.Thu.May.19.17.25.28.2011.htgm.txt.dir/generic.BP.NCI60.0.6.CCR7.express.genes.correlation.complete.Thu.May.19.17.25.28.2011.htgm.txt.change.gce.CIM.dir/xplclust.gif]

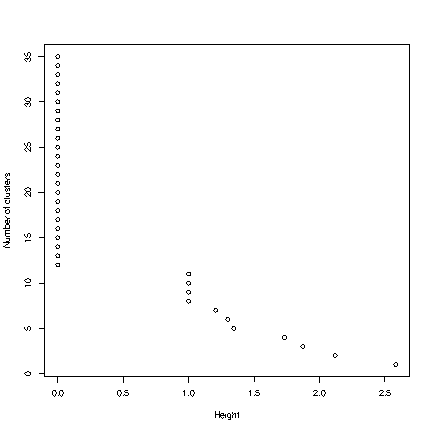

Supplement: Download S1 — Zip archive of HTGM results. (ZIP) [file pone.0040062.s007.zip › work2026406846/Generated_Total2026406846.dir/generic.BP.NCI60.0.6.CCR7.express.genes.correlation.complete.Thu.May.19.17.25.28.2011.htgm.txt.dir/generic.BP.NCI60.0.6.CCR7.express.genes.correlation.complete.Thu.May.19.17.25.28.2011.htgm.txt.change.gce.CIM.dir/xplot.png]

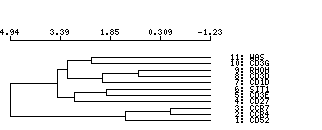

Supplement: Download S1 — Zip archive of HTGM results. (ZIP) [file pone.0040062.s007.zip › work2026406846/Generated_Total2026406846.dir/generic.BP.NCI60.0.6.CCR7.express.genes.correlation.complete.Thu.May.19.17.25.28.2011.htgm.txt.dir/generic.BP.NCI60.0.6.CCR7.express.genes.correlation.complete.Thu.May.19.17.25.28.2011.htgm.txt.change.gce.CIM.dir/yplclust.gif]

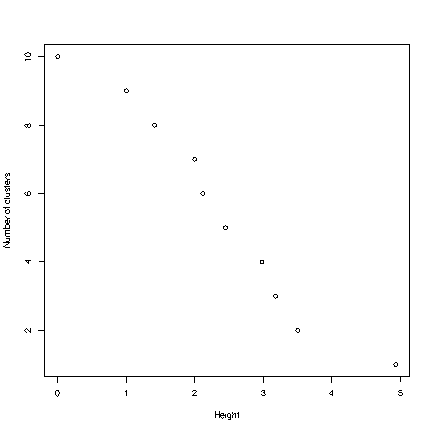

Supplement: Download S1 — Zip archive of HTGM results. (ZIP) [file pone.0040062.s007.zip › work2026406846/Generated_Total2026406846.dir/generic.BP.NCI60.0.6.CCR7.express.genes.correlation.complete.Thu.May.19.17.25.28.2011.htgm.txt.dir/generic.BP.NCI60.0.6.CCR7.express.genes.correlation.complete.Thu.May.19.17.25.28.2011.htgm.txt.change.gce.CIM.dir/yplot.png]

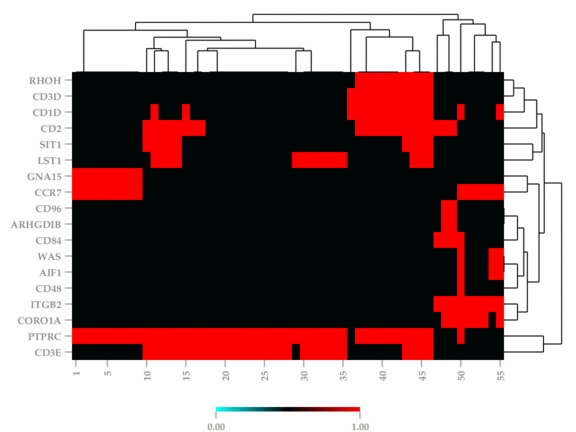

Supplement: Download S1 — Zip archive of HTGM results. (ZIP) [file pone.0040062.s007.zip › work2026406846/Generated_Total2026406846.dir/generic.BP.NCI60.0.6.CD1D.express.genes.correlation.complete.Thu.May.19.17.08.12.2011.htgm.txt.dir/generic.BP.NCI60.0.6.CD1D.express.genes.correlation.complete.Thu.May.19.17.08.12.2011.htgm.txt.change.gce.CIM.dir/cgi_user_matrix.gif]
