# Supplementary material for: Concordance of Gene Expression and Functional Correlation Patterns across the NCI-60 Cell Lines and the Cancer Genome Atlas Glioblastoma Samples
Source: PLoS One. 2012 Jul 26;7(7):e40062. doi: 10.1371/journal.pone.0040062 (PMC3406063; doi:10.1371/journal.pone.0040062)
Supplement: Download S1 — Zip archive of HTGM results. (ZIP) [file pone.0040062.s007.zip › work2026406846/Generated_Total2026406846.dir/Generated_Total.change.series.CIM.1.dir/cgi_user_x.html]

**X-axis Names**   
Cluster is based on euclidean distance  
Cluster method is: average  
plclust  
height plot  

|  |
| --- |
| 1.GO:0006952\_defense\_response |
| 2.GO:0051249\_regulation\_of\_lymphocyte\_activation |
| 3.GO:0002682\_regulation\_of\_immune\_system\_process |
| 4.GO:0050865\_regulation\_of\_cell\_activation |
| 5.GO:0002694\_regulation\_of\_leukocyte\_activation |
| 6.GO:0050863\_regulation\_of\_T\_cell\_activation |
| 7.GO:0002684\_positive\_regulation\_of\_immune\_system\_process |
| 8.GO:0050867\_positive\_regulation\_of\_cell\_activation |
| 9.GO:0002696\_positive\_regulation\_of\_leukocyte\_activation |
| 10.GO:0001775\_cell\_activation |
| 11.GO:0045321\_leukocyte\_activation |
| 12.GO:0046649\_lymphocyte\_activation |
| 13.GO:0042110\_T\_cell\_activation |
| 14.GO:0045058\_T\_cell\_selection |
| 15.GO:0002520\_immune\_system\_development |
| 16.GO:0030097\_hemopoiesis |
| 17.GO:0048534\_hemopoietic\_or\_lymphoid\_organ\_development |
| 18.GO:0002521\_leukocyte\_differentiation |
| 19.GO:0030098\_lymphocyte\_differentiation |
| 20.GO:0030217\_T\_cell\_differentiation |
| 21.GO:0006968\_cellular\_defense\_response |
| 22.GO:0007155\_cell\_adhesion |
| 23.GO:0022610\_biological\_adhesion |
| 24.GO:0043206\_fibril\_organization |
| 25.GO:0001501\_skeletal\_system\_development |
| 26.GO:0007398\_ectoderm\_development |
| 27.GO:0008544\_epidermis\_development |
| 28.GO:0009888\_tissue\_development |
| 29.GO:0042476\_odontogenesis |
| 30.GO:0048730\_epidermis\_morphogenesis |
| 31.GO:0030198\_extracellular\_matrix\_organization |
| 32.GO:0043062\_extracellular\_structure\_organization |
| 33.GO:0030199\_collagen\_fibril\_organization |
| 34.GO:0043588\_skin\_development |
| 35.GO:0044236\_multicellular\_organismal\_metabolic\_process |
| 36.GO:0032964\_collagen\_biosynthetic\_process |
| 37.GO:0032963\_collagen\_metabolic\_process |
| 38.GO:0044259\_multicellular\_organismal\_macromolecule\_metabolic\_process |
| 39.GO:0009653\_anatomical\_structure\_morphogenesis |
| 40.GO:0009887\_organ\_morphogenesis |
| 41.GO:0009605\_response\_to\_external\_stimulus |
| 42.GO:0032943\_mononuclear\_cell\_proliferation |
| 43.GO:0070661\_leukocyte\_proliferation |
| 44.GO:0032944\_regulation\_of\_mononuclear\_cell\_proliferation |
| 45.GO:0070663\_regulation\_of\_leukocyte\_proliferation |
| 46.GO:0050670\_regulation\_of\_lymphocyte\_proliferation |
| 47.GO:0046651\_lymphocyte\_proliferation |
| 48.GO:0051674\_localization\_of\_cell |
| 49.GO:0006928\_cell\_motion |
| 50.GO:0040011\_locomotion |
| 51.GO:0048870\_cell\_motility |
| 52.GO:0016477\_cell\_migration |
| 53.GO:0048878\_chemical\_homeostasis |
| 54.GO:0055082\_cellular\_chemical\_homeostasis |
| 55.GO:0006873\_cellular\_ion\_homeostasis |
| 56.GO:0050801\_ion\_homeostasis |
| 57.GO:0019725\_cellular\_homeostasis |
| 58.GO:0007204\_elevation\_of\_cytosolic\_calcium\_ion\_concentration |
| 59.GO:0051480\_cytosolic\_calcium\_ion\_homeostasis |
| 60.GO:0055080\_cation\_homeostasis |
| 61.GO:0030003\_cellular\_cation\_homeostasis |
| 62.GO:0055066\_di-\_\_tri-valent\_inorganic\_cation\_homeostasis |
| 63.GO:0030005\_cellular\_di-\_\_tri-valent\_inorganic\_cation\_homeostasis |
| 64.GO:0006875\_cellular\_metal\_ion\_homeostasis |
| 65.GO:0055065\_metal\_ion\_homeostasis |
| 66.GO:0006874\_cellular\_calcium\_ion\_homeostasis |
| 67.GO:0055074\_calcium\_ion\_homeostasis |
| 68.GO:0042554\_superoxide\_anion\_generation |
| 69.GO:0045730\_respiratory\_burst |
| 70.GO:0007162\_negative\_regulation\_of\_cell\_adhesion |
| 71.GO:0016044\_membrane\_organization |
| 72.GO:0045621\_positive\_regulation\_of\_lymphocyte\_differentiation |
| 73.GO:0051208\_sequestering\_of\_calcium\_ion |
| 74.GO:0051209\_release\_of\_sequestered\_calcium\_ion\_into\_cytosol |
| 75.GO:0051283\_negative\_regulation\_of\_sequestering\_of\_calcium\_ion |
| 76.GO:0051282\_regulation\_of\_sequestering\_of\_calcium\_ion |
| 77.GO:0060401\_cytosolic\_calcium\_ion\_transport |
| 78.GO:0060402\_calcium\_ion\_transport\_into\_cytosol |
| 79.GO:0051238\_sequestering\_of\_metal\_ion |
| 80.GO:0015674\_di-\_\_tri-valent\_inorganic\_cation\_transport |
| 81.GO:0006816\_calcium\_ion\_transport |
| 82.GO:0070838\_divalent\_metal\_ion\_transport |
| 83.GO:0042102\_positive\_regulation\_of\_T\_cell\_proliferation |
| 84.GO:0042098\_T\_cell\_proliferation |
| 85.GO:0070666\_regulation\_of\_mast\_cell\_proliferation |
| 86.GO:0070662\_mast\_cell\_proliferation |
| 87.GO:0042129\_regulation\_of\_T\_cell\_proliferation |
| 88.GO:0050671\_positive\_regulation\_of\_lymphocyte\_proliferation |
| 89.GO:0070668\_positive\_regulation\_of\_mast\_cell\_proliferation |
| 90.GO:0048583\_regulation\_of\_response\_to\_stimulus |
| 91.GO:0032946\_positive\_regulation\_of\_mononuclear\_cell\_proliferation |
| 92.GO:0070665\_positive\_regulation\_of\_leukocyte\_proliferation |
| 93.GO:0016337\_cell-cell\_adhesion |
| 94.GO:0010647\_positive\_regulation\_of\_cell\_communication |
| 95.GO:0009967\_positive\_regulation\_of\_signal\_transduction |
| 96.GO:0032570\_response\_to\_progesterone\_stimulus |
| 97.GO:0048545\_response\_to\_steroid\_hormone\_stimulus |
| 98.GO:0080134\_regulation\_of\_response\_to\_stress |
| 99.GO:0051241\_negative\_regulation\_of\_multicellular\_organismal\_process |
| 100.GO:0032101\_regulation\_of\_response\_to\_external\_stimulus |
| 101.GO:0048514\_blood\_vessel\_morphogenesis |
| 102.GO:0022603\_regulation\_of\_anatomical\_structure\_morphogenesis |
| 103.GO:0007179\_transforming\_growth\_factor\_beta\_receptor\_signaling\_pathway |
| 104.GO:0007178\_transmembrane\_receptor\_protein\_serine\_threonine\_kinase\_signaling\_pathway |
| 105.GO:0048729\_tissue\_morphogenesis |
| 106.GO:0007517\_muscle\_organ\_development |
| 107.GO:0007519\_skeletal\_muscle\_tissue\_development |
| 108.GO:0060538\_skeletal\_muscle\_organ\_development |
| 109.GO:0014706\_striated\_muscle\_tissue\_development |
| 110.GO:0060537\_muscle\_tissue\_development |
| 111.GO:0001666\_response\_to\_hypoxia |
| 112.GO:0070482\_response\_to\_oxygen\_levels |
| 113.GO:0017015\_regulation\_of\_transforming\_growth\_factor\_beta\_receptor\_signaling\_pathway |
| 114.GO:0010038\_response\_to\_metal\_ion |
| 115.GO:0010035\_response\_to\_inorganic\_substance |
| 116.GO:0001937\_negative\_regulation\_of\_endothelial\_cell\_proliferation |
| 117.GO:0051592\_response\_to\_calcium\_ion |
| 118.GO:0001935\_endothelial\_cell\_proliferation |
| 119.GO:0001936\_regulation\_of\_endothelial\_cell\_proliferation |
| 120.GO:0007626\_locomotory\_behavior |
| 121.GO:0042330\_taxis |
| 122.GO:0006935\_chemotaxis |
| 123.GO:0016311\_dephosphorylation |
| 124.GO:0006470\_protein\_amino\_acid\_dephosphorylation |
| 125.GO:0018149\_peptide\_cross-linking |
| 126.GO:0002683\_negative\_regulation\_of\_immune\_system\_process |
| 127.GO:0034621\_cellular\_macromolecular\_complex\_subunit\_organization |
| 128.GO:0006325\_chromatin\_organization |
| 129.GO:0043933\_macromolecular\_complex\_subunit\_organization |
| 130.GO:0031497\_chromatin\_assembly |
| 131.GO:0022607\_cellular\_component\_assembly |
| 132.GO:0006333\_chromatin\_assembly\_or\_disassembly |
| 133.GO:0034728\_nucleosome\_organization |
| 134.GO:0065003\_macromolecular\_complex\_assembly |
| 135.GO:0006323\_DNA\_packaging |
| 136.GO:0006334\_nucleosome\_assembly |
| 137.GO:0051276\_chromosome\_organization |
| 138.GO:0033044\_regulation\_of\_chromosome\_organization |
| 139.GO:0051128\_regulation\_of\_cellular\_component\_organization |
| 140.GO:0034622\_cellular\_macromolecular\_complex\_assembly |
| 141.GO:0065004\_protein-DNA\_complex\_assembly |
| 142.GO:0006297\_nucleotide-excision\_repair\_\_DNA\_gap\_filling |
| 143.GO:0006298\_mismatch\_repair |
| 144.GO:0051716\_cellular\_response\_to\_stimulus |
| 145.GO:0006289\_nucleotide-excision\_repair |
| 146.GO:0022616\_DNA\_strand\_elongation |
| 147.GO:0006271\_DNA\_strand\_elongation\_during\_DNA\_replication |
| 148.GO:0006261\_DNA-dependent\_DNA\_replication |
| 149.GO:0006259\_DNA\_metabolic\_process |
| 150.GO:0034984\_cellular\_response\_to\_DNA\_damage\_stimulus |
| 151.GO:0033554\_cellular\_response\_to\_stress |
| 152.GO:0006260\_DNA\_replication |
| 153.GO:0006281\_DNA\_repair |
| 154.GO:0006974\_response\_to\_DNA\_damage\_stimulus |
| 155.GO:0006302\_double-strand\_break\_repair |
| 156.GO:0048015\_phosphoinositide-mediated\_signaling |
| 157.GO:0043244\_regulation\_of\_protein\_complex\_disassembly |
| 158.GO:0043241\_protein\_complex\_disassembly |
| 159.GO:0045087\_innate\_immune\_response |
| 160.GO:0006801\_superoxide\_metabolic\_process |
| 161.GO:0006401\_RNA\_catabolic\_process |
| 162.GO:0007507\_heart\_development |
| 163.GO:0007423\_sensory\_organ\_development |
| 164.GO:0001654\_eye\_development |
| 165.GO:0048592\_eye\_morphogenesis |
| 166.GO:0012502\_induction\_of\_programmed\_cell\_death |
| 167.GO:0006917\_induction\_of\_apoptosis |
| 168.GO:0010941\_regulation\_of\_cell\_death |
| 169.GO:0043067\_regulation\_of\_programmed\_cell\_death |
| 170.GO:0042981\_regulation\_of\_apoptosis |
| 171.GO:0030155\_regulation\_of\_cell\_adhesion |
| 172.GO:0030224\_monocyte\_differentiation |
| 173.GO:0001952\_regulation\_of\_cell-matrix\_adhesion |
| 174.GO:0010810\_regulation\_of\_cell-substrate\_adhesion |
| 175.GO:0002274\_myeloid\_leukocyte\_activation |
| 176.GO:0051246\_regulation\_of\_protein\_metabolic\_process |
| 177.GO:0051247\_positive\_regulation\_of\_protein\_metabolic\_process |
| 178.GO:0032270\_positive\_regulation\_of\_cellular\_protein\_metabolic\_process |
| 179.GO:0030099\_myeloid\_cell\_differentiation |
| 180.GO:0051272\_positive\_regulation\_of\_cell\_motion |
| 181.GO:0030335\_positive\_regulation\_of\_cell\_migration |
| 182.GO:0009893\_positive\_regulation\_of\_metabolic\_process |
| 183.GO:0031325\_positive\_regulation\_of\_cellular\_metabolic\_process |
| 184.GO:0010604\_positive\_regulation\_of\_macromolecule\_metabolic\_process |
| 185.GO:0002573\_myeloid\_leukocyte\_differentiation |
| 186.GO:0042327\_positive\_regulation\_of\_phosphorylation |
| 187.GO:0010562\_positive\_regulation\_of\_phosphorus\_metabolic\_process |
| 188.GO:0045937\_positive\_regulation\_of\_phosphate\_metabolic\_process |
| 189.GO:0042517\_positive\_regulation\_of\_tyrosine\_phosphorylation\_of\_Stat3\_protein |
| 190.GO:0007260\_tyrosine\_phosphorylation\_of\_STAT\_protein |
| 191.GO:0042509\_regulation\_of\_tyrosine\_phosphorylation\_of\_STAT\_protein |
| 192.GO:0042516\_regulation\_of\_tyrosine\_phosphorylation\_of\_Stat3\_protein |
| 193.GO:0042503\_tyrosine\_phosphorylation\_of\_Stat3\_protein |
| 194.GO:0046427\_positive\_regulation\_of\_JAK-STAT\_cascade |
| 195.GO:0042531\_positive\_regulation\_of\_tyrosine\_phosphorylation\_of\_STAT\_protein |
| 196.GO:0001934\_positive\_regulation\_of\_protein\_amino\_acid\_phosphorylation |
| 197.GO:0007259\_JAK-STAT\_cascade |
| 198.GO:0018108\_peptidyl-tyrosine\_phosphorylation |
| 199.GO:0018212\_peptidyl-tyrosine\_modification |
| 200.GO:0050730\_regulation\_of\_peptidyl-tyrosine\_phosphorylation |
| 201.GO:0046425\_regulation\_of\_JAK-STAT\_cascade |
| 202.GO:0050731\_positive\_regulation\_of\_peptidyl-tyrosine\_phosphorylation |
| 203.GO:0051239\_regulation\_of\_multicellular\_organismal\_process |
| 204.GO:0050820\_positive\_regulation\_of\_coagulation |
| 205.GO:0051893\_regulation\_of\_focal\_adhesion\_formation |
| 206.GO:0031639\_plasminogen\_activation |
| 207.GO:0015911\_plasma\_membrane\_long-chain\_fatty\_acid\_transport |
| 208.GO:0030194\_positive\_regulation\_of\_blood\_coagulation |
| 209.GO:0030334\_regulation\_of\_cell\_migration |
| 210.GO:0010811\_positive\_regulation\_of\_cell-substrate\_adhesion |
| 211.GO:0042117\_monocyte\_activation |
| 212.GO:0001954\_positive\_regulation\_of\_cell-matrix\_adhesion |
| 213.GO:0043066\_negative\_regulation\_of\_apoptosis |
| 214.GO:0043069\_negative\_regulation\_of\_programmed\_cell\_death |
| 215.GO:0060548\_negative\_regulation\_of\_cell\_death |
| 216.GO:0045597\_positive\_regulation\_of\_cell\_differentiation |
| 217.GO:0006417\_regulation\_of\_translation |
| 218.GO:0045727\_positive\_regulation\_of\_translation |
| 219.GO:0019221\_cytokine-mediated\_signaling\_pathway |
| 220.GO:0032103\_positive\_regulation\_of\_response\_to\_external\_stimulus |
| 221.GO:0001819\_positive\_regulation\_of\_cytokine\_production |
| 222.GO:0031399\_regulation\_of\_protein\_modification\_process |
| 223.GO:0051174\_regulation\_of\_phosphorus\_metabolic\_process |
| 224.GO:0019220\_regulation\_of\_phosphate\_metabolic\_process |
| 225.GO:0042325\_regulation\_of\_phosphorylation |
| 226.GO:0032268\_regulation\_of\_cellular\_protein\_metabolic\_process |
| 227.GO:0050655\_dermatan\_sulfate\_proteoglycan\_metabolic\_process |
| 228.GO:0010573\_vascular\_endothelial\_growth\_factor\_production |
| 229.GO:0010574\_regulation\_of\_vascular\_endothelial\_growth\_factor\_production |
| 230.GO:0045073\_regulation\_of\_chemokine\_biosynthetic\_process |
| 231.GO:0051240\_positive\_regulation\_of\_multicellular\_organismal\_process |
| 232.GO:0031401\_positive\_regulation\_of\_protein\_modification\_process |
| 233.GO:0033028\_myeloid\_cell\_apoptosis |
| 234.GO:0032722\_positive\_regulation\_of\_chemokine\_production |
| 235.GO:0031018\_endocrine\_pancreas\_development |
| 236.GO:0050829\_defense\_response\_to\_Gram-negative\_bacterium |
| 237.GO:0009607\_response\_to\_biotic\_stimulus |
| 238.GO:0009615\_response\_to\_virus |
| 239.GO:0051707\_response\_to\_other\_organism |
| 240.GO:0007264\_small\_GTPase\_mediated\_signal\_transduction |
| 241.GO:0007266\_Rho\_protein\_signal\_transduction |
| 242.GO:0051270\_regulation\_of\_cell\_motion |
| 243.GO:0007229\_integrin-mediated\_signaling\_pathway |
| 244.GO:0016525\_negative\_regulation\_of\_angiogenesis |
| 245.GO:0030511\_positive\_regulation\_of\_transforming\_growth\_factor\_beta\_receptor\_signaling\_pathway |
| 246.GO:0032879\_regulation\_of\_localization |
| 247.GO:0007346\_regulation\_of\_mitotic\_cell\_cycle |
| 248.GO:0007093\_mitotic\_cell\_cycle\_checkpoint |
| 249.GO:0007059\_chromosome\_segregation |
| 250.GO:0051726\_regulation\_of\_cell\_cycle |
| 251.GO:0007049\_cell\_cycle |
| 252.GO:0000278\_mitotic\_cell\_cycle |
| 253.GO:0000075\_cell\_cycle\_checkpoint |
| 254.GO:0022402\_cell\_cycle\_process |
| 255.GO:0050848\_regulation\_of\_calcium-mediated\_signaling |
| 256.GO:0007159\_leukocyte\_adhesion |
| 257.GO:0060326\_cell\_chemotaxis |
| 258.GO:0030595\_leukocyte\_chemotaxis |
| 259.GO:0030029\_actin\_filament-based\_process |
| 260.GO:0030036\_actin\_cytoskeleton\_organization |
| 261.GO:0007167\_enzyme\_linked\_receptor\_protein\_signaling\_pathway |
| 262.GO:0032507\_maintenance\_of\_protein\_location\_in\_cell |
| 263.GO:0045185\_maintenance\_of\_protein\_location |
| 264.GO:0051651\_maintenance\_of\_location\_in\_cell |
| 265.GO:0030163\_protein\_catabolic\_process |
| 266.GO:0010324\_membrane\_invagination |
| 267.GO:0006897\_endocytosis |
| 268.GO:0008104\_protein\_localization |
| 269.GO:0042176\_regulation\_of\_protein\_catabolic\_process |
| 270.GO:0006954\_inflammatory\_response |
| 271.GO:0006606\_protein\_import\_into\_nucleus |
| 272.GO:0019935\_cyclic-nucleotide-mediated\_signaling |
| 273.GO:0051170\_nuclear\_import |
| 274.GO:0034504\_protein\_localization\_in\_nucleus |
| 275.GO:0051051\_negative\_regulation\_of\_transport |
| 276.GO:0050871\_positive\_regulation\_of\_B\_cell\_activation |
| 277.GO:0050864\_regulation\_of\_B\_cell\_activation |
| 278.GO:0032844\_regulation\_of\_homeostatic\_process |
| 279.GO:0051235\_maintenance\_of\_location |
| 280.GO:0030001\_metal\_ion\_transport |
| 281.GO:0007043\_cell-cell\_junction\_assembly |
| 282.GO:0045216\_cell-cell\_junction\_organization |
| 283.GO:0007169\_transmembrane\_receptor\_protein\_tyrosine\_kinase\_signaling\_pathway |
| 284.GO:0042592\_homeostatic\_process |
| 285.GO:0051094\_positive\_regulation\_of\_developmental\_process |
| 286.GO:0045595\_regulation\_of\_cell\_differentiation |
| 287.GO:0006509\_membrane\_protein\_ectodomain\_proteolysis |
| 288.GO:0033619\_membrane\_protein\_proteolysis |
| 289.GO:0043537\_negative\_regulation\_of\_blood\_vessel\_endothelial\_cell\_migration |
| 290.GO:0080010\_regulation\_of\_oxygen\_and\_reactive\_oxygen\_species\_metabolic\_process |
| 291.GO:0010744\_positive\_regulation\_of\_foam\_cell\_differentiation |
| 292.GO:0010812\_negative\_regulation\_of\_cell-substrate\_adhesion |
| 293.GO:0031638\_zymogen\_activation |
| 294.GO:0019934\_cGMP-mediated\_signaling |
| 295.GO:0001953\_negative\_regulation\_of\_cell-matrix\_adhesion |
| 296.GO:0040012\_regulation\_of\_locomotion |
| 297.GO:0006911\_phagocytosis\_\_engulfment |
| 298.GO:0070613\_regulation\_of\_protein\_processing |
| 299.GO:0032890\_regulation\_of\_organic\_acid\_transport |
| 300.GO:0051917\_regulation\_of\_fibrinolysis |
| 301.GO:0043536\_positive\_regulation\_of\_blood\_vessel\_endothelial\_cell\_migration |
| 302.GO:0051093\_negative\_regulation\_of\_developmental\_process |
| 303.GO:0032965\_regulation\_of\_collagen\_biosynthetic\_process |
| 304.GO:0042033\_chemokine\_biosynthetic\_process |
| 305.GO:0050755\_chemokine\_metabolic\_process |
| 306.GO:0001817\_regulation\_of\_cytokine\_production |
| 307.GO:0001932\_regulation\_of\_protein\_amino\_acid\_phosphorylation |
| 308.GO:0010608\_posttranscriptional\_regulation\_of\_gene\_expression |
| 309.GO:0018193\_peptidyl-amino\_acid\_modification |
| 310.GO:0032642\_regulation\_of\_chemokine\_production |
| 311.GO:0032655\_regulation\_of\_interleukin-12\_production |
| 312.GO:0045651\_positive\_regulation\_of\_macrophage\_differentiation |
| 313.GO:0032615\_interleukin-12\_production |
| 314.GO:0043030\_regulation\_of\_macrophage\_activation |
| 315.GO:0048661\_positive\_regulation\_of\_smooth\_muscle\_cell\_proliferation |
| 316.GO:0048706\_embryonic\_skeletal\_system\_development |
| 317.GO:0033135\_regulation\_of\_peptidyl-serine\_phosphorylation |
| 318.GO:0001776\_leukocyte\_homeostasis |
| 319.GO:0050650\_chondroitin\_sulfate\_proteoglycan\_biosynthetic\_process |
| 320.GO:0048246\_macrophage\_chemotaxis |
| 321.GO:0002688\_regulation\_of\_leukocyte\_chemotaxis |
| 322.GO:0002690\_positive\_regulation\_of\_leukocyte\_chemotaxis |
| 323.GO:0001816\_cytokine\_production |
| 324.GO:0006508\_proteolysis |
| 325.GO:0051384\_response\_to\_glucocorticoid\_stimulus |
| 326.GO:0031016\_pancreas\_development |
| 327.GO:0030204\_chondroitin\_sulfate\_metabolic\_process |
| 328.GO:0033138\_positive\_regulation\_of\_peptidyl-serine\_phosphorylation |
| 329.GO:0031960\_response\_to\_corticosteroid\_stimulus |
| 330.GO:0010712\_regulation\_of\_collagen\_metabolic\_process |
| 331.GO:0030206\_chondroitin\_sulfate\_biosynthetic\_process |
| 332.GO:0050729\_positive\_regulation\_of\_inflammatory\_response |
| 333.GO:0002637\_regulation\_of\_immunoglobulin\_production |
| 334.GO:0002673\_regulation\_of\_acute\_inflammatory\_response |
| 335.GO:0044246\_regulation\_of\_multicellular\_organismal\_metabolic\_process |
| 336.GO:0009749\_response\_to\_glucose\_stimulus |
| 337.GO:0007263\_nitric\_oxide\_mediated\_signal\_transduction |
| 338.GO:0034284\_response\_to\_monosaccharide\_stimulus |
| 339.GO:0009746\_response\_to\_hexose\_stimulus |
| 340.GO:0045766\_positive\_regulation\_of\_angiogenesis |
| 341.GO:0010595\_positive\_regulation\_of\_endothelial\_cell\_migration |
| 342.GO:0032602\_chemokine\_production |
| 343.GO:0050654\_chondroitin\_sulfate\_proteoglycan\_metabolic\_process |
| 344.GO:0033674\_positive\_regulation\_of\_kinase\_activity |
| 345.GO:0045860\_positive\_regulation\_of\_protein\_kinase\_activity |
| 346.GO:0009719\_response\_to\_endogenous\_stimulus |
| 347.GO:0002763\_positive\_regulation\_of\_myeloid\_leukocyte\_differentiation |
| 348.GO:0002685\_regulation\_of\_leukocyte\_migration |
| 349.GO:0010596\_negative\_regulation\_of\_endothelial\_cell\_migration |
| 350.GO:0019882\_antigen\_processing\_and\_presentation |
| 351.GO:0045669\_positive\_regulation\_of\_osteoblast\_differentiation |
| 352.GO:0010740\_positive\_regulation\_of\_protein\_kinase\_cascade |
| 353.GO:0009725\_response\_to\_hormone\_stimulus |
| 354.GO:0051347\_positive\_regulation\_of\_transferase\_activity |
| 355.GO:0050830\_defense\_response\_to\_Gram-positive\_bacterium |
| 356.GO:0043535\_regulation\_of\_blood\_vessel\_endothelial\_cell\_migration |
| 357.GO:0007009\_plasma\_membrane\_organization |
| 358.GO:0002687\_positive\_regulation\_of\_leukocyte\_migration |
| 359.GO:0022604\_regulation\_of\_cell\_morphogenesis |
| 360.GO:0045765\_regulation\_of\_angiogenesis |
| 361.GO:0030336\_negative\_regulation\_of\_cell\_migration |
| 362.GO:0007610\_behavior |
| 363.GO:0019722\_calcium-mediated\_signaling |
| 364.GO:0006800\_oxygen\_and\_reactive\_oxygen\_species\_metabolic\_process |
| 365.GO:0000768\_syncytium\_formation\_by\_plasma\_membrane\_fusion |
| 366.GO:0014902\_myotube\_differentiation |
| 367.GO:0051248\_negative\_regulation\_of\_protein\_metabolic\_process |
| 368.GO:0007212\_dopamine\_receptor\_signaling\_pathway |
| 369.GO:0006949\_syncytium\_formation |
| 370.GO:0033365\_protein\_localization\_in\_organelle |
| 371.GO:0015031\_protein\_transport |
| 372.GO:0017038\_protein\_import |
| 373.GO:0045184\_establishment\_of\_protein\_localization |
| 374.GO:0051704\_multi-organism\_process |
| 375.GO:0006869\_lipid\_transport |
| 376.GO:0009966\_regulation\_of\_signal\_transduction |
| 377.GO:0042127\_regulation\_of\_cell\_proliferation |
| 378.GO:0010627\_regulation\_of\_protein\_kinase\_cascade |
| 379.GO:0060193\_positive\_regulation\_of\_lipase\_activity |
| 380.GO:0010863\_positive\_regulation\_of\_phospholipase\_C\_activity |
| 381.GO:0007202\_activation\_of\_phospholipase\_C\_activity |
| 382.GO:0060191\_regulation\_of\_lipase\_activity |
| 383.GO:0010517\_regulation\_of\_phospholipase\_activity |
| 384.GO:0010518\_positive\_regulation\_of\_phospholipase\_activity |
| 385.GO:0042060\_wound\_healing |
| 386.GO:0009611\_response\_to\_wounding |
| 387.GO:0050878\_regulation\_of\_body\_fluid\_levels |
| 388.GO:0007599\_hemostasis |
| 389.GO:0007596\_blood\_coagulation |
| 390.GO:0050817\_coagulation |
| 391.GO:0031532\_actin\_cytoskeleton\_reorganization |
| 392.GO:0007568\_aging |
| 393.GO:0034330\_cell\_junction\_organization |
| 394.GO:0034329\_cell\_junction\_assembly |
| 395.GO:0051271\_negative\_regulation\_of\_cell\_motion |
| 396.GO:0048041\_focal\_adhesion\_formation |
| 397.GO:0007044\_cell-substrate\_junction\_assembly |
| 398.GO:0001944\_vasculature\_development |
| 399.GO:0001568\_blood\_vessel\_development |
| 400.GO:0050818\_regulation\_of\_coagulation |
| 401.GO:0030193\_regulation\_of\_blood\_coagulation |
| 402.GO:0042730\_fibrinolysis |
| 403.GO:0030195\_negative\_regulation\_of\_blood\_coagulation |
| 404.GO:0050819\_negative\_regulation\_of\_coagulation |
| 405.GO:0043534\_blood\_vessel\_endothelial\_cell\_migration |
| 406.GO:0031589\_cell-substrate\_adhesion |
| 407.GO:0007160\_cell-matrix\_adhesion |
| 408.GO:0050900\_leukocyte\_migration |
| 409.GO:0043542\_endothelial\_cell\_migration |
| 410.GO:0048646\_anatomical\_structure\_formation\_involved\_in\_morphogenesis |
| 411.GO:0001525\_angiogenesis |
| 412.GO:0033077\_T\_cell\_differentiation\_in\_the\_thymus |
| 413.GO:0045580\_regulation\_of\_T\_cell\_differentiation |
| 414.GO:0045619\_regulation\_of\_lymphocyte\_differentiation |
| 415.GO:0050870\_positive\_regulation\_of\_T\_cell\_activation |
| 416.GO:0051251\_positive\_regulation\_of\_lymphocyte\_activation |
| 417.GO:0002757\_immune\_response-activating\_signal\_transduction |
| 418.GO:0002764\_immune\_response-regulating\_signal\_transduction |
| 419.GO:0050851\_antigen\_receptor-mediated\_signaling\_pathway |
| 420.GO:0002768\_immune\_response-regulating\_cell\_surface\_receptor\_signaling\_pathway |
| 421.GO:0002429\_immune\_response-activating\_cell\_surface\_receptor\_signaling\_pathway |
| 422.GO:0050852\_T\_cell\_receptor\_signaling\_pathway |
| 423.GO:0050854\_regulation\_of\_antigen\_receptor-mediated\_signaling\_pathway |
| 424.GO:0050778\_positive\_regulation\_of\_immune\_response |
| 425.GO:0050776\_regulation\_of\_immune\_response |
| 426.GO:0048584\_positive\_regulation\_of\_response\_to\_stimulus |
| 427.GO:0002253\_activation\_of\_immune\_response |
