# Supplementary material for: Concordance of Gene Expression and Functional Correlation Patterns across the NCI-60 Cell Lines and the Cancer Genome Atlas Glioblastoma Samples
Source: PLoS One. 2012 Jul 26;7(7):e40062. doi: 10.1371/journal.pone.0040062 (PMC3406063; doi:10.1371/journal.pone.0040062)
Supplement: Download S1 — Zip archive of HTGM results. (ZIP) [file pone.0040062.s007.zip › work2026406846/Generated_Total2026406846.dir/Generated_Total.change.series.CIM.1.dir/cgi_user_y.html]

**Y-axis Names**   
Cluster is based on euclidean distance  
Cluster method is: average  
plclust  
height plot  

|  |
| --- |
| 1.generic.BP.NCI60.0.6.DSE.express.genes.correlation.complete.Thu.May.19.17.28.12.2011.htgm.txt |
| 2.generic.BP.NCI60.0.6.CD2.express.genes.correlation.complete.Thu.May.19.17.09.36.2011.htgm.txt |
| 3.generic.BP.NCI60.0.6.CD3D.express.genes.correlation.complete.Thu.May.19.17.05.42.2011.htgm.txt |
| 4.generic.BP.NCI60.0.6.LOX.express.genes.correlation.complete.Thu.May.19.17.16.37.2011.htgm.txt |
| 5.generic.BP.NCI60.0.6.SERPINE1.express.genes.correlation.complete.Thu.May.19.17.11.45.2011.htgm.txt |
| 6.generic.BP.NCI60.0.6.FOSL2.express.genes.correlation.complete.Thu.May.19.17.28.40.2011.htgm.txt |
| 7.generic.BP.NCI60.0.6.SIT1.express.genes.correlation.complete.Thu.May.19.17.09.08.2011.htgm.txt |
| 8.generic.BP.NCI60.0.6.CD7.express.genes.correlation.complete.Thu.May.19.17.20.43.2011.htgm.txt |
| 9.generic.BP.NCI60.0.6.ATP2A3.express.genes.correlation.complete.Thu.May.19.17.20.06.2011.htgm.txt |
| 10.generic.BP.NCI60.0.6.CD96.express.genes.correlation.complete.Thu.May.19.17.21.20.2011.htgm.txt |
| 11.generic.BP.NCI60.0.6.LCK.express.genes.correlation.complete.Thu.May.19.17.29.07.2011.htgm.txt |
| 12.generic.BP.NCI60.0.6.SH2D1A.express.genes.correlation.complete.Thu.May.19.17.15.13.2011.htgm.txt |
| 13.generic.BP.NCI60.0.6.IL2RG.express.genes.correlation.complete.Thu.May.19.16.57.41.2011.htgm.txt |
| 14.generic.BP.NCI60.0.6.RHOH.express.genes.correlation.complete.Thu.May.19.17.03.25.2011.htgm.txt |
| 15.generic.BP.NCI60.0.6.CCR7.express.genes.correlation.complete.Thu.May.19.17.25.28.2011.htgm.txt |
| 16.generic.BP.NCI60.0.6.CD5.express.genes.correlation.complete.Thu.May.19.17.17.40.2011.htgm.txt |
| 17.generic.BP.NCI60.0.6.CD48.express.genes.correlation.complete.Thu.May.19.16.58.12.2011.htgm.txt |
| 18.generic.BP.NCI60.0.6.CD1D.express.genes.correlation.complete.Thu.May.19.17.08.12.2011.htgm.txt |
| 19.generic.BP.NCI60.0.6.PTPRC.express.genes.correlation.complete.Thu.May.19.17.01.12.2011.htgm.txt |
| 20.generic.BP.NCI60.0.6.CTCF.express.genes.correlation.complete.Thu.May.19.17.32.17.2011.htgm.txt |
| 21.generic.BP.NCI60.0.6.MCM3.express.genes.correlation.complete.Thu.May.19.17.22.45.2011.htgm.txt |
| 22.generic.BP.NCI60.0.6.MYH9.express.genes.correlation.complete.Thu.May.19.17.10.13.2011.htgm.txt |
| 23.generic.BP.NCI60.0.6.CD52.express.genes.correlation.complete.Thu.May.19.17.15.40.2011.htgm.txt |
| 24.generic.BP.NCI60.0.6.GNA15.express.genes.correlation.complete.Thu.May.19.17.14.35.2011.htgm.txt |
| 25.generic.BP.NCI60.0.6.WAS.express.genes.correlation.complete.Thu.May.19.16.56.33.2011.htgm.txt |
| 26.generic.BP.NCI60.0.6.CORO1A.express.genes.correlation.complete.Thu.May.19.16.58.44.2011.htgm.txt |
| 27.generic.BP.NCI60.0.6.PTPN7.express.genes.correlation.complete.Thu.May.19.17.00.01.2011.htgm.txt |
| 28.generic.BP.NCI60.0.6.PLAUR.express.genes.correlation.complete.Thu.May.19.17.31.21.2011.htgm.txt |
| 29.generic.BP.NCI60.0.6.ITGB2.express.genes.correlation.complete.Thu.May.19.17.21.45.2011.htgm.txt |
| 30.generic.BP.NCI60.0.6.CTSW.express.genes.correlation.complete.Thu.May.19.17.30.28.2011.htgm.txt |
| 31.generic.BP.NCI60.0.6.LTB.express.genes.correlation.complete.Thu.May.19.17.30.53.2011.htgm.txt |
| 32.generic.BP.NCI60.0.6.FLNA.express.genes.correlation.complete.Thu.May.19.17.16.07.2011.htgm.txt |
| 33.generic.BP.NCI60.0.6.CYR61.express.genes.correlation.complete.Thu.May.19.17.10.46.2011.htgm.txt |
| 34.generic.BP.NCI60.0.6.PLK4.express.genes.correlation.complete.Thu.May.19.17.23.47.2011.htgm.txt |
| 35.generic.BP.NCI60.0.6.CLCF1.express.genes.correlation.complete.Thu.May.19.17.26.54.2011.htgm.txt |
| 36.generic.BP.NCI60.0.6.TNFRSF12A.express.genes.correlation.complete.Thu.May.19.17.24.37.2011.htgm.txt |
| 37.generic.BP.NCI60.0.6.CYBB.express.genes.correlation.complete.Thu.May.19.17.07.45.2011.htgm.txt |
| 38.generic.BP.NCI60.0.6.RNASE3.express.genes.correlation.complete.Thu.May.19.17.19.05.2011.htgm.txt |
| 39.generic.BP.NCI60.0.6.CD4.express.genes.correlation.complete.Thu.May.19.16.57.09.2011.htgm.txt |
| 40.generic.BP.NCI60.0.6.OAS2.express.genes.correlation.complete.Thu.May.19.17.23.19.2011.htgm.txt |
| 41.generic.BP.NCI60.0.6.DOCK2.express.genes.correlation.complete.Thu.May.19.17.08.40.2011.htgm.txt |
| 42.generic.BP.NCI60.0.6.HCLS1.express.genes.correlation.complete.Thu.May.19.16.59.22.2011.htgm.txt |
| 43.generic.BP.NCI60.0.6.BTK.express.genes.correlation.complete.Thu.May.19.17.12.30.2011.htgm.txt |
| 44.generic.BP.NCI60.0.6.CD37.express.genes.correlation.complete.Thu.May.19.17.02.14.2011.htgm.txt |
| 45.generic.BP.NCI60.0.6.LSP1.express.genes.correlation.complete.Thu.May.19.17.22.14.2011.htgm.txt |
| 46.generic.BP.NCI60.0.6.PTPN22.express.genes.correlation.complete.Thu.May.19.17.31.48.2011.htgm.txt |
| 47.generic.BP.NCI60.0.6.MED6.express.genes.correlation.complete.Thu.May.19.17.30.03.2011.htgm.txt |
| 48.generic.BP.NCI60.0.6.LGALS1.express.genes.correlation.complete.Thu.May.19.17.29.36.2011.htgm.txt |
| 49.generic.BP.NCI60.0.6.ANXA2.express.genes.correlation.complete.Thu.May.19.17.26.28.2011.htgm.txt |
| 50.generic.BP.NCI60.0.6.GRB7.express.genes.correlation.complete.Thu.May.19.17.33.00.2011.htgm.txt |
| 51.generic.BP.NCI60.0.6.LY86.express.genes.correlation.complete.Thu.May.19.17.17.05.2011.htgm.txt |
| 52.generic.BP.NCI60.0.6.LILRA2.express.genes.correlation.complete.Thu.May.19.17.04.11.2011.htgm.txt |
| 53.generic.BP.NCI60.0.6.ST8SIA4.express.genes.correlation.complete.Thu.May.19.17.24.12.2011.htgm.txt |
| 54.generic.BP.NCI60.0.6.RNASE6.express.genes.correlation.complete.Thu.May.19.17.06.49.2011.htgm.txt |
| 55.generic.BP.NCI60.0.6.PLCB2.express.genes.correlation.complete.Thu.May.19.17.02.52.2011.htgm.txt |
| 56.generic.BP.NCI60.0.6.LRMP.express.genes.correlation.complete.Thu.May.19.17.04.50.2011.htgm.txt |
| 57.generic.BP.NCI60.0.6.CSF2RB.express.genes.correlation.complete.Thu.May.19.17.18.11.2011.htgm.txt |
| 58.generic.BP.NCI60.0.6.LGALS9.express.genes.correlation.complete.Thu.May.19.17.18.39.2011.htgm.txt |
| 59.generic.BP.NCI60.0.6.CSF3R.express.genes.correlation.complete.Thu.May.19.17.07.17.2011.htgm.txt |
| 60.generic.BP.NCI60.0.6.NCF4.express.genes.correlation.complete.Thu.May.19.17.06.20.2011.htgm.txt |
| 61.generic.BP.NCI60.0.6.IRAK3.express.genes.correlation.complete.Thu.May.19.17.34.41.2011.htgm.txt |
| 62.generic.BP.NCI60.0.6.S100A8.express.genes.correlation.complete.Thu.May.19.17.19.34.2011.htgm.txt |
| 63.generic.BP.NCI60.0.6.LOXL2.express.genes.correlation.complete.Thu.May.19.17.11.15.2011.htgm.txt |
| 64.generic.BP.NCI60.0.6.COL6A3.express.genes.correlation.complete.Thu.May.19.17.27.41.2011.htgm.txt |
| 65.generic.BP.NCI60.0.6.COL1A1.express.genes.correlation.complete.Thu.May.19.17.27.18.2011.htgm.txt |
| 66.generic.BP.NCI60.0.6.COL1A2.express.genes.correlation.complete.Thu.May.19.17.25.56.2011.htgm.txt |
| 67.generic.BP.NCI60.0.6.ADAM12.express.genes.correlation.complete.Thu.May.19.17.25.03.2011.htgm.txt |
| 68.generic.BP.NCI60.0.6.COL5A1.express.genes.correlation.complete.Thu.May.19.17.13.34.2011.htgm.txt |
