# Supplementary material for: Concordance of Gene Expression and Functional Correlation Patterns across the NCI-60 Cell Lines and the Cancer Genome Atlas Glioblastoma Samples
Source: PLoS One. 2012 Jul 26;7(7):e40062. doi: 10.1371/journal.pone.0040062 (PMC3406063; doi:10.1371/journal.pone.0040062)
Supplement: Download S1 — Zip archive of HTGM results. (ZIP) [file pone.0040062.s007.zip › work2026406846/Generated_Total2026406846.dir/generic.BP.NCI60.0.6.ADAM12.express.genes.correlation.complete.Thu.May.19.17.25.03.2011.htgm.txt.dir/generic.BP.NCI60.0.6.ADAM12.express.genes.correlation.complete.Thu.May.19.17.25.03.2011.htgm.txt.change.gce.CIM.dir/cgi_user_info.html]

Input file: **generic.BP.NCI60.0.6.ADAM12.express.genes.correlation.complete.Thu.May.19.17.25.03.2011.htgm.txt.change.gce.CIM**
