# Supplementary material for: Concordance of Gene Expression and Functional Correlation Patterns across the NCI-60 Cell Lines and the Cancer Genome Atlas Glioblastoma Samples
Source: PLoS One. 2012 Jul 26;7(7):e40062. doi: 10.1371/journal.pone.0040062 (PMC3406063; doi:10.1371/journal.pone.0040062)
Supplement: Download S1 — Zip archive of HTGM results. (ZIP) [file pone.0040062.s007.zip › work2026406846/Generated_Total2026406846.dir/generic.BP.NCI60.0.6.ADAM12.express.genes.correlation.complete.Thu.May.19.17.25.03.2011.htgm.txt.dir/generic.BP.NCI60.0.6.ADAM12.express.genes.correlation.complete.Thu.May.19.17.25.03.2011.htgm.txt.change.gce.CIM.dir/cgi_user_x.html]

**X-axis Names**   
Cluster is based on euclidean distance  
Cluster method is: average  
plclust  
height plot  

|  |
| --- |
| 1.GO:0001501\_skeletal\_system\_development |
| 2.GO:0009887\_organ\_morphogenesis |
| 3.GO:0009653\_anatomical\_structure\_morphogenesis |
| 4.GO:0009888\_tissue\_development |
| 5.GO:0001568\_blood\_vessel\_development |
| 6.GO:0001944\_vasculature\_development |
| 7.GO:0044236\_multicellular\_organismal\_metabolic\_process |
| 8.GO:0032964\_collagen\_biosynthetic\_process |
| 9.GO:0044259\_multicellular\_organismal\_macromolecule\_metabolic\_process |
| 10.GO:0032963\_collagen\_metabolic\_process |
| 11.GO:0030198\_extracellular\_matrix\_organization |
| 12.GO:0008544\_epidermis\_development |
| 13.GO:0007398\_ectoderm\_development |
| 14.GO:0030199\_collagen\_fibril\_organization |
| 15.GO:0043588\_skin\_development |
| 16.GO:0043062\_extracellular\_structure\_organization |
| 17.GO:0048729\_tissue\_morphogenesis |
| 18.GO:0042476\_odontogenesis |
| 19.GO:0048730\_epidermis\_morphogenesis |
| 20.GO:0016044\_membrane\_organization |
| 21.GO:0030155\_regulation\_of\_cell\_adhesion |
| 22.GO:0007162\_negative\_regulation\_of\_cell\_adhesion |
| 23.GO:0022610\_biological\_adhesion |
| 24.GO:0007155\_cell\_adhesion |
