# Supplementary material for: Concordance of Gene Expression and Functional Correlation Patterns across the NCI-60 Cell Lines and the Cancer Genome Atlas Glioblastoma Samples
Source: PLoS One. 2012 Jul 26;7(7):e40062. doi: 10.1371/journal.pone.0040062 (PMC3406063; doi:10.1371/journal.pone.0040062)
Supplement: Download S1 — Zip archive of HTGM results. (ZIP) [file pone.0040062.s007.zip › work2026406846/Generated_Total2026406846.dir/generic.BP.NCI60.0.6.ADAM12.express.genes.correlation.complete.Thu.May.19.17.25.03.2011.htgm.txt.dir/generic.BP.NCI60.0.6.ADAM12.express.genes.correlation.complete.Thu.May.19.17.25.03.2011.htgm.txt.change.gce.html]

Gene Category Report for generic.BP.NCI60.0.6.ADAM12.express.genes.correlation.complete.Thu.May.19.17.25.03.2011.htgm.txt

# Gene Category Report for generic.BP.NCI60.0.6.ADAM12.express.genes.correlation.complete.Thu.May.19.17.25.03.2011.htgm.txt

| HYPERLINKED GO CATEGORY | HYPERLINKED GENE NAME | TOTAL GENES | CHANGED GENES | ENRICHMENT | LOG10(p) | CUMULATIVE NUMBER OF CATEGORIES | CUMULATIVE RANDOMS MEAN | FALSE DISCOVERY RATE |
| --- | --- | --- | --- | --- | --- | --- | --- | --- |
| GO:0043588\_skin\_development | COL5A1 | 10 | 3 | 163.200000 | -6.222923 | 1 | 0.0 | 0.000000 |
| GO:0043588\_skin\_development | COL1A1 | 10 | 3 | 163.200000 | -6.222923 | 1 | 0.0 | 0.000000 |
| GO:0043588\_skin\_development | COL1A2 | 10 | 3 | 163.200000 | -6.222923 | 1 | 0.0 | 0.000000 |
| GO:0043589\_skin\_morphogenesis | COL1A1 | 2 | 2 |  |  |  |  |  |  |
| GO:0043589\_skin\_morphogenesis | COL1A2 | 2 | 2 |  |  |  |  |  |  |
| GO:0030199\_collagen\_fibril\_organization | COL5A1 | 17 | 3 | 96.000000 | -5.472952 | 2 | 0.0 | 0.000000 |
| GO:0030199\_collagen\_fibril\_organization | COL1A1 | 17 | 3 | 96.000000 | -5.472952 | 2 | 0.0 | 0.000000 |
| GO:0030199\_collagen\_fibril\_organization | COL1A2 | 17 | 3 | 96.000000 | -5.472952 | 2 | 0.0 | 0.000000 |
| GO:0030198\_extracellular\_matrix\_organization | COL5A1 | 35 | 3 | 46.628571 | -4.498186 | 3 | 0.03 | 0.010000 |
| GO:0030198\_extracellular\_matrix\_organization | COL1A1 | 35 | 3 | 46.628571 | -4.498186 | 3 | 0.03 | 0.010000 |
| GO:0030198\_extracellular\_matrix\_organization | COL1A2 | 35 | 3 | 46.628571 | -4.498186 | 3 | 0.03 | 0.010000 |
| GO:0009887\_organ\_morphogenesis | COL5A1 | 246 | 5 | 11.056911 | -4.251434 | 4 | 0.03 | 0.007500 |
| GO:0009887\_organ\_morphogenesis | THBS1 | 246 | 5 | 11.056911 | -4.251434 | 4 | 0.03 | 0.007500 |
| GO:0009887\_organ\_morphogenesis | BMP1 | 246 | 5 | 11.056911 | -4.251434 | 4 | 0.03 | 0.007500 |
| GO:0009887\_organ\_morphogenesis | COL1A1 | 246 | 5 | 11.056911 | -4.251434 | 4 | 0.03 | 0.007500 |
| GO:0009887\_organ\_morphogenesis | COL1A2 | 246 | 5 | 11.056911 | -4.251434 | 4 | 0.03 | 0.007500 |
| GO:0048730\_epidermis\_morphogenesis | COL1A1 | 7 | 2 | 155.428571 | -4.181196 | 5 | 0.04 | 0.008000 |
| GO:0048730\_epidermis\_morphogenesis | COL1A2 | 7 | 2 | 155.428571 | -4.181196 | 5 | 0.04 | 0.008000 |
| GO:0001501\_skeletal\_system\_development | COL1A1 | 142 | 4 | 15.323944 | -3.985609 | 6 | 0.04 | 0.006667 |
| GO:0001501\_skeletal\_system\_development | BMP1 | 142 | 4 | 15.323944 | -3.985609 | 6 | 0.04 | 0.006667 |
| GO:0001501\_skeletal\_system\_development | FBN1 | 142 | 4 | 15.323944 | -3.985609 | 6 | 0.04 | 0.006667 |
| GO:0001501\_skeletal\_system\_development | COL1A2 | 142 | 4 | 15.323944 | -3.985609 | 6 | 0.04 | 0.006667 |
| GO:0009888\_tissue\_development | ADAM12 | 287 | 5 | 9.477352 | -3.932714 | 7 | 0.07 | 0.010000 |
| GO:0009888\_tissue\_development | COL5A1 | 287 | 5 | 9.477352 | -3.932714 | 7 | 0.07 | 0.010000 |
| GO:0009888\_tissue\_development | BMP1 | 287 | 5 | 9.477352 | -3.932714 | 7 | 0.07 | 0.010000 |
| GO:0009888\_tissue\_development | COL1A1 | 287 | 5 | 9.477352 | -3.932714 | 7 | 0.07 | 0.010000 |
| GO:0009888\_tissue\_development | COL1A2 | 287 | 5 | 9.477352 | -3.932714 | 7 | 0.07 | 0.010000 |
| GO:0032964\_collagen\_biosynthetic\_process | COL5A1 | 10 | 2 | 108.800000 | -3.851588 | 8 | 0.09 | 0.011250 |
| GO:0032964\_collagen\_biosynthetic\_process | COL1A1 | 10 | 2 | 108.800000 | -3.851588 | 8 | 0.09 | 0.011250 |
| GO:0009653\_anatomical\_structure\_morphogenesis | ADAM12 | 500 | 6 | 6.528000 | -3.796717 | 9 | 0.09 | 0.010000 |
| GO:0009653\_anatomical\_structure\_morphogenesis | COL5A1 | 500 | 6 | 6.528000 | -3.796717 | 9 | 0.09 | 0.010000 |
| GO:0009653\_anatomical\_structure\_morphogenesis | THBS1 | 500 | 6 | 6.528000 | -3.796717 | 9 | 0.09 | 0.010000 |
| GO:0009653\_anatomical\_structure\_morphogenesis | BMP1 | 500 | 6 | 6.528000 | -3.796717 | 9 | 0.09 | 0.010000 |
| GO:0009653\_anatomical\_structure\_morphogenesis | COL1A1 | 500 | 6 | 6.528000 | -3.796717 | 9 | 0.09 | 0.010000 |
| GO:0009653\_anatomical\_structure\_morphogenesis | COL1A2 | 500 | 6 | 6.528000 | -3.796717 | 9 | 0.09 | 0.010000 |
| GO:0048513\_organ\_development | ADAM12 | 741 | 7 | 5.139001 | -3.779512 | 10 | 0.09 | 0.009000 |
| GO:0048513\_organ\_development | COL5A1 | 741 | 7 | 5.139001 | -3.779512 | 10 | 0.09 | 0.009000 |
| GO:0048513\_organ\_development | THBS1 | 741 | 7 | 5.139001 | -3.779512 | 10 | 0.09 | 0.009000 |
| GO:0048513\_organ\_development | BMP1 | 741 | 7 | 5.139001 | -3.779512 | 10 | 0.09 | 0.009000 |
| GO:0048513\_organ\_development | COL1A1 | 741 | 7 | 5.139001 | -3.779512 | 10 | 0.09 | 0.009000 |
| GO:0048513\_organ\_development | FBN1 | 741 | 7 | 5.139001 | -3.779512 | 10 | 0.09 | 0.009000 |
| GO:0048513\_organ\_development | COL1A2 | 741 | 7 | 5.139001 | -3.779512 | 10 | 0.09 | 0.009000 |
| GO:0043062\_extracellular\_structure\_organization | COL5A1 | 68 | 3 | 24.000000 | -3.629959 | 11 | 0.1 | 0.009091 |
| GO:0043062\_extracellular\_structure\_organization | COL1A1 | 68 | 3 | 24.000000 | -3.629959 | 11 | 0.1 | 0.009091 |
| GO:0043062\_extracellular\_structure\_organization | COL1A2 | 68 | 3 | 24.000000 | -3.629959 | 11 | 0.1 | 0.009091 |
| GO:0032963\_collagen\_metabolic\_process | COL5A1 | 15 | 2 | 72.533333 | -3.485918 | 13 | 0.16 | 0.012308 |
| GO:0032963\_collagen\_metabolic\_process | COL1A1 | 15 | 2 | 72.533333 | -3.485918 | 13 | 0.16 | 0.012308 |
| GO:0044259\_multicellular\_organismal\_macromolecule\_metabolic\_process | COL5A1 | 15 | 2 | 72.533333 | -3.485918 | 13 | 0.16 | 0.012308 |
| GO:0044259\_multicellular\_organismal\_macromolecule\_metabolic\_process | COL1A1 | 15 | 2 | 72.533333 | -3.485918 | 13 | 0.16 | 0.012308 |
| GO:0042476\_odontogenesis | COL1A1 | 16 | 2 | 68.000000 | -3.428387 | 14 | 0.16 | 0.011429 |
| GO:0042476\_odontogenesis | COL1A2 | 16 | 2 | 68.000000 | -3.428387 | 14 | 0.16 | 0.011429 |
| GO:0001568\_blood\_vessel\_development | THBS1 | 85 | 3 | 19.200000 | -3.343475 | 15 | 0.22 | 0.014667 |
| GO:0001568\_blood\_vessel\_development | COL1A1 | 85 | 3 | 19.200000 | -3.343475 | 15 | 0.22 | 0.014667 |
| GO:0001568\_blood\_vessel\_development | COL1A2 | 85 | 3 | 19.200000 | -3.343475 | 15 | 0.22 | 0.014667 |
| GO:0001944\_vasculature\_development | THBS1 | 88 | 3 | 18.545455 | -3.299192 | 16 | 0.25 | 0.015625 |
| GO:0001944\_vasculature\_development | COL1A1 | 88 | 3 | 18.545455 | -3.299192 | 16 | 0.25 | 0.015625 |
| GO:0001944\_vasculature\_development | COL1A2 | 88 | 3 | 18.545455 | -3.299192 | 16 | 0.25 | 0.015625 |
| GO:0044236\_multicellular\_organismal\_metabolic\_process | COL5A1 | 19 | 2 | 57.263158 | -3.275957 | 17 | 0.28 | 0.016471 |
| GO:0044236\_multicellular\_organismal\_metabolic\_process | COL1A1 | 19 | 2 | 57.263158 | -3.275957 | 17 | 0.28 | 0.016471 |
| GO:0016044\_membrane\_organization | ADAM12 | 225 | 4 | 9.671111 | -3.218474 | 18 | 0.29 | 0.016111 |
| GO:0016044\_membrane\_organization | THBS1 | 225 | 4 | 9.671111 | -3.218474 | 18 | 0.29 | 0.016111 |
| GO:0016044\_membrane\_organization | COL5A1 | 225 | 4 | 9.671111 | -3.218474 | 18 | 0.29 | 0.016111 |
| GO:0016044\_membrane\_organization | SEC24D | 225 | 4 | 9.671111 | -3.218474 | 18 | 0.29 | 0.016111 |
| GO:0007155\_cell\_adhesion | COL5A1 | 428 | 5 | 6.355140 | -3.124152 | 19 | 0.33 | 0.017368 |
| GO:0007155\_cell\_adhesion | THBS1 | 428 | 5 | 6.355140 | -3.124152 | 19 | 0.33 | 0.017368 |
| GO:0007155\_cell\_adhesion | BMP1 | 428 | 5 | 6.355140 | -3.124152 | 19 | 0.33 | 0.017368 |
| GO:0007155\_cell\_adhesion | LOXL2 | 428 | 5 | 6.355140 | -3.124152 | 19 | 0.33 | 0.017368 |
| GO:0007155\_cell\_adhesion | TGFBI | 428 | 5 | 6.355140 | -3.124152 | 19 | 0.33 | 0.017368 |
| GO:0022610\_biological\_adhesion | COL5A1 | 429 | 5 | 6.340326 | -3.119519 | 20 | 0.33 | 0.016500 |
| GO:0022610\_biological\_adhesion | THBS1 | 429 | 5 | 6.340326 | -3.119519 | 20 | 0.33 | 0.016500 |
| GO:0022610\_biological\_adhesion | BMP1 | 429 | 5 | 6.340326 | -3.119519 | 20 | 0.33 | 0.016500 |
| GO:0022610\_biological\_adhesion | LOXL2 | 429 | 5 | 6.340326 | -3.119519 | 20 | 0.33 | 0.016500 |
| GO:0022610\_biological\_adhesion | TGFBI | 429 | 5 | 6.340326 | -3.119519 | 20 | 0.33 | 0.016500 |
| GO:0008544\_epidermis\_development | COL5A1 | 104 | 3 | 15.692308 | -3.086909 | 21 | 0.34 | 0.016190 |
| GO:0008544\_epidermis\_development | COL1A1 | 104 | 3 | 15.692308 | -3.086909 | 21 | 0.34 | 0.016190 |
| GO:0008544\_epidermis\_development | COL1A2 | 104 | 3 | 15.692308 | -3.086909 | 21 | 0.34 | 0.016190 |
| GO:0007162\_negative\_regulation\_of\_cell\_adhesion | THBS1 | 26 | 2 | 41.846154 | -3.000298 | 22 | 0.46 | 0.020909 |
| GO:0007162\_negative\_regulation\_of\_cell\_adhesion | TGFBI | 26 | 2 | 41.846154 | -3.000298 | 22 | 0.46 | 0.020909 |
| GO:0007398\_ectoderm\_development | COL5A1 | 112 | 3 | 14.571429 | -2.993290 | 23 | 0.46 | 0.020000 |
| GO:0007398\_ectoderm\_development | COL1A1 | 112 | 3 | 14.571429 | -2.993290 | 23 | 0.46 | 0.020000 |
| GO:0007398\_ectoderm\_development | COL1A2 | 112 | 3 | 14.571429 | -2.993290 | 23 | 0.46 | 0.020000 |
| GO:0002468\_dendritic\_cell\_antigen\_processing\_and\_presentation | THBS1 | 1 | 1 |  |  |  |  |  |  |
| GO:0002577\_regulation\_of\_antigen\_processing\_and\_presentation | THBS1 | 1 | 1 |  |  |  |  |  |  |
| GO:0002578\_negative\_regulation\_of\_antigen\_processing\_and\_presentation | THBS1 | 1 | 1 |  |  |  |  |  |  |
| GO:0002580\_regulation\_of\_antigen\_processing\_and\_presentation\_of\_peptide\_or\_polysaccharide\_antigen\_via\_MHC\_class\_II | THBS1 | 1 | 1 |  |  |  |  |  |  |
| GO:0002581\_negative\_regulation\_of\_antigen\_processing\_and\_presentation\_of\_peptide\_or\_polysaccharide\_antigen\_via\_MHC\_class\_II | THBS1 | 1 | 1 |  |  |  |  |  |  |
| GO:0002604\_regulation\_of\_dendritic\_cell\_antigen\_processing\_and\_presentation | THBS1 | 1 | 1 |  |  |  |  |  |  |
| GO:0002605\_negative\_regulation\_of\_dendritic\_cell\_antigen\_processing\_and\_presentation | THBS1 | 1 | 1 |  |  |  |  |  |  |
| GO:0010749\_regulation\_of\_nitric\_oxide\_mediated\_signal\_transduction | THBS1 | 1 | 1 |  |  |  |  |  |  |
| GO:0010751\_negative\_regulation\_of\_nitric\_oxide\_mediated\_signal\_transduction | THBS1 | 1 | 1 |  |  |  |  |  |  |
| GO:0010752\_regulation\_of\_cGMP-mediated\_signaling | THBS1 | 1 | 1 |  |  |  |  |  |  |
| GO:0010754\_negative\_regulation\_of\_cGMP-mediated\_signaling | THBS1 | 1 | 1 |  |  |  |  |  |  |
| GO:0010757\_negative\_regulation\_of\_plasminogen\_activation | THBS1 | 1 | 1 |  |  |  |  |  |  |
| GO:0010758\_regulation\_of\_macrophage\_chemotaxis | THBS1 | 1 | 1 |  |  |  |  |  |  |
| GO:0010759\_positive\_regulation\_of\_macrophage\_chemotaxis | THBS1 | 1 | 1 |  |  |  |  |  |  |
| GO:0018277\_protein\_amino\_acid\_deamination | LOXL1 | 1 | 1 |  |  |  |  |  |  |
| GO:0040037\_negative\_regulation\_of\_fibroblast\_growth\_factor\_receptor\_signaling\_pathway | THBS1 | 1 | 1 |  |  |  |  |  |  |
| GO:0043652\_engulfment\_of\_apoptotic\_cell | THBS1 | 1 | 1 |  |  |  |  |  |  |
| GO:0051764\_actin\_crosslink\_formation | FLNA | 1 | 1 |  |  |  |  |  |  |
| GO:0048731\_system\_development | ADAM12 | 1140 | 7 | 3.340351 | -2.628596 | 24 | 1.09 | 0.045417 |
| GO:0048731\_system\_development | COL5A1 | 1140 | 7 | 3.340351 | -2.628596 | 24 | 1.09 | 0.045417 |
| GO:0048731\_system\_development | THBS1 | 1140 | 7 | 3.340351 | -2.628596 | 24 | 1.09 | 0.045417 |
| GO:0048731\_system\_development | COL1A1 | 1140 | 7 | 3.340351 | -2.628596 | 24 | 1.09 | 0.045417 |
| GO:0048731\_system\_development | BMP1 | 1140 | 7 | 3.340351 | -2.628596 | 24 | 1.09 | 0.045417 |
| GO:0048731\_system\_development | FBN1 | 1140 | 7 | 3.340351 | -2.628596 | 24 | 1.09 | 0.045417 |
| GO:0048731\_system\_development | COL1A2 | 1140 | 7 | 3.340351 | -2.628596 | 24 | 1.09 | 0.045417 |
| GO:0001667\_ameboidal\_cell\_migration | THBS1 | 2 | 1 |  |  |  |  |  |  |
| GO:0002504\_antigen\_processing\_and\_presentation\_of\_peptide\_or\_polysaccharide\_antigen\_via\_MHC\_class\_II | THBS1 | 2 | 1 |  |  |  |  |  |  |
| GO:0002544\_chronic\_inflammatory\_response | THBS1 | 2 | 1 |  |  |  |  |  |  |
| GO:0010670\_positive\_regulation\_of\_oxygen\_and\_reactive\_oxygen\_species\_metabolic\_process | THBS1 | 2 | 1 |  |  |  |  |  |  |
| GO:0010755\_regulation\_of\_plasminogen\_activation | THBS1 | 2 | 1 |  |  |  |  |  |  |
| GO:0010761\_fibroblast\_migration | THBS1 | 2 | 1 |  |  |  |  |  |  |
| GO:0010762\_regulation\_of\_fibroblast\_migration | THBS1 | 2 | 1 |  |  |  |  |  |  |
| GO:0010763\_positive\_regulation\_of\_fibroblast\_migration | THBS1 | 2 | 1 |  |  |  |  |  |  |
| GO:0032026\_response\_to\_magnesium\_ion | THBS1 | 2 | 1 |  |  |  |  |  |  |
| GO:0032914\_positive\_regulation\_of\_transforming\_growth\_factor-beta1\_production | THBS1 | 2 | 1 |  |  |  |  |  |  |
| GO:0034605\_cellular\_response\_to\_heat | THBS1 | 2 | 1 |  |  |  |  |  |  |
| GO:0040036\_regulation\_of\_fibroblast\_growth\_factor\_receptor\_signaling\_pathway | THBS1 | 2 | 1 |  |  |  |  |  |  |
| GO:0043277\_apoptotic\_cell\_clearance | THBS1 | 2 | 1 |  |  |  |  |  |  |
| GO:0048856\_anatomical\_structure\_development | ADAM12 | 1289 | 7 | 2.954228 | -2.316784 | 25 | 2.08 | 0.083200 |
| GO:0048856\_anatomical\_structure\_development | COL5A1 | 1289 | 7 | 2.954228 | -2.316784 | 25 | 2.08 | 0.083200 |
| GO:0048856\_anatomical\_structure\_development | THBS1 | 1289 | 7 | 2.954228 | -2.316784 | 25 | 2.08 | 0.083200 |
| GO:0048856\_anatomical\_structure\_development | BMP1 | 1289 | 7 | 2.954228 | -2.316784 | 25 | 2.08 | 0.083200 |
| GO:0048856\_anatomical\_structure\_development | COL1A1 | 1289 | 7 | 2.954228 | -2.316784 | 25 | 2.08 | 0.083200 |
| GO:0048856\_anatomical\_structure\_development | FBN1 | 1289 | 7 | 2.954228 | -2.316784 | 25 | 2.08 | 0.083200 |
| GO:0048856\_anatomical\_structure\_development | COL1A2 | 1289 | 7 | 2.954228 | -2.316784 | 25 | 2.08 | 0.083200 |
| GO:0030155\_regulation\_of\_cell\_adhesion | THBS1 | 61 | 2 | 17.836066 | -2.265859 | 27 | 2.26 | 0.083704 |
| GO:0030155\_regulation\_of\_cell\_adhesion | TGFBI | 61 | 2 | 17.836066 | -2.265859 | 27 | 2.26 | 0.083704 |
| GO:0048729\_tissue\_morphogenesis | COL1A1 | 61 | 2 | 17.836066 | -2.265859 | 27 | 2.26 | 0.083704 |
| GO:0048729\_tissue\_morphogenesis | COL1A2 | 61 | 2 | 17.836066 | -2.265859 | 27 | 2.26 | 0.083704 |
| GO:0032695\_negative\_regulation\_of\_interleukin-12\_production | THBS1 | 3 | 1 |  |  |  |  |  |  |
| GO:0032905\_transforming\_growth\_factor-beta1\_production | THBS1 | 3 | 1 |  |  |  |  |  |  |
| GO:0032908\_regulation\_of\_transforming\_growth\_factor-beta1\_production | THBS1 | 3 | 1 |  |  |  |  |  |  |
| GO:0034505\_tooth\_mineralization | COL1A1 | 3 | 1 |  |  |  |  |  |  |
| GO:0045112\_integrin\_biosynthetic\_process | COL5A1 | 3 | 1 |  |  |  |  |  |  |
| GO:0051918\_negative\_regulation\_of\_fibrinolysis | THBS1 | 3 | 1 |  |  |  |  |  |  |
| GO:0016043\_cellular\_component\_organization | ADAM12 | 1366 | 7 | 2.787701 | -2.172691 | 28 | 2.82 | 0.100714 |
| GO:0016043\_cellular\_component\_organization | THBS1 | 1366 | 7 | 2.787701 | -2.172691 | 28 | 2.82 | 0.100714 |
| GO:0016043\_cellular\_component\_organization | COL5A1 | 1366 | 7 | 2.787701 | -2.172691 | 28 | 2.82 | 0.100714 |
| GO:0016043\_cellular\_component\_organization | COL1A1 | 1366 | 7 | 2.787701 | -2.172691 | 28 | 2.82 | 0.100714 |
| GO:0016043\_cellular\_component\_organization | FLNA | 1366 | 7 | 2.787701 | -2.172691 | 28 | 2.82 | 0.100714 |
| GO:0016043\_cellular\_component\_organization | COL1A2 | 1366 | 7 | 2.787701 | -2.172691 | 28 | 2.82 | 0.100714 |
| GO:0016043\_cellular\_component\_organization | SEC24D | 1366 | 7 | 2.787701 | -2.172691 | 28 | 2.82 | 0.100714 |
| GO:0007275\_multicellular\_organismal\_development | ADAM12 | 1372 | 7 | 2.775510 | -2.161895 | 29 | 2.84 | 0.097931 |
| GO:0007275\_multicellular\_organismal\_development | COL5A1 | 1372 | 7 | 2.775510 | -2.161895 | 29 | 2.84 | 0.097931 |
| GO:0007275\_multicellular\_organismal\_development | THBS1 | 1372 | 7 | 2.775510 | -2.161895 | 29 | 2.84 | 0.097931 |
| GO:0007275\_multicellular\_organismal\_development | BMP1 | 1372 | 7 | 2.775510 | -2.161895 | 29 | 2.84 | 0.097931 |
| GO:0007275\_multicellular\_organismal\_development | COL1A1 | 1372 | 7 | 2.775510 | -2.161895 | 29 | 2.84 | 0.097931 |
| GO:0007275\_multicellular\_organismal\_development | FBN1 | 1372 | 7 | 2.775510 | -2.161895 | 29 | 2.84 | 0.097931 |
| GO:0007275\_multicellular\_organismal\_development | COL1A2 | 1372 | 7 | 2.775510 | -2.161895 | 29 | 2.84 | 0.097931 |
| GO:0001502\_cartilage\_condensation | BMP1 | 4 | 1 |  |  |  |  |  |  |
| GO:0002040\_sprouting\_angiogenesis | THBS1 | 4 | 1 |  |  |  |  |  |  |
| GO:0007195\_inhibition\_of\_adenylate\_cyclase\_activity\_by\_dopamine\_receptor\_signaling\_pathway | FLNA | 4 | 1 |  |  |  |  |  |  |
| GO:0007520\_myoblast\_fusion | ADAM12 | 4 | 1 |  |  |  |  |  |  |
| GO:0010746\_regulation\_of\_plasma\_membrane\_long-chain\_fatty\_acid\_transport | THBS1 | 4 | 1 |  |  |  |  |  |  |
| GO:0010748\_negative\_regulation\_of\_plasma\_membrane\_long-chain\_fatty\_acid\_transport | THBS1 | 4 | 1 |  |  |  |  |  |  |
| GO:0032891\_negative\_regulation\_of\_organic\_acid\_transport | THBS1 | 4 | 1 |  |  |  |  |  |  |
| GO:0034394\_protein\_localization\_at\_cell\_surface | FLNA | 4 | 1 |  |  |  |  |  |  |
| GO:0035313\_wound\_healing\_\_spreading\_of\_epidermal\_cells | COL5A1 | 4 | 1 |  |  |  |  |  |  |
| GO:0043032\_positive\_regulation\_of\_macrophage\_activation | THBS1 | 4 | 1 |  |  |  |  |  |  |
| GO:0043113\_receptor\_clustering | FLNA | 4 | 1 |  |  |  |  |  |  |
| GO:0051895\_negative\_regulation\_of\_focal\_adhesion\_formation | THBS1 | 4 | 1 |  |  |  |  |  |  |
| GO:0007179\_transforming\_growth\_factor\_beta\_receptor\_signaling\_pathway | THBS1 | 72 | 2 | 15.111111 | -2.125813 | 30 | 3.06 | 0.102000 |
| GO:0007179\_transforming\_growth\_factor\_beta\_receptor\_signaling\_pathway | COL1A2 | 72 | 2 | 15.111111 | -2.125813 | 30 | 3.06 | 0.102000 |
| GO:0051051\_negative\_regulation\_of\_transport | THBS1 | 76 | 2 | 14.315789 | -2.080369 | 31 | 3.49 | 0.112581 |
| GO:0051051\_negative\_regulation\_of\_transport | FLNA | 76 | 2 | 14.315789 | -2.080369 | 31 | 3.49 | 0.112581 |
| GO:0000768\_syncytium\_formation\_by\_plasma\_membrane\_fusion | ADAM12 | 5 | 1 | 108.800000 | -2.038119 | 38 | 6.56 | 0.172632 |
| GO:0015911\_plasma\_membrane\_long-chain\_fatty\_acid\_transport | THBS1 | 5 | 1 | 108.800000 | -2.038119 | 38 | 6.56 | 0.172632 |
| GO:0030194\_positive\_regulation\_of\_blood\_coagulation | THBS1 | 5 | 1 | 108.800000 | -2.038119 | 38 | 6.56 | 0.172632 |
| GO:0031639\_plasminogen\_activation | THBS1 | 5 | 1 | 108.800000 | -2.038119 | 38 | 6.56 | 0.172632 |
| GO:0032570\_response\_to\_progesterone\_stimulus | THBS1 | 5 | 1 | 108.800000 | -2.038119 | 38 | 6.56 | 0.172632 |
| GO:0050820\_positive\_regulation\_of\_coagulation | THBS1 | 5 | 1 | 108.800000 | -2.038119 | 38 | 6.56 | 0.172632 |
| GO:0051893\_regulation\_of\_focal\_adhesion\_formation | THBS1 | 5 | 1 | 108.800000 | -2.038119 | 38 | 6.56 | 0.172632 |
| GO:0019935\_cyclic-nucleotide-mediated\_signaling | THBS1 | 82 | 2 | 13.268293 | -2.016706 | 39 | 6.79 | 0.174103 |
| GO:0019935\_cyclic-nucleotide-mediated\_signaling | FLNA | 82 | 2 | 13.268293 | -2.016706 | 39 | 6.79 | 0.174103 |
| GO:0007167\_enzyme\_linked\_receptor\_protein\_signaling\_pathway | THBS1 | 258 | 3 | 6.325581 | -1.969697 | 40 | 6.93 | 0.173250 |
| GO:0007167\_enzyme\_linked\_receptor\_protein\_signaling\_pathway | FLNA | 258 | 3 | 6.325581 | -1.969697 | 40 | 6.93 | 0.173250 |
| GO:0007167\_enzyme\_linked\_receptor\_protein\_signaling\_pathway | COL1A2 | 258 | 3 | 6.325581 | -1.969697 | 40 | 6.93 | 0.173250 |
| GO:0006911\_phagocytosis\_\_engulfment | THBS1 | 6 | 1 | 90.666667 | -1.959311 | 48 | 9.89 | 0.206042 |
| GO:0006949\_syncytium\_formation | ADAM12 | 6 | 1 | 90.666667 | -1.959311 | 48 | 9.89 | 0.206042 |
| GO:0007212\_dopamine\_receptor\_signaling\_pathway | FLNA | 6 | 1 | 90.666667 | -1.959311 | 48 | 9.89 | 0.206042 |
| GO:0032890\_regulation\_of\_organic\_acid\_transport | THBS1 | 6 | 1 | 90.666667 | -1.959311 | 48 | 9.89 | 0.206042 |
| GO:0043206\_fibril\_organization | COL5A1 | 6 | 1 | 90.666667 | -1.959311 | 48 | 9.89 | 0.206042 |
| GO:0043536\_positive\_regulation\_of\_blood\_vessel\_endothelial\_cell\_migration | THBS1 | 6 | 1 | 90.666667 | -1.959311 | 48 | 9.89 | 0.206042 |
| GO:0051917\_regulation\_of\_fibrinolysis | THBS1 | 6 | 1 | 90.666667 | -1.959311 | 48 | 9.89 | 0.206042 |
| GO:0070613\_regulation\_of\_protein\_processing | THBS1 | 6 | 1 | 90.666667 | -1.959311 | 48 | 9.89 | 0.206042 |
| GO:0032502\_developmental\_process | ADAM12 | 1919 | 8 | 2.267848 | -1.927402 | 49 | 10.2 | 0.208163 |
| GO:0032502\_developmental\_process | COL5A1 | 1919 | 8 | 2.267848 | -1.927402 | 49 | 10.2 | 0.208163 |
| GO:0032502\_developmental\_process | THBS1 | 1919 | 8 | 2.267848 | -1.927402 | 49 | 10.2 | 0.208163 |
| GO:0032502\_developmental\_process | BMP1 | 1919 | 8 | 2.267848 | -1.927402 | 49 | 10.2 | 0.208163 |
| GO:0032502\_developmental\_process | COL1A1 | 1919 | 8 | 2.267848 | -1.927402 | 49 | 10.2 | 0.208163 |
| GO:0032502\_developmental\_process | LOXL2 | 1919 | 8 | 2.267848 | -1.927402 | 49 | 10.2 | 0.208163 |
| GO:0032502\_developmental\_process | FBN1 | 1919 | 8 | 2.267848 | -1.927402 | 49 | 10.2 | 0.208163 |
| GO:0032502\_developmental\_process | COL1A2 | 1919 | 8 | 2.267848 | -1.927402 | 49 | 10.2 | 0.208163 |
| GO:0007178\_transmembrane\_receptor\_protein\_serine\_threonine\_kinase\_signaling\_pathway | THBS1 | 92 | 2 | 11.826087 | -1.920775 | 50 | 10.26 | 0.205200 |
| GO:0007178\_transmembrane\_receptor\_protein\_serine\_threonine\_kinase\_signaling\_pathway | COL1A2 | 92 | 2 | 11.826087 | -1.920775 | 50 | 10.26 | 0.205200 |
| GO:0001953\_negative\_regulation\_of\_cell-matrix\_adhesion | THBS1 | 7 | 1 | 77.714286 | -1.892736 | 57 | 12.9 | 0.226316 |
| GO:0010812\_negative\_regulation\_of\_cell-substrate\_adhesion | THBS1 | 7 | 1 | 77.714286 | -1.892736 | 57 | 12.9 | 0.226316 |
| GO:0014902\_myotube\_differentiation | ADAM12 | 7 | 1 | 77.714286 | -1.892736 | 57 | 12.9 | 0.226316 |
| GO:0019934\_cGMP-mediated\_signaling | THBS1 | 7 | 1 | 77.714286 | -1.892736 | 57 | 12.9 | 0.226316 |
| GO:0031638\_zymogen\_activation | THBS1 | 7 | 1 | 77.714286 | -1.892736 | 57 | 12.9 | 0.226316 |
| GO:0043537\_negative\_regulation\_of\_blood\_vessel\_endothelial\_cell\_migration | THBS1 | 7 | 1 | 77.714286 | -1.892736 | 57 | 12.9 | 0.226316 |
| GO:0080010\_regulation\_of\_oxygen\_and\_reactive\_oxygen\_species\_metabolic\_process | THBS1 | 7 | 1 | 77.714286 | -1.892736 | 57 | 12.9 | 0.226316 |
| GO:0051248\_negative\_regulation\_of\_protein\_metabolic\_process | THBS1 | 96 | 2 | 11.333333 | -1.885448 | 58 | 13.03 | 0.224655 |
| GO:0051248\_negative\_regulation\_of\_protein\_metabolic\_process | FLNA | 96 | 2 | 11.333333 | -1.885448 | 58 | 13.03 | 0.224655 |
| GO:0042060\_wound\_healing | COL5A1 | 98 | 2 | 11.102041 | -1.868364 | 59 | 13.22 | 0.224068 |
| GO:0042060\_wound\_healing | THBS1 | 98 | 2 | 11.102041 | -1.868364 | 59 | 13.22 | 0.224068 |
| GO:0002688\_regulation\_of\_leukocyte\_chemotaxis | THBS1 | 8 | 1 | 68.000000 | -1.835117 | 62 | 15.97 | 0.257581 |
| GO:0002690\_positive\_regulation\_of\_leukocyte\_chemotaxis | THBS1 | 8 | 1 | 68.000000 | -1.835117 | 62 | 15.97 | 0.257581 |
| GO:0048246\_macrophage\_chemotaxis | THBS1 | 8 | 1 | 68.000000 | -1.835117 | 62 | 15.97 | 0.257581 |
| GO:0032615\_interleukin-12\_production | THBS1 | 9 | 1 | 60.444444 | -1.784336 | 66 | 18.22 | 0.276061 |
| GO:0032655\_regulation\_of\_interleukin-12\_production | THBS1 | 9 | 1 | 60.444444 | -1.784336 | 66 | 18.22 | 0.276061 |
| GO:0043030\_regulation\_of\_macrophage\_activation | THBS1 | 9 | 1 | 60.444444 | -1.784336 | 66 | 18.22 | 0.276061 |
| GO:0048706\_embryonic\_skeletal\_system\_development | COL1A1 | 9 | 1 | 60.444444 | -1.784336 | 66 | 18.22 | 0.276061 |
| GO:0048646\_anatomical\_structure\_formation\_involved\_in\_morphogenesis | ADAM12 | 111 | 2 | 9.801802 | -1.765620 | 67 | 18.39 | 0.274478 |
| GO:0048646\_anatomical\_structure\_formation\_involved\_in\_morphogenesis | THBS1 | 111 | 2 | 9.801802 | -1.765620 | 67 | 18.39 | 0.274478 |
| GO:0010595\_positive\_regulation\_of\_endothelial\_cell\_migration | THBS1 | 10 | 1 | 54.400000 | -1.738951 | 71 | 20.87 | 0.293944 |
| GO:0030511\_positive\_regulation\_of\_transforming\_growth\_factor\_beta\_receptor\_signaling\_pathway | THBS1 | 10 | 1 | 54.400000 | -1.738951 | 71 | 20.87 | 0.293944 |
| GO:0042307\_positive\_regulation\_of\_protein\_import\_into\_nucleus | FLNA | 10 | 1 | 54.400000 | -1.738951 | 71 | 20.87 | 0.293944 |
| GO:0042993\_positive\_regulation\_of\_transcription\_factor\_import\_into\_nucleus | FLNA | 10 | 1 | 54.400000 | -1.738951 | 71 | 20.87 | 0.293944 |
| GO:0010608\_posttranscriptional\_regulation\_of\_gene\_expression | THBS1 | 118 | 2 | 9.220339 | -1.715483 | 72 | 21.15 | 0.293750 |
| GO:0010608\_posttranscriptional\_regulation\_of\_gene\_expression | FLNA | 118 | 2 | 9.220339 | -1.715483 | 72 | 21.15 | 0.293750 |
| GO:0032501\_multicellular\_organismal\_process | ADAM12 | 2082 | 8 | 2.090298 | -1.710686 | 73 | 21.17 | 0.290000 |
| GO:0032501\_multicellular\_organismal\_process | COL5A1 | 2082 | 8 | 2.090298 | -1.710686 | 73 | 21.17 | 0.290000 |
| GO:0032501\_multicellular\_organismal\_process | THBS1 | 2082 | 8 | 2.090298 | -1.710686 | 73 | 21.17 | 0.290000 |
| GO:0032501\_multicellular\_organismal\_process | BMP1 | 2082 | 8 | 2.090298 | -1.710686 | 73 | 21.17 | 0.290000 |
| GO:0032501\_multicellular\_organismal\_process | COL1A1 | 2082 | 8 | 2.090298 | -1.710686 | 73 | 21.17 | 0.290000 |
| GO:0032501\_multicellular\_organismal\_process | FBN1 | 2082 | 8 | 2.090298 | -1.710686 | 73 | 21.17 | 0.290000 |
| GO:0032501\_multicellular\_organismal\_process | TGFBI | 2082 | 8 | 2.090298 | -1.710686 | 73 | 21.17 | 0.290000 |
| GO:0032501\_multicellular\_organismal\_process | COL1A2 | 2082 | 8 | 2.090298 | -1.710686 | 73 | 21.17 | 0.290000 |
| GO:0002687\_positive\_regulation\_of\_leukocyte\_migration | THBS1 | 11 | 1 | 49.454545 | -1.697930 | 79 | 22.94 | 0.290380 |
| GO:0007009\_plasma\_membrane\_organization | COL5A1 | 11 | 1 | 49.454545 | -1.697930 | 79 | 22.94 | 0.290380 |
| GO:0007172\_signal\_complex\_assembly | FLNA | 11 | 1 | 49.454545 | -1.697930 | 79 | 22.94 | 0.290380 |
| GO:0042730\_fibrinolysis | THBS1 | 11 | 1 | 49.454545 | -1.697930 | 79 | 22.94 | 0.290380 |
| GO:0043535\_regulation\_of\_blood\_vessel\_endothelial\_cell\_migration | THBS1 | 11 | 1 | 49.454545 | -1.697930 | 79 | 22.94 | 0.290380 |
| GO:0051220\_cytoplasmic\_sequestering\_of\_protein | FLNA | 11 | 1 | 49.454545 | -1.697930 | 79 | 22.94 | 0.290380 |
| GO:0002685\_regulation\_of\_leukocyte\_migration | THBS1 | 12 | 1 | 45.333333 | -1.660514 | 86 | 25.2 | 0.293023 |
| GO:0010596\_negative\_regulation\_of\_endothelial\_cell\_migration | THBS1 | 12 | 1 | 45.333333 | -1.660514 | 86 | 25.2 | 0.293023 |
| GO:0019882\_antigen\_processing\_and\_presentation | THBS1 | 12 | 1 | 45.333333 | -1.660514 | 86 | 25.2 | 0.293023 |
| GO:0031532\_actin\_cytoskeleton\_reorganization | FLNA | 12 | 1 | 45.333333 | -1.660514 | 86 | 25.2 | 0.293023 |
| GO:0045026\_plasma\_membrane\_fusion | ADAM12 | 12 | 1 | 45.333333 | -1.660514 | 86 | 25.2 | 0.293023 |
| GO:0048705\_skeletal\_system\_morphogenesis | BMP1 | 12 | 1 | 45.333333 | -1.660514 | 86 | 25.2 | 0.293023 |
| GO:0051216\_cartilage\_development | BMP1 | 12 | 1 | 45.333333 | -1.660514 | 86 | 25.2 | 0.293023 |
| GO:0001952\_regulation\_of\_cell-matrix\_adhesion | THBS1 | 13 | 1 | 41.846154 | -1.626124 | 96 | 27.18 | 0.283125 |
| GO:0007193\_inhibition\_of\_adenylate\_cyclase\_activity\_by\_G-protein\_signaling | FLNA | 13 | 1 | 41.846154 | -1.626124 | 96 | 27.18 | 0.283125 |
| GO:0007263\_nitric\_oxide\_mediated\_signal\_transduction | THBS1 | 13 | 1 | 41.846154 | -1.626124 | 96 | 27.18 | 0.283125 |
| GO:0009746\_response\_to\_hexose\_stimulus | THBS1 | 13 | 1 | 41.846154 | -1.626124 | 96 | 27.18 | 0.283125 |
| GO:0009749\_response\_to\_glucose\_stimulus | THBS1 | 13 | 1 | 41.846154 | -1.626124 | 96 | 27.18 | 0.283125 |
| GO:0010810\_regulation\_of\_cell-substrate\_adhesion | THBS1 | 13 | 1 | 41.846154 | -1.626124 | 96 | 27.18 | 0.283125 |
| GO:0018149\_peptide\_cross-linking | THBS1 | 13 | 1 | 41.846154 | -1.626124 | 96 | 27.18 | 0.283125 |
| GO:0034284\_response\_to\_monosaccharide\_stimulus | THBS1 | 13 | 1 | 41.846154 | -1.626124 | 96 | 27.18 | 0.283125 |
| GO:0045766\_positive\_regulation\_of\_angiogenesis | THBS1 | 13 | 1 | 41.846154 | -1.626124 | 96 | 27.18 | 0.283125 |
| GO:0046824\_positive\_regulation\_of\_nucleocytoplasmic\_transport | FLNA | 13 | 1 | 41.846154 | -1.626124 | 96 | 27.18 | 0.283125 |
| GO:0007601\_visual\_perception | COL1A1 | 134 | 2 | 8.119403 | -1.611939 | 98 | 27.48 | 0.280408 |
| GO:0007601\_visual\_perception | TGFBI | 134 | 2 | 8.119403 | -1.611939 | 98 | 27.48 | 0.280408 |
| GO:0050953\_sensory\_perception\_of\_light\_stimulus | COL1A1 | 134 | 2 | 8.119403 | -1.611939 | 98 | 27.48 | 0.280408 |
| GO:0050953\_sensory\_perception\_of\_light\_stimulus | TGFBI | 134 | 2 | 8.119403 | -1.611939 | 98 | 27.48 | 0.280408 |
| GO:0032369\_negative\_regulation\_of\_lipid\_transport | THBS1 | 14 | 1 | 38.857143 | -1.594311 | 101 | 29.88 | 0.295842 |
| GO:0043534\_blood\_vessel\_endothelial\_cell\_migration | THBS1 | 14 | 1 | 38.857143 | -1.594311 | 101 | 29.88 | 0.295842 |
| GO:0045727\_positive\_regulation\_of\_translation | THBS1 | 14 | 1 | 38.857143 | -1.594311 | 101 | 29.88 | 0.295842 |
| GO:0001937\_negative\_regulation\_of\_endothelial\_cell\_proliferation | THBS1 | 15 | 1 | 36.266667 | -1.564719 | 105 | 32.4 | 0.308571 |
| GO:0042116\_macrophage\_activation | THBS1 | 15 | 1 | 36.266667 | -1.564719 | 105 | 32.4 | 0.308571 |
| GO:0048041\_focal\_adhesion\_formation | THBS1 | 15 | 1 | 36.266667 | -1.564719 | 105 | 32.4 | 0.308571 |
| GO:0048592\_eye\_morphogenesis | COL5A1 | 15 | 1 | 36.266667 | -1.564719 | 105 | 32.4 | 0.308571 |
| GO:0009408\_response\_to\_heat | THBS1 | 16 | 1 | 34.000000 | -1.537062 | 108 | 34.88 | 0.322963 |
| GO:0009743\_response\_to\_carbohydrate\_stimulus | THBS1 | 16 | 1 | 34.000000 | -1.537062 | 108 | 34.88 | 0.322963 |
| GO:0015909\_long-chain\_fatty\_acid\_transport | THBS1 | 16 | 1 | 34.000000 | -1.537062 | 108 | 34.88 | 0.322963 |
| GO:0007044\_cell-substrate\_junction\_assembly | THBS1 | 17 | 1 | 32.000000 | -1.511105 | 109 | 36.63 | 0.336055 |
| GO:0019932\_second-messenger-mediated\_signaling | THBS1 | 153 | 2 | 7.111111 | -1.505074 | 110 | 36.78 | 0.334364 |
| GO:0019932\_second-messenger-mediated\_signaling | FLNA | 153 | 2 | 7.111111 | -1.505074 | 110 | 36.78 | 0.334364 |
| GO:0001818\_negative\_regulation\_of\_cytokine\_production | THBS1 | 18 | 1 | 30.222222 | -1.486653 | 115 | 38.35 | 0.333478 |
| GO:0030195\_negative\_regulation\_of\_blood\_coagulation | THBS1 | 18 | 1 | 30.222222 | -1.486653 | 115 | 38.35 | 0.333478 |
| GO:0040017\_positive\_regulation\_of\_locomotion | THBS1 | 18 | 1 | 30.222222 | -1.486653 | 115 | 38.35 | 0.333478 |
| GO:0042177\_negative\_regulation\_of\_protein\_catabolic\_process | FLNA | 18 | 1 | 30.222222 | -1.486653 | 115 | 38.35 | 0.333478 |
| GO:0050921\_positive\_regulation\_of\_chemotaxis | THBS1 | 18 | 1 | 30.222222 | -1.486653 | 115 | 38.35 | 0.333478 |
| GO:0007169\_transmembrane\_receptor\_protein\_tyrosine\_kinase\_signaling\_pathway | THBS1 | 157 | 2 | 6.929936 | -1.484419 | 116 | 38.48 | 0.331724 |
| GO:0007169\_transmembrane\_receptor\_protein\_tyrosine\_kinase\_signaling\_pathway | FLNA | 157 | 2 | 6.929936 | -1.484419 | 116 | 38.48 | 0.331724 |
| GO:0016525\_negative\_regulation\_of\_angiogenesis | THBS1 | 19 | 1 | 28.631579 | -1.463543 | 120 | 40.27 | 0.335583 |
| GO:0032388\_positive\_regulation\_of\_intracellular\_transport | FLNA | 19 | 1 | 28.631579 | -1.463543 | 120 | 40.27 | 0.335583 |
| GO:0050819\_negative\_regulation\_of\_coagulation | THBS1 | 19 | 1 | 28.631579 | -1.463543 | 120 | 40.27 | 0.335583 |
| GO:0050920\_regulation\_of\_chemotaxis | THBS1 | 19 | 1 | 28.631579 | -1.463543 | 120 | 40.27 | 0.335583 |
| GO:0010594\_regulation\_of\_endothelial\_cell\_migration | THBS1 | 20 | 1 | 27.200000 | -1.441638 | 121 | 42.08 | 0.347769 |
| GO:0001654\_eye\_development | COL5A1 | 21 | 1 | 25.904762 | -1.420820 | 124 | 44.58 | 0.359516 |
| GO:0001936\_regulation\_of\_endothelial\_cell\_proliferation | THBS1 | 21 | 1 | 25.904762 | -1.420820 | 124 | 44.58 | 0.359516 |
| GO:0048520\_positive\_regulation\_of\_behavior | THBS1 | 21 | 1 | 25.904762 | -1.420820 | 124 | 44.58 | 0.359516 |
| GO:0015908\_fatty\_acid\_transport | THBS1 | 22 | 1 | 24.727273 | -1.400988 | 127 | 46.17 | 0.363543 |
| GO:0042990\_regulation\_of\_transcription\_factor\_import\_into\_nucleus | FLNA | 22 | 1 | 24.727273 | -1.400988 | 127 | 46.17 | 0.363543 |
| GO:0042991\_transcription\_factor\_import\_into\_nucleus | FLNA | 22 | 1 | 24.727273 | -1.400988 | 127 | 46.17 | 0.363543 |
| GO:0016477\_cell\_migration | COL5A1 | 177 | 2 | 6.146893 | -1.389118 | 128 | 46.47 | 0.363047 |
| GO:0016477\_cell\_migration | THBS1 | 177 | 2 | 6.146893 | -1.389118 | 128 | 46.47 | 0.363047 |
| GO:0007568\_aging | LOXL2 | 24 | 1 | 22.666667 | -1.363942 | 131 | 48.99 | 0.373969 |
| GO:0009266\_response\_to\_temperature\_stimulus | THBS1 | 24 | 1 | 22.666667 | -1.363942 | 131 | 48.99 | 0.373969 |
| GO:0030193\_regulation\_of\_blood\_coagulation | THBS1 | 24 | 1 | 22.666667 | -1.363942 | 131 | 48.99 | 0.373969 |
| GO:0009967\_positive\_regulation\_of\_signal\_transduction | THBS1 | 185 | 2 | 5.881081 | -1.354276 | 132 | 49.12 | 0.372121 |
| GO:0009967\_positive\_regulation\_of\_signal\_transduction | FLNA | 185 | 2 | 5.881081 | -1.354276 | 132 | 49.12 | 0.372121 |
| GO:0001935\_endothelial\_cell\_proliferation | THBS1 | 25 | 1 | 21.760000 | -1.346584 | 137 | 50.71 | 0.370146 |
| GO:0032368\_regulation\_of\_lipid\_transport | THBS1 | 25 | 1 | 21.760000 | -1.346584 | 137 | 50.71 | 0.370146 |
| GO:0050795\_regulation\_of\_behavior | THBS1 | 25 | 1 | 21.760000 | -1.346584 | 137 | 50.71 | 0.370146 |
| GO:0050818\_regulation\_of\_coagulation | THBS1 | 25 | 1 | 21.760000 | -1.346584 | 137 | 50.71 | 0.370146 |
| GO:0050821\_protein\_stabilization | FLNA | 25 | 1 | 21.760000 | -1.346584 | 137 | 50.71 | 0.370146 |
| GO:0010647\_positive\_regulation\_of\_cell\_communication | THBS1 | 189 | 2 | 5.756614 | -1.337476 | 138 | 50.89 | 0.368768 |
| GO:0010647\_positive\_regulation\_of\_cell\_communication | FLNA | 189 | 2 | 5.756614 | -1.337476 | 138 | 50.89 | 0.368768 |
| GO:0007194\_negative\_regulation\_of\_adenylate\_cyclase\_activity | FLNA | 26 | 1 | 20.923077 | -1.329922 | 141 | 52.39 | 0.371560 |
| GO:0031280\_negative\_regulation\_of\_cyclase\_activity | FLNA | 26 | 1 | 20.923077 | -1.329922 | 141 | 52.39 | 0.371560 |
| GO:0051350\_negative\_regulation\_of\_lyase\_activity | FLNA | 26 | 1 | 20.923077 | -1.329922 | 141 | 52.39 | 0.371560 |
| GO:0006909\_phagocytosis | THBS1 | 27 | 1 | 20.148148 | -1.313902 | 143 | 53.87 | 0.376713 |
| GO:0008543\_fibroblast\_growth\_factor\_receptor\_signaling\_pathway | THBS1 | 27 | 1 | 20.148148 | -1.313902 | 143 | 53.87 | 0.376713 |
| GO:0048870\_cell\_motility | COL5A1 | 197 | 2 | 5.522843 | -1.305033 | 144 | 54.14 | 0.375972 |
| GO:0048870\_cell\_motility | THBS1 | 197 | 2 | 5.522843 | -1.305033 | 144 | 54.14 | 0.375972 |
| GO:0009895\_negative\_regulation\_of\_catabolic\_process | FLNA | 28 | 1 | 19.428571 | -1.298479 | 147 | 55.81 | 0.379660 |
| GO:0031214\_biomineral\_formation | COL1A1 | 28 | 1 | 19.428571 | -1.298479 | 147 | 55.81 | 0.379660 |
| GO:0042306\_regulation\_of\_protein\_import\_into\_nucleus | FLNA | 28 | 1 | 19.428571 | -1.298479 | 147 | 55.81 | 0.379660 |
| GO:0019538\_protein\_metabolic\_process | THBS1 | 1569 | 6 | 2.080306 | -1.288586 | 148 | 56.07 | 0.378851 |
| GO:0019538\_protein\_metabolic\_process | LOX | 1569 | 6 | 2.080306 | -1.288586 | 148 | 56.07 | 0.378851 |
| GO:0019538\_protein\_metabolic\_process | FLNA | 1569 | 6 | 2.080306 | -1.288586 | 148 | 56.07 | 0.378851 |
| GO:0019538\_protein\_metabolic\_process | BMP1 | 1569 | 6 | 2.080306 | -1.288586 | 148 | 56.07 | 0.378851 |
| GO:0019538\_protein\_metabolic\_process | LOXL2 | 1569 | 6 | 2.080306 | -1.288586 | 148 | 56.07 | 0.378851 |
| GO:0019538\_protein\_metabolic\_process | LOXL1 | 1569 | 6 | 2.080306 | -1.288586 | 148 | 56.07 | 0.378851 |
| GO:0008285\_negative\_regulation\_of\_cell\_proliferation | THBS1 | 202 | 2 | 5.386139 | -1.285494 | 149 | 56.18 | 0.377047 |
| GO:0008285\_negative\_regulation\_of\_cell\_proliferation | LEPRE1 | 202 | 2 | 5.386139 | -1.285494 | 149 | 56.18 | 0.377047 |
| GO:0002274\_myeloid\_leukocyte\_activation | THBS1 | 29 | 1 | 18.758621 | -1.283609 | 155 | 57.28 | 0.369548 |
| GO:0032507\_maintenance\_of\_protein\_location\_in\_cell | FLNA | 29 | 1 | 18.758621 | -1.283609 | 155 | 57.28 | 0.369548 |
| GO:0043112\_receptor\_metabolic\_process | FLNA | 29 | 1 | 18.758621 | -1.283609 | 155 | 57.28 | 0.369548 |
| GO:0043433\_negative\_regulation\_of\_transcription\_factor\_activity | FLNA | 29 | 1 | 18.758621 | -1.283609 | 155 | 57.28 | 0.369548 |
| GO:0048741\_skeletal\_muscle\_fiber\_development | ADAM12 | 29 | 1 | 18.758621 | -1.283609 | 155 | 57.28 | 0.369548 |
| GO:0090048\_negative\_regulation\_of\_transcription\_regulator\_activity | FLNA | 29 | 1 | 18.758621 | -1.283609 | 155 | 57.28 | 0.369548 |
| GO:0002683\_negative\_regulation\_of\_immune\_system\_process | THBS1 | 30 | 1 | 18.133333 | -1.269257 | 160 | 58.49 | 0.365562 |
| GO:0032103\_positive\_regulation\_of\_response\_to\_external\_stimulus | THBS1 | 30 | 1 | 18.133333 | -1.269257 | 160 | 58.49 | 0.365562 |
| GO:0048747\_muscle\_fiber\_development | ADAM12 | 30 | 1 | 18.133333 | -1.269257 | 160 | 58.49 | 0.365562 |
| GO:0051224\_negative\_regulation\_of\_protein\_transport | FLNA | 30 | 1 | 18.133333 | -1.269257 | 160 | 58.49 | 0.365562 |
| GO:0051592\_response\_to\_calcium\_ion | THBS1 | 30 | 1 | 18.133333 | -1.269257 | 160 | 58.49 | 0.365562 |
| GO:0006888\_ER\_to\_Golgi\_vesicle-mediated\_transport | SEC24D | 31 | 1 | 17.548387 | -1.255387 | 163 | 59.82 | 0.366994 |
| GO:0007423\_sensory\_organ\_development | COL5A1 | 31 | 1 | 17.548387 | -1.255387 | 163 | 59.82 | 0.366994 |
| GO:0045185\_maintenance\_of\_protein\_location | FLNA | 31 | 1 | 17.548387 | -1.255387 | 163 | 59.82 | 0.366994 |
| GO:0030595\_leukocyte\_chemotaxis | THBS1 | 32 | 1 | 17.000000 | -1.241969 | 166 | 61.11 | 0.368133 |
| GO:0033157\_regulation\_of\_intracellular\_protein\_transport | FLNA | 32 | 1 | 17.000000 | -1.241969 | 166 | 61.11 | 0.368133 |
| GO:0043542\_endothelial\_cell\_migration | THBS1 | 32 | 1 | 17.000000 | -1.241969 | 166 | 61.11 | 0.368133 |
| GO:0034329\_cell\_junction\_assembly | THBS1 | 33 | 1 | 16.484848 | -1.228975 | 168 | 62.05 | 0.369345 |
| GO:0043392\_negative\_regulation\_of\_DNA\_binding | FLNA | 33 | 1 | 16.484848 | -1.228975 | 168 | 62.05 | 0.369345 |
| GO:0007188\_G-protein\_signaling\_\_coupled\_to\_cAMP\_nucleotide\_second\_messenger | FLNA | 34 | 1 | 16.000000 | -1.216381 | 175 | 64.03 | 0.365886 |
| GO:0015718\_monocarboxylic\_acid\_transport | THBS1 | 34 | 1 | 16.000000 | -1.216381 | 175 | 64.03 | 0.365886 |
| GO:0031400\_negative\_regulation\_of\_protein\_modification\_process | THBS1 | 34 | 1 | 16.000000 | -1.216381 | 175 | 64.03 | 0.365886 |
| GO:0031647\_regulation\_of\_protein\_stability | FLNA | 34 | 1 | 16.000000 | -1.216381 | 175 | 64.03 | 0.365886 |
| GO:0048545\_response\_to\_steroid\_hormone\_stimulus | THBS1 | 34 | 1 | 16.000000 | -1.216381 | 175 | 64.03 | 0.365886 |
| GO:0051651\_maintenance\_of\_location\_in\_cell | FLNA | 34 | 1 | 16.000000 | -1.216381 | 175 | 64.03 | 0.365886 |
| GO:0060326\_cell\_chemotaxis | THBS1 | 34 | 1 | 16.000000 | -1.216381 | 175 | 64.03 | 0.365886 |
| GO:0051049\_regulation\_of\_transport | THBS1 | 227 | 2 | 4.792952 | -1.195329 | 176 | 65.47 | 0.371989 |
| GO:0051049\_regulation\_of\_transport | FLNA | 227 | 2 | 4.792952 | -1.195329 | 176 | 65.47 | 0.371989 |
| GO:0001819\_positive\_regulation\_of\_cytokine\_production | THBS1 | 36 | 1 | 15.111111 | -1.192297 | 177 | 65.99 | 0.372825 |
| GO:0046822\_regulation\_of\_nucleocytoplasmic\_transport | FLNA | 37 | 1 | 14.702703 | -1.180768 | 178 | 67.13 | 0.377135 |
| GO:0019933\_cAMP-mediated\_signaling | FLNA | 38 | 1 | 14.315789 | -1.169556 | 181 | 68.93 | 0.380829 |
| GO:0030336\_negative\_regulation\_of\_cell\_migration | THBS1 | 38 | 1 | 14.315789 | -1.169556 | 181 | 68.93 | 0.380829 |
| GO:0045765\_regulation\_of\_angiogenesis | THBS1 | 38 | 1 | 14.315789 | -1.169556 | 181 | 68.93 | 0.380829 |
| GO:0009792\_embryonic\_development\_ending\_in\_birth\_or\_egg\_hatching | COL1A1 | 39 | 1 | 13.948718 | -1.158645 | 183 | 69.85 | 0.381694 |
| GO:0043009\_chordate\_embryonic\_development | COL1A1 | 39 | 1 | 13.948718 | -1.158645 | 183 | 69.85 | 0.381694 |
| GO:0043170\_macromolecule\_metabolic\_process | COL5A1 | 3103 | 9 | 1.577828 | -1.153015 | 184 | 69.94 | 0.380109 |
| GO:0043170\_macromolecule\_metabolic\_process | THBS1 | 3103 | 9 | 1.577828 | -1.153015 | 184 | 69.94 | 0.380109 |
| GO:0043170\_macromolecule\_metabolic\_process | LOX | 3103 | 9 | 1.577828 | -1.153015 | 184 | 69.94 | 0.380109 |
| GO:0043170\_macromolecule\_metabolic\_process | COL1A1 | 3103 | 9 | 1.577828 | -1.153015 | 184 | 69.94 | 0.380109 |
| GO:0043170\_macromolecule\_metabolic\_process | FLNA | 3103 | 9 | 1.577828 | -1.153015 | 184 | 69.94 | 0.380109 |
| GO:0043170\_macromolecule\_metabolic\_process | BMP1 | 3103 | 9 | 1.577828 | -1.153015 | 184 | 69.94 | 0.380109 |
| GO:0043170\_macromolecule\_metabolic\_process | LOXL2 | 3103 | 9 | 1.577828 | -1.153015 | 184 | 69.94 | 0.380109 |
| GO:0043170\_macromolecule\_metabolic\_process | FOSL2 | 3103 | 9 | 1.577828 | -1.153015 | 184 | 69.94 | 0.380109 |
| GO:0043170\_macromolecule\_metabolic\_process | LOXL1 | 3103 | 9 | 1.577828 | -1.153015 | 184 | 69.94 | 0.380109 |
| GO:0017015\_regulation\_of\_transforming\_growth\_factor\_beta\_receptor\_signaling\_pathway | THBS1 | 40 | 1 | 13.600000 | -1.148020 | 186 | 70.75 | 0.380376 |
| GO:0051100\_negative\_regulation\_of\_binding | FLNA | 40 | 1 | 13.600000 | -1.148020 | 186 | 70.75 | 0.380376 |
| GO:0006800\_oxygen\_and\_reactive\_oxygen\_species\_metabolic\_process | THBS1 | 41 | 1 | 13.268293 | -1.137666 | 191 | 71.78 | 0.375812 |
| GO:0007519\_skeletal\_muscle\_tissue\_development | ADAM12 | 41 | 1 | 13.268293 | -1.137666 | 191 | 71.78 | 0.375812 |
| GO:0034330\_cell\_junction\_organization | THBS1 | 41 | 1 | 13.268293 | -1.137666 | 191 | 71.78 | 0.375812 |
| GO:0051222\_positive\_regulation\_of\_protein\_transport | FLNA | 41 | 1 | 13.268293 | -1.137666 | 191 | 71.78 | 0.375812 |
| GO:0060538\_skeletal\_muscle\_organ\_development | ADAM12 | 41 | 1 | 13.268293 | -1.137666 | 191 | 71.78 | 0.375812 |
| GO:0007600\_sensory\_perception | COL1A1 | 245 | 2 | 4.440816 | -1.137121 | 192 | 71.9 | 0.374479 |
| GO:0007600\_sensory\_perception | TGFBI | 245 | 2 | 4.440816 | -1.137121 | 192 | 71.9 | 0.374479 |
| GO:0006944\_membrane\_fusion | ADAM12 | 42 | 1 | 12.952381 | -1.127570 | 195 | 73.39 | 0.376359 |
| GO:0042692\_muscle\_cell\_differentiation | ADAM12 | 42 | 1 | 12.952381 | -1.127570 | 195 | 73.39 | 0.376359 |
| GO:0051271\_negative\_regulation\_of\_cell\_motion | THBS1 | 42 | 1 | 12.952381 | -1.127570 | 195 | 73.39 | 0.376359 |
| GO:0050900\_leukocyte\_migration | THBS1 | 44 | 1 | 12.363636 | -1.108106 | 196 | 75.28 | 0.384082 |
| GO:0010926\_anatomical\_structure\_formation | ADAM12 | 560 | 3 | 2.914286 | -1.103006 | 197 | 75.37 | 0.382589 |
| GO:0010926\_anatomical\_structure\_formation | THBS1 | 560 | 3 | 2.914286 | -1.103006 | 197 | 75.37 | 0.382589 |
| GO:0010926\_anatomical\_structure\_formation | FLNA | 560 | 3 | 2.914286 | -1.103006 | 197 | 75.37 | 0.382589 |
| GO:0006464\_protein\_modification\_process | THBS1 | 922 | 4 | 2.360087 | -1.095873 | 198 | 76.26 | 0.385152 |
| GO:0006464\_protein\_modification\_process | LOX | 922 | 4 | 2.360087 | -1.095873 | 198 | 76.26 | 0.385152 |
| GO:0006464\_protein\_modification\_process | LOXL2 | 922 | 4 | 2.360087 | -1.095873 | 198 | 76.26 | 0.385152 |
| GO:0006464\_protein\_modification\_process | LOXL1 | 922 | 4 | 2.360087 | -1.095873 | 198 | 76.26 | 0.385152 |
| GO:0002696\_positive\_regulation\_of\_leukocyte\_activation | THBS1 | 46 | 1 | 11.826087 | -1.089539 | 201 | 77.64 | 0.386269 |
| GO:0010038\_response\_to\_metal\_ion | THBS1 | 46 | 1 | 11.826087 | -1.089539 | 201 | 77.64 | 0.386269 |
| GO:0030335\_positive\_regulation\_of\_cell\_migration | THBS1 | 46 | 1 | 11.826087 | -1.089539 | 201 | 77.64 | 0.386269 |
| GO:0010035\_response\_to\_inorganic\_substance | THBS1 | 47 | 1 | 11.574468 | -1.080569 | 204 | 79.1 | 0.387745 |
| GO:0032386\_regulation\_of\_intracellular\_transport | FLNA | 47 | 1 | 11.574468 | -1.080569 | 204 | 79.1 | 0.387745 |
| GO:0042176\_regulation\_of\_protein\_catabolic\_process | FLNA | 47 | 1 | 11.574468 | -1.080569 | 204 | 79.1 | 0.387745 |
| GO:0008217\_regulation\_of\_blood\_pressure | COL1A2 | 48 | 1 | 11.333333 | -1.071794 | 205 | 79.98 | 0.390146 |
| GO:0050890\_cognition | COL1A1 | 268 | 2 | 4.059701 | -1.069513 | 206 | 80.03 | 0.388495 |
| GO:0050890\_cognition | TGFBI | 268 | 2 | 4.059701 | -1.069513 | 206 | 80.03 | 0.388495 |
| GO:0050867\_positive\_regulation\_of\_cell\_activation | THBS1 | 49 | 1 | 11.102041 | -1.063209 | 207 | 81.19 | 0.392222 |
| GO:0000187\_activation\_of\_MAPK\_activity | THBS1 | 50 | 1 | 10.880000 | -1.054804 | 212 | 82.14 | 0.387453 |
| GO:0001666\_response\_to\_hypoxia | THBS1 | 50 | 1 | 10.880000 | -1.054804 | 212 | 82.14 | 0.387453 |
| GO:0007266\_Rho\_protein\_signal\_transduction | COL1A2 | 50 | 1 | 10.880000 | -1.054804 | 212 | 82.14 | 0.387453 |
| GO:0045761\_regulation\_of\_adenylate\_cyclase\_activity | FLNA | 50 | 1 | 10.880000 | -1.054804 | 212 | 82.14 | 0.387453 |
| GO:0051272\_positive\_regulation\_of\_cell\_motion | THBS1 | 50 | 1 | 10.880000 | -1.054804 | 212 | 82.14 | 0.387453 |
| GO:0031279\_regulation\_of\_cyclase\_activity | FLNA | 51 | 1 | 10.666667 | -1.046573 | 214 | 83.17 | 0.388645 |
| GO:0070482\_response\_to\_oxygen\_levels | THBS1 | 51 | 1 | 10.666667 | -1.046573 | 214 | 83.17 | 0.388645 |
| GO:0043412\_biopolymer\_modification | THBS1 | 960 | 4 | 2.266667 | -1.044223 | 215 | 83.35 | 0.387674 |
| GO:0043412\_biopolymer\_modification | LOX | 960 | 4 | 2.266667 | -1.044223 | 215 | 83.35 | 0.387674 |
| GO:0043412\_biopolymer\_modification | LOXL2 | 960 | 4 | 2.266667 | -1.044223 | 215 | 83.35 | 0.387674 |
| GO:0043412\_biopolymer\_modification | LOXL1 | 960 | 4 | 2.266667 | -1.044223 | 215 | 83.35 | 0.387674 |
| GO:0009611\_response\_to\_wounding | COL5A1 | 279 | 2 | 3.899642 | -1.039518 | 216 | 83.64 | 0.387222 |
| GO:0009611\_response\_to\_wounding | THBS1 | 279 | 2 | 3.899642 | -1.039518 | 216 | 83.64 | 0.387222 |
| GO:0016485\_protein\_processing | THBS1 | 53 | 1 | 10.264151 | -1.030605 | 218 | 85.57 | 0.392523 |
| GO:0051339\_regulation\_of\_lyase\_activity | FLNA | 53 | 1 | 10.264151 | -1.030605 | 218 | 85.57 | 0.392523 |
| GO:0007605\_sensory\_perception\_of\_sound | COL1A1 | 54 | 1 | 10.074074 | -1.022856 | 220 | 86.02 | 0.391000 |
| GO:0050954\_sensory\_perception\_of\_mechanical\_stimulus | COL1A1 | 54 | 1 | 10.074074 | -1.022856 | 220 | 86.02 | 0.391000 |
| GO:0030814\_regulation\_of\_cAMP\_metabolic\_process | FLNA | 55 | 1 | 9.890909 | -1.015255 | 222 | 86.9 | 0.391441 |
| GO:0030817\_regulation\_of\_cAMP\_biosynthetic\_process | FLNA | 55 | 1 | 9.890909 | -1.015255 | 222 | 86.9 | 0.391441 |
| GO:0040011\_locomotion | COL5A1 | 292 | 2 | 3.726027 | -1.005806 | 223 | 87.82 | 0.393812 |
| GO:0040011\_locomotion | THBS1 | 292 | 2 | 3.726027 | -1.005806 | 223 | 87.82 | 0.393812 |
| GO:0016192\_vesicle-mediated\_transport | THBS1 | 297 | 2 | 3.663300 | -0.993307 | 224 | 88.8 | 0.396429 |
| GO:0016192\_vesicle-mediated\_transport | SEC24D | 297 | 2 | 3.663300 | -0.993307 | 224 | 88.8 | 0.396429 |
| GO:0007160\_cell-matrix\_adhesion | THBS1 | 58 | 1 | 9.379310 | -0.993296 | 228 | 90.14 | 0.395351 |
| GO:0030799\_regulation\_of\_cyclic\_nucleotide\_metabolic\_process | FLNA | 58 | 1 | 9.379310 | -0.993296 | 228 | 90.14 | 0.395351 |
| GO:0030802\_regulation\_of\_cyclic\_nucleotide\_biosynthetic\_process | FLNA | 58 | 1 | 9.379310 | -0.993296 | 228 | 90.14 | 0.395351 |
| GO:0030808\_regulation\_of\_nucleotide\_biosynthetic\_process | FLNA | 58 | 1 | 9.379310 | -0.993296 | 228 | 90.14 | 0.395351 |
| GO:0006171\_cAMP\_biosynthetic\_process | FLNA | 59 | 1 | 9.220339 | -0.986240 | 230 | 91.01 | 0.395696 |
| GO:0051604\_protein\_maturation | THBS1 | 59 | 1 | 9.220339 | -0.986240 | 230 | 91.01 | 0.395696 |
| GO:0051246\_regulation\_of\_protein\_metabolic\_process | THBS1 | 301 | 2 | 3.614618 | -0.983485 | 231 | 91.12 | 0.394459 |
| GO:0051246\_regulation\_of\_protein\_metabolic\_process | FLNA | 301 | 2 | 3.614618 | -0.983485 | 231 | 91.12 | 0.394459 |
| GO:0006140\_regulation\_of\_nucleotide\_metabolic\_process | FLNA | 60 | 1 | 9.066667 | -0.979309 | 234 | 91.88 | 0.392650 |
| GO:0044087\_regulation\_of\_cellular\_component\_biogenesis | THBS1 | 60 | 1 | 9.066667 | -0.979309 | 234 | 91.88 | 0.392650 |
| GO:0046058\_cAMP\_metabolic\_process | FLNA | 60 | 1 | 9.066667 | -0.979309 | 234 | 91.88 | 0.392650 |
| GO:0048519\_negative\_regulation\_of\_biological\_process | THBS1 | 1013 | 4 | 2.148075 | -0.976744 | 235 | 92.06 | 0.391745 |
| GO:0048519\_negative\_regulation\_of\_biological\_process | FLNA | 1013 | 4 | 2.148075 | -0.976744 | 235 | 92.06 | 0.391745 |
| GO:0048519\_negative\_regulation\_of\_biological\_process | LEPRE1 | 1013 | 4 | 2.148075 | -0.976744 | 235 | 92.06 | 0.391745 |
| GO:0048519\_negative\_regulation\_of\_biological\_process | TGFBI | 1013 | 4 | 2.148075 | -0.976744 | 235 | 92.06 | 0.391745 |
| GO:0006928\_cell\_motion | COL5A1 | 308 | 2 | 3.532468 | -0.966665 | 237 | 93.31 | 0.393713 |
| GO:0006928\_cell\_motion | THBS1 | 308 | 2 | 3.532468 | -0.966665 | 237 | 93.31 | 0.393713 |
| GO:0051674\_localization\_of\_cell | COL5A1 | 308 | 2 | 3.532468 | -0.966665 | 237 | 93.31 | 0.393713 |
| GO:0051674\_localization\_of\_cell | THBS1 | 308 | 2 | 3.532468 | -0.966665 | 237 | 93.31 | 0.393713 |
| GO:0009190\_cyclic\_nucleotide\_biosynthetic\_process | FLNA | 62 | 1 | 8.774194 | -0.965805 | 239 | 94.55 | 0.395607 |
| GO:0043406\_positive\_regulation\_of\_MAP\_kinase\_activity | THBS1 | 62 | 1 | 8.774194 | -0.965805 | 239 | 94.55 | 0.395607 |
| GO:0008283\_cell\_proliferation | THBS1 | 647 | 3 | 2.522411 | -0.956567 | 240 | 95.07 | 0.396125 |
| GO:0008283\_cell\_proliferation | LEPRE1 | 647 | 3 | 2.522411 | -0.956567 | 240 | 95.07 | 0.396125 |
| GO:0008283\_cell\_proliferation | TGFBI | 647 | 3 | 2.522411 | -0.956567 | 240 | 95.07 | 0.396125 |
| GO:0042327\_positive\_regulation\_of\_phosphorylation | THBS1 | 64 | 1 | 8.500000 | -0.952753 | 241 | 96.33 | 0.399710 |
| GO:0051235\_maintenance\_of\_location | FLNA | 65 | 1 | 8.369231 | -0.946387 | 242 | 96.95 | 0.400620 |
| GO:0010562\_positive\_regulation\_of\_phosphorus\_metabolic\_process | THBS1 | 66 | 1 | 8.242424 | -0.940124 | 248 | 98.68 | 0.397903 |
| GO:0014706\_striated\_muscle\_tissue\_development | ADAM12 | 66 | 1 | 8.242424 | -0.940124 | 248 | 98.68 | 0.397903 |
| GO:0031589\_cell-substrate\_adhesion | THBS1 | 66 | 1 | 8.242424 | -0.940124 | 248 | 98.68 | 0.397903 |
| GO:0045937\_positive\_regulation\_of\_phosphate\_metabolic\_process | THBS1 | 66 | 1 | 8.242424 | -0.940124 | 248 | 98.68 | 0.397903 |
| GO:0051090\_regulation\_of\_transcription\_factor\_activity | FLNA | 66 | 1 | 8.242424 | -0.940124 | 248 | 98.68 | 0.397903 |
| GO:0090046\_regulation\_of\_transcription\_regulator\_activity | FLNA | 66 | 1 | 8.242424 | -0.940124 | 248 | 98.68 | 0.397903 |
| GO:0007015\_actin\_filament\_organization | FLNA | 67 | 1 | 8.119403 | -0.933961 | 251 | 99.32 | 0.395697 |
| GO:0009187\_cyclic\_nucleotide\_metabolic\_process | FLNA | 67 | 1 | 8.119403 | -0.933961 | 251 | 99.32 | 0.395697 |
| GO:0060537\_muscle\_tissue\_development | ADAM12 | 67 | 1 | 8.119403 | -0.933961 | 251 | 99.32 | 0.395697 |
| GO:0006606\_protein\_import\_into\_nucleus | FLNA | 68 | 1 | 8.000000 | -0.927895 | 253 | 100.14 | 0.395810 |
| GO:0007507\_heart\_development | FBN1 | 68 | 1 | 8.000000 | -0.927895 | 253 | 100.14 | 0.395810 |
| GO:0032879\_regulation\_of\_localization | THBS1 | 326 | 2 | 3.337423 | -0.925425 | 254 | 100.6 | 0.396063 |
| GO:0032879\_regulation\_of\_localization | FLNA | 326 | 2 | 3.337423 | -0.925425 | 254 | 100.6 | 0.396063 |
| GO:0001525\_angiogenesis | THBS1 | 69 | 1 | 7.884058 | -0.921922 | 256 | 101.57 | 0.396758 |
| GO:0051241\_negative\_regulation\_of\_multicellular\_organismal\_process | THBS1 | 69 | 1 | 7.884058 | -0.921922 | 256 | 101.57 | 0.396758 |
| GO:0030163\_protein\_catabolic\_process | FLNA | 330 | 2 | 3.296970 | -0.916631 | 257 | 101.65 | 0.395525 |
| GO:0030163\_protein\_catabolic\_process | BMP1 | 330 | 2 | 3.296970 | -0.916631 | 257 | 101.65 | 0.395525 |
| GO:0002694\_regulation\_of\_leukocyte\_activation | THBS1 | 70 | 1 | 7.771429 | -0.916041 | 260 | 102.42 | 0.393923 |
| GO:0009124\_nucleoside\_monophosphate\_biosynthetic\_process | FLNA | 70 | 1 | 7.771429 | -0.916041 | 260 | 102.42 | 0.393923 |
| GO:0051170\_nuclear\_import | FLNA | 70 | 1 | 7.771429 | -0.916041 | 260 | 102.42 | 0.393923 |
| GO:0006417\_regulation\_of\_translation | THBS1 | 71 | 1 | 7.661972 | -0.910248 | 264 | 103.3 | 0.391288 |
| GO:0007050\_cell\_cycle\_arrest | THBS1 | 71 | 1 | 7.661972 | -0.910248 | 264 | 103.3 | 0.391288 |
| GO:0032101\_regulation\_of\_response\_to\_external\_stimulus | THBS1 | 71 | 1 | 7.661972 | -0.910248 | 264 | 103.3 | 0.391288 |
| GO:0051223\_regulation\_of\_protein\_transport | FLNA | 71 | 1 | 7.661972 | -0.910248 | 264 | 103.3 | 0.391288 |
| GO:0007187\_G-protein\_signaling\_\_coupled\_to\_cyclic\_nucleotide\_second\_messenger | FLNA | 73 | 1 | 7.452055 | -0.898918 | 267 | 104.68 | 0.392060 |
| GO:0007596\_blood\_coagulation | THBS1 | 73 | 1 | 7.452055 | -0.898918 | 267 | 104.68 | 0.392060 |
| GO:0009894\_regulation\_of\_catabolic\_process | FLNA | 73 | 1 | 7.452055 | -0.898918 | 267 | 104.68 | 0.392060 |
| GO:0050817\_coagulation | THBS1 | 74 | 1 | 7.351351 | -0.893376 | 268 | 105.55 | 0.393843 |
| GO:0006869\_lipid\_transport | THBS1 | 75 | 1 | 7.253333 | -0.887914 | 270 | 106.36 | 0.393926 |
| GO:0070201\_regulation\_of\_establishment\_of\_protein\_localization | FLNA | 75 | 1 | 7.253333 | -0.887914 | 270 | 106.36 | 0.393926 |
| GO:0034504\_protein\_localization\_in\_nucleus | FLNA | 76 | 1 | 7.157895 | -0.882529 | 272 | 107.45 | 0.395037 |
| GO:0051101\_regulation\_of\_DNA\_binding | FLNA | 76 | 1 | 7.157895 | -0.882529 | 272 | 107.45 | 0.395037 |
| GO:0009123\_nucleoside\_monophosphate\_metabolic\_process | FLNA | 77 | 1 | 7.064935 | -0.877218 | 275 | 108.31 | 0.393855 |
| GO:0050865\_regulation\_of\_cell\_activation | THBS1 | 77 | 1 | 7.064935 | -0.877218 | 275 | 108.31 | 0.393855 |
| GO:0051129\_negative\_regulation\_of\_cellular\_component\_organization | THBS1 | 77 | 1 | 7.064935 | -0.877218 | 275 | 108.31 | 0.393855 |
| GO:0006164\_purine\_nucleotide\_biosynthetic\_process | FLNA | 79 | 1 | 6.886076 | -0.866816 | 279 | 109.61 | 0.392867 |
| GO:0007599\_hemostasis | THBS1 | 79 | 1 | 6.886076 | -0.866816 | 279 | 109.61 | 0.392867 |
| GO:0032880\_regulation\_of\_protein\_localization | FLNA | 79 | 1 | 6.886076 | -0.866816 | 279 | 109.61 | 0.392867 |
| GO:0046942\_carboxylic\_acid\_transport | THBS1 | 79 | 1 | 6.886076 | -0.866816 | 279 | 109.61 | 0.392867 |
| GO:0003008\_system\_process | COL1A1 | 710 | 3 | 2.298592 | -0.865750 | 280 | 109.86 | 0.392357 |
| GO:0003008\_system\_process | TGFBI | 710 | 3 | 2.298592 | -0.865750 | 280 | 109.86 | 0.392357 |
| GO:0003008\_system\_process | COL1A2 | 710 | 3 | 2.298592 | -0.865750 | 280 | 109.86 | 0.392357 |
| GO:0015849\_organic\_acid\_transport | THBS1 | 80 | 1 | 6.800000 | -0.861719 | 281 | 110.22 | 0.392242 |
| GO:0048193\_Golgi\_vesicle\_transport | SEC24D | 82 | 1 | 6.634146 | -0.851729 | 283 | 111.74 | 0.394841 |
| GO:0048514\_blood\_vessel\_morphogenesis | THBS1 | 82 | 1 | 6.634146 | -0.851729 | 283 | 111.74 | 0.394841 |
| GO:0043123\_positive\_regulation\_of\_I-kappaB\_kinase\_NF-kappaB\_cascade | FLNA | 84 | 1 | 6.476190 | -0.841996 | 284 | 113.39 | 0.399261 |
| GO:0007243\_protein\_kinase\_cascade | THBS1 | 377 | 2 | 2.885942 | -0.822100 | 285 | 116.0 | 0.407018 |
| GO:0007243\_protein\_kinase\_cascade | FLNA | 377 | 2 | 2.885942 | -0.822100 | 285 | 116.0 | 0.407018 |
| GO:0009966\_regulation\_of\_signal\_transduction | THBS1 | 378 | 2 | 2.878307 | -0.820248 | 286 | 116.28 | 0.406573 |
| GO:0009966\_regulation\_of\_signal\_transduction | FLNA | 378 | 2 | 2.878307 | -0.820248 | 286 | 116.28 | 0.406573 |
| GO:0030334\_regulation\_of\_cell\_migration | THBS1 | 89 | 1 | 6.112360 | -0.818716 | 289 | 117.25 | 0.405709 |
| GO:0043405\_regulation\_of\_MAP\_kinase\_activity | THBS1 | 89 | 1 | 6.112360 | -0.818716 | 289 | 117.25 | 0.405709 |
| GO:0051240\_positive\_regulation\_of\_multicellular\_organismal\_process | THBS1 | 89 | 1 | 6.112360 | -0.818716 | 289 | 117.25 | 0.405709 |
| GO:0009165\_nucleotide\_biosynthetic\_process | FLNA | 90 | 1 | 6.044444 | -0.814230 | 290 | 117.79 | 0.406172 |
| GO:0032269\_negative\_regulation\_of\_cellular\_protein\_metabolic\_process | THBS1 | 91 | 1 | 5.978022 | -0.809797 | 291 | 118.38 | 0.406804 |
| GO:0017038\_protein\_import | FLNA | 93 | 1 | 5.849462 | -0.801087 | 293 | 119.38 | 0.407440 |
| GO:0043122\_regulation\_of\_I-kappaB\_kinase\_NF-kappaB\_cascade | FLNA | 93 | 1 | 5.849462 | -0.801087 | 293 | 119.38 | 0.407440 |
| GO:0033365\_protein\_localization\_in\_organelle | FLNA | 95 | 1 | 5.726316 | -0.792577 | 295 | 120.09 | 0.407085 |
| GO:0050878\_regulation\_of\_body\_fluid\_levels | THBS1 | 95 | 1 | 5.726316 | -0.792577 | 295 | 120.09 | 0.407085 |
| GO:0009059\_macromolecule\_biosynthetic\_process | COL5A1 | 1626 | 5 | 1.672817 | -0.790078 | 296 | 120.37 | 0.406655 |
| GO:0009059\_macromolecule\_biosynthetic\_process | THBS1 | 1626 | 5 | 1.672817 | -0.790078 | 296 | 120.37 | 0.406655 |
| GO:0009059\_macromolecule\_biosynthetic\_process | COL1A1 | 1626 | 5 | 1.672817 | -0.790078 | 296 | 120.37 | 0.406655 |
| GO:0009059\_macromolecule\_biosynthetic\_process | FLNA | 1626 | 5 | 1.672817 | -0.790078 | 296 | 120.37 | 0.406655 |
| GO:0009059\_macromolecule\_biosynthetic\_process | FOSL2 | 1626 | 5 | 1.672817 | -0.790078 | 296 | 120.37 | 0.406655 |
| GO:0040012\_regulation\_of\_locomotion | THBS1 | 96 | 1 | 5.666667 | -0.788395 | 297 | 121.14 | 0.407879 |
| GO:0051098\_regulation\_of\_binding | FLNA | 97 | 1 | 5.608247 | -0.784260 | 298 | 121.6 | 0.408054 |
| GO:0051270\_regulation\_of\_cell\_motion | THBS1 | 98 | 1 | 5.551020 | -0.780171 | 299 | 122.37 | 0.409264 |
| GO:0006163\_purine\_nucleotide\_metabolic\_process | FLNA | 99 | 1 | 5.494949 | -0.776127 | 301 | 122.87 | 0.408206 |
| GO:0009968\_negative\_regulation\_of\_signal\_transduction | THBS1 | 99 | 1 | 5.494949 | -0.776127 | 301 | 122.87 | 0.408206 |
| GO:0022603\_regulation\_of\_anatomical\_structure\_morphogenesis | THBS1 | 100 | 1 | 5.440000 | -0.772127 | 303 | 123.73 | 0.408350 |
| GO:0055114\_oxidation\_reduction | LOXL1 | 100 | 1 | 5.440000 | -0.772127 | 303 | 123.73 | 0.408350 |
| GO:0043623\_cellular\_protein\_complex\_assembly | FLNA | 101 | 1 | 5.386139 | -0.768171 | 304 | 124.2 | 0.408553 |
| GO:0010648\_negative\_regulation\_of\_cell\_communication | THBS1 | 102 | 1 | 5.333333 | -0.764257 | 305 | 124.39 | 0.407836 |
| GO:0042127\_regulation\_of\_cell\_proliferation | THBS1 | 411 | 2 | 2.647202 | -0.762359 | 306 | 124.72 | 0.407582 |
| GO:0042127\_regulation\_of\_cell\_proliferation | LEPRE1 | 411 | 2 | 2.647202 | -0.762359 | 306 | 124.72 | 0.407582 |
| GO:0001817\_regulation\_of\_cytokine\_production | THBS1 | 103 | 1 | 5.281553 | -0.760385 | 307 | 124.93 | 0.406938 |
| GO:0010605\_negative\_regulation\_of\_macromolecule\_metabolic\_process | THBS1 | 413 | 2 | 2.634383 | -0.759040 | 308 | 125.23 | 0.406591 |
| GO:0010605\_negative\_regulation\_of\_macromolecule\_metabolic\_process | FLNA | 413 | 2 | 2.634383 | -0.759040 | 308 | 125.23 | 0.406591 |
| GO:0002684\_positive\_regulation\_of\_immune\_system\_process | THBS1 | 106 | 1 | 5.132075 | -0.749010 | 309 | 125.93 | 0.407540 |
| GO:0046907\_intracellular\_transport | FLNA | 420 | 2 | 2.590476 | -0.747580 | 310 | 126.08 | 0.406710 |
| GO:0046907\_intracellular\_transport | SEC24D | 420 | 2 | 2.590476 | -0.747580 | 310 | 126.08 | 0.406710 |
| GO:0010646\_regulation\_of\_cell\_communication | THBS1 | 423 | 2 | 2.572104 | -0.742743 | 311 | 126.29 | 0.406077 |
| GO:0010646\_regulation\_of\_cell\_communication | FLNA | 423 | 2 | 2.572104 | -0.742743 | 311 | 126.29 | 0.406077 |
| GO:0009790\_embryonic\_development | COL1A1 | 109 | 1 | 4.990826 | -0.737983 | 312 | 127.72 | 0.409359 |
| GO:0043285\_biopolymer\_catabolic\_process | FLNA | 426 | 2 | 2.553991 | -0.737949 | 313 | 127.84 | 0.408435 |
| GO:0043285\_biopolymer\_catabolic\_process | BMP1 | 426 | 2 | 2.553991 | -0.737949 | 313 | 127.84 | 0.408435 |
| GO:0007265\_Ras\_protein\_signal\_transduction | COL1A2 | 110 | 1 | 4.945455 | -0.734381 | 314 | 128.25 | 0.408439 |
| GO:0001816\_cytokine\_production | THBS1 | 112 | 1 | 4.857143 | -0.727284 | 315 | 129.19 | 0.410127 |
| GO:0007166\_cell\_surface\_receptor\_linked\_signal\_transduction | THBS1 | 828 | 3 | 1.971014 | -0.722117 | 316 | 129.66 | 0.410316 |
| GO:0007166\_cell\_surface\_receptor\_linked\_signal\_transduction | FLNA | 828 | 3 | 1.971014 | -0.722117 | 316 | 129.66 | 0.410316 |
| GO:0007166\_cell\_surface\_receptor\_linked\_signal\_transduction | COL1A2 | 828 | 3 | 1.971014 | -0.722117 | 316 | 129.66 | 0.410316 |
| GO:0048584\_positive\_regulation\_of\_response\_to\_stimulus | THBS1 | 114 | 1 | 4.771930 | -0.720326 | 317 | 130.05 | 0.410252 |
| GO:0009057\_macromolecule\_catabolic\_process | FLNA | 439 | 2 | 2.478360 | -0.717664 | 318 | 130.27 | 0.409654 |
| GO:0009057\_macromolecule\_catabolic\_process | BMP1 | 439 | 2 | 2.478360 | -0.717664 | 318 | 130.27 | 0.409654 |
| GO:0009892\_negative\_regulation\_of\_metabolic\_process | THBS1 | 440 | 2 | 2.472727 | -0.716136 | 319 | 131.0 | 0.410658 |
| GO:0009892\_negative\_regulation\_of\_metabolic\_process | FLNA | 440 | 2 | 2.472727 | -0.716136 | 319 | 131.0 | 0.410658 |
| GO:0051050\_positive\_regulation\_of\_transport | FLNA | 116 | 1 | 4.689655 | -0.713500 | 320 | 131.39 | 0.410594 |
| GO:0065008\_regulation\_of\_biological\_quality | THBS1 | 848 | 3 | 1.924528 | -0.700627 | 321 | 132.7 | 0.413396 |
| GO:0065008\_regulation\_of\_biological\_quality | FLNA | 848 | 3 | 1.924528 | -0.700627 | 321 | 132.7 | 0.413396 |
| GO:0065008\_regulation\_of\_biological\_quality | COL1A2 | 848 | 3 | 1.924528 | -0.700627 | 321 | 132.7 | 0.413396 |
| GO:0006913\_nucleocytoplasmic\_transport | FLNA | 121 | 1 | 4.495868 | -0.696991 | 322 | 133.06 | 0.413230 |
| GO:0007242\_intracellular\_signaling\_cascade | THBS1 | 853 | 3 | 1.913247 | -0.695369 | 323 | 133.18 | 0.412322 |
| GO:0007242\_intracellular\_signaling\_cascade | FLNA | 853 | 3 | 1.913247 | -0.695369 | 323 | 133.18 | 0.412322 |
| GO:0007242\_intracellular\_signaling\_cascade | COL1A2 | 853 | 3 | 1.913247 | -0.695369 | 323 | 133.18 | 0.412322 |
| GO:0033674\_positive\_regulation\_of\_kinase\_activity | THBS1 | 122 | 1 | 4.459016 | -0.693780 | 327 | 134.06 | 0.409969 |
| GO:0045860\_positive\_regulation\_of\_protein\_kinase\_activity | THBS1 | 122 | 1 | 4.459016 | -0.693780 | 327 | 134.06 | 0.409969 |
| GO:0051169\_nuclear\_transport | FLNA | 122 | 1 | 4.459016 | -0.693780 | 327 | 134.06 | 0.409969 |
| GO:0060341\_regulation\_of\_cellular\_localization | FLNA | 122 | 1 | 4.459016 | -0.693780 | 327 | 134.06 | 0.409969 |
| GO:0007517\_muscle\_organ\_development | ADAM12 | 123 | 1 | 4.422764 | -0.690598 | 328 | 134.52 | 0.410122 |
| GO:0006897\_endocytosis | THBS1 | 124 | 1 | 4.387097 | -0.687445 | 330 | 135.2 | 0.409697 |
| GO:0010324\_membrane\_invagination | THBS1 | 124 | 1 | 4.387097 | -0.687445 | 330 | 135.2 | 0.409697 |
| GO:0006935\_chemotaxis | THBS1 | 125 | 1 | 4.352000 | -0.684319 | 332 | 135.94 | 0.409458 |
| GO:0042330\_taxis | THBS1 | 125 | 1 | 4.352000 | -0.684319 | 332 | 135.94 | 0.409458 |
| GO:0009605\_response\_to\_external\_stimulus | COL5A1 | 464 | 2 | 2.344828 | -0.680747 | 333 | 136.1 | 0.408709 |
| GO:0009605\_response\_to\_external\_stimulus | THBS1 | 464 | 2 | 2.344828 | -0.680747 | 333 | 136.1 | 0.408709 |
| GO:0050877\_neurological\_system\_process | COL1A1 | 468 | 2 | 2.324786 | -0.675080 | 334 | 136.78 | 0.409521 |
| GO:0050877\_neurological\_system\_process | TGFBI | 468 | 2 | 2.324786 | -0.675080 | 334 | 136.78 | 0.409521 |
| GO:0009987\_cellular\_process | ADAM12 | 6671 | 14 | 1.141658 | -0.674738 | 335 | 136.91 | 0.408687 |
| GO:0009987\_cellular\_process | COL5A1 | 6671 | 14 | 1.141658 | -0.674738 | 335 | 136.91 | 0.408687 |
| GO:0009987\_cellular\_process | LOX | 6671 | 14 | 1.141658 | -0.674738 | 335 | 136.91 | 0.408687 |
| GO:0009987\_cellular\_process | FLNA | 6671 | 14 | 1.141658 | -0.674738 | 335 | 136.91 | 0.408687 |
| GO:0009987\_cellular\_process | COL1A1 | 6671 | 14 | 1.141658 | -0.674738 | 335 | 136.91 | 0.408687 |
| GO:0009987\_cellular\_process | LOXL2 | 6671 | 14 | 1.141658 | -0.674738 | 335 | 136.91 | 0.408687 |
| GO:0009987\_cellular\_process | LEPRE1 | 6671 | 14 | 1.141658 | -0.674738 | 335 | 136.91 | 0.408687 |
| GO:0009987\_cellular\_process | TGFBI | 6671 | 14 | 1.141658 | -0.674738 | 335 | 136.91 | 0.408687 |
| GO:0009987\_cellular\_process | COL1A2 | 6671 | 14 | 1.141658 | -0.674738 | 335 | 136.91 | 0.408687 |
| GO:0009987\_cellular\_process | LOXL1 | 6671 | 14 | 1.141658 | -0.674738 | 335 | 136.91 | 0.408687 |
| GO:0009987\_cellular\_process | THBS1 | 6671 | 14 | 1.141658 | -0.674738 | 335 | 136.91 | 0.408687 |
| GO:0009987\_cellular\_process | BMP1 | 6671 | 14 | 1.141658 | -0.674738 | 335 | 136.91 | 0.408687 |
| GO:0009987\_cellular\_process | FOSL2 | 6671 | 14 | 1.141658 | -0.674738 | 335 | 136.91 | 0.408687 |
| GO:0009987\_cellular\_process | SEC24D | 6671 | 14 | 1.141658 | -0.674738 | 335 | 136.91 | 0.408687 |
| GO:0009725\_response\_to\_hormone\_stimulus | THBS1 | 129 | 1 | 4.217054 | -0.672091 | 338 | 137.84 | 0.407811 |
| GO:0010740\_positive\_regulation\_of\_protein\_kinase\_cascade | FLNA | 129 | 1 | 4.217054 | -0.672091 | 338 | 137.84 | 0.407811 |
| GO:0051347\_positive\_regulation\_of\_transferase\_activity | THBS1 | 129 | 1 | 4.217054 | -0.672091 | 338 | 137.84 | 0.407811 |
| GO:0032270\_positive\_regulation\_of\_cellular\_protein\_metabolic\_process | THBS1 | 131 | 1 | 4.152672 | -0.666135 | 339 | 139.82 | 0.412448 |
| GO:0022607\_cellular\_component\_assembly | THBS1 | 478 | 2 | 2.276151 | -0.661187 | 340 | 141.01 | 0.414735 |
| GO:0022607\_cellular\_component\_assembly | FLNA | 478 | 2 | 2.276151 | -0.661187 | 340 | 141.01 | 0.414735 |
| GO:0003013\_circulatory\_system\_process | COL1A2 | 133 | 1 | 4.090226 | -0.660280 | 342 | 141.76 | 0.414503 |
| GO:0008015\_blood\_circulation | COL1A2 | 133 | 1 | 4.090226 | -0.660280 | 342 | 141.76 | 0.414503 |
| GO:0007249\_I-kappaB\_kinase\_NF-kappaB\_cascade | FLNA | 134 | 1 | 4.059701 | -0.657389 | 343 | 142.44 | 0.415277 |
| GO:0007264\_small\_GTPase\_mediated\_signal\_transduction | COL1A2 | 135 | 1 | 4.029630 | -0.654523 | 346 | 143.49 | 0.414711 |
| GO:0009719\_response\_to\_endogenous\_stimulus | THBS1 | 135 | 1 | 4.029630 | -0.654523 | 346 | 143.49 | 0.414711 |
| GO:0051247\_positive\_regulation\_of\_protein\_metabolic\_process | THBS1 | 135 | 1 | 4.029630 | -0.654523 | 346 | 143.49 | 0.414711 |
| GO:0009628\_response\_to\_abiotic\_stimulus | THBS1 | 140 | 1 | 3.885714 | -0.640539 | 347 | 145.12 | 0.418213 |
| GO:0007626\_locomotory\_behavior | THBS1 | 142 | 1 | 3.830986 | -0.635102 | 348 | 146.54 | 0.421092 |
| GO:0000165\_MAPKKK\_cascade | THBS1 | 143 | 1 | 3.804196 | -0.632416 | 349 | 146.78 | 0.420573 |
| GO:0031399\_regulation\_of\_protein\_modification\_process | THBS1 | 144 | 1 | 3.777778 | -0.629751 | 350 | 147.63 | 0.421800 |
| GO:0006605\_protein\_targeting | FLNA | 145 | 1 | 3.751724 | -0.627107 | 352 | 148.49 | 0.421847 |
| GO:0030036\_actin\_cytoskeleton\_organization | FLNA | 145 | 1 | 3.751724 | -0.627107 | 352 | 148.49 | 0.421847 |
| GO:0006753\_nucleoside\_phosphate\_metabolic\_process | FLNA | 146 | 1 | 3.726027 | -0.624484 | 354 | 149.58 | 0.422542 |
| GO:0009117\_nucleotide\_metabolic\_process | FLNA | 146 | 1 | 3.726027 | -0.624484 | 354 | 149.58 | 0.422542 |
| GO:0048523\_negative\_regulation\_of\_cellular\_process | THBS1 | 925 | 3 | 1.764324 | -0.624363 | 355 | 149.69 | 0.421662 |
| GO:0048523\_negative\_regulation\_of\_cellular\_process | LEPRE1 | 925 | 3 | 1.764324 | -0.624363 | 355 | 149.69 | 0.421662 |
| GO:0048523\_negative\_regulation\_of\_cellular\_process | TGFBI | 925 | 3 | 1.764324 | -0.624363 | 355 | 149.69 | 0.421662 |
| GO:0080134\_regulation\_of\_response\_to\_stress | THBS1 | 147 | 1 | 3.700680 | -0.621881 | 356 | 150.7 | 0.423315 |
| GO:0044267\_cellular\_protein\_metabolic\_process | THBS1 | 1382 | 4 | 1.574530 | -0.618674 | 357 | 150.82 | 0.422465 |
| GO:0044267\_cellular\_protein\_metabolic\_process | LOX | 1382 | 4 | 1.574530 | -0.618674 | 357 | 150.82 | 0.422465 |
| GO:0044267\_cellular\_protein\_metabolic\_process | LOXL2 | 1382 | 4 | 1.574530 | -0.618674 | 357 | 150.82 | 0.422465 |
| GO:0044267\_cellular\_protein\_metabolic\_process | LOXL1 | 1382 | 4 | 1.574530 | -0.618674 | 357 | 150.82 | 0.422465 |
| GO:0045321\_leukocyte\_activation | THBS1 | 150 | 1 | 3.626667 | -0.614190 | 358 | 151.29 | 0.422598 |
| GO:0006916\_anti-apoptosis | THBS1 | 155 | 1 | 3.509677 | -0.601754 | 359 | 152.53 | 0.424875 |
| GO:0050790\_regulation\_of\_catalytic\_activity | THBS1 | 525 | 2 | 2.072381 | -0.600697 | 360 | 152.78 | 0.424389 |
| GO:0050790\_regulation\_of\_catalytic\_activity | FLNA | 525 | 2 | 2.072381 | -0.600697 | 360 | 152.78 | 0.424389 |
| GO:0016337\_cell-cell\_adhesion | BMP1 | 156 | 1 | 3.487179 | -0.599322 | 361 | 153.17 | 0.424294 |
| GO:0070887\_cellular\_response\_to\_chemical\_stimulus | THBS1 | 157 | 1 | 3.464968 | -0.596908 | 362 | 153.68 | 0.424530 |
| GO:0030029\_actin\_filament-based\_process | FLNA | 165 | 1 | 3.296970 | -0.578205 | 363 | 155.31 | 0.427851 |
| GO:0055086\_nucleobase\_\_nucleoside\_and\_nucleotide\_metabolic\_process | FLNA | 167 | 1 | 3.257485 | -0.573692 | 364 | 155.73 | 0.427830 |
| GO:0046483\_heterocycle\_metabolic\_process | FLNA | 173 | 1 | 3.144509 | -0.560519 | 365 | 157.43 | 0.431315 |
| GO:0044085\_cellular\_component\_biogenesis | THBS1 | 560 | 2 | 1.942857 | -0.560215 | 366 | 157.79 | 0.431120 |
| GO:0044085\_cellular\_component\_biogenesis | FLNA | 560 | 2 | 1.942857 | -0.560215 | 366 | 157.79 | 0.431120 |
| GO:0001775\_cell\_activation | THBS1 | 175 | 1 | 3.108571 | -0.556245 | 367 | 158.7 | 0.432425 |
| GO:0051649\_establishment\_of\_localization\_in\_cell | FLNA | 573 | 2 | 1.898778 | -0.546056 | 368 | 161.14 | 0.437880 |
| GO:0051649\_establishment\_of\_localization\_in\_cell | SEC24D | 573 | 2 | 1.898778 | -0.546056 | 368 | 161.14 | 0.437880 |
| GO:0006954\_inflammatory\_response | THBS1 | 182 | 1 | 2.989011 | -0.541723 | 369 | 162.16 | 0.439458 |
| GO:0010627\_regulation\_of\_protein\_kinase\_cascade | FLNA | 184 | 1 | 2.956522 | -0.537694 | 370 | 162.43 | 0.439000 |
| GO:0009058\_biosynthetic\_process | COL5A1 | 1988 | 5 | 1.368209 | -0.533817 | 371 | 162.95 | 0.439218 |
| GO:0009058\_biosynthetic\_process | THBS1 | 1988 | 5 | 1.368209 | -0.533817 | 371 | 162.95 | 0.439218 |
| GO:0009058\_biosynthetic\_process | COL1A1 | 1988 | 5 | 1.368209 | -0.533817 | 371 | 162.95 | 0.439218 |
| GO:0009058\_biosynthetic\_process | FLNA | 1988 | 5 | 1.368209 | -0.533817 | 371 | 162.95 | 0.439218 |
| GO:0009058\_biosynthetic\_process | FOSL2 | 1988 | 5 | 1.368209 | -0.533817 | 371 | 162.95 | 0.439218 |
| GO:0034622\_cellular\_macromolecular\_complex\_assembly | FLNA | 186 | 1 | 2.924731 | -0.533715 | 372 | 163.43 | 0.439328 |
| GO:0008219\_cell\_death | THBS1 | 585 | 2 | 1.859829 | -0.533379 | 374 | 164.03 | 0.438583 |
| GO:0008219\_cell\_death | FOSL2 | 585 | 2 | 1.859829 | -0.533379 | 374 | 164.03 | 0.438583 |
| GO:0016265\_death | THBS1 | 585 | 2 | 1.859829 | -0.533379 | 374 | 164.03 | 0.438583 |
| GO:0016265\_death | FOSL2 | 585 | 2 | 1.859829 | -0.533379 | 374 | 164.03 | 0.438583 |
| GO:0043283\_biopolymer\_metabolic\_process | THBS1 | 3027 | 7 | 1.258011 | -0.518678 | 375 | 165.81 | 0.442160 |
| GO:0043283\_biopolymer\_metabolic\_process | LOX | 3027 | 7 | 1.258011 | -0.518678 | 375 | 165.81 | 0.442160 |
| GO:0043283\_biopolymer\_metabolic\_process | FLNA | 3027 | 7 | 1.258011 | -0.518678 | 375 | 165.81 | 0.442160 |
| GO:0043283\_biopolymer\_metabolic\_process | BMP1 | 3027 | 7 | 1.258011 | -0.518678 | 375 | 165.81 | 0.442160 |
| GO:0043283\_biopolymer\_metabolic\_process | LOXL2 | 3027 | 7 | 1.258011 | -0.518678 | 375 | 165.81 | 0.442160 |
| GO:0043283\_biopolymer\_metabolic\_process | FOSL2 | 3027 | 7 | 1.258011 | -0.518678 | 375 | 165.81 | 0.442160 |
| GO:0043283\_biopolymer\_metabolic\_process | LOXL1 | 3027 | 7 | 1.258011 | -0.518678 | 375 | 165.81 | 0.442160 |
| GO:0010556\_regulation\_of\_macromolecule\_biosynthetic\_process | THBS1 | 1055 | 3 | 1.546919 | -0.515364 | 376 | 166.41 | 0.442580 |
| GO:0010556\_regulation\_of\_macromolecule\_biosynthetic\_process | FLNA | 1055 | 3 | 1.546919 | -0.515364 | 376 | 166.41 | 0.442580 |
| GO:0010556\_regulation\_of\_macromolecule\_biosynthetic\_process | FOSL2 | 1055 | 3 | 1.546919 | -0.515364 | 376 | 166.41 | 0.442580 |
| GO:0002682\_regulation\_of\_immune\_system\_process | THBS1 | 196 | 1 | 2.775510 | -0.514549 | 378 | 167.21 | 0.442354 |
| GO:0043086\_negative\_regulation\_of\_catalytic\_activity | FLNA | 196 | 1 | 2.775510 | -0.514549 | 378 | 167.21 | 0.442354 |
| GO:0065009\_regulation\_of\_molecular\_function | THBS1 | 606 | 2 | 1.795380 | -0.512053 | 379 | 167.86 | 0.442902 |
| GO:0065009\_regulation\_of\_molecular\_function | FLNA | 606 | 2 | 1.795380 | -0.512053 | 379 | 167.86 | 0.442902 |
| GO:0010468\_regulation\_of\_gene\_expression | THBS1 | 1067 | 3 | 1.529522 | -0.506374 | 380 | 168.67 | 0.443868 |
| GO:0010468\_regulation\_of\_gene\_expression | FLNA | 1067 | 3 | 1.529522 | -0.506374 | 380 | 168.67 | 0.443868 |
| GO:0010468\_regulation\_of\_gene\_expression | FOSL2 | 1067 | 3 | 1.529522 | -0.506374 | 380 | 168.67 | 0.443868 |
| GO:0008152\_metabolic\_process | COL5A1 | 4111 | 9 | 1.190951 | -0.503097 | 381 | 169.47 | 0.444803 |
| GO:0008152\_metabolic\_process | THBS1 | 4111 | 9 | 1.190951 | -0.503097 | 381 | 169.47 | 0.444803 |
| GO:0008152\_metabolic\_process | LOX | 4111 | 9 | 1.190951 | -0.503097 | 381 | 169.47 | 0.444803 |
| GO:0008152\_metabolic\_process | COL1A1 | 4111 | 9 | 1.190951 | -0.503097 | 381 | 169.47 | 0.444803 |
| GO:0008152\_metabolic\_process | BMP1 | 4111 | 9 | 1.190951 | -0.503097 | 381 | 169.47 | 0.444803 |
| GO:0008152\_metabolic\_process | FLNA | 4111 | 9 | 1.190951 | -0.503097 | 381 | 169.47 | 0.444803 |
| GO:0008152\_metabolic\_process | LOXL2 | 4111 | 9 | 1.190951 | -0.503097 | 381 | 169.47 | 0.444803 |
| GO:0008152\_metabolic\_process | FOSL2 | 4111 | 9 | 1.190951 | -0.503097 | 381 | 169.47 | 0.444803 |
| GO:0008152\_metabolic\_process | LOXL1 | 4111 | 9 | 1.190951 | -0.503097 | 381 | 169.47 | 0.444803 |
| GO:0051641\_cellular\_localization | FLNA | 617 | 2 | 1.763371 | -0.501296 | 382 | 169.69 | 0.444215 |
| GO:0051641\_cellular\_localization | SEC24D | 617 | 2 | 1.763371 | -0.501296 | 382 | 169.69 | 0.444215 |
| GO:0006886\_intracellular\_protein\_transport | FLNA | 204 | 1 | 2.666667 | -0.500031 | 383 | 169.89 | 0.443577 |
| GO:0051179\_localization | COL5A1 | 1561 | 4 | 1.393978 | -0.495756 | 384 | 170.74 | 0.444635 |
| GO:0051179\_localization | THBS1 | 1561 | 4 | 1.393978 | -0.495756 | 384 | 170.74 | 0.444635 |
| GO:0051179\_localization | FLNA | 1561 | 4 | 1.393978 | -0.495756 | 384 | 170.74 | 0.444635 |
| GO:0051179\_localization | SEC24D | 1561 | 4 | 1.393978 | -0.495756 | 384 | 170.74 | 0.444635 |
| GO:0043066\_negative\_regulation\_of\_apoptosis | THBS1 | 207 | 1 | 2.628019 | -0.494760 | 385 | 171.31 | 0.444961 |
| GO:0043069\_negative\_regulation\_of\_programmed\_cell\_death | THBS1 | 209 | 1 | 2.602871 | -0.491297 | 387 | 172.51 | 0.445762 |
| GO:0060548\_negative\_regulation\_of\_cell\_death | THBS1 | 209 | 1 | 2.602871 | -0.491297 | 387 | 172.51 | 0.445762 |
| GO:0009056\_catabolic\_process | FLNA | 633 | 2 | 1.718799 | -0.486133 | 388 | 173.61 | 0.447448 |
| GO:0009056\_catabolic\_process | BMP1 | 633 | 2 | 1.718799 | -0.486133 | 388 | 173.61 | 0.447448 |
| GO:0045859\_regulation\_of\_protein\_kinase\_activity | THBS1 | 213 | 1 | 2.553991 | -0.484488 | 389 | 174.35 | 0.448201 |
| GO:0007610\_behavior | THBS1 | 214 | 1 | 2.542056 | -0.482810 | 390 | 174.65 | 0.447821 |
| GO:0043549\_regulation\_of\_kinase\_activity | THBS1 | 217 | 1 | 2.506912 | -0.477831 | 391 | 175.43 | 0.448670 |
| GO:0031326\_regulation\_of\_cellular\_biosynthetic\_process | THBS1 | 1125 | 3 | 1.450667 | -0.465157 | 392 | 176.86 | 0.451173 |
| GO:0031326\_regulation\_of\_cellular\_biosynthetic\_process | FLNA | 1125 | 3 | 1.450667 | -0.465157 | 392 | 176.86 | 0.451173 |
| GO:0031326\_regulation\_of\_cellular\_biosynthetic\_process | FOSL2 | 1125 | 3 | 1.450667 | -0.465157 | 392 | 176.86 | 0.451173 |
| GO:0034621\_cellular\_macromolecular\_complex\_subunit\_organization | FLNA | 227 | 1 | 2.396476 | -0.461817 | 394 | 178.31 | 0.452563 |
| GO:0051338\_regulation\_of\_transferase\_activity | THBS1 | 227 | 1 | 2.396476 | -0.461817 | 394 | 178.31 | 0.452563 |
| GO:0034613\_cellular\_protein\_localization | FLNA | 228 | 1 | 2.385965 | -0.460262 | 395 | 178.53 | 0.451975 |
| GO:0070727\_cellular\_macromolecule\_localization | FLNA | 229 | 1 | 2.375546 | -0.458716 | 396 | 178.74 | 0.451364 |
| GO:0009889\_regulation\_of\_biosynthetic\_process | THBS1 | 1135 | 3 | 1.437885 | -0.458406 | 397 | 178.94 | 0.450730 |
| GO:0009889\_regulation\_of\_biosynthetic\_process | FLNA | 1135 | 3 | 1.437885 | -0.458406 | 397 | 178.94 | 0.450730 |
| GO:0009889\_regulation\_of\_biosynthetic\_process | FOSL2 | 1135 | 3 | 1.437885 | -0.458406 | 397 | 178.94 | 0.450730 |
| GO:0006412\_translation | THBS1 | 233 | 1 | 2.334764 | -0.452614 | 399 | 180.61 | 0.452657 |
| GO:0044092\_negative\_regulation\_of\_molecular\_function | FLNA | 233 | 1 | 2.334764 | -0.452614 | 399 | 180.61 | 0.452657 |
| GO:0051128\_regulation\_of\_cellular\_component\_organization | THBS1 | 237 | 1 | 2.295359 | -0.446638 | 400 | 181.25 | 0.453125 |
| GO:0048583\_regulation\_of\_response\_to\_stimulus | THBS1 | 241 | 1 | 2.257261 | -0.440784 | 401 | 181.86 | 0.453516 |
| GO:0048468\_cell\_development | ADAM12 | 251 | 1 | 2.167331 | -0.426659 | 402 | 185.0 | 0.460199 |
| GO:0006461\_protein\_complex\_assembly | FLNA | 273 | 1 | 1.992674 | -0.397910 | 404 | 188.55 | 0.466708 |
| GO:0070271\_protein\_complex\_biogenesis | FLNA | 273 | 1 | 1.992674 | -0.397910 | 404 | 188.55 | 0.466708 |
| GO:0015031\_protein\_transport | FLNA | 274 | 1 | 1.985401 | -0.396673 | 405 | 189.06 | 0.466815 |
| GO:0007010\_cytoskeleton\_organization | FLNA | 275 | 1 | 1.978182 | -0.395442 | 406 | 189.39 | 0.466478 |
| GO:0010033\_response\_to\_organic\_substance | THBS1 | 276 | 1 | 1.971014 | -0.394216 | 407 | 189.72 | 0.466143 |
| GO:0006810\_transport | THBS1 | 1243 | 3 | 1.312953 | -0.391504 | 408 | 190.98 | 0.468088 |
| GO:0006810\_transport | FLNA | 1243 | 3 | 1.312953 | -0.391504 | 408 | 190.98 | 0.468088 |
| GO:0006810\_transport | SEC24D | 1243 | 3 | 1.312953 | -0.391504 | 408 | 190.98 | 0.468088 |
| GO:0045184\_establishment\_of\_protein\_localization | FLNA | 279 | 1 | 1.949821 | -0.390573 | 409 | 191.55 | 0.468337 |
| GO:0042325\_regulation\_of\_phosphorylation | THBS1 | 285 | 1 | 1.908772 | -0.383433 | 410 | 192.04 | 0.468390 |
| GO:0032268\_regulation\_of\_cellular\_protein\_metabolic\_process | THBS1 | 286 | 1 | 1.902098 | -0.382262 | 411 | 192.9 | 0.469343 |
| GO:0051234\_establishment\_of\_localization | THBS1 | 1260 | 3 | 1.295238 | -0.381898 | 412 | 193.2 | 0.468932 |
| GO:0051234\_establishment\_of\_localization | FLNA | 1260 | 3 | 1.295238 | -0.381898 | 412 | 193.2 | 0.468932 |
| GO:0051234\_establishment\_of\_localization | SEC24D | 1260 | 3 | 1.295238 | -0.381898 | 412 | 193.2 | 0.468932 |
| GO:0051093\_negative\_regulation\_of\_developmental\_process | THBS1 | 290 | 1 | 1.875862 | -0.377628 | 413 | 194.92 | 0.471961 |
| GO:0034960\_cellular\_biopolymer\_metabolic\_process | THBS1 | 2820 | 6 | 1.157447 | -0.375419 | 414 | 195.35 | 0.471860 |
| GO:0034960\_cellular\_biopolymer\_metabolic\_process | LOX | 2820 | 6 | 1.157447 | -0.375419 | 414 | 195.35 | 0.471860 |
| GO:0034960\_cellular\_biopolymer\_metabolic\_process | FLNA | 2820 | 6 | 1.157447 | -0.375419 | 414 | 195.35 | 0.471860 |
| GO:0034960\_cellular\_biopolymer\_metabolic\_process | LOXL2 | 2820 | 6 | 1.157447 | -0.375419 | 414 | 195.35 | 0.471860 |
| GO:0034960\_cellular\_biopolymer\_metabolic\_process | FOSL2 | 2820 | 6 | 1.157447 | -0.375419 | 414 | 195.35 | 0.471860 |
| GO:0034960\_cellular\_biopolymer\_metabolic\_process | LOXL1 | 2820 | 6 | 1.157447 | -0.375419 | 414 | 195.35 | 0.471860 |
| GO:0019220\_regulation\_of\_phosphate\_metabolic\_process | THBS1 | 297 | 1 | 1.831650 | -0.369714 | 416 | 197.49 | 0.474736 |
| GO:0051174\_regulation\_of\_phosphorus\_metabolic\_process | THBS1 | 297 | 1 | 1.831650 | -0.369714 | 416 | 197.49 | 0.474736 |
| GO:0080090\_regulation\_of\_primary\_metabolic\_process | THBS1 | 1311 | 3 | 1.244851 | -0.354446 | 417 | 200.19 | 0.480072 |
| GO:0080090\_regulation\_of\_primary\_metabolic\_process | FLNA | 1311 | 3 | 1.244851 | -0.354446 | 417 | 200.19 | 0.480072 |
| GO:0080090\_regulation\_of\_primary\_metabolic\_process | FOSL2 | 1311 | 3 | 1.244851 | -0.354446 | 417 | 200.19 | 0.480072 |
| GO:0006508\_proteolysis | BMP1 | 313 | 1 | 1.738019 | -0.352497 | 418 | 200.49 | 0.479641 |
| GO:0044260\_cellular\_macromolecule\_metabolic\_process | THBS1 | 2883 | 6 | 1.132154 | -0.350156 | 419 | 200.65 | 0.478878 |
| GO:0044260\_cellular\_macromolecule\_metabolic\_process | LOX | 2883 | 6 | 1.132154 | -0.350156 | 419 | 200.65 | 0.478878 |
| GO:0044260\_cellular\_macromolecule\_metabolic\_process | FLNA | 2883 | 6 | 1.132154 | -0.350156 | 419 | 200.65 | 0.478878 |
| GO:0044260\_cellular\_macromolecule\_metabolic\_process | LOXL2 | 2883 | 6 | 1.132154 | -0.350156 | 419 | 200.65 | 0.478878 |
| GO:0044260\_cellular\_macromolecule\_metabolic\_process | FOSL2 | 2883 | 6 | 1.132154 | -0.350156 | 419 | 200.65 | 0.478878 |
| GO:0044260\_cellular\_macromolecule\_metabolic\_process | LOXL1 | 2883 | 6 | 1.132154 | -0.350156 | 419 | 200.65 | 0.478878 |
| GO:0060255\_regulation\_of\_macromolecule\_metabolic\_process | THBS1 | 1328 | 3 | 1.228916 | -0.345730 | 420 | 201.74 | 0.480333 |
| GO:0060255\_regulation\_of\_macromolecule\_metabolic\_process | FLNA | 1328 | 3 | 1.228916 | -0.345730 | 420 | 201.74 | 0.480333 |
| GO:0060255\_regulation\_of\_macromolecule\_metabolic\_process | FOSL2 | 1328 | 3 | 1.228916 | -0.345730 | 420 | 201.74 | 0.480333 |
| GO:0010557\_positive\_regulation\_of\_macromolecule\_biosynthetic\_process | THBS1 | 334 | 1 | 1.628743 | -0.331574 | 421 | 203.84 | 0.484181 |
| GO:0008104\_protein\_localization | FLNA | 339 | 1 | 1.604720 | -0.326849 | 422 | 204.51 | 0.484621 |
| GO:0051094\_positive\_regulation\_of\_developmental\_process | THBS1 | 340 | 1 | 1.600000 | -0.325915 | 423 | 205.46 | 0.485721 |
| GO:0033554\_cellular\_response\_to\_stress | THBS1 | 341 | 1 | 1.595308 | -0.324984 | 424 | 205.8 | 0.485377 |
| GO:0006357\_regulation\_of\_transcription\_from\_RNA\_polymerase\_II\_promoter | FOSL2 | 351 | 1 | 1.549858 | -0.315878 | 425 | 206.91 | 0.486847 |
| GO:0031328\_positive\_regulation\_of\_cellular\_biosynthetic\_process | THBS1 | 352 | 1 | 1.545455 | -0.314987 | 426 | 207.23 | 0.486455 |
| GO:0043085\_positive\_regulation\_of\_catalytic\_activity | THBS1 | 354 | 1 | 1.536723 | -0.313215 | 427 | 207.76 | 0.486557 |
| GO:0009891\_positive\_regulation\_of\_biosynthetic\_process | THBS1 | 359 | 1 | 1.515320 | -0.308842 | 428 | 208.26 | 0.486589 |
| GO:0007186\_G-protein\_coupled\_receptor\_protein\_signaling\_pathway | FLNA | 363 | 1 | 1.498623 | -0.305404 | 429 | 209.3 | 0.487879 |
| GO:0065003\_macromolecular\_complex\_assembly | FLNA | 366 | 1 | 1.486339 | -0.302859 | 430 | 210.31 | 0.489093 |
| GO:0006952\_defense\_response | THBS1 | 369 | 1 | 1.474255 | -0.300342 | 431 | 211.13 | 0.489861 |
| GO:0022402\_cell\_cycle\_process | THBS1 | 370 | 1 | 1.470270 | -0.299509 | 432 | 211.46 | 0.489491 |
| GO:0045449\_regulation\_of\_transcription | FLNA | 900 | 2 | 1.208889 | -0.296938 | 433 | 212.03 | 0.489677 |
| GO:0045449\_regulation\_of\_transcription | FOSL2 | 900 | 2 | 1.208889 | -0.296938 | 433 | 212.03 | 0.489677 |
| GO:0051239\_regulation\_of\_multicellular\_organismal\_process | THBS1 | 378 | 1 | 1.439153 | -0.292957 | 434 | 213.06 | 0.490922 |
| GO:0034961\_cellular\_biopolymer\_biosynthetic\_process | THBS1 | 1448 | 3 | 1.127072 | -0.289802 | 435 | 213.88 | 0.491678 |
| GO:0034961\_cellular\_biopolymer\_biosynthetic\_process | FLNA | 1448 | 3 | 1.127072 | -0.289802 | 435 | 213.88 | 0.491678 |
| GO:0034961\_cellular\_biopolymer\_biosynthetic\_process | FOSL2 | 1448 | 3 | 1.127072 | -0.289802 | 435 | 213.88 | 0.491678 |
| GO:0043284\_biopolymer\_biosynthetic\_process | THBS1 | 1458 | 3 | 1.119342 | -0.285552 | 436 | 214.27 | 0.491445 |
| GO:0043284\_biopolymer\_biosynthetic\_process | FLNA | 1458 | 3 | 1.119342 | -0.285552 | 436 | 214.27 | 0.491445 |
| GO:0043284\_biopolymer\_biosynthetic\_process | FOSL2 | 1458 | 3 | 1.119342 | -0.285552 | 436 | 214.27 | 0.491445 |
| GO:0033036\_macromolecule\_localization | FLNA | 388 | 1 | 1.402062 | -0.285032 | 437 | 214.77 | 0.491465 |
| GO:0031323\_regulation\_of\_cellular\_metabolic\_process | THBS1 | 1466 | 3 | 1.113233 | -0.282194 | 438 | 215.31 | 0.491575 |
| GO:0031323\_regulation\_of\_cellular\_metabolic\_process | FLNA | 1466 | 3 | 1.113233 | -0.282194 | 438 | 215.31 | 0.491575 |
| GO:0031323\_regulation\_of\_cellular\_metabolic\_process | FOSL2 | 1466 | 3 | 1.113233 | -0.282194 | 438 | 215.31 | 0.491575 |
| GO:0006468\_protein\_amino\_acid\_phosphorylation | THBS1 | 393 | 1 | 1.384224 | -0.281175 | 439 | 215.65 | 0.491230 |
| GO:0044093\_positive\_regulation\_of\_molecular\_function | THBS1 | 394 | 1 | 1.380711 | -0.280412 | 440 | 215.99 | 0.490886 |
| GO:0031324\_negative\_regulation\_of\_cellular\_metabolic\_process | THBS1 | 404 | 1 | 1.346535 | -0.272928 | 441 | 216.74 | 0.491474 |
| GO:0006950\_response\_to\_stress | COL5A1 | 959 | 2 | 1.134515 | -0.267078 | 442 | 218.19 | 0.493643 |
| GO:0006950\_response\_to\_stress | THBS1 | 959 | 2 | 1.134515 | -0.267078 | 442 | 218.19 | 0.493643 |
| GO:0043933\_macromolecular\_complex\_subunit\_organization | FLNA | 424 | 1 | 1.283019 | -0.258719 | 443 | 220.18 | 0.497020 |
| GO:0019222\_regulation\_of\_metabolic\_process | THBS1 | 1538 | 3 | 1.061118 | -0.253591 | 444 | 221.96 | 0.499910 |
| GO:0019222\_regulation\_of\_metabolic\_process | FLNA | 1538 | 3 | 1.061118 | -0.253591 | 444 | 221.96 | 0.499910 |
| GO:0019222\_regulation\_of\_metabolic\_process | FOSL2 | 1538 | 3 | 1.061118 | -0.253591 | 444 | 221.96 | 0.499910 |
| GO:0044238\_primary\_metabolic\_process | THBS1 | 3719 | 7 | 1.023931 | -0.247273 | 445 | 222.94 | 0.500989 |
| GO:0044238\_primary\_metabolic\_process | LOX | 3719 | 7 | 1.023931 | -0.247273 | 445 | 222.94 | 0.500989 |
| GO:0044238\_primary\_metabolic\_process | BMP1 | 3719 | 7 | 1.023931 | -0.247273 | 445 | 222.94 | 0.500989 |
| GO:0044238\_primary\_metabolic\_process | FLNA | 3719 | 7 | 1.023931 | -0.247273 | 445 | 222.94 | 0.500989 |
| GO:0044238\_primary\_metabolic\_process | LOXL2 | 3719 | 7 | 1.023931 | -0.247273 | 445 | 222.94 | 0.500989 |
| GO:0044238\_primary\_metabolic\_process | FOSL2 | 3719 | 7 | 1.023931 | -0.247273 | 445 | 222.94 | 0.500989 |
| GO:0044238\_primary\_metabolic\_process | LOXL1 | 3719 | 7 | 1.023931 | -0.247273 | 445 | 222.94 | 0.500989 |
| GO:0048522\_positive\_regulation\_of\_cellular\_process | THBS1 | 1009 | 2 | 1.078295 | -0.244227 | 446 | 223.84 | 0.501883 |
| GO:0048522\_positive\_regulation\_of\_cellular\_process | FLNA | 1009 | 2 | 1.078295 | -0.244227 | 446 | 223.84 | 0.501883 |
| GO:0010604\_positive\_regulation\_of\_macromolecule\_metabolic\_process | THBS1 | 446 | 1 | 1.219731 | -0.244163 | 447 | 224.21 | 0.501588 |
| GO:0031325\_positive\_regulation\_of\_cellular\_metabolic\_process | THBS1 | 454 | 1 | 1.198238 | -0.239127 | 448 | 225.06 | 0.502366 |
| GO:0034645\_cellular\_macromolecule\_biosynthetic\_process | THBS1 | 1600 | 3 | 1.020000 | -0.231144 | 449 | 226.32 | 0.504053 |
| GO:0034645\_cellular\_macromolecule\_biosynthetic\_process | FLNA | 1600 | 3 | 1.020000 | -0.231144 | 449 | 226.32 | 0.504053 |
| GO:0034645\_cellular\_macromolecule\_biosynthetic\_process | FOSL2 | 1600 | 3 | 1.020000 | -0.231144 | 449 | 226.32 | 0.504053 |
| GO:0019219\_regulation\_of\_nucleobase\_\_nucleoside\_\_nucleotide\_and\_nucleic\_acid\_metabolic\_process | FLNA | 1041 | 2 | 1.045149 | -0.230666 | 450 | 226.63 | 0.503622 |
| GO:0019219\_regulation\_of\_nucleobase\_\_nucleoside\_\_nucleotide\_and\_nucleic\_acid\_metabolic\_process | FOSL2 | 1041 | 2 | 1.045149 | -0.230666 | 450 | 226.63 | 0.503622 |
| GO:0042981\_regulation\_of\_apoptosis | THBS1 | 471 | 1 | 1.154989 | -0.228849 | 451 | 227.81 | 0.505122 |
| GO:0051716\_cellular\_response\_to\_stimulus | THBS1 | 474 | 1 | 1.147679 | -0.227092 | 452 | 228.19 | 0.504845 |
| GO:0009893\_positive\_regulation\_of\_metabolic\_process | THBS1 | 476 | 1 | 1.142857 | -0.225930 | 454 | 228.92 | 0.504229 |
| GO:0043067\_regulation\_of\_programmed\_cell\_death | THBS1 | 476 | 1 | 1.142857 | -0.225930 | 454 | 228.92 | 0.504229 |
| GO:0051171\_regulation\_of\_nitrogen\_compound\_metabolic\_process | FLNA | 1055 | 2 | 1.031280 | -0.224976 | 455 | 229.54 | 0.504484 |
| GO:0051171\_regulation\_of\_nitrogen\_compound\_metabolic\_process | FOSL2 | 1055 | 2 | 1.031280 | -0.224976 | 455 | 229.54 | 0.504484 |
| GO:0010941\_regulation\_of\_cell\_death | THBS1 | 478 | 1 | 1.138075 | -0.224776 | 456 | 230.43 | 0.505329 |
| GO:0006350\_transcription | FLNA | 1069 | 2 | 1.017774 | -0.219429 | 457 | 231.38 | 0.506302 |
| GO:0006350\_transcription | FOSL2 | 1069 | 2 | 1.017774 | -0.219429 | 457 | 231.38 | 0.506302 |
| GO:0007049\_cell\_cycle | THBS1 | 494 | 1 | 1.101215 | -0.215796 | 458 | 231.7 | 0.505895 |
| GO:0010467\_gene\_expression | THBS1 | 1663 | 3 | 0.981359 | -0.210217 | 459 | 232.34 | 0.506187 |
| GO:0010467\_gene\_expression | FLNA | 1663 | 3 | 0.981359 | -0.210217 | 459 | 232.34 | 0.506187 |
| GO:0010467\_gene\_expression | FOSL2 | 1663 | 3 | 0.981359 | -0.210217 | 459 | 232.34 | 0.506187 |
| GO:0048518\_positive\_regulation\_of\_biological\_process | THBS1 | 1094 | 2 | 0.994516 | -0.209864 | 460 | 232.59 | 0.505630 |
| GO:0048518\_positive\_regulation\_of\_biological\_process | FLNA | 1094 | 2 | 0.994516 | -0.209864 | 460 | 232.59 | 0.505630 |
| GO:0006366\_transcription\_from\_RNA\_polymerase\_II\_promoter | FOSL2 | 506 | 1 | 1.075099 | -0.209346 | 462 | 233.43 | 0.505260 |
| GO:0030154\_cell\_differentiation | ADAM12 | 506 | 1 | 1.075099 | -0.209346 | 462 | 233.43 | 0.505260 |
| GO:0006955\_immune\_response | THBS1 | 529 | 1 | 1.028355 | -0.197623 | 463 | 234.39 | 0.506242 |
| GO:0048869\_cellular\_developmental\_process | ADAM12 | 555 | 1 | 0.980180 | -0.185299 | 464 | 235.69 | 0.507953 |
| GO:0006915\_apoptosis | THBS1 | 565 | 1 | 0.962832 | -0.180801 | 465 | 237.33 | 0.510387 |
| GO:0012501\_programmed\_cell\_death | THBS1 | 571 | 1 | 0.952715 | -0.178164 | 466 | 237.7 | 0.510086 |
| GO:0016310\_phosphorylation | THBS1 | 601 | 1 | 0.905158 | -0.165630 | 467 | 239.37 | 0.512570 |
| GO:0050794\_regulation\_of\_cellular\_process | THBS1 | 3515 | 6 | 0.928592 | -0.162458 | 468 | 240.06 | 0.512949 |
| GO:0050794\_regulation\_of\_cellular\_process | FLNA | 3515 | 6 | 0.928592 | -0.162458 | 468 | 240.06 | 0.512949 |
| GO:0050794\_regulation\_of\_cellular\_process | LEPRE1 | 3515 | 6 | 0.928592 | -0.162458 | 468 | 240.06 | 0.512949 |
| GO:0050794\_regulation\_of\_cellular\_process | TGFBI | 3515 | 6 | 0.928592 | -0.162458 | 468 | 240.06 | 0.512949 |
| GO:0050794\_regulation\_of\_cellular\_process | COL1A2 | 3515 | 6 | 0.928592 | -0.162458 | 468 | 240.06 | 0.512949 |
| GO:0050794\_regulation\_of\_cellular\_process | FOSL2 | 3515 | 6 | 0.928592 | -0.162458 | 468 | 240.06 | 0.512949 |
| GO:0042221\_response\_to\_chemical\_stimulus | THBS1 | 631 | 1 | 0.862124 | -0.154097 | 469 | 241.61 | 0.515160 |
| GO:0050793\_regulation\_of\_developmental\_process | THBS1 | 669 | 1 | 0.813154 | -0.140772 | 470 | 244.04 | 0.519234 |
| GO:0050789\_regulation\_of\_biological\_process | THBS1 | 3649 | 6 | 0.894492 | -0.135374 | 471 | 244.49 | 0.519087 |
| GO:0050789\_regulation\_of\_biological\_process | FLNA | 3649 | 6 | 0.894492 | -0.135374 | 471 | 244.49 | 0.519087 |
| GO:0050789\_regulation\_of\_biological\_process | LEPRE1 | 3649 | 6 | 0.894492 | -0.135374 | 471 | 244.49 | 0.519087 |
| GO:0050789\_regulation\_of\_biological\_process | COL1A2 | 3649 | 6 | 0.894492 | -0.135374 | 471 | 244.49 | 0.519087 |
| GO:0050789\_regulation\_of\_biological\_process | TGFBI | 3649 | 6 | 0.894492 | -0.135374 | 471 | 244.49 | 0.519087 |
| GO:0050789\_regulation\_of\_biological\_process | FOSL2 | 3649 | 6 | 0.894492 | -0.135374 | 471 | 244.49 | 0.519087 |
| GO:0044249\_cellular\_biosynthetic\_process | THBS1 | 1951 | 3 | 0.836494 | -0.134665 | 472 | 244.77 | 0.518581 |
| GO:0044249\_cellular\_biosynthetic\_process | FLNA | 1951 | 3 | 0.836494 | -0.134665 | 472 | 244.77 | 0.518581 |
| GO:0044249\_cellular\_biosynthetic\_process | FOSL2 | 1951 | 3 | 0.836494 | -0.134665 | 472 | 244.77 | 0.518581 |
| GO:0006793\_phosphorus\_metabolic\_process | THBS1 | 697 | 1 | 0.780488 | -0.131775 | 474 | 245.61 | 0.518165 |
| GO:0006796\_phosphate\_metabolic\_process | THBS1 | 697 | 1 | 0.780488 | -0.131775 | 474 | 245.61 | 0.518165 |
| GO:0002376\_immune\_system\_process | THBS1 | 718 | 1 | 0.757660 | -0.125444 | 475 | 247.08 | 0.520168 |
| GO:0006355\_regulation\_of\_transcription\_\_DNA-dependent | FOSL2 | 723 | 1 | 0.752420 | -0.123986 | 476 | 247.72 | 0.520420 |
| GO:0043687\_post-translational\_protein\_modification | THBS1 | 728 | 1 | 0.747253 | -0.122547 | 477 | 248.28 | 0.520503 |
| GO:0007165\_signal\_transduction | THBS1 | 2029 | 3 | 0.804337 | -0.118902 | 478 | 248.5 | 0.519874 |
| GO:0007165\_signal\_transduction | FLNA | 2029 | 3 | 0.804337 | -0.118902 | 478 | 248.5 | 0.519874 |
| GO:0007165\_signal\_transduction | COL1A2 | 2029 | 3 | 0.804337 | -0.118902 | 478 | 248.5 | 0.519874 |
| GO:0051252\_regulation\_of\_RNA\_metabolic\_process | FOSL2 | 746 | 1 | 0.729223 | -0.117514 | 479 | 249.18 | 0.520209 |
| GO:0044237\_cellular\_metabolic\_process | THBS1 | 3753 | 6 | 0.869704 | -0.116841 | 480 | 249.37 | 0.519521 |
| GO:0044237\_cellular\_metabolic\_process | LOX | 3753 | 6 | 0.869704 | -0.116841 | 480 | 249.37 | 0.519521 |
| GO:0044237\_cellular\_metabolic\_process | FLNA | 3753 | 6 | 0.869704 | -0.116841 | 480 | 249.37 | 0.519521 |
| GO:0044237\_cellular\_metabolic\_process | LOXL2 | 3753 | 6 | 0.869704 | -0.116841 | 480 | 249.37 | 0.519521 |
| GO:0044237\_cellular\_metabolic\_process | FOSL2 | 3753 | 6 | 0.869704 | -0.116841 | 480 | 249.37 | 0.519521 |
| GO:0044237\_cellular\_metabolic\_process | LOXL1 | 3753 | 6 | 0.869704 | -0.116841 | 480 | 249.37 | 0.519521 |
| GO:0006996\_organelle\_organization | FLNA | 764 | 1 | 0.712042 | -0.112705 | 481 | 250.07 | 0.519896 |
| GO:0006351\_transcription\_\_DNA-dependent | FOSL2 | 884 | 1 | 0.615385 | -0.085549 | 482 | 253.39 | 0.525705 |
| GO:0032774\_RNA\_biosynthetic\_process | FOSL2 | 887 | 1 | 0.613303 | -0.084966 | 483 | 253.68 | 0.525217 |
| GO:0065007\_biological\_regulation | THBS1 | 3971 | 6 | 0.821959 | -0.084309 | 484 | 253.81 | 0.524401 |
| GO:0065007\_biological\_regulation | FLNA | 3971 | 6 | 0.821959 | -0.084309 | 484 | 253.81 | 0.524401 |
| GO:0065007\_biological\_regulation | LEPRE1 | 3971 | 6 | 0.821959 | -0.084309 | 484 | 253.81 | 0.524401 |
| GO:0065007\_biological\_regulation | COL1A2 | 3971 | 6 | 0.821959 | -0.084309 | 484 | 253.81 | 0.524401 |
| GO:0065007\_biological\_regulation | TGFBI | 3971 | 6 | 0.821959 | -0.084309 | 484 | 253.81 | 0.524401 |
| GO:0065007\_biological\_regulation | FOSL2 | 3971 | 6 | 0.821959 | -0.084309 | 484 | 253.81 | 0.524401 |
| GO:0007154\_cell\_communication | THBS1 | 2272 | 3 | 0.718310 | -0.079654 | 485 | 254.83 | 0.525423 |
| GO:0007154\_cell\_communication | FLNA | 2272 | 3 | 0.718310 | -0.079654 | 485 | 254.83 | 0.525423 |
| GO:0007154\_cell\_communication | COL1A2 | 2272 | 3 | 0.718310 | -0.079654 | 485 | 254.83 | 0.525423 |
| GO:0050896\_response\_to\_stimulus | COL5A1 | 1775 | 2 | 0.612958 | -0.060603 | 486 | 257.88 | 0.530617 |
| GO:0050896\_response\_to\_stimulus | THBS1 | 1775 | 2 | 0.612958 | -0.060603 | 486 | 257.88 | 0.530617 |
| GO:0006139\_nucleobase\_\_nucleoside\_\_nucleotide\_and\_nucleic\_acid\_metabolic\_process | FLNA | 1845 | 2 | 0.589702 | -0.053015 | 487 | 260.01 | 0.533901 |
| GO:0006139\_nucleobase\_\_nucleoside\_\_nucleotide\_and\_nucleic\_acid\_metabolic\_process | FOSL2 | 1845 | 2 | 0.589702 | -0.053015 | 487 | 260.01 | 0.533901 |
| GO:0016070\_RNA\_metabolic\_process | FOSL2 | 1230 | 1 | 0.442276 | -0.039068 | 488 | 261.69 | 0.536250 |
| GO:0006807\_nitrogen\_compound\_metabolic\_process | FLNA | 2053 | 2 | 0.529956 | -0.035281 | 489 | 262.3 | 0.536401 |
| GO:0006807\_nitrogen\_compound\_metabolic\_process | FOSL2 | 2053 | 2 | 0.529956 | -0.035281 | 489 | 262.3 | 0.536401 |
| GO:0008150\_biological\_process | ADAM12 | 8160 | 15 | 1.000000 | 0.000000 | 655 | 458.59 | 0.700137 |
| GO:0008150\_biological\_process | COL5A1 | 8160 | 15 | 1.000000 | 0.000000 | 655 | 458.59 | 0.700137 |
| GO:0008150\_biological\_process | FLNA | 8160 | 15 | 1.000000 | 0.000000 | 655 | 458.59 | 0.700137 |
| GO:0008150\_biological\_process | COL1A1 | 8160 | 15 | 1.000000 | 0.000000 | 655 | 458.59 | 0.700137 |
| GO:0008150\_biological\_process | LOX | 8160 | 15 | 1.000000 | 0.000000 | 655 | 458.59 | 0.700137 |
| GO:0008150\_biological\_process | LOXL2 | 8160 | 15 | 1.000000 | 0.000000 | 655 | 458.59 | 0.700137 |
| GO:0008150\_biological\_process | FBN1 | 8160 | 15 | 1.000000 | 0.000000 | 655 | 458.59 | 0.700137 |
| GO:0008150\_biological\_process | LEPRE1 | 8160 | 15 | 1.000000 | 0.000000 | 655 | 458.59 | 0.700137 |
| GO:0008150\_biological\_process | TGFBI | 8160 | 15 | 1.000000 | 0.000000 | 655 | 458.59 | 0.700137 |
| GO:0008150\_biological\_process | COL1A2 | 8160 | 15 | 1.000000 | 0.000000 | 655 | 458.59 | 0.700137 |
| GO:0008150\_biological\_process | LOXL1 | 8160 | 15 | 1.000000 | 0.000000 | 655 | 458.59 | 0.700137 |
| GO:0008150\_biological\_process | THBS1 | 8160 | 15 | 1.000000 | 0.000000 | 655 | 458.59 | 0.700137 |
| GO:0008150\_biological\_process | BMP1 | 8160 | 15 | 1.000000 | 0.000000 | 655 | 458.59 | 0.700137 |
| GO:0008150\_biological\_process | FOSL2 | 8160 | 15 | 1.000000 | 0.000000 | 655 | 458.59 | 0.700137 |
| GO:0008150\_biological\_process | SEC24D | 8160 | 15 | 1.000000 | 0.000000 | 655 | 458.59 | 0.700137 |
