# Supplementary material for: Concordance of Gene Expression and Functional Correlation Patterns across the NCI-60 Cell Lines and the Cancer Genome Atlas Glioblastoma Samples
Source: PLoS One. 2012 Jul 26;7(7):e40062. doi: 10.1371/journal.pone.0040062 (PMC3406063; doi:10.1371/journal.pone.0040062)
Supplement: Download S1 — Zip archive of HTGM results. (ZIP) [file pone.0040062.s007.zip › work2026406846/Generated_Total2026406846.dir/generic.BP.NCI60.0.6.ADAM12.express.genes.correlation.complete.Thu.May.19.17.25.03.2011.htgm.txt.dir/generic.BP.NCI60.0.6.ADAM12.express.genes.correlation.complete.Thu.May.19.17.25.03.2011.htgm.txt.change.html]

Category Summary Report for generic.BP.NCI60.0.6.ADAM12.express.genes.correlation.complete.Thu.May.19.17.25.03.2011.htgm.txt

# Category Summary Report for generic.BP.NCI60.0.6.ADAM12.express.genes.correlation.complete.Thu.May.19.17.25.03.2011.htgm.txt

| HYPERLINKED GO CATEGORY | TOTAL GENES | CHANGED GENES | ENRICHMENT | LOG10(p) | CUMULATIVE NUMBER OF CATEGORIES | CUMULATIVE RANDOMS LOWER BOUND | CUMULATIVE RANDOMS MEAN | CUMULATIVE RANDOMS UPPER BOUND | FALSE DISCOVERY RATE |
| --- | --- | --- | --- | --- | --- | --- | --- | --- | --- |
| GO:0043588\_skin\_development | 10 | 3 | 163.200000 | -6.222923 | 1 | 0.000000 | 0.0 | 0.000000 | 0.000000 |
| GO:0043589\_skin\_morphogenesis | 2 | 2 |  |  |  |  |  |  |  |  |
| GO:0030199\_collagen\_fibril\_organization | 17 | 3 | 96.000000 | -5.472952 | 2 | 0.000000 | 0.0 | 0.000000 | 0.000000 |
| GO:0030198\_extracellular\_matrix\_organization | 35 | 3 | 46.628571 | -4.498186 | 3 | -0.270000 | 0.03 | 0.330000 | 0.010000 |
| GO:0009887\_organ\_morphogenesis | 246 | 5 | 11.056911 | -4.251434 | 4 | -0.270000 | 0.03 | 0.330000 | 0.007500 |
| GO:0048730\_epidermis\_morphogenesis | 7 | 2 | 155.428571 | -4.181196 | 5 | -0.275268 | 0.04 | 0.355268 | 0.008000 |
| GO:0001501\_skeletal\_system\_development | 142 | 4 | 15.323944 | -3.985609 | 6 | -0.275268 | 0.04 | 0.355268 | 0.006667 |
| GO:0009888\_tissue\_development | 287 | 5 | 9.477352 | -3.932714 | 7 | -0.285477 | 0.07 | 0.425477 | 0.010000 |
| GO:0032964\_collagen\_biosynthetic\_process | 10 | 2 | 108.800000 | -3.851588 | 8 | -0.314395 | 0.09 | 0.494395 | 0.011250 |
| GO:0009653\_anatomical\_structure\_morphogenesis | 500 | 6 | 6.528000 | -3.796717 | 9 | -0.314395 | 0.09 | 0.494395 | 0.010000 |
| GO:0048513\_organ\_development | 741 | 7 | 5.139001 | -3.779512 | 10 | -0.314395 | 0.09 | 0.494395 | 0.009000 |
| GO:0043062\_extracellular\_structure\_organization | 68 | 3 | 24.000000 | -3.629959 | 11 | -0.338086 | 0.1 | 0.538086 | 0.009091 |
| GO:0032963\_collagen\_metabolic\_process | 15 | 2 | 72.533333 | -3.485918 | 13 | -0.627786 | 0.16 | 0.947786 | 0.012308 |
| GO:0044259\_multicellular\_organismal\_macromolecule\_metabolic\_process | 15 | 2 | 72.533333 | -3.485918 | 13 | -0.627786 | 0.16 | 0.947786 | 0.012308 |
| GO:0042476\_odontogenesis | 16 | 2 | 68.000000 | -3.428387 | 14 | -0.627786 | 0.16 | 0.947786 | 0.011429 |
| GO:0001568\_blood\_vessel\_development | 85 | 3 | 19.200000 | -3.343475 | 15 | -0.662833 | 0.22 | 1.102833 | 0.014667 |
| GO:0001944\_vasculature\_development | 88 | 3 | 18.545455 | -3.299192 | 16 | -0.758800 | 0.25 | 1.258800 | 0.015625 |
| GO:0044236\_multicellular\_organismal\_metabolic\_process | 19 | 2 | 57.263158 | -3.275957 | 17 | -0.875400 | 0.28 | 1.435400 | 0.016471 |
| GO:0016044\_membrane\_organization | 225 | 4 | 9.671111 | -3.218474 | 18 | -0.918514 | 0.29 | 1.498514 | 0.016111 |
| GO:0007155\_cell\_adhesion | 428 | 5 | 6.355140 | -3.124152 | 19 | -0.949639 | 0.33 | 1.609639 | 0.017368 |
| GO:0022610\_biological\_adhesion | 429 | 5 | 6.340326 | -3.119519 | 20 | -0.949639 | 0.33 | 1.609639 | 0.016500 |
| GO:0008544\_epidermis\_development | 104 | 3 | 15.692308 | -3.086909 | 21 | -1.010047 | 0.34 | 1.690047 | 0.016190 |
| GO:0007162\_negative\_regulation\_of\_cell\_adhesion | 26 | 2 | 41.846154 | -3.000298 | 22 | -1.224271 | 0.46 | 2.144271 | 0.020909 |
| GO:0007398\_ectoderm\_development | 112 | 3 | 14.571429 | -2.993290 | 23 | -1.224271 | 0.46 | 2.144271 | 0.020000 |
| GO:0002468\_dendritic\_cell\_antigen\_processing\_and\_presentation | 1 | 1 |  |  |  |  |  |  |  |  |
| GO:0002577\_regulation\_of\_antigen\_processing\_and\_presentation | 1 | 1 |  |  |  |  |  |  |  |  |
| GO:0002578\_negative\_regulation\_of\_antigen\_processing\_and\_presentation | 1 | 1 |  |  |  |  |  |  |  |  |
| GO:0002580\_regulation\_of\_antigen\_processing\_and\_presentation\_of\_peptide\_or\_polysaccharide\_antigen\_via\_MHC\_class\_II | 1 | 1 |  |  |  |  |  |  |  |  |
| GO:0002581\_negative\_regulation\_of\_antigen\_processing\_and\_presentation\_of\_peptide\_or\_polysaccharide\_antigen\_via\_MHC\_class\_II | 1 | 1 |  |  |  |  |  |  |  |  |
| GO:0002604\_regulation\_of\_dendritic\_cell\_antigen\_processing\_and\_presentation | 1 | 1 |  |  |  |  |  |  |  |  |
| GO:0002605\_negative\_regulation\_of\_dendritic\_cell\_antigen\_processing\_and\_presentation | 1 | 1 |  |  |  |  |  |  |  |  |
| GO:0010749\_regulation\_of\_nitric\_oxide\_mediated\_signal\_transduction | 1 | 1 |  |  |  |  |  |  |  |  |
| GO:0010751\_negative\_regulation\_of\_nitric\_oxide\_mediated\_signal\_transduction | 1 | 1 |  |  |  |  |  |  |  |  |
| GO:0010752\_regulation\_of\_cGMP-mediated\_signaling | 1 | 1 |  |  |  |  |  |  |  |  |
| GO:0010754\_negative\_regulation\_of\_cGMP-mediated\_signaling | 1 | 1 |  |  |  |  |  |  |  |  |
| GO:0010757\_negative\_regulation\_of\_plasminogen\_activation | 1 | 1 |  |  |  |  |  |  |  |  |
| GO:0010758\_regulation\_of\_macrophage\_chemotaxis | 1 | 1 |  |  |  |  |  |  |  |  |
| GO:0010759\_positive\_regulation\_of\_macrophage\_chemotaxis | 1 | 1 |  |  |  |  |  |  |  |  |
| GO:0018277\_protein\_amino\_acid\_deamination | 1 | 1 |  |  |  |  |  |  |  |  |
| GO:0040037\_negative\_regulation\_of\_fibroblast\_growth\_factor\_receptor\_signaling\_pathway | 1 | 1 |  |  |  |  |  |  |  |  |
| GO:0043652\_engulfment\_of\_apoptotic\_cell | 1 | 1 |  |  |  |  |  |  |  |  |
| GO:0051764\_actin\_crosslink\_formation | 1 | 1 |  |  |  |  |  |  |  |  |
| GO:0048731\_system\_development | 1140 | 7 | 3.340351 | -2.628596 | 24 | -1.843936 | 1.09 | 4.023936 | 0.045417 |
| GO:0001667\_ameboidal\_cell\_migration | 2 | 1 |  |  |  |  |  |  |  |  |
| GO:0002504\_antigen\_processing\_and\_presentation\_of\_peptide\_or\_polysaccharide\_antigen\_via\_MHC\_class\_II | 2 | 1 |  |  |  |  |  |  |  |  |
| GO:0002544\_chronic\_inflammatory\_response | 2 | 1 |  |  |  |  |  |  |  |  |
| GO:0010670\_positive\_regulation\_of\_oxygen\_and\_reactive\_oxygen\_species\_metabolic\_process | 2 | 1 |  |  |  |  |  |  |  |  |
| GO:0010755\_regulation\_of\_plasminogen\_activation | 2 | 1 |  |  |  |  |  |  |  |  |
| GO:0010761\_fibroblast\_migration | 2 | 1 |  |  |  |  |  |  |  |  |
| GO:0010762\_regulation\_of\_fibroblast\_migration | 2 | 1 |  |  |  |  |  |  |  |  |
| GO:0010763\_positive\_regulation\_of\_fibroblast\_migration | 2 | 1 |  |  |  |  |  |  |  |  |
| GO:0032026\_response\_to\_magnesium\_ion | 2 | 1 |  |  |  |  |  |  |  |  |
| GO:0032914\_positive\_regulation\_of\_transforming\_growth\_factor-beta1\_production | 2 | 1 |  |  |  |  |  |  |  |  |
| GO:0034605\_cellular\_response\_to\_heat | 2 | 1 |  |  |  |  |  |  |  |  |
| GO:0040036\_regulation\_of\_fibroblast\_growth\_factor\_receptor\_signaling\_pathway | 2 | 1 |  |  |  |  |  |  |  |  |
| GO:0043277\_apoptotic\_cell\_clearance | 2 | 1 |  |  |  |  |  |  |  |  |
| GO:0048856\_anatomical\_structure\_development | 1289 | 7 | 2.954228 | -2.316784 | 25 | -2.489729 | 2.08 | 6.649729 | 0.083200 |
| GO:0030155\_regulation\_of\_cell\_adhesion | 61 | 2 | 17.836066 | -2.265859 | 27 | -2.691523 | 2.26 | 7.211523 | 0.083704 |
| GO:0048729\_tissue\_morphogenesis | 61 | 2 | 17.836066 | -2.265859 | 27 | -2.691523 | 2.26 | 7.211523 | 0.083704 |
| GO:0032695\_negative\_regulation\_of\_interleukin-12\_production | 3 | 1 |  |  |  |  |  |  |  |  |
| GO:0032905\_transforming\_growth\_factor-beta1\_production | 3 | 1 |  |  |  |  |  |  |  |  |
| GO:0032908\_regulation\_of\_transforming\_growth\_factor-beta1\_production | 3 | 1 |  |  |  |  |  |  |  |  |
| GO:0034505\_tooth\_mineralization | 3 | 1 |  |  |  |  |  |  |  |  |
| GO:0045112\_integrin\_biosynthetic\_process | 3 | 1 |  |  |  |  |  |  |  |  |
| GO:0051918\_negative\_regulation\_of\_fibrinolysis | 3 | 1 |  |  |  |  |  |  |  |  |
| GO:0016043\_cellular\_component\_organization | 1366 | 7 | 2.787701 | -2.172691 | 28 | -2.835747 | 2.82 | 8.475747 | 0.100714 |
| GO:0007275\_multicellular\_organismal\_development | 1372 | 7 | 2.775510 | -2.161895 | 29 | -2.889103 | 2.84 | 8.569103 | 0.097931 |
| GO:0001502\_cartilage\_condensation | 4 | 1 |  |  |  |  |  |  |  |  |
| GO:0002040\_sprouting\_angiogenesis | 4 | 1 |  |  |  |  |  |  |  |  |
| GO:0007195\_inhibition\_of\_adenylate\_cyclase\_activity\_by\_dopamine\_receptor\_signaling\_pathway | 4 | 1 |  |  |  |  |  |  |  |  |
| GO:0007520\_myoblast\_fusion | 4 | 1 |  |  |  |  |  |  |  |  |
| GO:0010746\_regulation\_of\_plasma\_membrane\_long-chain\_fatty\_acid\_transport | 4 | 1 |  |  |  |  |  |  |  |  |
| GO:0010748\_negative\_regulation\_of\_plasma\_membrane\_long-chain\_fatty\_acid\_transport | 4 | 1 |  |  |  |  |  |  |  |  |
| GO:0032891\_negative\_regulation\_of\_organic\_acid\_transport | 4 | 1 |  |  |  |  |  |  |  |  |
| GO:0034394\_protein\_localization\_at\_cell\_surface | 4 | 1 |  |  |  |  |  |  |  |  |
| GO:0035313\_wound\_healing\_\_spreading\_of\_epidermal\_cells | 4 | 1 |  |  |  |  |  |  |  |  |
| GO:0043032\_positive\_regulation\_of\_macrophage\_activation | 4 | 1 |  |  |  |  |  |  |  |  |
| GO:0043113\_receptor\_clustering | 4 | 1 |  |  |  |  |  |  |  |  |
| GO:0051895\_negative\_regulation\_of\_focal\_adhesion\_formation | 4 | 1 |  |  |  |  |  |  |  |  |
| GO:0007179\_transforming\_growth\_factor\_beta\_receptor\_signaling\_pathway | 72 | 2 | 15.111111 | -2.125813 | 30 | -3.102480 | 3.06 | 9.222480 | 0.102000 |
| GO:0051051\_negative\_regulation\_of\_transport | 76 | 2 | 14.315789 | -2.080369 | 31 | -3.246745 | 3.49 | 10.226745 | 0.112581 |
| GO:0000768\_syncytium\_formation\_by\_plasma\_membrane\_fusion | 5 | 1 | 108.800000 | -2.038119 | 38 | -2.740071 | 6.56 | 15.860071 | 0.172632 |
| GO:0015911\_plasma\_membrane\_long-chain\_fatty\_acid\_transport | 5 | 1 | 108.800000 | -2.038119 | 38 | -2.740071 | 6.56 | 15.860071 | 0.172632 |
| GO:0030194\_positive\_regulation\_of\_blood\_coagulation | 5 | 1 | 108.800000 | -2.038119 | 38 | -2.740071 | 6.56 | 15.860071 | 0.172632 |
| GO:0031639\_plasminogen\_activation | 5 | 1 | 108.800000 | -2.038119 | 38 | -2.740071 | 6.56 | 15.860071 | 0.172632 |
| GO:0032570\_response\_to\_progesterone\_stimulus | 5 | 1 | 108.800000 | -2.038119 | 38 | -2.740071 | 6.56 | 15.860071 | 0.172632 |
| GO:0050820\_positive\_regulation\_of\_coagulation | 5 | 1 | 108.800000 | -2.038119 | 38 | -2.740071 | 6.56 | 15.860071 | 0.172632 |
| GO:0051893\_regulation\_of\_focal\_adhesion\_formation | 5 | 1 | 108.800000 | -2.038119 | 38 | -2.740071 | 6.56 | 15.860071 | 0.172632 |
| GO:0019935\_cyclic-nucleotide-mediated\_signaling | 82 | 2 | 13.268293 | -2.016706 | 39 | -2.673695 | 6.79 | 16.253695 | 0.174103 |
| GO:0007167\_enzyme\_linked\_receptor\_protein\_signaling\_pathway | 258 | 3 | 6.325581 | -1.969697 | 40 | -2.638736 | 6.93 | 16.498736 | 0.173250 |
| GO:0006911\_phagocytosis\_\_engulfment | 6 | 1 | 90.666667 | -1.959311 | 48 | -1.656914 | 9.89 | 21.436914 | 0.206042 |
| GO:0006949\_syncytium\_formation | 6 | 1 | 90.666667 | -1.959311 | 48 | -1.656914 | 9.89 | 21.436914 | 0.206042 |
| GO:0007212\_dopamine\_receptor\_signaling\_pathway | 6 | 1 | 90.666667 | -1.959311 | 48 | -1.656914 | 9.89 | 21.436914 | 0.206042 |
| GO:0032890\_regulation\_of\_organic\_acid\_transport | 6 | 1 | 90.666667 | -1.959311 | 48 | -1.656914 | 9.89 | 21.436914 | 0.206042 |
| GO:0043206\_fibril\_organization | 6 | 1 | 90.666667 | -1.959311 | 48 | -1.656914 | 9.89 | 21.436914 | 0.206042 |
| GO:0043536\_positive\_regulation\_of\_blood\_vessel\_endothelial\_cell\_migration | 6 | 1 | 90.666667 | -1.959311 | 48 | -1.656914 | 9.89 | 21.436914 | 0.206042 |
| GO:0051917\_regulation\_of\_fibrinolysis | 6 | 1 | 90.666667 | -1.959311 | 48 | -1.656914 | 9.89 | 21.436914 | 0.206042 |
| GO:0070613\_regulation\_of\_protein\_processing | 6 | 1 | 90.666667 | -1.959311 | 48 | -1.656914 | 9.89 | 21.436914 | 0.206042 |
| GO:0032502\_developmental\_process | 1919 | 8 | 2.267848 | -1.927402 | 49 | -1.701956 | 10.2 | 22.101956 | 0.208163 |
| GO:0007178\_transmembrane\_receptor\_protein\_serine\_threonine\_kinase\_signaling\_pathway | 92 | 2 | 11.826087 | -1.920775 | 50 | -1.711026 | 10.26 | 22.231026 | 0.205200 |
| GO:0001953\_negative\_regulation\_of\_cell-matrix\_adhesion | 7 | 1 | 77.714286 | -1.892736 | 57 | -0.916620 | 12.9 | 26.716620 | 0.226316 |
| GO:0010812\_negative\_regulation\_of\_cell-substrate\_adhesion | 7 | 1 | 77.714286 | -1.892736 | 57 | -0.916620 | 12.9 | 26.716620 | 0.226316 |
| GO:0014902\_myotube\_differentiation | 7 | 1 | 77.714286 | -1.892736 | 57 | -0.916620 | 12.9 | 26.716620 | 0.226316 |
| GO:0019934\_cGMP-mediated\_signaling | 7 | 1 | 77.714286 | -1.892736 | 57 | -0.916620 | 12.9 | 26.716620 | 0.226316 |
| GO:0031638\_zymogen\_activation | 7 | 1 | 77.714286 | -1.892736 | 57 | -0.916620 | 12.9 | 26.716620 | 0.226316 |
| GO:0043537\_negative\_regulation\_of\_blood\_vessel\_endothelial\_cell\_migration | 7 | 1 | 77.714286 | -1.892736 | 57 | -0.916620 | 12.9 | 26.716620 | 0.226316 |
| GO:0080010\_regulation\_of\_oxygen\_and\_reactive\_oxygen\_species\_metabolic\_process | 7 | 1 | 77.714286 | -1.892736 | 57 | -0.916620 | 12.9 | 26.716620 | 0.226316 |
| GO:0051248\_negative\_regulation\_of\_protein\_metabolic\_process | 96 | 2 | 11.333333 | -1.885448 | 58 | -0.892190 | 13.03 | 26.952190 | 0.224655 |
| GO:0042060\_wound\_healing | 98 | 2 | 11.102041 | -1.868364 | 59 | -0.988818 | 13.22 | 27.428818 | 0.224068 |
| GO:0002688\_regulation\_of\_leukocyte\_chemotaxis | 8 | 1 | 68.000000 | -1.835117 | 62 | -0.197341 | 15.97 | 32.137341 | 0.257581 |
| GO:0002690\_positive\_regulation\_of\_leukocyte\_chemotaxis | 8 | 1 | 68.000000 | -1.835117 | 62 | -0.197341 | 15.97 | 32.137341 | 0.257581 |
| GO:0048246\_macrophage\_chemotaxis | 8 | 1 | 68.000000 | -1.835117 | 62 | -0.197341 | 15.97 | 32.137341 | 0.257581 |
| GO:0032615\_interleukin-12\_production | 9 | 1 | 60.444444 | -1.784336 | 66 | 0.549857 | 18.22 | 35.890143 | 0.276061 |
| GO:0032655\_regulation\_of\_interleukin-12\_production | 9 | 1 | 60.444444 | -1.784336 | 66 | 0.549857 | 18.22 | 35.890143 | 0.276061 |
| GO:0043030\_regulation\_of\_macrophage\_activation | 9 | 1 | 60.444444 | -1.784336 | 66 | 0.549857 | 18.22 | 35.890143 | 0.276061 |
| GO:0048706\_embryonic\_skeletal\_system\_development | 9 | 1 | 60.444444 | -1.784336 | 66 | 0.549857 | 18.22 | 35.890143 | 0.276061 |
| GO:0048646\_anatomical\_structure\_formation\_involved\_in\_morphogenesis | 111 | 2 | 9.801802 | -1.765620 | 67 | 0.694543 | 18.39 | 36.085457 | 0.274478 |
| GO:0010595\_positive\_regulation\_of\_endothelial\_cell\_migration | 10 | 1 | 54.400000 | -1.738951 | 71 | 1.755959 | 20.87 | 39.984041 | 0.293944 |
| GO:0030511\_positive\_regulation\_of\_transforming\_growth\_factor\_beta\_receptor\_signaling\_pathway | 10 | 1 | 54.400000 | -1.738951 | 71 | 1.755959 | 20.87 | 39.984041 | 0.293944 |
| GO:0042307\_positive\_regulation\_of\_protein\_import\_into\_nucleus | 10 | 1 | 54.400000 | -1.738951 | 71 | 1.755959 | 20.87 | 39.984041 | 0.293944 |
| GO:0042993\_positive\_regulation\_of\_transcription\_factor\_import\_into\_nucleus | 10 | 1 | 54.400000 | -1.738951 | 71 | 1.755959 | 20.87 | 39.984041 | 0.293944 |
| GO:0010608\_posttranscriptional\_regulation\_of\_gene\_expression | 118 | 2 | 9.220339 | -1.715483 | 72 | 1.892901 | 21.15 | 40.407099 | 0.293750 |
| GO:0032501\_multicellular\_organismal\_process | 2082 | 8 | 2.090298 | -1.710686 | 73 | 1.876386 | 21.17 | 40.463614 | 0.290000 |
| GO:0002687\_positive\_regulation\_of\_leukocyte\_migration | 11 | 1 | 49.454545 | -1.697930 | 79 | 2.616445 | 22.94 | 43.263555 | 0.290380 |
| GO:0007009\_plasma\_membrane\_organization | 11 | 1 | 49.454545 | -1.697930 | 79 | 2.616445 | 22.94 | 43.263555 | 0.290380 |
| GO:0007172\_signal\_complex\_assembly | 11 | 1 | 49.454545 | -1.697930 | 79 | 2.616445 | 22.94 | 43.263555 | 0.290380 |
| GO:0042730\_fibrinolysis | 11 | 1 | 49.454545 | -1.697930 | 79 | 2.616445 | 22.94 | 43.263555 | 0.290380 |
| GO:0043535\_regulation\_of\_blood\_vessel\_endothelial\_cell\_migration | 11 | 1 | 49.454545 | -1.697930 | 79 | 2.616445 | 22.94 | 43.263555 | 0.290380 |
| GO:0051220\_cytoplasmic\_sequestering\_of\_protein | 11 | 1 | 49.454545 | -1.697930 | 79 | 2.616445 | 22.94 | 43.263555 | 0.290380 |
| GO:0002685\_regulation\_of\_leukocyte\_migration | 12 | 1 | 45.333333 | -1.660514 | 86 | 3.356664 | 25.2 | 47.043336 | 0.293023 |
| GO:0010596\_negative\_regulation\_of\_endothelial\_cell\_migration | 12 | 1 | 45.333333 | -1.660514 | 86 | 3.356664 | 25.2 | 47.043336 | 0.293023 |
| GO:0019882\_antigen\_processing\_and\_presentation | 12 | 1 | 45.333333 | -1.660514 | 86 | 3.356664 | 25.2 | 47.043336 | 0.293023 |
| GO:0031532\_actin\_cytoskeleton\_reorganization | 12 | 1 | 45.333333 | -1.660514 | 86 | 3.356664 | 25.2 | 47.043336 | 0.293023 |
| GO:0045026\_plasma\_membrane\_fusion | 12 | 1 | 45.333333 | -1.660514 | 86 | 3.356664 | 25.2 | 47.043336 | 0.293023 |
| GO:0048705\_skeletal\_system\_morphogenesis | 12 | 1 | 45.333333 | -1.660514 | 86 | 3.356664 | 25.2 | 47.043336 | 0.293023 |
| GO:0051216\_cartilage\_development | 12 | 1 | 45.333333 | -1.660514 | 86 | 3.356664 | 25.2 | 47.043336 | 0.293023 |
| GO:0001952\_regulation\_of\_cell-matrix\_adhesion | 13 | 1 | 41.846154 | -1.626124 | 96 | 4.093697 | 27.18 | 50.266303 | 0.283125 |
| GO:0007193\_inhibition\_of\_adenylate\_cyclase\_activity\_by\_G-protein\_signaling | 13 | 1 | 41.846154 | -1.626124 | 96 | 4.093697 | 27.18 | 50.266303 | 0.283125 |
| GO:0007263\_nitric\_oxide\_mediated\_signal\_transduction | 13 | 1 | 41.846154 | -1.626124 | 96 | 4.093697 | 27.18 | 50.266303 | 0.283125 |
| GO:0009746\_response\_to\_hexose\_stimulus | 13 | 1 | 41.846154 | -1.626124 | 96 | 4.093697 | 27.18 | 50.266303 | 0.283125 |
| GO:0009749\_response\_to\_glucose\_stimulus | 13 | 1 | 41.846154 | -1.626124 | 96 | 4.093697 | 27.18 | 50.266303 | 0.283125 |
| GO:0010810\_regulation\_of\_cell-substrate\_adhesion | 13 | 1 | 41.846154 | -1.626124 | 96 | 4.093697 | 27.18 | 50.266303 | 0.283125 |
| GO:0018149\_peptide\_cross-linking | 13 | 1 | 41.846154 | -1.626124 | 96 | 4.093697 | 27.18 | 50.266303 | 0.283125 |
| GO:0034284\_response\_to\_monosaccharide\_stimulus | 13 | 1 | 41.846154 | -1.626124 | 96 | 4.093697 | 27.18 | 50.266303 | 0.283125 |
| GO:0045766\_positive\_regulation\_of\_angiogenesis | 13 | 1 | 41.846154 | -1.626124 | 96 | 4.093697 | 27.18 | 50.266303 | 0.283125 |
| GO:0046824\_positive\_regulation\_of\_nucleocytoplasmic\_transport | 13 | 1 | 41.846154 | -1.626124 | 96 | 4.093697 | 27.18 | 50.266303 | 0.283125 |
| GO:0007601\_visual\_perception | 134 | 2 | 8.119403 | -1.611939 | 98 | 4.139101 | 27.48 | 50.820899 | 0.280408 |
| GO:0050953\_sensory\_perception\_of\_light\_stimulus | 134 | 2 | 8.119403 | -1.611939 | 98 | 4.139101 | 27.48 | 50.820899 | 0.280408 |
| GO:0032369\_negative\_regulation\_of\_lipid\_transport | 14 | 1 | 38.857143 | -1.594311 | 101 | 4.441113 | 29.88 | 55.318887 | 0.295842 |
| GO:0043534\_blood\_vessel\_endothelial\_cell\_migration | 14 | 1 | 38.857143 | -1.594311 | 101 | 4.441113 | 29.88 | 55.318887 | 0.295842 |
| GO:0045727\_positive\_regulation\_of\_translation | 14 | 1 | 38.857143 | -1.594311 | 101 | 4.441113 | 29.88 | 55.318887 | 0.295842 |
| GO:0001937\_negative\_regulation\_of\_endothelial\_cell\_proliferation | 15 | 1 | 36.266667 | -1.564719 | 105 | 5.441371 | 32.4 | 59.358629 | 0.308571 |
| GO:0042116\_macrophage\_activation | 15 | 1 | 36.266667 | -1.564719 | 105 | 5.441371 | 32.4 | 59.358629 | 0.308571 |
| GO:0048041\_focal\_adhesion\_formation | 15 | 1 | 36.266667 | -1.564719 | 105 | 5.441371 | 32.4 | 59.358629 | 0.308571 |
| GO:0048592\_eye\_morphogenesis | 15 | 1 | 36.266667 | -1.564719 | 105 | 5.441371 | 32.4 | 59.358629 | 0.308571 |
| GO:0009408\_response\_to\_heat | 16 | 1 | 34.000000 | -1.537062 | 108 | 6.027789 | 34.88 | 63.732211 | 0.322963 |
| GO:0009743\_response\_to\_carbohydrate\_stimulus | 16 | 1 | 34.000000 | -1.537062 | 108 | 6.027789 | 34.88 | 63.732211 | 0.322963 |
| GO:0015909\_long-chain\_fatty\_acid\_transport | 16 | 1 | 34.000000 | -1.537062 | 108 | 6.027789 | 34.88 | 63.732211 | 0.322963 |
| GO:0007044\_cell-substrate\_junction\_assembly | 17 | 1 | 32.000000 | -1.511105 | 109 | 6.411718 | 36.63 | 66.848282 | 0.336055 |
| GO:0019932\_second-messenger-mediated\_signaling | 153 | 2 | 7.111111 | -1.505074 | 110 | 6.476625 | 36.78 | 67.083375 | 0.334364 |
| GO:0001818\_negative\_regulation\_of\_cytokine\_production | 18 | 1 | 30.222222 | -1.486653 | 115 | 6.809302 | 38.35 | 69.890698 | 0.333478 |
| GO:0030195\_negative\_regulation\_of\_blood\_coagulation | 18 | 1 | 30.222222 | -1.486653 | 115 | 6.809302 | 38.35 | 69.890698 | 0.333478 |
| GO:0040017\_positive\_regulation\_of\_locomotion | 18 | 1 | 30.222222 | -1.486653 | 115 | 6.809302 | 38.35 | 69.890698 | 0.333478 |
| GO:0042177\_negative\_regulation\_of\_protein\_catabolic\_process | 18 | 1 | 30.222222 | -1.486653 | 115 | 6.809302 | 38.35 | 69.890698 | 0.333478 |
| GO:0050921\_positive\_regulation\_of\_chemotaxis | 18 | 1 | 30.222222 | -1.486653 | 115 | 6.809302 | 38.35 | 69.890698 | 0.333478 |
| GO:0007169\_transmembrane\_receptor\_protein\_tyrosine\_kinase\_signaling\_pathway | 157 | 2 | 6.929936 | -1.484419 | 116 | 6.890947 | 38.48 | 70.069053 | 0.331724 |
| GO:0016525\_negative\_regulation\_of\_angiogenesis | 19 | 1 | 28.631579 | -1.463543 | 120 | 7.680464 | 40.27 | 72.859536 | 0.335583 |
| GO:0032388\_positive\_regulation\_of\_intracellular\_transport | 19 | 1 | 28.631579 | -1.463543 | 120 | 7.680464 | 40.27 | 72.859536 | 0.335583 |
| GO:0050819\_negative\_regulation\_of\_coagulation | 19 | 1 | 28.631579 | -1.463543 | 120 | 7.680464 | 40.27 | 72.859536 | 0.335583 |
| GO:0050920\_regulation\_of\_chemotaxis | 19 | 1 | 28.631579 | -1.463543 | 120 | 7.680464 | 40.27 | 72.859536 | 0.335583 |
| GO:0010594\_regulation\_of\_endothelial\_cell\_migration | 20 | 1 | 27.200000 | -1.441638 | 121 | 8.203015 | 42.08 | 75.956985 | 0.347769 |
| GO:0001654\_eye\_development | 21 | 1 | 25.904762 | -1.420820 | 124 | 8.815004 | 44.58 | 80.344996 | 0.359516 |
| GO:0001936\_regulation\_of\_endothelial\_cell\_proliferation | 21 | 1 | 25.904762 | -1.420820 | 124 | 8.815004 | 44.58 | 80.344996 | 0.359516 |
| GO:0048520\_positive\_regulation\_of\_behavior | 21 | 1 | 25.904762 | -1.420820 | 124 | 8.815004 | 44.58 | 80.344996 | 0.359516 |
| GO:0015908\_fatty\_acid\_transport | 22 | 1 | 24.727273 | -1.400988 | 127 | 9.470826 | 46.17 | 82.869174 | 0.363543 |
| GO:0042990\_regulation\_of\_transcription\_factor\_import\_into\_nucleus | 22 | 1 | 24.727273 | -1.400988 | 127 | 9.470826 | 46.17 | 82.869174 | 0.363543 |
| GO:0042991\_transcription\_factor\_import\_into\_nucleus | 22 | 1 | 24.727273 | -1.400988 | 127 | 9.470826 | 46.17 | 82.869174 | 0.363543 |
| GO:0016477\_cell\_migration | 177 | 2 | 6.146893 | -1.389118 | 128 | 9.675607 | 46.47 | 83.264393 | 0.363047 |
| GO:0007568\_aging | 24 | 1 | 22.666667 | -1.363942 | 131 | 11.300547 | 48.99 | 86.679453 | 0.373969 |
| GO:0009266\_response\_to\_temperature\_stimulus | 24 | 1 | 22.666667 | -1.363942 | 131 | 11.300547 | 48.99 | 86.679453 | 0.373969 |
| GO:0030193\_regulation\_of\_blood\_coagulation | 24 | 1 | 22.666667 | -1.363942 | 131 | 11.300547 | 48.99 | 86.679453 | 0.373969 |
| GO:0009967\_positive\_regulation\_of\_signal\_transduction | 185 | 2 | 5.881081 | -1.354276 | 132 | 11.377042 | 49.12 | 86.862958 | 0.372121 |
| GO:0001935\_endothelial\_cell\_proliferation | 25 | 1 | 21.760000 | -1.346584 | 137 | 12.138986 | 50.71 | 89.281014 | 0.370146 |
| GO:0032368\_regulation\_of\_lipid\_transport | 25 | 1 | 21.760000 | -1.346584 | 137 | 12.138986 | 50.71 | 89.281014 | 0.370146 |
| GO:0050795\_regulation\_of\_behavior | 25 | 1 | 21.760000 | -1.346584 | 137 | 12.138986 | 50.71 | 89.281014 | 0.370146 |
| GO:0050818\_regulation\_of\_coagulation | 25 | 1 | 21.760000 | -1.346584 | 137 | 12.138986 | 50.71 | 89.281014 | 0.370146 |
| GO:0050821\_protein\_stabilization | 25 | 1 | 21.760000 | -1.346584 | 137 | 12.138986 | 50.71 | 89.281014 | 0.370146 |
| GO:0010647\_positive\_regulation\_of\_cell\_communication | 189 | 2 | 5.756614 | -1.337476 | 138 | 12.291865 | 50.89 | 89.488135 | 0.368768 |
| GO:0007194\_negative\_regulation\_of\_adenylate\_cyclase\_activity | 26 | 1 | 20.923077 | -1.329922 | 141 | 12.953593 | 52.39 | 91.826407 | 0.371560 |
| GO:0031280\_negative\_regulation\_of\_cyclase\_activity | 26 | 1 | 20.923077 | -1.329922 | 141 | 12.953593 | 52.39 | 91.826407 | 0.371560 |
| GO:0051350\_negative\_regulation\_of\_lyase\_activity | 26 | 1 | 20.923077 | -1.329922 | 141 | 12.953593 | 52.39 | 91.826407 | 0.371560 |
| GO:0006909\_phagocytosis | 27 | 1 | 20.148148 | -1.313902 | 143 | 13.747296 | 53.87 | 93.992704 | 0.376713 |
| GO:0008543\_fibroblast\_growth\_factor\_receptor\_signaling\_pathway | 27 | 1 | 20.148148 | -1.313902 | 143 | 13.747296 | 53.87 | 93.992704 | 0.376713 |
| GO:0048870\_cell\_motility | 197 | 2 | 5.522843 | -1.305033 | 144 | 13.944262 | 54.14 | 94.335738 | 0.375972 |
| GO:0009895\_negative\_regulation\_of\_catabolic\_process | 28 | 1 | 19.428571 | -1.298479 | 147 | 14.401785 | 55.81 | 97.218215 | 0.379660 |
| GO:0031214\_biomineral\_formation | 28 | 1 | 19.428571 | -1.298479 | 147 | 14.401785 | 55.81 | 97.218215 | 0.379660 |
| GO:0042306\_regulation\_of\_protein\_import\_into\_nucleus | 28 | 1 | 19.428571 | -1.298479 | 147 | 14.401785 | 55.81 | 97.218215 | 0.379660 |
| GO:0019538\_protein\_metabolic\_process | 1569 | 6 | 2.080306 | -1.288586 | 148 | 14.547399 | 56.07 | 97.592601 | 0.378851 |
| GO:0008285\_negative\_regulation\_of\_cell\_proliferation | 202 | 2 | 5.386139 | -1.285494 | 149 | 14.580811 | 56.18 | 97.779189 | 0.377047 |
| GO:0002274\_myeloid\_leukocyte\_activation | 29 | 1 | 18.758621 | -1.283609 | 155 | 14.745147 | 57.28 | 99.814853 | 0.369548 |
| GO:0032507\_maintenance\_of\_protein\_location\_in\_cell | 29 | 1 | 18.758621 | -1.283609 | 155 | 14.745147 | 57.28 | 99.814853 | 0.369548 |
| GO:0043112\_receptor\_metabolic\_process | 29 | 1 | 18.758621 | -1.283609 | 155 | 14.745147 | 57.28 | 99.814853 | 0.369548 |
| GO:0043433\_negative\_regulation\_of\_transcription\_factor\_activity | 29 | 1 | 18.758621 | -1.283609 | 155 | 14.745147 | 57.28 | 99.814853 | 0.369548 |
| GO:0048741\_skeletal\_muscle\_fiber\_development | 29 | 1 | 18.758621 | -1.283609 | 155 | 14.745147 | 57.28 | 99.814853 | 0.369548 |
| GO:0090048\_negative\_regulation\_of\_transcription\_regulator\_activity | 29 | 1 | 18.758621 | -1.283609 | 155 | 14.745147 | 57.28 | 99.814853 | 0.369548 |
| GO:0002683\_negative\_regulation\_of\_immune\_system\_process | 30 | 1 | 18.133333 | -1.269257 | 160 | 14.870549 | 58.49 | 102.109451 | 0.365562 |
| GO:0032103\_positive\_regulation\_of\_response\_to\_external\_stimulus | 30 | 1 | 18.133333 | -1.269257 | 160 | 14.870549 | 58.49 | 102.109451 | 0.365562 |
| GO:0048747\_muscle\_fiber\_development | 30 | 1 | 18.133333 | -1.269257 | 160 | 14.870549 | 58.49 | 102.109451 | 0.365562 |
| GO:0051224\_negative\_regulation\_of\_protein\_transport | 30 | 1 | 18.133333 | -1.269257 | 160 | 14.870549 | 58.49 | 102.109451 | 0.365562 |
| GO:0051592\_response\_to\_calcium\_ion | 30 | 1 | 18.133333 | -1.269257 | 160 | 14.870549 | 58.49 | 102.109451 | 0.365562 |
| GO:0006888\_ER\_to\_Golgi\_vesicle-mediated\_transport | 31 | 1 | 17.548387 | -1.255387 | 163 | 15.565369 | 59.82 | 104.074631 | 0.366994 |
| GO:0007423\_sensory\_organ\_development | 31 | 1 | 17.548387 | -1.255387 | 163 | 15.565369 | 59.82 | 104.074631 | 0.366994 |
| GO:0045185\_maintenance\_of\_protein\_location | 31 | 1 | 17.548387 | -1.255387 | 163 | 15.565369 | 59.82 | 104.074631 | 0.366994 |
| GO:0030595\_leukocyte\_chemotaxis | 32 | 1 | 17.000000 | -1.241969 | 166 | 16.242992 | 61.11 | 105.977008 | 0.368133 |
| GO:0033157\_regulation\_of\_intracellular\_protein\_transport | 32 | 1 | 17.000000 | -1.241969 | 166 | 16.242992 | 61.11 | 105.977008 | 0.368133 |
| GO:0043542\_endothelial\_cell\_migration | 32 | 1 | 17.000000 | -1.241969 | 166 | 16.242992 | 61.11 | 105.977008 | 0.368133 |
| GO:0034329\_cell\_junction\_assembly | 33 | 1 | 16.484848 | -1.228975 | 168 | 16.769644 | 62.05 | 107.330356 | 0.369345 |
| GO:0043392\_negative\_regulation\_of\_DNA\_binding | 33 | 1 | 16.484848 | -1.228975 | 168 | 16.769644 | 62.05 | 107.330356 | 0.369345 |
| GO:0007188\_G-protein\_signaling\_\_coupled\_to\_cAMP\_nucleotide\_second\_messenger | 34 | 1 | 16.000000 | -1.216381 | 175 | 17.559535 | 64.03 | 110.500465 | 0.365886 |
| GO:0015718\_monocarboxylic\_acid\_transport | 34 | 1 | 16.000000 | -1.216381 | 175 | 17.559535 | 64.03 | 110.500465 | 0.365886 |
| GO:0031400\_negative\_regulation\_of\_protein\_modification\_process | 34 | 1 | 16.000000 | -1.216381 | 175 | 17.559535 | 64.03 | 110.500465 | 0.365886 |
| GO:0031647\_regulation\_of\_protein\_stability | 34 | 1 | 16.000000 | -1.216381 | 175 | 17.559535 | 64.03 | 110.500465 | 0.365886 |
| GO:0048545\_response\_to\_steroid\_hormone\_stimulus | 34 | 1 | 16.000000 | -1.216381 | 175 | 17.559535 | 64.03 | 110.500465 | 0.365886 |
| GO:0051651\_maintenance\_of\_location\_in\_cell | 34 | 1 | 16.000000 | -1.216381 | 175 | 17.559535 | 64.03 | 110.500465 | 0.365886 |
| GO:0060326\_cell\_chemotaxis | 34 | 1 | 16.000000 | -1.216381 | 175 | 17.559535 | 64.03 | 110.500465 | 0.365886 |
| GO:0051049\_regulation\_of\_transport | 227 | 2 | 4.792952 | -1.195329 | 176 | 18.362246 | 65.47 | 112.577754 | 0.371989 |
| GO:0001819\_positive\_regulation\_of\_cytokine\_production | 36 | 1 | 15.111111 | -1.192297 | 177 | 18.507415 | 65.99 | 113.472585 | 0.372825 |
| GO:0046822\_regulation\_of\_nucleocytoplasmic\_transport | 37 | 1 | 14.702703 | -1.180768 | 178 | 18.862510 | 67.13 | 115.397490 | 0.377135 |
| GO:0019933\_cAMP-mediated\_signaling | 38 | 1 | 14.315789 | -1.169556 | 181 | 19.603167 | 68.93 | 118.256833 | 0.380829 |
| GO:0030336\_negative\_regulation\_of\_cell\_migration | 38 | 1 | 14.315789 | -1.169556 | 181 | 19.603167 | 68.93 | 118.256833 | 0.380829 |
| GO:0045765\_regulation\_of\_angiogenesis | 38 | 1 | 14.315789 | -1.169556 | 181 | 19.603167 | 68.93 | 118.256833 | 0.380829 |
| GO:0009792\_embryonic\_development\_ending\_in\_birth\_or\_egg\_hatching | 39 | 1 | 13.948718 | -1.158645 | 183 | 20.176038 | 69.85 | 119.523962 | 0.381694 |
| GO:0043009\_chordate\_embryonic\_development | 39 | 1 | 13.948718 | -1.158645 | 183 | 20.176038 | 69.85 | 119.523962 | 0.381694 |
| GO:0043170\_macromolecule\_metabolic\_process | 3103 | 9 | 1.577828 | -1.153015 | 184 | 20.240942 | 69.94 | 119.639058 | 0.380109 |
| GO:0017015\_regulation\_of\_transforming\_growth\_factor\_beta\_receptor\_signaling\_pathway | 40 | 1 | 13.600000 | -1.148020 | 186 | 20.640146 | 70.75 | 120.859854 | 0.380376 |
| GO:0051100\_negative\_regulation\_of\_binding | 40 | 1 | 13.600000 | -1.148020 | 186 | 20.640146 | 70.75 | 120.859854 | 0.380376 |
| GO:0006800\_oxygen\_and\_reactive\_oxygen\_species\_metabolic\_process | 41 | 1 | 13.268293 | -1.137666 | 191 | 21.048865 | 71.78 | 122.511135 | 0.375812 |
| GO:0007519\_skeletal\_muscle\_tissue\_development | 41 | 1 | 13.268293 | -1.137666 | 191 | 21.048865 | 71.78 | 122.511135 | 0.375812 |
| GO:0034330\_cell\_junction\_organization | 41 | 1 | 13.268293 | -1.137666 | 191 | 21.048865 | 71.78 | 122.511135 | 0.375812 |
| GO:0051222\_positive\_regulation\_of\_protein\_transport | 41 | 1 | 13.268293 | -1.137666 | 191 | 21.048865 | 71.78 | 122.511135 | 0.375812 |
| GO:0060538\_skeletal\_muscle\_organ\_development | 41 | 1 | 13.268293 | -1.137666 | 191 | 21.048865 | 71.78 | 122.511135 | 0.375812 |
| GO:0007600\_sensory\_perception | 245 | 2 | 4.440816 | -1.137121 | 192 | 21.154945 | 71.9 | 122.645055 | 0.374479 |
| GO:0006944\_membrane\_fusion | 42 | 1 | 12.952381 | -1.127570 | 195 | 21.933459 | 73.39 | 124.846541 | 0.376359 |
| GO:0042692\_muscle\_cell\_differentiation | 42 | 1 | 12.952381 | -1.127570 | 195 | 21.933459 | 73.39 | 124.846541 | 0.376359 |
| GO:0051271\_negative\_regulation\_of\_cell\_motion | 42 | 1 | 12.952381 | -1.127570 | 195 | 21.933459 | 73.39 | 124.846541 | 0.376359 |
| GO:0050900\_leukocyte\_migration | 44 | 1 | 12.363636 | -1.108106 | 196 | 23.233773 | 75.28 | 127.326227 | 0.384082 |
| GO:0010926\_anatomical\_structure\_formation | 560 | 3 | 2.914286 | -1.103006 | 197 | 23.282340 | 75.37 | 127.457660 | 0.382589 |
| GO:0006464\_protein\_modification\_process | 922 | 4 | 2.360087 | -1.095873 | 198 | 23.947300 | 76.26 | 128.572700 | 0.385152 |
| GO:0002696\_positive\_regulation\_of\_leukocyte\_activation | 46 | 1 | 11.826087 | -1.089539 | 201 | 24.813140 | 77.64 | 130.466860 | 0.386269 |
| GO:0010038\_response\_to\_metal\_ion | 46 | 1 | 11.826087 | -1.089539 | 201 | 24.813140 | 77.64 | 130.466860 | 0.386269 |
| GO:0030335\_positive\_regulation\_of\_cell\_migration | 46 | 1 | 11.826087 | -1.089539 | 201 | 24.813140 | 77.64 | 130.466860 | 0.386269 |
| GO:0010035\_response\_to\_inorganic\_substance | 47 | 1 | 11.574468 | -1.080569 | 204 | 25.699380 | 79.1 | 132.500620 | 0.387745 |
| GO:0032386\_regulation\_of\_intracellular\_transport | 47 | 1 | 11.574468 | -1.080569 | 204 | 25.699380 | 79.1 | 132.500620 | 0.387745 |
| GO:0042176\_regulation\_of\_protein\_catabolic\_process | 47 | 1 | 11.574468 | -1.080569 | 204 | 25.699380 | 79.1 | 132.500620 | 0.387745 |
| GO:0008217\_regulation\_of\_blood\_pressure | 48 | 1 | 11.333333 | -1.071794 | 205 | 26.145615 | 79.98 | 133.814385 | 0.390146 |
| GO:0050890\_cognition | 268 | 2 | 4.059701 | -1.069513 | 206 | 26.173015 | 80.03 | 133.886985 | 0.388495 |
| GO:0050867\_positive\_regulation\_of\_cell\_activation | 49 | 1 | 11.102041 | -1.063209 | 207 | 26.590689 | 81.19 | 135.789311 | 0.392222 |
| GO:0000187\_activation\_of\_MAPK\_activity | 50 | 1 | 10.880000 | -1.054804 | 212 | 27.038987 | 82.14 | 137.241013 | 0.387453 |
| GO:0001666\_response\_to\_hypoxia | 50 | 1 | 10.880000 | -1.054804 | 212 | 27.038987 | 82.14 | 137.241013 | 0.387453 |
| GO:0007266\_Rho\_protein\_signal\_transduction | 50 | 1 | 10.880000 | -1.054804 | 212 | 27.038987 | 82.14 | 137.241013 | 0.387453 |
| GO:0045761\_regulation\_of\_adenylate\_cyclase\_activity | 50 | 1 | 10.880000 | -1.054804 | 212 | 27.038987 | 82.14 | 137.241013 | 0.387453 |
| GO:0051272\_positive\_regulation\_of\_cell\_motion | 50 | 1 | 10.880000 | -1.054804 | 212 | 27.038987 | 82.14 | 137.241013 | 0.387453 |
| GO:0031279\_regulation\_of\_cyclase\_activity | 51 | 1 | 10.666667 | -1.046573 | 214 | 27.541714 | 83.17 | 138.798286 | 0.388645 |
| GO:0070482\_response\_to\_oxygen\_levels | 51 | 1 | 10.666667 | -1.046573 | 214 | 27.541714 | 83.17 | 138.798286 | 0.388645 |
| GO:0043412\_biopolymer\_modification | 960 | 4 | 2.266667 | -1.044223 | 215 | 27.673195 | 83.35 | 139.026805 | 0.387674 |
| GO:0009611\_response\_to\_wounding | 279 | 2 | 3.899642 | -1.039518 | 216 | 27.835232 | 83.64 | 139.444768 | 0.387222 |
| GO:0016485\_protein\_processing | 53 | 1 | 10.264151 | -1.030605 | 218 | 28.958752 | 85.57 | 142.181248 | 0.392523 |
| GO:0051339\_regulation\_of\_lyase\_activity | 53 | 1 | 10.264151 | -1.030605 | 218 | 28.958752 | 85.57 | 142.181248 | 0.392523 |
| GO:0007605\_sensory\_perception\_of\_sound | 54 | 1 | 10.074074 | -1.022856 | 220 | 29.212869 | 86.02 | 142.827131 | 0.391000 |
| GO:0050954\_sensory\_perception\_of\_mechanical\_stimulus | 54 | 1 | 10.074074 | -1.022856 | 220 | 29.212869 | 86.02 | 142.827131 | 0.391000 |
| GO:0030814\_regulation\_of\_cAMP\_metabolic\_process | 55 | 1 | 9.890909 | -1.015255 | 222 | 29.762473 | 86.9 | 144.037527 | 0.391441 |
| GO:0030817\_regulation\_of\_cAMP\_biosynthetic\_process | 55 | 1 | 9.890909 | -1.015255 | 222 | 29.762473 | 86.9 | 144.037527 | 0.391441 |
| GO:0040011\_locomotion | 292 | 2 | 3.726027 | -1.005806 | 223 | 30.218552 | 87.82 | 145.421448 | 0.393812 |
| GO:0016192\_vesicle-mediated\_transport | 297 | 2 | 3.663300 | -0.993307 | 224 | 30.767268 | 88.8 | 146.832732 | 0.396429 |
| GO:0007160\_cell-matrix\_adhesion | 58 | 1 | 9.379310 | -0.993296 | 228 | 31.773512 | 90.14 | 148.506488 | 0.395351 |
| GO:0030799\_regulation\_of\_cyclic\_nucleotide\_metabolic\_process | 58 | 1 | 9.379310 | -0.993296 | 228 | 31.773512 | 90.14 | 148.506488 | 0.395351 |
| GO:0030802\_regulation\_of\_cyclic\_nucleotide\_biosynthetic\_process | 58 | 1 | 9.379310 | -0.993296 | 228 | 31.773512 | 90.14 | 148.506488 | 0.395351 |
| GO:0030808\_regulation\_of\_nucleotide\_biosynthetic\_process | 58 | 1 | 9.379310 | -0.993296 | 228 | 31.773512 | 90.14 | 148.506488 | 0.395351 |
| GO:0006171\_cAMP\_biosynthetic\_process | 59 | 1 | 9.220339 | -0.986240 | 230 | 32.365635 | 91.01 | 149.654365 | 0.395696 |
| GO:0051604\_protein\_maturation | 59 | 1 | 9.220339 | -0.986240 | 230 | 32.365635 | 91.01 | 149.654365 | 0.395696 |
| GO:0051246\_regulation\_of\_protein\_metabolic\_process | 301 | 2 | 3.614618 | -0.983485 | 231 | 32.369323 | 91.12 | 149.870677 | 0.394459 |
| GO:0006140\_regulation\_of\_nucleotide\_metabolic\_process | 60 | 1 | 9.066667 | -0.979309 | 234 | 32.672220 | 91.88 | 151.087780 | 0.392650 |
| GO:0044087\_regulation\_of\_cellular\_component\_biogenesis | 60 | 1 | 9.066667 | -0.979309 | 234 | 32.672220 | 91.88 | 151.087780 | 0.392650 |
| GO:0046058\_cAMP\_metabolic\_process | 60 | 1 | 9.066667 | -0.979309 | 234 | 32.672220 | 91.88 | 151.087780 | 0.392650 |
| GO:0048519\_negative\_regulation\_of\_biological\_process | 1013 | 4 | 2.148075 | -0.976744 | 235 | 32.765696 | 92.06 | 151.354304 | 0.391745 |
| GO:0006928\_cell\_motion | 308 | 2 | 3.532468 | -0.966665 | 237 | 33.444039 | 93.31 | 153.175961 | 0.393713 |
| GO:0051674\_localization\_of\_cell | 308 | 2 | 3.532468 | -0.966665 | 237 | 33.444039 | 93.31 | 153.175961 | 0.393713 |
| GO:0009190\_cyclic\_nucleotide\_biosynthetic\_process | 62 | 1 | 8.774194 | -0.965805 | 239 | 34.135711 | 94.55 | 154.964289 | 0.395607 |
| GO:0043406\_positive\_regulation\_of\_MAP\_kinase\_activity | 62 | 1 | 8.774194 | -0.965805 | 239 | 34.135711 | 94.55 | 154.964289 | 0.395607 |
| GO:0008283\_cell\_proliferation | 647 | 3 | 2.522411 | -0.956567 | 240 | 34.392469 | 95.07 | 155.747531 | 0.396125 |
| GO:0042327\_positive\_regulation\_of\_phosphorylation | 64 | 1 | 8.500000 | -0.952753 | 241 | 35.201876 | 96.33 | 157.458124 | 0.399710 |
| GO:0051235\_maintenance\_of\_location | 65 | 1 | 8.369231 | -0.946387 | 242 | 35.475792 | 96.95 | 158.424208 | 0.400620 |
| GO:0010562\_positive\_regulation\_of\_phosphorus\_metabolic\_process | 66 | 1 | 8.242424 | -0.940124 | 248 | 36.585923 | 98.68 | 160.774077 | 0.397903 |
| GO:0014706\_striated\_muscle\_tissue\_development | 66 | 1 | 8.242424 | -0.940124 | 248 | 36.585923 | 98.68 | 160.774077 | 0.397903 |
| GO:0031589\_cell-substrate\_adhesion | 66 | 1 | 8.242424 | -0.940124 | 248 | 36.585923 | 98.68 | 160.774077 | 0.397903 |
| GO:0045937\_positive\_regulation\_of\_phosphate\_metabolic\_process | 66 | 1 | 8.242424 | -0.940124 | 248 | 36.585923 | 98.68 | 160.774077 | 0.397903 |
| GO:0051090\_regulation\_of\_transcription\_factor\_activity | 66 | 1 | 8.242424 | -0.940124 | 248 | 36.585923 | 98.68 | 160.774077 | 0.397903 |
| GO:0090046\_regulation\_of\_transcription\_regulator\_activity | 66 | 1 | 8.242424 | -0.940124 | 248 | 36.585923 | 98.68 | 160.774077 | 0.397903 |
| GO:0007015\_actin\_filament\_organization | 67 | 1 | 8.119403 | -0.933961 | 251 | 36.969592 | 99.32 | 161.670408 | 0.395697 |
| GO:0009187\_cyclic\_nucleotide\_metabolic\_process | 67 | 1 | 8.119403 | -0.933961 | 251 | 36.969592 | 99.32 | 161.670408 | 0.395697 |
| GO:0060537\_muscle\_tissue\_development | 67 | 1 | 8.119403 | -0.933961 | 251 | 36.969592 | 99.32 | 161.670408 | 0.395697 |
| GO:0006606\_protein\_import\_into\_nucleus | 68 | 1 | 8.000000 | -0.927895 | 253 | 37.460437 | 100.14 | 162.819563 | 0.395810 |
| GO:0007507\_heart\_development | 68 | 1 | 8.000000 | -0.927895 | 253 | 37.460437 | 100.14 | 162.819563 | 0.395810 |
| GO:0032879\_regulation\_of\_localization | 326 | 2 | 3.337423 | -0.925425 | 254 | 37.685757 | 100.6 | 163.514243 | 0.396063 |
| GO:0001525\_angiogenesis | 69 | 1 | 7.884058 | -0.921922 | 256 | 38.199157 | 101.57 | 164.940843 | 0.396758 |
| GO:0051241\_negative\_regulation\_of\_multicellular\_organismal\_process | 69 | 1 | 7.884058 | -0.921922 | 256 | 38.199157 | 101.57 | 164.940843 | 0.396758 |
| GO:0030163\_protein\_catabolic\_process | 330 | 2 | 3.296970 | -0.916631 | 257 | 38.262244 | 101.65 | 165.037756 | 0.395525 |
| GO:0002694\_regulation\_of\_leukocyte\_activation | 70 | 1 | 7.771429 | -0.916041 | 260 | 38.527543 | 102.42 | 166.312457 | 0.393923 |
| GO:0009124\_nucleoside\_monophosphate\_biosynthetic\_process | 70 | 1 | 7.771429 | -0.916041 | 260 | 38.527543 | 102.42 | 166.312457 | 0.393923 |
| GO:0051170\_nuclear\_import | 70 | 1 | 7.771429 | -0.916041 | 260 | 38.527543 | 102.42 | 166.312457 | 0.393923 |
| GO:0006417\_regulation\_of\_translation | 71 | 1 | 7.661972 | -0.910248 | 264 | 39.091454 | 103.3 | 167.508546 | 0.391288 |
| GO:0007050\_cell\_cycle\_arrest | 71 | 1 | 7.661972 | -0.910248 | 264 | 39.091454 | 103.3 | 167.508546 | 0.391288 |
| GO:0032101\_regulation\_of\_response\_to\_external\_stimulus | 71 | 1 | 7.661972 | -0.910248 | 264 | 39.091454 | 103.3 | 167.508546 | 0.391288 |
| GO:0051223\_regulation\_of\_protein\_transport | 71 | 1 | 7.661972 | -0.910248 | 264 | 39.091454 | 103.3 | 167.508546 | 0.391288 |
| GO:0007187\_G-protein\_signaling\_\_coupled\_to\_cyclic\_nucleotide\_second\_messenger | 73 | 1 | 7.452055 | -0.898918 | 267 | 39.980870 | 104.68 | 169.379130 | 0.392060 |
| GO:0007596\_blood\_coagulation | 73 | 1 | 7.452055 | -0.898918 | 267 | 39.980870 | 104.68 | 169.379130 | 0.392060 |
| GO:0009894\_regulation\_of\_catabolic\_process | 73 | 1 | 7.452055 | -0.898918 | 267 | 39.980870 | 104.68 | 169.379130 | 0.392060 |
| GO:0050817\_coagulation | 74 | 1 | 7.351351 | -0.893376 | 268 | 40.367215 | 105.55 | 170.732785 | 0.393843 |
| GO:0006869\_lipid\_transport | 75 | 1 | 7.253333 | -0.887914 | 270 | 40.756582 | 106.36 | 171.963418 | 0.393926 |
| GO:0070201\_regulation\_of\_establishment\_of\_protein\_localization | 75 | 1 | 7.253333 | -0.887914 | 270 | 40.756582 | 106.36 | 171.963418 | 0.393926 |
| GO:0034504\_protein\_localization\_in\_nucleus | 76 | 1 | 7.157895 | -0.882529 | 272 | 41.109072 | 107.45 | 173.790928 | 0.395037 |
| GO:0051101\_regulation\_of\_DNA\_binding | 76 | 1 | 7.157895 | -0.882529 | 272 | 41.109072 | 107.45 | 173.790928 | 0.395037 |
| GO:0009123\_nucleoside\_monophosphate\_metabolic\_process | 77 | 1 | 7.064935 | -0.877218 | 275 | 41.634592 | 108.31 | 174.985408 | 0.393855 |
| GO:0050865\_regulation\_of\_cell\_activation | 77 | 1 | 7.064935 | -0.877218 | 275 | 41.634592 | 108.31 | 174.985408 | 0.393855 |
| GO:0051129\_negative\_regulation\_of\_cellular\_component\_organization | 77 | 1 | 7.064935 | -0.877218 | 275 | 41.634592 | 108.31 | 174.985408 | 0.393855 |
| GO:0006164\_purine\_nucleotide\_biosynthetic\_process | 79 | 1 | 6.886076 | -0.866816 | 279 | 42.270382 | 109.61 | 176.949618 | 0.392867 |
| GO:0007599\_hemostasis | 79 | 1 | 6.886076 | -0.866816 | 279 | 42.270382 | 109.61 | 176.949618 | 0.392867 |
| GO:0032880\_regulation\_of\_protein\_localization | 79 | 1 | 6.886076 | -0.866816 | 279 | 42.270382 | 109.61 | 176.949618 | 0.392867 |
| GO:0046942\_carboxylic\_acid\_transport | 79 | 1 | 6.886076 | -0.866816 | 279 | 42.270382 | 109.61 | 176.949618 | 0.392867 |
| GO:0003008\_system\_process | 710 | 3 | 2.298592 | -0.865750 | 280 | 42.441060 | 109.86 | 177.278940 | 0.392357 |
| GO:0015849\_organic\_acid\_transport | 80 | 1 | 6.800000 | -0.861719 | 281 | 42.581242 | 110.22 | 177.858758 | 0.392242 |
| GO:0048193\_Golgi\_vesicle\_transport | 82 | 1 | 6.634146 | -0.851729 | 283 | 43.432522 | 111.74 | 180.047478 | 0.394841 |
| GO:0048514\_blood\_vessel\_morphogenesis | 82 | 1 | 6.634146 | -0.851729 | 283 | 43.432522 | 111.74 | 180.047478 | 0.394841 |
| GO:0043123\_positive\_regulation\_of\_I-kappaB\_kinase\_NF-kappaB\_cascade | 84 | 1 | 6.476190 | -0.841996 | 284 | 44.496304 | 113.39 | 182.283696 | 0.399261 |
| GO:0007243\_protein\_kinase\_cascade | 377 | 2 | 2.885942 | -0.822100 | 285 | 45.905547 | 116.0 | 186.094453 | 0.407018 |
| GO:0009966\_regulation\_of\_signal\_transduction | 378 | 2 | 2.878307 | -0.820248 | 286 | 46.064879 | 116.28 | 186.495121 | 0.406573 |
| GO:0030334\_regulation\_of\_cell\_migration | 89 | 1 | 6.112360 | -0.818716 | 289 | 46.525699 | 117.25 | 187.974301 | 0.405709 |
| GO:0043405\_regulation\_of\_MAP\_kinase\_activity | 89 | 1 | 6.112360 | -0.818716 | 289 | 46.525699 | 117.25 | 187.974301 | 0.405709 |
| GO:0051240\_positive\_regulation\_of\_multicellular\_organismal\_process | 89 | 1 | 6.112360 | -0.818716 | 289 | 46.525699 | 117.25 | 187.974301 | 0.405709 |
| GO:0009165\_nucleotide\_biosynthetic\_process | 90 | 1 | 6.044444 | -0.814230 | 290 | 46.895813 | 117.79 | 188.684187 | 0.406172 |
| GO:0032269\_negative\_regulation\_of\_cellular\_protein\_metabolic\_process | 91 | 1 | 5.978022 | -0.809797 | 291 | 47.107897 | 118.38 | 189.652103 | 0.406804 |
| GO:0017038\_protein\_import | 93 | 1 | 5.849462 | -0.801087 | 293 | 47.693283 | 119.38 | 191.066717 | 0.407440 |
| GO:0043122\_regulation\_of\_I-kappaB\_kinase\_NF-kappaB\_cascade | 93 | 1 | 5.849462 | -0.801087 | 293 | 47.693283 | 119.38 | 191.066717 | 0.407440 |
| GO:0033365\_protein\_localization\_in\_organelle | 95 | 1 | 5.726316 | -0.792577 | 295 | 48.060391 | 120.09 | 192.119609 | 0.407085 |
| GO:0050878\_regulation\_of\_body\_fluid\_levels | 95 | 1 | 5.726316 | -0.792577 | 295 | 48.060391 | 120.09 | 192.119609 | 0.407085 |
| GO:0009059\_macromolecule\_biosynthetic\_process | 1626 | 5 | 1.672817 | -0.790078 | 296 | 48.219392 | 120.37 | 192.520608 | 0.406655 |
| GO:0040012\_regulation\_of\_locomotion | 96 | 1 | 5.666667 | -0.788395 | 297 | 48.647713 | 121.14 | 193.632287 | 0.407879 |
| GO:0051098\_regulation\_of\_binding | 97 | 1 | 5.608247 | -0.784260 | 298 | 48.801537 | 121.6 | 194.398463 | 0.408054 |
| GO:0051270\_regulation\_of\_cell\_motion | 98 | 1 | 5.551020 | -0.780171 | 299 | 49.260610 | 122.37 | 195.479390 | 0.409264 |
| GO:0006163\_purine\_nucleotide\_metabolic\_process | 99 | 1 | 5.494949 | -0.776127 | 301 | 49.569366 | 122.87 | 196.170634 | 0.408206 |
| GO:0009968\_negative\_regulation\_of\_signal\_transduction | 99 | 1 | 5.494949 | -0.776127 | 301 | 49.569366 | 122.87 | 196.170634 | 0.408206 |
| GO:0022603\_regulation\_of\_anatomical\_structure\_morphogenesis | 100 | 1 | 5.440000 | -0.772127 | 303 | 50.114996 | 123.73 | 197.345004 | 0.408350 |
| GO:0055114\_oxidation\_reduction | 100 | 1 | 5.440000 | -0.772127 | 303 | 50.114996 | 123.73 | 197.345004 | 0.408350 |
| GO:0043623\_cellular\_protein\_complex\_assembly | 101 | 1 | 5.386139 | -0.768171 | 304 | 50.351671 | 124.2 | 198.048329 | 0.408553 |
| GO:0010648\_negative\_regulation\_of\_cell\_communication | 102 | 1 | 5.333333 | -0.764257 | 305 | 50.452421 | 124.39 | 198.327579 | 0.407836 |
| GO:0042127\_regulation\_of\_cell\_proliferation | 411 | 2 | 2.647202 | -0.762359 | 306 | 50.661726 | 124.72 | 198.778274 | 0.407582 |
| GO:0001817\_regulation\_of\_cytokine\_production | 103 | 1 | 5.281553 | -0.760385 | 307 | 50.692019 | 124.93 | 199.167981 | 0.406938 |
| GO:0010605\_negative\_regulation\_of\_macromolecule\_metabolic\_process | 413 | 2 | 2.634383 | -0.759040 | 308 | 50.948682 | 125.23 | 199.511318 | 0.406591 |
| GO:0002684\_positive\_regulation\_of\_immune\_system\_process | 106 | 1 | 5.132075 | -0.749010 | 309 | 51.467854 | 125.93 | 200.392146 | 0.407540 |
| GO:0046907\_intracellular\_transport | 420 | 2 | 2.590476 | -0.747580 | 310 | 51.594332 | 126.08 | 200.565668 | 0.406710 |
| GO:0010646\_regulation\_of\_cell\_communication | 423 | 2 | 2.572104 | -0.742743 | 311 | 51.761679 | 126.29 | 200.818321 | 0.406077 |
| GO:0009790\_embryonic\_development | 109 | 1 | 4.990826 | -0.737983 | 312 | 52.864573 | 127.72 | 202.575427 | 0.409359 |
| GO:0043285\_biopolymer\_catabolic\_process | 426 | 2 | 2.553991 | -0.737949 | 313 | 52.970859 | 127.84 | 202.709141 | 0.408435 |
| GO:0007265\_Ras\_protein\_signal\_transduction | 110 | 1 | 4.945455 | -0.734381 | 314 | 53.184591 | 128.25 | 203.315409 | 0.408439 |
| GO:0001816\_cytokine\_production | 112 | 1 | 4.857143 | -0.727284 | 315 | 53.718326 | 129.19 | 204.661674 | 0.410127 |
| GO:0007166\_cell\_surface\_receptor\_linked\_signal\_transduction | 828 | 3 | 1.971014 | -0.722117 | 316 | 53.962407 | 129.66 | 205.357593 | 0.410316 |
| GO:0048584\_positive\_regulation\_of\_response\_to\_stimulus | 114 | 1 | 4.771930 | -0.720326 | 317 | 54.119759 | 130.05 | 205.980241 | 0.410252 |
| GO:0009057\_macromolecule\_catabolic\_process | 439 | 2 | 2.478360 | -0.717664 | 318 | 54.199345 | 130.27 | 206.340655 | 0.409654 |
| GO:0009892\_negative\_regulation\_of\_metabolic\_process | 440 | 2 | 2.472727 | -0.716136 | 319 | 54.706309 | 131.0 | 207.293691 | 0.410658 |
| GO:0051050\_positive\_regulation\_of\_transport | 116 | 1 | 4.689655 | -0.713500 | 320 | 54.779494 | 131.39 | 208.000506 | 0.410594 |
| GO:0065008\_regulation\_of\_biological\_quality | 848 | 3 | 1.924528 | -0.700627 | 321 | 55.501777 | 132.7 | 209.898223 | 0.413396 |
| GO:0006913\_nucleocytoplasmic\_transport | 121 | 1 | 4.495868 | -0.696991 | 322 | 55.650709 | 133.06 | 210.469291 | 0.413230 |
| GO:0007242\_intracellular\_signaling\_cascade | 853 | 3 | 1.913247 | -0.695369 | 323 | 55.708809 | 133.18 | 210.651191 | 0.412322 |
| GO:0033674\_positive\_regulation\_of\_kinase\_activity | 122 | 1 | 4.459016 | -0.693780 | 327 | 56.027784 | 134.06 | 212.092216 | 0.409969 |
| GO:0045860\_positive\_regulation\_of\_protein\_kinase\_activity | 122 | 1 | 4.459016 | -0.693780 | 327 | 56.027784 | 134.06 | 212.092216 | 0.409969 |
| GO:0051169\_nuclear\_transport | 122 | 1 | 4.459016 | -0.693780 | 327 | 56.027784 | 134.06 | 212.092216 | 0.409969 |
| GO:0060341\_regulation\_of\_cellular\_localization | 122 | 1 | 4.459016 | -0.693780 | 327 | 56.027784 | 134.06 | 212.092216 | 0.409969 |
| GO:0007517\_muscle\_organ\_development | 123 | 1 | 4.422764 | -0.690598 | 328 | 56.389640 | 134.52 | 212.650360 | 0.410122 |
| GO:0006897\_endocytosis | 124 | 1 | 4.387097 | -0.687445 | 330 | 56.935620 | 135.2 | 213.464380 | 0.409697 |
| GO:0010324\_membrane\_invagination | 124 | 1 | 4.387097 | -0.687445 | 330 | 56.935620 | 135.2 | 213.464380 | 0.409697 |
| GO:0006935\_chemotaxis | 125 | 1 | 4.352000 | -0.684319 | 332 | 57.367661 | 135.94 | 214.512339 | 0.409458 |
| GO:0042330\_taxis | 125 | 1 | 4.352000 | -0.684319 | 332 | 57.367661 | 135.94 | 214.512339 | 0.409458 |
| GO:0009605\_response\_to\_external\_stimulus | 464 | 2 | 2.344828 | -0.680747 | 333 | 57.417477 | 136.1 | 214.782523 | 0.408709 |
| GO:0050877\_neurological\_system\_process | 468 | 2 | 2.324786 | -0.675080 | 334 | 57.868391 | 136.78 | 215.691609 | 0.409521 |
| GO:0009987\_cellular\_process | 6671 | 14 | 1.141658 | -0.674738 | 335 | 57.932559 | 136.91 | 215.887441 | 0.408687 |
| GO:0009725\_response\_to\_hormone\_stimulus | 129 | 1 | 4.217054 | -0.672091 | 338 | 58.680052 | 137.84 | 216.999948 | 0.407811 |
| GO:0010740\_positive\_regulation\_of\_protein\_kinase\_cascade | 129 | 1 | 4.217054 | -0.672091 | 338 | 58.680052 | 137.84 | 216.999948 | 0.407811 |
| GO:0051347\_positive\_regulation\_of\_transferase\_activity | 129 | 1 | 4.217054 | -0.672091 | 338 | 58.680052 | 137.84 | 216.999948 | 0.407811 |
| GO:0032270\_positive\_regulation\_of\_cellular\_protein\_metabolic\_process | 131 | 1 | 4.152672 | -0.666135 | 339 | 59.996156 | 139.82 | 219.643844 | 0.412448 |
| GO:0022607\_cellular\_component\_assembly | 478 | 2 | 2.276151 | -0.661187 | 340 | 60.668620 | 141.01 | 221.351380 | 0.414735 |
| GO:0003013\_circulatory\_system\_process | 133 | 1 | 4.090226 | -0.660280 | 342 | 61.184028 | 141.76 | 222.335972 | 0.414503 |
| GO:0008015\_blood\_circulation | 133 | 1 | 4.090226 | -0.660280 | 342 | 61.184028 | 141.76 | 222.335972 | 0.414503 |
| GO:0007249\_I-kappaB\_kinase\_NF-kappaB\_cascade | 134 | 1 | 4.059701 | -0.657389 | 343 | 61.831165 | 142.44 | 223.048835 | 0.415277 |
| GO:0007264\_small\_GTPase\_mediated\_signal\_transduction | 135 | 1 | 4.029630 | -0.654523 | 346 | 62.562611 | 143.49 | 224.417389 | 0.414711 |
| GO:0009719\_response\_to\_endogenous\_stimulus | 135 | 1 | 4.029630 | -0.654523 | 346 | 62.562611 | 143.49 | 224.417389 | 0.414711 |
| GO:0051247\_positive\_regulation\_of\_protein\_metabolic\_process | 135 | 1 | 4.029630 | -0.654523 | 346 | 62.562611 | 143.49 | 224.417389 | 0.414711 |
| GO:0009628\_response\_to\_abiotic\_stimulus | 140 | 1 | 3.885714 | -0.640539 | 347 | 63.686470 | 145.12 | 226.553530 | 0.418213 |
| GO:0007626\_locomotory\_behavior | 142 | 1 | 3.830986 | -0.635102 | 348 | 64.636457 | 146.54 | 228.443543 | 0.421092 |
| GO:0000165\_MAPKKK\_cascade | 143 | 1 | 3.804196 | -0.632416 | 349 | 64.800749 | 146.78 | 228.759251 | 0.420573 |
| GO:0031399\_regulation\_of\_protein\_modification\_process | 144 | 1 | 3.777778 | -0.629751 | 350 | 65.370046 | 147.63 | 229.889954 | 0.421800 |
| GO:0006605\_protein\_targeting | 145 | 1 | 3.751724 | -0.627107 | 352 | 65.985455 | 148.49 | 230.994545 | 0.421847 |
| GO:0030036\_actin\_cytoskeleton\_organization | 145 | 1 | 3.751724 | -0.627107 | 352 | 65.985455 | 148.49 | 230.994545 | 0.421847 |
| GO:0006753\_nucleoside\_phosphate\_metabolic\_process | 146 | 1 | 3.726027 | -0.624484 | 354 | 66.842838 | 149.58 | 232.317162 | 0.422542 |
| GO:0009117\_nucleotide\_metabolic\_process | 146 | 1 | 3.726027 | -0.624484 | 354 | 66.842838 | 149.58 | 232.317162 | 0.422542 |
| GO:0048523\_negative\_regulation\_of\_cellular\_process | 925 | 3 | 1.764324 | -0.624363 | 355 | 66.884923 | 149.69 | 232.495077 | 0.421662 |
| GO:0080134\_regulation\_of\_response\_to\_stress | 147 | 1 | 3.700680 | -0.621881 | 356 | 67.503931 | 150.7 | 233.896069 | 0.423315 |
| GO:0044267\_cellular\_protein\_metabolic\_process | 1382 | 4 | 1.574530 | -0.618674 | 357 | 67.582797 | 150.82 | 234.057203 | 0.422465 |
| GO:0045321\_leukocyte\_activation | 150 | 1 | 3.626667 | -0.614190 | 358 | 67.805737 | 151.29 | 234.774263 | 0.422598 |
| GO:0006916\_anti-apoptosis | 155 | 1 | 3.509677 | -0.601754 | 359 | 68.742214 | 152.53 | 236.317786 | 0.424875 |
| GO:0050790\_regulation\_of\_catalytic\_activity | 525 | 2 | 2.072381 | -0.600697 | 360 | 68.856685 | 152.78 | 236.703315 | 0.424389 |
| GO:0016337\_cell-cell\_adhesion | 156 | 1 | 3.487179 | -0.599322 | 361 | 69.134767 | 153.17 | 237.205233 | 0.424294 |
| GO:0070887\_cellular\_response\_to\_chemical\_stimulus | 157 | 1 | 3.464968 | -0.596908 | 362 | 69.424990 | 153.68 | 237.935010 | 0.424530 |
| GO:0030029\_actin\_filament-based\_process | 165 | 1 | 3.296970 | -0.578205 | 363 | 70.592443 | 155.31 | 240.027557 | 0.427851 |
| GO:0055086\_nucleobase\_\_nucleoside\_and\_nucleotide\_metabolic\_process | 167 | 1 | 3.257485 | -0.573692 | 364 | 70.829481 | 155.73 | 240.630519 | 0.427830 |
| GO:0046483\_heterocycle\_metabolic\_process | 173 | 1 | 3.144509 | -0.560519 | 365 | 72.133339 | 157.43 | 242.726661 | 0.431315 |
| GO:0044085\_cellular\_component\_biogenesis | 560 | 2 | 1.942857 | -0.560215 | 366 | 72.391079 | 157.79 | 243.188921 | 0.431120 |
| GO:0001775\_cell\_activation | 175 | 1 | 3.108571 | -0.556245 | 367 | 72.888661 | 158.7 | 244.511339 | 0.432425 |
| GO:0051649\_establishment\_of\_localization\_in\_cell | 573 | 2 | 1.898778 | -0.546056 | 368 | 74.295541 | 161.14 | 247.984459 | 0.437880 |
| GO:0006954\_inflammatory\_response | 182 | 1 | 2.989011 | -0.541723 | 369 | 75.067257 | 162.16 | 249.252743 | 0.439458 |
| GO:0010627\_regulation\_of\_protein\_kinase\_cascade | 184 | 1 | 2.956522 | -0.537694 | 370 | 75.258017 | 162.43 | 249.601983 | 0.439000 |
| GO:0009058\_biosynthetic\_process | 1988 | 5 | 1.368209 | -0.533817 | 371 | 75.636288 | 162.95 | 250.263712 | 0.439218 |
| GO:0034622\_cellular\_macromolecular\_complex\_assembly | 186 | 1 | 2.924731 | -0.533715 | 372 | 76.025882 | 163.43 | 250.834118 | 0.439328 |
| GO:0008219\_cell\_death | 585 | 2 | 1.859829 | -0.533379 | 374 | 76.353545 | 164.03 | 251.706455 | 0.438583 |
| GO:0016265\_death | 585 | 2 | 1.859829 | -0.533379 | 374 | 76.353545 | 164.03 | 251.706455 | 0.438583 |
| GO:0043283\_biopolymer\_metabolic\_process | 3027 | 7 | 1.258011 | -0.518678 | 375 | 77.819103 | 165.81 | 253.800897 | 0.442160 |
| GO:0010556\_regulation\_of\_macromolecule\_biosynthetic\_process | 1055 | 3 | 1.546919 | -0.515364 | 376 | 78.179689 | 166.41 | 254.640311 | 0.442580 |
| GO:0002682\_regulation\_of\_immune\_system\_process | 196 | 1 | 2.775510 | -0.514549 | 378 | 78.803536 | 167.21 | 255.616464 | 0.442354 |
| GO:0043086\_negative\_regulation\_of\_catalytic\_activity | 196 | 1 | 2.775510 | -0.514549 | 378 | 78.803536 | 167.21 | 255.616464 | 0.442354 |
| GO:0065009\_regulation\_of\_molecular\_function | 606 | 2 | 1.795380 | -0.512053 | 379 | 79.102696 | 167.86 | 256.617304 | 0.442902 |
| GO:0010468\_regulation\_of\_gene\_expression | 1067 | 3 | 1.529522 | -0.506374 | 380 | 79.885725 | 168.67 | 257.454275 | 0.443868 |
| GO:0008152\_metabolic\_process | 4111 | 9 | 1.190951 | -0.503097 | 381 | 80.647347 | 169.47 | 258.292653 | 0.444803 |
| GO:0051641\_cellular\_localization | 617 | 2 | 1.763371 | -0.501296 | 382 | 80.791613 | 169.69 | 258.588387 | 0.444215 |
| GO:0006886\_intracellular\_protein\_transport | 204 | 1 | 2.666667 | -0.500031 | 383 | 80.967506 | 169.89 | 258.812494 | 0.443577 |
| GO:0051179\_localization | 1561 | 4 | 1.393978 | -0.495756 | 384 | 81.468468 | 170.74 | 260.011532 | 0.444635 |
| GO:0043066\_negative\_regulation\_of\_apoptosis | 207 | 1 | 2.628019 | -0.494760 | 385 | 81.970935 | 171.31 | 260.649065 | 0.444961 |
| GO:0043069\_negative\_regulation\_of\_programmed\_cell\_death | 209 | 1 | 2.602871 | -0.491297 | 387 | 82.840743 | 172.51 | 262.179257 | 0.445762 |
| GO:0060548\_negative\_regulation\_of\_cell\_death | 209 | 1 | 2.602871 | -0.491297 | 387 | 82.840743 | 172.51 | 262.179257 | 0.445762 |
| GO:0009056\_catabolic\_process | 633 | 2 | 1.718799 | -0.486133 | 388 | 83.580948 | 173.61 | 263.639052 | 0.447448 |
| GO:0045859\_regulation\_of\_protein\_kinase\_activity | 213 | 1 | 2.553991 | -0.484488 | 389 | 84.097226 | 174.35 | 264.602774 | 0.448201 |
| GO:0007610\_behavior | 214 | 1 | 2.542056 | -0.482810 | 390 | 84.382566 | 174.65 | 264.917434 | 0.447821 |
| GO:0043549\_regulation\_of\_kinase\_activity | 217 | 1 | 2.506912 | -0.477831 | 391 | 84.997006 | 175.43 | 265.862994 | 0.448670 |
| GO:0031326\_regulation\_of\_cellular\_biosynthetic\_process | 1125 | 3 | 1.450667 | -0.465157 | 392 | 85.942342 | 176.86 | 267.777658 | 0.451173 |
| GO:0034621\_cellular\_macromolecular\_complex\_subunit\_organization | 227 | 1 | 2.396476 | -0.461817 | 394 | 86.879208 | 178.31 | 269.740792 | 0.452563 |
| GO:0051338\_regulation\_of\_transferase\_activity | 227 | 1 | 2.396476 | -0.461817 | 394 | 86.879208 | 178.31 | 269.740792 | 0.452563 |
| GO:0034613\_cellular\_protein\_localization | 228 | 1 | 2.385965 | -0.460262 | 395 | 87.073828 | 178.53 | 269.986172 | 0.451975 |
| GO:0070727\_cellular\_macromolecule\_localization | 229 | 1 | 2.375546 | -0.458716 | 396 | 87.247038 | 178.74 | 270.232962 | 0.451364 |
| GO:0009889\_regulation\_of\_biosynthetic\_process | 1135 | 3 | 1.437885 | -0.458406 | 397 | 87.389515 | 178.94 | 270.490485 | 0.450730 |
| GO:0006412\_translation | 233 | 1 | 2.334764 | -0.452614 | 399 | 88.560508 | 180.61 | 272.659492 | 0.452657 |
| GO:0044092\_negative\_regulation\_of\_molecular\_function | 233 | 1 | 2.334764 | -0.452614 | 399 | 88.560508 | 180.61 | 272.659492 | 0.452657 |
| GO:0051128\_regulation\_of\_cellular\_component\_organization | 237 | 1 | 2.295359 | -0.446638 | 400 | 88.997232 | 181.25 | 273.502768 | 0.453125 |
| GO:0048583\_regulation\_of\_response\_to\_stimulus | 241 | 1 | 2.257261 | -0.440784 | 401 | 89.416950 | 181.86 | 274.303050 | 0.453516 |
| GO:0048468\_cell\_development | 251 | 1 | 2.167331 | -0.426659 | 402 | 91.687015 | 185.0 | 278.312985 | 0.460199 |
| GO:0006461\_protein\_complex\_assembly | 273 | 1 | 1.992674 | -0.397910 | 404 | 94.463281 | 188.55 | 282.636719 | 0.466708 |
| GO:0070271\_protein\_complex\_biogenesis | 273 | 1 | 1.992674 | -0.397910 | 404 | 94.463281 | 188.55 | 282.636719 | 0.466708 |
| GO:0015031\_protein\_transport | 274 | 1 | 1.985401 | -0.396673 | 405 | 94.891677 | 189.06 | 283.228323 | 0.466815 |
| GO:0007010\_cytoskeleton\_organization | 275 | 1 | 1.978182 | -0.395442 | 406 | 95.161190 | 189.39 | 283.618810 | 0.466478 |
| GO:0010033\_response\_to\_organic\_substance | 276 | 1 | 1.971014 | -0.394216 | 407 | 95.441659 | 189.72 | 283.998341 | 0.466143 |
| GO:0006810\_transport | 1243 | 3 | 1.312953 | -0.391504 | 408 | 96.265012 | 190.98 | 285.694988 | 0.468088 |
| GO:0045184\_establishment\_of\_protein\_localization | 279 | 1 | 1.949821 | -0.390573 | 409 | 96.729982 | 191.55 | 286.370018 | 0.468337 |
| GO:0042325\_regulation\_of\_phosphorylation | 285 | 1 | 1.908772 | -0.383433 | 410 | 97.148896 | 192.04 | 286.931104 | 0.468390 |
| GO:0032268\_regulation\_of\_cellular\_protein\_metabolic\_process | 286 | 1 | 1.902098 | -0.382262 | 411 | 97.806266 | 192.9 | 287.993734 | 0.469343 |
| GO:0051234\_establishment\_of\_localization | 1260 | 3 | 1.295238 | -0.381898 | 412 | 98.049189 | 193.2 | 288.350811 | 0.468932 |
| GO:0051093\_negative\_regulation\_of\_developmental\_process | 290 | 1 | 1.875862 | -0.377628 | 413 | 99.562440 | 194.92 | 290.277560 | 0.471961 |
| GO:0034960\_cellular\_biopolymer\_metabolic\_process | 2820 | 6 | 1.157447 | -0.375419 | 414 | 99.933277 | 195.35 | 290.766723 | 0.471860 |
| GO:0019220\_regulation\_of\_phosphate\_metabolic\_process | 297 | 1 | 1.831650 | -0.369714 | 416 | 101.439141 | 197.49 | 293.540859 | 0.474736 |
| GO:0051174\_regulation\_of\_phosphorus\_metabolic\_process | 297 | 1 | 1.831650 | -0.369714 | 416 | 101.439141 | 197.49 | 293.540859 | 0.474736 |
| GO:0080090\_regulation\_of\_primary\_metabolic\_process | 1311 | 3 | 1.244851 | -0.354446 | 417 | 103.475101 | 200.19 | 296.904899 | 0.480072 |
| GO:0006508\_proteolysis | 313 | 1 | 1.738019 | -0.352497 | 418 | 103.770945 | 200.49 | 297.209055 | 0.479641 |
| GO:0044260\_cellular\_macromolecule\_metabolic\_process | 2883 | 6 | 1.132154 | -0.350156 | 419 | 103.911116 | 200.65 | 297.388884 | 0.478878 |
| GO:0060255\_regulation\_of\_macromolecule\_metabolic\_process | 1328 | 3 | 1.228916 | -0.345730 | 420 | 104.764566 | 201.74 | 298.715434 | 0.480333 |
| GO:0010557\_positive\_regulation\_of\_macromolecule\_biosynthetic\_process | 334 | 1 | 1.628743 | -0.331574 | 421 | 106.356334 | 203.84 | 301.323666 | 0.484181 |
| GO:0008104\_protein\_localization | 339 | 1 | 1.604720 | -0.326849 | 422 | 106.816654 | 204.51 | 302.203346 | 0.484621 |
| GO:0051094\_positive\_regulation\_of\_developmental\_process | 340 | 1 | 1.600000 | -0.325915 | 423 | 107.594041 | 205.46 | 303.325959 | 0.485721 |
| GO:0033554\_cellular\_response\_to\_stress | 341 | 1 | 1.595308 | -0.324984 | 424 | 107.892705 | 205.8 | 303.707295 | 0.485377 |
| GO:0006357\_regulation\_of\_transcription\_from\_RNA\_polymerase\_II\_promoter | 351 | 1 | 1.549858 | -0.315878 | 425 | 108.863413 | 206.91 | 304.956587 | 0.486847 |
| GO:0031328\_positive\_regulation\_of\_cellular\_biosynthetic\_process | 352 | 1 | 1.545455 | -0.314987 | 426 | 109.074500 | 207.23 | 305.385500 | 0.486455 |
| GO:0043085\_positive\_regulation\_of\_catalytic\_activity | 354 | 1 | 1.536723 | -0.313215 | 427 | 109.568976 | 207.76 | 305.951024 | 0.486557 |
| GO:0009891\_positive\_regulation\_of\_biosynthetic\_process | 359 | 1 | 1.515320 | -0.308842 | 428 | 109.938263 | 208.26 | 306.581737 | 0.486589 |
| GO:0007186\_G-protein\_coupled\_receptor\_protein\_signaling\_pathway | 363 | 1 | 1.498623 | -0.305404 | 429 | 110.787375 | 209.3 | 307.812625 | 0.487879 |
| GO:0065003\_macromolecular\_complex\_assembly | 366 | 1 | 1.486339 | -0.302859 | 430 | 111.680739 | 210.31 | 308.939261 | 0.489093 |
| GO:0006952\_defense\_response | 369 | 1 | 1.474255 | -0.300342 | 431 | 112.423553 | 211.13 | 309.836447 | 0.489861 |
| GO:0022402\_cell\_cycle\_process | 370 | 1 | 1.470270 | -0.299509 | 432 | 112.762276 | 211.46 | 310.157724 | 0.489491 |
| GO:0045449\_regulation\_of\_transcription | 900 | 2 | 1.208889 | -0.296938 | 433 | 113.179286 | 212.03 | 310.880714 | 0.489677 |
| GO:0051239\_regulation\_of\_multicellular\_organismal\_process | 378 | 1 | 1.439153 | -0.292957 | 434 | 114.055580 | 213.06 | 312.064420 | 0.490922 |
| GO:0034961\_cellular\_biopolymer\_biosynthetic\_process | 1448 | 3 | 1.127072 | -0.289802 | 435 | 114.743192 | 213.88 | 313.016808 | 0.491678 |
| GO:0043284\_biopolymer\_biosynthetic\_process | 1458 | 3 | 1.119342 | -0.285552 | 436 | 115.035571 | 214.27 | 313.504429 | 0.491445 |
| GO:0033036\_macromolecule\_localization | 388 | 1 | 1.402062 | -0.285032 | 437 | 115.377821 | 214.77 | 314.162179 | 0.491465 |
| GO:0031323\_regulation\_of\_cellular\_metabolic\_process | 1466 | 3 | 1.113233 | -0.282194 | 438 | 115.798598 | 215.31 | 314.821402 | 0.491575 |
| GO:0006468\_protein\_amino\_acid\_phosphorylation | 393 | 1 | 1.384224 | -0.281175 | 439 | 116.034743 | 215.65 | 315.265257 | 0.491230 |
| GO:0044093\_positive\_regulation\_of\_molecular\_function | 394 | 1 | 1.380711 | -0.280412 | 440 | 116.339754 | 215.99 | 315.640246 | 0.490886 |
| GO:0031324\_negative\_regulation\_of\_cellular\_metabolic\_process | 404 | 1 | 1.346535 | -0.272928 | 441 | 117.017494 | 216.74 | 316.462506 | 0.491474 |
| GO:0006950\_response\_to\_stress | 959 | 2 | 1.134515 | -0.267078 | 442 | 118.506289 | 218.19 | 317.873711 | 0.493643 |
| GO:0043933\_macromolecular\_complex\_subunit\_organization | 424 | 1 | 1.283019 | -0.258719 | 443 | 120.286281 | 220.18 | 320.073719 | 0.497020 |
| GO:0019222\_regulation\_of\_metabolic\_process | 1538 | 3 | 1.061118 | -0.253591 | 444 | 122.046510 | 221.96 | 321.873490 | 0.499910 |
| GO:0044238\_primary\_metabolic\_process | 3719 | 7 | 1.023931 | -0.247273 | 445 | 123.122913 | 222.94 | 322.757087 | 0.500989 |
| GO:0048522\_positive\_regulation\_of\_cellular\_process | 1009 | 2 | 1.078295 | -0.244227 | 446 | 124.073128 | 223.84 | 323.606872 | 0.501883 |
| GO:0010604\_positive\_regulation\_of\_macromolecule\_metabolic\_process | 446 | 1 | 1.219731 | -0.244163 | 447 | 124.382847 | 224.21 | 324.037153 | 0.501588 |
| GO:0031325\_positive\_regulation\_of\_cellular\_metabolic\_process | 454 | 1 | 1.198238 | -0.239127 | 448 | 125.052039 | 225.06 | 325.067961 | 0.502366 |
| GO:0034645\_cellular\_macromolecule\_biosynthetic\_process | 1600 | 3 | 1.020000 | -0.231144 | 449 | 126.277597 | 226.32 | 326.362403 | 0.504053 |
| GO:0019219\_regulation\_of\_nucleobase\_\_nucleoside\_\_nucleotide\_and\_nucleic\_acid\_metabolic\_process | 1041 | 2 | 1.045149 | -0.230666 | 450 | 126.568841 | 226.63 | 326.691159 | 0.503622 |
| GO:0042981\_regulation\_of\_apoptosis | 471 | 1 | 1.154989 | -0.228849 | 451 | 127.743891 | 227.81 | 327.876109 | 0.505122 |
| GO:0051716\_cellular\_response\_to\_stimulus | 474 | 1 | 1.147679 | -0.227092 | 452 | 128.108852 | 228.19 | 328.271148 | 0.504845 |
| GO:0009893\_positive\_regulation\_of\_metabolic\_process | 476 | 1 | 1.142857 | -0.225930 | 454 | 128.782450 | 228.92 | 329.057550 | 0.504229 |
| GO:0043067\_regulation\_of\_programmed\_cell\_death | 476 | 1 | 1.142857 | -0.225930 | 454 | 128.782450 | 228.92 | 329.057550 | 0.504229 |
| GO:0051171\_regulation\_of\_nitrogen\_compound\_metabolic\_process | 1055 | 2 | 1.031280 | -0.224976 | 455 | 129.344593 | 229.54 | 329.735407 | 0.504484 |
| GO:0010941\_regulation\_of\_cell\_death | 478 | 1 | 1.138075 | -0.224776 | 456 | 130.244893 | 230.43 | 330.615107 | 0.505329 |
| GO:0006350\_transcription | 1069 | 2 | 1.017774 | -0.219429 | 457 | 130.967439 | 231.38 | 331.792561 | 0.506302 |
| GO:0007049\_cell\_cycle | 494 | 1 | 1.101215 | -0.215796 | 458 | 131.274893 | 231.7 | 332.125107 | 0.505895 |
| GO:0010467\_gene\_expression | 1663 | 3 | 0.981359 | -0.210217 | 459 | 131.866151 | 232.34 | 332.813849 | 0.506187 |
| GO:0048518\_positive\_regulation\_of\_biological\_process | 1094 | 2 | 0.994516 | -0.209864 | 460 | 132.143412 | 232.59 | 333.036588 | 0.505630 |
| GO:0006366\_transcription\_from\_RNA\_polymerase\_II\_promoter | 506 | 1 | 1.075099 | -0.209346 | 462 | 132.924886 | 233.43 | 333.935114 | 0.505260 |
| GO:0030154\_cell\_differentiation | 506 | 1 | 1.075099 | -0.209346 | 462 | 132.924886 | 233.43 | 333.935114 | 0.505260 |
| GO:0006955\_immune\_response | 529 | 1 | 1.028355 | -0.197623 | 463 | 133.854274 | 234.39 | 334.925726 | 0.506242 |
| GO:0048869\_cellular\_developmental\_process | 555 | 1 | 0.980180 | -0.185299 | 464 | 134.979825 | 235.69 | 336.400175 | 0.507953 |
| GO:0006915\_apoptosis | 565 | 1 | 0.962832 | -0.180801 | 465 | 136.673663 | 237.33 | 337.986337 | 0.510387 |
| GO:0012501\_programmed\_cell\_death | 571 | 1 | 0.952715 | -0.178164 | 466 | 137.062587 | 237.7 | 338.337413 | 0.510086 |
| GO:0016310\_phosphorylation | 601 | 1 | 0.905158 | -0.165630 | 467 | 138.830179 | 239.37 | 339.909821 | 0.512570 |
| GO:0050794\_regulation\_of\_cellular\_process | 3515 | 6 | 0.928592 | -0.162458 | 468 | 139.455482 | 240.06 | 340.664518 | 0.512949 |
| GO:0042221\_response\_to\_chemical\_stimulus | 631 | 1 | 0.862124 | -0.154097 | 469 | 141.276929 | 241.61 | 341.943071 | 0.515160 |
| GO:0050793\_regulation\_of\_developmental\_process | 669 | 1 | 0.813154 | -0.140772 | 470 | 144.103564 | 244.04 | 343.976436 | 0.519234 |
| GO:0050789\_regulation\_of\_biological\_process | 3649 | 6 | 0.894492 | -0.135374 | 471 | 144.619428 | 244.49 | 344.360572 | 0.519087 |
| GO:0044249\_cellular\_biosynthetic\_process | 1951 | 3 | 0.836494 | -0.134665 | 472 | 144.889884 | 244.77 | 344.650116 | 0.518581 |
| GO:0006793\_phosphorus\_metabolic\_process | 697 | 1 | 0.780488 | -0.131775 | 474 | 145.849692 | 245.61 | 345.370308 | 0.518165 |
| GO:0006796\_phosphate\_metabolic\_process | 697 | 1 | 0.780488 | -0.131775 | 474 | 145.849692 | 245.61 | 345.370308 | 0.518165 |
| GO:0002376\_immune\_system\_process | 718 | 1 | 0.757660 | -0.125444 | 475 | 147.454268 | 247.08 | 346.705732 | 0.520168 |
| GO:0006355\_regulation\_of\_transcription\_\_DNA-dependent | 723 | 1 | 0.752420 | -0.123986 | 476 | 148.196582 | 247.72 | 347.243418 | 0.520420 |
| GO:0043687\_post-translational\_protein\_modification | 728 | 1 | 0.747253 | -0.122547 | 477 | 148.745824 | 248.28 | 347.814176 | 0.520503 |
| GO:0007165\_signal\_transduction | 2029 | 3 | 0.804337 | -0.118902 | 478 | 149.031158 | 248.5 | 347.968842 | 0.519874 |
| GO:0051252\_regulation\_of\_RNA\_metabolic\_process | 746 | 1 | 0.729223 | -0.117514 | 479 | 149.807079 | 249.18 | 348.552921 | 0.520209 |
| GO:0044237\_cellular\_metabolic\_process | 3753 | 6 | 0.869704 | -0.116841 | 480 | 149.990444 | 249.37 | 348.749556 | 0.519521 |
| GO:0006996\_organelle\_organization | 764 | 1 | 0.712042 | -0.112705 | 481 | 150.634090 | 250.07 | 349.505910 | 0.519896 |
| GO:0006351\_transcription\_\_DNA-dependent | 884 | 1 | 0.615385 | -0.085549 | 482 | 154.760820 | 253.39 | 352.019180 | 0.525705 |
| GO:0032774\_RNA\_biosynthetic\_process | 887 | 1 | 0.613303 | -0.084966 | 483 | 155.078785 | 253.68 | 352.281215 | 0.525217 |
| GO:0065007\_biological\_regulation | 3971 | 6 | 0.821959 | -0.084309 | 484 | 155.263216 | 253.81 | 352.356784 | 0.524401 |
| GO:0007154\_cell\_communication | 2272 | 3 | 0.718310 | -0.079654 | 485 | 156.442079 | 254.83 | 353.217921 | 0.525423 |
| GO:0050896\_response\_to\_stimulus | 1775 | 2 | 0.612958 | -0.060603 | 486 | 160.159384 | 257.88 | 355.600616 | 0.530617 |
| GO:0006139\_nucleobase\_\_nucleoside\_\_nucleotide\_and\_nucleic\_acid\_metabolic\_process | 1845 | 2 | 0.589702 | -0.053015 | 487 | 163.089383 | 260.01 | 356.930617 | 0.533901 |
| GO:0016070\_RNA\_metabolic\_process | 1230 | 1 | 0.442276 | -0.039068 | 488 | 165.440986 | 261.69 | 357.939014 | 0.536250 |
| GO:0006807\_nitrogen\_compound\_metabolic\_process | 2053 | 2 | 0.529956 | -0.035281 | 489 | 166.145610 | 262.3 | 358.454390 | 0.536401 |
| GO:0045941\_positive\_regulation\_of\_transcription | 264 | 0 | 0.000000 | -0.000000 | 490 | 175.925336 | 269.08 | 362.234664 | 0.549143 |
| GO:0002697\_regulation\_of\_immune\_effector\_process | 49 | 0 | 0.000000 | -0.000000 | 500 | 186.521002 | 278.97 | 371.418998 | 0.557940 |
| GO:0006275\_regulation\_of\_DNA\_replication | 49 | 0 | 0.000000 | -0.000000 | 500 | 186.521002 | 278.97 | 371.418998 | 0.557940 |
| GO:0006643\_membrane\_lipid\_metabolic\_process | 49 | 0 | 0.000000 | -0.000000 | 500 | 186.521002 | 278.97 | 371.418998 | 0.557940 |
| GO:0009914\_hormone\_transport | 49 | 0 | 0.000000 | -0.000000 | 500 | 186.521002 | 278.97 | 371.418998 | 0.557940 |
| GO:0010517\_regulation\_of\_phospholipase\_activity | 49 | 0 | 0.000000 | -0.000000 | 500 | 186.521002 | 278.97 | 371.418998 | 0.557940 |
| GO:0010952\_positive\_regulation\_of\_peptidase\_activity | 49 | 0 | 0.000000 | -0.000000 | 500 | 186.521002 | 278.97 | 371.418998 | 0.557940 |
| GO:0018108\_peptidyl-tyrosine\_phosphorylation | 49 | 0 | 0.000000 | -0.000000 | 500 | 186.521002 | 278.97 | 371.418998 | 0.557940 |
| GO:0032984\_macromolecular\_complex\_disassembly | 49 | 0 | 0.000000 | -0.000000 | 500 | 186.521002 | 278.97 | 371.418998 | 0.557940 |
| GO:0043280\_positive\_regulation\_of\_caspase\_activity | 49 | 0 | 0.000000 | -0.000000 | 500 | 186.521002 | 278.97 | 371.418998 | 0.557940 |
| GO:0050863\_regulation\_of\_T\_cell\_activation | 49 | 0 | 0.000000 | -0.000000 | 500 | 186.521002 | 278.97 | 371.418998 | 0.557940 |
| GO:0009416\_response\_to\_light\_stimulus | 70 | 0 | 0.000000 | -0.000000 | 503 | 192.317816 | 284.14 | 375.962184 | 0.564891 |
| GO:0016032\_viral\_reproduction | 70 | 0 | 0.000000 | -0.000000 | 503 | 192.317816 | 284.14 | 375.962184 | 0.564891 |
| GO:0022411\_cellular\_component\_disassembly | 70 | 0 | 0.000000 | -0.000000 | 503 | 192.317816 | 284.14 | 375.962184 | 0.564891 |
| GO:0006519\_cellular\_amino\_acid\_and\_derivative\_metabolic\_process | 173 | 0 | 0.000000 | -0.000000 | 505 | 194.857940 | 286.41 | 377.962060 | 0.567149 |
| GO:0055080\_cation\_homeostasis | 173 | 0 | 0.000000 | -0.000000 | 505 | 194.857940 | 286.41 | 377.962060 | 0.567149 |
| GO:0000079\_regulation\_of\_cyclin-dependent\_protein\_kinase\_activity | 48 | 0 | 0.000000 | -0.000000 | 512 | 202.524578 | 293.59 | 384.655422 | 0.573418 |
| GO:0010518\_positive\_regulation\_of\_phospholipase\_activity | 48 | 0 | 0.000000 | -0.000000 | 512 | 202.524578 | 293.59 | 384.655422 | 0.573418 |
| GO:0010551\_regulation\_of\_specific\_transcription\_from\_RNA\_polymerase\_II\_promoter | 48 | 0 | 0.000000 | -0.000000 | 512 | 202.524578 | 293.59 | 384.655422 | 0.573418 |
| GO:0032569\_specific\_transcription\_from\_RNA\_polymerase\_II\_promoter | 48 | 0 | 0.000000 | -0.000000 | 512 | 202.524578 | 293.59 | 384.655422 | 0.573418 |
| GO:0042035\_regulation\_of\_cytokine\_biosynthetic\_process | 48 | 0 | 0.000000 | -0.000000 | 512 | 202.524578 | 293.59 | 384.655422 | 0.573418 |
| GO:0042773\_ATP\_synthesis\_coupled\_electron\_transport | 48 | 0 | 0.000000 | -0.000000 | 512 | 202.524578 | 293.59 | 384.655422 | 0.573418 |
| GO:0042775\_mitochondrial\_ATP\_synthesis\_coupled\_electron\_transport | 48 | 0 | 0.000000 | -0.000000 | 512 | 202.524578 | 293.59 | 384.655422 | 0.573418 |
| GO:0008361\_regulation\_of\_cell\_size | 149 | 0 | 0.000000 | -0.000000 | 513 | 203.358276 | 294.31 | 385.261724 | 0.573704 |
| GO:0000377\_RNA\_splicing\_\_via\_transesterification\_reactions\_with\_bulged\_adenosine\_as\_nucleophile | 151 | 0 | 0.000000 | -0.000000 | 515 | 204.960903 | 295.97 | 386.979097 | 0.574699 |
| GO:0000398\_nuclear\_mRNA\_splicing\_\_via\_spliceosome | 151 | 0 | 0.000000 | -0.000000 | 515 | 204.960903 | 295.97 | 386.979097 | 0.574699 |
| GO:0016569\_covalent\_chromatin\_modification | 99 | 0 | 0.000000 | -0.000000 | 516 | 207.678589 | 298.42 | 389.161411 | 0.578333 |
| GO:0030001\_metal\_ion\_transport | 186 | 0 | 0.000000 | -0.000000 | 517 | 209.263474 | 299.9 | 390.536526 | 0.580077 |
| GO:0000070\_mitotic\_sister\_chromatid\_segregation | 27 | 0 | 0.000000 | -0.000000 | 538 | 231.596894 | 321.77 | 411.943106 | 0.598086 |
| GO:0000245\_spliceosome\_assembly | 27 | 0 | 0.000000 | -0.000000 | 538 | 231.596894 | 321.77 | 411.943106 | 0.598086 |
| GO:0003002\_regionalization | 27 | 0 | 0.000000 | -0.000000 | 538 | 231.596894 | 321.77 | 411.943106 | 0.598086 |
| GO:0006638\_neutral\_lipid\_metabolic\_process | 27 | 0 | 0.000000 | -0.000000 | 538 | 231.596894 | 321.77 | 411.943106 | 0.598086 |
| GO:0006639\_acylglycerol\_metabolic\_process | 27 | 0 | 0.000000 | -0.000000 | 538 | 231.596894 | 321.77 | 411.943106 | 0.598086 |
| GO:0006690\_icosanoid\_metabolic\_process | 27 | 0 | 0.000000 | -0.000000 | 538 | 231.596894 | 321.77 | 411.943106 | 0.598086 |
| GO:0006836\_neurotransmitter\_transport | 27 | 0 | 0.000000 | -0.000000 | 538 | 231.596894 | 321.77 | 411.943106 | 0.598086 |
| GO:0007260\_tyrosine\_phosphorylation\_of\_STAT\_protein | 27 | 0 | 0.000000 | -0.000000 | 538 | 231.596894 | 321.77 | 411.943106 | 0.598086 |
| GO:0007631\_feeding\_behavior | 27 | 0 | 0.000000 | -0.000000 | 538 | 231.596894 | 321.77 | 411.943106 | 0.598086 |
| GO:0019079\_viral\_genome\_replication | 27 | 0 | 0.000000 | -0.000000 | 538 | 231.596894 | 321.77 | 411.943106 | 0.598086 |
| GO:0031349\_positive\_regulation\_of\_defense\_response | 27 | 0 | 0.000000 | -0.000000 | 538 | 231.596894 | 321.77 | 411.943106 | 0.598086 |
| GO:0031669\_cellular\_response\_to\_nutrient\_levels | 27 | 0 | 0.000000 | -0.000000 | 538 | 231.596894 | 321.77 | 411.943106 | 0.598086 |
| GO:0032200\_telomere\_organization | 27 | 0 | 0.000000 | -0.000000 | 538 | 231.596894 | 321.77 | 411.943106 | 0.598086 |
| GO:0035150\_regulation\_of\_tube\_size | 27 | 0 | 0.000000 | -0.000000 | 538 | 231.596894 | 321.77 | 411.943106 | 0.598086 |
| GO:0043254\_regulation\_of\_protein\_complex\_assembly | 27 | 0 | 0.000000 | -0.000000 | 538 | 231.596894 | 321.77 | 411.943106 | 0.598086 |
| GO:0044272\_sulfur\_compound\_biosynthetic\_process | 27 | 0 | 0.000000 | -0.000000 | 538 | 231.596894 | 321.77 | 411.943106 | 0.598086 |
| GO:0050880\_regulation\_of\_blood\_vessel\_size | 27 | 0 | 0.000000 | -0.000000 | 538 | 231.596894 | 321.77 | 411.943106 | 0.598086 |
| GO:0050906\_detection\_of\_stimulus\_involved\_in\_sensory\_perception | 27 | 0 | 0.000000 | -0.000000 | 538 | 231.596894 | 321.77 | 411.943106 | 0.598086 |
| GO:0051048\_negative\_regulation\_of\_secretion | 27 | 0 | 0.000000 | -0.000000 | 538 | 231.596894 | 321.77 | 411.943106 | 0.598086 |
| GO:0051092\_positive\_regulation\_of\_NF-kappaB\_transcription\_factor\_activity | 27 | 0 | 0.000000 | -0.000000 | 538 | 231.596894 | 321.77 | 411.943106 | 0.598086 |
| GO:0051607\_defense\_response\_to\_virus | 27 | 0 | 0.000000 | -0.000000 | 538 | 231.596894 | 321.77 | 411.943106 | 0.598086 |
| GO:0016568\_chromatin\_modification | 146 | 0 | 0.000000 | -0.000000 | 540 | 234.947343 | 324.79 | 414.632657 | 0.601463 |
| GO:0022008\_neurogenesis | 146 | 0 | 0.000000 | -0.000000 | 540 | 234.947343 | 324.79 | 414.632657 | 0.601463 |
| GO:0002757\_immune\_response-activating\_signal\_transduction | 25 | 0 | 0.000000 | -0.000000 | 565 | 264.379804 | 353.31 | 442.240196 | 0.625327 |
| GO:0002764\_immune\_response-regulating\_signal\_transduction | 25 | 0 | 0.000000 | -0.000000 | 565 | 264.379804 | 353.31 | 442.240196 | 0.625327 |
| GO:0006007\_glucose\_catabolic\_process | 25 | 0 | 0.000000 | -0.000000 | 565 | 264.379804 | 353.31 | 442.240196 | 0.625327 |
| GO:0006112\_energy\_reserve\_metabolic\_process | 25 | 0 | 0.000000 | -0.000000 | 565 | 264.379804 | 353.31 | 442.240196 | 0.625327 |
| GO:0006518\_peptide\_metabolic\_process | 25 | 0 | 0.000000 | -0.000000 | 565 | 264.379804 | 353.31 | 442.240196 | 0.625327 |
| GO:0006767\_water-soluble\_vitamin\_metabolic\_process | 25 | 0 | 0.000000 | -0.000000 | 565 | 264.379804 | 353.31 | 442.240196 | 0.625327 |
| GO:0007127\_meiosis\_I | 25 | 0 | 0.000000 | -0.000000 | 565 | 264.379804 | 353.31 | 442.240196 | 0.625327 |
| GO:0007416\_synaptogenesis | 25 | 0 | 0.000000 | -0.000000 | 565 | 264.379804 | 353.31 | 442.240196 | 0.625327 |
| GO:0009141\_nucleoside\_triphosphate\_metabolic\_process | 25 | 0 | 0.000000 | -0.000000 | 565 | 264.379804 | 353.31 | 442.240196 | 0.625327 |
| GO:0010876\_lipid\_localization | 25 | 0 | 0.000000 | -0.000000 | 565 | 264.379804 | 353.31 | 442.240196 | 0.625327 |
| GO:0015711\_organic\_anion\_transport | 25 | 0 | 0.000000 | -0.000000 | 565 | 264.379804 | 353.31 | 442.240196 | 0.625327 |
| GO:0019217\_regulation\_of\_fatty\_acid\_metabolic\_process | 25 | 0 | 0.000000 | -0.000000 | 565 | 264.379804 | 353.31 | 442.240196 | 0.625327 |
| GO:0019915\_lipid\_storage | 25 | 0 | 0.000000 | -0.000000 | 565 | 264.379804 | 353.31 | 442.240196 | 0.625327 |
| GO:0030168\_platelet\_activation | 25 | 0 | 0.000000 | -0.000000 | 565 | 264.379804 | 353.31 | 442.240196 | 0.625327 |
| GO:0030282\_bone\_mineralization | 25 | 0 | 0.000000 | -0.000000 | 565 | 264.379804 | 353.31 | 442.240196 | 0.625327 |
| GO:0031023\_microtubule\_organizing\_center\_organization | 25 | 0 | 0.000000 | -0.000000 | 565 | 264.379804 | 353.31 | 442.240196 | 0.625327 |
| GO:0031644\_regulation\_of\_neurological\_system\_process | 25 | 0 | 0.000000 | -0.000000 | 565 | 264.379804 | 353.31 | 442.240196 | 0.625327 |
| GO:0034101\_erythrocyte\_homeostasis | 25 | 0 | 0.000000 | -0.000000 | 565 | 264.379804 | 353.31 | 442.240196 | 0.625327 |
| GO:0042129\_regulation\_of\_T\_cell\_proliferation | 25 | 0 | 0.000000 | -0.000000 | 565 | 264.379804 | 353.31 | 442.240196 | 0.625327 |
| GO:0043087\_regulation\_of\_GTPase\_activity | 25 | 0 | 0.000000 | -0.000000 | 565 | 264.379804 | 353.31 | 442.240196 | 0.625327 |
| GO:0043624\_cellular\_protein\_complex\_disassembly | 25 | 0 | 0.000000 | -0.000000 | 565 | 264.379804 | 353.31 | 442.240196 | 0.625327 |
| GO:0043966\_histone\_H3\_acetylation | 25 | 0 | 0.000000 | -0.000000 | 565 | 264.379804 | 353.31 | 442.240196 | 0.625327 |
| GO:0045762\_positive\_regulation\_of\_adenylate\_cyclase\_activity | 25 | 0 | 0.000000 | -0.000000 | 565 | 264.379804 | 353.31 | 442.240196 | 0.625327 |
| GO:0050678\_regulation\_of\_epithelial\_cell\_proliferation | 25 | 0 | 0.000000 | -0.000000 | 565 | 264.379804 | 353.31 | 442.240196 | 0.625327 |
| GO:0050727\_regulation\_of\_inflammatory\_response | 25 | 0 | 0.000000 | -0.000000 | 565 | 264.379804 | 353.31 | 442.240196 | 0.625327 |
| GO:0000038\_very-long-chain\_fatty\_acid\_metabolic\_process | 11 | 0 | 0.000000 | -0.000000 | 654 | 358.722243 | 446.62 | 534.517757 | 0.682905 |
| GO:0000272\_polysaccharide\_catabolic\_process | 11 | 0 | 0.000000 | -0.000000 | 654 | 358.722243 | 446.62 | 534.517757 | 0.682905 |
| GO:0001570\_vasculogenesis | 11 | 0 | 0.000000 | -0.000000 | 654 | 358.722243 | 446.62 | 534.517757 | 0.682905 |
| GO:0001836\_release\_of\_cytochrome\_c\_from\_mitochondria | 11 | 0 | 0.000000 | -0.000000 | 654 | 358.722243 | 446.62 | 534.517757 | 0.682905 |
| GO:0002285\_lymphocyte\_activation\_during\_immune\_response | 11 | 0 | 0.000000 | -0.000000 | 654 | 358.722243 | 446.62 | 534.517757 | 0.682905 |
| GO:0006094\_gluconeogenesis | 11 | 0 | 0.000000 | -0.000000 | 654 | 358.722243 | 446.62 | 534.517757 | 0.682905 |
| GO:0006342\_chromatin\_silencing | 11 | 0 | 0.000000 | -0.000000 | 654 | 358.722243 | 446.62 | 534.517757 | 0.682905 |
| GO:0006400\_tRNA\_modification | 11 | 0 | 0.000000 | -0.000000 | 654 | 358.722243 | 446.62 | 534.517757 | 0.682905 |
| GO:0006515\_misfolded\_or\_incompletely\_synthesized\_protein\_catabolic\_process | 11 | 0 | 0.000000 | -0.000000 | 654 | 358.722243 | 446.62 | 534.517757 | 0.682905 |
| GO:0006706\_steroid\_catabolic\_process | 11 | 0 | 0.000000 | -0.000000 | 654 | 358.722243 | 446.62 | 534.517757 | 0.682905 |
| GO:0006749\_glutathione\_metabolic\_process | 11 | 0 | 0.000000 | -0.000000 | 654 | 358.722243 | 446.62 | 534.517757 | 0.682905 |
| GO:0006904\_vesicle\_docking\_during\_exocytosis | 11 | 0 | 0.000000 | -0.000000 | 654 | 358.722243 | 446.62 | 534.517757 | 0.682905 |
| GO:0007076\_mitotic\_chromosome\_condensation | 11 | 0 | 0.000000 | -0.000000 | 654 | 358.722243 | 446.62 | 534.517757 | 0.682905 |
| GO:0007090\_regulation\_of\_S\_phase\_of\_mitotic\_cell\_cycle | 11 | 0 | 0.000000 | -0.000000 | 654 | 358.722243 | 446.62 | 534.517757 | 0.682905 |
| GO:0008206\_bile\_acid\_metabolic\_process | 11 | 0 | 0.000000 | -0.000000 | 654 | 358.722243 | 446.62 | 534.517757 | 0.682905 |
| GO:0008333\_endosome\_to\_lysosome\_transport | 11 | 0 | 0.000000 | -0.000000 | 654 | 358.722243 | 446.62 | 534.517757 | 0.682905 |
| GO:0009072\_aromatic\_amino\_acid\_family\_metabolic\_process | 11 | 0 | 0.000000 | -0.000000 | 654 | 358.722243 | 446.62 | 534.517757 | 0.682905 |
| GO:0009143\_nucleoside\_triphosphate\_catabolic\_process | 11 | 0 | 0.000000 | -0.000000 | 654 | 358.722243 | 446.62 | 534.517757 | 0.682905 |
| GO:0009247\_glycolipid\_biosynthetic\_process | 11 | 0 | 0.000000 | -0.000000 | 654 | 358.722243 | 446.62 | 534.517757 | 0.682905 |
| GO:0009264\_deoxyribonucleotide\_catabolic\_process | 11 | 0 | 0.000000 | -0.000000 | 654 | 358.722243 | 446.62 | 534.517757 | 0.682905 |
| GO:0009394\_2'-deoxyribonucleotide\_metabolic\_process | 11 | 0 | 0.000000 | -0.000000 | 654 | 358.722243 | 446.62 | 534.517757 | 0.682905 |
| GO:0009395\_phospholipid\_catabolic\_process | 11 | 0 | 0.000000 | -0.000000 | 654 | 358.722243 | 446.62 | 534.517757 | 0.682905 |
| GO:0009953\_dorsal\_ventral\_pattern\_formation | 11 | 0 | 0.000000 | -0.000000 | 654 | 358.722243 | 446.62 | 534.517757 | 0.682905 |
| GO:0010458\_exit\_from\_mitosis | 11 | 0 | 0.000000 | -0.000000 | 654 | 358.722243 | 446.62 | 534.517757 | 0.682905 |
| GO:0010469\_regulation\_of\_receptor\_activity | 11 | 0 | 0.000000 | -0.000000 | 654 | 358.722243 | 446.62 | 534.517757 | 0.682905 |
| GO:0010878\_cholesterol\_storage | 11 | 0 | 0.000000 | -0.000000 | 654 | 358.722243 | 446.62 | 534.517757 | 0.682905 |
| GO:0010907\_positive\_regulation\_of\_glucose\_metabolic\_process | 11 | 0 | 0.000000 | -0.000000 | 654 | 358.722243 | 446.62 | 534.517757 | 0.682905 |
| GO:0015804\_neutral\_amino\_acid\_transport | 11 | 0 | 0.000000 | -0.000000 | 654 | 358.722243 | 446.62 | 534.517757 | 0.682905 |
| GO:0016445\_somatic\_diversification\_of\_immunoglobulins | 11 | 0 | 0.000000 | -0.000000 | 654 | 358.722243 | 446.62 | 534.517757 | 0.682905 |
| GO:0016575\_histone\_deacetylation | 11 | 0 | 0.000000 | -0.000000 | 654 | 358.722243 | 446.62 | 534.517757 | 0.682905 |
| GO:0018205\_peptidyl-lysine\_modification | 11 | 0 | 0.000000 | -0.000000 | 654 | 358.722243 | 446.62 | 534.517757 | 0.682905 |
| GO:0018958\_phenol\_metabolic\_process | 11 | 0 | 0.000000 | -0.000000 | 654 | 358.722243 | 446.62 | 534.517757 | 0.682905 |
| GO:0019692\_deoxyribose\_phosphate\_metabolic\_process | 11 | 0 | 0.000000 | -0.000000 | 654 | 358.722243 | 446.62 | 534.517757 | 0.682905 |
| GO:0021915\_neural\_tube\_development | 11 | 0 | 0.000000 | -0.000000 | 654 | 358.722243 | 446.62 | 534.517757 | 0.682905 |
| GO:0030035\_microspike\_assembly | 11 | 0 | 0.000000 | -0.000000 | 654 | 358.722243 | 446.62 | 534.517757 | 0.682905 |
| GO:0030049\_muscle\_filament\_sliding | 11 | 0 | 0.000000 | -0.000000 | 654 | 358.722243 | 446.62 | 534.517757 | 0.682905 |
| GO:0030317\_sperm\_motility | 11 | 0 | 0.000000 | -0.000000 | 654 | 358.722243 | 446.62 | 534.517757 | 0.682905 |
| GO:0030326\_embryonic\_limb\_morphogenesis | 11 | 0 | 0.000000 | -0.000000 | 654 | 358.722243 | 446.62 | 534.517757 | 0.682905 |
| GO:0030433\_ER-associated\_protein\_catabolic\_process | 11 | 0 | 0.000000 | -0.000000 | 654 | 358.722243 | 446.62 | 534.517757 | 0.682905 |
| GO:0031333\_negative\_regulation\_of\_protein\_complex\_assembly | 11 | 0 | 0.000000 | -0.000000 | 654 | 358.722243 | 446.62 | 534.517757 | 0.682905 |
| GO:0031343\_positive\_regulation\_of\_cell\_killing | 11 | 0 | 0.000000 | -0.000000 | 654 | 358.722243 | 446.62 | 534.517757 | 0.682905 |
| GO:0031348\_negative\_regulation\_of\_defense\_response | 11 | 0 | 0.000000 | -0.000000 | 654 | 358.722243 | 446.62 | 534.517757 | 0.682905 |
| GO:0031572\_G2\_M\_transition\_DNA\_damage\_checkpoint | 11 | 0 | 0.000000 | -0.000000 | 654 | 358.722243 | 446.62 | 534.517757 | 0.682905 |
| GO:0031576\_G2\_M\_transition\_checkpoint | 11 | 0 | 0.000000 | -0.000000 | 654 | 358.722243 | 446.62 | 534.517757 | 0.682905 |
| GO:0031929\_TOR\_signaling\_pathway | 11 | 0 | 0.000000 | -0.000000 | 654 | 358.722243 | 446.62 | 534.517757 | 0.682905 |
| GO:0032088\_negative\_regulation\_of\_NF-kappaB\_transcription\_factor\_activity | 11 | 0 | 0.000000 | -0.000000 | 654 | 358.722243 | 446.62 | 534.517757 | 0.682905 |
| GO:0032204\_regulation\_of\_telomere\_maintenance | 11 | 0 | 0.000000 | -0.000000 | 654 | 358.722243 | 446.62 | 534.517757 | 0.682905 |
| GO:0032231\_regulation\_of\_actin\_filament\_bundle\_formation | 11 | 0 | 0.000000 | -0.000000 | 654 | 358.722243 | 446.62 | 534.517757 | 0.682905 |
| GO:0032355\_response\_to\_estradiol\_stimulus | 11 | 0 | 0.000000 | -0.000000 | 654 | 358.722243 | 446.62 | 534.517757 | 0.682905 |
| GO:0032924\_activin\_receptor\_signaling\_pathway | 11 | 0 | 0.000000 | -0.000000 | 654 | 358.722243 | 446.62 | 534.517757 | 0.682905 |
| GO:0033275\_actin-myosin\_filament\_sliding | 11 | 0 | 0.000000 | -0.000000 | 654 | 358.722243 | 446.62 | 534.517757 | 0.682905 |
| GO:0034375\_high-density\_lipoprotein\_particle\_remodeling | 11 | 0 | 0.000000 | -0.000000 | 654 | 358.722243 | 446.62 | 534.517757 | 0.682905 |
| GO:0034377\_plasma\_lipoprotein\_particle\_assembly | 11 | 0 | 0.000000 | -0.000000 | 654 | 358.722243 | 446.62 | 534.517757 | 0.682905 |
| GO:0034433\_steroid\_esterification | 11 | 0 | 0.000000 | -0.000000 | 654 | 358.722243 | 446.62 | 534.517757 | 0.682905 |
| GO:0034434\_sterol\_esterification | 11 | 0 | 0.000000 | -0.000000 | 654 | 358.722243 | 446.62 | 534.517757 | 0.682905 |
| GO:0034435\_cholesterol\_esterification | 11 | 0 | 0.000000 | -0.000000 | 654 | 358.722243 | 446.62 | 534.517757 | 0.682905 |
| GO:0035113\_embryonic\_appendage\_morphogenesis | 11 | 0 | 0.000000 | -0.000000 | 654 | 358.722243 | 446.62 | 534.517757 | 0.682905 |
| GO:0042278\_purine\_nucleoside\_metabolic\_process | 11 | 0 | 0.000000 | -0.000000 | 654 | 358.722243 | 446.62 | 534.517757 | 0.682905 |
| GO:0042308\_negative\_regulation\_of\_protein\_import\_into\_nucleus | 11 | 0 | 0.000000 | -0.000000 | 654 | 358.722243 | 446.62 | 534.517757 | 0.682905 |
| GO:0042354\_L-fucose\_metabolic\_process | 11 | 0 | 0.000000 | -0.000000 | 654 | 358.722243 | 446.62 | 534.517757 | 0.682905 |
| GO:0042384\_cilium\_assembly | 11 | 0 | 0.000000 | -0.000000 | 654 | 358.722243 | 446.62 | 534.517757 | 0.682905 |
| GO:0042787\_protein\_ubiquitination\_during\_ubiquitin-dependent\_protein\_catabolic\_process | 11 | 0 | 0.000000 | -0.000000 | 654 | 358.722243 | 446.62 | 534.517757 | 0.682905 |
| GO:0042992\_negative\_regulation\_of\_transcription\_factor\_import\_into\_nucleus | 11 | 0 | 0.000000 | -0.000000 | 654 | 358.722243 | 446.62 | 534.517757 | 0.682905 |
| GO:0043149\_stress\_fiber\_formation | 11 | 0 | 0.000000 | -0.000000 | 654 | 358.722243 | 446.62 | 534.517757 | 0.682905 |
| GO:0043154\_negative\_regulation\_of\_caspase\_activity | 11 | 0 | 0.000000 | -0.000000 | 654 | 358.722243 | 446.62 | 534.517757 | 0.682905 |
| GO:0043524\_negative\_regulation\_of\_neuron\_apoptosis | 11 | 0 | 0.000000 | -0.000000 | 654 | 358.722243 | 446.62 | 534.517757 | 0.682905 |
| GO:0043547\_positive\_regulation\_of\_GTPase\_activity | 11 | 0 | 0.000000 | -0.000000 | 654 | 358.722243 | 446.62 | 534.517757 | 0.682905 |
| GO:0043583\_ear\_development | 11 | 0 | 0.000000 | -0.000000 | 654 | 358.722243 | 446.62 | 534.517757 | 0.682905 |
| GO:0045069\_regulation\_of\_viral\_genome\_replication | 11 | 0 | 0.000000 | -0.000000 | 654 | 358.722243 | 446.62 | 534.517757 | 0.682905 |
| GO:0045076\_regulation\_of\_interleukin-2\_biosynthetic\_process | 11 | 0 | 0.000000 | -0.000000 | 654 | 358.722243 | 446.62 | 534.517757 | 0.682905 |
| GO:0045453\_bone\_resorption | 11 | 0 | 0.000000 | -0.000000 | 654 | 358.722243 | 446.62 | 534.517757 | 0.682905 |
| GO:0045806\_negative\_regulation\_of\_endocytosis | 11 | 0 | 0.000000 | -0.000000 | 654 | 358.722243 | 446.62 | 534.517757 | 0.682905 |
| GO:0045923\_positive\_regulation\_of\_fatty\_acid\_metabolic\_process | 11 | 0 | 0.000000 | -0.000000 | 654 | 358.722243 | 446.62 | 534.517757 | 0.682905 |
| GO:0046128\_purine\_ribonucleoside\_metabolic\_process | 11 | 0 | 0.000000 | -0.000000 | 654 | 358.722243 | 446.62 | 534.517757 | 0.682905 |
| GO:0046504\_glycerol\_ether\_biosynthetic\_process | 11 | 0 | 0.000000 | -0.000000 | 654 | 358.722243 | 446.62 | 534.517757 | 0.682905 |
| GO:0048284\_organelle\_fusion | 11 | 0 | 0.000000 | -0.000000 | 654 | 358.722243 | 446.62 | 534.517757 | 0.682905 |
| GO:0050680\_negative\_regulation\_of\_epithelial\_cell\_proliferation | 11 | 0 | 0.000000 | -0.000000 | 654 | 358.722243 | 446.62 | 534.517757 | 0.682905 |
| GO:0050704\_regulation\_of\_interleukin-1\_secretion | 11 | 0 | 0.000000 | -0.000000 | 654 | 358.722243 | 446.62 | 534.517757 | 0.682905 |
| GO:0050777\_negative\_regulation\_of\_immune\_response | 11 | 0 | 0.000000 | -0.000000 | 654 | 358.722243 | 446.62 | 534.517757 | 0.682905 |
| GO:0050830\_defense\_response\_to\_Gram-positive\_bacterium | 11 | 0 | 0.000000 | -0.000000 | 654 | 358.722243 | 446.62 | 534.517757 | 0.682905 |
| GO:0050871\_positive\_regulation\_of\_B\_cell\_activation | 11 | 0 | 0.000000 | -0.000000 | 654 | 358.722243 | 446.62 | 534.517757 | 0.682905 |
| GO:0050918\_positive\_chemotaxis | 11 | 0 | 0.000000 | -0.000000 | 654 | 358.722243 | 446.62 | 534.517757 | 0.682905 |
| GO:0050996\_positive\_regulation\_of\_lipid\_catabolic\_process | 11 | 0 | 0.000000 | -0.000000 | 654 | 358.722243 | 446.62 | 534.517757 | 0.682905 |
| GO:0051043\_regulation\_of\_membrane\_protein\_ectodomain\_proteolysis | 11 | 0 | 0.000000 | -0.000000 | 654 | 358.722243 | 446.62 | 534.517757 | 0.682905 |
| GO:0051187\_cofactor\_catabolic\_process | 11 | 0 | 0.000000 | -0.000000 | 654 | 358.722243 | 446.62 | 534.517757 | 0.682905 |
| GO:0051453\_regulation\_of\_intracellular\_pH | 11 | 0 | 0.000000 | -0.000000 | 654 | 358.722243 | 446.62 | 534.517757 | 0.682905 |
| GO:0051668\_localization\_within\_membrane | 11 | 0 | 0.000000 | -0.000000 | 654 | 358.722243 | 446.62 | 534.517757 | 0.682905 |
| GO:0065005\_protein-lipid\_complex\_assembly | 11 | 0 | 0.000000 | -0.000000 | 654 | 358.722243 | 446.62 | 534.517757 | 0.682905 |
| GO:0070252\_actin-mediated\_cell\_contraction | 11 | 0 | 0.000000 | -0.000000 | 654 | 358.722243 | 446.62 | 534.517757 | 0.682905 |
| GO:0008150\_biological\_process | 8160 | 15 | 1.000000 | 0.000000 | 655 | 375.917701 | 458.59 | 541.262299 | 0.700137 |
| GO:0044057\_regulation\_of\_system\_process | 106 | 0 | 0.000000 | 0.000000 | 656 | 377.687257 | 460.12 | 542.552743 | 0.701402 |
| GO:0000387\_spliceosomal\_snRNP\_biogenesis | 28 | 0 | 0.000000 | 0.000000 | 681 | 405.336323 | 486.53 | 567.723677 | 0.714435 |
| GO:0000819\_sister\_chromatid\_segregation | 28 | 0 | 0.000000 | 0.000000 | 681 | 405.336323 | 486.53 | 567.723677 | 0.714435 |
| GO:0002440\_production\_of\_molecular\_mediator\_of\_immune\_response | 28 | 0 | 0.000000 | 0.000000 | 681 | 405.336323 | 486.53 | 567.723677 | 0.714435 |
| GO:0006304\_DNA\_modification | 28 | 0 | 0.000000 | 0.000000 | 681 | 405.336323 | 486.53 | 567.723677 | 0.714435 |
| GO:0006405\_RNA\_export\_from\_nucleus | 28 | 0 | 0.000000 | 0.000000 | 681 | 405.336323 | 486.53 | 567.723677 | 0.714435 |
| GO:0006662\_glycerol\_ether\_metabolic\_process | 28 | 0 | 0.000000 | 0.000000 | 681 | 405.336323 | 486.53 | 567.723677 | 0.714435 |
| GO:0006939\_smooth\_muscle\_contraction | 28 | 0 | 0.000000 | 0.000000 | 681 | 405.336323 | 486.53 | 567.723677 | 0.714435 |
| GO:0007156\_homophilic\_cell\_adhesion | 28 | 0 | 0.000000 | 0.000000 | 681 | 405.336323 | 486.53 | 567.723677 | 0.714435 |
| GO:0007281\_germ\_cell\_development | 28 | 0 | 0.000000 | 0.000000 | 681 | 405.336323 | 486.53 | 567.723677 | 0.714435 |
| GO:0007411\_axon\_guidance | 28 | 0 | 0.000000 | 0.000000 | 681 | 405.336323 | 486.53 | 567.723677 | 0.714435 |
| GO:0009062\_fatty\_acid\_catabolic\_process | 28 | 0 | 0.000000 | 0.000000 | 681 | 405.336323 | 486.53 | 567.723677 | 0.714435 |
| GO:0009593\_detection\_of\_chemical\_stimulus | 28 | 0 | 0.000000 | 0.000000 | 681 | 405.336323 | 486.53 | 567.723677 | 0.714435 |
| GO:0010563\_negative\_regulation\_of\_phosphorus\_metabolic\_process | 28 | 0 | 0.000000 | 0.000000 | 681 | 405.336323 | 486.53 | 567.723677 | 0.714435 |
| GO:0010565\_regulation\_of\_cellular\_ketone\_metabolic\_process | 28 | 0 | 0.000000 | 0.000000 | 681 | 405.336323 | 486.53 | 567.723677 | 0.714435 |
| GO:0016055\_Wnt\_receptor\_signaling\_pathway | 28 | 0 | 0.000000 | 0.000000 | 681 | 405.336323 | 486.53 | 567.723677 | 0.714435 |
| GO:0018904\_organic\_ether\_metabolic\_process | 28 | 0 | 0.000000 | 0.000000 | 681 | 405.336323 | 486.53 | 567.723677 | 0.714435 |
| GO:0033044\_regulation\_of\_chromosome\_organization | 28 | 0 | 0.000000 | 0.000000 | 681 | 405.336323 | 486.53 | 567.723677 | 0.714435 |
| GO:0045664\_regulation\_of\_neuron\_differentiation | 28 | 0 | 0.000000 | 0.000000 | 681 | 405.336323 | 486.53 | 567.723677 | 0.714435 |
| GO:0045834\_positive\_regulation\_of\_lipid\_metabolic\_process | 28 | 0 | 0.000000 | 0.000000 | 681 | 405.336323 | 486.53 | 567.723677 | 0.714435 |
| GO:0045936\_negative\_regulation\_of\_phosphate\_metabolic\_process | 28 | 0 | 0.000000 | 0.000000 | 681 | 405.336323 | 486.53 | 567.723677 | 0.714435 |
| GO:0051349\_positive\_regulation\_of\_lyase\_activity | 28 | 0 | 0.000000 | 0.000000 | 681 | 405.336323 | 486.53 | 567.723677 | 0.714435 |
| GO:0051650\_establishment\_of\_vesicle\_localization | 28 | 0 | 0.000000 | 0.000000 | 681 | 405.336323 | 486.53 | 567.723677 | 0.714435 |
| GO:0060402\_calcium\_ion\_transport\_into\_cytosol | 28 | 0 | 0.000000 | 0.000000 | 681 | 405.336323 | 486.53 | 567.723677 | 0.714435 |
| GO:0070662\_mast\_cell\_proliferation | 28 | 0 | 0.000000 | 0.000000 | 681 | 405.336323 | 486.53 | 567.723677 | 0.714435 |
| GO:0070666\_regulation\_of\_mast\_cell\_proliferation | 28 | 0 | 0.000000 | 0.000000 | 681 | 405.336323 | 486.53 | 567.723677 | 0.714435 |
| GO:0006974\_response\_to\_DNA\_damage\_stimulus | 234 | 0 | 0.000000 | 0.000000 | 682 | 407.503412 | 488.2 | 568.896588 | 0.715836 |
| GO:0006289\_nucleotide-excision\_repair | 45 | 0 | 0.000000 | 0.000000 | 692 | 417.460360 | 497.87 | 578.279640 | 0.719465 |
| GO:0006308\_DNA\_catabolic\_process | 45 | 0 | 0.000000 | 0.000000 | 692 | 417.460360 | 497.87 | 578.279640 | 0.719465 |
| GO:0006368\_RNA\_elongation\_from\_RNA\_polymerase\_II\_promoter | 45 | 0 | 0.000000 | 0.000000 | 692 | 417.460360 | 497.87 | 578.279640 | 0.719465 |
| GO:0006576\_biogenic\_amine\_metabolic\_process | 45 | 0 | 0.000000 | 0.000000 | 692 | 417.460360 | 497.87 | 578.279640 | 0.719465 |
| GO:0006919\_activation\_of\_caspase\_activity | 45 | 0 | 0.000000 | 0.000000 | 692 | 417.460360 | 497.87 | 578.279640 | 0.719465 |
| GO:0016197\_endosome\_transport | 45 | 0 | 0.000000 | 0.000000 | 692 | 417.460360 | 497.87 | 578.279640 | 0.719465 |
| GO:0016573\_histone\_acetylation | 45 | 0 | 0.000000 | 0.000000 | 692 | 417.460360 | 497.87 | 578.279640 | 0.719465 |
| GO:0034728\_nucleosome\_organization | 45 | 0 | 0.000000 | 0.000000 | 692 | 417.460360 | 497.87 | 578.279640 | 0.719465 |
| GO:0048771\_tissue\_remodeling | 45 | 0 | 0.000000 | 0.000000 | 692 | 417.460360 | 497.87 | 578.279640 | 0.719465 |
| GO:0051168\_nuclear\_export | 45 | 0 | 0.000000 | 0.000000 | 692 | 417.460360 | 497.87 | 578.279640 | 0.719465 |
| GO:0006396\_RNA\_processing | 306 | 0 | 0.000000 | 0.000000 | 693 | 418.557061 | 498.8 | 579.042939 | 0.719769 |
| GO:0000226\_microtubule\_cytoskeleton\_organization | 86 | 0 | 0.000000 | 0.000000 | 694 | 422.203142 | 501.78 | 581.356858 | 0.723026 |
| GO:0006029\_proteoglycan\_metabolic\_process | 32 | 0 | 0.000000 | 0.000000 | 709 | 439.554341 | 518.73 | 597.905659 | 0.731636 |
| GO:0006446\_regulation\_of\_translational\_initiation | 32 | 0 | 0.000000 | 0.000000 | 709 | 439.554341 | 518.73 | 597.905659 | 0.731636 |
| GO:0006487\_protein\_amino\_acid\_N-linked\_glycosylation | 32 | 0 | 0.000000 | 0.000000 | 709 | 439.554341 | 518.73 | 597.905659 | 0.731636 |
| GO:0006839\_mitochondrial\_transport | 32 | 0 | 0.000000 | 0.000000 | 709 | 439.554341 | 518.73 | 597.905659 | 0.731636 |
| GO:0008286\_insulin\_receptor\_signaling\_pathway | 32 | 0 | 0.000000 | 0.000000 | 709 | 439.554341 | 518.73 | 597.905659 | 0.731636 |
| GO:0015698\_inorganic\_anion\_transport | 32 | 0 | 0.000000 | 0.000000 | 709 | 439.554341 | 518.73 | 597.905659 | 0.731636 |
| GO:0015992\_proton\_transport | 32 | 0 | 0.000000 | 0.000000 | 709 | 439.554341 | 518.73 | 597.905659 | 0.731636 |
| GO:0030216\_keratinocyte\_differentiation | 32 | 0 | 0.000000 | 0.000000 | 709 | 439.554341 | 518.73 | 597.905659 | 0.731636 |
| GO:0031329\_regulation\_of\_cellular\_catabolic\_process | 32 | 0 | 0.000000 | 0.000000 | 709 | 439.554341 | 518.73 | 597.905659 | 0.731636 |
| GO:0034623\_cellular\_macromolecular\_complex\_disassembly | 32 | 0 | 0.000000 | 0.000000 | 709 | 439.554341 | 518.73 | 597.905659 | 0.731636 |
| GO:0046578\_regulation\_of\_Ras\_protein\_signal\_transduction | 32 | 0 | 0.000000 | 0.000000 | 709 | 439.554341 | 518.73 | 597.905659 | 0.731636 |
| GO:0050657\_nucleic\_acid\_transport | 32 | 0 | 0.000000 | 0.000000 | 709 | 439.554341 | 518.73 | 597.905659 | 0.731636 |
| GO:0050658\_RNA\_transport | 32 | 0 | 0.000000 | 0.000000 | 709 | 439.554341 | 518.73 | 597.905659 | 0.731636 |
| GO:0051056\_regulation\_of\_small\_GTPase\_mediated\_signal\_transduction | 32 | 0 | 0.000000 | 0.000000 | 709 | 439.554341 | 518.73 | 597.905659 | 0.731636 |
| GO:0051236\_establishment\_of\_RNA\_localization | 32 | 0 | 0.000000 | 0.000000 | 709 | 439.554341 | 518.73 | 597.905659 | 0.731636 |
| GO:0000019\_regulation\_of\_mitotic\_recombination | 2 | 0 |  |  |  |  |  |  |  |  |
| GO:0000022\_mitotic\_spindle\_elongation | 2 | 0 |  |  |  |  |  |  |  |  |
| GO:0000059\_protein\_import\_into\_nucleus\_\_docking | 2 | 0 |  |  |  |  |  |  |  |  |
| GO:0000066\_mitochondrial\_ornithine\_transport | 2 | 0 |  |  |  |  |  |  |  |  |
| GO:0000183\_chromatin\_silencing\_at\_rDNA | 2 | 0 |  |  |  |  |  |  |  |  |
| GO:0000305\_response\_to\_oxygen\_radical | 2 | 0 |  |  |  |  |  |  |  |  |
| GO:0000429\_regulation\_of\_transcription\_from\_RNA\_polymerase\_II\_promoter\_by\_carbon\_catabolites | 2 | 0 |  |  |  |  |  |  |  |  |
| GO:0000430\_regulation\_of\_transcription\_from\_RNA\_polymerase\_II\_promoter\_by\_glucose | 2 | 0 |  |  |  |  |  |  |  |  |
| GO:0000432\_positive\_regulation\_of\_transcription\_from\_RNA\_polymerase\_II\_promoter\_by\_glucose | 2 | 0 |  |  |  |  |  |  |  |  |
| GO:0000436\_positive\_regulation\_of\_transcription\_from\_RNA\_polymerase\_II\_promoter\_by\_carbon\_catabolites | 2 | 0 |  |  |  |  |  |  |  |  |
| GO:0000460\_maturation\_of\_5.8S\_rRNA | 2 | 0 |  |  |  |  |  |  |  |  |
| GO:0000466\_maturation\_of\_5.8S\_rRNA\_from\_tricistronic\_rRNA\_transcript\_(SSU-rRNA\_\_5.8S\_rRNA\_\_LSU-rRNA) | 2 | 0 |  |  |  |  |  |  |  |  |
| GO:0000729\_DNA\_double-strand\_break\_processing | 2 | 0 |  |  |  |  |  |  |  |  |
| GO:0000733\_DNA\_strand\_renaturation | 2 | 0 |  |  |  |  |  |  |  |  |
| GO:0000920\_cell\_separation\_during\_cytokinesis | 2 | 0 |  |  |  |  |  |  |  |  |
| GO:0001101\_response\_to\_acid | 2 | 0 |  |  |  |  |  |  |  |  |
| GO:0001300\_chronological\_cell\_aging | 2 | 0 |  |  |  |  |  |  |  |  |
| GO:0001301\_progressive\_alteration\_of\_chromatin\_during\_cell\_aging | 2 | 0 |  |  |  |  |  |  |  |  |
| GO:0001304\_progressive\_alteration\_of\_chromatin\_during\_replicative\_cell\_aging | 2 | 0 |  |  |  |  |  |  |  |  |
| GO:0001309\_age-dependent\_telomere\_shortening | 2 | 0 |  |  |  |  |  |  |  |  |
| GO:0001507\_acetylcholine\_catabolic\_process\_in\_synaptic\_cleft | 2 | 0 |  |  |  |  |  |  |  |  |
| GO:0001522\_pseudouridine\_synthesis | 2 | 0 |  |  |  |  |  |  |  |  |
| GO:0001547\_antral\_ovarian\_follicle\_growth | 2 | 0 |  |  |  |  |  |  |  |  |
| GO:0001550\_ovarian\_cumulus\_expansion | 2 | 0 |  |  |  |  |  |  |  |  |
| GO:0001556\_oocyte\_maturation | 2 | 0 |  |  |  |  |  |  |  |  |
| GO:0001562\_response\_to\_protozoan | 2 | 0 |  |  |  |  |  |  |  |  |
| GO:0001582\_detection\_of\_chemical\_stimulus\_involved\_in\_sensory\_perception\_of\_sweet\_taste | 2 | 0 |  |  |  |  |  |  |  |  |
| GO:0001678\_cellular\_glucose\_homeostasis | 2 | 0 |  |  |  |  |  |  |  |  |
| GO:0001702\_gastrulation\_with\_mouth\_forming\_second | 2 | 0 |  |  |  |  |  |  |  |  |
| GO:0001736\_establishment\_of\_planar\_polarity | 2 | 0 |  |  |  |  |  |  |  |  |
| GO:0001738\_morphogenesis\_of\_a\_polarized\_epithelium | 2 | 0 |  |  |  |  |  |  |  |  |
| GO:0001756\_somitogenesis | 2 | 0 |  |  |  |  |  |  |  |  |
| GO:0001766\_membrane\_raft\_polarization | 2 | 0 |  |  |  |  |  |  |  |  |
| GO:0001780\_neutrophil\_homeostasis | 2 | 0 |  |  |  |  |  |  |  |  |
| GO:0001781\_neutrophil\_apoptosis | 2 | 0 |  |  |  |  |  |  |  |  |
| GO:0001825\_blastocyst\_formation | 2 | 0 |  |  |  |  |  |  |  |  |
| GO:0001829\_trophectodermal\_cell\_differentiation | 2 | 0 |  |  |  |  |  |  |  |  |
| GO:0001840\_neural\_plate\_development | 2 | 0 |  |  |  |  |  |  |  |  |
| GO:0001868\_regulation\_of\_complement\_activation\_\_lectin\_pathway | 2 | 0 |  |  |  |  |  |  |  |  |
| GO:0001869\_negative\_regulation\_of\_complement\_activation\_\_lectin\_pathway | 2 | 0 |  |  |  |  |  |  |  |  |
| GO:0001880\_Mullerian\_duct\_regression | 2 | 0 |  |  |  |  |  |  |  |  |
| GO:0001885\_endothelial\_cell\_development | 2 | 0 |  |  |  |  |  |  |  |  |
| GO:0001897\_cytolysis\_by\_symbiont\_of\_host\_cells | 2 | 0 |  |  |  |  |  |  |  |  |
| GO:0001907\_killing\_by\_symbiont\_of\_host\_cells | 2 | 0 |  |  |  |  |  |  |  |  |
| GO:0001921\_positive\_regulation\_of\_receptor\_recycling | 2 | 0 |  |  |  |  |  |  |  |  |
| GO:0001967\_suckling\_behavior | 2 | 0 |  |  |  |  |  |  |  |  |
| GO:0001975\_response\_to\_amphetamine | 2 | 0 |  |  |  |  |  |  |  |  |
| GO:0002003\_angiotensin\_maturation | 2 | 0 |  |  |  |  |  |  |  |  |
| GO:0002016\_regulation\_of\_blood\_volume\_by\_renin-angiotensin | 2 | 0 |  |  |  |  |  |  |  |  |
| GO:0002032\_desensitization\_of\_G-protein\_coupled\_receptor\_protein\_signaling\_pathway\_by\_arrestin | 2 | 0 |  |  |  |  |  |  |  |  |
| GO:0002090\_regulation\_of\_receptor\_internalization | 2 | 0 |  |  |  |  |  |  |  |  |
| GO:0002092\_positive\_regulation\_of\_receptor\_internalization | 2 | 0 |  |  |  |  |  |  |  |  |
| GO:0002227\_innate\_immune\_response\_in\_mucosa | 2 | 0 |  |  |  |  |  |  |  |  |
| GO:0002248\_connective\_tissue\_replacement\_during\_inflammatory\_response | 2 | 0 |  |  |  |  |  |  |  |  |
| GO:0002254\_kinin\_cascade | 2 | 0 |  |  |  |  |  |  |  |  |
| GO:0002275\_myeloid\_cell\_activation\_during\_immune\_response | 2 | 0 |  |  |  |  |  |  |  |  |
| GO:0002281\_macrophage\_activation\_during\_immune\_response | 2 | 0 |  |  |  |  |  |  |  |  |
| GO:0002291\_T\_cell\_activation\_via\_T\_cell\_receptor\_contact\_with\_antigen\_bound\_to\_MHC\_molecule\_on\_antigen\_presenting\_cell | 2 | 0 |  |  |  |  |  |  |  |  |
| GO:0002353\_plasma\_kallikrein-kinin\_cascade | 2 | 0 |  |  |  |  |  |  |  |  |
| GO:0002378\_immunoglobulin\_biosynthetic\_process | 2 | 0 |  |  |  |  |  |  |  |  |
| GO:0002384\_hepatic\_immune\_response | 2 | 0 |  |  |  |  |  |  |  |  |
| GO:0002385\_mucosal\_immune\_response | 2 | 0 |  |  |  |  |  |  |  |  |
| GO:0002507\_tolerance\_induction | 2 | 0 |  |  |  |  |  |  |  |  |
| GO:0002532\_production\_of\_molecular\_mediator\_of\_acute\_inflammatory\_response | 2 | 0 |  |  |  |  |  |  |  |  |
| GO:0002536\_respiratory\_burst\_during\_acute\_inflammatory\_response | 2 | 0 |  |  |  |  |  |  |  |  |
| GO:0002542\_Factor\_XII\_activation | 2 | 0 |  |  |  |  |  |  |  |  |
| GO:0002548\_monocyte\_chemotaxis | 2 | 0 |  |  |  |  |  |  |  |  |
| GO:0002643\_regulation\_of\_tolerance\_induction | 2 | 0 |  |  |  |  |  |  |  |  |
| GO:0002645\_positive\_regulation\_of\_tolerance\_induction | 2 | 0 |  |  |  |  |  |  |  |  |
| GO:0002675\_positive\_regulation\_of\_acute\_inflammatory\_response | 2 | 0 |  |  |  |  |  |  |  |  |
| GO:0002679\_respiratory\_burst\_during\_defense\_response | 2 | 0 |  |  |  |  |  |  |  |  |
| GO:0002704\_negative\_regulation\_of\_leukocyte\_mediated\_immunity | 2 | 0 |  |  |  |  |  |  |  |  |
| GO:0002707\_negative\_regulation\_of\_lymphocyte\_mediated\_immunity | 2 | 0 |  |  |  |  |  |  |  |  |
| GO:0002710\_negative\_regulation\_of\_T\_cell\_mediated\_immunity | 2 | 0 |  |  |  |  |  |  |  |  |
| GO:0002714\_positive\_regulation\_of\_B\_cell\_mediated\_immunity | 2 | 0 |  |  |  |  |  |  |  |  |
| GO:0002820\_negative\_regulation\_of\_adaptive\_immune\_response | 2 | 0 |  |  |  |  |  |  |  |  |
| GO:0002823\_negative\_regulation\_of\_adaptive\_immune\_response\_based\_on\_somatic\_recombination\_of\_immune\_receptors\_built\_from\_immunoglobulin\_superfamily\_domains | 2 | 0 |  |  |  |  |  |  |  |  |
| GO:0002891\_positive\_regulation\_of\_immunoglobulin\_mediated\_immune\_response | 2 | 0 |  |  |  |  |  |  |  |  |
| GO:0003057\_regulation\_of\_the\_force\_of\_heart\_contraction\_by\_chemical\_signal | 2 | 0 |  |  |  |  |  |  |  |  |
| GO:0003078\_regulation\_of\_natriuresis | 2 | 0 |  |  |  |  |  |  |  |  |
| GO:0006005\_L-fucose\_biosynthetic\_process | 2 | 0 |  |  |  |  |  |  |  |  |
| GO:0006011\_UDP-glucose\_metabolic\_process | 2 | 0 |  |  |  |  |  |  |  |  |
| GO:0006030\_chitin\_metabolic\_process | 2 | 0 |  |  |  |  |  |  |  |  |
| GO:0006032\_chitin\_catabolic\_process | 2 | 0 |  |  |  |  |  |  |  |  |
| GO:0006046\_N-acetylglucosamine\_catabolic\_process | 2 | 0 |  |  |  |  |  |  |  |  |
| GO:0006047\_UDP-N-acetylglucosamine\_metabolic\_process | 2 | 0 |  |  |  |  |  |  |  |  |
| GO:0006054\_N-acetylneuraminate\_metabolic\_process | 2 | 0 |  |  |  |  |  |  |  |  |
| GO:0006063\_uronic\_acid\_metabolic\_process | 2 | 0 |  |  |  |  |  |  |  |  |
| GO:0006122\_mitochondrial\_electron\_transport\_\_ubiquinol\_to\_cytochrome\_c | 2 | 0 |  |  |  |  |  |  |  |  |
| GO:0006184\_GTP\_catabolic\_process | 2 | 0 |  |  |  |  |  |  |  |  |
| GO:0006208\_pyrimidine\_base\_catabolic\_process | 2 | 0 |  |  |  |  |  |  |  |  |
| GO:0006210\_thymine\_catabolic\_process | 2 | 0 |  |  |  |  |  |  |  |  |
| GO:0006212\_uracil\_catabolic\_process | 2 | 0 |  |  |  |  |  |  |  |  |
| GO:0006264\_mitochondrial\_DNA\_replication | 2 | 0 |  |  |  |  |  |  |  |  |
| GO:0006307\_DNA\_dealkylation | 2 | 0 |  |  |  |  |  |  |  |  |
| GO:0006335\_DNA\_replication-dependent\_nucleosome\_assembly | 2 | 0 |  |  |  |  |  |  |  |  |
| GO:0006345\_loss\_of\_chromatin\_silencing | 2 | 0 |  |  |  |  |  |  |  |  |
| GO:0006370\_mRNA\_capping | 2 | 0 |  |  |  |  |  |  |  |  |
| GO:0006398\_histone\_mRNA\_3'-end\_processing | 2 | 0 |  |  |  |  |  |  |  |  |
| GO:0006410\_transcription\_\_RNA-dependent | 2 | 0 |  |  |  |  |  |  |  |  |
| GO:0006422\_aspartyl-tRNA\_aminoacylation | 2 | 0 |  |  |  |  |  |  |  |  |
| GO:0006465\_signal\_peptide\_processing | 2 | 0 |  |  |  |  |  |  |  |  |
| GO:0006475\_internal\_protein\_amino\_acid\_acetylation | 2 | 0 |  |  |  |  |  |  |  |  |
| GO:0006478\_peptidyl-tyrosine\_sulfation | 2 | 0 |  |  |  |  |  |  |  |  |
| GO:0006481\_C-terminal\_protein\_amino\_acid\_methylation | 2 | 0 |  |  |  |  |  |  |  |  |
| GO:0006488\_dolichol-linked\_oligosaccharide\_biosynthetic\_process | 2 | 0 |  |  |  |  |  |  |  |  |
| GO:0006498\_N-terminal\_protein\_lipidation | 2 | 0 |  |  |  |  |  |  |  |  |
| GO:0006517\_protein\_deglycosylation | 2 | 0 |  |  |  |  |  |  |  |  |
| GO:0006521\_regulation\_of\_cellular\_amino\_acid\_metabolic\_process | 2 | 0 |  |  |  |  |  |  |  |  |
| GO:0006537\_glutamate\_biosynthetic\_process | 2 | 0 |  |  |  |  |  |  |  |  |
| GO:0006540\_glutamate\_decarboxylation\_to\_succinate | 2 | 0 |  |  |  |  |  |  |  |  |
| GO:0006541\_glutamine\_metabolic\_process | 2 | 0 |  |  |  |  |  |  |  |  |
| GO:0006551\_leucine\_metabolic\_process | 2 | 0 |  |  |  |  |  |  |  |  |
| GO:0006552\_leucine\_catabolic\_process | 2 | 0 |  |  |  |  |  |  |  |  |
| GO:0006561\_proline\_biosynthetic\_process | 2 | 0 |  |  |  |  |  |  |  |  |
| GO:0006569\_tryptophan\_catabolic\_process | 2 | 0 |  |  |  |  |  |  |  |  |
| GO:0006581\_acetylcholine\_catabolic\_process | 2 | 0 |  |  |  |  |  |  |  |  |
| GO:0006582\_melanin\_metabolic\_process | 2 | 0 |  |  |  |  |  |  |  |  |
| GO:0006583\_melanin\_biosynthetic\_process\_from\_tyrosine | 2 | 0 |  |  |  |  |  |  |  |  |
| GO:0006601\_creatine\_biosynthetic\_process | 2 | 0 |  |  |  |  |  |  |  |  |
| GO:0006608\_snRNP\_protein\_import\_into\_nucleus | 2 | 0 |  |  |  |  |  |  |  |  |
| GO:0006651\_diacylglycerol\_biosynthetic\_process | 2 | 0 |  |  |  |  |  |  |  |  |
| GO:0006679\_glucosylceramide\_biosynthetic\_process | 2 | 0 |  |  |  |  |  |  |  |  |
| GO:0006681\_galactosylceramide\_metabolic\_process | 2 | 0 |  |  |  |  |  |  |  |  |
| GO:0006685\_sphingomyelin\_catabolic\_process | 2 | 0 |  |  |  |  |  |  |  |  |
| GO:0006702\_androgen\_biosynthetic\_process | 2 | 0 |  |  |  |  |  |  |  |  |
| GO:0006703\_estrogen\_biosynthetic\_process | 2 | 0 |  |  |  |  |  |  |  |  |
| GO:0006710\_androgen\_catabolic\_process | 2 | 0 |  |  |  |  |  |  |  |  |
| GO:0006738\_nicotinamide\_riboside\_catabolic\_process | 2 | 0 |  |  |  |  |  |  |  |  |
| GO:0006772\_thiamin\_metabolic\_process | 2 | 0 |  |  |  |  |  |  |  |  |
| GO:0006780\_uroporphyrinogen\_III\_biosynthetic\_process | 2 | 0 |  |  |  |  |  |  |  |  |
| GO:0006784\_heme\_a\_biosynthetic\_process | 2 | 0 |  |  |  |  |  |  |  |  |
| GO:0006824\_cobalt\_ion\_transport | 2 | 0 |  |  |  |  |  |  |  |  |
| GO:0006828\_manganese\_ion\_transport | 2 | 0 |  |  |  |  |  |  |  |  |
| GO:0006880\_intracellular\_sequestering\_of\_iron\_ion | 2 | 0 |  |  |  |  |  |  |  |  |
| GO:0006883\_cellular\_sodium\_ion\_homeostasis | 2 | 0 |  |  |  |  |  |  |  |  |
| GO:0006924\_activation-induced\_cell\_death\_of\_T\_cells | 2 | 0 |  |  |  |  |  |  |  |  |
| GO:0006972\_hyperosmotic\_response | 2 | 0 |  |  |  |  |  |  |  |  |
| GO:0006975\_DNA\_damage\_induced\_protein\_phosphorylation | 2 | 0 |  |  |  |  |  |  |  |  |
| GO:0006982\_response\_to\_lipid\_hydroperoxide | 2 | 0 |  |  |  |  |  |  |  |  |
| GO:0006998\_nuclear\_envelope\_organization | 2 | 0 |  |  |  |  |  |  |  |  |
| GO:0007021\_tubulin\_complex\_assembly | 2 | 0 |  |  |  |  |  |  |  |  |
| GO:0007023\_post-chaperonin\_tubulin\_folding\_pathway | 2 | 0 |  |  |  |  |  |  |  |  |
| GO:0007063\_regulation\_of\_sister\_chromatid\_cohesion | 2 | 0 |  |  |  |  |  |  |  |  |
| GO:0007079\_mitotic\_chromosome\_movement\_towards\_spindle\_pole | 2 | 0 |  |  |  |  |  |  |  |  |
| GO:0007095\_mitotic\_cell\_cycle\_G2\_M\_transition\_DNA\_damage\_checkpoint | 2 | 0 |  |  |  |  |  |  |  |  |
| GO:0007128\_meiotic\_prophase\_I | 2 | 0 |  |  |  |  |  |  |  |  |
| GO:0007141\_male\_meiosis\_I | 2 | 0 |  |  |  |  |  |  |  |  |
| GO:0007143\_female\_meiosis | 2 | 0 |  |  |  |  |  |  |  |  |
| GO:0007191\_activation\_of\_adenylate\_cyclase\_activity\_by\_dopamine\_receptor\_signaling\_pathway | 2 | 0 |  |  |  |  |  |  |  |  |
| GO:0007206\_activation\_of\_phospholipase\_C\_activity\_by\_metabotropic\_glutamate\_receptor\_signaling\_pathway | 2 | 0 |  |  |  |  |  |  |  |  |
| GO:0007231\_osmosensory\_signaling\_pathway | 2 | 0 |  |  |  |  |  |  |  |  |
| GO:0007262\_STAT\_protein\_nuclear\_translocation | 2 | 0 |  |  |  |  |  |  |  |  |
| GO:0007290\_spermatid\_nucleus\_elongation | 2 | 0 |  |  |  |  |  |  |  |  |
| GO:0007308\_oocyte\_construction | 2 | 0 |  |  |  |  |  |  |  |  |
| GO:0007309\_oocyte\_axis\_specification | 2 | 0 |  |  |  |  |  |  |  |  |
| GO:0007341\_penetration\_of\_zona\_pellucida | 2 | 0 |  |  |  |  |  |  |  |  |
| GO:0007379\_segment\_specification | 2 | 0 |  |  |  |  |  |  |  |  |
| GO:0007418\_ventral\_midline\_development | 2 | 0 |  |  |  |  |  |  |  |  |
| GO:0007442\_hindgut\_morphogenesis | 2 | 0 |  |  |  |  |  |  |  |  |
| GO:0007492\_endoderm\_development | 2 | 0 |  |  |  |  |  |  |  |  |
| GO:0007525\_somatic\_muscle\_development | 2 | 0 |  |  |  |  |  |  |  |  |
| GO:0007549\_dosage\_compensation | 2 | 0 |  |  |  |  |  |  |  |  |
| GO:0007628\_adult\_walking\_behavior | 2 | 0 |  |  |  |  |  |  |  |  |
| GO:0008216\_spermidine\_metabolic\_process | 2 | 0 |  |  |  |  |  |  |  |  |
| GO:0008306\_associative\_learning | 2 | 0 |  |  |  |  |  |  |  |  |
| GO:0008582\_regulation\_of\_synaptic\_growth\_at\_neuromuscular\_junction | 2 | 0 |  |  |  |  |  |  |  |  |
| GO:0008593\_regulation\_of\_Notch\_signaling\_pathway | 2 | 0 |  |  |  |  |  |  |  |  |
| GO:0008608\_attachment\_of\_spindle\_microtubules\_to\_kinetochore | 2 | 0 |  |  |  |  |  |  |  |  |
| GO:0008616\_queuosine\_biosynthetic\_process | 2 | 0 |  |  |  |  |  |  |  |  |
| GO:0008618\_7-methylguanosine\_metabolic\_process | 2 | 0 |  |  |  |  |  |  |  |  |
| GO:0008653\_lipopolysaccharide\_metabolic\_process | 2 | 0 |  |  |  |  |  |  |  |  |
| GO:0009051\_pentose-phosphate\_shunt\_\_oxidative\_branch | 2 | 0 |  |  |  |  |  |  |  |  |
| GO:0009082\_branched\_chain\_family\_amino\_acid\_biosynthetic\_process | 2 | 0 |  |  |  |  |  |  |  |  |
| GO:0009103\_lipopolysaccharide\_biosynthetic\_process | 2 | 0 |  |  |  |  |  |  |  |  |
| GO:0009120\_deoxyribonucleoside\_metabolic\_process | 2 | 0 |  |  |  |  |  |  |  |  |
| GO:0009134\_nucleoside\_diphosphate\_catabolic\_process | 2 | 0 |  |  |  |  |  |  |  |  |
| GO:0009162\_deoxyribonucleoside\_monophosphate\_metabolic\_process | 2 | 0 |  |  |  |  |  |  |  |  |
| GO:0009191\_ribonucleoside\_diphosphate\_catabolic\_process | 2 | 0 |  |  |  |  |  |  |  |  |
| GO:0009263\_deoxyribonucleotide\_biosynthetic\_process | 2 | 0 |  |  |  |  |  |  |  |  |
| GO:0009439\_cyanate\_metabolic\_process | 2 | 0 |  |  |  |  |  |  |  |  |
| GO:0009440\_cyanate\_catabolic\_process | 2 | 0 |  |  |  |  |  |  |  |  |
| GO:0009448\_gamma-aminobutyric\_acid\_metabolic\_process | 2 | 0 |  |  |  |  |  |  |  |  |
| GO:0009450\_gamma-aminobutyric\_acid\_catabolic\_process | 2 | 0 |  |  |  |  |  |  |  |  |
| GO:0009452\_RNA\_capping | 2 | 0 |  |  |  |  |  |  |  |  |
| GO:0009586\_rhodopsin\_mediated\_phototransduction | 2 | 0 |  |  |  |  |  |  |  |  |
| GO:0009597\_detection\_of\_virus | 2 | 0 |  |  |  |  |  |  |  |  |
| GO:0009602\_detection\_of\_symbiont | 2 | 0 |  |  |  |  |  |  |  |  |
| GO:0009608\_response\_to\_symbiont | 2 | 0 |  |  |  |  |  |  |  |  |
| GO:0009649\_entrainment\_of\_circadian\_clock | 2 | 0 |  |  |  |  |  |  |  |  |
| GO:0009651\_response\_to\_salt\_stress | 2 | 0 |  |  |  |  |  |  |  |  |
| GO:0009756\_carbohydrate\_mediated\_signaling | 2 | 0 |  |  |  |  |  |  |  |  |
| GO:0010042\_response\_to\_manganese\_ion | 2 | 0 |  |  |  |  |  |  |  |  |
| GO:0010155\_regulation\_of\_proton\_transport | 2 | 0 |  |  |  |  |  |  |  |  |
| GO:0010216\_maintenance\_of\_DNA\_methylation | 2 | 0 |  |  |  |  |  |  |  |  |
| GO:0010248\_establishment\_or\_maintenance\_of\_transmembrane\_electrochemical\_gradient | 2 | 0 |  |  |  |  |  |  |  |  |
| GO:0010389\_regulation\_of\_G2\_M\_transition\_of\_mitotic\_cell\_cycle | 2 | 0 |  |  |  |  |  |  |  |  |
| GO:0010459\_negative\_regulation\_of\_heart\_rate | 2 | 0 |  |  |  |  |  |  |  |  |
| GO:0010470\_regulation\_of\_gastrulation | 2 | 0 |  |  |  |  |  |  |  |  |
| GO:0010506\_regulation\_of\_autophagy | 2 | 0 |  |  |  |  |  |  |  |  |
| GO:0010511\_regulation\_of\_phosphatidylinositol\_biosynthetic\_process | 2 | 0 |  |  |  |  |  |  |  |  |
| GO:0010512\_negative\_regulation\_of\_phosphatidylinositol\_biosynthetic\_process | 2 | 0 |  |  |  |  |  |  |  |  |
| GO:0010519\_negative\_regulation\_of\_phospholipase\_activity | 2 | 0 |  |  |  |  |  |  |  |  |
| GO:0010523\_negative\_regulation\_of\_calcium\_ion\_transport\_into\_cytosol | 2 | 0 |  |  |  |  |  |  |  |  |
| GO:0010533\_regulation\_of\_activation\_of\_Janus\_kinase\_activity | 2 | 0 |  |  |  |  |  |  |  |  |
| GO:0010536\_positive\_regulation\_of\_activation\_of\_Janus\_kinase\_activity | 2 | 0 |  |  |  |  |  |  |  |  |
| GO:0010614\_negative\_regulation\_of\_cardiac\_muscle\_hypertrophy | 2 | 0 |  |  |  |  |  |  |  |  |
| GO:0010616\_negative\_regulation\_of\_cardiac\_muscle\_adaptation | 2 | 0 |  |  |  |  |  |  |  |  |
| GO:0010640\_regulation\_of\_platelet-derived\_growth\_factor\_receptor\_signaling\_pathway | 2 | 0 |  |  |  |  |  |  |  |  |
| GO:0010641\_positive\_regulation\_of\_platelet-derived\_growth\_factor\_receptor\_signaling\_pathway | 2 | 0 |  |  |  |  |  |  |  |  |
| GO:0010815\_bradykinin\_catabolic\_process | 2 | 0 |  |  |  |  |  |  |  |  |
| GO:0010818\_T\_cell\_chemotaxis | 2 | 0 |  |  |  |  |  |  |  |  |
| GO:0010819\_regulation\_of\_T\_cell\_chemotaxis | 2 | 0 |  |  |  |  |  |  |  |  |
| GO:0010820\_positive\_regulation\_of\_T\_cell\_chemotaxis | 2 | 0 |  |  |  |  |  |  |  |  |
| GO:0010866\_regulation\_of\_triglyceride\_biosynthetic\_process | 2 | 0 |  |  |  |  |  |  |  |  |
| GO:0010867\_positive\_regulation\_of\_triglyceride\_biosynthetic\_process | 2 | 0 |  |  |  |  |  |  |  |  |
| GO:0010881\_regulation\_of\_cardiac\_muscle\_contraction\_by\_regulation\_of\_the\_release\_of\_sequestered\_calcium\_ion | 2 | 0 |  |  |  |  |  |  |  |  |
| GO:0010882\_regulation\_of\_cardiac\_muscle\_contraction\_by\_calcium\_ion\_signaling | 2 | 0 |  |  |  |  |  |  |  |  |
| GO:0010908\_regulation\_of\_heparan\_sulfate\_proteoglycan\_biosynthetic\_process | 2 | 0 |  |  |  |  |  |  |  |  |
| GO:0010909\_positive\_regulation\_of\_heparan\_sulfate\_proteoglycan\_biosynthetic\_process | 2 | 0 |  |  |  |  |  |  |  |  |
| GO:0010949\_negative\_regulation\_of\_intestinal\_phytosterol\_absorption | 2 | 0 |  |  |  |  |  |  |  |  |
| GO:0010954\_positive\_regulation\_of\_protein\_maturation\_by\_peptide\_bond\_cleavage | 2 | 0 |  |  |  |  |  |  |  |  |
| GO:0010979\_regulation\_of\_vitamin\_D\_24-hydroxylase\_activity | 2 | 0 |  |  |  |  |  |  |  |  |
| GO:0010980\_positive\_regulation\_of\_vitamin\_D\_24-hydroxylase\_activity | 2 | 0 |  |  |  |  |  |  |  |  |
| GO:0010982\_regulation\_of\_high-density\_lipoprotein\_particle\_clearance | 2 | 0 |  |  |  |  |  |  |  |  |
| GO:0014002\_astrocyte\_development | 2 | 0 |  |  |  |  |  |  |  |  |
| GO:0014013\_regulation\_of\_gliogenesis | 2 | 0 |  |  |  |  |  |  |  |  |
| GO:0014014\_negative\_regulation\_of\_gliogenesis | 2 | 0 |  |  |  |  |  |  |  |  |
| GO:0014037\_Schwann\_cell\_differentiation | 2 | 0 |  |  |  |  |  |  |  |  |
| GO:0014044\_Schwann\_cell\_development | 2 | 0 |  |  |  |  |  |  |  |  |
| GO:0014072\_response\_to\_isoquinoline\_alkaloid | 2 | 0 |  |  |  |  |  |  |  |  |
| GO:0014074\_response\_to\_purine | 2 | 0 |  |  |  |  |  |  |  |  |
| GO:0014741\_negative\_regulation\_of\_muscle\_hypertrophy | 2 | 0 |  |  |  |  |  |  |  |  |
| GO:0014745\_negative\_regulation\_of\_muscle\_adaptation | 2 | 0 |  |  |  |  |  |  |  |  |
| GO:0014819\_regulation\_of\_skeletal\_muscle\_contraction | 2 | 0 |  |  |  |  |  |  |  |  |
| GO:0014911\_positive\_regulation\_of\_smooth\_muscle\_cell\_migration | 2 | 0 |  |  |  |  |  |  |  |  |
| GO:0015677\_copper\_ion\_import | 2 | 0 |  |  |  |  |  |  |  |  |
| GO:0015691\_cadmium\_ion\_transport | 2 | 0 |  |  |  |  |  |  |  |  |
| GO:0015732\_prostaglandin\_transport | 2 | 0 |  |  |  |  |  |  |  |  |
| GO:0015788\_UDP-N-acetylglucosamine\_transport | 2 | 0 |  |  |  |  |  |  |  |  |
| GO:0015793\_glycerol\_transport | 2 | 0 |  |  |  |  |  |  |  |  |
| GO:0015801\_aromatic\_amino\_acid\_transport | 2 | 0 |  |  |  |  |  |  |  |  |
| GO:0015808\_L-alanine\_transport | 2 | 0 |  |  |  |  |  |  |  |  |
| GO:0015822\_ornithine\_transport | 2 | 0 |  |  |  |  |  |  |  |  |
| GO:0015824\_proline\_transport | 2 | 0 |  |  |  |  |  |  |  |  |
| GO:0015825\_L-serine\_transport | 2 | 0 |  |  |  |  |  |  |  |  |
| GO:0015860\_purine\_nucleoside\_transport | 2 | 0 |  |  |  |  |  |  |  |  |
| GO:0015870\_acetylcholine\_transport | 2 | 0 |  |  |  |  |  |  |  |  |
| GO:0015871\_choline\_transport | 2 | 0 |  |  |  |  |  |  |  |  |
| GO:0015893\_drug\_transport | 2 | 0 |  |  |  |  |  |  |  |  |
| GO:0015920\_lipopolysaccharide\_transport | 2 | 0 |  |  |  |  |  |  |  |  |
| GO:0015936\_coenzyme\_A\_metabolic\_process | 2 | 0 |  |  |  |  |  |  |  |  |
| GO:0015939\_pantothenate\_metabolic\_process | 2 | 0 |  |  |  |  |  |  |  |  |
| GO:0015942\_formate\_metabolic\_process | 2 | 0 |  |  |  |  |  |  |  |  |
| GO:0015988\_energy\_coupled\_proton\_transport\_\_against\_electrochemical\_gradient | 2 | 0 |  |  |  |  |  |  |  |  |
| GO:0015991\_ATP\_hydrolysis\_coupled\_proton\_transport | 2 | 0 |  |  |  |  |  |  |  |  |
| GO:0015993\_molecular\_hydrogen\_transport | 2 | 0 |  |  |  |  |  |  |  |  |
| GO:0016075\_rRNA\_catabolic\_process | 2 | 0 |  |  |  |  |  |  |  |  |
| GO:0016080\_synaptic\_vesicle\_targeting | 2 | 0 |  |  |  |  |  |  |  |  |
| GO:0016090\_prenol\_metabolic\_process | 2 | 0 |  |  |  |  |  |  |  |  |
| GO:0016093\_polyprenol\_metabolic\_process | 2 | 0 |  |  |  |  |  |  |  |  |
| GO:0016233\_telomere\_capping | 2 | 0 |  |  |  |  |  |  |  |  |
| GO:0016264\_gap\_junction\_assembly | 2 | 0 |  |  |  |  |  |  |  |  |
| GO:0016266\_O-glycan\_processing | 2 | 0 |  |  |  |  |  |  |  |  |
| GO:0016322\_neuron\_remodeling | 2 | 0 |  |  |  |  |  |  |  |  |
| GO:0016482\_cytoplasmic\_transport | 2 | 0 |  |  |  |  |  |  |  |  |
| GO:0016557\_peroxisome\_membrane\_biogenesis | 2 | 0 |  |  |  |  |  |  |  |  |
| GO:0016561\_protein\_import\_into\_peroxisome\_matrix\_\_translocation | 2 | 0 |  |  |  |  |  |  |  |  |
| GO:0016973\_poly(A)+\_mRNA\_export\_from\_nucleus | 2 | 0 |  |  |  |  |  |  |  |  |
| GO:0017004\_cytochrome\_complex\_assembly | 2 | 0 |  |  |  |  |  |  |  |  |
| GO:0017055\_negative\_regulation\_of\_transcriptional\_preinitiation\_complex\_assembly | 2 | 0 |  |  |  |  |  |  |  |  |
| GO:0017145\_stem\_cell\_division | 2 | 0 |  |  |  |  |  |  |  |  |
| GO:0017158\_regulation\_of\_calcium\_ion-dependent\_exocytosis | 2 | 0 |  |  |  |  |  |  |  |  |
| GO:0017182\_peptidyl-diphthamide\_metabolic\_process | 2 | 0 |  |  |  |  |  |  |  |  |
| GO:0017183\_peptidyl-diphthamide\_biosynthetic\_process\_from\_peptidyl-histidine | 2 | 0 |  |  |  |  |  |  |  |  |
| GO:0018198\_peptidyl-cysteine\_modification | 2 | 0 |  |  |  |  |  |  |  |  |
| GO:0018202\_peptidyl-histidine\_modification | 2 | 0 |  |  |  |  |  |  |  |  |
| GO:0018282\_metal\_incorporation\_into\_metallo-sulfur\_cluster | 2 | 0 |  |  |  |  |  |  |  |  |
| GO:0018283\_iron\_incorporation\_into\_metallo-sulfur\_cluster | 2 | 0 |  |  |  |  |  |  |  |  |
| GO:0018347\_protein\_amino\_acid\_farnesylation | 2 | 0 |  |  |  |  |  |  |  |  |
| GO:0018410\_peptide\_or\_protein\_carboxyl-terminal\_blocking | 2 | 0 |  |  |  |  |  |  |  |  |
| GO:0019042\_latent\_virus\_infection | 2 | 0 |  |  |  |  |  |  |  |  |
| GO:0019046\_reactivation\_of\_latent\_virus | 2 | 0 |  |  |  |  |  |  |  |  |
| GO:0019049\_evasion\_of\_host\_defenses\_by\_virus | 2 | 0 |  |  |  |  |  |  |  |  |
| GO:0019076\_release\_of\_virus\_from\_host | 2 | 0 |  |  |  |  |  |  |  |  |
| GO:0019348\_dolichol\_metabolic\_process | 2 | 0 |  |  |  |  |  |  |  |  |
| GO:0019359\_nicotinamide\_nucleotide\_biosynthetic\_process | 2 | 0 |  |  |  |  |  |  |  |  |
| GO:0019363\_pyridine\_nucleotide\_biosynthetic\_process | 2 | 0 |  |  |  |  |  |  |  |  |
| GO:0019374\_galactolipid\_metabolic\_process | 2 | 0 |  |  |  |  |  |  |  |  |
| GO:0019459\_glutamate\_deamidation | 2 | 0 |  |  |  |  |  |  |  |  |
| GO:0019509\_methionine\_salvage | 2 | 0 |  |  |  |  |  |  |  |  |
| GO:0019530\_taurine\_metabolic\_process | 2 | 0 |  |  |  |  |  |  |  |  |
| GO:0019532\_oxalate\_transport | 2 | 0 |  |  |  |  |  |  |  |  |
| GO:0019585\_glucuronate\_metabolic\_process | 2 | 0 |  |  |  |  |  |  |  |  |
| GO:0019605\_butyrate\_metabolic\_process | 2 | 0 |  |  |  |  |  |  |  |  |
| GO:0019626\_short-chain\_fatty\_acid\_catabolic\_process | 2 | 0 |  |  |  |  |  |  |  |  |
| GO:0019730\_antimicrobial\_humoral\_response | 2 | 0 |  |  |  |  |  |  |  |  |
| GO:0019731\_antibacterial\_humoral\_response | 2 | 0 |  |  |  |  |  |  |  |  |
| GO:0019805\_quinolinate\_biosynthetic\_process | 2 | 0 |  |  |  |  |  |  |  |  |
| GO:0019836\_hemolysis\_by\_symbiont\_of\_host\_erythrocytes | 2 | 0 |  |  |  |  |  |  |  |  |
| GO:0019860\_uracil\_metabolic\_process | 2 | 0 |  |  |  |  |  |  |  |  |
| GO:0019896\_axon\_transport\_of\_mitochondrion | 2 | 0 |  |  |  |  |  |  |  |  |
| GO:0019919\_peptidyl-arginine\_methylation\_\_to\_asymmetrical-dimethyl\_arginine | 2 | 0 |  |  |  |  |  |  |  |  |
| GO:0021511\_spinal\_cord\_patterning | 2 | 0 |  |  |  |  |  |  |  |  |
| GO:0021513\_spinal\_cord\_dorsal\_ventral\_patterning | 2 | 0 |  |  |  |  |  |  |  |  |
| GO:0021517\_ventral\_spinal\_cord\_development | 2 | 0 |  |  |  |  |  |  |  |  |
| GO:0021545\_cranial\_nerve\_development | 2 | 0 |  |  |  |  |  |  |  |  |
| GO:0021587\_cerebellum\_morphogenesis | 2 | 0 |  |  |  |  |  |  |  |  |
| GO:0021675\_nerve\_development | 2 | 0 |  |  |  |  |  |  |  |  |
| GO:0021695\_cerebellar\_cortex\_development | 2 | 0 |  |  |  |  |  |  |  |  |
| GO:0021696\_cerebellar\_cortex\_morphogenesis | 2 | 0 |  |  |  |  |  |  |  |  |
| GO:0021795\_cerebral\_cortex\_cell\_migration | 2 | 0 |  |  |  |  |  |  |  |  |
| GO:0021826\_substrate-independent\_telencephalic\_tangential\_migration | 2 | 0 |  |  |  |  |  |  |  |  |
| GO:0021830\_interneuron\_migration\_from\_the\_subpallium\_to\_the\_cortex | 2 | 0 |  |  |  |  |  |  |  |  |
| GO:0021843\_substrate-independent\_telencephalic\_tangential\_interneuron\_migration | 2 | 0 |  |  |  |  |  |  |  |  |
| GO:0021853\_cerebral\_cortex\_GABAergic\_interneuron\_migration | 2 | 0 |  |  |  |  |  |  |  |  |
| GO:0021892\_cerebral\_cortex\_GABAergic\_interneuron\_differentiation | 2 | 0 |  |  |  |  |  |  |  |  |
| GO:0021894\_cerebral\_cortex\_GABAergic\_interneuron\_development | 2 | 0 |  |  |  |  |  |  |  |  |
| GO:0021895\_cerebral\_cortex\_neuron\_differentiation | 2 | 0 |  |  |  |  |  |  |  |  |
| GO:0021910\_smoothened\_signaling\_pathway\_involved\_in\_ventral\_spinal\_cord\_patterning | 2 | 0 |  |  |  |  |  |  |  |  |
| GO:0022011\_myelination\_in\_the\_peripheral\_nervous\_system | 2 | 0 |  |  |  |  |  |  |  |  |
| GO:0022605\_oogenesis\_stage | 2 | 0 |  |  |  |  |  |  |  |  |
| GO:0030150\_protein\_import\_into\_mitochondrial\_matrix | 2 | 0 |  |  |  |  |  |  |  |  |
| GO:0030185\_nitric\_oxide\_transport | 2 | 0 |  |  |  |  |  |  |  |  |
| GO:0030202\_heparin\_metabolic\_process | 2 | 0 |  |  |  |  |  |  |  |  |
| GO:0030205\_dermatan\_sulfate\_metabolic\_process | 2 | 0 |  |  |  |  |  |  |  |  |
| GO:0030208\_dermatan\_sulfate\_biosynthetic\_process | 2 | 0 |  |  |  |  |  |  |  |  |
| GO:0030210\_heparin\_biosynthetic\_process | 2 | 0 |  |  |  |  |  |  |  |  |
| GO:0030264\_nuclear\_fragmentation\_during\_apoptosis | 2 | 0 |  |  |  |  |  |  |  |  |
| GO:0030300\_regulation\_of\_intestinal\_cholesterol\_absorption | 2 | 0 |  |  |  |  |  |  |  |  |
| GO:0030311\_poly-N-acetyllactosamine\_biosynthetic\_process | 2 | 0 |  |  |  |  |  |  |  |  |
| GO:0030318\_melanocyte\_differentiation | 2 | 0 |  |  |  |  |  |  |  |  |
| GO:0030321\_transepithelial\_chloride\_transport | 2 | 0 |  |  |  |  |  |  |  |  |
| GO:0030490\_maturation\_of\_SSU-rRNA | 2 | 0 |  |  |  |  |  |  |  |  |
| GO:0030505\_inorganic\_diphosphate\_transport | 2 | 0 |  |  |  |  |  |  |  |  |
| GO:0030513\_positive\_regulation\_of\_BMP\_signaling\_pathway | 2 | 0 |  |  |  |  |  |  |  |  |
| GO:0030540\_female\_genitalia\_development | 2 | 0 |  |  |  |  |  |  |  |  |
| GO:0030575\_nuclear\_body\_organization | 2 | 0 |  |  |  |  |  |  |  |  |
| GO:0030578\_PML\_body\_organization | 2 | 0 |  |  |  |  |  |  |  |  |
| GO:0030815\_negative\_regulation\_of\_cAMP\_metabolic\_process | 2 | 0 |  |  |  |  |  |  |  |  |
| GO:0030818\_negative\_regulation\_of\_cAMP\_biosynthetic\_process | 2 | 0 |  |  |  |  |  |  |  |  |
| GO:0030889\_negative\_regulation\_of\_B\_cell\_proliferation | 2 | 0 |  |  |  |  |  |  |  |  |
| GO:0030901\_midbrain\_development | 2 | 0 |  |  |  |  |  |  |  |  |
| GO:0030916\_otic\_vesicle\_formation | 2 | 0 |  |  |  |  |  |  |  |  |
| GO:0030949\_positive\_regulation\_of\_vascular\_endothelial\_growth\_factor\_receptor\_signaling\_pathway | 2 | 0 |  |  |  |  |  |  |  |  |
| GO:0030951\_establishment\_or\_maintenance\_of\_microtubule\_cytoskeleton\_polarity | 2 | 0 |  |  |  |  |  |  |  |  |
| GO:0030952\_establishment\_or\_maintenance\_of\_cytoskeleton\_polarity | 2 | 0 |  |  |  |  |  |  |  |  |
| GO:0030997\_regulation\_of\_centriole-centriole\_cohesion | 2 | 0 |  |  |  |  |  |  |  |  |
| GO:0031000\_response\_to\_caffeine | 2 | 0 |  |  |  |  |  |  |  |  |
| GO:0031061\_negative\_regulation\_of\_histone\_methylation | 2 | 0 |  |  |  |  |  |  |  |  |
| GO:0031062\_positive\_regulation\_of\_histone\_methylation | 2 | 0 |  |  |  |  |  |  |  |  |
| GO:0031122\_cytoplasmic\_microtubule\_organization | 2 | 0 |  |  |  |  |  |  |  |  |
| GO:0031294\_lymphocyte\_costimulation | 2 | 0 |  |  |  |  |  |  |  |  |
| GO:0031295\_T\_cell\_costimulation | 2 | 0 |  |  |  |  |  |  |  |  |
| GO:0031297\_replication\_fork\_processing | 2 | 0 |  |  |  |  |  |  |  |  |
| GO:0031440\_regulation\_of\_mRNA\_3'-end\_processing | 2 | 0 |  |  |  |  |  |  |  |  |
| GO:0031452\_negative\_regulation\_of\_heterochromatin\_formation | 2 | 0 |  |  |  |  |  |  |  |  |
| GO:0031508\_centromeric\_heterochromatin\_formation | 2 | 0 |  |  |  |  |  |  |  |  |
| GO:0031536\_positive\_regulation\_of\_exit\_from\_mitosis | 2 | 0 |  |  |  |  |  |  |  |  |
| GO:0031573\_intra-S\_DNA\_damage\_checkpoint | 2 | 0 |  |  |  |  |  |  |  |  |
| GO:0031580\_membrane\_raft\_distribution | 2 | 0 |  |  |  |  |  |  |  |  |
| GO:0031641\_regulation\_of\_myelination | 2 | 0 |  |  |  |  |  |  |  |  |
| GO:0031642\_negative\_regulation\_of\_myelination | 2 | 0 |  |  |  |  |  |  |  |  |
| GO:0031664\_regulation\_of\_lipopolysaccharide-mediated\_signaling\_pathway | 2 | 0 |  |  |  |  |  |  |  |  |
| GO:0031665\_negative\_regulation\_of\_lipopolysaccharide-mediated\_signaling\_pathway | 2 | 0 |  |  |  |  |  |  |  |  |
| GO:0031936\_negative\_regulation\_of\_chromatin\_silencing | 2 | 0 |  |  |  |  |  |  |  |  |
| GO:0032069\_regulation\_of\_nuclease\_activity | 2 | 0 |  |  |  |  |  |  |  |  |
| GO:0032096\_negative\_regulation\_of\_response\_to\_food | 2 | 0 |  |  |  |  |  |  |  |  |
| GO:0032099\_negative\_regulation\_of\_appetite | 2 | 0 |  |  |  |  |  |  |  |  |
| GO:0032201\_telomere\_maintenance\_via\_semi-conservative\_replication | 2 | 0 |  |  |  |  |  |  |  |  |
| GO:0032212\_positive\_regulation\_of\_telomere\_maintenance\_via\_telomerase | 2 | 0 |  |  |  |  |  |  |  |  |
| GO:0032213\_regulation\_of\_telomere\_maintenance\_via\_semi-conservative\_replication | 2 | 0 |  |  |  |  |  |  |  |  |
| GO:0032214\_negative\_regulation\_of\_telomere\_maintenance\_via\_semi-conservative\_replication | 2 | 0 |  |  |  |  |  |  |  |  |
| GO:0032225\_regulation\_of\_synaptic\_transmission\_\_dopaminergic | 2 | 0 |  |  |  |  |  |  |  |  |
| GO:0032228\_regulation\_of\_synaptic\_transmission\_\_GABAergic | 2 | 0 |  |  |  |  |  |  |  |  |
| GO:0032236\_positive\_regulation\_of\_calcium\_ion\_transport\_via\_store-operated\_calcium\_channel\_activity | 2 | 0 |  |  |  |  |  |  |  |  |
| GO:0032237\_activation\_of\_store-operated\_calcium\_channel\_activity | 2 | 0 |  |  |  |  |  |  |  |  |
| GO:0032292\_ensheathment\_of\_axons\_in\_the\_peripheral\_nervous\_system | 2 | 0 |  |  |  |  |  |  |  |  |
| GO:0032303\_regulation\_of\_icosanoid\_secretion | 2 | 0 |  |  |  |  |  |  |  |  |
| GO:0032305\_positive\_regulation\_of\_icosanoid\_secretion | 2 | 0 |  |  |  |  |  |  |  |  |
| GO:0032306\_regulation\_of\_prostaglandin\_secretion | 2 | 0 |  |  |  |  |  |  |  |  |
| GO:0032308\_positive\_regulation\_of\_prostaglandin\_secretion | 2 | 0 |  |  |  |  |  |  |  |  |
| GO:0032310\_prostaglandin\_secretion | 2 | 0 |  |  |  |  |  |  |  |  |
| GO:0032328\_alanine\_transport | 2 | 0 |  |  |  |  |  |  |  |  |
| GO:0032329\_serine\_transport | 2 | 0 |  |  |  |  |  |  |  |  |
| GO:0032344\_regulation\_of\_aldosterone\_metabolic\_process | 2 | 0 |  |  |  |  |  |  |  |  |
| GO:0032417\_positive\_regulation\_of\_sodium:hydrogen\_antiporter\_activity | 2 | 0 |  |  |  |  |  |  |  |  |
| GO:0032435\_negative\_regulation\_of\_proteasomal\_ubiquitin-dependent\_protein\_catabolic\_process | 2 | 0 |  |  |  |  |  |  |  |  |
| GO:0032506\_cytokinetic\_process | 2 | 0 |  |  |  |  |  |  |  |  |
| GO:0032510\_endosome\_to\_lysosome\_transport\_via\_multivesicular\_body\_sorting\_pathway | 2 | 0 |  |  |  |  |  |  |  |  |
| GO:0032512\_regulation\_of\_protein\_phosphatase\_type\_2B\_activity | 2 | 0 |  |  |  |  |  |  |  |  |
| GO:0032513\_negative\_regulation\_of\_protein\_phosphatase\_type\_2B\_activity | 2 | 0 |  |  |  |  |  |  |  |  |
| GO:0032516\_positive\_regulation\_of\_phosphoprotein\_phosphatase\_activity | 2 | 0 |  |  |  |  |  |  |  |  |
| GO:0032525\_somite\_rostral\_caudal\_axis\_specification | 2 | 0 |  |  |  |  |  |  |  |  |
| GO:0032620\_interleukin-17\_production | 2 | 0 |  |  |  |  |  |  |  |  |
| GO:0032634\_interleukin-5\_production | 2 | 0 |  |  |  |  |  |  |  |  |
| GO:0032645\_regulation\_of\_granulocyte\_macrophage\_colony-stimulating\_factor\_production | 2 | 0 |  |  |  |  |  |  |  |  |
| GO:0032656\_regulation\_of\_interleukin-13\_production | 2 | 0 |  |  |  |  |  |  |  |  |
| GO:0032660\_regulation\_of\_interleukin-17\_production | 2 | 0 |  |  |  |  |  |  |  |  |
| GO:0032674\_regulation\_of\_interleukin-5\_production | 2 | 0 |  |  |  |  |  |  |  |  |
| GO:0032689\_negative\_regulation\_of\_interferon-gamma\_production | 2 | 0 |  |  |  |  |  |  |  |  |
| GO:0032691\_negative\_regulation\_of\_interleukin-1\_beta\_production | 2 | 0 |  |  |  |  |  |  |  |  |
| GO:0032692\_negative\_regulation\_of\_interleukin-1\_production | 2 | 0 |  |  |  |  |  |  |  |  |
| GO:0032733\_positive\_regulation\_of\_interleukin-10\_production | 2 | 0 |  |  |  |  |  |  |  |  |
| GO:0032740\_positive\_regulation\_of\_interleukin-17\_production | 2 | 0 |  |  |  |  |  |  |  |  |
| GO:0032802\_low-density\_lipoprotein\_receptor\_catabolic\_process | 2 | 0 |  |  |  |  |  |  |  |  |
| GO:0032803\_regulation\_of\_low-density\_lipoprotein\_receptor\_catabolic\_process | 2 | 0 |  |  |  |  |  |  |  |  |
| GO:0032814\_regulation\_of\_natural\_killer\_cell\_activation | 2 | 0 |  |  |  |  |  |  |  |  |
| GO:0032816\_positive\_regulation\_of\_natural\_killer\_cell\_activation | 2 | 0 |  |  |  |  |  |  |  |  |
| GO:0032863\_activation\_of\_Rac\_GTPase\_activity | 2 | 0 |  |  |  |  |  |  |  |  |
| GO:0032892\_positive\_regulation\_of\_organic\_acid\_transport | 2 | 0 |  |  |  |  |  |  |  |  |
| GO:0032897\_negative\_regulation\_of\_viral\_transcription | 2 | 0 |  |  |  |  |  |  |  |  |
| GO:0032898\_neurotrophin\_production | 2 | 0 |  |  |  |  |  |  |  |  |
| GO:0032902\_nerve\_growth\_factor\_production | 2 | 0 |  |  |  |  |  |  |  |  |
| GO:0032966\_negative\_regulation\_of\_collagen\_biosynthetic\_process | 2 | 0 |  |  |  |  |  |  |  |  |
| GO:0032971\_regulation\_of\_muscle\_filament\_sliding | 2 | 0 |  |  |  |  |  |  |  |  |
| GO:0033003\_regulation\_of\_mast\_cell\_activation | 2 | 0 |  |  |  |  |  |  |  |  |
| GO:0033005\_positive\_regulation\_of\_mast\_cell\_activation | 2 | 0 |  |  |  |  |  |  |  |  |
| GO:0033033\_negative\_regulation\_of\_myeloid\_cell\_apoptosis | 2 | 0 |  |  |  |  |  |  |  |  |
| GO:0033034\_positive\_regulation\_of\_myeloid\_cell\_apoptosis | 2 | 0 |  |  |  |  |  |  |  |  |
| GO:0033144\_negative\_regulation\_of\_steroid\_hormone\_receptor\_signaling\_pathway | 2 | 0 |  |  |  |  |  |  |  |  |
| GO:0033194\_response\_to\_hydroperoxide | 2 | 0 |  |  |  |  |  |  |  |  |
| GO:0033364\_mast\_cell\_secretory\_granule\_organization | 2 | 0 |  |  |  |  |  |  |  |  |
| GO:0033567\_DNA\_replication\_\_Okazaki\_fragment\_processing | 2 | 0 |  |  |  |  |  |  |  |  |
| GO:0033598\_mammary\_gland\_epithelial\_cell\_proliferation | 2 | 0 |  |  |  |  |  |  |  |  |
| GO:0033599\_regulation\_of\_mammary\_gland\_epithelial\_cell\_proliferation | 2 | 0 |  |  |  |  |  |  |  |  |
| GO:0033603\_positive\_regulation\_of\_dopamine\_secretion | 2 | 0 |  |  |  |  |  |  |  |  |
| GO:0033615\_mitochondrial\_proton-transporting\_ATP\_synthase\_complex\_assembly | 2 | 0 |  |  |  |  |  |  |  |  |
| GO:0033630\_positive\_regulation\_of\_cell\_adhesion\_mediated\_by\_integrin | 2 | 0 |  |  |  |  |  |  |  |  |
| GO:0033632\_regulation\_of\_cell-cell\_adhesion\_mediated\_by\_integrin | 2 | 0 |  |  |  |  |  |  |  |  |
| GO:0033861\_negative\_regulation\_of\_NAD(P)H\_oxidase\_activity | 2 | 0 |  |  |  |  |  |  |  |  |
| GO:0033864\_positive\_regulation\_of\_NAD(P)H\_oxidase\_activity | 2 | 0 |  |  |  |  |  |  |  |  |
| GO:0034113\_heterotypic\_cell-cell\_adhesion | 2 | 0 |  |  |  |  |  |  |  |  |
| GO:0034114\_regulation\_of\_heterotypic\_cell-cell\_adhesion | 2 | 0 |  |  |  |  |  |  |  |  |
| GO:0034121\_regulation\_of\_toll-like\_receptor\_signaling\_pathway | 2 | 0 |  |  |  |  |  |  |  |  |
| GO:0034134\_toll-like\_receptor\_2\_signaling\_pathway | 2 | 0 |  |  |  |  |  |  |  |  |
| GO:0034214\_protein\_hexamerization | 2 | 0 |  |  |  |  |  |  |  |  |
| GO:0034259\_negative\_regulation\_of\_Rho\_GTPase\_activity | 2 | 0 |  |  |  |  |  |  |  |  |
| GO:0034260\_negative\_regulation\_of\_GTPase\_activity | 2 | 0 |  |  |  |  |  |  |  |  |
| GO:0034261\_negative\_regulation\_of\_Ras\_GTPase\_activity | 2 | 0 |  |  |  |  |  |  |  |  |
| GO:0034331\_cell\_junction\_maintenance | 2 | 0 |  |  |  |  |  |  |  |  |
| GO:0034392\_negative\_regulation\_of\_smooth\_muscle\_cell\_apoptosis | 2 | 0 |  |  |  |  |  |  |  |  |
| GO:0034418\_urate\_biosynthetic\_process | 2 | 0 |  |  |  |  |  |  |  |  |
| GO:0034441\_plasma\_lipoprotein\_oxidation | 2 | 0 |  |  |  |  |  |  |  |  |
| GO:0034444\_regulation\_of\_plasma\_lipoprotein\_oxidation | 2 | 0 |  |  |  |  |  |  |  |  |
| GO:0034445\_negative\_regulation\_of\_plasma\_lipoprotein\_oxidation | 2 | 0 |  |  |  |  |  |  |  |  |
| GO:0034616\_response\_to\_laminar\_fluid\_shear\_stress | 2 | 0 |  |  |  |  |  |  |  |  |
| GO:0034619\_cellular\_chaperone-mediated\_protein\_complex\_assembly | 2 | 0 |  |  |  |  |  |  |  |  |
| GO:0034638\_phosphatidylcholine\_catabolic\_process | 2 | 0 |  |  |  |  |  |  |  |  |
| GO:0034644\_cellular\_response\_to\_UV | 2 | 0 |  |  |  |  |  |  |  |  |
| GO:0034650\_cortisol\_metabolic\_process | 2 | 0 |  |  |  |  |  |  |  |  |
| GO:0034651\_cortisol\_biosynthetic\_process | 2 | 0 |  |  |  |  |  |  |  |  |
| GO:0034723\_DNA\_replication-dependent\_nucleosome\_organization | 2 | 0 |  |  |  |  |  |  |  |  |
| GO:0034970\_histone\_H3-R2\_methylation | 2 | 0 |  |  |  |  |  |  |  |  |
| GO:0035025\_positive\_regulation\_of\_Rho\_protein\_signal\_transduction | 2 | 0 |  |  |  |  |  |  |  |  |
| GO:0035039\_male\_pronucleus\_formation | 2 | 0 |  |  |  |  |  |  |  |  |
| GO:0035050\_embryonic\_heart\_tube\_development | 2 | 0 |  |  |  |  |  |  |  |  |
| GO:0035058\_sensory\_cilium\_assembly | 2 | 0 |  |  |  |  |  |  |  |  |
| GO:0035117\_embryonic\_arm\_morphogenesis | 2 | 0 |  |  |  |  |  |  |  |  |
| GO:0035140\_arm\_morphogenesis | 2 | 0 |  |  |  |  |  |  |  |  |
| GO:0035162\_embryonic\_hemopoiesis | 2 | 0 |  |  |  |  |  |  |  |  |
| GO:0035247\_peptidyl-arginine\_omega-N-methylation | 2 | 0 |  |  |  |  |  |  |  |  |
| GO:0035279\_gene\_silencing\_by\_miRNA\_\_mRNA\_cleavage | 2 | 0 |  |  |  |  |  |  |  |  |
| GO:0035305\_negative\_regulation\_of\_dephosphorylation | 2 | 0 |  |  |  |  |  |  |  |  |
| GO:0035308\_negative\_regulation\_of\_protein\_amino\_acid\_dephosphorylation | 2 | 0 |  |  |  |  |  |  |  |  |
| GO:0042062\_long-term\_strengthening\_of\_neuromuscular\_junction | 2 | 0 |  |  |  |  |  |  |  |  |
| GO:0042097\_interleukin-4\_biosynthetic\_process | 2 | 0 |  |  |  |  |  |  |  |  |
| GO:0042167\_heme\_catabolic\_process | 2 | 0 |  |  |  |  |  |  |  |  |
| GO:0042222\_interleukin-1\_biosynthetic\_process | 2 | 0 |  |  |  |  |  |  |  |  |
| GO:0042231\_interleukin-13\_biosynthetic\_process | 2 | 0 |  |  |  |  |  |  |  |  |
| GO:0042321\_negative\_regulation\_of\_circadian\_sleep\_wake\_cycle\_\_sleep | 2 | 0 |  |  |  |  |  |  |  |  |
| GO:0042322\_negative\_regulation\_of\_circadian\_sleep\_wake\_cycle\_\_REM\_sleep | 2 | 0 |  |  |  |  |  |  |  |  |
| GO:0042335\_cuticle\_development | 2 | 0 |  |  |  |  |  |  |  |  |
| GO:0042339\_keratan\_sulfate\_metabolic\_process | 2 | 0 |  |  |  |  |  |  |  |  |
| GO:0042350\_GDP-L-fucose\_biosynthetic\_process | 2 | 0 |  |  |  |  |  |  |  |  |
| GO:0042351\_'de\_novo'\_GDP-L-fucose\_biosynthetic\_process | 2 | 0 |  |  |  |  |  |  |  |  |
| GO:0042353\_fucose\_biosynthetic\_process | 2 | 0 |  |  |  |  |  |  |  |  |
| GO:0042396\_phosphagen\_biosynthetic\_process | 2 | 0 |  |  |  |  |  |  |  |  |
| GO:0042407\_cristae\_formation | 2 | 0 |  |  |  |  |  |  |  |  |
| GO:0042414\_epinephrine\_metabolic\_process | 2 | 0 |  |  |  |  |  |  |  |  |
| GO:0042415\_norepinephrine\_metabolic\_process | 2 | 0 |  |  |  |  |  |  |  |  |
| GO:0042436\_indole\_derivative\_catabolic\_process | 2 | 0 |  |  |  |  |  |  |  |  |
| GO:0042438\_melanin\_biosynthetic\_process | 2 | 0 |  |  |  |  |  |  |  |  |
| GO:0042481\_regulation\_of\_odontogenesis | 2 | 0 |  |  |  |  |  |  |  |  |
| GO:0042501\_serine\_phosphorylation\_of\_STAT\_protein | 2 | 0 |  |  |  |  |  |  |  |  |
| GO:0042508\_tyrosine\_phosphorylation\_of\_Stat1\_protein | 2 | 0 |  |  |  |  |  |  |  |  |
| GO:0042510\_regulation\_of\_tyrosine\_phosphorylation\_of\_Stat1\_protein | 2 | 0 |  |  |  |  |  |  |  |  |
| GO:0042511\_positive\_regulation\_of\_tyrosine\_phosphorylation\_of\_Stat1\_protein | 2 | 0 |  |  |  |  |  |  |  |  |
| GO:0042536\_negative\_regulation\_of\_tumor\_necrosis\_factor\_biosynthetic\_process | 2 | 0 |  |  |  |  |  |  |  |  |
| GO:0042634\_regulation\_of\_hair\_cycle | 2 | 0 |  |  |  |  |  |  |  |  |
| GO:0042754\_negative\_regulation\_of\_circadian\_rhythm | 2 | 0 |  |  |  |  |  |  |  |  |
| GO:0042776\_mitochondrial\_ATP\_synthesis\_coupled\_proton\_transport | 2 | 0 |  |  |  |  |  |  |  |  |
| GO:0042816\_vitamin\_B6\_metabolic\_process | 2 | 0 |  |  |  |  |  |  |  |  |
| GO:0042819\_vitamin\_B6\_biosynthetic\_process | 2 | 0 |  |  |  |  |  |  |  |  |
| GO:0042832\_defense\_response\_to\_protozoan | 2 | 0 |  |  |  |  |  |  |  |  |
| GO:0043000\_Golgi\_to\_plasma\_membrane\_CFTR\_protein\_transport | 2 | 0 |  |  |  |  |  |  |  |  |
| GO:0043006\_activation\_of\_phospholipase\_A2\_activity\_by\_calcium-mediated\_signaling | 2 | 0 |  |  |  |  |  |  |  |  |
| GO:0043011\_myeloid\_dendritic\_cell\_differentiation | 2 | 0 |  |  |  |  |  |  |  |  |
| GO:0043031\_negative\_regulation\_of\_macrophage\_activation | 2 | 0 |  |  |  |  |  |  |  |  |
| GO:0043045\_DNA\_methylation\_during\_embryonic\_development | 2 | 0 |  |  |  |  |  |  |  |  |
| GO:0043101\_purine\_salvage | 2 | 0 |  |  |  |  |  |  |  |  |
| GO:0043102\_amino\_acid\_salvage | 2 | 0 |  |  |  |  |  |  |  |  |
| GO:0043137\_DNA\_replication\_\_removal\_of\_RNA\_primer | 2 | 0 |  |  |  |  |  |  |  |  |
| GO:0043162\_ubiquitin-dependent\_protein\_catabolic\_process\_via\_the\_multivesicular\_body\_sorting\_pathway | 2 | 0 |  |  |  |  |  |  |  |  |
| GO:0043163\_cell\_envelope\_organization | 2 | 0 |  |  |  |  |  |  |  |  |
| GO:0043243\_positive\_regulation\_of\_protein\_complex\_disassembly | 2 | 0 |  |  |  |  |  |  |  |  |
| GO:0043266\_regulation\_of\_potassium\_ion\_transport | 2 | 0 |  |  |  |  |  |  |  |  |
| GO:0043278\_response\_to\_morphine | 2 | 0 |  |  |  |  |  |  |  |  |
| GO:0043297\_apical\_junction\_assembly | 2 | 0 |  |  |  |  |  |  |  |  |
| GO:0043400\_cortisol\_secretion | 2 | 0 |  |  |  |  |  |  |  |  |
| GO:0043476\_pigment\_accumulation | 2 | 0 |  |  |  |  |  |  |  |  |
| GO:0043482\_cellular\_pigment\_accumulation | 2 | 0 |  |  |  |  |  |  |  |  |
| GO:0043490\_malate-aspartate\_shuttle | 2 | 0 |  |  |  |  |  |  |  |  |
| GO:0043508\_negative\_regulation\_of\_JUN\_kinase\_activity | 2 | 0 |  |  |  |  |  |  |  |  |
| GO:0043516\_regulation\_of\_DNA\_damage\_response\_\_signal\_transduction\_by\_p53\_class\_mediator | 2 | 0 |  |  |  |  |  |  |  |  |
| GO:0043569\_negative\_regulation\_of\_insulin-like\_growth\_factor\_receptor\_signaling\_pathway | 2 | 0 |  |  |  |  |  |  |  |  |
| GO:0043619\_regulation\_of\_transcription\_from\_RNA\_polymerase\_II\_promoter\_in\_response\_to\_oxidative\_stress | 2 | 0 |  |  |  |  |  |  |  |  |
| GO:0043628\_ncRNA\_3'-end\_processing | 2 | 0 |  |  |  |  |  |  |  |  |
| GO:0043653\_mitochondrial\_fragmentation\_during\_apoptosis | 2 | 0 |  |  |  |  |  |  |  |  |
| GO:0043903\_regulation\_of\_symbiosis\_\_encompassing\_mutualism\_through\_parasitism | 2 | 0 |  |  |  |  |  |  |  |  |
| GO:0043932\_ossification\_involved\_in\_bone\_remodeling | 2 | 0 |  |  |  |  |  |  |  |  |
| GO:0043954\_cellular\_component\_maintenance | 2 | 0 |  |  |  |  |  |  |  |  |
| GO:0043985\_histone\_H4-R3\_methylation | 2 | 0 |  |  |  |  |  |  |  |  |
| GO:0044004\_disruption\_by\_symbiont\_of\_host\_cells | 2 | 0 |  |  |  |  |  |  |  |  |
| GO:0044030\_regulation\_of\_DNA\_methylation | 2 | 0 |  |  |  |  |  |  |  |  |
| GO:0044036\_cell\_wall\_macromolecule\_metabolic\_process | 2 | 0 |  |  |  |  |  |  |  |  |
| GO:0044058\_regulation\_of\_digestive\_system\_process | 2 | 0 |  |  |  |  |  |  |  |  |
| GO:0044062\_regulation\_of\_excretion | 2 | 0 |  |  |  |  |  |  |  |  |
| GO:0044091\_membrane\_biogenesis | 2 | 0 |  |  |  |  |  |  |  |  |
| GO:0044413\_avoidance\_of\_host\_defenses | 2 | 0 |  |  |  |  |  |  |  |  |
| GO:0044415\_evasion\_or\_tolerance\_of\_host\_defenses | 2 | 0 |  |  |  |  |  |  |  |  |
| GO:0045002\_double-strand\_break\_repair\_via\_single-strand\_annealing | 2 | 0 |  |  |  |  |  |  |  |  |
| GO:0045005\_maintenance\_of\_fidelity\_during\_DNA-dependent\_DNA\_replication | 2 | 0 |  |  |  |  |  |  |  |  |
| GO:0045019\_negative\_regulation\_of\_nitric\_oxide\_biosynthetic\_process | 2 | 0 |  |  |  |  |  |  |  |  |
| GO:0045046\_protein\_import\_into\_peroxisome\_membrane | 2 | 0 |  |  |  |  |  |  |  |  |
| GO:0045059\_positive\_thymic\_T\_cell\_selection | 2 | 0 |  |  |  |  |  |  |  |  |
| GO:0045066\_regulatory\_T\_cell\_differentiation | 2 | 0 |  |  |  |  |  |  |  |  |
| GO:0045077\_negative\_regulation\_of\_interferon-gamma\_biosynthetic\_process | 2 | 0 |  |  |  |  |  |  |  |  |
| GO:0045084\_positive\_regulation\_of\_interleukin-12\_biosynthetic\_process | 2 | 0 |  |  |  |  |  |  |  |  |
| GO:0045085\_negative\_regulation\_of\_interleukin-2\_biosynthetic\_process | 2 | 0 |  |  |  |  |  |  |  |  |
| GO:0045110\_intermediate\_filament\_bundle\_assembly | 2 | 0 |  |  |  |  |  |  |  |  |
| GO:0045113\_regulation\_of\_integrin\_biosynthetic\_process | 2 | 0 |  |  |  |  |  |  |  |  |
| GO:0045162\_clustering\_of\_voltage-gated\_sodium\_channels | 2 | 0 |  |  |  |  |  |  |  |  |
| GO:0045217\_cell-cell\_junction\_maintenance | 2 | 0 |  |  |  |  |  |  |  |  |
| GO:0045229\_external\_encapsulating\_structure\_organization | 2 | 0 |  |  |  |  |  |  |  |  |
| GO:0045341\_MHC\_class\_I\_biosynthetic\_process | 2 | 0 |  |  |  |  |  |  |  |  |
| GO:0045343\_regulation\_of\_MHC\_class\_I\_biosynthetic\_process | 2 | 0 |  |  |  |  |  |  |  |  |
| GO:0045347\_negative\_regulation\_of\_MHC\_class\_II\_biosynthetic\_process | 2 | 0 |  |  |  |  |  |  |  |  |
| GO:0045348\_positive\_regulation\_of\_MHC\_class\_II\_biosynthetic\_process | 2 | 0 |  |  |  |  |  |  |  |  |
| GO:0045402\_regulation\_of\_interleukin-4\_biosynthetic\_process | 2 | 0 |  |  |  |  |  |  |  |  |
| GO:0045404\_positive\_regulation\_of\_interleukin-4\_biosynthetic\_process | 2 | 0 |  |  |  |  |  |  |  |  |
| GO:0045415\_negative\_regulation\_of\_interleukin-8\_biosynthetic\_process | 2 | 0 |  |  |  |  |  |  |  |  |
| GO:0045423\_regulation\_of\_granulocyte\_macrophage\_colony-stimulating\_factor\_biosynthetic\_process | 2 | 0 |  |  |  |  |  |  |  |  |
| GO:0045542\_positive\_regulation\_of\_cholesterol\_biosynthetic\_process | 2 | 0 |  |  |  |  |  |  |  |  |
| GO:0045576\_mast\_cell\_activation | 2 | 0 |  |  |  |  |  |  |  |  |
| GO:0045578\_negative\_regulation\_of\_B\_cell\_differentiation | 2 | 0 |  |  |  |  |  |  |  |  |
| GO:0045599\_negative\_regulation\_of\_fat\_cell\_differentiation | 2 | 0 |  |  |  |  |  |  |  |  |
| GO:0045601\_regulation\_of\_endothelial\_cell\_differentiation | 2 | 0 |  |  |  |  |  |  |  |  |
| GO:0045627\_positive\_regulation\_of\_T-helper\_1\_cell\_differentiation | 2 | 0 |  |  |  |  |  |  |  |  |
| GO:0045630\_positive\_regulation\_of\_T-helper\_2\_cell\_differentiation | 2 | 0 |  |  |  |  |  |  |  |  |
| GO:0045647\_negative\_regulation\_of\_erythrocyte\_differentiation | 2 | 0 |  |  |  |  |  |  |  |  |
| GO:0045653\_negative\_regulation\_of\_megakaryocyte\_differentiation | 2 | 0 |  |  |  |  |  |  |  |  |
| GO:0045655\_regulation\_of\_monocyte\_differentiation | 2 | 0 |  |  |  |  |  |  |  |  |
| GO:0045657\_positive\_regulation\_of\_monocyte\_differentiation | 2 | 0 |  |  |  |  |  |  |  |  |
| GO:0045685\_regulation\_of\_glial\_cell\_differentiation | 2 | 0 |  |  |  |  |  |  |  |  |
| GO:0045686\_negative\_regulation\_of\_glial\_cell\_differentiation | 2 | 0 |  |  |  |  |  |  |  |  |
| GO:0045721\_negative\_regulation\_of\_gluconeogenesis | 2 | 0 |  |  |  |  |  |  |  |  |
| GO:0045722\_positive\_regulation\_of\_gluconeogenesis | 2 | 0 |  |  |  |  |  |  |  |  |
| GO:0045726\_positive\_regulation\_of\_integrin\_biosynthetic\_process | 2 | 0 |  |  |  |  |  |  |  |  |
| GO:0045780\_positive\_regulation\_of\_bone\_resorption | 2 | 0 |  |  |  |  |  |  |  |  |
| GO:0045796\_negative\_regulation\_of\_intestinal\_cholesterol\_absorption | 2 | 0 |  |  |  |  |  |  |  |  |
| GO:0045798\_negative\_regulation\_of\_chromatin\_assembly\_or\_disassembly | 2 | 0 |  |  |  |  |  |  |  |  |
| GO:0045842\_positive\_regulation\_of\_mitotic\_metaphase\_anaphase\_transition | 2 | 0 |  |  |  |  |  |  |  |  |
| GO:0045869\_negative\_regulation\_of\_retroviral\_genome\_replication | 2 | 0 |  |  |  |  |  |  |  |  |
| GO:0045906\_negative\_regulation\_of\_vasoconstriction | 2 | 0 |  |  |  |  |  |  |  |  |
| GO:0045908\_negative\_regulation\_of\_vasodilation | 2 | 0 |  |  |  |  |  |  |  |  |
| GO:0045918\_negative\_regulation\_of\_cytolysis | 2 | 0 |  |  |  |  |  |  |  |  |
| GO:0045919\_positive\_regulation\_of\_cytolysis | 2 | 0 |  |  |  |  |  |  |  |  |
| GO:0045938\_positive\_regulation\_of\_circadian\_sleep\_wake\_cycle\_\_sleep | 2 | 0 |  |  |  |  |  |  |  |  |
| GO:0045986\_negative\_regulation\_of\_smooth\_muscle\_contraction | 2 | 0 |  |  |  |  |  |  |  |  |
| GO:0045991\_positive\_regulation\_of\_transcription\_by\_carbon\_catabolites | 2 | 0 |  |  |  |  |  |  |  |  |
| GO:0046016\_positive\_regulation\_of\_transcription\_by\_glucose | 2 | 0 |  |  |  |  |  |  |  |  |
| GO:0046039\_GTP\_metabolic\_process | 2 | 0 |  |  |  |  |  |  |  |  |
| GO:0046100\_hypoxanthine\_metabolic\_process | 2 | 0 |  |  |  |  |  |  |  |  |
| GO:0046102\_inosine\_metabolic\_process | 2 | 0 |  |  |  |  |  |  |  |  |
| GO:0046113\_nucleobase\_catabolic\_process | 2 | 0 |  |  |  |  |  |  |  |  |
| GO:0046114\_guanosine\_biosynthetic\_process | 2 | 0 |  |  |  |  |  |  |  |  |
| GO:0046116\_queuosine\_metabolic\_process | 2 | 0 |  |  |  |  |  |  |  |  |
| GO:0046118\_7-methylguanosine\_biosynthetic\_process | 2 | 0 |  |  |  |  |  |  |  |  |
| GO:0046121\_deoxyribonucleoside\_catabolic\_process | 2 | 0 |  |  |  |  |  |  |  |  |
| GO:0046135\_pyrimidine\_nucleoside\_catabolic\_process | 2 | 0 |  |  |  |  |  |  |  |  |
| GO:0046136\_positive\_regulation\_of\_vitamin\_metabolic\_process | 2 | 0 |  |  |  |  |  |  |  |  |
| GO:0046149\_pigment\_catabolic\_process | 2 | 0 |  |  |  |  |  |  |  |  |
| GO:0046160\_heme\_a\_metabolic\_process | 2 | 0 |  |  |  |  |  |  |  |  |
| GO:0046174\_polyol\_catabolic\_process | 2 | 0 |  |  |  |  |  |  |  |  |
| GO:0046218\_indolalkylamine\_catabolic\_process | 2 | 0 |  |  |  |  |  |  |  |  |
| GO:0046349\_amino\_sugar\_biosynthetic\_process | 2 | 0 |  |  |  |  |  |  |  |  |
| GO:0046359\_butyrate\_catabolic\_process | 2 | 0 |  |  |  |  |  |  |  |  |
| GO:0046368\_GDP-L-fucose\_metabolic\_process | 2 | 0 |  |  |  |  |  |  |  |  |
| GO:0046398\_UDP-glucuronate\_metabolic\_process | 2 | 0 |  |  |  |  |  |  |  |  |
| GO:0046415\_urate\_metabolic\_process | 2 | 0 |  |  |  |  |  |  |  |  |
| GO:0046476\_glycosylceramide\_biosynthetic\_process | 2 | 0 |  |  |  |  |  |  |  |  |
| GO:0046479\_glycosphingolipid\_catabolic\_process | 2 | 0 |  |  |  |  |  |  |  |  |
| GO:0046495\_nicotinamide\_riboside\_metabolic\_process | 2 | 0 |  |  |  |  |  |  |  |  |
| GO:0046500\_S-adenosylmethionine\_metabolic\_process | 2 | 0 |  |  |  |  |  |  |  |  |
| GO:0046502\_uroporphyrinogen\_III\_metabolic\_process | 2 | 0 |  |  |  |  |  |  |  |  |
| GO:0046521\_sphingoid\_catabolic\_process | 2 | 0 |  |  |  |  |  |  |  |  |
| GO:0046541\_saliva\_secretion | 2 | 0 |  |  |  |  |  |  |  |  |
| GO:0046596\_regulation\_of\_virion\_penetration\_into\_host\_cell | 2 | 0 |  |  |  |  |  |  |  |  |
| GO:0046599\_regulation\_of\_centriole\_replication | 2 | 0 |  |  |  |  |  |  |  |  |
| GO:0046629\_gamma-delta\_T\_cell\_activation | 2 | 0 |  |  |  |  |  |  |  |  |
| GO:0046633\_alpha-beta\_T\_cell\_proliferation | 2 | 0 |  |  |  |  |  |  |  |  |
| GO:0046753\_non-lytic\_viral\_release | 2 | 0 |  |  |  |  |  |  |  |  |
| GO:0046755\_non-lytic\_virus\_budding | 2 | 0 |  |  |  |  |  |  |  |  |
| GO:0046794\_virion\_transport | 2 | 0 |  |  |  |  |  |  |  |  |
| GO:0046795\_intracellular\_virion\_transport | 2 | 0 |  |  |  |  |  |  |  |  |
| GO:0046836\_glycolipid\_transport | 2 | 0 |  |  |  |  |  |  |  |  |
| GO:0046839\_phospholipid\_dephosphorylation | 2 | 0 |  |  |  |  |  |  |  |  |
| GO:0046967\_cytosol\_to\_ER\_transport | 2 | 0 |  |  |  |  |  |  |  |  |
| GO:0046968\_peptide\_antigen\_transport | 2 | 0 |  |  |  |  |  |  |  |  |
| GO:0047496\_vesicle\_transport\_along\_microtubule | 2 | 0 |  |  |  |  |  |  |  |  |
| GO:0048025\_negative\_regulation\_of\_nuclear\_mRNA\_splicing\_\_via\_spliceosome | 2 | 0 |  |  |  |  |  |  |  |  |
| GO:0048102\_autophagic\_cell\_death | 2 | 0 |  |  |  |  |  |  |  |  |
| GO:0048103\_somatic\_stem\_cell\_division | 2 | 0 |  |  |  |  |  |  |  |  |
| GO:0048147\_negative\_regulation\_of\_fibroblast\_proliferation | 2 | 0 |  |  |  |  |  |  |  |  |
| GO:0048149\_behavioral\_response\_to\_ethanol | 2 | 0 |  |  |  |  |  |  |  |  |
| GO:0048165\_fused\_antrum\_stage\_\_oogenesis | 2 | 0 |  |  |  |  |  |  |  |  |
| GO:0048169\_regulation\_of\_long-term\_neuronal\_synaptic\_plasticity | 2 | 0 |  |  |  |  |  |  |  |  |
| GO:0048227\_plasma\_membrane\_to\_endosome\_transport | 2 | 0 |  |  |  |  |  |  |  |  |
| GO:0048290\_isotype\_switching\_to\_IgA\_isotypes | 2 | 0 |  |  |  |  |  |  |  |  |
| GO:0048291\_isotype\_switching\_to\_IgG\_isotypes | 2 | 0 |  |  |  |  |  |  |  |  |
| GO:0048296\_regulation\_of\_isotype\_switching\_to\_IgA\_isotypes | 2 | 0 |  |  |  |  |  |  |  |  |
| GO:0048298\_positive\_regulation\_of\_isotype\_switching\_to\_IgA\_isotypes | 2 | 0 |  |  |  |  |  |  |  |  |
| GO:0048386\_positive\_regulation\_of\_retinoic\_acid\_receptor\_signaling\_pathway | 2 | 0 |  |  |  |  |  |  |  |  |
| GO:0048550\_negative\_regulation\_of\_pinocytosis | 2 | 0 |  |  |  |  |  |  |  |  |
| GO:0048625\_myoblast\_cell\_fate\_commitment | 2 | 0 |  |  |  |  |  |  |  |  |
| GO:0048630\_skeletal\_muscle\_tissue\_growth | 2 | 0 |  |  |  |  |  |  |  |  |
| GO:0048708\_astrocyte\_differentiation | 2 | 0 |  |  |  |  |  |  |  |  |
| GO:0048713\_regulation\_of\_oligodendrocyte\_differentiation | 2 | 0 |  |  |  |  |  |  |  |  |
| GO:0048715\_negative\_regulation\_of\_oligodendrocyte\_differentiation | 2 | 0 |  |  |  |  |  |  |  |  |
| GO:0048820\_hair\_follicle\_maturation | 2 | 0 |  |  |  |  |  |  |  |  |
| GO:0048844\_artery\_morphogenesis | 2 | 0 |  |  |  |  |  |  |  |  |
| GO:0048854\_brain\_morphogenesis | 2 | 0 |  |  |  |  |  |  |  |  |
| GO:0048857\_neural\_nucleus\_development | 2 | 0 |  |  |  |  |  |  |  |  |
| GO:0050653\_chondroitin\_sulfate\_proteoglycan\_biosynthetic\_process\_\_polysaccharide\_chain\_biosynthetic\_process | 2 | 0 |  |  |  |  |  |  |  |  |
| GO:0050667\_homocysteine\_metabolic\_process | 2 | 0 |  |  |  |  |  |  |  |  |
| GO:0050711\_negative\_regulation\_of\_interleukin-1\_secretion | 2 | 0 |  |  |  |  |  |  |  |  |
| GO:0050720\_interleukin-1\_beta\_biosynthetic\_process | 2 | 0 |  |  |  |  |  |  |  |  |
| GO:0050805\_negative\_regulation\_of\_synaptic\_transmission | 2 | 0 |  |  |  |  |  |  |  |  |
| GO:0050847\_progesterone\_receptor\_signaling\_pathway | 2 | 0 |  |  |  |  |  |  |  |  |
| GO:0050849\_negative\_regulation\_of\_calcium-mediated\_signaling | 2 | 0 |  |  |  |  |  |  |  |  |
| GO:0050862\_positive\_regulation\_of\_T\_cell\_receptor\_signaling\_pathway | 2 | 0 |  |  |  |  |  |  |  |  |
| GO:0050872\_white\_fat\_cell\_differentiation | 2 | 0 |  |  |  |  |  |  |  |  |
| GO:0050916\_sensory\_perception\_of\_sweet\_taste | 2 | 0 |  |  |  |  |  |  |  |  |
| GO:0050917\_sensory\_perception\_of\_umami\_taste | 2 | 0 |  |  |  |  |  |  |  |  |
| GO:0050919\_negative\_chemotaxis | 2 | 0 |  |  |  |  |  |  |  |  |
| GO:0050931\_pigment\_cell\_differentiation | 2 | 0 |  |  |  |  |  |  |  |  |
| GO:0050982\_detection\_of\_mechanical\_stimulus | 2 | 0 |  |  |  |  |  |  |  |  |
| GO:0051088\_PMA-inducible\_membrane\_protein\_ectodomain\_proteolysis | 2 | 0 |  |  |  |  |  |  |  |  |
| GO:0051097\_negative\_regulation\_of\_helicase\_activity | 2 | 0 |  |  |  |  |  |  |  |  |
| GO:0051124\_synaptic\_growth\_at\_neuromuscular\_junction | 2 | 0 |  |  |  |  |  |  |  |  |
| GO:0051151\_negative\_regulation\_of\_smooth\_muscle\_cell\_differentiation | 2 | 0 |  |  |  |  |  |  |  |  |
| GO:0051176\_positive\_regulation\_of\_sulfur\_metabolic\_process | 2 | 0 |  |  |  |  |  |  |  |  |
| GO:0051225\_spindle\_assembly | 2 | 0 |  |  |  |  |  |  |  |  |
| GO:0051231\_spindle\_elongation | 2 | 0 |  |  |  |  |  |  |  |  |
| GO:0051313\_attachment\_of\_spindle\_microtubules\_to\_chromosome | 2 | 0 |  |  |  |  |  |  |  |  |
| GO:0051386\_regulation\_of\_nerve\_growth\_factor\_receptor\_signaling\_pathway | 2 | 0 |  |  |  |  |  |  |  |  |
| GO:0051387\_negative\_regulation\_of\_nerve\_growth\_factor\_receptor\_signaling\_pathway | 2 | 0 |  |  |  |  |  |  |  |  |
| GO:0051462\_regulation\_of\_cortisol\_secretion | 2 | 0 |  |  |  |  |  |  |  |  |
| GO:0051464\_positive\_regulation\_of\_cortisol\_secretion | 2 | 0 |  |  |  |  |  |  |  |  |
| GO:0051541\_elastin\_metabolic\_process | 2 | 0 |  |  |  |  |  |  |  |  |
| GO:0051547\_regulation\_of\_keratinocyte\_migration | 2 | 0 |  |  |  |  |  |  |  |  |
| GO:0051549\_positive\_regulation\_of\_keratinocyte\_migration | 2 | 0 |  |  |  |  |  |  |  |  |
| GO:0051570\_regulation\_of\_histone\_H3-K9\_methylation | 2 | 0 |  |  |  |  |  |  |  |  |
| GO:0051571\_positive\_regulation\_of\_histone\_H3-K4\_methylation | 2 | 0 |  |  |  |  |  |  |  |  |
| GO:0051573\_negative\_regulation\_of\_histone\_H3-K9\_methylation | 2 | 0 |  |  |  |  |  |  |  |  |
| GO:0051580\_regulation\_of\_neurotransmitter\_uptake | 2 | 0 |  |  |  |  |  |  |  |  |
| GO:0051583\_dopamine\_uptake | 2 | 0 |  |  |  |  |  |  |  |  |
| GO:0051584\_regulation\_of\_dopamine\_uptake | 2 | 0 |  |  |  |  |  |  |  |  |
| GO:0051657\_maintenance\_of\_organelle\_location | 2 | 0 |  |  |  |  |  |  |  |  |
| GO:0051659\_maintenance\_of\_mitochondrion\_location | 2 | 0 |  |  |  |  |  |  |  |  |
| GO:0051665\_membrane\_raft\_localization | 2 | 0 |  |  |  |  |  |  |  |  |
| GO:0051715\_cytolysis\_of\_cells\_of\_another\_organism | 2 | 0 |  |  |  |  |  |  |  |  |
| GO:0051797\_regulation\_of\_hair\_follicle\_development | 2 | 0 |  |  |  |  |  |  |  |  |
| GO:0051798\_positive\_regulation\_of\_hair\_follicle\_development | 2 | 0 |  |  |  |  |  |  |  |  |
| GO:0051801\_cytolysis\_of\_cells\_in\_other\_organism\_during\_symbiotic\_interaction | 2 | 0 |  |  |  |  |  |  |  |  |
| GO:0051818\_disruption\_of\_cells\_of\_other\_organism\_during\_symbiotic\_interaction | 2 | 0 |  |  |  |  |  |  |  |  |
| GO:0051832\_avoidance\_of\_defenses\_of\_other\_organism\_during\_symbiotic\_interaction | 2 | 0 |  |  |  |  |  |  |  |  |
| GO:0051834\_evasion\_or\_tolerance\_of\_defenses\_of\_other\_organism\_during\_symbiotic\_interaction | 2 | 0 |  |  |  |  |  |  |  |  |
| GO:0051883\_killing\_of\_cells\_in\_other\_organism\_during\_symbiotic\_interaction | 2 | 0 |  |  |  |  |  |  |  |  |
| GO:0051890\_regulation\_of\_cardioblast\_differentiation | 2 | 0 |  |  |  |  |  |  |  |  |
| GO:0051891\_positive\_regulation\_of\_cardioblast\_differentiation | 2 | 0 |  |  |  |  |  |  |  |  |
| GO:0051900\_regulation\_of\_mitochondrial\_depolarization | 2 | 0 |  |  |  |  |  |  |  |  |
| GO:0051902\_negative\_regulation\_of\_mitochondrial\_depolarization | 2 | 0 |  |  |  |  |  |  |  |  |
| GO:0051927\_negative\_regulation\_of\_calcium\_ion\_transport\_via\_voltage-gated\_calcium\_channel\_activity | 2 | 0 |  |  |  |  |  |  |  |  |
| GO:0051932\_synaptic\_transmission\_\_GABAergic | 2 | 0 |  |  |  |  |  |  |  |  |
| GO:0051934\_catecholamine\_uptake\_during\_transmission\_of\_nerve\_impulse | 2 | 0 |  |  |  |  |  |  |  |  |
| GO:0051940\_regulation\_of\_catecholamine\_uptake\_during\_transmission\_of\_nerve\_impulse | 2 | 0 |  |  |  |  |  |  |  |  |
| GO:0051967\_negative\_regulation\_of\_synaptic\_transmission\_\_glutamatergic | 2 | 0 |  |  |  |  |  |  |  |  |
| GO:0052025\_modification\_by\_symbiont\_of\_host\_cell\_membrane | 2 | 0 |  |  |  |  |  |  |  |  |
| GO:0052043\_modification\_by\_symbiont\_of\_host\_cellular\_component | 2 | 0 |  |  |  |  |  |  |  |  |
| GO:0052111\_modification\_by\_symbiont\_of\_host\_structure | 2 | 0 |  |  |  |  |  |  |  |  |
| GO:0052173\_response\_to\_defenses\_of\_other\_organism\_during\_symbiotic\_interaction | 2 | 0 |  |  |  |  |  |  |  |  |
| GO:0052185\_modification\_of\_structure\_of\_other\_organism\_during\_symbiotic\_interaction | 2 | 0 |  |  |  |  |  |  |  |  |
| GO:0052188\_modification\_of\_cellular\_component\_in\_other\_organism\_during\_symbiotic\_interaction | 2 | 0 |  |  |  |  |  |  |  |  |
| GO:0052200\_response\_to\_host\_defenses | 2 | 0 |  |  |  |  |  |  |  |  |
| GO:0052331\_hemolysis\_by\_organism\_of\_erythrocytes\_in\_other\_organism\_during\_symbiotic\_interaction | 2 | 0 |  |  |  |  |  |  |  |  |
| GO:0052332\_modification\_by\_organism\_of\_cell\_membrane\_in\_other\_organism\_during\_symbiotic\_interaction | 2 | 0 |  |  |  |  |  |  |  |  |
| GO:0055007\_cardiac\_muscle\_cell\_differentiation | 2 | 0 |  |  |  |  |  |  |  |  |
| GO:0055075\_potassium\_ion\_homeostasis | 2 | 0 |  |  |  |  |  |  |  |  |
| GO:0055090\_acylglycerol\_homeostasis | 2 | 0 |  |  |  |  |  |  |  |  |
| GO:0055098\_response\_to\_low\_density\_lipoprotein\_stimulus | 2 | 0 |  |  |  |  |  |  |  |  |
| GO:0060004\_reflex | 2 | 0 |  |  |  |  |  |  |  |  |
| GO:0060026\_convergent\_extension | 2 | 0 |  |  |  |  |  |  |  |  |
| GO:0060027\_convergent\_extension\_involved\_in\_gastrulation | 2 | 0 |  |  |  |  |  |  |  |  |
| GO:0060033\_anatomical\_structure\_regression | 2 | 0 |  |  |  |  |  |  |  |  |
| GO:0060037\_pharyngeal\_system\_development | 2 | 0 |  |  |  |  |  |  |  |  |
| GO:0060044\_negative\_regulation\_of\_cardiac\_muscle\_cell\_proliferation | 2 | 0 |  |  |  |  |  |  |  |  |
| GO:0060045\_positive\_regulation\_of\_cardiac\_muscle\_cell\_proliferation | 2 | 0 |  |  |  |  |  |  |  |  |
| GO:0060052\_neurofilament\_cytoskeleton\_organization | 2 | 0 |  |  |  |  |  |  |  |  |
| GO:0060056\_mammary\_gland\_involution | 2 | 0 |  |  |  |  |  |  |  |  |
| GO:0060079\_regulation\_of\_excitatory\_postsynaptic\_membrane\_potential | 2 | 0 |  |  |  |  |  |  |  |  |
| GO:0060080\_regulation\_of\_inhibitory\_postsynaptic\_membrane\_potential | 2 | 0 |  |  |  |  |  |  |  |  |
| GO:0060081\_membrane\_hyperpolarization | 2 | 0 |  |  |  |  |  |  |  |  |
| GO:0060087\_relaxation\_of\_vascular\_smooth\_muscle | 2 | 0 |  |  |  |  |  |  |  |  |
| GO:0060117\_auditory\_receptor\_cell\_development | 2 | 0 |  |  |  |  |  |  |  |  |
| GO:0060122\_inner\_ear\_receptor\_stereocilium\_organization | 2 | 0 |  |  |  |  |  |  |  |  |
| GO:0060151\_peroxisome\_localization | 2 | 0 |  |  |  |  |  |  |  |  |
| GO:0060152\_microtubule-based\_peroxisome\_localization | 2 | 0 |  |  |  |  |  |  |  |  |
| GO:0060159\_regulation\_of\_dopamine\_receptor\_signaling\_pathway | 2 | 0 |  |  |  |  |  |  |  |  |
| GO:0060261\_positive\_regulation\_of\_transcription\_initiation\_from\_RNA\_polymerase\_II\_promoter | 2 | 0 |  |  |  |  |  |  |  |  |
| GO:0060264\_regulation\_of\_respiratory\_burst\_during\_acute\_inflammatory\_response | 2 | 0 |  |  |  |  |  |  |  |  |
| GO:0060271\_cilium\_morphogenesis | 2 | 0 |  |  |  |  |  |  |  |  |
| GO:0060272\_embryonic\_skeletal\_joint\_morphogenesis | 2 | 0 |  |  |  |  |  |  |  |  |
| GO:0060285\_ciliary\_cell\_motility | 2 | 0 |  |  |  |  |  |  |  |  |
| GO:0060297\_regulation\_of\_sarcomere\_organization | 2 | 0 |  |  |  |  |  |  |  |  |
| GO:0060337\_type\_I\_interferon-mediated\_signaling\_pathway | 2 | 0 |  |  |  |  |  |  |  |  |
| GO:0060338\_regulation\_of\_type\_I\_interferon-mediated\_signaling\_pathway | 2 | 0 |  |  |  |  |  |  |  |  |
| GO:0060370\_susceptibility\_to\_T\_cell\_mediated\_cytotoxicity | 2 | 0 |  |  |  |  |  |  |  |  |
| GO:0060443\_mammary\_gland\_morphogenesis | 2 | 0 |  |  |  |  |  |  |  |  |
| GO:0060457\_negative\_regulation\_of\_digestive\_system\_process | 2 | 0 |  |  |  |  |  |  |  |  |
| GO:0060544\_regulation\_of\_necroptosis | 2 | 0 |  |  |  |  |  |  |  |  |
| GO:0060545\_positive\_regulation\_of\_necroptosis | 2 | 0 |  |  |  |  |  |  |  |  |
| GO:0060553\_induction\_of\_necroptosis | 2 | 0 |  |  |  |  |  |  |  |  |
| GO:0060555\_induction\_of\_necroptosis\_by\_extracellular\_signals | 2 | 0 |  |  |  |  |  |  |  |  |
| GO:0060556\_regulation\_of\_vitamin\_D\_biosynthetic\_process | 2 | 0 |  |  |  |  |  |  |  |  |
| GO:0060557\_positive\_regulation\_of\_vitamin\_D\_biosynthetic\_process | 2 | 0 |  |  |  |  |  |  |  |  |
| GO:0060584\_regulation\_of\_prostaglandin-endoperoxide\_synthase\_activity | 2 | 0 |  |  |  |  |  |  |  |  |
| GO:0060585\_positive\_regulation\_of\_prostaglandin-endoperoxidase\_synthase\_activity | 2 | 0 |  |  |  |  |  |  |  |  |
| GO:0060620\_regulation\_of\_cholesterol\_import | 2 | 0 |  |  |  |  |  |  |  |  |
| GO:0060621\_negative\_regulation\_of\_cholesterol\_import | 2 | 0 |  |  |  |  |  |  |  |  |
| GO:0060696\_regulation\_of\_phospholipid\_catabolic\_process | 2 | 0 |  |  |  |  |  |  |  |  |
| GO:0060752\_intestinal\_phytosterol\_absorption | 2 | 0 |  |  |  |  |  |  |  |  |
| GO:0060759\_regulation\_of\_response\_to\_cytokine\_stimulus | 2 | 0 |  |  |  |  |  |  |  |  |
| GO:0060840\_artery\_development | 2 | 0 |  |  |  |  |  |  |  |  |
| GO:0070059\_apoptosis\_in\_response\_to\_endoplasmic\_reticulum\_stress | 2 | 0 |  |  |  |  |  |  |  |  |
| GO:0070070\_proton-transporting\_V-type\_ATPase\_complex\_assembly | 2 | 0 |  |  |  |  |  |  |  |  |
| GO:0070072\_vacuolar\_proton-transporting\_V-type\_ATPase\_complex\_assembly | 2 | 0 |  |  |  |  |  |  |  |  |
| GO:0070166\_enamel\_mineralization | 2 | 0 |  |  |  |  |  |  |  |  |
| GO:0070170\_regulation\_of\_tooth\_mineralization | 2 | 0 |  |  |  |  |  |  |  |  |
| GO:0070230\_positive\_regulation\_of\_lymphocyte\_apoptosis | 2 | 0 |  |  |  |  |  |  |  |  |
| GO:0070266\_necroptosis | 2 | 0 |  |  |  |  |  |  |  |  |
| GO:0070483\_detection\_of\_hypoxia | 2 | 0 |  |  |  |  |  |  |  |  |
| GO:0070493\_thrombin\_receptor\_signaling\_pathway | 2 | 0 |  |  |  |  |  |  |  |  |
| GO:0070536\_protein\_K63-linked\_deubiquitination | 2 | 0 |  |  |  |  |  |  |  |  |
| GO:0070574\_cadmium\_ion\_transmembrane\_transport | 2 | 0 |  |  |  |  |  |  |  |  |
| GO:0070586\_cell-cell\_adhesion\_involved\_in\_gastrulation | 2 | 0 |  |  |  |  |  |  |  |  |
| GO:0070587\_regulation\_of\_cell-cell\_adhesion\_involved\_in\_gastrulation | 2 | 0 |  |  |  |  |  |  |  |  |
| GO:0070637\_pyridine\_nucleoside\_metabolic\_process | 2 | 0 |  |  |  |  |  |  |  |  |
| GO:0070638\_pyridine\_nucleoside\_catabolic\_process | 2 | 0 |  |  |  |  |  |  |  |  |
| GO:0070874\_negative\_regulation\_of\_glycogen\_metabolic\_process | 2 | 0 |  |  |  |  |  |  |  |  |
| GO:0075136\_response\_to\_host | 2 | 0 |  |  |  |  |  |  |  |  |
| GO:0006820\_anion\_transport | 59 | 0 | 0.000000 | 0.000000 | 714 | 446.819975 | 525.64 | 604.460025 | 0.736190 |
| GO:0007409\_axonogenesis | 59 | 0 | 0.000000 | 0.000000 | 714 | 446.819975 | 525.64 | 604.460025 | 0.736190 |
| GO:0033673\_negative\_regulation\_of\_kinase\_activity | 59 | 0 | 0.000000 | 0.000000 | 714 | 446.819975 | 525.64 | 604.460025 | 0.736190 |
| GO:0043281\_regulation\_of\_caspase\_activity | 59 | 0 | 0.000000 | 0.000000 | 714 | 446.819975 | 525.64 | 604.460025 | 0.736190 |
| GO:0043408\_regulation\_of\_MAPKKK\_cascade | 59 | 0 | 0.000000 | 0.000000 | 714 | 446.819975 | 525.64 | 604.460025 | 0.736190 |
| GO:0001822\_kidney\_development | 20 | 0 | 0.000000 | 0.000000 | 756 | 490.115406 | 567.53 | 644.944594 | 0.750701 |
| GO:0002703\_regulation\_of\_leukocyte\_mediated\_immunity | 20 | 0 | 0.000000 | 0.000000 | 756 | 490.115406 | 567.53 | 644.944594 | 0.750701 |
| GO:0002706\_regulation\_of\_lymphocyte\_mediated\_immunity | 20 | 0 | 0.000000 | 0.000000 | 756 | 490.115406 | 567.53 | 644.944594 | 0.750701 |
| GO:0005977\_glycogen\_metabolic\_process | 20 | 0 | 0.000000 | 0.000000 | 756 | 490.115406 | 567.53 | 644.944594 | 0.750701 |
| GO:0006096\_glycolysis | 20 | 0 | 0.000000 | 0.000000 | 756 | 490.115406 | 567.53 | 644.944594 | 0.750701 |
| GO:0006305\_DNA\_alkylation | 20 | 0 | 0.000000 | 0.000000 | 756 | 490.115406 | 567.53 | 644.944594 | 0.750701 |
| GO:0006306\_DNA\_methylation | 20 | 0 | 0.000000 | 0.000000 | 756 | 490.115406 | 567.53 | 644.944594 | 0.750701 |
| GO:0006733\_oxidoreduction\_coenzyme\_metabolic\_process | 20 | 0 | 0.000000 | 0.000000 | 756 | 490.115406 | 567.53 | 644.944594 | 0.750701 |
| GO:0006885\_regulation\_of\_pH | 20 | 0 | 0.000000 | 0.000000 | 756 | 490.115406 | 567.53 | 644.944594 | 0.750701 |
| GO:0007159\_leukocyte\_adhesion | 20 | 0 | 0.000000 | 0.000000 | 756 | 490.115406 | 567.53 | 644.944594 | 0.750701 |
| GO:0007215\_glutamate\_signaling\_pathway | 20 | 0 | 0.000000 | 0.000000 | 756 | 490.115406 | 567.53 | 644.944594 | 0.750701 |
| GO:0008633\_activation\_of\_pro-apoptotic\_gene\_products | 20 | 0 | 0.000000 | 0.000000 | 756 | 490.115406 | 567.53 | 644.944594 | 0.750701 |
| GO:0009064\_glutamine\_family\_amino\_acid\_metabolic\_process | 20 | 0 | 0.000000 | 0.000000 | 756 | 490.115406 | 567.53 | 644.944594 | 0.750701 |
| GO:0009144\_purine\_nucleoside\_triphosphate\_metabolic\_process | 20 | 0 | 0.000000 | 0.000000 | 756 | 490.115406 | 567.53 | 644.944594 | 0.750701 |
| GO:0009205\_purine\_ribonucleoside\_triphosphate\_metabolic\_process | 20 | 0 | 0.000000 | 0.000000 | 756 | 490.115406 | 567.53 | 644.944594 | 0.750701 |
| GO:0009583\_detection\_of\_light\_stimulus | 20 | 0 | 0.000000 | 0.000000 | 756 | 490.115406 | 567.53 | 644.944594 | 0.750701 |
| GO:0009584\_detection\_of\_visible\_light | 20 | 0 | 0.000000 | 0.000000 | 756 | 490.115406 | 567.53 | 644.944594 | 0.750701 |
| GO:0016202\_regulation\_of\_striated\_muscle\_tissue\_development | 20 | 0 | 0.000000 | 0.000000 | 756 | 490.115406 | 567.53 | 644.944594 | 0.750701 |
| GO:0021700\_developmental\_maturation | 20 | 0 | 0.000000 | 0.000000 | 756 | 490.115406 | 567.53 | 644.944594 | 0.750701 |
| GO:0031047\_gene\_silencing\_by\_RNA | 20 | 0 | 0.000000 | 0.000000 | 756 | 490.115406 | 567.53 | 644.944594 | 0.750701 |
| GO:0032409\_regulation\_of\_transporter\_activity | 20 | 0 | 0.000000 | 0.000000 | 756 | 490.115406 | 567.53 | 644.944594 | 0.750701 |
| GO:0032535\_regulation\_of\_cellular\_component\_size | 20 | 0 | 0.000000 | 0.000000 | 756 | 490.115406 | 567.53 | 644.944594 | 0.750701 |
| GO:0033692\_cellular\_polysaccharide\_biosynthetic\_process | 20 | 0 | 0.000000 | 0.000000 | 756 | 490.115406 | 567.53 | 644.944594 | 0.750701 |
| GO:0034367\_macromolecular\_complex\_remodeling | 20 | 0 | 0.000000 | 0.000000 | 756 | 490.115406 | 567.53 | 644.944594 | 0.750701 |
| GO:0034368\_protein-lipid\_complex\_remodeling | 20 | 0 | 0.000000 | 0.000000 | 756 | 490.115406 | 567.53 | 644.944594 | 0.750701 |
| GO:0034369\_plasma\_lipoprotein\_particle\_remodeling | 20 | 0 | 0.000000 | 0.000000 | 756 | 490.115406 | 567.53 | 644.944594 | 0.750701 |
| GO:0042594\_response\_to\_starvation | 20 | 0 | 0.000000 | 0.000000 | 756 | 490.115406 | 567.53 | 644.944594 | 0.750701 |
| GO:0043244\_regulation\_of\_protein\_complex\_disassembly | 20 | 0 | 0.000000 | 0.000000 | 756 | 490.115406 | 567.53 | 644.944594 | 0.750701 |
| GO:0043900\_regulation\_of\_multi-organism\_process | 20 | 0 | 0.000000 | 0.000000 | 756 | 490.115406 | 567.53 | 644.944594 | 0.750701 |
| GO:0045089\_positive\_regulation\_of\_innate\_immune\_response | 20 | 0 | 0.000000 | 0.000000 | 756 | 490.115406 | 567.53 | 644.944594 | 0.750701 |
| GO:0045165\_cell\_fate\_commitment | 20 | 0 | 0.000000 | 0.000000 | 756 | 490.115406 | 567.53 | 644.944594 | 0.750701 |
| GO:0045619\_regulation\_of\_lymphocyte\_differentiation | 20 | 0 | 0.000000 | 0.000000 | 756 | 490.115406 | 567.53 | 644.944594 | 0.750701 |
| GO:0045840\_positive\_regulation\_of\_mitosis | 20 | 0 | 0.000000 | 0.000000 | 756 | 490.115406 | 567.53 | 644.944594 | 0.750701 |
| GO:0046209\_nitric\_oxide\_metabolic\_process | 20 | 0 | 0.000000 | 0.000000 | 756 | 490.115406 | 567.53 | 644.944594 | 0.750701 |
| GO:0046323\_glucose\_import | 20 | 0 | 0.000000 | 0.000000 | 756 | 490.115406 | 567.53 | 644.944594 | 0.750701 |
| GO:0046519\_sphingoid\_metabolic\_process | 20 | 0 | 0.000000 | 0.000000 | 756 | 490.115406 | 567.53 | 644.944594 | 0.750701 |
| GO:0046661\_male\_sex\_differentiation | 20 | 0 | 0.000000 | 0.000000 | 756 | 490.115406 | 567.53 | 644.944594 | 0.750701 |
| GO:0048634\_regulation\_of\_muscle\_development | 20 | 0 | 0.000000 | 0.000000 | 756 | 490.115406 | 567.53 | 644.944594 | 0.750701 |
| GO:0050804\_regulation\_of\_synaptic\_transmission | 20 | 0 | 0.000000 | 0.000000 | 756 | 490.115406 | 567.53 | 644.944594 | 0.750701 |
| GO:0050866\_negative\_regulation\_of\_cell\_activation | 20 | 0 | 0.000000 | 0.000000 | 756 | 490.115406 | 567.53 | 644.944594 | 0.750701 |
| GO:0051320\_S\_phase | 20 | 0 | 0.000000 | 0.000000 | 756 | 490.115406 | 567.53 | 644.944594 | 0.750701 |
| GO:0051785\_positive\_regulation\_of\_nuclear\_division | 20 | 0 | 0.000000 | 0.000000 | 756 | 490.115406 | 567.53 | 644.944594 | 0.750701 |
| GO:0051172\_negative\_regulation\_of\_nitrogen\_compound\_metabolic\_process | 298 | 0 | 0.000000 | 0.000000 | 757 | 490.732193 | 568.07 | 645.407807 | 0.750423 |
| GO:0006575\_cellular\_amino\_acid\_derivative\_metabolic\_process | 76 | 0 | 0.000000 | 0.000000 | 760 | 497.296481 | 573.79 | 650.283519 | 0.754987 |
| GO:0006979\_response\_to\_oxidative\_stress | 76 | 0 | 0.000000 | 0.000000 | 760 | 497.296481 | 573.79 | 650.283519 | 0.754987 |
| GO:0070838\_divalent\_metal\_ion\_transport | 76 | 0 | 0.000000 | 0.000000 | 760 | 497.296481 | 573.79 | 650.283519 | 0.754987 |
| GO:0060249\_anatomical\_structure\_homeostasis | 72 | 0 | 0.000000 | 0.000000 | 761 | 500.522063 | 576.52 | 652.517937 | 0.757582 |
| GO:0008380\_RNA\_splicing | 192 | 0 | 0.000000 | 0.000000 | 762 | 501.953654 | 577.77 | 653.586346 | 0.758228 |
| GO:0010629\_negative\_regulation\_of\_gene\_expression | 289 | 0 | 0.000000 | 0.000000 | 763 | 502.619058 | 578.34 | 654.060942 | 0.757982 |
| GO:0009100\_glycoprotein\_metabolic\_process | 139 | 0 | 0.000000 | 0.000000 | 765 | 504.240734 | 579.88 | 655.519266 | 0.758013 |
| GO:0048534\_hemopoietic\_or\_lymphoid\_organ\_development | 139 | 0 | 0.000000 | 0.000000 | 765 | 504.240734 | 579.88 | 655.519266 | 0.758013 |
| GO:0000902\_cell\_morphogenesis | 144 | 0 | 0.000000 | 0.000000 | 766 | 506.055874 | 581.41 | 656.764126 | 0.759021 |
| GO:0051707\_response\_to\_other\_organism | 125 | 0 | 0.000000 | 0.000000 | 768 | 510.969599 | 585.62 | 660.270401 | 0.762526 |
| GO:0055065\_metal\_ion\_homeostasis | 125 | 0 | 0.000000 | 0.000000 | 768 | 510.969599 | 585.62 | 660.270401 | 0.762526 |
| GO:0000087\_M\_phase\_of\_mitotic\_cell\_cycle | 118 | 0 | 0.000000 | 0.000000 | 770 | 513.544752 | 588.19 | 662.835248 | 0.763883 |
| GO:0048285\_organelle\_fission | 118 | 0 | 0.000000 | 0.000000 | 770 | 513.544752 | 588.19 | 662.835248 | 0.763883 |
| GO:0008654\_phospholipid\_biosynthetic\_process | 63 | 0 | 0.000000 | 0.000000 | 774 | 517.206076 | 591.73 | 666.253924 | 0.764509 |
| GO:0030522\_intracellular\_receptor-mediated\_signaling\_pathway | 63 | 0 | 0.000000 | 0.000000 | 774 | 517.206076 | 591.73 | 666.253924 | 0.764509 |
| GO:0042445\_hormone\_metabolic\_process | 63 | 0 | 0.000000 | 0.000000 | 774 | 517.206076 | 591.73 | 666.253924 | 0.764509 |
| GO:0051348\_negative\_regulation\_of\_transferase\_activity | 63 | 0 | 0.000000 | 0.000000 | 774 | 517.206076 | 591.73 | 666.253924 | 0.764509 |
| GO:0000956\_nuclear-transcribed\_mRNA\_catabolic\_process | 15 | 0 | 0.000000 | 0.000000 | 831 | 579.484636 | 652.77 | 726.055364 | 0.785523 |
| GO:0001701\_in\_utero\_embryonic\_development | 15 | 0 | 0.000000 | 0.000000 | 831 | 579.484636 | 652.77 | 726.055364 | 0.785523 |
| GO:0006611\_protein\_export\_from\_nucleus | 15 | 0 | 0.000000 | 0.000000 | 831 | 579.484636 | 652.77 | 726.055364 | 0.785523 |
| GO:0006612\_protein\_targeting\_to\_membrane | 15 | 0 | 0.000000 | 0.000000 | 831 | 579.484636 | 652.77 | 726.055364 | 0.785523 |
| GO:0006692\_prostanoid\_metabolic\_process | 15 | 0 | 0.000000 | 0.000000 | 831 | 579.484636 | 652.77 | 726.055364 | 0.785523 |
| GO:0006693\_prostaglandin\_metabolic\_process | 15 | 0 | 0.000000 | 0.000000 | 831 | 579.484636 | 652.77 | 726.055364 | 0.785523 |
| GO:0006942\_regulation\_of\_striated\_muscle\_contraction | 15 | 0 | 0.000000 | 0.000000 | 831 | 579.484636 | 652.77 | 726.055364 | 0.785523 |
| GO:0006956\_complement\_activation | 15 | 0 | 0.000000 | 0.000000 | 831 | 579.484636 | 652.77 | 726.055364 | 0.785523 |
| GO:0007043\_cell-cell\_junction\_assembly | 15 | 0 | 0.000000 | 0.000000 | 831 | 579.484636 | 652.77 | 726.055364 | 0.785523 |
| GO:0007098\_centrosome\_cycle | 15 | 0 | 0.000000 | 0.000000 | 831 | 579.484636 | 652.77 | 726.055364 | 0.785523 |
| GO:0007219\_Notch\_signaling\_pathway | 15 | 0 | 0.000000 | 0.000000 | 831 | 579.484636 | 652.77 | 726.055364 | 0.785523 |
| GO:0007369\_gastrulation | 15 | 0 | 0.000000 | 0.000000 | 831 | 579.484636 | 652.77 | 726.055364 | 0.785523 |
| GO:0007569\_cell\_aging | 15 | 0 | 0.000000 | 0.000000 | 831 | 579.484636 | 652.77 | 726.055364 | 0.785523 |
| GO:0007585\_respiratory\_gaseous\_exchange | 15 | 0 | 0.000000 | 0.000000 | 831 | 579.484636 | 652.77 | 726.055364 | 0.785523 |
| GO:0007589\_body\_fluid\_secretion | 15 | 0 | 0.000000 | 0.000000 | 831 | 579.484636 | 652.77 | 726.055364 | 0.785523 |
| GO:0008584\_male\_gonad\_development | 15 | 0 | 0.000000 | 0.000000 | 831 | 579.484636 | 652.77 | 726.055364 | 0.785523 |
| GO:0008637\_apoptotic\_mitochondrial\_changes | 15 | 0 | 0.000000 | 0.000000 | 831 | 579.484636 | 652.77 | 726.055364 | 0.785523 |
| GO:0009060\_aerobic\_respiration | 15 | 0 | 0.000000 | 0.000000 | 831 | 579.484636 | 652.77 | 726.055364 | 0.785523 |
| GO:0009311\_oligosaccharide\_metabolic\_process | 15 | 0 | 0.000000 | 0.000000 | 831 | 579.484636 | 652.77 | 726.055364 | 0.785523 |
| GO:0009820\_alkaloid\_metabolic\_process | 15 | 0 | 0.000000 | 0.000000 | 831 | 579.484636 | 652.77 | 726.055364 | 0.785523 |
| GO:0009952\_anterior\_posterior\_pattern\_formation | 15 | 0 | 0.000000 | 0.000000 | 831 | 579.484636 | 652.77 | 726.055364 | 0.785523 |
| GO:0010720\_positive\_regulation\_of\_cell\_development | 15 | 0 | 0.000000 | 0.000000 | 831 | 579.484636 | 652.77 | 726.055364 | 0.785523 |
| GO:0010927\_cellular\_component\_assembly\_involved\_in\_morphogenesis | 15 | 0 | 0.000000 | 0.000000 | 831 | 579.484636 | 652.77 | 726.055364 | 0.785523 |
| GO:0016441\_posttranscriptional\_gene\_silencing | 15 | 0 | 0.000000 | 0.000000 | 831 | 579.484636 | 652.77 | 726.055364 | 0.785523 |
| GO:0016571\_histone\_methylation | 15 | 0 | 0.000000 | 0.000000 | 831 | 579.484636 | 652.77 | 726.055364 | 0.785523 |
| GO:0019321\_pentose\_metabolic\_process | 15 | 0 | 0.000000 | 0.000000 | 831 | 579.484636 | 652.77 | 726.055364 | 0.785523 |
| GO:0019724\_B\_cell\_mediated\_immunity | 15 | 0 | 0.000000 | 0.000000 | 831 | 579.484636 | 652.77 | 726.055364 | 0.785523 |
| GO:0022407\_regulation\_of\_cell-cell\_adhesion | 15 | 0 | 0.000000 | 0.000000 | 831 | 579.484636 | 652.77 | 726.055364 | 0.785523 |
| GO:0022898\_regulation\_of\_transmembrane\_transporter\_activity | 15 | 0 | 0.000000 | 0.000000 | 831 | 579.484636 | 652.77 | 726.055364 | 0.785523 |
| GO:0030101\_natural\_killer\_cell\_activation | 15 | 0 | 0.000000 | 0.000000 | 831 | 579.484636 | 652.77 | 726.055364 | 0.785523 |
| GO:0030512\_negative\_regulation\_of\_transforming\_growth\_factor\_beta\_receptor\_signaling\_pathway | 15 | 0 | 0.000000 | 0.000000 | 831 | 579.484636 | 652.77 | 726.055364 | 0.785523 |
| GO:0030641\_regulation\_of\_cellular\_pH | 15 | 0 | 0.000000 | 0.000000 | 831 | 579.484636 | 652.77 | 726.055364 | 0.785523 |
| GO:0031341\_regulation\_of\_cell\_killing | 15 | 0 | 0.000000 | 0.000000 | 831 | 579.484636 | 652.77 | 726.055364 | 0.785523 |
| GO:0031346\_positive\_regulation\_of\_cell\_projection\_organization | 15 | 0 | 0.000000 | 0.000000 | 831 | 579.484636 | 652.77 | 726.055364 | 0.785523 |
| GO:0032387\_negative\_regulation\_of\_intracellular\_transport | 15 | 0 | 0.000000 | 0.000000 | 831 | 579.484636 | 652.77 | 726.055364 | 0.785523 |
| GO:0032663\_regulation\_of\_interleukin-2\_production | 15 | 0 | 0.000000 | 0.000000 | 831 | 579.484636 | 652.77 | 726.055364 | 0.785523 |
| GO:0032677\_regulation\_of\_interleukin-8\_production | 15 | 0 | 0.000000 | 0.000000 | 831 | 579.484636 | 652.77 | 726.055364 | 0.785523 |
| GO:0033261\_regulation\_of\_S\_phase | 15 | 0 | 0.000000 | 0.000000 | 831 | 579.484636 | 652.77 | 726.055364 | 0.785523 |
| GO:0035194\_posttranscriptional\_gene\_silencing\_by\_RNA | 15 | 0 | 0.000000 | 0.000000 | 831 | 579.484636 | 652.77 | 726.055364 | 0.785523 |
| GO:0042100\_B\_cell\_proliferation | 15 | 0 | 0.000000 | 0.000000 | 831 | 579.484636 | 652.77 | 726.055364 | 0.785523 |
| GO:0042168\_heme\_metabolic\_process | 15 | 0 | 0.000000 | 0.000000 | 831 | 579.484636 | 652.77 | 726.055364 | 0.785523 |
| GO:0042503\_tyrosine\_phosphorylation\_of\_Stat3\_protein | 15 | 0 | 0.000000 | 0.000000 | 831 | 579.484636 | 652.77 | 726.055364 | 0.785523 |
| GO:0042516\_regulation\_of\_tyrosine\_phosphorylation\_of\_Stat3\_protein | 15 | 0 | 0.000000 | 0.000000 | 831 | 579.484636 | 652.77 | 726.055364 | 0.785523 |
| GO:0043242\_negative\_regulation\_of\_protein\_complex\_disassembly | 15 | 0 | 0.000000 | 0.000000 | 831 | 579.484636 | 652.77 | 726.055364 | 0.785523 |
| GO:0043484\_regulation\_of\_RNA\_splicing | 15 | 0 | 0.000000 | 0.000000 | 831 | 579.484636 | 652.77 | 726.055364 | 0.785523 |
| GO:0043574\_peroxisomal\_transport | 15 | 0 | 0.000000 | 0.000000 | 831 | 579.484636 | 652.77 | 726.055364 | 0.785523 |
| GO:0045739\_positive\_regulation\_of\_DNA\_repair | 15 | 0 | 0.000000 | 0.000000 | 831 | 579.484636 | 652.77 | 726.055364 | 0.785523 |
| GO:0045778\_positive\_regulation\_of\_ossification | 15 | 0 | 0.000000 | 0.000000 | 831 | 579.484636 | 652.77 | 726.055364 | 0.785523 |
| GO:0048146\_positive\_regulation\_of\_fibroblast\_proliferation | 15 | 0 | 0.000000 | 0.000000 | 831 | 579.484636 | 652.77 | 726.055364 | 0.785523 |
| GO:0048515\_spermatid\_differentiation | 15 | 0 | 0.000000 | 0.000000 | 831 | 579.484636 | 652.77 | 726.055364 | 0.785523 |
| GO:0048736\_appendage\_development | 15 | 0 | 0.000000 | 0.000000 | 831 | 579.484636 | 652.77 | 726.055364 | 0.785523 |
| GO:0050796\_regulation\_of\_insulin\_secretion | 15 | 0 | 0.000000 | 0.000000 | 831 | 579.484636 | 652.77 | 726.055364 | 0.785523 |
| GO:0051004\_regulation\_of\_lipoprotein\_lipase\_activity | 15 | 0 | 0.000000 | 0.000000 | 831 | 579.484636 | 652.77 | 726.055364 | 0.785523 |
| GO:0060048\_cardiac\_muscle\_contraction | 15 | 0 | 0.000000 | 0.000000 | 831 | 579.484636 | 652.77 | 726.055364 | 0.785523 |
| GO:0060173\_limb\_development | 15 | 0 | 0.000000 | 0.000000 | 831 | 579.484636 | 652.77 | 726.055364 | 0.785523 |
| GO:0060393\_regulation\_of\_pathway-restricted\_SMAD\_protein\_phosphorylation | 15 | 0 | 0.000000 | 0.000000 | 831 | 579.484636 | 652.77 | 726.055364 | 0.785523 |
| GO:0070169\_positive\_regulation\_of\_biomineral\_formation | 15 | 0 | 0.000000 | 0.000000 | 831 | 579.484636 | 652.77 | 726.055364 | 0.785523 |
| GO:0007204\_elevation\_of\_cytosolic\_calcium\_ion\_concentration | 80 | 0 | 0.000000 | 0.000000 | 833 | 582.356053 | 655.39 | 728.423947 | 0.786783 |
| GO:0031175\_neuron\_projection\_development | 80 | 0 | 0.000000 | 0.000000 | 833 | 582.356053 | 655.39 | 728.423947 | 0.786783 |
| GO:0006413\_translational\_initiation | 46 | 0 | 0.000000 | 0.000000 | 845 | 598.064023 | 670.29 | 742.515977 | 0.793243 |
| GO:0007202\_activation\_of\_phospholipase\_C\_activity | 46 | 0 | 0.000000 | 0.000000 | 845 | 598.064023 | 670.29 | 742.515977 | 0.793243 |
| GO:0007588\_excretion | 46 | 0 | 0.000000 | 0.000000 | 845 | 598.064023 | 670.29 | 742.515977 | 0.793243 |
| GO:0010638\_positive\_regulation\_of\_organelle\_organization | 46 | 0 | 0.000000 | 0.000000 | 845 | 598.064023 | 670.29 | 742.515977 | 0.793243 |
| GO:0010639\_negative\_regulation\_of\_organelle\_organization | 46 | 0 | 0.000000 | 0.000000 | 845 | 598.064023 | 670.29 | 742.515977 | 0.793243 |
| GO:0010863\_positive\_regulation\_of\_phospholipase\_C\_activity | 46 | 0 | 0.000000 | 0.000000 | 845 | 598.064023 | 670.29 | 742.515977 | 0.793243 |
| GO:0030384\_phosphoinositide\_metabolic\_process | 46 | 0 | 0.000000 | 0.000000 | 845 | 598.064023 | 670.29 | 742.515977 | 0.793243 |
| GO:0042157\_lipoprotein\_metabolic\_process | 46 | 0 | 0.000000 | 0.000000 | 845 | 598.064023 | 670.29 | 742.515977 | 0.793243 |
| GO:0042254\_ribosome\_biogenesis | 46 | 0 | 0.000000 | 0.000000 | 845 | 598.064023 | 670.29 | 742.515977 | 0.793243 |
| GO:0042391\_regulation\_of\_membrane\_potential | 46 | 0 | 0.000000 | 0.000000 | 845 | 598.064023 | 670.29 | 742.515977 | 0.793243 |
| GO:0046879\_hormone\_secretion | 46 | 0 | 0.000000 | 0.000000 | 845 | 598.064023 | 670.29 | 742.515977 | 0.793243 |
| GO:0065004\_protein-DNA\_complex\_assembly | 46 | 0 | 0.000000 | 0.000000 | 845 | 598.064023 | 670.29 | 742.515977 | 0.793243 |
| GO:0006260\_DNA\_replication | 153 | 0 | 0.000000 | 0.000000 | 846 | 599.754811 | 671.78 | 743.805189 | 0.794066 |
| GO:0030030\_cell\_projection\_organization | 127 | 0 | 0.000000 | 0.000000 | 847 | 600.620530 | 672.58 | 744.539470 | 0.794073 |
| GO:0051249\_regulation\_of\_lymphocyte\_activation | 60 | 0 | 0.000000 | 0.000000 | 848 | 604.387916 | 675.99 | 747.592084 | 0.797158 |
| GO:0006917\_induction\_of\_apoptosis | 190 | 0 | 0.000000 | 0.000000 | 849 | 605.159210 | 676.63 | 748.100790 | 0.796973 |
| GO:0002526\_acute\_inflammatory\_response | 31 | 0 | 0.000000 | 0.000000 | 861 | 619.706777 | 690.58 | 761.453223 | 0.802067 |
| GO:0006633\_fatty\_acid\_biosynthetic\_process | 31 | 0 | 0.000000 | 0.000000 | 861 | 619.706777 | 690.58 | 761.453223 | 0.802067 |
| GO:0007173\_epidermal\_growth\_factor\_receptor\_signaling\_pathway | 31 | 0 | 0.000000 | 0.000000 | 861 | 619.706777 | 690.58 | 761.453223 | 0.802067 |
| GO:0007200\_activation\_of\_phospholipase\_C\_activity\_by\_G-protein\_coupled\_receptor\_protein\_signaling\_pathway\_coupled\_to\_IP3\_second\_messenger | 31 | 0 | 0.000000 | 0.000000 | 861 | 619.706777 | 690.58 | 761.453223 | 0.802067 |
| GO:0008629\_induction\_of\_apoptosis\_by\_intracellular\_signals | 31 | 0 | 0.000000 | 0.000000 | 861 | 619.706777 | 690.58 | 761.453223 | 0.802067 |
| GO:0031668\_cellular\_response\_to\_extracellular\_stimulus | 31 | 0 | 0.000000 | 0.000000 | 861 | 619.706777 | 690.58 | 761.453223 | 0.802067 |
| GO:0032944\_regulation\_of\_mononuclear\_cell\_proliferation | 31 | 0 | 0.000000 | 0.000000 | 861 | 619.706777 | 690.58 | 761.453223 | 0.802067 |
| GO:0042742\_defense\_response\_to\_bacterium | 31 | 0 | 0.000000 | 0.000000 | 861 | 619.706777 | 690.58 | 761.453223 | 0.802067 |
| GO:0044403\_symbiosis\_\_encompassing\_mutualism\_through\_parasitism | 31 | 0 | 0.000000 | 0.000000 | 861 | 619.706777 | 690.58 | 761.453223 | 0.802067 |
| GO:0044419\_interspecies\_interaction\_between\_organisms | 31 | 0 | 0.000000 | 0.000000 | 861 | 619.706777 | 690.58 | 761.453223 | 0.802067 |
| GO:0046883\_regulation\_of\_hormone\_secretion | 31 | 0 | 0.000000 | 0.000000 | 861 | 619.706777 | 690.58 | 761.453223 | 0.802067 |
| GO:0070663\_regulation\_of\_leukocyte\_proliferation | 31 | 0 | 0.000000 | 0.000000 | 861 | 619.706777 | 690.58 | 761.453223 | 0.802067 |
| GO:0001655\_urogenital\_system\_development | 23 | 0 | 0.000000 | 0.000000 | 885 | 643.177335 | 713.52 | 783.862665 | 0.806237 |
| GO:0001906\_cell\_killing | 23 | 0 | 0.000000 | 0.000000 | 885 | 643.177335 | 713.52 | 783.862665 | 0.806237 |
| GO:0006023\_aminoglycan\_biosynthetic\_process | 23 | 0 | 0.000000 | 0.000000 | 885 | 643.177335 | 713.52 | 783.862665 | 0.806237 |
| GO:0006509\_membrane\_protein\_ectodomain\_proteolysis | 23 | 0 | 0.000000 | 0.000000 | 885 | 643.177335 | 713.52 | 783.862665 | 0.806237 |
| GO:0006641\_triglyceride\_metabolic\_process | 23 | 0 | 0.000000 | 0.000000 | 885 | 643.177335 | 713.52 | 783.862665 | 0.806237 |
| GO:0007190\_activation\_of\_adenylate\_cyclase\_activity | 23 | 0 | 0.000000 | 0.000000 | 885 | 643.177335 | 713.52 | 783.862665 | 0.806237 |
| GO:0007218\_neuropeptide\_signaling\_pathway | 23 | 0 | 0.000000 | 0.000000 | 885 | 643.177335 | 713.52 | 783.862665 | 0.806237 |
| GO:0007623\_circadian\_rhythm | 23 | 0 | 0.000000 | 0.000000 | 885 | 643.177335 | 713.52 | 783.862665 | 0.806237 |
| GO:0016579\_protein\_deubiquitination | 23 | 0 | 0.000000 | 0.000000 | 885 | 643.177335 | 713.52 | 783.862665 | 0.806237 |
| GO:0018130\_heterocycle\_biosynthetic\_process | 23 | 0 | 0.000000 | 0.000000 | 885 | 643.177335 | 713.52 | 783.862665 | 0.806237 |
| GO:0030166\_proteoglycan\_biosynthetic\_process | 23 | 0 | 0.000000 | 0.000000 | 885 | 643.177335 | 713.52 | 783.862665 | 0.806237 |
| GO:0033344\_cholesterol\_efflux | 23 | 0 | 0.000000 | 0.000000 | 885 | 643.177335 | 713.52 | 783.862665 | 0.806237 |
| GO:0033619\_membrane\_protein\_proteolysis | 23 | 0 | 0.000000 | 0.000000 | 885 | 643.177335 | 713.52 | 783.862665 | 0.806237 |
| GO:0034637\_cellular\_carbohydrate\_biosynthetic\_process | 23 | 0 | 0.000000 | 0.000000 | 885 | 643.177335 | 713.52 | 783.862665 | 0.806237 |
| GO:0043473\_pigmentation | 23 | 0 | 0.000000 | 0.000000 | 885 | 643.177335 | 713.52 | 783.862665 | 0.806237 |
| GO:0043627\_response\_to\_estrogen\_stimulus | 23 | 0 | 0.000000 | 0.000000 | 885 | 643.177335 | 713.52 | 783.862665 | 0.806237 |
| GO:0045740\_positive\_regulation\_of\_DNA\_replication | 23 | 0 | 0.000000 | 0.000000 | 885 | 643.177335 | 713.52 | 783.862665 | 0.806237 |
| GO:0046467\_membrane\_lipid\_biosynthetic\_process | 23 | 0 | 0.000000 | 0.000000 | 885 | 643.177335 | 713.52 | 783.862665 | 0.806237 |
| GO:0048871\_multicellular\_organismal\_homeostasis | 23 | 0 | 0.000000 | 0.000000 | 885 | 643.177335 | 713.52 | 783.862665 | 0.806237 |
| GO:0050671\_positive\_regulation\_of\_lymphocyte\_proliferation | 23 | 0 | 0.000000 | 0.000000 | 885 | 643.177335 | 713.52 | 783.862665 | 0.806237 |
| GO:0050707\_regulation\_of\_cytokine\_secretion | 23 | 0 | 0.000000 | 0.000000 | 885 | 643.177335 | 713.52 | 783.862665 | 0.806237 |
| GO:0051262\_protein\_tetramerization | 23 | 0 | 0.000000 | 0.000000 | 885 | 643.177335 | 713.52 | 783.862665 | 0.806237 |
| GO:0051353\_positive\_regulation\_of\_oxidoreductase\_activity | 23 | 0 | 0.000000 | 0.000000 | 885 | 643.177335 | 713.52 | 783.862665 | 0.806237 |
| GO:0070668\_positive\_regulation\_of\_mast\_cell\_proliferation | 23 | 0 | 0.000000 | 0.000000 | 885 | 643.177335 | 713.52 | 783.862665 | 0.806237 |
| GO:0042110\_T\_cell\_activation | 83 | 0 | 0.000000 | 0.000000 | 888 | 645.830103 | 715.97 | 786.109897 | 0.806273 |
| GO:0051259\_protein\_oligomerization | 83 | 0 | 0.000000 | 0.000000 | 888 | 645.830103 | 715.97 | 786.109897 | 0.806273 |
| GO:0051329\_interphase\_of\_mitotic\_cell\_cycle | 83 | 0 | 0.000000 | 0.000000 | 888 | 645.830103 | 715.97 | 786.109897 | 0.806273 |
| GO:0009101\_glycoprotein\_biosynthetic\_process | 109 | 0 | 0.000000 | 0.000000 | 890 | 648.506368 | 718.52 | 788.533632 | 0.807326 |
| GO:0010817\_regulation\_of\_hormone\_levels | 109 | 0 | 0.000000 | 0.000000 | 890 | 648.506368 | 718.52 | 788.533632 | 0.807326 |
| GO:0006066\_alcohol\_metabolic\_process | 206 | 0 | 0.000000 | 0.000000 | 892 | 653.753107 | 723.41 | 793.066893 | 0.810998 |
| GO:0006873\_cellular\_ion\_homeostasis | 206 | 0 | 0.000000 | 0.000000 | 892 | 653.753107 | 723.41 | 793.066893 | 0.810998 |
| GO:0042592\_homeostatic\_process | 397 | 0 | 0.000000 | 0.000000 | 893 | 655.157221 | 724.51 | 793.862779 | 0.811321 |
| GO:0051254\_positive\_regulation\_of\_RNA\_metabolic\_process | 213 | 0 | 0.000000 | 0.000000 | 895 | 657.669205 | 726.6 | 795.530795 | 0.811844 |
| GO:0051726\_regulation\_of\_cell\_cycle | 213 | 0 | 0.000000 | 0.000000 | 895 | 657.669205 | 726.6 | 795.530795 | 0.811844 |
| GO:0015672\_monovalent\_inorganic\_cation\_transport | 129 | 0 | 0.000000 | 0.000000 | 896 | 661.109390 | 729.72 | 798.330610 | 0.814420 |
| GO:0006812\_cation\_transport | 246 | 0 | 0.000000 | 0.000000 | 898 | 663.278948 | 731.48 | 799.681052 | 0.814566 |
| GO:0043068\_positive\_regulation\_of\_programmed\_cell\_death | 246 | 0 | 0.000000 | 0.000000 | 898 | 663.278948 | 731.48 | 799.681052 | 0.814566 |
| GO:0042180\_cellular\_ketone\_metabolic\_process | 291 | 0 | 0.000000 | 0.000000 | 899 | 666.901716 | 734.64 | 802.378284 | 0.817175 |
| GO:0046486\_glycerolipid\_metabolic\_process | 97 | 0 | 0.000000 | 0.000000 | 900 | 669.382879 | 736.82 | 804.257121 | 0.818689 |
| GO:0002520\_immune\_system\_development | 147 | 0 | 0.000000 | 0.000000 | 901 | 671.580774 | 738.64 | 805.699226 | 0.819800 |
| GO:0000082\_G1\_S\_transition\_of\_mitotic\_cell\_cycle | 36 | 0 | 0.000000 | 0.000000 | 908 | 679.424110 | 746.11 | 812.795890 | 0.821707 |
| GO:0000910\_cytokinesis | 36 | 0 | 0.000000 | 0.000000 | 908 | 679.424110 | 746.11 | 812.795890 | 0.821707 |
| GO:0006334\_nucleosome\_assembly | 36 | 0 | 0.000000 | 0.000000 | 908 | 679.424110 | 746.11 | 812.795890 | 0.821707 |
| GO:0006814\_sodium\_ion\_transport | 36 | 0 | 0.000000 | 0.000000 | 908 | 679.424110 | 746.11 | 812.795890 | 0.821707 |
| GO:0009566\_fertilization | 36 | 0 | 0.000000 | 0.000000 | 908 | 679.424110 | 746.11 | 812.795890 | 0.821707 |
| GO:0034097\_response\_to\_cytokine\_stimulus | 36 | 0 | 0.000000 | 0.000000 | 908 | 679.424110 | 746.11 | 812.795890 | 0.821707 |
| GO:0034103\_regulation\_of\_tissue\_remodeling | 36 | 0 | 0.000000 | 0.000000 | 908 | 679.424110 | 746.11 | 812.795890 | 0.821707 |
| GO:0000302\_response\_to\_reactive\_oxygen\_species | 21 | 0 | 0.000000 | 0.000000 | 951 | 725.662352 | 790.81 | 855.957648 | 0.831556 |
| GO:0000718\_nucleotide-excision\_repair\_\_DNA\_damage\_removal | 21 | 0 | 0.000000 | 0.000000 | 951 | 725.662352 | 790.81 | 855.957648 | 0.831556 |
| GO:0002819\_regulation\_of\_adaptive\_immune\_response | 21 | 0 | 0.000000 | 0.000000 | 951 | 725.662352 | 790.81 | 855.957648 | 0.831556 |
| GO:0002822\_regulation\_of\_adaptive\_immune\_response\_based\_on\_somatic\_recombination\_of\_immune\_receptors\_built\_from\_immunoglobulin\_superfamily\_domains | 21 | 0 | 0.000000 | 0.000000 | 951 | 725.662352 | 790.81 | 855.957648 | 0.831556 |
| GO:0002831\_regulation\_of\_response\_to\_biotic\_stimulus | 21 | 0 | 0.000000 | 0.000000 | 951 | 725.662352 | 790.81 | 855.957648 | 0.831556 |
| GO:0006024\_glycosaminoglycan\_biosynthetic\_process | 21 | 0 | 0.000000 | 0.000000 | 951 | 725.662352 | 790.81 | 855.957648 | 0.831556 |
| GO:0006040\_amino\_sugar\_metabolic\_process | 21 | 0 | 0.000000 | 0.000000 | 951 | 725.662352 | 790.81 | 855.957648 | 0.831556 |
| GO:0006073\_cellular\_glucan\_metabolic\_process | 21 | 0 | 0.000000 | 0.000000 | 951 | 725.662352 | 790.81 | 855.957648 | 0.831556 |
| GO:0006109\_regulation\_of\_carbohydrate\_metabolic\_process | 21 | 0 | 0.000000 | 0.000000 | 951 | 725.662352 | 790.81 | 855.957648 | 0.831556 |
| GO:0006282\_regulation\_of\_DNA\_repair | 21 | 0 | 0.000000 | 0.000000 | 951 | 725.662352 | 790.81 | 855.957648 | 0.831556 |
| GO:0006284\_base-excision\_repair | 21 | 0 | 0.000000 | 0.000000 | 951 | 725.662352 | 790.81 | 855.957648 | 0.831556 |
| GO:0006402\_mRNA\_catabolic\_process | 21 | 0 | 0.000000 | 0.000000 | 951 | 725.662352 | 790.81 | 855.957648 | 0.831556 |
| GO:0006493\_protein\_amino\_acid\_O-linked\_glycosylation | 21 | 0 | 0.000000 | 0.000000 | 951 | 725.662352 | 790.81 | 855.957648 | 0.831556 |
| GO:0006664\_glycolipid\_metabolic\_process | 21 | 0 | 0.000000 | 0.000000 | 951 | 725.662352 | 790.81 | 855.957648 | 0.831556 |
| GO:0006775\_fat-soluble\_vitamin\_metabolic\_process | 21 | 0 | 0.000000 | 0.000000 | 951 | 725.662352 | 790.81 | 855.957648 | 0.831556 |
| GO:0006892\_post-Golgi\_vesicle-mediated\_transport | 21 | 0 | 0.000000 | 0.000000 | 951 | 725.662352 | 790.81 | 855.957648 | 0.831556 |
| GO:0006903\_vesicle\_targeting | 21 | 0 | 0.000000 | 0.000000 | 951 | 725.662352 | 790.81 | 855.957648 | 0.831556 |
| GO:0007292\_female\_gamete\_generation | 21 | 0 | 0.000000 | 0.000000 | 951 | 725.662352 | 790.81 | 855.957648 | 0.831556 |
| GO:0008156\_negative\_regulation\_of\_DNA\_replication | 21 | 0 | 0.000000 | 0.000000 | 951 | 725.662352 | 790.81 | 855.957648 | 0.831556 |
| GO:0008360\_regulation\_of\_cell\_shape | 21 | 0 | 0.000000 | 0.000000 | 951 | 725.662352 | 790.81 | 855.957648 | 0.831556 |
| GO:0009199\_ribonucleoside\_triphosphate\_metabolic\_process | 21 | 0 | 0.000000 | 0.000000 | 951 | 725.662352 | 790.81 | 855.957648 | 0.831556 |
| GO:0010675\_regulation\_of\_cellular\_carbohydrate\_metabolic\_process | 21 | 0 | 0.000000 | 0.000000 | 951 | 725.662352 | 790.81 | 855.957648 | 0.831556 |
| GO:0014031\_mesenchymal\_cell\_development | 21 | 0 | 0.000000 | 0.000000 | 951 | 725.662352 | 790.81 | 855.957648 | 0.831556 |
| GO:0016338\_calcium-independent\_cell-cell\_adhesion | 21 | 0 | 0.000000 | 0.000000 | 951 | 725.662352 | 790.81 | 855.957648 | 0.831556 |
| GO:0030048\_actin\_filament-based\_movement | 21 | 0 | 0.000000 | 0.000000 | 951 | 725.662352 | 790.81 | 855.957648 | 0.831556 |
| GO:0030111\_regulation\_of\_Wnt\_receptor\_signaling\_pathway | 21 | 0 | 0.000000 | 0.000000 | 951 | 725.662352 | 790.81 | 855.957648 | 0.831556 |
| GO:0030148\_sphingolipid\_biosynthetic\_process | 21 | 0 | 0.000000 | 0.000000 | 951 | 725.662352 | 790.81 | 855.957648 | 0.831556 |
| GO:0032768\_regulation\_of\_monooxygenase\_activity | 21 | 0 | 0.000000 | 0.000000 | 951 | 725.662352 | 790.81 | 855.957648 | 0.831556 |
| GO:0032886\_regulation\_of\_microtubule-based\_process | 21 | 0 | 0.000000 | 0.000000 | 951 | 725.662352 | 790.81 | 855.957648 | 0.831556 |
| GO:0035295\_tube\_development | 21 | 0 | 0.000000 | 0.000000 | 951 | 725.662352 | 790.81 | 855.957648 | 0.831556 |
| GO:0042398\_cellular\_amino\_acid\_derivative\_biosynthetic\_process | 21 | 0 | 0.000000 | 0.000000 | 951 | 725.662352 | 790.81 | 855.957648 | 0.831556 |
| GO:0042439\_ethanolamine\_and\_derivative\_metabolic\_process | 21 | 0 | 0.000000 | 0.000000 | 951 | 725.662352 | 790.81 | 855.957648 | 0.831556 |
| GO:0043523\_regulation\_of\_neuron\_apoptosis | 21 | 0 | 0.000000 | 0.000000 | 951 | 725.662352 | 790.81 | 855.957648 | 0.831556 |
| GO:0044042\_glucan\_metabolic\_process | 21 | 0 | 0.000000 | 0.000000 | 951 | 725.662352 | 790.81 | 855.957648 | 0.831556 |
| GO:0045649\_regulation\_of\_macrophage\_differentiation | 21 | 0 | 0.000000 | 0.000000 | 951 | 725.662352 | 790.81 | 855.957648 | 0.831556 |
| GO:0046427\_positive\_regulation\_of\_JAK-STAT\_cascade | 21 | 0 | 0.000000 | 0.000000 | 951 | 725.662352 | 790.81 | 855.957648 | 0.831556 |
| GO:0048762\_mesenchymal\_cell\_differentiation | 21 | 0 | 0.000000 | 0.000000 | 951 | 725.662352 | 790.81 | 855.957648 | 0.831556 |
| GO:0051238\_sequestering\_of\_metal\_ion | 21 | 0 | 0.000000 | 0.000000 | 951 | 725.662352 | 790.81 | 855.957648 | 0.831556 |
| GO:0051297\_centrosome\_organization | 21 | 0 | 0.000000 | 0.000000 | 951 | 725.662352 | 790.81 | 855.957648 | 0.831556 |
| GO:0055072\_iron\_ion\_homeostasis | 21 | 0 | 0.000000 | 0.000000 | 951 | 725.662352 | 790.81 | 855.957648 | 0.831556 |
| GO:0060485\_mesenchyme\_development | 21 | 0 | 0.000000 | 0.000000 | 951 | 725.662352 | 790.81 | 855.957648 | 0.831556 |
| GO:0070167\_regulation\_of\_biomineral\_formation | 21 | 0 | 0.000000 | 0.000000 | 951 | 725.662352 | 790.81 | 855.957648 | 0.831556 |
| GO:0070507\_regulation\_of\_microtubule\_cytoskeleton\_organization | 21 | 0 | 0.000000 | 0.000000 | 951 | 725.662352 | 790.81 | 855.957648 | 0.831556 |
| GO:0019226\_transmission\_of\_nerve\_impulse | 209 | 0 | 0.000000 | 0.000000 | 952 | 730.124876 | 794.71 | 859.295124 | 0.834779 |
| GO:0032940\_secretion\_by\_cell | 155 | 0 | 0.000000 | 0.000000 | 953 | 731.765148 | 796.14 | 860.514852 | 0.835404 |
| GO:0034660\_ncRNA\_metabolic\_process | 89 | 0 | 0.000000 | 0.000000 | 955 | 736.407194 | 800.23 | 864.052806 | 0.837937 |
| GO:0051325\_interphase | 89 | 0 | 0.000000 | 0.000000 | 955 | 736.407194 | 800.23 | 864.052806 | 0.837937 |
| GO:0006082\_organic\_acid\_metabolic\_process | 290 | 0 | 0.000000 | 0.000000 | 956 | 737.830167 | 801.51 | 865.189833 | 0.838400 |
| GO:0009991\_response\_to\_extracellular\_stimulus | 68 | 0 | 0.000000 | 0.000000 | 959 | 743.420281 | 806.81 | 870.199719 | 0.841303 |
| GO:0048858\_cell\_projection\_morphogenesis | 68 | 0 | 0.000000 | 0.000000 | 959 | 743.420281 | 806.81 | 870.199719 | 0.841303 |
| GO:0051439\_regulation\_of\_ubiquitin-protein\_ligase\_activity\_during\_mitotic\_cell\_cycle | 68 | 0 | 0.000000 | 0.000000 | 959 | 743.420281 | 806.81 | 870.199719 | 0.841303 |
| GO:0045087\_innate\_immune\_response | 82 | 0 | 0.000000 | 0.000000 | 960 | 747.033972 | 810.08 | 873.126028 | 0.843833 |
| GO:0031327\_negative\_regulation\_of\_cellular\_biosynthetic\_process | 332 | 0 | 0.000000 | 0.000000 | 961 | 747.651220 | 810.57 | 873.488780 | 0.843465 |
| GO:0000077\_DNA\_damage\_checkpoint | 37 | 0 | 0.000000 | 0.000000 | 972 | 759.384124 | 821.75 | 884.115876 | 0.845422 |
| GO:0002443\_leukocyte\_mediated\_immunity | 37 | 0 | 0.000000 | 0.000000 | 972 | 759.384124 | 821.75 | 884.115876 | 0.845422 |
| GO:0002460\_adaptive\_immune\_response\_based\_on\_somatic\_recombination\_of\_immune\_receptors\_built\_from\_immunoglobulin\_superfamily\_domains | 37 | 0 | 0.000000 | 0.000000 | 972 | 759.384124 | 821.75 | 884.115876 | 0.845422 |
| GO:0006937\_regulation\_of\_muscle\_contraction | 37 | 0 | 0.000000 | 0.000000 | 972 | 759.384124 | 821.75 | 884.115876 | 0.845422 |
| GO:0007584\_response\_to\_nutrient | 37 | 0 | 0.000000 | 0.000000 | 972 | 759.384124 | 821.75 | 884.115876 | 0.845422 |
| GO:0009913\_epidermal\_cell\_differentiation | 37 | 0 | 0.000000 | 0.000000 | 972 | 759.384124 | 821.75 | 884.115876 | 0.845422 |
| GO:0015918\_sterol\_transport | 37 | 0 | 0.000000 | 0.000000 | 972 | 759.384124 | 821.75 | 884.115876 | 0.845422 |
| GO:0030301\_cholesterol\_transport | 37 | 0 | 0.000000 | 0.000000 | 972 | 759.384124 | 821.75 | 884.115876 | 0.845422 |
| GO:0032956\_regulation\_of\_actin\_cytoskeleton\_organization | 37 | 0 | 0.000000 | 0.000000 | 972 | 759.384124 | 821.75 | 884.115876 | 0.845422 |
| GO:0046489\_phosphoinositide\_biosynthetic\_process | 37 | 0 | 0.000000 | 0.000000 | 972 | 759.384124 | 821.75 | 884.115876 | 0.845422 |
| GO:0046700\_heterocycle\_catabolic\_process | 37 | 0 | 0.000000 | 0.000000 | 972 | 759.384124 | 821.75 | 884.115876 | 0.845422 |
| GO:0003006\_reproductive\_developmental\_process | 51 | 0 | 0.000000 | 0.000000 | 980 | 769.026159 | 830.97 | 892.913841 | 0.847929 |
| GO:0006898\_receptor-mediated\_endocytosis | 51 | 0 | 0.000000 | 0.000000 | 980 | 769.026159 | 830.97 | 892.913841 | 0.847929 |
| GO:0007059\_chromosome\_segregation | 51 | 0 | 0.000000 | 0.000000 | 980 | 769.026159 | 830.97 | 892.913841 | 0.847929 |
| GO:0016052\_carbohydrate\_catabolic\_process | 51 | 0 | 0.000000 | 0.000000 | 980 | 769.026159 | 830.97 | 892.913841 | 0.847929 |
| GO:0018212\_peptidyl-tyrosine\_modification | 51 | 0 | 0.000000 | 0.000000 | 980 | 769.026159 | 830.97 | 892.913841 | 0.847929 |
| GO:0030518\_steroid\_hormone\_receptor\_signaling\_pathway | 51 | 0 | 0.000000 | 0.000000 | 980 | 769.026159 | 830.97 | 892.913841 | 0.847929 |
| GO:0032147\_activation\_of\_protein\_kinase\_activity | 51 | 0 | 0.000000 | 0.000000 | 980 | 769.026159 | 830.97 | 892.913841 | 0.847929 |
| GO:0046474\_glycerophospholipid\_biosynthetic\_process | 51 | 0 | 0.000000 | 0.000000 | 980 | 769.026159 | 830.97 | 892.913841 | 0.847929 |
| GO:0006364\_rRNA\_processing | 39 | 0 | 0.000000 | 0.000000 | 990 | 780.540011 | 842.19 | 903.839989 | 0.850697 |
| GO:0006401\_RNA\_catabolic\_process | 39 | 0 | 0.000000 | 0.000000 | 990 | 780.540011 | 842.19 | 903.839989 | 0.850697 |
| GO:0006497\_protein\_amino\_acid\_lipidation | 39 | 0 | 0.000000 | 0.000000 | 990 | 780.540011 | 842.19 | 903.839989 | 0.850697 |
| GO:0009310\_amine\_catabolic\_process | 39 | 0 | 0.000000 | 0.000000 | 990 | 780.540011 | 842.19 | 903.839989 | 0.850697 |
| GO:0030509\_BMP\_signaling\_pathway | 39 | 0 | 0.000000 | 0.000000 | 990 | 780.540011 | 842.19 | 903.839989 | 0.850697 |
| GO:0032970\_regulation\_of\_actin\_filament-based\_process | 39 | 0 | 0.000000 | 0.000000 | 990 | 780.540011 | 842.19 | 903.839989 | 0.850697 |
| GO:0042158\_lipoprotein\_biosynthetic\_process | 39 | 0 | 0.000000 | 0.000000 | 990 | 780.540011 | 842.19 | 903.839989 | 0.850697 |
| GO:0046328\_regulation\_of\_JNK\_cascade | 39 | 0 | 0.000000 | 0.000000 | 990 | 780.540011 | 842.19 | 903.839989 | 0.850697 |
| GO:0046365\_monosaccharide\_catabolic\_process | 39 | 0 | 0.000000 | 0.000000 | 990 | 780.540011 | 842.19 | 903.839989 | 0.850697 |
| GO:0051960\_regulation\_of\_nervous\_system\_development | 39 | 0 | 0.000000 | 0.000000 | 990 | 780.540011 | 842.19 | 903.839989 | 0.850697 |
| GO:0006732\_coenzyme\_metabolic\_process | 65 | 0 | 0.000000 | 0.000000 | 997 | 788.287957 | 849.55 | 910.812043 | 0.852106 |
| GO:0016054\_organic\_acid\_catabolic\_process | 65 | 0 | 0.000000 | 0.000000 | 997 | 788.287957 | 849.55 | 910.812043 | 0.852106 |
| GO:0022415\_viral\_reproductive\_process | 65 | 0 | 0.000000 | 0.000000 | 997 | 788.287957 | 849.55 | 910.812043 | 0.852106 |
| GO:0045596\_negative\_regulation\_of\_cell\_differentiation | 65 | 0 | 0.000000 | 0.000000 | 997 | 788.287957 | 849.55 | 910.812043 | 0.852106 |
| GO:0046395\_carboxylic\_acid\_catabolic\_process | 65 | 0 | 0.000000 | 0.000000 | 997 | 788.287957 | 849.55 | 910.812043 | 0.852106 |
| GO:0051437\_positive\_regulation\_of\_ubiquitin-protein\_ligase\_activity\_during\_mitotic\_cell\_cycle | 65 | 0 | 0.000000 | 0.000000 | 997 | 788.287957 | 849.55 | 910.812043 | 0.852106 |
| GO:0080135\_regulation\_of\_cellular\_response\_to\_stress | 65 | 0 | 0.000000 | 0.000000 | 997 | 788.287957 | 849.55 | 910.812043 | 0.852106 |
| GO:0006397\_mRNA\_processing | 180 | 0 | 0.000000 | 0.000000 | 1000 | 791.692879 | 852.54 | 913.387121 | 0.852540 |
| GO:0051253\_negative\_regulation\_of\_RNA\_metabolic\_process | 180 | 0 | 0.000000 | 0.000000 | 1000 | 791.692879 | 852.54 | 913.387121 | 0.852540 |
| GO:0051336\_regulation\_of\_hydrolase\_activity | 180 | 0 | 0.000000 | 0.000000 | 1000 | 791.692879 | 852.54 | 913.387121 | 0.852540 |
| GO:0006520\_cellular\_amino\_acid\_metabolic\_process | 108 | 0 | 0.000000 | 0.000000 | 1007 | 797.894235 | 858.47 | 919.045765 | 0.852502 |
| GO:0006644\_phospholipid\_metabolic\_process | 108 | 0 | 0.000000 | 0.000000 | 1007 | 797.894235 | 858.47 | 919.045765 | 0.852502 |
| GO:0007346\_regulation\_of\_mitotic\_cell\_cycle | 108 | 0 | 0.000000 | 0.000000 | 1007 | 797.894235 | 858.47 | 919.045765 | 0.852502 |
| GO:0008202\_steroid\_metabolic\_process | 108 | 0 | 0.000000 | 0.000000 | 1007 | 797.894235 | 858.47 | 919.045765 | 0.852502 |
| GO:0019637\_organophosphate\_metabolic\_process | 108 | 0 | 0.000000 | 0.000000 | 1007 | 797.894235 | 858.47 | 919.045765 | 0.852502 |
| GO:0032446\_protein\_modification\_by\_small\_protein\_conjugation | 108 | 0 | 0.000000 | 0.000000 | 1007 | 797.894235 | 858.47 | 919.045765 | 0.852502 |
| GO:0044106\_cellular\_amine\_metabolic\_process | 108 | 0 | 0.000000 | 0.000000 | 1007 | 797.894235 | 858.47 | 919.045765 | 0.852502 |
| GO:0006790\_sulfur\_metabolic\_process | 62 | 0 | 0.000000 | 0.000000 | 1017 | 809.184458 | 869.14 | 929.095542 | 0.854612 |
| GO:0007420\_brain\_development | 62 | 0 | 0.000000 | 0.000000 | 1017 | 809.184458 | 869.14 | 929.095542 | 0.854612 |
| GO:0007586\_digestion | 62 | 0 | 0.000000 | 0.000000 | 1017 | 809.184458 | 869.14 | 929.095542 | 0.854612 |
| GO:0019216\_regulation\_of\_lipid\_metabolic\_process | 62 | 0 | 0.000000 | 0.000000 | 1017 | 809.184458 | 869.14 | 929.095542 | 0.854612 |
| GO:0031145\_anaphase-promoting\_complex-dependent\_proteasomal\_ubiquitin-dependent\_protein\_catabolic\_process | 62 | 0 | 0.000000 | 0.000000 | 1017 | 809.184458 | 869.14 | 929.095542 | 0.854612 |
| GO:0031667\_response\_to\_nutrient\_levels | 62 | 0 | 0.000000 | 0.000000 | 1017 | 809.184458 | 869.14 | 929.095542 | 0.854612 |
| GO:0044242\_cellular\_lipid\_catabolic\_process | 62 | 0 | 0.000000 | 0.000000 | 1017 | 809.184458 | 869.14 | 929.095542 | 0.854612 |
| GO:0051436\_negative\_regulation\_of\_ubiquitin-protein\_ligase\_activity\_during\_mitotic\_cell\_cycle | 62 | 0 | 0.000000 | 0.000000 | 1017 | 809.184458 | 869.14 | 929.095542 | 0.854612 |
| GO:0052548\_regulation\_of\_endopeptidase\_activity | 62 | 0 | 0.000000 | 0.000000 | 1017 | 809.184458 | 869.14 | 929.095542 | 0.854612 |
| GO:0060191\_regulation\_of\_lipase\_activity | 62 | 0 | 0.000000 | 0.000000 | 1017 | 809.184458 | 869.14 | 929.095542 | 0.854612 |
| GO:0003012\_muscle\_system\_process | 141 | 0 | 0.000000 | 0.000000 | 1020 | 812.442874 | 872.09 | 931.737126 | 0.854990 |
| GO:0016049\_cell\_growth | 141 | 0 | 0.000000 | 0.000000 | 1020 | 812.442874 | 872.09 | 931.737126 | 0.854990 |
| GO:0051603\_proteolysis\_involved\_in\_cellular\_protein\_catabolic\_process | 141 | 0 | 0.000000 | 0.000000 | 1020 | 812.442874 | 872.09 | 931.737126 | 0.854990 |
| GO:0006091\_generation\_of\_precursor\_metabolites\_and\_energy | 163 | 0 | 0.000000 | 0.000000 | 1021 | 813.245571 | 872.83 | 932.414429 | 0.854878 |
| GO:0000122\_negative\_regulation\_of\_transcription\_from\_RNA\_polymerase\_II\_promoter | 113 | 0 | 0.000000 | 0.000000 | 1023 | 815.897250 | 875.19 | 934.482750 | 0.855513 |
| GO:0006631\_fatty\_acid\_metabolic\_process | 113 | 0 | 0.000000 | 0.000000 | 1023 | 815.897250 | 875.19 | 934.482750 | 0.855513 |
| GO:0034984\_cellular\_response\_to\_DNA\_damage\_stimulus | 215 | 0 | 0.000000 | 0.000000 | 1024 | 816.625317 | 875.85 | 935.074683 | 0.855322 |
| GO:0001932\_regulation\_of\_protein\_amino\_acid\_phosphorylation | 101 | 0 | 0.000000 | 0.000000 | 1025 | 818.484523 | 877.49 | 936.495477 | 0.856088 |
| GO:0000028\_ribosomal\_small\_subunit\_assembly | 1 | 0 |  |  |  |  |  |  |  |  |
| GO:0000042\_protein\_targeting\_to\_Golgi | 1 | 0 |  |  |  |  |  |  |  |  |
| GO:0000046\_autophagic\_vacuole\_fusion | 1 | 0 |  |  |  |  |  |  |  |  |
| GO:0000052\_citrulline\_metabolic\_process | 1 | 0 |  |  |  |  |  |  |  |  |
| GO:0000054\_ribosome\_export\_from\_nucleus | 1 | 0 |  |  |  |  |  |  |  |  |
| GO:0000056\_ribosomal\_small\_subunit\_export\_from\_nucleus | 1 | 0 |  |  |  |  |  |  |  |  |
| GO:0000072\_M\_phase\_specific\_microtubule\_process | 1 | 0 |  |  |  |  |  |  |  |  |
| GO:0000093\_mitotic\_telophase | 1 | 0 |  |  |  |  |  |  |  |  |
| GO:0000098\_sulfur\_amino\_acid\_catabolic\_process | 1 | 0 |  |  |  |  |  |  |  |  |
| GO:0000114\_regulation\_of\_transcription\_during\_G1\_phase\_of\_mitotic\_cell\_cycle | 1 | 0 |  |  |  |  |  |  |  |  |
| GO:0000115\_regulation\_of\_transcription\_during\_S-phase\_of\_mitotic\_cell\_cycle | 1 | 0 |  |  |  |  |  |  |  |  |
| GO:0000117\_regulation\_of\_transcription\_during\_G2\_M-phase\_of\_mitotic\_cell\_cycle | 1 | 0 |  |  |  |  |  |  |  |  |
| GO:0000132\_establishment\_of\_mitotic\_spindle\_orientation | 1 | 0 |  |  |  |  |  |  |  |  |
| GO:0000154\_rRNA\_modification | 1 | 0 |  |  |  |  |  |  |  |  |
| GO:0000160\_two-component\_signal\_transduction\_system\_(phosphorelay) | 1 | 0 |  |  |  |  |  |  |  |  |
| GO:0000161\_MAPKKK\_cascade\_involved\_in\_osmosensory\_signaling\_pathway | 1 | 0 |  |  |  |  |  |  |  |  |
| GO:0000173\_inactivation\_of\_MAPK\_activity\_involved\_in\_osmosensory\_signaling\_pathway | 1 | 0 |  |  |  |  |  |  |  |  |
| GO:0000212\_meiotic\_spindle\_organization | 1 | 0 |  |  |  |  |  |  |  |  |
| GO:0000255\_allantoin\_metabolic\_process | 1 | 0 |  |  |  |  |  |  |  |  |
| GO:0000270\_peptidoglycan\_metabolic\_process | 1 | 0 |  |  |  |  |  |  |  |  |
| GO:0000296\_spermine\_transport | 1 | 0 |  |  |  |  |  |  |  |  |
| GO:0000301\_retrograde\_transport\_\_vesicle\_recycling\_within\_Golgi | 1 | 0 |  |  |  |  |  |  |  |  |
| GO:0000303\_response\_to\_superoxide | 1 | 0 |  |  |  |  |  |  |  |  |
| GO:0000320\_re-entry\_into\_mitotic\_cell\_cycle | 1 | 0 |  |  |  |  |  |  |  |  |
| GO:0000338\_protein\_deneddylation | 1 | 0 |  |  |  |  |  |  |  |  |
| GO:0000395\_nuclear\_mRNA\_5'-splice\_site\_recognition | 1 | 0 |  |  |  |  |  |  |  |  |
| GO:0000710\_meiotic\_mismatch\_repair | 1 | 0 |  |  |  |  |  |  |  |  |
| GO:0000717\_nucleotide-excision\_repair\_\_DNA\_duplex\_unwinding | 1 | 0 |  |  |  |  |  |  |  |  |
| GO:0000722\_telomere\_maintenance\_via\_recombination | 1 | 0 |  |  |  |  |  |  |  |  |
| GO:0000746\_conjugation | 1 | 0 |  |  |  |  |  |  |  |  |
| GO:0000747\_conjugation\_with\_cellular\_fusion | 1 | 0 |  |  |  |  |  |  |  |  |
| GO:0000912\_formation\_of\_actomyosin\_apparatus\_involved\_in\_cytokinesis | 1 | 0 |  |  |  |  |  |  |  |  |
| GO:0000915\_cytokinesis\_\_contractile\_ring\_formation | 1 | 0 |  |  |  |  |  |  |  |  |
| GO:0000921\_septin\_ring\_assembly | 1 | 0 |  |  |  |  |  |  |  |  |
| GO:0000966\_RNA\_5'-end\_processing | 1 | 0 |  |  |  |  |  |  |  |  |
| GO:0001315\_age-dependent\_response\_to\_reactive\_oxygen\_species | 1 | 0 |  |  |  |  |  |  |  |  |
| GO:0001519\_peptide\_amidation | 1 | 0 |  |  |  |  |  |  |  |  |
| GO:0001560\_regulation\_of\_cell\_growth\_by\_extracellular\_stimulus | 1 | 0 |  |  |  |  |  |  |  |  |
| GO:0001574\_ganglioside\_biosynthetic\_process | 1 | 0 |  |  |  |  |  |  |  |  |
| GO:0001575\_globoside\_metabolic\_process | 1 | 0 |  |  |  |  |  |  |  |  |
| GO:0001658\_branching\_involved\_in\_ureteric\_bud\_morphogenesis | 1 | 0 |  |  |  |  |  |  |  |  |
| GO:0001662\_behavioral\_fear\_response | 1 | 0 |  |  |  |  |  |  |  |  |
| GO:0001675\_acrosome\_assembly | 1 | 0 |  |  |  |  |  |  |  |  |
| GO:0001692\_histamine\_metabolic\_process | 1 | 0 |  |  |  |  |  |  |  |  |
| GO:0001694\_histamine\_biosynthetic\_process | 1 | 0 |  |  |  |  |  |  |  |  |
| GO:0001732\_formation\_of\_translation\_initiation\_complex | 1 | 0 |  |  |  |  |  |  |  |  |
| GO:0001757\_somite\_specification | 1 | 0 |  |  |  |  |  |  |  |  |
| GO:0001774\_microglial\_cell\_activation | 1 | 0 |  |  |  |  |  |  |  |  |
| GO:0001782\_B\_cell\_homeostasis | 1 | 0 |  |  |  |  |  |  |  |  |
| GO:0001787\_natural\_killer\_cell\_proliferation | 1 | 0 |  |  |  |  |  |  |  |  |
| GO:0001823\_mesonephros\_development | 1 | 0 |  |  |  |  |  |  |  |  |
| GO:0001832\_blastocyst\_growth | 1 | 0 |  |  |  |  |  |  |  |  |
| GO:0001833\_inner\_cell\_mass\_cell\_proliferation | 1 | 0 |  |  |  |  |  |  |  |  |
| GO:0001839\_neural\_plate\_morphogenesis | 1 | 0 |  |  |  |  |  |  |  |  |
| GO:0001845\_phagolysosome\_formation | 1 | 0 |  |  |  |  |  |  |  |  |
| GO:0001865\_NK\_T\_cell\_differentiation | 1 | 0 |  |  |  |  |  |  |  |  |
| GO:0001866\_NK\_T\_cell\_proliferation | 1 | 0 |  |  |  |  |  |  |  |  |
| GO:0001887\_selenium\_metabolic\_process | 1 | 0 |  |  |  |  |  |  |  |  |
| GO:0001892\_embryonic\_placenta\_development | 1 | 0 |  |  |  |  |  |  |  |  |
| GO:0001911\_negative\_regulation\_of\_leukocyte\_mediated\_cytotoxicity | 1 | 0 |  |  |  |  |  |  |  |  |
| GO:0001915\_negative\_regulation\_of\_T\_cell\_mediated\_cytotoxicity | 1 | 0 |  |  |  |  |  |  |  |  |
| GO:0001920\_negative\_regulation\_of\_receptor\_recycling | 1 | 0 |  |  |  |  |  |  |  |  |
| GO:0001941\_postsynaptic\_membrane\_organization | 1 | 0 |  |  |  |  |  |  |  |  |
| GO:0001958\_endochondral\_ossification | 1 | 0 |  |  |  |  |  |  |  |  |
| GO:0001973\_adenosine\_receptor\_signaling\_pathway | 1 | 0 |  |  |  |  |  |  |  |  |
| GO:0001977\_renal\_system\_process\_involved\_in\_regulation\_of\_blood\_volume | 1 | 0 |  |  |  |  |  |  |  |  |
| GO:0001980\_regulation\_of\_systemic\_arterial\_blood\_pressure\_by\_ischemic\_conditions | 1 | 0 |  |  |  |  |  |  |  |  |
| GO:0001993\_regulation\_of\_systemic\_arterial\_blood\_pressure\_by\_norepinephrine-epinephrine | 1 | 0 |  |  |  |  |  |  |  |  |
| GO:0001996\_positive\_regulation\_of\_heart\_rate\_by\_epinephrine-norepinephrine | 1 | 0 |  |  |  |  |  |  |  |  |
| GO:0001999\_renal\_response\_to\_blood\_flow\_during\_renin-angiotensin\_regulation\_of\_systemic\_arterial\_blood\_pressure | 1 | 0 |  |  |  |  |  |  |  |  |
| GO:0002001\_renin\_secretion\_into\_blood\_stream | 1 | 0 |  |  |  |  |  |  |  |  |
| GO:0002017\_regulation\_of\_blood\_volume\_by\_renal\_aldosterone | 1 | 0 |  |  |  |  |  |  |  |  |
| GO:0002018\_renin-angiotensin\_regulation\_of\_aldosterone\_production | 1 | 0 |  |  |  |  |  |  |  |  |
| GO:0002031\_G-protein\_coupled\_receptor\_internalization | 1 | 0 |  |  |  |  |  |  |  |  |
| GO:0002035\_brain\_renin-angiotensin\_system | 1 | 0 |  |  |  |  |  |  |  |  |
| GO:0002042\_cell\_migration\_involved\_in\_sprouting\_angiogenesis | 1 | 0 |  |  |  |  |  |  |  |  |
| GO:0002052\_positive\_regulation\_of\_neuroblast\_proliferation | 1 | 0 |  |  |  |  |  |  |  |  |
| GO:0002053\_positive\_regulation\_of\_mesenchymal\_cell\_proliferation | 1 | 0 |  |  |  |  |  |  |  |  |
| GO:0002063\_chondrocyte\_development | 1 | 0 |  |  |  |  |  |  |  |  |
| GO:0002064\_epithelial\_cell\_development | 1 | 0 |  |  |  |  |  |  |  |  |
| GO:0002074\_extraocular\_skeletal\_muscle\_development | 1 | 0 |  |  |  |  |  |  |  |  |
| GO:0002077\_acrosome\_matrix\_dispersal | 1 | 0 |  |  |  |  |  |  |  |  |
| GO:0002082\_regulation\_of\_oxidative\_phosphorylation | 1 | 0 |  |  |  |  |  |  |  |  |
| GO:0002084\_protein\_depalmitoylation | 1 | 0 |  |  |  |  |  |  |  |  |
| GO:0002088\_lens\_development\_in\_camera-type\_eye | 1 | 0 |  |  |  |  |  |  |  |  |
| GO:0002089\_lens\_morphogenesis\_in\_camera-type\_eye | 1 | 0 |  |  |  |  |  |  |  |  |
| GO:0002093\_auditory\_receptor\_cell\_morphogenesis | 1 | 0 |  |  |  |  |  |  |  |  |
| GO:0002209\_behavioral\_defense\_response | 1 | 0 |  |  |  |  |  |  |  |  |
| GO:0002220\_innate\_immune\_response\_activating\_cell\_surface\_receptor\_signaling\_pathway | 1 | 0 |  |  |  |  |  |  |  |  |
| GO:0002223\_stimulatory\_C-type\_lectin\_receptor\_signaling\_pathway | 1 | 0 |  |  |  |  |  |  |  |  |
| GO:0002312\_B\_cell\_activation\_during\_immune\_response | 1 | 0 |  |  |  |  |  |  |  |  |
| GO:0002313\_mature\_B\_cell\_differentiation\_during\_immune\_response | 1 | 0 |  |  |  |  |  |  |  |  |
| GO:0002318\_myeloid\_progenitor\_cell\_differentiation | 1 | 0 |  |  |  |  |  |  |  |  |
| GO:0002320\_lymphoid\_progenitor\_cell\_differentiation | 1 | 0 |  |  |  |  |  |  |  |  |
| GO:0002326\_B\_cell\_lineage\_commitment | 1 | 0 |  |  |  |  |  |  |  |  |
| GO:0002328\_pro-B\_cell\_differentiation | 1 | 0 |  |  |  |  |  |  |  |  |
| GO:0002335\_mature\_B\_cell\_differentiation | 1 | 0 |  |  |  |  |  |  |  |  |
| GO:0002355\_detection\_of\_tumor\_cell | 1 | 0 |  |  |  |  |  |  |  |  |
| GO:0002368\_B\_cell\_cytokine\_production | 1 | 0 |  |  |  |  |  |  |  |  |
| GO:0002424\_T\_cell\_mediated\_immune\_response\_to\_tumor\_cell | 1 | 0 |  |  |  |  |  |  |  |  |
| GO:0002431\_Fc\_receptor\_mediated\_stimulatory\_signaling\_pathway | 1 | 0 |  |  |  |  |  |  |  |  |
| GO:0002437\_inflammatory\_response\_to\_antigenic\_stimulus | 1 | 0 |  |  |  |  |  |  |  |  |
| GO:0002439\_chronic\_inflammatory\_response\_to\_antigenic\_stimulus | 1 | 0 |  |  |  |  |  |  |  |  |
| GO:0002447\_eosinophil\_mediated\_immunity | 1 | 0 |  |  |  |  |  |  |  |  |
| GO:0002455\_humoral\_immune\_response\_mediated\_by\_circulating\_immunoglobulin | 1 | 0 |  |  |  |  |  |  |  |  |
| GO:0002467\_germinal\_center\_formation | 1 | 0 |  |  |  |  |  |  |  |  |
| GO:0002475\_antigen\_processing\_and\_presentation\_via\_MHC\_class\_Ib | 1 | 0 |  |  |  |  |  |  |  |  |
| GO:0002478\_antigen\_processing\_and\_presentation\_of\_exogenous\_peptide\_antigen | 1 | 0 |  |  |  |  |  |  |  |  |
| GO:0002495\_antigen\_processing\_and\_presentation\_of\_peptide\_antigen\_via\_MHC\_class\_II | 1 | 0 |  |  |  |  |  |  |  |  |
| GO:0002513\_tolerance\_induction\_to\_self\_antigen | 1 | 0 |  |  |  |  |  |  |  |  |
| GO:0002514\_B\_cell\_tolerance\_induction | 1 | 0 |  |  |  |  |  |  |  |  |
| GO:0002517\_T\_cell\_tolerance\_induction | 1 | 0 |  |  |  |  |  |  |  |  |
| GO:0002523\_leukocyte\_migration\_during\_inflammatory\_response | 1 | 0 |  |  |  |  |  |  |  |  |
| GO:0002566\_somatic\_diversification\_of\_immune\_receptors\_via\_somatic\_mutation | 1 | 0 |  |  |  |  |  |  |  |  |
| GO:0002568\_somatic\_diversification\_of\_T\_cell\_receptor\_genes | 1 | 0 |  |  |  |  |  |  |  |  |
| GO:0002576\_platelet\_degranulation | 1 | 0 |  |  |  |  |  |  |  |  |
| GO:0002634\_regulation\_of\_germinal\_center\_formation | 1 | 0 |  |  |  |  |  |  |  |  |
| GO:0002649\_regulation\_of\_tolerance\_induction\_to\_self\_antigen | 1 | 0 |  |  |  |  |  |  |  |  |
| GO:0002651\_positive\_regulation\_of\_tolerance\_induction\_to\_self\_antigen | 1 | 0 |  |  |  |  |  |  |  |  |
| GO:0002661\_regulation\_of\_B\_cell\_tolerance\_induction | 1 | 0 |  |  |  |  |  |  |  |  |
| GO:0002663\_positive\_regulation\_of\_B\_cell\_tolerance\_induction | 1 | 0 |  |  |  |  |  |  |  |  |
| GO:0002664\_regulation\_of\_T\_cell\_tolerance\_induction | 1 | 0 |  |  |  |  |  |  |  |  |
| GO:0002666\_positive\_regulation\_of\_T\_cell\_tolerance\_induction | 1 | 0 |  |  |  |  |  |  |  |  |
| GO:0002674\_negative\_regulation\_of\_acute\_inflammatory\_response | 1 | 0 |  |  |  |  |  |  |  |  |
| GO:0002681\_somatic\_recombination\_of\_T\_cell\_receptor\_gene\_segments | 1 | 0 |  |  |  |  |  |  |  |  |
| GO:0002686\_negative\_regulation\_of\_leukocyte\_migration | 1 | 0 |  |  |  |  |  |  |  |  |
| GO:0002691\_regulation\_of\_cellular\_extravasation | 1 | 0 |  |  |  |  |  |  |  |  |
| GO:0002693\_positive\_regulation\_of\_cellular\_extravasation | 1 | 0 |  |  |  |  |  |  |  |  |
| GO:0002701\_negative\_regulation\_of\_production\_of\_molecular\_mediator\_of\_immune\_response | 1 | 0 |  |  |  |  |  |  |  |  |
| GO:0002719\_negative\_regulation\_of\_cytokine\_production\_during\_immune\_response | 1 | 0 |  |  |  |  |  |  |  |  |
| GO:0002725\_negative\_regulation\_of\_T\_cell\_cytokine\_production | 1 | 0 |  |  |  |  |  |  |  |  |
| GO:0002759\_regulation\_of\_antimicrobial\_humoral\_response | 1 | 0 |  |  |  |  |  |  |  |  |
| GO:0002775\_antimicrobial\_peptide\_production | 1 | 0 |  |  |  |  |  |  |  |  |
| GO:0002777\_antimicrobial\_peptide\_biosynthetic\_process | 1 | 0 |  |  |  |  |  |  |  |  |
| GO:0002778\_antibacterial\_peptide\_production | 1 | 0 |  |  |  |  |  |  |  |  |
| GO:0002780\_antibacterial\_peptide\_biosynthetic\_process | 1 | 0 |  |  |  |  |  |  |  |  |
| GO:0002784\_regulation\_of\_antimicrobial\_peptide\_production | 1 | 0 |  |  |  |  |  |  |  |  |
| GO:0002786\_regulation\_of\_antibacterial\_peptide\_production | 1 | 0 |  |  |  |  |  |  |  |  |
| GO:0002805\_regulation\_of\_antimicrobial\_peptide\_biosynthetic\_process | 1 | 0 |  |  |  |  |  |  |  |  |
| GO:0002807\_positive\_regulation\_of\_antimicrobial\_peptide\_biosynthetic\_process | 1 | 0 |  |  |  |  |  |  |  |  |
| GO:0002808\_regulation\_of\_antibacterial\_peptide\_biosynthetic\_process | 1 | 0 |  |  |  |  |  |  |  |  |
| GO:0002815\_biosynthetic\_process\_of\_antibacterial\_peptides\_active\_against\_Gram-positive\_bacteria | 1 | 0 |  |  |  |  |  |  |  |  |
| GO:0002816\_regulation\_of\_biosynthetic\_process\_of\_antibacterial\_peptides\_active\_against\_Gram-positive\_bacteria | 1 | 0 |  |  |  |  |  |  |  |  |
| GO:0002832\_negative\_regulation\_of\_response\_to\_biotic\_stimulus | 1 | 0 |  |  |  |  |  |  |  |  |
| GO:0002840\_regulation\_of\_T\_cell\_mediated\_immune\_response\_to\_tumor\_cell | 1 | 0 |  |  |  |  |  |  |  |  |
| GO:0002842\_positive\_regulation\_of\_T\_cell\_mediated\_immune\_response\_to\_tumor\_cell | 1 | 0 |  |  |  |  |  |  |  |  |
| GO:0002901\_mature\_B\_cell\_apoptosis | 1 | 0 |  |  |  |  |  |  |  |  |
| GO:0002904\_positive\_regulation\_of\_B\_cell\_apoptosis | 1 | 0 |  |  |  |  |  |  |  |  |
| GO:0002905\_regulation\_of\_mature\_B\_cell\_apoptosis | 1 | 0 |  |  |  |  |  |  |  |  |
| GO:0002906\_negative\_regulation\_of\_mature\_B\_cell\_apoptosis | 1 | 0 |  |  |  |  |  |  |  |  |
| GO:0003010\_voluntary\_skeletal\_muscle\_contraction | 1 | 0 |  |  |  |  |  |  |  |  |
| GO:0003051\_angiotensin-mediated\_drinking\_behavior | 1 | 0 |  |  |  |  |  |  |  |  |
| GO:0003058\_hormonal\_regulation\_of\_the\_force\_of\_heart\_contraction | 1 | 0 |  |  |  |  |  |  |  |  |
| GO:0003062\_regulation\_of\_heart\_rate\_by\_chemical\_signal | 1 | 0 |  |  |  |  |  |  |  |  |
| GO:0003065\_positive\_regulation\_of\_heart\_rate\_by\_epinephrine | 1 | 0 |  |  |  |  |  |  |  |  |
| GO:0003071\_renal\_system\_process\_involved\_in\_regulation\_of\_systemic\_arterial\_blood\_pressure | 1 | 0 |  |  |  |  |  |  |  |  |
| GO:0003085\_negative\_regulation\_of\_systemic\_arterial\_blood\_pressure | 1 | 0 |  |  |  |  |  |  |  |  |
| GO:0003099\_positive\_regulation\_of\_the\_force\_of\_heart\_contraction\_by\_chemical\_signal | 1 | 0 |  |  |  |  |  |  |  |  |
| GO:0003108\_negative\_regulation\_of\_the\_force\_of\_heart\_contraction\_by\_chemical\_signal | 1 | 0 |  |  |  |  |  |  |  |  |
| GO:0005981\_regulation\_of\_glycogen\_catabolic\_process | 1 | 0 |  |  |  |  |  |  |  |  |
| GO:0005982\_starch\_metabolic\_process | 1 | 0 |  |  |  |  |  |  |  |  |
| GO:0005983\_starch\_catabolic\_process | 1 | 0 |  |  |  |  |  |  |  |  |
| GO:0005988\_lactose\_metabolic\_process | 1 | 0 |  |  |  |  |  |  |  |  |
| GO:0005989\_lactose\_biosynthetic\_process | 1 | 0 |  |  |  |  |  |  |  |  |
| GO:0005991\_trehalose\_metabolic\_process | 1 | 0 |  |  |  |  |  |  |  |  |
| GO:0005993\_trehalose\_catabolic\_process | 1 | 0 |  |  |  |  |  |  |  |  |
| GO:0006010\_glucose\_6-phosphate\_utilization | 1 | 0 |  |  |  |  |  |  |  |  |
| GO:0006013\_mannose\_metabolic\_process | 1 | 0 |  |  |  |  |  |  |  |  |
| GO:0006021\_inositol\_biosynthetic\_process | 1 | 0 |  |  |  |  |  |  |  |  |
| GO:0006037\_cell\_wall\_chitin\_metabolic\_process | 1 | 0 |  |  |  |  |  |  |  |  |
| GO:0006042\_glucosamine\_biosynthetic\_process | 1 | 0 |  |  |  |  |  |  |  |  |
| GO:0006045\_N-acetylglucosamine\_biosynthetic\_process | 1 | 0 |  |  |  |  |  |  |  |  |
| GO:0006048\_UDP-N-acetylglucosamine\_biosynthetic\_process | 1 | 0 |  |  |  |  |  |  |  |  |
| GO:0006050\_mannosamine\_metabolic\_process | 1 | 0 |  |  |  |  |  |  |  |  |
| GO:0006051\_N-acetylmannosamine\_metabolic\_process | 1 | 0 |  |  |  |  |  |  |  |  |
| GO:0006059\_hexitol\_metabolic\_process | 1 | 0 |  |  |  |  |  |  |  |  |
| GO:0006060\_sorbitol\_metabolic\_process | 1 | 0 |  |  |  |  |  |  |  |  |
| GO:0006062\_sorbitol\_catabolic\_process | 1 | 0 |  |  |  |  |  |  |  |  |
| GO:0006065\_UDP-glucuronate\_biosynthetic\_process | 1 | 0 |  |  |  |  |  |  |  |  |
| GO:0006083\_acetate\_metabolic\_process | 1 | 0 |  |  |  |  |  |  |  |  |
| GO:0006085\_acetyl-CoA\_biosynthetic\_process | 1 | 0 |  |  |  |  |  |  |  |  |
| GO:0006103\_2-oxoglutarate\_metabolic\_process | 1 | 0 |  |  |  |  |  |  |  |  |
| GO:0006106\_fumarate\_metabolic\_process | 1 | 0 |  |  |  |  |  |  |  |  |
| GO:0006107\_oxaloacetate\_metabolic\_process | 1 | 0 |  |  |  |  |  |  |  |  |
| GO:0006116\_NADH\_oxidation | 1 | 0 |  |  |  |  |  |  |  |  |
| GO:0006145\_purine\_base\_catabolic\_process | 1 | 0 |  |  |  |  |  |  |  |  |
| GO:0006148\_inosine\_catabolic\_process | 1 | 0 |  |  |  |  |  |  |  |  |
| GO:0006154\_adenosine\_catabolic\_process | 1 | 0 |  |  |  |  |  |  |  |  |
| GO:0006166\_purine\_ribonucleoside\_salvage | 1 | 0 |  |  |  |  |  |  |  |  |
| GO:0006172\_ADP\_biosynthetic\_process | 1 | 0 |  |  |  |  |  |  |  |  |
| GO:0006173\_dADP\_biosynthetic\_process | 1 | 0 |  |  |  |  |  |  |  |  |
| GO:0006188\_IMP\_biosynthetic\_process | 1 | 0 |  |  |  |  |  |  |  |  |
| GO:0006189\_'de\_novo'\_IMP\_biosynthetic\_process | 1 | 0 |  |  |  |  |  |  |  |  |
| GO:0006196\_AMP\_catabolic\_process | 1 | 0 |  |  |  |  |  |  |  |  |
| GO:0006198\_cAMP\_catabolic\_process | 1 | 0 |  |  |  |  |  |  |  |  |
| GO:0006207\_'de\_novo'\_pyrimidine\_base\_biosynthetic\_process | 1 | 0 |  |  |  |  |  |  |  |  |
| GO:0006214\_thymidine\_catabolic\_process | 1 | 0 |  |  |  |  |  |  |  |  |
| GO:0006216\_cytidine\_catabolic\_process | 1 | 0 |  |  |  |  |  |  |  |  |
| GO:0006222\_UMP\_biosynthetic\_process | 1 | 0 |  |  |  |  |  |  |  |  |
| GO:0006241\_CTP\_biosynthetic\_process | 1 | 0 |  |  |  |  |  |  |  |  |
| GO:0006256\_UDP\_catabolic\_process | 1 | 0 |  |  |  |  |  |  |  |  |
| GO:0006265\_DNA\_topological\_change | 1 | 0 |  |  |  |  |  |  |  |  |
| GO:0006272\_leading\_strand\_elongation | 1 | 0 |  |  |  |  |  |  |  |  |
| GO:0006287\_base-excision\_repair\_\_gap-filling | 1 | 0 |  |  |  |  |  |  |  |  |
| GO:0006313\_transposition\_\_DNA-mediated | 1 | 0 |  |  |  |  |  |  |  |  |
| GO:0006336\_DNA\_replication-independent\_nucleosome\_assembly | 1 | 0 |  |  |  |  |  |  |  |  |
| GO:0006343\_establishment\_of\_chromatin\_silencing | 1 | 0 |  |  |  |  |  |  |  |  |
| GO:0006344\_maintenance\_of\_chromatin\_silencing | 1 | 0 |  |  |  |  |  |  |  |  |
| GO:0006346\_methylation-dependent\_chromatin\_silencing | 1 | 0 |  |  |  |  |  |  |  |  |
| GO:0006348\_chromatin\_silencing\_at\_telomere | 1 | 0 |  |  |  |  |  |  |  |  |
| GO:0006361\_transcription\_initiation\_from\_RNA\_polymerase\_I\_promoter | 1 | 0 |  |  |  |  |  |  |  |  |
| GO:0006369\_termination\_of\_RNA\_polymerase\_II\_transcription | 1 | 0 |  |  |  |  |  |  |  |  |
| GO:0006393\_termination\_of\_mitochondrial\_transcription | 1 | 0 |  |  |  |  |  |  |  |  |
| GO:0006407\_rRNA\_export\_from\_nucleus | 1 | 0 |  |  |  |  |  |  |  |  |
| GO:0006408\_snRNA\_export\_from\_nucleus | 1 | 0 |  |  |  |  |  |  |  |  |
| GO:0006409\_tRNA\_export\_from\_nucleus | 1 | 0 |  |  |  |  |  |  |  |  |
| GO:0006419\_alanyl-tRNA\_aminoacylation | 1 | 0 |  |  |  |  |  |  |  |  |
| GO:0006420\_arginyl-tRNA\_aminoacylation | 1 | 0 |  |  |  |  |  |  |  |  |
| GO:0006423\_cysteinyl-tRNA\_aminoacylation | 1 | 0 |  |  |  |  |  |  |  |  |
| GO:0006431\_methionyl-tRNA\_aminoacylation | 1 | 0 |  |  |  |  |  |  |  |  |
| GO:0006432\_phenylalanyl-tRNA\_aminoacylation | 1 | 0 |  |  |  |  |  |  |  |  |
| GO:0006434\_seryl-tRNA\_aminoacylation | 1 | 0 |  |  |  |  |  |  |  |  |
| GO:0006435\_threonyl-tRNA\_aminoacylation | 1 | 0 |  |  |  |  |  |  |  |  |
| GO:0006436\_tryptophanyl-tRNA\_aminoacylation | 1 | 0 |  |  |  |  |  |  |  |  |
| GO:0006437\_tyrosyl-tRNA\_aminoacylation | 1 | 0 |  |  |  |  |  |  |  |  |
| GO:0006447\_regulation\_of\_translational\_initiation\_by\_iron | 1 | 0 |  |  |  |  |  |  |  |  |
| GO:0006448\_regulation\_of\_translational\_elongation | 1 | 0 |  |  |  |  |  |  |  |  |
| GO:0006450\_regulation\_of\_translational\_fidelity | 1 | 0 |  |  |  |  |  |  |  |  |
| GO:0006494\_protein\_amino\_acid\_terminal\_glycosylation | 1 | 0 |  |  |  |  |  |  |  |  |
| GO:0006496\_protein\_amino\_acid\_terminal\_N-glycosylation | 1 | 0 |  |  |  |  |  |  |  |  |
| GO:0006499\_N-terminal\_protein\_myristoylation | 1 | 0 |  |  |  |  |  |  |  |  |
| GO:0006500\_N-terminal\_protein\_palmitoylation | 1 | 0 |  |  |  |  |  |  |  |  |
| GO:0006526\_arginine\_biosynthetic\_process | 1 | 0 |  |  |  |  |  |  |  |  |
| GO:0006528\_asparagine\_metabolic\_process | 1 | 0 |  |  |  |  |  |  |  |  |
| GO:0006530\_asparagine\_catabolic\_process | 1 | 0 |  |  |  |  |  |  |  |  |
| GO:0006534\_cysteine\_metabolic\_process | 1 | 0 |  |  |  |  |  |  |  |  |
| GO:0006543\_glutamine\_catabolic\_process | 1 | 0 |  |  |  |  |  |  |  |  |
| GO:0006545\_glycine\_biosynthetic\_process | 1 | 0 |  |  |  |  |  |  |  |  |
| GO:0006547\_histidine\_metabolic\_process | 1 | 0 |  |  |  |  |  |  |  |  |
| GO:0006549\_isoleucine\_metabolic\_process | 1 | 0 |  |  |  |  |  |  |  |  |
| GO:0006556\_S-adenosylmethionine\_biosynthetic\_process | 1 | 0 |  |  |  |  |  |  |  |  |
| GO:0006562\_proline\_catabolic\_process | 1 | 0 |  |  |  |  |  |  |  |  |
| GO:0006564\_L-serine\_biosynthetic\_process | 1 | 0 |  |  |  |  |  |  |  |  |
| GO:0006577\_betaine\_metabolic\_process | 1 | 0 |  |  |  |  |  |  |  |  |
| GO:0006580\_ethanolamine\_metabolic\_process | 1 | 0 |  |  |  |  |  |  |  |  |
| GO:0006585\_dopamine\_biosynthetic\_process\_from\_tyrosine | 1 | 0 |  |  |  |  |  |  |  |  |
| GO:0006591\_ornithine\_metabolic\_process | 1 | 0 |  |  |  |  |  |  |  |  |
| GO:0006597\_spermine\_biosynthetic\_process | 1 | 0 |  |  |  |  |  |  |  |  |
| GO:0006598\_polyamine\_catabolic\_process | 1 | 0 |  |  |  |  |  |  |  |  |
| GO:0006610\_ribosomal\_protein\_import\_into\_nucleus | 1 | 0 |  |  |  |  |  |  |  |  |
| GO:0006614\_SRP-dependent\_cotranslational\_protein\_targeting\_to\_membrane | 1 | 0 |  |  |  |  |  |  |  |  |
| GO:0006616\_SRP-dependent\_cotranslational\_protein\_targeting\_to\_membrane\_\_translocation | 1 | 0 |  |  |  |  |  |  |  |  |
| GO:0006617\_SRP-dependent\_cotranslational\_protein\_targeting\_to\_membrane\_\_signal\_sequence\_recognition | 1 | 0 |  |  |  |  |  |  |  |  |
| GO:0006627\_mitochondrial\_protein\_processing\_during\_import | 1 | 0 |  |  |  |  |  |  |  |  |
| GO:0006646\_phosphatidylethanolamine\_biosynthetic\_process | 1 | 0 |  |  |  |  |  |  |  |  |
| GO:0006655\_phosphatidylglycerol\_biosynthetic\_process | 1 | 0 |  |  |  |  |  |  |  |  |
| GO:0006657\_CDP-choline\_pathway | 1 | 0 |  |  |  |  |  |  |  |  |
| GO:0006667\_sphinganine\_metabolic\_process | 1 | 0 |  |  |  |  |  |  |  |  |
| GO:0006668\_sphinganine-1-phosphate\_metabolic\_process | 1 | 0 |  |  |  |  |  |  |  |  |
| GO:0006669\_sphinganine-1-phosphate\_biosynthetic\_process | 1 | 0 |  |  |  |  |  |  |  |  |
| GO:0006670\_sphingosine\_metabolic\_process | 1 | 0 |  |  |  |  |  |  |  |  |
| GO:0006689\_ganglioside\_catabolic\_process | 1 | 0 |  |  |  |  |  |  |  |  |
| GO:0006711\_estrogen\_catabolic\_process | 1 | 0 |  |  |  |  |  |  |  |  |
| GO:0006713\_glucocorticoid\_catabolic\_process | 1 | 0 |  |  |  |  |  |  |  |  |
| GO:0006734\_NADH\_metabolic\_process | 1 | 0 |  |  |  |  |  |  |  |  |
| GO:0006741\_NADP\_biosynthetic\_process | 1 | 0 |  |  |  |  |  |  |  |  |
| GO:0006746\_FADH2\_metabolic\_process | 1 | 0 |  |  |  |  |  |  |  |  |
| GO:0006768\_biotin\_metabolic\_process | 1 | 0 |  |  |  |  |  |  |  |  |
| GO:0006771\_riboflavin\_metabolic\_process | 1 | 0 |  |  |  |  |  |  |  |  |
| GO:0006781\_succinyl-CoA\_pathway | 1 | 0 |  |  |  |  |  |  |  |  |
| GO:0006789\_bilirubin\_conjugation | 1 | 0 |  |  |  |  |  |  |  |  |
| GO:0006797\_polyphosphate\_metabolic\_process | 1 | 0 |  |  |  |  |  |  |  |  |
| GO:0006837\_serotonin\_transport | 1 | 0 |  |  |  |  |  |  |  |  |
| GO:0006842\_tricarboxylic\_acid\_transport | 1 | 0 |  |  |  |  |  |  |  |  |
| GO:0006843\_mitochondrial\_citrate\_transport | 1 | 0 |  |  |  |  |  |  |  |  |
| GO:0006848\_pyruvate\_transport | 1 | 0 |  |  |  |  |  |  |  |  |
| GO:0006862\_nucleotide\_transport | 1 | 0 |  |  |  |  |  |  |  |  |
| GO:0006867\_asparagine\_transport | 1 | 0 |  |  |  |  |  |  |  |  |
| GO:0006868\_glutamine\_transport | 1 | 0 |  |  |  |  |  |  |  |  |
| GO:0006876\_cellular\_cadmium\_ion\_homeostasis | 1 | 0 |  |  |  |  |  |  |  |  |
| GO:0006926\_virus-infected\_cell\_apoptosis | 1 | 0 |  |  |  |  |  |  |  |  |
| GO:0006931\_substrate-bound\_cell\_migration\_\_cell\_attachment\_to\_substrate | 1 | 0 |  |  |  |  |  |  |  |  |
| GO:0006948\_induction\_by\_virus\_of\_host\_cell-cell\_fusion | 1 | 0 |  |  |  |  |  |  |  |  |
| GO:0006958\_complement\_activation\_\_classical\_pathway | 1 | 0 |  |  |  |  |  |  |  |  |
| GO:0006963\_positive\_regulation\_of\_antibacterial\_peptide\_biosynthetic\_process | 1 | 0 |  |  |  |  |  |  |  |  |
| GO:0006965\_positive\_regulation\_of\_biosynthetic\_process\_of\_antibacterial\_peptides\_active\_against\_Gram-positive\_bacteria | 1 | 0 |  |  |  |  |  |  |  |  |
| GO:0006987\_activation\_of\_signaling\_protein\_activity\_involved\_in\_unfolded\_protein\_response | 1 | 0 |  |  |  |  |  |  |  |  |
| GO:0006990\_positive\_regulation\_of\_gene-specific\_transcription\_involved\_in\_unfolded\_protein\_response | 1 | 0 |  |  |  |  |  |  |  |  |
| GO:0006991\_response\_to\_sterol\_depletion | 1 | 0 |  |  |  |  |  |  |  |  |
| GO:0006994\_positive\_regulation\_of\_transcription\_via\_sterol\_regulatory\_element\_binding\_involved\_in\_ER-nuclear\_sterol\_response\_pathway | 1 | 0 |  |  |  |  |  |  |  |  |
| GO:0007039\_vacuolar\_protein\_catabolic\_process | 1 | 0 |  |  |  |  |  |  |  |  |
| GO:0007068\_negative\_regulation\_of\_transcription\_\_mitotic | 1 | 0 |  |  |  |  |  |  |  |  |
| GO:0007097\_nuclear\_migration | 1 | 0 |  |  |  |  |  |  |  |  |
| GO:0007100\_mitotic\_centrosome\_separation | 1 | 0 |  |  |  |  |  |  |  |  |
| GO:0007108\_cytokinesis\_\_initiation\_of\_separation | 1 | 0 |  |  |  |  |  |  |  |  |
| GO:0007109\_cytokinesis\_\_completion\_of\_separation | 1 | 0 |  |  |  |  |  |  |  |  |
| GO:0007132\_meiotic\_metaphase\_I | 1 | 0 |  |  |  |  |  |  |  |  |
| GO:0007135\_meiosis\_II | 1 | 0 |  |  |  |  |  |  |  |  |
| GO:0007136\_meiotic\_prophase\_II | 1 | 0 |  |  |  |  |  |  |  |  |
| GO:0007161\_calcium-independent\_cell-matrix\_adhesion | 1 | 0 |  |  |  |  |  |  |  |  |
| GO:0007196\_inhibition\_of\_adenylate\_cyclase\_activity\_by\_metabotropic\_glutamate\_receptor\_signaling\_pathway | 1 | 0 |  |  |  |  |  |  |  |  |
| GO:0007197\_inhibition\_of\_adenylate\_cyclase\_activity\_by\_muscarinic\_acetylcholine\_receptor\_signaling\_pathway | 1 | 0 |  |  |  |  |  |  |  |  |
| GO:0007258\_JUN\_phosphorylation | 1 | 0 |  |  |  |  |  |  |  |  |
| GO:0007321\_sperm\_displacement | 1 | 0 |  |  |  |  |  |  |  |  |
| GO:0007343\_egg\_activation | 1 | 0 |  |  |  |  |  |  |  |  |
| GO:0007386\_compartment\_specification | 1 | 0 |  |  |  |  |  |  |  |  |
| GO:0007387\_anterior\_compartment\_specification | 1 | 0 |  |  |  |  |  |  |  |  |
| GO:0007388\_posterior\_compartment\_specification | 1 | 0 |  |  |  |  |  |  |  |  |
| GO:0007402\_ganglion\_mother\_cell\_fate\_determination | 1 | 0 |  |  |  |  |  |  |  |  |
| GO:0007406\_negative\_regulation\_of\_neuroblast\_proliferation | 1 | 0 |  |  |  |  |  |  |  |  |
| GO:0007424\_open\_tracheal\_system\_development | 1 | 0 |  |  |  |  |  |  |  |  |
| GO:0007440\_foregut\_morphogenesis | 1 | 0 |  |  |  |  |  |  |  |  |
| GO:0007443\_Malpighian\_tubule\_morphogenesis | 1 | 0 |  |  |  |  |  |  |  |  |
| GO:0007444\_imaginal\_disc\_development | 1 | 0 |  |  |  |  |  |  |  |  |
| GO:0007447\_imaginal\_disc\_pattern\_formation | 1 | 0 |  |  |  |  |  |  |  |  |
| GO:0007494\_midgut\_development | 1 | 0 |  |  |  |  |  |  |  |  |
| GO:0007497\_posterior\_midgut\_development | 1 | 0 |  |  |  |  |  |  |  |  |
| GO:0007499\_ectoderm\_and\_mesoderm\_interaction | 1 | 0 |  |  |  |  |  |  |  |  |
| GO:0007501\_mesodermal\_cell\_fate\_specification | 1 | 0 |  |  |  |  |  |  |  |  |
| GO:0007509\_mesoderm\_migration | 1 | 0 |  |  |  |  |  |  |  |  |
| GO:0007518\_myoblast\_cell\_fate\_determination | 1 | 0 |  |  |  |  |  |  |  |  |
| GO:0007538\_primary\_sex\_determination | 1 | 0 |  |  |  |  |  |  |  |  |
| GO:0007597\_blood\_coagulation\_\_intrinsic\_pathway | 1 | 0 |  |  |  |  |  |  |  |  |
| GO:0007616\_long-term\_memory | 1 | 0 |  |  |  |  |  |  |  |  |
| GO:0007617\_mating\_behavior | 1 | 0 |  |  |  |  |  |  |  |  |
| GO:0007624\_ultradian\_rhythm | 1 | 0 |  |  |  |  |  |  |  |  |
| GO:0007638\_mechanosensory\_behavior | 1 | 0 |  |  |  |  |  |  |  |  |
| GO:0008045\_motor\_axon\_guidance | 1 | 0 |  |  |  |  |  |  |  |  |
| GO:0008057\_eye\_pigment\_granule\_organization | 1 | 0 |  |  |  |  |  |  |  |  |
| GO:0008063\_Toll\_signaling\_pathway | 1 | 0 |  |  |  |  |  |  |  |  |
| GO:0008065\_establishment\_of\_blood-nerve\_barrier | 1 | 0 |  |  |  |  |  |  |  |  |
| GO:0008090\_retrograde\_axon\_cargo\_transport | 1 | 0 |  |  |  |  |  |  |  |  |
| GO:0008215\_spermine\_metabolic\_process | 1 | 0 |  |  |  |  |  |  |  |  |
| GO:0008292\_acetylcholine\_biosynthetic\_process | 1 | 0 |  |  |  |  |  |  |  |  |
| GO:0008295\_spermidine\_biosynthetic\_process | 1 | 0 |  |  |  |  |  |  |  |  |
| GO:0008298\_intracellular\_mRNA\_localization | 1 | 0 |  |  |  |  |  |  |  |  |
| GO:0008356\_asymmetric\_cell\_division | 1 | 0 |  |  |  |  |  |  |  |  |
| GO:0008592\_regulation\_of\_Toll\_signaling\_pathway | 1 | 0 |  |  |  |  |  |  |  |  |
| GO:0008611\_ether\_lipid\_biosynthetic\_process | 1 | 0 |  |  |  |  |  |  |  |  |
| GO:0008614\_pyridoxine\_metabolic\_process | 1 | 0 |  |  |  |  |  |  |  |  |
| GO:0008615\_pyridoxine\_biosynthetic\_process | 1 | 0 |  |  |  |  |  |  |  |  |
| GO:0008627\_induction\_of\_apoptosis\_by\_ionic\_changes | 1 | 0 |  |  |  |  |  |  |  |  |
| GO:0008655\_pyrimidine\_salvage | 1 | 0 |  |  |  |  |  |  |  |  |
| GO:0009052\_pentose-phosphate\_shunt\_\_non-oxidative\_branch | 1 | 0 |  |  |  |  |  |  |  |  |
| GO:0009067\_aspartate\_family\_amino\_acid\_biosynthetic\_process | 1 | 0 |  |  |  |  |  |  |  |  |
| GO:0009075\_histidine\_family\_amino\_acid\_metabolic\_process | 1 | 0 |  |  |  |  |  |  |  |  |
| GO:0009128\_purine\_nucleoside\_monophosphate\_catabolic\_process | 1 | 0 |  |  |  |  |  |  |  |  |
| GO:0009129\_pyrimidine\_nucleoside\_monophosphate\_metabolic\_process | 1 | 0 |  |  |  |  |  |  |  |  |
| GO:0009130\_pyrimidine\_nucleoside\_monophosphate\_biosynthetic\_process | 1 | 0 |  |  |  |  |  |  |  |  |
| GO:0009133\_nucleoside\_diphosphate\_biosynthetic\_process | 1 | 0 |  |  |  |  |  |  |  |  |
| GO:0009135\_purine\_nucleoside\_diphosphate\_metabolic\_process | 1 | 0 |  |  |  |  |  |  |  |  |
| GO:0009136\_purine\_nucleoside\_diphosphate\_biosynthetic\_process | 1 | 0 |  |  |  |  |  |  |  |  |
| GO:0009138\_pyrimidine\_nucleoside\_diphosphate\_metabolic\_process | 1 | 0 |  |  |  |  |  |  |  |  |
| GO:0009140\_pyrimidine\_nucleoside\_diphosphate\_catabolic\_process | 1 | 0 |  |  |  |  |  |  |  |  |
| GO:0009147\_pyrimidine\_nucleoside\_triphosphate\_metabolic\_process | 1 | 0 |  |  |  |  |  |  |  |  |
| GO:0009148\_pyrimidine\_nucleoside\_triphosphate\_biosynthetic\_process | 1 | 0 |  |  |  |  |  |  |  |  |
| GO:0009153\_purine\_deoxyribonucleotide\_biosynthetic\_process | 1 | 0 |  |  |  |  |  |  |  |  |
| GO:0009157\_deoxyribonucleoside\_monophosphate\_biosynthetic\_process | 1 | 0 |  |  |  |  |  |  |  |  |
| GO:0009158\_ribonucleoside\_monophosphate\_catabolic\_process | 1 | 0 |  |  |  |  |  |  |  |  |
| GO:0009159\_deoxyribonucleoside\_monophosphate\_catabolic\_process | 1 | 0 |  |  |  |  |  |  |  |  |
| GO:0009169\_purine\_ribonucleoside\_monophosphate\_catabolic\_process | 1 | 0 |  |  |  |  |  |  |  |  |
| GO:0009173\_pyrimidine\_ribonucleoside\_monophosphate\_metabolic\_process | 1 | 0 |  |  |  |  |  |  |  |  |
| GO:0009174\_pyrimidine\_ribonucleoside\_monophosphate\_biosynthetic\_process | 1 | 0 |  |  |  |  |  |  |  |  |
| GO:0009179\_purine\_ribonucleoside\_diphosphate\_metabolic\_process | 1 | 0 |  |  |  |  |  |  |  |  |
| GO:0009180\_purine\_ribonucleoside\_diphosphate\_biosynthetic\_process | 1 | 0 |  |  |  |  |  |  |  |  |
| GO:0009182\_purine\_deoxyribonucleoside\_diphosphate\_metabolic\_process | 1 | 0 |  |  |  |  |  |  |  |  |
| GO:0009183\_purine\_deoxyribonucleoside\_diphosphate\_biosynthetic\_process | 1 | 0 |  |  |  |  |  |  |  |  |
| GO:0009186\_deoxyribonucleoside\_diphosphate\_metabolic\_process | 1 | 0 |  |  |  |  |  |  |  |  |
| GO:0009188\_ribonucleoside\_diphosphate\_biosynthetic\_process | 1 | 0 |  |  |  |  |  |  |  |  |
| GO:0009189\_deoxyribonucleoside\_diphosphate\_biosynthetic\_process | 1 | 0 |  |  |  |  |  |  |  |  |
| GO:0009193\_pyrimidine\_ribonucleoside\_diphosphate\_metabolic\_process | 1 | 0 |  |  |  |  |  |  |  |  |
| GO:0009195\_pyrimidine\_ribonucleoside\_diphosphate\_catabolic\_process | 1 | 0 |  |  |  |  |  |  |  |  |
| GO:0009208\_pyrimidine\_ribonucleoside\_triphosphate\_metabolic\_process | 1 | 0 |  |  |  |  |  |  |  |  |
| GO:0009209\_pyrimidine\_ribonucleoside\_triphosphate\_biosynthetic\_process | 1 | 0 |  |  |  |  |  |  |  |  |
| GO:0009214\_cyclic\_nucleotide\_catabolic\_process | 1 | 0 |  |  |  |  |  |  |  |  |
| GO:0009222\_pyrimidine\_ribonucleotide\_catabolic\_process | 1 | 0 |  |  |  |  |  |  |  |  |
| GO:0009231\_riboflavin\_biosynthetic\_process | 1 | 0 |  |  |  |  |  |  |  |  |
| GO:0009253\_peptidoglycan\_catabolic\_process | 1 | 0 |  |  |  |  |  |  |  |  |
| GO:0009256\_10-formyltetrahydrofolate\_metabolic\_process | 1 | 0 |  |  |  |  |  |  |  |  |
| GO:0009258\_10-formyltetrahydrofolate\_catabolic\_process | 1 | 0 |  |  |  |  |  |  |  |  |
| GO:0009265\_2'-deoxyribonucleotide\_biosynthetic\_process | 1 | 0 |  |  |  |  |  |  |  |  |
| GO:0009292\_genetic\_transfer | 1 | 0 |  |  |  |  |  |  |  |  |
| GO:0009294\_DNA\_mediated\_transformation | 1 | 0 |  |  |  |  |  |  |  |  |
| GO:0009296\_flagellum\_assembly | 1 | 0 |  |  |  |  |  |  |  |  |
| GO:0009298\_GDP-mannose\_biosynthetic\_process | 1 | 0 |  |  |  |  |  |  |  |  |
| GO:0009304\_tRNA\_transcription | 1 | 0 |  |  |  |  |  |  |  |  |
| GO:0009313\_oligosaccharide\_catabolic\_process | 1 | 0 |  |  |  |  |  |  |  |  |
| GO:0009372\_quorum\_sensing | 1 | 0 |  |  |  |  |  |  |  |  |
| GO:0009386\_translational\_attenuation | 1 | 0 |  |  |  |  |  |  |  |  |
| GO:0009397\_folic\_acid\_and\_derivative\_catabolic\_process | 1 | 0 |  |  |  |  |  |  |  |  |
| GO:0009399\_nitrogen\_fixation | 1 | 0 |  |  |  |  |  |  |  |  |
| GO:0009404\_toxin\_metabolic\_process | 1 | 0 |  |  |  |  |  |  |  |  |
| GO:0009435\_NAD\_biosynthetic\_process | 1 | 0 |  |  |  |  |  |  |  |  |
| GO:0009437\_carnitine\_metabolic\_process | 1 | 0 |  |  |  |  |  |  |  |  |
| GO:0009441\_glycolate\_metabolic\_process | 1 | 0 |  |  |  |  |  |  |  |  |
| GO:0009624\_response\_to\_nematode | 1 | 0 |  |  |  |  |  |  |  |  |
| GO:0009642\_response\_to\_light\_intensity | 1 | 0 |  |  |  |  |  |  |  |  |
| GO:0009648\_photoperiodism | 1 | 0 |  |  |  |  |  |  |  |  |
| GO:0009720\_detection\_of\_hormone\_stimulus | 1 | 0 |  |  |  |  |  |  |  |  |
| GO:0009726\_detection\_of\_endogenous\_stimulus | 1 | 0 |  |  |  |  |  |  |  |  |
| GO:0009730\_detection\_of\_carbohydrate\_stimulus | 1 | 0 |  |  |  |  |  |  |  |  |
| GO:0009732\_detection\_of\_hexose\_stimulus | 1 | 0 |  |  |  |  |  |  |  |  |
| GO:0009826\_unidimensional\_cell\_growth | 1 | 0 |  |  |  |  |  |  |  |  |
| GO:0009912\_auditory\_receptor\_cell\_fate\_commitment | 1 | 0 |  |  |  |  |  |  |  |  |
| GO:0009954\_proximal\_distal\_pattern\_formation | 1 | 0 |  |  |  |  |  |  |  |  |
| GO:0009972\_cytidine\_deamination | 1 | 0 |  |  |  |  |  |  |  |  |
| GO:0010107\_potassium\_ion\_import | 1 | 0 |  |  |  |  |  |  |  |  |
| GO:0010259\_multicellular\_organismal\_aging | 1 | 0 |  |  |  |  |  |  |  |  |
| GO:0010269\_response\_to\_selenium\_ion | 1 | 0 |  |  |  |  |  |  |  |  |
| GO:0010273\_detoxification\_of\_copper\_ion | 1 | 0 |  |  |  |  |  |  |  |  |
| GO:0010383\_cell\_wall\_polysaccharide\_metabolic\_process | 1 | 0 |  |  |  |  |  |  |  |  |
| GO:0010430\_fatty\_acid\_omega-oxidation | 1 | 0 |  |  |  |  |  |  |  |  |
| GO:0010463\_mesenchymal\_cell\_proliferation | 1 | 0 |  |  |  |  |  |  |  |  |
| GO:0010464\_regulation\_of\_mesenchymal\_cell\_proliferation | 1 | 0 |  |  |  |  |  |  |  |  |
| GO:0010507\_negative\_regulation\_of\_autophagy | 1 | 0 |  |  |  |  |  |  |  |  |
| GO:0010509\_polyamine\_homeostasis | 1 | 0 |  |  |  |  |  |  |  |  |
| GO:0010534\_regulation\_of\_activation\_of\_JAK2\_kinase\_activity | 1 | 0 |  |  |  |  |  |  |  |  |
| GO:0010535\_positive\_regulation\_of\_activation\_of\_JAK2\_kinase\_activity | 1 | 0 |  |  |  |  |  |  |  |  |
| GO:0010561\_negative\_regulation\_of\_glycoprotein\_biosynthetic\_process | 1 | 0 |  |  |  |  |  |  |  |  |
| GO:0010569\_regulation\_of\_double-strand\_break\_repair\_via\_homologous\_recombination | 1 | 0 |  |  |  |  |  |  |  |  |
| GO:0010591\_regulation\_of\_lamellipodium\_assembly | 1 | 0 |  |  |  |  |  |  |  |  |
| GO:0010592\_positive\_regulation\_of\_lamellipodium\_assembly | 1 | 0 |  |  |  |  |  |  |  |  |
| GO:0010621\_negative\_regulation\_of\_transcription\_by\_transcription\_factor\_localization | 1 | 0 |  |  |  |  |  |  |  |  |
| GO:0010623\_developmental\_programmed\_cell\_death | 1 | 0 |  |  |  |  |  |  |  |  |
| GO:0010631\_epithelial\_cell\_migration | 1 | 0 |  |  |  |  |  |  |  |  |
| GO:0010632\_regulation\_of\_epithelial\_cell\_migration | 1 | 0 |  |  |  |  |  |  |  |  |
| GO:0010634\_positive\_regulation\_of\_epithelial\_cell\_migration | 1 | 0 |  |  |  |  |  |  |  |  |
| GO:0010658\_striated\_muscle\_cell\_apoptosis | 1 | 0 |  |  |  |  |  |  |  |  |
| GO:0010659\_cardiac\_muscle\_cell\_apoptosis | 1 | 0 |  |  |  |  |  |  |  |  |
| GO:0010662\_regulation\_of\_striated\_muscle\_cell\_apoptosis | 1 | 0 |  |  |  |  |  |  |  |  |
| GO:0010664\_negative\_regulation\_of\_striated\_muscle\_cell\_apoptosis | 1 | 0 |  |  |  |  |  |  |  |  |
| GO:0010665\_regulation\_of\_cardiac\_muscle\_cell\_apoptosis | 1 | 0 |  |  |  |  |  |  |  |  |
| GO:0010667\_negative\_regulation\_of\_cardiac\_muscle\_cell\_apoptosis | 1 | 0 |  |  |  |  |  |  |  |  |
| GO:0010669\_epithelial\_structure\_maintenance | 1 | 0 |  |  |  |  |  |  |  |  |
| GO:0010692\_regulation\_of\_alkaline\_phosphatase\_activity | 1 | 0 |  |  |  |  |  |  |  |  |
| GO:0010693\_negative\_regulation\_of\_alkaline\_phosphatase\_activity | 1 | 0 |  |  |  |  |  |  |  |  |
| GO:0010710\_regulation\_of\_collagen\_catabolic\_process | 1 | 0 |  |  |  |  |  |  |  |  |
| GO:0010711\_negative\_regulation\_of\_collagen\_catabolic\_process | 1 | 0 |  |  |  |  |  |  |  |  |
| GO:0010715\_regulation\_of\_extracellular\_matrix\_disassembly | 1 | 0 |  |  |  |  |  |  |  |  |
| GO:0010716\_negative\_regulation\_of\_extracellular\_matrix\_disassembly | 1 | 0 |  |  |  |  |  |  |  |  |
| GO:0010719\_negative\_regulation\_of\_epithelial\_to\_mesenchymal\_transition | 1 | 0 |  |  |  |  |  |  |  |  |
| GO:0010722\_regulation\_of\_ferrochelatase\_activity | 1 | 0 |  |  |  |  |  |  |  |  |
| GO:0010731\_protein\_amino\_acid\_glutathionylation | 1 | 0 |  |  |  |  |  |  |  |  |
| GO:0010732\_regulation\_of\_protein\_amino\_acid\_glutathionylation | 1 | 0 |  |  |  |  |  |  |  |  |
| GO:0010734\_negative\_regulation\_of\_protein\_amino\_acid\_glutathionylation | 1 | 0 |  |  |  |  |  |  |  |  |
| GO:0010735\_positive\_regulation\_of\_transcription\_via\_serum\_response\_element\_binding | 1 | 0 |  |  |  |  |  |  |  |  |
| GO:0010737\_protein\_kinase\_A\_signaling\_cascade | 1 | 0 |  |  |  |  |  |  |  |  |
| GO:0010738\_regulation\_of\_protein\_kinase\_A\_signaling\_cascade | 1 | 0 |  |  |  |  |  |  |  |  |
| GO:0010739\_positive\_regulation\_of\_protein\_kinase\_A\_signaling\_cascade | 1 | 0 |  |  |  |  |  |  |  |  |
| GO:0010756\_positive\_regulation\_of\_plasminogen\_activation | 1 | 0 |  |  |  |  |  |  |  |  |
| GO:0010766\_negative\_regulation\_of\_sodium\_ion\_transport | 1 | 0 |  |  |  |  |  |  |  |  |
| GO:0010767\_regulation\_of\_transcription\_from\_RNA\_polymerase\_II\_promoter\_in\_response\_to\_UV-induced\_DNA\_damage | 1 | 0 |  |  |  |  |  |  |  |  |
| GO:0010768\_negative\_regulation\_of\_transcription\_from\_RNA\_polymerase\_II\_promoter\_in\_response\_to\_UV-induced\_DNA\_damage | 1 | 0 |  |  |  |  |  |  |  |  |
| GO:0010771\_negative\_regulation\_of\_cell\_morphogenesis\_involved\_in\_differentiation | 1 | 0 |  |  |  |  |  |  |  |  |
| GO:0010793\_regulation\_of\_mRNA\_export\_from\_nucleus | 1 | 0 |  |  |  |  |  |  |  |  |
| GO:0010801\_negative\_regulation\_of\_peptidyl-threonine\_phosphorylation | 1 | 0 |  |  |  |  |  |  |  |  |
| GO:0010803\_regulation\_of\_tumor\_necrosis\_factor-mediated\_signaling\_pathway | 1 | 0 |  |  |  |  |  |  |  |  |
| GO:0010804\_negative\_regulation\_of\_tumor\_necrosis\_factor-mediated\_signaling\_pathway | 1 | 0 |  |  |  |  |  |  |  |  |
| GO:0010813\_neuropeptide\_catabolic\_process | 1 | 0 |  |  |  |  |  |  |  |  |
| GO:0010814\_substance\_P\_catabolic\_process | 1 | 0 |  |  |  |  |  |  |  |  |
| GO:0010816\_calcitonin\_catabolic\_process | 1 | 0 |  |  |  |  |  |  |  |  |
| GO:0010826\_negative\_regulation\_of\_centrosome\_duplication | 1 | 0 |  |  |  |  |  |  |  |  |
| GO:0010830\_regulation\_of\_myotube\_differentiation | 1 | 0 |  |  |  |  |  |  |  |  |
| GO:0010832\_negative\_regulation\_of\_myotube\_differentiation | 1 | 0 |  |  |  |  |  |  |  |  |
| GO:0010835\_regulation\_of\_protein\_amino\_acid\_ADP-ribosylation | 1 | 0 |  |  |  |  |  |  |  |  |
| GO:0010836\_negative\_regulation\_of\_protein\_amino\_acid\_ADP-ribosylation | 1 | 0 |  |  |  |  |  |  |  |  |
| GO:0010837\_regulation\_of\_keratinocyte\_proliferation | 1 | 0 |  |  |  |  |  |  |  |  |
| GO:0010839\_negative\_regulation\_of\_keratinocyte\_proliferation | 1 | 0 |  |  |  |  |  |  |  |  |
| GO:0010840\_regulation\_of\_circadian\_sleep\_wake\_cycle\_\_wakefulness | 1 | 0 |  |  |  |  |  |  |  |  |
| GO:0010841\_positive\_regulation\_of\_circadian\_sleep\_wake\_cycle\_\_wakefulness | 1 | 0 |  |  |  |  |  |  |  |  |
| GO:0010842\_retina\_layer\_formation | 1 | 0 |  |  |  |  |  |  |  |  |
| GO:0010897\_negative\_regulation\_of\_triglyceride\_catabolic\_process | 1 | 0 |  |  |  |  |  |  |  |  |
| GO:0010899\_regulation\_of\_phosphatidylcholine\_catabolic\_process | 1 | 0 |  |  |  |  |  |  |  |  |
| GO:0010900\_negative\_regulation\_of\_phosphatidylcholine\_catabolic\_process | 1 | 0 |  |  |  |  |  |  |  |  |
| GO:0010902\_positive\_regulation\_of\_very-low-density\_lipoprotein\_particle\_remodeling | 1 | 0 |  |  |  |  |  |  |  |  |
| GO:0010919\_regulation\_of\_inositol\_phosphate\_biosynthetic\_process | 1 | 0 |  |  |  |  |  |  |  |  |
| GO:0010920\_negative\_regulation\_of\_inositol\_phosphate\_biosynthetic\_process | 1 | 0 |  |  |  |  |  |  |  |  |
| GO:0010924\_regulation\_of\_inositol-polyphosphate\_5-phosphatase\_activity | 1 | 0 |  |  |  |  |  |  |  |  |
| GO:0010925\_positive\_regulation\_of\_inositol-polyphosphate\_5-phosphatase\_activity | 1 | 0 |  |  |  |  |  |  |  |  |
| GO:0010931\_macrophage\_tolerance\_induction | 1 | 0 |  |  |  |  |  |  |  |  |
| GO:0010932\_regulation\_of\_macrophage\_tolerance\_induction | 1 | 0 |  |  |  |  |  |  |  |  |
| GO:0010933\_positive\_regulation\_of\_macrophage\_tolerance\_induction | 1 | 0 |  |  |  |  |  |  |  |  |
| GO:0010934\_macrophage\_cytokine\_production | 1 | 0 |  |  |  |  |  |  |  |  |
| GO:0010935\_regulation\_of\_macrophage\_cytokine\_production | 1 | 0 |  |  |  |  |  |  |  |  |
| GO:0010936\_negative\_regulation\_of\_macrophage\_cytokine\_production | 1 | 0 |  |  |  |  |  |  |  |  |
| GO:0010944\_negative\_regulation\_of\_transcription\_by\_competitive\_promoter\_binding | 1 | 0 |  |  |  |  |  |  |  |  |
| GO:0010983\_positive\_regulation\_of\_high-density\_lipoprotein\_particle\_clearance | 1 | 0 |  |  |  |  |  |  |  |  |
| GO:0010986\_positive\_regulation\_of\_lipoprotein\_particle\_clearance | 1 | 0 |  |  |  |  |  |  |  |  |
| GO:0010987\_negative\_regulation\_of\_high-density\_lipoprotein\_particle\_clearance | 1 | 0 |  |  |  |  |  |  |  |  |
| GO:0010988\_regulation\_of\_low-density\_lipoprotein\_particle\_clearance | 1 | 0 |  |  |  |  |  |  |  |  |
| GO:0010989\_negative\_regulation\_of\_low-density\_lipoprotein\_particle\_clearance | 1 | 0 |  |  |  |  |  |  |  |  |
| GO:0010990\_regulation\_of\_SMAD\_protein\_complex\_assembly | 1 | 0 |  |  |  |  |  |  |  |  |
| GO:0010991\_negative\_regulation\_of\_SMAD\_protein\_complex\_assembly | 1 | 0 |  |  |  |  |  |  |  |  |
| GO:0014009\_glial\_cell\_proliferation | 1 | 0 |  |  |  |  |  |  |  |  |
| GO:0014010\_Schwann\_cell\_proliferation | 1 | 0 |  |  |  |  |  |  |  |  |
| GO:0014045\_establishment\_of\_endothelial\_blood-brain\_barrier | 1 | 0 |  |  |  |  |  |  |  |  |
| GO:0014055\_acetylcholine\_secretion | 1 | 0 |  |  |  |  |  |  |  |  |
| GO:0014056\_regulation\_of\_acetylcholine\_secretion | 1 | 0 |  |  |  |  |  |  |  |  |
| GO:0014060\_regulation\_of\_epinephrine\_secretion | 1 | 0 |  |  |  |  |  |  |  |  |
| GO:0014067\_negative\_regulation\_of\_phosphoinositide\_3-kinase\_cascade | 1 | 0 |  |  |  |  |  |  |  |  |
| GO:0014071\_response\_to\_cycloalkane | 1 | 0 |  |  |  |  |  |  |  |  |
| GO:0014721\_twitch\_skeletal\_muscle\_contraction | 1 | 0 |  |  |  |  |  |  |  |  |
| GO:0014724\_regulation\_of\_twitch\_skeletal\_muscle\_contraction | 1 | 0 |  |  |  |  |  |  |  |  |
| GO:0014806\_smooth\_muscle\_hyperplasia | 1 | 0 |  |  |  |  |  |  |  |  |
| GO:0014823\_response\_to\_activity | 1 | 0 |  |  |  |  |  |  |  |  |
| GO:0014832\_urinary\_bladder\_smooth\_muscle\_contraction | 1 | 0 |  |  |  |  |  |  |  |  |
| GO:0014834\_satellite\_cell\_maintenance\_involved\_in\_skeletal\_muscle\_regeneration | 1 | 0 |  |  |  |  |  |  |  |  |
| GO:0014848\_urinary\_tract\_smooth\_muscle\_contraction | 1 | 0 |  |  |  |  |  |  |  |  |
| GO:0014850\_response\_to\_muscle\_activity | 1 | 0 |  |  |  |  |  |  |  |  |
| GO:0014873\_response\_to\_muscle\_activity\_involved\_in\_regulation\_of\_muscle\_adaptation | 1 | 0 |  |  |  |  |  |  |  |  |
| GO:0014874\_response\_to\_stimulus\_involved\_in\_regulation\_of\_muscle\_adaptation | 1 | 0 |  |  |  |  |  |  |  |  |
| GO:0014895\_smooth\_muscle\_hypertrophy | 1 | 0 |  |  |  |  |  |  |  |  |
| GO:0014904\_myotube\_cell\_development | 1 | 0 |  |  |  |  |  |  |  |  |
| GO:0014916\_regulation\_of\_lung\_blood\_pressure | 1 | 0 |  |  |  |  |  |  |  |  |
| GO:0015675\_nickel\_ion\_transport | 1 | 0 |  |  |  |  |  |  |  |  |
| GO:0015676\_vanadium\_ion\_transport | 1 | 0 |  |  |  |  |  |  |  |  |
| GO:0015680\_intracellular\_copper\_ion\_transport | 1 | 0 |  |  |  |  |  |  |  |  |
| GO:0015684\_ferrous\_iron\_transport | 1 | 0 |  |  |  |  |  |  |  |  |
| GO:0015692\_lead\_ion\_transport | 1 | 0 |  |  |  |  |  |  |  |  |
| GO:0015693\_magnesium\_ion\_transport | 1 | 0 |  |  |  |  |  |  |  |  |
| GO:0015727\_lactate\_transport | 1 | 0 |  |  |  |  |  |  |  |  |
| GO:0015728\_mevalonate\_transport | 1 | 0 |  |  |  |  |  |  |  |  |
| GO:0015742\_alpha-ketoglutarate\_transport | 1 | 0 |  |  |  |  |  |  |  |  |
| GO:0015746\_citrate\_transport | 1 | 0 |  |  |  |  |  |  |  |  |
| GO:0015747\_urate\_transport | 1 | 0 |  |  |  |  |  |  |  |  |
| GO:0015755\_fructose\_transport | 1 | 0 |  |  |  |  |  |  |  |  |
| GO:0015760\_glucose-6-phosphate\_transport | 1 | 0 |  |  |  |  |  |  |  |  |
| GO:0015782\_CMP-sialic\_acid\_transport | 1 | 0 |  |  |  |  |  |  |  |  |
| GO:0015785\_UDP-galactose\_transport | 1 | 0 |  |  |  |  |  |  |  |  |
| GO:0015789\_UDP-N-acetylgalactosamine\_transport | 1 | 0 |  |  |  |  |  |  |  |  |
| GO:0015790\_UDP-xylose\_transport | 1 | 0 |  |  |  |  |  |  |  |  |
| GO:0015798\_myo-inositol\_transport | 1 | 0 |  |  |  |  |  |  |  |  |
| GO:0015803\_branched-chain\_aliphatic\_amino\_acid\_transport | 1 | 0 |  |  |  |  |  |  |  |  |
| GO:0015805\_S-adenosylmethionine\_transport | 1 | 0 |  |  |  |  |  |  |  |  |
| GO:0015809\_arginine\_transport | 1 | 0 |  |  |  |  |  |  |  |  |
| GO:0015817\_histidine\_transport | 1 | 0 |  |  |  |  |  |  |  |  |
| GO:0015820\_leucine\_transport | 1 | 0 |  |  |  |  |  |  |  |  |
| GO:0015826\_threonine\_transport | 1 | 0 |  |  |  |  |  |  |  |  |
| GO:0015827\_tryptophan\_transport | 1 | 0 |  |  |  |  |  |  |  |  |
| GO:0015846\_polyamine\_transport | 1 | 0 |  |  |  |  |  |  |  |  |
| GO:0015853\_adenine\_transport | 1 | 0 |  |  |  |  |  |  |  |  |
| GO:0015855\_pyrimidine\_transport | 1 | 0 |  |  |  |  |  |  |  |  |
| GO:0015886\_heme\_transport | 1 | 0 |  |  |  |  |  |  |  |  |
| GO:0015888\_thiamin\_transport | 1 | 0 |  |  |  |  |  |  |  |  |
| GO:0015910\_peroxisomal\_long-chain\_fatty\_acid\_import | 1 | 0 |  |  |  |  |  |  |  |  |
| GO:0015919\_peroxisomal\_membrane\_transport | 1 | 0 |  |  |  |  |  |  |  |  |
| GO:0015937\_coenzyme\_A\_biosynthetic\_process | 1 | 0 |  |  |  |  |  |  |  |  |
| GO:0015956\_bis(5'-nucleosidyl)\_oligophosphate\_metabolic\_process | 1 | 0 |  |  |  |  |  |  |  |  |
| GO:0015958\_bis(5'-nucleosidyl)\_oligophosphate\_catabolic\_process | 1 | 0 |  |  |  |  |  |  |  |  |
| GO:0015959\_diadenosine\_polyphosphate\_metabolic\_process | 1 | 0 |  |  |  |  |  |  |  |  |
| GO:0015961\_diadenosine\_polyphosphate\_catabolic\_process | 1 | 0 |  |  |  |  |  |  |  |  |
| GO:0016046\_detection\_of\_fungus | 1 | 0 |  |  |  |  |  |  |  |  |
| GO:0016078\_tRNA\_catabolic\_process | 1 | 0 |  |  |  |  |  |  |  |  |
| GO:0016091\_prenol\_biosynthetic\_process | 1 | 0 |  |  |  |  |  |  |  |  |
| GO:0016094\_polyprenol\_biosynthetic\_process | 1 | 0 |  |  |  |  |  |  |  |  |
| GO:0016108\_tetraterpenoid\_metabolic\_process | 1 | 0 |  |  |  |  |  |  |  |  |
| GO:0016116\_carotenoid\_metabolic\_process | 1 | 0 |  |  |  |  |  |  |  |  |
| GO:0016119\_carotene\_metabolic\_process | 1 | 0 |  |  |  |  |  |  |  |  |
| GO:0016140\_O-glycoside\_metabolic\_process | 1 | 0 |  |  |  |  |  |  |  |  |
| GO:0016142\_O-glycoside\_catabolic\_process | 1 | 0 |  |  |  |  |  |  |  |  |
| GO:0016188\_synaptic\_vesicle\_maturation | 1 | 0 |  |  |  |  |  |  |  |  |
| GO:0016189\_synaptic\_vesicle\_to\_endosome\_fusion | 1 | 0 |  |  |  |  |  |  |  |  |
| GO:0016241\_regulation\_of\_macroautophagy | 1 | 0 |  |  |  |  |  |  |  |  |
| GO:0016242\_negative\_regulation\_of\_macroautophagy | 1 | 0 |  |  |  |  |  |  |  |  |
| GO:0016259\_selenocysteine\_metabolic\_process | 1 | 0 |  |  |  |  |  |  |  |  |
| GO:0016260\_selenocysteine\_biosynthetic\_process | 1 | 0 |  |  |  |  |  |  |  |  |
| GO:0016269\_O-glycan\_processing\_\_core\_3 | 1 | 0 |  |  |  |  |  |  |  |  |
| GO:0016320\_endoplasmic\_reticulum\_membrane\_fusion | 1 | 0 |  |  |  |  |  |  |  |  |
| GO:0016344\_meiotic\_chromosome\_movement\_towards\_spindle\_pole | 1 | 0 |  |  |  |  |  |  |  |  |
| GO:0016446\_somatic\_hypermutation\_of\_immunoglobulin\_genes | 1 | 0 |  |  |  |  |  |  |  |  |
| GO:0016559\_peroxisome\_fission | 1 | 0 |  |  |  |  |  |  |  |  |
| GO:0016560\_protein\_import\_into\_peroxisome\_matrix\_\_docking | 1 | 0 |  |  |  |  |  |  |  |  |
| GO:0016598\_protein\_arginylation | 1 | 0 |  |  |  |  |  |  |  |  |
| GO:0016998\_cell\_wall\_macromolecule\_catabolic\_process | 1 | 0 |  |  |  |  |  |  |  |  |
| GO:0017062\_respiratory\_chain\_complex\_III\_assembly | 1 | 0 |  |  |  |  |  |  |  |  |
| GO:0017185\_peptidyl-lysine\_hydroxylation | 1 | 0 |  |  |  |  |  |  |  |  |
| GO:0018095\_protein\_polyglutamylation | 1 | 0 |  |  |  |  |  |  |  |  |
| GO:0018125\_peptidyl-cysteine\_methylation | 1 | 0 |  |  |  |  |  |  |  |  |
| GO:0018126\_protein\_amino\_acid\_hydroxylation | 1 | 0 |  |  |  |  |  |  |  |  |
| GO:0018146\_keratan\_sulfate\_biosynthetic\_process | 1 | 0 |  |  |  |  |  |  |  |  |
| GO:0018153\_isopeptide\_cross-linking\_via\_N6-(L-isoglutamyl)-L-lysine | 1 | 0 |  |  |  |  |  |  |  |  |
| GO:0018184\_protein\_amino\_acid\_polyamination | 1 | 0 |  |  |  |  |  |  |  |  |
| GO:0018190\_protein\_amino\_acid\_octanoylation | 1 | 0 |  |  |  |  |  |  |  |  |
| GO:0018191\_peptidyl-serine\_octanoylation | 1 | 0 |  |  |  |  |  |  |  |  |
| GO:0018192\_enzyme\_active\_site\_formation\_via\_L-cysteine\_persulfide | 1 | 0 |  |  |  |  |  |  |  |  |
| GO:0018199\_peptidyl-glutamine\_modification | 1 | 0 |  |  |  |  |  |  |  |  |
| GO:0018200\_peptidyl-glutamic\_acid\_modification | 1 | 0 |  |  |  |  |  |  |  |  |
| GO:0018208\_peptidyl-proline\_modification | 1 | 0 |  |  |  |  |  |  |  |  |
| GO:0018262\_isopeptide\_cross-linking | 1 | 0 |  |  |  |  |  |  |  |  |
| GO:0018307\_enzyme\_active\_site\_formation | 1 | 0 |  |  |  |  |  |  |  |  |
| GO:0018318\_protein\_amino\_acid\_palmitoylation | 1 | 0 |  |  |  |  |  |  |  |  |
| GO:0018319\_protein\_amino\_acid\_myristoylation | 1 | 0 |  |  |  |  |  |  |  |  |
| GO:0018345\_protein\_palmitoylation | 1 | 0 |  |  |  |  |  |  |  |  |
| GO:0018350\_protein\_amino\_acid\_esterification | 1 | 0 |  |  |  |  |  |  |  |  |
| GO:0018352\_protein-pyridoxal-5-phosphate\_linkage | 1 | 0 |  |  |  |  |  |  |  |  |
| GO:0018377\_protein\_myristoylation | 1 | 0 |  |  |  |  |  |  |  |  |
| GO:0018395\_peptidyl-lysine\_hydroxylation\_to\_5-hydroxy-L-lysine | 1 | 0 |  |  |  |  |  |  |  |  |
| GO:0018401\_peptidyl-proline\_hydroxylation\_to\_4-hydroxy-L-proline | 1 | 0 |  |  |  |  |  |  |  |  |
| GO:0018872\_arsonoacetate\_metabolic\_process | 1 | 0 |  |  |  |  |  |  |  |  |
| GO:0018874\_benzoate\_metabolic\_process | 1 | 0 |  |  |  |  |  |  |  |  |
| GO:0019060\_intracellular\_transport\_of\_viral\_proteins\_in\_host\_cell | 1 | 0 |  |  |  |  |  |  |  |  |
| GO:0019064\_viral\_envelope\_fusion\_with\_host\_membrane | 1 | 0 |  |  |  |  |  |  |  |  |
| GO:0019086\_late\_viral\_mRNA\_transcription | 1 | 0 |  |  |  |  |  |  |  |  |
| GO:0019087\_transformation\_of\_host\_cell\_by\_virus | 1 | 0 |  |  |  |  |  |  |  |  |
| GO:0019089\_transmission\_of\_virus | 1 | 0 |  |  |  |  |  |  |  |  |
| GO:0019098\_reproductive\_behavior | 1 | 0 |  |  |  |  |  |  |  |  |
| GO:0019240\_citrulline\_biosynthetic\_process | 1 | 0 |  |  |  |  |  |  |  |  |
| GO:0019302\_D-ribose\_biosynthetic\_process | 1 | 0 |  |  |  |  |  |  |  |  |
| GO:0019303\_D-ribose\_catabolic\_process | 1 | 0 |  |  |  |  |  |  |  |  |
| GO:0019307\_mannose\_biosynthetic\_process | 1 | 0 |  |  |  |  |  |  |  |  |
| GO:0019310\_inositol\_catabolic\_process | 1 | 0 |  |  |  |  |  |  |  |  |
| GO:0019322\_pentose\_biosynthetic\_process | 1 | 0 |  |  |  |  |  |  |  |  |
| GO:0019323\_pentose\_catabolic\_process | 1 | 0 |  |  |  |  |  |  |  |  |
| GO:0019371\_cyclooxygenase\_pathway | 1 | 0 |  |  |  |  |  |  |  |  |
| GO:0019372\_lipoxygenase\_pathway | 1 | 0 |  |  |  |  |  |  |  |  |
| GO:0019388\_galactose\_catabolic\_process | 1 | 0 |  |  |  |  |  |  |  |  |
| GO:0019405\_alditol\_catabolic\_process | 1 | 0 |  |  |  |  |  |  |  |  |
| GO:0019407\_hexitol\_catabolic\_process | 1 | 0 |  |  |  |  |  |  |  |  |
| GO:0019408\_dolichol\_biosynthetic\_process | 1 | 0 |  |  |  |  |  |  |  |  |
| GO:0019441\_tryptophan\_catabolic\_process\_to\_kynurenine | 1 | 0 |  |  |  |  |  |  |  |  |
| GO:0019471\_4-hydroxyproline\_metabolic\_process | 1 | 0 |  |  |  |  |  |  |  |  |
| GO:0019511\_peptidyl-proline\_hydroxylation | 1 | 0 |  |  |  |  |  |  |  |  |
| GO:0019519\_pentitol\_metabolic\_process | 1 | 0 |  |  |  |  |  |  |  |  |
| GO:0019527\_pentitol\_catabolic\_process | 1 | 0 |  |  |  |  |  |  |  |  |
| GO:0019614\_catechol\_catabolic\_process | 1 | 0 |  |  |  |  |  |  |  |  |
| GO:0019673\_GDP-mannose\_metabolic\_process | 1 | 0 |  |  |  |  |  |  |  |  |
| GO:0019693\_ribose\_phosphate\_metabolic\_process | 1 | 0 |  |  |  |  |  |  |  |  |
| GO:0019695\_choline\_metabolic\_process | 1 | 0 |  |  |  |  |  |  |  |  |
| GO:0019747\_regulation\_of\_isoprenoid\_metabolic\_process | 1 | 0 |  |  |  |  |  |  |  |  |
| GO:0019852\_L-ascorbic\_acid\_metabolic\_process | 1 | 0 |  |  |  |  |  |  |  |  |
| GO:0019856\_pyrimidine\_base\_biosynthetic\_process | 1 | 0 |  |  |  |  |  |  |  |  |
| GO:0019858\_cytosine\_metabolic\_process | 1 | 0 |  |  |  |  |  |  |  |  |
| GO:0019884\_antigen\_processing\_and\_presentation\_of\_exogenous\_antigen | 1 | 0 |  |  |  |  |  |  |  |  |
| GO:0019886\_antigen\_processing\_and\_presentation\_of\_exogenous\_peptide\_antigen\_via\_MHC\_class\_II | 1 | 0 |  |  |  |  |  |  |  |  |
| GO:0021508\_floor\_plate\_formation | 1 | 0 |  |  |  |  |  |  |  |  |
| GO:0021514\_ventral\_spinal\_cord\_interneuron\_differentiation | 1 | 0 |  |  |  |  |  |  |  |  |
| GO:0021521\_ventral\_spinal\_cord\_interneuron\_specification | 1 | 0 |  |  |  |  |  |  |  |  |
| GO:0021522\_spinal\_cord\_motor\_neuron\_differentiation | 1 | 0 |  |  |  |  |  |  |  |  |
| GO:0021527\_spinal\_cord\_association\_neuron\_differentiation | 1 | 0 |  |  |  |  |  |  |  |  |
| GO:0021528\_commissural\_neuron\_differentiation\_in\_the\_spinal\_cord | 1 | 0 |  |  |  |  |  |  |  |  |
| GO:0021533\_cell\_differentiation\_in\_hindbrain | 1 | 0 |  |  |  |  |  |  |  |  |
| GO:0021540\_corpus\_callosum\_morphogenesis | 1 | 0 |  |  |  |  |  |  |  |  |
| GO:0021544\_subpallium\_development | 1 | 0 |  |  |  |  |  |  |  |  |
| GO:0021554\_optic\_nerve\_development | 1 | 0 |  |  |  |  |  |  |  |  |
| GO:0021562\_vestibulocochlear\_nerve\_development | 1 | 0 |  |  |  |  |  |  |  |  |
| GO:0021602\_cranial\_nerve\_morphogenesis | 1 | 0 |  |  |  |  |  |  |  |  |
| GO:0021631\_optic\_nerve\_morphogenesis | 1 | 0 |  |  |  |  |  |  |  |  |
| GO:0021680\_cerebellar\_Purkinje\_cell\_layer\_development | 1 | 0 |  |  |  |  |  |  |  |  |
| GO:0021692\_cerebellar\_Purkinje\_cell\_layer\_morphogenesis | 1 | 0 |  |  |  |  |  |  |  |  |
| GO:0021694\_cerebellar\_Purkinje\_cell\_layer\_formation | 1 | 0 |  |  |  |  |  |  |  |  |
| GO:0021697\_cerebellar\_cortex\_formation | 1 | 0 |  |  |  |  |  |  |  |  |
| GO:0021702\_cerebellar\_Purkinje\_cell\_differentiation | 1 | 0 |  |  |  |  |  |  |  |  |
| GO:0021756\_striatum\_development | 1 | 0 |  |  |  |  |  |  |  |  |
| GO:0021757\_caudate\_nucleus\_development | 1 | 0 |  |  |  |  |  |  |  |  |
| GO:0021758\_putamen\_development | 1 | 0 |  |  |  |  |  |  |  |  |
| GO:0021761\_limbic\_system\_development | 1 | 0 |  |  |  |  |  |  |  |  |
| GO:0021771\_lateral\_geniculate\_nucleus\_development | 1 | 0 |  |  |  |  |  |  |  |  |
| GO:0021775\_smoothened\_signaling\_pathway\_involved\_in\_ventral\_spinal\_cord\_interneuron\_specification | 1 | 0 |  |  |  |  |  |  |  |  |
| GO:0021794\_thalamus\_development | 1 | 0 |  |  |  |  |  |  |  |  |
| GO:0021799\_cerebral\_cortex\_radially\_oriented\_cell\_migration | 1 | 0 |  |  |  |  |  |  |  |  |
| GO:0021800\_cerebral\_cortex\_tangential\_migration | 1 | 0 |  |  |  |  |  |  |  |  |
| GO:0021854\_hypothalamus\_development | 1 | 0 |  |  |  |  |  |  |  |  |
| GO:0021859\_pyramidal\_neuron\_differentiation | 1 | 0 |  |  |  |  |  |  |  |  |
| GO:0021860\_pyramidal\_neuron\_development | 1 | 0 |  |  |  |  |  |  |  |  |
| GO:0021872\_generation\_of\_neurons\_in\_the\_forebrain | 1 | 0 |  |  |  |  |  |  |  |  |
| GO:0021879\_forebrain\_neuron\_differentiation | 1 | 0 |  |  |  |  |  |  |  |  |
| GO:0021884\_forebrain\_neuron\_development | 1 | 0 |  |  |  |  |  |  |  |  |
| GO:0021896\_forebrain\_astrocyte\_differentiation | 1 | 0 |  |  |  |  |  |  |  |  |
| GO:0021897\_forebrain\_astrocyte\_development | 1 | 0 |  |  |  |  |  |  |  |  |
| GO:0021914\_negative\_regulation\_of\_smoothened\_signaling\_pathway\_involved\_in\_ventral\_spinal\_cord\_patterning | 1 | 0 |  |  |  |  |  |  |  |  |
| GO:0021919\_BMP\_signaling\_pathway\_in\_spinal\_cord\_dorsal\_ventral\_patterning | 1 | 0 |  |  |  |  |  |  |  |  |
| GO:0021965\_spinal\_cord\_ventral\_commissure\_morphogenesis | 1 | 0 |  |  |  |  |  |  |  |  |
| GO:0021984\_adenohypophysis\_development | 1 | 0 |  |  |  |  |  |  |  |  |
| GO:0021990\_neural\_plate\_formation | 1 | 0 |  |  |  |  |  |  |  |  |
| GO:0021997\_neural\_plate\_axis\_specification | 1 | 0 |  |  |  |  |  |  |  |  |
| GO:0021999\_neural\_plate\_anterior\_posterior\_pattern\_formation | 1 | 0 |  |  |  |  |  |  |  |  |
| GO:0022009\_central\_nervous\_system\_vasculogenesis | 1 | 0 |  |  |  |  |  |  |  |  |
| GO:0022038\_corpus\_callosum\_development | 1 | 0 |  |  |  |  |  |  |  |  |
| GO:0030007\_cellular\_potassium\_ion\_homeostasis | 1 | 0 |  |  |  |  |  |  |  |  |
| GO:0030011\_maintenance\_of\_cell\_polarity | 1 | 0 |  |  |  |  |  |  |  |  |
| GO:0030026\_cellular\_manganese\_ion\_homeostasis | 1 | 0 |  |  |  |  |  |  |  |  |
| GO:0030033\_microvillus\_assembly | 1 | 0 |  |  |  |  |  |  |  |  |
| GO:0030037\_actin\_filament\_reorganization\_during\_cell\_cycle | 1 | 0 |  |  |  |  |  |  |  |  |
| GO:0030047\_actin\_modification | 1 | 0 |  |  |  |  |  |  |  |  |
| GO:0030070\_insulin\_processing | 1 | 0 |  |  |  |  |  |  |  |  |
| GO:0030103\_vasopressin\_secretion | 1 | 0 |  |  |  |  |  |  |  |  |
| GO:0030186\_melatonin\_metabolic\_process | 1 | 0 |  |  |  |  |  |  |  |  |
| GO:0030187\_melatonin\_biosynthetic\_process | 1 | 0 |  |  |  |  |  |  |  |  |
| GO:0030212\_hyaluronan\_metabolic\_process | 1 | 0 |  |  |  |  |  |  |  |  |
| GO:0030220\_platelet\_formation | 1 | 0 |  |  |  |  |  |  |  |  |
| GO:0030238\_male\_sex\_determination | 1 | 0 |  |  |  |  |  |  |  |  |
| GO:0030259\_lipid\_glycosylation | 1 | 0 |  |  |  |  |  |  |  |  |
| GO:0030302\_deoxynucleotide\_transport | 1 | 0 |  |  |  |  |  |  |  |  |
| GO:0030327\_prenylated\_protein\_catabolic\_process | 1 | 0 |  |  |  |  |  |  |  |  |
| GO:0030389\_fructosamine\_metabolic\_process | 1 | 0 |  |  |  |  |  |  |  |  |
| GO:0030393\_fructoselysine\_metabolic\_process | 1 | 0 |  |  |  |  |  |  |  |  |
| GO:0030432\_peristalsis | 1 | 0 |  |  |  |  |  |  |  |  |
| GO:0030488\_tRNA\_methylation | 1 | 0 |  |  |  |  |  |  |  |  |
| GO:0030517\_negative\_regulation\_of\_axon\_extension | 1 | 0 |  |  |  |  |  |  |  |  |
| GO:0030581\_symbiont\_intracellular\_protein\_transport\_in\_host | 1 | 0 |  |  |  |  |  |  |  |  |
| GO:0030718\_germ-line\_stem\_cell\_maintenance | 1 | 0 |  |  |  |  |  |  |  |  |
| GO:0030728\_ovulation | 1 | 0 |  |  |  |  |  |  |  |  |
| GO:0030824\_negative\_regulation\_of\_cGMP\_metabolic\_process | 1 | 0 |  |  |  |  |  |  |  |  |
| GO:0030825\_positive\_regulation\_of\_cGMP\_metabolic\_process | 1 | 0 |  |  |  |  |  |  |  |  |
| GO:0030827\_negative\_regulation\_of\_cGMP\_biosynthetic\_process | 1 | 0 |  |  |  |  |  |  |  |  |
| GO:0030828\_positive\_regulation\_of\_cGMP\_biosynthetic\_process | 1 | 0 |  |  |  |  |  |  |  |  |
| GO:0030836\_positive\_regulation\_of\_actin\_filament\_depolymerization | 1 | 0 |  |  |  |  |  |  |  |  |
| GO:0030845\_inhibition\_of\_phospholipase\_C\_activity\_involved\_in\_G-protein\_coupled\_receptor\_signaling\_pathway | 1 | 0 |  |  |  |  |  |  |  |  |
| GO:0030854\_positive\_regulation\_of\_granulocyte\_differentiation | 1 | 0 |  |  |  |  |  |  |  |  |
| GO:0030857\_negative\_regulation\_of\_epithelial\_cell\_differentiation | 1 | 0 |  |  |  |  |  |  |  |  |
| GO:0030878\_thyroid\_gland\_development | 1 | 0 |  |  |  |  |  |  |  |  |
| GO:0030885\_regulation\_of\_myeloid\_dendritic\_cell\_activation | 1 | 0 |  |  |  |  |  |  |  |  |
| GO:0030887\_positive\_regulation\_of\_myeloid\_dendritic\_cell\_activation | 1 | 0 |  |  |  |  |  |  |  |  |
| GO:0030903\_notochord\_development | 1 | 0 |  |  |  |  |  |  |  |  |
| GO:0030910\_olfactory\_placode\_formation | 1 | 0 |  |  |  |  |  |  |  |  |
| GO:0030913\_paranodal\_junction\_assembly | 1 | 0 |  |  |  |  |  |  |  |  |
| GO:0030948\_negative\_regulation\_of\_vascular\_endothelial\_growth\_factor\_receptor\_signaling\_pathway | 1 | 0 |  |  |  |  |  |  |  |  |
| GO:0030967\_ER-nuclear\_sterol\_response\_pathway | 1 | 0 |  |  |  |  |  |  |  |  |
| GO:0031017\_exocrine\_pancreas\_development | 1 | 0 |  |  |  |  |  |  |  |  |
| GO:0031063\_regulation\_of\_histone\_deacetylation | 1 | 0 |  |  |  |  |  |  |  |  |
| GO:0031065\_positive\_regulation\_of\_histone\_deacetylation | 1 | 0 |  |  |  |  |  |  |  |  |
| GO:0031076\_embryonic\_camera-type\_eye\_development | 1 | 0 |  |  |  |  |  |  |  |  |
| GO:0031081\_nuclear\_pore\_distribution | 1 | 0 |  |  |  |  |  |  |  |  |
| GO:0031086\_nuclear-transcribed\_mRNA\_catabolic\_process\_\_deadenylation-independent\_decay | 1 | 0 |  |  |  |  |  |  |  |  |
| GO:0031087\_deadenylation-independent\_decapping\_of\_nuclear-transcribed\_mRNA | 1 | 0 |  |  |  |  |  |  |  |  |
| GO:0031106\_septin\_ring\_organization | 1 | 0 |  |  |  |  |  |  |  |  |
| GO:0031115\_negative\_regulation\_of\_microtubule\_polymerization | 1 | 0 |  |  |  |  |  |  |  |  |
| GO:0031117\_positive\_regulation\_of\_microtubule\_depolymerization | 1 | 0 |  |  |  |  |  |  |  |  |
| GO:0031118\_rRNA\_pseudouridine\_synthesis | 1 | 0 |  |  |  |  |  |  |  |  |
| GO:0031125\_rRNA\_3'-end\_processing | 1 | 0 |  |  |  |  |  |  |  |  |
| GO:0031146\_SCF-dependent\_proteasomal\_ubiquitin-dependent\_protein\_catabolic\_process | 1 | 0 |  |  |  |  |  |  |  |  |
| GO:0031179\_peptide\_modification | 1 | 0 |  |  |  |  |  |  |  |  |
| GO:0031282\_regulation\_of\_guanylate\_cyclase\_activity | 1 | 0 |  |  |  |  |  |  |  |  |
| GO:0031284\_positive\_regulation\_of\_guanylate\_cyclase\_activity | 1 | 0 |  |  |  |  |  |  |  |  |
| GO:0031290\_retinal\_ganglion\_cell\_axon\_guidance | 1 | 0 |  |  |  |  |  |  |  |  |
| GO:0031293\_membrane\_protein\_intracellular\_domain\_proteolysis | 1 | 0 |  |  |  |  |  |  |  |  |
| GO:0031335\_regulation\_of\_sulfur\_amino\_acid\_metabolic\_process | 1 | 0 |  |  |  |  |  |  |  |  |
| GO:0031342\_negative\_regulation\_of\_cell\_killing | 1 | 0 |  |  |  |  |  |  |  |  |
| GO:0031424\_keratinization | 1 | 0 |  |  |  |  |  |  |  |  |
| GO:0031441\_negative\_regulation\_of\_mRNA\_3'-end\_processing | 1 | 0 |  |  |  |  |  |  |  |  |
| GO:0031442\_positive\_regulation\_of\_mRNA\_3'-end\_processing | 1 | 0 |  |  |  |  |  |  |  |  |
| GO:0031443\_fast-twitch\_skeletal\_muscle\_fiber\_contraction | 1 | 0 |  |  |  |  |  |  |  |  |
| GO:0031446\_regulation\_of\_fast-twitch\_skeletal\_muscle\_fiber\_contraction | 1 | 0 |  |  |  |  |  |  |  |  |
| GO:0031448\_positive\_regulation\_of\_fast-twitch\_skeletal\_muscle\_fiber\_contraction | 1 | 0 |  |  |  |  |  |  |  |  |
| GO:0031453\_positive\_regulation\_of\_heterochromatin\_formation | 1 | 0 |  |  |  |  |  |  |  |  |
| GO:0031557\_induction\_of\_programmed\_cell\_death\_in\_response\_to\_chemical\_stimulus | 1 | 0 |  |  |  |  |  |  |  |  |
| GO:0031574\_S-M\_checkpoint | 1 | 0 |  |  |  |  |  |  |  |  |
| GO:0031581\_hemidesmosome\_assembly | 1 | 0 |  |  |  |  |  |  |  |  |
| GO:0031627\_telomeric\_loop\_formation | 1 | 0 |  |  |  |  |  |  |  |  |
| GO:0031848\_protection\_from\_non-homologous\_end\_joining\_at\_telomere | 1 | 0 |  |  |  |  |  |  |  |  |
| GO:0031937\_positive\_regulation\_of\_chromatin\_silencing | 1 | 0 |  |  |  |  |  |  |  |  |
| GO:0031943\_regulation\_of\_glucocorticoid\_metabolic\_process | 1 | 0 |  |  |  |  |  |  |  |  |
| GO:0031954\_positive\_regulation\_of\_protein\_amino\_acid\_autophosphorylation | 1 | 0 |  |  |  |  |  |  |  |  |
| GO:0031999\_negative\_regulation\_of\_fatty\_acid\_beta-oxidation | 1 | 0 |  |  |  |  |  |  |  |  |
| GO:0032011\_ARF\_protein\_signal\_transduction | 1 | 0 |  |  |  |  |  |  |  |  |
| GO:0032023\_trypsinogen\_activation | 1 | 0 |  |  |  |  |  |  |  |  |
| GO:0032025\_response\_to\_cobalt\_ion | 1 | 0 |  |  |  |  |  |  |  |  |
| GO:0032048\_cardiolipin\_metabolic\_process | 1 | 0 |  |  |  |  |  |  |  |  |
| GO:0032049\_cardiolipin\_biosynthetic\_process | 1 | 0 |  |  |  |  |  |  |  |  |
| GO:0032060\_bleb\_formation | 1 | 0 |  |  |  |  |  |  |  |  |
| GO:0032066\_nucleolus\_to\_nucleoplasm\_transport | 1 | 0 |  |  |  |  |  |  |  |  |
| GO:0032074\_negative\_regulation\_of\_nuclease\_activity | 1 | 0 |  |  |  |  |  |  |  |  |
| GO:0032075\_positive\_regulation\_of\_nuclease\_activity | 1 | 0 |  |  |  |  |  |  |  |  |
| GO:0032119\_sequestering\_of\_zinc\_ion | 1 | 0 |  |  |  |  |  |  |  |  |
| GO:0032185\_septin\_cytoskeleton\_organization | 1 | 0 |  |  |  |  |  |  |  |  |
| GO:0032196\_transposition | 1 | 0 |  |  |  |  |  |  |  |  |
| GO:0032235\_negative\_regulation\_of\_calcium\_ion\_transport\_via\_store-operated\_calcium\_channel\_activity | 1 | 0 |  |  |  |  |  |  |  |  |
| GO:0032241\_positive\_regulation\_of\_nucleobase\_\_nucleoside\_\_nucleotide\_and\_nucleic\_acid\_transport | 1 | 0 |  |  |  |  |  |  |  |  |
| GO:0032261\_purine\_nucleotide\_salvage | 1 | 0 |  |  |  |  |  |  |  |  |
| GO:0032275\_luteinizing\_hormone\_secretion | 1 | 0 |  |  |  |  |  |  |  |  |
| GO:0032287\_myelin\_maintenance\_in\_the\_peripheral\_nervous\_system | 1 | 0 |  |  |  |  |  |  |  |  |
| GO:0032288\_myelin\_assembly | 1 | 0 |  |  |  |  |  |  |  |  |
| GO:0032314\_regulation\_of\_Rac\_GTPase\_activity | 1 | 0 |  |  |  |  |  |  |  |  |
| GO:0032330\_regulation\_of\_chondrocyte\_differentiation | 1 | 0 |  |  |  |  |  |  |  |  |
| GO:0032331\_negative\_regulation\_of\_chondrocyte\_differentiation | 1 | 0 |  |  |  |  |  |  |  |  |
| GO:0032346\_positive\_regulation\_of\_aldosterone\_metabolic\_process | 1 | 0 |  |  |  |  |  |  |  |  |
| GO:0032347\_regulation\_of\_aldosterone\_biosynthetic\_process | 1 | 0 |  |  |  |  |  |  |  |  |
| GO:0032349\_positive\_regulation\_of\_aldosterone\_biosynthetic\_process | 1 | 0 |  |  |  |  |  |  |  |  |
| GO:0032354\_response\_to\_follicle-stimulating\_hormone\_stimulus | 1 | 0 |  |  |  |  |  |  |  |  |
| GO:0032377\_regulation\_of\_intracellular\_lipid\_transport | 1 | 0 |  |  |  |  |  |  |  |  |
| GO:0032380\_regulation\_of\_intracellular\_sterol\_transport | 1 | 0 |  |  |  |  |  |  |  |  |
| GO:0032383\_regulation\_of\_intracellular\_cholesterol\_transport | 1 | 0 |  |  |  |  |  |  |  |  |
| GO:0032423\_regulation\_of\_mismatch\_repair | 1 | 0 |  |  |  |  |  |  |  |  |
| GO:0032425\_positive\_regulation\_of\_mismatch\_repair | 1 | 0 |  |  |  |  |  |  |  |  |
| GO:0032459\_regulation\_of\_protein\_oligomerization | 1 | 0 |  |  |  |  |  |  |  |  |
| GO:0032460\_negative\_regulation\_of\_protein\_oligomerization | 1 | 0 |  |  |  |  |  |  |  |  |
| GO:0032462\_regulation\_of\_protein\_homooligomerization | 1 | 0 |  |  |  |  |  |  |  |  |
| GO:0032463\_negative\_regulation\_of\_protein\_homooligomerization | 1 | 0 |  |  |  |  |  |  |  |  |
| GO:0032467\_positive\_regulation\_of\_cytokinesis | 1 | 0 |  |  |  |  |  |  |  |  |
| GO:0032468\_Golgi\_calcium\_ion\_homeostasis | 1 | 0 |  |  |  |  |  |  |  |  |
| GO:0032470\_elevation\_of\_endoplasmic\_reticulum\_calcium\_ion\_concentration | 1 | 0 |  |  |  |  |  |  |  |  |
| GO:0032471\_reduction\_of\_endoplasmic\_reticulum\_calcium\_ion\_concentration | 1 | 0 |  |  |  |  |  |  |  |  |
| GO:0032472\_Golgi\_calcium\_ion\_transport | 1 | 0 |  |  |  |  |  |  |  |  |
| GO:0032486\_Rap\_protein\_signal\_transduction | 1 | 0 |  |  |  |  |  |  |  |  |
| GO:0032495\_response\_to\_muramyl\_dipeptide | 1 | 0 |  |  |  |  |  |  |  |  |
| GO:0032498\_detection\_of\_muramyl\_dipeptide | 1 | 0 |  |  |  |  |  |  |  |  |
| GO:0032499\_detection\_of\_peptidoglycan | 1 | 0 |  |  |  |  |  |  |  |  |
| GO:0032528\_microvillus\_organization | 1 | 0 |  |  |  |  |  |  |  |  |
| GO:0032581\_ER-dependent\_peroxisome\_biogenesis | 1 | 0 |  |  |  |  |  |  |  |  |
| GO:0032594\_protein\_transport\_within\_lipid\_bilayer | 1 | 0 |  |  |  |  |  |  |  |  |
| GO:0032595\_B\_cell\_receptor\_transport\_within\_lipid\_bilayer | 1 | 0 |  |  |  |  |  |  |  |  |
| GO:0032596\_protein\_transport\_into\_membrane\_raft | 1 | 0 |  |  |  |  |  |  |  |  |
| GO:0032597\_B\_cell\_receptor\_transport\_into\_membrane\_raft | 1 | 0 |  |  |  |  |  |  |  |  |
| GO:0032599\_protein\_transport\_out\_of\_membrane\_raft | 1 | 0 |  |  |  |  |  |  |  |  |
| GO:0032600\_chemokine\_receptor\_transport\_out\_of\_membrane\_raft | 1 | 0 |  |  |  |  |  |  |  |  |
| GO:0032601\_connective\_tissue\_growth\_factor\_production | 1 | 0 |  |  |  |  |  |  |  |  |
| GO:0032603\_fractalkine\_production | 1 | 0 |  |  |  |  |  |  |  |  |
| GO:0032605\_hepatocyte\_growth\_factor\_production | 1 | 0 |  |  |  |  |  |  |  |  |
| GO:0032610\_interleukin-1\_alpha\_production | 1 | 0 |  |  |  |  |  |  |  |  |
| GO:0032621\_interleukin-18\_production | 1 | 0 |  |  |  |  |  |  |  |  |
| GO:0032639\_TRAIL\_production | 1 | 0 |  |  |  |  |  |  |  |  |
| GO:0032644\_regulation\_of\_fractalkine\_production | 1 | 0 |  |  |  |  |  |  |  |  |
| GO:0032646\_regulation\_of\_hepatocyte\_growth\_factor\_production | 1 | 0 |  |  |  |  |  |  |  |  |
| GO:0032650\_regulation\_of\_interleukin-1\_alpha\_production | 1 | 0 |  |  |  |  |  |  |  |  |
| GO:0032661\_regulation\_of\_interleukin-18\_production | 1 | 0 |  |  |  |  |  |  |  |  |
| GO:0032679\_regulation\_of\_TRAIL\_production | 1 | 0 |  |  |  |  |  |  |  |  |
| GO:0032681\_regulation\_of\_lymphotoxin\_A\_production | 1 | 0 |  |  |  |  |  |  |  |  |
| GO:0032693\_negative\_regulation\_of\_interleukin-10\_production | 1 | 0 |  |  |  |  |  |  |  |  |
| GO:0032703\_negative\_regulation\_of\_interleukin-2\_production | 1 | 0 |  |  |  |  |  |  |  |  |
| GO:0032713\_negative\_regulation\_of\_interleukin-4\_production | 1 | 0 |  |  |  |  |  |  |  |  |
| GO:0032730\_positive\_regulation\_of\_interleukin-1\_alpha\_production | 1 | 0 |  |  |  |  |  |  |  |  |
| GO:0032732\_positive\_regulation\_of\_interleukin-1\_production | 1 | 0 |  |  |  |  |  |  |  |  |
| GO:0032736\_positive\_regulation\_of\_interleukin-13\_production | 1 | 0 |  |  |  |  |  |  |  |  |
| GO:0032753\_positive\_regulation\_of\_interleukin-4\_production | 1 | 0 |  |  |  |  |  |  |  |  |
| GO:0032754\_positive\_regulation\_of\_interleukin-5\_production | 1 | 0 |  |  |  |  |  |  |  |  |
| GO:0032762\_mast\_cell\_cytokine\_production | 1 | 0 |  |  |  |  |  |  |  |  |
| GO:0032763\_regulation\_of\_mast\_cell\_cytokine\_production | 1 | 0 |  |  |  |  |  |  |  |  |
| GO:0032765\_positive\_regulation\_of\_mast\_cell\_cytokine\_production | 1 | 0 |  |  |  |  |  |  |  |  |
| GO:0032784\_regulation\_of\_RNA\_elongation | 1 | 0 |  |  |  |  |  |  |  |  |
| GO:0032786\_positive\_regulation\_of\_RNA\_elongation | 1 | 0 |  |  |  |  |  |  |  |  |
| GO:0032788\_saturated\_monocarboxylic\_acid\_metabolic\_process | 1 | 0 |  |  |  |  |  |  |  |  |
| GO:0032789\_unsaturated\_monocarboxylic\_acid\_metabolic\_process | 1 | 0 |  |  |  |  |  |  |  |  |
| GO:0032790\_ribosome\_disassembly | 1 | 0 |  |  |  |  |  |  |  |  |
| GO:0032792\_negative\_regulation\_of\_CREB\_transcription\_factor\_activity | 1 | 0 |  |  |  |  |  |  |  |  |
| GO:0032793\_positive\_regulation\_of\_CREB\_transcription\_factor\_activity | 1 | 0 |  |  |  |  |  |  |  |  |
| GO:0032804\_negative\_regulation\_of\_low-density\_lipoprotein\_receptor\_catabolic\_process | 1 | 0 |  |  |  |  |  |  |  |  |
| GO:0032805\_positive\_regulation\_of\_low-density\_lipoprotein\_receptor\_catabolic\_process | 1 | 0 |  |  |  |  |  |  |  |  |
| GO:0032812\_positive\_regulation\_of\_epinephrine\_secretion | 1 | 0 |  |  |  |  |  |  |  |  |
| GO:0032835\_glomerulus\_development | 1 | 0 |  |  |  |  |  |  |  |  |
| GO:0032847\_regulation\_of\_cellular\_pH\_reduction | 1 | 0 |  |  |  |  |  |  |  |  |
| GO:0032848\_negative\_regulation\_of\_cellular\_pH\_reduction | 1 | 0 |  |  |  |  |  |  |  |  |
| GO:0032899\_regulation\_of\_neurotrophin\_production | 1 | 0 |  |  |  |  |  |  |  |  |
| GO:0032900\_negative\_regulation\_of\_neurotrophin\_production | 1 | 0 |  |  |  |  |  |  |  |  |
| GO:0032903\_regulation\_of\_nerve\_growth\_factor\_production | 1 | 0 |  |  |  |  |  |  |  |  |
| GO:0032904\_negative\_regulation\_of\_nerve\_growth\_factor\_production | 1 | 0 |  |  |  |  |  |  |  |  |
| GO:0032907\_transforming\_growth\_factor-beta3\_production | 1 | 0 |  |  |  |  |  |  |  |  |
| GO:0032910\_regulation\_of\_transforming\_growth\_factor-beta3\_production | 1 | 0 |  |  |  |  |  |  |  |  |
| GO:0032911\_negative\_regulation\_of\_transforming\_growth\_factor-beta1\_production | 1 | 0 |  |  |  |  |  |  |  |  |
| GO:0032913\_negative\_regulation\_of\_transforming\_growth\_factor-beta3\_production | 1 | 0 |  |  |  |  |  |  |  |  |
| GO:0032926\_negative\_regulation\_of\_activin\_receptor\_signaling\_pathway | 1 | 0 |  |  |  |  |  |  |  |  |
| GO:0032933\_SREBP-mediated\_signaling\_pathway | 1 | 0 |  |  |  |  |  |  |  |  |
| GO:0032938\_negative\_regulation\_of\_translation\_in\_response\_to\_oxidative\_stress | 1 | 0 |  |  |  |  |  |  |  |  |
| GO:0032958\_inositol\_phosphate\_biosynthetic\_process | 1 | 0 |  |  |  |  |  |  |  |  |
| GO:0032976\_release\_of\_matrix\_enzymes\_from\_mitochondria | 1 | 0 |  |  |  |  |  |  |  |  |
| GO:0032980\_keratinocyte\_activation | 1 | 0 |  |  |  |  |  |  |  |  |
| GO:0032988\_ribonucleoprotein\_complex\_disassembly | 1 | 0 |  |  |  |  |  |  |  |  |
| GO:0033029\_regulation\_of\_neutrophil\_apoptosis | 1 | 0 |  |  |  |  |  |  |  |  |
| GO:0033031\_positive\_regulation\_of\_neutrophil\_apoptosis | 1 | 0 |  |  |  |  |  |  |  |  |
| GO:0033079\_immature\_T\_cell\_proliferation | 1 | 0 |  |  |  |  |  |  |  |  |
| GO:0033080\_immature\_T\_cell\_proliferation\_in\_the\_thymus | 1 | 0 |  |  |  |  |  |  |  |  |
| GO:0033083\_regulation\_of\_immature\_T\_cell\_proliferation | 1 | 0 |  |  |  |  |  |  |  |  |
| GO:0033084\_regulation\_of\_immature\_T\_cell\_proliferation\_in\_the\_thymus | 1 | 0 |  |  |  |  |  |  |  |  |
| GO:0033085\_negative\_regulation\_of\_T\_cell\_differentiation\_in\_the\_thymus | 1 | 0 |  |  |  |  |  |  |  |  |
| GO:0033087\_negative\_regulation\_of\_immature\_T\_cell\_proliferation | 1 | 0 |  |  |  |  |  |  |  |  |
| GO:0033088\_negative\_regulation\_of\_immature\_T\_cell\_proliferation\_in\_the\_thymus | 1 | 0 |  |  |  |  |  |  |  |  |
| GO:0033136\_serine\_phosphorylation\_of\_STAT3\_protein | 1 | 0 |  |  |  |  |  |  |  |  |
| GO:0033137\_negative\_regulation\_of\_peptidyl-serine\_phosphorylation | 1 | 0 |  |  |  |  |  |  |  |  |
| GO:0033139\_regulation\_of\_peptidyl-serine\_phosphorylation\_of\_STAT\_protein | 1 | 0 |  |  |  |  |  |  |  |  |
| GO:0033141\_positive\_regulation\_of\_peptidyl-serine\_phosphorylation\_of\_STAT\_protein | 1 | 0 |  |  |  |  |  |  |  |  |
| GO:0033153\_T\_cell\_receptor\_V(D)J\_recombination | 1 | 0 |  |  |  |  |  |  |  |  |
| GO:0033169\_histone\_H3-K9\_demethylation | 1 | 0 |  |  |  |  |  |  |  |  |
| GO:0033173\_calcineurin-NFAT\_signaling\_pathway | 1 | 0 |  |  |  |  |  |  |  |  |
| GO:0033182\_regulation\_of\_histone\_ubiquitination | 1 | 0 |  |  |  |  |  |  |  |  |
| GO:0033206\_cytokinesis\_after\_meiosis | 1 | 0 |  |  |  |  |  |  |  |  |
| GO:0033239\_negative\_regulation\_of\_cellular\_amine\_metabolic\_process | 1 | 0 |  |  |  |  |  |  |  |  |
| GO:0033240\_positive\_regulation\_of\_cellular\_amine\_metabolic\_process | 1 | 0 |  |  |  |  |  |  |  |  |
| GO:0033260\_DNA\_replication\_during\_S\_phase | 1 | 0 |  |  |  |  |  |  |  |  |
| GO:0033262\_regulation\_of\_DNA\_replication\_during\_S\_phase | 1 | 0 |  |  |  |  |  |  |  |  |
| GO:0033292\_T-tubule\_organization | 1 | 0 |  |  |  |  |  |  |  |  |
| GO:0033341\_regulation\_of\_collagen\_binding | 1 | 0 |  |  |  |  |  |  |  |  |
| GO:0033342\_negative\_regulation\_of\_collagen\_binding | 1 | 0 |  |  |  |  |  |  |  |  |
| GO:0033345\_asparagine\_catabolic\_process\_via\_L-aspartate | 1 | 0 |  |  |  |  |  |  |  |  |
| GO:0033366\_protein\_localization\_in\_secretory\_granule | 1 | 0 |  |  |  |  |  |  |  |  |
| GO:0033367\_protein\_localization\_in\_mast\_cell\_secretory\_granule | 1 | 0 |  |  |  |  |  |  |  |  |
| GO:0033368\_protease\_localization\_in\_mast\_cell\_secretory\_granule | 1 | 0 |  |  |  |  |  |  |  |  |
| GO:0033370\_maintenance\_of\_protein\_location\_in\_mast\_cell\_secretory\_granule | 1 | 0 |  |  |  |  |  |  |  |  |
| GO:0033371\_T\_cell\_secretory\_granule\_organization | 1 | 0 |  |  |  |  |  |  |  |  |
| GO:0033373\_maintenance\_of\_protease\_location\_in\_mast\_cell\_secretory\_granule | 1 | 0 |  |  |  |  |  |  |  |  |
| GO:0033374\_protein\_localization\_in\_T\_cell\_secretory\_granule | 1 | 0 |  |  |  |  |  |  |  |  |
| GO:0033375\_protease\_localization\_in\_T\_cell\_secretory\_granule | 1 | 0 |  |  |  |  |  |  |  |  |
| GO:0033377\_maintenance\_of\_protein\_location\_in\_T\_cell\_secretory\_granule | 1 | 0 |  |  |  |  |  |  |  |  |
| GO:0033379\_maintenance\_of\_protease\_location\_in\_T\_cell\_secretory\_granule | 1 | 0 |  |  |  |  |  |  |  |  |
| GO:0033380\_granzyme\_B\_localization\_in\_T\_cell\_secretory\_granule | 1 | 0 |  |  |  |  |  |  |  |  |
| GO:0033382\_maintenance\_of\_granzyme\_B\_location\_in\_T\_cell\_secretory\_granule | 1 | 0 |  |  |  |  |  |  |  |  |
| GO:0033484\_nitric\_oxide\_homeostasis | 1 | 0 |  |  |  |  |  |  |  |  |
| GO:0033504\_floor\_plate\_development | 1 | 0 |  |  |  |  |  |  |  |  |
| GO:0033566\_gamma-tubulin\_complex\_localization | 1 | 0 |  |  |  |  |  |  |  |  |
| GO:0033577\_protein\_amino\_acid\_glycosylation\_in\_endoplasmic\_reticulum | 1 | 0 |  |  |  |  |  |  |  |  |
| GO:0033595\_response\_to\_genistein | 1 | 0 |  |  |  |  |  |  |  |  |
| GO:0033600\_negative\_regulation\_of\_mammary\_gland\_epithelial\_cell\_proliferation | 1 | 0 |  |  |  |  |  |  |  |  |
| GO:0033606\_chemokine\_receptor\_transport\_within\_lipid\_bilayer | 1 | 0 |  |  |  |  |  |  |  |  |
| GO:0033617\_mitochondrial\_respiratory\_chain\_complex\_IV\_assembly | 1 | 0 |  |  |  |  |  |  |  |  |
| GO:0033622\_integrin\_activation | 1 | 0 |  |  |  |  |  |  |  |  |
| GO:0033623\_regulation\_of\_integrin\_activation | 1 | 0 |  |  |  |  |  |  |  |  |
| GO:0033625\_positive\_regulation\_of\_integrin\_activation | 1 | 0 |  |  |  |  |  |  |  |  |
| GO:0033693\_neurofilament\_bundle\_assembly | 1 | 0 |  |  |  |  |  |  |  |  |
| GO:0033750\_ribosome\_localization | 1 | 0 |  |  |  |  |  |  |  |  |
| GO:0033753\_establishment\_of\_ribosome\_localization | 1 | 0 |  |  |  |  |  |  |  |  |
| GO:0033875\_ribonucleoside\_bisphosphate\_metabolic\_process | 1 | 0 |  |  |  |  |  |  |  |  |
| GO:0033962\_cytoplasmic\_mRNA\_processing\_body\_assembly | 1 | 0 |  |  |  |  |  |  |  |  |
| GO:0034032\_purine\_nucleoside\_bisphosphate\_metabolic\_process | 1 | 0 |  |  |  |  |  |  |  |  |
| GO:0034035\_purine\_ribonucleoside\_bisphosphate\_metabolic\_process | 1 | 0 |  |  |  |  |  |  |  |  |
| GO:0034063\_stress\_granule\_assembly | 1 | 0 |  |  |  |  |  |  |  |  |
| GO:0034080\_CenH3-containing\_nucleosome\_assembly\_at\_centromere | 1 | 0 |  |  |  |  |  |  |  |  |
| GO:0034109\_homotypic\_cell-cell\_adhesion | 1 | 0 |  |  |  |  |  |  |  |  |
| GO:0034115\_negative\_regulation\_of\_heterotypic\_cell-cell\_adhesion | 1 | 0 |  |  |  |  |  |  |  |  |
| GO:0034116\_positive\_regulation\_of\_heterotypic\_cell-cell\_adhesion | 1 | 0 |  |  |  |  |  |  |  |  |
| GO:0034122\_negative\_regulation\_of\_toll-like\_receptor\_signaling\_pathway | 1 | 0 |  |  |  |  |  |  |  |  |
| GO:0034123\_positive\_regulation\_of\_toll-like\_receptor\_signaling\_pathway | 1 | 0 |  |  |  |  |  |  |  |  |
| GO:0034142\_toll-like\_receptor\_4\_signaling\_pathway | 1 | 0 |  |  |  |  |  |  |  |  |
| GO:0034143\_regulation\_of\_toll-like\_receptor\_4\_signaling\_pathway | 1 | 0 |  |  |  |  |  |  |  |  |
| GO:0034145\_positive\_regulation\_of\_toll-like\_receptor\_4\_signaling\_pathway | 1 | 0 |  |  |  |  |  |  |  |  |
| GO:0034196\_acylglycerol\_transport | 1 | 0 |  |  |  |  |  |  |  |  |
| GO:0034197\_triglyceride\_transport | 1 | 0 |  |  |  |  |  |  |  |  |
| GO:0034205\_beta-amyloid\_formation | 1 | 0 |  |  |  |  |  |  |  |  |
| GO:0034213\_quinolinate\_catabolic\_process | 1 | 0 |  |  |  |  |  |  |  |  |
| GO:0034231\_islet\_amyloid\_polypeptide\_processing | 1 | 0 |  |  |  |  |  |  |  |  |
| GO:0034238\_macrophage\_fusion | 1 | 0 |  |  |  |  |  |  |  |  |
| GO:0034239\_regulation\_of\_macrophage\_fusion | 1 | 0 |  |  |  |  |  |  |  |  |
| GO:0034241\_positive\_regulation\_of\_macrophage\_fusion | 1 | 0 |  |  |  |  |  |  |  |  |
| GO:0034248\_regulation\_of\_amide\_metabolic\_process | 1 | 0 |  |  |  |  |  |  |  |  |
| GO:0034263\_autophagy\_in\_response\_to\_ER\_overload | 1 | 0 |  |  |  |  |  |  |  |  |
| GO:0034287\_detection\_of\_monosaccharide\_stimulus | 1 | 0 |  |  |  |  |  |  |  |  |
| GO:0034313\_diol\_catabolic\_process | 1 | 0 |  |  |  |  |  |  |  |  |
| GO:0034332\_adherens\_junction\_organization | 1 | 0 |  |  |  |  |  |  |  |  |
| GO:0034333\_adherens\_junction\_assembly | 1 | 0 |  |  |  |  |  |  |  |  |
| GO:0034340\_response\_to\_type\_I\_interferon | 1 | 0 |  |  |  |  |  |  |  |  |
| GO:0034356\_NAD\_biosynthesis\_via\_nicotinamide\_riboside\_salvage\_pathway | 1 | 0 |  |  |  |  |  |  |  |  |
| GO:0034373\_intermediate-density\_lipoprotein\_particle\_remodeling | 1 | 0 |  |  |  |  |  |  |  |  |
| GO:0034378\_chylomicron\_assembly | 1 | 0 |  |  |  |  |  |  |  |  |
| GO:0034436\_glycoprotein\_transport | 1 | 0 |  |  |  |  |  |  |  |  |
| GO:0034439\_lipoprotein\_lipid\_oxidation | 1 | 0 |  |  |  |  |  |  |  |  |
| GO:0034454\_microtubule\_anchoring\_at\_centrosome | 1 | 0 |  |  |  |  |  |  |  |  |
| GO:0034465\_response\_to\_carbon\_monoxide | 1 | 0 |  |  |  |  |  |  |  |  |
| GO:0034509\_centromeric\_core\_chromatin\_formation | 1 | 0 |  |  |  |  |  |  |  |  |
| GO:0034516\_response\_to\_vitamin\_B6 | 1 | 0 |  |  |  |  |  |  |  |  |
| GO:0034551\_mitochondrial\_respiratory\_chain\_complex\_III\_assembly | 1 | 0 |  |  |  |  |  |  |  |  |
| GO:0034552\_respiratory\_chain\_complex\_II\_assembly | 1 | 0 |  |  |  |  |  |  |  |  |
| GO:0034553\_mitochondrial\_respiratory\_chain\_complex\_II\_assembly | 1 | 0 |  |  |  |  |  |  |  |  |
| GO:0034589\_hydroxyproline\_transport | 1 | 0 |  |  |  |  |  |  |  |  |
| GO:0034694\_response\_to\_prostaglandin\_stimulus | 1 | 0 |  |  |  |  |  |  |  |  |
| GO:0034695\_response\_to\_prostaglandin\_E\_stimulus | 1 | 0 |  |  |  |  |  |  |  |  |
| GO:0034698\_response\_to\_gonadotropin\_stimulus | 1 | 0 |  |  |  |  |  |  |  |  |
| GO:0034699\_response\_to\_luteinizing\_hormone\_stimulus | 1 | 0 |  |  |  |  |  |  |  |  |
| GO:0034724\_DNA\_replication-independent\_nucleosome\_organization | 1 | 0 |  |  |  |  |  |  |  |  |
| GO:0034729\_histone\_H3-K79\_methylation | 1 | 0 |  |  |  |  |  |  |  |  |
| GO:0034755\_iron\_ion\_transmembrane\_transport | 1 | 0 |  |  |  |  |  |  |  |  |
| GO:0034764\_positive\_regulation\_of\_transmembrane\_transport | 1 | 0 |  |  |  |  |  |  |  |  |
| GO:0034765\_regulation\_of\_ion\_transmembrane\_transport | 1 | 0 |  |  |  |  |  |  |  |  |
| GO:0034767\_positive\_regulation\_of\_ion\_transmembrane\_transport | 1 | 0 |  |  |  |  |  |  |  |  |
| GO:0034959\_endothelin\_maturation | 1 | 0 |  |  |  |  |  |  |  |  |
| GO:0034982\_mitochondrial\_protein\_processing | 1 | 0 |  |  |  |  |  |  |  |  |
| GO:0034983\_peptidyl-lysine\_deacetylation | 1 | 0 |  |  |  |  |  |  |  |  |
| GO:0035021\_negative\_regulation\_of\_Rac\_protein\_signal\_transduction | 1 | 0 |  |  |  |  |  |  |  |  |
| GO:0035041\_sperm\_chromatin\_decondensation | 1 | 0 |  |  |  |  |  |  |  |  |
| GO:0035042\_fertilization\_\_exchange\_of\_chromosomal\_proteins | 1 | 0 |  |  |  |  |  |  |  |  |
| GO:0035054\_embryonic\_heart\_tube\_anterior\_posterior\_pattern\_formation | 1 | 0 |  |  |  |  |  |  |  |  |
| GO:0035066\_positive\_regulation\_of\_histone\_acetylation | 1 | 0 |  |  |  |  |  |  |  |  |
| GO:0035082\_axoneme\_assembly | 1 | 0 |  |  |  |  |  |  |  |  |
| GO:0035087\_RNA\_interference\_\_siRNA\_loading\_onto\_RISC | 1 | 0 |  |  |  |  |  |  |  |  |
| GO:0035090\_maintenance\_of\_apical\_basal\_cell\_polarity | 1 | 0 |  |  |  |  |  |  |  |  |
| GO:0035093\_spermatogenesis\_\_exchange\_of\_chromosomal\_proteins | 1 | 0 |  |  |  |  |  |  |  |  |
| GO:0035104\_positive\_regulation\_of\_transcription\_via\_sterol\_regulatory\_element\_binding | 1 | 0 |  |  |  |  |  |  |  |  |
| GO:0035110\_leg\_morphogenesis | 1 | 0 |  |  |  |  |  |  |  |  |
| GO:0035112\_genitalia\_morphogenesis | 1 | 0 |  |  |  |  |  |  |  |  |
| GO:0035116\_embryonic\_hindlimb\_morphogenesis | 1 | 0 |  |  |  |  |  |  |  |  |
| GO:0035137\_hindlimb\_morphogenesis | 1 | 0 |  |  |  |  |  |  |  |  |
| GO:0035238\_vitamin\_A\_biosynthetic\_process | 1 | 0 |  |  |  |  |  |  |  |  |
| GO:0035265\_organ\_growth | 1 | 0 |  |  |  |  |  |  |  |  |
| GO:0035280\_gene\_silencing\_by\_miRNA\_\_miRNA\_loading\_onto\_RISC | 1 | 0 |  |  |  |  |  |  |  |  |
| GO:0040009\_regulation\_of\_growth\_rate | 1 | 0 |  |  |  |  |  |  |  |  |
| GO:0040013\_negative\_regulation\_of\_locomotion | 1 | 0 |  |  |  |  |  |  |  |  |
| GO:0040015\_negative\_regulation\_of\_multicellular\_organism\_growth | 1 | 0 |  |  |  |  |  |  |  |  |
| GO:0040020\_regulation\_of\_meiosis | 1 | 0 |  |  |  |  |  |  |  |  |
| GO:0040023\_establishment\_of\_nucleus\_localization | 1 | 0 |  |  |  |  |  |  |  |  |
| GO:0040030\_regulation\_of\_molecular\_function\_\_epigenetic | 1 | 0 |  |  |  |  |  |  |  |  |
| GO:0040038\_polar\_body\_extrusion\_after\_meiotic\_divisions | 1 | 0 |  |  |  |  |  |  |  |  |
| GO:0042074\_cell\_migration\_involved\_in\_gastrulation | 1 | 0 |  |  |  |  |  |  |  |  |
| GO:0042091\_interleukin-10\_biosynthetic\_process | 1 | 0 |  |  |  |  |  |  |  |  |
| GO:0042118\_endothelial\_cell\_activation | 1 | 0 |  |  |  |  |  |  |  |  |
| GO:0042159\_lipoprotein\_catabolic\_process | 1 | 0 |  |  |  |  |  |  |  |  |
| GO:0042214\_terpene\_metabolic\_process | 1 | 0 |  |  |  |  |  |  |  |  |
| GO:0042225\_interleukin-5\_biosynthetic\_process | 1 | 0 |  |  |  |  |  |  |  |  |
| GO:0042241\_interleukin-18\_biosynthetic\_process | 1 | 0 |  |  |  |  |  |  |  |  |
| GO:0042257\_ribosomal\_subunit\_assembly | 1 | 0 |  |  |  |  |  |  |  |  |
| GO:0042262\_DNA\_protection | 1 | 0 |  |  |  |  |  |  |  |  |
| GO:0042276\_error-prone\_postreplication\_DNA\_repair | 1 | 0 |  |  |  |  |  |  |  |  |
| GO:0042313\_protein\_kinase\_C\_deactivation | 1 | 0 |  |  |  |  |  |  |  |  |
| GO:0042369\_vitamin\_D\_catabolic\_process | 1 | 0 |  |  |  |  |  |  |  |  |
| GO:0042412\_taurine\_biosynthetic\_process | 1 | 0 |  |  |  |  |  |  |  |  |
| GO:0042418\_epinephrine\_biosynthetic\_process | 1 | 0 |  |  |  |  |  |  |  |  |
| GO:0042421\_norepinephrine\_biosynthetic\_process | 1 | 0 |  |  |  |  |  |  |  |  |
| GO:0042424\_catecholamine\_catabolic\_process | 1 | 0 |  |  |  |  |  |  |  |  |
| GO:0042428\_serotonin\_metabolic\_process | 1 | 0 |  |  |  |  |  |  |  |  |
| GO:0042435\_indole\_derivative\_biosynthetic\_process | 1 | 0 |  |  |  |  |  |  |  |  |
| GO:0042474\_middle\_ear\_morphogenesis | 1 | 0 |  |  |  |  |  |  |  |  |
| GO:0042504\_tyrosine\_phosphorylation\_of\_Stat4\_protein | 1 | 0 |  |  |  |  |  |  |  |  |
| GO:0042519\_regulation\_of\_tyrosine\_phosphorylation\_of\_Stat4\_protein | 1 | 0 |  |  |  |  |  |  |  |  |
| GO:0042520\_positive\_regulation\_of\_tyrosine\_phosphorylation\_of\_Stat4\_protein | 1 | 0 |  |  |  |  |  |  |  |  |
| GO:0042524\_negative\_regulation\_of\_tyrosine\_phosphorylation\_of\_Stat5\_protein | 1 | 0 |  |  |  |  |  |  |  |  |
| GO:0042537\_benzene\_and\_derivative\_metabolic\_process | 1 | 0 |  |  |  |  |  |  |  |  |
| GO:0042560\_pteridine\_and\_derivative\_catabolic\_process | 1 | 0 |  |  |  |  |  |  |  |  |
| GO:0042596\_fear\_response | 1 | 0 |  |  |  |  |  |  |  |  |
| GO:0042637\_catagen | 1 | 0 |  |  |  |  |  |  |  |  |
| GO:0042640\_anagen | 1 | 0 |  |  |  |  |  |  |  |  |
| GO:0042670\_retinal\_cone\_cell\_differentiation | 1 | 0 |  |  |  |  |  |  |  |  |
| GO:0042700\_luteinizing\_hormone\_signaling\_pathway | 1 | 0 |  |  |  |  |  |  |  |  |
| GO:0042703\_menstruation | 1 | 0 |  |  |  |  |  |  |  |  |
| GO:0042726\_riboflavin\_and\_derivative\_metabolic\_process | 1 | 0 |  |  |  |  |  |  |  |  |
| GO:0042727\_riboflavin\_and\_derivative\_biosynthetic\_process | 1 | 0 |  |  |  |  |  |  |  |  |
| GO:0042746\_circadian\_sleep\_wake\_cycle\_\_wakefulness | 1 | 0 |  |  |  |  |  |  |  |  |
| GO:0042748\_circadian\_sleep\_wake\_cycle\_\_non-REM\_sleep | 1 | 0 |  |  |  |  |  |  |  |  |
| GO:0042755\_eating\_behavior | 1 | 0 |  |  |  |  |  |  |  |  |
| GO:0042756\_drinking\_behavior | 1 | 0 |  |  |  |  |  |  |  |  |
| GO:0042766\_nucleosome\_mobilization | 1 | 0 |  |  |  |  |  |  |  |  |
| GO:0042780\_tRNA\_3'-end\_processing | 1 | 0 |  |  |  |  |  |  |  |  |
| GO:0042789\_mRNA\_transcription\_from\_RNA\_polymerase\_II\_promoter | 1 | 0 |  |  |  |  |  |  |  |  |
| GO:0042795\_snRNA\_transcription\_from\_RNA\_polymerase\_II\_promoter | 1 | 0 |  |  |  |  |  |  |  |  |
| GO:0042796\_snRNA\_transcription\_from\_RNA\_polymerase\_III\_promoter | 1 | 0 |  |  |  |  |  |  |  |  |
| GO:0042822\_pyridoxal\_phosphate\_metabolic\_process | 1 | 0 |  |  |  |  |  |  |  |  |
| GO:0042823\_pyridoxal\_phosphate\_biosynthetic\_process | 1 | 0 |  |  |  |  |  |  |  |  |
| GO:0042866\_pyruvate\_biosynthetic\_process | 1 | 0 |  |  |  |  |  |  |  |  |
| GO:0042904\_9-cis-retinoic\_acid\_biosynthetic\_process | 1 | 0 |  |  |  |  |  |  |  |  |
| GO:0042905\_9-cis-retinoic\_acid\_metabolic\_process | 1 | 0 |  |  |  |  |  |  |  |  |
| GO:0042985\_negative\_regulation\_of\_amyloid\_precursor\_protein\_biosynthetic\_process | 1 | 0 |  |  |  |  |  |  |  |  |
| GO:0042986\_positive\_regulation\_of\_amyloid\_precursor\_protein\_biosynthetic\_process | 1 | 0 |  |  |  |  |  |  |  |  |
| GO:0042989\_sequestering\_of\_actin\_monomers | 1 | 0 |  |  |  |  |  |  |  |  |
| GO:0042996\_regulation\_of\_Golgi\_to\_plasma\_membrane\_protein\_transport | 1 | 0 |  |  |  |  |  |  |  |  |
| GO:0042997\_negative\_regulation\_of\_Golgi\_to\_plasma\_membrane\_protein\_transport | 1 | 0 |  |  |  |  |  |  |  |  |
| GO:0042999\_regulation\_of\_Golgi\_to\_plasma\_membrane\_CFTR\_protein\_transport | 1 | 0 |  |  |  |  |  |  |  |  |
| GO:0043002\_negative\_regulation\_of\_Golgi\_to\_plasma\_membrane\_CFTR\_protein\_transport | 1 | 0 |  |  |  |  |  |  |  |  |
| GO:0043004\_cytoplasmic\_sequestering\_of\_CFTR\_protein | 1 | 0 |  |  |  |  |  |  |  |  |
| GO:0043012\_regulation\_of\_fusion\_of\_sperm\_to\_egg\_plasma\_membrane | 1 | 0 |  |  |  |  |  |  |  |  |
| GO:0043016\_regulation\_of\_lymphotoxin\_A\_biosynthetic\_process | 1 | 0 |  |  |  |  |  |  |  |  |
| GO:0043017\_positive\_regulation\_of\_lymphotoxin\_A\_biosynthetic\_process | 1 | 0 |  |  |  |  |  |  |  |  |
| GO:0043049\_otic\_placode\_formation | 1 | 0 |  |  |  |  |  |  |  |  |
| GO:0043064\_flagellum\_organization | 1 | 0 |  |  |  |  |  |  |  |  |
| GO:0043116\_negative\_regulation\_of\_vascular\_permeability | 1 | 0 |  |  |  |  |  |  |  |  |
| GO:0043126\_regulation\_of\_1-phosphatidylinositol\_4-kinase\_activity | 1 | 0 |  |  |  |  |  |  |  |  |
| GO:0043128\_positive\_regulation\_of\_1-phosphatidylinositol\_4-kinase\_activity | 1 | 0 |  |  |  |  |  |  |  |  |
| GO:0043129\_surfactant\_homeostasis | 1 | 0 |  |  |  |  |  |  |  |  |
| GO:0043146\_spindle\_stabilization | 1 | 0 |  |  |  |  |  |  |  |  |
| GO:0043148\_mitotic\_spindle\_stabilization | 1 | 0 |  |  |  |  |  |  |  |  |
| GO:0043152\_induction\_of\_bacterial\_agglutination | 1 | 0 |  |  |  |  |  |  |  |  |
| GO:0043173\_nucleotide\_salvage | 1 | 0 |  |  |  |  |  |  |  |  |
| GO:0043174\_nucleoside\_salvage | 1 | 0 |  |  |  |  |  |  |  |  |
| GO:0043181\_vacuolar\_sequestering | 1 | 0 |  |  |  |  |  |  |  |  |
| GO:0043200\_response\_to\_amino\_acid\_stimulus | 1 | 0 |  |  |  |  |  |  |  |  |
| GO:0043217\_myelin\_maintenance | 1 | 0 |  |  |  |  |  |  |  |  |
| GO:0043247\_telomere\_maintenance\_in\_response\_to\_DNA\_damage | 1 | 0 |  |  |  |  |  |  |  |  |
| GO:0043249\_erythrocyte\_maturation | 1 | 0 |  |  |  |  |  |  |  |  |
| GO:0043268\_positive\_regulation\_of\_potassium\_ion\_transport | 1 | 0 |  |  |  |  |  |  |  |  |
| GO:0043299\_leukocyte\_degranulation | 1 | 0 |  |  |  |  |  |  |  |  |
| GO:0043307\_eosinophil\_activation | 1 | 0 |  |  |  |  |  |  |  |  |
| GO:0043308\_eosinophil\_degranulation | 1 | 0 |  |  |  |  |  |  |  |  |
| GO:0043312\_neutrophil\_degranulation | 1 | 0 |  |  |  |  |  |  |  |  |
| GO:0043330\_response\_to\_exogenous\_dsRNA | 1 | 0 |  |  |  |  |  |  |  |  |
| GO:0043353\_enucleate\_erythrocyte\_differentiation | 1 | 0 |  |  |  |  |  |  |  |  |
| GO:0043371\_negative\_regulation\_of\_CD4-positive\_\_alpha\_beta\_T\_cell\_differentiation | 1 | 0 |  |  |  |  |  |  |  |  |
| GO:0043383\_negative\_T\_cell\_selection | 1 | 0 |  |  |  |  |  |  |  |  |
| GO:0043403\_skeletal\_muscle\_regeneration | 1 | 0 |  |  |  |  |  |  |  |  |
| GO:0043418\_homocysteine\_catabolic\_process | 1 | 0 |  |  |  |  |  |  |  |  |
| GO:0043420\_anthranilate\_metabolic\_process | 1 | 0 |  |  |  |  |  |  |  |  |
| GO:0043437\_butanoic\_acid\_metabolic\_process | 1 | 0 |  |  |  |  |  |  |  |  |
| GO:0043455\_regulation\_of\_secondary\_metabolic\_process | 1 | 0 |  |  |  |  |  |  |  |  |
| GO:0043456\_regulation\_of\_pentose-phosphate\_shunt | 1 | 0 |  |  |  |  |  |  |  |  |
| GO:0043457\_regulation\_of\_cellular\_respiration | 1 | 0 |  |  |  |  |  |  |  |  |
| GO:0043517\_positive\_regulation\_of\_DNA\_damage\_response\_\_signal\_transduction\_by\_p53\_class\_mediator | 1 | 0 |  |  |  |  |  |  |  |  |
| GO:0043518\_negative\_regulation\_of\_DNA\_damage\_response\_\_signal\_transduction\_by\_p53\_class\_mediator | 1 | 0 |  |  |  |  |  |  |  |  |
| GO:0043551\_regulation\_of\_phosphoinositide\_3-kinase\_activity | 1 | 0 |  |  |  |  |  |  |  |  |
| GO:0043552\_positive\_regulation\_of\_phosphoinositide\_3-kinase\_activity | 1 | 0 |  |  |  |  |  |  |  |  |
| GO:0043556\_regulation\_of\_translation\_in\_response\_to\_oxidative\_stress | 1 | 0 |  |  |  |  |  |  |  |  |
| GO:0043584\_nose\_development | 1 | 0 |  |  |  |  |  |  |  |  |
| GO:0043586\_tongue\_development | 1 | 0 |  |  |  |  |  |  |  |  |
| GO:0043587\_tongue\_morphogenesis | 1 | 0 |  |  |  |  |  |  |  |  |
| GO:0043647\_inositol\_phosphate\_metabolic\_process | 1 | 0 |  |  |  |  |  |  |  |  |
| GO:0043654\_recognition\_of\_apoptotic\_cell | 1 | 0 |  |  |  |  |  |  |  |  |
| GO:0043696\_dedifferentiation | 1 | 0 |  |  |  |  |  |  |  |  |
| GO:0043697\_cell\_dedifferentiation | 1 | 0 |  |  |  |  |  |  |  |  |
| GO:0043901\_negative\_regulation\_of\_multi-organism\_process | 1 | 0 |  |  |  |  |  |  |  |  |
| GO:0043921\_modulation\_by\_host\_of\_viral\_transcription | 1 | 0 |  |  |  |  |  |  |  |  |
| GO:0043923\_positive\_regulation\_by\_host\_of\_viral\_transcription | 1 | 0 |  |  |  |  |  |  |  |  |
| GO:0044007\_dissemination\_or\_transmission\_of\_symbiont\_from\_host | 1 | 0 |  |  |  |  |  |  |  |  |
| GO:0044089\_positive\_regulation\_of\_cellular\_component\_biogenesis | 1 | 0 |  |  |  |  |  |  |  |  |
| GO:0044258\_intestinal\_lipid\_catabolic\_process | 1 | 0 |  |  |  |  |  |  |  |  |
| GO:0044273\_sulfur\_compound\_catabolic\_process | 1 | 0 |  |  |  |  |  |  |  |  |
| GO:0045013\_negative\_regulation\_of\_transcription\_by\_carbon\_catabolites | 1 | 0 |  |  |  |  |  |  |  |  |
| GO:0045014\_negative\_regulation\_of\_transcription\_by\_glucose | 1 | 0 |  |  |  |  |  |  |  |  |
| GO:0045020\_error-prone\_DNA\_repair | 1 | 0 |  |  |  |  |  |  |  |  |
| GO:0045023\_G0\_to\_G1\_transition | 1 | 0 |  |  |  |  |  |  |  |  |
| GO:0045047\_protein\_targeting\_to\_ER | 1 | 0 |  |  |  |  |  |  |  |  |
| GO:0045065\_cytotoxic\_T\_cell\_differentiation | 1 | 0 |  |  |  |  |  |  |  |  |
| GO:0045074\_regulation\_of\_interleukin-10\_biosynthetic\_process | 1 | 0 |  |  |  |  |  |  |  |  |
| GO:0045082\_positive\_regulation\_of\_interleukin-10\_biosynthetic\_process | 1 | 0 |  |  |  |  |  |  |  |  |
| GO:0045132\_meiotic\_chromosome\_segregation | 1 | 0 |  |  |  |  |  |  |  |  |
| GO:0045163\_clustering\_of\_voltage-gated\_potassium\_channels | 1 | 0 |  |  |  |  |  |  |  |  |
| GO:0045175\_basal\_protein\_localization | 1 | 0 |  |  |  |  |  |  |  |  |
| GO:0045188\_regulation\_of\_circadian\_sleep\_wake\_cycle\_\_non-REM\_sleep | 1 | 0 |  |  |  |  |  |  |  |  |
| GO:0045189\_connective\_tissue\_growth\_factor\_biosynthetic\_process | 1 | 0 |  |  |  |  |  |  |  |  |
| GO:0045196\_establishment\_or\_maintenance\_of\_neuroblast\_polarity | 1 | 0 |  |  |  |  |  |  |  |  |
| GO:0045199\_maintenance\_of\_epithelial\_cell\_apical\_basal\_polarity | 1 | 0 |  |  |  |  |  |  |  |  |
| GO:0045200\_establishment\_of\_neuroblast\_polarity | 1 | 0 |  |  |  |  |  |  |  |  |
| GO:0045204\_MAPK\_export\_from\_nucleus | 1 | 0 |  |  |  |  |  |  |  |  |
| GO:0045208\_MAPK\_phosphatase\_export\_from\_nucleus | 1 | 0 |  |  |  |  |  |  |  |  |
| GO:0045209\_MAPK\_phosphatase\_export\_from\_nucleus\_\_leptomycin\_B\_sensitive | 1 | 0 |  |  |  |  |  |  |  |  |
| GO:0045292\_nuclear\_mRNA\_cis\_splicing\_\_via\_spliceosome | 1 | 0 |  |  |  |  |  |  |  |  |
| GO:0045324\_late\_endosome\_to\_vacuole\_transport | 1 | 0 |  |  |  |  |  |  |  |  |
| GO:0045329\_carnitine\_biosynthetic\_process | 1 | 0 |  |  |  |  |  |  |  |  |
| GO:0045345\_positive\_regulation\_of\_MHC\_class\_I\_biosynthetic\_process | 1 | 0 |  |  |  |  |  |  |  |  |
| GO:0045355\_negative\_regulation\_of\_interferon-alpha\_biosynthetic\_process | 1 | 0 |  |  |  |  |  |  |  |  |
| GO:0045360\_regulation\_of\_interleukin-1\_biosynthetic\_process | 1 | 0 |  |  |  |  |  |  |  |  |
| GO:0045362\_positive\_regulation\_of\_interleukin-1\_biosynthetic\_process | 1 | 0 |  |  |  |  |  |  |  |  |
| GO:0045366\_regulation\_of\_interleukin-13\_biosynthetic\_process | 1 | 0 |  |  |  |  |  |  |  |  |
| GO:0045368\_positive\_regulation\_of\_interleukin-13\_biosynthetic\_process | 1 | 0 |  |  |  |  |  |  |  |  |
| GO:0045381\_regulation\_of\_interleukin-18\_biosynthetic\_process | 1 | 0 |  |  |  |  |  |  |  |  |
| GO:0045405\_regulation\_of\_interleukin-5\_biosynthetic\_process | 1 | 0 |  |  |  |  |  |  |  |  |
| GO:0045407\_positive\_regulation\_of\_interleukin-5\_biosynthetic\_process | 1 | 0 |  |  |  |  |  |  |  |  |
| GO:0045425\_positive\_regulation\_of\_granulocyte\_macrophage\_colony-stimulating\_factor\_biosynthetic\_process | 1 | 0 |  |  |  |  |  |  |  |  |
| GO:0045475\_locomotor\_rhythm | 1 | 0 |  |  |  |  |  |  |  |  |
| GO:0045553\_TRAIL\_biosynthetic\_process | 1 | 0 |  |  |  |  |  |  |  |  |
| GO:0045554\_regulation\_of\_TRAIL\_biosynthetic\_process | 1 | 0 |  |  |  |  |  |  |  |  |
| GO:0045556\_positive\_regulation\_of\_TRAIL\_biosynthetic\_process | 1 | 0 |  |  |  |  |  |  |  |  |
| GO:0045575\_basophil\_activation | 1 | 0 |  |  |  |  |  |  |  |  |
| GO:0045579\_positive\_regulation\_of\_B\_cell\_differentiation | 1 | 0 |  |  |  |  |  |  |  |  |
| GO:0045583\_regulation\_of\_cytotoxic\_T\_cell\_differentiation | 1 | 0 |  |  |  |  |  |  |  |  |
| GO:0045585\_positive\_regulation\_of\_cytotoxic\_T\_cell\_differentiation | 1 | 0 |  |  |  |  |  |  |  |  |
| GO:0045589\_regulation\_of\_regulatory\_T\_cell\_differentiation | 1 | 0 |  |  |  |  |  |  |  |  |
| GO:0045590\_negative\_regulation\_of\_regulatory\_T\_cell\_differentiation | 1 | 0 |  |  |  |  |  |  |  |  |
| GO:0045602\_negative\_regulation\_of\_endothelial\_cell\_differentiation | 1 | 0 |  |  |  |  |  |  |  |  |
| GO:0045603\_positive\_regulation\_of\_endothelial\_cell\_differentiation | 1 | 0 |  |  |  |  |  |  |  |  |
| GO:0045605\_negative\_regulation\_of\_epidermal\_cell\_differentiation | 1 | 0 |  |  |  |  |  |  |  |  |
| GO:0045617\_negative\_regulation\_of\_keratinocyte\_differentiation | 1 | 0 |  |  |  |  |  |  |  |  |
| GO:0045623\_negative\_regulation\_of\_T-helper\_cell\_differentiation | 1 | 0 |  |  |  |  |  |  |  |  |
| GO:0045629\_negative\_regulation\_of\_T-helper\_2\_cell\_differentiation | 1 | 0 |  |  |  |  |  |  |  |  |
| GO:0045654\_positive\_regulation\_of\_megakaryocyte\_differentiation | 1 | 0 |  |  |  |  |  |  |  |  |
| GO:0045672\_positive\_regulation\_of\_osteoclast\_differentiation | 1 | 0 |  |  |  |  |  |  |  |  |
| GO:0045683\_negative\_regulation\_of\_epidermis\_development | 1 | 0 |  |  |  |  |  |  |  |  |
| GO:0045716\_positive\_regulation\_of\_low-density\_lipoprotein\_receptor\_biosynthetic\_process | 1 | 0 |  |  |  |  |  |  |  |  |
| GO:0045719\_negative\_regulation\_of\_glycogen\_biosynthetic\_process | 1 | 0 |  |  |  |  |  |  |  |  |
| GO:0045738\_negative\_regulation\_of\_DNA\_repair | 1 | 0 |  |  |  |  |  |  |  |  |
| GO:0045747\_positive\_regulation\_of\_Notch\_signaling\_pathway | 1 | 0 |  |  |  |  |  |  |  |  |
| GO:0045750\_positive\_regulation\_of\_S\_phase\_of\_mitotic\_cell\_cycle | 1 | 0 |  |  |  |  |  |  |  |  |
| GO:0045751\_negative\_regulation\_of\_Toll\_signaling\_pathway | 1 | 0 |  |  |  |  |  |  |  |  |
| GO:0045759\_negative\_regulation\_of\_action\_potential | 1 | 0 |  |  |  |  |  |  |  |  |
| GO:0045773\_positive\_regulation\_of\_axon\_extension | 1 | 0 |  |  |  |  |  |  |  |  |
| GO:0045794\_negative\_regulation\_of\_cell\_volume | 1 | 0 |  |  |  |  |  |  |  |  |
| GO:0045799\_positive\_regulation\_of\_chromatin\_assembly\_or\_disassembly | 1 | 0 |  |  |  |  |  |  |  |  |
| GO:0045818\_negative\_regulation\_of\_glycogen\_catabolic\_process | 1 | 0 |  |  |  |  |  |  |  |  |
| GO:0045836\_positive\_regulation\_of\_meiosis | 1 | 0 |  |  |  |  |  |  |  |  |
| GO:0045837\_negative\_regulation\_of\_membrane\_potential | 1 | 0 |  |  |  |  |  |  |  |  |
| GO:0045844\_positive\_regulation\_of\_striated\_muscle\_development | 1 | 0 |  |  |  |  |  |  |  |  |
| GO:0045852\_pH\_elevation | 1 | 0 |  |  |  |  |  |  |  |  |
| GO:0045870\_positive\_regulation\_of\_retroviral\_genome\_replication | 1 | 0 |  |  |  |  |  |  |  |  |
| GO:0045875\_negative\_regulation\_of\_sister\_chromatid\_cohesion | 1 | 0 |  |  |  |  |  |  |  |  |
| GO:0045879\_negative\_regulation\_of\_smoothened\_signaling\_pathway | 1 | 0 |  |  |  |  |  |  |  |  |
| GO:0045896\_regulation\_of\_transcription\_\_mitotic | 1 | 0 |  |  |  |  |  |  |  |  |
| GO:0045907\_positive\_regulation\_of\_vasoconstriction | 1 | 0 |  |  |  |  |  |  |  |  |
| GO:0045910\_negative\_regulation\_of\_DNA\_recombination | 1 | 0 |  |  |  |  |  |  |  |  |
| GO:0045915\_positive\_regulation\_of\_catecholamine\_metabolic\_process | 1 | 0 |  |  |  |  |  |  |  |  |
| GO:0045921\_positive\_regulation\_of\_exocytosis | 1 | 0 |  |  |  |  |  |  |  |  |
| GO:0045945\_positive\_regulation\_of\_transcription\_from\_RNA\_polymerase\_III\_promoter | 1 | 0 |  |  |  |  |  |  |  |  |
| GO:0045956\_positive\_regulation\_of\_calcium\_ion-dependent\_exocytosis | 1 | 0 |  |  |  |  |  |  |  |  |
| GO:0045964\_positive\_regulation\_of\_dopamine\_metabolic\_process | 1 | 0 |  |  |  |  |  |  |  |  |
| GO:0045989\_positive\_regulation\_of\_striated\_muscle\_contraction | 1 | 0 |  |  |  |  |  |  |  |  |
| GO:0045993\_negative\_regulation\_of\_translational\_initiation\_by\_iron | 1 | 0 |  |  |  |  |  |  |  |  |
| GO:0046005\_positive\_regulation\_of\_circadian\_sleep\_wake\_cycle\_\_REM\_sleep | 1 | 0 |  |  |  |  |  |  |  |  |
| GO:0046007\_negative\_regulation\_of\_activated\_T\_cell\_proliferation | 1 | 0 |  |  |  |  |  |  |  |  |
| GO:0046010\_positive\_regulation\_of\_circadian\_sleep\_wake\_cycle\_\_non-REM\_sleep | 1 | 0 |  |  |  |  |  |  |  |  |
| GO:0046031\_ADP\_metabolic\_process | 1 | 0 |  |  |  |  |  |  |  |  |
| GO:0046036\_CTP\_metabolic\_process | 1 | 0 |  |  |  |  |  |  |  |  |
| GO:0046040\_IMP\_metabolic\_process | 1 | 0 |  |  |  |  |  |  |  |  |
| GO:0046048\_UDP\_metabolic\_process | 1 | 0 |  |  |  |  |  |  |  |  |
| GO:0046049\_UMP\_metabolic\_process | 1 | 0 |  |  |  |  |  |  |  |  |
| GO:0046056\_dADP\_metabolic\_process | 1 | 0 |  |  |  |  |  |  |  |  |
| GO:0046085\_adenosine\_metabolic\_process | 1 | 0 |  |  |  |  |  |  |  |  |
| GO:0046087\_cytidine\_metabolic\_process | 1 | 0 |  |  |  |  |  |  |  |  |
| GO:0046101\_hypoxanthine\_biosynthetic\_process | 1 | 0 |  |  |  |  |  |  |  |  |
| GO:0046103\_inosine\_biosynthetic\_process | 1 | 0 |  |  |  |  |  |  |  |  |
| GO:0046104\_thymidine\_metabolic\_process | 1 | 0 |  |  |  |  |  |  |  |  |
| GO:0046108\_uridine\_metabolic\_process | 1 | 0 |  |  |  |  |  |  |  |  |
| GO:0046125\_pyrimidine\_deoxyribonucleoside\_metabolic\_process | 1 | 0 |  |  |  |  |  |  |  |  |
| GO:0046127\_pyrimidine\_deoxyribonucleoside\_catabolic\_process | 1 | 0 |  |  |  |  |  |  |  |  |
| GO:0046133\_pyrimidine\_ribonucleoside\_catabolic\_process | 1 | 0 |  |  |  |  |  |  |  |  |
| GO:0046173\_polyol\_biosynthetic\_process | 1 | 0 |  |  |  |  |  |  |  |  |
| GO:0046184\_aldehyde\_biosynthetic\_process | 1 | 0 |  |  |  |  |  |  |  |  |
| GO:0046203\_spermidine\_catabolic\_process | 1 | 0 |  |  |  |  |  |  |  |  |
| GO:0046219\_indolalkylamine\_biosynthetic\_process | 1 | 0 |  |  |  |  |  |  |  |  |
| GO:0046292\_formaldehyde\_metabolic\_process | 1 | 0 |  |  |  |  |  |  |  |  |
| GO:0046293\_formaldehyde\_biosynthetic\_process | 1 | 0 |  |  |  |  |  |  |  |  |
| GO:0046317\_regulation\_of\_glucosylceramide\_biosynthetic\_process | 1 | 0 |  |  |  |  |  |  |  |  |
| GO:0046318\_negative\_regulation\_of\_glucosylceramide\_biosynthetic\_process | 1 | 0 |  |  |  |  |  |  |  |  |
| GO:0046322\_negative\_regulation\_of\_fatty\_acid\_oxidation | 1 | 0 |  |  |  |  |  |  |  |  |
| GO:0046335\_ethanolamine\_biosynthetic\_process | 1 | 0 |  |  |  |  |  |  |  |  |
| GO:0046337\_phosphatidylethanolamine\_metabolic\_process | 1 | 0 |  |  |  |  |  |  |  |  |
| GO:0046340\_diacylglycerol\_catabolic\_process | 1 | 0 |  |  |  |  |  |  |  |  |
| GO:0046351\_disaccharide\_biosynthetic\_process | 1 | 0 |  |  |  |  |  |  |  |  |
| GO:0046352\_disaccharide\_catabolic\_process | 1 | 0 |  |  |  |  |  |  |  |  |
| GO:0046370\_fructose\_biosynthetic\_process | 1 | 0 |  |  |  |  |  |  |  |  |
| GO:0046380\_N-acetylneuraminate\_biosynthetic\_process | 1 | 0 |  |  |  |  |  |  |  |  |
| GO:0046390\_ribose\_phosphate\_biosynthetic\_process | 1 | 0 |  |  |  |  |  |  |  |  |
| GO:0046399\_glucuronate\_biosynthetic\_process | 1 | 0 |  |  |  |  |  |  |  |  |
| GO:0046434\_organophosphate\_catabolic\_process | 1 | 0 |  |  |  |  |  |  |  |  |
| GO:0046448\_tropane\_alkaloid\_metabolic\_process | 1 | 0 |  |  |  |  |  |  |  |  |
| GO:0046449\_creatinine\_metabolic\_process | 1 | 0 |  |  |  |  |  |  |  |  |
| GO:0046471\_phosphatidylglycerol\_metabolic\_process | 1 | 0 |  |  |  |  |  |  |  |  |
| GO:0046477\_glycosylceramide\_catabolic\_process | 1 | 0 |  |  |  |  |  |  |  |  |
| GO:0046485\_ether\_lipid\_metabolic\_process | 1 | 0 |  |  |  |  |  |  |  |  |
| GO:0046487\_glyoxylate\_metabolic\_process | 1 | 0 |  |  |  |  |  |  |  |  |
| GO:0046491\_L-methylmalonyl-CoA\_metabolic\_process | 1 | 0 |  |  |  |  |  |  |  |  |
| GO:0046501\_protoporphyrinogen\_IX\_metabolic\_process | 1 | 0 |  |  |  |  |  |  |  |  |
| GO:0046511\_sphinganine\_biosynthetic\_process | 1 | 0 |  |  |  |  |  |  |  |  |
| GO:0046514\_ceramide\_catabolic\_process | 1 | 0 |  |  |  |  |  |  |  |  |
| GO:0046549\_retinal\_cone\_cell\_development | 1 | 0 |  |  |  |  |  |  |  |  |
| GO:0046586\_regulation\_of\_calcium-dependent\_cell-cell\_adhesion | 1 | 0 |  |  |  |  |  |  |  |  |
| GO:0046588\_negative\_regulation\_of\_calcium-dependent\_cell-cell\_adhesion | 1 | 0 |  |  |  |  |  |  |  |  |
| GO:0046597\_negative\_regulation\_of\_virion\_penetration\_into\_host\_cell | 1 | 0 |  |  |  |  |  |  |  |  |
| GO:0046600\_negative\_regulation\_of\_centriole\_replication | 1 | 0 |  |  |  |  |  |  |  |  |
| GO:0046606\_negative\_regulation\_of\_centrosome\_cycle | 1 | 0 |  |  |  |  |  |  |  |  |
| GO:0046620\_regulation\_of\_organ\_growth | 1 | 0 |  |  |  |  |  |  |  |  |
| GO:0046636\_negative\_regulation\_of\_alpha-beta\_T\_cell\_activation | 1 | 0 |  |  |  |  |  |  |  |  |
| GO:0046639\_negative\_regulation\_of\_alpha-beta\_T\_cell\_differentiation | 1 | 0 |  |  |  |  |  |  |  |  |
| GO:0046640\_regulation\_of\_alpha-beta\_T\_cell\_proliferation | 1 | 0 |  |  |  |  |  |  |  |  |
| GO:0046641\_positive\_regulation\_of\_alpha-beta\_T\_cell\_proliferation | 1 | 0 |  |  |  |  |  |  |  |  |
| GO:0046655\_folic\_acid\_metabolic\_process | 1 | 0 |  |  |  |  |  |  |  |  |
| GO:0046666\_retinal\_cell\_programmed\_cell\_death | 1 | 0 |  |  |  |  |  |  |  |  |
| GO:0046668\_regulation\_of\_retinal\_cell\_programmed\_cell\_death | 1 | 0 |  |  |  |  |  |  |  |  |
| GO:0046670\_positive\_regulation\_of\_retinal\_cell\_programmed\_cell\_death | 1 | 0 |  |  |  |  |  |  |  |  |
| GO:0046674\_induction\_of\_retinal\_programmed\_cell\_death | 1 | 0 |  |  |  |  |  |  |  |  |
| GO:0046685\_response\_to\_arsenic | 1 | 0 |  |  |  |  |  |  |  |  |
| GO:0046686\_response\_to\_cadmium\_ion | 1 | 0 |  |  |  |  |  |  |  |  |
| GO:0046689\_response\_to\_mercury\_ion | 1 | 0 |  |  |  |  |  |  |  |  |
| GO:0046692\_sperm\_competition | 1 | 0 |  |  |  |  |  |  |  |  |
| GO:0046713\_boron\_transport | 1 | 0 |  |  |  |  |  |  |  |  |
| GO:0046719\_regulation\_of\_viral\_protein\_levels\_in\_host\_cell | 1 | 0 |  |  |  |  |  |  |  |  |
| GO:0046814\_virion\_attachment\_\_binding\_of\_host\_cell\_surface\_coreceptor | 1 | 0 |  |  |  |  |  |  |  |  |
| GO:0046826\_negative\_regulation\_of\_protein\_export\_from\_nucleus | 1 | 0 |  |  |  |  |  |  |  |  |
| GO:0046827\_positive\_regulation\_of\_protein\_export\_from\_nucleus | 1 | 0 |  |  |  |  |  |  |  |  |
| GO:0046833\_positive\_regulation\_of\_RNA\_export\_from\_nucleus | 1 | 0 |  |  |  |  |  |  |  |  |
| GO:0046835\_carbohydrate\_phosphorylation | 1 | 0 |  |  |  |  |  |  |  |  |
| GO:0046838\_phosphorylated\_carbohydrate\_dephosphorylation | 1 | 0 |  |  |  |  |  |  |  |  |
| GO:0046853\_inositol\_and\_derivative\_phosphorylation | 1 | 0 |  |  |  |  |  |  |  |  |
| GO:0046855\_inositol\_phosphate\_dephosphorylation | 1 | 0 |  |  |  |  |  |  |  |  |
| GO:0046856\_phosphoinositide\_dephosphorylation | 1 | 0 |  |  |  |  |  |  |  |  |
| GO:0046898\_response\_to\_cycloheximide | 1 | 0 |  |  |  |  |  |  |  |  |
| GO:0046916\_cellular\_transition\_metal\_ion\_homeostasis | 1 | 0 |  |  |  |  |  |  |  |  |
| GO:0046931\_pore\_complex\_biogenesis | 1 | 0 |  |  |  |  |  |  |  |  |
| GO:0046939\_nucleotide\_phosphorylation | 1 | 0 |  |  |  |  |  |  |  |  |
| GO:0046946\_hydroxylysine\_metabolic\_process | 1 | 0 |  |  |  |  |  |  |  |  |
| GO:0046947\_hydroxylysine\_biosynthetic\_process | 1 | 0 |  |  |  |  |  |  |  |  |
| GO:0046963\_3'-phosphoadenosine\_5'-phosphosulfate\_transport | 1 | 0 |  |  |  |  |  |  |  |  |
| GO:0046984\_regulation\_of\_hemoglobin\_biosynthetic\_process | 1 | 0 |  |  |  |  |  |  |  |  |
| GO:0046986\_negative\_regulation\_of\_hemoglobin\_biosynthetic\_process | 1 | 0 |  |  |  |  |  |  |  |  |
| GO:0048003\_antigen\_processing\_and\_presentation\_of\_lipid\_antigen\_via\_MHC\_class\_Ib | 1 | 0 |  |  |  |  |  |  |  |  |
| GO:0048006\_antigen\_processing\_and\_presentation\_\_endogenous\_lipid\_antigen\_via\_MHC\_class\_Ib | 1 | 0 |  |  |  |  |  |  |  |  |
| GO:0048013\_ephrin\_receptor\_signaling\_pathway | 1 | 0 |  |  |  |  |  |  |  |  |
| GO:0048070\_regulation\_of\_pigmentation\_during\_development | 1 | 0 |  |  |  |  |  |  |  |  |
| GO:0048073\_regulation\_of\_eye\_pigmentation | 1 | 0 |  |  |  |  |  |  |  |  |
| GO:0048075\_positive\_regulation\_of\_eye\_pigmentation | 1 | 0 |  |  |  |  |  |  |  |  |
| GO:0048087\_positive\_regulation\_of\_pigmentation\_during\_development | 1 | 0 |  |  |  |  |  |  |  |  |
| GO:0048160\_primary\_follicle\_stage\_\_oogenesis | 1 | 0 |  |  |  |  |  |  |  |  |
| GO:0048170\_positive\_regulation\_of\_long-term\_neuronal\_synaptic\_plasticity | 1 | 0 |  |  |  |  |  |  |  |  |
| GO:0048172\_regulation\_of\_short-term\_neuronal\_synaptic\_plasticity | 1 | 0 |  |  |  |  |  |  |  |  |
| GO:0048175\_hepatocyte\_growth\_factor\_biosynthetic\_process | 1 | 0 |  |  |  |  |  |  |  |  |
| GO:0048176\_regulation\_of\_hepatocyte\_growth\_factor\_biosynthetic\_process | 1 | 0 |  |  |  |  |  |  |  |  |
| GO:0048178\_negative\_regulation\_of\_hepatocyte\_growth\_factor\_biosynthetic\_process | 1 | 0 |  |  |  |  |  |  |  |  |
| GO:0048203\_vesicle\_targeting\_\_trans-Golgi\_to\_endosome | 1 | 0 |  |  |  |  |  |  |  |  |
| GO:0048210\_Golgi\_vesicle\_fusion\_to\_target\_membrane | 1 | 0 |  |  |  |  |  |  |  |  |
| GO:0048241\_epinephrine\_transport | 1 | 0 |  |  |  |  |  |  |  |  |
| GO:0048242\_epinephrine\_secretion | 1 | 0 |  |  |  |  |  |  |  |  |
| GO:0048245\_eosinophil\_chemotaxis | 1 | 0 |  |  |  |  |  |  |  |  |
| GO:0048265\_response\_to\_pain | 1 | 0 |  |  |  |  |  |  |  |  |
| GO:0048289\_isotype\_switching\_to\_IgE\_isotypes | 1 | 0 |  |  |  |  |  |  |  |  |
| GO:0048293\_regulation\_of\_isotype\_switching\_to\_IgE\_isotypes | 1 | 0 |  |  |  |  |  |  |  |  |
| GO:0048295\_positive\_regulation\_of\_isotype\_switching\_to\_IgE\_isotypes | 1 | 0 |  |  |  |  |  |  |  |  |
| GO:0048302\_regulation\_of\_isotype\_switching\_to\_IgG\_isotypes | 1 | 0 |  |  |  |  |  |  |  |  |
| GO:0048304\_positive\_regulation\_of\_isotype\_switching\_to\_IgG\_isotypes | 1 | 0 |  |  |  |  |  |  |  |  |
| GO:0048311\_mitochondrion\_distribution | 1 | 0 |  |  |  |  |  |  |  |  |
| GO:0048339\_paraxial\_mesoderm\_development | 1 | 0 |  |  |  |  |  |  |  |  |
| GO:0048340\_paraxial\_mesoderm\_morphogenesis | 1 | 0 |  |  |  |  |  |  |  |  |
| GO:0048388\_endosomal\_lumen\_acidification | 1 | 0 |  |  |  |  |  |  |  |  |
| GO:0048478\_replication\_fork\_protection | 1 | 0 |  |  |  |  |  |  |  |  |
| GO:0048483\_autonomic\_nervous\_system\_development | 1 | 0 |  |  |  |  |  |  |  |  |
| GO:0048485\_sympathetic\_nervous\_system\_development | 1 | 0 |  |  |  |  |  |  |  |  |
| GO:0048499\_synaptic\_vesicle\_membrane\_organization | 1 | 0 |  |  |  |  |  |  |  |  |
| GO:0048535\_lymph\_node\_development | 1 | 0 |  |  |  |  |  |  |  |  |
| GO:0048539\_bone\_marrow\_development | 1 | 0 |  |  |  |  |  |  |  |  |
| GO:0048549\_positive\_regulation\_of\_pinocytosis | 1 | 0 |  |  |  |  |  |  |  |  |
| GO:0048553\_negative\_regulation\_of\_metalloenzyme\_activity | 1 | 0 |  |  |  |  |  |  |  |  |
| GO:0048566\_embryonic\_gut\_development | 1 | 0 |  |  |  |  |  |  |  |  |
| GO:0048596\_embryonic\_camera-type\_eye\_morphogenesis | 1 | 0 |  |  |  |  |  |  |  |  |
| GO:0048617\_embryonic\_foregut\_morphogenesis | 1 | 0 |  |  |  |  |  |  |  |  |
| GO:0048619\_embryonic\_hindgut\_morphogenesis | 1 | 0 |  |  |  |  |  |  |  |  |
| GO:0048636\_positive\_regulation\_of\_muscle\_development | 1 | 0 |  |  |  |  |  |  |  |  |
| GO:0048639\_positive\_regulation\_of\_developmental\_growth | 1 | 0 |  |  |  |  |  |  |  |  |
| GO:0048640\_negative\_regulation\_of\_developmental\_growth | 1 | 0 |  |  |  |  |  |  |  |  |
| GO:0048665\_neuron\_fate\_specification | 1 | 0 |  |  |  |  |  |  |  |  |
| GO:0048679\_regulation\_of\_axon\_regeneration | 1 | 0 |  |  |  |  |  |  |  |  |
| GO:0048681\_negative\_regulation\_of\_axon\_regeneration | 1 | 0 |  |  |  |  |  |  |  |  |
| GO:0048703\_embryonic\_viscerocranium\_morphogenesis | 1 | 0 |  |  |  |  |  |  |  |  |
| GO:0048745\_smooth\_muscle\_tissue\_development | 1 | 0 |  |  |  |  |  |  |  |  |
| GO:0048755\_branching\_morphogenesis\_of\_a\_nerve | 1 | 0 |  |  |  |  |  |  |  |  |
| GO:0048793\_pronephros\_development | 1 | 0 |  |  |  |  |  |  |  |  |
| GO:0048807\_female\_genitalia\_morphogenesis | 1 | 0 |  |  |  |  |  |  |  |  |
| GO:0048818\_positive\_regulation\_of\_hair\_follicle\_maturation | 1 | 0 |  |  |  |  |  |  |  |  |
| GO:0048819\_regulation\_of\_hair\_follicle\_maturation | 1 | 0 |  |  |  |  |  |  |  |  |
| GO:0048821\_erythrocyte\_development | 1 | 0 |  |  |  |  |  |  |  |  |
| GO:0048845\_venous\_blood\_vessel\_morphogenesis | 1 | 0 |  |  |  |  |  |  |  |  |
| GO:0048853\_forebrain\_morphogenesis | 1 | 0 |  |  |  |  |  |  |  |  |
| GO:0048865\_stem\_cell\_fate\_commitment | 1 | 0 |  |  |  |  |  |  |  |  |
| GO:0048867\_stem\_cell\_fate\_determination | 1 | 0 |  |  |  |  |  |  |  |  |
| GO:0048874\_homeostasis\_of\_number\_of\_cells\_in\_a\_free-living\_population | 1 | 0 |  |  |  |  |  |  |  |  |
| GO:0048875\_chemical\_homeostasis\_within\_a\_tissue | 1 | 0 |  |  |  |  |  |  |  |  |
| GO:0050427\_3'-phosphoadenosine\_5'-phosphosulfate\_metabolic\_process | 1 | 0 |  |  |  |  |  |  |  |  |
| GO:0050652\_dermatan\_sulfate\_proteoglycan\_biosynthetic\_process\_\_polysaccharide\_chain\_biosynthetic\_process | 1 | 0 |  |  |  |  |  |  |  |  |
| GO:0050666\_regulation\_of\_homocysteine\_metabolic\_process | 1 | 0 |  |  |  |  |  |  |  |  |
| GO:0050674\_urothelial\_cell\_proliferation | 1 | 0 |  |  |  |  |  |  |  |  |
| GO:0050675\_regulation\_of\_urothelial\_cell\_proliferation | 1 | 0 |  |  |  |  |  |  |  |  |
| GO:0050677\_positive\_regulation\_of\_urothelial\_cell\_proliferation | 1 | 0 |  |  |  |  |  |  |  |  |
| GO:0050685\_positive\_regulation\_of\_mRNA\_processing | 1 | 0 |  |  |  |  |  |  |  |  |
| GO:0050687\_negative\_regulation\_of\_defense\_response\_to\_virus | 1 | 0 |  |  |  |  |  |  |  |  |
| GO:0050689\_negative\_regulation\_of\_defense\_response\_to\_virus\_by\_host | 1 | 0 |  |  |  |  |  |  |  |  |
| GO:0050713\_negative\_regulation\_of\_interleukin-1\_beta\_secretion | 1 | 0 |  |  |  |  |  |  |  |  |
| GO:0050722\_regulation\_of\_interleukin-1\_beta\_biosynthetic\_process | 1 | 0 |  |  |  |  |  |  |  |  |
| GO:0050725\_positive\_regulation\_of\_interleukin-1\_beta\_biosynthetic\_process | 1 | 0 |  |  |  |  |  |  |  |  |
| GO:0050751\_fractalkine\_biosynthetic\_process | 1 | 0 |  |  |  |  |  |  |  |  |
| GO:0050752\_regulation\_of\_fractalkine\_biosynthetic\_process | 1 | 0 |  |  |  |  |  |  |  |  |
| GO:0050754\_positive\_regulation\_of\_fractalkine\_biosynthetic\_process | 1 | 0 |  |  |  |  |  |  |  |  |
| GO:0050756\_fractalkine\_metabolic\_process | 1 | 0 |  |  |  |  |  |  |  |  |
| GO:0050757\_thymidylate\_synthase\_biosynthetic\_process | 1 | 0 |  |  |  |  |  |  |  |  |
| GO:0050758\_regulation\_of\_thymidylate\_synthase\_biosynthetic\_process | 1 | 0 |  |  |  |  |  |  |  |  |
| GO:0050760\_negative\_regulation\_of\_thymidylate\_synthase\_biosynthetic\_process | 1 | 0 |  |  |  |  |  |  |  |  |
| GO:0050765\_negative\_regulation\_of\_phagocytosis | 1 | 0 |  |  |  |  |  |  |  |  |
| GO:0050774\_negative\_regulation\_of\_dendrite\_morphogenesis | 1 | 0 |  |  |  |  |  |  |  |  |
| GO:0050783\_cocaine\_metabolic\_process | 1 | 0 |  |  |  |  |  |  |  |  |
| GO:0050822\_peptide\_stabilization | 1 | 0 |  |  |  |  |  |  |  |  |
| GO:0050823\_peptide\_antigen\_stabilization | 1 | 0 |  |  |  |  |  |  |  |  |
| GO:0050832\_defense\_response\_to\_fungus | 1 | 0 |  |  |  |  |  |  |  |  |
| GO:0050855\_regulation\_of\_B\_cell\_receptor\_signaling\_pathway | 1 | 0 |  |  |  |  |  |  |  |  |
| GO:0050858\_negative\_regulation\_of\_antigen\_receptor-mediated\_signaling\_pathway | 1 | 0 |  |  |  |  |  |  |  |  |
| GO:0050860\_negative\_regulation\_of\_T\_cell\_receptor\_signaling\_pathway | 1 | 0 |  |  |  |  |  |  |  |  |
| GO:0050861\_positive\_regulation\_of\_B\_cell\_receptor\_signaling\_pathway | 1 | 0 |  |  |  |  |  |  |  |  |
| GO:0050883\_musculoskeletal\_movement\_\_spinal\_reflex\_action | 1 | 0 |  |  |  |  |  |  |  |  |
| GO:0050884\_neuromuscular\_process\_controlling\_posture | 1 | 0 |  |  |  |  |  |  |  |  |
| GO:0050893\_sensory\_processing | 1 | 0 |  |  |  |  |  |  |  |  |
| GO:0050902\_leukocyte\_adhesive\_activation | 1 | 0 |  |  |  |  |  |  |  |  |
| GO:0050910\_detection\_of\_mechanical\_stimulus\_involved\_in\_sensory\_perception\_of\_sound | 1 | 0 |  |  |  |  |  |  |  |  |
| GO:0050922\_negative\_regulation\_of\_chemotaxis | 1 | 0 |  |  |  |  |  |  |  |  |
| GO:0050923\_regulation\_of\_negative\_chemotaxis | 1 | 0 |  |  |  |  |  |  |  |  |
| GO:0050924\_positive\_regulation\_of\_negative\_chemotaxis | 1 | 0 |  |  |  |  |  |  |  |  |
| GO:0050929\_induction\_of\_negative\_chemotaxis | 1 | 0 |  |  |  |  |  |  |  |  |
| GO:0050951\_sensory\_perception\_of\_temperature\_stimulus | 1 | 0 |  |  |  |  |  |  |  |  |
| GO:0050955\_thermoception | 1 | 0 |  |  |  |  |  |  |  |  |
| GO:0050974\_detection\_of\_mechanical\_stimulus\_involved\_in\_sensory\_perception | 1 | 0 |  |  |  |  |  |  |  |  |
| GO:0050983\_spermidine\_catabolic\_process\_to\_deoxyhypusine\_\_using\_deoxyhypusine\_synthase | 1 | 0 |  |  |  |  |  |  |  |  |
| GO:0051013\_microtubule\_severing | 1 | 0 |  |  |  |  |  |  |  |  |
| GO:0051029\_rRNA\_transport | 1 | 0 |  |  |  |  |  |  |  |  |
| GO:0051030\_snRNA\_transport | 1 | 0 |  |  |  |  |  |  |  |  |
| GO:0051031\_tRNA\_transport | 1 | 0 |  |  |  |  |  |  |  |  |
| GO:0051036\_regulation\_of\_endosome\_size | 1 | 0 |  |  |  |  |  |  |  |  |
| GO:0051040\_regulation\_of\_calcium-independent\_cell-cell\_adhesion | 1 | 0 |  |  |  |  |  |  |  |  |
| GO:0051041\_positive\_regulation\_of\_calcium-independent\_cell-cell\_adhesion | 1 | 0 |  |  |  |  |  |  |  |  |
| GO:0051066\_dihydrobiopterin\_metabolic\_process | 1 | 0 |  |  |  |  |  |  |  |  |
| GO:0051085\_chaperone\_mediated\_protein\_folding\_requiring\_cofactor | 1 | 0 |  |  |  |  |  |  |  |  |
| GO:0051089\_constitutive\_protein\_ectodomain\_proteolysis | 1 | 0 |  |  |  |  |  |  |  |  |
| GO:0051102\_DNA\_ligation\_during\_DNA\_recombination | 1 | 0 |  |  |  |  |  |  |  |  |
| GO:0051105\_regulation\_of\_DNA\_ligation | 1 | 0 |  |  |  |  |  |  |  |  |
| GO:0051106\_positive\_regulation\_of\_DNA\_ligation | 1 | 0 |  |  |  |  |  |  |  |  |
| GO:0051125\_regulation\_of\_actin\_nucleation | 1 | 0 |  |  |  |  |  |  |  |  |
| GO:0051126\_negative\_regulation\_of\_actin\_nucleation | 1 | 0 |  |  |  |  |  |  |  |  |
| GO:0051136\_regulation\_of\_NK\_T\_cell\_differentiation | 1 | 0 |  |  |  |  |  |  |  |  |
| GO:0051138\_positive\_regulation\_of\_NK\_T\_cell\_differentiation | 1 | 0 |  |  |  |  |  |  |  |  |
| GO:0051155\_positive\_regulation\_of\_striated\_muscle\_cell\_differentiation | 1 | 0 |  |  |  |  |  |  |  |  |
| GO:0051156\_glucose\_6-phosphate\_metabolic\_process | 1 | 0 |  |  |  |  |  |  |  |  |
| GO:0051160\_L-xylitol\_catabolic\_process | 1 | 0 |  |  |  |  |  |  |  |  |
| GO:0051164\_L-xylitol\_metabolic\_process | 1 | 0 |  |  |  |  |  |  |  |  |
| GO:0051193\_regulation\_of\_cofactor\_metabolic\_process | 1 | 0 |  |  |  |  |  |  |  |  |
| GO:0051196\_regulation\_of\_coenzyme\_metabolic\_process | 1 | 0 |  |  |  |  |  |  |  |  |
| GO:0051204\_protein\_insertion\_into\_mitochondrial\_membrane | 1 | 0 |  |  |  |  |  |  |  |  |
| GO:0051290\_protein\_heterotetramerization | 1 | 0 |  |  |  |  |  |  |  |  |
| GO:0051292\_nuclear\_pore\_complex\_assembly | 1 | 0 |  |  |  |  |  |  |  |  |
| GO:0051294\_establishment\_of\_spindle\_orientation | 1 | 0 |  |  |  |  |  |  |  |  |
| GO:0051295\_establishment\_of\_meiotic\_spindle\_localization | 1 | 0 |  |  |  |  |  |  |  |  |
| GO:0051315\_attachment\_of\_spindle\_microtubules\_to\_kinetochore\_during\_mitosis | 1 | 0 |  |  |  |  |  |  |  |  |
| GO:0051326\_telophase | 1 | 0 |  |  |  |  |  |  |  |  |
| GO:0051342\_regulation\_of\_cyclic-nucleotide\_phosphodiesterase\_activity | 1 | 0 |  |  |  |  |  |  |  |  |
| GO:0051344\_negative\_regulation\_of\_cyclic-nucleotide\_phosphodiesterase\_activity | 1 | 0 |  |  |  |  |  |  |  |  |
| GO:0051445\_regulation\_of\_meiotic\_cell\_cycle | 1 | 0 |  |  |  |  |  |  |  |  |
| GO:0051450\_myoblast\_proliferation | 1 | 0 |  |  |  |  |  |  |  |  |
| GO:0051454\_intracellular\_pH\_elevation | 1 | 0 |  |  |  |  |  |  |  |  |
| GO:0051458\_adrenocorticotropin\_secretion | 1 | 0 |  |  |  |  |  |  |  |  |
| GO:0051459\_regulation\_of\_adrenocorticotropin\_secretion | 1 | 0 |  |  |  |  |  |  |  |  |
| GO:0051461\_positive\_regulation\_of\_adrenocorticotropin\_secretion | 1 | 0 |  |  |  |  |  |  |  |  |
| GO:0051531\_NFAT\_protein\_import\_into\_nucleus | 1 | 0 |  |  |  |  |  |  |  |  |
| GO:0051532\_regulation\_of\_NFAT\_protein\_import\_into\_nucleus | 1 | 0 |  |  |  |  |  |  |  |  |
| GO:0051533\_positive\_regulation\_of\_NFAT\_protein\_import\_into\_nucleus | 1 | 0 |  |  |  |  |  |  |  |  |
| GO:0051542\_elastin\_biosynthetic\_process | 1 | 0 |  |  |  |  |  |  |  |  |
| GO:0051560\_mitochondrial\_calcium\_ion\_homeostasis | 1 | 0 |  |  |  |  |  |  |  |  |
| GO:0051561\_elevation\_of\_mitochondrial\_calcium\_ion\_concentration | 1 | 0 |  |  |  |  |  |  |  |  |
| GO:0051582\_positive\_regulation\_of\_neurotransmitter\_uptake | 1 | 0 |  |  |  |  |  |  |  |  |
| GO:0051586\_positive\_regulation\_of\_dopamine\_uptake | 1 | 0 |  |  |  |  |  |  |  |  |
| GO:0051590\_positive\_regulation\_of\_neurotransmitter\_transport | 1 | 0 |  |  |  |  |  |  |  |  |
| GO:0051594\_detection\_of\_glucose | 1 | 0 |  |  |  |  |  |  |  |  |
| GO:0051642\_centrosome\_localization | 1 | 0 |  |  |  |  |  |  |  |  |
| GO:0051645\_Golgi\_localization | 1 | 0 |  |  |  |  |  |  |  |  |
| GO:0051647\_nucleus\_localization | 1 | 0 |  |  |  |  |  |  |  |  |
| GO:0051664\_nuclear\_pore\_localization | 1 | 0 |  |  |  |  |  |  |  |  |
| GO:0051708\_intracellular\_protein\_transport\_in\_other\_organism\_during\_symbiotic\_interaction | 1 | 0 |  |  |  |  |  |  |  |  |
| GO:0051767\_nitric-oxide\_synthase\_biosynthetic\_process | 1 | 0 |  |  |  |  |  |  |  |  |
| GO:0051768\_nitric-oxide\_synthase\_2\_biosynthetic\_process | 1 | 0 |  |  |  |  |  |  |  |  |
| GO:0051769\_regulation\_of\_nitric-oxide\_synthase\_biosynthetic\_process | 1 | 0 |  |  |  |  |  |  |  |  |
| GO:0051771\_negative\_regulation\_of\_nitric-oxide\_synthase\_biosynthetic\_process | 1 | 0 |  |  |  |  |  |  |  |  |
| GO:0051772\_regulation\_of\_nitric-oxide\_synthase\_2\_biosynthetic\_process | 1 | 0 |  |  |  |  |  |  |  |  |
| GO:0051773\_positive\_regulation\_of\_nitric-oxide\_synthase\_2\_biosynthetic\_process | 1 | 0 |  |  |  |  |  |  |  |  |
| GO:0051781\_positive\_regulation\_of\_cell\_division | 1 | 0 |  |  |  |  |  |  |  |  |
| GO:0051782\_negative\_regulation\_of\_cell\_division | 1 | 0 |  |  |  |  |  |  |  |  |
| GO:0051788\_response\_to\_misfolded\_protein | 1 | 0 |  |  |  |  |  |  |  |  |
| GO:0051790\_short-chain\_fatty\_acid\_biosynthetic\_process | 1 | 0 |  |  |  |  |  |  |  |  |
| GO:0051791\_medium-chain\_fatty\_acid\_metabolic\_process | 1 | 0 |  |  |  |  |  |  |  |  |
| GO:0051792\_medium-chain\_fatty\_acid\_biosynthetic\_process | 1 | 0 |  |  |  |  |  |  |  |  |
| GO:0051794\_regulation\_of\_catagen | 1 | 0 |  |  |  |  |  |  |  |  |
| GO:0051795\_positive\_regulation\_of\_catagen | 1 | 0 |  |  |  |  |  |  |  |  |
| GO:0051821\_dissemination\_or\_transmission\_of\_organism\_from\_other\_organism\_during\_symbiotic\_interaction | 1 | 0 |  |  |  |  |  |  |  |  |
| GO:0051894\_positive\_regulation\_of\_focal\_adhesion\_formation | 1 | 0 |  |  |  |  |  |  |  |  |
| GO:0051930\_regulation\_of\_sensory\_perception\_of\_pain | 1 | 0 |  |  |  |  |  |  |  |  |
| GO:0051931\_regulation\_of\_sensory\_perception | 1 | 0 |  |  |  |  |  |  |  |  |
| GO:0051944\_positive\_regulation\_of\_catecholamine\_uptake\_during\_transmission\_of\_nerve\_impulse | 1 | 0 |  |  |  |  |  |  |  |  |
| GO:0051962\_positive\_regulation\_of\_nervous\_system\_development | 1 | 0 |  |  |  |  |  |  |  |  |
| GO:0051965\_positive\_regulation\_of\_synaptogenesis | 1 | 0 |  |  |  |  |  |  |  |  |
| GO:0051977\_lysophospholipid\_transport | 1 | 0 |  |  |  |  |  |  |  |  |
| GO:0051988\_regulation\_of\_attachment\_of\_spindle\_microtubules\_to\_kinetochore | 1 | 0 |  |  |  |  |  |  |  |  |
| GO:0052097\_interspecies\_quorum\_sensing | 1 | 0 |  |  |  |  |  |  |  |  |
| GO:0052106\_quorum\_sensing\_during\_interaction\_with\_host | 1 | 0 |  |  |  |  |  |  |  |  |
| GO:0052312\_modulation\_of\_transcription\_in\_other\_organism\_during\_symbiotic\_interaction | 1 | 0 |  |  |  |  |  |  |  |  |
| GO:0052472\_modulation\_by\_host\_of\_symbiont\_transcription | 1 | 0 |  |  |  |  |  |  |  |  |
| GO:0055009\_atrial\_cardiac\_muscle\_morphogenesis | 1 | 0 |  |  |  |  |  |  |  |  |
| GO:0055012\_ventricular\_cardiac\_muscle\_cell\_differentiation | 1 | 0 |  |  |  |  |  |  |  |  |
| GO:0055071\_manganese\_ion\_homeostasis | 1 | 0 |  |  |  |  |  |  |  |  |
| GO:0055073\_cadmium\_ion\_homeostasis | 1 | 0 |  |  |  |  |  |  |  |  |
| GO:0055076\_transition\_metal\_ion\_homeostasis | 1 | 0 |  |  |  |  |  |  |  |  |
| GO:0055089\_fatty\_acid\_homeostasis | 1 | 0 |  |  |  |  |  |  |  |  |
| GO:0055095\_lipoprotein\_mediated\_signaling | 1 | 0 |  |  |  |  |  |  |  |  |
| GO:0055096\_low\_density\_lipoprotein\_mediated\_signaling | 1 | 0 |  |  |  |  |  |  |  |  |
| GO:0055099\_response\_to\_high\_density\_lipoprotein\_stimulus | 1 | 0 |  |  |  |  |  |  |  |  |
| GO:0055118\_negative\_regulation\_of\_cardiac\_muscle\_contraction | 1 | 0 |  |  |  |  |  |  |  |  |
| GO:0055119\_relaxation\_of\_cardiac\_muscle | 1 | 0 |  |  |  |  |  |  |  |  |
| GO:0060003\_copper\_ion\_export | 1 | 0 |  |  |  |  |  |  |  |  |
| GO:0060022\_hard\_palate\_development | 1 | 0 |  |  |  |  |  |  |  |  |
| GO:0060039\_pericardium\_development | 1 | 0 |  |  |  |  |  |  |  |  |
| GO:0060055\_angiogenesis\_involved\_in\_wound\_healing | 1 | 0 |  |  |  |  |  |  |  |  |
| GO:0060059\_embryonic\_retina\_morphogenesis\_in\_camera-type\_eye | 1 | 0 |  |  |  |  |  |  |  |  |
| GO:0060065\_uterus\_development | 1 | 0 |  |  |  |  |  |  |  |  |
| GO:0060068\_vagina\_development | 1 | 0 |  |  |  |  |  |  |  |  |
| GO:0060082\_eye\_blink\_reflex | 1 | 0 |  |  |  |  |  |  |  |  |
| GO:0060083\_smooth\_muscle\_contraction\_involved\_in\_micturition | 1 | 0 |  |  |  |  |  |  |  |  |
| GO:0060088\_auditory\_receptor\_cell\_stereocilium\_organization | 1 | 0 |  |  |  |  |  |  |  |  |
| GO:0060120\_inner\_ear\_receptor\_cell\_fate\_commitment | 1 | 0 |  |  |  |  |  |  |  |  |
| GO:0060135\_maternal\_process\_involved\_in\_female\_pregnancy | 1 | 0 |  |  |  |  |  |  |  |  |
| GO:0060142\_regulation\_of\_syncytium\_formation\_by\_plasma\_membrane\_fusion | 1 | 0 |  |  |  |  |  |  |  |  |
| GO:0060143\_positive\_regulation\_of\_syncytium\_formation\_by\_plasma\_membrane\_fusion | 1 | 0 |  |  |  |  |  |  |  |  |
| GO:0060157\_urinary\_bladder\_development | 1 | 0 |  |  |  |  |  |  |  |  |
| GO:0060160\_negative\_regulation\_of\_dopamine\_receptor\_signaling\_pathway | 1 | 0 |  |  |  |  |  |  |  |  |
| GO:0060161\_positive\_regulation\_of\_dopamine\_receptor\_signaling\_pathway | 1 | 0 |  |  |  |  |  |  |  |  |
| GO:0060167\_regulation\_of\_adenosine\_receptor\_signaling\_pathway | 1 | 0 |  |  |  |  |  |  |  |  |
| GO:0060169\_negative\_regulation\_of\_adenosine\_receptor\_signaling\_pathway | 1 | 0 |  |  |  |  |  |  |  |  |
| GO:0060216\_definitive\_hemopoiesis | 1 | 0 |  |  |  |  |  |  |  |  |
| GO:0060219\_camera-type\_eye\_photoreceptor\_cell\_differentiation | 1 | 0 |  |  |  |  |  |  |  |  |
| GO:0060231\_mesenchymal\_to\_epithelial\_transition | 1 | 0 |  |  |  |  |  |  |  |  |
| GO:0060254\_regulation\_of\_N-terminal\_protein\_palmitoylation | 1 | 0 |  |  |  |  |  |  |  |  |
| GO:0060259\_regulation\_of\_feeding\_behavior | 1 | 0 |  |  |  |  |  |  |  |  |
| GO:0060262\_negative\_regulation\_of\_N-terminal\_protein\_palmitoylation | 1 | 0 |  |  |  |  |  |  |  |  |
| GO:0060265\_positive\_regulation\_of\_respiratory\_burst\_during\_acute\_inflammatory\_response | 1 | 0 |  |  |  |  |  |  |  |  |
| GO:0060266\_negative\_regulation\_of\_respiratory\_burst\_during\_acute\_inflammatory\_response | 1 | 0 |  |  |  |  |  |  |  |  |
| GO:0060268\_negative\_regulation\_of\_respiratory\_burst | 1 | 0 |  |  |  |  |  |  |  |  |
| GO:0060286\_flagellar\_cell\_motility | 1 | 0 |  |  |  |  |  |  |  |  |
| GO:0060298\_positive\_regulation\_of\_sarcomere\_organization | 1 | 0 |  |  |  |  |  |  |  |  |
| GO:0060299\_negative\_regulation\_of\_sarcomere\_organization | 1 | 0 |  |  |  |  |  |  |  |  |
| GO:0060300\_regulation\_of\_cytokine\_activity | 1 | 0 |  |  |  |  |  |  |  |  |
| GO:0060302\_negative\_regulation\_of\_cytokine\_activity | 1 | 0 |  |  |  |  |  |  |  |  |
| GO:0060305\_regulation\_of\_cell\_diameter | 1 | 0 |  |  |  |  |  |  |  |  |
| GO:0060306\_regulation\_of\_membrane\_repolarization | 1 | 0 |  |  |  |  |  |  |  |  |
| GO:0060307\_regulation\_of\_ventricular\_cardiomyocyte\_membrane\_repolarization | 1 | 0 |  |  |  |  |  |  |  |  |
| GO:0060309\_elastin\_catabolic\_process | 1 | 0 |  |  |  |  |  |  |  |  |
| GO:0060310\_regulation\_of\_elastin\_catabolic\_process | 1 | 0 |  |  |  |  |  |  |  |  |
| GO:0060311\_negative\_regulation\_of\_elastin\_catabolic\_process | 1 | 0 |  |  |  |  |  |  |  |  |
| GO:0060312\_regulation\_of\_blood\_vessel\_remodeling | 1 | 0 |  |  |  |  |  |  |  |  |
| GO:0060313\_negative\_regulation\_of\_blood\_vessel\_remodeling | 1 | 0 |  |  |  |  |  |  |  |  |
| GO:0060315\_negative\_regulation\_of\_ryanodine-sensitive\_calcium-release\_channel\_activity | 1 | 0 |  |  |  |  |  |  |  |  |
| GO:0060316\_positive\_regulation\_of\_ryanodine-sensitive\_calcium-release\_channel\_activity | 1 | 0 |  |  |  |  |  |  |  |  |
| GO:0060318\_definitive\_erythrocyte\_differentiation | 1 | 0 |  |  |  |  |  |  |  |  |
| GO:0060322\_head\_development | 1 | 0 |  |  |  |  |  |  |  |  |
| GO:0060324\_face\_development | 1 | 0 |  |  |  |  |  |  |  |  |
| GO:0060336\_negative\_regulation\_of\_interferon-gamma-mediated\_signaling\_pathway | 1 | 0 |  |  |  |  |  |  |  |  |
| GO:0060349\_bone\_morphogenesis | 1 | 0 |  |  |  |  |  |  |  |  |
| GO:0060350\_endochondral\_bone\_morphogenesis | 1 | 0 |  |  |  |  |  |  |  |  |
| GO:0060356\_leucine\_import | 1 | 0 |  |  |  |  |  |  |  |  |
| GO:0060368\_regulation\_of\_Fc\_receptor\_mediated\_stimulatory\_signaling\_pathway | 1 | 0 |  |  |  |  |  |  |  |  |
| GO:0060369\_positive\_regulation\_of\_Fc\_receptor\_mediated\_stimulatory\_signaling\_pathway | 1 | 0 |  |  |  |  |  |  |  |  |
| GO:0060380\_regulation\_of\_single-stranded\_telomeric\_DNA\_binding | 1 | 0 |  |  |  |  |  |  |  |  |
| GO:0060381\_positive\_regulation\_of\_single-stranded\_telomeric\_DNA\_binding | 1 | 0 |  |  |  |  |  |  |  |  |
| GO:0060382\_regulation\_of\_DNA\_strand\_elongation | 1 | 0 |  |  |  |  |  |  |  |  |
| GO:0060383\_positive\_regulation\_of\_DNA\_strand\_elongation | 1 | 0 |  |  |  |  |  |  |  |  |
| GO:0060397\_JAK-STAT\_cascade\_involved\_in\_growth\_hormone\_signaling\_pathway | 1 | 0 |  |  |  |  |  |  |  |  |
| GO:0060398\_regulation\_of\_growth\_hormone\_receptor\_signaling\_pathway | 1 | 0 |  |  |  |  |  |  |  |  |
| GO:0060425\_lung\_morphogenesis | 1 | 0 |  |  |  |  |  |  |  |  |
| GO:0060433\_bronchus\_development | 1 | 0 |  |  |  |  |  |  |  |  |
| GO:0060438\_trachea\_development | 1 | 0 |  |  |  |  |  |  |  |  |
| GO:0060441\_branching\_involved\_in\_lung\_morphogenesis | 1 | 0 |  |  |  |  |  |  |  |  |
| GO:0060445\_branching\_involved\_in\_salivary\_gland\_morphogenesis | 1 | 0 |  |  |  |  |  |  |  |  |
| GO:0060502\_epithelial\_cell\_proliferation\_involved\_in\_lung\_morphogenesis | 1 | 0 |  |  |  |  |  |  |  |  |
| GO:0060503\_bud\_dilation\_involved\_in\_lung\_branching | 1 | 0 |  |  |  |  |  |  |  |  |
| GO:0060560\_developmental\_growth\_involved\_in\_morphogenesis | 1 | 0 |  |  |  |  |  |  |  |  |
| GO:0060579\_ventral\_spinal\_cord\_interneuron\_fate\_commitment | 1 | 0 |  |  |  |  |  |  |  |  |
| GO:0060586\_multicellular\_organismal\_iron\_ion\_homeostasis | 1 | 0 |  |  |  |  |  |  |  |  |
| GO:0060587\_regulation\_of\_lipoprotein\_lipid\_oxidation | 1 | 0 |  |  |  |  |  |  |  |  |
| GO:0060588\_negative\_regulation\_of\_lipoprotein\_lipid\_oxidation | 1 | 0 |  |  |  |  |  |  |  |  |
| GO:0060638\_mesenchymal-epithelial\_cell\_signaling | 1 | 0 |  |  |  |  |  |  |  |  |
| GO:0060665\_regulation\_of\_branching\_involved\_in\_salivary\_gland\_morphogenesis\_by\_mesenchymal-epithelial\_signaling | 1 | 0 |  |  |  |  |  |  |  |  |
| GO:0060675\_ureteric\_bud\_morphogenesis | 1 | 0 |  |  |  |  |  |  |  |  |
| GO:0060688\_regulation\_of\_morphogenesis\_of\_a\_branching\_structure | 1 | 0 |  |  |  |  |  |  |  |  |
| GO:0060693\_regulation\_of\_branching\_involved\_in\_salivary\_gland\_morphogenesis | 1 | 0 |  |  |  |  |  |  |  |  |
| GO:0060694\_regulation\_of\_cholesterol\_transporter\_activity | 1 | 0 |  |  |  |  |  |  |  |  |
| GO:0060695\_negative\_regulation\_of\_cholesterol\_transporter\_activity | 1 | 0 |  |  |  |  |  |  |  |  |
| GO:0060697\_positive\_regulation\_of\_phospholipid\_catabolic\_process | 1 | 0 |  |  |  |  |  |  |  |  |
| GO:0060729\_intestinal\_epithelial\_structure\_maintenance | 1 | 0 |  |  |  |  |  |  |  |  |
| GO:0060730\_regulation\_of\_intestinal\_epithelial\_structure\_maintenance | 1 | 0 |  |  |  |  |  |  |  |  |
| GO:0060731\_positive\_regulation\_of\_intestinal\_epithelial\_structure\_maintenance | 1 | 0 |  |  |  |  |  |  |  |  |
| GO:0060760\_positive\_regulation\_of\_response\_to\_cytokine\_stimulus | 1 | 0 |  |  |  |  |  |  |  |  |
| GO:0060761\_negative\_regulation\_of\_response\_to\_cytokine\_stimulus | 1 | 0 |  |  |  |  |  |  |  |  |
| GO:0060788\_ectodermal\_placode\_formation | 1 | 0 |  |  |  |  |  |  |  |  |
| GO:0060841\_venous\_blood\_vessel\_development | 1 | 0 |  |  |  |  |  |  |  |  |
| GO:0060856\_establishment\_of\_blood-brain\_barrier | 1 | 0 |  |  |  |  |  |  |  |  |
| GO:0060896\_neural\_plate\_pattern\_specification | 1 | 0 |  |  |  |  |  |  |  |  |
| GO:0065001\_specification\_of\_axis\_polarity | 1 | 0 |  |  |  |  |  |  |  |  |
| GO:0070075\_tear\_secretion | 1 | 0 |  |  |  |  |  |  |  |  |
| GO:0070076\_histone\_lysine\_demethylation | 1 | 0 |  |  |  |  |  |  |  |  |
| GO:0070077\_histone\_arginine\_demethylation | 1 | 0 |  |  |  |  |  |  |  |  |
| GO:0070078\_histone\_H3-R2\_demethylation | 1 | 0 |  |  |  |  |  |  |  |  |
| GO:0070079\_histone\_H4-R3\_demethylation | 1 | 0 |  |  |  |  |  |  |  |  |
| GO:0070086\_ubiquitin-dependent\_endocytosis | 1 | 0 |  |  |  |  |  |  |  |  |
| GO:0070091\_glucagon\_secretion | 1 | 0 |  |  |  |  |  |  |  |  |
| GO:0070103\_regulation\_of\_interleukin-6-mediated\_signaling\_pathway | 1 | 0 |  |  |  |  |  |  |  |  |
| GO:0070104\_negative\_regulation\_of\_interleukin-6-mediated\_signaling\_pathway | 1 | 0 |  |  |  |  |  |  |  |  |
| GO:0070106\_interleukin-27-mediated\_signaling\_pathway | 1 | 0 |  |  |  |  |  |  |  |  |
| GO:0070162\_adiponectin\_secretion | 1 | 0 |  |  |  |  |  |  |  |  |
| GO:0070163\_regulation\_of\_adiponectin\_secretion | 1 | 0 |  |  |  |  |  |  |  |  |
| GO:0070165\_positive\_regulation\_of\_adiponectin\_secretion | 1 | 0 |  |  |  |  |  |  |  |  |
| GO:0070172\_positive\_regulation\_of\_tooth\_mineralization | 1 | 0 |  |  |  |  |  |  |  |  |
| GO:0070173\_regulation\_of\_enamel\_mineralization | 1 | 0 |  |  |  |  |  |  |  |  |
| GO:0070189\_kynurenine\_metabolic\_process | 1 | 0 |  |  |  |  |  |  |  |  |
| GO:0070212\_protein\_amino\_acid\_poly-ADP-ribosylation | 1 | 0 |  |  |  |  |  |  |  |  |
| GO:0070213\_protein\_amino\_acid\_auto-ADP-ribosylation | 1 | 0 |  |  |  |  |  |  |  |  |
| GO:0070232\_regulation\_of\_T\_cell\_apoptosis | 1 | 0 |  |  |  |  |  |  |  |  |
| GO:0070234\_positive\_regulation\_of\_T\_cell\_apoptosis | 1 | 0 |  |  |  |  |  |  |  |  |
| GO:0070242\_thymocyte\_apoptosis | 1 | 0 |  |  |  |  |  |  |  |  |
| GO:0070243\_regulation\_of\_thymocyte\_apoptosis | 1 | 0 |  |  |  |  |  |  |  |  |
| GO:0070245\_positive\_regulation\_of\_thymocyte\_apoptosis | 1 | 0 |  |  |  |  |  |  |  |  |
| GO:0070267\_oncosis | 1 | 0 |  |  |  |  |  |  |  |  |
| GO:0070286\_axonemal\_dynein\_complex\_assembly | 1 | 0 |  |  |  |  |  |  |  |  |
| GO:0070314\_G1\_to\_G0\_transition | 1 | 0 |  |  |  |  |  |  |  |  |
| GO:0070327\_thyroid\_hormone\_transport | 1 | 0 |  |  |  |  |  |  |  |  |
| GO:0070407\_oxidation-dependent\_protein\_catabolic\_process | 1 | 0 |  |  |  |  |  |  |  |  |
| GO:0070408\_carbamoyl\_phosphate\_metabolic\_process | 1 | 0 |  |  |  |  |  |  |  |  |
| GO:0070409\_carbamoyl\_phosphate\_biosynthetic\_process | 1 | 0 |  |  |  |  |  |  |  |  |
| GO:0070509\_calcium\_ion\_import | 1 | 0 |  |  |  |  |  |  |  |  |
| GO:0070527\_platelet\_aggregation | 1 | 0 |  |  |  |  |  |  |  |  |
| GO:0070528\_protein\_kinase\_C\_signaling\_cascade | 1 | 0 |  |  |  |  |  |  |  |  |
| GO:0070534\_protein\_K63-linked\_ubiquitination | 1 | 0 |  |  |  |  |  |  |  |  |
| GO:0070535\_histone\_H2A\_K63-linked\_ubiquitination | 1 | 0 |  |  |  |  |  |  |  |  |
| GO:0070537\_histone\_H2A\_K63-linked\_deubiquitination | 1 | 0 |  |  |  |  |  |  |  |  |
| GO:0070560\_protein\_secretion\_by\_platelet | 1 | 0 |  |  |  |  |  |  |  |  |
| GO:0070562\_regulation\_of\_vitamin\_D\_receptor\_signaling\_pathway | 1 | 0 |  |  |  |  |  |  |  |  |
| GO:0070564\_positive\_regulation\_of\_vitamin\_D\_receptor\_signaling\_pathway | 1 | 0 |  |  |  |  |  |  |  |  |
| GO:0070570\_regulation\_of\_neuron\_projection\_regeneration | 1 | 0 |  |  |  |  |  |  |  |  |
| GO:0070571\_negative\_regulation\_of\_neuron\_projection\_regeneration | 1 | 0 |  |  |  |  |  |  |  |  |
| GO:0070601\_centromeric\_sister\_chromatid\_cohesion | 1 | 0 |  |  |  |  |  |  |  |  |
| GO:0070602\_regulation\_of\_centromeric\_sister\_chromatid\_cohesion | 1 | 0 |  |  |  |  |  |  |  |  |
| GO:0070625\_zymogen\_granule\_exocytosis | 1 | 0 |  |  |  |  |  |  |  |  |
| GO:0070684\_seminal\_clot\_liquefaction | 1 | 0 |  |  |  |  |  |  |  |  |
| GO:0070715\_sodium-dependent\_organic\_cation\_transport | 1 | 0 |  |  |  |  |  |  |  |  |
| GO:0070813\_hydrogen\_sulfide\_metabolic\_process | 1 | 0 |  |  |  |  |  |  |  |  |
| GO:0070814\_hydrogen\_sulfide\_biosynthetic\_process | 1 | 0 |  |  |  |  |  |  |  |  |
| GO:0070846\_Hsp90\_deacetylation | 1 | 0 |  |  |  |  |  |  |  |  |
| GO:0090030\_regulation\_of\_steroid\_hormone\_biosynthetic\_process | 1 | 0 |  |  |  |  |  |  |  |  |
| GO:0090031\_positive\_regulation\_of\_steroid\_hormone\_biosynthetic\_process | 1 | 0 |  |  |  |  |  |  |  |  |
| GO:0000085\_G2\_phase\_of\_mitotic\_cell\_cycle | 5 | 0 | 0.000000 | 0.000000 | 1298 | 1099.072643 | 1155.31 | 1211.547357 | 0.890069 |
| GO:0000389\_nuclear\_mRNA\_3'-splice\_site\_recognition | 5 | 0 | 0.000000 | 0.000000 | 1298 | 1099.072643 | 1155.31 | 1211.547357 | 0.890069 |
| GO:0001504\_neurotransmitter\_uptake | 5 | 0 | 0.000000 | 0.000000 | 1298 | 1099.072643 | 1155.31 | 1211.547357 | 0.890069 |
| GO:0001580\_detection\_of\_chemical\_stimulus\_involved\_in\_sensory\_perception\_of\_bitter\_taste | 5 | 0 | 0.000000 | 0.000000 | 1298 | 1099.072643 | 1155.31 | 1211.547357 | 0.890069 |
| GO:0001672\_regulation\_of\_chromatin\_assembly\_or\_disassembly | 5 | 0 | 0.000000 | 0.000000 | 1298 | 1099.072643 | 1155.31 | 1211.547357 | 0.890069 |
| GO:0001754\_eye\_photoreceptor\_cell\_differentiation | 5 | 0 | 0.000000 | 0.000000 | 1298 | 1099.072643 | 1155.31 | 1211.547357 | 0.890069 |
| GO:0001783\_B\_cell\_apoptosis | 5 | 0 | 0.000000 | 0.000000 | 1298 | 1099.072643 | 1155.31 | 1211.547357 | 0.890069 |
| GO:0001954\_positive\_regulation\_of\_cell-matrix\_adhesion | 5 | 0 | 0.000000 | 0.000000 | 1298 | 1099.072643 | 1155.31 | 1211.547357 | 0.890069 |
| GO:0001960\_negative\_regulation\_of\_cytokine-mediated\_signaling\_pathway | 5 | 0 | 0.000000 | 0.000000 | 1298 | 1099.072643 | 1155.31 | 1211.547357 | 0.890069 |
| GO:0001964\_startle\_response | 5 | 0 | 0.000000 | 0.000000 | 1298 | 1099.072643 | 1155.31 | 1211.547357 | 0.890069 |
| GO:0001991\_regulation\_of\_systemic\_arterial\_blood\_pressure\_by\_circulatory\_renin-angiotensin | 5 | 0 | 0.000000 | 0.000000 | 1298 | 1099.072643 | 1155.31 | 1211.547357 | 0.890069 |
| GO:0002369\_T\_cell\_cytokine\_production | 5 | 0 | 0.000000 | 0.000000 | 1298 | 1099.072643 | 1155.31 | 1211.547357 | 0.890069 |
| GO:0002702\_positive\_regulation\_of\_production\_of\_molecular\_mediator\_of\_immune\_response | 5 | 0 | 0.000000 | 0.000000 | 1298 | 1099.072643 | 1155.31 | 1211.547357 | 0.890069 |
| GO:0002720\_positive\_regulation\_of\_cytokine\_production\_during\_immune\_response | 5 | 0 | 0.000000 | 0.000000 | 1298 | 1099.072643 | 1155.31 | 1211.547357 | 0.890069 |
| GO:0002724\_regulation\_of\_T\_cell\_cytokine\_production | 5 | 0 | 0.000000 | 0.000000 | 1298 | 1099.072643 | 1155.31 | 1211.547357 | 0.890069 |
| GO:0003091\_renal\_water\_homeostasis | 5 | 0 | 0.000000 | 0.000000 | 1298 | 1099.072643 | 1155.31 | 1211.547357 | 0.890069 |
| GO:0006012\_galactose\_metabolic\_process | 5 | 0 | 0.000000 | 0.000000 | 1298 | 1099.072643 | 1155.31 | 1211.547357 | 0.890069 |
| GO:0006027\_glycosaminoglycan\_catabolic\_process | 5 | 0 | 0.000000 | 0.000000 | 1298 | 1099.072643 | 1155.31 | 1211.547357 | 0.890069 |
| GO:0006098\_pentose-phosphate\_shunt | 5 | 0 | 0.000000 | 0.000000 | 1298 | 1099.072643 | 1155.31 | 1211.547357 | 0.890069 |
| GO:0006105\_succinate\_metabolic\_process | 5 | 0 | 0.000000 | 0.000000 | 1298 | 1099.072643 | 1155.31 | 1211.547357 | 0.890069 |
| GO:0006206\_pyrimidine\_base\_metabolic\_process | 5 | 0 | 0.000000 | 0.000000 | 1298 | 1099.072643 | 1155.31 | 1211.547357 | 0.890069 |
| GO:0006221\_pyrimidine\_nucleotide\_biosynthetic\_process | 5 | 0 | 0.000000 | 0.000000 | 1298 | 1099.072643 | 1155.31 | 1211.547357 | 0.890069 |
| GO:0006268\_DNA\_unwinding\_during\_replication | 5 | 0 | 0.000000 | 0.000000 | 1298 | 1099.072643 | 1155.31 | 1211.547357 | 0.890069 |
| GO:0006271\_DNA\_strand\_elongation\_during\_DNA\_replication | 5 | 0 | 0.000000 | 0.000000 | 1298 | 1099.072643 | 1155.31 | 1211.547357 | 0.890069 |
| GO:0006544\_glycine\_metabolic\_process | 5 | 0 | 0.000000 | 0.000000 | 1298 | 1099.072643 | 1155.31 | 1211.547357 | 0.890069 |
| GO:0006563\_L-serine\_metabolic\_process | 5 | 0 | 0.000000 | 0.000000 | 1298 | 1099.072643 | 1155.31 | 1211.547357 | 0.890069 |
| GO:0006595\_polyamine\_metabolic\_process | 5 | 0 | 0.000000 | 0.000000 | 1298 | 1099.072643 | 1155.31 | 1211.547357 | 0.890069 |
| GO:0006613\_cotranslational\_protein\_targeting\_to\_membrane | 5 | 0 | 0.000000 | 0.000000 | 1298 | 1099.072643 | 1155.31 | 1211.547357 | 0.890069 |
| GO:0006677\_glycosylceramide\_metabolic\_process | 5 | 0 | 0.000000 | 0.000000 | 1298 | 1099.072643 | 1155.31 | 1211.547357 | 0.890069 |
| GO:0006740\_NADPH\_regeneration | 5 | 0 | 0.000000 | 0.000000 | 1298 | 1099.072643 | 1155.31 | 1211.547357 | 0.890069 |
| GO:0006743\_ubiquinone\_metabolic\_process | 5 | 0 | 0.000000 | 0.000000 | 1298 | 1099.072643 | 1155.31 | 1211.547357 | 0.890069 |
| GO:0006744\_ubiquinone\_biosynthetic\_process | 5 | 0 | 0.000000 | 0.000000 | 1298 | 1099.072643 | 1155.31 | 1211.547357 | 0.890069 |
| GO:0006760\_folic\_acid\_and\_derivative\_metabolic\_process | 5 | 0 | 0.000000 | 0.000000 | 1298 | 1099.072643 | 1155.31 | 1211.547357 | 0.890069 |
| GO:0006817\_phosphate\_transport | 5 | 0 | 0.000000 | 0.000000 | 1298 | 1099.072643 | 1155.31 | 1211.547357 | 0.890069 |
| GO:0006835\_dicarboxylic\_acid\_transport | 5 | 0 | 0.000000 | 0.000000 | 1298 | 1099.072643 | 1155.31 | 1211.547357 | 0.890069 |
| GO:0006884\_cell\_volume\_homeostasis | 5 | 0 | 0.000000 | 0.000000 | 1298 | 1099.072643 | 1155.31 | 1211.547357 | 0.890069 |
| GO:0006977\_DNA\_damage\_response\_\_signal\_transduction\_by\_p53\_class\_mediator\_resulting\_in\_cell\_cycle\_arrest | 5 | 0 | 0.000000 | 0.000000 | 1298 | 1099.072643 | 1155.31 | 1211.547357 | 0.890069 |
| GO:0007016\_cytoskeletal\_anchoring\_at\_plasma\_membrane | 5 | 0 | 0.000000 | 0.000000 | 1298 | 1099.072643 | 1155.31 | 1211.547357 | 0.890069 |
| GO:0007064\_mitotic\_sister\_chromatid\_cohesion | 5 | 0 | 0.000000 | 0.000000 | 1298 | 1099.072643 | 1155.31 | 1211.547357 | 0.890069 |
| GO:0007140\_male\_meiosis | 5 | 0 | 0.000000 | 0.000000 | 1298 | 1099.072643 | 1155.31 | 1211.547357 | 0.890069 |
| GO:0007171\_activation\_of\_transmembrane\_receptor\_protein\_tyrosine\_kinase\_activity | 5 | 0 | 0.000000 | 0.000000 | 1298 | 1099.072643 | 1155.31 | 1211.547357 | 0.890069 |
| GO:0007220\_Notch\_receptor\_processing | 5 | 0 | 0.000000 | 0.000000 | 1298 | 1099.072643 | 1155.31 | 1211.547357 | 0.890069 |
| GO:0007350\_blastoderm\_segmentation | 5 | 0 | 0.000000 | 0.000000 | 1298 | 1099.072643 | 1155.31 | 1211.547357 | 0.890069 |
| GO:0007351\_tripartite\_regional\_subdivision | 5 | 0 | 0.000000 | 0.000000 | 1298 | 1099.072643 | 1155.31 | 1211.547357 | 0.890069 |
| GO:0007431\_salivary\_gland\_development | 5 | 0 | 0.000000 | 0.000000 | 1298 | 1099.072643 | 1155.31 | 1211.547357 | 0.890069 |
| GO:0007435\_salivary\_gland\_morphogenesis | 5 | 0 | 0.000000 | 0.000000 | 1298 | 1099.072643 | 1155.31 | 1211.547357 | 0.890069 |
| GO:0007598\_blood\_coagulation\_\_extrinsic\_pathway | 5 | 0 | 0.000000 | 0.000000 | 1298 | 1099.072643 | 1155.31 | 1211.547357 | 0.890069 |
| GO:0007603\_phototransduction\_\_visible\_light | 5 | 0 | 0.000000 | 0.000000 | 1298 | 1099.072643 | 1155.31 | 1211.547357 | 0.890069 |
| GO:0007635\_chemosensory\_behavior | 5 | 0 | 0.000000 | 0.000000 | 1298 | 1099.072643 | 1155.31 | 1211.547357 | 0.890069 |
| GO:0008089\_anterograde\_axon\_cargo\_transport | 5 | 0 | 0.000000 | 0.000000 | 1298 | 1099.072643 | 1155.31 | 1211.547357 | 0.890069 |
| GO:0008210\_estrogen\_metabolic\_process | 5 | 0 | 0.000000 | 0.000000 | 1298 | 1099.072643 | 1155.31 | 1211.547357 | 0.890069 |
| GO:0008211\_glucocorticoid\_metabolic\_process | 5 | 0 | 0.000000 | 0.000000 | 1298 | 1099.072643 | 1155.31 | 1211.547357 | 0.890069 |
| GO:0008228\_opsonization | 5 | 0 | 0.000000 | 0.000000 | 1298 | 1099.072643 | 1155.31 | 1211.547357 | 0.890069 |
| GO:0008542\_visual\_learning | 5 | 0 | 0.000000 | 0.000000 | 1298 | 1099.072643 | 1155.31 | 1211.547357 | 0.890069 |
| GO:0008589\_regulation\_of\_smoothened\_signaling\_pathway | 5 | 0 | 0.000000 | 0.000000 | 1298 | 1099.072643 | 1155.31 | 1211.547357 | 0.890069 |
| GO:0008595\_determination\_of\_anterior\_posterior\_axis\_\_embryo | 5 | 0 | 0.000000 | 0.000000 | 1298 | 1099.072643 | 1155.31 | 1211.547357 | 0.890069 |
| GO:0008634\_negative\_regulation\_of\_survival\_gene\_product\_expression | 5 | 0 | 0.000000 | 0.000000 | 1298 | 1099.072643 | 1155.31 | 1211.547357 | 0.890069 |
| GO:0009068\_aspartate\_family\_amino\_acid\_catabolic\_process | 5 | 0 | 0.000000 | 0.000000 | 1298 | 1099.072643 | 1155.31 | 1211.547357 | 0.890069 |
| GO:0009083\_branched\_chain\_family\_amino\_acid\_catabolic\_process | 5 | 0 | 0.000000 | 0.000000 | 1298 | 1099.072643 | 1155.31 | 1211.547357 | 0.890069 |
| GO:0009084\_glutamine\_family\_amino\_acid\_biosynthetic\_process | 5 | 0 | 0.000000 | 0.000000 | 1298 | 1099.072643 | 1155.31 | 1211.547357 | 0.890069 |
| GO:0009126\_purine\_nucleoside\_monophosphate\_metabolic\_process | 5 | 0 | 0.000000 | 0.000000 | 1298 | 1099.072643 | 1155.31 | 1211.547357 | 0.890069 |
| GO:0009167\_purine\_ribonucleoside\_monophosphate\_metabolic\_process | 5 | 0 | 0.000000 | 0.000000 | 1298 | 1099.072643 | 1155.31 | 1211.547357 | 0.890069 |
| GO:0009218\_pyrimidine\_ribonucleotide\_metabolic\_process | 5 | 0 | 0.000000 | 0.000000 | 1298 | 1099.072643 | 1155.31 | 1211.547357 | 0.890069 |
| GO:0009988\_cell-cell\_recognition | 5 | 0 | 0.000000 | 0.000000 | 1298 | 1099.072643 | 1155.31 | 1211.547357 | 0.890069 |
| GO:0010043\_response\_to\_zinc\_ion | 5 | 0 | 0.000000 | 0.000000 | 1298 | 1099.072643 | 1155.31 | 1211.547357 | 0.890069 |
| GO:0010165\_response\_to\_X-ray | 5 | 0 | 0.000000 | 0.000000 | 1298 | 1099.072643 | 1155.31 | 1211.547357 | 0.890069 |
| GO:0010559\_regulation\_of\_glycoprotein\_biosynthetic\_process | 5 | 0 | 0.000000 | 0.000000 | 1298 | 1099.072643 | 1155.31 | 1211.547357 | 0.890069 |
| GO:0010575\_positive\_regulation\_vascular\_endothelial\_growth\_factor\_production | 5 | 0 | 0.000000 | 0.000000 | 1298 | 1099.072643 | 1155.31 | 1211.547357 | 0.890069 |
| GO:0010714\_positive\_regulation\_of\_collagen\_metabolic\_process | 5 | 0 | 0.000000 | 0.000000 | 1298 | 1099.072643 | 1155.31 | 1211.547357 | 0.890069 |
| GO:0010811\_positive\_regulation\_of\_cell-substrate\_adhesion | 5 | 0 | 0.000000 | 0.000000 | 1298 | 1099.072643 | 1155.31 | 1211.547357 | 0.890069 |
| GO:0010871\_negative\_regulation\_of\_receptor\_biosynthetic\_process | 5 | 0 | 0.000000 | 0.000000 | 1298 | 1099.072643 | 1155.31 | 1211.547357 | 0.890069 |
| GO:0010893\_positive\_regulation\_of\_steroid\_biosynthetic\_process | 5 | 0 | 0.000000 | 0.000000 | 1298 | 1099.072643 | 1155.31 | 1211.547357 | 0.890069 |
| GO:0010898\_positive\_regulation\_of\_triglyceride\_catabolic\_process | 5 | 0 | 0.000000 | 0.000000 | 1298 | 1099.072643 | 1155.31 | 1211.547357 | 0.890069 |
| GO:0010953\_regulation\_of\_protein\_maturation\_by\_peptide\_bond\_cleavage | 5 | 0 | 0.000000 | 0.000000 | 1298 | 1099.072643 | 1155.31 | 1211.547357 | 0.890069 |
| GO:0010984\_regulation\_of\_lipoprotein\_particle\_clearance | 5 | 0 | 0.000000 | 0.000000 | 1298 | 1099.072643 | 1155.31 | 1211.547357 | 0.890069 |
| GO:0014003\_oligodendrocyte\_development | 5 | 0 | 0.000000 | 0.000000 | 1298 | 1099.072643 | 1155.31 | 1211.547357 | 0.890069 |
| GO:0014073\_response\_to\_tropane | 5 | 0 | 0.000000 | 0.000000 | 1298 | 1099.072643 | 1155.31 | 1211.547357 | 0.890069 |
| GO:0014821\_phasic\_smooth\_muscle\_contraction | 5 | 0 | 0.000000 | 0.000000 | 1298 | 1099.072643 | 1155.31 | 1211.547357 | 0.890069 |
| GO:0014829\_vascular\_smooth\_muscle\_contraction | 5 | 0 | 0.000000 | 0.000000 | 1298 | 1099.072643 | 1155.31 | 1211.547357 | 0.890069 |
| GO:0014866\_skeletal\_myofibril\_assembly | 5 | 0 | 0.000000 | 0.000000 | 1298 | 1099.072643 | 1155.31 | 1211.547357 | 0.890069 |
| GO:0015696\_ammonium\_transport | 5 | 0 | 0.000000 | 0.000000 | 1298 | 1099.072643 | 1155.31 | 1211.547357 | 0.890069 |
| GO:0015780\_nucleotide-sugar\_transport | 5 | 0 | 0.000000 | 0.000000 | 1298 | 1099.072643 | 1155.31 | 1211.547357 | 0.890069 |
| GO:0015781\_pyrimidine\_nucleotide-sugar\_transport | 5 | 0 | 0.000000 | 0.000000 | 1298 | 1099.072643 | 1155.31 | 1211.547357 | 0.890069 |
| GO:0015851\_nucleobase\_transport | 5 | 0 | 0.000000 | 0.000000 | 1298 | 1099.072643 | 1155.31 | 1211.547357 | 0.890069 |
| GO:0015858\_nucleoside\_transport | 5 | 0 | 0.000000 | 0.000000 | 1298 | 1099.072643 | 1155.31 | 1211.547357 | 0.890069 |
| GO:0015872\_dopamine\_transport | 5 | 0 | 0.000000 | 0.000000 | 1298 | 1099.072643 | 1155.31 | 1211.547357 | 0.890069 |
| GO:0015985\_energy\_coupled\_proton\_transport\_\_down\_electrochemical\_gradient | 5 | 0 | 0.000000 | 0.000000 | 1298 | 1099.072643 | 1155.31 | 1211.547357 | 0.890069 |
| GO:0015986\_ATP\_synthesis\_coupled\_proton\_transport | 5 | 0 | 0.000000 | 0.000000 | 1298 | 1099.072643 | 1155.31 | 1211.547357 | 0.890069 |
| GO:0016246\_RNA\_interference | 5 | 0 | 0.000000 | 0.000000 | 1298 | 1099.072643 | 1155.31 | 1211.547357 | 0.890069 |
| GO:0018065\_protein-cofactor\_linkage | 5 | 0 | 0.000000 | 0.000000 | 1298 | 1099.072643 | 1155.31 | 1211.547357 | 0.890069 |
| GO:0018196\_peptidyl-asparagine\_modification | 5 | 0 | 0.000000 | 0.000000 | 1298 | 1099.072643 | 1155.31 | 1211.547357 | 0.890069 |
| GO:0018279\_protein\_amino\_acid\_N-linked\_glycosylation\_via\_asparagine | 5 | 0 | 0.000000 | 0.000000 | 1298 | 1099.072643 | 1155.31 | 1211.547357 | 0.890069 |
| GO:0018342\_protein\_prenylation | 5 | 0 | 0.000000 | 0.000000 | 1298 | 1099.072643 | 1155.31 | 1211.547357 | 0.890069 |
| GO:0019369\_arachidonic\_acid\_metabolic\_process | 5 | 0 | 0.000000 | 0.000000 | 1298 | 1099.072643 | 1155.31 | 1211.547357 | 0.890069 |
| GO:0019835\_cytolysis | 5 | 0 | 0.000000 | 0.000000 | 1298 | 1099.072643 | 1155.31 | 1211.547357 | 0.890069 |
| GO:0020027\_hemoglobin\_metabolic\_process | 5 | 0 | 0.000000 | 0.000000 | 1298 | 1099.072643 | 1155.31 | 1211.547357 | 0.890069 |
| GO:0022410\_circadian\_sleep\_wake\_cycle\_process | 5 | 0 | 0.000000 | 0.000000 | 1298 | 1099.072643 | 1155.31 | 1211.547357 | 0.890069 |
| GO:0022614\_membrane\_to\_membrane\_docking | 5 | 0 | 0.000000 | 0.000000 | 1298 | 1099.072643 | 1155.31 | 1211.547357 | 0.890069 |
| GO:0030219\_megakaryocyte\_differentiation | 5 | 0 | 0.000000 | 0.000000 | 1298 | 1099.072643 | 1155.31 | 1211.547357 | 0.890069 |
| GO:0030816\_positive\_regulation\_of\_cAMP\_metabolic\_process | 5 | 0 | 0.000000 | 0.000000 | 1298 | 1099.072643 | 1155.31 | 1211.547357 | 0.890069 |
| GO:0030819\_positive\_regulation\_of\_cAMP\_biosynthetic\_process | 5 | 0 | 0.000000 | 0.000000 | 1298 | 1099.072643 | 1155.31 | 1211.547357 | 0.890069 |
| GO:0030838\_positive\_regulation\_of\_actin\_filament\_polymerization | 5 | 0 | 0.000000 | 0.000000 | 1298 | 1099.072643 | 1155.31 | 1211.547357 | 0.890069 |
| GO:0031018\_endocrine\_pancreas\_development | 5 | 0 | 0.000000 | 0.000000 | 1298 | 1099.072643 | 1155.31 | 1211.547357 | 0.890069 |
| GO:0031057\_negative\_regulation\_of\_histone\_modification | 5 | 0 | 0.000000 | 0.000000 | 1298 | 1099.072643 | 1155.31 | 1211.547357 | 0.890069 |
| GO:0031058\_positive\_regulation\_of\_histone\_modification | 5 | 0 | 0.000000 | 0.000000 | 1298 | 1099.072643 | 1155.31 | 1211.547357 | 0.890069 |
| GO:0031112\_positive\_regulation\_of\_microtubule\_polymerization\_or\_depolymerization | 5 | 0 | 0.000000 | 0.000000 | 1298 | 1099.072643 | 1155.31 | 1211.547357 | 0.890069 |
| GO:0031113\_regulation\_of\_microtubule\_polymerization | 5 | 0 | 0.000000 | 0.000000 | 1298 | 1099.072643 | 1155.31 | 1211.547357 | 0.890069 |
| GO:0031345\_negative\_regulation\_of\_cell\_projection\_organization | 5 | 0 | 0.000000 | 0.000000 | 1298 | 1099.072643 | 1155.31 | 1211.547357 | 0.890069 |
| GO:0031397\_negative\_regulation\_of\_protein\_ubiquitination | 5 | 0 | 0.000000 | 0.000000 | 1298 | 1099.072643 | 1155.31 | 1211.547357 | 0.890069 |
| GO:0031507\_heterochromatin\_formation | 5 | 0 | 0.000000 | 0.000000 | 1298 | 1099.072643 | 1155.31 | 1211.547357 | 0.890069 |
| GO:0031645\_negative\_regulation\_of\_neurological\_system\_process | 5 | 0 | 0.000000 | 0.000000 | 1298 | 1099.072643 | 1155.31 | 1211.547357 | 0.890069 |
| GO:0031998\_regulation\_of\_fatty\_acid\_beta-oxidation | 5 | 0 | 0.000000 | 0.000000 | 1298 | 1099.072643 | 1155.31 | 1211.547357 | 0.890069 |
| GO:0032055\_negative\_regulation\_of\_translation\_in\_response\_to\_stress | 5 | 0 | 0.000000 | 0.000000 | 1298 | 1099.072643 | 1155.31 | 1211.547357 | 0.890069 |
| GO:0032094\_response\_to\_food | 5 | 0 | 0.000000 | 0.000000 | 1298 | 1099.072643 | 1155.31 | 1211.547357 | 0.890069 |
| GO:0032095\_regulation\_of\_response\_to\_food | 5 | 0 | 0.000000 | 0.000000 | 1298 | 1099.072643 | 1155.31 | 1211.547357 | 0.890069 |
| GO:0032098\_regulation\_of\_appetite | 5 | 0 | 0.000000 | 0.000000 | 1298 | 1099.072643 | 1155.31 | 1211.547357 | 0.890069 |
| GO:0032148\_activation\_of\_protein\_kinase\_B\_activity | 5 | 0 | 0.000000 | 0.000000 | 1298 | 1099.072643 | 1155.31 | 1211.547357 | 0.890069 |
| GO:0032202\_telomere\_assembly | 5 | 0 | 0.000000 | 0.000000 | 1298 | 1099.072643 | 1155.31 | 1211.547357 | 0.890069 |
| GO:0032210\_regulation\_of\_telomere\_maintenance\_via\_telomerase | 5 | 0 | 0.000000 | 0.000000 | 1298 | 1099.072643 | 1155.31 | 1211.547357 | 0.890069 |
| GO:0032274\_gonadotropin\_secretion | 5 | 0 | 0.000000 | 0.000000 | 1298 | 1099.072643 | 1155.31 | 1211.547357 | 0.890069 |
| GO:0032364\_oxygen\_homeostasis | 5 | 0 | 0.000000 | 0.000000 | 1298 | 1099.072643 | 1155.31 | 1211.547357 | 0.890069 |
| GO:0032366\_intracellular\_sterol\_transport | 5 | 0 | 0.000000 | 0.000000 | 1298 | 1099.072643 | 1155.31 | 1211.547357 | 0.890069 |
| GO:0032367\_intracellular\_cholesterol\_transport | 5 | 0 | 0.000000 | 0.000000 | 1298 | 1099.072643 | 1155.31 | 1211.547357 | 0.890069 |
| GO:0032414\_positive\_regulation\_of\_ion\_transmembrane\_transporter\_activity | 5 | 0 | 0.000000 | 0.000000 | 1298 | 1099.072643 | 1155.31 | 1211.547357 | 0.890069 |
| GO:0032429\_regulation\_of\_phospholipase\_A2\_activity | 5 | 0 | 0.000000 | 0.000000 | 1298 | 1099.072643 | 1155.31 | 1211.547357 | 0.890069 |
| GO:0032430\_positive\_regulation\_of\_phospholipase\_A2\_activity | 5 | 0 | 0.000000 | 0.000000 | 1298 | 1099.072643 | 1155.31 | 1211.547357 | 0.890069 |
| GO:0032438\_melanosome\_organization | 5 | 0 | 0.000000 | 0.000000 | 1298 | 1099.072643 | 1155.31 | 1211.547357 | 0.890069 |
| GO:0032465\_regulation\_of\_cytokinesis | 5 | 0 | 0.000000 | 0.000000 | 1298 | 1099.072643 | 1155.31 | 1211.547357 | 0.890069 |
| GO:0032479\_regulation\_of\_type\_I\_interferon\_production | 5 | 0 | 0.000000 | 0.000000 | 1298 | 1099.072643 | 1155.31 | 1211.547357 | 0.890069 |
| GO:0032490\_detection\_of\_molecule\_of\_bacterial\_origin | 5 | 0 | 0.000000 | 0.000000 | 1298 | 1099.072643 | 1155.31 | 1211.547357 | 0.890069 |
| GO:0032606\_type\_I\_interferon\_production | 5 | 0 | 0.000000 | 0.000000 | 1298 | 1099.072643 | 1155.31 | 1211.547357 | 0.890069 |
| GO:0032607\_interferon-alpha\_production | 5 | 0 | 0.000000 | 0.000000 | 1298 | 1099.072643 | 1155.31 | 1211.547357 | 0.890069 |
| GO:0032612\_interleukin-1\_production | 5 | 0 | 0.000000 | 0.000000 | 1298 | 1099.072643 | 1155.31 | 1211.547357 | 0.890069 |
| GO:0032647\_regulation\_of\_interferon-alpha\_production | 5 | 0 | 0.000000 | 0.000000 | 1298 | 1099.072643 | 1155.31 | 1211.547357 | 0.890069 |
| GO:0032722\_positive\_regulation\_of\_chemokine\_production | 5 | 0 | 0.000000 | 0.000000 | 1298 | 1099.072643 | 1155.31 | 1211.547357 | 0.890069 |
| GO:0032801\_receptor\_catabolic\_process | 5 | 0 | 0.000000 | 0.000000 | 1298 | 1099.072643 | 1155.31 | 1211.547357 | 0.890069 |
| GO:0032856\_activation\_of\_Ras\_GTPase\_activity | 5 | 0 | 0.000000 | 0.000000 | 1298 | 1099.072643 | 1155.31 | 1211.547357 | 0.890069 |
| GO:0032862\_activation\_of\_Rho\_GTPase\_activity | 5 | 0 | 0.000000 | 0.000000 | 1298 | 1099.072643 | 1155.31 | 1211.547357 | 0.890069 |
| GO:0032967\_positive\_regulation\_of\_collagen\_biosynthetic\_process | 5 | 0 | 0.000000 | 0.000000 | 1298 | 1099.072643 | 1155.31 | 1211.547357 | 0.890069 |
| GO:0033028\_myeloid\_cell\_apoptosis | 5 | 0 | 0.000000 | 0.000000 | 1298 | 1099.072643 | 1155.31 | 1211.547357 | 0.890069 |
| GO:0033160\_positive\_regulation\_of\_protein\_import\_into\_nucleus\_\_translocation | 5 | 0 | 0.000000 | 0.000000 | 1298 | 1099.072643 | 1155.31 | 1211.547357 | 0.890069 |
| GO:0034379\_very-low-density\_lipoprotein\_particle\_assembly | 5 | 0 | 0.000000 | 0.000000 | 1298 | 1099.072643 | 1155.31 | 1211.547357 | 0.890069 |
| GO:0034380\_high-density\_lipoprotein\_particle\_assembly | 5 | 0 | 0.000000 | 0.000000 | 1298 | 1099.072643 | 1155.31 | 1211.547357 | 0.890069 |
| GO:0034382\_chylomicron\_remnant\_clearance | 5 | 0 | 0.000000 | 0.000000 | 1298 | 1099.072643 | 1155.31 | 1211.547357 | 0.890069 |
| GO:0034383\_low-density\_lipoprotein\_particle\_clearance | 5 | 0 | 0.000000 | 0.000000 | 1298 | 1099.072643 | 1155.31 | 1211.547357 | 0.890069 |
| GO:0034390\_smooth\_muscle\_cell\_apoptosis | 5 | 0 | 0.000000 | 0.000000 | 1298 | 1099.072643 | 1155.31 | 1211.547357 | 0.890069 |
| GO:0034391\_regulation\_of\_smooth\_muscle\_cell\_apoptosis | 5 | 0 | 0.000000 | 0.000000 | 1298 | 1099.072643 | 1155.31 | 1211.547357 | 0.890069 |
| GO:0034643\_mitochondrion\_localization\_\_microtubule-mediated | 5 | 0 | 0.000000 | 0.000000 | 1298 | 1099.072643 | 1155.31 | 1211.547357 | 0.890069 |
| GO:0034968\_histone\_lysine\_methylation | 5 | 0 | 0.000000 | 0.000000 | 1298 | 1099.072643 | 1155.31 | 1211.547357 | 0.890069 |
| GO:0034969\_histone\_arginine\_methylation | 5 | 0 | 0.000000 | 0.000000 | 1298 | 1099.072643 | 1155.31 | 1211.547357 | 0.890069 |
| GO:0035095\_behavioral\_response\_to\_nicotine | 5 | 0 | 0.000000 | 0.000000 | 1298 | 1099.072643 | 1155.31 | 1211.547357 | 0.890069 |
| GO:0035176\_social\_behavior | 5 | 0 | 0.000000 | 0.000000 | 1298 | 1099.072643 | 1155.31 | 1211.547357 | 0.890069 |
| GO:0035278\_gene\_silencing\_by\_miRNA\_\_negative\_regulation\_of\_translation | 5 | 0 | 0.000000 | 0.000000 | 1298 | 1099.072643 | 1155.31 | 1211.547357 | 0.890069 |
| GO:0040018\_positive\_regulation\_of\_multicellular\_organism\_growth | 5 | 0 | 0.000000 | 0.000000 | 1298 | 1099.072643 | 1155.31 | 1211.547357 | 0.890069 |
| GO:0040033\_negative\_regulation\_of\_translation\_\_ncRNA-mediated | 5 | 0 | 0.000000 | 0.000000 | 1298 | 1099.072643 | 1155.31 | 1211.547357 | 0.890069 |
| GO:0042026\_protein\_refolding | 5 | 0 | 0.000000 | 0.000000 | 1298 | 1099.072643 | 1155.31 | 1211.547357 | 0.890069 |
| GO:0042104\_positive\_regulation\_of\_activated\_T\_cell\_proliferation | 5 | 0 | 0.000000 | 0.000000 | 1298 | 1099.072643 | 1155.31 | 1211.547357 | 0.890069 |
| GO:0042117\_monocyte\_activation | 5 | 0 | 0.000000 | 0.000000 | 1298 | 1099.072643 | 1155.31 | 1211.547357 | 0.890069 |
| GO:0042130\_negative\_regulation\_of\_T\_cell\_proliferation | 5 | 0 | 0.000000 | 0.000000 | 1298 | 1099.072643 | 1155.31 | 1211.547357 | 0.890069 |
| GO:0042220\_response\_to\_cocaine | 5 | 0 | 0.000000 | 0.000000 | 1298 | 1099.072643 | 1155.31 | 1211.547357 | 0.890069 |
| GO:0042347\_negative\_regulation\_of\_NF-kappaB\_import\_into\_nucleus | 5 | 0 | 0.000000 | 0.000000 | 1298 | 1099.072643 | 1155.31 | 1211.547357 | 0.890069 |
| GO:0042359\_vitamin\_D\_metabolic\_process | 5 | 0 | 0.000000 | 0.000000 | 1298 | 1099.072643 | 1155.31 | 1211.547357 | 0.890069 |
| GO:0042364\_water-soluble\_vitamin\_biosynthetic\_process | 5 | 0 | 0.000000 | 0.000000 | 1298 | 1099.072643 | 1155.31 | 1211.547357 | 0.890069 |
| GO:0042471\_ear\_morphogenesis | 5 | 0 | 0.000000 | 0.000000 | 1298 | 1099.072643 | 1155.31 | 1211.547357 | 0.890069 |
| GO:0042490\_mechanoreceptor\_differentiation | 5 | 0 | 0.000000 | 0.000000 | 1298 | 1099.072643 | 1155.31 | 1211.547357 | 0.890069 |
| GO:0042535\_positive\_regulation\_of\_tumor\_necrosis\_factor\_biosynthetic\_process | 5 | 0 | 0.000000 | 0.000000 | 1298 | 1099.072643 | 1155.31 | 1211.547357 | 0.890069 |
| GO:0042744\_hydrogen\_peroxide\_catabolic\_process | 5 | 0 | 0.000000 | 0.000000 | 1298 | 1099.072643 | 1155.31 | 1211.547357 | 0.890069 |
| GO:0042745\_circadian\_sleep\_wake\_cycle | 5 | 0 | 0.000000 | 0.000000 | 1298 | 1099.072643 | 1155.31 | 1211.547357 | 0.890069 |
| GO:0042749\_regulation\_of\_circadian\_sleep\_wake\_cycle | 5 | 0 | 0.000000 | 0.000000 | 1298 | 1099.072643 | 1155.31 | 1211.547357 | 0.890069 |
| GO:0042769\_DNA\_damage\_response\_\_detection\_of\_DNA\_damage | 5 | 0 | 0.000000 | 0.000000 | 1298 | 1099.072643 | 1155.31 | 1211.547357 | 0.890069 |
| GO:0043029\_T\_cell\_homeostasis | 5 | 0 | 0.000000 | 0.000000 | 1298 | 1099.072643 | 1155.31 | 1211.547357 | 0.890069 |
| GO:0043044\_ATP-dependent\_chromatin\_remodeling | 5 | 0 | 0.000000 | 0.000000 | 1298 | 1099.072643 | 1155.31 | 1211.547357 | 0.890069 |
| GO:0043094\_cellular\_metabolic\_compound\_salvage | 5 | 0 | 0.000000 | 0.000000 | 1298 | 1099.072643 | 1155.31 | 1211.547357 | 0.890069 |
| GO:0043171\_peptide\_catabolic\_process | 5 | 0 | 0.000000 | 0.000000 | 1298 | 1099.072643 | 1155.31 | 1211.547357 | 0.890069 |
| GO:0043487\_regulation\_of\_RNA\_stability | 5 | 0 | 0.000000 | 0.000000 | 1298 | 1099.072643 | 1155.31 | 1211.547357 | 0.890069 |
| GO:0043488\_regulation\_of\_mRNA\_stability | 5 | 0 | 0.000000 | 0.000000 | 1298 | 1099.072643 | 1155.31 | 1211.547357 | 0.890069 |
| GO:0043496\_regulation\_of\_protein\_homodimerization\_activity | 5 | 0 | 0.000000 | 0.000000 | 1298 | 1099.072643 | 1155.31 | 1211.547357 | 0.890069 |
| GO:0043550\_regulation\_of\_lipid\_kinase\_activity | 5 | 0 | 0.000000 | 0.000000 | 1298 | 1099.072643 | 1155.31 | 1211.547357 | 0.890069 |
| GO:0043555\_regulation\_of\_translation\_in\_response\_to\_stress | 5 | 0 | 0.000000 | 0.000000 | 1298 | 1099.072643 | 1155.31 | 1211.547357 | 0.890069 |
| GO:0043616\_keratinocyte\_proliferation | 5 | 0 | 0.000000 | 0.000000 | 1298 | 1099.072643 | 1155.31 | 1211.547357 | 0.890069 |
| GO:0044253\_positive\_regulation\_of\_multicellular\_organismal\_metabolic\_process | 5 | 0 | 0.000000 | 0.000000 | 1298 | 1099.072643 | 1155.31 | 1211.547357 | 0.890069 |
| GO:0045008\_depyrimidination | 5 | 0 | 0.000000 | 0.000000 | 1298 | 1099.072643 | 1155.31 | 1211.547357 | 0.890069 |
| GO:0045071\_negative\_regulation\_of\_viral\_genome\_replication | 5 | 0 | 0.000000 | 0.000000 | 1298 | 1099.072643 | 1155.31 | 1211.547357 | 0.890069 |
| GO:0045109\_intermediate\_filament\_organization | 5 | 0 | 0.000000 | 0.000000 | 1298 | 1099.072643 | 1155.31 | 1211.547357 | 0.890069 |
| GO:0045161\_neuronal\_ion\_channel\_clustering | 5 | 0 | 0.000000 | 0.000000 | 1298 | 1099.072643 | 1155.31 | 1211.547357 | 0.890069 |
| GO:0045176\_apical\_protein\_localization | 5 | 0 | 0.000000 | 0.000000 | 1298 | 1099.072643 | 1155.31 | 1211.547357 | 0.890069 |
| GO:0045187\_regulation\_of\_circadian\_sleep\_wake\_cycle\_\_sleep | 5 | 0 | 0.000000 | 0.000000 | 1298 | 1099.072643 | 1155.31 | 1211.547357 | 0.890069 |
| GO:0045349\_interferon-alpha\_biosynthetic\_process | 5 | 0 | 0.000000 | 0.000000 | 1298 | 1099.072643 | 1155.31 | 1211.547357 | 0.890069 |
| GO:0045351\_type\_I\_interferon\_biosynthetic\_process | 5 | 0 | 0.000000 | 0.000000 | 1298 | 1099.072643 | 1155.31 | 1211.547357 | 0.890069 |
| GO:0045354\_regulation\_of\_interferon-alpha\_biosynthetic\_process | 5 | 0 | 0.000000 | 0.000000 | 1298 | 1099.072643 | 1155.31 | 1211.547357 | 0.890069 |
| GO:0045426\_quinone\_cofactor\_biosynthetic\_process | 5 | 0 | 0.000000 | 0.000000 | 1298 | 1099.072643 | 1155.31 | 1211.547357 | 0.890069 |
| GO:0045620\_negative\_regulation\_of\_lymphocyte\_differentiation | 5 | 0 | 0.000000 | 0.000000 | 1298 | 1099.072643 | 1155.31 | 1211.547357 | 0.890069 |
| GO:0045648\_positive\_regulation\_of\_erythrocyte\_differentiation | 5 | 0 | 0.000000 | 0.000000 | 1298 | 1099.072643 | 1155.31 | 1211.547357 | 0.890069 |
| GO:0045713\_low-density\_lipoprotein\_receptor\_biosynthetic\_process | 5 | 0 | 0.000000 | 0.000000 | 1298 | 1099.072643 | 1155.31 | 1211.547357 | 0.890069 |
| GO:0045741\_positive\_regulation\_of\_epidermal\_growth\_factor\_receptor\_activity | 5 | 0 | 0.000000 | 0.000000 | 1298 | 1099.072643 | 1155.31 | 1211.547357 | 0.890069 |
| GO:0045777\_positive\_regulation\_of\_blood\_pressure | 5 | 0 | 0.000000 | 0.000000 | 1298 | 1099.072643 | 1155.31 | 1211.547357 | 0.890069 |
| GO:0045821\_positive\_regulation\_of\_glycolysis | 5 | 0 | 0.000000 | 0.000000 | 1298 | 1099.072643 | 1155.31 | 1211.547357 | 0.890069 |
| GO:0045843\_negative\_regulation\_of\_striated\_muscle\_development | 5 | 0 | 0.000000 | 0.000000 | 1298 | 1099.072643 | 1155.31 | 1211.547357 | 0.890069 |
| GO:0045922\_negative\_regulation\_of\_fatty\_acid\_metabolic\_process | 5 | 0 | 0.000000 | 0.000000 | 1298 | 1099.072643 | 1155.31 | 1211.547357 | 0.890069 |
| GO:0045933\_positive\_regulation\_of\_muscle\_contraction | 5 | 0 | 0.000000 | 0.000000 | 1298 | 1099.072643 | 1155.31 | 1211.547357 | 0.890069 |
| GO:0045948\_positive\_regulation\_of\_translational\_initiation | 5 | 0 | 0.000000 | 0.000000 | 1298 | 1099.072643 | 1155.31 | 1211.547357 | 0.890069 |
| GO:0045974\_regulation\_of\_translation\_\_ncRNA-mediated | 5 | 0 | 0.000000 | 0.000000 | 1298 | 1099.072643 | 1155.31 | 1211.547357 | 0.890069 |
| GO:0046112\_nucleobase\_biosynthetic\_process | 5 | 0 | 0.000000 | 0.000000 | 1298 | 1099.072643 | 1155.31 | 1211.547357 | 0.890069 |
| GO:0046131\_pyrimidine\_ribonucleoside\_metabolic\_process | 5 | 0 | 0.000000 | 0.000000 | 1298 | 1099.072643 | 1155.31 | 1211.547357 | 0.890069 |
| GO:0046321\_positive\_regulation\_of\_fatty\_acid\_oxidation | 5 | 0 | 0.000000 | 0.000000 | 1298 | 1099.072643 | 1155.31 | 1211.547357 | 0.890069 |
| GO:0046329\_negative\_regulation\_of\_JNK\_cascade | 5 | 0 | 0.000000 | 0.000000 | 1298 | 1099.072643 | 1155.31 | 1211.547357 | 0.890069 |
| GO:0046339\_diacylglycerol\_metabolic\_process | 5 | 0 | 0.000000 | 0.000000 | 1298 | 1099.072643 | 1155.31 | 1211.547357 | 0.890069 |
| GO:0046513\_ceramide\_biosynthetic\_process | 5 | 0 | 0.000000 | 0.000000 | 1298 | 1099.072643 | 1155.31 | 1211.547357 | 0.890069 |
| GO:0046530\_photoreceptor\_cell\_differentiation | 5 | 0 | 0.000000 | 0.000000 | 1298 | 1099.072643 | 1155.31 | 1211.547357 | 0.890069 |
| GO:0046580\_negative\_regulation\_of\_Ras\_protein\_signal\_transduction | 5 | 0 | 0.000000 | 0.000000 | 1298 | 1099.072643 | 1155.31 | 1211.547357 | 0.890069 |
| GO:0046782\_regulation\_of\_viral\_transcription | 5 | 0 | 0.000000 | 0.000000 | 1298 | 1099.072643 | 1155.31 | 1211.547357 | 0.890069 |
| GO:0046884\_follicle-stimulating\_hormone\_secretion | 5 | 0 | 0.000000 | 0.000000 | 1298 | 1099.072643 | 1155.31 | 1211.547357 | 0.890069 |
| GO:0046902\_regulation\_of\_mitochondrial\_membrane\_permeability | 5 | 0 | 0.000000 | 0.000000 | 1298 | 1099.072643 | 1155.31 | 1211.547357 | 0.890069 |
| GO:0047497\_mitochondrion\_transport\_along\_microtubule | 5 | 0 | 0.000000 | 0.000000 | 1298 | 1099.072643 | 1155.31 | 1211.547357 | 0.890069 |
| GO:0048010\_vascular\_endothelial\_growth\_factor\_receptor\_signaling\_pathway | 5 | 0 | 0.000000 | 0.000000 | 1298 | 1099.072643 | 1155.31 | 1211.547357 | 0.890069 |
| GO:0048011\_nerve\_growth\_factor\_receptor\_signaling\_pathway | 5 | 0 | 0.000000 | 0.000000 | 1298 | 1099.072643 | 1155.31 | 1211.547357 | 0.890069 |
| GO:0048069\_eye\_pigmentation | 5 | 0 | 0.000000 | 0.000000 | 1298 | 1099.072643 | 1155.31 | 1211.547357 | 0.890069 |
| GO:0048168\_regulation\_of\_neuronal\_synaptic\_plasticity | 5 | 0 | 0.000000 | 0.000000 | 1298 | 1099.072643 | 1155.31 | 1211.547357 | 0.890069 |
| GO:0048261\_negative\_regulation\_of\_receptor-mediated\_endocytosis | 5 | 0 | 0.000000 | 0.000000 | 1298 | 1099.072643 | 1155.31 | 1211.547357 | 0.890069 |
| GO:0048286\_lung\_alveolus\_development | 5 | 0 | 0.000000 | 0.000000 | 1298 | 1099.072643 | 1155.31 | 1211.547357 | 0.890069 |
| GO:0048488\_synaptic\_vesicle\_endocytosis | 5 | 0 | 0.000000 | 0.000000 | 1298 | 1099.072643 | 1155.31 | 1211.547357 | 0.890069 |
| GO:0048565\_gut\_development | 5 | 0 | 0.000000 | 0.000000 | 1298 | 1099.072643 | 1155.31 | 1211.547357 | 0.890069 |
| GO:0048599\_oocyte\_development | 5 | 0 | 0.000000 | 0.000000 | 1298 | 1099.072643 | 1155.31 | 1211.547357 | 0.890069 |
| GO:0048635\_negative\_regulation\_of\_muscle\_development | 5 | 0 | 0.000000 | 0.000000 | 1298 | 1099.072643 | 1155.31 | 1211.547357 | 0.890069 |
| GO:0048675\_axon\_extension | 5 | 0 | 0.000000 | 0.000000 | 1298 | 1099.072643 | 1155.31 | 1211.547357 | 0.890069 |
| GO:0048753\_pigment\_granule\_organization | 5 | 0 | 0.000000 | 0.000000 | 1298 | 1099.072643 | 1155.31 | 1211.547357 | 0.890069 |
| GO:0048754\_branching\_morphogenesis\_of\_a\_tube | 5 | 0 | 0.000000 | 0.000000 | 1298 | 1099.072643 | 1155.31 | 1211.547357 | 0.890069 |
| GO:0048813\_dendrite\_morphogenesis | 5 | 0 | 0.000000 | 0.000000 | 1298 | 1099.072643 | 1155.31 | 1211.547357 | 0.890069 |
| GO:0050655\_dermatan\_sulfate\_proteoglycan\_metabolic\_process | 5 | 0 | 0.000000 | 0.000000 | 1298 | 1099.072643 | 1155.31 | 1211.547357 | 0.890069 |
| GO:0050691\_regulation\_of\_defense\_response\_to\_virus\_by\_host | 5 | 0 | 0.000000 | 0.000000 | 1298 | 1099.072643 | 1155.31 | 1211.547357 | 0.890069 |
| GO:0050766\_positive\_regulation\_of\_phagocytosis | 5 | 0 | 0.000000 | 0.000000 | 1298 | 1099.072643 | 1155.31 | 1211.547357 | 0.890069 |
| GO:0050772\_positive\_regulation\_of\_axonogenesis | 5 | 0 | 0.000000 | 0.000000 | 1298 | 1099.072643 | 1155.31 | 1211.547357 | 0.890069 |
| GO:0050802\_circadian\_sleep\_wake\_cycle\_\_sleep | 5 | 0 | 0.000000 | 0.000000 | 1298 | 1099.072643 | 1155.31 | 1211.547357 | 0.890069 |
| GO:0050807\_regulation\_of\_synapse\_organization | 5 | 0 | 0.000000 | 0.000000 | 1298 | 1099.072643 | 1155.31 | 1211.547357 | 0.890069 |
| GO:0050829\_defense\_response\_to\_Gram-negative\_bacterium | 5 | 0 | 0.000000 | 0.000000 | 1298 | 1099.072643 | 1155.31 | 1211.547357 | 0.890069 |
| GO:0050853\_B\_cell\_receptor\_signaling\_pathway | 5 | 0 | 0.000000 | 0.000000 | 1298 | 1099.072643 | 1155.31 | 1211.547357 | 0.890069 |
| GO:0050854\_regulation\_of\_antigen\_receptor-mediated\_signaling\_pathway | 5 | 0 | 0.000000 | 0.000000 | 1298 | 1099.072643 | 1155.31 | 1211.547357 | 0.890069 |
| GO:0050869\_negative\_regulation\_of\_B\_cell\_activation | 5 | 0 | 0.000000 | 0.000000 | 1298 | 1099.072643 | 1155.31 | 1211.547357 | 0.890069 |
| GO:0050891\_multicellular\_organismal\_water\_homeostasis | 5 | 0 | 0.000000 | 0.000000 | 1298 | 1099.072643 | 1155.31 | 1211.547357 | 0.890069 |
| GO:0050930\_induction\_of\_positive\_chemotaxis | 5 | 0 | 0.000000 | 0.000000 | 1298 | 1099.072643 | 1155.31 | 1211.547357 | 0.890069 |
| GO:0051005\_negative\_regulation\_of\_lipoprotein\_lipase\_activity | 5 | 0 | 0.000000 | 0.000000 | 1298 | 1099.072643 | 1155.31 | 1211.547357 | 0.890069 |
| GO:0051016\_barbed-end\_actin\_filament\_capping | 5 | 0 | 0.000000 | 0.000000 | 1298 | 1099.072643 | 1155.31 | 1211.547357 | 0.890069 |
| GO:0051058\_negative\_regulation\_of\_small\_GTPase\_mediated\_signal\_transduction | 5 | 0 | 0.000000 | 0.000000 | 1298 | 1099.072643 | 1155.31 | 1211.547357 | 0.890069 |
| GO:0051096\_positive\_regulation\_of\_helicase\_activity | 5 | 0 | 0.000000 | 0.000000 | 1298 | 1099.072643 | 1155.31 | 1211.547357 | 0.890069 |
| GO:0051103\_DNA\_ligation\_during\_DNA\_repair | 5 | 0 | 0.000000 | 0.000000 | 1298 | 1099.072643 | 1155.31 | 1211.547357 | 0.890069 |
| GO:0051123\_transcriptional\_preinitiation\_complex\_assembly | 5 | 0 | 0.000000 | 0.000000 | 1298 | 1099.072643 | 1155.31 | 1211.547357 | 0.890069 |
| GO:0051281\_positive\_regulation\_of\_release\_of\_sequestered\_calcium\_ion\_into\_cytosol | 5 | 0 | 0.000000 | 0.000000 | 1298 | 1099.072643 | 1155.31 | 1211.547357 | 0.890069 |
| GO:0051310\_metaphase\_plate\_congression | 5 | 0 | 0.000000 | 0.000000 | 1298 | 1099.072643 | 1155.31 | 1211.547357 | 0.890069 |
| GO:0051319\_G2\_phase | 5 | 0 | 0.000000 | 0.000000 | 1298 | 1099.072643 | 1155.31 | 1211.547357 | 0.890069 |
| GO:0051403\_stress-activated\_MAPK\_cascade | 5 | 0 | 0.000000 | 0.000000 | 1298 | 1099.072643 | 1155.31 | 1211.547357 | 0.890069 |
| GO:0051496\_positive\_regulation\_of\_stress\_fiber\_formation | 5 | 0 | 0.000000 | 0.000000 | 1298 | 1099.072643 | 1155.31 | 1211.547357 | 0.890069 |
| GO:0051654\_establishment\_of\_mitochondrion\_localization | 5 | 0 | 0.000000 | 0.000000 | 1298 | 1099.072643 | 1155.31 | 1211.547357 | 0.890069 |
| GO:0051693\_actin\_filament\_capping | 5 | 0 | 0.000000 | 0.000000 | 1298 | 1099.072643 | 1155.31 | 1211.547357 | 0.890069 |
| GO:0051898\_negative\_regulation\_of\_protein\_kinase\_B\_signaling\_cascade | 5 | 0 | 0.000000 | 0.000000 | 1298 | 1099.072643 | 1155.31 | 1211.547357 | 0.890069 |
| GO:0051926\_negative\_regulation\_of\_calcium\_ion\_transport | 5 | 0 | 0.000000 | 0.000000 | 1298 | 1099.072643 | 1155.31 | 1211.547357 | 0.890069 |
| GO:0051954\_positive\_regulation\_of\_amine\_transport | 5 | 0 | 0.000000 | 0.000000 | 1298 | 1099.072643 | 1155.31 | 1211.547357 | 0.890069 |
| GO:0055091\_phospholipid\_homeostasis | 5 | 0 | 0.000000 | 0.000000 | 1298 | 1099.072643 | 1155.31 | 1211.547357 | 0.890069 |
| GO:0055117\_regulation\_of\_cardiac\_muscle\_contraction | 5 | 0 | 0.000000 | 0.000000 | 1298 | 1099.072643 | 1155.31 | 1211.547357 | 0.890069 |
| GO:0060041\_retina\_development\_in\_camera-type\_eye | 5 | 0 | 0.000000 | 0.000000 | 1298 | 1099.072643 | 1155.31 | 1211.547357 | 0.890069 |
| GO:0060113\_inner\_ear\_receptor\_cell\_differentiation | 5 | 0 | 0.000000 | 0.000000 | 1298 | 1099.072643 | 1155.31 | 1211.547357 | 0.890069 |
| GO:0060123\_regulation\_of\_growth\_hormone\_secretion | 5 | 0 | 0.000000 | 0.000000 | 1298 | 1099.072643 | 1155.31 | 1211.547357 | 0.890069 |
| GO:0060260\_regulation\_of\_transcription\_initiation\_from\_RNA\_polymerase\_II\_promoter | 5 | 0 | 0.000000 | 0.000000 | 1298 | 1099.072643 | 1155.31 | 1211.547357 | 0.890069 |
| GO:0060390\_regulation\_of\_SMAD\_protein\_nuclear\_translocation | 5 | 0 | 0.000000 | 0.000000 | 1298 | 1099.072643 | 1155.31 | 1211.547357 | 0.890069 |
| GO:0060391\_positive\_regulation\_of\_SMAD\_protein\_nuclear\_translocation | 5 | 0 | 0.000000 | 0.000000 | 1298 | 1099.072643 | 1155.31 | 1211.547357 | 0.890069 |
| GO:0065002\_intracellular\_protein\_transmembrane\_transport | 5 | 0 | 0.000000 | 0.000000 | 1298 | 1099.072643 | 1155.31 | 1211.547357 | 0.890069 |
| GO:0070071\_proton-transporting\_two-sector\_ATPase\_complex\_assembly | 5 | 0 | 0.000000 | 0.000000 | 1298 | 1099.072643 | 1155.31 | 1211.547357 | 0.890069 |
| GO:0070120\_ciliary\_neurotrophic\_factor-mediated\_signaling\_pathway | 5 | 0 | 0.000000 | 0.000000 | 1298 | 1099.072643 | 1155.31 | 1211.547357 | 0.890069 |
| GO:0070228\_regulation\_of\_lymphocyte\_apoptosis | 5 | 0 | 0.000000 | 0.000000 | 1298 | 1099.072643 | 1155.31 | 1211.547357 | 0.890069 |
| GO:0070301\_cellular\_response\_to\_hydrogen\_peroxide | 5 | 0 | 0.000000 | 0.000000 | 1298 | 1099.072643 | 1155.31 | 1211.547357 | 0.890069 |
| GO:0070303\_negative\_regulation\_of\_stress-activated\_protein\_kinase\_signaling\_pathway | 5 | 0 | 0.000000 | 0.000000 | 1298 | 1099.072643 | 1155.31 | 1211.547357 | 0.890069 |
| GO:0070584\_mitochondrion\_morphogenesis | 5 | 0 | 0.000000 | 0.000000 | 1298 | 1099.072643 | 1155.31 | 1211.547357 | 0.890069 |
| GO:0070667\_negative\_regulation\_of\_mast\_cell\_proliferation | 5 | 0 | 0.000000 | 0.000000 | 1298 | 1099.072643 | 1155.31 | 1211.547357 | 0.890069 |
[truncated: 286,712 more chars]
