# Supplementary material for: Concordance of Gene Expression and Functional Correlation Patterns across the NCI-60 Cell Lines and the Cancer Genome Atlas Glioblastoma Samples
Source: PLoS One. 2012 Jul 26;7(7):e40062. doi: 10.1371/journal.pone.0040062 (PMC3406063; doi:10.1371/journal.pone.0040062)
Supplement: Download S1 — Zip archive of HTGM results. (ZIP) [file pone.0040062.s007.zip › work2026406846/Generated_Total2026406846.dir/generic.BP.NCI60.0.6.ANXA2.express.genes.correlation.complete.Thu.May.19.17.26.28.2011.htgm.txt.dir/generic.BP.NCI60.0.6.ANXA2.express.genes.correlation.complete.Thu.May.19.17.26.28.2011.htgm.txt.change.gce.html]

Gene Category Report for generic.BP.NCI60.0.6.ANXA2.express.genes.correlation.complete.Thu.May.19.17.26.28.2011.htgm.txt

# Gene Category Report for generic.BP.NCI60.0.6.ANXA2.express.genes.correlation.complete.Thu.May.19.17.26.28.2011.htgm.txt

| HYPERLINKED GO CATEGORY | HYPERLINKED GENE NAME | TOTAL GENES | CHANGED GENES | ENRICHMENT | LOG10(p) | CUMULATIVE NUMBER OF CATEGORIES | CUMULATIVE RANDOMS MEAN | FALSE DISCOVERY RATE |
| --- | --- | --- | --- | --- | --- | --- | --- | --- |
| GO:0051592\_response\_to\_calcium\_ion | CAV1 | 30 | 2 | 38.857143 | -2.936692 | 1 | 0.71 | 0.710000 |
| GO:0051592\_response\_to\_calcium\_ion | ADAM9 | 30 | 2 | 38.857143 | -2.936692 | 1 | 0.71 | 0.710000 |
| GO:0030857\_negative\_regulation\_of\_epithelial\_cell\_differentiation | CAV1 | 1 | 1 |  |  |  |  |  |  |
| GO:0033484\_nitric\_oxide\_homeostasis | CAV1 | 1 | 1 |  |  |  |  |  |  |
| GO:0034238\_macrophage\_fusion | ADAM9 | 1 | 1 |  |  |  |  |  |  |
| GO:0034239\_regulation\_of\_macrophage\_fusion | ADAM9 | 1 | 1 |  |  |  |  |  |  |
| GO:0034241\_positive\_regulation\_of\_macrophage\_fusion | ADAM9 | 1 | 1 |  |  |  |  |  |  |
| GO:0045907\_positive\_regulation\_of\_vasoconstriction | CAV1 | 1 | 1 |  |  |  |  |  |  |
| GO:0060142\_regulation\_of\_syncytium\_formation\_by\_plasma\_membrane\_fusion | ADAM9 | 1 | 1 |  |  |  |  |  |  |
| GO:0060143\_positive\_regulation\_of\_syncytium\_formation\_by\_plasma\_membrane\_fusion | ADAM9 | 1 | 1 |  |  |  |  |  |  |
| GO:0007242\_intracellular\_signaling\_cascade | DCBLD2 | 853 | 6 | 4.099816 | -2.735353 | 2 | 1.08 | 0.540000 |
| GO:0007242\_intracellular\_signaling\_cascade | CAV1 | 853 | 6 | 4.099816 | -2.735353 | 2 | 1.08 | 0.540000 |
| GO:0007242\_intracellular\_signaling\_cascade | ADAM9 | 853 | 6 | 4.099816 | -2.735353 | 2 | 1.08 | 0.540000 |
| GO:0007242\_intracellular\_signaling\_cascade | RRAS | 853 | 6 | 4.099816 | -2.735353 | 2 | 1.08 | 0.540000 |
| GO:0007242\_intracellular\_signaling\_cascade | ARHGAP29 | 853 | 6 | 4.099816 | -2.735353 | 2 | 1.08 | 0.540000 |
| GO:0007242\_intracellular\_signaling\_cascade | HRH1 | 853 | 6 | 4.099816 | -2.735353 | 2 | 1.08 | 0.540000 |
| GO:0010038\_response\_to\_metal\_ion | CAV1 | 46 | 2 | 25.341615 | -2.567051 | 3 | 1.6 | 0.533333 |
| GO:0010038\_response\_to\_metal\_ion | ADAM9 | 46 | 2 | 25.341615 | -2.567051 | 3 | 1.6 | 0.533333 |
| GO:0010035\_response\_to\_inorganic\_substance | CAV1 | 47 | 2 | 24.802432 | -2.548591 | 4 | 1.65 | 0.412500 |
| GO:0010035\_response\_to\_inorganic\_substance | ADAM9 | 47 | 2 | 24.802432 | -2.548591 | 4 | 1.65 | 0.412500 |
| GO:0010042\_response\_to\_manganese\_ion | ADAM9 | 2 | 1 |  |  |  |  |  |  |
| GO:0033630\_positive\_regulation\_of\_cell\_adhesion\_mediated\_by\_integrin | ADAM9 | 2 | 1 |  |  |  |  |  |  |
| GO:0045019\_negative\_regulation\_of\_nitric\_oxide\_biosynthetic\_process | CAV1 | 2 | 1 |  |  |  |  |  |  |
| GO:0045908\_negative\_regulation\_of\_vasodilation | CAV1 | 2 | 1 |  |  |  |  |  |  |
| GO:0051088\_PMA-inducible\_membrane\_protein\_ectodomain\_proteolysis | ADAM9 | 2 | 1 |  |  |  |  |  |  |
| GO:0051547\_regulation\_of\_keratinocyte\_migration | ADAM9 | 2 | 1 |  |  |  |  |  |  |
| GO:0051549\_positive\_regulation\_of\_keratinocyte\_migration | ADAM9 | 2 | 1 |  |  |  |  |  |  |
| GO:0060056\_mammary\_gland\_involution | CAV1 | 2 | 1 |  |  |  |  |  |  |
| GO:0060443\_mammary\_gland\_morphogenesis | CAV1 | 2 | 1 |  |  |  |  |  |  |
| GO:0007160\_cell-matrix\_adhesion | ACTN1 | 58 | 2 | 20.098522 | -2.368823 | 5 | 2.08 | 0.416000 |
| GO:0007160\_cell-matrix\_adhesion | ADAM9 | 58 | 2 | 20.098522 | -2.368823 | 5 | 2.08 | 0.416000 |
| GO:0030856\_regulation\_of\_epithelial\_cell\_differentiation | CAV1 | 3 | 1 |  |  |  |  |  |  |
| GO:0048552\_regulation\_of\_metalloenzyme\_activity | CAV1 | 3 | 1 |  |  |  |  |  |  |
| GO:0048554\_positive\_regulation\_of\_metalloenzyme\_activity | CAV1 | 3 | 1 |  |  |  |  |  |  |
| GO:0051546\_keratinocyte\_migration | ADAM9 | 3 | 1 |  |  |  |  |  |  |
| GO:0031589\_cell-substrate\_adhesion | ACTN1 | 66 | 2 | 17.662338 | -2.259071 | 6 | 2.58 | 0.430000 |
| GO:0031589\_cell-substrate\_adhesion | ADAM9 | 66 | 2 | 17.662338 | -2.259071 | 6 | 2.58 | 0.430000 |
| GO:0009605\_response\_to\_external\_stimulus | DCBLD2 | 464 | 4 | 5.024631 | -2.184302 | 7 | 2.96 | 0.422857 |
| GO:0009605\_response\_to\_external\_stimulus | CAV1 | 464 | 4 | 5.024631 | -2.184302 | 7 | 2.96 | 0.422857 |
| GO:0009605\_response\_to\_external\_stimulus | FOSL1 | 464 | 4 | 5.024631 | -2.184302 | 7 | 2.96 | 0.422857 |
| GO:0009605\_response\_to\_external\_stimulus | HRH1 | 464 | 4 | 5.024631 | -2.184302 | 7 | 2.96 | 0.422857 |
| GO:0033628\_regulation\_of\_cell\_adhesion\_mediated\_by\_integrin | ADAM9 | 4 | 1 |  |  |  |  |  |  |
| GO:0033631\_cell-cell\_adhesion\_mediated\_by\_integrin | ADAM9 | 4 | 1 |  |  |  |  |  |  |
| GO:0000768\_syncytium\_formation\_by\_plasma\_membrane\_fusion | ADAM9 | 5 | 1 | 116.571429 | -2.067976 | 10 | 6.66 | 0.666000 |
| GO:0032570\_response\_to\_progesterone\_stimulus | CAV1 | 5 | 1 | 116.571429 | -2.067976 | 10 | 6.66 | 0.666000 |
| GO:0042117\_monocyte\_activation | ADAM9 | 5 | 1 | 116.571429 | -2.067976 | 10 | 6.66 | 0.666000 |
| GO:0006949\_syncytium\_formation | ADAM9 | 6 | 1 | 97.142857 | -1.989141 | 14 | 9.45 | 0.675000 |
| GO:0033483\_gas\_homeostasis | CAV1 | 6 | 1 | 97.142857 | -1.989141 | 14 | 9.45 | 0.675000 |
| GO:0033627\_cell\_adhesion\_mediated\_by\_integrin | ADAM9 | 6 | 1 | 97.142857 | -1.989141 | 14 | 9.45 | 0.675000 |
| GO:0046426\_negative\_regulation\_of\_JAK-STAT\_cascade | CAV1 | 6 | 1 | 97.142857 | -1.989141 | 14 | 9.45 | 0.675000 |
| GO:0009611\_response\_to\_wounding | DCBLD2 | 279 | 3 | 6.267281 | -1.963438 | 15 | 9.69 | 0.646000 |
| GO:0009611\_response\_to\_wounding | CAV1 | 279 | 3 | 6.267281 | -1.963438 | 15 | 9.69 | 0.646000 |
| GO:0009611\_response\_to\_wounding | HRH1 | 279 | 3 | 6.267281 | -1.963438 | 15 | 9.69 | 0.646000 |
| GO:0042060\_wound\_healing | DCBLD2 | 98 | 2 | 11.895044 | -1.927129 | 17 | 10.0 | 0.588235 |
| GO:0042060\_wound\_healing | CAV1 | 98 | 2 | 11.895044 | -1.927129 | 17 | 10.0 | 0.588235 |
| GO:0051270\_regulation\_of\_cell\_motion | ACTN1 | 98 | 2 | 11.895044 | -1.927129 | 17 | 10.0 | 0.588235 |
| GO:0051270\_regulation\_of\_cell\_motion | ADAM9 | 98 | 2 | 11.895044 | -1.927129 | 17 | 10.0 | 0.588235 |
| GO:0000186\_activation\_of\_MAPKK\_activity | ADAM9 | 7 | 1 | 83.265306 | -1.922540 | 19 | 12.52 | 0.658947 |
| GO:0022612\_gland\_morphogenesis | CAV1 | 7 | 1 | 83.265306 | -1.922540 | 19 | 12.52 | 0.658947 |
| GO:0022603\_regulation\_of\_anatomical\_structure\_morphogenesis | CAV1 | 100 | 2 | 11.657143 | -1.910341 | 20 | 12.65 | 0.632500 |
| GO:0022603\_regulation\_of\_anatomical\_structure\_morphogenesis | ADAM9 | 100 | 2 | 11.657143 | -1.910341 | 20 | 12.65 | 0.632500 |
| GO:0019229\_regulation\_of\_vasoconstriction | CAV1 | 8 | 1 | 72.857143 | -1.864894 | 22 | 15.27 | 0.694091 |
| GO:0043409\_negative\_regulation\_of\_MAPKKK\_cascade | CAV1 | 8 | 1 | 72.857143 | -1.864894 | 22 | 15.27 | 0.694091 |
| GO:0006928\_cell\_motion | ACTN1 | 308 | 3 | 5.677180 | -1.846998 | 24 | 15.42 | 0.642500 |
| GO:0006928\_cell\_motion | TNFRSF12A | 308 | 3 | 5.677180 | -1.846998 | 24 | 15.42 | 0.642500 |
| GO:0006928\_cell\_motion | ADAM9 | 308 | 3 | 5.677180 | -1.846998 | 24 | 15.42 | 0.642500 |
| GO:0051674\_localization\_of\_cell | ACTN1 | 308 | 3 | 5.677180 | -1.846998 | 24 | 15.42 | 0.642500 |
| GO:0051674\_localization\_of\_cell | TNFRSF12A | 308 | 3 | 5.677180 | -1.846998 | 24 | 15.42 | 0.642500 |
| GO:0051674\_localization\_of\_cell | ADAM9 | 308 | 3 | 5.677180 | -1.846998 | 24 | 15.42 | 0.642500 |
| GO:0007265\_Ras\_protein\_signal\_transduction | RRAS | 110 | 2 | 10.597403 | -1.831403 | 25 | 15.66 | 0.626400 |
| GO:0007265\_Ras\_protein\_signal\_transduction | ARHGAP29 | 110 | 2 | 10.597403 | -1.831403 | 25 | 15.66 | 0.626400 |
| GO:0030514\_negative\_regulation\_of\_BMP\_signaling\_pathway | CAV1 | 9 | 1 | 64.761905 | -1.814087 | 26 | 17.78 | 0.683846 |
| GO:0051050\_positive\_regulation\_of\_transport | CAV1 | 116 | 2 | 10.049261 | -1.787614 | 27 | 17.96 | 0.665185 |
| GO:0051050\_positive\_regulation\_of\_transport | ADAM9 | 116 | 2 | 10.049261 | -1.787614 | 27 | 17.96 | 0.665185 |
| GO:0032879\_regulation\_of\_localization | ACTN1 | 326 | 3 | 5.363716 | -1.780737 | 28 | 18.0 | 0.642857 |
| GO:0032879\_regulation\_of\_localization | CAV1 | 326 | 3 | 5.363716 | -1.780737 | 28 | 18.0 | 0.642857 |
| GO:0032879\_regulation\_of\_localization | ADAM9 | 326 | 3 | 5.363716 | -1.780737 | 28 | 18.0 | 0.642857 |
| GO:0034612\_response\_to\_tumor\_necrosis\_factor | ADAM9 | 10 | 1 | 58.285714 | -1.768675 | 31 | 20.46 | 0.660000 |
| GO:0042312\_regulation\_of\_vasodilation | CAV1 | 10 | 1 | 58.285714 | -1.768675 | 31 | 20.46 | 0.660000 |
| GO:0042542\_response\_to\_hydrogen\_peroxide | ADAM9 | 10 | 1 | 58.285714 | -1.768675 | 31 | 20.46 | 0.660000 |
| GO:0006950\_response\_to\_stress | DCBLD2 | 959 | 5 | 3.038880 | -1.747511 | 32 | 20.57 | 0.642812 |
| GO:0006950\_response\_to\_stress | CAV1 | 959 | 5 | 3.038880 | -1.747511 | 32 | 20.57 | 0.642812 |
| GO:0006950\_response\_to\_stress | ADAM9 | 959 | 5 | 3.038880 | -1.747511 | 32 | 20.57 | 0.642812 |
| GO:0006950\_response\_to\_stress | FOSL1 | 959 | 5 | 3.038880 | -1.747511 | 32 | 20.57 | 0.642812 |
| GO:0006950\_response\_to\_stress | HRH1 | 959 | 5 | 3.038880 | -1.747511 | 32 | 20.57 | 0.642812 |
| GO:0001570\_vasculogenesis | CAV1 | 11 | 1 | 52.987013 | -1.727628 | 33 | 22.71 | 0.688182 |
| GO:0010524\_positive\_regulation\_of\_calcium\_ion\_transport\_into\_cytosol | CAV1 | 12 | 1 | 48.571429 | -1.690185 | 37 | 24.57 | 0.664054 |
| GO:0030510\_regulation\_of\_BMP\_signaling\_pathway | CAV1 | 12 | 1 | 48.571429 | -1.690185 | 37 | 24.57 | 0.664054 |
| GO:0042311\_vasodilation | CAV1 | 12 | 1 | 48.571429 | -1.690185 | 37 | 24.57 | 0.664054 |
| GO:0045026\_plasma\_membrane\_fusion | ADAM9 | 12 | 1 | 48.571429 | -1.690185 | 37 | 24.57 | 0.664054 |
| GO:0043085\_positive\_regulation\_of\_catalytic\_activity | CAV1 | 354 | 3 | 4.939467 | -1.685472 | 38 | 24.8 | 0.652632 |
| GO:0043085\_positive\_regulation\_of\_catalytic\_activity | ADAM9 | 354 | 3 | 4.939467 | -1.685472 | 38 | 24.8 | 0.652632 |
| GO:0043085\_positive\_regulation\_of\_catalytic\_activity | HRH1 | 354 | 3 | 4.939467 | -1.685472 | 38 | 24.8 | 0.652632 |
| GO:0007264\_small\_GTPase\_mediated\_signal\_transduction | RRAS | 135 | 2 | 8.634921 | -1.663392 | 39 | 25.16 | 0.645128 |
| GO:0007264\_small\_GTPase\_mediated\_signal\_transduction | ARHGAP29 | 135 | 2 | 8.634921 | -1.663392 | 39 | 25.16 | 0.645128 |
| GO:0006940\_regulation\_of\_smooth\_muscle\_contraction | CAV1 | 13 | 1 | 44.835165 | -1.655768 | 41 | 26.96 | 0.657561 |
| GO:0051899\_membrane\_depolarization | CAV1 | 13 | 1 | 44.835165 | -1.655768 | 41 | 26.96 | 0.657561 |
| GO:0030879\_mammary\_gland\_development | CAV1 | 14 | 1 | 41.632653 | -1.623929 | 44 | 29.04 | 0.660000 |
| GO:0042310\_vasoconstriction | CAV1 | 14 | 1 | 41.632653 | -1.623929 | 44 | 29.04 | 0.660000 |
| GO:0045428\_regulation\_of\_nitric\_oxide\_biosynthetic\_process | CAV1 | 14 | 1 | 41.632653 | -1.623929 | 44 | 29.04 | 0.660000 |
| GO:0000165\_MAPKKK\_cascade | CAV1 | 143 | 2 | 8.151848 | -1.616599 | 45 | 29.11 | 0.646889 |
| GO:0000165\_MAPKKK\_cascade | ADAM9 | 143 | 2 | 8.151848 | -1.616599 | 45 | 29.11 | 0.646889 |
| GO:0001937\_negative\_regulation\_of\_endothelial\_cell\_proliferation | CAV1 | 15 | 1 | 38.857143 | -1.594311 | 48 | 31.19 | 0.649792 |
| GO:0030512\_negative\_regulation\_of\_transforming\_growth\_factor\_beta\_receptor\_signaling\_pathway | CAV1 | 15 | 1 | 38.857143 | -1.594311 | 48 | 31.19 | 0.649792 |
| GO:0048041\_focal\_adhesion\_formation | ACTN1 | 15 | 1 | 38.857143 | -1.594311 | 48 | 31.19 | 0.649792 |
| GO:0030855\_epithelial\_cell\_differentiation | CAV1 | 16 | 1 | 36.428571 | -1.566627 | 49 | 33.43 | 0.682245 |
| GO:0044093\_positive\_regulation\_of\_molecular\_function | CAV1 | 394 | 3 | 4.437999 | -1.563358 | 50 | 33.46 | 0.669200 |
| GO:0044093\_positive\_regulation\_of\_molecular\_function | ADAM9 | 394 | 3 | 4.437999 | -1.563358 | 50 | 33.46 | 0.669200 |
| GO:0044093\_positive\_regulation\_of\_molecular\_function | HRH1 | 394 | 3 | 4.437999 | -1.563358 | 50 | 33.46 | 0.669200 |
| GO:0000188\_inactivation\_of\_MAPK\_activity | CAV1 | 17 | 1 | 34.285714 | -1.540644 | 54 | 35.02 | 0.648519 |
| GO:0007044\_cell-substrate\_junction\_assembly | ACTN1 | 17 | 1 | 34.285714 | -1.540644 | 54 | 35.02 | 0.648519 |
| GO:0010522\_regulation\_of\_calcium\_ion\_transport\_into\_cytosol | CAV1 | 17 | 1 | 34.285714 | -1.540644 | 54 | 35.02 | 0.648519 |
| GO:0051928\_positive\_regulation\_of\_calcium\_ion\_transport | CAV1 | 17 | 1 | 34.285714 | -1.540644 | 54 | 35.02 | 0.648519 |
| GO:0032846\_positive\_regulation\_of\_homeostatic\_process | CAV1 | 18 | 1 | 32.380952 | -1.516165 | 56 | 36.64 | 0.654286 |
| GO:0043270\_positive\_regulation\_of\_ion\_transport | CAV1 | 18 | 1 | 32.380952 | -1.516165 | 56 | 36.64 | 0.654286 |
| GO:0006809\_nitric\_oxide\_biosynthetic\_process | CAV1 | 19 | 1 | 30.676692 | -1.493029 | 58 | 38.64 | 0.666207 |
| GO:0009267\_cellular\_response\_to\_starvation | CAV1 | 19 | 1 | 30.676692 | -1.493029 | 58 | 38.64 | 0.666207 |
| GO:0006096\_glycolysis | PKM2 | 20 | 1 | 29.142857 | -1.471098 | 61 | 40.65 | 0.666393 |
| GO:0042594\_response\_to\_starvation | CAV1 | 20 | 1 | 29.142857 | -1.471098 | 61 | 40.65 | 0.666393 |
| GO:0046209\_nitric\_oxide\_metabolic\_process | CAV1 | 20 | 1 | 29.142857 | -1.471098 | 61 | 40.65 | 0.666393 |
| GO:0007155\_cell\_adhesion | ACTN1 | 428 | 3 | 4.085447 | -1.470341 | 62 | 40.67 | 0.655968 |
| GO:0007155\_cell\_adhesion | ADAM9 | 428 | 3 | 4.085447 | -1.470341 | 62 | 40.67 | 0.655968 |
| GO:0007155\_cell\_adhesion | CD151 | 428 | 3 | 4.085447 | -1.470341 | 62 | 40.67 | 0.655968 |
| GO:0022610\_biological\_adhesion | ACTN1 | 429 | 3 | 4.075924 | -1.467737 | 63 | 40.7 | 0.646032 |
| GO:0022610\_biological\_adhesion | ADAM9 | 429 | 3 | 4.075924 | -1.467737 | 63 | 40.7 | 0.646032 |
| GO:0022610\_biological\_adhesion | CD151 | 429 | 3 | 4.075924 | -1.467737 | 63 | 40.7 | 0.646032 |
| GO:0000302\_response\_to\_reactive\_oxygen\_species | ADAM9 | 21 | 1 | 27.755102 | -1.450253 | 66 | 42.98 | 0.651212 |
| GO:0001936\_regulation\_of\_endothelial\_cell\_proliferation | CAV1 | 21 | 1 | 27.755102 | -1.450253 | 66 | 42.98 | 0.651212 |
| GO:0030048\_actin\_filament-based\_movement | MYO1E | 21 | 1 | 27.755102 | -1.450253 | 66 | 42.98 | 0.651212 |
| GO:0051336\_regulation\_of\_hydrolase\_activity | CAV1 | 180 | 2 | 6.476190 | -1.431774 | 67 | 43.43 | 0.648209 |
| GO:0051336\_regulation\_of\_hydrolase\_activity | HRH1 | 180 | 2 | 6.476190 | -1.431774 | 67 | 43.43 | 0.648209 |
| GO:0007165\_signal\_transduction | DCBLD2 | 2029 | 7 | 2.010843 | -1.430678 | 68 | 43.5 | 0.639706 |
| GO:0007165\_signal\_transduction | CAV1 | 2029 | 7 | 2.010843 | -1.430678 | 68 | 43.5 | 0.639706 |
| GO:0007165\_signal\_transduction | ADAM9 | 2029 | 7 | 2.010843 | -1.430678 | 68 | 43.5 | 0.639706 |
| GO:0007165\_signal\_transduction | RRAS | 2029 | 7 | 2.010843 | -1.430678 | 68 | 43.5 | 0.639706 |
| GO:0007165\_signal\_transduction | S100A10 | 2029 | 7 | 2.010843 | -1.430678 | 68 | 43.5 | 0.639706 |
| GO:0007165\_signal\_transduction | ARHGAP29 | 2029 | 7 | 2.010843 | -1.430678 | 68 | 43.5 | 0.639706 |
| GO:0007165\_signal\_transduction | HRH1 | 2029 | 7 | 2.010843 | -1.430678 | 68 | 43.5 | 0.639706 |
| GO:0042632\_cholesterol\_homeostasis | CAV1 | 22 | 1 | 26.493506 | -1.430395 | 70 | 44.64 | 0.637714 |
| GO:0055092\_sterol\_homeostasis | CAV1 | 22 | 1 | 26.493506 | -1.430395 | 70 | 44.64 | 0.637714 |
| GO:0006509\_membrane\_protein\_ectodomain\_proteolysis | ADAM9 | 23 | 1 | 25.341615 | -1.411434 | 74 | 45.82 | 0.619189 |
| GO:0006641\_triglyceride\_metabolic\_process | CAV1 | 23 | 1 | 25.341615 | -1.411434 | 74 | 45.82 | 0.619189 |
| GO:0033619\_membrane\_protein\_proteolysis | ADAM9 | 23 | 1 | 25.341615 | -1.411434 | 74 | 45.82 | 0.619189 |
| GO:0043627\_response\_to\_estrogen\_stimulus | CAV1 | 23 | 1 | 25.341615 | -1.411434 | 74 | 45.82 | 0.619189 |
| GO:0030193\_regulation\_of\_blood\_coagulation | CAV1 | 24 | 1 | 24.285714 | -1.393296 | 77 | 47.27 | 0.613896 |
| GO:0043407\_negative\_regulation\_of\_MAP\_kinase\_activity | CAV1 | 24 | 1 | 24.285714 | -1.393296 | 77 | 47.27 | 0.613896 |
| GO:0045785\_positive\_regulation\_of\_cell\_adhesion | ADAM9 | 24 | 1 | 24.285714 | -1.393296 | 77 | 47.27 | 0.613896 |
| GO:0001935\_endothelial\_cell\_proliferation | CAV1 | 25 | 1 | 23.314286 | -1.375911 | 83 | 49.22 | 0.593012 |
| GO:0006007\_glucose\_catabolic\_process | PKM2 | 25 | 1 | 23.314286 | -1.375911 | 83 | 49.22 | 0.593012 |
| GO:0010876\_lipid\_localization | CAV1 | 25 | 1 | 23.314286 | -1.375911 | 83 | 49.22 | 0.593012 |
| GO:0019217\_regulation\_of\_fatty\_acid\_metabolic\_process | CAV1 | 25 | 1 | 23.314286 | -1.375911 | 83 | 49.22 | 0.593012 |
| GO:0019915\_lipid\_storage | CAV1 | 25 | 1 | 23.314286 | -1.375911 | 83 | 49.22 | 0.593012 |
| GO:0050818\_regulation\_of\_coagulation | CAV1 | 25 | 1 | 23.314286 | -1.375911 | 83 | 49.22 | 0.593012 |
| GO:0010741\_negative\_regulation\_of\_protein\_kinase\_cascade | CAV1 | 26 | 1 | 22.417582 | -1.359223 | 84 | 50.9 | 0.605952 |
| GO:0006638\_neutral\_lipid\_metabolic\_process | CAV1 | 27 | 1 | 21.587302 | -1.343177 | 89 | 52.33 | 0.587978 |
| GO:0006639\_acylglycerol\_metabolic\_process | CAV1 | 27 | 1 | 21.587302 | -1.343177 | 89 | 52.33 | 0.587978 |
| GO:0031669\_cellular\_response\_to\_nutrient\_levels | CAV1 | 27 | 1 | 21.587302 | -1.343177 | 89 | 52.33 | 0.587978 |
| GO:0035150\_regulation\_of\_tube\_size | CAV1 | 27 | 1 | 21.587302 | -1.343177 | 89 | 52.33 | 0.587978 |
| GO:0050880\_regulation\_of\_blood\_vessel\_size | CAV1 | 27 | 1 | 21.587302 | -1.343177 | 89 | 52.33 | 0.587978 |
| GO:0006662\_glycerol\_ether\_metabolic\_process | CAV1 | 28 | 1 | 20.816327 | -1.327727 | 94 | 53.96 | 0.574043 |
| GO:0006939\_smooth\_muscle\_contraction | CAV1 | 28 | 1 | 20.816327 | -1.327727 | 94 | 53.96 | 0.574043 |
| GO:0010565\_regulation\_of\_cellular\_ketone\_metabolic\_process | CAV1 | 28 | 1 | 20.816327 | -1.327727 | 94 | 53.96 | 0.574043 |
| GO:0018904\_organic\_ether\_metabolic\_process | CAV1 | 28 | 1 | 20.816327 | -1.327727 | 94 | 53.96 | 0.574043 |
| GO:0060402\_calcium\_ion\_transport\_into\_cytosol | CAV1 | 28 | 1 | 20.816327 | -1.327727 | 94 | 53.96 | 0.574043 |
| GO:0002009\_morphogenesis\_of\_an\_epithelium | CAV1 | 29 | 1 | 20.098522 | -1.312831 | 104 | 55.27 | 0.531442 |
| GO:0002274\_myeloid\_leukocyte\_activation | ADAM9 | 29 | 1 | 20.098522 | -1.312831 | 104 | 55.27 | 0.531442 |
| GO:0003018\_vascular\_process\_in\_circulatory\_system | CAV1 | 29 | 1 | 20.098522 | -1.312831 | 104 | 55.27 | 0.531442 |
| GO:0032507\_maintenance\_of\_protein\_location\_in\_cell | CAV1 | 29 | 1 | 20.098522 | -1.312831 | 104 | 55.27 | 0.531442 |
| GO:0046425\_regulation\_of\_JAK-STAT\_cascade | CAV1 | 29 | 1 | 20.098522 | -1.312831 | 104 | 55.27 | 0.531442 |
| GO:0048732\_gland\_development | CAV1 | 29 | 1 | 20.098522 | -1.312831 | 104 | 55.27 | 0.531442 |
| GO:0050714\_positive\_regulation\_of\_protein\_secretion | ADAM9 | 29 | 1 | 20.098522 | -1.312831 | 104 | 55.27 | 0.531442 |
| GO:0051924\_regulation\_of\_calcium\_ion\_transport | CAV1 | 29 | 1 | 20.098522 | -1.312831 | 104 | 55.27 | 0.531442 |
| GO:0060401\_cytosolic\_calcium\_ion\_transport | CAV1 | 29 | 1 | 20.098522 | -1.312831 | 104 | 55.27 | 0.531442 |
| GO:0060429\_epithelium\_development | CAV1 | 29 | 1 | 20.098522 | -1.312831 | 104 | 55.27 | 0.531442 |
| GO:0045859\_regulation\_of\_protein\_kinase\_activity | CAV1 | 213 | 2 | 5.472837 | -1.299144 | 105 | 55.68 | 0.530286 |
| GO:0045859\_regulation\_of\_protein\_kinase\_activity | ADAM9 | 213 | 2 | 5.472837 | -1.299144 | 105 | 55.68 | 0.530286 |
| GO:0010959\_regulation\_of\_metal\_ion\_transport | CAV1 | 30 | 1 | 19.428571 | -1.298453 | 106 | 56.69 | 0.534811 |
| GO:0043549\_regulation\_of\_kinase\_activity | CAV1 | 217 | 2 | 5.371955 | -1.284637 | 107 | 56.99 | 0.532617 |
| GO:0043549\_regulation\_of\_kinase\_activity | ADAM9 | 217 | 2 | 5.371955 | -1.284637 | 107 | 56.99 | 0.532617 |
| GO:0007200\_activation\_of\_phospholipase\_C\_activity\_by\_G-protein\_coupled\_receptor\_protein\_signaling\_pathway\_coupled\_to\_IP3\_second\_messenger | HRH1 | 31 | 1 | 18.801843 | -1.284556 | 110 | 57.87 | 0.526091 |
| GO:0031668\_cellular\_response\_to\_extracellular\_stimulus | CAV1 | 31 | 1 | 18.801843 | -1.284556 | 110 | 57.87 | 0.526091 |
| GO:0045185\_maintenance\_of\_protein\_location | CAV1 | 31 | 1 | 18.801843 | -1.284556 | 110 | 57.87 | 0.526091 |
| GO:0030216\_keratinocyte\_differentiation | ADAM9 | 32 | 1 | 18.214286 | -1.271112 | 111 | 58.86 | 0.530270 |
| GO:0034329\_cell\_junction\_assembly | ACTN1 | 33 | 1 | 17.662338 | -1.258092 | 113 | 59.65 | 0.527876 |
| GO:0055088\_lipid\_homeostasis | CAV1 | 33 | 1 | 17.662338 | -1.258092 | 113 | 59.65 | 0.527876 |
| GO:0016044\_membrane\_organization | CAV1 | 225 | 2 | 5.180952 | -1.256501 | 114 | 59.72 | 0.523860 |
| GO:0016044\_membrane\_organization | ADAM9 | 225 | 2 | 5.180952 | -1.256501 | 114 | 59.72 | 0.523860 |
| GO:0051049\_regulation\_of\_transport | CAV1 | 227 | 2 | 5.135305 | -1.249642 | 116 | 59.95 | 0.516810 |
| GO:0051049\_regulation\_of\_transport | ADAM9 | 227 | 2 | 5.135305 | -1.249642 | 116 | 59.95 | 0.516810 |
| GO:0051338\_regulation\_of\_transferase\_activity | CAV1 | 227 | 2 | 5.135305 | -1.249642 | 116 | 59.95 | 0.516810 |
| GO:0051338\_regulation\_of\_transferase\_activity | ADAM9 | 227 | 2 | 5.135305 | -1.249642 | 116 | 59.95 | 0.516810 |
| GO:0050790\_regulation\_of\_catalytic\_activity | CAV1 | 525 | 3 | 3.330612 | -1.246722 | 117 | 60.07 | 0.513419 |
| GO:0050790\_regulation\_of\_catalytic\_activity | ADAM9 | 525 | 3 | 3.330612 | -1.246722 | 117 | 60.07 | 0.513419 |
| GO:0050790\_regulation\_of\_catalytic\_activity | HRH1 | 525 | 3 | 3.330612 | -1.246722 | 117 | 60.07 | 0.513419 |
| GO:0043269\_regulation\_of\_ion\_transport | CAV1 | 34 | 1 | 17.142857 | -1.245472 | 120 | 61.83 | 0.515250 |
| GO:0048545\_response\_to\_steroid\_hormone\_stimulus | CAV1 | 34 | 1 | 17.142857 | -1.245472 | 120 | 61.83 | 0.515250 |
| GO:0051651\_maintenance\_of\_location\_in\_cell | CAV1 | 34 | 1 | 17.142857 | -1.245472 | 120 | 61.83 | 0.515250 |
| GO:0019320\_hexose\_catabolic\_process | PKM2 | 35 | 1 | 16.653061 | -1.233226 | 122 | 62.99 | 0.516311 |
| GO:0030705\_cytoskeleton-dependent\_intracellular\_transport | MYO1E | 35 | 1 | 16.653061 | -1.233226 | 122 | 62.99 | 0.516311 |
| GO:0034097\_response\_to\_cytokine\_stimulus | ADAM9 | 36 | 1 | 16.190476 | -1.221336 | 123 | 63.74 | 0.518211 |
| GO:0006937\_regulation\_of\_muscle\_contraction | CAV1 | 37 | 1 | 15.752896 | -1.209781 | 127 | 64.69 | 0.509370 |
| GO:0009913\_epidermal\_cell\_differentiation | ADAM9 | 37 | 1 | 15.752896 | -1.209781 | 127 | 64.69 | 0.509370 |
| GO:0015918\_sterol\_transport | CAV1 | 37 | 1 | 15.752896 | -1.209781 | 127 | 64.69 | 0.509370 |
| GO:0030301\_cholesterol\_transport | CAV1 | 37 | 1 | 15.752896 | -1.209781 | 127 | 64.69 | 0.509370 |
| GO:0016050\_vesicle\_organization | CAV1 | 38 | 1 | 15.338346 | -1.198542 | 128 | 66.22 | 0.517344 |
| GO:0030509\_BMP\_signaling\_pathway | CAV1 | 39 | 1 | 14.945055 | -1.187605 | 130 | 67.15 | 0.516538 |
| GO:0046365\_monosaccharide\_catabolic\_process | PKM2 | 39 | 1 | 14.945055 | -1.187605 | 130 | 67.15 | 0.516538 |
| GO:0007154\_cell\_communication | DCBLD2 | 2272 | 7 | 1.795775 | -1.185068 | 131 | 67.32 | 0.513893 |
| GO:0007154\_cell\_communication | CAV1 | 2272 | 7 | 1.795775 | -1.185068 | 131 | 67.32 | 0.513893 |
| GO:0007154\_cell\_communication | ADAM9 | 2272 | 7 | 1.795775 | -1.185068 | 131 | 67.32 | 0.513893 |
| GO:0007154\_cell\_communication | RRAS | 2272 | 7 | 1.795775 | -1.185068 | 131 | 67.32 | 0.513893 |
| GO:0007154\_cell\_communication | S100A10 | 2272 | 7 | 1.795775 | -1.185068 | 131 | 67.32 | 0.513893 |
| GO:0007154\_cell\_communication | ARHGAP29 | 2272 | 7 | 1.795775 | -1.185068 | 131 | 67.32 | 0.513893 |
| GO:0007154\_cell\_communication | HRH1 | 2272 | 7 | 1.795775 | -1.185068 | 131 | 67.32 | 0.513893 |
| GO:0010926\_anatomical\_structure\_formation | ACTN1 | 560 | 3 | 3.122449 | -1.178042 | 132 | 67.38 | 0.510455 |
| GO:0010926\_anatomical\_structure\_formation | CAV1 | 560 | 3 | 3.122449 | -1.178042 | 132 | 67.38 | 0.510455 |
| GO:0010926\_anatomical\_structure\_formation | ADAM9 | 560 | 3 | 3.122449 | -1.178042 | 132 | 67.38 | 0.510455 |
| GO:0017015\_regulation\_of\_transforming\_growth\_factor\_beta\_receptor\_signaling\_pathway | CAV1 | 40 | 1 | 14.571429 | -1.176954 | 133 | 68.02 | 0.511429 |
| GO:0007519\_skeletal\_muscle\_tissue\_development | CAV1 | 41 | 1 | 14.216028 | -1.166573 | 138 | 69.09 | 0.500652 |
| GO:0034330\_cell\_junction\_organization | ACTN1 | 41 | 1 | 14.216028 | -1.166573 | 138 | 69.09 | 0.500652 |
| GO:0050708\_regulation\_of\_protein\_secretion | ADAM9 | 41 | 1 | 14.216028 | -1.166573 | 138 | 69.09 | 0.500652 |
| GO:0051222\_positive\_regulation\_of\_protein\_transport | ADAM9 | 41 | 1 | 14.216028 | -1.166573 | 138 | 69.09 | 0.500652 |
| GO:0060538\_skeletal\_muscle\_organ\_development | CAV1 | 41 | 1 | 14.216028 | -1.166573 | 138 | 69.09 | 0.500652 |
| GO:0006944\_membrane\_fusion | ADAM9 | 42 | 1 | 13.877551 | -1.156451 | 140 | 70.2 | 0.501429 |
| GO:0051271\_negative\_regulation\_of\_cell\_motion | ACTN1 | 42 | 1 | 13.877551 | -1.156451 | 140 | 70.2 | 0.501429 |
| GO:0046164\_alcohol\_catabolic\_process | PKM2 | 43 | 1 | 13.554817 | -1.146576 | 141 | 71.29 | 0.505603 |
| GO:0048771\_tissue\_remodeling | CAV1 | 45 | 1 | 12.952381 | -1.127518 | 142 | 72.95 | 0.513732 |
| GO:0007202\_activation\_of\_phospholipase\_C\_activity | HRH1 | 46 | 1 | 12.670807 | -1.118316 | 146 | 74.14 | 0.507808 |
| GO:0010863\_positive\_regulation\_of\_phospholipase\_C\_activity | HRH1 | 46 | 1 | 12.670807 | -1.118316 | 146 | 74.14 | 0.507808 |
| GO:0030335\_positive\_regulation\_of\_cell\_migration | ADAM9 | 46 | 1 | 12.670807 | -1.118316 | 146 | 74.14 | 0.507808 |
| GO:0042391\_regulation\_of\_membrane\_potential | CAV1 | 46 | 1 | 12.670807 | -1.118316 | 146 | 74.14 | 0.507808 |
| GO:0007259\_JAK-STAT\_cascade | CAV1 | 47 | 1 | 12.401216 | -1.109320 | 148 | 75.35 | 0.509122 |
| GO:0051260\_protein\_homooligomerization | CAV1 | 47 | 1 | 12.401216 | -1.109320 | 148 | 75.35 | 0.509122 |
| GO:0010518\_positive\_regulation\_of\_phospholipase\_activity | HRH1 | 48 | 1 | 12.142857 | -1.100519 | 149 | 76.49 | 0.513356 |
| GO:0010033\_response\_to\_organic\_substance | CAV1 | 276 | 2 | 4.223602 | -1.100213 | 150 | 76.59 | 0.510600 |
| GO:0010033\_response\_to\_organic\_substance | ADAM9 | 276 | 2 | 4.223602 | -1.100213 | 150 | 76.59 | 0.510600 |
| GO:0065009\_regulation\_of\_molecular\_function | CAV1 | 606 | 3 | 2.885431 | -1.095473 | 151 | 76.8 | 0.508609 |
| GO:0065009\_regulation\_of\_molecular\_function | ADAM9 | 606 | 3 | 2.885431 | -1.095473 | 151 | 76.8 | 0.508609 |
| GO:0065009\_regulation\_of\_molecular\_function | HRH1 | 606 | 3 | 2.885431 | -1.095473 | 151 | 76.8 | 0.508609 |
| GO:0010517\_regulation\_of\_phospholipase\_activity | HRH1 | 49 | 1 | 11.895044 | -1.091908 | 152 | 78.12 | 0.513947 |
| GO:0001666\_response\_to\_hypoxia | CAV1 | 50 | 1 | 11.657143 | -1.083477 | 155 | 78.84 | 0.508645 |
| GO:0007266\_Rho\_protein\_signal\_transduction | ARHGAP29 | 50 | 1 | 11.657143 | -1.083477 | 155 | 78.84 | 0.508645 |
| GO:0051272\_positive\_regulation\_of\_cell\_motion | ADAM9 | 50 | 1 | 11.657143 | -1.083477 | 155 | 78.84 | 0.508645 |
| GO:0051641\_cellular\_localization | CAV1 | 617 | 3 | 2.833989 | -1.076892 | 156 | 78.9 | 0.505769 |
| GO:0051641\_cellular\_localization | ADAM9 | 617 | 3 | 2.833989 | -1.076892 | 156 | 78.9 | 0.505769 |
| GO:0051641\_cellular\_localization | MYO1E | 617 | 3 | 2.833989 | -1.076892 | 156 | 78.9 | 0.505769 |
| GO:0042325\_regulation\_of\_phosphorylation | CAV1 | 285 | 2 | 4.090226 | -1.076084 | 157 | 78.99 | 0.503121 |
| GO:0042325\_regulation\_of\_phosphorylation | ADAM9 | 285 | 2 | 4.090226 | -1.076084 | 157 | 78.99 | 0.503121 |
| GO:0016052\_carbohydrate\_catabolic\_process | PKM2 | 51 | 1 | 11.428571 | -1.075220 | 160 | 79.87 | 0.499188 |
| GO:0032147\_activation\_of\_protein\_kinase\_activity | ADAM9 | 51 | 1 | 11.428571 | -1.075220 | 160 | 79.87 | 0.499188 |
| GO:0070482\_response\_to\_oxygen\_levels | CAV1 | 51 | 1 | 11.428571 | -1.075220 | 160 | 79.87 | 0.499188 |
| GO:0009888\_tissue\_development | CAV1 | 287 | 2 | 4.061722 | -1.070842 | 161 | 80.24 | 0.498385 |
| GO:0009888\_tissue\_development | ADAM9 | 287 | 2 | 4.061722 | -1.070842 | 161 | 80.24 | 0.498385 |
| GO:0006006\_glucose\_metabolic\_process | PKM2 | 53 | 1 | 10.997305 | -1.059200 | 165 | 82.29 | 0.498727 |
| GO:0048015\_phosphoinositide-mediated\_signaling | HRH1 | 53 | 1 | 10.997305 | -1.059200 | 165 | 82.29 | 0.498727 |
| GO:0051047\_positive\_regulation\_of\_secretion | ADAM9 | 53 | 1 | 10.997305 | -1.059200 | 165 | 82.29 | 0.498727 |
| GO:0060193\_positive\_regulation\_of\_lipase\_activity | HRH1 | 53 | 1 | 10.997305 | -1.059200 | 165 | 82.29 | 0.498727 |
| GO:0040011\_locomotion | ADAM9 | 292 | 2 | 3.992172 | -1.057920 | 166 | 82.5 | 0.496988 |
| GO:0040011\_locomotion | FOSL1 | 292 | 2 | 3.992172 | -1.057920 | 166 | 82.5 | 0.496988 |
| GO:0042221\_response\_to\_chemical\_stimulus | CAV1 | 631 | 3 | 2.771112 | -1.053842 | 167 | 82.6 | 0.494611 |
| GO:0042221\_response\_to\_chemical\_stimulus | ADAM9 | 631 | 3 | 2.771112 | -1.053842 | 167 | 82.6 | 0.494611 |
| GO:0042221\_response\_to\_chemical\_stimulus | FOSL1 | 631 | 3 | 2.771112 | -1.053842 | 167 | 82.6 | 0.494611 |
| GO:0019220\_regulation\_of\_phosphate\_metabolic\_process | CAV1 | 297 | 2 | 3.924964 | -1.045254 | 169 | 83.21 | 0.492367 |
| GO:0019220\_regulation\_of\_phosphate\_metabolic\_process | ADAM9 | 297 | 2 | 3.924964 | -1.045254 | 169 | 83.21 | 0.492367 |
| GO:0051174\_regulation\_of\_phosphorus\_metabolic\_process | CAV1 | 297 | 2 | 3.924964 | -1.045254 | 169 | 83.21 | 0.492367 |
| GO:0051174\_regulation\_of\_phosphorus\_metabolic\_process | ADAM9 | 297 | 2 | 3.924964 | -1.045254 | 169 | 83.21 | 0.492367 |
| GO:0006469\_negative\_regulation\_of\_protein\_kinase\_activity | CAV1 | 55 | 1 | 10.597403 | -1.043798 | 170 | 83.81 | 0.493000 |
| GO:0050794\_regulation\_of\_cellular\_process | ACTN1 | 3515 | 9 | 1.492380 | -1.037411 | 171 | 83.89 | 0.490585 |
| GO:0050794\_regulation\_of\_cellular\_process | CAV1 | 3515 | 9 | 1.492380 | -1.037411 | 171 | 83.89 | 0.490585 |
| GO:0050794\_regulation\_of\_cellular\_process | DCBLD2 | 3515 | 9 | 1.492380 | -1.037411 | 171 | 83.89 | 0.490585 |
| GO:0050794\_regulation\_of\_cellular\_process | ADAM9 | 3515 | 9 | 1.492380 | -1.037411 | 171 | 83.89 | 0.490585 |
| GO:0050794\_regulation\_of\_cellular\_process | FOSL1 | 3515 | 9 | 1.492380 | -1.037411 | 171 | 83.89 | 0.490585 |
| GO:0050794\_regulation\_of\_cellular\_process | RRAS | 3515 | 9 | 1.492380 | -1.037411 | 171 | 83.89 | 0.490585 |
| GO:0050794\_regulation\_of\_cellular\_process | S100A10 | 3515 | 9 | 1.492380 | -1.037411 | 171 | 83.89 | 0.490585 |
| GO:0050794\_regulation\_of\_cellular\_process | ARHGAP29 | 3515 | 9 | 1.492380 | -1.037411 | 171 | 83.89 | 0.490585 |
| GO:0050794\_regulation\_of\_cellular\_process | HRH1 | 3515 | 9 | 1.492380 | -1.037411 | 171 | 83.89 | 0.490585 |
| GO:0009306\_protein\_secretion | ADAM9 | 56 | 1 | 10.408163 | -1.036316 | 172 | 84.38 | 0.490581 |
| GO:0032844\_regulation\_of\_homeostatic\_process | CAV1 | 57 | 1 | 10.225564 | -1.028971 | 173 | 85.01 | 0.491387 |
| GO:0006968\_cellular\_defense\_response | FOSL1 | 58 | 1 | 10.049261 | -1.021761 | 175 | 86.19 | 0.492514 |
| GO:0030308\_negative\_regulation\_of\_cell\_growth | DCBLD2 | 58 | 1 | 10.049261 | -1.021761 | 175 | 86.19 | 0.492514 |
| GO:0033673\_negative\_regulation\_of\_kinase\_activity | CAV1 | 59 | 1 | 9.878935 | -1.014679 | 177 | 87.28 | 0.493107 |
| GO:0043408\_regulation\_of\_MAPKKK\_cascade | CAV1 | 59 | 1 | 9.878935 | -1.014679 | 177 | 87.28 | 0.493107 |
| GO:0030155\_regulation\_of\_cell\_adhesion | ADAM9 | 61 | 1 | 9.555035 | -1.000886 | 180 | 88.71 | 0.492833 |
| GO:0045792\_negative\_regulation\_of\_cell\_size | DCBLD2 | 61 | 1 | 9.555035 | -1.000886 | 180 | 88.71 | 0.492833 |
| GO:0048729\_tissue\_morphogenesis | CAV1 | 61 | 1 | 9.555035 | -1.000886 | 180 | 88.71 | 0.492833 |
| GO:0050793\_regulation\_of\_developmental\_process | ACTN1 | 669 | 3 | 2.613709 | -0.994447 | 181 | 89.11 | 0.492320 |
| GO:0050793\_regulation\_of\_developmental\_process | CAV1 | 669 | 3 | 2.613709 | -0.994447 | 181 | 89.11 | 0.492320 |
| GO:0050793\_regulation\_of\_developmental\_process | ADAM9 | 669 | 3 | 2.613709 | -0.994447 | 181 | 89.11 | 0.492320 |
| GO:0019216\_regulation\_of\_lipid\_metabolic\_process | CAV1 | 62 | 1 | 9.400922 | -0.994166 | 184 | 90.33 | 0.490924 |
| GO:0031667\_response\_to\_nutrient\_levels | CAV1 | 62 | 1 | 9.400922 | -0.994166 | 184 | 90.33 | 0.490924 |
| GO:0060191\_regulation\_of\_lipase\_activity | HRH1 | 62 | 1 | 9.400922 | -0.994166 | 184 | 90.33 | 0.490924 |
| GO:0030522\_intracellular\_receptor-mediated\_signaling\_pathway | DCBLD2 | 63 | 1 | 9.251701 | -0.987560 | 186 | 90.79 | 0.488118 |
| GO:0051348\_negative\_regulation\_of\_transferase\_activity | CAV1 | 63 | 1 | 9.251701 | -0.987560 | 186 | 90.79 | 0.488118 |
| GO:0045926\_negative\_regulation\_of\_growth | DCBLD2 | 64 | 1 | 9.107143 | -0.981062 | 187 | 91.87 | 0.491283 |
| GO:0045596\_negative\_regulation\_of\_cell\_differentiation | CAV1 | 65 | 1 | 8.967033 | -0.974671 | 189 | 92.77 | 0.490847 |
| GO:0051235\_maintenance\_of\_location | CAV1 | 65 | 1 | 8.967033 | -0.974671 | 189 | 92.77 | 0.490847 |
| GO:0014706\_striated\_muscle\_tissue\_development | CAV1 | 66 | 1 | 8.831169 | -0.968383 | 191 | 94.22 | 0.493298 |
| GO:0052547\_regulation\_of\_peptidase\_activity | CAV1 | 66 | 1 | 8.831169 | -0.968383 | 191 | 94.22 | 0.493298 |
| GO:0060537\_muscle\_tissue\_development | CAV1 | 67 | 1 | 8.699360 | -0.962194 | 192 | 94.75 | 0.493490 |
| GO:0009991\_response\_to\_extracellular\_stimulus | CAV1 | 68 | 1 | 8.571429 | -0.956101 | 193 | 95.57 | 0.495181 |
| GO:0051179\_localization | ACTN1 | 1561 | 5 | 1.866935 | -0.952056 | 194 | 95.76 | 0.493608 |
| GO:0051179\_localization | TNFRSF12A | 1561 | 5 | 1.866935 | -0.952056 | 194 | 95.76 | 0.493608 |
| GO:0051179\_localization | CAV1 | 1561 | 5 | 1.866935 | -0.952056 | 194 | 95.76 | 0.493608 |
| GO:0051179\_localization | ADAM9 | 1561 | 5 | 1.866935 | -0.952056 | 194 | 95.76 | 0.493608 |
| GO:0051179\_localization | MYO1E | 1561 | 5 | 1.866935 | -0.952056 | 194 | 95.76 | 0.493608 |
| GO:0051241\_negative\_regulation\_of\_multicellular\_organismal\_process | CAV1 | 69 | 1 | 8.447205 | -0.950103 | 195 | 96.38 | 0.494256 |
| GO:0008104\_protein\_localization | CAV1 | 339 | 2 | 3.438685 | -0.947851 | 196 | 96.52 | 0.492449 |
| GO:0008104\_protein\_localization | ADAM9 | 339 | 2 | 3.438685 | -0.947851 | 196 | 96.52 | 0.492449 |
| GO:0050789\_regulation\_of\_biological\_process | ACTN1 | 3649 | 9 | 1.437576 | -0.941511 | 197 | 97.68 | 0.495838 |
| GO:0050789\_regulation\_of\_biological\_process | DCBLD2 | 3649 | 9 | 1.437576 | -0.941511 | 197 | 97.68 | 0.495838 |
| GO:0050789\_regulation\_of\_biological\_process | CAV1 | 3649 | 9 | 1.437576 | -0.941511 | 197 | 97.68 | 0.495838 |
| GO:0050789\_regulation\_of\_biological\_process | ADAM9 | 3649 | 9 | 1.437576 | -0.941511 | 197 | 97.68 | 0.495838 |
| GO:0050789\_regulation\_of\_biological\_process | FOSL1 | 3649 | 9 | 1.437576 | -0.941511 | 197 | 97.68 | 0.495838 |
| GO:0050789\_regulation\_of\_biological\_process | RRAS | 3649 | 9 | 1.437576 | -0.941511 | 197 | 97.68 | 0.495838 |
| GO:0050789\_regulation\_of\_biological\_process | S100A10 | 3649 | 9 | 1.437576 | -0.941511 | 197 | 97.68 | 0.495838 |
| GO:0050789\_regulation\_of\_biological\_process | ARHGAP29 | 3649 | 9 | 1.437576 | -0.941511 | 197 | 97.68 | 0.495838 |
| GO:0050789\_regulation\_of\_biological\_process | HRH1 | 3649 | 9 | 1.437576 | -0.941511 | 197 | 97.68 | 0.495838 |
| GO:0009615\_response\_to\_virus | FOSL1 | 71 | 1 | 8.209256 | -0.938377 | 200 | 98.28 | 0.491400 |
| GO:0032101\_regulation\_of\_response\_to\_external\_stimulus | CAV1 | 71 | 1 | 8.209256 | -0.938377 | 200 | 98.28 | 0.491400 |
| GO:0051223\_regulation\_of\_protein\_transport | ADAM9 | 71 | 1 | 8.209256 | -0.938377 | 200 | 98.28 | 0.491400 |
| GO:0007179\_transforming\_growth\_factor\_beta\_receptor\_signaling\_pathway | CAV1 | 72 | 1 | 8.095238 | -0.932645 | 201 | 98.68 | 0.490945 |
| GO:0007596\_blood\_coagulation | CAV1 | 73 | 1 | 7.984344 | -0.926996 | 202 | 99.65 | 0.493317 |
| GO:0050817\_coagulation | CAV1 | 74 | 1 | 7.876448 | -0.921428 | 203 | 100.1 | 0.493103 |
| GO:0006816\_calcium\_ion\_transport | CAV1 | 75 | 1 | 7.771429 | -0.915940 | 207 | 101.32 | 0.489469 |
| GO:0006869\_lipid\_transport | CAV1 | 75 | 1 | 7.771429 | -0.915940 | 207 | 101.32 | 0.489469 |
| GO:0044271\_nitrogen\_compound\_biosynthetic\_process | CAV1 | 75 | 1 | 7.771429 | -0.915940 | 207 | 101.32 | 0.489469 |
| GO:0070201\_regulation\_of\_establishment\_of\_protein\_localization | ADAM9 | 75 | 1 | 7.771429 | -0.915940 | 207 | 101.32 | 0.489469 |
| GO:0006979\_response\_to\_oxidative\_stress | ADAM9 | 76 | 1 | 7.669173 | -0.910529 | 209 | 102.14 | 0.488708 |
| GO:0070838\_divalent\_metal\_ion\_transport | CAV1 | 76 | 1 | 7.669173 | -0.910529 | 209 | 102.14 | 0.488708 |
| GO:0007599\_hemostasis | CAV1 | 79 | 1 | 7.377939 | -0.894739 | 211 | 104.15 | 0.493602 |
| GO:0032880\_regulation\_of\_protein\_localization | ADAM9 | 79 | 1 | 7.377939 | -0.894739 | 211 | 104.15 | 0.493602 |
| GO:0007204\_elevation\_of\_cytosolic\_calcium\_ion\_concentration | CAV1 | 80 | 1 | 7.285714 | -0.889617 | 212 | 105.23 | 0.496368 |
| GO:0006952\_defense\_response | FOSL1 | 369 | 2 | 3.159117 | -0.886688 | 213 | 105.34 | 0.494554 |
| GO:0006952\_defense\_response | HRH1 | 369 | 2 | 3.159117 | -0.886688 | 213 | 105.34 | 0.494554 |
| GO:0019318\_hexose\_metabolic\_process | PKM2 | 81 | 1 | 7.195767 | -0.884563 | 215 | 106.46 | 0.495163 |
| GO:0051480\_cytosolic\_calcium\_ion\_homeostasis | CAV1 | 81 | 1 | 7.195767 | -0.884563 | 215 | 106.46 | 0.495163 |
| GO:0048514\_blood\_vessel\_morphogenesis | CAV1 | 82 | 1 | 7.108014 | -0.879575 | 216 | 107.02 | 0.495463 |
| GO:0051259\_protein\_oligomerization | CAV1 | 83 | 1 | 7.022375 | -0.874652 | 217 | 107.41 | 0.494977 |
| GO:0007243\_protein\_kinase\_cascade | CAV1 | 377 | 2 | 3.092080 | -0.871388 | 218 | 107.63 | 0.493716 |
| GO:0007243\_protein\_kinase\_cascade | ADAM9 | 377 | 2 | 3.092080 | -0.871388 | 218 | 107.63 | 0.493716 |
| GO:0015674\_di-\_\_tri-valent\_inorganic\_cation\_transport | CAV1 | 84 | 1 | 6.938776 | -0.869792 | 219 | 108.08 | 0.493516 |
| GO:0001568\_blood\_vessel\_development | CAV1 | 85 | 1 | 6.857143 | -0.864993 | 220 | 108.63 | 0.493773 |
| GO:0033036\_macromolecule\_localization | CAV1 | 388 | 2 | 3.004418 | -0.850986 | 221 | 109.56 | 0.495747 |
| GO:0033036\_macromolecule\_localization | ADAM9 | 388 | 2 | 3.004418 | -0.850986 | 221 | 109.56 | 0.495747 |
| GO:0001944\_vasculature\_development | CAV1 | 88 | 1 | 6.623377 | -0.850951 | 222 | 109.69 | 0.494099 |
| GO:0030334\_regulation\_of\_cell\_migration | ADAM9 | 89 | 1 | 6.548957 | -0.846384 | 225 | 110.22 | 0.489867 |
| GO:0043405\_regulation\_of\_MAP\_kinase\_activity | CAV1 | 89 | 1 | 6.548957 | -0.846384 | 225 | 110.22 | 0.489867 |
| GO:0051240\_positive\_regulation\_of\_multicellular\_organismal\_process | CAV1 | 89 | 1 | 6.548957 | -0.846384 | 225 | 110.22 | 0.489867 |
| GO:0051046\_regulation\_of\_secretion | ADAM9 | 91 | 1 | 6.405024 | -0.837413 | 227 | 111.34 | 0.490485 |
| GO:0051130\_positive\_regulation\_of\_cellular\_component\_organization | ADAM9 | 91 | 1 | 6.405024 | -0.837413 | 227 | 111.34 | 0.490485 |
| GO:0007178\_transmembrane\_receptor\_protein\_serine\_threonine\_kinase\_signaling\_pathway | CAV1 | 92 | 1 | 6.335404 | -0.833007 | 228 | 111.81 | 0.490395 |
| GO:0050878\_regulation\_of\_body\_fluid\_levels | CAV1 | 95 | 1 | 6.135338 | -0.820092 | 229 | 112.62 | 0.491790 |
| GO:0040012\_regulation\_of\_locomotion | ADAM9 | 96 | 1 | 6.071429 | -0.815884 | 230 | 113.52 | 0.493565 |
| GO:0046486\_glycerolipid\_metabolic\_process | CAV1 | 97 | 1 | 6.008837 | -0.811724 | 231 | 113.94 | 0.493247 |
| GO:0042127\_regulation\_of\_cell\_proliferation | CAV1 | 411 | 2 | 2.836288 | -0.810534 | 232 | 114.05 | 0.491595 |
| GO:0042127\_regulation\_of\_cell\_proliferation | FOSL1 | 411 | 2 | 2.836288 | -0.810534 | 232 | 114.05 | 0.491595 |
| GO:0009968\_negative\_regulation\_of\_signal\_transduction | CAV1 | 99 | 1 | 5.887446 | -0.803540 | 233 | 115.44 | 0.495451 |
| GO:0010648\_negative\_regulation\_of\_cell\_communication | CAV1 | 102 | 1 | 5.714286 | -0.791594 | 234 | 116.81 | 0.499188 |
| GO:0008544\_epidermis\_development | ADAM9 | 104 | 1 | 5.604396 | -0.783840 | 235 | 117.66 | 0.500681 |
| GO:0044057\_regulation\_of\_system\_process | CAV1 | 106 | 1 | 5.498652 | -0.776246 | 236 | 118.34 | 0.501441 |
| GO:0050896\_response\_to\_stimulus | DCBLD2 | 1775 | 5 | 1.641851 | -0.769198 | 237 | 118.6 | 0.500422 |
| GO:0050896\_response\_to\_stimulus | CAV1 | 1775 | 5 | 1.641851 | -0.769198 | 237 | 118.6 | 0.500422 |
| GO:0050896\_response\_to\_stimulus | ADAM9 | 1775 | 5 | 1.641851 | -0.769198 | 237 | 118.6 | 0.500422 |
| GO:0050896\_response\_to\_stimulus | FOSL1 | 1775 | 5 | 1.641851 | -0.769198 | 237 | 118.6 | 0.500422 |
| GO:0050896\_response\_to\_stimulus | HRH1 | 1775 | 5 | 1.641851 | -0.769198 | 237 | 118.6 | 0.500422 |
| GO:0001558\_regulation\_of\_cell\_growth | DCBLD2 | 110 | 1 | 5.298701 | -0.761516 | 238 | 120.7 | 0.507143 |
| GO:0048646\_anatomical\_structure\_formation\_involved\_in\_morphogenesis | ADAM9 | 111 | 1 | 5.250965 | -0.757925 | 239 | 121.1 | 0.506695 |
| GO:0007398\_ectoderm\_development | ADAM9 | 112 | 1 | 5.204082 | -0.754369 | 240 | 121.82 | 0.507583 |
| GO:0006631\_fatty\_acid\_metabolic\_process | CAV1 | 113 | 1 | 5.158028 | -0.750847 | 241 | 122.27 | 0.507344 |
| GO:0006874\_cellular\_calcium\_ion\_homeostasis | CAV1 | 114 | 1 | 5.112782 | -0.747359 | 242 | 122.6 | 0.506612 |
| GO:0005996\_monosaccharide\_metabolic\_process | PKM2 | 115 | 1 | 5.068323 | -0.743905 | 243 | 123.39 | 0.507778 |
| GO:0055074\_calcium\_ion\_homeostasis | CAV1 | 116 | 1 | 5.024631 | -0.740484 | 244 | 124.0 | 0.508197 |
| GO:0065007\_biological\_regulation | ACTN1 | 3971 | 9 | 1.321006 | -0.736182 | 245 | 124.36 | 0.507592 |
| GO:0065007\_biological\_regulation | DCBLD2 | 3971 | 9 | 1.321006 | -0.736182 | 245 | 124.36 | 0.507592 |
| GO:0065007\_biological\_regulation | CAV1 | 3971 | 9 | 1.321006 | -0.736182 | 245 | 124.36 | 0.507592 |
| GO:0065007\_biological\_regulation | ADAM9 | 3971 | 9 | 1.321006 | -0.736182 | 245 | 124.36 | 0.507592 |
| GO:0065007\_biological\_regulation | FOSL1 | 3971 | 9 | 1.321006 | -0.736182 | 245 | 124.36 | 0.507592 |
| GO:0065007\_biological\_regulation | RRAS | 3971 | 9 | 1.321006 | -0.736182 | 245 | 124.36 | 0.507592 |
| GO:0065007\_biological\_regulation | S100A10 | 3971 | 9 | 1.321006 | -0.736182 | 245 | 124.36 | 0.507592 |
| GO:0065007\_biological\_regulation | ARHGAP29 | 3971 | 9 | 1.321006 | -0.736182 | 245 | 124.36 | 0.507592 |
| GO:0065007\_biological\_regulation | HRH1 | 3971 | 9 | 1.321006 | -0.736182 | 245 | 124.36 | 0.507592 |
| GO:0006875\_cellular\_metal\_ion\_homeostasis | CAV1 | 121 | 1 | 4.817001 | -0.723849 | 246 | 125.81 | 0.511423 |
| GO:0033674\_positive\_regulation\_of\_kinase\_activity | ADAM9 | 122 | 1 | 4.777518 | -0.720613 | 249 | 126.92 | 0.509719 |
| GO:0045860\_positive\_regulation\_of\_protein\_kinase\_activity | ADAM9 | 122 | 1 | 4.777518 | -0.720613 | 249 | 126.92 | 0.509719 |
| GO:0060341\_regulation\_of\_cellular\_localization | ADAM9 | 122 | 1 | 4.777518 | -0.720613 | 249 | 126.92 | 0.509719 |
| GO:0007517\_muscle\_organ\_development | CAV1 | 123 | 1 | 4.738676 | -0.717406 | 250 | 127.12 | 0.508480 |
| GO:0006897\_endocytosis | CAV1 | 124 | 1 | 4.700461 | -0.714227 | 252 | 128.01 | 0.507976 |
| GO:0010324\_membrane\_invagination | CAV1 | 124 | 1 | 4.700461 | -0.714227 | 252 | 128.01 | 0.507976 |
| GO:0006935\_chemotaxis | FOSL1 | 125 | 1 | 4.662857 | -0.711077 | 256 | 128.94 | 0.503672 |
| GO:0042330\_taxis | FOSL1 | 125 | 1 | 4.662857 | -0.711077 | 256 | 128.94 | 0.503672 |
| GO:0051707\_response\_to\_other\_organism | FOSL1 | 125 | 1 | 4.662857 | -0.711077 | 256 | 128.94 | 0.503672 |
| GO:0055065\_metal\_ion\_homeostasis | CAV1 | 125 | 1 | 4.662857 | -0.711077 | 256 | 128.94 | 0.503672 |
| GO:0022607\_cellular\_component\_assembly | ACTN1 | 478 | 2 | 2.438733 | -0.707196 | 257 | 129.76 | 0.504903 |
| GO:0022607\_cellular\_component\_assembly | CAV1 | 478 | 2 | 2.438733 | -0.707196 | 257 | 129.76 | 0.504903 |
| GO:0007275\_multicellular\_organismal\_development | TNFRSF12A | 1372 | 4 | 1.699292 | -0.703358 | 258 | 130.16 | 0.504496 |
| GO:0007275\_multicellular\_organismal\_development | CAV1 | 1372 | 4 | 1.699292 | -0.703358 | 258 | 130.16 | 0.504496 |
| GO:0007275\_multicellular\_organismal\_development | ADAM9 | 1372 | 4 | 1.699292 | -0.703358 | 258 | 130.16 | 0.504496 |
| GO:0007275\_multicellular\_organismal\_development | ANXA2 | 1372 | 4 | 1.699292 | -0.703358 | 258 | 130.16 | 0.504496 |
| GO:0009725\_response\_to\_hormone\_stimulus | CAV1 | 129 | 1 | 4.518272 | -0.698749 | 260 | 130.95 | 0.503654 |
| GO:0051347\_positive\_regulation\_of\_transferase\_activity | ADAM9 | 129 | 1 | 4.518272 | -0.698749 | 260 | 130.95 | 0.503654 |
| GO:0040008\_regulation\_of\_growth | DCBLD2 | 131 | 1 | 4.449291 | -0.692743 | 262 | 132.41 | 0.505382 |
| GO:0051345\_positive\_regulation\_of\_hydrolase\_activity | HRH1 | 131 | 1 | 4.449291 | -0.692743 | 262 | 132.41 | 0.505382 |
| GO:0003013\_circulatory\_system\_process | CAV1 | 133 | 1 | 4.382385 | -0.686837 | 264 | 133.29 | 0.504886 |
| GO:0008015\_blood\_circulation | CAV1 | 133 | 1 | 4.382385 | -0.686837 | 264 | 133.29 | 0.504886 |
| GO:0048523\_negative\_regulation\_of\_cellular\_process | ACTN1 | 925 | 3 | 1.890347 | -0.685750 | 265 | 133.41 | 0.503434 |
| GO:0048523\_negative\_regulation\_of\_cellular\_process | DCBLD2 | 925 | 3 | 1.890347 | -0.685750 | 265 | 133.41 | 0.503434 |
| GO:0048523\_negative\_regulation\_of\_cellular\_process | CAV1 | 925 | 3 | 1.890347 | -0.685750 | 265 | 133.41 | 0.503434 |
| GO:0006936\_muscle\_contraction | CAV1 | 134 | 1 | 4.349680 | -0.683922 | 266 | 134.4 | 0.505263 |
| GO:0009719\_response\_to\_endogenous\_stimulus | CAV1 | 135 | 1 | 4.317460 | -0.681030 | 268 | 135.32 | 0.504925 |
| GO:0034641\_cellular\_nitrogen\_compound\_metabolic\_process | CAV1 | 135 | 1 | 4.317460 | -0.681030 | 268 | 135.32 | 0.504925 |
| GO:0009653\_anatomical\_structure\_morphogenesis | CAV1 | 500 | 2 | 2.331429 | -0.677234 | 269 | 135.92 | 0.505279 |
| GO:0009653\_anatomical\_structure\_morphogenesis | ADAM9 | 500 | 2 | 2.331429 | -0.677234 | 269 | 135.92 | 0.505279 |
| GO:0030154\_cell\_differentiation | CAV1 | 506 | 2 | 2.303783 | -0.669359 | 270 | 136.99 | 0.507370 |
| GO:0030154\_cell\_differentiation | ADAM9 | 506 | 2 | 2.303783 | -0.669359 | 270 | 136.99 | 0.507370 |
| GO:0030005\_cellular\_di-\_\_tri-valent\_inorganic\_cation\_homeostasis | CAV1 | 140 | 1 | 4.163265 | -0.666922 | 271 | 137.47 | 0.507269 |
| GO:0032502\_developmental\_process | ACTN1 | 1919 | 5 | 1.518648 | -0.665221 | 272 | 137.61 | 0.505919 |
| GO:0032502\_developmental\_process | TNFRSF12A | 1919 | 5 | 1.518648 | -0.665221 | 272 | 137.61 | 0.505919 |
| GO:0032502\_developmental\_process | CAV1 | 1919 | 5 | 1.518648 | -0.665221 | 272 | 137.61 | 0.505919 |
| GO:0032502\_developmental\_process | ADAM9 | 1919 | 5 | 1.518648 | -0.665221 | 272 | 137.61 | 0.505919 |
| GO:0032502\_developmental\_process | ANXA2 | 1919 | 5 | 1.518648 | -0.665221 | 272 | 137.61 | 0.505919 |
| GO:0003012\_muscle\_system\_process | CAV1 | 141 | 1 | 4.133739 | -0.664168 | 274 | 138.19 | 0.504343 |
| GO:0016049\_cell\_growth | DCBLD2 | 141 | 1 | 4.133739 | -0.664168 | 274 | 138.19 | 0.504343 |
| GO:0001501\_skeletal\_system\_development | ANXA2 | 142 | 1 | 4.104628 | -0.661435 | 276 | 138.75 | 0.502717 |
| GO:0007626\_locomotory\_behavior | FOSL1 | 142 | 1 | 4.104628 | -0.661435 | 276 | 138.75 | 0.502717 |
| GO:0055066\_di-\_\_tri-valent\_inorganic\_cation\_homeostasis | CAV1 | 145 | 1 | 4.019704 | -0.653366 | 277 | 140.7 | 0.507942 |
| GO:0080134\_regulation\_of\_response\_to\_stress | CAV1 | 147 | 1 | 3.965015 | -0.648090 | 278 | 142.38 | 0.512158 |
| GO:0008361\_regulation\_of\_cell\_size | DCBLD2 | 149 | 1 | 3.911793 | -0.642894 | 279 | 143.17 | 0.513154 |
| GO:0045321\_leukocyte\_activation | ADAM9 | 150 | 1 | 3.885714 | -0.640326 | 280 | 143.36 | 0.512000 |
| GO:0019932\_second-messenger-mediated\_signaling | HRH1 | 153 | 1 | 3.809524 | -0.632734 | 281 | 144.89 | 0.515623 |
| GO:0032940\_secretion\_by\_cell | ADAM9 | 155 | 1 | 3.760369 | -0.627766 | 282 | 145.43 | 0.515709 |
| GO:0016337\_cell-cell\_adhesion | ADAM9 | 156 | 1 | 3.736264 | -0.625309 | 284 | 145.85 | 0.513556 |
| GO:0032787\_monocarboxylic\_acid\_metabolic\_process | CAV1 | 156 | 1 | 3.736264 | -0.625309 | 284 | 145.85 | 0.513556 |
| GO:0030003\_cellular\_cation\_homeostasis | CAV1 | 161 | 1 | 3.620231 | -0.613287 | 285 | 146.93 | 0.515544 |
| GO:0009987\_cellular\_process | PKM2 | 6671 | 13 | 1.135833 | -0.610019 | 286 | 147.08 | 0.514266 |
| GO:0009987\_cellular\_process | FOSL1 | 6671 | 13 | 1.135833 | -0.610019 | 286 | 147.08 | 0.514266 |
| GO:0009987\_cellular\_process | CD151 | 6671 | 13 | 1.135833 | -0.610019 | 286 | 147.08 | 0.514266 |
| GO:0009987\_cellular\_process | ARHGAP29 | 6671 | 13 | 1.135833 | -0.610019 | 286 | 147.08 | 0.514266 |
| GO:0009987\_cellular\_process | MYO1E | 6671 | 13 | 1.135833 | -0.610019 | 286 | 147.08 | 0.514266 |
| GO:0009987\_cellular\_process | ACTN1 | 6671 | 13 | 1.135833 | -0.610019 | 286 | 147.08 | 0.514266 |
| GO:0009987\_cellular\_process | TNFRSF12A | 6671 | 13 | 1.135833 | -0.610019 | 286 | 147.08 | 0.514266 |
| GO:0009987\_cellular\_process | DCBLD2 | 6671 | 13 | 1.135833 | -0.610019 | 286 | 147.08 | 0.514266 |
| GO:0009987\_cellular\_process | CAV1 | 6671 | 13 | 1.135833 | -0.610019 | 286 | 147.08 | 0.514266 |
| GO:0009987\_cellular\_process | ADAM9 | 6671 | 13 | 1.135833 | -0.610019 | 286 | 147.08 | 0.514266 |
| GO:0009987\_cellular\_process | RRAS | 6671 | 13 | 1.135833 | -0.610019 | 286 | 147.08 | 0.514266 |
| GO:0009987\_cellular\_process | S100A10 | 6671 | 13 | 1.135833 | -0.610019 | 286 | 147.08 | 0.514266 |
| GO:0009987\_cellular\_process | HRH1 | 6671 | 13 | 1.135833 | -0.610019 | 286 | 147.08 | 0.514266 |
| GO:0048522\_positive\_regulation\_of\_cellular\_process | CAV1 | 1009 | 3 | 1.732975 | -0.609766 | 287 | 147.3 | 0.513240 |
| GO:0048522\_positive\_regulation\_of\_cellular\_process | ADAM9 | 1009 | 3 | 1.732975 | -0.609766 | 287 | 147.3 | 0.513240 |
| GO:0048522\_positive\_regulation\_of\_cellular\_process | FOSL1 | 1009 | 3 | 1.732975 | -0.609766 | 287 | 147.3 | 0.513240 |
| GO:0048869\_cellular\_developmental\_process | CAV1 | 555 | 2 | 2.100386 | -0.609351 | 288 | 147.49 | 0.512118 |
| GO:0048869\_cellular\_developmental\_process | ADAM9 | 555 | 2 | 2.100386 | -0.609351 | 288 | 147.49 | 0.512118 |
| GO:0006091\_generation\_of\_precursor\_metabolites\_and\_energy | PKM2 | 163 | 1 | 3.575811 | -0.608596 | 289 | 147.8 | 0.511419 |
| GO:0048519\_negative\_regulation\_of\_biological\_process | ACTN1 | 1013 | 3 | 1.726132 | -0.606385 | 290 | 147.95 | 0.510172 |
| GO:0048519\_negative\_regulation\_of\_biological\_process | DCBLD2 | 1013 | 3 | 1.726132 | -0.606385 | 290 | 147.95 | 0.510172 |
| GO:0048519\_negative\_regulation\_of\_biological\_process | CAV1 | 1013 | 3 | 1.726132 | -0.606385 | 290 | 147.95 | 0.510172 |
| GO:0030029\_actin\_filament-based\_process | MYO1E | 165 | 1 | 3.532468 | -0.603971 | 291 | 148.59 | 0.510619 |
| GO:0044085\_cellular\_component\_biogenesis | ACTN1 | 560 | 2 | 2.081633 | -0.603627 | 292 | 149.04 | 0.510411 |
| GO:0044085\_cellular\_component\_biogenesis | CAV1 | 560 | 2 | 2.081633 | -0.603627 | 292 | 149.04 | 0.510411 |
| GO:0045595\_regulation\_of\_cell\_differentiation | CAV1 | 170 | 1 | 3.428571 | -0.592681 | 293 | 150.23 | 0.512730 |
| GO:0012501\_programmed\_cell\_death | ACTN1 | 571 | 2 | 2.041531 | -0.591273 | 294 | 150.35 | 0.511395 |
| GO:0012501\_programmed\_cell\_death | PKM2 | 571 | 2 | 2.041531 | -0.591273 | 294 | 150.35 | 0.511395 |
| GO:0051649\_establishment\_of\_localization\_in\_cell | ADAM9 | 573 | 2 | 2.034405 | -0.589062 | 295 | 150.5 | 0.510169 |
| GO:0051649\_establishment\_of\_localization\_in\_cell | MYO1E | 573 | 2 | 2.034405 | -0.589062 | 295 | 150.5 | 0.510169 |
| GO:0055080\_cation\_homeostasis | CAV1 | 173 | 1 | 3.369116 | -0.586089 | 296 | 151.25 | 0.510980 |
| GO:0040007\_growth | DCBLD2 | 174 | 1 | 3.349754 | -0.583921 | 297 | 151.47 | 0.510000 |
| GO:0001775\_cell\_activation | ADAM9 | 175 | 1 | 3.330612 | -0.581766 | 298 | 152.03 | 0.510168 |
| GO:0009607\_response\_to\_biotic\_stimulus | FOSL1 | 177 | 1 | 3.292978 | -0.577500 | 300 | 153.05 | 0.510167 |
| GO:0016477\_cell\_migration | ADAM9 | 177 | 1 | 3.292978 | -0.577500 | 300 | 153.05 | 0.510167 |
| GO:0008219\_cell\_death | ACTN1 | 585 | 2 | 1.992674 | -0.576011 | 302 | 153.29 | 0.507583 |
| GO:0008219\_cell\_death | PKM2 | 585 | 2 | 1.992674 | -0.576011 | 302 | 153.29 | 0.507583 |
| GO:0016265\_death | ACTN1 | 585 | 2 | 1.992674 | -0.576011 | 302 | 153.29 | 0.507583 |
| GO:0016265\_death | PKM2 | 585 | 2 | 1.992674 | -0.576011 | 302 | 153.29 | 0.507583 |
| GO:0006954\_inflammatory\_response | HRH1 | 182 | 1 | 3.202512 | -0.567074 | 303 | 154.74 | 0.510693 |
| GO:0010627\_regulation\_of\_protein\_kinase\_cascade | CAV1 | 184 | 1 | 3.167702 | -0.562996 | 304 | 155.42 | 0.511250 |
| GO:0016310\_phosphorylation | CAV1 | 601 | 2 | 1.939624 | -0.559171 | 305 | 156.55 | 0.513279 |
| GO:0016310\_phosphorylation | ADAM9 | 601 | 2 | 1.939624 | -0.559171 | 305 | 156.55 | 0.513279 |
| GO:0030001\_metal\_ion\_transport | CAV1 | 186 | 1 | 3.133641 | -0.558969 | 306 | 157.03 | 0.513170 |
| GO:0048518\_positive\_regulation\_of\_biological\_process | CAV1 | 1094 | 3 | 1.598329 | -0.542080 | 307 | 159.33 | 0.518990 |
| GO:0048518\_positive\_regulation\_of\_biological\_process | ADAM9 | 1094 | 3 | 1.598329 | -0.542080 | 307 | 159.33 | 0.518990 |
| GO:0048518\_positive\_regulation\_of\_biological\_process | FOSL1 | 1094 | 3 | 1.598329 | -0.542080 | 307 | 159.33 | 0.518990 |
| GO:0043086\_negative\_regulation\_of\_catalytic\_activity | CAV1 | 196 | 1 | 2.973761 | -0.539561 | 308 | 160.2 | 0.520130 |
| GO:0048870\_cell\_motility | ADAM9 | 197 | 1 | 2.958666 | -0.537684 | 309 | 160.44 | 0.519223 |
| GO:0008284\_positive\_regulation\_of\_cell\_proliferation | FOSL1 | 200 | 1 | 2.914286 | -0.532119 | 310 | 160.73 | 0.518484 |
| GO:0008285\_negative\_regulation\_of\_cell\_proliferation | CAV1 | 202 | 1 | 2.885431 | -0.528464 | 311 | 161.52 | 0.519357 |
| GO:0009056\_catabolic\_process | ADAM9 | 633 | 2 | 1.841571 | -0.527287 | 312 | 161.71 | 0.518301 |
| GO:0009056\_catabolic\_process | PKM2 | 633 | 2 | 1.841571 | -0.527287 | 312 | 161.71 | 0.518301 |
| GO:0006066\_alcohol\_metabolic\_process | PKM2 | 206 | 1 | 2.829404 | -0.521279 | 314 | 162.8 | 0.518471 |
| GO:0006873\_cellular\_ion\_homeostasis | CAV1 | 206 | 1 | 2.829404 | -0.521279 | 314 | 162.8 | 0.518471 |
| GO:0055082\_cellular\_chemical\_homeostasis | CAV1 | 208 | 1 | 2.802198 | -0.517747 | 315 | 163.88 | 0.520254 |
| GO:0008283\_cell\_proliferation | CAV1 | 647 | 2 | 1.801722 | -0.514040 | 316 | 164.85 | 0.521677 |
| GO:0008283\_cell\_proliferation | FOSL1 | 647 | 2 | 1.801722 | -0.514040 | 316 | 164.85 | 0.521677 |
| GO:0048731\_system\_development | CAV1 | 1140 | 3 | 1.533835 | -0.508803 | 317 | 166.44 | 0.525047 |
| GO:0048731\_system\_development | ADAM9 | 1140 | 3 | 1.533835 | -0.508803 | 317 | 166.44 | 0.525047 |
| GO:0048731\_system\_development | ANXA2 | 1140 | 3 | 1.533835 | -0.508803 | 317 | 166.44 | 0.525047 |
| GO:0007610\_behavior | FOSL1 | 214 | 1 | 2.723632 | -0.507390 | 318 | 166.71 | 0.524245 |
| GO:0046903\_secretion | ADAM9 | 218 | 1 | 2.673657 | -0.500674 | 319 | 167.5 | 0.525078 |
| GO:0050801\_ion\_homeostasis | CAV1 | 221 | 1 | 2.637363 | -0.495733 | 320 | 167.84 | 0.524500 |
| GO:0019725\_cellular\_homeostasis | CAV1 | 231 | 1 | 2.523191 | -0.479825 | 322 | 170.58 | 0.529752 |
| GO:0051704\_multi-organism\_process | FOSL1 | 231 | 1 | 2.523191 | -0.479825 | 322 | 170.58 | 0.529752 |
| GO:0044092\_negative\_regulation\_of\_molecular\_function | CAV1 | 233 | 1 | 2.501533 | -0.476743 | 323 | 171.09 | 0.529690 |
| GO:0051128\_regulation\_of\_cellular\_component\_organization | ADAM9 | 237 | 1 | 2.459313 | -0.470672 | 324 | 171.66 | 0.529815 |
| GO:0006793\_phosphorus\_metabolic\_process | CAV1 | 697 | 2 | 1.672474 | -0.469890 | 326 | 172.22 | 0.528282 |
| GO:0006793\_phosphorus\_metabolic\_process | ADAM9 | 697 | 2 | 1.672474 | -0.469890 | 326 | 172.22 | 0.528282 |
| GO:0006796\_phosphate\_metabolic\_process | CAV1 | 697 | 2 | 1.672474 | -0.469890 | 326 | 172.22 | 0.528282 |
| GO:0006796\_phosphate\_metabolic\_process | ADAM9 | 697 | 2 | 1.672474 | -0.469890 | 326 | 172.22 | 0.528282 |
| GO:0048583\_regulation\_of\_response\_to\_stimulus | CAV1 | 241 | 1 | 2.418494 | -0.464724 | 327 | 172.8 | 0.528440 |
| GO:0006812\_cation\_transport | CAV1 | 246 | 1 | 2.369338 | -0.457456 | 329 | 175.04 | 0.532036 |
| GO:0009887\_organ\_morphogenesis | CAV1 | 246 | 1 | 2.369338 | -0.457456 | 329 | 175.04 | 0.532036 |
| GO:0006810\_transport | CAV1 | 1243 | 3 | 1.406735 | -0.441698 | 330 | 176.99 | 0.536333 |
| GO:0006810\_transport | ADAM9 | 1243 | 3 | 1.406735 | -0.441698 | 330 | 176.99 | 0.536333 |
| GO:0006810\_transport | MYO1E | 1243 | 3 | 1.406735 | -0.441698 | 330 | 176.99 | 0.536333 |
| GO:0007167\_enzyme\_linked\_receptor\_protein\_signaling\_pathway | CAV1 | 258 | 1 | 2.259136 | -0.440723 | 331 | 177.22 | 0.535408 |
| GO:0048513\_organ\_development | CAV1 | 741 | 2 | 1.573164 | -0.434702 | 332 | 177.92 | 0.535904 |
| GO:0048513\_organ\_development | ADAM9 | 741 | 2 | 1.573164 | -0.434702 | 332 | 177.92 | 0.535904 |
| GO:0051234\_establishment\_of\_localization | CAV1 | 1260 | 3 | 1.387755 | -0.431517 | 333 | 178.66 | 0.536517 |
| GO:0051234\_establishment\_of\_localization | ADAM9 | 1260 | 3 | 1.387755 | -0.431517 | 333 | 178.66 | 0.536517 |
| GO:0051234\_establishment\_of\_localization | MYO1E | 1260 | 3 | 1.387755 | -0.431517 | 333 | 178.66 | 0.536517 |
| GO:0006461\_protein\_complex\_assembly | CAV1 | 273 | 1 | 2.135008 | -0.421105 | 335 | 180.23 | 0.538000 |
| GO:0070271\_protein\_complex\_biogenesis | CAV1 | 273 | 1 | 2.135008 | -0.421105 | 335 | 180.23 | 0.538000 |
| GO:0015031\_protein\_transport | ADAM9 | 274 | 1 | 2.127216 | -0.419845 | 336 | 180.52 | 0.537262 |
| GO:0048878\_chemical\_homeostasis | CAV1 | 278 | 1 | 2.096608 | -0.414862 | 337 | 182.22 | 0.540712 |
| GO:0048856\_anatomical\_structure\_development | CAV1 | 1289 | 3 | 1.356533 | -0.414686 | 338 | 182.46 | 0.539822 |
| GO:0048856\_anatomical\_structure\_development | ADAM9 | 1289 | 3 | 1.356533 | -0.414686 | 338 | 182.46 | 0.539822 |
| GO:0048856\_anatomical\_structure\_development | ANXA2 | 1289 | 3 | 1.356533 | -0.414686 | 338 | 182.46 | 0.539822 |
| GO:0045184\_establishment\_of\_protein\_localization | ADAM9 | 279 | 1 | 2.089094 | -0.413630 | 339 | 182.99 | 0.539794 |
| GO:0019752\_carboxylic\_acid\_metabolic\_process | CAV1 | 286 | 1 | 2.037962 | -0.405158 | 341 | 184.12 | 0.539941 |
| GO:0043436\_oxoacid\_metabolic\_process | CAV1 | 286 | 1 | 2.037962 | -0.405158 | 341 | 184.12 | 0.539941 |
| GO:0006082\_organic\_acid\_metabolic\_process | CAV1 | 290 | 1 | 2.009852 | -0.400433 | 343 | 185.98 | 0.542216 |
| GO:0051093\_negative\_regulation\_of\_developmental\_process | CAV1 | 290 | 1 | 2.009852 | -0.400433 | 343 | 185.98 | 0.542216 |
| GO:0042180\_cellular\_ketone\_metabolic\_process | CAV1 | 291 | 1 | 2.002946 | -0.399265 | 344 | 186.22 | 0.541337 |
| GO:0005975\_carbohydrate\_metabolic\_process | PKM2 | 292 | 1 | 1.996086 | -0.398101 | 345 | 187.18 | 0.542551 |
| GO:0016192\_vesicle-mediated\_transport | CAV1 | 297 | 1 | 1.962482 | -0.392359 | 346 | 188.67 | 0.545289 |
| GO:0051172\_negative\_regulation\_of\_nitrogen\_compound\_metabolic\_process | CAV1 | 298 | 1 | 1.955896 | -0.391226 | 347 | 188.98 | 0.544611 |
| GO:0006508\_proteolysis | ADAM9 | 313 | 1 | 1.862163 | -0.374780 | 348 | 191.27 | 0.549626 |
| GO:0007166\_cell\_surface\_receptor\_linked\_signal\_transduction | CAV1 | 828 | 2 | 1.407867 | -0.373648 | 349 | 191.53 | 0.548797 |
| GO:0007166\_cell\_surface\_receptor\_linked\_signal\_transduction | HRH1 | 828 | 2 | 1.407867 | -0.373648 | 349 | 191.53 | 0.548797 |
| GO:0016043\_cellular\_component\_organization | ACTN1 | 1366 | 3 | 1.280067 | -0.373076 | 350 | 191.77 | 0.547914 |
| GO:0016043\_cellular\_component\_organization | CAV1 | 1366 | 3 | 1.280067 | -0.373076 | 350 | 191.77 | 0.547914 |
| GO:0016043\_cellular\_component\_organization | ADAM9 | 1366 | 3 | 1.280067 | -0.373076 | 350 | 191.77 | 0.547914 |
| GO:0006811\_ion\_transport | CAV1 | 317 | 1 | 1.838666 | -0.370565 | 351 | 192.65 | 0.548860 |
| GO:0065008\_regulation\_of\_biological\_quality | CAV1 | 848 | 2 | 1.374663 | -0.361011 | 352 | 194.3 | 0.551989 |
| GO:0065008\_regulation\_of\_biological\_quality | DCBLD2 | 848 | 2 | 1.374663 | -0.361011 | 352 | 194.3 | 0.551989 |
| GO:0030163\_protein\_catabolic\_process | ADAM9 | 330 | 1 | 1.766234 | -0.357325 | 353 | 194.86 | 0.552011 |
| GO:0031327\_negative\_regulation\_of\_cellular\_biosynthetic\_process | CAV1 | 332 | 1 | 1.755594 | -0.355348 | 354 | 195.36 | 0.551864 |
| GO:0009890\_negative\_regulation\_of\_biosynthetic\_process | CAV1 | 340 | 1 | 1.714286 | -0.347594 | 356 | 196.93 | 0.553174 |
| GO:0051094\_positive\_regulation\_of\_developmental\_process | ADAM9 | 340 | 1 | 1.714286 | -0.347594 | 356 | 196.93 | 0.553174 |
| GO:0033554\_cellular\_response\_to\_stress | CAV1 | 341 | 1 | 1.709258 | -0.346642 | 357 | 197.26 | 0.552549 |
| GO:0006357\_regulation\_of\_transcription\_from\_RNA\_polymerase\_II\_promoter | FOSL1 | 351 | 1 | 1.660562 | -0.337315 | 358 | 198.68 | 0.554972 |
| GO:0007186\_G-protein\_coupled\_receptor\_protein\_signaling\_pathway | HRH1 | 363 | 1 | 1.605667 | -0.326577 | 359 | 201.29 | 0.560696 |
| GO:0031323\_regulation\_of\_cellular\_metabolic\_process | CAV1 | 1466 | 3 | 1.192750 | -0.325045 | 360 | 201.53 | 0.559806 |
| GO:0031323\_regulation\_of\_cellular\_metabolic\_process | ADAM9 | 1466 | 3 | 1.192750 | -0.325045 | 360 | 201.53 | 0.559806 |
| GO:0031323\_regulation\_of\_cellular\_metabolic\_process | FOSL1 | 1466 | 3 | 1.192750 | -0.325045 | 360 | 201.53 | 0.559806 |
| GO:0065003\_macromolecular\_complex\_assembly | CAV1 | 366 | 1 | 1.592506 | -0.323967 | 361 | 202.59 | 0.561191 |
| GO:0009966\_regulation\_of\_signal\_transduction | CAV1 | 378 | 1 | 1.541950 | -0.313804 | 363 | 205.56 | 0.566281 |
| GO:0051239\_regulation\_of\_multicellular\_organismal\_process | CAV1 | 378 | 1 | 1.541950 | -0.313804 | 363 | 205.56 | 0.566281 |
| GO:0044255\_cellular\_lipid\_metabolic\_process | CAV1 | 381 | 1 | 1.529809 | -0.311332 | 364 | 205.84 | 0.565495 |
| GO:0032501\_multicellular\_organismal\_process | TNFRSF12A | 2082 | 4 | 1.119802 | -0.303823 | 365 | 206.32 | 0.565260 |
| GO:0032501\_multicellular\_organismal\_process | CAV1 | 2082 | 4 | 1.119802 | -0.303823 | 365 | 206.32 | 0.565260 |
| GO:0032501\_multicellular\_organismal\_process | ADAM9 | 2082 | 4 | 1.119802 | -0.303823 | 365 | 206.32 | 0.565260 |
| GO:0032501\_multicellular\_organismal\_process | ANXA2 | 2082 | 4 | 1.119802 | -0.303823 | 365 | 206.32 | 0.565260 |
| GO:0006468\_protein\_amino\_acid\_phosphorylation | ADAM9 | 393 | 1 | 1.483097 | -0.301700 | 366 | 206.62 | 0.564536 |
| GO:0042592\_homeostatic\_process | CAV1 | 397 | 1 | 1.468154 | -0.298578 | 367 | 207.59 | 0.565640 |
| GO:0019222\_regulation\_of\_metabolic\_process | CAV1 | 1538 | 3 | 1.136913 | -0.294175 | 368 | 208.11 | 0.565516 |
| GO:0019222\_regulation\_of\_metabolic\_process | ADAM9 | 1538 | 3 | 1.136913 | -0.294175 | 368 | 208.11 | 0.565516 |
| GO:0019222\_regulation\_of\_metabolic\_process | FOSL1 | 1538 | 3 | 1.136913 | -0.294175 | 368 | 208.11 | 0.565516 |
| GO:0031324\_negative\_regulation\_of\_cellular\_metabolic\_process | CAV1 | 404 | 1 | 1.442716 | -0.293218 | 369 | 208.44 | 0.564878 |
| GO:0046907\_intracellular\_transport | MYO1E | 420 | 1 | 1.387755 | -0.281434 | 370 | 210.45 | 0.568784 |
| GO:0010646\_regulation\_of\_cell\_communication | CAV1 | 423 | 1 | 1.377913 | -0.279294 | 371 | 210.82 | 0.568248 |
| GO:0043933\_macromolecular\_complex\_subunit\_organization | CAV1 | 424 | 1 | 1.374663 | -0.278586 | 372 | 211.12 | 0.567527 |
| GO:0043285\_biopolymer\_catabolic\_process | ADAM9 | 426 | 1 | 1.368209 | -0.277176 | 373 | 211.49 | 0.566997 |
| GO:0009057\_macromolecule\_catabolic\_process | ADAM9 | 439 | 1 | 1.327693 | -0.268232 | 374 | 213.29 | 0.570294 |
| GO:0009892\_negative\_regulation\_of\_metabolic\_process | CAV1 | 440 | 1 | 1.324675 | -0.267560 | 375 | 213.63 | 0.569680 |
| GO:0051171\_regulation\_of\_nitrogen\_compound\_metabolic\_process | CAV1 | 1055 | 2 | 1.104942 | -0.254171 | 376 | 216.37 | 0.575452 |
| GO:0051171\_regulation\_of\_nitrogen\_compound\_metabolic\_process | FOSL1 | 1055 | 2 | 1.104942 | -0.254171 | 376 | 216.37 | 0.575452 |
| GO:0006629\_lipid\_metabolic\_process | CAV1 | 468 | 1 | 1.245421 | -0.249578 | 377 | 217.38 | 0.576605 |
| GO:0042981\_regulation\_of\_apoptosis | ACTN1 | 471 | 1 | 1.237489 | -0.247743 | 378 | 218.72 | 0.578624 |
| GO:0051716\_cellular\_response\_to\_stimulus | CAV1 | 474 | 1 | 1.229656 | -0.245926 | 379 | 219.29 | 0.578602 |
| GO:0043067\_regulation\_of\_programmed\_cell\_death | ACTN1 | 476 | 1 | 1.224490 | -0.244724 | 380 | 220.02 | 0.579000 |
| GO:0010941\_regulation\_of\_cell\_death | ACTN1 | 478 | 1 | 1.219366 | -0.243529 | 381 | 220.73 | 0.579344 |
| GO:0006366\_transcription\_from\_RNA\_polymerase\_II\_promoter | FOSL1 | 506 | 1 | 1.151892 | -0.227537 | 382 | 223.11 | 0.584058 |
| GO:0031326\_regulation\_of\_cellular\_biosynthetic\_process | CAV1 | 1125 | 2 | 1.036190 | -0.225991 | 383 | 223.42 | 0.583342 |
| GO:0031326\_regulation\_of\_cellular\_biosynthetic\_process | FOSL1 | 1125 | 2 | 1.036190 | -0.225991 | 383 | 223.42 | 0.583342 |
| GO:0009889\_regulation\_of\_biosynthetic\_process | CAV1 | 1135 | 2 | 1.027061 | -0.222232 | 384 | 223.72 | 0.582604 |
| GO:0009889\_regulation\_of\_biosynthetic\_process | FOSL1 | 1135 | 2 | 1.027061 | -0.222232 | 384 | 223.72 | 0.582604 |
| GO:0006915\_apoptosis | ACTN1 | 565 | 1 | 1.031606 | -0.197844 | 385 | 227.04 | 0.589714 |
| GO:0080090\_regulation\_of\_primary\_metabolic\_process | CAV1 | 1311 | 2 | 0.889179 | -0.165374 | 386 | 233.05 | 0.603756 |
| GO:0080090\_regulation\_of\_primary\_metabolic\_process | FOSL1 | 1311 | 2 | 0.889179 | -0.165374 | 386 | 233.05 | 0.603756 |
| GO:0003008\_system\_process | CAV1 | 710 | 1 | 0.820926 | -0.142243 | 387 | 237.04 | 0.612506 |
| GO:0002376\_immune\_system\_process | ADAM9 | 718 | 1 | 0.811779 | -0.139735 | 388 | 237.75 | 0.612758 |
| GO:0006355\_regulation\_of\_transcription\_\_DNA-dependent | FOSL1 | 723 | 1 | 0.806165 | -0.138193 | 389 | 238.16 | 0.612237 |
| GO:0043687\_post-translational\_protein\_modification | ADAM9 | 728 | 1 | 0.800628 | -0.136669 | 390 | 238.53 | 0.611615 |
| GO:0051252\_regulation\_of\_RNA\_metabolic\_process | FOSL1 | 746 | 1 | 0.781310 | -0.131336 | 391 | 239.51 | 0.612558 |
| GO:0006996\_organelle\_organization | CAV1 | 764 | 1 | 0.762902 | -0.126231 | 392 | 240.68 | 0.613980 |
| GO:0006351\_transcription\_\_DNA-dependent | FOSL1 | 884 | 1 | 0.659341 | -0.097215 | 393 | 244.37 | 0.621807 |
| GO:0032774\_RNA\_biosynthetic\_process | FOSL1 | 887 | 1 | 0.657111 | -0.096587 | 394 | 244.95 | 0.621701 |
| GO:0045449\_regulation\_of\_transcription | FOSL1 | 900 | 1 | 0.647619 | -0.093917 | 395 | 245.35 | 0.621139 |
| GO:0006464\_protein\_modification\_process | ADAM9 | 922 | 1 | 0.632166 | -0.089574 | 396 | 246.01 | 0.621237 |
| GO:0043412\_biopolymer\_modification | ADAM9 | 960 | 1 | 0.607143 | -0.082557 | 397 | 247.3 | 0.622922 |
| GO:0019219\_regulation\_of\_nucleobase\_\_nucleoside\_\_nucleotide\_and\_nucleic\_acid\_metabolic\_process | FOSL1 | 1041 | 1 | 0.559901 | -0.069427 | 398 | 249.2 | 0.626131 |
| GO:0010556\_regulation\_of\_macromolecule\_biosynthetic\_process | FOSL1 | 1055 | 1 | 0.552471 | -0.067384 | 399 | 249.88 | 0.626266 |
| GO:0010468\_regulation\_of\_gene\_expression | FOSL1 | 1067 | 1 | 0.546258 | -0.065681 | 400 | 250.44 | 0.626100 |
| GO:0006350\_transcription | FOSL1 | 1069 | 1 | 0.545236 | -0.065401 | 401 | 250.81 | 0.625461 |
| GO:0044249\_cellular\_biosynthetic\_process | CAV1 | 1951 | 2 | 0.597496 | -0.054304 | 402 | 252.37 | 0.627786 |
| GO:0044249\_cellular\_biosynthetic\_process | FOSL1 | 1951 | 2 | 0.597496 | -0.054304 | 402 | 252.37 | 0.627786 |
| GO:0009058\_biosynthetic\_process | CAV1 | 1988 | 2 | 0.586375 | -0.050758 | 403 | 252.55 | 0.626675 |
| GO:0009058\_biosynthetic\_process | FOSL1 | 1988 | 2 | 0.586375 | -0.050758 | 403 | 252.55 | 0.626675 |
| GO:0016070\_RNA\_metabolic\_process | FOSL1 | 1230 | 1 | 0.473868 | -0.046400 | 404 | 253.08 | 0.626436 |
| GO:0006807\_nitrogen\_compound\_metabolic\_process | CAV1 | 2053 | 2 | 0.567810 | -0.045033 | 405 | 253.55 | 0.626049 |
| GO:0006807\_nitrogen\_compound\_metabolic\_process | FOSL1 | 2053 | 2 | 0.567810 | -0.045033 | 405 | 253.55 | 0.626049 |
| GO:0060255\_regulation\_of\_macromolecule\_metabolic\_process | FOSL1 | 1328 | 1 | 0.438898 | -0.037629 | 406 | 255.25 | 0.628695 |
| GO:0044267\_cellular\_protein\_metabolic\_process | ADAM9 | 1382 | 1 | 0.421749 | -0.033512 | 407 | 256.26 | 0.629631 |
| GO:0034961\_cellular\_biopolymer\_biosynthetic\_process | FOSL1 | 1448 | 1 | 0.402526 | -0.029071 | 408 | 256.74 | 0.629265 |
| GO:0043284\_biopolymer\_biosynthetic\_process | FOSL1 | 1458 | 1 | 0.399765 | -0.028450 | 409 | 256.97 | 0.628289 |
| GO:0044238\_primary\_metabolic\_process | CAV1 | 3719 | 4 | 0.626897 | -0.025908 | 410 | 257.45 | 0.627927 |
| GO:0044238\_primary\_metabolic\_process | ADAM9 | 3719 | 4 | 0.626897 | -0.025908 | 410 | 257.45 | 0.627927 |
| GO:0044238\_primary\_metabolic\_process | FOSL1 | 3719 | 4 | 0.626897 | -0.025908 | 410 | 257.45 | 0.627927 |
| GO:0044238\_primary\_metabolic\_process | PKM2 | 3719 | 4 | 0.626897 | -0.025908 | 410 | 257.45 | 0.627927 |
| GO:0044237\_cellular\_metabolic\_process | CAV1 | 3753 | 4 | 0.621217 | -0.024294 | 411 | 257.6 | 0.626764 |
| GO:0044237\_cellular\_metabolic\_process | ADAM9 | 3753 | 4 | 0.621217 | -0.024294 | 411 | 257.6 | 0.626764 |
| GO:0044237\_cellular\_metabolic\_process | FOSL1 | 3753 | 4 | 0.621217 | -0.024294 | 411 | 257.6 | 0.626764 |
| GO:0044237\_cellular\_metabolic\_process | PKM2 | 3753 | 4 | 0.621217 | -0.024294 | 411 | 257.6 | 0.626764 |
| GO:0019538\_protein\_metabolic\_process | ADAM9 | 1569 | 1 | 0.371483 | -0.022357 | 412 | 258.34 | 0.627039 |
| GO:0034645\_cellular\_macromolecule\_biosynthetic\_process | FOSL1 | 1600 | 1 | 0.364286 | -0.020893 | 413 | 258.54 | 0.626005 |
| GO:0009059\_macromolecule\_biosynthetic\_process | FOSL1 | 1626 | 1 | 0.358461 | -0.019736 | 414 | 258.74 | 0.624976 |
| GO:0010467\_gene\_expression | FOSL1 | 1663 | 1 | 0.350485 | -0.018194 | 415 | 258.93 | 0.623928 |
| GO:0006139\_nucleobase\_\_nucleoside\_\_nucleotide\_and\_nucleic\_acid\_metabolic\_process | FOSL1 | 1845 | 1 | 0.315912 | -0.012133 | 416 | 259.32 | 0.623365 |
| GO:0008152\_metabolic\_process | CAV1 | 4111 | 4 | 0.567120 | -0.011788 | 417 | 259.39 | 0.622038 |
| GO:0008152\_metabolic\_process | ADAM9 | 4111 | 4 | 0.567120 | -0.011788 | 417 | 259.39 | 0.622038 |
| GO:0008152\_metabolic\_process | FOSL1 | 4111 | 4 | 0.567120 | -0.011788 | 417 | 259.39 | 0.622038 |
| GO:0008152\_metabolic\_process | PKM2 | 4111 | 4 | 0.567120 | -0.011788 | 417 | 259.39 | 0.622038 |
| GO:0034960\_cellular\_biopolymer\_metabolic\_process | ADAM9 | 2820 | 2 | 0.413374 | -0.009702 | 418 | 259.73 | 0.621364 |
| GO:0034960\_cellular\_biopolymer\_metabolic\_process | FOSL1 | 2820 | 2 | 0.413374 | -0.009702 | 418 | 259.73 | 0.621364 |
| GO:0044260\_cellular\_macromolecule\_metabolic\_process | ADAM9 | 2883 | 2 | 0.404341 | -0.008453 | 419 | 259.99 | 0.620501 |
| GO:0044260\_cellular\_macromolecule\_metabolic\_process | FOSL1 | 2883 | 2 | 0.404341 | -0.008453 | 419 | 259.99 | 0.620501 |
| GO:0043283\_biopolymer\_metabolic\_process | ADAM9 | 3027 | 2 | 0.385105 | -0.006122 | 420 | 260.42 | 0.620048 |
| GO:0043283\_biopolymer\_metabolic\_process | FOSL1 | 3027 | 2 | 0.385105 | -0.006122 | 420 | 260.42 | 0.620048 |
| GO:0043170\_macromolecule\_metabolic\_process | ADAM9 | 3103 | 2 | 0.375673 | -0.005141 | 421 | 260.47 | 0.618694 |
| GO:0043170\_macromolecule\_metabolic\_process | FOSL1 | 3103 | 2 | 0.375673 | -0.005141 | 421 | 260.47 | 0.618694 |
| GO:0008150\_biological\_process | PKM2 | 8160 | 14 | 1.000000 | 0.000000 | 474 | 327.95 | 0.691878 |
| GO:0008150\_biological\_process | FOSL1 | 8160 | 14 | 1.000000 | 0.000000 | 474 | 327.95 | 0.691878 |
| GO:0008150\_biological\_process | CD151 | 8160 | 14 | 1.000000 | 0.000000 | 474 | 327.95 | 0.691878 |
| GO:0008150\_biological\_process | ANXA2 | 8160 | 14 | 1.000000 | 0.000000 | 474 | 327.95 | 0.691878 |
| GO:0008150\_biological\_process | ARHGAP29 | 8160 | 14 | 1.000000 | 0.000000 | 474 | 327.95 | 0.691878 |
| GO:0008150\_biological\_process | MYO1E | 8160 | 14 | 1.000000 | 0.000000 | 474 | 327.95 | 0.691878 |
| GO:0008150\_biological\_process | ACTN1 | 8160 | 14 | 1.000000 | 0.000000 | 474 | 327.95 | 0.691878 |
| GO:0008150\_biological\_process | TNFRSF12A | 8160 | 14 | 1.000000 | 0.000000 | 474 | 327.95 | 0.691878 |
| GO:0008150\_biological\_process | CAV1 | 8160 | 14 | 1.000000 | 0.000000 | 474 | 327.95 | 0.691878 |
| GO:0008150\_biological\_process | DCBLD2 | 8160 | 14 | 1.000000 | 0.000000 | 474 | 327.95 | 0.691878 |
| GO:0008150\_biological\_process | ADAM9 | 8160 | 14 | 1.000000 | 0.000000 | 474 | 327.95 | 0.691878 |
| GO:0008150\_biological\_process | RRAS | 8160 | 14 | 1.000000 | 0.000000 | 474 | 327.95 | 0.691878 |
| GO:0008150\_biological\_process | S100A10 | 8160 | 14 | 1.000000 | 0.000000 | 474 | 327.95 | 0.691878 |
| GO:0008150\_biological\_process | HRH1 | 8160 | 14 | 1.000000 | 0.000000 | 474 | 327.95 | 0.691878 |
