# Supplementary material for: Concordance of Gene Expression and Functional Correlation Patterns across the NCI-60 Cell Lines and the Cancer Genome Atlas Glioblastoma Samples
Source: PLoS One. 2012 Jul 26;7(7):e40062. doi: 10.1371/journal.pone.0040062 (PMC3406063; doi:10.1371/journal.pone.0040062)
Supplement: Download S1 — Zip archive of HTGM results. (ZIP) [file pone.0040062.s007.zip › work2026406846/Generated_Total2026406846.dir/generic.BP.NCI60.0.6.ANXA2.express.genes.correlation.complete.Thu.May.19.17.26.28.2011.htgm.txt.dir/generic.BP.NCI60.0.6.ANXA2.express.genes.correlation.complete.Thu.May.19.17.26.28.2011.htgm.txt.change.html]

Category Summary Report for generic.BP.NCI60.0.6.ANXA2.express.genes.correlation.complete.Thu.May.19.17.26.28.2011.htgm.txt

# Category Summary Report for generic.BP.NCI60.0.6.ANXA2.express.genes.correlation.complete.Thu.May.19.17.26.28.2011.htgm.txt

| HYPERLINKED GO CATEGORY | TOTAL GENES | CHANGED GENES | ENRICHMENT | LOG10(p) | CUMULATIVE NUMBER OF CATEGORIES | CUMULATIVE RANDOMS LOWER BOUND | CUMULATIVE RANDOMS MEAN | CUMULATIVE RANDOMS UPPER BOUND | FALSE DISCOVERY RATE |
| --- | --- | --- | --- | --- | --- | --- | --- | --- | --- |
| GO:0051592\_response\_to\_calcium\_ion | 30 | 2 | 38.857143 | -2.936692 | 1 | -0.789461 | 0.71 | 2.209461 | 0.710000 |
| GO:0030857\_negative\_regulation\_of\_epithelial\_cell\_differentiation | 1 | 1 |  |  |  |  |  |  |  |  |
| GO:0033484\_nitric\_oxide\_homeostasis | 1 | 1 |  |  |  |  |  |  |  |  |
| GO:0034238\_macrophage\_fusion | 1 | 1 |  |  |  |  |  |  |  |  |
| GO:0034239\_regulation\_of\_macrophage\_fusion | 1 | 1 |  |  |  |  |  |  |  |  |
| GO:0034241\_positive\_regulation\_of\_macrophage\_fusion | 1 | 1 |  |  |  |  |  |  |  |  |
| GO:0045907\_positive\_regulation\_of\_vasoconstriction | 1 | 1 |  |  |  |  |  |  |  |  |
| GO:0060142\_regulation\_of\_syncytium\_formation\_by\_plasma\_membrane\_fusion | 1 | 1 |  |  |  |  |  |  |  |  |
| GO:0060143\_positive\_regulation\_of\_syncytium\_formation\_by\_plasma\_membrane\_fusion | 1 | 1 |  |  |  |  |  |  |  |  |
| GO:0007242\_intracellular\_signaling\_cascade | 853 | 6 | 4.099816 | -2.735353 | 2 | -1.102020 | 1.08 | 3.262020 | 0.540000 |
| GO:0010038\_response\_to\_metal\_ion | 46 | 2 | 25.341615 | -2.567051 | 3 | -1.152409 | 1.6 | 4.352409 | 0.533333 |
| GO:0010035\_response\_to\_inorganic\_substance | 47 | 2 | 24.802432 | -2.548591 | 4 | -1.136602 | 1.65 | 4.436602 | 0.412500 |
| GO:0010042\_response\_to\_manganese\_ion | 2 | 1 |  |  |  |  |  |  |  |  |
| GO:0033630\_positive\_regulation\_of\_cell\_adhesion\_mediated\_by\_integrin | 2 | 1 |  |  |  |  |  |  |  |  |
| GO:0045019\_negative\_regulation\_of\_nitric\_oxide\_biosynthetic\_process | 2 | 1 |  |  |  |  |  |  |  |  |
| GO:0045908\_negative\_regulation\_of\_vasodilation | 2 | 1 |  |  |  |  |  |  |  |  |
| GO:0051088\_PMA-inducible\_membrane\_protein\_ectodomain\_proteolysis | 2 | 1 |  |  |  |  |  |  |  |  |
| GO:0051547\_regulation\_of\_keratinocyte\_migration | 2 | 1 |  |  |  |  |  |  |  |  |
| GO:0051549\_positive\_regulation\_of\_keratinocyte\_migration | 2 | 1 |  |  |  |  |  |  |  |  |
| GO:0060056\_mammary\_gland\_involution | 2 | 1 |  |  |  |  |  |  |  |  |
| GO:0060443\_mammary\_gland\_morphogenesis | 2 | 1 |  |  |  |  |  |  |  |  |
| GO:0007160\_cell-matrix\_adhesion | 58 | 2 | 20.098522 | -2.368823 | 5 | -1.166381 | 2.08 | 5.326381 | 0.416000 |
| GO:0030856\_regulation\_of\_epithelial\_cell\_differentiation | 3 | 1 |  |  |  |  |  |  |  |  |
| GO:0048552\_regulation\_of\_metalloenzyme\_activity | 3 | 1 |  |  |  |  |  |  |  |  |
| GO:0048554\_positive\_regulation\_of\_metalloenzyme\_activity | 3 | 1 |  |  |  |  |  |  |  |  |
| GO:0051546\_keratinocyte\_migration | 3 | 1 |  |  |  |  |  |  |  |  |
| GO:0031589\_cell-substrate\_adhesion | 66 | 2 | 17.662338 | -2.259071 | 6 | -1.252819 | 2.58 | 6.412819 | 0.430000 |
| GO:0009605\_response\_to\_external\_stimulus | 464 | 4 | 5.024631 | -2.184302 | 7 | -1.277686 | 2.96 | 7.197686 | 0.422857 |
| GO:0033628\_regulation\_of\_cell\_adhesion\_mediated\_by\_integrin | 4 | 1 |  |  |  |  |  |  |  |  |
| GO:0033631\_cell-cell\_adhesion\_mediated\_by\_integrin | 4 | 1 |  |  |  |  |  |  |  |  |
| GO:0000768\_syncytium\_formation\_by\_plasma\_membrane\_fusion | 5 | 1 | 116.571429 | -2.067976 | 10 | 0.127687 | 6.66 | 13.192313 | 0.666000 |
| GO:0032570\_response\_to\_progesterone\_stimulus | 5 | 1 | 116.571429 | -2.067976 | 10 | 0.127687 | 6.66 | 13.192313 | 0.666000 |
| GO:0042117\_monocyte\_activation | 5 | 1 | 116.571429 | -2.067976 | 10 | 0.127687 | 6.66 | 13.192313 | 0.666000 |
| GO:0006949\_syncytium\_formation | 6 | 1 | 97.142857 | -1.989141 | 14 | 1.124094 | 9.45 | 17.775906 | 0.675000 |
| GO:0033483\_gas\_homeostasis | 6 | 1 | 97.142857 | -1.989141 | 14 | 1.124094 | 9.45 | 17.775906 | 0.675000 |
| GO:0033627\_cell\_adhesion\_mediated\_by\_integrin | 6 | 1 | 97.142857 | -1.989141 | 14 | 1.124094 | 9.45 | 17.775906 | 0.675000 |
| GO:0046426\_negative\_regulation\_of\_JAK-STAT\_cascade | 6 | 1 | 97.142857 | -1.989141 | 14 | 1.124094 | 9.45 | 17.775906 | 0.675000 |
| GO:0009611\_response\_to\_wounding | 279 | 3 | 6.267281 | -1.963438 | 15 | 1.184868 | 9.69 | 18.195132 | 0.646000 |
| GO:0042060\_wound\_healing | 98 | 2 | 11.895044 | -1.927129 | 17 | 1.230217 | 10.0 | 18.769783 | 0.588235 |
| GO:0051270\_regulation\_of\_cell\_motion | 98 | 2 | 11.895044 | -1.927129 | 17 | 1.230217 | 10.0 | 18.769783 | 0.588235 |
| GO:0000186\_activation\_of\_MAPKK\_activity | 7 | 1 | 83.265306 | -1.922540 | 19 | 2.325448 | 12.52 | 22.714552 | 0.658947 |
| GO:0022612\_gland\_morphogenesis | 7 | 1 | 83.265306 | -1.922540 | 19 | 2.325448 | 12.52 | 22.714552 | 0.658947 |
| GO:0022603\_regulation\_of\_anatomical\_structure\_morphogenesis | 100 | 2 | 11.657143 | -1.910341 | 20 | 2.369711 | 12.65 | 22.930289 | 0.632500 |
| GO:0019229\_regulation\_of\_vasoconstriction | 8 | 1 | 72.857143 | -1.864894 | 22 | 3.434550 | 15.27 | 27.105450 | 0.694091 |
| GO:0043409\_negative\_regulation\_of\_MAPKKK\_cascade | 8 | 1 | 72.857143 | -1.864894 | 22 | 3.434550 | 15.27 | 27.105450 | 0.694091 |
| GO:0006928\_cell\_motion | 308 | 3 | 5.677180 | -1.846998 | 24 | 3.483146 | 15.42 | 27.356854 | 0.642500 |
| GO:0051674\_localization\_of\_cell | 308 | 3 | 5.677180 | -1.846998 | 24 | 3.483146 | 15.42 | 27.356854 | 0.642500 |
| GO:0007265\_Ras\_protein\_signal\_transduction | 110 | 2 | 10.597403 | -1.831403 | 25 | 3.523387 | 15.66 | 27.796613 | 0.626400 |
| GO:0030514\_negative\_regulation\_of\_BMP\_signaling\_pathway | 9 | 1 | 64.761905 | -1.814087 | 26 | 4.321812 | 17.78 | 31.238188 | 0.683846 |
| GO:0051050\_positive\_regulation\_of\_transport | 116 | 2 | 10.049261 | -1.787614 | 27 | 4.383490 | 17.96 | 31.536510 | 0.665185 |
| GO:0032879\_regulation\_of\_localization | 326 | 3 | 5.363716 | -1.780737 | 28 | 4.359597 | 18.0 | 31.640403 | 0.642857 |
| GO:0034612\_response\_to\_tumor\_necrosis\_factor | 10 | 1 | 58.285714 | -1.768675 | 31 | 5.668916 | 20.46 | 35.251084 | 0.660000 |
| GO:0042312\_regulation\_of\_vasodilation | 10 | 1 | 58.285714 | -1.768675 | 31 | 5.668916 | 20.46 | 35.251084 | 0.660000 |
| GO:0042542\_response\_to\_hydrogen\_peroxide | 10 | 1 | 58.285714 | -1.768675 | 31 | 5.668916 | 20.46 | 35.251084 | 0.660000 |
| GO:0006950\_response\_to\_stress | 959 | 5 | 3.038880 | -1.747511 | 32 | 5.693230 | 20.57 | 35.446770 | 0.642812 |
| GO:0001570\_vasculogenesis | 11 | 1 | 52.987013 | -1.727628 | 33 | 6.775605 | 22.71 | 38.644395 | 0.688182 |
| GO:0010524\_positive\_regulation\_of\_calcium\_ion\_transport\_into\_cytosol | 12 | 1 | 48.571429 | -1.690185 | 37 | 7.563017 | 24.57 | 41.576983 | 0.664054 |
| GO:0030510\_regulation\_of\_BMP\_signaling\_pathway | 12 | 1 | 48.571429 | -1.690185 | 37 | 7.563017 | 24.57 | 41.576983 | 0.664054 |
| GO:0042311\_vasodilation | 12 | 1 | 48.571429 | -1.690185 | 37 | 7.563017 | 24.57 | 41.576983 | 0.664054 |
| GO:0045026\_plasma\_membrane\_fusion | 12 | 1 | 48.571429 | -1.690185 | 37 | 7.563017 | 24.57 | 41.576983 | 0.664054 |
| GO:0043085\_positive\_regulation\_of\_catalytic\_activity | 354 | 3 | 4.939467 | -1.685472 | 38 | 7.674793 | 24.8 | 41.925207 | 0.652632 |
| GO:0007264\_small\_GTPase\_mediated\_signal\_transduction | 135 | 2 | 8.634921 | -1.663392 | 39 | 7.753557 | 25.16 | 42.566443 | 0.645128 |
| GO:0006940\_regulation\_of\_smooth\_muscle\_contraction | 13 | 1 | 44.835165 | -1.655768 | 41 | 8.453152 | 26.96 | 45.466848 | 0.657561 |
| GO:0051899\_membrane\_depolarization | 13 | 1 | 44.835165 | -1.655768 | 41 | 8.453152 | 26.96 | 45.466848 | 0.657561 |
| GO:0030879\_mammary\_gland\_development | 14 | 1 | 41.632653 | -1.623929 | 44 | 9.518492 | 29.04 | 48.561508 | 0.660000 |
| GO:0042310\_vasoconstriction | 14 | 1 | 41.632653 | -1.623929 | 44 | 9.518492 | 29.04 | 48.561508 | 0.660000 |
| GO:0045428\_regulation\_of\_nitric\_oxide\_biosynthetic\_process | 14 | 1 | 41.632653 | -1.623929 | 44 | 9.518492 | 29.04 | 48.561508 | 0.660000 |
| GO:0000165\_MAPKKK\_cascade | 143 | 2 | 8.151848 | -1.616599 | 45 | 9.528059 | 29.11 | 48.691941 | 0.646889 |
| GO:0001937\_negative\_regulation\_of\_endothelial\_cell\_proliferation | 15 | 1 | 38.857143 | -1.594311 | 48 | 10.702657 | 31.19 | 51.677343 | 0.649792 |
| GO:0030512\_negative\_regulation\_of\_transforming\_growth\_factor\_beta\_receptor\_signaling\_pathway | 15 | 1 | 38.857143 | -1.594311 | 48 | 10.702657 | 31.19 | 51.677343 | 0.649792 |
| GO:0048041\_focal\_adhesion\_formation | 15 | 1 | 38.857143 | -1.594311 | 48 | 10.702657 | 31.19 | 51.677343 | 0.649792 |
| GO:0030855\_epithelial\_cell\_differentiation | 16 | 1 | 36.428571 | -1.566627 | 49 | 11.724787 | 33.43 | 55.135213 | 0.682245 |
| GO:0044093\_positive\_regulation\_of\_molecular\_function | 394 | 3 | 4.437999 | -1.563358 | 50 | 11.740754 | 33.46 | 55.179246 | 0.669200 |
| GO:0000188\_inactivation\_of\_MAPK\_activity | 17 | 1 | 34.285714 | -1.540644 | 54 | 12.380988 | 35.02 | 57.659012 | 0.648519 |
| GO:0007044\_cell-substrate\_junction\_assembly | 17 | 1 | 34.285714 | -1.540644 | 54 | 12.380988 | 35.02 | 57.659012 | 0.648519 |
| GO:0010522\_regulation\_of\_calcium\_ion\_transport\_into\_cytosol | 17 | 1 | 34.285714 | -1.540644 | 54 | 12.380988 | 35.02 | 57.659012 | 0.648519 |
| GO:0051928\_positive\_regulation\_of\_calcium\_ion\_transport | 17 | 1 | 34.285714 | -1.540644 | 54 | 12.380988 | 35.02 | 57.659012 | 0.648519 |
| GO:0032846\_positive\_regulation\_of\_homeostatic\_process | 18 | 1 | 32.380952 | -1.516165 | 56 | 13.111586 | 36.64 | 60.168414 | 0.654286 |
| GO:0043270\_positive\_regulation\_of\_ion\_transport | 18 | 1 | 32.380952 | -1.516165 | 56 | 13.111586 | 36.64 | 60.168414 | 0.654286 |
| GO:0006809\_nitric\_oxide\_biosynthetic\_process | 19 | 1 | 30.676692 | -1.493029 | 58 | 14.156850 | 38.64 | 63.123150 | 0.666207 |
| GO:0009267\_cellular\_response\_to\_starvation | 19 | 1 | 30.676692 | -1.493029 | 58 | 14.156850 | 38.64 | 63.123150 | 0.666207 |
| GO:0006096\_glycolysis | 20 | 1 | 29.142857 | -1.471098 | 61 | 15.172985 | 40.65 | 66.127015 | 0.666393 |
| GO:0042594\_response\_to\_starvation | 20 | 1 | 29.142857 | -1.471098 | 61 | 15.172985 | 40.65 | 66.127015 | 0.666393 |
| GO:0046209\_nitric\_oxide\_metabolic\_process | 20 | 1 | 29.142857 | -1.471098 | 61 | 15.172985 | 40.65 | 66.127015 | 0.666393 |
| GO:0007155\_cell\_adhesion | 428 | 3 | 4.085447 | -1.470341 | 62 | 15.187958 | 40.67 | 66.152042 | 0.655968 |
| GO:0022610\_biological\_adhesion | 429 | 3 | 4.075924 | -1.467737 | 63 | 15.205893 | 40.7 | 66.194107 | 0.646032 |
| GO:0000302\_response\_to\_reactive\_oxygen\_species | 21 | 1 | 27.755102 | -1.450253 | 66 | 16.416571 | 42.98 | 69.543429 | 0.651212 |
| GO:0001936\_regulation\_of\_endothelial\_cell\_proliferation | 21 | 1 | 27.755102 | -1.450253 | 66 | 16.416571 | 42.98 | 69.543429 | 0.651212 |
| GO:0030048\_actin\_filament-based\_movement | 21 | 1 | 27.755102 | -1.450253 | 66 | 16.416571 | 42.98 | 69.543429 | 0.651212 |
| GO:0051336\_regulation\_of\_hydrolase\_activity | 180 | 2 | 6.476190 | -1.431774 | 67 | 16.772708 | 43.43 | 70.087292 | 0.648209 |
| GO:0007165\_signal\_transduction | 2029 | 7 | 2.010843 | -1.430678 | 68 | 16.859672 | 43.5 | 70.140328 | 0.639706 |
| GO:0042632\_cholesterol\_homeostasis | 22 | 1 | 26.493506 | -1.430395 | 70 | 17.354928 | 44.64 | 71.925072 | 0.637714 |
| GO:0055092\_sterol\_homeostasis | 22 | 1 | 26.493506 | -1.430395 | 70 | 17.354928 | 44.64 | 71.925072 | 0.637714 |
| GO:0006509\_membrane\_protein\_ectodomain\_proteolysis | 23 | 1 | 25.341615 | -1.411434 | 74 | 18.031339 | 45.82 | 73.608661 | 0.619189 |
| GO:0006641\_triglyceride\_metabolic\_process | 23 | 1 | 25.341615 | -1.411434 | 74 | 18.031339 | 45.82 | 73.608661 | 0.619189 |
| GO:0033619\_membrane\_protein\_proteolysis | 23 | 1 | 25.341615 | -1.411434 | 74 | 18.031339 | 45.82 | 73.608661 | 0.619189 |
| GO:0043627\_response\_to\_estrogen\_stimulus | 23 | 1 | 25.341615 | -1.411434 | 74 | 18.031339 | 45.82 | 73.608661 | 0.619189 |
| GO:0030193\_regulation\_of\_blood\_coagulation | 24 | 1 | 24.285714 | -1.393296 | 77 | 19.010433 | 47.27 | 75.529567 | 0.613896 |
| GO:0043407\_negative\_regulation\_of\_MAP\_kinase\_activity | 24 | 1 | 24.285714 | -1.393296 | 77 | 19.010433 | 47.27 | 75.529567 | 0.613896 |
| GO:0045785\_positive\_regulation\_of\_cell\_adhesion | 24 | 1 | 24.285714 | -1.393296 | 77 | 19.010433 | 47.27 | 75.529567 | 0.613896 |
| GO:0001935\_endothelial\_cell\_proliferation | 25 | 1 | 23.314286 | -1.375911 | 83 | 19.760019 | 49.22 | 78.679981 | 0.593012 |
| GO:0006007\_glucose\_catabolic\_process | 25 | 1 | 23.314286 | -1.375911 | 83 | 19.760019 | 49.22 | 78.679981 | 0.593012 |
| GO:0010876\_lipid\_localization | 25 | 1 | 23.314286 | -1.375911 | 83 | 19.760019 | 49.22 | 78.679981 | 0.593012 |
| GO:0019217\_regulation\_of\_fatty\_acid\_metabolic\_process | 25 | 1 | 23.314286 | -1.375911 | 83 | 19.760019 | 49.22 | 78.679981 | 0.593012 |
| GO:0019915\_lipid\_storage | 25 | 1 | 23.314286 | -1.375911 | 83 | 19.760019 | 49.22 | 78.679981 | 0.593012 |
| GO:0050818\_regulation\_of\_coagulation | 25 | 1 | 23.314286 | -1.375911 | 83 | 19.760019 | 49.22 | 78.679981 | 0.593012 |
| GO:0010741\_negative\_regulation\_of\_protein\_kinase\_cascade | 26 | 1 | 22.417582 | -1.359223 | 84 | 20.533718 | 50.9 | 81.266282 | 0.605952 |
| GO:0006638\_neutral\_lipid\_metabolic\_process | 27 | 1 | 21.587302 | -1.343177 | 89 | 21.420861 | 52.33 | 83.239139 | 0.587978 |
| GO:0006639\_acylglycerol\_metabolic\_process | 27 | 1 | 21.587302 | -1.343177 | 89 | 21.420861 | 52.33 | 83.239139 | 0.587978 |
| GO:0031669\_cellular\_response\_to\_nutrient\_levels | 27 | 1 | 21.587302 | -1.343177 | 89 | 21.420861 | 52.33 | 83.239139 | 0.587978 |
| GO:0035150\_regulation\_of\_tube\_size | 27 | 1 | 21.587302 | -1.343177 | 89 | 21.420861 | 52.33 | 83.239139 | 0.587978 |
| GO:0050880\_regulation\_of\_blood\_vessel\_size | 27 | 1 | 21.587302 | -1.343177 | 89 | 21.420861 | 52.33 | 83.239139 | 0.587978 |
| GO:0006662\_glycerol\_ether\_metabolic\_process | 28 | 1 | 20.816327 | -1.327727 | 94 | 22.241885 | 53.96 | 85.678115 | 0.574043 |
| GO:0006939\_smooth\_muscle\_contraction | 28 | 1 | 20.816327 | -1.327727 | 94 | 22.241885 | 53.96 | 85.678115 | 0.574043 |
| GO:0010565\_regulation\_of\_cellular\_ketone\_metabolic\_process | 28 | 1 | 20.816327 | -1.327727 | 94 | 22.241885 | 53.96 | 85.678115 | 0.574043 |
| GO:0018904\_organic\_ether\_metabolic\_process | 28 | 1 | 20.816327 | -1.327727 | 94 | 22.241885 | 53.96 | 85.678115 | 0.574043 |
| GO:0060402\_calcium\_ion\_transport\_into\_cytosol | 28 | 1 | 20.816327 | -1.327727 | 94 | 22.241885 | 53.96 | 85.678115 | 0.574043 |
| GO:0002009\_morphogenesis\_of\_an\_epithelium | 29 | 1 | 20.098522 | -1.312831 | 104 | 22.945617 | 55.27 | 87.594383 | 0.531442 |
| GO:0002274\_myeloid\_leukocyte\_activation | 29 | 1 | 20.098522 | -1.312831 | 104 | 22.945617 | 55.27 | 87.594383 | 0.531442 |
| GO:0003018\_vascular\_process\_in\_circulatory\_system | 29 | 1 | 20.098522 | -1.312831 | 104 | 22.945617 | 55.27 | 87.594383 | 0.531442 |
| GO:0032507\_maintenance\_of\_protein\_location\_in\_cell | 29 | 1 | 20.098522 | -1.312831 | 104 | 22.945617 | 55.27 | 87.594383 | 0.531442 |
| GO:0046425\_regulation\_of\_JAK-STAT\_cascade | 29 | 1 | 20.098522 | -1.312831 | 104 | 22.945617 | 55.27 | 87.594383 | 0.531442 |
| GO:0048732\_gland\_development | 29 | 1 | 20.098522 | -1.312831 | 104 | 22.945617 | 55.27 | 87.594383 | 0.531442 |
| GO:0050714\_positive\_regulation\_of\_protein\_secretion | 29 | 1 | 20.098522 | -1.312831 | 104 | 22.945617 | 55.27 | 87.594383 | 0.531442 |
| GO:0051924\_regulation\_of\_calcium\_ion\_transport | 29 | 1 | 20.098522 | -1.312831 | 104 | 22.945617 | 55.27 | 87.594383 | 0.531442 |
| GO:0060401\_cytosolic\_calcium\_ion\_transport | 29 | 1 | 20.098522 | -1.312831 | 104 | 22.945617 | 55.27 | 87.594383 | 0.531442 |
| GO:0060429\_epithelium\_development | 29 | 1 | 20.098522 | -1.312831 | 104 | 22.945617 | 55.27 | 87.594383 | 0.531442 |
| GO:0045859\_regulation\_of\_protein\_kinase\_activity | 213 | 2 | 5.472837 | -1.299144 | 105 | 23.053903 | 55.68 | 88.306097 | 0.530286 |
| GO:0010959\_regulation\_of\_metal\_ion\_transport | 30 | 1 | 19.428571 | -1.298453 | 106 | 23.511665 | 56.69 | 89.868335 | 0.534811 |
| GO:0043549\_regulation\_of\_kinase\_activity | 217 | 2 | 5.371955 | -1.284637 | 107 | 23.678645 | 56.99 | 90.301355 | 0.532617 |
| GO:0007200\_activation\_of\_phospholipase\_C\_activity\_by\_G-protein\_coupled\_receptor\_protein\_signaling\_pathway\_coupled\_to\_IP3\_second\_messenger | 31 | 1 | 18.801843 | -1.284556 | 110 | 24.106217 | 57.87 | 91.633783 | 0.526091 |
| GO:0031668\_cellular\_response\_to\_extracellular\_stimulus | 31 | 1 | 18.801843 | -1.284556 | 110 | 24.106217 | 57.87 | 91.633783 | 0.526091 |
| GO:0045185\_maintenance\_of\_protein\_location | 31 | 1 | 18.801843 | -1.284556 | 110 | 24.106217 | 57.87 | 91.633783 | 0.526091 |
| GO:0030216\_keratinocyte\_differentiation | 32 | 1 | 18.214286 | -1.271112 | 111 | 24.599554 | 58.86 | 93.120446 | 0.530270 |
| GO:0034329\_cell\_junction\_assembly | 33 | 1 | 17.662338 | -1.258092 | 113 | 25.026083 | 59.65 | 94.273917 | 0.527876 |
| GO:0055088\_lipid\_homeostasis | 33 | 1 | 17.662338 | -1.258092 | 113 | 25.026083 | 59.65 | 94.273917 | 0.527876 |
| GO:0016044\_membrane\_organization | 225 | 2 | 5.180952 | -1.256501 | 114 | 25.077503 | 59.72 | 94.362497 | 0.523860 |
| GO:0051049\_regulation\_of\_transport | 227 | 2 | 5.135305 | -1.249642 | 116 | 25.224125 | 59.95 | 94.675875 | 0.516810 |
| GO:0051338\_regulation\_of\_transferase\_activity | 227 | 2 | 5.135305 | -1.249642 | 116 | 25.224125 | 59.95 | 94.675875 | 0.516810 |
| GO:0050790\_regulation\_of\_catalytic\_activity | 525 | 3 | 3.330612 | -1.246722 | 117 | 25.283420 | 60.07 | 94.856580 | 0.513419 |
| GO:0043269\_regulation\_of\_ion\_transport | 34 | 1 | 17.142857 | -1.245472 | 120 | 26.106580 | 61.83 | 97.553420 | 0.515250 |
| GO:0048545\_response\_to\_steroid\_hormone\_stimulus | 34 | 1 | 17.142857 | -1.245472 | 120 | 26.106580 | 61.83 | 97.553420 | 0.515250 |
| GO:0051651\_maintenance\_of\_location\_in\_cell | 34 | 1 | 17.142857 | -1.245472 | 120 | 26.106580 | 61.83 | 97.553420 | 0.515250 |
| GO:0019320\_hexose\_catabolic\_process | 35 | 1 | 16.653061 | -1.233226 | 122 | 26.944435 | 62.99 | 99.035565 | 0.516311 |
| GO:0030705\_cytoskeleton-dependent\_intracellular\_transport | 35 | 1 | 16.653061 | -1.233226 | 122 | 26.944435 | 62.99 | 99.035565 | 0.516311 |
| GO:0034097\_response\_to\_cytokine\_stimulus | 36 | 1 | 16.190476 | -1.221336 | 123 | 27.389401 | 63.74 | 100.090599 | 0.518211 |
| GO:0006937\_regulation\_of\_muscle\_contraction | 37 | 1 | 15.752896 | -1.209781 | 127 | 27.785620 | 64.69 | 101.594380 | 0.509370 |
| GO:0009913\_epidermal\_cell\_differentiation | 37 | 1 | 15.752896 | -1.209781 | 127 | 27.785620 | 64.69 | 101.594380 | 0.509370 |
| GO:0015918\_sterol\_transport | 37 | 1 | 15.752896 | -1.209781 | 127 | 27.785620 | 64.69 | 101.594380 | 0.509370 |
| GO:0030301\_cholesterol\_transport | 37 | 1 | 15.752896 | -1.209781 | 127 | 27.785620 | 64.69 | 101.594380 | 0.509370 |
| GO:0016050\_vesicle\_organization | 38 | 1 | 15.338346 | -1.198542 | 128 | 28.374599 | 66.22 | 104.065401 | 0.517344 |
| GO:0030509\_BMP\_signaling\_pathway | 39 | 1 | 14.945055 | -1.187605 | 130 | 29.014046 | 67.15 | 105.285954 | 0.516538 |
| GO:0046365\_monosaccharide\_catabolic\_process | 39 | 1 | 14.945055 | -1.187605 | 130 | 29.014046 | 67.15 | 105.285954 | 0.516538 |
| GO:0007154\_cell\_communication | 2272 | 7 | 1.795775 | -1.185068 | 131 | 29.133885 | 67.32 | 105.506115 | 0.513893 |
| GO:0010926\_anatomical\_structure\_formation | 560 | 3 | 3.122449 | -1.178042 | 132 | 29.126254 | 67.38 | 105.633746 | 0.510455 |
| GO:0017015\_regulation\_of\_transforming\_growth\_factor\_beta\_receptor\_signaling\_pathway | 40 | 1 | 14.571429 | -1.176954 | 133 | 29.405621 | 68.02 | 106.634379 | 0.511429 |
| GO:0007519\_skeletal\_muscle\_tissue\_development | 41 | 1 | 14.216028 | -1.166573 | 138 | 29.920827 | 69.09 | 108.259173 | 0.500652 |
| GO:0034330\_cell\_junction\_organization | 41 | 1 | 14.216028 | -1.166573 | 138 | 29.920827 | 69.09 | 108.259173 | 0.500652 |
| GO:0050708\_regulation\_of\_protein\_secretion | 41 | 1 | 14.216028 | -1.166573 | 138 | 29.920827 | 69.09 | 108.259173 | 0.500652 |
| GO:0051222\_positive\_regulation\_of\_protein\_transport | 41 | 1 | 14.216028 | -1.166573 | 138 | 29.920827 | 69.09 | 108.259173 | 0.500652 |
| GO:0060538\_skeletal\_muscle\_organ\_development | 41 | 1 | 14.216028 | -1.166573 | 138 | 29.920827 | 69.09 | 108.259173 | 0.500652 |
| GO:0006944\_membrane\_fusion | 42 | 1 | 13.877551 | -1.156451 | 140 | 30.738217 | 70.2 | 109.661783 | 0.501429 |
| GO:0051271\_negative\_regulation\_of\_cell\_motion | 42 | 1 | 13.877551 | -1.156451 | 140 | 30.738217 | 70.2 | 109.661783 | 0.501429 |
| GO:0046164\_alcohol\_catabolic\_process | 43 | 1 | 13.554817 | -1.146576 | 141 | 31.383975 | 71.29 | 111.196025 | 0.505603 |
| GO:0048771\_tissue\_remodeling | 45 | 1 | 12.952381 | -1.127518 | 142 | 32.456735 | 72.95 | 113.443265 | 0.513732 |
| GO:0007202\_activation\_of\_phospholipase\_C\_activity | 46 | 1 | 12.670807 | -1.118316 | 146 | 33.350811 | 74.14 | 114.929189 | 0.507808 |
| GO:0010863\_positive\_regulation\_of\_phospholipase\_C\_activity | 46 | 1 | 12.670807 | -1.118316 | 146 | 33.350811 | 74.14 | 114.929189 | 0.507808 |
| GO:0030335\_positive\_regulation\_of\_cell\_migration | 46 | 1 | 12.670807 | -1.118316 | 146 | 33.350811 | 74.14 | 114.929189 | 0.507808 |
| GO:0042391\_regulation\_of\_membrane\_potential | 46 | 1 | 12.670807 | -1.118316 | 146 | 33.350811 | 74.14 | 114.929189 | 0.507808 |
| GO:0007259\_JAK-STAT\_cascade | 47 | 1 | 12.401216 | -1.109320 | 148 | 34.237596 | 75.35 | 116.462404 | 0.509122 |
| GO:0051260\_protein\_homooligomerization | 47 | 1 | 12.401216 | -1.109320 | 148 | 34.237596 | 75.35 | 116.462404 | 0.509122 |
| GO:0010518\_positive\_regulation\_of\_phospholipase\_activity | 48 | 1 | 12.142857 | -1.100519 | 149 | 34.819214 | 76.49 | 118.160786 | 0.513356 |
| GO:0010033\_response\_to\_organic\_substance | 276 | 2 | 4.223602 | -1.100213 | 150 | 34.820044 | 76.59 | 118.359956 | 0.510600 |
| GO:0065009\_regulation\_of\_molecular\_function | 606 | 3 | 2.885431 | -1.095473 | 151 | 34.849813 | 76.8 | 118.750187 | 0.508609 |
| GO:0010517\_regulation\_of\_phospholipase\_activity | 49 | 1 | 11.895044 | -1.091908 | 152 | 35.472447 | 78.12 | 120.767553 | 0.513947 |
| GO:0001666\_response\_to\_hypoxia | 50 | 1 | 11.657143 | -1.083477 | 155 | 35.906008 | 78.84 | 121.773992 | 0.508645 |
| GO:0007266\_Rho\_protein\_signal\_transduction | 50 | 1 | 11.657143 | -1.083477 | 155 | 35.906008 | 78.84 | 121.773992 | 0.508645 |
| GO:0051272\_positive\_regulation\_of\_cell\_motion | 50 | 1 | 11.657143 | -1.083477 | 155 | 35.906008 | 78.84 | 121.773992 | 0.508645 |
| GO:0051641\_cellular\_localization | 617 | 3 | 2.833989 | -1.076892 | 156 | 35.962060 | 78.9 | 121.837940 | 0.505769 |
| GO:0042325\_regulation\_of\_phosphorylation | 285 | 2 | 4.090226 | -1.076084 | 157 | 36.011148 | 78.99 | 121.968852 | 0.503121 |
| GO:0016052\_carbohydrate\_catabolic\_process | 51 | 1 | 11.428571 | -1.075220 | 160 | 36.607201 | 79.87 | 123.132799 | 0.499188 |
| GO:0032147\_activation\_of\_protein\_kinase\_activity | 51 | 1 | 11.428571 | -1.075220 | 160 | 36.607201 | 79.87 | 123.132799 | 0.499188 |
| GO:0070482\_response\_to\_oxygen\_levels | 51 | 1 | 11.428571 | -1.075220 | 160 | 36.607201 | 79.87 | 123.132799 | 0.499188 |
| GO:0009888\_tissue\_development | 287 | 2 | 4.061722 | -1.070842 | 161 | 36.782574 | 80.24 | 123.697426 | 0.498385 |
| GO:0006006\_glucose\_metabolic\_process | 53 | 1 | 10.997305 | -1.059200 | 165 | 37.867716 | 82.29 | 126.712284 | 0.498727 |
| GO:0048015\_phosphoinositide-mediated\_signaling | 53 | 1 | 10.997305 | -1.059200 | 165 | 37.867716 | 82.29 | 126.712284 | 0.498727 |
| GO:0051047\_positive\_regulation\_of\_secretion | 53 | 1 | 10.997305 | -1.059200 | 165 | 37.867716 | 82.29 | 126.712284 | 0.498727 |
| GO:0060193\_positive\_regulation\_of\_lipase\_activity | 53 | 1 | 10.997305 | -1.059200 | 165 | 37.867716 | 82.29 | 126.712284 | 0.498727 |
| GO:0040011\_locomotion | 292 | 2 | 3.992172 | -1.057920 | 166 | 38.035396 | 82.5 | 126.964604 | 0.496988 |
| GO:0042221\_response\_to\_chemical\_stimulus | 631 | 3 | 2.771112 | -1.053842 | 167 | 38.071720 | 82.6 | 127.128280 | 0.494611 |
| GO:0019220\_regulation\_of\_phosphate\_metabolic\_process | 297 | 2 | 3.924964 | -1.045254 | 169 | 38.400573 | 83.21 | 128.019427 | 0.492367 |
| GO:0051174\_regulation\_of\_phosphorus\_metabolic\_process | 297 | 2 | 3.924964 | -1.045254 | 169 | 38.400573 | 83.21 | 128.019427 | 0.492367 |
| GO:0006469\_negative\_regulation\_of\_protein\_kinase\_activity | 55 | 1 | 10.597403 | -1.043798 | 170 | 38.892411 | 83.81 | 128.727589 | 0.493000 |
| GO:0050794\_regulation\_of\_cellular\_process | 3515 | 9 | 1.492380 | -1.037411 | 171 | 38.906300 | 83.89 | 128.873700 | 0.490585 |
| GO:0009306\_protein\_secretion | 56 | 1 | 10.408163 | -1.036316 | 172 | 39.020876 | 84.38 | 129.739124 | 0.490581 |
| GO:0032844\_regulation\_of\_homeostatic\_process | 57 | 1 | 10.225564 | -1.028971 | 173 | 39.278351 | 85.01 | 130.741649 | 0.491387 |
| GO:0006968\_cellular\_defense\_response | 58 | 1 | 10.049261 | -1.021761 | 175 | 40.114152 | 86.19 | 132.265848 | 0.492514 |
| GO:0030308\_negative\_regulation\_of\_cell\_growth | 58 | 1 | 10.049261 | -1.021761 | 175 | 40.114152 | 86.19 | 132.265848 | 0.492514 |
| GO:0033673\_negative\_regulation\_of\_kinase\_activity | 59 | 1 | 9.878935 | -1.014679 | 177 | 40.869648 | 87.28 | 133.690352 | 0.493107 |
| GO:0043408\_regulation\_of\_MAPKKK\_cascade | 59 | 1 | 9.878935 | -1.014679 | 177 | 40.869648 | 87.28 | 133.690352 | 0.493107 |
| GO:0030155\_regulation\_of\_cell\_adhesion | 61 | 1 | 9.555035 | -1.000886 | 180 | 41.584915 | 88.71 | 135.835085 | 0.492833 |
| GO:0045792\_negative\_regulation\_of\_cell\_size | 61 | 1 | 9.555035 | -1.000886 | 180 | 41.584915 | 88.71 | 135.835085 | 0.492833 |
| GO:0048729\_tissue\_morphogenesis | 61 | 1 | 9.555035 | -1.000886 | 180 | 41.584915 | 88.71 | 135.835085 | 0.492833 |
| GO:0050793\_regulation\_of\_developmental\_process | 669 | 3 | 2.613709 | -0.994447 | 181 | 41.662869 | 89.11 | 136.557131 | 0.492320 |
| GO:0019216\_regulation\_of\_lipid\_metabolic\_process | 62 | 1 | 9.400922 | -0.994166 | 184 | 42.381363 | 90.33 | 138.278637 | 0.490924 |
| GO:0031667\_response\_to\_nutrient\_levels | 62 | 1 | 9.400922 | -0.994166 | 184 | 42.381363 | 90.33 | 138.278637 | 0.490924 |
| GO:0060191\_regulation\_of\_lipase\_activity | 62 | 1 | 9.400922 | -0.994166 | 184 | 42.381363 | 90.33 | 138.278637 | 0.490924 |
| GO:0030522\_intracellular\_receptor-mediated\_signaling\_pathway | 63 | 1 | 9.251701 | -0.987560 | 186 | 42.776893 | 90.79 | 138.803107 | 0.488118 |
| GO:0051348\_negative\_regulation\_of\_transferase\_activity | 63 | 1 | 9.251701 | -0.987560 | 186 | 42.776893 | 90.79 | 138.803107 | 0.488118 |
| GO:0045926\_negative\_regulation\_of\_growth | 64 | 1 | 9.107143 | -0.981062 | 187 | 43.471266 | 91.87 | 140.268734 | 0.491283 |
| GO:0045596\_negative\_regulation\_of\_cell\_differentiation | 65 | 1 | 8.967033 | -0.974671 | 189 | 43.821696 | 92.77 | 141.718304 | 0.490847 |
| GO:0051235\_maintenance\_of\_location | 65 | 1 | 8.967033 | -0.974671 | 189 | 43.821696 | 92.77 | 141.718304 | 0.490847 |
| GO:0014706\_striated\_muscle\_tissue\_development | 66 | 1 | 8.831169 | -0.968383 | 191 | 44.822503 | 94.22 | 143.617497 | 0.493298 |
| GO:0052547\_regulation\_of\_peptidase\_activity | 66 | 1 | 8.831169 | -0.968383 | 191 | 44.822503 | 94.22 | 143.617497 | 0.493298 |
| GO:0060537\_muscle\_tissue\_development | 67 | 1 | 8.699360 | -0.962194 | 192 | 45.083359 | 94.75 | 144.416641 | 0.493490 |
| GO:0009991\_response\_to\_extracellular\_stimulus | 68 | 1 | 8.571429 | -0.956101 | 193 | 45.624119 | 95.57 | 145.515881 | 0.495181 |
| GO:0051179\_localization | 1561 | 5 | 1.866935 | -0.952056 | 194 | 45.734326 | 95.76 | 145.785674 | 0.493608 |
| GO:0051241\_negative\_regulation\_of\_multicellular\_organismal\_process | 69 | 1 | 8.447205 | -0.950103 | 195 | 45.971411 | 96.38 | 146.788589 | 0.494256 |
| GO:0008104\_protein\_localization | 339 | 2 | 3.438685 | -0.947851 | 196 | 46.082023 | 96.52 | 146.957977 | 0.492449 |
| GO:0050789\_regulation\_of\_biological\_process | 3649 | 9 | 1.437576 | -0.941511 | 197 | 46.617970 | 97.68 | 148.742030 | 0.495838 |
| GO:0009615\_response\_to\_virus | 71 | 1 | 8.209256 | -0.938377 | 200 | 47.040795 | 98.28 | 149.519205 | 0.491400 |
| GO:0032101\_regulation\_of\_response\_to\_external\_stimulus | 71 | 1 | 8.209256 | -0.938377 | 200 | 47.040795 | 98.28 | 149.519205 | 0.491400 |
| GO:0051223\_regulation\_of\_protein\_transport | 71 | 1 | 8.209256 | -0.938377 | 200 | 47.040795 | 98.28 | 149.519205 | 0.491400 |
| GO:0007179\_transforming\_growth\_factor\_beta\_receptor\_signaling\_pathway | 72 | 1 | 8.095238 | -0.932645 | 201 | 47.226602 | 98.68 | 150.133398 | 0.490945 |
| GO:0007596\_blood\_coagulation | 73 | 1 | 7.984344 | -0.926996 | 202 | 47.890772 | 99.65 | 151.409228 | 0.493317 |
| GO:0050817\_coagulation | 74 | 1 | 7.876448 | -0.921428 | 203 | 48.052333 | 100.1 | 152.147667 | 0.493103 |
| GO:0006816\_calcium\_ion\_transport | 75 | 1 | 7.771429 | -0.915940 | 207 | 48.506075 | 101.32 | 154.133925 | 0.489469 |
| GO:0006869\_lipid\_transport | 75 | 1 | 7.771429 | -0.915940 | 207 | 48.506075 | 101.32 | 154.133925 | 0.489469 |
| GO:0044271\_nitrogen\_compound\_biosynthetic\_process | 75 | 1 | 7.771429 | -0.915940 | 207 | 48.506075 | 101.32 | 154.133925 | 0.489469 |
| GO:0070201\_regulation\_of\_establishment\_of\_protein\_localization | 75 | 1 | 7.771429 | -0.915940 | 207 | 48.506075 | 101.32 | 154.133925 | 0.489469 |
| GO:0006979\_response\_to\_oxidative\_stress | 76 | 1 | 7.669173 | -0.910529 | 209 | 48.796815 | 102.14 | 155.483185 | 0.488708 |
| GO:0070838\_divalent\_metal\_ion\_transport | 76 | 1 | 7.669173 | -0.910529 | 209 | 48.796815 | 102.14 | 155.483185 | 0.488708 |
| GO:0007599\_hemostasis | 79 | 1 | 7.377939 | -0.894739 | 211 | 49.938969 | 104.15 | 158.361031 | 0.493602 |
| GO:0032880\_regulation\_of\_protein\_localization | 79 | 1 | 7.377939 | -0.894739 | 211 | 49.938969 | 104.15 | 158.361031 | 0.493602 |
| GO:0007204\_elevation\_of\_cytosolic\_calcium\_ion\_concentration | 80 | 1 | 7.285714 | -0.889617 | 212 | 50.533804 | 105.23 | 159.926196 | 0.496368 |
| GO:0006952\_defense\_response | 369 | 2 | 3.159117 | -0.886688 | 213 | 50.604600 | 105.34 | 160.075400 | 0.494554 |
| GO:0019318\_hexose\_metabolic\_process | 81 | 1 | 7.195767 | -0.884563 | 215 | 51.380733 | 106.46 | 161.539267 | 0.495163 |
| GO:0051480\_cytosolic\_calcium\_ion\_homeostasis | 81 | 1 | 7.195767 | -0.884563 | 215 | 51.380733 | 106.46 | 161.539267 | 0.495163 |
| GO:0048514\_blood\_vessel\_morphogenesis | 82 | 1 | 7.108014 | -0.879575 | 216 | 51.706389 | 107.02 | 162.333611 | 0.495463 |
| GO:0051259\_protein\_oligomerization | 83 | 1 | 7.022375 | -0.874652 | 217 | 51.973423 | 107.41 | 162.846577 | 0.494977 |
| GO:0007243\_protein\_kinase\_cascade | 377 | 2 | 3.092080 | -0.871388 | 218 | 52.127947 | 107.63 | 163.132053 | 0.493716 |
| GO:0015674\_di-\_\_tri-valent\_inorganic\_cation\_transport | 84 | 1 | 6.938776 | -0.869792 | 219 | 52.363242 | 108.08 | 163.796758 | 0.493516 |
| GO:0001568\_blood\_vessel\_development | 85 | 1 | 6.857143 | -0.864993 | 220 | 52.503762 | 108.63 | 164.756238 | 0.493773 |
| GO:0033036\_macromolecule\_localization | 388 | 2 | 3.004418 | -0.850986 | 221 | 53.170603 | 109.56 | 165.949397 | 0.495747 |
| GO:0001944\_vasculature\_development | 88 | 1 | 6.623377 | -0.850951 | 222 | 53.177430 | 109.69 | 166.202570 | 0.494099 |
| GO:0030334\_regulation\_of\_cell\_migration | 89 | 1 | 6.548957 | -0.846384 | 225 | 53.503519 | 110.22 | 166.936481 | 0.489867 |
| GO:0043405\_regulation\_of\_MAP\_kinase\_activity | 89 | 1 | 6.548957 | -0.846384 | 225 | 53.503519 | 110.22 | 166.936481 | 0.489867 |
| GO:0051240\_positive\_regulation\_of\_multicellular\_organismal\_process | 89 | 1 | 6.548957 | -0.846384 | 225 | 53.503519 | 110.22 | 166.936481 | 0.489867 |
| GO:0051046\_regulation\_of\_secretion | 91 | 1 | 6.405024 | -0.837413 | 227 | 54.218259 | 111.34 | 168.461741 | 0.490485 |
| GO:0051130\_positive\_regulation\_of\_cellular\_component\_organization | 91 | 1 | 6.405024 | -0.837413 | 227 | 54.218259 | 111.34 | 168.461741 | 0.490485 |
| GO:0007178\_transmembrane\_receptor\_protein\_serine\_threonine\_kinase\_signaling\_pathway | 92 | 1 | 6.335404 | -0.833007 | 228 | 54.626493 | 111.81 | 168.993507 | 0.490395 |
| GO:0050878\_regulation\_of\_body\_fluid\_levels | 95 | 1 | 6.135338 | -0.820092 | 229 | 55.138201 | 112.62 | 170.101799 | 0.491790 |
| GO:0040012\_regulation\_of\_locomotion | 96 | 1 | 6.071429 | -0.815884 | 230 | 55.759351 | 113.52 | 171.280649 | 0.493565 |
| GO:0046486\_glycerolipid\_metabolic\_process | 97 | 1 | 6.008837 | -0.811724 | 231 | 56.018106 | 113.94 | 171.861894 | 0.493247 |
| GO:0042127\_regulation\_of\_cell\_proliferation | 411 | 2 | 2.836288 | -0.810534 | 232 | 56.102030 | 114.05 | 171.997970 | 0.491595 |
| GO:0009968\_negative\_regulation\_of\_signal\_transduction | 99 | 1 | 5.887446 | -0.803540 | 233 | 57.037648 | 115.44 | 173.842352 | 0.495451 |
| GO:0010648\_negative\_regulation\_of\_cell\_communication | 102 | 1 | 5.714286 | -0.791594 | 234 | 57.905404 | 116.81 | 175.714596 | 0.499188 |
| GO:0008544\_epidermis\_development | 104 | 1 | 5.604396 | -0.783840 | 235 | 58.496774 | 117.66 | 176.823226 | 0.500681 |
| GO:0044057\_regulation\_of\_system\_process | 106 | 1 | 5.498652 | -0.776246 | 236 | 58.912390 | 118.34 | 177.767610 | 0.501441 |
| GO:0050896\_response\_to\_stimulus | 1775 | 5 | 1.641851 | -0.769198 | 237 | 58.989480 | 118.6 | 178.210520 | 0.500422 |
| GO:0001558\_regulation\_of\_cell\_growth | 110 | 1 | 5.298701 | -0.761516 | 238 | 60.413730 | 120.7 | 180.986270 | 0.507143 |
| GO:0048646\_anatomical\_structure\_formation\_involved\_in\_morphogenesis | 111 | 1 | 5.250965 | -0.757925 | 239 | 60.590964 | 121.1 | 181.609036 | 0.506695 |
| GO:0007398\_ectoderm\_development | 112 | 1 | 5.204082 | -0.754369 | 240 | 61.136622 | 121.82 | 182.503378 | 0.507583 |
| GO:0006631\_fatty\_acid\_metabolic\_process | 113 | 1 | 5.158028 | -0.750847 | 241 | 61.262821 | 122.27 | 183.277179 | 0.507344 |
| GO:0006874\_cellular\_calcium\_ion\_homeostasis | 114 | 1 | 5.112782 | -0.747359 | 242 | 61.503124 | 122.6 | 183.696876 | 0.506612 |
| GO:0005996\_monosaccharide\_metabolic\_process | 115 | 1 | 5.068323 | -0.743905 | 243 | 62.064425 | 123.39 | 184.715575 | 0.507778 |
| GO:0055074\_calcium\_ion\_homeostasis | 116 | 1 | 5.024631 | -0.740484 | 244 | 62.322442 | 124.0 | 185.677558 | 0.508197 |
| GO:0065007\_biological\_regulation | 3971 | 9 | 1.321006 | -0.736182 | 245 | 62.533178 | 124.36 | 186.186822 | 0.507592 |
| GO:0006875\_cellular\_metal\_ion\_homeostasis | 121 | 1 | 4.817001 | -0.723849 | 246 | 63.522187 | 125.81 | 188.097813 | 0.511423 |
| GO:0033674\_positive\_regulation\_of\_kinase\_activity | 122 | 1 | 4.777518 | -0.720613 | 249 | 64.188138 | 126.92 | 189.651862 | 0.509719 |
| GO:0045860\_positive\_regulation\_of\_protein\_kinase\_activity | 122 | 1 | 4.777518 | -0.720613 | 249 | 64.188138 | 126.92 | 189.651862 | 0.509719 |
| GO:0060341\_regulation\_of\_cellular\_localization | 122 | 1 | 4.777518 | -0.720613 | 249 | 64.188138 | 126.92 | 189.651862 | 0.509719 |
| GO:0007517\_muscle\_organ\_development | 123 | 1 | 4.738676 | -0.717406 | 250 | 64.298097 | 127.12 | 189.941903 | 0.508480 |
| GO:0006897\_endocytosis | 124 | 1 | 4.700461 | -0.714227 | 252 | 65.066945 | 128.01 | 190.953055 | 0.507976 |
| GO:0010324\_membrane\_invagination | 124 | 1 | 4.700461 | -0.714227 | 252 | 65.066945 | 128.01 | 190.953055 | 0.507976 |
| GO:0006935\_chemotaxis | 125 | 1 | 4.662857 | -0.711077 | 256 | 65.725307 | 128.94 | 192.154693 | 0.503672 |
| GO:0042330\_taxis | 125 | 1 | 4.662857 | -0.711077 | 256 | 65.725307 | 128.94 | 192.154693 | 0.503672 |
| GO:0051707\_response\_to\_other\_organism | 125 | 1 | 4.662857 | -0.711077 | 256 | 65.725307 | 128.94 | 192.154693 | 0.503672 |
| GO:0055065\_metal\_ion\_homeostasis | 125 | 1 | 4.662857 | -0.711077 | 256 | 65.725307 | 128.94 | 192.154693 | 0.503672 |
| GO:0022607\_cellular\_component\_assembly | 478 | 2 | 2.438733 | -0.707196 | 257 | 66.211066 | 129.76 | 193.308934 | 0.504903 |
| GO:0007275\_multicellular\_organismal\_development | 1372 | 4 | 1.699292 | -0.703358 | 258 | 66.588245 | 130.16 | 193.731755 | 0.504496 |
| GO:0009725\_response\_to\_hormone\_stimulus | 129 | 1 | 4.518272 | -0.698749 | 260 | 67.172708 | 130.95 | 194.727292 | 0.503654 |
| GO:0051347\_positive\_regulation\_of\_transferase\_activity | 129 | 1 | 4.518272 | -0.698749 | 260 | 67.172708 | 130.95 | 194.727292 | 0.503654 |
| GO:0040008\_regulation\_of\_growth | 131 | 1 | 4.449291 | -0.692743 | 262 | 68.240387 | 132.41 | 196.579613 | 0.505382 |
| GO:0051345\_positive\_regulation\_of\_hydrolase\_activity | 131 | 1 | 4.449291 | -0.692743 | 262 | 68.240387 | 132.41 | 196.579613 | 0.505382 |
| GO:0003013\_circulatory\_system\_process | 133 | 1 | 4.382385 | -0.686837 | 264 | 68.897219 | 133.29 | 197.682781 | 0.504886 |
| GO:0008015\_blood\_circulation | 133 | 1 | 4.382385 | -0.686837 | 264 | 68.897219 | 133.29 | 197.682781 | 0.504886 |
| GO:0048523\_negative\_regulation\_of\_cellular\_process | 925 | 3 | 1.890347 | -0.685750 | 265 | 68.878730 | 133.41 | 197.941270 | 0.503434 |
| GO:0006936\_muscle\_contraction | 134 | 1 | 4.349680 | -0.683922 | 266 | 69.748018 | 134.4 | 199.051982 | 0.505263 |
| GO:0009719\_response\_to\_endogenous\_stimulus | 135 | 1 | 4.317460 | -0.681030 | 268 | 70.279084 | 135.32 | 200.360916 | 0.504925 |
| GO:0034641\_cellular\_nitrogen\_compound\_metabolic\_process | 135 | 1 | 4.317460 | -0.681030 | 268 | 70.279084 | 135.32 | 200.360916 | 0.504925 |
| GO:0009653\_anatomical\_structure\_morphogenesis | 500 | 2 | 2.331429 | -0.677234 | 269 | 70.783208 | 135.92 | 201.056792 | 0.505279 |
| GO:0030154\_cell\_differentiation | 506 | 2 | 2.303783 | -0.669359 | 270 | 71.623358 | 136.99 | 202.356642 | 0.507370 |
| GO:0030005\_cellular\_di-\_\_tri-valent\_inorganic\_cation\_homeostasis | 140 | 1 | 4.163265 | -0.666922 | 271 | 72.007781 | 137.47 | 202.932219 | 0.507269 |
| GO:0032502\_developmental\_process | 1919 | 5 | 1.518648 | -0.665221 | 272 | 72.122567 | 137.61 | 203.097433 | 0.505919 |
| GO:0003012\_muscle\_system\_process | 141 | 1 | 4.133739 | -0.664168 | 274 | 72.549920 | 138.19 | 203.830080 | 0.504343 |
| GO:0016049\_cell\_growth | 141 | 1 | 4.133739 | -0.664168 | 274 | 72.549920 | 138.19 | 203.830080 | 0.504343 |
| GO:0001501\_skeletal\_system\_development | 142 | 1 | 4.104628 | -0.661435 | 276 | 72.943830 | 138.75 | 204.556170 | 0.502717 |
| GO:0007626\_locomotory\_behavior | 142 | 1 | 4.104628 | -0.661435 | 276 | 72.943830 | 138.75 | 204.556170 | 0.502717 |
| GO:0055066\_di-\_\_tri-valent\_inorganic\_cation\_homeostasis | 145 | 1 | 4.019704 | -0.653366 | 277 | 74.410689 | 140.7 | 206.989311 | 0.507942 |
| GO:0080134\_regulation\_of\_response\_to\_stress | 147 | 1 | 3.965015 | -0.648090 | 278 | 75.588181 | 142.38 | 209.171819 | 0.512158 |
| GO:0008361\_regulation\_of\_cell\_size | 149 | 1 | 3.911793 | -0.642894 | 279 | 76.134949 | 143.17 | 210.205051 | 0.513154 |
| GO:0045321\_leukocyte\_activation | 150 | 1 | 3.885714 | -0.640326 | 280 | 76.231220 | 143.36 | 210.488780 | 0.512000 |
| GO:0019932\_second-messenger-mediated\_signaling | 153 | 1 | 3.809524 | -0.632734 | 281 | 77.633534 | 144.89 | 212.146466 | 0.515623 |
| GO:0032940\_secretion\_by\_cell | 155 | 1 | 3.760369 | -0.627766 | 282 | 78.017767 | 145.43 | 212.842233 | 0.515709 |
| GO:0016337\_cell-cell\_adhesion | 156 | 1 | 3.736264 | -0.625309 | 284 | 78.265504 | 145.85 | 213.434496 | 0.513556 |
| GO:0032787\_monocarboxylic\_acid\_metabolic\_process | 156 | 1 | 3.736264 | -0.625309 | 284 | 78.265504 | 145.85 | 213.434496 | 0.513556 |
| GO:0030003\_cellular\_cation\_homeostasis | 161 | 1 | 3.620231 | -0.613287 | 285 | 79.168646 | 146.93 | 214.691354 | 0.515544 |
| GO:0009987\_cellular\_process | 6671 | 13 | 1.135833 | -0.610019 | 286 | 79.256450 | 147.08 | 214.903550 | 0.514266 |
| GO:0048522\_positive\_regulation\_of\_cellular\_process | 1009 | 3 | 1.732975 | -0.609766 | 287 | 79.346436 | 147.3 | 215.253564 | 0.513240 |
| GO:0048869\_cellular\_developmental\_process | 555 | 2 | 2.100386 | -0.609351 | 288 | 79.503742 | 147.49 | 215.476258 | 0.512118 |
| GO:0006091\_generation\_of\_precursor\_metabolites\_and\_energy | 163 | 1 | 3.575811 | -0.608596 | 289 | 79.772079 | 147.8 | 215.827921 | 0.511419 |
| GO:0048519\_negative\_regulation\_of\_biological\_process | 1013 | 3 | 1.726132 | -0.606385 | 290 | 79.773258 | 147.95 | 216.126742 | 0.510172 |
| GO:0030029\_actin\_filament-based\_process | 165 | 1 | 3.532468 | -0.603971 | 291 | 80.287924 | 148.59 | 216.892076 | 0.510619 |
| GO:0044085\_cellular\_component\_biogenesis | 560 | 2 | 2.081633 | -0.603627 | 292 | 80.500854 | 149.04 | 217.579146 | 0.510411 |
| GO:0045595\_regulation\_of\_cell\_differentiation | 170 | 1 | 3.428571 | -0.592681 | 293 | 81.270364 | 150.23 | 219.189636 | 0.512730 |
| GO:0012501\_programmed\_cell\_death | 571 | 2 | 2.041531 | -0.591273 | 294 | 81.363927 | 150.35 | 219.336073 | 0.511395 |
| GO:0051649\_establishment\_of\_localization\_in\_cell | 573 | 2 | 2.034405 | -0.589062 | 295 | 81.473801 | 150.5 | 219.526199 | 0.510169 |
| GO:0055080\_cation\_homeostasis | 173 | 1 | 3.369116 | -0.586089 | 296 | 81.985557 | 151.25 | 220.514443 | 0.510980 |
| GO:0040007\_growth | 174 | 1 | 3.349754 | -0.583921 | 297 | 82.122761 | 151.47 | 220.817239 | 0.510000 |
| GO:0001775\_cell\_activation | 175 | 1 | 3.330612 | -0.581766 | 298 | 82.435002 | 152.03 | 221.624998 | 0.510168 |
| GO:0009607\_response\_to\_biotic\_stimulus | 177 | 1 | 3.292978 | -0.577500 | 300 | 83.264705 | 153.05 | 222.835295 | 0.510167 |
| GO:0016477\_cell\_migration | 177 | 1 | 3.292978 | -0.577500 | 300 | 83.264705 | 153.05 | 222.835295 | 0.510167 |
| GO:0008219\_cell\_death | 585 | 2 | 1.992674 | -0.576011 | 302 | 83.448000 | 153.29 | 223.132000 | 0.507583 |
| GO:0016265\_death | 585 | 2 | 1.992674 | -0.576011 | 302 | 83.448000 | 153.29 | 223.132000 | 0.507583 |
| GO:0006954\_inflammatory\_response | 182 | 1 | 3.202512 | -0.567074 | 303 | 84.606992 | 154.74 | 224.873008 | 0.510693 |
| GO:0010627\_regulation\_of\_protein\_kinase\_cascade | 184 | 1 | 3.167702 | -0.562996 | 304 | 85.076373 | 155.42 | 225.763627 | 0.511250 |
| GO:0016310\_phosphorylation | 601 | 2 | 1.939624 | -0.559171 | 305 | 85.908727 | 156.55 | 227.191273 | 0.513279 |
| GO:0030001\_metal\_ion\_transport | 186 | 1 | 3.133641 | -0.558969 | 306 | 86.320971 | 157.03 | 227.739029 | 0.513170 |
| GO:0048518\_positive\_regulation\_of\_biological\_process | 1094 | 3 | 1.598329 | -0.542080 | 307 | 88.302753 | 159.33 | 230.357247 | 0.518990 |
| GO:0043086\_negative\_regulation\_of\_catalytic\_activity | 196 | 1 | 2.973761 | -0.539561 | 308 | 88.909019 | 160.2 | 231.490981 | 0.520130 |
| GO:0048870\_cell\_motility | 197 | 1 | 2.958666 | -0.537684 | 309 | 89.115543 | 160.44 | 231.764457 | 0.519223 |
| GO:0008284\_positive\_regulation\_of\_cell\_proliferation | 200 | 1 | 2.914286 | -0.532119 | 310 | 89.366957 | 160.73 | 232.093043 | 0.518484 |
| GO:0008285\_negative\_regulation\_of\_cell\_proliferation | 202 | 1 | 2.885431 | -0.528464 | 311 | 89.989194 | 161.52 | 233.050806 | 0.519357 |
| GO:0009056\_catabolic\_process | 633 | 2 | 1.841571 | -0.527287 | 312 | 90.165807 | 161.71 | 233.254193 | 0.518301 |
| GO:0006066\_alcohol\_metabolic\_process | 206 | 1 | 2.829404 | -0.521279 | 314 | 91.071133 | 162.8 | 234.528867 | 0.518471 |
| GO:0006873\_cellular\_ion\_homeostasis | 206 | 1 | 2.829404 | -0.521279 | 314 | 91.071133 | 162.8 | 234.528867 | 0.518471 |
| GO:0055082\_cellular\_chemical\_homeostasis | 208 | 1 | 2.802198 | -0.517747 | 315 | 91.936240 | 163.88 | 235.823760 | 0.520254 |
| GO:0008283\_cell\_proliferation | 647 | 2 | 1.801722 | -0.514040 | 316 | 92.734440 | 164.85 | 236.965560 | 0.521677 |
| GO:0048731\_system\_development | 1140 | 3 | 1.533835 | -0.508803 | 317 | 93.639689 | 166.44 | 239.240311 | 0.525047 |
| GO:0007610\_behavior | 214 | 1 | 2.723632 | -0.507390 | 318 | 93.868634 | 166.71 | 239.551366 | 0.524245 |
| GO:0046903\_secretion | 218 | 1 | 2.673657 | -0.500674 | 319 | 94.448821 | 167.5 | 240.551179 | 0.525078 |
| GO:0050801\_ion\_homeostasis | 221 | 1 | 2.637363 | -0.495733 | 320 | 94.700211 | 167.84 | 240.979789 | 0.524500 |
| GO:0019725\_cellular\_homeostasis | 231 | 1 | 2.523191 | -0.479825 | 322 | 96.740811 | 170.58 | 244.419189 | 0.529752 |
| GO:0051704\_multi-organism\_process | 231 | 1 | 2.523191 | -0.479825 | 322 | 96.740811 | 170.58 | 244.419189 | 0.529752 |
| GO:0044092\_negative\_regulation\_of\_molecular\_function | 233 | 1 | 2.501533 | -0.476743 | 323 | 97.110738 | 171.09 | 245.069262 | 0.529690 |
| GO:0051128\_regulation\_of\_cellular\_component\_organization | 237 | 1 | 2.459313 | -0.470672 | 324 | 97.531635 | 171.66 | 245.788365 | 0.529815 |
| GO:0006793\_phosphorus\_metabolic\_process | 697 | 2 | 1.672474 | -0.469890 | 326 | 97.963472 | 172.22 | 246.476528 | 0.528282 |
| GO:0006796\_phosphate\_metabolic\_process | 697 | 2 | 1.672474 | -0.469890 | 326 | 97.963472 | 172.22 | 246.476528 | 0.528282 |
| GO:0048583\_regulation\_of\_response\_to\_stimulus | 241 | 1 | 2.418494 | -0.464724 | 327 | 98.411041 | 172.8 | 247.188959 | 0.528440 |
| GO:0006812\_cation\_transport | 246 | 1 | 2.369338 | -0.457456 | 329 | 100.285868 | 175.04 | 249.794132 | 0.532036 |
| GO:0009887\_organ\_morphogenesis | 246 | 1 | 2.369338 | -0.457456 | 329 | 100.285868 | 175.04 | 249.794132 | 0.532036 |
| GO:0006810\_transport | 1243 | 3 | 1.406735 | -0.441698 | 330 | 101.672692 | 176.99 | 252.307308 | 0.536333 |
| GO:0007167\_enzyme\_linked\_receptor\_protein\_signaling\_pathway | 258 | 1 | 2.259136 | -0.440723 | 331 | 101.847983 | 177.22 | 252.592017 | 0.535408 |
| GO:0048513\_organ\_development | 741 | 2 | 1.573164 | -0.434702 | 332 | 102.404709 | 177.92 | 253.435291 | 0.535904 |
| GO:0051234\_establishment\_of\_localization | 1260 | 3 | 1.387755 | -0.431517 | 333 | 102.935056 | 178.66 | 254.384944 | 0.536517 |
| GO:0006461\_protein\_complex\_assembly | 273 | 1 | 2.135008 | -0.421105 | 335 | 104.380887 | 180.23 | 256.079113 | 0.538000 |
| GO:0070271\_protein\_complex\_biogenesis | 273 | 1 | 2.135008 | -0.421105 | 335 | 104.380887 | 180.23 | 256.079113 | 0.538000 |
| GO:0015031\_protein\_transport | 274 | 1 | 2.127216 | -0.419845 | 336 | 104.582163 | 180.52 | 256.457837 | 0.537262 |
| GO:0048878\_chemical\_homeostasis | 278 | 1 | 2.096608 | -0.414862 | 337 | 106.018966 | 182.22 | 258.421034 | 0.540712 |
| GO:0048856\_anatomical\_structure\_development | 1289 | 3 | 1.356533 | -0.414686 | 338 | 106.223603 | 182.46 | 258.696397 | 0.539822 |
| GO:0045184\_establishment\_of\_protein\_localization | 279 | 1 | 2.089094 | -0.413630 | 339 | 106.614466 | 182.99 | 259.365534 | 0.539794 |
| GO:0019752\_carboxylic\_acid\_metabolic\_process | 286 | 1 | 2.037962 | -0.405158 | 341 | 107.440386 | 184.12 | 260.799614 | 0.539941 |
| GO:0043436\_oxoacid\_metabolic\_process | 286 | 1 | 2.037962 | -0.405158 | 341 | 107.440386 | 184.12 | 260.799614 | 0.539941 |
| GO:0006082\_organic\_acid\_metabolic\_process | 290 | 1 | 2.009852 | -0.400433 | 343 | 108.951738 | 185.98 | 263.008262 | 0.542216 |
| GO:0051093\_negative\_regulation\_of\_developmental\_process | 290 | 1 | 2.009852 | -0.400433 | 343 | 108.951738 | 185.98 | 263.008262 | 0.542216 |
| GO:0042180\_cellular\_ketone\_metabolic\_process | 291 | 1 | 2.002946 | -0.399265 | 344 | 109.091669 | 186.22 | 263.348331 | 0.541337 |
| GO:0005975\_carbohydrate\_metabolic\_process | 292 | 1 | 1.996086 | -0.398101 | 345 | 109.894045 | 187.18 | 264.465955 | 0.542551 |
| GO:0016192\_vesicle-mediated\_transport | 297 | 1 | 1.962482 | -0.392359 | 346 | 110.981935 | 188.67 | 266.358065 | 0.545289 |
| GO:0051172\_negative\_regulation\_of\_nitrogen\_compound\_metabolic\_process | 298 | 1 | 1.955896 | -0.391226 | 347 | 111.187479 | 188.98 | 266.772521 | 0.544611 |
| GO:0006508\_proteolysis | 313 | 1 | 1.862163 | -0.374780 | 348 | 113.282840 | 191.27 | 269.257160 | 0.549626 |
| GO:0007166\_cell\_surface\_receptor\_linked\_signal\_transduction | 828 | 2 | 1.407867 | -0.373648 | 349 | 113.522430 | 191.53 | 269.537570 | 0.548797 |
| GO:0016043\_cellular\_component\_organization | 1366 | 3 | 1.280067 | -0.373076 | 350 | 113.707883 | 191.77 | 269.832117 | 0.547914 |
| GO:0006811\_ion\_transport | 317 | 1 | 1.838666 | -0.370565 | 351 | 114.530514 | 192.65 | 270.769486 | 0.548860 |
| GO:0065008\_regulation\_of\_biological\_quality | 848 | 2 | 1.374663 | -0.361011 | 352 | 115.592704 | 194.3 | 273.007296 | 0.551989 |
| GO:0030163\_protein\_catabolic\_process | 330 | 1 | 1.766234 | -0.357325 | 353 | 116.141345 | 194.86 | 273.578655 | 0.552011 |
| GO:0031327\_negative\_regulation\_of\_cellular\_biosynthetic\_process | 332 | 1 | 1.755594 | -0.355348 | 354 | 116.521395 | 195.36 | 274.198605 | 0.551864 |
| GO:0009890\_negative\_regulation\_of\_biosynthetic\_process | 340 | 1 | 1.714286 | -0.347594 | 356 | 117.602619 | 196.93 | 276.257381 | 0.553174 |
| GO:0051094\_positive\_regulation\_of\_developmental\_process | 340 | 1 | 1.714286 | -0.347594 | 356 | 117.602619 | 196.93 | 276.257381 | 0.553174 |
| GO:0033554\_cellular\_response\_to\_stress | 341 | 1 | 1.709258 | -0.346642 | 357 | 117.877456 | 197.26 | 276.642544 | 0.552549 |
| GO:0006357\_regulation\_of\_transcription\_from\_RNA\_polymerase\_II\_promoter | 351 | 1 | 1.660562 | -0.337315 | 358 | 119.265873 | 198.68 | 278.094127 | 0.554972 |
| GO:0007186\_G-protein\_coupled\_receptor\_protein\_signaling\_pathway | 363 | 1 | 1.605667 | -0.326577 | 359 | 121.532629 | 201.29 | 281.047371 | 0.560696 |
| GO:0031323\_regulation\_of\_cellular\_metabolic\_process | 1466 | 3 | 1.192750 | -0.325045 | 360 | 121.752348 | 201.53 | 281.307652 | 0.559806 |
| GO:0065003\_macromolecular\_complex\_assembly | 366 | 1 | 1.592506 | -0.323967 | 361 | 122.750376 | 202.59 | 282.429624 | 0.561191 |
| GO:0009966\_regulation\_of\_signal\_transduction | 378 | 1 | 1.541950 | -0.313804 | 363 | 125.443126 | 205.56 | 285.676874 | 0.566281 |
| GO:0051239\_regulation\_of\_multicellular\_organismal\_process | 378 | 1 | 1.541950 | -0.313804 | 363 | 125.443126 | 205.56 | 285.676874 | 0.566281 |
| GO:0044255\_cellular\_lipid\_metabolic\_process | 381 | 1 | 1.529809 | -0.311332 | 364 | 125.651117 | 205.84 | 286.028883 | 0.565495 |
| GO:0032501\_multicellular\_organismal\_process | 2082 | 4 | 1.119802 | -0.303823 | 365 | 126.009381 | 206.32 | 286.630619 | 0.565260 |
| GO:0006468\_protein\_amino\_acid\_phosphorylation | 393 | 1 | 1.483097 | -0.301700 | 366 | 126.263499 | 206.62 | 286.976501 | 0.564536 |
| GO:0042592\_homeostatic\_process | 397 | 1 | 1.468154 | -0.298578 | 367 | 127.101078 | 207.59 | 288.078922 | 0.565640 |
| GO:0019222\_regulation\_of\_metabolic\_process | 1538 | 3 | 1.136913 | -0.294175 | 368 | 127.579198 | 208.11 | 288.640802 | 0.565516 |
| GO:0031324\_negative\_regulation\_of\_cellular\_metabolic\_process | 404 | 1 | 1.442716 | -0.293218 | 369 | 127.861997 | 208.44 | 289.018003 | 0.564878 |
| GO:0046907\_intracellular\_transport | 420 | 1 | 1.387755 | -0.281434 | 370 | 129.598050 | 210.45 | 291.301950 | 0.568784 |
| GO:0010646\_regulation\_of\_cell\_communication | 423 | 1 | 1.377913 | -0.279294 | 371 | 129.904109 | 210.82 | 291.735891 | 0.568248 |
| GO:0043933\_macromolecular\_complex\_subunit\_organization | 424 | 1 | 1.374663 | -0.278586 | 372 | 130.235336 | 211.12 | 292.004664 | 0.567527 |
| GO:0043285\_biopolymer\_catabolic\_process | 426 | 1 | 1.368209 | -0.277176 | 373 | 130.586579 | 211.49 | 292.393421 | 0.566997 |
| GO:0009057\_macromolecule\_catabolic\_process | 439 | 1 | 1.327693 | -0.268232 | 374 | 132.169900 | 213.29 | 294.410100 | 0.570294 |
| GO:0009892\_negative\_regulation\_of\_metabolic\_process | 440 | 1 | 1.324675 | -0.267560 | 375 | 132.427963 | 213.63 | 294.832037 | 0.569680 |
| GO:0051171\_regulation\_of\_nitrogen\_compound\_metabolic\_process | 1055 | 2 | 1.104942 | -0.254171 | 376 | 134.907403 | 216.37 | 297.832597 | 0.575452 |
| GO:0006629\_lipid\_metabolic\_process | 468 | 1 | 1.245421 | -0.249578 | 377 | 135.886271 | 217.38 | 298.873729 | 0.576605 |
| GO:0042981\_regulation\_of\_apoptosis | 471 | 1 | 1.237489 | -0.247743 | 378 | 136.998611 | 218.72 | 300.441389 | 0.578624 |
| GO:0051716\_cellular\_response\_to\_stimulus | 474 | 1 | 1.229656 | -0.245926 | 379 | 137.575877 | 219.29 | 301.004123 | 0.578602 |
| GO:0043067\_regulation\_of\_programmed\_cell\_death | 476 | 1 | 1.224490 | -0.244724 | 380 | 138.112195 | 220.02 | 301.927805 | 0.579000 |
| GO:0010941\_regulation\_of\_cell\_death | 478 | 1 | 1.219366 | -0.243529 | 381 | 138.689378 | 220.73 | 302.770622 | 0.579344 |
| GO:0006366\_transcription\_from\_RNA\_polymerase\_II\_promoter | 506 | 1 | 1.151892 | -0.227537 | 382 | 140.754170 | 223.11 | 305.465830 | 0.584058 |
| GO:0031326\_regulation\_of\_cellular\_biosynthetic\_process | 1125 | 2 | 1.036190 | -0.225991 | 383 | 141.043164 | 223.42 | 305.796836 | 0.583342 |
| GO:0009889\_regulation\_of\_biosynthetic\_process | 1135 | 2 | 1.027061 | -0.222232 | 384 | 141.318656 | 223.72 | 306.121344 | 0.582604 |
| GO:0006915\_apoptosis | 565 | 1 | 1.031606 | -0.197844 | 385 | 144.424923 | 227.04 | 309.655077 | 0.589714 |
| GO:0080090\_regulation\_of\_primary\_metabolic\_process | 1311 | 2 | 0.889179 | -0.165374 | 386 | 150.265337 | 233.05 | 315.834663 | 0.603756 |
| GO:0003008\_system\_process | 710 | 1 | 0.820926 | -0.142243 | 387 | 154.491218 | 237.04 | 319.588782 | 0.612506 |
| GO:0002376\_immune\_system\_process | 718 | 1 | 0.811779 | -0.139735 | 388 | 155.427234 | 237.75 | 320.072766 | 0.612758 |
| GO:0006355\_regulation\_of\_transcription\_\_DNA-dependent | 723 | 1 | 0.806165 | -0.138193 | 389 | 155.841731 | 238.16 | 320.478269 | 0.612237 |
| GO:0043687\_post-translational\_protein\_modification | 728 | 1 | 0.800628 | -0.136669 | 390 | 156.263562 | 238.53 | 320.796438 | 0.611615 |
| GO:0051252\_regulation\_of\_RNA\_metabolic\_process | 746 | 1 | 0.781310 | -0.131336 | 391 | 157.266890 | 239.51 | 321.753110 | 0.612558 |
| GO:0006996\_organelle\_organization | 764 | 1 | 0.762902 | -0.126231 | 392 | 158.472222 | 240.68 | 322.887778 | 0.613980 |
| GO:0006351\_transcription\_\_DNA-dependent | 884 | 1 | 0.659341 | -0.097215 | 393 | 162.606526 | 244.37 | 326.133474 | 0.621807 |
| GO:0032774\_RNA\_biosynthetic\_process | 887 | 1 | 0.657111 | -0.096587 | 394 | 163.260470 | 244.95 | 326.639530 | 0.621701 |
| GO:0045449\_regulation\_of\_transcription | 900 | 1 | 0.647619 | -0.093917 | 395 | 163.674691 | 245.35 | 327.025309 | 0.621139 |
| GO:0006464\_protein\_modification\_process | 922 | 1 | 0.632166 | -0.089574 | 396 | 164.374014 | 246.01 | 327.645986 | 0.621237 |
| GO:0043412\_biopolymer\_modification | 960 | 1 | 0.607143 | -0.082557 | 397 | 165.895970 | 247.3 | 328.704030 | 0.622922 |
| GO:0019219\_regulation\_of\_nucleobase\_\_nucleoside\_\_nucleotide\_and\_nucleic\_acid\_metabolic\_process | 1041 | 1 | 0.559901 | -0.069427 | 398 | 168.320866 | 249.2 | 330.079134 | 0.626131 |
| GO:0010556\_regulation\_of\_macromolecule\_biosynthetic\_process | 1055 | 1 | 0.552471 | -0.067384 | 399 | 169.127944 | 249.88 | 330.632056 | 0.626266 |
| GO:0010468\_regulation\_of\_gene\_expression | 1067 | 1 | 0.546258 | -0.065681 | 400 | 169.682310 | 250.44 | 331.197690 | 0.626100 |
| GO:0006350\_transcription | 1069 | 1 | 0.545236 | -0.065401 | 401 | 170.113450 | 250.81 | 331.506550 | 0.625461 |
| GO:0044249\_cellular\_biosynthetic\_process | 1951 | 2 | 0.597496 | -0.054304 | 402 | 172.153166 | 252.37 | 332.586834 | 0.627786 |
| GO:0009058\_biosynthetic\_process | 1988 | 2 | 0.586375 | -0.050758 | 403 | 172.390264 | 252.55 | 332.709736 | 0.626675 |
| GO:0016070\_RNA\_metabolic\_process | 1230 | 1 | 0.473868 | -0.046400 | 404 | 173.034977 | 253.08 | 333.125023 | 0.626436 |
| GO:0006807\_nitrogen\_compound\_metabolic\_process | 2053 | 2 | 0.567810 | -0.045033 | 405 | 173.469185 | 253.55 | 333.630815 | 0.626049 |
| GO:0060255\_regulation\_of\_macromolecule\_metabolic\_process | 1328 | 1 | 0.438898 | -0.037629 | 406 | 175.664283 | 255.25 | 334.835717 | 0.628695 |
| GO:0044267\_cellular\_protein\_metabolic\_process | 1382 | 1 | 0.421749 | -0.033512 | 407 | 176.983649 | 256.26 | 335.536351 | 0.629631 |
| GO:0034961\_cellular\_biopolymer\_biosynthetic\_process | 1448 | 1 | 0.402526 | -0.029071 | 408 | 177.657302 | 256.74 | 335.822698 | 0.629265 |
| GO:0043284\_biopolymer\_biosynthetic\_process | 1458 | 1 | 0.399765 | -0.028450 | 409 | 177.966809 | 256.97 | 335.973191 | 0.628289 |
| GO:0044238\_primary\_metabolic\_process | 3719 | 4 | 0.626897 | -0.025908 | 410 | 178.691916 | 257.45 | 336.208084 | 0.627927 |
| GO:0044237\_cellular\_metabolic\_process | 3753 | 4 | 0.621217 | -0.024294 | 411 | 178.915102 | 257.6 | 336.284898 | 0.626764 |
| GO:0019538\_protein\_metabolic\_process | 1569 | 1 | 0.371483 | -0.022357 | 412 | 179.957331 | 258.34 | 336.722669 | 0.627039 |
| GO:0034645\_cellular\_macromolecule\_biosynthetic\_process | 1600 | 1 | 0.364286 | -0.020893 | 413 | 180.248468 | 258.54 | 336.831532 | 0.626005 |
| GO:0009059\_macromolecule\_biosynthetic\_process | 1626 | 1 | 0.358461 | -0.019736 | 414 | 180.537644 | 258.74 | 336.942356 | 0.624976 |
| GO:0010467\_gene\_expression | 1663 | 1 | 0.350485 | -0.018194 | 415 | 180.783252 | 258.93 | 337.076748 | 0.623928 |
| GO:0006139\_nucleobase\_\_nucleoside\_\_nucleotide\_and\_nucleic\_acid\_metabolic\_process | 1845 | 1 | 0.315912 | -0.012133 | 416 | 181.375980 | 259.32 | 337.264020 | 0.623365 |
| GO:0008152\_metabolic\_process | 4111 | 4 | 0.567120 | -0.011788 | 417 | 181.503279 | 259.39 | 337.276721 | 0.622038 |
| GO:0034960\_cellular\_biopolymer\_metabolic\_process | 2820 | 2 | 0.413374 | -0.009702 | 418 | 182.012322 | 259.73 | 337.447678 | 0.621364 |
| GO:0044260\_cellular\_macromolecule\_metabolic\_process | 2883 | 2 | 0.404341 | -0.008453 | 419 | 182.391904 | 259.99 | 337.588096 | 0.620501 |
| GO:0043283\_biopolymer\_metabolic\_process | 3027 | 2 | 0.385105 | -0.006122 | 420 | 182.974916 | 260.42 | 337.865084 | 0.620048 |
| GO:0043170\_macromolecule\_metabolic\_process | 3103 | 2 | 0.375673 | -0.005141 | 421 | 183.038837 | 260.47 | 337.901163 | 0.618694 |
| GO:0000718\_nucleotide-excision\_repair\_\_DNA\_damage\_removal | 21 | 0 | 0.000000 | -0.000000 | 464 | 228.171525 | 304.65 | 381.128475 | 0.656573 |
| GO:0001654\_eye\_development | 21 | 0 | 0.000000 | -0.000000 | 464 | 228.171525 | 304.65 | 381.128475 | 0.656573 |
| GO:0002819\_regulation\_of\_adaptive\_immune\_response | 21 | 0 | 0.000000 | -0.000000 | 464 | 228.171525 | 304.65 | 381.128475 | 0.656573 |
| GO:0002822\_regulation\_of\_adaptive\_immune\_response\_based\_on\_somatic\_recombination\_of\_immune\_receptors\_built\_from\_immunoglobulin\_superfamily\_domains | 21 | 0 | 0.000000 | -0.000000 | 464 | 228.171525 | 304.65 | 381.128475 | 0.656573 |
| GO:0002831\_regulation\_of\_response\_to\_biotic\_stimulus | 21 | 0 | 0.000000 | -0.000000 | 464 | 228.171525 | 304.65 | 381.128475 | 0.656573 |
| GO:0006024\_glycosaminoglycan\_biosynthetic\_process | 21 | 0 | 0.000000 | -0.000000 | 464 | 228.171525 | 304.65 | 381.128475 | 0.656573 |
| GO:0006040\_amino\_sugar\_metabolic\_process | 21 | 0 | 0.000000 | -0.000000 | 464 | 228.171525 | 304.65 | 381.128475 | 0.656573 |
| GO:0006073\_cellular\_glucan\_metabolic\_process | 21 | 0 | 0.000000 | -0.000000 | 464 | 228.171525 | 304.65 | 381.128475 | 0.656573 |
| GO:0006109\_regulation\_of\_carbohydrate\_metabolic\_process | 21 | 0 | 0.000000 | -0.000000 | 464 | 228.171525 | 304.65 | 381.128475 | 0.656573 |
| GO:0006282\_regulation\_of\_DNA\_repair | 21 | 0 | 0.000000 | -0.000000 | 464 | 228.171525 | 304.65 | 381.128475 | 0.656573 |
| GO:0006284\_base-excision\_repair | 21 | 0 | 0.000000 | -0.000000 | 464 | 228.171525 | 304.65 | 381.128475 | 0.656573 |
| GO:0006402\_mRNA\_catabolic\_process | 21 | 0 | 0.000000 | -0.000000 | 464 | 228.171525 | 304.65 | 381.128475 | 0.656573 |
| GO:0006493\_protein\_amino\_acid\_O-linked\_glycosylation | 21 | 0 | 0.000000 | -0.000000 | 464 | 228.171525 | 304.65 | 381.128475 | 0.656573 |
| GO:0006664\_glycolipid\_metabolic\_process | 21 | 0 | 0.000000 | -0.000000 | 464 | 228.171525 | 304.65 | 381.128475 | 0.656573 |
| GO:0006775\_fat-soluble\_vitamin\_metabolic\_process | 21 | 0 | 0.000000 | -0.000000 | 464 | 228.171525 | 304.65 | 381.128475 | 0.656573 |
| GO:0006892\_post-Golgi\_vesicle-mediated\_transport | 21 | 0 | 0.000000 | -0.000000 | 464 | 228.171525 | 304.65 | 381.128475 | 0.656573 |
| GO:0006903\_vesicle\_targeting | 21 | 0 | 0.000000 | -0.000000 | 464 | 228.171525 | 304.65 | 381.128475 | 0.656573 |
| GO:0007292\_female\_gamete\_generation | 21 | 0 | 0.000000 | -0.000000 | 464 | 228.171525 | 304.65 | 381.128475 | 0.656573 |
| GO:0008156\_negative\_regulation\_of\_DNA\_replication | 21 | 0 | 0.000000 | -0.000000 | 464 | 228.171525 | 304.65 | 381.128475 | 0.656573 |
| GO:0008360\_regulation\_of\_cell\_shape | 21 | 0 | 0.000000 | -0.000000 | 464 | 228.171525 | 304.65 | 381.128475 | 0.656573 |
| GO:0009199\_ribonucleoside\_triphosphate\_metabolic\_process | 21 | 0 | 0.000000 | -0.000000 | 464 | 228.171525 | 304.65 | 381.128475 | 0.656573 |
| GO:0010675\_regulation\_of\_cellular\_carbohydrate\_metabolic\_process | 21 | 0 | 0.000000 | -0.000000 | 464 | 228.171525 | 304.65 | 381.128475 | 0.656573 |
| GO:0014031\_mesenchymal\_cell\_development | 21 | 0 | 0.000000 | -0.000000 | 464 | 228.171525 | 304.65 | 381.128475 | 0.656573 |
| GO:0016338\_calcium-independent\_cell-cell\_adhesion | 21 | 0 | 0.000000 | -0.000000 | 464 | 228.171525 | 304.65 | 381.128475 | 0.656573 |
| GO:0030111\_regulation\_of\_Wnt\_receptor\_signaling\_pathway | 21 | 0 | 0.000000 | -0.000000 | 464 | 228.171525 | 304.65 | 381.128475 | 0.656573 |
| GO:0030148\_sphingolipid\_biosynthetic\_process | 21 | 0 | 0.000000 | -0.000000 | 464 | 228.171525 | 304.65 | 381.128475 | 0.656573 |
| GO:0032768\_regulation\_of\_monooxygenase\_activity | 21 | 0 | 0.000000 | -0.000000 | 464 | 228.171525 | 304.65 | 381.128475 | 0.656573 |
| GO:0032886\_regulation\_of\_microtubule-based\_process | 21 | 0 | 0.000000 | -0.000000 | 464 | 228.171525 | 304.65 | 381.128475 | 0.656573 |
| GO:0035295\_tube\_development | 21 | 0 | 0.000000 | -0.000000 | 464 | 228.171525 | 304.65 | 381.128475 | 0.656573 |
| GO:0042398\_cellular\_amino\_acid\_derivative\_biosynthetic\_process | 21 | 0 | 0.000000 | -0.000000 | 464 | 228.171525 | 304.65 | 381.128475 | 0.656573 |
| GO:0042439\_ethanolamine\_and\_derivative\_metabolic\_process | 21 | 0 | 0.000000 | -0.000000 | 464 | 228.171525 | 304.65 | 381.128475 | 0.656573 |
| GO:0043523\_regulation\_of\_neuron\_apoptosis | 21 | 0 | 0.000000 | -0.000000 | 464 | 228.171525 | 304.65 | 381.128475 | 0.656573 |
| GO:0044042\_glucan\_metabolic\_process | 21 | 0 | 0.000000 | -0.000000 | 464 | 228.171525 | 304.65 | 381.128475 | 0.656573 |
| GO:0045649\_regulation\_of\_macrophage\_differentiation | 21 | 0 | 0.000000 | -0.000000 | 464 | 228.171525 | 304.65 | 381.128475 | 0.656573 |
| GO:0046427\_positive\_regulation\_of\_JAK-STAT\_cascade | 21 | 0 | 0.000000 | -0.000000 | 464 | 228.171525 | 304.65 | 381.128475 | 0.656573 |
| GO:0048520\_positive\_regulation\_of\_behavior | 21 | 0 | 0.000000 | -0.000000 | 464 | 228.171525 | 304.65 | 381.128475 | 0.656573 |
| GO:0048762\_mesenchymal\_cell\_differentiation | 21 | 0 | 0.000000 | -0.000000 | 464 | 228.171525 | 304.65 | 381.128475 | 0.656573 |
| GO:0051238\_sequestering\_of\_metal\_ion | 21 | 0 | 0.000000 | -0.000000 | 464 | 228.171525 | 304.65 | 381.128475 | 0.656573 |
| GO:0051297\_centrosome\_organization | 21 | 0 | 0.000000 | -0.000000 | 464 | 228.171525 | 304.65 | 381.128475 | 0.656573 |
| GO:0055072\_iron\_ion\_homeostasis | 21 | 0 | 0.000000 | -0.000000 | 464 | 228.171525 | 304.65 | 381.128475 | 0.656573 |
| GO:0060485\_mesenchyme\_development | 21 | 0 | 0.000000 | -0.000000 | 464 | 228.171525 | 304.65 | 381.128475 | 0.656573 |
| GO:0070167\_regulation\_of\_biomineral\_formation | 21 | 0 | 0.000000 | -0.000000 | 464 | 228.171525 | 304.65 | 381.128475 | 0.656573 |
| GO:0070507\_regulation\_of\_microtubule\_cytoskeleton\_organization | 21 | 0 | 0.000000 | -0.000000 | 464 | 228.171525 | 304.65 | 381.128475 | 0.656573 |
| GO:0006403\_RNA\_localization | 33 | 0 | 0.000000 | -0.000000 | 473 | 238.729142 | 314.93 | 391.130858 | 0.665814 |
| GO:0009259\_ribonucleotide\_metabolic\_process | 33 | 0 | 0.000000 | -0.000000 | 473 | 238.729142 | 314.93 | 391.130858 | 0.665814 |
| GO:0009581\_detection\_of\_external\_stimulus | 33 | 0 | 0.000000 | -0.000000 | 473 | 238.729142 | 314.93 | 391.130858 | 0.665814 |
| GO:0016458\_gene\_silencing | 33 | 0 | 0.000000 | -0.000000 | 473 | 238.729142 | 314.93 | 391.130858 | 0.665814 |
| GO:0030217\_T\_cell\_differentiation | 33 | 0 | 0.000000 | -0.000000 | 473 | 238.729142 | 314.93 | 391.130858 | 0.665814 |
| GO:0042108\_positive\_regulation\_of\_cytokine\_biosynthetic\_process | 33 | 0 | 0.000000 | -0.000000 | 473 | 238.729142 | 314.93 | 391.130858 | 0.665814 |
| GO:0043392\_negative\_regulation\_of\_DNA\_binding | 33 | 0 | 0.000000 | -0.000000 | 473 | 238.729142 | 314.93 | 391.130858 | 0.665814 |
| GO:0050870\_positive\_regulation\_of\_T\_cell\_activation | 33 | 0 | 0.000000 | -0.000000 | 473 | 238.729142 | 314.93 | 391.130858 | 0.665814 |
| GO:0051341\_regulation\_of\_oxidoreductase\_activity | 33 | 0 | 0.000000 | -0.000000 | 473 | 238.729142 | 314.93 | 391.130858 | 0.665814 |
| GO:0008150\_biological\_process | 8160 | 14 | 1.000000 | 0.000000 | 474 | 256.774960 | 327.95 | 399.125040 | 0.691878 |
| GO:0006396\_RNA\_processing | 306 | 0 | 0.000000 | 0.000000 | 475 | 257.307948 | 328.56 | 399.812052 | 0.691705 |
| GO:0045941\_positive\_regulation\_of\_transcription | 264 | 0 | 0.000000 | 0.000000 | 476 | 258.034168 | 329.09 | 400.145832 | 0.691366 |
| GO:0034622\_cellular\_macromolecular\_complex\_assembly | 186 | 0 | 0.000000 | 0.000000 | 477 | 259.617058 | 330.55 | 401.482942 | 0.692977 |
| GO:0000070\_mitotic\_sister\_chromatid\_segregation | 27 | 0 | 0.000000 | 0.000000 | 495 | 281.971247 | 352.32 | 422.668753 | 0.711758 |
| GO:0000245\_spliceosome\_assembly | 27 | 0 | 0.000000 | 0.000000 | 495 | 281.971247 | 352.32 | 422.668753 | 0.711758 |
| GO:0003002\_regionalization | 27 | 0 | 0.000000 | 0.000000 | 495 | 281.971247 | 352.32 | 422.668753 | 0.711758 |
| GO:0006690\_icosanoid\_metabolic\_process | 27 | 0 | 0.000000 | 0.000000 | 495 | 281.971247 | 352.32 | 422.668753 | 0.711758 |
| GO:0006836\_neurotransmitter\_transport | 27 | 0 | 0.000000 | 0.000000 | 495 | 281.971247 | 352.32 | 422.668753 | 0.711758 |
| GO:0006909\_phagocytosis | 27 | 0 | 0.000000 | 0.000000 | 495 | 281.971247 | 352.32 | 422.668753 | 0.711758 |
| GO:0007260\_tyrosine\_phosphorylation\_of\_STAT\_protein | 27 | 0 | 0.000000 | 0.000000 | 495 | 281.971247 | 352.32 | 422.668753 | 0.711758 |
| GO:0007631\_feeding\_behavior | 27 | 0 | 0.000000 | 0.000000 | 495 | 281.971247 | 352.32 | 422.668753 | 0.711758 |
| GO:0008543\_fibroblast\_growth\_factor\_receptor\_signaling\_pathway | 27 | 0 | 0.000000 | 0.000000 | 495 | 281.971247 | 352.32 | 422.668753 | 0.711758 |
| GO:0019079\_viral\_genome\_replication | 27 | 0 | 0.000000 | 0.000000 | 495 | 281.971247 | 352.32 | 422.668753 | 0.711758 |
| GO:0031349\_positive\_regulation\_of\_defense\_response | 27 | 0 | 0.000000 | 0.000000 | 495 | 281.971247 | 352.32 | 422.668753 | 0.711758 |
| GO:0032200\_telomere\_organization | 27 | 0 | 0.000000 | 0.000000 | 495 | 281.971247 | 352.32 | 422.668753 | 0.711758 |
| GO:0043254\_regulation\_of\_protein\_complex\_assembly | 27 | 0 | 0.000000 | 0.000000 | 495 | 281.971247 | 352.32 | 422.668753 | 0.711758 |
| GO:0044272\_sulfur\_compound\_biosynthetic\_process | 27 | 0 | 0.000000 | 0.000000 | 495 | 281.971247 | 352.32 | 422.668753 | 0.711758 |
| GO:0050906\_detection\_of\_stimulus\_involved\_in\_sensory\_perception | 27 | 0 | 0.000000 | 0.000000 | 495 | 281.971247 | 352.32 | 422.668753 | 0.711758 |
| GO:0051048\_negative\_regulation\_of\_secretion | 27 | 0 | 0.000000 | 0.000000 | 495 | 281.971247 | 352.32 | 422.668753 | 0.711758 |
| GO:0051092\_positive\_regulation\_of\_NF-kappaB\_transcription\_factor\_activity | 27 | 0 | 0.000000 | 0.000000 | 495 | 281.971247 | 352.32 | 422.668753 | 0.711758 |
| GO:0051607\_defense\_response\_to\_virus | 27 | 0 | 0.000000 | 0.000000 | 495 | 281.971247 | 352.32 | 422.668753 | 0.711758 |
| GO:0009891\_positive\_regulation\_of\_biosynthetic\_process | 359 | 0 | 0.000000 | 0.000000 | 496 | 282.626102 | 352.75 | 422.873898 | 0.711190 |
| GO:0003007\_heart\_morphogenesis | 29 | 0 | 0.000000 | 0.000000 | 506 | 302.249329 | 371.79 | 441.330671 | 0.734763 |
| GO:0006505\_GPI\_anchor\_metabolic\_process | 29 | 0 | 0.000000 | 0.000000 | 506 | 302.249329 | 371.79 | 441.330671 | 0.734763 |
| GO:0006506\_GPI\_anchor\_biosynthetic\_process | 29 | 0 | 0.000000 | 0.000000 | 506 | 302.249329 | 371.79 | 441.330671 | 0.734763 |
| GO:0030278\_regulation\_of\_ossification | 29 | 0 | 0.000000 | 0.000000 | 506 | 302.249329 | 371.79 | 441.330671 | 0.734763 |
| GO:0043112\_receptor\_metabolic\_process | 29 | 0 | 0.000000 | 0.000000 | 506 | 302.249329 | 371.79 | 441.330671 | 0.734763 |
| GO:0043433\_negative\_regulation\_of\_transcription\_factor\_activity | 29 | 0 | 0.000000 | 0.000000 | 506 | 302.249329 | 371.79 | 441.330671 | 0.734763 |
| GO:0048741\_skeletal\_muscle\_fiber\_development | 29 | 0 | 0.000000 | 0.000000 | 506 | 302.249329 | 371.79 | 441.330671 | 0.734763 |
| GO:0050731\_positive\_regulation\_of\_peptidyl-tyrosine\_phosphorylation | 29 | 0 | 0.000000 | 0.000000 | 506 | 302.249329 | 371.79 | 441.330671 | 0.734763 |
| GO:0051648\_vesicle\_localization | 29 | 0 | 0.000000 | 0.000000 | 506 | 302.249329 | 371.79 | 441.330671 | 0.734763 |
| GO:0090048\_negative\_regulation\_of\_transcription\_regulator\_activity | 29 | 0 | 0.000000 | 0.000000 | 506 | 302.249329 | 371.79 | 441.330671 | 0.734763 |
| GO:0031328\_positive\_regulation\_of\_cellular\_biosynthetic\_process | 352 | 0 | 0.000000 | 0.000000 | 507 | 302.900715 | 372.22 | 441.539285 | 0.734162 |
| GO:0006665\_sphingolipid\_metabolic\_process | 44 | 0 | 0.000000 | 0.000000 | 514 | 311.176657 | 380.04 | 448.903343 | 0.739377 |
| GO:0006730\_one-carbon\_metabolic\_process | 44 | 0 | 0.000000 | 0.000000 | 514 | 311.176657 | 380.04 | 448.903343 | 0.739377 |
| GO:0006959\_humoral\_immune\_response | 44 | 0 | 0.000000 | 0.000000 | 514 | 311.176657 | 380.04 | 448.903343 | 0.739377 |
| GO:0016125\_sterol\_metabolic\_process | 44 | 0 | 0.000000 | 0.000000 | 514 | 311.176657 | 380.04 | 448.903343 | 0.739377 |
| GO:0050900\_leukocyte\_migration | 44 | 0 | 0.000000 | 0.000000 | 514 | 311.176657 | 380.04 | 448.903343 | 0.739377 |
| GO:0051301\_cell\_division | 44 | 0 | 0.000000 | 0.000000 | 514 | 311.176657 | 380.04 | 448.903343 | 0.739377 |
| GO:0051321\_meiotic\_cell\_cycle | 44 | 0 | 0.000000 | 0.000000 | 514 | 311.176657 | 380.04 | 448.903343 | 0.739377 |
| GO:0000079\_regulation\_of\_cyclin-dependent\_protein\_kinase\_activity | 48 | 0 | 0.000000 | 0.000000 | 521 | 318.798679 | 387.25 | 455.701321 | 0.743282 |
| GO:0008217\_regulation\_of\_blood\_pressure | 48 | 0 | 0.000000 | 0.000000 | 521 | 318.798679 | 387.25 | 455.701321 | 0.743282 |
| GO:0010551\_regulation\_of\_specific\_transcription\_from\_RNA\_polymerase\_II\_promoter | 48 | 0 | 0.000000 | 0.000000 | 521 | 318.798679 | 387.25 | 455.701321 | 0.743282 |
| GO:0032569\_specific\_transcription\_from\_RNA\_polymerase\_II\_promoter | 48 | 0 | 0.000000 | 0.000000 | 521 | 318.798679 | 387.25 | 455.701321 | 0.743282 |
| GO:0042035\_regulation\_of\_cytokine\_biosynthetic\_process | 48 | 0 | 0.000000 | 0.000000 | 521 | 318.798679 | 387.25 | 455.701321 | 0.743282 |
| GO:0042773\_ATP\_synthesis\_coupled\_electron\_transport | 48 | 0 | 0.000000 | 0.000000 | 521 | 318.798679 | 387.25 | 455.701321 | 0.743282 |
| GO:0042775\_mitochondrial\_ATP\_synthesis\_coupled\_electron\_transport | 48 | 0 | 0.000000 | 0.000000 | 521 | 318.798679 | 387.25 | 455.701321 | 0.743282 |
| GO:0002697\_regulation\_of\_immune\_effector\_process | 49 | 0 | 0.000000 | 0.000000 | 531 | 329.319229 | 397.14 | 464.960771 | 0.747910 |
| GO:0006275\_regulation\_of\_DNA\_replication | 49 | 0 | 0.000000 | 0.000000 | 531 | 329.319229 | 397.14 | 464.960771 | 0.747910 |
| GO:0006643\_membrane\_lipid\_metabolic\_process | 49 | 0 | 0.000000 | 0.000000 | 531 | 329.319229 | 397.14 | 464.960771 | 0.747910 |
| GO:0009914\_hormone\_transport | 49 | 0 | 0.000000 | 0.000000 | 531 | 329.319229 | 397.14 | 464.960771 | 0.747910 |
| GO:0010952\_positive\_regulation\_of\_peptidase\_activity | 49 | 0 | 0.000000 | 0.000000 | 531 | 329.319229 | 397.14 | 464.960771 | 0.747910 |
| GO:0018108\_peptidyl-tyrosine\_phosphorylation | 49 | 0 | 0.000000 | 0.000000 | 531 | 329.319229 | 397.14 | 464.960771 | 0.747910 |
| GO:0032984\_macromolecular\_complex\_disassembly | 49 | 0 | 0.000000 | 0.000000 | 531 | 329.319229 | 397.14 | 464.960771 | 0.747910 |
| GO:0043280\_positive\_regulation\_of\_caspase\_activity | 49 | 0 | 0.000000 | 0.000000 | 531 | 329.319229 | 397.14 | 464.960771 | 0.747910 |
| GO:0050863\_regulation\_of\_T\_cell\_activation | 49 | 0 | 0.000000 | 0.000000 | 531 | 329.319229 | 397.14 | 464.960771 | 0.747910 |
| GO:0050867\_positive\_regulation\_of\_cell\_activation | 49 | 0 | 0.000000 | 0.000000 | 531 | 329.319229 | 397.14 | 464.960771 | 0.747910 |
| GO:0010740\_positive\_regulation\_of\_protein\_kinase\_cascade | 129 | 0 | 0.000000 | 0.000000 | 533 | 332.719666 | 400.27 | 467.820334 | 0.750976 |
| GO:0015672\_monovalent\_inorganic\_cation\_transport | 129 | 0 | 0.000000 | 0.000000 | 533 | 332.719666 | 400.27 | 467.820334 | 0.750976 |
| GO:0000377\_RNA\_splicing\_\_via\_transesterification\_reactions\_with\_bulged\_adenosine\_as\_nucleophile | 151 | 0 | 0.000000 | 0.000000 | 535 | 334.018363 | 401.73 | 469.441637 | 0.750897 |
| GO:0000398\_nuclear\_mRNA\_splicing\_\_via\_spliceosome | 151 | 0 | 0.000000 | 0.000000 | 535 | 334.018363 | 401.73 | 469.441637 | 0.750897 |
| GO:0031401\_positive\_regulation\_of\_protein\_modification\_process | 84 | 0 | 0.000000 | 0.000000 | 537 | 336.783522 | 404.23 | 471.676478 | 0.752756 |
| GO:0043123\_positive\_regulation\_of\_I-kappaB\_kinase\_NF-kappaB\_cascade | 84 | 0 | 0.000000 | 0.000000 | 537 | 336.783522 | 404.23 | 471.676478 | 0.752756 |
| GO:0000226\_microtubule\_cytoskeleton\_organization | 86 | 0 | 0.000000 | 0.000000 | 538 | 337.680805 | 405.06 | 472.439195 | 0.752900 |
| GO:0006417\_regulation\_of\_translation | 71 | 0 | 0.000000 | 0.000000 | 541 | 343.276225 | 410.42 | 477.563775 | 0.758632 |
| GO:0007050\_cell\_cycle\_arrest | 71 | 0 | 0.000000 | 0.000000 | 541 | 343.276225 | 410.42 | 477.563775 | 0.758632 |
| GO:0051606\_detection\_of\_stimulus | 71 | 0 | 0.000000 | 0.000000 | 541 | 343.276225 | 410.42 | 477.563775 | 0.758632 |
| GO:0070727\_cellular\_macromolecule\_localization | 229 | 0 | 0.000000 | 0.000000 | 542 | 344.002985 | 411.11 | 478.217015 | 0.758506 |
| GO:0010629\_negative\_regulation\_of\_gene\_expression | 289 | 0 | 0.000000 | 0.000000 | 543 | 344.755817 | 411.79 | 478.824183 | 0.758361 |
| GO:0006732\_coenzyme\_metabolic\_process | 65 | 0 | 0.000000 | 0.000000 | 549 | 353.166707 | 419.66 | 486.153293 | 0.764408 |
| GO:0016054\_organic\_acid\_catabolic\_process | 65 | 0 | 0.000000 | 0.000000 | 549 | 353.166707 | 419.66 | 486.153293 | 0.764408 |
| GO:0022415\_viral\_reproductive\_process | 65 | 0 | 0.000000 | 0.000000 | 549 | 353.166707 | 419.66 | 486.153293 | 0.764408 |
| GO:0046395\_carboxylic\_acid\_catabolic\_process | 65 | 0 | 0.000000 | 0.000000 | 549 | 353.166707 | 419.66 | 486.153293 | 0.764408 |
| GO:0051437\_positive\_regulation\_of\_ubiquitin-protein\_ligase\_activity\_during\_mitotic\_cell\_cycle | 65 | 0 | 0.000000 | 0.000000 | 549 | 353.166707 | 419.66 | 486.153293 | 0.764408 |
| GO:0080135\_regulation\_of\_cellular\_response\_to\_stress | 65 | 0 | 0.000000 | 0.000000 | 549 | 353.166707 | 419.66 | 486.153293 | 0.764408 |
| GO:0002684\_positive\_regulation\_of\_immune\_system\_process | 106 | 0 | 0.000000 | 0.000000 | 550 | 354.931093 | 421.24 | 487.548907 | 0.765891 |
| GO:0006605\_protein\_targeting | 145 | 0 | 0.000000 | 0.000000 | 553 | 358.147760 | 424.24 | 490.332240 | 0.767161 |
| GO:0007417\_central\_nervous\_system\_development | 145 | 0 | 0.000000 | 0.000000 | 553 | 358.147760 | 424.24 | 490.332240 | 0.767161 |
| GO:0030036\_actin\_cytoskeleton\_organization | 145 | 0 | 0.000000 | 0.000000 | 553 | 358.147760 | 424.24 | 490.332240 | 0.767161 |
| GO:0006916\_anti-apoptosis | 155 | 0 | 0.000000 | 0.000000 | 554 | 359.915358 | 425.82 | 491.724642 | 0.768628 |
| GO:0000084\_S\_phase\_of\_mitotic\_cell\_cycle | 16 | 0 | 0.000000 | 0.000000 | 610 | 416.904441 | 481.65 | 546.395559 | 0.789590 |
| GO:0001523\_retinoid\_metabolic\_process | 16 | 0 | 0.000000 | 0.000000 | 610 | 416.904441 | 481.65 | 546.395559 | 0.789590 |
| GO:0001933\_negative\_regulation\_of\_protein\_amino\_acid\_phosphorylation | 16 | 0 | 0.000000 | 0.000000 | 610 | 416.904441 | 481.65 | 546.395559 | 0.789590 |
| GO:0002695\_negative\_regulation\_of\_leukocyte\_activation | 16 | 0 | 0.000000 | 0.000000 | 610 | 416.904441 | 481.65 | 546.395559 | 0.789590 |
| GO:0002699\_positive\_regulation\_of\_immune\_effector\_process | 16 | 0 | 0.000000 | 0.000000 | 610 | 416.904441 | 481.65 | 546.395559 | 0.789590 |
| GO:0006220\_pyrimidine\_nucleotide\_metabolic\_process | 16 | 0 | 0.000000 | 0.000000 | 610 | 416.904441 | 481.65 | 546.395559 | 0.789590 |
| GO:0006298\_mismatch\_repair | 16 | 0 | 0.000000 | 0.000000 | 610 | 416.904441 | 481.65 | 546.395559 | 0.789590 |
| GO:0006471\_protein\_amino\_acid\_ADP-ribosylation | 16 | 0 | 0.000000 | 0.000000 | 610 | 416.904441 | 481.65 | 546.395559 | 0.789590 |
| GO:0006636\_unsaturated\_fatty\_acid\_biosynthetic\_process | 16 | 0 | 0.000000 | 0.000000 | 610 | 416.904441 | 481.65 | 546.395559 | 0.789590 |
| GO:0006687\_glycosphingolipid\_metabolic\_process | 16 | 0 | 0.000000 | 0.000000 | 610 | 416.904441 | 481.65 | 546.395559 | 0.789590 |
| GO:0006695\_cholesterol\_biosynthetic\_process | 16 | 0 | 0.000000 | 0.000000 | 610 | 416.904441 | 481.65 | 546.395559 | 0.789590 |
| GO:0006721\_terpenoid\_metabolic\_process | 16 | 0 | 0.000000 | 0.000000 | 610 | 416.904441 | 481.65 | 546.395559 | 0.789590 |
| GO:0006776\_vitamin\_A\_metabolic\_process | 16 | 0 | 0.000000 | 0.000000 | 610 | 416.904441 | 481.65 | 546.395559 | 0.789590 |
| GO:0006805\_xenobiotic\_metabolic\_process | 16 | 0 | 0.000000 | 0.000000 | 610 | 416.904441 | 481.65 | 546.395559 | 0.789590 |
| GO:0006891\_intra-Golgi\_vesicle-mediated\_transport | 16 | 0 | 0.000000 | 0.000000 | 610 | 416.904441 | 481.65 | 546.395559 | 0.789590 |
| GO:0006914\_autophagy | 16 | 0 | 0.000000 | 0.000000 | 610 | 416.904441 | 481.65 | 546.395559 | 0.789590 |
| GO:0007006\_mitochondrial\_membrane\_organization | 16 | 0 | 0.000000 | 0.000000 | 610 | 416.904441 | 481.65 | 546.395559 | 0.789590 |
| GO:0009119\_ribonucleoside\_metabolic\_process | 16 | 0 | 0.000000 | 0.000000 | 610 | 416.904441 | 481.65 | 546.395559 | 0.789590 |
| GO:0009408\_response\_to\_heat | 16 | 0 | 0.000000 | 0.000000 | 610 | 416.904441 | 481.65 | 546.395559 | 0.789590 |
| GO:0009595\_detection\_of\_biotic\_stimulus | 16 | 0 | 0.000000 | 0.000000 | 610 | 416.904441 | 481.65 | 546.395559 | 0.789590 |
| GO:0009743\_response\_to\_carbohydrate\_stimulus | 16 | 0 | 0.000000 | 0.000000 | 610 | 416.904441 | 481.65 | 546.395559 | 0.789590 |
| GO:0010553\_negative\_regulation\_of\_specific\_transcription\_from\_RNA\_polymerase\_II\_promoter | 16 | 0 | 0.000000 | 0.000000 | 610 | 416.904441 | 481.65 | 546.395559 | 0.789590 |
| GO:0010676\_positive\_regulation\_of\_cellular\_carbohydrate\_metabolic\_process | 16 | 0 | 0.000000 | 0.000000 | 610 | 416.904441 | 481.65 | 546.395559 | 0.789590 |
| GO:0010743\_regulation\_of\_foam\_cell\_differentiation | 16 | 0 | 0.000000 | 0.000000 | 610 | 416.904441 | 481.65 | 546.395559 | 0.789590 |
| GO:0010906\_regulation\_of\_glucose\_metabolic\_process | 16 | 0 | 0.000000 | 0.000000 | 610 | 416.904441 | 481.65 | 546.395559 | 0.789590 |
| GO:0010975\_regulation\_of\_neuron\_projection\_development | 16 | 0 | 0.000000 | 0.000000 | 610 | 416.904441 | 481.65 | 546.395559 | 0.789590 |
| GO:0014070\_response\_to\_organic\_cyclic\_substance | 16 | 0 | 0.000000 | 0.000000 | 610 | 416.904441 | 481.65 | 546.395559 | 0.789590 |
| GO:0015909\_long-chain\_fatty\_acid\_transport | 16 | 0 | 0.000000 | 0.000000 | 610 | 416.904441 | 481.65 | 546.395559 | 0.789590 |
| GO:0016101\_diterpenoid\_metabolic\_process | 16 | 0 | 0.000000 | 0.000000 | 610 | 416.904441 | 481.65 | 546.395559 | 0.789590 |
| GO:0016254\_preassembly\_of\_GPI\_anchor\_in\_ER\_membrane | 16 | 0 | 0.000000 | 0.000000 | 610 | 416.904441 | 481.65 | 546.395559 | 0.789590 |
| GO:0019319\_hexose\_biosynthetic\_process | 16 | 0 | 0.000000 | 0.000000 | 610 | 416.904441 | 481.65 | 546.395559 | 0.789590 |
| GO:0030261\_chromosome\_condensation | 16 | 0 | 0.000000 | 0.000000 | 610 | 416.904441 | 481.65 | 546.395559 | 0.789590 |
| GO:0031110\_regulation\_of\_microtubule\_polymerization\_or\_depolymerization | 16 | 0 | 0.000000 | 0.000000 | 610 | 416.904441 | 481.65 | 546.395559 | 0.789590 |
| GO:0032623\_interleukin-2\_production | 16 | 0 | 0.000000 | 0.000000 | 610 | 416.904441 | 481.65 | 546.395559 | 0.789590 |
| GO:0032640\_tumor\_necrosis\_factor\_production | 16 | 0 | 0.000000 | 0.000000 | 610 | 416.904441 | 481.65 | 546.395559 | 0.789590 |
| GO:0032649\_regulation\_of\_interferon-gamma\_production | 16 | 0 | 0.000000 | 0.000000 | 610 | 416.904441 | 481.65 | 546.395559 | 0.789590 |
| GO:0032675\_regulation\_of\_interleukin-6\_production | 16 | 0 | 0.000000 | 0.000000 | 610 | 416.904441 | 481.65 | 546.395559 | 0.789590 |
| GO:0032680\_regulation\_of\_tumor\_necrosis\_factor\_production | 16 | 0 | 0.000000 | 0.000000 | 610 | 416.904441 | 481.65 | 546.395559 | 0.789590 |
| GO:0034381\_lipoprotein\_particle\_clearance | 16 | 0 | 0.000000 | 0.000000 | 610 | 416.904441 | 481.65 | 546.395559 | 0.789590 |
| GO:0034762\_regulation\_of\_transmembrane\_transport | 16 | 0 | 0.000000 | 0.000000 | 610 | 416.904441 | 481.65 | 546.395559 | 0.789590 |
| GO:0042476\_odontogenesis | 16 | 0 | 0.000000 | 0.000000 | 610 | 416.904441 | 481.65 | 546.395559 | 0.789590 |
| GO:0044275\_cellular\_carbohydrate\_catabolic\_process | 16 | 0 | 0.000000 | 0.000000 | 610 | 416.904441 | 481.65 | 546.395559 | 0.789590 |
| GO:0045667\_regulation\_of\_osteoblast\_differentiation | 16 | 0 | 0.000000 | 0.000000 | 610 | 416.904441 | 481.65 | 546.395559 | 0.789590 |
| GO:0045730\_respiratory\_burst | 16 | 0 | 0.000000 | 0.000000 | 610 | 416.904441 | 481.65 | 546.395559 | 0.789590 |
| GO:0045807\_positive\_regulation\_of\_endocytosis | 16 | 0 | 0.000000 | 0.000000 | 610 | 416.904441 | 481.65 | 546.395559 | 0.789590 |
| GO:0045862\_positive\_regulation\_of\_proteolysis | 16 | 0 | 0.000000 | 0.000000 | 610 | 416.904441 | 481.65 | 546.395559 | 0.789590 |
| GO:0045913\_positive\_regulation\_of\_carbohydrate\_metabolic\_process | 16 | 0 | 0.000000 | 0.000000 | 610 | 416.904441 | 481.65 | 546.395559 | 0.789590 |
| GO:0045930\_negative\_regulation\_of\_mitotic\_cell\_cycle | 16 | 0 | 0.000000 | 0.000000 | 610 | 416.904441 | 481.65 | 546.395559 | 0.789590 |
| GO:0046545\_development\_of\_primary\_female\_sexual\_characteristics | 16 | 0 | 0.000000 | 0.000000 | 610 | 416.904441 | 481.65 | 546.395559 | 0.789590 |
| GO:0046660\_female\_sex\_differentiation | 16 | 0 | 0.000000 | 0.000000 | 610 | 416.904441 | 481.65 | 546.395559 | 0.789590 |
| GO:0048589\_developmental\_growth | 16 | 0 | 0.000000 | 0.000000 | 610 | 416.904441 | 481.65 | 546.395559 | 0.789590 |
| GO:0050851\_antigen\_receptor-mediated\_signaling\_pathway | 16 | 0 | 0.000000 | 0.000000 | 610 | 416.904441 | 481.65 | 546.395559 | 0.789590 |
| GO:0050864\_regulation\_of\_B\_cell\_activation | 16 | 0 | 0.000000 | 0.000000 | 610 | 416.904441 | 481.65 | 546.395559 | 0.789590 |
| GO:0051146\_striated\_muscle\_cell\_differentiation | 16 | 0 | 0.000000 | 0.000000 | 610 | 416.904441 | 481.65 | 546.395559 | 0.789590 |
| GO:0051181\_cofactor\_transport | 16 | 0 | 0.000000 | 0.000000 | 610 | 416.904441 | 481.65 | 546.395559 | 0.789590 |
| GO:0051261\_protein\_depolymerization | 16 | 0 | 0.000000 | 0.000000 | 610 | 416.904441 | 481.65 | 546.395559 | 0.789590 |
| GO:0006470\_protein\_amino\_acid\_dephosphorylation | 77 | 0 | 0.000000 | 0.000000 | 616 | 422.541121 | 486.92 | 551.298879 | 0.790455 |
| GO:0009123\_nucleoside\_monophosphate\_metabolic\_process | 77 | 0 | 0.000000 | 0.000000 | 616 | 422.541121 | 486.92 | 551.298879 | 0.790455 |
| GO:0034470\_ncRNA\_processing | 77 | 0 | 0.000000 | 0.000000 | 616 | 422.541121 | 486.92 | 551.298879 | 0.790455 |
| GO:0050865\_regulation\_of\_cell\_activation | 77 | 0 | 0.000000 | 0.000000 | 616 | 422.541121 | 486.92 | 551.298879 | 0.790455 |
| GO:0051129\_negative\_regulation\_of\_cellular\_component\_organization | 77 | 0 | 0.000000 | 0.000000 | 616 | 422.541121 | 486.92 | 551.298879 | 0.790455 |
| GO:0051340\_regulation\_of\_ligase\_activity | 77 | 0 | 0.000000 | 0.000000 | 616 | 422.541121 | 486.92 | 551.298879 | 0.790455 |
| GO:0019941\_modification-dependent\_protein\_catabolic\_process | 138 | 0 | 0.000000 | 0.000000 | 618 | 424.229678 | 488.56 | 552.890322 | 0.790550 |
| GO:0043632\_modification-dependent\_macromolecule\_catabolic\_process | 138 | 0 | 0.000000 | 0.000000 | 618 | 424.229678 | 488.56 | 552.890322 | 0.790550 |
| GO:0043065\_positive\_regulation\_of\_apoptosis | 243 | 0 | 0.000000 | 0.000000 | 619 | 426.565734 | 490.45 | 554.334266 | 0.792326 |
| GO:0009617\_response\_to\_bacterium | 53 | 0 | 0.000000 | 0.000000 | 627 | 438.008439 | 501.26 | 564.511561 | 0.799458 |
| GO:0016485\_protein\_processing | 53 | 0 | 0.000000 | 0.000000 | 627 | 438.008439 | 501.26 | 564.511561 | 0.799458 |
| GO:0022900\_electron\_transport\_chain | 53 | 0 | 0.000000 | 0.000000 | 627 | 438.008439 | 501.26 | 564.511561 | 0.799458 |
| GO:0030258\_lipid\_modification | 53 | 0 | 0.000000 | 0.000000 | 627 | 438.008439 | 501.26 | 564.511561 | 0.799458 |
| GO:0042107\_cytokine\_metabolic\_process | 53 | 0 | 0.000000 | 0.000000 | 627 | 438.008439 | 501.26 | 564.511561 | 0.799458 |
| GO:0043434\_response\_to\_peptide\_hormone\_stimulus | 53 | 0 | 0.000000 | 0.000000 | 627 | 438.008439 | 501.26 | 564.511561 | 0.799458 |
| GO:0045786\_negative\_regulation\_of\_cell\_cycle | 53 | 0 | 0.000000 | 0.000000 | 627 | 438.008439 | 501.26 | 564.511561 | 0.799458 |
| GO:0051339\_regulation\_of\_lyase\_activity | 53 | 0 | 0.000000 | 0.000000 | 627 | 438.008439 | 501.26 | 564.511561 | 0.799458 |
| GO:0006364\_rRNA\_processing | 39 | 0 | 0.000000 | 0.000000 | 637 | 449.553367 | 512.57 | 575.586633 | 0.804662 |
| GO:0006401\_RNA\_catabolic\_process | 39 | 0 | 0.000000 | 0.000000 | 637 | 449.553367 | 512.57 | 575.586633 | 0.804662 |
| GO:0006497\_protein\_amino\_acid\_lipidation | 39 | 0 | 0.000000 | 0.000000 | 637 | 449.553367 | 512.57 | 575.586633 | 0.804662 |
| GO:0009310\_amine\_catabolic\_process | 39 | 0 | 0.000000 | 0.000000 | 637 | 449.553367 | 512.57 | 575.586633 | 0.804662 |
| GO:0009792\_embryonic\_development\_ending\_in\_birth\_or\_egg\_hatching | 39 | 0 | 0.000000 | 0.000000 | 637 | 449.553367 | 512.57 | 575.586633 | 0.804662 |
| GO:0032970\_regulation\_of\_actin\_filament-based\_process | 39 | 0 | 0.000000 | 0.000000 | 637 | 449.553367 | 512.57 | 575.586633 | 0.804662 |
| GO:0042158\_lipoprotein\_biosynthetic\_process | 39 | 0 | 0.000000 | 0.000000 | 637 | 449.553367 | 512.57 | 575.586633 | 0.804662 |
| GO:0043009\_chordate\_embryonic\_development | 39 | 0 | 0.000000 | 0.000000 | 637 | 449.553367 | 512.57 | 575.586633 | 0.804662 |
| GO:0046328\_regulation\_of\_JNK\_cascade | 39 | 0 | 0.000000 | 0.000000 | 637 | 449.553367 | 512.57 | 575.586633 | 0.804662 |
| GO:0051960\_regulation\_of\_nervous\_system\_development | 39 | 0 | 0.000000 | 0.000000 | 637 | 449.553367 | 512.57 | 575.586633 | 0.804662 |
| GO:0006917\_induction\_of\_apoptosis | 190 | 0 | 0.000000 | 0.000000 | 638 | 450.340654 | 513.33 | 576.319346 | 0.804592 |
| GO:0009967\_positive\_regulation\_of\_signal\_transduction | 185 | 0 | 0.000000 | 0.000000 | 639 | 451.146805 | 514.04 | 576.933195 | 0.804444 |
| GO:0007049\_cell\_cycle | 494 | 0 | 0.000000 | 0.000000 | 640 | 452.545308 | 515.19 | 577.834692 | 0.804984 |
| GO:0010604\_positive\_regulation\_of\_macromolecule\_metabolic\_process | 446 | 0 | 0.000000 | 0.000000 | 641 | 453.145401 | 515.56 | 577.974599 | 0.804306 |
| GO:0000041\_transition\_metal\_ion\_transport | 18 | 0 | 0.000000 | 0.000000 | 677 | 491.648655 | 552.9 | 614.151345 | 0.816691 |
| GO:0000737\_DNA\_catabolic\_process\_\_endonucleolytic | 18 | 0 | 0.000000 | 0.000000 | 677 | 491.648655 | 552.9 | 614.151345 | 0.816691 |
| GO:0001818\_negative\_regulation\_of\_cytokine\_production | 18 | 0 | 0.000000 | 0.000000 | 677 | 491.648655 | 552.9 | 614.151345 | 0.816691 |
| GO:0002541\_activation\_of\_plasma\_proteins\_involved\_in\_acute\_inflammatory\_response | 18 | 0 | 0.000000 | 0.000000 | 677 | 491.648655 | 552.9 | 614.151345 | 0.816691 |
| GO:0002700\_regulation\_of\_production\_of\_molecular\_mediator\_of\_immune\_response | 18 | 0 | 0.000000 | 0.000000 | 677 | 491.648655 | 552.9 | 614.151345 | 0.816691 |
| GO:0003073\_regulation\_of\_systemic\_arterial\_blood\_pressure | 18 | 0 | 0.000000 | 0.000000 | 677 | 491.648655 | 552.9 | 614.151345 | 0.816691 |
| GO:0006672\_ceramide\_metabolic\_process | 18 | 0 | 0.000000 | 0.000000 | 677 | 491.648655 | 552.9 | 614.151345 | 0.816691 |
| GO:0007031\_peroxisome\_organization | 18 | 0 | 0.000000 | 0.000000 | 677 | 491.648655 | 552.9 | 614.151345 | 0.816691 |
| GO:0007033\_vacuole\_organization | 18 | 0 | 0.000000 | 0.000000 | 677 | 491.648655 | 552.9 | 614.151345 | 0.816691 |
| GO:0007041\_lysosomal\_transport | 18 | 0 | 0.000000 | 0.000000 | 677 | 491.648655 | 552.9 | 614.151345 | 0.816691 |
| GO:0007602\_phototransduction | 18 | 0 | 0.000000 | 0.000000 | 677 | 491.648655 | 552.9 | 614.151345 | 0.816691 |
| GO:0009636\_response\_to\_toxin | 18 | 0 | 0.000000 | 0.000000 | 677 | 491.648655 | 552.9 | 614.151345 | 0.816691 |
| GO:0010742\_foam\_cell\_differentiation | 18 | 0 | 0.000000 | 0.000000 | 677 | 491.648655 | 552.9 | 614.151345 | 0.816691 |
| GO:0010827\_regulation\_of\_glucose\_transport | 18 | 0 | 0.000000 | 0.000000 | 677 | 491.648655 | 552.9 | 614.151345 | 0.816691 |
| GO:0016126\_sterol\_biosynthetic\_process | 18 | 0 | 0.000000 | 0.000000 | 677 | 491.648655 | 552.9 | 614.151345 | 0.816691 |
| GO:0019218\_regulation\_of\_steroid\_metabolic\_process | 18 | 0 | 0.000000 | 0.000000 | 677 | 491.648655 | 552.9 | 614.151345 | 0.816691 |
| GO:0030004\_cellular\_monovalent\_inorganic\_cation\_homeostasis | 18 | 0 | 0.000000 | 0.000000 | 677 | 491.648655 | 552.9 | 614.151345 | 0.816691 |
| GO:0030183\_B\_cell\_differentiation | 18 | 0 | 0.000000 | 0.000000 | 677 | 491.648655 | 552.9 | 614.151345 | 0.816691 |
| GO:0030195\_negative\_regulation\_of\_blood\_coagulation | 18 | 0 | 0.000000 | 0.000000 | 677 | 491.648655 | 552.9 | 614.151345 | 0.816691 |
| GO:0030262\_apoptotic\_nuclear\_changes | 18 | 0 | 0.000000 | 0.000000 | 677 | 491.648655 | 552.9 | 614.151345 | 0.816691 |
| GO:0032319\_regulation\_of\_Rho\_GTPase\_activity | 18 | 0 | 0.000000 | 0.000000 | 677 | 491.648655 | 552.9 | 614.151345 | 0.816691 |
| GO:0034599\_cellular\_response\_to\_oxidative\_stress | 18 | 0 | 0.000000 | 0.000000 | 677 | 491.648655 | 552.9 | 614.151345 | 0.816691 |
| GO:0040017\_positive\_regulation\_of\_locomotion | 18 | 0 | 0.000000 | 0.000000 | 677 | 491.648655 | 552.9 | 614.151345 | 0.816691 |
| GO:0042177\_negative\_regulation\_of\_protein\_catabolic\_process | 18 | 0 | 0.000000 | 0.000000 | 677 | 491.648655 | 552.9 | 614.151345 | 0.816691 |
| GO:0043393\_regulation\_of\_protein\_binding | 18 | 0 | 0.000000 | 0.000000 | 677 | 491.648655 | 552.9 | 614.151345 | 0.816691 |
| GO:0045444\_fat\_cell\_differentiation | 18 | 0 | 0.000000 | 0.000000 | 677 | 491.648655 | 552.9 | 614.151345 | 0.816691 |
| GO:0045639\_positive\_regulation\_of\_myeloid\_cell\_differentiation | 18 | 0 | 0.000000 | 0.000000 | 677 | 491.648655 | 552.9 | 614.151345 | 0.816691 |
| GO:0045833\_negative\_regulation\_of\_lipid\_metabolic\_process | 18 | 0 | 0.000000 | 0.000000 | 677 | 491.648655 | 552.9 | 614.151345 | 0.816691 |
| GO:0046034\_ATP\_metabolic\_process | 18 | 0 | 0.000000 | 0.000000 | 677 | 491.648655 | 552.9 | 614.151345 | 0.816691 |
| GO:0046324\_regulation\_of\_glucose\_import | 18 | 0 | 0.000000 | 0.000000 | 677 | 491.648655 | 552.9 | 614.151345 | 0.816691 |
| GO:0046546\_development\_of\_primary\_male\_sexual\_characteristics | 18 | 0 | 0.000000 | 0.000000 | 677 | 491.648655 | 552.9 | 614.151345 | 0.816691 |
| GO:0048659\_smooth\_muscle\_cell\_proliferation | 18 | 0 | 0.000000 | 0.000000 | 677 | 491.648655 | 552.9 | 614.151345 | 0.816691 |
| GO:0050715\_positive\_regulation\_of\_cytokine\_secretion | 18 | 0 | 0.000000 | 0.000000 | 677 | 491.648655 | 552.9 | 614.151345 | 0.816691 |
| GO:0050905\_neuromuscular\_process | 18 | 0 | 0.000000 | 0.000000 | 677 | 491.648655 | 552.9 | 614.151345 | 0.816691 |
| GO:0050921\_positive\_regulation\_of\_chemotaxis | 18 | 0 | 0.000000 | 0.000000 | 677 | 491.648655 | 552.9 | 614.151345 | 0.816691 |
| GO:0060389\_pathway-restricted\_SMAD\_protein\_phosphorylation | 18 | 0 | 0.000000 | 0.000000 | 677 | 491.648655 | 552.9 | 614.151345 | 0.816691 |
| GO:0006171\_cAMP\_biosynthetic\_process | 59 | 0 | 0.000000 | 0.000000 | 682 | 498.015682 | 559.14 | 620.264318 | 0.819853 |
| GO:0006820\_anion\_transport | 59 | 0 | 0.000000 | 0.000000 | 682 | 498.015682 | 559.14 | 620.264318 | 0.819853 |
| GO:0007409\_axonogenesis | 59 | 0 | 0.000000 | 0.000000 | 682 | 498.015682 | 559.14 | 620.264318 | 0.819853 |
| GO:0043281\_regulation\_of\_caspase\_activity | 59 | 0 | 0.000000 | 0.000000 | 682 | 498.015682 | 559.14 | 620.264318 | 0.819853 |
| GO:0051604\_protein\_maturation | 59 | 0 | 0.000000 | 0.000000 | 682 | 498.015682 | 559.14 | 620.264318 | 0.819853 |
| GO:0022402\_cell\_cycle\_process | 370 | 0 | 0.000000 | 0.000000 | 683 | 498.696426 | 559.69 | 620.683574 | 0.819458 |
| GO:0022414\_reproductive\_process | 365 | 0 | 0.000000 | 0.000000 | 684 | 500.031114 | 560.85 | 621.668886 | 0.819956 |
| GO:0002520\_immune\_system\_development | 147 | 0 | 0.000000 | 0.000000 | 685 | 501.831099 | 562.36 | 622.888901 | 0.820964 |
| GO:0006511\_ubiquitin-dependent\_protein\_catabolic\_process | 136 | 0 | 0.000000 | 0.000000 | 687 | 503.416852 | 563.86 | 624.303148 | 0.820757 |
| GO:0048699\_generation\_of\_neurons | 136 | 0 | 0.000000 | 0.000000 | 687 | 503.416852 | 563.86 | 624.303148 | 0.820757 |
| GO:0000050\_urea\_cycle | 3 | 0 |  |  |  |  |  |  |  |  |
| GO:0000089\_mitotic\_metaphase | 3 | 0 |  |  |  |  |  |  |  |  |
| GO:0000097\_sulfur\_amino\_acid\_biosynthetic\_process | 3 | 0 |  |  |  |  |  |  |  |  |
| GO:0000266\_mitochondrial\_fission | 3 | 0 |  |  |  |  |  |  |  |  |
| GO:0000281\_cytokinesis\_after\_mitosis | 3 | 0 |  |  |  |  |  |  |  |  |
| GO:0000394\_RNA\_splicing\_\_via\_endonucleolytic\_cleavage\_and\_ligation | 3 | 0 |  |  |  |  |  |  |  |  |
| GO:0000463\_maturation\_of\_LSU-rRNA\_from\_tricistronic\_rRNA\_transcript\_(SSU-rRNA\_\_5.8S\_rRNA\_\_LSU-rRNA) | 3 | 0 |  |  |  |  |  |  |  |  |
| GO:0000470\_maturation\_of\_LSU-rRNA | 3 | 0 |  |  |  |  |  |  |  |  |
| GO:0000491\_small\_nucleolar\_ribonucleoprotein\_complex\_assembly | 3 | 0 |  |  |  |  |  |  |  |  |
| GO:0000492\_box\_C\_D\_snoRNP\_assembly | 3 | 0 |  |  |  |  |  |  |  |  |
| GO:0001302\_replicative\_cell\_aging | 3 | 0 |  |  |  |  |  |  |  |  |
| GO:0001510\_RNA\_methylation | 3 | 0 |  |  |  |  |  |  |  |  |
| GO:0001569\_patterning\_of\_blood\_vessels | 3 | 0 |  |  |  |  |  |  |  |  |
| GO:0001573\_ganglioside\_metabolic\_process | 3 | 0 |  |  |  |  |  |  |  |  |
| GO:0001659\_temperature\_homeostasis | 3 | 0 |  |  |  |  |  |  |  |  |
| GO:0001755\_neural\_crest\_cell\_migration | 3 | 0 |  |  |  |  |  |  |  |  |
| GO:0001773\_myeloid\_dendritic\_cell\_activation | 3 | 0 |  |  |  |  |  |  |  |  |
| GO:0001881\_receptor\_recycling | 3 | 0 |  |  |  |  |  |  |  |  |
| GO:0001895\_retina\_homeostasis | 3 | 0 |  |  |  |  |  |  |  |  |
| GO:0001916\_positive\_regulation\_of\_T\_cell\_mediated\_cytotoxicity | 3 | 0 |  |  |  |  |  |  |  |  |
| GO:0001919\_regulation\_of\_receptor\_recycling | 3 | 0 |  |  |  |  |  |  |  |  |
| GO:0001945\_lymph\_vessel\_development | 3 | 0 |  |  |  |  |  |  |  |  |
| GO:0001946\_lymphangiogenesis | 3 | 0 |  |  |  |  |  |  |  |  |
| GO:0002002\_regulation\_of\_angiotensin\_levels\_in\_blood | 3 | 0 |  |  |  |  |  |  |  |  |
| GO:0002029\_desensitization\_of\_G-protein\_coupled\_receptor\_protein\_signaling\_pathway | 3 | 0 |  |  |  |  |  |  |  |  |
| GO:0002062\_chondrocyte\_differentiation | 3 | 0 |  |  |  |  |  |  |  |  |
| GO:0002076\_osteoblast\_development | 3 | 0 |  |  |  |  |  |  |  |  |
| GO:0002230\_positive\_regulation\_of\_defense\_response\_to\_virus\_by\_host | 3 | 0 |  |  |  |  |  |  |  |  |
| GO:0002244\_hemopoietic\_progenitor\_cell\_differentiation | 3 | 0 |  |  |  |  |  |  |  |  |
| GO:0002246\_healing\_during\_inflammatory\_response | 3 | 0 |  |  |  |  |  |  |  |  |
| GO:0002262\_myeloid\_cell\_homeostasis | 3 | 0 |  |  |  |  |  |  |  |  |
| GO:0002444\_myeloid\_leukocyte\_mediated\_immunity | 3 | 0 |  |  |  |  |  |  |  |  |
| GO:0002446\_neutrophil\_mediated\_immunity | 3 | 0 |  |  |  |  |  |  |  |  |
| GO:0002792\_negative\_regulation\_of\_peptide\_secretion | 3 | 0 |  |  |  |  |  |  |  |  |
| GO:0002825\_regulation\_of\_T-helper\_1\_type\_immune\_response | 3 | 0 |  |  |  |  |  |  |  |  |
| GO:0002828\_regulation\_of\_T-helper\_2\_type\_immune\_response | 3 | 0 |  |  |  |  |  |  |  |  |
| GO:0002903\_negative\_regulation\_of\_B\_cell\_apoptosis | 3 | 0 |  |  |  |  |  |  |  |  |
| GO:0002921\_negative\_regulation\_of\_humoral\_immune\_response | 3 | 0 |  |  |  |  |  |  |  |  |
| GO:0003084\_positive\_regulation\_of\_systemic\_arterial\_blood\_pressure | 3 | 0 |  |  |  |  |  |  |  |  |
| GO:0005980\_glycogen\_catabolic\_process | 3 | 0 |  |  |  |  |  |  |  |  |
| GO:0005984\_disaccharide\_metabolic\_process | 3 | 0 |  |  |  |  |  |  |  |  |
| GO:0006002\_fructose\_6-phosphate\_metabolic\_process | 3 | 0 |  |  |  |  |  |  |  |  |
| GO:0006003\_fructose\_2\_6-bisphosphate\_metabolic\_process | 3 | 0 |  |  |  |  |  |  |  |  |
| GO:0006043\_glucosamine\_catabolic\_process | 3 | 0 |  |  |  |  |  |  |  |  |
| GO:0006071\_glycerol\_metabolic\_process | 3 | 0 |  |  |  |  |  |  |  |  |
| GO:0006102\_isocitrate\_metabolic\_process | 3 | 0 |  |  |  |  |  |  |  |  |
| GO:0006104\_succinyl-CoA\_metabolic\_process | 3 | 0 |  |  |  |  |  |  |  |  |
| GO:0006108\_malate\_metabolic\_process | 3 | 0 |  |  |  |  |  |  |  |  |
| GO:0006123\_mitochondrial\_electron\_transport\_\_cytochrome\_c\_to\_oxygen | 3 | 0 |  |  |  |  |  |  |  |  |
| GO:0006152\_purine\_nucleoside\_catabolic\_process | 3 | 0 |  |  |  |  |  |  |  |  |
| GO:0006167\_AMP\_biosynthetic\_process | 3 | 0 |  |  |  |  |  |  |  |  |
| GO:0006182\_cGMP\_biosynthetic\_process | 3 | 0 |  |  |  |  |  |  |  |  |
| GO:0006269\_DNA\_replication\_\_synthesis\_of\_RNA\_primer | 3 | 0 |  |  |  |  |  |  |  |  |
| GO:0006273\_lagging\_strand\_elongation | 3 | 0 |  |  |  |  |  |  |  |  |
| GO:0006288\_base-excision\_repair\_\_DNA\_ligation | 3 | 0 |  |  |  |  |  |  |  |  |
| GO:0006295\_nucleotide-excision\_repair\_\_DNA\_incision\_\_3'-to\_lesion | 3 | 0 |  |  |  |  |  |  |  |  |
| GO:0006296\_nucleotide-excision\_repair\_\_DNA\_incision\_\_5'-to\_lesion | 3 | 0 |  |  |  |  |  |  |  |  |
| GO:0006384\_transcription\_initiation\_from\_RNA\_polymerase\_III\_promoter | 3 | 0 |  |  |  |  |  |  |  |  |
| GO:0006388\_tRNA\_splicing\_\_via\_endonucleolytic\_cleavage\_and\_ligation | 3 | 0 |  |  |  |  |  |  |  |  |
| GO:0006467\_protein\_thiol-disulfide\_exchange | 3 | 0 |  |  |  |  |  |  |  |  |
| GO:0006546\_glycine\_catabolic\_process | 3 | 0 |  |  |  |  |  |  |  |  |
| GO:0006558\_L-phenylalanine\_metabolic\_process | 3 | 0 |  |  |  |  |  |  |  |  |
| GO:0006559\_L-phenylalanine\_catabolic\_process | 3 | 0 |  |  |  |  |  |  |  |  |
| GO:0006560\_proline\_metabolic\_process | 3 | 0 |  |  |  |  |  |  |  |  |
| GO:0006565\_L-serine\_catabolic\_process | 3 | 0 |  |  |  |  |  |  |  |  |
| GO:0006568\_tryptophan\_metabolic\_process | 3 | 0 |  |  |  |  |  |  |  |  |
| GO:0006573\_valine\_metabolic\_process | 3 | 0 |  |  |  |  |  |  |  |  |
| GO:0006590\_thyroid\_hormone\_generation | 3 | 0 |  |  |  |  |  |  |  |  |
| GO:0006596\_polyamine\_biosynthetic\_process | 3 | 0 |  |  |  |  |  |  |  |  |
| GO:0006621\_protein\_retention\_in\_ER\_lumen | 3 | 0 |  |  |  |  |  |  |  |  |
| GO:0006654\_phosphatidic\_acid\_biosynthetic\_process | 3 | 0 |  |  |  |  |  |  |  |  |
| GO:0006658\_phosphatidylserine\_metabolic\_process | 3 | 0 |  |  |  |  |  |  |  |  |
| GO:0006663\_platelet\_activating\_factor\_biosynthetic\_process | 3 | 0 |  |  |  |  |  |  |  |  |
| GO:0006678\_glucosylceramide\_metabolic\_process | 3 | 0 |  |  |  |  |  |  |  |  |
| GO:0006686\_sphingomyelin\_biosynthetic\_process | 3 | 0 |  |  |  |  |  |  |  |  |
| GO:0006701\_progesterone\_biosynthetic\_process | 3 | 0 |  |  |  |  |  |  |  |  |
| GO:0006704\_glucocorticoid\_biosynthetic\_process | 3 | 0 |  |  |  |  |  |  |  |  |
| GO:0006705\_mineralocorticoid\_biosynthetic\_process | 3 | 0 |  |  |  |  |  |  |  |  |
| GO:0006729\_tetrahydrobiopterin\_biosynthetic\_process | 3 | 0 |  |  |  |  |  |  |  |  |
| GO:0006777\_Mo-molybdopterin\_cofactor\_biosynthetic\_process | 3 | 0 |  |  |  |  |  |  |  |  |
| GO:0006787\_porphyrin\_catabolic\_process | 3 | 0 |  |  |  |  |  |  |  |  |
| GO:0006829\_zinc\_ion\_transport | 3 | 0 |  |  |  |  |  |  |  |  |
| GO:0006853\_carnitine\_shuttle | 3 | 0 |  |  |  |  |  |  |  |  |
| GO:0006863\_purine\_transport | 3 | 0 |  |  |  |  |  |  |  |  |
| GO:0006882\_cellular\_zinc\_ion\_homeostasis | 3 | 0 |  |  |  |  |  |  |  |  |
| GO:0006910\_phagocytosis\_\_recognition | 3 | 0 |  |  |  |  |  |  |  |  |
| GO:0006922\_cleavage\_of\_lamin | 3 | 0 |  |  |  |  |  |  |  |  |
| GO:0006923\_cleavage\_of\_cytoskeletal\_proteins\_during\_apoptosis | 3 | 0 |  |  |  |  |  |  |  |  |
| GO:0006927\_transformed\_cell\_apoptosis | 3 | 0 |  |  |  |  |  |  |  |  |
| GO:0007007\_inner\_mitochondrial\_membrane\_organization | 3 | 0 |  |  |  |  |  |  |  |  |
| GO:0007035\_vacuolar\_acidification | 3 | 0 |  |  |  |  |  |  |  |  |
| GO:0007175\_negative\_regulation\_of\_epidermal\_growth\_factor\_receptor\_activity | 3 | 0 |  |  |  |  |  |  |  |  |
| GO:0007181\_transforming\_growth\_factor\_beta\_receptor\_complex\_assembly | 3 | 0 |  |  |  |  |  |  |  |  |
| GO:0007199\_G-protein\_signaling\_\_coupled\_to\_cGMP\_nucleotide\_second\_messenger | 3 | 0 |  |  |  |  |  |  |  |  |
| GO:0007207\_activation\_of\_phospholipase\_C\_activity\_by\_muscarinic\_acetylcholine\_receptor\_signaling\_pathway | 3 | 0 |  |  |  |  |  |  |  |  |
| GO:0007252\_I-kappaB\_phosphorylation | 3 | 0 |  |  |  |  |  |  |  |  |
| GO:0007339\_binding\_of\_sperm\_to\_zona\_pellucida | 3 | 0 |  |  |  |  |  |  |  |  |
| GO:0007352\_zygotic\_determination\_of\_dorsal\_ventral\_axis | 3 | 0 |  |  |  |  |  |  |  |  |
| GO:0007439\_ectodermal\_gut\_development | 3 | 0 |  |  |  |  |  |  |  |  |
| GO:0007500\_mesodermal\_cell\_fate\_determination | 3 | 0 |  |  |  |  |  |  |  |  |
| GO:0007512\_adult\_heart\_development | 3 | 0 |  |  |  |  |  |  |  |  |
| GO:0008053\_mitochondrial\_fusion | 3 | 0 |  |  |  |  |  |  |  |  |
| GO:0008291\_acetylcholine\_metabolic\_process | 3 | 0 |  |  |  |  |  |  |  |  |
| GO:0008334\_histone\_mRNA\_metabolic\_process | 3 | 0 |  |  |  |  |  |  |  |  |
| GO:0008617\_guanosine\_metabolic\_process | 3 | 0 |  |  |  |  |  |  |  |  |
| GO:0008628\_induction\_of\_apoptosis\_by\_hormones | 3 | 0 |  |  |  |  |  |  |  |  |
| GO:0008631\_induction\_of\_apoptosis\_by\_oxidative\_stress | 3 | 0 |  |  |  |  |  |  |  |  |
| GO:0009070\_serine\_family\_amino\_acid\_biosynthetic\_process | 3 | 0 |  |  |  |  |  |  |  |  |
| GO:0009125\_nucleoside\_monophosphate\_catabolic\_process | 3 | 0 |  |  |  |  |  |  |  |  |
| GO:0009132\_nucleoside\_diphosphate\_metabolic\_process | 3 | 0 |  |  |  |  |  |  |  |  |
| GO:0009155\_purine\_deoxyribonucleotide\_catabolic\_process | 3 | 0 |  |  |  |  |  |  |  |  |
| GO:0009163\_nucleoside\_biosynthetic\_process | 3 | 0 |  |  |  |  |  |  |  |  |
| GO:0009185\_ribonucleoside\_diphosphate\_metabolic\_process | 3 | 0 |  |  |  |  |  |  |  |  |
| GO:0009226\_nucleotide-sugar\_biosynthetic\_process | 3 | 0 |  |  |  |  |  |  |  |  |
| GO:0009301\_snRNA\_transcription | 3 | 0 |  |  |  |  |  |  |  |  |
| GO:0009620\_response\_to\_fungus | 3 | 0 |  |  |  |  |  |  |  |  |
| GO:0009791\_post-embryonic\_development | 3 | 0 |  |  |  |  |  |  |  |  |
| GO:0010002\_cardioblast\_differentiation | 3 | 0 |  |  |  |  |  |  |  |  |
| GO:0010039\_response\_to\_iron\_ion | 3 | 0 |  |  |  |  |  |  |  |  |
| GO:0010332\_response\_to\_gamma\_radiation | 3 | 0 |  |  |  |  |  |  |  |  |
| GO:0010457\_centriole-centriole\_cohesion | 3 | 0 |  |  |  |  |  |  |  |  |
| GO:0010560\_positive\_regulation\_of\_glycoprotein\_biosynthetic\_process | 3 | 0 |  |  |  |  |  |  |  |  |
| GO:0010572\_positive\_regulation\_of\_platelet\_activation | 3 | 0 |  |  |  |  |  |  |  |  |
| GO:0010656\_negative\_regulation\_of\_muscle\_cell\_apoptosis | 3 | 0 |  |  |  |  |  |  |  |  |
| GO:0010661\_positive\_regulation\_of\_muscle\_cell\_apoptosis | 3 | 0 |  |  |  |  |  |  |  |  |
| GO:0010677\_negative\_regulation\_of\_cellular\_carbohydrate\_metabolic\_process | 3 | 0 |  |  |  |  |  |  |  |  |
| GO:0010713\_negative\_regulation\_of\_collagen\_metabolic\_process | 3 | 0 |  |  |  |  |  |  |  |  |
| GO:0010800\_positive\_regulation\_of\_peptidyl-threonine\_phosphorylation | 3 | 0 |  |  |  |  |  |  |  |  |
| GO:0010824\_regulation\_of\_centrosome\_duplication | 3 | 0 |  |  |  |  |  |  |  |  |
| GO:0010834\_telomere\_maintenance\_via\_telomere\_shortening | 3 | 0 |  |  |  |  |  |  |  |  |
| GO:0010847\_regulation\_of\_chromatin\_assembly | 3 | 0 |  |  |  |  |  |  |  |  |
| GO:0010880\_regulation\_of\_release\_of\_sequestered\_calcium\_ion\_into\_cytosol\_by\_sarcoplasmic\_reticulum | 3 | 0 |  |  |  |  |  |  |  |  |
| GO:0010890\_positive\_regulation\_of\_sequestering\_of\_triglyceride | 3 | 0 |  |  |  |  |  |  |  |  |
| GO:0010894\_negative\_regulation\_of\_steroid\_biosynthetic\_process | 3 | 0 |  |  |  |  |  |  |  |  |
| GO:0010903\_negative\_regulation\_of\_very-low-density\_lipoprotein\_particle\_remodeling | 3 | 0 |  |  |  |  |  |  |  |  |
| GO:0010922\_positive\_regulation\_of\_phosphatase\_activity | 3 | 0 |  |  |  |  |  |  |  |  |
| GO:0010939\_regulation\_of\_necrotic\_cell\_death | 3 | 0 |  |  |  |  |  |  |  |  |
| GO:0010940\_positive\_regulation\_of\_necrotic\_cell\_death | 3 | 0 |  |  |  |  |  |  |  |  |
| GO:0010955\_negative\_regulation\_of\_protein\_maturation\_by\_peptide\_bond\_cleavage | 3 | 0 |  |  |  |  |  |  |  |  |
| GO:0010956\_negative\_regulation\_of\_calcidiol\_1-monooxygenase\_activity | 3 | 0 |  |  |  |  |  |  |  |  |
| GO:0014805\_smooth\_muscle\_adaptation | 3 | 0 |  |  |  |  |  |  |  |  |
| GO:0014808\_release\_of\_sequestered\_calcium\_ion\_into\_cytosol\_by\_sarcoplasmic\_reticulum | 3 | 0 |  |  |  |  |  |  |  |  |
| GO:0014912\_negative\_regulation\_of\_smooth\_muscle\_cell\_migration | 3 | 0 |  |  |  |  |  |  |  |  |
| GO:0015014\_heparan\_sulfate\_proteoglycan\_biosynthetic\_process\_\_polysaccharide\_chain\_biosynthetic\_process | 3 | 0 |  |  |  |  |  |  |  |  |
| GO:0015015\_heparan\_sulfate\_proteoglycan\_biosynthetic\_process\_\_enzymatic\_modification | 3 | 0 |  |  |  |  |  |  |  |  |
| GO:0015670\_carbon\_dioxide\_transport | 3 | 0 |  |  |  |  |  |  |  |  |
| GO:0015721\_bile\_acid\_and\_bile\_salt\_transport | 3 | 0 |  |  |  |  |  |  |  |  |
| GO:0015802\_basic\_amino\_acid\_transport | 3 | 0 |  |  |  |  |  |  |  |  |
| GO:0015811\_L-cystine\_transport | 3 | 0 |  |  |  |  |  |  |  |  |
| GO:0015840\_urea\_transport | 3 | 0 |  |  |  |  |  |  |  |  |
| GO:0015889\_cobalamin\_transport | 3 | 0 |  |  |  |  |  |  |  |  |
| GO:0015917\_aminophospholipid\_transport | 3 | 0 |  |  |  |  |  |  |  |  |
| GO:0016056\_rhodopsin\_mediated\_signaling\_pathway | 3 | 0 |  |  |  |  |  |  |  |  |
| GO:0016081\_synaptic\_vesicle\_docking\_during\_exocytosis | 3 | 0 |  |  |  |  |  |  |  |  |
| GO:0016137\_glycoside\_metabolic\_process | 3 | 0 |  |  |  |  |  |  |  |  |
| GO:0016139\_glycoside\_catabolic\_process | 3 | 0 |  |  |  |  |  |  |  |  |
| GO:0016553\_base\_conversion\_or\_substitution\_editing | 3 | 0 |  |  |  |  |  |  |  |  |
| GO:0016572\_histone\_phosphorylation | 3 | 0 |  |  |  |  |  |  |  |  |
| GO:0016576\_histone\_dephosphorylation | 3 | 0 |  |  |  |  |  |  |  |  |
| GO:0016577\_histone\_demethylation | 3 | 0 |  |  |  |  |  |  |  |  |
| GO:0016584\_nucleosome\_positioning | 3 | 0 |  |  |  |  |  |  |  |  |
| GO:0018076\_N-terminal\_peptidyl-lysine\_acetylation | 3 | 0 |  |  |  |  |  |  |  |  |
| GO:0018094\_protein\_polyglycylation | 3 | 0 |  |  |  |  |  |  |  |  |
| GO:0018103\_protein\_amino\_acid\_C-linked\_glycosylation | 3 | 0 |  |  |  |  |  |  |  |  |
| GO:0018211\_peptidyl-tryptophan\_modification | 3 | 0 |  |  |  |  |  |  |  |  |
| GO:0018242\_protein\_amino\_acid\_O-linked\_glycosylation\_via\_serine | 3 | 0 |  |  |  |  |  |  |  |  |
| GO:0018243\_protein\_amino\_acid\_O-linked\_glycosylation\_via\_threonine | 3 | 0 |  |  |  |  |  |  |  |  |
| GO:0018317\_protein\_amino\_acid\_C-linked\_glycosylation\_via\_tryptophan | 3 | 0 |  |  |  |  |  |  |  |  |
| GO:0018343\_protein\_farnesylation | 3 | 0 |  |  |  |  |  |  |  |  |
| GO:0018344\_protein\_geranylgeranylation | 3 | 0 |  |  |  |  |  |  |  |  |
| GO:0018348\_protein\_amino\_acid\_geranylgeranylation | 3 | 0 |  |  |  |  |  |  |  |  |
| GO:0018394\_peptidyl-lysine\_acetylation | 3 | 0 |  |  |  |  |  |  |  |  |
| GO:0018406\_protein\_amino\_acid\_C-linked\_glycosylation\_via\_2'-alpha-mannosyl-L-tryptophan | 3 | 0 |  |  |  |  |  |  |  |  |
| GO:0019063\_virion\_penetration\_into\_host\_cell | 3 | 0 |  |  |  |  |  |  |  |  |
| GO:0019067\_viral\_assembly\_\_maturation\_\_egress\_\_and\_release | 3 | 0 |  |  |  |  |  |  |  |  |
| GO:0019276\_UDP-N-acetylgalactosamine\_metabolic\_process | 3 | 0 |  |  |  |  |  |  |  |  |
| GO:0019377\_glycolipid\_catabolic\_process | 3 | 0 |  |  |  |  |  |  |  |  |
| GO:0019627\_urea\_metabolic\_process | 3 | 0 |  |  |  |  |  |  |  |  |
| GO:0019720\_Mo-molybdopterin\_cofactor\_metabolic\_process | 3 | 0 |  |  |  |  |  |  |  |  |
| GO:0019794\_nonprotein\_amino\_acid\_metabolic\_process | 3 | 0 |  |  |  |  |  |  |  |  |
| GO:0019859\_thymine\_metabolic\_process | 3 | 0 |  |  |  |  |  |  |  |  |
| GO:0021516\_dorsal\_spinal\_cord\_development | 3 | 0 |  |  |  |  |  |  |  |  |
| GO:0021543\_pallium\_development | 3 | 0 |  |  |  |  |  |  |  |  |
| GO:0021575\_hindbrain\_morphogenesis | 3 | 0 |  |  |  |  |  |  |  |  |
| GO:0021885\_forebrain\_cell\_migration | 3 | 0 |  |  |  |  |  |  |  |  |
| GO:0021952\_central\_nervous\_system\_projection\_neuron\_axonogenesis | 3 | 0 |  |  |  |  |  |  |  |  |
| GO:0021955\_central\_nervous\_system\_neuron\_axonogenesis | 3 | 0 |  |  |  |  |  |  |  |  |
| GO:0021983\_pituitary\_gland\_development | 3 | 0 |  |  |  |  |  |  |  |  |
| GO:0021987\_cerebral\_cortex\_development | 3 | 0 |  |  |  |  |  |  |  |  |
| GO:0022029\_telencephalon\_cell\_migration | 3 | 0 |  |  |  |  |  |  |  |  |
| GO:0022401\_adaptation\_of\_signaling\_pathway | 3 | 0 |  |  |  |  |  |  |  |  |
| GO:0022601\_menstrual\_cycle\_phase | 3 | 0 |  |  |  |  |  |  |  |  |
| GO:0022617\_extracellular\_matrix\_disassembly | 3 | 0 |  |  |  |  |  |  |  |  |
| GO:0030091\_protein\_repair | 3 | 0 |  |  |  |  |  |  |  |  |
| GO:0030157\_pancreatic\_juice\_secretion | 3 | 0 |  |  |  |  |  |  |  |  |
| GO:0030241\_muscle\_thick\_filament\_assembly | 3 | 0 |  |  |  |  |  |  |  |  |
| GO:0030263\_apoptotic\_chromosome\_condensation | 3 | 0 |  |  |  |  |  |  |  |  |
| GO:0030277\_maintenance\_of\_gastrointestinal\_epithelium | 3 | 0 |  |  |  |  |  |  |  |  |
| GO:0030309\_poly-N-acetyllactosamine\_metabolic\_process | 3 | 0 |  |  |  |  |  |  |  |  |
| GO:0030325\_adrenal\_gland\_development | 3 | 0 |  |  |  |  |  |  |  |  |
| GO:0030388\_fructose\_1\_6-bisphosphate\_metabolic\_process | 3 | 0 |  |  |  |  |  |  |  |  |
| GO:0030422\_RNA\_interference\_\_production\_of\_siRNA | 3 | 0 |  |  |  |  |  |  |  |  |
| GO:0030423\_RNA\_interference\_\_targeting\_of\_mRNA\_for\_destruction | 3 | 0 |  |  |  |  |  |  |  |  |
| GO:0030449\_regulation\_of\_complement\_activation | 3 | 0 |  |  |  |  |  |  |  |  |
| GO:0030502\_negative\_regulation\_of\_bone\_mineralization | 3 | 0 |  |  |  |  |  |  |  |  |
| GO:0030643\_cellular\_phosphate\_ion\_homeostasis | 3 | 0 |  |  |  |  |  |  |  |  |
| GO:0030656\_regulation\_of\_vitamin\_metabolic\_process | 3 | 0 |  |  |  |  |  |  |  |  |
| GO:0030800\_negative\_regulation\_of\_cyclic\_nucleotide\_metabolic\_process | 3 | 0 |  |  |  |  |  |  |  |  |
| GO:0030803\_negative\_regulation\_of\_cyclic\_nucleotide\_biosynthetic\_process | 3 | 0 |  |  |  |  |  |  |  |  |
| GO:0030809\_negative\_regulation\_of\_nucleotide\_biosynthetic\_process | 3 | 0 |  |  |  |  |  |  |  |  |
| GO:0030823\_regulation\_of\_cGMP\_metabolic\_process | 3 | 0 |  |  |  |  |  |  |  |  |
| GO:0030826\_regulation\_of\_cGMP\_biosynthetic\_process | 3 | 0 |  |  |  |  |  |  |  |  |
| GO:0030850\_prostate\_gland\_development | 3 | 0 |  |  |  |  |  |  |  |  |
| GO:0030852\_regulation\_of\_granulocyte\_differentiation | 3 | 0 |  |  |  |  |  |  |  |  |
| GO:0030853\_negative\_regulation\_of\_granulocyte\_differentiation | 3 | 0 |  |  |  |  |  |  |  |  |
| GO:0030947\_regulation\_of\_vascular\_endothelial\_growth\_factor\_receptor\_signaling\_pathway | 3 | 0 |  |  |  |  |  |  |  |  |
| GO:0031033\_myosin\_filament\_assembly\_or\_disassembly | 3 | 0 |  |  |  |  |  |  |  |  |
| GO:0031034\_myosin\_filament\_assembly | 3 | 0 |  |  |  |  |  |  |  |  |
| GO:0031054\_pre-microRNA\_processing | 3 | 0 |  |  |  |  |  |  |  |  |
| GO:0031055\_chromatin\_remodeling\_at\_centromere | 3 | 0 |  |  |  |  |  |  |  |  |
| GO:0031060\_regulation\_of\_histone\_methylation | 3 | 0 |  |  |  |  |  |  |  |  |
| GO:0031102\_neuron\_projection\_regeneration | 3 | 0 |  |  |  |  |  |  |  |  |
| GO:0031103\_axon\_regeneration | 3 | 0 |  |  |  |  |  |  |  |  |
| GO:0031445\_regulation\_of\_heterochromatin\_formation | 3 | 0 |  |  |  |  |  |  |  |  |
| GO:0031579\_membrane\_raft\_organization | 3 | 0 |  |  |  |  |  |  |  |  |
| GO:0031648\_protein\_destabilization | 3 | 0 |  |  |  |  |  |  |  |  |
| GO:0031657\_regulation\_of\_cyclin-dependent\_protein\_kinase\_activity\_during\_G1\_S | 3 | 0 |  |  |  |  |  |  |  |  |
| GO:0031659\_positive\_regulation\_of\_cyclin-dependent\_protein\_kinase\_activity\_during\_G1\_S | 3 | 0 |  |  |  |  |  |  |  |  |
| GO:0031935\_regulation\_of\_chromatin\_silencing | 3 | 0 |  |  |  |  |  |  |  |  |
| GO:0031953\_negative\_regulation\_of\_protein\_amino\_acid\_autophosphorylation | 3 | 0 |  |  |  |  |  |  |  |  |
| GO:0032007\_negative\_regulation\_of\_TOR\_signaling\_pathway | 3 | 0 |  |  |  |  |  |  |  |  |
| GO:0032042\_mitochondrial\_DNA\_metabolic\_process | 3 | 0 |  |  |  |  |  |  |  |  |
| GO:0032097\_positive\_regulation\_of\_response\_to\_food | 3 | 0 |  |  |  |  |  |  |  |  |
| GO:0032100\_positive\_regulation\_of\_appetite | 3 | 0 |  |  |  |  |  |  |  |  |
| GO:0032105\_negative\_regulation\_of\_response\_to\_extracellular\_stimulus | 3 | 0 |  |  |  |  |  |  |  |  |
| GO:0032108\_negative\_regulation\_of\_response\_to\_nutrient\_levels | 3 | 0 |  |  |  |  |  |  |  |  |
| GO:0032203\_telomere\_formation\_via\_telomerase | 3 | 0 |  |  |  |  |  |  |  |  |
| GO:0032206\_positive\_regulation\_of\_telomere\_maintenance | 3 | 0 |  |  |  |  |  |  |  |  |
| GO:0032232\_negative\_regulation\_of\_actin\_filament\_bundle\_formation | 3 | 0 |  |  |  |  |  |  |  |  |
| GO:0032234\_regulation\_of\_calcium\_ion\_transport\_via\_store-operated\_calcium\_channel\_activity | 3 | 0 |  |  |  |  |  |  |  |  |
| GO:0032278\_positive\_regulation\_of\_gonadotropin\_secretion | 3 | 0 |  |  |  |  |  |  |  |  |
| GO:0032324\_molybdopterin\_cofactor\_biosynthetic\_process | 3 | 0 |  |  |  |  |  |  |  |  |
| GO:0032342\_aldosterone\_biosynthetic\_process | 3 | 0 |  |  |  |  |  |  |  |  |
| GO:0032352\_positive\_regulation\_of\_hormone\_metabolic\_process | 3 | 0 |  |  |  |  |  |  |  |  |
| GO:0032415\_regulation\_of\_sodium:hydrogen\_antiporter\_activity | 3 | 0 |  |  |  |  |  |  |  |  |
| GO:0032469\_endoplasmic\_reticulum\_calcium\_ion\_homeostasis | 3 | 0 |  |  |  |  |  |  |  |  |
| GO:0032494\_response\_to\_peptidoglycan | 3 | 0 |  |  |  |  |  |  |  |  |
| GO:0032497\_detection\_of\_lipopolysaccharide | 3 | 0 |  |  |  |  |  |  |  |  |
| GO:0032509\_endosome\_transport\_via\_multivesicular\_body\_sorting\_pathway | 3 | 0 |  |  |  |  |  |  |  |  |
| GO:0032515\_negative\_regulation\_of\_phosphoprotein\_phosphatase\_activity | 3 | 0 |  |  |  |  |  |  |  |  |
| GO:0032568\_general\_transcription\_from\_RNA\_polymerase\_II\_promoter | 3 | 0 |  |  |  |  |  |  |  |  |
| GO:0032604\_granulocyte\_macrophage\_colony-stimulating\_factor\_production | 3 | 0 |  |  |  |  |  |  |  |  |
| GO:0032616\_interleukin-13\_production | 3 | 0 |  |  |  |  |  |  |  |  |
| GO:0032641\_lymphotoxin\_A\_production | 3 | 0 |  |  |  |  |  |  |  |  |
| GO:0032651\_regulation\_of\_interleukin-1\_beta\_production | 3 | 0 |  |  |  |  |  |  |  |  |
| GO:0032695\_negative\_regulation\_of\_interleukin-12\_production | 3 | 0 |  |  |  |  |  |  |  |  |
| GO:0032717\_negative\_regulation\_of\_interleukin-8\_production | 3 | 0 |  |  |  |  |  |  |  |  |
| GO:0032729\_positive\_regulation\_of\_interferon-gamma\_production | 3 | 0 |  |  |  |  |  |  |  |  |
| GO:0032735\_positive\_regulation\_of\_interleukin-12\_production | 3 | 0 |  |  |  |  |  |  |  |  |
| GO:0032757\_positive\_regulation\_of\_interleukin-8\_production | 3 | 0 |  |  |  |  |  |  |  |  |
| GO:0032760\_positive\_regulation\_of\_tumor\_necrosis\_factor\_production | 3 | 0 |  |  |  |  |  |  |  |  |
| GO:0032855\_positive\_regulation\_of\_Rac\_GTPase\_activity | 3 | 0 |  |  |  |  |  |  |  |  |
| GO:0032872\_regulation\_of\_stress-activated\_MAPK\_cascade | 3 | 0 |  |  |  |  |  |  |  |  |
| GO:0032874\_positive\_regulation\_of\_stress-activated\_MAPK\_cascade | 3 | 0 |  |  |  |  |  |  |  |  |
| GO:0032905\_transforming\_growth\_factor-beta1\_production | 3 | 0 |  |  |  |  |  |  |  |  |
| GO:0032908\_regulation\_of\_transforming\_growth\_factor-beta1\_production | 3 | 0 |  |  |  |  |  |  |  |  |
| GO:0032922\_circadian\_regulation\_of\_gene\_expression | 3 | 0 |  |  |  |  |  |  |  |  |
| GO:0032927\_positive\_regulation\_of\_activin\_receptor\_signaling\_pathway | 3 | 0 |  |  |  |  |  |  |  |  |
| GO:0033015\_tetrapyrrole\_catabolic\_process | 3 | 0 |  |  |  |  |  |  |  |  |
| GO:0033081\_regulation\_of\_T\_cell\_differentiation\_in\_the\_thymus | 3 | 0 |  |  |  |  |  |  |  |  |
| GO:0033143\_regulation\_of\_steroid\_hormone\_receptor\_signaling\_pathway | 3 | 0 |  |  |  |  |  |  |  |  |
| GO:0033151\_V(D)J\_recombination | 3 | 0 |  |  |  |  |  |  |  |  |
| GO:0033198\_response\_to\_ATP | 3 | 0 |  |  |  |  |  |  |  |  |
| GO:0033555\_multicellular\_organismal\_response\_to\_stress | 3 | 0 |  |  |  |  |  |  |  |  |
| GO:0033865\_nucleoside\_bisphosphate\_metabolic\_process | 3 | 0 |  |  |  |  |  |  |  |  |
| GO:0034067\_protein\_localization\_in\_Golgi\_apparatus | 3 | 0 |  |  |  |  |  |  |  |  |
| GO:0034086\_maintenance\_of\_sister\_chromatid\_cohesion | 3 | 0 |  |  |  |  |  |  |  |  |
| GO:0034088\_maintenance\_of\_mitotic\_sister\_chromatid\_cohesion | 3 | 0 |  |  |  |  |  |  |  |  |
| GO:0034393\_positive\_regulation\_of\_smooth\_muscle\_cell\_apoptosis | 3 | 0 |  |  |  |  |  |  |  |  |
| GO:0034442\_regulation\_of\_lipoprotein\_oxidation | 3 | 0 |  |  |  |  |  |  |  |  |
| GO:0034443\_negative\_regulation\_of\_lipoprotein\_oxidation | 3 | 0 |  |  |  |  |  |  |  |  |
| GO:0034453\_microtubule\_anchoring | 3 | 0 |  |  |  |  |  |  |  |  |
| GO:0034505\_tooth\_mineralization | 3 | 0 |  |  |  |  |  |  |  |  |
| GO:0034629\_cellular\_protein\_complex\_localization | 3 | 0 |  |  |  |  |  |  |  |  |
| GO:0034653\_retinoic\_acid\_catabolic\_process | 3 | 0 |  |  |  |  |  |  |  |  |
| GO:0034661\_ncRNA\_catabolic\_process | 3 | 0 |  |  |  |  |  |  |  |  |
| GO:0034776\_response\_to\_histamine | 3 | 0 |  |  |  |  |  |  |  |  |
| GO:0035019\_somatic\_stem\_cell\_maintenance | 3 | 0 |  |  |  |  |  |  |  |  |
| GO:0035020\_regulation\_of\_Rac\_protein\_signal\_transduction | 3 | 0 |  |  |  |  |  |  |  |  |
| GO:0035024\_negative\_regulation\_of\_Rho\_protein\_signal\_transduction | 3 | 0 |  |  |  |  |  |  |  |  |
| GO:0035036\_sperm-egg\_recognition | 3 | 0 |  |  |  |  |  |  |  |  |
| GO:0035067\_negative\_regulation\_of\_histone\_acetylation | 3 | 0 |  |  |  |  |  |  |  |  |
| GO:0035081\_induction\_of\_programmed\_cell\_death\_by\_hormones | 3 | 0 |  |  |  |  |  |  |  |  |
| GO:0035092\_sperm\_chromatin\_condensation | 3 | 0 |  |  |  |  |  |  |  |  |
| GO:0035115\_embryonic\_forelimb\_morphogenesis | 3 | 0 |  |  |  |  |  |  |  |  |
| GO:0035136\_forelimb\_morphogenesis | 3 | 0 |  |  |  |  |  |  |  |  |
| GO:0035246\_peptidyl-arginine\_N-methylation | 3 | 0 |  |  |  |  |  |  |  |  |
| GO:0035268\_protein\_amino\_acid\_mannosylation | 3 | 0 |  |  |  |  |  |  |  |  |
| GO:0035269\_protein\_amino\_acid\_O-linked\_mannosylation | 3 | 0 |  |  |  |  |  |  |  |  |
| GO:0035306\_positive\_regulation\_of\_dephosphorylation | 3 | 0 |  |  |  |  |  |  |  |  |
| GO:0035307\_positive\_regulation\_of\_protein\_amino\_acid\_dephosphorylation | 3 | 0 |  |  |  |  |  |  |  |  |
| GO:0035315\_hair\_cell\_differentiation | 3 | 0 |  |  |  |  |  |  |  |  |
| GO:0040001\_establishment\_of\_mitotic\_spindle\_localization | 3 | 0 |  |  |  |  |  |  |  |  |
| GO:0042053\_regulation\_of\_dopamine\_metabolic\_process | 3 | 0 |  |  |  |  |  |  |  |  |
| GO:0042059\_negative\_regulation\_of\_epidermal\_growth\_factor\_receptor\_signaling\_pathway | 3 | 0 |  |  |  |  |  |  |  |  |
| GO:0042069\_regulation\_of\_catecholamine\_metabolic\_process | 3 | 0 |  |  |  |  |  |  |  |  |
| GO:0042090\_interleukin-12\_biosynthetic\_process | 3 | 0 |  |  |  |  |  |  |  |  |
| GO:0042109\_lymphotoxin\_A\_biosynthetic\_process | 3 | 0 |  |  |  |  |  |  |  |  |
| GO:0042160\_lipoprotein\_modification | 3 | 0 |  |  |  |  |  |  |  |  |
| GO:0042161\_lipoprotein\_oxidation | 3 | 0 |  |  |  |  |  |  |  |  |
| GO:0042246\_tissue\_regeneration | 3 | 0 |  |  |  |  |  |  |  |  |
| GO:0042253\_granulocyte\_macrophage\_colony-stimulating\_factor\_biosynthetic\_process | 3 | 0 |  |  |  |  |  |  |  |  |
| GO:0042271\_susceptibility\_to\_natural\_killer\_cell\_mediated\_cytotoxicity | 3 | 0 |  |  |  |  |  |  |  |  |
| GO:0042368\_vitamin\_D\_biosynthetic\_process | 3 | 0 |  |  |  |  |  |  |  |  |
| GO:0042416\_dopamine\_biosynthetic\_process | 3 | 0 |  |  |  |  |  |  |  |  |
| GO:0042451\_purine\_nucleoside\_biosynthetic\_process | 3 | 0 |  |  |  |  |  |  |  |  |
| GO:0042455\_ribonucleoside\_biosynthetic\_process | 3 | 0 |  |  |  |  |  |  |  |  |
| GO:0042574\_retinal\_metabolic\_process | 3 | 0 |  |  |  |  |  |  |  |  |
| GO:0042753\_positive\_regulation\_of\_circadian\_rhythm | 3 | 0 |  |  |  |  |  |  |  |  |
| GO:0042762\_regulation\_of\_sulfur\_metabolic\_process | 3 | 0 |  |  |  |  |  |  |  |  |
| GO:0042886\_amide\_transport | 3 | 0 |  |  |  |  |  |  |  |  |
| GO:0042940\_D-amino\_acid\_transport | 3 | 0 |  |  |  |  |  |  |  |  |
| GO:0042953\_lipoprotein\_transport | 3 | 0 |  |  |  |  |  |  |  |  |
| GO:0042977\_activation\_of\_JAK2\_kinase\_activity | 3 | 0 |  |  |  |  |  |  |  |  |
| GO:0042983\_amyloid\_precursor\_protein\_biosynthetic\_process | 3 | 0 |  |  |  |  |  |  |  |  |
| GO:0042984\_regulation\_of\_amyloid\_precursor\_protein\_biosynthetic\_process | 3 | 0 |  |  |  |  |  |  |  |  |
| GO:0043001\_Golgi\_to\_plasma\_membrane\_protein\_transport | 3 | 0 |  |  |  |  |  |  |  |  |
| GO:0043089\_positive\_regulation\_of\_Cdc42\_GTPase\_activity | 3 | 0 |  |  |  |  |  |  |  |  |
| GO:0043090\_amino\_acid\_import | 3 | 0 |  |  |  |  |  |  |  |  |
| GO:0043092\_L-amino\_acid\_import | 3 | 0 |  |  |  |  |  |  |  |  |
| GO:0043248\_proteasome\_assembly | 3 | 0 |  |  |  |  |  |  |  |  |
| GO:0043288\_apocarotenoid\_metabolic\_process | 3 | 0 |  |  |  |  |  |  |  |  |
| GO:0043368\_positive\_T\_cell\_selection | 3 | 0 |  |  |  |  |  |  |  |  |
| GO:0043461\_proton-transporting\_ATP\_synthase\_complex\_assembly | 3 | 0 |  |  |  |  |  |  |  |  |
| GO:0043489\_RNA\_stabilization | 3 | 0 |  |  |  |  |  |  |  |  |
| GO:0043570\_maintenance\_of\_DNA\_repeat\_elements | 3 | 0 |  |  |  |  |  |  |  |  |
| GO:0043604\_amide\_biosynthetic\_process | 3 | 0 |  |  |  |  |  |  |  |  |
| GO:0043618\_regulation\_of\_transcription\_from\_RNA\_polymerase\_II\_promoter\_in\_response\_to\_stress | 3 | 0 |  |  |  |  |  |  |  |  |
| GO:0043620\_regulation\_of\_transcription\_in\_response\_to\_stress | 3 | 0 |  |  |  |  |  |  |  |  |
| GO:0043902\_positive\_regulation\_of\_multi-organism\_process | 3 | 0 |  |  |  |  |  |  |  |  |
| GO:0044003\_modification\_by\_symbiont\_of\_host\_morphology\_or\_physiology | 3 | 0 |  |  |  |  |  |  |  |  |
| GO:0044240\_multicellular\_organismal\_lipid\_catabolic\_process | 3 | 0 |  |  |  |  |  |  |  |  |
| GO:0044252\_negative\_regulation\_of\_multicellular\_organismal\_metabolic\_process | 3 | 0 |  |  |  |  |  |  |  |  |
| GO:0045007\_depurination | 3 | 0 |  |  |  |  |  |  |  |  |
| GO:0045010\_actin\_nucleation | 3 | 0 |  |  |  |  |  |  |  |  |
| GO:0045061\_thymic\_T\_cell\_selection | 3 | 0 |  |  |  |  |  |  |  |  |
| GO:0045063\_T-helper\_1\_cell\_differentiation | 3 | 0 |  |  |  |  |  |  |  |  |
| GO:0045064\_T-helper\_2\_cell\_differentiation | 3 | 0 |  |  |  |  |  |  |  |  |
| GO:0045070\_positive\_regulation\_of\_viral\_genome\_replication | 3 | 0 |  |  |  |  |  |  |  |  |
| GO:0045075\_regulation\_of\_interleukin-12\_biosynthetic\_process | 3 | 0 |  |  |  |  |  |  |  |  |
| GO:0045079\_negative\_regulation\_of\_chemokine\_biosynthetic\_process | 3 | 0 |  |  |  |  |  |  |  |  |
| GO:0045080\_positive\_regulation\_of\_chemokine\_biosynthetic\_process | 3 | 0 |  |  |  |  |  |  |  |  |
| GO:0045112\_integrin\_biosynthetic\_process | 3 | 0 |  |  |  |  |  |  |  |  |
| GO:0045409\_negative\_regulation\_of\_interleukin-6\_biosynthetic\_process | 3 | 0 |  |  |  |  |  |  |  |  |
| GO:0045410\_positive\_regulation\_of\_interleukin-6\_biosynthetic\_process | 3 | 0 |  |  |  |  |  |  |  |  |
| GO:0045541\_negative\_regulation\_of\_cholesterol\_biosynthetic\_process | 3 | 0 |  |  |  |  |  |  |  |  |
| GO:0045577\_regulation\_of\_B\_cell\_differentiation | 3 | 0 |  |  |  |  |  |  |  |  |
| GO:0045581\_negative\_regulation\_of\_T\_cell\_differentiation | 3 | 0 |  |  |  |  |  |  |  |  |
| GO:0045625\_regulation\_of\_T-helper\_1\_cell\_differentiation | 3 | 0 |  |  |  |  |  |  |  |  |
| GO:0045628\_regulation\_of\_T-helper\_2\_cell\_differentiation | 3 | 0 |  |  |  |  |  |  |  |  |
| GO:0045662\_negative\_regulation\_of\_myoblast\_differentiation | 3 | 0 |  |  |  |  |  |  |  |  |
| GO:0045663\_positive\_regulation\_of\_myoblast\_differentiation | 3 | 0 |  |  |  |  |  |  |  |  |
| GO:0045665\_negative\_regulation\_of\_neuron\_differentiation | 3 | 0 |  |  |  |  |  |  |  |  |
| GO:0045715\_negative\_regulation\_of\_low-density\_lipoprotein\_receptor\_biosynthetic\_process | 3 | 0 |  |  |  |  |  |  |  |  |
| GO:0045717\_negative\_regulation\_of\_fatty\_acid\_biosynthetic\_process | 3 | 0 |  |  |  |  |  |  |  |  |
| GO:0045779\_negative\_regulation\_of\_bone\_resorption | 3 | 0 |  |  |  |  |  |  |  |  |
| GO:0045817\_positive\_regulation\_of\_transcription\_from\_RNA\_polymerase\_II\_promoter\_\_global | 3 | 0 |  |  |  |  |  |  |  |  |
| GO:0045822\_negative\_regulation\_of\_heart\_contraction | 3 | 0 |  |  |  |  |  |  |  |  |
| GO:0045830\_positive\_regulation\_of\_isotype\_switching | 3 | 0 |  |  |  |  |  |  |  |  |
| GO:0045898\_regulation\_of\_transcriptional\_preinitiation\_complex\_assembly | 3 | 0 |  |  |  |  |  |  |  |  |
| GO:0045911\_positive\_regulation\_of\_DNA\_recombination | 3 | 0 |  |  |  |  |  |  |  |  |
| GO:0045912\_negative\_regulation\_of\_carbohydrate\_metabolic\_process | 3 | 0 |  |  |  |  |  |  |  |  |
| GO:0045916\_negative\_regulation\_of\_complement\_activation | 3 | 0 |  |  |  |  |  |  |  |  |
| GO:0045939\_negative\_regulation\_of\_steroid\_metabolic\_process | 3 | 0 |  |  |  |  |  |  |  |  |
| GO:0045988\_negative\_regulation\_of\_striated\_muscle\_contraction | 3 | 0 |  |  |  |  |  |  |  |  |
| GO:0045990\_regulation\_of\_transcription\_by\_carbon\_catabolites | 3 | 0 |  |  |  |  |  |  |  |  |
| GO:0045995\_regulation\_of\_embryonic\_development | 3 | 0 |  |  |  |  |  |  |  |  |
| GO:0046015\_regulation\_of\_transcription\_by\_glucose | 3 | 0 |  |  |  |  |  |  |  |  |
| GO:0046068\_cGMP\_metabolic\_process | 3 | 0 |  |  |  |  |  |  |  |  |
| GO:0046129\_purine\_ribonucleoside\_biosynthetic\_process | 3 | 0 |  |  |  |  |  |  |  |  |
| GO:0046130\_purine\_ribonucleoside\_catabolic\_process | 3 | 0 |  |  |  |  |  |  |  |  |
| GO:0046146\_tetrahydrobiopterin\_metabolic\_process | 3 | 0 |  |  |  |  |  |  |  |  |
| GO:0046348\_amino\_sugar\_catabolic\_process | 3 | 0 |  |  |  |  |  |  |  |  |
| GO:0046605\_regulation\_of\_centrosome\_cycle | 3 | 0 |  |  |  |  |  |  |  |  |
| GO:0046628\_positive\_regulation\_of\_insulin\_receptor\_signaling\_pathway | 3 | 0 |  |  |  |  |  |  |  |  |
| GO:0046653\_tetrahydrofolate\_metabolic\_process | 3 | 0 |  |  |  |  |  |  |  |  |
| GO:0046676\_negative\_regulation\_of\_insulin\_secretion | 3 | 0 |  |  |  |  |  |  |  |  |
| GO:0046688\_response\_to\_copper\_ion | 3 | 0 |  |  |  |  |  |  |  |  |
| GO:0046813\_virion\_attachment\_\_binding\_of\_host\_cell\_surface\_receptor | 3 | 0 |  |  |  |  |  |  |  |  |
| GO:0046825\_regulation\_of\_protein\_export\_from\_nucleus | 3 | 0 |  |  |  |  |  |  |  |  |
| GO:0046881\_positive\_regulation\_of\_follicle-stimulating\_hormone\_secretion | 3 | 0 |  |  |  |  |  |  |  |  |
| GO:0046886\_positive\_regulation\_of\_hormone\_biosynthetic\_process | 3 | 0 |  |  |  |  |  |  |  |  |
| GO:0048048\_embryonic\_eye\_morphogenesis | 3 | 0 |  |  |  |  |  |  |  |  |
| GO:0048251\_elastic\_fiber\_assembly | 3 | 0 |  |  |  |  |  |  |  |  |
| GO:0048255\_mRNA\_stabilization | 3 | 0 |  |  |  |  |  |  |  |  |
| GO:0048268\_clathrin\_coat\_assembly | 3 | 0 |  |  |  |  |  |  |  |  |
| GO:0048521\_negative\_regulation\_of\_behavior | 3 | 0 |  |  |  |  |  |  |  |  |
| GO:0048538\_thymus\_development | 3 | 0 |  |  |  |  |  |  |  |  |
| GO:0048548\_regulation\_of\_pinocytosis | 3 | 0 |  |  |  |  |  |  |  |  |
| GO:0048567\_ectodermal\_gut\_morphogenesis | 3 | 0 |  |  |  |  |  |  |  |  |
| GO:0048701\_embryonic\_cranial\_skeleton\_morphogenesis | 3 | 0 |  |  |  |  |  |  |  |  |
| GO:0048739\_cardiac\_muscle\_fiber\_development | 3 | 0 |  |  |  |  |  |  |  |  |
| GO:0048814\_regulation\_of\_dendrite\_morphogenesis | 3 | 0 |  |  |  |  |  |  |  |  |
| GO:0048861\_leukemia\_inhibitory\_factor\_signaling\_pathway | 3 | 0 |  |  |  |  |  |  |  |  |
| GO:0050434\_positive\_regulation\_of\_viral\_transcription | 3 | 0 |  |  |  |  |  |  |  |  |
| GO:0050435\_beta-amyloid\_metabolic\_process | 3 | 0 |  |  |  |  |  |  |  |  |
| GO:0050665\_hydrogen\_peroxide\_biosynthetic\_process | 3 | 0 |  |  |  |  |  |  |  |  |
| GO:0050686\_negative\_regulation\_of\_mRNA\_processing | 3 | 0 |  |  |  |  |  |  |  |  |
| GO:0050806\_positive\_regulation\_of\_synaptic\_transmission | 3 | 0 |  |  |  |  |  |  |  |  |
| GO:0050856\_regulation\_of\_T\_cell\_receptor\_signaling\_pathway | 3 | 0 |  |  |  |  |  |  |  |  |
| GO:0050873\_brown\_fat\_cell\_differentiation | 3 | 0 |  |  |  |  |  |  |  |  |
| GO:0051001\_negative\_regulation\_of\_nitric-oxide\_synthase\_activity | 3 | 0 |  |  |  |  |  |  |  |  |
| GO:0051084\_'de\_novo'\_posttranslational\_protein\_folding | 3 | 0 |  |  |  |  |  |  |  |  |
| GO:0051153\_regulation\_of\_striated\_muscle\_cell\_differentiation | 3 | 0 |  |  |  |  |  |  |  |  |
| GO:0051299\_centrosome\_separation | 3 | 0 |  |  |  |  |  |  |  |  |
| GO:0051305\_chromosome\_movement\_towards\_spindle\_pole | 3 | 0 |  |  |  |  |  |  |  |  |
| GO:0051324\_prophase | 3 | 0 |  |  |  |  |  |  |  |  |
| GO:0051382\_kinetochore\_assembly | 3 | 0 |  |  |  |  |  |  |  |  |
| GO:0051489\_regulation\_of\_filopodium\_assembly | 3 | 0 |  |  |  |  |  |  |  |  |
| GO:0051491\_positive\_regulation\_of\_filopodium\_assembly | 3 | 0 |  |  |  |  |  |  |  |  |
| GO:0051497\_negative\_regulation\_of\_stress\_fiber\_formation | 3 | 0 |  |  |  |  |  |  |  |  |
| GO:0051567\_histone\_H3-K9\_methylation | 3 | 0 |  |  |  |  |  |  |  |  |
| GO:0051569\_regulation\_of\_histone\_H3-K4\_methylation | 3 | 0 |  |  |  |  |  |  |  |  |
| GO:0051712\_positive\_regulation\_of\_killing\_of\_cells\_of\_another\_organism | 3 | 0 |  |  |  |  |  |  |  |  |
| GO:0051825\_adhesion\_to\_other\_organism\_during\_symbiotic\_interaction | 3 | 0 |  |  |  |  |  |  |  |  |
| GO:0051851\_modification\_by\_host\_of\_symbiont\_morphology\_or\_physiology | 3 | 0 |  |  |  |  |  |  |  |  |
| GO:0051856\_adhesion\_to\_symbiont | 3 | 0 |  |  |  |  |  |  |  |  |
| GO:0051877\_pigment\_granule\_aggregation\_in\_cell\_center | 3 | 0 |  |  |  |  |  |  |  |  |
| GO:0051882\_mitochondrial\_depolarization | 3 | 0 |  |  |  |  |  |  |  |  |
| GO:0051918\_negative\_regulation\_of\_fibrinolysis | 3 | 0 |  |  |  |  |  |  |  |  |
| GO:0051925\_regulation\_of\_calcium\_ion\_transport\_via\_voltage-gated\_calcium\_channel\_activity | 3 | 0 |  |  |  |  |  |  |  |  |
| GO:0051938\_L-glutamate\_import | 3 | 0 |  |  |  |  |  |  |  |  |
| GO:0051966\_regulation\_of\_synaptic\_transmission\_\_glutamatergic | 3 | 0 |  |  |  |  |  |  |  |  |
| GO:0051970\_negative\_regulation\_of\_transmission\_of\_nerve\_impulse | 3 | 0 |  |  |  |  |  |  |  |  |
| GO:0051973\_positive\_regulation\_of\_telomerase\_activity | 3 | 0 |  |  |  |  |  |  |  |  |
| GO:0051983\_regulation\_of\_chromosome\_segregation | 3 | 0 |  |  |  |  |  |  |  |  |
| GO:0055062\_phosphate\_ion\_homeostasis | 3 | 0 |  |  |  |  |  |  |  |  |
| GO:0055069\_zinc\_ion\_homeostasis | 3 | 0 |  |  |  |  |  |  |  |  |
| GO:0055078\_sodium\_ion\_homeostasis | 3 | 0 |  |  |  |  |  |  |  |  |
| GO:0055094\_response\_to\_lipoprotein\_stimulus | 3 | 0 |  |  |  |  |  |  |  |  |
| GO:0060017\_parathyroid\_gland\_development | 3 | 0 |  |  |  |  |  |  |  |  |
| GO:0060023\_soft\_palate\_development | 3 | 0 |  |  |  |  |  |  |  |  |
| GO:0060084\_synaptic\_transmission\_involved\_in\_micturition | 3 | 0 |  |  |  |  |  |  |  |  |
| GO:0060119\_inner\_ear\_receptor\_cell\_development | 3 | 0 |  |  |  |  |  |  |  |  |
| GO:0060134\_prepulse\_inhibition | 3 | 0 |  |  |  |  |  |  |  |  |
| GO:0060158\_activation\_of\_phospholipase\_C\_activity\_by\_dopamine\_receptor\_signaling\_pathway | 3 | 0 |  |  |  |  |  |  |  |  |
| GO:0060177\_regulation\_of\_angiotensin\_metabolic\_process | 3 | 0 |  |  |  |  |  |  |  |  |
| GO:0060267\_positive\_regulation\_of\_respiratory\_burst | 3 | 0 |  |  |  |  |  |  |  |  |
| GO:0060317\_cardiac\_epithelial\_to\_mesenchymal\_transition | 3 | 0 |  |  |  |  |  |  |  |  |
| GO:0060333\_interferon-gamma-mediated\_signaling\_pathway | 3 | 0 |  |  |  |  |  |  |  |  |
| GO:0060334\_regulation\_of\_interferon-gamma-mediated\_signaling\_pathway | 3 | 0 |  |  |  |  |  |  |  |  |
| GO:0060343\_trabecula\_formation | 3 | 0 |  |  |  |  |  |  |  |  |
| GO:0060347\_heart\_trabecula\_formation | 3 | 0 |  |  |  |  |  |  |  |  |
| GO:0060394\_negative\_regulation\_of\_pathway-restricted\_SMAD\_protein\_phosphorylation | 3 | 0 |  |  |  |  |  |  |  |  |
| GO:0060559\_positive\_regulation\_of\_calcidiol\_1-monooxygenase\_activity | 3 | 0 |  |  |  |  |  |  |  |  |
| GO:0070050\_neuron\_maintenance | 3 | 0 |  |  |  |  |  |  |  |  |
| GO:0070141\_response\_to\_UV-A | 3 | 0 |  |  |  |  |  |  |  |  |
| GO:0070168\_negative\_regulation\_of\_biomineral\_formation | 3 | 0 |  |  |  |  |  |  |  |  |
| GO:0070207\_protein\_homotrimerization | 3 | 0 |  |  |  |  |  |  |  |  |
| GO:0070229\_negative\_regulation\_of\_lymphocyte\_apoptosis | 3 | 0 |  |  |  |  |  |  |  |  |
| GO:0070231\_T\_cell\_apoptosis | 3 | 0 |  |  |  |  |  |  |  |  |
| GO:0070272\_proton-transporting\_ATP\_synthase\_complex\_biogenesis | 3 | 0 |  |  |  |  |  |  |  |  |
| GO:0070561\_vitamin\_D\_receptor\_signaling\_pathway | 3 | 0 |  |  |  |  |  |  |  |  |
| GO:0070634\_transepithelial\_ammonium\_transport | 3 | 0 |  |  |  |  |  |  |  |  |
| GO:0070777\_D-aspartate\_transport | 3 | 0 |  |  |  |  |  |  |  |  |
| GO:0070779\_D-aspartate\_import | 3 | 0 |  |  |  |  |  |  |  |  |
| GO:0045892\_negative\_regulation\_of\_transcription\_\_DNA-dependent | 175 | 0 | 0.000000 | 0.000000 | 688 | 505.693878 | 565.88 | 626.066122 | 0.822500 |
| GO:0000387\_spliceosomal\_snRNP\_biogenesis | 28 | 0 | 0.000000 | 0.000000 | 711 | 533.489070 | 592.63 | 651.770930 | 0.833516 |
| GO:0000819\_sister\_chromatid\_segregation | 28 | 0 | 0.000000 | 0.000000 | 711 | 533.489070 | 592.63 | 651.770930 | 0.833516 |
| GO:0002440\_production\_of\_molecular\_mediator\_of\_immune\_response | 28 | 0 | 0.000000 | 0.000000 | 711 | 533.489070 | 592.63 | 651.770930 | 0.833516 |
| GO:0006304\_DNA\_modification | 28 | 0 | 0.000000 | 0.000000 | 711 | 533.489070 | 592.63 | 651.770930 | 0.833516 |
| GO:0006405\_RNA\_export\_from\_nucleus | 28 | 0 | 0.000000 | 0.000000 | 711 | 533.489070 | 592.63 | 651.770930 | 0.833516 |
| GO:0007156\_homophilic\_cell\_adhesion | 28 | 0 | 0.000000 | 0.000000 | 711 | 533.489070 | 592.63 | 651.770930 | 0.833516 |
| GO:0007281\_germ\_cell\_development | 28 | 0 | 0.000000 | 0.000000 | 711 | 533.489070 | 592.63 | 651.770930 | 0.833516 |
| GO:0007411\_axon\_guidance | 28 | 0 | 0.000000 | 0.000000 | 711 | 533.489070 | 592.63 | 651.770930 | 0.833516 |
| GO:0009062\_fatty\_acid\_catabolic\_process | 28 | 0 | 0.000000 | 0.000000 | 711 | 533.489070 | 592.63 | 651.770930 | 0.833516 |
| GO:0009593\_detection\_of\_chemical\_stimulus | 28 | 0 | 0.000000 | 0.000000 | 711 | 533.489070 | 592.63 | 651.770930 | 0.833516 |
| GO:0009895\_negative\_regulation\_of\_catabolic\_process | 28 | 0 | 0.000000 | 0.000000 | 711 | 533.489070 | 592.63 | 651.770930 | 0.833516 |
| GO:0010563\_negative\_regulation\_of\_phosphorus\_metabolic\_process | 28 | 0 | 0.000000 | 0.000000 | 711 | 533.489070 | 592.63 | 651.770930 | 0.833516 |
| GO:0016055\_Wnt\_receptor\_signaling\_pathway | 28 | 0 | 0.000000 | 0.000000 | 711 | 533.489070 | 592.63 | 651.770930 | 0.833516 |
| GO:0031214\_biomineral\_formation | 28 | 0 | 0.000000 | 0.000000 | 711 | 533.489070 | 592.63 | 651.770930 | 0.833516 |
| GO:0033044\_regulation\_of\_chromosome\_organization | 28 | 0 | 0.000000 | 0.000000 | 711 | 533.489070 | 592.63 | 651.770930 | 0.833516 |
| GO:0042306\_regulation\_of\_protein\_import\_into\_nucleus | 28 | 0 | 0.000000 | 0.000000 | 711 | 533.489070 | 592.63 | 651.770930 | 0.833516 |
| GO:0045664\_regulation\_of\_neuron\_differentiation | 28 | 0 | 0.000000 | 0.000000 | 711 | 533.489070 | 592.63 | 651.770930 | 0.833516 |
| GO:0045834\_positive\_regulation\_of\_lipid\_metabolic\_process | 28 | 0 | 0.000000 | 0.000000 | 711 | 533.489070 | 592.63 | 651.770930 | 0.833516 |
| GO:0045936\_negative\_regulation\_of\_phosphate\_metabolic\_process | 28 | 0 | 0.000000 | 0.000000 | 711 | 533.489070 | 592.63 | 651.770930 | 0.833516 |
| GO:0051349\_positive\_regulation\_of\_lyase\_activity | 28 | 0 | 0.000000 | 0.000000 | 711 | 533.489070 | 592.63 | 651.770930 | 0.833516 |
| GO:0051650\_establishment\_of\_vesicle\_localization | 28 | 0 | 0.000000 | 0.000000 | 711 | 533.489070 | 592.63 | 651.770930 | 0.833516 |
| GO:0070662\_mast\_cell\_proliferation | 28 | 0 | 0.000000 | 0.000000 | 711 | 533.489070 | 592.63 | 651.770930 | 0.833516 |
| GO:0070666\_regulation\_of\_mast\_cell\_proliferation | 28 | 0 | 0.000000 | 0.000000 | 711 | 533.489070 | 592.63 | 651.770930 | 0.833516 |
| GO:0019935\_cyclic-nucleotide-mediated\_signaling | 82 | 0 | 0.000000 | 0.000000 | 714 | 538.494622 | 597.12 | 655.745378 | 0.836303 |
| GO:0045087\_innate\_immune\_response | 82 | 0 | 0.000000 | 0.000000 | 714 | 538.494622 | 597.12 | 655.745378 | 0.836303 |
| GO:0048193\_Golgi\_vesicle\_transport | 82 | 0 | 0.000000 | 0.000000 | 714 | 538.494622 | 597.12 | 655.745378 | 0.836303 |
| GO:0007605\_sensory\_perception\_of\_sound | 54 | 0 | 0.000000 | 0.000000 | 716 | 540.435952 | 598.94 | 657.444048 | 0.836508 |
| GO:0050954\_sensory\_perception\_of\_mechanical\_stimulus | 54 | 0 | 0.000000 | 0.000000 | 716 | 540.435952 | 598.94 | 657.444048 | 0.836508 |
| GO:0009101\_glycoprotein\_biosynthetic\_process | 109 | 0 | 0.000000 | 0.000000 | 719 | 543.066885 | 601.44 | 659.813115 | 0.836495 |
| GO:0009790\_embryonic\_development | 109 | 0 | 0.000000 | 0.000000 | 719 | 543.066885 | 601.44 | 659.813115 | 0.836495 |
| GO:0010817\_regulation\_of\_hormone\_levels | 109 | 0 | 0.000000 | 0.000000 | 719 | 543.066885 | 601.44 | 659.813115 | 0.836495 |
| GO:0018193\_peptidyl-amino\_acid\_modification | 117 | 0 | 0.000000 | 0.000000 | 720 | 543.958087 | 602.29 | 660.621913 | 0.836514 |
| GO:0051254\_positive\_regulation\_of\_RNA\_metabolic\_process | 213 | 0 | 0.000000 | 0.000000 | 722 | 546.326425 | 604.24 | 662.153575 | 0.836898 |
| GO:0051726\_regulation\_of\_cell\_cycle | 213 | 0 | 0.000000 | 0.000000 | 722 | 546.326425 | 604.24 | 662.153575 | 0.836898 |
| GO:0001655\_urogenital\_system\_development | 23 | 0 | 0.000000 | 0.000000 | 742 | 571.886717 | 628.85 | 685.813283 | 0.847507 |
| GO:0001906\_cell\_killing | 23 | 0 | 0.000000 | 0.000000 | 742 | 571.886717 | 628.85 | 685.813283 | 0.847507 |
| GO:0006023\_aminoglycan\_biosynthetic\_process | 23 | 0 | 0.000000 | 0.000000 | 742 | 571.886717 | 628.85 | 685.813283 | 0.847507 |
| GO:0007190\_activation\_of\_adenylate\_cyclase\_activity | 23 | 0 | 0.000000 | 0.000000 | 742 | 571.886717 | 628.85 | 685.813283 | 0.847507 |
| GO:0007218\_neuropeptide\_signaling\_pathway | 23 | 0 | 0.000000 | 0.000000 | 742 | 571.886717 | 628.85 | 685.813283 | 0.847507 |
| GO:0007623\_circadian\_rhythm | 23 | 0 | 0.000000 | 0.000000 | 742 | 571.886717 | 628.85 | 685.813283 | 0.847507 |
| GO:0016579\_protein\_deubiquitination | 23 | 0 | 0.000000 | 0.000000 | 742 | 571.886717 | 628.85 | 685.813283 | 0.847507 |
| GO:0018130\_heterocycle\_biosynthetic\_process | 23 | 0 | 0.000000 | 0.000000 | 742 | 571.886717 | 628.85 | 685.813283 | 0.847507 |
| GO:0030166\_proteoglycan\_biosynthetic\_process | 23 | 0 | 0.000000 | 0.000000 | 742 | 571.886717 | 628.85 | 685.813283 | 0.847507 |
| GO:0033344\_cholesterol\_efflux | 23 | 0 | 0.000000 | 0.000000 | 742 | 571.886717 | 628.85 | 685.813283 | 0.847507 |
| GO:0034637\_cellular\_carbohydrate\_biosynthetic\_process | 23 | 0 | 0.000000 | 0.000000 | 742 | 571.886717 | 628.85 | 685.813283 | 0.847507 |
| GO:0043473\_pigmentation | 23 | 0 | 0.000000 | 0.000000 | 742 | 571.886717 | 628.85 | 685.813283 | 0.847507 |
| GO:0045740\_positive\_regulation\_of\_DNA\_replication | 23 | 0 | 0.000000 | 0.000000 | 742 | 571.886717 | 628.85 | 685.813283 | 0.847507 |
| GO:0046467\_membrane\_lipid\_biosynthetic\_process | 23 | 0 | 0.000000 | 0.000000 | 742 | 571.886717 | 628.85 | 685.813283 | 0.847507 |
| GO:0048871\_multicellular\_organismal\_homeostasis | 23 | 0 | 0.000000 | 0.000000 | 742 | 571.886717 | 628.85 | 685.813283 | 0.847507 |
| GO:0050671\_positive\_regulation\_of\_lymphocyte\_proliferation | 23 | 0 | 0.000000 | 0.000000 | 742 | 571.886717 | 628.85 | 685.813283 | 0.847507 |
| GO:0050707\_regulation\_of\_cytokine\_secretion | 23 | 0 | 0.000000 | 0.000000 | 742 | 571.886717 | 628.85 | 685.813283 | 0.847507 |
| GO:0051262\_protein\_tetramerization | 23 | 0 | 0.000000 | 0.000000 | 742 | 571.886717 | 628.85 | 685.813283 | 0.847507 |
| GO:0051353\_positive\_regulation\_of\_oxidoreductase\_activity | 23 | 0 | 0.000000 | 0.000000 | 742 | 571.886717 | 628.85 | 685.813283 | 0.847507 |
| GO:0070668\_positive\_regulation\_of\_mast\_cell\_proliferation | 23 | 0 | 0.000000 | 0.000000 | 742 | 571.886717 | 628.85 | 685.813283 | 0.847507 |
| GO:0015849\_organic\_acid\_transport | 80 | 0 | 0.000000 | 0.000000 | 744 | 574.521767 | 631.29 | 688.058233 | 0.848508 |
| GO:0031175\_neuron\_projection\_development | 80 | 0 | 0.000000 | 0.000000 | 744 | 574.521767 | 631.29 | 688.058233 | 0.848508 |
| GO:0000002\_mitochondrial\_genome\_maintenance | 6 | 0 | 0.000000 | 0.000000 | 960 | 793.499213 | 848.89 | 904.280787 | 0.884260 |
| GO:0000076\_DNA\_replication\_checkpoint | 6 | 0 | 0.000000 | 0.000000 | 960 | 793.499213 | 848.89 | 904.280787 | 0.884260 |
| GO:0000381\_regulation\_of\_alternative\_nuclear\_mRNA\_splicing\_\_via\_spliceosome | 6 | 0 | 0.000000 | 0.000000 | 960 | 793.499213 | 848.89 | 904.280787 | 0.884260 |
| GO:0001656\_metanephros\_development | 6 | 0 | 0.000000 | 0.000000 | 960 | 793.499213 | 848.89 | 904.280787 | 0.884260 |
| GO:0001838\_embryonic\_epithelial\_tube\_formation | 6 | 0 | 0.000000 | 0.000000 | 960 | 793.499213 | 848.89 | 904.280787 | 0.884260 |
| GO:0001841\_neural\_tube\_formation | 6 | 0 | 0.000000 | 0.000000 | 960 | 793.499213 | 848.89 | 904.280787 | 0.884260 |
| GO:0001843\_neural\_tube\_closure | 6 | 0 | 0.000000 | 0.000000 | 960 | 793.499213 | 848.89 | 904.280787 | 0.884260 |
| GO:0001893\_maternal\_placenta\_development | 6 | 0 | 0.000000 | 0.000000 | 960 | 793.499213 | 848.89 | 904.280787 | 0.884260 |
| GO:0001913\_T\_cell\_mediated\_cytotoxicity | 6 | 0 | 0.000000 | 0.000000 | 960 | 793.499213 | 848.89 | 904.280787 | 0.884260 |
| GO:0001942\_hair\_follicle\_development | 6 | 0 | 0.000000 | 0.000000 | 960 | 793.499213 | 848.89 | 904.280787 | 0.884260 |
| GO:0001947\_heart\_looping | 6 | 0 | 0.000000 | 0.000000 | 960 | 793.499213 | 848.89 | 904.280787 | 0.884260 |
| GO:0002221\_pattern\_recognition\_receptor\_signaling\_pathway | 6 | 0 | 0.000000 | 0.000000 | 960 | 793.499213 | 848.89 | 904.280787 | 0.884260 |
| GO:0002224\_toll-like\_receptor\_signaling\_pathway | 6 | 0 | 0.000000 | 0.000000 | 960 | 793.499213 | 848.89 | 904.280787 | 0.884260 |
| GO:0002260\_lymphocyte\_homeostasis | 6 | 0 | 0.000000 | 0.000000 | 960 | 793.499213 | 848.89 | 904.280787 | 0.884260 |
| GO:0002420\_natural\_killer\_cell\_mediated\_cytotoxicity\_directed\_against\_tumor\_cell\_target | 6 | 0 | 0.000000 | 0.000000 | 960 | 793.499213 | 848.89 | 904.280787 | 0.884260 |
| GO:0002423\_natural\_killer\_cell\_mediated\_immune\_response\_to\_tumor\_cell | 6 | 0 | 0.000000 | 0.000000 | 960 | 793.499213 | 848.89 | 904.280787 | 0.884260 |
| GO:0002712\_regulation\_of\_B\_cell\_mediated\_immunity | 6 | 0 | 0.000000 | 0.000000 | 960 | 793.499213 | 848.89 | 904.280787 | 0.884260 |
| GO:0002855\_regulation\_of\_natural\_killer\_cell\_mediated\_immune\_response\_to\_tumor\_cell | 6 | 0 | 0.000000 | 0.000000 | 960 | 793.499213 | 848.89 | 904.280787 | 0.884260 |
| GO:0002857\_positive\_regulation\_of\_natural\_killer\_cell\_mediated\_immune\_response\_to\_tumor\_cell | 6 | 0 | 0.000000 | 0.000000 | 960 | 793.499213 | 848.89 | 904.280787 | 0.884260 |
| GO:0002858\_regulation\_of\_natural\_killer\_cell\_mediated\_cytotoxicity\_directed\_against\_tumor\_cell\_target | 6 | 0 | 0.000000 | 0.000000 | 960 | 793.499213 | 848.89 | 904.280787 | 0.884260 |
| GO:0002860\_positive\_regulation\_of\_natural\_killer\_cell\_mediated\_cytotoxicity\_directed\_against\_tumor\_cell\_target | 6 | 0 | 0.000000 | 0.000000 | 960 | 793.499213 | 848.89 | 904.280787 | 0.884260 |
| GO:0002889\_regulation\_of\_immunoglobulin\_mediated\_immune\_response | 6 | 0 | 0.000000 | 0.000000 | 960 | 793.499213 | 848.89 | 904.280787 | 0.884260 |
| GO:0006020\_inositol\_metabolic\_process | 6 | 0 | 0.000000 | 0.000000 | 960 | 793.499213 | 848.89 | 904.280787 | 0.884260 |
| GO:0006144\_purine\_base\_metabolic\_process | 6 | 0 | 0.000000 | 0.000000 | 960 | 793.499213 | 848.89 | 904.280787 | 0.884260 |
| GO:0006283\_transcription-coupled\_nucleotide-excision\_repair | 6 | 0 | 0.000000 | 0.000000 | 960 | 793.499213 | 848.89 | 904.280787 | 0.884260 |
| GO:0006337\_nucleosome\_disassembly | 6 | 0 | 0.000000 | 0.000000 | 960 | 793.499213 | 848.89 | 904.280787 | 0.884260 |
| GO:0006390\_transcription\_from\_mitochondrial\_promoter | 6 | 0 | 0.000000 | 0.000000 | 960 | 793.499213 | 848.89 | 904.280787 | 0.884260 |
| GO:0006458\_'de\_novo'\_protein\_folding | 6 | 0 | 0.000000 | 0.000000 | 960 | 793.499213 | 848.89 | 904.280787 | 0.884260 |
| GO:0006477\_protein\_amino\_acid\_sulfation | 6 | 0 | 0.000000 | 0.000000 | 960 | 793.499213 | 848.89 | 904.280787 | 0.884260 |
| GO:0006527\_arginine\_catabolic\_process | 6 | 0 | 0.000000 | 0.000000 | 960 | 793.499213 | 848.89 | 904.280787 | 0.884260 |
| GO:0006700\_C21-steroid\_hormone\_biosynthetic\_process | 6 | 0 | 0.000000 | 0.000000 | 960 | 793.499213 | 848.89 | 904.280787 | 0.884260 |
| GO:0006739\_NADP\_metabolic\_process | 6 | 0 | 0.000000 | 0.000000 | 960 | 793.499213 | 848.89 | 904.280787 | 0.884260 |
| GO:0006826\_iron\_ion\_transport | 6 | 0 | 0.000000 | 0.000000 | 960 | 793.499213 | 848.89 | 904.280787 | 0.884260 |
| GO:0006878\_cellular\_copper\_ion\_homeostasis | 6 | 0 | 0.000000 | 0.000000 | 960 | 793.499213 | 848.89 | 904.280787 | 0.884260 |
| GO:0006893\_Golgi\_to\_plasma\_membrane\_transport | 6 | 0 | 0.000000 | 0.000000 | 960 | 793.499213 | 848.89 | 904.280787 | 0.884260 |
| GO:0006906\_vesicle\_fusion | 6 | 0 | 0.000000 | 0.000000 | 960 | 793.499213 | 848.89 | 904.280787 | 0.884260 |
| GO:0006911\_phagocytosis\_\_engulfment | 6 | 0 | 0.000000 | 0.000000 | 960 | 793.499213 | 848.89 | 904.280787 | 0.884260 |
| GO:0006929\_substrate-bound\_cell\_migration | 6 | 0 | 0.000000 | 0.000000 | 960 | 793.499213 | 848.89 | 904.280787 | 0.884260 |
| GO:0006978\_DNA\_damage\_response\_\_signal\_transduction\_by\_p53\_class\_mediator\_resulting\_in\_transcription\_of\_p21\_class\_mediator | 6 | 0 | 0.000000 | 0.000000 | 960 | 793.499213 | 848.89 | 904.280787 | 0.884260 |
| GO:0006983\_ER\_overload\_response | 6 | 0 | 0.000000 | 0.000000 | 960 | 793.499213 | 848.89 | 904.280787 | 0.884260 |
| GO:0007158\_neuron\_adhesion | 6 | 0 | 0.000000 | 0.000000 | 960 | 793.499213 | 848.89 | 904.280787 | 0.884260 |
| GO:0007185\_transmembrane\_receptor\_protein\_tyrosine\_phosphatase\_signaling\_pathway | 6 | 0 | 0.000000 | 0.000000 | 960 | 793.499213 | 848.89 | 904.280787 | 0.884260 |
| GO:0007189\_activation\_of\_adenylate\_cyclase\_activity\_by\_G-protein\_signaling\_pathway | 6 | 0 | 0.000000 | 0.000000 | 960 | 793.499213 | 848.89 | 904.280787 | 0.884260 |
| GO:0007212\_dopamine\_receptor\_signaling\_pathway | 6 | 0 | 0.000000 | 0.000000 | 960 | 793.499213 | 848.89 | 904.280787 | 0.884260 |
| GO:0007217\_tachykinin\_receptor\_signaling\_pathway | 6 | 0 | 0.000000 | 0.000000 | 960 | 793.499213 | 848.89 | 904.280787 | 0.884260 |
| GO:0007224\_smoothened\_signaling\_pathway | 6 | 0 | 0.000000 | 0.000000 | 960 | 793.499213 | 848.89 | 904.280787 | 0.884260 |
| GO:0007340\_acrosome\_reaction | 6 | 0 | 0.000000 | 0.000000 | 960 | 793.499213 | 848.89 | 904.280787 | 0.884260 |
| GO:0007530\_sex\_determination | 6 | 0 | 0.000000 | 0.000000 | 960 | 793.499213 | 848.89 | 904.280787 | 0.884260 |
| GO:0007613\_memory | 6 | 0 | 0.000000 | 0.000000 | 960 | 793.499213 | 848.89 | 904.280787 | 0.884260 |
| GO:0007618\_mating | 6 | 0 | 0.000000 | 0.000000 | 960 | 793.499213 | 848.89 | 904.280787 | 0.884260 |
| GO:0007622\_rhythmic\_behavior | 6 | 0 | 0.000000 | 0.000000 | 960 | 793.499213 | 848.89 | 904.280787 | 0.884260 |
| GO:0008343\_adult\_feeding\_behavior | 6 | 0 | 0.000000 | 0.000000 | 960 | 793.499213 | 848.89 | 904.280787 | 0.884260 |
| GO:0009071\_serine\_family\_amino\_acid\_catabolic\_process | 6 | 0 | 0.000000 | 0.000000 | 960 | 793.499213 | 848.89 | 904.280787 | 0.884260 |
| GO:0009164\_nucleoside\_catabolic\_process | 6 | 0 | 0.000000 | 0.000000 | 960 | 793.499213 | 848.89 | 904.280787 | 0.884260 |
| GO:0009312\_oligosaccharide\_biosynthetic\_process | 6 | 0 | 0.000000 | 0.000000 | 960 | 793.499213 | 848.89 | 904.280787 | 0.884260 |
| GO:0009994\_oocyte\_differentiation | 6 | 0 | 0.000000 | 0.000000 | 960 | 793.499213 | 848.89 | 904.280787 | 0.884260 |
| GO:0010149\_senescence | 6 | 0 | 0.000000 | 0.000000 | 960 | 793.499213 | 848.89 | 904.280787 | 0.884260 |
| GO:0010573\_vascular\_endothelial\_growth\_factor\_production | 6 | 0 | 0.000000 | 0.000000 | 960 | 793.499213 | 848.89 | 904.280787 | 0.884260 |
| GO:0010574\_regulation\_of\_vascular\_endothelial\_growth\_factor\_production | 6 | 0 | 0.000000 | 0.000000 | 960 | 793.499213 | 848.89 | 904.280787 | 0.884260 |
| GO:0010578\_regulation\_of\_adenylate\_cyclase\_activity\_involved\_in\_G-protein\_signaling | 6 | 0 | 0.000000 | 0.000000 | 960 | 793.499213 | 848.89 | 904.280787 | 0.884260 |
| GO:0010579\_positive\_regulation\_of\_adenylate\_cyclase\_activity\_by\_G-protein\_signaling\_pathway | 6 | 0 | 0.000000 | 0.000000 | 960 | 793.499213 | 848.89 | 904.280787 | 0.884260 |
| GO:0010611\_regulation\_of\_cardiac\_muscle\_hypertrophy | 6 | 0 | 0.000000 | 0.000000 | 960 | 793.499213 | 848.89 | 904.280787 | 0.884260 |
| GO:0010612\_regulation\_of\_cardiac\_muscle\_adaptation | 6 | 0 | 0.000000 | 0.000000 | 960 | 793.499213 | 848.89 | 904.280787 | 0.884260 |
| GO:0010657\_muscle\_cell\_apoptosis | 6 | 0 | 0.000000 | 0.000000 | 960 | 793.499213 | 848.89 | 904.280787 | 0.884260 |
| GO:0010660\_regulation\_of\_muscle\_cell\_apoptosis | 6 | 0 | 0.000000 | 0.000000 | 960 | 793.499213 | 848.89 | 904.280787 | 0.884260 |
| GO:0010718\_positive\_regulation\_of\_epithelial\_to\_mesenchymal\_transition | 6 | 0 | 0.000000 | 0.000000 | 960 | 793.499213 | 848.89 | 904.280787 | 0.884260 |
| GO:0010770\_positive\_regulation\_of\_cell\_morphogenesis\_involved\_in\_differentiation | 6 | 0 | 0.000000 | 0.000000 | 960 | 793.499213 | 848.89 | 904.280787 | 0.884260 |
| GO:0010875\_positive\_regulation\_of\_cholesterol\_efflux | 6 | 0 | 0.000000 | 0.000000 | 960 | 793.499213 | 848.89 | 904.280787 | 0.884260 |
| GO:0010887\_negative\_regulation\_of\_cholesterol\_storage | 6 | 0 | 0.000000 | 0.000000 | 960 | 793.499213 | 848.89 | 904.280787 | 0.884260 |
| GO:0010896\_regulation\_of\_triglyceride\_catabolic\_process | 6 | 0 | 0.000000 | 0.000000 | 960 | 793.499213 | 848.89 | 904.280787 | 0.884260 |
| GO:0014020\_primary\_neural\_tube\_formation | 6 | 0 | 0.000000 | 0.000000 | 960 | 793.499213 | 848.89 | 904.280787 | 0.884260 |
| GO:0014743\_regulation\_of\_muscle\_hypertrophy | 6 | 0 | 0.000000 | 0.000000 | 960 | 793.499213 | 848.89 | 904.280787 | 0.884260 |
| GO:0014855\_striated\_muscle\_cell\_proliferation | 6 | 0 | 0.000000 | 0.000000 | 960 | 793.499213 | 848.89 | 904.280787 | 0.884260 |
| GO:0014887\_cardiac\_muscle\_adaptation | 6 | 0 | 0.000000 | 0.000000 | 960 | 793.499213 | 848.89 | 904.280787 | 0.884260 |
| GO:0014888\_striated\_muscle\_adaptation | 6 | 0 | 0.000000 | 0.000000 | 960 | 793.499213 | 848.89 | 904.280787 | 0.884260 |
| GO:0014897\_striated\_muscle\_hypertrophy | 6 | 0 | 0.000000 | 0.000000 | 960 | 793.499213 | 848.89 | 904.280787 | 0.884260 |
| GO:0014898\_cardiac\_muscle\_hypertrophy | 6 | 0 | 0.000000 | 0.000000 | 960 | 793.499213 | 848.89 | 904.280787 | 0.884260 |
| GO:0015671\_oxygen\_transport | 6 | 0 | 0.000000 | 0.000000 | 960 | 793.499213 | 848.89 | 904.280787 | 0.884260 |
| GO:0015810\_aspartate\_transport | 6 | 0 | 0.000000 | 0.000000 | 960 | 793.499213 | 848.89 | 904.280787 | 0.884260 |
| GO:0015850\_organic\_alcohol\_transport | 6 | 0 | 0.000000 | 0.000000 | 960 | 793.499213 | 848.89 | 904.280787 | 0.884260 |
| GO:0017156\_calcium\_ion-dependent\_exocytosis | 6 | 0 | 0.000000 | 0.000000 | 960 | 793.499213 | 848.89 | 904.280787 | 0.884260 |
| GO:0019062\_virion\_attachment\_to\_host\_cell\_surface\_receptor | 6 | 0 | 0.000000 | 0.000000 | 960 | 793.499213 | 848.89 | 904.280787 | 0.884260 |
| GO:0019080\_viral\_genome\_expression | 6 | 0 | 0.000000 | 0.000000 | 960 | 793.499213 | 848.89 | 904.280787 | 0.884260 |
| GO:0019083\_viral\_transcription | 6 | 0 | 0.000000 | 0.000000 | 960 | 793.499213 | 848.89 | 904.280787 | 0.884260 |
| GO:0019827\_stem\_cell\_maintenance | 6 | 0 | 0.000000 | 0.000000 | 960 | 793.499213 | 848.89 | 904.280787 | 0.884260 |
| GO:0019883\_antigen\_processing\_and\_presentation\_of\_endogenous\_antigen | 6 | 0 | 0.000000 | 0.000000 | 960 | 793.499213 | 848.89 | 904.280787 | 0.884260 |
| GO:0021537\_telencephalon\_development | 6 | 0 | 0.000000 | 0.000000 | 960 | 793.499213 | 848.89 | 904.280787 | 0.884260 |
| GO:0021953\_central\_nervous\_system\_neuron\_differentiation | 6 | 0 | 0.000000 | 0.000000 | 960 | 793.499213 | 848.89 | 904.280787 | 0.884260 |
| GO:0021954\_central\_nervous\_system\_neuron\_development | 6 | 0 | 0.000000 | 0.000000 | 960 | 793.499213 | 848.89 | 904.280787 | 0.884260 |
| GO:0022404\_molting\_cycle\_process | 6 | 0 | 0.000000 | 0.000000 | 960 | 793.499213 | 848.89 | 904.280787 | 0.884260 |
| GO:0022405\_hair\_cycle\_process | 6 | 0 | 0.000000 | 0.000000 | 960 | 793.499213 | 848.89 | 904.280787 | 0.884260 |
| GO:0022408\_negative\_regulation\_of\_cell-cell\_adhesion | 6 | 0 | 0.000000 | 0.000000 | 960 | 793.499213 | 848.89 | 904.280787 | 0.884260 |
| GO:0022409\_positive\_regulation\_of\_cell-cell\_adhesion | 6 | 0 | 0.000000 | 0.000000 | 960 | 793.499213 | 848.89 | 904.280787 | 0.884260 |
| GO:0030032\_lamellipodium\_assembly | 6 | 0 | 0.000000 | 0.000000 | 960 | 793.499213 | 848.89 | 904.280787 | 0.884260 |
| GO:0030147\_natriuresis | 6 | 0 | 0.000000 | 0.000000 | 960 | 793.499213 | 848.89 | 904.280787 | 0.884260 |
| GO:0030224\_monocyte\_differentiation | 6 | 0 | 0.000000 | 0.000000 | 960 | 793.499213 | 848.89 | 904.280787 | 0.884260 |
| GO:0030299\_intestinal\_cholesterol\_absorption | 6 | 0 | 0.000000 | 0.000000 | 960 | 793.499213 | 848.89 | 904.280787 | 0.884260 |
| GO:0030801\_positive\_regulation\_of\_cyclic\_nucleotide\_metabolic\_process | 6 | 0 | 0.000000 | 0.000000 | 960 | 793.499213 | 848.89 | 904.280787 | 0.884260 |
| GO:0030804\_positive\_regulation\_of\_cyclic\_nucleotide\_biosynthetic\_process | 6 | 0 | 0.000000 | 0.000000 | 960 | 793.499213 | 848.89 | 904.280787 | 0.884260 |
| GO:0030810\_positive\_regulation\_of\_nucleotide\_biosynthetic\_process | 6 | 0 | 0.000000 | 0.000000 | 960 | 793.499213 | 848.89 | 904.280787 | 0.884260 |
| GO:0030835\_negative\_regulation\_of\_actin\_filament\_depolymerization | 6 | 0 | 0.000000 | 0.000000 | 960 | 793.499213 | 848.89 | 904.280787 | 0.884260 |
| GO:0030837\_negative\_regulation\_of\_actin\_filament\_polymerization | 6 | 0 | 0.000000 | 0.000000 | 960 | 793.499213 | 848.89 | 904.280787 | 0.884260 |
| GO:0031099\_regeneration | 6 | 0 | 0.000000 | 0.000000 | 960 | 793.499213 | 848.89 | 904.280787 | 0.884260 |
| GO:0031268\_pseudopodium\_organization | 6 | 0 | 0.000000 | 0.000000 | 960 | 793.499213 | 848.89 | 904.280787 | 0.884260 |
| GO:0031269\_pseudopodium\_assembly | 6 | 0 | 0.000000 | 0.000000 | 960 | 793.499213 | 848.89 | 904.280787 | 0.884260 |
| GO:0031272\_regulation\_of\_pseudopodium\_assembly | 6 | 0 | 0.000000 | 0.000000 | 960 | 793.499213 | 848.89 | 904.280787 | 0.884260 |
| GO:0031274\_positive\_regulation\_of\_pseudopodium\_assembly | 6 | 0 | 0.000000 | 0.000000 | 960 | 793.499213 | 848.89 | 904.280787 | 0.884260 |
| GO:0031958\_corticosteroid\_receptor\_signaling\_pathway | 6 | 0 | 0.000000 | 0.000000 | 960 | 793.499213 | 848.89 | 904.280787 | 0.884260 |
| GO:0032297\_negative\_regulation\_of\_DNA\_replication\_initiation | 6 | 0 | 0.000000 | 0.000000 | 960 | 793.499213 | 848.89 | 904.280787 | 0.884260 |
| GO:0032372\_negative\_regulation\_of\_sterol\_transport | 6 | 0 | 0.000000 | 0.000000 | 960 | 793.499213 | 848.89 | 904.280787 | 0.884260 |
| GO:0032375\_negative\_regulation\_of\_cholesterol\_transport | 6 | 0 | 0.000000 | 0.000000 | 960 | 793.499213 | 848.89 | 904.280787 | 0.884260 |
| GO:0032402\_melanosome\_transport | 6 | 0 | 0.000000 | 0.000000 | 960 | 793.499213 | 848.89 | 904.280787 | 0.884260 |
| GO:0032411\_positive\_regulation\_of\_transporter\_activity | 6 | 0 | 0.000000 | 0.000000 | 960 | 793.499213 | 848.89 | 904.280787 | 0.884260 |
| GO:0032434\_regulation\_of\_proteasomal\_ubiquitin-dependent\_protein\_catabolic\_process | 6 | 0 | 0.000000 | 0.000000 | 960 | 793.499213 | 848.89 | 904.280787 | 0.884260 |
| GO:0032890\_regulation\_of\_organic\_acid\_transport | 6 | 0 | 0.000000 | 0.000000 | 960 | 793.499213 | 848.89 | 904.280787 | 0.884260 |
| GO:0032945\_negative\_regulation\_of\_mononuclear\_cell\_proliferation | 6 | 0 | 0.000000 | 0.000000 | 960 | 793.499213 | 848.89 | 904.280787 | 0.884260 |
| GO:0032986\_protein-DNA\_complex\_disassembly | 6 | 0 | 0.000000 | 0.000000 | 960 | 793.499213 | 848.89 | 904.280787 | 0.884260 |
| GO:0033158\_regulation\_of\_protein\_import\_into\_nucleus\_\_translocation | 6 | 0 | 0.000000 | 0.000000 | 960 | 793.499213 | 848.89 | 904.280787 | 0.884260 |
| GO:0033238\_regulation\_of\_cellular\_amine\_metabolic\_process | 6 | 0 | 0.000000 | 0.000000 | 960 | 793.499213 | 848.89 | 904.280787 | 0.884260 |
| GO:0033363\_secretory\_granule\_organization | 6 | 0 | 0.000000 | 0.000000 | 960 | 793.499213 | 848.89 | 904.280787 | 0.884260 |
| GO:0033683\_nucleotide-excision\_repair\_\_DNA\_incision | 6 | 0 | 0.000000 | 0.000000 | 960 | 793.499213 | 848.89 | 904.280787 | 0.884260 |
| GO:0034405\_response\_to\_fluid\_shear\_stress | 6 | 0 | 0.000000 | 0.000000 | 960 | 793.499213 | 848.89 | 904.280787 | 0.884260 |
| GO:0034446\_substrate\_adhesion-dependent\_cell\_spreading | 6 | 0 | 0.000000 | 0.000000 | 960 | 793.499213 | 848.89 | 904.280787 | 0.884260 |
| GO:0034447\_very-low-density\_lipoprotein\_particle\_clearance | 6 | 0 | 0.000000 | 0.000000 | 960 | 793.499213 | 848.89 | 904.280787 | 0.884260 |
| GO:0034502\_protein\_localization\_to\_chromosome | 6 | 0 | 0.000000 | 0.000000 | 960 | 793.499213 | 848.89 | 904.280787 | 0.884260 |
| GO:0034508\_centromere\_complex\_assembly | 6 | 0 | 0.000000 | 0.000000 | 960 | 793.499213 | 848.89 | 904.280787 | 0.884260 |
| GO:0035148\_tube\_lumen\_formation | 6 | 0 | 0.000000 | 0.000000 | 960 | 793.499213 | 848.89 | 904.280787 | 0.884260 |
| GO:0042092\_T-helper\_2\_type\_immune\_response | 6 | 0 | 0.000000 | 0.000000 | 960 | 793.499213 | 848.89 | 904.280787 | 0.884260 |
| GO:0042119\_neutrophil\_activation | 6 | 0 | 0.000000 | 0.000000 | 960 | 793.499213 | 848.89 | 904.280787 | 0.884260 |
| GO:0042255\_ribosome\_assembly | 6 | 0 | 0.000000 | 0.000000 | 960 | 793.499213 | 848.89 | 904.280787 | 0.884260 |
| GO:0042273\_ribosomal\_large\_subunit\_biogenesis | 6 | 0 | 0.000000 | 0.000000 | 960 | 793.499213 | 848.89 | 904.280787 | 0.884260 |
| GO:0042303\_molting\_cycle | 6 | 0 | 0.000000 | 0.000000 | 960 | 793.499213 | 848.89 | 904.280787 | 0.884260 |
| GO:0042346\_positive\_regulation\_of\_NF-kappaB\_import\_into\_nucleus | 6 | 0 | 0.000000 | 0.000000 | 960 | 793.499213 | 848.89 | 904.280787 | 0.884260 |
| GO:0042402\_biogenic\_amine\_catabolic\_process | 6 | 0 | 0.000000 | 0.000000 | 960 | 793.499213 | 848.89 | 904.280787 | 0.884260 |
| GO:0042447\_hormone\_catabolic\_process | 6 | 0 | 0.000000 | 0.000000 | 960 | 793.499213 | 848.89 | 904.280787 | 0.884260 |
| GO:0042559\_pteridine\_and\_derivative\_biosynthetic\_process | 6 | 0 | 0.000000 | 0.000000 | 960 | 793.499213 | 848.89 | 904.280787 | 0.884260 |
| GO:0042633\_hair\_cycle | 6 | 0 | 0.000000 | 0.000000 | 960 | 793.499213 | 848.89 | 904.280787 | 0.884260 |
| GO:0042772\_DNA\_damage\_response\_\_signal\_transduction\_resulting\_in\_transcription | 6 | 0 | 0.000000 | 0.000000 | 960 | 793.499213 | 848.89 | 904.280787 | 0.884260 |
| GO:0042921\_glucocorticoid\_receptor\_signaling\_pathway | 6 | 0 | 0.000000 | 0.000000 | 960 | 793.499213 | 848.89 | 904.280787 | 0.884260 |
| GO:0043206\_fibril\_organization | 6 | 0 | 0.000000 | 0.000000 | 960 | 793.499213 | 848.89 | 904.280787 | 0.884260 |
| GO:0043370\_regulation\_of\_CD4-positive\_\_alpha\_beta\_T\_cell\_differentiation | 6 | 0 | 0.000000 | 0.000000 | 960 | 793.499213 | 848.89 | 904.280787 | 0.884260 |
| GO:0043502\_regulation\_of\_muscle\_adaptation | 6 | 0 | 0.000000 | 0.000000 | 960 | 793.499213 | 848.89 | 904.280787 | 0.884260 |
| GO:0043525\_positive\_regulation\_of\_neuron\_apoptosis | 6 | 0 | 0.000000 | 0.000000 | 960 | 793.499213 | 848.89 | 904.280787 | 0.884260 |
| GO:0043536\_positive\_regulation\_of\_blood\_vessel\_endothelial\_cell\_migration | 6 | 0 | 0.000000 | 0.000000 | 960 | 793.499213 | 848.89 | 904.280787 | 0.884260 |
| GO:0043666\_regulation\_of\_phosphoprotein\_phosphatase\_activity | 6 | 0 | 0.000000 | 0.000000 | 960 | 793.499213 | 848.89 | 904.280787 | 0.884260 |
| GO:0043981\_histone\_H4-K5\_acetylation | 6 | 0 | 0.000000 | 0.000000 | 960 | 793.499213 | 848.89 | 904.280787 | 0.884260 |
| GO:0043982\_histone\_H4-K8\_acetylation | 6 | 0 | 0.000000 | 0.000000 | 960 | 793.499213 | 848.89 | 904.280787 | 0.884260 |
| GO:0043983\_histone\_H4-K12\_acetylation | 6 | 0 | 0.000000 | 0.000000 | 960 | 793.499213 | 848.89 | 904.280787 | 0.884260 |
| GO:0043984\_histone\_H4-K16\_acetylation | 6 | 0 | 0.000000 | 0.000000 | 960 | 793.499213 | 848.89 | 904.280787 | 0.884260 |
| GO:0045073\_regulation\_of\_chemokine\_biosynthetic\_process | 6 | 0 | 0.000000 | 0.000000 | 960 | 793.499213 | 848.89 | 904.280787 | 0.884260 |
| GO:0045090\_retroviral\_genome\_replication | 6 | 0 | 0.000000 | 0.000000 | 960 | 793.499213 | 848.89 | 904.280787 | 0.884260 |
| GO:0045116\_protein\_neddylation | 6 | 0 | 0.000000 | 0.000000 | 960 | 793.499213 | 848.89 | 904.280787 | 0.884260 |
| GO:0045540\_regulation\_of\_cholesterol\_biosynthetic\_process | 6 | 0 | 0.000000 | 0.000000 | 960 | 793.499213 | 848.89 | 904.280787 | 0.884260 |
| GO:0045604\_regulation\_of\_epidermal\_cell\_differentiation | 6 | 0 | 0.000000 | 0.000000 | 960 | 793.499213 | 848.89 | 904.280787 | 0.884260 |
| GO:0045616\_regulation\_of\_keratinocyte\_differentiation | 6 | 0 | 0.000000 | 0.000000 | 960 | 793.499213 | 848.89 | 904.280787 | 0.884260 |
| GO:0045622\_regulation\_of\_T-helper\_cell\_differentiation | 6 | 0 | 0.000000 | 0.000000 | 960 | 793.499213 | 848.89 | 904.280787 | 0.884260 |
| GO:0045661\_regulation\_of\_myoblast\_differentiation | 6 | 0 | 0.000000 | 0.000000 | 960 | 793.499213 | 848.89 | 904.280787 | 0.884260 |
| GO:0045671\_negative\_regulation\_of\_osteoclast\_differentiation | 6 | 0 | 0.000000 | 0.000000 | 960 | 793.499213 | 848.89 | 904.280787 | 0.884260 |
| GO:0045684\_positive\_regulation\_of\_epidermis\_development | 6 | 0 | 0.000000 | 0.000000 | 960 | 793.499213 | 848.89 | 904.280787 | 0.884260 |
| GO:0045723\_positive\_regulation\_of\_fatty\_acid\_biosynthetic\_process | 6 | 0 | 0.000000 | 0.000000 | 960 | 793.499213 | 848.89 | 904.280787 | 0.884260 |
| GO:0045737\_positive\_regulation\_of\_cyclin-dependent\_protein\_kinase\_activity | 6 | 0 | 0.000000 | 0.000000 | 960 | 793.499213 | 848.89 | 904.280787 | 0.884260 |
| GO:0045823\_positive\_regulation\_of\_heart\_contraction | 6 | 0 | 0.000000 | 0.000000 | 960 | 793.499213 | 848.89 | 904.280787 | 0.884260 |
| GO:0045885\_positive\_regulation\_of\_survival\_gene\_product\_expression | 6 | 0 | 0.000000 | 0.000000 | 960 | 793.499213 | 848.89 | 904.280787 | 0.884260 |
| GO:0045931\_positive\_regulation\_of\_mitotic\_cell\_cycle | 6 | 0 | 0.000000 | 0.000000 | 960 | 793.499213 | 848.89 | 904.280787 | 0.884260 |
| GO:0045932\_negative\_regulation\_of\_muscle\_contraction | 6 | 0 | 0.000000 | 0.000000 | 960 | 793.499213 | 848.89 | 904.280787 | 0.884260 |
| GO:0045981\_positive\_regulation\_of\_nucleotide\_metabolic\_process | 6 | 0 | 0.000000 | 0.000000 | 960 | 793.499213 | 848.89 | 904.280787 | 0.884260 |
| GO:0046006\_regulation\_of\_activated\_T\_cell\_proliferation | 6 | 0 | 0.000000 | 0.000000 | 960 | 793.499213 | 848.89 | 904.280787 | 0.884260 |
| GO:0046520\_sphingoid\_biosynthetic\_process | 6 | 0 | 0.000000 | 0.000000 | 960 | 793.499213 | 848.89 | 904.280787 | 0.884260 |
| GO:0046579\_positive\_regulation\_of\_Ras\_protein\_signal\_transduction | 6 | 0 | 0.000000 | 0.000000 | 960 | 793.499213 | 848.89 | 904.280787 | 0.884260 |
| GO:0046638\_positive\_regulation\_of\_alpha-beta\_T\_cell\_differentiation | 6 | 0 | 0.000000 | 0.000000 | 960 | 793.499213 | 848.89 | 904.280787 | 0.884260 |
| GO:0046677\_response\_to\_antibiotic | 6 | 0 | 0.000000 | 0.000000 | 960 | 793.499213 | 848.89 | 904.280787 | 0.884260 |
| GO:0046697\_decidualization | 6 | 0 | 0.000000 | 0.000000 | 960 | 793.499213 | 848.89 | 904.280787 | 0.884260 |
| GO:0046716\_muscle\_maintenance | 6 | 0 | 0.000000 | 0.000000 | 960 | 793.499213 | 848.89 | 904.280787 | 0.884260 |
| GO:0048008\_platelet-derived\_growth\_factor\_receptor\_signaling\_pathway | 6 | 0 | 0.000000 | 0.000000 | 960 | 793.499213 | 848.89 | 904.280787 | 0.884260 |
| GO:0048247\_lymphocyte\_chemotaxis | 6 | 0 | 0.000000 | 0.000000 | 960 | 793.499213 | 848.89 | 904.280787 | 0.884260 |
| GO:0048385\_regulation\_of\_retinoic\_acid\_receptor\_signaling\_pathway | 6 | 0 | 0.000000 | 0.000000 | 960 | 793.499213 | 848.89 | 904.280787 | 0.884260 |
| GO:0048512\_circadian\_behavior | 6 | 0 | 0.000000 | 0.000000 | 960 | 793.499213 | 848.89 | 904.280787 | 0.884260 |
| GO:0048525\_negative\_regulation\_of\_viral\_reproduction | 6 | 0 | 0.000000 | 0.000000 | 960 | 793.499213 | 848.89 | 904.280787 | 0.884260 |
| GO:0048562\_embryonic\_organ\_morphogenesis | 6 | 0 | 0.000000 | 0.000000 | 960 | 793.499213 | 848.89 | 904.280787 | 0.884260 |
| GO:0048610\_reproductive\_cellular\_process | 6 | 0 | 0.000000 | 0.000000 | 960 | 793.499213 | 848.89 | 904.280787 | 0.884260 |
| GO:0048704\_embryonic\_skeletal\_system\_morphogenesis | 6 | 0 | 0.000000 | 0.000000 | 960 | 793.499213 | 848.89 | 904.280787 | 0.884260 |
| GO:0048738\_cardiac\_muscle\_tissue\_development | 6 | 0 | 0.000000 | 0.000000 | 960 | 793.499213 | 848.89 | 904.280787 | 0.884260 |
| GO:0048864\_stem\_cell\_development | 6 | 0 | 0.000000 | 0.000000 | 960 | 793.499213 | 848.89 | 904.280787 | 0.884260 |
| GO:0050432\_catecholamine\_secretion | 6 | 0 | 0.000000 | 0.000000 | 960 | 793.499213 | 848.89 | 904.280787 | 0.884260 |
| GO:0050433\_regulation\_of\_catecholamine\_secretion | 6 | 0 | 0.000000 | 0.000000 | 960 | 793.499213 | 848.89 | 904.280787 | 0.884260 |
| GO:0050672\_negative\_regulation\_of\_lymphocyte\_proliferation | 6 | 0 | 0.000000 | 0.000000 | 960 | 793.499213 | 848.89 | 904.280787 | 0.884260 |
| GO:0050732\_negative\_regulation\_of\_peptidyl-tyrosine\_phosphorylation | 6 | 0 | 0.000000 | 0.000000 | 960 | 793.499213 | 848.89 | 904.280787 | 0.884260 |
| GO:0050746\_regulation\_of\_lipoprotein\_metabolic\_process | 6 | 0 | 0.000000 | 0.000000 | 960 | 793.499213 | 848.89 | 904.280787 | 0.884260 |
| GO:0050748\_negative\_regulation\_of\_lipoprotein\_metabolic\_process | 6 | 0 | 0.000000 | 0.000000 | 960 | 793.499213 | 848.89 | 904.280787 | 0.884260 |
| GO:0050764\_regulation\_of\_phagocytosis | 6 | 0 | 0.000000 | 0.000000 | 960 | 793.499213 | 848.89 | 904.280787 | 0.884260 |
| GO:0050803\_regulation\_of\_synapse\_structure\_and\_activity | 6 | 0 | 0.000000 | 0.000000 | 960 | 793.499213 | 848.89 | 904.280787 | 0.884260 |
| GO:0050850\_positive\_regulation\_of\_calcium-mediated\_signaling | 6 | 0 | 0.000000 | 0.000000 | 960 | 793.499213 | 848.89 | 904.280787 | 0.884260 |
| GO:0050892\_intestinal\_absorption | 6 | 0 | 0.000000 | 0.000000 | 960 | 793.499213 | 848.89 | 904.280787 | 0.884260 |
| GO:0050913\_sensory\_perception\_of\_bitter\_taste | 6 | 0 | 0.000000 | 0.000000 | 960 | 793.499213 | 848.89 | 904.280787 | 0.884260 |
| GO:0050957\_equilibrioception | 6 | 0 | 0.000000 | 0.000000 | 960 | 793.499213 | 848.89 | 904.280787 | 0.884260 |
| GO:0051006\_positive\_regulation\_of\_lipoprotein\_lipase\_activity | 6 | 0 | 0.000000 | 0.000000 | 960 | 793.499213 | 848.89 | 904.280787 | 0.884260 |
| GO:0051057\_positive\_regulation\_of\_small\_GTPase\_mediated\_signal\_transduction | 6 | 0 | 0.000000 | 0.000000 | 960 | 793.499213 | 848.89 | 904.280787 | 0.884260 |
| GO:0051148\_negative\_regulation\_of\_muscle\_cell\_differentiation | 6 | 0 | 0.000000 | 0.000000 | 960 | 793.499213 | 848.89 | 904.280787 | 0.884260 |
| GO:0051205\_protein\_insertion\_into\_membrane | 6 | 0 | 0.000000 | 0.000000 | 960 | 793.499213 | 848.89 | 904.280787 | 0.884260 |
| GO:0051291\_protein\_heterooligomerization | 6 | 0 | 0.000000 | 0.000000 | 960 | 793.499213 | 848.89 | 904.280787 | 0.884260 |
| GO:0051302\_regulation\_of\_cell\_division | 6 | 0 | 0.000000 | 0.000000 | 960 | 793.499213 | 848.89 | 904.280787 | 0.884260 |
| GO:0051588\_regulation\_of\_neurotransmitter\_transport | 6 | 0 | 0.000000 | 0.000000 | 960 | 793.499213 | 848.89 | 904.280787 | 0.884260 |
| GO:0051702\_interaction\_with\_symbiont | 6 | 0 | 0.000000 | 0.000000 | 960 | 793.499213 | 848.89 | 904.280787 | 0.884260 |
| GO:0051865\_protein\_autoubiquitination | 6 | 0 | 0.000000 | 0.000000 | 960 | 793.499213 | 848.89 | 904.280787 | 0.884260 |
| GO:0051904\_pigment\_granule\_transport | 6 | 0 | 0.000000 | 0.000000 | 960 | 793.499213 | 848.89 | 904.280787 | 0.884260 |
| GO:0051917\_regulation\_of\_fibrinolysis | 6 | 0 | 0.000000 | 0.000000 | 960 | 793.499213 | 848.89 | 904.280787 | 0.884260 |
| GO:0051923\_sulfation | 6 | 0 | 0.000000 | 0.000000 | 960 | 793.499213 | 848.89 | 904.280787 | 0.884260 |
| GO:0051972\_regulation\_of\_telomerase\_activity | 6 | 0 | 0.000000 | 0.000000 | 960 | 793.499213 | 848.89 | 904.280787 | 0.884260 |
| GO:0060038\_cardiac\_muscle\_cell\_proliferation | 6 | 0 | 0.000000 | 0.000000 | 960 | 793.499213 | 848.89 | 904.280787 | 0.884260 |
| GO:0060192\_negative\_regulation\_of\_lipase\_activity | 6 | 0 | 0.000000 | 0.000000 | 960 | 793.499213 | 848.89 | 904.280787 | 0.884260 |
| GO:0060396\_growth\_hormone\_receptor\_signaling\_pathway | 6 | 0 | 0.000000 | 0.000000 | 960 | 793.499213 | 848.89 | 904.280787 | 0.884260 |
| GO:0060606\_tube\_closure | 6 | 0 | 0.000000 | 0.000000 | 960 | 793.499213 | 848.89 | 904.280787 | 0.884260 |
| GO:0070198\_protein\_localization\_to\_telomere | 6 | 0 | 0.000000 | 0.000000 | 960 | 793.499213 | 848.89 | 904.280787 | 0.884260 |
| GO:0070265\_necrotic\_cell\_death | 6 | 0 | 0.000000 | 0.000000 | 960 | 793.499213 | 848.89 | 904.280787 | 0.884260 |
| GO:0070613\_regulation\_of\_protein\_processing | 6 | 0 | 0.000000 | 0.000000 | 960 | 793.499213 | 848.89 | 904.280787 | 0.884260 |
| GO:0070664\_negative\_regulation\_of\_leukocyte\_proliferation | 6 | 0 | 0.000000 | 0.000000 | 960 | 793.499213 | 848.89 | 904.280787 | 0.884260 |
| GO:0000122\_negative\_regulation\_of\_transcription\_from\_RNA\_polymerase\_II\_promoter | 113 | 0 | 0.000000 | 0.000000 | 961 | 795.330759 | 850.56 | 905.789241 | 0.885078 |
| GO:0001508\_regulation\_of\_action\_potential | 24 | 0 | 0.000000 | 0.000000 | 987 | 823.501095 | 878.29 | 933.078905 | 0.889858 |
| GO:0001649\_osteoblast\_differentiation | 24 | 0 | 0.000000 | 0.000000 | 987 | 823.501095 | 878.29 | 933.078905 | 0.889858 |
| GO:0006479\_protein\_amino\_acid\_methylation | 24 | 0 | 0.000000 | 0.000000 | 987 | 823.501095 | 878.29 | 933.078905 | 0.889858 |
| GO:0007163\_establishment\_or\_maintenance\_of\_cell\_polarity | 24 | 0 | 0.000000 | 0.000000 | 987 | 823.501095 | 878.29 | 933.078905 | 0.889858 |
| GO:0007229\_integrin-mediated\_signaling\_pathway | 24 | 0 | 0.000000 | 0.000000 | 987 | 823.501095 | 878.29 | 933.078905 | 0.889858 |
| GO:0007568\_aging | 24 | 0 | 0.000000 | 0.000000 | 987 | 823.501095 | 878.29 | 933.078905 | 0.889858 |
| GO:0008213\_protein\_amino\_acid\_alkylation | 24 | 0 | 0.000000 | 0.000000 | 987 | 823.501095 | 878.29 | 933.078905 | 0.889858 |
| GO:0009116\_nucleoside\_metabolic\_process | 24 | 0 | 0.000000 | 0.000000 | 987 | 823.501095 | 878.29 | 933.078905 | 0.889858 |
| GO:0009266\_response\_to\_temperature\_stimulus | 24 | 0 | 0.000000 | 0.000000 | 987 | 823.501095 | 878.29 | 933.078905 | 0.889858 |
| GO:0017148\_negative\_regulation\_of\_translation | 24 | 0 | 0.000000 | 0.000000 | 987 | 823.501095 | 878.29 | 933.078905 | 0.889858 |
| GO:0019059\_initiation\_of\_viral\_infection | 24 | 0 | 0.000000 | 0.000000 | 987 | 823.501095 | 878.29 | 933.078905 | 0.889858 |
| GO:0030073\_insulin\_secretion | 24 | 0 | 0.000000 | 0.000000 | 987 | 823.501095 | 878.29 | 933.078905 | 0.889858 |
| GO:0032946\_positive\_regulation\_of\_mononuclear\_cell\_proliferation | 24 | 0 | 0.000000 | 0.000000 | 987 | 823.501095 | 878.29 | 933.078905 | 0.889858 |
| GO:0033002\_muscle\_cell\_proliferation | 24 | 0 | 0.000000 | 0.000000 | 987 | 823.501095 | 878.29 | 933.078905 | 0.889858 |
| GO:0035023\_regulation\_of\_Rho\_protein\_signal\_transduction | 24 | 0 | 0.000000 | 0.000000 | 987 | 823.501095 | 878.29 | 933.078905 | 0.889858 |
| GO:0042440\_pigment\_metabolic\_process | 24 | 0 | 0.000000 | 0.000000 | 987 | 823.501095 | 878.29 | 933.078905 | 0.889858 |
| GO:0042509\_regulation\_of\_tyrosine\_phosphorylation\_of\_STAT\_protein | 24 | 0 | 0.000000 | 0.000000 | 987 | 823.501095 | 878.29 | 933.078905 | 0.889858 |
| GO:0043506\_regulation\_of\_JUN\_kinase\_activity | 24 | 0 | 0.000000 | 0.000000 | 987 | 823.501095 | 878.29 | 933.078905 | 0.889858 |
| GO:0045216\_cell-cell\_junction\_organization | 24 | 0 | 0.000000 | 0.000000 | 987 | 823.501095 | 878.29 | 933.078905 | 0.889858 |
| GO:0045638\_negative\_regulation\_of\_myeloid\_cell\_differentiation | 24 | 0 | 0.000000 | 0.000000 | 987 | 823.501095 | 878.29 | 933.078905 | 0.889858 |
| GO:0046890\_regulation\_of\_lipid\_biosynthetic\_process | 24 | 0 | 0.000000 | 0.000000 | 987 | 823.501095 | 878.29 | 933.078905 | 0.889858 |
| GO:0051701\_interaction\_with\_host | 24 | 0 | 0.000000 | 0.000000 | 987 | 823.501095 | 878.29 | 933.078905 | 0.889858 |
| GO:0051969\_regulation\_of\_transmission\_of\_nerve\_impulse | 24 | 0 | 0.000000 | 0.000000 | 987 | 823.501095 | 878.29 | 933.078905 | 0.889858 |
| GO:0055067\_monovalent\_inorganic\_cation\_homeostasis | 24 | 0 | 0.000000 | 0.000000 | 987 | 823.501095 | 878.29 | 933.078905 | 0.889858 |
| GO:0070646\_protein\_modification\_by\_small\_protein\_removal | 24 | 0 | 0.000000 | 0.000000 | 987 | 823.501095 | 878.29 | 933.078905 | 0.889858 |
| GO:0070665\_positive\_regulation\_of\_leukocyte\_proliferation | 24 | 0 | 0.000000 | 0.000000 | 987 | 823.501095 | 878.29 | 933.078905 | 0.889858 |
| GO:0045934\_negative\_regulation\_of\_nucleobase\_\_nucleoside\_\_nucleotide\_and\_nucleic\_acid\_metabolic\_process | 295 | 0 | 0.000000 | 0.000000 | 988 | 824.292424 | 878.96 | 933.627576 | 0.889636 |
| GO:0006260\_DNA\_replication | 153 | 0 | 0.000000 | 0.000000 | 989 | 828.104703 | 882.29 | 936.475297 | 0.892103 |
| GO:0000087\_M\_phase\_of\_mitotic\_cell\_cycle | 118 | 0 | 0.000000 | 0.000000 | 992 | 830.803180 | 884.8 | 938.796820 | 0.891935 |
| GO:0010608\_posttranscriptional\_regulation\_of\_gene\_expression | 118 | 0 | 0.000000 | 0.000000 | 992 | 830.803180 | 884.8 | 938.796820 | 0.891935 |
| GO:0048285\_organelle\_fission | 118 | 0 | 0.000000 | 0.000000 | 992 | 830.803180 | 884.8 | 938.796820 | 0.891935 |
| GO:0000082\_G1\_S\_transition\_of\_mitotic\_cell\_cycle | 36 | 0 | 0.000000 | 0.000000 | 999 | 840.041372 | 893.33 | 946.618628 | 0.894224 |
| GO:0000910\_cytokinesis | 36 | 0 | 0.000000 | 0.000000 | 999 | 840.041372 | 893.33 | 946.618628 | 0.894224 |
| GO:0001819\_positive\_regulation\_of\_cytokine\_production | 36 | 0 | 0.000000 | 0.000000 | 999 | 840.041372 | 893.33 | 946.618628 | 0.894224 |
| GO:0006334\_nucleosome\_assembly | 36 | 0 | 0.000000 | 0.000000 | 999 | 840.041372 | 893.33 | 946.618628 | 0.894224 |
| GO:0006814\_sodium\_ion\_transport | 36 | 0 | 0.000000 | 0.000000 | 999 | 840.041372 | 893.33 | 946.618628 | 0.894224 |
| GO:0009566\_fertilization | 36 | 0 | 0.000000 | 0.000000 | 999 | 840.041372 | 893.33 | 946.618628 | 0.894224 |
| GO:0034103\_regulation\_of\_tissue\_remodeling | 36 | 0 | 0.000000 | 0.000000 | 999 | 840.041372 | 893.33 | 946.618628 | 0.894224 |
| GO:0031325\_positive\_regulation\_of\_cellular\_metabolic\_process | 454 | 0 | 0.000000 | 0.000000 | 1000 | 840.635103 | 893.69 | 946.744897 | 0.893690 |
| GO:0000028\_ribosomal\_small\_subunit\_assembly | 1 | 0 |  |  |  |  |  |  |  |  |
| GO:0000042\_protein\_targeting\_to\_Golgi | 1 | 0 |  |  |  |  |  |  |  |  |
| GO:0000046\_autophagic\_vacuole\_fusion | 1 | 0 |  |  |  |  |  |  |  |  |
| GO:0000052\_citrulline\_metabolic\_process | 1 | 0 |  |  |  |  |  |  |  |  |
| GO:0000054\_ribosome\_export\_from\_nucleus | 1 | 0 |  |  |  |  |  |  |  |  |
| GO:0000056\_ribosomal\_small\_subunit\_export\_from\_nucleus | 1 | 0 |  |  |  |  |  |  |  |  |
| GO:0000072\_M\_phase\_specific\_microtubule\_process | 1 | 0 |  |  |  |  |  |  |  |  |
| GO:0000093\_mitotic\_telophase | 1 | 0 |  |  |  |  |  |  |  |  |
| GO:0000098\_sulfur\_amino\_acid\_catabolic\_process | 1 | 0 |  |  |  |  |  |  |  |  |
| GO:0000114\_regulation\_of\_transcription\_during\_G1\_phase\_of\_mitotic\_cell\_cycle | 1 | 0 |  |  |  |  |  |  |  |  |
| GO:0000115\_regulation\_of\_transcription\_during\_S-phase\_of\_mitotic\_cell\_cycle | 1 | 0 |  |  |  |  |  |  |  |  |
| GO:0000117\_regulation\_of\_transcription\_during\_G2\_M-phase\_of\_mitotic\_cell\_cycle | 1 | 0 |  |  |  |  |  |  |  |  |
| GO:0000132\_establishment\_of\_mitotic\_spindle\_orientation | 1 | 0 |  |  |  |  |  |  |  |  |
| GO:0000154\_rRNA\_modification | 1 | 0 |  |  |  |  |  |  |  |  |
| GO:0000160\_two-component\_signal\_transduction\_system\_(phosphorelay) | 1 | 0 |  |  |  |  |  |  |  |  |
| GO:0000161\_MAPKKK\_cascade\_involved\_in\_osmosensory\_signaling\_pathway | 1 | 0 |  |  |  |  |  |  |  |  |
| GO:0000173\_inactivation\_of\_MAPK\_activity\_involved\_in\_osmosensory\_signaling\_pathway | 1 | 0 |  |  |  |  |  |  |  |  |
| GO:0000212\_meiotic\_spindle\_organization | 1 | 0 |  |  |  |  |  |  |  |  |
| GO:0000255\_allantoin\_metabolic\_process | 1 | 0 |  |  |  |  |  |  |  |  |
| GO:0000270\_peptidoglycan\_metabolic\_process | 1 | 0 |  |  |  |  |  |  |  |  |
| GO:0000296\_spermine\_transport | 1 | 0 |  |  |  |  |  |  |  |  |
| GO:0000301\_retrograde\_transport\_\_vesicle\_recycling\_within\_Golgi | 1 | 0 |  |  |  |  |  |  |  |  |
| GO:0000303\_response\_to\_superoxide | 1 | 0 |  |  |  |  |  |  |  |  |
| GO:0000320\_re-entry\_into\_mitotic\_cell\_cycle | 1 | 0 |  |  |  |  |  |  |  |  |
| GO:0000338\_protein\_deneddylation | 1 | 0 |  |  |  |  |  |  |  |  |
| GO:0000395\_nuclear\_mRNA\_5'-splice\_site\_recognition | 1 | 0 |  |  |  |  |  |  |  |  |
| GO:0000710\_meiotic\_mismatch\_repair | 1 | 0 |  |  |  |  |  |  |  |  |
| GO:0000717\_nucleotide-excision\_repair\_\_DNA\_duplex\_unwinding | 1 | 0 |  |  |  |  |  |  |  |  |
| GO:0000722\_telomere\_maintenance\_via\_recombination | 1 | 0 |  |  |  |  |  |  |  |  |
| GO:0000746\_conjugation | 1 | 0 |  |  |  |  |  |  |  |  |
| GO:0000747\_conjugation\_with\_cellular\_fusion | 1 | 0 |  |  |  |  |  |  |  |  |
| GO:0000912\_formation\_of\_actomyosin\_apparatus\_involved\_in\_cytokinesis | 1 | 0 |  |  |  |  |  |  |  |  |
| GO:0000915\_cytokinesis\_\_contractile\_ring\_formation | 1 | 0 |  |  |  |  |  |  |  |  |
| GO:0000921\_septin\_ring\_assembly | 1 | 0 |  |  |  |  |  |  |  |  |
| GO:0000966\_RNA\_5'-end\_processing | 1 | 0 |  |  |  |  |  |  |  |  |
| GO:0001315\_age-dependent\_response\_to\_reactive\_oxygen\_species | 1 | 0 |  |  |  |  |  |  |  |  |
| GO:0001519\_peptide\_amidation | 1 | 0 |  |  |  |  |  |  |  |  |
| GO:0001560\_regulation\_of\_cell\_growth\_by\_extracellular\_stimulus | 1 | 0 |  |  |  |  |  |  |  |  |
| GO:0001574\_ganglioside\_biosynthetic\_process | 1 | 0 |  |  |  |  |  |  |  |  |
| GO:0001575\_globoside\_metabolic\_process | 1 | 0 |  |  |  |  |  |  |  |  |
| GO:0001658\_branching\_involved\_in\_ureteric\_bud\_morphogenesis | 1 | 0 |  |  |  |  |  |  |  |  |
| GO:0001662\_behavioral\_fear\_response | 1 | 0 |  |  |  |  |  |  |  |  |
| GO:0001675\_acrosome\_assembly | 1 | 0 |  |  |  |  |  |  |  |  |
| GO:0001692\_histamine\_metabolic\_process | 1 | 0 |  |  |  |  |  |  |  |  |
| GO:0001694\_histamine\_biosynthetic\_process | 1 | 0 |  |  |  |  |  |  |  |  |
| GO:0001732\_formation\_of\_translation\_initiation\_complex | 1 | 0 |  |  |  |  |  |  |  |  |
| GO:0001757\_somite\_specification | 1 | 0 |  |  |  |  |  |  |  |  |
| GO:0001774\_microglial\_cell\_activation | 1 | 0 |  |  |  |  |  |  |  |  |
| GO:0001782\_B\_cell\_homeostasis | 1 | 0 |  |  |  |  |  |  |  |  |
| GO:0001787\_natural\_killer\_cell\_proliferation | 1 | 0 |  |  |  |  |  |  |  |  |
| GO:0001823\_mesonephros\_development | 1 | 0 |  |  |  |  |  |  |  |  |
| GO:0001832\_blastocyst\_growth | 1 | 0 |  |  |  |  |  |  |  |  |
| GO:0001833\_inner\_cell\_mass\_cell\_proliferation | 1 | 0 |  |  |  |  |  |  |  |  |
| GO:0001839\_neural\_plate\_morphogenesis | 1 | 0 |  |  |  |  |  |  |  |  |
| GO:0001845\_phagolysosome\_formation | 1 | 0 |  |  |  |  |  |  |  |  |
| GO:0001865\_NK\_T\_cell\_differentiation | 1 | 0 |  |  |  |  |  |  |  |  |
| GO:0001866\_NK\_T\_cell\_proliferation | 1 | 0 |  |  |  |  |  |  |  |  |
| GO:0001887\_selenium\_metabolic\_process | 1 | 0 |  |  |  |  |  |  |  |  |
| GO:0001892\_embryonic\_placenta\_development | 1 | 0 |  |  |  |  |  |  |  |  |
| GO:0001911\_negative\_regulation\_of\_leukocyte\_mediated\_cytotoxicity | 1 | 0 |  |  |  |  |  |  |  |  |
| GO:0001915\_negative\_regulation\_of\_T\_cell\_mediated\_cytotoxicity | 1 | 0 |  |  |  |  |  |  |  |  |
| GO:0001920\_negative\_regulation\_of\_receptor\_recycling | 1 | 0 |  |  |  |  |  |  |  |  |
| GO:0001941\_postsynaptic\_membrane\_organization | 1 | 0 |  |  |  |  |  |  |  |  |
| GO:0001958\_endochondral\_ossification | 1 | 0 |  |  |  |  |  |  |  |  |
| GO:0001973\_adenosine\_receptor\_signaling\_pathway | 1 | 0 |  |  |  |  |  |  |  |  |
| GO:0001977\_renal\_system\_process\_involved\_in\_regulation\_of\_blood\_volume | 1 | 0 |  |  |  |  |  |  |  |  |
| GO:0001980\_regulation\_of\_systemic\_arterial\_blood\_pressure\_by\_ischemic\_conditions | 1 | 0 |  |  |  |  |  |  |  |  |
| GO:0001993\_regulation\_of\_systemic\_arterial\_blood\_pressure\_by\_norepinephrine-epinephrine | 1 | 0 |  |  |  |  |  |  |  |  |
| GO:0001996\_positive\_regulation\_of\_heart\_rate\_by\_epinephrine-norepinephrine | 1 | 0 |  |  |  |  |  |  |  |  |
| GO:0001999\_renal\_response\_to\_blood\_flow\_during\_renin-angiotensin\_regulation\_of\_systemic\_arterial\_blood\_pressure | 1 | 0 |  |  |  |  |  |  |  |  |
| GO:0002001\_renin\_secretion\_into\_blood\_stream | 1 | 0 |  |  |  |  |  |  |  |  |
| GO:0002017\_regulation\_of\_blood\_volume\_by\_renal\_aldosterone | 1 | 0 |  |  |  |  |  |  |  |  |
| GO:0002018\_renin-angiotensin\_regulation\_of\_aldosterone\_production | 1 | 0 |  |  |  |  |  |  |  |  |
| GO:0002031\_G-protein\_coupled\_receptor\_internalization | 1 | 0 |  |  |  |  |  |  |  |  |
| GO:0002035\_brain\_renin-angiotensin\_system | 1 | 0 |  |  |  |  |  |  |  |  |
| GO:0002042\_cell\_migration\_involved\_in\_sprouting\_angiogenesis | 1 | 0 |  |  |  |  |  |  |  |  |
| GO:0002052\_positive\_regulation\_of\_neuroblast\_proliferation | 1 | 0 |  |  |  |  |  |  |  |  |
| GO:0002053\_positive\_regulation\_of\_mesenchymal\_cell\_proliferation | 1 | 0 |  |  |  |  |  |  |  |  |
| GO:0002063\_chondrocyte\_development | 1 | 0 |  |  |  |  |  |  |  |  |
| GO:0002064\_epithelial\_cell\_development | 1 | 0 |  |  |  |  |  |  |  |  |
| GO:0002074\_extraocular\_skeletal\_muscle\_development | 1 | 0 |  |  |  |  |  |  |  |  |
| GO:0002077\_acrosome\_matrix\_dispersal | 1 | 0 |  |  |  |  |  |  |  |  |
| GO:0002082\_regulation\_of\_oxidative\_phosphorylation | 1 | 0 |  |  |  |  |  |  |  |  |
| GO:0002084\_protein\_depalmitoylation | 1 | 0 |  |  |  |  |  |  |  |  |
| GO:0002088\_lens\_development\_in\_camera-type\_eye | 1 | 0 |  |  |  |  |  |  |  |  |
| GO:0002089\_lens\_morphogenesis\_in\_camera-type\_eye | 1 | 0 |  |  |  |  |  |  |  |  |
| GO:0002093\_auditory\_receptor\_cell\_morphogenesis | 1 | 0 |  |  |  |  |  |  |  |  |
| GO:0002209\_behavioral\_defense\_response | 1 | 0 |  |  |  |  |  |  |  |  |
| GO:0002220\_innate\_immune\_response\_activating\_cell\_surface\_receptor\_signaling\_pathway | 1 | 0 |  |  |  |  |  |  |  |  |
| GO:0002223\_stimulatory\_C-type\_lectin\_receptor\_signaling\_pathway | 1 | 0 |  |  |  |  |  |  |  |  |
| GO:0002312\_B\_cell\_activation\_during\_immune\_response | 1 | 0 |  |  |  |  |  |  |  |  |
| GO:0002313\_mature\_B\_cell\_differentiation\_during\_immune\_response | 1 | 0 |  |  |  |  |  |  |  |  |
| GO:0002318\_myeloid\_progenitor\_cell\_differentiation | 1 | 0 |  |  |  |  |  |  |  |  |
| GO:0002320\_lymphoid\_progenitor\_cell\_differentiation | 1 | 0 |  |  |  |  |  |  |  |  |
| GO:0002326\_B\_cell\_lineage\_commitment | 1 | 0 |  |  |  |  |  |  |  |  |
| GO:0002328\_pro-B\_cell\_differentiation | 1 | 0 |  |  |  |  |  |  |  |  |
| GO:0002335\_mature\_B\_cell\_differentiation | 1 | 0 |  |  |  |  |  |  |  |  |
| GO:0002355\_detection\_of\_tumor\_cell | 1 | 0 |  |  |  |  |  |  |  |  |
| GO:0002368\_B\_cell\_cytokine\_production | 1 | 0 |  |  |  |  |  |  |  |  |
| GO:0002424\_T\_cell\_mediated\_immune\_response\_to\_tumor\_cell | 1 | 0 |  |  |  |  |  |  |  |  |
| GO:0002431\_Fc\_receptor\_mediated\_stimulatory\_signaling\_pathway | 1 | 0 |  |  |  |  |  |  |  |  |
| GO:0002437\_inflammatory\_response\_to\_antigenic\_stimulus | 1 | 0 |  |  |  |  |  |  |  |  |
| GO:0002439\_chronic\_inflammatory\_response\_to\_antigenic\_stimulus | 1 | 0 |  |  |  |  |  |  |  |  |
| GO:0002447\_eosinophil\_mediated\_immunity | 1 | 0 |  |  |  |  |  |  |  |  |
| GO:0002455\_humoral\_immune\_response\_mediated\_by\_circulating\_immunoglobulin | 1 | 0 |  |  |  |  |  |  |  |  |
| GO:0002467\_germinal\_center\_formation | 1 | 0 |  |  |  |  |  |  |  |  |
| GO:0002468\_dendritic\_cell\_antigen\_processing\_and\_presentation | 1 | 0 |  |  |  |  |  |  |  |  |
| GO:0002475\_antigen\_processing\_and\_presentation\_via\_MHC\_class\_Ib | 1 | 0 |  |  |  |  |  |  |  |  |
| GO:0002478\_antigen\_processing\_and\_presentation\_of\_exogenous\_peptide\_antigen | 1 | 0 |  |  |  |  |  |  |  |  |
| GO:0002495\_antigen\_processing\_and\_presentation\_of\_peptide\_antigen\_via\_MHC\_class\_II | 1 | 0 |  |  |  |  |  |  |  |  |
| GO:0002513\_tolerance\_induction\_to\_self\_antigen | 1 | 0 |  |  |  |  |  |  |  |  |
| GO:0002514\_B\_cell\_tolerance\_induction | 1 | 0 |  |  |  |  |  |  |  |  |
| GO:0002517\_T\_cell\_tolerance\_induction | 1 | 0 |  |  |  |  |  |  |  |  |
| GO:0002523\_leukocyte\_migration\_during\_inflammatory\_response | 1 | 0 |  |  |  |  |  |  |  |  |
| GO:0002566\_somatic\_diversification\_of\_immune\_receptors\_via\_somatic\_mutation | 1 | 0 |  |  |  |  |  |  |  |  |
| GO:0002568\_somatic\_diversification\_of\_T\_cell\_receptor\_genes | 1 | 0 |  |  |  |  |  |  |  |  |
| GO:0002576\_platelet\_degranulation | 1 | 0 |  |  |  |  |  |  |  |  |
| GO:0002577\_regulation\_of\_antigen\_processing\_and\_presentation | 1 | 0 |  |  |  |  |  |  |  |  |
| GO:0002578\_negative\_regulation\_of\_antigen\_processing\_and\_presentation | 1 | 0 |  |  |  |  |  |  |  |  |
| GO:0002580\_regulation\_of\_antigen\_processing\_and\_presentation\_of\_peptide\_or\_polysaccharide\_antigen\_via\_MHC\_class\_II | 1 | 0 |  |  |  |  |  |  |  |  |
| GO:0002581\_negative\_regulation\_of\_antigen\_processing\_and\_presentation\_of\_peptide\_or\_polysaccharide\_antigen\_via\_MHC\_class\_II | 1 | 0 |  |  |  |  |  |  |  |  |
| GO:0002604\_regulation\_of\_dendritic\_cell\_antigen\_processing\_and\_presentation | 1 | 0 |  |  |  |  |  |  |  |  |
| GO:0002605\_negative\_regulation\_of\_dendritic\_cell\_antigen\_processing\_and\_presentation | 1 | 0 |  |  |  |  |  |  |  |  |
| GO:0002634\_regulation\_of\_germinal\_center\_formation | 1 | 0 |  |  |  |  |  |  |  |  |
| GO:0002649\_regulation\_of\_tolerance\_induction\_to\_self\_antigen | 1 | 0 |  |  |  |  |  |  |  |  |
| GO:0002651\_positive\_regulation\_of\_tolerance\_induction\_to\_self\_antigen | 1 | 0 |  |  |  |  |  |  |  |  |
| GO:0002661\_regulation\_of\_B\_cell\_tolerance\_induction | 1 | 0 |  |  |  |  |  |  |  |  |
| GO:0002663\_positive\_regulation\_of\_B\_cell\_tolerance\_induction | 1 | 0 |  |  |  |  |  |  |  |  |
| GO:0002664\_regulation\_of\_T\_cell\_tolerance\_induction | 1 | 0 |  |  |  |  |  |  |  |  |
| GO:0002666\_positive\_regulation\_of\_T\_cell\_tolerance\_induction | 1 | 0 |  |  |  |  |  |  |  |  |
| GO:0002674\_negative\_regulation\_of\_acute\_inflammatory\_response | 1 | 0 |  |  |  |  |  |  |  |  |
| GO:0002681\_somatic\_recombination\_of\_T\_cell\_receptor\_gene\_segments | 1 | 0 |  |  |  |  |  |  |  |  |
| GO:0002686\_negative\_regulation\_of\_leukocyte\_migration | 1 | 0 |  |  |  |  |  |  |  |  |
| GO:0002691\_regulation\_of\_cellular\_extravasation | 1 | 0 |  |  |  |  |  |  |  |  |
| GO:0002693\_positive\_regulation\_of\_cellular\_extravasation | 1 | 0 |  |  |  |  |  |  |  |  |
| GO:0002701\_negative\_regulation\_of\_production\_of\_molecular\_mediator\_of\_immune\_response | 1 | 0 |  |  |  |  |  |  |  |  |
| GO:0002719\_negative\_regulation\_of\_cytokine\_production\_during\_immune\_response | 1 | 0 |  |  |  |  |  |  |  |  |
| GO:0002725\_negative\_regulation\_of\_T\_cell\_cytokine\_production | 1 | 0 |  |  |  |  |  |  |  |  |
| GO:0002759\_regulation\_of\_antimicrobial\_humoral\_response | 1 | 0 |  |  |  |  |  |  |  |  |
| GO:0002775\_antimicrobial\_peptide\_production | 1 | 0 |  |  |  |  |  |  |  |  |
| GO:0002777\_antimicrobial\_peptide\_biosynthetic\_process | 1 | 0 |  |  |  |  |  |  |  |  |
| GO:0002778\_antibacterial\_peptide\_production | 1 | 0 |  |  |  |  |  |  |  |  |
| GO:0002780\_antibacterial\_peptide\_biosynthetic\_process | 1 | 0 |  |  |  |  |  |  |  |  |
| GO:0002784\_regulation\_of\_antimicrobial\_peptide\_production | 1 | 0 |  |  |  |  |  |  |  |  |
| GO:0002786\_regulation\_of\_antibacterial\_peptide\_production | 1 | 0 |  |  |  |  |  |  |  |  |
| GO:0002805\_regulation\_of\_antimicrobial\_peptide\_biosynthetic\_process | 1 | 0 |  |  |  |  |  |  |  |  |
| GO:0002807\_positive\_regulation\_of\_antimicrobial\_peptide\_biosynthetic\_process | 1 | 0 |  |  |  |  |  |  |  |  |
| GO:0002808\_regulation\_of\_antibacterial\_peptide\_biosynthetic\_process | 1 | 0 |  |  |  |  |  |  |  |  |
| GO:0002815\_biosynthetic\_process\_of\_antibacterial\_peptides\_active\_against\_Gram-positive\_bacteria | 1 | 0 |  |  |  |  |  |  |  |  |
| GO:0002816\_regulation\_of\_biosynthetic\_process\_of\_antibacterial\_peptides\_active\_against\_Gram-positive\_bacteria | 1 | 0 |  |  |  |  |  |  |  |  |
| GO:0002832\_negative\_regulation\_of\_response\_to\_biotic\_stimulus | 1 | 0 |  |  |  |  |  |  |  |  |
| GO:0002840\_regulation\_of\_T\_cell\_mediated\_immune\_response\_to\_tumor\_cell | 1 | 0 |  |  |  |  |  |  |  |  |
| GO:0002842\_positive\_regulation\_of\_T\_cell\_mediated\_immune\_response\_to\_tumor\_cell | 1 | 0 |  |  |  |  |  |  |  |  |
| GO:0002901\_mature\_B\_cell\_apoptosis | 1 | 0 |  |  |  |  |  |  |  |  |
| GO:0002904\_positive\_regulation\_of\_B\_cell\_apoptosis | 1 | 0 |  |  |  |  |  |  |  |  |
| GO:0002905\_regulation\_of\_mature\_B\_cell\_apoptosis | 1 | 0 |  |  |  |  |  |  |  |  |
| GO:0002906\_negative\_regulation\_of\_mature\_B\_cell\_apoptosis | 1 | 0 |  |  |  |  |  |  |  |  |
| GO:0003010\_voluntary\_skeletal\_muscle\_contraction | 1 | 0 |  |  |  |  |  |  |  |  |
| GO:0003051\_angiotensin-mediated\_drinking\_behavior | 1 | 0 |  |  |  |  |  |  |  |  |
| GO:0003058\_hormonal\_regulation\_of\_the\_force\_of\_heart\_contraction | 1 | 0 |  |  |  |  |  |  |  |  |
| GO:0003062\_regulation\_of\_heart\_rate\_by\_chemical\_signal | 1 | 0 |  |  |  |  |  |  |  |  |
| GO:0003065\_positive\_regulation\_of\_heart\_rate\_by\_epinephrine | 1 | 0 |  |  |  |  |  |  |  |  |
| GO:0003071\_renal\_system\_process\_involved\_in\_regulation\_of\_systemic\_arterial\_blood\_pressure | 1 | 0 |  |  |  |  |  |  |  |  |
| GO:0003085\_negative\_regulation\_of\_systemic\_arterial\_blood\_pressure | 1 | 0 |  |  |  |  |  |  |  |  |
| GO:0003099\_positive\_regulation\_of\_the\_force\_of\_heart\_contraction\_by\_chemical\_signal | 1 | 0 |  |  |  |  |  |  |  |  |
| GO:0003108\_negative\_regulation\_of\_the\_force\_of\_heart\_contraction\_by\_chemical\_signal | 1 | 0 |  |  |  |  |  |  |  |  |
| GO:0005981\_regulation\_of\_glycogen\_catabolic\_process | 1 | 0 |  |  |  |  |  |  |  |  |
| GO:0005982\_starch\_metabolic\_process | 1 | 0 |  |  |  |  |  |  |  |  |
| GO:0005983\_starch\_catabolic\_process | 1 | 0 |  |  |  |  |  |  |  |  |
| GO:0005988\_lactose\_metabolic\_process | 1 | 0 |  |  |  |  |  |  |  |  |
| GO:0005989\_lactose\_biosynthetic\_process | 1 | 0 |  |  |  |  |  |  |  |  |
| GO:0005991\_trehalose\_metabolic\_process | 1 | 0 |  |  |  |  |  |  |  |  |
| GO:0005993\_trehalose\_catabolic\_process | 1 | 0 |  |  |  |  |  |  |  |  |
| GO:0006010\_glucose\_6-phosphate\_utilization | 1 | 0 |  |  |  |  |  |  |  |  |
| GO:0006013\_mannose\_metabolic\_process | 1 | 0 |  |  |  |  |  |  |  |  |
| GO:0006021\_inositol\_biosynthetic\_process | 1 | 0 |  |  |  |  |  |  |  |  |
| GO:0006037\_cell\_wall\_chitin\_metabolic\_process | 1 | 0 |  |  |  |  |  |  |  |  |
| GO:0006042\_glucosamine\_biosynthetic\_process | 1 | 0 |  |  |  |  |  |  |  |  |
| GO:0006045\_N-acetylglucosamine\_biosynthetic\_process | 1 | 0 |  |  |  |  |  |  |  |  |
| GO:0006048\_UDP-N-acetylglucosamine\_biosynthetic\_process | 1 | 0 |  |  |  |  |  |  |  |  |
| GO:0006050\_mannosamine\_metabolic\_process | 1 | 0 |  |  |  |  |  |  |  |  |
| GO:0006051\_N-acetylmannosamine\_metabolic\_process | 1 | 0 |  |  |  |  |  |  |  |  |
| GO:0006059\_hexitol\_metabolic\_process | 1 | 0 |  |  |  |  |  |  |  |  |
| GO:0006060\_sorbitol\_metabolic\_process | 1 | 0 |  |  |  |  |  |  |  |  |
| GO:0006062\_sorbitol\_catabolic\_process | 1 | 0 |  |  |  |  |  |  |  |  |
| GO:0006065\_UDP-glucuronate\_biosynthetic\_process | 1 | 0 |  |  |  |  |  |  |  |  |
| GO:0006083\_acetate\_metabolic\_process | 1 | 0 |  |  |  |  |  |  |  |  |
| GO:0006085\_acetyl-CoA\_biosynthetic\_process | 1 | 0 |  |  |  |  |  |  |  |  |
| GO:0006103\_2-oxoglutarate\_metabolic\_process | 1 | 0 |  |  |  |  |  |  |  |  |
| GO:0006106\_fumarate\_metabolic\_process | 1 | 0 |  |  |  |  |  |  |  |  |
| GO:0006107\_oxaloacetate\_metabolic\_process | 1 | 0 |  |  |  |  |  |  |  |  |
| GO:0006116\_NADH\_oxidation | 1 | 0 |  |  |  |  |  |  |  |  |
| GO:0006145\_purine\_base\_catabolic\_process | 1 | 0 |  |  |  |  |  |  |  |  |
| GO:0006148\_inosine\_catabolic\_process | 1 | 0 |  |  |  |  |  |  |  |  |
| GO:0006154\_adenosine\_catabolic\_process | 1 | 0 |  |  |  |  |  |  |  |  |
| GO:0006166\_purine\_ribonucleoside\_salvage | 1 | 0 |  |  |  |  |  |  |  |  |
| GO:0006172\_ADP\_biosynthetic\_process | 1 | 0 |  |  |  |  |  |  |  |  |
| GO:0006173\_dADP\_biosynthetic\_process | 1 | 0 |  |  |  |  |  |  |  |  |
| GO:0006188\_IMP\_biosynthetic\_process | 1 | 0 |  |  |  |  |  |  |  |  |
| GO:0006189\_'de\_novo'\_IMP\_biosynthetic\_process | 1 | 0 |  |  |  |  |  |  |  |  |
| GO:0006196\_AMP\_catabolic\_process | 1 | 0 |  |  |  |  |  |  |  |  |
| GO:0006198\_cAMP\_catabolic\_process | 1 | 0 |  |  |  |  |  |  |  |  |
| GO:0006207\_'de\_novo'\_pyrimidine\_base\_biosynthetic\_process | 1 | 0 |  |  |  |  |  |  |  |  |
| GO:0006214\_thymidine\_catabolic\_process | 1 | 0 |  |  |  |  |  |  |  |  |
| GO:0006216\_cytidine\_catabolic\_process | 1 | 0 |  |  |  |  |  |  |  |  |
| GO:0006222\_UMP\_biosynthetic\_process | 1 | 0 |  |  |  |  |  |  |  |  |
| GO:0006241\_CTP\_biosynthetic\_process | 1 | 0 |  |  |  |  |  |  |  |  |
| GO:0006256\_UDP\_catabolic\_process | 1 | 0 |  |  |  |  |  |  |  |  |
| GO:0006265\_DNA\_topological\_change | 1 | 0 |  |  |  |  |  |  |  |  |
| GO:0006272\_leading\_strand\_elongation | 1 | 0 |  |  |  |  |  |  |  |  |
| GO:0006287\_base-excision\_repair\_\_gap-filling | 1 | 0 |  |  |  |  |  |  |  |  |
| GO:0006313\_transposition\_\_DNA-mediated | 1 | 0 |  |  |  |  |  |  |  |  |
| GO:0006336\_DNA\_replication-independent\_nucleosome\_assembly | 1 | 0 |  |  |  |  |  |  |  |  |
| GO:0006343\_establishment\_of\_chromatin\_silencing | 1 | 0 |  |  |  |  |  |  |  |  |
| GO:0006344\_maintenance\_of\_chromatin\_silencing | 1 | 0 |  |  |  |  |  |  |  |  |
| GO:0006346\_methylation-dependent\_chromatin\_silencing | 1 | 0 |  |  |  |  |  |  |  |  |
| GO:0006348\_chromatin\_silencing\_at\_telomere | 1 | 0 |  |  |  |  |  |  |  |  |
| GO:0006361\_transcription\_initiation\_from\_RNA\_polymerase\_I\_promoter | 1 | 0 |  |  |  |  |  |  |  |  |
| GO:0006369\_termination\_of\_RNA\_polymerase\_II\_transcription | 1 | 0 |  |  |  |  |  |  |  |  |
| GO:0006393\_termination\_of\_mitochondrial\_transcription | 1 | 0 |  |  |  |  |  |  |  |  |
| GO:0006407\_rRNA\_export\_from\_nucleus | 1 | 0 |  |  |  |  |  |  |  |  |
| GO:0006408\_snRNA\_export\_from\_nucleus | 1 | 0 |  |  |  |  |  |  |  |  |
| GO:0006409\_tRNA\_export\_from\_nucleus | 1 | 0 |  |  |  |  |  |  |  |  |
| GO:0006419\_alanyl-tRNA\_aminoacylation | 1 | 0 |  |  |  |  |  |  |  |  |
| GO:0006420\_arginyl-tRNA\_aminoacylation | 1 | 0 |  |  |  |  |  |  |  |  |
| GO:0006423\_cysteinyl-tRNA\_aminoacylation | 1 | 0 |  |  |  |  |  |  |  |  |
| GO:0006431\_methionyl-tRNA\_aminoacylation | 1 | 0 |  |  |  |  |  |  |  |  |
| GO:0006432\_phenylalanyl-tRNA\_aminoacylation | 1 | 0 |  |  |  |  |  |  |  |  |
| GO:0006434\_seryl-tRNA\_aminoacylation | 1 | 0 |  |  |  |  |  |  |  |  |
| GO:0006435\_threonyl-tRNA\_aminoacylation | 1 | 0 |  |  |  |  |  |  |  |  |
| GO:0006436\_tryptophanyl-tRNA\_aminoacylation | 1 | 0 |  |  |  |  |  |  |  |  |
| GO:0006437\_tyrosyl-tRNA\_aminoacylation | 1 | 0 |  |  |  |  |  |  |  |  |
| GO:0006447\_regulation\_of\_translational\_initiation\_by\_iron | 1 | 0 |  |  |  |  |  |  |  |  |
| GO:0006448\_regulation\_of\_translational\_elongation | 1 | 0 |  |  |  |  |  |  |  |  |
| GO:0006450\_regulation\_of\_translational\_fidelity | 1 | 0 |  |  |  |  |  |  |  |  |
| GO:0006494\_protein\_amino\_acid\_terminal\_glycosylation | 1 | 0 |  |  |  |  |  |  |  |  |
| GO:0006496\_protein\_amino\_acid\_terminal\_N-glycosylation | 1 | 0 |  |  |  |  |  |  |  |  |
| GO:0006499\_N-terminal\_protein\_myristoylation | 1 | 0 |  |  |  |  |  |  |  |  |
| GO:0006500\_N-terminal\_protein\_palmitoylation | 1 | 0 |  |  |  |  |  |  |  |  |
| GO:0006526\_arginine\_biosynthetic\_process | 1 | 0 |  |  |  |  |  |  |  |  |
| GO:0006528\_asparagine\_metabolic\_process | 1 | 0 |  |  |  |  |  |  |  |  |
| GO:0006530\_asparagine\_catabolic\_process | 1 | 0 |  |  |  |  |  |  |  |  |
| GO:0006534\_cysteine\_metabolic\_process | 1 | 0 |  |  |  |  |  |  |  |  |
| GO:0006543\_glutamine\_catabolic\_process | 1 | 0 |  |  |  |  |  |  |  |  |
| GO:0006545\_glycine\_biosynthetic\_process | 1 | 0 |  |  |  |  |  |  |  |  |
| GO:0006547\_histidine\_metabolic\_process | 1 | 0 |  |  |  |  |  |  |  |  |
| GO:0006549\_isoleucine\_metabolic\_process | 1 | 0 |  |  |  |  |  |  |  |  |
| GO:0006556\_S-adenosylmethionine\_biosynthetic\_process | 1 | 0 |  |  |  |  |  |  |  |  |
| GO:0006562\_proline\_catabolic\_process | 1 | 0 |  |  |  |  |  |  |  |  |
| GO:0006564\_L-serine\_biosynthetic\_process | 1 | 0 |  |  |  |  |  |  |  |  |
| GO:0006577\_betaine\_metabolic\_process | 1 | 0 |  |  |  |  |  |  |  |  |
| GO:0006580\_ethanolamine\_metabolic\_process | 1 | 0 |  |  |  |  |  |  |  |  |
| GO:0006585\_dopamine\_biosynthetic\_process\_from\_tyrosine | 1 | 0 |  |  |  |  |  |  |  |  |
| GO:0006591\_ornithine\_metabolic\_process | 1 | 0 |  |  |  |  |  |  |  |  |
| GO:0006597\_spermine\_biosynthetic\_process | 1 | 0 |  |  |  |  |  |  |  |  |
| GO:0006598\_polyamine\_catabolic\_process | 1 | 0 |  |  |  |  |  |  |  |  |
| GO:0006610\_ribosomal\_protein\_import\_into\_nucleus | 1 | 0 |  |  |  |  |  |  |  |  |
| GO:0006614\_SRP-dependent\_cotranslational\_protein\_targeting\_to\_membrane | 1 | 0 |  |  |  |  |  |  |  |  |
| GO:0006616\_SRP-dependent\_cotranslational\_protein\_targeting\_to\_membrane\_\_translocation | 1 | 0 |  |  |  |  |  |  |  |  |
| GO:0006617\_SRP-dependent\_cotranslational\_protein\_targeting\_to\_membrane\_\_signal\_sequence\_recognition | 1 | 0 |  |  |  |  |  |  |  |  |
| GO:0006627\_mitochondrial\_protein\_processing\_during\_import | 1 | 0 |  |  |  |  |  |  |  |  |
| GO:0006646\_phosphatidylethanolamine\_biosynthetic\_process | 1 | 0 |  |  |  |  |  |  |  |  |
| GO:0006655\_phosphatidylglycerol\_biosynthetic\_process | 1 | 0 |  |  |  |  |  |  |  |  |
| GO:0006657\_CDP-choline\_pathway | 1 | 0 |  |  |  |  |  |  |  |  |
| GO:0006667\_sphinganine\_metabolic\_process | 1 | 0 |  |  |  |  |  |  |  |  |
| GO:0006668\_sphinganine-1-phosphate\_metabolic\_process | 1 | 0 |  |  |  |  |  |  |  |  |
| GO:0006669\_sphinganine-1-phosphate\_biosynthetic\_process | 1 | 0 |  |  |  |  |  |  |  |  |
| GO:0006670\_sphingosine\_metabolic\_process | 1 | 0 |  |  |  |  |  |  |  |  |
| GO:0006689\_ganglioside\_catabolic\_process | 1 | 0 |  |  |  |  |  |  |  |  |
| GO:0006711\_estrogen\_catabolic\_process | 1 | 0 |  |  |  |  |  |  |  |  |
| GO:0006713\_glucocorticoid\_catabolic\_process | 1 | 0 |  |  |  |  |  |  |  |  |
| GO:0006734\_NADH\_metabolic\_process | 1 | 0 |  |  |  |  |  |  |  |  |
| GO:0006741\_NADP\_biosynthetic\_process | 1 | 0 |  |  |  |  |  |  |  |  |
| GO:0006746\_FADH2\_metabolic\_process | 1 | 0 |  |  |  |  |  |  |  |  |
| GO:0006768\_biotin\_metabolic\_process | 1 | 0 |  |  |  |  |  |  |  |  |
| GO:0006771\_riboflavin\_metabolic\_process | 1 | 0 |  |  |  |  |  |  |  |  |
| GO:0006781\_succinyl-CoA\_pathway | 1 | 0 |  |  |  |  |  |  |  |  |
| GO:0006789\_bilirubin\_conjugation | 1 | 0 |  |  |  |  |  |  |  |  |
| GO:0006797\_polyphosphate\_metabolic\_process | 1 | 0 |  |  |  |  |  |  |  |  |
| GO:0006837\_serotonin\_transport | 1 | 0 |  |  |  |  |  |  |  |  |
| GO:0006842\_tricarboxylic\_acid\_transport | 1 | 0 |  |  |  |  |  |  |  |  |
| GO:0006843\_mitochondrial\_citrate\_transport | 1 | 0 |  |  |  |  |  |  |  |  |
| GO:0006848\_pyruvate\_transport | 1 | 0 |  |  |  |  |  |  |  |  |
| GO:0006862\_nucleotide\_transport | 1 | 0 |  |  |  |  |  |  |  |  |
| GO:0006867\_asparagine\_transport | 1 | 0 |  |  |  |  |  |  |  |  |
| GO:0006868\_glutamine\_transport | 1 | 0 |  |  |  |  |  |  |  |  |
| GO:0006876\_cellular\_cadmium\_ion\_homeostasis | 1 | 0 |  |  |  |  |  |  |  |  |
| GO:0006926\_virus-infected\_cell\_apoptosis | 1 | 0 |  |  |  |  |  |  |  |  |
| GO:0006931\_substrate-bound\_cell\_migration\_\_cell\_attachment\_to\_substrate | 1 | 0 |  |  |  |  |  |  |  |  |
| GO:0006948\_induction\_by\_virus\_of\_host\_cell-cell\_fusion | 1 | 0 |  |  |  |  |  |  |  |  |
| GO:0006958\_complement\_activation\_\_classical\_pathway | 1 | 0 |  |  |  |  |  |  |  |  |
| GO:0006963\_positive\_regulation\_of\_antibacterial\_peptide\_biosynthetic\_process | 1 | 0 |  |  |  |  |  |  |  |  |
| GO:0006965\_positive\_regulation\_of\_biosynthetic\_process\_of\_antibacterial\_peptides\_active\_against\_Gram-positive\_bacteria | 1 | 0 |  |  |  |  |  |  |  |  |
| GO:0006987\_activation\_of\_signaling\_protein\_activity\_involved\_in\_unfolded\_protein\_response | 1 | 0 |  |  |  |  |  |  |  |  |
| GO:0006990\_positive\_regulation\_of\_gene-specific\_transcription\_involved\_in\_unfolded\_protein\_response | 1 | 0 |  |  |  |  |  |  |  |  |
| GO:0006991\_response\_to\_sterol\_depletion | 1 | 0 |  |  |  |  |  |  |  |  |
| GO:0006994\_positive\_regulation\_of\_transcription\_via\_sterol\_regulatory\_element\_binding\_involved\_in\_ER-nuclear\_sterol\_response\_pathway | 1 | 0 |  |  |  |  |  |  |  |  |
| GO:0007039\_vacuolar\_protein\_catabolic\_process | 1 | 0 |  |  |  |  |  |  |  |  |
| GO:0007068\_negative\_regulation\_of\_transcription\_\_mitotic | 1 | 0 |  |  |  |  |  |  |  |  |
| GO:0007097\_nuclear\_migration | 1 | 0 |  |  |  |  |  |  |  |  |
| GO:0007100\_mitotic\_centrosome\_separation | 1 | 0 |  |  |  |  |  |  |  |  |
| GO:0007108\_cytokinesis\_\_initiation\_of\_separation | 1 | 0 |  |  |  |  |  |  |  |  |
| GO:0007109\_cytokinesis\_\_completion\_of\_separation | 1 | 0 |  |  |  |  |  |  |  |  |
| GO:0007132\_meiotic\_metaphase\_I | 1 | 0 |  |  |  |  |  |  |  |  |
| GO:0007135\_meiosis\_II | 1 | 0 |  |  |  |  |  |  |  |  |
| GO:0007136\_meiotic\_prophase\_II | 1 | 0 |  |  |  |  |  |  |  |  |
| GO:0007161\_calcium-independent\_cell-matrix\_adhesion | 1 | 0 |  |  |  |  |  |  |  |  |
| GO:0007196\_inhibition\_of\_adenylate\_cyclase\_activity\_by\_metabotropic\_glutamate\_receptor\_signaling\_pathway | 1 | 0 |  |  |  |  |  |  |  |  |
| GO:0007197\_inhibition\_of\_adenylate\_cyclase\_activity\_by\_muscarinic\_acetylcholine\_receptor\_signaling\_pathway | 1 | 0 |  |  |  |  |  |  |  |  |
| GO:0007258\_JUN\_phosphorylation | 1 | 0 |  |  |  |  |  |  |  |  |
| GO:0007321\_sperm\_displacement | 1 | 0 |  |  |  |  |  |  |  |  |
| GO:0007343\_egg\_activation | 1 | 0 |  |  |  |  |  |  |  |  |
| GO:0007386\_compartment\_specification | 1 | 0 |  |  |  |  |  |  |  |  |
| GO:0007387\_anterior\_compartment\_specification | 1 | 0 |  |  |  |  |  |  |  |  |
| GO:0007388\_posterior\_compartment\_specification | 1 | 0 |  |  |  |  |  |  |  |  |
| GO:0007402\_ganglion\_mother\_cell\_fate\_determination | 1 | 0 |  |  |  |  |  |  |  |  |
| GO:0007406\_negative\_regulation\_of\_neuroblast\_proliferation | 1 | 0 |  |  |  |  |  |  |  |  |
| GO:0007424\_open\_tracheal\_system\_development | 1 | 0 |  |  |  |  |  |  |  |  |
| GO:0007440\_foregut\_morphogenesis | 1 | 0 |  |  |  |  |  |  |  |  |
| GO:0007443\_Malpighian\_tubule\_morphogenesis | 1 | 0 |  |  |  |  |  |  |  |  |
| GO:0007444\_imaginal\_disc\_development | 1 | 0 |  |  |  |  |  |  |  |  |
| GO:0007447\_imaginal\_disc\_pattern\_formation | 1 | 0 |  |  |  |  |  |  |  |  |
| GO:0007494\_midgut\_development | 1 | 0 |  |  |  |  |  |  |  |  |
| GO:0007497\_posterior\_midgut\_development | 1 | 0 |  |  |  |  |  |  |  |  |
| GO:0007499\_ectoderm\_and\_mesoderm\_interaction | 1 | 0 |  |  |  |  |  |  |  |  |
| GO:0007501\_mesodermal\_cell\_fate\_specification | 1 | 0 |  |  |  |  |  |  |  |  |
| GO:0007509\_mesoderm\_migration | 1 | 0 |  |  |  |  |  |  |  |  |
| GO:0007518\_myoblast\_cell\_fate\_determination | 1 | 0 |  |  |  |  |  |  |  |  |
| GO:0007538\_primary\_sex\_determination | 1 | 0 |  |  |  |  |  |  |  |  |
| GO:0007597\_blood\_coagulation\_\_intrinsic\_pathway | 1 | 0 |  |  |  |  |  |  |  |  |
| GO:0007616\_long-term\_memory | 1 | 0 |  |  |  |  |  |  |  |  |
| GO:0007617\_mating\_behavior | 1 | 0 |  |  |  |  |  |  |  |  |
| GO:0007624\_ultradian\_rhythm | 1 | 0 |  |  |  |  |  |  |  |  |
| GO:0007638\_mechanosensory\_behavior | 1 | 0 |  |  |  |  |  |  |  |  |
| GO:0008045\_motor\_axon\_guidance | 1 | 0 |  |  |  |  |  |  |  |  |
| GO:0008057\_eye\_pigment\_granule\_organization | 1 | 0 |  |  |  |  |  |  |  |  |
| GO:0008063\_Toll\_signaling\_pathway | 1 | 0 |  |  |  |  |  |  |  |  |
| GO:0008065\_establishment\_of\_blood-nerve\_barrier | 1 | 0 |  |  |  |  |  |  |  |  |
| GO:0008090\_retrograde\_axon\_cargo\_transport | 1 | 0 |  |  |  |  |  |  |  |  |
| GO:0008215\_spermine\_metabolic\_process | 1 | 0 |  |  |  |  |  |  |  |  |
| GO:0008292\_acetylcholine\_biosynthetic\_process | 1 | 0 |  |  |  |  |  |  |  |  |
| GO:0008295\_spermidine\_biosynthetic\_process | 1 | 0 |  |  |  |  |  |  |  |  |
| GO:0008298\_intracellular\_mRNA\_localization | 1 | 0 |  |  |  |  |  |  |  |  |
| GO:0008356\_asymmetric\_cell\_division | 1 | 0 |  |  |  |  |  |  |  |  |
| GO:0008592\_regulation\_of\_Toll\_signaling\_pathway | 1 | 0 |  |  |  |  |  |  |  |  |
| GO:0008611\_ether\_lipid\_biosynthetic\_process | 1 | 0 |  |  |  |  |  |  |  |  |
| GO:0008614\_pyridoxine\_metabolic\_process | 1 | 0 |  |  |  |  |  |  |  |  |
| GO:0008615\_pyridoxine\_biosynthetic\_process | 1 | 0 |  |  |  |  |  |  |  |  |
| GO:0008627\_induction\_of\_apoptosis\_by\_ionic\_changes | 1 | 0 |  |  |  |  |  |  |  |  |
| GO:0008655\_pyrimidine\_salvage | 1 | 0 |  |  |  |  |  |  |  |  |
| GO:0009052\_pentose-phosphate\_shunt\_\_non-oxidative\_branch | 1 | 0 |  |  |  |  |  |  |  |  |
| GO:0009067\_aspartate\_family\_amino\_acid\_biosynthetic\_process | 1 | 0 |  |  |  |  |  |  |  |  |
| GO:0009075\_histidine\_family\_amino\_acid\_metabolic\_process | 1 | 0 |  |  |  |  |  |  |  |  |
| GO:0009128\_purine\_nucleoside\_monophosphate\_catabolic\_process | 1 | 0 |  |  |  |  |  |  |  |  |
| GO:0009129\_pyrimidine\_nucleoside\_monophosphate\_metabolic\_process | 1 | 0 |  |  |  |  |  |  |  |  |
| GO:0009130\_pyrimidine\_nucleoside\_monophosphate\_biosynthetic\_process | 1 | 0 |  |  |  |  |  |  |  |  |
| GO:0009133\_nucleoside\_diphosphate\_biosynthetic\_process | 1 | 0 |  |  |  |  |  |  |  |  |
| GO:0009135\_purine\_nucleoside\_diphosphate\_metabolic\_process | 1 | 0 |  |  |  |  |  |  |  |  |
| GO:0009136\_purine\_nucleoside\_diphosphate\_biosynthetic\_process | 1 | 0 |  |  |  |  |  |  |  |  |
| GO:0009138\_pyrimidine\_nucleoside\_diphosphate\_metabolic\_process | 1 | 0 |  |  |  |  |  |  |  |  |
| GO:0009140\_pyrimidine\_nucleoside\_diphosphate\_catabolic\_process | 1 | 0 |  |  |  |  |  |  |  |  |
| GO:0009147\_pyrimidine\_nucleoside\_triphosphate\_metabolic\_process | 1 | 0 |  |  |  |  |  |  |  |  |
| GO:0009148\_pyrimidine\_nucleoside\_triphosphate\_biosynthetic\_process | 1 | 0 |  |  |  |  |  |  |  |  |
| GO:0009153\_purine\_deoxyribonucleotide\_biosynthetic\_process | 1 | 0 |  |  |  |  |  |  |  |  |
| GO:0009157\_deoxyribonucleoside\_monophosphate\_biosynthetic\_process | 1 | 0 |  |  |  |  |  |  |  |  |
| GO:0009158\_ribonucleoside\_monophosphate\_catabolic\_process | 1 | 0 |  |  |  |  |  |  |  |  |
| GO:0009159\_deoxyribonucleoside\_monophosphate\_catabolic\_process | 1 | 0 |  |  |  |  |  |  |  |  |
| GO:0009169\_purine\_ribonucleoside\_monophosphate\_catabolic\_process | 1 | 0 |  |  |  |  |  |  |  |  |
| GO:0009173\_pyrimidine\_ribonucleoside\_monophosphate\_metabolic\_process | 1 | 0 |  |  |  |  |  |  |  |  |
| GO:0009174\_pyrimidine\_ribonucleoside\_monophosphate\_biosynthetic\_process | 1 | 0 |  |  |  |  |  |  |  |  |
| GO:0009179\_purine\_ribonucleoside\_diphosphate\_metabolic\_process | 1 | 0 |  |  |  |  |  |  |  |  |
| GO:0009180\_purine\_ribonucleoside\_diphosphate\_biosynthetic\_process | 1 | 0 |  |  |  |  |  |  |  |  |
| GO:0009182\_purine\_deoxyribonucleoside\_diphosphate\_metabolic\_process | 1 | 0 |  |  |  |  |  |  |  |  |
| GO:0009183\_purine\_deoxyribonucleoside\_diphosphate\_biosynthetic\_process | 1 | 0 |  |  |  |  |  |  |  |  |
| GO:0009186\_deoxyribonucleoside\_diphosphate\_metabolic\_process | 1 | 0 |  |  |  |  |  |  |  |  |
| GO:0009188\_ribonucleoside\_diphosphate\_biosynthetic\_process | 1 | 0 |  |  |  |  |  |  |  |  |
| GO:0009189\_deoxyribonucleoside\_diphosphate\_biosynthetic\_process | 1 | 0 |  |  |  |  |  |  |  |  |
| GO:0009193\_pyrimidine\_ribonucleoside\_diphosphate\_metabolic\_process | 1 | 0 |  |  |  |  |  |  |  |  |
| GO:0009195\_pyrimidine\_ribonucleoside\_diphosphate\_catabolic\_process | 1 | 0 |  |  |  |  |  |  |  |  |
| GO:0009208\_pyrimidine\_ribonucleoside\_triphosphate\_metabolic\_process | 1 | 0 |  |  |  |  |  |  |  |  |
| GO:0009209\_pyrimidine\_ribonucleoside\_triphosphate\_biosynthetic\_process | 1 | 0 |  |  |  |  |  |  |  |  |
| GO:0009214\_cyclic\_nucleotide\_catabolic\_process | 1 | 0 |  |  |  |  |  |  |  |  |
| GO:0009222\_pyrimidine\_ribonucleotide\_catabolic\_process | 1 | 0 |  |  |  |  |  |  |  |  |
| GO:0009231\_riboflavin\_biosynthetic\_process | 1 | 0 |  |  |  |  |  |  |  |  |
| GO:0009253\_peptidoglycan\_catabolic\_process | 1 | 0 |  |  |  |  |  |  |  |  |
| GO:0009256\_10-formyltetrahydrofolate\_metabolic\_process | 1 | 0 |  |  |  |  |  |  |  |  |
| GO:0009258\_10-formyltetrahydrofolate\_catabolic\_process | 1 | 0 |  |  |  |  |  |  |  |  |
| GO:0009265\_2'-deoxyribonucleotide\_biosynthetic\_process | 1 | 0 |  |  |  |  |  |  |  |  |
| GO:0009292\_genetic\_transfer | 1 | 0 |  |  |  |  |  |  |  |  |
| GO:0009294\_DNA\_mediated\_transformation | 1 | 0 |  |  |  |  |  |  |  |  |
| GO:0009296\_flagellum\_assembly | 1 | 0 |  |  |  |  |  |  |  |  |
| GO:0009298\_GDP-mannose\_biosynthetic\_process | 1 | 0 |  |  |  |  |  |  |  |  |
| GO:0009304\_tRNA\_transcription | 1 | 0 |  |  |  |  |  |  |  |  |
| GO:0009313\_oligosaccharide\_catabolic\_process | 1 | 0 |  |  |  |  |  |  |  |  |
| GO:0009372\_quorum\_sensing | 1 | 0 |  |  |  |  |  |  |  |  |
| GO:0009386\_translational\_attenuation | 1 | 0 |  |  |  |  |  |  |  |  |
| GO:0009397\_folic\_acid\_and\_derivative\_catabolic\_process | 1 | 0 |  |  |  |  |  |  |  |  |
| GO:0009399\_nitrogen\_fixation | 1 | 0 |  |  |  |  |  |  |  |  |
| GO:0009404\_toxin\_metabolic\_process | 1 | 0 |  |  |  |  |  |  |  |  |
| GO:0009435\_NAD\_biosynthetic\_process | 1 | 0 |  |  |  |  |  |  |  |  |
| GO:0009437\_carnitine\_metabolic\_process | 1 | 0 |  |  |  |  |  |  |  |  |
| GO:0009441\_glycolate\_metabolic\_process | 1 | 0 |  |  |  |  |  |  |  |  |
| GO:0009624\_response\_to\_nematode | 1 | 0 |  |  |  |  |  |  |  |  |
| GO:0009642\_response\_to\_light\_intensity | 1 | 0 |  |  |  |  |  |  |  |  |
| GO:0009648\_photoperiodism | 1 | 0 |  |  |  |  |  |  |  |  |
| GO:0009720\_detection\_of\_hormone\_stimulus | 1 | 0 |  |  |  |  |  |  |  |  |
| GO:0009726\_detection\_of\_endogenous\_stimulus | 1 | 0 |  |  |  |  |  |  |  |  |
| GO:0009730\_detection\_of\_carbohydrate\_stimulus | 1 | 0 |  |  |  |  |  |  |  |  |
| GO:0009732\_detection\_of\_hexose\_stimulus | 1 | 0 |  |  |  |  |  |  |  |  |
| GO:0009826\_unidimensional\_cell\_growth | 1 | 0 |  |  |  |  |  |  |  |  |
| GO:0009912\_auditory\_receptor\_cell\_fate\_commitment | 1 | 0 |  |  |  |  |  |  |  |  |
| GO:0009954\_proximal\_distal\_pattern\_formation | 1 | 0 |  |  |  |  |  |  |  |  |
| GO:0009972\_cytidine\_deamination | 1 | 0 |  |  |  |  |  |  |  |  |
| GO:0010107\_potassium\_ion\_import | 1 | 0 |  |  |  |  |  |  |  |  |
| GO:0010259\_multicellular\_organismal\_aging | 1 | 0 |  |  |  |  |  |  |  |  |
| GO:0010269\_response\_to\_selenium\_ion | 1 | 0 |  |  |  |  |  |  |  |  |
| GO:0010273\_detoxification\_of\_copper\_ion | 1 | 0 |  |  |  |  |  |  |  |  |
| GO:0010383\_cell\_wall\_polysaccharide\_metabolic\_process | 1 | 0 |  |  |  |  |  |  |  |  |
| GO:0010430\_fatty\_acid\_omega-oxidation | 1 | 0 |  |  |  |  |  |  |  |  |
| GO:0010463\_mesenchymal\_cell\_proliferation | 1 | 0 |  |  |  |  |  |  |  |  |
| GO:0010464\_regulation\_of\_mesenchymal\_cell\_proliferation | 1 | 0 |  |  |  |  |  |  |  |  |
| GO:0010507\_negative\_regulation\_of\_autophagy | 1 | 0 |  |  |  |  |  |  |  |  |
| GO:0010509\_polyamine\_homeostasis | 1 | 0 |  |  |  |  |  |  |  |  |
| GO:0010534\_regulation\_of\_activation\_of\_JAK2\_kinase\_activity | 1 | 0 |  |  |  |  |  |  |  |  |
| GO:0010535\_positive\_regulation\_of\_activation\_of\_JAK2\_kinase\_activity | 1 | 0 |  |  |  |  |  |  |  |  |
| GO:0010561\_negative\_regulation\_of\_glycoprotein\_biosynthetic\_process | 1 | 0 |  |  |  |  |  |  |  |  |
| GO:0010569\_regulation\_of\_double-strand\_break\_repair\_via\_homologous\_recombination | 1 | 0 |  |  |  |  |  |  |  |  |
| GO:0010591\_regulation\_of\_lamellipodium\_assembly | 1 | 0 |  |  |  |  |  |  |  |  |
| GO:0010592\_positive\_regulation\_of\_lamellipodium\_assembly | 1 | 0 |  |  |  |  |  |  |  |  |
| GO:0010621\_negative\_regulation\_of\_transcription\_by\_transcription\_factor\_localization | 1 | 0 |  |  |  |  |  |  |  |  |
| GO:0010623\_developmental\_programmed\_cell\_death | 1 | 0 |  |  |  |  |  |  |  |  |
| GO:0010631\_epithelial\_cell\_migration | 1 | 0 |  |  |  |  |  |  |  |  |
| GO:0010632\_regulation\_of\_epithelial\_cell\_migration | 1 | 0 |  |  |  |  |  |  |  |  |
| GO:0010634\_positive\_regulation\_of\_epithelial\_cell\_migration | 1 | 0 |  |  |  |  |  |  |  |  |
| GO:0010658\_striated\_muscle\_cell\_apoptosis | 1 | 0 |  |  |  |  |  |  |  |  |
| GO:0010659\_cardiac\_muscle\_cell\_apoptosis | 1 | 0 |  |  |  |  |  |  |  |  |
| GO:0010662\_regulation\_of\_striated\_muscle\_cell\_apoptosis | 1 | 0 |  |  |  |  |  |  |  |  |
| GO:0010664\_negative\_regulation\_of\_striated\_muscle\_cell\_apoptosis | 1 | 0 |  |  |  |  |  |  |  |  |
| GO:0010665\_regulation\_of\_cardiac\_muscle\_cell\_apoptosis | 1 | 0 |  |  |  |  |  |  |  |  |
| GO:0010667\_negative\_regulation\_of\_cardiac\_muscle\_cell\_apoptosis | 1 | 0 |  |  |  |  |  |  |  |  |
| GO:0010669\_epithelial\_structure\_maintenance | 1 | 0 |  |  |  |  |  |  |  |  |
| GO:0010692\_regulation\_of\_alkaline\_phosphatase\_activity | 1 | 0 |  |  |  |  |  |  |  |  |
| GO:0010693\_negative\_regulation\_of\_alkaline\_phosphatase\_activity | 1 | 0 |  |  |  |  |  |  |  |  |
| GO:0010710\_regulation\_of\_collagen\_catabolic\_process | 1 | 0 |  |  |  |  |  |  |  |  |
| GO:0010711\_negative\_regulation\_of\_collagen\_catabolic\_process | 1 | 0 |  |  |  |  |  |  |  |  |
| GO:0010715\_regulation\_of\_extracellular\_matrix\_disassembly | 1 | 0 |  |  |  |  |  |  |  |  |
| GO:0010716\_negative\_regulation\_of\_extracellular\_matrix\_disassembly | 1 | 0 |  |  |  |  |  |  |  |  |
| GO:0010719\_negative\_regulation\_of\_epithelial\_to\_mesenchymal\_transition | 1 | 0 |  |  |  |  |  |  |  |  |
| GO:0010722\_regulation\_of\_ferrochelatase\_activity | 1 | 0 |  |  |  |  |  |  |  |  |
| GO:0010731\_protein\_amino\_acid\_glutathionylation | 1 | 0 |  |  |  |  |  |  |  |  |
| GO:0010732\_regulation\_of\_protein\_amino\_acid\_glutathionylation | 1 | 0 |  |  |  |  |  |  |  |  |
| GO:0010734\_negative\_regulation\_of\_protein\_amino\_acid\_glutathionylation | 1 | 0 |  |  |  |  |  |  |  |  |
| GO:0010735\_positive\_regulation\_of\_transcription\_via\_serum\_response\_element\_binding | 1 | 0 |  |  |  |  |  |  |  |  |
| GO:0010737\_protein\_kinase\_A\_signaling\_cascade | 1 | 0 |  |  |  |  |  |  |  |  |
| GO:0010738\_regulation\_of\_protein\_kinase\_A\_signaling\_cascade | 1 | 0 |  |  |  |  |  |  |  |  |
| GO:0010739\_positive\_regulation\_of\_protein\_kinase\_A\_signaling\_cascade | 1 | 0 |  |  |  |  |  |  |  |  |
| GO:0010749\_regulation\_of\_nitric\_oxide\_mediated\_signal\_transduction | 1 | 0 |  |  |  |  |  |  |  |  |
| GO:0010751\_negative\_regulation\_of\_nitric\_oxide\_mediated\_signal\_transduction | 1 | 0 |  |  |  |  |  |  |  |  |
| GO:0010752\_regulation\_of\_cGMP-mediated\_signaling | 1 | 0 |  |  |  |  |  |  |  |  |
| GO:0010754\_negative\_regulation\_of\_cGMP-mediated\_signaling | 1 | 0 |  |  |  |  |  |  |  |  |
| GO:0010756\_positive\_regulation\_of\_plasminogen\_activation | 1 | 0 |  |  |  |  |  |  |  |  |
| GO:0010757\_negative\_regulation\_of\_plasminogen\_activation | 1 | 0 |  |  |  |  |  |  |  |  |
| GO:0010758\_regulation\_of\_macrophage\_chemotaxis | 1 | 0 |  |  |  |  |  |  |  |  |
| GO:0010759\_positive\_regulation\_of\_macrophage\_chemotaxis | 1 | 0 |  |  |  |  |  |  |  |  |
| GO:0010766\_negative\_regulation\_of\_sodium\_ion\_transport | 1 | 0 |  |  |  |  |  |  |  |  |
| GO:0010767\_regulation\_of\_transcription\_from\_RNA\_polymerase\_II\_promoter\_in\_response\_to\_UV-induced\_DNA\_damage | 1 | 0 |  |  |  |  |  |  |  |  |
| GO:0010768\_negative\_regulation\_of\_transcription\_from\_RNA\_polymerase\_II\_promoter\_in\_response\_to\_UV-induced\_DNA\_damage | 1 | 0 |  |  |  |  |  |  |  |  |
| GO:0010771\_negative\_regulation\_of\_cell\_morphogenesis\_involved\_in\_differentiation | 1 | 0 |  |  |  |  |  |  |  |  |
| GO:0010793\_regulation\_of\_mRNA\_export\_from\_nucleus | 1 | 0 |  |  |  |  |  |  |  |  |
| GO:0010801\_negative\_regulation\_of\_peptidyl-threonine\_phosphorylation | 1 | 0 |  |  |  |  |  |  |  |  |
| GO:0010803\_regulation\_of\_tumor\_necrosis\_factor-mediated\_signaling\_pathway | 1 | 0 |  |  |  |  |  |  |  |  |
| GO:0010804\_negative\_regulation\_of\_tumor\_necrosis\_factor-mediated\_signaling\_pathway | 1 | 0 |  |  |  |  |  |  |  |  |
| GO:0010813\_neuropeptide\_catabolic\_process | 1 | 0 |  |  |  |  |  |  |  |  |
| GO:0010814\_substance\_P\_catabolic\_process | 1 | 0 |  |  |  |  |  |  |  |  |
| GO:0010816\_calcitonin\_catabolic\_process | 1 | 0 |  |  |  |  |  |  |  |  |
| GO:0010826\_negative\_regulation\_of\_centrosome\_duplication | 1 | 0 |  |  |  |  |  |  |  |  |
| GO:0010830\_regulation\_of\_myotube\_differentiation | 1 | 0 |  |  |  |  |  |  |  |  |
| GO:0010832\_negative\_regulation\_of\_myotube\_differentiation | 1 | 0 |  |  |  |  |  |  |  |  |
| GO:0010835\_regulation\_of\_protein\_amino\_acid\_ADP-ribosylation | 1 | 0 |  |  |  |  |  |  |  |  |
| GO:0010836\_negative\_regulation\_of\_protein\_amino\_acid\_ADP-ribosylation | 1 | 0 |  |  |  |  |  |  |  |  |
| GO:0010837\_regulation\_of\_keratinocyte\_proliferation | 1 | 0 |  |  |  |  |  |  |  |  |
| GO:0010839\_negative\_regulation\_of\_keratinocyte\_proliferation | 1 | 0 |  |  |  |  |  |  |  |  |
| GO:0010840\_regulation\_of\_circadian\_sleep\_wake\_cycle\_\_wakefulness | 1 | 0 |  |  |  |  |  |  |  |  |
| GO:0010841\_positive\_regulation\_of\_circadian\_sleep\_wake\_cycle\_\_wakefulness | 1 | 0 |  |  |  |  |  |  |  |  |
| GO:0010842\_retina\_layer\_formation | 1 | 0 |  |  |  |  |  |  |  |  |
| GO:0010897\_negative\_regulation\_of\_triglyceride\_catabolic\_process | 1 | 0 |  |  |  |  |  |  |  |  |
| GO:0010899\_regulation\_of\_phosphatidylcholine\_catabolic\_process | 1 | 0 |  |  |  |  |  |  |  |  |
| GO:0010900\_negative\_regulation\_of\_phosphatidylcholine\_catabolic\_process | 1 | 0 |  |  |  |  |  |  |  |  |
| GO:0010902\_positive\_regulation\_of\_very-low-density\_lipoprotein\_particle\_remodeling | 1 | 0 |  |  |  |  |  |  |  |  |
| GO:0010919\_regulation\_of\_inositol\_phosphate\_biosynthetic\_process | 1 | 0 |  |  |  |  |  |  |  |  |
| GO:0010920\_negative\_regulation\_of\_inositol\_phosphate\_biosynthetic\_process | 1 | 0 |  |  |  |  |  |  |  |  |
| GO:0010924\_regulation\_of\_inositol-polyphosphate\_5-phosphatase\_activity | 1 | 0 |  |  |  |  |  |  |  |  |
| GO:0010925\_positive\_regulation\_of\_inositol-polyphosphate\_5-phosphatase\_activity | 1 | 0 |  |  |  |  |  |  |  |  |
| GO:0010931\_macrophage\_tolerance\_induction | 1 | 0 |  |  |  |  |  |  |  |  |
| GO:0010932\_regulation\_of\_macrophage\_tolerance\_induction | 1 | 0 |  |  |  |  |  |  |  |  |
| GO:0010933\_positive\_regulation\_of\_macrophage\_tolerance\_induction | 1 | 0 |  |  |  |  |  |  |  |  |
| GO:0010934\_macrophage\_cytokine\_production | 1 | 0 |  |  |  |  |  |  |  |  |
| GO:0010935\_regulation\_of\_macrophage\_cytokine\_production | 1 | 0 |  |  |  |  |  |  |  |  |
| GO:0010936\_negative\_regulation\_of\_macrophage\_cytokine\_production | 1 | 0 |  |  |  |  |  |  |  |  |
| GO:0010944\_negative\_regulation\_of\_transcription\_by\_competitive\_promoter\_binding | 1 | 0 |  |  |  |  |  |  |  |  |
| GO:0010983\_positive\_regulation\_of\_high-density\_lipoprotein\_particle\_clearance | 1 | 0 |  |  |  |  |  |  |  |  |
| GO:0010986\_positive\_regulation\_of\_lipoprotein\_particle\_clearance | 1 | 0 |  |  |  |  |  |  |  |  |
| GO:0010987\_negative\_regulation\_of\_high-density\_lipoprotein\_particle\_clearance | 1 | 0 |  |  |  |  |  |  |  |  |
| GO:0010988\_regulation\_of\_low-density\_lipoprotein\_particle\_clearance | 1 | 0 |  |  |  |  |  |  |  |  |
| GO:0010989\_negative\_regulation\_of\_low-density\_lipoprotein\_particle\_clearance | 1 | 0 |  |  |  |  |  |  |  |  |
| GO:0010990\_regulation\_of\_SMAD\_protein\_complex\_assembly | 1 | 0 |  |  |  |  |  |  |  |  |
| GO:0010991\_negative\_regulation\_of\_SMAD\_protein\_complex\_assembly | 1 | 0 |  |  |  |  |  |  |  |  |
| GO:0014009\_glial\_cell\_proliferation | 1 | 0 |  |  |  |  |  |  |  |  |
| GO:0014010\_Schwann\_cell\_proliferation | 1 | 0 |  |  |  |  |  |  |  |  |
| GO:0014045\_establishment\_of\_endothelial\_blood-brain\_barrier | 1 | 0 |  |  |  |  |  |  |  |  |
| GO:0014055\_acetylcholine\_secretion | 1 | 0 |  |  |  |  |  |  |  |  |
| GO:0014056\_regulation\_of\_acetylcholine\_secretion | 1 | 0 |  |  |  |  |  |  |  |  |
| GO:0014060\_regulation\_of\_epinephrine\_secretion | 1 | 0 |  |  |  |  |  |  |  |  |
| GO:0014067\_negative\_regulation\_of\_phosphoinositide\_3-kinase\_cascade | 1 | 0 |  |  |  |  |  |  |  |  |
| GO:0014071\_response\_to\_cycloalkane | 1 | 0 |  |  |  |  |  |  |  |  |
| GO:0014721\_twitch\_skeletal\_muscle\_contraction | 1 | 0 |  |  |  |  |  |  |  |  |
| GO:0014724\_regulation\_of\_twitch\_skeletal\_muscle\_contraction | 1 | 0 |  |  |  |  |  |  |  |  |
| GO:0014806\_smooth\_muscle\_hyperplasia | 1 | 0 |  |  |  |  |  |  |  |  |
| GO:0014823\_response\_to\_activity | 1 | 0 |  |  |  |  |  |  |  |  |
| GO:0014832\_urinary\_bladder\_smooth\_muscle\_contraction | 1 | 0 |  |  |  |  |  |  |  |  |
| GO:0014834\_satellite\_cell\_maintenance\_involved\_in\_skeletal\_muscle\_regeneration | 1 | 0 |  |  |  |  |  |  |  |  |
| GO:0014848\_urinary\_tract\_smooth\_muscle\_contraction | 1 | 0 |  |  |  |  |  |  |  |  |
| GO:0014850\_response\_to\_muscle\_activity | 1 | 0 |  |  |  |  |  |  |  |  |
| GO:0014873\_response\_to\_muscle\_activity\_involved\_in\_regulation\_of\_muscle\_adaptation | 1 | 0 |  |  |  |  |  |  |  |  |
| GO:0014874\_response\_to\_stimulus\_involved\_in\_regulation\_of\_muscle\_adaptation | 1 | 0 |  |  |  |  |  |  |  |  |
| GO:0014895\_smooth\_muscle\_hypertrophy | 1 | 0 |  |  |  |  |  |  |  |  |
| GO:0014904\_myotube\_cell\_development | 1 | 0 |  |  |  |  |  |  |  |  |
| GO:0014916\_regulation\_of\_lung\_blood\_pressure | 1 | 0 |  |  |  |  |  |  |  |  |
| GO:0015675\_nickel\_ion\_transport | 1 | 0 |  |  |  |  |  |  |  |  |
| GO:0015676\_vanadium\_ion\_transport | 1 | 0 |  |  |  |  |  |  |  |  |
| GO:0015680\_intracellular\_copper\_ion\_transport | 1 | 0 |  |  |  |  |  |  |  |  |
| GO:0015684\_ferrous\_iron\_transport | 1 | 0 |  |  |  |  |  |  |  |  |
| GO:0015692\_lead\_ion\_transport | 1 | 0 |  |  |  |  |  |  |  |  |
| GO:0015693\_magnesium\_ion\_transport | 1 | 0 |  |  |  |  |  |  |  |  |
| GO:0015727\_lactate\_transport | 1 | 0 |  |  |  |  |  |  |  |  |
| GO:0015728\_mevalonate\_transport | 1 | 0 |  |  |  |  |  |  |  |  |
| GO:0015742\_alpha-ketoglutarate\_transport | 1 | 0 |  |  |  |  |  |  |  |  |
| GO:0015746\_citrate\_transport | 1 | 0 |  |  |  |  |  |  |  |  |
| GO:0015747\_urate\_transport | 1 | 0 |  |  |  |  |  |  |  |  |
| GO:0015755\_fructose\_transport | 1 | 0 |  |  |  |  |  |  |  |  |
| GO:0015760\_glucose-6-phosphate\_transport | 1 | 0 |  |  |  |  |  |  |  |  |
| GO:0015782\_CMP-sialic\_acid\_transport | 1 | 0 |  |  |  |  |  |  |  |  |
| GO:0015785\_UDP-galactose\_transport | 1 | 0 |  |  |  |  |  |  |  |  |
| GO:0015789\_UDP-N-acetylgalactosamine\_transport | 1 | 0 |  |  |  |  |  |  |  |  |
| GO:0015790\_UDP-xylose\_transport | 1 | 0 |  |  |  |  |  |  |  |  |
| GO:0015798\_myo-inositol\_transport | 1 | 0 |  |  |  |  |  |  |  |  |
| GO:0015803\_branched-chain\_aliphatic\_amino\_acid\_transport | 1 | 0 |  |  |  |  |  |  |  |  |
| GO:0015805\_S-adenosylmethionine\_transport | 1 | 0 |  |  |  |  |  |  |  |  |
| GO:0015809\_arginine\_transport | 1 | 0 |  |  |  |  |  |  |  |  |
| GO:0015817\_histidine\_transport | 1 | 0 |  |  |  |  |  |  |  |  |
| GO:0015820\_leucine\_transport | 1 | 0 |  |  |  |  |  |  |  |  |
| GO:0015826\_threonine\_transport | 1 | 0 |  |  |  |  |  |  |  |  |
| GO:0015827\_tryptophan\_transport | 1 | 0 |  |  |  |  |  |  |  |  |
| GO:0015846\_polyamine\_transport | 1 | 0 |  |  |  |  |  |  |  |  |
| GO:0015853\_adenine\_transport | 1 | 0 |  |  |  |  |  |  |  |  |
| GO:0015855\_pyrimidine\_transport | 1 | 0 |  |  |  |  |  |  |  |  |
| GO:0015886\_heme\_transport | 1 | 0 |  |  |  |  |  |  |  |  |
| GO:0015888\_thiamin\_transport | 1 | 0 |  |  |  |  |  |  |  |  |
| GO:0015910\_peroxisomal\_long-chain\_fatty\_acid\_import | 1 | 0 |  |  |  |  |  |  |  |  |
| GO:0015919\_peroxisomal\_membrane\_transport | 1 | 0 |  |  |  |  |  |  |  |  |
| GO:0015937\_coenzyme\_A\_biosynthetic\_process | 1 | 0 |  |  |  |  |  |  |  |  |
| GO:0015956\_bis(5'-nucleosidyl)\_oligophosphate\_metabolic\_process | 1 | 0 |  |  |  |  |  |  |  |  |
| GO:0015958\_bis(5'-nucleosidyl)\_oligophosphate\_catabolic\_process | 1 | 0 |  |  |  |  |  |  |  |  |
| GO:0015959\_diadenosine\_polyphosphate\_metabolic\_process | 1 | 0 |  |  |  |  |  |  |  |  |
| GO:0015961\_diadenosine\_polyphosphate\_catabolic\_process | 1 | 0 |  |  |  |  |  |  |  |  |
| GO:0016046\_detection\_of\_fungus | 1 | 0 |  |  |  |  |  |  |  |  |
| GO:0016078\_tRNA\_catabolic\_process | 1 | 0 |  |  |  |  |  |  |  |  |
| GO:0016091\_prenol\_biosynthetic\_process | 1 | 0 |  |  |  |  |  |  |  |  |
| GO:0016094\_polyprenol\_biosynthetic\_process | 1 | 0 |  |  |  |  |  |  |  |  |
| GO:0016108\_tetraterpenoid\_metabolic\_process | 1 | 0 |  |  |  |  |  |  |  |  |
| GO:0016116\_carotenoid\_metabolic\_process | 1 | 0 |  |  |  |  |  |  |  |  |
| GO:0016119\_carotene\_metabolic\_process | 1 | 0 |  |  |  |  |  |  |  |  |
| GO:0016140\_O-glycoside\_metabolic\_process | 1 | 0 |  |  |  |  |  |  |  |  |
| GO:0016142\_O-glycoside\_catabolic\_process | 1 | 0 |  |  |  |  |  |  |  |  |
| GO:0016188\_synaptic\_vesicle\_maturation | 1 | 0 |  |  |  |  |  |  |  |  |
| GO:0016189\_synaptic\_vesicle\_to\_endosome\_fusion | 1 | 0 |  |  |  |  |  |  |  |  |
| GO:0016241\_regulation\_of\_macroautophagy | 1 | 0 |  |  |  |  |  |  |  |  |
| GO:0016242\_negative\_regulation\_of\_macroautophagy | 1 | 0 |  |  |  |  |  |  |  |  |
| GO:0016259\_selenocysteine\_metabolic\_process | 1 | 0 |  |  |  |  |  |  |  |  |
| GO:0016260\_selenocysteine\_biosynthetic\_process | 1 | 0 |  |  |  |  |  |  |  |  |
| GO:0016269\_O-glycan\_processing\_\_core\_3 | 1 | 0 |  |  |  |  |  |  |  |  |
| GO:0016320\_endoplasmic\_reticulum\_membrane\_fusion | 1 | 0 |  |  |  |  |  |  |  |  |
| GO:0016344\_meiotic\_chromosome\_movement\_towards\_spindle\_pole | 1 | 0 |  |  |  |  |  |  |  |  |
| GO:0016446\_somatic\_hypermutation\_of\_immunoglobulin\_genes | 1 | 0 |  |  |  |  |  |  |  |  |
| GO:0016559\_peroxisome\_fission | 1 | 0 |  |  |  |  |  |  |  |  |
| GO:0016560\_protein\_import\_into\_peroxisome\_matrix\_\_docking | 1 | 0 |  |  |  |  |  |  |  |  |
| GO:0016598\_protein\_arginylation | 1 | 0 |  |  |  |  |  |  |  |  |
| GO:0016998\_cell\_wall\_macromolecule\_catabolic\_process | 1 | 0 |  |  |  |  |  |  |  |  |
| GO:0017062\_respiratory\_chain\_complex\_III\_assembly | 1 | 0 |  |  |  |  |  |  |  |  |
| GO:0017185\_peptidyl-lysine\_hydroxylation | 1 | 0 |  |  |  |  |  |  |  |  |
| GO:0018095\_protein\_polyglutamylation | 1 | 0 |  |  |  |  |  |  |  |  |
| GO:0018125\_peptidyl-cysteine\_methylation | 1 | 0 |  |  |  |  |  |  |  |  |
| GO:0018126\_protein\_amino\_acid\_hydroxylation | 1 | 0 |  |  |  |  |  |  |  |  |
| GO:0018146\_keratan\_sulfate\_biosynthetic\_process | 1 | 0 |  |  |  |  |  |  |  |  |
| GO:0018153\_isopeptide\_cross-linking\_via\_N6-(L-isoglutamyl)-L-lysine | 1 | 0 |  |  |  |  |  |  |  |  |
| GO:0018184\_protein\_amino\_acid\_polyamination | 1 | 0 |  |  |  |  |  |  |  |  |
| GO:0018190\_protein\_amino\_acid\_octanoylation | 1 | 0 |  |  |  |  |  |  |  |  |
| GO:0018191\_peptidyl-serine\_octanoylation | 1 | 0 |  |  |  |  |  |  |  |  |
| GO:0018192\_enzyme\_active\_site\_formation\_via\_L-cysteine\_persulfide | 1 | 0 |  |  |  |  |  |  |  |  |
| GO:0018199\_peptidyl-glutamine\_modification | 1 | 0 |  |  |  |  |  |  |  |  |
| GO:0018200\_peptidyl-glutamic\_acid\_modification | 1 | 0 |  |  |  |  |  |  |  |  |
| GO:0018208\_peptidyl-proline\_modification | 1 | 0 |  |  |  |  |  |  |  |  |
| GO:0018262\_isopeptide\_cross-linking | 1 | 0 |  |  |  |  |  |  |  |  |
| GO:0018277\_protein\_amino\_acid\_deamination | 1 | 0 |  |  |  |  |  |  |  |  |
| GO:0018307\_enzyme\_active\_site\_formation | 1 | 0 |  |  |  |  |  |  |  |  |
| GO:0018318\_protein\_amino\_acid\_palmitoylation | 1 | 0 |  |  |  |  |  |  |  |  |
| GO:0018319\_protein\_amino\_acid\_myristoylation | 1 | 0 |  |  |  |  |  |  |  |  |
| GO:0018345\_protein\_palmitoylation | 1 | 0 |  |  |  |  |  |  |  |  |
| GO:0018350\_protein\_amino\_acid\_esterification | 1 | 0 |  |  |  |  |  |  |  |  |
| GO:0018352\_protein-pyridoxal-5-phosphate\_linkage | 1 | 0 |  |  |  |  |  |  |  |  |
| GO:0018377\_protein\_myristoylation | 1 | 0 |  |  |  |  |  |  |  |  |
| GO:0018395\_peptidyl-lysine\_hydroxylation\_to\_5-hydroxy-L-lysine | 1 | 0 |  |  |  |  |  |  |  |  |
| GO:0018401\_peptidyl-proline\_hydroxylation\_to\_4-hydroxy-L-proline | 1 | 0 |  |  |  |  |  |  |  |  |
| GO:0018872\_arsonoacetate\_metabolic\_process | 1 | 0 |  |  |  |  |  |  |  |  |
| GO:0018874\_benzoate\_metabolic\_process | 1 | 0 |  |  |  |  |  |  |  |  |
| GO:0019060\_intracellular\_transport\_of\_viral\_proteins\_in\_host\_cell | 1 | 0 |  |  |  |  |  |  |  |  |
| GO:0019064\_viral\_envelope\_fusion\_with\_host\_membrane | 1 | 0 |  |  |  |  |  |  |  |  |
| GO:0019086\_late\_viral\_mRNA\_transcription | 1 | 0 |  |  |  |  |  |  |  |  |
| GO:0019087\_transformation\_of\_host\_cell\_by\_virus | 1 | 0 |  |  |  |  |  |  |  |  |
| GO:0019089\_transmission\_of\_virus | 1 | 0 |  |  |  |  |  |  |  |  |
| GO:0019098\_reproductive\_behavior | 1 | 0 |  |  |  |  |  |  |  |  |
| GO:0019240\_citrulline\_biosynthetic\_process | 1 | 0 |  |  |  |  |  |  |  |  |
| GO:0019302\_D-ribose\_biosynthetic\_process | 1 | 0 |  |  |  |  |  |  |  |  |
| GO:0019303\_D-ribose\_catabolic\_process | 1 | 0 |  |  |  |  |  |  |  |  |
| GO:0019307\_mannose\_biosynthetic\_process | 1 | 0 |  |  |  |  |  |  |  |  |
| GO:0019310\_inositol\_catabolic\_process | 1 | 0 |  |  |  |  |  |  |  |  |
| GO:0019322\_pentose\_biosynthetic\_process | 1 | 0 |  |  |  |  |  |  |  |  |
| GO:0019323\_pentose\_catabolic\_process | 1 | 0 |  |  |  |  |  |  |  |  |
| GO:0019371\_cyclooxygenase\_pathway | 1 | 0 |  |  |  |  |  |  |  |  |
| GO:0019372\_lipoxygenase\_pathway | 1 | 0 |  |  |  |  |  |  |  |  |
| GO:0019388\_galactose\_catabolic\_process | 1 | 0 |  |  |  |  |  |  |  |  |
| GO:0019405\_alditol\_catabolic\_process | 1 | 0 |  |  |  |  |  |  |  |  |
| GO:0019407\_hexitol\_catabolic\_process | 1 | 0 |  |  |  |  |  |  |  |  |
| GO:0019408\_dolichol\_biosynthetic\_process | 1 | 0 |  |  |  |  |  |  |  |  |
| GO:0019441\_tryptophan\_catabolic\_process\_to\_kynurenine | 1 | 0 |  |  |  |  |  |  |  |  |
| GO:0019471\_4-hydroxyproline\_metabolic\_process | 1 | 0 |  |  |  |  |  |  |  |  |
| GO:0019511\_peptidyl-proline\_hydroxylation | 1 | 0 |  |  |  |  |  |  |  |  |
| GO:0019519\_pentitol\_metabolic\_process | 1 | 0 |  |  |  |  |  |  |  |  |
| GO:0019527\_pentitol\_catabolic\_process | 1 | 0 |  |  |  |  |  |  |  |  |
| GO:0019614\_catechol\_catabolic\_process | 1 | 0 |  |  |  |  |  |  |  |  |
| GO:0019673\_GDP-mannose\_metabolic\_process | 1 | 0 |  |  |  |  |  |  |  |  |
| GO:0019693\_ribose\_phosphate\_metabolic\_process | 1 | 0 |  |  |  |  |  |  |  |  |
| GO:0019695\_choline\_metabolic\_process | 1 | 0 |  |  |  |  |  |  |  |  |
| GO:0019747\_regulation\_of\_isoprenoid\_metabolic\_process | 1 | 0 |  |  |  |  |  |  |  |  |
| GO:0019852\_L-ascorbic\_acid\_metabolic\_process | 1 | 0 |  |  |  |  |  |  |  |  |
| GO:0019856\_pyrimidine\_base\_biosynthetic\_process | 1 | 0 |  |  |  |  |  |  |  |  |
| GO:0019858\_cytosine\_metabolic\_process | 1 | 0 |  |  |  |  |  |  |  |  |
| GO:0019884\_antigen\_processing\_and\_presentation\_of\_exogenous\_antigen | 1 | 0 |  |  |  |  |  |  |  |  |
| GO:0019886\_antigen\_processing\_and\_presentation\_of\_exogenous\_peptide\_antigen\_via\_MHC\_class\_II | 1 | 0 |  |  |  |  |  |  |  |  |
| GO:0021508\_floor\_plate\_formation | 1 | 0 |  |  |  |  |  |  |  |  |
| GO:0021514\_ventral\_spinal\_cord\_interneuron\_differentiation | 1 | 0 |  |  |  |  |  |  |  |  |
| GO:0021521\_ventral\_spinal\_cord\_interneuron\_specification | 1 | 0 |  |  |  |  |  |  |  |  |
| GO:0021522\_spinal\_cord\_motor\_neuron\_differentiation | 1 | 0 |  |  |  |  |  |  |  |  |
| GO:0021527\_spinal\_cord\_association\_neuron\_differentiation | 1 | 0 |  |  |  |  |  |  |  |  |
| GO:0021528\_commissural\_neuron\_differentiation\_in\_the\_spinal\_cord | 1 | 0 |  |  |  |  |  |  |  |  |
| GO:0021533\_cell\_differentiation\_in\_hindbrain | 1 | 0 |  |  |  |  |  |  |  |  |
| GO:0021540\_corpus\_callosum\_morphogenesis | 1 | 0 |  |  |  |  |  |  |  |  |
| GO:0021544\_subpallium\_development | 1 | 0 |  |  |  |  |  |  |  |  |
| GO:0021554\_optic\_nerve\_development | 1 | 0 |  |  |  |  |  |  |  |  |
| GO:0021562\_vestibulocochlear\_nerve\_development | 1 | 0 |  |  |  |  |  |  |  |  |
| GO:0021602\_cranial\_nerve\_morphogenesis | 1 | 0 |  |  |  |  |  |  |  |  |
| GO:0021631\_optic\_nerve\_morphogenesis | 1 | 0 |  |  |  |  |  |  |  |  |
| GO:0021680\_cerebellar\_Purkinje\_cell\_layer\_development | 1 | 0 |  |  |  |  |  |  |  |  |
| GO:0021692\_cerebellar\_Purkinje\_cell\_layer\_morphogenesis | 1 | 0 |  |  |  |  |  |  |  |  |
| GO:0021694\_cerebellar\_Purkinje\_cell\_layer\_formation | 1 | 0 |  |  |  |  |  |  |  |  |
| GO:0021697\_cerebellar\_cortex\_formation | 1 | 0 |  |  |  |  |  |  |  |  |
| GO:0021702\_cerebellar\_Purkinje\_cell\_differentiation | 1 | 0 |  |  |  |  |  |  |  |  |
| GO:0021756\_striatum\_development | 1 | 0 |  |  |  |  |  |  |  |  |
| GO:0021757\_caudate\_nucleus\_development | 1 | 0 |  |  |  |  |  |  |  |  |
| GO:0021758\_putamen\_development | 1 | 0 |  |  |  |  |  |  |  |  |
| GO:0021761\_limbic\_system\_development | 1 | 0 |  |  |  |  |  |  |  |  |
| GO:0021771\_lateral\_geniculate\_nucleus\_development | 1 | 0 |  |  |  |  |  |  |  |  |
| GO:0021775\_smoothened\_signaling\_pathway\_involved\_in\_ventral\_spinal\_cord\_interneuron\_specification | 1 | 0 |  |  |  |  |  |  |  |  |
| GO:0021794\_thalamus\_development | 1 | 0 |  |  |  |  |  |  |  |  |
| GO:0021799\_cerebral\_cortex\_radially\_oriented\_cell\_migration | 1 | 0 |  |  |  |  |  |  |  |  |
| GO:0021800\_cerebral\_cortex\_tangential\_migration | 1 | 0 |  |  |  |  |  |  |  |  |
| GO:0021854\_hypothalamus\_development | 1 | 0 |  |  |  |  |  |  |  |  |
| GO:0021859\_pyramidal\_neuron\_differentiation | 1 | 0 |  |  |  |  |  |  |  |  |
| GO:0021860\_pyramidal\_neuron\_development | 1 | 0 |  |  |  |  |  |  |  |  |
| GO:0021872\_generation\_of\_neurons\_in\_the\_forebrain | 1 | 0 |  |  |  |  |  |  |  |  |
| GO:0021879\_forebrain\_neuron\_differentiation | 1 | 0 |  |  |  |  |  |  |  |  |
| GO:0021884\_forebrain\_neuron\_development | 1 | 0 |  |  |  |  |  |  |  |  |
| GO:0021896\_forebrain\_astrocyte\_differentiation | 1 | 0 |  |  |  |  |  |  |  |  |
| GO:0021897\_forebrain\_astrocyte\_development | 1 | 0 |  |  |  |  |  |  |  |  |
| GO:0021914\_negative\_regulation\_of\_smoothened\_signaling\_pathway\_involved\_in\_ventral\_spinal\_cord\_patterning | 1 | 0 |  |  |  |  |  |  |  |  |
| GO:0021919\_BMP\_signaling\_pathway\_in\_spinal\_cord\_dorsal\_ventral\_patterning | 1 | 0 |  |  |  |  |  |  |  |  |
| GO:0021965\_spinal\_cord\_ventral\_commissure\_morphogenesis | 1 | 0 |  |  |  |  |  |  |  |  |
| GO:0021984\_adenohypophysis\_development | 1 | 0 |  |  |  |  |  |  |  |  |
| GO:0021990\_neural\_plate\_formation | 1 | 0 |  |  |  |  |  |  |  |  |
| GO:0021997\_neural\_plate\_axis\_specification | 1 | 0 |  |  |  |  |  |  |  |  |
| GO:0021999\_neural\_plate\_anterior\_posterior\_pattern\_formation | 1 | 0 |  |  |  |  |  |  |  |  |
| GO:0022009\_central\_nervous\_system\_vasculogenesis | 1 | 0 |  |  |  |  |  |  |  |  |
| GO:0022038\_corpus\_callosum\_development | 1 | 0 |  |  |  |  |  |  |  |  |
| GO:0030007\_cellular\_potassium\_ion\_homeostasis | 1 | 0 |  |  |  |  |  |  |  |  |
| GO:0030011\_maintenance\_of\_cell\_polarity | 1 | 0 |  |  |  |  |  |  |  |  |
| GO:0030026\_cellular\_manganese\_ion\_homeostasis | 1 | 0 |  |  |  |  |  |  |  |  |
| GO:0030033\_microvillus\_assembly | 1 | 0 |  |  |  |  |  |  |  |  |
| GO:0030037\_actin\_filament\_reorganization\_during\_cell\_cycle | 1 | 0 |  |  |  |  |  |  |  |  |
| GO:0030047\_actin\_modification | 1 | 0 |  |  |  |  |  |  |  |  |
| GO:0030070\_insulin\_processing | 1 | 0 |  |  |  |  |  |  |  |  |
| GO:0030103\_vasopressin\_secretion | 1 | 0 |  |  |  |  |  |  |  |  |
| GO:0030186\_melatonin\_metabolic\_process | 1 | 0 |  |  |  |  |  |  |  |  |
| GO:0030187\_melatonin\_biosynthetic\_process | 1 | 0 |  |  |  |  |  |  |  |  |
| GO:0030212\_hyaluronan\_metabolic\_process | 1 | 0 |  |  |  |  |  |  |  |  |
| GO:0030220\_platelet\_formation | 1 | 0 |  |  |  |  |  |  |  |  |
| GO:0030238\_male\_sex\_determination | 1 | 0 |  |  |  |  |  |  |  |  |
| GO:0030259\_lipid\_glycosylation | 1 | 0 |  |  |  |  |  |  |  |  |
| GO:0030302\_deoxynucleotide\_transport | 1 | 0 |  |  |  |  |  |  |  |  |
| GO:0030327\_prenylated\_protein\_catabolic\_process | 1 | 0 |  |  |  |  |  |  |  |  |
| GO:0030389\_fructosamine\_metabolic\_process | 1 | 0 |  |  |  |  |  |  |  |  |
| GO:0030393\_fructoselysine\_metabolic\_process | 1 | 0 |  |  |  |  |  |  |  |  |
| GO:0030432\_peristalsis | 1 | 0 |  |  |  |  |  |  |  |  |
| GO:0030488\_tRNA\_methylation | 1 | 0 |  |  |  |  |  |  |  |  |
| GO:0030517\_negative\_regulation\_of\_axon\_extension | 1 | 0 |  |  |  |  |  |  |  |  |
| GO:0030581\_symbiont\_intracellular\_protein\_transport\_in\_host | 1 | 0 |  |  |  |  |  |  |  |  |
| GO:0030718\_germ-line\_stem\_cell\_maintenance | 1 | 0 |  |  |  |  |  |  |  |  |
| GO:0030728\_ovulation | 1 | 0 |  |  |  |  |  |  |  |  |
| GO:0030824\_negative\_regulation\_of\_cGMP\_metabolic\_process | 1 | 0 |  |  |  |  |  |  |  |  |
| GO:0030825\_positive\_regulation\_of\_cGMP\_metabolic\_process | 1 | 0 |  |  |  |  |  |  |  |  |
| GO:0030827\_negative\_regulation\_of\_cGMP\_biosynthetic\_process | 1 | 0 |  |  |  |  |  |  |  |  |
| GO:0030828\_positive\_regulation\_of\_cGMP\_biosynthetic\_process | 1 | 0 |  |  |  |  |  |  |  |  |
| GO:0030836\_positive\_regulation\_of\_actin\_filament\_depolymerization | 1 | 0 |  |  |  |  |  |  |  |  |
| GO:0030845\_inhibition\_of\_phospholipase\_C\_activity\_involved\_in\_G-protein\_coupled\_receptor\_signaling\_pathway | 1 | 0 |  |  |  |  |  |  |  |  |
| GO:0030854\_positive\_regulation\_of\_granulocyte\_differentiation | 1 | 0 |  |  |  |  |  |  |  |  |
| GO:0030878\_thyroid\_gland\_development | 1 | 0 |  |  |  |  |  |  |  |  |
| GO:0030885\_regulation\_of\_myeloid\_dendritic\_cell\_activation | 1 | 0 |  |  |  |  |  |  |  |  |
| GO:0030887\_positive\_regulation\_of\_myeloid\_dendritic\_cell\_activation | 1 | 0 |  |  |  |  |  |  |  |  |
| GO:0030903\_notochord\_development | 1 | 0 |  |  |  |  |  |  |  |  |
| GO:0030910\_olfactory\_placode\_formation | 1 | 0 |  |  |  |  |  |  |  |  |
| GO:0030913\_paranodal\_junction\_assembly | 1 | 0 |  |  |  |  |  |  |  |  |
| GO:0030948\_negative\_regulation\_of\_vascular\_endothelial\_growth\_factor\_receptor\_signaling\_pathway | 1 | 0 |  |  |  |  |  |  |  |  |
| GO:0030967\_ER-nuclear\_sterol\_response\_pathway | 1 | 0 |  |  |  |  |  |  |  |  |
| GO:0031017\_exocrine\_pancreas\_development | 1 | 0 |  |  |  |  |  |  |  |  |
| GO:0031063\_regulation\_of\_histone\_deacetylation | 1 | 0 |  |  |  |  |  |  |  |  |
| GO:0031065\_positive\_regulation\_of\_histone\_deacetylation | 1 | 0 |  |  |  |  |  |  |  |  |
| GO:0031076\_embryonic\_camera-type\_eye\_development | 1 | 0 |  |  |  |  |  |  |  |  |
| GO:0031081\_nuclear\_pore\_distribution | 1 | 0 |  |  |  |  |  |  |  |  |
| GO:0031086\_nuclear-transcribed\_mRNA\_catabolic\_process\_\_deadenylation-independent\_decay | 1 | 0 |  |  |  |  |  |  |  |  |
| GO:0031087\_deadenylation-independent\_decapping\_of\_nuclear-transcribed\_mRNA | 1 | 0 |  |  |  |  |  |  |  |  |
| GO:0031106\_septin\_ring\_organization | 1 | 0 |  |  |  |  |  |  |  |  |
| GO:0031115\_negative\_regulation\_of\_microtubule\_polymerization | 1 | 0 |  |  |  |  |  |  |  |  |
| GO:0031117\_positive\_regulation\_of\_microtubule\_depolymerization | 1 | 0 |  |  |  |  |  |  |  |  |
| GO:0031118\_rRNA\_pseudouridine\_synthesis | 1 | 0 |  |  |  |  |  |  |  |  |
| GO:0031125\_rRNA\_3'-end\_processing | 1 | 0 |  |  |  |  |  |  |  |  |
| GO:0031146\_SCF-dependent\_proteasomal\_ubiquitin-dependent\_protein\_catabolic\_process | 1 | 0 |  |  |  |  |  |  |  |  |
| GO:0031179\_peptide\_modification | 1 | 0 |  |  |  |  |  |  |  |  |
| GO:0031282\_regulation\_of\_guanylate\_cyclase\_activity | 1 | 0 |  |  |  |  |  |  |  |  |
| GO:0031284\_positive\_regulation\_of\_guanylate\_cyclase\_activity | 1 | 0 |  |  |  |  |  |  |  |  |
| GO:0031290\_retinal\_ganglion\_cell\_axon\_guidance | 1 | 0 |  |  |  |  |  |  |  |  |
| GO:0031293\_membrane\_protein\_intracellular\_domain\_proteolysis | 1 | 0 |  |  |  |  |  |  |  |  |
| GO:0031335\_regulation\_of\_sulfur\_amino\_acid\_metabolic\_process | 1 | 0 |  |  |  |  |  |  |  |  |
| GO:0031342\_negative\_regulation\_of\_cell\_killing | 1 | 0 |  |  |  |  |  |  |  |  |
| GO:0031424\_keratinization | 1 | 0 |  |  |  |  |  |  |  |  |
| GO:0031441\_negative\_regulation\_of\_mRNA\_3'-end\_processing | 1 | 0 |  |  |  |  |  |  |  |  |
| GO:0031442\_positive\_regulation\_of\_mRNA\_3'-end\_processing | 1 | 0 |  |  |  |  |  |  |  |  |
| GO:0031443\_fast-twitch\_skeletal\_muscle\_fiber\_contraction | 1 | 0 |  |  |  |  |  |  |  |  |
| GO:0031446\_regulation\_of\_fast-twitch\_skeletal\_muscle\_fiber\_contraction | 1 | 0 |  |  |  |  |  |  |  |  |
| GO:0031448\_positive\_regulation\_of\_fast-twitch\_skeletal\_muscle\_fiber\_contraction | 1 | 0 |  |  |  |  |  |  |  |  |
| GO:0031453\_positive\_regulation\_of\_heterochromatin\_formation | 1 | 0 |  |  |  |  |  |  |  |  |
| GO:0031557\_induction\_of\_programmed\_cell\_death\_in\_response\_to\_chemical\_stimulus | 1 | 0 |  |  |  |  |  |  |  |  |
| GO:0031574\_S-M\_checkpoint | 1 | 0 |  |  |  |  |  |  |  |  |
| GO:0031581\_hemidesmosome\_assembly | 1 | 0 |  |  |  |  |  |  |  |  |
| GO:0031627\_telomeric\_loop\_formation | 1 | 0 |  |  |  |  |  |  |  |  |
| GO:0031848\_protection\_from\_non-homologous\_end\_joining\_at\_telomere | 1 | 0 |  |  |  |  |  |  |  |  |
| GO:0031937\_positive\_regulation\_of\_chromatin\_silencing | 1 | 0 |  |  |  |  |  |  |  |  |
| GO:0031943\_regulation\_of\_glucocorticoid\_metabolic\_process | 1 | 0 |  |  |  |  |  |  |  |  |
| GO:0031954\_positive\_regulation\_of\_protein\_amino\_acid\_autophosphorylation | 1 | 0 |  |  |  |  |  |  |  |  |
| GO:0031999\_negative\_regulation\_of\_fatty\_acid\_beta-oxidation | 1 | 0 |  |  |  |  |  |  |  |  |
| GO:0032011\_ARF\_protein\_signal\_transduction | 1 | 0 |  |  |  |  |  |  |  |  |
| GO:0032023\_trypsinogen\_activation | 1 | 0 |  |  |  |  |  |  |  |  |
| GO:0032025\_response\_to\_cobalt\_ion | 1 | 0 |  |  |  |  |  |  |  |  |
| GO:0032048\_cardiolipin\_metabolic\_process | 1 | 0 |  |  |  |  |  |  |  |  |
| GO:0032049\_cardiolipin\_biosynthetic\_process | 1 | 0 |  |  |  |  |  |  |  |  |
| GO:0032060\_bleb\_formation | 1 | 0 |  |  |  |  |  |  |  |  |
| GO:0032066\_nucleolus\_to\_nucleoplasm\_transport | 1 | 0 |  |  |  |  |  |  |  |  |
| GO:0032074\_negative\_regulation\_of\_nuclease\_activity | 1 | 0 |  |  |  |  |  |  |  |  |
| GO:0032075\_positive\_regulation\_of\_nuclease\_activity | 1 | 0 |  |  |  |  |  |  |  |  |
| GO:0032119\_sequestering\_of\_zinc\_ion | 1 | 0 |  |  |  |  |  |  |  |  |
| GO:0032185\_septin\_cytoskeleton\_organization | 1 | 0 |  |  |  |  |  |  |  |  |
| GO:0032196\_transposition | 1 | 0 |  |  |  |  |  |  |  |  |
| GO:0032235\_negative\_regulation\_of\_calcium\_ion\_transport\_via\_store-operated\_calcium\_channel\_activity | 1 | 0 |  |  |  |  |  |  |  |  |
| GO:0032241\_positive\_regulation\_of\_nucleobase\_\_nucleoside\_\_nucleotide\_and\_nucleic\_acid\_transport | 1 | 0 |  |  |  |  |  |  |  |  |
| GO:0032261\_purine\_nucleotide\_salvage | 1 | 0 |  |  |  |  |  |  |  |  |
| GO:0032275\_luteinizing\_hormone\_secretion | 1 | 0 |  |  |  |  |  |  |  |  |
| GO:0032287\_myelin\_maintenance\_in\_the\_peripheral\_nervous\_system | 1 | 0 |  |  |  |  |  |  |  |  |
| GO:0032288\_myelin\_assembly | 1 | 0 |  |  |  |  |  |  |  |  |
| GO:0032314\_regulation\_of\_Rac\_GTPase\_activity | 1 | 0 |  |  |  |  |  |  |  |  |
| GO:0032330\_regulation\_of\_chondrocyte\_differentiation | 1 | 0 |  |  |  |  |  |  |  |  |
| GO:0032331\_negative\_regulation\_of\_chondrocyte\_differentiation | 1 | 0 |  |  |  |  |  |  |  |  |
| GO:0032346\_positive\_regulation\_of\_aldosterone\_metabolic\_process | 1 | 0 |  |  |  |  |  |  |  |  |
| GO:0032347\_regulation\_of\_aldosterone\_biosynthetic\_process | 1 | 0 |  |  |  |  |  |  |  |  |
| GO:0032349\_positive\_regulation\_of\_aldosterone\_biosynthetic\_process | 1 | 0 |  |  |  |  |  |  |  |  |
| GO:0032354\_response\_to\_follicle-stimulating\_hormone\_stimulus | 1 | 0 |  |  |  |  |  |  |  |  |
| GO:0032377\_regulation\_of\_intracellular\_lipid\_transport | 1 | 0 |  |  |  |  |  |  |  |  |
| GO:0032380\_regulation\_of\_intracellular\_sterol\_transport | 1 | 0 |  |  |  |  |  |  |  |  |
| GO:0032383\_regulation\_of\_intracellular\_cholesterol\_transport | 1 | 0 |  |  |  |  |  |  |  |  |
| GO:0032423\_regulation\_of\_mismatch\_repair | 1 | 0 |  |  |  |  |  |  |  |  |
| GO:0032425\_positive\_regulation\_of\_mismatch\_repair | 1 | 0 |  |  |  |  |  |  |  |  |
| GO:0032459\_regulation\_of\_protein\_oligomerization | 1 | 0 |  |  |  |  |  |  |  |  |
| GO:0032460\_negative\_regulation\_of\_protein\_oligomerization | 1 | 0 |  |  |  |  |  |  |  |  |
| GO:0032462\_regulation\_of\_protein\_homooligomerization | 1 | 0 |  |  |  |  |  |  |  |  |
| GO:0032463\_negative\_regulation\_of\_protein\_homooligomerization | 1 | 0 |  |  |  |  |  |  |  |  |
| GO:0032467\_positive\_regulation\_of\_cytokinesis | 1 | 0 |  |  |  |  |  |  |  |  |
| GO:0032468\_Golgi\_calcium\_ion\_homeostasis | 1 | 0 |  |  |  |  |  |  |  |  |
| GO:0032470\_elevation\_of\_endoplasmic\_reticulum\_calcium\_ion\_concentration | 1 | 0 |  |  |  |  |  |  |  |  |
| GO:0032471\_reduction\_of\_endoplasmic\_reticulum\_calcium\_ion\_concentration | 1 | 0 |  |  |  |  |  |  |  |  |
| GO:0032472\_Golgi\_calcium\_ion\_transport | 1 | 0 |  |  |  |  |  |  |  |  |
| GO:0032486\_Rap\_protein\_signal\_transduction | 1 | 0 |  |  |  |  |  |  |  |  |
| GO:0032495\_response\_to\_muramyl\_dipeptide | 1 | 0 |  |  |  |  |  |  |  |  |
| GO:0032498\_detection\_of\_muramyl\_dipeptide | 1 | 0 |  |  |  |  |  |  |  |  |
| GO:0032499\_detection\_of\_peptidoglycan | 1 | 0 |  |  |  |  |  |  |  |  |
| GO:0032528\_microvillus\_organization | 1 | 0 |  |  |  |  |  |  |  |  |
| GO:0032581\_ER-dependent\_peroxisome\_biogenesis | 1 | 0 |  |  |  |  |  |  |  |  |
| GO:0032594\_protein\_transport\_within\_lipid\_bilayer | 1 | 0 |  |  |  |  |  |  |  |  |
| GO:0032595\_B\_cell\_receptor\_transport\_within\_lipid\_bilayer | 1 | 0 |  |  |  |  |  |  |  |  |
| GO:0032596\_protein\_transport\_into\_membrane\_raft | 1 | 0 |  |  |  |  |  |  |  |  |
| GO:0032597\_B\_cell\_receptor\_transport\_into\_membrane\_raft | 1 | 0 |  |  |  |  |  |  |  |  |
| GO:0032599\_protein\_transport\_out\_of\_membrane\_raft | 1 | 0 |  |  |  |  |  |  |  |  |
| GO:0032600\_chemokine\_receptor\_transport\_out\_of\_membrane\_raft | 1 | 0 |  |  |  |  |  |  |  |  |
| GO:0032601\_connective\_tissue\_growth\_factor\_production | 1 | 0 |  |  |  |  |  |  |  |  |
| GO:0032603\_fractalkine\_production | 1 | 0 |  |  |  |  |  |  |  |  |
| GO:0032605\_hepatocyte\_growth\_factor\_production | 1 | 0 |  |  |  |  |  |  |  |  |
| GO:0032610\_interleukin-1\_alpha\_production | 1 | 0 |  |  |  |  |  |  |  |  |
| GO:0032621\_interleukin-18\_production | 1 | 0 |  |  |  |  |  |  |  |  |
| GO:0032639\_TRAIL\_production | 1 | 0 |  |  |  |  |  |  |  |  |
| GO:0032644\_regulation\_of\_fractalkine\_production | 1 | 0 |  |  |  |  |  |  |  |  |
| GO:0032646\_regulation\_of\_hepatocyte\_growth\_factor\_production | 1 | 0 |  |  |  |  |  |  |  |  |
| GO:0032650\_regulation\_of\_interleukin-1\_alpha\_production | 1 | 0 |  |  |  |  |  |  |  |  |
| GO:0032661\_regulation\_of\_interleukin-18\_production | 1 | 0 |  |  |  |  |  |  |  |  |
| GO:0032679\_regulation\_of\_TRAIL\_production | 1 | 0 |  |  |  |  |  |  |  |  |
| GO:0032681\_regulation\_of\_lymphotoxin\_A\_production | 1 | 0 |  |  |  |  |  |  |  |  |
| GO:0032693\_negative\_regulation\_of\_interleukin-10\_production | 1 | 0 |  |  |  |  |  |  |  |  |
| GO:0032703\_negative\_regulation\_of\_interleukin-2\_production | 1 | 0 |  |  |  |  |  |  |  |  |
| GO:0032713\_negative\_regulation\_of\_interleukin-4\_production | 1 | 0 |  |  |  |  |  |  |  |  |
| GO:0032730\_positive\_regulation\_of\_interleukin-1\_alpha\_production | 1 | 0 |  |  |  |  |  |  |  |  |
| GO:0032732\_positive\_regulation\_of\_interleukin-1\_production | 1 | 0 |  |  |  |  |  |  |  |  |
| GO:0032736\_positive\_regulation\_of\_interleukin-13\_production | 1 | 0 |  |  |  |  |  |  |  |  |
| GO:0032753\_positive\_regulation\_of\_interleukin-4\_production | 1 | 0 |  |  |  |  |  |  |  |  |
| GO:0032754\_positive\_regulation\_of\_interleukin-5\_production | 1 | 0 |  |  |  |  |  |  |  |  |
| GO:0032762\_mast\_cell\_cytokine\_production | 1 | 0 |  |  |  |  |  |  |  |  |
| GO:0032763\_regulation\_of\_mast\_cell\_cytokine\_production | 1 | 0 |  |  |  |  |  |  |  |  |
| GO:0032765\_positive\_regulation\_of\_mast\_cell\_cytokine\_production | 1 | 0 |  |  |  |  |  |  |  |  |
| GO:0032784\_regulation\_of\_RNA\_elongation | 1 | 0 |  |  |  |  |  |  |  |  |
| GO:0032786\_positive\_regulation\_of\_RNA\_elongation | 1 | 0 |  |  |  |  |  |  |  |  |
| GO:0032788\_saturated\_monocarboxylic\_acid\_metabolic\_process | 1 | 0 |  |  |  |  |  |  |  |  |
| GO:0032789\_unsaturated\_monocarboxylic\_acid\_metabolic\_process | 1 | 0 |  |  |  |  |  |  |  |  |
| GO:0032790\_ribosome\_disassembly | 1 | 0 |  |  |  |  |  |  |  |  |
| GO:0032792\_negative\_regulation\_of\_CREB\_transcription\_factor\_activity | 1 | 0 |  |  |  |  |  |  |  |  |
| GO:0032793\_positive\_regulation\_of\_CREB\_transcription\_factor\_activity | 1 | 0 |  |  |  |  |  |  |  |  |
| GO:0032804\_negative\_regulation\_of\_low-density\_lipoprotein\_receptor\_catabolic\_process | 1 | 0 |  |  |  |  |  |  |  |  |
| GO:0032805\_positive\_regulation\_of\_low-density\_lipoprotein\_receptor\_catabolic\_process | 1 | 0 |  |  |  |  |  |  |  |  |
| GO:0032812\_positive\_regulation\_of\_epinephrine\_secretion | 1 | 0 |  |  |  |  |  |  |  |  |
| GO:0032835\_glomerulus\_development | 1 | 0 |  |  |  |  |  |  |  |  |
| GO:0032847\_regulation\_of\_cellular\_pH\_reduction | 1 | 0 |  |  |  |  |  |  |  |  |
| GO:0032848\_negative\_regulation\_of\_cellular\_pH\_reduction | 1 | 0 |  |  |  |  |  |  |  |  |
| GO:0032899\_regulation\_of\_neurotrophin\_production | 1 | 0 |  |  |  |  |  |  |  |  |
| GO:0032900\_negative\_regulation\_of\_neurotrophin\_production | 1 | 0 |  |  |  |  |  |  |  |  |
| GO:0032903\_regulation\_of\_nerve\_growth\_factor\_production | 1 | 0 |  |  |  |  |  |  |  |  |
| GO:0032904\_negative\_regulation\_of\_nerve\_growth\_factor\_production | 1 | 0 |  |  |  |  |  |  |  |  |
| GO:0032907\_transforming\_growth\_factor-beta3\_production | 1 | 0 |  |  |  |  |  |  |  |  |
| GO:0032910\_regulation\_of\_transforming\_growth\_factor-beta3\_production | 1 | 0 |  |  |  |  |  |  |  |  |
| GO:0032911\_negative\_regulation\_of\_transforming\_growth\_factor-beta1\_production | 1 | 0 |  |  |  |  |  |  |  |  |
| GO:0032913\_negative\_regulation\_of\_transforming\_growth\_factor-beta3\_production | 1 | 0 |  |  |  |  |  |  |  |  |
| GO:0032926\_negative\_regulation\_of\_activin\_receptor\_signaling\_pathway | 1 | 0 |  |  |  |  |  |  |  |  |
| GO:0032933\_SREBP-mediated\_signaling\_pathway | 1 | 0 |  |  |  |  |  |  |  |  |
| GO:0032938\_negative\_regulation\_of\_translation\_in\_response\_to\_oxidative\_stress | 1 | 0 |  |  |  |  |  |  |  |  |
| GO:0032958\_inositol\_phosphate\_biosynthetic\_process | 1 | 0 |  |  |  |  |  |  |  |  |
| GO:0032976\_release\_of\_matrix\_enzymes\_from\_mitochondria | 1 | 0 |  |  |  |  |  |  |  |  |
| GO:0032980\_keratinocyte\_activation | 1 | 0 |  |  |  |  |  |  |  |  |
| GO:0032988\_ribonucleoprotein\_complex\_disassembly | 1 | 0 |  |  |  |  |  |  |  |  |
| GO:0033029\_regulation\_of\_neutrophil\_apoptosis | 1 | 0 |  |  |  |  |  |  |  |  |
| GO:0033031\_positive\_regulation\_of\_neutrophil\_apoptosis | 1 | 0 |  |  |  |  |  |  |  |  |
| GO:0033079\_immature\_T\_cell\_proliferation | 1 | 0 |  |  |  |  |  |  |  |  |
| GO:0033080\_immature\_T\_cell\_proliferation\_in\_the\_thymus | 1 | 0 |  |  |  |  |  |  |  |  |
| GO:0033083\_regulation\_of\_immature\_T\_cell\_proliferation | 1 | 0 |  |  |  |  |  |  |  |  |
| GO:0033084\_regulation\_of\_immature\_T\_cell\_proliferation\_in\_the\_thymus | 1 | 0 |  |  |  |  |  |  |  |  |
| GO:0033085\_negative\_regulation\_of\_T\_cell\_differentiation\_in\_the\_thymus | 1 | 0 |  |  |  |  |  |  |  |  |
| GO:0033087\_negative\_regulation\_of\_immature\_T\_cell\_proliferation | 1 | 0 |  |  |  |  |  |  |  |  |
| GO:0033088\_negative\_regulation\_of\_immature\_T\_cell\_proliferation\_in\_the\_thymus | 1 | 0 |  |  |  |  |  |  |  |  |
| GO:0033136\_serine\_phosphorylation\_of\_STAT3\_protein | 1 | 0 |  |  |  |  |  |  |  |  |
| GO:0033137\_negative\_regulation\_of\_peptidyl-serine\_phosphorylation | 1 | 0 |  |  |  |  |  |  |  |  |
| GO:0033139\_regulation\_of\_peptidyl-serine\_phosphorylation\_of\_STAT\_protein | 1 | 0 |  |  |  |  |  |  |  |  |
| GO:0033141\_positive\_regulation\_of\_peptidyl-serine\_phosphorylation\_of\_STAT\_protein | 1 | 0 |  |  |  |  |  |  |  |  |
| GO:0033153\_T\_cell\_receptor\_V(D)J\_recombination | 1 | 0 |  |  |  |  |  |  |  |  |
| GO:0033169\_histone\_H3-K9\_demethylation | 1 | 0 |  |  |  |  |  |  |  |  |
| GO:0033173\_calcineurin-NFAT\_signaling\_pathway | 1 | 0 |  |  |  |  |  |  |  |  |
| GO:0033182\_regulation\_of\_histone\_ubiquitination | 1 | 0 |  |  |  |  |  |  |  |  |
| GO:0033206\_cytokinesis\_after\_meiosis | 1 | 0 |  |  |  |  |  |  |  |  |
| GO:0033239\_negative\_regulation\_of\_cellular\_amine\_metabolic\_process | 1 | 0 |  |  |  |  |  |  |  |  |
| GO:0033240\_positive\_regulation\_of\_cellular\_amine\_metabolic\_process | 1 | 0 |  |  |  |  |  |  |  |  |
| GO:0033260\_DNA\_replication\_during\_S\_phase | 1 | 0 |  |  |  |  |  |  |  |  |
| GO:0033262\_regulation\_of\_DNA\_replication\_during\_S\_phase | 1 | 0 |  |  |  |  |  |  |  |  |
| GO:0033292\_T-tubule\_organization | 1 | 0 |  |  |  |  |  |  |  |  |
| GO:0033341\_regulation\_of\_collagen\_binding | 1 | 0 |  |  |  |  |  |  |  |  |
| GO:0033342\_negative\_regulation\_of\_collagen\_binding | 1 | 0 |  |  |  |  |  |  |  |  |
| GO:0033345\_asparagine\_catabolic\_process\_via\_L-aspartate | 1 | 0 |  |  |  |  |  |  |  |  |
| GO:0033366\_protein\_localization\_in\_secretory\_granule | 1 | 0 |  |  |  |  |  |  |  |  |
| GO:0033367\_protein\_localization\_in\_mast\_cell\_secretory\_granule | 1 | 0 |  |  |  |  |  |  |  |  |
| GO:0033368\_protease\_localization\_in\_mast\_cell\_secretory\_granule | 1 | 0 |  |  |  |  |  |  |  |  |
| GO:0033370\_maintenance\_of\_protein\_location\_in\_mast\_cell\_secretory\_granule | 1 | 0 |  |  |  |  |  |  |  |  |
| GO:0033371\_T\_cell\_secretory\_granule\_organization | 1 | 0 |  |  |  |  |  |  |  |  |
| GO:0033373\_maintenance\_of\_protease\_location\_in\_mast\_cell\_secretory\_granule | 1 | 0 |  |  |  |  |  |  |  |  |
| GO:0033374\_protein\_localization\_in\_T\_cell\_secretory\_granule | 1 | 0 |  |  |  |  |  |  |  |  |
| GO:0033375\_protease\_localization\_in\_T\_cell\_secretory\_granule | 1 | 0 |  |  |  |  |  |  |  |  |
| GO:0033377\_maintenance\_of\_protein\_location\_in\_T\_cell\_secretory\_granule | 1 | 0 |  |  |  |  |  |  |  |  |
| GO:0033379\_maintenance\_of\_protease\_location\_in\_T\_cell\_secretory\_granule | 1 | 0 |  |  |  |  |  |  |  |  |
| GO:0033380\_granzyme\_B\_localization\_in\_T\_cell\_secretory\_granule | 1 | 0 |  |  |  |  |  |  |  |  |
| GO:0033382\_maintenance\_of\_granzyme\_B\_location\_in\_T\_cell\_secretory\_granule | 1 | 0 |  |  |  |  |  |  |  |  |
| GO:0033504\_floor\_plate\_development | 1 | 0 |  |  |  |  |  |  |  |  |
| GO:0033566\_gamma-tubulin\_complex\_localization | 1 | 0 |  |  |  |  |  |  |  |  |
| GO:0033577\_protein\_amino\_acid\_glycosylation\_in\_endoplasmic\_reticulum | 1 | 0 |  |  |  |  |  |  |  |  |
| GO:0033595\_response\_to\_genistein | 1 | 0 |  |  |  |  |  |  |  |  |
| GO:0033600\_negative\_regulation\_of\_mammary\_gland\_epithelial\_cell\_proliferation | 1 | 0 |  |  |  |  |  |  |  |  |
| GO:0033606\_chemokine\_receptor\_transport\_within\_lipid\_bilayer | 1 | 0 |  |  |  |  |  |  |  |  |
| GO:0033617\_mitochondrial\_respiratory\_chain\_complex\_IV\_assembly | 1 | 0 |  |  |  |  |  |  |  |  |
| GO:0033622\_integrin\_activation | 1 | 0 |  |  |  |  |  |  |  |  |
| GO:0033623\_regulation\_of\_integrin\_activation | 1 | 0 |  |  |  |  |  |  |  |  |
| GO:0033625\_positive\_regulation\_of\_integrin\_activation | 1 | 0 |  |  |  |  |  |  |  |  |
| GO:0033693\_neurofilament\_bundle\_assembly | 1 | 0 |  |  |  |  |  |  |  |  |
| GO:0033750\_ribosome\_localization | 1 | 0 |  |  |  |  |  |  |  |  |
| GO:0033753\_establishment\_of\_ribosome\_localization | 1 | 0 |  |  |  |  |  |  |  |  |
| GO:0033875\_ribonucleoside\_bisphosphate\_metabolic\_process | 1 | 0 |  |  |  |  |  |  |  |  |
| GO:0033962\_cytoplasmic\_mRNA\_processing\_body\_assembly | 1 | 0 |  |  |  |  |  |  |  |  |
| GO:0034032\_purine\_nucleoside\_bisphosphate\_metabolic\_process | 1 | 0 |  |  |  |  |  |  |  |  |
| GO:0034035\_purine\_ribonucleoside\_bisphosphate\_metabolic\_process | 1 | 0 |  |  |  |  |  |  |  |  |
| GO:0034063\_stress\_granule\_assembly | 1 | 0 |  |  |  |  |  |  |  |  |
| GO:0034080\_CenH3-containing\_nucleosome\_assembly\_at\_centromere | 1 | 0 |  |  |  |  |  |  |  |  |
| GO:0034109\_homotypic\_cell-cell\_adhesion | 1 | 0 |  |  |  |  |  |  |  |  |
| GO:0034115\_negative\_regulation\_of\_heterotypic\_cell-cell\_adhesion | 1 | 0 |  |  |  |  |  |  |  |  |
| GO:0034116\_positive\_regulation\_of\_heterotypic\_cell-cell\_adhesion | 1 | 0 |  |  |  |  |  |  |  |  |
| GO:0034122\_negative\_regulation\_of\_toll-like\_receptor\_signaling\_pathway | 1 | 0 |  |  |  |  |  |  |  |  |
| GO:0034123\_positive\_regulation\_of\_toll-like\_receptor\_signaling\_pathway | 1 | 0 |  |  |  |  |  |  |  |  |
| GO:0034142\_toll-like\_receptor\_4\_signaling\_pathway | 1 | 0 |  |  |  |  |  |  |  |  |
| GO:0034143\_regulation\_of\_toll-like\_receptor\_4\_signaling\_pathway | 1 | 0 |  |  |  |  |  |  |  |  |
| GO:0034145\_positive\_regulation\_of\_toll-like\_receptor\_4\_signaling\_pathway | 1 | 0 |  |  |  |  |  |  |  |  |
| GO:0034196\_acylglycerol\_transport | 1 | 0 |  |  |  |  |  |  |  |  |
| GO:0034197\_triglyceride\_transport | 1 | 0 |  |  |  |  |  |  |  |  |
| GO:0034205\_beta-amyloid\_formation | 1 | 0 |  |  |  |  |  |  |  |  |
| GO:0034213\_quinolinate\_catabolic\_process | 1 | 0 |  |  |  |  |  |  |  |  |
| GO:0034231\_islet\_amyloid\_polypeptide\_processing | 1 | 0 |  |  |  |  |  |  |  |  |
| GO:0034248\_regulation\_of\_amide\_metabolic\_process | 1 | 0 |  |  |  |  |  |  |  |  |
| GO:0034263\_autophagy\_in\_response\_to\_ER\_overload | 1 | 0 |  |  |  |  |  |  |  |  |
| GO:0034287\_detection\_of\_monosaccharide\_stimulus | 1 | 0 |  |  |  |  |  |  |  |  |
| GO:0034313\_diol\_catabolic\_process | 1 | 0 |  |  |  |  |  |  |  |  |
| GO:0034332\_adherens\_junction\_organization | 1 | 0 |  |  |  |  |  |  |  |  |
| GO:0034333\_adherens\_junction\_assembly | 1 | 0 |  |  |  |  |  |  |  |  |
| GO:0034340\_response\_to\_type\_I\_interferon | 1 | 0 |  |  |  |  |  |  |  |  |
| GO:0034356\_NAD\_biosynthesis\_via\_nicotinamide\_riboside\_salvage\_pathway | 1 | 0 |  |  |  |  |  |  |  |  |
| GO:0034373\_intermediate-density\_lipoprotein\_particle\_remodeling | 1 | 0 |  |  |  |  |  |  |  |  |
| GO:0034378\_chylomicron\_assembly | 1 | 0 |  |  |  |  |  |  |  |  |
| GO:0034436\_glycoprotein\_transport | 1 | 0 |  |  |  |  |  |  |  |  |
| GO:0034439\_lipoprotein\_lipid\_oxidation | 1 | 0 |  |  |  |  |  |  |  |  |
| GO:0034454\_microtubule\_anchoring\_at\_centrosome | 1 | 0 |  |  |  |  |  |  |  |  |
| GO:0034465\_response\_to\_carbon\_monoxide | 1 | 0 |  |  |  |  |  |  |  |  |
| GO:0034509\_centromeric\_core\_chromatin\_formation | 1 | 0 |  |  |  |  |  |  |  |  |
| GO:0034516\_response\_to\_vitamin\_B6 | 1 | 0 |  |  |  |  |  |  |  |  |
| GO:0034551\_mitochondrial\_respiratory\_chain\_complex\_III\_assembly | 1 | 0 |  |  |  |  |  |  |  |  |
| GO:0034552\_respiratory\_chain\_complex\_II\_assembly | 1 | 0 |  |  |  |  |  |  |  |  |
| GO:0034553\_mitochondrial\_respiratory\_chain\_complex\_II\_assembly | 1 | 0 |  |  |  |  |  |  |  |  |
| GO:0034589\_hydroxyproline\_transport | 1 | 0 |  |  |  |  |  |  |  |  |
| GO:0034694\_response\_to\_prostaglandin\_stimulus | 1 | 0 |  |  |  |  |  |  |  |  |
| GO:0034695\_response\_to\_prostaglandin\_E\_stimulus | 1 | 0 |  |  |  |  |  |  |  |  |
| GO:0034698\_response\_to\_gonadotropin\_stimulus | 1 | 0 |  |  |  |  |  |  |  |  |
| GO:0034699\_response\_to\_luteinizing\_hormone\_stimulus | 1 | 0 |  |  |  |  |  |  |  |  |
| GO:0034724\_DNA\_replication-independent\_nucleosome\_organization | 1 | 0 |  |  |  |  |  |  |  |  |
| GO:0034729\_histone\_H3-K79\_methylation | 1 | 0 |  |  |  |  |  |  |  |  |
| GO:0034755\_iron\_ion\_transmembrane\_transport | 1 | 0 |  |  |  |  |  |  |  |  |
| GO:0034764\_positive\_regulation\_of\_transmembrane\_transport | 1 | 0 |  |  |  |  |  |  |  |  |
| GO:0034765\_regulation\_of\_ion\_transmembrane\_transport | 1 | 0 |  |  |  |  |  |  |  |  |
| GO:0034767\_positive\_regulation\_of\_ion\_transmembrane\_transport | 1 | 0 |  |  |  |  |  |  |  |  |
| GO:0034959\_endothelin\_maturation | 1 | 0 |  |  |  |  |  |  |  |  |
| GO:0034982\_mitochondrial\_protein\_processing | 1 | 0 |  |  |  |  |  |  |  |  |
| GO:0034983\_peptidyl-lysine\_deacetylation | 1 | 0 |  |  |  |  |  |  |  |  |
| GO:0035021\_negative\_regulation\_of\_Rac\_protein\_signal\_transduction | 1 | 0 |  |  |  |  |  |  |  |  |
| GO:0035041\_sperm\_chromatin\_decondensation | 1 | 0 |  |  |  |  |  |  |  |  |
| GO:0035042\_fertilization\_\_exchange\_of\_chromosomal\_proteins | 1 | 0 |  |  |  |  |  |  |  |  |
| GO:0035054\_embryonic\_heart\_tube\_anterior\_posterior\_pattern\_formation | 1 | 0 |  |  |  |  |  |  |  |  |
| GO:0035066\_positive\_regulation\_of\_histone\_acetylation | 1 | 0 |  |  |  |  |  |  |  |  |
| GO:0035082\_axoneme\_assembly | 1 | 0 |  |  |  |  |  |  |  |  |
| GO:0035087\_RNA\_interference\_\_siRNA\_loading\_onto\_RISC | 1 | 0 |  |  |  |  |  |  |  |  |
| GO:0035090\_maintenance\_of\_apical\_basal\_cell\_polarity | 1 | 0 |  |  |  |  |  |  |  |  |
| GO:0035093\_spermatogenesis\_\_exchange\_of\_chromosomal\_proteins | 1 | 0 |  |  |  |  |  |  |  |  |
| GO:0035104\_positive\_regulation\_of\_transcription\_via\_sterol\_regulatory\_element\_binding | 1 | 0 |  |  |  |  |  |  |  |  |
| GO:0035110\_leg\_morphogenesis | 1 | 0 |  |  |  |  |  |  |  |  |
| GO:0035112\_genitalia\_morphogenesis | 1 | 0 |  |  |  |  |  |  |  |  |
| GO:0035116\_embryonic\_hindlimb\_morphogenesis | 1 | 0 |  |  |  |  |  |  |  |  |
| GO:0035137\_hindlimb\_morphogenesis | 1 | 0 |  |  |  |  |  |  |  |  |
| GO:0035238\_vitamin\_A\_biosynthetic\_process | 1 | 0 |  |  |  |  |  |  |  |  |
| GO:0035265\_organ\_growth | 1 | 0 |  |  |  |  |  |  |  |  |
| GO:0035280\_gene\_silencing\_by\_miRNA\_\_miRNA\_loading\_onto\_RISC | 1 | 0 |  |  |  |  |  |  |  |  |
| GO:0040009\_regulation\_of\_growth\_rate | 1 | 0 |  |  |  |  |  |  |  |  |
| GO:0040013\_negative\_regulation\_of\_locomotion | 1 | 0 |  |  |  |  |  |  |  |  |
| GO:0040015\_negative\_regulation\_of\_multicellular\_organism\_growth | 1 | 0 |  |  |  |  |  |  |  |  |
| GO:0040020\_regulation\_of\_meiosis | 1 | 0 |  |  |  |  |  |  |  |  |
| GO:0040023\_establishment\_of\_nucleus\_localization | 1 | 0 |  |  |  |  |  |  |  |  |
| GO:0040030\_regulation\_of\_molecular\_function\_\_epigenetic | 1 | 0 |  |  |  |  |  |  |  |  |
| GO:0040037\_negative\_regulation\_of\_fibroblast\_growth\_factor\_receptor\_signaling\_pathway | 1 | 0 |  |  |  |  |  |  |  |  |
| GO:0040038\_polar\_body\_extrusion\_after\_meiotic\_divisions | 1 | 0 |  |  |  |  |  |  |  |  |
| GO:0042074\_cell\_migration\_involved\_in\_gastrulation | 1 | 0 |  |  |  |  |  |  |  |  |
| GO:0042091\_interleukin-10\_biosynthetic\_process | 1 | 0 |  |  |  |  |  |  |  |  |
| GO:0042118\_endothelial\_cell\_activation | 1 | 0 |  |  |  |  |  |  |  |  |
| GO:0042159\_lipoprotein\_catabolic\_process | 1 | 0 |  |  |  |  |  |  |  |  |
| GO:0042214\_terpene\_metabolic\_process | 1 | 0 |  |  |  |  |  |  |  |  |
| GO:0042225\_interleukin-5\_biosynthetic\_process | 1 | 0 |  |  |  |  |  |  |  |  |
| GO:0042241\_interleukin-18\_biosynthetic\_process | 1 | 0 |  |  |  |  |  |  |  |  |
| GO:0042257\_ribosomal\_subunit\_assembly | 1 | 0 |  |  |  |  |  |  |  |  |
| GO:0042262\_DNA\_protection | 1 | 0 |  |  |  |  |  |  |  |  |
| GO:0042276\_error-prone\_postreplication\_DNA\_repair | 1 | 0 |  |  |  |  |  |  |  |  |
| GO:0042313\_protein\_kinase\_C\_deactivation | 1 | 0 |  |  |  |  |  |  |  |  |
| GO:0042369\_vitamin\_D\_catabolic\_process | 1 | 0 |  |  |  |  |  |  |  |  |
| GO:0042412\_taurine\_biosynthetic\_process | 1 | 0 |  |  |  |  |  |  |  |  |
| GO:0042418\_epinephrine\_biosynthetic\_process | 1 | 0 |  |  |  |  |  |  |  |  |
| GO:0042421\_norepinephrine\_biosynthetic\_process | 1 | 0 |  |  |  |  |  |  |  |  |
| GO:0042424\_catecholamine\_catabolic\_process | 1 | 0 |  |  |  |  |  |  |  |  |
| GO:0042428\_serotonin\_metabolic\_process | 1 | 0 |  |  |  |  |  |  |  |  |
| GO:0042435\_indole\_derivative\_biosynthetic\_process | 1 | 0 |  |  |  |  |  |  |  |  |
| GO:0042474\_middle\_ear\_morphogenesis | 1 | 0 |  |  |  |  |  |  |  |  |
| GO:0042504\_tyrosine\_phosphorylation\_of\_Stat4\_protein | 1 | 0 |  |  |  |  |  |  |  |  |
| GO:0042519\_regulation\_of\_tyrosine\_phosphorylation\_of\_Stat4\_protein | 1 | 0 |  |  |  |  |  |  |  |  |
| GO:0042520\_positive\_regulation\_of\_tyrosine\_phosphorylation\_of\_Stat4\_protein | 1 | 0 |  |  |  |  |  |  |  |  |
| GO:0042524\_negative\_regulation\_of\_tyrosine\_phosphorylation\_of\_Stat5\_protein | 1 | 0 |  |  |  |  |  |  |  |  |
| GO:0042537\_benzene\_and\_derivative\_metabolic\_process | 1 | 0 |  |  |  |  |  |  |  |  |
| GO:0042560\_pteridine\_and\_derivative\_catabolic\_process | 1 | 0 |  |  |  |  |  |  |  |  |
| GO:0042596\_fear\_response | 1 | 0 |  |  |  |  |  |  |  |  |
| GO:0042637\_catagen | 1 | 0 |  |  |  |  |  |  |  |  |
| GO:0042640\_anagen | 1 | 0 |  |  |  |  |  |  |  |  |
| GO:0042670\_retinal\_cone\_cell\_differentiation | 1 | 0 |  |  |  |  |  |  |  |  |
| GO:0042700\_luteinizing\_hormone\_signaling\_pathway | 1 | 0 |  |  |  |  |  |  |  |  |
| GO:0042703\_menstruation | 1 | 0 |  |  |  |  |  |  |  |  |
| GO:0042726\_riboflavin\_and\_derivative\_metabolic\_process | 1 | 0 |  |  |  |  |  |  |  |  |
| GO:0042727\_riboflavin\_and\_derivative\_biosynthetic\_process | 1 | 0 |  |  |  |  |  |  |  |  |
| GO:0042746\_circadian\_sleep\_wake\_cycle\_\_wakefulness | 1 | 0 |  |  |  |  |  |  |  |  |
| GO:0042748\_circadian\_sleep\_wake\_cycle\_\_non-REM\_sleep | 1 | 0 |  |  |  |  |  |  |  |  |
| GO:0042755\_eating\_behavior | 1 | 0 |  |  |  |  |  |  |  |  |
| GO:0042756\_drinking\_behavior | 1 | 0 |  |  |  |  |  |  |  |  |
| GO:0042766\_nucleosome\_mobilization | 1 | 0 |  |  |  |  |  |  |  |  |
| GO:0042780\_tRNA\_3'-end\_processing | 1 | 0 |  |  |  |  |  |  |  |  |
| GO:0042789\_mRNA\_transcription\_from\_RNA\_polymerase\_II\_promoter | 1 | 0 |  |  |  |  |  |  |  |  |
| GO:0042795\_snRNA\_transcription\_from\_RNA\_polymerase\_II\_promoter | 1 | 0 |  |  |  |  |  |  |  |  |
| GO:0042796\_snRNA\_transcription\_from\_RNA\_polymerase\_III\_promoter | 1 | 0 |  |  |  |  |  |  |  |  |
| GO:0042822\_pyridoxal\_phosphate\_metabolic\_process | 1 | 0 |  |  |  |  |  |  |  |  |
| GO:0042823\_pyridoxal\_phosphate\_biosynthetic\_process | 1 | 0 |  |  |  |  |  |  |  |  |
| GO:0042866\_pyruvate\_biosynthetic\_process | 1 | 0 |  |  |  |  |  |  |  |  |
| GO:0042904\_9-cis-retinoic\_acid\_biosynthetic\_process | 1 | 0 |  |  |  |  |  |  |  |  |
| GO:0042905\_9-cis-retinoic\_acid\_metabolic\_process | 1 | 0 |  |  |  |  |  |  |  |  |
| GO:0042985\_negative\_regulation\_of\_amyloid\_precursor\_protein\_biosynthetic\_process | 1 | 0 |  |  |  |  |  |  |  |  |
| GO:0042986\_positive\_regulation\_of\_amyloid\_precursor\_protein\_biosynthetic\_process | 1 | 0 |  |  |  |  |  |  |  |  |
| GO:0042989\_sequestering\_of\_actin\_monomers | 1 | 0 |  |  |  |  |  |  |  |  |
| GO:0042996\_regulation\_of\_Golgi\_to\_plasma\_membrane\_protein\_transport | 1 | 0 |  |  |  |  |  |  |  |  |
| GO:0042997\_negative\_regulation\_of\_Golgi\_to\_plasma\_membrane\_protein\_transport | 1 | 0 |  |  |  |  |  |  |  |  |
| GO:0042999\_regulation\_of\_Golgi\_to\_plasma\_membrane\_CFTR\_protein\_transport | 1 | 0 |  |  |  |  |  |  |  |  |
| GO:0043002\_negative\_regulation\_of\_Golgi\_to\_plasma\_membrane\_CFTR\_protein\_transport | 1 | 0 |  |  |  |  |  |  |  |  |
| GO:0043004\_cytoplasmic\_sequestering\_of\_CFTR\_protein | 1 | 0 |  |  |  |  |  |  |  |  |
| GO:0043012\_regulation\_of\_fusion\_of\_sperm\_to\_egg\_plasma\_membrane | 1 | 0 |  |  |  |  |  |  |  |  |
| GO:0043016\_regulation\_of\_lymphotoxin\_A\_biosynthetic\_process | 1 | 0 |  |  |  |  |  |  |  |  |
| GO:0043017\_positive\_regulation\_of\_lymphotoxin\_A\_biosynthetic\_process | 1 | 0 |  |  |  |  |  |  |  |  |
| GO:0043049\_otic\_placode\_formation | 1 | 0 |  |  |  |  |  |  |  |  |
| GO:0043064\_flagellum\_organization | 1 | 0 |  |  |  |  |  |  |  |  |
| GO:0043116\_negative\_regulation\_of\_vascular\_permeability | 1 | 0 |  |  |  |  |  |  |  |  |
| GO:0043126\_regulation\_of\_1-phosphatidylinositol\_4-kinase\_activity | 1 | 0 |  |  |  |  |  |  |  |  |
| GO:0043128\_positive\_regulation\_of\_1-phosphatidylinositol\_4-kinase\_activity | 1 | 0 |  |  |  |  |  |  |  |  |
| GO:0043129\_surfactant\_homeostasis | 1 | 0 |  |  |  |  |  |  |  |  |
| GO:0043146\_spindle\_stabilization | 1 | 0 |  |  |  |  |  |  |  |  |
| GO:0043148\_mitotic\_spindle\_stabilization | 1 | 0 |  |  |  |  |  |  |  |  |
| GO:0043152\_induction\_of\_bacterial\_agglutination | 1 | 0 |  |  |  |  |  |  |  |  |
| GO:0043173\_nucleotide\_salvage | 1 | 0 |  |  |  |  |  |  |  |  |
| GO:0043174\_nucleoside\_salvage | 1 | 0 |  |  |  |  |  |  |  |  |
| GO:0043181\_vacuolar\_sequestering | 1 | 0 |  |  |  |  |  |  |  |  |
| GO:0043200\_response\_to\_amino\_acid\_stimulus | 1 | 0 |  |  |  |  |  |  |  |  |
| GO:0043217\_myelin\_maintenance | 1 | 0 |  |  |  |  |  |  |  |  |
| GO:0043247\_telomere\_maintenance\_in\_response\_to\_DNA\_damage | 1 | 0 |  |  |  |  |  |  |  |  |
| GO:0043249\_erythrocyte\_maturation | 1 | 0 |  |  |  |  |  |  |  |  |
| GO:0043268\_positive\_regulation\_of\_potassium\_ion\_transport | 1 | 0 |  |  |  |  |  |  |  |  |
| GO:0043299\_leukocyte\_degranulation | 1 | 0 |  |  |  |  |  |  |  |  |
| GO:0043307\_eosinophil\_activation | 1 | 0 |  |  |  |  |  |  |  |  |
| GO:0043308\_eosinophil\_degranulation | 1 | 0 |  |  |  |  |  |  |  |  |
| GO:0043312\_neutrophil\_degranulation | 1 | 0 |  |  |  |  |  |  |  |  |
| GO:0043330\_response\_to\_exogenous\_dsRNA | 1 | 0 |  |  |  |  |  |  |  |  |
| GO:0043353\_enucleate\_erythrocyte\_differentiation | 1 | 0 |  |  |  |  |  |  |  |  |
| GO:0043371\_negative\_regulation\_of\_CD4-positive\_\_alpha\_beta\_T\_cell\_differentiation | 1 | 0 |  |  |  |  |  |  |  |  |
| GO:0043383\_negative\_T\_cell\_selection | 1 | 0 |  |  |  |  |  |  |  |  |
| GO:0043403\_skeletal\_muscle\_regeneration | 1 | 0 |  |  |  |  |  |  |  |  |
| GO:0043418\_homocysteine\_catabolic\_process | 1 | 0 |  |  |  |  |  |  |  |  |
| GO:0043420\_anthranilate\_metabolic\_process | 1 | 0 |  |  |  |  |  |  |  |  |
| GO:0043437\_butanoic\_acid\_metabolic\_process | 1 | 0 |  |  |  |  |  |  |  |  |
| GO:0043455\_regulation\_of\_secondary\_metabolic\_process | 1 | 0 |  |  |  |  |  |  |  |  |
| GO:0043456\_regulation\_of\_pentose-phosphate\_shunt | 1 | 0 |  |  |  |  |  |  |  |  |
| GO:0043457\_regulation\_of\_cellular\_respiration | 1 | 0 |  |  |  |  |  |  |  |  |
| GO:0043517\_positive\_regulation\_of\_DNA\_damage\_response\_\_signal\_transduction\_by\_p53\_class\_mediator | 1 | 0 |  |  |  |  |  |  |  |  |
| GO:0043518\_negative\_regulation\_of\_DNA\_damage\_response\_\_signal\_transduction\_by\_p53\_class\_mediator | 1 | 0 |  |  |  |  |  |  |  |  |
| GO:0043551\_regulation\_of\_phosphoinositide\_3-kinase\_activity | 1 | 0 |  |  |  |  |  |  |  |  |
| GO:0043552\_positive\_regulation\_of\_phosphoinositide\_3-kinase\_activity | 1 | 0 |  |  |  |  |  |  |  |  |
| GO:0043556\_regulation\_of\_translation\_in\_response\_to\_oxidative\_stress | 1 | 0 |  |  |  |  |  |  |  |  |
| GO:0043584\_nose\_development | 1 | 0 |  |  |  |  |  |  |  |  |
| GO:0043586\_tongue\_development | 1 | 0 |  |  |  |  |  |  |  |  |
| GO:0043587\_tongue\_morphogenesis | 1 | 0 |  |  |  |  |  |  |  |  |
| GO:0043647\_inositol\_phosphate\_metabolic\_process | 1 | 0 |  |  |  |  |  |  |  |  |
| GO:0043652\_engulfment\_of\_apoptotic\_cell | 1 | 0 |  |  |  |  |  |  |  |  |
| GO:0043654\_recognition\_of\_apoptotic\_cell | 1 | 0 |  |  |  |  |  |  |  |  |
| GO:0043696\_dedifferentiation | 1 | 0 |  |  |  |  |  |  |  |  |
| GO:0043697\_cell\_dedifferentiation | 1 | 0 |  |  |  |  |  |  |  |  |
| GO:0043901\_negative\_regulation\_of\_multi-organism\_process | 1 | 0 |  |  |  |  |  |  |  |  |
| GO:0043921\_modulation\_by\_host\_of\_viral\_transcription | 1 | 0 |  |  |  |  |  |  |  |  |
| GO:0043923\_positive\_regulation\_by\_host\_of\_viral\_transcription | 1 | 0 |  |  |  |  |  |  |  |  |
| GO:0044007\_dissemination\_or\_transmission\_of\_symbiont\_from\_host | 1 | 0 |  |  |  |  |  |  |  |  |
| GO:0044089\_positive\_regulation\_of\_cellular\_component\_biogenesis | 1 | 0 |  |  |  |  |  |  |  |  |
| GO:0044258\_intestinal\_lipid\_catabolic\_process | 1 | 0 |  |  |  |  |  |  |  |  |
| GO:0044273\_sulfur\_compound\_catabolic\_process | 1 | 0 |  |  |  |  |  |  |  |  |
| GO:0045013\_negative\_regulation\_of\_transcription\_by\_carbon\_catabolites | 1 | 0 |  |  |  |  |  |  |  |  |
| GO:0045014\_negative\_regulation\_of\_transcription\_by\_glucose | 1 | 0 |  |  |  |  |  |  |  |  |
| GO:0045020\_error-prone\_DNA\_repair | 1 | 0 |  |  |  |  |  |  |  |  |
| GO:0045023\_G0\_to\_G1\_transition | 1 | 0 |  |  |  |  |  |  |  |  |
| GO:0045047\_protein\_targeting\_to\_ER | 1 | 0 |  |  |  |  |  |  |  |  |
| GO:0045065\_cytotoxic\_T\_cell\_differentiation | 1 | 0 |  |  |  |  |  |  |  |  |
| GO:0045074\_regulation\_of\_interleukin-10\_biosynthetic\_process | 1 | 0 |  |  |  |  |  |  |  |  |
| GO:0045082\_positive\_regulation\_of\_interleukin-10\_biosynthetic\_process | 1 | 0 |  |  |  |  |  |  |  |  |
| GO:0045132\_meiotic\_chromosome\_segregation | 1 | 0 |  |  |  |  |  |  |  |  |
| GO:0045163\_clustering\_of\_voltage-gated\_potassium\_channels | 1 | 0 |  |  |  |  |  |  |  |  |
| GO:0045175\_basal\_protein\_localization | 1 | 0 |  |  |  |  |  |  |  |  |
| GO:0045188\_regulation\_of\_circadian\_sleep\_wake\_cycle\_\_non-REM\_sleep | 1 | 0 |  |  |  |  |  |  |  |  |
| GO:0045189\_connective\_tissue\_growth\_factor\_biosynthetic\_process | 1 | 0 |  |  |  |  |  |  |  |  |
| GO:0045196\_establishment\_or\_maintenance\_of\_neuroblast\_polarity | 1 | 0 |  |  |  |  |  |  |  |  |
| GO:0045199\_maintenance\_of\_epithelial\_cell\_apical\_basal\_polarity | 1 | 0 |  |  |  |  |  |  |  |  |
| GO:0045200\_establishment\_of\_neuroblast\_polarity | 1 | 0 |  |  |  |  |  |  |  |  |
| GO:0045204\_MAPK\_export\_from\_nucleus | 1 | 0 |  |  |  |  |  |  |  |  |
| GO:0045208\_MAPK\_phosphatase\_export\_from\_nucleus | 1 | 0 |  |  |  |  |  |  |  |  |
| GO:0045209\_MAPK\_phosphatase\_export\_from\_nucleus\_\_leptomycin\_B\_sensitive | 1 | 0 |  |  |  |  |  |  |  |  |
| GO:0045292\_nuclear\_mRNA\_cis\_splicing\_\_via\_spliceosome | 1 | 0 |  |  |  |  |  |  |  |  |
| GO:0045324\_late\_endosome\_to\_vacuole\_transport | 1 | 0 |  |  |  |  |  |  |  |  |
| GO:0045329\_carnitine\_biosynthetic\_process | 1 | 0 |  |  |  |  |  |  |  |  |
| GO:0045345\_positive\_regulation\_of\_MHC\_class\_I\_biosynthetic\_process | 1 | 0 |  |  |  |  |  |  |  |  |
| GO:0045355\_negative\_regulation\_of\_interferon-alpha\_biosynthetic\_process | 1 | 0 |  |  |  |  |  |  |  |  |
| GO:0045360\_regulation\_of\_interleukin-1\_biosynthetic\_process | 1 | 0 |  |  |  |  |  |  |  |  |
| GO:0045362\_positive\_regulation\_of\_interleukin-1\_biosynthetic\_process | 1 | 0 |  |  |  |  |  |  |  |  |
| GO:0045366\_regulation\_of\_interleukin-13\_biosynthetic\_process | 1 | 0 |  |  |  |  |  |  |  |  |
| GO:0045368\_positive\_regulation\_of\_interleukin-13\_biosynthetic\_process | 1 | 0 |  |  |  |  |  |  |  |  |
| GO:0045381\_regulation\_of\_interleukin-18\_biosynthetic\_process | 1 | 0 |  |  |  |  |  |  |  |  |
| GO:0045405\_regulation\_of\_interleukin-5\_biosynthetic\_process | 1 | 0 |  |  |  |  |  |  |  |  |
| GO:0045407\_positive\_regulation\_of\_interleukin-5\_biosynthetic\_process | 1 | 0 |  |  |  |  |  |  |  |  |
| GO:0045425\_positive\_regulation\_of\_granulocyte\_macrophage\_colony-stimulating\_factor\_biosynthetic\_process | 1 | 0 |  |  |  |  |  |  |  |  |
| GO:0045475\_locomotor\_rhythm | 1 | 0 |  |  |  |  |  |  |  |  |
| GO:0045553\_TRAIL\_biosynthetic\_process | 1 | 0 |  |  |  |  |  |  |  |  |
| GO:0045554\_regulation\_of\_TRAIL\_biosynthetic\_process | 1 | 0 |  |  |  |  |  |  |  |  |
| GO:0045556\_positive\_regulation\_of\_TRAIL\_biosynthetic\_process | 1 | 0 |  |  |  |  |  |  |  |  |
| GO:0045575\_basophil\_activation | 1 | 0 |  |  |  |  |  |  |  |  |
| GO:0045579\_positive\_regulation\_of\_B\_cell\_differentiation | 1 | 0 |  |  |  |  |  |  |  |  |
| GO:0045583\_regulation\_of\_cytotoxic\_T\_cell\_differentiation | 1 | 0 |  |  |  |  |  |  |  |  |
| GO:0045585\_positive\_regulation\_of\_cytotoxic\_T\_cell\_differentiation | 1 | 0 |  |  |  |  |  |  |  |  |
| GO:0045589\_regulation\_of\_regulatory\_T\_cell\_differentiation | 1 | 0 |  |  |  |  |  |  |  |  |
| GO:0045590\_negative\_regulation\_of\_regulatory\_T\_cell\_differentiation | 1 | 0 |  |  |  |  |  |  |  |  |
| GO:0045602\_negative\_regulation\_of\_endothelial\_cell\_differentiation | 1 | 0 |  |  |  |  |  |  |  |  |
| GO:0045603\_positive\_regulation\_of\_endothelial\_cell\_differentiation | 1 | 0 |  |  |  |  |  |  |  |  |
| GO:0045605\_negative\_regulation\_of\_epidermal\_cell\_differentiation | 1 | 0 |  |  |  |  |  |  |  |  |
| GO:0045617\_negative\_regulation\_of\_keratinocyte\_differentiation | 1 | 0 |  |  |  |  |  |  |  |  |
| GO:0045623\_negative\_regulation\_of\_T-helper\_cell\_differentiation | 1 | 0 |  |  |  |  |  |  |  |  |
| GO:0045629\_negative\_regulation\_of\_T-helper\_2\_cell\_differentiation | 1 | 0 |  |  |  |  |  |  |  |  |
| GO:0045654\_positive\_regulation\_of\_megakaryocyte\_differentiation | 1 | 0 |  |  |  |  |  |  |  |  |
| GO:0045672\_positive\_regulation\_of\_osteoclast\_differentiation | 1 | 0 |  |  |  |  |  |  |  |  |
| GO:0045683\_negative\_regulation\_of\_epidermis\_development | 1 | 0 |  |  |  |  |  |  |  |  |
| GO:0045716\_positive\_regulation\_of\_low-density\_lipoprotein\_receptor\_biosynthetic\_process | 1 | 0 |  |  |  |  |  |  |  |  |
| GO:0045719\_negative\_regulation\_of\_glycogen\_biosynthetic\_process | 1 | 0 |  |  |  |  |  |  |  |  |
| GO:0045738\_negative\_regulation\_of\_DNA\_repair | 1 | 0 |  |  |  |  |  |  |  |  |
| GO:0045747\_positive\_regulation\_of\_Notch\_signaling\_pathway | 1 | 0 |  |  |  |  |  |  |  |  |
| GO:0045750\_positive\_regulation\_of\_S\_phase\_of\_mitotic\_cell\_cycle | 1 | 0 |  |  |  |  |  |  |  |  |
| GO:0045751\_negative\_regulation\_of\_Toll\_signaling\_pathway | 1 | 0 |  |  |  |  |  |  |  |  |
| GO:0045759\_negative\_regulation\_of\_action\_potential | 1 | 0 |  |  |  |  |  |  |  |  |
| GO:0045773\_positive\_regulation\_of\_axon\_extension | 1 | 0 |  |  |  |  |  |  |  |  |
| GO:0045794\_negative\_regulation\_of\_cell\_volume | 1 | 0 |  |  |  |  |  |  |  |  |
| GO:0045799\_positive\_regulation\_of\_chromatin\_assembly\_or\_disassembly | 1 | 0 |  |  |  |  |  |  |  |  |
| GO:0045818\_negative\_regulation\_of\_glycogen\_catabolic\_process | 1 | 0 |  |  |  |  |  |  |  |  |
| GO:0045836\_positive\_regulation\_of\_meiosis | 1 | 0 |  |  |  |  |  |  |  |  |
| GO:0045837\_negative\_regulation\_of\_membrane\_potential | 1 | 0 |  |  |  |  |  |  |  |  |
| GO:0045844\_positive\_regulation\_of\_striated\_muscle\_development | 1 | 0 |  |  |  |  |  |  |  |  |
| GO:0045852\_pH\_elevation | 1 | 0 |  |  |  |  |  |  |  |  |
| GO:0045870\_positive\_regulation\_of\_retroviral\_genome\_replication | 1 | 0 |  |  |  |  |  |  |  |  |
| GO:0045875\_negative\_regulation\_of\_sister\_chromatid\_cohesion | 1 | 0 |  |  |  |  |  |  |  |  |
| GO:0045879\_negative\_regulation\_of\_smoothened\_signaling\_pathway | 1 | 0 |  |  |  |  |  |  |  |  |
| GO:0045896\_regulation\_of\_transcription\_\_mitotic | 1 | 0 |  |  |  |  |  |  |  |  |
| GO:0045910\_negative\_regulation\_of\_DNA\_recombination | 1 | 0 |  |  |  |  |  |  |  |  |
| GO:0045915\_positive\_regulation\_of\_catecholamine\_metabolic\_process | 1 | 0 |  |  |  |  |  |  |  |  |
| GO:0045921\_positive\_regulation\_of\_exocytosis | 1 | 0 |  |  |  |  |  |  |  |  |
| GO:0045945\_positive\_regulation\_of\_transcription\_from\_RNA\_polymerase\_III\_promoter | 1 | 0 |  |  |  |  |  |  |  |  |
| GO:0045956\_positive\_regulation\_of\_calcium\_ion-dependent\_exocytosis | 1 | 0 |  |  |  |  |  |  |  |  |
| GO:0045964\_positive\_regulation\_of\_dopamine\_metabolic\_process | 1 | 0 |  |  |  |  |  |  |  |  |
| GO:0045989\_positive\_regulation\_of\_striated\_muscle\_contraction | 1 | 0 |  |  |  |  |  |  |  |  |
| GO:0045993\_negative\_regulation\_of\_translational\_initiation\_by\_iron | 1 | 0 |  |  |  |  |  |  |  |  |
| GO:0046005\_positive\_regulation\_of\_circadian\_sleep\_wake\_cycle\_\_REM\_sleep | 1 | 0 |  |  |  |  |  |  |  |  |
| GO:0046007\_negative\_regulation\_of\_activated\_T\_cell\_proliferation | 1 | 0 |  |  |  |  |  |  |  |  |
| GO:0046010\_positive\_regulation\_of\_circadian\_sleep\_wake\_cycle\_\_non-REM\_sleep | 1 | 0 |  |  |  |  |  |  |  |  |
| GO:0046031\_ADP\_metabolic\_process | 1 | 0 |  |  |  |  |  |  |  |  |
| GO:0046036\_CTP\_metabolic\_process | 1 | 0 |  |  |  |  |  |  |  |  |
| GO:0046040\_IMP\_metabolic\_process | 1 | 0 |  |  |  |  |  |  |  |  |
| GO:0046048\_UDP\_metabolic\_process | 1 | 0 |  |  |  |  |  |  |  |  |
| GO:0046049\_UMP\_metabolic\_process | 1 | 0 |  |  |  |  |  |  |  |  |
| GO:0046056\_dADP\_metabolic\_process | 1 | 0 |  |  |  |  |  |  |  |  |
| GO:0046085\_adenosine\_metabolic\_process | 1 | 0 |  |  |  |  |  |  |  |  |
| GO:0046087\_cytidine\_metabolic\_process | 1 | 0 |  |  |  |  |  |  |  |  |
| GO:0046101\_hypoxanthine\_biosynthetic\_process | 1 | 0 |  |  |  |  |  |  |  |  |
| GO:0046103\_inosine\_biosynthetic\_process | 1 | 0 |  |  |  |  |  |  |  |  |
| GO:0046104\_thymidine\_metabolic\_process | 1 | 0 |  |  |  |  |  |  |  |  |
| GO:0046108\_uridine\_metabolic\_process | 1 | 0 |  |  |  |  |  |  |  |  |
| GO:0046125\_pyrimidine\_deoxyribonucleoside\_metabolic\_process | 1 | 0 |  |  |  |  |  |  |  |  |
| GO:0046127\_pyrimidine\_deoxyribonucleoside\_catabolic\_process | 1 | 0 |  |  |  |  |  |  |  |  |
| GO:0046133\_pyrimidine\_ribonucleoside\_catabolic\_process | 1 | 0 |  |  |  |  |  |  |  |  |
| GO:0046173\_polyol\_biosynthetic\_process | 1 | 0 |  |  |  |  |  |  |  |  |
| GO:0046184\_aldehyde\_biosynthetic\_process | 1 | 0 |  |  |  |  |  |  |  |  |
| GO:0046203\_spermidine\_catabolic\_process | 1 | 0 |  |  |  |  |  |  |  |  |
| GO:0046219\_indolalkylamine\_biosynthetic\_process | 1 | 0 |  |  |  |  |  |  |  |  |
| GO:0046292\_formaldehyde\_metabolic\_process | 1 | 0 |  |  |  |  |  |  |  |  |
| GO:0046293\_formaldehyde\_biosynthetic\_process | 1 | 0 |  |  |  |  |  |  |  |  |
| GO:0046317\_regulation\_of\_glucosylceramide\_biosynthetic\_process | 1 | 0 |  |  |  |  |  |  |  |  |
| GO:0046318\_negative\_regulation\_of\_glucosylceramide\_biosynthetic\_process | 1 | 0 |  |  |  |  |  |  |  |  |
| GO:0046322\_negative\_regulation\_of\_fatty\_acid\_oxidation | 1 | 0 |  |  |  |  |  |  |  |  |
| GO:0046335\_ethanolamine\_biosynthetic\_process | 1 | 0 |  |  |  |  |  |  |  |  |
| GO:0046337\_phosphatidylethanolamine\_metabolic\_process | 1 | 0 |  |  |  |  |  |  |  |  |
| GO:0046340\_diacylglycerol\_catabolic\_process | 1 | 0 |  |  |  |  |  |  |  |  |
| GO:0046351\_disaccharide\_biosynthetic\_process | 1 | 0 |  |  |  |  |  |  |  |  |
| GO:0046352\_disaccharide\_catabolic\_process | 1 | 0 |  |  |  |  |  |  |  |  |
| GO:0046370\_fructose\_biosynthetic\_process | 1 | 0 |  |  |  |  |  |  |  |  |
| GO:0046380\_N-acetylneuraminate\_biosynthetic\_process | 1 | 0 |  |  |  |  |  |  |  |  |
| GO:0046390\_ribose\_phosphate\_biosynthetic\_process | 1 | 0 |  |  |  |  |  |  |  |  |
| GO:0046399\_glucuronate\_biosynthetic\_process | 1 | 0 |  |  |  |  |  |  |  |  |
| GO:0046434\_organophosphate\_catabolic\_process | 1 | 0 |  |  |  |  |  |  |  |  |
| GO:0046448\_tropane\_alkaloid\_metabolic\_process | 1 | 0 |  |  |  |  |  |  |  |  |
| GO:0046449\_creatinine\_metabolic\_process | 1 | 0 |  |  |  |  |  |  |  |  |
| GO:0046471\_phosphatidylglycerol\_metabolic\_process | 1 | 0 |  |  |  |  |  |  |  |  |
| GO:0046477\_glycosylceramide\_catabolic\_process | 1 | 0 |  |  |  |  |  |  |  |  |
| GO:0046485\_ether\_lipid\_metabolic\_process | 1 | 0 |  |  |  |  |  |  |  |  |
| GO:0046487\_glyoxylate\_metabolic\_process | 1 | 0 |  |  |  |  |  |  |  |  |
| GO:0046491\_L-methylmalonyl-CoA\_metabolic\_process | 1 | 0 |  |  |  |  |  |  |  |  |
| GO:0046501\_protoporphyrinogen\_IX\_metabolic\_process | 1 | 0 |  |  |  |  |  |  |  |  |
| GO:0046511\_sphinganine\_biosynthetic\_process | 1 | 0 |  |  |  |  |  |  |  |  |
| GO:0046514\_ceramide\_catabolic\_process | 1 | 0 |  |  |  |  |  |  |  |  |
| GO:0046549\_retinal\_cone\_cell\_development | 1 | 0 |  |  |  |  |  |  |  |  |
| GO:0046586\_regulation\_of\_calcium-dependent\_cell-cell\_adhesion | 1 | 0 |  |  |  |  |  |  |  |  |
| GO:0046588\_negative\_regulation\_of\_calcium-dependent\_cell-cell\_adhesion | 1 | 0 |  |  |  |  |  |  |  |  |
| GO:0046597\_negative\_regulation\_of\_virion\_penetration\_into\_host\_cell | 1 | 0 |  |  |  |  |  |  |  |  |
| GO:0046600\_negative\_regulation\_of\_centriole\_replication | 1 | 0 |  |  |  |  |  |  |  |  |
| GO:0046606\_negative\_regulation\_of\_centrosome\_cycle | 1 | 0 |  |  |  |  |  |  |  |  |
| GO:0046620\_regulation\_of\_organ\_growth | 1 | 0 |  |  |  |  |  |  |  |  |
| GO:0046636\_negative\_regulation\_of\_alpha-beta\_T\_cell\_activation | 1 | 0 |  |  |  |  |  |  |  |  |
| GO:0046639\_negative\_regulation\_of\_alpha-beta\_T\_cell\_differentiation | 1 | 0 |  |  |  |  |  |  |  |  |
| GO:0046640\_regulation\_of\_alpha-beta\_T\_cell\_proliferation | 1 | 0 |  |  |  |  |  |  |  |  |
| GO:0046641\_positive\_regulation\_of\_alpha-beta\_T\_cell\_proliferation | 1 | 0 |  |  |  |  |  |  |  |  |
| GO:0046655\_folic\_acid\_metabolic\_process | 1 | 0 |  |  |  |  |  |  |  |  |
| GO:0046666\_retinal\_cell\_programmed\_cell\_death | 1 | 0 |  |  |  |  |  |  |  |  |
| GO:0046668\_regulation\_of\_retinal\_cell\_programmed\_cell\_death | 1 | 0 |  |  |  |  |  |  |  |  |
| GO:0046670\_positive\_regulation\_of\_retinal\_cell\_programmed\_cell\_death | 1 | 0 |  |  |  |  |  |  |  |  |
| GO:0046674\_induction\_of\_retinal\_programmed\_cell\_death | 1 | 0 |  |  |  |  |  |  |  |  |
| GO:0046685\_response\_to\_arsenic | 1 | 0 |  |  |  |  |  |  |  |  |
| GO:0046686\_response\_to\_cadmium\_ion | 1 | 0 |  |  |  |  |  |  |  |  |
| GO:0046689\_response\_to\_mercury\_ion | 1 | 0 |  |  |  |  |  |  |  |  |
| GO:0046692\_sperm\_competition | 1 | 0 |  |  |  |  |  |  |  |  |
| GO:0046713\_boron\_transport | 1 | 0 |  |  |  |  |  |  |  |  |
| GO:0046719\_regulation\_of\_viral\_protein\_levels\_in\_host\_cell | 1 | 0 |  |  |  |  |  |  |  |  |
| GO:0046814\_virion\_attachment\_\_binding\_of\_host\_cell\_surface\_coreceptor | 1 | 0 |  |  |  |  |  |  |  |  |
| GO:0046826\_negative\_regulation\_of\_protein\_export\_from\_nucleus | 1 | 0 |  |  |  |  |  |  |  |  |
| GO:0046827\_positive\_regulation\_of\_protein\_export\_from\_nucleus | 1 | 0 |  |  |  |  |  |  |  |  |
| GO:0046833\_positive\_regulation\_of\_RNA\_export\_from\_nucleus | 1 | 0 |  |  |  |  |  |  |  |  |
| GO:0046835\_carbohydrate\_phosphorylation | 1 | 0 |  |  |  |  |  |  |  |  |
| GO:0046838\_phosphorylated\_carbohydrate\_dephosphorylation | 1 | 0 |  |  |  |  |  |  |  |  |
| GO:0046853\_inositol\_and\_derivative\_phosphorylation | 1 | 0 |  |  |  |  |  |  |  |  |
| GO:0046855\_inositol\_phosphate\_dephosphorylation | 1 | 0 |  |  |  |  |  |  |  |  |
| GO:0046856\_phosphoinositide\_dephosphorylation | 1 | 0 |  |  |  |  |  |  |  |  |
| GO:0046898\_response\_to\_cycloheximide | 1 | 0 |  |  |  |  |  |  |  |  |
| GO:0046916\_cellular\_transition\_metal\_ion\_homeostasis | 1 | 0 |  |  |  |  |  |  |  |  |
| GO:0046931\_pore\_complex\_biogenesis | 1 | 0 |  |  |  |  |  |  |  |  |
| GO:0046939\_nucleotide\_phosphorylation | 1 | 0 |  |  |  |  |  |  |  |  |
| GO:0046946\_hydroxylysine\_metabolic\_process | 1 | 0 |  |  |  |  |  |  |  |  |
| GO:0046947\_hydroxylysine\_biosynthetic\_process | 1 | 0 |  |  |  |  |  |  |  |  |
| GO:0046963\_3'-phosphoadenosine\_5'-phosphosulfate\_transport | 1 | 0 |  |  |  |  |  |  |  |  |
| GO:0046984\_regulation\_of\_hemoglobin\_biosynthetic\_process | 1 | 0 |  |  |  |  |  |  |  |  |
| GO:0046986\_negative\_regulation\_of\_hemoglobin\_biosynthetic\_process | 1 | 0 |  |  |  |  |  |  |  |  |
| GO:0048003\_antigen\_processing\_and\_presentation\_of\_lipid\_antigen\_via\_MHC\_class\_Ib | 1 | 0 |  |  |  |  |  |  |  |  |
| GO:0048006\_antigen\_processing\_and\_presentation\_\_endogenous\_lipid\_antigen\_via\_MHC\_class\_Ib | 1 | 0 |  |  |  |  |  |  |  |  |
| GO:0048013\_ephrin\_receptor\_signaling\_pathway | 1 | 0 |  |  |  |  |  |  |  |  |
| GO:0048070\_regulation\_of\_pigmentation\_during\_development | 1 | 0 |  |  |  |  |  |  |  |  |
| GO:0048073\_regulation\_of\_eye\_pigmentation | 1 | 0 |  |  |  |  |  |  |  |  |
| GO:0048075\_positive\_regulation\_of\_eye\_pigmentation | 1 | 0 |  |  |  |  |  |  |  |  |
| GO:0048087\_positive\_regulation\_of\_pigmentation\_during\_development | 1 | 0 |  |  |  |  |  |  |  |  |
| GO:0048160\_primary\_follicle\_stage\_\_oogenesis | 1 | 0 |  |  |  |  |  |  |  |  |
| GO:0048170\_positive\_regulation\_of\_long-term\_neuronal\_synaptic\_plasticity | 1 | 0 |  |  |  |  |  |  |  |  |
| GO:0048172\_regulation\_of\_short-term\_neuronal\_synaptic\_plasticity | 1 | 0 |  |  |  |  |  |  |  |  |
| GO:0048175\_hepatocyte\_growth\_factor\_biosynthetic\_process | 1 | 0 |  |  |  |  |  |  |  |  |
| GO:0048176\_regulation\_of\_hepatocyte\_growth\_factor\_biosynthetic\_process | 1 | 0 |  |  |  |  |  |  |  |  |
| GO:0048178\_negative\_regulation\_of\_hepatocyte\_growth\_factor\_biosynthetic\_process | 1 | 0 |  |  |  |  |  |  |  |  |
| GO:0048203\_vesicle\_targeting\_\_trans-Golgi\_to\_endosome | 1 | 0 |  |  |  |  |  |  |  |  |
| GO:0048210\_Golgi\_vesicle\_fusion\_to\_target\_membrane | 1 | 0 |  |  |  |  |  |  |  |  |
| GO:0048241\_epinephrine\_transport | 1 | 0 |  |  |  |  |  |  |  |  |
| GO:0048242\_epinephrine\_secretion | 1 | 0 |  |  |  |  |  |  |  |  |
| GO:0048245\_eosinophil\_chemotaxis | 1 | 0 |  |  |  |  |  |  |  |  |
| GO:0048265\_response\_to\_pain | 1 | 0 |  |  |  |  |  |  |  |  |
| GO:0048289\_isotype\_switching\_to\_IgE\_isotypes | 1 | 0 |  |  |  |  |  |  |  |  |
| GO:0048293\_regulation\_of\_isotype\_switching\_to\_IgE\_isotypes | 1 | 0 |  |  |  |  |  |  |  |  |
| GO:0048295\_positive\_regulation\_of\_isotype\_switching\_to\_IgE\_isotypes | 1 | 0 |  |  |  |  |  |  |  |  |
| GO:0048302\_regulation\_of\_isotype\_switching\_to\_IgG\_isotypes | 1 | 0 |  |  |  |  |  |  |  |  |
| GO:0048304\_positive\_regulation\_of\_isotype\_switching\_to\_IgG\_isotypes | 1 | 0 |  |  |  |  |  |  |  |  |
| GO:0048311\_mitochondrion\_distribution | 1 | 0 |  |  |  |  |  |  |  |  |
| GO:0048339\_paraxial\_mesoderm\_development | 1 | 0 |  |  |  |  |  |  |  |  |
| GO:0048340\_paraxial\_mesoderm\_morphogenesis | 1 | 0 |  |  |  |  |  |  |  |  |
| GO:0048388\_endosomal\_lumen\_acidification | 1 | 0 |  |  |  |  |  |  |  |  |
| GO:0048478\_replication\_fork\_protection | 1 | 0 |  |  |  |  |  |  |  |  |
| GO:0048483\_autonomic\_nervous\_system\_development | 1 | 0 |  |  |  |  |  |  |  |  |
| GO:0048485\_sympathetic\_nervous\_system\_development | 1 | 0 |  |  |  |  |  |  |  |  |
| GO:0048499\_synaptic\_vesicle\_membrane\_organization | 1 | 0 |  |  |  |  |  |  |  |  |
| GO:0048535\_lymph\_node\_development | 1 | 0 |  |  |  |  |  |  |  |  |
| GO:0048539\_bone\_marrow\_development | 1 | 0 |  |  |  |  |  |  |  |  |
| GO:0048549\_positive\_regulation\_of\_pinocytosis | 1 | 0 |  |  |  |  |  |  |  |  |
| GO:0048553\_negative\_regulation\_of\_metalloenzyme\_activity | 1 | 0 |  |  |  |  |  |  |  |  |
| GO:0048566\_embryonic\_gut\_development | 1 | 0 |  |  |  |  |  |  |  |  |
| GO:0048596\_embryonic\_camera-type\_eye\_morphogenesis | 1 | 0 |  |  |  |  |  |  |  |  |
| GO:0048617\_embryonic\_foregut\_morphogenesis | 1 | 0 |  |  |  |  |  |  |  |  |
| GO:0048619\_embryonic\_hindgut\_morphogenesis | 1 | 0 |  |  |  |  |  |  |  |  |
| GO:0048636\_positive\_regulation\_of\_muscle\_development | 1 | 0 |  |  |  |  |  |  |  |  |
| GO:0048639\_positive\_regulation\_of\_developmental\_growth | 1 | 0 |  |  |  |  |  |  |  |  |
| GO:0048640\_negative\_regulation\_of\_developmental\_growth | 1 | 0 |  |  |  |  |  |  |  |  |
| GO:0048665\_neuron\_fate\_specification | 1 | 0 |  |  |  |  |  |  |  |  |
| GO:0048679\_regulation\_of\_axon\_regeneration | 1 | 0 |  |  |  |  |  |  |  |  |
| GO:0048681\_negative\_regulation\_of\_axon\_regeneration | 1 | 0 |  |  |  |  |  |  |  |  |
| GO:0048703\_embryonic\_viscerocranium\_morphogenesis | 1 | 0 |  |  |  |  |  |  |  |  |
| GO:0048745\_smooth\_muscle\_tissue\_development | 1 | 0 |  |  |  |  |  |  |  |  |
| GO:0048755\_branching\_morphogenesis\_of\_a\_nerve | 1 | 0 |  |  |  |  |  |  |  |  |
| GO:0048793\_pronephros\_development | 1 | 0 |  |  |  |  |  |  |  |  |
| GO:0048807\_female\_genitalia\_morphogenesis | 1 | 0 |  |  |  |  |  |  |  |  |
| GO:0048818\_positive\_regulation\_of\_hair\_follicle\_maturation | 1 | 0 |  |  |  |  |  |  |  |  |
| GO:0048819\_regulation\_of\_hair\_follicle\_maturation | 1 | 0 |  |  |  |  |  |  |  |  |
| GO:0048821\_erythrocyte\_development | 1 | 0 |  |  |  |  |  |  |  |  |
| GO:0048845\_venous\_blood\_vessel\_morphogenesis | 1 | 0 |  |  |  |  |  |  |  |  |
| GO:0048853\_forebrain\_morphogenesis | 1 | 0 |  |  |  |  |  |  |  |  |
| GO:0048865\_stem\_cell\_fate\_commitment | 1 | 0 |  |  |  |  |  |  |  |  |
| GO:0048867\_stem\_cell\_fate\_determination | 1 | 0 |  |  |  |  |  |  |  |  |
| GO:0048874\_homeostasis\_of\_number\_of\_cells\_in\_a\_free-living\_population | 1 | 0 |  |  |  |  |  |  |  |  |
| GO:0048875\_chemical\_homeostasis\_within\_a\_tissue | 1 | 0 |  |  |  |  |  |  |  |  |
| GO:0050427\_3'-phosphoadenosine\_5'-phosphosulfate\_metabolic\_process | 1 | 0 |  |  |  |  |  |  |  |  |
| GO:0050652\_dermatan\_sulfate\_proteoglycan\_biosynthetic\_process\_\_polysaccharide\_chain\_biosynthetic\_process | 1 | 0 |  |  |  |  |  |  |  |  |
| GO:0050666\_regulation\_of\_homocysteine\_metabolic\_process | 1 | 0 |  |  |  |  |  |  |  |  |
| GO:0050674\_urothelial\_cell\_proliferation | 1 | 0 |  |  |  |  |  |  |  |  |
| GO:0050675\_regulation\_of\_urothelial\_cell\_proliferation | 1 | 0 |  |  |  |  |  |  |  |  |
| GO:0050677\_positive\_regulation\_of\_urothelial\_cell\_proliferation | 1 | 0 |  |  |  |  |  |  |  |  |
| GO:0050685\_positive\_regulation\_of\_mRNA\_processing | 1 | 0 |  |  |  |  |  |  |  |  |
| GO:0050687\_negative\_regulation\_of\_defense\_response\_to\_virus | 1 | 0 |  |  |  |  |  |  |  |  |
| GO:0050689\_negative\_regulation\_of\_defense\_response\_to\_virus\_by\_host | 1 | 0 |  |  |  |  |  |  |  |  |
| GO:0050713\_negative\_regulation\_of\_interleukin-1\_beta\_secretion | 1 | 0 |  |  |  |  |  |  |  |  |
| GO:0050722\_regulation\_of\_interleukin-1\_beta\_biosynthetic\_process | 1 | 0 |  |  |  |  |  |  |  |  |
| GO:0050725\_positive\_regulation\_of\_interleukin-1\_beta\_biosynthetic\_process | 1 | 0 |  |  |  |  |  |  |  |  |
| GO:0050751\_fractalkine\_biosynthetic\_process | 1 | 0 |  |  |  |  |  |  |  |  |
| GO:0050752\_regulation\_of\_fractalkine\_biosynthetic\_process | 1 | 0 |  |  |  |  |  |  |  |  |
| GO:0050754\_positive\_regulation\_of\_fractalkine\_biosynthetic\_process | 1 | 0 |  |  |  |  |  |  |  |  |
| GO:0050756\_fractalkine\_metabolic\_process | 1 | 0 |  |  |  |  |  |  |  |  |
| GO:0050757\_thymidylate\_synthase\_biosynthetic\_process | 1 | 0 |  |  |  |  |  |  |  |  |
| GO:0050758\_regulation\_of\_thymidylate\_synthase\_biosynthetic\_process | 1 | 0 |  |  |  |  |  |  |  |  |
| GO:0050760\_negative\_regulation\_of\_thymidylate\_synthase\_biosynthetic\_process | 1 | 0 |  |  |  |  |  |  |  |  |
| GO:0050765\_negative\_regulation\_of\_phagocytosis | 1 | 0 |  |  |  |  |  |  |  |  |
| GO:0050774\_negative\_regulation\_of\_dendrite\_morphogenesis | 1 | 0 |  |  |  |  |  |  |  |  |
| GO:0050783\_cocaine\_metabolic\_process | 1 | 0 |  |  |  |  |  |  |  |  |
| GO:0050822\_peptide\_stabilization | 1 | 0 |  |  |  |  |  |  |  |  |
| GO:0050823\_peptide\_antigen\_stabilization | 1 | 0 |  |  |  |  |  |  |  |  |
| GO:0050832\_defense\_response\_to\_fungus | 1 | 0 |  |  |  |  |  |  |  |  |
| GO:0050855\_regulation\_of\_B\_cell\_receptor\_signaling\_pathway | 1 | 0 |  |  |  |  |  |  |  |  |
| GO:0050858\_negative\_regulation\_of\_antigen\_receptor-mediated\_signaling\_pathway | 1 | 0 |  |  |  |  |  |  |  |  |
| GO:0050860\_negative\_regulation\_of\_T\_cell\_receptor\_signaling\_pathway | 1 | 0 |  |  |  |  |  |  |  |  |
| GO:0050861\_positive\_regulation\_of\_B\_cell\_receptor\_signaling\_pathway | 1 | 0 |  |  |  |  |  |  |  |  |
| GO:0050883\_musculoskeletal\_movement\_\_spinal\_reflex\_action | 1 | 0 |  |  |  |  |  |  |  |  |
| GO:0050884\_neuromuscular\_process\_controlling\_posture | 1 | 0 |  |  |  |  |  |  |  |  |
| GO:0050893\_sensory\_processing | 1 | 0 |  |  |  |  |  |  |  |  |
| GO:0050902\_leukocyte\_adhesive\_activation | 1 | 0 |  |  |  |  |  |  |  |  |
| GO:0050910\_detection\_of\_mechanical\_stimulus\_involved\_in\_sensory\_perception\_of\_sound | 1 | 0 |  |  |  |  |  |  |  |  |
| GO:0050922\_negative\_regulation\_of\_chemotaxis | 1 | 0 |  |  |  |  |  |  |  |  |
| GO:0050923\_regulation\_of\_negative\_chemotaxis | 1 | 0 |  |  |  |  |  |  |  |  |
| GO:0050924\_positive\_regulation\_of\_negative\_chemotaxis | 1 | 0 |  |  |  |  |  |  |  |  |
| GO:0050929\_induction\_of\_negative\_chemotaxis | 1 | 0 |  |  |  |  |  |  |  |  |
| GO:0050951\_sensory\_perception\_of\_temperature\_stimulus | 1 | 0 |  |  |  |  |  |  |  |  |
| GO:0050955\_thermoception | 1 | 0 |  |  |  |  |  |  |  |  |
| GO:0050974\_detection\_of\_mechanical\_stimulus\_involved\_in\_sensory\_perception | 1 | 0 |  |  |  |  |  |  |  |  |
| GO:0050983\_spermidine\_catabolic\_process\_to\_deoxyhypusine\_\_using\_deoxyhypusine\_synthase | 1 | 0 |  |  |  |  |  |  |  |  |
| GO:0051013\_microtubule\_severing | 1 | 0 |  |  |  |  |  |  |  |  |
| GO:0051029\_rRNA\_transport | 1 | 0 |  |  |  |  |  |  |  |  |
| GO:0051030\_snRNA\_transport | 1 | 0 |  |  |  |  |  |  |  |  |
| GO:0051031\_tRNA\_transport | 1 | 0 |  |  |  |  |  |  |  |  |
| GO:0051036\_regulation\_of\_endosome\_size | 1 | 0 |  |  |  |  |  |  |  |  |
| GO:0051040\_regulation\_of\_calcium-independent\_cell-cell\_adhesion | 1 | 0 |  |  |  |  |  |  |  |  |
| GO:0051041\_positive\_regulation\_of\_calcium-independent\_cell-cell\_adhesion | 1 | 0 |  |  |  |  |  |  |  |  |
| GO:0051066\_dihydrobiopterin\_metabolic\_process | 1 | 0 |  |  |  |  |  |  |  |  |
| GO:0051085\_chaperone\_mediated\_protein\_folding\_requiring\_cofactor | 1 | 0 |  |  |  |  |  |  |  |  |
| GO:0051089\_constitutive\_protein\_ectodomain\_proteolysis | 1 | 0 |  |  |  |  |  |  |  |  |
| GO:0051102\_DNA\_ligation\_during\_DNA\_recombination | 1 | 0 |  |  |  |  |  |  |  |  |
| GO:0051105\_regulation\_of\_DNA\_ligation | 1 | 0 |  |  |  |  |  |  |  |  |
| GO:0051106\_positive\_regulation\_of\_DNA\_ligation | 1 | 0 |  |  |  |  |  |  |  |  |
| GO:0051125\_regulation\_of\_actin\_nucleation | 1 | 0 |  |  |  |  |  |  |  |  |
| GO:0051126\_negative\_regulation\_of\_actin\_nucleation | 1 | 0 |  |  |  |  |  |  |  |  |
| GO:0051136\_regulation\_of\_NK\_T\_cell\_differentiation | 1 | 0 |  |  |  |  |  |  |  |  |
| GO:0051138\_positive\_regulation\_of\_NK\_T\_cell\_differentiation | 1 | 0 |  |  |  |  |  |  |  |  |
| GO:0051155\_positive\_regulation\_of\_striated\_muscle\_cell\_differentiation | 1 | 0 |  |  |  |  |  |  |  |  |
| GO:0051156\_glucose\_6-phosphate\_metabolic\_process | 1 | 0 |  |  |  |  |  |  |  |  |
| GO:0051160\_L-xylitol\_catabolic\_process | 1 | 0 |  |  |  |  |  |  |  |  |
| GO:0051164\_L-xylitol\_metabolic\_process | 1 | 0 |  |  |  |  |  |  |  |  |
| GO:0051193\_regulation\_of\_cofactor\_metabolic\_process | 1 | 0 |  |  |  |  |  |  |  |  |
| GO:0051196\_regulation\_of\_coenzyme\_metabolic\_process | 1 | 0 |  |  |  |  |  |  |  |  |
| GO:0051204\_protein\_insertion\_into\_mitochondrial\_membrane | 1 | 0 |  |  |  |  |  |  |  |  |
| GO:0051290\_protein\_heterotetramerization | 1 | 0 |  |  |  |  |  |  |  |  |
| GO:0051292\_nuclear\_pore\_complex\_assembly | 1 | 0 |  |  |  |  |  |  |  |  |
| GO:0051294\_establishment\_of\_spindle\_orientation | 1 | 0 |  |  |  |  |  |  |  |  |
| GO:0051295\_establishment\_of\_meiotic\_spindle\_localization | 1 | 0 |  |  |  |  |  |  |  |  |
| GO:0051315\_attachment\_of\_spindle\_microtubules\_to\_kinetochore\_during\_mitosis | 1 | 0 |  |  |  |  |  |  |  |  |
| GO:0051326\_telophase | 1 | 0 |  |  |  |  |  |  |  |  |
| GO:0051342\_regulation\_of\_cyclic-nucleotide\_phosphodiesterase\_activity | 1 | 0 |  |  |  |  |  |  |  |  |
| GO:0051344\_negative\_regulation\_of\_cyclic-nucleotide\_phosphodiesterase\_activity | 1 | 0 |  |  |  |  |  |  |  |  |
| GO:0051445\_regulation\_of\_meiotic\_cell\_cycle | 1 | 0 |  |  |  |  |  |  |  |  |
| GO:0051450\_myoblast\_proliferation | 1 | 0 |  |  |  |  |  |  |  |  |
| GO:0051454\_intracellular\_pH\_elevation | 1 | 0 |  |  |  |  |  |  |  |  |
| GO:0051458\_adrenocorticotropin\_secretion | 1 | 0 |  |  |  |  |  |  |  |  |
| GO:0051459\_regulation\_of\_adrenocorticotropin\_secretion | 1 | 0 |  |  |  |  |  |  |  |  |
| GO:0051461\_positive\_regulation\_of\_adrenocorticotropin\_secretion | 1 | 0 |  |  |  |  |  |  |  |  |
| GO:0051531\_NFAT\_protein\_import\_into\_nucleus | 1 | 0 |  |  |  |  |  |  |  |  |
| GO:0051532\_regulation\_of\_NFAT\_protein\_import\_into\_nucleus | 1 | 0 |  |  |  |  |  |  |  |  |
| GO:0051533\_positive\_regulation\_of\_NFAT\_protein\_import\_into\_nucleus | 1 | 0 |  |  |  |  |  |  |  |  |
| GO:0051542\_elastin\_biosynthetic\_process | 1 | 0 |  |  |  |  |  |  |  |  |
| GO:0051560\_mitochondrial\_calcium\_ion\_homeostasis | 1 | 0 |  |  |  |  |  |  |  |  |
| GO:0051561\_elevation\_of\_mitochondrial\_calcium\_ion\_concentration | 1 | 0 |  |  |  |  |  |  |  |  |
| GO:0051582\_positive\_regulation\_of\_neurotransmitter\_uptake | 1 | 0 |  |  |  |  |  |  |  |  |
| GO:0051586\_positive\_regulation\_of\_dopamine\_uptake | 1 | 0 |  |  |  |  |  |  |  |  |
| GO:0051590\_positive\_regulation\_of\_neurotransmitter\_transport | 1 | 0 |  |  |  |  |  |  |  |  |
| GO:0051594\_detection\_of\_glucose | 1 | 0 |  |  |  |  |  |  |  |  |
| GO:0051642\_centrosome\_localization | 1 | 0 |  |  |  |  |  |  |  |  |
| GO:0051645\_Golgi\_localization | 1 | 0 |  |  |  |  |  |  |  |  |
| GO:0051647\_nucleus\_localization | 1 | 0 |  |  |  |  |  |  |  |  |
| GO:0051664\_nuclear\_pore\_localization | 1 | 0 |  |  |  |  |  |  |  |  |
| GO:0051708\_intracellular\_protein\_transport\_in\_other\_organism\_during\_symbiotic\_interaction | 1 | 0 |  |  |  |  |  |  |  |  |
| GO:0051764\_actin\_crosslink\_formation | 1 | 0 |  |  |  |  |  |  |  |  |
| GO:0051767\_nitric-oxide\_synthase\_biosynthetic\_process | 1 | 0 |  |  |  |  |  |  |  |  |
| GO:0051768\_nitric-oxide\_synthase\_2\_biosynthetic\_process | 1 | 0 |  |  |  |  |  |  |  |  |
| GO:0051769\_regulation\_of\_nitric-oxide\_synthase\_biosynthetic\_process | 1 | 0 |  |  |  |  |  |  |  |  |
| GO:0051771\_negative\_regulation\_of\_nitric-oxide\_synthase\_biosynthetic\_process | 1 | 0 |  |  |  |  |  |  |  |  |
| GO:0051772\_regulation\_of\_nitric-oxide\_synthase\_2\_biosynthetic\_process | 1 | 0 |  |  |  |  |  |  |  |  |
| GO:0051773\_positive\_regulation\_of\_nitric-oxide\_synthase\_2\_biosynthetic\_process | 1 | 0 |  |  |  |  |  |  |  |  |
| GO:0051781\_positive\_regulation\_of\_cell\_division | 1 | 0 |  |  |  |  |  |  |  |  |
| GO:0051782\_negative\_regulation\_of\_cell\_division | 1 | 0 |  |  |  |  |  |  |  |  |
| GO:0051788\_response\_to\_misfolded\_protein | 1 | 0 |  |  |  |  |  |  |  |  |
| GO:0051790\_short-chain\_fatty\_acid\_biosynthetic\_process | 1 | 0 |  |  |  |  |  |  |  |  |
| GO:0051791\_medium-chain\_fatty\_acid\_metabolic\_process | 1 | 0 |  |  |  |  |  |  |  |  |
| GO:0051792\_medium-chain\_fatty\_acid\_biosynthetic\_process | 1 | 0 |  |  |  |  |  |  |  |  |
| GO:0051794\_regulation\_of\_catagen | 1 | 0 |  |  |  |  |  |  |  |  |
| GO:0051795\_positive\_regulation\_of\_catagen | 1 | 0 |  |  |  |  |  |  |  |  |
| GO:0051821\_dissemination\_or\_transmission\_of\_organism\_from\_other\_organism\_during\_symbiotic\_interaction | 1 | 0 |  |  |  |  |  |  |  |  |
| GO:0051894\_positive\_regulation\_of\_focal\_adhesion\_formation | 1 | 0 |  |  |  |  |  |  |  |  |
| GO:0051930\_regulation\_of\_sensory\_perception\_of\_pain | 1 | 0 |  |  |  |  |  |  |  |  |
| GO:0051931\_regulation\_of\_sensory\_perception | 1 | 0 |  |  |  |  |  |  |  |  |
| GO:0051944\_positive\_regulation\_of\_catecholamine\_uptake\_during\_transmission\_of\_nerve\_impulse | 1 | 0 |  |  |  |  |  |  |  |  |
| GO:0051962\_positive\_regulation\_of\_nervous\_system\_development | 1 | 0 |  |  |  |  |  |  |  |  |
| GO:0051965\_positive\_regulation\_of\_synaptogenesis | 1 | 0 |  |  |  |  |  |  |  |  |
| GO:0051977\_lysophospholipid\_transport | 1 | 0 |  |  |  |  |  |  |  |  |
| GO:0051988\_regulation\_of\_attachment\_of\_spindle\_microtubules\_to\_kinetochore | 1 | 0 |  |  |  |  |  |  |  |  |
| GO:0052097\_interspecies\_quorum\_sensing | 1 | 0 |  |  |  |  |  |  |  |  |
| GO:0052106\_quorum\_sensing\_during\_interaction\_with\_host | 1 | 0 |  |  |  |  |  |  |  |  |
| GO:0052312\_modulation\_of\_transcription\_in\_other\_organism\_during\_symbiotic\_interaction | 1 | 0 |  |  |  |  |  |  |  |  |
| GO:0052472\_modulation\_by\_host\_of\_symbiont\_transcription | 1 | 0 |  |  |  |  |  |  |  |  |
| GO:0055009\_atrial\_cardiac\_muscle\_morphogenesis | 1 | 0 |  |  |  |  |  |  |  |  |
| GO:0055012\_ventricular\_cardiac\_muscle\_cell\_differentiation | 1 | 0 |  |  |  |  |  |  |  |  |
| GO:0055071\_manganese\_ion\_homeostasis | 1 | 0 |  |  |  |  |  |  |  |  |
| GO:0055073\_cadmium\_ion\_homeostasis | 1 | 0 |  |  |  |  |  |  |  |  |
| GO:0055076\_transition\_metal\_ion\_homeostasis | 1 | 0 |  |  |  |  |  |  |  |  |
| GO:0055089\_fatty\_acid\_homeostasis | 1 | 0 |  |  |  |  |  |  |  |  |
| GO:0055095\_lipoprotein\_mediated\_signaling | 1 | 0 |  |  |  |  |  |  |  |  |
| GO:0055096\_low\_density\_lipoprotein\_mediated\_signaling | 1 | 0 |  |  |  |  |  |  |  |  |
| GO:0055099\_response\_to\_high\_density\_lipoprotein\_stimulus | 1 | 0 |  |  |  |  |  |  |  |  |
| GO:0055118\_negative\_regulation\_of\_cardiac\_muscle\_contraction | 1 | 0 |  |  |  |  |  |  |  |  |
| GO:0055119\_relaxation\_of\_cardiac\_muscle | 1 | 0 |  |  |  |  |  |  |  |  |
| GO:0060003\_copper\_ion\_export | 1 | 0 |  |  |  |  |  |  |  |  |
| GO:0060022\_hard\_palate\_development | 1 | 0 |  |  |  |  |  |  |  |  |
| GO:0060039\_pericardium\_development | 1 | 0 |  |  |  |  |  |  |  |  |
| GO:0060055\_angiogenesis\_involved\_in\_wound\_healing | 1 | 0 |  |  |  |  |  |  |  |  |
| GO:0060059\_embryonic\_retina\_morphogenesis\_in\_camera-type\_eye | 1 | 0 |  |  |  |  |  |  |  |  |
| GO:0060065\_uterus\_development | 1 | 0 |  |  |  |  |  |  |  |  |
| GO:0060068\_vagina\_development | 1 | 0 |  |  |  |  |  |  |  |  |
| GO:0060082\_eye\_blink\_reflex | 1 | 0 |  |  |  |  |  |  |  |  |
| GO:0060083\_smooth\_muscle\_contraction\_involved\_in\_micturition | 1 | 0 |  |  |  |  |  |  |  |  |
| GO:0060088\_auditory\_receptor\_cell\_stereocilium\_organization | 1 | 0 |  |  |  |  |  |  |  |  |
| GO:0060120\_inner\_ear\_receptor\_cell\_fate\_commitment | 1 | 0 |  |  |  |  |  |  |  |  |
| GO:0060135\_maternal\_process\_involved\_in\_female\_pregnancy | 1 | 0 |  |  |  |  |  |  |  |  |
| GO:0060157\_urinary\_bladder\_development | 1 | 0 |  |  |  |  |  |  |  |  |
| GO:0060160\_negative\_regulation\_of\_dopamine\_receptor\_signaling\_pathway | 1 | 0 |  |  |  |  |  |  |  |  |
| GO:0060161\_positive\_regulation\_of\_dopamine\_receptor\_signaling\_pathway | 1 | 0 |  |  |  |  |  |  |  |  |
| GO:0060167\_regulation\_of\_adenosine\_receptor\_signaling\_pathway | 1 | 0 |  |  |  |  |  |  |  |  |
| GO:0060169\_negative\_regulation\_of\_adenosine\_receptor\_signaling\_pathway | 1 | 0 |  |  |  |  |  |  |  |  |
| GO:0060216\_definitive\_hemopoiesis | 1 | 0 |  |  |  |  |  |  |  |  |
| GO:0060219\_camera-type\_eye\_photoreceptor\_cell\_differentiation | 1 | 0 |  |  |  |  |  |  |  |  |
| GO:0060231\_mesenchymal\_to\_epithelial\_transition | 1 | 0 |  |  |  |  |  |  |  |  |
| GO:0060254\_regulation\_of\_N-terminal\_protein\_palmitoylation | 1 | 0 |  |  |  |  |  |  |  |  |
| GO:0060259\_regulation\_of\_feeding\_behavior | 1 | 0 |  |  |  |  |  |  |  |  |
| GO:0060262\_negative\_regulation\_of\_N-terminal\_protein\_palmitoylation | 1 | 0 |  |  |  |  |  |  |  |  |
| GO:0060265\_positive\_regulation\_of\_respiratory\_burst\_during\_acute\_inflammatory\_response | 1 | 0 |  |  |  |  |  |  |  |  |
| GO:0060266\_negative\_regulation\_of\_respiratory\_burst\_during\_acute\_inflammatory\_response | 1 | 0 |  |  |  |  |  |  |  |  |
| GO:0060268\_negative\_regulation\_of\_respiratory\_burst | 1 | 0 |  |  |  |  |  |  |  |  |
| GO:0060286\_flagellar\_cell\_motility | 1 | 0 |  |  |  |  |  |  |  |  |
| GO:0060298\_positive\_regulation\_of\_sarcomere\_organization | 1 | 0 |  |  |  |  |  |  |  |  |
| GO:0060299\_negative\_regulation\_of\_sarcomere\_organization | 1 | 0 |  |  |  |  |  |  |  |  |
| GO:0060300\_regulation\_of\_cytokine\_activity | 1 | 0 |  |  |  |  |  |  |  |  |
| GO:0060302\_negative\_regulation\_of\_cytokine\_activity | 1 | 0 |  |  |  |  |  |  |  |  |
| GO:0060305\_regulation\_of\_cell\_diameter | 1 | 0 |  |  |  |  |  |  |  |  |
| GO:0060306\_regulation\_of\_membrane\_repolarization | 1 | 0 |  |  |  |  |  |  |  |  |
| GO:0060307\_regulation\_of\_ventricular\_cardiomyocyte\_membrane\_repolarization | 1 | 0 |  |  |  |  |  |  |  |  |
| GO:0060309\_elastin\_catabolic\_process | 1 | 0 |  |  |  |  |  |  |  |  |
| GO:0060310\_regulation\_of\_elastin\_catabolic\_process | 1 | 0 |  |  |  |  |  |  |  |  |
| GO:0060311\_negative\_regulation\_of\_elastin\_catabolic\_process | 1 | 0 |  |  |  |  |  |  |  |  |
| GO:0060312\_regulation\_of\_blood\_vessel\_remodeling | 1 | 0 |  |  |  |  |  |  |  |  |
| GO:0060313\_negative\_regulation\_of\_blood\_vessel\_remodeling | 1 | 0 |  |  |  |  |  |  |  |  |
| GO:0060315\_negative\_regulation\_of\_ryanodine-sensitive\_calcium-release\_channel\_activity | 1 | 0 |  |  |  |  |  |  |  |  |
| GO:0060316\_positive\_regulation\_of\_ryanodine-sensitive\_calcium-release\_channel\_activity | 1 | 0 |  |  |  |  |  |  |  |  |
| GO:0060318\_definitive\_erythrocyte\_differentiation | 1 | 0 |  |  |  |  |  |  |  |  |
| GO:0060322\_head\_development | 1 | 0 |  |  |  |  |  |  |  |  |
| GO:0060324\_face\_development | 1 | 0 |  |  |  |  |  |  |  |  |
| GO:0060336\_negative\_regulation\_of\_interferon-gamma-mediated\_signaling\_pathway | 1 | 0 |  |  |  |  |  |  |  |  |
| GO:0060349\_bone\_morphogenesis | 1 | 0 |  |  |  |  |  |  |  |  |
| GO:0060350\_endochondral\_bone\_morphogenesis | 1 | 0 |  |  |  |  |  |  |  |  |
| GO:0060356\_leucine\_import | 1 | 0 |  |  |  |  |  |  |  |  |
| GO:0060368\_regulation\_of\_Fc\_receptor\_mediated\_stimulatory\_signaling\_pathway | 1 | 0 |  |  |  |  |  |  |  |  |
| GO:0060369\_positive\_regulation\_of\_Fc\_receptor\_mediated\_stimulatory\_signaling\_pathway | 1 | 0 |  |  |  |  |  |  |  |  |
| GO:0060380\_regulation\_of\_single-stranded\_telomeric\_DNA\_binding | 1 | 0 |  |  |  |  |  |  |  |  |
| GO:0060381\_positive\_regulation\_of\_single-stranded\_telomeric\_DNA\_binding | 1 | 0 |  |  |  |  |  |  |  |  |
| GO:0060382\_regulation\_of\_DNA\_strand\_elongation | 1 | 0 |  |  |  |  |  |  |  |  |
| GO:0060383\_positive\_regulation\_of\_DNA\_strand\_elongation | 1 | 0 |  |  |  |  |  |  |  |  |
| GO:0060397\_JAK-STAT\_cascade\_involved\_in\_growth\_hormone\_signaling\_pathway | 1 | 0 |  |  |  |  |  |  |  |  |
| GO:0060398\_regulation\_of\_growth\_hormone\_receptor\_signaling\_pathway | 1 | 0 |  |  |  |  |  |  |  |  |
| GO:0060425\_lung\_morphogenesis | 1 | 0 |  |  |  |  |  |  |  |  |
| GO:0060433\_bronchus\_development | 1 | 0 |  |  |  |  |  |  |  |  |
| GO:0060438\_trachea\_development | 1 | 0 |  |  |  |  |  |  |  |  |
| GO:0060441\_branching\_involved\_in\_lung\_morphogenesis | 1 | 0 |  |  |  |  |  |  |  |  |
| GO:0060445\_branching\_involved\_in\_salivary\_gland\_morphogenesis | 1 | 0 |  |  |  |  |  |  |  |  |
| GO:0060502\_epithelial\_cell\_proliferation\_involved\_in\_lung\_morphogenesis | 1 | 0 |  |  |  |  |  |  |  |  |
| GO:0060503\_bud\_dilation\_involved\_in\_lung\_branching | 1 | 0 |  |  |  |  |  |  |  |  |
| GO:0060560\_developmental\_growth\_involved\_in\_morphogenesis | 1 | 0 |  |  |  |  |  |  |  |  |
| GO:0060579\_ventral\_spinal\_cord\_interneuron\_fate\_commitment | 1 | 0 |  |  |  |  |  |  |  |  |
| GO:0060586\_multicellular\_organismal\_iron\_ion\_homeostasis | 1 | 0 |  |  |  |  |  |  |  |  |
| GO:0060587\_regulation\_of\_lipoprotein\_lipid\_oxidation | 1 | 0 |  |  |  |  |  |  |  |  |
| GO:0060588\_negative\_regulation\_of\_lipoprotein\_lipid\_oxidation | 1 | 0 |  |  |  |  |  |  |  |  |
| GO:0060638\_mesenchymal-epithelial\_cell\_signaling | 1 | 0 |  |  |  |  |  |  |  |  |
| GO:0060665\_regulation\_of\_branching\_involved\_in\_salivary\_gland\_morphogenesis\_by\_mesenchymal-epithelial\_signaling | 1 | 0 |  |  |  |  |  |  |  |  |
| GO:0060675\_ureteric\_bud\_morphogenesis | 1 | 0 |  |  |  |  |  |  |  |  |
| GO:0060688\_regulation\_of\_morphogenesis\_of\_a\_branching\_structure | 1 | 0 |  |  |  |  |  |  |  |  |
| GO:0060693\_regulation\_of\_branching\_involved\_in\_salivary\_gland\_morphogenesis | 1 | 0 |  |  |  |  |  |  |  |  |
| GO:0060694\_regulation\_of\_cholesterol\_transporter\_activity | 1 | 0 |  |  |  |  |  |  |  |  |
| GO:0060695\_negative\_regulation\_of\_cholesterol\_transporter\_activity | 1 | 0 |  |  |  |  |  |  |  |  |
| GO:0060697\_positive\_regulation\_of\_phospholipid\_catabolic\_process | 1 | 0 |  |  |  |  |  |  |  |  |
| GO:0060729\_intestinal\_epithelial\_structure\_maintenance | 1 | 0 |  |  |  |  |  |  |  |  |
| GO:0060730\_regulation\_of\_intestinal\_epithelial\_structure\_maintenance | 1 | 0 |  |  |  |  |  |  |  |  |
| GO:0060731\_positive\_regulation\_of\_intestinal\_epithelial\_structure\_maintenance | 1 | 0 |  |  |  |  |  |  |  |  |
| GO:0060760\_positive\_regulation\_of\_response\_to\_cytokine\_stimulus | 1 | 0 |  |  |  |  |  |  |  |  |
| GO:0060761\_negative\_regulation\_of\_response\_to\_cytokine\_stimulus | 1 | 0 |  |  |  |  |  |  |  |  |
| GO:0060788\_ectodermal\_placode\_formation | 1 | 0 |  |  |  |  |  |  |  |  |
| GO:0060841\_venous\_blood\_vessel\_development | 1 | 0 |  |  |  |  |  |  |  |  |
| GO:0060856\_establishment\_of\_blood-brain\_barrier | 1 | 0 |  |  |  |  |  |  |  |  |
| GO:0060896\_neural\_plate\_pattern\_specification | 1 | 0 |  |  |  |  |  |  |  |  |
| GO:0065001\_specification\_of\_axis\_polarity | 1 | 0 |  |  |  |  |  |  |  |  |
| GO:0070075\_tear\_secretion | 1 | 0 |  |  |  |  |  |  |  |  |
| GO:0070076\_histone\_lysine\_demethylation | 1 | 0 |  |  |  |  |  |  |  |  |
| GO:0070077\_histone\_arginine\_demethylation | 1 | 0 |  |  |  |  |  |  |  |  |
| GO:0070078\_histone\_H3-R2\_demethylation | 1 | 0 |  |  |  |  |  |  |  |  |
| GO:0070079\_histone\_H4-R3\_demethylation | 1 | 0 |  |  |  |  |  |  |  |  |
| GO:0070086\_ubiquitin-dependent\_endocytosis | 1 | 0 |  |  |  |  |  |  |  |  |
| GO:0070091\_glucagon\_secretion | 1 | 0 |  |  |  |  |  |  |  |  |
| GO:0070103\_regulation\_of\_interleukin-6-mediated\_signaling\_pathway | 1 | 0 |  |  |  |  |  |  |  |  |
| GO:0070104\_negative\_regulation\_of\_interleukin-6-mediated\_signaling\_pathway | 1 | 0 |  |  |  |  |  |  |  |  |
| GO:0070106\_interleukin-27-mediated\_signaling\_pathway | 1 | 0 |  |  |  |  |  |  |  |  |
| GO:0070162\_adiponectin\_secretion | 1 | 0 |  |  |  |  |  |  |  |  |
| GO:0070163\_regulation\_of\_adiponectin\_secretion | 1 | 0 |  |  |  |  |  |  |  |  |
| GO:0070165\_positive\_regulation\_of\_adiponectin\_secretion | 1 | 0 |  |  |  |  |  |  |  |  |
| GO:0070172\_positive\_regulation\_of\_tooth\_mineralization | 1 | 0 |  |  |  |  |  |  |  |  |
| GO:0070173\_regulation\_of\_enamel\_mineralization | 1 | 0 |  |  |  |  |  |  |  |  |
| GO:0070189\_kynurenine\_metabolic\_process | 1 | 0 |  |  |  |  |  |  |  |  |
| GO:0070212\_protein\_amino\_acid\_poly-ADP-ribosylation | 1 | 0 |  |  |  |  |  |  |  |  |
| GO:0070213\_protein\_amino\_acid\_auto-ADP-ribosylation | 1 | 0 |  |  |  |  |  |  |  |  |
| GO:0070232\_regulation\_of\_T\_cell\_apoptosis | 1 | 0 |  |  |  |  |  |  |  |  |
| GO:0070234\_positive\_regulation\_of\_T\_cell\_apoptosis | 1 | 0 |  |  |  |  |  |  |  |  |
| GO:0070242\_thymocyte\_apoptosis | 1 | 0 |  |  |  |  |  |  |  |  |
| GO:0070243\_regulation\_of\_thymocyte\_apoptosis | 1 | 0 |  |  |  |  |  |  |  |  |
| GO:0070245\_positive\_regulation\_of\_thymocyte\_apoptosis | 1 | 0 |  |  |  |  |  |  |  |  |
| GO:0070267\_oncosis | 1 | 0 |  |  |  |  |  |  |  |  |
| GO:0070286\_axonemal\_dynein\_complex\_assembly | 1 | 0 |  |  |  |  |  |  |  |  |
| GO:0070314\_G1\_to\_G0\_transition | 1 | 0 |  |  |  |  |  |  |  |  |
| GO:0070327\_thyroid\_hormone\_transport | 1 | 0 |  |  |  |  |  |  |  |  |
| GO:0070407\_oxidation-dependent\_protein\_catabolic\_process | 1 | 0 |  |  |  |  |  |  |  |  |
| GO:0070408\_carbamoyl\_phosphate\_metabolic\_process | 1 | 0 |  |  |  |  |  |  |  |  |
| GO:0070409\_carbamoyl\_phosphate\_biosynthetic\_process | 1 | 0 |  |  |  |  |  |  |  |  |
| GO:0070509\_calcium\_ion\_import | 1 | 0 |  |  |  |  |  |  |  |  |
| GO:0070527\_platelet\_aggregation | 1 | 0 |  |  |  |  |  |  |  |  |
| GO:0070528\_protein\_kinase\_C\_signaling\_cascade | 1 | 0 |  |  |  |  |  |  |  |  |
| GO:0070534\_protein\_K63-linked\_ubiquitination | 1 | 0 |  |  |  |  |  |  |  |  |
| GO:0070535\_histone\_H2A\_K63-linked\_ubiquitination | 1 | 0 |  |  |  |  |  |  |  |  |
| GO:0070537\_histone\_H2A\_K63-linked\_deubiquitination | 1 | 0 |  |  |  |  |  |  |  |  |
| GO:0070560\_protein\_secretion\_by\_platelet | 1 | 0 |  |  |  |  |  |  |  |  |
| GO:0070562\_regulation\_of\_vitamin\_D\_receptor\_signaling\_pathway | 1 | 0 |  |  |  |  |  |  |  |  |
| GO:0070564\_positive\_regulation\_of\_vitamin\_D\_receptor\_signaling\_pathway | 1 | 0 |  |  |  |  |  |  |  |  |
| GO:0070570\_regulation\_of\_neuron\_projection\_regeneration | 1 | 0 |  |  |  |  |  |  |  |  |
| GO:0070571\_negative\_regulation\_of\_neuron\_projection\_regeneration | 1 | 0 |  |  |  |  |  |  |  |  |
| GO:0070601\_centromeric\_sister\_chromatid\_cohesion | 1 | 0 |  |  |  |  |  |  |  |  |
| GO:0070602\_regulation\_of\_centromeric\_sister\_chromatid\_cohesion | 1 | 0 |  |  |  |  |  |  |  |  |
| GO:0070625\_zymogen\_granule\_exocytosis | 1 | 0 |  |  |  |  |  |  |  |  |
| GO:0070684\_seminal\_clot\_liquefaction | 1 | 0 |  |  |  |  |  |  |  |  |
| GO:0070715\_sodium-dependent\_organic\_cation\_transport | 1 | 0 |  |  |  |  |  |  |  |  |
| GO:0070813\_hydrogen\_sulfide\_metabolic\_process | 1 | 0 |  |  |  |  |  |  |  |  |
| GO:0070814\_hydrogen\_sulfide\_biosynthetic\_process | 1 | 0 |  |  |  |  |  |  |  |  |
| GO:0070846\_Hsp90\_deacetylation | 1 | 0 |  |  |  |  |  |  |  |  |
| GO:0090030\_regulation\_of\_steroid\_hormone\_biosynthetic\_process | 1 | 0 |  |  |  |  |  |  |  |  |
| GO:0090031\_positive\_regulation\_of\_steroid\_hormone\_biosynthetic\_process | 1 | 0 |  |  |  |  |  |  |  |  |
| GO:0010557\_positive\_regulation\_of\_macromolecule\_biosynthetic\_process | 334 | 0 | 0.000000 | 0.000000 | 1001 | 842.653226 | 895.28 | 947.906774 | 0.894386 |
| GO:0002694\_regulation\_of\_leukocyte\_activation | 70 | 0 | 0.000000 | 0.000000 | 1007 | 848.281954 | 900.48 | 952.678046 | 0.894220 |
| GO:0009124\_nucleoside\_monophosphate\_biosynthetic\_process | 70 | 0 | 0.000000 | 0.000000 | 1007 | 848.281954 | 900.48 | 952.678046 | 0.894220 |
| GO:0009416\_response\_to\_light\_stimulus | 70 | 0 | 0.000000 | 0.000000 | 1007 | 848.281954 | 900.48 | 952.678046 | 0.894220 |
| GO:0016032\_viral\_reproduction | 70 | 0 | 0.000000 | 0.000000 | 1007 | 848.281954 | 900.48 | 952.678046 | 0.894220 |
| GO:0022411\_cellular\_component\_disassembly | 70 | 0 | 0.000000 | 0.000000 | 1007 | 848.281954 | 900.48 | 952.678046 | 0.894220 |
| GO:0051170\_nuclear\_import | 70 | 0 | 0.000000 | 0.000000 | 1007 | 848.281954 | 900.48 | 952.678046 | 0.894220 |
| GO:0002682\_regulation\_of\_immune\_system\_process | 196 | 0 | 0.000000 | 0.000000 | 1009 | 850.757741 | 902.57 | 954.382259 | 0.894519 |
| GO:0009308\_amine\_metabolic\_process | 196 | 0 | 0.000000 | 0.000000 | 1009 | 850.757741 | 902.57 | 954.382259 | 0.894519 |
| GO:0006519\_cellular\_amino\_acid\_and\_derivative\_metabolic\_process | 173 | 0 | 0.000000 | 0.000000 | 1011 | 853.935093 | 905.25 | 956.564907 | 0.895401 |
| GO:0046483\_heterocycle\_metabolic\_process | 173 | 0 | 0.000000 | 0.000000 | 1011 | 853.935093 | 905.25 | 956.564907 | 0.895401 |
| GO:0043068\_positive\_regulation\_of\_programmed\_cell\_death | 246 | 0 | 0.000000 | 0.000000 | 1012 | 856.242605 | 907.27 | 958.297395 | 0.896512 |
| GO:0009100\_glycoprotein\_metabolic\_process | 139 | 0 | 0.000000 | 0.000000 | 1014 | 858.585410 | 909.29 | 959.994590 | 0.896736 |
| GO:0048534\_hemopoietic\_or\_lymphoid\_organ\_development | 139 | 0 | 0.000000 | 0.000000 | 1014 | 858.585410 | 909.29 | 959.994590 | 0.896736 |
| GO:0006790\_sulfur\_metabolic\_process | 62 | 0 | 0.000000 | 0.000000 | 1023 | 871.918606 | 921.82 | 971.721394 | 0.901095 |
| GO:0007420\_brain\_development | 62 | 0 | 0.000000 | 0.000000 | 1023 | 871.918606 | 921.82 | 971.721394 | 0.901095 |
| GO:0007586\_digestion | 62 | 0 | 0.000000 | 0.000000 | 1023 | 871.918606 | 921.82 | 971.721394 | 0.901095 |
| GO:0009190\_cyclic\_nucleotide\_biosynthetic\_process | 62 | 0 | 0.000000 | 0.000000 | 1023 | 871.918606 | 921.82 | 971.721394 | 0.901095 |
| GO:0031145\_anaphase-promoting\_complex-dependent\_proteasomal\_ubiquitin-dependent\_protein\_catabolic\_process | 62 | 0 | 0.000000 | 0.000000 | 1023 | 871.918606 | 921.82 | 971.721394 | 0.901095 |
| GO:0043406\_positive\_regulation\_of\_MAP\_kinase\_activity | 62 | 0 | 0.000000 | 0.000000 | 1023 | 871.918606 | 921.82 | 971.721394 | 0.901095 |
| GO:0044242\_cellular\_lipid\_catabolic\_process | 62 | 0 | 0.000000 | 0.000000 | 1023 | 871.918606 | 921.82 | 971.721394 | 0.901095 |
| GO:0051436\_negative\_regulation\_of\_ubiquitin-protein\_ligase\_activity\_during\_mitotic\_cell\_cycle | 62 | 0 | 0.000000 | 0.000000 | 1023 | 871.918606 | 921.82 | 971.721394 | 0.901095 |
| GO:0052548\_regulation\_of\_endopeptidase\_activity | 62 | 0 | 0.000000 | 0.000000 | 1023 | 871.918606 | 921.82 | 971.721394 | 0.901095 |
| GO:0000077\_DNA\_damage\_checkpoint | 37 | 0 | 0.000000 | 0.000000 | 1031 | 884.382739 | 933.69 | 982.997261 | 0.905616 |
| GO:0002443\_leukocyte\_mediated\_immunity | 37 | 0 | 0.000000 | 0.000000 | 1031 | 884.382739 | 933.69 | 982.997261 | 0.905616 |
| GO:0002460\_adaptive\_immune\_response\_based\_on\_somatic\_recombination\_of\_immune\_receptors\_built\_from\_immunoglobulin\_superfamily\_domains | 37 | 0 | 0.000000 | 0.000000 | 1031 | 884.382739 | 933.69 | 982.997261 | 0.905616 |
| GO:0007584\_response\_to\_nutrient | 37 | 0 | 0.000000 | 0.000000 | 1031 | 884.382739 | 933.69 | 982.997261 | 0.905616 |
| GO:0032956\_regulation\_of\_actin\_cytoskeleton\_organization | 37 | 0 | 0.000000 | 0.000000 | 1031 | 884.382739 | 933.69 | 982.997261 | 0.905616 |
| GO:0046489\_phosphoinositide\_biosynthetic\_process | 37 | 0 | 0.000000 | 0.000000 | 1031 | 884.382739 | 933.69 | 982.997261 | 0.905616 |
| GO:0046700\_heterocycle\_catabolic\_process | 37 | 0 | 0.000000 | 0.000000 | 1031 | 884.382739 | 933.69 | 982.997261 | 0.905616 |
| GO:0046822\_regulation\_of\_nucleocytoplasmic\_transport | 37 | 0 | 0.000000 | 0.000000 | 1031 | 884.382739 | 933.69 | 982.997261 | 0.905616 |
| GO:0000187\_activation\_of\_MAPK\_activity | 50 | 0 | 0.000000 | 0.000000 | 1036 | 891.979529 | 940.94 | 989.900471 | 0.908243 |
| GO:0007389\_pattern\_specification\_process | 50 | 0 | 0.000000 | 0.000000 | 1036 | 891.979529 | 940.94 | 989.900471 | 0.908243 |
| GO:0030098\_lymphocyte\_differentiation | 50 | 0 | 0.000000 | 0.000000 | 1036 | 891.979529 | 940.94 | 989.900471 | 0.908243 |
| GO:0045761\_regulation\_of\_adenylate\_cyclase\_activity | 50 | 0 | 0.000000 | 0.000000 | 1036 | 891.979529 | 940.94 | 989.900471 | 0.908243 |
| GO:0048598\_embryonic\_morphogenesis | 50 | 0 | 0.000000 | 0.000000 | 1036 | 891.979529 | 940.94 | 989.900471 | 0.908243 |
| GO:0006163\_purine\_nucleotide\_metabolic\_process | 99 | 0 | 0.000000 | 0.000000 | 1038 | 894.625041 | 943.38 | 992.134959 | 0.908844 |
| GO:0016569\_covalent\_chromatin\_modification | 99 | 0 | 0.000000 | 0.000000 | 1038 | 894.625041 | 943.38 | 992.134959 | 0.908844 |
| GO:0006725\_cellular\_aromatic\_compound\_metabolic\_process | 55 | 0 | 0.000000 | 0.000000 | 1043 | 900.179104 | 948.76 | 997.340896 | 0.909645 |
| GO:0007606\_sensory\_perception\_of\_chemical\_stimulus | 55 | 0 | 0.000000 | 0.000000 | 1043 | 900.179104 | 948.76 | 997.340896 | 0.909645 |
| GO:0030814\_regulation\_of\_cAMP\_metabolic\_process | 55 | 0 | 0.000000 | 0.000000 | 1043 | 900.179104 | 948.76 | 997.340896 | 0.909645 |
| GO:0030817\_regulation\_of\_cAMP\_biosynthetic\_process | 55 | 0 | 0.000000 | 0.000000 | 1043 | 900.179104 | 948.76 | 997.340896 | 0.909645 |
| GO:0043543\_protein\_amino\_acid\_acylation | 55 | 0 | 0.000000 | 0.000000 | 1043 | 900.179104 | 948.76 | 997.340896 | 0.909645 |
| GO:0006333\_chromatin\_assembly\_or\_disassembly | 52 | 0 | 0.000000 | 0.000000 | 1049 | 906.612788 | 954.96 | 1003.307212 | 0.910353 |
| GO:0006473\_protein\_amino\_acid\_acetylation | 52 | 0 | 0.000000 | 0.000000 | 1049 | 906.612788 | 954.96 | 1003.307212 | 0.910353 |
| GO:0022904\_respiratory\_electron\_transport\_chain | 52 | 0 | 0.000000 | 0.000000 | 1049 | 906.612788 | 954.96 | 1003.307212 | 0.910353 |
| GO:0042089\_cytokine\_biosynthetic\_process | 52 | 0 | 0.000000 | 0.000000 | 1049 | 906.612788 | 954.96 | 1003.307212 | 0.910353 |
| GO:0044270\_nitrogen\_compound\_catabolic\_process | 52 | 0 | 0.000000 | 0.000000 | 1049 | 906.612788 | 954.96 | 1003.307212 | 0.910353 |
| GO:0060284\_regulation\_of\_cell\_development | 52 | 0 | 0.000000 | 0.000000 | 1049 | 906.612788 | 954.96 | 1003.307212 | 0.910353 |
| GO:0000075\_cell\_cycle\_checkpoint | 73 | 0 | 0.000000 | 0.000000 | 1056 | 914.437928 | 962.45 | 1010.462072 | 0.911411 |
| GO:0006352\_transcription\_initiation | 73 | 0 | 0.000000 | 0.000000 | 1056 | 914.437928 | 962.45 | 1010.462072 | 0.911411 |
| GO:0007187\_G-protein\_signaling\_\_coupled\_to\_cyclic\_nucleotide\_second\_messenger | 73 | 0 | 0.000000 | 0.000000 | 1056 | 914.437928 | 962.45 | 1010.462072 | 0.911411 |
| GO:0009894\_regulation\_of\_catabolic\_process | 73 | 0 | 0.000000 | 0.000000 | 1056 | 914.437928 | 962.45 | 1010.462072 | 0.911411 |
| GO:0030099\_myeloid\_cell\_differentiation | 73 | 0 | 0.000000 | 0.000000 | 1056 | 914.437928 | 962.45 | 1010.462072 | 0.911411 |
| GO:0032990\_cell\_part\_morphogenesis | 73 | 0 | 0.000000 | 0.000000 | 1056 | 914.437928 | 962.45 | 1010.462072 | 0.911411 |
| GO:0055085\_transmembrane\_transport | 73 | 0 | 0.000000 | 0.000000 | 1056 | 914.437928 | 962.45 | 1010.462072 | 0.911411 |
| GO:0019226\_transmission\_of\_nerve\_impulse | 209 | 0 | 0.000000 | 0.000000 | 1059 | 918.063436 | 965.67 | 1013.276564 | 0.911870 |
| GO:0043069\_negative\_regulation\_of\_programmed\_cell\_death | 209 | 0 | 0.000000 | 0.000000 | 1059 | 918.063436 | 965.67 | 1013.276564 | 0.911870 |
| GO:0060548\_negative\_regulation\_of\_cell\_death | 209 | 0 | 0.000000 | 0.000000 | 1059 | 918.063436 | 965.67 | 1013.276564 | 0.911870 |
| GO:0001525\_angiogenesis | 69 | 0 | 0.000000 | 0.000000 | 1064 | 924.382178 | 971.63 | 1018.877822 | 0.913186 |
| GO:0007005\_mitochondrion\_organization | 69 | 0 | 0.000000 | 0.000000 | 1064 | 924.382178 | 971.63 | 1018.877822 | 0.913186 |
| GO:0016042\_lipid\_catabolic\_process | 69 | 0 | 0.000000 | 0.000000 | 1064 | 924.382178 | 971.63 | 1018.877822 | 0.913186 |
| GO:0045333\_cellular\_respiration | 69 | 0 | 0.000000 | 0.000000 | 1064 | 924.382178 | 971.63 | 1018.877822 | 0.913186 |
| GO:0051351\_positive\_regulation\_of\_ligase\_activity | 69 | 0 | 0.000000 | 0.000000 | 1064 | 924.382178 | 971.63 | 1018.877822 | 0.913186 |
| GO:0007169\_transmembrane\_receptor\_protein\_tyrosine\_kinase\_signaling\_pathway | 157 | 0 | 0.000000 | 0.000000 | 1066 | 926.913757 | 973.9 | 1020.886243 | 0.913602 |
| GO:0070887\_cellular\_response\_to\_chemical\_stimulus | 157 | 0 | 0.000000 | 0.000000 | 1066 | 926.913757 | 973.9 | 1020.886243 | 0.913602 |
| GO:0000271\_polysaccharide\_biosynthetic\_process | 38 | 0 | 0.000000 | 0.000000 | 1084 | 945.307557 | 991.49 | 1037.672443 | 0.914659 |
| GO:0002250\_adaptive\_immune\_response | 38 | 0 | 0.000000 | 0.000000 | 1084 | 945.307557 | 991.49 | 1037.672443 | 0.914659 |
| GO:0008016\_regulation\_of\_heart\_contraction | 38 | 0 | 0.000000 | 0.000000 | 1084 | 945.307557 | 991.49 | 1037.672443 | 0.914659 |
| GO:0009411\_response\_to\_UV | 38 | 0 | 0.000000 | 0.000000 | 1084 | 945.307557 | 991.49 | 1037.672443 | 0.914659 |
| GO:0019933\_cAMP-mediated\_signaling | 38 | 0 | 0.000000 | 0.000000 | 1084 | 945.307557 | 991.49 | 1037.672443 | 0.914659 |
| GO:0030336\_negative\_regulation\_of\_cell\_migration | 38 | 0 | 0.000000 | 0.000000 | 1084 | 945.307557 | 991.49 | 1037.672443 | 0.914659 |
| GO:0032869\_cellular\_response\_to\_insulin\_stimulus | 38 | 0 | 0.000000 | 0.000000 | 1084 | 945.307557 | 991.49 | 1037.672443 | 0.914659 |
| GO:0042113\_B\_cell\_activation | 38 | 0 | 0.000000 | 0.000000 | 1084 | 945.307557 | 991.49 | 1037.672443 | 0.914659 |
| GO:0042493\_response\_to\_drug | 38 | 0 | 0.000000 | 0.000000 | 1084 | 945.307557 | 991.49 | 1037.672443 | 0.914659 |
| GO:0045765\_regulation\_of\_angiogenesis | 38 | 0 | 0.000000 | 0.000000 | 1084 | 945.307557 | 991.49 | 1037.672443 | 0.914659 |
| GO:0045787\_positive\_regulation\_of\_cell\_cycle | 38 | 0 | 0.000000 | 0.000000 | 1084 | 945.307557 | 991.49 | 1037.672443 | 0.914659 |
| GO:0046849\_bone\_remodeling | 38 | 0 | 0.000000 | 0.000000 | 1084 | 945.307557 | 991.49 | 1037.672443 | 0.914659 |
| GO:0048511\_rhythmic\_process | 38 | 0 | 0.000000 | 0.000000 | 1084 | 945.307557 | 991.49 | 1037.672443 | 0.914659 |
| GO:0050730\_regulation\_of\_peptidyl-tyrosine\_phosphorylation | 38 | 0 | 0.000000 | 0.000000 | 1084 | 945.307557 | 991.49 | 1037.672443 | 0.914659 |
| GO:0051091\_positive\_regulation\_of\_transcription\_factor\_activity | 38 | 0 | 0.000000 | 0.000000 | 1084 | 945.307557 | 991.49 | 1037.672443 | 0.914659 |
| GO:0051188\_cofactor\_biosynthetic\_process | 38 | 0 | 0.000000 | 0.000000 | 1084 | 945.307557 | 991.49 | 1037.672443 | 0.914659 |
| GO:0060627\_regulation\_of\_vesicle-mediated\_transport | 38 | 0 | 0.000000 | 0.000000 | 1084 | 945.307557 | 991.49 | 1037.672443 | 0.914659 |
| GO:0090047\_positive\_regulation\_of\_transcription\_regulator\_activity | 38 | 0 | 0.000000 | 0.000000 | 1084 | 945.307557 | 991.49 | 1037.672443 | 0.914659 |
| GO:0044265\_cellular\_macromolecule\_catabolic\_process | 239 | 0 | 0.000000 | 0.000000 | 1085 | 946.020691 | 992.17 | 1038.319309 | 0.914442 |
| GO:0007015\_actin\_filament\_organization | 67 | 0 | 0.000000 | 0.000000 | 1088 | 949.756951 | 995.71 | 1041.663049 | 0.915175 |
| GO:0009187\_cyclic\_nucleotide\_metabolic\_process | 67 | 0 | 0.000000 | 0.000000 | 1088 | 949.756951 | 995.71 | 1041.663049 | 0.915175 |
| GO:0051443\_positive\_regulation\_of\_ubiquitin-protein\_ligase\_activity | 67 | 0 | 0.000000 | 0.000000 | 1088 | 949.756951 | 995.71 | 1041.663049 | 0.915175 |
| GO:0006575\_cellular\_amino\_acid\_derivative\_metabolic\_process | 76 | 0 | 0.000000 | 0.000000 | 1092 | 955.509923 | 1000.89 | 1046.270077 | 0.916566 |
| GO:0034504\_protein\_localization\_in\_nucleus | 76 | 0 | 0.000000 | 0.000000 | 1092 | 955.509923 | 1000.89 | 1046.270077 | 0.916566 |
| GO:0051051\_negative\_regulation\_of\_transport | 76 | 0 | 0.000000 | 0.000000 | 1092 | 955.509923 | 1000.89 | 1046.270077 | 0.916566 |
| GO:0051101\_regulation\_of\_DNA\_binding | 76 | 0 | 0.000000 | 0.000000 | 1092 | 955.509923 | 1000.89 | 1046.270077 | 0.916566 |
| GO:0051276\_chromosome\_organization | 272 | 0 | 0.000000 | 0.000000 | 1093 | 957.167019 | 1002.44 | 1047.712981 | 0.917145 |
| GO:0002696\_positive\_regulation\_of\_leukocyte\_activation | 46 | 0 | 0.000000 | 0.000000 | 1103 | 971.402669 | 1016.24 | 1061.077331 | 0.921342 |
| GO:0006413\_translational\_initiation | 46 | 0 | 0.000000 | 0.000000 | 1103 | 971.402669 | 1016.24 | 1061.077331 | 0.921342 |
| GO:0007588\_excretion | 46 | 0 | 0.000000 | 0.000000 | 1103 | 971.402669 | 1016.24 | 1061.077331 | 0.921342 |
| GO:0010638\_positive\_regulation\_of\_organelle\_organization | 46 | 0 | 0.000000 | 0.000000 | 1103 | 971.402669 | 1016.24 | 1061.077331 | 0.921342 |
| GO:0010639\_negative\_regulation\_of\_organelle\_organization | 46 | 0 | 0.000000 | 0.000000 | 1103 | 971.402669 | 1016.24 | 1061.077331 | 0.921342 |
| GO:0030384\_phosphoinositide\_metabolic\_process | 46 | 0 | 0.000000 | 0.000000 | 1103 | 971.402669 | 1016.24 | 1061.077331 | 0.921342 |
| GO:0042157\_lipoprotein\_metabolic\_process | 46 | 0 | 0.000000 | 0.000000 | 1103 | 971.402669 | 1016.24 | 1061.077331 | 0.921342 |
| GO:0042254\_ribosome\_biogenesis | 46 | 0 | 0.000000 | 0.000000 | 1103 | 971.402669 | 1016.24 | 1061.077331 | 0.921342 |
| GO:0046879\_hormone\_secretion | 46 | 0 | 0.000000 | 0.000000 | 1103 | 971.402669 | 1016.24 | 1061.077331 | 0.921342 |
| GO:0065004\_protein-DNA\_complex\_assembly | 46 | 0 | 0.000000 | 0.000000 | 1103 | 971.402669 | 1016.24 | 1061.077331 | 0.921342 |
| GO:0007283\_spermatogenesis | 130 | 0 | 0.000000 | 0.000000 | 1105 | 974.170913 | 1018.73 | 1063.289087 | 0.921928 |
| GO:0048232\_male\_gamete\_generation | 130 | 0 | 0.000000 | 0.000000 | 1105 | 974.170913 | 1018.73 | 1063.289087 | 0.921928 |
| GO:0003001\_generation\_of\_a\_signal\_involved\_in\_cell-cell\_signaling | 64 | 0 | 0.000000 | 0.000000 | 1114 | 983.395543 | 1027.58 | 1071.764457 | 0.922424 |
| GO:0006310\_DNA\_recombination | 64 | 0 | 0.000000 | 0.000000 | 1114 | 983.395543 | 1027.58 | 1071.764457 | 0.922424 |
| GO:0006323\_DNA\_packaging | 64 | 0 | 0.000000 | 0.000000 | 1114 | 983.395543 | 1027.58 | 1071.764457 | 0.922424 |
| GO:0015837\_amine\_transport | 64 | 0 | 0.000000 | 0.000000 | 1114 | 983.395543 | 1027.58 | 1071.764457 | 0.922424 |
| GO:0016051\_carbohydrate\_biosynthetic\_process | 64 | 0 | 0.000000 | 0.000000 | 1114 | 983.395543 | 1027.58 | 1071.764457 | 0.922424 |
| GO:0031098\_stress-activated\_protein\_kinase\_signaling\_pathway | 64 | 0 | 0.000000 | 0.000000 | 1114 | 983.395543 | 1027.58 | 1071.764457 | 0.922424 |
| GO:0042327\_positive\_regulation\_of\_phosphorylation | 64 | 0 | 0.000000 | 0.000000 | 1114 | 983.395543 | 1027.58 | 1071.764457 | 0.922424 |
| GO:0051352\_negative\_regulation\_of\_ligase\_activity | 64 | 0 | 0.000000 | 0.000000 | 1114 | 983.395543 | 1027.58 | 1071.764457 | 0.922424 |
| GO:0051444\_negative\_regulation\_of\_ubiquitin-protein\_ligase\_activity | 64 | 0 | 0.000000 | 0.000000 | 1114 | 983.395543 | 1027.58 | 1071.764457 | 0.922424 |
| GO:0008610\_lipid\_biosynthetic\_process | 179 | 0 | 0.000000 | 0.000000 | 1115 | 984.159959 | 1028.29 | 1072.420041 | 0.922233 |
| GO:0002521\_leukocyte\_differentiation | 87 | 0 | 0.000000 | 0.000000 | 1117 | 986.047546 | 1030.0 | 1073.952454 | 0.922113 |
| GO:0051052\_regulation\_of\_DNA\_metabolic\_process | 87 | 0 | 0.000000 | 0.000000 | 1117 | 986.047546 | 1030.0 | 1073.952454 | 0.922113 |
| GO:0000019\_regulation\_of\_mitotic\_recombination | 2 | 0 |  |  |  |  |  |  |  |  |
| GO:0000022\_mitotic\_spindle\_elongation | 2 | 0 |  |  |  |  |  |  |  |  |
| GO:0000059\_protein\_import\_into\_nucleus\_\_docking | 2 | 0 |  |  |  |  |  |  |  |  |
| GO:0000066\_mitochondrial\_ornithine\_transport | 2 | 0 |  |  |  |  |  |  |  |  |
| GO:0000183\_chromatin\_silencing\_at\_rDNA | 2 | 0 |  |  |  |  |  |  |  |  |
| GO:0000305\_response\_to\_oxygen\_radical | 2 | 0 |  |  |  |  |  |  |  |  |
| GO:0000429\_regulation\_of\_transcription\_from\_RNA\_polymerase\_II\_promoter\_by\_carbon\_catabolites | 2 | 0 |  |  |  |  |  |  |  |  |
| GO:0000430\_regulation\_of\_transcription\_from\_RNA\_polymerase\_II\_promoter\_by\_glucose | 2 | 0 |  |  |  |  |  |  |  |  |
| GO:0000432\_positive\_regulation\_of\_transcription\_from\_RNA\_polymerase\_II\_promoter\_by\_glucose | 2 | 0 |  |  |  |  |  |  |  |  |
| GO:0000436\_positive\_regulation\_of\_transcription\_from\_RNA\_polymerase\_II\_promoter\_by\_carbon\_catabolites | 2 | 0 |  |  |  |  |  |  |  |  |
| GO:0000460\_maturation\_of\_5.8S\_rRNA | 2 | 0 |  |  |  |  |  |  |  |  |
| GO:0000466\_maturation\_of\_5.8S\_rRNA\_from\_tricistronic\_rRNA\_transcript\_(SSU-rRNA\_\_5.8S\_rRNA\_\_LSU-rRNA) | 2 | 0 |  |  |  |  |  |  |  |  |
| GO:0000729\_DNA\_double-strand\_break\_processing | 2 | 0 |  |  |  |  |  |  |  |  |
| GO:0000733\_DNA\_strand\_renaturation | 2 | 0 |  |  |  |  |  |  |  |  |
| GO:0000920\_cell\_separation\_during\_cytokinesis | 2 | 0 |  |  |  |  |  |  |  |  |
| GO:0001101\_response\_to\_acid | 2 | 0 |  |  |  |  |  |  |  |  |
| GO:0001300\_chronological\_cell\_aging | 2 | 0 |  |  |  |  |  |  |  |  |
| GO:0001301\_progressive\_alteration\_of\_chromatin\_during\_cell\_aging | 2 | 0 |  |  |  |  |  |  |  |  |
| GO:0001304\_progressive\_alteration\_of\_chromatin\_during\_replicative\_cell\_aging | 2 | 0 |  |  |  |  |  |  |  |  |
| GO:0001309\_age-dependent\_telomere\_shortening | 2 | 0 |  |  |  |  |  |  |  |  |
| GO:0001507\_acetylcholine\_catabolic\_process\_in\_synaptic\_cleft | 2 | 0 |  |  |  |  |  |  |  |  |
| GO:0001522\_pseudouridine\_synthesis | 2 | 0 |  |  |  |  |  |  |  |  |
| GO:0001547\_antral\_ovarian\_follicle\_growth | 2 | 0 |  |  |  |  |  |  |  |  |
| GO:0001550\_ovarian\_cumulus\_expansion | 2 | 0 |  |  |  |  |  |  |  |  |
| GO:0001556\_oocyte\_maturation | 2 | 0 |  |  |  |  |  |  |  |  |
| GO:0001562\_response\_to\_protozoan | 2 | 0 |  |  |  |  |  |  |  |  |
| GO:0001582\_detection\_of\_chemical\_stimulus\_involved\_in\_sensory\_perception\_of\_sweet\_taste | 2 | 0 |  |  |  |  |  |  |  |  |
| GO:0001667\_ameboidal\_cell\_migration | 2 | 0 |  |  |  |  |  |  |  |  |
| GO:0001678\_cellular\_glucose\_homeostasis | 2 | 0 |  |  |  |  |  |  |  |  |
| GO:0001702\_gastrulation\_with\_mouth\_forming\_second | 2 | 0 |  |  |  |  |  |  |  |  |
| GO:0001736\_establishment\_of\_planar\_polarity | 2 | 0 |  |  |  |  |  |  |  |  |
| GO:0001738\_morphogenesis\_of\_a\_polarized\_epithelium | 2 | 0 |  |  |  |  |  |  |  |  |
| GO:0001756\_somitogenesis | 2 | 0 |  |  |  |  |  |  |  |  |
| GO:0001766\_membrane\_raft\_polarization | 2 | 0 |  |  |  |  |  |  |  |  |
| GO:0001780\_neutrophil\_homeostasis | 2 | 0 |  |  |  |  |  |  |  |  |
| GO:0001781\_neutrophil\_apoptosis | 2 | 0 |  |  |  |  |  |  |  |  |
| GO:0001825\_blastocyst\_formation | 2 | 0 |  |  |  |  |  |  |  |  |
| GO:0001829\_trophectodermal\_cell\_differentiation | 2 | 0 |  |  |  |  |  |  |  |  |
| GO:0001840\_neural\_plate\_development | 2 | 0 |  |  |  |  |  |  |  |  |
| GO:0001868\_regulation\_of\_complement\_activation\_\_lectin\_pathway | 2 | 0 |  |  |  |  |  |  |  |  |
| GO:0001869\_negative\_regulation\_of\_complement\_activation\_\_lectin\_pathway | 2 | 0 |  |  |  |  |  |  |  |  |
| GO:0001880\_Mullerian\_duct\_regression | 2 | 0 |  |  |  |  |  |  |  |  |
| GO:0001885\_endothelial\_cell\_development | 2 | 0 |  |  |  |  |  |  |  |  |
| GO:0001897\_cytolysis\_by\_symbiont\_of\_host\_cells | 2 | 0 |  |  |  |  |  |  |  |  |
| GO:0001907\_killing\_by\_symbiont\_of\_host\_cells | 2 | 0 |  |  |  |  |  |  |  |  |
| GO:0001921\_positive\_regulation\_of\_receptor\_recycling | 2 | 0 |  |  |  |  |  |  |  |  |
| GO:0001967\_suckling\_behavior | 2 | 0 |  |  |  |  |  |  |  |  |
| GO:0001975\_response\_to\_amphetamine | 2 | 0 |  |  |  |  |  |  |  |  |
| GO:0002003\_angiotensin\_maturation | 2 | 0 |  |  |  |  |  |  |  |  |
| GO:0002016\_regulation\_of\_blood\_volume\_by\_renin-angiotensin | 2 | 0 |  |  |  |  |  |  |  |  |
| GO:0002032\_desensitization\_of\_G-protein\_coupled\_receptor\_protein\_signaling\_pathway\_by\_arrestin | 2 | 0 |  |  |  |  |  |  |  |  |
| GO:0002090\_regulation\_of\_receptor\_internalization | 2 | 0 |  |  |  |  |  |  |  |  |
| GO:0002092\_positive\_regulation\_of\_receptor\_internalization | 2 | 0 |  |  |  |  |  |  |  |  |
| GO:0002227\_innate\_immune\_response\_in\_mucosa | 2 | 0 |  |  |  |  |  |  |  |  |
| GO:0002248\_connective\_tissue\_replacement\_during\_inflammatory\_response | 2 | 0 |  |  |  |  |  |  |  |  |
| GO:0002254\_kinin\_cascade | 2 | 0 |  |  |  |  |  |  |  |  |
| GO:0002275\_myeloid\_cell\_activation\_during\_immune\_response | 2 | 0 |  |  |  |  |  |  |  |  |
| GO:0002281\_macrophage\_activation\_during\_immune\_response | 2 | 0 |  |  |  |  |  |  |  |  |
| GO:0002291\_T\_cell\_activation\_via\_T\_cell\_receptor\_contact\_with\_antigen\_bound\_to\_MHC\_molecule\_on\_antigen\_presenting\_cell | 2 | 0 |  |  |  |  |  |  |  |  |
| GO:0002353\_plasma\_kallikrein-kinin\_cascade | 2 | 0 |  |  |  |  |  |  |  |  |
| GO:0002378\_immunoglobulin\_biosynthetic\_process | 2 | 0 |  |  |  |  |  |  |  |  |
| GO:0002384\_hepatic\_immune\_response | 2 | 0 |  |  |  |  |  |  |  |  |
| GO:0002385\_mucosal\_immune\_response | 2 | 0 |  |  |  |  |  |  |  |  |
| GO:0002504\_antigen\_processing\_and\_presentation\_of\_peptide\_or\_polysaccharide\_antigen\_via\_MHC\_class\_II | 2 | 0 |  |  |  |  |  |  |  |  |
| GO:0002507\_tolerance\_induction | 2 | 0 |  |  |  |  |  |  |  |  |
| GO:0002532\_production\_of\_molecular\_mediator\_of\_acute\_inflammatory\_response | 2 | 0 |  |  |  |  |  |  |  |  |
| GO:0002536\_respiratory\_burst\_during\_acute\_inflammatory\_response | 2 | 0 |  |  |  |  |  |  |  |  |
| GO:0002542\_Factor\_XII\_activation | 2 | 0 |  |  |  |  |  |  |  |  |
| GO:0002544\_chronic\_inflammatory\_response | 2 | 0 |  |  |  |  |  |  |  |  |
| GO:0002548\_monocyte\_chemotaxis | 2 | 0 |  |  |  |  |  |  |  |  |
| GO:0002643\_regulation\_of\_tolerance\_induction | 2 | 0 |  |  |  |  |  |  |  |  |
| GO:0002645\_positive\_regulation\_of\_tolerance\_induction | 2 | 0 |  |  |  |  |  |  |  |  |
| GO:0002675\_positive\_regulation\_of\_acute\_inflammatory\_response | 2 | 0 |  |  |  |  |  |  |  |  |
| GO:0002679\_respiratory\_burst\_during\_defense\_response | 2 | 0 |  |  |  |  |  |  |  |  |
| GO:0002704\_negative\_regulation\_of\_leukocyte\_mediated\_immunity | 2 | 0 |  |  |  |  |  |  |  |  |
| GO:0002707\_negative\_regulation\_of\_lymphocyte\_mediated\_immunity | 2 | 0 |  |  |  |  |  |  |  |  |
| GO:0002710\_negative\_regulation\_of\_T\_cell\_mediated\_immunity | 2 | 0 |  |  |  |  |  |  |  |  |
| GO:0002714\_positive\_regulation\_of\_B\_cell\_mediated\_immunity | 2 | 0 |  |  |  |  |  |  |  |  |
| GO:0002820\_negative\_regulation\_of\_adaptive\_immune\_response | 2 | 0 |  |  |  |  |  |  |  |  |
| GO:0002823\_negative\_regulation\_of\_adaptive\_immune\_response\_based\_on\_somatic\_recombination\_of\_immune\_receptors\_built\_from\_immunoglobulin\_superfamily\_domains | 2 | 0 |  |  |  |  |  |  |  |  |
| GO:0002891\_positive\_regulation\_of\_immunoglobulin\_mediated\_immune\_response | 2 | 0 |  |  |  |  |  |  |  |  |
| GO:0003057\_regulation\_of\_the\_force\_of\_heart\_contraction\_by\_chemical\_signal | 2 | 0 |  |  |  |  |  |  |  |  |
| GO:0003078\_regulation\_of\_natriuresis | 2 | 0 |  |  |  |  |  |  |  |  |
| GO:0006005\_L-fucose\_biosynthetic\_process | 2 | 0 |  |  |  |  |  |  |  |  |
| GO:0006011\_UDP-glucose\_metabolic\_process | 2 | 0 |  |  |  |  |  |  |  |  |
| GO:0006030\_chitin\_metabolic\_process | 2 | 0 |  |  |  |  |  |  |  |  |
| GO:0006032\_chitin\_catabolic\_process | 2 | 0 |  |  |  |  |  |  |  |  |
| GO:0006046\_N-acetylglucosamine\_catabolic\_process | 2 | 0 |  |  |  |  |  |  |  |  |
| GO:0006047\_UDP-N-acetylglucosamine\_metabolic\_process | 2 | 0 |  |  |  |  |  |  |  |  |
| GO:0006054\_N-acetylneuraminate\_metabolic\_process | 2 | 0 |  |  |  |  |  |  |  |  |
| GO:0006063\_uronic\_acid\_metabolic\_process | 2 | 0 |  |  |  |  |  |  |  |  |
| GO:0006122\_mitochondrial\_electron\_transport\_\_ubiquinol\_to\_cytochrome\_c | 2 | 0 |  |  |  |  |  |  |  |  |
| GO:0006184\_GTP\_catabolic\_process | 2 | 0 |  |  |  |  |  |  |  |  |
| GO:0006208\_pyrimidine\_base\_catabolic\_process | 2 | 0 |  |  |  |  |  |  |  |  |
| GO:0006210\_thymine\_catabolic\_process | 2 | 0 |  |  |  |  |  |  |  |  |
| GO:0006212\_uracil\_catabolic\_process | 2 | 0 |  |  |  |  |  |  |  |  |
| GO:0006264\_mitochondrial\_DNA\_replication | 2 | 0 |  |  |  |  |  |  |  |  |
| GO:0006307\_DNA\_dealkylation | 2 | 0 |  |  |  |  |  |  |  |  |
| GO:0006335\_DNA\_replication-dependent\_nucleosome\_assembly | 2 | 0 |  |  |  |  |  |  |  |  |
| GO:0006345\_loss\_of\_chromatin\_silencing | 2 | 0 |  |  |  |  |  |  |  |  |
| GO:0006370\_mRNA\_capping | 2 | 0 |  |  |  |  |  |  |  |  |
| GO:0006398\_histone\_mRNA\_3'-end\_processing | 2 | 0 |  |  |  |  |  |  |  |  |
| GO:0006410\_transcription\_\_RNA-dependent | 2 | 0 |  |  |  |  |  |  |  |  |
| GO:0006422\_aspartyl-tRNA\_aminoacylation | 2 | 0 |  |  |  |  |  |  |  |  |
| GO:0006465\_signal\_peptide\_processing | 2 | 0 |  |  |  |  |  |  |  |  |
| GO:0006475\_internal\_protein\_amino\_acid\_acetylation | 2 | 0 |  |  |  |  |  |  |  |  |
| GO:0006478\_peptidyl-tyrosine\_sulfation | 2 | 0 |  |  |  |  |  |  |  |  |
| GO:0006481\_C-terminal\_protein\_amino\_acid\_methylation | 2 | 0 |  |  |  |  |  |  |  |  |
| GO:0006488\_dolichol-linked\_oligosaccharide\_biosynthetic\_process | 2 | 0 |  |  |  |  |  |  |  |  |
| GO:0006498\_N-terminal\_protein\_lipidation | 2 | 0 |  |  |  |  |  |  |  |  |
| GO:0006517\_protein\_deglycosylation | 2 | 0 |  |  |  |  |  |  |  |  |
| GO:0006521\_regulation\_of\_cellular\_amino\_acid\_metabolic\_process | 2 | 0 |  |  |  |  |  |  |  |  |
| GO:0006537\_glutamate\_biosynthetic\_process | 2 | 0 |  |  |  |  |  |  |  |  |
| GO:0006540\_glutamate\_decarboxylation\_to\_succinate | 2 | 0 |  |  |  |  |  |  |  |  |
| GO:0006541\_glutamine\_metabolic\_process | 2 | 0 |  |  |  |  |  |  |  |  |
| GO:0006551\_leucine\_metabolic\_process | 2 | 0 |  |  |  |  |  |  |  |  |
| GO:0006552\_leucine\_catabolic\_process | 2 | 0 |  |  |  |  |  |  |  |  |
| GO:0006561\_proline\_biosynthetic\_process | 2 | 0 |  |  |  |  |  |  |  |  |
| GO:0006569\_tryptophan\_catabolic\_process | 2 | 0 |  |  |  |  |  |  |  |  |
| GO:0006581\_acetylcholine\_catabolic\_process | 2 | 0 |  |  |  |  |  |  |  |  |
| GO:0006582\_melanin\_metabolic\_process | 2 | 0 |  |  |  |  |  |  |  |  |
| GO:0006583\_melanin\_biosynthetic\_process\_from\_tyrosine | 2 | 0 |  |  |  |  |  |  |  |  |
| GO:0006601\_creatine\_biosynthetic\_process | 2 | 0 |  |  |  |  |  |  |  |  |
| GO:0006608\_snRNP\_protein\_import\_into\_nucleus | 2 | 0 |  |  |  |  |  |  |  |  |
| GO:0006651\_diacylglycerol\_biosynthetic\_process | 2 | 0 |  |  |  |  |  |  |  |  |
| GO:0006679\_glucosylceramide\_biosynthetic\_process | 2 | 0 |  |  |  |  |  |  |  |  |
| GO:0006681\_galactosylceramide\_metabolic\_process | 2 | 0 |  |  |  |  |  |  |  |  |
| GO:0006685\_sphingomyelin\_catabolic\_process | 2 | 0 |  |  |  |  |  |  |  |  |
| GO:0006702\_androgen\_biosynthetic\_process | 2 | 0 |  |  |  |  |  |  |  |  |
| GO:0006703\_estrogen\_biosynthetic\_process | 2 | 0 |  |  |  |  |  |  |  |  |
| GO:0006710\_androgen\_catabolic\_process | 2 | 0 |  |  |  |  |  |  |  |  |
| GO:0006738\_nicotinamide\_riboside\_catabolic\_process | 2 | 0 |  |  |  |  |  |  |  |  |
| GO:0006772\_thiamin\_metabolic\_process | 2 | 0 |  |  |  |  |  |  |  |  |
| GO:0006780\_uroporphyrinogen\_III\_biosynthetic\_process | 2 | 0 |  |  |  |  |  |  |  |  |
| GO:0006784\_heme\_a\_biosynthetic\_process | 2 | 0 |  |  |  |  |  |  |  |  |
| GO:0006824\_cobalt\_ion\_transport | 2 | 0 |  |  |  |  |  |  |  |  |
| GO:0006828\_manganese\_ion\_transport | 2 | 0 |  |  |  |  |  |  |  |  |
| GO:0006880\_intracellular\_sequestering\_of\_iron\_ion | 2 | 0 |  |  |  |  |  |  |  |  |
| GO:0006883\_cellular\_sodium\_ion\_homeostasis | 2 | 0 |  |  |  |  |  |  |  |  |
| GO:0006924\_activation-induced\_cell\_death\_of\_T\_cells | 2 | 0 |  |  |  |  |  |  |  |  |
| GO:0006972\_hyperosmotic\_response | 2 | 0 |  |  |  |  |  |  |  |  |
| GO:0006975\_DNA\_damage\_induced\_protein\_phosphorylation | 2 | 0 |  |  |  |  |  |  |  |  |
| GO:0006982\_response\_to\_lipid\_hydroperoxide | 2 | 0 |  |  |  |  |  |  |  |  |
| GO:0006998\_nuclear\_envelope\_organization | 2 | 0 |  |  |  |  |  |  |  |  |
| GO:0007021\_tubulin\_complex\_assembly | 2 | 0 |  |  |  |  |  |  |  |  |
| GO:0007023\_post-chaperonin\_tubulin\_folding\_pathway | 2 | 0 |  |  |  |  |  |  |  |  |
| GO:0007063\_regulation\_of\_sister\_chromatid\_cohesion | 2 | 0 |  |  |  |  |  |  |  |  |
| GO:0007079\_mitotic\_chromosome\_movement\_towards\_spindle\_pole | 2 | 0 |  |  |  |  |  |  |  |  |
| GO:0007095\_mitotic\_cell\_cycle\_G2\_M\_transition\_DNA\_damage\_checkpoint | 2 | 0 |  |  |  |  |  |  |  |  |
| GO:0007128\_meiotic\_prophase\_I | 2 | 0 |  |  |  |  |  |  |  |  |
| GO:0007141\_male\_meiosis\_I | 2 | 0 |  |  |  |  |  |  |  |  |
| GO:0007143\_female\_meiosis | 2 | 0 |  |  |  |  |  |  |  |  |
| GO:0007191\_activation\_of\_adenylate\_cyclase\_activity\_by\_dopamine\_receptor\_signaling\_pathway | 2 | 0 |  |  |  |  |  |  |  |  |
| GO:0007206\_activation\_of\_phospholipase\_C\_activity\_by\_metabotropic\_glutamate\_receptor\_signaling\_pathway | 2 | 0 |  |  |  |  |  |  |  |  |
| GO:0007231\_osmosensory\_signaling\_pathway | 2 | 0 |  |  |  |  |  |  |  |  |
| GO:0007262\_STAT\_protein\_nuclear\_translocation | 2 | 0 |  |  |  |  |  |  |  |  |
| GO:0007290\_spermatid\_nucleus\_elongation | 2 | 0 |  |  |  |  |  |  |  |  |
| GO:0007308\_oocyte\_construction | 2 | 0 |  |  |  |  |  |  |  |  |
| GO:0007309\_oocyte\_axis\_specification | 2 | 0 |  |  |  |  |  |  |  |  |
| GO:0007341\_penetration\_of\_zona\_pellucida | 2 | 0 |  |  |  |  |  |  |  |  |
| GO:0007379\_segment\_specification | 2 | 0 |  |  |  |  |  |  |  |  |
| GO:0007418\_ventral\_midline\_development | 2 | 0 |  |  |  |  |  |  |  |  |
| GO:0007442\_hindgut\_morphogenesis | 2 | 0 |  |  |  |  |  |  |  |  |
| GO:0007492\_endoderm\_development | 2 | 0 |  |  |  |  |  |  |  |  |
| GO:0007525\_somatic\_muscle\_development | 2 | 0 |  |  |  |  |  |  |  |  |
| GO:0007549\_dosage\_compensation | 2 | 0 |  |  |  |  |  |  |  |  |
| GO:0007628\_adult\_walking\_behavior | 2 | 0 |  |  |  |  |  |  |  |  |
| GO:0008216\_spermidine\_metabolic\_process | 2 | 0 |  |  |  |  |  |  |  |  |
| GO:0008306\_associative\_learning | 2 | 0 |  |  |  |  |  |  |  |  |
| GO:0008582\_regulation\_of\_synaptic\_growth\_at\_neuromuscular\_junction | 2 | 0 |  |  |  |  |  |  |  |  |
| GO:0008593\_regulation\_of\_Notch\_signaling\_pathway | 2 | 0 |  |  |  |  |  |  |  |  |
| GO:0008608\_attachment\_of\_spindle\_microtubules\_to\_kinetochore | 2 | 0 |  |  |  |  |  |  |  |  |
| GO:0008616\_queuosine\_biosynthetic\_process | 2 | 0 |  |  |  |  |  |  |  |  |
| GO:0008618\_7-methylguanosine\_metabolic\_process | 2 | 0 |  |  |  |  |  |  |  |  |
| GO:0008653\_lipopolysaccharide\_metabolic\_process | 2 | 0 |  |  |  |  |  |  |  |  |
| GO:0009051\_pentose-phosphate\_shunt\_\_oxidative\_branch | 2 | 0 |  |  |  |  |  |  |  |  |
| GO:0009082\_branched\_chain\_family\_amino\_acid\_biosynthetic\_process | 2 | 0 |  |  |  |  |  |  |  |  |
| GO:0009103\_lipopolysaccharide\_biosynthetic\_process | 2 | 0 |  |  |  |  |  |  |  |  |
| GO:0009120\_deoxyribonucleoside\_metabolic\_process | 2 | 0 |  |  |  |  |  |  |  |  |
| GO:0009134\_nucleoside\_diphosphate\_catabolic\_process | 2 | 0 |  |  |  |  |  |  |  |  |
| GO:0009162\_deoxyribonucleoside\_monophosphate\_metabolic\_process | 2 | 0 |  |  |  |  |  |  |  |  |
| GO:0009191\_ribonucleoside\_diphosphate\_catabolic\_process | 2 | 0 |  |  |  |  |  |  |  |  |
| GO:0009263\_deoxyribonucleotide\_biosynthetic\_process | 2 | 0 |  |  |  |  |  |  |  |  |
| GO:0009439\_cyanate\_metabolic\_process | 2 | 0 |  |  |  |  |  |  |  |  |
| GO:0009440\_cyanate\_catabolic\_process | 2 | 0 |  |  |  |  |  |  |  |  |
| GO:0009448\_gamma-aminobutyric\_acid\_metabolic\_process | 2 | 0 |  |  |  |  |  |  |  |  |
| GO:0009450\_gamma-aminobutyric\_acid\_catabolic\_process | 2 | 0 |  |  |  |  |  |  |  |  |
| GO:0009452\_RNA\_capping | 2 | 0 |  |  |  |  |  |  |  |  |
| GO:0009586\_rhodopsin\_mediated\_phototransduction | 2 | 0 |  |  |  |  |  |  |  |  |
| GO:0009597\_detection\_of\_virus | 2 | 0 |  |  |  |  |  |  |  |  |
| GO:0009602\_detection\_of\_symbiont | 2 | 0 |  |  |  |  |  |  |  |  |
| GO:0009608\_response\_to\_symbiont | 2 | 0 |  |  |  |  |  |  |  |  |
| GO:0009649\_entrainment\_of\_circadian\_clock | 2 | 0 |  |  |  |  |  |  |  |  |
| GO:0009651\_response\_to\_salt\_stress | 2 | 0 |  |  |  |  |  |  |  |  |
| GO:0009756\_carbohydrate\_mediated\_signaling | 2 | 0 |  |  |  |  |  |  |  |  |
| GO:0010155\_regulation\_of\_proton\_transport | 2 | 0 |  |  |  |  |  |  |  |  |
| GO:0010216\_maintenance\_of\_DNA\_methylation | 2 | 0 |  |  |  |  |  |  |  |  |
| GO:0010248\_establishment\_or\_maintenance\_of\_transmembrane\_electrochemical\_gradient | 2 | 0 |  |  |  |  |  |  |  |  |
| GO:0010389\_regulation\_of\_G2\_M\_transition\_of\_mitotic\_cell\_cycle | 2 | 0 |  |  |  |  |  |  |  |  |
| GO:0010459\_negative\_regulation\_of\_heart\_rate | 2 | 0 |  |  |  |  |  |  |  |  |
| GO:0010470\_regulation\_of\_gastrulation | 2 | 0 |  |  |  |  |  |  |  |  |
| GO:0010506\_regulation\_of\_autophagy | 2 | 0 |  |  |  |  |  |  |  |  |
| GO:0010511\_regulation\_of\_phosphatidylinositol\_biosynthetic\_process | 2 | 0 |  |  |  |  |  |  |  |  |
| GO:0010512\_negative\_regulation\_of\_phosphatidylinositol\_biosynthetic\_process | 2 | 0 |  |  |  |  |  |  |  |  |
| GO:0010519\_negative\_regulation\_of\_phospholipase\_activity | 2 | 0 |  |  |  |  |  |  |  |  |
| GO:0010523\_negative\_regulation\_of\_calcium\_ion\_transport\_into\_cytosol | 2 | 0 |  |  |  |  |  |  |  |  |
| GO:0010533\_regulation\_of\_activation\_of\_Janus\_kinase\_activity | 2 | 0 |  |  |  |  |  |  |  |  |
| GO:0010536\_positive\_regulation\_of\_activation\_of\_Janus\_kinase\_activity | 2 | 0 |  |  |  |  |  |  |  |  |
| GO:0010614\_negative\_regulation\_of\_cardiac\_muscle\_hypertrophy | 2 | 0 |  |  |  |  |  |  |  |  |
| GO:0010616\_negative\_regulation\_of\_cardiac\_muscle\_adaptation | 2 | 0 |  |  |  |  |  |  |  |  |
| GO:0010640\_regulation\_of\_platelet-derived\_growth\_factor\_receptor\_signaling\_pathway | 2 | 0 |  |  |  |  |  |  |  |  |
| GO:0010641\_positive\_regulation\_of\_platelet-derived\_growth\_factor\_receptor\_signaling\_pathway | 2 | 0 |  |  |  |  |  |  |  |  |
| GO:0010670\_positive\_regulation\_of\_oxygen\_and\_reactive\_oxygen\_species\_metabolic\_process | 2 | 0 |  |  |  |  |  |  |  |  |
| GO:0010755\_regulation\_of\_plasminogen\_activation | 2 | 0 |  |  |  |  |  |  |  |  |
| GO:0010761\_fibroblast\_migration | 2 | 0 |  |  |  |  |  |  |  |  |
| GO:0010762\_regulation\_of\_fibroblast\_migration | 2 | 0 |  |  |  |  |  |  |  |  |
| GO:0010763\_positive\_regulation\_of\_fibroblast\_migration | 2 | 0 |  |  |  |  |  |  |  |  |
| GO:0010815\_bradykinin\_catabolic\_process | 2 | 0 |  |  |  |  |  |  |  |  |
| GO:0010818\_T\_cell\_chemotaxis | 2 | 0 |  |  |  |  |  |  |  |  |
| GO:0010819\_regulation\_of\_T\_cell\_chemotaxis | 2 | 0 |  |  |  |  |  |  |  |  |
| GO:0010820\_positive\_regulation\_of\_T\_cell\_chemotaxis | 2 | 0 |  |  |  |  |  |  |  |  |
| GO:0010866\_regulation\_of\_triglyceride\_biosynthetic\_process | 2 | 0 |  |  |  |  |  |  |  |  |
| GO:0010867\_positive\_regulation\_of\_triglyceride\_biosynthetic\_process | 2 | 0 |  |  |  |  |  |  |  |  |
| GO:0010881\_regulation\_of\_cardiac\_muscle\_contraction\_by\_regulation\_of\_the\_release\_of\_sequestered\_calcium\_ion | 2 | 0 |  |  |  |  |  |  |  |  |
| GO:0010882\_regulation\_of\_cardiac\_muscle\_contraction\_by\_calcium\_ion\_signaling | 2 | 0 |  |  |  |  |  |  |  |  |
| GO:0010908\_regulation\_of\_heparan\_sulfate\_proteoglycan\_biosynthetic\_process | 2 | 0 |  |  |  |  |  |  |  |  |
| GO:0010909\_positive\_regulation\_of\_heparan\_sulfate\_proteoglycan\_biosynthetic\_process | 2 | 0 |  |  |  |  |  |  |  |  |
| GO:0010949\_negative\_regulation\_of\_intestinal\_phytosterol\_absorption | 2 | 0 |  |  |  |  |  |  |  |  |
| GO:0010954\_positive\_regulation\_of\_protein\_maturation\_by\_peptide\_bond\_cleavage | 2 | 0 |  |  |  |  |  |  |  |  |
| GO:0010979\_regulation\_of\_vitamin\_D\_24-hydroxylase\_activity | 2 | 0 |  |  |  |  |  |  |  |  |
| GO:0010980\_positive\_regulation\_of\_vitamin\_D\_24-hydroxylase\_activity | 2 | 0 |  |  |  |  |  |  |  |  |
| GO:0010982\_regulation\_of\_high-density\_lipoprotein\_particle\_clearance | 2 | 0 |  |  |  |  |  |  |  |  |
| GO:0014002\_astrocyte\_development | 2 | 0 |  |  |  |  |  |  |  |  |
| GO:0014013\_regulation\_of\_gliogenesis | 2 | 0 |  |  |  |  |  |  |  |  |
| GO:0014014\_negative\_regulation\_of\_gliogenesis | 2 | 0 |  |  |  |  |  |  |  |  |
| GO:0014037\_Schwann\_cell\_differentiation | 2 | 0 |  |  |  |  |  |  |  |  |
| GO:0014044\_Schwann\_cell\_development | 2 | 0 |  |  |  |  |  |  |  |  |
| GO:0014072\_response\_to\_isoquinoline\_alkaloid | 2 | 0 |  |  |  |  |  |  |  |  |
| GO:0014074\_response\_to\_purine | 2 | 0 |  |  |  |  |  |  |  |  |
| GO:0014741\_negative\_regulation\_of\_muscle\_hypertrophy | 2 | 0 |  |  |  |  |  |  |  |  |
| GO:0014745\_negative\_regulation\_of\_muscle\_adaptation | 2 | 0 |  |  |  |  |  |  |  |  |
| GO:0014819\_regulation\_of\_skeletal\_muscle\_contraction | 2 | 0 |  |  |  |  |  |  |  |  |
| GO:0014911\_positive\_regulation\_of\_smooth\_muscle\_cell\_migration | 2 | 0 |  |  |  |  |  |  |  |  |
| GO:0015677\_copper\_ion\_import | 2 | 0 |  |  |  |  |  |  |  |  |
| GO:0015691\_cadmium\_ion\_transport | 2 | 0 |  |  |  |  |  |  |  |  |
| GO:0015732\_prostaglandin\_transport | 2 | 0 |  |  |  |  |  |  |  |  |
| GO:0015788\_UDP-N-acetylglucosamine\_transport | 2 | 0 |  |  |  |  |  |  |  |  |
| GO:0015793\_glycerol\_transport | 2 | 0 |  |  |  |  |  |  |  |  |
| GO:0015801\_aromatic\_amino\_acid\_transport | 2 | 0 |  |  |  |  |  |  |  |  |
| GO:0015808\_L-alanine\_transport | 2 | 0 |  |  |  |  |  |  |  |  |
| GO:0015822\_ornithine\_transport | 2 | 0 |  |  |  |  |  |  |  |  |
| GO:0015824\_proline\_transport | 2 | 0 |  |  |  |  |  |  |  |  |
| GO:0015825\_L-serine\_transport | 2 | 0 |  |  |  |  |  |  |  |  |
| GO:0015860\_purine\_nucleoside\_transport | 2 | 0 |  |  |  |  |  |  |  |  |
| GO:0015870\_acetylcholine\_transport | 2 | 0 |  |  |  |  |  |  |  |  |
| GO:0015871\_choline\_transport | 2 | 0 |  |  |  |  |  |  |  |  |
| GO:0015893\_drug\_transport | 2 | 0 |  |  |  |  |  |  |  |  |
| GO:0015920\_lipopolysaccharide\_transport | 2 | 0 |  |  |  |  |  |  |  |  |
| GO:0015936\_coenzyme\_A\_metabolic\_process | 2 | 0 |  |  |  |  |  |  |  |  |
| GO:0015939\_pantothenate\_metabolic\_process | 2 | 0 |  |  |  |  |  |  |  |  |
| GO:0015942\_formate\_metabolic\_process | 2 | 0 |  |  |  |  |  |  |  |  |
| GO:0015988\_energy\_coupled\_proton\_transport\_\_against\_electrochemical\_gradient | 2 | 0 |  |  |  |  |  |  |  |  |
| GO:0015991\_ATP\_hydrolysis\_coupled\_proton\_transport | 2 | 0 |  |  |  |  |  |  |  |  |
| GO:0015993\_molecular\_hydrogen\_transport | 2 | 0 |  |  |  |  |  |  |  |  |
| GO:0016075\_rRNA\_catabolic\_process | 2 | 0 |  |  |  |  |  |  |  |  |
| GO:0016080\_synaptic\_vesicle\_targeting | 2 | 0 |  |  |  |  |  |  |  |  |
| GO:0016090\_prenol\_metabolic\_process | 2 | 0 |  |  |  |  |  |  |  |  |
| GO:0016093\_polyprenol\_metabolic\_process | 2 | 0 |  |  |  |  |  |  |  |  |
| GO:0016233\_telomere\_capping | 2 | 0 |  |  |  |  |  |  |  |  |
| GO:0016264\_gap\_junction\_assembly | 2 | 0 |  |  |  |  |  |  |  |  |
| GO:0016266\_O-glycan\_processing | 2 | 0 |  |  |  |  |  |  |  |  |
| GO:0016322\_neuron\_remodeling | 2 | 0 |  |  |  |  |  |  |  |  |
| GO:0016482\_cytoplasmic\_transport | 2 | 0 |  |  |  |  |  |  |  |  |
| GO:0016557\_peroxisome\_membrane\_biogenesis | 2 | 0 |  |  |  |  |  |  |  |  |
| GO:0016561\_protein\_import\_into\_peroxisome\_matrix\_\_translocation | 2 | 0 |  |  |  |  |  |  |  |  |
| GO:0016973\_poly(A)+\_mRNA\_export\_from\_nucleus | 2 | 0 |  |  |  |  |  |  |  |  |
| GO:0017004\_cytochrome\_complex\_assembly | 2 | 0 |  |  |  |  |  |  |  |  |
| GO:0017055\_negative\_regulation\_of\_transcriptional\_preinitiation\_complex\_assembly | 2 | 0 |  |  |  |  |  |  |  |  |
| GO:0017145\_stem\_cell\_division | 2 | 0 |  |  |  |  |  |  |  |  |
| GO:0017158\_regulation\_of\_calcium\_ion-dependent\_exocytosis | 2 | 0 |  |  |  |  |  |  |  |  |
| GO:0017182\_peptidyl-diphthamide\_metabolic\_process | 2 | 0 |  |  |  |  |  |  |  |  |
| GO:0017183\_peptidyl-diphthamide\_biosynthetic\_process\_from\_peptidyl-histidine | 2 | 0 |  |  |  |  |  |  |  |  |
| GO:0018198\_peptidyl-cysteine\_modification | 2 | 0 |  |  |  |  |  |  |  |  |
| GO:0018202\_peptidyl-histidine\_modification | 2 | 0 |  |  |  |  |  |  |  |  |
| GO:0018282\_metal\_incorporation\_into\_metallo-sulfur\_cluster | 2 | 0 |  |  |  |  |  |  |  |  |
| GO:0018283\_iron\_incorporation\_into\_metallo-sulfur\_cluster | 2 | 0 |  |  |  |  |  |  |  |  |
| GO:0018347\_protein\_amino\_acid\_farnesylation | 2 | 0 |  |  |  |  |  |  |  |  |
| GO:0018410\_peptide\_or\_protein\_carboxyl-terminal\_blocking | 2 | 0 |  |  |  |  |  |  |  |  |
| GO:0019042\_latent\_virus\_infection | 2 | 0 |  |  |  |  |  |  |  |  |
| GO:0019046\_reactivation\_of\_latent\_virus | 2 | 0 |  |  |  |  |  |  |  |  |
| GO:0019049\_evasion\_of\_host\_defenses\_by\_virus | 2 | 0 |  |  |  |  |  |  |  |  |
| GO:0019076\_release\_of\_virus\_from\_host | 2 | 0 |  |  |  |  |  |  |  |  |
| GO:0019348\_dolichol\_metabolic\_process | 2 | 0 |  |  |  |  |  |  |  |  |
| GO:0019359\_nicotinamide\_nucleotide\_biosynthetic\_process | 2 | 0 |  |  |  |  |  |  |  |  |
| GO:0019363\_pyridine\_nucleotide\_biosynthetic\_process | 2 | 0 |  |  |  |  |  |  |  |  |
| GO:0019374\_galactolipid\_metabolic\_process | 2 | 0 |  |  |  |  |  |  |  |  |
| GO:0019459\_glutamate\_deamidation | 2 | 0 |  |  |  |  |  |  |  |  |
| GO:0019509\_methionine\_salvage | 2 | 0 |  |  |  |  |  |  |  |  |
| GO:0019530\_taurine\_metabolic\_process | 2 | 0 |  |  |  |  |  |  |  |  |
| GO:0019532\_oxalate\_transport | 2 | 0 |  |  |  |  |  |  |  |  |
| GO:0019585\_glucuronate\_metabolic\_process | 2 | 0 |  |  |  |  |  |  |  |  |
| GO:0019605\_butyrate\_metabolic\_process | 2 | 0 |  |  |  |  |  |  |  |  |
| GO:0019626\_short-chain\_fatty\_acid\_catabolic\_process | 2 | 0 |  |  |  |  |  |  |  |  |
| GO:0019730\_antimicrobial\_humoral\_response | 2 | 0 |  |  |  |  |  |  |  |  |
| GO:0019731\_antibacterial\_humoral\_response | 2 | 0 |  |  |  |  |  |  |  |  |
| GO:0019805\_quinolinate\_biosynthetic\_process | 2 | 0 |  |  |  |  |  |  |  |  |
| GO:0019836\_hemolysis\_by\_symbiont\_of\_host\_erythrocytes | 2 | 0 |  |  |  |  |  |  |  |  |
| GO:0019860\_uracil\_metabolic\_process | 2 | 0 |  |  |  |  |  |  |  |  |
| GO:0019896\_axon\_transport\_of\_mitochondrion | 2 | 0 |  |  |  |  |  |  |  |  |
| GO:0019919\_peptidyl-arginine\_methylation\_\_to\_asymmetrical-dimethyl\_arginine | 2 | 0 |  |  |  |  |  |  |  |  |
| GO:0021511\_spinal\_cord\_patterning | 2 | 0 |  |  |  |  |  |  |  |  |
| GO:0021513\_spinal\_cord\_dorsal\_ventral\_patterning | 2 | 0 |  |  |  |  |  |  |  |  |
| GO:0021517\_ventral\_spinal\_cord\_development | 2 | 0 |  |  |  |  |  |  |  |  |
| GO:0021545\_cranial\_nerve\_development | 2 | 0 |  |  |  |  |  |  |  |  |
| GO:0021587\_cerebellum\_morphogenesis | 2 | 0 |  |  |  |  |  |  |  |  |
| GO:0021675\_nerve\_development | 2 | 0 |  |  |  |  |  |  |  |  |
| GO:0021695\_cerebellar\_cortex\_development | 2 | 0 |  |  |  |  |  |  |  |  |
| GO:0021696\_cerebellar\_cortex\_morphogenesis | 2 | 0 |  |  |  |  |  |  |  |  |
| GO:0021795\_cerebral\_cortex\_cell\_migration | 2 | 0 |  |  |  |  |  |  |  |  |
| GO:0021826\_substrate-independent\_telencephalic\_tangential\_migration | 2 | 0 |  |  |  |  |  |  |  |  |
| GO:0021830\_interneuron\_migration\_from\_the\_subpallium\_to\_the\_cortex | 2 | 0 |  |  |  |  |  |  |  |  |
| GO:0021843\_substrate-independent\_telencephalic\_tangential\_interneuron\_migration | 2 | 0 |  |  |  |  |  |  |  |  |
| GO:0021853\_cerebral\_cortex\_GABAergic\_interneuron\_migration | 2 | 0 |  |  |  |  |  |  |  |  |
| GO:0021892\_cerebral\_cortex\_GABAergic\_interneuron\_differentiation | 2 | 0 |  |  |  |  |  |  |  |  |
| GO:0021894\_cerebral\_cortex\_GABAergic\_interneuron\_development | 2 | 0 |  |  |  |  |  |  |  |  |
| GO:0021895\_cerebral\_cortex\_neuron\_differentiation | 2 | 0 |  |  |  |  |  |  |  |  |
| GO:0021910\_smoothened\_signaling\_pathway\_involved\_in\_ventral\_spinal\_cord\_patterning | 2 | 0 |  |  |  |  |  |  |  |  |
| GO:0022011\_myelination\_in\_the\_peripheral\_nervous\_system | 2 | 0 |  |  |  |  |  |  |  |  |
| GO:0022605\_oogenesis\_stage | 2 | 0 |  |  |  |  |  |  |  |  |
| GO:0030150\_protein\_import\_into\_mitochondrial\_matrix | 2 | 0 |  |  |  |  |  |  |  |  |
| GO:0030185\_nitric\_oxide\_transport | 2 | 0 |  |  |  |  |  |  |  |  |
| GO:0030202\_heparin\_metabolic\_process | 2 | 0 |  |  |  |  |  |  |  |  |
| GO:0030205\_dermatan\_sulfate\_metabolic\_process | 2 | 0 |  |  |  |  |  |  |  |  |
| GO:0030208\_dermatan\_sulfate\_biosynthetic\_process | 2 | 0 |  |  |  |  |  |  |  |  |
| GO:0030210\_heparin\_biosynthetic\_process | 2 | 0 |  |  |  |  |  |  |  |  |
| GO:0030264\_nuclear\_fragmentation\_during\_apoptosis | 2 | 0 |  |  |  |  |  |  |  |  |
| GO:0030300\_regulation\_of\_intestinal\_cholesterol\_absorption | 2 | 0 |  |  |  |  |  |  |  |  |
| GO:0030311\_poly-N-acetyllactosamine\_biosynthetic\_process | 2 | 0 |  |  |  |  |  |  |  |  |
| GO:0030318\_melanocyte\_differentiation | 2 | 0 |  |  |  |  |  |  |  |  |
| GO:0030321\_transepithelial\_chloride\_transport | 2 | 0 |  |  |  |  |  |  |  |  |
| GO:0030490\_maturation\_of\_SSU-rRNA | 2 | 0 |  |  |  |  |  |  |  |  |
| GO:0030505\_inorganic\_diphosphate\_transport | 2 | 0 |  |  |  |  |  |  |  |  |
| GO:0030513\_positive\_regulation\_of\_BMP\_signaling\_pathway | 2 | 0 |  |  |  |  |  |  |  |  |
| GO:0030540\_female\_genitalia\_development | 2 | 0 |  |  |  |  |  |  |  |  |
| GO:0030575\_nuclear\_body\_organization | 2 | 0 |  |  |  |  |  |  |  |  |
| GO:0030578\_PML\_body\_organization | 2 | 0 |  |  |  |  |  |  |  |  |
| GO:0030815\_negative\_regulation\_of\_cAMP\_metabolic\_process | 2 | 0 |  |  |  |  |  |  |  |  |
| GO:0030818\_negative\_regulation\_of\_cAMP\_biosynthetic\_process | 2 | 0 |  |  |  |  |  |  |  |  |
| GO:0030889\_negative\_regulation\_of\_B\_cell\_proliferation | 2 | 0 |  |  |  |  |  |  |  |  |
| GO:0030901\_midbrain\_development | 2 | 0 |  |  |  |  |  |  |  |  |
| GO:0030916\_otic\_vesicle\_formation | 2 | 0 |  |  |  |  |  |  |  |  |
| GO:0030949\_positive\_regulation\_of\_vascular\_endothelial\_growth\_factor\_receptor\_signaling\_pathway | 2 | 0 |  |  |  |  |  |  |  |  |
| GO:0030951\_establishment\_or\_maintenance\_of\_microtubule\_cytoskeleton\_polarity | 2 | 0 |  |  |  |  |  |  |  |  |
| GO:0030952\_establishment\_or\_maintenance\_of\_cytoskeleton\_polarity | 2 | 0 |  |  |  |  |  |  |  |  |
| GO:0030997\_regulation\_of\_centriole-centriole\_cohesion | 2 | 0 |  |  |  |  |  |  |  |  |
| GO:0031000\_response\_to\_caffeine | 2 | 0 |  |  |  |  |  |  |  |  |
| GO:0031061\_negative\_regulation\_of\_histone\_methylation | 2 | 0 |  |  |  |  |  |  |  |  |
| GO:0031062\_positive\_regulation\_of\_histone\_methylation | 2 | 0 |  |  |  |  |  |  |  |  |
| GO:0031122\_cytoplasmic\_microtubule\_organization | 2 | 0 |  |  |  |  |  |  |  |  |
| GO:0031294\_lymphocyte\_costimulation | 2 | 0 |  |  |  |  |  |  |  |  |
| GO:0031295\_T\_cell\_costimulation | 2 | 0 |  |  |  |  |  |  |  |  |
| GO:0031297\_replication\_fork\_processing | 2 | 0 |  |  |  |  |  |  |  |  |
| GO:0031440\_regulation\_of\_mRNA\_3'-end\_processing | 2 | 0 |  |  |  |  |  |  |  |  |
| GO:0031452\_negative\_regulation\_of\_heterochromatin\_formation | 2 | 0 |  |  |  |  |  |  |  |  |
| GO:0031508\_centromeric\_heterochromatin\_formation | 2 | 0 |  |  |  |  |  |  |  |  |
| GO:0031536\_positive\_regulation\_of\_exit\_from\_mitosis | 2 | 0 |  |  |  |  |  |  |  |  |
| GO:0031573\_intra-S\_DNA\_damage\_checkpoint | 2 | 0 |  |  |  |  |  |  |  |  |
| GO:0031580\_membrane\_raft\_distribution | 2 | 0 |  |  |  |  |  |  |  |  |
| GO:0031641\_regulation\_of\_myelination | 2 | 0 |  |  |  |  |  |  |  |  |
| GO:0031642\_negative\_regulation\_of\_myelination | 2 | 0 |  |  |  |  |  |  |  |  |
| GO:0031664\_regulation\_of\_lipopolysaccharide-mediated\_signaling\_pathway | 2 | 0 |  |  |  |  |  |  |  |  |
| GO:0031665\_negative\_regulation\_of\_lipopolysaccharide-mediated\_signaling\_pathway | 2 | 0 |  |  |  |  |  |  |  |  |
| GO:0031936\_negative\_regulation\_of\_chromatin\_silencing | 2 | 0 |  |  |  |  |  |  |  |  |
| GO:0032026\_response\_to\_magnesium\_ion | 2 | 0 |  |  |  |  |  |  |  |  |
| GO:0032069\_regulation\_of\_nuclease\_activity | 2 | 0 |  |  |  |  |  |  |  |  |
| GO:0032096\_negative\_regulation\_of\_response\_to\_food | 2 | 0 |  |  |  |  |  |  |  |  |
| GO:0032099\_negative\_regulation\_of\_appetite | 2 | 0 |  |  |  |  |  |  |  |  |
| GO:0032201\_telomere\_maintenance\_via\_semi-conservative\_replication | 2 | 0 |  |  |  |  |  |  |  |  |
| GO:0032212\_positive\_regulation\_of\_telomere\_maintenance\_via\_telomerase | 2 | 0 |  |  |  |  |  |  |  |  |
| GO:0032213\_regulation\_of\_telomere\_maintenance\_via\_semi-conservative\_replication | 2 | 0 |  |  |  |  |  |  |  |  |
| GO:0032214\_negative\_regulation\_of\_telomere\_maintenance\_via\_semi-conservative\_replication | 2 | 0 |  |  |  |  |  |  |  |  |
| GO:0032225\_regulation\_of\_synaptic\_transmission\_\_dopaminergic | 2 | 0 |  |  |  |  |  |  |  |  |
| GO:0032228\_regulation\_of\_synaptic\_transmission\_\_GABAergic | 2 | 0 |  |  |  |  |  |  |  |  |
| GO:0032236\_positive\_regulation\_of\_calcium\_ion\_transport\_via\_store-operated\_calcium\_channel\_activity | 2 | 0 |  |  |  |  |  |  |  |  |
| GO:0032237\_activation\_of\_store-operated\_calcium\_channel\_activity | 2 | 0 |  |  |  |  |  |  |  |  |
| GO:0032292\_ensheathment\_of\_axons\_in\_the\_peripheral\_nervous\_system | 2 | 0 |  |  |  |  |  |  |  |  |
| GO:0032303\_regulation\_of\_icosanoid\_secretion | 2 | 0 |  |  |  |  |  |  |  |  |
| GO:0032305\_positive\_regulation\_of\_icosanoid\_secretion | 2 | 0 |  |  |  |  |  |  |  |  |
| GO:0032306\_regulation\_of\_prostaglandin\_secretion | 2 | 0 |  |  |  |  |  |  |  |  |
| GO:0032308\_positive\_regulation\_of\_prostaglandin\_secretion | 2 | 0 |  |  |  |  |  |  |  |  |
| GO:0032310\_prostaglandin\_secretion | 2 | 0 |  |  |  |  |  |  |  |  |
| GO:0032328\_alanine\_transport | 2 | 0 |  |  |  |  |  |  |  |  |
| GO:0032329\_serine\_transport | 2 | 0 |  |  |  |  |  |  |  |  |
| GO:0032344\_regulation\_of\_aldosterone\_metabolic\_process | 2 | 0 |  |  |  |  |  |  |  |  |
| GO:0032417\_positive\_regulation\_of\_sodium:hydrogen\_antiporter\_activity | 2 | 0 |  |  |  |  |  |  |  |  |
| GO:0032435\_negative\_regulation\_of\_proteasomal\_ubiquitin-dependent\_protein\_catabolic\_process | 2 | 0 |  |  |  |  |  |  |  |  |
| GO:0032506\_cytokinetic\_process | 2 | 0 |  |  |  |  |  |  |  |  |
| GO:0032510\_endosome\_to\_lysosome\_transport\_via\_multivesicular\_body\_sorting\_pathway | 2 | 0 |  |  |  |  |  |  |  |  |
| GO:0032512\_regulation\_of\_protein\_phosphatase\_type\_2B\_activity | 2 | 0 |  |  |  |  |  |  |  |  |
| GO:0032513\_negative\_regulation\_of\_protein\_phosphatase\_type\_2B\_activity | 2 | 0 |  |  |  |  |  |  |  |  |
| GO:0032516\_positive\_regulation\_of\_phosphoprotein\_phosphatase\_activity | 2 | 0 |  |  |  |  |  |  |  |  |
| GO:0032525\_somite\_rostral\_caudal\_axis\_specification | 2 | 0 |  |  |  |  |  |  |  |  |
| GO:0032620\_interleukin-17\_production | 2 | 0 |  |  |  |  |  |  |  |  |
| GO:0032634\_interleukin-5\_production | 2 | 0 |  |  |  |  |  |  |  |  |
| GO:0032645\_regulation\_of\_granulocyte\_macrophage\_colony-stimulating\_factor\_production | 2 | 0 |  |  |  |  |  |  |  |  |
| GO:0032656\_regulation\_of\_interleukin-13\_production | 2 | 0 |  |  |  |  |  |  |  |  |
| GO:0032660\_regulation\_of\_interleukin-17\_production | 2 | 0 |  |  |  |  |  |  |  |  |
| GO:0032674\_regulation\_of\_interleukin-5\_production | 2 | 0 |  |  |  |  |  |  |  |  |
| GO:0032689\_negative\_regulation\_of\_interferon-gamma\_production | 2 | 0 |  |  |  |  |  |  |  |  |
| GO:0032691\_negative\_regulation\_of\_interleukin-1\_beta\_production | 2 | 0 |  |  |  |  |  |  |  |  |
| GO:0032692\_negative\_regulation\_of\_interleukin-1\_production | 2 | 0 |  |  |  |  |  |  |  |  |
| GO:0032733\_positive\_regulation\_of\_interleukin-10\_production | 2 | 0 |  |  |  |  |  |  |  |  |
| GO:0032740\_positive\_regulation\_of\_interleukin-17\_production | 2 | 0 |  |  |  |  |  |  |  |  |
| GO:0032802\_low-density\_lipoprotein\_receptor\_catabolic\_process | 2 | 0 |  |  |  |  |  |  |  |  |
| GO:0032803\_regulation\_of\_low-density\_lipoprotein\_receptor\_catabolic\_process | 2 | 0 |  |  |  |  |  |  |  |  |
| GO:0032814\_regulation\_of\_natural\_killer\_cell\_activation | 2 | 0 |  |  |  |  |  |  |  |  |
| GO:0032816\_positive\_regulation\_of\_natural\_killer\_cell\_activation | 2 | 0 |  |  |  |  |  |  |  |  |
| GO:0032863\_activation\_of\_Rac\_GTPase\_activity | 2 | 0 |  |  |  |  |  |  |  |  |
| GO:0032892\_positive\_regulation\_of\_organic\_acid\_transport | 2 | 0 |  |  |  |  |  |  |  |  |
| GO:0032897\_negative\_regulation\_of\_viral\_transcription | 2 | 0 |  |  |  |  |  |  |  |  |
| GO:0032898\_neurotrophin\_production | 2 | 0 |  |  |  |  |  |  |  |  |
| GO:0032902\_nerve\_growth\_factor\_production | 2 | 0 |  |  |  |  |  |  |  |  |
| GO:0032914\_positive\_regulation\_of\_transforming\_growth\_factor-beta1\_production | 2 | 0 |  |  |  |  |  |  |  |  |
| GO:0032966\_negative\_regulation\_of\_collagen\_biosynthetic\_process | 2 | 0 |  |  |  |  |  |  |  |  |
| GO:0032971\_regulation\_of\_muscle\_filament\_sliding | 2 | 0 |  |  |  |  |  |  |  |  |
| GO:0033003\_regulation\_of\_mast\_cell\_activation | 2 | 0 |  |  |  |  |  |  |  |  |
| GO:0033005\_positive\_regulation\_of\_mast\_cell\_activation | 2 | 0 |  |  |  |  |  |  |  |  |
| GO:0033033\_negative\_regulation\_of\_myeloid\_cell\_apoptosis | 2 | 0 |  |  |  |  |  |  |  |  |
| GO:0033034\_positive\_regulation\_of\_myeloid\_cell\_apoptosis | 2 | 0 |  |  |  |  |  |  |  |  |
| GO:0033144\_negative\_regulation\_of\_steroid\_hormone\_receptor\_signaling\_pathway | 2 | 0 |  |  |  |  |  |  |  |  |
| GO:0033194\_response\_to\_hydroperoxide | 2 | 0 |  |  |  |  |  |  |  |  |
| GO:0033364\_mast\_cell\_secretory\_granule\_organization | 2 | 0 |  |  |  |  |  |  |  |  |
| GO:0033567\_DNA\_replication\_\_Okazaki\_fragment\_processing | 2 | 0 |  |  |  |  |  |  |  |  |
| GO:0033598\_mammary\_gland\_epithelial\_cell\_proliferation | 2 | 0 |  |  |  |  |  |  |  |  |
| GO:0033599\_regulation\_of\_mammary\_gland\_epithelial\_cell\_proliferation | 2 | 0 |  |  |  |  |  |  |  |  |
| GO:0033603\_positive\_regulation\_of\_dopamine\_secretion | 2 | 0 |  |  |  |  |  |  |  |  |
| GO:0033615\_mitochondrial\_proton-transporting\_ATP\_synthase\_complex\_assembly | 2 | 0 |  |  |  |  |  |  |  |  |
| GO:0033632\_regulation\_of\_cell-cell\_adhesion\_mediated\_by\_integrin | 2 | 0 |  |  |  |  |  |  |  |  |
| GO:0033861\_negative\_regulation\_of\_NAD(P)H\_oxidase\_activity | 2 | 0 |  |  |  |  |  |  |  |  |
| GO:0033864\_positive\_regulation\_of\_NAD(P)H\_oxidase\_activity | 2 | 0 |  |  |  |  |  |  |  |  |
| GO:0034113\_heterotypic\_cell-cell\_adhesion | 2 | 0 |  |  |  |  |  |  |  |  |
| GO:0034114\_regulation\_of\_heterotypic\_cell-cell\_adhesion | 2 | 0 |  |  |  |  |  |  |  |  |
| GO:0034121\_regulation\_of\_toll-like\_receptor\_signaling\_pathway | 2 | 0 |  |  |  |  |  |  |  |  |
| GO:0034134\_toll-like\_receptor\_2\_signaling\_pathway | 2 | 0 |  |  |  |  |  |  |  |  |
| GO:0034214\_protein\_hexamerization | 2 | 0 |  |  |  |  |  |  |  |  |
| GO:0034259\_negative\_regulation\_of\_Rho\_GTPase\_activity | 2 | 0 |  |  |  |  |  |  |  |  |
| GO:0034260\_negative\_regulation\_of\_GTPase\_activity | 2 | 0 |  |  |  |  |  |  |  |  |
| GO:0034261\_negative\_regulation\_of\_Ras\_GTPase\_activity | 2 | 0 |  |  |  |  |  |  |  |  |
| GO:0034331\_cell\_junction\_maintenance | 2 | 0 |  |  |  |  |  |  |  |  |
| GO:0034392\_negative\_regulation\_of\_smooth\_muscle\_cell\_apoptosis | 2 | 0 |  |  |  |  |  |  |  |  |
| GO:0034418\_urate\_biosynthetic\_process | 2 | 0 |  |  |  |  |  |  |  |  |
| GO:0034441\_plasma\_lipoprotein\_oxidation | 2 | 0 |  |  |  |  |  |  |  |  |
| GO:0034444\_regulation\_of\_plasma\_lipoprotein\_oxidation | 2 | 0 |  |  |  |  |  |  |  |  |
| GO:0034445\_negative\_regulation\_of\_plasma\_lipoprotein\_oxidation | 2 | 0 |  |  |  |  |  |  |  |  |
| GO:0034605\_cellular\_response\_to\_heat | 2 | 0 |  |  |  |  |  |  |  |  |
| GO:0034616\_response\_to\_laminar\_fluid\_shear\_stress | 2 | 0 |  |  |  |  |  |  |  |  |
| GO:0034619\_cellular\_chaperone-mediated\_protein\_complex\_assembly | 2 | 0 |  |  |  |  |  |  |  |  |
| GO:0034638\_phosphatidylcholine\_catabolic\_process | 2 | 0 |  |  |  |  |  |  |  |  |
| GO:0034644\_cellular\_response\_to\_UV | 2 | 0 |  |  |  |  |  |  |  |  |
| GO:0034650\_cortisol\_metabolic\_process | 2 | 0 |  |  |  |  |  |  |  |  |
| GO:0034651\_cortisol\_biosynthetic\_process | 2 | 0 |  |  |  |  |  |  |  |  |
| GO:0034723\_DNA\_replication-dependent\_nucleosome\_organization | 2 | 0 |  |  |  |  |  |  |  |  |
| GO:0034970\_histone\_H3-R2\_methylation | 2 | 0 |  |  |  |  |  |  |  |  |
| GO:0035025\_positive\_regulation\_of\_Rho\_protein\_signal\_transduction | 2 | 0 |  |  |  |  |  |  |  |  |
| GO:0035039\_male\_pronucleus\_formation | 2 | 0 |  |  |  |  |  |  |  |  |
| GO:0035050\_embryonic\_heart\_tube\_development | 2 | 0 |  |  |  |  |  |  |  |  |
| GO:0035058\_sensory\_cilium\_assembly | 2 | 0 |  |  |  |  |  |  |  |  |
| GO:0035117\_embryonic\_arm\_morphogenesis | 2 | 0 |  |  |  |  |  |  |  |  |
| GO:0035140\_arm\_morphogenesis | 2 | 0 |  |  |  |  |  |  |  |  |
| GO:0035162\_embryonic\_hemopoiesis | 2 | 0 |  |  |  |  |  |  |  |  |
| GO:0035247\_peptidyl-arginine\_omega-N-methylation | 2 | 0 |  |  |  |  |  |  |  |  |
| GO:0035279\_gene\_silencing\_by\_miRNA\_\_mRNA\_cleavage | 2 | 0 |  |  |  |  |  |  |  |  |
| GO:0035305\_negative\_regulation\_of\_dephosphorylation | 2 | 0 |  |  |  |  |  |  |  |  |
| GO:0035308\_negative\_regulation\_of\_protein\_amino\_acid\_dephosphorylation | 2 | 0 |  |  |  |  |  |  |  |  |
| GO:0040036\_regulation\_of\_fibroblast\_growth\_factor\_receptor\_signaling\_pathway | 2 | 0 |  |  |  |  |  |  |  |  |
| GO:0042062\_long-term\_strengthening\_of\_neuromuscular\_junction | 2 | 0 |  |  |  |  |  |  |  |  |
| GO:0042097\_interleukin-4\_biosynthetic\_process | 2 | 0 |  |  |  |  |  |  |  |  |
| GO:0042167\_heme\_catabolic\_process | 2 | 0 |  |  |  |  |  |  |  |  |
| GO:0042222\_interleukin-1\_biosynthetic\_process | 2 | 0 |  |  |  |  |  |  |  |  |
| GO:0042231\_interleukin-13\_biosynthetic\_process | 2 | 0 |  |  |  |  |  |  |  |  |
| GO:0042321\_negative\_regulation\_of\_circadian\_sleep\_wake\_cycle\_\_sleep | 2 | 0 |  |  |  |  |  |  |  |  |
| GO:0042322\_negative\_regulation\_of\_circadian\_sleep\_wake\_cycle\_\_REM\_sleep | 2 | 0 |  |  |  |  |  |  |  |  |
| GO:0042335\_cuticle\_development | 2 | 0 |  |  |  |  |  |  |  |  |
| GO:0042339\_keratan\_sulfate\_metabolic\_process | 2 | 0 |  |  |  |  |  |  |  |  |
| GO:0042350\_GDP-L-fucose\_biosynthetic\_process | 2 | 0 |  |  |  |  |  |  |  |  |
| GO:0042351\_'de\_novo'\_GDP-L-fucose\_biosynthetic\_process | 2 | 0 |  |  |  |  |  |  |  |  |
| GO:0042353\_fucose\_biosynthetic\_process | 2 | 0 |  |  |  |  |  |  |  |  |
| GO:0042396\_phosphagen\_biosynthetic\_process | 2 | 0 |  |  |  |  |  |  |  |  |
| GO:0042407\_cristae\_formation | 2 | 0 |  |  |  |  |  |  |  |  |
| GO:0042414\_epinephrine\_metabolic\_process | 2 | 0 |  |  |  |  |  |  |  |  |
| GO:0042415\_norepinephrine\_metabolic\_process | 2 | 0 |  |  |  |  |  |  |  |  |
| GO:0042436\_indole\_derivative\_catabolic\_process | 2 | 0 |  |  |  |  |  |  |  |  |
| GO:0042438\_melanin\_biosynthetic\_process | 2 | 0 |  |  |  |  |  |  |  |  |
| GO:0042481\_regulation\_of\_odontogenesis | 2 | 0 |  |  |  |  |  |  |  |  |
| GO:0042501\_serine\_phosphorylation\_of\_STAT\_protein | 2 | 0 |  |  |  |  |  |  |  |  |
| GO:0042508\_tyrosine\_phosphorylation\_of\_Stat1\_protein | 2 | 0 |  |  |  |  |  |  |  |  |
| GO:0042510\_regulation\_of\_tyrosine\_phosphorylation\_of\_Stat1\_protein | 2 | 0 |  |  |  |  |  |  |  |  |
| GO:0042511\_positive\_regulation\_of\_tyrosine\_phosphorylation\_of\_Stat1\_protein | 2 | 0 |  |  |  |  |  |  |  |  |
| GO:0042536\_negative\_regulation\_of\_tumor\_necrosis\_factor\_biosynthetic\_process | 2 | 0 |  |  |  |  |  |  |  |  |
| GO:0042634\_regulation\_of\_hair\_cycle | 2 | 0 |  |  |  |  |  |  |  |  |
| GO:0042754\_negative\_regulation\_of\_circadian\_rhythm | 2 | 0 |  |  |  |  |  |  |  |  |
| GO:0042776\_mitochondrial\_ATP\_synthesis\_coupled\_proton\_transport | 2 | 0 |  |  |  |  |  |  |  |  |
| GO:0042816\_vitamin\_B6\_metabolic\_process | 2 | 0 |  |  |  |  |  |  |  |  |
| GO:0042819\_vitamin\_B6\_biosynthetic\_process | 2 | 0 |  |  |  |  |  |  |  |  |
| GO:0042832\_defense\_response\_to\_protozoan | 2 | 0 |  |  |  |  |  |  |  |  |
| GO:0043000\_Golgi\_to\_plasma\_membrane\_CFTR\_protein\_transport | 2 | 0 |  |  |  |  |  |  |  |  |
| GO:0043006\_activation\_of\_phospholipase\_A2\_activity\_by\_calcium-mediated\_signaling | 2 | 0 |  |  |  |  |  |  |  |  |
| GO:0043011\_myeloid\_dendritic\_cell\_differentiation | 2 | 0 |  |  |  |  |  |  |  |  |
| GO:0043031\_negative\_regulation\_of\_macrophage\_activation | 2 | 0 |  |  |  |  |  |  |  |  |
| GO:0043045\_DNA\_methylation\_during\_embryonic\_development | 2 | 0 |  |  |  |  |  |  |  |  |
| GO:0043101\_purine\_salvage | 2 | 0 |  |  |  |  |  |  |  |  |
| GO:0043102\_amino\_acid\_salvage | 2 | 0 |  |  |  |  |  |  |  |  |
| GO:0043137\_DNA\_replication\_\_removal\_of\_RNA\_primer | 2 | 0 |  |  |  |  |  |  |  |  |
| GO:0043162\_ubiquitin-dependent\_protein\_catabolic\_process\_via\_the\_multivesicular\_body\_sorting\_pathway | 2 | 0 |  |  |  |  |  |  |  |  |
| GO:0043163\_cell\_envelope\_organization | 2 | 0 |  |  |  |  |  |  |  |  |
| GO:0043243\_positive\_regulation\_of\_protein\_complex\_disassembly | 2 | 0 |  |  |  |  |  |  |  |  |
| GO:0043266\_regulation\_of\_potassium\_ion\_transport | 2 | 0 |  |  |  |  |  |  |  |  |
| GO:0043277\_apoptotic\_cell\_clearance | 2 | 0 |  |  |  |  |  |  |  |  |
| GO:0043278\_response\_to\_morphine | 2 | 0 |  |  |  |  |  |  |  |  |
| GO:0043297\_apical\_junction\_assembly | 2 | 0 |  |  |  |  |  |  |  |  |
| GO:0043400\_cortisol\_secretion | 2 | 0 |  |  |  |  |  |  |  |  |
| GO:0043476\_pigment\_accumulation | 2 | 0 |  |  |  |  |  |  |  |  |
| GO:0043482\_cellular\_pigment\_accumulation | 2 | 0 |  |  |  |  |  |  |  |  |
| GO:0043490\_malate-aspartate\_shuttle | 2 | 0 |  |  |  |  |  |  |  |  |
| GO:0043508\_negative\_regulation\_of\_JUN\_kinase\_activity | 2 | 0 |  |  |  |  |  |  |  |  |
| GO:0043516\_regulation\_of\_DNA\_damage\_response\_\_signal\_transduction\_by\_p53\_class\_mediator | 2 | 0 |  |  |  |  |  |  |  |  |
| GO:0043569\_negative\_regulation\_of\_insulin-like\_growth\_factor\_receptor\_signaling\_pathway | 2 | 0 |  |  |  |  |  |  |  |  |
| GO:0043589\_skin\_morphogenesis | 2 | 0 |  |  |  |  |  |  |  |  |
| GO:0043619\_regulation\_of\_transcription\_from\_RNA\_polymerase\_II\_promoter\_in\_response\_to\_oxidative\_stress | 2 | 0 |  |  |  |  |  |  |  |  |
| GO:0043628\_ncRNA\_3'-end\_processing | 2 | 0 |  |  |  |  |  |  |  |  |
| GO:0043653\_mitochondrial\_fragmentation\_during\_apoptosis | 2 | 0 |  |  |  |  |  |  |  |  |
| GO:0043903\_regulation\_of\_symbiosis\_\_encompassing\_mutualism\_through\_parasitism | 2 | 0 |  |  |  |  |  |  |  |  |
| GO:0043932\_ossification\_involved\_in\_bone\_remodeling | 2 | 0 |  |  |  |  |  |  |  |  |
| GO:0043954\_cellular\_component\_maintenance | 2 | 0 |  |  |  |  |  |  |  |  |
| GO:0043985\_histone\_H4-R3\_methylation | 2 | 0 |  |  |  |  |  |  |  |  |
| GO:0044004\_disruption\_by\_symbiont\_of\_host\_cells | 2 | 0 |  |  |  |  |  |  |  |  |
| GO:0044030\_regulation\_of\_DNA\_methylation | 2 | 0 |  |  |  |  |  |  |  |  |
| GO:0044036\_cell\_wall\_macromolecule\_metabolic\_process | 2 | 0 |  |  |  |  |  |  |  |  |
| GO:0044058\_regulation\_of\_digestive\_system\_process | 2 | 0 |  |  |  |  |  |  |  |  |
| GO:0044062\_regulation\_of\_excretion | 2 | 0 |  |  |  |  |  |  |  |  |
| GO:0044091\_membrane\_biogenesis | 2 | 0 |  |  |  |  |  |  |  |  |
| GO:0044413\_avoidance\_of\_host\_defenses | 2 | 0 |  |  |  |  |  |  |  |  |
| GO:0044415\_evasion\_or\_tolerance\_of\_host\_defenses | 2 | 0 |  |  |  |  |  |  |  |  |
| GO:0045002\_double-strand\_break\_repair\_via\_single-strand\_annealing | 2 | 0 |  |  |  |  |  |  |  |  |
| GO:0045005\_maintenance\_of\_fidelity\_during\_DNA-dependent\_DNA\_replication | 2 | 0 |  |  |  |  |  |  |  |  |
| GO:0045046\_protein\_import\_into\_peroxisome\_membrane | 2 | 0 |  |  |  |  |  |  |  |  |
| GO:0045059\_positive\_thymic\_T\_cell\_selection | 2 | 0 |  |  |  |  |  |  |  |  |
| GO:0045066\_regulatory\_T\_cell\_differentiation | 2 | 0 |  |  |  |  |  |  |  |  |
| GO:0045077\_negative\_regulation\_of\_interferon-gamma\_biosynthetic\_process | 2 | 0 |  |  |  |  |  |  |  |  |
| GO:0045084\_positive\_regulation\_of\_interleukin-12\_biosynthetic\_process | 2 | 0 |  |  |  |  |  |  |  |  |
| GO:0045085\_negative\_regulation\_of\_interleukin-2\_biosynthetic\_process | 2 | 0 |  |  |  |  |  |  |  |  |
| GO:0045110\_intermediate\_filament\_bundle\_assembly | 2 | 0 |  |  |  |  |  |  |  |  |
| GO:0045113\_regulation\_of\_integrin\_biosynthetic\_process | 2 | 0 |  |  |  |  |  |  |  |  |
| GO:0045162\_clustering\_of\_voltage-gated\_sodium\_channels | 2 | 0 |  |  |  |  |  |  |  |  |
| GO:0045217\_cell-cell\_junction\_maintenance | 2 | 0 |  |  |  |  |  |  |  |  |
| GO:0045229\_external\_encapsulating\_structure\_organization | 2 | 0 |  |  |  |  |  |  |  |  |
| GO:0045341\_MHC\_class\_I\_biosynthetic\_process | 2 | 0 |  |  |  |  |  |  |  |  |
| GO:0045343\_regulation\_of\_MHC\_class\_I\_biosynthetic\_process | 2 | 0 |  |  |  |  |  |  |  |  |
| GO:0045347\_negative\_regulation\_of\_MHC\_class\_II\_biosynthetic\_process | 2 | 0 |  |  |  |  |  |  |  |  |
| GO:0045348\_positive\_regulation\_of\_MHC\_class\_II\_biosynthetic\_process | 2 | 0 |  |  |  |  |  |  |  |  |
| GO:0045402\_regulation\_of\_interleukin-4\_biosynthetic\_process | 2 | 0 |  |  |  |  |  |  |  |  |
| GO:0045404\_positive\_regulation\_of\_interleukin-4\_biosynthetic\_process | 2 | 0 |  |  |  |  |  |  |  |  |
| GO:0045415\_negative\_regulation\_of\_interleukin-8\_biosynthetic\_process | 2 | 0 |  |  |  |  |  |  |  |  |
| GO:0045423\_regulation\_of\_granulocyte\_macrophage\_colony-stimulating\_factor\_biosynthetic\_process | 2 | 0 |  |  |  |  |  |  |  |  |
| GO:0045542\_positive\_regulation\_of\_cholesterol\_biosynthetic\_process | 2 | 0 |  |  |  |  |  |  |  |  |
| GO:0045576\_mast\_cell\_activation | 2 | 0 |  |  |  |  |  |  |  |  |
| GO:0045578\_negative\_regulation\_of\_B\_cell\_differentiation | 2 | 0 |  |  |  |  |  |  |  |  |
| GO:0045599\_negative\_regulation\_of\_fat\_cell\_differentiation | 2 | 0 |  |  |  |  |  |  |  |  |
| GO:0045601\_regulation\_of\_endothelial\_cell\_differentiation | 2 | 0 |  |  |  |  |  |  |  |  |
| GO:0045627\_positive\_regulation\_of\_T-helper\_1\_cell\_differentiation | 2 | 0 |  |  |  |  |  |  |  |  |
| GO:0045630\_positive\_regulation\_of\_T-helper\_2\_cell\_differentiation | 2 | 0 |  |  |  |  |  |  |  |  |
| GO:0045647\_negative\_regulation\_of\_erythrocyte\_differentiation | 2 | 0 |  |  |  |  |  |  |  |  |
| GO:0045653\_negative\_regulation\_of\_megakaryocyte\_differentiation | 2 | 0 |  |  |  |  |  |  |  |  |
| GO:0045655\_regulation\_of\_monocyte\_differentiation | 2 | 0 |  |  |  |  |  |  |  |  |
| GO:0045657\_positive\_regulation\_of\_monocyte\_differentiation | 2 | 0 |  |  |  |  |  |  |  |  |
| GO:0045685\_regulation\_of\_glial\_cell\_differentiation | 2 | 0 |  |  |  |  |  |  |  |  |
| GO:0045686\_negative\_regulation\_of\_glial\_cell\_differentiation | 2 | 0 |  |  |  |  |  |  |  |  |
| GO:0045721\_negative\_regulation\_of\_gluconeogenesis | 2 | 0 |  |  |  |  |  |  |  |  |
| GO:0045722\_positive\_regulation\_of\_gluconeogenesis | 2 | 0 |  |  |  |  |  |  |  |  |
| GO:0045726\_positive\_regulation\_of\_integrin\_biosynthetic\_process | 2 | 0 |  |  |  |  |  |  |  |  |
| GO:0045780\_positive\_regulation\_of\_bone\_resorption | 2 | 0 |  |  |  |  |  |  |  |  |
| GO:0045796\_negative\_regulation\_of\_intestinal\_cholesterol\_absorption | 2 | 0 |  |  |  |  |  |  |  |  |
| GO:0045798\_negative\_regulation\_of\_chromatin\_assembly\_or\_disassembly | 2 | 0 |  |  |  |  |  |  |  |  |
| GO:0045842\_positive\_regulation\_of\_mitotic\_metaphase\_anaphase\_transition | 2 | 0 |  |  |  |  |  |  |  |  |
| GO:0045869\_negative\_regulation\_of\_retroviral\_genome\_replication | 2 | 0 |  |  |  |  |  |  |  |  |
| GO:0045906\_negative\_regulation\_of\_vasoconstriction | 2 | 0 |  |  |  |  |  |  |  |  |
| GO:0045918\_negative\_regulation\_of\_cytolysis | 2 | 0 |  |  |  |  |  |  |  |  |
| GO:0045919\_positive\_regulation\_of\_cytolysis | 2 | 0 |  |  |  |  |  |  |  |  |
| GO:0045938\_positive\_regulation\_of\_circadian\_sleep\_wake\_cycle\_\_sleep | 2 | 0 |  |  |  |  |  |  |  |  |
| GO:0045986\_negative\_regulation\_of\_smooth\_muscle\_contraction | 2 | 0 |  |  |  |  |  |  |  |  |
| GO:0045991\_positive\_regulation\_of\_transcription\_by\_carbon\_catabolites | 2 | 0 |  |  |  |  |  |  |  |  |
| GO:0046016\_positive\_regulation\_of\_transcription\_by\_glucose | 2 | 0 |  |  |  |  |  |  |  |  |
| GO:0046039\_GTP\_metabolic\_process | 2 | 0 |  |  |  |  |  |  |  |  |
| GO:0046100\_hypoxanthine\_metabolic\_process | 2 | 0 |  |  |  |  |  |  |  |  |
| GO:0046102\_inosine\_metabolic\_process | 2 | 0 |  |  |  |  |  |  |  |  |
| GO:0046113\_nucleobase\_catabolic\_process | 2 | 0 |  |  |  |  |  |  |  |  |
| GO:0046114\_guanosine\_biosynthetic\_process | 2 | 0 |  |  |  |  |  |  |  |  |
| GO:0046116\_queuosine\_metabolic\_process | 2 | 0 |  |  |  |  |  |  |  |  |
| GO:0046118\_7-methylguanosine\_biosynthetic\_process | 2 | 0 |  |  |  |  |  |  |  |  |
| GO:0046121\_deoxyribonucleoside\_catabolic\_process | 2 | 0 |  |  |  |  |  |  |  |  |
| GO:0046135\_pyrimidine\_nucleoside\_catabolic\_process | 2 | 0 |  |  |  |  |  |  |  |  |
| GO:0046136\_positive\_regulation\_of\_vitamin\_metabolic\_process | 2 | 0 |  |  |  |  |  |  |  |  |
| GO:0046149\_pigment\_catabolic\_process | 2 | 0 |  |  |  |  |  |  |  |  |
| GO:0046160\_heme\_a\_metabolic\_process | 2 | 0 |  |  |  |  |  |  |  |  |
| GO:0046174\_polyol\_catabolic\_process | 2 | 0 |  |  |  |  |  |  |  |  |
| GO:0046218\_indolalkylamine\_catabolic\_process | 2 | 0 |  |  |  |  |  |  |  |  |
| GO:0046349\_amino\_sugar\_biosynthetic\_process | 2 | 0 |  |  |  |  |  |  |  |  |
| GO:0046359\_butyrate\_catabolic\_process | 2 | 0 |  |  |  |  |  |  |  |  |
| GO:0046368\_GDP-L-fucose\_metabolic\_process | 2 | 0 |  |  |  |  |  |  |  |  |
| GO:0046398\_UDP-glucuronate\_metabolic\_process | 2 | 0 |  |  |  |  |  |  |  |  |
| GO:0046415\_urate\_metabolic\_process | 2 | 0 |  |  |  |  |  |  |  |  |
| GO:0046476\_glycosylceramide\_biosynthetic\_process | 2 | 0 |  |  |  |  |  |  |  |  |
| GO:0046479\_glycosphingolipid\_catabolic\_process | 2 | 0 |  |  |  |  |  |  |  |  |
| GO:0046495\_nicotinamide\_riboside\_metabolic\_process | 2 | 0 |  |  |  |  |  |  |  |  |
| GO:0046500\_S-adenosylmethionine\_metabolic\_process | 2 | 0 |  |  |  |  |  |  |  |  |
| GO:0046502\_uroporphyrinogen\_III\_metabolic\_process | 2 | 0 |  |  |  |  |  |  |  |  |
| GO:0046521\_sphingoid\_catabolic\_process | 2 | 0 |  |  |  |  |  |  |  |  |
| GO:0046541\_saliva\_secretion | 2 | 0 |  |  |  |  |  |  |  |  |
| GO:0046596\_regulation\_of\_virion\_penetration\_into\_host\_cell | 2 | 0 |  |  |  |  |  |  |  |  |
| GO:0046599\_regulation\_of\_centriole\_replication | 2 | 0 |  |  |  |  |  |  |  |  |
| GO:0046629\_gamma-delta\_T\_cell\_activation | 2 | 0 |  |  |  |  |  |  |  |  |
[truncated: 286,559 more chars]
