# Supplementary material for: Concordance of Gene Expression and Functional Correlation Patterns across the NCI-60 Cell Lines and the Cancer Genome Atlas Glioblastoma Samples
Source: PLoS One. 2012 Jul 26;7(7):e40062. doi: 10.1371/journal.pone.0040062 (PMC3406063; doi:10.1371/journal.pone.0040062)
Supplement: Download S1 — Zip archive of HTGM results. (ZIP) [file pone.0040062.s007.zip › work2026406846/Generated_Total2026406846.dir/generic.BP.NCI60.0.6.ATP2A3.express.genes.correlation.complete.Thu.May.19.17.20.06.2011.htgm.txt.dir/generic.BP.NCI60.0.6.ATP2A3.express.genes.correlation.complete.Thu.May.19.17.20.06.2011.htgm.txt.change.gce.CIM.dir/cgi_user_y.html]

**Y-axis Names**   
Cluster is based on euclidean distance  
Cluster method is: average  
plclust  
height plot  

|  |
| --- |
| 1.LCK |
| 2.CD2 |
| 3.ZAP70 |
| 4.CD3D |
| 5.CD3E |
| 6.SIT1 |
| 7.ATP2A3 |
| 8.CD3G |
| 9.CD7 |
