# Supplementary material for: Concordance of Gene Expression and Functional Correlation Patterns across the NCI-60 Cell Lines and the Cancer Genome Atlas Glioblastoma Samples
Source: PLoS One. 2012 Jul 26;7(7):e40062. doi: 10.1371/journal.pone.0040062 (PMC3406063; doi:10.1371/journal.pone.0040062)
Supplement: Download S1 — Zip archive of HTGM results. (ZIP) [file pone.0040062.s007.zip › work2026406846/Generated_Total2026406846.dir/generic.BP.NCI60.0.6.ATP2A3.express.genes.correlation.complete.Thu.May.19.17.20.06.2011.htgm.txt.dir/generic.BP.NCI60.0.6.ATP2A3.express.genes.correlation.complete.Thu.May.19.17.20.06.2011.htgm.txt.change.gce.html]

Gene Category Report for generic.BP.NCI60.0.6.ATP2A3.express.genes.correlation.complete.Thu.May.19.17.20.06.2011.htgm.txt

# Gene Category Report for generic.BP.NCI60.0.6.ATP2A3.express.genes.correlation.complete.Thu.May.19.17.20.06.2011.htgm.txt

| HYPERLINKED GO CATEGORY | HYPERLINKED GENE NAME | TOTAL GENES | CHANGED GENES | ENRICHMENT | LOG10(p) | CUMULATIVE NUMBER OF CATEGORIES | CUMULATIVE RANDOMS MEAN | FALSE DISCOVERY RATE |
| --- | --- | --- | --- | --- | --- | --- | --- | --- |
| GO:0042110\_T\_cell\_activation | CD3E | 83 | 8 | 49.156627 | -12.009490 | 1 | 0.0 | 0.000000 |
| GO:0042110\_T\_cell\_activation | ZAP70 | 83 | 8 | 49.156627 | -12.009490 | 1 | 0.0 | 0.000000 |
| GO:0042110\_T\_cell\_activation | CD3G | 83 | 8 | 49.156627 | -12.009490 | 1 | 0.0 | 0.000000 |
| GO:0042110\_T\_cell\_activation | CD7 | 83 | 8 | 49.156627 | -12.009490 | 1 | 0.0 | 0.000000 |
| GO:0042110\_T\_cell\_activation | CD2 | 83 | 8 | 49.156627 | -12.009490 | 1 | 0.0 | 0.000000 |
| GO:0042110\_T\_cell\_activation | LCK | 83 | 8 | 49.156627 | -12.009490 | 1 | 0.0 | 0.000000 |
| GO:0042110\_T\_cell\_activation | CD3D | 83 | 8 | 49.156627 | -12.009490 | 1 | 0.0 | 0.000000 |
| GO:0042110\_T\_cell\_activation | SIT1 | 83 | 8 | 49.156627 | -12.009490 | 1 | 0.0 | 0.000000 |
| GO:0046649\_lymphocyte\_activation | CD3E | 119 | 8 | 34.285714 | -10.724782 | 2 | 0.0 | 0.000000 |
| GO:0046649\_lymphocyte\_activation | ZAP70 | 119 | 8 | 34.285714 | -10.724782 | 2 | 0.0 | 0.000000 |
| GO:0046649\_lymphocyte\_activation | CD3G | 119 | 8 | 34.285714 | -10.724782 | 2 | 0.0 | 0.000000 |
| GO:0046649\_lymphocyte\_activation | CD7 | 119 | 8 | 34.285714 | -10.724782 | 2 | 0.0 | 0.000000 |
| GO:0046649\_lymphocyte\_activation | CD2 | 119 | 8 | 34.285714 | -10.724782 | 2 | 0.0 | 0.000000 |
| GO:0046649\_lymphocyte\_activation | LCK | 119 | 8 | 34.285714 | -10.724782 | 2 | 0.0 | 0.000000 |
| GO:0046649\_lymphocyte\_activation | CD3D | 119 | 8 | 34.285714 | -10.724782 | 2 | 0.0 | 0.000000 |
| GO:0046649\_lymphocyte\_activation | SIT1 | 119 | 8 | 34.285714 | -10.724782 | 2 | 0.0 | 0.000000 |
| GO:0045321\_leukocyte\_activation | CD3E | 150 | 8 | 27.200000 | -9.910376 | 3 | 0.0 | 0.000000 |
| GO:0045321\_leukocyte\_activation | ZAP70 | 150 | 8 | 27.200000 | -9.910376 | 3 | 0.0 | 0.000000 |
| GO:0045321\_leukocyte\_activation | CD3G | 150 | 8 | 27.200000 | -9.910376 | 3 | 0.0 | 0.000000 |
| GO:0045321\_leukocyte\_activation | CD7 | 150 | 8 | 27.200000 | -9.910376 | 3 | 0.0 | 0.000000 |
| GO:0045321\_leukocyte\_activation | CD2 | 150 | 8 | 27.200000 | -9.910376 | 3 | 0.0 | 0.000000 |
| GO:0045321\_leukocyte\_activation | CD3D | 150 | 8 | 27.200000 | -9.910376 | 3 | 0.0 | 0.000000 |
| GO:0045321\_leukocyte\_activation | LCK | 150 | 8 | 27.200000 | -9.910376 | 3 | 0.0 | 0.000000 |
| GO:0045321\_leukocyte\_activation | SIT1 | 150 | 8 | 27.200000 | -9.910376 | 3 | 0.0 | 0.000000 |
| GO:0001775\_cell\_activation | CD3E | 175 | 8 | 23.314286 | -9.372471 | 4 | 0.0 | 0.000000 |
| GO:0001775\_cell\_activation | ZAP70 | 175 | 8 | 23.314286 | -9.372471 | 4 | 0.0 | 0.000000 |
| GO:0001775\_cell\_activation | CD3G | 175 | 8 | 23.314286 | -9.372471 | 4 | 0.0 | 0.000000 |
| GO:0001775\_cell\_activation | CD7 | 175 | 8 | 23.314286 | -9.372471 | 4 | 0.0 | 0.000000 |
| GO:0001775\_cell\_activation | CD2 | 175 | 8 | 23.314286 | -9.372471 | 4 | 0.0 | 0.000000 |
| GO:0001775\_cell\_activation | CD3D | 175 | 8 | 23.314286 | -9.372471 | 4 | 0.0 | 0.000000 |
| GO:0001775\_cell\_activation | LCK | 175 | 8 | 23.314286 | -9.372471 | 4 | 0.0 | 0.000000 |
| GO:0001775\_cell\_activation | SIT1 | 175 | 8 | 23.314286 | -9.372471 | 4 | 0.0 | 0.000000 |
| GO:0050863\_regulation\_of\_T\_cell\_activation | CD3E | 49 | 5 | 52.040816 | -7.579656 | 5 | 0.0 | 0.000000 |
| GO:0050863\_regulation\_of\_T\_cell\_activation | ZAP70 | 49 | 5 | 52.040816 | -7.579656 | 5 | 0.0 | 0.000000 |
| GO:0050863\_regulation\_of\_T\_cell\_activation | CD2 | 49 | 5 | 52.040816 | -7.579656 | 5 | 0.0 | 0.000000 |
| GO:0050863\_regulation\_of\_T\_cell\_activation | LCK | 49 | 5 | 52.040816 | -7.579656 | 5 | 0.0 | 0.000000 |
| GO:0050863\_regulation\_of\_T\_cell\_activation | SIT1 | 49 | 5 | 52.040816 | -7.579656 | 5 | 0.0 | 0.000000 |
| GO:0051249\_regulation\_of\_lymphocyte\_activation | CD3E | 60 | 5 | 42.500000 | -7.128059 | 6 | 0.0 | 0.000000 |
| GO:0051249\_regulation\_of\_lymphocyte\_activation | ZAP70 | 60 | 5 | 42.500000 | -7.128059 | 6 | 0.0 | 0.000000 |
| GO:0051249\_regulation\_of\_lymphocyte\_activation | CD2 | 60 | 5 | 42.500000 | -7.128059 | 6 | 0.0 | 0.000000 |
| GO:0051249\_regulation\_of\_lymphocyte\_activation | LCK | 60 | 5 | 42.500000 | -7.128059 | 6 | 0.0 | 0.000000 |
| GO:0051249\_regulation\_of\_lymphocyte\_activation | SIT1 | 60 | 5 | 42.500000 | -7.128059 | 6 | 0.0 | 0.000000 |
| GO:0002694\_regulation\_of\_leukocyte\_activation | CD3E | 70 | 5 | 36.428571 | -6.787384 | 7 | 0.0 | 0.000000 |
| GO:0002694\_regulation\_of\_leukocyte\_activation | ZAP70 | 70 | 5 | 36.428571 | -6.787384 | 7 | 0.0 | 0.000000 |
| GO:0002694\_regulation\_of\_leukocyte\_activation | CD2 | 70 | 5 | 36.428571 | -6.787384 | 7 | 0.0 | 0.000000 |
| GO:0002694\_regulation\_of\_leukocyte\_activation | LCK | 70 | 5 | 36.428571 | -6.787384 | 7 | 0.0 | 0.000000 |
| GO:0002694\_regulation\_of\_leukocyte\_activation | SIT1 | 70 | 5 | 36.428571 | -6.787384 | 7 | 0.0 | 0.000000 |
| GO:0050865\_regulation\_of\_cell\_activation | CD3E | 77 | 5 | 33.116883 | -6.577975 | 8 | 0.0 | 0.000000 |
| GO:0050865\_regulation\_of\_cell\_activation | ZAP70 | 77 | 5 | 33.116883 | -6.577975 | 8 | 0.0 | 0.000000 |
| GO:0050865\_regulation\_of\_cell\_activation | CD2 | 77 | 5 | 33.116883 | -6.577975 | 8 | 0.0 | 0.000000 |
| GO:0050865\_regulation\_of\_cell\_activation | LCK | 77 | 5 | 33.116883 | -6.577975 | 8 | 0.0 | 0.000000 |
| GO:0050865\_regulation\_of\_cell\_activation | SIT1 | 77 | 5 | 33.116883 | -6.577975 | 8 | 0.0 | 0.000000 |
| GO:0030217\_T\_cell\_differentiation | ZAP70 | 33 | 4 | 61.818182 | -6.409067 | 9 | 0.0 | 0.000000 |
| GO:0030217\_T\_cell\_differentiation | CD2 | 33 | 4 | 61.818182 | -6.409067 | 9 | 0.0 | 0.000000 |
| GO:0030217\_T\_cell\_differentiation | LCK | 33 | 4 | 61.818182 | -6.409067 | 9 | 0.0 | 0.000000 |
| GO:0030217\_T\_cell\_differentiation | CD3D | 33 | 4 | 61.818182 | -6.409067 | 9 | 0.0 | 0.000000 |
| GO:0002696\_positive\_regulation\_of\_leukocyte\_activation | CD3E | 46 | 4 | 44.347826 | -5.814984 | 10 | 0.0 | 0.000000 |
| GO:0002696\_positive\_regulation\_of\_leukocyte\_activation | ZAP70 | 46 | 4 | 44.347826 | -5.814984 | 10 | 0.0 | 0.000000 |
| GO:0002696\_positive\_regulation\_of\_leukocyte\_activation | CD2 | 46 | 4 | 44.347826 | -5.814984 | 10 | 0.0 | 0.000000 |
| GO:0002696\_positive\_regulation\_of\_leukocyte\_activation | LCK | 46 | 4 | 44.347826 | -5.814984 | 10 | 0.0 | 0.000000 |
| GO:0002376\_immune\_system\_process | CD3E | 718 | 9 | 6.392758 | -5.708840 | 11 | 0.0 | 0.000000 |
| GO:0002376\_immune\_system\_process | CCR4 | 718 | 9 | 6.392758 | -5.708840 | 11 | 0.0 | 0.000000 |
| GO:0002376\_immune\_system\_process | ZAP70 | 718 | 9 | 6.392758 | -5.708840 | 11 | 0.0 | 0.000000 |
| GO:0002376\_immune\_system\_process | CD3G | 718 | 9 | 6.392758 | -5.708840 | 11 | 0.0 | 0.000000 |
| GO:0002376\_immune\_system\_process | CD7 | 718 | 9 | 6.392758 | -5.708840 | 11 | 0.0 | 0.000000 |
| GO:0002376\_immune\_system\_process | CD2 | 718 | 9 | 6.392758 | -5.708840 | 11 | 0.0 | 0.000000 |
| GO:0002376\_immune\_system\_process | CD3D | 718 | 9 | 6.392758 | -5.708840 | 11 | 0.0 | 0.000000 |
| GO:0002376\_immune\_system\_process | LCK | 718 | 9 | 6.392758 | -5.708840 | 11 | 0.0 | 0.000000 |
| GO:0002376\_immune\_system\_process | SIT1 | 718 | 9 | 6.392758 | -5.708840 | 11 | 0.0 | 0.000000 |
| GO:0050867\_positive\_regulation\_of\_cell\_activation | CD3E | 49 | 4 | 41.632653 | -5.703121 | 12 | 0.0 | 0.000000 |
| GO:0050867\_positive\_regulation\_of\_cell\_activation | ZAP70 | 49 | 4 | 41.632653 | -5.703121 | 12 | 0.0 | 0.000000 |
| GO:0050867\_positive\_regulation\_of\_cell\_activation | CD2 | 49 | 4 | 41.632653 | -5.703121 | 12 | 0.0 | 0.000000 |
| GO:0050867\_positive\_regulation\_of\_cell\_activation | LCK | 49 | 4 | 41.632653 | -5.703121 | 12 | 0.0 | 0.000000 |
| GO:0030098\_lymphocyte\_differentiation | ZAP70 | 50 | 4 | 40.800000 | -5.667421 | 13 | 0.0 | 0.000000 |
| GO:0030098\_lymphocyte\_differentiation | CD2 | 50 | 4 | 40.800000 | -5.667421 | 13 | 0.0 | 0.000000 |
| GO:0030098\_lymphocyte\_differentiation | LCK | 50 | 4 | 40.800000 | -5.667421 | 13 | 0.0 | 0.000000 |
| GO:0030098\_lymphocyte\_differentiation | CD3D | 50 | 4 | 40.800000 | -5.667421 | 13 | 0.0 | 0.000000 |
| GO:0045059\_positive\_thymic\_T\_cell\_selection | ZAP70 | 2 | 2 |  |  |  |  |  |  |
| GO:0045059\_positive\_thymic\_T\_cell\_selection | CD3D | 2 | 2 |  |  |  |  |  |  |
| GO:0043368\_positive\_T\_cell\_selection | ZAP70 | 3 | 2 |  |  |  |  |  |  |
| GO:0043368\_positive\_T\_cell\_selection | CD3D | 3 | 2 |  |  |  |  |  |  |
| GO:0045061\_thymic\_T\_cell\_selection | ZAP70 | 3 | 2 |  |  |  |  |  |  |
| GO:0045061\_thymic\_T\_cell\_selection | CD3D | 3 | 2 |  |  |  |  |  |  |
| GO:0002521\_leukocyte\_differentiation | ZAP70 | 87 | 4 | 23.448276 | -4.701199 | 14 | 0.0 | 0.000000 |
| GO:0002521\_leukocyte\_differentiation | CD2 | 87 | 4 | 23.448276 | -4.701199 | 14 | 0.0 | 0.000000 |
| GO:0002521\_leukocyte\_differentiation | LCK | 87 | 4 | 23.448276 | -4.701199 | 14 | 0.0 | 0.000000 |
| GO:0002521\_leukocyte\_differentiation | CD3D | 87 | 4 | 23.448276 | -4.701199 | 14 | 0.0 | 0.000000 |
| GO:0002682\_regulation\_of\_immune\_system\_process | CD3E | 196 | 5 | 13.010204 | -4.572623 | 15 | 0.0 | 0.000000 |
| GO:0002682\_regulation\_of\_immune\_system\_process | ZAP70 | 196 | 5 | 13.010204 | -4.572623 | 15 | 0.0 | 0.000000 |
| GO:0002682\_regulation\_of\_immune\_system\_process | CD2 | 196 | 5 | 13.010204 | -4.572623 | 15 | 0.0 | 0.000000 |
| GO:0002682\_regulation\_of\_immune\_system\_process | LCK | 196 | 5 | 13.010204 | -4.572623 | 15 | 0.0 | 0.000000 |
| GO:0002682\_regulation\_of\_immune\_system\_process | SIT1 | 196 | 5 | 13.010204 | -4.572623 | 15 | 0.0 | 0.000000 |
| GO:0050870\_positive\_regulation\_of\_T\_cell\_activation | CD3E | 33 | 3 | 46.363636 | -4.487284 | 16 | 0.0 | 0.000000 |
| GO:0050870\_positive\_regulation\_of\_T\_cell\_activation | ZAP70 | 33 | 3 | 46.363636 | -4.487284 | 16 | 0.0 | 0.000000 |
| GO:0050870\_positive\_regulation\_of\_T\_cell\_activation | LCK | 33 | 3 | 46.363636 | -4.487284 | 16 | 0.0 | 0.000000 |
| GO:0002684\_positive\_regulation\_of\_immune\_system\_process | CD3E | 106 | 4 | 19.245283 | -4.362314 | 17 | 0.03 | 0.001765 |
| GO:0002684\_positive\_regulation\_of\_immune\_system\_process | ZAP70 | 106 | 4 | 19.245283 | -4.362314 | 17 | 0.03 | 0.001765 |
| GO:0002684\_positive\_regulation\_of\_immune\_system\_process | CD2 | 106 | 4 | 19.245283 | -4.362314 | 17 | 0.03 | 0.001765 |
| GO:0002684\_positive\_regulation\_of\_immune\_system\_process | LCK | 106 | 4 | 19.245283 | -4.362314 | 17 | 0.03 | 0.001765 |
| GO:0051251\_positive\_regulation\_of\_lymphocyte\_activation | CD3E | 40 | 3 | 38.250000 | -4.233039 | 18 | 0.03 | 0.001667 |
| GO:0051251\_positive\_regulation\_of\_lymphocyte\_activation | ZAP70 | 40 | 3 | 38.250000 | -4.233039 | 18 | 0.03 | 0.001667 |
| GO:0051251\_positive\_regulation\_of\_lymphocyte\_activation | LCK | 40 | 3 | 38.250000 | -4.233039 | 18 | 0.03 | 0.001667 |
| GO:0033077\_T\_cell\_differentiation\_in\_the\_thymus | ZAP70 | 7 | 2 | 145.714286 | -4.123382 | 20 | 0.03 | 0.001500 |
| GO:0033077\_T\_cell\_differentiation\_in\_the\_thymus | CD3D | 7 | 2 | 145.714286 | -4.123382 | 20 | 0.03 | 0.001500 |
| GO:0045058\_T\_cell\_selection | ZAP70 | 7 | 2 | 145.714286 | -4.123382 | 20 | 0.03 | 0.001500 |
| GO:0045058\_T\_cell\_selection | CD3D | 7 | 2 | 145.714286 | -4.123382 | 20 | 0.03 | 0.001500 |
| GO:0030097\_hemopoiesis | ZAP70 | 135 | 4 | 15.111111 | -3.951738 | 21 | 0.05 | 0.002381 |
| GO:0030097\_hemopoiesis | CD2 | 135 | 4 | 15.111111 | -3.951738 | 21 | 0.05 | 0.002381 |
| GO:0030097\_hemopoiesis | LCK | 135 | 4 | 15.111111 | -3.951738 | 21 | 0.05 | 0.002381 |
| GO:0030097\_hemopoiesis | CD3D | 135 | 4 | 15.111111 | -3.951738 | 21 | 0.05 | 0.002381 |
| GO:0048534\_hemopoietic\_or\_lymphoid\_organ\_development | ZAP70 | 139 | 4 | 14.676259 | -3.902509 | 22 | 0.05 | 0.002273 |
| GO:0048534\_hemopoietic\_or\_lymphoid\_organ\_development | CD2 | 139 | 4 | 14.676259 | -3.902509 | 22 | 0.05 | 0.002273 |
| GO:0048534\_hemopoietic\_or\_lymphoid\_organ\_development | LCK | 139 | 4 | 14.676259 | -3.902509 | 22 | 0.05 | 0.002273 |
| GO:0048534\_hemopoietic\_or\_lymphoid\_organ\_development | CD3D | 139 | 4 | 14.676259 | -3.902509 | 22 | 0.05 | 0.002273 |
| GO:0002520\_immune\_system\_development | ZAP70 | 147 | 4 | 13.877551 | -3.808383 | 23 | 0.05 | 0.002174 |
| GO:0002520\_immune\_system\_development | CD2 | 147 | 4 | 13.877551 | -3.808383 | 23 | 0.05 | 0.002174 |
| GO:0002520\_immune\_system\_development | LCK | 147 | 4 | 13.877551 | -3.808383 | 23 | 0.05 | 0.002174 |
| GO:0002520\_immune\_system\_development | CD3D | 147 | 4 | 13.877551 | -3.808383 | 23 | 0.05 | 0.002174 |
| GO:0045580\_regulation\_of\_T\_cell\_differentiation | ZAP70 | 17 | 2 | 60.000000 | -3.317031 | 24 | 0.16 | 0.006667 |
| GO:0045580\_regulation\_of\_T\_cell\_differentiation | CD2 | 17 | 2 | 60.000000 | -3.317031 | 24 | 0.16 | 0.006667 |
| GO:0045619\_regulation\_of\_lymphocyte\_differentiation | ZAP70 | 20 | 2 | 51.000000 | -3.173307 | 25 | 0.25 | 0.010000 |
| GO:0045619\_regulation\_of\_lymphocyte\_differentiation | CD2 | 20 | 2 | 51.000000 | -3.173307 | 25 | 0.25 | 0.010000 |
| GO:0030885\_regulation\_of\_myeloid\_dendritic\_cell\_activation | CD2 | 1 | 1 |  |  |  |  |  |  |
| GO:0030887\_positive\_regulation\_of\_myeloid\_dendritic\_cell\_activation | CD2 | 1 | 1 |  |  |  |  |  |  |
| GO:0048869\_cellular\_developmental\_process | ZAP70 | 555 | 5 | 4.594595 | -2.477989 | 26 | 1.01 | 0.038846 |
| GO:0048869\_cellular\_developmental\_process | CD3G | 555 | 5 | 4.594595 | -2.477989 | 26 | 1.01 | 0.038846 |
| GO:0048869\_cellular\_developmental\_process | CD2 | 555 | 5 | 4.594595 | -2.477989 | 26 | 1.01 | 0.038846 |
| GO:0048869\_cellular\_developmental\_process | LCK | 555 | 5 | 4.594595 | -2.477989 | 26 | 1.01 | 0.038846 |
| GO:0048869\_cellular\_developmental\_process | CD3D | 555 | 5 | 4.594595 | -2.477989 | 26 | 1.01 | 0.038846 |
| GO:0001766\_membrane\_raft\_polarization | CD2 | 2 | 1 |  |  |  |  |  |  |
| GO:0031580\_membrane\_raft\_distribution | CD2 | 2 | 1 |  |  |  |  |  |  |
| GO:0050862\_positive\_regulation\_of\_T\_cell\_receptor\_signaling\_pathway | LCK | 2 | 1 |  |  |  |  |  |  |
| GO:0051665\_membrane\_raft\_localization | CD2 | 2 | 1 |  |  |  |  |  |  |
| GO:0007154\_cell\_communication | SH2D1A | 2272 | 10 | 2.244718 | -2.406877 | 27 | 1.19 | 0.044074 |
| GO:0007154\_cell\_communication | CD3E | 2272 | 10 | 2.244718 | -2.406877 | 27 | 1.19 | 0.044074 |
| GO:0007154\_cell\_communication | MAP4K1 | 2272 | 10 | 2.244718 | -2.406877 | 27 | 1.19 | 0.044074 |
| GO:0007154\_cell\_communication | ZAP70 | 2272 | 10 | 2.244718 | -2.406877 | 27 | 1.19 | 0.044074 |
| GO:0007154\_cell\_communication | CD3G | 2272 | 10 | 2.244718 | -2.406877 | 27 | 1.19 | 0.044074 |
| GO:0007154\_cell\_communication | CD2 | 2272 | 10 | 2.244718 | -2.406877 | 27 | 1.19 | 0.044074 |
| GO:0007154\_cell\_communication | CD7 | 2272 | 10 | 2.244718 | -2.406877 | 27 | 1.19 | 0.044074 |
| GO:0007154\_cell\_communication | GRAP2 | 2272 | 10 | 2.244718 | -2.406877 | 27 | 1.19 | 0.044074 |
| GO:0007154\_cell\_communication | LCK | 2272 | 10 | 2.244718 | -2.406877 | 27 | 1.19 | 0.044074 |
| GO:0007154\_cell\_communication | SIT1 | 2272 | 10 | 2.244718 | -2.406877 | 27 | 1.19 | 0.044074 |
| GO:0001773\_myeloid\_dendritic\_cell\_activation | CD2 | 3 | 1 |  |  |  |  |  |  |
| GO:0006882\_cellular\_zinc\_ion\_homeostasis | LCK | 3 | 1 |  |  |  |  |  |  |
| GO:0010956\_negative\_regulation\_of\_calcidiol\_1-monooxygenase\_activity | GFI1 | 3 | 1 |  |  |  |  |  |  |
| GO:0031579\_membrane\_raft\_organization | CD2 | 3 | 1 |  |  |  |  |  |  |
| GO:0050856\_regulation\_of\_T\_cell\_receptor\_signaling\_pathway | LCK | 3 | 1 |  |  |  |  |  |  |
| GO:0055069\_zinc\_ion\_homeostasis | LCK | 3 | 1 |  |  |  |  |  |  |
| GO:0007165\_signal\_transduction | CD3E | 2029 | 9 | 2.262198 | -2.146413 | 28 | 1.92 | 0.068571 |
| GO:0007165\_signal\_transduction | MAP4K1 | 2029 | 9 | 2.262198 | -2.146413 | 28 | 1.92 | 0.068571 |
| GO:0007165\_signal\_transduction | ZAP70 | 2029 | 9 | 2.262198 | -2.146413 | 28 | 1.92 | 0.068571 |
| GO:0007165\_signal\_transduction | CD3G | 2029 | 9 | 2.262198 | -2.146413 | 28 | 1.92 | 0.068571 |
| GO:0007165\_signal\_transduction | CD2 | 2029 | 9 | 2.262198 | -2.146413 | 28 | 1.92 | 0.068571 |
| GO:0007165\_signal\_transduction | CD7 | 2029 | 9 | 2.262198 | -2.146413 | 28 | 1.92 | 0.068571 |
| GO:0007165\_signal\_transduction | GRAP2 | 2029 | 9 | 2.262198 | -2.146413 | 28 | 1.92 | 0.068571 |
| GO:0007165\_signal\_transduction | LCK | 2029 | 9 | 2.262198 | -2.146413 | 28 | 1.92 | 0.068571 |
| GO:0007165\_signal\_transduction | SIT1 | 2029 | 9 | 2.262198 | -2.146413 | 28 | 1.92 | 0.068571 |
| GO:0050793\_regulation\_of\_developmental\_process | CD3E | 669 | 5 | 3.811659 | -2.129080 | 29 | 2.02 | 0.069655 |
| GO:0050793\_regulation\_of\_developmental\_process | ZAP70 | 669 | 5 | 3.811659 | -2.129080 | 29 | 2.02 | 0.069655 |
| GO:0050793\_regulation\_of\_developmental\_process | CD3G | 669 | 5 | 3.811659 | -2.129080 | 29 | 2.02 | 0.069655 |
| GO:0050793\_regulation\_of\_developmental\_process | CD2 | 669 | 5 | 3.811659 | -2.129080 | 29 | 2.02 | 0.069655 |
| GO:0050793\_regulation\_of\_developmental\_process | LCK | 669 | 5 | 3.811659 | -2.129080 | 29 | 2.02 | 0.069655 |
| GO:0000083\_regulation\_of\_transcription\_of\_G1\_S-phase\_of\_mitotic\_cell\_cycle | GFI1 | 4 | 1 |  |  |  |  |  |  |
| GO:0050857\_positive\_regulation\_of\_antigen\_receptor-mediated\_signaling\_pathway | LCK | 4 | 1 |  |  |  |  |  |  |
| GO:0006816\_calcium\_ion\_transport | LCK | 75 | 2 | 13.600000 | -2.036075 | 30 | 2.44 | 0.081333 |
| GO:0006816\_calcium\_ion\_transport | ATP2A3 | 75 | 2 | 13.600000 | -2.036075 | 30 | 2.44 | 0.081333 |
| GO:0070838\_divalent\_metal\_ion\_transport | LCK | 76 | 2 | 13.421053 | -2.024989 | 31 | 2.55 | 0.082258 |
| GO:0070838\_divalent\_metal\_ion\_transport | ATP2A3 | 76 | 2 | 13.421053 | -2.024989 | 31 | 2.55 | 0.082258 |
| GO:0050854\_regulation\_of\_antigen\_receptor-mediated\_signaling\_pathway | LCK | 5 | 1 | 102.000000 | -2.010197 | 32 | 5.45 | 0.170312 |
| GO:0007204\_elevation\_of\_cytosolic\_calcium\_ion\_concentration | CCR4 | 80 | 2 | 12.750000 | -1.982127 | 33 | 5.51 | 0.166970 |
| GO:0007204\_elevation\_of\_cytosolic\_calcium\_ion\_concentration | LCK | 80 | 2 | 12.750000 | -1.982127 | 33 | 5.51 | 0.166970 |
| GO:0051480\_cytosolic\_calcium\_ion\_homeostasis | CCR4 | 81 | 2 | 12.592593 | -1.971764 | 34 | 5.59 | 0.164412 |
| GO:0051480\_cytosolic\_calcium\_ion\_homeostasis | LCK | 81 | 2 | 12.592593 | -1.971764 | 34 | 5.59 | 0.164412 |
| GO:0042981\_regulation\_of\_apoptosis | CD3E | 471 | 4 | 4.331210 | -1.941485 | 35 | 5.78 | 0.165143 |
| GO:0042981\_regulation\_of\_apoptosis | CD3G | 471 | 4 | 4.331210 | -1.941485 | 35 | 5.78 | 0.165143 |
| GO:0042981\_regulation\_of\_apoptosis | CD2 | 471 | 4 | 4.331210 | -1.941485 | 35 | 5.78 | 0.165143 |
| GO:0042981\_regulation\_of\_apoptosis | LCK | 471 | 4 | 4.331210 | -1.941485 | 35 | 5.78 | 0.165143 |
| GO:0015674\_di-\_\_tri-valent\_inorganic\_cation\_transport | LCK | 84 | 2 | 12.142857 | -1.941466 | 36 | 5.85 | 0.162500 |
| GO:0015674\_di-\_\_tri-valent\_inorganic\_cation\_transport | ATP2A3 | 84 | 2 | 12.142857 | -1.941466 | 36 | 5.85 | 0.162500 |
| GO:0043067\_regulation\_of\_programmed\_cell\_death | CD3E | 476 | 4 | 4.285714 | -1.925698 | 37 | 8.21 | 0.221892 |
| GO:0043067\_regulation\_of\_programmed\_cell\_death | CD3G | 476 | 4 | 4.285714 | -1.925698 | 37 | 8.21 | 0.221892 |
| GO:0043067\_regulation\_of\_programmed\_cell\_death | CD2 | 476 | 4 | 4.285714 | -1.925698 | 37 | 8.21 | 0.221892 |
| GO:0043067\_regulation\_of\_programmed\_cell\_death | LCK | 476 | 4 | 4.285714 | -1.925698 | 37 | 8.21 | 0.221892 |
| GO:0010941\_regulation\_of\_cell\_death | CD3E | 478 | 4 | 4.267782 | -1.919438 | 38 | 8.24 | 0.216842 |
| GO:0010941\_regulation\_of\_cell\_death | CD3G | 478 | 4 | 4.267782 | -1.919438 | 38 | 8.24 | 0.216842 |
| GO:0010941\_regulation\_of\_cell\_death | CD2 | 478 | 4 | 4.267782 | -1.919438 | 38 | 8.24 | 0.216842 |
| GO:0010941\_regulation\_of\_cell\_death | LCK | 478 | 4 | 4.267782 | -1.919438 | 38 | 8.24 | 0.216842 |
| GO:0032769\_negative\_regulation\_of\_monooxygenase\_activity | GFI1 | 7 | 1 | 72.857143 | -1.864867 | 40 | 10.63 | 0.265750 |
| GO:0060558\_regulation\_of\_calcidiol\_1-monooxygenase\_activity | GFI1 | 7 | 1 | 72.857143 | -1.864867 | 40 | 10.63 | 0.265750 |
| GO:0030154\_cell\_differentiation | ZAP70 | 506 | 4 | 4.031621 | -1.834902 | 41 | 10.79 | 0.263171 |
| GO:0030154\_cell\_differentiation | CD2 | 506 | 4 | 4.031621 | -1.834902 | 41 | 10.79 | 0.263171 |
| GO:0030154\_cell\_differentiation | LCK | 506 | 4 | 4.031621 | -1.834902 | 41 | 10.79 | 0.263171 |
| GO:0030154\_cell\_differentiation | CD3D | 506 | 4 | 4.031621 | -1.834902 | 41 | 10.79 | 0.263171 |
| GO:0006955\_immune\_response | CCR4 | 529 | 4 | 3.856333 | -1.769509 | 42 | 13.33 | 0.317381 |
| GO:0006955\_immune\_response | ZAP70 | 529 | 4 | 3.856333 | -1.769509 | 42 | 13.33 | 0.317381 |
| GO:0006955\_immune\_response | CD7 | 529 | 4 | 3.856333 | -1.769509 | 42 | 13.33 | 0.317381 |
| GO:0006955\_immune\_response | LCK | 529 | 4 | 3.856333 | -1.769509 | 42 | 13.33 | 0.317381 |
| GO:0045582\_positive\_regulation\_of\_T\_cell\_differentiation | ZAP70 | 9 | 1 | 56.666667 | -1.756520 | 43 | 15.49 | 0.360233 |
| GO:0007166\_cell\_surface\_receptor\_linked\_signal\_transduction | CD3E | 828 | 5 | 3.079710 | -1.746559 | 44 | 15.59 | 0.354318 |
| GO:0007166\_cell\_surface\_receptor\_linked\_signal\_transduction | CD3G | 828 | 5 | 3.079710 | -1.746559 | 44 | 15.59 | 0.354318 |
| GO:0007166\_cell\_surface\_receptor\_linked\_signal\_transduction | CD2 | 828 | 5 | 3.079710 | -1.746559 | 44 | 15.59 | 0.354318 |
| GO:0007166\_cell\_surface\_receptor\_linked\_signal\_transduction | CD7 | 828 | 5 | 3.079710 | -1.746559 | 44 | 15.59 | 0.354318 |
| GO:0007166\_cell\_surface\_receptor\_linked\_signal\_transduction | LCK | 828 | 5 | 3.079710 | -1.746559 | 44 | 15.59 | 0.354318 |
| GO:0045621\_positive\_regulation\_of\_lymphocyte\_differentiation | ZAP70 | 10 | 1 | 51.000000 | -1.711162 | 46 | 17.82 | 0.387391 |
| GO:0051354\_negative\_regulation\_of\_oxidoreductase\_activity | GFI1 | 10 | 1 | 51.000000 | -1.711162 | 46 | 17.82 | 0.387391 |
| GO:0006874\_cellular\_calcium\_ion\_homeostasis | CCR4 | 114 | 2 | 8.947368 | -1.689676 | 47 | 18.0 | 0.382979 |
| GO:0006874\_cellular\_calcium\_ion\_homeostasis | LCK | 114 | 2 | 8.947368 | -1.689676 | 47 | 18.0 | 0.382979 |
| GO:0055074\_calcium\_ion\_homeostasis | CCR4 | 116 | 2 | 8.793103 | -1.675491 | 48 | 18.16 | 0.378333 |
| GO:0055074\_calcium\_ion\_homeostasis | LCK | 116 | 2 | 8.793103 | -1.675491 | 48 | 18.16 | 0.378333 |
| GO:0006915\_apoptosis | CD3E | 565 | 4 | 3.610619 | -1.673711 | 49 | 18.17 | 0.370816 |
| GO:0006915\_apoptosis | CD3G | 565 | 4 | 3.610619 | -1.673711 | 49 | 18.17 | 0.370816 |
| GO:0006915\_apoptosis | CD2 | 565 | 4 | 3.610619 | -1.673711 | 49 | 18.17 | 0.370816 |
| GO:0006915\_apoptosis | LCK | 565 | 4 | 3.610619 | -1.673711 | 49 | 18.17 | 0.370816 |
| GO:0007172\_signal\_complex\_assembly | CD3E | 11 | 1 | 46.363636 | -1.670167 | 51 | 20.05 | 0.393137 |
| GO:0051668\_localization\_within\_membrane | CD2 | 11 | 1 | 46.363636 | -1.670167 | 51 | 20.05 | 0.393137 |
| GO:0012501\_programmed\_cell\_death | CD3E | 571 | 4 | 3.572680 | -1.658462 | 52 | 20.16 | 0.387692 |
| GO:0012501\_programmed\_cell\_death | CD3G | 571 | 4 | 3.572680 | -1.658462 | 52 | 20.16 | 0.387692 |
| GO:0012501\_programmed\_cell\_death | CD2 | 571 | 4 | 3.572680 | -1.658462 | 52 | 20.16 | 0.387692 |
| GO:0012501\_programmed\_cell\_death | LCK | 571 | 4 | 3.572680 | -1.658462 | 52 | 20.16 | 0.387692 |
| GO:0006875\_cellular\_metal\_ion\_homeostasis | CCR4 | 121 | 2 | 8.429752 | -1.641150 | 53 | 20.27 | 0.382453 |
| GO:0006875\_cellular\_metal\_ion\_homeostasis | LCK | 121 | 2 | 8.429752 | -1.641150 | 53 | 20.27 | 0.382453 |
| GO:0050852\_T\_cell\_receptor\_signaling\_pathway | LCK | 12 | 1 | 42.500000 | -1.632777 | 54 | 22.22 | 0.411481 |
| GO:0008219\_cell\_death | CD3E | 585 | 4 | 3.487179 | -1.623627 | 56 | 22.27 | 0.397679 |
| GO:0008219\_cell\_death | CD3G | 585 | 4 | 3.487179 | -1.623627 | 56 | 22.27 | 0.397679 |
| GO:0008219\_cell\_death | CD2 | 585 | 4 | 3.487179 | -1.623627 | 56 | 22.27 | 0.397679 |
| GO:0008219\_cell\_death | LCK | 585 | 4 | 3.487179 | -1.623627 | 56 | 22.27 | 0.397679 |
| GO:0016265\_death | CD3E | 585 | 4 | 3.487179 | -1.623627 | 56 | 22.27 | 0.397679 |
| GO:0016265\_death | CD3G | 585 | 4 | 3.487179 | -1.623627 | 56 | 22.27 | 0.397679 |
| GO:0016265\_death | CD2 | 585 | 4 | 3.487179 | -1.623627 | 56 | 22.27 | 0.397679 |
| GO:0016265\_death | LCK | 585 | 4 | 3.487179 | -1.623627 | 56 | 22.27 | 0.397679 |
| GO:0055065\_metal\_ion\_homeostasis | CCR4 | 125 | 2 | 8.160000 | -1.614760 | 57 | 22.37 | 0.392456 |
| GO:0055065\_metal\_ion\_homeostasis | LCK | 125 | 2 | 8.160000 | -1.614760 | 57 | 22.37 | 0.392456 |
| GO:0051094\_positive\_regulation\_of\_developmental\_process | ZAP70 | 340 | 3 | 4.500000 | -1.571568 | 58 | 24.43 | 0.421207 |
| GO:0051094\_positive\_regulation\_of\_developmental\_process | CD2 | 340 | 3 | 4.500000 | -1.571568 | 58 | 24.43 | 0.421207 |
| GO:0051094\_positive\_regulation\_of\_developmental\_process | LCK | 340 | 3 | 4.500000 | -1.571568 | 58 | 24.43 | 0.421207 |
| GO:0051209\_release\_of\_sequestered\_calcium\_ion\_into\_cytosol | LCK | 14 | 1 | 36.428571 | -1.566627 | 61 | 26.22 | 0.429836 |
| GO:0051282\_regulation\_of\_sequestering\_of\_calcium\_ion | LCK | 14 | 1 | 36.428571 | -1.566627 | 61 | 26.22 | 0.429836 |
| GO:0051283\_negative\_regulation\_of\_sequestering\_of\_calcium\_ion | LCK | 14 | 1 | 36.428571 | -1.566627 | 61 | 26.22 | 0.429836 |
| GO:0030101\_natural\_killer\_cell\_activation | CD2 | 15 | 1 | 34.000000 | -1.537062 | 62 | 28.6 | 0.461290 |
| GO:0030005\_cellular\_di-\_\_tri-valent\_inorganic\_cation\_homeostasis | CCR4 | 140 | 2 | 7.285714 | -1.523352 | 63 | 28.82 | 0.457460 |
| GO:0030005\_cellular\_di-\_\_tri-valent\_inorganic\_cation\_homeostasis | LCK | 140 | 2 | 7.285714 | -1.523352 | 63 | 28.82 | 0.457460 |
| GO:0010553\_negative\_regulation\_of\_specific\_transcription\_from\_RNA\_polymerase\_II\_promoter | GFI1 | 16 | 1 | 31.875000 | -1.509432 | 65 | 30.57 | 0.470308 |
| GO:0050851\_antigen\_receptor-mediated\_signaling\_pathway | LCK | 16 | 1 | 31.875000 | -1.509432 | 65 | 30.57 | 0.470308 |
| GO:0055066\_di-\_\_tri-valent\_inorganic\_cation\_homeostasis | CCR4 | 145 | 2 | 7.034483 | -1.495230 | 66 | 30.79 | 0.466515 |
| GO:0055066\_di-\_\_tri-valent\_inorganic\_cation\_homeostasis | LCK | 145 | 2 | 7.034483 | -1.495230 | 66 | 30.79 | 0.466515 |
| GO:0051208\_sequestering\_of\_calcium\_ion | LCK | 17 | 1 | 30.000000 | -1.483501 | 67 | 32.65 | 0.487313 |
| GO:0006952\_defense\_response | SH2D1A | 369 | 3 | 4.146341 | -1.479742 | 68 | 32.7 | 0.480882 |
| GO:0006952\_defense\_response | PTPRCAP | 369 | 3 | 4.146341 | -1.479742 | 68 | 32.7 | 0.480882 |
| GO:0006952\_defense\_response | CCR4 | 369 | 3 | 4.146341 | -1.479742 | 68 | 32.7 | 0.480882 |
| GO:0002429\_immune\_response-activating\_cell\_surface\_receptor\_signaling\_pathway | LCK | 19 | 1 | 26.842105 | -1.435992 | 72 | 36.24 | 0.503333 |
| GO:0002768\_immune\_response-regulating\_cell\_surface\_receptor\_signaling\_pathway | LCK | 19 | 1 | 26.842105 | -1.435992 | 72 | 36.24 | 0.503333 |
| GO:0007257\_activation\_of\_JUN\_kinase\_activity | MAP4K1 | 19 | 1 | 26.842105 | -1.435992 | 72 | 36.24 | 0.503333 |
| GO:0042102\_positive\_regulation\_of\_T\_cell\_proliferation | CD3E | 19 | 1 | 26.842105 | -1.435992 | 72 | 36.24 | 0.503333 |
| GO:0007169\_transmembrane\_receptor\_protein\_tyrosine\_kinase\_signaling\_pathway | CD3E | 157 | 2 | 6.496815 | -1.431851 | 73 | 36.32 | 0.497534 |
| GO:0007169\_transmembrane\_receptor\_protein\_tyrosine\_kinase\_signaling\_pathway | CD7 | 157 | 2 | 6.496815 | -1.431851 | 73 | 36.32 | 0.497534 |
| GO:0030003\_cellular\_cation\_homeostasis | CCR4 | 161 | 2 | 6.335404 | -1.411900 | 74 | 37.89 | 0.512027 |
| GO:0030003\_cellular\_cation\_homeostasis | LCK | 161 | 2 | 6.335404 | -1.411900 | 74 | 37.89 | 0.512027 |
| GO:0032768\_regulation\_of\_monooxygenase\_activity | GFI1 | 21 | 1 | 24.285714 | -1.393322 | 76 | 40.02 | 0.526579 |
| GO:0051238\_sequestering\_of\_metal\_ion | LCK | 21 | 1 | 24.285714 | -1.393322 | 76 | 40.02 | 0.526579 |
| GO:0050896\_response\_to\_stimulus | SH2D1A | 1775 | 7 | 2.011268 | -1.390959 | 77 | 40.04 | 0.520000 |
| GO:0050896\_response\_to\_stimulus | MAP4K1 | 1775 | 7 | 2.011268 | -1.390959 | 77 | 40.04 | 0.520000 |
| GO:0050896\_response\_to\_stimulus | PTPRCAP | 1775 | 7 | 2.011268 | -1.390959 | 77 | 40.04 | 0.520000 |
| GO:0050896\_response\_to\_stimulus | CCR4 | 1775 | 7 | 2.011268 | -1.390959 | 77 | 40.04 | 0.520000 |
| GO:0050896\_response\_to\_stimulus | ZAP70 | 1775 | 7 | 2.011268 | -1.390959 | 77 | 40.04 | 0.520000 |
| GO:0050896\_response\_to\_stimulus | CD7 | 1775 | 7 | 2.011268 | -1.390959 | 77 | 40.04 | 0.520000 |
| GO:0050896\_response\_to\_stimulus | LCK | 1775 | 7 | 2.011268 | -1.390959 | 77 | 40.04 | 0.520000 |
| GO:0032582\_negative\_regulation\_of\_gene-specific\_transcription | GFI1 | 22 | 1 | 23.181818 | -1.373516 | 79 | 41.29 | 0.522658 |
| GO:0043507\_positive\_regulation\_of\_JUN\_kinase\_activity | MAP4K1 | 22 | 1 | 23.181818 | -1.373516 | 79 | 41.29 | 0.522658 |
| GO:0045595\_regulation\_of\_cell\_differentiation | ZAP70 | 170 | 2 | 6.000000 | -1.368943 | 80 | 41.38 | 0.517250 |
| GO:0045595\_regulation\_of\_cell\_differentiation | CD2 | 170 | 2 | 6.000000 | -1.368943 | 80 | 41.38 | 0.517250 |
| GO:0055080\_cation\_homeostasis | CCR4 | 173 | 2 | 5.895954 | -1.355181 | 81 | 41.59 | 0.513457 |
| GO:0055080\_cation\_homeostasis | LCK | 173 | 2 | 5.895954 | -1.355181 | 81 | 41.59 | 0.513457 |
| GO:0050671\_positive\_regulation\_of\_lymphocyte\_proliferation | CD3E | 23 | 1 | 22.173913 | -1.354608 | 83 | 42.46 | 0.511566 |
| GO:0070668\_positive\_regulation\_of\_mast\_cell\_proliferation | CD3E | 23 | 1 | 22.173913 | -1.354608 | 83 | 42.46 | 0.511566 |
| GO:0007163\_establishment\_or\_maintenance\_of\_cell\_polarity | CD3G | 24 | 1 | 21.250000 | -1.336522 | 87 | 44.21 | 0.508161 |
| GO:0032946\_positive\_regulation\_of\_mononuclear\_cell\_proliferation | CD3E | 24 | 1 | 21.250000 | -1.336522 | 87 | 44.21 | 0.508161 |
| GO:0043506\_regulation\_of\_JUN\_kinase\_activity | MAP4K1 | 24 | 1 | 21.250000 | -1.336522 | 87 | 44.21 | 0.508161 |
| GO:0070665\_positive\_regulation\_of\_leukocyte\_proliferation | CD3E | 24 | 1 | 21.250000 | -1.336522 | 87 | 44.21 | 0.508161 |
| GO:0002757\_immune\_response-activating\_signal\_transduction | LCK | 25 | 1 | 20.400000 | -1.319191 | 90 | 45.93 | 0.510333 |
| GO:0002764\_immune\_response-regulating\_signal\_transduction | LCK | 25 | 1 | 20.400000 | -1.319191 | 90 | 45.93 | 0.510333 |
| GO:0042129\_regulation\_of\_T\_cell\_proliferation | CD3E | 25 | 1 | 20.400000 | -1.319191 | 90 | 45.93 | 0.510333 |
| GO:0008037\_cell\_recognition | CD5 | 26 | 1 | 19.615385 | -1.302555 | 92 | 47.42 | 0.515435 |
| GO:0018105\_peptidyl-serine\_phosphorylation | MAP4K1 | 26 | 1 | 19.615385 | -1.302555 | 92 | 47.42 | 0.515435 |
| GO:0030001\_metal\_ion\_transport | LCK | 186 | 2 | 5.483871 | -1.298467 | 93 | 47.51 | 0.510860 |
| GO:0030001\_metal\_ion\_transport | ATP2A3 | 186 | 2 | 5.483871 | -1.298467 | 93 | 47.51 | 0.510860 |
| GO:0048513\_organ\_development | ZAP70 | 741 | 4 | 2.753036 | -1.294242 | 94 | 47.59 | 0.506277 |
| GO:0048513\_organ\_development | CD2 | 741 | 4 | 2.753036 | -1.294242 | 94 | 47.59 | 0.506277 |
| GO:0048513\_organ\_development | LCK | 741 | 4 | 2.753036 | -1.294242 | 94 | 47.59 | 0.506277 |
| GO:0048513\_organ\_development | CD3D | 741 | 4 | 2.753036 | -1.294242 | 94 | 47.59 | 0.506277 |
| GO:0006917\_induction\_of\_apoptosis | CD2 | 190 | 2 | 5.368421 | -1.281903 | 95 | 48.99 | 0.515684 |
| GO:0006917\_induction\_of\_apoptosis | LCK | 190 | 2 | 5.368421 | -1.281903 | 95 | 48.99 | 0.515684 |
| GO:0012502\_induction\_of\_programmed\_cell\_death | CD2 | 191 | 2 | 5.340314 | -1.277823 | 96 | 49.08 | 0.511250 |
| GO:0012502\_induction\_of\_programmed\_cell\_death | LCK | 191 | 2 | 5.340314 | -1.277823 | 96 | 49.08 | 0.511250 |
| GO:0060402\_calcium\_ion\_transport\_into\_cytosol | LCK | 28 | 1 | 18.214286 | -1.271164 | 99 | 50.57 | 0.510808 |
| GO:0070662\_mast\_cell\_proliferation | CD3E | 28 | 1 | 18.214286 | -1.271164 | 99 | 50.57 | 0.510808 |
| GO:0070666\_regulation\_of\_mast\_cell\_proliferation | CD3E | 28 | 1 | 18.214286 | -1.271164 | 99 | 50.57 | 0.510808 |
| GO:0002274\_myeloid\_leukocyte\_activation | CD2 | 29 | 1 | 17.586207 | -1.256321 | 101 | 52.02 | 0.515050 |
| GO:0060401\_cytosolic\_calcium\_ion\_transport | LCK | 29 | 1 | 17.586207 | -1.256321 | 101 | 52.02 | 0.515050 |
| GO:0018209\_peptidyl-serine\_modification | MAP4K1 | 30 | 1 | 17.000000 | -1.241995 | 104 | 53.18 | 0.511346 |
| GO:0042098\_T\_cell\_proliferation | CD3E | 30 | 1 | 17.000000 | -1.241995 | 104 | 53.18 | 0.511346 |
| GO:0050670\_regulation\_of\_lymphocyte\_proliferation | CD3E | 30 | 1 | 17.000000 | -1.241995 | 104 | 53.18 | 0.511346 |
| GO:0032944\_regulation\_of\_mononuclear\_cell\_proliferation | CD3E | 31 | 1 | 16.451613 | -1.228151 | 106 | 54.24 | 0.511698 |
| GO:0070663\_regulation\_of\_leukocyte\_proliferation | CD3E | 31 | 1 | 16.451613 | -1.228151 | 106 | 54.24 | 0.511698 |
| GO:0006873\_cellular\_ion\_homeostasis | CCR4 | 206 | 2 | 4.951456 | -1.219360 | 107 | 54.54 | 0.509720 |
| GO:0006873\_cellular\_ion\_homeostasis | LCK | 206 | 2 | 4.951456 | -1.219360 | 107 | 54.54 | 0.509720 |
| GO:0055082\_cellular\_chemical\_homeostasis | CCR4 | 208 | 2 | 4.903846 | -1.211929 | 108 | 55.8 | 0.516667 |
| GO:0055082\_cellular\_chemical\_homeostasis | LCK | 208 | 2 | 4.903846 | -1.211929 | 108 | 55.8 | 0.516667 |
| GO:0051341\_regulation\_of\_oxidoreductase\_activity | GFI1 | 33 | 1 | 15.454545 | -1.201792 | 109 | 56.8 | 0.521101 |
| GO:0050801\_ion\_homeostasis | CCR4 | 221 | 2 | 4.615385 | -1.165529 | 110 | 59.98 | 0.545273 |
| GO:0050801\_ion\_homeostasis | LCK | 221 | 2 | 4.615385 | -1.165529 | 110 | 59.98 | 0.545273 |
| GO:0000082\_G1\_S\_transition\_of\_mitotic\_cell\_cycle | GFI1 | 36 | 1 | 14.166667 | -1.165193 | 111 | 60.56 | 0.545586 |
| GO:0042493\_response\_to\_drug | LCK | 38 | 1 | 13.421053 | -1.142504 | 112 | 62.99 | 0.562411 |
| GO:0019725\_cellular\_homeostasis | CCR4 | 231 | 2 | 4.415584 | -1.131909 | 113 | 63.3 | 0.560177 |
| GO:0019725\_cellular\_homeostasis | LCK | 231 | 2 | 4.415584 | -1.131909 | 113 | 63.3 | 0.560177 |
| GO:0046328\_regulation\_of\_JNK\_cascade | MAP4K1 | 39 | 1 | 13.076923 | -1.131619 | 114 | 64.2 | 0.563158 |
| GO:0002253\_activation\_of\_immune\_response | LCK | 40 | 1 | 12.750000 | -1.121020 | 116 | 65.02 | 0.560517 |
| GO:0070302\_regulation\_of\_stress-activated\_protein\_kinase\_signaling\_pathway | MAP4K1 | 40 | 1 | 12.750000 | -1.121020 | 116 | 65.02 | 0.560517 |
| GO:0046651\_lymphocyte\_proliferation | CD3E | 41 | 1 | 12.439024 | -1.110692 | 117 | 66.12 | 0.565128 |
| GO:0032943\_mononuclear\_cell\_proliferation | CD3E | 42 | 1 | 12.142857 | -1.100622 | 119 | 67.31 | 0.565630 |
| GO:0070661\_leukocyte\_proliferation | CD3E | 42 | 1 | 12.142857 | -1.100622 | 119 | 67.31 | 0.565630 |
| GO:0050790\_regulation\_of\_catalytic\_activity | MAP4K1 | 525 | 3 | 2.914286 | -1.100614 | 120 | 67.39 | 0.561583 |
| GO:0050790\_regulation\_of\_catalytic\_activity | GFI1 | 525 | 3 | 2.914286 | -1.100614 | 120 | 67.39 | 0.561583 |
| GO:0050790\_regulation\_of\_catalytic\_activity | LCK | 525 | 3 | 2.914286 | -1.100614 | 120 | 67.39 | 0.561583 |
| GO:0048583\_regulation\_of\_response\_to\_stimulus | MAP4K1 | 241 | 2 | 4.232365 | -1.099922 | 121 | 67.43 | 0.557273 |
| GO:0048583\_regulation\_of\_response\_to\_stimulus | LCK | 241 | 2 | 4.232365 | -1.099922 | 121 | 67.43 | 0.557273 |
| GO:0043065\_positive\_regulation\_of\_apoptosis | CD2 | 243 | 2 | 4.197531 | -1.093708 | 122 | 67.6 | 0.554098 |
| GO:0043065\_positive\_regulation\_of\_apoptosis | LCK | 243 | 2 | 4.197531 | -1.093708 | 122 | 67.6 | 0.554098 |
| GO:0006812\_cation\_transport | LCK | 246 | 2 | 4.146341 | -1.084498 | 124 | 68.93 | 0.555887 |
| GO:0006812\_cation\_transport | ATP2A3 | 246 | 2 | 4.146341 | -1.084498 | 124 | 68.93 | 0.555887 |
| GO:0043068\_positive\_regulation\_of\_programmed\_cell\_death | CD2 | 246 | 2 | 4.146341 | -1.084498 | 124 | 68.93 | 0.555887 |
| GO:0043068\_positive\_regulation\_of\_programmed\_cell\_death | LCK | 246 | 2 | 4.146341 | -1.084498 | 124 | 68.93 | 0.555887 |
| GO:0010942\_positive\_regulation\_of\_cell\_death | CD2 | 250 | 2 | 4.080000 | -1.072419 | 125 | 69.53 | 0.556240 |
| GO:0010942\_positive\_regulation\_of\_cell\_death | LCK | 250 | 2 | 4.080000 | -1.072419 | 125 | 69.53 | 0.556240 |
| GO:0006919\_activation\_of\_caspase\_activity | LCK | 45 | 1 | 11.333333 | -1.071846 | 126 | 70.42 | 0.558889 |
| GO:0065007\_biological\_regulation | CD3E | 3971 | 11 | 1.412742 | -1.062771 | 127 | 70.56 | 0.555591 |
| GO:0065007\_biological\_regulation | MAP4K1 | 3971 | 11 | 1.412742 | -1.062771 | 127 | 70.56 | 0.555591 |
| GO:0065007\_biological\_regulation | CCR4 | 3971 | 11 | 1.412742 | -1.062771 | 127 | 70.56 | 0.555591 |
| GO:0065007\_biological\_regulation | ZAP70 | 3971 | 11 | 1.412742 | -1.062771 | 127 | 70.56 | 0.555591 |
| GO:0065007\_biological\_regulation | CD3G | 3971 | 11 | 1.412742 | -1.062771 | 127 | 70.56 | 0.555591 |
| GO:0065007\_biological\_regulation | CD7 | 3971 | 11 | 1.412742 | -1.062771 | 127 | 70.56 | 0.555591 |
| GO:0065007\_biological\_regulation | CD2 | 3971 | 11 | 1.412742 | -1.062771 | 127 | 70.56 | 0.555591 |
| GO:0065007\_biological\_regulation | GRAP2 | 3971 | 11 | 1.412742 | -1.062771 | 127 | 70.56 | 0.555591 |
| GO:0065007\_biological\_regulation | GFI1 | 3971 | 11 | 1.412742 | -1.062771 | 127 | 70.56 | 0.555591 |
| GO:0065007\_biological\_regulation | LCK | 3971 | 11 | 1.412742 | -1.062771 | 127 | 70.56 | 0.555591 |
| GO:0065007\_biological\_regulation | SIT1 | 3971 | 11 | 1.412742 | -1.062771 | 127 | 70.56 | 0.555591 |
| GO:0007167\_enzyme\_linked\_receptor\_protein\_signaling\_pathway | CD3E | 258 | 2 | 3.953488 | -1.048921 | 128 | 73.62 | 0.575156 |
| GO:0007167\_enzyme\_linked\_receptor\_protein\_signaling\_pathway | CD7 | 258 | 2 | 3.953488 | -1.048921 | 128 | 73.62 | 0.575156 |
| GO:0010551\_regulation\_of\_specific\_transcription\_from\_RNA\_polymerase\_II\_promoter | GFI1 | 48 | 1 | 10.625000 | -1.045003 | 130 | 74.4 | 0.572308 |
| GO:0032569\_specific\_transcription\_from\_RNA\_polymerase\_II\_promoter | GFI1 | 48 | 1 | 10.625000 | -1.045003 | 130 | 74.4 | 0.572308 |
| GO:0010952\_positive\_regulation\_of\_peptidase\_activity | LCK | 49 | 1 | 10.408163 | -1.036443 | 132 | 75.56 | 0.572424 |
| GO:0043280\_positive\_regulation\_of\_caspase\_activity | LCK | 49 | 1 | 10.408163 | -1.036443 | 132 | 75.56 | 0.572424 |
| GO:0000187\_activation\_of\_MAPK\_activity | MAP4K1 | 50 | 1 | 10.200000 | -1.028064 | 133 | 76.44 | 0.574737 |
| GO:0048856\_anatomical\_structure\_development | ZAP70 | 1289 | 5 | 1.978278 | -1.025081 | 134 | 76.62 | 0.571791 |
| GO:0048856\_anatomical\_structure\_development | CD3G | 1289 | 5 | 1.978278 | -1.025081 | 134 | 76.62 | 0.571791 |
| GO:0048856\_anatomical\_structure\_development | CD2 | 1289 | 5 | 1.978278 | -1.025081 | 134 | 76.62 | 0.571791 |
| GO:0048856\_anatomical\_structure\_development | LCK | 1289 | 5 | 1.978278 | -1.025081 | 134 | 76.62 | 0.571791 |
| GO:0048856\_anatomical\_structure\_development | CD3D | 1289 | 5 | 1.978278 | -1.025081 | 134 | 76.62 | 0.571791 |
| GO:0050794\_regulation\_of\_cellular\_process | CD3E | 3515 | 10 | 1.450925 | -1.025063 | 135 | 76.66 | 0.567852 |
| GO:0050794\_regulation\_of\_cellular\_process | MAP4K1 | 3515 | 10 | 1.450925 | -1.025063 | 135 | 76.66 | 0.567852 |
| GO:0050794\_regulation\_of\_cellular\_process | ZAP70 | 3515 | 10 | 1.450925 | -1.025063 | 135 | 76.66 | 0.567852 |
| GO:0050794\_regulation\_of\_cellular\_process | CD3G | 3515 | 10 | 1.450925 | -1.025063 | 135 | 76.66 | 0.567852 |
| GO:0050794\_regulation\_of\_cellular\_process | CD2 | 3515 | 10 | 1.450925 | -1.025063 | 135 | 76.66 | 0.567852 |
| GO:0050794\_regulation\_of\_cellular\_process | CD7 | 3515 | 10 | 1.450925 | -1.025063 | 135 | 76.66 | 0.567852 |
| GO:0050794\_regulation\_of\_cellular\_process | GRAP2 | 3515 | 10 | 1.450925 | -1.025063 | 135 | 76.66 | 0.567852 |
| GO:0050794\_regulation\_of\_cellular\_process | GFI1 | 3515 | 10 | 1.450925 | -1.025063 | 135 | 76.66 | 0.567852 |
| GO:0050794\_regulation\_of\_cellular\_process | LCK | 3515 | 10 | 1.450925 | -1.025063 | 135 | 76.66 | 0.567852 |
| GO:0050794\_regulation\_of\_cellular\_process | SIT1 | 3515 | 10 | 1.450925 | -1.025063 | 135 | 76.66 | 0.567852 |
| GO:0006461\_protein\_complex\_assembly | CD3E | 273 | 2 | 3.736264 | -1.007078 | 137 | 78.57 | 0.573504 |
| GO:0006461\_protein\_complex\_assembly | CD3G | 273 | 2 | 3.736264 | -1.007078 | 137 | 78.57 | 0.573504 |
| GO:0070271\_protein\_complex\_biogenesis | CD3E | 273 | 2 | 3.736264 | -1.007078 | 137 | 78.57 | 0.573504 |
| GO:0070271\_protein\_complex\_biogenesis | CD3G | 273 | 2 | 3.736264 | -1.007078 | 137 | 78.57 | 0.573504 |
| GO:0048878\_chemical\_homeostasis | CCR4 | 278 | 2 | 3.669065 | -0.993729 | 138 | 80.44 | 0.582899 |
| GO:0048878\_chemical\_homeostasis | LCK | 278 | 2 | 3.669065 | -0.993729 | 138 | 80.44 | 0.582899 |
| GO:0050778\_positive\_regulation\_of\_immune\_response | LCK | 56 | 1 | 9.107143 | -0.981215 | 139 | 81.85 | 0.588849 |
| GO:0032844\_regulation\_of\_homeostatic\_process | LCK | 57 | 1 | 8.947368 | -0.973922 | 140 | 82.42 | 0.588714 |
| GO:0006968\_cellular\_defense\_response | SH2D1A | 58 | 1 | 8.793103 | -0.966763 | 141 | 84.03 | 0.595957 |
| GO:0006950\_response\_to\_stress | SH2D1A | 959 | 4 | 2.127216 | -0.961014 | 142 | 84.47 | 0.594859 |
| GO:0006950\_response\_to\_stress | MAP4K1 | 959 | 4 | 2.127216 | -0.961014 | 142 | 84.47 | 0.594859 |
| GO:0006950\_response\_to\_stress | PTPRCAP | 959 | 4 | 2.127216 | -0.961014 | 142 | 84.47 | 0.594859 |
| GO:0006950\_response\_to\_stress | CCR4 | 959 | 4 | 2.127216 | -0.961014 | 142 | 84.47 | 0.594859 |
| GO:0043281\_regulation\_of\_caspase\_activity | LCK | 59 | 1 | 8.644068 | -0.959734 | 144 | 85.27 | 0.592153 |
| GO:0043408\_regulation\_of\_MAPKKK\_cascade | MAP4K1 | 59 | 1 | 8.644068 | -0.959734 | 144 | 85.27 | 0.592153 |
| GO:0065009\_regulation\_of\_molecular\_function | MAP4K1 | 606 | 3 | 2.524752 | -0.955539 | 145 | 85.76 | 0.591448 |
| GO:0065009\_regulation\_of\_molecular\_function | LCK | 606 | 3 | 2.524752 | -0.955539 | 145 | 85.76 | 0.591448 |
| GO:0065009\_regulation\_of\_molecular\_function | GFI1 | 606 | 3 | 2.524752 | -0.955539 | 145 | 85.76 | 0.591448 |
| GO:0007254\_JNK\_cascade | MAP4K1 | 61 | 1 | 8.360656 | -0.946044 | 146 | 86.92 | 0.595342 |
| GO:0043406\_positive\_regulation\_of\_MAP\_kinase\_activity | MAP4K1 | 62 | 1 | 8.225806 | -0.939376 | 148 | 88.68 | 0.599189 |
| GO:0052548\_regulation\_of\_endopeptidase\_activity | LCK | 62 | 1 | 8.225806 | -0.939376 | 148 | 88.68 | 0.599189 |
| GO:0031098\_stress-activated\_protein\_kinase\_signaling\_pathway | MAP4K1 | 64 | 1 | 7.968750 | -0.926375 | 149 | 90.44 | 0.606980 |
| GO:0050789\_regulation\_of\_biological\_process | CD3E | 3649 | 10 | 1.397643 | -0.923702 | 150 | 90.58 | 0.603867 |
| GO:0050789\_regulation\_of\_biological\_process | MAP4K1 | 3649 | 10 | 1.397643 | -0.923702 | 150 | 90.58 | 0.603867 |
| GO:0050789\_regulation\_of\_biological\_process | ZAP70 | 3649 | 10 | 1.397643 | -0.923702 | 150 | 90.58 | 0.603867 |
| GO:0050789\_regulation\_of\_biological\_process | CD3G | 3649 | 10 | 1.397643 | -0.923702 | 150 | 90.58 | 0.603867 |
| GO:0050789\_regulation\_of\_biological\_process | CD7 | 3649 | 10 | 1.397643 | -0.923702 | 150 | 90.58 | 0.603867 |
| GO:0050789\_regulation\_of\_biological\_process | CD2 | 3649 | 10 | 1.397643 | -0.923702 | 150 | 90.58 | 0.603867 |
| GO:0050789\_regulation\_of\_biological\_process | GRAP2 | 3649 | 10 | 1.397643 | -0.923702 | 150 | 90.58 | 0.603867 |
| GO:0050789\_regulation\_of\_biological\_process | GFI1 | 3649 | 10 | 1.397643 | -0.923702 | 150 | 90.58 | 0.603867 |
| GO:0050789\_regulation\_of\_biological\_process | LCK | 3649 | 10 | 1.397643 | -0.923702 | 150 | 90.58 | 0.603867 |
| GO:0050789\_regulation\_of\_biological\_process | SIT1 | 3649 | 10 | 1.397643 | -0.923702 | 150 | 90.58 | 0.603867 |
| GO:0051235\_maintenance\_of\_location | LCK | 65 | 1 | 7.846154 | -0.920036 | 152 | 91.81 | 0.604013 |
| GO:0080135\_regulation\_of\_cellular\_response\_to\_stress | MAP4K1 | 65 | 1 | 7.846154 | -0.920036 | 152 | 91.81 | 0.604013 |
| GO:0052547\_regulation\_of\_peptidase\_activity | LCK | 66 | 1 | 7.727273 | -0.913799 | 153 | 93.39 | 0.610392 |
| GO:0048522\_positive\_regulation\_of\_cellular\_process | CD3E | 1009 | 4 | 2.021804 | -0.899193 | 154 | 94.99 | 0.616818 |
| GO:0048522\_positive\_regulation\_of\_cellular\_process | ZAP70 | 1009 | 4 | 2.021804 | -0.899193 | 154 | 94.99 | 0.616818 |
| GO:0048522\_positive\_regulation\_of\_cellular\_process | CD2 | 1009 | 4 | 2.021804 | -0.899193 | 154 | 94.99 | 0.616818 |
| GO:0048522\_positive\_regulation\_of\_cellular\_process | LCK | 1009 | 4 | 2.021804 | -0.899193 | 154 | 94.99 | 0.616818 |
| GO:0006811\_ion\_transport | LCK | 317 | 2 | 3.217666 | -0.898534 | 155 | 95.18 | 0.614065 |
| GO:0006811\_ion\_transport | ATP2A3 | 317 | 2 | 3.217666 | -0.898534 | 155 | 95.18 | 0.614065 |
| GO:0016032\_viral\_reproduction | GFI1 | 70 | 1 | 7.285714 | -0.889818 | 156 | 96.93 | 0.621346 |
| GO:0045597\_positive\_regulation\_of\_cell\_differentiation | ZAP70 | 74 | 1 | 6.891892 | -0.867256 | 157 | 99.8 | 0.635669 |
| GO:0032583\_regulation\_of\_gene-specific\_transcription | GFI1 | 81 | 1 | 6.296296 | -0.830750 | 158 | 105.86 | 0.670000 |
| GO:0051329\_interphase\_of\_mitotic\_cell\_cycle | GFI1 | 83 | 1 | 6.144578 | -0.820941 | 159 | 107.42 | 0.675597 |
| GO:0043085\_positive\_regulation\_of\_catalytic\_activity | MAP4K1 | 354 | 2 | 2.881356 | -0.820501 | 160 | 107.53 | 0.672062 |
| GO:0043085\_positive\_regulation\_of\_catalytic\_activity | LCK | 354 | 2 | 2.881356 | -0.820501 | 160 | 107.53 | 0.672062 |
| GO:0032502\_developmental\_process | CD3E | 1919 | 6 | 1.594581 | -0.817396 | 161 | 107.83 | 0.669752 |
| GO:0032502\_developmental\_process | ZAP70 | 1919 | 6 | 1.594581 | -0.817396 | 161 | 107.83 | 0.669752 |
| GO:0032502\_developmental\_process | CD3G | 1919 | 6 | 1.594581 | -0.817396 | 161 | 107.83 | 0.669752 |
| GO:0032502\_developmental\_process | CD2 | 1919 | 6 | 1.594581 | -0.817396 | 161 | 107.83 | 0.669752 |
| GO:0032502\_developmental\_process | LCK | 1919 | 6 | 1.594581 | -0.817396 | 161 | 107.83 | 0.669752 |
| GO:0032502\_developmental\_process | CD3D | 1919 | 6 | 1.594581 | -0.817396 | 161 | 107.83 | 0.669752 |
| GO:0048518\_positive\_regulation\_of\_biological\_process | CD3E | 1094 | 4 | 1.864717 | -0.803784 | 162 | 109.28 | 0.674568 |
| GO:0048518\_positive\_regulation\_of\_biological\_process | ZAP70 | 1094 | 4 | 1.864717 | -0.803784 | 162 | 109.28 | 0.674568 |
| GO:0048518\_positive\_regulation\_of\_biological\_process | CD2 | 1094 | 4 | 1.864717 | -0.803784 | 162 | 109.28 | 0.674568 |
| GO:0048518\_positive\_regulation\_of\_biological\_process | LCK | 1094 | 4 | 1.864717 | -0.803784 | 162 | 109.28 | 0.674568 |
| GO:0065003\_macromolecular\_complex\_assembly | CD3E | 366 | 2 | 2.786885 | -0.797330 | 163 | 110.22 | 0.676196 |
| GO:0065003\_macromolecular\_complex\_assembly | CD3G | 366 | 2 | 2.786885 | -0.797330 | 163 | 110.22 | 0.676196 |
| GO:0043405\_regulation\_of\_MAP\_kinase\_activity | MAP4K1 | 89 | 1 | 5.730337 | -0.792979 | 165 | 111.06 | 0.673091 |
| GO:0051325\_interphase | GFI1 | 89 | 1 | 5.730337 | -0.792979 | 165 | 111.06 | 0.673091 |
| GO:0007243\_protein\_kinase\_cascade | MAP4K1 | 377 | 2 | 2.705570 | -0.776910 | 166 | 113.21 | 0.681988 |
| GO:0007243\_protein\_kinase\_cascade | ZAP70 | 377 | 2 | 2.705570 | -0.776910 | 166 | 113.21 | 0.681988 |
| GO:0009966\_regulation\_of\_signal\_transduction | MAP4K1 | 378 | 2 | 2.698413 | -0.775091 | 168 | 113.92 | 0.678095 |
| GO:0009966\_regulation\_of\_signal\_transduction | LCK | 378 | 2 | 2.698413 | -0.775091 | 168 | 113.92 | 0.678095 |
| GO:0051239\_regulation\_of\_multicellular\_organismal\_process | ZAP70 | 378 | 2 | 2.698413 | -0.775091 | 168 | 113.92 | 0.678095 |
| GO:0051239\_regulation\_of\_multicellular\_organismal\_process | CD2 | 378 | 2 | 2.698413 | -0.775091 | 168 | 113.92 | 0.678095 |
| GO:0048731\_system\_development | ZAP70 | 1140 | 4 | 1.789474 | -0.756682 | 169 | 116.32 | 0.688284 |
| GO:0048731\_system\_development | CD2 | 1140 | 4 | 1.789474 | -0.756682 | 169 | 116.32 | 0.688284 |
| GO:0048731\_system\_development | LCK | 1140 | 4 | 1.789474 | -0.756682 | 169 | 116.32 | 0.688284 |
| GO:0048731\_system\_development | CD3D | 1140 | 4 | 1.789474 | -0.756682 | 169 | 116.32 | 0.688284 |
| GO:0006468\_protein\_amino\_acid\_phosphorylation | MAP4K1 | 393 | 2 | 2.595420 | -0.748512 | 170 | 117.89 | 0.693471 |
| GO:0006468\_protein\_amino\_acid\_phosphorylation | ZAP70 | 393 | 2 | 2.595420 | -0.748512 | 170 | 117.89 | 0.693471 |
| GO:0044093\_positive\_regulation\_of\_molecular\_function | MAP4K1 | 394 | 2 | 2.588832 | -0.746786 | 171 | 118.14 | 0.690877 |
| GO:0044093\_positive\_regulation\_of\_molecular\_function | LCK | 394 | 2 | 2.588832 | -0.746786 | 171 | 118.14 | 0.690877 |
| GO:0050776\_regulation\_of\_immune\_response | LCK | 100 | 1 | 5.100000 | -0.746669 | 172 | 118.81 | 0.690756 |
| GO:0001932\_regulation\_of\_protein\_amino\_acid\_phosphorylation | MAP4K1 | 101 | 1 | 5.049505 | -0.742738 | 174 | 119.09 | 0.684425 |
| GO:0043623\_cellular\_protein\_complex\_assembly | CD3E | 101 | 1 | 5.049505 | -0.742738 | 174 | 119.09 | 0.684425 |
| GO:0042592\_homeostatic\_process | CCR4 | 397 | 2 | 2.569270 | -0.741641 | 175 | 119.2 | 0.681143 |
| GO:0042592\_homeostatic\_process | LCK | 397 | 2 | 2.569270 | -0.741641 | 175 | 119.2 | 0.681143 |
| GO:0007265\_Ras\_protein\_signal\_transduction | GRAP2 | 110 | 1 | 4.636364 | -0.709175 | 176 | 123.18 | 0.699886 |
| GO:0010646\_regulation\_of\_cell\_communication | MAP4K1 | 423 | 2 | 2.411348 | -0.699042 | 177 | 124.33 | 0.702429 |
| GO:0010646\_regulation\_of\_cell\_communication | LCK | 423 | 2 | 2.411348 | -0.699042 | 177 | 124.33 | 0.702429 |
| GO:0000122\_negative\_regulation\_of\_transcription\_from\_RNA\_polymerase\_II\_promoter | GFI1 | 113 | 1 | 4.513274 | -0.698657 | 178 | 124.64 | 0.700225 |
| GO:0043933\_macromolecular\_complex\_subunit\_organization | CD3E | 424 | 2 | 2.405660 | -0.697471 | 179 | 124.78 | 0.697095 |
| GO:0043933\_macromolecular\_complex\_subunit\_organization | CD3G | 424 | 2 | 2.405660 | -0.697471 | 179 | 124.78 | 0.697095 |
| GO:0048584\_positive\_regulation\_of\_response\_to\_stimulus | LCK | 114 | 1 | 4.473684 | -0.695220 | 180 | 125.15 | 0.695278 |
| GO:0018193\_peptidyl-amino\_acid\_modification | MAP4K1 | 117 | 1 | 4.358974 | -0.685105 | 181 | 127.0 | 0.701657 |
| GO:0033674\_positive\_regulation\_of\_kinase\_activity | MAP4K1 | 122 | 1 | 4.180328 | -0.668874 | 183 | 129.68 | 0.708634 |
| GO:0045860\_positive\_regulation\_of\_protein\_kinase\_activity | MAP4K1 | 122 | 1 | 4.180328 | -0.668874 | 183 | 129.68 | 0.708634 |
| GO:0007267\_cell-cell\_signaling | SH2D1A | 445 | 2 | 2.292135 | -0.665564 | 184 | 130.1 | 0.707065 |
| GO:0007267\_cell-cell\_signaling | GRAP2 | 445 | 2 | 2.292135 | -0.665564 | 184 | 130.1 | 0.707065 |
| GO:0006935\_chemotaxis | CCR4 | 125 | 1 | 4.080000 | -0.659488 | 186 | 131.93 | 0.709301 |
| GO:0042330\_taxis | CCR4 | 125 | 1 | 4.080000 | -0.659488 | 186 | 131.93 | 0.709301 |
| GO:0051347\_positive\_regulation\_of\_transferase\_activity | MAP4K1 | 129 | 1 | 3.953488 | -0.647360 | 187 | 132.94 | 0.710909 |
| GO:0051345\_positive\_regulation\_of\_hydrolase\_activity | LCK | 131 | 1 | 3.893130 | -0.641453 | 188 | 134.8 | 0.717021 |
| GO:0007242\_intracellular\_signaling\_cascade | MAP4K1 | 853 | 3 | 1.793669 | -0.637839 | 189 | 135.18 | 0.715238 |
| GO:0007242\_intracellular\_signaling\_cascade | ZAP70 | 853 | 3 | 1.793669 | -0.637839 | 189 | 135.18 | 0.715238 |
| GO:0007242\_intracellular\_signaling\_cascade | GRAP2 | 853 | 3 | 1.793669 | -0.637839 | 189 | 135.18 | 0.715238 |
| GO:0007264\_small\_GTPase\_mediated\_signal\_transduction | GRAP2 | 135 | 1 | 3.777778 | -0.629940 | 190 | 137.88 | 0.725684 |
| GO:0022607\_cellular\_component\_assembly | CD3E | 478 | 2 | 2.133891 | -0.619239 | 191 | 139.8 | 0.731937 |
| GO:0022607\_cellular\_component\_assembly | CD3G | 478 | 2 | 2.133891 | -0.619239 | 191 | 139.8 | 0.731937 |
| GO:0007626\_locomotory\_behavior | CCR4 | 142 | 1 | 3.591549 | -0.610692 | 192 | 141.95 | 0.739323 |
| GO:0000165\_MAPKKK\_cascade | MAP4K1 | 143 | 1 | 3.566434 | -0.608031 | 193 | 142.14 | 0.736477 |
| GO:0000902\_cell\_morphogenesis | CD3G | 144 | 1 | 3.541667 | -0.605391 | 195 | 142.66 | 0.731590 |
| GO:0031399\_regulation\_of\_protein\_modification\_process | MAP4K1 | 144 | 1 | 3.541667 | -0.605391 | 195 | 142.66 | 0.731590 |
| GO:0080134\_regulation\_of\_response\_to\_stress | MAP4K1 | 147 | 1 | 3.469388 | -0.597594 | 196 | 145.39 | 0.741786 |
| GO:0016337\_cell-cell\_adhesion | CD2 | 156 | 1 | 3.269231 | -0.575256 | 197 | 148.65 | 0.754569 |
| GO:0007275\_multicellular\_organismal\_development | ZAP70 | 1372 | 4 | 1.486880 | -0.558529 | 198 | 151.01 | 0.762677 |
| GO:0007275\_multicellular\_organismal\_development | CD2 | 1372 | 4 | 1.486880 | -0.558529 | 198 | 151.01 | 0.762677 |
| GO:0007275\_multicellular\_organismal\_development | LCK | 1372 | 4 | 1.486880 | -0.558529 | 198 | 151.01 | 0.762677 |
| GO:0007275\_multicellular\_organismal\_development | CD3D | 1372 | 4 | 1.486880 | -0.558529 | 198 | 151.01 | 0.762677 |
| GO:0032989\_cellular\_component\_morphogenesis | CD3G | 164 | 1 | 3.109756 | -0.556615 | 199 | 151.23 | 0.759950 |
| GO:0045892\_negative\_regulation\_of\_transcription\_\_DNA-dependent | GFI1 | 175 | 1 | 2.914286 | -0.532642 | 200 | 154.51 | 0.772550 |
| GO:0051253\_negative\_regulation\_of\_RNA\_metabolic\_process | GFI1 | 180 | 1 | 2.833333 | -0.522323 | 202 | 156.67 | 0.775594 |
| GO:0051336\_regulation\_of\_hydrolase\_activity | LCK | 180 | 1 | 2.833333 | -0.522323 | 202 | 156.67 | 0.775594 |
| GO:0010926\_anatomical\_structure\_formation | CD3E | 560 | 2 | 1.821429 | -0.520825 | 204 | 157.07 | 0.769951 |
| GO:0010926\_anatomical\_structure\_formation | CD3G | 560 | 2 | 1.821429 | -0.520825 | 204 | 157.07 | 0.769951 |
| GO:0044085\_cellular\_component\_biogenesis | CD3E | 560 | 2 | 1.821429 | -0.520825 | 204 | 157.07 | 0.769951 |
| GO:0044085\_cellular\_component\_biogenesis | CD3G | 560 | 2 | 1.821429 | -0.520825 | 204 | 157.07 | 0.769951 |
| GO:0006954\_inflammatory\_response | CCR4 | 182 | 1 | 2.802198 | -0.518289 | 205 | 157.3 | 0.767317 |
| GO:0010627\_regulation\_of\_protein\_kinase\_cascade | MAP4K1 | 184 | 1 | 2.771739 | -0.514308 | 206 | 157.71 | 0.765583 |
| GO:0009967\_positive\_regulation\_of\_signal\_transduction | LCK | 185 | 1 | 2.756757 | -0.512336 | 207 | 158.06 | 0.763575 |
| GO:0034622\_cellular\_macromolecular\_complex\_assembly | CD3E | 186 | 1 | 2.741935 | -0.510377 | 208 | 158.57 | 0.762356 |
| GO:0010647\_positive\_regulation\_of\_cell\_communication | LCK | 189 | 1 | 2.698413 | -0.504574 | 209 | 159.8 | 0.764593 |
| GO:0043086\_negative\_regulation\_of\_catalytic\_activity | GFI1 | 196 | 1 | 2.602041 | -0.491451 | 210 | 163.22 | 0.777238 |
| GO:0008284\_positive\_regulation\_of\_cell\_proliferation | CD3E | 200 | 1 | 2.550000 | -0.484201 | 211 | 163.75 | 0.776066 |
| GO:0016310\_phosphorylation | MAP4K1 | 601 | 2 | 1.697171 | -0.478897 | 212 | 164.67 | 0.776745 |
| GO:0016310\_phosphorylation | ZAP70 | 601 | 2 | 1.697171 | -0.478897 | 212 | 164.67 | 0.776745 |
| GO:0045859\_regulation\_of\_protein\_kinase\_activity | MAP4K1 | 213 | 1 | 2.394366 | -0.461794 | 213 | 169.11 | 0.793944 |
| GO:0007610\_behavior | CCR4 | 214 | 1 | 2.383178 | -0.460140 | 214 | 169.45 | 0.791822 |
| GO:0043549\_regulation\_of\_kinase\_activity | MAP4K1 | 217 | 1 | 2.350230 | -0.455232 | 215 | 170.31 | 0.792140 |
| GO:0042221\_response\_to\_chemical\_stimulus | CCR4 | 631 | 2 | 1.616482 | -0.450767 | 216 | 171.04 | 0.791852 |
| GO:0042221\_response\_to\_chemical\_stimulus | LCK | 631 | 2 | 1.616482 | -0.450767 | 216 | 171.04 | 0.791852 |
| GO:0016044\_membrane\_organization | CD2 | 225 | 1 | 2.266667 | -0.442539 | 217 | 172.38 | 0.794378 |
| GO:0034621\_cellular\_macromolecular\_complex\_subunit\_organization | CD3E | 227 | 1 | 2.246696 | -0.439452 | 219 | 173.54 | 0.792420 |
| GO:0051338\_regulation\_of\_transferase\_activity | MAP4K1 | 227 | 1 | 2.246696 | -0.439452 | 219 | 173.54 | 0.792420 |
| GO:0008283\_cell\_proliferation | CD3E | 647 | 2 | 1.576507 | -0.436561 | 220 | 174.29 | 0.792227 |
| GO:0008283\_cell\_proliferation | CD5 | 647 | 2 | 1.576507 | -0.436561 | 220 | 174.29 | 0.792227 |
| GO:0051179\_localization | CD3G | 1561 | 4 | 1.306855 | -0.435272 | 221 | 174.73 | 0.790633 |
| GO:0051179\_localization | CD2 | 1561 | 4 | 1.306855 | -0.435272 | 221 | 174.73 | 0.790633 |
| GO:0051179\_localization | LCK | 1561 | 4 | 1.306855 | -0.435272 | 221 | 174.73 | 0.790633 |
| GO:0051179\_localization | ATP2A3 | 1561 | 4 | 1.306855 | -0.435272 | 221 | 174.73 | 0.790633 |
| GO:0044092\_negative\_regulation\_of\_molecular\_function | GFI1 | 233 | 1 | 2.188841 | -0.430390 | 222 | 176.42 | 0.794685 |
| GO:0022403\_cell\_cycle\_phase | GFI1 | 245 | 1 | 2.081633 | -0.413104 | 223 | 179.58 | 0.805291 |
| GO:0006793\_phosphorus\_metabolic\_process | MAP4K1 | 697 | 2 | 1.463415 | -0.395398 | 225 | 182.71 | 0.812044 |
| GO:0006793\_phosphorus\_metabolic\_process | ZAP70 | 697 | 2 | 1.463415 | -0.395398 | 225 | 182.71 | 0.812044 |
| GO:0006796\_phosphate\_metabolic\_process | MAP4K1 | 697 | 2 | 1.463415 | -0.395398 | 225 | 182.71 | 0.812044 |
| GO:0006796\_phosphate\_metabolic\_process | ZAP70 | 697 | 2 | 1.463415 | -0.395398 | 225 | 182.71 | 0.812044 |
| GO:0016481\_negative\_regulation\_of\_transcription | GFI1 | 261 | 1 | 1.954023 | -0.391640 | 226 | 183.02 | 0.809823 |
| GO:0009987\_cellular\_process | CD3E | 6671 | 14 | 1.070304 | -0.376937 | 227 | 185.21 | 0.815903 |
| GO:0009987\_cellular\_process | SH2D1A | 6671 | 14 | 1.070304 | -0.376937 | 227 | 185.21 | 0.815903 |
| GO:0009987\_cellular\_process | ZAP70 | 6671 | 14 | 1.070304 | -0.376937 | 227 | 185.21 | 0.815903 |
| GO:0009987\_cellular\_process | CD2 | 6671 | 14 | 1.070304 | -0.376937 | 227 | 185.21 | 0.815903 |
| GO:0009987\_cellular\_process | CD7 | 6671 | 14 | 1.070304 | -0.376937 | 227 | 185.21 | 0.815903 |
| GO:0009987\_cellular\_process | GRAP2 | 6671 | 14 | 1.070304 | -0.376937 | 227 | 185.21 | 0.815903 |
| GO:0009987\_cellular\_process | LCK | 6671 | 14 | 1.070304 | -0.376937 | 227 | 185.21 | 0.815903 |
| GO:0009987\_cellular\_process | SIT1 | 6671 | 14 | 1.070304 | -0.376937 | 227 | 185.21 | 0.815903 |
| GO:0009987\_cellular\_process | MAP4K1 | 6671 | 14 | 1.070304 | -0.376937 | 227 | 185.21 | 0.815903 |
| GO:0009987\_cellular\_process | CCR4 | 6671 | 14 | 1.070304 | -0.376937 | 227 | 185.21 | 0.815903 |
| GO:0009987\_cellular\_process | CD5 | 6671 | 14 | 1.070304 | -0.376937 | 227 | 185.21 | 0.815903 |
| GO:0009987\_cellular\_process | CD3G | 6671 | 14 | 1.070304 | -0.376937 | 227 | 185.21 | 0.815903 |
| GO:0009987\_cellular\_process | CD3D | 6671 | 14 | 1.070304 | -0.376937 | 227 | 185.21 | 0.815903 |
| GO:0009987\_cellular\_process | GFI1 | 6671 | 14 | 1.070304 | -0.376937 | 227 | 185.21 | 0.815903 |
| GO:0015031\_protein\_transport | CD3G | 274 | 1 | 1.861314 | -0.375395 | 228 | 186.38 | 0.817456 |
| GO:0043687\_post-translational\_protein\_modification | MAP4K1 | 728 | 2 | 1.401099 | -0.372103 | 229 | 187.14 | 0.817205 |
| GO:0043687\_post-translational\_protein\_modification | ZAP70 | 728 | 2 | 1.401099 | -0.372103 | 229 | 187.14 | 0.817205 |
| GO:0009611\_response\_to\_wounding | CCR4 | 279 | 1 | 1.827957 | -0.369408 | 231 | 188.34 | 0.815325 |
| GO:0045184\_establishment\_of\_protein\_localization | CD3G | 279 | 1 | 1.827957 | -0.369408 | 231 | 188.34 | 0.815325 |
| GO:0042325\_regulation\_of\_phosphorylation | MAP4K1 | 285 | 1 | 1.789474 | -0.362404 | 232 | 189.09 | 0.815043 |
| GO:0032268\_regulation\_of\_cellular\_protein\_metabolic\_process | MAP4K1 | 286 | 1 | 1.783217 | -0.361255 | 233 | 190.13 | 0.816009 |
| GO:0010629\_negative\_regulation\_of\_gene\_expression | GFI1 | 289 | 1 | 1.764706 | -0.357840 | 234 | 191.05 | 0.816453 |
| GO:0000278\_mitotic\_cell\_cycle | GFI1 | 292 | 1 | 1.746575 | -0.354471 | 236 | 193.44 | 0.819661 |
| GO:0040011\_locomotion | CCR4 | 292 | 1 | 1.746575 | -0.354471 | 236 | 193.44 | 0.819661 |
| GO:0045934\_negative\_regulation\_of\_nucleobase\_\_nucleoside\_\_nucleotide\_and\_nucleic\_acid\_metabolic\_process | GFI1 | 295 | 1 | 1.728814 | -0.351147 | 237 | 193.79 | 0.817679 |
| GO:0019220\_regulation\_of\_phosphate\_metabolic\_process | MAP4K1 | 297 | 1 | 1.717172 | -0.348955 | 239 | 194.87 | 0.815356 |
| GO:0051174\_regulation\_of\_phosphorus\_metabolic\_process | MAP4K1 | 297 | 1 | 1.717172 | -0.348955 | 239 | 194.87 | 0.815356 |
| GO:0051172\_negative\_regulation\_of\_nitrogen\_compound\_metabolic\_process | GFI1 | 298 | 1 | 1.711409 | -0.347867 | 240 | 195.22 | 0.813417 |
| GO:0006810\_transport | CD3G | 1243 | 3 | 1.230893 | -0.347448 | 241 | 195.43 | 0.810913 |
| GO:0006810\_transport | LCK | 1243 | 3 | 1.230893 | -0.347448 | 241 | 195.43 | 0.810913 |
| GO:0006810\_transport | ATP2A3 | 1243 | 3 | 1.230893 | -0.347448 | 241 | 195.43 | 0.810913 |
| GO:0051246\_regulation\_of\_protein\_metabolic\_process | MAP4K1 | 301 | 1 | 1.694352 | -0.344630 | 242 | 196.01 | 0.809959 |
| GO:0051234\_establishment\_of\_localization | CD3G | 1260 | 3 | 1.214286 | -0.338398 | 243 | 196.76 | 0.809712 |
| GO:0051234\_establishment\_of\_localization | LCK | 1260 | 3 | 1.214286 | -0.338398 | 243 | 196.76 | 0.809712 |
| GO:0051234\_establishment\_of\_localization | ATP2A3 | 1260 | 3 | 1.214286 | -0.338398 | 243 | 196.76 | 0.809712 |
| GO:0010558\_negative\_regulation\_of\_macromolecule\_biosynthetic\_process | GFI1 | 324 | 1 | 1.574074 | -0.321155 | 244 | 199.93 | 0.819385 |
| GO:0032879\_regulation\_of\_localization | LCK | 326 | 1 | 1.564417 | -0.319220 | 245 | 200.23 | 0.817265 |
| GO:0031327\_negative\_regulation\_of\_cellular\_biosynthetic\_process | GFI1 | 332 | 1 | 1.536145 | -0.313508 | 246 | 200.95 | 0.816870 |
| GO:0008104\_protein\_localization | CD3G | 339 | 1 | 1.504425 | -0.307018 | 247 | 201.7 | 0.816599 |
| GO:0009890\_negative\_regulation\_of\_biosynthetic\_process | GFI1 | 340 | 1 | 1.500000 | -0.306106 | 248 | 202.6 | 0.816935 |
| GO:0033554\_cellular\_response\_to\_stress | MAP4K1 | 341 | 1 | 1.495601 | -0.305198 | 249 | 203.09 | 0.815622 |
| GO:0006357\_regulation\_of\_transcription\_from\_RNA\_polymerase\_II\_promoter | GFI1 | 351 | 1 | 1.452991 | -0.296309 | 250 | 203.87 | 0.815480 |
| GO:0065008\_regulation\_of\_biological\_quality | CCR4 | 848 | 2 | 1.202830 | -0.295208 | 251 | 204.54 | 0.814900 |
| GO:0065008\_regulation\_of\_biological\_quality | LCK | 848 | 2 | 1.202830 | -0.295208 | 251 | 204.54 | 0.814900 |
| GO:0016043\_cellular\_component\_organization | CD3E | 1366 | 3 | 1.120059 | -0.286889 | 252 | 206.25 | 0.818452 |
| GO:0016043\_cellular\_component\_organization | CD3G | 1366 | 3 | 1.120059 | -0.286889 | 252 | 206.25 | 0.818452 |
| GO:0016043\_cellular\_component\_organization | CD2 | 1366 | 3 | 1.120059 | -0.286889 | 252 | 206.25 | 0.818452 |
| GO:0007186\_G-protein\_coupled\_receptor\_protein\_signaling\_pathway | CD3E | 363 | 1 | 1.404959 | -0.286092 | 253 | 206.61 | 0.816640 |
| GO:0022402\_cell\_cycle\_process | GFI1 | 370 | 1 | 1.378378 | -0.280347 | 254 | 208.83 | 0.822165 |
| GO:0033036\_macromolecule\_localization | CD3G | 388 | 1 | 1.314433 | -0.266252 | 255 | 212.08 | 0.831686 |
| GO:0006464\_protein\_modification\_process | MAP4K1 | 922 | 2 | 1.106291 | -0.256423 | 256 | 213.7 | 0.834766 |
| GO:0006464\_protein\_modification\_process | ZAP70 | 922 | 2 | 1.106291 | -0.256423 | 256 | 213.7 | 0.834766 |
| GO:0031324\_negative\_regulation\_of\_cellular\_metabolic\_process | GFI1 | 404 | 1 | 1.262376 | -0.254483 | 257 | 214.81 | 0.835837 |
| GO:0042127\_regulation\_of\_cell\_proliferation | CD3E | 411 | 1 | 1.240876 | -0.249544 | 258 | 215.98 | 0.837132 |
| GO:0010605\_negative\_regulation\_of\_macromolecule\_metabolic\_process | GFI1 | 413 | 1 | 1.234867 | -0.248155 | 259 | 216.62 | 0.836371 |
| GO:0043412\_biopolymer\_modification | MAP4K1 | 960 | 2 | 1.062500 | -0.238610 | 260 | 219.5 | 0.844231 |
| GO:0043412\_biopolymer\_modification | ZAP70 | 960 | 2 | 1.062500 | -0.238610 | 260 | 219.5 | 0.844231 |
| GO:0007155\_cell\_adhesion | CD2 | 428 | 1 | 1.191589 | -0.238043 | 261 | 219.84 | 0.842299 |
| GO:0022610\_biological\_adhesion | CD2 | 429 | 1 | 1.188811 | -0.237388 | 262 | 220.18 | 0.840382 |
| GO:0009892\_negative\_regulation\_of\_metabolic\_process | GFI1 | 440 | 1 | 1.159091 | -0.230323 | 263 | 221.07 | 0.840570 |
| GO:0048519\_negative\_regulation\_of\_biological\_process | LCK | 1013 | 2 | 1.006910 | -0.215861 | 264 | 223.82 | 0.847803 |
| GO:0048519\_negative\_regulation\_of\_biological\_process | GFI1 | 1013 | 2 | 1.006910 | -0.215861 | 264 | 223.82 | 0.847803 |
| GO:0009605\_response\_to\_external\_stimulus | CCR4 | 464 | 1 | 1.099138 | -0.215792 | 265 | 224.13 | 0.845774 |
| GO:0032501\_multicellular\_organismal\_process | ZAP70 | 2082 | 4 | 0.979827 | -0.212155 | 266 | 225.62 | 0.848195 |
| GO:0032501\_multicellular\_organismal\_process | CD2 | 2082 | 4 | 0.979827 | -0.212155 | 266 | 225.62 | 0.848195 |
| GO:0032501\_multicellular\_organismal\_process | LCK | 2082 | 4 | 0.979827 | -0.212155 | 266 | 225.62 | 0.848195 |
| GO:0032501\_multicellular\_organismal\_process | CD3D | 2082 | 4 | 0.979827 | -0.212155 | 266 | 225.62 | 0.848195 |
| GO:0051716\_cellular\_response\_to\_stimulus | MAP4K1 | 474 | 1 | 1.075949 | -0.210069 | 267 | 226.6 | 0.848689 |
| GO:0007049\_cell\_cycle | GFI1 | 494 | 1 | 1.032389 | -0.199166 | 268 | 230.06 | 0.858433 |
| GO:0009653\_anatomical\_structure\_morphogenesis | CD3G | 500 | 1 | 1.020000 | -0.196028 | 269 | 230.76 | 0.857844 |
| GO:0006366\_transcription\_from\_RNA\_polymerase\_II\_promoter | GFI1 | 506 | 1 | 1.007905 | -0.192949 | 270 | 232.38 | 0.860667 |
| GO:0051641\_cellular\_localization | CD2 | 617 | 1 | 0.826580 | -0.145016 | 271 | 240.69 | 0.888155 |
| GO:0080090\_regulation\_of\_primary\_metabolic\_process | MAP4K1 | 1311 | 2 | 0.778032 | -0.122746 | 272 | 244.47 | 0.898787 |
| GO:0080090\_regulation\_of\_primary\_metabolic\_process | GFI1 | 1311 | 2 | 0.778032 | -0.122746 | 272 | 244.47 | 0.898787 |
| GO:0060255\_regulation\_of\_macromolecule\_metabolic\_process | MAP4K1 | 1328 | 2 | 0.768072 | -0.118821 | 273 | 245.02 | 0.897509 |
| GO:0060255\_regulation\_of\_macromolecule\_metabolic\_process | GFI1 | 1328 | 2 | 0.768072 | -0.118821 | 273 | 245.02 | 0.897509 |
| GO:0006355\_regulation\_of\_transcription\_\_DNA-dependent | GFI1 | 723 | 1 | 0.705394 | -0.111432 | 274 | 247.27 | 0.902445 |
| GO:0044267\_cellular\_protein\_metabolic\_process | MAP4K1 | 1382 | 2 | 0.738061 | -0.107130 | 275 | 248.85 | 0.904909 |
| GO:0044267\_cellular\_protein\_metabolic\_process | ZAP70 | 1382 | 2 | 0.738061 | -0.107130 | 275 | 248.85 | 0.904909 |
| GO:0051252\_regulation\_of\_RNA\_metabolic\_process | GFI1 | 746 | 1 | 0.683646 | -0.105327 | 276 | 249.8 | 0.905072 |
| GO:0031323\_regulation\_of\_cellular\_metabolic\_process | MAP4K1 | 1466 | 2 | 0.695771 | -0.091078 | 277 | 252.35 | 0.911011 |
| GO:0031323\_regulation\_of\_cellular\_metabolic\_process | GFI1 | 1466 | 2 | 0.695771 | -0.091078 | 277 | 252.35 | 0.911011 |
| GO:0019222\_regulation\_of\_metabolic\_process | MAP4K1 | 1538 | 2 | 0.663199 | -0.079143 | 278 | 253.96 | 0.913525 |
| GO:0019222\_regulation\_of\_metabolic\_process | GFI1 | 1538 | 2 | 0.663199 | -0.079143 | 278 | 253.96 | 0.913525 |
| GO:0006351\_transcription\_\_DNA-dependent | GFI1 | 884 | 1 | 0.576923 | -0.075406 | 279 | 254.53 | 0.912294 |
| GO:0032774\_RNA\_biosynthetic\_process | GFI1 | 887 | 1 | 0.574972 | -0.074864 | 280 | 254.88 | 0.910286 |
| GO:0019538\_protein\_metabolic\_process | MAP4K1 | 1569 | 2 | 0.650096 | -0.074467 | 281 | 255.15 | 0.908007 |
| GO:0019538\_protein\_metabolic\_process | ZAP70 | 1569 | 2 | 0.650096 | -0.074467 | 281 | 255.15 | 0.908007 |
| GO:0045449\_regulation\_of\_transcription | GFI1 | 900 | 1 | 0.566667 | -0.072562 | 282 | 255.51 | 0.906064 |
| GO:0048523\_negative\_regulation\_of\_cellular\_process | GFI1 | 925 | 1 | 0.551351 | -0.068338 | 283 | 256.64 | 0.906855 |
| GO:0019219\_regulation\_of\_nucleobase\_\_nucleoside\_\_nucleotide\_and\_nucleic\_acid\_metabolic\_process | GFI1 | 1041 | 1 | 0.489914 | -0.051778 | 284 | 259.49 | 0.913697 |
| GO:0010556\_regulation\_of\_macromolecule\_biosynthetic\_process | GFI1 | 1055 | 1 | 0.483412 | -0.050074 | 286 | 260.17 | 0.909685 |
| GO:0051171\_regulation\_of\_nitrogen\_compound\_metabolic\_process | GFI1 | 1055 | 1 | 0.483412 | -0.050074 | 286 | 260.17 | 0.909685 |
| GO:0010468\_regulation\_of\_gene\_expression | GFI1 | 1067 | 1 | 0.477976 | -0.048659 | 287 | 260.65 | 0.908188 |
| GO:0006350\_transcription | GFI1 | 1069 | 1 | 0.477081 | -0.048427 | 288 | 260.93 | 0.906007 |
| GO:0031326\_regulation\_of\_cellular\_biosynthetic\_process | GFI1 | 1125 | 1 | 0.453333 | -0.042360 | 289 | 261.9 | 0.906228 |
| GO:0009889\_regulation\_of\_biosynthetic\_process | GFI1 | 1135 | 1 | 0.449339 | -0.041359 | 290 | 262.13 | 0.903897 |
| GO:0016070\_RNA\_metabolic\_process | GFI1 | 1230 | 1 | 0.414634 | -0.032938 | 291 | 263.28 | 0.904742 |
| GO:0034960\_cellular\_biopolymer\_metabolic\_process | MAP4K1 | 2820 | 3 | 0.542553 | -0.021544 | 292 | 265.38 | 0.908836 |
| GO:0034960\_cellular\_biopolymer\_metabolic\_process | ZAP70 | 2820 | 3 | 0.542553 | -0.021544 | 292 | 265.38 | 0.908836 |
| GO:0034960\_cellular\_biopolymer\_metabolic\_process | GFI1 | 2820 | 3 | 0.542553 | -0.021544 | 292 | 265.38 | 0.908836 |
| GO:0034961\_cellular\_biopolymer\_biosynthetic\_process | GFI1 | 1448 | 1 | 0.352210 | -0.019439 | 293 | 265.58 | 0.906416 |
| GO:0043284\_biopolymer\_biosynthetic\_process | GFI1 | 1458 | 1 | 0.349794 | -0.018970 | 294 | 265.69 | 0.903707 |
| GO:0044260\_cellular\_macromolecule\_metabolic\_process | MAP4K1 | 2883 | 3 | 0.530697 | -0.018854 | 295 | 265.78 | 0.900949 |
| GO:0044260\_cellular\_macromolecule\_metabolic\_process | ZAP70 | 2883 | 3 | 0.530697 | -0.018854 | 295 | 265.78 | 0.900949 |
| GO:0044260\_cellular\_macromolecule\_metabolic\_process | GFI1 | 2883 | 3 | 0.530697 | -0.018854 | 295 | 265.78 | 0.900949 |
| GO:0043283\_biopolymer\_metabolic\_process | MAP4K1 | 3027 | 3 | 0.505451 | -0.013780 | 296 | 266.58 | 0.900608 |
| GO:0043283\_biopolymer\_metabolic\_process | ZAP70 | 3027 | 3 | 0.505451 | -0.013780 | 296 | 266.58 | 0.900608 |
| GO:0043283\_biopolymer\_metabolic\_process | GFI1 | 3027 | 3 | 0.505451 | -0.013780 | 296 | 266.58 | 0.900608 |
| GO:0034645\_cellular\_macromolecule\_biosynthetic\_process | GFI1 | 1600 | 1 | 0.318750 | -0.013376 | 297 | 266.66 | 0.897845 |
| GO:0009059\_macromolecule\_biosynthetic\_process | GFI1 | 1626 | 1 | 0.313653 | -0.012539 | 298 | 266.8 | 0.895302 |
| GO:0043170\_macromolecule\_metabolic\_process | MAP4K1 | 3103 | 3 | 0.493071 | -0.011620 | 299 | 266.87 | 0.892542 |
| GO:0043170\_macromolecule\_metabolic\_process | ZAP70 | 3103 | 3 | 0.493071 | -0.011620 | 299 | 266.87 | 0.892542 |
| GO:0043170\_macromolecule\_metabolic\_process | GFI1 | 3103 | 3 | 0.493071 | -0.011620 | 299 | 266.87 | 0.892542 |
| GO:0010467\_gene\_expression | GFI1 | 1663 | 1 | 0.306675 | -0.011434 | 300 | 266.94 | 0.889800 |
| GO:0006139\_nucleobase\_\_nucleoside\_\_nucleotide\_and\_nucleic\_acid\_metabolic\_process | GFI1 | 1845 | 1 | 0.276423 | -0.007219 | 301 | 267.18 | 0.887641 |
| GO:0044249\_cellular\_biosynthetic\_process | GFI1 | 1951 | 1 | 0.261404 | -0.005493 | 302 | 267.33 | 0.885199 |
| GO:0009058\_biosynthetic\_process | GFI1 | 1988 | 1 | 0.256539 | -0.004989 | 303 | 267.37 | 0.882409 |
| GO:0006807\_nitrogen\_compound\_metabolic\_process | GFI1 | 2053 | 1 | 0.248417 | -0.004207 | 304 | 267.6 | 0.880263 |
| GO:0044238\_primary\_metabolic\_process | MAP4K1 | 3719 | 3 | 0.411401 | -0.002527 | 305 | 267.75 | 0.877869 |
| GO:0044238\_primary\_metabolic\_process | ZAP70 | 3719 | 3 | 0.411401 | -0.002527 | 305 | 267.75 | 0.877869 |
| GO:0044238\_primary\_metabolic\_process | GFI1 | 3719 | 3 | 0.411401 | -0.002527 | 305 | 267.75 | 0.877869 |
| GO:0044237\_cellular\_metabolic\_process | MAP4K1 | 3753 | 3 | 0.407674 | -0.002304 | 306 | 267.82 | 0.875229 |
| GO:0044237\_cellular\_metabolic\_process | ZAP70 | 3753 | 3 | 0.407674 | -0.002304 | 306 | 267.82 | 0.875229 |
| GO:0044237\_cellular\_metabolic\_process | GFI1 | 3753 | 3 | 0.407674 | -0.002304 | 306 | 267.82 | 0.875229 |
| GO:0008152\_metabolic\_process | MAP4K1 | 4111 | 3 | 0.372172 | -0.000821 | 307 | 267.9 | 0.872638 |
| GO:0008152\_metabolic\_process | ZAP70 | 4111 | 3 | 0.372172 | -0.000821 | 307 | 267.9 | 0.872638 |
| GO:0008152\_metabolic\_process | GFI1 | 4111 | 3 | 0.372172 | -0.000821 | 307 | 267.9 | 0.872638 |
| GO:0008150\_biological\_process | CD3E | 8160 | 16 | 1.000000 | 0.000000 | 478 | 453.79 | 0.949351 |
| GO:0008150\_biological\_process | SH2D1A | 8160 | 16 | 1.000000 | 0.000000 | 478 | 453.79 | 0.949351 |
| GO:0008150\_biological\_process | PTPRCAP | 8160 | 16 | 1.000000 | 0.000000 | 478 | 453.79 | 0.949351 |
| GO:0008150\_biological\_process | ZAP70 | 8160 | 16 | 1.000000 | 0.000000 | 478 | 453.79 | 0.949351 |
| GO:0008150\_biological\_process | CD2 | 8160 | 16 | 1.000000 | 0.000000 | 478 | 453.79 | 0.949351 |
| GO:0008150\_biological\_process | CD7 | 8160 | 16 | 1.000000 | 0.000000 | 478 | 453.79 | 0.949351 |
| GO:0008150\_biological\_process | GRAP2 | 8160 | 16 | 1.000000 | 0.000000 | 478 | 453.79 | 0.949351 |
| GO:0008150\_biological\_process | LCK | 8160 | 16 | 1.000000 | 0.000000 | 478 | 453.79 | 0.949351 |
| GO:0008150\_biological\_process | SIT1 | 8160 | 16 | 1.000000 | 0.000000 | 478 | 453.79 | 0.949351 |
| GO:0008150\_biological\_process | ATP2A3 | 8160 | 16 | 1.000000 | 0.000000 | 478 | 453.79 | 0.949351 |
| GO:0008150\_biological\_process | MAP4K1 | 8160 | 16 | 1.000000 | 0.000000 | 478 | 453.79 | 0.949351 |
| GO:0008150\_biological\_process | CCR4 | 8160 | 16 | 1.000000 | 0.000000 | 478 | 453.79 | 0.949351 |
| GO:0008150\_biological\_process | CD5 | 8160 | 16 | 1.000000 | 0.000000 | 478 | 453.79 | 0.949351 |
| GO:0008150\_biological\_process | CD3G | 8160 | 16 | 1.000000 | 0.000000 | 478 | 453.79 | 0.949351 |
| GO:0008150\_biological\_process | GFI1 | 8160 | 16 | 1.000000 | 0.000000 | 478 | 453.79 | 0.949351 |
| GO:0008150\_biological\_process | CD3D | 8160 | 16 | 1.000000 | 0.000000 | 478 | 453.79 | 0.949351 |
