# Supplementary material for: Concordance of Gene Expression and Functional Correlation Patterns across the NCI-60 Cell Lines and the Cancer Genome Atlas Glioblastoma Samples
Source: PLoS One. 2012 Jul 26;7(7):e40062. doi: 10.1371/journal.pone.0040062 (PMC3406063; doi:10.1371/journal.pone.0040062)
Supplement: Download S1 — Zip archive of HTGM results. (ZIP) [file pone.0040062.s007.zip › work2026406846/Generated_Total2026406846.dir/generic.BP.NCI60.0.6.ATP2A3.express.genes.correlation.complete.Thu.May.19.17.20.06.2011.htgm.txt.dir/generic.BP.NCI60.0.6.ATP2A3.express.genes.correlation.complete.Thu.May.19.17.20.06.2011.htgm.txt.change.html]

Category Summary Report for generic.BP.NCI60.0.6.ATP2A3.express.genes.correlation.complete.Thu.May.19.17.20.06.2011.htgm.txt

# Category Summary Report for generic.BP.NCI60.0.6.ATP2A3.express.genes.correlation.complete.Thu.May.19.17.20.06.2011.htgm.txt

| HYPERLINKED GO CATEGORY | TOTAL GENES | CHANGED GENES | ENRICHMENT | LOG10(p) | CUMULATIVE NUMBER OF CATEGORIES | CUMULATIVE RANDOMS LOWER BOUND | CUMULATIVE RANDOMS MEAN | CUMULATIVE RANDOMS UPPER BOUND | FALSE DISCOVERY RATE |
| --- | --- | --- | --- | --- | --- | --- | --- | --- | --- |
| GO:0042110\_T\_cell\_activation | 83 | 8 | 49.156627 | -12.009490 | 1 | 0.000000 | 0.0 | 0.000000 | 0.000000 |
| GO:0046649\_lymphocyte\_activation | 119 | 8 | 34.285714 | -10.724782 | 2 | 0.000000 | 0.0 | 0.000000 | 0.000000 |
| GO:0045321\_leukocyte\_activation | 150 | 8 | 27.200000 | -9.910376 | 3 | 0.000000 | 0.0 | 0.000000 | 0.000000 |
| GO:0001775\_cell\_activation | 175 | 8 | 23.314286 | -9.372471 | 4 | 0.000000 | 0.0 | 0.000000 | 0.000000 |
| GO:0050863\_regulation\_of\_T\_cell\_activation | 49 | 5 | 52.040816 | -7.579656 | 5 | 0.000000 | 0.0 | 0.000000 | 0.000000 |
| GO:0051249\_regulation\_of\_lymphocyte\_activation | 60 | 5 | 42.500000 | -7.128059 | 6 | 0.000000 | 0.0 | 0.000000 | 0.000000 |
| GO:0002694\_regulation\_of\_leukocyte\_activation | 70 | 5 | 36.428571 | -6.787384 | 7 | 0.000000 | 0.0 | 0.000000 | 0.000000 |
| GO:0050865\_regulation\_of\_cell\_activation | 77 | 5 | 33.116883 | -6.577975 | 8 | 0.000000 | 0.0 | 0.000000 | 0.000000 |
| GO:0030217\_T\_cell\_differentiation | 33 | 4 | 61.818182 | -6.409067 | 9 | 0.000000 | 0.0 | 0.000000 | 0.000000 |
| GO:0002696\_positive\_regulation\_of\_leukocyte\_activation | 46 | 4 | 44.347826 | -5.814984 | 10 | 0.000000 | 0.0 | 0.000000 | 0.000000 |
| GO:0002376\_immune\_system\_process | 718 | 9 | 6.392758 | -5.708840 | 11 | 0.000000 | 0.0 | 0.000000 | 0.000000 |
| GO:0050867\_positive\_regulation\_of\_cell\_activation | 49 | 4 | 41.632653 | -5.703121 | 12 | 0.000000 | 0.0 | 0.000000 | 0.000000 |
| GO:0030098\_lymphocyte\_differentiation | 50 | 4 | 40.800000 | -5.667421 | 13 | 0.000000 | 0.0 | 0.000000 | 0.000000 |
| GO:0045059\_positive\_thymic\_T\_cell\_selection | 2 | 2 |  |  |  |  |  |  |  |  |
| GO:0043368\_positive\_T\_cell\_selection | 3 | 2 |  |  |  |  |  |  |  |  |
| GO:0045061\_thymic\_T\_cell\_selection | 3 | 2 |  |  |  |  |  |  |  |  |
| GO:0002521\_leukocyte\_differentiation | 87 | 4 | 23.448276 | -4.701199 | 14 | 0.000000 | 0.0 | 0.000000 | 0.000000 |
| GO:0002682\_regulation\_of\_immune\_system\_process | 196 | 5 | 13.010204 | -4.572623 | 15 | 0.000000 | 0.0 | 0.000000 | 0.000000 |
| GO:0050870\_positive\_regulation\_of\_T\_cell\_activation | 33 | 3 | 46.363636 | -4.487284 | 16 | 0.000000 | 0.0 | 0.000000 | 0.000000 |
| GO:0002684\_positive\_regulation\_of\_immune\_system\_process | 106 | 4 | 19.245283 | -4.362314 | 17 | -0.141447 | 0.03 | 0.201447 | 0.001765 |
| GO:0051251\_positive\_regulation\_of\_lymphocyte\_activation | 40 | 3 | 38.250000 | -4.233039 | 18 | -0.141447 | 0.03 | 0.201447 | 0.001667 |
| GO:0033077\_T\_cell\_differentiation\_in\_the\_thymus | 7 | 2 | 145.714286 | -4.123382 | 20 | -0.141447 | 0.03 | 0.201447 | 0.001500 |
| GO:0045058\_T\_cell\_selection | 7 | 2 | 145.714286 | -4.123382 | 20 | -0.141447 | 0.03 | 0.201447 | 0.001500 |
| GO:0030097\_hemopoiesis | 135 | 4 | 15.111111 | -3.951738 | 21 | -0.211116 | 0.05 | 0.311116 | 0.002381 |
| GO:0048534\_hemopoietic\_or\_lymphoid\_organ\_development | 139 | 4 | 14.676259 | -3.902509 | 22 | -0.211116 | 0.05 | 0.311116 | 0.002273 |
| GO:0002520\_immune\_system\_development | 147 | 4 | 13.877551 | -3.808383 | 23 | -0.211116 | 0.05 | 0.311116 | 0.002174 |
| GO:0045580\_regulation\_of\_T\_cell\_differentiation | 17 | 2 | 60.000000 | -3.317031 | 24 | -0.326588 | 0.16 | 0.646588 | 0.006667 |
| GO:0045619\_regulation\_of\_lymphocyte\_differentiation | 20 | 2 | 51.000000 | -3.173307 | 25 | -0.479951 | 0.25 | 0.979951 | 0.010000 |
| GO:0030885\_regulation\_of\_myeloid\_dendritic\_cell\_activation | 1 | 1 |  |  |  |  |  |  |  |  |
| GO:0030887\_positive\_regulation\_of\_myeloid\_dendritic\_cell\_activation | 1 | 1 |  |  |  |  |  |  |  |  |
| GO:0048869\_cellular\_developmental\_process | 555 | 5 | 4.594595 | -2.477989 | 26 | -1.037393 | 1.01 | 3.057393 | 0.038846 |
| GO:0001766\_membrane\_raft\_polarization | 2 | 1 |  |  |  |  |  |  |  |  |
| GO:0031580\_membrane\_raft\_distribution | 2 | 1 |  |  |  |  |  |  |  |  |
| GO:0050862\_positive\_regulation\_of\_T\_cell\_receptor\_signaling\_pathway | 2 | 1 |  |  |  |  |  |  |  |  |
| GO:0051665\_membrane\_raft\_localization | 2 | 1 |  |  |  |  |  |  |  |  |
| GO:0007154\_cell\_communication | 2272 | 10 | 2.244718 | -2.406877 | 27 | -1.233350 | 1.19 | 3.613350 | 0.044074 |
| GO:0001773\_myeloid\_dendritic\_cell\_activation | 3 | 1 |  |  |  |  |  |  |  |  |
| GO:0006882\_cellular\_zinc\_ion\_homeostasis | 3 | 1 |  |  |  |  |  |  |  |  |
| GO:0010956\_negative\_regulation\_of\_calcidiol\_1-monooxygenase\_activity | 3 | 1 |  |  |  |  |  |  |  |  |
| GO:0031579\_membrane\_raft\_organization | 3 | 1 |  |  |  |  |  |  |  |  |
| GO:0050856\_regulation\_of\_T\_cell\_receptor\_signaling\_pathway | 3 | 1 |  |  |  |  |  |  |  |  |
| GO:0055069\_zinc\_ion\_homeostasis | 3 | 1 |  |  |  |  |  |  |  |  |
| GO:0007165\_signal\_transduction | 2029 | 9 | 2.262198 | -2.146413 | 28 | -1.260369 | 1.92 | 5.100369 | 0.068571 |
| GO:0050793\_regulation\_of\_developmental\_process | 669 | 5 | 3.811659 | -2.129080 | 29 | -1.405926 | 2.02 | 5.445926 | 0.069655 |
| GO:0000083\_regulation\_of\_transcription\_of\_G1\_S-phase\_of\_mitotic\_cell\_cycle | 4 | 1 |  |  |  |  |  |  |  |  |
| GO:0050857\_positive\_regulation\_of\_antigen\_receptor-mediated\_signaling\_pathway | 4 | 1 |  |  |  |  |  |  |  |  |
| GO:0006816\_calcium\_ion\_transport | 75 | 2 | 13.600000 | -2.036075 | 30 | -1.385275 | 2.44 | 6.265275 | 0.081333 |
| GO:0070838\_divalent\_metal\_ion\_transport | 76 | 2 | 13.421053 | -2.024989 | 31 | -1.364167 | 2.55 | 6.464167 | 0.082258 |
| GO:0050854\_regulation\_of\_antigen\_receptor-mediated\_signaling\_pathway | 5 | 1 | 102.000000 | -2.010197 | 32 | -0.122978 | 5.45 | 11.022978 | 0.170312 |
| GO:0007204\_elevation\_of\_cytosolic\_calcium\_ion\_concentration | 80 | 2 | 12.750000 | -1.982127 | 33 | -0.113714 | 5.51 | 11.133714 | 0.166970 |
| GO:0051480\_cytosolic\_calcium\_ion\_homeostasis | 81 | 2 | 12.592593 | -1.971764 | 34 | -0.100849 | 5.59 | 11.280849 | 0.164412 |
| GO:0042981\_regulation\_of\_apoptosis | 471 | 4 | 4.331210 | -1.941485 | 35 | -0.011983 | 5.78 | 11.571983 | 0.165143 |
| GO:0015674\_di-\_\_tri-valent\_inorganic\_cation\_transport | 84 | 2 | 12.142857 | -1.941466 | 36 | -0.012697 | 5.85 | 11.712697 | 0.162500 |
| GO:0043067\_regulation\_of\_programmed\_cell\_death | 476 | 4 | 4.285714 | -1.925698 | 37 | 0.394844 | 8.21 | 16.025156 | 0.221892 |
| GO:0010941\_regulation\_of\_cell\_death | 478 | 4 | 4.267782 | -1.919438 | 38 | 0.377392 | 8.24 | 16.102608 | 0.216842 |
| GO:0032769\_negative\_regulation\_of\_monooxygenase\_activity | 7 | 1 | 72.857143 | -1.864867 | 40 | 1.140341 | 10.63 | 20.119659 | 0.265750 |
| GO:0060558\_regulation\_of\_calcidiol\_1-monooxygenase\_activity | 7 | 1 | 72.857143 | -1.864867 | 40 | 1.140341 | 10.63 | 20.119659 | 0.265750 |
| GO:0030154\_cell\_differentiation | 506 | 4 | 4.031621 | -1.834902 | 41 | 1.128774 | 10.79 | 20.451226 | 0.263171 |
| GO:0006955\_immune\_response | 529 | 4 | 3.856333 | -1.769509 | 42 | 1.999292 | 13.33 | 24.660708 | 0.317381 |
| GO:0045582\_positive\_regulation\_of\_T\_cell\_differentiation | 9 | 1 | 56.666667 | -1.756520 | 43 | 2.808593 | 15.49 | 28.171407 | 0.360233 |
| GO:0007166\_cell\_surface\_receptor\_linked\_signal\_transduction | 828 | 5 | 3.079710 | -1.746559 | 44 | 2.712885 | 15.59 | 28.467115 | 0.354318 |
| GO:0045621\_positive\_regulation\_of\_lymphocyte\_differentiation | 10 | 1 | 51.000000 | -1.711162 | 46 | 3.688311 | 17.82 | 31.951689 | 0.387391 |
| GO:0051354\_negative\_regulation\_of\_oxidoreductase\_activity | 10 | 1 | 51.000000 | -1.711162 | 46 | 3.688311 | 17.82 | 31.951689 | 0.387391 |
| GO:0006874\_cellular\_calcium\_ion\_homeostasis | 114 | 2 | 8.947368 | -1.689676 | 47 | 3.683943 | 18.0 | 32.316057 | 0.382979 |
| GO:0055074\_calcium\_ion\_homeostasis | 116 | 2 | 8.793103 | -1.675491 | 48 | 3.726089 | 18.16 | 32.593911 | 0.378333 |
| GO:0006915\_apoptosis | 565 | 4 | 3.610619 | -1.673711 | 49 | 3.709985 | 18.17 | 32.630015 | 0.370816 |
| GO:0007172\_signal\_complex\_assembly | 11 | 1 | 46.363636 | -1.670167 | 51 | 5.124342 | 20.05 | 34.975658 | 0.393137 |
| GO:0051668\_localization\_within\_membrane | 11 | 1 | 46.363636 | -1.670167 | 51 | 5.124342 | 20.05 | 34.975658 | 0.393137 |
| GO:0012501\_programmed\_cell\_death | 571 | 4 | 3.572680 | -1.658462 | 52 | 5.189511 | 20.16 | 35.130489 | 0.387692 |
| GO:0006875\_cellular\_metal\_ion\_homeostasis | 121 | 2 | 8.429752 | -1.641150 | 53 | 5.168421 | 20.27 | 35.371579 | 0.382453 |
| GO:0050852\_T\_cell\_receptor\_signaling\_pathway | 12 | 1 | 42.500000 | -1.632777 | 54 | 5.965347 | 22.22 | 38.474653 | 0.411481 |
| GO:0008219\_cell\_death | 585 | 4 | 3.487179 | -1.623627 | 56 | 5.967397 | 22.27 | 38.572603 | 0.397679 |
| GO:0016265\_death | 585 | 4 | 3.487179 | -1.623627 | 56 | 5.967397 | 22.27 | 38.572603 | 0.397679 |
| GO:0055065\_metal\_ion\_homeostasis | 125 | 2 | 8.160000 | -1.614760 | 57 | 5.983473 | 22.37 | 38.756527 | 0.392456 |
| GO:0051094\_positive\_regulation\_of\_developmental\_process | 340 | 3 | 4.500000 | -1.571568 | 58 | 6.768620 | 24.43 | 42.091380 | 0.421207 |
| GO:0051209\_release\_of\_sequestered\_calcium\_ion\_into\_cytosol | 14 | 1 | 36.428571 | -1.566627 | 61 | 7.539496 | 26.22 | 44.900504 | 0.429836 |
| GO:0051282\_regulation\_of\_sequestering\_of\_calcium\_ion | 14 | 1 | 36.428571 | -1.566627 | 61 | 7.539496 | 26.22 | 44.900504 | 0.429836 |
| GO:0051283\_negative\_regulation\_of\_sequestering\_of\_calcium\_ion | 14 | 1 | 36.428571 | -1.566627 | 61 | 7.539496 | 26.22 | 44.900504 | 0.429836 |
| GO:0030101\_natural\_killer\_cell\_activation | 15 | 1 | 34.000000 | -1.537062 | 62 | 8.628303 | 28.6 | 48.571697 | 0.461290 |
| GO:0030005\_cellular\_di-\_\_tri-valent\_inorganic\_cation\_homeostasis | 140 | 2 | 7.285714 | -1.523352 | 63 | 8.612801 | 28.82 | 49.027199 | 0.457460 |
| GO:0010553\_negative\_regulation\_of\_specific\_transcription\_from\_RNA\_polymerase\_II\_promoter | 16 | 1 | 31.875000 | -1.509432 | 65 | 9.356675 | 30.57 | 51.783325 | 0.470308 |
| GO:0050851\_antigen\_receptor-mediated\_signaling\_pathway | 16 | 1 | 31.875000 | -1.509432 | 65 | 9.356675 | 30.57 | 51.783325 | 0.470308 |
| GO:0055066\_di-\_\_tri-valent\_inorganic\_cation\_homeostasis | 145 | 2 | 7.034483 | -1.495230 | 66 | 9.306017 | 30.79 | 52.273983 | 0.466515 |
| GO:0051208\_sequestering\_of\_calcium\_ion | 17 | 1 | 30.000000 | -1.483501 | 67 | 10.299091 | 32.65 | 55.000909 | 0.487313 |
| GO:0006952\_defense\_response | 369 | 3 | 4.146341 | -1.479742 | 68 | 10.309300 | 32.7 | 55.090700 | 0.480882 |
| GO:0002429\_immune\_response-activating\_cell\_surface\_receptor\_signaling\_pathway | 19 | 1 | 26.842105 | -1.435992 | 72 | 11.621242 | 36.24 | 60.858758 | 0.503333 |
| GO:0002768\_immune\_response-regulating\_cell\_surface\_receptor\_signaling\_pathway | 19 | 1 | 26.842105 | -1.435992 | 72 | 11.621242 | 36.24 | 60.858758 | 0.503333 |
| GO:0007257\_activation\_of\_JUN\_kinase\_activity | 19 | 1 | 26.842105 | -1.435992 | 72 | 11.621242 | 36.24 | 60.858758 | 0.503333 |
| GO:0042102\_positive\_regulation\_of\_T\_cell\_proliferation | 19 | 1 | 26.842105 | -1.435992 | 72 | 11.621242 | 36.24 | 60.858758 | 0.503333 |
| GO:0007169\_transmembrane\_receptor\_protein\_tyrosine\_kinase\_signaling\_pathway | 157 | 2 | 6.496815 | -1.431851 | 73 | 11.607970 | 36.32 | 61.032030 | 0.497534 |
| GO:0030003\_cellular\_cation\_homeostasis | 161 | 2 | 6.335404 | -1.411900 | 74 | 12.444120 | 37.89 | 63.335880 | 0.512027 |
| GO:0032768\_regulation\_of\_monooxygenase\_activity | 21 | 1 | 24.285714 | -1.393322 | 76 | 13.301497 | 40.02 | 66.738503 | 0.526579 |
| GO:0051238\_sequestering\_of\_metal\_ion | 21 | 1 | 24.285714 | -1.393322 | 76 | 13.301497 | 40.02 | 66.738503 | 0.526579 |
| GO:0050896\_response\_to\_stimulus | 1775 | 7 | 2.011268 | -1.390959 | 77 | 13.317362 | 40.04 | 66.762638 | 0.520000 |
| GO:0032582\_negative\_regulation\_of\_gene-specific\_transcription | 22 | 1 | 23.181818 | -1.373516 | 79 | 13.978370 | 41.29 | 68.601630 | 0.522658 |
| GO:0043507\_positive\_regulation\_of\_JUN\_kinase\_activity | 22 | 1 | 23.181818 | -1.373516 | 79 | 13.978370 | 41.29 | 68.601630 | 0.522658 |
| GO:0045595\_regulation\_of\_cell\_differentiation | 170 | 2 | 6.000000 | -1.368943 | 80 | 14.027169 | 41.38 | 68.732831 | 0.517250 |
| GO:0055080\_cation\_homeostasis | 173 | 2 | 5.895954 | -1.355181 | 81 | 14.126123 | 41.59 | 69.053877 | 0.513457 |
| GO:0050671\_positive\_regulation\_of\_lymphocyte\_proliferation | 23 | 1 | 22.173913 | -1.354608 | 83 | 14.793728 | 42.46 | 70.126272 | 0.511566 |
| GO:0070668\_positive\_regulation\_of\_mast\_cell\_proliferation | 23 | 1 | 22.173913 | -1.354608 | 83 | 14.793728 | 42.46 | 70.126272 | 0.511566 |
| GO:0007163\_establishment\_or\_maintenance\_of\_cell\_polarity | 24 | 1 | 21.250000 | -1.336522 | 87 | 15.641707 | 44.21 | 72.778293 | 0.508161 |
| GO:0032946\_positive\_regulation\_of\_mononuclear\_cell\_proliferation | 24 | 1 | 21.250000 | -1.336522 | 87 | 15.641707 | 44.21 | 72.778293 | 0.508161 |
| GO:0043506\_regulation\_of\_JUN\_kinase\_activity | 24 | 1 | 21.250000 | -1.336522 | 87 | 15.641707 | 44.21 | 72.778293 | 0.508161 |
| GO:0070665\_positive\_regulation\_of\_leukocyte\_proliferation | 24 | 1 | 21.250000 | -1.336522 | 87 | 15.641707 | 44.21 | 72.778293 | 0.508161 |
| GO:0002757\_immune\_response-activating\_signal\_transduction | 25 | 1 | 20.400000 | -1.319191 | 90 | 16.352075 | 45.93 | 75.507925 | 0.510333 |
| GO:0002764\_immune\_response-regulating\_signal\_transduction | 25 | 1 | 20.400000 | -1.319191 | 90 | 16.352075 | 45.93 | 75.507925 | 0.510333 |
| GO:0042129\_regulation\_of\_T\_cell\_proliferation | 25 | 1 | 20.400000 | -1.319191 | 90 | 16.352075 | 45.93 | 75.507925 | 0.510333 |
| GO:0008037\_cell\_recognition | 26 | 1 | 19.615385 | -1.302555 | 92 | 16.938285 | 47.42 | 77.901715 | 0.515435 |
| GO:0018105\_peptidyl-serine\_phosphorylation | 26 | 1 | 19.615385 | -1.302555 | 92 | 16.938285 | 47.42 | 77.901715 | 0.515435 |
| GO:0030001\_metal\_ion\_transport | 186 | 2 | 5.483871 | -1.298467 | 93 | 17.044754 | 47.51 | 77.975246 | 0.510860 |
| GO:0048513\_organ\_development | 741 | 4 | 2.753036 | -1.294242 | 94 | 17.081483 | 47.59 | 78.098517 | 0.506277 |
| GO:0006917\_induction\_of\_apoptosis | 190 | 2 | 5.368421 | -1.281903 | 95 | 18.024560 | 48.99 | 79.955440 | 0.515684 |
| GO:0012502\_induction\_of\_programmed\_cell\_death | 191 | 2 | 5.340314 | -1.277823 | 96 | 18.067888 | 49.08 | 80.092112 | 0.511250 |
| GO:0060402\_calcium\_ion\_transport\_into\_cytosol | 28 | 1 | 18.214286 | -1.271164 | 99 | 18.667290 | 50.57 | 82.472710 | 0.510808 |
| GO:0070662\_mast\_cell\_proliferation | 28 | 1 | 18.214286 | -1.271164 | 99 | 18.667290 | 50.57 | 82.472710 | 0.510808 |
| GO:0070666\_regulation\_of\_mast\_cell\_proliferation | 28 | 1 | 18.214286 | -1.271164 | 99 | 18.667290 | 50.57 | 82.472710 | 0.510808 |
| GO:0002274\_myeloid\_leukocyte\_activation | 29 | 1 | 17.586207 | -1.256321 | 101 | 19.055685 | 52.02 | 84.984315 | 0.515050 |
| GO:0060401\_cytosolic\_calcium\_ion\_transport | 29 | 1 | 17.586207 | -1.256321 | 101 | 19.055685 | 52.02 | 84.984315 | 0.515050 |
| GO:0018209\_peptidyl-serine\_modification | 30 | 1 | 17.000000 | -1.241995 | 104 | 19.630284 | 53.18 | 86.729716 | 0.511346 |
| GO:0042098\_T\_cell\_proliferation | 30 | 1 | 17.000000 | -1.241995 | 104 | 19.630284 | 53.18 | 86.729716 | 0.511346 |
| GO:0050670\_regulation\_of\_lymphocyte\_proliferation | 30 | 1 | 17.000000 | -1.241995 | 104 | 19.630284 | 53.18 | 86.729716 | 0.511346 |
| GO:0032944\_regulation\_of\_mononuclear\_cell\_proliferation | 31 | 1 | 16.451613 | -1.228151 | 106 | 20.179116 | 54.24 | 88.300884 | 0.511698 |
| GO:0070663\_regulation\_of\_leukocyte\_proliferation | 31 | 1 | 16.451613 | -1.228151 | 106 | 20.179116 | 54.24 | 88.300884 | 0.511698 |
| GO:0006873\_cellular\_ion\_homeostasis | 206 | 2 | 4.951456 | -1.219360 | 107 | 20.337566 | 54.54 | 88.742434 | 0.509720 |
| GO:0055082\_cellular\_chemical\_homeostasis | 208 | 2 | 4.903846 | -1.211929 | 108 | 21.012913 | 55.8 | 90.587087 | 0.516667 |
| GO:0051341\_regulation\_of\_oxidoreductase\_activity | 33 | 1 | 15.454545 | -1.201792 | 109 | 21.425810 | 56.8 | 92.174190 | 0.521101 |
| GO:0050801\_ion\_homeostasis | 221 | 2 | 4.615385 | -1.165529 | 110 | 23.118265 | 59.98 | 96.841735 | 0.545273 |
| GO:0000082\_G1\_S\_transition\_of\_mitotic\_cell\_cycle | 36 | 1 | 14.166667 | -1.165193 | 111 | 23.412649 | 60.56 | 97.707351 | 0.545586 |
| GO:0042493\_response\_to\_drug | 38 | 1 | 13.421053 | -1.142504 | 112 | 24.552199 | 62.99 | 101.427801 | 0.562411 |
| GO:0019725\_cellular\_homeostasis | 231 | 2 | 4.415584 | -1.131909 | 113 | 24.724219 | 63.3 | 101.875781 | 0.560177 |
| GO:0046328\_regulation\_of\_JNK\_cascade | 39 | 1 | 13.076923 | -1.131619 | 114 | 25.612306 | 64.2 | 102.787694 | 0.563158 |
| GO:0002253\_activation\_of\_immune\_response | 40 | 1 | 12.750000 | -1.121020 | 116 | 26.014696 | 65.02 | 104.025304 | 0.560517 |
| GO:0070302\_regulation\_of\_stress-activated\_protein\_kinase\_signaling\_pathway | 40 | 1 | 12.750000 | -1.121020 | 116 | 26.014696 | 65.02 | 104.025304 | 0.560517 |
| GO:0046651\_lymphocyte\_proliferation | 41 | 1 | 12.439024 | -1.110692 | 117 | 26.614654 | 66.12 | 105.625346 | 0.565128 |
| GO:0032943\_mononuclear\_cell\_proliferation | 42 | 1 | 12.142857 | -1.100622 | 119 | 27.303512 | 67.31 | 107.316488 | 0.565630 |
| GO:0070661\_leukocyte\_proliferation | 42 | 1 | 12.142857 | -1.100622 | 119 | 27.303512 | 67.31 | 107.316488 | 0.565630 |
| GO:0050790\_regulation\_of\_catalytic\_activity | 525 | 3 | 2.914286 | -1.100614 | 120 | 27.293930 | 67.39 | 107.486070 | 0.561583 |
| GO:0048583\_regulation\_of\_response\_to\_stimulus | 241 | 2 | 4.232365 | -1.099922 | 121 | 27.281977 | 67.43 | 107.578023 | 0.557273 |
| GO:0043065\_positive\_regulation\_of\_apoptosis | 243 | 2 | 4.197531 | -1.093708 | 122 | 27.391704 | 67.6 | 107.808296 | 0.554098 |
| GO:0006812\_cation\_transport | 246 | 2 | 4.146341 | -1.084498 | 124 | 28.362497 | 68.93 | 109.497503 | 0.555887 |
| GO:0043068\_positive\_regulation\_of\_programmed\_cell\_death | 246 | 2 | 4.146341 | -1.084498 | 124 | 28.362497 | 68.93 | 109.497503 | 0.555887 |
| GO:0010942\_positive\_regulation\_of\_cell\_death | 250 | 2 | 4.080000 | -1.072419 | 125 | 28.754325 | 69.53 | 110.305675 | 0.556240 |
| GO:0006919\_activation\_of\_caspase\_activity | 45 | 1 | 11.333333 | -1.071846 | 126 | 29.291431 | 70.42 | 111.548569 | 0.558889 |
| GO:0065007\_biological\_regulation | 3971 | 11 | 1.412742 | -1.062771 | 127 | 29.365629 | 70.56 | 111.754371 | 0.555591 |
| GO:0007167\_enzyme\_linked\_receptor\_protein\_signaling\_pathway | 258 | 2 | 3.953488 | -1.048921 | 128 | 31.527562 | 73.62 | 115.712438 | 0.575156 |
| GO:0010551\_regulation\_of\_specific\_transcription\_from\_RNA\_polymerase\_II\_promoter | 48 | 1 | 10.625000 | -1.045003 | 130 | 32.289995 | 74.4 | 116.510005 | 0.572308 |
| GO:0032569\_specific\_transcription\_from\_RNA\_polymerase\_II\_promoter | 48 | 1 | 10.625000 | -1.045003 | 130 | 32.289995 | 74.4 | 116.510005 | 0.572308 |
| GO:0010952\_positive\_regulation\_of\_peptidase\_activity | 49 | 1 | 10.408163 | -1.036443 | 132 | 32.833404 | 75.56 | 118.286596 | 0.572424 |
| GO:0043280\_positive\_regulation\_of\_caspase\_activity | 49 | 1 | 10.408163 | -1.036443 | 132 | 32.833404 | 75.56 | 118.286596 | 0.572424 |
| GO:0000187\_activation\_of\_MAPK\_activity | 50 | 1 | 10.200000 | -1.028064 | 133 | 33.332120 | 76.44 | 119.547880 | 0.574737 |
| GO:0048856\_anatomical\_structure\_development | 1289 | 5 | 1.978278 | -1.025081 | 134 | 33.349467 | 76.62 | 119.890533 | 0.571791 |
| GO:0050794\_regulation\_of\_cellular\_process | 3515 | 10 | 1.450925 | -1.025063 | 135 | 33.308437 | 76.66 | 120.011563 | 0.567852 |
| GO:0006461\_protein\_complex\_assembly | 273 | 2 | 3.736264 | -1.007078 | 137 | 34.598647 | 78.57 | 122.541353 | 0.573504 |
| GO:0070271\_protein\_complex\_biogenesis | 273 | 2 | 3.736264 | -1.007078 | 137 | 34.598647 | 78.57 | 122.541353 | 0.573504 |
| GO:0048878\_chemical\_homeostasis | 278 | 2 | 3.669065 | -0.993729 | 138 | 35.763538 | 80.44 | 125.116462 | 0.582899 |
| GO:0050778\_positive\_regulation\_of\_immune\_response | 56 | 1 | 9.107143 | -0.981215 | 139 | 36.594413 | 81.85 | 127.105587 | 0.588849 |
| GO:0032844\_regulation\_of\_homeostatic\_process | 57 | 1 | 8.947368 | -0.973922 | 140 | 36.690408 | 82.42 | 128.149592 | 0.588714 |
| GO:0006968\_cellular\_defense\_response | 58 | 1 | 8.793103 | -0.966763 | 141 | 38.020020 | 84.03 | 130.039980 | 0.595957 |
| GO:0006950\_response\_to\_stress | 959 | 4 | 2.127216 | -0.961014 | 142 | 38.196886 | 84.47 | 130.743114 | 0.594859 |
| GO:0043281\_regulation\_of\_caspase\_activity | 59 | 1 | 8.644068 | -0.959734 | 144 | 38.667695 | 85.27 | 131.872305 | 0.592153 |
| GO:0043408\_regulation\_of\_MAPKKK\_cascade | 59 | 1 | 8.644068 | -0.959734 | 144 | 38.667695 | 85.27 | 131.872305 | 0.592153 |
| GO:0065009\_regulation\_of\_molecular\_function | 606 | 3 | 2.524752 | -0.955539 | 145 | 38.781793 | 85.76 | 132.738207 | 0.591448 |
| GO:0007254\_JNK\_cascade | 61 | 1 | 8.360656 | -0.946044 | 146 | 39.282336 | 86.92 | 134.557664 | 0.595342 |
| GO:0043406\_positive\_regulation\_of\_MAP\_kinase\_activity | 62 | 1 | 8.225806 | -0.939376 | 148 | 40.365400 | 88.68 | 136.994600 | 0.599189 |
| GO:0052548\_regulation\_of\_endopeptidase\_activity | 62 | 1 | 8.225806 | -0.939376 | 148 | 40.365400 | 88.68 | 136.994600 | 0.599189 |
| GO:0031098\_stress-activated\_protein\_kinase\_signaling\_pathway | 64 | 1 | 7.968750 | -0.926375 | 149 | 41.556647 | 90.44 | 139.323353 | 0.606980 |
| GO:0050789\_regulation\_of\_biological\_process | 3649 | 10 | 1.397643 | -0.923702 | 150 | 41.645663 | 90.58 | 139.514337 | 0.603867 |
| GO:0051235\_maintenance\_of\_location | 65 | 1 | 7.846154 | -0.920036 | 152 | 42.473863 | 91.81 | 141.146137 | 0.604013 |
| GO:0080135\_regulation\_of\_cellular\_response\_to\_stress | 65 | 1 | 7.846154 | -0.920036 | 152 | 42.473863 | 91.81 | 141.146137 | 0.604013 |
| GO:0052547\_regulation\_of\_peptidase\_activity | 66 | 1 | 7.727273 | -0.913799 | 153 | 43.111203 | 93.39 | 143.668797 | 0.610392 |
| GO:0048522\_positive\_regulation\_of\_cellular\_process | 1009 | 4 | 2.021804 | -0.899193 | 154 | 44.037756 | 94.99 | 145.942244 | 0.616818 |
| GO:0006811\_ion\_transport | 317 | 2 | 3.217666 | -0.898534 | 155 | 44.194880 | 95.18 | 146.165120 | 0.614065 |
| GO:0016032\_viral\_reproduction | 70 | 1 | 7.285714 | -0.889818 | 156 | 45.250089 | 96.93 | 148.609911 | 0.621346 |
| GO:0045597\_positive\_regulation\_of\_cell\_differentiation | 74 | 1 | 6.891892 | -0.867256 | 157 | 46.664192 | 99.8 | 152.935808 | 0.635669 |
| GO:0032583\_regulation\_of\_gene-specific\_transcription | 81 | 1 | 6.296296 | -0.830750 | 158 | 49.916843 | 105.86 | 161.803157 | 0.670000 |
| GO:0051329\_interphase\_of\_mitotic\_cell\_cycle | 83 | 1 | 6.144578 | -0.820941 | 159 | 51.055712 | 107.42 | 163.784288 | 0.675597 |
| GO:0043085\_positive\_regulation\_of\_catalytic\_activity | 354 | 2 | 2.881356 | -0.820501 | 160 | 51.095278 | 107.53 | 163.964722 | 0.672062 |
| GO:0032502\_developmental\_process | 1919 | 6 | 1.594581 | -0.817396 | 161 | 51.224140 | 107.83 | 164.435860 | 0.669752 |
| GO:0048518\_positive\_regulation\_of\_biological\_process | 1094 | 4 | 1.864717 | -0.803784 | 162 | 52.097309 | 109.28 | 166.462691 | 0.674568 |
| GO:0065003\_macromolecular\_complex\_assembly | 366 | 2 | 2.786885 | -0.797330 | 163 | 52.753527 | 110.22 | 167.686473 | 0.676196 |
| GO:0043405\_regulation\_of\_MAP\_kinase\_activity | 89 | 1 | 5.730337 | -0.792979 | 165 | 53.250347 | 111.06 | 168.869653 | 0.673091 |
| GO:0051325\_interphase | 89 | 1 | 5.730337 | -0.792979 | 165 | 53.250347 | 111.06 | 168.869653 | 0.673091 |
| GO:0007243\_protein\_kinase\_cascade | 377 | 2 | 2.705570 | -0.776910 | 166 | 54.359686 | 113.21 | 172.060314 | 0.681988 |
| GO:0009966\_regulation\_of\_signal\_transduction | 378 | 2 | 2.698413 | -0.775091 | 168 | 54.843491 | 113.92 | 172.996509 | 0.678095 |
| GO:0051239\_regulation\_of\_multicellular\_organismal\_process | 378 | 2 | 2.698413 | -0.775091 | 168 | 54.843491 | 113.92 | 172.996509 | 0.678095 |
| GO:0048731\_system\_development | 1140 | 4 | 1.789474 | -0.756682 | 169 | 55.839286 | 116.32 | 176.800714 | 0.688284 |
| GO:0006468\_protein\_amino\_acid\_phosphorylation | 393 | 2 | 2.595420 | -0.748512 | 170 | 56.744719 | 117.89 | 179.035281 | 0.693471 |
| GO:0044093\_positive\_regulation\_of\_molecular\_function | 394 | 2 | 2.588832 | -0.746786 | 171 | 56.824947 | 118.14 | 179.455053 | 0.690877 |
| GO:0050776\_regulation\_of\_immune\_response | 100 | 1 | 5.100000 | -0.746669 | 172 | 57.348886 | 118.81 | 180.271114 | 0.690756 |
| GO:0001932\_regulation\_of\_protein\_amino\_acid\_phosphorylation | 101 | 1 | 5.049505 | -0.742738 | 174 | 57.520938 | 119.09 | 180.659062 | 0.684425 |
| GO:0043623\_cellular\_protein\_complex\_assembly | 101 | 1 | 5.049505 | -0.742738 | 174 | 57.520938 | 119.09 | 180.659062 | 0.684425 |
| GO:0042592\_homeostatic\_process | 397 | 2 | 2.569270 | -0.741641 | 175 | 57.595035 | 119.2 | 180.804965 | 0.681143 |
| GO:0007265\_Ras\_protein\_signal\_transduction | 110 | 1 | 4.636364 | -0.709175 | 176 | 60.345380 | 123.18 | 186.014620 | 0.699886 |
| GO:0010646\_regulation\_of\_cell\_communication | 423 | 2 | 2.411348 | -0.699042 | 177 | 61.104884 | 124.33 | 187.555116 | 0.702429 |
| GO:0000122\_negative\_regulation\_of\_transcription\_from\_RNA\_polymerase\_II\_promoter | 113 | 1 | 4.513274 | -0.698657 | 178 | 61.285375 | 124.64 | 187.994625 | 0.700225 |
| GO:0043933\_macromolecular\_complex\_subunit\_organization | 424 | 2 | 2.405660 | -0.697471 | 179 | 61.403527 | 124.78 | 188.156473 | 0.697095 |
| GO:0048584\_positive\_regulation\_of\_response\_to\_stimulus | 114 | 1 | 4.473684 | -0.695220 | 180 | 61.627416 | 125.15 | 188.672584 | 0.695278 |
| GO:0018193\_peptidyl-amino\_acid\_modification | 117 | 1 | 4.358974 | -0.685105 | 181 | 63.160555 | 127.0 | 190.839445 | 0.701657 |
| GO:0033674\_positive\_regulation\_of\_kinase\_activity | 122 | 1 | 4.180328 | -0.668874 | 183 | 64.852041 | 129.68 | 194.507959 | 0.708634 |
| GO:0045860\_positive\_regulation\_of\_protein\_kinase\_activity | 122 | 1 | 4.180328 | -0.668874 | 183 | 64.852041 | 129.68 | 194.507959 | 0.708634 |
| GO:0007267\_cell-cell\_signaling | 445 | 2 | 2.292135 | -0.665564 | 184 | 65.160168 | 130.1 | 195.039832 | 0.707065 |
| GO:0006935\_chemotaxis | 125 | 1 | 4.080000 | -0.659488 | 186 | 66.405966 | 131.93 | 197.454034 | 0.709301 |
| GO:0042330\_taxis | 125 | 1 | 4.080000 | -0.659488 | 186 | 66.405966 | 131.93 | 197.454034 | 0.709301 |
| GO:0051347\_positive\_regulation\_of\_transferase\_activity | 129 | 1 | 3.953488 | -0.647360 | 187 | 67.123939 | 132.94 | 198.756061 | 0.710909 |
| GO:0051345\_positive\_regulation\_of\_hydrolase\_activity | 131 | 1 | 3.893130 | -0.641453 | 188 | 68.560002 | 134.8 | 201.039998 | 0.717021 |
| GO:0007242\_intracellular\_signaling\_cascade | 853 | 3 | 1.793669 | -0.637839 | 189 | 68.844401 | 135.18 | 201.515599 | 0.715238 |
| GO:0007264\_small\_GTPase\_mediated\_signal\_transduction | 135 | 1 | 3.777778 | -0.629940 | 190 | 70.911399 | 137.88 | 204.848601 | 0.725684 |
| GO:0022607\_cellular\_component\_assembly | 478 | 2 | 2.133891 | -0.619239 | 191 | 72.293622 | 139.8 | 207.306378 | 0.731937 |
| GO:0007626\_locomotory\_behavior | 142 | 1 | 3.591549 | -0.610692 | 192 | 73.849899 | 141.95 | 210.050101 | 0.739323 |
| GO:0000165\_MAPKKK\_cascade | 143 | 1 | 3.566434 | -0.608031 | 193 | 73.932352 | 142.14 | 210.347648 | 0.736477 |
| GO:0000902\_cell\_morphogenesis | 144 | 1 | 3.541667 | -0.605391 | 195 | 74.172558 | 142.66 | 211.147442 | 0.731590 |
| GO:0031399\_regulation\_of\_protein\_modification\_process | 144 | 1 | 3.541667 | -0.605391 | 195 | 74.172558 | 142.66 | 211.147442 | 0.731590 |
| GO:0080134\_regulation\_of\_response\_to\_stress | 147 | 1 | 3.469388 | -0.597594 | 196 | 75.870307 | 145.39 | 214.909693 | 0.741786 |
| GO:0016337\_cell-cell\_adhesion | 156 | 1 | 3.269231 | -0.575256 | 197 | 78.480022 | 148.65 | 218.819978 | 0.754569 |
| GO:0007275\_multicellular\_organismal\_development | 1372 | 4 | 1.486880 | -0.558529 | 198 | 80.242705 | 151.01 | 221.777295 | 0.762677 |
| GO:0032989\_cellular\_component\_morphogenesis | 164 | 1 | 3.109756 | -0.556615 | 199 | 80.393461 | 151.23 | 222.066539 | 0.759950 |
| GO:0045892\_negative\_regulation\_of\_transcription\_\_DNA-dependent | 175 | 1 | 2.914286 | -0.532642 | 200 | 82.745585 | 154.51 | 226.274415 | 0.772550 |
| GO:0051253\_negative\_regulation\_of\_RNA\_metabolic\_process | 180 | 1 | 2.833333 | -0.522323 | 202 | 84.416371 | 156.67 | 228.923629 | 0.775594 |
| GO:0051336\_regulation\_of\_hydrolase\_activity | 180 | 1 | 2.833333 | -0.522323 | 202 | 84.416371 | 156.67 | 228.923629 | 0.775594 |
| GO:0010926\_anatomical\_structure\_formation | 560 | 2 | 1.821429 | -0.520825 | 204 | 84.693145 | 157.07 | 229.446855 | 0.769951 |
| GO:0044085\_cellular\_component\_biogenesis | 560 | 2 | 1.821429 | -0.520825 | 204 | 84.693145 | 157.07 | 229.446855 | 0.769951 |
| GO:0006954\_inflammatory\_response | 182 | 1 | 2.802198 | -0.518289 | 205 | 84.820327 | 157.3 | 229.779673 | 0.767317 |
| GO:0010627\_regulation\_of\_protein\_kinase\_cascade | 184 | 1 | 2.771739 | -0.514308 | 206 | 85.074853 | 157.71 | 230.345147 | 0.765583 |
| GO:0009967\_positive\_regulation\_of\_signal\_transduction | 185 | 1 | 2.756757 | -0.512336 | 207 | 85.299035 | 158.06 | 230.820965 | 0.763575 |
| GO:0034622\_cellular\_macromolecular\_complex\_assembly | 186 | 1 | 2.741935 | -0.510377 | 208 | 85.693703 | 158.57 | 231.446297 | 0.762356 |
| GO:0010647\_positive\_regulation\_of\_cell\_communication | 189 | 1 | 2.698413 | -0.504574 | 209 | 86.546738 | 159.8 | 233.053262 | 0.764593 |
| GO:0043086\_negative\_regulation\_of\_catalytic\_activity | 196 | 1 | 2.602041 | -0.491451 | 210 | 89.032879 | 163.22 | 237.407121 | 0.777238 |
| GO:0008284\_positive\_regulation\_of\_cell\_proliferation | 200 | 1 | 2.550000 | -0.484201 | 211 | 89.426468 | 163.75 | 238.073532 | 0.776066 |
| GO:0016310\_phosphorylation | 601 | 2 | 1.697171 | -0.478897 | 212 | 90.131412 | 164.67 | 239.208588 | 0.776745 |
| GO:0045859\_regulation\_of\_protein\_kinase\_activity | 213 | 1 | 2.394366 | -0.461794 | 213 | 93.541729 | 169.11 | 244.678271 | 0.793944 |
| GO:0007610\_behavior | 214 | 1 | 2.383178 | -0.460140 | 214 | 93.905729 | 169.45 | 244.994271 | 0.791822 |
| GO:0043549\_regulation\_of\_kinase\_activity | 217 | 1 | 2.350230 | -0.455232 | 215 | 94.525258 | 170.31 | 246.094742 | 0.792140 |
| GO:0042221\_response\_to\_chemical\_stimulus | 631 | 2 | 1.616482 | -0.450767 | 216 | 95.125386 | 171.04 | 246.954614 | 0.791852 |
| GO:0016044\_membrane\_organization | 225 | 1 | 2.266667 | -0.442539 | 217 | 96.079322 | 172.38 | 248.680678 | 0.794378 |
| GO:0034621\_cellular\_macromolecular\_complex\_subunit\_organization | 227 | 1 | 2.246696 | -0.439452 | 219 | 96.951183 | 173.54 | 250.128817 | 0.792420 |
| GO:0051338\_regulation\_of\_transferase\_activity | 227 | 1 | 2.246696 | -0.439452 | 219 | 96.951183 | 173.54 | 250.128817 | 0.792420 |
| GO:0008283\_cell\_proliferation | 647 | 2 | 1.576507 | -0.436561 | 220 | 97.531518 | 174.29 | 251.048482 | 0.792227 |
| GO:0051179\_localization | 1561 | 4 | 1.306855 | -0.435272 | 221 | 97.861773 | 174.73 | 251.598227 | 0.790633 |
| GO:0044092\_negative\_regulation\_of\_molecular\_function | 233 | 1 | 2.188841 | -0.430390 | 222 | 99.300235 | 176.42 | 253.539765 | 0.794685 |
| GO:0022403\_cell\_cycle\_phase | 245 | 1 | 2.081633 | -0.413104 | 223 | 101.719962 | 179.58 | 257.440038 | 0.805291 |
| GO:0006793\_phosphorus\_metabolic\_process | 697 | 2 | 1.463415 | -0.395398 | 225 | 104.363284 | 182.71 | 261.056716 | 0.812044 |
| GO:0006796\_phosphate\_metabolic\_process | 697 | 2 | 1.463415 | -0.395398 | 225 | 104.363284 | 182.71 | 261.056716 | 0.812044 |
| GO:0016481\_negative\_regulation\_of\_transcription | 261 | 1 | 1.954023 | -0.391640 | 226 | 104.702983 | 183.02 | 261.337017 | 0.809823 |
| GO:0009987\_cellular\_process | 6671 | 14 | 1.070304 | -0.376937 | 227 | 106.362840 | 185.21 | 264.057160 | 0.815903 |
| GO:0015031\_protein\_transport | 274 | 1 | 1.861314 | -0.375395 | 228 | 107.325386 | 186.38 | 265.434614 | 0.817456 |
| GO:0043687\_post-translational\_protein\_modification | 728 | 2 | 1.401099 | -0.372103 | 229 | 108.077179 | 187.14 | 266.202821 | 0.817205 |
| GO:0009611\_response\_to\_wounding | 279 | 1 | 1.827957 | -0.369408 | 231 | 108.910186 | 188.34 | 267.769814 | 0.815325 |
| GO:0045184\_establishment\_of\_protein\_localization | 279 | 1 | 1.827957 | -0.369408 | 231 | 108.910186 | 188.34 | 267.769814 | 0.815325 |
| GO:0042325\_regulation\_of\_phosphorylation | 285 | 1 | 1.789474 | -0.362404 | 232 | 109.450269 | 189.09 | 268.729731 | 0.815043 |
| GO:0032268\_regulation\_of\_cellular\_protein\_metabolic\_process | 286 | 1 | 1.783217 | -0.361255 | 233 | 110.415908 | 190.13 | 269.844092 | 0.816009 |
| GO:0010629\_negative\_regulation\_of\_gene\_expression | 289 | 1 | 1.764706 | -0.357840 | 234 | 111.361164 | 191.05 | 270.738836 | 0.816453 |
| GO:0000278\_mitotic\_cell\_cycle | 292 | 1 | 1.746575 | -0.354471 | 236 | 113.500968 | 193.44 | 273.379032 | 0.819661 |
| GO:0040011\_locomotion | 292 | 1 | 1.746575 | -0.354471 | 236 | 113.500968 | 193.44 | 273.379032 | 0.819661 |
| GO:0045934\_negative\_regulation\_of\_nucleobase\_\_nucleoside\_\_nucleotide\_and\_nucleic\_acid\_metabolic\_process | 295 | 1 | 1.728814 | -0.351147 | 237 | 113.866009 | 193.79 | 273.713991 | 0.817679 |
| GO:0019220\_regulation\_of\_phosphate\_metabolic\_process | 297 | 1 | 1.717172 | -0.348955 | 239 | 114.559786 | 194.87 | 275.180214 | 0.815356 |
| GO:0051174\_regulation\_of\_phosphorus\_metabolic\_process | 297 | 1 | 1.717172 | -0.348955 | 239 | 114.559786 | 194.87 | 275.180214 | 0.815356 |
| GO:0051172\_negative\_regulation\_of\_nitrogen\_compound\_metabolic\_process | 298 | 1 | 1.711409 | -0.347867 | 240 | 114.920991 | 195.22 | 275.519009 | 0.813417 |
| GO:0006810\_transport | 1243 | 3 | 1.230893 | -0.347448 | 241 | 115.175197 | 195.43 | 275.684803 | 0.810913 |
| GO:0051246\_regulation\_of\_protein\_metabolic\_process | 301 | 1 | 1.694352 | -0.344630 | 242 | 115.792556 | 196.01 | 276.227444 | 0.809959 |
| GO:0051234\_establishment\_of\_localization | 1260 | 3 | 1.214286 | -0.338398 | 243 | 116.601808 | 196.76 | 276.918192 | 0.809712 |
| GO:0010558\_negative\_regulation\_of\_macromolecule\_biosynthetic\_process | 324 | 1 | 1.574074 | -0.321155 | 244 | 119.519405 | 199.93 | 280.340595 | 0.819385 |
| GO:0032879\_regulation\_of\_localization | 326 | 1 | 1.564417 | -0.319220 | 245 | 119.712124 | 200.23 | 280.747876 | 0.817265 |
| GO:0031327\_negative\_regulation\_of\_cellular\_biosynthetic\_process | 332 | 1 | 1.536145 | -0.313508 | 246 | 120.415501 | 200.95 | 281.484499 | 0.816870 |
| GO:0008104\_protein\_localization | 339 | 1 | 1.504425 | -0.307018 | 247 | 121.052306 | 201.7 | 282.347694 | 0.816599 |
| GO:0009890\_negative\_regulation\_of\_biosynthetic\_process | 340 | 1 | 1.500000 | -0.306106 | 248 | 121.891020 | 202.6 | 283.308980 | 0.816935 |
| GO:0033554\_cellular\_response\_to\_stress | 341 | 1 | 1.495601 | -0.305198 | 249 | 122.361862 | 203.09 | 283.818138 | 0.815622 |
| GO:0006357\_regulation\_of\_transcription\_from\_RNA\_polymerase\_II\_promoter | 351 | 1 | 1.452991 | -0.296309 | 250 | 123.127404 | 203.87 | 284.612596 | 0.815480 |
| GO:0065008\_regulation\_of\_biological\_quality | 848 | 2 | 1.202830 | -0.295208 | 251 | 123.654694 | 204.54 | 285.425306 | 0.814900 |
| GO:0016043\_cellular\_component\_organization | 1366 | 3 | 1.120059 | -0.286889 | 252 | 125.174107 | 206.25 | 287.325893 | 0.818452 |
| GO:0007186\_G-protein\_coupled\_receptor\_protein\_signaling\_pathway | 363 | 1 | 1.404959 | -0.286092 | 253 | 125.582022 | 206.61 | 287.637978 | 0.816640 |
| GO:0022402\_cell\_cycle\_process | 370 | 1 | 1.378378 | -0.280347 | 254 | 127.729856 | 208.83 | 289.930144 | 0.822165 |
| GO:0033036\_macromolecule\_localization | 388 | 1 | 1.314433 | -0.266252 | 255 | 130.811685 | 212.08 | 293.348315 | 0.831686 |
| GO:0006464\_protein\_modification\_process | 922 | 2 | 1.106291 | -0.256423 | 256 | 132.199737 | 213.7 | 295.200263 | 0.834766 |
| GO:0031324\_negative\_regulation\_of\_cellular\_metabolic\_process | 404 | 1 | 1.262376 | -0.254483 | 257 | 133.454105 | 214.81 | 296.165895 | 0.835837 |
| GO:0042127\_regulation\_of\_cell\_proliferation | 411 | 1 | 1.240876 | -0.249544 | 258 | 134.494179 | 215.98 | 297.465821 | 0.837132 |
| GO:0010605\_negative\_regulation\_of\_macromolecule\_metabolic\_process | 413 | 1 | 1.234867 | -0.248155 | 259 | 135.124784 | 216.62 | 298.115216 | 0.836371 |
| GO:0043412\_biopolymer\_modification | 960 | 2 | 1.062500 | -0.238610 | 260 | 137.804025 | 219.5 | 301.195975 | 0.844231 |
| GO:0007155\_cell\_adhesion | 428 | 1 | 1.191589 | -0.238043 | 261 | 138.133984 | 219.84 | 301.546016 | 0.842299 |
| GO:0022610\_biological\_adhesion | 429 | 1 | 1.188811 | -0.237388 | 262 | 138.461169 | 220.18 | 301.898831 | 0.840382 |
| GO:0009892\_negative\_regulation\_of\_metabolic\_process | 440 | 1 | 1.159091 | -0.230323 | 263 | 139.419073 | 221.07 | 302.720927 | 0.840570 |
| GO:0048519\_negative\_regulation\_of\_biological\_process | 1013 | 2 | 1.006910 | -0.215861 | 264 | 141.946807 | 223.82 | 305.693193 | 0.847803 |
| GO:0009605\_response\_to\_external\_stimulus | 464 | 1 | 1.099138 | -0.215792 | 265 | 142.276021 | 224.13 | 305.983979 | 0.845774 |
| GO:0032501\_multicellular\_organismal\_process | 2082 | 4 | 0.979827 | -0.212155 | 266 | 143.987938 | 225.62 | 307.252062 | 0.848195 |
| GO:0051716\_cellular\_response\_to\_stimulus | 474 | 1 | 1.075949 | -0.210069 | 267 | 144.868733 | 226.6 | 308.331267 | 0.848689 |
| GO:0007049\_cell\_cycle | 494 | 1 | 1.032389 | -0.199166 | 268 | 148.040438 | 230.06 | 312.079562 | 0.858433 |
| GO:0009653\_anatomical\_structure\_morphogenesis | 500 | 1 | 1.020000 | -0.196028 | 269 | 148.672203 | 230.76 | 312.847797 | 0.857844 |
| GO:0006366\_transcription\_from\_RNA\_polymerase\_II\_promoter | 506 | 1 | 1.007905 | -0.192949 | 270 | 150.461985 | 232.38 | 314.298015 | 0.860667 |
| GO:0051641\_cellular\_localization | 617 | 1 | 0.826580 | -0.145016 | 271 | 158.755474 | 240.69 | 322.624526 | 0.888155 |
| GO:0080090\_regulation\_of\_primary\_metabolic\_process | 1311 | 2 | 0.778032 | -0.122746 | 272 | 162.753756 | 244.47 | 326.186244 | 0.898787 |
| GO:0060255\_regulation\_of\_macromolecule\_metabolic\_process | 1328 | 2 | 0.768072 | -0.118821 | 273 | 163.413160 | 245.02 | 326.626840 | 0.897509 |
| GO:0006355\_regulation\_of\_transcription\_\_DNA-dependent | 723 | 1 | 0.705394 | -0.111432 | 274 | 165.863816 | 247.27 | 328.676184 | 0.902445 |
| GO:0044267\_cellular\_protein\_metabolic\_process | 1382 | 2 | 0.738061 | -0.107130 | 275 | 167.547177 | 248.85 | 330.152823 | 0.904909 |
| GO:0051252\_regulation\_of\_RNA\_metabolic\_process | 746 | 1 | 0.683646 | -0.105327 | 276 | 168.628029 | 249.8 | 330.971971 | 0.905072 |
| GO:0031323\_regulation\_of\_cellular\_metabolic\_process | 1466 | 2 | 0.695771 | -0.091078 | 277 | 171.529539 | 252.35 | 333.170461 | 0.911011 |
| GO:0019222\_regulation\_of\_metabolic\_process | 1538 | 2 | 0.663199 | -0.079143 | 278 | 173.415148 | 253.96 | 334.504852 | 0.913525 |
| GO:0006351\_transcription\_\_DNA-dependent | 884 | 1 | 0.576923 | -0.075406 | 279 | 174.096521 | 254.53 | 334.963479 | 0.912294 |
| GO:0032774\_RNA\_biosynthetic\_process | 887 | 1 | 0.574972 | -0.074864 | 280 | 174.559898 | 254.88 | 335.200102 | 0.910286 |
| GO:0019538\_protein\_metabolic\_process | 1569 | 2 | 0.650096 | -0.074467 | 281 | 174.808761 | 255.15 | 335.491239 | 0.908007 |
| GO:0045449\_regulation\_of\_transcription | 900 | 1 | 0.566667 | -0.072562 | 282 | 175.277195 | 255.51 | 335.742805 | 0.906064 |
| GO:0048523\_negative\_regulation\_of\_cellular\_process | 925 | 1 | 0.551351 | -0.068338 | 283 | 176.630086 | 256.64 | 336.649914 | 0.906855 |
| GO:0019219\_regulation\_of\_nucleobase\_\_nucleoside\_\_nucleotide\_and\_nucleic\_acid\_metabolic\_process | 1041 | 1 | 0.489914 | -0.051778 | 284 | 180.390843 | 259.49 | 338.589157 | 0.913697 |
| GO:0010556\_regulation\_of\_macromolecule\_biosynthetic\_process | 1055 | 1 | 0.483412 | -0.050074 | 286 | 181.312495 | 260.17 | 339.027505 | 0.909685 |
| GO:0051171\_regulation\_of\_nitrogen\_compound\_metabolic\_process | 1055 | 1 | 0.483412 | -0.050074 | 286 | 181.312495 | 260.17 | 339.027505 | 0.909685 |
| GO:0010468\_regulation\_of\_gene\_expression | 1067 | 1 | 0.477976 | -0.048659 | 287 | 181.881143 | 260.65 | 339.418857 | 0.908188 |
| GO:0006350\_transcription | 1069 | 1 | 0.477081 | -0.048427 | 288 | 182.257009 | 260.93 | 339.602991 | 0.906007 |
| GO:0031326\_regulation\_of\_cellular\_biosynthetic\_process | 1125 | 1 | 0.453333 | -0.042360 | 289 | 183.757744 | 261.9 | 340.042256 | 0.906228 |
| GO:0009889\_regulation\_of\_biosynthetic\_process | 1135 | 1 | 0.449339 | -0.041359 | 290 | 184.132635 | 262.13 | 340.127365 | 0.903897 |
| GO:0016070\_RNA\_metabolic\_process | 1230 | 1 | 0.414634 | -0.032938 | 291 | 185.537732 | 263.28 | 341.022268 | 0.904742 |
| GO:0034960\_cellular\_biopolymer\_metabolic\_process | 2820 | 3 | 0.542553 | -0.021544 | 292 | 188.411978 | 265.38 | 342.348022 | 0.908836 |
| GO:0034961\_cellular\_biopolymer\_biosynthetic\_process | 1448 | 1 | 0.352210 | -0.019439 | 293 | 188.704897 | 265.58 | 342.455103 | 0.906416 |
| GO:0043284\_biopolymer\_biosynthetic\_process | 1458 | 1 | 0.349794 | -0.018970 | 294 | 188.848606 | 265.69 | 342.531394 | 0.903707 |
| GO:0044260\_cellular\_macromolecule\_metabolic\_process | 2883 | 3 | 0.530697 | -0.018854 | 295 | 188.970833 | 265.78 | 342.589167 | 0.900949 |
| GO:0043283\_biopolymer\_metabolic\_process | 3027 | 3 | 0.505451 | -0.013780 | 296 | 190.285226 | 266.58 | 342.874774 | 0.900608 |
| GO:0034645\_cellular\_macromolecule\_biosynthetic\_process | 1600 | 1 | 0.318750 | -0.013376 | 297 | 190.405347 | 266.66 | 342.914653 | 0.897845 |
| GO:0009059\_macromolecule\_biosynthetic\_process | 1626 | 1 | 0.313653 | -0.012539 | 298 | 190.654737 | 266.8 | 342.945263 | 0.895302 |
| GO:0043170\_macromolecule\_metabolic\_process | 3103 | 3 | 0.493071 | -0.011620 | 299 | 190.746409 | 266.87 | 342.993591 | 0.892542 |
| GO:0010467\_gene\_expression | 1663 | 1 | 0.306675 | -0.011434 | 300 | 190.802721 | 266.94 | 343.077279 | 0.889800 |
| GO:0006139\_nucleobase\_\_nucleoside\_\_nucleotide\_and\_nucleic\_acid\_metabolic\_process | 1845 | 1 | 0.276423 | -0.007219 | 301 | 191.240314 | 267.18 | 343.119686 | 0.887641 |
| GO:0044249\_cellular\_biosynthetic\_process | 1951 | 1 | 0.261404 | -0.005493 | 302 | 191.514027 | 267.33 | 343.145973 | 0.885199 |
| GO:0009058\_biosynthetic\_process | 1988 | 1 | 0.256539 | -0.004989 | 303 | 191.574601 | 267.37 | 343.165399 | 0.882409 |
| GO:0006807\_nitrogen\_compound\_metabolic\_process | 2053 | 1 | 0.248417 | -0.004207 | 304 | 191.942478 | 267.6 | 343.257522 | 0.880263 |
| GO:0044238\_primary\_metabolic\_process | 3719 | 3 | 0.411401 | -0.002527 | 305 | 192.119936 | 267.75 | 343.380064 | 0.877869 |
| GO:0044237\_cellular\_metabolic\_process | 3753 | 3 | 0.407674 | -0.002304 | 306 | 192.259150 | 267.82 | 343.380850 | 0.875229 |
| GO:0008152\_metabolic\_process | 4111 | 3 | 0.372172 | -0.000821 | 307 | 192.404265 | 267.9 | 343.395735 | 0.872638 |
| GO:0000079\_regulation\_of\_cyclin-dependent\_protein\_kinase\_activity | 48 | 0 | 0.000000 | -0.000000 | 313 | 199.869892 | 275.16 | 350.450108 | 0.879105 |
| GO:0008217\_regulation\_of\_blood\_pressure | 48 | 0 | 0.000000 | -0.000000 | 313 | 199.869892 | 275.16 | 350.450108 | 0.879105 |
| GO:0010518\_positive\_regulation\_of\_phospholipase\_activity | 48 | 0 | 0.000000 | -0.000000 | 313 | 199.869892 | 275.16 | 350.450108 | 0.879105 |
| GO:0042035\_regulation\_of\_cytokine\_biosynthetic\_process | 48 | 0 | 0.000000 | -0.000000 | 313 | 199.869892 | 275.16 | 350.450108 | 0.879105 |
| GO:0042773\_ATP\_synthesis\_coupled\_electron\_transport | 48 | 0 | 0.000000 | -0.000000 | 313 | 199.869892 | 275.16 | 350.450108 | 0.879105 |
| GO:0042775\_mitochondrial\_ATP\_synthesis\_coupled\_electron\_transport | 48 | 0 | 0.000000 | -0.000000 | 313 | 199.869892 | 275.16 | 350.450108 | 0.879105 |
| GO:0000070\_mitotic\_sister\_chromatid\_segregation | 27 | 0 | 0.000000 | -0.000000 | 336 | 222.112745 | 297.0 | 371.887255 | 0.883929 |
| GO:0000245\_spliceosome\_assembly | 27 | 0 | 0.000000 | -0.000000 | 336 | 222.112745 | 297.0 | 371.887255 | 0.883929 |
| GO:0003002\_regionalization | 27 | 0 | 0.000000 | -0.000000 | 336 | 222.112745 | 297.0 | 371.887255 | 0.883929 |
| GO:0006638\_neutral\_lipid\_metabolic\_process | 27 | 0 | 0.000000 | -0.000000 | 336 | 222.112745 | 297.0 | 371.887255 | 0.883929 |
| GO:0006639\_acylglycerol\_metabolic\_process | 27 | 0 | 0.000000 | -0.000000 | 336 | 222.112745 | 297.0 | 371.887255 | 0.883929 |
| GO:0006690\_icosanoid\_metabolic\_process | 27 | 0 | 0.000000 | -0.000000 | 336 | 222.112745 | 297.0 | 371.887255 | 0.883929 |
| GO:0006836\_neurotransmitter\_transport | 27 | 0 | 0.000000 | -0.000000 | 336 | 222.112745 | 297.0 | 371.887255 | 0.883929 |
| GO:0006909\_phagocytosis | 27 | 0 | 0.000000 | -0.000000 | 336 | 222.112745 | 297.0 | 371.887255 | 0.883929 |
| GO:0007260\_tyrosine\_phosphorylation\_of\_STAT\_protein | 27 | 0 | 0.000000 | -0.000000 | 336 | 222.112745 | 297.0 | 371.887255 | 0.883929 |
| GO:0007631\_feeding\_behavior | 27 | 0 | 0.000000 | -0.000000 | 336 | 222.112745 | 297.0 | 371.887255 | 0.883929 |
| GO:0008543\_fibroblast\_growth\_factor\_receptor\_signaling\_pathway | 27 | 0 | 0.000000 | -0.000000 | 336 | 222.112745 | 297.0 | 371.887255 | 0.883929 |
| GO:0019079\_viral\_genome\_replication | 27 | 0 | 0.000000 | -0.000000 | 336 | 222.112745 | 297.0 | 371.887255 | 0.883929 |
| GO:0031349\_positive\_regulation\_of\_defense\_response | 27 | 0 | 0.000000 | -0.000000 | 336 | 222.112745 | 297.0 | 371.887255 | 0.883929 |
| GO:0031669\_cellular\_response\_to\_nutrient\_levels | 27 | 0 | 0.000000 | -0.000000 | 336 | 222.112745 | 297.0 | 371.887255 | 0.883929 |
| GO:0032200\_telomere\_organization | 27 | 0 | 0.000000 | -0.000000 | 336 | 222.112745 | 297.0 | 371.887255 | 0.883929 |
| GO:0035150\_regulation\_of\_tube\_size | 27 | 0 | 0.000000 | -0.000000 | 336 | 222.112745 | 297.0 | 371.887255 | 0.883929 |
| GO:0043254\_regulation\_of\_protein\_complex\_assembly | 27 | 0 | 0.000000 | -0.000000 | 336 | 222.112745 | 297.0 | 371.887255 | 0.883929 |
| GO:0044272\_sulfur\_compound\_biosynthetic\_process | 27 | 0 | 0.000000 | -0.000000 | 336 | 222.112745 | 297.0 | 371.887255 | 0.883929 |
| GO:0050880\_regulation\_of\_blood\_vessel\_size | 27 | 0 | 0.000000 | -0.000000 | 336 | 222.112745 | 297.0 | 371.887255 | 0.883929 |
| GO:0050906\_detection\_of\_stimulus\_involved\_in\_sensory\_perception | 27 | 0 | 0.000000 | -0.000000 | 336 | 222.112745 | 297.0 | 371.887255 | 0.883929 |
| GO:0051048\_negative\_regulation\_of\_secretion | 27 | 0 | 0.000000 | -0.000000 | 336 | 222.112745 | 297.0 | 371.887255 | 0.883929 |
| GO:0051092\_positive\_regulation\_of\_NF-kappaB\_transcription\_factor\_activity | 27 | 0 | 0.000000 | -0.000000 | 336 | 222.112745 | 297.0 | 371.887255 | 0.883929 |
| GO:0051607\_defense\_response\_to\_virus | 27 | 0 | 0.000000 | -0.000000 | 336 | 222.112745 | 297.0 | 371.887255 | 0.883929 |
| GO:0045941\_positive\_regulation\_of\_transcription | 264 | 0 | 0.000000 | -0.000000 | 337 | 222.828577 | 297.66 | 372.491423 | 0.883264 |
| GO:0006171\_cAMP\_biosynthetic\_process | 59 | 0 | 0.000000 | -0.000000 | 342 | 229.437451 | 303.92 | 378.402549 | 0.888655 |
| GO:0006820\_anion\_transport | 59 | 0 | 0.000000 | -0.000000 | 342 | 229.437451 | 303.92 | 378.402549 | 0.888655 |
| GO:0007409\_axonogenesis | 59 | 0 | 0.000000 | -0.000000 | 342 | 229.437451 | 303.92 | 378.402549 | 0.888655 |
| GO:0033673\_negative\_regulation\_of\_kinase\_activity | 59 | 0 | 0.000000 | -0.000000 | 342 | 229.437451 | 303.92 | 378.402549 | 0.888655 |
| GO:0051604\_protein\_maturation | 59 | 0 | 0.000000 | -0.000000 | 342 | 229.437451 | 303.92 | 378.402549 | 0.888655 |
| GO:0070727\_cellular\_macromolecule\_localization | 229 | 0 | 0.000000 | -0.000000 | 343 | 230.207992 | 304.58 | 378.952008 | 0.887988 |
| GO:0001935\_endothelial\_cell\_proliferation | 25 | 0 | 0.000000 | -0.000000 | 370 | 259.684459 | 333.19 | 406.695541 | 0.900514 |
| GO:0006007\_glucose\_catabolic\_process | 25 | 0 | 0.000000 | -0.000000 | 370 | 259.684459 | 333.19 | 406.695541 | 0.900514 |
| GO:0006112\_energy\_reserve\_metabolic\_process | 25 | 0 | 0.000000 | -0.000000 | 370 | 259.684459 | 333.19 | 406.695541 | 0.900514 |
| GO:0006518\_peptide\_metabolic\_process | 25 | 0 | 0.000000 | -0.000000 | 370 | 259.684459 | 333.19 | 406.695541 | 0.900514 |
| GO:0006767\_water-soluble\_vitamin\_metabolic\_process | 25 | 0 | 0.000000 | -0.000000 | 370 | 259.684459 | 333.19 | 406.695541 | 0.900514 |
| GO:0007127\_meiosis\_I | 25 | 0 | 0.000000 | -0.000000 | 370 | 259.684459 | 333.19 | 406.695541 | 0.900514 |
| GO:0007416\_synaptogenesis | 25 | 0 | 0.000000 | -0.000000 | 370 | 259.684459 | 333.19 | 406.695541 | 0.900514 |
| GO:0009141\_nucleoside\_triphosphate\_metabolic\_process | 25 | 0 | 0.000000 | -0.000000 | 370 | 259.684459 | 333.19 | 406.695541 | 0.900514 |
| GO:0010876\_lipid\_localization | 25 | 0 | 0.000000 | -0.000000 | 370 | 259.684459 | 333.19 | 406.695541 | 0.900514 |
| GO:0015711\_organic\_anion\_transport | 25 | 0 | 0.000000 | -0.000000 | 370 | 259.684459 | 333.19 | 406.695541 | 0.900514 |
| GO:0019217\_regulation\_of\_fatty\_acid\_metabolic\_process | 25 | 0 | 0.000000 | -0.000000 | 370 | 259.684459 | 333.19 | 406.695541 | 0.900514 |
| GO:0019915\_lipid\_storage | 25 | 0 | 0.000000 | -0.000000 | 370 | 259.684459 | 333.19 | 406.695541 | 0.900514 |
| GO:0030168\_platelet\_activation | 25 | 0 | 0.000000 | -0.000000 | 370 | 259.684459 | 333.19 | 406.695541 | 0.900514 |
| GO:0030282\_bone\_mineralization | 25 | 0 | 0.000000 | -0.000000 | 370 | 259.684459 | 333.19 | 406.695541 | 0.900514 |
| GO:0031023\_microtubule\_organizing\_center\_organization | 25 | 0 | 0.000000 | -0.000000 | 370 | 259.684459 | 333.19 | 406.695541 | 0.900514 |
| GO:0031644\_regulation\_of\_neurological\_system\_process | 25 | 0 | 0.000000 | -0.000000 | 370 | 259.684459 | 333.19 | 406.695541 | 0.900514 |
| GO:0032368\_regulation\_of\_lipid\_transport | 25 | 0 | 0.000000 | -0.000000 | 370 | 259.684459 | 333.19 | 406.695541 | 0.900514 |
| GO:0034101\_erythrocyte\_homeostasis | 25 | 0 | 0.000000 | -0.000000 | 370 | 259.684459 | 333.19 | 406.695541 | 0.900514 |
| GO:0043087\_regulation\_of\_GTPase\_activity | 25 | 0 | 0.000000 | -0.000000 | 370 | 259.684459 | 333.19 | 406.695541 | 0.900514 |
| GO:0043624\_cellular\_protein\_complex\_disassembly | 25 | 0 | 0.000000 | -0.000000 | 370 | 259.684459 | 333.19 | 406.695541 | 0.900514 |
| GO:0043966\_histone\_H3\_acetylation | 25 | 0 | 0.000000 | -0.000000 | 370 | 259.684459 | 333.19 | 406.695541 | 0.900514 |
| GO:0045762\_positive\_regulation\_of\_adenylate\_cyclase\_activity | 25 | 0 | 0.000000 | -0.000000 | 370 | 259.684459 | 333.19 | 406.695541 | 0.900514 |
| GO:0050678\_regulation\_of\_epithelial\_cell\_proliferation | 25 | 0 | 0.000000 | -0.000000 | 370 | 259.684459 | 333.19 | 406.695541 | 0.900514 |
| GO:0050727\_regulation\_of\_inflammatory\_response | 25 | 0 | 0.000000 | -0.000000 | 370 | 259.684459 | 333.19 | 406.695541 | 0.900514 |
| GO:0050795\_regulation\_of\_behavior | 25 | 0 | 0.000000 | -0.000000 | 370 | 259.684459 | 333.19 | 406.695541 | 0.900514 |
| GO:0050818\_regulation\_of\_coagulation | 25 | 0 | 0.000000 | -0.000000 | 370 | 259.684459 | 333.19 | 406.695541 | 0.900514 |
| GO:0050821\_protein\_stabilization | 25 | 0 | 0.000000 | -0.000000 | 370 | 259.684459 | 333.19 | 406.695541 | 0.900514 |
| GO:0051128\_regulation\_of\_cellular\_component\_organization | 237 | 0 | 0.000000 | -0.000000 | 371 | 260.510412 | 333.86 | 407.209588 | 0.899892 |
| GO:0006511\_ubiquitin-dependent\_protein\_catabolic\_process | 136 | 0 | 0.000000 | -0.000000 | 373 | 262.208215 | 335.39 | 408.571785 | 0.899169 |
| GO:0048699\_generation\_of\_neurons | 136 | 0 | 0.000000 | -0.000000 | 373 | 262.208215 | 335.39 | 408.571785 | 0.899169 |
| GO:0006289\_nucleotide-excision\_repair | 45 | 0 | 0.000000 | -0.000000 | 382 | 271.686196 | 344.49 | 417.293804 | 0.901806 |
| GO:0006308\_DNA\_catabolic\_process | 45 | 0 | 0.000000 | -0.000000 | 382 | 271.686196 | 344.49 | 417.293804 | 0.901806 |
| GO:0006368\_RNA\_elongation\_from\_RNA\_polymerase\_II\_promoter | 45 | 0 | 0.000000 | -0.000000 | 382 | 271.686196 | 344.49 | 417.293804 | 0.901806 |
| GO:0006576\_biogenic\_amine\_metabolic\_process | 45 | 0 | 0.000000 | -0.000000 | 382 | 271.686196 | 344.49 | 417.293804 | 0.901806 |
| GO:0016197\_endosome\_transport | 45 | 0 | 0.000000 | -0.000000 | 382 | 271.686196 | 344.49 | 417.293804 | 0.901806 |
| GO:0016573\_histone\_acetylation | 45 | 0 | 0.000000 | -0.000000 | 382 | 271.686196 | 344.49 | 417.293804 | 0.901806 |
| GO:0034728\_nucleosome\_organization | 45 | 0 | 0.000000 | -0.000000 | 382 | 271.686196 | 344.49 | 417.293804 | 0.901806 |
| GO:0048771\_tissue\_remodeling | 45 | 0 | 0.000000 | -0.000000 | 382 | 271.686196 | 344.49 | 417.293804 | 0.901806 |
| GO:0051168\_nuclear\_export | 45 | 0 | 0.000000 | -0.000000 | 382 | 271.686196 | 344.49 | 417.293804 | 0.901806 |
| GO:0002526\_acute\_inflammatory\_response | 31 | 0 | 0.000000 | -0.000000 | 395 | 286.137033 | 358.58 | 431.022967 | 0.907797 |
| GO:0006633\_fatty\_acid\_biosynthetic\_process | 31 | 0 | 0.000000 | -0.000000 | 395 | 286.137033 | 358.58 | 431.022967 | 0.907797 |
| GO:0006888\_ER\_to\_Golgi\_vesicle-mediated\_transport | 31 | 0 | 0.000000 | -0.000000 | 395 | 286.137033 | 358.58 | 431.022967 | 0.907797 |
| GO:0007173\_epidermal\_growth\_factor\_receptor\_signaling\_pathway | 31 | 0 | 0.000000 | -0.000000 | 395 | 286.137033 | 358.58 | 431.022967 | 0.907797 |
| GO:0007200\_activation\_of\_phospholipase\_C\_activity\_by\_G-protein\_coupled\_receptor\_protein\_signaling\_pathway\_coupled\_to\_IP3\_second\_messenger | 31 | 0 | 0.000000 | -0.000000 | 395 | 286.137033 | 358.58 | 431.022967 | 0.907797 |
| GO:0007423\_sensory\_organ\_development | 31 | 0 | 0.000000 | -0.000000 | 395 | 286.137033 | 358.58 | 431.022967 | 0.907797 |
| GO:0008629\_induction\_of\_apoptosis\_by\_intracellular\_signals | 31 | 0 | 0.000000 | -0.000000 | 395 | 286.137033 | 358.58 | 431.022967 | 0.907797 |
| GO:0031668\_cellular\_response\_to\_extracellular\_stimulus | 31 | 0 | 0.000000 | -0.000000 | 395 | 286.137033 | 358.58 | 431.022967 | 0.907797 |
| GO:0042742\_defense\_response\_to\_bacterium | 31 | 0 | 0.000000 | -0.000000 | 395 | 286.137033 | 358.58 | 431.022967 | 0.907797 |
| GO:0044403\_symbiosis\_\_encompassing\_mutualism\_through\_parasitism | 31 | 0 | 0.000000 | -0.000000 | 395 | 286.137033 | 358.58 | 431.022967 | 0.907797 |
| GO:0044419\_interspecies\_interaction\_between\_organisms | 31 | 0 | 0.000000 | -0.000000 | 395 | 286.137033 | 358.58 | 431.022967 | 0.907797 |
| GO:0045185\_maintenance\_of\_protein\_location | 31 | 0 | 0.000000 | -0.000000 | 395 | 286.137033 | 358.58 | 431.022967 | 0.907797 |
| GO:0046883\_regulation\_of\_hormone\_secretion | 31 | 0 | 0.000000 | -0.000000 | 395 | 286.137033 | 358.58 | 431.022967 | 0.907797 |
| GO:0006732\_coenzyme\_metabolic\_process | 65 | 0 | 0.000000 | -0.000000 | 401 | 293.368400 | 365.49 | 437.611600 | 0.911446 |
| GO:0016054\_organic\_acid\_catabolic\_process | 65 | 0 | 0.000000 | -0.000000 | 401 | 293.368400 | 365.49 | 437.611600 | 0.911446 |
| GO:0022415\_viral\_reproductive\_process | 65 | 0 | 0.000000 | -0.000000 | 401 | 293.368400 | 365.49 | 437.611600 | 0.911446 |
| GO:0045596\_negative\_regulation\_of\_cell\_differentiation | 65 | 0 | 0.000000 | -0.000000 | 401 | 293.368400 | 365.49 | 437.611600 | 0.911446 |
| GO:0046395\_carboxylic\_acid\_catabolic\_process | 65 | 0 | 0.000000 | -0.000000 | 401 | 293.368400 | 365.49 | 437.611600 | 0.911446 |
| GO:0051437\_positive\_regulation\_of\_ubiquitin-protein\_ligase\_activity\_during\_mitotic\_cell\_cycle | 65 | 0 | 0.000000 | -0.000000 | 401 | 293.368400 | 365.49 | 437.611600 | 0.911446 |
| GO:0000086\_G2\_M\_transition\_of\_mitotic\_cell\_cycle | 19 | 0 | 0.000000 | -0.000000 | 446 | 341.700315 | 412.79 | 483.879685 | 0.925538 |
| GO:0002237\_response\_to\_molecule\_of\_bacterial\_origin | 19 | 0 | 0.000000 | -0.000000 | 446 | 341.700315 | 412.79 | 483.879685 | 0.925538 |
| GO:0002762\_negative\_regulation\_of\_myeloid\_leukocyte\_differentiation | 19 | 0 | 0.000000 | -0.000000 | 446 | 341.700315 | 412.79 | 483.879685 | 0.925538 |
| GO:0002791\_regulation\_of\_peptide\_secretion | 19 | 0 | 0.000000 | -0.000000 | 446 | 341.700315 | 412.79 | 483.879685 | 0.925538 |
| GO:0006635\_fatty\_acid\_beta-oxidation | 19 | 0 | 0.000000 | -0.000000 | 446 | 341.700315 | 412.79 | 483.879685 | 0.925538 |
| GO:0006809\_nitric\_oxide\_biosynthetic\_process | 19 | 0 | 0.000000 | -0.000000 | 446 | 341.700315 | 412.79 | 483.879685 | 0.925538 |
| GO:0006879\_cellular\_iron\_ion\_homeostasis | 19 | 0 | 0.000000 | -0.000000 | 446 | 341.700315 | 412.79 | 483.879685 | 0.925538 |
| GO:0006890\_retrograde\_vesicle-mediated\_transport\_\_Golgi\_to\_ER | 19 | 0 | 0.000000 | -0.000000 | 446 | 341.700315 | 412.79 | 483.879685 | 0.925538 |
| GO:0007131\_reciprocal\_meiotic\_recombination | 19 | 0 | 0.000000 | -0.000000 | 446 | 341.700315 | 412.79 | 483.879685 | 0.925538 |
| GO:0007269\_neurotransmitter\_secretion | 19 | 0 | 0.000000 | -0.000000 | 446 | 341.700315 | 412.79 | 483.879685 | 0.925538 |
| GO:0009260\_ribonucleotide\_biosynthetic\_process | 19 | 0 | 0.000000 | -0.000000 | 446 | 341.700315 | 412.79 | 483.879685 | 0.925538 |
| GO:0009267\_cellular\_response\_to\_starvation | 19 | 0 | 0.000000 | -0.000000 | 446 | 341.700315 | 412.79 | 483.879685 | 0.925538 |
| GO:0009451\_RNA\_modification | 19 | 0 | 0.000000 | -0.000000 | 446 | 341.700315 | 412.79 | 483.879685 | 0.925538 |
| GO:0010883\_regulation\_of\_lipid\_storage | 19 | 0 | 0.000000 | -0.000000 | 446 | 341.700315 | 412.79 | 483.879685 | 0.925538 |
| GO:0010948\_negative\_regulation\_of\_cell\_cycle\_process | 19 | 0 | 0.000000 | -0.000000 | 446 | 341.700315 | 412.79 | 483.879685 | 0.925538 |
| GO:0015695\_organic\_cation\_transport | 19 | 0 | 0.000000 | -0.000000 | 446 | 341.700315 | 412.79 | 483.879685 | 0.925538 |
| GO:0016525\_negative\_regulation\_of\_angiogenesis | 19 | 0 | 0.000000 | -0.000000 | 446 | 341.700315 | 412.79 | 483.879685 | 0.925538 |
| GO:0019228\_regulation\_of\_action\_potential\_in\_neuron | 19 | 0 | 0.000000 | -0.000000 | 446 | 341.700315 | 412.79 | 483.879685 | 0.925538 |
| GO:0030041\_actin\_filament\_polymerization | 19 | 0 | 0.000000 | -0.000000 | 446 | 341.700315 | 412.79 | 483.879685 | 0.925538 |
| GO:0030307\_positive\_regulation\_of\_cell\_growth | 19 | 0 | 0.000000 | -0.000000 | 446 | 341.700315 | 412.79 | 483.879685 | 0.925538 |
| GO:0030500\_regulation\_of\_bone\_mineralization | 19 | 0 | 0.000000 | -0.000000 | 446 | 341.700315 | 412.79 | 483.879685 | 0.925538 |
| GO:0030832\_regulation\_of\_actin\_filament\_length | 19 | 0 | 0.000000 | -0.000000 | 446 | 341.700315 | 412.79 | 483.879685 | 0.925538 |
| GO:0031109\_microtubule\_polymerization\_or\_depolymerization | 19 | 0 | 0.000000 | -0.000000 | 446 | 341.700315 | 412.79 | 483.879685 | 0.925538 |
| GO:0031331\_positive\_regulation\_of\_cellular\_catabolic\_process | 19 | 0 | 0.000000 | -0.000000 | 446 | 341.700315 | 412.79 | 483.879685 | 0.925538 |
| GO:0031396\_regulation\_of\_protein\_ubiquitination | 19 | 0 | 0.000000 | -0.000000 | 446 | 341.700315 | 412.79 | 483.879685 | 0.925538 |
| GO:0032271\_regulation\_of\_protein\_polymerization | 19 | 0 | 0.000000 | -0.000000 | 446 | 341.700315 | 412.79 | 483.879685 | 0.925538 |
| GO:0032318\_regulation\_of\_Ras\_GTPase\_activity | 19 | 0 | 0.000000 | -0.000000 | 446 | 341.700315 | 412.79 | 483.879685 | 0.925538 |
| GO:0032388\_positive\_regulation\_of\_intracellular\_transport | 19 | 0 | 0.000000 | -0.000000 | 446 | 341.700315 | 412.79 | 483.879685 | 0.925538 |
| GO:0033273\_response\_to\_vitamin | 19 | 0 | 0.000000 | -0.000000 | 446 | 341.700315 | 412.79 | 483.879685 | 0.925538 |
| GO:0034976\_response\_to\_endoplasmic\_reticulum\_stress | 19 | 0 | 0.000000 | -0.000000 | 446 | 341.700315 | 412.79 | 483.879685 | 0.925538 |
| GO:0042531\_positive\_regulation\_of\_tyrosine\_phosphorylation\_of\_STAT\_protein | 19 | 0 | 0.000000 | -0.000000 | 446 | 341.700315 | 412.79 | 483.879685 | 0.925538 |
| GO:0043467\_regulation\_of\_generation\_of\_precursor\_metabolites\_and\_energy | 19 | 0 | 0.000000 | -0.000000 | 446 | 341.700315 | 412.79 | 483.879685 | 0.925538 |
| GO:0043603\_cellular\_amide\_metabolic\_process | 19 | 0 | 0.000000 | -0.000000 | 446 | 341.700315 | 412.79 | 483.879685 | 0.925538 |
| GO:0043967\_histone\_H4\_acetylation | 19 | 0 | 0.000000 | -0.000000 | 446 | 341.700315 | 412.79 | 483.879685 | 0.925538 |
| GO:0044236\_multicellular\_organismal\_metabolic\_process | 19 | 0 | 0.000000 | -0.000000 | 446 | 341.700315 | 412.79 | 483.879685 | 0.925538 |
| GO:0045732\_positive\_regulation\_of\_protein\_catabolic\_process | 19 | 0 | 0.000000 | -0.000000 | 446 | 341.700315 | 412.79 | 483.879685 | 0.925538 |
| GO:0045767\_regulation\_of\_anti-apoptosis | 19 | 0 | 0.000000 | -0.000000 | 446 | 341.700315 | 412.79 | 483.879685 | 0.925538 |
| GO:0046364\_monosaccharide\_biosynthetic\_process | 19 | 0 | 0.000000 | -0.000000 | 446 | 341.700315 | 412.79 | 483.879685 | 0.925538 |
| GO:0050819\_negative\_regulation\_of\_coagulation | 19 | 0 | 0.000000 | -0.000000 | 446 | 341.700315 | 412.79 | 483.879685 | 0.925538 |
| GO:0050908\_detection\_of\_light\_stimulus\_involved\_in\_visual\_perception | 19 | 0 | 0.000000 | -0.000000 | 446 | 341.700315 | 412.79 | 483.879685 | 0.925538 |
| GO:0050920\_regulation\_of\_chemotaxis | 19 | 0 | 0.000000 | -0.000000 | 446 | 341.700315 | 412.79 | 483.879685 | 0.925538 |
| GO:0050962\_detection\_of\_light\_stimulus\_involved\_in\_sensory\_perception | 19 | 0 | 0.000000 | -0.000000 | 446 | 341.700315 | 412.79 | 483.879685 | 0.925538 |
| GO:0050994\_regulation\_of\_lipid\_catabolic\_process | 19 | 0 | 0.000000 | -0.000000 | 446 | 341.700315 | 412.79 | 483.879685 | 0.925538 |
| GO:0051180\_vitamin\_transport | 19 | 0 | 0.000000 | -0.000000 | 446 | 341.700315 | 412.79 | 483.879685 | 0.925538 |
| GO:0051495\_positive\_regulation\_of\_cytoskeleton\_organization | 19 | 0 | 0.000000 | -0.000000 | 446 | 341.700315 | 412.79 | 483.879685 | 0.925538 |
| GO:0006605\_protein\_targeting | 145 | 0 | 0.000000 | -0.000000 | 449 | 346.346812 | 416.89 | 487.433188 | 0.928486 |
| GO:0007417\_central\_nervous\_system\_development | 145 | 0 | 0.000000 | -0.000000 | 449 | 346.346812 | 416.89 | 487.433188 | 0.928486 |
| GO:0030036\_actin\_cytoskeleton\_organization | 145 | 0 | 0.000000 | -0.000000 | 449 | 346.346812 | 416.89 | 487.433188 | 0.928486 |
| GO:0006519\_cellular\_amino\_acid\_and\_derivative\_metabolic\_process | 173 | 0 | 0.000000 | -0.000000 | 451 | 349.500866 | 419.59 | 489.679134 | 0.930355 |
| GO:0046483\_heterocycle\_metabolic\_process | 173 | 0 | 0.000000 | -0.000000 | 451 | 349.500866 | 419.59 | 489.679134 | 0.930355 |
| GO:0042180\_cellular\_ketone\_metabolic\_process | 291 | 0 | 0.000000 | -0.000000 | 452 | 350.568461 | 420.63 | 490.691539 | 0.930597 |
| GO:0031401\_positive\_regulation\_of\_protein\_modification\_process | 84 | 0 | 0.000000 | -0.000000 | 454 | 353.331824 | 423.16 | 492.988176 | 0.932070 |
| GO:0043123\_positive\_regulation\_of\_I-kappaB\_kinase\_NF-kappaB\_cascade | 84 | 0 | 0.000000 | -0.000000 | 454 | 353.331824 | 423.16 | 492.988176 | 0.932070 |
| GO:0006665\_sphingolipid\_metabolic\_process | 44 | 0 | 0.000000 | -0.000000 | 461 | 360.504210 | 430.07 | 499.635790 | 0.932907 |
| GO:0006730\_one-carbon\_metabolic\_process | 44 | 0 | 0.000000 | -0.000000 | 461 | 360.504210 | 430.07 | 499.635790 | 0.932907 |
| GO:0006959\_humoral\_immune\_response | 44 | 0 | 0.000000 | -0.000000 | 461 | 360.504210 | 430.07 | 499.635790 | 0.932907 |
| GO:0016125\_sterol\_metabolic\_process | 44 | 0 | 0.000000 | -0.000000 | 461 | 360.504210 | 430.07 | 499.635790 | 0.932907 |
| GO:0050900\_leukocyte\_migration | 44 | 0 | 0.000000 | -0.000000 | 461 | 360.504210 | 430.07 | 499.635790 | 0.932907 |
| GO:0051301\_cell\_division | 44 | 0 | 0.000000 | -0.000000 | 461 | 360.504210 | 430.07 | 499.635790 | 0.932907 |
| GO:0051321\_meiotic\_cell\_cycle | 44 | 0 | 0.000000 | -0.000000 | 461 | 360.504210 | 430.07 | 499.635790 | 0.932907 |
| GO:0006396\_RNA\_processing | 306 | 0 | 0.000000 | -0.000000 | 462 | 361.453421 | 430.93 | 500.406579 | 0.932749 |
| GO:0000377\_RNA\_splicing\_\_via\_transesterification\_reactions\_with\_bulged\_adenosine\_as\_nucleophile | 151 | 0 | 0.000000 | -0.000000 | 464 | 362.864710 | 432.41 | 501.955290 | 0.931918 |
| GO:0000398\_nuclear\_mRNA\_splicing\_\_via\_spliceosome | 151 | 0 | 0.000000 | -0.000000 | 464 | 362.864710 | 432.41 | 501.955290 | 0.931918 |
| GO:0019935\_cyclic-nucleotide-mediated\_signaling | 82 | 0 | 0.000000 | -0.000000 | 468 | 366.322275 | 435.74 | 505.157725 | 0.931068 |
| GO:0045087\_innate\_immune\_response | 82 | 0 | 0.000000 | -0.000000 | 468 | 366.322275 | 435.74 | 505.157725 | 0.931068 |
| GO:0048193\_Golgi\_vesicle\_transport | 82 | 0 | 0.000000 | -0.000000 | 468 | 366.322275 | 435.74 | 505.157725 | 0.931068 |
| GO:0048514\_blood\_vessel\_morphogenesis | 82 | 0 | 0.000000 | -0.000000 | 468 | 366.322275 | 435.74 | 505.157725 | 0.931068 |
| GO:0006753\_nucleoside\_phosphate\_metabolic\_process | 146 | 0 | 0.000000 | -0.000000 | 472 | 369.730141 | 438.77 | 507.809859 | 0.929597 |
| GO:0009117\_nucleotide\_metabolic\_process | 146 | 0 | 0.000000 | -0.000000 | 472 | 369.730141 | 438.77 | 507.809859 | 0.929597 |
| GO:0016568\_chromatin\_modification | 146 | 0 | 0.000000 | -0.000000 | 472 | 369.730141 | 438.77 | 507.809859 | 0.929597 |
| GO:0022008\_neurogenesis | 146 | 0 | 0.000000 | -0.000000 | 472 | 369.730141 | 438.77 | 507.809859 | 0.929597 |
| GO:0001817\_regulation\_of\_cytokine\_production | 103 | 0 | 0.000000 | -0.000000 | 473 | 370.661550 | 439.56 | 508.458450 | 0.929302 |
| GO:0000028\_ribosomal\_small\_subunit\_assembly | 1 | 0 |  |  |  |  |  |  |  |  |
| GO:0000042\_protein\_targeting\_to\_Golgi | 1 | 0 |  |  |  |  |  |  |  |  |
| GO:0000046\_autophagic\_vacuole\_fusion | 1 | 0 |  |  |  |  |  |  |  |  |
| GO:0000052\_citrulline\_metabolic\_process | 1 | 0 |  |  |  |  |  |  |  |  |
| GO:0000054\_ribosome\_export\_from\_nucleus | 1 | 0 |  |  |  |  |  |  |  |  |
| GO:0000056\_ribosomal\_small\_subunit\_export\_from\_nucleus | 1 | 0 |  |  |  |  |  |  |  |  |
| GO:0000072\_M\_phase\_specific\_microtubule\_process | 1 | 0 |  |  |  |  |  |  |  |  |
| GO:0000093\_mitotic\_telophase | 1 | 0 |  |  |  |  |  |  |  |  |
| GO:0000098\_sulfur\_amino\_acid\_catabolic\_process | 1 | 0 |  |  |  |  |  |  |  |  |
| GO:0000114\_regulation\_of\_transcription\_during\_G1\_phase\_of\_mitotic\_cell\_cycle | 1 | 0 |  |  |  |  |  |  |  |  |
| GO:0000115\_regulation\_of\_transcription\_during\_S-phase\_of\_mitotic\_cell\_cycle | 1 | 0 |  |  |  |  |  |  |  |  |
| GO:0000117\_regulation\_of\_transcription\_during\_G2\_M-phase\_of\_mitotic\_cell\_cycle | 1 | 0 |  |  |  |  |  |  |  |  |
| GO:0000132\_establishment\_of\_mitotic\_spindle\_orientation | 1 | 0 |  |  |  |  |  |  |  |  |
| GO:0000154\_rRNA\_modification | 1 | 0 |  |  |  |  |  |  |  |  |
| GO:0000160\_two-component\_signal\_transduction\_system\_(phosphorelay) | 1 | 0 |  |  |  |  |  |  |  |  |
| GO:0000161\_MAPKKK\_cascade\_involved\_in\_osmosensory\_signaling\_pathway | 1 | 0 |  |  |  |  |  |  |  |  |
| GO:0000173\_inactivation\_of\_MAPK\_activity\_involved\_in\_osmosensory\_signaling\_pathway | 1 | 0 |  |  |  |  |  |  |  |  |
| GO:0000212\_meiotic\_spindle\_organization | 1 | 0 |  |  |  |  |  |  |  |  |
| GO:0000255\_allantoin\_metabolic\_process | 1 | 0 |  |  |  |  |  |  |  |  |
| GO:0000270\_peptidoglycan\_metabolic\_process | 1 | 0 |  |  |  |  |  |  |  |  |
| GO:0000296\_spermine\_transport | 1 | 0 |  |  |  |  |  |  |  |  |
| GO:0000301\_retrograde\_transport\_\_vesicle\_recycling\_within\_Golgi | 1 | 0 |  |  |  |  |  |  |  |  |
| GO:0000303\_response\_to\_superoxide | 1 | 0 |  |  |  |  |  |  |  |  |
| GO:0000320\_re-entry\_into\_mitotic\_cell\_cycle | 1 | 0 |  |  |  |  |  |  |  |  |
| GO:0000338\_protein\_deneddylation | 1 | 0 |  |  |  |  |  |  |  |  |
| GO:0000395\_nuclear\_mRNA\_5'-splice\_site\_recognition | 1 | 0 |  |  |  |  |  |  |  |  |
| GO:0000710\_meiotic\_mismatch\_repair | 1 | 0 |  |  |  |  |  |  |  |  |
| GO:0000717\_nucleotide-excision\_repair\_\_DNA\_duplex\_unwinding | 1 | 0 |  |  |  |  |  |  |  |  |
| GO:0000722\_telomere\_maintenance\_via\_recombination | 1 | 0 |  |  |  |  |  |  |  |  |
| GO:0000746\_conjugation | 1 | 0 |  |  |  |  |  |  |  |  |
| GO:0000747\_conjugation\_with\_cellular\_fusion | 1 | 0 |  |  |  |  |  |  |  |  |
| GO:0000912\_formation\_of\_actomyosin\_apparatus\_involved\_in\_cytokinesis | 1 | 0 |  |  |  |  |  |  |  |  |
| GO:0000915\_cytokinesis\_\_contractile\_ring\_formation | 1 | 0 |  |  |  |  |  |  |  |  |
| GO:0000921\_septin\_ring\_assembly | 1 | 0 |  |  |  |  |  |  |  |  |
| GO:0000966\_RNA\_5'-end\_processing | 1 | 0 |  |  |  |  |  |  |  |  |
| GO:0001315\_age-dependent\_response\_to\_reactive\_oxygen\_species | 1 | 0 |  |  |  |  |  |  |  |  |
| GO:0001519\_peptide\_amidation | 1 | 0 |  |  |  |  |  |  |  |  |
| GO:0001560\_regulation\_of\_cell\_growth\_by\_extracellular\_stimulus | 1 | 0 |  |  |  |  |  |  |  |  |
| GO:0001574\_ganglioside\_biosynthetic\_process | 1 | 0 |  |  |  |  |  |  |  |  |
| GO:0001575\_globoside\_metabolic\_process | 1 | 0 |  |  |  |  |  |  |  |  |
| GO:0001658\_branching\_involved\_in\_ureteric\_bud\_morphogenesis | 1 | 0 |  |  |  |  |  |  |  |  |
| GO:0001662\_behavioral\_fear\_response | 1 | 0 |  |  |  |  |  |  |  |  |
| GO:0001675\_acrosome\_assembly | 1 | 0 |  |  |  |  |  |  |  |  |
| GO:0001692\_histamine\_metabolic\_process | 1 | 0 |  |  |  |  |  |  |  |  |
| GO:0001694\_histamine\_biosynthetic\_process | 1 | 0 |  |  |  |  |  |  |  |  |
| GO:0001732\_formation\_of\_translation\_initiation\_complex | 1 | 0 |  |  |  |  |  |  |  |  |
| GO:0001757\_somite\_specification | 1 | 0 |  |  |  |  |  |  |  |  |
| GO:0001774\_microglial\_cell\_activation | 1 | 0 |  |  |  |  |  |  |  |  |
| GO:0001782\_B\_cell\_homeostasis | 1 | 0 |  |  |  |  |  |  |  |  |
| GO:0001787\_natural\_killer\_cell\_proliferation | 1 | 0 |  |  |  |  |  |  |  |  |
| GO:0001823\_mesonephros\_development | 1 | 0 |  |  |  |  |  |  |  |  |
| GO:0001832\_blastocyst\_growth | 1 | 0 |  |  |  |  |  |  |  |  |
| GO:0001833\_inner\_cell\_mass\_cell\_proliferation | 1 | 0 |  |  |  |  |  |  |  |  |
| GO:0001839\_neural\_plate\_morphogenesis | 1 | 0 |  |  |  |  |  |  |  |  |
| GO:0001845\_phagolysosome\_formation | 1 | 0 |  |  |  |  |  |  |  |  |
| GO:0001865\_NK\_T\_cell\_differentiation | 1 | 0 |  |  |  |  |  |  |  |  |
| GO:0001866\_NK\_T\_cell\_proliferation | 1 | 0 |  |  |  |  |  |  |  |  |
| GO:0001887\_selenium\_metabolic\_process | 1 | 0 |  |  |  |  |  |  |  |  |
| GO:0001892\_embryonic\_placenta\_development | 1 | 0 |  |  |  |  |  |  |  |  |
| GO:0001911\_negative\_regulation\_of\_leukocyte\_mediated\_cytotoxicity | 1 | 0 |  |  |  |  |  |  |  |  |
| GO:0001915\_negative\_regulation\_of\_T\_cell\_mediated\_cytotoxicity | 1 | 0 |  |  |  |  |  |  |  |  |
| GO:0001920\_negative\_regulation\_of\_receptor\_recycling | 1 | 0 |  |  |  |  |  |  |  |  |
| GO:0001941\_postsynaptic\_membrane\_organization | 1 | 0 |  |  |  |  |  |  |  |  |
| GO:0001958\_endochondral\_ossification | 1 | 0 |  |  |  |  |  |  |  |  |
| GO:0001973\_adenosine\_receptor\_signaling\_pathway | 1 | 0 |  |  |  |  |  |  |  |  |
| GO:0001977\_renal\_system\_process\_involved\_in\_regulation\_of\_blood\_volume | 1 | 0 |  |  |  |  |  |  |  |  |
| GO:0001980\_regulation\_of\_systemic\_arterial\_blood\_pressure\_by\_ischemic\_conditions | 1 | 0 |  |  |  |  |  |  |  |  |
| GO:0001993\_regulation\_of\_systemic\_arterial\_blood\_pressure\_by\_norepinephrine-epinephrine | 1 | 0 |  |  |  |  |  |  |  |  |
| GO:0001996\_positive\_regulation\_of\_heart\_rate\_by\_epinephrine-norepinephrine | 1 | 0 |  |  |  |  |  |  |  |  |
| GO:0001999\_renal\_response\_to\_blood\_flow\_during\_renin-angiotensin\_regulation\_of\_systemic\_arterial\_blood\_pressure | 1 | 0 |  |  |  |  |  |  |  |  |
| GO:0002001\_renin\_secretion\_into\_blood\_stream | 1 | 0 |  |  |  |  |  |  |  |  |
| GO:0002017\_regulation\_of\_blood\_volume\_by\_renal\_aldosterone | 1 | 0 |  |  |  |  |  |  |  |  |
| GO:0002018\_renin-angiotensin\_regulation\_of\_aldosterone\_production | 1 | 0 |  |  |  |  |  |  |  |  |
| GO:0002031\_G-protein\_coupled\_receptor\_internalization | 1 | 0 |  |  |  |  |  |  |  |  |
| GO:0002035\_brain\_renin-angiotensin\_system | 1 | 0 |  |  |  |  |  |  |  |  |
| GO:0002042\_cell\_migration\_involved\_in\_sprouting\_angiogenesis | 1 | 0 |  |  |  |  |  |  |  |  |
| GO:0002052\_positive\_regulation\_of\_neuroblast\_proliferation | 1 | 0 |  |  |  |  |  |  |  |  |
| GO:0002053\_positive\_regulation\_of\_mesenchymal\_cell\_proliferation | 1 | 0 |  |  |  |  |  |  |  |  |
| GO:0002063\_chondrocyte\_development | 1 | 0 |  |  |  |  |  |  |  |  |
| GO:0002064\_epithelial\_cell\_development | 1 | 0 |  |  |  |  |  |  |  |  |
| GO:0002074\_extraocular\_skeletal\_muscle\_development | 1 | 0 |  |  |  |  |  |  |  |  |
| GO:0002077\_acrosome\_matrix\_dispersal | 1 | 0 |  |  |  |  |  |  |  |  |
| GO:0002082\_regulation\_of\_oxidative\_phosphorylation | 1 | 0 |  |  |  |  |  |  |  |  |
| GO:0002084\_protein\_depalmitoylation | 1 | 0 |  |  |  |  |  |  |  |  |
| GO:0002088\_lens\_development\_in\_camera-type\_eye | 1 | 0 |  |  |  |  |  |  |  |  |
| GO:0002089\_lens\_morphogenesis\_in\_camera-type\_eye | 1 | 0 |  |  |  |  |  |  |  |  |
| GO:0002093\_auditory\_receptor\_cell\_morphogenesis | 1 | 0 |  |  |  |  |  |  |  |  |
| GO:0002209\_behavioral\_defense\_response | 1 | 0 |  |  |  |  |  |  |  |  |
| GO:0002220\_innate\_immune\_response\_activating\_cell\_surface\_receptor\_signaling\_pathway | 1 | 0 |  |  |  |  |  |  |  |  |
| GO:0002223\_stimulatory\_C-type\_lectin\_receptor\_signaling\_pathway | 1 | 0 |  |  |  |  |  |  |  |  |
| GO:0002312\_B\_cell\_activation\_during\_immune\_response | 1 | 0 |  |  |  |  |  |  |  |  |
| GO:0002313\_mature\_B\_cell\_differentiation\_during\_immune\_response | 1 | 0 |  |  |  |  |  |  |  |  |
| GO:0002318\_myeloid\_progenitor\_cell\_differentiation | 1 | 0 |  |  |  |  |  |  |  |  |
| GO:0002320\_lymphoid\_progenitor\_cell\_differentiation | 1 | 0 |  |  |  |  |  |  |  |  |
| GO:0002326\_B\_cell\_lineage\_commitment | 1 | 0 |  |  |  |  |  |  |  |  |
| GO:0002328\_pro-B\_cell\_differentiation | 1 | 0 |  |  |  |  |  |  |  |  |
| GO:0002335\_mature\_B\_cell\_differentiation | 1 | 0 |  |  |  |  |  |  |  |  |
| GO:0002355\_detection\_of\_tumor\_cell | 1 | 0 |  |  |  |  |  |  |  |  |
| GO:0002368\_B\_cell\_cytokine\_production | 1 | 0 |  |  |  |  |  |  |  |  |
| GO:0002424\_T\_cell\_mediated\_immune\_response\_to\_tumor\_cell | 1 | 0 |  |  |  |  |  |  |  |  |
| GO:0002431\_Fc\_receptor\_mediated\_stimulatory\_signaling\_pathway | 1 | 0 |  |  |  |  |  |  |  |  |
| GO:0002437\_inflammatory\_response\_to\_antigenic\_stimulus | 1 | 0 |  |  |  |  |  |  |  |  |
| GO:0002439\_chronic\_inflammatory\_response\_to\_antigenic\_stimulus | 1 | 0 |  |  |  |  |  |  |  |  |
| GO:0002447\_eosinophil\_mediated\_immunity | 1 | 0 |  |  |  |  |  |  |  |  |
| GO:0002455\_humoral\_immune\_response\_mediated\_by\_circulating\_immunoglobulin | 1 | 0 |  |  |  |  |  |  |  |  |
| GO:0002467\_germinal\_center\_formation | 1 | 0 |  |  |  |  |  |  |  |  |
| GO:0002468\_dendritic\_cell\_antigen\_processing\_and\_presentation | 1 | 0 |  |  |  |  |  |  |  |  |
| GO:0002475\_antigen\_processing\_and\_presentation\_via\_MHC\_class\_Ib | 1 | 0 |  |  |  |  |  |  |  |  |
| GO:0002478\_antigen\_processing\_and\_presentation\_of\_exogenous\_peptide\_antigen | 1 | 0 |  |  |  |  |  |  |  |  |
| GO:0002495\_antigen\_processing\_and\_presentation\_of\_peptide\_antigen\_via\_MHC\_class\_II | 1 | 0 |  |  |  |  |  |  |  |  |
| GO:0002513\_tolerance\_induction\_to\_self\_antigen | 1 | 0 |  |  |  |  |  |  |  |  |
| GO:0002514\_B\_cell\_tolerance\_induction | 1 | 0 |  |  |  |  |  |  |  |  |
| GO:0002517\_T\_cell\_tolerance\_induction | 1 | 0 |  |  |  |  |  |  |  |  |
| GO:0002523\_leukocyte\_migration\_during\_inflammatory\_response | 1 | 0 |  |  |  |  |  |  |  |  |
| GO:0002566\_somatic\_diversification\_of\_immune\_receptors\_via\_somatic\_mutation | 1 | 0 |  |  |  |  |  |  |  |  |
| GO:0002568\_somatic\_diversification\_of\_T\_cell\_receptor\_genes | 1 | 0 |  |  |  |  |  |  |  |  |
| GO:0002576\_platelet\_degranulation | 1 | 0 |  |  |  |  |  |  |  |  |
| GO:0002577\_regulation\_of\_antigen\_processing\_and\_presentation | 1 | 0 |  |  |  |  |  |  |  |  |
| GO:0002578\_negative\_regulation\_of\_antigen\_processing\_and\_presentation | 1 | 0 |  |  |  |  |  |  |  |  |
| GO:0002580\_regulation\_of\_antigen\_processing\_and\_presentation\_of\_peptide\_or\_polysaccharide\_antigen\_via\_MHC\_class\_II | 1 | 0 |  |  |  |  |  |  |  |  |
| GO:0002581\_negative\_regulation\_of\_antigen\_processing\_and\_presentation\_of\_peptide\_or\_polysaccharide\_antigen\_via\_MHC\_class\_II | 1 | 0 |  |  |  |  |  |  |  |  |
| GO:0002604\_regulation\_of\_dendritic\_cell\_antigen\_processing\_and\_presentation | 1 | 0 |  |  |  |  |  |  |  |  |
| GO:0002605\_negative\_regulation\_of\_dendritic\_cell\_antigen\_processing\_and\_presentation | 1 | 0 |  |  |  |  |  |  |  |  |
| GO:0002634\_regulation\_of\_germinal\_center\_formation | 1 | 0 |  |  |  |  |  |  |  |  |
| GO:0002649\_regulation\_of\_tolerance\_induction\_to\_self\_antigen | 1 | 0 |  |  |  |  |  |  |  |  |
| GO:0002651\_positive\_regulation\_of\_tolerance\_induction\_to\_self\_antigen | 1 | 0 |  |  |  |  |  |  |  |  |
| GO:0002661\_regulation\_of\_B\_cell\_tolerance\_induction | 1 | 0 |  |  |  |  |  |  |  |  |
| GO:0002663\_positive\_regulation\_of\_B\_cell\_tolerance\_induction | 1 | 0 |  |  |  |  |  |  |  |  |
| GO:0002664\_regulation\_of\_T\_cell\_tolerance\_induction | 1 | 0 |  |  |  |  |  |  |  |  |
| GO:0002666\_positive\_regulation\_of\_T\_cell\_tolerance\_induction | 1 | 0 |  |  |  |  |  |  |  |  |
| GO:0002674\_negative\_regulation\_of\_acute\_inflammatory\_response | 1 | 0 |  |  |  |  |  |  |  |  |
| GO:0002681\_somatic\_recombination\_of\_T\_cell\_receptor\_gene\_segments | 1 | 0 |  |  |  |  |  |  |  |  |
| GO:0002686\_negative\_regulation\_of\_leukocyte\_migration | 1 | 0 |  |  |  |  |  |  |  |  |
| GO:0002691\_regulation\_of\_cellular\_extravasation | 1 | 0 |  |  |  |  |  |  |  |  |
| GO:0002693\_positive\_regulation\_of\_cellular\_extravasation | 1 | 0 |  |  |  |  |  |  |  |  |
| GO:0002701\_negative\_regulation\_of\_production\_of\_molecular\_mediator\_of\_immune\_response | 1 | 0 |  |  |  |  |  |  |  |  |
| GO:0002719\_negative\_regulation\_of\_cytokine\_production\_during\_immune\_response | 1 | 0 |  |  |  |  |  |  |  |  |
| GO:0002725\_negative\_regulation\_of\_T\_cell\_cytokine\_production | 1 | 0 |  |  |  |  |  |  |  |  |
| GO:0002759\_regulation\_of\_antimicrobial\_humoral\_response | 1 | 0 |  |  |  |  |  |  |  |  |
| GO:0002775\_antimicrobial\_peptide\_production | 1 | 0 |  |  |  |  |  |  |  |  |
| GO:0002777\_antimicrobial\_peptide\_biosynthetic\_process | 1 | 0 |  |  |  |  |  |  |  |  |
| GO:0002778\_antibacterial\_peptide\_production | 1 | 0 |  |  |  |  |  |  |  |  |
| GO:0002780\_antibacterial\_peptide\_biosynthetic\_process | 1 | 0 |  |  |  |  |  |  |  |  |
| GO:0002784\_regulation\_of\_antimicrobial\_peptide\_production | 1 | 0 |  |  |  |  |  |  |  |  |
| GO:0002786\_regulation\_of\_antibacterial\_peptide\_production | 1 | 0 |  |  |  |  |  |  |  |  |
| GO:0002805\_regulation\_of\_antimicrobial\_peptide\_biosynthetic\_process | 1 | 0 |  |  |  |  |  |  |  |  |
| GO:0002807\_positive\_regulation\_of\_antimicrobial\_peptide\_biosynthetic\_process | 1 | 0 |  |  |  |  |  |  |  |  |
| GO:0002808\_regulation\_of\_antibacterial\_peptide\_biosynthetic\_process | 1 | 0 |  |  |  |  |  |  |  |  |
| GO:0002815\_biosynthetic\_process\_of\_antibacterial\_peptides\_active\_against\_Gram-positive\_bacteria | 1 | 0 |  |  |  |  |  |  |  |  |
| GO:0002816\_regulation\_of\_biosynthetic\_process\_of\_antibacterial\_peptides\_active\_against\_Gram-positive\_bacteria | 1 | 0 |  |  |  |  |  |  |  |  |
| GO:0002832\_negative\_regulation\_of\_response\_to\_biotic\_stimulus | 1 | 0 |  |  |  |  |  |  |  |  |
| GO:0002840\_regulation\_of\_T\_cell\_mediated\_immune\_response\_to\_tumor\_cell | 1 | 0 |  |  |  |  |  |  |  |  |
| GO:0002842\_positive\_regulation\_of\_T\_cell\_mediated\_immune\_response\_to\_tumor\_cell | 1 | 0 |  |  |  |  |  |  |  |  |
| GO:0002901\_mature\_B\_cell\_apoptosis | 1 | 0 |  |  |  |  |  |  |  |  |
| GO:0002904\_positive\_regulation\_of\_B\_cell\_apoptosis | 1 | 0 |  |  |  |  |  |  |  |  |
| GO:0002905\_regulation\_of\_mature\_B\_cell\_apoptosis | 1 | 0 |  |  |  |  |  |  |  |  |
| GO:0002906\_negative\_regulation\_of\_mature\_B\_cell\_apoptosis | 1 | 0 |  |  |  |  |  |  |  |  |
| GO:0003010\_voluntary\_skeletal\_muscle\_contraction | 1 | 0 |  |  |  |  |  |  |  |  |
| GO:0003051\_angiotensin-mediated\_drinking\_behavior | 1 | 0 |  |  |  |  |  |  |  |  |
| GO:0003058\_hormonal\_regulation\_of\_the\_force\_of\_heart\_contraction | 1 | 0 |  |  |  |  |  |  |  |  |
| GO:0003062\_regulation\_of\_heart\_rate\_by\_chemical\_signal | 1 | 0 |  |  |  |  |  |  |  |  |
| GO:0003065\_positive\_regulation\_of\_heart\_rate\_by\_epinephrine | 1 | 0 |  |  |  |  |  |  |  |  |
| GO:0003071\_renal\_system\_process\_involved\_in\_regulation\_of\_systemic\_arterial\_blood\_pressure | 1 | 0 |  |  |  |  |  |  |  |  |
| GO:0003085\_negative\_regulation\_of\_systemic\_arterial\_blood\_pressure | 1 | 0 |  |  |  |  |  |  |  |  |
| GO:0003099\_positive\_regulation\_of\_the\_force\_of\_heart\_contraction\_by\_chemical\_signal | 1 | 0 |  |  |  |  |  |  |  |  |
| GO:0003108\_negative\_regulation\_of\_the\_force\_of\_heart\_contraction\_by\_chemical\_signal | 1 | 0 |  |  |  |  |  |  |  |  |
| GO:0005981\_regulation\_of\_glycogen\_catabolic\_process | 1 | 0 |  |  |  |  |  |  |  |  |
| GO:0005982\_starch\_metabolic\_process | 1 | 0 |  |  |  |  |  |  |  |  |
| GO:0005983\_starch\_catabolic\_process | 1 | 0 |  |  |  |  |  |  |  |  |
| GO:0005988\_lactose\_metabolic\_process | 1 | 0 |  |  |  |  |  |  |  |  |
| GO:0005989\_lactose\_biosynthetic\_process | 1 | 0 |  |  |  |  |  |  |  |  |
| GO:0005991\_trehalose\_metabolic\_process | 1 | 0 |  |  |  |  |  |  |  |  |
| GO:0005993\_trehalose\_catabolic\_process | 1 | 0 |  |  |  |  |  |  |  |  |
| GO:0006010\_glucose\_6-phosphate\_utilization | 1 | 0 |  |  |  |  |  |  |  |  |
| GO:0006013\_mannose\_metabolic\_process | 1 | 0 |  |  |  |  |  |  |  |  |
| GO:0006021\_inositol\_biosynthetic\_process | 1 | 0 |  |  |  |  |  |  |  |  |
| GO:0006037\_cell\_wall\_chitin\_metabolic\_process | 1 | 0 |  |  |  |  |  |  |  |  |
| GO:0006042\_glucosamine\_biosynthetic\_process | 1 | 0 |  |  |  |  |  |  |  |  |
| GO:0006045\_N-acetylglucosamine\_biosynthetic\_process | 1 | 0 |  |  |  |  |  |  |  |  |
| GO:0006048\_UDP-N-acetylglucosamine\_biosynthetic\_process | 1 | 0 |  |  |  |  |  |  |  |  |
| GO:0006050\_mannosamine\_metabolic\_process | 1 | 0 |  |  |  |  |  |  |  |  |
| GO:0006051\_N-acetylmannosamine\_metabolic\_process | 1 | 0 |  |  |  |  |  |  |  |  |
| GO:0006059\_hexitol\_metabolic\_process | 1 | 0 |  |  |  |  |  |  |  |  |
| GO:0006060\_sorbitol\_metabolic\_process | 1 | 0 |  |  |  |  |  |  |  |  |
| GO:0006062\_sorbitol\_catabolic\_process | 1 | 0 |  |  |  |  |  |  |  |  |
| GO:0006065\_UDP-glucuronate\_biosynthetic\_process | 1 | 0 |  |  |  |  |  |  |  |  |
| GO:0006083\_acetate\_metabolic\_process | 1 | 0 |  |  |  |  |  |  |  |  |
| GO:0006085\_acetyl-CoA\_biosynthetic\_process | 1 | 0 |  |  |  |  |  |  |  |  |
| GO:0006103\_2-oxoglutarate\_metabolic\_process | 1 | 0 |  |  |  |  |  |  |  |  |
| GO:0006106\_fumarate\_metabolic\_process | 1 | 0 |  |  |  |  |  |  |  |  |
| GO:0006107\_oxaloacetate\_metabolic\_process | 1 | 0 |  |  |  |  |  |  |  |  |
| GO:0006116\_NADH\_oxidation | 1 | 0 |  |  |  |  |  |  |  |  |
| GO:0006145\_purine\_base\_catabolic\_process | 1 | 0 |  |  |  |  |  |  |  |  |
| GO:0006148\_inosine\_catabolic\_process | 1 | 0 |  |  |  |  |  |  |  |  |
| GO:0006154\_adenosine\_catabolic\_process | 1 | 0 |  |  |  |  |  |  |  |  |
| GO:0006166\_purine\_ribonucleoside\_salvage | 1 | 0 |  |  |  |  |  |  |  |  |
| GO:0006172\_ADP\_biosynthetic\_process | 1 | 0 |  |  |  |  |  |  |  |  |
| GO:0006173\_dADP\_biosynthetic\_process | 1 | 0 |  |  |  |  |  |  |  |  |
| GO:0006188\_IMP\_biosynthetic\_process | 1 | 0 |  |  |  |  |  |  |  |  |
| GO:0006189\_'de\_novo'\_IMP\_biosynthetic\_process | 1 | 0 |  |  |  |  |  |  |  |  |
| GO:0006196\_AMP\_catabolic\_process | 1 | 0 |  |  |  |  |  |  |  |  |
| GO:0006198\_cAMP\_catabolic\_process | 1 | 0 |  |  |  |  |  |  |  |  |
| GO:0006207\_'de\_novo'\_pyrimidine\_base\_biosynthetic\_process | 1 | 0 |  |  |  |  |  |  |  |  |
| GO:0006214\_thymidine\_catabolic\_process | 1 | 0 |  |  |  |  |  |  |  |  |
| GO:0006216\_cytidine\_catabolic\_process | 1 | 0 |  |  |  |  |  |  |  |  |
| GO:0006222\_UMP\_biosynthetic\_process | 1 | 0 |  |  |  |  |  |  |  |  |
| GO:0006241\_CTP\_biosynthetic\_process | 1 | 0 |  |  |  |  |  |  |  |  |
| GO:0006256\_UDP\_catabolic\_process | 1 | 0 |  |  |  |  |  |  |  |  |
| GO:0006265\_DNA\_topological\_change | 1 | 0 |  |  |  |  |  |  |  |  |
| GO:0006272\_leading\_strand\_elongation | 1 | 0 |  |  |  |  |  |  |  |  |
| GO:0006287\_base-excision\_repair\_\_gap-filling | 1 | 0 |  |  |  |  |  |  |  |  |
| GO:0006313\_transposition\_\_DNA-mediated | 1 | 0 |  |  |  |  |  |  |  |  |
| GO:0006336\_DNA\_replication-independent\_nucleosome\_assembly | 1 | 0 |  |  |  |  |  |  |  |  |
| GO:0006343\_establishment\_of\_chromatin\_silencing | 1 | 0 |  |  |  |  |  |  |  |  |
| GO:0006344\_maintenance\_of\_chromatin\_silencing | 1 | 0 |  |  |  |  |  |  |  |  |
| GO:0006346\_methylation-dependent\_chromatin\_silencing | 1 | 0 |  |  |  |  |  |  |  |  |
| GO:0006348\_chromatin\_silencing\_at\_telomere | 1 | 0 |  |  |  |  |  |  |  |  |
| GO:0006361\_transcription\_initiation\_from\_RNA\_polymerase\_I\_promoter | 1 | 0 |  |  |  |  |  |  |  |  |
| GO:0006369\_termination\_of\_RNA\_polymerase\_II\_transcription | 1 | 0 |  |  |  |  |  |  |  |  |
| GO:0006393\_termination\_of\_mitochondrial\_transcription | 1 | 0 |  |  |  |  |  |  |  |  |
| GO:0006407\_rRNA\_export\_from\_nucleus | 1 | 0 |  |  |  |  |  |  |  |  |
| GO:0006408\_snRNA\_export\_from\_nucleus | 1 | 0 |  |  |  |  |  |  |  |  |
| GO:0006409\_tRNA\_export\_from\_nucleus | 1 | 0 |  |  |  |  |  |  |  |  |
| GO:0006419\_alanyl-tRNA\_aminoacylation | 1 | 0 |  |  |  |  |  |  |  |  |
| GO:0006420\_arginyl-tRNA\_aminoacylation | 1 | 0 |  |  |  |  |  |  |  |  |
| GO:0006423\_cysteinyl-tRNA\_aminoacylation | 1 | 0 |  |  |  |  |  |  |  |  |
| GO:0006431\_methionyl-tRNA\_aminoacylation | 1 | 0 |  |  |  |  |  |  |  |  |
| GO:0006432\_phenylalanyl-tRNA\_aminoacylation | 1 | 0 |  |  |  |  |  |  |  |  |
| GO:0006434\_seryl-tRNA\_aminoacylation | 1 | 0 |  |  |  |  |  |  |  |  |
| GO:0006435\_threonyl-tRNA\_aminoacylation | 1 | 0 |  |  |  |  |  |  |  |  |
| GO:0006436\_tryptophanyl-tRNA\_aminoacylation | 1 | 0 |  |  |  |  |  |  |  |  |
| GO:0006437\_tyrosyl-tRNA\_aminoacylation | 1 | 0 |  |  |  |  |  |  |  |  |
| GO:0006447\_regulation\_of\_translational\_initiation\_by\_iron | 1 | 0 |  |  |  |  |  |  |  |  |
| GO:0006448\_regulation\_of\_translational\_elongation | 1 | 0 |  |  |  |  |  |  |  |  |
| GO:0006450\_regulation\_of\_translational\_fidelity | 1 | 0 |  |  |  |  |  |  |  |  |
| GO:0006494\_protein\_amino\_acid\_terminal\_glycosylation | 1 | 0 |  |  |  |  |  |  |  |  |
| GO:0006496\_protein\_amino\_acid\_terminal\_N-glycosylation | 1 | 0 |  |  |  |  |  |  |  |  |
| GO:0006499\_N-terminal\_protein\_myristoylation | 1 | 0 |  |  |  |  |  |  |  |  |
| GO:0006500\_N-terminal\_protein\_palmitoylation | 1 | 0 |  |  |  |  |  |  |  |  |
| GO:0006526\_arginine\_biosynthetic\_process | 1 | 0 |  |  |  |  |  |  |  |  |
| GO:0006528\_asparagine\_metabolic\_process | 1 | 0 |  |  |  |  |  |  |  |  |
| GO:0006530\_asparagine\_catabolic\_process | 1 | 0 |  |  |  |  |  |  |  |  |
| GO:0006534\_cysteine\_metabolic\_process | 1 | 0 |  |  |  |  |  |  |  |  |
| GO:0006543\_glutamine\_catabolic\_process | 1 | 0 |  |  |  |  |  |  |  |  |
| GO:0006545\_glycine\_biosynthetic\_process | 1 | 0 |  |  |  |  |  |  |  |  |
| GO:0006547\_histidine\_metabolic\_process | 1 | 0 |  |  |  |  |  |  |  |  |
| GO:0006549\_isoleucine\_metabolic\_process | 1 | 0 |  |  |  |  |  |  |  |  |
| GO:0006556\_S-adenosylmethionine\_biosynthetic\_process | 1 | 0 |  |  |  |  |  |  |  |  |
| GO:0006562\_proline\_catabolic\_process | 1 | 0 |  |  |  |  |  |  |  |  |
| GO:0006564\_L-serine\_biosynthetic\_process | 1 | 0 |  |  |  |  |  |  |  |  |
| GO:0006577\_betaine\_metabolic\_process | 1 | 0 |  |  |  |  |  |  |  |  |
| GO:0006580\_ethanolamine\_metabolic\_process | 1 | 0 |  |  |  |  |  |  |  |  |
| GO:0006585\_dopamine\_biosynthetic\_process\_from\_tyrosine | 1 | 0 |  |  |  |  |  |  |  |  |
| GO:0006591\_ornithine\_metabolic\_process | 1 | 0 |  |  |  |  |  |  |  |  |
| GO:0006597\_spermine\_biosynthetic\_process | 1 | 0 |  |  |  |  |  |  |  |  |
| GO:0006598\_polyamine\_catabolic\_process | 1 | 0 |  |  |  |  |  |  |  |  |
| GO:0006610\_ribosomal\_protein\_import\_into\_nucleus | 1 | 0 |  |  |  |  |  |  |  |  |
| GO:0006614\_SRP-dependent\_cotranslational\_protein\_targeting\_to\_membrane | 1 | 0 |  |  |  |  |  |  |  |  |
| GO:0006616\_SRP-dependent\_cotranslational\_protein\_targeting\_to\_membrane\_\_translocation | 1 | 0 |  |  |  |  |  |  |  |  |
| GO:0006617\_SRP-dependent\_cotranslational\_protein\_targeting\_to\_membrane\_\_signal\_sequence\_recognition | 1 | 0 |  |  |  |  |  |  |  |  |
| GO:0006627\_mitochondrial\_protein\_processing\_during\_import | 1 | 0 |  |  |  |  |  |  |  |  |
| GO:0006646\_phosphatidylethanolamine\_biosynthetic\_process | 1 | 0 |  |  |  |  |  |  |  |  |
| GO:0006655\_phosphatidylglycerol\_biosynthetic\_process | 1 | 0 |  |  |  |  |  |  |  |  |
| GO:0006657\_CDP-choline\_pathway | 1 | 0 |  |  |  |  |  |  |  |  |
| GO:0006667\_sphinganine\_metabolic\_process | 1 | 0 |  |  |  |  |  |  |  |  |
| GO:0006668\_sphinganine-1-phosphate\_metabolic\_process | 1 | 0 |  |  |  |  |  |  |  |  |
| GO:0006669\_sphinganine-1-phosphate\_biosynthetic\_process | 1 | 0 |  |  |  |  |  |  |  |  |
| GO:0006670\_sphingosine\_metabolic\_process | 1 | 0 |  |  |  |  |  |  |  |  |
| GO:0006689\_ganglioside\_catabolic\_process | 1 | 0 |  |  |  |  |  |  |  |  |
| GO:0006711\_estrogen\_catabolic\_process | 1 | 0 |  |  |  |  |  |  |  |  |
| GO:0006713\_glucocorticoid\_catabolic\_process | 1 | 0 |  |  |  |  |  |  |  |  |
| GO:0006734\_NADH\_metabolic\_process | 1 | 0 |  |  |  |  |  |  |  |  |
| GO:0006741\_NADP\_biosynthetic\_process | 1 | 0 |  |  |  |  |  |  |  |  |
| GO:0006746\_FADH2\_metabolic\_process | 1 | 0 |  |  |  |  |  |  |  |  |
| GO:0006768\_biotin\_metabolic\_process | 1 | 0 |  |  |  |  |  |  |  |  |
| GO:0006771\_riboflavin\_metabolic\_process | 1 | 0 |  |  |  |  |  |  |  |  |
| GO:0006781\_succinyl-CoA\_pathway | 1 | 0 |  |  |  |  |  |  |  |  |
| GO:0006789\_bilirubin\_conjugation | 1 | 0 |  |  |  |  |  |  |  |  |
| GO:0006797\_polyphosphate\_metabolic\_process | 1 | 0 |  |  |  |  |  |  |  |  |
| GO:0006837\_serotonin\_transport | 1 | 0 |  |  |  |  |  |  |  |  |
| GO:0006842\_tricarboxylic\_acid\_transport | 1 | 0 |  |  |  |  |  |  |  |  |
| GO:0006843\_mitochondrial\_citrate\_transport | 1 | 0 |  |  |  |  |  |  |  |  |
| GO:0006848\_pyruvate\_transport | 1 | 0 |  |  |  |  |  |  |  |  |
| GO:0006862\_nucleotide\_transport | 1 | 0 |  |  |  |  |  |  |  |  |
| GO:0006867\_asparagine\_transport | 1 | 0 |  |  |  |  |  |  |  |  |
| GO:0006868\_glutamine\_transport | 1 | 0 |  |  |  |  |  |  |  |  |
| GO:0006876\_cellular\_cadmium\_ion\_homeostasis | 1 | 0 |  |  |  |  |  |  |  |  |
| GO:0006926\_virus-infected\_cell\_apoptosis | 1 | 0 |  |  |  |  |  |  |  |  |
| GO:0006931\_substrate-bound\_cell\_migration\_\_cell\_attachment\_to\_substrate | 1 | 0 |  |  |  |  |  |  |  |  |
| GO:0006948\_induction\_by\_virus\_of\_host\_cell-cell\_fusion | 1 | 0 |  |  |  |  |  |  |  |  |
| GO:0006958\_complement\_activation\_\_classical\_pathway | 1 | 0 |  |  |  |  |  |  |  |  |
| GO:0006963\_positive\_regulation\_of\_antibacterial\_peptide\_biosynthetic\_process | 1 | 0 |  |  |  |  |  |  |  |  |
| GO:0006965\_positive\_regulation\_of\_biosynthetic\_process\_of\_antibacterial\_peptides\_active\_against\_Gram-positive\_bacteria | 1 | 0 |  |  |  |  |  |  |  |  |
| GO:0006987\_activation\_of\_signaling\_protein\_activity\_involved\_in\_unfolded\_protein\_response | 1 | 0 |  |  |  |  |  |  |  |  |
| GO:0006990\_positive\_regulation\_of\_gene-specific\_transcription\_involved\_in\_unfolded\_protein\_response | 1 | 0 |  |  |  |  |  |  |  |  |
| GO:0006991\_response\_to\_sterol\_depletion | 1 | 0 |  |  |  |  |  |  |  |  |
| GO:0006994\_positive\_regulation\_of\_transcription\_via\_sterol\_regulatory\_element\_binding\_involved\_in\_ER-nuclear\_sterol\_response\_pathway | 1 | 0 |  |  |  |  |  |  |  |  |
| GO:0007039\_vacuolar\_protein\_catabolic\_process | 1 | 0 |  |  |  |  |  |  |  |  |
| GO:0007068\_negative\_regulation\_of\_transcription\_\_mitotic | 1 | 0 |  |  |  |  |  |  |  |  |
| GO:0007097\_nuclear\_migration | 1 | 0 |  |  |  |  |  |  |  |  |
| GO:0007100\_mitotic\_centrosome\_separation | 1 | 0 |  |  |  |  |  |  |  |  |
| GO:0007108\_cytokinesis\_\_initiation\_of\_separation | 1 | 0 |  |  |  |  |  |  |  |  |
| GO:0007109\_cytokinesis\_\_completion\_of\_separation | 1 | 0 |  |  |  |  |  |  |  |  |
| GO:0007132\_meiotic\_metaphase\_I | 1 | 0 |  |  |  |  |  |  |  |  |
| GO:0007135\_meiosis\_II | 1 | 0 |  |  |  |  |  |  |  |  |
| GO:0007136\_meiotic\_prophase\_II | 1 | 0 |  |  |  |  |  |  |  |  |
| GO:0007161\_calcium-independent\_cell-matrix\_adhesion | 1 | 0 |  |  |  |  |  |  |  |  |
| GO:0007196\_inhibition\_of\_adenylate\_cyclase\_activity\_by\_metabotropic\_glutamate\_receptor\_signaling\_pathway | 1 | 0 |  |  |  |  |  |  |  |  |
| GO:0007197\_inhibition\_of\_adenylate\_cyclase\_activity\_by\_muscarinic\_acetylcholine\_receptor\_signaling\_pathway | 1 | 0 |  |  |  |  |  |  |  |  |
| GO:0007258\_JUN\_phosphorylation | 1 | 0 |  |  |  |  |  |  |  |  |
| GO:0007321\_sperm\_displacement | 1 | 0 |  |  |  |  |  |  |  |  |
| GO:0007343\_egg\_activation | 1 | 0 |  |  |  |  |  |  |  |  |
| GO:0007386\_compartment\_specification | 1 | 0 |  |  |  |  |  |  |  |  |
| GO:0007387\_anterior\_compartment\_specification | 1 | 0 |  |  |  |  |  |  |  |  |
| GO:0007388\_posterior\_compartment\_specification | 1 | 0 |  |  |  |  |  |  |  |  |
| GO:0007402\_ganglion\_mother\_cell\_fate\_determination | 1 | 0 |  |  |  |  |  |  |  |  |
| GO:0007406\_negative\_regulation\_of\_neuroblast\_proliferation | 1 | 0 |  |  |  |  |  |  |  |  |
| GO:0007424\_open\_tracheal\_system\_development | 1 | 0 |  |  |  |  |  |  |  |  |
| GO:0007440\_foregut\_morphogenesis | 1 | 0 |  |  |  |  |  |  |  |  |
| GO:0007443\_Malpighian\_tubule\_morphogenesis | 1 | 0 |  |  |  |  |  |  |  |  |
| GO:0007444\_imaginal\_disc\_development | 1 | 0 |  |  |  |  |  |  |  |  |
| GO:0007447\_imaginal\_disc\_pattern\_formation | 1 | 0 |  |  |  |  |  |  |  |  |
| GO:0007494\_midgut\_development | 1 | 0 |  |  |  |  |  |  |  |  |
| GO:0007497\_posterior\_midgut\_development | 1 | 0 |  |  |  |  |  |  |  |  |
| GO:0007499\_ectoderm\_and\_mesoderm\_interaction | 1 | 0 |  |  |  |  |  |  |  |  |
| GO:0007501\_mesodermal\_cell\_fate\_specification | 1 | 0 |  |  |  |  |  |  |  |  |
| GO:0007509\_mesoderm\_migration | 1 | 0 |  |  |  |  |  |  |  |  |
| GO:0007518\_myoblast\_cell\_fate\_determination | 1 | 0 |  |  |  |  |  |  |  |  |
| GO:0007538\_primary\_sex\_determination | 1 | 0 |  |  |  |  |  |  |  |  |
| GO:0007597\_blood\_coagulation\_\_intrinsic\_pathway | 1 | 0 |  |  |  |  |  |  |  |  |
| GO:0007616\_long-term\_memory | 1 | 0 |  |  |  |  |  |  |  |  |
| GO:0007617\_mating\_behavior | 1 | 0 |  |  |  |  |  |  |  |  |
| GO:0007624\_ultradian\_rhythm | 1 | 0 |  |  |  |  |  |  |  |  |
| GO:0007638\_mechanosensory\_behavior | 1 | 0 |  |  |  |  |  |  |  |  |
| GO:0008045\_motor\_axon\_guidance | 1 | 0 |  |  |  |  |  |  |  |  |
| GO:0008057\_eye\_pigment\_granule\_organization | 1 | 0 |  |  |  |  |  |  |  |  |
| GO:0008063\_Toll\_signaling\_pathway | 1 | 0 |  |  |  |  |  |  |  |  |
| GO:0008065\_establishment\_of\_blood-nerve\_barrier | 1 | 0 |  |  |  |  |  |  |  |  |
| GO:0008090\_retrograde\_axon\_cargo\_transport | 1 | 0 |  |  |  |  |  |  |  |  |
| GO:0008215\_spermine\_metabolic\_process | 1 | 0 |  |  |  |  |  |  |  |  |
| GO:0008292\_acetylcholine\_biosynthetic\_process | 1 | 0 |  |  |  |  |  |  |  |  |
| GO:0008295\_spermidine\_biosynthetic\_process | 1 | 0 |  |  |  |  |  |  |  |  |
| GO:0008298\_intracellular\_mRNA\_localization | 1 | 0 |  |  |  |  |  |  |  |  |
| GO:0008356\_asymmetric\_cell\_division | 1 | 0 |  |  |  |  |  |  |  |  |
| GO:0008592\_regulation\_of\_Toll\_signaling\_pathway | 1 | 0 |  |  |  |  |  |  |  |  |
| GO:0008611\_ether\_lipid\_biosynthetic\_process | 1 | 0 |  |  |  |  |  |  |  |  |
| GO:0008614\_pyridoxine\_metabolic\_process | 1 | 0 |  |  |  |  |  |  |  |  |
| GO:0008615\_pyridoxine\_biosynthetic\_process | 1 | 0 |  |  |  |  |  |  |  |  |
| GO:0008627\_induction\_of\_apoptosis\_by\_ionic\_changes | 1 | 0 |  |  |  |  |  |  |  |  |
| GO:0008655\_pyrimidine\_salvage | 1 | 0 |  |  |  |  |  |  |  |  |
| GO:0009052\_pentose-phosphate\_shunt\_\_non-oxidative\_branch | 1 | 0 |  |  |  |  |  |  |  |  |
| GO:0009067\_aspartate\_family\_amino\_acid\_biosynthetic\_process | 1 | 0 |  |  |  |  |  |  |  |  |
| GO:0009075\_histidine\_family\_amino\_acid\_metabolic\_process | 1 | 0 |  |  |  |  |  |  |  |  |
| GO:0009128\_purine\_nucleoside\_monophosphate\_catabolic\_process | 1 | 0 |  |  |  |  |  |  |  |  |
| GO:0009129\_pyrimidine\_nucleoside\_monophosphate\_metabolic\_process | 1 | 0 |  |  |  |  |  |  |  |  |
| GO:0009130\_pyrimidine\_nucleoside\_monophosphate\_biosynthetic\_process | 1 | 0 |  |  |  |  |  |  |  |  |
| GO:0009133\_nucleoside\_diphosphate\_biosynthetic\_process | 1 | 0 |  |  |  |  |  |  |  |  |
| GO:0009135\_purine\_nucleoside\_diphosphate\_metabolic\_process | 1 | 0 |  |  |  |  |  |  |  |  |
| GO:0009136\_purine\_nucleoside\_diphosphate\_biosynthetic\_process | 1 | 0 |  |  |  |  |  |  |  |  |
| GO:0009138\_pyrimidine\_nucleoside\_diphosphate\_metabolic\_process | 1 | 0 |  |  |  |  |  |  |  |  |
| GO:0009140\_pyrimidine\_nucleoside\_diphosphate\_catabolic\_process | 1 | 0 |  |  |  |  |  |  |  |  |
| GO:0009147\_pyrimidine\_nucleoside\_triphosphate\_metabolic\_process | 1 | 0 |  |  |  |  |  |  |  |  |
| GO:0009148\_pyrimidine\_nucleoside\_triphosphate\_biosynthetic\_process | 1 | 0 |  |  |  |  |  |  |  |  |
| GO:0009153\_purine\_deoxyribonucleotide\_biosynthetic\_process | 1 | 0 |  |  |  |  |  |  |  |  |
| GO:0009157\_deoxyribonucleoside\_monophosphate\_biosynthetic\_process | 1 | 0 |  |  |  |  |  |  |  |  |
| GO:0009158\_ribonucleoside\_monophosphate\_catabolic\_process | 1 | 0 |  |  |  |  |  |  |  |  |
| GO:0009159\_deoxyribonucleoside\_monophosphate\_catabolic\_process | 1 | 0 |  |  |  |  |  |  |  |  |
| GO:0009169\_purine\_ribonucleoside\_monophosphate\_catabolic\_process | 1 | 0 |  |  |  |  |  |  |  |  |
| GO:0009173\_pyrimidine\_ribonucleoside\_monophosphate\_metabolic\_process | 1 | 0 |  |  |  |  |  |  |  |  |
| GO:0009174\_pyrimidine\_ribonucleoside\_monophosphate\_biosynthetic\_process | 1 | 0 |  |  |  |  |  |  |  |  |
| GO:0009179\_purine\_ribonucleoside\_diphosphate\_metabolic\_process | 1 | 0 |  |  |  |  |  |  |  |  |
| GO:0009180\_purine\_ribonucleoside\_diphosphate\_biosynthetic\_process | 1 | 0 |  |  |  |  |  |  |  |  |
| GO:0009182\_purine\_deoxyribonucleoside\_diphosphate\_metabolic\_process | 1 | 0 |  |  |  |  |  |  |  |  |
| GO:0009183\_purine\_deoxyribonucleoside\_diphosphate\_biosynthetic\_process | 1 | 0 |  |  |  |  |  |  |  |  |
| GO:0009186\_deoxyribonucleoside\_diphosphate\_metabolic\_process | 1 | 0 |  |  |  |  |  |  |  |  |
| GO:0009188\_ribonucleoside\_diphosphate\_biosynthetic\_process | 1 | 0 |  |  |  |  |  |  |  |  |
| GO:0009189\_deoxyribonucleoside\_diphosphate\_biosynthetic\_process | 1 | 0 |  |  |  |  |  |  |  |  |
| GO:0009193\_pyrimidine\_ribonucleoside\_diphosphate\_metabolic\_process | 1 | 0 |  |  |  |  |  |  |  |  |
| GO:0009195\_pyrimidine\_ribonucleoside\_diphosphate\_catabolic\_process | 1 | 0 |  |  |  |  |  |  |  |  |
| GO:0009208\_pyrimidine\_ribonucleoside\_triphosphate\_metabolic\_process | 1 | 0 |  |  |  |  |  |  |  |  |
| GO:0009209\_pyrimidine\_ribonucleoside\_triphosphate\_biosynthetic\_process | 1 | 0 |  |  |  |  |  |  |  |  |
| GO:0009214\_cyclic\_nucleotide\_catabolic\_process | 1 | 0 |  |  |  |  |  |  |  |  |
| GO:0009222\_pyrimidine\_ribonucleotide\_catabolic\_process | 1 | 0 |  |  |  |  |  |  |  |  |
| GO:0009231\_riboflavin\_biosynthetic\_process | 1 | 0 |  |  |  |  |  |  |  |  |
| GO:0009253\_peptidoglycan\_catabolic\_process | 1 | 0 |  |  |  |  |  |  |  |  |
| GO:0009256\_10-formyltetrahydrofolate\_metabolic\_process | 1 | 0 |  |  |  |  |  |  |  |  |
| GO:0009258\_10-formyltetrahydrofolate\_catabolic\_process | 1 | 0 |  |  |  |  |  |  |  |  |
| GO:0009265\_2'-deoxyribonucleotide\_biosynthetic\_process | 1 | 0 |  |  |  |  |  |  |  |  |
| GO:0009292\_genetic\_transfer | 1 | 0 |  |  |  |  |  |  |  |  |
| GO:0009294\_DNA\_mediated\_transformation | 1 | 0 |  |  |  |  |  |  |  |  |
| GO:0009296\_flagellum\_assembly | 1 | 0 |  |  |  |  |  |  |  |  |
| GO:0009298\_GDP-mannose\_biosynthetic\_process | 1 | 0 |  |  |  |  |  |  |  |  |
| GO:0009304\_tRNA\_transcription | 1 | 0 |  |  |  |  |  |  |  |  |
| GO:0009313\_oligosaccharide\_catabolic\_process | 1 | 0 |  |  |  |  |  |  |  |  |
| GO:0009372\_quorum\_sensing | 1 | 0 |  |  |  |  |  |  |  |  |
| GO:0009386\_translational\_attenuation | 1 | 0 |  |  |  |  |  |  |  |  |
| GO:0009397\_folic\_acid\_and\_derivative\_catabolic\_process | 1 | 0 |  |  |  |  |  |  |  |  |
| GO:0009399\_nitrogen\_fixation | 1 | 0 |  |  |  |  |  |  |  |  |
| GO:0009404\_toxin\_metabolic\_process | 1 | 0 |  |  |  |  |  |  |  |  |
| GO:0009435\_NAD\_biosynthetic\_process | 1 | 0 |  |  |  |  |  |  |  |  |
| GO:0009437\_carnitine\_metabolic\_process | 1 | 0 |  |  |  |  |  |  |  |  |
| GO:0009441\_glycolate\_metabolic\_process | 1 | 0 |  |  |  |  |  |  |  |  |
| GO:0009624\_response\_to\_nematode | 1 | 0 |  |  |  |  |  |  |  |  |
| GO:0009642\_response\_to\_light\_intensity | 1 | 0 |  |  |  |  |  |  |  |  |
| GO:0009648\_photoperiodism | 1 | 0 |  |  |  |  |  |  |  |  |
| GO:0009720\_detection\_of\_hormone\_stimulus | 1 | 0 |  |  |  |  |  |  |  |  |
| GO:0009726\_detection\_of\_endogenous\_stimulus | 1 | 0 |  |  |  |  |  |  |  |  |
| GO:0009730\_detection\_of\_carbohydrate\_stimulus | 1 | 0 |  |  |  |  |  |  |  |  |
| GO:0009732\_detection\_of\_hexose\_stimulus | 1 | 0 |  |  |  |  |  |  |  |  |
| GO:0009826\_unidimensional\_cell\_growth | 1 | 0 |  |  |  |  |  |  |  |  |
| GO:0009912\_auditory\_receptor\_cell\_fate\_commitment | 1 | 0 |  |  |  |  |  |  |  |  |
| GO:0009954\_proximal\_distal\_pattern\_formation | 1 | 0 |  |  |  |  |  |  |  |  |
| GO:0009972\_cytidine\_deamination | 1 | 0 |  |  |  |  |  |  |  |  |
| GO:0010107\_potassium\_ion\_import | 1 | 0 |  |  |  |  |  |  |  |  |
| GO:0010259\_multicellular\_organismal\_aging | 1 | 0 |  |  |  |  |  |  |  |  |
| GO:0010269\_response\_to\_selenium\_ion | 1 | 0 |  |  |  |  |  |  |  |  |
| GO:0010273\_detoxification\_of\_copper\_ion | 1 | 0 |  |  |  |  |  |  |  |  |
| GO:0010383\_cell\_wall\_polysaccharide\_metabolic\_process | 1 | 0 |  |  |  |  |  |  |  |  |
| GO:0010430\_fatty\_acid\_omega-oxidation | 1 | 0 |  |  |  |  |  |  |  |  |
| GO:0010463\_mesenchymal\_cell\_proliferation | 1 | 0 |  |  |  |  |  |  |  |  |
| GO:0010464\_regulation\_of\_mesenchymal\_cell\_proliferation | 1 | 0 |  |  |  |  |  |  |  |  |
| GO:0010507\_negative\_regulation\_of\_autophagy | 1 | 0 |  |  |  |  |  |  |  |  |
| GO:0010509\_polyamine\_homeostasis | 1 | 0 |  |  |  |  |  |  |  |  |
| GO:0010534\_regulation\_of\_activation\_of\_JAK2\_kinase\_activity | 1 | 0 |  |  |  |  |  |  |  |  |
| GO:0010535\_positive\_regulation\_of\_activation\_of\_JAK2\_kinase\_activity | 1 | 0 |  |  |  |  |  |  |  |  |
| GO:0010561\_negative\_regulation\_of\_glycoprotein\_biosynthetic\_process | 1 | 0 |  |  |  |  |  |  |  |  |
| GO:0010569\_regulation\_of\_double-strand\_break\_repair\_via\_homologous\_recombination | 1 | 0 |  |  |  |  |  |  |  |  |
| GO:0010591\_regulation\_of\_lamellipodium\_assembly | 1 | 0 |  |  |  |  |  |  |  |  |
| GO:0010592\_positive\_regulation\_of\_lamellipodium\_assembly | 1 | 0 |  |  |  |  |  |  |  |  |
| GO:0010621\_negative\_regulation\_of\_transcription\_by\_transcription\_factor\_localization | 1 | 0 |  |  |  |  |  |  |  |  |
| GO:0010623\_developmental\_programmed\_cell\_death | 1 | 0 |  |  |  |  |  |  |  |  |
| GO:0010631\_epithelial\_cell\_migration | 1 | 0 |  |  |  |  |  |  |  |  |
| GO:0010632\_regulation\_of\_epithelial\_cell\_migration | 1 | 0 |  |  |  |  |  |  |  |  |
| GO:0010634\_positive\_regulation\_of\_epithelial\_cell\_migration | 1 | 0 |  |  |  |  |  |  |  |  |
| GO:0010658\_striated\_muscle\_cell\_apoptosis | 1 | 0 |  |  |  |  |  |  |  |  |
| GO:0010659\_cardiac\_muscle\_cell\_apoptosis | 1 | 0 |  |  |  |  |  |  |  |  |
| GO:0010662\_regulation\_of\_striated\_muscle\_cell\_apoptosis | 1 | 0 |  |  |  |  |  |  |  |  |
| GO:0010664\_negative\_regulation\_of\_striated\_muscle\_cell\_apoptosis | 1 | 0 |  |  |  |  |  |  |  |  |
| GO:0010665\_regulation\_of\_cardiac\_muscle\_cell\_apoptosis | 1 | 0 |  |  |  |  |  |  |  |  |
| GO:0010667\_negative\_regulation\_of\_cardiac\_muscle\_cell\_apoptosis | 1 | 0 |  |  |  |  |  |  |  |  |
| GO:0010669\_epithelial\_structure\_maintenance | 1 | 0 |  |  |  |  |  |  |  |  |
| GO:0010692\_regulation\_of\_alkaline\_phosphatase\_activity | 1 | 0 |  |  |  |  |  |  |  |  |
| GO:0010693\_negative\_regulation\_of\_alkaline\_phosphatase\_activity | 1 | 0 |  |  |  |  |  |  |  |  |
| GO:0010710\_regulation\_of\_collagen\_catabolic\_process | 1 | 0 |  |  |  |  |  |  |  |  |
| GO:0010711\_negative\_regulation\_of\_collagen\_catabolic\_process | 1 | 0 |  |  |  |  |  |  |  |  |
| GO:0010715\_regulation\_of\_extracellular\_matrix\_disassembly | 1 | 0 |  |  |  |  |  |  |  |  |
| GO:0010716\_negative\_regulation\_of\_extracellular\_matrix\_disassembly | 1 | 0 |  |  |  |  |  |  |  |  |
| GO:0010719\_negative\_regulation\_of\_epithelial\_to\_mesenchymal\_transition | 1 | 0 |  |  |  |  |  |  |  |  |
| GO:0010722\_regulation\_of\_ferrochelatase\_activity | 1 | 0 |  |  |  |  |  |  |  |  |
| GO:0010731\_protein\_amino\_acid\_glutathionylation | 1 | 0 |  |  |  |  |  |  |  |  |
| GO:0010732\_regulation\_of\_protein\_amino\_acid\_glutathionylation | 1 | 0 |  |  |  |  |  |  |  |  |
| GO:0010734\_negative\_regulation\_of\_protein\_amino\_acid\_glutathionylation | 1 | 0 |  |  |  |  |  |  |  |  |
| GO:0010735\_positive\_regulation\_of\_transcription\_via\_serum\_response\_element\_binding | 1 | 0 |  |  |  |  |  |  |  |  |
| GO:0010737\_protein\_kinase\_A\_signaling\_cascade | 1 | 0 |  |  |  |  |  |  |  |  |
| GO:0010738\_regulation\_of\_protein\_kinase\_A\_signaling\_cascade | 1 | 0 |  |  |  |  |  |  |  |  |
| GO:0010739\_positive\_regulation\_of\_protein\_kinase\_A\_signaling\_cascade | 1 | 0 |  |  |  |  |  |  |  |  |
| GO:0010749\_regulation\_of\_nitric\_oxide\_mediated\_signal\_transduction | 1 | 0 |  |  |  |  |  |  |  |  |
| GO:0010751\_negative\_regulation\_of\_nitric\_oxide\_mediated\_signal\_transduction | 1 | 0 |  |  |  |  |  |  |  |  |
| GO:0010752\_regulation\_of\_cGMP-mediated\_signaling | 1 | 0 |  |  |  |  |  |  |  |  |
| GO:0010754\_negative\_regulation\_of\_cGMP-mediated\_signaling | 1 | 0 |  |  |  |  |  |  |  |  |
| GO:0010756\_positive\_regulation\_of\_plasminogen\_activation | 1 | 0 |  |  |  |  |  |  |  |  |
| GO:0010757\_negative\_regulation\_of\_plasminogen\_activation | 1 | 0 |  |  |  |  |  |  |  |  |
| GO:0010758\_regulation\_of\_macrophage\_chemotaxis | 1 | 0 |  |  |  |  |  |  |  |  |
| GO:0010759\_positive\_regulation\_of\_macrophage\_chemotaxis | 1 | 0 |  |  |  |  |  |  |  |  |
| GO:0010766\_negative\_regulation\_of\_sodium\_ion\_transport | 1 | 0 |  |  |  |  |  |  |  |  |
| GO:0010767\_regulation\_of\_transcription\_from\_RNA\_polymerase\_II\_promoter\_in\_response\_to\_UV-induced\_DNA\_damage | 1 | 0 |  |  |  |  |  |  |  |  |
| GO:0010768\_negative\_regulation\_of\_transcription\_from\_RNA\_polymerase\_II\_promoter\_in\_response\_to\_UV-induced\_DNA\_damage | 1 | 0 |  |  |  |  |  |  |  |  |
| GO:0010771\_negative\_regulation\_of\_cell\_morphogenesis\_involved\_in\_differentiation | 1 | 0 |  |  |  |  |  |  |  |  |
| GO:0010793\_regulation\_of\_mRNA\_export\_from\_nucleus | 1 | 0 |  |  |  |  |  |  |  |  |
| GO:0010801\_negative\_regulation\_of\_peptidyl-threonine\_phosphorylation | 1 | 0 |  |  |  |  |  |  |  |  |
| GO:0010803\_regulation\_of\_tumor\_necrosis\_factor-mediated\_signaling\_pathway | 1 | 0 |  |  |  |  |  |  |  |  |
| GO:0010804\_negative\_regulation\_of\_tumor\_necrosis\_factor-mediated\_signaling\_pathway | 1 | 0 |  |  |  |  |  |  |  |  |
| GO:0010813\_neuropeptide\_catabolic\_process | 1 | 0 |  |  |  |  |  |  |  |  |
| GO:0010814\_substance\_P\_catabolic\_process | 1 | 0 |  |  |  |  |  |  |  |  |
| GO:0010816\_calcitonin\_catabolic\_process | 1 | 0 |  |  |  |  |  |  |  |  |
| GO:0010826\_negative\_regulation\_of\_centrosome\_duplication | 1 | 0 |  |  |  |  |  |  |  |  |
| GO:0010830\_regulation\_of\_myotube\_differentiation | 1 | 0 |  |  |  |  |  |  |  |  |
| GO:0010832\_negative\_regulation\_of\_myotube\_differentiation | 1 | 0 |  |  |  |  |  |  |  |  |
| GO:0010835\_regulation\_of\_protein\_amino\_acid\_ADP-ribosylation | 1 | 0 |  |  |  |  |  |  |  |  |
| GO:0010836\_negative\_regulation\_of\_protein\_amino\_acid\_ADP-ribosylation | 1 | 0 |  |  |  |  |  |  |  |  |
| GO:0010837\_regulation\_of\_keratinocyte\_proliferation | 1 | 0 |  |  |  |  |  |  |  |  |
| GO:0010839\_negative\_regulation\_of\_keratinocyte\_proliferation | 1 | 0 |  |  |  |  |  |  |  |  |
| GO:0010840\_regulation\_of\_circadian\_sleep\_wake\_cycle\_\_wakefulness | 1 | 0 |  |  |  |  |  |  |  |  |
| GO:0010841\_positive\_regulation\_of\_circadian\_sleep\_wake\_cycle\_\_wakefulness | 1 | 0 |  |  |  |  |  |  |  |  |
| GO:0010842\_retina\_layer\_formation | 1 | 0 |  |  |  |  |  |  |  |  |
| GO:0010897\_negative\_regulation\_of\_triglyceride\_catabolic\_process | 1 | 0 |  |  |  |  |  |  |  |  |
| GO:0010899\_regulation\_of\_phosphatidylcholine\_catabolic\_process | 1 | 0 |  |  |  |  |  |  |  |  |
| GO:0010900\_negative\_regulation\_of\_phosphatidylcholine\_catabolic\_process | 1 | 0 |  |  |  |  |  |  |  |  |
| GO:0010902\_positive\_regulation\_of\_very-low-density\_lipoprotein\_particle\_remodeling | 1 | 0 |  |  |  |  |  |  |  |  |
| GO:0010919\_regulation\_of\_inositol\_phosphate\_biosynthetic\_process | 1 | 0 |  |  |  |  |  |  |  |  |
| GO:0010920\_negative\_regulation\_of\_inositol\_phosphate\_biosynthetic\_process | 1 | 0 |  |  |  |  |  |  |  |  |
| GO:0010924\_regulation\_of\_inositol-polyphosphate\_5-phosphatase\_activity | 1 | 0 |  |  |  |  |  |  |  |  |
| GO:0010925\_positive\_regulation\_of\_inositol-polyphosphate\_5-phosphatase\_activity | 1 | 0 |  |  |  |  |  |  |  |  |
| GO:0010931\_macrophage\_tolerance\_induction | 1 | 0 |  |  |  |  |  |  |  |  |
| GO:0010932\_regulation\_of\_macrophage\_tolerance\_induction | 1 | 0 |  |  |  |  |  |  |  |  |
| GO:0010933\_positive\_regulation\_of\_macrophage\_tolerance\_induction | 1 | 0 |  |  |  |  |  |  |  |  |
| GO:0010934\_macrophage\_cytokine\_production | 1 | 0 |  |  |  |  |  |  |  |  |
| GO:0010935\_regulation\_of\_macrophage\_cytokine\_production | 1 | 0 |  |  |  |  |  |  |  |  |
| GO:0010936\_negative\_regulation\_of\_macrophage\_cytokine\_production | 1 | 0 |  |  |  |  |  |  |  |  |
| GO:0010944\_negative\_regulation\_of\_transcription\_by\_competitive\_promoter\_binding | 1 | 0 |  |  |  |  |  |  |  |  |
| GO:0010983\_positive\_regulation\_of\_high-density\_lipoprotein\_particle\_clearance | 1 | 0 |  |  |  |  |  |  |  |  |
| GO:0010986\_positive\_regulation\_of\_lipoprotein\_particle\_clearance | 1 | 0 |  |  |  |  |  |  |  |  |
| GO:0010987\_negative\_regulation\_of\_high-density\_lipoprotein\_particle\_clearance | 1 | 0 |  |  |  |  |  |  |  |  |
| GO:0010988\_regulation\_of\_low-density\_lipoprotein\_particle\_clearance | 1 | 0 |  |  |  |  |  |  |  |  |
| GO:0010989\_negative\_regulation\_of\_low-density\_lipoprotein\_particle\_clearance | 1 | 0 |  |  |  |  |  |  |  |  |
| GO:0010990\_regulation\_of\_SMAD\_protein\_complex\_assembly | 1 | 0 |  |  |  |  |  |  |  |  |
| GO:0010991\_negative\_regulation\_of\_SMAD\_protein\_complex\_assembly | 1 | 0 |  |  |  |  |  |  |  |  |
| GO:0014009\_glial\_cell\_proliferation | 1 | 0 |  |  |  |  |  |  |  |  |
| GO:0014010\_Schwann\_cell\_proliferation | 1 | 0 |  |  |  |  |  |  |  |  |
| GO:0014045\_establishment\_of\_endothelial\_blood-brain\_barrier | 1 | 0 |  |  |  |  |  |  |  |  |
| GO:0014055\_acetylcholine\_secretion | 1 | 0 |  |  |  |  |  |  |  |  |
| GO:0014056\_regulation\_of\_acetylcholine\_secretion | 1 | 0 |  |  |  |  |  |  |  |  |
| GO:0014060\_regulation\_of\_epinephrine\_secretion | 1 | 0 |  |  |  |  |  |  |  |  |
| GO:0014067\_negative\_regulation\_of\_phosphoinositide\_3-kinase\_cascade | 1 | 0 |  |  |  |  |  |  |  |  |
| GO:0014071\_response\_to\_cycloalkane | 1 | 0 |  |  |  |  |  |  |  |  |
| GO:0014721\_twitch\_skeletal\_muscle\_contraction | 1 | 0 |  |  |  |  |  |  |  |  |
| GO:0014724\_regulation\_of\_twitch\_skeletal\_muscle\_contraction | 1 | 0 |  |  |  |  |  |  |  |  |
| GO:0014806\_smooth\_muscle\_hyperplasia | 1 | 0 |  |  |  |  |  |  |  |  |
| GO:0014823\_response\_to\_activity | 1 | 0 |  |  |  |  |  |  |  |  |
| GO:0014832\_urinary\_bladder\_smooth\_muscle\_contraction | 1 | 0 |  |  |  |  |  |  |  |  |
| GO:0014834\_satellite\_cell\_maintenance\_involved\_in\_skeletal\_muscle\_regeneration | 1 | 0 |  |  |  |  |  |  |  |  |
| GO:0014848\_urinary\_tract\_smooth\_muscle\_contraction | 1 | 0 |  |  |  |  |  |  |  |  |
| GO:0014850\_response\_to\_muscle\_activity | 1 | 0 |  |  |  |  |  |  |  |  |
| GO:0014873\_response\_to\_muscle\_activity\_involved\_in\_regulation\_of\_muscle\_adaptation | 1 | 0 |  |  |  |  |  |  |  |  |
| GO:0014874\_response\_to\_stimulus\_involved\_in\_regulation\_of\_muscle\_adaptation | 1 | 0 |  |  |  |  |  |  |  |  |
| GO:0014895\_smooth\_muscle\_hypertrophy | 1 | 0 |  |  |  |  |  |  |  |  |
| GO:0014904\_myotube\_cell\_development | 1 | 0 |  |  |  |  |  |  |  |  |
| GO:0014916\_regulation\_of\_lung\_blood\_pressure | 1 | 0 |  |  |  |  |  |  |  |  |
| GO:0015675\_nickel\_ion\_transport | 1 | 0 |  |  |  |  |  |  |  |  |
| GO:0015676\_vanadium\_ion\_transport | 1 | 0 |  |  |  |  |  |  |  |  |
| GO:0015680\_intracellular\_copper\_ion\_transport | 1 | 0 |  |  |  |  |  |  |  |  |
| GO:0015684\_ferrous\_iron\_transport | 1 | 0 |  |  |  |  |  |  |  |  |
| GO:0015692\_lead\_ion\_transport | 1 | 0 |  |  |  |  |  |  |  |  |
| GO:0015693\_magnesium\_ion\_transport | 1 | 0 |  |  |  |  |  |  |  |  |
| GO:0015727\_lactate\_transport | 1 | 0 |  |  |  |  |  |  |  |  |
| GO:0015728\_mevalonate\_transport | 1 | 0 |  |  |  |  |  |  |  |  |
| GO:0015742\_alpha-ketoglutarate\_transport | 1 | 0 |  |  |  |  |  |  |  |  |
| GO:0015746\_citrate\_transport | 1 | 0 |  |  |  |  |  |  |  |  |
| GO:0015747\_urate\_transport | 1 | 0 |  |  |  |  |  |  |  |  |
| GO:0015755\_fructose\_transport | 1 | 0 |  |  |  |  |  |  |  |  |
| GO:0015760\_glucose-6-phosphate\_transport | 1 | 0 |  |  |  |  |  |  |  |  |
| GO:0015782\_CMP-sialic\_acid\_transport | 1 | 0 |  |  |  |  |  |  |  |  |
| GO:0015785\_UDP-galactose\_transport | 1 | 0 |  |  |  |  |  |  |  |  |
| GO:0015789\_UDP-N-acetylgalactosamine\_transport | 1 | 0 |  |  |  |  |  |  |  |  |
| GO:0015790\_UDP-xylose\_transport | 1 | 0 |  |  |  |  |  |  |  |  |
| GO:0015798\_myo-inositol\_transport | 1 | 0 |  |  |  |  |  |  |  |  |
| GO:0015803\_branched-chain\_aliphatic\_amino\_acid\_transport | 1 | 0 |  |  |  |  |  |  |  |  |
| GO:0015805\_S-adenosylmethionine\_transport | 1 | 0 |  |  |  |  |  |  |  |  |
| GO:0015809\_arginine\_transport | 1 | 0 |  |  |  |  |  |  |  |  |
| GO:0015817\_histidine\_transport | 1 | 0 |  |  |  |  |  |  |  |  |
| GO:0015820\_leucine\_transport | 1 | 0 |  |  |  |  |  |  |  |  |
| GO:0015826\_threonine\_transport | 1 | 0 |  |  |  |  |  |  |  |  |
| GO:0015827\_tryptophan\_transport | 1 | 0 |  |  |  |  |  |  |  |  |
| GO:0015846\_polyamine\_transport | 1 | 0 |  |  |  |  |  |  |  |  |
| GO:0015853\_adenine\_transport | 1 | 0 |  |  |  |  |  |  |  |  |
| GO:0015855\_pyrimidine\_transport | 1 | 0 |  |  |  |  |  |  |  |  |
| GO:0015886\_heme\_transport | 1 | 0 |  |  |  |  |  |  |  |  |
| GO:0015888\_thiamin\_transport | 1 | 0 |  |  |  |  |  |  |  |  |
| GO:0015910\_peroxisomal\_long-chain\_fatty\_acid\_import | 1 | 0 |  |  |  |  |  |  |  |  |
| GO:0015919\_peroxisomal\_membrane\_transport | 1 | 0 |  |  |  |  |  |  |  |  |
| GO:0015937\_coenzyme\_A\_biosynthetic\_process | 1 | 0 |  |  |  |  |  |  |  |  |
| GO:0015956\_bis(5'-nucleosidyl)\_oligophosphate\_metabolic\_process | 1 | 0 |  |  |  |  |  |  |  |  |
| GO:0015958\_bis(5'-nucleosidyl)\_oligophosphate\_catabolic\_process | 1 | 0 |  |  |  |  |  |  |  |  |
| GO:0015959\_diadenosine\_polyphosphate\_metabolic\_process | 1 | 0 |  |  |  |  |  |  |  |  |
| GO:0015961\_diadenosine\_polyphosphate\_catabolic\_process | 1 | 0 |  |  |  |  |  |  |  |  |
| GO:0016046\_detection\_of\_fungus | 1 | 0 |  |  |  |  |  |  |  |  |
| GO:0016078\_tRNA\_catabolic\_process | 1 | 0 |  |  |  |  |  |  |  |  |
| GO:0016091\_prenol\_biosynthetic\_process | 1 | 0 |  |  |  |  |  |  |  |  |
| GO:0016094\_polyprenol\_biosynthetic\_process | 1 | 0 |  |  |  |  |  |  |  |  |
| GO:0016108\_tetraterpenoid\_metabolic\_process | 1 | 0 |  |  |  |  |  |  |  |  |
| GO:0016116\_carotenoid\_metabolic\_process | 1 | 0 |  |  |  |  |  |  |  |  |
| GO:0016119\_carotene\_metabolic\_process | 1 | 0 |  |  |  |  |  |  |  |  |
| GO:0016140\_O-glycoside\_metabolic\_process | 1 | 0 |  |  |  |  |  |  |  |  |
| GO:0016142\_O-glycoside\_catabolic\_process | 1 | 0 |  |  |  |  |  |  |  |  |
| GO:0016188\_synaptic\_vesicle\_maturation | 1 | 0 |  |  |  |  |  |  |  |  |
| GO:0016189\_synaptic\_vesicle\_to\_endosome\_fusion | 1 | 0 |  |  |  |  |  |  |  |  |
| GO:0016241\_regulation\_of\_macroautophagy | 1 | 0 |  |  |  |  |  |  |  |  |
| GO:0016242\_negative\_regulation\_of\_macroautophagy | 1 | 0 |  |  |  |  |  |  |  |  |
| GO:0016259\_selenocysteine\_metabolic\_process | 1 | 0 |  |  |  |  |  |  |  |  |
| GO:0016260\_selenocysteine\_biosynthetic\_process | 1 | 0 |  |  |  |  |  |  |  |  |
| GO:0016269\_O-glycan\_processing\_\_core\_3 | 1 | 0 |  |  |  |  |  |  |  |  |
| GO:0016320\_endoplasmic\_reticulum\_membrane\_fusion | 1 | 0 |  |  |  |  |  |  |  |  |
| GO:0016344\_meiotic\_chromosome\_movement\_towards\_spindle\_pole | 1 | 0 |  |  |  |  |  |  |  |  |
| GO:0016446\_somatic\_hypermutation\_of\_immunoglobulin\_genes | 1 | 0 |  |  |  |  |  |  |  |  |
| GO:0016559\_peroxisome\_fission | 1 | 0 |  |  |  |  |  |  |  |  |
| GO:0016560\_protein\_import\_into\_peroxisome\_matrix\_\_docking | 1 | 0 |  |  |  |  |  |  |  |  |
| GO:0016598\_protein\_arginylation | 1 | 0 |  |  |  |  |  |  |  |  |
| GO:0016998\_cell\_wall\_macromolecule\_catabolic\_process | 1 | 0 |  |  |  |  |  |  |  |  |
| GO:0017062\_respiratory\_chain\_complex\_III\_assembly | 1 | 0 |  |  |  |  |  |  |  |  |
| GO:0017185\_peptidyl-lysine\_hydroxylation | 1 | 0 |  |  |  |  |  |  |  |  |
| GO:0018095\_protein\_polyglutamylation | 1 | 0 |  |  |  |  |  |  |  |  |
| GO:0018125\_peptidyl-cysteine\_methylation | 1 | 0 |  |  |  |  |  |  |  |  |
| GO:0018126\_protein\_amino\_acid\_hydroxylation | 1 | 0 |  |  |  |  |  |  |  |  |
| GO:0018146\_keratan\_sulfate\_biosynthetic\_process | 1 | 0 |  |  |  |  |  |  |  |  |
| GO:0018153\_isopeptide\_cross-linking\_via\_N6-(L-isoglutamyl)-L-lysine | 1 | 0 |  |  |  |  |  |  |  |  |
| GO:0018184\_protein\_amino\_acid\_polyamination | 1 | 0 |  |  |  |  |  |  |  |  |
| GO:0018190\_protein\_amino\_acid\_octanoylation | 1 | 0 |  |  |  |  |  |  |  |  |
| GO:0018191\_peptidyl-serine\_octanoylation | 1 | 0 |  |  |  |  |  |  |  |  |
| GO:0018192\_enzyme\_active\_site\_formation\_via\_L-cysteine\_persulfide | 1 | 0 |  |  |  |  |  |  |  |  |
| GO:0018199\_peptidyl-glutamine\_modification | 1 | 0 |  |  |  |  |  |  |  |  |
| GO:0018200\_peptidyl-glutamic\_acid\_modification | 1 | 0 |  |  |  |  |  |  |  |  |
| GO:0018208\_peptidyl-proline\_modification | 1 | 0 |  |  |  |  |  |  |  |  |
| GO:0018262\_isopeptide\_cross-linking | 1 | 0 |  |  |  |  |  |  |  |  |
| GO:0018277\_protein\_amino\_acid\_deamination | 1 | 0 |  |  |  |  |  |  |  |  |
| GO:0018307\_enzyme\_active\_site\_formation | 1 | 0 |  |  |  |  |  |  |  |  |
| GO:0018318\_protein\_amino\_acid\_palmitoylation | 1 | 0 |  |  |  |  |  |  |  |  |
| GO:0018319\_protein\_amino\_acid\_myristoylation | 1 | 0 |  |  |  |  |  |  |  |  |
| GO:0018345\_protein\_palmitoylation | 1 | 0 |  |  |  |  |  |  |  |  |
| GO:0018350\_protein\_amino\_acid\_esterification | 1 | 0 |  |  |  |  |  |  |  |  |
| GO:0018352\_protein-pyridoxal-5-phosphate\_linkage | 1 | 0 |  |  |  |  |  |  |  |  |
| GO:0018377\_protein\_myristoylation | 1 | 0 |  |  |  |  |  |  |  |  |
| GO:0018395\_peptidyl-lysine\_hydroxylation\_to\_5-hydroxy-L-lysine | 1 | 0 |  |  |  |  |  |  |  |  |
| GO:0018401\_peptidyl-proline\_hydroxylation\_to\_4-hydroxy-L-proline | 1 | 0 |  |  |  |  |  |  |  |  |
| GO:0018872\_arsonoacetate\_metabolic\_process | 1 | 0 |  |  |  |  |  |  |  |  |
| GO:0018874\_benzoate\_metabolic\_process | 1 | 0 |  |  |  |  |  |  |  |  |
| GO:0019060\_intracellular\_transport\_of\_viral\_proteins\_in\_host\_cell | 1 | 0 |  |  |  |  |  |  |  |  |
| GO:0019064\_viral\_envelope\_fusion\_with\_host\_membrane | 1 | 0 |  |  |  |  |  |  |  |  |
| GO:0019086\_late\_viral\_mRNA\_transcription | 1 | 0 |  |  |  |  |  |  |  |  |
| GO:0019087\_transformation\_of\_host\_cell\_by\_virus | 1 | 0 |  |  |  |  |  |  |  |  |
| GO:0019089\_transmission\_of\_virus | 1 | 0 |  |  |  |  |  |  |  |  |
| GO:0019098\_reproductive\_behavior | 1 | 0 |  |  |  |  |  |  |  |  |
| GO:0019240\_citrulline\_biosynthetic\_process | 1 | 0 |  |  |  |  |  |  |  |  |
| GO:0019302\_D-ribose\_biosynthetic\_process | 1 | 0 |  |  |  |  |  |  |  |  |
| GO:0019303\_D-ribose\_catabolic\_process | 1 | 0 |  |  |  |  |  |  |  |  |
| GO:0019307\_mannose\_biosynthetic\_process | 1 | 0 |  |  |  |  |  |  |  |  |
| GO:0019310\_inositol\_catabolic\_process | 1 | 0 |  |  |  |  |  |  |  |  |
| GO:0019322\_pentose\_biosynthetic\_process | 1 | 0 |  |  |  |  |  |  |  |  |
| GO:0019323\_pentose\_catabolic\_process | 1 | 0 |  |  |  |  |  |  |  |  |
| GO:0019371\_cyclooxygenase\_pathway | 1 | 0 |  |  |  |  |  |  |  |  |
| GO:0019372\_lipoxygenase\_pathway | 1 | 0 |  |  |  |  |  |  |  |  |
| GO:0019388\_galactose\_catabolic\_process | 1 | 0 |  |  |  |  |  |  |  |  |
| GO:0019405\_alditol\_catabolic\_process | 1 | 0 |  |  |  |  |  |  |  |  |
| GO:0019407\_hexitol\_catabolic\_process | 1 | 0 |  |  |  |  |  |  |  |  |
| GO:0019408\_dolichol\_biosynthetic\_process | 1 | 0 |  |  |  |  |  |  |  |  |
| GO:0019441\_tryptophan\_catabolic\_process\_to\_kynurenine | 1 | 0 |  |  |  |  |  |  |  |  |
| GO:0019471\_4-hydroxyproline\_metabolic\_process | 1 | 0 |  |  |  |  |  |  |  |  |
| GO:0019511\_peptidyl-proline\_hydroxylation | 1 | 0 |  |  |  |  |  |  |  |  |
| GO:0019519\_pentitol\_metabolic\_process | 1 | 0 |  |  |  |  |  |  |  |  |
| GO:0019527\_pentitol\_catabolic\_process | 1 | 0 |  |  |  |  |  |  |  |  |
| GO:0019614\_catechol\_catabolic\_process | 1 | 0 |  |  |  |  |  |  |  |  |
| GO:0019673\_GDP-mannose\_metabolic\_process | 1 | 0 |  |  |  |  |  |  |  |  |
| GO:0019693\_ribose\_phosphate\_metabolic\_process | 1 | 0 |  |  |  |  |  |  |  |  |
| GO:0019695\_choline\_metabolic\_process | 1 | 0 |  |  |  |  |  |  |  |  |
| GO:0019747\_regulation\_of\_isoprenoid\_metabolic\_process | 1 | 0 |  |  |  |  |  |  |  |  |
| GO:0019852\_L-ascorbic\_acid\_metabolic\_process | 1 | 0 |  |  |  |  |  |  |  |  |
| GO:0019856\_pyrimidine\_base\_biosynthetic\_process | 1 | 0 |  |  |  |  |  |  |  |  |
| GO:0019858\_cytosine\_metabolic\_process | 1 | 0 |  |  |  |  |  |  |  |  |
| GO:0019884\_antigen\_processing\_and\_presentation\_of\_exogenous\_antigen | 1 | 0 |  |  |  |  |  |  |  |  |
| GO:0019886\_antigen\_processing\_and\_presentation\_of\_exogenous\_peptide\_antigen\_via\_MHC\_class\_II | 1 | 0 |  |  |  |  |  |  |  |  |
| GO:0021508\_floor\_plate\_formation | 1 | 0 |  |  |  |  |  |  |  |  |
| GO:0021514\_ventral\_spinal\_cord\_interneuron\_differentiation | 1 | 0 |  |  |  |  |  |  |  |  |
| GO:0021521\_ventral\_spinal\_cord\_interneuron\_specification | 1 | 0 |  |  |  |  |  |  |  |  |
| GO:0021522\_spinal\_cord\_motor\_neuron\_differentiation | 1 | 0 |  |  |  |  |  |  |  |  |
| GO:0021527\_spinal\_cord\_association\_neuron\_differentiation | 1 | 0 |  |  |  |  |  |  |  |  |
| GO:0021528\_commissural\_neuron\_differentiation\_in\_the\_spinal\_cord | 1 | 0 |  |  |  |  |  |  |  |  |
| GO:0021533\_cell\_differentiation\_in\_hindbrain | 1 | 0 |  |  |  |  |  |  |  |  |
| GO:0021540\_corpus\_callosum\_morphogenesis | 1 | 0 |  |  |  |  |  |  |  |  |
| GO:0021544\_subpallium\_development | 1 | 0 |  |  |  |  |  |  |  |  |
| GO:0021554\_optic\_nerve\_development | 1 | 0 |  |  |  |  |  |  |  |  |
| GO:0021562\_vestibulocochlear\_nerve\_development | 1 | 0 |  |  |  |  |  |  |  |  |
| GO:0021602\_cranial\_nerve\_morphogenesis | 1 | 0 |  |  |  |  |  |  |  |  |
| GO:0021631\_optic\_nerve\_morphogenesis | 1 | 0 |  |  |  |  |  |  |  |  |
| GO:0021680\_cerebellar\_Purkinje\_cell\_layer\_development | 1 | 0 |  |  |  |  |  |  |  |  |
| GO:0021692\_cerebellar\_Purkinje\_cell\_layer\_morphogenesis | 1 | 0 |  |  |  |  |  |  |  |  |
| GO:0021694\_cerebellar\_Purkinje\_cell\_layer\_formation | 1 | 0 |  |  |  |  |  |  |  |  |
| GO:0021697\_cerebellar\_cortex\_formation | 1 | 0 |  |  |  |  |  |  |  |  |
| GO:0021702\_cerebellar\_Purkinje\_cell\_differentiation | 1 | 0 |  |  |  |  |  |  |  |  |
| GO:0021756\_striatum\_development | 1 | 0 |  |  |  |  |  |  |  |  |
| GO:0021757\_caudate\_nucleus\_development | 1 | 0 |  |  |  |  |  |  |  |  |
| GO:0021758\_putamen\_development | 1 | 0 |  |  |  |  |  |  |  |  |
| GO:0021761\_limbic\_system\_development | 1 | 0 |  |  |  |  |  |  |  |  |
| GO:0021771\_lateral\_geniculate\_nucleus\_development | 1 | 0 |  |  |  |  |  |  |  |  |
| GO:0021775\_smoothened\_signaling\_pathway\_involved\_in\_ventral\_spinal\_cord\_interneuron\_specification | 1 | 0 |  |  |  |  |  |  |  |  |
| GO:0021794\_thalamus\_development | 1 | 0 |  |  |  |  |  |  |  |  |
| GO:0021799\_cerebral\_cortex\_radially\_oriented\_cell\_migration | 1 | 0 |  |  |  |  |  |  |  |  |
| GO:0021800\_cerebral\_cortex\_tangential\_migration | 1 | 0 |  |  |  |  |  |  |  |  |
| GO:0021854\_hypothalamus\_development | 1 | 0 |  |  |  |  |  |  |  |  |
| GO:0021859\_pyramidal\_neuron\_differentiation | 1 | 0 |  |  |  |  |  |  |  |  |
| GO:0021860\_pyramidal\_neuron\_development | 1 | 0 |  |  |  |  |  |  |  |  |
| GO:0021872\_generation\_of\_neurons\_in\_the\_forebrain | 1 | 0 |  |  |  |  |  |  |  |  |
| GO:0021879\_forebrain\_neuron\_differentiation | 1 | 0 |  |  |  |  |  |  |  |  |
| GO:0021884\_forebrain\_neuron\_development | 1 | 0 |  |  |  |  |  |  |  |  |
| GO:0021896\_forebrain\_astrocyte\_differentiation | 1 | 0 |  |  |  |  |  |  |  |  |
| GO:0021897\_forebrain\_astrocyte\_development | 1 | 0 |  |  |  |  |  |  |  |  |
| GO:0021914\_negative\_regulation\_of\_smoothened\_signaling\_pathway\_involved\_in\_ventral\_spinal\_cord\_patterning | 1 | 0 |  |  |  |  |  |  |  |  |
| GO:0021919\_BMP\_signaling\_pathway\_in\_spinal\_cord\_dorsal\_ventral\_patterning | 1 | 0 |  |  |  |  |  |  |  |  |
| GO:0021965\_spinal\_cord\_ventral\_commissure\_morphogenesis | 1 | 0 |  |  |  |  |  |  |  |  |
| GO:0021984\_adenohypophysis\_development | 1 | 0 |  |  |  |  |  |  |  |  |
| GO:0021990\_neural\_plate\_formation | 1 | 0 |  |  |  |  |  |  |  |  |
| GO:0021997\_neural\_plate\_axis\_specification | 1 | 0 |  |  |  |  |  |  |  |  |
| GO:0021999\_neural\_plate\_anterior\_posterior\_pattern\_formation | 1 | 0 |  |  |  |  |  |  |  |  |
| GO:0022009\_central\_nervous\_system\_vasculogenesis | 1 | 0 |  |  |  |  |  |  |  |  |
| GO:0022038\_corpus\_callosum\_development | 1 | 0 |  |  |  |  |  |  |  |  |
| GO:0030007\_cellular\_potassium\_ion\_homeostasis | 1 | 0 |  |  |  |  |  |  |  |  |
| GO:0030011\_maintenance\_of\_cell\_polarity | 1 | 0 |  |  |  |  |  |  |  |  |
| GO:0030026\_cellular\_manganese\_ion\_homeostasis | 1 | 0 |  |  |  |  |  |  |  |  |
| GO:0030033\_microvillus\_assembly | 1 | 0 |  |  |  |  |  |  |  |  |
| GO:0030037\_actin\_filament\_reorganization\_during\_cell\_cycle | 1 | 0 |  |  |  |  |  |  |  |  |
| GO:0030047\_actin\_modification | 1 | 0 |  |  |  |  |  |  |  |  |
| GO:0030070\_insulin\_processing | 1 | 0 |  |  |  |  |  |  |  |  |
| GO:0030103\_vasopressin\_secretion | 1 | 0 |  |  |  |  |  |  |  |  |
| GO:0030186\_melatonin\_metabolic\_process | 1 | 0 |  |  |  |  |  |  |  |  |
| GO:0030187\_melatonin\_biosynthetic\_process | 1 | 0 |  |  |  |  |  |  |  |  |
| GO:0030212\_hyaluronan\_metabolic\_process | 1 | 0 |  |  |  |  |  |  |  |  |
| GO:0030220\_platelet\_formation | 1 | 0 |  |  |  |  |  |  |  |  |
| GO:0030238\_male\_sex\_determination | 1 | 0 |  |  |  |  |  |  |  |  |
| GO:0030259\_lipid\_glycosylation | 1 | 0 |  |  |  |  |  |  |  |  |
| GO:0030302\_deoxynucleotide\_transport | 1 | 0 |  |  |  |  |  |  |  |  |
| GO:0030327\_prenylated\_protein\_catabolic\_process | 1 | 0 |  |  |  |  |  |  |  |  |
| GO:0030389\_fructosamine\_metabolic\_process | 1 | 0 |  |  |  |  |  |  |  |  |
| GO:0030393\_fructoselysine\_metabolic\_process | 1 | 0 |  |  |  |  |  |  |  |  |
| GO:0030432\_peristalsis | 1 | 0 |  |  |  |  |  |  |  |  |
| GO:0030488\_tRNA\_methylation | 1 | 0 |  |  |  |  |  |  |  |  |
| GO:0030517\_negative\_regulation\_of\_axon\_extension | 1 | 0 |  |  |  |  |  |  |  |  |
| GO:0030581\_symbiont\_intracellular\_protein\_transport\_in\_host | 1 | 0 |  |  |  |  |  |  |  |  |
| GO:0030718\_germ-line\_stem\_cell\_maintenance | 1 | 0 |  |  |  |  |  |  |  |  |
| GO:0030728\_ovulation | 1 | 0 |  |  |  |  |  |  |  |  |
| GO:0030824\_negative\_regulation\_of\_cGMP\_metabolic\_process | 1 | 0 |  |  |  |  |  |  |  |  |
| GO:0030825\_positive\_regulation\_of\_cGMP\_metabolic\_process | 1 | 0 |  |  |  |  |  |  |  |  |
| GO:0030827\_negative\_regulation\_of\_cGMP\_biosynthetic\_process | 1 | 0 |  |  |  |  |  |  |  |  |
| GO:0030828\_positive\_regulation\_of\_cGMP\_biosynthetic\_process | 1 | 0 |  |  |  |  |  |  |  |  |
| GO:0030836\_positive\_regulation\_of\_actin\_filament\_depolymerization | 1 | 0 |  |  |  |  |  |  |  |  |
| GO:0030845\_inhibition\_of\_phospholipase\_C\_activity\_involved\_in\_G-protein\_coupled\_receptor\_signaling\_pathway | 1 | 0 |  |  |  |  |  |  |  |  |
| GO:0030854\_positive\_regulation\_of\_granulocyte\_differentiation | 1 | 0 |  |  |  |  |  |  |  |  |
| GO:0030857\_negative\_regulation\_of\_epithelial\_cell\_differentiation | 1 | 0 |  |  |  |  |  |  |  |  |
| GO:0030878\_thyroid\_gland\_development | 1 | 0 |  |  |  |  |  |  |  |  |
| GO:0030903\_notochord\_development | 1 | 0 |  |  |  |  |  |  |  |  |
| GO:0030910\_olfactory\_placode\_formation | 1 | 0 |  |  |  |  |  |  |  |  |
| GO:0030913\_paranodal\_junction\_assembly | 1 | 0 |  |  |  |  |  |  |  |  |
| GO:0030948\_negative\_regulation\_of\_vascular\_endothelial\_growth\_factor\_receptor\_signaling\_pathway | 1 | 0 |  |  |  |  |  |  |  |  |
| GO:0030967\_ER-nuclear\_sterol\_response\_pathway | 1 | 0 |  |  |  |  |  |  |  |  |
| GO:0031017\_exocrine\_pancreas\_development | 1 | 0 |  |  |  |  |  |  |  |  |
| GO:0031063\_regulation\_of\_histone\_deacetylation | 1 | 0 |  |  |  |  |  |  |  |  |
| GO:0031065\_positive\_regulation\_of\_histone\_deacetylation | 1 | 0 |  |  |  |  |  |  |  |  |
| GO:0031076\_embryonic\_camera-type\_eye\_development | 1 | 0 |  |  |  |  |  |  |  |  |
| GO:0031081\_nuclear\_pore\_distribution | 1 | 0 |  |  |  |  |  |  |  |  |
| GO:0031086\_nuclear-transcribed\_mRNA\_catabolic\_process\_\_deadenylation-independent\_decay | 1 | 0 |  |  |  |  |  |  |  |  |
| GO:0031087\_deadenylation-independent\_decapping\_of\_nuclear-transcribed\_mRNA | 1 | 0 |  |  |  |  |  |  |  |  |
| GO:0031106\_septin\_ring\_organization | 1 | 0 |  |  |  |  |  |  |  |  |
| GO:0031115\_negative\_regulation\_of\_microtubule\_polymerization | 1 | 0 |  |  |  |  |  |  |  |  |
| GO:0031117\_positive\_regulation\_of\_microtubule\_depolymerization | 1 | 0 |  |  |  |  |  |  |  |  |
| GO:0031118\_rRNA\_pseudouridine\_synthesis | 1 | 0 |  |  |  |  |  |  |  |  |
| GO:0031125\_rRNA\_3'-end\_processing | 1 | 0 |  |  |  |  |  |  |  |  |
| GO:0031146\_SCF-dependent\_proteasomal\_ubiquitin-dependent\_protein\_catabolic\_process | 1 | 0 |  |  |  |  |  |  |  |  |
| GO:0031179\_peptide\_modification | 1 | 0 |  |  |  |  |  |  |  |  |
| GO:0031282\_regulation\_of\_guanylate\_cyclase\_activity | 1 | 0 |  |  |  |  |  |  |  |  |
| GO:0031284\_positive\_regulation\_of\_guanylate\_cyclase\_activity | 1 | 0 |  |  |  |  |  |  |  |  |
| GO:0031290\_retinal\_ganglion\_cell\_axon\_guidance | 1 | 0 |  |  |  |  |  |  |  |  |
| GO:0031293\_membrane\_protein\_intracellular\_domain\_proteolysis | 1 | 0 |  |  |  |  |  |  |  |  |
| GO:0031335\_regulation\_of\_sulfur\_amino\_acid\_metabolic\_process | 1 | 0 |  |  |  |  |  |  |  |  |
| GO:0031342\_negative\_regulation\_of\_cell\_killing | 1 | 0 |  |  |  |  |  |  |  |  |
| GO:0031424\_keratinization | 1 | 0 |  |  |  |  |  |  |  |  |
| GO:0031441\_negative\_regulation\_of\_mRNA\_3'-end\_processing | 1 | 0 |  |  |  |  |  |  |  |  |
| GO:0031442\_positive\_regulation\_of\_mRNA\_3'-end\_processing | 1 | 0 |  |  |  |  |  |  |  |  |
| GO:0031443\_fast-twitch\_skeletal\_muscle\_fiber\_contraction | 1 | 0 |  |  |  |  |  |  |  |  |
| GO:0031446\_regulation\_of\_fast-twitch\_skeletal\_muscle\_fiber\_contraction | 1 | 0 |  |  |  |  |  |  |  |  |
| GO:0031448\_positive\_regulation\_of\_fast-twitch\_skeletal\_muscle\_fiber\_contraction | 1 | 0 |  |  |  |  |  |  |  |  |
| GO:0031453\_positive\_regulation\_of\_heterochromatin\_formation | 1 | 0 |  |  |  |  |  |  |  |  |
| GO:0031557\_induction\_of\_programmed\_cell\_death\_in\_response\_to\_chemical\_stimulus | 1 | 0 |  |  |  |  |  |  |  |  |
| GO:0031574\_S-M\_checkpoint | 1 | 0 |  |  |  |  |  |  |  |  |
| GO:0031581\_hemidesmosome\_assembly | 1 | 0 |  |  |  |  |  |  |  |  |
| GO:0031627\_telomeric\_loop\_formation | 1 | 0 |  |  |  |  |  |  |  |  |
| GO:0031848\_protection\_from\_non-homologous\_end\_joining\_at\_telomere | 1 | 0 |  |  |  |  |  |  |  |  |
| GO:0031937\_positive\_regulation\_of\_chromatin\_silencing | 1 | 0 |  |  |  |  |  |  |  |  |
| GO:0031943\_regulation\_of\_glucocorticoid\_metabolic\_process | 1 | 0 |  |  |  |  |  |  |  |  |
| GO:0031954\_positive\_regulation\_of\_protein\_amino\_acid\_autophosphorylation | 1 | 0 |  |  |  |  |  |  |  |  |
| GO:0031999\_negative\_regulation\_of\_fatty\_acid\_beta-oxidation | 1 | 0 |  |  |  |  |  |  |  |  |
| GO:0032011\_ARF\_protein\_signal\_transduction | 1 | 0 |  |  |  |  |  |  |  |  |
| GO:0032023\_trypsinogen\_activation | 1 | 0 |  |  |  |  |  |  |  |  |
| GO:0032025\_response\_to\_cobalt\_ion | 1 | 0 |  |  |  |  |  |  |  |  |
| GO:0032048\_cardiolipin\_metabolic\_process | 1 | 0 |  |  |  |  |  |  |  |  |
| GO:0032049\_cardiolipin\_biosynthetic\_process | 1 | 0 |  |  |  |  |  |  |  |  |
| GO:0032060\_bleb\_formation | 1 | 0 |  |  |  |  |  |  |  |  |
| GO:0032066\_nucleolus\_to\_nucleoplasm\_transport | 1 | 0 |  |  |  |  |  |  |  |  |
| GO:0032074\_negative\_regulation\_of\_nuclease\_activity | 1 | 0 |  |  |  |  |  |  |  |  |
| GO:0032075\_positive\_regulation\_of\_nuclease\_activity | 1 | 0 |  |  |  |  |  |  |  |  |
| GO:0032119\_sequestering\_of\_zinc\_ion | 1 | 0 |  |  |  |  |  |  |  |  |
| GO:0032185\_septin\_cytoskeleton\_organization | 1 | 0 |  |  |  |  |  |  |  |  |
| GO:0032196\_transposition | 1 | 0 |  |  |  |  |  |  |  |  |
| GO:0032235\_negative\_regulation\_of\_calcium\_ion\_transport\_via\_store-operated\_calcium\_channel\_activity | 1 | 0 |  |  |  |  |  |  |  |  |
| GO:0032241\_positive\_regulation\_of\_nucleobase\_\_nucleoside\_\_nucleotide\_and\_nucleic\_acid\_transport | 1 | 0 |  |  |  |  |  |  |  |  |
| GO:0032261\_purine\_nucleotide\_salvage | 1 | 0 |  |  |  |  |  |  |  |  |
| GO:0032275\_luteinizing\_hormone\_secretion | 1 | 0 |  |  |  |  |  |  |  |  |
| GO:0032287\_myelin\_maintenance\_in\_the\_peripheral\_nervous\_system | 1 | 0 |  |  |  |  |  |  |  |  |
| GO:0032288\_myelin\_assembly | 1 | 0 |  |  |  |  |  |  |  |  |
| GO:0032314\_regulation\_of\_Rac\_GTPase\_activity | 1 | 0 |  |  |  |  |  |  |  |  |
| GO:0032330\_regulation\_of\_chondrocyte\_differentiation | 1 | 0 |  |  |  |  |  |  |  |  |
| GO:0032331\_negative\_regulation\_of\_chondrocyte\_differentiation | 1 | 0 |  |  |  |  |  |  |  |  |
| GO:0032346\_positive\_regulation\_of\_aldosterone\_metabolic\_process | 1 | 0 |  |  |  |  |  |  |  |  |
| GO:0032347\_regulation\_of\_aldosterone\_biosynthetic\_process | 1 | 0 |  |  |  |  |  |  |  |  |
| GO:0032349\_positive\_regulation\_of\_aldosterone\_biosynthetic\_process | 1 | 0 |  |  |  |  |  |  |  |  |
| GO:0032354\_response\_to\_follicle-stimulating\_hormone\_stimulus | 1 | 0 |  |  |  |  |  |  |  |  |
| GO:0032377\_regulation\_of\_intracellular\_lipid\_transport | 1 | 0 |  |  |  |  |  |  |  |  |
| GO:0032380\_regulation\_of\_intracellular\_sterol\_transport | 1 | 0 |  |  |  |  |  |  |  |  |
| GO:0032383\_regulation\_of\_intracellular\_cholesterol\_transport | 1 | 0 |  |  |  |  |  |  |  |  |
| GO:0032423\_regulation\_of\_mismatch\_repair | 1 | 0 |  |  |  |  |  |  |  |  |
| GO:0032425\_positive\_regulation\_of\_mismatch\_repair | 1 | 0 |  |  |  |  |  |  |  |  |
| GO:0032459\_regulation\_of\_protein\_oligomerization | 1 | 0 |  |  |  |  |  |  |  |  |
| GO:0032460\_negative\_regulation\_of\_protein\_oligomerization | 1 | 0 |  |  |  |  |  |  |  |  |
| GO:0032462\_regulation\_of\_protein\_homooligomerization | 1 | 0 |  |  |  |  |  |  |  |  |
| GO:0032463\_negative\_regulation\_of\_protein\_homooligomerization | 1 | 0 |  |  |  |  |  |  |  |  |
| GO:0032467\_positive\_regulation\_of\_cytokinesis | 1 | 0 |  |  |  |  |  |  |  |  |
| GO:0032468\_Golgi\_calcium\_ion\_homeostasis | 1 | 0 |  |  |  |  |  |  |  |  |
| GO:0032470\_elevation\_of\_endoplasmic\_reticulum\_calcium\_ion\_concentration | 1 | 0 |  |  |  |  |  |  |  |  |
| GO:0032471\_reduction\_of\_endoplasmic\_reticulum\_calcium\_ion\_concentration | 1 | 0 |  |  |  |  |  |  |  |  |
| GO:0032472\_Golgi\_calcium\_ion\_transport | 1 | 0 |  |  |  |  |  |  |  |  |
| GO:0032486\_Rap\_protein\_signal\_transduction | 1 | 0 |  |  |  |  |  |  |  |  |
| GO:0032495\_response\_to\_muramyl\_dipeptide | 1 | 0 |  |  |  |  |  |  |  |  |
| GO:0032498\_detection\_of\_muramyl\_dipeptide | 1 | 0 |  |  |  |  |  |  |  |  |
| GO:0032499\_detection\_of\_peptidoglycan | 1 | 0 |  |  |  |  |  |  |  |  |
| GO:0032528\_microvillus\_organization | 1 | 0 |  |  |  |  |  |  |  |  |
| GO:0032581\_ER-dependent\_peroxisome\_biogenesis | 1 | 0 |  |  |  |  |  |  |  |  |
| GO:0032594\_protein\_transport\_within\_lipid\_bilayer | 1 | 0 |  |  |  |  |  |  |  |  |
| GO:0032595\_B\_cell\_receptor\_transport\_within\_lipid\_bilayer | 1 | 0 |  |  |  |  |  |  |  |  |
| GO:0032596\_protein\_transport\_into\_membrane\_raft | 1 | 0 |  |  |  |  |  |  |  |  |
| GO:0032597\_B\_cell\_receptor\_transport\_into\_membrane\_raft | 1 | 0 |  |  |  |  |  |  |  |  |
| GO:0032599\_protein\_transport\_out\_of\_membrane\_raft | 1 | 0 |  |  |  |  |  |  |  |  |
| GO:0032600\_chemokine\_receptor\_transport\_out\_of\_membrane\_raft | 1 | 0 |  |  |  |  |  |  |  |  |
| GO:0032601\_connective\_tissue\_growth\_factor\_production | 1 | 0 |  |  |  |  |  |  |  |  |
| GO:0032603\_fractalkine\_production | 1 | 0 |  |  |  |  |  |  |  |  |
| GO:0032605\_hepatocyte\_growth\_factor\_production | 1 | 0 |  |  |  |  |  |  |  |  |
| GO:0032610\_interleukin-1\_alpha\_production | 1 | 0 |  |  |  |  |  |  |  |  |
| GO:0032621\_interleukin-18\_production | 1 | 0 |  |  |  |  |  |  |  |  |
| GO:0032639\_TRAIL\_production | 1 | 0 |  |  |  |  |  |  |  |  |
| GO:0032644\_regulation\_of\_fractalkine\_production | 1 | 0 |  |  |  |  |  |  |  |  |
| GO:0032646\_regulation\_of\_hepatocyte\_growth\_factor\_production | 1 | 0 |  |  |  |  |  |  |  |  |
| GO:0032650\_regulation\_of\_interleukin-1\_alpha\_production | 1 | 0 |  |  |  |  |  |  |  |  |
| GO:0032661\_regulation\_of\_interleukin-18\_production | 1 | 0 |  |  |  |  |  |  |  |  |
| GO:0032679\_regulation\_of\_TRAIL\_production | 1 | 0 |  |  |  |  |  |  |  |  |
| GO:0032681\_regulation\_of\_lymphotoxin\_A\_production | 1 | 0 |  |  |  |  |  |  |  |  |
| GO:0032693\_negative\_regulation\_of\_interleukin-10\_production | 1 | 0 |  |  |  |  |  |  |  |  |
| GO:0032703\_negative\_regulation\_of\_interleukin-2\_production | 1 | 0 |  |  |  |  |  |  |  |  |
| GO:0032713\_negative\_regulation\_of\_interleukin-4\_production | 1 | 0 |  |  |  |  |  |  |  |  |
| GO:0032730\_positive\_regulation\_of\_interleukin-1\_alpha\_production | 1 | 0 |  |  |  |  |  |  |  |  |
| GO:0032732\_positive\_regulation\_of\_interleukin-1\_production | 1 | 0 |  |  |  |  |  |  |  |  |
| GO:0032736\_positive\_regulation\_of\_interleukin-13\_production | 1 | 0 |  |  |  |  |  |  |  |  |
| GO:0032753\_positive\_regulation\_of\_interleukin-4\_production | 1 | 0 |  |  |  |  |  |  |  |  |
| GO:0032754\_positive\_regulation\_of\_interleukin-5\_production | 1 | 0 |  |  |  |  |  |  |  |  |
| GO:0032762\_mast\_cell\_cytokine\_production | 1 | 0 |  |  |  |  |  |  |  |  |
| GO:0032763\_regulation\_of\_mast\_cell\_cytokine\_production | 1 | 0 |  |  |  |  |  |  |  |  |
| GO:0032765\_positive\_regulation\_of\_mast\_cell\_cytokine\_production | 1 | 0 |  |  |  |  |  |  |  |  |
| GO:0032784\_regulation\_of\_RNA\_elongation | 1 | 0 |  |  |  |  |  |  |  |  |
| GO:0032786\_positive\_regulation\_of\_RNA\_elongation | 1 | 0 |  |  |  |  |  |  |  |  |
| GO:0032788\_saturated\_monocarboxylic\_acid\_metabolic\_process | 1 | 0 |  |  |  |  |  |  |  |  |
| GO:0032789\_unsaturated\_monocarboxylic\_acid\_metabolic\_process | 1 | 0 |  |  |  |  |  |  |  |  |
| GO:0032790\_ribosome\_disassembly | 1 | 0 |  |  |  |  |  |  |  |  |
| GO:0032792\_negative\_regulation\_of\_CREB\_transcription\_factor\_activity | 1 | 0 |  |  |  |  |  |  |  |  |
| GO:0032793\_positive\_regulation\_of\_CREB\_transcription\_factor\_activity | 1 | 0 |  |  |  |  |  |  |  |  |
| GO:0032804\_negative\_regulation\_of\_low-density\_lipoprotein\_receptor\_catabolic\_process | 1 | 0 |  |  |  |  |  |  |  |  |
| GO:0032805\_positive\_regulation\_of\_low-density\_lipoprotein\_receptor\_catabolic\_process | 1 | 0 |  |  |  |  |  |  |  |  |
| GO:0032812\_positive\_regulation\_of\_epinephrine\_secretion | 1 | 0 |  |  |  |  |  |  |  |  |
| GO:0032835\_glomerulus\_development | 1 | 0 |  |  |  |  |  |  |  |  |
| GO:0032847\_regulation\_of\_cellular\_pH\_reduction | 1 | 0 |  |  |  |  |  |  |  |  |
| GO:0032848\_negative\_regulation\_of\_cellular\_pH\_reduction | 1 | 0 |  |  |  |  |  |  |  |  |
| GO:0032899\_regulation\_of\_neurotrophin\_production | 1 | 0 |  |  |  |  |  |  |  |  |
| GO:0032900\_negative\_regulation\_of\_neurotrophin\_production | 1 | 0 |  |  |  |  |  |  |  |  |
| GO:0032903\_regulation\_of\_nerve\_growth\_factor\_production | 1 | 0 |  |  |  |  |  |  |  |  |
| GO:0032904\_negative\_regulation\_of\_nerve\_growth\_factor\_production | 1 | 0 |  |  |  |  |  |  |  |  |
| GO:0032907\_transforming\_growth\_factor-beta3\_production | 1 | 0 |  |  |  |  |  |  |  |  |
| GO:0032910\_regulation\_of\_transforming\_growth\_factor-beta3\_production | 1 | 0 |  |  |  |  |  |  |  |  |
| GO:0032911\_negative\_regulation\_of\_transforming\_growth\_factor-beta1\_production | 1 | 0 |  |  |  |  |  |  |  |  |
| GO:0032913\_negative\_regulation\_of\_transforming\_growth\_factor-beta3\_production | 1 | 0 |  |  |  |  |  |  |  |  |
| GO:0032926\_negative\_regulation\_of\_activin\_receptor\_signaling\_pathway | 1 | 0 |  |  |  |  |  |  |  |  |
| GO:0032933\_SREBP-mediated\_signaling\_pathway | 1 | 0 |  |  |  |  |  |  |  |  |
| GO:0032938\_negative\_regulation\_of\_translation\_in\_response\_to\_oxidative\_stress | 1 | 0 |  |  |  |  |  |  |  |  |
| GO:0032958\_inositol\_phosphate\_biosynthetic\_process | 1 | 0 |  |  |  |  |  |  |  |  |
| GO:0032976\_release\_of\_matrix\_enzymes\_from\_mitochondria | 1 | 0 |  |  |  |  |  |  |  |  |
| GO:0032980\_keratinocyte\_activation | 1 | 0 |  |  |  |  |  |  |  |  |
| GO:0032988\_ribonucleoprotein\_complex\_disassembly | 1 | 0 |  |  |  |  |  |  |  |  |
| GO:0033029\_regulation\_of\_neutrophil\_apoptosis | 1 | 0 |  |  |  |  |  |  |  |  |
| GO:0033031\_positive\_regulation\_of\_neutrophil\_apoptosis | 1 | 0 |  |  |  |  |  |  |  |  |
| GO:0033079\_immature\_T\_cell\_proliferation | 1 | 0 |  |  |  |  |  |  |  |  |
| GO:0033080\_immature\_T\_cell\_proliferation\_in\_the\_thymus | 1 | 0 |  |  |  |  |  |  |  |  |
| GO:0033083\_regulation\_of\_immature\_T\_cell\_proliferation | 1 | 0 |  |  |  |  |  |  |  |  |
| GO:0033084\_regulation\_of\_immature\_T\_cell\_proliferation\_in\_the\_thymus | 1 | 0 |  |  |  |  |  |  |  |  |
| GO:0033085\_negative\_regulation\_of\_T\_cell\_differentiation\_in\_the\_thymus | 1 | 0 |  |  |  |  |  |  |  |  |
| GO:0033087\_negative\_regulation\_of\_immature\_T\_cell\_proliferation | 1 | 0 |  |  |  |  |  |  |  |  |
| GO:0033088\_negative\_regulation\_of\_immature\_T\_cell\_proliferation\_in\_the\_thymus | 1 | 0 |  |  |  |  |  |  |  |  |
| GO:0033136\_serine\_phosphorylation\_of\_STAT3\_protein | 1 | 0 |  |  |  |  |  |  |  |  |
| GO:0033137\_negative\_regulation\_of\_peptidyl-serine\_phosphorylation | 1 | 0 |  |  |  |  |  |  |  |  |
| GO:0033139\_regulation\_of\_peptidyl-serine\_phosphorylation\_of\_STAT\_protein | 1 | 0 |  |  |  |  |  |  |  |  |
| GO:0033141\_positive\_regulation\_of\_peptidyl-serine\_phosphorylation\_of\_STAT\_protein | 1 | 0 |  |  |  |  |  |  |  |  |
| GO:0033153\_T\_cell\_receptor\_V(D)J\_recombination | 1 | 0 |  |  |  |  |  |  |  |  |
| GO:0033169\_histone\_H3-K9\_demethylation | 1 | 0 |  |  |  |  |  |  |  |  |
| GO:0033173\_calcineurin-NFAT\_signaling\_pathway | 1 | 0 |  |  |  |  |  |  |  |  |
| GO:0033182\_regulation\_of\_histone\_ubiquitination | 1 | 0 |  |  |  |  |  |  |  |  |
| GO:0033206\_cytokinesis\_after\_meiosis | 1 | 0 |  |  |  |  |  |  |  |  |
| GO:0033239\_negative\_regulation\_of\_cellular\_amine\_metabolic\_process | 1 | 0 |  |  |  |  |  |  |  |  |
| GO:0033240\_positive\_regulation\_of\_cellular\_amine\_metabolic\_process | 1 | 0 |  |  |  |  |  |  |  |  |
| GO:0033260\_DNA\_replication\_during\_S\_phase | 1 | 0 |  |  |  |  |  |  |  |  |
| GO:0033262\_regulation\_of\_DNA\_replication\_during\_S\_phase | 1 | 0 |  |  |  |  |  |  |  |  |
| GO:0033292\_T-tubule\_organization | 1 | 0 |  |  |  |  |  |  |  |  |
| GO:0033341\_regulation\_of\_collagen\_binding | 1 | 0 |  |  |  |  |  |  |  |  |
| GO:0033342\_negative\_regulation\_of\_collagen\_binding | 1 | 0 |  |  |  |  |  |  |  |  |
| GO:0033345\_asparagine\_catabolic\_process\_via\_L-aspartate | 1 | 0 |  |  |  |  |  |  |  |  |
| GO:0033366\_protein\_localization\_in\_secretory\_granule | 1 | 0 |  |  |  |  |  |  |  |  |
| GO:0033367\_protein\_localization\_in\_mast\_cell\_secretory\_granule | 1 | 0 |  |  |  |  |  |  |  |  |
| GO:0033368\_protease\_localization\_in\_mast\_cell\_secretory\_granule | 1 | 0 |  |  |  |  |  |  |  |  |
| GO:0033370\_maintenance\_of\_protein\_location\_in\_mast\_cell\_secretory\_granule | 1 | 0 |  |  |  |  |  |  |  |  |
| GO:0033371\_T\_cell\_secretory\_granule\_organization | 1 | 0 |  |  |  |  |  |  |  |  |
| GO:0033373\_maintenance\_of\_protease\_location\_in\_mast\_cell\_secretory\_granule | 1 | 0 |  |  |  |  |  |  |  |  |
| GO:0033374\_protein\_localization\_in\_T\_cell\_secretory\_granule | 1 | 0 |  |  |  |  |  |  |  |  |
| GO:0033375\_protease\_localization\_in\_T\_cell\_secretory\_granule | 1 | 0 |  |  |  |  |  |  |  |  |
| GO:0033377\_maintenance\_of\_protein\_location\_in\_T\_cell\_secretory\_granule | 1 | 0 |  |  |  |  |  |  |  |  |
| GO:0033379\_maintenance\_of\_protease\_location\_in\_T\_cell\_secretory\_granule | 1 | 0 |  |  |  |  |  |  |  |  |
| GO:0033380\_granzyme\_B\_localization\_in\_T\_cell\_secretory\_granule | 1 | 0 |  |  |  |  |  |  |  |  |
| GO:0033382\_maintenance\_of\_granzyme\_B\_location\_in\_T\_cell\_secretory\_granule | 1 | 0 |  |  |  |  |  |  |  |  |
| GO:0033484\_nitric\_oxide\_homeostasis | 1 | 0 |  |  |  |  |  |  |  |  |
| GO:0033504\_floor\_plate\_development | 1 | 0 |  |  |  |  |  |  |  |  |
| GO:0033566\_gamma-tubulin\_complex\_localization | 1 | 0 |  |  |  |  |  |  |  |  |
| GO:0033577\_protein\_amino\_acid\_glycosylation\_in\_endoplasmic\_reticulum | 1 | 0 |  |  |  |  |  |  |  |  |
| GO:0033595\_response\_to\_genistein | 1 | 0 |  |  |  |  |  |  |  |  |
| GO:0033600\_negative\_regulation\_of\_mammary\_gland\_epithelial\_cell\_proliferation | 1 | 0 |  |  |  |  |  |  |  |  |
| GO:0033606\_chemokine\_receptor\_transport\_within\_lipid\_bilayer | 1 | 0 |  |  |  |  |  |  |  |  |
| GO:0033617\_mitochondrial\_respiratory\_chain\_complex\_IV\_assembly | 1 | 0 |  |  |  |  |  |  |  |  |
| GO:0033622\_integrin\_activation | 1 | 0 |  |  |  |  |  |  |  |  |
| GO:0033623\_regulation\_of\_integrin\_activation | 1 | 0 |  |  |  |  |  |  |  |  |
| GO:0033625\_positive\_regulation\_of\_integrin\_activation | 1 | 0 |  |  |  |  |  |  |  |  |
| GO:0033693\_neurofilament\_bundle\_assembly | 1 | 0 |  |  |  |  |  |  |  |  |
| GO:0033750\_ribosome\_localization | 1 | 0 |  |  |  |  |  |  |  |  |
| GO:0033753\_establishment\_of\_ribosome\_localization | 1 | 0 |  |  |  |  |  |  |  |  |
| GO:0033875\_ribonucleoside\_bisphosphate\_metabolic\_process | 1 | 0 |  |  |  |  |  |  |  |  |
| GO:0033962\_cytoplasmic\_mRNA\_processing\_body\_assembly | 1 | 0 |  |  |  |  |  |  |  |  |
| GO:0034032\_purine\_nucleoside\_bisphosphate\_metabolic\_process | 1 | 0 |  |  |  |  |  |  |  |  |
| GO:0034035\_purine\_ribonucleoside\_bisphosphate\_metabolic\_process | 1 | 0 |  |  |  |  |  |  |  |  |
| GO:0034063\_stress\_granule\_assembly | 1 | 0 |  |  |  |  |  |  |  |  |
| GO:0034080\_CenH3-containing\_nucleosome\_assembly\_at\_centromere | 1 | 0 |  |  |  |  |  |  |  |  |
| GO:0034109\_homotypic\_cell-cell\_adhesion | 1 | 0 |  |  |  |  |  |  |  |  |
| GO:0034115\_negative\_regulation\_of\_heterotypic\_cell-cell\_adhesion | 1 | 0 |  |  |  |  |  |  |  |  |
| GO:0034116\_positive\_regulation\_of\_heterotypic\_cell-cell\_adhesion | 1 | 0 |  |  |  |  |  |  |  |  |
| GO:0034122\_negative\_regulation\_of\_toll-like\_receptor\_signaling\_pathway | 1 | 0 |  |  |  |  |  |  |  |  |
| GO:0034123\_positive\_regulation\_of\_toll-like\_receptor\_signaling\_pathway | 1 | 0 |  |  |  |  |  |  |  |  |
| GO:0034142\_toll-like\_receptor\_4\_signaling\_pathway | 1 | 0 |  |  |  |  |  |  |  |  |
| GO:0034143\_regulation\_of\_toll-like\_receptor\_4\_signaling\_pathway | 1 | 0 |  |  |  |  |  |  |  |  |
| GO:0034145\_positive\_regulation\_of\_toll-like\_receptor\_4\_signaling\_pathway | 1 | 0 |  |  |  |  |  |  |  |  |
| GO:0034196\_acylglycerol\_transport | 1 | 0 |  |  |  |  |  |  |  |  |
| GO:0034197\_triglyceride\_transport | 1 | 0 |  |  |  |  |  |  |  |  |
| GO:0034205\_beta-amyloid\_formation | 1 | 0 |  |  |  |  |  |  |  |  |
| GO:0034213\_quinolinate\_catabolic\_process | 1 | 0 |  |  |  |  |  |  |  |  |
| GO:0034231\_islet\_amyloid\_polypeptide\_processing | 1 | 0 |  |  |  |  |  |  |  |  |
| GO:0034238\_macrophage\_fusion | 1 | 0 |  |  |  |  |  |  |  |  |
| GO:0034239\_regulation\_of\_macrophage\_fusion | 1 | 0 |  |  |  |  |  |  |  |  |
| GO:0034241\_positive\_regulation\_of\_macrophage\_fusion | 1 | 0 |  |  |  |  |  |  |  |  |
| GO:0034248\_regulation\_of\_amide\_metabolic\_process | 1 | 0 |  |  |  |  |  |  |  |  |
| GO:0034263\_autophagy\_in\_response\_to\_ER\_overload | 1 | 0 |  |  |  |  |  |  |  |  |
| GO:0034287\_detection\_of\_monosaccharide\_stimulus | 1 | 0 |  |  |  |  |  |  |  |  |
| GO:0034313\_diol\_catabolic\_process | 1 | 0 |  |  |  |  |  |  |  |  |
| GO:0034332\_adherens\_junction\_organization | 1 | 0 |  |  |  |  |  |  |  |  |
| GO:0034333\_adherens\_junction\_assembly | 1 | 0 |  |  |  |  |  |  |  |  |
| GO:0034340\_response\_to\_type\_I\_interferon | 1 | 0 |  |  |  |  |  |  |  |  |
| GO:0034356\_NAD\_biosynthesis\_via\_nicotinamide\_riboside\_salvage\_pathway | 1 | 0 |  |  |  |  |  |  |  |  |
| GO:0034373\_intermediate-density\_lipoprotein\_particle\_remodeling | 1 | 0 |  |  |  |  |  |  |  |  |
| GO:0034378\_chylomicron\_assembly | 1 | 0 |  |  |  |  |  |  |  |  |
| GO:0034436\_glycoprotein\_transport | 1 | 0 |  |  |  |  |  |  |  |  |
| GO:0034439\_lipoprotein\_lipid\_oxidation | 1 | 0 |  |  |  |  |  |  |  |  |
| GO:0034454\_microtubule\_anchoring\_at\_centrosome | 1 | 0 |  |  |  |  |  |  |  |  |
| GO:0034465\_response\_to\_carbon\_monoxide | 1 | 0 |  |  |  |  |  |  |  |  |
| GO:0034509\_centromeric\_core\_chromatin\_formation | 1 | 0 |  |  |  |  |  |  |  |  |
| GO:0034516\_response\_to\_vitamin\_B6 | 1 | 0 |  |  |  |  |  |  |  |  |
| GO:0034551\_mitochondrial\_respiratory\_chain\_complex\_III\_assembly | 1 | 0 |  |  |  |  |  |  |  |  |
| GO:0034552\_respiratory\_chain\_complex\_II\_assembly | 1 | 0 |  |  |  |  |  |  |  |  |
| GO:0034553\_mitochondrial\_respiratory\_chain\_complex\_II\_assembly | 1 | 0 |  |  |  |  |  |  |  |  |
| GO:0034589\_hydroxyproline\_transport | 1 | 0 |  |  |  |  |  |  |  |  |
| GO:0034694\_response\_to\_prostaglandin\_stimulus | 1 | 0 |  |  |  |  |  |  |  |  |
| GO:0034695\_response\_to\_prostaglandin\_E\_stimulus | 1 | 0 |  |  |  |  |  |  |  |  |
| GO:0034698\_response\_to\_gonadotropin\_stimulus | 1 | 0 |  |  |  |  |  |  |  |  |
| GO:0034699\_response\_to\_luteinizing\_hormone\_stimulus | 1 | 0 |  |  |  |  |  |  |  |  |
| GO:0034724\_DNA\_replication-independent\_nucleosome\_organization | 1 | 0 |  |  |  |  |  |  |  |  |
| GO:0034729\_histone\_H3-K79\_methylation | 1 | 0 |  |  |  |  |  |  |  |  |
| GO:0034755\_iron\_ion\_transmembrane\_transport | 1 | 0 |  |  |  |  |  |  |  |  |
| GO:0034764\_positive\_regulation\_of\_transmembrane\_transport | 1 | 0 |  |  |  |  |  |  |  |  |
| GO:0034765\_regulation\_of\_ion\_transmembrane\_transport | 1 | 0 |  |  |  |  |  |  |  |  |
| GO:0034767\_positive\_regulation\_of\_ion\_transmembrane\_transport | 1 | 0 |  |  |  |  |  |  |  |  |
| GO:0034959\_endothelin\_maturation | 1 | 0 |  |  |  |  |  |  |  |  |
| GO:0034982\_mitochondrial\_protein\_processing | 1 | 0 |  |  |  |  |  |  |  |  |
| GO:0034983\_peptidyl-lysine\_deacetylation | 1 | 0 |  |  |  |  |  |  |  |  |
| GO:0035021\_negative\_regulation\_of\_Rac\_protein\_signal\_transduction | 1 | 0 |  |  |  |  |  |  |  |  |
| GO:0035041\_sperm\_chromatin\_decondensation | 1 | 0 |  |  |  |  |  |  |  |  |
| GO:0035042\_fertilization\_\_exchange\_of\_chromosomal\_proteins | 1 | 0 |  |  |  |  |  |  |  |  |
| GO:0035054\_embryonic\_heart\_tube\_anterior\_posterior\_pattern\_formation | 1 | 0 |  |  |  |  |  |  |  |  |
| GO:0035066\_positive\_regulation\_of\_histone\_acetylation | 1 | 0 |  |  |  |  |  |  |  |  |
| GO:0035082\_axoneme\_assembly | 1 | 0 |  |  |  |  |  |  |  |  |
| GO:0035087\_RNA\_interference\_\_siRNA\_loading\_onto\_RISC | 1 | 0 |  |  |  |  |  |  |  |  |
| GO:0035090\_maintenance\_of\_apical\_basal\_cell\_polarity | 1 | 0 |  |  |  |  |  |  |  |  |
| GO:0035093\_spermatogenesis\_\_exchange\_of\_chromosomal\_proteins | 1 | 0 |  |  |  |  |  |  |  |  |
| GO:0035104\_positive\_regulation\_of\_transcription\_via\_sterol\_regulatory\_element\_binding | 1 | 0 |  |  |  |  |  |  |  |  |
| GO:0035110\_leg\_morphogenesis | 1 | 0 |  |  |  |  |  |  |  |  |
| GO:0035112\_genitalia\_morphogenesis | 1 | 0 |  |  |  |  |  |  |  |  |
| GO:0035116\_embryonic\_hindlimb\_morphogenesis | 1 | 0 |  |  |  |  |  |  |  |  |
| GO:0035137\_hindlimb\_morphogenesis | 1 | 0 |  |  |  |  |  |  |  |  |
| GO:0035238\_vitamin\_A\_biosynthetic\_process | 1 | 0 |  |  |  |  |  |  |  |  |
| GO:0035265\_organ\_growth | 1 | 0 |  |  |  |  |  |  |  |  |
| GO:0035280\_gene\_silencing\_by\_miRNA\_\_miRNA\_loading\_onto\_RISC | 1 | 0 |  |  |  |  |  |  |  |  |
| GO:0040009\_regulation\_of\_growth\_rate | 1 | 0 |  |  |  |  |  |  |  |  |
| GO:0040013\_negative\_regulation\_of\_locomotion | 1 | 0 |  |  |  |  |  |  |  |  |
| GO:0040015\_negative\_regulation\_of\_multicellular\_organism\_growth | 1 | 0 |  |  |  |  |  |  |  |  |
| GO:0040020\_regulation\_of\_meiosis | 1 | 0 |  |  |  |  |  |  |  |  |
| GO:0040023\_establishment\_of\_nucleus\_localization | 1 | 0 |  |  |  |  |  |  |  |  |
| GO:0040030\_regulation\_of\_molecular\_function\_\_epigenetic | 1 | 0 |  |  |  |  |  |  |  |  |
| GO:0040037\_negative\_regulation\_of\_fibroblast\_growth\_factor\_receptor\_signaling\_pathway | 1 | 0 |  |  |  |  |  |  |  |  |
| GO:0040038\_polar\_body\_extrusion\_after\_meiotic\_divisions | 1 | 0 |  |  |  |  |  |  |  |  |
| GO:0042074\_cell\_migration\_involved\_in\_gastrulation | 1 | 0 |  |  |  |  |  |  |  |  |
| GO:0042091\_interleukin-10\_biosynthetic\_process | 1 | 0 |  |  |  |  |  |  |  |  |
| GO:0042118\_endothelial\_cell\_activation | 1 | 0 |  |  |  |  |  |  |  |  |
| GO:0042159\_lipoprotein\_catabolic\_process | 1 | 0 |  |  |  |  |  |  |  |  |
| GO:0042214\_terpene\_metabolic\_process | 1 | 0 |  |  |  |  |  |  |  |  |
| GO:0042225\_interleukin-5\_biosynthetic\_process | 1 | 0 |  |  |  |  |  |  |  |  |
| GO:0042241\_interleukin-18\_biosynthetic\_process | 1 | 0 |  |  |  |  |  |  |  |  |
| GO:0042257\_ribosomal\_subunit\_assembly | 1 | 0 |  |  |  |  |  |  |  |  |
| GO:0042262\_DNA\_protection | 1 | 0 |  |  |  |  |  |  |  |  |
| GO:0042276\_error-prone\_postreplication\_DNA\_repair | 1 | 0 |  |  |  |  |  |  |  |  |
| GO:0042313\_protein\_kinase\_C\_deactivation | 1 | 0 |  |  |  |  |  |  |  |  |
| GO:0042369\_vitamin\_D\_catabolic\_process | 1 | 0 |  |  |  |  |  |  |  |  |
| GO:0042412\_taurine\_biosynthetic\_process | 1 | 0 |  |  |  |  |  |  |  |  |
| GO:0042418\_epinephrine\_biosynthetic\_process | 1 | 0 |  |  |  |  |  |  |  |  |
| GO:0042421\_norepinephrine\_biosynthetic\_process | 1 | 0 |  |  |  |  |  |  |  |  |
| GO:0042424\_catecholamine\_catabolic\_process | 1 | 0 |  |  |  |  |  |  |  |  |
| GO:0042428\_serotonin\_metabolic\_process | 1 | 0 |  |  |  |  |  |  |  |  |
| GO:0042435\_indole\_derivative\_biosynthetic\_process | 1 | 0 |  |  |  |  |  |  |  |  |
| GO:0042474\_middle\_ear\_morphogenesis | 1 | 0 |  |  |  |  |  |  |  |  |
| GO:0042504\_tyrosine\_phosphorylation\_of\_Stat4\_protein | 1 | 0 |  |  |  |  |  |  |  |  |
| GO:0042519\_regulation\_of\_tyrosine\_phosphorylation\_of\_Stat4\_protein | 1 | 0 |  |  |  |  |  |  |  |  |
| GO:0042520\_positive\_regulation\_of\_tyrosine\_phosphorylation\_of\_Stat4\_protein | 1 | 0 |  |  |  |  |  |  |  |  |
| GO:0042524\_negative\_regulation\_of\_tyrosine\_phosphorylation\_of\_Stat5\_protein | 1 | 0 |  |  |  |  |  |  |  |  |
| GO:0042537\_benzene\_and\_derivative\_metabolic\_process | 1 | 0 |  |  |  |  |  |  |  |  |
| GO:0042560\_pteridine\_and\_derivative\_catabolic\_process | 1 | 0 |  |  |  |  |  |  |  |  |
| GO:0042596\_fear\_response | 1 | 0 |  |  |  |  |  |  |  |  |
| GO:0042637\_catagen | 1 | 0 |  |  |  |  |  |  |  |  |
| GO:0042640\_anagen | 1 | 0 |  |  |  |  |  |  |  |  |
| GO:0042670\_retinal\_cone\_cell\_differentiation | 1 | 0 |  |  |  |  |  |  |  |  |
| GO:0042700\_luteinizing\_hormone\_signaling\_pathway | 1 | 0 |  |  |  |  |  |  |  |  |
| GO:0042703\_menstruation | 1 | 0 |  |  |  |  |  |  |  |  |
| GO:0042726\_riboflavin\_and\_derivative\_metabolic\_process | 1 | 0 |  |  |  |  |  |  |  |  |
| GO:0042727\_riboflavin\_and\_derivative\_biosynthetic\_process | 1 | 0 |  |  |  |  |  |  |  |  |
| GO:0042746\_circadian\_sleep\_wake\_cycle\_\_wakefulness | 1 | 0 |  |  |  |  |  |  |  |  |
| GO:0042748\_circadian\_sleep\_wake\_cycle\_\_non-REM\_sleep | 1 | 0 |  |  |  |  |  |  |  |  |
| GO:0042755\_eating\_behavior | 1 | 0 |  |  |  |  |  |  |  |  |
| GO:0042756\_drinking\_behavior | 1 | 0 |  |  |  |  |  |  |  |  |
| GO:0042766\_nucleosome\_mobilization | 1 | 0 |  |  |  |  |  |  |  |  |
| GO:0042780\_tRNA\_3'-end\_processing | 1 | 0 |  |  |  |  |  |  |  |  |
| GO:0042789\_mRNA\_transcription\_from\_RNA\_polymerase\_II\_promoter | 1 | 0 |  |  |  |  |  |  |  |  |
| GO:0042795\_snRNA\_transcription\_from\_RNA\_polymerase\_II\_promoter | 1 | 0 |  |  |  |  |  |  |  |  |
| GO:0042796\_snRNA\_transcription\_from\_RNA\_polymerase\_III\_promoter | 1 | 0 |  |  |  |  |  |  |  |  |
| GO:0042822\_pyridoxal\_phosphate\_metabolic\_process | 1 | 0 |  |  |  |  |  |  |  |  |
| GO:0042823\_pyridoxal\_phosphate\_biosynthetic\_process | 1 | 0 |  |  |  |  |  |  |  |  |
| GO:0042866\_pyruvate\_biosynthetic\_process | 1 | 0 |  |  |  |  |  |  |  |  |
| GO:0042904\_9-cis-retinoic\_acid\_biosynthetic\_process | 1 | 0 |  |  |  |  |  |  |  |  |
| GO:0042905\_9-cis-retinoic\_acid\_metabolic\_process | 1 | 0 |  |  |  |  |  |  |  |  |
| GO:0042985\_negative\_regulation\_of\_amyloid\_precursor\_protein\_biosynthetic\_process | 1 | 0 |  |  |  |  |  |  |  |  |
| GO:0042986\_positive\_regulation\_of\_amyloid\_precursor\_protein\_biosynthetic\_process | 1 | 0 |  |  |  |  |  |  |  |  |
| GO:0042989\_sequestering\_of\_actin\_monomers | 1 | 0 |  |  |  |  |  |  |  |  |
| GO:0042996\_regulation\_of\_Golgi\_to\_plasma\_membrane\_protein\_transport | 1 | 0 |  |  |  |  |  |  |  |  |
| GO:0042997\_negative\_regulation\_of\_Golgi\_to\_plasma\_membrane\_protein\_transport | 1 | 0 |  |  |  |  |  |  |  |  |
| GO:0042999\_regulation\_of\_Golgi\_to\_plasma\_membrane\_CFTR\_protein\_transport | 1 | 0 |  |  |  |  |  |  |  |  |
| GO:0043002\_negative\_regulation\_of\_Golgi\_to\_plasma\_membrane\_CFTR\_protein\_transport | 1 | 0 |  |  |  |  |  |  |  |  |
| GO:0043004\_cytoplasmic\_sequestering\_of\_CFTR\_protein | 1 | 0 |  |  |  |  |  |  |  |  |
| GO:0043012\_regulation\_of\_fusion\_of\_sperm\_to\_egg\_plasma\_membrane | 1 | 0 |  |  |  |  |  |  |  |  |
| GO:0043016\_regulation\_of\_lymphotoxin\_A\_biosynthetic\_process | 1 | 0 |  |  |  |  |  |  |  |  |
| GO:0043017\_positive\_regulation\_of\_lymphotoxin\_A\_biosynthetic\_process | 1 | 0 |  |  |  |  |  |  |  |  |
| GO:0043049\_otic\_placode\_formation | 1 | 0 |  |  |  |  |  |  |  |  |
| GO:0043064\_flagellum\_organization | 1 | 0 |  |  |  |  |  |  |  |  |
| GO:0043116\_negative\_regulation\_of\_vascular\_permeability | 1 | 0 |  |  |  |  |  |  |  |  |
| GO:0043126\_regulation\_of\_1-phosphatidylinositol\_4-kinase\_activity | 1 | 0 |  |  |  |  |  |  |  |  |
| GO:0043128\_positive\_regulation\_of\_1-phosphatidylinositol\_4-kinase\_activity | 1 | 0 |  |  |  |  |  |  |  |  |
| GO:0043129\_surfactant\_homeostasis | 1 | 0 |  |  |  |  |  |  |  |  |
| GO:0043146\_spindle\_stabilization | 1 | 0 |  |  |  |  |  |  |  |  |
| GO:0043148\_mitotic\_spindle\_stabilization | 1 | 0 |  |  |  |  |  |  |  |  |
| GO:0043152\_induction\_of\_bacterial\_agglutination | 1 | 0 |  |  |  |  |  |  |  |  |
| GO:0043173\_nucleotide\_salvage | 1 | 0 |  |  |  |  |  |  |  |  |
| GO:0043174\_nucleoside\_salvage | 1 | 0 |  |  |  |  |  |  |  |  |
| GO:0043181\_vacuolar\_sequestering | 1 | 0 |  |  |  |  |  |  |  |  |
| GO:0043200\_response\_to\_amino\_acid\_stimulus | 1 | 0 |  |  |  |  |  |  |  |  |
| GO:0043217\_myelin\_maintenance | 1 | 0 |  |  |  |  |  |  |  |  |
| GO:0043247\_telomere\_maintenance\_in\_response\_to\_DNA\_damage | 1 | 0 |  |  |  |  |  |  |  |  |
| GO:0043249\_erythrocyte\_maturation | 1 | 0 |  |  |  |  |  |  |  |  |
| GO:0043268\_positive\_regulation\_of\_potassium\_ion\_transport | 1 | 0 |  |  |  |  |  |  |  |  |
| GO:0043299\_leukocyte\_degranulation | 1 | 0 |  |  |  |  |  |  |  |  |
| GO:0043307\_eosinophil\_activation | 1 | 0 |  |  |  |  |  |  |  |  |
| GO:0043308\_eosinophil\_degranulation | 1 | 0 |  |  |  |  |  |  |  |  |
| GO:0043312\_neutrophil\_degranulation | 1 | 0 |  |  |  |  |  |  |  |  |
| GO:0043330\_response\_to\_exogenous\_dsRNA | 1 | 0 |  |  |  |  |  |  |  |  |
| GO:0043353\_enucleate\_erythrocyte\_differentiation | 1 | 0 |  |  |  |  |  |  |  |  |
| GO:0043371\_negative\_regulation\_of\_CD4-positive\_\_alpha\_beta\_T\_cell\_differentiation | 1 | 0 |  |  |  |  |  |  |  |  |
| GO:0043383\_negative\_T\_cell\_selection | 1 | 0 |  |  |  |  |  |  |  |  |
| GO:0043403\_skeletal\_muscle\_regeneration | 1 | 0 |  |  |  |  |  |  |  |  |
| GO:0043418\_homocysteine\_catabolic\_process | 1 | 0 |  |  |  |  |  |  |  |  |
| GO:0043420\_anthranilate\_metabolic\_process | 1 | 0 |  |  |  |  |  |  |  |  |
| GO:0043437\_butanoic\_acid\_metabolic\_process | 1 | 0 |  |  |  |  |  |  |  |  |
| GO:0043455\_regulation\_of\_secondary\_metabolic\_process | 1 | 0 |  |  |  |  |  |  |  |  |
| GO:0043456\_regulation\_of\_pentose-phosphate\_shunt | 1 | 0 |  |  |  |  |  |  |  |  |
| GO:0043457\_regulation\_of\_cellular\_respiration | 1 | 0 |  |  |  |  |  |  |  |  |
| GO:0043517\_positive\_regulation\_of\_DNA\_damage\_response\_\_signal\_transduction\_by\_p53\_class\_mediator | 1 | 0 |  |  |  |  |  |  |  |  |
| GO:0043518\_negative\_regulation\_of\_DNA\_damage\_response\_\_signal\_transduction\_by\_p53\_class\_mediator | 1 | 0 |  |  |  |  |  |  |  |  |
| GO:0043551\_regulation\_of\_phosphoinositide\_3-kinase\_activity | 1 | 0 |  |  |  |  |  |  |  |  |
| GO:0043552\_positive\_regulation\_of\_phosphoinositide\_3-kinase\_activity | 1 | 0 |  |  |  |  |  |  |  |  |
| GO:0043556\_regulation\_of\_translation\_in\_response\_to\_oxidative\_stress | 1 | 0 |  |  |  |  |  |  |  |  |
| GO:0043584\_nose\_development | 1 | 0 |  |  |  |  |  |  |  |  |
| GO:0043586\_tongue\_development | 1 | 0 |  |  |  |  |  |  |  |  |
| GO:0043587\_tongue\_morphogenesis | 1 | 0 |  |  |  |  |  |  |  |  |
| GO:0043647\_inositol\_phosphate\_metabolic\_process | 1 | 0 |  |  |  |  |  |  |  |  |
| GO:0043652\_engulfment\_of\_apoptotic\_cell | 1 | 0 |  |  |  |  |  |  |  |  |
| GO:0043654\_recognition\_of\_apoptotic\_cell | 1 | 0 |  |  |  |  |  |  |  |  |
| GO:0043696\_dedifferentiation | 1 | 0 |  |  |  |  |  |  |  |  |
| GO:0043697\_cell\_dedifferentiation | 1 | 0 |  |  |  |  |  |  |  |  |
| GO:0043901\_negative\_regulation\_of\_multi-organism\_process | 1 | 0 |  |  |  |  |  |  |  |  |
| GO:0043921\_modulation\_by\_host\_of\_viral\_transcription | 1 | 0 |  |  |  |  |  |  |  |  |
| GO:0043923\_positive\_regulation\_by\_host\_of\_viral\_transcription | 1 | 0 |  |  |  |  |  |  |  |  |
| GO:0044007\_dissemination\_or\_transmission\_of\_symbiont\_from\_host | 1 | 0 |  |  |  |  |  |  |  |  |
| GO:0044089\_positive\_regulation\_of\_cellular\_component\_biogenesis | 1 | 0 |  |  |  |  |  |  |  |  |
| GO:0044258\_intestinal\_lipid\_catabolic\_process | 1 | 0 |  |  |  |  |  |  |  |  |
| GO:0044273\_sulfur\_compound\_catabolic\_process | 1 | 0 |  |  |  |  |  |  |  |  |
| GO:0045013\_negative\_regulation\_of\_transcription\_by\_carbon\_catabolites | 1 | 0 |  |  |  |  |  |  |  |  |
| GO:0045014\_negative\_regulation\_of\_transcription\_by\_glucose | 1 | 0 |  |  |  |  |  |  |  |  |
| GO:0045020\_error-prone\_DNA\_repair | 1 | 0 |  |  |  |  |  |  |  |  |
| GO:0045023\_G0\_to\_G1\_transition | 1 | 0 |  |  |  |  |  |  |  |  |
| GO:0045047\_protein\_targeting\_to\_ER | 1 | 0 |  |  |  |  |  |  |  |  |
| GO:0045065\_cytotoxic\_T\_cell\_differentiation | 1 | 0 |  |  |  |  |  |  |  |  |
| GO:0045074\_regulation\_of\_interleukin-10\_biosynthetic\_process | 1 | 0 |  |  |  |  |  |  |  |  |
| GO:0045082\_positive\_regulation\_of\_interleukin-10\_biosynthetic\_process | 1 | 0 |  |  |  |  |  |  |  |  |
| GO:0045132\_meiotic\_chromosome\_segregation | 1 | 0 |  |  |  |  |  |  |  |  |
| GO:0045163\_clustering\_of\_voltage-gated\_potassium\_channels | 1 | 0 |  |  |  |  |  |  |  |  |
| GO:0045175\_basal\_protein\_localization | 1 | 0 |  |  |  |  |  |  |  |  |
| GO:0045188\_regulation\_of\_circadian\_sleep\_wake\_cycle\_\_non-REM\_sleep | 1 | 0 |  |  |  |  |  |  |  |  |
| GO:0045189\_connective\_tissue\_growth\_factor\_biosynthetic\_process | 1 | 0 |  |  |  |  |  |  |  |  |
| GO:0045196\_establishment\_or\_maintenance\_of\_neuroblast\_polarity | 1 | 0 |  |  |  |  |  |  |  |  |
| GO:0045199\_maintenance\_of\_epithelial\_cell\_apical\_basal\_polarity | 1 | 0 |  |  |  |  |  |  |  |  |
| GO:0045200\_establishment\_of\_neuroblast\_polarity | 1 | 0 |  |  |  |  |  |  |  |  |
| GO:0045204\_MAPK\_export\_from\_nucleus | 1 | 0 |  |  |  |  |  |  |  |  |
| GO:0045208\_MAPK\_phosphatase\_export\_from\_nucleus | 1 | 0 |  |  |  |  |  |  |  |  |
| GO:0045209\_MAPK\_phosphatase\_export\_from\_nucleus\_\_leptomycin\_B\_sensitive | 1 | 0 |  |  |  |  |  |  |  |  |
| GO:0045292\_nuclear\_mRNA\_cis\_splicing\_\_via\_spliceosome | 1 | 0 |  |  |  |  |  |  |  |  |
| GO:0045324\_late\_endosome\_to\_vacuole\_transport | 1 | 0 |  |  |  |  |  |  |  |  |
| GO:0045329\_carnitine\_biosynthetic\_process | 1 | 0 |  |  |  |  |  |  |  |  |
| GO:0045345\_positive\_regulation\_of\_MHC\_class\_I\_biosynthetic\_process | 1 | 0 |  |  |  |  |  |  |  |  |
| GO:0045355\_negative\_regulation\_of\_interferon-alpha\_biosynthetic\_process | 1 | 0 |  |  |  |  |  |  |  |  |
| GO:0045360\_regulation\_of\_interleukin-1\_biosynthetic\_process | 1 | 0 |  |  |  |  |  |  |  |  |
| GO:0045362\_positive\_regulation\_of\_interleukin-1\_biosynthetic\_process | 1 | 0 |  |  |  |  |  |  |  |  |
| GO:0045366\_regulation\_of\_interleukin-13\_biosynthetic\_process | 1 | 0 |  |  |  |  |  |  |  |  |
| GO:0045368\_positive\_regulation\_of\_interleukin-13\_biosynthetic\_process | 1 | 0 |  |  |  |  |  |  |  |  |
| GO:0045381\_regulation\_of\_interleukin-18\_biosynthetic\_process | 1 | 0 |  |  |  |  |  |  |  |  |
| GO:0045405\_regulation\_of\_interleukin-5\_biosynthetic\_process | 1 | 0 |  |  |  |  |  |  |  |  |
| GO:0045407\_positive\_regulation\_of\_interleukin-5\_biosynthetic\_process | 1 | 0 |  |  |  |  |  |  |  |  |
| GO:0045425\_positive\_regulation\_of\_granulocyte\_macrophage\_colony-stimulating\_factor\_biosynthetic\_process | 1 | 0 |  |  |  |  |  |  |  |  |
| GO:0045475\_locomotor\_rhythm | 1 | 0 |  |  |  |  |  |  |  |  |
| GO:0045553\_TRAIL\_biosynthetic\_process | 1 | 0 |  |  |  |  |  |  |  |  |
| GO:0045554\_regulation\_of\_TRAIL\_biosynthetic\_process | 1 | 0 |  |  |  |  |  |  |  |  |
| GO:0045556\_positive\_regulation\_of\_TRAIL\_biosynthetic\_process | 1 | 0 |  |  |  |  |  |  |  |  |
| GO:0045575\_basophil\_activation | 1 | 0 |  |  |  |  |  |  |  |  |
| GO:0045579\_positive\_regulation\_of\_B\_cell\_differentiation | 1 | 0 |  |  |  |  |  |  |  |  |
| GO:0045583\_regulation\_of\_cytotoxic\_T\_cell\_differentiation | 1 | 0 |  |  |  |  |  |  |  |  |
| GO:0045585\_positive\_regulation\_of\_cytotoxic\_T\_cell\_differentiation | 1 | 0 |  |  |  |  |  |  |  |  |
| GO:0045589\_regulation\_of\_regulatory\_T\_cell\_differentiation | 1 | 0 |  |  |  |  |  |  |  |  |
| GO:0045590\_negative\_regulation\_of\_regulatory\_T\_cell\_differentiation | 1 | 0 |  |  |  |  |  |  |  |  |
| GO:0045602\_negative\_regulation\_of\_endothelial\_cell\_differentiation | 1 | 0 |  |  |  |  |  |  |  |  |
| GO:0045603\_positive\_regulation\_of\_endothelial\_cell\_differentiation | 1 | 0 |  |  |  |  |  |  |  |  |
| GO:0045605\_negative\_regulation\_of\_epidermal\_cell\_differentiation | 1 | 0 |  |  |  |  |  |  |  |  |
| GO:0045617\_negative\_regulation\_of\_keratinocyte\_differentiation | 1 | 0 |  |  |  |  |  |  |  |  |
| GO:0045623\_negative\_regulation\_of\_T-helper\_cell\_differentiation | 1 | 0 |  |  |  |  |  |  |  |  |
| GO:0045629\_negative\_regulation\_of\_T-helper\_2\_cell\_differentiation | 1 | 0 |  |  |  |  |  |  |  |  |
| GO:0045654\_positive\_regulation\_of\_megakaryocyte\_differentiation | 1 | 0 |  |  |  |  |  |  |  |  |
| GO:0045672\_positive\_regulation\_of\_osteoclast\_differentiation | 1 | 0 |  |  |  |  |  |  |  |  |
| GO:0045683\_negative\_regulation\_of\_epidermis\_development | 1 | 0 |  |  |  |  |  |  |  |  |
| GO:0045716\_positive\_regulation\_of\_low-density\_lipoprotein\_receptor\_biosynthetic\_process | 1 | 0 |  |  |  |  |  |  |  |  |
| GO:0045719\_negative\_regulation\_of\_glycogen\_biosynthetic\_process | 1 | 0 |  |  |  |  |  |  |  |  |
| GO:0045738\_negative\_regulation\_of\_DNA\_repair | 1 | 0 |  |  |  |  |  |  |  |  |
| GO:0045747\_positive\_regulation\_of\_Notch\_signaling\_pathway | 1 | 0 |  |  |  |  |  |  |  |  |
| GO:0045750\_positive\_regulation\_of\_S\_phase\_of\_mitotic\_cell\_cycle | 1 | 0 |  |  |  |  |  |  |  |  |
| GO:0045751\_negative\_regulation\_of\_Toll\_signaling\_pathway | 1 | 0 |  |  |  |  |  |  |  |  |
| GO:0045759\_negative\_regulation\_of\_action\_potential | 1 | 0 |  |  |  |  |  |  |  |  |
| GO:0045773\_positive\_regulation\_of\_axon\_extension | 1 | 0 |  |  |  |  |  |  |  |  |
| GO:0045794\_negative\_regulation\_of\_cell\_volume | 1 | 0 |  |  |  |  |  |  |  |  |
| GO:0045799\_positive\_regulation\_of\_chromatin\_assembly\_or\_disassembly | 1 | 0 |  |  |  |  |  |  |  |  |
| GO:0045818\_negative\_regulation\_of\_glycogen\_catabolic\_process | 1 | 0 |  |  |  |  |  |  |  |  |
| GO:0045836\_positive\_regulation\_of\_meiosis | 1 | 0 |  |  |  |  |  |  |  |  |
| GO:0045837\_negative\_regulation\_of\_membrane\_potential | 1 | 0 |  |  |  |  |  |  |  |  |
| GO:0045844\_positive\_regulation\_of\_striated\_muscle\_development | 1 | 0 |  |  |  |  |  |  |  |  |
| GO:0045852\_pH\_elevation | 1 | 0 |  |  |  |  |  |  |  |  |
| GO:0045870\_positive\_regulation\_of\_retroviral\_genome\_replication | 1 | 0 |  |  |  |  |  |  |  |  |
| GO:0045875\_negative\_regulation\_of\_sister\_chromatid\_cohesion | 1 | 0 |  |  |  |  |  |  |  |  |
| GO:0045879\_negative\_regulation\_of\_smoothened\_signaling\_pathway | 1 | 0 |  |  |  |  |  |  |  |  |
| GO:0045896\_regulation\_of\_transcription\_\_mitotic | 1 | 0 |  |  |  |  |  |  |  |  |
| GO:0045907\_positive\_regulation\_of\_vasoconstriction | 1 | 0 |  |  |  |  |  |  |  |  |
| GO:0045910\_negative\_regulation\_of\_DNA\_recombination | 1 | 0 |  |  |  |  |  |  |  |  |
| GO:0045915\_positive\_regulation\_of\_catecholamine\_metabolic\_process | 1 | 0 |  |  |  |  |  |  |  |  |
| GO:0045921\_positive\_regulation\_of\_exocytosis | 1 | 0 |  |  |  |  |  |  |  |  |
| GO:0045945\_positive\_regulation\_of\_transcription\_from\_RNA\_polymerase\_III\_promoter | 1 | 0 |  |  |  |  |  |  |  |  |
| GO:0045956\_positive\_regulation\_of\_calcium\_ion-dependent\_exocytosis | 1 | 0 |  |  |  |  |  |  |  |  |
| GO:0045964\_positive\_regulation\_of\_dopamine\_metabolic\_process | 1 | 0 |  |  |  |  |  |  |  |  |
| GO:0045989\_positive\_regulation\_of\_striated\_muscle\_contraction | 1 | 0 |  |  |  |  |  |  |  |  |
| GO:0045993\_negative\_regulation\_of\_translational\_initiation\_by\_iron | 1 | 0 |  |  |  |  |  |  |  |  |
| GO:0046005\_positive\_regulation\_of\_circadian\_sleep\_wake\_cycle\_\_REM\_sleep | 1 | 0 |  |  |  |  |  |  |  |  |
| GO:0046007\_negative\_regulation\_of\_activated\_T\_cell\_proliferation | 1 | 0 |  |  |  |  |  |  |  |  |
| GO:0046010\_positive\_regulation\_of\_circadian\_sleep\_wake\_cycle\_\_non-REM\_sleep | 1 | 0 |  |  |  |  |  |  |  |  |
| GO:0046031\_ADP\_metabolic\_process | 1 | 0 |  |  |  |  |  |  |  |  |
| GO:0046036\_CTP\_metabolic\_process | 1 | 0 |  |  |  |  |  |  |  |  |
| GO:0046040\_IMP\_metabolic\_process | 1 | 0 |  |  |  |  |  |  |  |  |
| GO:0046048\_UDP\_metabolic\_process | 1 | 0 |  |  |  |  |  |  |  |  |
| GO:0046049\_UMP\_metabolic\_process | 1 | 0 |  |  |  |  |  |  |  |  |
| GO:0046056\_dADP\_metabolic\_process | 1 | 0 |  |  |  |  |  |  |  |  |
| GO:0046085\_adenosine\_metabolic\_process | 1 | 0 |  |  |  |  |  |  |  |  |
| GO:0046087\_cytidine\_metabolic\_process | 1 | 0 |  |  |  |  |  |  |  |  |
| GO:0046101\_hypoxanthine\_biosynthetic\_process | 1 | 0 |  |  |  |  |  |  |  |  |
| GO:0046103\_inosine\_biosynthetic\_process | 1 | 0 |  |  |  |  |  |  |  |  |
| GO:0046104\_thymidine\_metabolic\_process | 1 | 0 |  |  |  |  |  |  |  |  |
| GO:0046108\_uridine\_metabolic\_process | 1 | 0 |  |  |  |  |  |  |  |  |
| GO:0046125\_pyrimidine\_deoxyribonucleoside\_metabolic\_process | 1 | 0 |  |  |  |  |  |  |  |  |
| GO:0046127\_pyrimidine\_deoxyribonucleoside\_catabolic\_process | 1 | 0 |  |  |  |  |  |  |  |  |
| GO:0046133\_pyrimidine\_ribonucleoside\_catabolic\_process | 1 | 0 |  |  |  |  |  |  |  |  |
| GO:0046173\_polyol\_biosynthetic\_process | 1 | 0 |  |  |  |  |  |  |  |  |
| GO:0046184\_aldehyde\_biosynthetic\_process | 1 | 0 |  |  |  |  |  |  |  |  |
| GO:0046203\_spermidine\_catabolic\_process | 1 | 0 |  |  |  |  |  |  |  |  |
| GO:0046219\_indolalkylamine\_biosynthetic\_process | 1 | 0 |  |  |  |  |  |  |  |  |
| GO:0046292\_formaldehyde\_metabolic\_process | 1 | 0 |  |  |  |  |  |  |  |  |
| GO:0046293\_formaldehyde\_biosynthetic\_process | 1 | 0 |  |  |  |  |  |  |  |  |
| GO:0046317\_regulation\_of\_glucosylceramide\_biosynthetic\_process | 1 | 0 |  |  |  |  |  |  |  |  |
| GO:0046318\_negative\_regulation\_of\_glucosylceramide\_biosynthetic\_process | 1 | 0 |  |  |  |  |  |  |  |  |
| GO:0046322\_negative\_regulation\_of\_fatty\_acid\_oxidation | 1 | 0 |  |  |  |  |  |  |  |  |
| GO:0046335\_ethanolamine\_biosynthetic\_process | 1 | 0 |  |  |  |  |  |  |  |  |
| GO:0046337\_phosphatidylethanolamine\_metabolic\_process | 1 | 0 |  |  |  |  |  |  |  |  |
| GO:0046340\_diacylglycerol\_catabolic\_process | 1 | 0 |  |  |  |  |  |  |  |  |
| GO:0046351\_disaccharide\_biosynthetic\_process | 1 | 0 |  |  |  |  |  |  |  |  |
| GO:0046352\_disaccharide\_catabolic\_process | 1 | 0 |  |  |  |  |  |  |  |  |
| GO:0046370\_fructose\_biosynthetic\_process | 1 | 0 |  |  |  |  |  |  |  |  |
| GO:0046380\_N-acetylneuraminate\_biosynthetic\_process | 1 | 0 |  |  |  |  |  |  |  |  |
| GO:0046390\_ribose\_phosphate\_biosynthetic\_process | 1 | 0 |  |  |  |  |  |  |  |  |
| GO:0046399\_glucuronate\_biosynthetic\_process | 1 | 0 |  |  |  |  |  |  |  |  |
| GO:0046434\_organophosphate\_catabolic\_process | 1 | 0 |  |  |  |  |  |  |  |  |
| GO:0046448\_tropane\_alkaloid\_metabolic\_process | 1 | 0 |  |  |  |  |  |  |  |  |
| GO:0046449\_creatinine\_metabolic\_process | 1 | 0 |  |  |  |  |  |  |  |  |
| GO:0046471\_phosphatidylglycerol\_metabolic\_process | 1 | 0 |  |  |  |  |  |  |  |  |
| GO:0046477\_glycosylceramide\_catabolic\_process | 1 | 0 |  |  |  |  |  |  |  |  |
| GO:0046485\_ether\_lipid\_metabolic\_process | 1 | 0 |  |  |  |  |  |  |  |  |
| GO:0046487\_glyoxylate\_metabolic\_process | 1 | 0 |  |  |  |  |  |  |  |  |
| GO:0046491\_L-methylmalonyl-CoA\_metabolic\_process | 1 | 0 |  |  |  |  |  |  |  |  |
| GO:0046501\_protoporphyrinogen\_IX\_metabolic\_process | 1 | 0 |  |  |  |  |  |  |  |  |
| GO:0046511\_sphinganine\_biosynthetic\_process | 1 | 0 |  |  |  |  |  |  |  |  |
| GO:0046514\_ceramide\_catabolic\_process | 1 | 0 |  |  |  |  |  |  |  |  |
| GO:0046549\_retinal\_cone\_cell\_development | 1 | 0 |  |  |  |  |  |  |  |  |
| GO:0046586\_regulation\_of\_calcium-dependent\_cell-cell\_adhesion | 1 | 0 |  |  |  |  |  |  |  |  |
| GO:0046588\_negative\_regulation\_of\_calcium-dependent\_cell-cell\_adhesion | 1 | 0 |  |  |  |  |  |  |  |  |
| GO:0046597\_negative\_regulation\_of\_virion\_penetration\_into\_host\_cell | 1 | 0 |  |  |  |  |  |  |  |  |
| GO:0046600\_negative\_regulation\_of\_centriole\_replication | 1 | 0 |  |  |  |  |  |  |  |  |
| GO:0046606\_negative\_regulation\_of\_centrosome\_cycle | 1 | 0 |  |  |  |  |  |  |  |  |
| GO:0046620\_regulation\_of\_organ\_growth | 1 | 0 |  |  |  |  |  |  |  |  |
| GO:0046636\_negative\_regulation\_of\_alpha-beta\_T\_cell\_activation | 1 | 0 |  |  |  |  |  |  |  |  |
| GO:0046639\_negative\_regulation\_of\_alpha-beta\_T\_cell\_differentiation | 1 | 0 |  |  |  |  |  |  |  |  |
| GO:0046640\_regulation\_of\_alpha-beta\_T\_cell\_proliferation | 1 | 0 |  |  |  |  |  |  |  |  |
| GO:0046641\_positive\_regulation\_of\_alpha-beta\_T\_cell\_proliferation | 1 | 0 |  |  |  |  |  |  |  |  |
| GO:0046655\_folic\_acid\_metabolic\_process | 1 | 0 |  |  |  |  |  |  |  |  |
| GO:0046666\_retinal\_cell\_programmed\_cell\_death | 1 | 0 |  |  |  |  |  |  |  |  |
| GO:0046668\_regulation\_of\_retinal\_cell\_programmed\_cell\_death | 1 | 0 |  |  |  |  |  |  |  |  |
| GO:0046670\_positive\_regulation\_of\_retinal\_cell\_programmed\_cell\_death | 1 | 0 |  |  |  |  |  |  |  |  |
| GO:0046674\_induction\_of\_retinal\_programmed\_cell\_death | 1 | 0 |  |  |  |  |  |  |  |  |
| GO:0046685\_response\_to\_arsenic | 1 | 0 |  |  |  |  |  |  |  |  |
| GO:0046686\_response\_to\_cadmium\_ion | 1 | 0 |  |  |  |  |  |  |  |  |
| GO:0046689\_response\_to\_mercury\_ion | 1 | 0 |  |  |  |  |  |  |  |  |
| GO:0046692\_sperm\_competition | 1 | 0 |  |  |  |  |  |  |  |  |
| GO:0046713\_boron\_transport | 1 | 0 |  |  |  |  |  |  |  |  |
| GO:0046719\_regulation\_of\_viral\_protein\_levels\_in\_host\_cell | 1 | 0 |  |  |  |  |  |  |  |  |
| GO:0046814\_virion\_attachment\_\_binding\_of\_host\_cell\_surface\_coreceptor | 1 | 0 |  |  |  |  |  |  |  |  |
| GO:0046826\_negative\_regulation\_of\_protein\_export\_from\_nucleus | 1 | 0 |  |  |  |  |  |  |  |  |
| GO:0046827\_positive\_regulation\_of\_protein\_export\_from\_nucleus | 1 | 0 |  |  |  |  |  |  |  |  |
| GO:0046833\_positive\_regulation\_of\_RNA\_export\_from\_nucleus | 1 | 0 |  |  |  |  |  |  |  |  |
| GO:0046835\_carbohydrate\_phosphorylation | 1 | 0 |  |  |  |  |  |  |  |  |
| GO:0046838\_phosphorylated\_carbohydrate\_dephosphorylation | 1 | 0 |  |  |  |  |  |  |  |  |
| GO:0046853\_inositol\_and\_derivative\_phosphorylation | 1 | 0 |  |  |  |  |  |  |  |  |
| GO:0046855\_inositol\_phosphate\_dephosphorylation | 1 | 0 |  |  |  |  |  |  |  |  |
| GO:0046856\_phosphoinositide\_dephosphorylation | 1 | 0 |  |  |  |  |  |  |  |  |
| GO:0046898\_response\_to\_cycloheximide | 1 | 0 |  |  |  |  |  |  |  |  |
| GO:0046916\_cellular\_transition\_metal\_ion\_homeostasis | 1 | 0 |  |  |  |  |  |  |  |  |
| GO:0046931\_pore\_complex\_biogenesis | 1 | 0 |  |  |  |  |  |  |  |  |
| GO:0046939\_nucleotide\_phosphorylation | 1 | 0 |  |  |  |  |  |  |  |  |
| GO:0046946\_hydroxylysine\_metabolic\_process | 1 | 0 |  |  |  |  |  |  |  |  |
| GO:0046947\_hydroxylysine\_biosynthetic\_process | 1 | 0 |  |  |  |  |  |  |  |  |
| GO:0046963\_3'-phosphoadenosine\_5'-phosphosulfate\_transport | 1 | 0 |  |  |  |  |  |  |  |  |
| GO:0046984\_regulation\_of\_hemoglobin\_biosynthetic\_process | 1 | 0 |  |  |  |  |  |  |  |  |
| GO:0046986\_negative\_regulation\_of\_hemoglobin\_biosynthetic\_process | 1 | 0 |  |  |  |  |  |  |  |  |
| GO:0048003\_antigen\_processing\_and\_presentation\_of\_lipid\_antigen\_via\_MHC\_class\_Ib | 1 | 0 |  |  |  |  |  |  |  |  |
| GO:0048006\_antigen\_processing\_and\_presentation\_\_endogenous\_lipid\_antigen\_via\_MHC\_class\_Ib | 1 | 0 |  |  |  |  |  |  |  |  |
| GO:0048013\_ephrin\_receptor\_signaling\_pathway | 1 | 0 |  |  |  |  |  |  |  |  |
| GO:0048070\_regulation\_of\_pigmentation\_during\_development | 1 | 0 |  |  |  |  |  |  |  |  |
| GO:0048073\_regulation\_of\_eye\_pigmentation | 1 | 0 |  |  |  |  |  |  |  |  |
| GO:0048075\_positive\_regulation\_of\_eye\_pigmentation | 1 | 0 |  |  |  |  |  |  |  |  |
| GO:0048087\_positive\_regulation\_of\_pigmentation\_during\_development | 1 | 0 |  |  |  |  |  |  |  |  |
| GO:0048160\_primary\_follicle\_stage\_\_oogenesis | 1 | 0 |  |  |  |  |  |  |  |  |
| GO:0048170\_positive\_regulation\_of\_long-term\_neuronal\_synaptic\_plasticity | 1 | 0 |  |  |  |  |  |  |  |  |
| GO:0048172\_regulation\_of\_short-term\_neuronal\_synaptic\_plasticity | 1 | 0 |  |  |  |  |  |  |  |  |
| GO:0048175\_hepatocyte\_growth\_factor\_biosynthetic\_process | 1 | 0 |  |  |  |  |  |  |  |  |
| GO:0048176\_regulation\_of\_hepatocyte\_growth\_factor\_biosynthetic\_process | 1 | 0 |  |  |  |  |  |  |  |  |
| GO:0048178\_negative\_regulation\_of\_hepatocyte\_growth\_factor\_biosynthetic\_process | 1 | 0 |  |  |  |  |  |  |  |  |
| GO:0048203\_vesicle\_targeting\_\_trans-Golgi\_to\_endosome | 1 | 0 |  |  |  |  |  |  |  |  |
| GO:0048210\_Golgi\_vesicle\_fusion\_to\_target\_membrane | 1 | 0 |  |  |  |  |  |  |  |  |
| GO:0048241\_epinephrine\_transport | 1 | 0 |  |  |  |  |  |  |  |  |
| GO:0048242\_epinephrine\_secretion | 1 | 0 |  |  |  |  |  |  |  |  |
| GO:0048245\_eosinophil\_chemotaxis | 1 | 0 |  |  |  |  |  |  |  |  |
| GO:0048265\_response\_to\_pain | 1 | 0 |  |  |  |  |  |  |  |  |
| GO:0048289\_isotype\_switching\_to\_IgE\_isotypes | 1 | 0 |  |  |  |  |  |  |  |  |
| GO:0048293\_regulation\_of\_isotype\_switching\_to\_IgE\_isotypes | 1 | 0 |  |  |  |  |  |  |  |  |
| GO:0048295\_positive\_regulation\_of\_isotype\_switching\_to\_IgE\_isotypes | 1 | 0 |  |  |  |  |  |  |  |  |
| GO:0048302\_regulation\_of\_isotype\_switching\_to\_IgG\_isotypes | 1 | 0 |  |  |  |  |  |  |  |  |
| GO:0048304\_positive\_regulation\_of\_isotype\_switching\_to\_IgG\_isotypes | 1 | 0 |  |  |  |  |  |  |  |  |
| GO:0048311\_mitochondrion\_distribution | 1 | 0 |  |  |  |  |  |  |  |  |
| GO:0048339\_paraxial\_mesoderm\_development | 1 | 0 |  |  |  |  |  |  |  |  |
| GO:0048340\_paraxial\_mesoderm\_morphogenesis | 1 | 0 |  |  |  |  |  |  |  |  |
| GO:0048388\_endosomal\_lumen\_acidification | 1 | 0 |  |  |  |  |  |  |  |  |
| GO:0048478\_replication\_fork\_protection | 1 | 0 |  |  |  |  |  |  |  |  |
| GO:0048483\_autonomic\_nervous\_system\_development | 1 | 0 |  |  |  |  |  |  |  |  |
| GO:0048485\_sympathetic\_nervous\_system\_development | 1 | 0 |  |  |  |  |  |  |  |  |
| GO:0048499\_synaptic\_vesicle\_membrane\_organization | 1 | 0 |  |  |  |  |  |  |  |  |
| GO:0048535\_lymph\_node\_development | 1 | 0 |  |  |  |  |  |  |  |  |
| GO:0048539\_bone\_marrow\_development | 1 | 0 |  |  |  |  |  |  |  |  |
| GO:0048549\_positive\_regulation\_of\_pinocytosis | 1 | 0 |  |  |  |  |  |  |  |  |
| GO:0048553\_negative\_regulation\_of\_metalloenzyme\_activity | 1 | 0 |  |  |  |  |  |  |  |  |
| GO:0048566\_embryonic\_gut\_development | 1 | 0 |  |  |  |  |  |  |  |  |
| GO:0048596\_embryonic\_camera-type\_eye\_morphogenesis | 1 | 0 |  |  |  |  |  |  |  |  |
| GO:0048617\_embryonic\_foregut\_morphogenesis | 1 | 0 |  |  |  |  |  |  |  |  |
| GO:0048619\_embryonic\_hindgut\_morphogenesis | 1 | 0 |  |  |  |  |  |  |  |  |
| GO:0048636\_positive\_regulation\_of\_muscle\_development | 1 | 0 |  |  |  |  |  |  |  |  |
| GO:0048639\_positive\_regulation\_of\_developmental\_growth | 1 | 0 |  |  |  |  |  |  |  |  |
| GO:0048640\_negative\_regulation\_of\_developmental\_growth | 1 | 0 |  |  |  |  |  |  |  |  |
| GO:0048665\_neuron\_fate\_specification | 1 | 0 |  |  |  |  |  |  |  |  |
| GO:0048679\_regulation\_of\_axon\_regeneration | 1 | 0 |  |  |  |  |  |  |  |  |
| GO:0048681\_negative\_regulation\_of\_axon\_regeneration | 1 | 0 |  |  |  |  |  |  |  |  |
| GO:0048703\_embryonic\_viscerocranium\_morphogenesis | 1 | 0 |  |  |  |  |  |  |  |  |
| GO:0048745\_smooth\_muscle\_tissue\_development | 1 | 0 |  |  |  |  |  |  |  |  |
| GO:0048755\_branching\_morphogenesis\_of\_a\_nerve | 1 | 0 |  |  |  |  |  |  |  |  |
| GO:0048793\_pronephros\_development | 1 | 0 |  |  |  |  |  |  |  |  |
| GO:0048807\_female\_genitalia\_morphogenesis | 1 | 0 |  |  |  |  |  |  |  |  |
| GO:0048818\_positive\_regulation\_of\_hair\_follicle\_maturation | 1 | 0 |  |  |  |  |  |  |  |  |
| GO:0048819\_regulation\_of\_hair\_follicle\_maturation | 1 | 0 |  |  |  |  |  |  |  |  |
| GO:0048821\_erythrocyte\_development | 1 | 0 |  |  |  |  |  |  |  |  |
| GO:0048845\_venous\_blood\_vessel\_morphogenesis | 1 | 0 |  |  |  |  |  |  |  |  |
| GO:0048853\_forebrain\_morphogenesis | 1 | 0 |  |  |  |  |  |  |  |  |
| GO:0048865\_stem\_cell\_fate\_commitment | 1 | 0 |  |  |  |  |  |  |  |  |
| GO:0048867\_stem\_cell\_fate\_determination | 1 | 0 |  |  |  |  |  |  |  |  |
| GO:0048874\_homeostasis\_of\_number\_of\_cells\_in\_a\_free-living\_population | 1 | 0 |  |  |  |  |  |  |  |  |
| GO:0048875\_chemical\_homeostasis\_within\_a\_tissue | 1 | 0 |  |  |  |  |  |  |  |  |
| GO:0050427\_3'-phosphoadenosine\_5'-phosphosulfate\_metabolic\_process | 1 | 0 |  |  |  |  |  |  |  |  |
| GO:0050652\_dermatan\_sulfate\_proteoglycan\_biosynthetic\_process\_\_polysaccharide\_chain\_biosynthetic\_process | 1 | 0 |  |  |  |  |  |  |  |  |
| GO:0050666\_regulation\_of\_homocysteine\_metabolic\_process | 1 | 0 |  |  |  |  |  |  |  |  |
| GO:0050674\_urothelial\_cell\_proliferation | 1 | 0 |  |  |  |  |  |  |  |  |
| GO:0050675\_regulation\_of\_urothelial\_cell\_proliferation | 1 | 0 |  |  |  |  |  |  |  |  |
| GO:0050677\_positive\_regulation\_of\_urothelial\_cell\_proliferation | 1 | 0 |  |  |  |  |  |  |  |  |
| GO:0050685\_positive\_regulation\_of\_mRNA\_processing | 1 | 0 |  |  |  |  |  |  |  |  |
| GO:0050687\_negative\_regulation\_of\_defense\_response\_to\_virus | 1 | 0 |  |  |  |  |  |  |  |  |
| GO:0050689\_negative\_regulation\_of\_defense\_response\_to\_virus\_by\_host | 1 | 0 |  |  |  |  |  |  |  |  |
| GO:0050713\_negative\_regulation\_of\_interleukin-1\_beta\_secretion | 1 | 0 |  |  |  |  |  |  |  |  |
| GO:0050722\_regulation\_of\_interleukin-1\_beta\_biosynthetic\_process | 1 | 0 |  |  |  |  |  |  |  |  |
| GO:0050725\_positive\_regulation\_of\_interleukin-1\_beta\_biosynthetic\_process | 1 | 0 |  |  |  |  |  |  |  |  |
| GO:0050751\_fractalkine\_biosynthetic\_process | 1 | 0 |  |  |  |  |  |  |  |  |
| GO:0050752\_regulation\_of\_fractalkine\_biosynthetic\_process | 1 | 0 |  |  |  |  |  |  |  |  |
| GO:0050754\_positive\_regulation\_of\_fractalkine\_biosynthetic\_process | 1 | 0 |  |  |  |  |  |  |  |  |
| GO:0050756\_fractalkine\_metabolic\_process | 1 | 0 |  |  |  |  |  |  |  |  |
| GO:0050757\_thymidylate\_synthase\_biosynthetic\_process | 1 | 0 |  |  |  |  |  |  |  |  |
| GO:0050758\_regulation\_of\_thymidylate\_synthase\_biosynthetic\_process | 1 | 0 |  |  |  |  |  |  |  |  |
| GO:0050760\_negative\_regulation\_of\_thymidylate\_synthase\_biosynthetic\_process | 1 | 0 |  |  |  |  |  |  |  |  |
| GO:0050765\_negative\_regulation\_of\_phagocytosis | 1 | 0 |  |  |  |  |  |  |  |  |
| GO:0050774\_negative\_regulation\_of\_dendrite\_morphogenesis | 1 | 0 |  |  |  |  |  |  |  |  |
| GO:0050783\_cocaine\_metabolic\_process | 1 | 0 |  |  |  |  |  |  |  |  |
| GO:0050822\_peptide\_stabilization | 1 | 0 |  |  |  |  |  |  |  |  |
| GO:0050823\_peptide\_antigen\_stabilization | 1 | 0 |  |  |  |  |  |  |  |  |
| GO:0050832\_defense\_response\_to\_fungus | 1 | 0 |  |  |  |  |  |  |  |  |
| GO:0050855\_regulation\_of\_B\_cell\_receptor\_signaling\_pathway | 1 | 0 |  |  |  |  |  |  |  |  |
| GO:0050858\_negative\_regulation\_of\_antigen\_receptor-mediated\_signaling\_pathway | 1 | 0 |  |  |  |  |  |  |  |  |
| GO:0050860\_negative\_regulation\_of\_T\_cell\_receptor\_signaling\_pathway | 1 | 0 |  |  |  |  |  |  |  |  |
| GO:0050861\_positive\_regulation\_of\_B\_cell\_receptor\_signaling\_pathway | 1 | 0 |  |  |  |  |  |  |  |  |
| GO:0050883\_musculoskeletal\_movement\_\_spinal\_reflex\_action | 1 | 0 |  |  |  |  |  |  |  |  |
| GO:0050884\_neuromuscular\_process\_controlling\_posture | 1 | 0 |  |  |  |  |  |  |  |  |
| GO:0050893\_sensory\_processing | 1 | 0 |  |  |  |  |  |  |  |  |
| GO:0050902\_leukocyte\_adhesive\_activation | 1 | 0 |  |  |  |  |  |  |  |  |
| GO:0050910\_detection\_of\_mechanical\_stimulus\_involved\_in\_sensory\_perception\_of\_sound | 1 | 0 |  |  |  |  |  |  |  |  |
| GO:0050922\_negative\_regulation\_of\_chemotaxis | 1 | 0 |  |  |  |  |  |  |  |  |
| GO:0050923\_regulation\_of\_negative\_chemotaxis | 1 | 0 |  |  |  |  |  |  |  |  |
| GO:0050924\_positive\_regulation\_of\_negative\_chemotaxis | 1 | 0 |  |  |  |  |  |  |  |  |
| GO:0050929\_induction\_of\_negative\_chemotaxis | 1 | 0 |  |  |  |  |  |  |  |  |
| GO:0050951\_sensory\_perception\_of\_temperature\_stimulus | 1 | 0 |  |  |  |  |  |  |  |  |
| GO:0050955\_thermoception | 1 | 0 |  |  |  |  |  |  |  |  |
| GO:0050974\_detection\_of\_mechanical\_stimulus\_involved\_in\_sensory\_perception | 1 | 0 |  |  |  |  |  |  |  |  |
| GO:0050983\_spermidine\_catabolic\_process\_to\_deoxyhypusine\_\_using\_deoxyhypusine\_synthase | 1 | 0 |  |  |  |  |  |  |  |  |
| GO:0051013\_microtubule\_severing | 1 | 0 |  |  |  |  |  |  |  |  |
| GO:0051029\_rRNA\_transport | 1 | 0 |  |  |  |  |  |  |  |  |
| GO:0051030\_snRNA\_transport | 1 | 0 |  |  |  |  |  |  |  |  |
| GO:0051031\_tRNA\_transport | 1 | 0 |  |  |  |  |  |  |  |  |
| GO:0051036\_regulation\_of\_endosome\_size | 1 | 0 |  |  |  |  |  |  |  |  |
| GO:0051040\_regulation\_of\_calcium-independent\_cell-cell\_adhesion | 1 | 0 |  |  |  |  |  |  |  |  |
| GO:0051041\_positive\_regulation\_of\_calcium-independent\_cell-cell\_adhesion | 1 | 0 |  |  |  |  |  |  |  |  |
| GO:0051066\_dihydrobiopterin\_metabolic\_process | 1 | 0 |  |  |  |  |  |  |  |  |
| GO:0051085\_chaperone\_mediated\_protein\_folding\_requiring\_cofactor | 1 | 0 |  |  |  |  |  |  |  |  |
| GO:0051089\_constitutive\_protein\_ectodomain\_proteolysis | 1 | 0 |  |  |  |  |  |  |  |  |
| GO:0051102\_DNA\_ligation\_during\_DNA\_recombination | 1 | 0 |  |  |  |  |  |  |  |  |
| GO:0051105\_regulation\_of\_DNA\_ligation | 1 | 0 |  |  |  |  |  |  |  |  |
| GO:0051106\_positive\_regulation\_of\_DNA\_ligation | 1 | 0 |  |  |  |  |  |  |  |  |
| GO:0051125\_regulation\_of\_actin\_nucleation | 1 | 0 |  |  |  |  |  |  |  |  |
| GO:0051126\_negative\_regulation\_of\_actin\_nucleation | 1 | 0 |  |  |  |  |  |  |  |  |
| GO:0051136\_regulation\_of\_NK\_T\_cell\_differentiation | 1 | 0 |  |  |  |  |  |  |  |  |
| GO:0051138\_positive\_regulation\_of\_NK\_T\_cell\_differentiation | 1 | 0 |  |  |  |  |  |  |  |  |
| GO:0051155\_positive\_regulation\_of\_striated\_muscle\_cell\_differentiation | 1 | 0 |  |  |  |  |  |  |  |  |
| GO:0051156\_glucose\_6-phosphate\_metabolic\_process | 1 | 0 |  |  |  |  |  |  |  |  |
| GO:0051160\_L-xylitol\_catabolic\_process | 1 | 0 |  |  |  |  |  |  |  |  |
| GO:0051164\_L-xylitol\_metabolic\_process | 1 | 0 |  |  |  |  |  |  |  |  |
| GO:0051193\_regulation\_of\_cofactor\_metabolic\_process | 1 | 0 |  |  |  |  |  |  |  |  |
| GO:0051196\_regulation\_of\_coenzyme\_metabolic\_process | 1 | 0 |  |  |  |  |  |  |  |  |
| GO:0051204\_protein\_insertion\_into\_mitochondrial\_membrane | 1 | 0 |  |  |  |  |  |  |  |  |
| GO:0051290\_protein\_heterotetramerization | 1 | 0 |  |  |  |  |  |  |  |  |
| GO:0051292\_nuclear\_pore\_complex\_assembly | 1 | 0 |  |  |  |  |  |  |  |  |
| GO:0051294\_establishment\_of\_spindle\_orientation | 1 | 0 |  |  |  |  |  |  |  |  |
| GO:0051295\_establishment\_of\_meiotic\_spindle\_localization | 1 | 0 |  |  |  |  |  |  |  |  |
| GO:0051315\_attachment\_of\_spindle\_microtubules\_to\_kinetochore\_during\_mitosis | 1 | 0 |  |  |  |  |  |  |  |  |
| GO:0051326\_telophase | 1 | 0 |  |  |  |  |  |  |  |  |
| GO:0051342\_regulation\_of\_cyclic-nucleotide\_phosphodiesterase\_activity | 1 | 0 |  |  |  |  |  |  |  |  |
| GO:0051344\_negative\_regulation\_of\_cyclic-nucleotide\_phosphodiesterase\_activity | 1 | 0 |  |  |  |  |  |  |  |  |
| GO:0051445\_regulation\_of\_meiotic\_cell\_cycle | 1 | 0 |  |  |  |  |  |  |  |  |
| GO:0051450\_myoblast\_proliferation | 1 | 0 |  |  |  |  |  |  |  |  |
| GO:0051454\_intracellular\_pH\_elevation | 1 | 0 |  |  |  |  |  |  |  |  |
| GO:0051458\_adrenocorticotropin\_secretion | 1 | 0 |  |  |  |  |  |  |  |  |
| GO:0051459\_regulation\_of\_adrenocorticotropin\_secretion | 1 | 0 |  |  |  |  |  |  |  |  |
| GO:0051461\_positive\_regulation\_of\_adrenocorticotropin\_secretion | 1 | 0 |  |  |  |  |  |  |  |  |
| GO:0051531\_NFAT\_protein\_import\_into\_nucleus | 1 | 0 |  |  |  |  |  |  |  |  |
| GO:0051532\_regulation\_of\_NFAT\_protein\_import\_into\_nucleus | 1 | 0 |  |  |  |  |  |  |  |  |
| GO:0051533\_positive\_regulation\_of\_NFAT\_protein\_import\_into\_nucleus | 1 | 0 |  |  |  |  |  |  |  |  |
| GO:0051542\_elastin\_biosynthetic\_process | 1 | 0 |  |  |  |  |  |  |  |  |
| GO:0051560\_mitochondrial\_calcium\_ion\_homeostasis | 1 | 0 |  |  |  |  |  |  |  |  |
| GO:0051561\_elevation\_of\_mitochondrial\_calcium\_ion\_concentration | 1 | 0 |  |  |  |  |  |  |  |  |
| GO:0051582\_positive\_regulation\_of\_neurotransmitter\_uptake | 1 | 0 |  |  |  |  |  |  |  |  |
| GO:0051586\_positive\_regulation\_of\_dopamine\_uptake | 1 | 0 |  |  |  |  |  |  |  |  |
| GO:0051590\_positive\_regulation\_of\_neurotransmitter\_transport | 1 | 0 |  |  |  |  |  |  |  |  |
| GO:0051594\_detection\_of\_glucose | 1 | 0 |  |  |  |  |  |  |  |  |
| GO:0051642\_centrosome\_localization | 1 | 0 |  |  |  |  |  |  |  |  |
| GO:0051645\_Golgi\_localization | 1 | 0 |  |  |  |  |  |  |  |  |
| GO:0051647\_nucleus\_localization | 1 | 0 |  |  |  |  |  |  |  |  |
| GO:0051664\_nuclear\_pore\_localization | 1 | 0 |  |  |  |  |  |  |  |  |
| GO:0051708\_intracellular\_protein\_transport\_in\_other\_organism\_during\_symbiotic\_interaction | 1 | 0 |  |  |  |  |  |  |  |  |
| GO:0051764\_actin\_crosslink\_formation | 1 | 0 |  |  |  |  |  |  |  |  |
| GO:0051767\_nitric-oxide\_synthase\_biosynthetic\_process | 1 | 0 |  |  |  |  |  |  |  |  |
| GO:0051768\_nitric-oxide\_synthase\_2\_biosynthetic\_process | 1 | 0 |  |  |  |  |  |  |  |  |
| GO:0051769\_regulation\_of\_nitric-oxide\_synthase\_biosynthetic\_process | 1 | 0 |  |  |  |  |  |  |  |  |
| GO:0051771\_negative\_regulation\_of\_nitric-oxide\_synthase\_biosynthetic\_process | 1 | 0 |  |  |  |  |  |  |  |  |
| GO:0051772\_regulation\_of\_nitric-oxide\_synthase\_2\_biosynthetic\_process | 1 | 0 |  |  |  |  |  |  |  |  |
| GO:0051773\_positive\_regulation\_of\_nitric-oxide\_synthase\_2\_biosynthetic\_process | 1 | 0 |  |  |  |  |  |  |  |  |
| GO:0051781\_positive\_regulation\_of\_cell\_division | 1 | 0 |  |  |  |  |  |  |  |  |
| GO:0051782\_negative\_regulation\_of\_cell\_division | 1 | 0 |  |  |  |  |  |  |  |  |
| GO:0051788\_response\_to\_misfolded\_protein | 1 | 0 |  |  |  |  |  |  |  |  |
| GO:0051790\_short-chain\_fatty\_acid\_biosynthetic\_process | 1 | 0 |  |  |  |  |  |  |  |  |
| GO:0051791\_medium-chain\_fatty\_acid\_metabolic\_process | 1 | 0 |  |  |  |  |  |  |  |  |
| GO:0051792\_medium-chain\_fatty\_acid\_biosynthetic\_process | 1 | 0 |  |  |  |  |  |  |  |  |
| GO:0051794\_regulation\_of\_catagen | 1 | 0 |  |  |  |  |  |  |  |  |
| GO:0051795\_positive\_regulation\_of\_catagen | 1 | 0 |  |  |  |  |  |  |  |  |
| GO:0051821\_dissemination\_or\_transmission\_of\_organism\_from\_other\_organism\_during\_symbiotic\_interaction | 1 | 0 |  |  |  |  |  |  |  |  |
| GO:0051894\_positive\_regulation\_of\_focal\_adhesion\_formation | 1 | 0 |  |  |  |  |  |  |  |  |
| GO:0051930\_regulation\_of\_sensory\_perception\_of\_pain | 1 | 0 |  |  |  |  |  |  |  |  |
| GO:0051931\_regulation\_of\_sensory\_perception | 1 | 0 |  |  |  |  |  |  |  |  |
| GO:0051944\_positive\_regulation\_of\_catecholamine\_uptake\_during\_transmission\_of\_nerve\_impulse | 1 | 0 |  |  |  |  |  |  |  |  |
| GO:0051962\_positive\_regulation\_of\_nervous\_system\_development | 1 | 0 |  |  |  |  |  |  |  |  |
| GO:0051965\_positive\_regulation\_of\_synaptogenesis | 1 | 0 |  |  |  |  |  |  |  |  |
| GO:0051977\_lysophospholipid\_transport | 1 | 0 |  |  |  |  |  |  |  |  |
| GO:0051988\_regulation\_of\_attachment\_of\_spindle\_microtubules\_to\_kinetochore | 1 | 0 |  |  |  |  |  |  |  |  |
| GO:0052097\_interspecies\_quorum\_sensing | 1 | 0 |  |  |  |  |  |  |  |  |
| GO:0052106\_quorum\_sensing\_during\_interaction\_with\_host | 1 | 0 |  |  |  |  |  |  |  |  |
| GO:0052312\_modulation\_of\_transcription\_in\_other\_organism\_during\_symbiotic\_interaction | 1 | 0 |  |  |  |  |  |  |  |  |
| GO:0052472\_modulation\_by\_host\_of\_symbiont\_transcription | 1 | 0 |  |  |  |  |  |  |  |  |
| GO:0055009\_atrial\_cardiac\_muscle\_morphogenesis | 1 | 0 |  |  |  |  |  |  |  |  |
| GO:0055012\_ventricular\_cardiac\_muscle\_cell\_differentiation | 1 | 0 |  |  |  |  |  |  |  |  |
| GO:0055071\_manganese\_ion\_homeostasis | 1 | 0 |  |  |  |  |  |  |  |  |
| GO:0055073\_cadmium\_ion\_homeostasis | 1 | 0 |  |  |  |  |  |  |  |  |
| GO:0055076\_transition\_metal\_ion\_homeostasis | 1 | 0 |  |  |  |  |  |  |  |  |
| GO:0055089\_fatty\_acid\_homeostasis | 1 | 0 |  |  |  |  |  |  |  |  |
| GO:0055095\_lipoprotein\_mediated\_signaling | 1 | 0 |  |  |  |  |  |  |  |  |
| GO:0055096\_low\_density\_lipoprotein\_mediated\_signaling | 1 | 0 |  |  |  |  |  |  |  |  |
| GO:0055099\_response\_to\_high\_density\_lipoprotein\_stimulus | 1 | 0 |  |  |  |  |  |  |  |  |
| GO:0055118\_negative\_regulation\_of\_cardiac\_muscle\_contraction | 1 | 0 |  |  |  |  |  |  |  |  |
| GO:0055119\_relaxation\_of\_cardiac\_muscle | 1 | 0 |  |  |  |  |  |  |  |  |
| GO:0060003\_copper\_ion\_export | 1 | 0 |  |  |  |  |  |  |  |  |
| GO:0060022\_hard\_palate\_development | 1 | 0 |  |  |  |  |  |  |  |  |
| GO:0060039\_pericardium\_development | 1 | 0 |  |  |  |  |  |  |  |  |
| GO:0060055\_angiogenesis\_involved\_in\_wound\_healing | 1 | 0 |  |  |  |  |  |  |  |  |
| GO:0060059\_embryonic\_retina\_morphogenesis\_in\_camera-type\_eye | 1 | 0 |  |  |  |  |  |  |  |  |
| GO:0060065\_uterus\_development | 1 | 0 |  |  |  |  |  |  |  |  |
| GO:0060068\_vagina\_development | 1 | 0 |  |  |  |  |  |  |  |  |
| GO:0060082\_eye\_blink\_reflex | 1 | 0 |  |  |  |  |  |  |  |  |
| GO:0060083\_smooth\_muscle\_contraction\_involved\_in\_micturition | 1 | 0 |  |  |  |  |  |  |  |  |
| GO:0060088\_auditory\_receptor\_cell\_stereocilium\_organization | 1 | 0 |  |  |  |  |  |  |  |  |
| GO:0060120\_inner\_ear\_receptor\_cell\_fate\_commitment | 1 | 0 |  |  |  |  |  |  |  |  |
| GO:0060135\_maternal\_process\_involved\_in\_female\_pregnancy | 1 | 0 |  |  |  |  |  |  |  |  |
| GO:0060142\_regulation\_of\_syncytium\_formation\_by\_plasma\_membrane\_fusion | 1 | 0 |  |  |  |  |  |  |  |  |
| GO:0060143\_positive\_regulation\_of\_syncytium\_formation\_by\_plasma\_membrane\_fusion | 1 | 0 |  |  |  |  |  |  |  |  |
| GO:0060157\_urinary\_bladder\_development | 1 | 0 |  |  |  |  |  |  |  |  |
| GO:0060160\_negative\_regulation\_of\_dopamine\_receptor\_signaling\_pathway | 1 | 0 |  |  |  |  |  |  |  |  |
| GO:0060161\_positive\_regulation\_of\_dopamine\_receptor\_signaling\_pathway | 1 | 0 |  |  |  |  |  |  |  |  |
| GO:0060167\_regulation\_of\_adenosine\_receptor\_signaling\_pathway | 1 | 0 |  |  |  |  |  |  |  |  |
| GO:0060169\_negative\_regulation\_of\_adenosine\_receptor\_signaling\_pathway | 1 | 0 |  |  |  |  |  |  |  |  |
| GO:0060216\_definitive\_hemopoiesis | 1 | 0 |  |  |  |  |  |  |  |  |
| GO:0060219\_camera-type\_eye\_photoreceptor\_cell\_differentiation | 1 | 0 |  |  |  |  |  |  |  |  |
| GO:0060231\_mesenchymal\_to\_epithelial\_transition | 1 | 0 |  |  |  |  |  |  |  |  |
| GO:0060254\_regulation\_of\_N-terminal\_protein\_palmitoylation | 1 | 0 |  |  |  |  |  |  |  |  |
| GO:0060259\_regulation\_of\_feeding\_behavior | 1 | 0 |  |  |  |  |  |  |  |  |
| GO:0060262\_negative\_regulation\_of\_N-terminal\_protein\_palmitoylation | 1 | 0 |  |  |  |  |  |  |  |  |
| GO:0060265\_positive\_regulation\_of\_respiratory\_burst\_during\_acute\_inflammatory\_response | 1 | 0 |  |  |  |  |  |  |  |  |
| GO:0060266\_negative\_regulation\_of\_respiratory\_burst\_during\_acute\_inflammatory\_response | 1 | 0 |  |  |  |  |  |  |  |  |
| GO:0060268\_negative\_regulation\_of\_respiratory\_burst | 1 | 0 |  |  |  |  |  |  |  |  |
| GO:0060286\_flagellar\_cell\_motility | 1 | 0 |  |  |  |  |  |  |  |  |
| GO:0060298\_positive\_regulation\_of\_sarcomere\_organization | 1 | 0 |  |  |  |  |  |  |  |  |
| GO:0060299\_negative\_regulation\_of\_sarcomere\_organization | 1 | 0 |  |  |  |  |  |  |  |  |
| GO:0060300\_regulation\_of\_cytokine\_activity | 1 | 0 |  |  |  |  |  |  |  |  |
| GO:0060302\_negative\_regulation\_of\_cytokine\_activity | 1 | 0 |  |  |  |  |  |  |  |  |
| GO:0060305\_regulation\_of\_cell\_diameter | 1 | 0 |  |  |  |  |  |  |  |  |
| GO:0060306\_regulation\_of\_membrane\_repolarization | 1 | 0 |  |  |  |  |  |  |  |  |
| GO:0060307\_regulation\_of\_ventricular\_cardiomyocyte\_membrane\_repolarization | 1 | 0 |  |  |  |  |  |  |  |  |
| GO:0060309\_elastin\_catabolic\_process | 1 | 0 |  |  |  |  |  |  |  |  |
| GO:0060310\_regulation\_of\_elastin\_catabolic\_process | 1 | 0 |  |  |  |  |  |  |  |  |
| GO:0060311\_negative\_regulation\_of\_elastin\_catabolic\_process | 1 | 0 |  |  |  |  |  |  |  |  |
| GO:0060312\_regulation\_of\_blood\_vessel\_remodeling | 1 | 0 |  |  |  |  |  |  |  |  |
| GO:0060313\_negative\_regulation\_of\_blood\_vessel\_remodeling | 1 | 0 |  |  |  |  |  |  |  |  |
| GO:0060315\_negative\_regulation\_of\_ryanodine-sensitive\_calcium-release\_channel\_activity | 1 | 0 |  |  |  |  |  |  |  |  |
| GO:0060316\_positive\_regulation\_of\_ryanodine-sensitive\_calcium-release\_channel\_activity | 1 | 0 |  |  |  |  |  |  |  |  |
| GO:0060318\_definitive\_erythrocyte\_differentiation | 1 | 0 |  |  |  |  |  |  |  |  |
| GO:0060322\_head\_development | 1 | 0 |  |  |  |  |  |  |  |  |
| GO:0060324\_face\_development | 1 | 0 |  |  |  |  |  |  |  |  |
| GO:0060336\_negative\_regulation\_of\_interferon-gamma-mediated\_signaling\_pathway | 1 | 0 |  |  |  |  |  |  |  |  |
| GO:0060349\_bone\_morphogenesis | 1 | 0 |  |  |  |  |  |  |  |  |
| GO:0060350\_endochondral\_bone\_morphogenesis | 1 | 0 |  |  |  |  |  |  |  |  |
| GO:0060356\_leucine\_import | 1 | 0 |  |  |  |  |  |  |  |  |
| GO:0060368\_regulation\_of\_Fc\_receptor\_mediated\_stimulatory\_signaling\_pathway | 1 | 0 |  |  |  |  |  |  |  |  |
| GO:0060369\_positive\_regulation\_of\_Fc\_receptor\_mediated\_stimulatory\_signaling\_pathway | 1 | 0 |  |  |  |  |  |  |  |  |
| GO:0060380\_regulation\_of\_single-stranded\_telomeric\_DNA\_binding | 1 | 0 |  |  |  |  |  |  |  |  |
| GO:0060381\_positive\_regulation\_of\_single-stranded\_telomeric\_DNA\_binding | 1 | 0 |  |  |  |  |  |  |  |  |
| GO:0060382\_regulation\_of\_DNA\_strand\_elongation | 1 | 0 |  |  |  |  |  |  |  |  |
| GO:0060383\_positive\_regulation\_of\_DNA\_strand\_elongation | 1 | 0 |  |  |  |  |  |  |  |  |
| GO:0060397\_JAK-STAT\_cascade\_involved\_in\_growth\_hormone\_signaling\_pathway | 1 | 0 |  |  |  |  |  |  |  |  |
| GO:0060398\_regulation\_of\_growth\_hormone\_receptor\_signaling\_pathway | 1 | 0 |  |  |  |  |  |  |  |  |
| GO:0060425\_lung\_morphogenesis | 1 | 0 |  |  |  |  |  |  |  |  |
| GO:0060433\_bronchus\_development | 1 | 0 |  |  |  |  |  |  |  |  |
| GO:0060438\_trachea\_development | 1 | 0 |  |  |  |  |  |  |  |  |
| GO:0060441\_branching\_involved\_in\_lung\_morphogenesis | 1 | 0 |  |  |  |  |  |  |  |  |
| GO:0060445\_branching\_involved\_in\_salivary\_gland\_morphogenesis | 1 | 0 |  |  |  |  |  |  |  |  |
| GO:0060502\_epithelial\_cell\_proliferation\_involved\_in\_lung\_morphogenesis | 1 | 0 |  |  |  |  |  |  |  |  |
| GO:0060503\_bud\_dilation\_involved\_in\_lung\_branching | 1 | 0 |  |  |  |  |  |  |  |  |
| GO:0060560\_developmental\_growth\_involved\_in\_morphogenesis | 1 | 0 |  |  |  |  |  |  |  |  |
| GO:0060579\_ventral\_spinal\_cord\_interneuron\_fate\_commitment | 1 | 0 |  |  |  |  |  |  |  |  |
| GO:0060586\_multicellular\_organismal\_iron\_ion\_homeostasis | 1 | 0 |  |  |  |  |  |  |  |  |
| GO:0060587\_regulation\_of\_lipoprotein\_lipid\_oxidation | 1 | 0 |  |  |  |  |  |  |  |  |
| GO:0060588\_negative\_regulation\_of\_lipoprotein\_lipid\_oxidation | 1 | 0 |  |  |  |  |  |  |  |  |
| GO:0060638\_mesenchymal-epithelial\_cell\_signaling | 1 | 0 |  |  |  |  |  |  |  |  |
| GO:0060665\_regulation\_of\_branching\_involved\_in\_salivary\_gland\_morphogenesis\_by\_mesenchymal-epithelial\_signaling | 1 | 0 |  |  |  |  |  |  |  |  |
| GO:0060675\_ureteric\_bud\_morphogenesis | 1 | 0 |  |  |  |  |  |  |  |  |
| GO:0060688\_regulation\_of\_morphogenesis\_of\_a\_branching\_structure | 1 | 0 |  |  |  |  |  |  |  |  |
| GO:0060693\_regulation\_of\_branching\_involved\_in\_salivary\_gland\_morphogenesis | 1 | 0 |  |  |  |  |  |  |  |  |
| GO:0060694\_regulation\_of\_cholesterol\_transporter\_activity | 1 | 0 |  |  |  |  |  |  |  |  |
| GO:0060695\_negative\_regulation\_of\_cholesterol\_transporter\_activity | 1 | 0 |  |  |  |  |  |  |  |  |
| GO:0060697\_positive\_regulation\_of\_phospholipid\_catabolic\_process | 1 | 0 |  |  |  |  |  |  |  |  |
| GO:0060729\_intestinal\_epithelial\_structure\_maintenance | 1 | 0 |  |  |  |  |  |  |  |  |
| GO:0060730\_regulation\_of\_intestinal\_epithelial\_structure\_maintenance | 1 | 0 |  |  |  |  |  |  |  |  |
| GO:0060731\_positive\_regulation\_of\_intestinal\_epithelial\_structure\_maintenance | 1 | 0 |  |  |  |  |  |  |  |  |
| GO:0060760\_positive\_regulation\_of\_response\_to\_cytokine\_stimulus | 1 | 0 |  |  |  |  |  |  |  |  |
| GO:0060761\_negative\_regulation\_of\_response\_to\_cytokine\_stimulus | 1 | 0 |  |  |  |  |  |  |  |  |
| GO:0060788\_ectodermal\_placode\_formation | 1 | 0 |  |  |  |  |  |  |  |  |
| GO:0060841\_venous\_blood\_vessel\_development | 1 | 0 |  |  |  |  |  |  |  |  |
| GO:0060856\_establishment\_of\_blood-brain\_barrier | 1 | 0 |  |  |  |  |  |  |  |  |
| GO:0060896\_neural\_plate\_pattern\_specification | 1 | 0 |  |  |  |  |  |  |  |  |
| GO:0065001\_specification\_of\_axis\_polarity | 1 | 0 |  |  |  |  |  |  |  |  |
| GO:0070075\_tear\_secretion | 1 | 0 |  |  |  |  |  |  |  |  |
| GO:0070076\_histone\_lysine\_demethylation | 1 | 0 |  |  |  |  |  |  |  |  |
| GO:0070077\_histone\_arginine\_demethylation | 1 | 0 |  |  |  |  |  |  |  |  |
| GO:0070078\_histone\_H3-R2\_demethylation | 1 | 0 |  |  |  |  |  |  |  |  |
| GO:0070079\_histone\_H4-R3\_demethylation | 1 | 0 |  |  |  |  |  |  |  |  |
| GO:0070086\_ubiquitin-dependent\_endocytosis | 1 | 0 |  |  |  |  |  |  |  |  |
| GO:0070091\_glucagon\_secretion | 1 | 0 |  |  |  |  |  |  |  |  |
| GO:0070103\_regulation\_of\_interleukin-6-mediated\_signaling\_pathway | 1 | 0 |  |  |  |  |  |  |  |  |
| GO:0070104\_negative\_regulation\_of\_interleukin-6-mediated\_signaling\_pathway | 1 | 0 |  |  |  |  |  |  |  |  |
| GO:0070106\_interleukin-27-mediated\_signaling\_pathway | 1 | 0 |  |  |  |  |  |  |  |  |
| GO:0070162\_adiponectin\_secretion | 1 | 0 |  |  |  |  |  |  |  |  |
| GO:0070163\_regulation\_of\_adiponectin\_secretion | 1 | 0 |  |  |  |  |  |  |  |  |
| GO:0070165\_positive\_regulation\_of\_adiponectin\_secretion | 1 | 0 |  |  |  |  |  |  |  |  |
| GO:0070172\_positive\_regulation\_of\_tooth\_mineralization | 1 | 0 |  |  |  |  |  |  |  |  |
| GO:0070173\_regulation\_of\_enamel\_mineralization | 1 | 0 |  |  |  |  |  |  |  |  |
| GO:0070189\_kynurenine\_metabolic\_process | 1 | 0 |  |  |  |  |  |  |  |  |
| GO:0070212\_protein\_amino\_acid\_poly-ADP-ribosylation | 1 | 0 |  |  |  |  |  |  |  |  |
| GO:0070213\_protein\_amino\_acid\_auto-ADP-ribosylation | 1 | 0 |  |  |  |  |  |  |  |  |
| GO:0070232\_regulation\_of\_T\_cell\_apoptosis | 1 | 0 |  |  |  |  |  |  |  |  |
| GO:0070234\_positive\_regulation\_of\_T\_cell\_apoptosis | 1 | 0 |  |  |  |  |  |  |  |  |
| GO:0070242\_thymocyte\_apoptosis | 1 | 0 |  |  |  |  |  |  |  |  |
| GO:0070243\_regulation\_of\_thymocyte\_apoptosis | 1 | 0 |  |  |  |  |  |  |  |  |
| GO:0070245\_positive\_regulation\_of\_thymocyte\_apoptosis | 1 | 0 |  |  |  |  |  |  |  |  |
| GO:0070267\_oncosis | 1 | 0 |  |  |  |  |  |  |  |  |
| GO:0070286\_axonemal\_dynein\_complex\_assembly | 1 | 0 |  |  |  |  |  |  |  |  |
| GO:0070314\_G1\_to\_G0\_transition | 1 | 0 |  |  |  |  |  |  |  |  |
| GO:0070327\_thyroid\_hormone\_transport | 1 | 0 |  |  |  |  |  |  |  |  |
| GO:0070407\_oxidation-dependent\_protein\_catabolic\_process | 1 | 0 |  |  |  |  |  |  |  |  |
| GO:0070408\_carbamoyl\_phosphate\_metabolic\_process | 1 | 0 |  |  |  |  |  |  |  |  |
| GO:0070409\_carbamoyl\_phosphate\_biosynthetic\_process | 1 | 0 |  |  |  |  |  |  |  |  |
| GO:0070509\_calcium\_ion\_import | 1 | 0 |  |  |  |  |  |  |  |  |
| GO:0070527\_platelet\_aggregation | 1 | 0 |  |  |  |  |  |  |  |  |
| GO:0070528\_protein\_kinase\_C\_signaling\_cascade | 1 | 0 |  |  |  |  |  |  |  |  |
| GO:0070534\_protein\_K63-linked\_ubiquitination | 1 | 0 |  |  |  |  |  |  |  |  |
| GO:0070535\_histone\_H2A\_K63-linked\_ubiquitination | 1 | 0 |  |  |  |  |  |  |  |  |
| GO:0070537\_histone\_H2A\_K63-linked\_deubiquitination | 1 | 0 |  |  |  |  |  |  |  |  |
| GO:0070560\_protein\_secretion\_by\_platelet | 1 | 0 |  |  |  |  |  |  |  |  |
| GO:0070562\_regulation\_of\_vitamin\_D\_receptor\_signaling\_pathway | 1 | 0 |  |  |  |  |  |  |  |  |
| GO:0070564\_positive\_regulation\_of\_vitamin\_D\_receptor\_signaling\_pathway | 1 | 0 |  |  |  |  |  |  |  |  |
| GO:0070570\_regulation\_of\_neuron\_projection\_regeneration | 1 | 0 |  |  |  |  |  |  |  |  |
| GO:0070571\_negative\_regulation\_of\_neuron\_projection\_regeneration | 1 | 0 |  |  |  |  |  |  |  |  |
| GO:0070601\_centromeric\_sister\_chromatid\_cohesion | 1 | 0 |  |  |  |  |  |  |  |  |
| GO:0070602\_regulation\_of\_centromeric\_sister\_chromatid\_cohesion | 1 | 0 |  |  |  |  |  |  |  |  |
| GO:0070625\_zymogen\_granule\_exocytosis | 1 | 0 |  |  |  |  |  |  |  |  |
| GO:0070684\_seminal\_clot\_liquefaction | 1 | 0 |  |  |  |  |  |  |  |  |
| GO:0070715\_sodium-dependent\_organic\_cation\_transport | 1 | 0 |  |  |  |  |  |  |  |  |
| GO:0070813\_hydrogen\_sulfide\_metabolic\_process | 1 | 0 |  |  |  |  |  |  |  |  |
| GO:0070814\_hydrogen\_sulfide\_biosynthetic\_process | 1 | 0 |  |  |  |  |  |  |  |  |
| GO:0070846\_Hsp90\_deacetylation | 1 | 0 |  |  |  |  |  |  |  |  |
| GO:0090030\_regulation\_of\_steroid\_hormone\_biosynthetic\_process | 1 | 0 |  |  |  |  |  |  |  |  |
| GO:0090031\_positive\_regulation\_of\_steroid\_hormone\_biosynthetic\_process | 1 | 0 |  |  |  |  |  |  |  |  |
| GO:0003008\_system\_process | 710 | 0 | 0.000000 | 0.000000 | 478 | 389.957945 | 453.79 | 517.622055 | 0.949351 |
| GO:0006996\_organelle\_organization | 764 | 0 | 0.000000 | 0.000000 | 478 | 389.957945 | 453.79 | 517.622055 | 0.949351 |
| GO:0008150\_biological\_process | 8160 | 16 | 1.000000 | 0.000000 | 478 | 389.957945 | 453.79 | 517.622055 | 0.949351 |
| GO:0009056\_catabolic\_process | 633 | 0 | 0.000000 | 0.000000 | 478 | 389.957945 | 453.79 | 517.622055 | 0.949351 |
| GO:0051649\_establishment\_of\_localization\_in\_cell | 573 | 0 | 0.000000 | 0.000000 | 478 | 389.957945 | 453.79 | 517.622055 | 0.949351 |
| GO:0000087\_M\_phase\_of\_mitotic\_cell\_cycle | 118 | 0 | 0.000000 | 0.000000 | 481 | 392.503484 | 456.08 | 519.656516 | 0.948191 |
| GO:0010608\_posttranscriptional\_regulation\_of\_gene\_expression | 118 | 0 | 0.000000 | 0.000000 | 481 | 392.503484 | 456.08 | 519.656516 | 0.948191 |
| GO:0048285\_organelle\_fission | 118 | 0 | 0.000000 | 0.000000 | 481 | 392.503484 | 456.08 | 519.656516 | 0.948191 |
| GO:0001505\_regulation\_of\_neurotransmitter\_levels | 34 | 0 | 0.000000 | 0.000000 | 509 | 420.328894 | 482.97 | 545.611106 | 0.948861 |
| GO:0002449\_lymphocyte\_mediated\_immunity | 34 | 0 | 0.000000 | 0.000000 | 509 | 420.328894 | 482.97 | 545.611106 | 0.948861 |
| GO:0006399\_tRNA\_metabolic\_process | 34 | 0 | 0.000000 | 0.000000 | 509 | 420.328894 | 482.97 | 545.611106 | 0.948861 |
| GO:0006818\_hydrogen\_transport | 34 | 0 | 0.000000 | 0.000000 | 509 | 420.328894 | 482.97 | 545.611106 | 0.948861 |
| GO:0006997\_nucleus\_organization | 34 | 0 | 0.000000 | 0.000000 | 509 | 420.328894 | 482.97 | 545.611106 | 0.948861 |
| GO:0007188\_G-protein\_signaling\_\_coupled\_to\_cAMP\_nucleotide\_second\_messenger | 34 | 0 | 0.000000 | 0.000000 | 509 | 420.328894 | 482.97 | 545.611106 | 0.948861 |
| GO:0007338\_single\_fertilization | 34 | 0 | 0.000000 | 0.000000 | 509 | 420.328894 | 482.97 | 545.611106 | 0.948861 |
| GO:0008277\_regulation\_of\_G-protein\_coupled\_receptor\_protein\_signaling\_pathway | 34 | 0 | 0.000000 | 0.000000 | 509 | 420.328894 | 482.97 | 545.611106 | 0.948861 |
| GO:0008624\_induction\_of\_apoptosis\_by\_extracellular\_signals | 34 | 0 | 0.000000 | 0.000000 | 509 | 420.328894 | 482.97 | 545.611106 | 0.948861 |
| GO:0008645\_hexose\_transport | 34 | 0 | 0.000000 | 0.000000 | 509 | 420.328894 | 482.97 | 545.611106 | 0.948861 |
| GO:0015718\_monocarboxylic\_acid\_transport | 34 | 0 | 0.000000 | 0.000000 | 509 | 420.328894 | 482.97 | 545.611106 | 0.948861 |
| GO:0015749\_monosaccharide\_transport | 34 | 0 | 0.000000 | 0.000000 | 509 | 420.328894 | 482.97 | 545.611106 | 0.948861 |
| GO:0015758\_glucose\_transport | 34 | 0 | 0.000000 | 0.000000 | 509 | 420.328894 | 482.97 | 545.611106 | 0.948861 |
| GO:0019395\_fatty\_acid\_oxidation | 34 | 0 | 0.000000 | 0.000000 | 509 | 420.328894 | 482.97 | 545.611106 | 0.948861 |
| GO:0019748\_secondary\_metabolic\_process | 34 | 0 | 0.000000 | 0.000000 | 509 | 420.328894 | 482.97 | 545.611106 | 0.948861 |
| GO:0030100\_regulation\_of\_endocytosis | 34 | 0 | 0.000000 | 0.000000 | 509 | 420.328894 | 482.97 | 545.611106 | 0.948861 |
| GO:0030521\_androgen\_receptor\_signaling\_pathway | 34 | 0 | 0.000000 | 0.000000 | 509 | 420.328894 | 482.97 | 545.611106 | 0.948861 |
| GO:0031400\_negative\_regulation\_of\_protein\_modification\_process | 34 | 0 | 0.000000 | 0.000000 | 509 | 420.328894 | 482.97 | 545.611106 | 0.948861 |
| GO:0031647\_regulation\_of\_protein\_stability | 34 | 0 | 0.000000 | 0.000000 | 509 | 420.328894 | 482.97 | 545.611106 | 0.948861 |
| GO:0043269\_regulation\_of\_ion\_transport | 34 | 0 | 0.000000 | 0.000000 | 509 | 420.328894 | 482.97 | 545.611106 | 0.948861 |
| GO:0045137\_development\_of\_primary\_sexual\_characteristics | 34 | 0 | 0.000000 | 0.000000 | 509 | 420.328894 | 482.97 | 545.611106 | 0.948861 |
| GO:0048545\_response\_to\_steroid\_hormone\_stimulus | 34 | 0 | 0.000000 | 0.000000 | 509 | 420.328894 | 482.97 | 545.611106 | 0.948861 |
| GO:0048608\_reproductive\_structure\_development | 34 | 0 | 0.000000 | 0.000000 | 509 | 420.328894 | 482.97 | 545.611106 | 0.948861 |
| GO:0050767\_regulation\_of\_neurogenesis | 34 | 0 | 0.000000 | 0.000000 | 509 | 420.328894 | 482.97 | 545.611106 | 0.948861 |
| GO:0050808\_synapse\_organization | 34 | 0 | 0.000000 | 0.000000 | 509 | 420.328894 | 482.97 | 545.611106 | 0.948861 |
| GO:0051605\_protein\_maturation\_by\_peptide\_bond\_cleavage | 34 | 0 | 0.000000 | 0.000000 | 509 | 420.328894 | 482.97 | 545.611106 | 0.948861 |
| GO:0051651\_maintenance\_of\_location\_in\_cell | 34 | 0 | 0.000000 | 0.000000 | 509 | 420.328894 | 482.97 | 545.611106 | 0.948861 |
| GO:0060326\_cell\_chemotaxis | 34 | 0 | 0.000000 | 0.000000 | 509 | 420.328894 | 482.97 | 545.611106 | 0.948861 |
| GO:0006974\_response\_to\_DNA\_damage\_stimulus | 234 | 0 | 0.000000 | 0.000000 | 510 | 421.066969 | 483.64 | 546.213031 | 0.948314 |
| GO:0006790\_sulfur\_metabolic\_process | 62 | 0 | 0.000000 | 0.000000 | 520 | 432.059637 | 494.22 | 556.380363 | 0.950423 |
| GO:0007420\_brain\_development | 62 | 0 | 0.000000 | 0.000000 | 520 | 432.059637 | 494.22 | 556.380363 | 0.950423 |
| GO:0007586\_digestion | 62 | 0 | 0.000000 | 0.000000 | 520 | 432.059637 | 494.22 | 556.380363 | 0.950423 |
| GO:0009190\_cyclic\_nucleotide\_biosynthetic\_process | 62 | 0 | 0.000000 | 0.000000 | 520 | 432.059637 | 494.22 | 556.380363 | 0.950423 |
| GO:0019216\_regulation\_of\_lipid\_metabolic\_process | 62 | 0 | 0.000000 | 0.000000 | 520 | 432.059637 | 494.22 | 556.380363 | 0.950423 |
| GO:0031145\_anaphase-promoting\_complex-dependent\_proteasomal\_ubiquitin-dependent\_protein\_catabolic\_process | 62 | 0 | 0.000000 | 0.000000 | 520 | 432.059637 | 494.22 | 556.380363 | 0.950423 |
| GO:0031667\_response\_to\_nutrient\_levels | 62 | 0 | 0.000000 | 0.000000 | 520 | 432.059637 | 494.22 | 556.380363 | 0.950423 |
| GO:0044242\_cellular\_lipid\_catabolic\_process | 62 | 0 | 0.000000 | 0.000000 | 520 | 432.059637 | 494.22 | 556.380363 | 0.950423 |
| GO:0051436\_negative\_regulation\_of\_ubiquitin-protein\_ligase\_activity\_during\_mitotic\_cell\_cycle | 62 | 0 | 0.000000 | 0.000000 | 520 | 432.059637 | 494.22 | 556.380363 | 0.950423 |
| GO:0060191\_regulation\_of\_lipase\_activity | 62 | 0 | 0.000000 | 0.000000 | 520 | 432.059637 | 494.22 | 556.380363 | 0.950423 |
| GO:0008361\_regulation\_of\_cell\_size | 149 | 0 | 0.000000 | 0.000000 | 521 | 432.876481 | 494.97 | 557.063519 | 0.950038 |
| GO:0001525\_angiogenesis | 69 | 0 | 0.000000 | 0.000000 | 527 | 438.195673 | 500.03 | 561.864327 | 0.948824 |
| GO:0007005\_mitochondrion\_organization | 69 | 0 | 0.000000 | 0.000000 | 527 | 438.195673 | 500.03 | 561.864327 | 0.948824 |
| GO:0016042\_lipid\_catabolic\_process | 69 | 0 | 0.000000 | 0.000000 | 527 | 438.195673 | 500.03 | 561.864327 | 0.948824 |
| GO:0045333\_cellular\_respiration | 69 | 0 | 0.000000 | 0.000000 | 527 | 438.195673 | 500.03 | 561.864327 | 0.948824 |
| GO:0051241\_negative\_regulation\_of\_multicellular\_organismal\_process | 69 | 0 | 0.000000 | 0.000000 | 527 | 438.195673 | 500.03 | 561.864327 | 0.948824 |
| GO:0051351\_positive\_regulation\_of\_ligase\_activity | 69 | 0 | 0.000000 | 0.000000 | 527 | 438.195673 | 500.03 | 561.864327 | 0.948824 |
| GO:0006260\_DNA\_replication | 153 | 0 | 0.000000 | 0.000000 | 529 | 439.833866 | 501.59 | 563.346134 | 0.948185 |
| GO:0019932\_second-messenger-mediated\_signaling | 153 | 0 | 0.000000 | 0.000000 | 529 | 439.833866 | 501.59 | 563.346134 | 0.948185 |
| GO:0031328\_positive\_regulation\_of\_cellular\_biosynthetic\_process | 352 | 0 | 0.000000 | 0.000000 | 530 | 441.107987 | 502.56 | 564.012013 | 0.948226 |
| GO:0010604\_positive\_regulation\_of\_macromolecule\_metabolic\_process | 446 | 0 | 0.000000 | 0.000000 | 531 | 441.712828 | 503.01 | 564.307172 | 0.947288 |
| GO:0044057\_regulation\_of\_system\_process | 106 | 0 | 0.000000 | 0.000000 | 532 | 443.566680 | 504.7 | 565.833320 | 0.948684 |
| GO:0050817\_coagulation | 74 | 0 | 0.000000 | 0.000000 | 533 | 445.470299 | 506.39 | 567.309701 | 0.950075 |
| GO:0006066\_alcohol\_metabolic\_process | 206 | 0 | 0.000000 | 0.000000 | 534 | 448.254199 | 508.76 | 569.265801 | 0.952734 |
| GO:0009888\_tissue\_development | 287 | 0 | 0.000000 | 0.000000 | 535 | 448.962352 | 509.37 | 569.777648 | 0.952093 |
| GO:0048468\_cell\_development | 251 | 0 | 0.000000 | 0.000000 | 536 | 449.722630 | 510.03 | 570.337370 | 0.951549 |
| GO:0000096\_sulfur\_amino\_acid\_metabolic\_process | 10 | 0 | 0.000000 | 0.000000 | 640 | 555.519806 | 614.8 | 674.080194 | 0.960625 |
| GO:0001890\_placenta\_development | 10 | 0 | 0.000000 | 0.000000 | 640 | 555.519806 | 614.8 | 674.080194 | 0.960625 |
| GO:0001959\_regulation\_of\_cytokine-mediated\_signaling\_pathway | 10 | 0 | 0.000000 | 0.000000 | 640 | 555.519806 | 614.8 | 674.080194 | 0.960625 |
| GO:0002286\_T\_cell\_activation\_during\_immune\_response | 10 | 0 | 0.000000 | 0.000000 | 640 | 555.519806 | 614.8 | 674.080194 | 0.960625 |
| GO:0002709\_regulation\_of\_T\_cell\_mediated\_immunity | 10 | 0 | 0.000000 | 0.000000 | 640 | 555.519806 | 614.8 | 674.080194 | 0.960625 |
| GO:0002718\_regulation\_of\_cytokine\_production\_during\_immune\_response | 10 | 0 | 0.000000 | 0.000000 | 640 | 555.519806 | 614.8 | 674.080194 | 0.960625 |
| GO:0002793\_positive\_regulation\_of\_peptide\_secretion | 10 | 0 | 0.000000 | 0.000000 | 640 | 555.519806 | 614.8 | 674.080194 | 0.960625 |
| GO:0005979\_regulation\_of\_glycogen\_biosynthetic\_process | 10 | 0 | 0.000000 | 0.000000 | 640 | 555.519806 | 614.8 | 674.080194 | 0.960625 |
| GO:0006379\_mRNA\_cleavage | 10 | 0 | 0.000000 | 0.000000 | 640 | 555.519806 | 614.8 | 674.080194 | 0.960625 |
| GO:0006584\_catecholamine\_metabolic\_process | 10 | 0 | 0.000000 | 0.000000 | 640 | 555.519806 | 614.8 | 674.080194 | 0.960625 |
| GO:0006637\_acyl-CoA\_metabolic\_process | 10 | 0 | 0.000000 | 0.000000 | 640 | 555.519806 | 614.8 | 674.080194 | 0.960625 |
| GO:0006833\_water\_transport | 10 | 0 | 0.000000 | 0.000000 | 640 | 555.519806 | 614.8 | 674.080194 | 0.960625 |
| GO:0007032\_endosome\_organization | 10 | 0 | 0.000000 | 0.000000 | 640 | 555.519806 | 614.8 | 674.080194 | 0.960625 |
| GO:0007062\_sister\_chromatid\_cohesion | 10 | 0 | 0.000000 | 0.000000 | 640 | 555.519806 | 614.8 | 674.080194 | 0.960625 |
| GO:0007096\_regulation\_of\_exit\_from\_mitosis | 10 | 0 | 0.000000 | 0.000000 | 640 | 555.519806 | 614.8 | 674.080194 | 0.960625 |
| GO:0007176\_regulation\_of\_epidermal\_growth\_factor\_receptor\_activity | 10 | 0 | 0.000000 | 0.000000 | 640 | 555.519806 | 614.8 | 674.080194 | 0.960625 |
| GO:0007205\_activation\_of\_protein\_kinase\_C\_activity\_by\_G-protein\_coupled\_receptor\_protein\_signaling\_pathway | 10 | 0 | 0.000000 | 0.000000 | 640 | 555.519806 | 614.8 | 674.080194 | 0.960625 |
| GO:0007270\_nerve-nerve\_synaptic\_transmission | 10 | 0 | 0.000000 | 0.000000 | 640 | 555.519806 | 614.8 | 674.080194 | 0.960625 |
| GO:0007566\_embryo\_implantation | 10 | 0 | 0.000000 | 0.000000 | 640 | 555.519806 | 614.8 | 674.080194 | 0.960625 |
| GO:0007612\_learning | 10 | 0 | 0.000000 | 0.000000 | 640 | 555.519806 | 614.8 | 674.080194 | 0.960625 |
| GO:0008625\_induction\_of\_apoptosis\_via\_death\_domain\_receptors | 10 | 0 | 0.000000 | 0.000000 | 640 | 555.519806 | 614.8 | 674.080194 | 0.960625 |
| GO:0009066\_aspartate\_family\_amino\_acid\_metabolic\_process | 10 | 0 | 0.000000 | 0.000000 | 640 | 555.519806 | 614.8 | 674.080194 | 0.960625 |
| GO:0009081\_branched\_chain\_family\_amino\_acid\_metabolic\_process | 10 | 0 | 0.000000 | 0.000000 | 640 | 555.519806 | 614.8 | 674.080194 | 0.960625 |
| GO:0009112\_nucleobase\_metabolic\_process | 10 | 0 | 0.000000 | 0.000000 | 640 | 555.519806 | 614.8 | 674.080194 | 0.960625 |
| GO:0009154\_purine\_ribonucleotide\_catabolic\_process | 10 | 0 | 0.000000 | 0.000000 | 640 | 555.519806 | 614.8 | 674.080194 | 0.960625 |
| GO:0009201\_ribonucleoside\_triphosphate\_biosynthetic\_process | 10 | 0 | 0.000000 | 0.000000 | 640 | 555.519806 | 614.8 | 674.080194 | 0.960625 |
| GO:0009225\_nucleotide-sugar\_metabolic\_process | 10 | 0 | 0.000000 | 0.000000 | 640 | 555.519806 | 614.8 | 674.080194 | 0.960625 |
| GO:0009712\_catechol\_metabolic\_process | 10 | 0 | 0.000000 | 0.000000 | 640 | 555.519806 | 614.8 | 674.080194 | 0.960625 |
| GO:0010595\_positive\_regulation\_of\_endothelial\_cell\_migration | 10 | 0 | 0.000000 | 0.000000 | 640 | 555.519806 | 614.8 | 674.080194 | 0.960625 |
| GO:0010885\_regulation\_of\_cholesterol\_storage | 10 | 0 | 0.000000 | 0.000000 | 640 | 555.519806 | 614.8 | 674.080194 | 0.960625 |
| GO:0010962\_regulation\_of\_glucan\_biosynthetic\_process | 10 | 0 | 0.000000 | 0.000000 | 640 | 555.519806 | 614.8 | 674.080194 | 0.960625 |
| GO:0015012\_heparan\_sulfate\_proteoglycan\_biosynthetic\_process | 10 | 0 | 0.000000 | 0.000000 | 640 | 555.519806 | 614.8 | 674.080194 | 0.960625 |
| GO:0015669\_gas\_transport | 10 | 0 | 0.000000 | 0.000000 | 640 | 555.519806 | 614.8 | 674.080194 | 0.960625 |
| GO:0015697\_quaternary\_ammonium\_group\_transport | 10 | 0 | 0.000000 | 0.000000 | 640 | 555.519806 | 614.8 | 674.080194 | 0.960625 |
| GO:0016073\_snRNA\_metabolic\_process | 10 | 0 | 0.000000 | 0.000000 | 640 | 555.519806 | 614.8 | 674.080194 | 0.960625 |
| GO:0016180\_snRNA\_processing | 10 | 0 | 0.000000 | 0.000000 | 640 | 555.519806 | 614.8 | 674.080194 | 0.960625 |
| GO:0016558\_protein\_import\_into\_peroxisome\_matrix | 10 | 0 | 0.000000 | 0.000000 | 640 | 555.519806 | 614.8 | 674.080194 | 0.960625 |
| GO:0019048\_virus-host\_interaction | 10 | 0 | 0.000000 | 0.000000 | 640 | 555.519806 | 614.8 | 674.080194 | 0.960625 |
| GO:0019751\_polyol\_metabolic\_process | 10 | 0 | 0.000000 | 0.000000 | 640 | 555.519806 | 614.8 | 674.080194 | 0.960625 |
| GO:0030323\_respiratory\_tube\_development | 10 | 0 | 0.000000 | 0.000000 | 640 | 555.519806 | 614.8 | 674.080194 | 0.960625 |
| GO:0030511\_positive\_regulation\_of\_transforming\_growth\_factor\_beta\_receptor\_signaling\_pathway | 10 | 0 | 0.000000 | 0.000000 | 640 | 555.519806 | 614.8 | 674.080194 | 0.960625 |
| GO:0030520\_estrogen\_receptor\_signaling\_pathway | 10 | 0 | 0.000000 | 0.000000 | 640 | 555.519806 | 614.8 | 674.080194 | 0.960625 |
| GO:0030730\_sequestering\_of\_triglyceride | 10 | 0 | 0.000000 | 0.000000 | 640 | 555.519806 | 614.8 | 674.080194 | 0.960625 |
| GO:0030968\_endoplasmic\_reticulum\_unfolded\_protein\_response | 10 | 0 | 0.000000 | 0.000000 | 640 | 555.519806 | 614.8 | 674.080194 | 0.960625 |
| GO:0032273\_positive\_regulation\_of\_protein\_polymerization | 10 | 0 | 0.000000 | 0.000000 | 640 | 555.519806 | 614.8 | 674.080194 | 0.960625 |
| GO:0032320\_positive\_regulation\_of\_Ras\_GTPase\_activity | 10 | 0 | 0.000000 | 0.000000 | 640 | 555.519806 | 614.8 | 674.080194 | 0.960625 |
| GO:0032602\_chemokine\_production | 10 | 0 | 0.000000 | 0.000000 | 640 | 555.519806 | 614.8 | 674.080194 | 0.960625 |
| GO:0032885\_regulation\_of\_polysaccharide\_biosynthetic\_process | 10 | 0 | 0.000000 | 0.000000 | 640 | 555.519806 | 614.8 | 674.080194 | 0.960625 |
| GO:0032964\_collagen\_biosynthetic\_process | 10 | 0 | 0.000000 | 0.000000 | 640 | 555.519806 | 614.8 | 674.080194 | 0.960625 |
| GO:0033108\_mitochondrial\_respiratory\_chain\_complex\_assembly | 10 | 0 | 0.000000 | 0.000000 | 640 | 555.519806 | 614.8 | 674.080194 | 0.960625 |
| GO:0034311\_diol\_metabolic\_process | 10 | 0 | 0.000000 | 0.000000 | 640 | 555.519806 | 614.8 | 674.080194 | 0.960625 |
| GO:0034370\_triglyceride-rich\_lipoprotein\_particle\_remodeling | 10 | 0 | 0.000000 | 0.000000 | 640 | 555.519806 | 614.8 | 674.080194 | 0.960625 |
| GO:0034372\_very-low-density\_lipoprotein\_particle\_remodeling | 10 | 0 | 0.000000 | 0.000000 | 640 | 555.519806 | 614.8 | 674.080194 | 0.960625 |
| GO:0034612\_response\_to\_tumor\_necrosis\_factor | 10 | 0 | 0.000000 | 0.000000 | 640 | 555.519806 | 614.8 | 674.080194 | 0.960625 |
| GO:0034620\_cellular\_response\_to\_unfolded\_protein | 10 | 0 | 0.000000 | 0.000000 | 640 | 555.519806 | 614.8 | 674.080194 | 0.960625 |
| GO:0035264\_multicellular\_organism\_growth | 10 | 0 | 0.000000 | 0.000000 | 640 | 555.519806 | 614.8 | 674.080194 | 0.960625 |
| GO:0040014\_regulation\_of\_multicellular\_organism\_growth | 10 | 0 | 0.000000 | 0.000000 | 640 | 555.519806 | 614.8 | 674.080194 | 0.960625 |
| GO:0042044\_fluid\_transport | 10 | 0 | 0.000000 | 0.000000 | 640 | 555.519806 | 614.8 | 674.080194 | 0.960625 |
| GO:0042133\_neurotransmitter\_metabolic\_process | 10 | 0 | 0.000000 | 0.000000 | 640 | 555.519806 | 614.8 | 674.080194 | 0.960625 |
| GO:0042147\_retrograde\_transport\_\_endosome\_to\_Golgi | 10 | 0 | 0.000000 | 0.000000 | 640 | 555.519806 | 614.8 | 674.080194 | 0.960625 |
| GO:0042228\_interleukin-8\_biosynthetic\_process | 10 | 0 | 0.000000 | 0.000000 | 640 | 555.519806 | 614.8 | 674.080194 | 0.960625 |
| GO:0042274\_ribosomal\_small\_subunit\_biogenesis | 10 | 0 | 0.000000 | 0.000000 | 640 | 555.519806 | 614.8 | 674.080194 | 0.960625 |
| GO:0042307\_positive\_regulation\_of\_protein\_import\_into\_nucleus | 10 | 0 | 0.000000 | 0.000000 | 640 | 555.519806 | 614.8 | 674.080194 | 0.960625 |
| GO:0042312\_regulation\_of\_vasodilation | 10 | 0 | 0.000000 | 0.000000 | 640 | 555.519806 | 614.8 | 674.080194 | 0.960625 |
| GO:0042517\_positive\_regulation\_of\_tyrosine\_phosphorylation\_of\_Stat3\_protein | 10 | 0 | 0.000000 | 0.000000 | 640 | 555.519806 | 614.8 | 674.080194 | 0.960625 |
| GO:0042542\_response\_to\_hydrogen\_peroxide | 10 | 0 | 0.000000 | 0.000000 | 640 | 555.519806 | 614.8 | 674.080194 | 0.960625 |
| GO:0042993\_positive\_regulation\_of\_transcription\_factor\_import\_into\_nucleus | 10 | 0 | 0.000000 | 0.000000 | 640 | 555.519806 | 614.8 | 674.080194 | 0.960625 |
| GO:0043010\_camera-type\_eye\_development | 10 | 0 | 0.000000 | 0.000000 | 640 | 555.519806 | 614.8 | 674.080194 | 0.960625 |
| GO:0043331\_response\_to\_dsRNA | 10 | 0 | 0.000000 | 0.000000 | 640 | 555.519806 | 614.8 | 674.080194 | 0.960625 |
| GO:0043500\_muscle\_adaptation | 10 | 0 | 0.000000 | 0.000000 | 640 | 555.519806 | 614.8 | 674.080194 | 0.960625 |
| GO:0043588\_skin\_development | 10 | 0 | 0.000000 | 0.000000 | 640 | 555.519806 | 614.8 | 674.080194 | 0.960625 |
| GO:0043631\_RNA\_polyadenylation | 10 | 0 | 0.000000 | 0.000000 | 640 | 555.519806 | 614.8 | 674.080194 | 0.960625 |
| GO:0043691\_reverse\_cholesterol\_transport | 10 | 0 | 0.000000 | 0.000000 | 640 | 555.519806 | 614.8 | 674.080194 | 0.960625 |
| GO:0044269\_glycerol\_ether\_catabolic\_process | 10 | 0 | 0.000000 | 0.000000 | 640 | 555.519806 | 614.8 | 674.080194 | 0.960625 |
| GO:0045078\_positive\_regulation\_of\_interferon-gamma\_biosynthetic\_process | 10 | 0 | 0.000000 | 0.000000 | 640 | 555.519806 | 614.8 | 674.080194 | 0.960625 |
| GO:0045103\_intermediate\_filament-based\_process | 10 | 0 | 0.000000 | 0.000000 | 640 | 555.519806 | 614.8 | 674.080194 | 0.960625 |
| GO:0045104\_intermediate\_filament\_cytoskeleton\_organization | 10 | 0 | 0.000000 | 0.000000 | 640 | 555.519806 | 614.8 | 674.080194 | 0.960625 |
| GO:0045947\_negative\_regulation\_of\_translational\_initiation | 10 | 0 | 0.000000 | 0.000000 | 640 | 555.519806 | 614.8 | 674.080194 | 0.960625 |
| GO:0046460\_neutral\_lipid\_biosynthetic\_process | 10 | 0 | 0.000000 | 0.000000 | 640 | 555.519806 | 614.8 | 674.080194 | 0.960625 |
| GO:0046461\_neutral\_lipid\_catabolic\_process | 10 | 0 | 0.000000 | 0.000000 | 640 | 555.519806 | 614.8 | 674.080194 | 0.960625 |
| GO:0046463\_acylglycerol\_biosynthetic\_process | 10 | 0 | 0.000000 | 0.000000 | 640 | 555.519806 | 614.8 | 674.080194 | 0.960625 |
| GO:0046464\_acylglycerol\_catabolic\_process | 10 | 0 | 0.000000 | 0.000000 | 640 | 555.519806 | 614.8 | 674.080194 | 0.960625 |
| GO:0046632\_alpha-beta\_T\_cell\_differentiation | 10 | 0 | 0.000000 | 0.000000 | 640 | 555.519806 | 614.8 | 674.080194 | 0.960625 |
| GO:0046847\_filopodium\_assembly | 10 | 0 | 0.000000 | 0.000000 | 640 | 555.519806 | 614.8 | 674.080194 | 0.960625 |
| GO:0046888\_negative\_regulation\_of\_hormone\_secretion | 10 | 0 | 0.000000 | 0.000000 | 640 | 555.519806 | 614.8 | 674.080194 | 0.960625 |
| GO:0048167\_regulation\_of\_synaptic\_plasticity | 10 | 0 | 0.000000 | 0.000000 | 640 | 555.519806 | 614.8 | 674.080194 | 0.960625 |
| GO:0048194\_Golgi\_vesicle\_budding | 10 | 0 | 0.000000 | 0.000000 | 640 | 555.519806 | 614.8 | 674.080194 | 0.960625 |
| GO:0048200\_Golgi\_transport\_vesicle\_coating | 10 | 0 | 0.000000 | 0.000000 | 640 | 555.519806 | 614.8 | 674.080194 | 0.960625 |
| GO:0048205\_COPI\_coating\_of\_Golgi\_vesicle | 10 | 0 | 0.000000 | 0.000000 | 640 | 555.519806 | 614.8 | 674.080194 | 0.960625 |
| GO:0048384\_retinoic\_acid\_receptor\_signaling\_pathway | 10 | 0 | 0.000000 | 0.000000 | 640 | 555.519806 | 614.8 | 674.080194 | 0.960625 |
| GO:0050654\_chondroitin\_sulfate\_proteoglycan\_metabolic\_process | 10 | 0 | 0.000000 | 0.000000 | 640 | 555.519806 | 614.8 | 674.080194 | 0.960625 |
| GO:0050706\_regulation\_of\_interleukin-1\_beta\_secretion | 10 | 0 | 0.000000 | 0.000000 | 640 | 555.519806 | 614.8 | 674.080194 | 0.960625 |
| GO:0050716\_positive\_regulation\_of\_interleukin-1\_secretion | 10 | 0 | 0.000000 | 0.000000 | 640 | 555.519806 | 614.8 | 674.080194 | 0.960625 |
| GO:0050810\_regulation\_of\_steroid\_biosynthetic\_process | 10 | 0 | 0.000000 | 0.000000 | 640 | 555.519806 | 614.8 | 674.080194 | 0.960625 |
| GO:0050868\_negative\_regulation\_of\_T\_cell\_activation | 10 | 0 | 0.000000 | 0.000000 | 640 | 555.519806 | 614.8 | 674.080194 | 0.960625 |
| GO:0050879\_multicellular\_organismal\_movement | 10 | 0 | 0.000000 | 0.000000 | 640 | 555.519806 | 614.8 | 674.080194 | 0.960625 |
| GO:0050881\_musculoskeletal\_movement | 10 | 0 | 0.000000 | 0.000000 | 640 | 555.519806 | 614.8 | 674.080194 | 0.960625 |
| GO:0051492\_regulation\_of\_stress\_fiber\_formation | 10 | 0 | 0.000000 | 0.000000 | 640 | 555.519806 | 614.8 | 674.080194 | 0.960625 |
| GO:0051646\_mitochondrion\_localization | 10 | 0 | 0.000000 | 0.000000 | 640 | 555.519806 | 614.8 | 674.080194 | 0.960625 |
| GO:0060021\_palate\_development | 10 | 0 | 0.000000 | 0.000000 | 640 | 555.519806 | 614.8 | 674.080194 | 0.960625 |
| GO:0060491\_regulation\_of\_cell\_projection\_assembly | 10 | 0 | 0.000000 | 0.000000 | 640 | 555.519806 | 614.8 | 674.080194 | 0.960625 |
| GO:0060541\_respiratory\_system\_development | 10 | 0 | 0.000000 | 0.000000 | 640 | 555.519806 | 614.8 | 674.080194 | 0.960625 |
| GO:0070206\_protein\_trimerization | 10 | 0 | 0.000000 | 0.000000 | 640 | 555.519806 | 614.8 | 674.080194 | 0.960625 |
| GO:0070873\_regulation\_of\_glycogen\_metabolic\_process | 10 | 0 | 0.000000 | 0.000000 | 640 | 555.519806 | 614.8 | 674.080194 | 0.960625 |
| GO:0030163\_protein\_catabolic\_process | 330 | 0 | 0.000000 | 0.000000 | 641 | 556.133105 | 615.32 | 674.506895 | 0.959938 |
| GO:0006417\_regulation\_of\_translation | 71 | 0 | 0.000000 | 0.000000 | 647 | 561.647209 | 620.44 | 679.232791 | 0.958949 |
| GO:0007050\_cell\_cycle\_arrest | 71 | 0 | 0.000000 | 0.000000 | 647 | 561.647209 | 620.44 | 679.232791 | 0.958949 |
| GO:0009615\_response\_to\_virus | 71 | 0 | 0.000000 | 0.000000 | 647 | 561.647209 | 620.44 | 679.232791 | 0.958949 |
| GO:0032101\_regulation\_of\_response\_to\_external\_stimulus | 71 | 0 | 0.000000 | 0.000000 | 647 | 561.647209 | 620.44 | 679.232791 | 0.958949 |
| GO:0051223\_regulation\_of\_protein\_transport | 71 | 0 | 0.000000 | 0.000000 | 647 | 561.647209 | 620.44 | 679.232791 | 0.958949 |
| GO:0051606\_detection\_of\_stimulus | 71 | 0 | 0.000000 | 0.000000 | 647 | 561.647209 | 620.44 | 679.232791 | 0.958949 |
| GO:0010557\_positive\_regulation\_of\_macromolecule\_biosynthetic\_process | 334 | 0 | 0.000000 | 0.000000 | 648 | 562.309304 | 620.99 | 679.670696 | 0.958318 |
| GO:0034984\_cellular\_response\_to\_DNA\_damage\_stimulus | 215 | 0 | 0.000000 | 0.000000 | 649 | 563.036086 | 621.68 | 680.323914 | 0.957904 |
| GO:0006163\_purine\_nucleotide\_metabolic\_process | 99 | 0 | 0.000000 | 0.000000 | 652 | 565.806199 | 624.16 | 682.513801 | 0.957301 |
| GO:0009968\_negative\_regulation\_of\_signal\_transduction | 99 | 0 | 0.000000 | 0.000000 | 652 | 565.806199 | 624.16 | 682.513801 | 0.957301 |
| GO:0016569\_covalent\_chromatin\_modification | 99 | 0 | 0.000000 | 0.000000 | 652 | 565.806199 | 624.16 | 682.513801 | 0.957301 |
| GO:0030334\_regulation\_of\_cell\_migration | 89 | 0 | 0.000000 | 0.000000 | 655 | 572.998524 | 630.34 | 687.681476 | 0.962351 |
| GO:0034660\_ncRNA\_metabolic\_process | 89 | 0 | 0.000000 | 0.000000 | 655 | 572.998524 | 630.34 | 687.681476 | 0.962351 |
| GO:0051240\_positive\_regulation\_of\_multicellular\_organismal\_process | 89 | 0 | 0.000000 | 0.000000 | 655 | 572.998524 | 630.34 | 687.681476 | 0.962351 |
| GO:0022414\_reproductive\_process | 365 | 0 | 0.000000 | 0.000000 | 656 | 573.526149 | 630.92 | 688.313851 | 0.961768 |
| GO:0006364\_rRNA\_processing | 39 | 0 | 0.000000 | 0.000000 | 667 | 585.552001 | 642.8 | 700.047999 | 0.963718 |
| GO:0006401\_RNA\_catabolic\_process | 39 | 0 | 0.000000 | 0.000000 | 667 | 585.552001 | 642.8 | 700.047999 | 0.963718 |
| GO:0006497\_protein\_amino\_acid\_lipidation | 39 | 0 | 0.000000 | 0.000000 | 667 | 585.552001 | 642.8 | 700.047999 | 0.963718 |
| GO:0009310\_amine\_catabolic\_process | 39 | 0 | 0.000000 | 0.000000 | 667 | 585.552001 | 642.8 | 700.047999 | 0.963718 |
| GO:0009792\_embryonic\_development\_ending\_in\_birth\_or\_egg\_hatching | 39 | 0 | 0.000000 | 0.000000 | 667 | 585.552001 | 642.8 | 700.047999 | 0.963718 |
| GO:0030509\_BMP\_signaling\_pathway | 39 | 0 | 0.000000 | 0.000000 | 667 | 585.552001 | 642.8 | 700.047999 | 0.963718 |
| GO:0032970\_regulation\_of\_actin\_filament-based\_process | 39 | 0 | 0.000000 | 0.000000 | 667 | 585.552001 | 642.8 | 700.047999 | 0.963718 |
| GO:0042158\_lipoprotein\_biosynthetic\_process | 39 | 0 | 0.000000 | 0.000000 | 667 | 585.552001 | 642.8 | 700.047999 | 0.963718 |
| GO:0043009\_chordate\_embryonic\_development | 39 | 0 | 0.000000 | 0.000000 | 667 | 585.552001 | 642.8 | 700.047999 | 0.963718 |
| GO:0046365\_monosaccharide\_catabolic\_process | 39 | 0 | 0.000000 | 0.000000 | 667 | 585.552001 | 642.8 | 700.047999 | 0.963718 |
| GO:0051960\_regulation\_of\_nervous\_system\_development | 39 | 0 | 0.000000 | 0.000000 | 667 | 585.552001 | 642.8 | 700.047999 | 0.963718 |
| GO:0001944\_vasculature\_development | 88 | 0 | 0.000000 | 0.000000 | 668 | 586.446018 | 643.62 | 700.793982 | 0.963503 |
| GO:0001508\_regulation\_of\_action\_potential | 24 | 0 | 0.000000 | 0.000000 | 693 | 614.647961 | 671.22 | 727.792039 | 0.968571 |
| GO:0001649\_osteoblast\_differentiation | 24 | 0 | 0.000000 | 0.000000 | 693 | 614.647961 | 671.22 | 727.792039 | 0.968571 |
| GO:0006479\_protein\_amino\_acid\_methylation | 24 | 0 | 0.000000 | 0.000000 | 693 | 614.647961 | 671.22 | 727.792039 | 0.968571 |
| GO:0007229\_integrin-mediated\_signaling\_pathway | 24 | 0 | 0.000000 | 0.000000 | 693 | 614.647961 | 671.22 | 727.792039 | 0.968571 |
| GO:0007568\_aging | 24 | 0 | 0.000000 | 0.000000 | 693 | 614.647961 | 671.22 | 727.792039 | 0.968571 |
| GO:0008213\_protein\_amino\_acid\_alkylation | 24 | 0 | 0.000000 | 0.000000 | 693 | 614.647961 | 671.22 | 727.792039 | 0.968571 |
| GO:0009116\_nucleoside\_metabolic\_process | 24 | 0 | 0.000000 | 0.000000 | 693 | 614.647961 | 671.22 | 727.792039 | 0.968571 |
| GO:0009266\_response\_to\_temperature\_stimulus | 24 | 0 | 0.000000 | 0.000000 | 693 | 614.647961 | 671.22 | 727.792039 | 0.968571 |
| GO:0017148\_negative\_regulation\_of\_translation | 24 | 0 | 0.000000 | 0.000000 | 693 | 614.647961 | 671.22 | 727.792039 | 0.968571 |
| GO:0019059\_initiation\_of\_viral\_infection | 24 | 0 | 0.000000 | 0.000000 | 693 | 614.647961 | 671.22 | 727.792039 | 0.968571 |
| GO:0030073\_insulin\_secretion | 24 | 0 | 0.000000 | 0.000000 | 693 | 614.647961 | 671.22 | 727.792039 | 0.968571 |
| GO:0030193\_regulation\_of\_blood\_coagulation | 24 | 0 | 0.000000 | 0.000000 | 693 | 614.647961 | 671.22 | 727.792039 | 0.968571 |
| GO:0033002\_muscle\_cell\_proliferation | 24 | 0 | 0.000000 | 0.000000 | 693 | 614.647961 | 671.22 | 727.792039 | 0.968571 |
| GO:0035023\_regulation\_of\_Rho\_protein\_signal\_transduction | 24 | 0 | 0.000000 | 0.000000 | 693 | 614.647961 | 671.22 | 727.792039 | 0.968571 |
| GO:0042440\_pigment\_metabolic\_process | 24 | 0 | 0.000000 | 0.000000 | 693 | 614.647961 | 671.22 | 727.792039 | 0.968571 |
| GO:0042509\_regulation\_of\_tyrosine\_phosphorylation\_of\_STAT\_protein | 24 | 0 | 0.000000 | 0.000000 | 693 | 614.647961 | 671.22 | 727.792039 | 0.968571 |
| GO:0043407\_negative\_regulation\_of\_MAP\_kinase\_activity | 24 | 0 | 0.000000 | 0.000000 | 693 | 614.647961 | 671.22 | 727.792039 | 0.968571 |
| GO:0045216\_cell-cell\_junction\_organization | 24 | 0 | 0.000000 | 0.000000 | 693 | 614.647961 | 671.22 | 727.792039 | 0.968571 |
| GO:0045638\_negative\_regulation\_of\_myeloid\_cell\_differentiation | 24 | 0 | 0.000000 | 0.000000 | 693 | 614.647961 | 671.22 | 727.792039 | 0.968571 |
| GO:0045785\_positive\_regulation\_of\_cell\_adhesion | 24 | 0 | 0.000000 | 0.000000 | 693 | 614.647961 | 671.22 | 727.792039 | 0.968571 |
| GO:0046890\_regulation\_of\_lipid\_biosynthetic\_process | 24 | 0 | 0.000000 | 0.000000 | 693 | 614.647961 | 671.22 | 727.792039 | 0.968571 |
| GO:0051701\_interaction\_with\_host | 24 | 0 | 0.000000 | 0.000000 | 693 | 614.647961 | 671.22 | 727.792039 | 0.968571 |
| GO:0051969\_regulation\_of\_transmission\_of\_nerve\_impulse | 24 | 0 | 0.000000 | 0.000000 | 693 | 614.647961 | 671.22 | 727.792039 | 0.968571 |
| GO:0055067\_monovalent\_inorganic\_cation\_homeostasis | 24 | 0 | 0.000000 | 0.000000 | 693 | 614.647961 | 671.22 | 727.792039 | 0.968571 |
| GO:0070646\_protein\_modification\_by\_small\_protein\_removal | 24 | 0 | 0.000000 | 0.000000 | 693 | 614.647961 | 671.22 | 727.792039 | 0.968571 |
| GO:0006916\_anti-apoptosis | 155 | 0 | 0.000000 | 0.000000 | 695 | 616.342664 | 672.67 | 728.997336 | 0.967871 |
| GO:0032940\_secretion\_by\_cell | 155 | 0 | 0.000000 | 0.000000 | 695 | 616.342664 | 672.67 | 728.997336 | 0.967871 |
| GO:0009124\_nucleoside\_monophosphate\_biosynthetic\_process | 70 | 0 | 0.000000 | 0.000000 | 699 | 622.069268 | 678.02 | 733.970732 | 0.969986 |
| GO:0009416\_response\_to\_light\_stimulus | 70 | 0 | 0.000000 | 0.000000 | 699 | 622.069268 | 678.02 | 733.970732 | 0.969986 |
| GO:0022411\_cellular\_component\_disassembly | 70 | 0 | 0.000000 | 0.000000 | 699 | 622.069268 | 678.02 | 733.970732 | 0.969986 |
| GO:0051170\_nuclear\_import | 70 | 0 | 0.000000 | 0.000000 | 699 | 622.069268 | 678.02 | 733.970732 | 0.969986 |
| GO:0006928\_cell\_motion | 308 | 0 | 0.000000 | 0.000000 | 701 | 624.150467 | 679.77 | 735.389533 | 0.969715 |
| GO:0051674\_localization\_of\_cell | 308 | 0 | 0.000000 | 0.000000 | 701 | 624.150467 | 679.77 | 735.389533 | 0.969715 |
| GO:0006650\_glycerophospholipid\_metabolic\_process | 75 | 0 | 0.000000 | 0.000000 | 706 | 629.681818 | 684.78 | 739.878182 | 0.969943 |
| GO:0006869\_lipid\_transport | 75 | 0 | 0.000000 | 0.000000 | 706 | 629.681818 | 684.78 | 739.878182 | 0.969943 |
| GO:0044271\_nitrogen\_compound\_biosynthetic\_process | 75 | 0 | 0.000000 | 0.000000 | 706 | 629.681818 | 684.78 | 739.878182 | 0.969943 |
| GO:0051438\_regulation\_of\_ubiquitin-protein\_ligase\_activity | 75 | 0 | 0.000000 | 0.000000 | 706 | 629.681818 | 684.78 | 739.878182 | 0.969943 |
| GO:0070201\_regulation\_of\_establishment\_of\_protein\_localization | 75 | 0 | 0.000000 | 0.000000 | 706 | 629.681818 | 684.78 | 739.878182 | 0.969943 |
| GO:0019941\_modification-dependent\_protein\_catabolic\_process | 138 | 0 | 0.000000 | 0.000000 | 708 | 631.340349 | 686.28 | 741.219651 | 0.969322 |
| GO:0043632\_modification-dependent\_macromolecule\_catabolic\_process | 138 | 0 | 0.000000 | 0.000000 | 708 | 631.340349 | 686.28 | 741.219651 | 0.969322 |
| GO:0048646\_anatomical\_structure\_formation\_involved\_in\_morphogenesis | 111 | 0 | 0.000000 | 0.000000 | 709 | 634.290103 | 688.84 | 743.389897 | 0.971566 |
| GO:0010648\_negative\_regulation\_of\_cell\_communication | 102 | 0 | 0.000000 | 0.000000 | 710 | 636.169823 | 690.43 | 744.690177 | 0.972437 |
| GO:0000280\_nuclear\_division | 115 | 0 | 0.000000 | 0.000000 | 714 | 639.542469 | 693.62 | 747.697531 | 0.971457 |
| GO:0005996\_monosaccharide\_metabolic\_process | 115 | 0 | 0.000000 | 0.000000 | 714 | 639.542469 | 693.62 | 747.697531 | 0.971457 |
| GO:0007067\_mitosis | 115 | 0 | 0.000000 | 0.000000 | 714 | 639.542469 | 693.62 | 747.697531 | 0.971457 |
| GO:0045944\_positive\_regulation\_of\_transcription\_from\_RNA\_polymerase\_II\_promoter | 115 | 0 | 0.000000 | 0.000000 | 714 | 639.542469 | 693.62 | 747.697531 | 0.971457 |
| GO:0030030\_cell\_projection\_organization | 127 | 0 | 0.000000 | 0.000000 | 715 | 640.470252 | 694.44 | 748.409748 | 0.971245 |
| GO:0046907\_intracellular\_transport | 420 | 0 | 0.000000 | 0.000000 | 716 | 640.980220 | 694.86 | 748.739780 | 0.970475 |
| GO:0002697\_regulation\_of\_immune\_effector\_process | 49 | 0 | 0.000000 | 0.000000 | 723 | 651.495389 | 704.93 | 758.364611 | 0.975007 |
| GO:0006275\_regulation\_of\_DNA\_replication | 49 | 0 | 0.000000 | 0.000000 | 723 | 651.495389 | 704.93 | 758.364611 | 0.975007 |
| GO:0006643\_membrane\_lipid\_metabolic\_process | 49 | 0 | 0.000000 | 0.000000 | 723 | 651.495389 | 704.93 | 758.364611 | 0.975007 |
| GO:0009914\_hormone\_transport | 49 | 0 | 0.000000 | 0.000000 | 723 | 651.495389 | 704.93 | 758.364611 | 0.975007 |
| GO:0010517\_regulation\_of\_phospholipase\_activity | 49 | 0 | 0.000000 | 0.000000 | 723 | 651.495389 | 704.93 | 758.364611 | 0.975007 |
| GO:0018108\_peptidyl-tyrosine\_phosphorylation | 49 | 0 | 0.000000 | 0.000000 | 723 | 651.495389 | 704.93 | 758.364611 | 0.975007 |
| GO:0032984\_macromolecular\_complex\_disassembly | 49 | 0 | 0.000000 | 0.000000 | 723 | 651.495389 | 704.93 | 758.364611 | 0.975007 |
| GO:0019226\_transmission\_of\_nerve\_impulse | 209 | 0 | 0.000000 | 0.000000 | 726 | 654.544116 | 707.55 | 760.555884 | 0.974587 |
| GO:0043069\_negative\_regulation\_of\_programmed\_cell\_death | 209 | 0 | 0.000000 | 0.000000 | 726 | 654.544116 | 707.55 | 760.555884 | 0.974587 |
| GO:0060548\_negative\_regulation\_of\_cell\_death | 209 | 0 | 0.000000 | 0.000000 | 726 | 654.544116 | 707.55 | 760.555884 | 0.974587 |
| GO:0007179\_transforming\_growth\_factor\_beta\_receptor\_signaling\_pathway | 72 | 0 | 0.000000 | 0.000000 | 728 | 656.461844 | 709.29 | 762.118156 | 0.974299 |
| GO:0060249\_anatomical\_structure\_homeostasis | 72 | 0 | 0.000000 | 0.000000 | 728 | 656.461844 | 709.29 | 762.118156 | 0.974299 |
| GO:0006140\_regulation\_of\_nucleotide\_metabolic\_process | 60 | 0 | 0.000000 | 0.000000 | 731 | 660.371272 | 712.94 | 765.508728 | 0.975294 |
| GO:0044087\_regulation\_of\_cellular\_component\_biogenesis | 60 | 0 | 0.000000 | 0.000000 | 731 | 660.371272 | 712.94 | 765.508728 | 0.975294 |
| GO:0046058\_cAMP\_metabolic\_process | 60 | 0 | 0.000000 | 0.000000 | 731 | 660.371272 | 712.94 | 765.508728 | 0.975294 |
| GO:0051707\_response\_to\_other\_organism | 125 | 0 | 0.000000 | 0.000000 | 732 | 663.911025 | 716.13 | 768.348975 | 0.978320 |
| GO:0010033\_response\_to\_organic\_substance | 276 | 0 | 0.000000 | 0.000000 | 733 | 664.683342 | 716.75 | 768.816658 | 0.977831 |
| GO:0000910\_cytokinesis | 36 | 0 | 0.000000 | 0.000000 | 740 | 672.389567 | 724.17 | 775.950433 | 0.978608 |
| GO:0001819\_positive\_regulation\_of\_cytokine\_production | 36 | 0 | 0.000000 | 0.000000 | 740 | 672.389567 | 724.17 | 775.950433 | 0.978608 |
| GO:0006334\_nucleosome\_assembly | 36 | 0 | 0.000000 | 0.000000 | 740 | 672.389567 | 724.17 | 775.950433 | 0.978608 |
| GO:0006814\_sodium\_ion\_transport | 36 | 0 | 0.000000 | 0.000000 | 740 | 672.389567 | 724.17 | 775.950433 | 0.978608 |
| GO:0009566\_fertilization | 36 | 0 | 0.000000 | 0.000000 | 740 | 672.389567 | 724.17 | 775.950433 | 0.978608 |
| GO:0034097\_response\_to\_cytokine\_stimulus | 36 | 0 | 0.000000 | 0.000000 | 740 | 672.389567 | 724.17 | 775.950433 | 0.978608 |
| GO:0034103\_regulation\_of\_tissue\_remodeling | 36 | 0 | 0.000000 | 0.000000 | 740 | 672.389567 | 724.17 | 775.950433 | 0.978608 |
| GO:0045893\_positive\_regulation\_of\_transcription\_\_DNA-dependent | 211 | 0 | 0.000000 | 0.000000 | 741 | 673.696108 | 725.31 | 776.923892 | 0.978826 |
| GO:0008544\_epidermis\_development | 104 | 0 | 0.000000 | 0.000000 | 742 | 674.599646 | 726.18 | 777.760354 | 0.978679 |
| GO:0000302\_response\_to\_reactive\_oxygen\_species | 21 | 0 | 0.000000 | 0.000000 | 786 | 719.676110 | 770.38 | 821.083890 | 0.980127 |
| GO:0000718\_nucleotide-excision\_repair\_\_DNA\_damage\_removal | 21 | 0 | 0.000000 | 0.000000 | 786 | 719.676110 | 770.38 | 821.083890 | 0.980127 |
| GO:0001654\_eye\_development | 21 | 0 | 0.000000 | 0.000000 | 786 | 719.676110 | 770.38 | 821.083890 | 0.980127 |
| GO:0001936\_regulation\_of\_endothelial\_cell\_proliferation | 21 | 0 | 0.000000 | 0.000000 | 786 | 719.676110 | 770.38 | 821.083890 | 0.980127 |
| GO:0002819\_regulation\_of\_adaptive\_immune\_response | 21 | 0 | 0.000000 | 0.000000 | 786 | 719.676110 | 770.38 | 821.083890 | 0.980127 |
| GO:0002822\_regulation\_of\_adaptive\_immune\_response\_based\_on\_somatic\_recombination\_of\_immune\_receptors\_built\_from\_immunoglobulin\_superfamily\_domains | 21 | 0 | 0.000000 | 0.000000 | 786 | 719.676110 | 770.38 | 821.083890 | 0.980127 |
| GO:0002831\_regulation\_of\_response\_to\_biotic\_stimulus | 21 | 0 | 0.000000 | 0.000000 | 786 | 719.676110 | 770.38 | 821.083890 | 0.980127 |
| GO:0006024\_glycosaminoglycan\_biosynthetic\_process | 21 | 0 | 0.000000 | 0.000000 | 786 | 719.676110 | 770.38 | 821.083890 | 0.980127 |
| GO:0006040\_amino\_sugar\_metabolic\_process | 21 | 0 | 0.000000 | 0.000000 | 786 | 719.676110 | 770.38 | 821.083890 | 0.980127 |
| GO:0006073\_cellular\_glucan\_metabolic\_process | 21 | 0 | 0.000000 | 0.000000 | 786 | 719.676110 | 770.38 | 821.083890 | 0.980127 |
| GO:0006109\_regulation\_of\_carbohydrate\_metabolic\_process | 21 | 0 | 0.000000 | 0.000000 | 786 | 719.676110 | 770.38 | 821.083890 | 0.980127 |
| GO:0006282\_regulation\_of\_DNA\_repair | 21 | 0 | 0.000000 | 0.000000 | 786 | 719.676110 | 770.38 | 821.083890 | 0.980127 |
| GO:0006284\_base-excision\_repair | 21 | 0 | 0.000000 | 0.000000 | 786 | 719.676110 | 770.38 | 821.083890 | 0.980127 |
| GO:0006402\_mRNA\_catabolic\_process | 21 | 0 | 0.000000 | 0.000000 | 786 | 719.676110 | 770.38 | 821.083890 | 0.980127 |
| GO:0006493\_protein\_amino\_acid\_O-linked\_glycosylation | 21 | 0 | 0.000000 | 0.000000 | 786 | 719.676110 | 770.38 | 821.083890 | 0.980127 |
| GO:0006664\_glycolipid\_metabolic\_process | 21 | 0 | 0.000000 | 0.000000 | 786 | 719.676110 | 770.38 | 821.083890 | 0.980127 |
| GO:0006775\_fat-soluble\_vitamin\_metabolic\_process | 21 | 0 | 0.000000 | 0.000000 | 786 | 719.676110 | 770.38 | 821.083890 | 0.980127 |
| GO:0006892\_post-Golgi\_vesicle-mediated\_transport | 21 | 0 | 0.000000 | 0.000000 | 786 | 719.676110 | 770.38 | 821.083890 | 0.980127 |
| GO:0006903\_vesicle\_targeting | 21 | 0 | 0.000000 | 0.000000 | 786 | 719.676110 | 770.38 | 821.083890 | 0.980127 |
| GO:0007292\_female\_gamete\_generation | 21 | 0 | 0.000000 | 0.000000 | 786 | 719.676110 | 770.38 | 821.083890 | 0.980127 |
| GO:0008156\_negative\_regulation\_of\_DNA\_replication | 21 | 0 | 0.000000 | 0.000000 | 786 | 719.676110 | 770.38 | 821.083890 | 0.980127 |
| GO:0008360\_regulation\_of\_cell\_shape | 21 | 0 | 0.000000 | 0.000000 | 786 | 719.676110 | 770.38 | 821.083890 | 0.980127 |
| GO:0009199\_ribonucleoside\_triphosphate\_metabolic\_process | 21 | 0 | 0.000000 | 0.000000 | 786 | 719.676110 | 770.38 | 821.083890 | 0.980127 |
| GO:0010675\_regulation\_of\_cellular\_carbohydrate\_metabolic\_process | 21 | 0 | 0.000000 | 0.000000 | 786 | 719.676110 | 770.38 | 821.083890 | 0.980127 |
| GO:0014031\_mesenchymal\_cell\_development | 21 | 0 | 0.000000 | 0.000000 | 786 | 719.676110 | 770.38 | 821.083890 | 0.980127 |
| GO:0016338\_calcium-independent\_cell-cell\_adhesion | 21 | 0 | 0.000000 | 0.000000 | 786 | 719.676110 | 770.38 | 821.083890 | 0.980127 |
| GO:0030048\_actin\_filament-based\_movement | 21 | 0 | 0.000000 | 0.000000 | 786 | 719.676110 | 770.38 | 821.083890 | 0.980127 |
| GO:0030111\_regulation\_of\_Wnt\_receptor\_signaling\_pathway | 21 | 0 | 0.000000 | 0.000000 | 786 | 719.676110 | 770.38 | 821.083890 | 0.980127 |
| GO:0030148\_sphingolipid\_biosynthetic\_process | 21 | 0 | 0.000000 | 0.000000 | 786 | 719.676110 | 770.38 | 821.083890 | 0.980127 |
| GO:0032886\_regulation\_of\_microtubule-based\_process | 21 | 0 | 0.000000 | 0.000000 | 786 | 719.676110 | 770.38 | 821.083890 | 0.980127 |
| GO:0035295\_tube\_development | 21 | 0 | 0.000000 | 0.000000 | 786 | 719.676110 | 770.38 | 821.083890 | 0.980127 |
| GO:0042398\_cellular\_amino\_acid\_derivative\_biosynthetic\_process | 21 | 0 | 0.000000 | 0.000000 | 786 | 719.676110 | 770.38 | 821.083890 | 0.980127 |
| GO:0042439\_ethanolamine\_and\_derivative\_metabolic\_process | 21 | 0 | 0.000000 | 0.000000 | 786 | 719.676110 | 770.38 | 821.083890 | 0.980127 |
| GO:0043523\_regulation\_of\_neuron\_apoptosis | 21 | 0 | 0.000000 | 0.000000 | 786 | 719.676110 | 770.38 | 821.083890 | 0.980127 |
| GO:0044042\_glucan\_metabolic\_process | 21 | 0 | 0.000000 | 0.000000 | 786 | 719.676110 | 770.38 | 821.083890 | 0.980127 |
| GO:0045649\_regulation\_of\_macrophage\_differentiation | 21 | 0 | 0.000000 | 0.000000 | 786 | 719.676110 | 770.38 | 821.083890 | 0.980127 |
| GO:0046427\_positive\_regulation\_of\_JAK-STAT\_cascade | 21 | 0 | 0.000000 | 0.000000 | 786 | 719.676110 | 770.38 | 821.083890 | 0.980127 |
| GO:0048520\_positive\_regulation\_of\_behavior | 21 | 0 | 0.000000 | 0.000000 | 786 | 719.676110 | 770.38 | 821.083890 | 0.980127 |
| GO:0048762\_mesenchymal\_cell\_differentiation | 21 | 0 | 0.000000 | 0.000000 | 786 | 719.676110 | 770.38 | 821.083890 | 0.980127 |
| GO:0051297\_centrosome\_organization | 21 | 0 | 0.000000 | 0.000000 | 786 | 719.676110 | 770.38 | 821.083890 | 0.980127 |
| GO:0055072\_iron\_ion\_homeostasis | 21 | 0 | 0.000000 | 0.000000 | 786 | 719.676110 | 770.38 | 821.083890 | 0.980127 |
| GO:0060485\_mesenchyme\_development | 21 | 0 | 0.000000 | 0.000000 | 786 | 719.676110 | 770.38 | 821.083890 | 0.980127 |
| GO:0070167\_regulation\_of\_biomineral\_formation | 21 | 0 | 0.000000 | 0.000000 | 786 | 719.676110 | 770.38 | 821.083890 | 0.980127 |
| GO:0070507\_regulation\_of\_microtubule\_cytoskeleton\_organization | 21 | 0 | 0.000000 | 0.000000 | 786 | 719.676110 | 770.38 | 821.083890 | 0.980127 |
| GO:0044265\_cellular\_macromolecule\_catabolic\_process | 239 | 0 | 0.000000 | 0.000000 | 787 | 720.375217 | 771.0 | 821.624783 | 0.979670 |
| GO:0009891\_positive\_regulation\_of\_biosynthetic\_process | 359 | 0 | 0.000000 | 0.000000 | 788 | 721.863739 | 772.25 | 822.636261 | 0.980013 |
| GO:0006325\_chromatin\_organization | 201 | 0 | 0.000000 | 0.000000 | 789 | 725.177424 | 775.02 | 824.862576 | 0.982281 |
| GO:0015849\_organic\_acid\_transport | 80 | 0 | 0.000000 | 0.000000 | 791 | 729.627915 | 779.08 | 828.532085 | 0.984930 |
| GO:0031175\_neuron\_projection\_development | 80 | 0 | 0.000000 | 0.000000 | 791 | 729.627915 | 779.08 | 828.532085 | 0.984930 |
| GO:0008380\_RNA\_splicing | 192 | 0 | 0.000000 | 0.000000 | 792 | 730.233216 | 779.74 | 829.246784 | 0.984520 |
| GO:0006520\_cellular\_amino\_acid\_metabolic\_process | 108 | 0 | 0.000000 | 0.000000 | 799 | 737.329498 | 786.64 | 835.950502 | 0.984531 |
| GO:0006644\_phospholipid\_metabolic\_process | 108 | 0 | 0.000000 | 0.000000 | 799 | 737.329498 | 786.64 | 835.950502 | 0.984531 |
| GO:0007346\_regulation\_of\_mitotic\_cell\_cycle | 108 | 0 | 0.000000 | 0.000000 | 799 | 737.329498 | 786.64 | 835.950502 | 0.984531 |
| GO:0008202\_steroid\_metabolic\_process | 108 | 0 | 0.000000 | 0.000000 | 799 | 737.329498 | 786.64 | 835.950502 | 0.984531 |
| GO:0019637\_organophosphate\_metabolic\_process | 108 | 0 | 0.000000 | 0.000000 | 799 | 737.329498 | 786.64 | 835.950502 | 0.984531 |
| GO:0032446\_protein\_modification\_by\_small\_protein\_conjugation | 108 | 0 | 0.000000 | 0.000000 | 799 | 737.329498 | 786.64 | 835.950502 | 0.984531 |
| GO:0044106\_cellular\_amine\_metabolic\_process | 108 | 0 | 0.000000 | 0.000000 | 799 | 737.329498 | 786.64 | 835.950502 | 0.984531 |
| GO:0006120\_mitochondrial\_electron\_transport\_\_NADH\_to\_ubiquinone | 42 | 0 | 0.000000 | 0.000000 | 812 | 751.558164 | 800.45 | 849.341836 | 0.985776 |
| GO:0006865\_amino\_acid\_transport | 42 | 0 | 0.000000 | 0.000000 | 812 | 751.558164 | 800.45 | 849.341836 | 0.985776 |
| GO:0006887\_exocytosis | 42 | 0 | 0.000000 | 0.000000 | 812 | 751.558164 | 800.45 | 849.341836 | 0.985776 |
| GO:0006944\_membrane\_fusion | 42 | 0 | 0.000000 | 0.000000 | 812 | 751.558164 | 800.45 | 849.341836 | 0.985776 |
| GO:0007608\_sensory\_perception\_of\_smell | 42 | 0 | 0.000000 | 0.000000 | 812 | 751.558164 | 800.45 | 849.341836 | 0.985776 |
| GO:0008203\_cholesterol\_metabolic\_process | 42 | 0 | 0.000000 | 0.000000 | 812 | 751.558164 | 800.45 | 849.341836 | 0.985776 |
| GO:0016072\_rRNA\_metabolic\_process | 42 | 0 | 0.000000 | 0.000000 | 812 | 751.558164 | 800.45 | 849.341836 | 0.985776 |
| GO:0032259\_methylation | 42 | 0 | 0.000000 | 0.000000 | 812 | 751.558164 | 800.45 | 849.341836 | 0.985776 |
| GO:0032868\_response\_to\_insulin\_stimulus | 42 | 0 | 0.000000 | 0.000000 | 812 | 751.558164 | 800.45 | 849.341836 | 0.985776 |
| GO:0042692\_muscle\_cell\_differentiation | 42 | 0 | 0.000000 | 0.000000 | 812 | 751.558164 | 800.45 | 849.341836 | 0.985776 |
| GO:0043414\_biopolymer\_methylation | 42 | 0 | 0.000000 | 0.000000 | 812 | 751.558164 | 800.45 | 849.341836 | 0.985776 |
| GO:0048585\_negative\_regulation\_of\_response\_to\_stimulus | 42 | 0 | 0.000000 | 0.000000 | 812 | 751.558164 | 800.45 | 849.341836 | 0.985776 |
| GO:0051271\_negative\_regulation\_of\_cell\_motion | 42 | 0 | 0.000000 | 0.000000 | 812 | 751.558164 | 800.45 | 849.341836 | 0.985776 |
| GO:0009725\_response\_to\_hormone\_stimulus | 129 | 0 | 0.000000 | 0.000000 | 815 | 755.079121 | 803.7 | 852.320879 | 0.986135 |
| GO:0010740\_positive\_regulation\_of\_protein\_kinase\_cascade | 129 | 0 | 0.000000 | 0.000000 | 815 | 755.079121 | 803.7 | 852.320879 | 0.986135 |
| GO:0015672\_monovalent\_inorganic\_cation\_transport | 129 | 0 | 0.000000 | 0.000000 | 815 | 755.079121 | 803.7 | 852.320879 | 0.986135 |
| GO:0031325\_positive\_regulation\_of\_cellular\_metabolic\_process | 454 | 0 | 0.000000 | 0.000000 | 816 | 755.710435 | 804.15 | 852.589565 | 0.985478 |
| GO:0001666\_response\_to\_hypoxia | 50 | 0 | 0.000000 | 0.000000 | 822 | 763.353043 | 811.47 | 859.586957 | 0.987190 |
| GO:0007266\_Rho\_protein\_signal\_transduction | 50 | 0 | 0.000000 | 0.000000 | 822 | 763.353043 | 811.47 | 859.586957 | 0.987190 |
| GO:0007389\_pattern\_specification\_process | 50 | 0 | 0.000000 | 0.000000 | 822 | 763.353043 | 811.47 | 859.586957 | 0.987190 |
| GO:0045761\_regulation\_of\_adenylate\_cyclase\_activity | 50 | 0 | 0.000000 | 0.000000 | 822 | 763.353043 | 811.47 | 859.586957 | 0.987190 |
| GO:0048598\_embryonic\_morphogenesis | 50 | 0 | 0.000000 | 0.000000 | 822 | 763.353043 | 811.47 | 859.586957 | 0.987190 |
| GO:0051272\_positive\_regulation\_of\_cell\_motion | 50 | 0 | 0.000000 | 0.000000 | 822 | 763.353043 | 811.47 | 859.586957 | 0.987190 |
| GO:0000041\_transition\_metal\_ion\_transport | 18 | 0 | 0.000000 | 0.000000 | 860 | 800.944171 | 848.09 | 895.235829 | 0.986151 |
| GO:0000737\_DNA\_catabolic\_process\_\_endonucleolytic | 18 | 0 | 0.000000 | 0.000000 | 860 | 800.944171 | 848.09 | 895.235829 | 0.986151 |
| GO:0001818\_negative\_regulation\_of\_cytokine\_production | 18 | 0 | 0.000000 | 0.000000 | 860 | 800.944171 | 848.09 | 895.235829 | 0.986151 |
| GO:0002541\_activation\_of\_plasma\_proteins\_involved\_in\_acute\_inflammatory\_response | 18 | 0 | 0.000000 | 0.000000 | 860 | 800.944171 | 848.09 | 895.235829 | 0.986151 |
| GO:0002700\_regulation\_of\_production\_of\_molecular\_mediator\_of\_immune\_response | 18 | 0 | 0.000000 | 0.000000 | 860 | 800.944171 | 848.09 | 895.235829 | 0.986151 |
| GO:0003073\_regulation\_of\_systemic\_arterial\_blood\_pressure | 18 | 0 | 0.000000 | 0.000000 | 860 | 800.944171 | 848.09 | 895.235829 | 0.986151 |
| GO:0006672\_ceramide\_metabolic\_process | 18 | 0 | 0.000000 | 0.000000 | 860 | 800.944171 | 848.09 | 895.235829 | 0.986151 |
| GO:0007031\_peroxisome\_organization | 18 | 0 | 0.000000 | 0.000000 | 860 | 800.944171 | 848.09 | 895.235829 | 0.986151 |
| GO:0007033\_vacuole\_organization | 18 | 0 | 0.000000 | 0.000000 | 860 | 800.944171 | 848.09 | 895.235829 | 0.986151 |
| GO:0007041\_lysosomal\_transport | 18 | 0 | 0.000000 | 0.000000 | 860 | 800.944171 | 848.09 | 895.235829 | 0.986151 |
| GO:0007602\_phototransduction | 18 | 0 | 0.000000 | 0.000000 | 860 | 800.944171 | 848.09 | 895.235829 | 0.986151 |
| GO:0009636\_response\_to\_toxin | 18 | 0 | 0.000000 | 0.000000 | 860 | 800.944171 | 848.09 | 895.235829 | 0.986151 |
| GO:0010742\_foam\_cell\_differentiation | 18 | 0 | 0.000000 | 0.000000 | 860 | 800.944171 | 848.09 | 895.235829 | 0.986151 |
| GO:0010827\_regulation\_of\_glucose\_transport | 18 | 0 | 0.000000 | 0.000000 | 860 | 800.944171 | 848.09 | 895.235829 | 0.986151 |
| GO:0016126\_sterol\_biosynthetic\_process | 18 | 0 | 0.000000 | 0.000000 | 860 | 800.944171 | 848.09 | 895.235829 | 0.986151 |
| GO:0019218\_regulation\_of\_steroid\_metabolic\_process | 18 | 0 | 0.000000 | 0.000000 | 860 | 800.944171 | 848.09 | 895.235829 | 0.986151 |
| GO:0030004\_cellular\_monovalent\_inorganic\_cation\_homeostasis | 18 | 0 | 0.000000 | 0.000000 | 860 | 800.944171 | 848.09 | 895.235829 | 0.986151 |
| GO:0030183\_B\_cell\_differentiation | 18 | 0 | 0.000000 | 0.000000 | 860 | 800.944171 | 848.09 | 895.235829 | 0.986151 |
| GO:0030195\_negative\_regulation\_of\_blood\_coagulation | 18 | 0 | 0.000000 | 0.000000 | 860 | 800.944171 | 848.09 | 895.235829 | 0.986151 |
| GO:0030262\_apoptotic\_nuclear\_changes | 18 | 0 | 0.000000 | 0.000000 | 860 | 800.944171 | 848.09 | 895.235829 | 0.986151 |
| GO:0032319\_regulation\_of\_Rho\_GTPase\_activity | 18 | 0 | 0.000000 | 0.000000 | 860 | 800.944171 | 848.09 | 895.235829 | 0.986151 |
| GO:0032846\_positive\_regulation\_of\_homeostatic\_process | 18 | 0 | 0.000000 | 0.000000 | 860 | 800.944171 | 848.09 | 895.235829 | 0.986151 |
| GO:0034599\_cellular\_response\_to\_oxidative\_stress | 18 | 0 | 0.000000 | 0.000000 | 860 | 800.944171 | 848.09 | 895.235829 | 0.986151 |
| GO:0040017\_positive\_regulation\_of\_locomotion | 18 | 0 | 0.000000 | 0.000000 | 860 | 800.944171 | 848.09 | 895.235829 | 0.986151 |
| GO:0042177\_negative\_regulation\_of\_protein\_catabolic\_process | 18 | 0 | 0.000000 | 0.000000 | 860 | 800.944171 | 848.09 | 895.235829 | 0.986151 |
| GO:0043270\_positive\_regulation\_of\_ion\_transport | 18 | 0 | 0.000000 | 0.000000 | 860 | 800.944171 | 848.09 | 895.235829 | 0.986151 |
| GO:0043393\_regulation\_of\_protein\_binding | 18 | 0 | 0.000000 | 0.000000 | 860 | 800.944171 | 848.09 | 895.235829 | 0.986151 |
| GO:0045444\_fat\_cell\_differentiation | 18 | 0 | 0.000000 | 0.000000 | 860 | 800.944171 | 848.09 | 895.235829 | 0.986151 |
| GO:0045639\_positive\_regulation\_of\_myeloid\_cell\_differentiation | 18 | 0 | 0.000000 | 0.000000 | 860 | 800.944171 | 848.09 | 895.235829 | 0.986151 |
| GO:0045833\_negative\_regulation\_of\_lipid\_metabolic\_process | 18 | 0 | 0.000000 | 0.000000 | 860 | 800.944171 | 848.09 | 895.235829 | 0.986151 |
| GO:0046034\_ATP\_metabolic\_process | 18 | 0 | 0.000000 | 0.000000 | 860 | 800.944171 | 848.09 | 895.235829 | 0.986151 |
| GO:0046324\_regulation\_of\_glucose\_import | 18 | 0 | 0.000000 | 0.000000 | 860 | 800.944171 | 848.09 | 895.235829 | 0.986151 |
| GO:0046546\_development\_of\_primary\_male\_sexual\_characteristics | 18 | 0 | 0.000000 | 0.000000 | 860 | 800.944171 | 848.09 | 895.235829 | 0.986151 |
| GO:0048659\_smooth\_muscle\_cell\_proliferation | 18 | 0 | 0.000000 | 0.000000 | 860 | 800.944171 | 848.09 | 895.235829 | 0.986151 |
| GO:0050715\_positive\_regulation\_of\_cytokine\_secretion | 18 | 0 | 0.000000 | 0.000000 | 860 | 800.944171 | 848.09 | 895.235829 | 0.986151 |
| GO:0050905\_neuromuscular\_process | 18 | 0 | 0.000000 | 0.000000 | 860 | 800.944171 | 848.09 | 895.235829 | 0.986151 |
| GO:0050921\_positive\_regulation\_of\_chemotaxis | 18 | 0 | 0.000000 | 0.000000 | 860 | 800.944171 | 848.09 | 895.235829 | 0.986151 |
| GO:0060389\_pathway-restricted\_SMAD\_protein\_phosphorylation | 18 | 0 | 0.000000 | 0.000000 | 860 | 800.944171 | 848.09 | 895.235829 | 0.986151 |
| GO:0000080\_G1\_phase\_of\_mitotic\_cell\_cycle | 14 | 0 | 0.000000 | 0.000000 | 928 | 872.088139 | 918.0 | 963.911861 | 0.989224 |
| GO:0000184\_nuclear-transcribed\_mRNA\_catabolic\_process\_\_nonsense-mediated\_decay | 14 | 0 | 0.000000 | 0.000000 | 928 | 872.088139 | 918.0 | 963.911861 | 0.989224 |
| GO:0002228\_natural\_killer\_cell\_mediated\_immunity | 14 | 0 | 0.000000 | 0.000000 | 928 | 872.088139 | 918.0 | 963.911861 | 0.989224 |
| GO:0006041\_glucosamine\_metabolic\_process | 14 | 0 | 0.000000 | 0.000000 | 928 | 872.088139 | 918.0 | 963.911861 | 0.989224 |
| GO:0006195\_purine\_nucleotide\_catabolic\_process | 14 | 0 | 0.000000 | 0.000000 | 928 | 872.088139 | 918.0 | 963.911861 | 0.989224 |
| GO:0006516\_glycoprotein\_catabolic\_process | 14 | 0 | 0.000000 | 0.000000 | 928 | 872.088139 | 918.0 | 963.911861 | 0.989224 |
| GO:0006625\_protein\_targeting\_to\_peroxisome | 14 | 0 | 0.000000 | 0.000000 | 928 | 872.088139 | 918.0 | 963.911861 | 0.989224 |
| GO:0006626\_protein\_targeting\_to\_mitochondrion | 14 | 0 | 0.000000 | 0.000000 | 928 | 872.088139 | 918.0 | 963.911861 | 0.989224 |
| GO:0006769\_nicotinamide\_metabolic\_process | 14 | 0 | 0.000000 | 0.000000 | 928 | 872.088139 | 918.0 | 963.911861 | 0.989224 |
| GO:0006779\_porphyrin\_biosynthetic\_process | 14 | 0 | 0.000000 | 0.000000 | 928 | 872.088139 | 918.0 | 963.911861 | 0.989224 |
| GO:0007157\_heterophilic\_cell\_adhesion | 14 | 0 | 0.000000 | 0.000000 | 928 | 872.088139 | 918.0 | 963.911861 | 0.989224 |
| GO:0007272\_ensheathment\_of\_neurons | 14 | 0 | 0.000000 | 0.000000 | 928 | 872.088139 | 918.0 | 963.911861 | 0.989224 |
| GO:0007286\_spermatid\_development | 14 | 0 | 0.000000 | 0.000000 | 928 | 872.088139 | 918.0 | 963.911861 | 0.989224 |
| GO:0008366\_axon\_ensheathment | 14 | 0 | 0.000000 | 0.000000 | 928 | 872.088139 | 918.0 | 963.911861 | 0.989224 |
| GO:0008585\_female\_gonad\_development | 14 | 0 | 0.000000 | 0.000000 | 928 | 872.088139 | 918.0 | 963.911861 | 0.989224 |
| GO:0008652\_cellular\_amino\_acid\_biosynthetic\_process | 14 | 0 | 0.000000 | 0.000000 | 928 | 872.088139 | 918.0 | 963.911861 | 0.989224 |
| GO:0009152\_purine\_ribonucleotide\_biosynthetic\_process | 14 | 0 | 0.000000 | 0.000000 | 928 | 872.088139 | 918.0 | 963.911861 | 0.989224 |
| GO:0009798\_axis\_specification | 14 | 0 | 0.000000 | 0.000000 | 928 | 872.088139 | 918.0 | 963.911861 | 0.989224 |
| GO:0010466\_negative\_regulation\_of\_peptidase\_activity | 14 | 0 | 0.000000 | 0.000000 | 928 | 872.088139 | 918.0 | 963.911861 | 0.989224 |
| GO:0010970\_microtubule-based\_transport | 14 | 0 | 0.000000 | 0.000000 | 928 | 872.088139 | 918.0 | 963.911861 | 0.989224 |
| GO:0015844\_monoamine\_transport | 14 | 0 | 0.000000 | 0.000000 | 928 | 872.088139 | 918.0 | 963.911861 | 0.989224 |
| GO:0016064\_immunoglobulin\_mediated\_immune\_response | 14 | 0 | 0.000000 | 0.000000 | 928 | 872.088139 | 918.0 | 963.911861 | 0.989224 |
| GO:0016574\_histone\_ubiquitination | 14 | 0 | 0.000000 | 0.000000 | 928 | 872.088139 | 918.0 | 963.911861 | 0.989224 |
| GO:0018107\_peptidyl-threonine\_phosphorylation | 14 | 0 | 0.000000 | 0.000000 | 928 | 872.088139 | 918.0 | 963.911861 | 0.989224 |
| GO:0019362\_pyridine\_nucleotide\_metabolic\_process | 14 | 0 | 0.000000 | 0.000000 | 928 | 872.088139 | 918.0 | 963.911861 | 0.989224 |
| GO:0022600\_digestive\_system\_process | 14 | 0 | 0.000000 | 0.000000 | 928 | 872.088139 | 918.0 | 963.911861 | 0.989224 |
| GO:0022602\_ovulation\_cycle\_process | 14 | 0 | 0.000000 | 0.000000 | 928 | 872.088139 | 918.0 | 963.911861 | 0.989224 |
| GO:0030501\_positive\_regulation\_of\_bone\_mineralization | 14 | 0 | 0.000000 | 0.000000 | 928 | 872.088139 | 918.0 | 963.911861 | 0.989224 |
| GO:0030879\_mammary\_gland\_development | 14 | 0 | 0.000000 | 0.000000 | 928 | 872.088139 | 918.0 | 963.911861 | 0.989224 |
| GO:0030900\_forebrain\_development | 14 | 0 | 0.000000 | 0.000000 | 928 | 872.088139 | 918.0 | 963.911861 | 0.989224 |
| GO:0031032\_actomyosin\_structure\_organization | 14 | 0 | 0.000000 | 0.000000 | 928 | 872.088139 | 918.0 | 963.911861 | 0.989224 |
| GO:0031124\_mRNA\_3'-end\_processing | 14 | 0 | 0.000000 | 0.000000 | 928 | 872.088139 | 918.0 | 963.911861 | 0.989224 |
| GO:0032102\_negative\_regulation\_of\_response\_to\_external\_stimulus | 14 | 0 | 0.000000 | 0.000000 | 928 | 872.088139 | 918.0 | 963.911861 | 0.989224 |
| GO:0032369\_negative\_regulation\_of\_lipid\_transport | 14 | 0 | 0.000000 | 0.000000 | 928 | 872.088139 | 918.0 | 963.911861 | 0.989224 |
| GO:0032412\_regulation\_of\_ion\_transmembrane\_transporter\_activity | 14 | 0 | 0.000000 | 0.000000 | 928 | 872.088139 | 918.0 | 963.911861 | 0.989224 |
| GO:0032496\_response\_to\_lipopolysaccharide | 14 | 0 | 0.000000 | 0.000000 | 928 | 872.088139 | 918.0 | 963.911861 | 0.989224 |
| GO:0032845\_negative\_regulation\_of\_homeostatic\_process | 14 | 0 | 0.000000 | 0.000000 | 928 | 872.088139 | 918.0 | 963.911861 | 0.989224 |
| GO:0033014\_tetrapyrrole\_biosynthetic\_process | 14 | 0 | 0.000000 | 0.000000 | 928 | 872.088139 | 918.0 | 963.911861 | 0.989224 |
| GO:0035270\_endocrine\_system\_development | 14 | 0 | 0.000000 | 0.000000 | 928 | 872.088139 | 918.0 | 963.911861 | 0.989224 |
| GO:0035303\_regulation\_of\_dephosphorylation | 14 | 0 | 0.000000 | 0.000000 | 928 | 872.088139 | 918.0 | 963.911861 | 0.989224 |
| GO:0042036\_negative\_regulation\_of\_cytokine\_biosynthetic\_process | 14 | 0 | 0.000000 | 0.000000 | 928 | 872.088139 | 918.0 | 963.911861 | 0.989224 |
| GO:0042058\_regulation\_of\_epidermal\_growth\_factor\_receptor\_signaling\_pathway | 14 | 0 | 0.000000 | 0.000000 | 928 | 872.088139 | 918.0 | 963.911861 | 0.989224 |
| GO:0042063\_gliogenesis | 14 | 0 | 0.000000 | 0.000000 | 928 | 872.088139 | 918.0 | 963.911861 | 0.989224 |
| GO:0042267\_natural\_killer\_cell\_mediated\_cytotoxicity | 14 | 0 | 0.000000 | 0.000000 | 928 | 872.088139 | 918.0 | 963.911861 | 0.989224 |
| GO:0042310\_vasoconstriction | 14 | 0 | 0.000000 | 0.000000 | 928 | 872.088139 | 918.0 | 963.911861 | 0.989224 |
| GO:0042698\_ovulation\_cycle | 14 | 0 | 0.000000 | 0.000000 | 928 | 872.088139 | 918.0 | 963.911861 | 0.989224 |
| GO:0043255\_regulation\_of\_carbohydrate\_biosynthetic\_process | 14 | 0 | 0.000000 | 0.000000 | 928 | 872.088139 | 918.0 | 963.911861 | 0.989224 |
| GO:0043279\_response\_to\_alkaloid | 14 | 0 | 0.000000 | 0.000000 | 928 | 872.088139 | 918.0 | 963.911861 | 0.989224 |
| GO:0043491\_protein\_kinase\_B\_signaling\_cascade | 14 | 0 | 0.000000 | 0.000000 | 928 | 872.088139 | 918.0 | 963.911861 | 0.989224 |
| GO:0043534\_blood\_vessel\_endothelial\_cell\_migration | 14 | 0 | 0.000000 | 0.000000 | 928 | 872.088139 | 918.0 | 963.911861 | 0.989224 |
| GO:0043648\_dicarboxylic\_acid\_metabolic\_process | 14 | 0 | 0.000000 | 0.000000 | 928 | 872.088139 | 918.0 | 963.911861 | 0.989224 |
| GO:0045428\_regulation\_of\_nitric\_oxide\_biosynthetic\_process | 14 | 0 | 0.000000 | 0.000000 | 928 | 872.088139 | 918.0 | 963.911861 | 0.989224 |
| GO:0045445\_myoblast\_differentiation | 14 | 0 | 0.000000 | 0.000000 | 928 | 872.088139 | 918.0 | 963.911861 | 0.989224 |
| GO:0045471\_response\_to\_ethanol | 14 | 0 | 0.000000 | 0.000000 | 928 | 872.088139 | 918.0 | 963.911861 | 0.989224 |
| GO:0045727\_positive\_regulation\_of\_translation | 14 | 0 | 0.000000 | 0.000000 | 928 | 872.088139 | 918.0 | 963.911861 | 0.989224 |
| GO:0045768\_positive\_regulation\_of\_anti-apoptosis | 14 | 0 | 0.000000 | 0.000000 | 928 | 872.088139 | 918.0 | 963.911861 | 0.989224 |
| GO:0045814\_negative\_regulation\_of\_gene\_expression\_\_epigenetic | 14 | 0 | 0.000000 | 0.000000 | 928 | 872.088139 | 918.0 | 963.911861 | 0.989224 |
| GO:0046320\_regulation\_of\_fatty\_acid\_oxidation | 14 | 0 | 0.000000 | 0.000000 | 928 | 872.088139 | 918.0 | 963.911861 | 0.989224 |
| GO:0046496\_nicotinamide\_nucleotide\_metabolic\_process | 14 | 0 | 0.000000 | 0.000000 | 928 | 872.088139 | 918.0 | 963.911861 | 0.989224 |
| GO:0046889\_positive\_regulation\_of\_lipid\_biosynthetic\_process | 14 | 0 | 0.000000 | 0.000000 | 928 | 872.088139 | 918.0 | 963.911861 | 0.989224 |
| GO:0048009\_insulin-like\_growth\_factor\_receptor\_signaling\_pathway | 14 | 0 | 0.000000 | 0.000000 | 928 | 872.088139 | 918.0 | 963.911861 | 0.989224 |
| GO:0050709\_negative\_regulation\_of\_protein\_secretion | 14 | 0 | 0.000000 | 0.000000 | 928 | 872.088139 | 918.0 | 963.911861 | 0.989224 |
| GO:0051147\_regulation\_of\_muscle\_cell\_differentiation | 14 | 0 | 0.000000 | 0.000000 | 928 | 872.088139 | 918.0 | 963.911861 | 0.989224 |
| GO:0051250\_negative\_regulation\_of\_lymphocyte\_activation | 14 | 0 | 0.000000 | 0.000000 | 928 | 872.088139 | 918.0 | 963.911861 | 0.989224 |
| GO:0051896\_regulation\_of\_protein\_kinase\_B\_signaling\_cascade | 14 | 0 | 0.000000 | 0.000000 | 928 | 872.088139 | 918.0 | 963.911861 | 0.989224 |
| GO:0055001\_muscle\_cell\_development | 14 | 0 | 0.000000 | 0.000000 | 928 | 872.088139 | 918.0 | 963.911861 | 0.989224 |
| GO:0055002\_striated\_muscle\_cell\_development | 14 | 0 | 0.000000 | 0.000000 | 928 | 872.088139 | 918.0 | 963.911861 | 0.989224 |
| GO:0070585\_protein\_localization\_in\_mitochondrion | 14 | 0 | 0.000000 | 0.000000 | 928 | 872.088139 | 918.0 | 963.911861 | 0.989224 |
| GO:0008654\_phospholipid\_biosynthetic\_process | 63 | 0 | 0.000000 | 0.000000 | 932 | 875.865012 | 921.61 | 967.354988 | 0.988852 |
| GO:0030522\_intracellular\_receptor-mediated\_signaling\_pathway | 63 | 0 | 0.000000 | 0.000000 | 932 | 875.865012 | 921.61 | 967.354988 | 0.988852 |
| GO:0042445\_hormone\_metabolic\_process | 63 | 0 | 0.000000 | 0.000000 | 932 | 875.865012 | 921.61 | 967.354988 | 0.988852 |
| GO:0051348\_negative\_regulation\_of\_transferase\_activity | 63 | 0 | 0.000000 | 0.000000 | 932 | 875.865012 | 921.61 | 967.354988 | 0.988852 |
| GO:0000012\_single\_strand\_break\_repair | 4 | 0 |  |  |  |  |  |  |  |  |
| GO:0000101\_sulfur\_amino\_acid\_transport | 4 | 0 |  |  |  |  |  |  |  |  |
| GO:0000185\_activation\_of\_MAPKKK\_activity | 4 | 0 |  |  |  |  |  |  |  |  |
| GO:0000244\_assembly\_of\_spliceosomal\_tri-snRNP | 4 | 0 |  |  |  |  |  |  |  |  |
| GO:0000738\_DNA\_catabolic\_process\_\_exonucleolytic | 4 | 0 |  |  |  |  |  |  |  |  |
| GO:0001502\_cartilage\_condensation | 4 | 0 |  |  |  |  |  |  |  |  |
| GO:0001514\_selenocysteine\_incorporation | 4 | 0 |  |  |  |  |  |  |  |  |
| GO:0001561\_fatty\_acid\_alpha-oxidation | 4 | 0 |  |  |  |  |  |  |  |  |
| GO:0001657\_ureteric\_bud\_development | 4 | 0 |  |  |  |  |  |  |  |  |
| GO:0001708\_cell\_fate\_specification | 4 | 0 |  |  |  |  |  |  |  |  |
| GO:0001710\_mesodermal\_cell\_fate\_commitment | 4 | 0 |  |  |  |  |  |  |  |  |
| GO:0001824\_blastocyst\_development | 4 | 0 |  |  |  |  |  |  |  |  |
| GO:0001914\_regulation\_of\_T\_cell\_mediated\_cytotoxicity | 4 | 0 |  |  |  |  |  |  |  |  |
| GO:0001963\_synaptic\_transmission\_\_dopaminergic | 4 | 0 |  |  |  |  |  |  |  |  |
| GO:0001974\_blood\_vessel\_remodeling | 4 | 0 |  |  |  |  |  |  |  |  |
| GO:0001976\_neurological\_system\_process\_involved\_in\_regulation\_of\_systemic\_arterial\_blood\_pressure | 4 | 0 |  |  |  |  |  |  |  |  |
| GO:0002026\_regulation\_of\_the\_force\_of\_heart\_contraction | 4 | 0 |  |  |  |  |  |  |  |  |
| GO:0002028\_regulation\_of\_sodium\_ion\_transport | 4 | 0 |  |  |  |  |  |  |  |  |
| GO:0002040\_sprouting\_angiogenesis | 4 | 0 |  |  |  |  |  |  |  |  |
| GO:0002097\_tRNA\_wobble\_base\_modification | 4 | 0 |  |  |  |  |  |  |  |  |
| GO:0002098\_tRNA\_wobble\_uridine\_modification | 4 | 0 |  |  |  |  |  |  |  |  |
| GO:0002251\_organ\_or\_tissue\_specific\_immune\_response | 4 | 0 |  |  |  |  |  |  |  |  |
| GO:0002374\_cytokine\_secretion\_during\_immune\_response | 4 | 0 |  |  |  |  |  |  |  |  |
| GO:0002474\_antigen\_processing\_and\_presentation\_of\_peptide\_antigen\_via\_MHC\_class\_I | 4 | 0 |  |  |  |  |  |  |  |  |
| GO:0002483\_antigen\_processing\_and\_presentation\_of\_endogenous\_peptide\_antigen | 4 | 0 |  |  |  |  |  |  |  |  |
| GO:0002726\_positive\_regulation\_of\_T\_cell\_cytokine\_production | 4 | 0 |  |  |  |  |  |  |  |  |
| GO:0002739\_regulation\_of\_cytokine\_secretion\_during\_immune\_response | 4 | 0 |  |  |  |  |  |  |  |  |
| GO:0002740\_negative\_regulation\_of\_cytokine\_secretion\_during\_immune\_response | 4 | 0 |  |  |  |  |  |  |  |  |
| GO:0002755\_MyD88-dependent\_toll-like\_receptor\_signaling\_pathway | 4 | 0 |  |  |  |  |  |  |  |  |
| GO:0002902\_regulation\_of\_B\_cell\_apoptosis | 4 | 0 |  |  |  |  |  |  |  |  |
| GO:0002920\_regulation\_of\_humoral\_immune\_response | 4 | 0 |  |  |  |  |  |  |  |  |
| GO:0003032\_detection\_of\_oxygen | 4 | 0 |  |  |  |  |  |  |  |  |
| GO:0003100\_regulation\_of\_systemic\_arterial\_blood\_pressure\_by\_endothelin | 4 | 0 |  |  |  |  |  |  |  |  |
| GO:0006067\_ethanol\_metabolic\_process | 4 | 0 |  |  |  |  |  |  |  |  |
| GO:0006069\_ethanol\_oxidation | 4 | 0 |  |  |  |  |  |  |  |  |
| GO:0006101\_citrate\_metabolic\_process | 4 | 0 |  |  |  |  |  |  |  |  |
| GO:0006111\_regulation\_of\_gluconeogenesis | 4 | 0 |  |  |  |  |  |  |  |  |
| GO:0006353\_transcription\_termination | 4 | 0 |  |  |  |  |  |  |  |  |
| GO:0006356\_regulation\_of\_transcription\_from\_RNA\_polymerase\_I\_promoter | 4 | 0 |  |  |  |  |  |  |  |  |
| GO:0006358\_regulation\_of\_transcription\_from\_RNA\_polymerase\_II\_promoter\_\_global | 4 | 0 |  |  |  |  |  |  |  |  |
| GO:0006415\_translational\_termination | 4 | 0 |  |  |  |  |  |  |  |  |
| GO:0006449\_regulation\_of\_translational\_termination | 4 | 0 |  |  |  |  |  |  |  |  |
| GO:0006451\_translational\_readthrough | 4 | 0 |  |  |  |  |  |  |  |  |
| GO:0006482\_protein\_amino\_acid\_demethylation | 4 | 0 |  |  |  |  |  |  |  |  |
| GO:0006531\_aspartate\_metabolic\_process | 4 | 0 |  |  |  |  |  |  |  |  |
| GO:0006533\_aspartate\_catabolic\_process | 4 | 0 |  |  |  |  |  |  |  |  |
| GO:0006538\_glutamate\_catabolic\_process | 4 | 0 |  |  |  |  |  |  |  |  |
| GO:0006555\_methionine\_metabolic\_process | 4 | 0 |  |  |  |  |  |  |  |  |
| GO:0006572\_tyrosine\_catabolic\_process | 4 | 0 |  |  |  |  |  |  |  |  |
| GO:0006586\_indolalkylamine\_metabolic\_process | 4 | 0 |  |  |  |  |  |  |  |  |
| GO:0006622\_protein\_targeting\_to\_lysosome | 4 | 0 |  |  |  |  |  |  |  |  |
| GO:0006699\_bile\_acid\_biosynthetic\_process | 4 | 0 |  |  |  |  |  |  |  |  |
| GO:0006707\_cholesterol\_catabolic\_process | 4 | 0 |  |  |  |  |  |  |  |  |
| GO:0006726\_eye\_pigment\_biosynthetic\_process | 4 | 0 |  |  |  |  |  |  |  |  |
| GO:0006750\_glutathione\_biosynthetic\_process | 4 | 0 |  |  |  |  |  |  |  |  |
| GO:0006907\_pinocytosis | 4 | 0 |  |  |  |  |  |  |  |  |
| GO:0006930\_substrate-bound\_cell\_migration\_\_cell\_extension | 4 | 0 |  |  |  |  |  |  |  |  |
| GO:0006980\_redox\_signal\_response | 4 | 0 |  |  |  |  |  |  |  |  |
| GO:0006999\_nuclear\_pore\_organization | 4 | 0 |  |  |  |  |  |  |  |  |
| GO:0007008\_outer\_mitochondrial\_membrane\_organization | 4 | 0 |  |  |  |  |  |  |  |  |
| GO:0007029\_endoplasmic\_reticulum\_organization | 4 | 0 |  |  |  |  |  |  |  |  |
| GO:0007042\_lysosomal\_lumen\_acidification | 4 | 0 |  |  |  |  |  |  |  |  |
| GO:0007080\_mitotic\_metaphase\_plate\_congression | 4 | 0 |  |  |  |  |  |  |  |  |
| GO:0007089\_traversing\_start\_control\_point\_of\_mitotic\_cell\_cycle | 4 | 0 |  |  |  |  |  |  |  |  |
| GO:0007099\_centriole\_replication | 4 | 0 |  |  |  |  |  |  |  |  |
| GO:0007129\_synapsis | 4 | 0 |  |  |  |  |  |  |  |  |
| GO:0007130\_synaptonemal\_complex\_assembly | 4 | 0 |  |  |  |  |  |  |  |  |
| GO:0007164\_establishment\_of\_tissue\_polarity | 4 | 0 |  |  |  |  |  |  |  |  |
| GO:0007168\_receptor\_guanylyl\_cyclase\_signaling\_pathway | 4 | 0 |  |  |  |  |  |  |  |  |
| GO:0007195\_inhibition\_of\_adenylate\_cyclase\_activity\_by\_dopamine\_receptor\_signaling\_pathway | 4 | 0 |  |  |  |  |  |  |  |  |
| GO:0007253\_cytoplasmic\_sequestering\_of\_NF-kappaB | 4 | 0 |  |  |  |  |  |  |  |  |
| GO:0007289\_spermatid\_nucleus\_differentiation | 4 | 0 |  |  |  |  |  |  |  |  |
| GO:0007320\_insemination | 4 | 0 |  |  |  |  |  |  |  |  |
| GO:0007405\_neuroblast\_proliferation | 4 | 0 |  |  |  |  |  |  |  |  |
| GO:0007413\_axonal\_fasciculation | 4 | 0 |  |  |  |  |  |  |  |  |
| GO:0007520\_myoblast\_fusion | 4 | 0 |  |  |  |  |  |  |  |  |
| GO:0007595\_lactation | 4 | 0 |  |  |  |  |  |  |  |  |
| GO:0007620\_copulation | 4 | 0 |  |  |  |  |  |  |  |  |
| GO:0008054\_cyclin\_catabolic\_process | 4 | 0 |  |  |  |  |  |  |  |  |
| GO:0008212\_mineralocorticoid\_metabolic\_process | 4 | 0 |  |  |  |  |  |  |  |  |
| GO:0008214\_protein\_amino\_acid\_dealkylation | 4 | 0 |  |  |  |  |  |  |  |  |
| GO:0008344\_adult\_locomotory\_behavior | 4 | 0 |  |  |  |  |  |  |  |  |
| GO:0008347\_glial\_cell\_migration | 4 | 0 |  |  |  |  |  |  |  |  |
| GO:0008354\_germ\_cell\_migration | 4 | 0 |  |  |  |  |  |  |  |  |
| GO:0008588\_release\_of\_cytoplasmic\_sequestered\_NF-kappaB | 4 | 0 |  |  |  |  |  |  |  |  |
| GO:0008612\_peptidyl-lysine\_modification\_to\_hypusine | 4 | 0 |  |  |  |  |  |  |  |  |
| GO:0009111\_vitamin\_catabolic\_process | 4 | 0 |  |  |  |  |  |  |  |  |
| GO:0009113\_purine\_base\_biosynthetic\_process | 4 | 0 |  |  |  |  |  |  |  |  |
| GO:0009127\_purine\_nucleoside\_monophosphate\_biosynthetic\_process | 4 | 0 |  |  |  |  |  |  |  |  |
| GO:0009151\_purine\_deoxyribonucleotide\_metabolic\_process | 4 | 0 |  |  |  |  |  |  |  |  |
| GO:0009168\_purine\_ribonucleoside\_monophosphate\_biosynthetic\_process | 4 | 0 |  |  |  |  |  |  |  |  |
| GO:0009220\_pyrimidine\_ribonucleotide\_biosynthetic\_process | 4 | 0 |  |  |  |  |  |  |  |  |
| GO:0009249\_protein\_lipoylation | 4 | 0 |  |  |  |  |  |  |  |  |
| GO:0009251\_glucan\_catabolic\_process | 4 | 0 |  |  |  |  |  |  |  |  |
| GO:0009268\_response\_to\_pH | 4 | 0 |  |  |  |  |  |  |  |  |
| GO:0009409\_response\_to\_cold | 4 | 0 |  |  |  |  |  |  |  |  |
| GO:0009612\_response\_to\_mechanical\_stimulus | 4 | 0 |  |  |  |  |  |  |  |  |
| GO:0009950\_dorsal\_ventral\_axis\_specification | 4 | 0 |  |  |  |  |  |  |  |  |
| GO:0010224\_response\_to\_UV-B | 4 | 0 |  |  |  |  |  |  |  |  |
| GO:0010225\_response\_to\_UV-C | 4 | 0 |  |  |  |  |  |  |  |  |
| GO:0010243\_response\_to\_organic\_nitrogen | 4 | 0 |  |  |  |  |  |  |  |  |
| GO:0010310\_regulation\_of\_hydrogen\_peroxide\_metabolic\_process | 4 | 0 |  |  |  |  |  |  |  |  |
| GO:0010460\_positive\_regulation\_of\_heart\_rate | 4 | 0 |  |  |  |  |  |  |  |  |
| GO:0010544\_negative\_regulation\_of\_platelet\_activation | 4 | 0 |  |  |  |  |  |  |  |  |
| GO:0010613\_positive\_regulation\_of\_cardiac\_muscle\_hypertrophy | 4 | 0 |  |  |  |  |  |  |  |  |
| GO:0010615\_positive\_regulation\_of\_cardiac\_muscle\_adaptation | 4 | 0 |  |  |  |  |  |  |  |  |
| GO:0010746\_regulation\_of\_plasma\_membrane\_long-chain\_fatty\_acid\_transport | 4 | 0 |  |  |  |  |  |  |  |  |
| GO:0010748\_negative\_regulation\_of\_plasma\_membrane\_long-chain\_fatty\_acid\_transport | 4 | 0 |  |  |  |  |  |  |  |  |
| GO:0010799\_regulation\_of\_peptidyl-threonine\_phosphorylation | 4 | 0 |  |  |  |  |  |  |  |  |
| GO:0010829\_negative\_regulation\_of\_glucose\_transport | 4 | 0 |  |  |  |  |  |  |  |  |
| GO:0010870\_positive\_regulation\_of\_receptor\_biosynthetic\_process | 4 | 0 |  |  |  |  |  |  |  |  |
| GO:0010886\_positive\_regulation\_of\_cholesterol\_storage | 4 | 0 |  |  |  |  |  |  |  |  |
| GO:0010891\_negative\_regulation\_of\_sequestering\_of\_triglyceride | 4 | 0 |  |  |  |  |  |  |  |  |
| GO:0010901\_regulation\_of\_very-low-density\_lipoprotein\_particle\_remodeling | 4 | 0 |  |  |  |  |  |  |  |  |
| GO:0010915\_regulation\_of\_very-low-density\_lipoprotein\_particle\_clearance | 4 | 0 |  |  |  |  |  |  |  |  |
| GO:0010916\_negative\_regulation\_of\_very-low-density\_lipoprotein\_particle\_clearance | 4 | 0 |  |  |  |  |  |  |  |  |
| GO:0010923\_negative\_regulation\_of\_phosphatase\_activity | 4 | 0 |  |  |  |  |  |  |  |  |
| GO:0010985\_negative\_regulation\_of\_lipoprotein\_particle\_clearance | 4 | 0 |  |  |  |  |  |  |  |  |
| GO:0014032\_neural\_crest\_cell\_development | 4 | 0 |  |  |  |  |  |  |  |  |
| GO:0014033\_neural\_crest\_cell\_differentiation | 4 | 0 |  |  |  |  |  |  |  |  |
| GO:0014046\_dopamine\_secretion | 4 | 0 |  |  |  |  |  |  |  |  |
| GO:0014059\_regulation\_of\_dopamine\_secretion | 4 | 0 |  |  |  |  |  |  |  |  |
| GO:0014075\_response\_to\_amine\_stimulus | 4 | 0 |  |  |  |  |  |  |  |  |
| GO:0014742\_positive\_regulation\_of\_muscle\_hypertrophy | 4 | 0 |  |  |  |  |  |  |  |  |
| GO:0014744\_positive\_regulation\_of\_muscle\_adaptation | 4 | 0 |  |  |  |  |  |  |  |  |
| GO:0014820\_tonic\_smooth\_muscle\_contraction | 4 | 0 |  |  |  |  |  |  |  |  |
| GO:0014824\_artery\_smooth\_muscle\_contraction | 4 | 0 |  |  |  |  |  |  |  |  |
| GO:0014826\_vein\_smooth\_muscle\_contraction | 4 | 0 |  |  |  |  |  |  |  |  |
| GO:0015701\_bicarbonate\_transport | 4 | 0 |  |  |  |  |  |  |  |  |
| GO:0015791\_polyol\_transport | 4 | 0 |  |  |  |  |  |  |  |  |
| GO:0016127\_sterol\_catabolic\_process | 4 | 0 |  |  |  |  |  |  |  |  |
| GO:0016540\_protein\_autoprocessing | 4 | 0 |  |  |  |  |  |  |  |  |
| GO:0017085\_response\_to\_insecticide | 4 | 0 |  |  |  |  |  |  |  |  |
| GO:0017121\_phospholipid\_scrambling | 4 | 0 |  |  |  |  |  |  |  |  |
| GO:0017157\_regulation\_of\_exocytosis | 4 | 0 |  |  |  |  |  |  |  |  |
| GO:0018195\_peptidyl-arginine\_modification | 4 | 0 |  |  |  |  |  |  |  |  |
| GO:0018206\_peptidyl-methionine\_modification | 4 | 0 |  |  |  |  |  |  |  |  |
| GO:0018216\_peptidyl-arginine\_methylation | 4 | 0 |  |  |  |  |  |  |  |  |
| GO:0018346\_protein\_amino\_acid\_prenylation | 4 | 0 |  |  |  |  |  |  |  |  |
| GO:0019370\_leukotriene\_biosynthetic\_process | 4 | 0 |  |  |  |  |  |  |  |  |
| GO:0019400\_alditol\_metabolic\_process | 4 | 0 |  |  |  |  |  |  |  |  |
| GO:0019430\_removal\_of\_superoxide\_radicals | 4 | 0 |  |  |  |  |  |  |  |  |
| GO:0019885\_antigen\_processing\_and\_presentation\_of\_endogenous\_peptide\_antigen\_via\_MHC\_class\_I | 4 | 0 |  |  |  |  |  |  |  |  |
| GO:0019987\_negative\_regulation\_of\_anti-apoptosis | 4 | 0 |  |  |  |  |  |  |  |  |
| GO:0021515\_cell\_differentiation\_in\_spinal\_cord | 4 | 0 |  |  |  |  |  |  |  |  |
| GO:0021536\_diencephalon\_development | 4 | 0 |  |  |  |  |  |  |  |  |
| GO:0021549\_cerebellum\_development | 4 | 0 |  |  |  |  |  |  |  |  |
| GO:0022037\_metencephalon\_development | 4 | 0 |  |  |  |  |  |  |  |  |
| GO:0022417\_protein\_maturation\_by\_protein\_folding | 4 | 0 |  |  |  |  |  |  |  |  |
| GO:0030002\_cellular\_anion\_homeostasis | 4 | 0 |  |  |  |  |  |  |  |  |
| GO:0030010\_establishment\_of\_cell\_polarity | 4 | 0 |  |  |  |  |  |  |  |  |
| GO:0030146\_diuresis | 4 | 0 |  |  |  |  |  |  |  |  |
| GO:0030177\_positive\_regulation\_of\_Wnt\_receptor\_signaling\_pathway | 4 | 0 |  |  |  |  |  |  |  |  |
| GO:0030240\_muscle\_thin\_filament\_assembly | 4 | 0 |  |  |  |  |  |  |  |  |
| GO:0030279\_negative\_regulation\_of\_ossification | 4 | 0 |  |  |  |  |  |  |  |  |
| GO:0030319\_cellular\_di-\_\_tri-valent\_inorganic\_anion\_homeostasis | 4 | 0 |  |  |  |  |  |  |  |  |
| GO:0030516\_regulation\_of\_axon\_extension | 4 | 0 |  |  |  |  |  |  |  |  |
| GO:0030539\_male\_genitalia\_development | 4 | 0 |  |  |  |  |  |  |  |  |
| GO:0030574\_collagen\_catabolic\_process | 4 | 0 |  |  |  |  |  |  |  |  |
| GO:0030579\_ubiquitin-dependent\_SMAD\_protein\_catabolic\_process | 4 | 0 |  |  |  |  |  |  |  |  |
| GO:0030851\_granulocyte\_differentiation | 4 | 0 |  |  |  |  |  |  |  |  |
| GO:0030865\_cortical\_cytoskeleton\_organization | 4 | 0 |  |  |  |  |  |  |  |  |
| GO:0030866\_cortical\_actin\_cytoskeleton\_organization | 4 | 0 |  |  |  |  |  |  |  |  |
| GO:0030970\_retrograde\_protein\_transport\_\_ER\_to\_cytosol | 4 | 0 |  |  |  |  |  |  |  |  |
| GO:0031053\_primary\_microRNA\_processing | 4 | 0 |  |  |  |  |  |  |  |  |
| GO:0031069\_hair\_follicle\_morphogenesis | 4 | 0 |  |  |  |  |  |  |  |  |
| GO:0031116\_positive\_regulation\_of\_microtubule\_polymerization | 4 | 0 |  |  |  |  |  |  |  |  |
| GO:0031503\_protein\_complex\_localization | 4 | 0 |  |  |  |  |  |  |  |  |
| GO:0031646\_positive\_regulation\_of\_neurological\_system\_process | 4 | 0 |  |  |  |  |  |  |  |  |
| GO:0031663\_lipopolysaccharide-mediated\_signaling\_pathway | 4 | 0 |  |  |  |  |  |  |  |  |
| GO:0031952\_regulation\_of\_protein\_amino\_acid\_autophosphorylation | 4 | 0 |  |  |  |  |  |  |  |  |
| GO:0032000\_positive\_regulation\_of\_fatty\_acid\_beta-oxidation | 4 | 0 |  |  |  |  |  |  |  |  |
| GO:0032008\_positive\_regulation\_of\_TOR\_signaling\_pathway | 4 | 0 |  |  |  |  |  |  |  |  |
| GO:0032020\_ISG15-protein\_conjugation | 4 | 0 |  |  |  |  |  |  |  |  |
| GO:0032057\_negative\_regulation\_of\_translational\_initiation\_in\_response\_to\_stress | 4 | 0 |  |  |  |  |  |  |  |  |
| GO:0032092\_positive\_regulation\_of\_protein\_binding | 4 | 0 |  |  |  |  |  |  |  |  |
| GO:0032106\_positive\_regulation\_of\_response\_to\_extracellular\_stimulus | 4 | 0 |  |  |  |  |  |  |  |  |
| GO:0032109\_positive\_regulation\_of\_response\_to\_nutrient\_levels | 4 | 0 |  |  |  |  |  |  |  |  |
| GO:0032211\_negative\_regulation\_of\_telomere\_maintenance\_via\_telomerase | 4 | 0 |  |  |  |  |  |  |  |  |
| GO:0032239\_regulation\_of\_nucleobase\_\_nucleoside\_\_nucleotide\_and\_nucleic\_acid\_transport | 4 | 0 |  |  |  |  |  |  |  |  |
| GO:0032276\_regulation\_of\_gonadotropin\_secretion | 4 | 0 |  |  |  |  |  |  |  |  |
| GO:0032277\_negative\_regulation\_of\_gonadotropin\_secretion | 4 | 0 |  |  |  |  |  |  |  |  |
| GO:0032341\_aldosterone\_metabolic\_process | 4 | 0 |  |  |  |  |  |  |  |  |
| GO:0032413\_negative\_regulation\_of\_ion\_transmembrane\_transporter\_activity | 4 | 0 |  |  |  |  |  |  |  |  |
| GO:0032431\_activation\_of\_phospholipase\_A2\_activity | 4 | 0 |  |  |  |  |  |  |  |  |
| GO:0032436\_positive\_regulation\_of\_proteasomal\_ubiquitin-dependent\_protein\_catabolic\_process | 4 | 0 |  |  |  |  |  |  |  |  |
| GO:0032455\_nerve\_growth\_factor\_processing | 4 | 0 |  |  |  |  |  |  |  |  |
| GO:0032608\_interferon-beta\_production | 4 | 0 |  |  |  |  |  |  |  |  |
| GO:0032611\_interleukin-1\_beta\_production | 4 | 0 |  |  |  |  |  |  |  |  |
| GO:0032613\_interleukin-10\_production | 4 | 0 |  |  |  |  |  |  |  |  |
| GO:0032633\_interleukin-4\_production | 4 | 0 |  |  |  |  |  |  |  |  |
| GO:0032648\_regulation\_of\_interferon-beta\_production | 4 | 0 |  |  |  |  |  |  |  |  |
| GO:0032652\_regulation\_of\_interleukin-1\_production | 4 | 0 |  |  |  |  |  |  |  |  |
| GO:0032653\_regulation\_of\_interleukin-10\_production | 4 | 0 |  |  |  |  |  |  |  |  |
| GO:0032673\_regulation\_of\_interleukin-4\_production | 4 | 0 |  |  |  |  |  |  |  |  |
| GO:0032715\_negative\_regulation\_of\_interleukin-6\_production | 4 | 0 |  |  |  |  |  |  |  |  |
| GO:0032743\_positive\_regulation\_of\_interleukin-2\_production | 4 | 0 |  |  |  |  |  |  |  |  |
| GO:0032755\_positive\_regulation\_of\_interleukin-6\_production | 4 | 0 |  |  |  |  |  |  |  |  |
| GO:0032780\_negative\_regulation\_of\_ATPase\_activity | 4 | 0 |  |  |  |  |  |  |  |  |
| GO:0032891\_negative\_regulation\_of\_organic\_acid\_transport | 4 | 0 |  |  |  |  |  |  |  |  |
| GO:0032906\_transforming\_growth\_factor-beta2\_production | 4 | 0 |  |  |  |  |  |  |  |  |
| GO:0032909\_regulation\_of\_transforming\_growth\_factor-beta2\_production | 4 | 0 |  |  |  |  |  |  |  |  |
| GO:0033032\_regulation\_of\_myeloid\_cell\_apoptosis | 4 | 0 |  |  |  |  |  |  |  |  |
| GO:0033119\_negative\_regulation\_of\_RNA\_splicing | 4 | 0 |  |  |  |  |  |  |  |  |
| GO:0033233\_regulation\_of\_protein\_sumoylation | 4 | 0 |  |  |  |  |  |  |  |  |
| GO:0033235\_positive\_regulation\_of\_protein\_sumoylation | 4 | 0 |  |  |  |  |  |  |  |  |
| GO:0033522\_histone\_H2A\_ubiquitination | 4 | 0 |  |  |  |  |  |  |  |  |
| GO:0033605\_positive\_regulation\_of\_catecholamine\_secretion | 4 | 0 |  |  |  |  |  |  |  |  |
| GO:0033628\_regulation\_of\_cell\_adhesion\_mediated\_by\_integrin | 4 | 0 |  |  |  |  |  |  |  |  |
| GO:0033631\_cell-cell\_adhesion\_mediated\_by\_integrin | 4 | 0 |  |  |  |  |  |  |  |  |
| GO:0033860\_regulation\_of\_NAD(P)H\_oxidase\_activity | 4 | 0 |  |  |  |  |  |  |  |  |
| GO:0034227\_tRNA\_thio-modification | 4 | 0 |  |  |  |  |  |  |  |  |
| GO:0034308\_monohydric\_alcohol\_metabolic\_process | 4 | 0 |  |  |  |  |  |  |  |  |
| GO:0034394\_protein\_localization\_at\_cell\_surface | 4 | 0 |  |  |  |  |  |  |  |  |
| GO:0034587\_piRNA\_metabolic\_process | 4 | 0 |  |  |  |  |  |  |  |  |
| GO:0035065\_regulation\_of\_histone\_acetylation | 4 | 0 |  |  |  |  |  |  |  |  |
| GO:0035088\_establishment\_or\_maintenance\_of\_apical\_basal\_cell\_polarity | 4 | 0 |  |  |  |  |  |  |  |  |
| GO:0035235\_ionotropic\_glutamate\_receptor\_signaling\_pathway | 4 | 0 |  |  |  |  |  |  |  |  |
| GO:0035249\_synaptic\_transmission\_\_glutamatergic | 4 | 0 |  |  |  |  |  |  |  |  |
| GO:0035304\_regulation\_of\_protein\_amino\_acid\_dephosphorylation | 4 | 0 |  |  |  |  |  |  |  |  |
| GO:0035313\_wound\_healing\_\_spreading\_of\_epidermal\_cells | 4 | 0 |  |  |  |  |  |  |  |  |
| GO:0042135\_neurotransmitter\_catabolic\_process | 4 | 0 |  |  |  |  |  |  |  |  |
| GO:0042136\_neurotransmitter\_biosynthetic\_process | 4 | 0 |  |  |  |  |  |  |  |  |
| GO:0042149\_cellular\_response\_to\_glucose\_starvation | 4 | 0 |  |  |  |  |  |  |  |  |
| GO:0042268\_regulation\_of\_cytolysis | 4 | 0 |  |  |  |  |  |  |  |  |
| GO:0042320\_regulation\_of\_circadian\_sleep\_wake\_cycle\_\_REM\_sleep | 4 | 0 |  |  |  |  |  |  |  |  |
| GO:0042362\_fat-soluble\_vitamin\_biosynthetic\_process | 4 | 0 |  |  |  |  |  |  |  |  |
| GO:0042363\_fat-soluble\_vitamin\_catabolic\_process | 4 | 0 |  |  |  |  |  |  |  |  |
| GO:0042403\_thyroid\_hormone\_metabolic\_process | 4 | 0 |  |  |  |  |  |  |  |  |
| GO:0042423\_catecholamine\_biosynthetic\_process | 4 | 0 |  |  |  |  |  |  |  |  |
| GO:0042430\_indole\_and\_derivative\_metabolic\_process | 4 | 0 |  |  |  |  |  |  |  |  |
| GO:0042434\_indole\_derivative\_metabolic\_process | 4 | 0 |  |  |  |  |  |  |  |  |
| GO:0042441\_eye\_pigment\_metabolic\_process | 4 | 0 |  |  |  |  |  |  |  |  |
| GO:0042448\_progesterone\_metabolic\_process | 4 | 0 |  |  |  |  |  |  |  |  |
| GO:0042454\_ribonucleoside\_catabolic\_process | 4 | 0 |  |  |  |  |  |  |  |  |
| GO:0042461\_photoreceptor\_cell\_development | 4 | 0 |  |  |  |  |  |  |  |  |
| GO:0042462\_eye\_photoreceptor\_cell\_development | 4 | 0 |  |  |  |  |  |  |  |  |
| GO:0042472\_inner\_ear\_morphogenesis | 4 | 0 |  |  |  |  |  |  |  |  |
| GO:0042475\_odontogenesis\_of\_dentine-containing\_tooth | 4 | 0 |  |  |  |  |  |  |  |  |
| GO:0042491\_auditory\_receptor\_cell\_differentiation | 4 | 0 |  |  |  |  |  |  |  |  |
| GO:0042518\_negative\_regulation\_of\_tyrosine\_phosphorylation\_of\_Stat3\_protein | 4 | 0 |  |  |  |  |  |  |  |  |
| GO:0042532\_negative\_regulation\_of\_tyrosine\_phosphorylation\_of\_STAT\_protein | 4 | 0 |  |  |  |  |  |  |  |  |
| GO:0042541\_hemoglobin\_biosynthetic\_process | 4 | 0 |  |  |  |  |  |  |  |  |
| GO:0042723\_thiamin\_and\_derivative\_metabolic\_process | 4 | 0 |  |  |  |  |  |  |  |  |
| GO:0042733\_embryonic\_digit\_morphogenesis | 4 | 0 |  |  |  |  |  |  |  |  |
| GO:0042747\_circadian\_sleep\_wake\_cycle\_\_REM\_sleep | 4 | 0 |  |  |  |  |  |  |  |  |
| GO:0042976\_activation\_of\_Janus\_kinase\_activity | 4 | 0 |  |  |  |  |  |  |  |  |
| GO:0043032\_positive\_regulation\_of\_macrophage\_activation | 4 | 0 |  |  |  |  |  |  |  |  |
| GO:0043113\_receptor\_clustering | 4 | 0 |  |  |  |  |  |  |  |  |
| GO:0043114\_regulation\_of\_vascular\_permeability | 4 | 0 |  |  |  |  |  |  |  |  |
| GO:0043372\_positive\_regulation\_of\_CD4-positive\_\_alpha\_beta\_T\_cell\_differentiation | 4 | 0 |  |  |  |  |  |  |  |  |
| GO:0043450\_alkene\_biosynthetic\_process | 4 | 0 |  |  |  |  |  |  |  |  |
| GO:0043486\_histone\_exchange | 4 | 0 |  |  |  |  |  |  |  |  |
| GO:0043497\_regulation\_of\_protein\_heterodimerization\_activity | 4 | 0 |  |  |  |  |  |  |  |  |
| GO:0043526\_neuroprotection | 4 | 0 |  |  |  |  |  |  |  |  |
| GO:0043545\_molybdopterin\_cofactor\_metabolic\_process | 4 | 0 |  |  |  |  |  |  |  |  |
| GO:0043558\_regulation\_of\_translational\_initiation\_in\_response\_to\_stress | 4 | 0 |  |  |  |  |  |  |  |  |
| GO:0043568\_positive\_regulation\_of\_insulin-like\_growth\_factor\_receptor\_signaling\_pathway | 4 | 0 |  |  |  |  |  |  |  |  |
| GO:0044247\_cellular\_polysaccharide\_catabolic\_process | 4 | 0 |  |  |  |  |  |  |  |  |
| GO:0045055\_regulated\_secretory\_pathway | 4 | 0 |  |  |  |  |  |  |  |  |
| GO:0045091\_regulation\_of\_retroviral\_genome\_replication | 4 | 0 |  |  |  |  |  |  |  |  |
| GO:0045191\_regulation\_of\_isotype\_switching | 4 | 0 |  |  |  |  |  |  |  |  |
| GO:0045197\_establishment\_or\_maintenance\_of\_epithelial\_cell\_apical\_basal\_polarity | 4 | 0 |  |  |  |  |  |  |  |  |
| GO:0045342\_MHC\_class\_II\_biosynthetic\_process | 4 | 0 |  |  |  |  |  |  |  |  |
| GO:0045346\_regulation\_of\_MHC\_class\_II\_biosynthetic\_process | 4 | 0 |  |  |  |  |  |  |  |  |
| GO:0045350\_interferon-beta\_biosynthetic\_process | 4 | 0 |  |  |  |  |  |  |  |  |
| GO:0045356\_positive\_regulation\_of\_interferon-alpha\_biosynthetic\_process | 4 | 0 |  |  |  |  |  |  |  |  |
| GO:0045357\_regulation\_of\_interferon-beta\_biosynthetic\_process | 4 | 0 |  |  |  |  |  |  |  |  |
| GO:0045359\_positive\_regulation\_of\_interferon-beta\_biosynthetic\_process | 4 | 0 |  |  |  |  |  |  |  |  |
| GO:0045600\_positive\_regulation\_of\_fat\_cell\_differentiation | 4 | 0 |  |  |  |  |  |  |  |  |
| GO:0045606\_positive\_regulation\_of\_epidermal\_cell\_differentiation | 4 | 0 |  |  |  |  |  |  |  |  |
| GO:0045618\_positive\_regulation\_of\_keratinocyte\_differentiation | 4 | 0 |  |  |  |  |  |  |  |  |
| GO:0045624\_positive\_regulation\_of\_T-helper\_cell\_differentiation | 4 | 0 |  |  |  |  |  |  |  |  |
| GO:0045652\_regulation\_of\_megakaryocyte\_differentiation | 4 | 0 |  |  |  |  |  |  |  |  |
| GO:0045666\_positive\_regulation\_of\_neuron\_differentiation | 4 | 0 |  |  |  |  |  |  |  |  |
| GO:0045668\_negative\_regulation\_of\_osteoblast\_differentiation | 4 | 0 |  |  |  |  |  |  |  |  |
| GO:0045714\_regulation\_of\_low-density\_lipoprotein\_receptor\_biosynthetic\_process | 4 | 0 |  |  |  |  |  |  |  |  |
| GO:0045745\_positive\_regulation\_of\_G-protein\_coupled\_receptor\_protein\_signaling\_pathway | 4 | 0 |  |  |  |  |  |  |  |  |
| GO:0045776\_negative\_regulation\_of\_blood\_pressure | 4 | 0 |  |  |  |  |  |  |  |  |
| GO:0045824\_negative\_regulation\_of\_innate\_immune\_response | 4 | 0 |  |  |  |  |  |  |  |  |
| GO:0045980\_negative\_regulation\_of\_nucleotide\_metabolic\_process | 4 | 0 |  |  |  |  |  |  |  |  |
| GO:0045987\_positive\_regulation\_of\_smooth\_muscle\_contraction | 4 | 0 |  |  |  |  |  |  |  |  |
| GO:0046033\_AMP\_metabolic\_process | 4 | 0 |  |  |  |  |  |  |  |  |
| GO:0046325\_negative\_regulation\_of\_glucose\_import | 4 | 0 |  |  |  |  |  |  |  |  |
| GO:0046459\_short-chain\_fatty\_acid\_metabolic\_process | 4 | 0 |  |  |  |  |  |  |  |  |
| GO:0046469\_platelet\_activating\_factor\_metabolic\_process | 4 | 0 |  |  |  |  |  |  |  |  |
| GO:0046473\_phosphatidic\_acid\_metabolic\_process | 4 | 0 |  |  |  |  |  |  |  |  |
| GO:0046475\_glycerophospholipid\_catabolic\_process | 4 | 0 |  |  |  |  |  |  |  |  |
| GO:0046516\_hypusine\_metabolic\_process | 4 | 0 |  |  |  |  |  |  |  |  |
| GO:0046683\_response\_to\_organophosphorus | 4 | 0 |  |  |  |  |  |  |  |  |
| GO:0046831\_regulation\_of\_RNA\_export\_from\_nucleus | 4 | 0 |  |  |  |  |  |  |  |  |
| GO:0046834\_lipid\_phosphorylation | 4 | 0 |  |  |  |  |  |  |  |  |
| GO:0046854\_phosphoinositide\_phosphorylation | 4 | 0 |  |  |  |  |  |  |  |  |
| GO:0046874\_quinolinate\_metabolic\_process | 4 | 0 |  |  |  |  |  |  |  |  |
| GO:0046880\_regulation\_of\_follicle-stimulating\_hormone\_secretion | 4 | 0 |  |  |  |  |  |  |  |  |
| GO:0046882\_negative\_regulation\_of\_follicle-stimulating\_hormone\_secretion | 4 | 0 |  |  |  |  |  |  |  |  |
| GO:0046885\_regulation\_of\_hormone\_biosynthetic\_process | 4 | 0 |  |  |  |  |  |  |  |  |
| GO:0046928\_regulation\_of\_neurotransmitter\_secretion | 4 | 0 |  |  |  |  |  |  |  |  |
| GO:0048096\_chromatin-mediated\_maintenance\_of\_transcription | 4 | 0 |  |  |  |  |  |  |  |  |
| GO:0048148\_behavioral\_response\_to\_cocaine | 4 | 0 |  |  |  |  |  |  |  |  |
| GO:0048305\_immunoglobulin\_secretion | 4 | 0 |  |  |  |  |  |  |  |  |
| GO:0048333\_mesodermal\_cell\_differentiation | 4 | 0 |  |  |  |  |  |  |  |  |
| GO:0048387\_negative\_regulation\_of\_retinoic\_acid\_receptor\_signaling\_pathway | 4 | 0 |  |  |  |  |  |  |  |  |
| GO:0048496\_maintenance\_of\_organ\_identity | 4 | 0 |  |  |  |  |  |  |  |  |
| GO:0048546\_digestive\_tract\_morphogenesis | 4 | 0 |  |  |  |  |  |  |  |  |
| GO:0048547\_gut\_morphogenesis | 4 | 0 |  |  |  |  |  |  |  |  |
| GO:0048638\_regulation\_of\_developmental\_growth | 4 | 0 |  |  |  |  |  |  |  |  |
| GO:0048663\_neuron\_fate\_commitment | 4 | 0 |  |  |  |  |  |  |  |  |
| GO:0048678\_response\_to\_axon\_injury | 4 | 0 |  |  |  |  |  |  |  |  |
| GO:0050651\_dermatan\_sulfate\_proteoglycan\_biosynthetic\_process | 4 | 0 |  |  |  |  |  |  |  |  |
| GO:0050771\_negative\_regulation\_of\_axonogenesis | 4 | 0 |  |  |  |  |  |  |  |  |
| GO:0050773\_regulation\_of\_dendrite\_development | 4 | 0 |  |  |  |  |  |  |  |  |
| GO:0050901\_leukocyte\_tethering\_or\_rolling | 4 | 0 |  |  |  |  |  |  |  |  |
| GO:0051014\_actin\_filament\_severing | 4 | 0 |  |  |  |  |  |  |  |  |
| GO:0051023\_regulation\_of\_immunoglobulin\_secretion | 4 | 0 |  |  |  |  |  |  |  |  |
| GO:0051024\_positive\_regulation\_of\_immunoglobulin\_secretion | 4 | 0 |  |  |  |  |  |  |  |  |
| GO:0051045\_negative\_regulation\_of\_membrane\_protein\_ectodomain\_proteolysis | 4 | 0 |  |  |  |  |  |  |  |  |
| GO:0051145\_smooth\_muscle\_cell\_differentiation | 4 | 0 |  |  |  |  |  |  |  |  |
| GO:0051149\_positive\_regulation\_of\_muscle\_cell\_differentiation | 4 | 0 |  |  |  |  |  |  |  |  |
| GO:0051150\_regulation\_of\_smooth\_muscle\_cell\_differentiation | 4 | 0 |  |  |  |  |  |  |  |  |
| GO:0051189\_prosthetic\_group\_metabolic\_process | 4 | 0 |  |  |  |  |  |  |  |  |
| GO:0051293\_establishment\_of\_spindle\_localization | 4 | 0 |  |  |  |  |  |  |  |  |
| GO:0051300\_spindle\_pole\_body\_organization | 4 | 0 |  |  |  |  |  |  |  |  |
| GO:0051323\_metaphase | 4 | 0 |  |  |  |  |  |  |  |  |
| GO:0051383\_kinetochore\_organization | 4 | 0 |  |  |  |  |  |  |  |  |
| GO:0051482\_elevation\_of\_cytosolic\_calcium\_ion\_concentration\_during\_G-protein\_signaling\_\_coupled\_to\_IP3\_second\_messenger\_(phospholipase\_C\_activating) | 4 | 0 |  |  |  |  |  |  |  |  |
| GO:0051568\_histone\_H3-K4\_methylation | 4 | 0 |  |  |  |  |  |  |  |  |
| GO:0051591\_response\_to\_cAMP | 4 | 0 |  |  |  |  |  |  |  |  |
| GO:0051653\_spindle\_localization | 4 | 0 |  |  |  |  |  |  |  |  |
| GO:0051709\_regulation\_of\_killing\_of\_cells\_of\_another\_organism | 4 | 0 |  |  |  |  |  |  |  |  |
| GO:0051817\_modification\_of\_morphology\_or\_physiology\_of\_other\_organism\_during\_symbiotic\_interaction | 4 | 0 |  |  |  |  |  |  |  |  |
| GO:0051895\_negative\_regulation\_of\_focal\_adhesion\_formation | 4 | 0 |  |  |  |  |  |  |  |  |
| GO:0051919\_positive\_regulation\_of\_fibrinolysis | 4 | 0 |  |  |  |  |  |  |  |  |
| GO:0051963\_regulation\_of\_synaptogenesis | 4 | 0 |  |  |  |  |  |  |  |  |
| GO:0051971\_positive\_regulation\_of\_transmission\_of\_nerve\_impulse | 4 | 0 |  |  |  |  |  |  |  |  |
| GO:0051974\_negative\_regulation\_of\_telomerase\_activity | 4 | 0 |  |  |  |  |  |  |  |  |
| GO:0055003\_cardiac\_myofibril\_assembly | 4 | 0 |  |  |  |  |  |  |  |  |
| GO:0055013\_cardiac\_muscle\_cell\_development | 4 | 0 |  |  |  |  |  |  |  |  |
| GO:0055061\_di-\_\_tri-valent\_inorganic\_anion\_homeostasis | 4 | 0 |  |  |  |  |  |  |  |  |
| GO:0055081\_anion\_homeostasis | 4 | 0 |  |  |  |  |  |  |  |  |
| GO:0055123\_digestive\_system\_development | 4 | 0 |  |  |  |  |  |  |  |  |
| GO:0060042\_retina\_morphogenesis\_in\_camera-type\_eye | 4 | 0 |  |  |  |  |  |  |  |  |
| GO:0060043\_regulation\_of\_cardiac\_muscle\_cell\_proliferation | 4 | 0 |  |  |  |  |  |  |  |  |
| GO:0060073\_micturition | 4 | 0 |  |  |  |  |  |  |  |  |
| GO:0060078\_regulation\_of\_postsynaptic\_membrane\_potential | 4 | 0 |  |  |  |  |  |  |  |  |
| GO:0060124\_positive\_regulation\_of\_growth\_hormone\_secretion | 4 | 0 |  |  |  |  |  |  |  |  |
| GO:0060155\_platelet\_dense\_granule\_organization | 4 | 0 |  |  |  |  |  |  |  |  |
| GO:0060395\_SMAD\_protein\_signal\_transduction | 4 | 0 |  |  |  |  |  |  |  |  |
| GO:0060795\_cell\_fate\_commitment\_involved\_in\_the\_formation\_of\_primary\_germ\_layers | 4 | 0 |  |  |  |  |  |  |  |  |
| GO:0070096\_mitochondrial\_outer\_membrane\_translocase\_complex\_assembly | 4 | 0 |  |  |  |  |  |  |  |  |
| GO:0070102\_interleukin-6-mediated\_signaling\_pathway | 4 | 0 |  |  |  |  |  |  |  |  |
| GO:0070192\_chromosome\_organization\_involved\_in\_meiosis | 4 | 0 |  |  |  |  |  |  |  |  |
| GO:0070193\_synaptonemal\_complex\_organization | 4 | 0 |  |  |  |  |  |  |  |  |
| GO:0070296\_sarcoplasmic\_reticulum\_calcium\_ion\_transport | 4 | 0 |  |  |  |  |  |  |  |  |
| GO:0070498\_interleukin-1-mediated\_signaling\_pathway | 4 | 0 |  |  |  |  |  |  |  |  |
| GO:0070508\_cholesterol\_import | 4 | 0 |  |  |  |  |  |  |  |  |
| GO:0070633\_transepithelial\_transport | 4 | 0 |  |  |  |  |  |  |  |  |
| GO:0070723\_response\_to\_cholesterol | 4 | 0 |  |  |  |  |  |  |  |  |
| GO:0006470\_protein\_amino\_acid\_dephosphorylation | 77 | 0 | 0.000000 | 0.000000 | 937 | 881.794330 | 926.96 | 972.125670 | 0.989285 |
| GO:0009123\_nucleoside\_monophosphate\_metabolic\_process | 77 | 0 | 0.000000 | 0.000000 | 937 | 881.794330 | 926.96 | 972.125670 | 0.989285 |
| GO:0034470\_ncRNA\_processing | 77 | 0 | 0.000000 | 0.000000 | 937 | 881.794330 | 926.96 | 972.125670 | 0.989285 |
| GO:0051129\_negative\_regulation\_of\_cellular\_component\_organization | 77 | 0 | 0.000000 | 0.000000 | 937 | 881.794330 | 926.96 | 972.125670 | 0.989285 |
| GO:0051340\_regulation\_of\_ligase\_activity | 77 | 0 | 0.000000 | 0.000000 | 937 | 881.794330 | 926.96 | 972.125670 | 0.989285 |
| GO:0007399\_nervous\_system\_development | 459 | 0 | 0.000000 | 0.000000 | 938 | 882.133224 | 927.33 | 972.526776 | 0.988625 |
| GO:0000002\_mitochondrial\_genome\_maintenance | 6 | 0 | 0.000000 | 0.000000 | 1158 | 1102.457020 | 1145.61 | 1188.762980 | 0.989301 |
| GO:0000076\_DNA\_replication\_checkpoint | 6 | 0 | 0.000000 | 0.000000 | 1158 | 1102.457020 | 1145.61 | 1188.762980 | 0.989301 |
| GO:0000381\_regulation\_of\_alternative\_nuclear\_mRNA\_splicing\_\_via\_spliceosome | 6 | 0 | 0.000000 | 0.000000 | 1158 | 1102.457020 | 1145.61 | 1188.762980 | 0.989301 |
| GO:0001656\_metanephros\_development | 6 | 0 | 0.000000 | 0.000000 | 1158 | 1102.457020 | 1145.61 | 1188.762980 | 0.989301 |
| GO:0001838\_embryonic\_epithelial\_tube\_formation | 6 | 0 | 0.000000 | 0.000000 | 1158 | 1102.457020 | 1145.61 | 1188.762980 | 0.989301 |
| GO:0001841\_neural\_tube\_formation | 6 | 0 | 0.000000 | 0.000000 | 1158 | 1102.457020 | 1145.61 | 1188.762980 | 0.989301 |
| GO:0001843\_neural\_tube\_closure | 6 | 0 | 0.000000 | 0.000000 | 1158 | 1102.457020 | 1145.61 | 1188.762980 | 0.989301 |
| GO:0001893\_maternal\_placenta\_development | 6 | 0 | 0.000000 | 0.000000 | 1158 | 1102.457020 | 1145.61 | 1188.762980 | 0.989301 |
| GO:0001913\_T\_cell\_mediated\_cytotoxicity | 6 | 0 | 0.000000 | 0.000000 | 1158 | 1102.457020 | 1145.61 | 1188.762980 | 0.989301 |
| GO:0001942\_hair\_follicle\_development | 6 | 0 | 0.000000 | 0.000000 | 1158 | 1102.457020 | 1145.61 | 1188.762980 | 0.989301 |
| GO:0001947\_heart\_looping | 6 | 0 | 0.000000 | 0.000000 | 1158 | 1102.457020 | 1145.61 | 1188.762980 | 0.989301 |
| GO:0002221\_pattern\_recognition\_receptor\_signaling\_pathway | 6 | 0 | 0.000000 | 0.000000 | 1158 | 1102.457020 | 1145.61 | 1188.762980 | 0.989301 |
| GO:0002224\_toll-like\_receptor\_signaling\_pathway | 6 | 0 | 0.000000 | 0.000000 | 1158 | 1102.457020 | 1145.61 | 1188.762980 | 0.989301 |
| GO:0002260\_lymphocyte\_homeostasis | 6 | 0 | 0.000000 | 0.000000 | 1158 | 1102.457020 | 1145.61 | 1188.762980 | 0.989301 |
| GO:0002420\_natural\_killer\_cell\_mediated\_cytotoxicity\_directed\_against\_tumor\_cell\_target | 6 | 0 | 0.000000 | 0.000000 | 1158 | 1102.457020 | 1145.61 | 1188.762980 | 0.989301 |
| GO:0002423\_natural\_killer\_cell\_mediated\_immune\_response\_to\_tumor\_cell | 6 | 0 | 0.000000 | 0.000000 | 1158 | 1102.457020 | 1145.61 | 1188.762980 | 0.989301 |
| GO:0002712\_regulation\_of\_B\_cell\_mediated\_immunity | 6 | 0 | 0.000000 | 0.000000 | 1158 | 1102.457020 | 1145.61 | 1188.762980 | 0.989301 |
| GO:0002855\_regulation\_of\_natural\_killer\_cell\_mediated\_immune\_response\_to\_tumor\_cell | 6 | 0 | 0.000000 | 0.000000 | 1158 | 1102.457020 | 1145.61 | 1188.762980 | 0.989301 |
| GO:0002857\_positive\_regulation\_of\_natural\_killer\_cell\_mediated\_immune\_response\_to\_tumor\_cell | 6 | 0 | 0.000000 | 0.000000 | 1158 | 1102.457020 | 1145.61 | 1188.762980 | 0.989301 |
| GO:0002858\_regulation\_of\_natural\_killer\_cell\_mediated\_cytotoxicity\_directed\_against\_tumor\_cell\_target | 6 | 0 | 0.000000 | 0.000000 | 1158 | 1102.457020 | 1145.61 | 1188.762980 | 0.989301 |
| GO:0002860\_positive\_regulation\_of\_natural\_killer\_cell\_mediated\_cytotoxicity\_directed\_against\_tumor\_cell\_target | 6 | 0 | 0.000000 | 0.000000 | 1158 | 1102.457020 | 1145.61 | 1188.762980 | 0.989301 |
| GO:0002889\_regulation\_of\_immunoglobulin\_mediated\_immune\_response | 6 | 0 | 0.000000 | 0.000000 | 1158 | 1102.457020 | 1145.61 | 1188.762980 | 0.989301 |
| GO:0006020\_inositol\_metabolic\_process | 6 | 0 | 0.000000 | 0.000000 | 1158 | 1102.457020 | 1145.61 | 1188.762980 | 0.989301 |
| GO:0006144\_purine\_base\_metabolic\_process | 6 | 0 | 0.000000 | 0.000000 | 1158 | 1102.457020 | 1145.61 | 1188.762980 | 0.989301 |
| GO:0006283\_transcription-coupled\_nucleotide-excision\_repair | 6 | 0 | 0.000000 | 0.000000 | 1158 | 1102.457020 | 1145.61 | 1188.762980 | 0.989301 |
| GO:0006337\_nucleosome\_disassembly | 6 | 0 | 0.000000 | 0.000000 | 1158 | 1102.457020 | 1145.61 | 1188.762980 | 0.989301 |
| GO:0006390\_transcription\_from\_mitochondrial\_promoter | 6 | 0 | 0.000000 | 0.000000 | 1158 | 1102.457020 | 1145.61 | 1188.762980 | 0.989301 |
| GO:0006458\_'de\_novo'\_protein\_folding | 6 | 0 | 0.000000 | 0.000000 | 1158 | 1102.457020 | 1145.61 | 1188.762980 | 0.989301 |
| GO:0006477\_protein\_amino\_acid\_sulfation | 6 | 0 | 0.000000 | 0.000000 | 1158 | 1102.457020 | 1145.61 | 1188.762980 | 0.989301 |
| GO:0006527\_arginine\_catabolic\_process | 6 | 0 | 0.000000 | 0.000000 | 1158 | 1102.457020 | 1145.61 | 1188.762980 | 0.989301 |
| GO:0006700\_C21-steroid\_hormone\_biosynthetic\_process | 6 | 0 | 0.000000 | 0.000000 | 1158 | 1102.457020 | 1145.61 | 1188.762980 | 0.989301 |
| GO:0006739\_NADP\_metabolic\_process | 6 | 0 | 0.000000 | 0.000000 | 1158 | 1102.457020 | 1145.61 | 1188.762980 | 0.989301 |
| GO:0006826\_iron\_ion\_transport | 6 | 0 | 0.000000 | 0.000000 | 1158 | 1102.457020 | 1145.61 | 1188.762980 | 0.989301 |
| GO:0006878\_cellular\_copper\_ion\_homeostasis | 6 | 0 | 0.000000 | 0.000000 | 1158 | 1102.457020 | 1145.61 | 1188.762980 | 0.989301 |
| GO:0006893\_Golgi\_to\_plasma\_membrane\_transport | 6 | 0 | 0.000000 | 0.000000 | 1158 | 1102.457020 | 1145.61 | 1188.762980 | 0.989301 |
| GO:0006906\_vesicle\_fusion | 6 | 0 | 0.000000 | 0.000000 | 1158 | 1102.457020 | 1145.61 | 1188.762980 | 0.989301 |
| GO:0006911\_phagocytosis\_\_engulfment | 6 | 0 | 0.000000 | 0.000000 | 1158 | 1102.457020 | 1145.61 | 1188.762980 | 0.989301 |
| GO:0006929\_substrate-bound\_cell\_migration | 6 | 0 | 0.000000 | 0.000000 | 1158 | 1102.457020 | 1145.61 | 1188.762980 | 0.989301 |
| GO:0006949\_syncytium\_formation | 6 | 0 | 0.000000 | 0.000000 | 1158 | 1102.457020 | 1145.61 | 1188.762980 | 0.989301 |
| GO:0006978\_DNA\_damage\_response\_\_signal\_transduction\_by\_p53\_class\_mediator\_resulting\_in\_transcription\_of\_p21\_class\_mediator | 6 | 0 | 0.000000 | 0.000000 | 1158 | 1102.457020 | 1145.61 | 1188.762980 | 0.989301 |
| GO:0006983\_ER\_overload\_response | 6 | 0 | 0.000000 | 0.000000 | 1158 | 1102.457020 | 1145.61 | 1188.762980 | 0.989301 |
| GO:0007158\_neuron\_adhesion | 6 | 0 | 0.000000 | 0.000000 | 1158 | 1102.457020 | 1145.61 | 1188.762980 | 0.989301 |
| GO:0007185\_transmembrane\_receptor\_protein\_tyrosine\_phosphatase\_signaling\_pathway | 6 | 0 | 0.000000 | 0.000000 | 1158 | 1102.457020 | 1145.61 | 1188.762980 | 0.989301 |
| GO:0007189\_activation\_of\_adenylate\_cyclase\_activity\_by\_G-protein\_signaling\_pathway | 6 | 0 | 0.000000 | 0.000000 | 1158 | 1102.457020 | 1145.61 | 1188.762980 | 0.989301 |
| GO:0007212\_dopamine\_receptor\_signaling\_pathway | 6 | 0 | 0.000000 | 0.000000 | 1158 | 1102.457020 | 1145.61 | 1188.762980 | 0.989301 |
| GO:0007217\_tachykinin\_receptor\_signaling\_pathway | 6 | 0 | 0.000000 | 0.000000 | 1158 | 1102.457020 | 1145.61 | 1188.762980 | 0.989301 |
| GO:0007224\_smoothened\_signaling\_pathway | 6 | 0 | 0.000000 | 0.000000 | 1158 | 1102.457020 | 1145.61 | 1188.762980 | 0.989301 |
| GO:0007340\_acrosome\_reaction | 6 | 0 | 0.000000 | 0.000000 | 1158 | 1102.457020 | 1145.61 | 1188.762980 | 0.989301 |
| GO:0007530\_sex\_determination | 6 | 0 | 0.000000 | 0.000000 | 1158 | 1102.457020 | 1145.61 | 1188.762980 | 0.989301 |
| GO:0007613\_memory | 6 | 0 | 0.000000 | 0.000000 | 1158 | 1102.457020 | 1145.61 | 1188.762980 | 0.989301 |
| GO:0007618\_mating | 6 | 0 | 0.000000 | 0.000000 | 1158 | 1102.457020 | 1145.61 | 1188.762980 | 0.989301 |
| GO:0007622\_rhythmic\_behavior | 6 | 0 | 0.000000 | 0.000000 | 1158 | 1102.457020 | 1145.61 | 1188.762980 | 0.989301 |
| GO:0008343\_adult\_feeding\_behavior | 6 | 0 | 0.000000 | 0.000000 | 1158 | 1102.457020 | 1145.61 | 1188.762980 | 0.989301 |
| GO:0009071\_serine\_family\_amino\_acid\_catabolic\_process | 6 | 0 | 0.000000 | 0.000000 | 1158 | 1102.457020 | 1145.61 | 1188.762980 | 0.989301 |
| GO:0009164\_nucleoside\_catabolic\_process | 6 | 0 | 0.000000 | 0.000000 | 1158 | 1102.457020 | 1145.61 | 1188.762980 | 0.989301 |
| GO:0009312\_oligosaccharide\_biosynthetic\_process | 6 | 0 | 0.000000 | 0.000000 | 1158 | 1102.457020 | 1145.61 | 1188.762980 | 0.989301 |
| GO:0009994\_oocyte\_differentiation | 6 | 0 | 0.000000 | 0.000000 | 1158 | 1102.457020 | 1145.61 | 1188.762980 | 0.989301 |
| GO:0010149\_senescence | 6 | 0 | 0.000000 | 0.000000 | 1158 | 1102.457020 | 1145.61 | 1188.762980 | 0.989301 |
| GO:0010573\_vascular\_endothelial\_growth\_factor\_production | 6 | 0 | 0.000000 | 0.000000 | 1158 | 1102.457020 | 1145.61 | 1188.762980 | 0.989301 |
| GO:0010574\_regulation\_of\_vascular\_endothelial\_growth\_factor\_production | 6 | 0 | 0.000000 | 0.000000 | 1158 | 1102.457020 | 1145.61 | 1188.762980 | 0.989301 |
| GO:0010578\_regulation\_of\_adenylate\_cyclase\_activity\_involved\_in\_G-protein\_signaling | 6 | 0 | 0.000000 | 0.000000 | 1158 | 1102.457020 | 1145.61 | 1188.762980 | 0.989301 |
| GO:0010579\_positive\_regulation\_of\_adenylate\_cyclase\_activity\_by\_G-protein\_signaling\_pathway | 6 | 0 | 0.000000 | 0.000000 | 1158 | 1102.457020 | 1145.61 | 1188.762980 | 0.989301 |
| GO:0010611\_regulation\_of\_cardiac\_muscle\_hypertrophy | 6 | 0 | 0.000000 | 0.000000 | 1158 | 1102.457020 | 1145.61 | 1188.762980 | 0.989301 |
| GO:0010612\_regulation\_of\_cardiac\_muscle\_adaptation | 6 | 0 | 0.000000 | 0.000000 | 1158 | 1102.457020 | 1145.61 | 1188.762980 | 0.989301 |
| GO:0010657\_muscle\_cell\_apoptosis | 6 | 0 | 0.000000 | 0.000000 | 1158 | 1102.457020 | 1145.61 | 1188.762980 | 0.989301 |
| GO:0010660\_regulation\_of\_muscle\_cell\_apoptosis | 6 | 0 | 0.000000 | 0.000000 | 1158 | 1102.457020 | 1145.61 | 1188.762980 | 0.989301 |
| GO:0010718\_positive\_regulation\_of\_epithelial\_to\_mesenchymal\_transition | 6 | 0 | 0.000000 | 0.000000 | 1158 | 1102.457020 | 1145.61 | 1188.762980 | 0.989301 |
| GO:0010770\_positive\_regulation\_of\_cell\_morphogenesis\_involved\_in\_differentiation | 6 | 0 | 0.000000 | 0.000000 | 1158 | 1102.457020 | 1145.61 | 1188.762980 | 0.989301 |
| GO:0010875\_positive\_regulation\_of\_cholesterol\_efflux | 6 | 0 | 0.000000 | 0.000000 | 1158 | 1102.457020 | 1145.61 | 1188.762980 | 0.989301 |
| GO:0010887\_negative\_regulation\_of\_cholesterol\_storage | 6 | 0 | 0.000000 | 0.000000 | 1158 | 1102.457020 | 1145.61 | 1188.762980 | 0.989301 |
| GO:0010896\_regulation\_of\_triglyceride\_catabolic\_process | 6 | 0 | 0.000000 | 0.000000 | 1158 | 1102.457020 | 1145.61 | 1188.762980 | 0.989301 |
| GO:0014020\_primary\_neural\_tube\_formation | 6 | 0 | 0.000000 | 0.000000 | 1158 | 1102.457020 | 1145.61 | 1188.762980 | 0.989301 |
| GO:0014743\_regulation\_of\_muscle\_hypertrophy | 6 | 0 | 0.000000 | 0.000000 | 1158 | 1102.457020 | 1145.61 | 1188.762980 | 0.989301 |
| GO:0014855\_striated\_muscle\_cell\_proliferation | 6 | 0 | 0.000000 | 0.000000 | 1158 | 1102.457020 | 1145.61 | 1188.762980 | 0.989301 |
| GO:0014887\_cardiac\_muscle\_adaptation | 6 | 0 | 0.000000 | 0.000000 | 1158 | 1102.457020 | 1145.61 | 1188.762980 | 0.989301 |
| GO:0014888\_striated\_muscle\_adaptation | 6 | 0 | 0.000000 | 0.000000 | 1158 | 1102.457020 | 1145.61 | 1188.762980 | 0.989301 |
| GO:0014897\_striated\_muscle\_hypertrophy | 6 | 0 | 0.000000 | 0.000000 | 1158 | 1102.457020 | 1145.61 | 1188.762980 | 0.989301 |
| GO:0014898\_cardiac\_muscle\_hypertrophy | 6 | 0 | 0.000000 | 0.000000 | 1158 | 1102.457020 | 1145.61 | 1188.762980 | 0.989301 |
| GO:0015671\_oxygen\_transport | 6 | 0 | 0.000000 | 0.000000 | 1158 | 1102.457020 | 1145.61 | 1188.762980 | 0.989301 |
| GO:0015810\_aspartate\_transport | 6 | 0 | 0.000000 | 0.000000 | 1158 | 1102.457020 | 1145.61 | 1188.762980 | 0.989301 |
| GO:0015850\_organic\_alcohol\_transport | 6 | 0 | 0.000000 | 0.000000 | 1158 | 1102.457020 | 1145.61 | 1188.762980 | 0.989301 |
| GO:0017156\_calcium\_ion-dependent\_exocytosis | 6 | 0 | 0.000000 | 0.000000 | 1158 | 1102.457020 | 1145.61 | 1188.762980 | 0.989301 |
| GO:0019062\_virion\_attachment\_to\_host\_cell\_surface\_receptor | 6 | 0 | 0.000000 | 0.000000 | 1158 | 1102.457020 | 1145.61 | 1188.762980 | 0.989301 |
| GO:0019080\_viral\_genome\_expression | 6 | 0 | 0.000000 | 0.000000 | 1158 | 1102.457020 | 1145.61 | 1188.762980 | 0.989301 |
| GO:0019083\_viral\_transcription | 6 | 0 | 0.000000 | 0.000000 | 1158 | 1102.457020 | 1145.61 | 1188.762980 | 0.989301 |
| GO:0019827\_stem\_cell\_maintenance | 6 | 0 | 0.000000 | 0.000000 | 1158 | 1102.457020 | 1145.61 | 1188.762980 | 0.989301 |
| GO:0019883\_antigen\_processing\_and\_presentation\_of\_endogenous\_antigen | 6 | 0 | 0.000000 | 0.000000 | 1158 | 1102.457020 | 1145.61 | 1188.762980 | 0.989301 |
| GO:0021537\_telencephalon\_development | 6 | 0 | 0.000000 | 0.000000 | 1158 | 1102.457020 | 1145.61 | 1188.762980 | 0.989301 |
| GO:0021953\_central\_nervous\_system\_neuron\_differentiation | 6 | 0 | 0.000000 | 0.000000 | 1158 | 1102.457020 | 1145.61 | 1188.762980 | 0.989301 |
| GO:0021954\_central\_nervous\_system\_neuron\_development | 6 | 0 | 0.000000 | 0.000000 | 1158 | 1102.457020 | 1145.61 | 1188.762980 | 0.989301 |
| GO:0022404\_molting\_cycle\_process | 6 | 0 | 0.000000 | 0.000000 | 1158 | 1102.457020 | 1145.61 | 1188.762980 | 0.989301 |
| GO:0022405\_hair\_cycle\_process | 6 | 0 | 0.000000 | 0.000000 | 1158 | 1102.457020 | 1145.61 | 1188.762980 | 0.989301 |
| GO:0022408\_negative\_regulation\_of\_cell-cell\_adhesion | 6 | 0 | 0.000000 | 0.000000 | 1158 | 1102.457020 | 1145.61 | 1188.762980 | 0.989301 |
| GO:0022409\_positive\_regulation\_of\_cell-cell\_adhesion | 6 | 0 | 0.000000 | 0.000000 | 1158 | 1102.457020 | 1145.61 | 1188.762980 | 0.989301 |
| GO:0030032\_lamellipodium\_assembly | 6 | 0 | 0.000000 | 0.000000 | 1158 | 1102.457020 | 1145.61 | 1188.762980 | 0.989301 |
| GO:0030147\_natriuresis | 6 | 0 | 0.000000 | 0.000000 | 1158 | 1102.457020 | 1145.61 | 1188.762980 | 0.989301 |
| GO:0030224\_monocyte\_differentiation | 6 | 0 | 0.000000 | 0.000000 | 1158 | 1102.457020 | 1145.61 | 1188.762980 | 0.989301 |
| GO:0030299\_intestinal\_cholesterol\_absorption | 6 | 0 | 0.000000 | 0.000000 | 1158 | 1102.457020 | 1145.61 | 1188.762980 | 0.989301 |
| GO:0030801\_positive\_regulation\_of\_cyclic\_nucleotide\_metabolic\_process | 6 | 0 | 0.000000 | 0.000000 | 1158 | 1102.457020 | 1145.61 | 1188.762980 | 0.989301 |
| GO:0030804\_positive\_regulation\_of\_cyclic\_nucleotide\_biosynthetic\_process | 6 | 0 | 0.000000 | 0.000000 | 1158 | 1102.457020 | 1145.61 | 1188.762980 | 0.989301 |
| GO:0030810\_positive\_regulation\_of\_nucleotide\_biosynthetic\_process | 6 | 0 | 0.000000 | 0.000000 | 1158 | 1102.457020 | 1145.61 | 1188.762980 | 0.989301 |
| GO:0030835\_negative\_regulation\_of\_actin\_filament\_depolymerization | 6 | 0 | 0.000000 | 0.000000 | 1158 | 1102.457020 | 1145.61 | 1188.762980 | 0.989301 |
| GO:0030837\_negative\_regulation\_of\_actin\_filament\_polymerization | 6 | 0 | 0.000000 | 0.000000 | 1158 | 1102.457020 | 1145.61 | 1188.762980 | 0.989301 |
| GO:0031099\_regeneration | 6 | 0 | 0.000000 | 0.000000 | 1158 | 1102.457020 | 1145.61 | 1188.762980 | 0.989301 |
| GO:0031268\_pseudopodium\_organization | 6 | 0 | 0.000000 | 0.000000 | 1158 | 1102.457020 | 1145.61 | 1188.762980 | 0.989301 |
| GO:0031269\_pseudopodium\_assembly | 6 | 0 | 0.000000 | 0.000000 | 1158 | 1102.457020 | 1145.61 | 1188.762980 | 0.989301 |
| GO:0031272\_regulation\_of\_pseudopodium\_assembly | 6 | 0 | 0.000000 | 0.000000 | 1158 | 1102.457020 | 1145.61 | 1188.762980 | 0.989301 |
| GO:0031274\_positive\_regulation\_of\_pseudopodium\_assembly | 6 | 0 | 0.000000 | 0.000000 | 1158 | 1102.457020 | 1145.61 | 1188.762980 | 0.989301 |
| GO:0031958\_corticosteroid\_receptor\_signaling\_pathway | 6 | 0 | 0.000000 | 0.000000 | 1158 | 1102.457020 | 1145.61 | 1188.762980 | 0.989301 |
| GO:0032297\_negative\_regulation\_of\_DNA\_replication\_initiation | 6 | 0 | 0.000000 | 0.000000 | 1158 | 1102.457020 | 1145.61 | 1188.762980 | 0.989301 |
| GO:0032372\_negative\_regulation\_of\_sterol\_transport | 6 | 0 | 0.000000 | 0.000000 | 1158 | 1102.457020 | 1145.61 | 1188.762980 | 0.989301 |
| GO:0032375\_negative\_regulation\_of\_cholesterol\_transport | 6 | 0 | 0.000000 | 0.000000 | 1158 | 1102.457020 | 1145.61 | 1188.762980 | 0.989301 |
| GO:0032402\_melanosome\_transport | 6 | 0 | 0.000000 | 0.000000 | 1158 | 1102.457020 | 1145.61 | 1188.762980 | 0.989301 |
| GO:0032411\_positive\_regulation\_of\_transporter\_activity | 6 | 0 | 0.000000 | 0.000000 | 1158 | 1102.457020 | 1145.61 | 1188.762980 | 0.989301 |
| GO:0032434\_regulation\_of\_proteasomal\_ubiquitin-dependent\_protein\_catabolic\_process | 6 | 0 | 0.000000 | 0.000000 | 1158 | 1102.457020 | 1145.61 | 1188.762980 | 0.989301 |
| GO:0032890\_regulation\_of\_organic\_acid\_transport | 6 | 0 | 0.000000 | 0.000000 | 1158 | 1102.457020 | 1145.61 | 1188.762980 | 0.989301 |
| GO:0032945\_negative\_regulation\_of\_mononuclear\_cell\_proliferation | 6 | 0 | 0.000000 | 0.000000 | 1158 | 1102.457020 | 1145.61 | 1188.762980 | 0.989301 |
| GO:0032986\_protein-DNA\_complex\_disassembly | 6 | 0 | 0.000000 | 0.000000 | 1158 | 1102.457020 | 1145.61 | 1188.762980 | 0.989301 |
| GO:0033158\_regulation\_of\_protein\_import\_into\_nucleus\_\_translocation | 6 | 0 | 0.000000 | 0.000000 | 1158 | 1102.457020 | 1145.61 | 1188.762980 | 0.989301 |
| GO:0033238\_regulation\_of\_cellular\_amine\_metabolic\_process | 6 | 0 | 0.000000 | 0.000000 | 1158 | 1102.457020 | 1145.61 | 1188.762980 | 0.989301 |
| GO:0033363\_secretory\_granule\_organization | 6 | 0 | 0.000000 | 0.000000 | 1158 | 1102.457020 | 1145.61 | 1188.762980 | 0.989301 |
| GO:0033483\_gas\_homeostasis | 6 | 0 | 0.000000 | 0.000000 | 1158 | 1102.457020 | 1145.61 | 1188.762980 | 0.989301 |
| GO:0033627\_cell\_adhesion\_mediated\_by\_integrin | 6 | 0 | 0.000000 | 0.000000 | 1158 | 1102.457020 | 1145.61 | 1188.762980 | 0.989301 |
| GO:0033683\_nucleotide-excision\_repair\_\_DNA\_incision | 6 | 0 | 0.000000 | 0.000000 | 1158 | 1102.457020 | 1145.61 | 1188.762980 | 0.989301 |
| GO:0034405\_response\_to\_fluid\_shear\_stress | 6 | 0 | 0.000000 | 0.000000 | 1158 | 1102.457020 | 1145.61 | 1188.762980 | 0.989301 |
| GO:0034446\_substrate\_adhesion-dependent\_cell\_spreading | 6 | 0 | 0.000000 | 0.000000 | 1158 | 1102.457020 | 1145.61 | 1188.762980 | 0.989301 |
| GO:0034447\_very-low-density\_lipoprotein\_particle\_clearance | 6 | 0 | 0.000000 | 0.000000 | 1158 | 1102.457020 | 1145.61 | 1188.762980 | 0.989301 |
| GO:0034502\_protein\_localization\_to\_chromosome | 6 | 0 | 0.000000 | 0.000000 | 1158 | 1102.457020 | 1145.61 | 1188.762980 | 0.989301 |
| GO:0034508\_centromere\_complex\_assembly | 6 | 0 | 0.000000 | 0.000000 | 1158 | 1102.457020 | 1145.61 | 1188.762980 | 0.989301 |
| GO:0035148\_tube\_lumen\_formation | 6 | 0 | 0.000000 | 0.000000 | 1158 | 1102.457020 | 1145.61 | 1188.762980 | 0.989301 |
| GO:0042092\_T-helper\_2\_type\_immune\_response | 6 | 0 | 0.000000 | 0.000000 | 1158 | 1102.457020 | 1145.61 | 1188.762980 | 0.989301 |
| GO:0042119\_neutrophil\_activation | 6 | 0 | 0.000000 | 0.000000 | 1158 | 1102.457020 | 1145.61 | 1188.762980 | 0.989301 |
| GO:0042255\_ribosome\_assembly | 6 | 0 | 0.000000 | 0.000000 | 1158 | 1102.457020 | 1145.61 | 1188.762980 | 0.989301 |
| GO:0042273\_ribosomal\_large\_subunit\_biogenesis | 6 | 0 | 0.000000 | 0.000000 | 1158 | 1102.457020 | 1145.61 | 1188.762980 | 0.989301 |
| GO:0042303\_molting\_cycle | 6 | 0 | 0.000000 | 0.000000 | 1158 | 1102.457020 | 1145.61 | 1188.762980 | 0.989301 |
| GO:0042346\_positive\_regulation\_of\_NF-kappaB\_import\_into\_nucleus | 6 | 0 | 0.000000 | 0.000000 | 1158 | 1102.457020 | 1145.61 | 1188.762980 | 0.989301 |
| GO:0042402\_biogenic\_amine\_catabolic\_process | 6 | 0 | 0.000000 | 0.000000 | 1158 | 1102.457020 | 1145.61 | 1188.762980 | 0.989301 |
| GO:0042447\_hormone\_catabolic\_process | 6 | 0 | 0.000000 | 0.000000 | 1158 | 1102.457020 | 1145.61 | 1188.762980 | 0.989301 |
| GO:0042559\_pteridine\_and\_derivative\_biosynthetic\_process | 6 | 0 | 0.000000 | 0.000000 | 1158 | 1102.457020 | 1145.61 | 1188.762980 | 0.989301 |
| GO:0042633\_hair\_cycle | 6 | 0 | 0.000000 | 0.000000 | 1158 | 1102.457020 | 1145.61 | 1188.762980 | 0.989301 |
| GO:0042772\_DNA\_damage\_response\_\_signal\_transduction\_resulting\_in\_transcription | 6 | 0 | 0.000000 | 0.000000 | 1158 | 1102.457020 | 1145.61 | 1188.762980 | 0.989301 |
| GO:0042921\_glucocorticoid\_receptor\_signaling\_pathway | 6 | 0 | 0.000000 | 0.000000 | 1158 | 1102.457020 | 1145.61 | 1188.762980 | 0.989301 |
| GO:0043206\_fibril\_organization | 6 | 0 | 0.000000 | 0.000000 | 1158 | 1102.457020 | 1145.61 | 1188.762980 | 0.989301 |
| GO:0043370\_regulation\_of\_CD4-positive\_\_alpha\_beta\_T\_cell\_differentiation | 6 | 0 | 0.000000 | 0.000000 | 1158 | 1102.457020 | 1145.61 | 1188.762980 | 0.989301 |
| GO:0043502\_regulation\_of\_muscle\_adaptation | 6 | 0 | 0.000000 | 0.000000 | 1158 | 1102.457020 | 1145.61 | 1188.762980 | 0.989301 |
| GO:0043525\_positive\_regulation\_of\_neuron\_apoptosis | 6 | 0 | 0.000000 | 0.000000 | 1158 | 1102.457020 | 1145.61 | 1188.762980 | 0.989301 |
| GO:0043536\_positive\_regulation\_of\_blood\_vessel\_endothelial\_cell\_migration | 6 | 0 | 0.000000 | 0.000000 | 1158 | 1102.457020 | 1145.61 | 1188.762980 | 0.989301 |
| GO:0043666\_regulation\_of\_phosphoprotein\_phosphatase\_activity | 6 | 0 | 0.000000 | 0.000000 | 1158 | 1102.457020 | 1145.61 | 1188.762980 | 0.989301 |
| GO:0043981\_histone\_H4-K5\_acetylation | 6 | 0 | 0.000000 | 0.000000 | 1158 | 1102.457020 | 1145.61 | 1188.762980 | 0.989301 |
| GO:0043982\_histone\_H4-K8\_acetylation | 6 | 0 | 0.000000 | 0.000000 | 1158 | 1102.457020 | 1145.61 | 1188.762980 | 0.989301 |
| GO:0043983\_histone\_H4-K12\_acetylation | 6 | 0 | 0.000000 | 0.000000 | 1158 | 1102.457020 | 1145.61 | 1188.762980 | 0.989301 |
| GO:0043984\_histone\_H4-K16\_acetylation | 6 | 0 | 0.000000 | 0.000000 | 1158 | 1102.457020 | 1145.61 | 1188.762980 | 0.989301 |
| GO:0045073\_regulation\_of\_chemokine\_biosynthetic\_process | 6 | 0 | 0.000000 | 0.000000 | 1158 | 1102.457020 | 1145.61 | 1188.762980 | 0.989301 |
| GO:0045090\_retroviral\_genome\_replication | 6 | 0 | 0.000000 | 0.000000 | 1158 | 1102.457020 | 1145.61 | 1188.762980 | 0.989301 |
| GO:0045116\_protein\_neddylation | 6 | 0 | 0.000000 | 0.000000 | 1158 | 1102.457020 | 1145.61 | 1188.762980 | 0.989301 |
| GO:0045540\_regulation\_of\_cholesterol\_biosynthetic\_process | 6 | 0 | 0.000000 | 0.000000 | 1158 | 1102.457020 | 1145.61 | 1188.762980 | 0.989301 |
| GO:0045604\_regulation\_of\_epidermal\_cell\_differentiation | 6 | 0 | 0.000000 | 0.000000 | 1158 | 1102.457020 | 1145.61 | 1188.762980 | 0.989301 |
| GO:0045616\_regulation\_of\_keratinocyte\_differentiation | 6 | 0 | 0.000000 | 0.000000 | 1158 | 1102.457020 | 1145.61 | 1188.762980 | 0.989301 |
| GO:0045622\_regulation\_of\_T-helper\_cell\_differentiation | 6 | 0 | 0.000000 | 0.000000 | 1158 | 1102.457020 | 1145.61 | 1188.762980 | 0.989301 |
| GO:0045661\_regulation\_of\_myoblast\_differentiation | 6 | 0 | 0.000000 | 0.000000 | 1158 | 1102.457020 | 1145.61 | 1188.762980 | 0.989301 |
| GO:0045671\_negative\_regulation\_of\_osteoclast\_differentiation | 6 | 0 | 0.000000 | 0.000000 | 1158 | 1102.457020 | 1145.61 | 1188.762980 | 0.989301 |
| GO:0045684\_positive\_regulation\_of\_epidermis\_development | 6 | 0 | 0.000000 | 0.000000 | 1158 | 1102.457020 | 1145.61 | 1188.762980 | 0.989301 |
| GO:0045723\_positive\_regulation\_of\_fatty\_acid\_biosynthetic\_process | 6 | 0 | 0.000000 | 0.000000 | 1158 | 1102.457020 | 1145.61 | 1188.762980 | 0.989301 |
| GO:0045737\_positive\_regulation\_of\_cyclin-dependent\_protein\_kinase\_activity | 6 | 0 | 0.000000 | 0.000000 | 1158 | 1102.457020 | 1145.61 | 1188.762980 | 0.989301 |
| GO:0045823\_positive\_regulation\_of\_heart\_contraction | 6 | 0 | 0.000000 | 0.000000 | 1158 | 1102.457020 | 1145.61 | 1188.762980 | 0.989301 |
| GO:0045885\_positive\_regulation\_of\_survival\_gene\_product\_expression | 6 | 0 | 0.000000 | 0.000000 | 1158 | 1102.457020 | 1145.61 | 1188.762980 | 0.989301 |
| GO:0045931\_positive\_regulation\_of\_mitotic\_cell\_cycle | 6 | 0 | 0.000000 | 0.000000 | 1158 | 1102.457020 | 1145.61 | 1188.762980 | 0.989301 |
| GO:0045932\_negative\_regulation\_of\_muscle\_contraction | 6 | 0 | 0.000000 | 0.000000 | 1158 | 1102.457020 | 1145.61 | 1188.762980 | 0.989301 |
| GO:0045981\_positive\_regulation\_of\_nucleotide\_metabolic\_process | 6 | 0 | 0.000000 | 0.000000 | 1158 | 1102.457020 | 1145.61 | 1188.762980 | 0.989301 |
| GO:0046006\_regulation\_of\_activated\_T\_cell\_proliferation | 6 | 0 | 0.000000 | 0.000000 | 1158 | 1102.457020 | 1145.61 | 1188.762980 | 0.989301 |
| GO:0046426\_negative\_regulation\_of\_JAK-STAT\_cascade | 6 | 0 | 0.000000 | 0.000000 | 1158 | 1102.457020 | 1145.61 | 1188.762980 | 0.989301 |
| GO:0046520\_sphingoid\_biosynthetic\_process | 6 | 0 | 0.000000 | 0.000000 | 1158 | 1102.457020 | 1145.61 | 1188.762980 | 0.989301 |
| GO:0046579\_positive\_regulation\_of\_Ras\_protein\_signal\_transduction | 6 | 0 | 0.000000 | 0.000000 | 1158 | 1102.457020 | 1145.61 | 1188.762980 | 0.989301 |
| GO:0046638\_positive\_regulation\_of\_alpha-beta\_T\_cell\_differentiation | 6 | 0 | 0.000000 | 0.000000 | 1158 | 1102.457020 | 1145.61 | 1188.762980 | 0.989301 |
| GO:0046677\_response\_to\_antibiotic | 6 | 0 | 0.000000 | 0.000000 | 1158 | 1102.457020 | 1145.61 | 1188.762980 | 0.989301 |
| GO:0046697\_decidualization | 6 | 0 | 0.000000 | 0.000000 | 1158 | 1102.457020 | 1145.61 | 1188.762980 | 0.989301 |
| GO:0046716\_muscle\_maintenance | 6 | 0 | 0.000000 | 0.000000 | 1158 | 1102.457020 | 1145.61 | 1188.762980 | 0.989301 |
| GO:0048008\_platelet-derived\_growth\_factor\_receptor\_signaling\_pathway | 6 | 0 | 0.000000 | 0.000000 | 1158 | 1102.457020 | 1145.61 | 1188.762980 | 0.989301 |
| GO:0048247\_lymphocyte\_chemotaxis | 6 | 0 | 0.000000 | 0.000000 | 1158 | 1102.457020 | 1145.61 | 1188.762980 | 0.989301 |
| GO:0048385\_regulation\_of\_retinoic\_acid\_receptor\_signaling\_pathway | 6 | 0 | 0.000000 | 0.000000 | 1158 | 1102.457020 | 1145.61 | 1188.762980 | 0.989301 |
| GO:0048512\_circadian\_behavior | 6 | 0 | 0.000000 | 0.000000 | 1158 | 1102.457020 | 1145.61 | 1188.762980 | 0.989301 |
| GO:0048525\_negative\_regulation\_of\_viral\_reproduction | 6 | 0 | 0.000000 | 0.000000 | 1158 | 1102.457020 | 1145.61 | 1188.762980 | 0.989301 |
| GO:0048562\_embryonic\_organ\_morphogenesis | 6 | 0 | 0.000000 | 0.000000 | 1158 | 1102.457020 | 1145.61 | 1188.762980 | 0.989301 |
| GO:0048610\_reproductive\_cellular\_process | 6 | 0 | 0.000000 | 0.000000 | 1158 | 1102.457020 | 1145.61 | 1188.762980 | 0.989301 |
| GO:0048704\_embryonic\_skeletal\_system\_morphogenesis | 6 | 0 | 0.000000 | 0.000000 | 1158 | 1102.457020 | 1145.61 | 1188.762980 | 0.989301 |
| GO:0048738\_cardiac\_muscle\_tissue\_development | 6 | 0 | 0.000000 | 0.000000 | 1158 | 1102.457020 | 1145.61 | 1188.762980 | 0.989301 |
| GO:0048864\_stem\_cell\_development | 6 | 0 | 0.000000 | 0.000000 | 1158 | 1102.457020 | 1145.61 | 1188.762980 | 0.989301 |
| GO:0050432\_catecholamine\_secretion | 6 | 0 | 0.000000 | 0.000000 | 1158 | 1102.457020 | 1145.61 | 1188.762980 | 0.989301 |
| GO:0050433\_regulation\_of\_catecholamine\_secretion | 6 | 0 | 0.000000 | 0.000000 | 1158 | 1102.457020 | 1145.61 | 1188.762980 | 0.989301 |
| GO:0050672\_negative\_regulation\_of\_lymphocyte\_proliferation | 6 | 0 | 0.000000 | 0.000000 | 1158 | 1102.457020 | 1145.61 | 1188.762980 | 0.989301 |
| GO:0050732\_negative\_regulation\_of\_peptidyl-tyrosine\_phosphorylation | 6 | 0 | 0.000000 | 0.000000 | 1158 | 1102.457020 | 1145.61 | 1188.762980 | 0.989301 |
| GO:0050746\_regulation\_of\_lipoprotein\_metabolic\_process | 6 | 0 | 0.000000 | 0.000000 | 1158 | 1102.457020 | 1145.61 | 1188.762980 | 0.989301 |
| GO:0050748\_negative\_regulation\_of\_lipoprotein\_metabolic\_process | 6 | 0 | 0.000000 | 0.000000 | 1158 | 1102.457020 | 1145.61 | 1188.762980 | 0.989301 |
| GO:0050764\_regulation\_of\_phagocytosis | 6 | 0 | 0.000000 | 0.000000 | 1158 | 1102.457020 | 1145.61 | 1188.762980 | 0.989301 |
| GO:0050803\_regulation\_of\_synapse\_structure\_and\_activity | 6 | 0 | 0.000000 | 0.000000 | 1158 | 1102.457020 | 1145.61 | 1188.762980 | 0.989301 |
| GO:0050850\_positive\_regulation\_of\_calcium-mediated\_signaling | 6 | 0 | 0.000000 | 0.000000 | 1158 | 1102.457020 | 1145.61 | 1188.762980 | 0.989301 |
| GO:0050892\_intestinal\_absorption | 6 | 0 | 0.000000 | 0.000000 | 1158 | 1102.457020 | 1145.61 | 1188.762980 | 0.989301 |
| GO:0050913\_sensory\_perception\_of\_bitter\_taste | 6 | 0 | 0.000000 | 0.000000 | 1158 | 1102.457020 | 1145.61 | 1188.762980 | 0.989301 |
| GO:0050957\_equilibrioception | 6 | 0 | 0.000000 | 0.000000 | 1158 | 1102.457020 | 1145.61 | 1188.762980 | 0.989301 |
| GO:0051006\_positive\_regulation\_of\_lipoprotein\_lipase\_activity | 6 | 0 | 0.000000 | 0.000000 | 1158 | 1102.457020 | 1145.61 | 1188.762980 | 0.989301 |
| GO:0051057\_positive\_regulation\_of\_small\_GTPase\_mediated\_signal\_transduction | 6 | 0 | 0.000000 | 0.000000 | 1158 | 1102.457020 | 1145.61 | 1188.762980 | 0.989301 |
| GO:0051148\_negative\_regulation\_of\_muscle\_cell\_differentiation | 6 | 0 | 0.000000 | 0.000000 | 1158 | 1102.457020 | 1145.61 | 1188.762980 | 0.989301 |
| GO:0051205\_protein\_insertion\_into\_membrane | 6 | 0 | 0.000000 | 0.000000 | 1158 | 1102.457020 | 1145.61 | 1188.762980 | 0.989301 |
| GO:0051291\_protein\_heterooligomerization | 6 | 0 | 0.000000 | 0.000000 | 1158 | 1102.457020 | 1145.61 | 1188.762980 | 0.989301 |
| GO:0051302\_regulation\_of\_cell\_division | 6 | 0 | 0.000000 | 0.000000 | 1158 | 1102.457020 | 1145.61 | 1188.762980 | 0.989301 |
| GO:0051588\_regulation\_of\_neurotransmitter\_transport | 6 | 0 | 0.000000 | 0.000000 | 1158 | 1102.457020 | 1145.61 | 1188.762980 | 0.989301 |
| GO:0051702\_interaction\_with\_symbiont | 6 | 0 | 0.000000 | 0.000000 | 1158 | 1102.457020 | 1145.61 | 1188.762980 | 0.989301 |
| GO:0051865\_protein\_autoubiquitination | 6 | 0 | 0.000000 | 0.000000 | 1158 | 1102.457020 | 1145.61 | 1188.762980 | 0.989301 |
| GO:0051904\_pigment\_granule\_transport | 6 | 0 | 0.000000 | 0.000000 | 1158 | 1102.457020 | 1145.61 | 1188.762980 | 0.989301 |
| GO:0051917\_regulation\_of\_fibrinolysis | 6 | 0 | 0.000000 | 0.000000 | 1158 | 1102.457020 | 1145.61 | 1188.762980 | 0.989301 |
| GO:0051923\_sulfation | 6 | 0 | 0.000000 | 0.000000 | 1158 | 1102.457020 | 1145.61 | 1188.762980 | 0.989301 |
| GO:0051972\_regulation\_of\_telomerase\_activity | 6 | 0 | 0.000000 | 0.000000 | 1158 | 1102.457020 | 1145.61 | 1188.762980 | 0.989301 |
| GO:0060038\_cardiac\_muscle\_cell\_proliferation | 6 | 0 | 0.000000 | 0.000000 | 1158 | 1102.457020 | 1145.61 | 1188.762980 | 0.989301 |
| GO:0060192\_negative\_regulation\_of\_lipase\_activity | 6 | 0 | 0.000000 | 0.000000 | 1158 | 1102.457020 | 1145.61 | 1188.762980 | 0.989301 |
| GO:0060396\_growth\_hormone\_receptor\_signaling\_pathway | 6 | 0 | 0.000000 | 0.000000 | 1158 | 1102.457020 | 1145.61 | 1188.762980 | 0.989301 |
| GO:0060606\_tube\_closure | 6 | 0 | 0.000000 | 0.000000 | 1158 | 1102.457020 | 1145.61 | 1188.762980 | 0.989301 |
| GO:0070198\_protein\_localization\_to\_telomere | 6 | 0 | 0.000000 | 0.000000 | 1158 | 1102.457020 | 1145.61 | 1188.762980 | 0.989301 |
| GO:0070265\_necrotic\_cell\_death | 6 | 0 | 0.000000 | 0.000000 | 1158 | 1102.457020 | 1145.61 | 1188.762980 | 0.989301 |
| GO:0070613\_regulation\_of\_protein\_processing | 6 | 0 | 0.000000 | 0.000000 | 1158 | 1102.457020 | 1145.61 | 1188.762980 | 0.989301 |
| GO:0070664\_negative\_regulation\_of\_leukocyte\_proliferation | 6 | 0 | 0.000000 | 0.000000 | 1158 | 1102.457020 | 1145.61 | 1188.762980 | 0.989301 |
| GO:0006469\_negative\_regulation\_of\_protein\_kinase\_activity | 55 | 0 | 0.000000 | 0.000000 | 1164 | 1108.610301 | 1151.42 | 1194.229699 | 0.989192 |
| GO:0006725\_cellular\_aromatic\_compound\_metabolic\_process | 55 | 0 | 0.000000 | 0.000000 | 1164 | 1108.610301 | 1151.42 | 1194.229699 | 0.989192 |
| GO:0007606\_sensory\_perception\_of\_chemical\_stimulus | 55 | 0 | 0.000000 | 0.000000 | 1164 | 1108.610301 | 1151.42 | 1194.229699 | 0.989192 |
| GO:0030814\_regulation\_of\_cAMP\_metabolic\_process | 55 | 0 | 0.000000 | 0.000000 | 1164 | 1108.610301 | 1151.42 | 1194.229699 | 0.989192 |
| GO:0030817\_regulation\_of\_cAMP\_biosynthetic\_process | 55 | 0 | 0.000000 | 0.000000 | 1164 | 1108.610301 | 1151.42 | 1194.229699 | 0.989192 |
| GO:0043543\_protein\_amino\_acid\_acylation | 55 | 0 | 0.000000 | 0.000000 | 1164 | 1108.610301 | 1151.42 | 1194.229699 | 0.989192 |
| GO:0006281\_DNA\_repair | 177 | 0 | 0.000000 | 0.000000 | 1167 | 1111.101515 | 1153.6 | 1196.098485 | 0.988518 |
| GO:0009607\_response\_to\_biotic\_stimulus | 177 | 0 | 0.000000 | 0.000000 | 1167 | 1111.101515 | 1153.6 | 1196.098485 | 0.988518 |
| GO:0016477\_cell\_migration | 177 | 0 | 0.000000 | 0.000000 | 1167 | 1111.101515 | 1153.6 | 1196.098485 | 0.988518 |
| GO:0003006\_reproductive\_developmental\_process | 51 | 0 | 0.000000 | 0.000000 | 1177 | 1120.598435 | 1162.78 | 1204.961565 | 0.987918 |
| GO:0006898\_receptor-mediated\_endocytosis | 51 | 0 | 0.000000 | 0.000000 | 1177 | 1120.598435 | 1162.78 | 1204.961565 | 0.987918 |
| GO:0007059\_chromosome\_segregation | 51 | 0 | 0.000000 | 0.000000 | 1177 | 1120.598435 | 1162.78 | 1204.961565 | 0.987918 |
| GO:0016052\_carbohydrate\_catabolic\_process | 51 | 0 | 0.000000 | 0.000000 | 1177 | 1120.598435 | 1162.78 | 1204.961565 | 0.987918 |
| GO:0018212\_peptidyl-tyrosine\_modification | 51 | 0 | 0.000000 | 0.000000 | 1177 | 1120.598435 | 1162.78 | 1204.961565 | 0.987918 |
| GO:0030518\_steroid\_hormone\_receptor\_signaling\_pathway | 51 | 0 | 0.000000 | 0.000000 | 1177 | 1120.598435 | 1162.78 | 1204.961565 | 0.987918 |
| GO:0031279\_regulation\_of\_cyclase\_activity | 51 | 0 | 0.000000 | 0.000000 | 1177 | 1120.598435 | 1162.78 | 1204.961565 | 0.987918 |
| GO:0032147\_activation\_of\_protein\_kinase\_activity | 51 | 0 | 0.000000 | 0.000000 | 1177 | 1120.598435 | 1162.78 | 1204.961565 | 0.987918 |
| GO:0046474\_glycerophospholipid\_biosynthetic\_process | 51 | 0 | 0.000000 | 0.000000 | 1177 | 1120.598435 | 1162.78 | 1204.961565 | 0.987918 |
| GO:0070482\_response\_to\_oxygen\_levels | 51 | 0 | 0.000000 | 0.000000 | 1177 | 1120.598435 | 1162.78 | 1204.961565 | 0.987918 |
| GO:0006413\_translational\_initiation | 46 | 0 | 0.000000 | 0.000000 | 1191 | 1134.550993 | 1176.42 | 1218.289007 | 0.987758 |
| GO:0007202\_activation\_of\_phospholipase\_C\_activity | 46 | 0 | 0.000000 | 0.000000 | 1191 | 1134.550993 | 1176.42 | 1218.289007 | 0.987758 |
| GO:0007588\_excretion | 46 | 0 | 0.000000 | 0.000000 | 1191 | 1134.550993 | 1176.42 | 1218.289007 | 0.987758 |
| GO:0010038\_response\_to\_metal\_ion | 46 | 0 | 0.000000 | 0.000000 | 1191 | 1134.550993 | 1176.42 | 1218.289007 | 0.987758 |
| GO:0010638\_positive\_regulation\_of\_organelle\_organization | 46 | 0 | 0.000000 | 0.000000 | 1191 | 1134.550993 | 1176.42 | 1218.289007 | 0.987758 |
| GO:0010639\_negative\_regulation\_of\_organelle\_organization | 46 | 0 | 0.000000 | 0.000000 | 1191 | 1134.550993 | 1176.42 | 1218.289007 | 0.987758 |
| GO:0010863\_positive\_regulation\_of\_phospholipase\_C\_activity | 46 | 0 | 0.000000 | 0.000000 | 1191 | 1134.550993 | 1176.42 | 1218.289007 | 0.987758 |
| GO:0030335\_positive\_regulation\_of\_cell\_migration | 46 | 0 | 0.000000 | 0.000000 | 1191 | 1134.550993 | 1176.42 | 1218.289007 | 0.987758 |
| GO:0030384\_phosphoinositide\_metabolic\_process | 46 | 0 | 0.000000 | 0.000000 | 1191 | 1134.550993 | 1176.42 | 1218.289007 | 0.987758 |
| GO:0042157\_lipoprotein\_metabolic\_process | 46 | 0 | 0.000000 | 0.000000 | 1191 | 1134.550993 | 1176.42 | 1218.289007 | 0.987758 |
| GO:0042254\_ribosome\_biogenesis | 46 | 0 | 0.000000 | 0.000000 | 1191 | 1134.550993 | 1176.42 | 1218.289007 | 0.987758 |
| GO:0042391\_regulation\_of\_membrane\_potential | 46 | 0 | 0.000000 | 0.000000 | 1191 | 1134.550993 | 1176.42 | 1218.289007 | 0.987758 |
| GO:0046879\_hormone\_secretion | 46 | 0 | 0.000000 | 0.000000 | 1191 | 1134.550993 | 1176.42 | 1218.289007 | 0.987758 |
| GO:0065004\_protein-DNA\_complex\_assembly | 46 | 0 | 0.000000 | 0.000000 | 1191 | 1134.550993 | 1176.42 | 1218.289007 | 0.987758 |
| GO:0002009\_morphogenesis\_of\_an\_epithelium | 29 | 0 | 0.000000 | 0.000000 | 1209 | 1154.719879 | 1195.57 | 1236.420121 | 0.988892 |
| GO:0003007\_heart\_morphogenesis | 29 | 0 | 0.000000 | 0.000000 | 1209 | 1154.719879 | 1195.57 | 1236.420121 | 0.988892 |
| GO:0003018\_vascular\_process\_in\_circulatory\_system | 29 | 0 | 0.000000 | 0.000000 | 1209 | 1154.719879 | 1195.57 | 1236.420121 | 0.988892 |
| GO:0006505\_GPI\_anchor\_metabolic\_process | 29 | 0 | 0.000000 | 0.000000 | 1209 | 1154.719879 | 1195.57 | 1236.420121 | 0.988892 |
| GO:0006506\_GPI\_anchor\_biosynthetic\_process | 29 | 0 | 0.000000 | 0.000000 | 1209 | 1154.719879 | 1195.57 | 1236.420121 | 0.988892 |
| GO:0030278\_regulation\_of\_ossification | 29 | 0 | 0.000000 | 0.000000 | 1209 | 1154.719879 | 1195.57 | 1236.420121 | 0.988892 |
| GO:0032507\_maintenance\_of\_protein\_location\_in\_cell | 29 | 0 | 0.000000 | 0.000000 | 1209 | 1154.719879 | 1195.57 | 1236.420121 | 0.988892 |
| GO:0043112\_receptor\_metabolic\_process | 29 | 0 | 0.000000 | 0.000000 | 1209 | 1154.719879 | 1195.57 | 1236.420121 | 0.988892 |
| GO:0043433\_negative\_regulation\_of\_transcription\_factor\_activity | 29 | 0 | 0.000000 | 0.000000 | 1209 | 1154.719879 | 1195.57 | 1236.420121 | 0.988892 |
| GO:0046425\_regulation\_of\_JAK-STAT\_cascade | 29 | 0 | 0.000000 | 0.000000 | 1209 | 1154.719879 | 1195.57 | 1236.420121 | 0.988892 |
| GO:0048732\_gland\_development | 29 | 0 | 0.000000 | 0.000000 | 1209 | 1154.719879 | 1195.57 | 1236.420121 | 0.988892 |
| GO:0048741\_skeletal\_muscle\_fiber\_development | 29 | 0 | 0.000000 | 0.000000 | 1209 | 1154.719879 | 1195.57 | 1236.420121 | 0.988892 |
| GO:0050714\_positive\_regulation\_of\_protein\_secretion | 29 | 0 | 0.000000 | 0.000000 | 1209 | 1154.719879 | 1195.57 | 1236.420121 | 0.988892 |
| GO:0050731\_positive\_regulation\_of\_peptidyl-tyrosine\_phosphorylation | 29 | 0 | 0.000000 | 0.000000 | 1209 | 1154.719879 | 1195.57 | 1236.420121 | 0.988892 |
| GO:0051648\_vesicle\_localization | 29 | 0 | 0.000000 | 0.000000 | 1209 | 1154.719879 | 1195.57 | 1236.420121 | 0.988892 |
| GO:0051924\_regulation\_of\_calcium\_ion\_transport | 29 | 0 | 0.000000 | 0.000000 | 1209 | 1154.719879 | 1195.57 | 1236.420121 | 0.988892 |
| GO:0060429\_epithelium\_development | 29 | 0 | 0.000000 | 0.000000 | 1209 | 1154.719879 | 1195.57 | 1236.420121 | 0.988892 |
| GO:0090048\_negative\_regulation\_of\_transcription\_regulator\_activity | 29 | 0 | 0.000000 | 0.000000 | 1209 | 1154.719879 | 1195.57 | 1236.420121 | 0.988892 |
| GO:0008285\_negative\_regulation\_of\_cell\_proliferation | 202 | 0 | 0.000000 | 0.000000 | 1210 | 1155.517519 | 1196.27 | 1237.022481 | 0.988653 |
| GO:0043285\_biopolymer\_catabolic\_process | 426 | 0 | 0.000000 | 0.000000 | 1211 | 1156.030974 | 1196.7 | 1237.369026 | 0.988192 |
| GO:0006629\_lipid\_metabolic\_process | 468 | 0 | 0.000000 | 0.000000 | 1213 | 1156.910578 | 1197.51 | 1238.109422 | 0.987230 |
| GO:0050877\_neurological\_system\_process | 468 | 0 | 0.000000 | 0.000000 | 1213 | 1156.910578 | 1197.51 | 1238.109422 | 0.987230 |
| GO:0008610\_lipid\_biosynthetic\_process | 179 | 0 | 0.000000 | 0.000000 | 1214 | 1157.679531 | 1198.23 | 1238.780469 | 0.987010 |
| GO:0009887\_organ\_morphogenesis | 246 | 0 | 0.000000 | 0.000000 | 1215 | 1161.216403 | 1201.23 | 1241.243597 | 0.988667 |
| GO:0000084\_S\_phase\_of\_mitotic\_cell\_cycle | 16 | 0 | 0.000000 | 0.000000 | 1270 | 1218.164478 | 1257.14 | 1296.115522 | 0.989874 |
| GO:0001523\_retinoid\_metabolic\_process | 16 | 0 | 0.000000 | 0.000000 | 1270 | 1218.164478 | 1257.14 | 1296.115522 | 0.989874 |
| GO:0001933\_negative\_regulation\_of\_protein\_amino\_acid\_phosphorylation | 16 | 0 | 0.000000 | 0.000000 | 1270 | 1218.164478 | 1257.14 | 1296.115522 | 0.989874 |
| GO:0002695\_negative\_regulation\_of\_leukocyte\_activation | 16 | 0 | 0.000000 | 0.000000 | 1270 | 1218.164478 | 1257.14 | 1296.115522 | 0.989874 |
| GO:0002699\_positive\_regulation\_of\_immune\_effector\_process | 16 | 0 | 0.000000 | 0.000000 | 1270 | 1218.164478 | 1257.14 | 1296.115522 | 0.989874 |
| GO:0006220\_pyrimidine\_nucleotide\_metabolic\_process | 16 | 0 | 0.000000 | 0.000000 | 1270 | 1218.164478 | 1257.14 | 1296.115522 | 0.989874 |
| GO:0006298\_mismatch\_repair | 16 | 0 | 0.000000 | 0.000000 | 1270 | 1218.164478 | 1257.14 | 1296.115522 | 0.989874 |
| GO:0006471\_protein\_amino\_acid\_ADP-ribosylation | 16 | 0 | 0.000000 | 0.000000 | 1270 | 1218.164478 | 1257.14 | 1296.115522 | 0.989874 |
| GO:0006636\_unsaturated\_fatty\_acid\_biosynthetic\_process | 16 | 0 | 0.000000 | 0.000000 | 1270 | 1218.164478 | 1257.14 | 1296.115522 | 0.989874 |
| GO:0006687\_glycosphingolipid\_metabolic\_process | 16 | 0 | 0.000000 | 0.000000 | 1270 | 1218.164478 | 1257.14 | 1296.115522 | 0.989874 |
| GO:0006695\_cholesterol\_biosynthetic\_process | 16 | 0 | 0.000000 | 0.000000 | 1270 | 1218.164478 | 1257.14 | 1296.115522 | 0.989874 |
| GO:0006721\_terpenoid\_metabolic\_process | 16 | 0 | 0.000000 | 0.000000 | 1270 | 1218.164478 | 1257.14 | 1296.115522 | 0.989874 |
| GO:0006776\_vitamin\_A\_metabolic\_process | 16 | 0 | 0.000000 | 0.000000 | 1270 | 1218.164478 | 1257.14 | 1296.115522 | 0.989874 |
| GO:0006805\_xenobiotic\_metabolic\_process | 16 | 0 | 0.000000 | 0.000000 | 1270 | 1218.164478 | 1257.14 | 1296.115522 | 0.989874 |
| GO:0006891\_intra-Golgi\_vesicle-mediated\_transport | 16 | 0 | 0.000000 | 0.000000 | 1270 | 1218.164478 | 1257.14 | 1296.115522 | 0.989874 |
| GO:0006914\_autophagy | 16 | 0 | 0.000000 | 0.000000 | 1270 | 1218.164478 | 1257.14 | 1296.115522 | 0.989874 |
| GO:0007006\_mitochondrial\_membrane\_organization | 16 | 0 | 0.000000 | 0.000000 | 1270 | 1218.164478 | 1257.14 | 1296.115522 | 0.989874 |
| GO:0009119\_ribonucleoside\_metabolic\_process | 16 | 0 | 0.000000 | 0.000000 | 1270 | 1218.164478 | 1257.14 | 1296.115522 | 0.989874 |
| GO:0009408\_response\_to\_heat | 16 | 0 | 0.000000 | 0.000000 | 1270 | 1218.164478 | 1257.14 | 1296.115522 | 0.989874 |
| GO:0009595\_detection\_of\_biotic\_stimulus | 16 | 0 | 0.000000 | 0.000000 | 1270 | 1218.164478 | 1257.14 | 1296.115522 | 0.989874 |
| GO:0009743\_response\_to\_carbohydrate\_stimulus | 16 | 0 | 0.000000 | 0.000000 | 1270 | 1218.164478 | 1257.14 | 1296.115522 | 0.989874 |
| GO:0010676\_positive\_regulation\_of\_cellular\_carbohydrate\_metabolic\_process | 16 | 0 | 0.000000 | 0.000000 | 1270 | 1218.164478 | 1257.14 | 1296.115522 | 0.989874 |
| GO:0010743\_regulation\_of\_foam\_cell\_differentiation | 16 | 0 | 0.000000 | 0.000000 | 1270 | 1218.164478 | 1257.14 | 1296.115522 | 0.989874 |
| GO:0010906\_regulation\_of\_glucose\_metabolic\_process | 16 | 0 | 0.000000 | 0.000000 | 1270 | 1218.164478 | 1257.14 | 1296.115522 | 0.989874 |
| GO:0010975\_regulation\_of\_neuron\_projection\_development | 16 | 0 | 0.000000 | 0.000000 | 1270 | 1218.164478 | 1257.14 | 1296.115522 | 0.989874 |
| GO:0014070\_response\_to\_organic\_cyclic\_substance | 16 | 0 | 0.000000 | 0.000000 | 1270 | 1218.164478 | 1257.14 | 1296.115522 | 0.989874 |
| GO:0015909\_long-chain\_fatty\_acid\_transport | 16 | 0 | 0.000000 | 0.000000 | 1270 | 1218.164478 | 1257.14 | 1296.115522 | 0.989874 |
| GO:0016101\_diterpenoid\_metabolic\_process | 16 | 0 | 0.000000 | 0.000000 | 1270 | 1218.164478 | 1257.14 | 1296.115522 | 0.989874 |
| GO:0016254\_preassembly\_of\_GPI\_anchor\_in\_ER\_membrane | 16 | 0 | 0.000000 | 0.000000 | 1270 | 1218.164478 | 1257.14 | 1296.115522 | 0.989874 |
| GO:0019319\_hexose\_biosynthetic\_process | 16 | 0 | 0.000000 | 0.000000 | 1270 | 1218.164478 | 1257.14 | 1296.115522 | 0.989874 |
| GO:0030261\_chromosome\_condensation | 16 | 0 | 0.000000 | 0.000000 | 1270 | 1218.164478 | 1257.14 | 1296.115522 | 0.989874 |
| GO:0030855\_epithelial\_cell\_differentiation | 16 | 0 | 0.000000 | 0.000000 | 1270 | 1218.164478 | 1257.14 | 1296.115522 | 0.989874 |
| GO:0031110\_regulation\_of\_microtubule\_polymerization\_or\_depolymerization | 16 | 0 | 0.000000 | 0.000000 | 1270 | 1218.164478 | 1257.14 | 1296.115522 | 0.989874 |
| GO:0032623\_interleukin-2\_production | 16 | 0 | 0.000000 | 0.000000 | 1270 | 1218.164478 | 1257.14 | 1296.115522 | 0.989874 |
| GO:0032640\_tumor\_necrosis\_factor\_production | 16 | 0 | 0.000000 | 0.000000 | 1270 | 1218.164478 | 1257.14 | 1296.115522 | 0.989874 |
| GO:0032649\_regulation\_of\_interferon-gamma\_production | 16 | 0 | 0.000000 | 0.000000 | 1270 | 1218.164478 | 1257.14 | 1296.115522 | 0.989874 |
| GO:0032675\_regulation\_of\_interleukin-6\_production | 16 | 0 | 0.000000 | 0.000000 | 1270 | 1218.164478 | 1257.14 | 1296.115522 | 0.989874 |
| GO:0032680\_regulation\_of\_tumor\_necrosis\_factor\_production | 16 | 0 | 0.000000 | 0.000000 | 1270 | 1218.164478 | 1257.14 | 1296.115522 | 0.989874 |
| GO:0034381\_lipoprotein\_particle\_clearance | 16 | 0 | 0.000000 | 0.000000 | 1270 | 1218.164478 | 1257.14 | 1296.115522 | 0.989874 |
| GO:0034762\_regulation\_of\_transmembrane\_transport | 16 | 0 | 0.000000 | 0.000000 | 1270 | 1218.164478 | 1257.14 | 1296.115522 | 0.989874 |
| GO:0042476\_odontogenesis | 16 | 0 | 0.000000 | 0.000000 | 1270 | 1218.164478 | 1257.14 | 1296.115522 | 0.989874 |
| GO:0044275\_cellular\_carbohydrate\_catabolic\_process | 16 | 0 | 0.000000 | 0.000000 | 1270 | 1218.164478 | 1257.14 | 1296.115522 | 0.989874 |
| GO:0045667\_regulation\_of\_osteoblast\_differentiation | 16 | 0 | 0.000000 | 0.000000 | 1270 | 1218.164478 | 1257.14 | 1296.115522 | 0.989874 |
| GO:0045730\_respiratory\_burst | 16 | 0 | 0.000000 | 0.000000 | 1270 | 1218.164478 | 1257.14 | 1296.115522 | 0.989874 |
| GO:0045807\_positive\_regulation\_of\_endocytosis | 16 | 0 | 0.000000 | 0.000000 | 1270 | 1218.164478 | 1257.14 | 1296.115522 | 0.989874 |
| GO:0045862\_positive\_regulation\_of\_proteolysis | 16 | 0 | 0.000000 | 0.000000 | 1270 | 1218.164478 | 1257.14 | 1296.115522 | 0.989874 |
| GO:0045913\_positive\_regulation\_of\_carbohydrate\_metabolic\_process | 16 | 0 | 0.000000 | 0.000000 | 1270 | 1218.164478 | 1257.14 | 1296.115522 | 0.989874 |
| GO:0045930\_negative\_regulation\_of\_mitotic\_cell\_cycle | 16 | 0 | 0.000000 | 0.000000 | 1270 | 1218.164478 | 1257.14 | 1296.115522 | 0.989874 |
| GO:0046545\_development\_of\_primary\_female\_sexual\_characteristics | 16 | 0 | 0.000000 | 0.000000 | 1270 | 1218.164478 | 1257.14 | 1296.115522 | 0.989874 |
| GO:0046660\_female\_sex\_differentiation | 16 | 0 | 0.000000 | 0.000000 | 1270 | 1218.164478 | 1257.14 | 1296.115522 | 0.989874 |
| GO:0048589\_developmental\_growth | 16 | 0 | 0.000000 | 0.000000 | 1270 | 1218.164478 | 1257.14 | 1296.115522 | 0.989874 |
| GO:0050864\_regulation\_of\_B\_cell\_activation | 16 | 0 | 0.000000 | 0.000000 | 1270 | 1218.164478 | 1257.14 | 1296.115522 | 0.989874 |
| GO:0051146\_striated\_muscle\_cell\_differentiation | 16 | 0 | 0.000000 | 0.000000 | 1270 | 1218.164478 | 1257.14 | 1296.115522 | 0.989874 |
| GO:0051181\_cofactor\_transport | 16 | 0 | 0.000000 | 0.000000 | 1270 | 1218.164478 | 1257.14 | 1296.115522 | 0.989874 |
| GO:0051261\_protein\_depolymerization | 16 | 0 | 0.000000 | 0.000000 | 1270 | 1218.164478 | 1257.14 | 1296.115522 | 0.989874 |
| GO:0006006\_glucose\_metabolic\_process | 53 | 0 | 0.000000 | 0.000000 | 1282 | 1229.331247 | 1267.93 | 1306.528753 | 0.989025 |
| GO:0009617\_response\_to\_bacterium | 53 | 0 | 0.000000 | 0.000000 | 1282 | 1229.331247 | 1267.93 | 1306.528753 | 0.989025 |
| GO:0016485\_protein\_processing | 53 | 0 | 0.000000 | 0.000000 | 1282 | 1229.331247 | 1267.93 | 1306.528753 | 0.989025 |
| GO:0022900\_electron\_transport\_chain | 53 | 0 | 0.000000 | 0.000000 | 1282 | 1229.331247 | 1267.93 | 1306.528753 | 0.989025 |
| GO:0030258\_lipid\_modification | 53 | 0 | 0.000000 | 0.000000 | 1282 | 1229.331247 | 1267.93 | 1306.528753 | 0.989025 |
| GO:0042107\_cytokine\_metabolic\_process | 53 | 0 | 0.000000 | 0.000000 | 1282 | 1229.331247 | 1267.93 | 1306.528753 | 0.989025 |
| GO:0043434\_response\_to\_peptide\_hormone\_stimulus | 53 | 0 | 0.000000 | 0.000000 | 1282 | 1229.331247 | 1267.93 | 1306.528753 | 0.989025 |
| GO:0045786\_negative\_regulation\_of\_cell\_cycle | 53 | 0 | 0.000000 | 0.000000 | 1282 | 1229.331247 | 1267.93 | 1306.528753 | 0.989025 |
| GO:0048015\_phosphoinositide-mediated\_signaling | 53 | 0 | 0.000000 | 0.000000 | 1282 | 1229.331247 | 1267.93 | 1306.528753 | 0.989025 |
| GO:0051047\_positive\_regulation\_of\_secretion | 53 | 0 | 0.000000 | 0.000000 | 1282 | 1229.331247 | 1267.93 | 1306.528753 | 0.989025 |
| GO:0051339\_regulation\_of\_lyase\_activity | 53 | 0 | 0.000000 | 0.000000 | 1282 | 1229.331247 | 1267.93 | 1306.528753 | 0.989025 |
| GO:0060193\_positive\_regulation\_of\_lipase\_activity | 53 | 0 | 0.000000 | 0.000000 | 1282 | 1229.331247 | 1267.93 | 1306.528753 | 0.989025 |
| GO:0006631\_fatty\_acid\_metabolic\_process | 113 | 0 | 0.000000 | 0.000000 | 1283 | 1231.080003 | 1269.57 | 1308.059997 | 0.989532 |
| GO:0000050\_urea\_cycle | 3 | 0 |  |  |  |  |  |  |  |  |
| GO:0000089\_mitotic\_metaphase | 3 | 0 |  |  |  |  |  |  |  |  |
| GO:0000097\_sulfur\_amino\_acid\_biosynthetic\_process | 3 | 0 |  |  |  |  |  |  |  |  |
| GO:0000266\_mitochondrial\_fission | 3 | 0 |  |  |  |  |  |  |  |  |
| GO:0000281\_cytokinesis\_after\_mitosis | 3 | 0 |  |  |  |  |  |  |  |  |
| GO:0000394\_RNA\_splicing\_\_via\_endonucleolytic\_cleavage\_and\_ligation | 3 | 0 |  |  |  |  |  |  |  |  |
| GO:0000463\_maturation\_of\_LSU-rRNA\_from\_tricistronic\_rRNA\_transcript\_(SSU-rRNA\_\_5.8S\_rRNA\_\_LSU-rRNA) | 3 | 0 |  |  |  |  |  |  |  |  |
| GO:0000470\_maturation\_of\_LSU-rRNA | 3 | 0 |  |  |  |  |  |  |  |  |
| GO:0000491\_small\_nucleolar\_ribonucleoprotein\_complex\_assembly | 3 | 0 |  |  |  |  |  |  |  |  |
| GO:0000492\_box\_C\_D\_snoRNP\_assembly | 3 | 0 |  |  |  |  |  |  |  |  |
| GO:0001302\_replicative\_cell\_aging | 3 | 0 |  |  |  |  |  |  |  |  |
| GO:0001510\_RNA\_methylation | 3 | 0 |  |  |  |  |  |  |  |  |
| GO:0001569\_patterning\_of\_blood\_vessels | 3 | 0 |  |  |  |  |  |  |  |  |
| GO:0001573\_ganglioside\_metabolic\_process | 3 | 0 |  |  |  |  |  |  |  |  |
| GO:0001659\_temperature\_homeostasis | 3 | 0 |  |  |  |  |  |  |  |  |
| GO:0001755\_neural\_crest\_cell\_migration | 3 | 0 |  |  |  |  |  |  |  |  |
| GO:0001881\_receptor\_recycling | 3 | 0 |  |  |  |  |  |  |  |  |
| GO:0001895\_retina\_homeostasis | 3 | 0 |  |  |  |  |  |  |  |  |
| GO:0001916\_positive\_regulation\_of\_T\_cell\_mediated\_cytotoxicity | 3 | 0 |  |  |  |  |  |  |  |  |
| GO:0001919\_regulation\_of\_receptor\_recycling | 3 | 0 |  |  |  |  |  |  |  |  |
| GO:0001945\_lymph\_vessel\_development | 3 | 0 |  |  |  |  |  |  |  |  |
| GO:0001946\_lymphangiogenesis | 3 | 0 |  |  |  |  |  |  |  |  |
| GO:0002002\_regulation\_of\_angiotensin\_levels\_in\_blood | 3 | 0 |  |  |  |  |  |  |  |  |
| GO:0002029\_desensitization\_of\_G-protein\_coupled\_receptor\_protein\_signaling\_pathway | 3 | 0 |  |  |  |  |  |  |  |  |
| GO:0002062\_chondrocyte\_differentiation | 3 | 0 |  |  |  |  |  |  |  |  |
| GO:0002076\_osteoblast\_development | 3 | 0 |  |  |  |  |  |  |  |  |
| GO:0002230\_positive\_regulation\_of\_defense\_response\_to\_virus\_by\_host | 3 | 0 |  |  |  |  |  |  |  |  |
| GO:0002244\_hemopoietic\_progenitor\_cell\_differentiation | 3 | 0 |  |  |  |  |  |  |  |  |
| GO:0002246\_healing\_during\_inflammatory\_response | 3 | 0 |  |  |  |  |  |  |  |  |
| GO:0002262\_myeloid\_cell\_homeostasis | 3 | 0 |  |  |  |  |  |  |  |  |
| GO:0002444\_myeloid\_leukocyte\_mediated\_immunity | 3 | 0 |  |  |  |  |  |  |  |  |
| GO:0002446\_neutrophil\_mediated\_immunity | 3 | 0 |  |  |  |  |  |  |  |  |
| GO:0002792\_negative\_regulation\_of\_peptide\_secretion | 3 | 0 |  |  |  |  |  |  |  |  |
| GO:0002825\_regulation\_of\_T-helper\_1\_type\_immune\_response | 3 | 0 |  |  |  |  |  |  |  |  |
| GO:0002828\_regulation\_of\_T-helper\_2\_type\_immune\_response | 3 | 0 |  |  |  |  |  |  |  |  |
| GO:0002903\_negative\_regulation\_of\_B\_cell\_apoptosis | 3 | 0 |  |  |  |  |  |  |  |  |
| GO:0002921\_negative\_regulation\_of\_humoral\_immune\_response | 3 | 0 |  |  |  |  |  |  |  |  |
| GO:0003084\_positive\_regulation\_of\_systemic\_arterial\_blood\_pressure | 3 | 0 |  |  |  |  |  |  |  |  |
| GO:0005980\_glycogen\_catabolic\_process | 3 | 0 |  |  |  |  |  |  |  |  |
| GO:0005984\_disaccharide\_metabolic\_process | 3 | 0 |  |  |  |  |  |  |  |  |
| GO:0006002\_fructose\_6-phosphate\_metabolic\_process | 3 | 0 |  |  |  |  |  |  |  |  |
| GO:0006003\_fructose\_2\_6-bisphosphate\_metabolic\_process | 3 | 0 |  |  |  |  |  |  |  |  |
| GO:0006043\_glucosamine\_catabolic\_process | 3 | 0 |  |  |  |  |  |  |  |  |
| GO:0006071\_glycerol\_metabolic\_process | 3 | 0 |  |  |  |  |  |  |  |  |
| GO:0006102\_isocitrate\_metabolic\_process | 3 | 0 |  |  |  |  |  |  |  |  |
| GO:0006104\_succinyl-CoA\_metabolic\_process | 3 | 0 |  |  |  |  |  |  |  |  |
| GO:0006108\_malate\_metabolic\_process | 3 | 0 |  |  |  |  |  |  |  |  |
| GO:0006123\_mitochondrial\_electron\_transport\_\_cytochrome\_c\_to\_oxygen | 3 | 0 |  |  |  |  |  |  |  |  |
| GO:0006152\_purine\_nucleoside\_catabolic\_process | 3 | 0 |  |  |  |  |  |  |  |  |
| GO:0006167\_AMP\_biosynthetic\_process | 3 | 0 |  |  |  |  |  |  |  |  |
| GO:0006182\_cGMP\_biosynthetic\_process | 3 | 0 |  |  |  |  |  |  |  |  |
| GO:0006269\_DNA\_replication\_\_synthesis\_of\_RNA\_primer | 3 | 0 |  |  |  |  |  |  |  |  |
| GO:0006273\_lagging\_strand\_elongation | 3 | 0 |  |  |  |  |  |  |  |  |
| GO:0006288\_base-excision\_repair\_\_DNA\_ligation | 3 | 0 |  |  |  |  |  |  |  |  |
| GO:0006295\_nucleotide-excision\_repair\_\_DNA\_incision\_\_3'-to\_lesion | 3 | 0 |  |  |  |  |  |  |  |  |
| GO:0006296\_nucleotide-excision\_repair\_\_DNA\_incision\_\_5'-to\_lesion | 3 | 0 |  |  |  |  |  |  |  |  |
| GO:0006384\_transcription\_initiation\_from\_RNA\_polymerase\_III\_promoter | 3 | 0 |  |  |  |  |  |  |  |  |
| GO:0006388\_tRNA\_splicing\_\_via\_endonucleolytic\_cleavage\_and\_ligation | 3 | 0 |  |  |  |  |  |  |  |  |
| GO:0006467\_protein\_thiol-disulfide\_exchange | 3 | 0 |  |  |  |  |  |  |  |  |
| GO:0006546\_glycine\_catabolic\_process | 3 | 0 |  |  |  |  |  |  |  |  |
| GO:0006558\_L-phenylalanine\_metabolic\_process | 3 | 0 |  |  |  |  |  |  |  |  |
| GO:0006559\_L-phenylalanine\_catabolic\_process | 3 | 0 |  |  |  |  |  |  |  |  |
| GO:0006560\_proline\_metabolic\_process | 3 | 0 |  |  |  |  |  |  |  |  |
| GO:0006565\_L-serine\_catabolic\_process | 3 | 0 |  |  |  |  |  |  |  |  |
| GO:0006568\_tryptophan\_metabolic\_process | 3 | 0 |  |  |  |  |  |  |  |  |
| GO:0006573\_valine\_metabolic\_process | 3 | 0 |  |  |  |  |  |  |  |  |
| GO:0006590\_thyroid\_hormone\_generation | 3 | 0 |  |  |  |  |  |  |  |  |
| GO:0006596\_polyamine\_biosynthetic\_process | 3 | 0 |  |  |  |  |  |  |  |  |
| GO:0006621\_protein\_retention\_in\_ER\_lumen | 3 | 0 |  |  |  |  |  |  |  |  |
| GO:0006654\_phosphatidic\_acid\_biosynthetic\_process | 3 | 0 |  |  |  |  |  |  |  |  |
| GO:0006658\_phosphatidylserine\_metabolic\_process | 3 | 0 |  |  |  |  |  |  |  |  |
| GO:0006663\_platelet\_activating\_factor\_biosynthetic\_process | 3 | 0 |  |  |  |  |  |  |  |  |
| GO:0006678\_glucosylceramide\_metabolic\_process | 3 | 0 |  |  |  |  |  |  |  |  |
| GO:0006686\_sphingomyelin\_biosynthetic\_process | 3 | 0 |  |  |  |  |  |  |  |  |
| GO:0006701\_progesterone\_biosynthetic\_process | 3 | 0 |  |  |  |  |  |  |  |  |
| GO:0006704\_glucocorticoid\_biosynthetic\_process | 3 | 0 |  |  |  |  |  |  |  |  |
| GO:0006705\_mineralocorticoid\_biosynthetic\_process | 3 | 0 |  |  |  |  |  |  |  |  |
| GO:0006729\_tetrahydrobiopterin\_biosynthetic\_process | 3 | 0 |  |  |  |  |  |  |  |  |
| GO:0006777\_Mo-molybdopterin\_cofactor\_biosynthetic\_process | 3 | 0 |  |  |  |  |  |  |  |  |
| GO:0006787\_porphyrin\_catabolic\_process | 3 | 0 |  |  |  |  |  |  |  |  |
| GO:0006829\_zinc\_ion\_transport | 3 | 0 |  |  |  |  |  |  |  |  |
| GO:0006853\_carnitine\_shuttle | 3 | 0 |  |  |  |  |  |  |  |  |
| GO:0006863\_purine\_transport | 3 | 0 |  |  |  |  |  |  |  |  |
| GO:0006910\_phagocytosis\_\_recognition | 3 | 0 |  |  |  |  |  |  |  |  |
| GO:0006922\_cleavage\_of\_lamin | 3 | 0 |  |  |  |  |  |  |  |  |
| GO:0006923\_cleavage\_of\_cytoskeletal\_proteins\_during\_apoptosis | 3 | 0 |  |  |  |  |  |  |  |  |
| GO:0006927\_transformed\_cell\_apoptosis | 3 | 0 |  |  |  |  |  |  |  |  |
| GO:0007007\_inner\_mitochondrial\_membrane\_organization | 3 | 0 |  |  |  |  |  |  |  |  |
| GO:0007035\_vacuolar\_acidification | 3 | 0 |  |  |  |  |  |  |  |  |
| GO:0007175\_negative\_regulation\_of\_epidermal\_growth\_factor\_receptor\_activity | 3 | 0 |  |  |  |  |  |  |  |  |
| GO:0007181\_transforming\_growth\_factor\_beta\_receptor\_complex\_assembly | 3 | 0 |  |  |  |  |  |  |  |  |
| GO:0007199\_G-protein\_signaling\_\_coupled\_to\_cGMP\_nucleotide\_second\_messenger | 3 | 0 |  |  |  |  |  |  |  |  |
| GO:0007207\_activation\_of\_phospholipase\_C\_activity\_by\_muscarinic\_acetylcholine\_receptor\_signaling\_pathway | 3 | 0 |  |  |  |  |  |  |  |  |
| GO:0007252\_I-kappaB\_phosphorylation | 3 | 0 |  |  |  |  |  |  |  |  |
| GO:0007339\_binding\_of\_sperm\_to\_zona\_pellucida | 3 | 0 |  |  |  |  |  |  |  |  |
| GO:0007352\_zygotic\_determination\_of\_dorsal\_ventral\_axis | 3 | 0 |  |  |  |  |  |  |  |  |
| GO:0007439\_ectodermal\_gut\_development | 3 | 0 |  |  |  |  |  |  |  |  |
| GO:0007500\_mesodermal\_cell\_fate\_determination | 3 | 0 |  |  |  |  |  |  |  |  |
| GO:0007512\_adult\_heart\_development | 3 | 0 |  |  |  |  |  |  |  |  |
| GO:0008053\_mitochondrial\_fusion | 3 | 0 |  |  |  |  |  |  |  |  |
| GO:0008291\_acetylcholine\_metabolic\_process | 3 | 0 |  |  |  |  |  |  |  |  |
| GO:0008334\_histone\_mRNA\_metabolic\_process | 3 | 0 |  |  |  |  |  |  |  |  |
| GO:0008617\_guanosine\_metabolic\_process | 3 | 0 |  |  |  |  |  |  |  |  |
| GO:0008628\_induction\_of\_apoptosis\_by\_hormones | 3 | 0 |  |  |  |  |  |  |  |  |
| GO:0008631\_induction\_of\_apoptosis\_by\_oxidative\_stress | 3 | 0 |  |  |  |  |  |  |  |  |
| GO:0009070\_serine\_family\_amino\_acid\_biosynthetic\_process | 3 | 0 |  |  |  |  |  |  |  |  |
| GO:0009125\_nucleoside\_monophosphate\_catabolic\_process | 3 | 0 |  |  |  |  |  |  |  |  |
| GO:0009132\_nucleoside\_diphosphate\_metabolic\_process | 3 | 0 |  |  |  |  |  |  |  |  |
| GO:0009155\_purine\_deoxyribonucleotide\_catabolic\_process | 3 | 0 |  |  |  |  |  |  |  |  |
| GO:0009163\_nucleoside\_biosynthetic\_process | 3 | 0 |  |  |  |  |  |  |  |  |
| GO:0009185\_ribonucleoside\_diphosphate\_metabolic\_process | 3 | 0 |  |  |  |  |  |  |  |  |
| GO:0009226\_nucleotide-sugar\_biosynthetic\_process | 3 | 0 |  |  |  |  |  |  |  |  |
| GO:0009301\_snRNA\_transcription | 3 | 0 |  |  |  |  |  |  |  |  |
| GO:0009620\_response\_to\_fungus | 3 | 0 |  |  |  |  |  |  |  |  |
| GO:0009791\_post-embryonic\_development | 3 | 0 |  |  |  |  |  |  |  |  |
| GO:0010002\_cardioblast\_differentiation | 3 | 0 |  |  |  |  |  |  |  |  |
| GO:0010039\_response\_to\_iron\_ion | 3 | 0 |  |  |  |  |  |  |  |  |
| GO:0010332\_response\_to\_gamma\_radiation | 3 | 0 |  |  |  |  |  |  |  |  |
| GO:0010457\_centriole-centriole\_cohesion | 3 | 0 |  |  |  |  |  |  |  |  |
| GO:0010560\_positive\_regulation\_of\_glycoprotein\_biosynthetic\_process | 3 | 0 |  |  |  |  |  |  |  |  |
| GO:0010572\_positive\_regulation\_of\_platelet\_activation | 3 | 0 |  |  |  |  |  |  |  |  |
| GO:0010656\_negative\_regulation\_of\_muscle\_cell\_apoptosis | 3 | 0 |  |  |  |  |  |  |  |  |
| GO:0010661\_positive\_regulation\_of\_muscle\_cell\_apoptosis | 3 | 0 |  |  |  |  |  |  |  |  |
| GO:0010677\_negative\_regulation\_of\_cellular\_carbohydrate\_metabolic\_process | 3 | 0 |  |  |  |  |  |  |  |  |
| GO:0010713\_negative\_regulation\_of\_collagen\_metabolic\_process | 3 | 0 |  |  |  |  |  |  |  |  |
| GO:0010800\_positive\_regulation\_of\_peptidyl-threonine\_phosphorylation | 3 | 0 |  |  |  |  |  |  |  |  |
| GO:0010824\_regulation\_of\_centrosome\_duplication | 3 | 0 |  |  |  |  |  |  |  |  |
| GO:0010834\_telomere\_maintenance\_via\_telomere\_shortening | 3 | 0 |  |  |  |  |  |  |  |  |
| GO:0010847\_regulation\_of\_chromatin\_assembly | 3 | 0 |  |  |  |  |  |  |  |  |
| GO:0010880\_regulation\_of\_release\_of\_sequestered\_calcium\_ion\_into\_cytosol\_by\_sarcoplasmic\_reticulum | 3 | 0 |  |  |  |  |  |  |  |  |
| GO:0010890\_positive\_regulation\_of\_sequestering\_of\_triglyceride | 3 | 0 |  |  |  |  |  |  |  |  |
| GO:0010894\_negative\_regulation\_of\_steroid\_biosynthetic\_process | 3 | 0 |  |  |  |  |  |  |  |  |
| GO:0010903\_negative\_regulation\_of\_very-low-density\_lipoprotein\_particle\_remodeling | 3 | 0 |  |  |  |  |  |  |  |  |
| GO:0010922\_positive\_regulation\_of\_phosphatase\_activity | 3 | 0 |  |  |  |  |  |  |  |  |
| GO:0010939\_regulation\_of\_necrotic\_cell\_death | 3 | 0 |  |  |  |  |  |  |  |  |
| GO:0010940\_positive\_regulation\_of\_necrotic\_cell\_death | 3 | 0 |  |  |  |  |  |  |  |  |
| GO:0010955\_negative\_regulation\_of\_protein\_maturation\_by\_peptide\_bond\_cleavage | 3 | 0 |  |  |  |  |  |  |  |  |
| GO:0014805\_smooth\_muscle\_adaptation | 3 | 0 |  |  |  |  |  |  |  |  |
| GO:0014808\_release\_of\_sequestered\_calcium\_ion\_into\_cytosol\_by\_sarcoplasmic\_reticulum | 3 | 0 |  |  |  |  |  |  |  |  |
| GO:0014912\_negative\_regulation\_of\_smooth\_muscle\_cell\_migration | 3 | 0 |  |  |  |  |  |  |  |  |
| GO:0015014\_heparan\_sulfate\_proteoglycan\_biosynthetic\_process\_\_polysaccharide\_chain\_biosynthetic\_process | 3 | 0 |  |  |  |  |  |  |  |  |
| GO:0015015\_heparan\_sulfate\_proteoglycan\_biosynthetic\_process\_\_enzymatic\_modification | 3 | 0 |  |  |  |  |  |  |  |  |
| GO:0015670\_carbon\_dioxide\_transport | 3 | 0 |  |  |  |  |  |  |  |  |
| GO:0015721\_bile\_acid\_and\_bile\_salt\_transport | 3 | 0 |  |  |  |  |  |  |  |  |
| GO:0015802\_basic\_amino\_acid\_transport | 3 | 0 |  |  |  |  |  |  |  |  |
| GO:0015811\_L-cystine\_transport | 3 | 0 |  |  |  |  |  |  |  |  |
| GO:0015840\_urea\_transport | 3 | 0 |  |  |  |  |  |  |  |  |
| GO:0015889\_cobalamin\_transport | 3 | 0 |  |  |  |  |  |  |  |  |
| GO:0015917\_aminophospholipid\_transport | 3 | 0 |  |  |  |  |  |  |  |  |
| GO:0016056\_rhodopsin\_mediated\_signaling\_pathway | 3 | 0 |  |  |  |  |  |  |  |  |
| GO:0016081\_synaptic\_vesicle\_docking\_during\_exocytosis | 3 | 0 |  |  |  |  |  |  |  |  |
| GO:0016137\_glycoside\_metabolic\_process | 3 | 0 |  |  |  |  |  |  |  |  |
| GO:0016139\_glycoside\_catabolic\_process | 3 | 0 |  |  |  |  |  |  |  |  |
| GO:0016553\_base\_conversion\_or\_substitution\_editing | 3 | 0 |  |  |  |  |  |  |  |  |
| GO:0016572\_histone\_phosphorylation | 3 | 0 |  |  |  |  |  |  |  |  |
| GO:0016576\_histone\_dephosphorylation | 3 | 0 |  |  |  |  |  |  |  |  |
| GO:0016577\_histone\_demethylation | 3 | 0 |  |  |  |  |  |  |  |  |
| GO:0016584\_nucleosome\_positioning | 3 | 0 |  |  |  |  |  |  |  |  |
| GO:0018076\_N-terminal\_peptidyl-lysine\_acetylation | 3 | 0 |  |  |  |  |  |  |  |  |
| GO:0018094\_protein\_polyglycylation | 3 | 0 |  |  |  |  |  |  |  |  |
| GO:0018103\_protein\_amino\_acid\_C-linked\_glycosylation | 3 | 0 |  |  |  |  |  |  |  |  |
| GO:0018211\_peptidyl-tryptophan\_modification | 3 | 0 |  |  |  |  |  |  |  |  |
| GO:0018242\_protein\_amino\_acid\_O-linked\_glycosylation\_via\_serine | 3 | 0 |  |  |  |  |  |  |  |  |
| GO:0018243\_protein\_amino\_acid\_O-linked\_glycosylation\_via\_threonine | 3 | 0 |  |  |  |  |  |  |  |  |
| GO:0018317\_protein\_amino\_acid\_C-linked\_glycosylation\_via\_tryptophan | 3 | 0 |  |  |  |  |  |  |  |  |
| GO:0018343\_protein\_farnesylation | 3 | 0 |  |  |  |  |  |  |  |  |
| GO:0018344\_protein\_geranylgeranylation | 3 | 0 |  |  |  |  |  |  |  |  |
| GO:0018348\_protein\_amino\_acid\_geranylgeranylation | 3 | 0 |  |  |  |  |  |  |  |  |
| GO:0018394\_peptidyl-lysine\_acetylation | 3 | 0 |  |  |  |  |  |  |  |  |
| GO:0018406\_protein\_amino\_acid\_C-linked\_glycosylation\_via\_2'-alpha-mannosyl-L-tryptophan | 3 | 0 |  |  |  |  |  |  |  |  |
| GO:0019063\_virion\_penetration\_into\_host\_cell | 3 | 0 |  |  |  |  |  |  |  |  |
| GO:0019067\_viral\_assembly\_\_maturation\_\_egress\_\_and\_release | 3 | 0 |  |  |  |  |  |  |  |  |
| GO:0019276\_UDP-N-acetylgalactosamine\_metabolic\_process | 3 | 0 |  |  |  |  |  |  |  |  |
| GO:0019377\_glycolipid\_catabolic\_process | 3 | 0 |  |  |  |  |  |  |  |  |
| GO:0019627\_urea\_metabolic\_process | 3 | 0 |  |  |  |  |  |  |  |  |
| GO:0019720\_Mo-molybdopterin\_cofactor\_metabolic\_process | 3 | 0 |  |  |  |  |  |  |  |  |
| GO:0019794\_nonprotein\_amino\_acid\_metabolic\_process | 3 | 0 |  |  |  |  |  |  |  |  |
| GO:0019859\_thymine\_metabolic\_process | 3 | 0 |  |  |  |  |  |  |  |  |
| GO:0021516\_dorsal\_spinal\_cord\_development | 3 | 0 |  |  |  |  |  |  |  |  |
| GO:0021543\_pallium\_development | 3 | 0 |  |  |  |  |  |  |  |  |
| GO:0021575\_hindbrain\_morphogenesis | 3 | 0 |  |  |  |  |  |  |  |  |
| GO:0021885\_forebrain\_cell\_migration | 3 | 0 |  |  |  |  |  |  |  |  |
| GO:0021952\_central\_nervous\_system\_projection\_neuron\_axonogenesis | 3 | 0 |  |  |  |  |  |  |  |  |
| GO:0021955\_central\_nervous\_system\_neuron\_axonogenesis | 3 | 0 |  |  |  |  |  |  |  |  |
| GO:0021983\_pituitary\_gland\_development | 3 | 0 |  |  |  |  |  |  |  |  |
| GO:0021987\_cerebral\_cortex\_development | 3 | 0 |  |  |  |  |  |  |  |  |
| GO:0022029\_telencephalon\_cell\_migration | 3 | 0 |  |  |  |  |  |  |  |  |
| GO:0022401\_adaptation\_of\_signaling\_pathway | 3 | 0 |  |  |  |  |  |  |  |  |
| GO:0022601\_menstrual\_cycle\_phase | 3 | 0 |  |  |  |  |  |  |  |  |
| GO:0022617\_extracellular\_matrix\_disassembly | 3 | 0 |  |  |  |  |  |  |  |  |
| GO:0030091\_protein\_repair | 3 | 0 |  |  |  |  |  |  |  |  |
| GO:0030157\_pancreatic\_juice\_secretion | 3 | 0 |  |  |  |  |  |  |  |  |
| GO:0030241\_muscle\_thick\_filament\_assembly | 3 | 0 |  |  |  |  |  |  |  |  |
| GO:0030263\_apoptotic\_chromosome\_condensation | 3 | 0 |  |  |  |  |  |  |  |  |
| GO:0030277\_maintenance\_of\_gastrointestinal\_epithelium | 3 | 0 |  |  |  |  |  |  |  |  |
| GO:0030309\_poly-N-acetyllactosamine\_metabolic\_process | 3 | 0 |  |  |  |  |  |  |  |  |
| GO:0030325\_adrenal\_gland\_development | 3 | 0 |  |  |  |  |  |  |  |  |
| GO:0030388\_fructose\_1\_6-bisphosphate\_metabolic\_process | 3 | 0 |  |  |  |  |  |  |  |  |
| GO:0030422\_RNA\_interference\_\_production\_of\_siRNA | 3 | 0 |  |  |  |  |  |  |  |  |
| GO:0030423\_RNA\_interference\_\_targeting\_of\_mRNA\_for\_destruction | 3 | 0 |  |  |  |  |  |  |  |  |
| GO:0030449\_regulation\_of\_complement\_activation | 3 | 0 |  |  |  |  |  |  |  |  |
| GO:0030502\_negative\_regulation\_of\_bone\_mineralization | 3 | 0 |  |  |  |  |  |  |  |  |
| GO:0030643\_cellular\_phosphate\_ion\_homeostasis | 3 | 0 |  |  |  |  |  |  |  |  |
| GO:0030656\_regulation\_of\_vitamin\_metabolic\_process | 3 | 0 |  |  |  |  |  |  |  |  |
| GO:0030800\_negative\_regulation\_of\_cyclic\_nucleotide\_metabolic\_process | 3 | 0 |  |  |  |  |  |  |  |  |
| GO:0030803\_negative\_regulation\_of\_cyclic\_nucleotide\_biosynthetic\_process | 3 | 0 |  |  |  |  |  |  |  |  |
| GO:0030809\_negative\_regulation\_of\_nucleotide\_biosynthetic\_process | 3 | 0 |  |  |  |  |  |  |  |  |
| GO:0030823\_regulation\_of\_cGMP\_metabolic\_process | 3 | 0 |  |  |  |  |  |  |  |  |
| GO:0030826\_regulation\_of\_cGMP\_biosynthetic\_process | 3 | 0 |  |  |  |  |  |  |  |  |
| GO:0030850\_prostate\_gland\_development | 3 | 0 |  |  |  |  |  |  |  |  |
| GO:0030852\_regulation\_of\_granulocyte\_differentiation | 3 | 0 |  |  |  |  |  |  |  |  |
| GO:0030853\_negative\_regulation\_of\_granulocyte\_differentiation | 3 | 0 |  |  |  |  |  |  |  |  |
| GO:0030856\_regulation\_of\_epithelial\_cell\_differentiation | 3 | 0 |  |  |  |  |  |  |  |  |
| GO:0030947\_regulation\_of\_vascular\_endothelial\_growth\_factor\_receptor\_signaling\_pathway | 3 | 0 |  |  |  |  |  |  |  |  |
| GO:0031033\_myosin\_filament\_assembly\_or\_disassembly | 3 | 0 |  |  |  |  |  |  |  |  |
| GO:0031034\_myosin\_filament\_assembly | 3 | 0 |  |  |  |  |  |  |  |  |
| GO:0031054\_pre-microRNA\_processing | 3 | 0 |  |  |  |  |  |  |  |  |
| GO:0031055\_chromatin\_remodeling\_at\_centromere | 3 | 0 |  |  |  |  |  |  |  |  |
| GO:0031060\_regulation\_of\_histone\_methylation | 3 | 0 |  |  |  |  |  |  |  |  |
| GO:0031102\_neuron\_projection\_regeneration | 3 | 0 |  |  |  |  |  |  |  |  |
| GO:0031103\_axon\_regeneration | 3 | 0 |  |  |  |  |  |  |  |  |
| GO:0031445\_regulation\_of\_heterochromatin\_formation | 3 | 0 |  |  |  |  |  |  |  |  |
| GO:0031648\_protein\_destabilization | 3 | 0 |  |  |  |  |  |  |  |  |
| GO:0031657\_regulation\_of\_cyclin-dependent\_protein\_kinase\_activity\_during\_G1\_S | 3 | 0 |  |  |  |  |  |  |  |  |
| GO:0031659\_positive\_regulation\_of\_cyclin-dependent\_protein\_kinase\_activity\_during\_G1\_S | 3 | 0 |  |  |  |  |  |  |  |  |
| GO:0031935\_regulation\_of\_chromatin\_silencing | 3 | 0 |  |  |  |  |  |  |  |  |
| GO:0031953\_negative\_regulation\_of\_protein\_amino\_acid\_autophosphorylation | 3 | 0 |  |  |  |  |  |  |  |  |
| GO:0032007\_negative\_regulation\_of\_TOR\_signaling\_pathway | 3 | 0 |  |  |  |  |  |  |  |  |
| GO:0032042\_mitochondrial\_DNA\_metabolic\_process | 3 | 0 |  |  |  |  |  |  |  |  |
| GO:0032097\_positive\_regulation\_of\_response\_to\_food | 3 | 0 |  |  |  |  |  |  |  |  |
| GO:0032100\_positive\_regulation\_of\_appetite | 3 | 0 |  |  |  |  |  |  |  |  |
| GO:0032105\_negative\_regulation\_of\_response\_to\_extracellular\_stimulus | 3 | 0 |  |  |  |  |  |  |  |  |
| GO:0032108\_negative\_regulation\_of\_response\_to\_nutrient\_levels | 3 | 0 |  |  |  |  |  |  |  |  |
| GO:0032203\_telomere\_formation\_via\_telomerase | 3 | 0 |  |  |  |  |  |  |  |  |
| GO:0032206\_positive\_regulation\_of\_telomere\_maintenance | 3 | 0 |  |  |  |  |  |  |  |  |
| GO:0032232\_negative\_regulation\_of\_actin\_filament\_bundle\_formation | 3 | 0 |  |  |  |  |  |  |  |  |
| GO:0032234\_regulation\_of\_calcium\_ion\_transport\_via\_store-operated\_calcium\_channel\_activity | 3 | 0 |  |  |  |  |  |  |  |  |
| GO:0032278\_positive\_regulation\_of\_gonadotropin\_secretion | 3 | 0 |  |  |  |  |  |  |  |  |
| GO:0032324\_molybdopterin\_cofactor\_biosynthetic\_process | 3 | 0 |  |  |  |  |  |  |  |  |
| GO:0032342\_aldosterone\_biosynthetic\_process | 3 | 0 |  |  |  |  |  |  |  |  |
| GO:0032352\_positive\_regulation\_of\_hormone\_metabolic\_process | 3 | 0 |  |  |  |  |  |  |  |  |
| GO:0032415\_regulation\_of\_sodium:hydrogen\_antiporter\_activity | 3 | 0 |  |  |  |  |  |  |  |  |
| GO:0032469\_endoplasmic\_reticulum\_calcium\_ion\_homeostasis | 3 | 0 |  |  |  |  |  |  |  |  |
| GO:0032494\_response\_to\_peptidoglycan | 3 | 0 |  |  |  |  |  |  |  |  |
| GO:0032497\_detection\_of\_lipopolysaccharide | 3 | 0 |  |  |  |  |  |  |  |  |
| GO:0032509\_endosome\_transport\_via\_multivesicular\_body\_sorting\_pathway | 3 | 0 |  |  |  |  |  |  |  |  |
| GO:0032515\_negative\_regulation\_of\_phosphoprotein\_phosphatase\_activity | 3 | 0 |  |  |  |  |  |  |  |  |
| GO:0032568\_general\_transcription\_from\_RNA\_polymerase\_II\_promoter | 3 | 0 |  |  |  |  |  |  |  |  |
| GO:0032604\_granulocyte\_macrophage\_colony-stimulating\_factor\_production | 3 | 0 |  |  |  |  |  |  |  |  |
| GO:0032616\_interleukin-13\_production | 3 | 0 |  |  |  |  |  |  |  |  |
| GO:0032641\_lymphotoxin\_A\_production | 3 | 0 |  |  |  |  |  |  |  |  |
| GO:0032651\_regulation\_of\_interleukin-1\_beta\_production | 3 | 0 |  |  |  |  |  |  |  |  |
| GO:0032695\_negative\_regulation\_of\_interleukin-12\_production | 3 | 0 |  |  |  |  |  |  |  |  |
| GO:0032717\_negative\_regulation\_of\_interleukin-8\_production | 3 | 0 |  |  |  |  |  |  |  |  |
| GO:0032729\_positive\_regulation\_of\_interferon-gamma\_production | 3 | 0 |  |  |  |  |  |  |  |  |
| GO:0032735\_positive\_regulation\_of\_interleukin-12\_production | 3 | 0 |  |  |  |  |  |  |  |  |
| GO:0032757\_positive\_regulation\_of\_interleukin-8\_production | 3 | 0 |  |  |  |  |  |  |  |  |
| GO:0032760\_positive\_regulation\_of\_tumor\_necrosis\_factor\_production | 3 | 0 |  |  |  |  |  |  |  |  |
| GO:0032855\_positive\_regulation\_of\_Rac\_GTPase\_activity | 3 | 0 |  |  |  |  |  |  |  |  |
| GO:0032872\_regulation\_of\_stress-activated\_MAPK\_cascade | 3 | 0 |  |  |  |  |  |  |  |  |
| GO:0032874\_positive\_regulation\_of\_stress-activated\_MAPK\_cascade | 3 | 0 |  |  |  |  |  |  |  |  |
| GO:0032905\_transforming\_growth\_factor-beta1\_production | 3 | 0 |  |  |  |  |  |  |  |  |
| GO:0032908\_regulation\_of\_transforming\_growth\_factor-beta1\_production | 3 | 0 |  |  |  |  |  |  |  |  |
| GO:0032922\_circadian\_regulation\_of\_gene\_expression | 3 | 0 |  |  |  |  |  |  |  |  |
| GO:0032927\_positive\_regulation\_of\_activin\_receptor\_signaling\_pathway | 3 | 0 |  |  |  |  |  |  |  |  |
| GO:0033015\_tetrapyrrole\_catabolic\_process | 3 | 0 |  |  |  |  |  |  |  |  |
| GO:0033081\_regulation\_of\_T\_cell\_differentiation\_in\_the\_thymus | 3 | 0 |  |  |  |  |  |  |  |  |
| GO:0033143\_regulation\_of\_steroid\_hormone\_receptor\_signaling\_pathway | 3 | 0 |  |  |  |  |  |  |  |  |
| GO:0033151\_V(D)J\_recombination | 3 | 0 |  |  |  |  |  |  |  |  |
| GO:0033198\_response\_to\_ATP | 3 | 0 |  |  |  |  |  |  |  |  |
| GO:0033555\_multicellular\_organismal\_response\_to\_stress | 3 | 0 |  |  |  |  |  |  |  |  |
| GO:0033865\_nucleoside\_bisphosphate\_metabolic\_process | 3 | 0 |  |  |  |  |  |  |  |  |
| GO:0034067\_protein\_localization\_in\_Golgi\_apparatus | 3 | 0 |  |  |  |  |  |  |  |  |
| GO:0034086\_maintenance\_of\_sister\_chromatid\_cohesion | 3 | 0 |  |  |  |  |  |  |  |  |
| GO:0034088\_maintenance\_of\_mitotic\_sister\_chromatid\_cohesion | 3 | 0 |  |  |  |  |  |  |  |  |
| GO:0034393\_positive\_regulation\_of\_smooth\_muscle\_cell\_apoptosis | 3 | 0 |  |  |  |  |  |  |  |  |
| GO:0034442\_regulation\_of\_lipoprotein\_oxidation | 3 | 0 |  |  |  |  |  |  |  |  |
| GO:0034443\_negative\_regulation\_of\_lipoprotein\_oxidation | 3 | 0 |  |  |  |  |  |  |  |  |
| GO:0034453\_microtubule\_anchoring | 3 | 0 |  |  |  |  |  |  |  |  |
| GO:0034505\_tooth\_mineralization | 3 | 0 |  |  |  |  |  |  |  |  |
| GO:0034629\_cellular\_protein\_complex\_localization | 3 | 0 |  |  |  |  |  |  |  |  |
| GO:0034653\_retinoic\_acid\_catabolic\_process | 3 | 0 |  |  |  |  |  |  |  |  |
| GO:0034661\_ncRNA\_catabolic\_process | 3 | 0 |  |  |  |  |  |  |  |  |
| GO:0034776\_response\_to\_histamine | 3 | 0 |  |  |  |  |  |  |  |  |
| GO:0035019\_somatic\_stem\_cell\_maintenance | 3 | 0 |  |  |  |  |  |  |  |  |
| GO:0035020\_regulation\_of\_Rac\_protein\_signal\_transduction | 3 | 0 |  |  |  |  |  |  |  |  |
| GO:0035024\_negative\_regulation\_of\_Rho\_protein\_signal\_transduction | 3 | 0 |  |  |  |  |  |  |  |  |
| GO:0035036\_sperm-egg\_recognition | 3 | 0 |  |  |  |  |  |  |  |  |
| GO:0035067\_negative\_regulation\_of\_histone\_acetylation | 3 | 0 |  |  |  |  |  |  |  |  |
| GO:0035081\_induction\_of\_programmed\_cell\_death\_by\_hormones | 3 | 0 |  |  |  |  |  |  |  |  |
| GO:0035092\_sperm\_chromatin\_condensation | 3 | 0 |  |  |  |  |  |  |  |  |
| GO:0035115\_embryonic\_forelimb\_morphogenesis | 3 | 0 |  |  |  |  |  |  |  |  |
| GO:0035136\_forelimb\_morphogenesis | 3 | 0 |  |  |  |  |  |  |  |  |
| GO:0035246\_peptidyl-arginine\_N-methylation | 3 | 0 |  |  |  |  |  |  |  |  |
| GO:0035268\_protein\_amino\_acid\_mannosylation | 3 | 0 |  |  |  |  |  |  |  |  |
| GO:0035269\_protein\_amino\_acid\_O-linked\_mannosylation | 3 | 0 |  |  |  |  |  |  |  |  |
| GO:0035306\_positive\_regulation\_of\_dephosphorylation | 3 | 0 |  |  |  |  |  |  |  |  |
| GO:0035307\_positive\_regulation\_of\_protein\_amino\_acid\_dephosphorylation | 3 | 0 |  |  |  |  |  |  |  |  |
| GO:0035315\_hair\_cell\_differentiation | 3 | 0 |  |  |  |  |  |  |  |  |
| GO:0040001\_establishment\_of\_mitotic\_spindle\_localization | 3 | 0 |  |  |  |  |  |  |  |  |
| GO:0042053\_regulation\_of\_dopamine\_metabolic\_process | 3 | 0 |  |  |  |  |  |  |  |  |
| GO:0042059\_negative\_regulation\_of\_epidermal\_growth\_factor\_receptor\_signaling\_pathway | 3 | 0 |  |  |  |  |  |  |  |  |
| GO:0042069\_regulation\_of\_catecholamine\_metabolic\_process | 3 | 0 |  |  |  |  |  |  |  |  |
| GO:0042090\_interleukin-12\_biosynthetic\_process | 3 | 0 |  |  |  |  |  |  |  |  |
| GO:0042109\_lymphotoxin\_A\_biosynthetic\_process | 3 | 0 |  |  |  |  |  |  |  |  |
| GO:0042160\_lipoprotein\_modification | 3 | 0 |  |  |  |  |  |  |  |  |
| GO:0042161\_lipoprotein\_oxidation | 3 | 0 |  |  |  |  |  |  |  |  |
| GO:0042246\_tissue\_regeneration | 3 | 0 |  |  |  |  |  |  |  |  |
| GO:0042253\_granulocyte\_macrophage\_colony-stimulating\_factor\_biosynthetic\_process | 3 | 0 |  |  |  |  |  |  |  |  |
| GO:0042271\_susceptibility\_to\_natural\_killer\_cell\_mediated\_cytotoxicity | 3 | 0 |  |  |  |  |  |  |  |  |
| GO:0042368\_vitamin\_D\_biosynthetic\_process | 3 | 0 |  |  |  |  |  |  |  |  |
| GO:0042416\_dopamine\_biosynthetic\_process | 3 | 0 |  |  |  |  |  |  |  |  |
| GO:0042451\_purine\_nucleoside\_biosynthetic\_process | 3 | 0 |  |  |  |  |  |  |  |  |
| GO:0042455\_ribonucleoside\_biosynthetic\_process | 3 | 0 |  |  |  |  |  |  |  |  |
| GO:0042574\_retinal\_metabolic\_process | 3 | 0 |  |  |  |  |  |  |  |  |
| GO:0042753\_positive\_regulation\_of\_circadian\_rhythm | 3 | 0 |  |  |  |  |  |  |  |  |
| GO:0042762\_regulation\_of\_sulfur\_metabolic\_process | 3 | 0 |  |  |  |  |  |  |  |  |
| GO:0042886\_amide\_transport | 3 | 0 |  |  |  |  |  |  |  |  |
| GO:0042940\_D-amino\_acid\_transport | 3 | 0 |  |  |  |  |  |  |  |  |
| GO:0042953\_lipoprotein\_transport | 3 | 0 |  |  |  |  |  |  |  |  |
| GO:0042977\_activation\_of\_JAK2\_kinase\_activity | 3 | 0 |  |  |  |  |  |  |  |  |
| GO:0042983\_amyloid\_precursor\_protein\_biosynthetic\_process | 3 | 0 |  |  |  |  |  |  |  |  |
| GO:0042984\_regulation\_of\_amyloid\_precursor\_protein\_biosynthetic\_process | 3 | 0 |  |  |  |  |  |  |  |  |
| GO:0043001\_Golgi\_to\_plasma\_membrane\_protein\_transport | 3 | 0 |  |  |  |  |  |  |  |  |
| GO:0043089\_positive\_regulation\_of\_Cdc42\_GTPase\_activity | 3 | 0 |  |  |  |  |  |  |  |  |
| GO:0043090\_amino\_acid\_import | 3 | 0 |  |  |  |  |  |  |  |  |
| GO:0043092\_L-amino\_acid\_import | 3 | 0 |  |  |  |  |  |  |  |  |
| GO:0043248\_proteasome\_assembly | 3 | 0 |  |  |  |  |  |  |  |  |
| GO:0043288\_apocarotenoid\_metabolic\_process | 3 | 0 |  |  |  |  |  |  |  |  |
| GO:0043461\_proton-transporting\_ATP\_synthase\_complex\_assembly | 3 | 0 |  |  |  |  |  |  |  |  |
| GO:0043489\_RNA\_stabilization | 3 | 0 |  |  |  |  |  |  |  |  |
| GO:0043570\_maintenance\_of\_DNA\_repeat\_elements | 3 | 0 |  |  |  |  |  |  |  |  |
| GO:0043604\_amide\_biosynthetic\_process | 3 | 0 |  |  |  |  |  |  |  |  |
| GO:0043618\_regulation\_of\_transcription\_from\_RNA\_polymerase\_II\_promoter\_in\_response\_to\_stress | 3 | 0 |  |  |  |  |  |  |  |  |
| GO:0043620\_regulation\_of\_transcription\_in\_response\_to\_stress | 3 | 0 |  |  |  |  |  |  |  |  |
| GO:0043902\_positive\_regulation\_of\_multi-organism\_process | 3 | 0 |  |  |  |  |  |  |  |  |
| GO:0044003\_modification\_by\_symbiont\_of\_host\_morphology\_or\_physiology | 3 | 0 |  |  |  |  |  |  |  |  |
| GO:0044240\_multicellular\_organismal\_lipid\_catabolic\_process | 3 | 0 |  |  |  |  |  |  |  |  |
| GO:0044252\_negative\_regulation\_of\_multicellular\_organismal\_metabolic\_process | 3 | 0 |  |  |  |  |  |  |  |  |
| GO:0045007\_depurination | 3 | 0 |  |  |  |  |  |  |  |  |
| GO:0045010\_actin\_nucleation | 3 | 0 |  |  |  |  |  |  |  |  |
| GO:0045063\_T-helper\_1\_cell\_differentiation | 3 | 0 |  |  |  |  |  |  |  |  |
| GO:0045064\_T-helper\_2\_cell\_differentiation | 3 | 0 |  |  |  |  |  |  |  |  |
| GO:0045070\_positive\_regulation\_of\_viral\_genome\_replication | 3 | 0 |  |  |  |  |  |  |  |  |
| GO:0045075\_regulation\_of\_interleukin-12\_biosynthetic\_process | 3 | 0 |  |  |  |  |  |  |  |  |
| GO:0045079\_negative\_regulation\_of\_chemokine\_biosynthetic\_process | 3 | 0 |  |  |  |  |  |  |  |  |
| GO:0045080\_positive\_regulation\_of\_chemokine\_biosynthetic\_process | 3 | 0 |  |  |  |  |  |  |  |  |
| GO:0045112\_integrin\_biosynthetic\_process | 3 | 0 |  |  |  |  |  |  |  |  |
| GO:0045409\_negative\_regulation\_of\_interleukin-6\_biosynthetic\_process | 3 | 0 |  |  |  |  |  |  |  |  |
| GO:0045410\_positive\_regulation\_of\_interleukin-6\_biosynthetic\_process | 3 | 0 |  |  |  |  |  |  |  |  |
| GO:0045541\_negative\_regulation\_of\_cholesterol\_biosynthetic\_process | 3 | 0 |  |  |  |  |  |  |  |  |
| GO:0045577\_regulation\_of\_B\_cell\_differentiation | 3 | 0 |  |  |  |  |  |  |  |  |
| GO:0045581\_negative\_regulation\_of\_T\_cell\_differentiation | 3 | 0 |  |  |  |  |  |  |  |  |
| GO:0045625\_regulation\_of\_T-helper\_1\_cell\_differentiation | 3 | 0 |  |  |  |  |  |  |  |  |
| GO:0045628\_regulation\_of\_T-helper\_2\_cell\_differentiation | 3 | 0 |  |  |  |  |  |  |  |  |
| GO:0045662\_negative\_regulation\_of\_myoblast\_differentiation | 3 | 0 |  |  |  |  |  |  |  |  |
| GO:0045663\_positive\_regulation\_of\_myoblast\_differentiation | 3 | 0 |  |  |  |  |  |  |  |  |
| GO:0045665\_negative\_regulation\_of\_neuron\_differentiation | 3 | 0 |  |  |  |  |  |  |  |  |
| GO:0045715\_negative\_regulation\_of\_low-density\_lipoprotein\_receptor\_biosynthetic\_process | 3 | 0 |  |  |  |  |  |  |  |  |
| GO:0045717\_negative\_regulation\_of\_fatty\_acid\_biosynthetic\_process | 3 | 0 |  |  |  |  |  |  |  |  |
| GO:0045779\_negative\_regulation\_of\_bone\_resorption | 3 | 0 |  |  |  |  |  |  |  |  |
| GO:0045817\_positive\_regulation\_of\_transcription\_from\_RNA\_polymerase\_II\_promoter\_\_global | 3 | 0 |  |  |  |  |  |  |  |  |
| GO:0045822\_negative\_regulation\_of\_heart\_contraction | 3 | 0 |  |  |  |  |  |  |  |  |
| GO:0045830\_positive\_regulation\_of\_isotype\_switching | 3 | 0 |  |  |  |  |  |  |  |  |
| GO:0045898\_regulation\_of\_transcriptional\_preinitiation\_complex\_assembly | 3 | 0 |  |  |  |  |  |  |  |  |
| GO:0045911\_positive\_regulation\_of\_DNA\_recombination | 3 | 0 |  |  |  |  |  |  |  |  |
| GO:0045912\_negative\_regulation\_of\_carbohydrate\_metabolic\_process | 3 | 0 |  |  |  |  |  |  |  |  |
| GO:0045916\_negative\_regulation\_of\_complement\_activation | 3 | 0 |  |  |  |  |  |  |  |  |
| GO:0045939\_negative\_regulation\_of\_steroid\_metabolic\_process | 3 | 0 |  |  |  |  |  |  |  |  |
| GO:0045988\_negative\_regulation\_of\_striated\_muscle\_contraction | 3 | 0 |  |  |  |  |  |  |  |  |
| GO:0045990\_regulation\_of\_transcription\_by\_carbon\_catabolites | 3 | 0 |  |  |  |  |  |  |  |  |
| GO:0045995\_regulation\_of\_embryonic\_development | 3 | 0 |  |  |  |  |  |  |  |  |
| GO:0046015\_regulation\_of\_transcription\_by\_glucose | 3 | 0 |  |  |  |  |  |  |  |  |
| GO:0046068\_cGMP\_metabolic\_process | 3 | 0 |  |  |  |  |  |  |  |  |
| GO:0046129\_purine\_ribonucleoside\_biosynthetic\_process | 3 | 0 |  |  |  |  |  |  |  |  |
| GO:0046130\_purine\_ribonucleoside\_catabolic\_process | 3 | 0 |  |  |  |  |  |  |  |  |
| GO:0046146\_tetrahydrobiopterin\_metabolic\_process | 3 | 0 |  |  |  |  |  |  |  |  |
| GO:0046348\_amino\_sugar\_catabolic\_process | 3 | 0 |  |  |  |  |  |  |  |  |
| GO:0046605\_regulation\_of\_centrosome\_cycle | 3 | 0 |  |  |  |  |  |  |  |  |
| GO:0046628\_positive\_regulation\_of\_insulin\_receptor\_signaling\_pathway | 3 | 0 |  |  |  |  |  |  |  |  |
| GO:0046653\_tetrahydrofolate\_metabolic\_process | 3 | 0 |  |  |  |  |  |  |  |  |
| GO:0046676\_negative\_regulation\_of\_insulin\_secretion | 3 | 0 |  |  |  |  |  |  |  |  |
| GO:0046688\_response\_to\_copper\_ion | 3 | 0 |  |  |  |  |  |  |  |  |
| GO:0046813\_virion\_attachment\_\_binding\_of\_host\_cell\_surface\_receptor | 3 | 0 |  |  |  |  |  |  |  |  |
| GO:0046825\_regulation\_of\_protein\_export\_from\_nucleus | 3 | 0 |  |  |  |  |  |  |  |  |
| GO:0046881\_positive\_regulation\_of\_follicle-stimulating\_hormone\_secretion | 3 | 0 |  |  |  |  |  |  |  |  |
| GO:0046886\_positive\_regulation\_of\_hormone\_biosynthetic\_process | 3 | 0 |  |  |  |  |  |  |  |  |
| GO:0048048\_embryonic\_eye\_morphogenesis | 3 | 0 |  |  |  |  |  |  |  |  |
| GO:0048251\_elastic\_fiber\_assembly | 3 | 0 |  |  |  |  |  |  |  |  |
| GO:0048255\_mRNA\_stabilization | 3 | 0 |  |  |  |  |  |  |  |  |
| GO:0048268\_clathrin\_coat\_assembly | 3 | 0 |  |  |  |  |  |  |  |  |
| GO:0048521\_negative\_regulation\_of\_behavior | 3 | 0 |  |  |  |  |  |  |  |  |
| GO:0048538\_thymus\_development | 3 | 0 |  |  |  |  |  |  |  |  |
| GO:0048548\_regulation\_of\_pinocytosis | 3 | 0 |  |  |  |  |  |  |  |  |
| GO:0048552\_regulation\_of\_metalloenzyme\_activity | 3 | 0 |  |  |  |  |  |  |  |  |
| GO:0048554\_positive\_regulation\_of\_metalloenzyme\_activity | 3 | 0 |  |  |  |  |  |  |  |  |
| GO:0048567\_ectodermal\_gut\_morphogenesis | 3 | 0 |  |  |  |  |  |  |  |  |
| GO:0048701\_embryonic\_cranial\_skeleton\_morphogenesis | 3 | 0 |  |  |  |  |  |  |  |  |
| GO:0048739\_cardiac\_muscle\_fiber\_development | 3 | 0 |  |  |  |  |  |  |  |  |
| GO:0048814\_regulation\_of\_dendrite\_morphogenesis | 3 | 0 |  |  |  |  |  |  |  |  |
| GO:0048861\_leukemia\_inhibitory\_factor\_signaling\_pathway | 3 | 0 |  |  |  |  |  |  |  |  |
| GO:0050434\_positive\_regulation\_of\_viral\_transcription | 3 | 0 |  |  |  |  |  |  |  |  |
| GO:0050435\_beta-amyloid\_metabolic\_process | 3 | 0 |  |  |  |  |  |  |  |  |
| GO:0050665\_hydrogen\_peroxide\_biosynthetic\_process | 3 | 0 |  |  |  |  |  |  |  |  |
| GO:0050686\_negative\_regulation\_of\_mRNA\_processing | 3 | 0 |  |  |  |  |  |  |  |  |
| GO:0050806\_positive\_regulation\_of\_synaptic\_transmission | 3 | 0 |  |  |  |  |  |  |  |  |
| GO:0050873\_brown\_fat\_cell\_differentiation | 3 | 0 |  |  |  |  |  |  |  |  |
| GO:0051001\_negative\_regulation\_of\_nitric-oxide\_synthase\_activity | 3 | 0 |  |  |  |  |  |  |  |  |
| GO:0051084\_'de\_novo'\_posttranslational\_protein\_folding | 3 | 0 |  |  |  |  |  |  |  |  |
| GO:0051153\_regulation\_of\_striated\_muscle\_cell\_differentiation | 3 | 0 |  |  |  |  |  |  |  |  |
| GO:0051299\_centrosome\_separation | 3 | 0 |  |  |  |  |  |  |  |  |
| GO:0051305\_chromosome\_movement\_towards\_spindle\_pole | 3 | 0 |  |  |  |  |  |  |  |  |
| GO:0051324\_prophase | 3 | 0 |  |  |  |  |  |  |  |  |
| GO:0051382\_kinetochore\_assembly | 3 | 0 |  |  |  |  |  |  |  |  |
| GO:0051489\_regulation\_of\_filopodium\_assembly | 3 | 0 |  |  |  |  |  |  |  |  |
| GO:0051491\_positive\_regulation\_of\_filopodium\_assembly | 3 | 0 |  |  |  |  |  |  |  |  |
| GO:0051497\_negative\_regulation\_of\_stress\_fiber\_formation | 3 | 0 |  |  |  |  |  |  |  |  |
| GO:0051546\_keratinocyte\_migration | 3 | 0 |  |  |  |  |  |  |  |  |
| GO:0051567\_histone\_H3-K9\_methylation | 3 | 0 |  |  |  |  |  |  |  |  |
| GO:0051569\_regulation\_of\_histone\_H3-K4\_methylation | 3 | 0 |  |  |  |  |  |  |  |  |
| GO:0051712\_positive\_regulation\_of\_killing\_of\_cells\_of\_another\_organism | 3 | 0 |  |  |  |  |  |  |  |  |
| GO:0051825\_adhesion\_to\_other\_organism\_during\_symbiotic\_interaction | 3 | 0 |  |  |  |  |  |  |  |  |
| GO:0051851\_modification\_by\_host\_of\_symbiont\_morphology\_or\_physiology | 3 | 0 |  |  |  |  |  |  |  |  |
| GO:0051856\_adhesion\_to\_symbiont | 3 | 0 |  |  |  |  |  |  |  |  |
| GO:0051877\_pigment\_granule\_aggregation\_in\_cell\_center | 3 | 0 |  |  |  |  |  |  |  |  |
| GO:0051882\_mitochondrial\_depolarization | 3 | 0 |  |  |  |  |  |  |  |  |
| GO:0051918\_negative\_regulation\_of\_fibrinolysis | 3 | 0 |  |  |  |  |  |  |  |  |
| GO:0051925\_regulation\_of\_calcium\_ion\_transport\_via\_voltage-gated\_calcium\_channel\_activity | 3 | 0 |  |  |  |  |  |  |  |  |
| GO:0051938\_L-glutamate\_import | 3 | 0 |  |  |  |  |  |  |  |  |
| GO:0051966\_regulation\_of\_synaptic\_transmission\_\_glutamatergic | 3 | 0 |  |  |  |  |  |  |  |  |
| GO:0051970\_negative\_regulation\_of\_transmission\_of\_nerve\_impulse | 3 | 0 |  |  |  |  |  |  |  |  |
| GO:0051973\_positive\_regulation\_of\_telomerase\_activity | 3 | 0 |  |  |  |  |  |  |  |  |
| GO:0051983\_regulation\_of\_chromosome\_segregation | 3 | 0 |  |  |  |  |  |  |  |  |
| GO:0055062\_phosphate\_ion\_homeostasis | 3 | 0 |  |  |  |  |  |  |  |  |
| GO:0055078\_sodium\_ion\_homeostasis | 3 | 0 |  |  |  |  |  |  |  |  |
| GO:0055094\_response\_to\_lipoprotein\_stimulus | 3 | 0 |  |  |  |  |  |  |  |  |
| GO:0060017\_parathyroid\_gland\_development | 3 | 0 |  |  |  |  |  |  |  |  |
| GO:0060023\_soft\_palate\_development | 3 | 0 |  |  |  |  |  |  |  |  |
| GO:0060084\_synaptic\_transmission\_involved\_in\_micturition | 3 | 0 |  |  |  |  |  |  |  |  |
| GO:0060119\_inner\_ear\_receptor\_cell\_development | 3 | 0 |  |  |  |  |  |  |  |  |
| GO:0060134\_prepulse\_inhibition | 3 | 0 |  |  |  |  |  |  |  |  |
| GO:0060158\_activation\_of\_phospholipase\_C\_activity\_by\_dopamine\_receptor\_signaling\_pathway | 3 | 0 |  |  |  |  |  |  |  |  |
| GO:0060177\_regulation\_of\_angiotensin\_metabolic\_process | 3 | 0 |  |  |  |  |  |  |  |  |
| GO:0060267\_positive\_regulation\_of\_respiratory\_burst | 3 | 0 |  |  |  |  |  |  |  |  |
| GO:0060317\_cardiac\_epithelial\_to\_mesenchymal\_transition | 3 | 0 |  |  |  |  |  |  |  |  |
| GO:0060333\_interferon-gamma-mediated\_signaling\_pathway | 3 | 0 |  |  |  |  |  |  |  |  |
| GO:0060334\_regulation\_of\_interferon-gamma-mediated\_signaling\_pathway | 3 | 0 |  |  |  |  |  |  |  |  |
| GO:0060343\_trabecula\_formation | 3 | 0 |  |  |  |  |  |  |  |  |
| GO:0060347\_heart\_trabecula\_formation | 3 | 0 |  |  |  |  |  |  |  |  |
| GO:0060394\_negative\_regulation\_of\_pathway-restricted\_SMAD\_protein\_phosphorylation | 3 | 0 |  |  |  |  |  |  |  |  |
| GO:0060559\_positive\_regulation\_of\_calcidiol\_1-monooxygenase\_activity | 3 | 0 |  |  |  |  |  |  |  |  |
| GO:0070050\_neuron\_maintenance | 3 | 0 |  |  |  |  |  |  |  |  |
| GO:0070141\_response\_to\_UV-A | 3 | 0 |  |  |  |  |  |  |  |  |
| GO:0070168\_negative\_regulation\_of\_biomineral\_formation | 3 | 0 |  |  |  |  |  |  |  |  |
| GO:0070207\_protein\_homotrimerization | 3 | 0 |  |  |  |  |  |  |  |  |
| GO:0070229\_negative\_regulation\_of\_lymphocyte\_apoptosis | 3 | 0 |  |  |  |  |  |  |  |  |
| GO:0070231\_T\_cell\_apoptosis | 3 | 0 |  |  |  |  |  |  |  |  |
| GO:0070272\_proton-transporting\_ATP\_synthase\_complex\_biogenesis | 3 | 0 |  |  |  |  |  |  |  |  |
| GO:0070561\_vitamin\_D\_receptor\_signaling\_pathway | 3 | 0 |  |  |  |  |  |  |  |  |
| GO:0070634\_transepithelial\_ammonium\_transport | 3 | 0 |  |  |  |  |  |  |  |  |
| GO:0070777\_D-aspartate\_transport | 3 | 0 |  |  |  |  |  |  |  |  |
[truncated: 287,054 more chars]
