# Supplementary material for: Concordance of Gene Expression and Functional Correlation Patterns across the NCI-60 Cell Lines and the Cancer Genome Atlas Glioblastoma Samples
Source: PLoS One. 2012 Jul 26;7(7):e40062. doi: 10.1371/journal.pone.0040062 (PMC3406063; doi:10.1371/journal.pone.0040062)
Supplement: Download S1 — Zip archive of HTGM results. (ZIP) [file pone.0040062.s007.zip › work2026406846/Generated_Total2026406846.dir/generic.BP.NCI60.0.6.ATP2A3.express.genes.correlation.complete.Thu.May.19.17.20.06.2011.htgm.txt.dir/index.html]

Results for generic.BP.NCI60.0.6.ATP2A3.express.genes.correlation.complete.Thu.May.19.17.20.06.2011.htgm.txt

# Results for generic.BP.NCI60.0.6.ATP2A3.express.genes.correlation.complete.Thu.May.19.17.20.06.2011.htgm.txt

| Input Files | | | |
| --- | --- | --- | --- |
| Type | File |
| Total File | Generated |
| Changed File (Text) | generic.BP.NCI60.0.6.ATP2A3.express.genes.correlation.complete.Thu.May.19.17.20.06.2011.htgm.txt |
|  |
| --- | | | ||| Results files | | | |
| --- | --- | --- | --- |
| Type | Text | Excel | HTML |
| Gene Category Summary (Changed Genes) | generic.BP.NCI60.0.6.ATP2A3.express.genes.correlation.complete.Thu.May.19.17.20.06.2011.htgm.txt.change | generic.BP.NCI60.0.6.ATP2A3.express.genes.correlation.complete.Thu.May.19.17.20.06.2011.htgm.txt.change.xls | generic.BP.NCI60.0.6.ATP2A3.express.genes.correlation.complete.Thu.May.19.17.20.06.2011.htgm.txt.change.html |
| Gene Category Mapping (Changed Genes) | generic.BP.NCI60.0.6.ATP2A3.express.genes.correlation.complete.Thu.May.19.17.20.06.2011.htgm.txt.change.gce | generic.BP.NCI60.0.6.ATP2A3.express.genes.correlation.complete.Thu.May.19.17.20.06.2011.htgm.txt.change.gce.xls | generic.BP.NCI60.0.6.ATP2A3.express.genes.correlation.complete.Thu.May.19.17.20.06.2011.htgm.txt.change.gce.html |
| CIM (Changed) | generic.BP.NCI60.0.6.ATP2A3.express.genes.correlation.complete.Thu.May.19.17.20.06.2011.htgm.txt.change.gce.CIM |  | generic.BP.NCI60.0.6.ATP2A3.express.genes.correlation.complete.Thu.May.19.17.20.06.2011.htgm.txt.change.gce.CIM.html |
| --- | | | ||| Navigation | | | |
| --- | --- | --- | --- |
| Up to Parent(Results for Total File) | | | |
