# Supplementary material for: Concordance of Gene Expression and Functional Correlation Patterns across the NCI-60 Cell Lines and the Cancer Genome Atlas Glioblastoma Samples
Source: PLoS One. 2012 Jul 26;7(7):e40062. doi: 10.1371/journal.pone.0040062 (PMC3406063; doi:10.1371/journal.pone.0040062)
Supplement: Download S1 — Zip archive of HTGM results. (ZIP) [file pone.0040062.s007.zip › work2026406846/Generated_Total2026406846.dir/generic.BP.NCI60.0.6.BTK.express.genes.correlation.complete.Thu.May.19.17.12.30.2011.htgm.txt.dir/generic.BP.NCI60.0.6.BTK.express.genes.correlation.complete.Thu.May.19.17.12.30.2011.htgm.txt.change.gce.html]

Gene Category Report for generic.BP.NCI60.0.6.BTK.express.genes.correlation.complete.Thu.May.19.17.12.30.2011.htgm.txt

# Gene Category Report for generic.BP.NCI60.0.6.BTK.express.genes.correlation.complete.Thu.May.19.17.12.30.2011.htgm.txt

| HYPERLINKED GO CATEGORY | HYPERLINKED GENE NAME | TOTAL GENES | CHANGED GENES | ENRICHMENT | LOG10(p) | CUMULATIVE NUMBER OF CATEGORIES | CUMULATIVE RANDOMS MEAN | FALSE DISCOVERY RATE |
| --- | --- | --- | --- | --- | --- | --- | --- | --- |
| GO:0045730\_respiratory\_burst | CD52 | 16 | 3 | 80.526316 | -5.230560 | 1 | 0.01 | 0.010000 |
| GO:0045730\_respiratory\_burst | CYBB | 16 | 3 | 80.526316 | -5.230560 | 1 | 0.01 | 0.010000 |
| GO:0045730\_respiratory\_burst | NCF1 | 16 | 3 | 80.526316 | -5.230560 | 1 | 0.01 | 0.010000 |
| GO:0050896\_response\_to\_stimulus | RNASE2 | 1775 | 13 | 3.145441 | -4.776036 | 2 | 0.03 | 0.015000 |
| GO:0050896\_response\_to\_stimulus | CYBB | 1775 | 13 | 3.145441 | -4.776036 | 2 | 0.03 | 0.015000 |
| GO:0050896\_response\_to\_stimulus | MNDA | 1775 | 13 | 3.145441 | -4.776036 | 2 | 0.03 | 0.015000 |
| GO:0050896\_response\_to\_stimulus | NCF1 | 1775 | 13 | 3.145441 | -4.776036 | 2 | 0.03 | 0.015000 |
| GO:0050896\_response\_to\_stimulus | CD4 | 1775 | 13 | 3.145441 | -4.776036 | 2 | 0.03 | 0.015000 |
| GO:0050896\_response\_to\_stimulus | NCF4 | 1775 | 13 | 3.145441 | -4.776036 | 2 | 0.03 | 0.015000 |
| GO:0050896\_response\_to\_stimulus | TYROBP | 1775 | 13 | 3.145441 | -4.776036 | 2 | 0.03 | 0.015000 |
| GO:0050896\_response\_to\_stimulus | CSF3R | 1775 | 13 | 3.145441 | -4.776036 | 2 | 0.03 | 0.015000 |
| GO:0050896\_response\_to\_stimulus | IGF1 | 1775 | 13 | 3.145441 | -4.776036 | 2 | 0.03 | 0.015000 |
| GO:0050896\_response\_to\_stimulus | LILRA2 | 1775 | 13 | 3.145441 | -4.776036 | 2 | 0.03 | 0.015000 |
| GO:0050896\_response\_to\_stimulus | FGR | 1775 | 13 | 3.145441 | -4.776036 | 2 | 0.03 | 0.015000 |
| GO:0050896\_response\_to\_stimulus | CARD9 | 1775 | 13 | 3.145441 | -4.776036 | 2 | 0.03 | 0.015000 |
| GO:0050896\_response\_to\_stimulus | PLEK | 1775 | 13 | 3.145441 | -4.776036 | 2 | 0.03 | 0.015000 |
| GO:0006950\_response\_to\_stress | IGF1 | 959 | 9 | 4.030514 | -3.896380 | 3 | 0.16 | 0.053333 |
| GO:0006950\_response\_to\_stress | LILRA2 | 959 | 9 | 4.030514 | -3.896380 | 3 | 0.16 | 0.053333 |
| GO:0006950\_response\_to\_stress | MNDA | 959 | 9 | 4.030514 | -3.896380 | 3 | 0.16 | 0.053333 |
| GO:0006950\_response\_to\_stress | CYBB | 959 | 9 | 4.030514 | -3.896380 | 3 | 0.16 | 0.053333 |
| GO:0006950\_response\_to\_stress | NCF1 | 959 | 9 | 4.030514 | -3.896380 | 3 | 0.16 | 0.053333 |
| GO:0006950\_response\_to\_stress | CARD9 | 959 | 9 | 4.030514 | -3.896380 | 3 | 0.16 | 0.053333 |
| GO:0006950\_response\_to\_stress | PLEK | 959 | 9 | 4.030514 | -3.896380 | 3 | 0.16 | 0.053333 |
| GO:0006950\_response\_to\_stress | CSF3R | 959 | 9 | 4.030514 | -3.896380 | 3 | 0.16 | 0.053333 |
| GO:0006950\_response\_to\_stress | TYROBP | 959 | 9 | 4.030514 | -3.896380 | 3 | 0.16 | 0.053333 |
| GO:0006952\_defense\_response | LILRA2 | 369 | 6 | 6.983312 | -3.870098 | 4 | 0.16 | 0.040000 |
| GO:0006952\_defense\_response | MNDA | 369 | 6 | 6.983312 | -3.870098 | 4 | 0.16 | 0.040000 |
| GO:0006952\_defense\_response | CYBB | 369 | 6 | 6.983312 | -3.870098 | 4 | 0.16 | 0.040000 |
| GO:0006952\_defense\_response | NCF1 | 369 | 6 | 6.983312 | -3.870098 | 4 | 0.16 | 0.040000 |
| GO:0006952\_defense\_response | CSF3R | 369 | 6 | 6.983312 | -3.870098 | 4 | 0.16 | 0.040000 |
| GO:0006952\_defense\_response | TYROBP | 369 | 6 | 6.983312 | -3.870098 | 4 | 0.16 | 0.040000 |
| GO:0042554\_superoxide\_anion\_generation | CYBB | 9 | 2 | 95.438596 | -3.737224 | 5 | 0.22 | 0.044000 |
| GO:0042554\_superoxide\_anion\_generation | NCF1 | 9 | 2 | 95.438596 | -3.737224 | 5 | 0.22 | 0.044000 |
| GO:0006968\_cellular\_defense\_response | MNDA | 58 | 3 | 22.214156 | -3.516260 | 6 | 0.3 | 0.050000 |
| GO:0006968\_cellular\_defense\_response | NCF1 | 58 | 3 | 22.214156 | -3.516260 | 6 | 0.3 | 0.050000 |
| GO:0006968\_cellular\_defense\_response | TYROBP | 58 | 3 | 22.214156 | -3.516260 | 6 | 0.3 | 0.050000 |
| GO:0006801\_superoxide\_metabolic\_process | CYBB | 17 | 2 | 50.526316 | -3.164813 | 7 | 0.56 | 0.080000 |
| GO:0006801\_superoxide\_metabolic\_process | NCF1 | 17 | 2 | 50.526316 | -3.164813 | 7 | 0.56 | 0.080000 |
| GO:0019722\_calcium-mediated\_signaling | BTK | 30 | 2 | 28.631579 | -2.667698 | 8 | 1.32 | 0.165000 |
| GO:0019722\_calcium-mediated\_signaling | PLEK | 30 | 2 | 28.631579 | -2.667698 | 8 | 1.32 | 0.165000 |
| GO:0000746\_conjugation | CD4 | 1 | 1 |  |  |  |  |  |  |
| GO:0000747\_conjugation\_with\_cellular\_fusion | CD4 | 1 | 1 |  |  |  |  |  |  |
| GO:0002576\_platelet\_degranulation | PLEK | 1 | 1 |  |  |  |  |  |  |
| GO:0006021\_inositol\_biosynthetic\_process | PLEK | 1 | 1 |  |  |  |  |  |  |
| GO:0009441\_glycolate\_metabolic\_process | IGF1 | 1 | 1 |  |  |  |  |  |  |
| GO:0010919\_regulation\_of\_inositol\_phosphate\_biosynthetic\_process | PLEK | 1 | 1 |  |  |  |  |  |  |
| GO:0010920\_negative\_regulation\_of\_inositol\_phosphate\_biosynthetic\_process | PLEK | 1 | 1 |  |  |  |  |  |  |
| GO:0010924\_regulation\_of\_inositol-polyphosphate\_5-phosphatase\_activity | PLEK | 1 | 1 |  |  |  |  |  |  |
| GO:0010925\_positive\_regulation\_of\_inositol-polyphosphate\_5-phosphatase\_activity | PLEK | 1 | 1 |  |  |  |  |  |  |
| GO:0014834\_satellite\_cell\_maintenance\_involved\_in\_skeletal\_muscle\_regeneration | IGF1 | 1 | 1 |  |  |  |  |  |  |
| GO:0014904\_myotube\_cell\_development | IGF1 | 1 | 1 |  |  |  |  |  |  |
| GO:0030836\_positive\_regulation\_of\_actin\_filament\_depolymerization | PLEK | 1 | 1 |  |  |  |  |  |  |
| GO:0030845\_inhibition\_of\_phospholipase\_C\_activity\_involved\_in\_G-protein\_coupled\_receptor\_signaling\_pathway | PLEK | 1 | 1 |  |  |  |  |  |  |
| GO:0032958\_inositol\_phosphate\_biosynthetic\_process | PLEK | 1 | 1 |  |  |  |  |  |  |
| GO:0033622\_integrin\_activation | PLEK | 1 | 1 |  |  |  |  |  |  |
| GO:0033623\_regulation\_of\_integrin\_activation | PLEK | 1 | 1 |  |  |  |  |  |  |
| GO:0033625\_positive\_regulation\_of\_integrin\_activation | PLEK | 1 | 1 |  |  |  |  |  |  |
| GO:0034109\_homotypic\_cell-cell\_adhesion | PLEK | 1 | 1 |  |  |  |  |  |  |
| GO:0043403\_skeletal\_muscle\_regeneration | IGF1 | 1 | 1 |  |  |  |  |  |  |
| GO:0043647\_inositol\_phosphate\_metabolic\_process | PLEK | 1 | 1 |  |  |  |  |  |  |
| GO:0046173\_polyol\_biosynthetic\_process | PLEK | 1 | 1 |  |  |  |  |  |  |
| GO:0051450\_myoblast\_proliferation | IGF1 | 1 | 1 |  |  |  |  |  |  |
| GO:0060305\_regulation\_of\_cell\_diameter | PLEK | 1 | 1 |  |  |  |  |  |  |
| GO:0070527\_platelet\_aggregation | PLEK | 1 | 1 |  |  |  |  |  |  |
| GO:0070528\_protein\_kinase\_C\_signaling\_cascade | PLEK | 1 | 1 |  |  |  |  |  |  |
| GO:0070560\_protein\_secretion\_by\_platelet | PLEK | 1 | 1 |  |  |  |  |  |  |
| GO:0030097\_hemopoiesis | SPI1 | 135 | 3 | 9.543860 | -2.451678 | 9 | 2.08 | 0.231111 |
| GO:0030097\_hemopoiesis | CD4 | 135 | 3 | 9.543860 | -2.451678 | 9 | 2.08 | 0.231111 |
| GO:0030097\_hemopoiesis | PLEK | 135 | 3 | 9.543860 | -2.451678 | 9 | 2.08 | 0.231111 |
| GO:0048534\_hemopoietic\_or\_lymphoid\_organ\_development | SPI1 | 139 | 3 | 9.269216 | -2.415910 | 10 | 2.18 | 0.218000 |
| GO:0048534\_hemopoietic\_or\_lymphoid\_organ\_development | CD4 | 139 | 3 | 9.269216 | -2.415910 | 10 | 2.18 | 0.218000 |
| GO:0048534\_hemopoietic\_or\_lymphoid\_organ\_development | PLEK | 139 | 3 | 9.269216 | -2.415910 | 10 | 2.18 | 0.218000 |
| GO:0006793\_phosphorus\_metabolic\_process | IGF1 | 697 | 6 | 3.697047 | -2.407301 | 12 | 2.2 | 0.183333 |
| GO:0006793\_phosphorus\_metabolic\_process | PTPN7 | 697 | 6 | 3.697047 | -2.407301 | 12 | 2.2 | 0.183333 |
| GO:0006793\_phosphorus\_metabolic\_process | FGR | 697 | 6 | 3.697047 | -2.407301 | 12 | 2.2 | 0.183333 |
| GO:0006793\_phosphorus\_metabolic\_process | CD4 | 697 | 6 | 3.697047 | -2.407301 | 12 | 2.2 | 0.183333 |
| GO:0006793\_phosphorus\_metabolic\_process | PLEK | 697 | 6 | 3.697047 | -2.407301 | 12 | 2.2 | 0.183333 |
| GO:0006793\_phosphorus\_metabolic\_process | BTK | 697 | 6 | 3.697047 | -2.407301 | 12 | 2.2 | 0.183333 |
| GO:0006796\_phosphate\_metabolic\_process | IGF1 | 697 | 6 | 3.697047 | -2.407301 | 12 | 2.2 | 0.183333 |
| GO:0006796\_phosphate\_metabolic\_process | PTPN7 | 697 | 6 | 3.697047 | -2.407301 | 12 | 2.2 | 0.183333 |
| GO:0006796\_phosphate\_metabolic\_process | FGR | 697 | 6 | 3.697047 | -2.407301 | 12 | 2.2 | 0.183333 |
| GO:0006796\_phosphate\_metabolic\_process | CD4 | 697 | 6 | 3.697047 | -2.407301 | 12 | 2.2 | 0.183333 |
| GO:0006796\_phosphate\_metabolic\_process | PLEK | 697 | 6 | 3.697047 | -2.407301 | 12 | 2.2 | 0.183333 |
| GO:0006796\_phosphate\_metabolic\_process | BTK | 697 | 6 | 3.697047 | -2.407301 | 12 | 2.2 | 0.183333 |
| GO:0006800\_oxygen\_and\_reactive\_oxygen\_species\_metabolic\_process | CYBB | 41 | 2 | 20.949936 | -2.398996 | 13 | 2.29 | 0.176154 |
| GO:0006800\_oxygen\_and\_reactive\_oxygen\_species\_metabolic\_process | NCF1 | 41 | 2 | 20.949936 | -2.398996 | 13 | 2.29 | 0.176154 |
| GO:0002520\_immune\_system\_development | SPI1 | 147 | 3 | 8.764769 | -2.347598 | 14 | 2.59 | 0.185000 |
| GO:0002520\_immune\_system\_development | CD4 | 147 | 3 | 8.764769 | -2.347598 | 14 | 2.59 | 0.185000 |
| GO:0002520\_immune\_system\_development | PLEK | 147 | 3 | 8.764769 | -2.347598 | 14 | 2.59 | 0.185000 |
| GO:0002376\_immune\_system\_process | CYBB | 718 | 6 | 3.588917 | -2.342827 | 15 | 2.59 | 0.172667 |
| GO:0002376\_immune\_system\_process | NCF1 | 718 | 6 | 3.588917 | -2.342827 | 15 | 2.59 | 0.172667 |
| GO:0002376\_immune\_system\_process | SPI1 | 718 | 6 | 3.588917 | -2.342827 | 15 | 2.59 | 0.172667 |
| GO:0002376\_immune\_system\_process | CD4 | 718 | 6 | 3.588917 | -2.342827 | 15 | 2.59 | 0.172667 |
| GO:0002376\_immune\_system\_process | PLEK | 718 | 6 | 3.588917 | -2.342827 | 15 | 2.59 | 0.172667 |
| GO:0002376\_immune\_system\_process | NCF4 | 718 | 6 | 3.588917 | -2.342827 | 15 | 2.59 | 0.172667 |
| GO:0010519\_negative\_regulation\_of\_phospholipase\_activity | PLEK | 2 | 1 |  |  |  |  |  |  |
| GO:0014911\_positive\_regulation\_of\_smooth\_muscle\_cell\_migration | IGF1 | 2 | 1 |  |  |  |  |  |  |
| GO:0034392\_negative\_regulation\_of\_smooth\_muscle\_cell\_apoptosis | IGF1 | 2 | 1 |  |  |  |  |  |  |
| GO:0043243\_positive\_regulation\_of\_protein\_complex\_disassembly | PLEK | 2 | 1 |  |  |  |  |  |  |
| GO:0050849\_negative\_regulation\_of\_calcium-mediated\_signaling | PLEK | 2 | 1 |  |  |  |  |  |  |
| GO:0070493\_thrombin\_receptor\_signaling\_pathway | PLEK | 2 | 1 |  |  |  |  |  |  |
| GO:0010638\_positive\_regulation\_of\_organelle\_organization | IGF1 | 46 | 2 | 18.672769 | -2.300878 | 16 | 2.78 | 0.173750 |
| GO:0010638\_positive\_regulation\_of\_organelle\_organization | PLEK | 46 | 2 | 18.672769 | -2.300878 | 16 | 2.78 | 0.173750 |
| GO:0019932\_second-messenger-mediated\_signaling | IGF1 | 153 | 3 | 8.421053 | -2.298958 | 17 | 2.79 | 0.164118 |
| GO:0019932\_second-messenger-mediated\_signaling | BTK | 153 | 3 | 8.421053 | -2.298958 | 17 | 2.79 | 0.164118 |
| GO:0019932\_second-messenger-mediated\_signaling | PLEK | 153 | 3 | 8.421053 | -2.298958 | 17 | 2.79 | 0.164118 |
| GO:0048015\_phosphoinositide-mediated\_signaling | IGF1 | 53 | 2 | 16.206554 | -2.180779 | 18 | 3.44 | 0.191111 |
| GO:0048015\_phosphoinositide-mediated\_signaling | PLEK | 53 | 2 | 16.206554 | -2.180779 | 18 | 3.44 | 0.191111 |
| GO:0002244\_hemopoietic\_progenitor\_cell\_differentiation | PLEK | 3 | 1 |  |  |  |  |  |  |
| GO:0010572\_positive\_regulation\_of\_platelet\_activation | PLEK | 3 | 1 |  |  |  |  |  |  |
| GO:0010656\_negative\_regulation\_of\_muscle\_cell\_apoptosis | IGF1 | 3 | 1 |  |  |  |  |  |  |
| GO:0010922\_positive\_regulation\_of\_phosphatase\_activity | PLEK | 3 | 1 |  |  |  |  |  |  |
| GO:0032872\_regulation\_of\_stress-activated\_MAPK\_cascade | CARD9 | 3 | 1 |  |  |  |  |  |  |
| GO:0032874\_positive\_regulation\_of\_stress-activated\_MAPK\_cascade | CARD9 | 3 | 1 |  |  |  |  |  |  |
| GO:0033143\_regulation\_of\_steroid\_hormone\_receptor\_signaling\_pathway | IGF1 | 3 | 1 |  |  |  |  |  |  |
| GO:0035019\_somatic\_stem\_cell\_maintenance | IGF1 | 3 | 1 |  |  |  |  |  |  |
| GO:0042246\_tissue\_regeneration | IGF1 | 3 | 1 |  |  |  |  |  |  |
| GO:0043193\_positive\_regulation\_of\_gene-specific\_transcription | IGF1 | 57 | 2 | 15.069252 | -2.119399 | 19 | 3.61 | 0.190000 |
| GO:0043193\_positive\_regulation\_of\_gene-specific\_transcription | SPI1 | 57 | 2 | 15.069252 | -2.119399 | 19 | 3.61 | 0.190000 |
| GO:0030865\_cortical\_cytoskeleton\_organization | PLEK | 4 | 1 |  |  |  |  |  |  |
| GO:0030866\_cortical\_actin\_cytoskeleton\_organization | PLEK | 4 | 1 |  |  |  |  |  |  |
| GO:0043568\_positive\_regulation\_of\_insulin-like\_growth\_factor\_receptor\_signaling\_pathway | IGF1 | 4 | 1 |  |  |  |  |  |  |
| GO:0051239\_regulation\_of\_multicellular\_organismal\_process | IGF1 | 378 | 4 | 4.544695 | -1.995795 | 20 | 4.6 | 0.230000 |
| GO:0051239\_regulation\_of\_multicellular\_organismal\_process | SPI1 | 378 | 4 | 4.544695 | -1.995795 | 20 | 4.6 | 0.230000 |
| GO:0051239\_regulation\_of\_multicellular\_organismal\_process | CD4 | 378 | 4 | 4.544695 | -1.995795 | 20 | 4.6 | 0.230000 |
| GO:0051239\_regulation\_of\_multicellular\_organismal\_process | PLEK | 378 | 4 | 4.544695 | -1.995795 | 20 | 4.6 | 0.230000 |
| GO:0034390\_smooth\_muscle\_cell\_apoptosis | IGF1 | 5 | 1 | 85.894737 | -1.935883 | 24 | 7.52 | 0.313333 |
| GO:0034391\_regulation\_of\_smooth\_muscle\_cell\_apoptosis | IGF1 | 5 | 1 | 85.894737 | -1.935883 | 24 | 7.52 | 0.313333 |
| GO:0045821\_positive\_regulation\_of\_glycolysis | IGF1 | 5 | 1 | 85.894737 | -1.935883 | 24 | 7.52 | 0.313333 |
| GO:0051403\_stress-activated\_MAPK\_cascade | CARD9 | 5 | 1 | 85.894737 | -1.935883 | 24 | 7.52 | 0.313333 |
| GO:0006020\_inositol\_metabolic\_process | PLEK | 6 | 1 | 71.578947 | -1.857181 | 33 | 10.88 | 0.329697 |
| GO:0010657\_muscle\_cell\_apoptosis | IGF1 | 6 | 1 | 71.578947 | -1.857181 | 33 | 10.88 | 0.329697 |
| GO:0010660\_regulation\_of\_muscle\_cell\_apoptosis | IGF1 | 6 | 1 | 71.578947 | -1.857181 | 33 | 10.88 | 0.329697 |
| GO:0019827\_stem\_cell\_maintenance | IGF1 | 6 | 1 | 71.578947 | -1.857181 | 33 | 10.88 | 0.329697 |
| GO:0031099\_regeneration | IGF1 | 6 | 1 | 71.578947 | -1.857181 | 33 | 10.88 | 0.329697 |
| GO:0046579\_positive\_regulation\_of\_Ras\_protein\_signal\_transduction | IGF1 | 6 | 1 | 71.578947 | -1.857181 | 33 | 10.88 | 0.329697 |
| GO:0048864\_stem\_cell\_development | IGF1 | 6 | 1 | 71.578947 | -1.857181 | 33 | 10.88 | 0.329697 |
| GO:0051057\_positive\_regulation\_of\_small\_GTPase\_mediated\_signal\_transduction | IGF1 | 6 | 1 | 71.578947 | -1.857181 | 33 | 10.88 | 0.329697 |
| GO:0060192\_negative\_regulation\_of\_lipase\_activity | PLEK | 6 | 1 | 71.578947 | -1.857181 | 33 | 10.88 | 0.329697 |
| GO:0032583\_regulation\_of\_gene-specific\_transcription | IGF1 | 81 | 2 | 10.604288 | -1.826295 | 34 | 11.21 | 0.329706 |
| GO:0032583\_regulation\_of\_gene-specific\_transcription | SPI1 | 81 | 2 | 10.604288 | -1.826295 | 34 | 11.21 | 0.329706 |
| GO:0045087\_innate\_immune\_response | CYBB | 82 | 2 | 10.474968 | -1.816171 | 35 | 11.3 | 0.322857 |
| GO:0045087\_innate\_immune\_response | NCF1 | 82 | 2 | 10.474968 | -1.816171 | 35 | 11.3 | 0.322857 |
| GO:0010543\_regulation\_of\_platelet\_activation | PLEK | 7 | 1 | 61.353383 | -1.790712 | 48 | 13.96 | 0.290833 |
| GO:0014068\_positive\_regulation\_of\_phosphoinositide\_3-kinase\_cascade | IGF1 | 7 | 1 | 61.353383 | -1.790712 | 48 | 13.96 | 0.290833 |
| GO:0014902\_myotube\_differentiation | IGF1 | 7 | 1 | 61.353383 | -1.790712 | 48 | 13.96 | 0.290833 |
| GO:0014910\_regulation\_of\_smooth\_muscle\_cell\_migration | IGF1 | 7 | 1 | 61.353383 | -1.790712 | 48 | 13.96 | 0.290833 |
| GO:0030042\_actin\_filament\_depolymerization | PLEK | 7 | 1 | 61.353383 | -1.790712 | 48 | 13.96 | 0.290833 |
| GO:0030834\_regulation\_of\_actin\_filament\_depolymerization | PLEK | 7 | 1 | 61.353383 | -1.790712 | 48 | 13.96 | 0.290833 |
| GO:0031529\_ruffle\_organization | PLEK | 7 | 1 | 61.353383 | -1.790712 | 48 | 13.96 | 0.290833 |
| GO:0032233\_positive\_regulation\_of\_actin\_filament\_bundle\_formation | PLEK | 7 | 1 | 61.353383 | -1.790712 | 48 | 13.96 | 0.290833 |
| GO:0042523\_positive\_regulation\_of\_tyrosine\_phosphorylation\_of\_Stat5\_protein | IGF1 | 7 | 1 | 61.353383 | -1.790712 | 48 | 13.96 | 0.290833 |
| GO:0043567\_regulation\_of\_insulin-like\_growth\_factor\_receptor\_signaling\_pathway | IGF1 | 7 | 1 | 61.353383 | -1.790712 | 48 | 13.96 | 0.290833 |
| GO:0045058\_T\_cell\_selection | CD4 | 7 | 1 | 61.353383 | -1.790712 | 48 | 13.96 | 0.290833 |
| GO:0045744\_negative\_regulation\_of\_G-protein\_coupled\_receptor\_protein\_signaling\_pathway | PLEK | 7 | 1 | 61.353383 | -1.790712 | 48 | 13.96 | 0.290833 |
| GO:0048863\_stem\_cell\_differentiation | IGF1 | 7 | 1 | 61.353383 | -1.790712 | 48 | 13.96 | 0.290833 |
| GO:0010604\_positive\_regulation\_of\_macromolecule\_metabolic\_process | IGF1 | 446 | 4 | 3.851782 | -1.751446 | 49 | 14.32 | 0.292245 |
| GO:0010604\_positive\_regulation\_of\_macromolecule\_metabolic\_process | SPI1 | 446 | 4 | 3.851782 | -1.751446 | 49 | 14.32 | 0.292245 |
| GO:0010604\_positive\_regulation\_of\_macromolecule\_metabolic\_process | CD4 | 446 | 4 | 3.851782 | -1.751446 | 49 | 14.32 | 0.292245 |
| GO:0010604\_positive\_regulation\_of\_macromolecule\_metabolic\_process | PLEK | 446 | 4 | 3.851782 | -1.751446 | 49 | 14.32 | 0.292245 |
| GO:0010921\_regulation\_of\_phosphatase\_activity | PLEK | 8 | 1 | 53.684211 | -1.733199 | 58 | 17.14 | 0.295517 |
| GO:0014066\_regulation\_of\_phosphoinositide\_3-kinase\_cascade | IGF1 | 8 | 1 | 53.684211 | -1.733199 | 58 | 17.14 | 0.295517 |
| GO:0014812\_muscle\_cell\_migration | IGF1 | 8 | 1 | 53.684211 | -1.733199 | 58 | 17.14 | 0.295517 |
| GO:0014896\_muscle\_hypertrophy | IGF1 | 8 | 1 | 53.684211 | -1.733199 | 58 | 17.14 | 0.295517 |
| GO:0014909\_smooth\_muscle\_cell\_migration | IGF1 | 8 | 1 | 53.684211 | -1.733199 | 58 | 17.14 | 0.295517 |
| GO:0042506\_tyrosine\_phosphorylation\_of\_Stat5\_protein | IGF1 | 8 | 1 | 53.684211 | -1.733199 | 58 | 17.14 | 0.295517 |
| GO:0042522\_regulation\_of\_tyrosine\_phosphorylation\_of\_Stat5\_protein | IGF1 | 8 | 1 | 53.684211 | -1.733199 | 58 | 17.14 | 0.295517 |
| GO:0045086\_positive\_regulation\_of\_interleukin-2\_biosynthetic\_process | CD4 | 8 | 1 | 53.684211 | -1.733199 | 58 | 17.14 | 0.295517 |
| GO:0046330\_positive\_regulation\_of\_JNK\_cascade | CARD9 | 8 | 1 | 53.684211 | -1.733199 | 58 | 17.14 | 0.295517 |
| GO:0051130\_positive\_regulation\_of\_cellular\_component\_organization | IGF1 | 91 | 2 | 9.438982 | -1.730580 | 59 | 17.16 | 0.290847 |
| GO:0051130\_positive\_regulation\_of\_cellular\_component\_organization | PLEK | 91 | 2 | 9.438982 | -1.730580 | 59 | 17.16 | 0.290847 |
| GO:0031325\_positive\_regulation\_of\_cellular\_metabolic\_process | IGF1 | 454 | 4 | 3.783909 | -1.725649 | 60 | 17.2 | 0.286667 |
| GO:0031325\_positive\_regulation\_of\_cellular\_metabolic\_process | SPI1 | 454 | 4 | 3.783909 | -1.725649 | 60 | 17.2 | 0.286667 |
| GO:0031325\_positive\_regulation\_of\_cellular\_metabolic\_process | CD4 | 454 | 4 | 3.783909 | -1.725649 | 60 | 17.2 | 0.286667 |
| GO:0031325\_positive\_regulation\_of\_cellular\_metabolic\_process | PLEK | 454 | 4 | 3.783909 | -1.725649 | 60 | 17.2 | 0.286667 |
| GO:0016311\_dephosphorylation | PTPN7 | 92 | 2 | 9.336384 | -1.721634 | 61 | 17.25 | 0.282787 |
| GO:0016311\_dephosphorylation | PLEK | 92 | 2 | 9.336384 | -1.721634 | 61 | 17.25 | 0.282787 |
| GO:0009605\_response\_to\_external\_stimulus | RNASE2 | 464 | 4 | 3.702359 | -1.694166 | 62 | 17.41 | 0.280806 |
| GO:0009605\_response\_to\_external\_stimulus | IGF1 | 464 | 4 | 3.702359 | -1.694166 | 62 | 17.41 | 0.280806 |
| GO:0009605\_response\_to\_external\_stimulus | CYBB | 464 | 4 | 3.702359 | -1.694166 | 62 | 17.41 | 0.280806 |
| GO:0009605\_response\_to\_external\_stimulus | PLEK | 464 | 4 | 3.702359 | -1.694166 | 62 | 17.41 | 0.280806 |
| GO:0006110\_regulation\_of\_glycolysis | IGF1 | 9 | 1 | 47.719298 | -1.682525 | 69 | 19.67 | 0.285072 |
| GO:0043470\_regulation\_of\_carbohydrate\_catabolic\_process | IGF1 | 9 | 1 | 47.719298 | -1.682525 | 69 | 19.67 | 0.285072 |
| GO:0043471\_regulation\_of\_cellular\_carbohydrate\_catabolic\_process | IGF1 | 9 | 1 | 47.719298 | -1.682525 | 69 | 19.67 | 0.285072 |
| GO:0045646\_regulation\_of\_erythrocyte\_differentiation | SPI1 | 9 | 1 | 47.719298 | -1.682525 | 69 | 19.67 | 0.285072 |
| GO:0048661\_positive\_regulation\_of\_smooth\_muscle\_cell\_proliferation | IGF1 | 9 | 1 | 47.719298 | -1.682525 | 69 | 19.67 | 0.285072 |
| GO:0050848\_regulation\_of\_calcium-mediated\_signaling | PLEK | 9 | 1 | 47.719298 | -1.682525 | 69 | 19.67 | 0.285072 |
| GO:0070304\_positive\_regulation\_of\_stress-activated\_protein\_kinase\_signaling\_pathway | CARD9 | 9 | 1 | 47.719298 | -1.682525 | 69 | 19.67 | 0.285072 |
| GO:0042060\_wound\_healing | IGF1 | 98 | 2 | 8.764769 | -1.670058 | 70 | 19.88 | 0.284000 |
| GO:0042060\_wound\_healing | PLEK | 98 | 2 | 8.764769 | -1.670058 | 70 | 19.88 | 0.284000 |
| GO:0009893\_positive\_regulation\_of\_metabolic\_process | IGF1 | 476 | 4 | 3.609023 | -1.657458 | 71 | 20.06 | 0.282535 |
| GO:0009893\_positive\_regulation\_of\_metabolic\_process | SPI1 | 476 | 4 | 3.609023 | -1.657458 | 71 | 20.06 | 0.282535 |
| GO:0009893\_positive\_regulation\_of\_metabolic\_process | CD4 | 476 | 4 | 3.609023 | -1.657458 | 71 | 20.06 | 0.282535 |
| GO:0009893\_positive\_regulation\_of\_metabolic\_process | PLEK | 476 | 4 | 3.609023 | -1.657458 | 71 | 20.06 | 0.282535 |
| GO:0048522\_positive\_regulation\_of\_cellular\_process | IGF1 | 1009 | 6 | 2.553857 | -1.637830 | 72 | 20.31 | 0.282083 |
| GO:0048522\_positive\_regulation\_of\_cellular\_process | SPI1 | 1009 | 6 | 2.553857 | -1.637830 | 72 | 20.31 | 0.282083 |
| GO:0048522\_positive\_regulation\_of\_cellular\_process | CD4 | 1009 | 6 | 2.553857 | -1.637830 | 72 | 20.31 | 0.282083 |
| GO:0048522\_positive\_regulation\_of\_cellular\_process | CARD9 | 1009 | 6 | 2.553857 | -1.637830 | 72 | 20.31 | 0.282083 |
| GO:0048522\_positive\_regulation\_of\_cellular\_process | BTK | 1009 | 6 | 2.553857 | -1.637830 | 72 | 20.31 | 0.282083 |
| GO:0048522\_positive\_regulation\_of\_cellular\_process | PLEK | 1009 | 6 | 2.553857 | -1.637830 | 72 | 20.31 | 0.282083 |
| GO:0019751\_polyol\_metabolic\_process | PLEK | 10 | 1 | 42.947368 | -1.637246 | 76 | 22.68 | 0.298421 |
| GO:0035264\_multicellular\_organism\_growth | IGF1 | 10 | 1 | 42.947368 | -1.637246 | 76 | 22.68 | 0.298421 |
| GO:0040014\_regulation\_of\_multicellular\_organism\_growth | IGF1 | 10 | 1 | 42.947368 | -1.637246 | 76 | 22.68 | 0.298421 |
| GO:0043500\_muscle\_adaptation | IGF1 | 10 | 1 | 42.947368 | -1.637246 | 76 | 22.68 | 0.298421 |
| GO:0048513\_organ\_development | IGF1 | 741 | 5 | 2.897933 | -1.616950 | 77 | 22.91 | 0.297532 |
| GO:0048513\_organ\_development | SPI1 | 741 | 5 | 2.897933 | -1.616950 | 77 | 22.91 | 0.297532 |
| GO:0048513\_organ\_development | CD4 | 741 | 5 | 2.897933 | -1.616950 | 77 | 22.91 | 0.297532 |
| GO:0048513\_organ\_development | PLEK | 741 | 5 | 2.897933 | -1.616950 | 77 | 22.91 | 0.297532 |
| GO:0048513\_organ\_development | BTK | 741 | 5 | 2.897933 | -1.616950 | 77 | 22.91 | 0.297532 |
| GO:0006904\_vesicle\_docking\_during\_exocytosis | PLEK | 11 | 1 | 39.043062 | -1.596331 | 81 | 25.54 | 0.315309 |
| GO:0010907\_positive\_regulation\_of\_glucose\_metabolic\_process | IGF1 | 11 | 1 | 39.043062 | -1.596331 | 81 | 25.54 | 0.315309 |
| GO:0032231\_regulation\_of\_actin\_filament\_bundle\_formation | PLEK | 11 | 1 | 39.043062 | -1.596331 | 81 | 25.54 | 0.315309 |
| GO:0045076\_regulation\_of\_interleukin-2\_biosynthetic\_process | CD4 | 11 | 1 | 39.043062 | -1.596331 | 81 | 25.54 | 0.315309 |
| GO:0009611\_response\_to\_wounding | IGF1 | 279 | 3 | 4.617997 | -1.592780 | 82 | 25.59 | 0.312073 |
| GO:0009611\_response\_to\_wounding | CYBB | 279 | 3 | 4.617997 | -1.592780 | 82 | 25.59 | 0.312073 |
| GO:0009611\_response\_to\_wounding | PLEK | 279 | 3 | 4.617997 | -1.592780 | 82 | 25.59 | 0.312073 |
| GO:0009101\_glycoprotein\_biosynthetic\_process | IGF1 | 109 | 2 | 7.880251 | -1.583788 | 83 | 25.95 | 0.312651 |
| GO:0009101\_glycoprotein\_biosynthetic\_process | CD37 | 109 | 2 | 7.880251 | -1.583788 | 83 | 25.95 | 0.312651 |
| GO:0030154\_cell\_differentiation | IGF1 | 506 | 4 | 3.395049 | -1.570441 | 84 | 26.1 | 0.310714 |
| GO:0030154\_cell\_differentiation | SPI1 | 506 | 4 | 3.395049 | -1.570441 | 84 | 26.1 | 0.310714 |
| GO:0030154\_cell\_differentiation | CD4 | 506 | 4 | 3.395049 | -1.570441 | 84 | 26.1 | 0.310714 |
| GO:0030154\_cell\_differentiation | PLEK | 506 | 4 | 3.395049 | -1.570441 | 84 | 26.1 | 0.310714 |
| GO:0031532\_actin\_cytoskeleton\_reorganization | PLEK | 12 | 1 | 35.789474 | -1.559020 | 87 | 28.32 | 0.325517 |
| GO:0042094\_interleukin-2\_biosynthetic\_process | CD4 | 12 | 1 | 35.789474 | -1.559020 | 87 | 28.32 | 0.325517 |
| GO:0048278\_vesicle\_docking | PLEK | 12 | 1 | 35.789474 | -1.559020 | 87 | 28.32 | 0.325517 |
| GO:0007165\_signal\_transduction | IGF1 | 2029 | 9 | 1.905009 | -1.558361 | 88 | 28.32 | 0.321818 |
| GO:0007165\_signal\_transduction | LILRA2 | 2029 | 9 | 1.905009 | -1.558361 | 88 | 28.32 | 0.321818 |
| GO:0007165\_signal\_transduction | CD33 | 2029 | 9 | 1.905009 | -1.558361 | 88 | 28.32 | 0.321818 |
| GO:0007165\_signal\_transduction | CARD9 | 2029 | 9 | 1.905009 | -1.558361 | 88 | 28.32 | 0.321818 |
| GO:0007165\_signal\_transduction | CD4 | 2029 | 9 | 1.905009 | -1.558361 | 88 | 28.32 | 0.321818 |
| GO:0007165\_signal\_transduction | BTK | 2029 | 9 | 1.905009 | -1.558361 | 88 | 28.32 | 0.321818 |
| GO:0007165\_signal\_transduction | PLEK | 2029 | 9 | 1.905009 | -1.558361 | 88 | 28.32 | 0.321818 |
| GO:0007165\_signal\_transduction | CSF3R | 2029 | 9 | 1.905009 | -1.558361 | 88 | 28.32 | 0.321818 |
| GO:0007165\_signal\_transduction | TYROBP | 2029 | 9 | 1.905009 | -1.558361 | 88 | 28.32 | 0.321818 |
| GO:0014065\_phosphoinositide\_3-kinase\_cascade | IGF1 | 13 | 1 | 33.036437 | -1.524736 | 90 | 31.25 | 0.347222 |
| GO:0050679\_positive\_regulation\_of\_epithelial\_cell\_proliferation | IGF1 | 13 | 1 | 33.036437 | -1.524736 | 90 | 31.25 | 0.347222 |
| GO:0019220\_regulation\_of\_phosphate\_metabolic\_process | IGF1 | 297 | 3 | 4.338118 | -1.522517 | 92 | 31.28 | 0.340000 |
| GO:0019220\_regulation\_of\_phosphate\_metabolic\_process | CD4 | 297 | 3 | 4.338118 | -1.522517 | 92 | 31.28 | 0.340000 |
| GO:0019220\_regulation\_of\_phosphate\_metabolic\_process | PLEK | 297 | 3 | 4.338118 | -1.522517 | 92 | 31.28 | 0.340000 |
| GO:0051174\_regulation\_of\_phosphorus\_metabolic\_process | IGF1 | 297 | 3 | 4.338118 | -1.522517 | 92 | 31.28 | 0.340000 |
| GO:0051174\_regulation\_of\_phosphorus\_metabolic\_process | CD4 | 297 | 3 | 4.338118 | -1.522517 | 92 | 31.28 | 0.340000 |
| GO:0051174\_regulation\_of\_phosphorus\_metabolic\_process | PLEK | 297 | 3 | 4.338118 | -1.522517 | 92 | 31.28 | 0.340000 |
| GO:0006955\_immune\_response | CYBB | 529 | 4 | 3.247438 | -1.507941 | 93 | 31.43 | 0.337957 |
| GO:0006955\_immune\_response | NCF1 | 529 | 4 | 3.247438 | -1.507941 | 93 | 31.43 | 0.337957 |
| GO:0006955\_immune\_response | CD4 | 529 | 4 | 3.247438 | -1.507941 | 93 | 31.43 | 0.337957 |
| GO:0006955\_immune\_response | NCF4 | 529 | 4 | 3.247438 | -1.507941 | 93 | 31.43 | 0.337957 |
| GO:0035303\_regulation\_of\_dephosphorylation | PLEK | 14 | 1 | 30.676692 | -1.493029 | 96 | 33.91 | 0.353229 |
| GO:0045445\_myoblast\_differentiation | IGF1 | 14 | 1 | 30.676692 | -1.493029 | 96 | 33.91 | 0.353229 |
| GO:0048009\_insulin-like\_growth\_factor\_receptor\_signaling\_pathway | IGF1 | 14 | 1 | 30.676692 | -1.493029 | 96 | 33.91 | 0.353229 |
| GO:0048518\_positive\_regulation\_of\_biological\_process | IGF1 | 1094 | 6 | 2.355432 | -1.481066 | 97 | 34.09 | 0.351443 |
| GO:0048518\_positive\_regulation\_of\_biological\_process | SPI1 | 1094 | 6 | 2.355432 | -1.481066 | 97 | 34.09 | 0.351443 |
| GO:0048518\_positive\_regulation\_of\_biological\_process | CARD9 | 1094 | 6 | 2.355432 | -1.481066 | 97 | 34.09 | 0.351443 |
| GO:0048518\_positive\_regulation\_of\_biological\_process | CD4 | 1094 | 6 | 2.355432 | -1.481066 | 97 | 34.09 | 0.351443 |
| GO:0048518\_positive\_regulation\_of\_biological\_process | BTK | 1094 | 6 | 2.355432 | -1.481066 | 97 | 34.09 | 0.351443 |
| GO:0048518\_positive\_regulation\_of\_biological\_process | PLEK | 1094 | 6 | 2.355432 | -1.481066 | 97 | 34.09 | 0.351443 |
| GO:0032663\_regulation\_of\_interleukin-2\_production | CD4 | 15 | 1 | 28.631579 | -1.463543 | 99 | 36.69 | 0.370606 |
| GO:0048146\_positive\_regulation\_of\_fibroblast\_proliferation | IGF1 | 15 | 1 | 28.631579 | -1.463543 | 99 | 36.69 | 0.370606 |
| GO:0010740\_positive\_regulation\_of\_protein\_kinase\_cascade | IGF1 | 129 | 2 | 6.658507 | -1.448790 | 100 | 36.91 | 0.369100 |
| GO:0010740\_positive\_regulation\_of\_protein\_kinase\_cascade | CARD9 | 129 | 2 | 6.658507 | -1.448790 | 100 | 36.91 | 0.369100 |
| GO:0048869\_cellular\_developmental\_process | IGF1 | 555 | 4 | 3.095306 | -1.441263 | 101 | 36.97 | 0.366040 |
| GO:0048869\_cellular\_developmental\_process | SPI1 | 555 | 4 | 3.095306 | -1.441263 | 101 | 36.97 | 0.366040 |
| GO:0048869\_cellular\_developmental\_process | CD4 | 555 | 4 | 3.095306 | -1.441263 | 101 | 36.97 | 0.366040 |
| GO:0048869\_cellular\_developmental\_process | PLEK | 555 | 4 | 3.095306 | -1.441263 | 101 | 36.97 | 0.366040 |
| GO:0032270\_positive\_regulation\_of\_cellular\_protein\_metabolic\_process | IGF1 | 131 | 2 | 6.556850 | -1.436569 | 103 | 37.08 | 0.360000 |
| GO:0032270\_positive\_regulation\_of\_cellular\_protein\_metabolic\_process | PLEK | 131 | 2 | 6.556850 | -1.436569 | 103 | 37.08 | 0.360000 |
| GO:0033043\_regulation\_of\_organelle\_organization | IGF1 | 131 | 2 | 6.556850 | -1.436569 | 103 | 37.08 | 0.360000 |
| GO:0033043\_regulation\_of\_organelle\_organization | PLEK | 131 | 2 | 6.556850 | -1.436569 | 103 | 37.08 | 0.360000 |
| GO:0010676\_positive\_regulation\_of\_cellular\_carbohydrate\_metabolic\_process | IGF1 | 16 | 1 | 26.842105 | -1.435992 | 110 | 38.68 | 0.351636 |
| GO:0010906\_regulation\_of\_glucose\_metabolic\_process | IGF1 | 16 | 1 | 26.842105 | -1.435992 | 110 | 38.68 | 0.351636 |
| GO:0032623\_interleukin-2\_production | CD4 | 16 | 1 | 26.842105 | -1.435992 | 110 | 38.68 | 0.351636 |
| GO:0044275\_cellular\_carbohydrate\_catabolic\_process | IGF1 | 16 | 1 | 26.842105 | -1.435992 | 110 | 38.68 | 0.351636 |
| GO:0045913\_positive\_regulation\_of\_carbohydrate\_metabolic\_process | IGF1 | 16 | 1 | 26.842105 | -1.435992 | 110 | 38.68 | 0.351636 |
| GO:0048589\_developmental\_growth | IGF1 | 16 | 1 | 26.842105 | -1.435992 | 110 | 38.68 | 0.351636 |
| GO:0051261\_protein\_depolymerization | PLEK | 16 | 1 | 26.842105 | -1.435992 | 110 | 38.68 | 0.351636 |
| GO:0044262\_cellular\_carbohydrate\_metabolic\_process | IGF1 | 133 | 2 | 6.458251 | -1.424551 | 111 | 38.98 | 0.351171 |
| GO:0044262\_cellular\_carbohydrate\_metabolic\_process | CD37 | 133 | 2 | 6.458251 | -1.424551 | 111 | 38.98 | 0.351171 |
| GO:0051247\_positive\_regulation\_of\_protein\_metabolic\_process | IGF1 | 135 | 2 | 6.362573 | -1.412732 | 112 | 39.47 | 0.352411 |
| GO:0051247\_positive\_regulation\_of\_protein\_metabolic\_process | PLEK | 135 | 2 | 6.362573 | -1.412732 | 112 | 39.47 | 0.352411 |
| GO:0008064\_regulation\_of\_actin\_polymerization\_or\_depolymerization | PLEK | 17 | 1 | 25.263158 | -1.410140 | 117 | 41.04 | 0.350769 |
| GO:0022406\_membrane\_docking | PLEK | 17 | 1 | 25.263158 | -1.410140 | 117 | 41.04 | 0.350769 |
| GO:0048144\_fibroblast\_proliferation | IGF1 | 17 | 1 | 25.263158 | -1.410140 | 117 | 41.04 | 0.350769 |
| GO:0048145\_regulation\_of\_fibroblast\_proliferation | IGF1 | 17 | 1 | 25.263158 | -1.410140 | 117 | 41.04 | 0.350769 |
| GO:0048660\_regulation\_of\_smooth\_muscle\_cell\_proliferation | IGF1 | 17 | 1 | 25.263158 | -1.410140 | 117 | 41.04 | 0.350769 |
| GO:0010557\_positive\_regulation\_of\_macromolecule\_biosynthetic\_process | IGF1 | 334 | 3 | 3.857548 | -1.392639 | 118 | 41.57 | 0.352288 |
| GO:0010557\_positive\_regulation\_of\_macromolecule\_biosynthetic\_process | SPI1 | 334 | 3 | 3.857548 | -1.392639 | 118 | 41.57 | 0.352288 |
| GO:0010557\_positive\_regulation\_of\_macromolecule\_biosynthetic\_process | CD4 | 334 | 3 | 3.857548 | -1.392639 | 118 | 41.57 | 0.352288 |
| GO:0009100\_glycoprotein\_metabolic\_process | IGF1 | 139 | 2 | 6.179477 | -1.389662 | 119 | 41.62 | 0.349748 |
| GO:0009100\_glycoprotein\_metabolic\_process | CD37 | 139 | 2 | 6.179477 | -1.389662 | 119 | 41.62 | 0.349748 |
| GO:0048659\_smooth\_muscle\_cell\_proliferation | IGF1 | 18 | 1 | 23.859649 | -1.385794 | 120 | 43.06 | 0.358833 |
| GO:0007242\_intracellular\_signaling\_cascade | IGF1 | 853 | 5 | 2.517431 | -1.383308 | 121 | 43.22 | 0.357190 |
| GO:0007242\_intracellular\_signaling\_cascade | CARD9 | 853 | 5 | 2.517431 | -1.383308 | 121 | 43.22 | 0.357190 |
| GO:0007242\_intracellular\_signaling\_cascade | BTK | 853 | 5 | 2.517431 | -1.383308 | 121 | 43.22 | 0.357190 |
| GO:0007242\_intracellular\_signaling\_cascade | PLEK | 853 | 5 | 2.517431 | -1.383308 | 121 | 43.22 | 0.357190 |
| GO:0007242\_intracellular\_signaling\_cascade | TYROBP | 853 | 5 | 2.517431 | -1.383308 | 121 | 43.22 | 0.357190 |
| GO:0030832\_regulation\_of\_actin\_filament\_length | PLEK | 19 | 1 | 22.603878 | -1.362790 | 126 | 45.58 | 0.361746 |
| GO:0031331\_positive\_regulation\_of\_cellular\_catabolic\_process | IGF1 | 19 | 1 | 22.603878 | -1.362790 | 126 | 45.58 | 0.361746 |
| GO:0042531\_positive\_regulation\_of\_tyrosine\_phosphorylation\_of\_STAT\_protein | IGF1 | 19 | 1 | 22.603878 | -1.362790 | 126 | 45.58 | 0.361746 |
| GO:0043467\_regulation\_of\_generation\_of\_precursor\_metabolites\_and\_energy | IGF1 | 19 | 1 | 22.603878 | -1.362790 | 126 | 45.58 | 0.361746 |
| GO:0051495\_positive\_regulation\_of\_cytoskeleton\_organization | PLEK | 19 | 1 | 22.603878 | -1.362790 | 126 | 45.58 | 0.361746 |
| GO:0080134\_regulation\_of\_response\_to\_stress | CARD9 | 147 | 2 | 5.843179 | -1.345655 | 127 | 46.06 | 0.362677 |
| GO:0080134\_regulation\_of\_response\_to\_stress | PLEK | 147 | 2 | 5.843179 | -1.345655 | 127 | 46.06 | 0.362677 |
| GO:0006096\_glycolysis | IGF1 | 20 | 1 | 21.473684 | -1.340990 | 132 | 47.89 | 0.362803 |
| GO:0032535\_regulation\_of\_cellular\_component\_size | PLEK | 20 | 1 | 21.473684 | -1.340990 | 132 | 47.89 | 0.362803 |
| GO:0043244\_regulation\_of\_protein\_complex\_disassembly | PLEK | 20 | 1 | 21.473684 | -1.340990 | 132 | 47.89 | 0.362803 |
| GO:0045840\_positive\_regulation\_of\_mitosis | IGF1 | 20 | 1 | 21.473684 | -1.340990 | 132 | 47.89 | 0.362803 |
| GO:0051785\_positive\_regulation\_of\_nuclear\_division | IGF1 | 20 | 1 | 21.473684 | -1.340990 | 132 | 47.89 | 0.362803 |
| GO:0031328\_positive\_regulation\_of\_cellular\_biosynthetic\_process | IGF1 | 352 | 3 | 3.660287 | -1.335515 | 133 | 47.99 | 0.360827 |
| GO:0031328\_positive\_regulation\_of\_cellular\_biosynthetic\_process | SPI1 | 352 | 3 | 3.660287 | -1.335515 | 133 | 47.99 | 0.360827 |
| GO:0031328\_positive\_regulation\_of\_cellular\_biosynthetic\_process | CD4 | 352 | 3 | 3.660287 | -1.335515 | 133 | 47.99 | 0.360827 |
| GO:0016310\_phosphorylation | IGF1 | 601 | 4 | 2.858394 | -1.332501 | 134 | 48.03 | 0.358433 |
| GO:0016310\_phosphorylation | FGR | 601 | 4 | 2.858394 | -1.332501 | 134 | 48.03 | 0.358433 |
| GO:0016310\_phosphorylation | CD4 | 601 | 4 | 2.858394 | -1.332501 | 134 | 48.03 | 0.358433 |
| GO:0016310\_phosphorylation | BTK | 601 | 4 | 2.858394 | -1.332501 | 134 | 48.03 | 0.358433 |
| GO:0006109\_regulation\_of\_carbohydrate\_metabolic\_process | IGF1 | 21 | 1 | 20.451128 | -1.320278 | 137 | 50.39 | 0.367810 |
| GO:0010675\_regulation\_of\_cellular\_carbohydrate\_metabolic\_process | IGF1 | 21 | 1 | 20.451128 | -1.320278 | 137 | 50.39 | 0.367810 |
| GO:0046427\_positive\_regulation\_of\_JAK-STAT\_cascade | IGF1 | 21 | 1 | 20.451128 | -1.320278 | 137 | 50.39 | 0.367810 |
| GO:0009891\_positive\_regulation\_of\_biosynthetic\_process | IGF1 | 359 | 3 | 3.588917 | -1.314244 | 138 | 50.55 | 0.366304 |
| GO:0009891\_positive\_regulation\_of\_biosynthetic\_process | SPI1 | 359 | 3 | 3.588917 | -1.314244 | 138 | 50.55 | 0.366304 |
| GO:0009891\_positive\_regulation\_of\_biosynthetic\_process | CD4 | 359 | 3 | 3.588917 | -1.314244 | 138 | 50.55 | 0.366304 |
| GO:0030218\_erythrocyte\_differentiation | SPI1 | 22 | 1 | 19.521531 | -1.300551 | 142 | 52.27 | 0.368099 |
| GO:0043410\_positive\_regulation\_of\_MAPKKK\_cascade | CARD9 | 22 | 1 | 19.521531 | -1.300551 | 142 | 52.27 | 0.368099 |
| GO:0046165\_alcohol\_biosynthetic\_process | PLEK | 22 | 1 | 19.521531 | -1.300551 | 142 | 52.27 | 0.368099 |
| GO:0051017\_actin\_filament\_bundle\_formation | PLEK | 22 | 1 | 19.521531 | -1.300551 | 142 | 52.27 | 0.368099 |
| GO:0007169\_transmembrane\_receptor\_protein\_tyrosine\_kinase\_signaling\_pathway | IGF1 | 157 | 2 | 5.471002 | -1.294257 | 143 | 52.55 | 0.367483 |
| GO:0007169\_transmembrane\_receptor\_protein\_tyrosine\_kinase\_signaling\_pathway | CD4 | 157 | 2 | 5.471002 | -1.294257 | 143 | 52.55 | 0.367483 |
| GO:0030166\_proteoglycan\_biosynthetic\_process | IGF1 | 23 | 1 | 18.672769 | -1.281722 | 145 | 53.8 | 0.371034 |
| GO:0045740\_positive\_regulation\_of\_DNA\_replication | IGF1 | 23 | 1 | 18.672769 | -1.281722 | 145 | 53.8 | 0.371034 |
| GO:0007229\_integrin-mediated\_signaling\_pathway | PLEK | 24 | 1 | 17.894737 | -1.263715 | 150 | 55.45 | 0.369667 |
| GO:0019059\_initiation\_of\_viral\_infection | CD4 | 24 | 1 | 17.894737 | -1.263715 | 150 | 55.45 | 0.369667 |
| GO:0030193\_regulation\_of\_blood\_coagulation | PLEK | 24 | 1 | 17.894737 | -1.263715 | 150 | 55.45 | 0.369667 |
| GO:0033002\_muscle\_cell\_proliferation | IGF1 | 24 | 1 | 17.894737 | -1.263715 | 150 | 55.45 | 0.369667 |
| GO:0042509\_regulation\_of\_tyrosine\_phosphorylation\_of\_STAT\_protein | IGF1 | 24 | 1 | 17.894737 | -1.263715 | 150 | 55.45 | 0.369667 |
| GO:0007243\_protein\_kinase\_cascade | IGF1 | 377 | 3 | 3.417562 | -1.261788 | 151 | 55.59 | 0.368146 |
| GO:0007243\_protein\_kinase\_cascade | CARD9 | 377 | 3 | 3.417562 | -1.261788 | 151 | 55.59 | 0.368146 |
| GO:0007243\_protein\_kinase\_cascade | PLEK | 377 | 3 | 3.417562 | -1.261788 | 151 | 55.59 | 0.368146 |
| GO:0007154\_cell\_communication | IGF1 | 2272 | 9 | 1.701260 | -1.261431 | 152 | 55.67 | 0.366250 |
| GO:0007154\_cell\_communication | LILRA2 | 2272 | 9 | 1.701260 | -1.261431 | 152 | 55.67 | 0.366250 |
| GO:0007154\_cell\_communication | CD33 | 2272 | 9 | 1.701260 | -1.261431 | 152 | 55.67 | 0.366250 |
| GO:0007154\_cell\_communication | CARD9 | 2272 | 9 | 1.701260 | -1.261431 | 152 | 55.67 | 0.366250 |
| GO:0007154\_cell\_communication | CD4 | 2272 | 9 | 1.701260 | -1.261431 | 152 | 55.67 | 0.366250 |
| GO:0007154\_cell\_communication | BTK | 2272 | 9 | 1.701260 | -1.261431 | 152 | 55.67 | 0.366250 |
| GO:0007154\_cell\_communication | PLEK | 2272 | 9 | 1.701260 | -1.261431 | 152 | 55.67 | 0.366250 |
| GO:0007154\_cell\_communication | CSF3R | 2272 | 9 | 1.701260 | -1.261431 | 152 | 55.67 | 0.366250 |
| GO:0007154\_cell\_communication | TYROBP | 2272 | 9 | 1.701260 | -1.261431 | 152 | 55.67 | 0.366250 |
| GO:0006464\_protein\_modification\_process | IGF1 | 922 | 5 | 2.329033 | -1.259035 | 153 | 55.76 | 0.364444 |
| GO:0006464\_protein\_modification\_process | PTPN7 | 922 | 5 | 2.329033 | -1.259035 | 153 | 55.76 | 0.364444 |
| GO:0006464\_protein\_modification\_process | FGR | 922 | 5 | 2.329033 | -1.259035 | 153 | 55.76 | 0.364444 |
| GO:0006464\_protein\_modification\_process | BTK | 922 | 5 | 2.329033 | -1.259035 | 153 | 55.76 | 0.364444 |
| GO:0006464\_protein\_modification\_process | CD37 | 922 | 5 | 2.329033 | -1.259035 | 153 | 55.76 | 0.364444 |
| GO:0009966\_regulation\_of\_signal\_transduction | IGF1 | 378 | 3 | 3.408521 | -1.258963 | 154 | 55.83 | 0.362532 |
| GO:0009966\_regulation\_of\_signal\_transduction | CARD9 | 378 | 3 | 3.408521 | -1.258963 | 154 | 55.83 | 0.362532 |
| GO:0009966\_regulation\_of\_signal\_transduction | PLEK | 378 | 3 | 3.408521 | -1.258963 | 154 | 55.83 | 0.362532 |
| GO:0006007\_glucose\_catabolic\_process | IGF1 | 25 | 1 | 17.178947 | -1.246462 | 160 | 57.62 | 0.360125 |
| GO:0030168\_platelet\_activation | PLEK | 25 | 1 | 17.178947 | -1.246462 | 160 | 57.62 | 0.360125 |
| GO:0034101\_erythrocyte\_homeostasis | SPI1 | 25 | 1 | 17.178947 | -1.246462 | 160 | 57.62 | 0.360125 |
| GO:0043624\_cellular\_protein\_complex\_disassembly | PLEK | 25 | 1 | 17.178947 | -1.246462 | 160 | 57.62 | 0.360125 |
| GO:0050678\_regulation\_of\_epithelial\_cell\_proliferation | IGF1 | 25 | 1 | 17.178947 | -1.246462 | 160 | 57.62 | 0.360125 |
| GO:0050818\_regulation\_of\_coagulation | PLEK | 25 | 1 | 17.178947 | -1.246462 | 160 | 57.62 | 0.360125 |
| GO:0045595\_regulation\_of\_cell\_differentiation | IGF1 | 170 | 2 | 5.052632 | -1.232680 | 161 | 57.9 | 0.359627 |
| GO:0045595\_regulation\_of\_cell\_differentiation | SPI1 | 170 | 2 | 5.052632 | -1.232680 | 161 | 57.9 | 0.359627 |
| GO:0007498\_mesoderm\_development | BTK | 26 | 1 | 16.518219 | -1.229905 | 164 | 59.41 | 0.362256 |
| GO:0043241\_protein\_complex\_disassembly | PLEK | 26 | 1 | 16.518219 | -1.229905 | 164 | 59.41 | 0.362256 |
| GO:0050673\_epithelial\_cell\_proliferation | IGF1 | 26 | 1 | 16.518219 | -1.229905 | 164 | 59.41 | 0.362256 |
| GO:0006468\_protein\_amino\_acid\_phosphorylation | IGF1 | 393 | 3 | 3.278425 | -1.217673 | 165 | 59.72 | 0.361939 |
| GO:0006468\_protein\_amino\_acid\_phosphorylation | FGR | 393 | 3 | 3.278425 | -1.217673 | 165 | 59.72 | 0.361939 |
| GO:0006468\_protein\_amino\_acid\_phosphorylation | BTK | 393 | 3 | 3.278425 | -1.217673 | 165 | 59.72 | 0.361939 |
| GO:0007260\_tyrosine\_phosphorylation\_of\_STAT\_protein | IGF1 | 27 | 1 | 15.906433 | -1.213990 | 166 | 61.16 | 0.368434 |
| GO:0001775\_cell\_activation | CD4 | 175 | 2 | 4.908271 | -1.210400 | 167 | 61.36 | 0.367425 |
| GO:0001775\_cell\_activation | PLEK | 175 | 2 | 4.908271 | -1.210400 | 167 | 61.36 | 0.367425 |
| GO:0042592\_homeostatic\_process | IGF1 | 397 | 3 | 3.245393 | -1.206990 | 168 | 61.38 | 0.365357 |
| GO:0042592\_homeostatic\_process | CD52 | 397 | 3 | 3.245393 | -1.206990 | 168 | 61.38 | 0.365357 |
| GO:0042592\_homeostatic\_process | SPI1 | 397 | 3 | 3.245393 | -1.206990 | 168 | 61.38 | 0.365357 |
| GO:0043412\_biopolymer\_modification | IGF1 | 960 | 5 | 2.236842 | -1.195993 | 169 | 63.38 | 0.375030 |
| GO:0043412\_biopolymer\_modification | PTPN7 | 960 | 5 | 2.236842 | -1.195993 | 169 | 63.38 | 0.375030 |
| GO:0043412\_biopolymer\_modification | FGR | 960 | 5 | 2.236842 | -1.195993 | 169 | 63.38 | 0.375030 |
| GO:0043412\_biopolymer\_modification | BTK | 960 | 5 | 2.236842 | -1.195993 | 169 | 63.38 | 0.375030 |
| GO:0043412\_biopolymer\_modification | CD37 | 960 | 5 | 2.236842 | -1.195993 | 169 | 63.38 | 0.375030 |
| GO:0046425\_regulation\_of\_JAK-STAT\_cascade | IGF1 | 29 | 1 | 14.809437 | -1.183908 | 172 | 65.19 | 0.379012 |
| GO:0048741\_skeletal\_muscle\_fiber\_development | IGF1 | 29 | 1 | 14.809437 | -1.183908 | 172 | 65.19 | 0.379012 |
| GO:0050731\_positive\_regulation\_of\_peptidyl-tyrosine\_phosphorylation | IGF1 | 29 | 1 | 14.809437 | -1.183908 | 172 | 65.19 | 0.379012 |
| GO:0010627\_regulation\_of\_protein\_kinase\_cascade | IGF1 | 184 | 2 | 4.668192 | -1.172064 | 173 | 65.55 | 0.378902 |
| GO:0010627\_regulation\_of\_protein\_kinase\_cascade | CARD9 | 184 | 2 | 4.668192 | -1.172064 | 173 | 65.55 | 0.378902 |
| GO:0008154\_actin\_polymerization\_or\_depolymerization | PLEK | 30 | 1 | 14.315789 | -1.169660 | 175 | 66.61 | 0.380629 |
| GO:0048747\_muscle\_fiber\_development | IGF1 | 30 | 1 | 14.315789 | -1.169660 | 175 | 66.61 | 0.380629 |
| GO:0009967\_positive\_regulation\_of\_signal\_transduction | IGF1 | 185 | 2 | 4.642959 | -1.167937 | 176 | 66.75 | 0.379261 |
| GO:0009967\_positive\_regulation\_of\_signal\_transduction | CARD9 | 185 | 2 | 4.642959 | -1.167937 | 176 | 66.75 | 0.379261 |
| GO:0007200\_activation\_of\_phospholipase\_C\_activity\_by\_G-protein\_coupled\_receptor\_protein\_signaling\_pathway\_coupled\_to\_IP3\_second\_messenger | PLEK | 31 | 1 | 13.853990 | -1.155895 | 177 | 67.79 | 0.382994 |
| GO:0010647\_positive\_regulation\_of\_cell\_communication | IGF1 | 189 | 2 | 4.544695 | -1.151681 | 178 | 67.85 | 0.381180 |
| GO:0010647\_positive\_regulation\_of\_cell\_communication | CARD9 | 189 | 2 | 4.544695 | -1.151681 | 178 | 67.85 | 0.381180 |
| GO:0006029\_proteoglycan\_metabolic\_process | IGF1 | 32 | 1 | 13.421053 | -1.142581 | 184 | 69.41 | 0.377228 |
| GO:0006487\_protein\_amino\_acid\_N-linked\_glycosylation | CD37 | 32 | 1 | 13.421053 | -1.142581 | 184 | 69.41 | 0.377228 |
| GO:0031329\_regulation\_of\_cellular\_catabolic\_process | IGF1 | 32 | 1 | 13.421053 | -1.142581 | 184 | 69.41 | 0.377228 |
| GO:0034623\_cellular\_macromolecular\_complex\_disassembly | PLEK | 32 | 1 | 13.421053 | -1.142581 | 184 | 69.41 | 0.377228 |
| GO:0046578\_regulation\_of\_Ras\_protein\_signal\_transduction | IGF1 | 32 | 1 | 13.421053 | -1.142581 | 184 | 69.41 | 0.377228 |
| GO:0051056\_regulation\_of\_small\_GTPase\_mediated\_signal\_transduction | IGF1 | 32 | 1 | 13.421053 | -1.142581 | 184 | 69.41 | 0.377228 |
| GO:0010646\_regulation\_of\_cell\_communication | IGF1 | 423 | 3 | 3.045913 | -1.140669 | 185 | 69.44 | 0.375351 |
| GO:0010646\_regulation\_of\_cell\_communication | CARD9 | 423 | 3 | 3.045913 | -1.140669 | 185 | 69.44 | 0.375351 |
| GO:0010646\_regulation\_of\_cell\_communication | PLEK | 423 | 3 | 3.045913 | -1.140669 | 185 | 69.44 | 0.375351 |
| GO:0030217\_T\_cell\_differentiation | CD4 | 33 | 1 | 13.014354 | -1.129693 | 187 | 70.49 | 0.376952 |
| GO:0042108\_positive\_regulation\_of\_cytokine\_biosynthetic\_process | CD4 | 33 | 1 | 13.014354 | -1.129693 | 187 | 70.49 | 0.376952 |
| GO:0008277\_regulation\_of\_G-protein\_coupled\_receptor\_protein\_signaling\_pathway | PLEK | 34 | 1 | 12.631579 | -1.117202 | 189 | 72.94 | 0.385926 |
| GO:0008624\_induction\_of\_apoptosis\_by\_extracellular\_signals | BTK | 34 | 1 | 12.631579 | -1.117202 | 189 | 72.94 | 0.385926 |
| GO:0009896\_positive\_regulation\_of\_catabolic\_process | IGF1 | 35 | 1 | 12.270677 | -1.105088 | 193 | 74.22 | 0.384560 |
| GO:0019320\_hexose\_catabolic\_process | IGF1 | 35 | 1 | 12.270677 | -1.105088 | 193 | 74.22 | 0.384560 |
| GO:0048872\_homeostasis\_of\_number\_of\_cells | SPI1 | 35 | 1 | 12.270677 | -1.105088 | 193 | 74.22 | 0.384560 |
| GO:0051346\_negative\_regulation\_of\_hydrolase\_activity | PLEK | 35 | 1 | 12.270677 | -1.105088 | 193 | 74.22 | 0.384560 |
| GO:0006066\_alcohol\_metabolic\_process | IGF1 | 206 | 2 | 4.169647 | -1.086758 | 194 | 75.51 | 0.389227 |
| GO:0006066\_alcohol\_metabolic\_process | PLEK | 206 | 2 | 4.169647 | -1.086758 | 194 | 75.51 | 0.389227 |
| GO:0032956\_regulation\_of\_actin\_cytoskeleton\_organization | PLEK | 37 | 1 | 11.607397 | -1.081904 | 195 | 76.58 | 0.392718 |
| GO:0043687\_post-translational\_protein\_modification | IGF1 | 728 | 4 | 2.359746 | -1.081439 | 196 | 76.66 | 0.391122 |
| GO:0043687\_post-translational\_protein\_modification | PTPN7 | 728 | 4 | 2.359746 | -1.081439 | 196 | 76.66 | 0.391122 |
| GO:0043687\_post-translational\_protein\_modification | FGR | 728 | 4 | 2.359746 | -1.081439 | 196 | 76.66 | 0.391122 |
| GO:0043687\_post-translational\_protein\_modification | BTK | 728 | 4 | 2.359746 | -1.081439 | 196 | 76.66 | 0.391122 |
| GO:0045787\_positive\_regulation\_of\_cell\_cycle | IGF1 | 38 | 1 | 11.301939 | -1.070796 | 198 | 78.37 | 0.395808 |
| GO:0050730\_regulation\_of\_peptidyl-tyrosine\_phosphorylation | IGF1 | 38 | 1 | 11.301939 | -1.070796 | 198 | 78.37 | 0.395808 |
| GO:0045893\_positive\_regulation\_of\_transcription\_\_DNA-dependent | IGF1 | 211 | 2 | 4.070841 | -1.068838 | 199 | 78.49 | 0.394422 |
| GO:0045893\_positive\_regulation\_of\_transcription\_\_DNA-dependent | SPI1 | 211 | 2 | 4.070841 | -1.068838 | 199 | 78.49 | 0.394422 |
| GO:0051254\_positive\_regulation\_of\_RNA\_metabolic\_process | IGF1 | 213 | 2 | 4.032617 | -1.061809 | 200 | 78.77 | 0.393850 |
| GO:0051254\_positive\_regulation\_of\_RNA\_metabolic\_process | SPI1 | 213 | 2 | 4.032617 | -1.061809 | 200 | 78.77 | 0.393850 |
| GO:0006401\_RNA\_catabolic\_process | RNASE2 | 39 | 1 | 11.012146 | -1.059989 | 204 | 79.82 | 0.391275 |
| GO:0032970\_regulation\_of\_actin\_filament-based\_process | PLEK | 39 | 1 | 11.012146 | -1.059989 | 204 | 79.82 | 0.391275 |
| GO:0046328\_regulation\_of\_JNK\_cascade | CARD9 | 39 | 1 | 11.012146 | -1.059989 | 204 | 79.82 | 0.391275 |
| GO:0046365\_monosaccharide\_catabolic\_process | IGF1 | 39 | 1 | 11.012146 | -1.059989 | 204 | 79.82 | 0.391275 |
| GO:0044267\_cellular\_protein\_metabolic\_process | IGF1 | 1382 | 6 | 1.864575 | -1.057677 | 205 | 80.02 | 0.390341 |
| GO:0044267\_cellular\_protein\_metabolic\_process | PTPN7 | 1382 | 6 | 1.864575 | -1.057677 | 205 | 80.02 | 0.390341 |
| GO:0044267\_cellular\_protein\_metabolic\_process | FGR | 1382 | 6 | 1.864575 | -1.057677 | 205 | 80.02 | 0.390341 |
| GO:0044267\_cellular\_protein\_metabolic\_process | PLEK | 1382 | 6 | 1.864575 | -1.057677 | 205 | 80.02 | 0.390341 |
| GO:0044267\_cellular\_protein\_metabolic\_process | BTK | 1382 | 6 | 1.864575 | -1.057677 | 205 | 80.02 | 0.390341 |
| GO:0044267\_cellular\_protein\_metabolic\_process | CD37 | 1382 | 6 | 1.864575 | -1.057677 | 205 | 80.02 | 0.390341 |
| GO:0008152\_metabolic\_process | RNASE2 | 4111 | 13 | 1.358102 | -1.053007 | 206 | 80.25 | 0.389563 |
| GO:0008152\_metabolic\_process | CD52 | 4111 | 13 | 1.358102 | -1.053007 | 206 | 80.25 | 0.389563 |
| GO:0008152\_metabolic\_process | PTPN7 | 4111 | 13 | 1.358102 | -1.053007 | 206 | 80.25 | 0.389563 |
| GO:0008152\_metabolic\_process | CYBB | 4111 | 13 | 1.358102 | -1.053007 | 206 | 80.25 | 0.389563 |
| GO:0008152\_metabolic\_process | NCF1 | 4111 | 13 | 1.358102 | -1.053007 | 206 | 80.25 | 0.389563 |
| GO:0008152\_metabolic\_process | CD4 | 4111 | 13 | 1.358102 | -1.053007 | 206 | 80.25 | 0.389563 |
| GO:0008152\_metabolic\_process | BTK | 4111 | 13 | 1.358102 | -1.053007 | 206 | 80.25 | 0.389563 |
| GO:0008152\_metabolic\_process | NCF4 | 4111 | 13 | 1.358102 | -1.053007 | 206 | 80.25 | 0.389563 |
| GO:0008152\_metabolic\_process | CD37 | 4111 | 13 | 1.358102 | -1.053007 | 206 | 80.25 | 0.389563 |
| GO:0008152\_metabolic\_process | IGF1 | 4111 | 13 | 1.358102 | -1.053007 | 206 | 80.25 | 0.389563 |
| GO:0008152\_metabolic\_process | FGR | 4111 | 13 | 1.358102 | -1.053007 | 206 | 80.25 | 0.389563 |
| GO:0008152\_metabolic\_process | SPI1 | 4111 | 13 | 1.358102 | -1.053007 | 206 | 80.25 | 0.389563 |
| GO:0008152\_metabolic\_process | PLEK | 4111 | 13 | 1.358102 | -1.053007 | 206 | 80.25 | 0.389563 |
| GO:0070302\_regulation\_of\_stress-activated\_protein\_kinase\_signaling\_pathway | CARD9 | 40 | 1 | 10.736842 | -1.049468 | 207 | 81.18 | 0.392174 |
| GO:0007519\_skeletal\_muscle\_tissue\_development | IGF1 | 41 | 1 | 10.474968 | -1.039218 | 209 | 82.5 | 0.394737 |
| GO:0060538\_skeletal\_muscle\_organ\_development | IGF1 | 41 | 1 | 10.474968 | -1.039218 | 209 | 82.5 | 0.394737 |
| GO:0006887\_exocytosis | PLEK | 42 | 1 | 10.225564 | -1.029226 | 211 | 84.2 | 0.399052 |
| GO:0042692\_muscle\_cell\_differentiation | IGF1 | 42 | 1 | 10.225564 | -1.029226 | 211 | 84.2 | 0.399052 |
| GO:0045637\_regulation\_of\_myeloid\_cell\_differentiation | SPI1 | 43 | 1 | 9.987760 | -1.019481 | 214 | 85.82 | 0.401028 |
| GO:0046164\_alcohol\_catabolic\_process | IGF1 | 43 | 1 | 9.987760 | -1.019481 | 214 | 85.82 | 0.401028 |
| GO:0051054\_positive\_regulation\_of\_DNA\_metabolic\_process | IGF1 | 43 | 1 | 9.987760 | -1.019481 | 214 | 85.82 | 0.401028 |
| GO:0051704\_multi-organism\_process | FGR | 231 | 2 | 3.718387 | -1.001828 | 215 | 87.42 | 0.406605 |
| GO:0051704\_multi-organism\_process | CD4 | 231 | 2 | 3.718387 | -1.001828 | 215 | 87.42 | 0.406605 |
| GO:0007202\_activation\_of\_phospholipase\_C\_activity | PLEK | 46 | 1 | 9.336384 | -0.991612 | 219 | 90.09 | 0.411370 |
| GO:0010863\_positive\_regulation\_of\_phospholipase\_C\_activity | PLEK | 46 | 1 | 9.336384 | -0.991612 | 219 | 90.09 | 0.411370 |
| GO:0030335\_positive\_regulation\_of\_cell\_migration | IGF1 | 46 | 1 | 9.336384 | -0.991612 | 219 | 90.09 | 0.411370 |
| GO:0030384\_phosphoinositide\_metabolic\_process | PLEK | 46 | 1 | 9.336384 | -0.991612 | 219 | 90.09 | 0.411370 |
| GO:0051128\_regulation\_of\_cellular\_component\_organization | IGF1 | 237 | 2 | 3.624250 | -0.983050 | 220 | 90.22 | 0.410091 |
| GO:0051128\_regulation\_of\_cellular\_component\_organization | PLEK | 237 | 2 | 3.624250 | -0.983050 | 220 | 90.22 | 0.410091 |
| GO:0007088\_regulation\_of\_mitosis | IGF1 | 47 | 1 | 9.137738 | -0.982745 | 223 | 91.59 | 0.410717 |
| GO:0007259\_JAK-STAT\_cascade | IGF1 | 47 | 1 | 9.137738 | -0.982745 | 223 | 91.59 | 0.410717 |
| GO:0051783\_regulation\_of\_nuclear\_division | IGF1 | 47 | 1 | 9.137738 | -0.982745 | 223 | 91.59 | 0.410717 |
| GO:0044265\_cellular\_macromolecule\_catabolic\_process | RNASE2 | 239 | 2 | 3.593922 | -0.976916 | 224 | 91.82 | 0.409911 |
| GO:0044265\_cellular\_macromolecule\_catabolic\_process | IGF1 | 239 | 2 | 3.593922 | -0.976916 | 224 | 91.82 | 0.409911 |
| GO:0010518\_positive\_regulation\_of\_phospholipase\_activity | PLEK | 48 | 1 | 8.947368 | -0.974074 | 226 | 92.51 | 0.409336 |
| GO:0042035\_regulation\_of\_cytokine\_biosynthetic\_process | CD4 | 48 | 1 | 8.947368 | -0.974074 | 226 | 92.51 | 0.409336 |
| GO:0048583\_regulation\_of\_response\_to\_stimulus | CARD9 | 241 | 2 | 3.564097 | -0.970843 | 227 | 92.7 | 0.408370 |
| GO:0048583\_regulation\_of\_response\_to\_stimulus | PLEK | 241 | 2 | 3.564097 | -0.970843 | 227 | 92.7 | 0.408370 |
| GO:0006275\_regulation\_of\_DNA\_replication | IGF1 | 49 | 1 | 8.764769 | -0.965592 | 232 | 93.81 | 0.404353 |
| GO:0010517\_regulation\_of\_phospholipase\_activity | PLEK | 49 | 1 | 8.764769 | -0.965592 | 232 | 93.81 | 0.404353 |
| GO:0018108\_peptidyl-tyrosine\_phosphorylation | IGF1 | 49 | 1 | 8.764769 | -0.965592 | 232 | 93.81 | 0.404353 |
| GO:0032984\_macromolecular\_complex\_disassembly | PLEK | 49 | 1 | 8.764769 | -0.965592 | 232 | 93.81 | 0.404353 |
| GO:0050867\_positive\_regulation\_of\_cell\_activation | PLEK | 49 | 1 | 8.764769 | -0.965592 | 232 | 93.81 | 0.404353 |
| GO:0030098\_lymphocyte\_differentiation | CD4 | 50 | 1 | 8.589474 | -0.957291 | 234 | 95.4 | 0.407692 |
| GO:0051272\_positive\_regulation\_of\_cell\_motion | IGF1 | 50 | 1 | 8.589474 | -0.957291 | 234 | 95.4 | 0.407692 |
| GO:0016052\_carbohydrate\_catabolic\_process | IGF1 | 51 | 1 | 8.421053 | -0.949163 | 237 | 96.81 | 0.408481 |
| GO:0018212\_peptidyl-tyrosine\_modification | IGF1 | 51 | 1 | 8.421053 | -0.949163 | 237 | 96.81 | 0.408481 |
| GO:0030518\_steroid\_hormone\_receptor\_signaling\_pathway | IGF1 | 51 | 1 | 8.421053 | -0.949163 | 237 | 96.81 | 0.408481 |
| GO:0042089\_cytokine\_biosynthetic\_process | CD4 | 52 | 1 | 8.259109 | -0.941202 | 238 | 97.88 | 0.411261 |
| GO:0048731\_system\_development | IGF1 | 1140 | 5 | 1.883657 | -0.940287 | 239 | 97.94 | 0.409791 |
| GO:0048731\_system\_development | SPI1 | 1140 | 5 | 1.883657 | -0.940287 | 239 | 97.94 | 0.409791 |
| GO:0048731\_system\_development | CD4 | 1140 | 5 | 1.883657 | -0.940287 | 239 | 97.94 | 0.409791 |
| GO:0048731\_system\_development | BTK | 1140 | 5 | 1.883657 | -0.940287 | 239 | 97.94 | 0.409791 |
| GO:0048731\_system\_development | PLEK | 1140 | 5 | 1.883657 | -0.940287 | 239 | 97.94 | 0.409791 |
| GO:0006006\_glucose\_metabolic\_process | IGF1 | 53 | 1 | 8.103277 | -0.933402 | 242 | 99.21 | 0.409959 |
| GO:0042107\_cytokine\_metabolic\_process | CD4 | 53 | 1 | 8.103277 | -0.933402 | 242 | 99.21 | 0.409959 |
| GO:0060193\_positive\_regulation\_of\_lipase\_activity | PLEK | 53 | 1 | 8.103277 | -0.933402 | 242 | 99.21 | 0.409959 |
| GO:0007167\_enzyme\_linked\_receptor\_protein\_signaling\_pathway | IGF1 | 258 | 2 | 3.329253 | -0.921540 | 243 | 99.65 | 0.410082 |
| GO:0007167\_enzyme\_linked\_receptor\_protein\_signaling\_pathway | CD4 | 258 | 2 | 3.329253 | -0.921540 | 243 | 99.65 | 0.410082 |
| GO:0009306\_protein\_secretion | PLEK | 56 | 1 | 7.669173 | -0.910906 | 245 | 100.93 | 0.411959 |
| GO:0019058\_viral\_infectious\_cycle | CD4 | 56 | 1 | 7.669173 | -0.910906 | 245 | 100.93 | 0.411959 |
| GO:0045941\_positive\_regulation\_of\_transcription | IGF1 | 264 | 2 | 3.253589 | -0.905066 | 246 | 101.12 | 0.411057 |
| GO:0045941\_positive\_regulation\_of\_transcription | SPI1 | 264 | 2 | 3.253589 | -0.905066 | 246 | 101.12 | 0.411057 |
| GO:0001934\_positive\_regulation\_of\_protein\_amino\_acid\_phosphorylation | IGF1 | 57 | 1 | 7.534626 | -0.903691 | 248 | 101.58 | 0.409597 |
| GO:0032844\_regulation\_of\_homeostatic\_process | SPI1 | 57 | 1 | 7.534626 | -0.903691 | 248 | 101.58 | 0.409597 |
| GO:0065008\_regulation\_of\_biological\_quality | IGF1 | 848 | 4 | 2.025819 | -0.894130 | 249 | 103.16 | 0.414297 |
| GO:0065008\_regulation\_of\_biological\_quality | CD52 | 848 | 4 | 2.025819 | -0.894130 | 249 | 103.16 | 0.414297 |
| GO:0065008\_regulation\_of\_biological\_quality | SPI1 | 848 | 4 | 2.025819 | -0.894130 | 249 | 103.16 | 0.414297 |
| GO:0065008\_regulation\_of\_biological\_quality | PLEK | 848 | 4 | 2.025819 | -0.894130 | 249 | 103.16 | 0.414297 |
| GO:0043408\_regulation\_of\_MAPKKK\_cascade | CARD9 | 59 | 1 | 7.279215 | -0.889657 | 250 | 104.02 | 0.416080 |
| GO:0044087\_regulation\_of\_cellular\_component\_biogenesis | PLEK | 60 | 1 | 7.157895 | -0.882829 | 251 | 104.71 | 0.417171 |
| GO:0007254\_JNK\_cascade | CARD9 | 61 | 1 | 7.040552 | -0.876121 | 253 | 106.13 | 0.419486 |
| GO:0051493\_regulation\_of\_cytoskeleton\_organization | PLEK | 61 | 1 | 7.040552 | -0.876121 | 253 | 106.13 | 0.419486 |
| GO:0010628\_positive\_regulation\_of\_gene\_expression | IGF1 | 277 | 2 | 3.100893 | -0.870882 | 254 | 106.47 | 0.419173 |
| GO:0010628\_positive\_regulation\_of\_gene\_expression | SPI1 | 277 | 2 | 3.100893 | -0.870882 | 254 | 106.47 | 0.419173 |
| GO:0060191\_regulation\_of\_lipase\_activity | PLEK | 62 | 1 | 6.926995 | -0.869530 | 255 | 108.12 | 0.424000 |
| GO:0030522\_intracellular\_receptor-mediated\_signaling\_pathway | IGF1 | 63 | 1 | 6.817043 | -0.863052 | 256 | 109.01 | 0.425820 |
| GO:0031098\_stress-activated\_protein\_kinase\_signaling\_pathway | CARD9 | 64 | 1 | 6.710526 | -0.856683 | 258 | 110.72 | 0.429147 |
| GO:0042327\_positive\_regulation\_of\_phosphorylation | IGF1 | 64 | 1 | 6.710526 | -0.856683 | 258 | 110.72 | 0.429147 |
| GO:0042325\_regulation\_of\_phosphorylation | IGF1 | 285 | 2 | 3.013850 | -0.850807 | 259 | 111.02 | 0.428649 |
| GO:0042325\_regulation\_of\_phosphorylation | CD4 | 285 | 2 | 3.013850 | -0.850807 | 259 | 111.02 | 0.428649 |
| GO:0022415\_viral\_reproductive\_process | CD4 | 65 | 1 | 6.607287 | -0.850421 | 262 | 112.0 | 0.427481 |
| GO:0045596\_negative\_regulation\_of\_cell\_differentiation | IGF1 | 65 | 1 | 6.607287 | -0.850421 | 262 | 112.0 | 0.427481 |
| GO:0080135\_regulation\_of\_cellular\_response\_to\_stress | CARD9 | 65 | 1 | 6.607287 | -0.850421 | 262 | 112.0 | 0.427481 |
| GO:0019538\_protein\_metabolic\_process | IGF1 | 1569 | 6 | 1.642347 | -0.849363 | 263 | 112.19 | 0.426578 |
| GO:0019538\_protein\_metabolic\_process | PTPN7 | 1569 | 6 | 1.642347 | -0.849363 | 263 | 112.19 | 0.426578 |
| GO:0019538\_protein\_metabolic\_process | FGR | 1569 | 6 | 1.642347 | -0.849363 | 263 | 112.19 | 0.426578 |
| GO:0019538\_protein\_metabolic\_process | PLEK | 1569 | 6 | 1.642347 | -0.849363 | 263 | 112.19 | 0.426578 |
| GO:0019538\_protein\_metabolic\_process | BTK | 1569 | 6 | 1.642347 | -0.849363 | 263 | 112.19 | 0.426578 |
| GO:0019538\_protein\_metabolic\_process | CD37 | 1569 | 6 | 1.642347 | -0.849363 | 263 | 112.19 | 0.426578 |
| GO:0032268\_regulation\_of\_cellular\_protein\_metabolic\_process | IGF1 | 286 | 2 | 3.003312 | -0.848346 | 264 | 112.64 | 0.426667 |
| GO:0032268\_regulation\_of\_cellular\_protein\_metabolic\_process | PLEK | 286 | 2 | 3.003312 | -0.848346 | 264 | 112.64 | 0.426667 |
| GO:0009888\_tissue\_development | IGF1 | 287 | 2 | 2.992848 | -0.845896 | 265 | 112.77 | 0.425547 |
| GO:0009888\_tissue\_development | BTK | 287 | 2 | 2.992848 | -0.845896 | 265 | 112.77 | 0.425547 |
| GO:0010562\_positive\_regulation\_of\_phosphorus\_metabolic\_process | IGF1 | 66 | 1 | 6.507177 | -0.844260 | 269 | 114.46 | 0.425502 |
| GO:0010564\_regulation\_of\_cell\_cycle\_process | IGF1 | 66 | 1 | 6.507177 | -0.844260 | 269 | 114.46 | 0.425502 |
| GO:0014706\_striated\_muscle\_tissue\_development | IGF1 | 66 | 1 | 6.507177 | -0.844260 | 269 | 114.46 | 0.425502 |
| GO:0045937\_positive\_regulation\_of\_phosphate\_metabolic\_process | IGF1 | 66 | 1 | 6.507177 | -0.844260 | 269 | 114.46 | 0.425502 |
| GO:0007015\_actin\_filament\_organization | PLEK | 67 | 1 | 6.410055 | -0.838200 | 271 | 115.75 | 0.427122 |
| GO:0060537\_muscle\_tissue\_development | IGF1 | 67 | 1 | 6.410055 | -0.838200 | 271 | 115.75 | 0.427122 |
| GO:0005975\_carbohydrate\_metabolic\_process | IGF1 | 292 | 2 | 2.941601 | -0.833802 | 273 | 116.33 | 0.426117 |
| GO:0005975\_carbohydrate\_metabolic\_process | CD37 | 292 | 2 | 2.941601 | -0.833802 | 273 | 116.33 | 0.426117 |
| GO:0040011\_locomotion | RNASE2 | 292 | 2 | 2.941601 | -0.833802 | 273 | 116.33 | 0.426117 |
| GO:0040011\_locomotion | IGF1 | 292 | 2 | 2.941601 | -0.833802 | 273 | 116.33 | 0.426117 |
| GO:0016032\_viral\_reproduction | CD4 | 70 | 1 | 6.135338 | -0.820587 | 275 | 119.62 | 0.434982 |
| GO:0022411\_cellular\_component\_disassembly | PLEK | 70 | 1 | 6.135338 | -0.820587 | 275 | 119.62 | 0.434982 |
| GO:0009615\_response\_to\_virus | FGR | 71 | 1 | 6.048925 | -0.814896 | 277 | 120.94 | 0.436606 |
| GO:0032101\_regulation\_of\_response\_to\_external\_stimulus | PLEK | 71 | 1 | 6.048925 | -0.814896 | 277 | 120.94 | 0.436606 |
| GO:0051246\_regulation\_of\_protein\_metabolic\_process | IGF1 | 301 | 2 | 2.853646 | -0.812667 | 278 | 121.05 | 0.435432 |
| GO:0051246\_regulation\_of\_protein\_metabolic\_process | PLEK | 301 | 2 | 2.853646 | -0.812667 | 278 | 121.05 | 0.435432 |
| GO:0060249\_anatomical\_structure\_homeostasis | IGF1 | 72 | 1 | 5.964912 | -0.809291 | 279 | 121.35 | 0.434946 |
| GO:0007596\_blood\_coagulation | PLEK | 73 | 1 | 5.883201 | -0.803771 | 282 | 122.42 | 0.434113 |
| GO:0009894\_regulation\_of\_catabolic\_process | IGF1 | 73 | 1 | 5.883201 | -0.803771 | 282 | 122.42 | 0.434113 |
| GO:0030099\_myeloid\_cell\_differentiation | SPI1 | 73 | 1 | 5.883201 | -0.803771 | 282 | 122.42 | 0.434113 |
| GO:0045935\_positive\_regulation\_of\_nucleobase\_\_nucleoside\_\_nucleotide\_and\_nucleic\_acid\_metabolic\_process | IGF1 | 307 | 2 | 2.797874 | -0.799010 | 283 | 122.82 | 0.433993 |
| GO:0045935\_positive\_regulation\_of\_nucleobase\_\_nucleoside\_\_nucleotide\_and\_nucleic\_acid\_metabolic\_process | SPI1 | 307 | 2 | 2.797874 | -0.799010 | 283 | 122.82 | 0.433993 |
| GO:0050817\_coagulation | PLEK | 74 | 1 | 5.803698 | -0.798331 | 284 | 123.17 | 0.433697 |
| GO:0048523\_negative\_regulation\_of\_cellular\_process | IGF1 | 925 | 4 | 1.857183 | -0.793119 | 285 | 123.81 | 0.434421 |
| GO:0048523\_negative\_regulation\_of\_cellular\_process | SPI1 | 925 | 4 | 1.857183 | -0.793119 | 285 | 123.81 | 0.434421 |
| GO:0048523\_negative\_regulation\_of\_cellular\_process | CD33 | 925 | 4 | 1.857183 | -0.793119 | 285 | 123.81 | 0.434421 |
| GO:0048523\_negative\_regulation\_of\_cellular\_process | PLEK | 925 | 4 | 1.857183 | -0.793119 | 285 | 123.81 | 0.434421 |
| GO:0006650\_glycerophospholipid\_metabolic\_process | PLEK | 75 | 1 | 5.726316 | -0.792971 | 286 | 124.82 | 0.436434 |
| GO:0006470\_protein\_amino\_acid\_dephosphorylation | PTPN7 | 77 | 1 | 5.577580 | -0.782479 | 288 | 127.04 | 0.441111 |
| GO:0050865\_regulation\_of\_cell\_activation | PLEK | 77 | 1 | 5.577580 | -0.782479 | 288 | 127.04 | 0.441111 |
| GO:0051173\_positive\_regulation\_of\_nitrogen\_compound\_metabolic\_process | IGF1 | 317 | 2 | 2.709613 | -0.776976 | 289 | 127.48 | 0.441107 |
| GO:0051173\_positive\_regulation\_of\_nitrogen\_compound\_metabolic\_process | SPI1 | 317 | 2 | 2.709613 | -0.776976 | 289 | 127.48 | 0.441107 |
| GO:0007599\_hemostasis | PLEK | 79 | 1 | 5.436376 | -0.772280 | 290 | 128.29 | 0.442379 |
| GO:0048856\_anatomical\_structure\_development | IGF1 | 1289 | 5 | 1.665918 | -0.771449 | 291 | 128.36 | 0.441100 |
| GO:0048856\_anatomical\_structure\_development | SPI1 | 1289 | 5 | 1.665918 | -0.771449 | 291 | 128.36 | 0.441100 |
| GO:0048856\_anatomical\_structure\_development | CD4 | 1289 | 5 | 1.665918 | -0.771449 | 291 | 128.36 | 0.441100 |
| GO:0048856\_anatomical\_structure\_development | PLEK | 1289 | 5 | 1.665918 | -0.771449 | 291 | 128.36 | 0.441100 |
| GO:0048856\_anatomical\_structure\_development | BTK | 1289 | 5 | 1.665918 | -0.771449 | 291 | 128.36 | 0.441100 |
| GO:0007204\_elevation\_of\_cytosolic\_calcium\_ion\_concentration | CD52 | 80 | 1 | 5.368421 | -0.767285 | 292 | 128.9 | 0.441438 |
| GO:0006486\_protein\_amino\_acid\_glycosylation | CD37 | 81 | 1 | 5.302144 | -0.762359 | 297 | 129.91 | 0.437407 |
| GO:0019318\_hexose\_metabolic\_process | IGF1 | 81 | 1 | 5.302144 | -0.762359 | 297 | 129.91 | 0.437407 |
| GO:0043413\_biopolymer\_glycosylation | CD37 | 81 | 1 | 5.302144 | -0.762359 | 297 | 129.91 | 0.437407 |
| GO:0051480\_cytosolic\_calcium\_ion\_homeostasis | CD52 | 81 | 1 | 5.302144 | -0.762359 | 297 | 129.91 | 0.437407 |
| GO:0070085\_glycosylation | CD37 | 81 | 1 | 5.302144 | -0.762359 | 297 | 129.91 | 0.437407 |
| GO:0042110\_T\_cell\_activation | CD4 | 83 | 1 | 5.174382 | -0.752702 | 298 | 131.21 | 0.440302 |
| GO:0031401\_positive\_regulation\_of\_protein\_modification\_process | IGF1 | 84 | 1 | 5.112782 | -0.747969 | 300 | 132.41 | 0.441367 |
| GO:0043123\_positive\_regulation\_of\_I-kappaB\_kinase\_NF-kappaB\_cascade | CARD9 | 84 | 1 | 5.112782 | -0.747969 | 300 | 132.41 | 0.441367 |
| GO:0031327\_negative\_regulation\_of\_cellular\_biosynthetic\_process | SPI1 | 332 | 2 | 2.587191 | -0.745522 | 301 | 132.66 | 0.440731 |
| GO:0031327\_negative\_regulation\_of\_cellular\_biosynthetic\_process | PLEK | 332 | 2 | 2.587191 | -0.745522 | 301 | 132.66 | 0.440731 |
| GO:0002521\_leukocyte\_differentiation | CD4 | 87 | 1 | 4.936479 | -0.734131 | 303 | 133.79 | 0.441551 |
| GO:0051052\_regulation\_of\_DNA\_metabolic\_process | IGF1 | 87 | 1 | 4.936479 | -0.734131 | 303 | 133.79 | 0.441551 |
| GO:0009890\_negative\_regulation\_of\_biosynthetic\_process | SPI1 | 340 | 2 | 2.526316 | -0.729478 | 304 | 134.42 | 0.442171 |
| GO:0009890\_negative\_regulation\_of\_biosynthetic\_process | PLEK | 340 | 2 | 2.526316 | -0.729478 | 304 | 134.42 | 0.442171 |
| GO:0030334\_regulation\_of\_cell\_migration | IGF1 | 89 | 1 | 4.825547 | -0.725195 | 305 | 135.57 | 0.444492 |
| GO:0044260\_cellular\_macromolecule\_metabolic\_process | RNASE2 | 2883 | 9 | 1.340709 | -0.712924 | 306 | 136.54 | 0.446209 |
| GO:0044260\_cellular\_macromolecule\_metabolic\_process | IGF1 | 2883 | 9 | 1.340709 | -0.712924 | 306 | 136.54 | 0.446209 |
| GO:0044260\_cellular\_macromolecule\_metabolic\_process | PTPN7 | 2883 | 9 | 1.340709 | -0.712924 | 306 | 136.54 | 0.446209 |
| GO:0044260\_cellular\_macromolecule\_metabolic\_process | FGR | 2883 | 9 | 1.340709 | -0.712924 | 306 | 136.54 | 0.446209 |
| GO:0044260\_cellular\_macromolecule\_metabolic\_process | SPI1 | 2883 | 9 | 1.340709 | -0.712924 | 306 | 136.54 | 0.446209 |
| GO:0044260\_cellular\_macromolecule\_metabolic\_process | CD4 | 2883 | 9 | 1.340709 | -0.712924 | 306 | 136.54 | 0.446209 |
| GO:0044260\_cellular\_macromolecule\_metabolic\_process | PLEK | 2883 | 9 | 1.340709 | -0.712924 | 306 | 136.54 | 0.446209 |
| GO:0044260\_cellular\_macromolecule\_metabolic\_process | BTK | 2883 | 9 | 1.340709 | -0.712924 | 306 | 136.54 | 0.446209 |
| GO:0044260\_cellular\_macromolecule\_metabolic\_process | CD37 | 2883 | 9 | 1.340709 | -0.712924 | 306 | 136.54 | 0.446209 |
| GO:0043122\_regulation\_of\_I-kappaB\_kinase\_NF-kappaB\_cascade | CARD9 | 93 | 1 | 4.617997 | -0.707969 | 307 | 137.78 | 0.448795 |
| GO:0043085\_positive\_regulation\_of\_catalytic\_activity | CD4 | 354 | 2 | 2.426405 | -0.702537 | 308 | 138.17 | 0.448604 |
| GO:0043085\_positive\_regulation\_of\_catalytic\_activity | PLEK | 354 | 2 | 2.426405 | -0.702537 | 308 | 138.17 | 0.448604 |
| GO:0050878\_regulation\_of\_body\_fluid\_levels | PLEK | 95 | 1 | 4.520776 | -0.699661 | 309 | 138.58 | 0.448479 |
| GO:0050793\_regulation\_of\_developmental\_process | IGF1 | 669 | 3 | 1.925891 | -0.697933 | 310 | 138.74 | 0.447548 |
| GO:0050793\_regulation\_of\_developmental\_process | SPI1 | 669 | 3 | 1.925891 | -0.697933 | 310 | 138.74 | 0.447548 |
| GO:0050793\_regulation\_of\_developmental\_process | BTK | 669 | 3 | 1.925891 | -0.697933 | 310 | 138.74 | 0.447548 |
| GO:0040012\_regulation\_of\_locomotion | IGF1 | 96 | 1 | 4.473684 | -0.695580 | 311 | 139.83 | 0.449614 |
| GO:0048519\_negative\_regulation\_of\_biological\_process | IGF1 | 1013 | 4 | 1.695849 | -0.692395 | 312 | 140.02 | 0.448782 |
| GO:0048519\_negative\_regulation\_of\_biological\_process | CD33 | 1013 | 4 | 1.695849 | -0.692395 | 312 | 140.02 | 0.448782 |
| GO:0048519\_negative\_regulation\_of\_biological\_process | SPI1 | 1013 | 4 | 1.695849 | -0.692395 | 312 | 140.02 | 0.448782 |
| GO:0048519\_negative\_regulation\_of\_biological\_process | PLEK | 1013 | 4 | 1.695849 | -0.692395 | 312 | 140.02 | 0.448782 |
| GO:0046486\_glycerolipid\_metabolic\_process | PLEK | 97 | 1 | 4.427564 | -0.691546 | 313 | 140.43 | 0.448658 |
| GO:0007275\_multicellular\_organismal\_development | IGF1 | 1372 | 5 | 1.565137 | -0.690684 | 314 | 140.75 | 0.448248 |
| GO:0007275\_multicellular\_organismal\_development | SPI1 | 1372 | 5 | 1.565137 | -0.690684 | 314 | 140.75 | 0.448248 |
| GO:0007275\_multicellular\_organismal\_development | CD4 | 1372 | 5 | 1.565137 | -0.690684 | 314 | 140.75 | 0.448248 |
| GO:0007275\_multicellular\_organismal\_development | BTK | 1372 | 5 | 1.565137 | -0.690684 | 314 | 140.75 | 0.448248 |
| GO:0007275\_multicellular\_organismal\_development | PLEK | 1372 | 5 | 1.565137 | -0.690684 | 314 | 140.75 | 0.448248 |
| GO:0051270\_regulation\_of\_cell\_motion | IGF1 | 98 | 1 | 4.382385 | -0.687557 | 315 | 141.49 | 0.449175 |
| GO:0009968\_negative\_regulation\_of\_signal\_transduction | PLEK | 99 | 1 | 4.338118 | -0.683614 | 316 | 142.11 | 0.449715 |
| GO:0044237\_cellular\_metabolic\_process | RNASE2 | 3753 | 11 | 1.258782 | -0.681412 | 317 | 142.44 | 0.449338 |
| GO:0044237\_cellular\_metabolic\_process | IGF1 | 3753 | 11 | 1.258782 | -0.681412 | 317 | 142.44 | 0.449338 |
| GO:0044237\_cellular\_metabolic\_process | CYBB | 3753 | 11 | 1.258782 | -0.681412 | 317 | 142.44 | 0.449338 |
| GO:0044237\_cellular\_metabolic\_process | PTPN7 | 3753 | 11 | 1.258782 | -0.681412 | 317 | 142.44 | 0.449338 |
| GO:0044237\_cellular\_metabolic\_process | FGR | 3753 | 11 | 1.258782 | -0.681412 | 317 | 142.44 | 0.449338 |
| GO:0044237\_cellular\_metabolic\_process | NCF1 | 3753 | 11 | 1.258782 | -0.681412 | 317 | 142.44 | 0.449338 |
| GO:0044237\_cellular\_metabolic\_process | SPI1 | 3753 | 11 | 1.258782 | -0.681412 | 317 | 142.44 | 0.449338 |
| GO:0044237\_cellular\_metabolic\_process | CD4 | 3753 | 11 | 1.258782 | -0.681412 | 317 | 142.44 | 0.449338 |
| GO:0044237\_cellular\_metabolic\_process | PLEK | 3753 | 11 | 1.258782 | -0.681412 | 317 | 142.44 | 0.449338 |
| GO:0044237\_cellular\_metabolic\_process | BTK | 3753 | 11 | 1.258782 | -0.681412 | 317 | 142.44 | 0.449338 |
| GO:0044237\_cellular\_metabolic\_process | CD37 | 3753 | 11 | 1.258782 | -0.681412 | 317 | 142.44 | 0.449338 |
| GO:0055114\_oxidation\_reduction | NCF4 | 100 | 1 | 4.294737 | -0.679714 | 318 | 143.33 | 0.450723 |
| GO:0001932\_regulation\_of\_protein\_amino\_acid\_phosphorylation | IGF1 | 101 | 1 | 4.252215 | -0.675859 | 319 | 144.01 | 0.451442 |
| GO:0010648\_negative\_regulation\_of\_cell\_communication | PLEK | 102 | 1 | 4.210526 | -0.672045 | 320 | 144.53 | 0.451656 |
| GO:0001817\_regulation\_of\_cytokine\_production | CD4 | 103 | 1 | 4.169647 | -0.668273 | 321 | 144.72 | 0.450841 |
| GO:0006644\_phospholipid\_metabolic\_process | PLEK | 108 | 1 | 3.976608 | -0.650010 | 324 | 147.71 | 0.455895 |
| GO:0007346\_regulation\_of\_mitotic\_cell\_cycle | IGF1 | 108 | 1 | 3.976608 | -0.650010 | 324 | 147.71 | 0.455895 |
| GO:0019637\_organophosphate\_metabolic\_process | PLEK | 108 | 1 | 3.976608 | -0.650010 | 324 | 147.71 | 0.455895 |
| GO:0007265\_Ras\_protein\_signal\_transduction | IGF1 | 110 | 1 | 3.904306 | -0.642970 | 325 | 149.16 | 0.458954 |
| GO:0001816\_cytokine\_production | CD4 | 112 | 1 | 3.834586 | -0.636073 | 326 | 150.34 | 0.461166 |
| GO:0000122\_negative\_regulation\_of\_transcription\_from\_RNA\_polymerase\_II\_promoter | SPI1 | 113 | 1 | 3.800652 | -0.632676 | 327 | 151.05 | 0.461927 |
| GO:0044093\_positive\_regulation\_of\_molecular\_function | CD4 | 394 | 2 | 2.180069 | -0.632657 | 328 | 151.31 | 0.461311 |
| GO:0044093\_positive\_regulation\_of\_molecular\_function | PLEK | 394 | 2 | 2.180069 | -0.632657 | 328 | 151.31 | 0.461311 |
| GO:0006874\_cellular\_calcium\_ion\_homeostasis | CD52 | 114 | 1 | 3.767313 | -0.629313 | 330 | 151.82 | 0.460061 |
| GO:0048584\_positive\_regulation\_of\_response\_to\_stimulus | CARD9 | 114 | 1 | 3.767313 | -0.629313 | 330 | 151.82 | 0.460061 |
| GO:0000280\_nuclear\_division | IGF1 | 115 | 1 | 3.734554 | -0.625983 | 333 | 153.02 | 0.459520 |
| GO:0005996\_monosaccharide\_metabolic\_process | IGF1 | 115 | 1 | 3.734554 | -0.625983 | 333 | 153.02 | 0.459520 |
| GO:0007067\_mitosis | IGF1 | 115 | 1 | 3.734554 | -0.625983 | 333 | 153.02 | 0.459520 |
| GO:0055074\_calcium\_ion\_homeostasis | CD52 | 116 | 1 | 3.702359 | -0.622687 | 334 | 153.63 | 0.459970 |
| GO:0018193\_peptidyl-amino\_acid\_modification | IGF1 | 117 | 1 | 3.670715 | -0.619422 | 335 | 153.9 | 0.459403 |
| GO:0031324\_negative\_regulation\_of\_cellular\_metabolic\_process | SPI1 | 404 | 2 | 2.126107 | -0.616648 | 336 | 154.03 | 0.458423 |
| GO:0031324\_negative\_regulation\_of\_cellular\_metabolic\_process | PLEK | 404 | 2 | 2.126107 | -0.616648 | 336 | 154.03 | 0.458423 |
| GO:0000087\_M\_phase\_of\_mitotic\_cell\_cycle | IGF1 | 118 | 1 | 3.639607 | -0.616189 | 338 | 154.76 | 0.457870 |
| GO:0048285\_organelle\_fission | IGF1 | 118 | 1 | 3.639607 | -0.616189 | 338 | 154.76 | 0.457870 |
| GO:0046649\_lymphocyte\_activation | CD4 | 119 | 1 | 3.609023 | -0.612987 | 339 | 155.1 | 0.457522 |
| GO:0044248\_cellular\_catabolic\_process | RNASE2 | 410 | 2 | 2.094994 | -0.607297 | 340 | 155.86 | 0.458412 |
| GO:0044248\_cellular\_catabolic\_process | IGF1 | 410 | 2 | 2.094994 | -0.607297 | 340 | 155.86 | 0.458412 |
| GO:0006875\_cellular\_metal\_ion\_homeostasis | CD52 | 121 | 1 | 3.549369 | -0.606674 | 341 | 156.28 | 0.458299 |
| GO:0042127\_regulation\_of\_cell\_proliferation | IGF1 | 411 | 2 | 2.089896 | -0.605756 | 342 | 156.51 | 0.457632 |
| GO:0042127\_regulation\_of\_cell\_proliferation | CD33 | 411 | 2 | 2.089896 | -0.605756 | 342 | 156.51 | 0.457632 |
| GO:0033674\_positive\_regulation\_of\_kinase\_activity | CD4 | 122 | 1 | 3.520276 | -0.603562 | 344 | 157.55 | 0.457994 |
| GO:0045860\_positive\_regulation\_of\_protein\_kinase\_activity | CD4 | 122 | 1 | 3.520276 | -0.603562 | 344 | 157.55 | 0.457994 |
| GO:0007517\_muscle\_organ\_development | IGF1 | 123 | 1 | 3.491656 | -0.600479 | 345 | 157.94 | 0.457797 |
| GO:0006935\_chemotaxis | RNASE2 | 125 | 1 | 3.435789 | -0.594398 | 349 | 159.26 | 0.456332 |
| GO:0042330\_taxis | RNASE2 | 125 | 1 | 3.435789 | -0.594398 | 349 | 159.26 | 0.456332 |
| GO:0051707\_response\_to\_other\_organism | FGR | 125 | 1 | 3.435789 | -0.594398 | 349 | 159.26 | 0.456332 |
| GO:0055065\_metal\_ion\_homeostasis | CD52 | 125 | 1 | 3.435789 | -0.594398 | 349 | 159.26 | 0.456332 |
| GO:0030030\_cell\_projection\_organization | PLEK | 127 | 1 | 3.381683 | -0.588428 | 350 | 159.61 | 0.456029 |
| GO:0031326\_regulation\_of\_cellular\_biosynthetic\_process | IGF1 | 1125 | 4 | 1.527018 | -0.582942 | 351 | 160.64 | 0.457664 |
| GO:0031326\_regulation\_of\_cellular\_biosynthetic\_process | SPI1 | 1125 | 4 | 1.527018 | -0.582942 | 351 | 160.64 | 0.457664 |
| GO:0031326\_regulation\_of\_cellular\_biosynthetic\_process | CD4 | 1125 | 4 | 1.527018 | -0.582942 | 351 | 160.64 | 0.457664 |
| GO:0031326\_regulation\_of\_cellular\_biosynthetic\_process | PLEK | 1125 | 4 | 1.527018 | -0.582942 | 351 | 160.64 | 0.457664 |
| GO:0051347\_positive\_regulation\_of\_transferase\_activity | CD4 | 129 | 1 | 3.329253 | -0.582565 | 352 | 161.55 | 0.458949 |
| GO:0007155\_cell\_adhesion | CD33 | 428 | 2 | 2.006886 | -0.580325 | 353 | 161.75 | 0.458215 |
| GO:0007155\_cell\_adhesion | PLEK | 428 | 2 | 2.006886 | -0.580325 | 353 | 161.75 | 0.458215 |
| GO:0022610\_biological\_adhesion | CD33 | 429 | 2 | 2.002208 | -0.578872 | 354 | 162.37 | 0.458672 |
| GO:0022610\_biological\_adhesion | PLEK | 429 | 2 | 2.002208 | -0.578872 | 354 | 162.37 | 0.458672 |
| GO:0040008\_regulation\_of\_growth | IGF1 | 131 | 1 | 3.278425 | -0.576806 | 356 | 163.55 | 0.459410 |
| GO:0051345\_positive\_regulation\_of\_hydrolase\_activity | PLEK | 131 | 1 | 3.278425 | -0.576806 | 356 | 163.55 | 0.459410 |
| GO:0009889\_regulation\_of\_biosynthetic\_process | IGF1 | 1135 | 4 | 1.513564 | -0.574059 | 357 | 163.67 | 0.458459 |
| GO:0009889\_regulation\_of\_biosynthetic\_process | SPI1 | 1135 | 4 | 1.513564 | -0.574059 | 357 | 163.67 | 0.458459 |
| GO:0009889\_regulation\_of\_biosynthetic\_process | CD4 | 1135 | 4 | 1.513564 | -0.574059 | 357 | 163.67 | 0.458459 |
| GO:0009889\_regulation\_of\_biosynthetic\_process | PLEK | 1135 | 4 | 1.513564 | -0.574059 | 357 | 163.67 | 0.458459 |
| GO:0050794\_regulation\_of\_cellular\_process | IGF1 | 3515 | 10 | 1.221831 | -0.569946 | 358 | 164.38 | 0.459162 |
| GO:0050794\_regulation\_of\_cellular\_process | LILRA2 | 3515 | 10 | 1.221831 | -0.569946 | 358 | 164.38 | 0.459162 |
| GO:0050794\_regulation\_of\_cellular\_process | SPI1 | 3515 | 10 | 1.221831 | -0.569946 | 358 | 164.38 | 0.459162 |
| GO:0050794\_regulation\_of\_cellular\_process | CD33 | 3515 | 10 | 1.221831 | -0.569946 | 358 | 164.38 | 0.459162 |
| GO:0050794\_regulation\_of\_cellular\_process | CARD9 | 3515 | 10 | 1.221831 | -0.569946 | 358 | 164.38 | 0.459162 |
| GO:0050794\_regulation\_of\_cellular\_process | CD4 | 3515 | 10 | 1.221831 | -0.569946 | 358 | 164.38 | 0.459162 |
| GO:0050794\_regulation\_of\_cellular\_process | BTK | 3515 | 10 | 1.221831 | -0.569946 | 358 | 164.38 | 0.459162 |
| GO:0050794\_regulation\_of\_cellular\_process | PLEK | 3515 | 10 | 1.221831 | -0.569946 | 358 | 164.38 | 0.459162 |
| GO:0050794\_regulation\_of\_cellular\_process | CSF3R | 3515 | 10 | 1.221831 | -0.569946 | 358 | 164.38 | 0.459162 |
| GO:0050794\_regulation\_of\_cellular\_process | TYROBP | 3515 | 10 | 1.221831 | -0.569946 | 358 | 164.38 | 0.459162 |
| GO:0043170\_macromolecule\_metabolic\_process | RNASE2 | 3103 | 9 | 1.245654 | -0.569110 | 359 | 164.57 | 0.458412 |
| GO:0043170\_macromolecule\_metabolic\_process | IGF1 | 3103 | 9 | 1.245654 | -0.569110 | 359 | 164.57 | 0.458412 |
| GO:0043170\_macromolecule\_metabolic\_process | PTPN7 | 3103 | 9 | 1.245654 | -0.569110 | 359 | 164.57 | 0.458412 |
| GO:0043170\_macromolecule\_metabolic\_process | FGR | 3103 | 9 | 1.245654 | -0.569110 | 359 | 164.57 | 0.458412 |
| GO:0043170\_macromolecule\_metabolic\_process | SPI1 | 3103 | 9 | 1.245654 | -0.569110 | 359 | 164.57 | 0.458412 |
| GO:0043170\_macromolecule\_metabolic\_process | CD4 | 3103 | 9 | 1.245654 | -0.569110 | 359 | 164.57 | 0.458412 |
| GO:0043170\_macromolecule\_metabolic\_process | BTK | 3103 | 9 | 1.245654 | -0.569110 | 359 | 164.57 | 0.458412 |
| GO:0043170\_macromolecule\_metabolic\_process | PLEK | 3103 | 9 | 1.245654 | -0.569110 | 359 | 164.57 | 0.458412 |
| GO:0043170\_macromolecule\_metabolic\_process | CD37 | 3103 | 9 | 1.245654 | -0.569110 | 359 | 164.57 | 0.458412 |
| GO:0007249\_I-kappaB\_kinase\_NF-kappaB\_cascade | CARD9 | 134 | 1 | 3.205027 | -0.568355 | 360 | 165.39 | 0.459417 |
| GO:0007264\_small\_GTPase\_mediated\_signal\_transduction | IGF1 | 135 | 1 | 3.181287 | -0.565587 | 361 | 166.6 | 0.461496 |
| GO:0009057\_macromolecule\_catabolic\_process | RNASE2 | 439 | 2 | 1.956600 | -0.564596 | 362 | 166.79 | 0.460746 |
| GO:0009057\_macromolecule\_catabolic\_process | IGF1 | 439 | 2 | 1.956600 | -0.564596 | 362 | 166.79 | 0.460746 |
| GO:0009892\_negative\_regulation\_of\_metabolic\_process | SPI1 | 440 | 2 | 1.952153 | -0.563193 | 363 | 166.93 | 0.459862 |
| GO:0009892\_negative\_regulation\_of\_metabolic\_process | PLEK | 440 | 2 | 1.952153 | -0.563193 | 363 | 166.93 | 0.459862 |
| GO:0030005\_cellular\_di-\_\_tri-valent\_inorganic\_cation\_homeostasis | CD52 | 140 | 1 | 3.067669 | -0.552093 | 364 | 169.59 | 0.465907 |
| GO:0003012\_muscle\_system\_process | IGF1 | 141 | 1 | 3.045913 | -0.549462 | 365 | 170.34 | 0.466685 |
| GO:0065007\_biological\_regulation | IGF1 | 3971 | 11 | 1.189678 | -0.549121 | 366 | 170.45 | 0.465710 |
| GO:0065007\_biological\_regulation | LILRA2 | 3971 | 11 | 1.189678 | -0.549121 | 366 | 170.45 | 0.465710 |
| GO:0065007\_biological\_regulation | CD52 | 3971 | 11 | 1.189678 | -0.549121 | 366 | 170.45 | 0.465710 |
| GO:0065007\_biological\_regulation | CD33 | 3971 | 11 | 1.189678 | -0.549121 | 366 | 170.45 | 0.465710 |
| GO:0065007\_biological\_regulation | SPI1 | 3971 | 11 | 1.189678 | -0.549121 | 366 | 170.45 | 0.465710 |
| GO:0065007\_biological\_regulation | CARD9 | 3971 | 11 | 1.189678 | -0.549121 | 366 | 170.45 | 0.465710 |
| GO:0065007\_biological\_regulation | CD4 | 3971 | 11 | 1.189678 | -0.549121 | 366 | 170.45 | 0.465710 |
| GO:0065007\_biological\_regulation | BTK | 3971 | 11 | 1.189678 | -0.549121 | 366 | 170.45 | 0.465710 |
| GO:0065007\_biological\_regulation | PLEK | 3971 | 11 | 1.189678 | -0.549121 | 366 | 170.45 | 0.465710 |
| GO:0065007\_biological\_regulation | CSF3R | 3971 | 11 | 1.189678 | -0.549121 | 366 | 170.45 | 0.465710 |
| GO:0065007\_biological\_regulation | TYROBP | 3971 | 11 | 1.189678 | -0.549121 | 366 | 170.45 | 0.465710 |
| GO:0001501\_skeletal\_system\_development | IGF1 | 142 | 1 | 3.024463 | -0.546852 | 368 | 171.29 | 0.465462 |
| GO:0007626\_locomotory\_behavior | RNASE2 | 142 | 1 | 3.024463 | -0.546852 | 368 | 171.29 | 0.465462 |
| GO:0000165\_MAPKKK\_cascade | CARD9 | 143 | 1 | 3.003312 | -0.544264 | 369 | 171.59 | 0.465014 |
| GO:0031399\_regulation\_of\_protein\_modification\_process | IGF1 | 144 | 1 | 2.982456 | -0.541696 | 370 | 172.3 | 0.465676 |
| GO:0030036\_actin\_cytoskeleton\_organization | PLEK | 145 | 1 | 2.961887 | -0.539150 | 372 | 173.3 | 0.465860 |
| GO:0055066\_di-\_\_tri-valent\_inorganic\_cation\_homeostasis | CD52 | 145 | 1 | 2.961887 | -0.539150 | 372 | 173.3 | 0.465860 |
| GO:0008361\_regulation\_of\_cell\_size | PLEK | 149 | 1 | 2.882374 | -0.529167 | 373 | 175.79 | 0.471287 |
| GO:0045321\_leukocyte\_activation | CD4 | 150 | 1 | 2.863158 | -0.526720 | 374 | 175.95 | 0.470455 |
| GO:0009987\_cellular\_process | RNASE2 | 6671 | 17 | 1.094447 | -0.524302 | 375 | 176.48 | 0.470613 |
| GO:0009987\_cellular\_process | CD52 | 6671 | 17 | 1.094447 | -0.524302 | 375 | 176.48 | 0.470613 |
| GO:0009987\_cellular\_process | CYBB | 6671 | 17 | 1.094447 | -0.524302 | 375 | 176.48 | 0.470613 |
| GO:0009987\_cellular\_process | PTPN7 | 6671 | 17 | 1.094447 | -0.524302 | 375 | 176.48 | 0.470613 |
| GO:0009987\_cellular\_process | NCF1 | 6671 | 17 | 1.094447 | -0.524302 | 375 | 176.48 | 0.470613 |
| GO:0009987\_cellular\_process | CD33 | 6671 | 17 | 1.094447 | -0.524302 | 375 | 176.48 | 0.470613 |
| GO:0009987\_cellular\_process | CD4 | 6671 | 17 | 1.094447 | -0.524302 | 375 | 176.48 | 0.470613 |
| GO:0009987\_cellular\_process | BTK | 6671 | 17 | 1.094447 | -0.524302 | 375 | 176.48 | 0.470613 |
| GO:0009987\_cellular\_process | CD37 | 6671 | 17 | 1.094447 | -0.524302 | 375 | 176.48 | 0.470613 |
| GO:0009987\_cellular\_process | TYROBP | 6671 | 17 | 1.094447 | -0.524302 | 375 | 176.48 | 0.470613 |
| GO:0009987\_cellular\_process | CSF3R | 6671 | 17 | 1.094447 | -0.524302 | 375 | 176.48 | 0.470613 |
| GO:0009987\_cellular\_process | IGF1 | 6671 | 17 | 1.094447 | -0.524302 | 375 | 176.48 | 0.470613 |
| GO:0009987\_cellular\_process | LILRA2 | 6671 | 17 | 1.094447 | -0.524302 | 375 | 176.48 | 0.470613 |
| GO:0009987\_cellular\_process | FGR | 6671 | 17 | 1.094447 | -0.524302 | 375 | 176.48 | 0.470613 |
| GO:0009987\_cellular\_process | SPI1 | 6671 | 17 | 1.094447 | -0.524302 | 375 | 176.48 | 0.470613 |
| GO:0009987\_cellular\_process | CARD9 | 6671 | 17 | 1.094447 | -0.524302 | 375 | 176.48 | 0.470613 |
| GO:0009987\_cellular\_process | PLEK | 6671 | 17 | 1.094447 | -0.524302 | 375 | 176.48 | 0.470613 |
| GO:0042981\_regulation\_of\_apoptosis | IGF1 | 471 | 2 | 1.823667 | -0.521800 | 376 | 177.46 | 0.471968 |
| GO:0042981\_regulation\_of\_apoptosis | BTK | 471 | 2 | 1.823667 | -0.521800 | 376 | 177.46 | 0.471968 |
| GO:0006260\_DNA\_replication | IGF1 | 153 | 1 | 2.807018 | -0.519495 | 377 | 178.02 | 0.472202 |
| GO:0007166\_cell\_surface\_receptor\_linked\_signal\_transduction | IGF1 | 828 | 3 | 1.556064 | -0.519373 | 378 | 178.18 | 0.471376 |
| GO:0007166\_cell\_surface\_receptor\_linked\_signal\_transduction | CD4 | 828 | 3 | 1.556064 | -0.519373 | 378 | 178.18 | 0.471376 |
| GO:0007166\_cell\_surface\_receptor\_linked\_signal\_transduction | PLEK | 828 | 3 | 1.556064 | -0.519373 | 378 | 178.18 | 0.471376 |
| GO:0043067\_regulation\_of\_programmed\_cell\_death | IGF1 | 476 | 2 | 1.804511 | -0.515484 | 379 | 178.83 | 0.471847 |
| GO:0043067\_regulation\_of\_programmed\_cell\_death | BTK | 476 | 2 | 1.804511 | -0.515484 | 379 | 178.83 | 0.471847 |
| GO:0032940\_secretion\_by\_cell | PLEK | 155 | 1 | 2.770798 | -0.514770 | 380 | 179.23 | 0.471658 |
| GO:0010941\_regulation\_of\_cell\_death | IGF1 | 478 | 2 | 1.796961 | -0.512983 | 381 | 179.67 | 0.471575 |
| GO:0010941\_regulation\_of\_cell\_death | BTK | 478 | 2 | 1.796961 | -0.512983 | 381 | 179.67 | 0.471575 |
| GO:0016337\_cell-cell\_adhesion | PLEK | 156 | 1 | 2.753036 | -0.512434 | 383 | 180.13 | 0.470313 |
| GO:0032787\_monocarboxylic\_acid\_metabolic\_process | IGF1 | 156 | 1 | 2.753036 | -0.512434 | 383 | 180.13 | 0.470313 |
| GO:0030003\_cellular\_cation\_homeostasis | CD52 | 161 | 1 | 2.667538 | -0.501019 | 384 | 181.26 | 0.472031 |
| GO:0006091\_generation\_of\_precursor\_metabolites\_and\_energy | IGF1 | 163 | 1 | 2.634808 | -0.496570 | 385 | 181.75 | 0.472078 |
| GO:0034960\_cellular\_biopolymer\_metabolic\_process | RNASE2 | 2820 | 8 | 1.218365 | -0.495721 | 386 | 182.3 | 0.472280 |
| GO:0034960\_cellular\_biopolymer\_metabolic\_process | IGF1 | 2820 | 8 | 1.218365 | -0.495721 | 386 | 182.3 | 0.472280 |
| GO:0034960\_cellular\_biopolymer\_metabolic\_process | PTPN7 | 2820 | 8 | 1.218365 | -0.495721 | 386 | 182.3 | 0.472280 |
| GO:0034960\_cellular\_biopolymer\_metabolic\_process | FGR | 2820 | 8 | 1.218365 | -0.495721 | 386 | 182.3 | 0.472280 |
| GO:0034960\_cellular\_biopolymer\_metabolic\_process | SPI1 | 2820 | 8 | 1.218365 | -0.495721 | 386 | 182.3 | 0.472280 |
| GO:0034960\_cellular\_biopolymer\_metabolic\_process | PLEK | 2820 | 8 | 1.218365 | -0.495721 | 386 | 182.3 | 0.472280 |
| GO:0034960\_cellular\_biopolymer\_metabolic\_process | BTK | 2820 | 8 | 1.218365 | -0.495721 | 386 | 182.3 | 0.472280 |
| GO:0034960\_cellular\_biopolymer\_metabolic\_process | CD37 | 2820 | 8 | 1.218365 | -0.495721 | 386 | 182.3 | 0.472280 |
| GO:0050789\_regulation\_of\_biological\_process | IGF1 | 3649 | 10 | 1.176963 | -0.495187 | 387 | 182.43 | 0.471395 |
| GO:0050789\_regulation\_of\_biological\_process | LILRA2 | 3649 | 10 | 1.176963 | -0.495187 | 387 | 182.43 | 0.471395 |
| GO:0050789\_regulation\_of\_biological\_process | CD33 | 3649 | 10 | 1.176963 | -0.495187 | 387 | 182.43 | 0.471395 |
| GO:0050789\_regulation\_of\_biological\_process | SPI1 | 3649 | 10 | 1.176963 | -0.495187 | 387 | 182.43 | 0.471395 |
| GO:0050789\_regulation\_of\_biological\_process | CARD9 | 3649 | 10 | 1.176963 | -0.495187 | 387 | 182.43 | 0.471395 |
| GO:0050789\_regulation\_of\_biological\_process | CD4 | 3649 | 10 | 1.176963 | -0.495187 | 387 | 182.43 | 0.471395 |
| GO:0050789\_regulation\_of\_biological\_process | BTK | 3649 | 10 | 1.176963 | -0.495187 | 387 | 182.43 | 0.471395 |
| GO:0050789\_regulation\_of\_biological\_process | PLEK | 3649 | 10 | 1.176963 | -0.495187 | 387 | 182.43 | 0.471395 |
| GO:0050789\_regulation\_of\_biological\_process | CSF3R | 3649 | 10 | 1.176963 | -0.495187 | 387 | 182.43 | 0.471395 |
| GO:0050789\_regulation\_of\_biological\_process | TYROBP | 3649 | 10 | 1.176963 | -0.495187 | 387 | 182.43 | 0.471395 |
| GO:0030029\_actin\_filament-based\_process | PLEK | 165 | 1 | 2.602871 | -0.492186 | 388 | 183.19 | 0.472139 |
| GO:0000279\_M\_phase | IGF1 | 170 | 1 | 2.526316 | -0.481500 | 389 | 184.62 | 0.474602 |
| GO:0055080\_cation\_homeostasis | CD52 | 173 | 1 | 2.482507 | -0.475269 | 390 | 185.72 | 0.476205 |
| GO:0040007\_growth | IGF1 | 174 | 1 | 2.468240 | -0.473221 | 391 | 186.32 | 0.476522 |
| GO:0045892\_negative\_regulation\_of\_transcription\_\_DNA-dependent | SPI1 | 175 | 1 | 2.454135 | -0.471187 | 392 | 186.77 | 0.476454 |
| GO:0009607\_response\_to\_biotic\_stimulus | FGR | 177 | 1 | 2.426405 | -0.467160 | 394 | 187.93 | 0.476980 |
| GO:0016477\_cell\_migration | IGF1 | 177 | 1 | 2.426405 | -0.467160 | 394 | 187.93 | 0.476980 |
| GO:0051253\_negative\_regulation\_of\_RNA\_metabolic\_process | SPI1 | 180 | 1 | 2.385965 | -0.461224 | 396 | 189.49 | 0.478510 |
| GO:0051336\_regulation\_of\_hydrolase\_activity | PLEK | 180 | 1 | 2.385965 | -0.461224 | 396 | 189.49 | 0.478510 |
| GO:0050790\_regulation\_of\_catalytic\_activity | CD4 | 525 | 2 | 1.636090 | -0.458263 | 397 | 189.91 | 0.478363 |
| GO:0050790\_regulation\_of\_catalytic\_activity | PLEK | 525 | 2 | 1.636090 | -0.458263 | 397 | 189.91 | 0.478363 |
| GO:0006954\_inflammatory\_response | CYBB | 182 | 1 | 2.359746 | -0.457333 | 398 | 190.15 | 0.477764 |
| GO:0006917\_induction\_of\_apoptosis | BTK | 190 | 1 | 2.260388 | -0.442275 | 399 | 192.98 | 0.483659 |
| GO:0012502\_induction\_of\_programmed\_cell\_death | BTK | 191 | 1 | 2.248553 | -0.440448 | 400 | 193.44 | 0.483600 |
| GO:0019953\_sexual\_reproduction | CD4 | 193 | 1 | 2.225252 | -0.436828 | 401 | 194.4 | 0.484788 |
| GO:0002682\_regulation\_of\_immune\_system\_process | SPI1 | 196 | 1 | 2.191192 | -0.431485 | 403 | 195.44 | 0.484963 |
| GO:0043086\_negative\_regulation\_of\_catalytic\_activity | PLEK | 196 | 1 | 2.191192 | -0.431485 | 403 | 195.44 | 0.484963 |
| GO:0048870\_cell\_motility | IGF1 | 197 | 1 | 2.180069 | -0.429726 | 404 | 195.73 | 0.484480 |
| GO:0060255\_regulation\_of\_macromolecule\_metabolic\_process | IGF1 | 1328 | 4 | 1.293595 | -0.426282 | 405 | 196.18 | 0.484395 |
| GO:0060255\_regulation\_of\_macromolecule\_metabolic\_process | SPI1 | 1328 | 4 | 1.293595 | -0.426282 | 405 | 196.18 | 0.484395 |
| GO:0060255\_regulation\_of\_macromolecule\_metabolic\_process | CD4 | 1328 | 4 | 1.293595 | -0.426282 | 405 | 196.18 | 0.484395 |
| GO:0060255\_regulation\_of\_macromolecule\_metabolic\_process | PLEK | 1328 | 4 | 1.293595 | -0.426282 | 405 | 196.18 | 0.484395 |
| GO:0008284\_positive\_regulation\_of\_cell\_proliferation | IGF1 | 200 | 1 | 2.147368 | -0.424516 | 406 | 196.47 | 0.483916 |
| GO:0008285\_negative\_regulation\_of\_cell\_proliferation | CD33 | 202 | 1 | 2.126107 | -0.421097 | 407 | 197.55 | 0.485381 |
| GO:0006915\_apoptosis | IGF1 | 565 | 2 | 1.520261 | -0.417107 | 408 | 198.09 | 0.485515 |
| GO:0006915\_apoptosis | BTK | 565 | 2 | 1.520261 | -0.417107 | 408 | 198.09 | 0.485515 |
| GO:0006873\_cellular\_ion\_homeostasis | CD52 | 206 | 1 | 2.084824 | -0.414383 | 409 | 198.66 | 0.485721 |
| GO:0043066\_negative\_regulation\_of\_apoptosis | IGF1 | 207 | 1 | 2.074752 | -0.412730 | 410 | 199.17 | 0.485780 |
| GO:0012501\_programmed\_cell\_death | IGF1 | 571 | 2 | 1.504286 | -0.411314 | 411 | 199.41 | 0.485182 |
| GO:0012501\_programmed\_cell\_death | BTK | 571 | 2 | 1.504286 | -0.411314 | 411 | 199.41 | 0.485182 |
| GO:0055082\_cellular\_chemical\_homeostasis | CD52 | 208 | 1 | 2.064777 | -0.411087 | 412 | 199.69 | 0.484684 |
| GO:0043069\_negative\_regulation\_of\_programmed\_cell\_death | IGF1 | 209 | 1 | 2.054898 | -0.409453 | 414 | 200.37 | 0.483986 |
| GO:0060548\_negative\_regulation\_of\_cell\_death | IGF1 | 209 | 1 | 2.054898 | -0.409453 | 414 | 200.37 | 0.483986 |
| GO:0045859\_regulation\_of\_protein\_kinase\_activity | CD4 | 213 | 1 | 2.016308 | -0.403018 | 416 | 202.21 | 0.486082 |
| GO:0051726\_regulation\_of\_cell\_cycle | IGF1 | 213 | 1 | 2.016308 | -0.403018 | 416 | 202.21 | 0.486082 |
| GO:0007610\_behavior | RNASE2 | 214 | 1 | 2.006886 | -0.401433 | 417 | 202.65 | 0.485971 |
| GO:0008219\_cell\_death | IGF1 | 585 | 2 | 1.468286 | -0.398155 | 419 | 203.65 | 0.486038 |
| GO:0008219\_cell\_death | BTK | 585 | 2 | 1.468286 | -0.398155 | 419 | 203.65 | 0.486038 |
| GO:0016265\_death | IGF1 | 585 | 2 | 1.468286 | -0.398155 | 419 | 203.65 | 0.486038 |
| GO:0016265\_death | BTK | 585 | 2 | 1.468286 | -0.398155 | 419 | 203.65 | 0.486038 |
| GO:0043549\_regulation\_of\_kinase\_activity | CD4 | 217 | 1 | 1.979141 | -0.396733 | 420 | 204.11 | 0.485976 |
| GO:0046903\_secretion | PLEK | 218 | 1 | 1.970063 | -0.395185 | 421 | 204.42 | 0.485558 |
| GO:0050801\_ion\_homeostasis | CD52 | 221 | 1 | 1.943320 | -0.390593 | 422 | 204.9 | 0.485545 |
| GO:0043283\_biopolymer\_metabolic\_process | RNASE2 | 3027 | 8 | 1.135048 | -0.389759 | 423 | 205.06 | 0.484775 |
| GO:0043283\_biopolymer\_metabolic\_process | IGF1 | 3027 | 8 | 1.135048 | -0.389759 | 423 | 205.06 | 0.484775 |
| GO:0043283\_biopolymer\_metabolic\_process | PTPN7 | 3027 | 8 | 1.135048 | -0.389759 | 423 | 205.06 | 0.484775 |
| GO:0043283\_biopolymer\_metabolic\_process | FGR | 3027 | 8 | 1.135048 | -0.389759 | 423 | 205.06 | 0.484775 |
| GO:0043283\_biopolymer\_metabolic\_process | SPI1 | 3027 | 8 | 1.135048 | -0.389759 | 423 | 205.06 | 0.484775 |
| GO:0043283\_biopolymer\_metabolic\_process | PLEK | 3027 | 8 | 1.135048 | -0.389759 | 423 | 205.06 | 0.484775 |
| GO:0043283\_biopolymer\_metabolic\_process | BTK | 3027 | 8 | 1.135048 | -0.389759 | 423 | 205.06 | 0.484775 |
| GO:0043283\_biopolymer\_metabolic\_process | CD37 | 3027 | 8 | 1.135048 | -0.389759 | 423 | 205.06 | 0.484775 |
| GO:0034621\_cellular\_macromolecular\_complex\_subunit\_organization | PLEK | 227 | 1 | 1.891955 | -0.381645 | 426 | 206.96 | 0.485822 |
| GO:0034962\_cellular\_biopolymer\_catabolic\_process | RNASE2 | 227 | 1 | 1.891955 | -0.381645 | 426 | 206.96 | 0.485822 |
| GO:0051338\_regulation\_of\_transferase\_activity | CD4 | 227 | 1 | 1.891955 | -0.381645 | 426 | 206.96 | 0.485822 |
| GO:0065009\_regulation\_of\_molecular\_function | CD4 | 606 | 2 | 1.417405 | -0.379311 | 427 | 207.52 | 0.485995 |
| GO:0065009\_regulation\_of\_molecular\_function | PLEK | 606 | 2 | 1.417405 | -0.379311 | 427 | 207.52 | 0.485995 |
| GO:0019725\_cellular\_homeostasis | CD52 | 231 | 1 | 1.859193 | -0.375846 | 428 | 208.46 | 0.487056 |
| GO:0044092\_negative\_regulation\_of\_molecular\_function | PLEK | 233 | 1 | 1.843235 | -0.372994 | 429 | 209.35 | 0.487995 |
| GO:0043065\_positive\_regulation\_of\_apoptosis | BTK | 243 | 1 | 1.767381 | -0.359198 | 430 | 211.7 | 0.492326 |
| GO:0009056\_catabolic\_process | RNASE2 | 633 | 2 | 1.356947 | -0.356550 | 431 | 212.39 | 0.492784 |
| GO:0009056\_catabolic\_process | IGF1 | 633 | 2 | 1.356947 | -0.356550 | 431 | 212.39 | 0.492784 |
| GO:0022403\_cell\_cycle\_phase | IGF1 | 245 | 1 | 1.752954 | -0.356527 | 432 | 213.01 | 0.493079 |
| GO:0043068\_positive\_regulation\_of\_programmed\_cell\_death | BTK | 246 | 1 | 1.745828 | -0.355203 | 433 | 214.17 | 0.494619 |
| GO:0010942\_positive\_regulation\_of\_cell\_death | BTK | 250 | 1 | 1.717895 | -0.349973 | 434 | 215.02 | 0.495438 |
| GO:0048468\_cell\_development | IGF1 | 251 | 1 | 1.711051 | -0.348683 | 435 | 215.31 | 0.494966 |
| GO:0008283\_cell\_proliferation | IGF1 | 647 | 2 | 1.327585 | -0.345354 | 436 | 215.79 | 0.494931 |
| GO:0008283\_cell\_proliferation | CD33 | 647 | 2 | 1.327585 | -0.345354 | 436 | 215.79 | 0.494931 |
| GO:0010556\_regulation\_of\_macromolecule\_biosynthetic\_process | IGF1 | 1055 | 3 | 1.221252 | -0.343735 | 437 | 216.26 | 0.494874 |
| GO:0010556\_regulation\_of\_macromolecule\_biosynthetic\_process | SPI1 | 1055 | 3 | 1.221252 | -0.343735 | 437 | 216.26 | 0.494874 |
| GO:0010556\_regulation\_of\_macromolecule\_biosynthetic\_process | CD4 | 1055 | 3 | 1.221252 | -0.343735 | 437 | 216.26 | 0.494874 |
| GO:0031323\_regulation\_of\_cellular\_metabolic\_process | IGF1 | 1466 | 4 | 1.171825 | -0.343533 | 438 | 216.42 | 0.494110 |
| GO:0031323\_regulation\_of\_cellular\_metabolic\_process | SPI1 | 1466 | 4 | 1.171825 | -0.343533 | 438 | 216.42 | 0.494110 |
| GO:0031323\_regulation\_of\_cellular\_metabolic\_process | CD4 | 1466 | 4 | 1.171825 | -0.343533 | 438 | 216.42 | 0.494110 |
| GO:0031323\_regulation\_of\_cellular\_metabolic\_process | PLEK | 1466 | 4 | 1.171825 | -0.343533 | 438 | 216.42 | 0.494110 |
| GO:0016481\_negative\_regulation\_of\_transcription | SPI1 | 261 | 1 | 1.645493 | -0.336144 | 439 | 217.37 | 0.495148 |
| GO:0032502\_developmental\_process | IGF1 | 1919 | 5 | 1.119004 | -0.324558 | 440 | 218.54 | 0.496682 |
| GO:0032502\_developmental\_process | SPI1 | 1919 | 5 | 1.119004 | -0.324558 | 440 | 218.54 | 0.496682 |
| GO:0032502\_developmental\_process | CD4 | 1919 | 5 | 1.119004 | -0.324558 | 440 | 218.54 | 0.496682 |
| GO:0032502\_developmental\_process | BTK | 1919 | 5 | 1.119004 | -0.324558 | 440 | 218.54 | 0.496682 |
| GO:0032502\_developmental\_process | PLEK | 1919 | 5 | 1.119004 | -0.324558 | 440 | 218.54 | 0.496682 |
| GO:0015031\_protein\_transport | PLEK | 274 | 1 | 1.567422 | -0.320767 | 441 | 219.91 | 0.498662 |
| GO:0007010\_cytoskeleton\_organization | PLEK | 275 | 1 | 1.561722 | -0.319625 | 442 | 220.51 | 0.498891 |
| GO:0048878\_chemical\_homeostasis | CD52 | 278 | 1 | 1.544869 | -0.316232 | 443 | 221.4 | 0.499774 |
| GO:0045184\_establishment\_of\_protein\_localization | PLEK | 279 | 1 | 1.539332 | -0.315112 | 444 | 222.1 | 0.500225 |
| GO:0044249\_cellular\_biosynthetic\_process | IGF1 | 1951 | 5 | 1.100650 | -0.309849 | 445 | 222.29 | 0.499528 |
| GO:0044249\_cellular\_biosynthetic\_process | SPI1 | 1951 | 5 | 1.100650 | -0.309849 | 445 | 222.29 | 0.499528 |
| GO:0044249\_cellular\_biosynthetic\_process | CD4 | 1951 | 5 | 1.100650 | -0.309849 | 445 | 222.29 | 0.499528 |
| GO:0044249\_cellular\_biosynthetic\_process | PLEK | 1951 | 5 | 1.100650 | -0.309849 | 445 | 222.29 | 0.499528 |
| GO:0044249\_cellular\_biosynthetic\_process | CD37 | 1951 | 5 | 1.100650 | -0.309849 | 445 | 222.29 | 0.499528 |
| GO:0019752\_carboxylic\_acid\_metabolic\_process | IGF1 | 286 | 1 | 1.501656 | -0.307421 | 447 | 224.2 | 0.501566 |
| GO:0043436\_oxoacid\_metabolic\_process | IGF1 | 286 | 1 | 1.501656 | -0.307421 | 447 | 224.2 | 0.501566 |
| GO:0019222\_regulation\_of\_metabolic\_process | IGF1 | 1538 | 4 | 1.116967 | -0.306482 | 448 | 224.39 | 0.500871 |
| GO:0019222\_regulation\_of\_metabolic\_process | SPI1 | 1538 | 4 | 1.116967 | -0.306482 | 448 | 224.39 | 0.500871 |
| GO:0019222\_regulation\_of\_metabolic\_process | CD4 | 1538 | 4 | 1.116967 | -0.306482 | 448 | 224.39 | 0.500871 |
| GO:0019222\_regulation\_of\_metabolic\_process | PLEK | 1538 | 4 | 1.116967 | -0.306482 | 448 | 224.39 | 0.500871 |
| GO:0010629\_negative\_regulation\_of\_gene\_expression | SPI1 | 289 | 1 | 1.486068 | -0.304204 | 449 | 225.26 | 0.501693 |
| GO:0006082\_organic\_acid\_metabolic\_process | IGF1 | 290 | 1 | 1.480944 | -0.303141 | 451 | 225.82 | 0.500710 |
| GO:0051093\_negative\_regulation\_of\_developmental\_process | IGF1 | 290 | 1 | 1.480944 | -0.303141 | 451 | 225.82 | 0.500710 |
| GO:0042180\_cellular\_ketone\_metabolic\_process | IGF1 | 291 | 1 | 1.475855 | -0.302084 | 452 | 226.3 | 0.500664 |
| GO:0000278\_mitotic\_cell\_cycle | IGF1 | 292 | 1 | 1.470800 | -0.301031 | 453 | 227.47 | 0.502141 |
| GO:0045934\_negative\_regulation\_of\_nucleobase\_\_nucleoside\_\_nucleotide\_and\_nucleic\_acid\_metabolic\_process | SPI1 | 295 | 1 | 1.455843 | -0.297904 | 454 | 228.25 | 0.502753 |
| GO:0016192\_vesicle-mediated\_transport | PLEK | 297 | 1 | 1.446039 | -0.295843 | 455 | 229.42 | 0.504220 |
| GO:0051172\_negative\_regulation\_of\_nitrogen\_compound\_metabolic\_process | SPI1 | 298 | 1 | 1.441187 | -0.294819 | 456 | 229.96 | 0.504298 |
| GO:0009058\_biosynthetic\_process | IGF1 | 1988 | 5 | 1.080165 | -0.293554 | 457 | 230.84 | 0.505120 |
| GO:0009058\_biosynthetic\_process | SPI1 | 1988 | 5 | 1.080165 | -0.293554 | 457 | 230.84 | 0.505120 |
| GO:0009058\_biosynthetic\_process | CD4 | 1988 | 5 | 1.080165 | -0.293554 | 457 | 230.84 | 0.505120 |
| GO:0009058\_biosynthetic\_process | PLEK | 1988 | 5 | 1.080165 | -0.293554 | 457 | 230.84 | 0.505120 |
| GO:0009058\_biosynthetic\_process | CD37 | 1988 | 5 | 1.080165 | -0.293554 | 457 | 230.84 | 0.505120 |
| GO:0006355\_regulation\_of\_transcription\_\_DNA-dependent | IGF1 | 723 | 2 | 1.188032 | -0.290947 | 458 | 231.69 | 0.505873 |
| GO:0006355\_regulation\_of\_transcription\_\_DNA-dependent | SPI1 | 723 | 2 | 1.188032 | -0.290947 | 458 | 231.69 | 0.505873 |
| GO:0006928\_cell\_motion | IGF1 | 308 | 1 | 1.394395 | -0.284842 | 460 | 233.61 | 0.507848 |
| GO:0051674\_localization\_of\_cell | IGF1 | 308 | 1 | 1.394395 | -0.284842 | 460 | 233.61 | 0.507848 |
| GO:0034645\_cellular\_macromolecule\_biosynthetic\_process | IGF1 | 1600 | 4 | 1.073684 | -0.277515 | 461 | 234.71 | 0.509132 |
| GO:0034645\_cellular\_macromolecule\_biosynthetic\_process | SPI1 | 1600 | 4 | 1.073684 | -0.277515 | 461 | 234.71 | 0.509132 |
| GO:0034645\_cellular\_macromolecule\_biosynthetic\_process | CD4 | 1600 | 4 | 1.073684 | -0.277515 | 461 | 234.71 | 0.509132 |
| GO:0034645\_cellular\_macromolecule\_biosynthetic\_process | CD37 | 1600 | 4 | 1.073684 | -0.277515 | 461 | 234.71 | 0.509132 |
| GO:0051252\_regulation\_of\_RNA\_metabolic\_process | IGF1 | 746 | 2 | 1.151404 | -0.276367 | 462 | 234.98 | 0.508615 |
| GO:0051252\_regulation\_of\_RNA\_metabolic\_process | SPI1 | 746 | 2 | 1.151404 | -0.276367 | 462 | 234.98 | 0.508615 |
| GO:0010558\_negative\_regulation\_of\_macromolecule\_biosynthetic\_process | SPI1 | 324 | 1 | 1.325536 | -0.269787 | 463 | 236.66 | 0.511145 |
| GO:0032879\_regulation\_of\_localization | IGF1 | 326 | 1 | 1.317404 | -0.267979 | 464 | 237.0 | 0.510776 |
| GO:0009059\_macromolecule\_biosynthetic\_process | IGF1 | 1626 | 4 | 1.056516 | -0.266116 | 465 | 237.45 | 0.510645 |
| GO:0009059\_macromolecule\_biosynthetic\_process | SPI1 | 1626 | 4 | 1.056516 | -0.266116 | 465 | 237.45 | 0.510645 |
| GO:0009059\_macromolecule\_biosynthetic\_process | CD4 | 1626 | 4 | 1.056516 | -0.266116 | 465 | 237.45 | 0.510645 |
| GO:0009059\_macromolecule\_biosynthetic\_process | CD37 | 1626 | 4 | 1.056516 | -0.266116 | 465 | 237.45 | 0.510645 |
| GO:0006996\_organelle\_organization | IGF1 | 764 | 2 | 1.124277 | -0.265501 | 466 | 237.67 | 0.510021 |
| GO:0006996\_organelle\_organization | PLEK | 764 | 2 | 1.124277 | -0.265501 | 466 | 237.67 | 0.510021 |
| GO:0008104\_protein\_localization | PLEK | 339 | 1 | 1.266884 | -0.256603 | 467 | 239.09 | 0.511970 |
| GO:0051094\_positive\_regulation\_of\_developmental\_process | BTK | 340 | 1 | 1.263158 | -0.255754 | 468 | 239.82 | 0.512436 |
| GO:0032501\_multicellular\_organismal\_process | IGF1 | 2082 | 5 | 1.031397 | -0.255395 | 469 | 240.03 | 0.511791 |
| GO:0032501\_multicellular\_organismal\_process | SPI1 | 2082 | 5 | 1.031397 | -0.255395 | 469 | 240.03 | 0.511791 |
| GO:0032501\_multicellular\_organismal\_process | CD4 | 2082 | 5 | 1.031397 | -0.255395 | 469 | 240.03 | 0.511791 |
| GO:0032501\_multicellular\_organismal\_process | BTK | 2082 | 5 | 1.031397 | -0.255395 | 469 | 240.03 | 0.511791 |
| GO:0032501\_multicellular\_organismal\_process | PLEK | 2082 | 5 | 1.031397 | -0.255395 | 469 | 240.03 | 0.511791 |
| GO:0033554\_cellular\_response\_to\_stress | CARD9 | 341 | 1 | 1.259454 | -0.254909 | 470 | 240.37 | 0.511426 |
| GO:0016070\_RNA\_metabolic\_process | RNASE2 | 1230 | 3 | 1.047497 | -0.249912 | 471 | 240.79 | 0.511231 |
| GO:0016070\_RNA\_metabolic\_process | IGF1 | 1230 | 3 | 1.047497 | -0.249912 | 471 | 240.79 | 0.511231 |
| GO:0016070\_RNA\_metabolic\_process | SPI1 | 1230 | 3 | 1.047497 | -0.249912 | 471 | 240.79 | 0.511231 |
| GO:0006357\_regulation\_of\_transcription\_from\_RNA\_polymerase\_II\_promoter | SPI1 | 351 | 1 | 1.223572 | -0.246648 | 472 | 241.17 | 0.510953 |
| GO:0006259\_DNA\_metabolic\_process | IGF1 | 360 | 1 | 1.192982 | -0.239502 | 473 | 242.78 | 0.513277 |
| GO:0007186\_G-protein\_coupled\_receptor\_protein\_signaling\_pathway | PLEK | 363 | 1 | 1.183123 | -0.237179 | 474 | 243.17 | 0.513017 |
| GO:0022414\_reproductive\_process | CD4 | 365 | 1 | 1.176640 | -0.235645 | 475 | 243.7 | 0.513053 |
| GO:0000003\_reproduction | CD4 | 367 | 1 | 1.170228 | -0.234125 | 476 | 244.44 | 0.513529 |
| GO:0022402\_cell\_cycle\_process | IGF1 | 370 | 1 | 1.160740 | -0.231866 | 477 | 245.21 | 0.514067 |
| GO:0044255\_cellular\_lipid\_metabolic\_process | PLEK | 381 | 1 | 1.127228 | -0.223815 | 478 | 247.44 | 0.517657 |
| GO:0033036\_macromolecule\_localization | PLEK | 388 | 1 | 1.106891 | -0.218871 | 479 | 248.06 | 0.517871 |
| GO:0080090\_regulation\_of\_primary\_metabolic\_process | IGF1 | 1311 | 3 | 0.982777 | -0.215297 | 480 | 249.06 | 0.518875 |
| GO:0080090\_regulation\_of\_primary\_metabolic\_process | SPI1 | 1311 | 3 | 0.982777 | -0.215297 | 480 | 249.06 | 0.518875 |
| GO:0080090\_regulation\_of\_primary\_metabolic\_process | PLEK | 1311 | 3 | 0.982777 | -0.215297 | 480 | 249.06 | 0.518875 |
| GO:0006351\_transcription\_\_DNA-dependent | IGF1 | 884 | 2 | 0.971660 | -0.203569 | 481 | 251.28 | 0.522412 |
| GO:0006351\_transcription\_\_DNA-dependent | SPI1 | 884 | 2 | 0.971660 | -0.203569 | 481 | 251.28 | 0.522412 |
| GO:0010605\_negative\_regulation\_of\_macromolecule\_metabolic\_process | SPI1 | 413 | 1 | 1.039888 | -0.202275 | 482 | 252.2 | 0.523237 |
| GO:0032774\_RNA\_biosynthetic\_process | IGF1 | 887 | 2 | 0.968374 | -0.202227 | 483 | 252.5 | 0.522774 |
| GO:0032774\_RNA\_biosynthetic\_process | SPI1 | 887 | 2 | 0.968374 | -0.202227 | 483 | 252.5 | 0.522774 |
| GO:0045449\_regulation\_of\_transcription | IGF1 | 900 | 2 | 0.954386 | -0.196514 | 484 | 253.44 | 0.523636 |
| GO:0045449\_regulation\_of\_transcription | SPI1 | 900 | 2 | 0.954386 | -0.196514 | 484 | 253.44 | 0.523636 |
| GO:0043933\_macromolecular\_complex\_subunit\_organization | PLEK | 424 | 1 | 1.012910 | -0.195458 | 485 | 254.2 | 0.524124 |
| GO:0043285\_biopolymer\_catabolic\_process | RNASE2 | 426 | 1 | 1.008154 | -0.194249 | 486 | 254.85 | 0.524383 |
| GO:0007267\_cell-cell\_signaling | CD33 | 445 | 1 | 0.965109 | -0.183193 | 487 | 258.47 | 0.530739 |
| GO:0006629\_lipid\_metabolic\_process | PLEK | 468 | 1 | 0.917679 | -0.170790 | 488 | 261.21 | 0.535266 |
| GO:0051716\_cellular\_response\_to\_stimulus | CARD9 | 474 | 1 | 0.906063 | -0.167718 | 489 | 261.82 | 0.535419 |
| GO:0022607\_cellular\_component\_assembly | PLEK | 478 | 1 | 0.898481 | -0.165706 | 490 | 263.31 | 0.537367 |
| GO:0007049\_cell\_cycle | IGF1 | 494 | 1 | 0.869380 | -0.157931 | 491 | 264.82 | 0.539348 |
| GO:0009653\_anatomical\_structure\_morphogenesis | IGF1 | 500 | 1 | 0.858947 | -0.155124 | 492 | 265.19 | 0.539004 |
| GO:0044238\_primary\_metabolic\_process | RNASE2 | 3719 | 8 | 0.923848 | -0.154162 | 493 | 265.75 | 0.539047 |
| GO:0044238\_primary\_metabolic\_process | IGF1 | 3719 | 8 | 0.923848 | -0.154162 | 493 | 265.75 | 0.539047 |
| GO:0044238\_primary\_metabolic\_process | PTPN7 | 3719 | 8 | 0.923848 | -0.154162 | 493 | 265.75 | 0.539047 |
| GO:0044238\_primary\_metabolic\_process | FGR | 3719 | 8 | 0.923848 | -0.154162 | 493 | 265.75 | 0.539047 |
| GO:0044238\_primary\_metabolic\_process | SPI1 | 3719 | 8 | 0.923848 | -0.154162 | 493 | 265.75 | 0.539047 |
| GO:0044238\_primary\_metabolic\_process | BTK | 3719 | 8 | 0.923848 | -0.154162 | 493 | 265.75 | 0.539047 |
| GO:0044238\_primary\_metabolic\_process | PLEK | 3719 | 8 | 0.923848 | -0.154162 | 493 | 265.75 | 0.539047 |
| GO:0044238\_primary\_metabolic\_process | CD37 | 3719 | 8 | 0.923848 | -0.154162 | 493 | 265.75 | 0.539047 |
| GO:0006366\_transcription\_from\_RNA\_polymerase\_II\_promoter | SPI1 | 506 | 1 | 0.848762 | -0.152374 | 494 | 266.8 | 0.540081 |
| GO:0019219\_regulation\_of\_nucleobase\_\_nucleoside\_\_nucleotide\_and\_nucleic\_acid\_metabolic\_process | IGF1 | 1041 | 2 | 0.825118 | -0.144023 | 495 | 267.92 | 0.541253 |
| GO:0019219\_regulation\_of\_nucleobase\_\_nucleoside\_\_nucleotide\_and\_nucleic\_acid\_metabolic\_process | SPI1 | 1041 | 2 | 0.825118 | -0.144023 | 495 | 267.92 | 0.541253 |
| GO:0051171\_regulation\_of\_nitrogen\_compound\_metabolic\_process | IGF1 | 1055 | 2 | 0.814168 | -0.139638 | 496 | 269.13 | 0.542601 |
| GO:0051171\_regulation\_of\_nitrogen\_compound\_metabolic\_process | SPI1 | 1055 | 2 | 0.814168 | -0.139638 | 496 | 269.13 | 0.542601 |
| GO:0010468\_regulation\_of\_gene\_expression | IGF1 | 1067 | 2 | 0.805012 | -0.135984 | 497 | 269.41 | 0.542072 |
| GO:0010468\_regulation\_of\_gene\_expression | SPI1 | 1067 | 2 | 0.805012 | -0.135984 | 497 | 269.41 | 0.542072 |
| GO:0006350\_transcription | IGF1 | 1069 | 2 | 0.803505 | -0.135384 | 498 | 269.7 | 0.541566 |
| GO:0006350\_transcription | SPI1 | 1069 | 2 | 0.803505 | -0.135384 | 498 | 269.7 | 0.541566 |
| GO:0010926\_anatomical\_structure\_formation | PLEK | 560 | 1 | 0.766917 | -0.129963 | 500 | 271.54 | 0.543080 |
| GO:0044085\_cellular\_component\_biogenesis | PLEK | 560 | 1 | 0.766917 | -0.129963 | 500 | 271.54 | 0.543080 |
| GO:0051649\_establishment\_of\_localization\_in\_cell | PLEK | 573 | 1 | 0.749518 | -0.125136 | 501 | 273.06 | 0.545030 |
| GO:0051641\_cellular\_localization | PLEK | 617 | 1 | 0.696068 | -0.110205 | 502 | 276.41 | 0.550618 |
| GO:0042221\_response\_to\_chemical\_stimulus | RNASE2 | 631 | 1 | 0.680624 | -0.105871 | 503 | 276.97 | 0.550636 |
| GO:0003008\_system\_process | IGF1 | 710 | 1 | 0.604893 | -0.084603 | 504 | 280.41 | 0.556369 |
| GO:0006139\_nucleobase\_\_nucleoside\_\_nucleotide\_and\_nucleic\_acid\_metabolic\_process | RNASE2 | 1845 | 3 | 0.698331 | -0.076734 | 505 | 282.4 | 0.559208 |
| GO:0006139\_nucleobase\_\_nucleoside\_\_nucleotide\_and\_nucleic\_acid\_metabolic\_process | IGF1 | 1845 | 3 | 0.698331 | -0.076734 | 505 | 282.4 | 0.559208 |
| GO:0006139\_nucleobase\_\_nucleoside\_\_nucleotide\_and\_nucleic\_acid\_metabolic\_process | SPI1 | 1845 | 3 | 0.698331 | -0.076734 | 505 | 282.4 | 0.559208 |
| GO:0016043\_cellular\_component\_organization | IGF1 | 1366 | 2 | 0.628805 | -0.069587 | 506 | 283.83 | 0.560929 |
| GO:0016043\_cellular\_component\_organization | PLEK | 1366 | 2 | 0.628805 | -0.069587 | 506 | 283.83 | 0.560929 |
| GO:0034961\_cellular\_biopolymer\_biosynthetic\_process | IGF1 | 1448 | 2 | 0.593196 | -0.057652 | 507 | 285.17 | 0.562465 |
| GO:0034961\_cellular\_biopolymer\_biosynthetic\_process | SPI1 | 1448 | 2 | 0.593196 | -0.057652 | 507 | 285.17 | 0.562465 |
| GO:0043284\_biopolymer\_biosynthetic\_process | IGF1 | 1458 | 2 | 0.589127 | -0.056335 | 508 | 286.02 | 0.563031 |
| GO:0043284\_biopolymer\_biosynthetic\_process | SPI1 | 1458 | 2 | 0.589127 | -0.056335 | 508 | 286.02 | 0.563031 |
| GO:0006807\_nitrogen\_compound\_metabolic\_process | RNASE2 | 2053 | 3 | 0.627580 | -0.049732 | 509 | 287.65 | 0.565128 |
| GO:0006807\_nitrogen\_compound\_metabolic\_process | IGF1 | 2053 | 3 | 0.627580 | -0.049732 | 509 | 287.65 | 0.565128 |
| GO:0006807\_nitrogen\_compound\_metabolic\_process | SPI1 | 2053 | 3 | 0.627580 | -0.049732 | 509 | 287.65 | 0.565128 |
| GO:0051179\_localization | IGF1 | 1561 | 2 | 0.550255 | -0.044306 | 510 | 288.89 | 0.566451 |
| GO:0051179\_localization | PLEK | 1561 | 2 | 0.550255 | -0.044306 | 510 | 288.89 | 0.566451 |
| GO:0010467\_gene\_expression | IGF1 | 1663 | 2 | 0.516505 | -0.034777 | 511 | 290.91 | 0.569295 |
| GO:0010467\_gene\_expression | SPI1 | 1663 | 2 | 0.516505 | -0.034777 | 511 | 290.91 | 0.569295 |
| GO:0006810\_transport | PLEK | 1243 | 1 | 0.345514 | -0.019143 | 512 | 293.96 | 0.574141 |
| GO:0051234\_establishment\_of\_localization | PLEK | 1260 | 1 | 0.340852 | -0.018249 | 513 | 294.24 | 0.573567 |
| GO:0008150\_biological\_process | RNASE2 | 8160 | 19 | 1.000000 | 0.000000 | 761 | 575.22 | 0.755874 |
| GO:0008150\_biological\_process | CD52 | 8160 | 19 | 1.000000 | 0.000000 | 761 | 575.22 | 0.755874 |
| GO:0008150\_biological\_process | MNDA | 8160 | 19 | 1.000000 | 0.000000 | 761 | 575.22 | 0.755874 |
| GO:0008150\_biological\_process | PTPN7 | 8160 | 19 | 1.000000 | 0.000000 | 761 | 575.22 | 0.755874 |
| GO:0008150\_biological\_process | CYBB | 8160 | 19 | 1.000000 | 0.000000 | 761 | 575.22 | 0.755874 |
| GO:0008150\_biological\_process | NCF1 | 8160 | 19 | 1.000000 | 0.000000 | 761 | 575.22 | 0.755874 |
| GO:0008150\_biological\_process | CD33 | 8160 | 19 | 1.000000 | 0.000000 | 761 | 575.22 | 0.755874 |
| GO:0008150\_biological\_process | CD4 | 8160 | 19 | 1.000000 | 0.000000 | 761 | 575.22 | 0.755874 |
| GO:0008150\_biological\_process | BTK | 8160 | 19 | 1.000000 | 0.000000 | 761 | 575.22 | 0.755874 |
| GO:0008150\_biological\_process | NCF4 | 8160 | 19 | 1.000000 | 0.000000 | 761 | 575.22 | 0.755874 |
| GO:0008150\_biological\_process | CSF3R | 8160 | 19 | 1.000000 | 0.000000 | 761 | 575.22 | 0.755874 |
| GO:0008150\_biological\_process | TYROBP | 8160 | 19 | 1.000000 | 0.000000 | 761 | 575.22 | 0.755874 |
| GO:0008150\_biological\_process | CD37 | 8160 | 19 | 1.000000 | 0.000000 | 761 | 575.22 | 0.755874 |
| GO:0008150\_biological\_process | IGF1 | 8160 | 19 | 1.000000 | 0.000000 | 761 | 575.22 | 0.755874 |
| GO:0008150\_biological\_process | LILRA2 | 8160 | 19 | 1.000000 | 0.000000 | 761 | 575.22 | 0.755874 |
| GO:0008150\_biological\_process | FGR | 8160 | 19 | 1.000000 | 0.000000 | 761 | 575.22 | 0.755874 |
| GO:0008150\_biological\_process | SPI1 | 8160 | 19 | 1.000000 | 0.000000 | 761 | 575.22 | 0.755874 |
| GO:0008150\_biological\_process | CARD9 | 8160 | 19 | 1.000000 | 0.000000 | 761 | 575.22 | 0.755874 |
| GO:0008150\_biological\_process | PLEK | 8160 | 19 | 1.000000 | 0.000000 | 761 | 575.22 | 0.755874 |
