# Supplementary material for: Concordance of Gene Expression and Functional Correlation Patterns across the NCI-60 Cell Lines and the Cancer Genome Atlas Glioblastoma Samples
Source: PLoS One. 2012 Jul 26;7(7):e40062. doi: 10.1371/journal.pone.0040062 (PMC3406063; doi:10.1371/journal.pone.0040062)
Supplement: Download S1 — Zip archive of HTGM results. (ZIP) [file pone.0040062.s007.zip › work2026406846/Generated_Total2026406846.dir/generic.BP.NCI60.0.6.BTK.express.genes.correlation.complete.Thu.May.19.17.12.30.2011.htgm.txt.dir/generic.BP.NCI60.0.6.BTK.express.genes.correlation.complete.Thu.May.19.17.12.30.2011.htgm.txt.change.html]

Category Summary Report for generic.BP.NCI60.0.6.BTK.express.genes.correlation.complete.Thu.May.19.17.12.30.2011.htgm.txt

# Category Summary Report for generic.BP.NCI60.0.6.BTK.express.genes.correlation.complete.Thu.May.19.17.12.30.2011.htgm.txt

| HYPERLINKED GO CATEGORY | TOTAL GENES | CHANGED GENES | ENRICHMENT | LOG10(p) | CUMULATIVE NUMBER OF CATEGORIES | CUMULATIVE RANDOMS LOWER BOUND | CUMULATIVE RANDOMS MEAN | CUMULATIVE RANDOMS UPPER BOUND | FALSE DISCOVERY RATE |
| --- | --- | --- | --- | --- | --- | --- | --- | --- | --- |
| GO:0045730\_respiratory\_burst | 16 | 3 | 80.526316 | -5.230560 | 1 | -0.090000 | 0.01 | 0.110000 | 0.010000 |
| GO:0050896\_response\_to\_stimulus | 1775 | 13 | 3.145441 | -4.776036 | 2 | -0.141447 | 0.03 | 0.201447 | 0.015000 |
| GO:0006950\_response\_to\_stress | 959 | 9 | 4.030514 | -3.896380 | 3 | -0.454965 | 0.16 | 0.774965 | 0.053333 |
| GO:0006952\_defense\_response | 369 | 6 | 6.983312 | -3.870098 | 4 | -0.454965 | 0.16 | 0.774965 | 0.040000 |
| GO:0042554\_superoxide\_anion\_generation | 9 | 2 | 95.438596 | -3.737224 | 5 | -0.553030 | 0.22 | 0.993030 | 0.044000 |
| GO:0006968\_cellular\_defense\_response | 58 | 3 | 22.214156 | -3.516260 | 6 | -0.679590 | 0.3 | 1.279590 | 0.050000 |
| GO:0006801\_superoxide\_metabolic\_process | 17 | 2 | 50.526316 | -3.164813 | 7 | -1.062568 | 0.56 | 2.182568 | 0.080000 |
| GO:0019722\_calcium-mediated\_signaling | 30 | 2 | 28.631579 | -2.667698 | 8 | -1.711418 | 1.32 | 4.351418 | 0.165000 |
| GO:0000746\_conjugation | 1 | 1 |  |  |  |  |  |  |  |  |
| GO:0000747\_conjugation\_with\_cellular\_fusion | 1 | 1 |  |  |  |  |  |  |  |  |
| GO:0002576\_platelet\_degranulation | 1 | 1 |  |  |  |  |  |  |  |  |
| GO:0006021\_inositol\_biosynthetic\_process | 1 | 1 |  |  |  |  |  |  |  |  |
| GO:0009441\_glycolate\_metabolic\_process | 1 | 1 |  |  |  |  |  |  |  |  |
| GO:0010919\_regulation\_of\_inositol\_phosphate\_biosynthetic\_process | 1 | 1 |  |  |  |  |  |  |  |  |
| GO:0010920\_negative\_regulation\_of\_inositol\_phosphate\_biosynthetic\_process | 1 | 1 |  |  |  |  |  |  |  |  |
| GO:0010924\_regulation\_of\_inositol-polyphosphate\_5-phosphatase\_activity | 1 | 1 |  |  |  |  |  |  |  |  |
| GO:0010925\_positive\_regulation\_of\_inositol-polyphosphate\_5-phosphatase\_activity | 1 | 1 |  |  |  |  |  |  |  |  |
| GO:0014834\_satellite\_cell\_maintenance\_involved\_in\_skeletal\_muscle\_regeneration | 1 | 1 |  |  |  |  |  |  |  |  |
| GO:0014904\_myotube\_cell\_development | 1 | 1 |  |  |  |  |  |  |  |  |
| GO:0030836\_positive\_regulation\_of\_actin\_filament\_depolymerization | 1 | 1 |  |  |  |  |  |  |  |  |
| GO:0030845\_inhibition\_of\_phospholipase\_C\_activity\_involved\_in\_G-protein\_coupled\_receptor\_signaling\_pathway | 1 | 1 |  |  |  |  |  |  |  |  |
| GO:0032958\_inositol\_phosphate\_biosynthetic\_process | 1 | 1 |  |  |  |  |  |  |  |  |
| GO:0033622\_integrin\_activation | 1 | 1 |  |  |  |  |  |  |  |  |
| GO:0033623\_regulation\_of\_integrin\_activation | 1 | 1 |  |  |  |  |  |  |  |  |
| GO:0033625\_positive\_regulation\_of\_integrin\_activation | 1 | 1 |  |  |  |  |  |  |  |  |
| GO:0034109\_homotypic\_cell-cell\_adhesion | 1 | 1 |  |  |  |  |  |  |  |  |
| GO:0043403\_skeletal\_muscle\_regeneration | 1 | 1 |  |  |  |  |  |  |  |  |
| GO:0043647\_inositol\_phosphate\_metabolic\_process | 1 | 1 |  |  |  |  |  |  |  |  |
| GO:0046173\_polyol\_biosynthetic\_process | 1 | 1 |  |  |  |  |  |  |  |  |
| GO:0051450\_myoblast\_proliferation | 1 | 1 |  |  |  |  |  |  |  |  |
| GO:0060305\_regulation\_of\_cell\_diameter | 1 | 1 |  |  |  |  |  |  |  |  |
| GO:0070527\_platelet\_aggregation | 1 | 1 |  |  |  |  |  |  |  |  |
| GO:0070528\_protein\_kinase\_C\_signaling\_cascade | 1 | 1 |  |  |  |  |  |  |  |  |
| GO:0070560\_protein\_secretion\_by\_platelet | 1 | 1 |  |  |  |  |  |  |  |  |
| GO:0030097\_hemopoiesis | 135 | 3 | 9.543860 | -2.451678 | 9 | -1.803870 | 2.08 | 5.963870 | 0.231111 |
| GO:0048534\_hemopoietic\_or\_lymphoid\_organ\_development | 139 | 3 | 9.269216 | -2.415910 | 10 | -1.788003 | 2.18 | 6.148003 | 0.218000 |
| GO:0006793\_phosphorus\_metabolic\_process | 697 | 6 | 3.697047 | -2.407301 | 12 | -1.772125 | 2.2 | 6.172125 | 0.183333 |
| GO:0006796\_phosphate\_metabolic\_process | 697 | 6 | 3.697047 | -2.407301 | 12 | -1.772125 | 2.2 | 6.172125 | 0.183333 |
| GO:0006800\_oxygen\_and\_reactive\_oxygen\_species\_metabolic\_process | 41 | 2 | 20.949936 | -2.398996 | 13 | -1.803096 | 2.29 | 6.383096 | 0.176154 |
| GO:0002520\_immune\_system\_development | 147 | 3 | 8.764769 | -2.347598 | 14 | -1.989478 | 2.59 | 7.169478 | 0.185000 |
| GO:0002376\_immune\_system\_process | 718 | 6 | 3.588917 | -2.342827 | 15 | -1.989478 | 2.59 | 7.169478 | 0.172667 |
| GO:0010519\_negative\_regulation\_of\_phospholipase\_activity | 2 | 1 |  |  |  |  |  |  |  |  |
| GO:0014911\_positive\_regulation\_of\_smooth\_muscle\_cell\_migration | 2 | 1 |  |  |  |  |  |  |  |  |
| GO:0034392\_negative\_regulation\_of\_smooth\_muscle\_cell\_apoptosis | 2 | 1 |  |  |  |  |  |  |  |  |
| GO:0043243\_positive\_regulation\_of\_protein\_complex\_disassembly | 2 | 1 |  |  |  |  |  |  |  |  |
| GO:0050849\_negative\_regulation\_of\_calcium-mediated\_signaling | 2 | 1 |  |  |  |  |  |  |  |  |
| GO:0070493\_thrombin\_receptor\_signaling\_pathway | 2 | 1 |  |  |  |  |  |  |  |  |
| GO:0010638\_positive\_regulation\_of\_organelle\_organization | 46 | 2 | 18.672769 | -2.300878 | 16 | -2.001234 | 2.78 | 7.561234 | 0.173750 |
| GO:0019932\_second-messenger-mediated\_signaling | 153 | 3 | 8.421053 | -2.298958 | 17 | -1.996967 | 2.79 | 7.576967 | 0.164118 |
| GO:0048015\_phosphoinositide-mediated\_signaling | 53 | 2 | 16.206554 | -2.180779 | 18 | -1.965422 | 3.44 | 8.845422 | 0.191111 |
| GO:0002244\_hemopoietic\_progenitor\_cell\_differentiation | 3 | 1 |  |  |  |  |  |  |  |  |
| GO:0010572\_positive\_regulation\_of\_platelet\_activation | 3 | 1 |  |  |  |  |  |  |  |  |
| GO:0010656\_negative\_regulation\_of\_muscle\_cell\_apoptosis | 3 | 1 |  |  |  |  |  |  |  |  |
| GO:0010922\_positive\_regulation\_of\_phosphatase\_activity | 3 | 1 |  |  |  |  |  |  |  |  |
| GO:0032872\_regulation\_of\_stress-activated\_MAPK\_cascade | 3 | 1 |  |  |  |  |  |  |  |  |
| GO:0032874\_positive\_regulation\_of\_stress-activated\_MAPK\_cascade | 3 | 1 |  |  |  |  |  |  |  |  |
| GO:0033143\_regulation\_of\_steroid\_hormone\_receptor\_signaling\_pathway | 3 | 1 |  |  |  |  |  |  |  |  |
| GO:0035019\_somatic\_stem\_cell\_maintenance | 3 | 1 |  |  |  |  |  |  |  |  |
| GO:0042246\_tissue\_regeneration | 3 | 1 |  |  |  |  |  |  |  |  |
| GO:0043193\_positive\_regulation\_of\_gene-specific\_transcription | 57 | 2 | 15.069252 | -2.119399 | 19 | -1.929382 | 3.61 | 9.149382 | 0.190000 |
| GO:0030865\_cortical\_cytoskeleton\_organization | 4 | 1 |  |  |  |  |  |  |  |  |
| GO:0030866\_cortical\_actin\_cytoskeleton\_organization | 4 | 1 |  |  |  |  |  |  |  |  |
| GO:0043568\_positive\_regulation\_of\_insulin-like\_growth\_factor\_receptor\_signaling\_pathway | 4 | 1 |  |  |  |  |  |  |  |  |
| GO:0051239\_regulation\_of\_multicellular\_organismal\_process | 378 | 4 | 4.544695 | -1.995795 | 20 | -1.727749 | 4.6 | 10.927749 | 0.230000 |
| GO:0034390\_smooth\_muscle\_cell\_apoptosis | 5 | 1 | 85.894737 | -1.935883 | 24 | -0.091103 | 7.52 | 15.131103 | 0.313333 |
| GO:0034391\_regulation\_of\_smooth\_muscle\_cell\_apoptosis | 5 | 1 | 85.894737 | -1.935883 | 24 | -0.091103 | 7.52 | 15.131103 | 0.313333 |
| GO:0045821\_positive\_regulation\_of\_glycolysis | 5 | 1 | 85.894737 | -1.935883 | 24 | -0.091103 | 7.52 | 15.131103 | 0.313333 |
| GO:0051403\_stress-activated\_MAPK\_cascade | 5 | 1 | 85.894737 | -1.935883 | 24 | -0.091103 | 7.52 | 15.131103 | 0.313333 |
| GO:0006020\_inositol\_metabolic\_process | 6 | 1 | 71.578947 | -1.857181 | 33 | 1.296470 | 10.88 | 20.463530 | 0.329697 |
| GO:0010657\_muscle\_cell\_apoptosis | 6 | 1 | 71.578947 | -1.857181 | 33 | 1.296470 | 10.88 | 20.463530 | 0.329697 |
| GO:0010660\_regulation\_of\_muscle\_cell\_apoptosis | 6 | 1 | 71.578947 | -1.857181 | 33 | 1.296470 | 10.88 | 20.463530 | 0.329697 |
| GO:0019827\_stem\_cell\_maintenance | 6 | 1 | 71.578947 | -1.857181 | 33 | 1.296470 | 10.88 | 20.463530 | 0.329697 |
| GO:0031099\_regeneration | 6 | 1 | 71.578947 | -1.857181 | 33 | 1.296470 | 10.88 | 20.463530 | 0.329697 |
| GO:0046579\_positive\_regulation\_of\_Ras\_protein\_signal\_transduction | 6 | 1 | 71.578947 | -1.857181 | 33 | 1.296470 | 10.88 | 20.463530 | 0.329697 |
| GO:0048864\_stem\_cell\_development | 6 | 1 | 71.578947 | -1.857181 | 33 | 1.296470 | 10.88 | 20.463530 | 0.329697 |
| GO:0051057\_positive\_regulation\_of\_small\_GTPase\_mediated\_signal\_transduction | 6 | 1 | 71.578947 | -1.857181 | 33 | 1.296470 | 10.88 | 20.463530 | 0.329697 |
| GO:0060192\_negative\_regulation\_of\_lipase\_activity | 6 | 1 | 71.578947 | -1.857181 | 33 | 1.296470 | 10.88 | 20.463530 | 0.329697 |
| GO:0032583\_regulation\_of\_gene-specific\_transcription | 81 | 2 | 10.604288 | -1.826295 | 34 | 1.334722 | 11.21 | 21.085278 | 0.329706 |
| GO:0045087\_innate\_immune\_response | 82 | 2 | 10.474968 | -1.816171 | 35 | 1.300505 | 11.3 | 21.299495 | 0.322857 |
| GO:0010543\_regulation\_of\_platelet\_activation | 7 | 1 | 61.353383 | -1.790712 | 48 | 2.915627 | 13.96 | 25.004373 | 0.290833 |
| GO:0014068\_positive\_regulation\_of\_phosphoinositide\_3-kinase\_cascade | 7 | 1 | 61.353383 | -1.790712 | 48 | 2.915627 | 13.96 | 25.004373 | 0.290833 |
| GO:0014902\_myotube\_differentiation | 7 | 1 | 61.353383 | -1.790712 | 48 | 2.915627 | 13.96 | 25.004373 | 0.290833 |
| GO:0014910\_regulation\_of\_smooth\_muscle\_cell\_migration | 7 | 1 | 61.353383 | -1.790712 | 48 | 2.915627 | 13.96 | 25.004373 | 0.290833 |
| GO:0030042\_actin\_filament\_depolymerization | 7 | 1 | 61.353383 | -1.790712 | 48 | 2.915627 | 13.96 | 25.004373 | 0.290833 |
| GO:0030834\_regulation\_of\_actin\_filament\_depolymerization | 7 | 1 | 61.353383 | -1.790712 | 48 | 2.915627 | 13.96 | 25.004373 | 0.290833 |
| GO:0031529\_ruffle\_organization | 7 | 1 | 61.353383 | -1.790712 | 48 | 2.915627 | 13.96 | 25.004373 | 0.290833 |
| GO:0032233\_positive\_regulation\_of\_actin\_filament\_bundle\_formation | 7 | 1 | 61.353383 | -1.790712 | 48 | 2.915627 | 13.96 | 25.004373 | 0.290833 |
| GO:0042523\_positive\_regulation\_of\_tyrosine\_phosphorylation\_of\_Stat5\_protein | 7 | 1 | 61.353383 | -1.790712 | 48 | 2.915627 | 13.96 | 25.004373 | 0.290833 |
| GO:0043567\_regulation\_of\_insulin-like\_growth\_factor\_receptor\_signaling\_pathway | 7 | 1 | 61.353383 | -1.790712 | 48 | 2.915627 | 13.96 | 25.004373 | 0.290833 |
| GO:0045058\_T\_cell\_selection | 7 | 1 | 61.353383 | -1.790712 | 48 | 2.915627 | 13.96 | 25.004373 | 0.290833 |
| GO:0045744\_negative\_regulation\_of\_G-protein\_coupled\_receptor\_protein\_signaling\_pathway | 7 | 1 | 61.353383 | -1.790712 | 48 | 2.915627 | 13.96 | 25.004373 | 0.290833 |
| GO:0048863\_stem\_cell\_differentiation | 7 | 1 | 61.353383 | -1.790712 | 48 | 2.915627 | 13.96 | 25.004373 | 0.290833 |
| GO:0010604\_positive\_regulation\_of\_macromolecule\_metabolic\_process | 446 | 4 | 3.851782 | -1.751446 | 49 | 2.872377 | 14.32 | 25.767623 | 0.292245 |
| GO:0010921\_regulation\_of\_phosphatase\_activity | 8 | 1 | 53.684211 | -1.733199 | 58 | 4.206589 | 17.14 | 30.073411 | 0.295517 |
| GO:0014066\_regulation\_of\_phosphoinositide\_3-kinase\_cascade | 8 | 1 | 53.684211 | -1.733199 | 58 | 4.206589 | 17.14 | 30.073411 | 0.295517 |
| GO:0014812\_muscle\_cell\_migration | 8 | 1 | 53.684211 | -1.733199 | 58 | 4.206589 | 17.14 | 30.073411 | 0.295517 |
| GO:0014896\_muscle\_hypertrophy | 8 | 1 | 53.684211 | -1.733199 | 58 | 4.206589 | 17.14 | 30.073411 | 0.295517 |
| GO:0014909\_smooth\_muscle\_cell\_migration | 8 | 1 | 53.684211 | -1.733199 | 58 | 4.206589 | 17.14 | 30.073411 | 0.295517 |
| GO:0042506\_tyrosine\_phosphorylation\_of\_Stat5\_protein | 8 | 1 | 53.684211 | -1.733199 | 58 | 4.206589 | 17.14 | 30.073411 | 0.295517 |
| GO:0042522\_regulation\_of\_tyrosine\_phosphorylation\_of\_Stat5\_protein | 8 | 1 | 53.684211 | -1.733199 | 58 | 4.206589 | 17.14 | 30.073411 | 0.295517 |
| GO:0045086\_positive\_regulation\_of\_interleukin-2\_biosynthetic\_process | 8 | 1 | 53.684211 | -1.733199 | 58 | 4.206589 | 17.14 | 30.073411 | 0.295517 |
| GO:0046330\_positive\_regulation\_of\_JNK\_cascade | 8 | 1 | 53.684211 | -1.733199 | 58 | 4.206589 | 17.14 | 30.073411 | 0.295517 |
| GO:0051130\_positive\_regulation\_of\_cellular\_component\_organization | 91 | 2 | 9.438982 | -1.730580 | 59 | 4.216674 | 17.16 | 30.103326 | 0.290847 |
| GO:0031325\_positive\_regulation\_of\_cellular\_metabolic\_process | 454 | 4 | 3.783909 | -1.725649 | 60 | 4.196504 | 17.2 | 30.203496 | 0.286667 |
| GO:0016311\_dephosphorylation | 92 | 2 | 9.336384 | -1.721634 | 61 | 4.206657 | 17.25 | 30.293343 | 0.282787 |
| GO:0009605\_response\_to\_external\_stimulus | 464 | 4 | 3.702359 | -1.694166 | 62 | 4.301983 | 17.41 | 30.518017 | 0.280806 |
| GO:0006110\_regulation\_of\_glycolysis | 9 | 1 | 47.719298 | -1.682525 | 69 | 5.102806 | 19.67 | 34.237194 | 0.285072 |
| GO:0043470\_regulation\_of\_carbohydrate\_catabolic\_process | 9 | 1 | 47.719298 | -1.682525 | 69 | 5.102806 | 19.67 | 34.237194 | 0.285072 |
| GO:0043471\_regulation\_of\_cellular\_carbohydrate\_catabolic\_process | 9 | 1 | 47.719298 | -1.682525 | 69 | 5.102806 | 19.67 | 34.237194 | 0.285072 |
| GO:0045646\_regulation\_of\_erythrocyte\_differentiation | 9 | 1 | 47.719298 | -1.682525 | 69 | 5.102806 | 19.67 | 34.237194 | 0.285072 |
| GO:0048661\_positive\_regulation\_of\_smooth\_muscle\_cell\_proliferation | 9 | 1 | 47.719298 | -1.682525 | 69 | 5.102806 | 19.67 | 34.237194 | 0.285072 |
| GO:0050848\_regulation\_of\_calcium-mediated\_signaling | 9 | 1 | 47.719298 | -1.682525 | 69 | 5.102806 | 19.67 | 34.237194 | 0.285072 |
| GO:0070304\_positive\_regulation\_of\_stress-activated\_protein\_kinase\_signaling\_pathway | 9 | 1 | 47.719298 | -1.682525 | 69 | 5.102806 | 19.67 | 34.237194 | 0.285072 |
| GO:0042060\_wound\_healing | 98 | 2 | 8.764769 | -1.670058 | 70 | 5.191806 | 19.88 | 34.568194 | 0.284000 |
| GO:0009893\_positive\_regulation\_of\_metabolic\_process | 476 | 4 | 3.609023 | -1.657458 | 71 | 5.272058 | 20.06 | 34.847942 | 0.282535 |
| GO:0048522\_positive\_regulation\_of\_cellular\_process | 1009 | 6 | 2.553857 | -1.637830 | 72 | 5.230617 | 20.31 | 35.389383 | 0.282083 |
| GO:0019751\_polyol\_metabolic\_process | 10 | 1 | 42.947368 | -1.637246 | 76 | 6.633426 | 22.68 | 38.726574 | 0.298421 |
| GO:0035264\_multicellular\_organism\_growth | 10 | 1 | 42.947368 | -1.637246 | 76 | 6.633426 | 22.68 | 38.726574 | 0.298421 |
| GO:0040014\_regulation\_of\_multicellular\_organism\_growth | 10 | 1 | 42.947368 | -1.637246 | 76 | 6.633426 | 22.68 | 38.726574 | 0.298421 |
| GO:0043500\_muscle\_adaptation | 10 | 1 | 42.947368 | -1.637246 | 76 | 6.633426 | 22.68 | 38.726574 | 0.298421 |
| GO:0048513\_organ\_development | 741 | 5 | 2.897933 | -1.616950 | 77 | 6.774153 | 22.91 | 39.045847 | 0.297532 |
| GO:0006904\_vesicle\_docking\_during\_exocytosis | 11 | 1 | 39.043062 | -1.596331 | 81 | 8.077532 | 25.54 | 43.002468 | 0.315309 |
| GO:0010907\_positive\_regulation\_of\_glucose\_metabolic\_process | 11 | 1 | 39.043062 | -1.596331 | 81 | 8.077532 | 25.54 | 43.002468 | 0.315309 |
| GO:0032231\_regulation\_of\_actin\_filament\_bundle\_formation | 11 | 1 | 39.043062 | -1.596331 | 81 | 8.077532 | 25.54 | 43.002468 | 0.315309 |
| GO:0045076\_regulation\_of\_interleukin-2\_biosynthetic\_process | 11 | 1 | 39.043062 | -1.596331 | 81 | 8.077532 | 25.54 | 43.002468 | 0.315309 |
| GO:0009611\_response\_to\_wounding | 279 | 3 | 4.617997 | -1.592780 | 82 | 8.027934 | 25.59 | 43.152066 | 0.312073 |
| GO:0009101\_glycoprotein\_biosynthetic\_process | 109 | 2 | 7.880251 | -1.583788 | 83 | 8.374549 | 25.95 | 43.525451 | 0.312651 |
| GO:0030154\_cell\_differentiation | 506 | 4 | 3.395049 | -1.570441 | 84 | 8.469391 | 26.1 | 43.730609 | 0.310714 |
| GO:0031532\_actin\_cytoskeleton\_reorganization | 12 | 1 | 35.789474 | -1.559020 | 87 | 9.598824 | 28.32 | 47.041176 | 0.325517 |
| GO:0042094\_interleukin-2\_biosynthetic\_process | 12 | 1 | 35.789474 | -1.559020 | 87 | 9.598824 | 28.32 | 47.041176 | 0.325517 |
| GO:0048278\_vesicle\_docking | 12 | 1 | 35.789474 | -1.559020 | 87 | 9.598824 | 28.32 | 47.041176 | 0.325517 |
| GO:0007165\_signal\_transduction | 2029 | 9 | 1.905009 | -1.558361 | 88 | 9.598824 | 28.32 | 47.041176 | 0.321818 |
| GO:0014065\_phosphoinositide\_3-kinase\_cascade | 13 | 1 | 33.036437 | -1.524736 | 90 | 11.028312 | 31.25 | 51.471688 | 0.347222 |
| GO:0050679\_positive\_regulation\_of\_epithelial\_cell\_proliferation | 13 | 1 | 33.036437 | -1.524736 | 90 | 11.028312 | 31.25 | 51.471688 | 0.347222 |
| GO:0019220\_regulation\_of\_phosphate\_metabolic\_process | 297 | 3 | 4.338118 | -1.522517 | 92 | 11.022526 | 31.28 | 51.537474 | 0.340000 |
| GO:0051174\_regulation\_of\_phosphorus\_metabolic\_process | 297 | 3 | 4.338118 | -1.522517 | 92 | 11.022526 | 31.28 | 51.537474 | 0.340000 |
| GO:0006955\_immune\_response | 529 | 4 | 3.247438 | -1.507941 | 93 | 11.142543 | 31.43 | 51.717457 | 0.337957 |
| GO:0035303\_regulation\_of\_dephosphorylation | 14 | 1 | 30.676692 | -1.493029 | 96 | 12.124037 | 33.91 | 55.695963 | 0.353229 |
| GO:0045445\_myoblast\_differentiation | 14 | 1 | 30.676692 | -1.493029 | 96 | 12.124037 | 33.91 | 55.695963 | 0.353229 |
| GO:0048009\_insulin-like\_growth\_factor\_receptor\_signaling\_pathway | 14 | 1 | 30.676692 | -1.493029 | 96 | 12.124037 | 33.91 | 55.695963 | 0.353229 |
| GO:0048518\_positive\_regulation\_of\_biological\_process | 1094 | 6 | 2.355432 | -1.481066 | 97 | 12.181515 | 34.09 | 55.998485 | 0.351443 |
| GO:0032663\_regulation\_of\_interleukin-2\_production | 15 | 1 | 28.631579 | -1.463543 | 99 | 13.651738 | 36.69 | 59.728262 | 0.370606 |
| GO:0048146\_positive\_regulation\_of\_fibroblast\_proliferation | 15 | 1 | 28.631579 | -1.463543 | 99 | 13.651738 | 36.69 | 59.728262 | 0.370606 |
| GO:0010740\_positive\_regulation\_of\_protein\_kinase\_cascade | 129 | 2 | 6.658507 | -1.448790 | 100 | 13.796711 | 36.91 | 60.023289 | 0.369100 |
| GO:0048869\_cellular\_developmental\_process | 555 | 4 | 3.095306 | -1.441263 | 101 | 13.824673 | 36.97 | 60.115327 | 0.366040 |
| GO:0032270\_positive\_regulation\_of\_cellular\_protein\_metabolic\_process | 131 | 2 | 6.556850 | -1.436569 | 103 | 13.945488 | 37.08 | 60.214512 | 0.360000 |
| GO:0033043\_regulation\_of\_organelle\_organization | 131 | 2 | 6.556850 | -1.436569 | 103 | 13.945488 | 37.08 | 60.214512 | 0.360000 |
| GO:0010676\_positive\_regulation\_of\_cellular\_carbohydrate\_metabolic\_process | 16 | 1 | 26.842105 | -1.435992 | 110 | 14.731452 | 38.68 | 62.628548 | 0.351636 |
| GO:0010906\_regulation\_of\_glucose\_metabolic\_process | 16 | 1 | 26.842105 | -1.435992 | 110 | 14.731452 | 38.68 | 62.628548 | 0.351636 |
| GO:0032623\_interleukin-2\_production | 16 | 1 | 26.842105 | -1.435992 | 110 | 14.731452 | 38.68 | 62.628548 | 0.351636 |
| GO:0044275\_cellular\_carbohydrate\_catabolic\_process | 16 | 1 | 26.842105 | -1.435992 | 110 | 14.731452 | 38.68 | 62.628548 | 0.351636 |
| GO:0045913\_positive\_regulation\_of\_carbohydrate\_metabolic\_process | 16 | 1 | 26.842105 | -1.435992 | 110 | 14.731452 | 38.68 | 62.628548 | 0.351636 |
| GO:0048589\_developmental\_growth | 16 | 1 | 26.842105 | -1.435992 | 110 | 14.731452 | 38.68 | 62.628548 | 0.351636 |
| GO:0051261\_protein\_depolymerization | 16 | 1 | 26.842105 | -1.435992 | 110 | 14.731452 | 38.68 | 62.628548 | 0.351636 |
| GO:0044262\_cellular\_carbohydrate\_metabolic\_process | 133 | 2 | 6.458251 | -1.424551 | 111 | 14.896400 | 38.98 | 63.063600 | 0.351171 |
| GO:0051247\_positive\_regulation\_of\_protein\_metabolic\_process | 135 | 2 | 6.362573 | -1.412732 | 112 | 15.242353 | 39.47 | 63.697647 | 0.352411 |
| GO:0008064\_regulation\_of\_actin\_polymerization\_or\_depolymerization | 17 | 1 | 25.263158 | -1.410140 | 117 | 16.033770 | 41.04 | 66.046230 | 0.350769 |
| GO:0022406\_membrane\_docking | 17 | 1 | 25.263158 | -1.410140 | 117 | 16.033770 | 41.04 | 66.046230 | 0.350769 |
| GO:0048144\_fibroblast\_proliferation | 17 | 1 | 25.263158 | -1.410140 | 117 | 16.033770 | 41.04 | 66.046230 | 0.350769 |
| GO:0048145\_regulation\_of\_fibroblast\_proliferation | 17 | 1 | 25.263158 | -1.410140 | 117 | 16.033770 | 41.04 | 66.046230 | 0.350769 |
| GO:0048660\_regulation\_of\_smooth\_muscle\_cell\_proliferation | 17 | 1 | 25.263158 | -1.410140 | 117 | 16.033770 | 41.04 | 66.046230 | 0.350769 |
| GO:0010557\_positive\_regulation\_of\_macromolecule\_biosynthetic\_process | 334 | 3 | 3.857548 | -1.392639 | 118 | 16.271677 | 41.57 | 66.868323 | 0.352288 |
| GO:0009100\_glycoprotein\_metabolic\_process | 139 | 2 | 6.179477 | -1.389662 | 119 | 16.317874 | 41.62 | 66.922126 | 0.349748 |
| GO:0048659\_smooth\_muscle\_cell\_proliferation | 18 | 1 | 23.859649 | -1.385794 | 120 | 17.091169 | 43.06 | 69.028831 | 0.358833 |
| GO:0007242\_intracellular\_signaling\_cascade | 853 | 5 | 2.517431 | -1.383308 | 121 | 17.157689 | 43.22 | 69.282311 | 0.357190 |
| GO:0030832\_regulation\_of\_actin\_filament\_length | 19 | 1 | 22.603878 | -1.362790 | 126 | 18.226652 | 45.58 | 72.933348 | 0.361746 |
| GO:0031331\_positive\_regulation\_of\_cellular\_catabolic\_process | 19 | 1 | 22.603878 | -1.362790 | 126 | 18.226652 | 45.58 | 72.933348 | 0.361746 |
| GO:0042531\_positive\_regulation\_of\_tyrosine\_phosphorylation\_of\_STAT\_protein | 19 | 1 | 22.603878 | -1.362790 | 126 | 18.226652 | 45.58 | 72.933348 | 0.361746 |
| GO:0043467\_regulation\_of\_generation\_of\_precursor\_metabolites\_and\_energy | 19 | 1 | 22.603878 | -1.362790 | 126 | 18.226652 | 45.58 | 72.933348 | 0.361746 |
| GO:0051495\_positive\_regulation\_of\_cytoskeleton\_organization | 19 | 1 | 22.603878 | -1.362790 | 126 | 18.226652 | 45.58 | 72.933348 | 0.361746 |
| GO:0080134\_regulation\_of\_response\_to\_stress | 147 | 2 | 5.843179 | -1.345655 | 127 | 18.509928 | 46.06 | 73.610072 | 0.362677 |
| GO:0006096\_glycolysis | 20 | 1 | 21.473684 | -1.340990 | 132 | 19.521254 | 47.89 | 76.258746 | 0.362803 |
| GO:0032535\_regulation\_of\_cellular\_component\_size | 20 | 1 | 21.473684 | -1.340990 | 132 | 19.521254 | 47.89 | 76.258746 | 0.362803 |
| GO:0043244\_regulation\_of\_protein\_complex\_disassembly | 20 | 1 | 21.473684 | -1.340990 | 132 | 19.521254 | 47.89 | 76.258746 | 0.362803 |
| GO:0045840\_positive\_regulation\_of\_mitosis | 20 | 1 | 21.473684 | -1.340990 | 132 | 19.521254 | 47.89 | 76.258746 | 0.362803 |
| GO:0051785\_positive\_regulation\_of\_nuclear\_division | 20 | 1 | 21.473684 | -1.340990 | 132 | 19.521254 | 47.89 | 76.258746 | 0.362803 |
| GO:0031328\_positive\_regulation\_of\_cellular\_biosynthetic\_process | 352 | 3 | 3.660287 | -1.335515 | 133 | 19.468346 | 47.99 | 76.511654 | 0.360827 |
| GO:0016310\_phosphorylation | 601 | 4 | 2.858394 | -1.332501 | 134 | 19.490658 | 48.03 | 76.569342 | 0.358433 |
| GO:0006109\_regulation\_of\_carbohydrate\_metabolic\_process | 21 | 1 | 20.451128 | -1.320278 | 137 | 20.707541 | 50.39 | 80.072459 | 0.367810 |
| GO:0010675\_regulation\_of\_cellular\_carbohydrate\_metabolic\_process | 21 | 1 | 20.451128 | -1.320278 | 137 | 20.707541 | 50.39 | 80.072459 | 0.367810 |
| GO:0046427\_positive\_regulation\_of\_JAK-STAT\_cascade | 21 | 1 | 20.451128 | -1.320278 | 137 | 20.707541 | 50.39 | 80.072459 | 0.367810 |
| GO:0009891\_positive\_regulation\_of\_biosynthetic\_process | 359 | 3 | 3.588917 | -1.314244 | 138 | 20.764784 | 50.55 | 80.335216 | 0.366304 |
| GO:0030218\_erythrocyte\_differentiation | 22 | 1 | 19.521531 | -1.300551 | 142 | 21.861052 | 52.27 | 82.678948 | 0.368099 |
| GO:0043410\_positive\_regulation\_of\_MAPKKK\_cascade | 22 | 1 | 19.521531 | -1.300551 | 142 | 21.861052 | 52.27 | 82.678948 | 0.368099 |
| GO:0046165\_alcohol\_biosynthetic\_process | 22 | 1 | 19.521531 | -1.300551 | 142 | 21.861052 | 52.27 | 82.678948 | 0.368099 |
| GO:0051017\_actin\_filament\_bundle\_formation | 22 | 1 | 19.521531 | -1.300551 | 142 | 21.861052 | 52.27 | 82.678948 | 0.368099 |
| GO:0007169\_transmembrane\_receptor\_protein\_tyrosine\_kinase\_signaling\_pathway | 157 | 2 | 5.471002 | -1.294257 | 143 | 22.015907 | 52.55 | 83.084093 | 0.367483 |
| GO:0030166\_proteoglycan\_biosynthetic\_process | 23 | 1 | 18.672769 | -1.281722 | 145 | 22.536377 | 53.8 | 85.063623 | 0.371034 |
| GO:0045740\_positive\_regulation\_of\_DNA\_replication | 23 | 1 | 18.672769 | -1.281722 | 145 | 22.536377 | 53.8 | 85.063623 | 0.371034 |
| GO:0007229\_integrin-mediated\_signaling\_pathway | 24 | 1 | 17.894737 | -1.263715 | 150 | 23.223098 | 55.45 | 87.676902 | 0.369667 |
| GO:0019059\_initiation\_of\_viral\_infection | 24 | 1 | 17.894737 | -1.263715 | 150 | 23.223098 | 55.45 | 87.676902 | 0.369667 |
| GO:0030193\_regulation\_of\_blood\_coagulation | 24 | 1 | 17.894737 | -1.263715 | 150 | 23.223098 | 55.45 | 87.676902 | 0.369667 |
| GO:0033002\_muscle\_cell\_proliferation | 24 | 1 | 17.894737 | -1.263715 | 150 | 23.223098 | 55.45 | 87.676902 | 0.369667 |
| GO:0042509\_regulation\_of\_tyrosine\_phosphorylation\_of\_STAT\_protein | 24 | 1 | 17.894737 | -1.263715 | 150 | 23.223098 | 55.45 | 87.676902 | 0.369667 |
| GO:0007243\_protein\_kinase\_cascade | 377 | 3 | 3.417562 | -1.261788 | 151 | 23.286798 | 55.59 | 87.893202 | 0.368146 |
| GO:0007154\_cell\_communication | 2272 | 9 | 1.701260 | -1.261431 | 152 | 23.360557 | 55.67 | 87.979443 | 0.366250 |
| GO:0006464\_protein\_modification\_process | 922 | 5 | 2.329033 | -1.259035 | 153 | 23.449599 | 55.76 | 88.070401 | 0.364444 |
| GO:0009966\_regulation\_of\_signal\_transduction | 378 | 3 | 3.408521 | -1.258963 | 154 | 23.457467 | 55.83 | 88.202533 | 0.362532 |
| GO:0006007\_glucose\_catabolic\_process | 25 | 1 | 17.178947 | -1.246462 | 160 | 24.358933 | 57.62 | 90.881067 | 0.360125 |
| GO:0030168\_platelet\_activation | 25 | 1 | 17.178947 | -1.246462 | 160 | 24.358933 | 57.62 | 90.881067 | 0.360125 |
| GO:0034101\_erythrocyte\_homeostasis | 25 | 1 | 17.178947 | -1.246462 | 160 | 24.358933 | 57.62 | 90.881067 | 0.360125 |
| GO:0043624\_cellular\_protein\_complex\_disassembly | 25 | 1 | 17.178947 | -1.246462 | 160 | 24.358933 | 57.62 | 90.881067 | 0.360125 |
| GO:0050678\_regulation\_of\_epithelial\_cell\_proliferation | 25 | 1 | 17.178947 | -1.246462 | 160 | 24.358933 | 57.62 | 90.881067 | 0.360125 |
| GO:0050818\_regulation\_of\_coagulation | 25 | 1 | 17.178947 | -1.246462 | 160 | 24.358933 | 57.62 | 90.881067 | 0.360125 |
| GO:0045595\_regulation\_of\_cell\_differentiation | 170 | 2 | 5.052632 | -1.232680 | 161 | 24.492960 | 57.9 | 91.307040 | 0.359627 |
| GO:0007498\_mesoderm\_development | 26 | 1 | 16.518219 | -1.229905 | 164 | 25.470930 | 59.41 | 93.349070 | 0.362256 |
| GO:0043241\_protein\_complex\_disassembly | 26 | 1 | 16.518219 | -1.229905 | 164 | 25.470930 | 59.41 | 93.349070 | 0.362256 |
| GO:0050673\_epithelial\_cell\_proliferation | 26 | 1 | 16.518219 | -1.229905 | 164 | 25.470930 | 59.41 | 93.349070 | 0.362256 |
| GO:0006468\_protein\_amino\_acid\_phosphorylation | 393 | 3 | 3.278425 | -1.217673 | 165 | 25.596315 | 59.72 | 93.843685 | 0.361939 |
| GO:0007260\_tyrosine\_phosphorylation\_of\_STAT\_protein | 27 | 1 | 15.906433 | -1.213990 | 166 | 26.391002 | 61.16 | 95.928998 | 0.368434 |
| GO:0001775\_cell\_activation | 175 | 2 | 4.908271 | -1.210400 | 167 | 26.459414 | 61.36 | 96.260586 | 0.367425 |
| GO:0042592\_homeostatic\_process | 397 | 3 | 3.245393 | -1.206990 | 168 | 26.424970 | 61.38 | 96.335030 | 0.365357 |
| GO:0043412\_biopolymer\_modification | 960 | 5 | 2.236842 | -1.195993 | 169 | 27.302704 | 63.38 | 99.457296 | 0.375030 |
| GO:0046425\_regulation\_of\_JAK-STAT\_cascade | 29 | 1 | 14.809437 | -1.183908 | 172 | 28.126665 | 65.19 | 102.253335 | 0.379012 |
| GO:0048741\_skeletal\_muscle\_fiber\_development | 29 | 1 | 14.809437 | -1.183908 | 172 | 28.126665 | 65.19 | 102.253335 | 0.379012 |
| GO:0050731\_positive\_regulation\_of\_peptidyl-tyrosine\_phosphorylation | 29 | 1 | 14.809437 | -1.183908 | 172 | 28.126665 | 65.19 | 102.253335 | 0.379012 |
| GO:0010627\_regulation\_of\_protein\_kinase\_cascade | 184 | 2 | 4.668192 | -1.172064 | 173 | 28.369475 | 65.55 | 102.730525 | 0.378902 |
| GO:0008154\_actin\_polymerization\_or\_depolymerization | 30 | 1 | 14.315789 | -1.169660 | 175 | 28.800030 | 66.61 | 104.419970 | 0.380629 |
| GO:0048747\_muscle\_fiber\_development | 30 | 1 | 14.315789 | -1.169660 | 175 | 28.800030 | 66.61 | 104.419970 | 0.380629 |
| GO:0009967\_positive\_regulation\_of\_signal\_transduction | 185 | 2 | 4.642959 | -1.167937 | 176 | 28.840385 | 66.75 | 104.659615 | 0.379261 |
| GO:0007200\_activation\_of\_phospholipase\_C\_activity\_by\_G-protein\_coupled\_receptor\_protein\_signaling\_pathway\_coupled\_to\_IP3\_second\_messenger | 31 | 1 | 13.853990 | -1.155895 | 177 | 29.409582 | 67.79 | 106.170418 | 0.382994 |
| GO:0010647\_positive\_regulation\_of\_cell\_communication | 189 | 2 | 4.544695 | -1.151681 | 178 | 29.459035 | 67.85 | 106.240965 | 0.381180 |
| GO:0006029\_proteoglycan\_metabolic\_process | 32 | 1 | 13.421053 | -1.142581 | 184 | 30.481136 | 69.41 | 108.338864 | 0.377228 |
| GO:0006487\_protein\_amino\_acid\_N-linked\_glycosylation | 32 | 1 | 13.421053 | -1.142581 | 184 | 30.481136 | 69.41 | 108.338864 | 0.377228 |
| GO:0031329\_regulation\_of\_cellular\_catabolic\_process | 32 | 1 | 13.421053 | -1.142581 | 184 | 30.481136 | 69.41 | 108.338864 | 0.377228 |
| GO:0034623\_cellular\_macromolecular\_complex\_disassembly | 32 | 1 | 13.421053 | -1.142581 | 184 | 30.481136 | 69.41 | 108.338864 | 0.377228 |
| GO:0046578\_regulation\_of\_Ras\_protein\_signal\_transduction | 32 | 1 | 13.421053 | -1.142581 | 184 | 30.481136 | 69.41 | 108.338864 | 0.377228 |
| GO:0051056\_regulation\_of\_small\_GTPase\_mediated\_signal\_transduction | 32 | 1 | 13.421053 | -1.142581 | 184 | 30.481136 | 69.41 | 108.338864 | 0.377228 |
| GO:0010646\_regulation\_of\_cell\_communication | 423 | 3 | 3.045913 | -1.140669 | 185 | 30.502775 | 69.44 | 108.377225 | 0.375351 |
| GO:0030217\_T\_cell\_differentiation | 33 | 1 | 13.014354 | -1.129693 | 187 | 31.290305 | 70.49 | 109.689695 | 0.376952 |
| GO:0042108\_positive\_regulation\_of\_cytokine\_biosynthetic\_process | 33 | 1 | 13.014354 | -1.129693 | 187 | 31.290305 | 70.49 | 109.689695 | 0.376952 |
| GO:0008277\_regulation\_of\_G-protein\_coupled\_receptor\_protein\_signaling\_pathway | 34 | 1 | 12.631579 | -1.117202 | 189 | 32.887555 | 72.94 | 112.992445 | 0.385926 |
| GO:0008624\_induction\_of\_apoptosis\_by\_extracellular\_signals | 34 | 1 | 12.631579 | -1.117202 | 189 | 32.887555 | 72.94 | 112.992445 | 0.385926 |
| GO:0009896\_positive\_regulation\_of\_catabolic\_process | 35 | 1 | 12.270677 | -1.105088 | 193 | 33.683552 | 74.22 | 114.756448 | 0.384560 |
| GO:0019320\_hexose\_catabolic\_process | 35 | 1 | 12.270677 | -1.105088 | 193 | 33.683552 | 74.22 | 114.756448 | 0.384560 |
| GO:0048872\_homeostasis\_of\_number\_of\_cells | 35 | 1 | 12.270677 | -1.105088 | 193 | 33.683552 | 74.22 | 114.756448 | 0.384560 |
| GO:0051346\_negative\_regulation\_of\_hydrolase\_activity | 35 | 1 | 12.270677 | -1.105088 | 193 | 33.683552 | 74.22 | 114.756448 | 0.384560 |
| GO:0006066\_alcohol\_metabolic\_process | 206 | 2 | 4.169647 | -1.086758 | 194 | 34.412310 | 75.51 | 116.607690 | 0.389227 |
| GO:0032956\_regulation\_of\_actin\_cytoskeleton\_organization | 37 | 1 | 11.607397 | -1.081904 | 195 | 34.946394 | 76.58 | 118.213606 | 0.392718 |
| GO:0043687\_post-translational\_protein\_modification | 728 | 4 | 2.359746 | -1.081439 | 196 | 35.047740 | 76.66 | 118.272260 | 0.391122 |
| GO:0045787\_positive\_regulation\_of\_cell\_cycle | 38 | 1 | 11.301939 | -1.070796 | 198 | 35.706493 | 78.37 | 121.033507 | 0.395808 |
| GO:0050730\_regulation\_of\_peptidyl-tyrosine\_phosphorylation | 38 | 1 | 11.301939 | -1.070796 | 198 | 35.706493 | 78.37 | 121.033507 | 0.395808 |
| GO:0045893\_positive\_regulation\_of\_transcription\_\_DNA-dependent | 211 | 2 | 4.070841 | -1.068838 | 199 | 35.823216 | 78.49 | 121.156784 | 0.394422 |
| GO:0051254\_positive\_regulation\_of\_RNA\_metabolic\_process | 213 | 2 | 4.032617 | -1.061809 | 200 | 36.090585 | 78.77 | 121.449415 | 0.393850 |
| GO:0006401\_RNA\_catabolic\_process | 39 | 1 | 11.012146 | -1.059989 | 204 | 37.193035 | 79.82 | 122.446965 | 0.391275 |
| GO:0032970\_regulation\_of\_actin\_filament-based\_process | 39 | 1 | 11.012146 | -1.059989 | 204 | 37.193035 | 79.82 | 122.446965 | 0.391275 |
| GO:0046328\_regulation\_of\_JNK\_cascade | 39 | 1 | 11.012146 | -1.059989 | 204 | 37.193035 | 79.82 | 122.446965 | 0.391275 |
| GO:0046365\_monosaccharide\_catabolic\_process | 39 | 1 | 11.012146 | -1.059989 | 204 | 37.193035 | 79.82 | 122.446965 | 0.391275 |
| GO:0044267\_cellular\_protein\_metabolic\_process | 1382 | 6 | 1.864575 | -1.057677 | 205 | 37.300577 | 80.02 | 122.739423 | 0.390341 |
| GO:0008152\_metabolic\_process | 4111 | 13 | 1.358102 | -1.053007 | 206 | 37.482748 | 80.25 | 123.017252 | 0.389563 |
| GO:0070302\_regulation\_of\_stress-activated\_protein\_kinase\_signaling\_pathway | 40 | 1 | 10.736842 | -1.049468 | 207 | 38.040016 | 81.18 | 124.319984 | 0.392174 |
| GO:0007519\_skeletal\_muscle\_tissue\_development | 41 | 1 | 10.474968 | -1.039218 | 209 | 38.743673 | 82.5 | 126.256327 | 0.394737 |
| GO:0060538\_skeletal\_muscle\_organ\_development | 41 | 1 | 10.474968 | -1.039218 | 209 | 38.743673 | 82.5 | 126.256327 | 0.394737 |
| GO:0006887\_exocytosis | 42 | 1 | 10.225564 | -1.029226 | 211 | 39.869515 | 84.2 | 128.530485 | 0.399052 |
| GO:0042692\_muscle\_cell\_differentiation | 42 | 1 | 10.225564 | -1.029226 | 211 | 39.869515 | 84.2 | 128.530485 | 0.399052 |
| GO:0045637\_regulation\_of\_myeloid\_cell\_differentiation | 43 | 1 | 9.987760 | -1.019481 | 214 | 40.977880 | 85.82 | 130.662120 | 0.401028 |
| GO:0046164\_alcohol\_catabolic\_process | 43 | 1 | 9.987760 | -1.019481 | 214 | 40.977880 | 85.82 | 130.662120 | 0.401028 |
| GO:0051054\_positive\_regulation\_of\_DNA\_metabolic\_process | 43 | 1 | 9.987760 | -1.019481 | 214 | 40.977880 | 85.82 | 130.662120 | 0.401028 |
| GO:0051704\_multi-organism\_process | 231 | 2 | 3.718387 | -1.001828 | 215 | 41.872338 | 87.42 | 132.967662 | 0.406605 |
| GO:0007202\_activation\_of\_phospholipase\_C\_activity | 46 | 1 | 9.336384 | -0.991612 | 219 | 43.462073 | 90.09 | 136.717927 | 0.411370 |
| GO:0010863\_positive\_regulation\_of\_phospholipase\_C\_activity | 46 | 1 | 9.336384 | -0.991612 | 219 | 43.462073 | 90.09 | 136.717927 | 0.411370 |
| GO:0030335\_positive\_regulation\_of\_cell\_migration | 46 | 1 | 9.336384 | -0.991612 | 219 | 43.462073 | 90.09 | 136.717927 | 0.411370 |
| GO:0030384\_phosphoinositide\_metabolic\_process | 46 | 1 | 9.336384 | -0.991612 | 219 | 43.462073 | 90.09 | 136.717927 | 0.411370 |
| GO:0051128\_regulation\_of\_cellular\_component\_organization | 237 | 2 | 3.624250 | -0.983050 | 220 | 43.510587 | 90.22 | 136.929413 | 0.410091 |
| GO:0007088\_regulation\_of\_mitosis | 47 | 1 | 9.137738 | -0.982745 | 223 | 44.427891 | 91.59 | 138.752109 | 0.410717 |
| GO:0007259\_JAK-STAT\_cascade | 47 | 1 | 9.137738 | -0.982745 | 223 | 44.427891 | 91.59 | 138.752109 | 0.410717 |
| GO:0051783\_regulation\_of\_nuclear\_division | 47 | 1 | 9.137738 | -0.982745 | 223 | 44.427891 | 91.59 | 138.752109 | 0.410717 |
| GO:0044265\_cellular\_macromolecule\_catabolic\_process | 239 | 2 | 3.593922 | -0.976916 | 224 | 44.528431 | 91.82 | 139.111569 | 0.409911 |
| GO:0010518\_positive\_regulation\_of\_phospholipase\_activity | 48 | 1 | 8.947368 | -0.974074 | 226 | 45.109174 | 92.51 | 139.910826 | 0.409336 |
| GO:0042035\_regulation\_of\_cytokine\_biosynthetic\_process | 48 | 1 | 8.947368 | -0.974074 | 226 | 45.109174 | 92.51 | 139.910826 | 0.409336 |
| GO:0048583\_regulation\_of\_response\_to\_stimulus | 241 | 2 | 3.564097 | -0.970843 | 227 | 45.161286 | 92.7 | 140.238714 | 0.408370 |
| GO:0006275\_regulation\_of\_DNA\_replication | 49 | 1 | 8.764769 | -0.965592 | 232 | 45.588982 | 93.81 | 142.031018 | 0.404353 |
| GO:0010517\_regulation\_of\_phospholipase\_activity | 49 | 1 | 8.764769 | -0.965592 | 232 | 45.588982 | 93.81 | 142.031018 | 0.404353 |
| GO:0018108\_peptidyl-tyrosine\_phosphorylation | 49 | 1 | 8.764769 | -0.965592 | 232 | 45.588982 | 93.81 | 142.031018 | 0.404353 |
| GO:0032984\_macromolecular\_complex\_disassembly | 49 | 1 | 8.764769 | -0.965592 | 232 | 45.588982 | 93.81 | 142.031018 | 0.404353 |
| GO:0050867\_positive\_regulation\_of\_cell\_activation | 49 | 1 | 8.764769 | -0.965592 | 232 | 45.588982 | 93.81 | 142.031018 | 0.404353 |
| GO:0030098\_lymphocyte\_differentiation | 50 | 1 | 8.589474 | -0.957291 | 234 | 46.743712 | 95.4 | 144.056288 | 0.407692 |
| GO:0051272\_positive\_regulation\_of\_cell\_motion | 50 | 1 | 8.589474 | -0.957291 | 234 | 46.743712 | 95.4 | 144.056288 | 0.407692 |
| GO:0016052\_carbohydrate\_catabolic\_process | 51 | 1 | 8.421053 | -0.949163 | 237 | 47.949304 | 96.81 | 145.670696 | 0.408481 |
| GO:0018212\_peptidyl-tyrosine\_modification | 51 | 1 | 8.421053 | -0.949163 | 237 | 47.949304 | 96.81 | 145.670696 | 0.408481 |
| GO:0030518\_steroid\_hormone\_receptor\_signaling\_pathway | 51 | 1 | 8.421053 | -0.949163 | 237 | 47.949304 | 96.81 | 145.670696 | 0.408481 |
| GO:0042089\_cytokine\_biosynthetic\_process | 52 | 1 | 8.259109 | -0.941202 | 238 | 48.658940 | 97.88 | 147.101060 | 0.411261 |
| GO:0048731\_system\_development | 1140 | 5 | 1.883657 | -0.940287 | 239 | 48.703646 | 97.94 | 147.176354 | 0.409791 |
| GO:0006006\_glucose\_metabolic\_process | 53 | 1 | 8.103277 | -0.933402 | 242 | 49.597299 | 99.21 | 148.822701 | 0.409959 |
| GO:0042107\_cytokine\_metabolic\_process | 53 | 1 | 8.103277 | -0.933402 | 242 | 49.597299 | 99.21 | 148.822701 | 0.409959 |
| GO:0060193\_positive\_regulation\_of\_lipase\_activity | 53 | 1 | 8.103277 | -0.933402 | 242 | 49.597299 | 99.21 | 148.822701 | 0.409959 |
| GO:0007167\_enzyme\_linked\_receptor\_protein\_signaling\_pathway | 258 | 2 | 3.329253 | -0.921540 | 243 | 49.766423 | 99.65 | 149.533577 | 0.410082 |
| GO:0009306\_protein\_secretion | 56 | 1 | 7.669173 | -0.910906 | 245 | 50.566021 | 100.93 | 151.293979 | 0.411959 |
| GO:0019058\_viral\_infectious\_cycle | 56 | 1 | 7.669173 | -0.910906 | 245 | 50.566021 | 100.93 | 151.293979 | 0.411959 |
| GO:0045941\_positive\_regulation\_of\_transcription | 264 | 2 | 3.253589 | -0.905066 | 246 | 50.719527 | 101.12 | 151.520473 | 0.411057 |
| GO:0001934\_positive\_regulation\_of\_protein\_amino\_acid\_phosphorylation | 57 | 1 | 7.534626 | -0.903691 | 248 | 50.940019 | 101.58 | 152.219981 | 0.409597 |
| GO:0032844\_regulation\_of\_homeostatic\_process | 57 | 1 | 7.534626 | -0.903691 | 248 | 50.940019 | 101.58 | 152.219981 | 0.409597 |
| GO:0065008\_regulation\_of\_biological\_quality | 848 | 4 | 2.025819 | -0.894130 | 249 | 52.122141 | 103.16 | 154.197859 | 0.414297 |
| GO:0043408\_regulation\_of\_MAPKKK\_cascade | 59 | 1 | 7.279215 | -0.889657 | 250 | 52.808026 | 104.02 | 155.231974 | 0.416080 |
| GO:0044087\_regulation\_of\_cellular\_component\_biogenesis | 60 | 1 | 7.157895 | -0.882829 | 251 | 53.265749 | 104.71 | 156.154251 | 0.417171 |
| GO:0007254\_JNK\_cascade | 61 | 1 | 7.040552 | -0.876121 | 253 | 54.179041 | 106.13 | 158.080959 | 0.419486 |
| GO:0051493\_regulation\_of\_cytoskeleton\_organization | 61 | 1 | 7.040552 | -0.876121 | 253 | 54.179041 | 106.13 | 158.080959 | 0.419486 |
| GO:0010628\_positive\_regulation\_of\_gene\_expression | 277 | 2 | 3.100893 | -0.870882 | 254 | 54.355818 | 106.47 | 158.584182 | 0.419173 |
| GO:0060191\_regulation\_of\_lipase\_activity | 62 | 1 | 6.926995 | -0.869530 | 255 | 55.686824 | 108.12 | 160.553176 | 0.424000 |
| GO:0030522\_intracellular\_receptor-mediated\_signaling\_pathway | 63 | 1 | 6.817043 | -0.863052 | 256 | 56.359351 | 109.01 | 161.660649 | 0.425820 |
| GO:0031098\_stress-activated\_protein\_kinase\_signaling\_pathway | 64 | 1 | 6.710526 | -0.856683 | 258 | 57.535913 | 110.72 | 163.904087 | 0.429147 |
| GO:0042327\_positive\_regulation\_of\_phosphorylation | 64 | 1 | 6.710526 | -0.856683 | 258 | 57.535913 | 110.72 | 163.904087 | 0.429147 |
| GO:0042325\_regulation\_of\_phosphorylation | 285 | 2 | 3.013850 | -0.850807 | 259 | 57.659215 | 111.02 | 164.380785 | 0.428649 |
| GO:0022415\_viral\_reproductive\_process | 65 | 1 | 6.607287 | -0.850421 | 262 | 58.436293 | 112.0 | 165.563707 | 0.427481 |
| GO:0045596\_negative\_regulation\_of\_cell\_differentiation | 65 | 1 | 6.607287 | -0.850421 | 262 | 58.436293 | 112.0 | 165.563707 | 0.427481 |
| GO:0080135\_regulation\_of\_cellular\_response\_to\_stress | 65 | 1 | 6.607287 | -0.850421 | 262 | 58.436293 | 112.0 | 165.563707 | 0.427481 |
| GO:0019538\_protein\_metabolic\_process | 1569 | 6 | 1.642347 | -0.849363 | 263 | 58.614283 | 112.19 | 165.765717 | 0.426578 |
| GO:0032268\_regulation\_of\_cellular\_protein\_metabolic\_process | 286 | 2 | 3.003312 | -0.848346 | 264 | 59.013435 | 112.64 | 166.266565 | 0.426667 |
| GO:0009888\_tissue\_development | 287 | 2 | 2.992848 | -0.845896 | 265 | 59.093104 | 112.77 | 166.446896 | 0.425547 |
| GO:0010562\_positive\_regulation\_of\_phosphorus\_metabolic\_process | 66 | 1 | 6.507177 | -0.844260 | 269 | 60.210405 | 114.46 | 168.709595 | 0.425502 |
| GO:0010564\_regulation\_of\_cell\_cycle\_process | 66 | 1 | 6.507177 | -0.844260 | 269 | 60.210405 | 114.46 | 168.709595 | 0.425502 |
| GO:0014706\_striated\_muscle\_tissue\_development | 66 | 1 | 6.507177 | -0.844260 | 269 | 60.210405 | 114.46 | 168.709595 | 0.425502 |
| GO:0045937\_positive\_regulation\_of\_phosphate\_metabolic\_process | 66 | 1 | 6.507177 | -0.844260 | 269 | 60.210405 | 114.46 | 168.709595 | 0.425502 |
| GO:0007015\_actin\_filament\_organization | 67 | 1 | 6.410055 | -0.838200 | 271 | 61.163515 | 115.75 | 170.336485 | 0.427122 |
| GO:0060537\_muscle\_tissue\_development | 67 | 1 | 6.410055 | -0.838200 | 271 | 61.163515 | 115.75 | 170.336485 | 0.427122 |
| GO:0005975\_carbohydrate\_metabolic\_process | 292 | 2 | 2.941601 | -0.833802 | 273 | 61.590570 | 116.33 | 171.069430 | 0.426117 |
| GO:0040011\_locomotion | 292 | 2 | 2.941601 | -0.833802 | 273 | 61.590570 | 116.33 | 171.069430 | 0.426117 |
| GO:0016032\_viral\_reproduction | 70 | 1 | 6.135338 | -0.820587 | 275 | 63.835824 | 119.62 | 175.404176 | 0.434982 |
| GO:0022411\_cellular\_component\_disassembly | 70 | 1 | 6.135338 | -0.820587 | 275 | 63.835824 | 119.62 | 175.404176 | 0.434982 |
| GO:0009615\_response\_to\_virus | 71 | 1 | 6.048925 | -0.814896 | 277 | 64.458516 | 120.94 | 177.421484 | 0.436606 |
| GO:0032101\_regulation\_of\_response\_to\_external\_stimulus | 71 | 1 | 6.048925 | -0.814896 | 277 | 64.458516 | 120.94 | 177.421484 | 0.436606 |
| GO:0051246\_regulation\_of\_protein\_metabolic\_process | 301 | 2 | 2.853646 | -0.812667 | 278 | 64.542134 | 121.05 | 177.557866 | 0.435432 |
| GO:0060249\_anatomical\_structure\_homeostasis | 72 | 1 | 5.964912 | -0.809291 | 279 | 64.766572 | 121.35 | 177.933428 | 0.434946 |
| GO:0007596\_blood\_coagulation | 73 | 1 | 5.883201 | -0.803771 | 282 | 65.673144 | 122.42 | 179.166856 | 0.434113 |
| GO:0009894\_regulation\_of\_catabolic\_process | 73 | 1 | 5.883201 | -0.803771 | 282 | 65.673144 | 122.42 | 179.166856 | 0.434113 |
| GO:0030099\_myeloid\_cell\_differentiation | 73 | 1 | 5.883201 | -0.803771 | 282 | 65.673144 | 122.42 | 179.166856 | 0.434113 |
| GO:0045935\_positive\_regulation\_of\_nucleobase\_\_nucleoside\_\_nucleotide\_and\_nucleic\_acid\_metabolic\_process | 307 | 2 | 2.797874 | -0.799010 | 283 | 65.961789 | 122.82 | 179.678211 | 0.433993 |
| GO:0050817\_coagulation | 74 | 1 | 5.803698 | -0.798331 | 284 | 66.113754 | 123.17 | 180.226246 | 0.433697 |
| GO:0048523\_negative\_regulation\_of\_cellular\_process | 925 | 4 | 1.857183 | -0.793119 | 285 | 66.610245 | 123.81 | 181.009755 | 0.434421 |
| GO:0006650\_glycerophospholipid\_metabolic\_process | 75 | 1 | 5.726316 | -0.792971 | 286 | 67.334230 | 124.82 | 182.305770 | 0.436434 |
| GO:0006470\_protein\_amino\_acid\_dephosphorylation | 77 | 1 | 5.577580 | -0.782479 | 288 | 68.915452 | 127.04 | 185.164548 | 0.441111 |
| GO:0050865\_regulation\_of\_cell\_activation | 77 | 1 | 5.577580 | -0.782479 | 288 | 68.915452 | 127.04 | 185.164548 | 0.441111 |
| GO:0051173\_positive\_regulation\_of\_nitrogen\_compound\_metabolic\_process | 317 | 2 | 2.709613 | -0.776976 | 289 | 69.214762 | 127.48 | 185.745238 | 0.441107 |
| GO:0007599\_hemostasis | 79 | 1 | 5.436376 | -0.772280 | 290 | 69.596773 | 128.29 | 186.983227 | 0.442379 |
| GO:0048856\_anatomical\_structure\_development | 1289 | 5 | 1.665918 | -0.771449 | 291 | 69.617879 | 128.36 | 187.102121 | 0.441100 |
| GO:0007204\_elevation\_of\_cytosolic\_calcium\_ion\_concentration | 80 | 1 | 5.368421 | -0.767285 | 292 | 69.913698 | 128.9 | 187.886302 | 0.441438 |
| GO:0006486\_protein\_amino\_acid\_glycosylation | 81 | 1 | 5.302144 | -0.762359 | 297 | 70.873614 | 129.91 | 188.946386 | 0.437407 |
| GO:0019318\_hexose\_metabolic\_process | 81 | 1 | 5.302144 | -0.762359 | 297 | 70.873614 | 129.91 | 188.946386 | 0.437407 |
| GO:0043413\_biopolymer\_glycosylation | 81 | 1 | 5.302144 | -0.762359 | 297 | 70.873614 | 129.91 | 188.946386 | 0.437407 |
| GO:0051480\_cytosolic\_calcium\_ion\_homeostasis | 81 | 1 | 5.302144 | -0.762359 | 297 | 70.873614 | 129.91 | 188.946386 | 0.437407 |
| GO:0070085\_glycosylation | 81 | 1 | 5.302144 | -0.762359 | 297 | 70.873614 | 129.91 | 188.946386 | 0.437407 |
| GO:0042110\_T\_cell\_activation | 83 | 1 | 5.174382 | -0.752702 | 298 | 71.721389 | 131.21 | 190.698611 | 0.440302 |
| GO:0031401\_positive\_regulation\_of\_protein\_modification\_process | 84 | 1 | 5.112782 | -0.747969 | 300 | 72.635154 | 132.41 | 192.184846 | 0.441367 |
| GO:0043123\_positive\_regulation\_of\_I-kappaB\_kinase\_NF-kappaB\_cascade | 84 | 1 | 5.112782 | -0.747969 | 300 | 72.635154 | 132.41 | 192.184846 | 0.441367 |
| GO:0031327\_negative\_regulation\_of\_cellular\_biosynthetic\_process | 332 | 2 | 2.587191 | -0.745522 | 301 | 72.759540 | 132.66 | 192.560460 | 0.440731 |
| GO:0002521\_leukocyte\_differentiation | 87 | 1 | 4.936479 | -0.734131 | 303 | 73.732234 | 133.79 | 193.847766 | 0.441551 |
| GO:0051052\_regulation\_of\_DNA\_metabolic\_process | 87 | 1 | 4.936479 | -0.734131 | 303 | 73.732234 | 133.79 | 193.847766 | 0.441551 |
| GO:0009890\_negative\_regulation\_of\_biosynthetic\_process | 340 | 2 | 2.526316 | -0.729478 | 304 | 74.126914 | 134.42 | 194.713086 | 0.442171 |
| GO:0030334\_regulation\_of\_cell\_migration | 89 | 1 | 4.825547 | -0.725195 | 305 | 74.851032 | 135.57 | 196.288968 | 0.444492 |
| GO:0044260\_cellular\_macromolecule\_metabolic\_process | 2883 | 9 | 1.340709 | -0.712924 | 306 | 75.377950 | 136.54 | 197.702050 | 0.446209 |
| GO:0043122\_regulation\_of\_I-kappaB\_kinase\_NF-kappaB\_cascade | 93 | 1 | 4.617997 | -0.707969 | 307 | 76.445748 | 137.78 | 199.114252 | 0.448795 |
| GO:0043085\_positive\_regulation\_of\_catalytic\_activity | 354 | 2 | 2.426405 | -0.702537 | 308 | 76.770981 | 138.17 | 199.569019 | 0.448604 |
| GO:0050878\_regulation\_of\_body\_fluid\_levels | 95 | 1 | 4.520776 | -0.699661 | 309 | 77.038821 | 138.58 | 200.121179 | 0.448479 |
| GO:0050793\_regulation\_of\_developmental\_process | 669 | 3 | 1.925891 | -0.697933 | 310 | 77.123621 | 138.74 | 200.356379 | 0.447548 |
| GO:0040012\_regulation\_of\_locomotion | 96 | 1 | 4.473684 | -0.695580 | 311 | 77.904327 | 139.83 | 201.755673 | 0.449614 |
| GO:0048519\_negative\_regulation\_of\_biological\_process | 1013 | 4 | 1.695849 | -0.692395 | 312 | 78.033201 | 140.02 | 202.006799 | 0.448782 |
| GO:0046486\_glycerolipid\_metabolic\_process | 97 | 1 | 4.427564 | -0.691546 | 313 | 78.348878 | 140.43 | 202.511122 | 0.448658 |
| GO:0007275\_multicellular\_organismal\_development | 1372 | 5 | 1.565137 | -0.690684 | 314 | 78.560752 | 140.75 | 202.939248 | 0.448248 |
| GO:0051270\_regulation\_of\_cell\_motion | 98 | 1 | 4.382385 | -0.687557 | 315 | 79.064206 | 141.49 | 203.915794 | 0.449175 |
| GO:0009968\_negative\_regulation\_of\_signal\_transduction | 99 | 1 | 4.338118 | -0.683614 | 316 | 79.528837 | 142.11 | 204.691163 | 0.449715 |
| GO:0044237\_cellular\_metabolic\_process | 3753 | 11 | 1.258782 | -0.681412 | 317 | 79.704493 | 142.44 | 205.175507 | 0.449338 |
| GO:0055114\_oxidation\_reduction | 100 | 1 | 4.294737 | -0.679714 | 318 | 80.256041 | 143.33 | 206.403959 | 0.450723 |
| GO:0001932\_regulation\_of\_protein\_amino\_acid\_phosphorylation | 101 | 1 | 4.252215 | -0.675859 | 319 | 80.786891 | 144.01 | 207.233109 | 0.451442 |
| GO:0010648\_negative\_regulation\_of\_cell\_communication | 102 | 1 | 4.210526 | -0.672045 | 320 | 81.278305 | 144.53 | 207.781695 | 0.451656 |
| GO:0001817\_regulation\_of\_cytokine\_production | 103 | 1 | 4.169647 | -0.668273 | 321 | 81.306641 | 144.72 | 208.133359 | 0.450841 |
| GO:0006644\_phospholipid\_metabolic\_process | 108 | 1 | 3.976608 | -0.650010 | 324 | 83.676976 | 147.71 | 211.743024 | 0.455895 |
| GO:0007346\_regulation\_of\_mitotic\_cell\_cycle | 108 | 1 | 3.976608 | -0.650010 | 324 | 83.676976 | 147.71 | 211.743024 | 0.455895 |
| GO:0019637\_organophosphate\_metabolic\_process | 108 | 1 | 3.976608 | -0.650010 | 324 | 83.676976 | 147.71 | 211.743024 | 0.455895 |
| GO:0007265\_Ras\_protein\_signal\_transduction | 110 | 1 | 3.904306 | -0.642970 | 325 | 84.887581 | 149.16 | 213.432419 | 0.458954 |
| GO:0001816\_cytokine\_production | 112 | 1 | 3.834586 | -0.636073 | 326 | 85.705328 | 150.34 | 214.974672 | 0.461166 |
| GO:0000122\_negative\_regulation\_of\_transcription\_from\_RNA\_polymerase\_II\_promoter | 113 | 1 | 3.800652 | -0.632676 | 327 | 86.313647 | 151.05 | 215.786353 | 0.461927 |
| GO:0044093\_positive\_regulation\_of\_molecular\_function | 394 | 2 | 2.180069 | -0.632657 | 328 | 86.579682 | 151.31 | 216.040318 | 0.461311 |
| GO:0006874\_cellular\_calcium\_ion\_homeostasis | 114 | 1 | 3.767313 | -0.629313 | 330 | 86.929513 | 151.82 | 216.710487 | 0.460061 |
| GO:0048584\_positive\_regulation\_of\_response\_to\_stimulus | 114 | 1 | 3.767313 | -0.629313 | 330 | 86.929513 | 151.82 | 216.710487 | 0.460061 |
| GO:0000280\_nuclear\_division | 115 | 1 | 3.734554 | -0.625983 | 333 | 88.048214 | 153.02 | 217.991786 | 0.459520 |
| GO:0005996\_monosaccharide\_metabolic\_process | 115 | 1 | 3.734554 | -0.625983 | 333 | 88.048214 | 153.02 | 217.991786 | 0.459520 |
| GO:0007067\_mitosis | 115 | 1 | 3.734554 | -0.625983 | 333 | 88.048214 | 153.02 | 217.991786 | 0.459520 |
| GO:0055074\_calcium\_ion\_homeostasis | 116 | 1 | 3.702359 | -0.622687 | 334 | 88.506860 | 153.63 | 218.753140 | 0.459970 |
| GO:0018193\_peptidyl-amino\_acid\_modification | 117 | 1 | 3.670715 | -0.619422 | 335 | 88.763546 | 153.9 | 219.036454 | 0.459403 |
| GO:0031324\_negative\_regulation\_of\_cellular\_metabolic\_process | 404 | 2 | 2.126107 | -0.616648 | 336 | 88.865491 | 154.03 | 219.194509 | 0.458423 |
| GO:0000087\_M\_phase\_of\_mitotic\_cell\_cycle | 118 | 1 | 3.639607 | -0.616189 | 338 | 89.546269 | 154.76 | 219.973731 | 0.457870 |
| GO:0048285\_organelle\_fission | 118 | 1 | 3.639607 | -0.616189 | 338 | 89.546269 | 154.76 | 219.973731 | 0.457870 |
| GO:0046649\_lymphocyte\_activation | 119 | 1 | 3.609023 | -0.612987 | 339 | 89.817011 | 155.1 | 220.382989 | 0.457522 |
| GO:0044248\_cellular\_catabolic\_process | 410 | 2 | 2.094994 | -0.607297 | 340 | 90.282531 | 155.86 | 221.437469 | 0.458412 |
| GO:0006875\_cellular\_metal\_ion\_homeostasis | 121 | 1 | 3.549369 | -0.606674 | 341 | 90.602170 | 156.28 | 221.957830 | 0.458299 |
| GO:0042127\_regulation\_of\_cell\_proliferation | 411 | 2 | 2.089896 | -0.605756 | 342 | 90.833336 | 156.51 | 222.186664 | 0.457632 |
| GO:0033674\_positive\_regulation\_of\_kinase\_activity | 122 | 1 | 3.520276 | -0.603562 | 344 | 91.577190 | 157.55 | 223.522810 | 0.457994 |
| GO:0045860\_positive\_regulation\_of\_protein\_kinase\_activity | 122 | 1 | 3.520276 | -0.603562 | 344 | 91.577190 | 157.55 | 223.522810 | 0.457994 |
| GO:0007517\_muscle\_organ\_development | 123 | 1 | 3.491656 | -0.600479 | 345 | 91.899330 | 157.94 | 223.980670 | 0.457797 |
| GO:0006935\_chemotaxis | 125 | 1 | 3.435789 | -0.594398 | 349 | 93.104136 | 159.26 | 225.415864 | 0.456332 |
| GO:0042330\_taxis | 125 | 1 | 3.435789 | -0.594398 | 349 | 93.104136 | 159.26 | 225.415864 | 0.456332 |
| GO:0051707\_response\_to\_other\_organism | 125 | 1 | 3.435789 | -0.594398 | 349 | 93.104136 | 159.26 | 225.415864 | 0.456332 |
| GO:0055065\_metal\_ion\_homeostasis | 125 | 1 | 3.435789 | -0.594398 | 349 | 93.104136 | 159.26 | 225.415864 | 0.456332 |
| GO:0030030\_cell\_projection\_organization | 127 | 1 | 3.381683 | -0.588428 | 350 | 93.281480 | 159.61 | 225.938520 | 0.456029 |
| GO:0031326\_regulation\_of\_cellular\_biosynthetic\_process | 1125 | 4 | 1.527018 | -0.582942 | 351 | 94.216729 | 160.64 | 227.063271 | 0.457664 |
| GO:0051347\_positive\_regulation\_of\_transferase\_activity | 129 | 1 | 3.329253 | -0.582565 | 352 | 94.833901 | 161.55 | 228.266099 | 0.458949 |
| GO:0007155\_cell\_adhesion | 428 | 2 | 2.006886 | -0.580325 | 353 | 95.009832 | 161.75 | 228.490168 | 0.458215 |
| GO:0022610\_biological\_adhesion | 429 | 2 | 2.002208 | -0.578872 | 354 | 95.718539 | 162.37 | 229.021461 | 0.458672 |
| GO:0040008\_regulation\_of\_growth | 131 | 1 | 3.278425 | -0.576806 | 356 | 96.459924 | 163.55 | 230.640076 | 0.459410 |
| GO:0051345\_positive\_regulation\_of\_hydrolase\_activity | 131 | 1 | 3.278425 | -0.576806 | 356 | 96.459924 | 163.55 | 230.640076 | 0.459410 |
| GO:0009889\_regulation\_of\_biosynthetic\_process | 1135 | 4 | 1.513564 | -0.574059 | 357 | 96.561607 | 163.67 | 230.778393 | 0.458459 |
| GO:0050794\_regulation\_of\_cellular\_process | 3515 | 10 | 1.221831 | -0.569946 | 358 | 97.140975 | 164.38 | 231.619025 | 0.459162 |
| GO:0043170\_macromolecule\_metabolic\_process | 3103 | 9 | 1.245654 | -0.569110 | 359 | 97.303268 | 164.57 | 231.836732 | 0.458412 |
| GO:0007249\_I-kappaB\_kinase\_NF-kappaB\_cascade | 134 | 1 | 3.205027 | -0.568355 | 360 | 98.087893 | 165.39 | 232.692107 | 0.459417 |
| GO:0007264\_small\_GTPase\_mediated\_signal\_transduction | 135 | 1 | 3.181287 | -0.565587 | 361 | 98.986422 | 166.6 | 234.213578 | 0.461496 |
| GO:0009057\_macromolecule\_catabolic\_process | 439 | 2 | 1.956600 | -0.564596 | 362 | 99.166817 | 166.79 | 234.413183 | 0.460746 |
| GO:0009892\_negative\_regulation\_of\_metabolic\_process | 440 | 2 | 1.952153 | -0.563193 | 363 | 99.260235 | 166.93 | 234.599765 | 0.459862 |
| GO:0030005\_cellular\_di-\_\_tri-valent\_inorganic\_cation\_homeostasis | 140 | 1 | 3.067669 | -0.552093 | 364 | 101.583747 | 169.59 | 237.596253 | 0.465907 |
| GO:0003012\_muscle\_system\_process | 141 | 1 | 3.045913 | -0.549462 | 365 | 102.189362 | 170.34 | 238.490638 | 0.466685 |
| GO:0065007\_biological\_regulation | 3971 | 11 | 1.189678 | -0.549121 | 366 | 102.284667 | 170.45 | 238.615333 | 0.465710 |
| GO:0001501\_skeletal\_system\_development | 142 | 1 | 3.024463 | -0.546852 | 368 | 103.062618 | 171.29 | 239.517382 | 0.465462 |
| GO:0007626\_locomotory\_behavior | 142 | 1 | 3.024463 | -0.546852 | 368 | 103.062618 | 171.29 | 239.517382 | 0.465462 |
| GO:0000165\_MAPKKK\_cascade | 143 | 1 | 3.003312 | -0.544264 | 369 | 103.316472 | 171.59 | 239.863528 | 0.465014 |
| GO:0031399\_regulation\_of\_protein\_modification\_process | 144 | 1 | 2.982456 | -0.541696 | 370 | 103.730268 | 172.3 | 240.869732 | 0.465676 |
| GO:0030036\_actin\_cytoskeleton\_organization | 145 | 1 | 2.961887 | -0.539150 | 372 | 104.576943 | 173.3 | 242.023057 | 0.465860 |
| GO:0055066\_di-\_\_tri-valent\_inorganic\_cation\_homeostasis | 145 | 1 | 2.961887 | -0.539150 | 372 | 104.576943 | 173.3 | 242.023057 | 0.465860 |
| GO:0008361\_regulation\_of\_cell\_size | 149 | 1 | 2.882374 | -0.529167 | 373 | 106.506029 | 175.79 | 245.073971 | 0.471287 |
| GO:0045321\_leukocyte\_activation | 150 | 1 | 2.863158 | -0.526720 | 374 | 106.587335 | 175.95 | 245.312665 | 0.470455 |
| GO:0009987\_cellular\_process | 6671 | 17 | 1.094447 | -0.524302 | 375 | 106.952521 | 176.48 | 246.007479 | 0.470613 |
| GO:0042981\_regulation\_of\_apoptosis | 471 | 2 | 1.823667 | -0.521800 | 376 | 107.775803 | 177.46 | 247.144197 | 0.471968 |
| GO:0006260\_DNA\_replication | 153 | 1 | 2.807018 | -0.519495 | 377 | 108.327460 | 178.02 | 247.712540 | 0.472202 |
| GO:0007166\_cell\_surface\_receptor\_linked\_signal\_transduction | 828 | 3 | 1.556064 | -0.519373 | 378 | 108.469867 | 178.18 | 247.890133 | 0.471376 |
| GO:0043067\_regulation\_of\_programmed\_cell\_death | 476 | 2 | 1.804511 | -0.515484 | 379 | 108.939168 | 178.83 | 248.720832 | 0.471847 |
| GO:0032940\_secretion\_by\_cell | 155 | 1 | 2.770798 | -0.514770 | 380 | 109.171748 | 179.23 | 249.288252 | 0.471658 |
| GO:0010941\_regulation\_of\_cell\_death | 478 | 2 | 1.796961 | -0.512983 | 381 | 109.460781 | 179.67 | 249.879219 | 0.471575 |
| GO:0016337\_cell-cell\_adhesion | 156 | 1 | 2.753036 | -0.512434 | 383 | 109.840030 | 180.13 | 250.419970 | 0.470313 |
| GO:0032787\_monocarboxylic\_acid\_metabolic\_process | 156 | 1 | 2.753036 | -0.512434 | 383 | 109.840030 | 180.13 | 250.419970 | 0.470313 |
| GO:0030003\_cellular\_cation\_homeostasis | 161 | 1 | 2.667538 | -0.501019 | 384 | 110.876115 | 181.26 | 251.643885 | 0.472031 |
| GO:0006091\_generation\_of\_precursor\_metabolites\_and\_energy | 163 | 1 | 2.634808 | -0.496570 | 385 | 111.266049 | 181.75 | 252.233951 | 0.472078 |
| GO:0034960\_cellular\_biopolymer\_metabolic\_process | 2820 | 8 | 1.218365 | -0.495721 | 386 | 111.650560 | 182.3 | 252.949440 | 0.472280 |
| GO:0050789\_regulation\_of\_biological\_process | 3649 | 10 | 1.176963 | -0.495187 | 387 | 111.806478 | 182.43 | 253.053522 | 0.471395 |
| GO:0030029\_actin\_filament-based\_process | 165 | 1 | 2.602871 | -0.492186 | 388 | 112.417967 | 183.19 | 253.962033 | 0.472139 |
| GO:0000279\_M\_phase | 170 | 1 | 2.526316 | -0.481500 | 389 | 113.686145 | 184.62 | 255.553855 | 0.474602 |
| GO:0055080\_cation\_homeostasis | 173 | 1 | 2.482507 | -0.475269 | 390 | 114.562744 | 185.72 | 256.877256 | 0.476205 |
| GO:0040007\_growth | 174 | 1 | 2.468240 | -0.473221 | 391 | 114.975639 | 186.32 | 257.664361 | 0.476522 |
| GO:0045892\_negative\_regulation\_of\_transcription\_\_DNA-dependent | 175 | 1 | 2.454135 | -0.471187 | 392 | 115.270921 | 186.77 | 258.269079 | 0.476454 |
| GO:0009607\_response\_to\_biotic\_stimulus | 177 | 1 | 2.426405 | -0.467160 | 394 | 116.261677 | 187.93 | 259.598323 | 0.476980 |
| GO:0016477\_cell\_migration | 177 | 1 | 2.426405 | -0.467160 | 394 | 116.261677 | 187.93 | 259.598323 | 0.476980 |
| GO:0051253\_negative\_regulation\_of\_RNA\_metabolic\_process | 180 | 1 | 2.385965 | -0.461224 | 396 | 117.414493 | 189.49 | 261.565507 | 0.478510 |
| GO:0051336\_regulation\_of\_hydrolase\_activity | 180 | 1 | 2.385965 | -0.461224 | 396 | 117.414493 | 189.49 | 261.565507 | 0.478510 |
| GO:0050790\_regulation\_of\_catalytic\_activity | 525 | 2 | 1.636090 | -0.458263 | 397 | 117.748691 | 189.91 | 262.071309 | 0.478363 |
| GO:0006954\_inflammatory\_response | 182 | 1 | 2.359746 | -0.457333 | 398 | 117.953107 | 190.15 | 262.346893 | 0.477764 |
| GO:0006917\_induction\_of\_apoptosis | 190 | 1 | 2.260388 | -0.442275 | 399 | 120.234285 | 192.98 | 265.725715 | 0.483659 |
| GO:0012502\_induction\_of\_programmed\_cell\_death | 191 | 1 | 2.248553 | -0.440448 | 400 | 120.616660 | 193.44 | 266.263340 | 0.483600 |
| GO:0019953\_sexual\_reproduction | 193 | 1 | 2.225252 | -0.436828 | 401 | 121.510711 | 194.4 | 267.289289 | 0.484788 |
| GO:0002682\_regulation\_of\_immune\_system\_process | 196 | 1 | 2.191192 | -0.431485 | 403 | 122.270718 | 195.44 | 268.609282 | 0.484963 |
| GO:0043086\_negative\_regulation\_of\_catalytic\_activity | 196 | 1 | 2.191192 | -0.431485 | 403 | 122.270718 | 195.44 | 268.609282 | 0.484963 |
| GO:0048870\_cell\_motility | 197 | 1 | 2.180069 | -0.429726 | 404 | 122.560782 | 195.73 | 268.899218 | 0.484480 |
| GO:0060255\_regulation\_of\_macromolecule\_metabolic\_process | 1328 | 4 | 1.293595 | -0.426282 | 405 | 122.858737 | 196.18 | 269.501263 | 0.484395 |
| GO:0008284\_positive\_regulation\_of\_cell\_proliferation | 200 | 1 | 2.147368 | -0.424516 | 406 | 123.108923 | 196.47 | 269.831077 | 0.483916 |
| GO:0008285\_negative\_regulation\_of\_cell\_proliferation | 202 | 1 | 2.126107 | -0.421097 | 407 | 124.070755 | 197.55 | 271.029245 | 0.485381 |
| GO:0006915\_apoptosis | 565 | 2 | 1.520261 | -0.417107 | 408 | 124.529457 | 198.09 | 271.650543 | 0.485515 |
| GO:0006873\_cellular\_ion\_homeostasis | 206 | 1 | 2.084824 | -0.414383 | 409 | 124.936216 | 198.66 | 272.383784 | 0.485721 |
| GO:0043066\_negative\_regulation\_of\_apoptosis | 207 | 1 | 2.074752 | -0.412730 | 410 | 125.297730 | 199.17 | 273.042270 | 0.485780 |
| GO:0012501\_programmed\_cell\_death | 571 | 2 | 1.504286 | -0.411314 | 411 | 125.460100 | 199.41 | 273.359900 | 0.485182 |
| GO:0055082\_cellular\_chemical\_homeostasis | 208 | 1 | 2.064777 | -0.411087 | 412 | 125.639963 | 199.69 | 273.740037 | 0.484684 |
| GO:0043069\_negative\_regulation\_of\_programmed\_cell\_death | 209 | 1 | 2.054898 | -0.409453 | 414 | 126.217733 | 200.37 | 274.522267 | 0.483986 |
| GO:0060548\_negative\_regulation\_of\_cell\_death | 209 | 1 | 2.054898 | -0.409453 | 414 | 126.217733 | 200.37 | 274.522267 | 0.483986 |
| GO:0045859\_regulation\_of\_protein\_kinase\_activity | 213 | 1 | 2.016308 | -0.403018 | 416 | 127.630465 | 202.21 | 276.789535 | 0.486082 |
| GO:0051726\_regulation\_of\_cell\_cycle | 213 | 1 | 2.016308 | -0.403018 | 416 | 127.630465 | 202.21 | 276.789535 | 0.486082 |
| GO:0007610\_behavior | 214 | 1 | 2.006886 | -0.401433 | 417 | 127.992076 | 202.65 | 277.307924 | 0.485971 |
| GO:0008219\_cell\_death | 585 | 2 | 1.468286 | -0.398155 | 419 | 128.823551 | 203.65 | 278.476449 | 0.486038 |
| GO:0016265\_death | 585 | 2 | 1.468286 | -0.398155 | 419 | 128.823551 | 203.65 | 278.476449 | 0.486038 |
| GO:0043549\_regulation\_of\_kinase\_activity | 217 | 1 | 1.979141 | -0.396733 | 420 | 129.189046 | 204.11 | 279.030954 | 0.485976 |
| GO:0046903\_secretion | 218 | 1 | 1.970063 | -0.395185 | 421 | 129.394862 | 204.42 | 279.445138 | 0.485558 |
| GO:0050801\_ion\_homeostasis | 221 | 1 | 1.943320 | -0.390593 | 422 | 129.798789 | 204.9 | 280.001211 | 0.485545 |
| GO:0043283\_biopolymer\_metabolic\_process | 3027 | 8 | 1.135048 | -0.389759 | 423 | 129.906847 | 205.06 | 280.213153 | 0.484775 |
| GO:0034621\_cellular\_macromolecular\_complex\_subunit\_organization | 227 | 1 | 1.891955 | -0.381645 | 426 | 131.574001 | 206.96 | 282.345999 | 0.485822 |
| GO:0034962\_cellular\_biopolymer\_catabolic\_process | 227 | 1 | 1.891955 | -0.381645 | 426 | 131.574001 | 206.96 | 282.345999 | 0.485822 |
| GO:0051338\_regulation\_of\_transferase\_activity | 227 | 1 | 1.891955 | -0.381645 | 426 | 131.574001 | 206.96 | 282.345999 | 0.485822 |
| GO:0065009\_regulation\_of\_molecular\_function | 606 | 2 | 1.417405 | -0.379311 | 427 | 132.203767 | 207.52 | 282.836233 | 0.485995 |
| GO:0019725\_cellular\_homeostasis | 231 | 1 | 1.859193 | -0.375846 | 428 | 132.965212 | 208.46 | 283.954788 | 0.487056 |
| GO:0044092\_negative\_regulation\_of\_molecular\_function | 233 | 1 | 1.843235 | -0.372994 | 429 | 133.697368 | 209.35 | 285.002632 | 0.487995 |
| GO:0043065\_positive\_regulation\_of\_apoptosis | 243 | 1 | 1.767381 | -0.359198 | 430 | 135.797950 | 211.7 | 287.602050 | 0.492326 |
| GO:0009056\_catabolic\_process | 633 | 2 | 1.356947 | -0.356550 | 431 | 136.439605 | 212.39 | 288.340395 | 0.492784 |
| GO:0022403\_cell\_cycle\_phase | 245 | 1 | 1.752954 | -0.356527 | 432 | 137.094511 | 213.01 | 288.925489 | 0.493079 |
| GO:0043068\_positive\_regulation\_of\_programmed\_cell\_death | 246 | 1 | 1.745828 | -0.355203 | 433 | 138.013721 | 214.17 | 290.326279 | 0.494619 |
| GO:0010942\_positive\_regulation\_of\_cell\_death | 250 | 1 | 1.717895 | -0.349973 | 434 | 138.782866 | 215.02 | 291.257134 | 0.495438 |
| GO:0048468\_cell\_development | 251 | 1 | 1.711051 | -0.348683 | 435 | 139.000596 | 215.31 | 291.619404 | 0.494966 |
| GO:0008283\_cell\_proliferation | 647 | 2 | 1.327585 | -0.345354 | 436 | 139.477604 | 215.79 | 292.102396 | 0.494931 |
| GO:0010556\_regulation\_of\_macromolecule\_biosynthetic\_process | 1055 | 3 | 1.221252 | -0.343735 | 437 | 139.957886 | 216.26 | 292.562114 | 0.494874 |
| GO:0031323\_regulation\_of\_cellular\_metabolic\_process | 1466 | 4 | 1.171825 | -0.343533 | 438 | 140.097164 | 216.42 | 292.742836 | 0.494110 |
| GO:0016481\_negative\_regulation\_of\_transcription | 261 | 1 | 1.645493 | -0.336144 | 439 | 140.882376 | 217.37 | 293.857624 | 0.495148 |
| GO:0032502\_developmental\_process | 1919 | 5 | 1.119004 | -0.324558 | 440 | 141.705478 | 218.54 | 295.374522 | 0.496682 |
| GO:0015031\_protein\_transport | 274 | 1 | 1.567422 | -0.320767 | 441 | 143.071839 | 219.91 | 296.748161 | 0.498662 |
| GO:0007010\_cytoskeleton\_organization | 275 | 1 | 1.561722 | -0.319625 | 442 | 143.598862 | 220.51 | 297.421138 | 0.498891 |
| GO:0048878\_chemical\_homeostasis | 278 | 1 | 1.544869 | -0.316232 | 443 | 144.305934 | 221.4 | 298.494066 | 0.499774 |
| GO:0045184\_establishment\_of\_protein\_localization | 279 | 1 | 1.539332 | -0.315112 | 444 | 144.988183 | 222.1 | 299.211817 | 0.500225 |
| GO:0044249\_cellular\_biosynthetic\_process | 1951 | 5 | 1.100650 | -0.309849 | 445 | 145.101879 | 222.29 | 299.478121 | 0.499528 |
| GO:0019752\_carboxylic\_acid\_metabolic\_process | 286 | 1 | 1.501656 | -0.307421 | 447 | 146.388521 | 224.2 | 302.011479 | 0.501566 |
| GO:0043436\_oxoacid\_metabolic\_process | 286 | 1 | 1.501656 | -0.307421 | 447 | 146.388521 | 224.2 | 302.011479 | 0.501566 |
| GO:0019222\_regulation\_of\_metabolic\_process | 1538 | 4 | 1.116967 | -0.306482 | 448 | 146.503538 | 224.39 | 302.276462 | 0.500871 |
| GO:0010629\_negative\_regulation\_of\_gene\_expression | 289 | 1 | 1.486068 | -0.304204 | 449 | 147.238167 | 225.26 | 303.281833 | 0.501693 |
| GO:0006082\_organic\_acid\_metabolic\_process | 290 | 1 | 1.480944 | -0.303141 | 451 | 147.626459 | 225.82 | 304.013541 | 0.500710 |
| GO:0051093\_negative\_regulation\_of\_developmental\_process | 290 | 1 | 1.480944 | -0.303141 | 451 | 147.626459 | 225.82 | 304.013541 | 0.500710 |
| GO:0042180\_cellular\_ketone\_metabolic\_process | 291 | 1 | 1.475855 | -0.302084 | 452 | 148.037492 | 226.3 | 304.562508 | 0.500664 |
| GO:0000278\_mitotic\_cell\_cycle | 292 | 1 | 1.470800 | -0.301031 | 453 | 149.265599 | 227.47 | 305.674401 | 0.502141 |
| GO:0045934\_negative\_regulation\_of\_nucleobase\_\_nucleoside\_\_nucleotide\_and\_nucleic\_acid\_metabolic\_process | 295 | 1 | 1.455843 | -0.297904 | 454 | 149.956667 | 228.25 | 306.543333 | 0.502753 |
| GO:0016192\_vesicle-mediated\_transport | 297 | 1 | 1.446039 | -0.295843 | 455 | 150.942160 | 229.42 | 307.897840 | 0.504220 |
| GO:0051172\_negative\_regulation\_of\_nitrogen\_compound\_metabolic\_process | 298 | 1 | 1.441187 | -0.294819 | 456 | 151.412592 | 229.96 | 308.507408 | 0.504298 |
| GO:0009058\_biosynthetic\_process | 1988 | 5 | 1.080165 | -0.293554 | 457 | 152.094056 | 230.84 | 309.585944 | 0.505120 |
| GO:0006355\_regulation\_of\_transcription\_\_DNA-dependent | 723 | 2 | 1.188032 | -0.290947 | 458 | 152.842661 | 231.69 | 310.537339 | 0.505873 |
| GO:0006928\_cell\_motion | 308 | 1 | 1.394395 | -0.284842 | 460 | 154.552816 | 233.61 | 312.667184 | 0.507848 |
| GO:0051674\_localization\_of\_cell | 308 | 1 | 1.394395 | -0.284842 | 460 | 154.552816 | 233.61 | 312.667184 | 0.507848 |
| GO:0034645\_cellular\_macromolecule\_biosynthetic\_process | 1600 | 4 | 1.073684 | -0.277515 | 461 | 155.486713 | 234.71 | 313.933287 | 0.509132 |
| GO:0051252\_regulation\_of\_RNA\_metabolic\_process | 746 | 2 | 1.151404 | -0.276367 | 462 | 155.758666 | 234.98 | 314.201334 | 0.508615 |
| GO:0010558\_negative\_regulation\_of\_macromolecule\_biosynthetic\_process | 324 | 1 | 1.325536 | -0.269787 | 463 | 157.051714 | 236.66 | 316.268286 | 0.511145 |
| GO:0032879\_regulation\_of\_localization | 326 | 1 | 1.317404 | -0.267979 | 464 | 157.275601 | 237.0 | 316.724399 | 0.510776 |
| GO:0009059\_macromolecule\_biosynthetic\_process | 1626 | 4 | 1.056516 | -0.266116 | 465 | 157.668053 | 237.45 | 317.231947 | 0.510645 |
| GO:0006996\_organelle\_organization | 764 | 2 | 1.124277 | -0.265501 | 466 | 157.877206 | 237.67 | 317.462794 | 0.510021 |
| GO:0008104\_protein\_localization | 339 | 1 | 1.266884 | -0.256603 | 467 | 159.077868 | 239.09 | 319.102132 | 0.511970 |
| GO:0051094\_positive\_regulation\_of\_developmental\_process | 340 | 1 | 1.263158 | -0.255754 | 468 | 159.656987 | 239.82 | 319.983013 | 0.512436 |
| GO:0032501\_multicellular\_organismal\_process | 2082 | 5 | 1.031397 | -0.255395 | 469 | 159.862063 | 240.03 | 320.197937 | 0.511791 |
| GO:0033554\_cellular\_response\_to\_stress | 341 | 1 | 1.259454 | -0.254909 | 470 | 160.244511 | 240.37 | 320.495489 | 0.511426 |
| GO:0016070\_RNA\_metabolic\_process | 1230 | 3 | 1.047497 | -0.249912 | 471 | 160.523865 | 240.79 | 321.056135 | 0.511231 |
| GO:0006357\_regulation\_of\_transcription\_from\_RNA\_polymerase\_II\_promoter | 351 | 1 | 1.223572 | -0.246648 | 472 | 160.818995 | 241.17 | 321.521005 | 0.510953 |
| GO:0006259\_DNA\_metabolic\_process | 360 | 1 | 1.192982 | -0.239502 | 473 | 162.107376 | 242.78 | 323.452624 | 0.513277 |
| GO:0007186\_G-protein\_coupled\_receptor\_protein\_signaling\_pathway | 363 | 1 | 1.183123 | -0.237179 | 474 | 162.582880 | 243.17 | 323.757120 | 0.513017 |
| GO:0022414\_reproductive\_process | 365 | 1 | 1.176640 | -0.235645 | 475 | 163.118339 | 243.7 | 324.281661 | 0.513053 |
| GO:0000003\_reproduction | 367 | 1 | 1.170228 | -0.234125 | 476 | 163.865131 | 244.44 | 325.014869 | 0.513529 |
| GO:0022402\_cell\_cycle\_process | 370 | 1 | 1.160740 | -0.231866 | 477 | 164.653690 | 245.21 | 325.766310 | 0.514067 |
| GO:0044255\_cellular\_lipid\_metabolic\_process | 381 | 1 | 1.127228 | -0.223815 | 478 | 166.606923 | 247.44 | 328.273077 | 0.517657 |
| GO:0033036\_macromolecule\_localization | 388 | 1 | 1.106891 | -0.218871 | 479 | 167.237483 | 248.06 | 328.882517 | 0.517871 |
| GO:0080090\_regulation\_of\_primary\_metabolic\_process | 1311 | 3 | 0.982777 | -0.215297 | 480 | 168.268859 | 249.06 | 329.851141 | 0.518875 |
| GO:0006351\_transcription\_\_DNA-dependent | 884 | 2 | 0.971660 | -0.203569 | 481 | 170.378379 | 251.28 | 332.181621 | 0.522412 |
| GO:0010605\_negative\_regulation\_of\_macromolecule\_metabolic\_process | 413 | 1 | 1.039888 | -0.202275 | 482 | 171.262938 | 252.2 | 333.137062 | 0.523237 |
| GO:0032774\_RNA\_biosynthetic\_process | 887 | 2 | 0.968374 | -0.202227 | 483 | 171.573234 | 252.5 | 333.426766 | 0.522774 |
| GO:0045449\_regulation\_of\_transcription | 900 | 2 | 0.954386 | -0.196514 | 484 | 172.574940 | 253.44 | 334.305060 | 0.523636 |
| GO:0043933\_macromolecular\_complex\_subunit\_organization | 424 | 1 | 1.012910 | -0.195458 | 485 | 173.166150 | 254.2 | 335.233850 | 0.524124 |
| GO:0043285\_biopolymer\_catabolic\_process | 426 | 1 | 1.008154 | -0.194249 | 486 | 173.857249 | 254.85 | 335.842751 | 0.524383 |
| GO:0007267\_cell-cell\_signaling | 445 | 1 | 0.965109 | -0.183193 | 487 | 177.029875 | 258.47 | 339.910125 | 0.530739 |
| GO:0006629\_lipid\_metabolic\_process | 468 | 1 | 0.917679 | -0.170790 | 488 | 179.688943 | 261.21 | 342.731057 | 0.535266 |
| GO:0051716\_cellular\_response\_to\_stimulus | 474 | 1 | 0.906063 | -0.167718 | 489 | 180.273907 | 261.82 | 343.366093 | 0.535419 |
| GO:0022607\_cellular\_component\_assembly | 478 | 1 | 0.898481 | -0.165706 | 490 | 181.753835 | 263.31 | 344.866165 | 0.537367 |
| GO:0007049\_cell\_cycle | 494 | 1 | 0.869380 | -0.157931 | 491 | 183.448377 | 264.82 | 346.191623 | 0.539348 |
| GO:0009653\_anatomical\_structure\_morphogenesis | 500 | 1 | 0.858947 | -0.155124 | 492 | 183.863163 | 265.19 | 346.516837 | 0.539004 |
| GO:0044238\_primary\_metabolic\_process | 3719 | 8 | 0.923848 | -0.154162 | 493 | 184.569770 | 265.75 | 346.930230 | 0.539047 |
| GO:0006366\_transcription\_from\_RNA\_polymerase\_II\_promoter | 506 | 1 | 0.848762 | -0.152374 | 494 | 185.495360 | 266.8 | 348.104640 | 0.540081 |
| GO:0019219\_regulation\_of\_nucleobase\_\_nucleoside\_\_nucleotide\_and\_nucleic\_acid\_metabolic\_process | 1041 | 2 | 0.825118 | -0.144023 | 495 | 186.580621 | 267.92 | 349.259379 | 0.541253 |
| GO:0051171\_regulation\_of\_nitrogen\_compound\_metabolic\_process | 1055 | 2 | 0.814168 | -0.139638 | 496 | 187.781063 | 269.13 | 350.478937 | 0.542601 |
| GO:0010468\_regulation\_of\_gene\_expression | 1067 | 2 | 0.805012 | -0.135984 | 497 | 188.133679 | 269.41 | 350.686321 | 0.542072 |
| GO:0006350\_transcription | 1069 | 2 | 0.803505 | -0.135384 | 498 | 188.472983 | 269.7 | 350.927017 | 0.541566 |
| GO:0010926\_anatomical\_structure\_formation | 560 | 1 | 0.766917 | -0.129963 | 500 | 190.291358 | 271.54 | 352.788642 | 0.543080 |
| GO:0044085\_cellular\_component\_biogenesis | 560 | 1 | 0.766917 | -0.129963 | 500 | 190.291358 | 271.54 | 352.788642 | 0.543080 |
| GO:0051649\_establishment\_of\_localization\_in\_cell | 573 | 1 | 0.749518 | -0.125136 | 501 | 192.301372 | 273.06 | 353.818628 | 0.545030 |
| GO:0051641\_cellular\_localization | 617 | 1 | 0.696068 | -0.110205 | 502 | 195.907149 | 276.41 | 356.912851 | 0.550618 |
| GO:0042221\_response\_to\_chemical\_stimulus | 631 | 1 | 0.680624 | -0.105871 | 503 | 196.607883 | 276.97 | 357.332117 | 0.550636 |
| GO:0003008\_system\_process | 710 | 1 | 0.604893 | -0.084603 | 504 | 200.677354 | 280.41 | 360.142646 | 0.556369 |
| GO:0006139\_nucleobase\_\_nucleoside\_\_nucleotide\_and\_nucleic\_acid\_metabolic\_process | 1845 | 3 | 0.698331 | -0.076734 | 505 | 202.840099 | 282.4 | 361.959901 | 0.559208 |
| GO:0016043\_cellular\_component\_organization | 1366 | 2 | 0.628805 | -0.069587 | 506 | 204.623703 | 283.83 | 363.036297 | 0.560929 |
| GO:0034961\_cellular\_biopolymer\_biosynthetic\_process | 1448 | 2 | 0.593196 | -0.057652 | 507 | 206.431838 | 285.17 | 363.908162 | 0.562465 |
| GO:0043284\_biopolymer\_biosynthetic\_process | 1458 | 2 | 0.589127 | -0.056335 | 508 | 207.454068 | 286.02 | 364.585932 | 0.563031 |
| GO:0006807\_nitrogen\_compound\_metabolic\_process | 2053 | 3 | 0.627580 | -0.049732 | 509 | 209.572808 | 287.65 | 365.727192 | 0.565128 |
| GO:0051179\_localization | 1561 | 2 | 0.550255 | -0.044306 | 510 | 211.167118 | 288.89 | 366.612882 | 0.566451 |
| GO:0010467\_gene\_expression | 1663 | 2 | 0.516505 | -0.034777 | 511 | 213.897330 | 290.91 | 367.922670 | 0.569295 |
| GO:0006810\_transport | 1243 | 1 | 0.345514 | -0.019143 | 512 | 217.869421 | 293.96 | 370.050579 | 0.574141 |
| GO:0051234\_establishment\_of\_localization | 1260 | 1 | 0.340852 | -0.018249 | 513 | 218.234934 | 294.24 | 370.245066 | 0.573567 |
| GO:0007179\_transforming\_growth\_factor\_beta\_receptor\_signaling\_pathway | 72 | 0 | 0.000000 | -0.000000 | 514 | 224.926020 | 299.27 | 373.613980 | 0.582237 |
| GO:0006171\_cAMP\_biosynthetic\_process | 59 | 0 | 0.000000 | -0.000000 | 520 | 231.260689 | 305.38 | 379.499311 | 0.587269 |
| GO:0006820\_anion\_transport | 59 | 0 | 0.000000 | -0.000000 | 520 | 231.260689 | 305.38 | 379.499311 | 0.587269 |
| GO:0007409\_axonogenesis | 59 | 0 | 0.000000 | -0.000000 | 520 | 231.260689 | 305.38 | 379.499311 | 0.587269 |
| GO:0033673\_negative\_regulation\_of\_kinase\_activity | 59 | 0 | 0.000000 | -0.000000 | 520 | 231.260689 | 305.38 | 379.499311 | 0.587269 |
| GO:0043281\_regulation\_of\_caspase\_activity | 59 | 0 | 0.000000 | -0.000000 | 520 | 231.260689 | 305.38 | 379.499311 | 0.587269 |
| GO:0051604\_protein\_maturation | 59 | 0 | 0.000000 | -0.000000 | 520 | 231.260689 | 305.38 | 379.499311 | 0.587269 |
| GO:0000038\_very-long-chain\_fatty\_acid\_metabolic\_process | 11 | 0 | 0.000000 | -0.000000 | 611 | 324.824489 | 397.9 | 470.975511 | 0.651227 |
| GO:0000272\_polysaccharide\_catabolic\_process | 11 | 0 | 0.000000 | -0.000000 | 611 | 324.824489 | 397.9 | 470.975511 | 0.651227 |
| GO:0001570\_vasculogenesis | 11 | 0 | 0.000000 | -0.000000 | 611 | 324.824489 | 397.9 | 470.975511 | 0.651227 |
| GO:0001836\_release\_of\_cytochrome\_c\_from\_mitochondria | 11 | 0 | 0.000000 | -0.000000 | 611 | 324.824489 | 397.9 | 470.975511 | 0.651227 |
| GO:0002285\_lymphocyte\_activation\_during\_immune\_response | 11 | 0 | 0.000000 | -0.000000 | 611 | 324.824489 | 397.9 | 470.975511 | 0.651227 |
| GO:0002687\_positive\_regulation\_of\_leukocyte\_migration | 11 | 0 | 0.000000 | -0.000000 | 611 | 324.824489 | 397.9 | 470.975511 | 0.651227 |
| GO:0006094\_gluconeogenesis | 11 | 0 | 0.000000 | -0.000000 | 611 | 324.824489 | 397.9 | 470.975511 | 0.651227 |
| GO:0006342\_chromatin\_silencing | 11 | 0 | 0.000000 | -0.000000 | 611 | 324.824489 | 397.9 | 470.975511 | 0.651227 |
| GO:0006400\_tRNA\_modification | 11 | 0 | 0.000000 | -0.000000 | 611 | 324.824489 | 397.9 | 470.975511 | 0.651227 |
| GO:0006515\_misfolded\_or\_incompletely\_synthesized\_protein\_catabolic\_process | 11 | 0 | 0.000000 | -0.000000 | 611 | 324.824489 | 397.9 | 470.975511 | 0.651227 |
| GO:0006706\_steroid\_catabolic\_process | 11 | 0 | 0.000000 | -0.000000 | 611 | 324.824489 | 397.9 | 470.975511 | 0.651227 |
| GO:0006749\_glutathione\_metabolic\_process | 11 | 0 | 0.000000 | -0.000000 | 611 | 324.824489 | 397.9 | 470.975511 | 0.651227 |
| GO:0007009\_plasma\_membrane\_organization | 11 | 0 | 0.000000 | -0.000000 | 611 | 324.824489 | 397.9 | 470.975511 | 0.651227 |
| GO:0007076\_mitotic\_chromosome\_condensation | 11 | 0 | 0.000000 | -0.000000 | 611 | 324.824489 | 397.9 | 470.975511 | 0.651227 |
| GO:0007090\_regulation\_of\_S\_phase\_of\_mitotic\_cell\_cycle | 11 | 0 | 0.000000 | -0.000000 | 611 | 324.824489 | 397.9 | 470.975511 | 0.651227 |
| GO:0007172\_signal\_complex\_assembly | 11 | 0 | 0.000000 | -0.000000 | 611 | 324.824489 | 397.9 | 470.975511 | 0.651227 |
| GO:0008206\_bile\_acid\_metabolic\_process | 11 | 0 | 0.000000 | -0.000000 | 611 | 324.824489 | 397.9 | 470.975511 | 0.651227 |
| GO:0008333\_endosome\_to\_lysosome\_transport | 11 | 0 | 0.000000 | -0.000000 | 611 | 324.824489 | 397.9 | 470.975511 | 0.651227 |
| GO:0009072\_aromatic\_amino\_acid\_family\_metabolic\_process | 11 | 0 | 0.000000 | -0.000000 | 611 | 324.824489 | 397.9 | 470.975511 | 0.651227 |
| GO:0009143\_nucleoside\_triphosphate\_catabolic\_process | 11 | 0 | 0.000000 | -0.000000 | 611 | 324.824489 | 397.9 | 470.975511 | 0.651227 |
| GO:0009247\_glycolipid\_biosynthetic\_process | 11 | 0 | 0.000000 | -0.000000 | 611 | 324.824489 | 397.9 | 470.975511 | 0.651227 |
| GO:0009264\_deoxyribonucleotide\_catabolic\_process | 11 | 0 | 0.000000 | -0.000000 | 611 | 324.824489 | 397.9 | 470.975511 | 0.651227 |
| GO:0009394\_2'-deoxyribonucleotide\_metabolic\_process | 11 | 0 | 0.000000 | -0.000000 | 611 | 324.824489 | 397.9 | 470.975511 | 0.651227 |
| GO:0009395\_phospholipid\_catabolic\_process | 11 | 0 | 0.000000 | -0.000000 | 611 | 324.824489 | 397.9 | 470.975511 | 0.651227 |
| GO:0009953\_dorsal\_ventral\_pattern\_formation | 11 | 0 | 0.000000 | -0.000000 | 611 | 324.824489 | 397.9 | 470.975511 | 0.651227 |
| GO:0010458\_exit\_from\_mitosis | 11 | 0 | 0.000000 | -0.000000 | 611 | 324.824489 | 397.9 | 470.975511 | 0.651227 |
| GO:0010469\_regulation\_of\_receptor\_activity | 11 | 0 | 0.000000 | -0.000000 | 611 | 324.824489 | 397.9 | 470.975511 | 0.651227 |
| GO:0010878\_cholesterol\_storage | 11 | 0 | 0.000000 | -0.000000 | 611 | 324.824489 | 397.9 | 470.975511 | 0.651227 |
| GO:0015804\_neutral\_amino\_acid\_transport | 11 | 0 | 0.000000 | -0.000000 | 611 | 324.824489 | 397.9 | 470.975511 | 0.651227 |
| GO:0016445\_somatic\_diversification\_of\_immunoglobulins | 11 | 0 | 0.000000 | -0.000000 | 611 | 324.824489 | 397.9 | 470.975511 | 0.651227 |
| GO:0016575\_histone\_deacetylation | 11 | 0 | 0.000000 | -0.000000 | 611 | 324.824489 | 397.9 | 470.975511 | 0.651227 |
| GO:0018205\_peptidyl-lysine\_modification | 11 | 0 | 0.000000 | -0.000000 | 611 | 324.824489 | 397.9 | 470.975511 | 0.651227 |
| GO:0018958\_phenol\_metabolic\_process | 11 | 0 | 0.000000 | -0.000000 | 611 | 324.824489 | 397.9 | 470.975511 | 0.651227 |
| GO:0019692\_deoxyribose\_phosphate\_metabolic\_process | 11 | 0 | 0.000000 | -0.000000 | 611 | 324.824489 | 397.9 | 470.975511 | 0.651227 |
| GO:0021915\_neural\_tube\_development | 11 | 0 | 0.000000 | -0.000000 | 611 | 324.824489 | 397.9 | 470.975511 | 0.651227 |
| GO:0030035\_microspike\_assembly | 11 | 0 | 0.000000 | -0.000000 | 611 | 324.824489 | 397.9 | 470.975511 | 0.651227 |
| GO:0030049\_muscle\_filament\_sliding | 11 | 0 | 0.000000 | -0.000000 | 611 | 324.824489 | 397.9 | 470.975511 | 0.651227 |
| GO:0030317\_sperm\_motility | 11 | 0 | 0.000000 | -0.000000 | 611 | 324.824489 | 397.9 | 470.975511 | 0.651227 |
| GO:0030326\_embryonic\_limb\_morphogenesis | 11 | 0 | 0.000000 | -0.000000 | 611 | 324.824489 | 397.9 | 470.975511 | 0.651227 |
| GO:0030433\_ER-associated\_protein\_catabolic\_process | 11 | 0 | 0.000000 | -0.000000 | 611 | 324.824489 | 397.9 | 470.975511 | 0.651227 |
| GO:0031333\_negative\_regulation\_of\_protein\_complex\_assembly | 11 | 0 | 0.000000 | -0.000000 | 611 | 324.824489 | 397.9 | 470.975511 | 0.651227 |
| GO:0031343\_positive\_regulation\_of\_cell\_killing | 11 | 0 | 0.000000 | -0.000000 | 611 | 324.824489 | 397.9 | 470.975511 | 0.651227 |
| GO:0031348\_negative\_regulation\_of\_defense\_response | 11 | 0 | 0.000000 | -0.000000 | 611 | 324.824489 | 397.9 | 470.975511 | 0.651227 |
| GO:0031572\_G2\_M\_transition\_DNA\_damage\_checkpoint | 11 | 0 | 0.000000 | -0.000000 | 611 | 324.824489 | 397.9 | 470.975511 | 0.651227 |
| GO:0031576\_G2\_M\_transition\_checkpoint | 11 | 0 | 0.000000 | -0.000000 | 611 | 324.824489 | 397.9 | 470.975511 | 0.651227 |
| GO:0031929\_TOR\_signaling\_pathway | 11 | 0 | 0.000000 | -0.000000 | 611 | 324.824489 | 397.9 | 470.975511 | 0.651227 |
| GO:0032088\_negative\_regulation\_of\_NF-kappaB\_transcription\_factor\_activity | 11 | 0 | 0.000000 | -0.000000 | 611 | 324.824489 | 397.9 | 470.975511 | 0.651227 |
| GO:0032204\_regulation\_of\_telomere\_maintenance | 11 | 0 | 0.000000 | -0.000000 | 611 | 324.824489 | 397.9 | 470.975511 | 0.651227 |
| GO:0032355\_response\_to\_estradiol\_stimulus | 11 | 0 | 0.000000 | -0.000000 | 611 | 324.824489 | 397.9 | 470.975511 | 0.651227 |
| GO:0032924\_activin\_receptor\_signaling\_pathway | 11 | 0 | 0.000000 | -0.000000 | 611 | 324.824489 | 397.9 | 470.975511 | 0.651227 |
| GO:0033275\_actin-myosin\_filament\_sliding | 11 | 0 | 0.000000 | -0.000000 | 611 | 324.824489 | 397.9 | 470.975511 | 0.651227 |
| GO:0034375\_high-density\_lipoprotein\_particle\_remodeling | 11 | 0 | 0.000000 | -0.000000 | 611 | 324.824489 | 397.9 | 470.975511 | 0.651227 |
| GO:0034377\_plasma\_lipoprotein\_particle\_assembly | 11 | 0 | 0.000000 | -0.000000 | 611 | 324.824489 | 397.9 | 470.975511 | 0.651227 |
| GO:0034433\_steroid\_esterification | 11 | 0 | 0.000000 | -0.000000 | 611 | 324.824489 | 397.9 | 470.975511 | 0.651227 |
| GO:0034434\_sterol\_esterification | 11 | 0 | 0.000000 | -0.000000 | 611 | 324.824489 | 397.9 | 470.975511 | 0.651227 |
| GO:0034435\_cholesterol\_esterification | 11 | 0 | 0.000000 | -0.000000 | 611 | 324.824489 | 397.9 | 470.975511 | 0.651227 |
| GO:0035113\_embryonic\_appendage\_morphogenesis | 11 | 0 | 0.000000 | -0.000000 | 611 | 324.824489 | 397.9 | 470.975511 | 0.651227 |
| GO:0042278\_purine\_nucleoside\_metabolic\_process | 11 | 0 | 0.000000 | -0.000000 | 611 | 324.824489 | 397.9 | 470.975511 | 0.651227 |
| GO:0042308\_negative\_regulation\_of\_protein\_import\_into\_nucleus | 11 | 0 | 0.000000 | -0.000000 | 611 | 324.824489 | 397.9 | 470.975511 | 0.651227 |
| GO:0042354\_L-fucose\_metabolic\_process | 11 | 0 | 0.000000 | -0.000000 | 611 | 324.824489 | 397.9 | 470.975511 | 0.651227 |
| GO:0042384\_cilium\_assembly | 11 | 0 | 0.000000 | -0.000000 | 611 | 324.824489 | 397.9 | 470.975511 | 0.651227 |
| GO:0042730\_fibrinolysis | 11 | 0 | 0.000000 | -0.000000 | 611 | 324.824489 | 397.9 | 470.975511 | 0.651227 |
| GO:0042787\_protein\_ubiquitination\_during\_ubiquitin-dependent\_protein\_catabolic\_process | 11 | 0 | 0.000000 | -0.000000 | 611 | 324.824489 | 397.9 | 470.975511 | 0.651227 |
| GO:0042992\_negative\_regulation\_of\_transcription\_factor\_import\_into\_nucleus | 11 | 0 | 0.000000 | -0.000000 | 611 | 324.824489 | 397.9 | 470.975511 | 0.651227 |
| GO:0043149\_stress\_fiber\_formation | 11 | 0 | 0.000000 | -0.000000 | 611 | 324.824489 | 397.9 | 470.975511 | 0.651227 |
| GO:0043154\_negative\_regulation\_of\_caspase\_activity | 11 | 0 | 0.000000 | -0.000000 | 611 | 324.824489 | 397.9 | 470.975511 | 0.651227 |
| GO:0043524\_negative\_regulation\_of\_neuron\_apoptosis | 11 | 0 | 0.000000 | -0.000000 | 611 | 324.824489 | 397.9 | 470.975511 | 0.651227 |
| GO:0043535\_regulation\_of\_blood\_vessel\_endothelial\_cell\_migration | 11 | 0 | 0.000000 | -0.000000 | 611 | 324.824489 | 397.9 | 470.975511 | 0.651227 |
| GO:0043547\_positive\_regulation\_of\_GTPase\_activity | 11 | 0 | 0.000000 | -0.000000 | 611 | 324.824489 | 397.9 | 470.975511 | 0.651227 |
| GO:0043583\_ear\_development | 11 | 0 | 0.000000 | -0.000000 | 611 | 324.824489 | 397.9 | 470.975511 | 0.651227 |
| GO:0045069\_regulation\_of\_viral\_genome\_replication | 11 | 0 | 0.000000 | -0.000000 | 611 | 324.824489 | 397.9 | 470.975511 | 0.651227 |
| GO:0045453\_bone\_resorption | 11 | 0 | 0.000000 | -0.000000 | 611 | 324.824489 | 397.9 | 470.975511 | 0.651227 |
| GO:0045806\_negative\_regulation\_of\_endocytosis | 11 | 0 | 0.000000 | -0.000000 | 611 | 324.824489 | 397.9 | 470.975511 | 0.651227 |
| GO:0045923\_positive\_regulation\_of\_fatty\_acid\_metabolic\_process | 11 | 0 | 0.000000 | -0.000000 | 611 | 324.824489 | 397.9 | 470.975511 | 0.651227 |
| GO:0046128\_purine\_ribonucleoside\_metabolic\_process | 11 | 0 | 0.000000 | -0.000000 | 611 | 324.824489 | 397.9 | 470.975511 | 0.651227 |
| GO:0046504\_glycerol\_ether\_biosynthetic\_process | 11 | 0 | 0.000000 | -0.000000 | 611 | 324.824489 | 397.9 | 470.975511 | 0.651227 |
| GO:0048284\_organelle\_fusion | 11 | 0 | 0.000000 | -0.000000 | 611 | 324.824489 | 397.9 | 470.975511 | 0.651227 |
| GO:0050680\_negative\_regulation\_of\_epithelial\_cell\_proliferation | 11 | 0 | 0.000000 | -0.000000 | 611 | 324.824489 | 397.9 | 470.975511 | 0.651227 |
| GO:0050704\_regulation\_of\_interleukin-1\_secretion | 11 | 0 | 0.000000 | -0.000000 | 611 | 324.824489 | 397.9 | 470.975511 | 0.651227 |
| GO:0050777\_negative\_regulation\_of\_immune\_response | 11 | 0 | 0.000000 | -0.000000 | 611 | 324.824489 | 397.9 | 470.975511 | 0.651227 |
| GO:0050830\_defense\_response\_to\_Gram-positive\_bacterium | 11 | 0 | 0.000000 | -0.000000 | 611 | 324.824489 | 397.9 | 470.975511 | 0.651227 |
| GO:0050871\_positive\_regulation\_of\_B\_cell\_activation | 11 | 0 | 0.000000 | -0.000000 | 611 | 324.824489 | 397.9 | 470.975511 | 0.651227 |
| GO:0050918\_positive\_chemotaxis | 11 | 0 | 0.000000 | -0.000000 | 611 | 324.824489 | 397.9 | 470.975511 | 0.651227 |
| GO:0050996\_positive\_regulation\_of\_lipid\_catabolic\_process | 11 | 0 | 0.000000 | -0.000000 | 611 | 324.824489 | 397.9 | 470.975511 | 0.651227 |
| GO:0051043\_regulation\_of\_membrane\_protein\_ectodomain\_proteolysis | 11 | 0 | 0.000000 | -0.000000 | 611 | 324.824489 | 397.9 | 470.975511 | 0.651227 |
| GO:0051187\_cofactor\_catabolic\_process | 11 | 0 | 0.000000 | -0.000000 | 611 | 324.824489 | 397.9 | 470.975511 | 0.651227 |
| GO:0051220\_cytoplasmic\_sequestering\_of\_protein | 11 | 0 | 0.000000 | -0.000000 | 611 | 324.824489 | 397.9 | 470.975511 | 0.651227 |
| GO:0051453\_regulation\_of\_intracellular\_pH | 11 | 0 | 0.000000 | -0.000000 | 611 | 324.824489 | 397.9 | 470.975511 | 0.651227 |
| GO:0051668\_localization\_within\_membrane | 11 | 0 | 0.000000 | -0.000000 | 611 | 324.824489 | 397.9 | 470.975511 | 0.651227 |
| GO:0065005\_protein-lipid\_complex\_assembly | 11 | 0 | 0.000000 | -0.000000 | 611 | 324.824489 | 397.9 | 470.975511 | 0.651227 |
| GO:0070252\_actin-mediated\_cell\_contraction | 11 | 0 | 0.000000 | -0.000000 | 611 | 324.824489 | 397.9 | 470.975511 | 0.651227 |
| GO:0000079\_regulation\_of\_cyclin-dependent\_protein\_kinase\_activity | 48 | 0 | 0.000000 | -0.000000 | 617 | 332.281119 | 405.17 | 478.058881 | 0.656677 |
| GO:0008217\_regulation\_of\_blood\_pressure | 48 | 0 | 0.000000 | -0.000000 | 617 | 332.281119 | 405.17 | 478.058881 | 0.656677 |
| GO:0010551\_regulation\_of\_specific\_transcription\_from\_RNA\_polymerase\_II\_promoter | 48 | 0 | 0.000000 | -0.000000 | 617 | 332.281119 | 405.17 | 478.058881 | 0.656677 |
| GO:0032569\_specific\_transcription\_from\_RNA\_polymerase\_II\_promoter | 48 | 0 | 0.000000 | -0.000000 | 617 | 332.281119 | 405.17 | 478.058881 | 0.656677 |
| GO:0042773\_ATP\_synthesis\_coupled\_electron\_transport | 48 | 0 | 0.000000 | -0.000000 | 617 | 332.281119 | 405.17 | 478.058881 | 0.656677 |
| GO:0042775\_mitochondrial\_ATP\_synthesis\_coupled\_electron\_transport | 48 | 0 | 0.000000 | -0.000000 | 617 | 332.281119 | 405.17 | 478.058881 | 0.656677 |
| GO:0006974\_response\_to\_DNA\_damage\_stimulus | 234 | 0 | 0.000000 | -0.000000 | 618 | 332.869528 | 405.75 | 478.630472 | 0.656553 |
| GO:0000084\_S\_phase\_of\_mitotic\_cell\_cycle | 16 | 0 | 0.000000 | -0.000000 | 667 | 389.100313 | 461.13 | 533.159687 | 0.691349 |
| GO:0001523\_retinoid\_metabolic\_process | 16 | 0 | 0.000000 | -0.000000 | 667 | 389.100313 | 461.13 | 533.159687 | 0.691349 |
| GO:0001933\_negative\_regulation\_of\_protein\_amino\_acid\_phosphorylation | 16 | 0 | 0.000000 | -0.000000 | 667 | 389.100313 | 461.13 | 533.159687 | 0.691349 |
| GO:0002695\_negative\_regulation\_of\_leukocyte\_activation | 16 | 0 | 0.000000 | -0.000000 | 667 | 389.100313 | 461.13 | 533.159687 | 0.691349 |
| GO:0002699\_positive\_regulation\_of\_immune\_effector\_process | 16 | 0 | 0.000000 | -0.000000 | 667 | 389.100313 | 461.13 | 533.159687 | 0.691349 |
| GO:0006220\_pyrimidine\_nucleotide\_metabolic\_process | 16 | 0 | 0.000000 | -0.000000 | 667 | 389.100313 | 461.13 | 533.159687 | 0.691349 |
| GO:0006298\_mismatch\_repair | 16 | 0 | 0.000000 | -0.000000 | 667 | 389.100313 | 461.13 | 533.159687 | 0.691349 |
| GO:0006471\_protein\_amino\_acid\_ADP-ribosylation | 16 | 0 | 0.000000 | -0.000000 | 667 | 389.100313 | 461.13 | 533.159687 | 0.691349 |
| GO:0006636\_unsaturated\_fatty\_acid\_biosynthetic\_process | 16 | 0 | 0.000000 | -0.000000 | 667 | 389.100313 | 461.13 | 533.159687 | 0.691349 |
| GO:0006687\_glycosphingolipid\_metabolic\_process | 16 | 0 | 0.000000 | -0.000000 | 667 | 389.100313 | 461.13 | 533.159687 | 0.691349 |
| GO:0006695\_cholesterol\_biosynthetic\_process | 16 | 0 | 0.000000 | -0.000000 | 667 | 389.100313 | 461.13 | 533.159687 | 0.691349 |
| GO:0006721\_terpenoid\_metabolic\_process | 16 | 0 | 0.000000 | -0.000000 | 667 | 389.100313 | 461.13 | 533.159687 | 0.691349 |
| GO:0006776\_vitamin\_A\_metabolic\_process | 16 | 0 | 0.000000 | -0.000000 | 667 | 389.100313 | 461.13 | 533.159687 | 0.691349 |
| GO:0006805\_xenobiotic\_metabolic\_process | 16 | 0 | 0.000000 | -0.000000 | 667 | 389.100313 | 461.13 | 533.159687 | 0.691349 |
| GO:0006891\_intra-Golgi\_vesicle-mediated\_transport | 16 | 0 | 0.000000 | -0.000000 | 667 | 389.100313 | 461.13 | 533.159687 | 0.691349 |
| GO:0006914\_autophagy | 16 | 0 | 0.000000 | -0.000000 | 667 | 389.100313 | 461.13 | 533.159687 | 0.691349 |
| GO:0007006\_mitochondrial\_membrane\_organization | 16 | 0 | 0.000000 | -0.000000 | 667 | 389.100313 | 461.13 | 533.159687 | 0.691349 |
| GO:0009119\_ribonucleoside\_metabolic\_process | 16 | 0 | 0.000000 | -0.000000 | 667 | 389.100313 | 461.13 | 533.159687 | 0.691349 |
| GO:0009408\_response\_to\_heat | 16 | 0 | 0.000000 | -0.000000 | 667 | 389.100313 | 461.13 | 533.159687 | 0.691349 |
| GO:0009595\_detection\_of\_biotic\_stimulus | 16 | 0 | 0.000000 | -0.000000 | 667 | 389.100313 | 461.13 | 533.159687 | 0.691349 |
| GO:0009743\_response\_to\_carbohydrate\_stimulus | 16 | 0 | 0.000000 | -0.000000 | 667 | 389.100313 | 461.13 | 533.159687 | 0.691349 |
| GO:0010553\_negative\_regulation\_of\_specific\_transcription\_from\_RNA\_polymerase\_II\_promoter | 16 | 0 | 0.000000 | -0.000000 | 667 | 389.100313 | 461.13 | 533.159687 | 0.691349 |
| GO:0010743\_regulation\_of\_foam\_cell\_differentiation | 16 | 0 | 0.000000 | -0.000000 | 667 | 389.100313 | 461.13 | 533.159687 | 0.691349 |
| GO:0010975\_regulation\_of\_neuron\_projection\_development | 16 | 0 | 0.000000 | -0.000000 | 667 | 389.100313 | 461.13 | 533.159687 | 0.691349 |
| GO:0014070\_response\_to\_organic\_cyclic\_substance | 16 | 0 | 0.000000 | -0.000000 | 667 | 389.100313 | 461.13 | 533.159687 | 0.691349 |
| GO:0015909\_long-chain\_fatty\_acid\_transport | 16 | 0 | 0.000000 | -0.000000 | 667 | 389.100313 | 461.13 | 533.159687 | 0.691349 |
| GO:0016101\_diterpenoid\_metabolic\_process | 16 | 0 | 0.000000 | -0.000000 | 667 | 389.100313 | 461.13 | 533.159687 | 0.691349 |
| GO:0016254\_preassembly\_of\_GPI\_anchor\_in\_ER\_membrane | 16 | 0 | 0.000000 | -0.000000 | 667 | 389.100313 | 461.13 | 533.159687 | 0.691349 |
| GO:0019319\_hexose\_biosynthetic\_process | 16 | 0 | 0.000000 | -0.000000 | 667 | 389.100313 | 461.13 | 533.159687 | 0.691349 |
| GO:0030261\_chromosome\_condensation | 16 | 0 | 0.000000 | -0.000000 | 667 | 389.100313 | 461.13 | 533.159687 | 0.691349 |
| GO:0030855\_epithelial\_cell\_differentiation | 16 | 0 | 0.000000 | -0.000000 | 667 | 389.100313 | 461.13 | 533.159687 | 0.691349 |
| GO:0031110\_regulation\_of\_microtubule\_polymerization\_or\_depolymerization | 16 | 0 | 0.000000 | -0.000000 | 667 | 389.100313 | 461.13 | 533.159687 | 0.691349 |
| GO:0032640\_tumor\_necrosis\_factor\_production | 16 | 0 | 0.000000 | -0.000000 | 667 | 389.100313 | 461.13 | 533.159687 | 0.691349 |
| GO:0032649\_regulation\_of\_interferon-gamma\_production | 16 | 0 | 0.000000 | -0.000000 | 667 | 389.100313 | 461.13 | 533.159687 | 0.691349 |
| GO:0032675\_regulation\_of\_interleukin-6\_production | 16 | 0 | 0.000000 | -0.000000 | 667 | 389.100313 | 461.13 | 533.159687 | 0.691349 |
| GO:0032680\_regulation\_of\_tumor\_necrosis\_factor\_production | 16 | 0 | 0.000000 | -0.000000 | 667 | 389.100313 | 461.13 | 533.159687 | 0.691349 |
| GO:0034381\_lipoprotein\_particle\_clearance | 16 | 0 | 0.000000 | -0.000000 | 667 | 389.100313 | 461.13 | 533.159687 | 0.691349 |
| GO:0034762\_regulation\_of\_transmembrane\_transport | 16 | 0 | 0.000000 | -0.000000 | 667 | 389.100313 | 461.13 | 533.159687 | 0.691349 |
| GO:0042476\_odontogenesis | 16 | 0 | 0.000000 | -0.000000 | 667 | 389.100313 | 461.13 | 533.159687 | 0.691349 |
| GO:0045667\_regulation\_of\_osteoblast\_differentiation | 16 | 0 | 0.000000 | -0.000000 | 667 | 389.100313 | 461.13 | 533.159687 | 0.691349 |
| GO:0045807\_positive\_regulation\_of\_endocytosis | 16 | 0 | 0.000000 | -0.000000 | 667 | 389.100313 | 461.13 | 533.159687 | 0.691349 |
| GO:0045862\_positive\_regulation\_of\_proteolysis | 16 | 0 | 0.000000 | -0.000000 | 667 | 389.100313 | 461.13 | 533.159687 | 0.691349 |
| GO:0045930\_negative\_regulation\_of\_mitotic\_cell\_cycle | 16 | 0 | 0.000000 | -0.000000 | 667 | 389.100313 | 461.13 | 533.159687 | 0.691349 |
| GO:0046545\_development\_of\_primary\_female\_sexual\_characteristics | 16 | 0 | 0.000000 | -0.000000 | 667 | 389.100313 | 461.13 | 533.159687 | 0.691349 |
| GO:0046660\_female\_sex\_differentiation | 16 | 0 | 0.000000 | -0.000000 | 667 | 389.100313 | 461.13 | 533.159687 | 0.691349 |
| GO:0050851\_antigen\_receptor-mediated\_signaling\_pathway | 16 | 0 | 0.000000 | -0.000000 | 667 | 389.100313 | 461.13 | 533.159687 | 0.691349 |
| GO:0050864\_regulation\_of\_B\_cell\_activation | 16 | 0 | 0.000000 | -0.000000 | 667 | 389.100313 | 461.13 | 533.159687 | 0.691349 |
| GO:0051146\_striated\_muscle\_cell\_differentiation | 16 | 0 | 0.000000 | -0.000000 | 667 | 389.100313 | 461.13 | 533.159687 | 0.691349 |
| GO:0051181\_cofactor\_transport | 16 | 0 | 0.000000 | -0.000000 | 667 | 389.100313 | 461.13 | 533.159687 | 0.691349 |
| GO:0006289\_nucleotide-excision\_repair | 45 | 0 | 0.000000 | -0.000000 | 677 | 398.246677 | 470.04 | 541.833323 | 0.694298 |
| GO:0006308\_DNA\_catabolic\_process | 45 | 0 | 0.000000 | -0.000000 | 677 | 398.246677 | 470.04 | 541.833323 | 0.694298 |
| GO:0006368\_RNA\_elongation\_from\_RNA\_polymerase\_II\_promoter | 45 | 0 | 0.000000 | -0.000000 | 677 | 398.246677 | 470.04 | 541.833323 | 0.694298 |
| GO:0006576\_biogenic\_amine\_metabolic\_process | 45 | 0 | 0.000000 | -0.000000 | 677 | 398.246677 | 470.04 | 541.833323 | 0.694298 |
| GO:0006919\_activation\_of\_caspase\_activity | 45 | 0 | 0.000000 | -0.000000 | 677 | 398.246677 | 470.04 | 541.833323 | 0.694298 |
| GO:0016197\_endosome\_transport | 45 | 0 | 0.000000 | -0.000000 | 677 | 398.246677 | 470.04 | 541.833323 | 0.694298 |
| GO:0016573\_histone\_acetylation | 45 | 0 | 0.000000 | -0.000000 | 677 | 398.246677 | 470.04 | 541.833323 | 0.694298 |
| GO:0034728\_nucleosome\_organization | 45 | 0 | 0.000000 | -0.000000 | 677 | 398.246677 | 470.04 | 541.833323 | 0.694298 |
| GO:0048771\_tissue\_remodeling | 45 | 0 | 0.000000 | -0.000000 | 677 | 398.246677 | 470.04 | 541.833323 | 0.694298 |
| GO:0051168\_nuclear\_export | 45 | 0 | 0.000000 | -0.000000 | 677 | 398.246677 | 470.04 | 541.833323 | 0.694298 |
| GO:0006364\_rRNA\_processing | 39 | 0 | 0.000000 | -0.000000 | 685 | 409.151422 | 480.97 | 552.788578 | 0.702146 |
| GO:0006497\_protein\_amino\_acid\_lipidation | 39 | 0 | 0.000000 | -0.000000 | 685 | 409.151422 | 480.97 | 552.788578 | 0.702146 |
| GO:0009310\_amine\_catabolic\_process | 39 | 0 | 0.000000 | -0.000000 | 685 | 409.151422 | 480.97 | 552.788578 | 0.702146 |
| GO:0009792\_embryonic\_development\_ending\_in\_birth\_or\_egg\_hatching | 39 | 0 | 0.000000 | -0.000000 | 685 | 409.151422 | 480.97 | 552.788578 | 0.702146 |
| GO:0030509\_BMP\_signaling\_pathway | 39 | 0 | 0.000000 | -0.000000 | 685 | 409.151422 | 480.97 | 552.788578 | 0.702146 |
| GO:0042158\_lipoprotein\_biosynthetic\_process | 39 | 0 | 0.000000 | -0.000000 | 685 | 409.151422 | 480.97 | 552.788578 | 0.702146 |
| GO:0043009\_chordate\_embryonic\_development | 39 | 0 | 0.000000 | -0.000000 | 685 | 409.151422 | 480.97 | 552.788578 | 0.702146 |
| GO:0051960\_regulation\_of\_nervous\_system\_development | 39 | 0 | 0.000000 | -0.000000 | 685 | 409.151422 | 480.97 | 552.788578 | 0.702146 |
| GO:0000387\_spliceosomal\_snRNP\_biogenesis | 28 | 0 | 0.000000 | -0.000000 | 713 | 436.276202 | 507.23 | 578.183798 | 0.711403 |
| GO:0000819\_sister\_chromatid\_segregation | 28 | 0 | 0.000000 | -0.000000 | 713 | 436.276202 | 507.23 | 578.183798 | 0.711403 |
| GO:0002440\_production\_of\_molecular\_mediator\_of\_immune\_response | 28 | 0 | 0.000000 | -0.000000 | 713 | 436.276202 | 507.23 | 578.183798 | 0.711403 |
| GO:0006304\_DNA\_modification | 28 | 0 | 0.000000 | -0.000000 | 713 | 436.276202 | 507.23 | 578.183798 | 0.711403 |
| GO:0006405\_RNA\_export\_from\_nucleus | 28 | 0 | 0.000000 | -0.000000 | 713 | 436.276202 | 507.23 | 578.183798 | 0.711403 |
| GO:0006662\_glycerol\_ether\_metabolic\_process | 28 | 0 | 0.000000 | -0.000000 | 713 | 436.276202 | 507.23 | 578.183798 | 0.711403 |
| GO:0006939\_smooth\_muscle\_contraction | 28 | 0 | 0.000000 | -0.000000 | 713 | 436.276202 | 507.23 | 578.183798 | 0.711403 |
| GO:0007156\_homophilic\_cell\_adhesion | 28 | 0 | 0.000000 | -0.000000 | 713 | 436.276202 | 507.23 | 578.183798 | 0.711403 |
| GO:0007281\_germ\_cell\_development | 28 | 0 | 0.000000 | -0.000000 | 713 | 436.276202 | 507.23 | 578.183798 | 0.711403 |
| GO:0007411\_axon\_guidance | 28 | 0 | 0.000000 | -0.000000 | 713 | 436.276202 | 507.23 | 578.183798 | 0.711403 |
| GO:0009062\_fatty\_acid\_catabolic\_process | 28 | 0 | 0.000000 | -0.000000 | 713 | 436.276202 | 507.23 | 578.183798 | 0.711403 |
| GO:0009593\_detection\_of\_chemical\_stimulus | 28 | 0 | 0.000000 | -0.000000 | 713 | 436.276202 | 507.23 | 578.183798 | 0.711403 |
| GO:0009895\_negative\_regulation\_of\_catabolic\_process | 28 | 0 | 0.000000 | -0.000000 | 713 | 436.276202 | 507.23 | 578.183798 | 0.711403 |
| GO:0010563\_negative\_regulation\_of\_phosphorus\_metabolic\_process | 28 | 0 | 0.000000 | -0.000000 | 713 | 436.276202 | 507.23 | 578.183798 | 0.711403 |
| GO:0010565\_regulation\_of\_cellular\_ketone\_metabolic\_process | 28 | 0 | 0.000000 | -0.000000 | 713 | 436.276202 | 507.23 | 578.183798 | 0.711403 |
| GO:0016055\_Wnt\_receptor\_signaling\_pathway | 28 | 0 | 0.000000 | -0.000000 | 713 | 436.276202 | 507.23 | 578.183798 | 0.711403 |
| GO:0018904\_organic\_ether\_metabolic\_process | 28 | 0 | 0.000000 | -0.000000 | 713 | 436.276202 | 507.23 | 578.183798 | 0.711403 |
| GO:0031214\_biomineral\_formation | 28 | 0 | 0.000000 | -0.000000 | 713 | 436.276202 | 507.23 | 578.183798 | 0.711403 |
| GO:0033044\_regulation\_of\_chromosome\_organization | 28 | 0 | 0.000000 | -0.000000 | 713 | 436.276202 | 507.23 | 578.183798 | 0.711403 |
| GO:0042306\_regulation\_of\_protein\_import\_into\_nucleus | 28 | 0 | 0.000000 | -0.000000 | 713 | 436.276202 | 507.23 | 578.183798 | 0.711403 |
| GO:0045664\_regulation\_of\_neuron\_differentiation | 28 | 0 | 0.000000 | -0.000000 | 713 | 436.276202 | 507.23 | 578.183798 | 0.711403 |
| GO:0045834\_positive\_regulation\_of\_lipid\_metabolic\_process | 28 | 0 | 0.000000 | -0.000000 | 713 | 436.276202 | 507.23 | 578.183798 | 0.711403 |
| GO:0045936\_negative\_regulation\_of\_phosphate\_metabolic\_process | 28 | 0 | 0.000000 | -0.000000 | 713 | 436.276202 | 507.23 | 578.183798 | 0.711403 |
| GO:0051349\_positive\_regulation\_of\_lyase\_activity | 28 | 0 | 0.000000 | -0.000000 | 713 | 436.276202 | 507.23 | 578.183798 | 0.711403 |
| GO:0051650\_establishment\_of\_vesicle\_localization | 28 | 0 | 0.000000 | -0.000000 | 713 | 436.276202 | 507.23 | 578.183798 | 0.711403 |
| GO:0060402\_calcium\_ion\_transport\_into\_cytosol | 28 | 0 | 0.000000 | -0.000000 | 713 | 436.276202 | 507.23 | 578.183798 | 0.711403 |
| GO:0070662\_mast\_cell\_proliferation | 28 | 0 | 0.000000 | -0.000000 | 713 | 436.276202 | 507.23 | 578.183798 | 0.711403 |
| GO:0070666\_regulation\_of\_mast\_cell\_proliferation | 28 | 0 | 0.000000 | -0.000000 | 713 | 436.276202 | 507.23 | 578.183798 | 0.711403 |
| GO:0006665\_sphingolipid\_metabolic\_process | 44 | 0 | 0.000000 | -0.000000 | 720 | 444.263812 | 514.89 | 585.516188 | 0.715125 |
| GO:0006730\_one-carbon\_metabolic\_process | 44 | 0 | 0.000000 | -0.000000 | 720 | 444.263812 | 514.89 | 585.516188 | 0.715125 |
| GO:0006959\_humoral\_immune\_response | 44 | 0 | 0.000000 | -0.000000 | 720 | 444.263812 | 514.89 | 585.516188 | 0.715125 |
| GO:0016125\_sterol\_metabolic\_process | 44 | 0 | 0.000000 | -0.000000 | 720 | 444.263812 | 514.89 | 585.516188 | 0.715125 |
| GO:0050900\_leukocyte\_migration | 44 | 0 | 0.000000 | -0.000000 | 720 | 444.263812 | 514.89 | 585.516188 | 0.715125 |
| GO:0051301\_cell\_division | 44 | 0 | 0.000000 | -0.000000 | 720 | 444.263812 | 514.89 | 585.516188 | 0.715125 |
| GO:0051321\_meiotic\_cell\_cycle | 44 | 0 | 0.000000 | -0.000000 | 720 | 444.263812 | 514.89 | 585.516188 | 0.715125 |
| GO:0006605\_protein\_targeting | 145 | 0 | 0.000000 | -0.000000 | 722 | 447.355828 | 517.74 | 588.124172 | 0.717091 |
| GO:0007417\_central\_nervous\_system\_development | 145 | 0 | 0.000000 | -0.000000 | 722 | 447.355828 | 517.74 | 588.124172 | 0.717091 |
| GO:0006403\_RNA\_localization | 33 | 0 | 0.000000 | -0.000000 | 731 | 457.955948 | 528.03 | 598.104052 | 0.722339 |
| GO:0009259\_ribonucleotide\_metabolic\_process | 33 | 0 | 0.000000 | -0.000000 | 731 | 457.955948 | 528.03 | 598.104052 | 0.722339 |
| GO:0009581\_detection\_of\_external\_stimulus | 33 | 0 | 0.000000 | -0.000000 | 731 | 457.955948 | 528.03 | 598.104052 | 0.722339 |
| GO:0016458\_gene\_silencing | 33 | 0 | 0.000000 | -0.000000 | 731 | 457.955948 | 528.03 | 598.104052 | 0.722339 |
| GO:0034329\_cell\_junction\_assembly | 33 | 0 | 0.000000 | -0.000000 | 731 | 457.955948 | 528.03 | 598.104052 | 0.722339 |
| GO:0043392\_negative\_regulation\_of\_DNA\_binding | 33 | 0 | 0.000000 | -0.000000 | 731 | 457.955948 | 528.03 | 598.104052 | 0.722339 |
| GO:0050870\_positive\_regulation\_of\_T\_cell\_activation | 33 | 0 | 0.000000 | -0.000000 | 731 | 457.955948 | 528.03 | 598.104052 | 0.722339 |
| GO:0051341\_regulation\_of\_oxidoreductase\_activity | 33 | 0 | 0.000000 | -0.000000 | 731 | 457.955948 | 528.03 | 598.104052 | 0.722339 |
| GO:0055088\_lipid\_homeostasis | 33 | 0 | 0.000000 | -0.000000 | 731 | 457.955948 | 528.03 | 598.104052 | 0.722339 |
| GO:0006417\_regulation\_of\_translation | 71 | 0 | 0.000000 | -0.000000 | 735 | 464.114462 | 533.72 | 603.325538 | 0.726150 |
| GO:0007050\_cell\_cycle\_arrest | 71 | 0 | 0.000000 | -0.000000 | 735 | 464.114462 | 533.72 | 603.325538 | 0.726150 |
| GO:0051223\_regulation\_of\_protein\_transport | 71 | 0 | 0.000000 | -0.000000 | 735 | 464.114462 | 533.72 | 603.325538 | 0.726150 |
| GO:0051606\_detection\_of\_stimulus | 71 | 0 | 0.000000 | -0.000000 | 735 | 464.114462 | 533.72 | 603.325538 | 0.726150 |
| GO:0000070\_mitotic\_sister\_chromatid\_segregation | 27 | 0 | 0.000000 | -0.000000 | 757 | 486.277853 | 555.34 | 624.402147 | 0.733606 |
| GO:0000245\_spliceosome\_assembly | 27 | 0 | 0.000000 | -0.000000 | 757 | 486.277853 | 555.34 | 624.402147 | 0.733606 |
| GO:0003002\_regionalization | 27 | 0 | 0.000000 | -0.000000 | 757 | 486.277853 | 555.34 | 624.402147 | 0.733606 |
| GO:0006638\_neutral\_lipid\_metabolic\_process | 27 | 0 | 0.000000 | -0.000000 | 757 | 486.277853 | 555.34 | 624.402147 | 0.733606 |
| GO:0006639\_acylglycerol\_metabolic\_process | 27 | 0 | 0.000000 | -0.000000 | 757 | 486.277853 | 555.34 | 624.402147 | 0.733606 |
| GO:0006690\_icosanoid\_metabolic\_process | 27 | 0 | 0.000000 | -0.000000 | 757 | 486.277853 | 555.34 | 624.402147 | 0.733606 |
| GO:0006836\_neurotransmitter\_transport | 27 | 0 | 0.000000 | -0.000000 | 757 | 486.277853 | 555.34 | 624.402147 | 0.733606 |
| GO:0006909\_phagocytosis | 27 | 0 | 0.000000 | -0.000000 | 757 | 486.277853 | 555.34 | 624.402147 | 0.733606 |
| GO:0007631\_feeding\_behavior | 27 | 0 | 0.000000 | -0.000000 | 757 | 486.277853 | 555.34 | 624.402147 | 0.733606 |
| GO:0008543\_fibroblast\_growth\_factor\_receptor\_signaling\_pathway | 27 | 0 | 0.000000 | -0.000000 | 757 | 486.277853 | 555.34 | 624.402147 | 0.733606 |
| GO:0019079\_viral\_genome\_replication | 27 | 0 | 0.000000 | -0.000000 | 757 | 486.277853 | 555.34 | 624.402147 | 0.733606 |
| GO:0031349\_positive\_regulation\_of\_defense\_response | 27 | 0 | 0.000000 | -0.000000 | 757 | 486.277853 | 555.34 | 624.402147 | 0.733606 |
| GO:0031669\_cellular\_response\_to\_nutrient\_levels | 27 | 0 | 0.000000 | -0.000000 | 757 | 486.277853 | 555.34 | 624.402147 | 0.733606 |
| GO:0032200\_telomere\_organization | 27 | 0 | 0.000000 | -0.000000 | 757 | 486.277853 | 555.34 | 624.402147 | 0.733606 |
| GO:0035150\_regulation\_of\_tube\_size | 27 | 0 | 0.000000 | -0.000000 | 757 | 486.277853 | 555.34 | 624.402147 | 0.733606 |
| GO:0043254\_regulation\_of\_protein\_complex\_assembly | 27 | 0 | 0.000000 | -0.000000 | 757 | 486.277853 | 555.34 | 624.402147 | 0.733606 |
| GO:0044272\_sulfur\_compound\_biosynthetic\_process | 27 | 0 | 0.000000 | -0.000000 | 757 | 486.277853 | 555.34 | 624.402147 | 0.733606 |
| GO:0050880\_regulation\_of\_blood\_vessel\_size | 27 | 0 | 0.000000 | -0.000000 | 757 | 486.277853 | 555.34 | 624.402147 | 0.733606 |
| GO:0050906\_detection\_of\_stimulus\_involved\_in\_sensory\_perception | 27 | 0 | 0.000000 | -0.000000 | 757 | 486.277853 | 555.34 | 624.402147 | 0.733606 |
| GO:0051048\_negative\_regulation\_of\_secretion | 27 | 0 | 0.000000 | -0.000000 | 757 | 486.277853 | 555.34 | 624.402147 | 0.733606 |
| GO:0051092\_positive\_regulation\_of\_NF-kappaB\_transcription\_factor\_activity | 27 | 0 | 0.000000 | -0.000000 | 757 | 486.277853 | 555.34 | 624.402147 | 0.733606 |
| GO:0051607\_defense\_response\_to\_virus | 27 | 0 | 0.000000 | -0.000000 | 757 | 486.277853 | 555.34 | 624.402147 | 0.733606 |
| GO:0007399\_nervous\_system\_development | 459 | 0 | 0.000000 | 0.000000 | 761 | 513.035917 | 575.22 | 637.404083 | 0.755874 |
| GO:0008150\_biological\_process | 8160 | 19 | 1.000000 | 0.000000 | 761 | 513.035917 | 575.22 | 637.404083 | 0.755874 |
| GO:0046907\_intracellular\_transport | 420 | 0 | 0.000000 | 0.000000 | 761 | 513.035917 | 575.22 | 637.404083 | 0.755874 |
| GO:0050877\_neurological\_system\_process | 468 | 0 | 0.000000 | 0.000000 | 761 | 513.035917 | 575.22 | 637.404083 | 0.755874 |
| GO:0030001\_metal\_ion\_transport | 186 | 0 | 0.000000 | 0.000000 | 763 | 514.376869 | 576.43 | 638.483131 | 0.755478 |
| GO:0034622\_cellular\_macromolecular\_complex\_assembly | 186 | 0 | 0.000000 | 0.000000 | 763 | 514.376869 | 576.43 | 638.483131 | 0.755478 |
| GO:0006916\_anti-apoptosis | 155 | 0 | 0.000000 | 0.000000 | 764 | 516.128846 | 577.91 | 639.691154 | 0.756427 |
| GO:0019226\_transmission\_of\_nerve\_impulse | 209 | 0 | 0.000000 | 0.000000 | 765 | 519.136002 | 580.49 | 641.843998 | 0.758810 |
| GO:0002526\_acute\_inflammatory\_response | 31 | 0 | 0.000000 | 0.000000 | 779 | 533.616092 | 594.57 | 655.523908 | 0.763248 |
| GO:0006633\_fatty\_acid\_biosynthetic\_process | 31 | 0 | 0.000000 | 0.000000 | 779 | 533.616092 | 594.57 | 655.523908 | 0.763248 |
| GO:0006888\_ER\_to\_Golgi\_vesicle-mediated\_transport | 31 | 0 | 0.000000 | 0.000000 | 779 | 533.616092 | 594.57 | 655.523908 | 0.763248 |
| GO:0007173\_epidermal\_growth\_factor\_receptor\_signaling\_pathway | 31 | 0 | 0.000000 | 0.000000 | 779 | 533.616092 | 594.57 | 655.523908 | 0.763248 |
| GO:0007423\_sensory\_organ\_development | 31 | 0 | 0.000000 | 0.000000 | 779 | 533.616092 | 594.57 | 655.523908 | 0.763248 |
| GO:0008629\_induction\_of\_apoptosis\_by\_intracellular\_signals | 31 | 0 | 0.000000 | 0.000000 | 779 | 533.616092 | 594.57 | 655.523908 | 0.763248 |
| GO:0031668\_cellular\_response\_to\_extracellular\_stimulus | 31 | 0 | 0.000000 | 0.000000 | 779 | 533.616092 | 594.57 | 655.523908 | 0.763248 |
| GO:0032944\_regulation\_of\_mononuclear\_cell\_proliferation | 31 | 0 | 0.000000 | 0.000000 | 779 | 533.616092 | 594.57 | 655.523908 | 0.763248 |
| GO:0042742\_defense\_response\_to\_bacterium | 31 | 0 | 0.000000 | 0.000000 | 779 | 533.616092 | 594.57 | 655.523908 | 0.763248 |
| GO:0044403\_symbiosis\_\_encompassing\_mutualism\_through\_parasitism | 31 | 0 | 0.000000 | 0.000000 | 779 | 533.616092 | 594.57 | 655.523908 | 0.763248 |
| GO:0044419\_interspecies\_interaction\_between\_organisms | 31 | 0 | 0.000000 | 0.000000 | 779 | 533.616092 | 594.57 | 655.523908 | 0.763248 |
| GO:0045185\_maintenance\_of\_protein\_location | 31 | 0 | 0.000000 | 0.000000 | 779 | 533.616092 | 594.57 | 655.523908 | 0.763248 |
| GO:0046883\_regulation\_of\_hormone\_secretion | 31 | 0 | 0.000000 | 0.000000 | 779 | 533.616092 | 594.57 | 655.523908 | 0.763248 |
| GO:0070663\_regulation\_of\_leukocyte\_proliferation | 31 | 0 | 0.000000 | 0.000000 | 779 | 533.616092 | 594.57 | 655.523908 | 0.763248 |
| GO:0001508\_regulation\_of\_action\_potential | 24 | 0 | 0.000000 | 0.000000 | 803 | 562.539524 | 622.7 | 682.860476 | 0.775467 |
| GO:0001649\_osteoblast\_differentiation | 24 | 0 | 0.000000 | 0.000000 | 803 | 562.539524 | 622.7 | 682.860476 | 0.775467 |
| GO:0006479\_protein\_amino\_acid\_methylation | 24 | 0 | 0.000000 | 0.000000 | 803 | 562.539524 | 622.7 | 682.860476 | 0.775467 |
| GO:0007163\_establishment\_or\_maintenance\_of\_cell\_polarity | 24 | 0 | 0.000000 | 0.000000 | 803 | 562.539524 | 622.7 | 682.860476 | 0.775467 |
| GO:0007568\_aging | 24 | 0 | 0.000000 | 0.000000 | 803 | 562.539524 | 622.7 | 682.860476 | 0.775467 |
| GO:0008213\_protein\_amino\_acid\_alkylation | 24 | 0 | 0.000000 | 0.000000 | 803 | 562.539524 | 622.7 | 682.860476 | 0.775467 |
| GO:0009116\_nucleoside\_metabolic\_process | 24 | 0 | 0.000000 | 0.000000 | 803 | 562.539524 | 622.7 | 682.860476 | 0.775467 |
| GO:0009266\_response\_to\_temperature\_stimulus | 24 | 0 | 0.000000 | 0.000000 | 803 | 562.539524 | 622.7 | 682.860476 | 0.775467 |
| GO:0017148\_negative\_regulation\_of\_translation | 24 | 0 | 0.000000 | 0.000000 | 803 | 562.539524 | 622.7 | 682.860476 | 0.775467 |
| GO:0030073\_insulin\_secretion | 24 | 0 | 0.000000 | 0.000000 | 803 | 562.539524 | 622.7 | 682.860476 | 0.775467 |
| GO:0032946\_positive\_regulation\_of\_mononuclear\_cell\_proliferation | 24 | 0 | 0.000000 | 0.000000 | 803 | 562.539524 | 622.7 | 682.860476 | 0.775467 |
| GO:0035023\_regulation\_of\_Rho\_protein\_signal\_transduction | 24 | 0 | 0.000000 | 0.000000 | 803 | 562.539524 | 622.7 | 682.860476 | 0.775467 |
| GO:0042440\_pigment\_metabolic\_process | 24 | 0 | 0.000000 | 0.000000 | 803 | 562.539524 | 622.7 | 682.860476 | 0.775467 |
| GO:0043407\_negative\_regulation\_of\_MAP\_kinase\_activity | 24 | 0 | 0.000000 | 0.000000 | 803 | 562.539524 | 622.7 | 682.860476 | 0.775467 |
| GO:0043506\_regulation\_of\_JUN\_kinase\_activity | 24 | 0 | 0.000000 | 0.000000 | 803 | 562.539524 | 622.7 | 682.860476 | 0.775467 |
| GO:0045216\_cell-cell\_junction\_organization | 24 | 0 | 0.000000 | 0.000000 | 803 | 562.539524 | 622.7 | 682.860476 | 0.775467 |
| GO:0045638\_negative\_regulation\_of\_myeloid\_cell\_differentiation | 24 | 0 | 0.000000 | 0.000000 | 803 | 562.539524 | 622.7 | 682.860476 | 0.775467 |
| GO:0045785\_positive\_regulation\_of\_cell\_adhesion | 24 | 0 | 0.000000 | 0.000000 | 803 | 562.539524 | 622.7 | 682.860476 | 0.775467 |
| GO:0046890\_regulation\_of\_lipid\_biosynthetic\_process | 24 | 0 | 0.000000 | 0.000000 | 803 | 562.539524 | 622.7 | 682.860476 | 0.775467 |
| GO:0051701\_interaction\_with\_host | 24 | 0 | 0.000000 | 0.000000 | 803 | 562.539524 | 622.7 | 682.860476 | 0.775467 |
| GO:0051969\_regulation\_of\_transmission\_of\_nerve\_impulse | 24 | 0 | 0.000000 | 0.000000 | 803 | 562.539524 | 622.7 | 682.860476 | 0.775467 |
| GO:0055067\_monovalent\_inorganic\_cation\_homeostasis | 24 | 0 | 0.000000 | 0.000000 | 803 | 562.539524 | 622.7 | 682.860476 | 0.775467 |
| GO:0070646\_protein\_modification\_by\_small\_protein\_removal | 24 | 0 | 0.000000 | 0.000000 | 803 | 562.539524 | 622.7 | 682.860476 | 0.775467 |
| GO:0070665\_positive\_regulation\_of\_leukocyte\_proliferation | 24 | 0 | 0.000000 | 0.000000 | 803 | 562.539524 | 622.7 | 682.860476 | 0.775467 |
| GO:0000226\_microtubule\_cytoskeleton\_organization | 86 | 0 | 0.000000 | 0.000000 | 804 | 564.633782 | 624.51 | 684.386218 | 0.776754 |
| GO:0000028\_ribosomal\_small\_subunit\_assembly | 1 | 0 |  |  |  |  |  |  |  |  |
| GO:0000042\_protein\_targeting\_to\_Golgi | 1 | 0 |  |  |  |  |  |  |  |  |
| GO:0000046\_autophagic\_vacuole\_fusion | 1 | 0 |  |  |  |  |  |  |  |  |
| GO:0000052\_citrulline\_metabolic\_process | 1 | 0 |  |  |  |  |  |  |  |  |
| GO:0000054\_ribosome\_export\_from\_nucleus | 1 | 0 |  |  |  |  |  |  |  |  |
| GO:0000056\_ribosomal\_small\_subunit\_export\_from\_nucleus | 1 | 0 |  |  |  |  |  |  |  |  |
| GO:0000072\_M\_phase\_specific\_microtubule\_process | 1 | 0 |  |  |  |  |  |  |  |  |
| GO:0000093\_mitotic\_telophase | 1 | 0 |  |  |  |  |  |  |  |  |
| GO:0000098\_sulfur\_amino\_acid\_catabolic\_process | 1 | 0 |  |  |  |  |  |  |  |  |
| GO:0000114\_regulation\_of\_transcription\_during\_G1\_phase\_of\_mitotic\_cell\_cycle | 1 | 0 |  |  |  |  |  |  |  |  |
| GO:0000115\_regulation\_of\_transcription\_during\_S-phase\_of\_mitotic\_cell\_cycle | 1 | 0 |  |  |  |  |  |  |  |  |
| GO:0000117\_regulation\_of\_transcription\_during\_G2\_M-phase\_of\_mitotic\_cell\_cycle | 1 | 0 |  |  |  |  |  |  |  |  |
| GO:0000132\_establishment\_of\_mitotic\_spindle\_orientation | 1 | 0 |  |  |  |  |  |  |  |  |
| GO:0000154\_rRNA\_modification | 1 | 0 |  |  |  |  |  |  |  |  |
| GO:0000160\_two-component\_signal\_transduction\_system\_(phosphorelay) | 1 | 0 |  |  |  |  |  |  |  |  |
| GO:0000161\_MAPKKK\_cascade\_involved\_in\_osmosensory\_signaling\_pathway | 1 | 0 |  |  |  |  |  |  |  |  |
| GO:0000173\_inactivation\_of\_MAPK\_activity\_involved\_in\_osmosensory\_signaling\_pathway | 1 | 0 |  |  |  |  |  |  |  |  |
| GO:0000212\_meiotic\_spindle\_organization | 1 | 0 |  |  |  |  |  |  |  |  |
| GO:0000255\_allantoin\_metabolic\_process | 1 | 0 |  |  |  |  |  |  |  |  |
| GO:0000270\_peptidoglycan\_metabolic\_process | 1 | 0 |  |  |  |  |  |  |  |  |
| GO:0000296\_spermine\_transport | 1 | 0 |  |  |  |  |  |  |  |  |
| GO:0000301\_retrograde\_transport\_\_vesicle\_recycling\_within\_Golgi | 1 | 0 |  |  |  |  |  |  |  |  |
| GO:0000303\_response\_to\_superoxide | 1 | 0 |  |  |  |  |  |  |  |  |
| GO:0000320\_re-entry\_into\_mitotic\_cell\_cycle | 1 | 0 |  |  |  |  |  |  |  |  |
| GO:0000338\_protein\_deneddylation | 1 | 0 |  |  |  |  |  |  |  |  |
| GO:0000395\_nuclear\_mRNA\_5'-splice\_site\_recognition | 1 | 0 |  |  |  |  |  |  |  |  |
| GO:0000710\_meiotic\_mismatch\_repair | 1 | 0 |  |  |  |  |  |  |  |  |
| GO:0000717\_nucleotide-excision\_repair\_\_DNA\_duplex\_unwinding | 1 | 0 |  |  |  |  |  |  |  |  |
| GO:0000722\_telomere\_maintenance\_via\_recombination | 1 | 0 |  |  |  |  |  |  |  |  |
| GO:0000912\_formation\_of\_actomyosin\_apparatus\_involved\_in\_cytokinesis | 1 | 0 |  |  |  |  |  |  |  |  |
| GO:0000915\_cytokinesis\_\_contractile\_ring\_formation | 1 | 0 |  |  |  |  |  |  |  |  |
| GO:0000921\_septin\_ring\_assembly | 1 | 0 |  |  |  |  |  |  |  |  |
| GO:0000966\_RNA\_5'-end\_processing | 1 | 0 |  |  |  |  |  |  |  |  |
| GO:0001315\_age-dependent\_response\_to\_reactive\_oxygen\_species | 1 | 0 |  |  |  |  |  |  |  |  |
| GO:0001519\_peptide\_amidation | 1 | 0 |  |  |  |  |  |  |  |  |
| GO:0001560\_regulation\_of\_cell\_growth\_by\_extracellular\_stimulus | 1 | 0 |  |  |  |  |  |  |  |  |
| GO:0001574\_ganglioside\_biosynthetic\_process | 1 | 0 |  |  |  |  |  |  |  |  |
| GO:0001575\_globoside\_metabolic\_process | 1 | 0 |  |  |  |  |  |  |  |  |
| GO:0001658\_branching\_involved\_in\_ureteric\_bud\_morphogenesis | 1 | 0 |  |  |  |  |  |  |  |  |
| GO:0001662\_behavioral\_fear\_response | 1 | 0 |  |  |  |  |  |  |  |  |
| GO:0001675\_acrosome\_assembly | 1 | 0 |  |  |  |  |  |  |  |  |
| GO:0001692\_histamine\_metabolic\_process | 1 | 0 |  |  |  |  |  |  |  |  |
| GO:0001694\_histamine\_biosynthetic\_process | 1 | 0 |  |  |  |  |  |  |  |  |
| GO:0001732\_formation\_of\_translation\_initiation\_complex | 1 | 0 |  |  |  |  |  |  |  |  |
| GO:0001757\_somite\_specification | 1 | 0 |  |  |  |  |  |  |  |  |
| GO:0001774\_microglial\_cell\_activation | 1 | 0 |  |  |  |  |  |  |  |  |
| GO:0001782\_B\_cell\_homeostasis | 1 | 0 |  |  |  |  |  |  |  |  |
| GO:0001787\_natural\_killer\_cell\_proliferation | 1 | 0 |  |  |  |  |  |  |  |  |
| GO:0001823\_mesonephros\_development | 1 | 0 |  |  |  |  |  |  |  |  |
| GO:0001832\_blastocyst\_growth | 1 | 0 |  |  |  |  |  |  |  |  |
| GO:0001833\_inner\_cell\_mass\_cell\_proliferation | 1 | 0 |  |  |  |  |  |  |  |  |
| GO:0001839\_neural\_plate\_morphogenesis | 1 | 0 |  |  |  |  |  |  |  |  |
| GO:0001845\_phagolysosome\_formation | 1 | 0 |  |  |  |  |  |  |  |  |
| GO:0001865\_NK\_T\_cell\_differentiation | 1 | 0 |  |  |  |  |  |  |  |  |
| GO:0001866\_NK\_T\_cell\_proliferation | 1 | 0 |  |  |  |  |  |  |  |  |
| GO:0001887\_selenium\_metabolic\_process | 1 | 0 |  |  |  |  |  |  |  |  |
| GO:0001892\_embryonic\_placenta\_development | 1 | 0 |  |  |  |  |  |  |  |  |
| GO:0001911\_negative\_regulation\_of\_leukocyte\_mediated\_cytotoxicity | 1 | 0 |  |  |  |  |  |  |  |  |
| GO:0001915\_negative\_regulation\_of\_T\_cell\_mediated\_cytotoxicity | 1 | 0 |  |  |  |  |  |  |  |  |
| GO:0001920\_negative\_regulation\_of\_receptor\_recycling | 1 | 0 |  |  |  |  |  |  |  |  |
| GO:0001941\_postsynaptic\_membrane\_organization | 1 | 0 |  |  |  |  |  |  |  |  |
| GO:0001958\_endochondral\_ossification | 1 | 0 |  |  |  |  |  |  |  |  |
| GO:0001973\_adenosine\_receptor\_signaling\_pathway | 1 | 0 |  |  |  |  |  |  |  |  |
| GO:0001977\_renal\_system\_process\_involved\_in\_regulation\_of\_blood\_volume | 1 | 0 |  |  |  |  |  |  |  |  |
| GO:0001980\_regulation\_of\_systemic\_arterial\_blood\_pressure\_by\_ischemic\_conditions | 1 | 0 |  |  |  |  |  |  |  |  |
| GO:0001993\_regulation\_of\_systemic\_arterial\_blood\_pressure\_by\_norepinephrine-epinephrine | 1 | 0 |  |  |  |  |  |  |  |  |
| GO:0001996\_positive\_regulation\_of\_heart\_rate\_by\_epinephrine-norepinephrine | 1 | 0 |  |  |  |  |  |  |  |  |
| GO:0001999\_renal\_response\_to\_blood\_flow\_during\_renin-angiotensin\_regulation\_of\_systemic\_arterial\_blood\_pressure | 1 | 0 |  |  |  |  |  |  |  |  |
| GO:0002001\_renin\_secretion\_into\_blood\_stream | 1 | 0 |  |  |  |  |  |  |  |  |
| GO:0002017\_regulation\_of\_blood\_volume\_by\_renal\_aldosterone | 1 | 0 |  |  |  |  |  |  |  |  |
| GO:0002018\_renin-angiotensin\_regulation\_of\_aldosterone\_production | 1 | 0 |  |  |  |  |  |  |  |  |
| GO:0002031\_G-protein\_coupled\_receptor\_internalization | 1 | 0 |  |  |  |  |  |  |  |  |
| GO:0002035\_brain\_renin-angiotensin\_system | 1 | 0 |  |  |  |  |  |  |  |  |
| GO:0002042\_cell\_migration\_involved\_in\_sprouting\_angiogenesis | 1 | 0 |  |  |  |  |  |  |  |  |
| GO:0002052\_positive\_regulation\_of\_neuroblast\_proliferation | 1 | 0 |  |  |  |  |  |  |  |  |
| GO:0002053\_positive\_regulation\_of\_mesenchymal\_cell\_proliferation | 1 | 0 |  |  |  |  |  |  |  |  |
| GO:0002063\_chondrocyte\_development | 1 | 0 |  |  |  |  |  |  |  |  |
| GO:0002064\_epithelial\_cell\_development | 1 | 0 |  |  |  |  |  |  |  |  |
| GO:0002074\_extraocular\_skeletal\_muscle\_development | 1 | 0 |  |  |  |  |  |  |  |  |
| GO:0002077\_acrosome\_matrix\_dispersal | 1 | 0 |  |  |  |  |  |  |  |  |
| GO:0002082\_regulation\_of\_oxidative\_phosphorylation | 1 | 0 |  |  |  |  |  |  |  |  |
| GO:0002084\_protein\_depalmitoylation | 1 | 0 |  |  |  |  |  |  |  |  |
| GO:0002088\_lens\_development\_in\_camera-type\_eye | 1 | 0 |  |  |  |  |  |  |  |  |
| GO:0002089\_lens\_morphogenesis\_in\_camera-type\_eye | 1 | 0 |  |  |  |  |  |  |  |  |
| GO:0002093\_auditory\_receptor\_cell\_morphogenesis | 1 | 0 |  |  |  |  |  |  |  |  |
| GO:0002209\_behavioral\_defense\_response | 1 | 0 |  |  |  |  |  |  |  |  |
| GO:0002220\_innate\_immune\_response\_activating\_cell\_surface\_receptor\_signaling\_pathway | 1 | 0 |  |  |  |  |  |  |  |  |
| GO:0002223\_stimulatory\_C-type\_lectin\_receptor\_signaling\_pathway | 1 | 0 |  |  |  |  |  |  |  |  |
| GO:0002312\_B\_cell\_activation\_during\_immune\_response | 1 | 0 |  |  |  |  |  |  |  |  |
| GO:0002313\_mature\_B\_cell\_differentiation\_during\_immune\_response | 1 | 0 |  |  |  |  |  |  |  |  |
| GO:0002318\_myeloid\_progenitor\_cell\_differentiation | 1 | 0 |  |  |  |  |  |  |  |  |
| GO:0002320\_lymphoid\_progenitor\_cell\_differentiation | 1 | 0 |  |  |  |  |  |  |  |  |
| GO:0002326\_B\_cell\_lineage\_commitment | 1 | 0 |  |  |  |  |  |  |  |  |
| GO:0002328\_pro-B\_cell\_differentiation | 1 | 0 |  |  |  |  |  |  |  |  |
| GO:0002335\_mature\_B\_cell\_differentiation | 1 | 0 |  |  |  |  |  |  |  |  |
| GO:0002355\_detection\_of\_tumor\_cell | 1 | 0 |  |  |  |  |  |  |  |  |
| GO:0002368\_B\_cell\_cytokine\_production | 1 | 0 |  |  |  |  |  |  |  |  |
| GO:0002424\_T\_cell\_mediated\_immune\_response\_to\_tumor\_cell | 1 | 0 |  |  |  |  |  |  |  |  |
| GO:0002431\_Fc\_receptor\_mediated\_stimulatory\_signaling\_pathway | 1 | 0 |  |  |  |  |  |  |  |  |
| GO:0002437\_inflammatory\_response\_to\_antigenic\_stimulus | 1 | 0 |  |  |  |  |  |  |  |  |
| GO:0002439\_chronic\_inflammatory\_response\_to\_antigenic\_stimulus | 1 | 0 |  |  |  |  |  |  |  |  |
| GO:0002447\_eosinophil\_mediated\_immunity | 1 | 0 |  |  |  |  |  |  |  |  |
| GO:0002455\_humoral\_immune\_response\_mediated\_by\_circulating\_immunoglobulin | 1 | 0 |  |  |  |  |  |  |  |  |
| GO:0002467\_germinal\_center\_formation | 1 | 0 |  |  |  |  |  |  |  |  |
| GO:0002468\_dendritic\_cell\_antigen\_processing\_and\_presentation | 1 | 0 |  |  |  |  |  |  |  |  |
| GO:0002475\_antigen\_processing\_and\_presentation\_via\_MHC\_class\_Ib | 1 | 0 |  |  |  |  |  |  |  |  |
| GO:0002478\_antigen\_processing\_and\_presentation\_of\_exogenous\_peptide\_antigen | 1 | 0 |  |  |  |  |  |  |  |  |
| GO:0002495\_antigen\_processing\_and\_presentation\_of\_peptide\_antigen\_via\_MHC\_class\_II | 1 | 0 |  |  |  |  |  |  |  |  |
| GO:0002513\_tolerance\_induction\_to\_self\_antigen | 1 | 0 |  |  |  |  |  |  |  |  |
| GO:0002514\_B\_cell\_tolerance\_induction | 1 | 0 |  |  |  |  |  |  |  |  |
| GO:0002517\_T\_cell\_tolerance\_induction | 1 | 0 |  |  |  |  |  |  |  |  |
| GO:0002523\_leukocyte\_migration\_during\_inflammatory\_response | 1 | 0 |  |  |  |  |  |  |  |  |
| GO:0002566\_somatic\_diversification\_of\_immune\_receptors\_via\_somatic\_mutation | 1 | 0 |  |  |  |  |  |  |  |  |
| GO:0002568\_somatic\_diversification\_of\_T\_cell\_receptor\_genes | 1 | 0 |  |  |  |  |  |  |  |  |
| GO:0002577\_regulation\_of\_antigen\_processing\_and\_presentation | 1 | 0 |  |  |  |  |  |  |  |  |
| GO:0002578\_negative\_regulation\_of\_antigen\_processing\_and\_presentation | 1 | 0 |  |  |  |  |  |  |  |  |
| GO:0002580\_regulation\_of\_antigen\_processing\_and\_presentation\_of\_peptide\_or\_polysaccharide\_antigen\_via\_MHC\_class\_II | 1 | 0 |  |  |  |  |  |  |  |  |
| GO:0002581\_negative\_regulation\_of\_antigen\_processing\_and\_presentation\_of\_peptide\_or\_polysaccharide\_antigen\_via\_MHC\_class\_II | 1 | 0 |  |  |  |  |  |  |  |  |
| GO:0002604\_regulation\_of\_dendritic\_cell\_antigen\_processing\_and\_presentation | 1 | 0 |  |  |  |  |  |  |  |  |
| GO:0002605\_negative\_regulation\_of\_dendritic\_cell\_antigen\_processing\_and\_presentation | 1 | 0 |  |  |  |  |  |  |  |  |
| GO:0002634\_regulation\_of\_germinal\_center\_formation | 1 | 0 |  |  |  |  |  |  |  |  |
| GO:0002649\_regulation\_of\_tolerance\_induction\_to\_self\_antigen | 1 | 0 |  |  |  |  |  |  |  |  |
| GO:0002651\_positive\_regulation\_of\_tolerance\_induction\_to\_self\_antigen | 1 | 0 |  |  |  |  |  |  |  |  |
| GO:0002661\_regulation\_of\_B\_cell\_tolerance\_induction | 1 | 0 |  |  |  |  |  |  |  |  |
| GO:0002663\_positive\_regulation\_of\_B\_cell\_tolerance\_induction | 1 | 0 |  |  |  |  |  |  |  |  |
| GO:0002664\_regulation\_of\_T\_cell\_tolerance\_induction | 1 | 0 |  |  |  |  |  |  |  |  |
| GO:0002666\_positive\_regulation\_of\_T\_cell\_tolerance\_induction | 1 | 0 |  |  |  |  |  |  |  |  |
| GO:0002674\_negative\_regulation\_of\_acute\_inflammatory\_response | 1 | 0 |  |  |  |  |  |  |  |  |
| GO:0002681\_somatic\_recombination\_of\_T\_cell\_receptor\_gene\_segments | 1 | 0 |  |  |  |  |  |  |  |  |
| GO:0002686\_negative\_regulation\_of\_leukocyte\_migration | 1 | 0 |  |  |  |  |  |  |  |  |
| GO:0002691\_regulation\_of\_cellular\_extravasation | 1 | 0 |  |  |  |  |  |  |  |  |
| GO:0002693\_positive\_regulation\_of\_cellular\_extravasation | 1 | 0 |  |  |  |  |  |  |  |  |
| GO:0002701\_negative\_regulation\_of\_production\_of\_molecular\_mediator\_of\_immune\_response | 1 | 0 |  |  |  |  |  |  |  |  |
| GO:0002719\_negative\_regulation\_of\_cytokine\_production\_during\_immune\_response | 1 | 0 |  |  |  |  |  |  |  |  |
| GO:0002725\_negative\_regulation\_of\_T\_cell\_cytokine\_production | 1 | 0 |  |  |  |  |  |  |  |  |
| GO:0002759\_regulation\_of\_antimicrobial\_humoral\_response | 1 | 0 |  |  |  |  |  |  |  |  |
| GO:0002775\_antimicrobial\_peptide\_production | 1 | 0 |  |  |  |  |  |  |  |  |
| GO:0002777\_antimicrobial\_peptide\_biosynthetic\_process | 1 | 0 |  |  |  |  |  |  |  |  |
| GO:0002778\_antibacterial\_peptide\_production | 1 | 0 |  |  |  |  |  |  |  |  |
| GO:0002780\_antibacterial\_peptide\_biosynthetic\_process | 1 | 0 |  |  |  |  |  |  |  |  |
| GO:0002784\_regulation\_of\_antimicrobial\_peptide\_production | 1 | 0 |  |  |  |  |  |  |  |  |
| GO:0002786\_regulation\_of\_antibacterial\_peptide\_production | 1 | 0 |  |  |  |  |  |  |  |  |
| GO:0002805\_regulation\_of\_antimicrobial\_peptide\_biosynthetic\_process | 1 | 0 |  |  |  |  |  |  |  |  |
| GO:0002807\_positive\_regulation\_of\_antimicrobial\_peptide\_biosynthetic\_process | 1 | 0 |  |  |  |  |  |  |  |  |
| GO:0002808\_regulation\_of\_antibacterial\_peptide\_biosynthetic\_process | 1 | 0 |  |  |  |  |  |  |  |  |
| GO:0002815\_biosynthetic\_process\_of\_antibacterial\_peptides\_active\_against\_Gram-positive\_bacteria | 1 | 0 |  |  |  |  |  |  |  |  |
| GO:0002816\_regulation\_of\_biosynthetic\_process\_of\_antibacterial\_peptides\_active\_against\_Gram-positive\_bacteria | 1 | 0 |  |  |  |  |  |  |  |  |
| GO:0002832\_negative\_regulation\_of\_response\_to\_biotic\_stimulus | 1 | 0 |  |  |  |  |  |  |  |  |
| GO:0002840\_regulation\_of\_T\_cell\_mediated\_immune\_response\_to\_tumor\_cell | 1 | 0 |  |  |  |  |  |  |  |  |
| GO:0002842\_positive\_regulation\_of\_T\_cell\_mediated\_immune\_response\_to\_tumor\_cell | 1 | 0 |  |  |  |  |  |  |  |  |
| GO:0002901\_mature\_B\_cell\_apoptosis | 1 | 0 |  |  |  |  |  |  |  |  |
| GO:0002904\_positive\_regulation\_of\_B\_cell\_apoptosis | 1 | 0 |  |  |  |  |  |  |  |  |
| GO:0002905\_regulation\_of\_mature\_B\_cell\_apoptosis | 1 | 0 |  |  |  |  |  |  |  |  |
| GO:0002906\_negative\_regulation\_of\_mature\_B\_cell\_apoptosis | 1 | 0 |  |  |  |  |  |  |  |  |
| GO:0003010\_voluntary\_skeletal\_muscle\_contraction | 1 | 0 |  |  |  |  |  |  |  |  |
| GO:0003051\_angiotensin-mediated\_drinking\_behavior | 1 | 0 |  |  |  |  |  |  |  |  |
| GO:0003058\_hormonal\_regulation\_of\_the\_force\_of\_heart\_contraction | 1 | 0 |  |  |  |  |  |  |  |  |
| GO:0003062\_regulation\_of\_heart\_rate\_by\_chemical\_signal | 1 | 0 |  |  |  |  |  |  |  |  |
| GO:0003065\_positive\_regulation\_of\_heart\_rate\_by\_epinephrine | 1 | 0 |  |  |  |  |  |  |  |  |
| GO:0003071\_renal\_system\_process\_involved\_in\_regulation\_of\_systemic\_arterial\_blood\_pressure | 1 | 0 |  |  |  |  |  |  |  |  |
| GO:0003085\_negative\_regulation\_of\_systemic\_arterial\_blood\_pressure | 1 | 0 |  |  |  |  |  |  |  |  |
| GO:0003099\_positive\_regulation\_of\_the\_force\_of\_heart\_contraction\_by\_chemical\_signal | 1 | 0 |  |  |  |  |  |  |  |  |
| GO:0003108\_negative\_regulation\_of\_the\_force\_of\_heart\_contraction\_by\_chemical\_signal | 1 | 0 |  |  |  |  |  |  |  |  |
| GO:0005981\_regulation\_of\_glycogen\_catabolic\_process | 1 | 0 |  |  |  |  |  |  |  |  |
| GO:0005982\_starch\_metabolic\_process | 1 | 0 |  |  |  |  |  |  |  |  |
| GO:0005983\_starch\_catabolic\_process | 1 | 0 |  |  |  |  |  |  |  |  |
| GO:0005988\_lactose\_metabolic\_process | 1 | 0 |  |  |  |  |  |  |  |  |
| GO:0005989\_lactose\_biosynthetic\_process | 1 | 0 |  |  |  |  |  |  |  |  |
| GO:0005991\_trehalose\_metabolic\_process | 1 | 0 |  |  |  |  |  |  |  |  |
| GO:0005993\_trehalose\_catabolic\_process | 1 | 0 |  |  |  |  |  |  |  |  |
| GO:0006010\_glucose\_6-phosphate\_utilization | 1 | 0 |  |  |  |  |  |  |  |  |
| GO:0006013\_mannose\_metabolic\_process | 1 | 0 |  |  |  |  |  |  |  |  |
| GO:0006037\_cell\_wall\_chitin\_metabolic\_process | 1 | 0 |  |  |  |  |  |  |  |  |
| GO:0006042\_glucosamine\_biosynthetic\_process | 1 | 0 |  |  |  |  |  |  |  |  |
| GO:0006045\_N-acetylglucosamine\_biosynthetic\_process | 1 | 0 |  |  |  |  |  |  |  |  |
| GO:0006048\_UDP-N-acetylglucosamine\_biosynthetic\_process | 1 | 0 |  |  |  |  |  |  |  |  |
| GO:0006050\_mannosamine\_metabolic\_process | 1 | 0 |  |  |  |  |  |  |  |  |
| GO:0006051\_N-acetylmannosamine\_metabolic\_process | 1 | 0 |  |  |  |  |  |  |  |  |
| GO:0006059\_hexitol\_metabolic\_process | 1 | 0 |  |  |  |  |  |  |  |  |
| GO:0006060\_sorbitol\_metabolic\_process | 1 | 0 |  |  |  |  |  |  |  |  |
| GO:0006062\_sorbitol\_catabolic\_process | 1 | 0 |  |  |  |  |  |  |  |  |
| GO:0006065\_UDP-glucuronate\_biosynthetic\_process | 1 | 0 |  |  |  |  |  |  |  |  |
| GO:0006083\_acetate\_metabolic\_process | 1 | 0 |  |  |  |  |  |  |  |  |
| GO:0006085\_acetyl-CoA\_biosynthetic\_process | 1 | 0 |  |  |  |  |  |  |  |  |
| GO:0006103\_2-oxoglutarate\_metabolic\_process | 1 | 0 |  |  |  |  |  |  |  |  |
| GO:0006106\_fumarate\_metabolic\_process | 1 | 0 |  |  |  |  |  |  |  |  |
| GO:0006107\_oxaloacetate\_metabolic\_process | 1 | 0 |  |  |  |  |  |  |  |  |
| GO:0006116\_NADH\_oxidation | 1 | 0 |  |  |  |  |  |  |  |  |
| GO:0006145\_purine\_base\_catabolic\_process | 1 | 0 |  |  |  |  |  |  |  |  |
| GO:0006148\_inosine\_catabolic\_process | 1 | 0 |  |  |  |  |  |  |  |  |
| GO:0006154\_adenosine\_catabolic\_process | 1 | 0 |  |  |  |  |  |  |  |  |
| GO:0006166\_purine\_ribonucleoside\_salvage | 1 | 0 |  |  |  |  |  |  |  |  |
| GO:0006172\_ADP\_biosynthetic\_process | 1 | 0 |  |  |  |  |  |  |  |  |
| GO:0006173\_dADP\_biosynthetic\_process | 1 | 0 |  |  |  |  |  |  |  |  |
| GO:0006188\_IMP\_biosynthetic\_process | 1 | 0 |  |  |  |  |  |  |  |  |
| GO:0006189\_'de\_novo'\_IMP\_biosynthetic\_process | 1 | 0 |  |  |  |  |  |  |  |  |
| GO:0006196\_AMP\_catabolic\_process | 1 | 0 |  |  |  |  |  |  |  |  |
| GO:0006198\_cAMP\_catabolic\_process | 1 | 0 |  |  |  |  |  |  |  |  |
| GO:0006207\_'de\_novo'\_pyrimidine\_base\_biosynthetic\_process | 1 | 0 |  |  |  |  |  |  |  |  |
| GO:0006214\_thymidine\_catabolic\_process | 1 | 0 |  |  |  |  |  |  |  |  |
| GO:0006216\_cytidine\_catabolic\_process | 1 | 0 |  |  |  |  |  |  |  |  |
| GO:0006222\_UMP\_biosynthetic\_process | 1 | 0 |  |  |  |  |  |  |  |  |
| GO:0006241\_CTP\_biosynthetic\_process | 1 | 0 |  |  |  |  |  |  |  |  |
| GO:0006256\_UDP\_catabolic\_process | 1 | 0 |  |  |  |  |  |  |  |  |
| GO:0006265\_DNA\_topological\_change | 1 | 0 |  |  |  |  |  |  |  |  |
| GO:0006272\_leading\_strand\_elongation | 1 | 0 |  |  |  |  |  |  |  |  |
| GO:0006287\_base-excision\_repair\_\_gap-filling | 1 | 0 |  |  |  |  |  |  |  |  |
| GO:0006313\_transposition\_\_DNA-mediated | 1 | 0 |  |  |  |  |  |  |  |  |
| GO:0006336\_DNA\_replication-independent\_nucleosome\_assembly | 1 | 0 |  |  |  |  |  |  |  |  |
| GO:0006343\_establishment\_of\_chromatin\_silencing | 1 | 0 |  |  |  |  |  |  |  |  |
| GO:0006344\_maintenance\_of\_chromatin\_silencing | 1 | 0 |  |  |  |  |  |  |  |  |
| GO:0006346\_methylation-dependent\_chromatin\_silencing | 1 | 0 |  |  |  |  |  |  |  |  |
| GO:0006348\_chromatin\_silencing\_at\_telomere | 1 | 0 |  |  |  |  |  |  |  |  |
| GO:0006361\_transcription\_initiation\_from\_RNA\_polymerase\_I\_promoter | 1 | 0 |  |  |  |  |  |  |  |  |
| GO:0006369\_termination\_of\_RNA\_polymerase\_II\_transcription | 1 | 0 |  |  |  |  |  |  |  |  |
| GO:0006393\_termination\_of\_mitochondrial\_transcription | 1 | 0 |  |  |  |  |  |  |  |  |
| GO:0006407\_rRNA\_export\_from\_nucleus | 1 | 0 |  |  |  |  |  |  |  |  |
| GO:0006408\_snRNA\_export\_from\_nucleus | 1 | 0 |  |  |  |  |  |  |  |  |
| GO:0006409\_tRNA\_export\_from\_nucleus | 1 | 0 |  |  |  |  |  |  |  |  |
| GO:0006419\_alanyl-tRNA\_aminoacylation | 1 | 0 |  |  |  |  |  |  |  |  |
| GO:0006420\_arginyl-tRNA\_aminoacylation | 1 | 0 |  |  |  |  |  |  |  |  |
| GO:0006423\_cysteinyl-tRNA\_aminoacylation | 1 | 0 |  |  |  |  |  |  |  |  |
| GO:0006431\_methionyl-tRNA\_aminoacylation | 1 | 0 |  |  |  |  |  |  |  |  |
| GO:0006432\_phenylalanyl-tRNA\_aminoacylation | 1 | 0 |  |  |  |  |  |  |  |  |
| GO:0006434\_seryl-tRNA\_aminoacylation | 1 | 0 |  |  |  |  |  |  |  |  |
| GO:0006435\_threonyl-tRNA\_aminoacylation | 1 | 0 |  |  |  |  |  |  |  |  |
| GO:0006436\_tryptophanyl-tRNA\_aminoacylation | 1 | 0 |  |  |  |  |  |  |  |  |
| GO:0006437\_tyrosyl-tRNA\_aminoacylation | 1 | 0 |  |  |  |  |  |  |  |  |
| GO:0006447\_regulation\_of\_translational\_initiation\_by\_iron | 1 | 0 |  |  |  |  |  |  |  |  |
| GO:0006448\_regulation\_of\_translational\_elongation | 1 | 0 |  |  |  |  |  |  |  |  |
| GO:0006450\_regulation\_of\_translational\_fidelity | 1 | 0 |  |  |  |  |  |  |  |  |
| GO:0006494\_protein\_amino\_acid\_terminal\_glycosylation | 1 | 0 |  |  |  |  |  |  |  |  |
| GO:0006496\_protein\_amino\_acid\_terminal\_N-glycosylation | 1 | 0 |  |  |  |  |  |  |  |  |
| GO:0006499\_N-terminal\_protein\_myristoylation | 1 | 0 |  |  |  |  |  |  |  |  |
| GO:0006500\_N-terminal\_protein\_palmitoylation | 1 | 0 |  |  |  |  |  |  |  |  |
| GO:0006526\_arginine\_biosynthetic\_process | 1 | 0 |  |  |  |  |  |  |  |  |
| GO:0006528\_asparagine\_metabolic\_process | 1 | 0 |  |  |  |  |  |  |  |  |
| GO:0006530\_asparagine\_catabolic\_process | 1 | 0 |  |  |  |  |  |  |  |  |
| GO:0006534\_cysteine\_metabolic\_process | 1 | 0 |  |  |  |  |  |  |  |  |
| GO:0006543\_glutamine\_catabolic\_process | 1 | 0 |  |  |  |  |  |  |  |  |
| GO:0006545\_glycine\_biosynthetic\_process | 1 | 0 |  |  |  |  |  |  |  |  |
| GO:0006547\_histidine\_metabolic\_process | 1 | 0 |  |  |  |  |  |  |  |  |
| GO:0006549\_isoleucine\_metabolic\_process | 1 | 0 |  |  |  |  |  |  |  |  |
| GO:0006556\_S-adenosylmethionine\_biosynthetic\_process | 1 | 0 |  |  |  |  |  |  |  |  |
| GO:0006562\_proline\_catabolic\_process | 1 | 0 |  |  |  |  |  |  |  |  |
| GO:0006564\_L-serine\_biosynthetic\_process | 1 | 0 |  |  |  |  |  |  |  |  |
| GO:0006577\_betaine\_metabolic\_process | 1 | 0 |  |  |  |  |  |  |  |  |
| GO:0006580\_ethanolamine\_metabolic\_process | 1 | 0 |  |  |  |  |  |  |  |  |
| GO:0006585\_dopamine\_biosynthetic\_process\_from\_tyrosine | 1 | 0 |  |  |  |  |  |  |  |  |
| GO:0006591\_ornithine\_metabolic\_process | 1 | 0 |  |  |  |  |  |  |  |  |
| GO:0006597\_spermine\_biosynthetic\_process | 1 | 0 |  |  |  |  |  |  |  |  |
| GO:0006598\_polyamine\_catabolic\_process | 1 | 0 |  |  |  |  |  |  |  |  |
| GO:0006610\_ribosomal\_protein\_import\_into\_nucleus | 1 | 0 |  |  |  |  |  |  |  |  |
| GO:0006614\_SRP-dependent\_cotranslational\_protein\_targeting\_to\_membrane | 1 | 0 |  |  |  |  |  |  |  |  |
| GO:0006616\_SRP-dependent\_cotranslational\_protein\_targeting\_to\_membrane\_\_translocation | 1 | 0 |  |  |  |  |  |  |  |  |
| GO:0006617\_SRP-dependent\_cotranslational\_protein\_targeting\_to\_membrane\_\_signal\_sequence\_recognition | 1 | 0 |  |  |  |  |  |  |  |  |
| GO:0006627\_mitochondrial\_protein\_processing\_during\_import | 1 | 0 |  |  |  |  |  |  |  |  |
| GO:0006646\_phosphatidylethanolamine\_biosynthetic\_process | 1 | 0 |  |  |  |  |  |  |  |  |
| GO:0006655\_phosphatidylglycerol\_biosynthetic\_process | 1 | 0 |  |  |  |  |  |  |  |  |
| GO:0006657\_CDP-choline\_pathway | 1 | 0 |  |  |  |  |  |  |  |  |
| GO:0006667\_sphinganine\_metabolic\_process | 1 | 0 |  |  |  |  |  |  |  |  |
| GO:0006668\_sphinganine-1-phosphate\_metabolic\_process | 1 | 0 |  |  |  |  |  |  |  |  |
| GO:0006669\_sphinganine-1-phosphate\_biosynthetic\_process | 1 | 0 |  |  |  |  |  |  |  |  |
| GO:0006670\_sphingosine\_metabolic\_process | 1 | 0 |  |  |  |  |  |  |  |  |
| GO:0006689\_ganglioside\_catabolic\_process | 1 | 0 |  |  |  |  |  |  |  |  |
| GO:0006711\_estrogen\_catabolic\_process | 1 | 0 |  |  |  |  |  |  |  |  |
| GO:0006713\_glucocorticoid\_catabolic\_process | 1 | 0 |  |  |  |  |  |  |  |  |
| GO:0006734\_NADH\_metabolic\_process | 1 | 0 |  |  |  |  |  |  |  |  |
| GO:0006741\_NADP\_biosynthetic\_process | 1 | 0 |  |  |  |  |  |  |  |  |
| GO:0006746\_FADH2\_metabolic\_process | 1 | 0 |  |  |  |  |  |  |  |  |
| GO:0006768\_biotin\_metabolic\_process | 1 | 0 |  |  |  |  |  |  |  |  |
| GO:0006771\_riboflavin\_metabolic\_process | 1 | 0 |  |  |  |  |  |  |  |  |
| GO:0006781\_succinyl-CoA\_pathway | 1 | 0 |  |  |  |  |  |  |  |  |
| GO:0006789\_bilirubin\_conjugation | 1 | 0 |  |  |  |  |  |  |  |  |
| GO:0006797\_polyphosphate\_metabolic\_process | 1 | 0 |  |  |  |  |  |  |  |  |
| GO:0006837\_serotonin\_transport | 1 | 0 |  |  |  |  |  |  |  |  |
| GO:0006842\_tricarboxylic\_acid\_transport | 1 | 0 |  |  |  |  |  |  |  |  |
| GO:0006843\_mitochondrial\_citrate\_transport | 1 | 0 |  |  |  |  |  |  |  |  |
| GO:0006848\_pyruvate\_transport | 1 | 0 |  |  |  |  |  |  |  |  |
| GO:0006862\_nucleotide\_transport | 1 | 0 |  |  |  |  |  |  |  |  |
| GO:0006867\_asparagine\_transport | 1 | 0 |  |  |  |  |  |  |  |  |
| GO:0006868\_glutamine\_transport | 1 | 0 |  |  |  |  |  |  |  |  |
| GO:0006876\_cellular\_cadmium\_ion\_homeostasis | 1 | 0 |  |  |  |  |  |  |  |  |
| GO:0006926\_virus-infected\_cell\_apoptosis | 1 | 0 |  |  |  |  |  |  |  |  |
| GO:0006931\_substrate-bound\_cell\_migration\_\_cell\_attachment\_to\_substrate | 1 | 0 |  |  |  |  |  |  |  |  |
| GO:0006948\_induction\_by\_virus\_of\_host\_cell-cell\_fusion | 1 | 0 |  |  |  |  |  |  |  |  |
| GO:0006958\_complement\_activation\_\_classical\_pathway | 1 | 0 |  |  |  |  |  |  |  |  |
| GO:0006963\_positive\_regulation\_of\_antibacterial\_peptide\_biosynthetic\_process | 1 | 0 |  |  |  |  |  |  |  |  |
| GO:0006965\_positive\_regulation\_of\_biosynthetic\_process\_of\_antibacterial\_peptides\_active\_against\_Gram-positive\_bacteria | 1 | 0 |  |  |  |  |  |  |  |  |
| GO:0006987\_activation\_of\_signaling\_protein\_activity\_involved\_in\_unfolded\_protein\_response | 1 | 0 |  |  |  |  |  |  |  |  |
| GO:0006990\_positive\_regulation\_of\_gene-specific\_transcription\_involved\_in\_unfolded\_protein\_response | 1 | 0 |  |  |  |  |  |  |  |  |
| GO:0006991\_response\_to\_sterol\_depletion | 1 | 0 |  |  |  |  |  |  |  |  |
| GO:0006994\_positive\_regulation\_of\_transcription\_via\_sterol\_regulatory\_element\_binding\_involved\_in\_ER-nuclear\_sterol\_response\_pathway | 1 | 0 |  |  |  |  |  |  |  |  |
| GO:0007039\_vacuolar\_protein\_catabolic\_process | 1 | 0 |  |  |  |  |  |  |  |  |
| GO:0007068\_negative\_regulation\_of\_transcription\_\_mitotic | 1 | 0 |  |  |  |  |  |  |  |  |
| GO:0007097\_nuclear\_migration | 1 | 0 |  |  |  |  |  |  |  |  |
| GO:0007100\_mitotic\_centrosome\_separation | 1 | 0 |  |  |  |  |  |  |  |  |
| GO:0007108\_cytokinesis\_\_initiation\_of\_separation | 1 | 0 |  |  |  |  |  |  |  |  |
| GO:0007109\_cytokinesis\_\_completion\_of\_separation | 1 | 0 |  |  |  |  |  |  |  |  |
| GO:0007132\_meiotic\_metaphase\_I | 1 | 0 |  |  |  |  |  |  |  |  |
| GO:0007135\_meiosis\_II | 1 | 0 |  |  |  |  |  |  |  |  |
| GO:0007136\_meiotic\_prophase\_II | 1 | 0 |  |  |  |  |  |  |  |  |
| GO:0007161\_calcium-independent\_cell-matrix\_adhesion | 1 | 0 |  |  |  |  |  |  |  |  |
| GO:0007196\_inhibition\_of\_adenylate\_cyclase\_activity\_by\_metabotropic\_glutamate\_receptor\_signaling\_pathway | 1 | 0 |  |  |  |  |  |  |  |  |
| GO:0007197\_inhibition\_of\_adenylate\_cyclase\_activity\_by\_muscarinic\_acetylcholine\_receptor\_signaling\_pathway | 1 | 0 |  |  |  |  |  |  |  |  |
| GO:0007258\_JUN\_phosphorylation | 1 | 0 |  |  |  |  |  |  |  |  |
| GO:0007321\_sperm\_displacement | 1 | 0 |  |  |  |  |  |  |  |  |
| GO:0007343\_egg\_activation | 1 | 0 |  |  |  |  |  |  |  |  |
| GO:0007386\_compartment\_specification | 1 | 0 |  |  |  |  |  |  |  |  |
| GO:0007387\_anterior\_compartment\_specification | 1 | 0 |  |  |  |  |  |  |  |  |
| GO:0007388\_posterior\_compartment\_specification | 1 | 0 |  |  |  |  |  |  |  |  |
| GO:0007402\_ganglion\_mother\_cell\_fate\_determination | 1 | 0 |  |  |  |  |  |  |  |  |
| GO:0007406\_negative\_regulation\_of\_neuroblast\_proliferation | 1 | 0 |  |  |  |  |  |  |  |  |
| GO:0007424\_open\_tracheal\_system\_development | 1 | 0 |  |  |  |  |  |  |  |  |
| GO:0007440\_foregut\_morphogenesis | 1 | 0 |  |  |  |  |  |  |  |  |
| GO:0007443\_Malpighian\_tubule\_morphogenesis | 1 | 0 |  |  |  |  |  |  |  |  |
| GO:0007444\_imaginal\_disc\_development | 1 | 0 |  |  |  |  |  |  |  |  |
| GO:0007447\_imaginal\_disc\_pattern\_formation | 1 | 0 |  |  |  |  |  |  |  |  |
| GO:0007494\_midgut\_development | 1 | 0 |  |  |  |  |  |  |  |  |
| GO:0007497\_posterior\_midgut\_development | 1 | 0 |  |  |  |  |  |  |  |  |
| GO:0007499\_ectoderm\_and\_mesoderm\_interaction | 1 | 0 |  |  |  |  |  |  |  |  |
| GO:0007501\_mesodermal\_cell\_fate\_specification | 1 | 0 |  |  |  |  |  |  |  |  |
| GO:0007509\_mesoderm\_migration | 1 | 0 |  |  |  |  |  |  |  |  |
| GO:0007518\_myoblast\_cell\_fate\_determination | 1 | 0 |  |  |  |  |  |  |  |  |
| GO:0007538\_primary\_sex\_determination | 1 | 0 |  |  |  |  |  |  |  |  |
| GO:0007597\_blood\_coagulation\_\_intrinsic\_pathway | 1 | 0 |  |  |  |  |  |  |  |  |
| GO:0007616\_long-term\_memory | 1 | 0 |  |  |  |  |  |  |  |  |
| GO:0007617\_mating\_behavior | 1 | 0 |  |  |  |  |  |  |  |  |
| GO:0007624\_ultradian\_rhythm | 1 | 0 |  |  |  |  |  |  |  |  |
| GO:0007638\_mechanosensory\_behavior | 1 | 0 |  |  |  |  |  |  |  |  |
| GO:0008045\_motor\_axon\_guidance | 1 | 0 |  |  |  |  |  |  |  |  |
| GO:0008057\_eye\_pigment\_granule\_organization | 1 | 0 |  |  |  |  |  |  |  |  |
| GO:0008063\_Toll\_signaling\_pathway | 1 | 0 |  |  |  |  |  |  |  |  |
| GO:0008065\_establishment\_of\_blood-nerve\_barrier | 1 | 0 |  |  |  |  |  |  |  |  |
| GO:0008090\_retrograde\_axon\_cargo\_transport | 1 | 0 |  |  |  |  |  |  |  |  |
| GO:0008215\_spermine\_metabolic\_process | 1 | 0 |  |  |  |  |  |  |  |  |
| GO:0008292\_acetylcholine\_biosynthetic\_process | 1 | 0 |  |  |  |  |  |  |  |  |
| GO:0008295\_spermidine\_biosynthetic\_process | 1 | 0 |  |  |  |  |  |  |  |  |
| GO:0008298\_intracellular\_mRNA\_localization | 1 | 0 |  |  |  |  |  |  |  |  |
| GO:0008356\_asymmetric\_cell\_division | 1 | 0 |  |  |  |  |  |  |  |  |
| GO:0008592\_regulation\_of\_Toll\_signaling\_pathway | 1 | 0 |  |  |  |  |  |  |  |  |
| GO:0008611\_ether\_lipid\_biosynthetic\_process | 1 | 0 |  |  |  |  |  |  |  |  |
| GO:0008614\_pyridoxine\_metabolic\_process | 1 | 0 |  |  |  |  |  |  |  |  |
| GO:0008615\_pyridoxine\_biosynthetic\_process | 1 | 0 |  |  |  |  |  |  |  |  |
| GO:0008627\_induction\_of\_apoptosis\_by\_ionic\_changes | 1 | 0 |  |  |  |  |  |  |  |  |
| GO:0008655\_pyrimidine\_salvage | 1 | 0 |  |  |  |  |  |  |  |  |
| GO:0009052\_pentose-phosphate\_shunt\_\_non-oxidative\_branch | 1 | 0 |  |  |  |  |  |  |  |  |
| GO:0009067\_aspartate\_family\_amino\_acid\_biosynthetic\_process | 1 | 0 |  |  |  |  |  |  |  |  |
| GO:0009075\_histidine\_family\_amino\_acid\_metabolic\_process | 1 | 0 |  |  |  |  |  |  |  |  |
| GO:0009128\_purine\_nucleoside\_monophosphate\_catabolic\_process | 1 | 0 |  |  |  |  |  |  |  |  |
| GO:0009129\_pyrimidine\_nucleoside\_monophosphate\_metabolic\_process | 1 | 0 |  |  |  |  |  |  |  |  |
| GO:0009130\_pyrimidine\_nucleoside\_monophosphate\_biosynthetic\_process | 1 | 0 |  |  |  |  |  |  |  |  |
| GO:0009133\_nucleoside\_diphosphate\_biosynthetic\_process | 1 | 0 |  |  |  |  |  |  |  |  |
| GO:0009135\_purine\_nucleoside\_diphosphate\_metabolic\_process | 1 | 0 |  |  |  |  |  |  |  |  |
| GO:0009136\_purine\_nucleoside\_diphosphate\_biosynthetic\_process | 1 | 0 |  |  |  |  |  |  |  |  |
| GO:0009138\_pyrimidine\_nucleoside\_diphosphate\_metabolic\_process | 1 | 0 |  |  |  |  |  |  |  |  |
| GO:0009140\_pyrimidine\_nucleoside\_diphosphate\_catabolic\_process | 1 | 0 |  |  |  |  |  |  |  |  |
| GO:0009147\_pyrimidine\_nucleoside\_triphosphate\_metabolic\_process | 1 | 0 |  |  |  |  |  |  |  |  |
| GO:0009148\_pyrimidine\_nucleoside\_triphosphate\_biosynthetic\_process | 1 | 0 |  |  |  |  |  |  |  |  |
| GO:0009153\_purine\_deoxyribonucleotide\_biosynthetic\_process | 1 | 0 |  |  |  |  |  |  |  |  |
| GO:0009157\_deoxyribonucleoside\_monophosphate\_biosynthetic\_process | 1 | 0 |  |  |  |  |  |  |  |  |
| GO:0009158\_ribonucleoside\_monophosphate\_catabolic\_process | 1 | 0 |  |  |  |  |  |  |  |  |
| GO:0009159\_deoxyribonucleoside\_monophosphate\_catabolic\_process | 1 | 0 |  |  |  |  |  |  |  |  |
| GO:0009169\_purine\_ribonucleoside\_monophosphate\_catabolic\_process | 1 | 0 |  |  |  |  |  |  |  |  |
| GO:0009173\_pyrimidine\_ribonucleoside\_monophosphate\_metabolic\_process | 1 | 0 |  |  |  |  |  |  |  |  |
| GO:0009174\_pyrimidine\_ribonucleoside\_monophosphate\_biosynthetic\_process | 1 | 0 |  |  |  |  |  |  |  |  |
| GO:0009179\_purine\_ribonucleoside\_diphosphate\_metabolic\_process | 1 | 0 |  |  |  |  |  |  |  |  |
| GO:0009180\_purine\_ribonucleoside\_diphosphate\_biosynthetic\_process | 1 | 0 |  |  |  |  |  |  |  |  |
| GO:0009182\_purine\_deoxyribonucleoside\_diphosphate\_metabolic\_process | 1 | 0 |  |  |  |  |  |  |  |  |
| GO:0009183\_purine\_deoxyribonucleoside\_diphosphate\_biosynthetic\_process | 1 | 0 |  |  |  |  |  |  |  |  |
| GO:0009186\_deoxyribonucleoside\_diphosphate\_metabolic\_process | 1 | 0 |  |  |  |  |  |  |  |  |
| GO:0009188\_ribonucleoside\_diphosphate\_biosynthetic\_process | 1 | 0 |  |  |  |  |  |  |  |  |
| GO:0009189\_deoxyribonucleoside\_diphosphate\_biosynthetic\_process | 1 | 0 |  |  |  |  |  |  |  |  |
| GO:0009193\_pyrimidine\_ribonucleoside\_diphosphate\_metabolic\_process | 1 | 0 |  |  |  |  |  |  |  |  |
| GO:0009195\_pyrimidine\_ribonucleoside\_diphosphate\_catabolic\_process | 1 | 0 |  |  |  |  |  |  |  |  |
| GO:0009208\_pyrimidine\_ribonucleoside\_triphosphate\_metabolic\_process | 1 | 0 |  |  |  |  |  |  |  |  |
| GO:0009209\_pyrimidine\_ribonucleoside\_triphosphate\_biosynthetic\_process | 1 | 0 |  |  |  |  |  |  |  |  |
| GO:0009214\_cyclic\_nucleotide\_catabolic\_process | 1 | 0 |  |  |  |  |  |  |  |  |
| GO:0009222\_pyrimidine\_ribonucleotide\_catabolic\_process | 1 | 0 |  |  |  |  |  |  |  |  |
| GO:0009231\_riboflavin\_biosynthetic\_process | 1 | 0 |  |  |  |  |  |  |  |  |
| GO:0009253\_peptidoglycan\_catabolic\_process | 1 | 0 |  |  |  |  |  |  |  |  |
| GO:0009256\_10-formyltetrahydrofolate\_metabolic\_process | 1 | 0 |  |  |  |  |  |  |  |  |
| GO:0009258\_10-formyltetrahydrofolate\_catabolic\_process | 1 | 0 |  |  |  |  |  |  |  |  |
| GO:0009265\_2'-deoxyribonucleotide\_biosynthetic\_process | 1 | 0 |  |  |  |  |  |  |  |  |
| GO:0009292\_genetic\_transfer | 1 | 0 |  |  |  |  |  |  |  |  |
| GO:0009294\_DNA\_mediated\_transformation | 1 | 0 |  |  |  |  |  |  |  |  |
| GO:0009296\_flagellum\_assembly | 1 | 0 |  |  |  |  |  |  |  |  |
| GO:0009298\_GDP-mannose\_biosynthetic\_process | 1 | 0 |  |  |  |  |  |  |  |  |
| GO:0009304\_tRNA\_transcription | 1 | 0 |  |  |  |  |  |  |  |  |
| GO:0009313\_oligosaccharide\_catabolic\_process | 1 | 0 |  |  |  |  |  |  |  |  |
| GO:0009372\_quorum\_sensing | 1 | 0 |  |  |  |  |  |  |  |  |
| GO:0009386\_translational\_attenuation | 1 | 0 |  |  |  |  |  |  |  |  |
| GO:0009397\_folic\_acid\_and\_derivative\_catabolic\_process | 1 | 0 |  |  |  |  |  |  |  |  |
| GO:0009399\_nitrogen\_fixation | 1 | 0 |  |  |  |  |  |  |  |  |
| GO:0009404\_toxin\_metabolic\_process | 1 | 0 |  |  |  |  |  |  |  |  |
| GO:0009435\_NAD\_biosynthetic\_process | 1 | 0 |  |  |  |  |  |  |  |  |
| GO:0009437\_carnitine\_metabolic\_process | 1 | 0 |  |  |  |  |  |  |  |  |
| GO:0009624\_response\_to\_nematode | 1 | 0 |  |  |  |  |  |  |  |  |
| GO:0009642\_response\_to\_light\_intensity | 1 | 0 |  |  |  |  |  |  |  |  |
| GO:0009648\_photoperiodism | 1 | 0 |  |  |  |  |  |  |  |  |
| GO:0009720\_detection\_of\_hormone\_stimulus | 1 | 0 |  |  |  |  |  |  |  |  |
| GO:0009726\_detection\_of\_endogenous\_stimulus | 1 | 0 |  |  |  |  |  |  |  |  |
| GO:0009730\_detection\_of\_carbohydrate\_stimulus | 1 | 0 |  |  |  |  |  |  |  |  |
| GO:0009732\_detection\_of\_hexose\_stimulus | 1 | 0 |  |  |  |  |  |  |  |  |
| GO:0009826\_unidimensional\_cell\_growth | 1 | 0 |  |  |  |  |  |  |  |  |
| GO:0009912\_auditory\_receptor\_cell\_fate\_commitment | 1 | 0 |  |  |  |  |  |  |  |  |
| GO:0009954\_proximal\_distal\_pattern\_formation | 1 | 0 |  |  |  |  |  |  |  |  |
| GO:0009972\_cytidine\_deamination | 1 | 0 |  |  |  |  |  |  |  |  |
| GO:0010107\_potassium\_ion\_import | 1 | 0 |  |  |  |  |  |  |  |  |
| GO:0010259\_multicellular\_organismal\_aging | 1 | 0 |  |  |  |  |  |  |  |  |
| GO:0010269\_response\_to\_selenium\_ion | 1 | 0 |  |  |  |  |  |  |  |  |
| GO:0010273\_detoxification\_of\_copper\_ion | 1 | 0 |  |  |  |  |  |  |  |  |
| GO:0010383\_cell\_wall\_polysaccharide\_metabolic\_process | 1 | 0 |  |  |  |  |  |  |  |  |
| GO:0010430\_fatty\_acid\_omega-oxidation | 1 | 0 |  |  |  |  |  |  |  |  |
| GO:0010463\_mesenchymal\_cell\_proliferation | 1 | 0 |  |  |  |  |  |  |  |  |
| GO:0010464\_regulation\_of\_mesenchymal\_cell\_proliferation | 1 | 0 |  |  |  |  |  |  |  |  |
| GO:0010507\_negative\_regulation\_of\_autophagy | 1 | 0 |  |  |  |  |  |  |  |  |
| GO:0010509\_polyamine\_homeostasis | 1 | 0 |  |  |  |  |  |  |  |  |
| GO:0010534\_regulation\_of\_activation\_of\_JAK2\_kinase\_activity | 1 | 0 |  |  |  |  |  |  |  |  |
| GO:0010535\_positive\_regulation\_of\_activation\_of\_JAK2\_kinase\_activity | 1 | 0 |  |  |  |  |  |  |  |  |
| GO:0010561\_negative\_regulation\_of\_glycoprotein\_biosynthetic\_process | 1 | 0 |  |  |  |  |  |  |  |  |
| GO:0010569\_regulation\_of\_double-strand\_break\_repair\_via\_homologous\_recombination | 1 | 0 |  |  |  |  |  |  |  |  |
| GO:0010591\_regulation\_of\_lamellipodium\_assembly | 1 | 0 |  |  |  |  |  |  |  |  |
| GO:0010592\_positive\_regulation\_of\_lamellipodium\_assembly | 1 | 0 |  |  |  |  |  |  |  |  |
| GO:0010621\_negative\_regulation\_of\_transcription\_by\_transcription\_factor\_localization | 1 | 0 |  |  |  |  |  |  |  |  |
| GO:0010623\_developmental\_programmed\_cell\_death | 1 | 0 |  |  |  |  |  |  |  |  |
| GO:0010631\_epithelial\_cell\_migration | 1 | 0 |  |  |  |  |  |  |  |  |
| GO:0010632\_regulation\_of\_epithelial\_cell\_migration | 1 | 0 |  |  |  |  |  |  |  |  |
| GO:0010634\_positive\_regulation\_of\_epithelial\_cell\_migration | 1 | 0 |  |  |  |  |  |  |  |  |
| GO:0010658\_striated\_muscle\_cell\_apoptosis | 1 | 0 |  |  |  |  |  |  |  |  |
| GO:0010659\_cardiac\_muscle\_cell\_apoptosis | 1 | 0 |  |  |  |  |  |  |  |  |
| GO:0010662\_regulation\_of\_striated\_muscle\_cell\_apoptosis | 1 | 0 |  |  |  |  |  |  |  |  |
| GO:0010664\_negative\_regulation\_of\_striated\_muscle\_cell\_apoptosis | 1 | 0 |  |  |  |  |  |  |  |  |
| GO:0010665\_regulation\_of\_cardiac\_muscle\_cell\_apoptosis | 1 | 0 |  |  |  |  |  |  |  |  |
| GO:0010667\_negative\_regulation\_of\_cardiac\_muscle\_cell\_apoptosis | 1 | 0 |  |  |  |  |  |  |  |  |
| GO:0010669\_epithelial\_structure\_maintenance | 1 | 0 |  |  |  |  |  |  |  |  |
| GO:0010692\_regulation\_of\_alkaline\_phosphatase\_activity | 1 | 0 |  |  |  |  |  |  |  |  |
| GO:0010693\_negative\_regulation\_of\_alkaline\_phosphatase\_activity | 1 | 0 |  |  |  |  |  |  |  |  |
| GO:0010710\_regulation\_of\_collagen\_catabolic\_process | 1 | 0 |  |  |  |  |  |  |  |  |
| GO:0010711\_negative\_regulation\_of\_collagen\_catabolic\_process | 1 | 0 |  |  |  |  |  |  |  |  |
| GO:0010715\_regulation\_of\_extracellular\_matrix\_disassembly | 1 | 0 |  |  |  |  |  |  |  |  |
| GO:0010716\_negative\_regulation\_of\_extracellular\_matrix\_disassembly | 1 | 0 |  |  |  |  |  |  |  |  |
| GO:0010719\_negative\_regulation\_of\_epithelial\_to\_mesenchymal\_transition | 1 | 0 |  |  |  |  |  |  |  |  |
| GO:0010722\_regulation\_of\_ferrochelatase\_activity | 1 | 0 |  |  |  |  |  |  |  |  |
| GO:0010731\_protein\_amino\_acid\_glutathionylation | 1 | 0 |  |  |  |  |  |  |  |  |
| GO:0010732\_regulation\_of\_protein\_amino\_acid\_glutathionylation | 1 | 0 |  |  |  |  |  |  |  |  |
| GO:0010734\_negative\_regulation\_of\_protein\_amino\_acid\_glutathionylation | 1 | 0 |  |  |  |  |  |  |  |  |
| GO:0010735\_positive\_regulation\_of\_transcription\_via\_serum\_response\_element\_binding | 1 | 0 |  |  |  |  |  |  |  |  |
| GO:0010737\_protein\_kinase\_A\_signaling\_cascade | 1 | 0 |  |  |  |  |  |  |  |  |
| GO:0010738\_regulation\_of\_protein\_kinase\_A\_signaling\_cascade | 1 | 0 |  |  |  |  |  |  |  |  |
| GO:0010739\_positive\_regulation\_of\_protein\_kinase\_A\_signaling\_cascade | 1 | 0 |  |  |  |  |  |  |  |  |
| GO:0010749\_regulation\_of\_nitric\_oxide\_mediated\_signal\_transduction | 1 | 0 |  |  |  |  |  |  |  |  |
| GO:0010751\_negative\_regulation\_of\_nitric\_oxide\_mediated\_signal\_transduction | 1 | 0 |  |  |  |  |  |  |  |  |
| GO:0010752\_regulation\_of\_cGMP-mediated\_signaling | 1 | 0 |  |  |  |  |  |  |  |  |
| GO:0010754\_negative\_regulation\_of\_cGMP-mediated\_signaling | 1 | 0 |  |  |  |  |  |  |  |  |
| GO:0010756\_positive\_regulation\_of\_plasminogen\_activation | 1 | 0 |  |  |  |  |  |  |  |  |
| GO:0010757\_negative\_regulation\_of\_plasminogen\_activation | 1 | 0 |  |  |  |  |  |  |  |  |
| GO:0010758\_regulation\_of\_macrophage\_chemotaxis | 1 | 0 |  |  |  |  |  |  |  |  |
| GO:0010759\_positive\_regulation\_of\_macrophage\_chemotaxis | 1 | 0 |  |  |  |  |  |  |  |  |
| GO:0010766\_negative\_regulation\_of\_sodium\_ion\_transport | 1 | 0 |  |  |  |  |  |  |  |  |
| GO:0010767\_regulation\_of\_transcription\_from\_RNA\_polymerase\_II\_promoter\_in\_response\_to\_UV-induced\_DNA\_damage | 1 | 0 |  |  |  |  |  |  |  |  |
| GO:0010768\_negative\_regulation\_of\_transcription\_from\_RNA\_polymerase\_II\_promoter\_in\_response\_to\_UV-induced\_DNA\_damage | 1 | 0 |  |  |  |  |  |  |  |  |
| GO:0010771\_negative\_regulation\_of\_cell\_morphogenesis\_involved\_in\_differentiation | 1 | 0 |  |  |  |  |  |  |  |  |
| GO:0010793\_regulation\_of\_mRNA\_export\_from\_nucleus | 1 | 0 |  |  |  |  |  |  |  |  |
| GO:0010801\_negative\_regulation\_of\_peptidyl-threonine\_phosphorylation | 1 | 0 |  |  |  |  |  |  |  |  |
| GO:0010803\_regulation\_of\_tumor\_necrosis\_factor-mediated\_signaling\_pathway | 1 | 0 |  |  |  |  |  |  |  |  |
| GO:0010804\_negative\_regulation\_of\_tumor\_necrosis\_factor-mediated\_signaling\_pathway | 1 | 0 |  |  |  |  |  |  |  |  |
| GO:0010813\_neuropeptide\_catabolic\_process | 1 | 0 |  |  |  |  |  |  |  |  |
| GO:0010814\_substance\_P\_catabolic\_process | 1 | 0 |  |  |  |  |  |  |  |  |
| GO:0010816\_calcitonin\_catabolic\_process | 1 | 0 |  |  |  |  |  |  |  |  |
| GO:0010826\_negative\_regulation\_of\_centrosome\_duplication | 1 | 0 |  |  |  |  |  |  |  |  |
| GO:0010830\_regulation\_of\_myotube\_differentiation | 1 | 0 |  |  |  |  |  |  |  |  |
| GO:0010832\_negative\_regulation\_of\_myotube\_differentiation | 1 | 0 |  |  |  |  |  |  |  |  |
| GO:0010835\_regulation\_of\_protein\_amino\_acid\_ADP-ribosylation | 1 | 0 |  |  |  |  |  |  |  |  |
| GO:0010836\_negative\_regulation\_of\_protein\_amino\_acid\_ADP-ribosylation | 1 | 0 |  |  |  |  |  |  |  |  |
| GO:0010837\_regulation\_of\_keratinocyte\_proliferation | 1 | 0 |  |  |  |  |  |  |  |  |
| GO:0010839\_negative\_regulation\_of\_keratinocyte\_proliferation | 1 | 0 |  |  |  |  |  |  |  |  |
| GO:0010840\_regulation\_of\_circadian\_sleep\_wake\_cycle\_\_wakefulness | 1 | 0 |  |  |  |  |  |  |  |  |
| GO:0010841\_positive\_regulation\_of\_circadian\_sleep\_wake\_cycle\_\_wakefulness | 1 | 0 |  |  |  |  |  |  |  |  |
| GO:0010842\_retina\_layer\_formation | 1 | 0 |  |  |  |  |  |  |  |  |
| GO:0010897\_negative\_regulation\_of\_triglyceride\_catabolic\_process | 1 | 0 |  |  |  |  |  |  |  |  |
| GO:0010899\_regulation\_of\_phosphatidylcholine\_catabolic\_process | 1 | 0 |  |  |  |  |  |  |  |  |
| GO:0010900\_negative\_regulation\_of\_phosphatidylcholine\_catabolic\_process | 1 | 0 |  |  |  |  |  |  |  |  |
| GO:0010902\_positive\_regulation\_of\_very-low-density\_lipoprotein\_particle\_remodeling | 1 | 0 |  |  |  |  |  |  |  |  |
| GO:0010931\_macrophage\_tolerance\_induction | 1 | 0 |  |  |  |  |  |  |  |  |
| GO:0010932\_regulation\_of\_macrophage\_tolerance\_induction | 1 | 0 |  |  |  |  |  |  |  |  |
| GO:0010933\_positive\_regulation\_of\_macrophage\_tolerance\_induction | 1 | 0 |  |  |  |  |  |  |  |  |
| GO:0010934\_macrophage\_cytokine\_production | 1 | 0 |  |  |  |  |  |  |  |  |
| GO:0010935\_regulation\_of\_macrophage\_cytokine\_production | 1 | 0 |  |  |  |  |  |  |  |  |
| GO:0010936\_negative\_regulation\_of\_macrophage\_cytokine\_production | 1 | 0 |  |  |  |  |  |  |  |  |
| GO:0010944\_negative\_regulation\_of\_transcription\_by\_competitive\_promoter\_binding | 1 | 0 |  |  |  |  |  |  |  |  |
| GO:0010983\_positive\_regulation\_of\_high-density\_lipoprotein\_particle\_clearance | 1 | 0 |  |  |  |  |  |  |  |  |
| GO:0010986\_positive\_regulation\_of\_lipoprotein\_particle\_clearance | 1 | 0 |  |  |  |  |  |  |  |  |
| GO:0010987\_negative\_regulation\_of\_high-density\_lipoprotein\_particle\_clearance | 1 | 0 |  |  |  |  |  |  |  |  |
| GO:0010988\_regulation\_of\_low-density\_lipoprotein\_particle\_clearance | 1 | 0 |  |  |  |  |  |  |  |  |
| GO:0010989\_negative\_regulation\_of\_low-density\_lipoprotein\_particle\_clearance | 1 | 0 |  |  |  |  |  |  |  |  |
| GO:0010990\_regulation\_of\_SMAD\_protein\_complex\_assembly | 1 | 0 |  |  |  |  |  |  |  |  |
| GO:0010991\_negative\_regulation\_of\_SMAD\_protein\_complex\_assembly | 1 | 0 |  |  |  |  |  |  |  |  |
| GO:0014009\_glial\_cell\_proliferation | 1 | 0 |  |  |  |  |  |  |  |  |
| GO:0014010\_Schwann\_cell\_proliferation | 1 | 0 |  |  |  |  |  |  |  |  |
| GO:0014045\_establishment\_of\_endothelial\_blood-brain\_barrier | 1 | 0 |  |  |  |  |  |  |  |  |
| GO:0014055\_acetylcholine\_secretion | 1 | 0 |  |  |  |  |  |  |  |  |
| GO:0014056\_regulation\_of\_acetylcholine\_secretion | 1 | 0 |  |  |  |  |  |  |  |  |
| GO:0014060\_regulation\_of\_epinephrine\_secretion | 1 | 0 |  |  |  |  |  |  |  |  |
| GO:0014067\_negative\_regulation\_of\_phosphoinositide\_3-kinase\_cascade | 1 | 0 |  |  |  |  |  |  |  |  |
| GO:0014071\_response\_to\_cycloalkane | 1 | 0 |  |  |  |  |  |  |  |  |
| GO:0014721\_twitch\_skeletal\_muscle\_contraction | 1 | 0 |  |  |  |  |  |  |  |  |
| GO:0014724\_regulation\_of\_twitch\_skeletal\_muscle\_contraction | 1 | 0 |  |  |  |  |  |  |  |  |
| GO:0014806\_smooth\_muscle\_hyperplasia | 1 | 0 |  |  |  |  |  |  |  |  |
| GO:0014823\_response\_to\_activity | 1 | 0 |  |  |  |  |  |  |  |  |
| GO:0014832\_urinary\_bladder\_smooth\_muscle\_contraction | 1 | 0 |  |  |  |  |  |  |  |  |
| GO:0014848\_urinary\_tract\_smooth\_muscle\_contraction | 1 | 0 |  |  |  |  |  |  |  |  |
| GO:0014850\_response\_to\_muscle\_activity | 1 | 0 |  |  |  |  |  |  |  |  |
| GO:0014873\_response\_to\_muscle\_activity\_involved\_in\_regulation\_of\_muscle\_adaptation | 1 | 0 |  |  |  |  |  |  |  |  |
| GO:0014874\_response\_to\_stimulus\_involved\_in\_regulation\_of\_muscle\_adaptation | 1 | 0 |  |  |  |  |  |  |  |  |
| GO:0014895\_smooth\_muscle\_hypertrophy | 1 | 0 |  |  |  |  |  |  |  |  |
| GO:0014916\_regulation\_of\_lung\_blood\_pressure | 1 | 0 |  |  |  |  |  |  |  |  |
| GO:0015675\_nickel\_ion\_transport | 1 | 0 |  |  |  |  |  |  |  |  |
| GO:0015676\_vanadium\_ion\_transport | 1 | 0 |  |  |  |  |  |  |  |  |
| GO:0015680\_intracellular\_copper\_ion\_transport | 1 | 0 |  |  |  |  |  |  |  |  |
| GO:0015684\_ferrous\_iron\_transport | 1 | 0 |  |  |  |  |  |  |  |  |
| GO:0015692\_lead\_ion\_transport | 1 | 0 |  |  |  |  |  |  |  |  |
| GO:0015693\_magnesium\_ion\_transport | 1 | 0 |  |  |  |  |  |  |  |  |
| GO:0015727\_lactate\_transport | 1 | 0 |  |  |  |  |  |  |  |  |
| GO:0015728\_mevalonate\_transport | 1 | 0 |  |  |  |  |  |  |  |  |
| GO:0015742\_alpha-ketoglutarate\_transport | 1 | 0 |  |  |  |  |  |  |  |  |
| GO:0015746\_citrate\_transport | 1 | 0 |  |  |  |  |  |  |  |  |
| GO:0015747\_urate\_transport | 1 | 0 |  |  |  |  |  |  |  |  |
| GO:0015755\_fructose\_transport | 1 | 0 |  |  |  |  |  |  |  |  |
| GO:0015760\_glucose-6-phosphate\_transport | 1 | 0 |  |  |  |  |  |  |  |  |
| GO:0015782\_CMP-sialic\_acid\_transport | 1 | 0 |  |  |  |  |  |  |  |  |
| GO:0015785\_UDP-galactose\_transport | 1 | 0 |  |  |  |  |  |  |  |  |
| GO:0015789\_UDP-N-acetylgalactosamine\_transport | 1 | 0 |  |  |  |  |  |  |  |  |
| GO:0015790\_UDP-xylose\_transport | 1 | 0 |  |  |  |  |  |  |  |  |
| GO:0015798\_myo-inositol\_transport | 1 | 0 |  |  |  |  |  |  |  |  |
| GO:0015803\_branched-chain\_aliphatic\_amino\_acid\_transport | 1 | 0 |  |  |  |  |  |  |  |  |
| GO:0015805\_S-adenosylmethionine\_transport | 1 | 0 |  |  |  |  |  |  |  |  |
| GO:0015809\_arginine\_transport | 1 | 0 |  |  |  |  |  |  |  |  |
| GO:0015817\_histidine\_transport | 1 | 0 |  |  |  |  |  |  |  |  |
| GO:0015820\_leucine\_transport | 1 | 0 |  |  |  |  |  |  |  |  |
| GO:0015826\_threonine\_transport | 1 | 0 |  |  |  |  |  |  |  |  |
| GO:0015827\_tryptophan\_transport | 1 | 0 |  |  |  |  |  |  |  |  |
| GO:0015846\_polyamine\_transport | 1 | 0 |  |  |  |  |  |  |  |  |
| GO:0015853\_adenine\_transport | 1 | 0 |  |  |  |  |  |  |  |  |
| GO:0015855\_pyrimidine\_transport | 1 | 0 |  |  |  |  |  |  |  |  |
| GO:0015886\_heme\_transport | 1 | 0 |  |  |  |  |  |  |  |  |
| GO:0015888\_thiamin\_transport | 1 | 0 |  |  |  |  |  |  |  |  |
| GO:0015910\_peroxisomal\_long-chain\_fatty\_acid\_import | 1 | 0 |  |  |  |  |  |  |  |  |
| GO:0015919\_peroxisomal\_membrane\_transport | 1 | 0 |  |  |  |  |  |  |  |  |
| GO:0015937\_coenzyme\_A\_biosynthetic\_process | 1 | 0 |  |  |  |  |  |  |  |  |
| GO:0015956\_bis(5'-nucleosidyl)\_oligophosphate\_metabolic\_process | 1 | 0 |  |  |  |  |  |  |  |  |
| GO:0015958\_bis(5'-nucleosidyl)\_oligophosphate\_catabolic\_process | 1 | 0 |  |  |  |  |  |  |  |  |
| GO:0015959\_diadenosine\_polyphosphate\_metabolic\_process | 1 | 0 |  |  |  |  |  |  |  |  |
| GO:0015961\_diadenosine\_polyphosphate\_catabolic\_process | 1 | 0 |  |  |  |  |  |  |  |  |
| GO:0016046\_detection\_of\_fungus | 1 | 0 |  |  |  |  |  |  |  |  |
| GO:0016078\_tRNA\_catabolic\_process | 1 | 0 |  |  |  |  |  |  |  |  |
| GO:0016091\_prenol\_biosynthetic\_process | 1 | 0 |  |  |  |  |  |  |  |  |
| GO:0016094\_polyprenol\_biosynthetic\_process | 1 | 0 |  |  |  |  |  |  |  |  |
| GO:0016108\_tetraterpenoid\_metabolic\_process | 1 | 0 |  |  |  |  |  |  |  |  |
| GO:0016116\_carotenoid\_metabolic\_process | 1 | 0 |  |  |  |  |  |  |  |  |
| GO:0016119\_carotene\_metabolic\_process | 1 | 0 |  |  |  |  |  |  |  |  |
| GO:0016140\_O-glycoside\_metabolic\_process | 1 | 0 |  |  |  |  |  |  |  |  |
| GO:0016142\_O-glycoside\_catabolic\_process | 1 | 0 |  |  |  |  |  |  |  |  |
| GO:0016188\_synaptic\_vesicle\_maturation | 1 | 0 |  |  |  |  |  |  |  |  |
| GO:0016189\_synaptic\_vesicle\_to\_endosome\_fusion | 1 | 0 |  |  |  |  |  |  |  |  |
| GO:0016241\_regulation\_of\_macroautophagy | 1 | 0 |  |  |  |  |  |  |  |  |
| GO:0016242\_negative\_regulation\_of\_macroautophagy | 1 | 0 |  |  |  |  |  |  |  |  |
| GO:0016259\_selenocysteine\_metabolic\_process | 1 | 0 |  |  |  |  |  |  |  |  |
| GO:0016260\_selenocysteine\_biosynthetic\_process | 1 | 0 |  |  |  |  |  |  |  |  |
| GO:0016269\_O-glycan\_processing\_\_core\_3 | 1 | 0 |  |  |  |  |  |  |  |  |
| GO:0016320\_endoplasmic\_reticulum\_membrane\_fusion | 1 | 0 |  |  |  |  |  |  |  |  |
| GO:0016344\_meiotic\_chromosome\_movement\_towards\_spindle\_pole | 1 | 0 |  |  |  |  |  |  |  |  |
| GO:0016446\_somatic\_hypermutation\_of\_immunoglobulin\_genes | 1 | 0 |  |  |  |  |  |  |  |  |
| GO:0016559\_peroxisome\_fission | 1 | 0 |  |  |  |  |  |  |  |  |
| GO:0016560\_protein\_import\_into\_peroxisome\_matrix\_\_docking | 1 | 0 |  |  |  |  |  |  |  |  |
| GO:0016598\_protein\_arginylation | 1 | 0 |  |  |  |  |  |  |  |  |
| GO:0016998\_cell\_wall\_macromolecule\_catabolic\_process | 1 | 0 |  |  |  |  |  |  |  |  |
| GO:0017062\_respiratory\_chain\_complex\_III\_assembly | 1 | 0 |  |  |  |  |  |  |  |  |
| GO:0017185\_peptidyl-lysine\_hydroxylation | 1 | 0 |  |  |  |  |  |  |  |  |
| GO:0018095\_protein\_polyglutamylation | 1 | 0 |  |  |  |  |  |  |  |  |
| GO:0018125\_peptidyl-cysteine\_methylation | 1 | 0 |  |  |  |  |  |  |  |  |
| GO:0018126\_protein\_amino\_acid\_hydroxylation | 1 | 0 |  |  |  |  |  |  |  |  |
| GO:0018146\_keratan\_sulfate\_biosynthetic\_process | 1 | 0 |  |  |  |  |  |  |  |  |
| GO:0018153\_isopeptide\_cross-linking\_via\_N6-(L-isoglutamyl)-L-lysine | 1 | 0 |  |  |  |  |  |  |  |  |
| GO:0018184\_protein\_amino\_acid\_polyamination | 1 | 0 |  |  |  |  |  |  |  |  |
| GO:0018190\_protein\_amino\_acid\_octanoylation | 1 | 0 |  |  |  |  |  |  |  |  |
| GO:0018191\_peptidyl-serine\_octanoylation | 1 | 0 |  |  |  |  |  |  |  |  |
| GO:0018192\_enzyme\_active\_site\_formation\_via\_L-cysteine\_persulfide | 1 | 0 |  |  |  |  |  |  |  |  |
| GO:0018199\_peptidyl-glutamine\_modification | 1 | 0 |  |  |  |  |  |  |  |  |
| GO:0018200\_peptidyl-glutamic\_acid\_modification | 1 | 0 |  |  |  |  |  |  |  |  |
| GO:0018208\_peptidyl-proline\_modification | 1 | 0 |  |  |  |  |  |  |  |  |
| GO:0018262\_isopeptide\_cross-linking | 1 | 0 |  |  |  |  |  |  |  |  |
| GO:0018277\_protein\_amino\_acid\_deamination | 1 | 0 |  |  |  |  |  |  |  |  |
| GO:0018307\_enzyme\_active\_site\_formation | 1 | 0 |  |  |  |  |  |  |  |  |
| GO:0018318\_protein\_amino\_acid\_palmitoylation | 1 | 0 |  |  |  |  |  |  |  |  |
| GO:0018319\_protein\_amino\_acid\_myristoylation | 1 | 0 |  |  |  |  |  |  |  |  |
| GO:0018345\_protein\_palmitoylation | 1 | 0 |  |  |  |  |  |  |  |  |
| GO:0018350\_protein\_amino\_acid\_esterification | 1 | 0 |  |  |  |  |  |  |  |  |
| GO:0018352\_protein-pyridoxal-5-phosphate\_linkage | 1 | 0 |  |  |  |  |  |  |  |  |
| GO:0018377\_protein\_myristoylation | 1 | 0 |  |  |  |  |  |  |  |  |
| GO:0018395\_peptidyl-lysine\_hydroxylation\_to\_5-hydroxy-L-lysine | 1 | 0 |  |  |  |  |  |  |  |  |
| GO:0018401\_peptidyl-proline\_hydroxylation\_to\_4-hydroxy-L-proline | 1 | 0 |  |  |  |  |  |  |  |  |
| GO:0018872\_arsonoacetate\_metabolic\_process | 1 | 0 |  |  |  |  |  |  |  |  |
| GO:0018874\_benzoate\_metabolic\_process | 1 | 0 |  |  |  |  |  |  |  |  |
| GO:0019060\_intracellular\_transport\_of\_viral\_proteins\_in\_host\_cell | 1 | 0 |  |  |  |  |  |  |  |  |
| GO:0019064\_viral\_envelope\_fusion\_with\_host\_membrane | 1 | 0 |  |  |  |  |  |  |  |  |
| GO:0019086\_late\_viral\_mRNA\_transcription | 1 | 0 |  |  |  |  |  |  |  |  |
| GO:0019087\_transformation\_of\_host\_cell\_by\_virus | 1 | 0 |  |  |  |  |  |  |  |  |
| GO:0019089\_transmission\_of\_virus | 1 | 0 |  |  |  |  |  |  |  |  |
| GO:0019098\_reproductive\_behavior | 1 | 0 |  |  |  |  |  |  |  |  |
| GO:0019240\_citrulline\_biosynthetic\_process | 1 | 0 |  |  |  |  |  |  |  |  |
| GO:0019302\_D-ribose\_biosynthetic\_process | 1 | 0 |  |  |  |  |  |  |  |  |
| GO:0019303\_D-ribose\_catabolic\_process | 1 | 0 |  |  |  |  |  |  |  |  |
| GO:0019307\_mannose\_biosynthetic\_process | 1 | 0 |  |  |  |  |  |  |  |  |
| GO:0019310\_inositol\_catabolic\_process | 1 | 0 |  |  |  |  |  |  |  |  |
| GO:0019322\_pentose\_biosynthetic\_process | 1 | 0 |  |  |  |  |  |  |  |  |
| GO:0019323\_pentose\_catabolic\_process | 1 | 0 |  |  |  |  |  |  |  |  |
| GO:0019371\_cyclooxygenase\_pathway | 1 | 0 |  |  |  |  |  |  |  |  |
| GO:0019372\_lipoxygenase\_pathway | 1 | 0 |  |  |  |  |  |  |  |  |
| GO:0019388\_galactose\_catabolic\_process | 1 | 0 |  |  |  |  |  |  |  |  |
| GO:0019405\_alditol\_catabolic\_process | 1 | 0 |  |  |  |  |  |  |  |  |
| GO:0019407\_hexitol\_catabolic\_process | 1 | 0 |  |  |  |  |  |  |  |  |
| GO:0019408\_dolichol\_biosynthetic\_process | 1 | 0 |  |  |  |  |  |  |  |  |
| GO:0019441\_tryptophan\_catabolic\_process\_to\_kynurenine | 1 | 0 |  |  |  |  |  |  |  |  |
| GO:0019471\_4-hydroxyproline\_metabolic\_process | 1 | 0 |  |  |  |  |  |  |  |  |
| GO:0019511\_peptidyl-proline\_hydroxylation | 1 | 0 |  |  |  |  |  |  |  |  |
| GO:0019519\_pentitol\_metabolic\_process | 1 | 0 |  |  |  |  |  |  |  |  |
| GO:0019527\_pentitol\_catabolic\_process | 1 | 0 |  |  |  |  |  |  |  |  |
| GO:0019614\_catechol\_catabolic\_process | 1 | 0 |  |  |  |  |  |  |  |  |
| GO:0019673\_GDP-mannose\_metabolic\_process | 1 | 0 |  |  |  |  |  |  |  |  |
| GO:0019693\_ribose\_phosphate\_metabolic\_process | 1 | 0 |  |  |  |  |  |  |  |  |
| GO:0019695\_choline\_metabolic\_process | 1 | 0 |  |  |  |  |  |  |  |  |
| GO:0019747\_regulation\_of\_isoprenoid\_metabolic\_process | 1 | 0 |  |  |  |  |  |  |  |  |
| GO:0019852\_L-ascorbic\_acid\_metabolic\_process | 1 | 0 |  |  |  |  |  |  |  |  |
| GO:0019856\_pyrimidine\_base\_biosynthetic\_process | 1 | 0 |  |  |  |  |  |  |  |  |
| GO:0019858\_cytosine\_metabolic\_process | 1 | 0 |  |  |  |  |  |  |  |  |
| GO:0019884\_antigen\_processing\_and\_presentation\_of\_exogenous\_antigen | 1 | 0 |  |  |  |  |  |  |  |  |
| GO:0019886\_antigen\_processing\_and\_presentation\_of\_exogenous\_peptide\_antigen\_via\_MHC\_class\_II | 1 | 0 |  |  |  |  |  |  |  |  |
| GO:0021508\_floor\_plate\_formation | 1 | 0 |  |  |  |  |  |  |  |  |
| GO:0021514\_ventral\_spinal\_cord\_interneuron\_differentiation | 1 | 0 |  |  |  |  |  |  |  |  |
| GO:0021521\_ventral\_spinal\_cord\_interneuron\_specification | 1 | 0 |  |  |  |  |  |  |  |  |
| GO:0021522\_spinal\_cord\_motor\_neuron\_differentiation | 1 | 0 |  |  |  |  |  |  |  |  |
| GO:0021527\_spinal\_cord\_association\_neuron\_differentiation | 1 | 0 |  |  |  |  |  |  |  |  |
| GO:0021528\_commissural\_neuron\_differentiation\_in\_the\_spinal\_cord | 1 | 0 |  |  |  |  |  |  |  |  |
| GO:0021533\_cell\_differentiation\_in\_hindbrain | 1 | 0 |  |  |  |  |  |  |  |  |
| GO:0021540\_corpus\_callosum\_morphogenesis | 1 | 0 |  |  |  |  |  |  |  |  |
| GO:0021544\_subpallium\_development | 1 | 0 |  |  |  |  |  |  |  |  |
| GO:0021554\_optic\_nerve\_development | 1 | 0 |  |  |  |  |  |  |  |  |
| GO:0021562\_vestibulocochlear\_nerve\_development | 1 | 0 |  |  |  |  |  |  |  |  |
| GO:0021602\_cranial\_nerve\_morphogenesis | 1 | 0 |  |  |  |  |  |  |  |  |
| GO:0021631\_optic\_nerve\_morphogenesis | 1 | 0 |  |  |  |  |  |  |  |  |
| GO:0021680\_cerebellar\_Purkinje\_cell\_layer\_development | 1 | 0 |  |  |  |  |  |  |  |  |
| GO:0021692\_cerebellar\_Purkinje\_cell\_layer\_morphogenesis | 1 | 0 |  |  |  |  |  |  |  |  |
| GO:0021694\_cerebellar\_Purkinje\_cell\_layer\_formation | 1 | 0 |  |  |  |  |  |  |  |  |
| GO:0021697\_cerebellar\_cortex\_formation | 1 | 0 |  |  |  |  |  |  |  |  |
| GO:0021702\_cerebellar\_Purkinje\_cell\_differentiation | 1 | 0 |  |  |  |  |  |  |  |  |
| GO:0021756\_striatum\_development | 1 | 0 |  |  |  |  |  |  |  |  |
| GO:0021757\_caudate\_nucleus\_development | 1 | 0 |  |  |  |  |  |  |  |  |
| GO:0021758\_putamen\_development | 1 | 0 |  |  |  |  |  |  |  |  |
| GO:0021761\_limbic\_system\_development | 1 | 0 |  |  |  |  |  |  |  |  |
| GO:0021771\_lateral\_geniculate\_nucleus\_development | 1 | 0 |  |  |  |  |  |  |  |  |
| GO:0021775\_smoothened\_signaling\_pathway\_involved\_in\_ventral\_spinal\_cord\_interneuron\_specification | 1 | 0 |  |  |  |  |  |  |  |  |
| GO:0021794\_thalamus\_development | 1 | 0 |  |  |  |  |  |  |  |  |
| GO:0021799\_cerebral\_cortex\_radially\_oriented\_cell\_migration | 1 | 0 |  |  |  |  |  |  |  |  |
| GO:0021800\_cerebral\_cortex\_tangential\_migration | 1 | 0 |  |  |  |  |  |  |  |  |
| GO:0021854\_hypothalamus\_development | 1 | 0 |  |  |  |  |  |  |  |  |
| GO:0021859\_pyramidal\_neuron\_differentiation | 1 | 0 |  |  |  |  |  |  |  |  |
| GO:0021860\_pyramidal\_neuron\_development | 1 | 0 |  |  |  |  |  |  |  |  |
| GO:0021872\_generation\_of\_neurons\_in\_the\_forebrain | 1 | 0 |  |  |  |  |  |  |  |  |
| GO:0021879\_forebrain\_neuron\_differentiation | 1 | 0 |  |  |  |  |  |  |  |  |
| GO:0021884\_forebrain\_neuron\_development | 1 | 0 |  |  |  |  |  |  |  |  |
| GO:0021896\_forebrain\_astrocyte\_differentiation | 1 | 0 |  |  |  |  |  |  |  |  |
| GO:0021897\_forebrain\_astrocyte\_development | 1 | 0 |  |  |  |  |  |  |  |  |
| GO:0021914\_negative\_regulation\_of\_smoothened\_signaling\_pathway\_involved\_in\_ventral\_spinal\_cord\_patterning | 1 | 0 |  |  |  |  |  |  |  |  |
| GO:0021919\_BMP\_signaling\_pathway\_in\_spinal\_cord\_dorsal\_ventral\_patterning | 1 | 0 |  |  |  |  |  |  |  |  |
| GO:0021965\_spinal\_cord\_ventral\_commissure\_morphogenesis | 1 | 0 |  |  |  |  |  |  |  |  |
| GO:0021984\_adenohypophysis\_development | 1 | 0 |  |  |  |  |  |  |  |  |
| GO:0021990\_neural\_plate\_formation | 1 | 0 |  |  |  |  |  |  |  |  |
| GO:0021997\_neural\_plate\_axis\_specification | 1 | 0 |  |  |  |  |  |  |  |  |
| GO:0021999\_neural\_plate\_anterior\_posterior\_pattern\_formation | 1 | 0 |  |  |  |  |  |  |  |  |
| GO:0022009\_central\_nervous\_system\_vasculogenesis | 1 | 0 |  |  |  |  |  |  |  |  |
| GO:0022038\_corpus\_callosum\_development | 1 | 0 |  |  |  |  |  |  |  |  |
| GO:0030007\_cellular\_potassium\_ion\_homeostasis | 1 | 0 |  |  |  |  |  |  |  |  |
| GO:0030011\_maintenance\_of\_cell\_polarity | 1 | 0 |  |  |  |  |  |  |  |  |
| GO:0030026\_cellular\_manganese\_ion\_homeostasis | 1 | 0 |  |  |  |  |  |  |  |  |
| GO:0030033\_microvillus\_assembly | 1 | 0 |  |  |  |  |  |  |  |  |
| GO:0030037\_actin\_filament\_reorganization\_during\_cell\_cycle | 1 | 0 |  |  |  |  |  |  |  |  |
| GO:0030047\_actin\_modification | 1 | 0 |  |  |  |  |  |  |  |  |
| GO:0030070\_insulin\_processing | 1 | 0 |  |  |  |  |  |  |  |  |
| GO:0030103\_vasopressin\_secretion | 1 | 0 |  |  |  |  |  |  |  |  |
| GO:0030186\_melatonin\_metabolic\_process | 1 | 0 |  |  |  |  |  |  |  |  |
| GO:0030187\_melatonin\_biosynthetic\_process | 1 | 0 |  |  |  |  |  |  |  |  |
| GO:0030212\_hyaluronan\_metabolic\_process | 1 | 0 |  |  |  |  |  |  |  |  |
| GO:0030220\_platelet\_formation | 1 | 0 |  |  |  |  |  |  |  |  |
| GO:0030238\_male\_sex\_determination | 1 | 0 |  |  |  |  |  |  |  |  |
| GO:0030259\_lipid\_glycosylation | 1 | 0 |  |  |  |  |  |  |  |  |
| GO:0030302\_deoxynucleotide\_transport | 1 | 0 |  |  |  |  |  |  |  |  |
| GO:0030327\_prenylated\_protein\_catabolic\_process | 1 | 0 |  |  |  |  |  |  |  |  |
| GO:0030389\_fructosamine\_metabolic\_process | 1 | 0 |  |  |  |  |  |  |  |  |
| GO:0030393\_fructoselysine\_metabolic\_process | 1 | 0 |  |  |  |  |  |  |  |  |
| GO:0030432\_peristalsis | 1 | 0 |  |  |  |  |  |  |  |  |
| GO:0030488\_tRNA\_methylation | 1 | 0 |  |  |  |  |  |  |  |  |
| GO:0030517\_negative\_regulation\_of\_axon\_extension | 1 | 0 |  |  |  |  |  |  |  |  |
| GO:0030581\_symbiont\_intracellular\_protein\_transport\_in\_host | 1 | 0 |  |  |  |  |  |  |  |  |
| GO:0030718\_germ-line\_stem\_cell\_maintenance | 1 | 0 |  |  |  |  |  |  |  |  |
| GO:0030728\_ovulation | 1 | 0 |  |  |  |  |  |  |  |  |
| GO:0030824\_negative\_regulation\_of\_cGMP\_metabolic\_process | 1 | 0 |  |  |  |  |  |  |  |  |
| GO:0030825\_positive\_regulation\_of\_cGMP\_metabolic\_process | 1 | 0 |  |  |  |  |  |  |  |  |
| GO:0030827\_negative\_regulation\_of\_cGMP\_biosynthetic\_process | 1 | 0 |  |  |  |  |  |  |  |  |
| GO:0030828\_positive\_regulation\_of\_cGMP\_biosynthetic\_process | 1 | 0 |  |  |  |  |  |  |  |  |
| GO:0030854\_positive\_regulation\_of\_granulocyte\_differentiation | 1 | 0 |  |  |  |  |  |  |  |  |
| GO:0030857\_negative\_regulation\_of\_epithelial\_cell\_differentiation | 1 | 0 |  |  |  |  |  |  |  |  |
| GO:0030878\_thyroid\_gland\_development | 1 | 0 |  |  |  |  |  |  |  |  |
| GO:0030885\_regulation\_of\_myeloid\_dendritic\_cell\_activation | 1 | 0 |  |  |  |  |  |  |  |  |
| GO:0030887\_positive\_regulation\_of\_myeloid\_dendritic\_cell\_activation | 1 | 0 |  |  |  |  |  |  |  |  |
| GO:0030903\_notochord\_development | 1 | 0 |  |  |  |  |  |  |  |  |
| GO:0030910\_olfactory\_placode\_formation | 1 | 0 |  |  |  |  |  |  |  |  |
| GO:0030913\_paranodal\_junction\_assembly | 1 | 0 |  |  |  |  |  |  |  |  |
| GO:0030948\_negative\_regulation\_of\_vascular\_endothelial\_growth\_factor\_receptor\_signaling\_pathway | 1 | 0 |  |  |  |  |  |  |  |  |
| GO:0030967\_ER-nuclear\_sterol\_response\_pathway | 1 | 0 |  |  |  |  |  |  |  |  |
| GO:0031017\_exocrine\_pancreas\_development | 1 | 0 |  |  |  |  |  |  |  |  |
| GO:0031063\_regulation\_of\_histone\_deacetylation | 1 | 0 |  |  |  |  |  |  |  |  |
| GO:0031065\_positive\_regulation\_of\_histone\_deacetylation | 1 | 0 |  |  |  |  |  |  |  |  |
| GO:0031076\_embryonic\_camera-type\_eye\_development | 1 | 0 |  |  |  |  |  |  |  |  |
| GO:0031081\_nuclear\_pore\_distribution | 1 | 0 |  |  |  |  |  |  |  |  |
| GO:0031086\_nuclear-transcribed\_mRNA\_catabolic\_process\_\_deadenylation-independent\_decay | 1 | 0 |  |  |  |  |  |  |  |  |
| GO:0031087\_deadenylation-independent\_decapping\_of\_nuclear-transcribed\_mRNA | 1 | 0 |  |  |  |  |  |  |  |  |
| GO:0031106\_septin\_ring\_organization | 1 | 0 |  |  |  |  |  |  |  |  |
| GO:0031115\_negative\_regulation\_of\_microtubule\_polymerization | 1 | 0 |  |  |  |  |  |  |  |  |
| GO:0031117\_positive\_regulation\_of\_microtubule\_depolymerization | 1 | 0 |  |  |  |  |  |  |  |  |
| GO:0031118\_rRNA\_pseudouridine\_synthesis | 1 | 0 |  |  |  |  |  |  |  |  |
| GO:0031125\_rRNA\_3'-end\_processing | 1 | 0 |  |  |  |  |  |  |  |  |
| GO:0031146\_SCF-dependent\_proteasomal\_ubiquitin-dependent\_protein\_catabolic\_process | 1 | 0 |  |  |  |  |  |  |  |  |
| GO:0031179\_peptide\_modification | 1 | 0 |  |  |  |  |  |  |  |  |
| GO:0031282\_regulation\_of\_guanylate\_cyclase\_activity | 1 | 0 |  |  |  |  |  |  |  |  |
| GO:0031284\_positive\_regulation\_of\_guanylate\_cyclase\_activity | 1 | 0 |  |  |  |  |  |  |  |  |
| GO:0031290\_retinal\_ganglion\_cell\_axon\_guidance | 1 | 0 |  |  |  |  |  |  |  |  |
| GO:0031293\_membrane\_protein\_intracellular\_domain\_proteolysis | 1 | 0 |  |  |  |  |  |  |  |  |
| GO:0031335\_regulation\_of\_sulfur\_amino\_acid\_metabolic\_process | 1 | 0 |  |  |  |  |  |  |  |  |
| GO:0031342\_negative\_regulation\_of\_cell\_killing | 1 | 0 |  |  |  |  |  |  |  |  |
| GO:0031424\_keratinization | 1 | 0 |  |  |  |  |  |  |  |  |
| GO:0031441\_negative\_regulation\_of\_mRNA\_3'-end\_processing | 1 | 0 |  |  |  |  |  |  |  |  |
| GO:0031442\_positive\_regulation\_of\_mRNA\_3'-end\_processing | 1 | 0 |  |  |  |  |  |  |  |  |
| GO:0031443\_fast-twitch\_skeletal\_muscle\_fiber\_contraction | 1 | 0 |  |  |  |  |  |  |  |  |
| GO:0031446\_regulation\_of\_fast-twitch\_skeletal\_muscle\_fiber\_contraction | 1 | 0 |  |  |  |  |  |  |  |  |
| GO:0031448\_positive\_regulation\_of\_fast-twitch\_skeletal\_muscle\_fiber\_contraction | 1 | 0 |  |  |  |  |  |  |  |  |
| GO:0031453\_positive\_regulation\_of\_heterochromatin\_formation | 1 | 0 |  |  |  |  |  |  |  |  |
| GO:0031557\_induction\_of\_programmed\_cell\_death\_in\_response\_to\_chemical\_stimulus | 1 | 0 |  |  |  |  |  |  |  |  |
| GO:0031574\_S-M\_checkpoint | 1 | 0 |  |  |  |  |  |  |  |  |
| GO:0031581\_hemidesmosome\_assembly | 1 | 0 |  |  |  |  |  |  |  |  |
| GO:0031627\_telomeric\_loop\_formation | 1 | 0 |  |  |  |  |  |  |  |  |
| GO:0031848\_protection\_from\_non-homologous\_end\_joining\_at\_telomere | 1 | 0 |  |  |  |  |  |  |  |  |
| GO:0031937\_positive\_regulation\_of\_chromatin\_silencing | 1 | 0 |  |  |  |  |  |  |  |  |
| GO:0031943\_regulation\_of\_glucocorticoid\_metabolic\_process | 1 | 0 |  |  |  |  |  |  |  |  |
| GO:0031954\_positive\_regulation\_of\_protein\_amino\_acid\_autophosphorylation | 1 | 0 |  |  |  |  |  |  |  |  |
| GO:0031999\_negative\_regulation\_of\_fatty\_acid\_beta-oxidation | 1 | 0 |  |  |  |  |  |  |  |  |
| GO:0032011\_ARF\_protein\_signal\_transduction | 1 | 0 |  |  |  |  |  |  |  |  |
| GO:0032023\_trypsinogen\_activation | 1 | 0 |  |  |  |  |  |  |  |  |
| GO:0032025\_response\_to\_cobalt\_ion | 1 | 0 |  |  |  |  |  |  |  |  |
| GO:0032048\_cardiolipin\_metabolic\_process | 1 | 0 |  |  |  |  |  |  |  |  |
| GO:0032049\_cardiolipin\_biosynthetic\_process | 1 | 0 |  |  |  |  |  |  |  |  |
| GO:0032060\_bleb\_formation | 1 | 0 |  |  |  |  |  |  |  |  |
| GO:0032066\_nucleolus\_to\_nucleoplasm\_transport | 1 | 0 |  |  |  |  |  |  |  |  |
| GO:0032074\_negative\_regulation\_of\_nuclease\_activity | 1 | 0 |  |  |  |  |  |  |  |  |
| GO:0032075\_positive\_regulation\_of\_nuclease\_activity | 1 | 0 |  |  |  |  |  |  |  |  |
| GO:0032119\_sequestering\_of\_zinc\_ion | 1 | 0 |  |  |  |  |  |  |  |  |
| GO:0032185\_septin\_cytoskeleton\_organization | 1 | 0 |  |  |  |  |  |  |  |  |
| GO:0032196\_transposition | 1 | 0 |  |  |  |  |  |  |  |  |
| GO:0032235\_negative\_regulation\_of\_calcium\_ion\_transport\_via\_store-operated\_calcium\_channel\_activity | 1 | 0 |  |  |  |  |  |  |  |  |
| GO:0032241\_positive\_regulation\_of\_nucleobase\_\_nucleoside\_\_nucleotide\_and\_nucleic\_acid\_transport | 1 | 0 |  |  |  |  |  |  |  |  |
| GO:0032261\_purine\_nucleotide\_salvage | 1 | 0 |  |  |  |  |  |  |  |  |
| GO:0032275\_luteinizing\_hormone\_secretion | 1 | 0 |  |  |  |  |  |  |  |  |
| GO:0032287\_myelin\_maintenance\_in\_the\_peripheral\_nervous\_system | 1 | 0 |  |  |  |  |  |  |  |  |
| GO:0032288\_myelin\_assembly | 1 | 0 |  |  |  |  |  |  |  |  |
| GO:0032314\_regulation\_of\_Rac\_GTPase\_activity | 1 | 0 |  |  |  |  |  |  |  |  |
| GO:0032330\_regulation\_of\_chondrocyte\_differentiation | 1 | 0 |  |  |  |  |  |  |  |  |
| GO:0032331\_negative\_regulation\_of\_chondrocyte\_differentiation | 1 | 0 |  |  |  |  |  |  |  |  |
| GO:0032346\_positive\_regulation\_of\_aldosterone\_metabolic\_process | 1 | 0 |  |  |  |  |  |  |  |  |
| GO:0032347\_regulation\_of\_aldosterone\_biosynthetic\_process | 1 | 0 |  |  |  |  |  |  |  |  |
| GO:0032349\_positive\_regulation\_of\_aldosterone\_biosynthetic\_process | 1 | 0 |  |  |  |  |  |  |  |  |
| GO:0032354\_response\_to\_follicle-stimulating\_hormone\_stimulus | 1 | 0 |  |  |  |  |  |  |  |  |
| GO:0032377\_regulation\_of\_intracellular\_lipid\_transport | 1 | 0 |  |  |  |  |  |  |  |  |
| GO:0032380\_regulation\_of\_intracellular\_sterol\_transport | 1 | 0 |  |  |  |  |  |  |  |  |
| GO:0032383\_regulation\_of\_intracellular\_cholesterol\_transport | 1 | 0 |  |  |  |  |  |  |  |  |
| GO:0032423\_regulation\_of\_mismatch\_repair | 1 | 0 |  |  |  |  |  |  |  |  |
| GO:0032425\_positive\_regulation\_of\_mismatch\_repair | 1 | 0 |  |  |  |  |  |  |  |  |
| GO:0032459\_regulation\_of\_protein\_oligomerization | 1 | 0 |  |  |  |  |  |  |  |  |
| GO:0032460\_negative\_regulation\_of\_protein\_oligomerization | 1 | 0 |  |  |  |  |  |  |  |  |
| GO:0032462\_regulation\_of\_protein\_homooligomerization | 1 | 0 |  |  |  |  |  |  |  |  |
| GO:0032463\_negative\_regulation\_of\_protein\_homooligomerization | 1 | 0 |  |  |  |  |  |  |  |  |
| GO:0032467\_positive\_regulation\_of\_cytokinesis | 1 | 0 |  |  |  |  |  |  |  |  |
| GO:0032468\_Golgi\_calcium\_ion\_homeostasis | 1 | 0 |  |  |  |  |  |  |  |  |
| GO:0032470\_elevation\_of\_endoplasmic\_reticulum\_calcium\_ion\_concentration | 1 | 0 |  |  |  |  |  |  |  |  |
| GO:0032471\_reduction\_of\_endoplasmic\_reticulum\_calcium\_ion\_concentration | 1 | 0 |  |  |  |  |  |  |  |  |
| GO:0032472\_Golgi\_calcium\_ion\_transport | 1 | 0 |  |  |  |  |  |  |  |  |
| GO:0032486\_Rap\_protein\_signal\_transduction | 1 | 0 |  |  |  |  |  |  |  |  |
| GO:0032495\_response\_to\_muramyl\_dipeptide | 1 | 0 |  |  |  |  |  |  |  |  |
| GO:0032498\_detection\_of\_muramyl\_dipeptide | 1 | 0 |  |  |  |  |  |  |  |  |
| GO:0032499\_detection\_of\_peptidoglycan | 1 | 0 |  |  |  |  |  |  |  |  |
| GO:0032528\_microvillus\_organization | 1 | 0 |  |  |  |  |  |  |  |  |
| GO:0032581\_ER-dependent\_peroxisome\_biogenesis | 1 | 0 |  |  |  |  |  |  |  |  |
| GO:0032594\_protein\_transport\_within\_lipid\_bilayer | 1 | 0 |  |  |  |  |  |  |  |  |
| GO:0032595\_B\_cell\_receptor\_transport\_within\_lipid\_bilayer | 1 | 0 |  |  |  |  |  |  |  |  |
| GO:0032596\_protein\_transport\_into\_membrane\_raft | 1 | 0 |  |  |  |  |  |  |  |  |
| GO:0032597\_B\_cell\_receptor\_transport\_into\_membrane\_raft | 1 | 0 |  |  |  |  |  |  |  |  |
| GO:0032599\_protein\_transport\_out\_of\_membrane\_raft | 1 | 0 |  |  |  |  |  |  |  |  |
| GO:0032600\_chemokine\_receptor\_transport\_out\_of\_membrane\_raft | 1 | 0 |  |  |  |  |  |  |  |  |
| GO:0032601\_connective\_tissue\_growth\_factor\_production | 1 | 0 |  |  |  |  |  |  |  |  |
| GO:0032603\_fractalkine\_production | 1 | 0 |  |  |  |  |  |  |  |  |
| GO:0032605\_hepatocyte\_growth\_factor\_production | 1 | 0 |  |  |  |  |  |  |  |  |
| GO:0032610\_interleukin-1\_alpha\_production | 1 | 0 |  |  |  |  |  |  |  |  |
| GO:0032621\_interleukin-18\_production | 1 | 0 |  |  |  |  |  |  |  |  |
| GO:0032639\_TRAIL\_production | 1 | 0 |  |  |  |  |  |  |  |  |
| GO:0032644\_regulation\_of\_fractalkine\_production | 1 | 0 |  |  |  |  |  |  |  |  |
| GO:0032646\_regulation\_of\_hepatocyte\_growth\_factor\_production | 1 | 0 |  |  |  |  |  |  |  |  |
| GO:0032650\_regulation\_of\_interleukin-1\_alpha\_production | 1 | 0 |  |  |  |  |  |  |  |  |
| GO:0032661\_regulation\_of\_interleukin-18\_production | 1 | 0 |  |  |  |  |  |  |  |  |
| GO:0032679\_regulation\_of\_TRAIL\_production | 1 | 0 |  |  |  |  |  |  |  |  |
| GO:0032681\_regulation\_of\_lymphotoxin\_A\_production | 1 | 0 |  |  |  |  |  |  |  |  |
| GO:0032693\_negative\_regulation\_of\_interleukin-10\_production | 1 | 0 |  |  |  |  |  |  |  |  |
| GO:0032703\_negative\_regulation\_of\_interleukin-2\_production | 1 | 0 |  |  |  |  |  |  |  |  |
| GO:0032713\_negative\_regulation\_of\_interleukin-4\_production | 1 | 0 |  |  |  |  |  |  |  |  |
| GO:0032730\_positive\_regulation\_of\_interleukin-1\_alpha\_production | 1 | 0 |  |  |  |  |  |  |  |  |
| GO:0032732\_positive\_regulation\_of\_interleukin-1\_production | 1 | 0 |  |  |  |  |  |  |  |  |
| GO:0032736\_positive\_regulation\_of\_interleukin-13\_production | 1 | 0 |  |  |  |  |  |  |  |  |
| GO:0032753\_positive\_regulation\_of\_interleukin-4\_production | 1 | 0 |  |  |  |  |  |  |  |  |
| GO:0032754\_positive\_regulation\_of\_interleukin-5\_production | 1 | 0 |  |  |  |  |  |  |  |  |
| GO:0032762\_mast\_cell\_cytokine\_production | 1 | 0 |  |  |  |  |  |  |  |  |
| GO:0032763\_regulation\_of\_mast\_cell\_cytokine\_production | 1 | 0 |  |  |  |  |  |  |  |  |
| GO:0032765\_positive\_regulation\_of\_mast\_cell\_cytokine\_production | 1 | 0 |  |  |  |  |  |  |  |  |
| GO:0032784\_regulation\_of\_RNA\_elongation | 1 | 0 |  |  |  |  |  |  |  |  |
| GO:0032786\_positive\_regulation\_of\_RNA\_elongation | 1 | 0 |  |  |  |  |  |  |  |  |
| GO:0032788\_saturated\_monocarboxylic\_acid\_metabolic\_process | 1 | 0 |  |  |  |  |  |  |  |  |
| GO:0032789\_unsaturated\_monocarboxylic\_acid\_metabolic\_process | 1 | 0 |  |  |  |  |  |  |  |  |
| GO:0032790\_ribosome\_disassembly | 1 | 0 |  |  |  |  |  |  |  |  |
| GO:0032792\_negative\_regulation\_of\_CREB\_transcription\_factor\_activity | 1 | 0 |  |  |  |  |  |  |  |  |
| GO:0032793\_positive\_regulation\_of\_CREB\_transcription\_factor\_activity | 1 | 0 |  |  |  |  |  |  |  |  |
| GO:0032804\_negative\_regulation\_of\_low-density\_lipoprotein\_receptor\_catabolic\_process | 1 | 0 |  |  |  |  |  |  |  |  |
| GO:0032805\_positive\_regulation\_of\_low-density\_lipoprotein\_receptor\_catabolic\_process | 1 | 0 |  |  |  |  |  |  |  |  |
| GO:0032812\_positive\_regulation\_of\_epinephrine\_secretion | 1 | 0 |  |  |  |  |  |  |  |  |
| GO:0032835\_glomerulus\_development | 1 | 0 |  |  |  |  |  |  |  |  |
| GO:0032847\_regulation\_of\_cellular\_pH\_reduction | 1 | 0 |  |  |  |  |  |  |  |  |
| GO:0032848\_negative\_regulation\_of\_cellular\_pH\_reduction | 1 | 0 |  |  |  |  |  |  |  |  |
| GO:0032899\_regulation\_of\_neurotrophin\_production | 1 | 0 |  |  |  |  |  |  |  |  |
| GO:0032900\_negative\_regulation\_of\_neurotrophin\_production | 1 | 0 |  |  |  |  |  |  |  |  |
| GO:0032903\_regulation\_of\_nerve\_growth\_factor\_production | 1 | 0 |  |  |  |  |  |  |  |  |
| GO:0032904\_negative\_regulation\_of\_nerve\_growth\_factor\_production | 1 | 0 |  |  |  |  |  |  |  |  |
| GO:0032907\_transforming\_growth\_factor-beta3\_production | 1 | 0 |  |  |  |  |  |  |  |  |
| GO:0032910\_regulation\_of\_transforming\_growth\_factor-beta3\_production | 1 | 0 |  |  |  |  |  |  |  |  |
| GO:0032911\_negative\_regulation\_of\_transforming\_growth\_factor-beta1\_production | 1 | 0 |  |  |  |  |  |  |  |  |
| GO:0032913\_negative\_regulation\_of\_transforming\_growth\_factor-beta3\_production | 1 | 0 |  |  |  |  |  |  |  |  |
| GO:0032926\_negative\_regulation\_of\_activin\_receptor\_signaling\_pathway | 1 | 0 |  |  |  |  |  |  |  |  |
| GO:0032933\_SREBP-mediated\_signaling\_pathway | 1 | 0 |  |  |  |  |  |  |  |  |
| GO:0032938\_negative\_regulation\_of\_translation\_in\_response\_to\_oxidative\_stress | 1 | 0 |  |  |  |  |  |  |  |  |
| GO:0032976\_release\_of\_matrix\_enzymes\_from\_mitochondria | 1 | 0 |  |  |  |  |  |  |  |  |
| GO:0032980\_keratinocyte\_activation | 1 | 0 |  |  |  |  |  |  |  |  |
| GO:0032988\_ribonucleoprotein\_complex\_disassembly | 1 | 0 |  |  |  |  |  |  |  |  |
| GO:0033029\_regulation\_of\_neutrophil\_apoptosis | 1 | 0 |  |  |  |  |  |  |  |  |
| GO:0033031\_positive\_regulation\_of\_neutrophil\_apoptosis | 1 | 0 |  |  |  |  |  |  |  |  |
| GO:0033079\_immature\_T\_cell\_proliferation | 1 | 0 |  |  |  |  |  |  |  |  |
| GO:0033080\_immature\_T\_cell\_proliferation\_in\_the\_thymus | 1 | 0 |  |  |  |  |  |  |  |  |
| GO:0033083\_regulation\_of\_immature\_T\_cell\_proliferation | 1 | 0 |  |  |  |  |  |  |  |  |
| GO:0033084\_regulation\_of\_immature\_T\_cell\_proliferation\_in\_the\_thymus | 1 | 0 |  |  |  |  |  |  |  |  |
| GO:0033085\_negative\_regulation\_of\_T\_cell\_differentiation\_in\_the\_thymus | 1 | 0 |  |  |  |  |  |  |  |  |
| GO:0033087\_negative\_regulation\_of\_immature\_T\_cell\_proliferation | 1 | 0 |  |  |  |  |  |  |  |  |
| GO:0033088\_negative\_regulation\_of\_immature\_T\_cell\_proliferation\_in\_the\_thymus | 1 | 0 |  |  |  |  |  |  |  |  |
| GO:0033136\_serine\_phosphorylation\_of\_STAT3\_protein | 1 | 0 |  |  |  |  |  |  |  |  |
| GO:0033137\_negative\_regulation\_of\_peptidyl-serine\_phosphorylation | 1 | 0 |  |  |  |  |  |  |  |  |
| GO:0033139\_regulation\_of\_peptidyl-serine\_phosphorylation\_of\_STAT\_protein | 1 | 0 |  |  |  |  |  |  |  |  |
| GO:0033141\_positive\_regulation\_of\_peptidyl-serine\_phosphorylation\_of\_STAT\_protein | 1 | 0 |  |  |  |  |  |  |  |  |
| GO:0033153\_T\_cell\_receptor\_V(D)J\_recombination | 1 | 0 |  |  |  |  |  |  |  |  |
| GO:0033169\_histone\_H3-K9\_demethylation | 1 | 0 |  |  |  |  |  |  |  |  |
| GO:0033173\_calcineurin-NFAT\_signaling\_pathway | 1 | 0 |  |  |  |  |  |  |  |  |
| GO:0033182\_regulation\_of\_histone\_ubiquitination | 1 | 0 |  |  |  |  |  |  |  |  |
| GO:0033206\_cytokinesis\_after\_meiosis | 1 | 0 |  |  |  |  |  |  |  |  |
| GO:0033239\_negative\_regulation\_of\_cellular\_amine\_metabolic\_process | 1 | 0 |  |  |  |  |  |  |  |  |
| GO:0033240\_positive\_regulation\_of\_cellular\_amine\_metabolic\_process | 1 | 0 |  |  |  |  |  |  |  |  |
| GO:0033260\_DNA\_replication\_during\_S\_phase | 1 | 0 |  |  |  |  |  |  |  |  |
| GO:0033262\_regulation\_of\_DNA\_replication\_during\_S\_phase | 1 | 0 |  |  |  |  |  |  |  |  |
| GO:0033292\_T-tubule\_organization | 1 | 0 |  |  |  |  |  |  |  |  |
| GO:0033341\_regulation\_of\_collagen\_binding | 1 | 0 |  |  |  |  |  |  |  |  |
| GO:0033342\_negative\_regulation\_of\_collagen\_binding | 1 | 0 |  |  |  |  |  |  |  |  |
| GO:0033345\_asparagine\_catabolic\_process\_via\_L-aspartate | 1 | 0 |  |  |  |  |  |  |  |  |
| GO:0033366\_protein\_localization\_in\_secretory\_granule | 1 | 0 |  |  |  |  |  |  |  |  |
| GO:0033367\_protein\_localization\_in\_mast\_cell\_secretory\_granule | 1 | 0 |  |  |  |  |  |  |  |  |
| GO:0033368\_protease\_localization\_in\_mast\_cell\_secretory\_granule | 1 | 0 |  |  |  |  |  |  |  |  |
| GO:0033370\_maintenance\_of\_protein\_location\_in\_mast\_cell\_secretory\_granule | 1 | 0 |  |  |  |  |  |  |  |  |
| GO:0033371\_T\_cell\_secretory\_granule\_organization | 1 | 0 |  |  |  |  |  |  |  |  |
| GO:0033373\_maintenance\_of\_protease\_location\_in\_mast\_cell\_secretory\_granule | 1 | 0 |  |  |  |  |  |  |  |  |
| GO:0033374\_protein\_localization\_in\_T\_cell\_secretory\_granule | 1 | 0 |  |  |  |  |  |  |  |  |
| GO:0033375\_protease\_localization\_in\_T\_cell\_secretory\_granule | 1 | 0 |  |  |  |  |  |  |  |  |
| GO:0033377\_maintenance\_of\_protein\_location\_in\_T\_cell\_secretory\_granule | 1 | 0 |  |  |  |  |  |  |  |  |
| GO:0033379\_maintenance\_of\_protease\_location\_in\_T\_cell\_secretory\_granule | 1 | 0 |  |  |  |  |  |  |  |  |
| GO:0033380\_granzyme\_B\_localization\_in\_T\_cell\_secretory\_granule | 1 | 0 |  |  |  |  |  |  |  |  |
| GO:0033382\_maintenance\_of\_granzyme\_B\_location\_in\_T\_cell\_secretory\_granule | 1 | 0 |  |  |  |  |  |  |  |  |
| GO:0033484\_nitric\_oxide\_homeostasis | 1 | 0 |  |  |  |  |  |  |  |  |
| GO:0033504\_floor\_plate\_development | 1 | 0 |  |  |  |  |  |  |  |  |
| GO:0033566\_gamma-tubulin\_complex\_localization | 1 | 0 |  |  |  |  |  |  |  |  |
| GO:0033577\_protein\_amino\_acid\_glycosylation\_in\_endoplasmic\_reticulum | 1 | 0 |  |  |  |  |  |  |  |  |
| GO:0033595\_response\_to\_genistein | 1 | 0 |  |  |  |  |  |  |  |  |
| GO:0033600\_negative\_regulation\_of\_mammary\_gland\_epithelial\_cell\_proliferation | 1 | 0 |  |  |  |  |  |  |  |  |
| GO:0033606\_chemokine\_receptor\_transport\_within\_lipid\_bilayer | 1 | 0 |  |  |  |  |  |  |  |  |
| GO:0033617\_mitochondrial\_respiratory\_chain\_complex\_IV\_assembly | 1 | 0 |  |  |  |  |  |  |  |  |
| GO:0033693\_neurofilament\_bundle\_assembly | 1 | 0 |  |  |  |  |  |  |  |  |
| GO:0033750\_ribosome\_localization | 1 | 0 |  |  |  |  |  |  |  |  |
| GO:0033753\_establishment\_of\_ribosome\_localization | 1 | 0 |  |  |  |  |  |  |  |  |
| GO:0033875\_ribonucleoside\_bisphosphate\_metabolic\_process | 1 | 0 |  |  |  |  |  |  |  |  |
| GO:0033962\_cytoplasmic\_mRNA\_processing\_body\_assembly | 1 | 0 |  |  |  |  |  |  |  |  |
| GO:0034032\_purine\_nucleoside\_bisphosphate\_metabolic\_process | 1 | 0 |  |  |  |  |  |  |  |  |
| GO:0034035\_purine\_ribonucleoside\_bisphosphate\_metabolic\_process | 1 | 0 |  |  |  |  |  |  |  |  |
| GO:0034063\_stress\_granule\_assembly | 1 | 0 |  |  |  |  |  |  |  |  |
| GO:0034080\_CenH3-containing\_nucleosome\_assembly\_at\_centromere | 1 | 0 |  |  |  |  |  |  |  |  |
| GO:0034115\_negative\_regulation\_of\_heterotypic\_cell-cell\_adhesion | 1 | 0 |  |  |  |  |  |  |  |  |
| GO:0034116\_positive\_regulation\_of\_heterotypic\_cell-cell\_adhesion | 1 | 0 |  |  |  |  |  |  |  |  |
| GO:0034122\_negative\_regulation\_of\_toll-like\_receptor\_signaling\_pathway | 1 | 0 |  |  |  |  |  |  |  |  |
| GO:0034123\_positive\_regulation\_of\_toll-like\_receptor\_signaling\_pathway | 1 | 0 |  |  |  |  |  |  |  |  |
| GO:0034142\_toll-like\_receptor\_4\_signaling\_pathway | 1 | 0 |  |  |  |  |  |  |  |  |
| GO:0034143\_regulation\_of\_toll-like\_receptor\_4\_signaling\_pathway | 1 | 0 |  |  |  |  |  |  |  |  |
| GO:0034145\_positive\_regulation\_of\_toll-like\_receptor\_4\_signaling\_pathway | 1 | 0 |  |  |  |  |  |  |  |  |
| GO:0034196\_acylglycerol\_transport | 1 | 0 |  |  |  |  |  |  |  |  |
| GO:0034197\_triglyceride\_transport | 1 | 0 |  |  |  |  |  |  |  |  |
| GO:0034205\_beta-amyloid\_formation | 1 | 0 |  |  |  |  |  |  |  |  |
| GO:0034213\_quinolinate\_catabolic\_process | 1 | 0 |  |  |  |  |  |  |  |  |
| GO:0034231\_islet\_amyloid\_polypeptide\_processing | 1 | 0 |  |  |  |  |  |  |  |  |
| GO:0034238\_macrophage\_fusion | 1 | 0 |  |  |  |  |  |  |  |  |
| GO:0034239\_regulation\_of\_macrophage\_fusion | 1 | 0 |  |  |  |  |  |  |  |  |
| GO:0034241\_positive\_regulation\_of\_macrophage\_fusion | 1 | 0 |  |  |  |  |  |  |  |  |
| GO:0034248\_regulation\_of\_amide\_metabolic\_process | 1 | 0 |  |  |  |  |  |  |  |  |
| GO:0034263\_autophagy\_in\_response\_to\_ER\_overload | 1 | 0 |  |  |  |  |  |  |  |  |
| GO:0034287\_detection\_of\_monosaccharide\_stimulus | 1 | 0 |  |  |  |  |  |  |  |  |
| GO:0034313\_diol\_catabolic\_process | 1 | 0 |  |  |  |  |  |  |  |  |
| GO:0034332\_adherens\_junction\_organization | 1 | 0 |  |  |  |  |  |  |  |  |
| GO:0034333\_adherens\_junction\_assembly | 1 | 0 |  |  |  |  |  |  |  |  |
| GO:0034340\_response\_to\_type\_I\_interferon | 1 | 0 |  |  |  |  |  |  |  |  |
| GO:0034356\_NAD\_biosynthesis\_via\_nicotinamide\_riboside\_salvage\_pathway | 1 | 0 |  |  |  |  |  |  |  |  |
| GO:0034373\_intermediate-density\_lipoprotein\_particle\_remodeling | 1 | 0 |  |  |  |  |  |  |  |  |
| GO:0034378\_chylomicron\_assembly | 1 | 0 |  |  |  |  |  |  |  |  |
| GO:0034436\_glycoprotein\_transport | 1 | 0 |  |  |  |  |  |  |  |  |
| GO:0034439\_lipoprotein\_lipid\_oxidation | 1 | 0 |  |  |  |  |  |  |  |  |
| GO:0034454\_microtubule\_anchoring\_at\_centrosome | 1 | 0 |  |  |  |  |  |  |  |  |
| GO:0034465\_response\_to\_carbon\_monoxide | 1 | 0 |  |  |  |  |  |  |  |  |
| GO:0034509\_centromeric\_core\_chromatin\_formation | 1 | 0 |  |  |  |  |  |  |  |  |
| GO:0034516\_response\_to\_vitamin\_B6 | 1 | 0 |  |  |  |  |  |  |  |  |
| GO:0034551\_mitochondrial\_respiratory\_chain\_complex\_III\_assembly | 1 | 0 |  |  |  |  |  |  |  |  |
| GO:0034552\_respiratory\_chain\_complex\_II\_assembly | 1 | 0 |  |  |  |  |  |  |  |  |
| GO:0034553\_mitochondrial\_respiratory\_chain\_complex\_II\_assembly | 1 | 0 |  |  |  |  |  |  |  |  |
| GO:0034589\_hydroxyproline\_transport | 1 | 0 |  |  |  |  |  |  |  |  |
| GO:0034694\_response\_to\_prostaglandin\_stimulus | 1 | 0 |  |  |  |  |  |  |  |  |
| GO:0034695\_response\_to\_prostaglandin\_E\_stimulus | 1 | 0 |  |  |  |  |  |  |  |  |
| GO:0034698\_response\_to\_gonadotropin\_stimulus | 1 | 0 |  |  |  |  |  |  |  |  |
| GO:0034699\_response\_to\_luteinizing\_hormone\_stimulus | 1 | 0 |  |  |  |  |  |  |  |  |
| GO:0034724\_DNA\_replication-independent\_nucleosome\_organization | 1 | 0 |  |  |  |  |  |  |  |  |
| GO:0034729\_histone\_H3-K79\_methylation | 1 | 0 |  |  |  |  |  |  |  |  |
| GO:0034755\_iron\_ion\_transmembrane\_transport | 1 | 0 |  |  |  |  |  |  |  |  |
| GO:0034764\_positive\_regulation\_of\_transmembrane\_transport | 1 | 0 |  |  |  |  |  |  |  |  |
| GO:0034765\_regulation\_of\_ion\_transmembrane\_transport | 1 | 0 |  |  |  |  |  |  |  |  |
| GO:0034767\_positive\_regulation\_of\_ion\_transmembrane\_transport | 1 | 0 |  |  |  |  |  |  |  |  |
| GO:0034959\_endothelin\_maturation | 1 | 0 |  |  |  |  |  |  |  |  |
| GO:0034982\_mitochondrial\_protein\_processing | 1 | 0 |  |  |  |  |  |  |  |  |
| GO:0034983\_peptidyl-lysine\_deacetylation | 1 | 0 |  |  |  |  |  |  |  |  |
| GO:0035021\_negative\_regulation\_of\_Rac\_protein\_signal\_transduction | 1 | 0 |  |  |  |  |  |  |  |  |
| GO:0035041\_sperm\_chromatin\_decondensation | 1 | 0 |  |  |  |  |  |  |  |  |
| GO:0035042\_fertilization\_\_exchange\_of\_chromosomal\_proteins | 1 | 0 |  |  |  |  |  |  |  |  |
| GO:0035054\_embryonic\_heart\_tube\_anterior\_posterior\_pattern\_formation | 1 | 0 |  |  |  |  |  |  |  |  |
| GO:0035066\_positive\_regulation\_of\_histone\_acetylation | 1 | 0 |  |  |  |  |  |  |  |  |
| GO:0035082\_axoneme\_assembly | 1 | 0 |  |  |  |  |  |  |  |  |
| GO:0035087\_RNA\_interference\_\_siRNA\_loading\_onto\_RISC | 1 | 0 |  |  |  |  |  |  |  |  |
| GO:0035090\_maintenance\_of\_apical\_basal\_cell\_polarity | 1 | 0 |  |  |  |  |  |  |  |  |
| GO:0035093\_spermatogenesis\_\_exchange\_of\_chromosomal\_proteins | 1 | 0 |  |  |  |  |  |  |  |  |
| GO:0035104\_positive\_regulation\_of\_transcription\_via\_sterol\_regulatory\_element\_binding | 1 | 0 |  |  |  |  |  |  |  |  |
| GO:0035110\_leg\_morphogenesis | 1 | 0 |  |  |  |  |  |  |  |  |
| GO:0035112\_genitalia\_morphogenesis | 1 | 0 |  |  |  |  |  |  |  |  |
| GO:0035116\_embryonic\_hindlimb\_morphogenesis | 1 | 0 |  |  |  |  |  |  |  |  |
| GO:0035137\_hindlimb\_morphogenesis | 1 | 0 |  |  |  |  |  |  |  |  |
| GO:0035238\_vitamin\_A\_biosynthetic\_process | 1 | 0 |  |  |  |  |  |  |  |  |
| GO:0035265\_organ\_growth | 1 | 0 |  |  |  |  |  |  |  |  |
| GO:0035280\_gene\_silencing\_by\_miRNA\_\_miRNA\_loading\_onto\_RISC | 1 | 0 |  |  |  |  |  |  |  |  |
| GO:0040009\_regulation\_of\_growth\_rate | 1 | 0 |  |  |  |  |  |  |  |  |
| GO:0040013\_negative\_regulation\_of\_locomotion | 1 | 0 |  |  |  |  |  |  |  |  |
| GO:0040015\_negative\_regulation\_of\_multicellular\_organism\_growth | 1 | 0 |  |  |  |  |  |  |  |  |
| GO:0040020\_regulation\_of\_meiosis | 1 | 0 |  |  |  |  |  |  |  |  |
| GO:0040023\_establishment\_of\_nucleus\_localization | 1 | 0 |  |  |  |  |  |  |  |  |
| GO:0040030\_regulation\_of\_molecular\_function\_\_epigenetic | 1 | 0 |  |  |  |  |  |  |  |  |
| GO:0040037\_negative\_regulation\_of\_fibroblast\_growth\_factor\_receptor\_signaling\_pathway | 1 | 0 |  |  |  |  |  |  |  |  |
| GO:0040038\_polar\_body\_extrusion\_after\_meiotic\_divisions | 1 | 0 |  |  |  |  |  |  |  |  |
| GO:0042074\_cell\_migration\_involved\_in\_gastrulation | 1 | 0 |  |  |  |  |  |  |  |  |
| GO:0042091\_interleukin-10\_biosynthetic\_process | 1 | 0 |  |  |  |  |  |  |  |  |
| GO:0042118\_endothelial\_cell\_activation | 1 | 0 |  |  |  |  |  |  |  |  |
| GO:0042159\_lipoprotein\_catabolic\_process | 1 | 0 |  |  |  |  |  |  |  |  |
| GO:0042214\_terpene\_metabolic\_process | 1 | 0 |  |  |  |  |  |  |  |  |
| GO:0042225\_interleukin-5\_biosynthetic\_process | 1 | 0 |  |  |  |  |  |  |  |  |
| GO:0042241\_interleukin-18\_biosynthetic\_process | 1 | 0 |  |  |  |  |  |  |  |  |
| GO:0042257\_ribosomal\_subunit\_assembly | 1 | 0 |  |  |  |  |  |  |  |  |
| GO:0042262\_DNA\_protection | 1 | 0 |  |  |  |  |  |  |  |  |
| GO:0042276\_error-prone\_postreplication\_DNA\_repair | 1 | 0 |  |  |  |  |  |  |  |  |
| GO:0042313\_protein\_kinase\_C\_deactivation | 1 | 0 |  |  |  |  |  |  |  |  |
| GO:0042369\_vitamin\_D\_catabolic\_process | 1 | 0 |  |  |  |  |  |  |  |  |
| GO:0042412\_taurine\_biosynthetic\_process | 1 | 0 |  |  |  |  |  |  |  |  |
| GO:0042418\_epinephrine\_biosynthetic\_process | 1 | 0 |  |  |  |  |  |  |  |  |
| GO:0042421\_norepinephrine\_biosynthetic\_process | 1 | 0 |  |  |  |  |  |  |  |  |
| GO:0042424\_catecholamine\_catabolic\_process | 1 | 0 |  |  |  |  |  |  |  |  |
| GO:0042428\_serotonin\_metabolic\_process | 1 | 0 |  |  |  |  |  |  |  |  |
| GO:0042435\_indole\_derivative\_biosynthetic\_process | 1 | 0 |  |  |  |  |  |  |  |  |
| GO:0042474\_middle\_ear\_morphogenesis | 1 | 0 |  |  |  |  |  |  |  |  |
| GO:0042504\_tyrosine\_phosphorylation\_of\_Stat4\_protein | 1 | 0 |  |  |  |  |  |  |  |  |
| GO:0042519\_regulation\_of\_tyrosine\_phosphorylation\_of\_Stat4\_protein | 1 | 0 |  |  |  |  |  |  |  |  |
| GO:0042520\_positive\_regulation\_of\_tyrosine\_phosphorylation\_of\_Stat4\_protein | 1 | 0 |  |  |  |  |  |  |  |  |
| GO:0042524\_negative\_regulation\_of\_tyrosine\_phosphorylation\_of\_Stat5\_protein | 1 | 0 |  |  |  |  |  |  |  |  |
| GO:0042537\_benzene\_and\_derivative\_metabolic\_process | 1 | 0 |  |  |  |  |  |  |  |  |
| GO:0042560\_pteridine\_and\_derivative\_catabolic\_process | 1 | 0 |  |  |  |  |  |  |  |  |
| GO:0042596\_fear\_response | 1 | 0 |  |  |  |  |  |  |  |  |
| GO:0042637\_catagen | 1 | 0 |  |  |  |  |  |  |  |  |
| GO:0042640\_anagen | 1 | 0 |  |  |  |  |  |  |  |  |
| GO:0042670\_retinal\_cone\_cell\_differentiation | 1 | 0 |  |  |  |  |  |  |  |  |
| GO:0042700\_luteinizing\_hormone\_signaling\_pathway | 1 | 0 |  |  |  |  |  |  |  |  |
| GO:0042703\_menstruation | 1 | 0 |  |  |  |  |  |  |  |  |
| GO:0042726\_riboflavin\_and\_derivative\_metabolic\_process | 1 | 0 |  |  |  |  |  |  |  |  |
| GO:0042727\_riboflavin\_and\_derivative\_biosynthetic\_process | 1 | 0 |  |  |  |  |  |  |  |  |
| GO:0042746\_circadian\_sleep\_wake\_cycle\_\_wakefulness | 1 | 0 |  |  |  |  |  |  |  |  |
| GO:0042748\_circadian\_sleep\_wake\_cycle\_\_non-REM\_sleep | 1 | 0 |  |  |  |  |  |  |  |  |
| GO:0042755\_eating\_behavior | 1 | 0 |  |  |  |  |  |  |  |  |
| GO:0042756\_drinking\_behavior | 1 | 0 |  |  |  |  |  |  |  |  |
| GO:0042766\_nucleosome\_mobilization | 1 | 0 |  |  |  |  |  |  |  |  |
| GO:0042780\_tRNA\_3'-end\_processing | 1 | 0 |  |  |  |  |  |  |  |  |
| GO:0042789\_mRNA\_transcription\_from\_RNA\_polymerase\_II\_promoter | 1 | 0 |  |  |  |  |  |  |  |  |
| GO:0042795\_snRNA\_transcription\_from\_RNA\_polymerase\_II\_promoter | 1 | 0 |  |  |  |  |  |  |  |  |
| GO:0042796\_snRNA\_transcription\_from\_RNA\_polymerase\_III\_promoter | 1 | 0 |  |  |  |  |  |  |  |  |
| GO:0042822\_pyridoxal\_phosphate\_metabolic\_process | 1 | 0 |  |  |  |  |  |  |  |  |
| GO:0042823\_pyridoxal\_phosphate\_biosynthetic\_process | 1 | 0 |  |  |  |  |  |  |  |  |
| GO:0042866\_pyruvate\_biosynthetic\_process | 1 | 0 |  |  |  |  |  |  |  |  |
| GO:0042904\_9-cis-retinoic\_acid\_biosynthetic\_process | 1 | 0 |  |  |  |  |  |  |  |  |
| GO:0042905\_9-cis-retinoic\_acid\_metabolic\_process | 1 | 0 |  |  |  |  |  |  |  |  |
| GO:0042985\_negative\_regulation\_of\_amyloid\_precursor\_protein\_biosynthetic\_process | 1 | 0 |  |  |  |  |  |  |  |  |
| GO:0042986\_positive\_regulation\_of\_amyloid\_precursor\_protein\_biosynthetic\_process | 1 | 0 |  |  |  |  |  |  |  |  |
| GO:0042989\_sequestering\_of\_actin\_monomers | 1 | 0 |  |  |  |  |  |  |  |  |
| GO:0042996\_regulation\_of\_Golgi\_to\_plasma\_membrane\_protein\_transport | 1 | 0 |  |  |  |  |  |  |  |  |
| GO:0042997\_negative\_regulation\_of\_Golgi\_to\_plasma\_membrane\_protein\_transport | 1 | 0 |  |  |  |  |  |  |  |  |
| GO:0042999\_regulation\_of\_Golgi\_to\_plasma\_membrane\_CFTR\_protein\_transport | 1 | 0 |  |  |  |  |  |  |  |  |
| GO:0043002\_negative\_regulation\_of\_Golgi\_to\_plasma\_membrane\_CFTR\_protein\_transport | 1 | 0 |  |  |  |  |  |  |  |  |
| GO:0043004\_cytoplasmic\_sequestering\_of\_CFTR\_protein | 1 | 0 |  |  |  |  |  |  |  |  |
| GO:0043012\_regulation\_of\_fusion\_of\_sperm\_to\_egg\_plasma\_membrane | 1 | 0 |  |  |  |  |  |  |  |  |
| GO:0043016\_regulation\_of\_lymphotoxin\_A\_biosynthetic\_process | 1 | 0 |  |  |  |  |  |  |  |  |
| GO:0043017\_positive\_regulation\_of\_lymphotoxin\_A\_biosynthetic\_process | 1 | 0 |  |  |  |  |  |  |  |  |
| GO:0043049\_otic\_placode\_formation | 1 | 0 |  |  |  |  |  |  |  |  |
| GO:0043064\_flagellum\_organization | 1 | 0 |  |  |  |  |  |  |  |  |
| GO:0043116\_negative\_regulation\_of\_vascular\_permeability | 1 | 0 |  |  |  |  |  |  |  |  |
| GO:0043126\_regulation\_of\_1-phosphatidylinositol\_4-kinase\_activity | 1 | 0 |  |  |  |  |  |  |  |  |
| GO:0043128\_positive\_regulation\_of\_1-phosphatidylinositol\_4-kinase\_activity | 1 | 0 |  |  |  |  |  |  |  |  |
| GO:0043129\_surfactant\_homeostasis | 1 | 0 |  |  |  |  |  |  |  |  |
| GO:0043146\_spindle\_stabilization | 1 | 0 |  |  |  |  |  |  |  |  |
| GO:0043148\_mitotic\_spindle\_stabilization | 1 | 0 |  |  |  |  |  |  |  |  |
| GO:0043152\_induction\_of\_bacterial\_agglutination | 1 | 0 |  |  |  |  |  |  |  |  |
| GO:0043173\_nucleotide\_salvage | 1 | 0 |  |  |  |  |  |  |  |  |
| GO:0043174\_nucleoside\_salvage | 1 | 0 |  |  |  |  |  |  |  |  |
| GO:0043181\_vacuolar\_sequestering | 1 | 0 |  |  |  |  |  |  |  |  |
| GO:0043200\_response\_to\_amino\_acid\_stimulus | 1 | 0 |  |  |  |  |  |  |  |  |
| GO:0043217\_myelin\_maintenance | 1 | 0 |  |  |  |  |  |  |  |  |
| GO:0043247\_telomere\_maintenance\_in\_response\_to\_DNA\_damage | 1 | 0 |  |  |  |  |  |  |  |  |
| GO:0043249\_erythrocyte\_maturation | 1 | 0 |  |  |  |  |  |  |  |  |
| GO:0043268\_positive\_regulation\_of\_potassium\_ion\_transport | 1 | 0 |  |  |  |  |  |  |  |  |
| GO:0043299\_leukocyte\_degranulation | 1 | 0 |  |  |  |  |  |  |  |  |
| GO:0043307\_eosinophil\_activation | 1 | 0 |  |  |  |  |  |  |  |  |
| GO:0043308\_eosinophil\_degranulation | 1 | 0 |  |  |  |  |  |  |  |  |
| GO:0043312\_neutrophil\_degranulation | 1 | 0 |  |  |  |  |  |  |  |  |
| GO:0043330\_response\_to\_exogenous\_dsRNA | 1 | 0 |  |  |  |  |  |  |  |  |
| GO:0043353\_enucleate\_erythrocyte\_differentiation | 1 | 0 |  |  |  |  |  |  |  |  |
| GO:0043371\_negative\_regulation\_of\_CD4-positive\_\_alpha\_beta\_T\_cell\_differentiation | 1 | 0 |  |  |  |  |  |  |  |  |
| GO:0043383\_negative\_T\_cell\_selection | 1 | 0 |  |  |  |  |  |  |  |  |
| GO:0043418\_homocysteine\_catabolic\_process | 1 | 0 |  |  |  |  |  |  |  |  |
| GO:0043420\_anthranilate\_metabolic\_process | 1 | 0 |  |  |  |  |  |  |  |  |
| GO:0043437\_butanoic\_acid\_metabolic\_process | 1 | 0 |  |  |  |  |  |  |  |  |
| GO:0043455\_regulation\_of\_secondary\_metabolic\_process | 1 | 0 |  |  |  |  |  |  |  |  |
| GO:0043456\_regulation\_of\_pentose-phosphate\_shunt | 1 | 0 |  |  |  |  |  |  |  |  |
| GO:0043457\_regulation\_of\_cellular\_respiration | 1 | 0 |  |  |  |  |  |  |  |  |
| GO:0043517\_positive\_regulation\_of\_DNA\_damage\_response\_\_signal\_transduction\_by\_p53\_class\_mediator | 1 | 0 |  |  |  |  |  |  |  |  |
| GO:0043518\_negative\_regulation\_of\_DNA\_damage\_response\_\_signal\_transduction\_by\_p53\_class\_mediator | 1 | 0 |  |  |  |  |  |  |  |  |
| GO:0043551\_regulation\_of\_phosphoinositide\_3-kinase\_activity | 1 | 0 |  |  |  |  |  |  |  |  |
| GO:0043552\_positive\_regulation\_of\_phosphoinositide\_3-kinase\_activity | 1 | 0 |  |  |  |  |  |  |  |  |
| GO:0043556\_regulation\_of\_translation\_in\_response\_to\_oxidative\_stress | 1 | 0 |  |  |  |  |  |  |  |  |
| GO:0043584\_nose\_development | 1 | 0 |  |  |  |  |  |  |  |  |
| GO:0043586\_tongue\_development | 1 | 0 |  |  |  |  |  |  |  |  |
| GO:0043587\_tongue\_morphogenesis | 1 | 0 |  |  |  |  |  |  |  |  |
| GO:0043652\_engulfment\_of\_apoptotic\_cell | 1 | 0 |  |  |  |  |  |  |  |  |
| GO:0043654\_recognition\_of\_apoptotic\_cell | 1 | 0 |  |  |  |  |  |  |  |  |
| GO:0043696\_dedifferentiation | 1 | 0 |  |  |  |  |  |  |  |  |
| GO:0043697\_cell\_dedifferentiation | 1 | 0 |  |  |  |  |  |  |  |  |
| GO:0043901\_negative\_regulation\_of\_multi-organism\_process | 1 | 0 |  |  |  |  |  |  |  |  |
| GO:0043921\_modulation\_by\_host\_of\_viral\_transcription | 1 | 0 |  |  |  |  |  |  |  |  |
| GO:0043923\_positive\_regulation\_by\_host\_of\_viral\_transcription | 1 | 0 |  |  |  |  |  |  |  |  |
| GO:0044007\_dissemination\_or\_transmission\_of\_symbiont\_from\_host | 1 | 0 |  |  |  |  |  |  |  |  |
| GO:0044089\_positive\_regulation\_of\_cellular\_component\_biogenesis | 1 | 0 |  |  |  |  |  |  |  |  |
| GO:0044258\_intestinal\_lipid\_catabolic\_process | 1 | 0 |  |  |  |  |  |  |  |  |
| GO:0044273\_sulfur\_compound\_catabolic\_process | 1 | 0 |  |  |  |  |  |  |  |  |
| GO:0045013\_negative\_regulation\_of\_transcription\_by\_carbon\_catabolites | 1 | 0 |  |  |  |  |  |  |  |  |
| GO:0045014\_negative\_regulation\_of\_transcription\_by\_glucose | 1 | 0 |  |  |  |  |  |  |  |  |
| GO:0045020\_error-prone\_DNA\_repair | 1 | 0 |  |  |  |  |  |  |  |  |
| GO:0045023\_G0\_to\_G1\_transition | 1 | 0 |  |  |  |  |  |  |  |  |
| GO:0045047\_protein\_targeting\_to\_ER | 1 | 0 |  |  |  |  |  |  |  |  |
| GO:0045065\_cytotoxic\_T\_cell\_differentiation | 1 | 0 |  |  |  |  |  |  |  |  |
| GO:0045074\_regulation\_of\_interleukin-10\_biosynthetic\_process | 1 | 0 |  |  |  |  |  |  |  |  |
| GO:0045082\_positive\_regulation\_of\_interleukin-10\_biosynthetic\_process | 1 | 0 |  |  |  |  |  |  |  |  |
| GO:0045132\_meiotic\_chromosome\_segregation | 1 | 0 |  |  |  |  |  |  |  |  |
| GO:0045163\_clustering\_of\_voltage-gated\_potassium\_channels | 1 | 0 |  |  |  |  |  |  |  |  |
| GO:0045175\_basal\_protein\_localization | 1 | 0 |  |  |  |  |  |  |  |  |
| GO:0045188\_regulation\_of\_circadian\_sleep\_wake\_cycle\_\_non-REM\_sleep | 1 | 0 |  |  |  |  |  |  |  |  |
| GO:0045189\_connective\_tissue\_growth\_factor\_biosynthetic\_process | 1 | 0 |  |  |  |  |  |  |  |  |
| GO:0045196\_establishment\_or\_maintenance\_of\_neuroblast\_polarity | 1 | 0 |  |  |  |  |  |  |  |  |
| GO:0045199\_maintenance\_of\_epithelial\_cell\_apical\_basal\_polarity | 1 | 0 |  |  |  |  |  |  |  |  |
| GO:0045200\_establishment\_of\_neuroblast\_polarity | 1 | 0 |  |  |  |  |  |  |  |  |
| GO:0045204\_MAPK\_export\_from\_nucleus | 1 | 0 |  |  |  |  |  |  |  |  |
| GO:0045208\_MAPK\_phosphatase\_export\_from\_nucleus | 1 | 0 |  |  |  |  |  |  |  |  |
| GO:0045209\_MAPK\_phosphatase\_export\_from\_nucleus\_\_leptomycin\_B\_sensitive | 1 | 0 |  |  |  |  |  |  |  |  |
| GO:0045292\_nuclear\_mRNA\_cis\_splicing\_\_via\_spliceosome | 1 | 0 |  |  |  |  |  |  |  |  |
| GO:0045324\_late\_endosome\_to\_vacuole\_transport | 1 | 0 |  |  |  |  |  |  |  |  |
| GO:0045329\_carnitine\_biosynthetic\_process | 1 | 0 |  |  |  |  |  |  |  |  |
| GO:0045345\_positive\_regulation\_of\_MHC\_class\_I\_biosynthetic\_process | 1 | 0 |  |  |  |  |  |  |  |  |
| GO:0045355\_negative\_regulation\_of\_interferon-alpha\_biosynthetic\_process | 1 | 0 |  |  |  |  |  |  |  |  |
| GO:0045360\_regulation\_of\_interleukin-1\_biosynthetic\_process | 1 | 0 |  |  |  |  |  |  |  |  |
| GO:0045362\_positive\_regulation\_of\_interleukin-1\_biosynthetic\_process | 1 | 0 |  |  |  |  |  |  |  |  |
| GO:0045366\_regulation\_of\_interleukin-13\_biosynthetic\_process | 1 | 0 |  |  |  |  |  |  |  |  |
| GO:0045368\_positive\_regulation\_of\_interleukin-13\_biosynthetic\_process | 1 | 0 |  |  |  |  |  |  |  |  |
| GO:0045381\_regulation\_of\_interleukin-18\_biosynthetic\_process | 1 | 0 |  |  |  |  |  |  |  |  |
| GO:0045405\_regulation\_of\_interleukin-5\_biosynthetic\_process | 1 | 0 |  |  |  |  |  |  |  |  |
| GO:0045407\_positive\_regulation\_of\_interleukin-5\_biosynthetic\_process | 1 | 0 |  |  |  |  |  |  |  |  |
| GO:0045425\_positive\_regulation\_of\_granulocyte\_macrophage\_colony-stimulating\_factor\_biosynthetic\_process | 1 | 0 |  |  |  |  |  |  |  |  |
| GO:0045475\_locomotor\_rhythm | 1 | 0 |  |  |  |  |  |  |  |  |
| GO:0045553\_TRAIL\_biosynthetic\_process | 1 | 0 |  |  |  |  |  |  |  |  |
| GO:0045554\_regulation\_of\_TRAIL\_biosynthetic\_process | 1 | 0 |  |  |  |  |  |  |  |  |
| GO:0045556\_positive\_regulation\_of\_TRAIL\_biosynthetic\_process | 1 | 0 |  |  |  |  |  |  |  |  |
| GO:0045575\_basophil\_activation | 1 | 0 |  |  |  |  |  |  |  |  |
| GO:0045579\_positive\_regulation\_of\_B\_cell\_differentiation | 1 | 0 |  |  |  |  |  |  |  |  |
| GO:0045583\_regulation\_of\_cytotoxic\_T\_cell\_differentiation | 1 | 0 |  |  |  |  |  |  |  |  |
| GO:0045585\_positive\_regulation\_of\_cytotoxic\_T\_cell\_differentiation | 1 | 0 |  |  |  |  |  |  |  |  |
| GO:0045589\_regulation\_of\_regulatory\_T\_cell\_differentiation | 1 | 0 |  |  |  |  |  |  |  |  |
| GO:0045590\_negative\_regulation\_of\_regulatory\_T\_cell\_differentiation | 1 | 0 |  |  |  |  |  |  |  |  |
| GO:0045602\_negative\_regulation\_of\_endothelial\_cell\_differentiation | 1 | 0 |  |  |  |  |  |  |  |  |
| GO:0045603\_positive\_regulation\_of\_endothelial\_cell\_differentiation | 1 | 0 |  |  |  |  |  |  |  |  |
| GO:0045605\_negative\_regulation\_of\_epidermal\_cell\_differentiation | 1 | 0 |  |  |  |  |  |  |  |  |
| GO:0045617\_negative\_regulation\_of\_keratinocyte\_differentiation | 1 | 0 |  |  |  |  |  |  |  |  |
| GO:0045623\_negative\_regulation\_of\_T-helper\_cell\_differentiation | 1 | 0 |  |  |  |  |  |  |  |  |
| GO:0045629\_negative\_regulation\_of\_T-helper\_2\_cell\_differentiation | 1 | 0 |  |  |  |  |  |  |  |  |
| GO:0045654\_positive\_regulation\_of\_megakaryocyte\_differentiation | 1 | 0 |  |  |  |  |  |  |  |  |
| GO:0045672\_positive\_regulation\_of\_osteoclast\_differentiation | 1 | 0 |  |  |  |  |  |  |  |  |
| GO:0045683\_negative\_regulation\_of\_epidermis\_development | 1 | 0 |  |  |  |  |  |  |  |  |
| GO:0045716\_positive\_regulation\_of\_low-density\_lipoprotein\_receptor\_biosynthetic\_process | 1 | 0 |  |  |  |  |  |  |  |  |
| GO:0045719\_negative\_regulation\_of\_glycogen\_biosynthetic\_process | 1 | 0 |  |  |  |  |  |  |  |  |
| GO:0045738\_negative\_regulation\_of\_DNA\_repair | 1 | 0 |  |  |  |  |  |  |  |  |
| GO:0045747\_positive\_regulation\_of\_Notch\_signaling\_pathway | 1 | 0 |  |  |  |  |  |  |  |  |
| GO:0045750\_positive\_regulation\_of\_S\_phase\_of\_mitotic\_cell\_cycle | 1 | 0 |  |  |  |  |  |  |  |  |
| GO:0045751\_negative\_regulation\_of\_Toll\_signaling\_pathway | 1 | 0 |  |  |  |  |  |  |  |  |
| GO:0045759\_negative\_regulation\_of\_action\_potential | 1 | 0 |  |  |  |  |  |  |  |  |
| GO:0045773\_positive\_regulation\_of\_axon\_extension | 1 | 0 |  |  |  |  |  |  |  |  |
| GO:0045794\_negative\_regulation\_of\_cell\_volume | 1 | 0 |  |  |  |  |  |  |  |  |
| GO:0045799\_positive\_regulation\_of\_chromatin\_assembly\_or\_disassembly | 1 | 0 |  |  |  |  |  |  |  |  |
| GO:0045818\_negative\_regulation\_of\_glycogen\_catabolic\_process | 1 | 0 |  |  |  |  |  |  |  |  |
| GO:0045836\_positive\_regulation\_of\_meiosis | 1 | 0 |  |  |  |  |  |  |  |  |
| GO:0045837\_negative\_regulation\_of\_membrane\_potential | 1 | 0 |  |  |  |  |  |  |  |  |
| GO:0045844\_positive\_regulation\_of\_striated\_muscle\_development | 1 | 0 |  |  |  |  |  |  |  |  |
| GO:0045852\_pH\_elevation | 1 | 0 |  |  |  |  |  |  |  |  |
| GO:0045870\_positive\_regulation\_of\_retroviral\_genome\_replication | 1 | 0 |  |  |  |  |  |  |  |  |
| GO:0045875\_negative\_regulation\_of\_sister\_chromatid\_cohesion | 1 | 0 |  |  |  |  |  |  |  |  |
| GO:0045879\_negative\_regulation\_of\_smoothened\_signaling\_pathway | 1 | 0 |  |  |  |  |  |  |  |  |
| GO:0045896\_regulation\_of\_transcription\_\_mitotic | 1 | 0 |  |  |  |  |  |  |  |  |
| GO:0045907\_positive\_regulation\_of\_vasoconstriction | 1 | 0 |  |  |  |  |  |  |  |  |
| GO:0045910\_negative\_regulation\_of\_DNA\_recombination | 1 | 0 |  |  |  |  |  |  |  |  |
| GO:0045915\_positive\_regulation\_of\_catecholamine\_metabolic\_process | 1 | 0 |  |  |  |  |  |  |  |  |
| GO:0045921\_positive\_regulation\_of\_exocytosis | 1 | 0 |  |  |  |  |  |  |  |  |
| GO:0045945\_positive\_regulation\_of\_transcription\_from\_RNA\_polymerase\_III\_promoter | 1 | 0 |  |  |  |  |  |  |  |  |
| GO:0045956\_positive\_regulation\_of\_calcium\_ion-dependent\_exocytosis | 1 | 0 |  |  |  |  |  |  |  |  |
| GO:0045964\_positive\_regulation\_of\_dopamine\_metabolic\_process | 1 | 0 |  |  |  |  |  |  |  |  |
| GO:0045989\_positive\_regulation\_of\_striated\_muscle\_contraction | 1 | 0 |  |  |  |  |  |  |  |  |
| GO:0045993\_negative\_regulation\_of\_translational\_initiation\_by\_iron | 1 | 0 |  |  |  |  |  |  |  |  |
| GO:0046005\_positive\_regulation\_of\_circadian\_sleep\_wake\_cycle\_\_REM\_sleep | 1 | 0 |  |  |  |  |  |  |  |  |
| GO:0046007\_negative\_regulation\_of\_activated\_T\_cell\_proliferation | 1 | 0 |  |  |  |  |  |  |  |  |
| GO:0046010\_positive\_regulation\_of\_circadian\_sleep\_wake\_cycle\_\_non-REM\_sleep | 1 | 0 |  |  |  |  |  |  |  |  |
| GO:0046031\_ADP\_metabolic\_process | 1 | 0 |  |  |  |  |  |  |  |  |
| GO:0046036\_CTP\_metabolic\_process | 1 | 0 |  |  |  |  |  |  |  |  |
| GO:0046040\_IMP\_metabolic\_process | 1 | 0 |  |  |  |  |  |  |  |  |
| GO:0046048\_UDP\_metabolic\_process | 1 | 0 |  |  |  |  |  |  |  |  |
| GO:0046049\_UMP\_metabolic\_process | 1 | 0 |  |  |  |  |  |  |  |  |
| GO:0046056\_dADP\_metabolic\_process | 1 | 0 |  |  |  |  |  |  |  |  |
| GO:0046085\_adenosine\_metabolic\_process | 1 | 0 |  |  |  |  |  |  |  |  |
| GO:0046087\_cytidine\_metabolic\_process | 1 | 0 |  |  |  |  |  |  |  |  |
| GO:0046101\_hypoxanthine\_biosynthetic\_process | 1 | 0 |  |  |  |  |  |  |  |  |
| GO:0046103\_inosine\_biosynthetic\_process | 1 | 0 |  |  |  |  |  |  |  |  |
| GO:0046104\_thymidine\_metabolic\_process | 1 | 0 |  |  |  |  |  |  |  |  |
| GO:0046108\_uridine\_metabolic\_process | 1 | 0 |  |  |  |  |  |  |  |  |
| GO:0046125\_pyrimidine\_deoxyribonucleoside\_metabolic\_process | 1 | 0 |  |  |  |  |  |  |  |  |
| GO:0046127\_pyrimidine\_deoxyribonucleoside\_catabolic\_process | 1 | 0 |  |  |  |  |  |  |  |  |
| GO:0046133\_pyrimidine\_ribonucleoside\_catabolic\_process | 1 | 0 |  |  |  |  |  |  |  |  |
| GO:0046184\_aldehyde\_biosynthetic\_process | 1 | 0 |  |  |  |  |  |  |  |  |
| GO:0046203\_spermidine\_catabolic\_process | 1 | 0 |  |  |  |  |  |  |  |  |
| GO:0046219\_indolalkylamine\_biosynthetic\_process | 1 | 0 |  |  |  |  |  |  |  |  |
| GO:0046292\_formaldehyde\_metabolic\_process | 1 | 0 |  |  |  |  |  |  |  |  |
| GO:0046293\_formaldehyde\_biosynthetic\_process | 1 | 0 |  |  |  |  |  |  |  |  |
| GO:0046317\_regulation\_of\_glucosylceramide\_biosynthetic\_process | 1 | 0 |  |  |  |  |  |  |  |  |
| GO:0046318\_negative\_regulation\_of\_glucosylceramide\_biosynthetic\_process | 1 | 0 |  |  |  |  |  |  |  |  |
| GO:0046322\_negative\_regulation\_of\_fatty\_acid\_oxidation | 1 | 0 |  |  |  |  |  |  |  |  |
| GO:0046335\_ethanolamine\_biosynthetic\_process | 1 | 0 |  |  |  |  |  |  |  |  |
| GO:0046337\_phosphatidylethanolamine\_metabolic\_process | 1 | 0 |  |  |  |  |  |  |  |  |
| GO:0046340\_diacylglycerol\_catabolic\_process | 1 | 0 |  |  |  |  |  |  |  |  |
| GO:0046351\_disaccharide\_biosynthetic\_process | 1 | 0 |  |  |  |  |  |  |  |  |
| GO:0046352\_disaccharide\_catabolic\_process | 1 | 0 |  |  |  |  |  |  |  |  |
| GO:0046370\_fructose\_biosynthetic\_process | 1 | 0 |  |  |  |  |  |  |  |  |
| GO:0046380\_N-acetylneuraminate\_biosynthetic\_process | 1 | 0 |  |  |  |  |  |  |  |  |
| GO:0046390\_ribose\_phosphate\_biosynthetic\_process | 1 | 0 |  |  |  |  |  |  |  |  |
| GO:0046399\_glucuronate\_biosynthetic\_process | 1 | 0 |  |  |  |  |  |  |  |  |
| GO:0046434\_organophosphate\_catabolic\_process | 1 | 0 |  |  |  |  |  |  |  |  |
| GO:0046448\_tropane\_alkaloid\_metabolic\_process | 1 | 0 |  |  |  |  |  |  |  |  |
| GO:0046449\_creatinine\_metabolic\_process | 1 | 0 |  |  |  |  |  |  |  |  |
| GO:0046471\_phosphatidylglycerol\_metabolic\_process | 1 | 0 |  |  |  |  |  |  |  |  |
| GO:0046477\_glycosylceramide\_catabolic\_process | 1 | 0 |  |  |  |  |  |  |  |  |
| GO:0046485\_ether\_lipid\_metabolic\_process | 1 | 0 |  |  |  |  |  |  |  |  |
| GO:0046487\_glyoxylate\_metabolic\_process | 1 | 0 |  |  |  |  |  |  |  |  |
| GO:0046491\_L-methylmalonyl-CoA\_metabolic\_process | 1 | 0 |  |  |  |  |  |  |  |  |
| GO:0046501\_protoporphyrinogen\_IX\_metabolic\_process | 1 | 0 |  |  |  |  |  |  |  |  |
| GO:0046511\_sphinganine\_biosynthetic\_process | 1 | 0 |  |  |  |  |  |  |  |  |
| GO:0046514\_ceramide\_catabolic\_process | 1 | 0 |  |  |  |  |  |  |  |  |
| GO:0046549\_retinal\_cone\_cell\_development | 1 | 0 |  |  |  |  |  |  |  |  |
| GO:0046586\_regulation\_of\_calcium-dependent\_cell-cell\_adhesion | 1 | 0 |  |  |  |  |  |  |  |  |
| GO:0046588\_negative\_regulation\_of\_calcium-dependent\_cell-cell\_adhesion | 1 | 0 |  |  |  |  |  |  |  |  |
| GO:0046597\_negative\_regulation\_of\_virion\_penetration\_into\_host\_cell | 1 | 0 |  |  |  |  |  |  |  |  |
| GO:0046600\_negative\_regulation\_of\_centriole\_replication | 1 | 0 |  |  |  |  |  |  |  |  |
| GO:0046606\_negative\_regulation\_of\_centrosome\_cycle | 1 | 0 |  |  |  |  |  |  |  |  |
| GO:0046620\_regulation\_of\_organ\_growth | 1 | 0 |  |  |  |  |  |  |  |  |
| GO:0046636\_negative\_regulation\_of\_alpha-beta\_T\_cell\_activation | 1 | 0 |  |  |  |  |  |  |  |  |
| GO:0046639\_negative\_regulation\_of\_alpha-beta\_T\_cell\_differentiation | 1 | 0 |  |  |  |  |  |  |  |  |
| GO:0046640\_regulation\_of\_alpha-beta\_T\_cell\_proliferation | 1 | 0 |  |  |  |  |  |  |  |  |
| GO:0046641\_positive\_regulation\_of\_alpha-beta\_T\_cell\_proliferation | 1 | 0 |  |  |  |  |  |  |  |  |
| GO:0046655\_folic\_acid\_metabolic\_process | 1 | 0 |  |  |  |  |  |  |  |  |
| GO:0046666\_retinal\_cell\_programmed\_cell\_death | 1 | 0 |  |  |  |  |  |  |  |  |
| GO:0046668\_regulation\_of\_retinal\_cell\_programmed\_cell\_death | 1 | 0 |  |  |  |  |  |  |  |  |
| GO:0046670\_positive\_regulation\_of\_retinal\_cell\_programmed\_cell\_death | 1 | 0 |  |  |  |  |  |  |  |  |
| GO:0046674\_induction\_of\_retinal\_programmed\_cell\_death | 1 | 0 |  |  |  |  |  |  |  |  |
| GO:0046685\_response\_to\_arsenic | 1 | 0 |  |  |  |  |  |  |  |  |
| GO:0046686\_response\_to\_cadmium\_ion | 1 | 0 |  |  |  |  |  |  |  |  |
| GO:0046689\_response\_to\_mercury\_ion | 1 | 0 |  |  |  |  |  |  |  |  |
| GO:0046692\_sperm\_competition | 1 | 0 |  |  |  |  |  |  |  |  |
| GO:0046713\_boron\_transport | 1 | 0 |  |  |  |  |  |  |  |  |
| GO:0046719\_regulation\_of\_viral\_protein\_levels\_in\_host\_cell | 1 | 0 |  |  |  |  |  |  |  |  |
| GO:0046814\_virion\_attachment\_\_binding\_of\_host\_cell\_surface\_coreceptor | 1 | 0 |  |  |  |  |  |  |  |  |
| GO:0046826\_negative\_regulation\_of\_protein\_export\_from\_nucleus | 1 | 0 |  |  |  |  |  |  |  |  |
| GO:0046827\_positive\_regulation\_of\_protein\_export\_from\_nucleus | 1 | 0 |  |  |  |  |  |  |  |  |
| GO:0046833\_positive\_regulation\_of\_RNA\_export\_from\_nucleus | 1 | 0 |  |  |  |  |  |  |  |  |
| GO:0046835\_carbohydrate\_phosphorylation | 1 | 0 |  |  |  |  |  |  |  |  |
| GO:0046838\_phosphorylated\_carbohydrate\_dephosphorylation | 1 | 0 |  |  |  |  |  |  |  |  |
| GO:0046853\_inositol\_and\_derivative\_phosphorylation | 1 | 0 |  |  |  |  |  |  |  |  |
| GO:0046855\_inositol\_phosphate\_dephosphorylation | 1 | 0 |  |  |  |  |  |  |  |  |
| GO:0046856\_phosphoinositide\_dephosphorylation | 1 | 0 |  |  |  |  |  |  |  |  |
| GO:0046898\_response\_to\_cycloheximide | 1 | 0 |  |  |  |  |  |  |  |  |
| GO:0046916\_cellular\_transition\_metal\_ion\_homeostasis | 1 | 0 |  |  |  |  |  |  |  |  |
| GO:0046931\_pore\_complex\_biogenesis | 1 | 0 |  |  |  |  |  |  |  |  |
| GO:0046939\_nucleotide\_phosphorylation | 1 | 0 |  |  |  |  |  |  |  |  |
| GO:0046946\_hydroxylysine\_metabolic\_process | 1 | 0 |  |  |  |  |  |  |  |  |
| GO:0046947\_hydroxylysine\_biosynthetic\_process | 1 | 0 |  |  |  |  |  |  |  |  |
| GO:0046963\_3'-phosphoadenosine\_5'-phosphosulfate\_transport | 1 | 0 |  |  |  |  |  |  |  |  |
| GO:0046984\_regulation\_of\_hemoglobin\_biosynthetic\_process | 1 | 0 |  |  |  |  |  |  |  |  |
| GO:0046986\_negative\_regulation\_of\_hemoglobin\_biosynthetic\_process | 1 | 0 |  |  |  |  |  |  |  |  |
| GO:0048003\_antigen\_processing\_and\_presentation\_of\_lipid\_antigen\_via\_MHC\_class\_Ib | 1 | 0 |  |  |  |  |  |  |  |  |
| GO:0048006\_antigen\_processing\_and\_presentation\_\_endogenous\_lipid\_antigen\_via\_MHC\_class\_Ib | 1 | 0 |  |  |  |  |  |  |  |  |
| GO:0048013\_ephrin\_receptor\_signaling\_pathway | 1 | 0 |  |  |  |  |  |  |  |  |
| GO:0048070\_regulation\_of\_pigmentation\_during\_development | 1 | 0 |  |  |  |  |  |  |  |  |
| GO:0048073\_regulation\_of\_eye\_pigmentation | 1 | 0 |  |  |  |  |  |  |  |  |
| GO:0048075\_positive\_regulation\_of\_eye\_pigmentation | 1 | 0 |  |  |  |  |  |  |  |  |
| GO:0048087\_positive\_regulation\_of\_pigmentation\_during\_development | 1 | 0 |  |  |  |  |  |  |  |  |
| GO:0048160\_primary\_follicle\_stage\_\_oogenesis | 1 | 0 |  |  |  |  |  |  |  |  |
| GO:0048170\_positive\_regulation\_of\_long-term\_neuronal\_synaptic\_plasticity | 1 | 0 |  |  |  |  |  |  |  |  |
| GO:0048172\_regulation\_of\_short-term\_neuronal\_synaptic\_plasticity | 1 | 0 |  |  |  |  |  |  |  |  |
| GO:0048175\_hepatocyte\_growth\_factor\_biosynthetic\_process | 1 | 0 |  |  |  |  |  |  |  |  |
| GO:0048176\_regulation\_of\_hepatocyte\_growth\_factor\_biosynthetic\_process | 1 | 0 |  |  |  |  |  |  |  |  |
| GO:0048178\_negative\_regulation\_of\_hepatocyte\_growth\_factor\_biosynthetic\_process | 1 | 0 |  |  |  |  |  |  |  |  |
| GO:0048203\_vesicle\_targeting\_\_trans-Golgi\_to\_endosome | 1 | 0 |  |  |  |  |  |  |  |  |
| GO:0048210\_Golgi\_vesicle\_fusion\_to\_target\_membrane | 1 | 0 |  |  |  |  |  |  |  |  |
| GO:0048241\_epinephrine\_transport | 1 | 0 |  |  |  |  |  |  |  |  |
| GO:0048242\_epinephrine\_secretion | 1 | 0 |  |  |  |  |  |  |  |  |
| GO:0048245\_eosinophil\_chemotaxis | 1 | 0 |  |  |  |  |  |  |  |  |
| GO:0048265\_response\_to\_pain | 1 | 0 |  |  |  |  |  |  |  |  |
| GO:0048289\_isotype\_switching\_to\_IgE\_isotypes | 1 | 0 |  |  |  |  |  |  |  |  |
| GO:0048293\_regulation\_of\_isotype\_switching\_to\_IgE\_isotypes | 1 | 0 |  |  |  |  |  |  |  |  |
| GO:0048295\_positive\_regulation\_of\_isotype\_switching\_to\_IgE\_isotypes | 1 | 0 |  |  |  |  |  |  |  |  |
| GO:0048302\_regulation\_of\_isotype\_switching\_to\_IgG\_isotypes | 1 | 0 |  |  |  |  |  |  |  |  |
| GO:0048304\_positive\_regulation\_of\_isotype\_switching\_to\_IgG\_isotypes | 1 | 0 |  |  |  |  |  |  |  |  |
| GO:0048311\_mitochondrion\_distribution | 1 | 0 |  |  |  |  |  |  |  |  |
| GO:0048339\_paraxial\_mesoderm\_development | 1 | 0 |  |  |  |  |  |  |  |  |
| GO:0048340\_paraxial\_mesoderm\_morphogenesis | 1 | 0 |  |  |  |  |  |  |  |  |
| GO:0048388\_endosomal\_lumen\_acidification | 1 | 0 |  |  |  |  |  |  |  |  |
| GO:0048478\_replication\_fork\_protection | 1 | 0 |  |  |  |  |  |  |  |  |
| GO:0048483\_autonomic\_nervous\_system\_development | 1 | 0 |  |  |  |  |  |  |  |  |
| GO:0048485\_sympathetic\_nervous\_system\_development | 1 | 0 |  |  |  |  |  |  |  |  |
| GO:0048499\_synaptic\_vesicle\_membrane\_organization | 1 | 0 |  |  |  |  |  |  |  |  |
| GO:0048535\_lymph\_node\_development | 1 | 0 |  |  |  |  |  |  |  |  |
| GO:0048539\_bone\_marrow\_development | 1 | 0 |  |  |  |  |  |  |  |  |
| GO:0048549\_positive\_regulation\_of\_pinocytosis | 1 | 0 |  |  |  |  |  |  |  |  |
| GO:0048553\_negative\_regulation\_of\_metalloenzyme\_activity | 1 | 0 |  |  |  |  |  |  |  |  |
| GO:0048566\_embryonic\_gut\_development | 1 | 0 |  |  |  |  |  |  |  |  |
| GO:0048596\_embryonic\_camera-type\_eye\_morphogenesis | 1 | 0 |  |  |  |  |  |  |  |  |
| GO:0048617\_embryonic\_foregut\_morphogenesis | 1 | 0 |  |  |  |  |  |  |  |  |
| GO:0048619\_embryonic\_hindgut\_morphogenesis | 1 | 0 |  |  |  |  |  |  |  |  |
| GO:0048636\_positive\_regulation\_of\_muscle\_development | 1 | 0 |  |  |  |  |  |  |  |  |
| GO:0048639\_positive\_regulation\_of\_developmental\_growth | 1 | 0 |  |  |  |  |  |  |  |  |
| GO:0048640\_negative\_regulation\_of\_developmental\_growth | 1 | 0 |  |  |  |  |  |  |  |  |
| GO:0048665\_neuron\_fate\_specification | 1 | 0 |  |  |  |  |  |  |  |  |
| GO:0048679\_regulation\_of\_axon\_regeneration | 1 | 0 |  |  |  |  |  |  |  |  |
| GO:0048681\_negative\_regulation\_of\_axon\_regeneration | 1 | 0 |  |  |  |  |  |  |  |  |
| GO:0048703\_embryonic\_viscerocranium\_morphogenesis | 1 | 0 |  |  |  |  |  |  |  |  |
| GO:0048745\_smooth\_muscle\_tissue\_development | 1 | 0 |  |  |  |  |  |  |  |  |
| GO:0048755\_branching\_morphogenesis\_of\_a\_nerve | 1 | 0 |  |  |  |  |  |  |  |  |
| GO:0048793\_pronephros\_development | 1 | 0 |  |  |  |  |  |  |  |  |
| GO:0048807\_female\_genitalia\_morphogenesis | 1 | 0 |  |  |  |  |  |  |  |  |
| GO:0048818\_positive\_regulation\_of\_hair\_follicle\_maturation | 1 | 0 |  |  |  |  |  |  |  |  |
| GO:0048819\_regulation\_of\_hair\_follicle\_maturation | 1 | 0 |  |  |  |  |  |  |  |  |
| GO:0048821\_erythrocyte\_development | 1 | 0 |  |  |  |  |  |  |  |  |
| GO:0048845\_venous\_blood\_vessel\_morphogenesis | 1 | 0 |  |  |  |  |  |  |  |  |
| GO:0048853\_forebrain\_morphogenesis | 1 | 0 |  |  |  |  |  |  |  |  |
| GO:0048865\_stem\_cell\_fate\_commitment | 1 | 0 |  |  |  |  |  |  |  |  |
| GO:0048867\_stem\_cell\_fate\_determination | 1 | 0 |  |  |  |  |  |  |  |  |
| GO:0048874\_homeostasis\_of\_number\_of\_cells\_in\_a\_free-living\_population | 1 | 0 |  |  |  |  |  |  |  |  |
| GO:0048875\_chemical\_homeostasis\_within\_a\_tissue | 1 | 0 |  |  |  |  |  |  |  |  |
| GO:0050427\_3'-phosphoadenosine\_5'-phosphosulfate\_metabolic\_process | 1 | 0 |  |  |  |  |  |  |  |  |
| GO:0050652\_dermatan\_sulfate\_proteoglycan\_biosynthetic\_process\_\_polysaccharide\_chain\_biosynthetic\_process | 1 | 0 |  |  |  |  |  |  |  |  |
| GO:0050666\_regulation\_of\_homocysteine\_metabolic\_process | 1 | 0 |  |  |  |  |  |  |  |  |
| GO:0050674\_urothelial\_cell\_proliferation | 1 | 0 |  |  |  |  |  |  |  |  |
| GO:0050675\_regulation\_of\_urothelial\_cell\_proliferation | 1 | 0 |  |  |  |  |  |  |  |  |
| GO:0050677\_positive\_regulation\_of\_urothelial\_cell\_proliferation | 1 | 0 |  |  |  |  |  |  |  |  |
| GO:0050685\_positive\_regulation\_of\_mRNA\_processing | 1 | 0 |  |  |  |  |  |  |  |  |
| GO:0050687\_negative\_regulation\_of\_defense\_response\_to\_virus | 1 | 0 |  |  |  |  |  |  |  |  |
| GO:0050689\_negative\_regulation\_of\_defense\_response\_to\_virus\_by\_host | 1 | 0 |  |  |  |  |  |  |  |  |
| GO:0050713\_negative\_regulation\_of\_interleukin-1\_beta\_secretion | 1 | 0 |  |  |  |  |  |  |  |  |
| GO:0050722\_regulation\_of\_interleukin-1\_beta\_biosynthetic\_process | 1 | 0 |  |  |  |  |  |  |  |  |
| GO:0050725\_positive\_regulation\_of\_interleukin-1\_beta\_biosynthetic\_process | 1 | 0 |  |  |  |  |  |  |  |  |
| GO:0050751\_fractalkine\_biosynthetic\_process | 1 | 0 |  |  |  |  |  |  |  |  |
| GO:0050752\_regulation\_of\_fractalkine\_biosynthetic\_process | 1 | 0 |  |  |  |  |  |  |  |  |
| GO:0050754\_positive\_regulation\_of\_fractalkine\_biosynthetic\_process | 1 | 0 |  |  |  |  |  |  |  |  |
| GO:0050756\_fractalkine\_metabolic\_process | 1 | 0 |  |  |  |  |  |  |  |  |
| GO:0050757\_thymidylate\_synthase\_biosynthetic\_process | 1 | 0 |  |  |  |  |  |  |  |  |
| GO:0050758\_regulation\_of\_thymidylate\_synthase\_biosynthetic\_process | 1 | 0 |  |  |  |  |  |  |  |  |
| GO:0050760\_negative\_regulation\_of\_thymidylate\_synthase\_biosynthetic\_process | 1 | 0 |  |  |  |  |  |  |  |  |
| GO:0050765\_negative\_regulation\_of\_phagocytosis | 1 | 0 |  |  |  |  |  |  |  |  |
| GO:0050774\_negative\_regulation\_of\_dendrite\_morphogenesis | 1 | 0 |  |  |  |  |  |  |  |  |
| GO:0050783\_cocaine\_metabolic\_process | 1 | 0 |  |  |  |  |  |  |  |  |
| GO:0050822\_peptide\_stabilization | 1 | 0 |  |  |  |  |  |  |  |  |
| GO:0050823\_peptide\_antigen\_stabilization | 1 | 0 |  |  |  |  |  |  |  |  |
| GO:0050832\_defense\_response\_to\_fungus | 1 | 0 |  |  |  |  |  |  |  |  |
| GO:0050855\_regulation\_of\_B\_cell\_receptor\_signaling\_pathway | 1 | 0 |  |  |  |  |  |  |  |  |
| GO:0050858\_negative\_regulation\_of\_antigen\_receptor-mediated\_signaling\_pathway | 1 | 0 |  |  |  |  |  |  |  |  |
| GO:0050860\_negative\_regulation\_of\_T\_cell\_receptor\_signaling\_pathway | 1 | 0 |  |  |  |  |  |  |  |  |
| GO:0050861\_positive\_regulation\_of\_B\_cell\_receptor\_signaling\_pathway | 1 | 0 |  |  |  |  |  |  |  |  |
| GO:0050883\_musculoskeletal\_movement\_\_spinal\_reflex\_action | 1 | 0 |  |  |  |  |  |  |  |  |
| GO:0050884\_neuromuscular\_process\_controlling\_posture | 1 | 0 |  |  |  |  |  |  |  |  |
| GO:0050893\_sensory\_processing | 1 | 0 |  |  |  |  |  |  |  |  |
| GO:0050902\_leukocyte\_adhesive\_activation | 1 | 0 |  |  |  |  |  |  |  |  |
| GO:0050910\_detection\_of\_mechanical\_stimulus\_involved\_in\_sensory\_perception\_of\_sound | 1 | 0 |  |  |  |  |  |  |  |  |
| GO:0050922\_negative\_regulation\_of\_chemotaxis | 1 | 0 |  |  |  |  |  |  |  |  |
| GO:0050923\_regulation\_of\_negative\_chemotaxis | 1 | 0 |  |  |  |  |  |  |  |  |
| GO:0050924\_positive\_regulation\_of\_negative\_chemotaxis | 1 | 0 |  |  |  |  |  |  |  |  |
| GO:0050929\_induction\_of\_negative\_chemotaxis | 1 | 0 |  |  |  |  |  |  |  |  |
| GO:0050951\_sensory\_perception\_of\_temperature\_stimulus | 1 | 0 |  |  |  |  |  |  |  |  |
| GO:0050955\_thermoception | 1 | 0 |  |  |  |  |  |  |  |  |
| GO:0050974\_detection\_of\_mechanical\_stimulus\_involved\_in\_sensory\_perception | 1 | 0 |  |  |  |  |  |  |  |  |
| GO:0050983\_spermidine\_catabolic\_process\_to\_deoxyhypusine\_\_using\_deoxyhypusine\_synthase | 1 | 0 |  |  |  |  |  |  |  |  |
| GO:0051013\_microtubule\_severing | 1 | 0 |  |  |  |  |  |  |  |  |
| GO:0051029\_rRNA\_transport | 1 | 0 |  |  |  |  |  |  |  |  |
| GO:0051030\_snRNA\_transport | 1 | 0 |  |  |  |  |  |  |  |  |
| GO:0051031\_tRNA\_transport | 1 | 0 |  |  |  |  |  |  |  |  |
| GO:0051036\_regulation\_of\_endosome\_size | 1 | 0 |  |  |  |  |  |  |  |  |
| GO:0051040\_regulation\_of\_calcium-independent\_cell-cell\_adhesion | 1 | 0 |  |  |  |  |  |  |  |  |
| GO:0051041\_positive\_regulation\_of\_calcium-independent\_cell-cell\_adhesion | 1 | 0 |  |  |  |  |  |  |  |  |
| GO:0051066\_dihydrobiopterin\_metabolic\_process | 1 | 0 |  |  |  |  |  |  |  |  |
| GO:0051085\_chaperone\_mediated\_protein\_folding\_requiring\_cofactor | 1 | 0 |  |  |  |  |  |  |  |  |
| GO:0051089\_constitutive\_protein\_ectodomain\_proteolysis | 1 | 0 |  |  |  |  |  |  |  |  |
| GO:0051102\_DNA\_ligation\_during\_DNA\_recombination | 1 | 0 |  |  |  |  |  |  |  |  |
| GO:0051105\_regulation\_of\_DNA\_ligation | 1 | 0 |  |  |  |  |  |  |  |  |
| GO:0051106\_positive\_regulation\_of\_DNA\_ligation | 1 | 0 |  |  |  |  |  |  |  |  |
| GO:0051125\_regulation\_of\_actin\_nucleation | 1 | 0 |  |  |  |  |  |  |  |  |
| GO:0051126\_negative\_regulation\_of\_actin\_nucleation | 1 | 0 |  |  |  |  |  |  |  |  |
| GO:0051136\_regulation\_of\_NK\_T\_cell\_differentiation | 1 | 0 |  |  |  |  |  |  |  |  |
| GO:0051138\_positive\_regulation\_of\_NK\_T\_cell\_differentiation | 1 | 0 |  |  |  |  |  |  |  |  |
| GO:0051155\_positive\_regulation\_of\_striated\_muscle\_cell\_differentiation | 1 | 0 |  |  |  |  |  |  |  |  |
| GO:0051156\_glucose\_6-phosphate\_metabolic\_process | 1 | 0 |  |  |  |  |  |  |  |  |
| GO:0051160\_L-xylitol\_catabolic\_process | 1 | 0 |  |  |  |  |  |  |  |  |
| GO:0051164\_L-xylitol\_metabolic\_process | 1 | 0 |  |  |  |  |  |  |  |  |
| GO:0051193\_regulation\_of\_cofactor\_metabolic\_process | 1 | 0 |  |  |  |  |  |  |  |  |
| GO:0051196\_regulation\_of\_coenzyme\_metabolic\_process | 1 | 0 |  |  |  |  |  |  |  |  |
| GO:0051204\_protein\_insertion\_into\_mitochondrial\_membrane | 1 | 0 |  |  |  |  |  |  |  |  |
| GO:0051290\_protein\_heterotetramerization | 1 | 0 |  |  |  |  |  |  |  |  |
| GO:0051292\_nuclear\_pore\_complex\_assembly | 1 | 0 |  |  |  |  |  |  |  |  |
| GO:0051294\_establishment\_of\_spindle\_orientation | 1 | 0 |  |  |  |  |  |  |  |  |
| GO:0051295\_establishment\_of\_meiotic\_spindle\_localization | 1 | 0 |  |  |  |  |  |  |  |  |
| GO:0051315\_attachment\_of\_spindle\_microtubules\_to\_kinetochore\_during\_mitosis | 1 | 0 |  |  |  |  |  |  |  |  |
| GO:0051326\_telophase | 1 | 0 |  |  |  |  |  |  |  |  |
| GO:0051342\_regulation\_of\_cyclic-nucleotide\_phosphodiesterase\_activity | 1 | 0 |  |  |  |  |  |  |  |  |
| GO:0051344\_negative\_regulation\_of\_cyclic-nucleotide\_phosphodiesterase\_activity | 1 | 0 |  |  |  |  |  |  |  |  |
| GO:0051445\_regulation\_of\_meiotic\_cell\_cycle | 1 | 0 |  |  |  |  |  |  |  |  |
| GO:0051454\_intracellular\_pH\_elevation | 1 | 0 |  |  |  |  |  |  |  |  |
| GO:0051458\_adrenocorticotropin\_secretion | 1 | 0 |  |  |  |  |  |  |  |  |
| GO:0051459\_regulation\_of\_adrenocorticotropin\_secretion | 1 | 0 |  |  |  |  |  |  |  |  |
| GO:0051461\_positive\_regulation\_of\_adrenocorticotropin\_secretion | 1 | 0 |  |  |  |  |  |  |  |  |
| GO:0051531\_NFAT\_protein\_import\_into\_nucleus | 1 | 0 |  |  |  |  |  |  |  |  |
| GO:0051532\_regulation\_of\_NFAT\_protein\_import\_into\_nucleus | 1 | 0 |  |  |  |  |  |  |  |  |
| GO:0051533\_positive\_regulation\_of\_NFAT\_protein\_import\_into\_nucleus | 1 | 0 |  |  |  |  |  |  |  |  |
| GO:0051542\_elastin\_biosynthetic\_process | 1 | 0 |  |  |  |  |  |  |  |  |
| GO:0051560\_mitochondrial\_calcium\_ion\_homeostasis | 1 | 0 |  |  |  |  |  |  |  |  |
| GO:0051561\_elevation\_of\_mitochondrial\_calcium\_ion\_concentration | 1 | 0 |  |  |  |  |  |  |  |  |
| GO:0051582\_positive\_regulation\_of\_neurotransmitter\_uptake | 1 | 0 |  |  |  |  |  |  |  |  |
| GO:0051586\_positive\_regulation\_of\_dopamine\_uptake | 1 | 0 |  |  |  |  |  |  |  |  |
| GO:0051590\_positive\_regulation\_of\_neurotransmitter\_transport | 1 | 0 |  |  |  |  |  |  |  |  |
| GO:0051594\_detection\_of\_glucose | 1 | 0 |  |  |  |  |  |  |  |  |
| GO:0051642\_centrosome\_localization | 1 | 0 |  |  |  |  |  |  |  |  |
| GO:0051645\_Golgi\_localization | 1 | 0 |  |  |  |  |  |  |  |  |
| GO:0051647\_nucleus\_localization | 1 | 0 |  |  |  |  |  |  |  |  |
| GO:0051664\_nuclear\_pore\_localization | 1 | 0 |  |  |  |  |  |  |  |  |
| GO:0051708\_intracellular\_protein\_transport\_in\_other\_organism\_during\_symbiotic\_interaction | 1 | 0 |  |  |  |  |  |  |  |  |
| GO:0051764\_actin\_crosslink\_formation | 1 | 0 |  |  |  |  |  |  |  |  |
| GO:0051767\_nitric-oxide\_synthase\_biosynthetic\_process | 1 | 0 |  |  |  |  |  |  |  |  |
| GO:0051768\_nitric-oxide\_synthase\_2\_biosynthetic\_process | 1 | 0 |  |  |  |  |  |  |  |  |
| GO:0051769\_regulation\_of\_nitric-oxide\_synthase\_biosynthetic\_process | 1 | 0 |  |  |  |  |  |  |  |  |
| GO:0051771\_negative\_regulation\_of\_nitric-oxide\_synthase\_biosynthetic\_process | 1 | 0 |  |  |  |  |  |  |  |  |
| GO:0051772\_regulation\_of\_nitric-oxide\_synthase\_2\_biosynthetic\_process | 1 | 0 |  |  |  |  |  |  |  |  |
| GO:0051773\_positive\_regulation\_of\_nitric-oxide\_synthase\_2\_biosynthetic\_process | 1 | 0 |  |  |  |  |  |  |  |  |
| GO:0051781\_positive\_regulation\_of\_cell\_division | 1 | 0 |  |  |  |  |  |  |  |  |
| GO:0051782\_negative\_regulation\_of\_cell\_division | 1 | 0 |  |  |  |  |  |  |  |  |
| GO:0051788\_response\_to\_misfolded\_protein | 1 | 0 |  |  |  |  |  |  |  |  |
| GO:0051790\_short-chain\_fatty\_acid\_biosynthetic\_process | 1 | 0 |  |  |  |  |  |  |  |  |
| GO:0051791\_medium-chain\_fatty\_acid\_metabolic\_process | 1 | 0 |  |  |  |  |  |  |  |  |
| GO:0051792\_medium-chain\_fatty\_acid\_biosynthetic\_process | 1 | 0 |  |  |  |  |  |  |  |  |
| GO:0051794\_regulation\_of\_catagen | 1 | 0 |  |  |  |  |  |  |  |  |
| GO:0051795\_positive\_regulation\_of\_catagen | 1 | 0 |  |  |  |  |  |  |  |  |
| GO:0051821\_dissemination\_or\_transmission\_of\_organism\_from\_other\_organism\_during\_symbiotic\_interaction | 1 | 0 |  |  |  |  |  |  |  |  |
| GO:0051894\_positive\_regulation\_of\_focal\_adhesion\_formation | 1 | 0 |  |  |  |  |  |  |  |  |
| GO:0051930\_regulation\_of\_sensory\_perception\_of\_pain | 1 | 0 |  |  |  |  |  |  |  |  |
| GO:0051931\_regulation\_of\_sensory\_perception | 1 | 0 |  |  |  |  |  |  |  |  |
| GO:0051944\_positive\_regulation\_of\_catecholamine\_uptake\_during\_transmission\_of\_nerve\_impulse | 1 | 0 |  |  |  |  |  |  |  |  |
| GO:0051962\_positive\_regulation\_of\_nervous\_system\_development | 1 | 0 |  |  |  |  |  |  |  |  |
| GO:0051965\_positive\_regulation\_of\_synaptogenesis | 1 | 0 |  |  |  |  |  |  |  |  |
| GO:0051977\_lysophospholipid\_transport | 1 | 0 |  |  |  |  |  |  |  |  |
| GO:0051988\_regulation\_of\_attachment\_of\_spindle\_microtubules\_to\_kinetochore | 1 | 0 |  |  |  |  |  |  |  |  |
| GO:0052097\_interspecies\_quorum\_sensing | 1 | 0 |  |  |  |  |  |  |  |  |
| GO:0052106\_quorum\_sensing\_during\_interaction\_with\_host | 1 | 0 |  |  |  |  |  |  |  |  |
| GO:0052312\_modulation\_of\_transcription\_in\_other\_organism\_during\_symbiotic\_interaction | 1 | 0 |  |  |  |  |  |  |  |  |
| GO:0052472\_modulation\_by\_host\_of\_symbiont\_transcription | 1 | 0 |  |  |  |  |  |  |  |  |
| GO:0055009\_atrial\_cardiac\_muscle\_morphogenesis | 1 | 0 |  |  |  |  |  |  |  |  |
| GO:0055012\_ventricular\_cardiac\_muscle\_cell\_differentiation | 1 | 0 |  |  |  |  |  |  |  |  |
| GO:0055071\_manganese\_ion\_homeostasis | 1 | 0 |  |  |  |  |  |  |  |  |
| GO:0055073\_cadmium\_ion\_homeostasis | 1 | 0 |  |  |  |  |  |  |  |  |
| GO:0055076\_transition\_metal\_ion\_homeostasis | 1 | 0 |  |  |  |  |  |  |  |  |
| GO:0055089\_fatty\_acid\_homeostasis | 1 | 0 |  |  |  |  |  |  |  |  |
| GO:0055095\_lipoprotein\_mediated\_signaling | 1 | 0 |  |  |  |  |  |  |  |  |
| GO:0055096\_low\_density\_lipoprotein\_mediated\_signaling | 1 | 0 |  |  |  |  |  |  |  |  |
| GO:0055099\_response\_to\_high\_density\_lipoprotein\_stimulus | 1 | 0 |  |  |  |  |  |  |  |  |
| GO:0055118\_negative\_regulation\_of\_cardiac\_muscle\_contraction | 1 | 0 |  |  |  |  |  |  |  |  |
| GO:0055119\_relaxation\_of\_cardiac\_muscle | 1 | 0 |  |  |  |  |  |  |  |  |
| GO:0060003\_copper\_ion\_export | 1 | 0 |  |  |  |  |  |  |  |  |
| GO:0060022\_hard\_palate\_development | 1 | 0 |  |  |  |  |  |  |  |  |
| GO:0060039\_pericardium\_development | 1 | 0 |  |  |  |  |  |  |  |  |
| GO:0060055\_angiogenesis\_involved\_in\_wound\_healing | 1 | 0 |  |  |  |  |  |  |  |  |
| GO:0060059\_embryonic\_retina\_morphogenesis\_in\_camera-type\_eye | 1 | 0 |  |  |  |  |  |  |  |  |
| GO:0060065\_uterus\_development | 1 | 0 |  |  |  |  |  |  |  |  |
| GO:0060068\_vagina\_development | 1 | 0 |  |  |  |  |  |  |  |  |
| GO:0060082\_eye\_blink\_reflex | 1 | 0 |  |  |  |  |  |  |  |  |
| GO:0060083\_smooth\_muscle\_contraction\_involved\_in\_micturition | 1 | 0 |  |  |  |  |  |  |  |  |
| GO:0060088\_auditory\_receptor\_cell\_stereocilium\_organization | 1 | 0 |  |  |  |  |  |  |  |  |
| GO:0060120\_inner\_ear\_receptor\_cell\_fate\_commitment | 1 | 0 |  |  |  |  |  |  |  |  |
| GO:0060135\_maternal\_process\_involved\_in\_female\_pregnancy | 1 | 0 |  |  |  |  |  |  |  |  |
| GO:0060142\_regulation\_of\_syncytium\_formation\_by\_plasma\_membrane\_fusion | 1 | 0 |  |  |  |  |  |  |  |  |
| GO:0060143\_positive\_regulation\_of\_syncytium\_formation\_by\_plasma\_membrane\_fusion | 1 | 0 |  |  |  |  |  |  |  |  |
| GO:0060157\_urinary\_bladder\_development | 1 | 0 |  |  |  |  |  |  |  |  |
| GO:0060160\_negative\_regulation\_of\_dopamine\_receptor\_signaling\_pathway | 1 | 0 |  |  |  |  |  |  |  |  |
| GO:0060161\_positive\_regulation\_of\_dopamine\_receptor\_signaling\_pathway | 1 | 0 |  |  |  |  |  |  |  |  |
| GO:0060167\_regulation\_of\_adenosine\_receptor\_signaling\_pathway | 1 | 0 |  |  |  |  |  |  |  |  |
| GO:0060169\_negative\_regulation\_of\_adenosine\_receptor\_signaling\_pathway | 1 | 0 |  |  |  |  |  |  |  |  |
| GO:0060216\_definitive\_hemopoiesis | 1 | 0 |  |  |  |  |  |  |  |  |
| GO:0060219\_camera-type\_eye\_photoreceptor\_cell\_differentiation | 1 | 0 |  |  |  |  |  |  |  |  |
| GO:0060231\_mesenchymal\_to\_epithelial\_transition | 1 | 0 |  |  |  |  |  |  |  |  |
| GO:0060254\_regulation\_of\_N-terminal\_protein\_palmitoylation | 1 | 0 |  |  |  |  |  |  |  |  |
| GO:0060259\_regulation\_of\_feeding\_behavior | 1 | 0 |  |  |  |  |  |  |  |  |
| GO:0060262\_negative\_regulation\_of\_N-terminal\_protein\_palmitoylation | 1 | 0 |  |  |  |  |  |  |  |  |
| GO:0060265\_positive\_regulation\_of\_respiratory\_burst\_during\_acute\_inflammatory\_response | 1 | 0 |  |  |  |  |  |  |  |  |
| GO:0060266\_negative\_regulation\_of\_respiratory\_burst\_during\_acute\_inflammatory\_response | 1 | 0 |  |  |  |  |  |  |  |  |
| GO:0060268\_negative\_regulation\_of\_respiratory\_burst | 1 | 0 |  |  |  |  |  |  |  |  |
| GO:0060286\_flagellar\_cell\_motility | 1 | 0 |  |  |  |  |  |  |  |  |
| GO:0060298\_positive\_regulation\_of\_sarcomere\_organization | 1 | 0 |  |  |  |  |  |  |  |  |
| GO:0060299\_negative\_regulation\_of\_sarcomere\_organization | 1 | 0 |  |  |  |  |  |  |  |  |
| GO:0060300\_regulation\_of\_cytokine\_activity | 1 | 0 |  |  |  |  |  |  |  |  |
| GO:0060302\_negative\_regulation\_of\_cytokine\_activity | 1 | 0 |  |  |  |  |  |  |  |  |
| GO:0060306\_regulation\_of\_membrane\_repolarization | 1 | 0 |  |  |  |  |  |  |  |  |
| GO:0060307\_regulation\_of\_ventricular\_cardiomyocyte\_membrane\_repolarization | 1 | 0 |  |  |  |  |  |  |  |  |
| GO:0060309\_elastin\_catabolic\_process | 1 | 0 |  |  |  |  |  |  |  |  |
| GO:0060310\_regulation\_of\_elastin\_catabolic\_process | 1 | 0 |  |  |  |  |  |  |  |  |
| GO:0060311\_negative\_regulation\_of\_elastin\_catabolic\_process | 1 | 0 |  |  |  |  |  |  |  |  |
| GO:0060312\_regulation\_of\_blood\_vessel\_remodeling | 1 | 0 |  |  |  |  |  |  |  |  |
| GO:0060313\_negative\_regulation\_of\_blood\_vessel\_remodeling | 1 | 0 |  |  |  |  |  |  |  |  |
| GO:0060315\_negative\_regulation\_of\_ryanodine-sensitive\_calcium-release\_channel\_activity | 1 | 0 |  |  |  |  |  |  |  |  |
| GO:0060316\_positive\_regulation\_of\_ryanodine-sensitive\_calcium-release\_channel\_activity | 1 | 0 |  |  |  |  |  |  |  |  |
| GO:0060318\_definitive\_erythrocyte\_differentiation | 1 | 0 |  |  |  |  |  |  |  |  |
| GO:0060322\_head\_development | 1 | 0 |  |  |  |  |  |  |  |  |
| GO:0060324\_face\_development | 1 | 0 |  |  |  |  |  |  |  |  |
| GO:0060336\_negative\_regulation\_of\_interferon-gamma-mediated\_signaling\_pathway | 1 | 0 |  |  |  |  |  |  |  |  |
| GO:0060349\_bone\_morphogenesis | 1 | 0 |  |  |  |  |  |  |  |  |
| GO:0060350\_endochondral\_bone\_morphogenesis | 1 | 0 |  |  |  |  |  |  |  |  |
| GO:0060356\_leucine\_import | 1 | 0 |  |  |  |  |  |  |  |  |
| GO:0060368\_regulation\_of\_Fc\_receptor\_mediated\_stimulatory\_signaling\_pathway | 1 | 0 |  |  |  |  |  |  |  |  |
| GO:0060369\_positive\_regulation\_of\_Fc\_receptor\_mediated\_stimulatory\_signaling\_pathway | 1 | 0 |  |  |  |  |  |  |  |  |
| GO:0060380\_regulation\_of\_single-stranded\_telomeric\_DNA\_binding | 1 | 0 |  |  |  |  |  |  |  |  |
| GO:0060381\_positive\_regulation\_of\_single-stranded\_telomeric\_DNA\_binding | 1 | 0 |  |  |  |  |  |  |  |  |
| GO:0060382\_regulation\_of\_DNA\_strand\_elongation | 1 | 0 |  |  |  |  |  |  |  |  |
| GO:0060383\_positive\_regulation\_of\_DNA\_strand\_elongation | 1 | 0 |  |  |  |  |  |  |  |  |
| GO:0060397\_JAK-STAT\_cascade\_involved\_in\_growth\_hormone\_signaling\_pathway | 1 | 0 |  |  |  |  |  |  |  |  |
| GO:0060398\_regulation\_of\_growth\_hormone\_receptor\_signaling\_pathway | 1 | 0 |  |  |  |  |  |  |  |  |
| GO:0060425\_lung\_morphogenesis | 1 | 0 |  |  |  |  |  |  |  |  |
| GO:0060433\_bronchus\_development | 1 | 0 |  |  |  |  |  |  |  |  |
| GO:0060438\_trachea\_development | 1 | 0 |  |  |  |  |  |  |  |  |
| GO:0060441\_branching\_involved\_in\_lung\_morphogenesis | 1 | 0 |  |  |  |  |  |  |  |  |
| GO:0060445\_branching\_involved\_in\_salivary\_gland\_morphogenesis | 1 | 0 |  |  |  |  |  |  |  |  |
| GO:0060502\_epithelial\_cell\_proliferation\_involved\_in\_lung\_morphogenesis | 1 | 0 |  |  |  |  |  |  |  |  |
| GO:0060503\_bud\_dilation\_involved\_in\_lung\_branching | 1 | 0 |  |  |  |  |  |  |  |  |
| GO:0060560\_developmental\_growth\_involved\_in\_morphogenesis | 1 | 0 |  |  |  |  |  |  |  |  |
| GO:0060579\_ventral\_spinal\_cord\_interneuron\_fate\_commitment | 1 | 0 |  |  |  |  |  |  |  |  |
| GO:0060586\_multicellular\_organismal\_iron\_ion\_homeostasis | 1 | 0 |  |  |  |  |  |  |  |  |
| GO:0060587\_regulation\_of\_lipoprotein\_lipid\_oxidation | 1 | 0 |  |  |  |  |  |  |  |  |
| GO:0060588\_negative\_regulation\_of\_lipoprotein\_lipid\_oxidation | 1 | 0 |  |  |  |  |  |  |  |  |
| GO:0060638\_mesenchymal-epithelial\_cell\_signaling | 1 | 0 |  |  |  |  |  |  |  |  |
| GO:0060665\_regulation\_of\_branching\_involved\_in\_salivary\_gland\_morphogenesis\_by\_mesenchymal-epithelial\_signaling | 1 | 0 |  |  |  |  |  |  |  |  |
| GO:0060675\_ureteric\_bud\_morphogenesis | 1 | 0 |  |  |  |  |  |  |  |  |
| GO:0060688\_regulation\_of\_morphogenesis\_of\_a\_branching\_structure | 1 | 0 |  |  |  |  |  |  |  |  |
| GO:0060693\_regulation\_of\_branching\_involved\_in\_salivary\_gland\_morphogenesis | 1 | 0 |  |  |  |  |  |  |  |  |
| GO:0060694\_regulation\_of\_cholesterol\_transporter\_activity | 1 | 0 |  |  |  |  |  |  |  |  |
| GO:0060695\_negative\_regulation\_of\_cholesterol\_transporter\_activity | 1 | 0 |  |  |  |  |  |  |  |  |
| GO:0060697\_positive\_regulation\_of\_phospholipid\_catabolic\_process | 1 | 0 |  |  |  |  |  |  |  |  |
| GO:0060729\_intestinal\_epithelial\_structure\_maintenance | 1 | 0 |  |  |  |  |  |  |  |  |
| GO:0060730\_regulation\_of\_intestinal\_epithelial\_structure\_maintenance | 1 | 0 |  |  |  |  |  |  |  |  |
| GO:0060731\_positive\_regulation\_of\_intestinal\_epithelial\_structure\_maintenance | 1 | 0 |  |  |  |  |  |  |  |  |
| GO:0060760\_positive\_regulation\_of\_response\_to\_cytokine\_stimulus | 1 | 0 |  |  |  |  |  |  |  |  |
| GO:0060761\_negative\_regulation\_of\_response\_to\_cytokine\_stimulus | 1 | 0 |  |  |  |  |  |  |  |  |
| GO:0060788\_ectodermal\_placode\_formation | 1 | 0 |  |  |  |  |  |  |  |  |
| GO:0060841\_venous\_blood\_vessel\_development | 1 | 0 |  |  |  |  |  |  |  |  |
| GO:0060856\_establishment\_of\_blood-brain\_barrier | 1 | 0 |  |  |  |  |  |  |  |  |
| GO:0060896\_neural\_plate\_pattern\_specification | 1 | 0 |  |  |  |  |  |  |  |  |
| GO:0065001\_specification\_of\_axis\_polarity | 1 | 0 |  |  |  |  |  |  |  |  |
| GO:0070075\_tear\_secretion | 1 | 0 |  |  |  |  |  |  |  |  |
| GO:0070076\_histone\_lysine\_demethylation | 1 | 0 |  |  |  |  |  |  |  |  |
| GO:0070077\_histone\_arginine\_demethylation | 1 | 0 |  |  |  |  |  |  |  |  |
| GO:0070078\_histone\_H3-R2\_demethylation | 1 | 0 |  |  |  |  |  |  |  |  |
| GO:0070079\_histone\_H4-R3\_demethylation | 1 | 0 |  |  |  |  |  |  |  |  |
| GO:0070086\_ubiquitin-dependent\_endocytosis | 1 | 0 |  |  |  |  |  |  |  |  |
| GO:0070091\_glucagon\_secretion | 1 | 0 |  |  |  |  |  |  |  |  |
| GO:0070103\_regulation\_of\_interleukin-6-mediated\_signaling\_pathway | 1 | 0 |  |  |  |  |  |  |  |  |
| GO:0070104\_negative\_regulation\_of\_interleukin-6-mediated\_signaling\_pathway | 1 | 0 |  |  |  |  |  |  |  |  |
| GO:0070106\_interleukin-27-mediated\_signaling\_pathway | 1 | 0 |  |  |  |  |  |  |  |  |
| GO:0070162\_adiponectin\_secretion | 1 | 0 |  |  |  |  |  |  |  |  |
| GO:0070163\_regulation\_of\_adiponectin\_secretion | 1 | 0 |  |  |  |  |  |  |  |  |
| GO:0070165\_positive\_regulation\_of\_adiponectin\_secretion | 1 | 0 |  |  |  |  |  |  |  |  |
| GO:0070172\_positive\_regulation\_of\_tooth\_mineralization | 1 | 0 |  |  |  |  |  |  |  |  |
| GO:0070173\_regulation\_of\_enamel\_mineralization | 1 | 0 |  |  |  |  |  |  |  |  |
| GO:0070189\_kynurenine\_metabolic\_process | 1 | 0 |  |  |  |  |  |  |  |  |
| GO:0070212\_protein\_amino\_acid\_poly-ADP-ribosylation | 1 | 0 |  |  |  |  |  |  |  |  |
| GO:0070213\_protein\_amino\_acid\_auto-ADP-ribosylation | 1 | 0 |  |  |  |  |  |  |  |  |
| GO:0070232\_regulation\_of\_T\_cell\_apoptosis | 1 | 0 |  |  |  |  |  |  |  |  |
| GO:0070234\_positive\_regulation\_of\_T\_cell\_apoptosis | 1 | 0 |  |  |  |  |  |  |  |  |
| GO:0070242\_thymocyte\_apoptosis | 1 | 0 |  |  |  |  |  |  |  |  |
| GO:0070243\_regulation\_of\_thymocyte\_apoptosis | 1 | 0 |  |  |  |  |  |  |  |  |
| GO:0070245\_positive\_regulation\_of\_thymocyte\_apoptosis | 1 | 0 |  |  |  |  |  |  |  |  |
| GO:0070267\_oncosis | 1 | 0 |  |  |  |  |  |  |  |  |
| GO:0070286\_axonemal\_dynein\_complex\_assembly | 1 | 0 |  |  |  |  |  |  |  |  |
| GO:0070314\_G1\_to\_G0\_transition | 1 | 0 |  |  |  |  |  |  |  |  |
| GO:0070327\_thyroid\_hormone\_transport | 1 | 0 |  |  |  |  |  |  |  |  |
| GO:0070407\_oxidation-dependent\_protein\_catabolic\_process | 1 | 0 |  |  |  |  |  |  |  |  |
| GO:0070408\_carbamoyl\_phosphate\_metabolic\_process | 1 | 0 |  |  |  |  |  |  |  |  |
| GO:0070409\_carbamoyl\_phosphate\_biosynthetic\_process | 1 | 0 |  |  |  |  |  |  |  |  |
| GO:0070509\_calcium\_ion\_import | 1 | 0 |  |  |  |  |  |  |  |  |
| GO:0070534\_protein\_K63-linked\_ubiquitination | 1 | 0 |  |  |  |  |  |  |  |  |
| GO:0070535\_histone\_H2A\_K63-linked\_ubiquitination | 1 | 0 |  |  |  |  |  |  |  |  |
| GO:0070537\_histone\_H2A\_K63-linked\_deubiquitination | 1 | 0 |  |  |  |  |  |  |  |  |
| GO:0070562\_regulation\_of\_vitamin\_D\_receptor\_signaling\_pathway | 1 | 0 |  |  |  |  |  |  |  |  |
| GO:0070564\_positive\_regulation\_of\_vitamin\_D\_receptor\_signaling\_pathway | 1 | 0 |  |  |  |  |  |  |  |  |
| GO:0070570\_regulation\_of\_neuron\_projection\_regeneration | 1 | 0 |  |  |  |  |  |  |  |  |
| GO:0070571\_negative\_regulation\_of\_neuron\_projection\_regeneration | 1 | 0 |  |  |  |  |  |  |  |  |
| GO:0070601\_centromeric\_sister\_chromatid\_cohesion | 1 | 0 |  |  |  |  |  |  |  |  |
| GO:0070602\_regulation\_of\_centromeric\_sister\_chromatid\_cohesion | 1 | 0 |  |  |  |  |  |  |  |  |
| GO:0070625\_zymogen\_granule\_exocytosis | 1 | 0 |  |  |  |  |  |  |  |  |
| GO:0070684\_seminal\_clot\_liquefaction | 1 | 0 |  |  |  |  |  |  |  |  |
| GO:0070715\_sodium-dependent\_organic\_cation\_transport | 1 | 0 |  |  |  |  |  |  |  |  |
| GO:0070813\_hydrogen\_sulfide\_metabolic\_process | 1 | 0 |  |  |  |  |  |  |  |  |
| GO:0070814\_hydrogen\_sulfide\_biosynthetic\_process | 1 | 0 |  |  |  |  |  |  |  |  |
| GO:0070846\_Hsp90\_deacetylation | 1 | 0 |  |  |  |  |  |  |  |  |
| GO:0090030\_regulation\_of\_steroid\_hormone\_biosynthetic\_process | 1 | 0 |  |  |  |  |  |  |  |  |
| GO:0090031\_positive\_regulation\_of\_steroid\_hormone\_biosynthetic\_process | 1 | 0 |  |  |  |  |  |  |  |  |
| GO:0019935\_cyclic-nucleotide-mediated\_signaling | 82 | 0 | 0.000000 | 0.000000 | 807 | 570.325056 | 629.71 | 689.094944 | 0.780310 |
| GO:0048193\_Golgi\_vesicle\_transport | 82 | 0 | 0.000000 | 0.000000 | 807 | 570.325056 | 629.71 | 689.094944 | 0.780310 |
| GO:0048514\_blood\_vessel\_morphogenesis | 82 | 0 | 0.000000 | 0.000000 | 807 | 570.325056 | 629.71 | 689.094944 | 0.780310 |
| GO:0000041\_transition\_metal\_ion\_transport | 18 | 0 | 0.000000 | 0.000000 | 844 | 607.552951 | 666.26 | 724.967049 | 0.789408 |
| GO:0000737\_DNA\_catabolic\_process\_\_endonucleolytic | 18 | 0 | 0.000000 | 0.000000 | 844 | 607.552951 | 666.26 | 724.967049 | 0.789408 |
| GO:0001818\_negative\_regulation\_of\_cytokine\_production | 18 | 0 | 0.000000 | 0.000000 | 844 | 607.552951 | 666.26 | 724.967049 | 0.789408 |
| GO:0002541\_activation\_of\_plasma\_proteins\_involved\_in\_acute\_inflammatory\_response | 18 | 0 | 0.000000 | 0.000000 | 844 | 607.552951 | 666.26 | 724.967049 | 0.789408 |
| GO:0002700\_regulation\_of\_production\_of\_molecular\_mediator\_of\_immune\_response | 18 | 0 | 0.000000 | 0.000000 | 844 | 607.552951 | 666.26 | 724.967049 | 0.789408 |
| GO:0003073\_regulation\_of\_systemic\_arterial\_blood\_pressure | 18 | 0 | 0.000000 | 0.000000 | 844 | 607.552951 | 666.26 | 724.967049 | 0.789408 |
| GO:0006672\_ceramide\_metabolic\_process | 18 | 0 | 0.000000 | 0.000000 | 844 | 607.552951 | 666.26 | 724.967049 | 0.789408 |
| GO:0007031\_peroxisome\_organization | 18 | 0 | 0.000000 | 0.000000 | 844 | 607.552951 | 666.26 | 724.967049 | 0.789408 |
| GO:0007033\_vacuole\_organization | 18 | 0 | 0.000000 | 0.000000 | 844 | 607.552951 | 666.26 | 724.967049 | 0.789408 |
| GO:0007041\_lysosomal\_transport | 18 | 0 | 0.000000 | 0.000000 | 844 | 607.552951 | 666.26 | 724.967049 | 0.789408 |
| GO:0007602\_phototransduction | 18 | 0 | 0.000000 | 0.000000 | 844 | 607.552951 | 666.26 | 724.967049 | 0.789408 |
| GO:0009636\_response\_to\_toxin | 18 | 0 | 0.000000 | 0.000000 | 844 | 607.552951 | 666.26 | 724.967049 | 0.789408 |
| GO:0010742\_foam\_cell\_differentiation | 18 | 0 | 0.000000 | 0.000000 | 844 | 607.552951 | 666.26 | 724.967049 | 0.789408 |
| GO:0010827\_regulation\_of\_glucose\_transport | 18 | 0 | 0.000000 | 0.000000 | 844 | 607.552951 | 666.26 | 724.967049 | 0.789408 |
| GO:0016126\_sterol\_biosynthetic\_process | 18 | 0 | 0.000000 | 0.000000 | 844 | 607.552951 | 666.26 | 724.967049 | 0.789408 |
| GO:0019218\_regulation\_of\_steroid\_metabolic\_process | 18 | 0 | 0.000000 | 0.000000 | 844 | 607.552951 | 666.26 | 724.967049 | 0.789408 |
| GO:0030004\_cellular\_monovalent\_inorganic\_cation\_homeostasis | 18 | 0 | 0.000000 | 0.000000 | 844 | 607.552951 | 666.26 | 724.967049 | 0.789408 |
| GO:0030183\_B\_cell\_differentiation | 18 | 0 | 0.000000 | 0.000000 | 844 | 607.552951 | 666.26 | 724.967049 | 0.789408 |
| GO:0030195\_negative\_regulation\_of\_blood\_coagulation | 18 | 0 | 0.000000 | 0.000000 | 844 | 607.552951 | 666.26 | 724.967049 | 0.789408 |
| GO:0030262\_apoptotic\_nuclear\_changes | 18 | 0 | 0.000000 | 0.000000 | 844 | 607.552951 | 666.26 | 724.967049 | 0.789408 |
| GO:0032319\_regulation\_of\_Rho\_GTPase\_activity | 18 | 0 | 0.000000 | 0.000000 | 844 | 607.552951 | 666.26 | 724.967049 | 0.789408 |
| GO:0032846\_positive\_regulation\_of\_homeostatic\_process | 18 | 0 | 0.000000 | 0.000000 | 844 | 607.552951 | 666.26 | 724.967049 | 0.789408 |
| GO:0034599\_cellular\_response\_to\_oxidative\_stress | 18 | 0 | 0.000000 | 0.000000 | 844 | 607.552951 | 666.26 | 724.967049 | 0.789408 |
| GO:0040017\_positive\_regulation\_of\_locomotion | 18 | 0 | 0.000000 | 0.000000 | 844 | 607.552951 | 666.26 | 724.967049 | 0.789408 |
| GO:0042177\_negative\_regulation\_of\_protein\_catabolic\_process | 18 | 0 | 0.000000 | 0.000000 | 844 | 607.552951 | 666.26 | 724.967049 | 0.789408 |
| GO:0043270\_positive\_regulation\_of\_ion\_transport | 18 | 0 | 0.000000 | 0.000000 | 844 | 607.552951 | 666.26 | 724.967049 | 0.789408 |
| GO:0043393\_regulation\_of\_protein\_binding | 18 | 0 | 0.000000 | 0.000000 | 844 | 607.552951 | 666.26 | 724.967049 | 0.789408 |
| GO:0045444\_fat\_cell\_differentiation | 18 | 0 | 0.000000 | 0.000000 | 844 | 607.552951 | 666.26 | 724.967049 | 0.789408 |
| GO:0045639\_positive\_regulation\_of\_myeloid\_cell\_differentiation | 18 | 0 | 0.000000 | 0.000000 | 844 | 607.552951 | 666.26 | 724.967049 | 0.789408 |
| GO:0045833\_negative\_regulation\_of\_lipid\_metabolic\_process | 18 | 0 | 0.000000 | 0.000000 | 844 | 607.552951 | 666.26 | 724.967049 | 0.789408 |
| GO:0046034\_ATP\_metabolic\_process | 18 | 0 | 0.000000 | 0.000000 | 844 | 607.552951 | 666.26 | 724.967049 | 0.789408 |
| GO:0046324\_regulation\_of\_glucose\_import | 18 | 0 | 0.000000 | 0.000000 | 844 | 607.552951 | 666.26 | 724.967049 | 0.789408 |
| GO:0046546\_development\_of\_primary\_male\_sexual\_characteristics | 18 | 0 | 0.000000 | 0.000000 | 844 | 607.552951 | 666.26 | 724.967049 | 0.789408 |
| GO:0050715\_positive\_regulation\_of\_cytokine\_secretion | 18 | 0 | 0.000000 | 0.000000 | 844 | 607.552951 | 666.26 | 724.967049 | 0.789408 |
| GO:0050905\_neuromuscular\_process | 18 | 0 | 0.000000 | 0.000000 | 844 | 607.552951 | 666.26 | 724.967049 | 0.789408 |
| GO:0050921\_positive\_regulation\_of\_chemotaxis | 18 | 0 | 0.000000 | 0.000000 | 844 | 607.552951 | 666.26 | 724.967049 | 0.789408 |
| GO:0060389\_pathway-restricted\_SMAD\_protein\_phosphorylation | 18 | 0 | 0.000000 | 0.000000 | 844 | 607.552951 | 666.26 | 724.967049 | 0.789408 |
| GO:0000377\_RNA\_splicing\_\_via\_transesterification\_reactions\_with\_bulged\_adenosine\_as\_nucleophile | 151 | 0 | 0.000000 | 0.000000 | 846 | 609.065654 | 667.66 | 726.254346 | 0.789196 |
| GO:0000398\_nuclear\_mRNA\_splicing\_\_via\_spliceosome | 151 | 0 | 0.000000 | 0.000000 | 846 | 609.065654 | 667.66 | 726.254346 | 0.789196 |
| GO:0002694\_regulation\_of\_leukocyte\_activation | 70 | 0 | 0.000000 | 0.000000 | 850 | 614.663223 | 672.92 | 731.176777 | 0.791671 |
| GO:0009124\_nucleoside\_monophosphate\_biosynthetic\_process | 70 | 0 | 0.000000 | 0.000000 | 850 | 614.663223 | 672.92 | 731.176777 | 0.791671 |
| GO:0009416\_response\_to\_light\_stimulus | 70 | 0 | 0.000000 | 0.000000 | 850 | 614.663223 | 672.92 | 731.176777 | 0.791671 |
| GO:0051170\_nuclear\_import | 70 | 0 | 0.000000 | 0.000000 | 850 | 614.663223 | 672.92 | 731.176777 | 0.791671 |
| GO:0002697\_regulation\_of\_immune\_effector\_process | 49 | 0 | 0.000000 | 0.000000 | 856 | 625.218223 | 682.85 | 740.481777 | 0.797722 |
| GO:0006643\_membrane\_lipid\_metabolic\_process | 49 | 0 | 0.000000 | 0.000000 | 856 | 625.218223 | 682.85 | 740.481777 | 0.797722 |
| GO:0009914\_hormone\_transport | 49 | 0 | 0.000000 | 0.000000 | 856 | 625.218223 | 682.85 | 740.481777 | 0.797722 |
| GO:0010952\_positive\_regulation\_of\_peptidase\_activity | 49 | 0 | 0.000000 | 0.000000 | 856 | 625.218223 | 682.85 | 740.481777 | 0.797722 |
| GO:0043280\_positive\_regulation\_of\_caspase\_activity | 49 | 0 | 0.000000 | 0.000000 | 856 | 625.218223 | 682.85 | 740.481777 | 0.797722 |
| GO:0050863\_regulation\_of\_T\_cell\_activation | 49 | 0 | 0.000000 | 0.000000 | 856 | 625.218223 | 682.85 | 740.481777 | 0.797722 |
| GO:0009725\_response\_to\_hormone\_stimulus | 129 | 0 | 0.000000 | 0.000000 | 858 | 628.499289 | 685.75 | 743.000711 | 0.799242 |
| GO:0015672\_monovalent\_inorganic\_cation\_transport | 129 | 0 | 0.000000 | 0.000000 | 858 | 628.499289 | 685.75 | 743.000711 | 0.799242 |
| GO:0006790\_sulfur\_metabolic\_process | 62 | 0 | 0.000000 | 0.000000 | 869 | 640.067079 | 696.64 | 753.212921 | 0.801657 |
| GO:0007420\_brain\_development | 62 | 0 | 0.000000 | 0.000000 | 869 | 640.067079 | 696.64 | 753.212921 | 0.801657 |
| GO:0007586\_digestion | 62 | 0 | 0.000000 | 0.000000 | 869 | 640.067079 | 696.64 | 753.212921 | 0.801657 |
| GO:0009190\_cyclic\_nucleotide\_biosynthetic\_process | 62 | 0 | 0.000000 | 0.000000 | 869 | 640.067079 | 696.64 | 753.212921 | 0.801657 |
| GO:0019216\_regulation\_of\_lipid\_metabolic\_process | 62 | 0 | 0.000000 | 0.000000 | 869 | 640.067079 | 696.64 | 753.212921 | 0.801657 |
| GO:0031145\_anaphase-promoting\_complex-dependent\_proteasomal\_ubiquitin-dependent\_protein\_catabolic\_process | 62 | 0 | 0.000000 | 0.000000 | 869 | 640.067079 | 696.64 | 753.212921 | 0.801657 |
| GO:0031667\_response\_to\_nutrient\_levels | 62 | 0 | 0.000000 | 0.000000 | 869 | 640.067079 | 696.64 | 753.212921 | 0.801657 |
| GO:0043406\_positive\_regulation\_of\_MAP\_kinase\_activity | 62 | 0 | 0.000000 | 0.000000 | 869 | 640.067079 | 696.64 | 753.212921 | 0.801657 |
| GO:0044242\_cellular\_lipid\_catabolic\_process | 62 | 0 | 0.000000 | 0.000000 | 869 | 640.067079 | 696.64 | 753.212921 | 0.801657 |
| GO:0051436\_negative\_regulation\_of\_ubiquitin-protein\_ligase\_activity\_during\_mitotic\_cell\_cycle | 62 | 0 | 0.000000 | 0.000000 | 869 | 640.067079 | 696.64 | 753.212921 | 0.801657 |
| GO:0052548\_regulation\_of\_endopeptidase\_activity | 62 | 0 | 0.000000 | 0.000000 | 869 | 640.067079 | 696.64 | 753.212921 | 0.801657 |
| GO:0001503\_ossification | 56 | 0 | 0.000000 | 0.000000 | 872 | 644.805767 | 701.02 | 757.234233 | 0.803922 |
| GO:0050778\_positive\_regulation\_of\_immune\_response | 56 | 0 | 0.000000 | 0.000000 | 872 | 644.805767 | 701.02 | 757.234233 | 0.803922 |
| GO:0060348\_bone\_development | 56 | 0 | 0.000000 | 0.000000 | 872 | 644.805767 | 701.02 | 757.234233 | 0.803922 |
| GO:0003013\_circulatory\_system\_process | 133 | 0 | 0.000000 | 0.000000 | 874 | 648.064421 | 703.81 | 759.555579 | 0.805275 |
| GO:0008015\_blood\_circulation | 133 | 0 | 0.000000 | 0.000000 | 874 | 648.064421 | 703.81 | 759.555579 | 0.805275 |
| GO:0007605\_sensory\_perception\_of\_sound | 54 | 0 | 0.000000 | 0.000000 | 876 | 649.944169 | 705.61 | 761.275831 | 0.805491 |
| GO:0050954\_sensory\_perception\_of\_mechanical\_stimulus | 54 | 0 | 0.000000 | 0.000000 | 876 | 649.944169 | 705.61 | 761.275831 | 0.805491 |
| GO:0006396\_RNA\_processing | 306 | 0 | 0.000000 | 0.000000 | 877 | 650.547992 | 706.12 | 761.692008 | 0.805154 |
| GO:0006469\_negative\_regulation\_of\_protein\_kinase\_activity | 55 | 0 | 0.000000 | 0.000000 | 883 | 656.072257 | 711.51 | 766.947743 | 0.805787 |
| GO:0006725\_cellular\_aromatic\_compound\_metabolic\_process | 55 | 0 | 0.000000 | 0.000000 | 883 | 656.072257 | 711.51 | 766.947743 | 0.805787 |
| GO:0007606\_sensory\_perception\_of\_chemical\_stimulus | 55 | 0 | 0.000000 | 0.000000 | 883 | 656.072257 | 711.51 | 766.947743 | 0.805787 |
| GO:0030814\_regulation\_of\_cAMP\_metabolic\_process | 55 | 0 | 0.000000 | 0.000000 | 883 | 656.072257 | 711.51 | 766.947743 | 0.805787 |
| GO:0030817\_regulation\_of\_cAMP\_biosynthetic\_process | 55 | 0 | 0.000000 | 0.000000 | 883 | 656.072257 | 711.51 | 766.947743 | 0.805787 |
| GO:0043543\_protein\_amino\_acid\_acylation | 55 | 0 | 0.000000 | 0.000000 | 883 | 656.072257 | 711.51 | 766.947743 | 0.805787 |
| GO:0006519\_cellular\_amino\_acid\_and\_derivative\_metabolic\_process | 173 | 0 | 0.000000 | 0.000000 | 885 | 659.199787 | 714.22 | 769.240213 | 0.807028 |
| GO:0046483\_heterocycle\_metabolic\_process | 173 | 0 | 0.000000 | 0.000000 | 885 | 659.199787 | 714.22 | 769.240213 | 0.807028 |
| GO:0006753\_nucleoside\_phosphate\_metabolic\_process | 146 | 0 | 0.000000 | 0.000000 | 889 | 662.875237 | 717.52 | 772.164763 | 0.807109 |
| GO:0009117\_nucleotide\_metabolic\_process | 146 | 0 | 0.000000 | 0.000000 | 889 | 662.875237 | 717.52 | 772.164763 | 0.807109 |
| GO:0016568\_chromatin\_modification | 146 | 0 | 0.000000 | 0.000000 | 889 | 662.875237 | 717.52 | 772.164763 | 0.807109 |
| GO:0022008\_neurogenesis | 146 | 0 | 0.000000 | 0.000000 | 889 | 662.875237 | 717.52 | 772.164763 | 0.807109 |
| GO:0001655\_urogenital\_system\_development | 23 | 0 | 0.000000 | 0.000000 | 911 | 687.013941 | 740.95 | 794.886059 | 0.813337 |
| GO:0001906\_cell\_killing | 23 | 0 | 0.000000 | 0.000000 | 911 | 687.013941 | 740.95 | 794.886059 | 0.813337 |
| GO:0006023\_aminoglycan\_biosynthetic\_process | 23 | 0 | 0.000000 | 0.000000 | 911 | 687.013941 | 740.95 | 794.886059 | 0.813337 |
| GO:0006509\_membrane\_protein\_ectodomain\_proteolysis | 23 | 0 | 0.000000 | 0.000000 | 911 | 687.013941 | 740.95 | 794.886059 | 0.813337 |
| GO:0006641\_triglyceride\_metabolic\_process | 23 | 0 | 0.000000 | 0.000000 | 911 | 687.013941 | 740.95 | 794.886059 | 0.813337 |
| GO:0007190\_activation\_of\_adenylate\_cyclase\_activity | 23 | 0 | 0.000000 | 0.000000 | 911 | 687.013941 | 740.95 | 794.886059 | 0.813337 |
| GO:0007218\_neuropeptide\_signaling\_pathway | 23 | 0 | 0.000000 | 0.000000 | 911 | 687.013941 | 740.95 | 794.886059 | 0.813337 |
| GO:0007623\_circadian\_rhythm | 23 | 0 | 0.000000 | 0.000000 | 911 | 687.013941 | 740.95 | 794.886059 | 0.813337 |
| GO:0016579\_protein\_deubiquitination | 23 | 0 | 0.000000 | 0.000000 | 911 | 687.013941 | 740.95 | 794.886059 | 0.813337 |
| GO:0018130\_heterocycle\_biosynthetic\_process | 23 | 0 | 0.000000 | 0.000000 | 911 | 687.013941 | 740.95 | 794.886059 | 0.813337 |
| GO:0033344\_cholesterol\_efflux | 23 | 0 | 0.000000 | 0.000000 | 911 | 687.013941 | 740.95 | 794.886059 | 0.813337 |
| GO:0033619\_membrane\_protein\_proteolysis | 23 | 0 | 0.000000 | 0.000000 | 911 | 687.013941 | 740.95 | 794.886059 | 0.813337 |
| GO:0034637\_cellular\_carbohydrate\_biosynthetic\_process | 23 | 0 | 0.000000 | 0.000000 | 911 | 687.013941 | 740.95 | 794.886059 | 0.813337 |
| GO:0043473\_pigmentation | 23 | 0 | 0.000000 | 0.000000 | 911 | 687.013941 | 740.95 | 794.886059 | 0.813337 |
| GO:0043627\_response\_to\_estrogen\_stimulus | 23 | 0 | 0.000000 | 0.000000 | 911 | 687.013941 | 740.95 | 794.886059 | 0.813337 |
| GO:0046467\_membrane\_lipid\_biosynthetic\_process | 23 | 0 | 0.000000 | 0.000000 | 911 | 687.013941 | 740.95 | 794.886059 | 0.813337 |
| GO:0048871\_multicellular\_organismal\_homeostasis | 23 | 0 | 0.000000 | 0.000000 | 911 | 687.013941 | 740.95 | 794.886059 | 0.813337 |
| GO:0050671\_positive\_regulation\_of\_lymphocyte\_proliferation | 23 | 0 | 0.000000 | 0.000000 | 911 | 687.013941 | 740.95 | 794.886059 | 0.813337 |
| GO:0050707\_regulation\_of\_cytokine\_secretion | 23 | 0 | 0.000000 | 0.000000 | 911 | 687.013941 | 740.95 | 794.886059 | 0.813337 |
| GO:0051262\_protein\_tetramerization | 23 | 0 | 0.000000 | 0.000000 | 911 | 687.013941 | 740.95 | 794.886059 | 0.813337 |
| GO:0051353\_positive\_regulation\_of\_oxidoreductase\_activity | 23 | 0 | 0.000000 | 0.000000 | 911 | 687.013941 | 740.95 | 794.886059 | 0.813337 |
| GO:0070668\_positive\_regulation\_of\_mast\_cell\_proliferation | 23 | 0 | 0.000000 | 0.000000 | 911 | 687.013941 | 740.95 | 794.886059 | 0.813337 |
| GO:0045597\_positive\_regulation\_of\_cell\_differentiation | 74 | 0 | 0.000000 | 0.000000 | 912 | 690.213090 | 743.64 | 797.066910 | 0.815395 |
| GO:0070887\_cellular\_response\_to\_chemical\_stimulus | 157 | 0 | 0.000000 | 0.000000 | 913 | 692.320547 | 745.58 | 798.839453 | 0.816627 |
| GO:0045944\_positive\_regulation\_of\_transcription\_from\_RNA\_polymerase\_II\_promoter | 115 | 0 | 0.000000 | 0.000000 | 914 | 696.398207 | 749.47 | 802.541793 | 0.819989 |
| GO:0008380\_RNA\_splicing | 192 | 0 | 0.000000 | 0.000000 | 915 | 697.076535 | 750.08 | 803.083465 | 0.819760 |
| GO:0010033\_response\_to\_organic\_substance | 276 | 0 | 0.000000 | 0.000000 | 916 | 700.988794 | 753.06 | 805.131206 | 0.822118 |
| GO:0001952\_regulation\_of\_cell-matrix\_adhesion | 13 | 0 | 0.000000 | 0.000000 | 987 | 774.646482 | 825.45 | 876.253518 | 0.836322 |
| GO:0002200\_somatic\_diversification\_of\_immune\_receptors | 13 | 0 | 0.000000 | 0.000000 | 987 | 774.646482 | 825.45 | 876.253518 | 0.836322 |
| GO:0002263\_cell\_activation\_during\_immune\_response | 13 | 0 | 0.000000 | 0.000000 | 987 | 774.646482 | 825.45 | 876.253518 | 0.836322 |
| GO:0002366\_leukocyte\_activation\_during\_immune\_response | 13 | 0 | 0.000000 | 0.000000 | 987 | 774.646482 | 825.45 | 876.253518 | 0.836322 |
| GO:0002456\_T\_cell\_mediated\_immunity | 13 | 0 | 0.000000 | 0.000000 | 987 | 774.646482 | 825.45 | 876.253518 | 0.836322 |
| GO:0002705\_positive\_regulation\_of\_leukocyte\_mediated\_immunity | 13 | 0 | 0.000000 | 0.000000 | 987 | 774.646482 | 825.45 | 876.253518 | 0.836322 |
| GO:0002708\_positive\_regulation\_of\_lymphocyte\_mediated\_immunity | 13 | 0 | 0.000000 | 0.000000 | 987 | 774.646482 | 825.45 | 876.253518 | 0.836322 |
| GO:0003014\_renal\_system\_process | 13 | 0 | 0.000000 | 0.000000 | 987 | 774.646482 | 825.45 | 876.253518 | 0.836322 |
| GO:0003044\_regulation\_of\_systemic\_arterial\_blood\_pressure\_mediated\_by\_a\_chemical\_signal | 13 | 0 | 0.000000 | 0.000000 | 987 | 774.646482 | 825.45 | 876.253518 | 0.836322 |
| GO:0006014\_D-ribose\_metabolic\_process | 13 | 0 | 0.000000 | 0.000000 | 987 | 774.646482 | 825.45 | 876.253518 | 0.836322 |
| GO:0006081\_cellular\_aldehyde\_metabolic\_process | 13 | 0 | 0.000000 | 0.000000 | 987 | 774.646482 | 825.45 | 876.253518 | 0.836322 |
| GO:0006090\_pyruvate\_metabolic\_process | 13 | 0 | 0.000000 | 0.000000 | 987 | 774.646482 | 825.45 | 876.253518 | 0.836322 |
| GO:0006278\_RNA-dependent\_DNA\_replication | 13 | 0 | 0.000000 | 0.000000 | 987 | 774.646482 | 825.45 | 876.253518 | 0.836322 |
| GO:0006376\_mRNA\_splice\_site\_selection | 13 | 0 | 0.000000 | 0.000000 | 987 | 774.646482 | 825.45 | 876.253518 | 0.836322 |
| GO:0006607\_NLS-bearing\_substrate\_import\_into\_nucleus | 13 | 0 | 0.000000 | 0.000000 | 987 | 774.646482 | 825.45 | 876.253518 | 0.836322 |
| GO:0006783\_heme\_biosynthetic\_process | 13 | 0 | 0.000000 | 0.000000 | 987 | 774.646482 | 825.45 | 876.253518 | 0.836322 |
| GO:0006900\_membrane\_budding | 13 | 0 | 0.000000 | 0.000000 | 987 | 774.646482 | 825.45 | 876.253518 | 0.836322 |
| GO:0006901\_vesicle\_coating | 13 | 0 | 0.000000 | 0.000000 | 987 | 774.646482 | 825.45 | 876.253518 | 0.836322 |
| GO:0006940\_regulation\_of\_smooth\_muscle\_contraction | 13 | 0 | 0.000000 | 0.000000 | 987 | 774.646482 | 825.45 | 876.253518 | 0.836322 |
| GO:0006953\_acute-phase\_response | 13 | 0 | 0.000000 | 0.000000 | 987 | 774.646482 | 825.45 | 876.253518 | 0.836322 |
| GO:0007004\_telomere\_maintenance\_via\_telomerase | 13 | 0 | 0.000000 | 0.000000 | 987 | 774.646482 | 825.45 | 876.253518 | 0.836322 |
| GO:0007052\_mitotic\_spindle\_organization | 13 | 0 | 0.000000 | 0.000000 | 987 | 774.646482 | 825.45 | 876.253518 | 0.836322 |
| GO:0007091\_mitotic\_metaphase\_anaphase\_transition | 13 | 0 | 0.000000 | 0.000000 | 987 | 774.646482 | 825.45 | 876.253518 | 0.836322 |
| GO:0007193\_inhibition\_of\_adenylate\_cyclase\_activity\_by\_G-protein\_signaling | 13 | 0 | 0.000000 | 0.000000 | 987 | 774.646482 | 825.45 | 876.253518 | 0.836322 |
| GO:0007263\_nitric\_oxide\_mediated\_signal\_transduction | 13 | 0 | 0.000000 | 0.000000 | 987 | 774.646482 | 825.45 | 876.253518 | 0.836322 |
| GO:0009065\_glutamine\_family\_amino\_acid\_catabolic\_process | 13 | 0 | 0.000000 | 0.000000 | 987 | 774.646482 | 825.45 | 876.253518 | 0.836322 |
| GO:0009262\_deoxyribonucleotide\_metabolic\_process | 13 | 0 | 0.000000 | 0.000000 | 987 | 774.646482 | 825.45 | 876.253518 | 0.836322 |
| GO:0009746\_response\_to\_hexose\_stimulus | 13 | 0 | 0.000000 | 0.000000 | 987 | 774.646482 | 825.45 | 876.253518 | 0.836322 |
| GO:0009749\_response\_to\_glucose\_stimulus | 13 | 0 | 0.000000 | 0.000000 | 987 | 774.646482 | 825.45 | 876.253518 | 0.836322 |
| GO:0010810\_regulation\_of\_cell-substrate\_adhesion | 13 | 0 | 0.000000 | 0.000000 | 987 | 774.646482 | 825.45 | 876.253518 | 0.836322 |
| GO:0010833\_telomere\_maintenance\_via\_telomere\_lengthening | 13 | 0 | 0.000000 | 0.000000 | 987 | 774.646482 | 825.45 | 876.253518 | 0.836322 |
| GO:0015914\_phospholipid\_transport | 13 | 0 | 0.000000 | 0.000000 | 987 | 774.646482 | 825.45 | 876.253518 | 0.836322 |
| GO:0018149\_peptide\_cross-linking | 13 | 0 | 0.000000 | 0.000000 | 987 | 774.646482 | 825.45 | 876.253518 | 0.836322 |
| GO:0019439\_aromatic\_compound\_catabolic\_process | 13 | 0 | 0.000000 | 0.000000 | 987 | 774.646482 | 825.45 | 876.253518 | 0.836322 |
| GO:0030201\_heparan\_sulfate\_proteoglycan\_metabolic\_process | 13 | 0 | 0.000000 | 0.000000 | 987 | 774.646482 | 825.45 | 876.253518 | 0.836322 |
| GO:0030239\_myofibril\_assembly | 13 | 0 | 0.000000 | 0.000000 | 987 | 774.646482 | 825.45 | 876.253518 | 0.836322 |
| GO:0030260\_entry\_into\_host\_cell | 13 | 0 | 0.000000 | 0.000000 | 987 | 774.646482 | 825.45 | 876.253518 | 0.836322 |
| GO:0030833\_regulation\_of\_actin\_filament\_polymerization | 13 | 0 | 0.000000 | 0.000000 | 987 | 774.646482 | 825.45 | 876.253518 | 0.836322 |
| GO:0031334\_positive\_regulation\_of\_protein\_complex\_assembly | 13 | 0 | 0.000000 | 0.000000 | 987 | 774.646482 | 825.45 | 876.253518 | 0.836322 |
| GO:0031365\_N-terminal\_protein\_amino\_acid\_modification | 13 | 0 | 0.000000 | 0.000000 | 987 | 774.646482 | 825.45 | 876.253518 | 0.836322 |
| GO:0031398\_positive\_regulation\_of\_protein\_ubiquitination | 13 | 0 | 0.000000 | 0.000000 | 987 | 774.646482 | 825.45 | 876.253518 | 0.836322 |
| GO:0031575\_G1\_S\_transition\_checkpoint | 13 | 0 | 0.000000 | 0.000000 | 987 | 774.646482 | 825.45 | 876.253518 | 0.836322 |
| GO:0032488\_Cdc42\_protein\_signal\_transduction | 13 | 0 | 0.000000 | 0.000000 | 987 | 774.646482 | 825.45 | 876.253518 | 0.836322 |
| GO:0034284\_response\_to\_monosaccharide\_stimulus | 13 | 0 | 0.000000 | 0.000000 | 987 | 774.646482 | 825.45 | 876.253518 | 0.836322 |
| GO:0035107\_appendage\_morphogenesis | 13 | 0 | 0.000000 | 0.000000 | 987 | 774.646482 | 825.45 | 876.253518 | 0.836322 |
| GO:0035108\_limb\_morphogenesis | 13 | 0 | 0.000000 | 0.000000 | 987 | 774.646482 | 825.45 | 876.253518 | 0.836322 |
| GO:0042095\_interferon-gamma\_biosynthetic\_process | 13 | 0 | 0.000000 | 0.000000 | 987 | 774.646482 | 825.45 | 876.253518 | 0.836322 |
| GO:0042743\_hydrogen\_peroxide\_metabolic\_process | 13 | 0 | 0.000000 | 0.000000 | 987 | 774.646482 | 825.45 | 876.253518 | 0.836322 |
| GO:0042982\_amyloid\_precursor\_protein\_metabolic\_process | 13 | 0 | 0.000000 | 0.000000 | 987 | 774.646482 | 825.45 | 876.253518 | 0.836322 |
| GO:0043462\_regulation\_of\_ATPase\_activity | 13 | 0 | 0.000000 | 0.000000 | 987 | 774.646482 | 825.45 | 876.253518 | 0.836322 |
| GO:0044409\_entry\_into\_host | 13 | 0 | 0.000000 | 0.000000 | 987 | 774.646482 | 825.45 | 876.253518 | 0.836322 |
| GO:0045766\_positive\_regulation\_of\_angiogenesis | 13 | 0 | 0.000000 | 0.000000 | 987 | 774.646482 | 825.45 | 876.253518 | 0.836322 |
| GO:0045861\_negative\_regulation\_of\_proteolysis | 13 | 0 | 0.000000 | 0.000000 | 987 | 774.646482 | 825.45 | 876.253518 | 0.836322 |
| GO:0046456\_icosanoid\_biosynthetic\_process | 13 | 0 | 0.000000 | 0.000000 | 987 | 774.646482 | 825.45 | 876.253518 | 0.836322 |
| GO:0046470\_phosphatidylcholine\_metabolic\_process | 13 | 0 | 0.000000 | 0.000000 | 987 | 774.646482 | 825.45 | 876.253518 | 0.836322 |
| GO:0046503\_glycerolipid\_catabolic\_process | 13 | 0 | 0.000000 | 0.000000 | 987 | 774.646482 | 825.45 | 876.253518 | 0.836322 |
| GO:0046626\_regulation\_of\_insulin\_receptor\_signaling\_pathway | 13 | 0 | 0.000000 | 0.000000 | 987 | 774.646482 | 825.45 | 876.253518 | 0.836322 |
| GO:0046631\_alpha-beta\_T\_cell\_activation | 13 | 0 | 0.000000 | 0.000000 | 987 | 774.646482 | 825.45 | 876.253518 | 0.836322 |
| GO:0046718\_entry\_of\_virus\_into\_host\_cell | 13 | 0 | 0.000000 | 0.000000 | 987 | 774.646482 | 825.45 | 876.253518 | 0.836322 |
| GO:0046824\_positive\_regulation\_of\_nucleocytoplasmic\_transport | 13 | 0 | 0.000000 | 0.000000 | 987 | 774.646482 | 825.45 | 876.253518 | 0.836322 |
| GO:0048259\_regulation\_of\_receptor-mediated\_endocytosis | 13 | 0 | 0.000000 | 0.000000 | 987 | 774.646482 | 825.45 | 876.253518 | 0.836322 |
| GO:0048489\_synaptic\_vesicle\_transport | 13 | 0 | 0.000000 | 0.000000 | 987 | 774.646482 | 825.45 | 876.253518 | 0.836322 |
| GO:0050684\_regulation\_of\_mRNA\_processing | 13 | 0 | 0.000000 | 0.000000 | 987 | 774.646482 | 825.45 | 876.253518 | 0.836322 |
| GO:0050688\_regulation\_of\_defense\_response\_to\_virus | 13 | 0 | 0.000000 | 0.000000 | 987 | 774.646482 | 825.45 | 876.253518 | 0.836322 |
| GO:0050701\_interleukin-1\_secretion | 13 | 0 | 0.000000 | 0.000000 | 987 | 774.646482 | 825.45 | 876.253518 | 0.836322 |
| GO:0051289\_protein\_homotetramerization | 13 | 0 | 0.000000 | 0.000000 | 987 | 774.646482 | 825.45 | 876.253518 | 0.836322 |
| GO:0051806\_entry\_into\_cell\_of\_other\_organism\_during\_symbiotic\_interaction | 13 | 0 | 0.000000 | 0.000000 | 987 | 774.646482 | 825.45 | 876.253518 | 0.836322 |
| GO:0051828\_entry\_into\_other\_organism\_during\_symbiotic\_interaction | 13 | 0 | 0.000000 | 0.000000 | 987 | 774.646482 | 825.45 | 876.253518 | 0.836322 |
| GO:0051899\_membrane\_depolarization | 13 | 0 | 0.000000 | 0.000000 | 987 | 774.646482 | 825.45 | 876.253518 | 0.836322 |
| GO:0052126\_movement\_in\_host\_environment | 13 | 0 | 0.000000 | 0.000000 | 987 | 774.646482 | 825.45 | 876.253518 | 0.836322 |
| GO:0052192\_movement\_in\_environment\_of\_other\_organism\_during\_symbiotic\_interaction | 13 | 0 | 0.000000 | 0.000000 | 987 | 774.646482 | 825.45 | 876.253518 | 0.836322 |
| GO:0006120\_mitochondrial\_electron\_transport\_\_NADH\_to\_ubiquinone | 42 | 0 | 0.000000 | 0.000000 | 1000 | 788.756865 | 839.18 | 889.603135 | 0.839180 |
| GO:0006865\_amino\_acid\_transport | 42 | 0 | 0.000000 | 0.000000 | 1000 | 788.756865 | 839.18 | 889.603135 | 0.839180 |
| GO:0006944\_membrane\_fusion | 42 | 0 | 0.000000 | 0.000000 | 1000 | 788.756865 | 839.18 | 889.603135 | 0.839180 |
| GO:0007608\_sensory\_perception\_of\_smell | 42 | 0 | 0.000000 | 0.000000 | 1000 | 788.756865 | 839.18 | 889.603135 | 0.839180 |
| GO:0008203\_cholesterol\_metabolic\_process | 42 | 0 | 0.000000 | 0.000000 | 1000 | 788.756865 | 839.18 | 889.603135 | 0.839180 |
| GO:0016072\_rRNA\_metabolic\_process | 42 | 0 | 0.000000 | 0.000000 | 1000 | 788.756865 | 839.18 | 889.603135 | 0.839180 |
| GO:0032259\_methylation | 42 | 0 | 0.000000 | 0.000000 | 1000 | 788.756865 | 839.18 | 889.603135 | 0.839180 |
| GO:0032868\_response\_to\_insulin\_stimulus | 42 | 0 | 0.000000 | 0.000000 | 1000 | 788.756865 | 839.18 | 889.603135 | 0.839180 |
| GO:0032943\_mononuclear\_cell\_proliferation | 42 | 0 | 0.000000 | 0.000000 | 1000 | 788.756865 | 839.18 | 889.603135 | 0.839180 |
| GO:0043414\_biopolymer\_methylation | 42 | 0 | 0.000000 | 0.000000 | 1000 | 788.756865 | 839.18 | 889.603135 | 0.839180 |
| GO:0048585\_negative\_regulation\_of\_response\_to\_stimulus | 42 | 0 | 0.000000 | 0.000000 | 1000 | 788.756865 | 839.18 | 889.603135 | 0.839180 |
| GO:0051271\_negative\_regulation\_of\_cell\_motion | 42 | 0 | 0.000000 | 0.000000 | 1000 | 788.756865 | 839.18 | 889.603135 | 0.839180 |
| GO:0070661\_leukocyte\_proliferation | 42 | 0 | 0.000000 | 0.000000 | 1000 | 788.756865 | 839.18 | 889.603135 | 0.839180 |
| GO:0051098\_regulation\_of\_binding | 97 | 0 | 0.000000 | 0.000000 | 1001 | 791.088238 | 841.32 | 891.551762 | 0.840480 |
| GO:0006461\_protein\_complex\_assembly | 273 | 0 | 0.000000 | 0.000000 | 1003 | 792.298155 | 842.34 | 892.381845 | 0.839821 |
| GO:0070271\_protein\_complex\_biogenesis | 273 | 0 | 0.000000 | 0.000000 | 1003 | 792.298155 | 842.34 | 892.381845 | 0.839821 |
| GO:0006446\_regulation\_of\_translational\_initiation | 32 | 0 | 0.000000 | 0.000000 | 1015 | 809.350999 | 859.02 | 908.689001 | 0.846325 |
| GO:0006839\_mitochondrial\_transport | 32 | 0 | 0.000000 | 0.000000 | 1015 | 809.350999 | 859.02 | 908.689001 | 0.846325 |
| GO:0008286\_insulin\_receptor\_signaling\_pathway | 32 | 0 | 0.000000 | 0.000000 | 1015 | 809.350999 | 859.02 | 908.689001 | 0.846325 |
| GO:0015698\_inorganic\_anion\_transport | 32 | 0 | 0.000000 | 0.000000 | 1015 | 809.350999 | 859.02 | 908.689001 | 0.846325 |
| GO:0015992\_proton\_transport | 32 | 0 | 0.000000 | 0.000000 | 1015 | 809.350999 | 859.02 | 908.689001 | 0.846325 |
| GO:0030216\_keratinocyte\_differentiation | 32 | 0 | 0.000000 | 0.000000 | 1015 | 809.350999 | 859.02 | 908.689001 | 0.846325 |
| GO:0030595\_leukocyte\_chemotaxis | 32 | 0 | 0.000000 | 0.000000 | 1015 | 809.350999 | 859.02 | 908.689001 | 0.846325 |
| GO:0033157\_regulation\_of\_intracellular\_protein\_transport | 32 | 0 | 0.000000 | 0.000000 | 1015 | 809.350999 | 859.02 | 908.689001 | 0.846325 |
| GO:0043542\_endothelial\_cell\_migration | 32 | 0 | 0.000000 | 0.000000 | 1015 | 809.350999 | 859.02 | 908.689001 | 0.846325 |
| GO:0050657\_nucleic\_acid\_transport | 32 | 0 | 0.000000 | 0.000000 | 1015 | 809.350999 | 859.02 | 908.689001 | 0.846325 |
| GO:0050658\_RNA\_transport | 32 | 0 | 0.000000 | 0.000000 | 1015 | 809.350999 | 859.02 | 908.689001 | 0.846325 |
| GO:0051236\_establishment\_of\_RNA\_localization | 32 | 0 | 0.000000 | 0.000000 | 1015 | 809.350999 | 859.02 | 908.689001 | 0.846325 |
| GO:0070727\_cellular\_macromolecule\_localization | 229 | 0 | 0.000000 | 0.000000 | 1016 | 810.023001 | 859.6 | 909.176999 | 0.846063 |
| GO:0000302\_response\_to\_reactive\_oxygen\_species | 21 | 0 | 0.000000 | 0.000000 | 1059 | 857.192768 | 905.46 | 953.727232 | 0.855014 |
| GO:0000718\_nucleotide-excision\_repair\_\_DNA\_damage\_removal | 21 | 0 | 0.000000 | 0.000000 | 1059 | 857.192768 | 905.46 | 953.727232 | 0.855014 |
| GO:0001654\_eye\_development | 21 | 0 | 0.000000 | 0.000000 | 1059 | 857.192768 | 905.46 | 953.727232 | 0.855014 |
| GO:0001936\_regulation\_of\_endothelial\_cell\_proliferation | 21 | 0 | 0.000000 | 0.000000 | 1059 | 857.192768 | 905.46 | 953.727232 | 0.855014 |
| GO:0002819\_regulation\_of\_adaptive\_immune\_response | 21 | 0 | 0.000000 | 0.000000 | 1059 | 857.192768 | 905.46 | 953.727232 | 0.855014 |
| GO:0002822\_regulation\_of\_adaptive\_immune\_response\_based\_on\_somatic\_recombination\_of\_immune\_receptors\_built\_from\_immunoglobulin\_superfamily\_domains | 21 | 0 | 0.000000 | 0.000000 | 1059 | 857.192768 | 905.46 | 953.727232 | 0.855014 |
| GO:0002831\_regulation\_of\_response\_to\_biotic\_stimulus | 21 | 0 | 0.000000 | 0.000000 | 1059 | 857.192768 | 905.46 | 953.727232 | 0.855014 |
| GO:0006024\_glycosaminoglycan\_biosynthetic\_process | 21 | 0 | 0.000000 | 0.000000 | 1059 | 857.192768 | 905.46 | 953.727232 | 0.855014 |
| GO:0006040\_amino\_sugar\_metabolic\_process | 21 | 0 | 0.000000 | 0.000000 | 1059 | 857.192768 | 905.46 | 953.727232 | 0.855014 |
| GO:0006073\_cellular\_glucan\_metabolic\_process | 21 | 0 | 0.000000 | 0.000000 | 1059 | 857.192768 | 905.46 | 953.727232 | 0.855014 |
| GO:0006282\_regulation\_of\_DNA\_repair | 21 | 0 | 0.000000 | 0.000000 | 1059 | 857.192768 | 905.46 | 953.727232 | 0.855014 |
| GO:0006284\_base-excision\_repair | 21 | 0 | 0.000000 | 0.000000 | 1059 | 857.192768 | 905.46 | 953.727232 | 0.855014 |
| GO:0006402\_mRNA\_catabolic\_process | 21 | 0 | 0.000000 | 0.000000 | 1059 | 857.192768 | 905.46 | 953.727232 | 0.855014 |
| GO:0006493\_protein\_amino\_acid\_O-linked\_glycosylation | 21 | 0 | 0.000000 | 0.000000 | 1059 | 857.192768 | 905.46 | 953.727232 | 0.855014 |
| GO:0006664\_glycolipid\_metabolic\_process | 21 | 0 | 0.000000 | 0.000000 | 1059 | 857.192768 | 905.46 | 953.727232 | 0.855014 |
| GO:0006775\_fat-soluble\_vitamin\_metabolic\_process | 21 | 0 | 0.000000 | 0.000000 | 1059 | 857.192768 | 905.46 | 953.727232 | 0.855014 |
| GO:0006892\_post-Golgi\_vesicle-mediated\_transport | 21 | 0 | 0.000000 | 0.000000 | 1059 | 857.192768 | 905.46 | 953.727232 | 0.855014 |
| GO:0006903\_vesicle\_targeting | 21 | 0 | 0.000000 | 0.000000 | 1059 | 857.192768 | 905.46 | 953.727232 | 0.855014 |
| GO:0007292\_female\_gamete\_generation | 21 | 0 | 0.000000 | 0.000000 | 1059 | 857.192768 | 905.46 | 953.727232 | 0.855014 |
| GO:0008156\_negative\_regulation\_of\_DNA\_replication | 21 | 0 | 0.000000 | 0.000000 | 1059 | 857.192768 | 905.46 | 953.727232 | 0.855014 |
| GO:0008360\_regulation\_of\_cell\_shape | 21 | 0 | 0.000000 | 0.000000 | 1059 | 857.192768 | 905.46 | 953.727232 | 0.855014 |
| GO:0009199\_ribonucleoside\_triphosphate\_metabolic\_process | 21 | 0 | 0.000000 | 0.000000 | 1059 | 857.192768 | 905.46 | 953.727232 | 0.855014 |
| GO:0014031\_mesenchymal\_cell\_development | 21 | 0 | 0.000000 | 0.000000 | 1059 | 857.192768 | 905.46 | 953.727232 | 0.855014 |
| GO:0016338\_calcium-independent\_cell-cell\_adhesion | 21 | 0 | 0.000000 | 0.000000 | 1059 | 857.192768 | 905.46 | 953.727232 | 0.855014 |
| GO:0030048\_actin\_filament-based\_movement | 21 | 0 | 0.000000 | 0.000000 | 1059 | 857.192768 | 905.46 | 953.727232 | 0.855014 |
| GO:0030111\_regulation\_of\_Wnt\_receptor\_signaling\_pathway | 21 | 0 | 0.000000 | 0.000000 | 1059 | 857.192768 | 905.46 | 953.727232 | 0.855014 |
| GO:0030148\_sphingolipid\_biosynthetic\_process | 21 | 0 | 0.000000 | 0.000000 | 1059 | 857.192768 | 905.46 | 953.727232 | 0.855014 |
| GO:0032768\_regulation\_of\_monooxygenase\_activity | 21 | 0 | 0.000000 | 0.000000 | 1059 | 857.192768 | 905.46 | 953.727232 | 0.855014 |
| GO:0032886\_regulation\_of\_microtubule-based\_process | 21 | 0 | 0.000000 | 0.000000 | 1059 | 857.192768 | 905.46 | 953.727232 | 0.855014 |
| GO:0035295\_tube\_development | 21 | 0 | 0.000000 | 0.000000 | 1059 | 857.192768 | 905.46 | 953.727232 | 0.855014 |
| GO:0042398\_cellular\_amino\_acid\_derivative\_biosynthetic\_process | 21 | 0 | 0.000000 | 0.000000 | 1059 | 857.192768 | 905.46 | 953.727232 | 0.855014 |
| GO:0042439\_ethanolamine\_and\_derivative\_metabolic\_process | 21 | 0 | 0.000000 | 0.000000 | 1059 | 857.192768 | 905.46 | 953.727232 | 0.855014 |
| GO:0043523\_regulation\_of\_neuron\_apoptosis | 21 | 0 | 0.000000 | 0.000000 | 1059 | 857.192768 | 905.46 | 953.727232 | 0.855014 |
| GO:0044042\_glucan\_metabolic\_process | 21 | 0 | 0.000000 | 0.000000 | 1059 | 857.192768 | 905.46 | 953.727232 | 0.855014 |
| GO:0045649\_regulation\_of\_macrophage\_differentiation | 21 | 0 | 0.000000 | 0.000000 | 1059 | 857.192768 | 905.46 | 953.727232 | 0.855014 |
| GO:0048520\_positive\_regulation\_of\_behavior | 21 | 0 | 0.000000 | 0.000000 | 1059 | 857.192768 | 905.46 | 953.727232 | 0.855014 |
| GO:0048762\_mesenchymal\_cell\_differentiation | 21 | 0 | 0.000000 | 0.000000 | 1059 | 857.192768 | 905.46 | 953.727232 | 0.855014 |
| GO:0051238\_sequestering\_of\_metal\_ion | 21 | 0 | 0.000000 | 0.000000 | 1059 | 857.192768 | 905.46 | 953.727232 | 0.855014 |
| GO:0051297\_centrosome\_organization | 21 | 0 | 0.000000 | 0.000000 | 1059 | 857.192768 | 905.46 | 953.727232 | 0.855014 |
| GO:0055072\_iron\_ion\_homeostasis | 21 | 0 | 0.000000 | 0.000000 | 1059 | 857.192768 | 905.46 | 953.727232 | 0.855014 |
| GO:0060485\_mesenchyme\_development | 21 | 0 | 0.000000 | 0.000000 | 1059 | 857.192768 | 905.46 | 953.727232 | 0.855014 |
| GO:0070167\_regulation\_of\_biomineral\_formation | 21 | 0 | 0.000000 | 0.000000 | 1059 | 857.192768 | 905.46 | 953.727232 | 0.855014 |
| GO:0070507\_regulation\_of\_microtubule\_cytoskeleton\_organization | 21 | 0 | 0.000000 | 0.000000 | 1059 | 857.192768 | 905.46 | 953.727232 | 0.855014 |
| GO:0006140\_regulation\_of\_nucleotide\_metabolic\_process | 60 | 0 | 0.000000 | 0.000000 | 1062 | 860.955864 | 909.02 | 957.084136 | 0.855951 |
| GO:0046058\_cAMP\_metabolic\_process | 60 | 0 | 0.000000 | 0.000000 | 1062 | 860.955864 | 909.02 | 957.084136 | 0.855951 |
| GO:0051249\_regulation\_of\_lymphocyte\_activation | 60 | 0 | 0.000000 | 0.000000 | 1062 | 860.955864 | 909.02 | 957.084136 | 0.855951 |
| GO:0002573\_myeloid\_leukocyte\_differentiation | 41 | 0 | 0.000000 | 0.000000 | 1072 | 874.074976 | 921.29 | 968.505024 | 0.859412 |
| GO:0006338\_chromatin\_remodeling | 41 | 0 | 0.000000 | 0.000000 | 1072 | 874.074976 | 921.29 | 968.505024 | 0.859412 |
| GO:0008643\_carbohydrate\_transport | 41 | 0 | 0.000000 | 0.000000 | 1072 | 874.074976 | 921.29 | 968.505024 | 0.859412 |
| GO:0030031\_cell\_projection\_assembly | 41 | 0 | 0.000000 | 0.000000 | 1072 | 874.074976 | 921.29 | 968.505024 | 0.859412 |
| GO:0031497\_chromatin\_assembly | 41 | 0 | 0.000000 | 0.000000 | 1072 | 874.074976 | 921.29 | 968.505024 | 0.859412 |
| GO:0034330\_cell\_junction\_organization | 41 | 0 | 0.000000 | 0.000000 | 1072 | 874.074976 | 921.29 | 968.505024 | 0.859412 |
| GO:0043388\_positive\_regulation\_of\_DNA\_binding | 41 | 0 | 0.000000 | 0.000000 | 1072 | 874.074976 | 921.29 | 968.505024 | 0.859412 |
| GO:0046651\_lymphocyte\_proliferation | 41 | 0 | 0.000000 | 0.000000 | 1072 | 874.074976 | 921.29 | 968.505024 | 0.859412 |
| GO:0050708\_regulation\_of\_protein\_secretion | 41 | 0 | 0.000000 | 0.000000 | 1072 | 874.074976 | 921.29 | 968.505024 | 0.859412 |
| GO:0051222\_positive\_regulation\_of\_protein\_transport | 41 | 0 | 0.000000 | 0.000000 | 1072 | 874.074976 | 921.29 | 968.505024 | 0.859412 |
| GO:0034660\_ncRNA\_metabolic\_process | 89 | 0 | 0.000000 | 0.000000 | 1076 | 878.581524 | 925.5 | 972.418476 | 0.860130 |
| GO:0043405\_regulation\_of\_MAP\_kinase\_activity | 89 | 0 | 0.000000 | 0.000000 | 1076 | 878.581524 | 925.5 | 972.418476 | 0.860130 |
| GO:0051240\_positive\_regulation\_of\_multicellular\_organismal\_process | 89 | 0 | 0.000000 | 0.000000 | 1076 | 878.581524 | 925.5 | 972.418476 | 0.860130 |
| GO:0051325\_interphase | 89 | 0 | 0.000000 | 0.000000 | 1076 | 878.581524 | 925.5 | 972.418476 | 0.860130 |
| GO:0006606\_protein\_import\_into\_nucleus | 68 | 0 | 0.000000 | 0.000000 | 1082 | 886.745760 | 932.96 | 979.174240 | 0.862255 |
| GO:0007507\_heart\_development | 68 | 0 | 0.000000 | 0.000000 | 1082 | 886.745760 | 932.96 | 979.174240 | 0.862255 |
| GO:0009991\_response\_to\_extracellular\_stimulus | 68 | 0 | 0.000000 | 0.000000 | 1082 | 886.745760 | 932.96 | 979.174240 | 0.862255 |
| GO:0043062\_extracellular\_structure\_organization | 68 | 0 | 0.000000 | 0.000000 | 1082 | 886.745760 | 932.96 | 979.174240 | 0.862255 |
| GO:0048858\_cell\_projection\_morphogenesis | 68 | 0 | 0.000000 | 0.000000 | 1082 | 886.745760 | 932.96 | 979.174240 | 0.862255 |
| GO:0051439\_regulation\_of\_ubiquitin-protein\_ligase\_activity\_during\_mitotic\_cell\_cycle | 68 | 0 | 0.000000 | 0.000000 | 1082 | 886.745760 | 932.96 | 979.174240 | 0.862255 |
| GO:0051640\_organelle\_localization | 57 | 0 | 0.000000 | 0.000000 | 1083 | 890.993031 | 936.92 | 982.846969 | 0.865115 |
| GO:0000902\_cell\_morphogenesis | 144 | 0 | 0.000000 | 0.000000 | 1084 | 892.593966 | 938.28 | 983.966034 | 0.865572 |
| GO:0006397\_mRNA\_processing | 180 | 0 | 0.000000 | 0.000000 | 1085 | 898.811545 | 943.72 | 988.628455 | 0.869788 |
| GO:0001505\_regulation\_of\_neurotransmitter\_levels | 34 | 0 | 0.000000 | 0.000000 | 1111 | 926.172021 | 970.26 | 1014.347979 | 0.873321 |
| GO:0002449\_lymphocyte\_mediated\_immunity | 34 | 0 | 0.000000 | 0.000000 | 1111 | 926.172021 | 970.26 | 1014.347979 | 0.873321 |
| GO:0006399\_tRNA\_metabolic\_process | 34 | 0 | 0.000000 | 0.000000 | 1111 | 926.172021 | 970.26 | 1014.347979 | 0.873321 |
| GO:0006818\_hydrogen\_transport | 34 | 0 | 0.000000 | 0.000000 | 1111 | 926.172021 | 970.26 | 1014.347979 | 0.873321 |
| GO:0006997\_nucleus\_organization | 34 | 0 | 0.000000 | 0.000000 | 1111 | 926.172021 | 970.26 | 1014.347979 | 0.873321 |
| GO:0007188\_G-protein\_signaling\_\_coupled\_to\_cAMP\_nucleotide\_second\_messenger | 34 | 0 | 0.000000 | 0.000000 | 1111 | 926.172021 | 970.26 | 1014.347979 | 0.873321 |
| GO:0007338\_single\_fertilization | 34 | 0 | 0.000000 | 0.000000 | 1111 | 926.172021 | 970.26 | 1014.347979 | 0.873321 |
| GO:0008645\_hexose\_transport | 34 | 0 | 0.000000 | 0.000000 | 1111 | 926.172021 | 970.26 | 1014.347979 | 0.873321 |
| GO:0015718\_monocarboxylic\_acid\_transport | 34 | 0 | 0.000000 | 0.000000 | 1111 | 926.172021 | 970.26 | 1014.347979 | 0.873321 |
| GO:0015749\_monosaccharide\_transport | 34 | 0 | 0.000000 | 0.000000 | 1111 | 926.172021 | 970.26 | 1014.347979 | 0.873321 |
| GO:0015758\_glucose\_transport | 34 | 0 | 0.000000 | 0.000000 | 1111 | 926.172021 | 970.26 | 1014.347979 | 0.873321 |
| GO:0019395\_fatty\_acid\_oxidation | 34 | 0 | 0.000000 | 0.000000 | 1111 | 926.172021 | 970.26 | 1014.347979 | 0.873321 |
| GO:0019748\_secondary\_metabolic\_process | 34 | 0 | 0.000000 | 0.000000 | 1111 | 926.172021 | 970.26 | 1014.347979 | 0.873321 |
| GO:0030100\_regulation\_of\_endocytosis | 34 | 0 | 0.000000 | 0.000000 | 1111 | 926.172021 | 970.26 | 1014.347979 | 0.873321 |
| GO:0030521\_androgen\_receptor\_signaling\_pathway | 34 | 0 | 0.000000 | 0.000000 | 1111 | 926.172021 | 970.26 | 1014.347979 | 0.873321 |
| GO:0031400\_negative\_regulation\_of\_protein\_modification\_process | 34 | 0 | 0.000000 | 0.000000 | 1111 | 926.172021 | 970.26 | 1014.347979 | 0.873321 |
| GO:0031647\_regulation\_of\_protein\_stability | 34 | 0 | 0.000000 | 0.000000 | 1111 | 926.172021 | 970.26 | 1014.347979 | 0.873321 |
| GO:0043269\_regulation\_of\_ion\_transport | 34 | 0 | 0.000000 | 0.000000 | 1111 | 926.172021 | 970.26 | 1014.347979 | 0.873321 |
| GO:0045137\_development\_of\_primary\_sexual\_characteristics | 34 | 0 | 0.000000 | 0.000000 | 1111 | 926.172021 | 970.26 | 1014.347979 | 0.873321 |
| GO:0048545\_response\_to\_steroid\_hormone\_stimulus | 34 | 0 | 0.000000 | 0.000000 | 1111 | 926.172021 | 970.26 | 1014.347979 | 0.873321 |
| GO:0048608\_reproductive\_structure\_development | 34 | 0 | 0.000000 | 0.000000 | 1111 | 926.172021 | 970.26 | 1014.347979 | 0.873321 |
| GO:0050767\_regulation\_of\_neurogenesis | 34 | 0 | 0.000000 | 0.000000 | 1111 | 926.172021 | 970.26 | 1014.347979 | 0.873321 |
| GO:0050808\_synapse\_organization | 34 | 0 | 0.000000 | 0.000000 | 1111 | 926.172021 | 970.26 | 1014.347979 | 0.873321 |
| GO:0051605\_protein\_maturation\_by\_peptide\_bond\_cleavage | 34 | 0 | 0.000000 | 0.000000 | 1111 | 926.172021 | 970.26 | 1014.347979 | 0.873321 |
| GO:0051651\_maintenance\_of\_location\_in\_cell | 34 | 0 | 0.000000 | 0.000000 | 1111 | 926.172021 | 970.26 | 1014.347979 | 0.873321 |
| GO:0060326\_cell\_chemotaxis | 34 | 0 | 0.000000 | 0.000000 | 1111 | 926.172021 | 970.26 | 1014.347979 | 0.873321 |
| GO:0008544\_epidermis\_development | 104 | 0 | 0.000000 | 0.000000 | 1112 | 927.010340 | 971.08 | 1015.149660 | 0.873273 |
| GO:0008610\_lipid\_biosynthetic\_process | 179 | 0 | 0.000000 | 0.000000 | 1113 | 932.280539 | 975.73 | 1019.179461 | 0.876667 |
| GO:0000050\_urea\_cycle | 3 | 0 |  |  |  |  |  |  |  |  |
| GO:0000089\_mitotic\_metaphase | 3 | 0 |  |  |  |  |  |  |  |  |
| GO:0000097\_sulfur\_amino\_acid\_biosynthetic\_process | 3 | 0 |  |  |  |  |  |  |  |  |
| GO:0000266\_mitochondrial\_fission | 3 | 0 |  |  |  |  |  |  |  |  |
| GO:0000281\_cytokinesis\_after\_mitosis | 3 | 0 |  |  |  |  |  |  |  |  |
| GO:0000394\_RNA\_splicing\_\_via\_endonucleolytic\_cleavage\_and\_ligation | 3 | 0 |  |  |  |  |  |  |  |  |
| GO:0000463\_maturation\_of\_LSU-rRNA\_from\_tricistronic\_rRNA\_transcript\_(SSU-rRNA\_\_5.8S\_rRNA\_\_LSU-rRNA) | 3 | 0 |  |  |  |  |  |  |  |  |
| GO:0000470\_maturation\_of\_LSU-rRNA | 3 | 0 |  |  |  |  |  |  |  |  |
| GO:0000491\_small\_nucleolar\_ribonucleoprotein\_complex\_assembly | 3 | 0 |  |  |  |  |  |  |  |  |
| GO:0000492\_box\_C\_D\_snoRNP\_assembly | 3 | 0 |  |  |  |  |  |  |  |  |
| GO:0001302\_replicative\_cell\_aging | 3 | 0 |  |  |  |  |  |  |  |  |
| GO:0001510\_RNA\_methylation | 3 | 0 |  |  |  |  |  |  |  |  |
| GO:0001569\_patterning\_of\_blood\_vessels | 3 | 0 |  |  |  |  |  |  |  |  |
| GO:0001573\_ganglioside\_metabolic\_process | 3 | 0 |  |  |  |  |  |  |  |  |
| GO:0001659\_temperature\_homeostasis | 3 | 0 |  |  |  |  |  |  |  |  |
| GO:0001755\_neural\_crest\_cell\_migration | 3 | 0 |  |  |  |  |  |  |  |  |
| GO:0001773\_myeloid\_dendritic\_cell\_activation | 3 | 0 |  |  |  |  |  |  |  |  |
| GO:0001881\_receptor\_recycling | 3 | 0 |  |  |  |  |  |  |  |  |
| GO:0001895\_retina\_homeostasis | 3 | 0 |  |  |  |  |  |  |  |  |
| GO:0001916\_positive\_regulation\_of\_T\_cell\_mediated\_cytotoxicity | 3 | 0 |  |  |  |  |  |  |  |  |
| GO:0001919\_regulation\_of\_receptor\_recycling | 3 | 0 |  |  |  |  |  |  |  |  |
| GO:0001945\_lymph\_vessel\_development | 3 | 0 |  |  |  |  |  |  |  |  |
| GO:0001946\_lymphangiogenesis | 3 | 0 |  |  |  |  |  |  |  |  |
| GO:0002002\_regulation\_of\_angiotensin\_levels\_in\_blood | 3 | 0 |  |  |  |  |  |  |  |  |
| GO:0002029\_desensitization\_of\_G-protein\_coupled\_receptor\_protein\_signaling\_pathway | 3 | 0 |  |  |  |  |  |  |  |  |
| GO:0002062\_chondrocyte\_differentiation | 3 | 0 |  |  |  |  |  |  |  |  |
| GO:0002076\_osteoblast\_development | 3 | 0 |  |  |  |  |  |  |  |  |
| GO:0002230\_positive\_regulation\_of\_defense\_response\_to\_virus\_by\_host | 3 | 0 |  |  |  |  |  |  |  |  |
| GO:0002246\_healing\_during\_inflammatory\_response | 3 | 0 |  |  |  |  |  |  |  |  |
| GO:0002262\_myeloid\_cell\_homeostasis | 3 | 0 |  |  |  |  |  |  |  |  |
| GO:0002444\_myeloid\_leukocyte\_mediated\_immunity | 3 | 0 |  |  |  |  |  |  |  |  |
| GO:0002446\_neutrophil\_mediated\_immunity | 3 | 0 |  |  |  |  |  |  |  |  |
| GO:0002792\_negative\_regulation\_of\_peptide\_secretion | 3 | 0 |  |  |  |  |  |  |  |  |
| GO:0002825\_regulation\_of\_T-helper\_1\_type\_immune\_response | 3 | 0 |  |  |  |  |  |  |  |  |
| GO:0002828\_regulation\_of\_T-helper\_2\_type\_immune\_response | 3 | 0 |  |  |  |  |  |  |  |  |
| GO:0002903\_negative\_regulation\_of\_B\_cell\_apoptosis | 3 | 0 |  |  |  |  |  |  |  |  |
| GO:0002921\_negative\_regulation\_of\_humoral\_immune\_response | 3 | 0 |  |  |  |  |  |  |  |  |
| GO:0003084\_positive\_regulation\_of\_systemic\_arterial\_blood\_pressure | 3 | 0 |  |  |  |  |  |  |  |  |
| GO:0005980\_glycogen\_catabolic\_process | 3 | 0 |  |  |  |  |  |  |  |  |
| GO:0005984\_disaccharide\_metabolic\_process | 3 | 0 |  |  |  |  |  |  |  |  |
| GO:0006002\_fructose\_6-phosphate\_metabolic\_process | 3 | 0 |  |  |  |  |  |  |  |  |
| GO:0006003\_fructose\_2\_6-bisphosphate\_metabolic\_process | 3 | 0 |  |  |  |  |  |  |  |  |
| GO:0006043\_glucosamine\_catabolic\_process | 3 | 0 |  |  |  |  |  |  |  |  |
| GO:0006071\_glycerol\_metabolic\_process | 3 | 0 |  |  |  |  |  |  |  |  |
| GO:0006102\_isocitrate\_metabolic\_process | 3 | 0 |  |  |  |  |  |  |  |  |
| GO:0006104\_succinyl-CoA\_metabolic\_process | 3 | 0 |  |  |  |  |  |  |  |  |
| GO:0006108\_malate\_metabolic\_process | 3 | 0 |  |  |  |  |  |  |  |  |
| GO:0006123\_mitochondrial\_electron\_transport\_\_cytochrome\_c\_to\_oxygen | 3 | 0 |  |  |  |  |  |  |  |  |
| GO:0006152\_purine\_nucleoside\_catabolic\_process | 3 | 0 |  |  |  |  |  |  |  |  |
| GO:0006167\_AMP\_biosynthetic\_process | 3 | 0 |  |  |  |  |  |  |  |  |
| GO:0006182\_cGMP\_biosynthetic\_process | 3 | 0 |  |  |  |  |  |  |  |  |
| GO:0006269\_DNA\_replication\_\_synthesis\_of\_RNA\_primer | 3 | 0 |  |  |  |  |  |  |  |  |
| GO:0006273\_lagging\_strand\_elongation | 3 | 0 |  |  |  |  |  |  |  |  |
| GO:0006288\_base-excision\_repair\_\_DNA\_ligation | 3 | 0 |  |  |  |  |  |  |  |  |
| GO:0006295\_nucleotide-excision\_repair\_\_DNA\_incision\_\_3'-to\_lesion | 3 | 0 |  |  |  |  |  |  |  |  |
| GO:0006296\_nucleotide-excision\_repair\_\_DNA\_incision\_\_5'-to\_lesion | 3 | 0 |  |  |  |  |  |  |  |  |
| GO:0006384\_transcription\_initiation\_from\_RNA\_polymerase\_III\_promoter | 3 | 0 |  |  |  |  |  |  |  |  |
| GO:0006388\_tRNA\_splicing\_\_via\_endonucleolytic\_cleavage\_and\_ligation | 3 | 0 |  |  |  |  |  |  |  |  |
| GO:0006467\_protein\_thiol-disulfide\_exchange | 3 | 0 |  |  |  |  |  |  |  |  |
| GO:0006546\_glycine\_catabolic\_process | 3 | 0 |  |  |  |  |  |  |  |  |
| GO:0006558\_L-phenylalanine\_metabolic\_process | 3 | 0 |  |  |  |  |  |  |  |  |
| GO:0006559\_L-phenylalanine\_catabolic\_process | 3 | 0 |  |  |  |  |  |  |  |  |
| GO:0006560\_proline\_metabolic\_process | 3 | 0 |  |  |  |  |  |  |  |  |
| GO:0006565\_L-serine\_catabolic\_process | 3 | 0 |  |  |  |  |  |  |  |  |
| GO:0006568\_tryptophan\_metabolic\_process | 3 | 0 |  |  |  |  |  |  |  |  |
| GO:0006573\_valine\_metabolic\_process | 3 | 0 |  |  |  |  |  |  |  |  |
| GO:0006590\_thyroid\_hormone\_generation | 3 | 0 |  |  |  |  |  |  |  |  |
| GO:0006596\_polyamine\_biosynthetic\_process | 3 | 0 |  |  |  |  |  |  |  |  |
| GO:0006621\_protein\_retention\_in\_ER\_lumen | 3 | 0 |  |  |  |  |  |  |  |  |
| GO:0006654\_phosphatidic\_acid\_biosynthetic\_process | 3 | 0 |  |  |  |  |  |  |  |  |
| GO:0006658\_phosphatidylserine\_metabolic\_process | 3 | 0 |  |  |  |  |  |  |  |  |
| GO:0006663\_platelet\_activating\_factor\_biosynthetic\_process | 3 | 0 |  |  |  |  |  |  |  |  |
| GO:0006678\_glucosylceramide\_metabolic\_process | 3 | 0 |  |  |  |  |  |  |  |  |
| GO:0006686\_sphingomyelin\_biosynthetic\_process | 3 | 0 |  |  |  |  |  |  |  |  |
| GO:0006701\_progesterone\_biosynthetic\_process | 3 | 0 |  |  |  |  |  |  |  |  |
| GO:0006704\_glucocorticoid\_biosynthetic\_process | 3 | 0 |  |  |  |  |  |  |  |  |
| GO:0006705\_mineralocorticoid\_biosynthetic\_process | 3 | 0 |  |  |  |  |  |  |  |  |
| GO:0006729\_tetrahydrobiopterin\_biosynthetic\_process | 3 | 0 |  |  |  |  |  |  |  |  |
| GO:0006777\_Mo-molybdopterin\_cofactor\_biosynthetic\_process | 3 | 0 |  |  |  |  |  |  |  |  |
| GO:0006787\_porphyrin\_catabolic\_process | 3 | 0 |  |  |  |  |  |  |  |  |
| GO:0006829\_zinc\_ion\_transport | 3 | 0 |  |  |  |  |  |  |  |  |
| GO:0006853\_carnitine\_shuttle | 3 | 0 |  |  |  |  |  |  |  |  |
| GO:0006863\_purine\_transport | 3 | 0 |  |  |  |  |  |  |  |  |
| GO:0006882\_cellular\_zinc\_ion\_homeostasis | 3 | 0 |  |  |  |  |  |  |  |  |
| GO:0006910\_phagocytosis\_\_recognition | 3 | 0 |  |  |  |  |  |  |  |  |
| GO:0006922\_cleavage\_of\_lamin | 3 | 0 |  |  |  |  |  |  |  |  |
| GO:0006923\_cleavage\_of\_cytoskeletal\_proteins\_during\_apoptosis | 3 | 0 |  |  |  |  |  |  |  |  |
| GO:0006927\_transformed\_cell\_apoptosis | 3 | 0 |  |  |  |  |  |  |  |  |
| GO:0007007\_inner\_mitochondrial\_membrane\_organization | 3 | 0 |  |  |  |  |  |  |  |  |
| GO:0007035\_vacuolar\_acidification | 3 | 0 |  |  |  |  |  |  |  |  |
| GO:0007175\_negative\_regulation\_of\_epidermal\_growth\_factor\_receptor\_activity | 3 | 0 |  |  |  |  |  |  |  |  |
| GO:0007181\_transforming\_growth\_factor\_beta\_receptor\_complex\_assembly | 3 | 0 |  |  |  |  |  |  |  |  |
| GO:0007199\_G-protein\_signaling\_\_coupled\_to\_cGMP\_nucleotide\_second\_messenger | 3 | 0 |  |  |  |  |  |  |  |  |
| GO:0007207\_activation\_of\_phospholipase\_C\_activity\_by\_muscarinic\_acetylcholine\_receptor\_signaling\_pathway | 3 | 0 |  |  |  |  |  |  |  |  |
| GO:0007252\_I-kappaB\_phosphorylation | 3 | 0 |  |  |  |  |  |  |  |  |
| GO:0007339\_binding\_of\_sperm\_to\_zona\_pellucida | 3 | 0 |  |  |  |  |  |  |  |  |
| GO:0007352\_zygotic\_determination\_of\_dorsal\_ventral\_axis | 3 | 0 |  |  |  |  |  |  |  |  |
| GO:0007439\_ectodermal\_gut\_development | 3 | 0 |  |  |  |  |  |  |  |  |
| GO:0007500\_mesodermal\_cell\_fate\_determination | 3 | 0 |  |  |  |  |  |  |  |  |
| GO:0007512\_adult\_heart\_development | 3 | 0 |  |  |  |  |  |  |  |  |
| GO:0008053\_mitochondrial\_fusion | 3 | 0 |  |  |  |  |  |  |  |  |
| GO:0008291\_acetylcholine\_metabolic\_process | 3 | 0 |  |  |  |  |  |  |  |  |
| GO:0008334\_histone\_mRNA\_metabolic\_process | 3 | 0 |  |  |  |  |  |  |  |  |
| GO:0008617\_guanosine\_metabolic\_process | 3 | 0 |  |  |  |  |  |  |  |  |
| GO:0008628\_induction\_of\_apoptosis\_by\_hormones | 3 | 0 |  |  |  |  |  |  |  |  |
| GO:0008631\_induction\_of\_apoptosis\_by\_oxidative\_stress | 3 | 0 |  |  |  |  |  |  |  |  |
| GO:0009070\_serine\_family\_amino\_acid\_biosynthetic\_process | 3 | 0 |  |  |  |  |  |  |  |  |
| GO:0009125\_nucleoside\_monophosphate\_catabolic\_process | 3 | 0 |  |  |  |  |  |  |  |  |
| GO:0009132\_nucleoside\_diphosphate\_metabolic\_process | 3 | 0 |  |  |  |  |  |  |  |  |
| GO:0009155\_purine\_deoxyribonucleotide\_catabolic\_process | 3 | 0 |  |  |  |  |  |  |  |  |
| GO:0009163\_nucleoside\_biosynthetic\_process | 3 | 0 |  |  |  |  |  |  |  |  |
| GO:0009185\_ribonucleoside\_diphosphate\_metabolic\_process | 3 | 0 |  |  |  |  |  |  |  |  |
| GO:0009226\_nucleotide-sugar\_biosynthetic\_process | 3 | 0 |  |  |  |  |  |  |  |  |
| GO:0009301\_snRNA\_transcription | 3 | 0 |  |  |  |  |  |  |  |  |
| GO:0009620\_response\_to\_fungus | 3 | 0 |  |  |  |  |  |  |  |  |
| GO:0009791\_post-embryonic\_development | 3 | 0 |  |  |  |  |  |  |  |  |
| GO:0010002\_cardioblast\_differentiation | 3 | 0 |  |  |  |  |  |  |  |  |
| GO:0010039\_response\_to\_iron\_ion | 3 | 0 |  |  |  |  |  |  |  |  |
| GO:0010332\_response\_to\_gamma\_radiation | 3 | 0 |  |  |  |  |  |  |  |  |
| GO:0010457\_centriole-centriole\_cohesion | 3 | 0 |  |  |  |  |  |  |  |  |
| GO:0010560\_positive\_regulation\_of\_glycoprotein\_biosynthetic\_process | 3 | 0 |  |  |  |  |  |  |  |  |
| GO:0010661\_positive\_regulation\_of\_muscle\_cell\_apoptosis | 3 | 0 |  |  |  |  |  |  |  |  |
| GO:0010677\_negative\_regulation\_of\_cellular\_carbohydrate\_metabolic\_process | 3 | 0 |  |  |  |  |  |  |  |  |
| GO:0010713\_negative\_regulation\_of\_collagen\_metabolic\_process | 3 | 0 |  |  |  |  |  |  |  |  |
| GO:0010800\_positive\_regulation\_of\_peptidyl-threonine\_phosphorylation | 3 | 0 |  |  |  |  |  |  |  |  |
| GO:0010824\_regulation\_of\_centrosome\_duplication | 3 | 0 |  |  |  |  |  |  |  |  |
| GO:0010834\_telomere\_maintenance\_via\_telomere\_shortening | 3 | 0 |  |  |  |  |  |  |  |  |
| GO:0010847\_regulation\_of\_chromatin\_assembly | 3 | 0 |  |  |  |  |  |  |  |  |
| GO:0010880\_regulation\_of\_release\_of\_sequestered\_calcium\_ion\_into\_cytosol\_by\_sarcoplasmic\_reticulum | 3 | 0 |  |  |  |  |  |  |  |  |
| GO:0010890\_positive\_regulation\_of\_sequestering\_of\_triglyceride | 3 | 0 |  |  |  |  |  |  |  |  |
| GO:0010894\_negative\_regulation\_of\_steroid\_biosynthetic\_process | 3 | 0 |  |  |  |  |  |  |  |  |
| GO:0010903\_negative\_regulation\_of\_very-low-density\_lipoprotein\_particle\_remodeling | 3 | 0 |  |  |  |  |  |  |  |  |
| GO:0010939\_regulation\_of\_necrotic\_cell\_death | 3 | 0 |  |  |  |  |  |  |  |  |
| GO:0010940\_positive\_regulation\_of\_necrotic\_cell\_death | 3 | 0 |  |  |  |  |  |  |  |  |
| GO:0010955\_negative\_regulation\_of\_protein\_maturation\_by\_peptide\_bond\_cleavage | 3 | 0 |  |  |  |  |  |  |  |  |
| GO:0010956\_negative\_regulation\_of\_calcidiol\_1-monooxygenase\_activity | 3 | 0 |  |  |  |  |  |  |  |  |
| GO:0014805\_smooth\_muscle\_adaptation | 3 | 0 |  |  |  |  |  |  |  |  |
| GO:0014808\_release\_of\_sequestered\_calcium\_ion\_into\_cytosol\_by\_sarcoplasmic\_reticulum | 3 | 0 |  |  |  |  |  |  |  |  |
| GO:0014912\_negative\_regulation\_of\_smooth\_muscle\_cell\_migration | 3 | 0 |  |  |  |  |  |  |  |  |
| GO:0015014\_heparan\_sulfate\_proteoglycan\_biosynthetic\_process\_\_polysaccharide\_chain\_biosynthetic\_process | 3 | 0 |  |  |  |  |  |  |  |  |
| GO:0015015\_heparan\_sulfate\_proteoglycan\_biosynthetic\_process\_\_enzymatic\_modification | 3 | 0 |  |  |  |  |  |  |  |  |
| GO:0015670\_carbon\_dioxide\_transport | 3 | 0 |  |  |  |  |  |  |  |  |
| GO:0015721\_bile\_acid\_and\_bile\_salt\_transport | 3 | 0 |  |  |  |  |  |  |  |  |
| GO:0015802\_basic\_amino\_acid\_transport | 3 | 0 |  |  |  |  |  |  |  |  |
| GO:0015811\_L-cystine\_transport | 3 | 0 |  |  |  |  |  |  |  |  |
| GO:0015840\_urea\_transport | 3 | 0 |  |  |  |  |  |  |  |  |
| GO:0015889\_cobalamin\_transport | 3 | 0 |  |  |  |  |  |  |  |  |
| GO:0015917\_aminophospholipid\_transport | 3 | 0 |  |  |  |  |  |  |  |  |
| GO:0016056\_rhodopsin\_mediated\_signaling\_pathway | 3 | 0 |  |  |  |  |  |  |  |  |
| GO:0016081\_synaptic\_vesicle\_docking\_during\_exocytosis | 3 | 0 |  |  |  |  |  |  |  |  |
| GO:0016137\_glycoside\_metabolic\_process | 3 | 0 |  |  |  |  |  |  |  |  |
| GO:0016139\_glycoside\_catabolic\_process | 3 | 0 |  |  |  |  |  |  |  |  |
| GO:0016553\_base\_conversion\_or\_substitution\_editing | 3 | 0 |  |  |  |  |  |  |  |  |
| GO:0016572\_histone\_phosphorylation | 3 | 0 |  |  |  |  |  |  |  |  |
| GO:0016576\_histone\_dephosphorylation | 3 | 0 |  |  |  |  |  |  |  |  |
| GO:0016577\_histone\_demethylation | 3 | 0 |  |  |  |  |  |  |  |  |
| GO:0016584\_nucleosome\_positioning | 3 | 0 |  |  |  |  |  |  |  |  |
| GO:0018076\_N-terminal\_peptidyl-lysine\_acetylation | 3 | 0 |  |  |  |  |  |  |  |  |
| GO:0018094\_protein\_polyglycylation | 3 | 0 |  |  |  |  |  |  |  |  |
| GO:0018103\_protein\_amino\_acid\_C-linked\_glycosylation | 3 | 0 |  |  |  |  |  |  |  |  |
| GO:0018211\_peptidyl-tryptophan\_modification | 3 | 0 |  |  |  |  |  |  |  |  |
| GO:0018242\_protein\_amino\_acid\_O-linked\_glycosylation\_via\_serine | 3 | 0 |  |  |  |  |  |  |  |  |
| GO:0018243\_protein\_amino\_acid\_O-linked\_glycosylation\_via\_threonine | 3 | 0 |  |  |  |  |  |  |  |  |
| GO:0018317\_protein\_amino\_acid\_C-linked\_glycosylation\_via\_tryptophan | 3 | 0 |  |  |  |  |  |  |  |  |
| GO:0018343\_protein\_farnesylation | 3 | 0 |  |  |  |  |  |  |  |  |
| GO:0018344\_protein\_geranylgeranylation | 3 | 0 |  |  |  |  |  |  |  |  |
| GO:0018348\_protein\_amino\_acid\_geranylgeranylation | 3 | 0 |  |  |  |  |  |  |  |  |
| GO:0018394\_peptidyl-lysine\_acetylation | 3 | 0 |  |  |  |  |  |  |  |  |
| GO:0018406\_protein\_amino\_acid\_C-linked\_glycosylation\_via\_2'-alpha-mannosyl-L-tryptophan | 3 | 0 |  |  |  |  |  |  |  |  |
| GO:0019063\_virion\_penetration\_into\_host\_cell | 3 | 0 |  |  |  |  |  |  |  |  |
| GO:0019067\_viral\_assembly\_\_maturation\_\_egress\_\_and\_release | 3 | 0 |  |  |  |  |  |  |  |  |
| GO:0019276\_UDP-N-acetylgalactosamine\_metabolic\_process | 3 | 0 |  |  |  |  |  |  |  |  |
| GO:0019377\_glycolipid\_catabolic\_process | 3 | 0 |  |  |  |  |  |  |  |  |
| GO:0019627\_urea\_metabolic\_process | 3 | 0 |  |  |  |  |  |  |  |  |
| GO:0019720\_Mo-molybdopterin\_cofactor\_metabolic\_process | 3 | 0 |  |  |  |  |  |  |  |  |
| GO:0019794\_nonprotein\_amino\_acid\_metabolic\_process | 3 | 0 |  |  |  |  |  |  |  |  |
| GO:0019859\_thymine\_metabolic\_process | 3 | 0 |  |  |  |  |  |  |  |  |
| GO:0021516\_dorsal\_spinal\_cord\_development | 3 | 0 |  |  |  |  |  |  |  |  |
| GO:0021543\_pallium\_development | 3 | 0 |  |  |  |  |  |  |  |  |
| GO:0021575\_hindbrain\_morphogenesis | 3 | 0 |  |  |  |  |  |  |  |  |
| GO:0021885\_forebrain\_cell\_migration | 3 | 0 |  |  |  |  |  |  |  |  |
| GO:0021952\_central\_nervous\_system\_projection\_neuron\_axonogenesis | 3 | 0 |  |  |  |  |  |  |  |  |
| GO:0021955\_central\_nervous\_system\_neuron\_axonogenesis | 3 | 0 |  |  |  |  |  |  |  |  |
| GO:0021983\_pituitary\_gland\_development | 3 | 0 |  |  |  |  |  |  |  |  |
| GO:0021987\_cerebral\_cortex\_development | 3 | 0 |  |  |  |  |  |  |  |  |
| GO:0022029\_telencephalon\_cell\_migration | 3 | 0 |  |  |  |  |  |  |  |  |
| GO:0022401\_adaptation\_of\_signaling\_pathway | 3 | 0 |  |  |  |  |  |  |  |  |
| GO:0022601\_menstrual\_cycle\_phase | 3 | 0 |  |  |  |  |  |  |  |  |
| GO:0022617\_extracellular\_matrix\_disassembly | 3 | 0 |  |  |  |  |  |  |  |  |
| GO:0030091\_protein\_repair | 3 | 0 |  |  |  |  |  |  |  |  |
| GO:0030157\_pancreatic\_juice\_secretion | 3 | 0 |  |  |  |  |  |  |  |  |
| GO:0030241\_muscle\_thick\_filament\_assembly | 3 | 0 |  |  |  |  |  |  |  |  |
| GO:0030263\_apoptotic\_chromosome\_condensation | 3 | 0 |  |  |  |  |  |  |  |  |
| GO:0030277\_maintenance\_of\_gastrointestinal\_epithelium | 3 | 0 |  |  |  |  |  |  |  |  |
| GO:0030309\_poly-N-acetyllactosamine\_metabolic\_process | 3 | 0 |  |  |  |  |  |  |  |  |
| GO:0030325\_adrenal\_gland\_development | 3 | 0 |  |  |  |  |  |  |  |  |
| GO:0030388\_fructose\_1\_6-bisphosphate\_metabolic\_process | 3 | 0 |  |  |  |  |  |  |  |  |
| GO:0030422\_RNA\_interference\_\_production\_of\_siRNA | 3 | 0 |  |  |  |  |  |  |  |  |
| GO:0030423\_RNA\_interference\_\_targeting\_of\_mRNA\_for\_destruction | 3 | 0 |  |  |  |  |  |  |  |  |
| GO:0030449\_regulation\_of\_complement\_activation | 3 | 0 |  |  |  |  |  |  |  |  |
| GO:0030502\_negative\_regulation\_of\_bone\_mineralization | 3 | 0 |  |  |  |  |  |  |  |  |
| GO:0030643\_cellular\_phosphate\_ion\_homeostasis | 3 | 0 |  |  |  |  |  |  |  |  |
| GO:0030656\_regulation\_of\_vitamin\_metabolic\_process | 3 | 0 |  |  |  |  |  |  |  |  |
| GO:0030800\_negative\_regulation\_of\_cyclic\_nucleotide\_metabolic\_process | 3 | 0 |  |  |  |  |  |  |  |  |
| GO:0030803\_negative\_regulation\_of\_cyclic\_nucleotide\_biosynthetic\_process | 3 | 0 |  |  |  |  |  |  |  |  |
| GO:0030809\_negative\_regulation\_of\_nucleotide\_biosynthetic\_process | 3 | 0 |  |  |  |  |  |  |  |  |
| GO:0030823\_regulation\_of\_cGMP\_metabolic\_process | 3 | 0 |  |  |  |  |  |  |  |  |
| GO:0030826\_regulation\_of\_cGMP\_biosynthetic\_process | 3 | 0 |  |  |  |  |  |  |  |  |
| GO:0030850\_prostate\_gland\_development | 3 | 0 |  |  |  |  |  |  |  |  |
| GO:0030852\_regulation\_of\_granulocyte\_differentiation | 3 | 0 |  |  |  |  |  |  |  |  |
| GO:0030853\_negative\_regulation\_of\_granulocyte\_differentiation | 3 | 0 |  |  |  |  |  |  |  |  |
| GO:0030856\_regulation\_of\_epithelial\_cell\_differentiation | 3 | 0 |  |  |  |  |  |  |  |  |
| GO:0030947\_regulation\_of\_vascular\_endothelial\_growth\_factor\_receptor\_signaling\_pathway | 3 | 0 |  |  |  |  |  |  |  |  |
| GO:0031033\_myosin\_filament\_assembly\_or\_disassembly | 3 | 0 |  |  |  |  |  |  |  |  |
| GO:0031034\_myosin\_filament\_assembly | 3 | 0 |  |  |  |  |  |  |  |  |
| GO:0031054\_pre-microRNA\_processing | 3 | 0 |  |  |  |  |  |  |  |  |
| GO:0031055\_chromatin\_remodeling\_at\_centromere | 3 | 0 |  |  |  |  |  |  |  |  |
| GO:0031060\_regulation\_of\_histone\_methylation | 3 | 0 |  |  |  |  |  |  |  |  |
| GO:0031102\_neuron\_projection\_regeneration | 3 | 0 |  |  |  |  |  |  |  |  |
| GO:0031103\_axon\_regeneration | 3 | 0 |  |  |  |  |  |  |  |  |
| GO:0031445\_regulation\_of\_heterochromatin\_formation | 3 | 0 |  |  |  |  |  |  |  |  |
| GO:0031579\_membrane\_raft\_organization | 3 | 0 |  |  |  |  |  |  |  |  |
| GO:0031648\_protein\_destabilization | 3 | 0 |  |  |  |  |  |  |  |  |
| GO:0031657\_regulation\_of\_cyclin-dependent\_protein\_kinase\_activity\_during\_G1\_S | 3 | 0 |  |  |  |  |  |  |  |  |
| GO:0031659\_positive\_regulation\_of\_cyclin-dependent\_protein\_kinase\_activity\_during\_G1\_S | 3 | 0 |  |  |  |  |  |  |  |  |
| GO:0031935\_regulation\_of\_chromatin\_silencing | 3 | 0 |  |  |  |  |  |  |  |  |
| GO:0031953\_negative\_regulation\_of\_protein\_amino\_acid\_autophosphorylation | 3 | 0 |  |  |  |  |  |  |  |  |
| GO:0032007\_negative\_regulation\_of\_TOR\_signaling\_pathway | 3 | 0 |  |  |  |  |  |  |  |  |
| GO:0032042\_mitochondrial\_DNA\_metabolic\_process | 3 | 0 |  |  |  |  |  |  |  |  |
| GO:0032097\_positive\_regulation\_of\_response\_to\_food | 3 | 0 |  |  |  |  |  |  |  |  |
| GO:0032100\_positive\_regulation\_of\_appetite | 3 | 0 |  |  |  |  |  |  |  |  |
| GO:0032105\_negative\_regulation\_of\_response\_to\_extracellular\_stimulus | 3 | 0 |  |  |  |  |  |  |  |  |
| GO:0032108\_negative\_regulation\_of\_response\_to\_nutrient\_levels | 3 | 0 |  |  |  |  |  |  |  |  |
| GO:0032203\_telomere\_formation\_via\_telomerase | 3 | 0 |  |  |  |  |  |  |  |  |
| GO:0032206\_positive\_regulation\_of\_telomere\_maintenance | 3 | 0 |  |  |  |  |  |  |  |  |
| GO:0032232\_negative\_regulation\_of\_actin\_filament\_bundle\_formation | 3 | 0 |  |  |  |  |  |  |  |  |
| GO:0032234\_regulation\_of\_calcium\_ion\_transport\_via\_store-operated\_calcium\_channel\_activity | 3 | 0 |  |  |  |  |  |  |  |  |
| GO:0032278\_positive\_regulation\_of\_gonadotropin\_secretion | 3 | 0 |  |  |  |  |  |  |  |  |
| GO:0032324\_molybdopterin\_cofactor\_biosynthetic\_process | 3 | 0 |  |  |  |  |  |  |  |  |
| GO:0032342\_aldosterone\_biosynthetic\_process | 3 | 0 |  |  |  |  |  |  |  |  |
| GO:0032352\_positive\_regulation\_of\_hormone\_metabolic\_process | 3 | 0 |  |  |  |  |  |  |  |  |
| GO:0032415\_regulation\_of\_sodium:hydrogen\_antiporter\_activity | 3 | 0 |  |  |  |  |  |  |  |  |
| GO:0032469\_endoplasmic\_reticulum\_calcium\_ion\_homeostasis | 3 | 0 |  |  |  |  |  |  |  |  |
| GO:0032494\_response\_to\_peptidoglycan | 3 | 0 |  |  |  |  |  |  |  |  |
| GO:0032497\_detection\_of\_lipopolysaccharide | 3 | 0 |  |  |  |  |  |  |  |  |
| GO:0032509\_endosome\_transport\_via\_multivesicular\_body\_sorting\_pathway | 3 | 0 |  |  |  |  |  |  |  |  |
| GO:0032515\_negative\_regulation\_of\_phosphoprotein\_phosphatase\_activity | 3 | 0 |  |  |  |  |  |  |  |  |
| GO:0032568\_general\_transcription\_from\_RNA\_polymerase\_II\_promoter | 3 | 0 |  |  |  |  |  |  |  |  |
| GO:0032604\_granulocyte\_macrophage\_colony-stimulating\_factor\_production | 3 | 0 |  |  |  |  |  |  |  |  |
| GO:0032616\_interleukin-13\_production | 3 | 0 |  |  |  |  |  |  |  |  |
| GO:0032641\_lymphotoxin\_A\_production | 3 | 0 |  |  |  |  |  |  |  |  |
| GO:0032651\_regulation\_of\_interleukin-1\_beta\_production | 3 | 0 |  |  |  |  |  |  |  |  |
| GO:0032695\_negative\_regulation\_of\_interleukin-12\_production | 3 | 0 |  |  |  |  |  |  |  |  |
| GO:0032717\_negative\_regulation\_of\_interleukin-8\_production | 3 | 0 |  |  |  |  |  |  |  |  |
| GO:0032729\_positive\_regulation\_of\_interferon-gamma\_production | 3 | 0 |  |  |  |  |  |  |  |  |
| GO:0032735\_positive\_regulation\_of\_interleukin-12\_production | 3 | 0 |  |  |  |  |  |  |  |  |
| GO:0032757\_positive\_regulation\_of\_interleukin-8\_production | 3 | 0 |  |  |  |  |  |  |  |  |
| GO:0032760\_positive\_regulation\_of\_tumor\_necrosis\_factor\_production | 3 | 0 |  |  |  |  |  |  |  |  |
| GO:0032855\_positive\_regulation\_of\_Rac\_GTPase\_activity | 3 | 0 |  |  |  |  |  |  |  |  |
| GO:0032905\_transforming\_growth\_factor-beta1\_production | 3 | 0 |  |  |  |  |  |  |  |  |
| GO:0032908\_regulation\_of\_transforming\_growth\_factor-beta1\_production | 3 | 0 |  |  |  |  |  |  |  |  |
| GO:0032922\_circadian\_regulation\_of\_gene\_expression | 3 | 0 |  |  |  |  |  |  |  |  |
| GO:0032927\_positive\_regulation\_of\_activin\_receptor\_signaling\_pathway | 3 | 0 |  |  |  |  |  |  |  |  |
| GO:0033015\_tetrapyrrole\_catabolic\_process | 3 | 0 |  |  |  |  |  |  |  |  |
| GO:0033081\_regulation\_of\_T\_cell\_differentiation\_in\_the\_thymus | 3 | 0 |  |  |  |  |  |  |  |  |
| GO:0033151\_V(D)J\_recombination | 3 | 0 |  |  |  |  |  |  |  |  |
| GO:0033198\_response\_to\_ATP | 3 | 0 |  |  |  |  |  |  |  |  |
| GO:0033555\_multicellular\_organismal\_response\_to\_stress | 3 | 0 |  |  |  |  |  |  |  |  |
| GO:0033865\_nucleoside\_bisphosphate\_metabolic\_process | 3 | 0 |  |  |  |  |  |  |  |  |
| GO:0034067\_protein\_localization\_in\_Golgi\_apparatus | 3 | 0 |  |  |  |  |  |  |  |  |
| GO:0034086\_maintenance\_of\_sister\_chromatid\_cohesion | 3 | 0 |  |  |  |  |  |  |  |  |
| GO:0034088\_maintenance\_of\_mitotic\_sister\_chromatid\_cohesion | 3 | 0 |  |  |  |  |  |  |  |  |
| GO:0034393\_positive\_regulation\_of\_smooth\_muscle\_cell\_apoptosis | 3 | 0 |  |  |  |  |  |  |  |  |
| GO:0034442\_regulation\_of\_lipoprotein\_oxidation | 3 | 0 |  |  |  |  |  |  |  |  |
| GO:0034443\_negative\_regulation\_of\_lipoprotein\_oxidation | 3 | 0 |  |  |  |  |  |  |  |  |
| GO:0034453\_microtubule\_anchoring | 3 | 0 |  |  |  |  |  |  |  |  |
| GO:0034505\_tooth\_mineralization | 3 | 0 |  |  |  |  |  |  |  |  |
| GO:0034629\_cellular\_protein\_complex\_localization | 3 | 0 |  |  |  |  |  |  |  |  |
| GO:0034653\_retinoic\_acid\_catabolic\_process | 3 | 0 |  |  |  |  |  |  |  |  |
| GO:0034661\_ncRNA\_catabolic\_process | 3 | 0 |  |  |  |  |  |  |  |  |
| GO:0034776\_response\_to\_histamine | 3 | 0 |  |  |  |  |  |  |  |  |
| GO:0035020\_regulation\_of\_Rac\_protein\_signal\_transduction | 3 | 0 |  |  |  |  |  |  |  |  |
| GO:0035024\_negative\_regulation\_of\_Rho\_protein\_signal\_transduction | 3 | 0 |  |  |  |  |  |  |  |  |
| GO:0035036\_sperm-egg\_recognition | 3 | 0 |  |  |  |  |  |  |  |  |
| GO:0035067\_negative\_regulation\_of\_histone\_acetylation | 3 | 0 |  |  |  |  |  |  |  |  |
| GO:0035081\_induction\_of\_programmed\_cell\_death\_by\_hormones | 3 | 0 |  |  |  |  |  |  |  |  |
| GO:0035092\_sperm\_chromatin\_condensation | 3 | 0 |  |  |  |  |  |  |  |  |
| GO:0035115\_embryonic\_forelimb\_morphogenesis | 3 | 0 |  |  |  |  |  |  |  |  |
| GO:0035136\_forelimb\_morphogenesis | 3 | 0 |  |  |  |  |  |  |  |  |
| GO:0035246\_peptidyl-arginine\_N-methylation | 3 | 0 |  |  |  |  |  |  |  |  |
| GO:0035268\_protein\_amino\_acid\_mannosylation | 3 | 0 |  |  |  |  |  |  |  |  |
| GO:0035269\_protein\_amino\_acid\_O-linked\_mannosylation | 3 | 0 |  |  |  |  |  |  |  |  |
| GO:0035306\_positive\_regulation\_of\_dephosphorylation | 3 | 0 |  |  |  |  |  |  |  |  |
| GO:0035307\_positive\_regulation\_of\_protein\_amino\_acid\_dephosphorylation | 3 | 0 |  |  |  |  |  |  |  |  |
| GO:0035315\_hair\_cell\_differentiation | 3 | 0 |  |  |  |  |  |  |  |  |
| GO:0040001\_establishment\_of\_mitotic\_spindle\_localization | 3 | 0 |  |  |  |  |  |  |  |  |
| GO:0042053\_regulation\_of\_dopamine\_metabolic\_process | 3 | 0 |  |  |  |  |  |  |  |  |
| GO:0042059\_negative\_regulation\_of\_epidermal\_growth\_factor\_receptor\_signaling\_pathway | 3 | 0 |  |  |  |  |  |  |  |  |
| GO:0042069\_regulation\_of\_catecholamine\_metabolic\_process | 3 | 0 |  |  |  |  |  |  |  |  |
| GO:0042090\_interleukin-12\_biosynthetic\_process | 3 | 0 |  |  |  |  |  |  |  |  |
| GO:0042109\_lymphotoxin\_A\_biosynthetic\_process | 3 | 0 |  |  |  |  |  |  |  |  |
| GO:0042160\_lipoprotein\_modification | 3 | 0 |  |  |  |  |  |  |  |  |
| GO:0042161\_lipoprotein\_oxidation | 3 | 0 |  |  |  |  |  |  |  |  |
| GO:0042253\_granulocyte\_macrophage\_colony-stimulating\_factor\_biosynthetic\_process | 3 | 0 |  |  |  |  |  |  |  |  |
| GO:0042271\_susceptibility\_to\_natural\_killer\_cell\_mediated\_cytotoxicity | 3 | 0 |  |  |  |  |  |  |  |  |
| GO:0042368\_vitamin\_D\_biosynthetic\_process | 3 | 0 |  |  |  |  |  |  |  |  |
| GO:0042416\_dopamine\_biosynthetic\_process | 3 | 0 |  |  |  |  |  |  |  |  |
| GO:0042451\_purine\_nucleoside\_biosynthetic\_process | 3 | 0 |  |  |  |  |  |  |  |  |
| GO:0042455\_ribonucleoside\_biosynthetic\_process | 3 | 0 |  |  |  |  |  |  |  |  |
| GO:0042574\_retinal\_metabolic\_process | 3 | 0 |  |  |  |  |  |  |  |  |
| GO:0042753\_positive\_regulation\_of\_circadian\_rhythm | 3 | 0 |  |  |  |  |  |  |  |  |
| GO:0042762\_regulation\_of\_sulfur\_metabolic\_process | 3 | 0 |  |  |  |  |  |  |  |  |
| GO:0042886\_amide\_transport | 3 | 0 |  |  |  |  |  |  |  |  |
| GO:0042940\_D-amino\_acid\_transport | 3 | 0 |  |  |  |  |  |  |  |  |
| GO:0042953\_lipoprotein\_transport | 3 | 0 |  |  |  |  |  |  |  |  |
| GO:0042977\_activation\_of\_JAK2\_kinase\_activity | 3 | 0 |  |  |  |  |  |  |  |  |
| GO:0042983\_amyloid\_precursor\_protein\_biosynthetic\_process | 3 | 0 |  |  |  |  |  |  |  |  |
| GO:0042984\_regulation\_of\_amyloid\_precursor\_protein\_biosynthetic\_process | 3 | 0 |  |  |  |  |  |  |  |  |
| GO:0043001\_Golgi\_to\_plasma\_membrane\_protein\_transport | 3 | 0 |  |  |  |  |  |  |  |  |
| GO:0043089\_positive\_regulation\_of\_Cdc42\_GTPase\_activity | 3 | 0 |  |  |  |  |  |  |  |  |
| GO:0043090\_amino\_acid\_import | 3 | 0 |  |  |  |  |  |  |  |  |
| GO:0043092\_L-amino\_acid\_import | 3 | 0 |  |  |  |  |  |  |  |  |
| GO:0043248\_proteasome\_assembly | 3 | 0 |  |  |  |  |  |  |  |  |
| GO:0043288\_apocarotenoid\_metabolic\_process | 3 | 0 |  |  |  |  |  |  |  |  |
| GO:0043368\_positive\_T\_cell\_selection | 3 | 0 |  |  |  |  |  |  |  |  |
| GO:0043461\_proton-transporting\_ATP\_synthase\_complex\_assembly | 3 | 0 |  |  |  |  |  |  |  |  |
| GO:0043489\_RNA\_stabilization | 3 | 0 |  |  |  |  |  |  |  |  |
| GO:0043570\_maintenance\_of\_DNA\_repeat\_elements | 3 | 0 |  |  |  |  |  |  |  |  |
| GO:0043604\_amide\_biosynthetic\_process | 3 | 0 |  |  |  |  |  |  |  |  |
| GO:0043618\_regulation\_of\_transcription\_from\_RNA\_polymerase\_II\_promoter\_in\_response\_to\_stress | 3 | 0 |  |  |  |  |  |  |  |  |
| GO:0043620\_regulation\_of\_transcription\_in\_response\_to\_stress | 3 | 0 |  |  |  |  |  |  |  |  |
| GO:0043902\_positive\_regulation\_of\_multi-organism\_process | 3 | 0 |  |  |  |  |  |  |  |  |
| GO:0044003\_modification\_by\_symbiont\_of\_host\_morphology\_or\_physiology | 3 | 0 |  |  |  |  |  |  |  |  |
| GO:0044240\_multicellular\_organismal\_lipid\_catabolic\_process | 3 | 0 |  |  |  |  |  |  |  |  |
| GO:0044252\_negative\_regulation\_of\_multicellular\_organismal\_metabolic\_process | 3 | 0 |  |  |  |  |  |  |  |  |
| GO:0045007\_depurination | 3 | 0 |  |  |  |  |  |  |  |  |
| GO:0045010\_actin\_nucleation | 3 | 0 |  |  |  |  |  |  |  |  |
| GO:0045061\_thymic\_T\_cell\_selection | 3 | 0 |  |  |  |  |  |  |  |  |
| GO:0045063\_T-helper\_1\_cell\_differentiation | 3 | 0 |  |  |  |  |  |  |  |  |
| GO:0045064\_T-helper\_2\_cell\_differentiation | 3 | 0 |  |  |  |  |  |  |  |  |
| GO:0045070\_positive\_regulation\_of\_viral\_genome\_replication | 3 | 0 |  |  |  |  |  |  |  |  |
| GO:0045075\_regulation\_of\_interleukin-12\_biosynthetic\_process | 3 | 0 |  |  |  |  |  |  |  |  |
| GO:0045079\_negative\_regulation\_of\_chemokine\_biosynthetic\_process | 3 | 0 |  |  |  |  |  |  |  |  |
| GO:0045080\_positive\_regulation\_of\_chemokine\_biosynthetic\_process | 3 | 0 |  |  |  |  |  |  |  |  |
| GO:0045112\_integrin\_biosynthetic\_process | 3 | 0 |  |  |  |  |  |  |  |  |
| GO:0045409\_negative\_regulation\_of\_interleukin-6\_biosynthetic\_process | 3 | 0 |  |  |  |  |  |  |  |  |
| GO:0045410\_positive\_regulation\_of\_interleukin-6\_biosynthetic\_process | 3 | 0 |  |  |  |  |  |  |  |  |
| GO:0045541\_negative\_regulation\_of\_cholesterol\_biosynthetic\_process | 3 | 0 |  |  |  |  |  |  |  |  |
| GO:0045577\_regulation\_of\_B\_cell\_differentiation | 3 | 0 |  |  |  |  |  |  |  |  |
| GO:0045581\_negative\_regulation\_of\_T\_cell\_differentiation | 3 | 0 |  |  |  |  |  |  |  |  |
| GO:0045625\_regulation\_of\_T-helper\_1\_cell\_differentiation | 3 | 0 |  |  |  |  |  |  |  |  |
| GO:0045628\_regulation\_of\_T-helper\_2\_cell\_differentiation | 3 | 0 |  |  |  |  |  |  |  |  |
| GO:0045662\_negative\_regulation\_of\_myoblast\_differentiation | 3 | 0 |  |  |  |  |  |  |  |  |
| GO:0045663\_positive\_regulation\_of\_myoblast\_differentiation | 3 | 0 |  |  |  |  |  |  |  |  |
| GO:0045665\_negative\_regulation\_of\_neuron\_differentiation | 3 | 0 |  |  |  |  |  |  |  |  |
| GO:0045715\_negative\_regulation\_of\_low-density\_lipoprotein\_receptor\_biosynthetic\_process | 3 | 0 |  |  |  |  |  |  |  |  |
| GO:0045717\_negative\_regulation\_of\_fatty\_acid\_biosynthetic\_process | 3 | 0 |  |  |  |  |  |  |  |  |
| GO:0045779\_negative\_regulation\_of\_bone\_resorption | 3 | 0 |  |  |  |  |  |  |  |  |
| GO:0045817\_positive\_regulation\_of\_transcription\_from\_RNA\_polymerase\_II\_promoter\_\_global | 3 | 0 |  |  |  |  |  |  |  |  |
| GO:0045822\_negative\_regulation\_of\_heart\_contraction | 3 | 0 |  |  |  |  |  |  |  |  |
| GO:0045830\_positive\_regulation\_of\_isotype\_switching | 3 | 0 |  |  |  |  |  |  |  |  |
| GO:0045898\_regulation\_of\_transcriptional\_preinitiation\_complex\_assembly | 3 | 0 |  |  |  |  |  |  |  |  |
| GO:0045911\_positive\_regulation\_of\_DNA\_recombination | 3 | 0 |  |  |  |  |  |  |  |  |
| GO:0045912\_negative\_regulation\_of\_carbohydrate\_metabolic\_process | 3 | 0 |  |  |  |  |  |  |  |  |
| GO:0045916\_negative\_regulation\_of\_complement\_activation | 3 | 0 |  |  |  |  |  |  |  |  |
| GO:0045939\_negative\_regulation\_of\_steroid\_metabolic\_process | 3 | 0 |  |  |  |  |  |  |  |  |
| GO:0045988\_negative\_regulation\_of\_striated\_muscle\_contraction | 3 | 0 |  |  |  |  |  |  |  |  |
| GO:0045990\_regulation\_of\_transcription\_by\_carbon\_catabolites | 3 | 0 |  |  |  |  |  |  |  |  |
| GO:0045995\_regulation\_of\_embryonic\_development | 3 | 0 |  |  |  |  |  |  |  |  |
| GO:0046015\_regulation\_of\_transcription\_by\_glucose | 3 | 0 |  |  |  |  |  |  |  |  |
| GO:0046068\_cGMP\_metabolic\_process | 3 | 0 |  |  |  |  |  |  |  |  |
| GO:0046129\_purine\_ribonucleoside\_biosynthetic\_process | 3 | 0 |  |  |  |  |  |  |  |  |
| GO:0046130\_purine\_ribonucleoside\_catabolic\_process | 3 | 0 |  |  |  |  |  |  |  |  |
| GO:0046146\_tetrahydrobiopterin\_metabolic\_process | 3 | 0 |  |  |  |  |  |  |  |  |
| GO:0046348\_amino\_sugar\_catabolic\_process | 3 | 0 |  |  |  |  |  |  |  |  |
| GO:0046605\_regulation\_of\_centrosome\_cycle | 3 | 0 |  |  |  |  |  |  |  |  |
| GO:0046628\_positive\_regulation\_of\_insulin\_receptor\_signaling\_pathway | 3 | 0 |  |  |  |  |  |  |  |  |
| GO:0046653\_tetrahydrofolate\_metabolic\_process | 3 | 0 |  |  |  |  |  |  |  |  |
| GO:0046676\_negative\_regulation\_of\_insulin\_secretion | 3 | 0 |  |  |  |  |  |  |  |  |
| GO:0046688\_response\_to\_copper\_ion | 3 | 0 |  |  |  |  |  |  |  |  |
| GO:0046813\_virion\_attachment\_\_binding\_of\_host\_cell\_surface\_receptor | 3 | 0 |  |  |  |  |  |  |  |  |
| GO:0046825\_regulation\_of\_protein\_export\_from\_nucleus | 3 | 0 |  |  |  |  |  |  |  |  |
| GO:0046881\_positive\_regulation\_of\_follicle-stimulating\_hormone\_secretion | 3 | 0 |  |  |  |  |  |  |  |  |
| GO:0046886\_positive\_regulation\_of\_hormone\_biosynthetic\_process | 3 | 0 |  |  |  |  |  |  |  |  |
| GO:0048048\_embryonic\_eye\_morphogenesis | 3 | 0 |  |  |  |  |  |  |  |  |
| GO:0048251\_elastic\_fiber\_assembly | 3 | 0 |  |  |  |  |  |  |  |  |
| GO:0048255\_mRNA\_stabilization | 3 | 0 |  |  |  |  |  |  |  |  |
| GO:0048268\_clathrin\_coat\_assembly | 3 | 0 |  |  |  |  |  |  |  |  |
| GO:0048521\_negative\_regulation\_of\_behavior | 3 | 0 |  |  |  |  |  |  |  |  |
| GO:0048538\_thymus\_development | 3 | 0 |  |  |  |  |  |  |  |  |
| GO:0048548\_regulation\_of\_pinocytosis | 3 | 0 |  |  |  |  |  |  |  |  |
| GO:0048552\_regulation\_of\_metalloenzyme\_activity | 3 | 0 |  |  |  |  |  |  |  |  |
| GO:0048554\_positive\_regulation\_of\_metalloenzyme\_activity | 3 | 0 |  |  |  |  |  |  |  |  |
| GO:0048567\_ectodermal\_gut\_morphogenesis | 3 | 0 |  |  |  |  |  |  |  |  |
| GO:0048701\_embryonic\_cranial\_skeleton\_morphogenesis | 3 | 0 |  |  |  |  |  |  |  |  |
| GO:0048739\_cardiac\_muscle\_fiber\_development | 3 | 0 |  |  |  |  |  |  |  |  |
| GO:0048814\_regulation\_of\_dendrite\_morphogenesis | 3 | 0 |  |  |  |  |  |  |  |  |
| GO:0048861\_leukemia\_inhibitory\_factor\_signaling\_pathway | 3 | 0 |  |  |  |  |  |  |  |  |
| GO:0050434\_positive\_regulation\_of\_viral\_transcription | 3 | 0 |  |  |  |  |  |  |  |  |
| GO:0050435\_beta-amyloid\_metabolic\_process | 3 | 0 |  |  |  |  |  |  |  |  |
| GO:0050665\_hydrogen\_peroxide\_biosynthetic\_process | 3 | 0 |  |  |  |  |  |  |  |  |
| GO:0050686\_negative\_regulation\_of\_mRNA\_processing | 3 | 0 |  |  |  |  |  |  |  |  |
| GO:0050806\_positive\_regulation\_of\_synaptic\_transmission | 3 | 0 |  |  |  |  |  |  |  |  |
| GO:0050856\_regulation\_of\_T\_cell\_receptor\_signaling\_pathway | 3 | 0 |  |  |  |  |  |  |  |  |
| GO:0050873\_brown\_fat\_cell\_differentiation | 3 | 0 |  |  |  |  |  |  |  |  |
| GO:0051001\_negative\_regulation\_of\_nitric-oxide\_synthase\_activity | 3 | 0 |  |  |  |  |  |  |  |  |
| GO:0051084\_'de\_novo'\_posttranslational\_protein\_folding | 3 | 0 |  |  |  |  |  |  |  |  |
| GO:0051153\_regulation\_of\_striated\_muscle\_cell\_differentiation | 3 | 0 |  |  |  |  |  |  |  |  |
| GO:0051299\_centrosome\_separation | 3 | 0 |  |  |  |  |  |  |  |  |
| GO:0051305\_chromosome\_movement\_towards\_spindle\_pole | 3 | 0 |  |  |  |  |  |  |  |  |
| GO:0051324\_prophase | 3 | 0 |  |  |  |  |  |  |  |  |
| GO:0051382\_kinetochore\_assembly | 3 | 0 |  |  |  |  |  |  |  |  |
| GO:0051489\_regulation\_of\_filopodium\_assembly | 3 | 0 |  |  |  |  |  |  |  |  |
| GO:0051491\_positive\_regulation\_of\_filopodium\_assembly | 3 | 0 |  |  |  |  |  |  |  |  |
| GO:0051497\_negative\_regulation\_of\_stress\_fiber\_formation | 3 | 0 |  |  |  |  |  |  |  |  |
| GO:0051546\_keratinocyte\_migration | 3 | 0 |  |  |  |  |  |  |  |  |
| GO:0051567\_histone\_H3-K9\_methylation | 3 | 0 |  |  |  |  |  |  |  |  |
| GO:0051569\_regulation\_of\_histone\_H3-K4\_methylation | 3 | 0 |  |  |  |  |  |  |  |  |
| GO:0051712\_positive\_regulation\_of\_killing\_of\_cells\_of\_another\_organism | 3 | 0 |  |  |  |  |  |  |  |  |
| GO:0051825\_adhesion\_to\_other\_organism\_during\_symbiotic\_interaction | 3 | 0 |  |  |  |  |  |  |  |  |
| GO:0051851\_modification\_by\_host\_of\_symbiont\_morphology\_or\_physiology | 3 | 0 |  |  |  |  |  |  |  |  |
| GO:0051856\_adhesion\_to\_symbiont | 3 | 0 |  |  |  |  |  |  |  |  |
| GO:0051877\_pigment\_granule\_aggregation\_in\_cell\_center | 3 | 0 |  |  |  |  |  |  |  |  |
| GO:0051882\_mitochondrial\_depolarization | 3 | 0 |  |  |  |  |  |  |  |  |
| GO:0051918\_negative\_regulation\_of\_fibrinolysis | 3 | 0 |  |  |  |  |  |  |  |  |
| GO:0051925\_regulation\_of\_calcium\_ion\_transport\_via\_voltage-gated\_calcium\_channel\_activity | 3 | 0 |  |  |  |  |  |  |  |  |
| GO:0051938\_L-glutamate\_import | 3 | 0 |  |  |  |  |  |  |  |  |
| GO:0051966\_regulation\_of\_synaptic\_transmission\_\_glutamatergic | 3 | 0 |  |  |  |  |  |  |  |  |
| GO:0051970\_negative\_regulation\_of\_transmission\_of\_nerve\_impulse | 3 | 0 |  |  |  |  |  |  |  |  |
| GO:0051973\_positive\_regulation\_of\_telomerase\_activity | 3 | 0 |  |  |  |  |  |  |  |  |
| GO:0051983\_regulation\_of\_chromosome\_segregation | 3 | 0 |  |  |  |  |  |  |  |  |
| GO:0055062\_phosphate\_ion\_homeostasis | 3 | 0 |  |  |  |  |  |  |  |  |
| GO:0055069\_zinc\_ion\_homeostasis | 3 | 0 |  |  |  |  |  |  |  |  |
| GO:0055078\_sodium\_ion\_homeostasis | 3 | 0 |  |  |  |  |  |  |  |  |
| GO:0055094\_response\_to\_lipoprotein\_stimulus | 3 | 0 |  |  |  |  |  |  |  |  |
| GO:0060017\_parathyroid\_gland\_development | 3 | 0 |  |  |  |  |  |  |  |  |
| GO:0060023\_soft\_palate\_development | 3 | 0 |  |  |  |  |  |  |  |  |
| GO:0060084\_synaptic\_transmission\_involved\_in\_micturition | 3 | 0 |  |  |  |  |  |  |  |  |
| GO:0060119\_inner\_ear\_receptor\_cell\_development | 3 | 0 |  |  |  |  |  |  |  |  |
| GO:0060134\_prepulse\_inhibition | 3 | 0 |  |  |  |  |  |  |  |  |
| GO:0060158\_activation\_of\_phospholipase\_C\_activity\_by\_dopamine\_receptor\_signaling\_pathway | 3 | 0 |  |  |  |  |  |  |  |  |
| GO:0060177\_regulation\_of\_angiotensin\_metabolic\_process | 3 | 0 |  |  |  |  |  |  |  |  |
| GO:0060267\_positive\_regulation\_of\_respiratory\_burst | 3 | 0 |  |  |  |  |  |  |  |  |
| GO:0060317\_cardiac\_epithelial\_to\_mesenchymal\_transition | 3 | 0 |  |  |  |  |  |  |  |  |
| GO:0060333\_interferon-gamma-mediated\_signaling\_pathway | 3 | 0 |  |  |  |  |  |  |  |  |
| GO:0060334\_regulation\_of\_interferon-gamma-mediated\_signaling\_pathway | 3 | 0 |  |  |  |  |  |  |  |  |
| GO:0060343\_trabecula\_formation | 3 | 0 |  |  |  |  |  |  |  |  |
| GO:0060347\_heart\_trabecula\_formation | 3 | 0 |  |  |  |  |  |  |  |  |
| GO:0060394\_negative\_regulation\_of\_pathway-restricted\_SMAD\_protein\_phosphorylation | 3 | 0 |  |  |  |  |  |  |  |  |
| GO:0060559\_positive\_regulation\_of\_calcidiol\_1-monooxygenase\_activity | 3 | 0 |  |  |  |  |  |  |  |  |
| GO:0070050\_neuron\_maintenance | 3 | 0 |  |  |  |  |  |  |  |  |
| GO:0070141\_response\_to\_UV-A | 3 | 0 |  |  |  |  |  |  |  |  |
| GO:0070168\_negative\_regulation\_of\_biomineral\_formation | 3 | 0 |  |  |  |  |  |  |  |  |
| GO:0070207\_protein\_homotrimerization | 3 | 0 |  |  |  |  |  |  |  |  |
| GO:0070229\_negative\_regulation\_of\_lymphocyte\_apoptosis | 3 | 0 |  |  |  |  |  |  |  |  |
| GO:0070231\_T\_cell\_apoptosis | 3 | 0 |  |  |  |  |  |  |  |  |
| GO:0070272\_proton-transporting\_ATP\_synthase\_complex\_biogenesis | 3 | 0 |  |  |  |  |  |  |  |  |
| GO:0070561\_vitamin\_D\_receptor\_signaling\_pathway | 3 | 0 |  |  |  |  |  |  |  |  |
| GO:0070634\_transepithelial\_ammonium\_transport | 3 | 0 |  |  |  |  |  |  |  |  |
| GO:0070777\_D-aspartate\_transport | 3 | 0 |  |  |  |  |  |  |  |  |
| GO:0070779\_D-aspartate\_import | 3 | 0 |  |  |  |  |  |  |  |  |
| GO:0015674\_di-\_\_tri-valent\_inorganic\_cation\_transport | 84 | 0 | 0.000000 | 0.000000 | 1114 | 935.041378 | 978.2 | 1021.358622 | 0.878097 |
| GO:0006897\_endocytosis | 124 | 0 | 0.000000 | 0.000000 | 1117 | 940.975870 | 983.78 | 1026.584130 | 0.880734 |
| GO:0010324\_membrane\_invagination | 124 | 0 | 0.000000 | 0.000000 | 1117 | 940.975870 | 983.78 | 1026.584130 | 0.880734 |
| GO:0030182\_neuron\_differentiation | 124 | 0 | 0.000000 | 0.000000 | 1117 | 940.975870 | 983.78 | 1026.584130 | 0.880734 |
| GO:0001935\_endothelial\_cell\_proliferation | 25 | 0 | 0.000000 | 0.000000 | 1141 | 970.203259 | 1012.13 | 1054.056741 | 0.887055 |
| GO:0002757\_immune\_response-activating\_signal\_transduction | 25 | 0 | 0.000000 | 0.000000 | 1141 | 970.203259 | 1012.13 | 1054.056741 | 0.887055 |
| GO:0002764\_immune\_response-regulating\_signal\_transduction | 25 | 0 | 0.000000 | 0.000000 | 1141 | 970.203259 | 1012.13 | 1054.056741 | 0.887055 |
| GO:0006112\_energy\_reserve\_metabolic\_process | 25 | 0 | 0.000000 | 0.000000 | 1141 | 970.203259 | 1012.13 | 1054.056741 | 0.887055 |
| GO:0006518\_peptide\_metabolic\_process | 25 | 0 | 0.000000 | 0.000000 | 1141 | 970.203259 | 1012.13 | 1054.056741 | 0.887055 |
| GO:0006767\_water-soluble\_vitamin\_metabolic\_process | 25 | 0 | 0.000000 | 0.000000 | 1141 | 970.203259 | 1012.13 | 1054.056741 | 0.887055 |
| GO:0007127\_meiosis\_I | 25 | 0 | 0.000000 | 0.000000 | 1141 | 970.203259 | 1012.13 | 1054.056741 | 0.887055 |
| GO:0007416\_synaptogenesis | 25 | 0 | 0.000000 | 0.000000 | 1141 | 970.203259 | 1012.13 | 1054.056741 | 0.887055 |
| GO:0009141\_nucleoside\_triphosphate\_metabolic\_process | 25 | 0 | 0.000000 | 0.000000 | 1141 | 970.203259 | 1012.13 | 1054.056741 | 0.887055 |
| GO:0010876\_lipid\_localization | 25 | 0 | 0.000000 | 0.000000 | 1141 | 970.203259 | 1012.13 | 1054.056741 | 0.887055 |
| GO:0015711\_organic\_anion\_transport | 25 | 0 | 0.000000 | 0.000000 | 1141 | 970.203259 | 1012.13 | 1054.056741 | 0.887055 |
| GO:0019217\_regulation\_of\_fatty\_acid\_metabolic\_process | 25 | 0 | 0.000000 | 0.000000 | 1141 | 970.203259 | 1012.13 | 1054.056741 | 0.887055 |
| GO:0019915\_lipid\_storage | 25 | 0 | 0.000000 | 0.000000 | 1141 | 970.203259 | 1012.13 | 1054.056741 | 0.887055 |
| GO:0030282\_bone\_mineralization | 25 | 0 | 0.000000 | 0.000000 | 1141 | 970.203259 | 1012.13 | 1054.056741 | 0.887055 |
| GO:0031023\_microtubule\_organizing\_center\_organization | 25 | 0 | 0.000000 | 0.000000 | 1141 | 970.203259 | 1012.13 | 1054.056741 | 0.887055 |
| GO:0031644\_regulation\_of\_neurological\_system\_process | 25 | 0 | 0.000000 | 0.000000 | 1141 | 970.203259 | 1012.13 | 1054.056741 | 0.887055 |
| GO:0032368\_regulation\_of\_lipid\_transport | 25 | 0 | 0.000000 | 0.000000 | 1141 | 970.203259 | 1012.13 | 1054.056741 | 0.887055 |
| GO:0042129\_regulation\_of\_T\_cell\_proliferation | 25 | 0 | 0.000000 | 0.000000 | 1141 | 970.203259 | 1012.13 | 1054.056741 | 0.887055 |
| GO:0043087\_regulation\_of\_GTPase\_activity | 25 | 0 | 0.000000 | 0.000000 | 1141 | 970.203259 | 1012.13 | 1054.056741 | 0.887055 |
| GO:0043966\_histone\_H3\_acetylation | 25 | 0 | 0.000000 | 0.000000 | 1141 | 970.203259 | 1012.13 | 1054.056741 | 0.887055 |
| GO:0045762\_positive\_regulation\_of\_adenylate\_cyclase\_activity | 25 | 0 | 0.000000 | 0.000000 | 1141 | 970.203259 | 1012.13 | 1054.056741 | 0.887055 |
| GO:0050727\_regulation\_of\_inflammatory\_response | 25 | 0 | 0.000000 | 0.000000 | 1141 | 970.203259 | 1012.13 | 1054.056741 | 0.887055 |
| GO:0050795\_regulation\_of\_behavior | 25 | 0 | 0.000000 | 0.000000 | 1141 | 970.203259 | 1012.13 | 1054.056741 | 0.887055 |
| GO:0050821\_protein\_stabilization | 25 | 0 | 0.000000 | 0.000000 | 1141 | 970.203259 | 1012.13 | 1054.056741 | 0.887055 |
| GO:0006631\_fatty\_acid\_metabolic\_process | 113 | 0 | 0.000000 | 0.000000 | 1142 | 971.922790 | 1013.77 | 1055.617210 | 0.887715 |
| GO:0006163\_purine\_nucleotide\_metabolic\_process | 99 | 0 | 0.000000 | 0.000000 | 1144 | 975.671438 | 1017.26 | 1058.848562 | 0.889213 |
| GO:0016569\_covalent\_chromatin\_modification | 99 | 0 | 0.000000 | 0.000000 | 1144 | 975.671438 | 1017.26 | 1058.848562 | 0.889213 |
| GO:0002684\_positive\_regulation\_of\_immune\_system\_process | 106 | 0 | 0.000000 | 0.000000 | 1146 | 978.842463 | 1020.03 | 1061.217537 | 0.890079 |
| GO:0044057\_regulation\_of\_system\_process | 106 | 0 | 0.000000 | 0.000000 | 1146 | 978.842463 | 1020.03 | 1061.217537 | 0.890079 |
| GO:0048646\_anatomical\_structure\_formation\_involved\_in\_morphogenesis | 111 | 0 | 0.000000 | 0.000000 | 1147 | 979.724551 | 1020.82 | 1061.915449 | 0.889991 |
| GO:0043623\_cellular\_protein\_complex\_assembly | 101 | 0 | 0.000000 | 0.000000 | 1148 | 982.041496 | 1022.88 | 1063.718504 | 0.891010 |
| GO:0000075\_cell\_cycle\_checkpoint | 73 | 0 | 0.000000 | 0.000000 | 1153 | 989.124065 | 1029.71 | 1070.295935 | 0.893070 |
| GO:0006352\_transcription\_initiation | 73 | 0 | 0.000000 | 0.000000 | 1153 | 989.124065 | 1029.71 | 1070.295935 | 0.893070 |
| GO:0007187\_G-protein\_signaling\_\_coupled\_to\_cyclic\_nucleotide\_second\_messenger | 73 | 0 | 0.000000 | 0.000000 | 1153 | 989.124065 | 1029.71 | 1070.295935 | 0.893070 |
| GO:0032990\_cell\_part\_morphogenesis | 73 | 0 | 0.000000 | 0.000000 | 1153 | 989.124065 | 1029.71 | 1070.295935 | 0.893070 |
| GO:0055085\_transmembrane\_transport | 73 | 0 | 0.000000 | 0.000000 | 1153 | 989.124065 | 1029.71 | 1070.295935 | 0.893070 |
| GO:0006511\_ubiquitin-dependent\_protein\_catabolic\_process | 136 | 0 | 0.000000 | 0.000000 | 1155 | 991.354002 | 1031.8 | 1072.245998 | 0.893333 |
| GO:0048699\_generation\_of\_neurons | 136 | 0 | 0.000000 | 0.000000 | 1155 | 991.354002 | 1031.8 | 1072.245998 | 0.893333 |
| GO:0010608\_posttranscriptional\_regulation\_of\_gene\_expression | 118 | 0 | 0.000000 | 0.000000 | 1156 | 993.660643 | 1034.01 | 1074.359357 | 0.894472 |
| GO:0009628\_response\_to\_abiotic\_stimulus | 140 | 0 | 0.000000 | 0.000000 | 1157 | 995.301077 | 1035.51 | 1075.718923 | 0.894996 |
| GO:0030163\_protein\_catabolic\_process | 330 | 0 | 0.000000 | 0.000000 | 1158 | 995.820023 | 1035.94 | 1076.059977 | 0.894594 |
| GO:0000271\_polysaccharide\_biosynthetic\_process | 38 | 0 | 0.000000 | 0.000000 | 1175 | 1014.237770 | 1053.64 | 1093.042230 | 0.896715 |
| GO:0002250\_adaptive\_immune\_response | 38 | 0 | 0.000000 | 0.000000 | 1175 | 1014.237770 | 1053.64 | 1093.042230 | 0.896715 |
| GO:0008016\_regulation\_of\_heart\_contraction | 38 | 0 | 0.000000 | 0.000000 | 1175 | 1014.237770 | 1053.64 | 1093.042230 | 0.896715 |
| GO:0009411\_response\_to\_UV | 38 | 0 | 0.000000 | 0.000000 | 1175 | 1014.237770 | 1053.64 | 1093.042230 | 0.896715 |
| GO:0016050\_vesicle\_organization | 38 | 0 | 0.000000 | 0.000000 | 1175 | 1014.237770 | 1053.64 | 1093.042230 | 0.896715 |
| GO:0019933\_cAMP-mediated\_signaling | 38 | 0 | 0.000000 | 0.000000 | 1175 | 1014.237770 | 1053.64 | 1093.042230 | 0.896715 |
| GO:0030336\_negative\_regulation\_of\_cell\_migration | 38 | 0 | 0.000000 | 0.000000 | 1175 | 1014.237770 | 1053.64 | 1093.042230 | 0.896715 |
| GO:0032869\_cellular\_response\_to\_insulin\_stimulus | 38 | 0 | 0.000000 | 0.000000 | 1175 | 1014.237770 | 1053.64 | 1093.042230 | 0.896715 |
| GO:0042113\_B\_cell\_activation | 38 | 0 | 0.000000 | 0.000000 | 1175 | 1014.237770 | 1053.64 | 1093.042230 | 0.896715 |
| GO:0042493\_response\_to\_drug | 38 | 0 | 0.000000 | 0.000000 | 1175 | 1014.237770 | 1053.64 | 1093.042230 | 0.896715 |
| GO:0045765\_regulation\_of\_angiogenesis | 38 | 0 | 0.000000 | 0.000000 | 1175 | 1014.237770 | 1053.64 | 1093.042230 | 0.896715 |
| GO:0046849\_bone\_remodeling | 38 | 0 | 0.000000 | 0.000000 | 1175 | 1014.237770 | 1053.64 | 1093.042230 | 0.896715 |
| GO:0048511\_rhythmic\_process | 38 | 0 | 0.000000 | 0.000000 | 1175 | 1014.237770 | 1053.64 | 1093.042230 | 0.896715 |
| GO:0051091\_positive\_regulation\_of\_transcription\_factor\_activity | 38 | 0 | 0.000000 | 0.000000 | 1175 | 1014.237770 | 1053.64 | 1093.042230 | 0.896715 |
| GO:0051188\_cofactor\_biosynthetic\_process | 38 | 0 | 0.000000 | 0.000000 | 1175 | 1014.237770 | 1053.64 | 1093.042230 | 0.896715 |
| GO:0060627\_regulation\_of\_vesicle-mediated\_transport | 38 | 0 | 0.000000 | 0.000000 | 1175 | 1014.237770 | 1053.64 | 1093.042230 | 0.896715 |
| GO:0090047\_positive\_regulation\_of\_transcription\_regulator\_activity | 38 | 0 | 0.000000 | 0.000000 | 1175 | 1014.237770 | 1053.64 | 1093.042230 | 0.896715 |
| GO:0034984\_cellular\_response\_to\_DNA\_damage\_stimulus | 215 | 0 | 0.000000 | 0.000000 | 1176 | 1014.850179 | 1054.24 | 1093.629821 | 0.896463 |
| GO:0019941\_modification-dependent\_protein\_catabolic\_process | 138 | 0 | 0.000000 | 0.000000 | 1178 | 1016.170947 | 1055.48 | 1094.789053 | 0.895993 |
| GO:0043632\_modification-dependent\_macromolecule\_catabolic\_process | 138 | 0 | 0.000000 | 0.000000 | 1178 | 1016.170947 | 1055.48 | 1094.789053 | 0.895993 |
| GO:0033365\_protein\_localization\_in\_organelle | 95 | 0 | 0.000000 | 0.000000 | 1179 | 1017.913377 | 1057.03 | 1096.146623 | 0.896548 |
| GO:0006575\_cellular\_amino\_acid\_derivative\_metabolic\_process | 76 | 0 | 0.000000 | 0.000000 | 1185 | 1023.731285 | 1062.45 | 1101.168715 | 0.896582 |
| GO:0006979\_response\_to\_oxidative\_stress | 76 | 0 | 0.000000 | 0.000000 | 1185 | 1023.731285 | 1062.45 | 1101.168715 | 0.896582 |
| GO:0034504\_protein\_localization\_in\_nucleus | 76 | 0 | 0.000000 | 0.000000 | 1185 | 1023.731285 | 1062.45 | 1101.168715 | 0.896582 |
| GO:0051051\_negative\_regulation\_of\_transport | 76 | 0 | 0.000000 | 0.000000 | 1185 | 1023.731285 | 1062.45 | 1101.168715 | 0.896582 |
| GO:0051101\_regulation\_of\_DNA\_binding | 76 | 0 | 0.000000 | 0.000000 | 1185 | 1023.731285 | 1062.45 | 1101.168715 | 0.896582 |
| GO:0070838\_divalent\_metal\_ion\_transport | 76 | 0 | 0.000000 | 0.000000 | 1185 | 1023.731285 | 1062.45 | 1101.168715 | 0.896582 |
| GO:0009719\_response\_to\_endogenous\_stimulus | 135 | 0 | 0.000000 | 0.000000 | 1187 | 1028.545214 | 1066.73 | 1104.914786 | 0.898677 |
| GO:0034641\_cellular\_nitrogen\_compound\_metabolic\_process | 135 | 0 | 0.000000 | 0.000000 | 1187 | 1028.545214 | 1066.73 | 1104.914786 | 0.898677 |
| GO:0000060\_protein\_import\_into\_nucleus\_\_translocation | 22 | 0 | 0.000000 | 0.000000 | 1213 | 1057.578418 | 1095.15 | 1132.721582 | 0.902844 |
| GO:0001894\_tissue\_homeostasis | 22 | 0 | 0.000000 | 0.000000 | 1213 | 1057.578418 | 1095.15 | 1132.721582 | 0.902844 |
| GO:0006383\_transcription\_from\_RNA\_polymerase\_III\_promoter | 22 | 0 | 0.000000 | 0.000000 | 1213 | 1057.578418 | 1095.15 | 1132.721582 | 0.902844 |
| GO:0006406\_mRNA\_export\_from\_nucleus | 22 | 0 | 0.000000 | 0.000000 | 1213 | 1057.578418 | 1095.15 | 1132.721582 | 0.902844 |
| GO:0007018\_microtubule-based\_movement | 22 | 0 | 0.000000 | 0.000000 | 1213 | 1057.578418 | 1095.15 | 1132.721582 | 0.902844 |
| GO:0007034\_vacuolar\_transport | 22 | 0 | 0.000000 | 0.000000 | 1213 | 1057.578418 | 1095.15 | 1132.721582 | 0.902844 |
| GO:0007051\_spindle\_organization | 22 | 0 | 0.000000 | 0.000000 | 1213 | 1057.578418 | 1095.15 | 1132.721582 | 0.902844 |
| GO:0007611\_learning\_or\_memory | 22 | 0 | 0.000000 | 0.000000 | 1213 | 1057.578418 | 1095.15 | 1132.721582 | 0.902844 |
| GO:0008033\_tRNA\_processing | 22 | 0 | 0.000000 | 0.000000 | 1213 | 1057.578418 | 1095.15 | 1132.721582 | 0.902844 |
| GO:0010212\_response\_to\_ionizing\_radiation | 22 | 0 | 0.000000 | 0.000000 | 1213 | 1057.578418 | 1095.15 | 1132.721582 | 0.902844 |
| GO:0010769\_regulation\_of\_cell\_morphogenesis\_involved\_in\_differentiation | 22 | 0 | 0.000000 | 0.000000 | 1213 | 1057.578418 | 1095.15 | 1132.721582 | 0.902844 |
| GO:0015908\_fatty\_acid\_transport | 22 | 0 | 0.000000 | 0.000000 | 1213 | 1057.578418 | 1095.15 | 1132.721582 | 0.902844 |
| GO:0016339\_calcium-dependent\_cell-cell\_adhesion | 22 | 0 | 0.000000 | 0.000000 | 1213 | 1057.578418 | 1095.15 | 1132.721582 | 0.902844 |
| GO:0030330\_DNA\_damage\_response\_\_signal\_transduction\_by\_p53\_class\_mediator | 22 | 0 | 0.000000 | 0.000000 | 1213 | 1057.578418 | 1095.15 | 1132.721582 | 0.902844 |
| GO:0032582\_negative\_regulation\_of\_gene-specific\_transcription | 22 | 0 | 0.000000 | 0.000000 | 1213 | 1057.578418 | 1095.15 | 1132.721582 | 0.902844 |
| GO:0033500\_carbohydrate\_homeostasis | 22 | 0 | 0.000000 | 0.000000 | 1213 | 1057.578418 | 1095.15 | 1132.721582 | 0.902844 |
| GO:0042593\_glucose\_homeostasis | 22 | 0 | 0.000000 | 0.000000 | 1213 | 1057.578418 | 1095.15 | 1132.721582 | 0.902844 |
| GO:0042632\_cholesterol\_homeostasis | 22 | 0 | 0.000000 | 0.000000 | 1213 | 1057.578418 | 1095.15 | 1132.721582 | 0.902844 |
| GO:0042990\_regulation\_of\_transcription\_factor\_import\_into\_nucleus | 22 | 0 | 0.000000 | 0.000000 | 1213 | 1057.578418 | 1095.15 | 1132.721582 | 0.902844 |
| GO:0042991\_transcription\_factor\_import\_into\_nucleus | 22 | 0 | 0.000000 | 0.000000 | 1213 | 1057.578418 | 1095.15 | 1132.721582 | 0.902844 |
| GO:0043507\_positive\_regulation\_of\_JUN\_kinase\_activity | 22 | 0 | 0.000000 | 0.000000 | 1213 | 1057.578418 | 1095.15 | 1132.721582 | 0.902844 |
| GO:0045793\_positive\_regulation\_of\_cell\_size | 22 | 0 | 0.000000 | 0.000000 | 1213 | 1057.578418 | 1095.15 | 1132.721582 | 0.902844 |
| GO:0046148\_pigment\_biosynthetic\_process | 22 | 0 | 0.000000 | 0.000000 | 1213 | 1057.578418 | 1095.15 | 1132.721582 | 0.902844 |
| GO:0051028\_mRNA\_transport | 22 | 0 | 0.000000 | 0.000000 | 1213 | 1057.578418 | 1095.15 | 1132.721582 | 0.902844 |
| GO:0051494\_negative\_regulation\_of\_cytoskeleton\_organization | 22 | 0 | 0.000000 | 0.000000 | 1213 | 1057.578418 | 1095.15 | 1132.721582 | 0.902844 |
| GO:0055092\_sterol\_homeostasis | 22 | 0 | 0.000000 | 0.000000 | 1213 | 1057.578418 | 1095.15 | 1132.721582 | 0.902844 |
| GO:0000086\_G2\_M\_transition\_of\_mitotic\_cell\_cycle | 19 | 0 | 0.000000 | 0.000000 | 1257 | 1105.652209 | 1142.3 | 1178.947791 | 0.908751 |
| GO:0002237\_response\_to\_molecule\_of\_bacterial\_origin | 19 | 0 | 0.000000 | 0.000000 | 1257 | 1105.652209 | 1142.3 | 1178.947791 | 0.908751 |
| GO:0002429\_immune\_response-activating\_cell\_surface\_receptor\_signaling\_pathway | 19 | 0 | 0.000000 | 0.000000 | 1257 | 1105.652209 | 1142.3 | 1178.947791 | 0.908751 |
| GO:0002762\_negative\_regulation\_of\_myeloid\_leukocyte\_differentiation | 19 | 0 | 0.000000 | 0.000000 | 1257 | 1105.652209 | 1142.3 | 1178.947791 | 0.908751 |
| GO:0002768\_immune\_response-regulating\_cell\_surface\_receptor\_signaling\_pathway | 19 | 0 | 0.000000 | 0.000000 | 1257 | 1105.652209 | 1142.3 | 1178.947791 | 0.908751 |
| GO:0002791\_regulation\_of\_peptide\_secretion | 19 | 0 | 0.000000 | 0.000000 | 1257 | 1105.652209 | 1142.3 | 1178.947791 | 0.908751 |
| GO:0006635\_fatty\_acid\_beta-oxidation | 19 | 0 | 0.000000 | 0.000000 | 1257 | 1105.652209 | 1142.3 | 1178.947791 | 0.908751 |
| GO:0006809\_nitric\_oxide\_biosynthetic\_process | 19 | 0 | 0.000000 | 0.000000 | 1257 | 1105.652209 | 1142.3 | 1178.947791 | 0.908751 |
| GO:0006879\_cellular\_iron\_ion\_homeostasis | 19 | 0 | 0.000000 | 0.000000 | 1257 | 1105.652209 | 1142.3 | 1178.947791 | 0.908751 |
| GO:0006890\_retrograde\_vesicle-mediated\_transport\_\_Golgi\_to\_ER | 19 | 0 | 0.000000 | 0.000000 | 1257 | 1105.652209 | 1142.3 | 1178.947791 | 0.908751 |
| GO:0007131\_reciprocal\_meiotic\_recombination | 19 | 0 | 0.000000 | 0.000000 | 1257 | 1105.652209 | 1142.3 | 1178.947791 | 0.908751 |
| GO:0007257\_activation\_of\_JUN\_kinase\_activity | 19 | 0 | 0.000000 | 0.000000 | 1257 | 1105.652209 | 1142.3 | 1178.947791 | 0.908751 |
| GO:0007269\_neurotransmitter\_secretion | 19 | 0 | 0.000000 | 0.000000 | 1257 | 1105.652209 | 1142.3 | 1178.947791 | 0.908751 |
| GO:0009260\_ribonucleotide\_biosynthetic\_process | 19 | 0 | 0.000000 | 0.000000 | 1257 | 1105.652209 | 1142.3 | 1178.947791 | 0.908751 |
| GO:0009267\_cellular\_response\_to\_starvation | 19 | 0 | 0.000000 | 0.000000 | 1257 | 1105.652209 | 1142.3 | 1178.947791 | 0.908751 |
| GO:0009451\_RNA\_modification | 19 | 0 | 0.000000 | 0.000000 | 1257 | 1105.652209 | 1142.3 | 1178.947791 | 0.908751 |
| GO:0010883\_regulation\_of\_lipid\_storage | 19 | 0 | 0.000000 | 0.000000 | 1257 | 1105.652209 | 1142.3 | 1178.947791 | 0.908751 |
| GO:0010948\_negative\_regulation\_of\_cell\_cycle\_process | 19 | 0 | 0.000000 | 0.000000 | 1257 | 1105.652209 | 1142.3 | 1178.947791 | 0.908751 |
| GO:0015695\_organic\_cation\_transport | 19 | 0 | 0.000000 | 0.000000 | 1257 | 1105.652209 | 1142.3 | 1178.947791 | 0.908751 |
| GO:0016525\_negative\_regulation\_of\_angiogenesis | 19 | 0 | 0.000000 | 0.000000 | 1257 | 1105.652209 | 1142.3 | 1178.947791 | 0.908751 |
| GO:0019228\_regulation\_of\_action\_potential\_in\_neuron | 19 | 0 | 0.000000 | 0.000000 | 1257 | 1105.652209 | 1142.3 | 1178.947791 | 0.908751 |
| GO:0030041\_actin\_filament\_polymerization | 19 | 0 | 0.000000 | 0.000000 | 1257 | 1105.652209 | 1142.3 | 1178.947791 | 0.908751 |
| GO:0030307\_positive\_regulation\_of\_cell\_growth | 19 | 0 | 0.000000 | 0.000000 | 1257 | 1105.652209 | 1142.3 | 1178.947791 | 0.908751 |
| GO:0030500\_regulation\_of\_bone\_mineralization | 19 | 0 | 0.000000 | 0.000000 | 1257 | 1105.652209 | 1142.3 | 1178.947791 | 0.908751 |
| GO:0031109\_microtubule\_polymerization\_or\_depolymerization | 19 | 0 | 0.000000 | 0.000000 | 1257 | 1105.652209 | 1142.3 | 1178.947791 | 0.908751 |
| GO:0031396\_regulation\_of\_protein\_ubiquitination | 19 | 0 | 0.000000 | 0.000000 | 1257 | 1105.652209 | 1142.3 | 1178.947791 | 0.908751 |
| GO:0032271\_regulation\_of\_protein\_polymerization | 19 | 0 | 0.000000 | 0.000000 | 1257 | 1105.652209 | 1142.3 | 1178.947791 | 0.908751 |
| GO:0032318\_regulation\_of\_Ras\_GTPase\_activity | 19 | 0 | 0.000000 | 0.000000 | 1257 | 1105.652209 | 1142.3 | 1178.947791 | 0.908751 |
| GO:0032388\_positive\_regulation\_of\_intracellular\_transport | 19 | 0 | 0.000000 | 0.000000 | 1257 | 1105.652209 | 1142.3 | 1178.947791 | 0.908751 |
| GO:0033273\_response\_to\_vitamin | 19 | 0 | 0.000000 | 0.000000 | 1257 | 1105.652209 | 1142.3 | 1178.947791 | 0.908751 |
| GO:0034976\_response\_to\_endoplasmic\_reticulum\_stress | 19 | 0 | 0.000000 | 0.000000 | 1257 | 1105.652209 | 1142.3 | 1178.947791 | 0.908751 |
| GO:0042102\_positive\_regulation\_of\_T\_cell\_proliferation | 19 | 0 | 0.000000 | 0.000000 | 1257 | 1105.652209 | 1142.3 | 1178.947791 | 0.908751 |
| GO:0043603\_cellular\_amide\_metabolic\_process | 19 | 0 | 0.000000 | 0.000000 | 1257 | 1105.652209 | 1142.3 | 1178.947791 | 0.908751 |
| GO:0043967\_histone\_H4\_acetylation | 19 | 0 | 0.000000 | 0.000000 | 1257 | 1105.652209 | 1142.3 | 1178.947791 | 0.908751 |
| GO:0044236\_multicellular\_organismal\_metabolic\_process | 19 | 0 | 0.000000 | 0.000000 | 1257 | 1105.652209 | 1142.3 | 1178.947791 | 0.908751 |
| GO:0045732\_positive\_regulation\_of\_protein\_catabolic\_process | 19 | 0 | 0.000000 | 0.000000 | 1257 | 1105.652209 | 1142.3 | 1178.947791 | 0.908751 |
| GO:0045767\_regulation\_of\_anti-apoptosis | 19 | 0 | 0.000000 | 0.000000 | 1257 | 1105.652209 | 1142.3 | 1178.947791 | 0.908751 |
| GO:0046364\_monosaccharide\_biosynthetic\_process | 19 | 0 | 0.000000 | 0.000000 | 1257 | 1105.652209 | 1142.3 | 1178.947791 | 0.908751 |
| GO:0050819\_negative\_regulation\_of\_coagulation | 19 | 0 | 0.000000 | 0.000000 | 1257 | 1105.652209 | 1142.3 | 1178.947791 | 0.908751 |
| GO:0050908\_detection\_of\_light\_stimulus\_involved\_in\_visual\_perception | 19 | 0 | 0.000000 | 0.000000 | 1257 | 1105.652209 | 1142.3 | 1178.947791 | 0.908751 |
| GO:0050920\_regulation\_of\_chemotaxis | 19 | 0 | 0.000000 | 0.000000 | 1257 | 1105.652209 | 1142.3 | 1178.947791 | 0.908751 |
| GO:0050962\_detection\_of\_light\_stimulus\_involved\_in\_sensory\_perception | 19 | 0 | 0.000000 | 0.000000 | 1257 | 1105.652209 | 1142.3 | 1178.947791 | 0.908751 |
| GO:0050994\_regulation\_of\_lipid\_catabolic\_process | 19 | 0 | 0.000000 | 0.000000 | 1257 | 1105.652209 | 1142.3 | 1178.947791 | 0.908751 |
| GO:0051180\_vitamin\_transport | 19 | 0 | 0.000000 | 0.000000 | 1257 | 1105.652209 | 1142.3 | 1178.947791 | 0.908751 |
| GO:0000077\_DNA\_damage\_checkpoint | 37 | 0 | 0.000000 | 0.000000 | 1268 | 1117.141170 | 1153.39 | 1189.638830 | 0.909614 |
| GO:0002443\_leukocyte\_mediated\_immunity | 37 | 0 | 0.000000 | 0.000000 | 1268 | 1117.141170 | 1153.39 | 1189.638830 | 0.909614 |
| GO:0002460\_adaptive\_immune\_response\_based\_on\_somatic\_recombination\_of\_immune\_receptors\_built\_from\_immunoglobulin\_superfamily\_domains | 37 | 0 | 0.000000 | 0.000000 | 1268 | 1117.141170 | 1153.39 | 1189.638830 | 0.909614 |
| GO:0006937\_regulation\_of\_muscle\_contraction | 37 | 0 | 0.000000 | 0.000000 | 1268 | 1117.141170 | 1153.39 | 1189.638830 | 0.909614 |
| GO:0007584\_response\_to\_nutrient | 37 | 0 | 0.000000 | 0.000000 | 1268 | 1117.141170 | 1153.39 | 1189.638830 | 0.909614 |
| GO:0009913\_epidermal\_cell\_differentiation | 37 | 0 | 0.000000 | 0.000000 | 1268 | 1117.141170 | 1153.39 | 1189.638830 | 0.909614 |
| GO:0015918\_sterol\_transport | 37 | 0 | 0.000000 | 0.000000 | 1268 | 1117.141170 | 1153.39 | 1189.638830 | 0.909614 |
| GO:0030301\_cholesterol\_transport | 37 | 0 | 0.000000 | 0.000000 | 1268 | 1117.141170 | 1153.39 | 1189.638830 | 0.909614 |
| GO:0046489\_phosphoinositide\_biosynthetic\_process | 37 | 0 | 0.000000 | 0.000000 | 1268 | 1117.141170 | 1153.39 | 1189.638830 | 0.909614 |
| GO:0046700\_heterocycle\_catabolic\_process | 37 | 0 | 0.000000 | 0.000000 | 1268 | 1117.141170 | 1153.39 | 1189.638830 | 0.909614 |
| GO:0046822\_regulation\_of\_nucleocytoplasmic\_transport | 37 | 0 | 0.000000 | 0.000000 | 1268 | 1117.141170 | 1153.39 | 1189.638830 | 0.909614 |
| GO:0001525\_angiogenesis | 69 | 0 | 0.000000 | 0.000000 | 1274 | 1122.509984 | 1158.43 | 1194.350016 | 0.909286 |
| GO:0007005\_mitochondrion\_organization | 69 | 0 | 0.000000 | 0.000000 | 1274 | 1122.509984 | 1158.43 | 1194.350016 | 0.909286 |
| GO:0016042\_lipid\_catabolic\_process | 69 | 0 | 0.000000 | 0.000000 | 1274 | 1122.509984 | 1158.43 | 1194.350016 | 0.909286 |
| GO:0045333\_cellular\_respiration | 69 | 0 | 0.000000 | 0.000000 | 1274 | 1122.509984 | 1158.43 | 1194.350016 | 0.909286 |
| GO:0051241\_negative\_regulation\_of\_multicellular\_organismal\_process | 69 | 0 | 0.000000 | 0.000000 | 1274 | 1122.509984 | 1158.43 | 1194.350016 | 0.909286 |
| GO:0051351\_positive\_regulation\_of\_ligase\_activity | 69 | 0 | 0.000000 | 0.000000 | 1274 | 1122.509984 | 1158.43 | 1194.350016 | 0.909286 |
| GO:0006164\_purine\_nucleotide\_biosynthetic\_process | 79 | 0 | 0.000000 | 0.000000 | 1278 | 1127.144877 | 1162.55 | 1197.955123 | 0.909664 |
| GO:0032870\_cellular\_response\_to\_hormone\_stimulus | 79 | 0 | 0.000000 | 0.000000 | 1278 | 1127.144877 | 1162.55 | 1197.955123 | 0.909664 |
| GO:0032880\_regulation\_of\_protein\_localization | 79 | 0 | 0.000000 | 0.000000 | 1278 | 1127.144877 | 1162.55 | 1197.955123 | 0.909664 |
| GO:0046942\_carboxylic\_acid\_transport | 79 | 0 | 0.000000 | 0.000000 | 1278 | 1127.144877 | 1162.55 | 1197.955123 | 0.909664 |
| GO:0022603\_regulation\_of\_anatomical\_structure\_morphogenesis | 100 | 0 | 0.000000 | 0.000000 | 1281 | 1130.612348 | 1165.74 | 1200.867652 | 0.910023 |
| GO:0022613\_ribonucleoprotein\_complex\_biogenesis | 100 | 0 | 0.000000 | 0.000000 | 1281 | 1130.612348 | 1165.74 | 1200.867652 | 0.910023 |
| GO:0050776\_regulation\_of\_immune\_response | 100 | 0 | 0.000000 | 0.000000 | 1281 | 1130.612348 | 1165.74 | 1200.867652 | 0.910023 |
| GO:0009790\_embryonic\_development | 109 | 0 | 0.000000 | 0.000000 | 1283 | 1133.013721 | 1168.09 | 1203.166279 | 0.910436 |
| GO:0010817\_regulation\_of\_hormone\_levels | 109 | 0 | 0.000000 | 0.000000 | 1283 | 1133.013721 | 1168.09 | 1203.166279 | 0.910436 |
| GO:0006732\_coenzyme\_metabolic\_process | 65 | 0 | 0.000000 | 0.000000 | 1288 | 1140.858255 | 1175.58 | 1210.301745 | 0.912717 |
| GO:0016054\_organic\_acid\_catabolic\_process | 65 | 0 | 0.000000 | 0.000000 | 1288 | 1140.858255 | 1175.58 | 1210.301745 | 0.912717 |
| GO:0046395\_carboxylic\_acid\_catabolic\_process | 65 | 0 | 0.000000 | 0.000000 | 1288 | 1140.858255 | 1175.58 | 1210.301745 | 0.912717 |
| GO:0051235\_maintenance\_of\_location | 65 | 0 | 0.000000 | 0.000000 | 1288 | 1140.858255 | 1175.58 | 1210.301745 | 0.912717 |
| GO:0051437\_positive\_regulation\_of\_ubiquitin-protein\_ligase\_activity\_during\_mitotic\_cell\_cycle | 65 | 0 | 0.000000 | 0.000000 | 1288 | 1140.858255 | 1175.58 | 1210.301745 | 0.912717 |
| GO:0006520\_cellular\_amino\_acid\_metabolic\_process | 108 | 0 | 0.000000 | 0.000000 | 1292 | 1146.436711 | 1181.09 | 1215.743289 | 0.914156 |
| GO:0008202\_steroid\_metabolic\_process | 108 | 0 | 0.000000 | 0.000000 | 1292 | 1146.436711 | 1181.09 | 1215.743289 | 0.914156 |
| GO:0032446\_protein\_modification\_by\_small\_protein\_conjugation | 108 | 0 | 0.000000 | 0.000000 | 1292 | 1146.436711 | 1181.09 | 1215.743289 | 0.914156 |
| GO:0044106\_cellular\_amine\_metabolic\_process | 108 | 0 | 0.000000 | 0.000000 | 1292 | 1146.436711 | 1181.09 | 1215.743289 | 0.914156 |
| GO:0070647\_protein\_modification\_by\_small\_protein\_conjugation\_or\_removal | 131 | 0 | 0.000000 | 0.000000 | 1293 | 1151.847917 | 1185.66 | 1219.472083 | 0.916984 |
| GO:0006119\_oxidative\_phosphorylation | 58 | 0 | 0.000000 | 0.000000 | 1304 | 1162.913685 | 1196.23 | 1229.546315 | 0.917354 |
| GO:0007160\_cell-matrix\_adhesion | 58 | 0 | 0.000000 | 0.000000 | 1304 | 1162.913685 | 1196.23 | 1229.546315 | 0.917354 |
| GO:0007565\_female\_pregnancy | 58 | 0 | 0.000000 | 0.000000 | 1304 | 1162.913685 | 1196.23 | 1229.546315 | 0.917354 |
| GO:0016053\_organic\_acid\_biosynthetic\_process | 58 | 0 | 0.000000 | 0.000000 | 1304 | 1162.913685 | 1196.23 | 1229.546315 | 0.917354 |
| GO:0030308\_negative\_regulation\_of\_cell\_growth | 58 | 0 | 0.000000 | 0.000000 | 1304 | 1162.913685 | 1196.23 | 1229.546315 | 0.917354 |
| GO:0030799\_regulation\_of\_cyclic\_nucleotide\_metabolic\_process | 58 | 0 | 0.000000 | 0.000000 | 1304 | 1162.913685 | 1196.23 | 1229.546315 | 0.917354 |
| GO:0030802\_regulation\_of\_cyclic\_nucleotide\_biosynthetic\_process | 58 | 0 | 0.000000 | 0.000000 | 1304 | 1162.913685 | 1196.23 | 1229.546315 | 0.917354 |
| GO:0030808\_regulation\_of\_nucleotide\_biosynthetic\_process | 58 | 0 | 0.000000 | 0.000000 | 1304 | 1162.913685 | 1196.23 | 1229.546315 | 0.917354 |
| GO:0040029\_regulation\_of\_gene\_expression\_\_epigenetic | 58 | 0 | 0.000000 | 0.000000 | 1304 | 1162.913685 | 1196.23 | 1229.546315 | 0.917354 |
| GO:0042770\_DNA\_damage\_response\_\_signal\_transduction | 58 | 0 | 0.000000 | 0.000000 | 1304 | 1162.913685 | 1196.23 | 1229.546315 | 0.917354 |
| GO:0046394\_carboxylic\_acid\_biosynthetic\_process | 58 | 0 | 0.000000 | 0.000000 | 1304 | 1162.913685 | 1196.23 | 1229.546315 | 0.917354 |
| GO:0001944\_vasculature\_development | 88 | 0 | 0.000000 | 0.000000 | 1305 | 1163.859524 | 1197.09 | 1230.320476 | 0.917310 |
| GO:0006367\_transcription\_initiation\_from\_RNA\_polymerase\_II\_promoter | 66 | 0 | 0.000000 | 0.000000 | 1313 | 1175.174265 | 1207.73 | 1240.285735 | 0.919825 |
| GO:0006813\_potassium\_ion\_transport | 66 | 0 | 0.000000 | 0.000000 | 1313 | 1175.174265 | 1207.73 | 1240.285735 | 0.919825 |
| GO:0031589\_cell-substrate\_adhesion | 66 | 0 | 0.000000 | 0.000000 | 1313 | 1175.174265 | 1207.73 | 1240.285735 | 0.919825 |
| GO:0048667\_cell\_morphogenesis\_involved\_in\_neuron\_differentiation | 66 | 0 | 0.000000 | 0.000000 | 1313 | 1175.174265 | 1207.73 | 1240.285735 | 0.919825 |
| GO:0048812\_neuron\_projection\_morphogenesis | 66 | 0 | 0.000000 | 0.000000 | 1313 | 1175.174265 | 1207.73 | 1240.285735 | 0.919825 |
| GO:0051090\_regulation\_of\_transcription\_factor\_activity | 66 | 0 | 0.000000 | 0.000000 | 1313 | 1175.174265 | 1207.73 | 1240.285735 | 0.919825 |
| GO:0052547\_regulation\_of\_peptidase\_activity | 66 | 0 | 0.000000 | 0.000000 | 1313 | 1175.174265 | 1207.73 | 1240.285735 | 0.919825 |
| GO:0090046\_regulation\_of\_transcription\_regulator\_activity | 66 | 0 | 0.000000 | 0.000000 | 1313 | 1175.174265 | 1207.73 | 1240.285735 | 0.919825 |
| GO:0016071\_mRNA\_metabolic\_process | 207 | 0 | 0.000000 | 0.000000 | 1314 | 1176.702617 | 1209.03 | 1241.357383 | 0.920114 |
| GO:0051169\_nuclear\_transport | 122 | 0 | 0.000000 | 0.000000 | 1316 | 1180.071007 | 1211.94 | 1243.808993 | 0.920927 |
| GO:0060341\_regulation\_of\_cellular\_localization | 122 | 0 | 0.000000 | 0.000000 | 1316 | 1180.071007 | 1211.94 | 1243.808993 | 0.920927 |
| GO:0000019\_regulation\_of\_mitotic\_recombination | 2 | 0 |  |  |  |  |  |  |  |  |
| GO:0000022\_mitotic\_spindle\_elongation | 2 | 0 |  |  |  |  |  |  |  |  |
| GO:0000059\_protein\_import\_into\_nucleus\_\_docking | 2 | 0 |  |  |  |  |  |  |  |  |
| GO:0000066\_mitochondrial\_ornithine\_transport | 2 | 0 |  |  |  |  |  |  |  |  |
| GO:0000183\_chromatin\_silencing\_at\_rDNA | 2 | 0 |  |  |  |  |  |  |  |  |
| GO:0000305\_response\_to\_oxygen\_radical | 2 | 0 |  |  |  |  |  |  |  |  |
| GO:0000429\_regulation\_of\_transcription\_from\_RNA\_polymerase\_II\_promoter\_by\_carbon\_catabolites | 2 | 0 |  |  |  |  |  |  |  |  |
| GO:0000430\_regulation\_of\_transcription\_from\_RNA\_polymerase\_II\_promoter\_by\_glucose | 2 | 0 |  |  |  |  |  |  |  |  |
| GO:0000432\_positive\_regulation\_of\_transcription\_from\_RNA\_polymerase\_II\_promoter\_by\_glucose | 2 | 0 |  |  |  |  |  |  |  |  |
| GO:0000436\_positive\_regulation\_of\_transcription\_from\_RNA\_polymerase\_II\_promoter\_by\_carbon\_catabolites | 2 | 0 |  |  |  |  |  |  |  |  |
| GO:0000460\_maturation\_of\_5.8S\_rRNA | 2 | 0 |  |  |  |  |  |  |  |  |
| GO:0000466\_maturation\_of\_5.8S\_rRNA\_from\_tricistronic\_rRNA\_transcript\_(SSU-rRNA\_\_5.8S\_rRNA\_\_LSU-rRNA) | 2 | 0 |  |  |  |  |  |  |  |  |
| GO:0000729\_DNA\_double-strand\_break\_processing | 2 | 0 |  |  |  |  |  |  |  |  |
| GO:0000733\_DNA\_strand\_renaturation | 2 | 0 |  |  |  |  |  |  |  |  |
| GO:0000920\_cell\_separation\_during\_cytokinesis | 2 | 0 |  |  |  |  |  |  |  |  |
| GO:0001101\_response\_to\_acid | 2 | 0 |  |  |  |  |  |  |  |  |
| GO:0001300\_chronological\_cell\_aging | 2 | 0 |  |  |  |  |  |  |  |  |
| GO:0001301\_progressive\_alteration\_of\_chromatin\_during\_cell\_aging | 2 | 0 |  |  |  |  |  |  |  |  |
| GO:0001304\_progressive\_alteration\_of\_chromatin\_during\_replicative\_cell\_aging | 2 | 0 |  |  |  |  |  |  |  |  |
| GO:0001309\_age-dependent\_telomere\_shortening | 2 | 0 |  |  |  |  |  |  |  |  |
| GO:0001507\_acetylcholine\_catabolic\_process\_in\_synaptic\_cleft | 2 | 0 |  |  |  |  |  |  |  |  |
| GO:0001522\_pseudouridine\_synthesis | 2 | 0 |  |  |  |  |  |  |  |  |
| GO:0001547\_antral\_ovarian\_follicle\_growth | 2 | 0 |  |  |  |  |  |  |  |  |
| GO:0001550\_ovarian\_cumulus\_expansion | 2 | 0 |  |  |  |  |  |  |  |  |
| GO:0001556\_oocyte\_maturation | 2 | 0 |  |  |  |  |  |  |  |  |
| GO:0001562\_response\_to\_protozoan | 2 | 0 |  |  |  |  |  |  |  |  |
| GO:0001582\_detection\_of\_chemical\_stimulus\_involved\_in\_sensory\_perception\_of\_sweet\_taste | 2 | 0 |  |  |  |  |  |  |  |  |
| GO:0001667\_ameboidal\_cell\_migration | 2 | 0 |  |  |  |  |  |  |  |  |
| GO:0001678\_cellular\_glucose\_homeostasis | 2 | 0 |  |  |  |  |  |  |  |  |
| GO:0001702\_gastrulation\_with\_mouth\_forming\_second | 2 | 0 |  |  |  |  |  |  |  |  |
| GO:0001736\_establishment\_of\_planar\_polarity | 2 | 0 |  |  |  |  |  |  |  |  |
| GO:0001738\_morphogenesis\_of\_a\_polarized\_epithelium | 2 | 0 |  |  |  |  |  |  |  |  |
| GO:0001756\_somitogenesis | 2 | 0 |  |  |  |  |  |  |  |  |
| GO:0001766\_membrane\_raft\_polarization | 2 | 0 |  |  |  |  |  |  |  |  |
| GO:0001780\_neutrophil\_homeostasis | 2 | 0 |  |  |  |  |  |  |  |  |
| GO:0001781\_neutrophil\_apoptosis | 2 | 0 |  |  |  |  |  |  |  |  |
| GO:0001825\_blastocyst\_formation | 2 | 0 |  |  |  |  |  |  |  |  |
| GO:0001829\_trophectodermal\_cell\_differentiation | 2 | 0 |  |  |  |  |  |  |  |  |
| GO:0001840\_neural\_plate\_development | 2 | 0 |  |  |  |  |  |  |  |  |
| GO:0001868\_regulation\_of\_complement\_activation\_\_lectin\_pathway | 2 | 0 |  |  |  |  |  |  |  |  |
| GO:0001869\_negative\_regulation\_of\_complement\_activation\_\_lectin\_pathway | 2 | 0 |  |  |  |  |  |  |  |  |
| GO:0001880\_Mullerian\_duct\_regression | 2 | 0 |  |  |  |  |  |  |  |  |
| GO:0001885\_endothelial\_cell\_development | 2 | 0 |  |  |  |  |  |  |  |  |
| GO:0001897\_cytolysis\_by\_symbiont\_of\_host\_cells | 2 | 0 |  |  |  |  |  |  |  |  |
| GO:0001907\_killing\_by\_symbiont\_of\_host\_cells | 2 | 0 |  |  |  |  |  |  |  |  |
| GO:0001921\_positive\_regulation\_of\_receptor\_recycling | 2 | 0 |  |  |  |  |  |  |  |  |
| GO:0001967\_suckling\_behavior | 2 | 0 |  |  |  |  |  |  |  |  |
| GO:0001975\_response\_to\_amphetamine | 2 | 0 |  |  |  |  |  |  |  |  |
| GO:0002003\_angiotensin\_maturation | 2 | 0 |  |  |  |  |  |  |  |  |
| GO:0002016\_regulation\_of\_blood\_volume\_by\_renin-angiotensin | 2 | 0 |  |  |  |  |  |  |  |  |
| GO:0002032\_desensitization\_of\_G-protein\_coupled\_receptor\_protein\_signaling\_pathway\_by\_arrestin | 2 | 0 |  |  |  |  |  |  |  |  |
| GO:0002090\_regulation\_of\_receptor\_internalization | 2 | 0 |  |  |  |  |  |  |  |  |
| GO:0002092\_positive\_regulation\_of\_receptor\_internalization | 2 | 0 |  |  |  |  |  |  |  |  |
| GO:0002227\_innate\_immune\_response\_in\_mucosa | 2 | 0 |  |  |  |  |  |  |  |  |
| GO:0002248\_connective\_tissue\_replacement\_during\_inflammatory\_response | 2 | 0 |  |  |  |  |  |  |  |  |
| GO:0002254\_kinin\_cascade | 2 | 0 |  |  |  |  |  |  |  |  |
| GO:0002275\_myeloid\_cell\_activation\_during\_immune\_response | 2 | 0 |  |  |  |  |  |  |  |  |
| GO:0002281\_macrophage\_activation\_during\_immune\_response | 2 | 0 |  |  |  |  |  |  |  |  |
| GO:0002291\_T\_cell\_activation\_via\_T\_cell\_receptor\_contact\_with\_antigen\_bound\_to\_MHC\_molecule\_on\_antigen\_presenting\_cell | 2 | 0 |  |  |  |  |  |  |  |  |
| GO:0002353\_plasma\_kallikrein-kinin\_cascade | 2 | 0 |  |  |  |  |  |  |  |  |
| GO:0002378\_immunoglobulin\_biosynthetic\_process | 2 | 0 |  |  |  |  |  |  |  |  |
| GO:0002384\_hepatic\_immune\_response | 2 | 0 |  |  |  |  |  |  |  |  |
| GO:0002385\_mucosal\_immune\_response | 2 | 0 |  |  |  |  |  |  |  |  |
| GO:0002504\_antigen\_processing\_and\_presentation\_of\_peptide\_or\_polysaccharide\_antigen\_via\_MHC\_class\_II | 2 | 0 |  |  |  |  |  |  |  |  |
| GO:0002507\_tolerance\_induction | 2 | 0 |  |  |  |  |  |  |  |  |
| GO:0002532\_production\_of\_molecular\_mediator\_of\_acute\_inflammatory\_response | 2 | 0 |  |  |  |  |  |  |  |  |
| GO:0002536\_respiratory\_burst\_during\_acute\_inflammatory\_response | 2 | 0 |  |  |  |  |  |  |  |  |
| GO:0002542\_Factor\_XII\_activation | 2 | 0 |  |  |  |  |  |  |  |  |
| GO:0002544\_chronic\_inflammatory\_response | 2 | 0 |  |  |  |  |  |  |  |  |
| GO:0002548\_monocyte\_chemotaxis | 2 | 0 |  |  |  |  |  |  |  |  |
| GO:0002643\_regulation\_of\_tolerance\_induction | 2 | 0 |  |  |  |  |  |  |  |  |
| GO:0002645\_positive\_regulation\_of\_tolerance\_induction | 2 | 0 |  |  |  |  |  |  |  |  |
| GO:0002675\_positive\_regulation\_of\_acute\_inflammatory\_response | 2 | 0 |  |  |  |  |  |  |  |  |
| GO:0002679\_respiratory\_burst\_during\_defense\_response | 2 | 0 |  |  |  |  |  |  |  |  |
| GO:0002704\_negative\_regulation\_of\_leukocyte\_mediated\_immunity | 2 | 0 |  |  |  |  |  |  |  |  |
| GO:0002707\_negative\_regulation\_of\_lymphocyte\_mediated\_immunity | 2 | 0 |  |  |  |  |  |  |  |  |
| GO:0002710\_negative\_regulation\_of\_T\_cell\_mediated\_immunity | 2 | 0 |  |  |  |  |  |  |  |  |
| GO:0002714\_positive\_regulation\_of\_B\_cell\_mediated\_immunity | 2 | 0 |  |  |  |  |  |  |  |  |
| GO:0002820\_negative\_regulation\_of\_adaptive\_immune\_response | 2 | 0 |  |  |  |  |  |  |  |  |
| GO:0002823\_negative\_regulation\_of\_adaptive\_immune\_response\_based\_on\_somatic\_recombination\_of\_immune\_receptors\_built\_from\_immunoglobulin\_superfamily\_domains | 2 | 0 |  |  |  |  |  |  |  |  |
| GO:0002891\_positive\_regulation\_of\_immunoglobulin\_mediated\_immune\_response | 2 | 0 |  |  |  |  |  |  |  |  |
| GO:0003057\_regulation\_of\_the\_force\_of\_heart\_contraction\_by\_chemical\_signal | 2 | 0 |  |  |  |  |  |  |  |  |
| GO:0003078\_regulation\_of\_natriuresis | 2 | 0 |  |  |  |  |  |  |  |  |
| GO:0006005\_L-fucose\_biosynthetic\_process | 2 | 0 |  |  |  |  |  |  |  |  |
| GO:0006011\_UDP-glucose\_metabolic\_process | 2 | 0 |  |  |  |  |  |  |  |  |
| GO:0006030\_chitin\_metabolic\_process | 2 | 0 |  |  |  |  |  |  |  |  |
| GO:0006032\_chitin\_catabolic\_process | 2 | 0 |  |  |  |  |  |  |  |  |
| GO:0006046\_N-acetylglucosamine\_catabolic\_process | 2 | 0 |  |  |  |  |  |  |  |  |
| GO:0006047\_UDP-N-acetylglucosamine\_metabolic\_process | 2 | 0 |  |  |  |  |  |  |  |  |
| GO:0006054\_N-acetylneuraminate\_metabolic\_process | 2 | 0 |  |  |  |  |  |  |  |  |
| GO:0006063\_uronic\_acid\_metabolic\_process | 2 | 0 |  |  |  |  |  |  |  |  |
| GO:0006122\_mitochondrial\_electron\_transport\_\_ubiquinol\_to\_cytochrome\_c | 2 | 0 |  |  |  |  |  |  |  |  |
| GO:0006184\_GTP\_catabolic\_process | 2 | 0 |  |  |  |  |  |  |  |  |
| GO:0006208\_pyrimidine\_base\_catabolic\_process | 2 | 0 |  |  |  |  |  |  |  |  |
| GO:0006210\_thymine\_catabolic\_process | 2 | 0 |  |  |  |  |  |  |  |  |
| GO:0006212\_uracil\_catabolic\_process | 2 | 0 |  |  |  |  |  |  |  |  |
| GO:0006264\_mitochondrial\_DNA\_replication | 2 | 0 |  |  |  |  |  |  |  |  |
| GO:0006307\_DNA\_dealkylation | 2 | 0 |  |  |  |  |  |  |  |  |
| GO:0006335\_DNA\_replication-dependent\_nucleosome\_assembly | 2 | 0 |  |  |  |  |  |  |  |  |
| GO:0006345\_loss\_of\_chromatin\_silencing | 2 | 0 |  |  |  |  |  |  |  |  |
| GO:0006370\_mRNA\_capping | 2 | 0 |  |  |  |  |  |  |  |  |
| GO:0006398\_histone\_mRNA\_3'-end\_processing | 2 | 0 |  |  |  |  |  |  |  |  |
| GO:0006410\_transcription\_\_RNA-dependent | 2 | 0 |  |  |  |  |  |  |  |  |
| GO:0006422\_aspartyl-tRNA\_aminoacylation | 2 | 0 |  |  |  |  |  |  |  |  |
| GO:0006465\_signal\_peptide\_processing | 2 | 0 |  |  |  |  |  |  |  |  |
| GO:0006475\_internal\_protein\_amino\_acid\_acetylation | 2 | 0 |  |  |  |  |  |  |  |  |
| GO:0006478\_peptidyl-tyrosine\_sulfation | 2 | 0 |  |  |  |  |  |  |  |  |
| GO:0006481\_C-terminal\_protein\_amino\_acid\_methylation | 2 | 0 |  |  |  |  |  |  |  |  |
| GO:0006488\_dolichol-linked\_oligosaccharide\_biosynthetic\_process | 2 | 0 |  |  |  |  |  |  |  |  |
| GO:0006498\_N-terminal\_protein\_lipidation | 2 | 0 |  |  |  |  |  |  |  |  |
| GO:0006517\_protein\_deglycosylation | 2 | 0 |  |  |  |  |  |  |  |  |
| GO:0006521\_regulation\_of\_cellular\_amino\_acid\_metabolic\_process | 2 | 0 |  |  |  |  |  |  |  |  |
| GO:0006537\_glutamate\_biosynthetic\_process | 2 | 0 |  |  |  |  |  |  |  |  |
| GO:0006540\_glutamate\_decarboxylation\_to\_succinate | 2 | 0 |  |  |  |  |  |  |  |  |
| GO:0006541\_glutamine\_metabolic\_process | 2 | 0 |  |  |  |  |  |  |  |  |
| GO:0006551\_leucine\_metabolic\_process | 2 | 0 |  |  |  |  |  |  |  |  |
| GO:0006552\_leucine\_catabolic\_process | 2 | 0 |  |  |  |  |  |  |  |  |
| GO:0006561\_proline\_biosynthetic\_process | 2 | 0 |  |  |  |  |  |  |  |  |
| GO:0006569\_tryptophan\_catabolic\_process | 2 | 0 |  |  |  |  |  |  |  |  |
| GO:0006581\_acetylcholine\_catabolic\_process | 2 | 0 |  |  |  |  |  |  |  |  |
| GO:0006582\_melanin\_metabolic\_process | 2 | 0 |  |  |  |  |  |  |  |  |
| GO:0006583\_melanin\_biosynthetic\_process\_from\_tyrosine | 2 | 0 |  |  |  |  |  |  |  |  |
| GO:0006601\_creatine\_biosynthetic\_process | 2 | 0 |  |  |  |  |  |  |  |  |
| GO:0006608\_snRNP\_protein\_import\_into\_nucleus | 2 | 0 |  |  |  |  |  |  |  |  |
| GO:0006651\_diacylglycerol\_biosynthetic\_process | 2 | 0 |  |  |  |  |  |  |  |  |
| GO:0006679\_glucosylceramide\_biosynthetic\_process | 2 | 0 |  |  |  |  |  |  |  |  |
| GO:0006681\_galactosylceramide\_metabolic\_process | 2 | 0 |  |  |  |  |  |  |  |  |
| GO:0006685\_sphingomyelin\_catabolic\_process | 2 | 0 |  |  |  |  |  |  |  |  |
| GO:0006702\_androgen\_biosynthetic\_process | 2 | 0 |  |  |  |  |  |  |  |  |
| GO:0006703\_estrogen\_biosynthetic\_process | 2 | 0 |  |  |  |  |  |  |  |  |
| GO:0006710\_androgen\_catabolic\_process | 2 | 0 |  |  |  |  |  |  |  |  |
| GO:0006738\_nicotinamide\_riboside\_catabolic\_process | 2 | 0 |  |  |  |  |  |  |  |  |
| GO:0006772\_thiamin\_metabolic\_process | 2 | 0 |  |  |  |  |  |  |  |  |
| GO:0006780\_uroporphyrinogen\_III\_biosynthetic\_process | 2 | 0 |  |  |  |  |  |  |  |  |
| GO:0006784\_heme\_a\_biosynthetic\_process | 2 | 0 |  |  |  |  |  |  |  |  |
| GO:0006824\_cobalt\_ion\_transport | 2 | 0 |  |  |  |  |  |  |  |  |
| GO:0006828\_manganese\_ion\_transport | 2 | 0 |  |  |  |  |  |  |  |  |
| GO:0006880\_intracellular\_sequestering\_of\_iron\_ion | 2 | 0 |  |  |  |  |  |  |  |  |
| GO:0006883\_cellular\_sodium\_ion\_homeostasis | 2 | 0 |  |  |  |  |  |  |  |  |
| GO:0006924\_activation-induced\_cell\_death\_of\_T\_cells | 2 | 0 |  |  |  |  |  |  |  |  |
| GO:0006972\_hyperosmotic\_response | 2 | 0 |  |  |  |  |  |  |  |  |
| GO:0006975\_DNA\_damage\_induced\_protein\_phosphorylation | 2 | 0 |  |  |  |  |  |  |  |  |
| GO:0006982\_response\_to\_lipid\_hydroperoxide | 2 | 0 |  |  |  |  |  |  |  |  |
| GO:0006998\_nuclear\_envelope\_organization | 2 | 0 |  |  |  |  |  |  |  |  |
| GO:0007021\_tubulin\_complex\_assembly | 2 | 0 |  |  |  |  |  |  |  |  |
| GO:0007023\_post-chaperonin\_tubulin\_folding\_pathway | 2 | 0 |  |  |  |  |  |  |  |  |
| GO:0007063\_regulation\_of\_sister\_chromatid\_cohesion | 2 | 0 |  |  |  |  |  |  |  |  |
| GO:0007079\_mitotic\_chromosome\_movement\_towards\_spindle\_pole | 2 | 0 |  |  |  |  |  |  |  |  |
| GO:0007095\_mitotic\_cell\_cycle\_G2\_M\_transition\_DNA\_damage\_checkpoint | 2 | 0 |  |  |  |  |  |  |  |  |
| GO:0007128\_meiotic\_prophase\_I | 2 | 0 |  |  |  |  |  |  |  |  |
| GO:0007141\_male\_meiosis\_I | 2 | 0 |  |  |  |  |  |  |  |  |
| GO:0007143\_female\_meiosis | 2 | 0 |  |  |  |  |  |  |  |  |
| GO:0007191\_activation\_of\_adenylate\_cyclase\_activity\_by\_dopamine\_receptor\_signaling\_pathway | 2 | 0 |  |  |  |  |  |  |  |  |
| GO:0007206\_activation\_of\_phospholipase\_C\_activity\_by\_metabotropic\_glutamate\_receptor\_signaling\_pathway | 2 | 0 |  |  |  |  |  |  |  |  |
| GO:0007231\_osmosensory\_signaling\_pathway | 2 | 0 |  |  |  |  |  |  |  |  |
| GO:0007262\_STAT\_protein\_nuclear\_translocation | 2 | 0 |  |  |  |  |  |  |  |  |
| GO:0007290\_spermatid\_nucleus\_elongation | 2 | 0 |  |  |  |  |  |  |  |  |
| GO:0007308\_oocyte\_construction | 2 | 0 |  |  |  |  |  |  |  |  |
| GO:0007309\_oocyte\_axis\_specification | 2 | 0 |  |  |  |  |  |  |  |  |
| GO:0007341\_penetration\_of\_zona\_pellucida | 2 | 0 |  |  |  |  |  |  |  |  |
| GO:0007379\_segment\_specification | 2 | 0 |  |  |  |  |  |  |  |  |
| GO:0007418\_ventral\_midline\_development | 2 | 0 |  |  |  |  |  |  |  |  |
| GO:0007442\_hindgut\_morphogenesis | 2 | 0 |  |  |  |  |  |  |  |  |
| GO:0007492\_endoderm\_development | 2 | 0 |  |  |  |  |  |  |  |  |
| GO:0007525\_somatic\_muscle\_development | 2 | 0 |  |  |  |  |  |  |  |  |
| GO:0007549\_dosage\_compensation | 2 | 0 |  |  |  |  |  |  |  |  |
| GO:0007628\_adult\_walking\_behavior | 2 | 0 |  |  |  |  |  |  |  |  |
| GO:0008216\_spermidine\_metabolic\_process | 2 | 0 |  |  |  |  |  |  |  |  |
| GO:0008306\_associative\_learning | 2 | 0 |  |  |  |  |  |  |  |  |
| GO:0008582\_regulation\_of\_synaptic\_growth\_at\_neuromuscular\_junction | 2 | 0 |  |  |  |  |  |  |  |  |
| GO:0008593\_regulation\_of\_Notch\_signaling\_pathway | 2 | 0 |  |  |  |  |  |  |  |  |
| GO:0008608\_attachment\_of\_spindle\_microtubules\_to\_kinetochore | 2 | 0 |  |  |  |  |  |  |  |  |
| GO:0008616\_queuosine\_biosynthetic\_process | 2 | 0 |  |  |  |  |  |  |  |  |
| GO:0008618\_7-methylguanosine\_metabolic\_process | 2 | 0 |  |  |  |  |  |  |  |  |
| GO:0008653\_lipopolysaccharide\_metabolic\_process | 2 | 0 |  |  |  |  |  |  |  |  |
| GO:0009051\_pentose-phosphate\_shunt\_\_oxidative\_branch | 2 | 0 |  |  |  |  |  |  |  |  |
| GO:0009082\_branched\_chain\_family\_amino\_acid\_biosynthetic\_process | 2 | 0 |  |  |  |  |  |  |  |  |
| GO:0009103\_lipopolysaccharide\_biosynthetic\_process | 2 | 0 |  |  |  |  |  |  |  |  |
| GO:0009120\_deoxyribonucleoside\_metabolic\_process | 2 | 0 |  |  |  |  |  |  |  |  |
| GO:0009134\_nucleoside\_diphosphate\_catabolic\_process | 2 | 0 |  |  |  |  |  |  |  |  |
| GO:0009162\_deoxyribonucleoside\_monophosphate\_metabolic\_process | 2 | 0 |  |  |  |  |  |  |  |  |
| GO:0009191\_ribonucleoside\_diphosphate\_catabolic\_process | 2 | 0 |  |  |  |  |  |  |  |  |
| GO:0009263\_deoxyribonucleotide\_biosynthetic\_process | 2 | 0 |  |  |  |  |  |  |  |  |
| GO:0009439\_cyanate\_metabolic\_process | 2 | 0 |  |  |  |  |  |  |  |  |
| GO:0009440\_cyanate\_catabolic\_process | 2 | 0 |  |  |  |  |  |  |  |  |
| GO:0009448\_gamma-aminobutyric\_acid\_metabolic\_process | 2 | 0 |  |  |  |  |  |  |  |  |
| GO:0009450\_gamma-aminobutyric\_acid\_catabolic\_process | 2 | 0 |  |  |  |  |  |  |  |  |
| GO:0009452\_RNA\_capping | 2 | 0 |  |  |  |  |  |  |  |  |
| GO:0009586\_rhodopsin\_mediated\_phototransduction | 2 | 0 |  |  |  |  |  |  |  |  |
| GO:0009597\_detection\_of\_virus | 2 | 0 |  |  |  |  |  |  |  |  |
| GO:0009602\_detection\_of\_symbiont | 2 | 0 |  |  |  |  |  |  |  |  |
| GO:0009608\_response\_to\_symbiont | 2 | 0 |  |  |  |  |  |  |  |  |
| GO:0009649\_entrainment\_of\_circadian\_clock | 2 | 0 |  |  |  |  |  |  |  |  |
| GO:0009651\_response\_to\_salt\_stress | 2 | 0 |  |  |  |  |  |  |  |  |
| GO:0009756\_carbohydrate\_mediated\_signaling | 2 | 0 |  |  |  |  |  |  |  |  |
| GO:0010042\_response\_to\_manganese\_ion | 2 | 0 |  |  |  |  |  |  |  |  |
| GO:0010155\_regulation\_of\_proton\_transport | 2 | 0 |  |  |  |  |  |  |  |  |
| GO:0010216\_maintenance\_of\_DNA\_methylation | 2 | 0 |  |  |  |  |  |  |  |  |
| GO:0010248\_establishment\_or\_maintenance\_of\_transmembrane\_electrochemical\_gradient | 2 | 0 |  |  |  |  |  |  |  |  |
| GO:0010389\_regulation\_of\_G2\_M\_transition\_of\_mitotic\_cell\_cycle | 2 | 0 |  |  |  |  |  |  |  |  |
| GO:0010459\_negative\_regulation\_of\_heart\_rate | 2 | 0 |  |  |  |  |  |  |  |  |
| GO:0010470\_regulation\_of\_gastrulation | 2 | 0 |  |  |  |  |  |  |  |  |
| GO:0010506\_regulation\_of\_autophagy | 2 | 0 |  |  |  |  |  |  |  |  |
| GO:0010511\_regulation\_of\_phosphatidylinositol\_biosynthetic\_process | 2 | 0 |  |  |  |  |  |  |  |  |
| GO:0010512\_negative\_regulation\_of\_phosphatidylinositol\_biosynthetic\_process | 2 | 0 |  |  |  |  |  |  |  |  |
| GO:0010523\_negative\_regulation\_of\_calcium\_ion\_transport\_into\_cytosol | 2 | 0 |  |  |  |  |  |  |  |  |
| GO:0010533\_regulation\_of\_activation\_of\_Janus\_kinase\_activity | 2 | 0 |  |  |  |  |  |  |  |  |
| GO:0010536\_positive\_regulation\_of\_activation\_of\_Janus\_kinase\_activity | 2 | 0 |  |  |  |  |  |  |  |  |
| GO:0010614\_negative\_regulation\_of\_cardiac\_muscle\_hypertrophy | 2 | 0 |  |  |  |  |  |  |  |  |
| GO:0010616\_negative\_regulation\_of\_cardiac\_muscle\_adaptation | 2 | 0 |  |  |  |  |  |  |  |  |
| GO:0010640\_regulation\_of\_platelet-derived\_growth\_factor\_receptor\_signaling\_pathway | 2 | 0 |  |  |  |  |  |  |  |  |
| GO:0010641\_positive\_regulation\_of\_platelet-derived\_growth\_factor\_receptor\_signaling\_pathway | 2 | 0 |  |  |  |  |  |  |  |  |
| GO:0010670\_positive\_regulation\_of\_oxygen\_and\_reactive\_oxygen\_species\_metabolic\_process | 2 | 0 |  |  |  |  |  |  |  |  |
| GO:0010755\_regulation\_of\_plasminogen\_activation | 2 | 0 |  |  |  |  |  |  |  |  |
| GO:0010761\_fibroblast\_migration | 2 | 0 |  |  |  |  |  |  |  |  |
| GO:0010762\_regulation\_of\_fibroblast\_migration | 2 | 0 |  |  |  |  |  |  |  |  |
| GO:0010763\_positive\_regulation\_of\_fibroblast\_migration | 2 | 0 |  |  |  |  |  |  |  |  |
| GO:0010815\_bradykinin\_catabolic\_process | 2 | 0 |  |  |  |  |  |  |  |  |
| GO:0010818\_T\_cell\_chemotaxis | 2 | 0 |  |  |  |  |  |  |  |  |
| GO:0010819\_regulation\_of\_T\_cell\_chemotaxis | 2 | 0 |  |  |  |  |  |  |  |  |
| GO:0010820\_positive\_regulation\_of\_T\_cell\_chemotaxis | 2 | 0 |  |  |  |  |  |  |  |  |
| GO:0010866\_regulation\_of\_triglyceride\_biosynthetic\_process | 2 | 0 |  |  |  |  |  |  |  |  |
| GO:0010867\_positive\_regulation\_of\_triglyceride\_biosynthetic\_process | 2 | 0 |  |  |  |  |  |  |  |  |
| GO:0010881\_regulation\_of\_cardiac\_muscle\_contraction\_by\_regulation\_of\_the\_release\_of\_sequestered\_calcium\_ion | 2 | 0 |  |  |  |  |  |  |  |  |
| GO:0010882\_regulation\_of\_cardiac\_muscle\_contraction\_by\_calcium\_ion\_signaling | 2 | 0 |  |  |  |  |  |  |  |  |
| GO:0010908\_regulation\_of\_heparan\_sulfate\_proteoglycan\_biosynthetic\_process | 2 | 0 |  |  |  |  |  |  |  |  |
| GO:0010909\_positive\_regulation\_of\_heparan\_sulfate\_proteoglycan\_biosynthetic\_process | 2 | 0 |  |  |  |  |  |  |  |  |
| GO:0010949\_negative\_regulation\_of\_intestinal\_phytosterol\_absorption | 2 | 0 |  |  |  |  |  |  |  |  |
| GO:0010954\_positive\_regulation\_of\_protein\_maturation\_by\_peptide\_bond\_cleavage | 2 | 0 |  |  |  |  |  |  |  |  |
| GO:0010979\_regulation\_of\_vitamin\_D\_24-hydroxylase\_activity | 2 | 0 |  |  |  |  |  |  |  |  |
| GO:0010980\_positive\_regulation\_of\_vitamin\_D\_24-hydroxylase\_activity | 2 | 0 |  |  |  |  |  |  |  |  |
| GO:0010982\_regulation\_of\_high-density\_lipoprotein\_particle\_clearance | 2 | 0 |  |  |  |  |  |  |  |  |
| GO:0014002\_astrocyte\_development | 2 | 0 |  |  |  |  |  |  |  |  |
| GO:0014013\_regulation\_of\_gliogenesis | 2 | 0 |  |  |  |  |  |  |  |  |
| GO:0014014\_negative\_regulation\_of\_gliogenesis | 2 | 0 |  |  |  |  |  |  |  |  |
| GO:0014037\_Schwann\_cell\_differentiation | 2 | 0 |  |  |  |  |  |  |  |  |
| GO:0014044\_Schwann\_cell\_development | 2 | 0 |  |  |  |  |  |  |  |  |
| GO:0014072\_response\_to\_isoquinoline\_alkaloid | 2 | 0 |  |  |  |  |  |  |  |  |
| GO:0014074\_response\_to\_purine | 2 | 0 |  |  |  |  |  |  |  |  |
| GO:0014741\_negative\_regulation\_of\_muscle\_hypertrophy | 2 | 0 |  |  |  |  |  |  |  |  |
| GO:0014745\_negative\_regulation\_of\_muscle\_adaptation | 2 | 0 |  |  |  |  |  |  |  |  |
| GO:0014819\_regulation\_of\_skeletal\_muscle\_contraction | 2 | 0 |  |  |  |  |  |  |  |  |
| GO:0015677\_copper\_ion\_import | 2 | 0 |  |  |  |  |  |  |  |  |
| GO:0015691\_cadmium\_ion\_transport | 2 | 0 |  |  |  |  |  |  |  |  |
| GO:0015732\_prostaglandin\_transport | 2 | 0 |  |  |  |  |  |  |  |  |
| GO:0015788\_UDP-N-acetylglucosamine\_transport | 2 | 0 |  |  |  |  |  |  |  |  |
| GO:0015793\_glycerol\_transport | 2 | 0 |  |  |  |  |  |  |  |  |
| GO:0015801\_aromatic\_amino\_acid\_transport | 2 | 0 |  |  |  |  |  |  |  |  |
| GO:0015808\_L-alanine\_transport | 2 | 0 |  |  |  |  |  |  |  |  |
| GO:0015822\_ornithine\_transport | 2 | 0 |  |  |  |  |  |  |  |  |
| GO:0015824\_proline\_transport | 2 | 0 |  |  |  |  |  |  |  |  |
| GO:0015825\_L-serine\_transport | 2 | 0 |  |  |  |  |  |  |  |  |
| GO:0015860\_purine\_nucleoside\_transport | 2 | 0 |  |  |  |  |  |  |  |  |
| GO:0015870\_acetylcholine\_transport | 2 | 0 |  |  |  |  |  |  |  |  |
| GO:0015871\_choline\_transport | 2 | 0 |  |  |  |  |  |  |  |  |
| GO:0015893\_drug\_transport | 2 | 0 |  |  |  |  |  |  |  |  |
| GO:0015920\_lipopolysaccharide\_transport | 2 | 0 |  |  |  |  |  |  |  |  |
| GO:0015936\_coenzyme\_A\_metabolic\_process | 2 | 0 |  |  |  |  |  |  |  |  |
| GO:0015939\_pantothenate\_metabolic\_process | 2 | 0 |  |  |  |  |  |  |  |  |
| GO:0015942\_formate\_metabolic\_process | 2 | 0 |  |  |  |  |  |  |  |  |
| GO:0015988\_energy\_coupled\_proton\_transport\_\_against\_electrochemical\_gradient | 2 | 0 |  |  |  |  |  |  |  |  |
| GO:0015991\_ATP\_hydrolysis\_coupled\_proton\_transport | 2 | 0 |  |  |  |  |  |  |  |  |
| GO:0015993\_molecular\_hydrogen\_transport | 2 | 0 |  |  |  |  |  |  |  |  |
| GO:0016075\_rRNA\_catabolic\_process | 2 | 0 |  |  |  |  |  |  |  |  |
| GO:0016080\_synaptic\_vesicle\_targeting | 2 | 0 |  |  |  |  |  |  |  |  |
| GO:0016090\_prenol\_metabolic\_process | 2 | 0 |  |  |  |  |  |  |  |  |
| GO:0016093\_polyprenol\_metabolic\_process | 2 | 0 |  |  |  |  |  |  |  |  |
| GO:0016233\_telomere\_capping | 2 | 0 |  |  |  |  |  |  |  |  |
| GO:0016264\_gap\_junction\_assembly | 2 | 0 |  |  |  |  |  |  |  |  |
| GO:0016266\_O-glycan\_processing | 2 | 0 |  |  |  |  |  |  |  |  |
| GO:0016322\_neuron\_remodeling | 2 | 0 |  |  |  |  |  |  |  |  |
| GO:0016482\_cytoplasmic\_transport | 2 | 0 |  |  |  |  |  |  |  |  |
| GO:0016557\_peroxisome\_membrane\_biogenesis | 2 | 0 |  |  |  |  |  |  |  |  |
| GO:0016561\_protein\_import\_into\_peroxisome\_matrix\_\_translocation | 2 | 0 |  |  |  |  |  |  |  |  |
| GO:0016973\_poly(A)+\_mRNA\_export\_from\_nucleus | 2 | 0 |  |  |  |  |  |  |  |  |
| GO:0017004\_cytochrome\_complex\_assembly | 2 | 0 |  |  |  |  |  |  |  |  |
| GO:0017055\_negative\_regulation\_of\_transcriptional\_preinitiation\_complex\_assembly | 2 | 0 |  |  |  |  |  |  |  |  |
| GO:0017145\_stem\_cell\_division | 2 | 0 |  |  |  |  |  |  |  |  |
| GO:0017158\_regulation\_of\_calcium\_ion-dependent\_exocytosis | 2 | 0 |  |  |  |  |  |  |  |  |
| GO:0017182\_peptidyl-diphthamide\_metabolic\_process | 2 | 0 |  |  |  |  |  |  |  |  |
| GO:0017183\_peptidyl-diphthamide\_biosynthetic\_process\_from\_peptidyl-histidine | 2 | 0 |  |  |  |  |  |  |  |  |
| GO:0018198\_peptidyl-cysteine\_modification | 2 | 0 |  |  |  |  |  |  |  |  |
| GO:0018202\_peptidyl-histidine\_modification | 2 | 0 |  |  |  |  |  |  |  |  |
| GO:0018282\_metal\_incorporation\_into\_metallo-sulfur\_cluster | 2 | 0 |  |  |  |  |  |  |  |  |
| GO:0018283\_iron\_incorporation\_into\_metallo-sulfur\_cluster | 2 | 0 |  |  |  |  |  |  |  |  |
| GO:0018347\_protein\_amino\_acid\_farnesylation | 2 | 0 |  |  |  |  |  |  |  |  |
| GO:0018410\_peptide\_or\_protein\_carboxyl-terminal\_blocking | 2 | 0 |  |  |  |  |  |  |  |  |
| GO:0019042\_latent\_virus\_infection | 2 | 0 |  |  |  |  |  |  |  |  |
| GO:0019046\_reactivation\_of\_latent\_virus | 2 | 0 |  |  |  |  |  |  |  |  |
| GO:0019049\_evasion\_of\_host\_defenses\_by\_virus | 2 | 0 |  |  |  |  |  |  |  |  |
| GO:0019076\_release\_of\_virus\_from\_host | 2 | 0 |  |  |  |  |  |  |  |  |
| GO:0019348\_dolichol\_metabolic\_process | 2 | 0 |  |  |  |  |  |  |  |  |
| GO:0019359\_nicotinamide\_nucleotide\_biosynthetic\_process | 2 | 0 |  |  |  |  |  |  |  |  |
| GO:0019363\_pyridine\_nucleotide\_biosynthetic\_process | 2 | 0 |  |  |  |  |  |  |  |  |
| GO:0019374\_galactolipid\_metabolic\_process | 2 | 0 |  |  |  |  |  |  |  |  |
| GO:0019459\_glutamate\_deamidation | 2 | 0 |  |  |  |  |  |  |  |  |
| GO:0019509\_methionine\_salvage | 2 | 0 |  |  |  |  |  |  |  |  |
| GO:0019530\_taurine\_metabolic\_process | 2 | 0 |  |  |  |  |  |  |  |  |
| GO:0019532\_oxalate\_transport | 2 | 0 |  |  |  |  |  |  |  |  |
| GO:0019585\_glucuronate\_metabolic\_process | 2 | 0 |  |  |  |  |  |  |  |  |
| GO:0019605\_butyrate\_metabolic\_process | 2 | 0 |  |  |  |  |  |  |  |  |
| GO:0019626\_short-chain\_fatty\_acid\_catabolic\_process | 2 | 0 |  |  |  |  |  |  |  |  |
| GO:0019730\_antimicrobial\_humoral\_response | 2 | 0 |  |  |  |  |  |  |  |  |
| GO:0019731\_antibacterial\_humoral\_response | 2 | 0 |  |  |  |  |  |  |  |  |
| GO:0019805\_quinolinate\_biosynthetic\_process | 2 | 0 |  |  |  |  |  |  |  |  |
| GO:0019836\_hemolysis\_by\_symbiont\_of\_host\_erythrocytes | 2 | 0 |  |  |  |  |  |  |  |  |
| GO:0019860\_uracil\_metabolic\_process | 2 | 0 |  |  |  |  |  |  |  |  |
| GO:0019896\_axon\_transport\_of\_mitochondrion | 2 | 0 |  |  |  |  |  |  |  |  |
| GO:0019919\_peptidyl-arginine\_methylation\_\_to\_asymmetrical-dimethyl\_arginine | 2 | 0 |  |  |  |  |  |  |  |  |
| GO:0021511\_spinal\_cord\_patterning | 2 | 0 |  |  |  |  |  |  |  |  |
| GO:0021513\_spinal\_cord\_dorsal\_ventral\_patterning | 2 | 0 |  |  |  |  |  |  |  |  |
| GO:0021517\_ventral\_spinal\_cord\_development | 2 | 0 |  |  |  |  |  |  |  |  |
| GO:0021545\_cranial\_nerve\_development | 2 | 0 |  |  |  |  |  |  |  |  |
| GO:0021587\_cerebellum\_morphogenesis | 2 | 0 |  |  |  |  |  |  |  |  |
| GO:0021675\_nerve\_development | 2 | 0 |  |  |  |  |  |  |  |  |
| GO:0021695\_cerebellar\_cortex\_development | 2 | 0 |  |  |  |  |  |  |  |  |
| GO:0021696\_cerebellar\_cortex\_morphogenesis | 2 | 0 |  |  |  |  |  |  |  |  |
| GO:0021795\_cerebral\_cortex\_cell\_migration | 2 | 0 |  |  |  |  |  |  |  |  |
| GO:0021826\_substrate-independent\_telencephalic\_tangential\_migration | 2 | 0 |  |  |  |  |  |  |  |  |
| GO:0021830\_interneuron\_migration\_from\_the\_subpallium\_to\_the\_cortex | 2 | 0 |  |  |  |  |  |  |  |  |
| GO:0021843\_substrate-independent\_telencephalic\_tangential\_interneuron\_migration | 2 | 0 |  |  |  |  |  |  |  |  |
[truncated: 286,592 more chars]
