# Supplementary material for: Concordance of Gene Expression and Functional Correlation Patterns across the NCI-60 Cell Lines and the Cancer Genome Atlas Glioblastoma Samples
Source: PLoS One. 2012 Jul 26;7(7):e40062. doi: 10.1371/journal.pone.0040062 (PMC3406063; doi:10.1371/journal.pone.0040062)
Supplement: Download S1 — Zip archive of HTGM results. (ZIP) [file pone.0040062.s007.zip › work2026406846/Generated_Total2026406846.dir/generic.BP.NCI60.0.6.CCR7.express.genes.correlation.complete.Thu.May.19.17.25.28.2011.htgm.txt.dir/generic.BP.NCI60.0.6.CCR7.express.genes.correlation.complete.Thu.May.19.17.25.28.2011.htgm.txt.change.gce.CIM.dir/cgi_user_x.html]

**X-axis Names**   
Cluster is based on euclidean distance  
Cluster method is: average  
plclust  
height plot  

|  |
| --- |
| 1.GO:0030005\_cellular\_di-\_\_tri-valent\_inorganic\_cation\_homeostasis |
| 2.GO:0050801\_ion\_homeostasis |
| 3.GO:0055080\_cation\_homeostasis |
| 4.GO:0006874\_cellular\_calcium\_ion\_homeostasis |
| 5.GO:0055082\_cellular\_chemical\_homeostasis |
| 6.GO:0006873\_cellular\_ion\_homeostasis |
| 7.GO:0030003\_cellular\_cation\_homeostasis |
| 8.GO:0051480\_cytosolic\_calcium\_ion\_homeostasis |
| 9.GO:0055074\_calcium\_ion\_homeostasis |
| 10.GO:0055066\_di-\_\_tri-valent\_inorganic\_cation\_homeostasis |
| 11.GO:0019725\_cellular\_homeostasis |
| 12.GO:0007204\_elevation\_of\_cytosolic\_calcium\_ion\_concentration |
| 13.GO:0055065\_metal\_ion\_homeostasis |
| 14.GO:0006875\_cellular\_metal\_ion\_homeostasis |
| 15.GO:0009605\_response\_to\_external\_stimulus |
| 16.GO:0006952\_defense\_response |
| 17.GO:0050863\_regulation\_of\_T\_cell\_activation |
| 18.GO:0050865\_regulation\_of\_cell\_activation |
| 19.GO:0002694\_regulation\_of\_leukocyte\_activation |
| 20.GO:0051249\_regulation\_of\_lymphocyte\_activation |
| 21.GO:0002696\_positive\_regulation\_of\_leukocyte\_activation |
| 22.GO:0050867\_positive\_regulation\_of\_cell\_activation |
| 23.GO:0051251\_positive\_regulation\_of\_lymphocyte\_activation |
| 24.GO:0002682\_regulation\_of\_immune\_system\_process |
| 25.GO:0002684\_positive\_regulation\_of\_immune\_system\_process |
| 26.GO:0042110\_T\_cell\_activation |
| 27.GO:0046649\_lymphocyte\_activation |
| 28.GO:0001775\_cell\_activation |
| 29.GO:0045321\_leukocyte\_activation |
| 30.GO:0045058\_T\_cell\_selection |
| 31.GO:0030217\_T\_cell\_differentiation |
| 32.GO:0030097\_hemopoiesis |
| 33.GO:0002520\_immune\_system\_development |
| 34.GO:0002521\_leukocyte\_differentiation |
| 35.GO:0030098\_lymphocyte\_differentiation |
| 36.GO:0048534\_hemopoietic\_or\_lymphoid\_organ\_development |
