# Supplementary material for: Concordance of Gene Expression and Functional Correlation Patterns across the NCI-60 Cell Lines and the Cancer Genome Atlas Glioblastoma Samples
Source: PLoS One. 2012 Jul 26;7(7):e40062. doi: 10.1371/journal.pone.0040062 (PMC3406063; doi:10.1371/journal.pone.0040062)
Supplement: Download S1 — Zip archive of HTGM results. (ZIP) [file pone.0040062.s007.zip › work2026406846/Generated_Total2026406846.dir/generic.BP.NCI60.0.6.CCR7.express.genes.correlation.complete.Thu.May.19.17.25.28.2011.htgm.txt.dir/generic.BP.NCI60.0.6.CCR7.express.genes.correlation.complete.Thu.May.19.17.25.28.2011.htgm.txt.change.gce.html]

Gene Category Report for generic.BP.NCI60.0.6.CCR7.express.genes.correlation.complete.Thu.May.19.17.25.28.2011.htgm.txt

# Gene Category Report for generic.BP.NCI60.0.6.CCR7.express.genes.correlation.complete.Thu.May.19.17.25.28.2011.htgm.txt

| HYPERLINKED GO CATEGORY | HYPERLINKED GENE NAME | TOTAL GENES | CHANGED GENES | ENRICHMENT | LOG10(p) | CUMULATIVE NUMBER OF CATEGORIES | CUMULATIVE RANDOMS MEAN | FALSE DISCOVERY RATE |
| --- | --- | --- | --- | --- | --- | --- | --- | --- |
| GO:0046649\_lymphocyte\_activation | CD3E | 119 | 7 | 32.000000 | -9.163413 | 1 | 0.0 | 0.000000 |
| GO:0046649\_lymphocyte\_activation | RHOH | 119 | 7 | 32.000000 | -9.163413 | 1 | 0.0 | 0.000000 |
| GO:0046649\_lymphocyte\_activation | CD3G | 119 | 7 | 32.000000 | -9.163413 | 1 | 0.0 | 0.000000 |
| GO:0046649\_lymphocyte\_activation | CD27 | 119 | 7 | 32.000000 | -9.163413 | 1 | 0.0 | 0.000000 |
| GO:0046649\_lymphocyte\_activation | CD1D | 119 | 7 | 32.000000 | -9.163413 | 1 | 0.0 | 0.000000 |
| GO:0046649\_lymphocyte\_activation | CD3D | 119 | 7 | 32.000000 | -9.163413 | 1 | 0.0 | 0.000000 |
| GO:0046649\_lymphocyte\_activation | SIT1 | 119 | 7 | 32.000000 | -9.163413 | 1 | 0.0 | 0.000000 |
| GO:0045321\_leukocyte\_activation | CD3E | 150 | 7 | 25.386667 | -8.454933 | 2 | 0.0 | 0.000000 |
| GO:0045321\_leukocyte\_activation | RHOH | 150 | 7 | 25.386667 | -8.454933 | 2 | 0.0 | 0.000000 |
| GO:0045321\_leukocyte\_activation | CD3G | 150 | 7 | 25.386667 | -8.454933 | 2 | 0.0 | 0.000000 |
| GO:0045321\_leukocyte\_activation | CD27 | 150 | 7 | 25.386667 | -8.454933 | 2 | 0.0 | 0.000000 |
| GO:0045321\_leukocyte\_activation | CD1D | 150 | 7 | 25.386667 | -8.454933 | 2 | 0.0 | 0.000000 |
| GO:0045321\_leukocyte\_activation | CD3D | 150 | 7 | 25.386667 | -8.454933 | 2 | 0.0 | 0.000000 |
| GO:0045321\_leukocyte\_activation | SIT1 | 150 | 7 | 25.386667 | -8.454933 | 2 | 0.0 | 0.000000 |
| GO:0042110\_T\_cell\_activation | CD3E | 83 | 6 | 39.325301 | -8.367508 | 3 | 0.0 | 0.000000 |
| GO:0042110\_T\_cell\_activation | RHOH | 83 | 6 | 39.325301 | -8.367508 | 3 | 0.0 | 0.000000 |
| GO:0042110\_T\_cell\_activation | CD3G | 83 | 6 | 39.325301 | -8.367508 | 3 | 0.0 | 0.000000 |
| GO:0042110\_T\_cell\_activation | CD1D | 83 | 6 | 39.325301 | -8.367508 | 3 | 0.0 | 0.000000 |
| GO:0042110\_T\_cell\_activation | CD3D | 83 | 6 | 39.325301 | -8.367508 | 3 | 0.0 | 0.000000 |
| GO:0042110\_T\_cell\_activation | SIT1 | 83 | 6 | 39.325301 | -8.367508 | 3 | 0.0 | 0.000000 |
| GO:0001775\_cell\_activation | CD3E | 175 | 7 | 21.760000 | -7.986845 | 4 | 0.0 | 0.000000 |
| GO:0001775\_cell\_activation | RHOH | 175 | 7 | 21.760000 | -7.986845 | 4 | 0.0 | 0.000000 |
| GO:0001775\_cell\_activation | CD3G | 175 | 7 | 21.760000 | -7.986845 | 4 | 0.0 | 0.000000 |
| GO:0001775\_cell\_activation | CD27 | 175 | 7 | 21.760000 | -7.986845 | 4 | 0.0 | 0.000000 |
| GO:0001775\_cell\_activation | CD1D | 175 | 7 | 21.760000 | -7.986845 | 4 | 0.0 | 0.000000 |
| GO:0001775\_cell\_activation | CD3D | 175 | 7 | 21.760000 | -7.986845 | 4 | 0.0 | 0.000000 |
| GO:0001775\_cell\_activation | SIT1 | 175 | 7 | 21.760000 | -7.986845 | 4 | 0.0 | 0.000000 |
| GO:0002376\_immune\_system\_process | CD3E | 718 | 10 | 7.576602 | -7.281458 | 5 | 0.0 | 0.000000 |
| GO:0002376\_immune\_system\_process | CCR4 | 718 | 10 | 7.576602 | -7.281458 | 5 | 0.0 | 0.000000 |
| GO:0002376\_immune\_system\_process | RHOH | 718 | 10 | 7.576602 | -7.281458 | 5 | 0.0 | 0.000000 |
| GO:0002376\_immune\_system\_process | CD3G | 718 | 10 | 7.576602 | -7.281458 | 5 | 0.0 | 0.000000 |
| GO:0002376\_immune\_system\_process | CD27 | 718 | 10 | 7.576602 | -7.281458 | 5 | 0.0 | 0.000000 |
| GO:0002376\_immune\_system\_process | CD1D | 718 | 10 | 7.576602 | -7.281458 | 5 | 0.0 | 0.000000 |
| GO:0002376\_immune\_system\_process | CD3D | 718 | 10 | 7.576602 | -7.281458 | 5 | 0.0 | 0.000000 |
| GO:0002376\_immune\_system\_process | IL2RG | 718 | 10 | 7.576602 | -7.281458 | 5 | 0.0 | 0.000000 |
| GO:0002376\_immune\_system\_process | SIT1 | 718 | 10 | 7.576602 | -7.281458 | 5 | 0.0 | 0.000000 |
| GO:0002376\_immune\_system\_process | WAS | 718 | 10 | 7.576602 | -7.281458 | 5 | 0.0 | 0.000000 |
| GO:0030098\_lymphocyte\_differentiation | RHOH | 50 | 4 | 43.520000 | -5.790398 | 6 | 0.01 | 0.001667 |
| GO:0030098\_lymphocyte\_differentiation | CD27 | 50 | 4 | 43.520000 | -5.790398 | 6 | 0.01 | 0.001667 |
| GO:0030098\_lymphocyte\_differentiation | CD1D | 50 | 4 | 43.520000 | -5.790398 | 6 | 0.01 | 0.001667 |
| GO:0030098\_lymphocyte\_differentiation | CD3D | 50 | 4 | 43.520000 | -5.790398 | 6 | 0.01 | 0.001667 |
| GO:0002521\_leukocyte\_differentiation | RHOH | 87 | 4 | 25.011494 | -4.822597 | 7 | 0.02 | 0.002857 |
| GO:0002521\_leukocyte\_differentiation | CD27 | 87 | 4 | 25.011494 | -4.822597 | 7 | 0.02 | 0.002857 |
| GO:0002521\_leukocyte\_differentiation | CD1D | 87 | 4 | 25.011494 | -4.822597 | 7 | 0.02 | 0.002857 |
| GO:0002521\_leukocyte\_differentiation | CD3D | 87 | 4 | 25.011494 | -4.822597 | 7 | 0.02 | 0.002857 |
| GO:0030217\_T\_cell\_differentiation | RHOH | 33 | 3 | 49.454545 | -4.576262 | 8 | 0.02 | 0.002500 |
| GO:0030217\_T\_cell\_differentiation | CD1D | 33 | 3 | 49.454545 | -4.576262 | 8 | 0.02 | 0.002500 |
| GO:0030217\_T\_cell\_differentiation | CD3D | 33 | 3 | 49.454545 | -4.576262 | 8 | 0.02 | 0.002500 |
| GO:0045058\_T\_cell\_selection | CD1D | 7 | 2 | 155.428571 | -4.181196 | 9 | 0.1 | 0.011111 |
| GO:0045058\_T\_cell\_selection | CD3D | 7 | 2 | 155.428571 | -4.181196 | 9 | 0.1 | 0.011111 |
| GO:0030097\_hemopoiesis | RHOH | 135 | 4 | 16.118519 | -4.071088 | 10 | 0.1 | 0.010000 |
| GO:0030097\_hemopoiesis | CD27 | 135 | 4 | 16.118519 | -4.071088 | 10 | 0.1 | 0.010000 |
| GO:0030097\_hemopoiesis | CD1D | 135 | 4 | 16.118519 | -4.071088 | 10 | 0.1 | 0.010000 |
| GO:0030097\_hemopoiesis | CD3D | 135 | 4 | 16.118519 | -4.071088 | 10 | 0.1 | 0.010000 |
| GO:0048534\_hemopoietic\_or\_lymphoid\_organ\_development | RHOH | 139 | 4 | 15.654676 | -4.021689 | 11 | 0.15 | 0.013636 |
| GO:0048534\_hemopoietic\_or\_lymphoid\_organ\_development | CD27 | 139 | 4 | 15.654676 | -4.021689 | 11 | 0.15 | 0.013636 |
| GO:0048534\_hemopoietic\_or\_lymphoid\_organ\_development | CD1D | 139 | 4 | 15.654676 | -4.021689 | 11 | 0.15 | 0.013636 |
| GO:0048534\_hemopoietic\_or\_lymphoid\_organ\_development | CD3D | 139 | 4 | 15.654676 | -4.021689 | 11 | 0.15 | 0.013636 |
| GO:0002520\_immune\_system\_development | RHOH | 147 | 4 | 14.802721 | -3.927221 | 12 | 0.16 | 0.013333 |
| GO:0002520\_immune\_system\_development | CD27 | 147 | 4 | 14.802721 | -3.927221 | 12 | 0.16 | 0.013333 |
| GO:0002520\_immune\_system\_development | CD1D | 147 | 4 | 14.802721 | -3.927221 | 12 | 0.16 | 0.013333 |
| GO:0002520\_immune\_system\_development | CD3D | 147 | 4 | 14.802721 | -3.927221 | 12 | 0.16 | 0.013333 |
| GO:0051249\_regulation\_of\_lymphocyte\_activation | CD3E | 60 | 3 | 27.200000 | -3.791815 | 13 | 0.16 | 0.012308 |
| GO:0051249\_regulation\_of\_lymphocyte\_activation | CD27 | 60 | 3 | 27.200000 | -3.791815 | 13 | 0.16 | 0.012308 |
| GO:0051249\_regulation\_of\_lymphocyte\_activation | SIT1 | 60 | 3 | 27.200000 | -3.791815 | 13 | 0.16 | 0.012308 |
| GO:0002694\_regulation\_of\_leukocyte\_activation | CD3E | 70 | 3 | 23.314286 | -3.592591 | 14 | 0.24 | 0.017143 |
| GO:0002694\_regulation\_of\_leukocyte\_activation | CD27 | 70 | 3 | 23.314286 | -3.592591 | 14 | 0.24 | 0.017143 |
| GO:0002694\_regulation\_of\_leukocyte\_activation | SIT1 | 70 | 3 | 23.314286 | -3.592591 | 14 | 0.24 | 0.017143 |
| GO:0050865\_regulation\_of\_cell\_activation | CD3E | 77 | 3 | 21.194805 | -3.470043 | 15 | 0.36 | 0.024000 |
| GO:0050865\_regulation\_of\_cell\_activation | CD27 | 77 | 3 | 21.194805 | -3.470043 | 15 | 0.36 | 0.024000 |
| GO:0050865\_regulation\_of\_cell\_activation | SIT1 | 77 | 3 | 21.194805 | -3.470043 | 15 | 0.36 | 0.024000 |
| GO:0002682\_regulation\_of\_immune\_system\_process | CD3E | 196 | 4 | 11.102041 | -3.446160 | 16 | 0.36 | 0.022500 |
| GO:0002682\_regulation\_of\_immune\_system\_process | CD27 | 196 | 4 | 11.102041 | -3.446160 | 16 | 0.36 | 0.022500 |
| GO:0002682\_regulation\_of\_immune\_system\_process | CD1D | 196 | 4 | 11.102041 | -3.446160 | 16 | 0.36 | 0.022500 |
| GO:0002682\_regulation\_of\_immune\_system\_process | SIT1 | 196 | 4 | 11.102041 | -3.446160 | 16 | 0.36 | 0.022500 |
| GO:0007204\_elevation\_of\_cytosolic\_calcium\_ion\_concentration | CCR4 | 80 | 3 | 20.400000 | -3.421038 | 17 | 0.41 | 0.024118 |
| GO:0007204\_elevation\_of\_cytosolic\_calcium\_ion\_concentration | CD52 | 80 | 3 | 20.400000 | -3.421038 | 17 | 0.41 | 0.024118 |
| GO:0007204\_elevation\_of\_cytosolic\_calcium\_ion\_concentration | CCR7 | 80 | 3 | 20.400000 | -3.421038 | 17 | 0.41 | 0.024118 |
| GO:0051480\_cytosolic\_calcium\_ion\_homeostasis | CCR4 | 81 | 3 | 20.148148 | -3.405128 | 18 | 0.42 | 0.023333 |
| GO:0051480\_cytosolic\_calcium\_ion\_homeostasis | CD52 | 81 | 3 | 20.148148 | -3.405128 | 18 | 0.42 | 0.023333 |
| GO:0051480\_cytosolic\_calcium\_ion\_homeostasis | CCR7 | 81 | 3 | 20.148148 | -3.405128 | 18 | 0.42 | 0.023333 |
| GO:0002684\_positive\_regulation\_of\_immune\_system\_process | CD3E | 106 | 3 | 15.396226 | -3.062812 | 19 | 0.62 | 0.032632 |
| GO:0002684\_positive\_regulation\_of\_immune\_system\_process | CD27 | 106 | 3 | 15.396226 | -3.062812 | 19 | 0.62 | 0.032632 |
| GO:0002684\_positive\_regulation\_of\_immune\_system\_process | CD1D | 106 | 3 | 15.396226 | -3.062812 | 19 | 0.62 | 0.032632 |
| GO:0006874\_cellular\_calcium\_ion\_homeostasis | CCR4 | 114 | 3 | 14.315789 | -2.970984 | 20 | 0.67 | 0.033500 |
| GO:0006874\_cellular\_calcium\_ion\_homeostasis | CD52 | 114 | 3 | 14.315789 | -2.970984 | 20 | 0.67 | 0.033500 |
| GO:0006874\_cellular\_calcium\_ion\_homeostasis | CCR7 | 114 | 3 | 14.315789 | -2.970984 | 20 | 0.67 | 0.033500 |
| GO:0055074\_calcium\_ion\_homeostasis | CCR4 | 116 | 3 | 14.068966 | -2.949086 | 21 | 0.7 | 0.033333 |
| GO:0055074\_calcium\_ion\_homeostasis | CD52 | 116 | 3 | 14.068966 | -2.949086 | 21 | 0.7 | 0.033333 |
| GO:0055074\_calcium\_ion\_homeostasis | CCR7 | 116 | 3 | 14.068966 | -2.949086 | 21 | 0.7 | 0.033333 |
| GO:0006875\_cellular\_metal\_ion\_homeostasis | CCR4 | 121 | 3 | 13.487603 | -2.896036 | 22 | 0.75 | 0.034091 |
| GO:0006875\_cellular\_metal\_ion\_homeostasis | CD52 | 121 | 3 | 13.487603 | -2.896036 | 22 | 0.75 | 0.034091 |
| GO:0006875\_cellular\_metal\_ion\_homeostasis | CCR7 | 121 | 3 | 13.487603 | -2.896036 | 22 | 0.75 | 0.034091 |
| GO:0055065\_metal\_ion\_homeostasis | CCR4 | 125 | 3 | 13.056000 | -2.855236 | 23 | 0.77 | 0.033478 |
| GO:0055065\_metal\_ion\_homeostasis | CD52 | 125 | 3 | 13.056000 | -2.855236 | 23 | 0.77 | 0.033478 |
| GO:0055065\_metal\_ion\_homeostasis | CCR7 | 125 | 3 | 13.056000 | -2.855236 | 23 | 0.77 | 0.033478 |
| GO:0002475\_antigen\_processing\_and\_presentation\_via\_MHC\_class\_Ib | CD1D | 1 | 1 |  |  |  |  |  |  |
| GO:0045579\_positive\_regulation\_of\_B\_cell\_differentiation | CD27 | 1 | 1 |  |  |  |  |  |  |
| GO:0048003\_antigen\_processing\_and\_presentation\_of\_lipid\_antigen\_via\_MHC\_class\_Ib | CD1D | 1 | 1 |  |  |  |  |  |  |
| GO:0048006\_antigen\_processing\_and\_presentation\_\_endogenous\_lipid\_antigen\_via\_MHC\_class\_Ib | CD1D | 1 | 1 |  |  |  |  |  |  |
| GO:0030005\_cellular\_di-\_\_tri-valent\_inorganic\_cation\_homeostasis | CCR4 | 140 | 3 | 11.657143 | -2.713663 | 24 | 1.02 | 0.042500 |
| GO:0030005\_cellular\_di-\_\_tri-valent\_inorganic\_cation\_homeostasis | CD52 | 140 | 3 | 11.657143 | -2.713663 | 24 | 1.02 | 0.042500 |
| GO:0030005\_cellular\_di-\_\_tri-valent\_inorganic\_cation\_homeostasis | CCR7 | 140 | 3 | 11.657143 | -2.713663 | 24 | 1.02 | 0.042500 |
| GO:0006955\_immune\_response | CCR4 | 529 | 5 | 5.141777 | -2.708594 | 25 | 1.02 | 0.040800 |
| GO:0006955\_immune\_response | CD27 | 529 | 5 | 5.141777 | -2.708594 | 25 | 1.02 | 0.040800 |
| GO:0006955\_immune\_response | CD1D | 529 | 5 | 5.141777 | -2.708594 | 25 | 1.02 | 0.040800 |
| GO:0006955\_immune\_response | IL2RG | 529 | 5 | 5.141777 | -2.708594 | 25 | 1.02 | 0.040800 |
| GO:0006955\_immune\_response | WAS | 529 | 5 | 5.141777 | -2.708594 | 25 | 1.02 | 0.040800 |
| GO:0055066\_di-\_\_tri-valent\_inorganic\_cation\_homeostasis | CCR4 | 145 | 3 | 11.255172 | -2.670023 | 26 | 1.13 | 0.043462 |
| GO:0055066\_di-\_\_tri-valent\_inorganic\_cation\_homeostasis | CD52 | 145 | 3 | 11.255172 | -2.670023 | 26 | 1.13 | 0.043462 |
| GO:0055066\_di-\_\_tri-valent\_inorganic\_cation\_homeostasis | CCR7 | 145 | 3 | 11.255172 | -2.670023 | 26 | 1.13 | 0.043462 |
| GO:0051251\_positive\_regulation\_of\_lymphocyte\_activation | CD3E | 40 | 2 | 27.200000 | -2.626541 | 27 | 1.21 | 0.044815 |
| GO:0051251\_positive\_regulation\_of\_lymphocyte\_activation | CD27 | 40 | 2 | 27.200000 | -2.626541 | 27 | 1.21 | 0.044815 |
| GO:0048869\_cellular\_developmental\_process | RHOH | 555 | 5 | 4.900901 | -2.616033 | 28 | 1.21 | 0.043214 |
| GO:0048869\_cellular\_developmental\_process | CD3G | 555 | 5 | 4.900901 | -2.616033 | 28 | 1.21 | 0.043214 |
| GO:0048869\_cellular\_developmental\_process | CD27 | 555 | 5 | 4.900901 | -2.616033 | 28 | 1.21 | 0.043214 |
| GO:0048869\_cellular\_developmental\_process | CD1D | 555 | 5 | 4.900901 | -2.616033 | 28 | 1.21 | 0.043214 |
| GO:0048869\_cellular\_developmental\_process | CD3D | 555 | 5 | 4.900901 | -2.616033 | 28 | 1.21 | 0.043214 |
| GO:0030003\_cellular\_cation\_homeostasis | CCR4 | 161 | 3 | 10.136646 | -2.540443 | 29 | 1.41 | 0.048621 |
| GO:0030003\_cellular\_cation\_homeostasis | CD52 | 161 | 3 | 10.136646 | -2.540443 | 29 | 1.41 | 0.048621 |
| GO:0030003\_cellular\_cation\_homeostasis | CCR7 | 161 | 3 | 10.136646 | -2.540443 | 29 | 1.41 | 0.048621 |
| GO:0002696\_positive\_regulation\_of\_leukocyte\_activation | CD3E | 46 | 2 | 23.652174 | -2.506460 | 30 | 1.49 | 0.049667 |
| GO:0002696\_positive\_regulation\_of\_leukocyte\_activation | CD27 | 46 | 2 | 23.652174 | -2.506460 | 30 | 1.49 | 0.049667 |
| GO:0050863\_regulation\_of\_T\_cell\_activation | CD3E | 49 | 2 | 22.204082 | -2.452376 | 32 | 1.6 | 0.050000 |
| GO:0050863\_regulation\_of\_T\_cell\_activation | SIT1 | 49 | 2 | 22.204082 | -2.452376 | 32 | 1.6 | 0.050000 |
| GO:0050867\_positive\_regulation\_of\_cell\_activation | CD3E | 49 | 2 | 22.204082 | -2.452376 | 32 | 1.6 | 0.050000 |
| GO:0050867\_positive\_regulation\_of\_cell\_activation | CD27 | 49 | 2 | 22.204082 | -2.452376 | 32 | 1.6 | 0.050000 |
| GO:0055080\_cation\_homeostasis | CCR4 | 173 | 3 | 9.433526 | -2.451988 | 33 | 1.61 | 0.048788 |
| GO:0055080\_cation\_homeostasis | CD52 | 173 | 3 | 9.433526 | -2.451988 | 33 | 1.61 | 0.048788 |
| GO:0055080\_cation\_homeostasis | CCR7 | 173 | 3 | 9.433526 | -2.451988 | 33 | 1.61 | 0.048788 |
| GO:0045059\_positive\_thymic\_T\_cell\_selection | CD3D | 2 | 1 |  |  |  |  |  |  |
| GO:0006952\_defense\_response | CCR4 | 369 | 4 | 5.897019 | -2.423141 | 34 | 1.68 | 0.049412 |
| GO:0006952\_defense\_response | CD1D | 369 | 4 | 5.897019 | -2.423141 | 34 | 1.68 | 0.049412 |
| GO:0006952\_defense\_response | WAS | 369 | 4 | 5.897019 | -2.423141 | 34 | 1.68 | 0.049412 |
| GO:0006952\_defense\_response | CCR7 | 369 | 4 | 5.897019 | -2.423141 | 34 | 1.68 | 0.049412 |
| GO:0043368\_positive\_T\_cell\_selection | CD3D | 3 | 1 |  |  |  |  |  |  |
| GO:0045061\_thymic\_T\_cell\_selection | CD3D | 3 | 1 |  |  |  |  |  |  |
| GO:0045577\_regulation\_of\_B\_cell\_differentiation | CD27 | 3 | 1 |  |  |  |  |  |  |
| GO:0006873\_cellular\_ion\_homeostasis | CCR4 | 206 | 3 | 7.922330 | -2.239192 | 35 | 2.25 | 0.064286 |
| GO:0006873\_cellular\_ion\_homeostasis | CD52 | 206 | 3 | 7.922330 | -2.239192 | 35 | 2.25 | 0.064286 |
| GO:0006873\_cellular\_ion\_homeostasis | CCR7 | 206 | 3 | 7.922330 | -2.239192 | 35 | 2.25 | 0.064286 |
| GO:0055082\_cellular\_chemical\_homeostasis | CCR4 | 208 | 3 | 7.846154 | -2.227505 | 36 | 2.31 | 0.064167 |
| GO:0055082\_cellular\_chemical\_homeostasis | CD52 | 208 | 3 | 7.846154 | -2.227505 | 36 | 2.31 | 0.064167 |
| GO:0055082\_cellular\_chemical\_homeostasis | CCR7 | 208 | 3 | 7.846154 | -2.227505 | 36 | 2.31 | 0.064167 |
| GO:0050801\_ion\_homeostasis | CCR4 | 221 | 3 | 7.384615 | -2.154407 | 37 | 2.81 | 0.075946 |
| GO:0050801\_ion\_homeostasis | CD52 | 221 | 3 | 7.384615 | -2.154407 | 37 | 2.81 | 0.075946 |
| GO:0050801\_ion\_homeostasis | CCR7 | 221 | 3 | 7.384615 | -2.154407 | 37 | 2.81 | 0.075946 |
| GO:0008588\_release\_of\_cytoplasmic\_sequestered\_NF-kappaB | CD27 | 4 | 1 |  |  |  |  |  |  |
| GO:0019725\_cellular\_homeostasis | CCR4 | 231 | 3 | 7.064935 | -2.101307 | 38 | 3.13 | 0.082368 |
| GO:0019725\_cellular\_homeostasis | CD52 | 231 | 3 | 7.064935 | -2.101307 | 38 | 3.13 | 0.082368 |
| GO:0019725\_cellular\_homeostasis | CCR7 | 231 | 3 | 7.064935 | -2.101307 | 38 | 3.13 | 0.082368 |
| GO:0048513\_organ\_development | RHOH | 741 | 5 | 3.670715 | -2.073019 | 39 | 3.28 | 0.084103 |
| GO:0048513\_organ\_development | CD27 | 741 | 5 | 3.670715 | -2.073019 | 39 | 3.28 | 0.084103 |
| GO:0048513\_organ\_development | CD1D | 741 | 5 | 3.670715 | -2.073019 | 39 | 3.28 | 0.084103 |
| GO:0048513\_organ\_development | CD3D | 741 | 5 | 3.670715 | -2.073019 | 39 | 3.28 | 0.084103 |
| GO:0048513\_organ\_development | WAS | 741 | 5 | 3.670715 | -2.073019 | 39 | 3.28 | 0.084103 |
| GO:0009605\_response\_to\_external\_stimulus | CCR4 | 464 | 4 | 4.689655 | -2.069253 | 40 | 3.37 | 0.084250 |
| GO:0009605\_response\_to\_external\_stimulus | CD1D | 464 | 4 | 4.689655 | -2.069253 | 40 | 3.37 | 0.084250 |
| GO:0009605\_response\_to\_external\_stimulus | WAS | 464 | 4 | 4.689655 | -2.069253 | 40 | 3.37 | 0.084250 |
| GO:0009605\_response\_to\_external\_stimulus | CCR7 | 464 | 4 | 4.689655 | -2.069253 | 40 | 3.37 | 0.084250 |
| GO:0019883\_antigen\_processing\_and\_presentation\_of\_endogenous\_antigen | CD1D | 6 | 1 | 90.666667 | -1.959311 | 42 | 8.45 | 0.201190 |
| GO:0042346\_positive\_regulation\_of\_NF-kappaB\_import\_into\_nucleus | CD27 | 6 | 1 | 90.666667 | -1.959311 | 42 | 8.45 | 0.201190 |
| GO:0030154\_cell\_differentiation | RHOH | 506 | 4 | 4.300395 | -1.938451 | 43 | 8.58 | 0.199535 |
| GO:0030154\_cell\_differentiation | CD27 | 506 | 4 | 4.300395 | -1.938451 | 43 | 8.58 | 0.199535 |
| GO:0030154\_cell\_differentiation | CD1D | 506 | 4 | 4.300395 | -1.938451 | 43 | 8.58 | 0.199535 |
| GO:0030154\_cell\_differentiation | CD3D | 506 | 4 | 4.300395 | -1.938451 | 43 | 8.58 | 0.199535 |
| GO:0006461\_protein\_complex\_assembly | CD3E | 273 | 3 | 5.978022 | -1.903017 | 45 | 8.72 | 0.193778 |
| GO:0006461\_protein\_complex\_assembly | CD3G | 273 | 3 | 5.978022 | -1.903017 | 45 | 8.72 | 0.193778 |
| GO:0006461\_protein\_complex\_assembly | WAS | 273 | 3 | 5.978022 | -1.903017 | 45 | 8.72 | 0.193778 |
| GO:0070271\_protein\_complex\_biogenesis | CD3E | 273 | 3 | 5.978022 | -1.903017 | 45 | 8.72 | 0.193778 |
| GO:0070271\_protein\_complex\_biogenesis | CD3G | 273 | 3 | 5.978022 | -1.903017 | 45 | 8.72 | 0.193778 |
| GO:0070271\_protein\_complex\_biogenesis | WAS | 273 | 3 | 5.978022 | -1.903017 | 45 | 8.72 | 0.193778 |
| GO:0033077\_T\_cell\_differentiation\_in\_the\_thymus | CD3D | 7 | 1 | 77.714286 | -1.892736 | 46 | 11.01 | 0.239348 |
| GO:0048878\_chemical\_homeostasis | CCR4 | 278 | 3 | 5.870504 | -1.881694 | 47 | 11.12 | 0.236596 |
| GO:0048878\_chemical\_homeostasis | CD52 | 278 | 3 | 5.870504 | -1.881694 | 47 | 11.12 | 0.236596 |
| GO:0048878\_chemical\_homeostasis | CCR7 | 278 | 3 | 5.870504 | -1.881694 | 47 | 11.12 | 0.236596 |
| GO:0009611\_response\_to\_wounding | CCR4 | 279 | 3 | 5.849462 | -1.877481 | 48 | 11.16 | 0.232500 |
| GO:0009611\_response\_to\_wounding | WAS | 279 | 3 | 5.849462 | -1.877481 | 48 | 11.16 | 0.232500 |
| GO:0009611\_response\_to\_wounding | CCR7 | 279 | 3 | 5.849462 | -1.877481 | 48 | 11.16 | 0.232500 |
| GO:0065007\_biological\_regulation | CD3E | 3971 | 12 | 1.643918 | -1.869413 | 49 | 11.19 | 0.228367 |
| GO:0065007\_biological\_regulation | CCR4 | 3971 | 12 | 1.643918 | -1.869413 | 49 | 11.19 | 0.228367 |
| GO:0065007\_biological\_regulation | CD52 | 3971 | 12 | 1.643918 | -1.869413 | 49 | 11.19 | 0.228367 |
| GO:0065007\_biological\_regulation | RHOH | 3971 | 12 | 1.643918 | -1.869413 | 49 | 11.19 | 0.228367 |
| GO:0065007\_biological\_regulation | CD3G | 3971 | 12 | 1.643918 | -1.869413 | 49 | 11.19 | 0.228367 |
| GO:0065007\_biological\_regulation | CD27 | 3971 | 12 | 1.643918 | -1.869413 | 49 | 11.19 | 0.228367 |
| GO:0065007\_biological\_regulation | CD1D | 3971 | 12 | 1.643918 | -1.869413 | 49 | 11.19 | 0.228367 |
| GO:0065007\_biological\_regulation | GRAP2 | 3971 | 12 | 1.643918 | -1.869413 | 49 | 11.19 | 0.228367 |
| GO:0065007\_biological\_regulation | IL2RG | 3971 | 12 | 1.643918 | -1.869413 | 49 | 11.19 | 0.228367 |
| GO:0065007\_biological\_regulation | SIT1 | 3971 | 12 | 1.643918 | -1.869413 | 49 | 11.19 | 0.228367 |
| GO:0065007\_biological\_regulation | WAS | 3971 | 12 | 1.643918 | -1.869413 | 49 | 11.19 | 0.228367 |
| GO:0065007\_biological\_regulation | CCR7 | 3971 | 12 | 1.643918 | -1.869413 | 49 | 11.19 | 0.228367 |
| GO:0016045\_detection\_of\_bacterium | CD1D | 8 | 1 | 68.000000 | -1.835117 | 52 | 13.67 | 0.262885 |
| GO:0043124\_negative\_regulation\_of\_I-kappaB\_kinase\_NF-kappaB\_cascade | RHOH | 8 | 1 | 68.000000 | -1.835117 | 52 | 13.67 | 0.262885 |
| GO:0046330\_positive\_regulation\_of\_JNK\_cascade | CD27 | 8 | 1 | 68.000000 | -1.835117 | 52 | 13.67 | 0.262885 |
| GO:0070304\_positive\_regulation\_of\_stress-activated\_protein\_kinase\_signaling\_pathway | CD27 | 9 | 1 | 60.444444 | -1.784336 | 53 | 15.82 | 0.298491 |
| GO:0048584\_positive\_regulation\_of\_response\_to\_stimulus | CD27 | 114 | 2 | 9.543860 | -1.743731 | 54 | 16.27 | 0.301296 |
| GO:0048584\_positive\_regulation\_of\_response\_to\_stimulus | CD1D | 114 | 2 | 9.543860 | -1.743731 | 54 | 16.27 | 0.301296 |
| GO:0042307\_positive\_regulation\_of\_protein\_import\_into\_nucleus | CD27 | 10 | 1 | 54.400000 | -1.738951 | 57 | 18.18 | 0.318947 |
| GO:0042993\_positive\_regulation\_of\_transcription\_factor\_import\_into\_nucleus | CD27 | 10 | 1 | 54.400000 | -1.738951 | 57 | 18.18 | 0.318947 |
| GO:0045621\_positive\_regulation\_of\_lymphocyte\_differentiation | CD27 | 10 | 1 | 54.400000 | -1.738951 | 57 | 18.18 | 0.318947 |
| GO:0007172\_signal\_complex\_assembly | CD3E | 11 | 1 | 49.454545 | -1.697930 | 59 | 20.38 | 0.345424 |
| GO:0050871\_positive\_regulation\_of\_B\_cell\_activation | CD27 | 11 | 1 | 49.454545 | -1.697930 | 59 | 20.38 | 0.345424 |
| GO:0048856\_anatomical\_structure\_development | RHOH | 1289 | 6 | 2.532196 | -1.671547 | 60 | 20.67 | 0.344500 |
| GO:0048856\_anatomical\_structure\_development | CD3G | 1289 | 6 | 2.532196 | -1.671547 | 60 | 20.67 | 0.344500 |
| GO:0048856\_anatomical\_structure\_development | CD27 | 1289 | 6 | 2.532196 | -1.671547 | 60 | 20.67 | 0.344500 |
| GO:0048856\_anatomical\_structure\_development | CD1D | 1289 | 6 | 2.532196 | -1.671547 | 60 | 20.67 | 0.344500 |
| GO:0048856\_anatomical\_structure\_development | CD3D | 1289 | 6 | 2.532196 | -1.671547 | 60 | 20.67 | 0.344500 |
| GO:0048856\_anatomical\_structure\_development | WAS | 1289 | 6 | 2.532196 | -1.671547 | 60 | 20.67 | 0.344500 |
| GO:0006935\_chemotaxis | CCR4 | 125 | 2 | 8.704000 | -1.668433 | 62 | 20.74 | 0.334516 |
| GO:0006935\_chemotaxis | CCR7 | 125 | 2 | 8.704000 | -1.668433 | 62 | 20.74 | 0.334516 |
| GO:0042330\_taxis | CCR4 | 125 | 2 | 8.704000 | -1.668433 | 62 | 20.74 | 0.334516 |
| GO:0042330\_taxis | CCR7 | 125 | 2 | 8.704000 | -1.668433 | 62 | 20.74 | 0.334516 |
| GO:0019882\_antigen\_processing\_and\_presentation | CD1D | 12 | 1 | 45.333333 | -1.660514 | 65 | 22.35 | 0.343846 |
| GO:0042345\_regulation\_of\_NF-kappaB\_import\_into\_nucleus | CD27 | 12 | 1 | 45.333333 | -1.660514 | 65 | 22.35 | 0.343846 |
| GO:0042348\_NF-kappaB\_import\_into\_nucleus | CD27 | 12 | 1 | 45.333333 | -1.660514 | 65 | 22.35 | 0.343846 |
| GO:0046824\_positive\_regulation\_of\_nucleocytoplasmic\_transport | CD27 | 13 | 1 | 41.846154 | -1.626124 | 66 | 24.26 | 0.367576 |
| GO:0006950\_response\_to\_stress | CCR4 | 959 | 5 | 2.836288 | -1.614593 | 67 | 24.36 | 0.363582 |
| GO:0006950\_response\_to\_stress | CD27 | 959 | 5 | 2.836288 | -1.614593 | 67 | 24.36 | 0.363582 |
| GO:0006950\_response\_to\_stress | CD1D | 959 | 5 | 2.836288 | -1.614593 | 67 | 24.36 | 0.363582 |
| GO:0006950\_response\_to\_stress | WAS | 959 | 5 | 2.836288 | -1.614593 | 67 | 24.36 | 0.363582 |
| GO:0006950\_response\_to\_stress | CCR7 | 959 | 5 | 2.836288 | -1.614593 | 67 | 24.36 | 0.363582 |
| GO:0007249\_I-kappaB\_kinase\_NF-kappaB\_cascade | RHOH | 134 | 2 | 8.119403 | -1.611939 | 68 | 24.53 | 0.360735 |
| GO:0007249\_I-kappaB\_kinase\_NF-kappaB\_cascade | CD27 | 134 | 2 | 8.119403 | -1.611939 | 68 | 24.53 | 0.360735 |
| GO:0016064\_immunoglobulin\_mediated\_immune\_response | CD27 | 14 | 1 | 38.857143 | -1.594311 | 69 | 26.16 | 0.379130 |
| GO:0007626\_locomotory\_behavior | CCR4 | 142 | 2 | 7.661972 | -1.565058 | 70 | 26.62 | 0.380286 |
| GO:0007626\_locomotory\_behavior | CCR7 | 142 | 2 | 7.661972 | -1.565058 | 70 | 26.62 | 0.380286 |
| GO:0019724\_B\_cell\_mediated\_immunity | CD27 | 15 | 1 | 36.266667 | -1.564719 | 71 | 28.45 | 0.400704 |
| GO:0065003\_macromolecular\_complex\_assembly | CD3E | 366 | 3 | 4.459016 | -1.564692 | 72 | 28.47 | 0.395417 |
| GO:0065003\_macromolecular\_complex\_assembly | CD3G | 366 | 3 | 4.459016 | -1.564692 | 72 | 28.47 | 0.395417 |
| GO:0065003\_macromolecular\_complex\_assembly | WAS | 366 | 3 | 4.459016 | -1.564692 | 72 | 28.47 | 0.395417 |
| GO:0080134\_regulation\_of\_response\_to\_stress | CD27 | 147 | 2 | 7.401361 | -1.537188 | 73 | 28.96 | 0.396712 |
| GO:0080134\_regulation\_of\_response\_to\_stress | CD1D | 147 | 2 | 7.401361 | -1.537188 | 73 | 28.96 | 0.396712 |
| GO:0009595\_detection\_of\_biotic\_stimulus | CD1D | 16 | 1 | 34.000000 | -1.537062 | 76 | 30.45 | 0.400658 |
| GO:0045730\_respiratory\_burst | CD52 | 16 | 1 | 34.000000 | -1.537062 | 76 | 30.45 | 0.400658 |
| GO:0050864\_regulation\_of\_B\_cell\_activation | CD27 | 16 | 1 | 34.000000 | -1.537062 | 76 | 30.45 | 0.400658 |
| GO:0030183\_B\_cell\_differentiation | CD27 | 18 | 1 | 30.222222 | -1.486653 | 77 | 33.24 | 0.431688 |
| GO:0042592\_homeostatic\_process | CCR4 | 397 | 3 | 4.110831 | -1.473445 | 78 | 33.41 | 0.428333 |
| GO:0042592\_homeostatic\_process | CD52 | 397 | 3 | 4.110831 | -1.473445 | 78 | 33.41 | 0.428333 |
| GO:0042592\_homeostatic\_process | CCR7 | 397 | 3 | 4.110831 | -1.473445 | 78 | 33.41 | 0.428333 |
| GO:0032388\_positive\_regulation\_of\_intracellular\_transport | CD27 | 19 | 1 | 28.631579 | -1.463543 | 80 | 34.97 | 0.437125 |
| GO:0042102\_positive\_regulation\_of\_T\_cell\_proliferation | CD3E | 19 | 1 | 28.631579 | -1.463543 | 80 | 34.97 | 0.437125 |
| GO:0045089\_positive\_regulation\_of\_innate\_immune\_response | CD1D | 20 | 1 | 27.200000 | -1.441638 | 82 | 36.94 | 0.450488 |
| GO:0045619\_regulation\_of\_lymphocyte\_differentiation | CD27 | 20 | 1 | 27.200000 | -1.441638 | 82 | 36.94 | 0.450488 |
| GO:0042990\_regulation\_of\_transcription\_factor\_import\_into\_nucleus | CD27 | 22 | 1 | 24.727273 | -1.400988 | 85 | 40.32 | 0.474353 |
| GO:0042991\_transcription\_factor\_import\_into\_nucleus | CD27 | 22 | 1 | 24.727273 | -1.400988 | 85 | 40.32 | 0.474353 |
| GO:0043410\_positive\_regulation\_of\_MAPKKK\_cascade | CD27 | 22 | 1 | 24.727273 | -1.400988 | 85 | 40.32 | 0.474353 |
| GO:0043933\_macromolecular\_complex\_subunit\_organization | CD3E | 424 | 3 | 3.849057 | -1.400542 | 86 | 40.34 | 0.469070 |
| GO:0043933\_macromolecular\_complex\_subunit\_organization | CD3G | 424 | 3 | 3.849057 | -1.400542 | 86 | 40.34 | 0.469070 |
| GO:0043933\_macromolecular\_complex\_subunit\_organization | WAS | 424 | 3 | 3.849057 | -1.400542 | 86 | 40.34 | 0.469070 |
| GO:0050671\_positive\_regulation\_of\_lymphocyte\_proliferation | CD3E | 23 | 1 | 23.652174 | -1.382054 | 88 | 41.73 | 0.474205 |
| GO:0070668\_positive\_regulation\_of\_mast\_cell\_proliferation | CD3E | 23 | 1 | 23.652174 | -1.382054 | 88 | 41.73 | 0.474205 |
| GO:0032502\_developmental\_process | CD3E | 1919 | 7 | 1.984367 | -1.379684 | 89 | 41.79 | 0.469551 |
| GO:0032502\_developmental\_process | RHOH | 1919 | 7 | 1.984367 | -1.379684 | 89 | 41.79 | 0.469551 |
| GO:0032502\_developmental\_process | CD3G | 1919 | 7 | 1.984367 | -1.379684 | 89 | 41.79 | 0.469551 |
| GO:0032502\_developmental\_process | CD27 | 1919 | 7 | 1.984367 | -1.379684 | 89 | 41.79 | 0.469551 |
| GO:0032502\_developmental\_process | CD1D | 1919 | 7 | 1.984367 | -1.379684 | 89 | 41.79 | 0.469551 |
| GO:0032502\_developmental\_process | CD3D | 1919 | 7 | 1.984367 | -1.379684 | 89 | 41.79 | 0.469551 |
| GO:0032502\_developmental\_process | WAS | 1919 | 7 | 1.984367 | -1.379684 | 89 | 41.79 | 0.469551 |
| GO:0006954\_inflammatory\_response | CCR4 | 182 | 2 | 5.978022 | -1.367143 | 90 | 41.96 | 0.466222 |
| GO:0006954\_inflammatory\_response | CCR7 | 182 | 2 | 5.978022 | -1.367143 | 90 | 41.96 | 0.466222 |
| GO:0007163\_establishment\_or\_maintenance\_of\_cell\_polarity | CD3G | 24 | 1 | 22.666667 | -1.363942 | 93 | 43.19 | 0.464409 |
| GO:0032946\_positive\_regulation\_of\_mononuclear\_cell\_proliferation | CD3E | 24 | 1 | 22.666667 | -1.363942 | 93 | 43.19 | 0.464409 |
| GO:0070665\_positive\_regulation\_of\_leukocyte\_proliferation | CD3E | 24 | 1 | 22.666667 | -1.363942 | 93 | 43.19 | 0.464409 |
| GO:0010627\_regulation\_of\_protein\_kinase\_cascade | RHOH | 184 | 2 | 5.913043 | -1.358539 | 94 | 43.25 | 0.460106 |
| GO:0010627\_regulation\_of\_protein\_kinase\_cascade | CD27 | 184 | 2 | 5.913043 | -1.358539 | 94 | 43.25 | 0.460106 |
| GO:0042129\_regulation\_of\_T\_cell\_proliferation | CD3E | 25 | 1 | 21.760000 | -1.346584 | 95 | 44.52 | 0.468632 |
| GO:0008037\_cell\_recognition | CD5 | 26 | 1 | 20.923077 | -1.329922 | 98 | 46.37 | 0.473163 |
| GO:0010741\_negative\_regulation\_of\_protein\_kinase\_cascade | RHOH | 26 | 1 | 20.923077 | -1.329922 | 98 | 46.37 | 0.473163 |
| GO:0045088\_regulation\_of\_innate\_immune\_response | CD1D | 26 | 1 | 20.923077 | -1.329922 | 98 | 46.37 | 0.473163 |
| GO:0048731\_system\_development | RHOH | 1140 | 5 | 2.385965 | -1.325027 | 99 | 46.6 | 0.470707 |
| GO:0048731\_system\_development | CD27 | 1140 | 5 | 2.385965 | -1.325027 | 99 | 46.6 | 0.470707 |
| GO:0048731\_system\_development | CD1D | 1140 | 5 | 2.385965 | -1.325027 | 99 | 46.6 | 0.470707 |
| GO:0048731\_system\_development | CD3D | 1140 | 5 | 2.385965 | -1.325027 | 99 | 46.6 | 0.470707 |
| GO:0048731\_system\_development | WAS | 1140 | 5 | 2.385965 | -1.325027 | 99 | 46.6 | 0.470707 |
| GO:0031349\_positive\_regulation\_of\_defense\_response | CD1D | 27 | 1 | 20.148148 | -1.313902 | 100 | 47.57 | 0.475700 |
| GO:0042306\_regulation\_of\_protein\_import\_into\_nucleus | CD27 | 28 | 1 | 19.428571 | -1.298479 | 103 | 49.3 | 0.478641 |
| GO:0070662\_mast\_cell\_proliferation | CD3E | 28 | 1 | 19.428571 | -1.298479 | 103 | 49.3 | 0.478641 |
| GO:0070666\_regulation\_of\_mast\_cell\_proliferation | CD3E | 28 | 1 | 19.428571 | -1.298479 | 103 | 49.3 | 0.478641 |
| GO:0042981\_regulation\_of\_apoptosis | CD3E | 471 | 3 | 3.464968 | -1.285962 | 104 | 49.58 | 0.476731 |
| GO:0042981\_regulation\_of\_apoptosis | CD3G | 471 | 3 | 3.464968 | -1.285962 | 104 | 49.58 | 0.476731 |
| GO:0042981\_regulation\_of\_apoptosis | CD27 | 471 | 3 | 3.464968 | -1.285962 | 104 | 49.58 | 0.476731 |
| GO:0043067\_regulation\_of\_programmed\_cell\_death | CD3E | 476 | 3 | 3.428571 | -1.274589 | 105 | 50.86 | 0.484381 |
| GO:0043067\_regulation\_of\_programmed\_cell\_death | CD3G | 476 | 3 | 3.428571 | -1.274589 | 105 | 50.86 | 0.484381 |
| GO:0043067\_regulation\_of\_programmed\_cell\_death | CD27 | 476 | 3 | 3.428571 | -1.274589 | 105 | 50.86 | 0.484381 |
| GO:0010941\_regulation\_of\_cell\_death | CD3E | 478 | 3 | 3.414226 | -1.270081 | 107 | 51.09 | 0.477477 |
| GO:0010941\_regulation\_of\_cell\_death | CD3G | 478 | 3 | 3.414226 | -1.270081 | 107 | 51.09 | 0.477477 |
| GO:0010941\_regulation\_of\_cell\_death | CD27 | 478 | 3 | 3.414226 | -1.270081 | 107 | 51.09 | 0.477477 |
| GO:0022607\_cellular\_component\_assembly | CD3E | 478 | 3 | 3.414226 | -1.270081 | 107 | 51.09 | 0.477477 |
| GO:0022607\_cellular\_component\_assembly | CD3G | 478 | 3 | 3.414226 | -1.270081 | 107 | 51.09 | 0.477477 |
| GO:0022607\_cellular\_component\_assembly | WAS | 478 | 3 | 3.414226 | -1.270081 | 107 | 51.09 | 0.477477 |
| GO:0042098\_T\_cell\_proliferation | CD3E | 30 | 1 | 18.133333 | -1.269257 | 109 | 51.84 | 0.475596 |
| GO:0050670\_regulation\_of\_lymphocyte\_proliferation | CD3E | 30 | 1 | 18.133333 | -1.269257 | 109 | 51.84 | 0.475596 |
| GO:0007165\_signal\_transduction | CD3E | 2029 | 7 | 1.876787 | -1.259776 | 110 | 52.01 | 0.472818 |
| GO:0007165\_signal\_transduction | RHOH | 2029 | 7 | 1.876787 | -1.259776 | 110 | 52.01 | 0.472818 |
| GO:0007165\_signal\_transduction | CD3G | 2029 | 7 | 1.876787 | -1.259776 | 110 | 52.01 | 0.472818 |
| GO:0007165\_signal\_transduction | CD27 | 2029 | 7 | 1.876787 | -1.259776 | 110 | 52.01 | 0.472818 |
| GO:0007165\_signal\_transduction | GRAP2 | 2029 | 7 | 1.876787 | -1.259776 | 110 | 52.01 | 0.472818 |
| GO:0007165\_signal\_transduction | IL2RG | 2029 | 7 | 1.876787 | -1.259776 | 110 | 52.01 | 0.472818 |
| GO:0007165\_signal\_transduction | SIT1 | 2029 | 7 | 1.876787 | -1.259776 | 110 | 52.01 | 0.472818 |
| GO:0032944\_regulation\_of\_mononuclear\_cell\_proliferation | CD3E | 31 | 1 | 17.548387 | -1.255387 | 112 | 52.95 | 0.472768 |
| GO:0070663\_regulation\_of\_leukocyte\_proliferation | CD3E | 31 | 1 | 17.548387 | -1.255387 | 112 | 52.95 | 0.472768 |
| GO:0033157\_regulation\_of\_intracellular\_protein\_transport | CD27 | 32 | 1 | 17.000000 | -1.241969 | 113 | 54.19 | 0.479558 |
| GO:0007610\_behavior | CCR4 | 214 | 2 | 5.084112 | -1.240733 | 114 | 54.22 | 0.475614 |
| GO:0007610\_behavior | CCR7 | 214 | 2 | 5.084112 | -1.240733 | 114 | 54.22 | 0.475614 |
| GO:0009581\_detection\_of\_external\_stimulus | CD1D | 33 | 1 | 16.484848 | -1.228975 | 116 | 55.23 | 0.476121 |
| GO:0050870\_positive\_regulation\_of\_T\_cell\_activation | CD3E | 33 | 1 | 16.484848 | -1.228975 | 116 | 55.23 | 0.476121 |
| GO:0002449\_lymphocyte\_mediated\_immunity | CD27 | 34 | 1 | 16.000000 | -1.216381 | 117 | 57.14 | 0.488376 |
| GO:0065008\_regulation\_of\_biological\_quality | CCR4 | 848 | 4 | 2.566038 | -1.205266 | 118 | 57.37 | 0.486186 |
| GO:0065008\_regulation\_of\_biological\_quality | CD52 | 848 | 4 | 2.566038 | -1.205266 | 118 | 57.37 | 0.486186 |
| GO:0065008\_regulation\_of\_biological\_quality | WAS | 848 | 4 | 2.566038 | -1.205266 | 118 | 57.37 | 0.486186 |
| GO:0065008\_regulation\_of\_biological\_quality | CCR7 | 848 | 4 | 2.566038 | -1.205266 | 118 | 57.37 | 0.486186 |
| GO:0002443\_leukocyte\_mediated\_immunity | CD27 | 37 | 1 | 14.702703 | -1.180768 | 121 | 60.26 | 0.498017 |
| GO:0002460\_adaptive\_immune\_response\_based\_on\_somatic\_recombination\_of\_immune\_receptors\_built\_from\_immunoglobulin\_superfamily\_domains | CD27 | 37 | 1 | 14.702703 | -1.180768 | 121 | 60.26 | 0.498017 |
| GO:0046822\_regulation\_of\_nucleocytoplasmic\_transport | CD27 | 37 | 1 | 14.702703 | -1.180768 | 121 | 60.26 | 0.498017 |
| GO:0002250\_adaptive\_immune\_response | CD27 | 38 | 1 | 14.315789 | -1.169556 | 123 | 61.55 | 0.500407 |
| GO:0042113\_B\_cell\_activation | CD27 | 38 | 1 | 14.315789 | -1.169556 | 123 | 61.55 | 0.500407 |
| GO:0046328\_regulation\_of\_JNK\_cascade | CD27 | 39 | 1 | 13.948718 | -1.158645 | 124 | 62.42 | 0.503387 |
| GO:0048583\_regulation\_of\_response\_to\_stimulus | CD27 | 241 | 2 | 4.514523 | -1.149624 | 125 | 62.59 | 0.500720 |
| GO:0048583\_regulation\_of\_response\_to\_stimulus | CD1D | 241 | 2 | 4.514523 | -1.149624 | 125 | 62.59 | 0.500720 |
| GO:0070302\_regulation\_of\_stress-activated\_protein\_kinase\_signaling\_pathway | CD27 | 40 | 1 | 13.600000 | -1.148020 | 126 | 63.21 | 0.501667 |
| GO:0046651\_lymphocyte\_proliferation | CD3E | 41 | 1 | 13.268293 | -1.137666 | 128 | 64.11 | 0.500859 |
| GO:0051222\_positive\_regulation\_of\_protein\_transport | CD27 | 41 | 1 | 13.268293 | -1.137666 | 128 | 64.11 | 0.500859 |
| GO:0032943\_mononuclear\_cell\_proliferation | CD3E | 42 | 1 | 12.952381 | -1.127570 | 130 | 65.48 | 0.503692 |
| GO:0070661\_leukocyte\_proliferation | CD3E | 42 | 1 | 12.952381 | -1.127570 | 130 | 65.48 | 0.503692 |
| GO:0010926\_anatomical\_structure\_formation | CD3E | 560 | 3 | 2.914286 | -1.103006 | 132 | 67.42 | 0.510758 |
| GO:0010926\_anatomical\_structure\_formation | CD3G | 560 | 3 | 2.914286 | -1.103006 | 132 | 67.42 | 0.510758 |
| GO:0010926\_anatomical\_structure\_formation | WAS | 560 | 3 | 2.914286 | -1.103006 | 132 | 67.42 | 0.510758 |
| GO:0044085\_cellular\_component\_biogenesis | CD3E | 560 | 3 | 2.914286 | -1.103006 | 132 | 67.42 | 0.510758 |
| GO:0044085\_cellular\_component\_biogenesis | CD3G | 560 | 3 | 2.914286 | -1.103006 | 132 | 67.42 | 0.510758 |
| GO:0044085\_cellular\_component\_biogenesis | WAS | 560 | 3 | 2.914286 | -1.103006 | 132 | 67.42 | 0.510758 |
| GO:0006915\_apoptosis | CD3E | 565 | 3 | 2.888496 | -1.093819 | 133 | 68.27 | 0.513308 |
| GO:0006915\_apoptosis | CD3G | 565 | 3 | 2.888496 | -1.093819 | 133 | 68.27 | 0.513308 |
| GO:0006915\_apoptosis | CD27 | 565 | 3 | 2.888496 | -1.093819 | 133 | 68.27 | 0.513308 |
| GO:0012501\_programmed\_cell\_death | CD3E | 571 | 3 | 2.858144 | -1.082930 | 134 | 69.62 | 0.519552 |
| GO:0012501\_programmed\_cell\_death | CD3G | 571 | 3 | 2.858144 | -1.082930 | 134 | 69.62 | 0.519552 |
| GO:0012501\_programmed\_cell\_death | CD27 | 571 | 3 | 2.858144 | -1.082930 | 134 | 69.62 | 0.519552 |
| GO:0032386\_regulation\_of\_intracellular\_transport | CD27 | 47 | 1 | 11.574468 | -1.080569 | 135 | 70.78 | 0.524296 |
| GO:0050896\_response\_to\_stimulus | CCR4 | 1775 | 6 | 1.838873 | -1.064329 | 136 | 71.57 | 0.526250 |
| GO:0050896\_response\_to\_stimulus | CD27 | 1775 | 6 | 1.838873 | -1.064329 | 136 | 71.57 | 0.526250 |
| GO:0050896\_response\_to\_stimulus | CD1D | 1775 | 6 | 1.838873 | -1.064329 | 136 | 71.57 | 0.526250 |
| GO:0050896\_response\_to\_stimulus | IL2RG | 1775 | 6 | 1.838873 | -1.064329 | 136 | 71.57 | 0.526250 |
| GO:0050896\_response\_to\_stimulus | WAS | 1775 | 6 | 1.838873 | -1.064329 | 136 | 71.57 | 0.526250 |
| GO:0050896\_response\_to\_stimulus | CCR7 | 1775 | 6 | 1.838873 | -1.064329 | 136 | 71.57 | 0.526250 |
| GO:0008219\_cell\_death | CD3E | 585 | 3 | 2.789744 | -1.058079 | 138 | 73.02 | 0.529130 |
| GO:0008219\_cell\_death | CD3G | 585 | 3 | 2.789744 | -1.058079 | 138 | 73.02 | 0.529130 |
| GO:0008219\_cell\_death | CD27 | 585 | 3 | 2.789744 | -1.058079 | 138 | 73.02 | 0.529130 |
| GO:0016265\_death | CD3E | 585 | 3 | 2.789744 | -1.058079 | 138 | 73.02 | 0.529130 |
| GO:0016265\_death | CD3G | 585 | 3 | 2.789744 | -1.058079 | 138 | 73.02 | 0.529130 |
| GO:0016265\_death | CD27 | 585 | 3 | 2.789744 | -1.058079 | 138 | 73.02 | 0.529130 |
| GO:0015031\_protein\_transport | CD3G | 274 | 2 | 3.970803 | -1.052978 | 139 | 73.98 | 0.532230 |
| GO:0015031\_protein\_transport | CD27 | 274 | 2 | 3.970803 | -1.052978 | 139 | 73.98 | 0.532230 |
| GO:0045184\_establishment\_of\_protein\_localization | CD3G | 279 | 2 | 3.899642 | -1.039518 | 140 | 75.6 | 0.540000 |
| GO:0045184\_establishment\_of\_protein\_localization | CD27 | 279 | 2 | 3.899642 | -1.039518 | 140 | 75.6 | 0.540000 |
| GO:0007275\_multicellular\_organismal\_development | RHOH | 1372 | 5 | 1.982507 | -1.034566 | 141 | 76.11 | 0.539787 |
| GO:0007275\_multicellular\_organismal\_development | CD27 | 1372 | 5 | 1.982507 | -1.034566 | 141 | 76.11 | 0.539787 |
| GO:0007275\_multicellular\_organismal\_development | CD1D | 1372 | 5 | 1.982507 | -1.034566 | 141 | 76.11 | 0.539787 |
| GO:0007275\_multicellular\_organismal\_development | CD3D | 1372 | 5 | 1.982507 | -1.034566 | 141 | 76.11 | 0.539787 |
| GO:0007275\_multicellular\_organismal\_development | WAS | 1372 | 5 | 1.982507 | -1.034566 | 141 | 76.11 | 0.539787 |
| GO:0009617\_response\_to\_bacterium | CD1D | 53 | 1 | 10.264151 | -1.030605 | 142 | 77.09 | 0.542887 |
| GO:0007154\_cell\_communication | CD3E | 2272 | 7 | 1.676056 | -1.027338 | 143 | 77.25 | 0.540210 |
| GO:0007154\_cell\_communication | RHOH | 2272 | 7 | 1.676056 | -1.027338 | 143 | 77.25 | 0.540210 |
| GO:0007154\_cell\_communication | CD3G | 2272 | 7 | 1.676056 | -1.027338 | 143 | 77.25 | 0.540210 |
| GO:0007154\_cell\_communication | CD27 | 2272 | 7 | 1.676056 | -1.027338 | 143 | 77.25 | 0.540210 |
| GO:0007154\_cell\_communication | GRAP2 | 2272 | 7 | 1.676056 | -1.027338 | 143 | 77.25 | 0.540210 |
| GO:0007154\_cell\_communication | IL2RG | 2272 | 7 | 1.676056 | -1.027338 | 143 | 77.25 | 0.540210 |
| GO:0007154\_cell\_communication | SIT1 | 2272 | 7 | 1.676056 | -1.027338 | 143 | 77.25 | 0.540210 |
| GO:0050778\_positive\_regulation\_of\_immune\_response | CD1D | 56 | 1 | 9.714286 | -1.007799 | 144 | 79.53 | 0.552292 |
| GO:0040011\_locomotion | CCR4 | 292 | 2 | 3.726027 | -1.005806 | 145 | 79.77 | 0.550138 |
| GO:0040011\_locomotion | CCR7 | 292 | 2 | 3.726027 | -1.005806 | 145 | 79.77 | 0.550138 |
| GO:0043408\_regulation\_of\_MAPKKK\_cascade | CD27 | 59 | 1 | 9.220339 | -0.986240 | 146 | 82.58 | 0.565616 |
| GO:0007254\_JNK\_cascade | CD27 | 61 | 1 | 8.918033 | -0.972499 | 148 | 84.24 | 0.569189 |
| GO:0031347\_regulation\_of\_defense\_response | CD1D | 61 | 1 | 8.918033 | -0.972499 | 148 | 84.24 | 0.569189 |
| GO:0031098\_stress-activated\_protein\_kinase\_signaling\_pathway | CD27 | 64 | 1 | 8.500000 | -0.952753 | 149 | 87.37 | 0.586376 |
| GO:0080135\_regulation\_of\_cellular\_response\_to\_stress | CD27 | 65 | 1 | 8.369231 | -0.946387 | 150 | 88.18 | 0.587867 |
| GO:0006606\_protein\_import\_into\_nucleus | CD27 | 68 | 1 | 8.000000 | -0.927895 | 151 | 90.98 | 0.602517 |
| GO:0050793\_regulation\_of\_developmental\_process | CD3E | 669 | 3 | 2.439462 | -0.923561 | 152 | 91.39 | 0.601250 |
| GO:0050793\_regulation\_of\_developmental\_process | CD3G | 669 | 3 | 2.439462 | -0.923561 | 152 | 91.39 | 0.601250 |
| GO:0050793\_regulation\_of\_developmental\_process | CD27 | 669 | 3 | 2.439462 | -0.923561 | 152 | 91.39 | 0.601250 |
| GO:0051170\_nuclear\_import | CD27 | 70 | 1 | 7.771429 | -0.916041 | 153 | 92.84 | 0.606797 |
| GO:0051223\_regulation\_of\_protein\_transport | CD27 | 71 | 1 | 7.661972 | -0.910248 | 155 | 93.64 | 0.604129 |
| GO:0051606\_detection\_of\_stimulus | CD1D | 71 | 1 | 7.661972 | -0.910248 | 155 | 93.64 | 0.604129 |
| GO:0007596\_blood\_coagulation | WAS | 73 | 1 | 7.452055 | -0.898918 | 156 | 94.87 | 0.608141 |
| GO:0008104\_protein\_localization | CD3G | 339 | 2 | 3.209440 | -0.897306 | 157 | 95.01 | 0.605159 |
| GO:0008104\_protein\_localization | CD27 | 339 | 2 | 3.209440 | -0.897306 | 157 | 95.01 | 0.605159 |
| GO:0045597\_positive\_regulation\_of\_cell\_differentiation | CD27 | 74 | 1 | 7.351351 | -0.893376 | 159 | 95.4 | 0.600000 |
| GO:0050817\_coagulation | WAS | 74 | 1 | 7.351351 | -0.893376 | 159 | 95.4 | 0.600000 |
| GO:0070201\_regulation\_of\_establishment\_of\_protein\_localization | CD27 | 75 | 1 | 7.253333 | -0.887914 | 160 | 96.13 | 0.600812 |
| GO:0034504\_protein\_localization\_in\_nucleus | CD27 | 76 | 1 | 7.157895 | -0.882529 | 161 | 97.12 | 0.603230 |
| GO:0007599\_hemostasis | WAS | 79 | 1 | 6.886076 | -0.866816 | 163 | 99.02 | 0.607485 |
| GO:0032880\_regulation\_of\_protein\_localization | CD27 | 79 | 1 | 6.886076 | -0.866816 | 163 | 99.02 | 0.607485 |
| GO:0045087\_innate\_immune\_response | CD1D | 82 | 1 | 6.634146 | -0.851729 | 164 | 101.25 | 0.617378 |
| GO:0007243\_protein\_kinase\_cascade | RHOH | 377 | 2 | 2.885942 | -0.822100 | 165 | 104.65 | 0.634242 |
| GO:0007243\_protein\_kinase\_cascade | CD27 | 377 | 2 | 2.885942 | -0.822100 | 165 | 104.65 | 0.634242 |
| GO:0009966\_regulation\_of\_signal\_transduction | RHOH | 378 | 2 | 2.878307 | -0.820248 | 166 | 104.83 | 0.631506 |
| GO:0009966\_regulation\_of\_signal\_transduction | CD27 | 378 | 2 | 2.878307 | -0.820248 | 166 | 104.83 | 0.631506 |
| GO:0033036\_macromolecule\_localization | CD3G | 388 | 2 | 2.804124 | -0.802058 | 167 | 107.28 | 0.642395 |
| GO:0033036\_macromolecule\_localization | CD27 | 388 | 2 | 2.804124 | -0.802058 | 167 | 107.28 | 0.642395 |
| GO:0017038\_protein\_import | CD27 | 93 | 1 | 5.849462 | -0.801087 | 169 | 107.66 | 0.637041 |
| GO:0043122\_regulation\_of\_I-kappaB\_kinase\_NF-kappaB\_cascade | RHOH | 93 | 1 | 5.849462 | -0.801087 | 169 | 107.66 | 0.637041 |
| GO:0033365\_protein\_localization\_in\_organelle | CD27 | 95 | 1 | 5.726316 | -0.792577 | 171 | 108.22 | 0.632865 |
| GO:0050878\_regulation\_of\_body\_fluid\_levels | WAS | 95 | 1 | 5.726316 | -0.792577 | 171 | 108.22 | 0.632865 |
| GO:0002252\_immune\_effector\_process | CD27 | 96 | 1 | 5.666667 | -0.788395 | 172 | 109.2 | 0.634884 |
| GO:0042060\_wound\_healing | WAS | 98 | 1 | 5.551020 | -0.780171 | 173 | 110.22 | 0.637110 |
| GO:0009968\_negative\_regulation\_of\_signal\_transduction | RHOH | 99 | 1 | 5.494949 | -0.776127 | 174 | 110.69 | 0.636149 |
| GO:0050776\_regulation\_of\_immune\_response | CD1D | 100 | 1 | 5.440000 | -0.772127 | 175 | 111.51 | 0.637200 |
| GO:0043623\_cellular\_protein\_complex\_assembly | CD3E | 101 | 1 | 5.386139 | -0.768171 | 176 | 111.95 | 0.636080 |
| GO:0010648\_negative\_regulation\_of\_cell\_communication | RHOH | 102 | 1 | 5.333333 | -0.764257 | 177 | 112.13 | 0.633503 |
| GO:0008544\_epidermis\_development | WAS | 104 | 1 | 5.230769 | -0.756553 | 178 | 112.97 | 0.634663 |
| GO:0010646\_regulation\_of\_cell\_communication | RHOH | 423 | 2 | 2.572104 | -0.742743 | 179 | 113.74 | 0.635419 |
| GO:0010646\_regulation\_of\_cell\_communication | CD27 | 423 | 2 | 2.572104 | -0.742743 | 179 | 113.74 | 0.635419 |
| GO:0007265\_Ras\_protein\_signal\_transduction | GRAP2 | 110 | 1 | 4.945455 | -0.734381 | 180 | 116.06 | 0.644778 |
| GO:0007398\_ectoderm\_development | WAS | 112 | 1 | 4.857143 | -0.727284 | 181 | 116.98 | 0.646298 |
| GO:0007166\_cell\_surface\_receptor\_linked\_signal\_transduction | CD3E | 828 | 3 | 1.971014 | -0.722117 | 182 | 117.39 | 0.645000 |
| GO:0007166\_cell\_surface\_receptor\_linked\_signal\_transduction | CD3G | 828 | 3 | 1.971014 | -0.722117 | 182 | 117.39 | 0.645000 |
| GO:0007166\_cell\_surface\_receptor\_linked\_signal\_transduction | CD27 | 828 | 3 | 1.971014 | -0.722117 | 182 | 117.39 | 0.645000 |
| GO:0051050\_positive\_regulation\_of\_transport | CD27 | 116 | 1 | 4.689655 | -0.713500 | 183 | 118.93 | 0.649891 |
| GO:0006913\_nucleocytoplasmic\_transport | CD27 | 121 | 1 | 4.495868 | -0.696991 | 184 | 120.79 | 0.656467 |
| GO:0007242\_intracellular\_signaling\_cascade | RHOH | 853 | 3 | 1.913247 | -0.695369 | 185 | 120.92 | 0.653622 |
| GO:0007242\_intracellular\_signaling\_cascade | CD27 | 853 | 3 | 1.913247 | -0.695369 | 185 | 120.92 | 0.653622 |
| GO:0007242\_intracellular\_signaling\_cascade | GRAP2 | 853 | 3 | 1.913247 | -0.695369 | 185 | 120.92 | 0.653622 |
| GO:0051169\_nuclear\_transport | CD27 | 122 | 1 | 4.459016 | -0.693780 | 187 | 121.93 | 0.652032 |
| GO:0060341\_regulation\_of\_cellular\_localization | CD27 | 122 | 1 | 4.459016 | -0.693780 | 187 | 121.93 | 0.652032 |
| GO:0051707\_response\_to\_other\_organism | CD1D | 125 | 1 | 4.352000 | -0.684319 | 188 | 123.76 | 0.658298 |
| GO:0009987\_cellular\_process | CD3E | 6671 | 14 | 1.141658 | -0.674738 | 189 | 124.91 | 0.660899 |
| GO:0009987\_cellular\_process | CD52 | 6671 | 14 | 1.141658 | -0.674738 | 189 | 124.91 | 0.660899 |
| GO:0009987\_cellular\_process | CD72 | 6671 | 14 | 1.141658 | -0.674738 | 189 | 124.91 | 0.660899 |
| GO:0009987\_cellular\_process | GRAP2 | 6671 | 14 | 1.141658 | -0.674738 | 189 | 124.91 | 0.660899 |
| GO:0009987\_cellular\_process | SIT1 | 6671 | 14 | 1.141658 | -0.674738 | 189 | 124.91 | 0.660899 |
| GO:0009987\_cellular\_process | IL2RG | 6671 | 14 | 1.141658 | -0.674738 | 189 | 124.91 | 0.660899 |
| GO:0009987\_cellular\_process | CCR7 | 6671 | 14 | 1.141658 | -0.674738 | 189 | 124.91 | 0.660899 |
| GO:0009987\_cellular\_process | CCR4 | 6671 | 14 | 1.141658 | -0.674738 | 189 | 124.91 | 0.660899 |
| GO:0009987\_cellular\_process | RHOH | 6671 | 14 | 1.141658 | -0.674738 | 189 | 124.91 | 0.660899 |
| GO:0009987\_cellular\_process | CD5 | 6671 | 14 | 1.141658 | -0.674738 | 189 | 124.91 | 0.660899 |
| GO:0009987\_cellular\_process | CD3G | 6671 | 14 | 1.141658 | -0.674738 | 189 | 124.91 | 0.660899 |
| GO:0009987\_cellular\_process | CD27 | 6671 | 14 | 1.141658 | -0.674738 | 189 | 124.91 | 0.660899 |
| GO:0009987\_cellular\_process | CD1D | 6671 | 14 | 1.141658 | -0.674738 | 189 | 124.91 | 0.660899 |
| GO:0009987\_cellular\_process | CD3D | 6671 | 14 | 1.141658 | -0.674738 | 189 | 124.91 | 0.660899 |
| GO:0010740\_positive\_regulation\_of\_protein\_kinase\_cascade | CD27 | 129 | 1 | 4.217054 | -0.672091 | 190 | 125.92 | 0.662737 |
| GO:0007264\_small\_GTPase\_mediated\_signal\_transduction | GRAP2 | 135 | 1 | 4.029630 | -0.654523 | 191 | 131.04 | 0.686073 |
| GO:0000165\_MAPKKK\_cascade | CD27 | 143 | 1 | 3.804196 | -0.632416 | 192 | 134.04 | 0.698125 |
| GO:0000902\_cell\_morphogenesis | CD3G | 144 | 1 | 3.777778 | -0.629751 | 193 | 134.9 | 0.698964 |
| GO:0006605\_protein\_targeting | CD27 | 145 | 1 | 3.751724 | -0.627107 | 194 | 135.86 | 0.700309 |
| GO:0006916\_anti-apoptosis | CD27 | 155 | 1 | 3.509677 | -0.601754 | 195 | 139.98 | 0.717846 |
| GO:0007169\_transmembrane\_receptor\_protein\_tyrosine\_kinase\_signaling\_pathway | CD3E | 157 | 1 | 3.464968 | -0.596908 | 196 | 141.05 | 0.719643 |
| GO:0032989\_cellular\_component\_morphogenesis | CD3G | 164 | 1 | 3.317073 | -0.580485 | 197 | 142.57 | 0.723706 |
| GO:0045595\_regulation\_of\_cell\_differentiation | CD27 | 170 | 1 | 3.200000 | -0.567038 | 198 | 143.82 | 0.726364 |
| GO:0009607\_response\_to\_biotic\_stimulus | CD1D | 177 | 1 | 3.073446 | -0.552028 | 199 | 147.41 | 0.740754 |
| GO:0009967\_positive\_regulation\_of\_signal\_transduction | CD27 | 185 | 1 | 2.940541 | -0.535698 | 200 | 149.91 | 0.749550 |
| GO:0034622\_cellular\_macromolecular\_complex\_assembly | CD3E | 186 | 1 | 2.924731 | -0.533715 | 201 | 150.61 | 0.749303 |
| GO:0010647\_positive\_regulation\_of\_cell\_communication | CD27 | 189 | 1 | 2.878307 | -0.527840 | 202 | 151.47 | 0.749851 |
| GO:0006917\_induction\_of\_apoptosis | CD27 | 190 | 1 | 2.863158 | -0.525906 | 203 | 151.98 | 0.748670 |
| GO:0012502\_induction\_of\_programmed\_cell\_death | CD27 | 191 | 1 | 2.848168 | -0.523984 | 204 | 152.27 | 0.746422 |
| GO:0008284\_positive\_regulation\_of\_cell\_proliferation | CD3E | 200 | 1 | 2.720000 | -0.507204 | 205 | 155.42 | 0.758146 |
| GO:0006886\_intracellular\_protein\_transport | CD27 | 204 | 1 | 2.666667 | -0.500031 | 206 | 156.92 | 0.761748 |
| GO:0043066\_negative\_regulation\_of\_apoptosis | CD27 | 207 | 1 | 2.628019 | -0.494760 | 207 | 158.4 | 0.765217 |
| GO:0043069\_negative\_regulation\_of\_programmed\_cell\_death | CD27 | 209 | 1 | 2.602871 | -0.491297 | 209 | 159.43 | 0.762823 |
| GO:0060548\_negative\_regulation\_of\_cell\_death | CD27 | 209 | 1 | 2.602871 | -0.491297 | 209 | 159.43 | 0.762823 |
| GO:0042221\_response\_to\_chemical\_stimulus | CCR4 | 631 | 2 | 1.724247 | -0.487998 | 210 | 159.85 | 0.761190 |
| GO:0042221\_response\_to\_chemical\_stimulus | CCR7 | 631 | 2 | 1.724247 | -0.487998 | 210 | 159.85 | 0.761190 |
| GO:0048518\_positive\_regulation\_of\_biological\_process | CD3E | 1094 | 3 | 1.491773 | -0.486737 | 211 | 160.21 | 0.759289 |
| GO:0048518\_positive\_regulation\_of\_biological\_process | CD27 | 1094 | 3 | 1.491773 | -0.486737 | 211 | 160.21 | 0.759289 |
| GO:0048518\_positive\_regulation\_of\_biological\_process | CD1D | 1094 | 3 | 1.491773 | -0.486737 | 211 | 160.21 | 0.759289 |
| GO:0032501\_multicellular\_organismal\_process | RHOH | 2082 | 5 | 1.306436 | -0.480779 | 212 | 162.15 | 0.764858 |
| GO:0032501\_multicellular\_organismal\_process | CD27 | 2082 | 5 | 1.306436 | -0.480779 | 212 | 162.15 | 0.764858 |
| GO:0032501\_multicellular\_organismal\_process | CD1D | 2082 | 5 | 1.306436 | -0.480779 | 212 | 162.15 | 0.764858 |
| GO:0032501\_multicellular\_organismal\_process | CD3D | 2082 | 5 | 1.306436 | -0.480779 | 212 | 162.15 | 0.764858 |
| GO:0032501\_multicellular\_organismal\_process | WAS | 2082 | 5 | 1.306436 | -0.480779 | 212 | 162.15 | 0.764858 |
| GO:0008283\_cell\_proliferation | CD3E | 647 | 2 | 1.681607 | -0.473314 | 213 | 162.91 | 0.764836 |
| GO:0008283\_cell\_proliferation | CD5 | 647 | 2 | 1.681607 | -0.473314 | 213 | 162.91 | 0.764836 |
| GO:0050789\_regulation\_of\_biological\_process | CD3E | 3649 | 8 | 1.192656 | -0.470900 | 214 | 163.47 | 0.763879 |
| GO:0050789\_regulation\_of\_biological\_process | RHOH | 3649 | 8 | 1.192656 | -0.470900 | 214 | 163.47 | 0.763879 |
| GO:0050789\_regulation\_of\_biological\_process | CD3G | 3649 | 8 | 1.192656 | -0.470900 | 214 | 163.47 | 0.763879 |
| GO:0050789\_regulation\_of\_biological\_process | CD27 | 3649 | 8 | 1.192656 | -0.470900 | 214 | 163.47 | 0.763879 |
| GO:0050789\_regulation\_of\_biological\_process | CD1D | 3649 | 8 | 1.192656 | -0.470900 | 214 | 163.47 | 0.763879 |
| GO:0050789\_regulation\_of\_biological\_process | GRAP2 | 3649 | 8 | 1.192656 | -0.470900 | 214 | 163.47 | 0.763879 |
| GO:0050789\_regulation\_of\_biological\_process | IL2RG | 3649 | 8 | 1.192656 | -0.470900 | 214 | 163.47 | 0.763879 |
| GO:0050789\_regulation\_of\_biological\_process | SIT1 | 3649 | 8 | 1.192656 | -0.470900 | 214 | 163.47 | 0.763879 |
| GO:0034621\_cellular\_macromolecular\_complex\_subunit\_organization | CD3E | 227 | 1 | 2.396476 | -0.461817 | 216 | 165.05 | 0.764120 |
| GO:0051049\_regulation\_of\_transport | CD27 | 227 | 1 | 2.396476 | -0.461817 | 216 | 165.05 | 0.764120 |
| GO:0034613\_cellular\_protein\_localization | CD27 | 228 | 1 | 2.385965 | -0.460262 | 217 | 165.33 | 0.761889 |
| GO:0070727\_cellular\_macromolecule\_localization | CD27 | 229 | 1 | 2.375546 | -0.458716 | 218 | 165.62 | 0.759725 |
| GO:0051704\_multi-organism\_process | CD1D | 231 | 1 | 2.354978 | -0.455649 | 219 | 166.61 | 0.760776 |
| GO:0043065\_positive\_regulation\_of\_apoptosis | CD27 | 243 | 1 | 2.238683 | -0.437902 | 220 | 169.27 | 0.769409 |
| GO:0043068\_positive\_regulation\_of\_programmed\_cell\_death | CD27 | 246 | 1 | 2.211382 | -0.433633 | 221 | 170.84 | 0.773032 |
| GO:0010942\_positive\_regulation\_of\_cell\_death | CD27 | 250 | 1 | 2.176000 | -0.428040 | 222 | 171.64 | 0.773153 |
| GO:0007167\_enzyme\_linked\_receptor\_protein\_signaling\_pathway | CD3E | 258 | 1 | 2.108527 | -0.417180 | 223 | 172.61 | 0.774036 |
| GO:0009888\_tissue\_development | WAS | 287 | 1 | 1.895470 | -0.381096 | 224 | 180.07 | 0.803884 |
| GO:0051093\_negative\_regulation\_of\_developmental\_process | CD27 | 290 | 1 | 1.875862 | -0.377628 | 225 | 181.42 | 0.806311 |
| GO:0032879\_regulation\_of\_localization | CD27 | 326 | 1 | 1.668712 | -0.339335 | 226 | 189.14 | 0.836903 |
| GO:0016043\_cellular\_component\_organization | CD3E | 1366 | 3 | 1.194729 | -0.326989 | 227 | 191.03 | 0.841542 |
| GO:0016043\_cellular\_component\_organization | CD3G | 1366 | 3 | 1.194729 | -0.326989 | 227 | 191.03 | 0.841542 |
| GO:0016043\_cellular\_component\_organization | WAS | 1366 | 3 | 1.194729 | -0.326989 | 227 | 191.03 | 0.841542 |
| GO:0051094\_positive\_regulation\_of\_developmental\_process | CD27 | 340 | 1 | 1.600000 | -0.325915 | 228 | 192.47 | 0.844167 |
| GO:0033554\_cellular\_response\_to\_stress | CD27 | 341 | 1 | 1.595308 | -0.324984 | 229 | 192.87 | 0.842227 |
| GO:0050794\_regulation\_of\_cellular\_process | CD3E | 3515 | 7 | 1.083357 | -0.312392 | 230 | 195.16 | 0.848522 |
| GO:0050794\_regulation\_of\_cellular\_process | RHOH | 3515 | 7 | 1.083357 | -0.312392 | 230 | 195.16 | 0.848522 |
| GO:0050794\_regulation\_of\_cellular\_process | CD3G | 3515 | 7 | 1.083357 | -0.312392 | 230 | 195.16 | 0.848522 |
| GO:0050794\_regulation\_of\_cellular\_process | CD27 | 3515 | 7 | 1.083357 | -0.312392 | 230 | 195.16 | 0.848522 |
| GO:0050794\_regulation\_of\_cellular\_process | GRAP2 | 3515 | 7 | 1.083357 | -0.312392 | 230 | 195.16 | 0.848522 |
| GO:0050794\_regulation\_of\_cellular\_process | IL2RG | 3515 | 7 | 1.083357 | -0.312392 | 230 | 195.16 | 0.848522 |
| GO:0050794\_regulation\_of\_cellular\_process | SIT1 | 3515 | 7 | 1.083357 | -0.312392 | 230 | 195.16 | 0.848522 |
| GO:0007186\_G-protein\_coupled\_receptor\_protein\_signaling\_pathway | CD3E | 363 | 1 | 1.498623 | -0.305404 | 231 | 196.45 | 0.850433 |
| GO:0051239\_regulation\_of\_multicellular\_organismal\_process | CD27 | 378 | 1 | 1.439153 | -0.292957 | 232 | 200.17 | 0.862802 |
| GO:0048523\_negative\_regulation\_of\_cellular\_process | RHOH | 925 | 2 | 1.176216 | -0.283878 | 233 | 202.55 | 0.869313 |
| GO:0048523\_negative\_regulation\_of\_cellular\_process | CD27 | 925 | 2 | 1.176216 | -0.283878 | 233 | 202.55 | 0.869313 |
| GO:0042127\_regulation\_of\_cell\_proliferation | CD3E | 411 | 1 | 1.323601 | -0.267843 | 234 | 205.59 | 0.878590 |
| GO:0046907\_intracellular\_transport | CD27 | 420 | 1 | 1.295238 | -0.261484 | 235 | 207.24 | 0.881872 |
| GO:0007155\_cell\_adhesion | CD72 | 428 | 1 | 1.271028 | -0.255992 | 236 | 208.74 | 0.884492 |
| GO:0022610\_biological\_adhesion | CD72 | 429 | 1 | 1.268065 | -0.255316 | 237 | 209.17 | 0.882574 |
| GO:0007267\_cell-cell\_signaling | GRAP2 | 445 | 1 | 1.222472 | -0.244802 | 238 | 211.25 | 0.887605 |
| GO:0048522\_positive\_regulation\_of\_cellular\_process | CD3E | 1009 | 2 | 1.078295 | -0.244227 | 239 | 211.51 | 0.884979 |
| GO:0048522\_positive\_regulation\_of\_cellular\_process | CD27 | 1009 | 2 | 1.078295 | -0.244227 | 239 | 211.51 | 0.884979 |
| GO:0048519\_negative\_regulation\_of\_biological\_process | RHOH | 1013 | 2 | 1.074038 | -0.242488 | 240 | 212.13 | 0.883875 |
| GO:0048519\_negative\_regulation\_of\_biological\_process | CD27 | 1013 | 2 | 1.074038 | -0.242488 | 240 | 212.13 | 0.883875 |
| GO:0051716\_cellular\_response\_to\_stimulus | CD27 | 474 | 1 | 1.147679 | -0.227092 | 241 | 215.47 | 0.894066 |
| GO:0009653\_anatomical\_structure\_morphogenesis | CD3G | 500 | 1 | 1.088000 | -0.212541 | 242 | 218.7 | 0.903719 |
| GO:0051649\_establishment\_of\_localization\_in\_cell | CD27 | 573 | 1 | 0.949389 | -0.177295 | 243 | 224.13 | 0.922346 |
| GO:0006810\_transport | CD3G | 1243 | 2 | 0.875302 | -0.160866 | 244 | 226.7 | 0.929098 |
| GO:0006810\_transport | CD27 | 1243 | 2 | 0.875302 | -0.160866 | 244 | 226.7 | 0.929098 |
| GO:0051641\_cellular\_localization | CD27 | 617 | 1 | 0.881686 | -0.159361 | 245 | 227.11 | 0.926980 |
| GO:0051234\_establishment\_of\_localization | CD3G | 1260 | 2 | 0.863492 | -0.156046 | 246 | 227.66 | 0.925447 |
| GO:0051234\_establishment\_of\_localization | CD27 | 1260 | 2 | 0.863492 | -0.156046 | 246 | 227.66 | 0.925447 |
| GO:0051179\_localization | CD3G | 1561 | 2 | 0.696989 | -0.090422 | 247 | 239.0 | 0.967611 |
| GO:0051179\_localization | CD27 | 1561 | 2 | 0.696989 | -0.090422 | 247 | 239.0 | 0.967611 |
| GO:0045449\_regulation\_of\_transcription | RHOH | 900 | 1 | 0.604444 | -0.082486 | 248 | 240.74 | 0.970726 |
| GO:0019219\_regulation\_of\_nucleobase\_\_nucleoside\_\_nucleotide\_and\_nucleic\_acid\_metabolic\_process | RHOH | 1041 | 1 | 0.522574 | -0.059911 | 249 | 244.44 | 0.981687 |
| GO:0010556\_regulation\_of\_macromolecule\_biosynthetic\_process | RHOH | 1055 | 1 | 0.515640 | -0.058045 | 251 | 245.07 | 0.976375 |
| GO:0051171\_regulation\_of\_nitrogen\_compound\_metabolic\_process | RHOH | 1055 | 1 | 0.515640 | -0.058045 | 251 | 245.07 | 0.976375 |
| GO:0010468\_regulation\_of\_gene\_expression | RHOH | 1067 | 1 | 0.509841 | -0.056491 | 252 | 245.59 | 0.974563 |
| GO:0006350\_transcription | RHOH | 1069 | 1 | 0.508887 | -0.056236 | 253 | 246.03 | 0.972451 |
| GO:0031326\_regulation\_of\_cellular\_biosynthetic\_process | RHOH | 1125 | 1 | 0.483556 | -0.049549 | 254 | 247.2 | 0.973228 |
| GO:0009889\_regulation\_of\_biosynthetic\_process | RHOH | 1135 | 1 | 0.479295 | -0.048442 | 255 | 247.55 | 0.970784 |
| GO:0080090\_regulation\_of\_primary\_metabolic\_process | RHOH | 1311 | 1 | 0.414950 | -0.032503 | 256 | 250.26 | 0.977578 |
| GO:0060255\_regulation\_of\_macromolecule\_metabolic\_process | RHOH | 1328 | 1 | 0.409639 | -0.031268 | 257 | 250.49 | 0.974669 |
| GO:0034961\_cellular\_biopolymer\_biosynthetic\_process | RHOH | 1448 | 1 | 0.375691 | -0.023759 | 258 | 251.79 | 0.975930 |
| GO:0043284\_biopolymer\_biosynthetic\_process | RHOH | 1458 | 1 | 0.373114 | -0.023219 | 259 | 251.98 | 0.972896 |
| GO:0031323\_regulation\_of\_cellular\_metabolic\_process | RHOH | 1466 | 1 | 0.371078 | -0.022795 | 260 | 252.13 | 0.969731 |
| GO:0019222\_regulation\_of\_metabolic\_process | RHOH | 1538 | 1 | 0.353706 | -0.019301 | 261 | 252.6 | 0.967816 |
| GO:0034645\_cellular\_macromolecule\_biosynthetic\_process | RHOH | 1600 | 1 | 0.340000 | -0.016709 | 262 | 253.27 | 0.966679 |
| GO:0009059\_macromolecule\_biosynthetic\_process | RHOH | 1626 | 1 | 0.334563 | -0.015724 | 263 | 253.44 | 0.963650 |
| GO:0010467\_gene\_expression | RHOH | 1663 | 1 | 0.327120 | -0.014417 | 264 | 253.56 | 0.960455 |
| GO:0006139\_nucleobase\_\_nucleoside\_\_nucleotide\_and\_nucleic\_acid\_metabolic\_process | RHOH | 1845 | 1 | 0.294851 | -0.009355 | 265 | 253.85 | 0.957925 |
| GO:0044249\_cellular\_biosynthetic\_process | RHOH | 1951 | 1 | 0.278831 | -0.007238 | 266 | 254.04 | 0.955038 |
| GO:0009058\_biosynthetic\_process | RHOH | 1988 | 1 | 0.273642 | -0.006612 | 267 | 254.19 | 0.952022 |
| GO:0006807\_nitrogen\_compound\_metabolic\_process | RHOH | 2053 | 1 | 0.264978 | -0.005634 | 268 | 254.36 | 0.949104 |
| GO:0034960\_cellular\_biopolymer\_metabolic\_process | RHOH | 2820 | 1 | 0.192908 | -0.000746 | 269 | 254.69 | 0.946803 |
| GO:0044260\_cellular\_macromolecule\_metabolic\_process | RHOH | 2883 | 1 | 0.188692 | -0.000624 | 270 | 254.7 | 0.943333 |
| GO:0043283\_biopolymer\_metabolic\_process | RHOH | 3027 | 1 | 0.179716 | -0.000412 | 271 | 254.71 | 0.939889 |
| GO:0043170\_macromolecule\_metabolic\_process | RHOH | 3103 | 1 | 0.175314 | -0.000329 | 272 | 254.72 | 0.936471 |
| GO:0008152\_metabolic\_process | CD52 | 4111 | 2 | 0.264656 | -0.000190 | 273 | 254.74 | 0.933114 |
| GO:0008152\_metabolic\_process | RHOH | 4111 | 2 | 0.264656 | -0.000190 | 273 | 254.74 | 0.933114 |
| GO:0044238\_primary\_metabolic\_process | RHOH | 3719 | 1 | 0.146276 | -0.000047 | 274 | 254.74 | 0.929708 |
| GO:0044237\_cellular\_metabolic\_process | RHOH | 3753 | 1 | 0.144951 | -0.000042 | 275 | 254.74 | 0.926327 |
| GO:0008150\_biological\_process | CD3E | 8160 | 15 | 1.000000 | 0.000000 | 474 | 447.05 | 0.943143 |
| GO:0008150\_biological\_process | CD52 | 8160 | 15 | 1.000000 | 0.000000 | 474 | 447.05 | 0.943143 |
| GO:0008150\_biological\_process | CD72 | 8160 | 15 | 1.000000 | 0.000000 | 474 | 447.05 | 0.943143 |
| GO:0008150\_biological\_process | GRAP2 | 8160 | 15 | 1.000000 | 0.000000 | 474 | 447.05 | 0.943143 |
| GO:0008150\_biological\_process | SIT1 | 8160 | 15 | 1.000000 | 0.000000 | 474 | 447.05 | 0.943143 |
| GO:0008150\_biological\_process | IL2RG | 8160 | 15 | 1.000000 | 0.000000 | 474 | 447.05 | 0.943143 |
| GO:0008150\_biological\_process | WAS | 8160 | 15 | 1.000000 | 0.000000 | 474 | 447.05 | 0.943143 |
| GO:0008150\_biological\_process | CCR7 | 8160 | 15 | 1.000000 | 0.000000 | 474 | 447.05 | 0.943143 |
| GO:0008150\_biological\_process | CCR4 | 8160 | 15 | 1.000000 | 0.000000 | 474 | 447.05 | 0.943143 |
| GO:0008150\_biological\_process | CD5 | 8160 | 15 | 1.000000 | 0.000000 | 474 | 447.05 | 0.943143 |
| GO:0008150\_biological\_process | RHOH | 8160 | 15 | 1.000000 | 0.000000 | 474 | 447.05 | 0.943143 |
| GO:0008150\_biological\_process | CD3G | 8160 | 15 | 1.000000 | 0.000000 | 474 | 447.05 | 0.943143 |
| GO:0008150\_biological\_process | CD27 | 8160 | 15 | 1.000000 | 0.000000 | 474 | 447.05 | 0.943143 |
| GO:0008150\_biological\_process | CD1D | 8160 | 15 | 1.000000 | 0.000000 | 474 | 447.05 | 0.943143 |
| GO:0008150\_biological\_process | CD3D | 8160 | 15 | 1.000000 | 0.000000 | 474 | 447.05 | 0.943143 |
