# Supplementary material for: Concordance of Gene Expression and Functional Correlation Patterns across the NCI-60 Cell Lines and the Cancer Genome Atlas Glioblastoma Samples
Source: PLoS One. 2012 Jul 26;7(7):e40062. doi: 10.1371/journal.pone.0040062 (PMC3406063; doi:10.1371/journal.pone.0040062)
Supplement: Download S1 — Zip archive of HTGM results. (ZIP) [file pone.0040062.s007.zip › work2026406846/Generated_Total2026406846.dir/generic.BP.NCI60.0.6.CCR7.express.genes.correlation.complete.Thu.May.19.17.25.28.2011.htgm.txt.dir/generic.BP.NCI60.0.6.CCR7.express.genes.correlation.complete.Thu.May.19.17.25.28.2011.htgm.txt.change.html]

Category Summary Report for generic.BP.NCI60.0.6.CCR7.express.genes.correlation.complete.Thu.May.19.17.25.28.2011.htgm.txt

# Category Summary Report for generic.BP.NCI60.0.6.CCR7.express.genes.correlation.complete.Thu.May.19.17.25.28.2011.htgm.txt

| HYPERLINKED GO CATEGORY | TOTAL GENES | CHANGED GENES | ENRICHMENT | LOG10(p) | CUMULATIVE NUMBER OF CATEGORIES | CUMULATIVE RANDOMS LOWER BOUND | CUMULATIVE RANDOMS MEAN | CUMULATIVE RANDOMS UPPER BOUND | FALSE DISCOVERY RATE |
| --- | --- | --- | --- | --- | --- | --- | --- | --- | --- |
| GO:0046649\_lymphocyte\_activation | 119 | 7 | 32.000000 | -9.163413 | 1 | 0.000000 | 0.0 | 0.000000 | 0.000000 |
| GO:0045321\_leukocyte\_activation | 150 | 7 | 25.386667 | -8.454933 | 2 | 0.000000 | 0.0 | 0.000000 | 0.000000 |
| GO:0042110\_T\_cell\_activation | 83 | 6 | 39.325301 | -8.367508 | 3 | 0.000000 | 0.0 | 0.000000 | 0.000000 |
| GO:0001775\_cell\_activation | 175 | 7 | 21.760000 | -7.986845 | 4 | 0.000000 | 0.0 | 0.000000 | 0.000000 |
| GO:0002376\_immune\_system\_process | 718 | 10 | 7.576602 | -7.281458 | 5 | 0.000000 | 0.0 | 0.000000 | 0.000000 |
| GO:0030098\_lymphocyte\_differentiation | 50 | 4 | 43.520000 | -5.790398 | 6 | -0.090000 | 0.01 | 0.110000 | 0.001667 |
| GO:0002521\_leukocyte\_differentiation | 87 | 4 | 25.011494 | -4.822597 | 7 | -0.180000 | 0.02 | 0.220000 | 0.002857 |
| GO:0030217\_T\_cell\_differentiation | 33 | 3 | 49.454545 | -4.576262 | 8 | -0.180000 | 0.02 | 0.220000 | 0.002500 |
| GO:0045058\_T\_cell\_selection | 7 | 2 | 155.428571 | -4.181196 | 9 | -0.494588 | 0.1 | 0.694588 | 0.011111 |
| GO:0030097\_hemopoiesis | 135 | 4 | 16.118519 | -4.071088 | 10 | -0.494588 | 0.1 | 0.694588 | 0.010000 |
| GO:0048534\_hemopoietic\_or\_lymphoid\_organ\_development | 139 | 4 | 15.654676 | -4.021689 | 11 | -0.507129 | 0.15 | 0.807129 | 0.013636 |
| GO:0002520\_immune\_system\_development | 147 | 4 | 14.802721 | -3.927221 | 12 | -0.546678 | 0.16 | 0.866678 | 0.013333 |
| GO:0051249\_regulation\_of\_lymphocyte\_activation | 60 | 3 | 27.200000 | -3.791815 | 13 | -0.546678 | 0.16 | 0.866678 | 0.012308 |
| GO:0002694\_regulation\_of\_leukocyte\_activation | 70 | 3 | 23.314286 | -3.592591 | 14 | -0.671431 | 0.24 | 1.151431 | 0.017143 |
| GO:0050865\_regulation\_of\_cell\_activation | 77 | 3 | 21.194805 | -3.470043 | 15 | -0.799240 | 0.36 | 1.519240 | 0.024000 |
| GO:0002682\_regulation\_of\_immune\_system\_process | 196 | 4 | 11.102041 | -3.446160 | 16 | -0.799240 | 0.36 | 1.519240 | 0.022500 |
| GO:0007204\_elevation\_of\_cytosolic\_calcium\_ion\_concentration | 80 | 3 | 20.400000 | -3.421038 | 17 | -0.877822 | 0.41 | 1.697822 | 0.024118 |
| GO:0051480\_cytosolic\_calcium\_ion\_homeostasis | 81 | 3 | 20.148148 | -3.405128 | 18 | -0.899473 | 0.42 | 1.739473 | 0.023333 |
| GO:0002684\_positive\_regulation\_of\_immune\_system\_process | 106 | 3 | 15.396226 | -3.062812 | 19 | -1.107847 | 0.62 | 2.347847 | 0.032632 |
| GO:0006874\_cellular\_calcium\_ion\_homeostasis | 114 | 3 | 14.315789 | -2.970984 | 20 | -1.167076 | 0.67 | 2.507076 | 0.033500 |
| GO:0055074\_calcium\_ion\_homeostasis | 116 | 3 | 14.068966 | -2.949086 | 21 | -1.251430 | 0.7 | 2.651430 | 0.033333 |
| GO:0006875\_cellular\_metal\_ion\_homeostasis | 121 | 3 | 13.487603 | -2.896036 | 22 | -1.266973 | 0.75 | 2.766973 | 0.034091 |
| GO:0055065\_metal\_ion\_homeostasis | 125 | 3 | 13.056000 | -2.855236 | 23 | -1.303668 | 0.77 | 2.843668 | 0.033478 |
| GO:0002475\_antigen\_processing\_and\_presentation\_via\_MHC\_class\_Ib | 1 | 1 |  |  |  |  |  |  |  |  |
| GO:0045579\_positive\_regulation\_of\_B\_cell\_differentiation | 1 | 1 |  |  |  |  |  |  |  |  |
| GO:0048003\_antigen\_processing\_and\_presentation\_of\_lipid\_antigen\_via\_MHC\_class\_Ib | 1 | 1 |  |  |  |  |  |  |  |  |
| GO:0048006\_antigen\_processing\_and\_presentation\_\_endogenous\_lipid\_antigen\_via\_MHC\_class\_Ib | 1 | 1 |  |  |  |  |  |  |  |  |
| GO:0030005\_cellular\_di-\_\_tri-valent\_inorganic\_cation\_homeostasis | 140 | 3 | 11.657143 | -2.713663 | 24 | -1.366748 | 1.02 | 3.406748 | 0.042500 |
| GO:0006955\_immune\_response | 529 | 5 | 5.141777 | -2.708594 | 25 | -1.366748 | 1.02 | 3.406748 | 0.040800 |
| GO:0055066\_di-\_\_tri-valent\_inorganic\_cation\_homeostasis | 145 | 3 | 11.255172 | -2.670023 | 26 | -1.407218 | 1.13 | 3.667218 | 0.043462 |
| GO:0051251\_positive\_regulation\_of\_lymphocyte\_activation | 40 | 2 | 27.200000 | -2.626541 | 27 | -1.427320 | 1.21 | 3.847320 | 0.044815 |
| GO:0048869\_cellular\_developmental\_process | 555 | 5 | 4.900901 | -2.616033 | 28 | -1.427320 | 1.21 | 3.847320 | 0.043214 |
| GO:0030003\_cellular\_cation\_homeostasis | 161 | 3 | 10.136646 | -2.540443 | 29 | -1.404447 | 1.41 | 4.224447 | 0.048621 |
| GO:0002696\_positive\_regulation\_of\_leukocyte\_activation | 46 | 2 | 23.652174 | -2.506460 | 30 | -1.506614 | 1.49 | 4.486614 | 0.049667 |
| GO:0050863\_regulation\_of\_T\_cell\_activation | 49 | 2 | 22.204082 | -2.452376 | 32 | -1.539839 | 1.6 | 4.739839 | 0.050000 |
| GO:0050867\_positive\_regulation\_of\_cell\_activation | 49 | 2 | 22.204082 | -2.452376 | 32 | -1.539839 | 1.6 | 4.739839 | 0.050000 |
| GO:0055080\_cation\_homeostasis | 173 | 3 | 9.433526 | -2.451988 | 33 | -1.539138 | 1.61 | 4.759138 | 0.048788 |
| GO:0045059\_positive\_thymic\_T\_cell\_selection | 2 | 1 |  |  |  |  |  |  |  |  |
| GO:0006952\_defense\_response | 369 | 4 | 5.897019 | -2.423141 | 34 | -1.469090 | 1.68 | 4.829090 | 0.049412 |
| GO:0043368\_positive\_T\_cell\_selection | 3 | 1 |  |  |  |  |  |  |  |  |
| GO:0045061\_thymic\_T\_cell\_selection | 3 | 1 |  |  |  |  |  |  |  |  |
| GO:0045577\_regulation\_of\_B\_cell\_differentiation | 3 | 1 |  |  |  |  |  |  |  |  |
| GO:0006873\_cellular\_ion\_homeostasis | 206 | 3 | 7.922330 | -2.239192 | 35 | -1.530265 | 2.25 | 6.030265 | 0.064286 |
| GO:0055082\_cellular\_chemical\_homeostasis | 208 | 3 | 7.846154 | -2.227505 | 36 | -1.584298 | 2.31 | 6.204298 | 0.064167 |
| GO:0050801\_ion\_homeostasis | 221 | 3 | 7.384615 | -2.154407 | 37 | -1.540073 | 2.81 | 7.160073 | 0.075946 |
| GO:0008588\_release\_of\_cytoplasmic\_sequestered\_NF-kappaB | 4 | 1 |  |  |  |  |  |  |  |  |
| GO:0019725\_cellular\_homeostasis | 231 | 3 | 7.064935 | -2.101307 | 38 | -1.395249 | 3.13 | 7.655249 | 0.082368 |
| GO:0048513\_organ\_development | 741 | 5 | 3.670715 | -2.073019 | 39 | -1.371664 | 3.28 | 7.931664 | 0.084103 |
| GO:0009605\_response\_to\_external\_stimulus | 464 | 4 | 4.689655 | -2.069253 | 40 | -1.364688 | 3.37 | 8.104688 | 0.084250 |
| GO:0019883\_antigen\_processing\_and\_presentation\_of\_endogenous\_antigen | 6 | 1 | 90.666667 | -1.959311 | 42 | 0.590919 | 8.45 | 16.309081 | 0.201190 |
| GO:0042346\_positive\_regulation\_of\_NF-kappaB\_import\_into\_nucleus | 6 | 1 | 90.666667 | -1.959311 | 42 | 0.590919 | 8.45 | 16.309081 | 0.201190 |
| GO:0030154\_cell\_differentiation | 506 | 4 | 4.300395 | -1.938451 | 43 | 0.620278 | 8.58 | 16.539722 | 0.199535 |
| GO:0006461\_protein\_complex\_assembly | 273 | 3 | 5.978022 | -1.903017 | 45 | 0.635747 | 8.72 | 16.804253 | 0.193778 |
| GO:0070271\_protein\_complex\_biogenesis | 273 | 3 | 5.978022 | -1.903017 | 45 | 0.635747 | 8.72 | 16.804253 | 0.193778 |
| GO:0033077\_T\_cell\_differentiation\_in\_the\_thymus | 7 | 1 | 77.714286 | -1.892736 | 46 | 1.696715 | 11.01 | 20.323285 | 0.239348 |
| GO:0048878\_chemical\_homeostasis | 278 | 3 | 5.870504 | -1.881694 | 47 | 1.660592 | 11.12 | 20.579408 | 0.236596 |
| GO:0009611\_response\_to\_wounding | 279 | 3 | 5.849462 | -1.877481 | 48 | 1.667079 | 11.16 | 20.652921 | 0.232500 |
| GO:0065007\_biological\_regulation | 3971 | 12 | 1.643918 | -1.869413 | 49 | 1.644051 | 11.19 | 20.735949 | 0.228367 |
| GO:0016045\_detection\_of\_bacterium | 8 | 1 | 68.000000 | -1.835117 | 52 | 2.687870 | 13.67 | 24.652130 | 0.262885 |
| GO:0043124\_negative\_regulation\_of\_I-kappaB\_kinase\_NF-kappaB\_cascade | 8 | 1 | 68.000000 | -1.835117 | 52 | 2.687870 | 13.67 | 24.652130 | 0.262885 |
| GO:0046330\_positive\_regulation\_of\_JNK\_cascade | 8 | 1 | 68.000000 | -1.835117 | 52 | 2.687870 | 13.67 | 24.652130 | 0.262885 |
| GO:0070304\_positive\_regulation\_of\_stress-activated\_protein\_kinase\_signaling\_pathway | 9 | 1 | 60.444444 | -1.784336 | 53 | 3.377502 | 15.82 | 28.262498 | 0.298491 |
| GO:0048584\_positive\_regulation\_of\_response\_to\_stimulus | 114 | 2 | 9.543860 | -1.743731 | 54 | 3.615416 | 16.27 | 28.924584 | 0.301296 |
| GO:0042307\_positive\_regulation\_of\_protein\_import\_into\_nucleus | 10 | 1 | 54.400000 | -1.738951 | 57 | 4.150176 | 18.18 | 32.209824 | 0.318947 |
| GO:0042993\_positive\_regulation\_of\_transcription\_factor\_import\_into\_nucleus | 10 | 1 | 54.400000 | -1.738951 | 57 | 4.150176 | 18.18 | 32.209824 | 0.318947 |
| GO:0045621\_positive\_regulation\_of\_lymphocyte\_differentiation | 10 | 1 | 54.400000 | -1.738951 | 57 | 4.150176 | 18.18 | 32.209824 | 0.318947 |
| GO:0007172\_signal\_complex\_assembly | 11 | 1 | 49.454545 | -1.697930 | 59 | 5.284493 | 20.38 | 35.475507 | 0.345424 |
| GO:0050871\_positive\_regulation\_of\_B\_cell\_activation | 11 | 1 | 49.454545 | -1.697930 | 59 | 5.284493 | 20.38 | 35.475507 | 0.345424 |
| GO:0048856\_anatomical\_structure\_development | 1289 | 6 | 2.532196 | -1.671547 | 60 | 5.257782 | 20.67 | 36.082218 | 0.344500 |
| GO:0006935\_chemotaxis | 125 | 2 | 8.704000 | -1.668433 | 62 | 5.332000 | 20.74 | 36.148000 | 0.334516 |
| GO:0042330\_taxis | 125 | 2 | 8.704000 | -1.668433 | 62 | 5.332000 | 20.74 | 36.148000 | 0.334516 |
| GO:0019882\_antigen\_processing\_and\_presentation | 12 | 1 | 45.333333 | -1.660514 | 65 | 6.056372 | 22.35 | 38.643628 | 0.343846 |
| GO:0042345\_regulation\_of\_NF-kappaB\_import\_into\_nucleus | 12 | 1 | 45.333333 | -1.660514 | 65 | 6.056372 | 22.35 | 38.643628 | 0.343846 |
| GO:0042348\_NF-kappaB\_import\_into\_nucleus | 12 | 1 | 45.333333 | -1.660514 | 65 | 6.056372 | 22.35 | 38.643628 | 0.343846 |
| GO:0046824\_positive\_regulation\_of\_nucleocytoplasmic\_transport | 13 | 1 | 41.846154 | -1.626124 | 66 | 6.879168 | 24.26 | 41.640832 | 0.367576 |
| GO:0006950\_response\_to\_stress | 959 | 5 | 2.836288 | -1.614593 | 67 | 6.916573 | 24.36 | 41.803427 | 0.363582 |
| GO:0007249\_I-kappaB\_kinase\_NF-kappaB\_cascade | 134 | 2 | 8.119403 | -1.611939 | 68 | 7.079085 | 24.53 | 41.980915 | 0.360735 |
| GO:0016064\_immunoglobulin\_mediated\_immune\_response | 14 | 1 | 38.857143 | -1.594311 | 69 | 7.638530 | 26.16 | 44.681470 | 0.379130 |
| GO:0007626\_locomotory\_behavior | 142 | 2 | 7.661972 | -1.565058 | 70 | 7.951288 | 26.62 | 45.288712 | 0.380286 |
| GO:0019724\_B\_cell\_mediated\_immunity | 15 | 1 | 36.266667 | -1.564719 | 71 | 8.788534 | 28.45 | 48.111466 | 0.400704 |
| GO:0065003\_macromolecular\_complex\_assembly | 366 | 3 | 4.459016 | -1.564692 | 72 | 8.795654 | 28.47 | 48.144346 | 0.395417 |
| GO:0080134\_regulation\_of\_response\_to\_stress | 147 | 2 | 7.401361 | -1.537188 | 73 | 8.980253 | 28.96 | 48.939747 | 0.396712 |
| GO:0009595\_detection\_of\_biotic\_stimulus | 16 | 1 | 34.000000 | -1.537062 | 76 | 9.427946 | 30.45 | 51.472054 | 0.400658 |
| GO:0045730\_respiratory\_burst | 16 | 1 | 34.000000 | -1.537062 | 76 | 9.427946 | 30.45 | 51.472054 | 0.400658 |
| GO:0050864\_regulation\_of\_B\_cell\_activation | 16 | 1 | 34.000000 | -1.537062 | 76 | 9.427946 | 30.45 | 51.472054 | 0.400658 |
| GO:0030183\_B\_cell\_differentiation | 18 | 1 | 30.222222 | -1.486653 | 77 | 10.524758 | 33.24 | 55.955242 | 0.431688 |
| GO:0042592\_homeostatic\_process | 397 | 3 | 4.110831 | -1.473445 | 78 | 10.609993 | 33.41 | 56.210007 | 0.428333 |
| GO:0032388\_positive\_regulation\_of\_intracellular\_transport | 19 | 1 | 28.631579 | -1.463543 | 80 | 11.485014 | 34.97 | 58.454986 | 0.437125 |
| GO:0042102\_positive\_regulation\_of\_T\_cell\_proliferation | 19 | 1 | 28.631579 | -1.463543 | 80 | 11.485014 | 34.97 | 58.454986 | 0.437125 |
| GO:0045089\_positive\_regulation\_of\_innate\_immune\_response | 20 | 1 | 27.200000 | -1.441638 | 82 | 12.538551 | 36.94 | 61.341449 | 0.450488 |
| GO:0045619\_regulation\_of\_lymphocyte\_differentiation | 20 | 1 | 27.200000 | -1.441638 | 82 | 12.538551 | 36.94 | 61.341449 | 0.450488 |
| GO:0042990\_regulation\_of\_transcription\_factor\_import\_into\_nucleus | 22 | 1 | 24.727273 | -1.400988 | 85 | 14.344534 | 40.32 | 66.295466 | 0.474353 |
| GO:0042991\_transcription\_factor\_import\_into\_nucleus | 22 | 1 | 24.727273 | -1.400988 | 85 | 14.344534 | 40.32 | 66.295466 | 0.474353 |
| GO:0043410\_positive\_regulation\_of\_MAPKKK\_cascade | 22 | 1 | 24.727273 | -1.400988 | 85 | 14.344534 | 40.32 | 66.295466 | 0.474353 |
| GO:0043933\_macromolecular\_complex\_subunit\_organization | 424 | 3 | 3.849057 | -1.400542 | 86 | 14.349629 | 40.34 | 66.330371 | 0.469070 |
| GO:0050671\_positive\_regulation\_of\_lymphocyte\_proliferation | 23 | 1 | 23.652174 | -1.382054 | 88 | 14.803293 | 41.73 | 68.656707 | 0.474205 |
| GO:0070668\_positive\_regulation\_of\_mast\_cell\_proliferation | 23 | 1 | 23.652174 | -1.382054 | 88 | 14.803293 | 41.73 | 68.656707 | 0.474205 |
| GO:0032502\_developmental\_process | 1919 | 7 | 1.984367 | -1.379684 | 89 | 14.812533 | 41.79 | 68.767467 | 0.469551 |
| GO:0006954\_inflammatory\_response | 182 | 2 | 5.978022 | -1.367143 | 90 | 14.922458 | 41.96 | 68.997542 | 0.466222 |
| GO:0007163\_establishment\_or\_maintenance\_of\_cell\_polarity | 24 | 1 | 22.666667 | -1.363942 | 93 | 15.560527 | 43.19 | 70.819473 | 0.464409 |
| GO:0032946\_positive\_regulation\_of\_mononuclear\_cell\_proliferation | 24 | 1 | 22.666667 | -1.363942 | 93 | 15.560527 | 43.19 | 70.819473 | 0.464409 |
| GO:0070665\_positive\_regulation\_of\_leukocyte\_proliferation | 24 | 1 | 22.666667 | -1.363942 | 93 | 15.560527 | 43.19 | 70.819473 | 0.464409 |
| GO:0010627\_regulation\_of\_protein\_kinase\_cascade | 184 | 2 | 5.913043 | -1.358539 | 94 | 15.578633 | 43.25 | 70.921367 | 0.460106 |
| GO:0042129\_regulation\_of\_T\_cell\_proliferation | 25 | 1 | 21.760000 | -1.346584 | 95 | 16.288103 | 44.52 | 72.751897 | 0.468632 |
| GO:0008037\_cell\_recognition | 26 | 1 | 20.923077 | -1.329922 | 98 | 17.318790 | 46.37 | 75.421210 | 0.473163 |
| GO:0010741\_negative\_regulation\_of\_protein\_kinase\_cascade | 26 | 1 | 20.923077 | -1.329922 | 98 | 17.318790 | 46.37 | 75.421210 | 0.473163 |
| GO:0045088\_regulation\_of\_innate\_immune\_response | 26 | 1 | 20.923077 | -1.329922 | 98 | 17.318790 | 46.37 | 75.421210 | 0.473163 |
| GO:0048731\_system\_development | 1140 | 5 | 2.385965 | -1.325027 | 99 | 17.416498 | 46.6 | 75.783502 | 0.470707 |
| GO:0031349\_positive\_regulation\_of\_defense\_response | 27 | 1 | 20.148148 | -1.313902 | 100 | 18.185659 | 47.57 | 76.954341 | 0.475700 |
| GO:0042306\_regulation\_of\_protein\_import\_into\_nucleus | 28 | 1 | 19.428571 | -1.298479 | 103 | 18.977658 | 49.3 | 79.622342 | 0.478641 |
| GO:0070662\_mast\_cell\_proliferation | 28 | 1 | 19.428571 | -1.298479 | 103 | 18.977658 | 49.3 | 79.622342 | 0.478641 |
| GO:0070666\_regulation\_of\_mast\_cell\_proliferation | 28 | 1 | 19.428571 | -1.298479 | 103 | 18.977658 | 49.3 | 79.622342 | 0.478641 |
| GO:0042981\_regulation\_of\_apoptosis | 471 | 3 | 3.464968 | -1.285962 | 104 | 19.167290 | 49.58 | 79.992710 | 0.476731 |
| GO:0043067\_regulation\_of\_programmed\_cell\_death | 476 | 3 | 3.428571 | -1.274589 | 105 | 19.649079 | 50.86 | 82.070921 | 0.484381 |
| GO:0010941\_regulation\_of\_cell\_death | 478 | 3 | 3.414226 | -1.270081 | 107 | 19.655895 | 51.09 | 82.524105 | 0.477477 |
| GO:0022607\_cellular\_component\_assembly | 478 | 3 | 3.414226 | -1.270081 | 107 | 19.655895 | 51.09 | 82.524105 | 0.477477 |
| GO:0042098\_T\_cell\_proliferation | 30 | 1 | 18.133333 | -1.269257 | 109 | 19.829989 | 51.84 | 83.850011 | 0.475596 |
| GO:0050670\_regulation\_of\_lymphocyte\_proliferation | 30 | 1 | 18.133333 | -1.269257 | 109 | 19.829989 | 51.84 | 83.850011 | 0.475596 |
| GO:0007165\_signal\_transduction | 2029 | 7 | 1.876787 | -1.259776 | 110 | 19.965683 | 52.01 | 84.054317 | 0.472818 |
| GO:0032944\_regulation\_of\_mononuclear\_cell\_proliferation | 31 | 1 | 17.548387 | -1.255387 | 112 | 20.593596 | 52.95 | 85.306404 | 0.472768 |
| GO:0070663\_regulation\_of\_leukocyte\_proliferation | 31 | 1 | 17.548387 | -1.255387 | 112 | 20.593596 | 52.95 | 85.306404 | 0.472768 |
| GO:0033157\_regulation\_of\_intracellular\_protein\_transport | 32 | 1 | 17.000000 | -1.241969 | 113 | 21.269927 | 54.19 | 87.110073 | 0.479558 |
| GO:0007610\_behavior | 214 | 2 | 5.084112 | -1.240733 | 114 | 21.290758 | 54.22 | 87.149242 | 0.475614 |
| GO:0009581\_detection\_of\_external\_stimulus | 33 | 1 | 16.484848 | -1.228975 | 116 | 21.763039 | 55.23 | 88.696961 | 0.476121 |
| GO:0050870\_positive\_regulation\_of\_T\_cell\_activation | 33 | 1 | 16.484848 | -1.228975 | 116 | 21.763039 | 55.23 | 88.696961 | 0.476121 |
| GO:0002449\_lymphocyte\_mediated\_immunity | 34 | 1 | 16.000000 | -1.216381 | 117 | 22.679946 | 57.14 | 91.600054 | 0.488376 |
| GO:0065008\_regulation\_of\_biological\_quality | 848 | 4 | 2.566038 | -1.205266 | 118 | 22.743375 | 57.37 | 91.996625 | 0.486186 |
| GO:0002443\_leukocyte\_mediated\_immunity | 37 | 1 | 14.702703 | -1.180768 | 121 | 24.380961 | 60.26 | 96.139039 | 0.498017 |
| GO:0002460\_adaptive\_immune\_response\_based\_on\_somatic\_recombination\_of\_immune\_receptors\_built\_from\_immunoglobulin\_superfamily\_domains | 37 | 1 | 14.702703 | -1.180768 | 121 | 24.380961 | 60.26 | 96.139039 | 0.498017 |
| GO:0046822\_regulation\_of\_nucleocytoplasmic\_transport | 37 | 1 | 14.702703 | -1.180768 | 121 | 24.380961 | 60.26 | 96.139039 | 0.498017 |
| GO:0002250\_adaptive\_immune\_response | 38 | 1 | 14.315789 | -1.169556 | 123 | 24.754805 | 61.55 | 98.345195 | 0.500407 |
| GO:0042113\_B\_cell\_activation | 38 | 1 | 14.315789 | -1.169556 | 123 | 24.754805 | 61.55 | 98.345195 | 0.500407 |
| GO:0046328\_regulation\_of\_JNK\_cascade | 39 | 1 | 13.948718 | -1.158645 | 124 | 25.601258 | 62.42 | 99.238742 | 0.503387 |
| GO:0048583\_regulation\_of\_response\_to\_stimulus | 241 | 2 | 4.514523 | -1.149624 | 125 | 25.674291 | 62.59 | 99.505709 | 0.500720 |
| GO:0070302\_regulation\_of\_stress-activated\_protein\_kinase\_signaling\_pathway | 40 | 1 | 13.600000 | -1.148020 | 126 | 25.924236 | 63.21 | 100.495764 | 0.501667 |
| GO:0046651\_lymphocyte\_proliferation | 41 | 1 | 13.268293 | -1.137666 | 128 | 26.114281 | 64.11 | 102.105719 | 0.500859 |
| GO:0051222\_positive\_regulation\_of\_protein\_transport | 41 | 1 | 13.268293 | -1.137666 | 128 | 26.114281 | 64.11 | 102.105719 | 0.500859 |
| GO:0032943\_mononuclear\_cell\_proliferation | 42 | 1 | 12.952381 | -1.127570 | 130 | 26.982859 | 65.48 | 103.977141 | 0.503692 |
| GO:0070661\_leukocyte\_proliferation | 42 | 1 | 12.952381 | -1.127570 | 130 | 26.982859 | 65.48 | 103.977141 | 0.503692 |
| GO:0010926\_anatomical\_structure\_formation | 560 | 3 | 2.914286 | -1.103006 | 132 | 28.118734 | 67.42 | 106.721266 | 0.510758 |
| GO:0044085\_cellular\_component\_biogenesis | 560 | 3 | 2.914286 | -1.103006 | 132 | 28.118734 | 67.42 | 106.721266 | 0.510758 |
| GO:0006915\_apoptosis | 565 | 3 | 2.888496 | -1.093819 | 133 | 28.634067 | 68.27 | 107.905933 | 0.513308 |
| GO:0012501\_programmed\_cell\_death | 571 | 3 | 2.858144 | -1.082930 | 134 | 29.501544 | 69.62 | 109.738456 | 0.519552 |
| GO:0032386\_regulation\_of\_intracellular\_transport | 47 | 1 | 11.574468 | -1.080569 | 135 | 30.181801 | 70.78 | 111.378199 | 0.524296 |
| GO:0050896\_response\_to\_stimulus | 1775 | 6 | 1.838873 | -1.064329 | 136 | 30.615910 | 71.57 | 112.524090 | 0.526250 |
| GO:0008219\_cell\_death | 585 | 3 | 2.789744 | -1.058079 | 138 | 31.229522 | 73.02 | 114.810478 | 0.529130 |
| GO:0016265\_death | 585 | 3 | 2.789744 | -1.058079 | 138 | 31.229522 | 73.02 | 114.810478 | 0.529130 |
| GO:0015031\_protein\_transport | 274 | 2 | 3.970803 | -1.052978 | 139 | 31.704334 | 73.98 | 116.255666 | 0.532230 |
| GO:0045184\_establishment\_of\_protein\_localization | 279 | 2 | 3.899642 | -1.039518 | 140 | 32.714906 | 75.6 | 118.485094 | 0.540000 |
| GO:0007275\_multicellular\_organismal\_development | 1372 | 5 | 1.982507 | -1.034566 | 141 | 32.994254 | 76.11 | 119.225746 | 0.539787 |
| GO:0009617\_response\_to\_bacterium | 53 | 1 | 10.264151 | -1.030605 | 142 | 33.548288 | 77.09 | 120.631712 | 0.542887 |
| GO:0007154\_cell\_communication | 2272 | 7 | 1.676056 | -1.027338 | 143 | 33.728410 | 77.25 | 120.771590 | 0.540210 |
| GO:0050778\_positive\_regulation\_of\_immune\_response | 56 | 1 | 9.714286 | -1.007799 | 144 | 35.057002 | 79.53 | 124.002998 | 0.552292 |
| GO:0040011\_locomotion | 292 | 2 | 3.726027 | -1.005806 | 145 | 35.284648 | 79.77 | 124.255352 | 0.550138 |
| GO:0043408\_regulation\_of\_MAPKKK\_cascade | 59 | 1 | 9.220339 | -0.986240 | 146 | 36.905000 | 82.58 | 128.255000 | 0.565616 |
| GO:0007254\_JNK\_cascade | 61 | 1 | 8.918033 | -0.972499 | 148 | 37.815712 | 84.24 | 130.664288 | 0.569189 |
| GO:0031347\_regulation\_of\_defense\_response | 61 | 1 | 8.918033 | -0.972499 | 148 | 37.815712 | 84.24 | 130.664288 | 0.569189 |
| GO:0031098\_stress-activated\_protein\_kinase\_signaling\_pathway | 64 | 1 | 8.500000 | -0.952753 | 149 | 40.093112 | 87.37 | 134.646888 | 0.586376 |
| GO:0080135\_regulation\_of\_cellular\_response\_to\_stress | 65 | 1 | 8.369231 | -0.946387 | 150 | 40.738940 | 88.18 | 135.621060 | 0.587867 |
| GO:0006606\_protein\_import\_into\_nucleus | 68 | 1 | 8.000000 | -0.927895 | 151 | 42.426379 | 90.98 | 139.533621 | 0.602517 |
| GO:0050793\_regulation\_of\_developmental\_process | 669 | 3 | 2.439462 | -0.923561 | 152 | 42.550354 | 91.39 | 140.229646 | 0.601250 |
| GO:0051170\_nuclear\_import | 70 | 1 | 7.771429 | -0.916041 | 153 | 43.532939 | 92.84 | 142.147061 | 0.606797 |
| GO:0051223\_regulation\_of\_protein\_transport | 71 | 1 | 7.661972 | -0.910248 | 155 | 44.024199 | 93.64 | 143.255801 | 0.604129 |
| GO:0051606\_detection\_of\_stimulus | 71 | 1 | 7.661972 | -0.910248 | 155 | 44.024199 | 93.64 | 143.255801 | 0.604129 |
| GO:0007596\_blood\_coagulation | 73 | 1 | 7.452055 | -0.898918 | 156 | 44.794571 | 94.87 | 144.945429 | 0.608141 |
| GO:0008104\_protein\_localization | 339 | 2 | 3.209440 | -0.897306 | 157 | 44.900525 | 95.01 | 145.119475 | 0.605159 |
| GO:0045597\_positive\_regulation\_of\_cell\_differentiation | 74 | 1 | 7.351351 | -0.893376 | 159 | 45.057938 | 95.4 | 145.742062 | 0.600000 |
| GO:0050817\_coagulation | 74 | 1 | 7.351351 | -0.893376 | 159 | 45.057938 | 95.4 | 145.742062 | 0.600000 |
| GO:0070201\_regulation\_of\_establishment\_of\_protein\_localization | 75 | 1 | 7.253333 | -0.887914 | 160 | 45.617344 | 96.13 | 146.642656 | 0.600812 |
| GO:0034504\_protein\_localization\_in\_nucleus | 76 | 1 | 7.157895 | -0.882529 | 161 | 46.169979 | 97.12 | 148.070021 | 0.603230 |
| GO:0007599\_hemostasis | 79 | 1 | 6.886076 | -0.866816 | 163 | 47.287397 | 99.02 | 150.752603 | 0.607485 |
| GO:0032880\_regulation\_of\_protein\_localization | 79 | 1 | 6.886076 | -0.866816 | 163 | 47.287397 | 99.02 | 150.752603 | 0.607485 |
| GO:0045087\_innate\_immune\_response | 82 | 1 | 6.634146 | -0.851729 | 164 | 48.816227 | 101.25 | 153.683773 | 0.617378 |
| GO:0007243\_protein\_kinase\_cascade | 377 | 2 | 2.885942 | -0.822100 | 165 | 51.192120 | 104.65 | 158.107880 | 0.634242 |
| GO:0009966\_regulation\_of\_signal\_transduction | 378 | 2 | 2.878307 | -0.820248 | 166 | 51.248559 | 104.83 | 158.411441 | 0.631506 |
| GO:0033036\_macromolecule\_localization | 388 | 2 | 2.804124 | -0.802058 | 167 | 53.034937 | 107.28 | 161.525063 | 0.642395 |
| GO:0017038\_protein\_import | 93 | 1 | 5.849462 | -0.801087 | 169 | 53.361123 | 107.66 | 161.958877 | 0.637041 |
| GO:0043122\_regulation\_of\_I-kappaB\_kinase\_NF-kappaB\_cascade | 93 | 1 | 5.849462 | -0.801087 | 169 | 53.361123 | 107.66 | 161.958877 | 0.637041 |
| GO:0033365\_protein\_localization\_in\_organelle | 95 | 1 | 5.726316 | -0.792577 | 171 | 53.778004 | 108.22 | 162.661996 | 0.632865 |
| GO:0050878\_regulation\_of\_body\_fluid\_levels | 95 | 1 | 5.726316 | -0.792577 | 171 | 53.778004 | 108.22 | 162.661996 | 0.632865 |
| GO:0002252\_immune\_effector\_process | 96 | 1 | 5.666667 | -0.788395 | 172 | 54.288317 | 109.2 | 164.111683 | 0.634884 |
| GO:0042060\_wound\_healing | 98 | 1 | 5.551020 | -0.780171 | 173 | 55.107520 | 110.22 | 165.332480 | 0.637110 |
| GO:0009968\_negative\_regulation\_of\_signal\_transduction | 99 | 1 | 5.494949 | -0.776127 | 174 | 55.433812 | 110.69 | 165.946188 | 0.636149 |
| GO:0050776\_regulation\_of\_immune\_response | 100 | 1 | 5.440000 | -0.772127 | 175 | 55.918689 | 111.51 | 167.101311 | 0.637200 |
| GO:0043623\_cellular\_protein\_complex\_assembly | 101 | 1 | 5.386139 | -0.768171 | 176 | 56.213721 | 111.95 | 167.686279 | 0.636080 |
| GO:0010648\_negative\_regulation\_of\_cell\_communication | 102 | 1 | 5.333333 | -0.764257 | 177 | 56.327199 | 112.13 | 167.932801 | 0.633503 |
| GO:0008544\_epidermis\_development | 104 | 1 | 5.230769 | -0.756553 | 178 | 56.890609 | 112.97 | 169.049391 | 0.634663 |
| GO:0010646\_regulation\_of\_cell\_communication | 423 | 2 | 2.572104 | -0.742743 | 179 | 57.298805 | 113.74 | 170.181195 | 0.635419 |
| GO:0007265\_Ras\_protein\_signal\_transduction | 110 | 1 | 4.945455 | -0.734381 | 180 | 59.337065 | 116.06 | 172.782935 | 0.644778 |
| GO:0007398\_ectoderm\_development | 112 | 1 | 4.857143 | -0.727284 | 181 | 60.074269 | 116.98 | 173.885731 | 0.646298 |
| GO:0007166\_cell\_surface\_receptor\_linked\_signal\_transduction | 828 | 3 | 1.971014 | -0.722117 | 182 | 60.453407 | 117.39 | 174.326593 | 0.645000 |
| GO:0051050\_positive\_regulation\_of\_transport | 116 | 1 | 4.689655 | -0.713500 | 183 | 61.388051 | 118.93 | 176.471949 | 0.649891 |
| GO:0006913\_nucleocytoplasmic\_transport | 121 | 1 | 4.495868 | -0.696991 | 184 | 62.405815 | 120.79 | 179.174185 | 0.656467 |
| GO:0007242\_intracellular\_signaling\_cascade | 853 | 3 | 1.913247 | -0.695369 | 185 | 62.549937 | 120.92 | 179.290063 | 0.653622 |
| GO:0051169\_nuclear\_transport | 122 | 1 | 4.459016 | -0.693780 | 187 | 63.005763 | 121.93 | 180.854237 | 0.652032 |
| GO:0060341\_regulation\_of\_cellular\_localization | 122 | 1 | 4.459016 | -0.693780 | 187 | 63.005763 | 121.93 | 180.854237 | 0.652032 |
| GO:0051707\_response\_to\_other\_organism | 125 | 1 | 4.352000 | -0.684319 | 188 | 64.263778 | 123.76 | 183.256222 | 0.658298 |
| GO:0009987\_cellular\_process | 6671 | 14 | 1.141658 | -0.674738 | 189 | 65.244418 | 124.91 | 184.575582 | 0.660899 |
| GO:0010740\_positive\_regulation\_of\_protein\_kinase\_cascade | 129 | 1 | 4.217054 | -0.672091 | 190 | 66.154519 | 125.92 | 185.685481 | 0.662737 |
| GO:0007264\_small\_GTPase\_mediated\_signal\_transduction | 135 | 1 | 4.029630 | -0.654523 | 191 | 69.593313 | 131.04 | 192.486687 | 0.686073 |
| GO:0000165\_MAPKKK\_cascade | 143 | 1 | 3.804196 | -0.632416 | 192 | 71.994575 | 134.04 | 196.085425 | 0.698125 |
| GO:0000902\_cell\_morphogenesis | 144 | 1 | 3.777778 | -0.629751 | 193 | 72.557593 | 134.9 | 197.242407 | 0.698964 |
| GO:0006605\_protein\_targeting | 145 | 1 | 3.751724 | -0.627107 | 194 | 73.355371 | 135.86 | 198.364629 | 0.700309 |
| GO:0006916\_anti-apoptosis | 155 | 1 | 3.509677 | -0.601754 | 195 | 76.408839 | 139.98 | 203.551161 | 0.717846 |
| GO:0007169\_transmembrane\_receptor\_protein\_tyrosine\_kinase\_signaling\_pathway | 157 | 1 | 3.464968 | -0.596908 | 196 | 77.249273 | 141.05 | 204.850727 | 0.719643 |
| GO:0032989\_cellular\_component\_morphogenesis | 164 | 1 | 3.317073 | -0.580485 | 197 | 78.490148 | 142.57 | 206.649852 | 0.723706 |
| GO:0045595\_regulation\_of\_cell\_differentiation | 170 | 1 | 3.200000 | -0.567038 | 198 | 79.435834 | 143.82 | 208.204166 | 0.726364 |
| GO:0009607\_response\_to\_biotic\_stimulus | 177 | 1 | 3.073446 | -0.552028 | 199 | 82.268027 | 147.41 | 212.551973 | 0.740754 |
| GO:0009967\_positive\_regulation\_of\_signal\_transduction | 185 | 1 | 2.940541 | -0.535698 | 200 | 84.094203 | 149.91 | 215.725797 | 0.749550 |
| GO:0034622\_cellular\_macromolecular\_complex\_assembly | 186 | 1 | 2.924731 | -0.533715 | 201 | 84.765080 | 150.61 | 216.454920 | 0.749303 |
| GO:0010647\_positive\_regulation\_of\_cell\_communication | 189 | 1 | 2.878307 | -0.527840 | 202 | 85.434586 | 151.47 | 217.505414 | 0.749851 |
| GO:0006917\_induction\_of\_apoptosis | 190 | 1 | 2.863158 | -0.525906 | 203 | 85.826676 | 151.98 | 218.133324 | 0.748670 |
| GO:0012502\_induction\_of\_programmed\_cell\_death | 191 | 1 | 2.848168 | -0.523984 | 204 | 86.059766 | 152.27 | 218.480234 | 0.746422 |
| GO:0008284\_positive\_regulation\_of\_cell\_proliferation | 200 | 1 | 2.720000 | -0.507204 | 205 | 88.512378 | 155.42 | 222.327622 | 0.758146 |
| GO:0006886\_intracellular\_protein\_transport | 204 | 1 | 2.666667 | -0.500031 | 206 | 89.615224 | 156.92 | 224.224776 | 0.761748 |
| GO:0043066\_negative\_regulation\_of\_apoptosis | 207 | 1 | 2.628019 | -0.494760 | 207 | 90.988254 | 158.4 | 225.811746 | 0.765217 |
| GO:0043069\_negative\_regulation\_of\_programmed\_cell\_death | 209 | 1 | 2.602871 | -0.491297 | 209 | 91.805927 | 159.43 | 227.054073 | 0.762823 |
| GO:0060548\_negative\_regulation\_of\_cell\_death | 209 | 1 | 2.602871 | -0.491297 | 209 | 91.805927 | 159.43 | 227.054073 | 0.762823 |
| GO:0042221\_response\_to\_chemical\_stimulus | 631 | 2 | 1.724247 | -0.487998 | 210 | 92.119492 | 159.85 | 227.580508 | 0.761190 |
| GO:0048518\_positive\_regulation\_of\_biological\_process | 1094 | 3 | 1.491773 | -0.486737 | 211 | 92.301521 | 160.21 | 228.118479 | 0.759289 |
| GO:0032501\_multicellular\_organismal\_process | 2082 | 5 | 1.306436 | -0.480779 | 212 | 93.938005 | 162.15 | 230.361995 | 0.764858 |
| GO:0008283\_cell\_proliferation | 647 | 2 | 1.681607 | -0.473314 | 213 | 94.483959 | 162.91 | 231.336041 | 0.764836 |
| GO:0050789\_regulation\_of\_biological\_process | 3649 | 8 | 1.192656 | -0.470900 | 214 | 95.012470 | 163.47 | 231.927530 | 0.763879 |
| GO:0034621\_cellular\_macromolecular\_complex\_subunit\_organization | 227 | 1 | 2.396476 | -0.461817 | 216 | 96.123546 | 165.05 | 233.976454 | 0.764120 |
| GO:0051049\_regulation\_of\_transport | 227 | 1 | 2.396476 | -0.461817 | 216 | 96.123546 | 165.05 | 233.976454 | 0.764120 |
| GO:0034613\_cellular\_protein\_localization | 228 | 1 | 2.385965 | -0.460262 | 217 | 96.295818 | 165.33 | 234.364182 | 0.761889 |
| GO:0070727\_cellular\_macromolecule\_localization | 229 | 1 | 2.375546 | -0.458716 | 218 | 96.485848 | 165.62 | 234.754152 | 0.759725 |
| GO:0051704\_multi-organism\_process | 231 | 1 | 2.354978 | -0.455649 | 219 | 97.248861 | 166.61 | 235.971139 | 0.760776 |
| GO:0043065\_positive\_regulation\_of\_apoptosis | 243 | 1 | 2.238683 | -0.437902 | 220 | 99.318378 | 169.27 | 239.221622 | 0.769409 |
| GO:0043068\_positive\_regulation\_of\_programmed\_cell\_death | 246 | 1 | 2.211382 | -0.433633 | 221 | 100.788977 | 170.84 | 240.891023 | 0.773032 |
| GO:0010942\_positive\_regulation\_of\_cell\_death | 250 | 1 | 2.176000 | -0.428040 | 222 | 101.374627 | 171.64 | 241.905373 | 0.773153 |
| GO:0007167\_enzyme\_linked\_receptor\_protein\_signaling\_pathway | 258 | 1 | 2.108527 | -0.417180 | 223 | 102.346586 | 172.61 | 242.873414 | 0.774036 |
| GO:0009888\_tissue\_development | 287 | 1 | 1.895470 | -0.381096 | 224 | 108.672230 | 180.07 | 251.467770 | 0.803884 |
| GO:0051093\_negative\_regulation\_of\_developmental\_process | 290 | 1 | 1.875862 | -0.377628 | 225 | 109.831081 | 181.42 | 253.008919 | 0.806311 |
| GO:0032879\_regulation\_of\_localization | 326 | 1 | 1.668712 | -0.339335 | 226 | 116.137437 | 189.14 | 262.142563 | 0.836903 |
| GO:0016043\_cellular\_component\_organization | 1366 | 3 | 1.194729 | -0.326989 | 227 | 117.868983 | 191.03 | 264.191017 | 0.841542 |
| GO:0051094\_positive\_regulation\_of\_developmental\_process | 340 | 1 | 1.600000 | -0.325915 | 228 | 119.086208 | 192.47 | 265.853792 | 0.844167 |
| GO:0033554\_cellular\_response\_to\_stress | 341 | 1 | 1.595308 | -0.324984 | 229 | 119.489347 | 192.87 | 266.250653 | 0.842227 |
| GO:0050794\_regulation\_of\_cellular\_process | 3515 | 7 | 1.083357 | -0.312392 | 230 | 121.416694 | 195.16 | 268.903306 | 0.848522 |
| GO:0007186\_G-protein\_coupled\_receptor\_protein\_signaling\_pathway | 363 | 1 | 1.498623 | -0.305404 | 231 | 122.458412 | 196.45 | 270.441588 | 0.850433 |
| GO:0051239\_regulation\_of\_multicellular\_organismal\_process | 378 | 1 | 1.439153 | -0.292957 | 232 | 125.669772 | 200.17 | 274.670228 | 0.862802 |
| GO:0048523\_negative\_regulation\_of\_cellular\_process | 925 | 2 | 1.176216 | -0.283878 | 233 | 128.003374 | 202.55 | 277.096626 | 0.869313 |
| GO:0042127\_regulation\_of\_cell\_proliferation | 411 | 1 | 1.323601 | -0.267843 | 234 | 130.728364 | 205.59 | 280.451636 | 0.878590 |
| GO:0046907\_intracellular\_transport | 420 | 1 | 1.295238 | -0.261484 | 235 | 132.056034 | 207.24 | 282.423966 | 0.881872 |
| GO:0007155\_cell\_adhesion | 428 | 1 | 1.271028 | -0.255992 | 236 | 133.548175 | 208.74 | 283.931825 | 0.884492 |
| GO:0022610\_biological\_adhesion | 429 | 1 | 1.268065 | -0.255316 | 237 | 133.915542 | 209.17 | 284.424458 | 0.882574 |
| GO:0007267\_cell-cell\_signaling | 445 | 1 | 1.222472 | -0.244802 | 238 | 136.015770 | 211.25 | 286.484230 | 0.887605 |
| GO:0048522\_positive\_regulation\_of\_cellular\_process | 1009 | 2 | 1.078295 | -0.244227 | 239 | 136.257631 | 211.51 | 286.762369 | 0.884979 |
| GO:0048519\_negative\_regulation\_of\_biological\_process | 1013 | 2 | 1.074038 | -0.242488 | 240 | 136.774458 | 212.13 | 287.485542 | 0.883875 |
| GO:0051716\_cellular\_response\_to\_stimulus | 474 | 1 | 1.147679 | -0.227092 | 241 | 140.065173 | 215.47 | 290.874827 | 0.894066 |
| GO:0009653\_anatomical\_structure\_morphogenesis | 500 | 1 | 1.088000 | -0.212541 | 242 | 142.955680 | 218.7 | 294.444320 | 0.903719 |
| GO:0051649\_establishment\_of\_localization\_in\_cell | 573 | 1 | 0.949389 | -0.177295 | 243 | 148.153840 | 224.13 | 300.106160 | 0.922346 |
| GO:0006810\_transport | 1243 | 2 | 0.875302 | -0.160866 | 244 | 150.962481 | 226.7 | 302.437519 | 0.929098 |
| GO:0051641\_cellular\_localization | 617 | 1 | 0.881686 | -0.159361 | 245 | 151.374029 | 227.11 | 302.845971 | 0.926980 |
| GO:0051234\_establishment\_of\_localization | 1260 | 2 | 0.863492 | -0.156046 | 246 | 151.865059 | 227.66 | 303.454941 | 0.925447 |
| GO:0051179\_localization | 1561 | 2 | 0.696989 | -0.090422 | 247 | 163.751187 | 239.0 | 314.248813 | 0.967611 |
| GO:0045449\_regulation\_of\_transcription | 900 | 1 | 0.604444 | -0.082486 | 248 | 165.745371 | 240.74 | 315.734629 | 0.970726 |
| GO:0019219\_regulation\_of\_nucleobase\_\_nucleoside\_\_nucleotide\_and\_nucleic\_acid\_metabolic\_process | 1041 | 1 | 0.522574 | -0.059911 | 249 | 169.893246 | 244.44 | 318.986754 | 0.981687 |
| GO:0010556\_regulation\_of\_macromolecule\_biosynthetic\_process | 1055 | 1 | 0.515640 | -0.058045 | 251 | 170.752057 | 245.07 | 319.387943 | 0.976375 |
| GO:0051171\_regulation\_of\_nitrogen\_compound\_metabolic\_process | 1055 | 1 | 0.515640 | -0.058045 | 251 | 170.752057 | 245.07 | 319.387943 | 0.976375 |
| GO:0010468\_regulation\_of\_gene\_expression | 1067 | 1 | 0.509841 | -0.056491 | 252 | 171.495317 | 245.59 | 319.684683 | 0.974563 |
| GO:0006350\_transcription | 1069 | 1 | 0.508887 | -0.056236 | 253 | 172.028709 | 246.03 | 320.031291 | 0.972451 |
| GO:0031326\_regulation\_of\_cellular\_biosynthetic\_process | 1125 | 1 | 0.483556 | -0.049549 | 254 | 173.456656 | 247.2 | 320.943344 | 0.973228 |
| GO:0009889\_regulation\_of\_biosynthetic\_process | 1135 | 1 | 0.479295 | -0.048442 | 255 | 173.965393 | 247.55 | 321.134607 | 0.970784 |
| GO:0080090\_regulation\_of\_primary\_metabolic\_process | 1311 | 1 | 0.414950 | -0.032503 | 256 | 177.575874 | 250.26 | 322.944126 | 0.977578 |
| GO:0060255\_regulation\_of\_macromolecule\_metabolic\_process | 1328 | 1 | 0.409639 | -0.031268 | 257 | 177.961288 | 250.49 | 323.018712 | 0.974669 |
| GO:0034961\_cellular\_biopolymer\_biosynthetic\_process | 1448 | 1 | 0.375691 | -0.023759 | 258 | 179.762607 | 251.79 | 323.817393 | 0.975930 |
| GO:0043284\_biopolymer\_biosynthetic\_process | 1458 | 1 | 0.373114 | -0.023219 | 259 | 180.048077 | 251.98 | 323.911923 | 0.972896 |
| GO:0031323\_regulation\_of\_cellular\_metabolic\_process | 1466 | 1 | 0.371078 | -0.022795 | 260 | 180.321802 | 252.13 | 323.938198 | 0.969731 |
| GO:0019222\_regulation\_of\_metabolic\_process | 1538 | 1 | 0.353706 | -0.019301 | 261 | 181.043284 | 252.6 | 324.156716 | 0.967816 |
| GO:0034645\_cellular\_macromolecule\_biosynthetic\_process | 1600 | 1 | 0.340000 | -0.016709 | 262 | 181.908655 | 253.27 | 324.631345 | 0.966679 |
| GO:0009059\_macromolecule\_biosynthetic\_process | 1626 | 1 | 0.334563 | -0.015724 | 263 | 182.140756 | 253.44 | 324.739244 | 0.963650 |
| GO:0010467\_gene\_expression | 1663 | 1 | 0.327120 | -0.014417 | 264 | 182.301994 | 253.56 | 324.818006 | 0.960455 |
| GO:0006139\_nucleobase\_\_nucleoside\_\_nucleotide\_and\_nucleic\_acid\_metabolic\_process | 1845 | 1 | 0.294851 | -0.009355 | 265 | 182.717832 | 253.85 | 324.982168 | 0.957925 |
| GO:0044249\_cellular\_biosynthetic\_process | 1951 | 1 | 0.278831 | -0.007238 | 266 | 183.013910 | 254.04 | 325.066090 | 0.955038 |
| GO:0009058\_biosynthetic\_process | 1988 | 1 | 0.273642 | -0.006612 | 267 | 183.198509 | 254.19 | 325.181491 | 0.952022 |
| GO:0006807\_nitrogen\_compound\_metabolic\_process | 2053 | 1 | 0.264978 | -0.005634 | 268 | 183.440566 | 254.36 | 325.279434 | 0.949104 |
| GO:0034960\_cellular\_biopolymer\_metabolic\_process | 2820 | 1 | 0.192908 | -0.000746 | 269 | 183.967367 | 254.69 | 325.412633 | 0.946803 |
| GO:0044260\_cellular\_macromolecule\_metabolic\_process | 2883 | 1 | 0.188692 | -0.000624 | 270 | 183.990393 | 254.7 | 325.409607 | 0.943333 |
| GO:0043283\_biopolymer\_metabolic\_process | 3027 | 1 | 0.179716 | -0.000412 | 271 | 184.013280 | 254.71 | 325.406720 | 0.939889 |
| GO:0043170\_macromolecule\_metabolic\_process | 3103 | 1 | 0.175314 | -0.000329 | 272 | 184.036028 | 254.72 | 325.403972 | 0.936471 |
| GO:0008152\_metabolic\_process | 4111 | 2 | 0.264656 | -0.000190 | 273 | 184.067099 | 254.74 | 325.412901 | 0.933114 |
| GO:0044238\_primary\_metabolic\_process | 3719 | 1 | 0.146276 | -0.000047 | 274 | 184.067099 | 254.74 | 325.412901 | 0.929708 |
| GO:0044237\_cellular\_metabolic\_process | 3753 | 1 | 0.144951 | -0.000042 | 275 | 184.067099 | 254.74 | 325.412901 | 0.926327 |
| GO:0031328\_positive\_regulation\_of\_cellular\_biosynthetic\_process | 352 | 0 | 0.000000 | -0.000000 | 276 | 184.894263 | 255.33 | 325.765737 | 0.925109 |
| GO:0007049\_cell\_cycle | 494 | 0 | 0.000000 | -0.000000 | 277 | 185.446199 | 255.84 | 326.233801 | 0.923610 |
| GO:0045941\_positive\_regulation\_of\_transcription | 264 | 0 | 0.000000 | -0.000000 | 278 | 186.270368 | 256.51 | 326.749632 | 0.922698 |
| GO:0002697\_regulation\_of\_immune\_effector\_process | 49 | 0 | 0.000000 | -0.000000 | 287 | 196.693676 | 266.3 | 335.906324 | 0.927875 |
| GO:0006275\_regulation\_of\_DNA\_replication | 49 | 0 | 0.000000 | -0.000000 | 287 | 196.693676 | 266.3 | 335.906324 | 0.927875 |
| GO:0006643\_membrane\_lipid\_metabolic\_process | 49 | 0 | 0.000000 | -0.000000 | 287 | 196.693676 | 266.3 | 335.906324 | 0.927875 |
| GO:0009914\_hormone\_transport | 49 | 0 | 0.000000 | -0.000000 | 287 | 196.693676 | 266.3 | 335.906324 | 0.927875 |
| GO:0010517\_regulation\_of\_phospholipase\_activity | 49 | 0 | 0.000000 | -0.000000 | 287 | 196.693676 | 266.3 | 335.906324 | 0.927875 |
| GO:0010952\_positive\_regulation\_of\_peptidase\_activity | 49 | 0 | 0.000000 | -0.000000 | 287 | 196.693676 | 266.3 | 335.906324 | 0.927875 |
| GO:0018108\_peptidyl-tyrosine\_phosphorylation | 49 | 0 | 0.000000 | -0.000000 | 287 | 196.693676 | 266.3 | 335.906324 | 0.927875 |
| GO:0032984\_macromolecular\_complex\_disassembly | 49 | 0 | 0.000000 | -0.000000 | 287 | 196.693676 | 266.3 | 335.906324 | 0.927875 |
| GO:0043280\_positive\_regulation\_of\_caspase\_activity | 49 | 0 | 0.000000 | -0.000000 | 287 | 196.693676 | 266.3 | 335.906324 | 0.927875 |
| GO:0009124\_nucleoside\_monophosphate\_biosynthetic\_process | 70 | 0 | 0.000000 | -0.000000 | 291 | 202.252086 | 271.49 | 340.727914 | 0.932955 |
| GO:0009416\_response\_to\_light\_stimulus | 70 | 0 | 0.000000 | -0.000000 | 291 | 202.252086 | 271.49 | 340.727914 | 0.932955 |
| GO:0016032\_viral\_reproduction | 70 | 0 | 0.000000 | -0.000000 | 291 | 202.252086 | 271.49 | 340.727914 | 0.932955 |
| GO:0022411\_cellular\_component\_disassembly | 70 | 0 | 0.000000 | -0.000000 | 291 | 202.252086 | 271.49 | 340.727914 | 0.932955 |
| GO:0006519\_cellular\_amino\_acid\_and\_derivative\_metabolic\_process | 173 | 0 | 0.000000 | -0.000000 | 293 | 204.412109 | 273.55 | 342.687891 | 0.933618 |
| GO:0046483\_heterocycle\_metabolic\_process | 173 | 0 | 0.000000 | -0.000000 | 293 | 204.412109 | 273.55 | 342.687891 | 0.933618 |
| GO:0000079\_regulation\_of\_cyclin-dependent\_protein\_kinase\_activity | 48 | 0 | 0.000000 | -0.000000 | 301 | 212.103210 | 281.0 | 349.896790 | 0.933555 |
| GO:0008217\_regulation\_of\_blood\_pressure | 48 | 0 | 0.000000 | -0.000000 | 301 | 212.103210 | 281.0 | 349.896790 | 0.933555 |
| GO:0010518\_positive\_regulation\_of\_phospholipase\_activity | 48 | 0 | 0.000000 | -0.000000 | 301 | 212.103210 | 281.0 | 349.896790 | 0.933555 |
| GO:0010551\_regulation\_of\_specific\_transcription\_from\_RNA\_polymerase\_II\_promoter | 48 | 0 | 0.000000 | -0.000000 | 301 | 212.103210 | 281.0 | 349.896790 | 0.933555 |
| GO:0032569\_specific\_transcription\_from\_RNA\_polymerase\_II\_promoter | 48 | 0 | 0.000000 | -0.000000 | 301 | 212.103210 | 281.0 | 349.896790 | 0.933555 |
| GO:0042035\_regulation\_of\_cytokine\_biosynthetic\_process | 48 | 0 | 0.000000 | -0.000000 | 301 | 212.103210 | 281.0 | 349.896790 | 0.933555 |
| GO:0042773\_ATP\_synthesis\_coupled\_electron\_transport | 48 | 0 | 0.000000 | -0.000000 | 301 | 212.103210 | 281.0 | 349.896790 | 0.933555 |
| GO:0042775\_mitochondrial\_ATP\_synthesis\_coupled\_electron\_transport | 48 | 0 | 0.000000 | -0.000000 | 301 | 212.103210 | 281.0 | 349.896790 | 0.933555 |
| GO:0008361\_regulation\_of\_cell\_size | 149 | 0 | 0.000000 | -0.000000 | 302 | 212.924389 | 281.74 | 350.555611 | 0.932914 |
| GO:0000377\_RNA\_splicing\_\_via\_transesterification\_reactions\_with\_bulged\_adenosine\_as\_nucleophile | 151 | 0 | 0.000000 | -0.000000 | 304 | 214.342997 | 283.18 | 352.017003 | 0.931513 |
| GO:0000398\_nuclear\_mRNA\_splicing\_\_via\_spliceosome | 151 | 0 | 0.000000 | -0.000000 | 304 | 214.342997 | 283.18 | 352.017003 | 0.931513 |
| GO:0006163\_purine\_nucleotide\_metabolic\_process | 99 | 0 | 0.000000 | -0.000000 | 306 | 216.965063 | 285.66 | 354.354937 | 0.933529 |
| GO:0016569\_covalent\_chromatin\_modification | 99 | 0 | 0.000000 | -0.000000 | 306 | 216.965063 | 285.66 | 354.354937 | 0.933529 |
| GO:0030001\_metal\_ion\_transport | 186 | 0 | 0.000000 | -0.000000 | 307 | 218.530495 | 287.13 | 355.729505 | 0.935277 |
| GO:0000070\_mitotic\_sister\_chromatid\_segregation | 27 | 0 | 0.000000 | -0.000000 | 329 | 240.843568 | 309.23 | 377.616432 | 0.939909 |
| GO:0000245\_spliceosome\_assembly | 27 | 0 | 0.000000 | -0.000000 | 329 | 240.843568 | 309.23 | 377.616432 | 0.939909 |
| GO:0003002\_regionalization | 27 | 0 | 0.000000 | -0.000000 | 329 | 240.843568 | 309.23 | 377.616432 | 0.939909 |
| GO:0006638\_neutral\_lipid\_metabolic\_process | 27 | 0 | 0.000000 | -0.000000 | 329 | 240.843568 | 309.23 | 377.616432 | 0.939909 |
| GO:0006639\_acylglycerol\_metabolic\_process | 27 | 0 | 0.000000 | -0.000000 | 329 | 240.843568 | 309.23 | 377.616432 | 0.939909 |
| GO:0006690\_icosanoid\_metabolic\_process | 27 | 0 | 0.000000 | -0.000000 | 329 | 240.843568 | 309.23 | 377.616432 | 0.939909 |
| GO:0006836\_neurotransmitter\_transport | 27 | 0 | 0.000000 | -0.000000 | 329 | 240.843568 | 309.23 | 377.616432 | 0.939909 |
| GO:0006909\_phagocytosis | 27 | 0 | 0.000000 | -0.000000 | 329 | 240.843568 | 309.23 | 377.616432 | 0.939909 |
| GO:0007260\_tyrosine\_phosphorylation\_of\_STAT\_protein | 27 | 0 | 0.000000 | -0.000000 | 329 | 240.843568 | 309.23 | 377.616432 | 0.939909 |
| GO:0007631\_feeding\_behavior | 27 | 0 | 0.000000 | -0.000000 | 329 | 240.843568 | 309.23 | 377.616432 | 0.939909 |
| GO:0008543\_fibroblast\_growth\_factor\_receptor\_signaling\_pathway | 27 | 0 | 0.000000 | -0.000000 | 329 | 240.843568 | 309.23 | 377.616432 | 0.939909 |
| GO:0019079\_viral\_genome\_replication | 27 | 0 | 0.000000 | -0.000000 | 329 | 240.843568 | 309.23 | 377.616432 | 0.939909 |
| GO:0031669\_cellular\_response\_to\_nutrient\_levels | 27 | 0 | 0.000000 | -0.000000 | 329 | 240.843568 | 309.23 | 377.616432 | 0.939909 |
| GO:0032200\_telomere\_organization | 27 | 0 | 0.000000 | -0.000000 | 329 | 240.843568 | 309.23 | 377.616432 | 0.939909 |
| GO:0035150\_regulation\_of\_tube\_size | 27 | 0 | 0.000000 | -0.000000 | 329 | 240.843568 | 309.23 | 377.616432 | 0.939909 |
| GO:0043254\_regulation\_of\_protein\_complex\_assembly | 27 | 0 | 0.000000 | -0.000000 | 329 | 240.843568 | 309.23 | 377.616432 | 0.939909 |
| GO:0044272\_sulfur\_compound\_biosynthetic\_process | 27 | 0 | 0.000000 | -0.000000 | 329 | 240.843568 | 309.23 | 377.616432 | 0.939909 |
| GO:0050880\_regulation\_of\_blood\_vessel\_size | 27 | 0 | 0.000000 | -0.000000 | 329 | 240.843568 | 309.23 | 377.616432 | 0.939909 |
| GO:0050906\_detection\_of\_stimulus\_involved\_in\_sensory\_perception | 27 | 0 | 0.000000 | -0.000000 | 329 | 240.843568 | 309.23 | 377.616432 | 0.939909 |
| GO:0051048\_negative\_regulation\_of\_secretion | 27 | 0 | 0.000000 | -0.000000 | 329 | 240.843568 | 309.23 | 377.616432 | 0.939909 |
| GO:0051092\_positive\_regulation\_of\_NF-kappaB\_transcription\_factor\_activity | 27 | 0 | 0.000000 | -0.000000 | 329 | 240.843568 | 309.23 | 377.616432 | 0.939909 |
| GO:0051607\_defense\_response\_to\_virus | 27 | 0 | 0.000000 | -0.000000 | 329 | 240.843568 | 309.23 | 377.616432 | 0.939909 |
| GO:0006753\_nucleoside\_phosphate\_metabolic\_process | 146 | 0 | 0.000000 | -0.000000 | 333 | 244.040671 | 312.25 | 380.459329 | 0.937688 |
| GO:0009117\_nucleotide\_metabolic\_process | 146 | 0 | 0.000000 | -0.000000 | 333 | 244.040671 | 312.25 | 380.459329 | 0.937688 |
| GO:0016568\_chromatin\_modification | 146 | 0 | 0.000000 | -0.000000 | 333 | 244.040671 | 312.25 | 380.459329 | 0.937688 |
| GO:0022008\_neurogenesis | 146 | 0 | 0.000000 | -0.000000 | 333 | 244.040671 | 312.25 | 380.459329 | 0.937688 |
| GO:0001935\_endothelial\_cell\_proliferation | 25 | 0 | 0.000000 | -0.000000 | 362 | 273.372164 | 341.13 | 408.887836 | 0.942348 |
| GO:0002757\_immune\_response-activating\_signal\_transduction | 25 | 0 | 0.000000 | -0.000000 | 362 | 273.372164 | 341.13 | 408.887836 | 0.942348 |
| GO:0002764\_immune\_response-regulating\_signal\_transduction | 25 | 0 | 0.000000 | -0.000000 | 362 | 273.372164 | 341.13 | 408.887836 | 0.942348 |
| GO:0006007\_glucose\_catabolic\_process | 25 | 0 | 0.000000 | -0.000000 | 362 | 273.372164 | 341.13 | 408.887836 | 0.942348 |
| GO:0006112\_energy\_reserve\_metabolic\_process | 25 | 0 | 0.000000 | -0.000000 | 362 | 273.372164 | 341.13 | 408.887836 | 0.942348 |
| GO:0006518\_peptide\_metabolic\_process | 25 | 0 | 0.000000 | -0.000000 | 362 | 273.372164 | 341.13 | 408.887836 | 0.942348 |
| GO:0006767\_water-soluble\_vitamin\_metabolic\_process | 25 | 0 | 0.000000 | -0.000000 | 362 | 273.372164 | 341.13 | 408.887836 | 0.942348 |
| GO:0007127\_meiosis\_I | 25 | 0 | 0.000000 | -0.000000 | 362 | 273.372164 | 341.13 | 408.887836 | 0.942348 |
| GO:0007416\_synaptogenesis | 25 | 0 | 0.000000 | -0.000000 | 362 | 273.372164 | 341.13 | 408.887836 | 0.942348 |
| GO:0009141\_nucleoside\_triphosphate\_metabolic\_process | 25 | 0 | 0.000000 | -0.000000 | 362 | 273.372164 | 341.13 | 408.887836 | 0.942348 |
| GO:0010876\_lipid\_localization | 25 | 0 | 0.000000 | -0.000000 | 362 | 273.372164 | 341.13 | 408.887836 | 0.942348 |
| GO:0015711\_organic\_anion\_transport | 25 | 0 | 0.000000 | -0.000000 | 362 | 273.372164 | 341.13 | 408.887836 | 0.942348 |
| GO:0019217\_regulation\_of\_fatty\_acid\_metabolic\_process | 25 | 0 | 0.000000 | -0.000000 | 362 | 273.372164 | 341.13 | 408.887836 | 0.942348 |
| GO:0019915\_lipid\_storage | 25 | 0 | 0.000000 | -0.000000 | 362 | 273.372164 | 341.13 | 408.887836 | 0.942348 |
| GO:0030168\_platelet\_activation | 25 | 0 | 0.000000 | -0.000000 | 362 | 273.372164 | 341.13 | 408.887836 | 0.942348 |
| GO:0030282\_bone\_mineralization | 25 | 0 | 0.000000 | -0.000000 | 362 | 273.372164 | 341.13 | 408.887836 | 0.942348 |
| GO:0031023\_microtubule\_organizing\_center\_organization | 25 | 0 | 0.000000 | -0.000000 | 362 | 273.372164 | 341.13 | 408.887836 | 0.942348 |
| GO:0031644\_regulation\_of\_neurological\_system\_process | 25 | 0 | 0.000000 | -0.000000 | 362 | 273.372164 | 341.13 | 408.887836 | 0.942348 |
| GO:0032368\_regulation\_of\_lipid\_transport | 25 | 0 | 0.000000 | -0.000000 | 362 | 273.372164 | 341.13 | 408.887836 | 0.942348 |
| GO:0034101\_erythrocyte\_homeostasis | 25 | 0 | 0.000000 | -0.000000 | 362 | 273.372164 | 341.13 | 408.887836 | 0.942348 |
| GO:0043087\_regulation\_of\_GTPase\_activity | 25 | 0 | 0.000000 | -0.000000 | 362 | 273.372164 | 341.13 | 408.887836 | 0.942348 |
| GO:0043624\_cellular\_protein\_complex\_disassembly | 25 | 0 | 0.000000 | -0.000000 | 362 | 273.372164 | 341.13 | 408.887836 | 0.942348 |
| GO:0043966\_histone\_H3\_acetylation | 25 | 0 | 0.000000 | -0.000000 | 362 | 273.372164 | 341.13 | 408.887836 | 0.942348 |
| GO:0045762\_positive\_regulation\_of\_adenylate\_cyclase\_activity | 25 | 0 | 0.000000 | -0.000000 | 362 | 273.372164 | 341.13 | 408.887836 | 0.942348 |
| GO:0050678\_regulation\_of\_epithelial\_cell\_proliferation | 25 | 0 | 0.000000 | -0.000000 | 362 | 273.372164 | 341.13 | 408.887836 | 0.942348 |
| GO:0050727\_regulation\_of\_inflammatory\_response | 25 | 0 | 0.000000 | -0.000000 | 362 | 273.372164 | 341.13 | 408.887836 | 0.942348 |
| GO:0050795\_regulation\_of\_behavior | 25 | 0 | 0.000000 | -0.000000 | 362 | 273.372164 | 341.13 | 408.887836 | 0.942348 |
| GO:0050818\_regulation\_of\_coagulation | 25 | 0 | 0.000000 | -0.000000 | 362 | 273.372164 | 341.13 | 408.887836 | 0.942348 |
| GO:0050821\_protein\_stabilization | 25 | 0 | 0.000000 | -0.000000 | 362 | 273.372164 | 341.13 | 408.887836 | 0.942348 |
| GO:0000038\_very-long-chain\_fatty\_acid\_metabolic\_process | 11 | 0 | 0.000000 | -0.000000 | 455 | 367.418631 | 434.25 | 501.081369 | 0.954396 |
| GO:0000272\_polysaccharide\_catabolic\_process | 11 | 0 | 0.000000 | -0.000000 | 455 | 367.418631 | 434.25 | 501.081369 | 0.954396 |
| GO:0001570\_vasculogenesis | 11 | 0 | 0.000000 | -0.000000 | 455 | 367.418631 | 434.25 | 501.081369 | 0.954396 |
| GO:0001836\_release\_of\_cytochrome\_c\_from\_mitochondria | 11 | 0 | 0.000000 | -0.000000 | 455 | 367.418631 | 434.25 | 501.081369 | 0.954396 |
| GO:0002285\_lymphocyte\_activation\_during\_immune\_response | 11 | 0 | 0.000000 | -0.000000 | 455 | 367.418631 | 434.25 | 501.081369 | 0.954396 |
| GO:0002687\_positive\_regulation\_of\_leukocyte\_migration | 11 | 0 | 0.000000 | -0.000000 | 455 | 367.418631 | 434.25 | 501.081369 | 0.954396 |
| GO:0006094\_gluconeogenesis | 11 | 0 | 0.000000 | -0.000000 | 455 | 367.418631 | 434.25 | 501.081369 | 0.954396 |
| GO:0006342\_chromatin\_silencing | 11 | 0 | 0.000000 | -0.000000 | 455 | 367.418631 | 434.25 | 501.081369 | 0.954396 |
| GO:0006400\_tRNA\_modification | 11 | 0 | 0.000000 | -0.000000 | 455 | 367.418631 | 434.25 | 501.081369 | 0.954396 |
| GO:0006515\_misfolded\_or\_incompletely\_synthesized\_protein\_catabolic\_process | 11 | 0 | 0.000000 | -0.000000 | 455 | 367.418631 | 434.25 | 501.081369 | 0.954396 |
| GO:0006706\_steroid\_catabolic\_process | 11 | 0 | 0.000000 | -0.000000 | 455 | 367.418631 | 434.25 | 501.081369 | 0.954396 |
| GO:0006749\_glutathione\_metabolic\_process | 11 | 0 | 0.000000 | -0.000000 | 455 | 367.418631 | 434.25 | 501.081369 | 0.954396 |
| GO:0006904\_vesicle\_docking\_during\_exocytosis | 11 | 0 | 0.000000 | -0.000000 | 455 | 367.418631 | 434.25 | 501.081369 | 0.954396 |
| GO:0007009\_plasma\_membrane\_organization | 11 | 0 | 0.000000 | -0.000000 | 455 | 367.418631 | 434.25 | 501.081369 | 0.954396 |
| GO:0007076\_mitotic\_chromosome\_condensation | 11 | 0 | 0.000000 | -0.000000 | 455 | 367.418631 | 434.25 | 501.081369 | 0.954396 |
| GO:0007090\_regulation\_of\_S\_phase\_of\_mitotic\_cell\_cycle | 11 | 0 | 0.000000 | -0.000000 | 455 | 367.418631 | 434.25 | 501.081369 | 0.954396 |
| GO:0008206\_bile\_acid\_metabolic\_process | 11 | 0 | 0.000000 | -0.000000 | 455 | 367.418631 | 434.25 | 501.081369 | 0.954396 |
| GO:0008333\_endosome\_to\_lysosome\_transport | 11 | 0 | 0.000000 | -0.000000 | 455 | 367.418631 | 434.25 | 501.081369 | 0.954396 |
| GO:0009072\_aromatic\_amino\_acid\_family\_metabolic\_process | 11 | 0 | 0.000000 | -0.000000 | 455 | 367.418631 | 434.25 | 501.081369 | 0.954396 |
| GO:0009143\_nucleoside\_triphosphate\_catabolic\_process | 11 | 0 | 0.000000 | -0.000000 | 455 | 367.418631 | 434.25 | 501.081369 | 0.954396 |
| GO:0009247\_glycolipid\_biosynthetic\_process | 11 | 0 | 0.000000 | -0.000000 | 455 | 367.418631 | 434.25 | 501.081369 | 0.954396 |
| GO:0009264\_deoxyribonucleotide\_catabolic\_process | 11 | 0 | 0.000000 | -0.000000 | 455 | 367.418631 | 434.25 | 501.081369 | 0.954396 |
| GO:0009394\_2'-deoxyribonucleotide\_metabolic\_process | 11 | 0 | 0.000000 | -0.000000 | 455 | 367.418631 | 434.25 | 501.081369 | 0.954396 |
| GO:0009395\_phospholipid\_catabolic\_process | 11 | 0 | 0.000000 | -0.000000 | 455 | 367.418631 | 434.25 | 501.081369 | 0.954396 |
| GO:0009953\_dorsal\_ventral\_pattern\_formation | 11 | 0 | 0.000000 | -0.000000 | 455 | 367.418631 | 434.25 | 501.081369 | 0.954396 |
| GO:0010458\_exit\_from\_mitosis | 11 | 0 | 0.000000 | -0.000000 | 455 | 367.418631 | 434.25 | 501.081369 | 0.954396 |
| GO:0010469\_regulation\_of\_receptor\_activity | 11 | 0 | 0.000000 | -0.000000 | 455 | 367.418631 | 434.25 | 501.081369 | 0.954396 |
| GO:0010878\_cholesterol\_storage | 11 | 0 | 0.000000 | -0.000000 | 455 | 367.418631 | 434.25 | 501.081369 | 0.954396 |
| GO:0010907\_positive\_regulation\_of\_glucose\_metabolic\_process | 11 | 0 | 0.000000 | -0.000000 | 455 | 367.418631 | 434.25 | 501.081369 | 0.954396 |
| GO:0015804\_neutral\_amino\_acid\_transport | 11 | 0 | 0.000000 | -0.000000 | 455 | 367.418631 | 434.25 | 501.081369 | 0.954396 |
| GO:0016445\_somatic\_diversification\_of\_immunoglobulins | 11 | 0 | 0.000000 | -0.000000 | 455 | 367.418631 | 434.25 | 501.081369 | 0.954396 |
| GO:0016575\_histone\_deacetylation | 11 | 0 | 0.000000 | -0.000000 | 455 | 367.418631 | 434.25 | 501.081369 | 0.954396 |
| GO:0018205\_peptidyl-lysine\_modification | 11 | 0 | 0.000000 | -0.000000 | 455 | 367.418631 | 434.25 | 501.081369 | 0.954396 |
| GO:0018958\_phenol\_metabolic\_process | 11 | 0 | 0.000000 | -0.000000 | 455 | 367.418631 | 434.25 | 501.081369 | 0.954396 |
| GO:0019692\_deoxyribose\_phosphate\_metabolic\_process | 11 | 0 | 0.000000 | -0.000000 | 455 | 367.418631 | 434.25 | 501.081369 | 0.954396 |
| GO:0021915\_neural\_tube\_development | 11 | 0 | 0.000000 | -0.000000 | 455 | 367.418631 | 434.25 | 501.081369 | 0.954396 |
| GO:0030035\_microspike\_assembly | 11 | 0 | 0.000000 | -0.000000 | 455 | 367.418631 | 434.25 | 501.081369 | 0.954396 |
| GO:0030049\_muscle\_filament\_sliding | 11 | 0 | 0.000000 | -0.000000 | 455 | 367.418631 | 434.25 | 501.081369 | 0.954396 |
| GO:0030317\_sperm\_motility | 11 | 0 | 0.000000 | -0.000000 | 455 | 367.418631 | 434.25 | 501.081369 | 0.954396 |
| GO:0030326\_embryonic\_limb\_morphogenesis | 11 | 0 | 0.000000 | -0.000000 | 455 | 367.418631 | 434.25 | 501.081369 | 0.954396 |
| GO:0030433\_ER-associated\_protein\_catabolic\_process | 11 | 0 | 0.000000 | -0.000000 | 455 | 367.418631 | 434.25 | 501.081369 | 0.954396 |
| GO:0031333\_negative\_regulation\_of\_protein\_complex\_assembly | 11 | 0 | 0.000000 | -0.000000 | 455 | 367.418631 | 434.25 | 501.081369 | 0.954396 |
| GO:0031343\_positive\_regulation\_of\_cell\_killing | 11 | 0 | 0.000000 | -0.000000 | 455 | 367.418631 | 434.25 | 501.081369 | 0.954396 |
| GO:0031348\_negative\_regulation\_of\_defense\_response | 11 | 0 | 0.000000 | -0.000000 | 455 | 367.418631 | 434.25 | 501.081369 | 0.954396 |
| GO:0031572\_G2\_M\_transition\_DNA\_damage\_checkpoint | 11 | 0 | 0.000000 | -0.000000 | 455 | 367.418631 | 434.25 | 501.081369 | 0.954396 |
| GO:0031576\_G2\_M\_transition\_checkpoint | 11 | 0 | 0.000000 | -0.000000 | 455 | 367.418631 | 434.25 | 501.081369 | 0.954396 |
| GO:0031929\_TOR\_signaling\_pathway | 11 | 0 | 0.000000 | -0.000000 | 455 | 367.418631 | 434.25 | 501.081369 | 0.954396 |
| GO:0032088\_negative\_regulation\_of\_NF-kappaB\_transcription\_factor\_activity | 11 | 0 | 0.000000 | -0.000000 | 455 | 367.418631 | 434.25 | 501.081369 | 0.954396 |
| GO:0032204\_regulation\_of\_telomere\_maintenance | 11 | 0 | 0.000000 | -0.000000 | 455 | 367.418631 | 434.25 | 501.081369 | 0.954396 |
| GO:0032231\_regulation\_of\_actin\_filament\_bundle\_formation | 11 | 0 | 0.000000 | -0.000000 | 455 | 367.418631 | 434.25 | 501.081369 | 0.954396 |
| GO:0032355\_response\_to\_estradiol\_stimulus | 11 | 0 | 0.000000 | -0.000000 | 455 | 367.418631 | 434.25 | 501.081369 | 0.954396 |
| GO:0032924\_activin\_receptor\_signaling\_pathway | 11 | 0 | 0.000000 | -0.000000 | 455 | 367.418631 | 434.25 | 501.081369 | 0.954396 |
| GO:0033275\_actin-myosin\_filament\_sliding | 11 | 0 | 0.000000 | -0.000000 | 455 | 367.418631 | 434.25 | 501.081369 | 0.954396 |
| GO:0034375\_high-density\_lipoprotein\_particle\_remodeling | 11 | 0 | 0.000000 | -0.000000 | 455 | 367.418631 | 434.25 | 501.081369 | 0.954396 |
| GO:0034377\_plasma\_lipoprotein\_particle\_assembly | 11 | 0 | 0.000000 | -0.000000 | 455 | 367.418631 | 434.25 | 501.081369 | 0.954396 |
| GO:0034433\_steroid\_esterification | 11 | 0 | 0.000000 | -0.000000 | 455 | 367.418631 | 434.25 | 501.081369 | 0.954396 |
| GO:0034434\_sterol\_esterification | 11 | 0 | 0.000000 | -0.000000 | 455 | 367.418631 | 434.25 | 501.081369 | 0.954396 |
| GO:0034435\_cholesterol\_esterification | 11 | 0 | 0.000000 | -0.000000 | 455 | 367.418631 | 434.25 | 501.081369 | 0.954396 |
| GO:0035113\_embryonic\_appendage\_morphogenesis | 11 | 0 | 0.000000 | -0.000000 | 455 | 367.418631 | 434.25 | 501.081369 | 0.954396 |
| GO:0042278\_purine\_nucleoside\_metabolic\_process | 11 | 0 | 0.000000 | -0.000000 | 455 | 367.418631 | 434.25 | 501.081369 | 0.954396 |
| GO:0042308\_negative\_regulation\_of\_protein\_import\_into\_nucleus | 11 | 0 | 0.000000 | -0.000000 | 455 | 367.418631 | 434.25 | 501.081369 | 0.954396 |
| GO:0042354\_L-fucose\_metabolic\_process | 11 | 0 | 0.000000 | -0.000000 | 455 | 367.418631 | 434.25 | 501.081369 | 0.954396 |
| GO:0042384\_cilium\_assembly | 11 | 0 | 0.000000 | -0.000000 | 455 | 367.418631 | 434.25 | 501.081369 | 0.954396 |
| GO:0042730\_fibrinolysis | 11 | 0 | 0.000000 | -0.000000 | 455 | 367.418631 | 434.25 | 501.081369 | 0.954396 |
| GO:0042787\_protein\_ubiquitination\_during\_ubiquitin-dependent\_protein\_catabolic\_process | 11 | 0 | 0.000000 | -0.000000 | 455 | 367.418631 | 434.25 | 501.081369 | 0.954396 |
| GO:0042992\_negative\_regulation\_of\_transcription\_factor\_import\_into\_nucleus | 11 | 0 | 0.000000 | -0.000000 | 455 | 367.418631 | 434.25 | 501.081369 | 0.954396 |
| GO:0043149\_stress\_fiber\_formation | 11 | 0 | 0.000000 | -0.000000 | 455 | 367.418631 | 434.25 | 501.081369 | 0.954396 |
| GO:0043154\_negative\_regulation\_of\_caspase\_activity | 11 | 0 | 0.000000 | -0.000000 | 455 | 367.418631 | 434.25 | 501.081369 | 0.954396 |
| GO:0043524\_negative\_regulation\_of\_neuron\_apoptosis | 11 | 0 | 0.000000 | -0.000000 | 455 | 367.418631 | 434.25 | 501.081369 | 0.954396 |
| GO:0043535\_regulation\_of\_blood\_vessel\_endothelial\_cell\_migration | 11 | 0 | 0.000000 | -0.000000 | 455 | 367.418631 | 434.25 | 501.081369 | 0.954396 |
| GO:0043547\_positive\_regulation\_of\_GTPase\_activity | 11 | 0 | 0.000000 | -0.000000 | 455 | 367.418631 | 434.25 | 501.081369 | 0.954396 |
| GO:0043583\_ear\_development | 11 | 0 | 0.000000 | -0.000000 | 455 | 367.418631 | 434.25 | 501.081369 | 0.954396 |
| GO:0045069\_regulation\_of\_viral\_genome\_replication | 11 | 0 | 0.000000 | -0.000000 | 455 | 367.418631 | 434.25 | 501.081369 | 0.954396 |
| GO:0045076\_regulation\_of\_interleukin-2\_biosynthetic\_process | 11 | 0 | 0.000000 | -0.000000 | 455 | 367.418631 | 434.25 | 501.081369 | 0.954396 |
| GO:0045453\_bone\_resorption | 11 | 0 | 0.000000 | -0.000000 | 455 | 367.418631 | 434.25 | 501.081369 | 0.954396 |
| GO:0045806\_negative\_regulation\_of\_endocytosis | 11 | 0 | 0.000000 | -0.000000 | 455 | 367.418631 | 434.25 | 501.081369 | 0.954396 |
| GO:0045923\_positive\_regulation\_of\_fatty\_acid\_metabolic\_process | 11 | 0 | 0.000000 | -0.000000 | 455 | 367.418631 | 434.25 | 501.081369 | 0.954396 |
| GO:0046128\_purine\_ribonucleoside\_metabolic\_process | 11 | 0 | 0.000000 | -0.000000 | 455 | 367.418631 | 434.25 | 501.081369 | 0.954396 |
| GO:0046504\_glycerol\_ether\_biosynthetic\_process | 11 | 0 | 0.000000 | -0.000000 | 455 | 367.418631 | 434.25 | 501.081369 | 0.954396 |
| GO:0048284\_organelle\_fusion | 11 | 0 | 0.000000 | -0.000000 | 455 | 367.418631 | 434.25 | 501.081369 | 0.954396 |
| GO:0050680\_negative\_regulation\_of\_epithelial\_cell\_proliferation | 11 | 0 | 0.000000 | -0.000000 | 455 | 367.418631 | 434.25 | 501.081369 | 0.954396 |
| GO:0050704\_regulation\_of\_interleukin-1\_secretion | 11 | 0 | 0.000000 | -0.000000 | 455 | 367.418631 | 434.25 | 501.081369 | 0.954396 |
| GO:0050777\_negative\_regulation\_of\_immune\_response | 11 | 0 | 0.000000 | -0.000000 | 455 | 367.418631 | 434.25 | 501.081369 | 0.954396 |
| GO:0050830\_defense\_response\_to\_Gram-positive\_bacterium | 11 | 0 | 0.000000 | -0.000000 | 455 | 367.418631 | 434.25 | 501.081369 | 0.954396 |
| GO:0050918\_positive\_chemotaxis | 11 | 0 | 0.000000 | -0.000000 | 455 | 367.418631 | 434.25 | 501.081369 | 0.954396 |
| GO:0050996\_positive\_regulation\_of\_lipid\_catabolic\_process | 11 | 0 | 0.000000 | -0.000000 | 455 | 367.418631 | 434.25 | 501.081369 | 0.954396 |
| GO:0051043\_regulation\_of\_membrane\_protein\_ectodomain\_proteolysis | 11 | 0 | 0.000000 | -0.000000 | 455 | 367.418631 | 434.25 | 501.081369 | 0.954396 |
| GO:0051187\_cofactor\_catabolic\_process | 11 | 0 | 0.000000 | -0.000000 | 455 | 367.418631 | 434.25 | 501.081369 | 0.954396 |
| GO:0051220\_cytoplasmic\_sequestering\_of\_protein | 11 | 0 | 0.000000 | -0.000000 | 455 | 367.418631 | 434.25 | 501.081369 | 0.954396 |
| GO:0051453\_regulation\_of\_intracellular\_pH | 11 | 0 | 0.000000 | -0.000000 | 455 | 367.418631 | 434.25 | 501.081369 | 0.954396 |
| GO:0051668\_localization\_within\_membrane | 11 | 0 | 0.000000 | -0.000000 | 455 | 367.418631 | 434.25 | 501.081369 | 0.954396 |
| GO:0065005\_protein-lipid\_complex\_assembly | 11 | 0 | 0.000000 | -0.000000 | 455 | 367.418631 | 434.25 | 501.081369 | 0.954396 |
| GO:0070252\_actin-mediated\_cell\_contraction | 11 | 0 | 0.000000 | -0.000000 | 455 | 367.418631 | 434.25 | 501.081369 | 0.954396 |
| GO:0003008\_system\_process | 710 | 0 | 0.000000 | 0.000000 | 474 | 384.308044 | 447.05 | 509.791956 | 0.943143 |
| GO:0006351\_transcription\_\_DNA-dependent | 884 | 0 | 0.000000 | 0.000000 | 474 | 384.308044 | 447.05 | 509.791956 | 0.943143 |
| GO:0006355\_regulation\_of\_transcription\_\_DNA-dependent | 723 | 0 | 0.000000 | 0.000000 | 474 | 384.308044 | 447.05 | 509.791956 | 0.943143 |
| GO:0006464\_protein\_modification\_process | 922 | 0 | 0.000000 | 0.000000 | 474 | 384.308044 | 447.05 | 509.791956 | 0.943143 |
| GO:0006793\_phosphorus\_metabolic\_process | 697 | 0 | 0.000000 | 0.000000 | 474 | 384.308044 | 447.05 | 509.791956 | 0.943143 |
| GO:0006796\_phosphate\_metabolic\_process | 697 | 0 | 0.000000 | 0.000000 | 474 | 384.308044 | 447.05 | 509.791956 | 0.943143 |
| GO:0006996\_organelle\_organization | 764 | 0 | 0.000000 | 0.000000 | 474 | 384.308044 | 447.05 | 509.791956 | 0.943143 |
| GO:0008150\_biological\_process | 8160 | 15 | 1.000000 | 0.000000 | 474 | 384.308044 | 447.05 | 509.791956 | 0.943143 |
| GO:0009056\_catabolic\_process | 633 | 0 | 0.000000 | 0.000000 | 474 | 384.308044 | 447.05 | 509.791956 | 0.943143 |
| GO:0016070\_RNA\_metabolic\_process | 1230 | 0 | 0.000000 | 0.000000 | 474 | 384.308044 | 447.05 | 509.791956 | 0.943143 |
| GO:0016310\_phosphorylation | 601 | 0 | 0.000000 | 0.000000 | 474 | 384.308044 | 447.05 | 509.791956 | 0.943143 |
| GO:0019538\_protein\_metabolic\_process | 1569 | 0 | 0.000000 | 0.000000 | 474 | 384.308044 | 447.05 | 509.791956 | 0.943143 |
| GO:0032774\_RNA\_biosynthetic\_process | 887 | 0 | 0.000000 | 0.000000 | 474 | 384.308044 | 447.05 | 509.791956 | 0.943143 |
| GO:0043412\_biopolymer\_modification | 960 | 0 | 0.000000 | 0.000000 | 474 | 384.308044 | 447.05 | 509.791956 | 0.943143 |
| GO:0043687\_post-translational\_protein\_modification | 728 | 0 | 0.000000 | 0.000000 | 474 | 384.308044 | 447.05 | 509.791956 | 0.943143 |
| GO:0044267\_cellular\_protein\_metabolic\_process | 1382 | 0 | 0.000000 | 0.000000 | 474 | 384.308044 | 447.05 | 509.791956 | 0.943143 |
| GO:0050790\_regulation\_of\_catalytic\_activity | 525 | 0 | 0.000000 | 0.000000 | 474 | 384.308044 | 447.05 | 509.791956 | 0.943143 |
| GO:0051252\_regulation\_of\_RNA\_metabolic\_process | 746 | 0 | 0.000000 | 0.000000 | 474 | 384.308044 | 447.05 | 509.791956 | 0.943143 |
| GO:0065009\_regulation\_of\_molecular\_function | 606 | 0 | 0.000000 | 0.000000 | 474 | 384.308044 | 447.05 | 509.791956 | 0.943143 |
| GO:0044057\_regulation\_of\_system\_process | 106 | 0 | 0.000000 | 0.000000 | 475 | 386.243470 | 448.7 | 511.156530 | 0.944632 |
| GO:0000387\_spliceosomal\_snRNP\_biogenesis | 28 | 0 | 0.000000 | 0.000000 | 500 | 413.677607 | 475.36 | 537.042393 | 0.950720 |
| GO:0000819\_sister\_chromatid\_segregation | 28 | 0 | 0.000000 | 0.000000 | 500 | 413.677607 | 475.36 | 537.042393 | 0.950720 |
| GO:0002440\_production\_of\_molecular\_mediator\_of\_immune\_response | 28 | 0 | 0.000000 | 0.000000 | 500 | 413.677607 | 475.36 | 537.042393 | 0.950720 |
| GO:0006304\_DNA\_modification | 28 | 0 | 0.000000 | 0.000000 | 500 | 413.677607 | 475.36 | 537.042393 | 0.950720 |
| GO:0006405\_RNA\_export\_from\_nucleus | 28 | 0 | 0.000000 | 0.000000 | 500 | 413.677607 | 475.36 | 537.042393 | 0.950720 |
| GO:0006662\_glycerol\_ether\_metabolic\_process | 28 | 0 | 0.000000 | 0.000000 | 500 | 413.677607 | 475.36 | 537.042393 | 0.950720 |
| GO:0006939\_smooth\_muscle\_contraction | 28 | 0 | 0.000000 | 0.000000 | 500 | 413.677607 | 475.36 | 537.042393 | 0.950720 |
| GO:0007156\_homophilic\_cell\_adhesion | 28 | 0 | 0.000000 | 0.000000 | 500 | 413.677607 | 475.36 | 537.042393 | 0.950720 |
| GO:0007281\_germ\_cell\_development | 28 | 0 | 0.000000 | 0.000000 | 500 | 413.677607 | 475.36 | 537.042393 | 0.950720 |
| GO:0007411\_axon\_guidance | 28 | 0 | 0.000000 | 0.000000 | 500 | 413.677607 | 475.36 | 537.042393 | 0.950720 |
| GO:0009062\_fatty\_acid\_catabolic\_process | 28 | 0 | 0.000000 | 0.000000 | 500 | 413.677607 | 475.36 | 537.042393 | 0.950720 |
| GO:0009593\_detection\_of\_chemical\_stimulus | 28 | 0 | 0.000000 | 0.000000 | 500 | 413.677607 | 475.36 | 537.042393 | 0.950720 |
| GO:0009895\_negative\_regulation\_of\_catabolic\_process | 28 | 0 | 0.000000 | 0.000000 | 500 | 413.677607 | 475.36 | 537.042393 | 0.950720 |
| GO:0010563\_negative\_regulation\_of\_phosphorus\_metabolic\_process | 28 | 0 | 0.000000 | 0.000000 | 500 | 413.677607 | 475.36 | 537.042393 | 0.950720 |
| GO:0010565\_regulation\_of\_cellular\_ketone\_metabolic\_process | 28 | 0 | 0.000000 | 0.000000 | 500 | 413.677607 | 475.36 | 537.042393 | 0.950720 |
| GO:0016055\_Wnt\_receptor\_signaling\_pathway | 28 | 0 | 0.000000 | 0.000000 | 500 | 413.677607 | 475.36 | 537.042393 | 0.950720 |
| GO:0018904\_organic\_ether\_metabolic\_process | 28 | 0 | 0.000000 | 0.000000 | 500 | 413.677607 | 475.36 | 537.042393 | 0.950720 |
| GO:0031214\_biomineral\_formation | 28 | 0 | 0.000000 | 0.000000 | 500 | 413.677607 | 475.36 | 537.042393 | 0.950720 |
| GO:0033044\_regulation\_of\_chromosome\_organization | 28 | 0 | 0.000000 | 0.000000 | 500 | 413.677607 | 475.36 | 537.042393 | 0.950720 |
| GO:0045664\_regulation\_of\_neuron\_differentiation | 28 | 0 | 0.000000 | 0.000000 | 500 | 413.677607 | 475.36 | 537.042393 | 0.950720 |
| GO:0045834\_positive\_regulation\_of\_lipid\_metabolic\_process | 28 | 0 | 0.000000 | 0.000000 | 500 | 413.677607 | 475.36 | 537.042393 | 0.950720 |
| GO:0045936\_negative\_regulation\_of\_phosphate\_metabolic\_process | 28 | 0 | 0.000000 | 0.000000 | 500 | 413.677607 | 475.36 | 537.042393 | 0.950720 |
| GO:0051349\_positive\_regulation\_of\_lyase\_activity | 28 | 0 | 0.000000 | 0.000000 | 500 | 413.677607 | 475.36 | 537.042393 | 0.950720 |
| GO:0051650\_establishment\_of\_vesicle\_localization | 28 | 0 | 0.000000 | 0.000000 | 500 | 413.677607 | 475.36 | 537.042393 | 0.950720 |
| GO:0060402\_calcium\_ion\_transport\_into\_cytosol | 28 | 0 | 0.000000 | 0.000000 | 500 | 413.677607 | 475.36 | 537.042393 | 0.950720 |
| GO:0044093\_positive\_regulation\_of\_molecular\_function | 394 | 0 | 0.000000 | 0.000000 | 501 | 414.999949 | 476.38 | 537.760051 | 0.950858 |
| GO:0006974\_response\_to\_DNA\_damage\_stimulus | 234 | 0 | 0.000000 | 0.000000 | 502 | 415.747207 | 477.04 | 538.332793 | 0.950279 |
| GO:0043285\_biopolymer\_catabolic\_process | 426 | 0 | 0.000000 | 0.000000 | 503 | 416.176351 | 477.49 | 538.803649 | 0.949284 |
| GO:0006289\_nucleotide-excision\_repair | 45 | 0 | 0.000000 | 0.000000 | 513 | 425.714543 | 486.76 | 547.805457 | 0.948850 |
| GO:0006308\_DNA\_catabolic\_process | 45 | 0 | 0.000000 | 0.000000 | 513 | 425.714543 | 486.76 | 547.805457 | 0.948850 |
| GO:0006368\_RNA\_elongation\_from\_RNA\_polymerase\_II\_promoter | 45 | 0 | 0.000000 | 0.000000 | 513 | 425.714543 | 486.76 | 547.805457 | 0.948850 |
| GO:0006576\_biogenic\_amine\_metabolic\_process | 45 | 0 | 0.000000 | 0.000000 | 513 | 425.714543 | 486.76 | 547.805457 | 0.948850 |
| GO:0006919\_activation\_of\_caspase\_activity | 45 | 0 | 0.000000 | 0.000000 | 513 | 425.714543 | 486.76 | 547.805457 | 0.948850 |
| GO:0016197\_endosome\_transport | 45 | 0 | 0.000000 | 0.000000 | 513 | 425.714543 | 486.76 | 547.805457 | 0.948850 |
| GO:0016573\_histone\_acetylation | 45 | 0 | 0.000000 | 0.000000 | 513 | 425.714543 | 486.76 | 547.805457 | 0.948850 |
| GO:0034728\_nucleosome\_organization | 45 | 0 | 0.000000 | 0.000000 | 513 | 425.714543 | 486.76 | 547.805457 | 0.948850 |
| GO:0048771\_tissue\_remodeling | 45 | 0 | 0.000000 | 0.000000 | 513 | 425.714543 | 486.76 | 547.805457 | 0.948850 |
| GO:0051168\_nuclear\_export | 45 | 0 | 0.000000 | 0.000000 | 513 | 425.714543 | 486.76 | 547.805457 | 0.948850 |
| GO:0010604\_positive\_regulation\_of\_macromolecule\_metabolic\_process | 446 | 0 | 0.000000 | 0.000000 | 514 | 426.450537 | 487.29 | 548.129463 | 0.948035 |
| GO:0006396\_RNA\_processing | 306 | 0 | 0.000000 | 0.000000 | 515 | 426.923812 | 487.79 | 548.656188 | 0.947165 |
| GO:0048468\_cell\_development | 251 | 0 | 0.000000 | 0.000000 | 516 | 428.368823 | 489.02 | 549.671177 | 0.947713 |
| GO:0000226\_microtubule\_cytoskeleton\_organization | 86 | 0 | 0.000000 | 0.000000 | 517 | 430.687380 | 490.95 | 551.212620 | 0.949613 |
| GO:0006029\_proteoglycan\_metabolic\_process | 32 | 0 | 0.000000 | 0.000000 | 534 | 448.119897 | 507.98 | 567.840103 | 0.951273 |
| GO:0006446\_regulation\_of\_translational\_initiation | 32 | 0 | 0.000000 | 0.000000 | 534 | 448.119897 | 507.98 | 567.840103 | 0.951273 |
| GO:0006487\_protein\_amino\_acid\_N-linked\_glycosylation | 32 | 0 | 0.000000 | 0.000000 | 534 | 448.119897 | 507.98 | 567.840103 | 0.951273 |
| GO:0006839\_mitochondrial\_transport | 32 | 0 | 0.000000 | 0.000000 | 534 | 448.119897 | 507.98 | 567.840103 | 0.951273 |
| GO:0008286\_insulin\_receptor\_signaling\_pathway | 32 | 0 | 0.000000 | 0.000000 | 534 | 448.119897 | 507.98 | 567.840103 | 0.951273 |
| GO:0015698\_inorganic\_anion\_transport | 32 | 0 | 0.000000 | 0.000000 | 534 | 448.119897 | 507.98 | 567.840103 | 0.951273 |
| GO:0015992\_proton\_transport | 32 | 0 | 0.000000 | 0.000000 | 534 | 448.119897 | 507.98 | 567.840103 | 0.951273 |
| GO:0030216\_keratinocyte\_differentiation | 32 | 0 | 0.000000 | 0.000000 | 534 | 448.119897 | 507.98 | 567.840103 | 0.951273 |
| GO:0030595\_leukocyte\_chemotaxis | 32 | 0 | 0.000000 | 0.000000 | 534 | 448.119897 | 507.98 | 567.840103 | 0.951273 |
| GO:0031329\_regulation\_of\_cellular\_catabolic\_process | 32 | 0 | 0.000000 | 0.000000 | 534 | 448.119897 | 507.98 | 567.840103 | 0.951273 |
| GO:0034623\_cellular\_macromolecular\_complex\_disassembly | 32 | 0 | 0.000000 | 0.000000 | 534 | 448.119897 | 507.98 | 567.840103 | 0.951273 |
| GO:0043542\_endothelial\_cell\_migration | 32 | 0 | 0.000000 | 0.000000 | 534 | 448.119897 | 507.98 | 567.840103 | 0.951273 |
| GO:0046578\_regulation\_of\_Ras\_protein\_signal\_transduction | 32 | 0 | 0.000000 | 0.000000 | 534 | 448.119897 | 507.98 | 567.840103 | 0.951273 |
| GO:0050657\_nucleic\_acid\_transport | 32 | 0 | 0.000000 | 0.000000 | 534 | 448.119897 | 507.98 | 567.840103 | 0.951273 |
| GO:0050658\_RNA\_transport | 32 | 0 | 0.000000 | 0.000000 | 534 | 448.119897 | 507.98 | 567.840103 | 0.951273 |
| GO:0051056\_regulation\_of\_small\_GTPase\_mediated\_signal\_transduction | 32 | 0 | 0.000000 | 0.000000 | 534 | 448.119897 | 507.98 | 567.840103 | 0.951273 |
| GO:0051236\_establishment\_of\_RNA\_localization | 32 | 0 | 0.000000 | 0.000000 | 534 | 448.119897 | 507.98 | 567.840103 | 0.951273 |
| GO:0000019\_regulation\_of\_mitotic\_recombination | 2 | 0 |  |  |  |  |  |  |  |  |
| GO:0000022\_mitotic\_spindle\_elongation | 2 | 0 |  |  |  |  |  |  |  |  |
| GO:0000059\_protein\_import\_into\_nucleus\_\_docking | 2 | 0 |  |  |  |  |  |  |  |  |
| GO:0000066\_mitochondrial\_ornithine\_transport | 2 | 0 |  |  |  |  |  |  |  |  |
| GO:0000183\_chromatin\_silencing\_at\_rDNA | 2 | 0 |  |  |  |  |  |  |  |  |
| GO:0000305\_response\_to\_oxygen\_radical | 2 | 0 |  |  |  |  |  |  |  |  |
| GO:0000429\_regulation\_of\_transcription\_from\_RNA\_polymerase\_II\_promoter\_by\_carbon\_catabolites | 2 | 0 |  |  |  |  |  |  |  |  |
| GO:0000430\_regulation\_of\_transcription\_from\_RNA\_polymerase\_II\_promoter\_by\_glucose | 2 | 0 |  |  |  |  |  |  |  |  |
| GO:0000432\_positive\_regulation\_of\_transcription\_from\_RNA\_polymerase\_II\_promoter\_by\_glucose | 2 | 0 |  |  |  |  |  |  |  |  |
| GO:0000436\_positive\_regulation\_of\_transcription\_from\_RNA\_polymerase\_II\_promoter\_by\_carbon\_catabolites | 2 | 0 |  |  |  |  |  |  |  |  |
| GO:0000460\_maturation\_of\_5.8S\_rRNA | 2 | 0 |  |  |  |  |  |  |  |  |
| GO:0000466\_maturation\_of\_5.8S\_rRNA\_from\_tricistronic\_rRNA\_transcript\_(SSU-rRNA\_\_5.8S\_rRNA\_\_LSU-rRNA) | 2 | 0 |  |  |  |  |  |  |  |  |
| GO:0000729\_DNA\_double-strand\_break\_processing | 2 | 0 |  |  |  |  |  |  |  |  |
| GO:0000733\_DNA\_strand\_renaturation | 2 | 0 |  |  |  |  |  |  |  |  |
| GO:0000920\_cell\_separation\_during\_cytokinesis | 2 | 0 |  |  |  |  |  |  |  |  |
| GO:0001101\_response\_to\_acid | 2 | 0 |  |  |  |  |  |  |  |  |
| GO:0001300\_chronological\_cell\_aging | 2 | 0 |  |  |  |  |  |  |  |  |
| GO:0001301\_progressive\_alteration\_of\_chromatin\_during\_cell\_aging | 2 | 0 |  |  |  |  |  |  |  |  |
| GO:0001304\_progressive\_alteration\_of\_chromatin\_during\_replicative\_cell\_aging | 2 | 0 |  |  |  |  |  |  |  |  |
| GO:0001309\_age-dependent\_telomere\_shortening | 2 | 0 |  |  |  |  |  |  |  |  |
| GO:0001507\_acetylcholine\_catabolic\_process\_in\_synaptic\_cleft | 2 | 0 |  |  |  |  |  |  |  |  |
| GO:0001522\_pseudouridine\_synthesis | 2 | 0 |  |  |  |  |  |  |  |  |
| GO:0001547\_antral\_ovarian\_follicle\_growth | 2 | 0 |  |  |  |  |  |  |  |  |
| GO:0001550\_ovarian\_cumulus\_expansion | 2 | 0 |  |  |  |  |  |  |  |  |
| GO:0001556\_oocyte\_maturation | 2 | 0 |  |  |  |  |  |  |  |  |
| GO:0001562\_response\_to\_protozoan | 2 | 0 |  |  |  |  |  |  |  |  |
| GO:0001582\_detection\_of\_chemical\_stimulus\_involved\_in\_sensory\_perception\_of\_sweet\_taste | 2 | 0 |  |  |  |  |  |  |  |  |
| GO:0001667\_ameboidal\_cell\_migration | 2 | 0 |  |  |  |  |  |  |  |  |
| GO:0001678\_cellular\_glucose\_homeostasis | 2 | 0 |  |  |  |  |  |  |  |  |
| GO:0001702\_gastrulation\_with\_mouth\_forming\_second | 2 | 0 |  |  |  |  |  |  |  |  |
| GO:0001736\_establishment\_of\_planar\_polarity | 2 | 0 |  |  |  |  |  |  |  |  |
| GO:0001738\_morphogenesis\_of\_a\_polarized\_epithelium | 2 | 0 |  |  |  |  |  |  |  |  |
| GO:0001756\_somitogenesis | 2 | 0 |  |  |  |  |  |  |  |  |
| GO:0001766\_membrane\_raft\_polarization | 2 | 0 |  |  |  |  |  |  |  |  |
| GO:0001780\_neutrophil\_homeostasis | 2 | 0 |  |  |  |  |  |  |  |  |
| GO:0001781\_neutrophil\_apoptosis | 2 | 0 |  |  |  |  |  |  |  |  |
| GO:0001825\_blastocyst\_formation | 2 | 0 |  |  |  |  |  |  |  |  |
| GO:0001829\_trophectodermal\_cell\_differentiation | 2 | 0 |  |  |  |  |  |  |  |  |
| GO:0001840\_neural\_plate\_development | 2 | 0 |  |  |  |  |  |  |  |  |
| GO:0001868\_regulation\_of\_complement\_activation\_\_lectin\_pathway | 2 | 0 |  |  |  |  |  |  |  |  |
| GO:0001869\_negative\_regulation\_of\_complement\_activation\_\_lectin\_pathway | 2 | 0 |  |  |  |  |  |  |  |  |
| GO:0001880\_Mullerian\_duct\_regression | 2 | 0 |  |  |  |  |  |  |  |  |
| GO:0001885\_endothelial\_cell\_development | 2 | 0 |  |  |  |  |  |  |  |  |
| GO:0001897\_cytolysis\_by\_symbiont\_of\_host\_cells | 2 | 0 |  |  |  |  |  |  |  |  |
| GO:0001907\_killing\_by\_symbiont\_of\_host\_cells | 2 | 0 |  |  |  |  |  |  |  |  |
| GO:0001921\_positive\_regulation\_of\_receptor\_recycling | 2 | 0 |  |  |  |  |  |  |  |  |
| GO:0001967\_suckling\_behavior | 2 | 0 |  |  |  |  |  |  |  |  |
| GO:0001975\_response\_to\_amphetamine | 2 | 0 |  |  |  |  |  |  |  |  |
| GO:0002003\_angiotensin\_maturation | 2 | 0 |  |  |  |  |  |  |  |  |
| GO:0002016\_regulation\_of\_blood\_volume\_by\_renin-angiotensin | 2 | 0 |  |  |  |  |  |  |  |  |
| GO:0002032\_desensitization\_of\_G-protein\_coupled\_receptor\_protein\_signaling\_pathway\_by\_arrestin | 2 | 0 |  |  |  |  |  |  |  |  |
| GO:0002090\_regulation\_of\_receptor\_internalization | 2 | 0 |  |  |  |  |  |  |  |  |
| GO:0002092\_positive\_regulation\_of\_receptor\_internalization | 2 | 0 |  |  |  |  |  |  |  |  |
| GO:0002227\_innate\_immune\_response\_in\_mucosa | 2 | 0 |  |  |  |  |  |  |  |  |
| GO:0002248\_connective\_tissue\_replacement\_during\_inflammatory\_response | 2 | 0 |  |  |  |  |  |  |  |  |
| GO:0002254\_kinin\_cascade | 2 | 0 |  |  |  |  |  |  |  |  |
| GO:0002275\_myeloid\_cell\_activation\_during\_immune\_response | 2 | 0 |  |  |  |  |  |  |  |  |
| GO:0002281\_macrophage\_activation\_during\_immune\_response | 2 | 0 |  |  |  |  |  |  |  |  |
| GO:0002291\_T\_cell\_activation\_via\_T\_cell\_receptor\_contact\_with\_antigen\_bound\_to\_MHC\_molecule\_on\_antigen\_presenting\_cell | 2 | 0 |  |  |  |  |  |  |  |  |
| GO:0002353\_plasma\_kallikrein-kinin\_cascade | 2 | 0 |  |  |  |  |  |  |  |  |
| GO:0002378\_immunoglobulin\_biosynthetic\_process | 2 | 0 |  |  |  |  |  |  |  |  |
| GO:0002384\_hepatic\_immune\_response | 2 | 0 |  |  |  |  |  |  |  |  |
| GO:0002385\_mucosal\_immune\_response | 2 | 0 |  |  |  |  |  |  |  |  |
| GO:0002504\_antigen\_processing\_and\_presentation\_of\_peptide\_or\_polysaccharide\_antigen\_via\_MHC\_class\_II | 2 | 0 |  |  |  |  |  |  |  |  |
| GO:0002507\_tolerance\_induction | 2 | 0 |  |  |  |  |  |  |  |  |
| GO:0002532\_production\_of\_molecular\_mediator\_of\_acute\_inflammatory\_response | 2 | 0 |  |  |  |  |  |  |  |  |
| GO:0002536\_respiratory\_burst\_during\_acute\_inflammatory\_response | 2 | 0 |  |  |  |  |  |  |  |  |
| GO:0002542\_Factor\_XII\_activation | 2 | 0 |  |  |  |  |  |  |  |  |
| GO:0002544\_chronic\_inflammatory\_response | 2 | 0 |  |  |  |  |  |  |  |  |
| GO:0002548\_monocyte\_chemotaxis | 2 | 0 |  |  |  |  |  |  |  |  |
| GO:0002643\_regulation\_of\_tolerance\_induction | 2 | 0 |  |  |  |  |  |  |  |  |
| GO:0002645\_positive\_regulation\_of\_tolerance\_induction | 2 | 0 |  |  |  |  |  |  |  |  |
| GO:0002675\_positive\_regulation\_of\_acute\_inflammatory\_response | 2 | 0 |  |  |  |  |  |  |  |  |
| GO:0002679\_respiratory\_burst\_during\_defense\_response | 2 | 0 |  |  |  |  |  |  |  |  |
| GO:0002704\_negative\_regulation\_of\_leukocyte\_mediated\_immunity | 2 | 0 |  |  |  |  |  |  |  |  |
| GO:0002707\_negative\_regulation\_of\_lymphocyte\_mediated\_immunity | 2 | 0 |  |  |  |  |  |  |  |  |
| GO:0002710\_negative\_regulation\_of\_T\_cell\_mediated\_immunity | 2 | 0 |  |  |  |  |  |  |  |  |
| GO:0002714\_positive\_regulation\_of\_B\_cell\_mediated\_immunity | 2 | 0 |  |  |  |  |  |  |  |  |
| GO:0002820\_negative\_regulation\_of\_adaptive\_immune\_response | 2 | 0 |  |  |  |  |  |  |  |  |
| GO:0002823\_negative\_regulation\_of\_adaptive\_immune\_response\_based\_on\_somatic\_recombination\_of\_immune\_receptors\_built\_from\_immunoglobulin\_superfamily\_domains | 2 | 0 |  |  |  |  |  |  |  |  |
| GO:0002891\_positive\_regulation\_of\_immunoglobulin\_mediated\_immune\_response | 2 | 0 |  |  |  |  |  |  |  |  |
| GO:0003057\_regulation\_of\_the\_force\_of\_heart\_contraction\_by\_chemical\_signal | 2 | 0 |  |  |  |  |  |  |  |  |
| GO:0003078\_regulation\_of\_natriuresis | 2 | 0 |  |  |  |  |  |  |  |  |
| GO:0006005\_L-fucose\_biosynthetic\_process | 2 | 0 |  |  |  |  |  |  |  |  |
| GO:0006011\_UDP-glucose\_metabolic\_process | 2 | 0 |  |  |  |  |  |  |  |  |
| GO:0006030\_chitin\_metabolic\_process | 2 | 0 |  |  |  |  |  |  |  |  |
| GO:0006032\_chitin\_catabolic\_process | 2 | 0 |  |  |  |  |  |  |  |  |
| GO:0006046\_N-acetylglucosamine\_catabolic\_process | 2 | 0 |  |  |  |  |  |  |  |  |
| GO:0006047\_UDP-N-acetylglucosamine\_metabolic\_process | 2 | 0 |  |  |  |  |  |  |  |  |
| GO:0006054\_N-acetylneuraminate\_metabolic\_process | 2 | 0 |  |  |  |  |  |  |  |  |
| GO:0006063\_uronic\_acid\_metabolic\_process | 2 | 0 |  |  |  |  |  |  |  |  |
| GO:0006122\_mitochondrial\_electron\_transport\_\_ubiquinol\_to\_cytochrome\_c | 2 | 0 |  |  |  |  |  |  |  |  |
| GO:0006184\_GTP\_catabolic\_process | 2 | 0 |  |  |  |  |  |  |  |  |
| GO:0006208\_pyrimidine\_base\_catabolic\_process | 2 | 0 |  |  |  |  |  |  |  |  |
| GO:0006210\_thymine\_catabolic\_process | 2 | 0 |  |  |  |  |  |  |  |  |
| GO:0006212\_uracil\_catabolic\_process | 2 | 0 |  |  |  |  |  |  |  |  |
| GO:0006264\_mitochondrial\_DNA\_replication | 2 | 0 |  |  |  |  |  |  |  |  |
| GO:0006307\_DNA\_dealkylation | 2 | 0 |  |  |  |  |  |  |  |  |
| GO:0006335\_DNA\_replication-dependent\_nucleosome\_assembly | 2 | 0 |  |  |  |  |  |  |  |  |
| GO:0006345\_loss\_of\_chromatin\_silencing | 2 | 0 |  |  |  |  |  |  |  |  |
| GO:0006370\_mRNA\_capping | 2 | 0 |  |  |  |  |  |  |  |  |
| GO:0006398\_histone\_mRNA\_3'-end\_processing | 2 | 0 |  |  |  |  |  |  |  |  |
| GO:0006410\_transcription\_\_RNA-dependent | 2 | 0 |  |  |  |  |  |  |  |  |
| GO:0006422\_aspartyl-tRNA\_aminoacylation | 2 | 0 |  |  |  |  |  |  |  |  |
| GO:0006465\_signal\_peptide\_processing | 2 | 0 |  |  |  |  |  |  |  |  |
| GO:0006475\_internal\_protein\_amino\_acid\_acetylation | 2 | 0 |  |  |  |  |  |  |  |  |
| GO:0006478\_peptidyl-tyrosine\_sulfation | 2 | 0 |  |  |  |  |  |  |  |  |
| GO:0006481\_C-terminal\_protein\_amino\_acid\_methylation | 2 | 0 |  |  |  |  |  |  |  |  |
| GO:0006488\_dolichol-linked\_oligosaccharide\_biosynthetic\_process | 2 | 0 |  |  |  |  |  |  |  |  |
| GO:0006498\_N-terminal\_protein\_lipidation | 2 | 0 |  |  |  |  |  |  |  |  |
| GO:0006517\_protein\_deglycosylation | 2 | 0 |  |  |  |  |  |  |  |  |
| GO:0006521\_regulation\_of\_cellular\_amino\_acid\_metabolic\_process | 2 | 0 |  |  |  |  |  |  |  |  |
| GO:0006537\_glutamate\_biosynthetic\_process | 2 | 0 |  |  |  |  |  |  |  |  |
| GO:0006540\_glutamate\_decarboxylation\_to\_succinate | 2 | 0 |  |  |  |  |  |  |  |  |
| GO:0006541\_glutamine\_metabolic\_process | 2 | 0 |  |  |  |  |  |  |  |  |
| GO:0006551\_leucine\_metabolic\_process | 2 | 0 |  |  |  |  |  |  |  |  |
| GO:0006552\_leucine\_catabolic\_process | 2 | 0 |  |  |  |  |  |  |  |  |
| GO:0006561\_proline\_biosynthetic\_process | 2 | 0 |  |  |  |  |  |  |  |  |
| GO:0006569\_tryptophan\_catabolic\_process | 2 | 0 |  |  |  |  |  |  |  |  |
| GO:0006581\_acetylcholine\_catabolic\_process | 2 | 0 |  |  |  |  |  |  |  |  |
| GO:0006582\_melanin\_metabolic\_process | 2 | 0 |  |  |  |  |  |  |  |  |
| GO:0006583\_melanin\_biosynthetic\_process\_from\_tyrosine | 2 | 0 |  |  |  |  |  |  |  |  |
| GO:0006601\_creatine\_biosynthetic\_process | 2 | 0 |  |  |  |  |  |  |  |  |
| GO:0006608\_snRNP\_protein\_import\_into\_nucleus | 2 | 0 |  |  |  |  |  |  |  |  |
| GO:0006651\_diacylglycerol\_biosynthetic\_process | 2 | 0 |  |  |  |  |  |  |  |  |
| GO:0006679\_glucosylceramide\_biosynthetic\_process | 2 | 0 |  |  |  |  |  |  |  |  |
| GO:0006681\_galactosylceramide\_metabolic\_process | 2 | 0 |  |  |  |  |  |  |  |  |
| GO:0006685\_sphingomyelin\_catabolic\_process | 2 | 0 |  |  |  |  |  |  |  |  |
| GO:0006702\_androgen\_biosynthetic\_process | 2 | 0 |  |  |  |  |  |  |  |  |
| GO:0006703\_estrogen\_biosynthetic\_process | 2 | 0 |  |  |  |  |  |  |  |  |
| GO:0006710\_androgen\_catabolic\_process | 2 | 0 |  |  |  |  |  |  |  |  |
| GO:0006738\_nicotinamide\_riboside\_catabolic\_process | 2 | 0 |  |  |  |  |  |  |  |  |
| GO:0006772\_thiamin\_metabolic\_process | 2 | 0 |  |  |  |  |  |  |  |  |
| GO:0006780\_uroporphyrinogen\_III\_biosynthetic\_process | 2 | 0 |  |  |  |  |  |  |  |  |
| GO:0006784\_heme\_a\_biosynthetic\_process | 2 | 0 |  |  |  |  |  |  |  |  |
| GO:0006824\_cobalt\_ion\_transport | 2 | 0 |  |  |  |  |  |  |  |  |
| GO:0006828\_manganese\_ion\_transport | 2 | 0 |  |  |  |  |  |  |  |  |
| GO:0006880\_intracellular\_sequestering\_of\_iron\_ion | 2 | 0 |  |  |  |  |  |  |  |  |
| GO:0006883\_cellular\_sodium\_ion\_homeostasis | 2 | 0 |  |  |  |  |  |  |  |  |
| GO:0006924\_activation-induced\_cell\_death\_of\_T\_cells | 2 | 0 |  |  |  |  |  |  |  |  |
| GO:0006972\_hyperosmotic\_response | 2 | 0 |  |  |  |  |  |  |  |  |
| GO:0006975\_DNA\_damage\_induced\_protein\_phosphorylation | 2 | 0 |  |  |  |  |  |  |  |  |
| GO:0006982\_response\_to\_lipid\_hydroperoxide | 2 | 0 |  |  |  |  |  |  |  |  |
| GO:0006998\_nuclear\_envelope\_organization | 2 | 0 |  |  |  |  |  |  |  |  |
| GO:0007021\_tubulin\_complex\_assembly | 2 | 0 |  |  |  |  |  |  |  |  |
| GO:0007023\_post-chaperonin\_tubulin\_folding\_pathway | 2 | 0 |  |  |  |  |  |  |  |  |
| GO:0007063\_regulation\_of\_sister\_chromatid\_cohesion | 2 | 0 |  |  |  |  |  |  |  |  |
| GO:0007079\_mitotic\_chromosome\_movement\_towards\_spindle\_pole | 2 | 0 |  |  |  |  |  |  |  |  |
| GO:0007095\_mitotic\_cell\_cycle\_G2\_M\_transition\_DNA\_damage\_checkpoint | 2 | 0 |  |  |  |  |  |  |  |  |
| GO:0007128\_meiotic\_prophase\_I | 2 | 0 |  |  |  |  |  |  |  |  |
| GO:0007141\_male\_meiosis\_I | 2 | 0 |  |  |  |  |  |  |  |  |
| GO:0007143\_female\_meiosis | 2 | 0 |  |  |  |  |  |  |  |  |
| GO:0007191\_activation\_of\_adenylate\_cyclase\_activity\_by\_dopamine\_receptor\_signaling\_pathway | 2 | 0 |  |  |  |  |  |  |  |  |
| GO:0007206\_activation\_of\_phospholipase\_C\_activity\_by\_metabotropic\_glutamate\_receptor\_signaling\_pathway | 2 | 0 |  |  |  |  |  |  |  |  |
| GO:0007231\_osmosensory\_signaling\_pathway | 2 | 0 |  |  |  |  |  |  |  |  |
| GO:0007262\_STAT\_protein\_nuclear\_translocation | 2 | 0 |  |  |  |  |  |  |  |  |
| GO:0007290\_spermatid\_nucleus\_elongation | 2 | 0 |  |  |  |  |  |  |  |  |
| GO:0007308\_oocyte\_construction | 2 | 0 |  |  |  |  |  |  |  |  |
| GO:0007309\_oocyte\_axis\_specification | 2 | 0 |  |  |  |  |  |  |  |  |
| GO:0007341\_penetration\_of\_zona\_pellucida | 2 | 0 |  |  |  |  |  |  |  |  |
| GO:0007379\_segment\_specification | 2 | 0 |  |  |  |  |  |  |  |  |
| GO:0007418\_ventral\_midline\_development | 2 | 0 |  |  |  |  |  |  |  |  |
| GO:0007442\_hindgut\_morphogenesis | 2 | 0 |  |  |  |  |  |  |  |  |
| GO:0007492\_endoderm\_development | 2 | 0 |  |  |  |  |  |  |  |  |
| GO:0007525\_somatic\_muscle\_development | 2 | 0 |  |  |  |  |  |  |  |  |
| GO:0007549\_dosage\_compensation | 2 | 0 |  |  |  |  |  |  |  |  |
| GO:0007628\_adult\_walking\_behavior | 2 | 0 |  |  |  |  |  |  |  |  |
| GO:0008216\_spermidine\_metabolic\_process | 2 | 0 |  |  |  |  |  |  |  |  |
| GO:0008306\_associative\_learning | 2 | 0 |  |  |  |  |  |  |  |  |
| GO:0008582\_regulation\_of\_synaptic\_growth\_at\_neuromuscular\_junction | 2 | 0 |  |  |  |  |  |  |  |  |
| GO:0008593\_regulation\_of\_Notch\_signaling\_pathway | 2 | 0 |  |  |  |  |  |  |  |  |
| GO:0008608\_attachment\_of\_spindle\_microtubules\_to\_kinetochore | 2 | 0 |  |  |  |  |  |  |  |  |
| GO:0008616\_queuosine\_biosynthetic\_process | 2 | 0 |  |  |  |  |  |  |  |  |
| GO:0008618\_7-methylguanosine\_metabolic\_process | 2 | 0 |  |  |  |  |  |  |  |  |
| GO:0008653\_lipopolysaccharide\_metabolic\_process | 2 | 0 |  |  |  |  |  |  |  |  |
| GO:0009051\_pentose-phosphate\_shunt\_\_oxidative\_branch | 2 | 0 |  |  |  |  |  |  |  |  |
| GO:0009082\_branched\_chain\_family\_amino\_acid\_biosynthetic\_process | 2 | 0 |  |  |  |  |  |  |  |  |
| GO:0009103\_lipopolysaccharide\_biosynthetic\_process | 2 | 0 |  |  |  |  |  |  |  |  |
| GO:0009120\_deoxyribonucleoside\_metabolic\_process | 2 | 0 |  |  |  |  |  |  |  |  |
| GO:0009134\_nucleoside\_diphosphate\_catabolic\_process | 2 | 0 |  |  |  |  |  |  |  |  |
| GO:0009162\_deoxyribonucleoside\_monophosphate\_metabolic\_process | 2 | 0 |  |  |  |  |  |  |  |  |
| GO:0009191\_ribonucleoside\_diphosphate\_catabolic\_process | 2 | 0 |  |  |  |  |  |  |  |  |
| GO:0009263\_deoxyribonucleotide\_biosynthetic\_process | 2 | 0 |  |  |  |  |  |  |  |  |
| GO:0009439\_cyanate\_metabolic\_process | 2 | 0 |  |  |  |  |  |  |  |  |
| GO:0009440\_cyanate\_catabolic\_process | 2 | 0 |  |  |  |  |  |  |  |  |
| GO:0009448\_gamma-aminobutyric\_acid\_metabolic\_process | 2 | 0 |  |  |  |  |  |  |  |  |
| GO:0009450\_gamma-aminobutyric\_acid\_catabolic\_process | 2 | 0 |  |  |  |  |  |  |  |  |
| GO:0009452\_RNA\_capping | 2 | 0 |  |  |  |  |  |  |  |  |
| GO:0009586\_rhodopsin\_mediated\_phototransduction | 2 | 0 |  |  |  |  |  |  |  |  |
| GO:0009597\_detection\_of\_virus | 2 | 0 |  |  |  |  |  |  |  |  |
| GO:0009602\_detection\_of\_symbiont | 2 | 0 |  |  |  |  |  |  |  |  |
| GO:0009608\_response\_to\_symbiont | 2 | 0 |  |  |  |  |  |  |  |  |
| GO:0009649\_entrainment\_of\_circadian\_clock | 2 | 0 |  |  |  |  |  |  |  |  |
| GO:0009651\_response\_to\_salt\_stress | 2 | 0 |  |  |  |  |  |  |  |  |
| GO:0009756\_carbohydrate\_mediated\_signaling | 2 | 0 |  |  |  |  |  |  |  |  |
| GO:0010042\_response\_to\_manganese\_ion | 2 | 0 |  |  |  |  |  |  |  |  |
| GO:0010155\_regulation\_of\_proton\_transport | 2 | 0 |  |  |  |  |  |  |  |  |
| GO:0010216\_maintenance\_of\_DNA\_methylation | 2 | 0 |  |  |  |  |  |  |  |  |
| GO:0010248\_establishment\_or\_maintenance\_of\_transmembrane\_electrochemical\_gradient | 2 | 0 |  |  |  |  |  |  |  |  |
| GO:0010389\_regulation\_of\_G2\_M\_transition\_of\_mitotic\_cell\_cycle | 2 | 0 |  |  |  |  |  |  |  |  |
| GO:0010459\_negative\_regulation\_of\_heart\_rate | 2 | 0 |  |  |  |  |  |  |  |  |
| GO:0010470\_regulation\_of\_gastrulation | 2 | 0 |  |  |  |  |  |  |  |  |
| GO:0010506\_regulation\_of\_autophagy | 2 | 0 |  |  |  |  |  |  |  |  |
| GO:0010511\_regulation\_of\_phosphatidylinositol\_biosynthetic\_process | 2 | 0 |  |  |  |  |  |  |  |  |
| GO:0010512\_negative\_regulation\_of\_phosphatidylinositol\_biosynthetic\_process | 2 | 0 |  |  |  |  |  |  |  |  |
| GO:0010519\_negative\_regulation\_of\_phospholipase\_activity | 2 | 0 |  |  |  |  |  |  |  |  |
| GO:0010523\_negative\_regulation\_of\_calcium\_ion\_transport\_into\_cytosol | 2 | 0 |  |  |  |  |  |  |  |  |
| GO:0010533\_regulation\_of\_activation\_of\_Janus\_kinase\_activity | 2 | 0 |  |  |  |  |  |  |  |  |
| GO:0010536\_positive\_regulation\_of\_activation\_of\_Janus\_kinase\_activity | 2 | 0 |  |  |  |  |  |  |  |  |
| GO:0010614\_negative\_regulation\_of\_cardiac\_muscle\_hypertrophy | 2 | 0 |  |  |  |  |  |  |  |  |
| GO:0010616\_negative\_regulation\_of\_cardiac\_muscle\_adaptation | 2 | 0 |  |  |  |  |  |  |  |  |
| GO:0010640\_regulation\_of\_platelet-derived\_growth\_factor\_receptor\_signaling\_pathway | 2 | 0 |  |  |  |  |  |  |  |  |
| GO:0010641\_positive\_regulation\_of\_platelet-derived\_growth\_factor\_receptor\_signaling\_pathway | 2 | 0 |  |  |  |  |  |  |  |  |
| GO:0010670\_positive\_regulation\_of\_oxygen\_and\_reactive\_oxygen\_species\_metabolic\_process | 2 | 0 |  |  |  |  |  |  |  |  |
| GO:0010755\_regulation\_of\_plasminogen\_activation | 2 | 0 |  |  |  |  |  |  |  |  |
| GO:0010761\_fibroblast\_migration | 2 | 0 |  |  |  |  |  |  |  |  |
| GO:0010762\_regulation\_of\_fibroblast\_migration | 2 | 0 |  |  |  |  |  |  |  |  |
| GO:0010763\_positive\_regulation\_of\_fibroblast\_migration | 2 | 0 |  |  |  |  |  |  |  |  |
| GO:0010815\_bradykinin\_catabolic\_process | 2 | 0 |  |  |  |  |  |  |  |  |
| GO:0010818\_T\_cell\_chemotaxis | 2 | 0 |  |  |  |  |  |  |  |  |
| GO:0010819\_regulation\_of\_T\_cell\_chemotaxis | 2 | 0 |  |  |  |  |  |  |  |  |
| GO:0010820\_positive\_regulation\_of\_T\_cell\_chemotaxis | 2 | 0 |  |  |  |  |  |  |  |  |
| GO:0010866\_regulation\_of\_triglyceride\_biosynthetic\_process | 2 | 0 |  |  |  |  |  |  |  |  |
| GO:0010867\_positive\_regulation\_of\_triglyceride\_biosynthetic\_process | 2 | 0 |  |  |  |  |  |  |  |  |
| GO:0010881\_regulation\_of\_cardiac\_muscle\_contraction\_by\_regulation\_of\_the\_release\_of\_sequestered\_calcium\_ion | 2 | 0 |  |  |  |  |  |  |  |  |
| GO:0010882\_regulation\_of\_cardiac\_muscle\_contraction\_by\_calcium\_ion\_signaling | 2 | 0 |  |  |  |  |  |  |  |  |
| GO:0010908\_regulation\_of\_heparan\_sulfate\_proteoglycan\_biosynthetic\_process | 2 | 0 |  |  |  |  |  |  |  |  |
| GO:0010909\_positive\_regulation\_of\_heparan\_sulfate\_proteoglycan\_biosynthetic\_process | 2 | 0 |  |  |  |  |  |  |  |  |
| GO:0010949\_negative\_regulation\_of\_intestinal\_phytosterol\_absorption | 2 | 0 |  |  |  |  |  |  |  |  |
| GO:0010954\_positive\_regulation\_of\_protein\_maturation\_by\_peptide\_bond\_cleavage | 2 | 0 |  |  |  |  |  |  |  |  |
| GO:0010979\_regulation\_of\_vitamin\_D\_24-hydroxylase\_activity | 2 | 0 |  |  |  |  |  |  |  |  |
| GO:0010980\_positive\_regulation\_of\_vitamin\_D\_24-hydroxylase\_activity | 2 | 0 |  |  |  |  |  |  |  |  |
| GO:0010982\_regulation\_of\_high-density\_lipoprotein\_particle\_clearance | 2 | 0 |  |  |  |  |  |  |  |  |
| GO:0014002\_astrocyte\_development | 2 | 0 |  |  |  |  |  |  |  |  |
| GO:0014013\_regulation\_of\_gliogenesis | 2 | 0 |  |  |  |  |  |  |  |  |
| GO:0014014\_negative\_regulation\_of\_gliogenesis | 2 | 0 |  |  |  |  |  |  |  |  |
| GO:0014037\_Schwann\_cell\_differentiation | 2 | 0 |  |  |  |  |  |  |  |  |
| GO:0014044\_Schwann\_cell\_development | 2 | 0 |  |  |  |  |  |  |  |  |
| GO:0014072\_response\_to\_isoquinoline\_alkaloid | 2 | 0 |  |  |  |  |  |  |  |  |
| GO:0014074\_response\_to\_purine | 2 | 0 |  |  |  |  |  |  |  |  |
| GO:0014741\_negative\_regulation\_of\_muscle\_hypertrophy | 2 | 0 |  |  |  |  |  |  |  |  |
| GO:0014745\_negative\_regulation\_of\_muscle\_adaptation | 2 | 0 |  |  |  |  |  |  |  |  |
| GO:0014819\_regulation\_of\_skeletal\_muscle\_contraction | 2 | 0 |  |  |  |  |  |  |  |  |
| GO:0014911\_positive\_regulation\_of\_smooth\_muscle\_cell\_migration | 2 | 0 |  |  |  |  |  |  |  |  |
| GO:0015677\_copper\_ion\_import | 2 | 0 |  |  |  |  |  |  |  |  |
| GO:0015691\_cadmium\_ion\_transport | 2 | 0 |  |  |  |  |  |  |  |  |
| GO:0015732\_prostaglandin\_transport | 2 | 0 |  |  |  |  |  |  |  |  |
| GO:0015788\_UDP-N-acetylglucosamine\_transport | 2 | 0 |  |  |  |  |  |  |  |  |
| GO:0015793\_glycerol\_transport | 2 | 0 |  |  |  |  |  |  |  |  |
| GO:0015801\_aromatic\_amino\_acid\_transport | 2 | 0 |  |  |  |  |  |  |  |  |
| GO:0015808\_L-alanine\_transport | 2 | 0 |  |  |  |  |  |  |  |  |
| GO:0015822\_ornithine\_transport | 2 | 0 |  |  |  |  |  |  |  |  |
| GO:0015824\_proline\_transport | 2 | 0 |  |  |  |  |  |  |  |  |
| GO:0015825\_L-serine\_transport | 2 | 0 |  |  |  |  |  |  |  |  |
| GO:0015860\_purine\_nucleoside\_transport | 2 | 0 |  |  |  |  |  |  |  |  |
| GO:0015870\_acetylcholine\_transport | 2 | 0 |  |  |  |  |  |  |  |  |
| GO:0015871\_choline\_transport | 2 | 0 |  |  |  |  |  |  |  |  |
| GO:0015893\_drug\_transport | 2 | 0 |  |  |  |  |  |  |  |  |
| GO:0015920\_lipopolysaccharide\_transport | 2 | 0 |  |  |  |  |  |  |  |  |
| GO:0015936\_coenzyme\_A\_metabolic\_process | 2 | 0 |  |  |  |  |  |  |  |  |
| GO:0015939\_pantothenate\_metabolic\_process | 2 | 0 |  |  |  |  |  |  |  |  |
| GO:0015942\_formate\_metabolic\_process | 2 | 0 |  |  |  |  |  |  |  |  |
| GO:0015988\_energy\_coupled\_proton\_transport\_\_against\_electrochemical\_gradient | 2 | 0 |  |  |  |  |  |  |  |  |
| GO:0015991\_ATP\_hydrolysis\_coupled\_proton\_transport | 2 | 0 |  |  |  |  |  |  |  |  |
| GO:0015993\_molecular\_hydrogen\_transport | 2 | 0 |  |  |  |  |  |  |  |  |
| GO:0016075\_rRNA\_catabolic\_process | 2 | 0 |  |  |  |  |  |  |  |  |
| GO:0016080\_synaptic\_vesicle\_targeting | 2 | 0 |  |  |  |  |  |  |  |  |
| GO:0016090\_prenol\_metabolic\_process | 2 | 0 |  |  |  |  |  |  |  |  |
| GO:0016093\_polyprenol\_metabolic\_process | 2 | 0 |  |  |  |  |  |  |  |  |
| GO:0016233\_telomere\_capping | 2 | 0 |  |  |  |  |  |  |  |  |
| GO:0016264\_gap\_junction\_assembly | 2 | 0 |  |  |  |  |  |  |  |  |
| GO:0016266\_O-glycan\_processing | 2 | 0 |  |  |  |  |  |  |  |  |
| GO:0016322\_neuron\_remodeling | 2 | 0 |  |  |  |  |  |  |  |  |
| GO:0016482\_cytoplasmic\_transport | 2 | 0 |  |  |  |  |  |  |  |  |
| GO:0016557\_peroxisome\_membrane\_biogenesis | 2 | 0 |  |  |  |  |  |  |  |  |
| GO:0016561\_protein\_import\_into\_peroxisome\_matrix\_\_translocation | 2 | 0 |  |  |  |  |  |  |  |  |
| GO:0016973\_poly(A)+\_mRNA\_export\_from\_nucleus | 2 | 0 |  |  |  |  |  |  |  |  |
| GO:0017004\_cytochrome\_complex\_assembly | 2 | 0 |  |  |  |  |  |  |  |  |
| GO:0017055\_negative\_regulation\_of\_transcriptional\_preinitiation\_complex\_assembly | 2 | 0 |  |  |  |  |  |  |  |  |
| GO:0017145\_stem\_cell\_division | 2 | 0 |  |  |  |  |  |  |  |  |
| GO:0017158\_regulation\_of\_calcium\_ion-dependent\_exocytosis | 2 | 0 |  |  |  |  |  |  |  |  |
| GO:0017182\_peptidyl-diphthamide\_metabolic\_process | 2 | 0 |  |  |  |  |  |  |  |  |
| GO:0017183\_peptidyl-diphthamide\_biosynthetic\_process\_from\_peptidyl-histidine | 2 | 0 |  |  |  |  |  |  |  |  |
| GO:0018198\_peptidyl-cysteine\_modification | 2 | 0 |  |  |  |  |  |  |  |  |
| GO:0018202\_peptidyl-histidine\_modification | 2 | 0 |  |  |  |  |  |  |  |  |
| GO:0018282\_metal\_incorporation\_into\_metallo-sulfur\_cluster | 2 | 0 |  |  |  |  |  |  |  |  |
| GO:0018283\_iron\_incorporation\_into\_metallo-sulfur\_cluster | 2 | 0 |  |  |  |  |  |  |  |  |
| GO:0018347\_protein\_amino\_acid\_farnesylation | 2 | 0 |  |  |  |  |  |  |  |  |
| GO:0018410\_peptide\_or\_protein\_carboxyl-terminal\_blocking | 2 | 0 |  |  |  |  |  |  |  |  |
| GO:0019042\_latent\_virus\_infection | 2 | 0 |  |  |  |  |  |  |  |  |
| GO:0019046\_reactivation\_of\_latent\_virus | 2 | 0 |  |  |  |  |  |  |  |  |
| GO:0019049\_evasion\_of\_host\_defenses\_by\_virus | 2 | 0 |  |  |  |  |  |  |  |  |
| GO:0019076\_release\_of\_virus\_from\_host | 2 | 0 |  |  |  |  |  |  |  |  |
| GO:0019348\_dolichol\_metabolic\_process | 2 | 0 |  |  |  |  |  |  |  |  |
| GO:0019359\_nicotinamide\_nucleotide\_biosynthetic\_process | 2 | 0 |  |  |  |  |  |  |  |  |
| GO:0019363\_pyridine\_nucleotide\_biosynthetic\_process | 2 | 0 |  |  |  |  |  |  |  |  |
| GO:0019374\_galactolipid\_metabolic\_process | 2 | 0 |  |  |  |  |  |  |  |  |
| GO:0019459\_glutamate\_deamidation | 2 | 0 |  |  |  |  |  |  |  |  |
| GO:0019509\_methionine\_salvage | 2 | 0 |  |  |  |  |  |  |  |  |
| GO:0019530\_taurine\_metabolic\_process | 2 | 0 |  |  |  |  |  |  |  |  |
| GO:0019532\_oxalate\_transport | 2 | 0 |  |  |  |  |  |  |  |  |
| GO:0019585\_glucuronate\_metabolic\_process | 2 | 0 |  |  |  |  |  |  |  |  |
| GO:0019605\_butyrate\_metabolic\_process | 2 | 0 |  |  |  |  |  |  |  |  |
| GO:0019626\_short-chain\_fatty\_acid\_catabolic\_process | 2 | 0 |  |  |  |  |  |  |  |  |
| GO:0019730\_antimicrobial\_humoral\_response | 2 | 0 |  |  |  |  |  |  |  |  |
| GO:0019731\_antibacterial\_humoral\_response | 2 | 0 |  |  |  |  |  |  |  |  |
| GO:0019805\_quinolinate\_biosynthetic\_process | 2 | 0 |  |  |  |  |  |  |  |  |
| GO:0019836\_hemolysis\_by\_symbiont\_of\_host\_erythrocytes | 2 | 0 |  |  |  |  |  |  |  |  |
| GO:0019860\_uracil\_metabolic\_process | 2 | 0 |  |  |  |  |  |  |  |  |
| GO:0019896\_axon\_transport\_of\_mitochondrion | 2 | 0 |  |  |  |  |  |  |  |  |
| GO:0019919\_peptidyl-arginine\_methylation\_\_to\_asymmetrical-dimethyl\_arginine | 2 | 0 |  |  |  |  |  |  |  |  |
| GO:0021511\_spinal\_cord\_patterning | 2 | 0 |  |  |  |  |  |  |  |  |
| GO:0021513\_spinal\_cord\_dorsal\_ventral\_patterning | 2 | 0 |  |  |  |  |  |  |  |  |
| GO:0021517\_ventral\_spinal\_cord\_development | 2 | 0 |  |  |  |  |  |  |  |  |
| GO:0021545\_cranial\_nerve\_development | 2 | 0 |  |  |  |  |  |  |  |  |
| GO:0021587\_cerebellum\_morphogenesis | 2 | 0 |  |  |  |  |  |  |  |  |
| GO:0021675\_nerve\_development | 2 | 0 |  |  |  |  |  |  |  |  |
| GO:0021695\_cerebellar\_cortex\_development | 2 | 0 |  |  |  |  |  |  |  |  |
| GO:0021696\_cerebellar\_cortex\_morphogenesis | 2 | 0 |  |  |  |  |  |  |  |  |
| GO:0021795\_cerebral\_cortex\_cell\_migration | 2 | 0 |  |  |  |  |  |  |  |  |
| GO:0021826\_substrate-independent\_telencephalic\_tangential\_migration | 2 | 0 |  |  |  |  |  |  |  |  |
| GO:0021830\_interneuron\_migration\_from\_the\_subpallium\_to\_the\_cortex | 2 | 0 |  |  |  |  |  |  |  |  |
| GO:0021843\_substrate-independent\_telencephalic\_tangential\_interneuron\_migration | 2 | 0 |  |  |  |  |  |  |  |  |
| GO:0021853\_cerebral\_cortex\_GABAergic\_interneuron\_migration | 2 | 0 |  |  |  |  |  |  |  |  |
| GO:0021892\_cerebral\_cortex\_GABAergic\_interneuron\_differentiation | 2 | 0 |  |  |  |  |  |  |  |  |
| GO:0021894\_cerebral\_cortex\_GABAergic\_interneuron\_development | 2 | 0 |  |  |  |  |  |  |  |  |
| GO:0021895\_cerebral\_cortex\_neuron\_differentiation | 2 | 0 |  |  |  |  |  |  |  |  |
| GO:0021910\_smoothened\_signaling\_pathway\_involved\_in\_ventral\_spinal\_cord\_patterning | 2 | 0 |  |  |  |  |  |  |  |  |
| GO:0022011\_myelination\_in\_the\_peripheral\_nervous\_system | 2 | 0 |  |  |  |  |  |  |  |  |
| GO:0022605\_oogenesis\_stage | 2 | 0 |  |  |  |  |  |  |  |  |
| GO:0030150\_protein\_import\_into\_mitochondrial\_matrix | 2 | 0 |  |  |  |  |  |  |  |  |
| GO:0030185\_nitric\_oxide\_transport | 2 | 0 |  |  |  |  |  |  |  |  |
| GO:0030202\_heparin\_metabolic\_process | 2 | 0 |  |  |  |  |  |  |  |  |
| GO:0030205\_dermatan\_sulfate\_metabolic\_process | 2 | 0 |  |  |  |  |  |  |  |  |
| GO:0030208\_dermatan\_sulfate\_biosynthetic\_process | 2 | 0 |  |  |  |  |  |  |  |  |
| GO:0030210\_heparin\_biosynthetic\_process | 2 | 0 |  |  |  |  |  |  |  |  |
| GO:0030264\_nuclear\_fragmentation\_during\_apoptosis | 2 | 0 |  |  |  |  |  |  |  |  |
| GO:0030300\_regulation\_of\_intestinal\_cholesterol\_absorption | 2 | 0 |  |  |  |  |  |  |  |  |
| GO:0030311\_poly-N-acetyllactosamine\_biosynthetic\_process | 2 | 0 |  |  |  |  |  |  |  |  |
| GO:0030318\_melanocyte\_differentiation | 2 | 0 |  |  |  |  |  |  |  |  |
| GO:0030321\_transepithelial\_chloride\_transport | 2 | 0 |  |  |  |  |  |  |  |  |
| GO:0030490\_maturation\_of\_SSU-rRNA | 2 | 0 |  |  |  |  |  |  |  |  |
| GO:0030505\_inorganic\_diphosphate\_transport | 2 | 0 |  |  |  |  |  |  |  |  |
| GO:0030513\_positive\_regulation\_of\_BMP\_signaling\_pathway | 2 | 0 |  |  |  |  |  |  |  |  |
| GO:0030540\_female\_genitalia\_development | 2 | 0 |  |  |  |  |  |  |  |  |
| GO:0030575\_nuclear\_body\_organization | 2 | 0 |  |  |  |  |  |  |  |  |
| GO:0030578\_PML\_body\_organization | 2 | 0 |  |  |  |  |  |  |  |  |
| GO:0030815\_negative\_regulation\_of\_cAMP\_metabolic\_process | 2 | 0 |  |  |  |  |  |  |  |  |
| GO:0030818\_negative\_regulation\_of\_cAMP\_biosynthetic\_process | 2 | 0 |  |  |  |  |  |  |  |  |
| GO:0030889\_negative\_regulation\_of\_B\_cell\_proliferation | 2 | 0 |  |  |  |  |  |  |  |  |
| GO:0030901\_midbrain\_development | 2 | 0 |  |  |  |  |  |  |  |  |
| GO:0030916\_otic\_vesicle\_formation | 2 | 0 |  |  |  |  |  |  |  |  |
| GO:0030949\_positive\_regulation\_of\_vascular\_endothelial\_growth\_factor\_receptor\_signaling\_pathway | 2 | 0 |  |  |  |  |  |  |  |  |
| GO:0030951\_establishment\_or\_maintenance\_of\_microtubule\_cytoskeleton\_polarity | 2 | 0 |  |  |  |  |  |  |  |  |
| GO:0030952\_establishment\_or\_maintenance\_of\_cytoskeleton\_polarity | 2 | 0 |  |  |  |  |  |  |  |  |
| GO:0030997\_regulation\_of\_centriole-centriole\_cohesion | 2 | 0 |  |  |  |  |  |  |  |  |
| GO:0031000\_response\_to\_caffeine | 2 | 0 |  |  |  |  |  |  |  |  |
| GO:0031061\_negative\_regulation\_of\_histone\_methylation | 2 | 0 |  |  |  |  |  |  |  |  |
| GO:0031062\_positive\_regulation\_of\_histone\_methylation | 2 | 0 |  |  |  |  |  |  |  |  |
| GO:0031122\_cytoplasmic\_microtubule\_organization | 2 | 0 |  |  |  |  |  |  |  |  |
| GO:0031294\_lymphocyte\_costimulation | 2 | 0 |  |  |  |  |  |  |  |  |
| GO:0031295\_T\_cell\_costimulation | 2 | 0 |  |  |  |  |  |  |  |  |
| GO:0031297\_replication\_fork\_processing | 2 | 0 |  |  |  |  |  |  |  |  |
| GO:0031440\_regulation\_of\_mRNA\_3'-end\_processing | 2 | 0 |  |  |  |  |  |  |  |  |
| GO:0031452\_negative\_regulation\_of\_heterochromatin\_formation | 2 | 0 |  |  |  |  |  |  |  |  |
| GO:0031508\_centromeric\_heterochromatin\_formation | 2 | 0 |  |  |  |  |  |  |  |  |
| GO:0031536\_positive\_regulation\_of\_exit\_from\_mitosis | 2 | 0 |  |  |  |  |  |  |  |  |
| GO:0031573\_intra-S\_DNA\_damage\_checkpoint | 2 | 0 |  |  |  |  |  |  |  |  |
| GO:0031580\_membrane\_raft\_distribution | 2 | 0 |  |  |  |  |  |  |  |  |
| GO:0031641\_regulation\_of\_myelination | 2 | 0 |  |  |  |  |  |  |  |  |
| GO:0031642\_negative\_regulation\_of\_myelination | 2 | 0 |  |  |  |  |  |  |  |  |
| GO:0031664\_regulation\_of\_lipopolysaccharide-mediated\_signaling\_pathway | 2 | 0 |  |  |  |  |  |  |  |  |
| GO:0031665\_negative\_regulation\_of\_lipopolysaccharide-mediated\_signaling\_pathway | 2 | 0 |  |  |  |  |  |  |  |  |
| GO:0031936\_negative\_regulation\_of\_chromatin\_silencing | 2 | 0 |  |  |  |  |  |  |  |  |
| GO:0032026\_response\_to\_magnesium\_ion | 2 | 0 |  |  |  |  |  |  |  |  |
| GO:0032069\_regulation\_of\_nuclease\_activity | 2 | 0 |  |  |  |  |  |  |  |  |
| GO:0032096\_negative\_regulation\_of\_response\_to\_food | 2 | 0 |  |  |  |  |  |  |  |  |
| GO:0032099\_negative\_regulation\_of\_appetite | 2 | 0 |  |  |  |  |  |  |  |  |
| GO:0032201\_telomere\_maintenance\_via\_semi-conservative\_replication | 2 | 0 |  |  |  |  |  |  |  |  |
| GO:0032212\_positive\_regulation\_of\_telomere\_maintenance\_via\_telomerase | 2 | 0 |  |  |  |  |  |  |  |  |
| GO:0032213\_regulation\_of\_telomere\_maintenance\_via\_semi-conservative\_replication | 2 | 0 |  |  |  |  |  |  |  |  |
| GO:0032214\_negative\_regulation\_of\_telomere\_maintenance\_via\_semi-conservative\_replication | 2 | 0 |  |  |  |  |  |  |  |  |
| GO:0032225\_regulation\_of\_synaptic\_transmission\_\_dopaminergic | 2 | 0 |  |  |  |  |  |  |  |  |
| GO:0032228\_regulation\_of\_synaptic\_transmission\_\_GABAergic | 2 | 0 |  |  |  |  |  |  |  |  |
| GO:0032236\_positive\_regulation\_of\_calcium\_ion\_transport\_via\_store-operated\_calcium\_channel\_activity | 2 | 0 |  |  |  |  |  |  |  |  |
| GO:0032237\_activation\_of\_store-operated\_calcium\_channel\_activity | 2 | 0 |  |  |  |  |  |  |  |  |
| GO:0032292\_ensheathment\_of\_axons\_in\_the\_peripheral\_nervous\_system | 2 | 0 |  |  |  |  |  |  |  |  |
| GO:0032303\_regulation\_of\_icosanoid\_secretion | 2 | 0 |  |  |  |  |  |  |  |  |
| GO:0032305\_positive\_regulation\_of\_icosanoid\_secretion | 2 | 0 |  |  |  |  |  |  |  |  |
| GO:0032306\_regulation\_of\_prostaglandin\_secretion | 2 | 0 |  |  |  |  |  |  |  |  |
| GO:0032308\_positive\_regulation\_of\_prostaglandin\_secretion | 2 | 0 |  |  |  |  |  |  |  |  |
| GO:0032310\_prostaglandin\_secretion | 2 | 0 |  |  |  |  |  |  |  |  |
| GO:0032328\_alanine\_transport | 2 | 0 |  |  |  |  |  |  |  |  |
| GO:0032329\_serine\_transport | 2 | 0 |  |  |  |  |  |  |  |  |
| GO:0032344\_regulation\_of\_aldosterone\_metabolic\_process | 2 | 0 |  |  |  |  |  |  |  |  |
| GO:0032417\_positive\_regulation\_of\_sodium:hydrogen\_antiporter\_activity | 2 | 0 |  |  |  |  |  |  |  |  |
| GO:0032435\_negative\_regulation\_of\_proteasomal\_ubiquitin-dependent\_protein\_catabolic\_process | 2 | 0 |  |  |  |  |  |  |  |  |
| GO:0032506\_cytokinetic\_process | 2 | 0 |  |  |  |  |  |  |  |  |
| GO:0032510\_endosome\_to\_lysosome\_transport\_via\_multivesicular\_body\_sorting\_pathway | 2 | 0 |  |  |  |  |  |  |  |  |
| GO:0032512\_regulation\_of\_protein\_phosphatase\_type\_2B\_activity | 2 | 0 |  |  |  |  |  |  |  |  |
| GO:0032513\_negative\_regulation\_of\_protein\_phosphatase\_type\_2B\_activity | 2 | 0 |  |  |  |  |  |  |  |  |
| GO:0032516\_positive\_regulation\_of\_phosphoprotein\_phosphatase\_activity | 2 | 0 |  |  |  |  |  |  |  |  |
| GO:0032525\_somite\_rostral\_caudal\_axis\_specification | 2 | 0 |  |  |  |  |  |  |  |  |
| GO:0032620\_interleukin-17\_production | 2 | 0 |  |  |  |  |  |  |  |  |
| GO:0032634\_interleukin-5\_production | 2 | 0 |  |  |  |  |  |  |  |  |
| GO:0032645\_regulation\_of\_granulocyte\_macrophage\_colony-stimulating\_factor\_production | 2 | 0 |  |  |  |  |  |  |  |  |
| GO:0032656\_regulation\_of\_interleukin-13\_production | 2 | 0 |  |  |  |  |  |  |  |  |
| GO:0032660\_regulation\_of\_interleukin-17\_production | 2 | 0 |  |  |  |  |  |  |  |  |
| GO:0032674\_regulation\_of\_interleukin-5\_production | 2 | 0 |  |  |  |  |  |  |  |  |
| GO:0032689\_negative\_regulation\_of\_interferon-gamma\_production | 2 | 0 |  |  |  |  |  |  |  |  |
| GO:0032691\_negative\_regulation\_of\_interleukin-1\_beta\_production | 2 | 0 |  |  |  |  |  |  |  |  |
| GO:0032692\_negative\_regulation\_of\_interleukin-1\_production | 2 | 0 |  |  |  |  |  |  |  |  |
| GO:0032733\_positive\_regulation\_of\_interleukin-10\_production | 2 | 0 |  |  |  |  |  |  |  |  |
| GO:0032740\_positive\_regulation\_of\_interleukin-17\_production | 2 | 0 |  |  |  |  |  |  |  |  |
| GO:0032802\_low-density\_lipoprotein\_receptor\_catabolic\_process | 2 | 0 |  |  |  |  |  |  |  |  |
| GO:0032803\_regulation\_of\_low-density\_lipoprotein\_receptor\_catabolic\_process | 2 | 0 |  |  |  |  |  |  |  |  |
| GO:0032814\_regulation\_of\_natural\_killer\_cell\_activation | 2 | 0 |  |  |  |  |  |  |  |  |
| GO:0032816\_positive\_regulation\_of\_natural\_killer\_cell\_activation | 2 | 0 |  |  |  |  |  |  |  |  |
| GO:0032863\_activation\_of\_Rac\_GTPase\_activity | 2 | 0 |  |  |  |  |  |  |  |  |
| GO:0032892\_positive\_regulation\_of\_organic\_acid\_transport | 2 | 0 |  |  |  |  |  |  |  |  |
| GO:0032897\_negative\_regulation\_of\_viral\_transcription | 2 | 0 |  |  |  |  |  |  |  |  |
| GO:0032898\_neurotrophin\_production | 2 | 0 |  |  |  |  |  |  |  |  |
| GO:0032902\_nerve\_growth\_factor\_production | 2 | 0 |  |  |  |  |  |  |  |  |
| GO:0032914\_positive\_regulation\_of\_transforming\_growth\_factor-beta1\_production | 2 | 0 |  |  |  |  |  |  |  |  |
| GO:0032966\_negative\_regulation\_of\_collagen\_biosynthetic\_process | 2 | 0 |  |  |  |  |  |  |  |  |
| GO:0032971\_regulation\_of\_muscle\_filament\_sliding | 2 | 0 |  |  |  |  |  |  |  |  |
| GO:0033003\_regulation\_of\_mast\_cell\_activation | 2 | 0 |  |  |  |  |  |  |  |  |
| GO:0033005\_positive\_regulation\_of\_mast\_cell\_activation | 2 | 0 |  |  |  |  |  |  |  |  |
| GO:0033033\_negative\_regulation\_of\_myeloid\_cell\_apoptosis | 2 | 0 |  |  |  |  |  |  |  |  |
| GO:0033034\_positive\_regulation\_of\_myeloid\_cell\_apoptosis | 2 | 0 |  |  |  |  |  |  |  |  |
| GO:0033144\_negative\_regulation\_of\_steroid\_hormone\_receptor\_signaling\_pathway | 2 | 0 |  |  |  |  |  |  |  |  |
| GO:0033194\_response\_to\_hydroperoxide | 2 | 0 |  |  |  |  |  |  |  |  |
| GO:0033364\_mast\_cell\_secretory\_granule\_organization | 2 | 0 |  |  |  |  |  |  |  |  |
| GO:0033567\_DNA\_replication\_\_Okazaki\_fragment\_processing | 2 | 0 |  |  |  |  |  |  |  |  |
| GO:0033598\_mammary\_gland\_epithelial\_cell\_proliferation | 2 | 0 |  |  |  |  |  |  |  |  |
| GO:0033599\_regulation\_of\_mammary\_gland\_epithelial\_cell\_proliferation | 2 | 0 |  |  |  |  |  |  |  |  |
| GO:0033603\_positive\_regulation\_of\_dopamine\_secretion | 2 | 0 |  |  |  |  |  |  |  |  |
| GO:0033615\_mitochondrial\_proton-transporting\_ATP\_synthase\_complex\_assembly | 2 | 0 |  |  |  |  |  |  |  |  |
| GO:0033630\_positive\_regulation\_of\_cell\_adhesion\_mediated\_by\_integrin | 2 | 0 |  |  |  |  |  |  |  |  |
| GO:0033632\_regulation\_of\_cell-cell\_adhesion\_mediated\_by\_integrin | 2 | 0 |  |  |  |  |  |  |  |  |
| GO:0033861\_negative\_regulation\_of\_NAD(P)H\_oxidase\_activity | 2 | 0 |  |  |  |  |  |  |  |  |
| GO:0033864\_positive\_regulation\_of\_NAD(P)H\_oxidase\_activity | 2 | 0 |  |  |  |  |  |  |  |  |
| GO:0034113\_heterotypic\_cell-cell\_adhesion | 2 | 0 |  |  |  |  |  |  |  |  |
| GO:0034114\_regulation\_of\_heterotypic\_cell-cell\_adhesion | 2 | 0 |  |  |  |  |  |  |  |  |
| GO:0034121\_regulation\_of\_toll-like\_receptor\_signaling\_pathway | 2 | 0 |  |  |  |  |  |  |  |  |
| GO:0034134\_toll-like\_receptor\_2\_signaling\_pathway | 2 | 0 |  |  |  |  |  |  |  |  |
| GO:0034214\_protein\_hexamerization | 2 | 0 |  |  |  |  |  |  |  |  |
| GO:0034259\_negative\_regulation\_of\_Rho\_GTPase\_activity | 2 | 0 |  |  |  |  |  |  |  |  |
| GO:0034260\_negative\_regulation\_of\_GTPase\_activity | 2 | 0 |  |  |  |  |  |  |  |  |
| GO:0034261\_negative\_regulation\_of\_Ras\_GTPase\_activity | 2 | 0 |  |  |  |  |  |  |  |  |
| GO:0034331\_cell\_junction\_maintenance | 2 | 0 |  |  |  |  |  |  |  |  |
| GO:0034392\_negative\_regulation\_of\_smooth\_muscle\_cell\_apoptosis | 2 | 0 |  |  |  |  |  |  |  |  |
| GO:0034418\_urate\_biosynthetic\_process | 2 | 0 |  |  |  |  |  |  |  |  |
| GO:0034441\_plasma\_lipoprotein\_oxidation | 2 | 0 |  |  |  |  |  |  |  |  |
| GO:0034444\_regulation\_of\_plasma\_lipoprotein\_oxidation | 2 | 0 |  |  |  |  |  |  |  |  |
| GO:0034445\_negative\_regulation\_of\_plasma\_lipoprotein\_oxidation | 2 | 0 |  |  |  |  |  |  |  |  |
| GO:0034605\_cellular\_response\_to\_heat | 2 | 0 |  |  |  |  |  |  |  |  |
| GO:0034616\_response\_to\_laminar\_fluid\_shear\_stress | 2 | 0 |  |  |  |  |  |  |  |  |
| GO:0034619\_cellular\_chaperone-mediated\_protein\_complex\_assembly | 2 | 0 |  |  |  |  |  |  |  |  |
| GO:0034638\_phosphatidylcholine\_catabolic\_process | 2 | 0 |  |  |  |  |  |  |  |  |
| GO:0034644\_cellular\_response\_to\_UV | 2 | 0 |  |  |  |  |  |  |  |  |
| GO:0034650\_cortisol\_metabolic\_process | 2 | 0 |  |  |  |  |  |  |  |  |
| GO:0034651\_cortisol\_biosynthetic\_process | 2 | 0 |  |  |  |  |  |  |  |  |
| GO:0034723\_DNA\_replication-dependent\_nucleosome\_organization | 2 | 0 |  |  |  |  |  |  |  |  |
| GO:0034970\_histone\_H3-R2\_methylation | 2 | 0 |  |  |  |  |  |  |  |  |
| GO:0035025\_positive\_regulation\_of\_Rho\_protein\_signal\_transduction | 2 | 0 |  |  |  |  |  |  |  |  |
| GO:0035039\_male\_pronucleus\_formation | 2 | 0 |  |  |  |  |  |  |  |  |
| GO:0035050\_embryonic\_heart\_tube\_development | 2 | 0 |  |  |  |  |  |  |  |  |
| GO:0035058\_sensory\_cilium\_assembly | 2 | 0 |  |  |  |  |  |  |  |  |
| GO:0035117\_embryonic\_arm\_morphogenesis | 2 | 0 |  |  |  |  |  |  |  |  |
| GO:0035140\_arm\_morphogenesis | 2 | 0 |  |  |  |  |  |  |  |  |
| GO:0035162\_embryonic\_hemopoiesis | 2 | 0 |  |  |  |  |  |  |  |  |
| GO:0035247\_peptidyl-arginine\_omega-N-methylation | 2 | 0 |  |  |  |  |  |  |  |  |
| GO:0035279\_gene\_silencing\_by\_miRNA\_\_mRNA\_cleavage | 2 | 0 |  |  |  |  |  |  |  |  |
| GO:0035305\_negative\_regulation\_of\_dephosphorylation | 2 | 0 |  |  |  |  |  |  |  |  |
| GO:0035308\_negative\_regulation\_of\_protein\_amino\_acid\_dephosphorylation | 2 | 0 |  |  |  |  |  |  |  |  |
| GO:0040036\_regulation\_of\_fibroblast\_growth\_factor\_receptor\_signaling\_pathway | 2 | 0 |  |  |  |  |  |  |  |  |
| GO:0042062\_long-term\_strengthening\_of\_neuromuscular\_junction | 2 | 0 |  |  |  |  |  |  |  |  |
| GO:0042097\_interleukin-4\_biosynthetic\_process | 2 | 0 |  |  |  |  |  |  |  |  |
| GO:0042167\_heme\_catabolic\_process | 2 | 0 |  |  |  |  |  |  |  |  |
| GO:0042222\_interleukin-1\_biosynthetic\_process | 2 | 0 |  |  |  |  |  |  |  |  |
| GO:0042231\_interleukin-13\_biosynthetic\_process | 2 | 0 |  |  |  |  |  |  |  |  |
| GO:0042321\_negative\_regulation\_of\_circadian\_sleep\_wake\_cycle\_\_sleep | 2 | 0 |  |  |  |  |  |  |  |  |
| GO:0042322\_negative\_regulation\_of\_circadian\_sleep\_wake\_cycle\_\_REM\_sleep | 2 | 0 |  |  |  |  |  |  |  |  |
| GO:0042335\_cuticle\_development | 2 | 0 |  |  |  |  |  |  |  |  |
| GO:0042339\_keratan\_sulfate\_metabolic\_process | 2 | 0 |  |  |  |  |  |  |  |  |
| GO:0042350\_GDP-L-fucose\_biosynthetic\_process | 2 | 0 |  |  |  |  |  |  |  |  |
| GO:0042351\_'de\_novo'\_GDP-L-fucose\_biosynthetic\_process | 2 | 0 |  |  |  |  |  |  |  |  |
| GO:0042353\_fucose\_biosynthetic\_process | 2 | 0 |  |  |  |  |  |  |  |  |
| GO:0042396\_phosphagen\_biosynthetic\_process | 2 | 0 |  |  |  |  |  |  |  |  |
| GO:0042407\_cristae\_formation | 2 | 0 |  |  |  |  |  |  |  |  |
| GO:0042414\_epinephrine\_metabolic\_process | 2 | 0 |  |  |  |  |  |  |  |  |
| GO:0042415\_norepinephrine\_metabolic\_process | 2 | 0 |  |  |  |  |  |  |  |  |
| GO:0042436\_indole\_derivative\_catabolic\_process | 2 | 0 |  |  |  |  |  |  |  |  |
| GO:0042438\_melanin\_biosynthetic\_process | 2 | 0 |  |  |  |  |  |  |  |  |
| GO:0042481\_regulation\_of\_odontogenesis | 2 | 0 |  |  |  |  |  |  |  |  |
| GO:0042501\_serine\_phosphorylation\_of\_STAT\_protein | 2 | 0 |  |  |  |  |  |  |  |  |
| GO:0042508\_tyrosine\_phosphorylation\_of\_Stat1\_protein | 2 | 0 |  |  |  |  |  |  |  |  |
| GO:0042510\_regulation\_of\_tyrosine\_phosphorylation\_of\_Stat1\_protein | 2 | 0 |  |  |  |  |  |  |  |  |
| GO:0042511\_positive\_regulation\_of\_tyrosine\_phosphorylation\_of\_Stat1\_protein | 2 | 0 |  |  |  |  |  |  |  |  |
| GO:0042536\_negative\_regulation\_of\_tumor\_necrosis\_factor\_biosynthetic\_process | 2 | 0 |  |  |  |  |  |  |  |  |
| GO:0042634\_regulation\_of\_hair\_cycle | 2 | 0 |  |  |  |  |  |  |  |  |
| GO:0042754\_negative\_regulation\_of\_circadian\_rhythm | 2 | 0 |  |  |  |  |  |  |  |  |
| GO:0042776\_mitochondrial\_ATP\_synthesis\_coupled\_proton\_transport | 2 | 0 |  |  |  |  |  |  |  |  |
| GO:0042816\_vitamin\_B6\_metabolic\_process | 2 | 0 |  |  |  |  |  |  |  |  |
| GO:0042819\_vitamin\_B6\_biosynthetic\_process | 2 | 0 |  |  |  |  |  |  |  |  |
| GO:0042832\_defense\_response\_to\_protozoan | 2 | 0 |  |  |  |  |  |  |  |  |
| GO:0043000\_Golgi\_to\_plasma\_membrane\_CFTR\_protein\_transport | 2 | 0 |  |  |  |  |  |  |  |  |
| GO:0043006\_activation\_of\_phospholipase\_A2\_activity\_by\_calcium-mediated\_signaling | 2 | 0 |  |  |  |  |  |  |  |  |
| GO:0043011\_myeloid\_dendritic\_cell\_differentiation | 2 | 0 |  |  |  |  |  |  |  |  |
| GO:0043031\_negative\_regulation\_of\_macrophage\_activation | 2 | 0 |  |  |  |  |  |  |  |  |
| GO:0043045\_DNA\_methylation\_during\_embryonic\_development | 2 | 0 |  |  |  |  |  |  |  |  |
| GO:0043101\_purine\_salvage | 2 | 0 |  |  |  |  |  |  |  |  |
| GO:0043102\_amino\_acid\_salvage | 2 | 0 |  |  |  |  |  |  |  |  |
| GO:0043137\_DNA\_replication\_\_removal\_of\_RNA\_primer | 2 | 0 |  |  |  |  |  |  |  |  |
| GO:0043162\_ubiquitin-dependent\_protein\_catabolic\_process\_via\_the\_multivesicular\_body\_sorting\_pathway | 2 | 0 |  |  |  |  |  |  |  |  |
| GO:0043163\_cell\_envelope\_organization | 2 | 0 |  |  |  |  |  |  |  |  |
| GO:0043243\_positive\_regulation\_of\_protein\_complex\_disassembly | 2 | 0 |  |  |  |  |  |  |  |  |
| GO:0043266\_regulation\_of\_potassium\_ion\_transport | 2 | 0 |  |  |  |  |  |  |  |  |
| GO:0043277\_apoptotic\_cell\_clearance | 2 | 0 |  |  |  |  |  |  |  |  |
| GO:0043278\_response\_to\_morphine | 2 | 0 |  |  |  |  |  |  |  |  |
| GO:0043297\_apical\_junction\_assembly | 2 | 0 |  |  |  |  |  |  |  |  |
| GO:0043400\_cortisol\_secretion | 2 | 0 |  |  |  |  |  |  |  |  |
| GO:0043476\_pigment\_accumulation | 2 | 0 |  |  |  |  |  |  |  |  |
| GO:0043482\_cellular\_pigment\_accumulation | 2 | 0 |  |  |  |  |  |  |  |  |
| GO:0043490\_malate-aspartate\_shuttle | 2 | 0 |  |  |  |  |  |  |  |  |
| GO:0043508\_negative\_regulation\_of\_JUN\_kinase\_activity | 2 | 0 |  |  |  |  |  |  |  |  |
| GO:0043516\_regulation\_of\_DNA\_damage\_response\_\_signal\_transduction\_by\_p53\_class\_mediator | 2 | 0 |  |  |  |  |  |  |  |  |
| GO:0043569\_negative\_regulation\_of\_insulin-like\_growth\_factor\_receptor\_signaling\_pathway | 2 | 0 |  |  |  |  |  |  |  |  |
| GO:0043589\_skin\_morphogenesis | 2 | 0 |  |  |  |  |  |  |  |  |
| GO:0043619\_regulation\_of\_transcription\_from\_RNA\_polymerase\_II\_promoter\_in\_response\_to\_oxidative\_stress | 2 | 0 |  |  |  |  |  |  |  |  |
| GO:0043628\_ncRNA\_3'-end\_processing | 2 | 0 |  |  |  |  |  |  |  |  |
| GO:0043653\_mitochondrial\_fragmentation\_during\_apoptosis | 2 | 0 |  |  |  |  |  |  |  |  |
| GO:0043903\_regulation\_of\_symbiosis\_\_encompassing\_mutualism\_through\_parasitism | 2 | 0 |  |  |  |  |  |  |  |  |
| GO:0043932\_ossification\_involved\_in\_bone\_remodeling | 2 | 0 |  |  |  |  |  |  |  |  |
| GO:0043954\_cellular\_component\_maintenance | 2 | 0 |  |  |  |  |  |  |  |  |
| GO:0043985\_histone\_H4-R3\_methylation | 2 | 0 |  |  |  |  |  |  |  |  |
| GO:0044004\_disruption\_by\_symbiont\_of\_host\_cells | 2 | 0 |  |  |  |  |  |  |  |  |
| GO:0044030\_regulation\_of\_DNA\_methylation | 2 | 0 |  |  |  |  |  |  |  |  |
| GO:0044036\_cell\_wall\_macromolecule\_metabolic\_process | 2 | 0 |  |  |  |  |  |  |  |  |
| GO:0044058\_regulation\_of\_digestive\_system\_process | 2 | 0 |  |  |  |  |  |  |  |  |
| GO:0044062\_regulation\_of\_excretion | 2 | 0 |  |  |  |  |  |  |  |  |
| GO:0044091\_membrane\_biogenesis | 2 | 0 |  |  |  |  |  |  |  |  |
| GO:0044413\_avoidance\_of\_host\_defenses | 2 | 0 |  |  |  |  |  |  |  |  |
| GO:0044415\_evasion\_or\_tolerance\_of\_host\_defenses | 2 | 0 |  |  |  |  |  |  |  |  |
| GO:0045002\_double-strand\_break\_repair\_via\_single-strand\_annealing | 2 | 0 |  |  |  |  |  |  |  |  |
| GO:0045005\_maintenance\_of\_fidelity\_during\_DNA-dependent\_DNA\_replication | 2 | 0 |  |  |  |  |  |  |  |  |
| GO:0045019\_negative\_regulation\_of\_nitric\_oxide\_biosynthetic\_process | 2 | 0 |  |  |  |  |  |  |  |  |
| GO:0045046\_protein\_import\_into\_peroxisome\_membrane | 2 | 0 |  |  |  |  |  |  |  |  |
| GO:0045066\_regulatory\_T\_cell\_differentiation | 2 | 0 |  |  |  |  |  |  |  |  |
| GO:0045077\_negative\_regulation\_of\_interferon-gamma\_biosynthetic\_process | 2 | 0 |  |  |  |  |  |  |  |  |
| GO:0045084\_positive\_regulation\_of\_interleukin-12\_biosynthetic\_process | 2 | 0 |  |  |  |  |  |  |  |  |
| GO:0045085\_negative\_regulation\_of\_interleukin-2\_biosynthetic\_process | 2 | 0 |  |  |  |  |  |  |  |  |
| GO:0045110\_intermediate\_filament\_bundle\_assembly | 2 | 0 |  |  |  |  |  |  |  |  |
| GO:0045113\_regulation\_of\_integrin\_biosynthetic\_process | 2 | 0 |  |  |  |  |  |  |  |  |
| GO:0045162\_clustering\_of\_voltage-gated\_sodium\_channels | 2 | 0 |  |  |  |  |  |  |  |  |
| GO:0045217\_cell-cell\_junction\_maintenance | 2 | 0 |  |  |  |  |  |  |  |  |
| GO:0045229\_external\_encapsulating\_structure\_organization | 2 | 0 |  |  |  |  |  |  |  |  |
| GO:0045341\_MHC\_class\_I\_biosynthetic\_process | 2 | 0 |  |  |  |  |  |  |  |  |
| GO:0045343\_regulation\_of\_MHC\_class\_I\_biosynthetic\_process | 2 | 0 |  |  |  |  |  |  |  |  |
| GO:0045347\_negative\_regulation\_of\_MHC\_class\_II\_biosynthetic\_process | 2 | 0 |  |  |  |  |  |  |  |  |
| GO:0045348\_positive\_regulation\_of\_MHC\_class\_II\_biosynthetic\_process | 2 | 0 |  |  |  |  |  |  |  |  |
| GO:0045402\_regulation\_of\_interleukin-4\_biosynthetic\_process | 2 | 0 |  |  |  |  |  |  |  |  |
| GO:0045404\_positive\_regulation\_of\_interleukin-4\_biosynthetic\_process | 2 | 0 |  |  |  |  |  |  |  |  |
| GO:0045415\_negative\_regulation\_of\_interleukin-8\_biosynthetic\_process | 2 | 0 |  |  |  |  |  |  |  |  |
| GO:0045423\_regulation\_of\_granulocyte\_macrophage\_colony-stimulating\_factor\_biosynthetic\_process | 2 | 0 |  |  |  |  |  |  |  |  |
| GO:0045542\_positive\_regulation\_of\_cholesterol\_biosynthetic\_process | 2 | 0 |  |  |  |  |  |  |  |  |
| GO:0045576\_mast\_cell\_activation | 2 | 0 |  |  |  |  |  |  |  |  |
| GO:0045578\_negative\_regulation\_of\_B\_cell\_differentiation | 2 | 0 |  |  |  |  |  |  |  |  |
| GO:0045599\_negative\_regulation\_of\_fat\_cell\_differentiation | 2 | 0 |  |  |  |  |  |  |  |  |
| GO:0045601\_regulation\_of\_endothelial\_cell\_differentiation | 2 | 0 |  |  |  |  |  |  |  |  |
| GO:0045627\_positive\_regulation\_of\_T-helper\_1\_cell\_differentiation | 2 | 0 |  |  |  |  |  |  |  |  |
| GO:0045630\_positive\_regulation\_of\_T-helper\_2\_cell\_differentiation | 2 | 0 |  |  |  |  |  |  |  |  |
| GO:0045647\_negative\_regulation\_of\_erythrocyte\_differentiation | 2 | 0 |  |  |  |  |  |  |  |  |
| GO:0045653\_negative\_regulation\_of\_megakaryocyte\_differentiation | 2 | 0 |  |  |  |  |  |  |  |  |
| GO:0045655\_regulation\_of\_monocyte\_differentiation | 2 | 0 |  |  |  |  |  |  |  |  |
| GO:0045657\_positive\_regulation\_of\_monocyte\_differentiation | 2 | 0 |  |  |  |  |  |  |  |  |
| GO:0045685\_regulation\_of\_glial\_cell\_differentiation | 2 | 0 |  |  |  |  |  |  |  |  |
| GO:0045686\_negative\_regulation\_of\_glial\_cell\_differentiation | 2 | 0 |  |  |  |  |  |  |  |  |
| GO:0045721\_negative\_regulation\_of\_gluconeogenesis | 2 | 0 |  |  |  |  |  |  |  |  |
| GO:0045722\_positive\_regulation\_of\_gluconeogenesis | 2 | 0 |  |  |  |  |  |  |  |  |
| GO:0045726\_positive\_regulation\_of\_integrin\_biosynthetic\_process | 2 | 0 |  |  |  |  |  |  |  |  |
| GO:0045780\_positive\_regulation\_of\_bone\_resorption | 2 | 0 |  |  |  |  |  |  |  |  |
| GO:0045796\_negative\_regulation\_of\_intestinal\_cholesterol\_absorption | 2 | 0 |  |  |  |  |  |  |  |  |
| GO:0045798\_negative\_regulation\_of\_chromatin\_assembly\_or\_disassembly | 2 | 0 |  |  |  |  |  |  |  |  |
| GO:0045842\_positive\_regulation\_of\_mitotic\_metaphase\_anaphase\_transition | 2 | 0 |  |  |  |  |  |  |  |  |
| GO:0045869\_negative\_regulation\_of\_retroviral\_genome\_replication | 2 | 0 |  |  |  |  |  |  |  |  |
| GO:0045906\_negative\_regulation\_of\_vasoconstriction | 2 | 0 |  |  |  |  |  |  |  |  |
| GO:0045908\_negative\_regulation\_of\_vasodilation | 2 | 0 |  |  |  |  |  |  |  |  |
| GO:0045918\_negative\_regulation\_of\_cytolysis | 2 | 0 |  |  |  |  |  |  |  |  |
| GO:0045919\_positive\_regulation\_of\_cytolysis | 2 | 0 |  |  |  |  |  |  |  |  |
| GO:0045938\_positive\_regulation\_of\_circadian\_sleep\_wake\_cycle\_\_sleep | 2 | 0 |  |  |  |  |  |  |  |  |
| GO:0045986\_negative\_regulation\_of\_smooth\_muscle\_contraction | 2 | 0 |  |  |  |  |  |  |  |  |
| GO:0045991\_positive\_regulation\_of\_transcription\_by\_carbon\_catabolites | 2 | 0 |  |  |  |  |  |  |  |  |
| GO:0046016\_positive\_regulation\_of\_transcription\_by\_glucose | 2 | 0 |  |  |  |  |  |  |  |  |
| GO:0046039\_GTP\_metabolic\_process | 2 | 0 |  |  |  |  |  |  |  |  |
| GO:0046100\_hypoxanthine\_metabolic\_process | 2 | 0 |  |  |  |  |  |  |  |  |
| GO:0046102\_inosine\_metabolic\_process | 2 | 0 |  |  |  |  |  |  |  |  |
| GO:0046113\_nucleobase\_catabolic\_process | 2 | 0 |  |  |  |  |  |  |  |  |
| GO:0046114\_guanosine\_biosynthetic\_process | 2 | 0 |  |  |  |  |  |  |  |  |
| GO:0046116\_queuosine\_metabolic\_process | 2 | 0 |  |  |  |  |  |  |  |  |
| GO:0046118\_7-methylguanosine\_biosynthetic\_process | 2 | 0 |  |  |  |  |  |  |  |  |
| GO:0046121\_deoxyribonucleoside\_catabolic\_process | 2 | 0 |  |  |  |  |  |  |  |  |
| GO:0046135\_pyrimidine\_nucleoside\_catabolic\_process | 2 | 0 |  |  |  |  |  |  |  |  |
| GO:0046136\_positive\_regulation\_of\_vitamin\_metabolic\_process | 2 | 0 |  |  |  |  |  |  |  |  |
| GO:0046149\_pigment\_catabolic\_process | 2 | 0 |  |  |  |  |  |  |  |  |
| GO:0046160\_heme\_a\_metabolic\_process | 2 | 0 |  |  |  |  |  |  |  |  |
| GO:0046174\_polyol\_catabolic\_process | 2 | 0 |  |  |  |  |  |  |  |  |
| GO:0046218\_indolalkylamine\_catabolic\_process | 2 | 0 |  |  |  |  |  |  |  |  |
| GO:0046349\_amino\_sugar\_biosynthetic\_process | 2 | 0 |  |  |  |  |  |  |  |  |
| GO:0046359\_butyrate\_catabolic\_process | 2 | 0 |  |  |  |  |  |  |  |  |
| GO:0046368\_GDP-L-fucose\_metabolic\_process | 2 | 0 |  |  |  |  |  |  |  |  |
| GO:0046398\_UDP-glucuronate\_metabolic\_process | 2 | 0 |  |  |  |  |  |  |  |  |
| GO:0046415\_urate\_metabolic\_process | 2 | 0 |  |  |  |  |  |  |  |  |
| GO:0046476\_glycosylceramide\_biosynthetic\_process | 2 | 0 |  |  |  |  |  |  |  |  |
| GO:0046479\_glycosphingolipid\_catabolic\_process | 2 | 0 |  |  |  |  |  |  |  |  |
| GO:0046495\_nicotinamide\_riboside\_metabolic\_process | 2 | 0 |  |  |  |  |  |  |  |  |
| GO:0046500\_S-adenosylmethionine\_metabolic\_process | 2 | 0 |  |  |  |  |  |  |  |  |
| GO:0046502\_uroporphyrinogen\_III\_metabolic\_process | 2 | 0 |  |  |  |  |  |  |  |  |
| GO:0046521\_sphingoid\_catabolic\_process | 2 | 0 |  |  |  |  |  |  |  |  |
| GO:0046541\_saliva\_secretion | 2 | 0 |  |  |  |  |  |  |  |  |
| GO:0046596\_regulation\_of\_virion\_penetration\_into\_host\_cell | 2 | 0 |  |  |  |  |  |  |  |  |
| GO:0046599\_regulation\_of\_centriole\_replication | 2 | 0 |  |  |  |  |  |  |  |  |
| GO:0046629\_gamma-delta\_T\_cell\_activation | 2 | 0 |  |  |  |  |  |  |  |  |
| GO:0046633\_alpha-beta\_T\_cell\_proliferation | 2 | 0 |  |  |  |  |  |  |  |  |
| GO:0046753\_non-lytic\_viral\_release | 2 | 0 |  |  |  |  |  |  |  |  |
| GO:0046755\_non-lytic\_virus\_budding | 2 | 0 |  |  |  |  |  |  |  |  |
| GO:0046794\_virion\_transport | 2 | 0 |  |  |  |  |  |  |  |  |
| GO:0046795\_intracellular\_virion\_transport | 2 | 0 |  |  |  |  |  |  |  |  |
| GO:0046836\_glycolipid\_transport | 2 | 0 |  |  |  |  |  |  |  |  |
| GO:0046839\_phospholipid\_dephosphorylation | 2 | 0 |  |  |  |  |  |  |  |  |
| GO:0046967\_cytosol\_to\_ER\_transport | 2 | 0 |  |  |  |  |  |  |  |  |
| GO:0046968\_peptide\_antigen\_transport | 2 | 0 |  |  |  |  |  |  |  |  |
| GO:0047496\_vesicle\_transport\_along\_microtubule | 2 | 0 |  |  |  |  |  |  |  |  |
| GO:0048025\_negative\_regulation\_of\_nuclear\_mRNA\_splicing\_\_via\_spliceosome | 2 | 0 |  |  |  |  |  |  |  |  |
| GO:0048102\_autophagic\_cell\_death | 2 | 0 |  |  |  |  |  |  |  |  |
| GO:0048103\_somatic\_stem\_cell\_division | 2 | 0 |  |  |  |  |  |  |  |  |
| GO:0048147\_negative\_regulation\_of\_fibroblast\_proliferation | 2 | 0 |  |  |  |  |  |  |  |  |
| GO:0048149\_behavioral\_response\_to\_ethanol | 2 | 0 |  |  |  |  |  |  |  |  |
| GO:0048165\_fused\_antrum\_stage\_\_oogenesis | 2 | 0 |  |  |  |  |  |  |  |  |
| GO:0048169\_regulation\_of\_long-term\_neuronal\_synaptic\_plasticity | 2 | 0 |  |  |  |  |  |  |  |  |
| GO:0048227\_plasma\_membrane\_to\_endosome\_transport | 2 | 0 |  |  |  |  |  |  |  |  |
| GO:0048290\_isotype\_switching\_to\_IgA\_isotypes | 2 | 0 |  |  |  |  |  |  |  |  |
| GO:0048291\_isotype\_switching\_to\_IgG\_isotypes | 2 | 0 |  |  |  |  |  |  |  |  |
| GO:0048296\_regulation\_of\_isotype\_switching\_to\_IgA\_isotypes | 2 | 0 |  |  |  |  |  |  |  |  |
| GO:0048298\_positive\_regulation\_of\_isotype\_switching\_to\_IgA\_isotypes | 2 | 0 |  |  |  |  |  |  |  |  |
| GO:0048386\_positive\_regulation\_of\_retinoic\_acid\_receptor\_signaling\_pathway | 2 | 0 |  |  |  |  |  |  |  |  |
| GO:0048550\_negative\_regulation\_of\_pinocytosis | 2 | 0 |  |  |  |  |  |  |  |  |
| GO:0048625\_myoblast\_cell\_fate\_commitment | 2 | 0 |  |  |  |  |  |  |  |  |
| GO:0048630\_skeletal\_muscle\_tissue\_growth | 2 | 0 |  |  |  |  |  |  |  |  |
| GO:0048708\_astrocyte\_differentiation | 2 | 0 |  |  |  |  |  |  |  |  |
| GO:0048713\_regulation\_of\_oligodendrocyte\_differentiation | 2 | 0 |  |  |  |  |  |  |  |  |
| GO:0048715\_negative\_regulation\_of\_oligodendrocyte\_differentiation | 2 | 0 |  |  |  |  |  |  |  |  |
| GO:0048820\_hair\_follicle\_maturation | 2 | 0 |  |  |  |  |  |  |  |  |
| GO:0048844\_artery\_morphogenesis | 2 | 0 |  |  |  |  |  |  |  |  |
| GO:0048854\_brain\_morphogenesis | 2 | 0 |  |  |  |  |  |  |  |  |
| GO:0048857\_neural\_nucleus\_development | 2 | 0 |  |  |  |  |  |  |  |  |
| GO:0050653\_chondroitin\_sulfate\_proteoglycan\_biosynthetic\_process\_\_polysaccharide\_chain\_biosynthetic\_process | 2 | 0 |  |  |  |  |  |  |  |  |
| GO:0050667\_homocysteine\_metabolic\_process | 2 | 0 |  |  |  |  |  |  |  |  |
| GO:0050711\_negative\_regulation\_of\_interleukin-1\_secretion | 2 | 0 |  |  |  |  |  |  |  |  |
| GO:0050720\_interleukin-1\_beta\_biosynthetic\_process | 2 | 0 |  |  |  |  |  |  |  |  |
| GO:0050805\_negative\_regulation\_of\_synaptic\_transmission | 2 | 0 |  |  |  |  |  |  |  |  |
| GO:0050847\_progesterone\_receptor\_signaling\_pathway | 2 | 0 |  |  |  |  |  |  |  |  |
| GO:0050849\_negative\_regulation\_of\_calcium-mediated\_signaling | 2 | 0 |  |  |  |  |  |  |  |  |
| GO:0050862\_positive\_regulation\_of\_T\_cell\_receptor\_signaling\_pathway | 2 | 0 |  |  |  |  |  |  |  |  |
| GO:0050872\_white\_fat\_cell\_differentiation | 2 | 0 |  |  |  |  |  |  |  |  |
| GO:0050916\_sensory\_perception\_of\_sweet\_taste | 2 | 0 |  |  |  |  |  |  |  |  |
| GO:0050917\_sensory\_perception\_of\_umami\_taste | 2 | 0 |  |  |  |  |  |  |  |  |
| GO:0050919\_negative\_chemotaxis | 2 | 0 |  |  |  |  |  |  |  |  |
| GO:0050931\_pigment\_cell\_differentiation | 2 | 0 |  |  |  |  |  |  |  |  |
| GO:0050982\_detection\_of\_mechanical\_stimulus | 2 | 0 |  |  |  |  |  |  |  |  |
| GO:0051088\_PMA-inducible\_membrane\_protein\_ectodomain\_proteolysis | 2 | 0 |  |  |  |  |  |  |  |  |
| GO:0051097\_negative\_regulation\_of\_helicase\_activity | 2 | 0 |  |  |  |  |  |  |  |  |
| GO:0051124\_synaptic\_growth\_at\_neuromuscular\_junction | 2 | 0 |  |  |  |  |  |  |  |  |
| GO:0051151\_negative\_regulation\_of\_smooth\_muscle\_cell\_differentiation | 2 | 0 |  |  |  |  |  |  |  |  |
| GO:0051176\_positive\_regulation\_of\_sulfur\_metabolic\_process | 2 | 0 |  |  |  |  |  |  |  |  |
| GO:0051225\_spindle\_assembly | 2 | 0 |  |  |  |  |  |  |  |  |
| GO:0051231\_spindle\_elongation | 2 | 0 |  |  |  |  |  |  |  |  |
| GO:0051313\_attachment\_of\_spindle\_microtubules\_to\_chromosome | 2 | 0 |  |  |  |  |  |  |  |  |
| GO:0051386\_regulation\_of\_nerve\_growth\_factor\_receptor\_signaling\_pathway | 2 | 0 |  |  |  |  |  |  |  |  |
| GO:0051387\_negative\_regulation\_of\_nerve\_growth\_factor\_receptor\_signaling\_pathway | 2 | 0 |  |  |  |  |  |  |  |  |
| GO:0051462\_regulation\_of\_cortisol\_secretion | 2 | 0 |  |  |  |  |  |  |  |  |
| GO:0051464\_positive\_regulation\_of\_cortisol\_secretion | 2 | 0 |  |  |  |  |  |  |  |  |
| GO:0051541\_elastin\_metabolic\_process | 2 | 0 |  |  |  |  |  |  |  |  |
| GO:0051547\_regulation\_of\_keratinocyte\_migration | 2 | 0 |  |  |  |  |  |  |  |  |
| GO:0051549\_positive\_regulation\_of\_keratinocyte\_migration | 2 | 0 |  |  |  |  |  |  |  |  |
| GO:0051570\_regulation\_of\_histone\_H3-K9\_methylation | 2 | 0 |  |  |  |  |  |  |  |  |
| GO:0051571\_positive\_regulation\_of\_histone\_H3-K4\_methylation | 2 | 0 |  |  |  |  |  |  |  |  |
| GO:0051573\_negative\_regulation\_of\_histone\_H3-K9\_methylation | 2 | 0 |  |  |  |  |  |  |  |  |
| GO:0051580\_regulation\_of\_neurotransmitter\_uptake | 2 | 0 |  |  |  |  |  |  |  |  |
| GO:0051583\_dopamine\_uptake | 2 | 0 |  |  |  |  |  |  |  |  |
| GO:0051584\_regulation\_of\_dopamine\_uptake | 2 | 0 |  |  |  |  |  |  |  |  |
| GO:0051657\_maintenance\_of\_organelle\_location | 2 | 0 |  |  |  |  |  |  |  |  |
| GO:0051659\_maintenance\_of\_mitochondrion\_location | 2 | 0 |  |  |  |  |  |  |  |  |
| GO:0051665\_membrane\_raft\_localization | 2 | 0 |  |  |  |  |  |  |  |  |
| GO:0051715\_cytolysis\_of\_cells\_of\_another\_organism | 2 | 0 |  |  |  |  |  |  |  |  |
| GO:0051797\_regulation\_of\_hair\_follicle\_development | 2 | 0 |  |  |  |  |  |  |  |  |
| GO:0051798\_positive\_regulation\_of\_hair\_follicle\_development | 2 | 0 |  |  |  |  |  |  |  |  |
| GO:0051801\_cytolysis\_of\_cells\_in\_other\_organism\_during\_symbiotic\_interaction | 2 | 0 |  |  |  |  |  |  |  |  |
| GO:0051818\_disruption\_of\_cells\_of\_other\_organism\_during\_symbiotic\_interaction | 2 | 0 |  |  |  |  |  |  |  |  |
| GO:0051832\_avoidance\_of\_defenses\_of\_other\_organism\_during\_symbiotic\_interaction | 2 | 0 |  |  |  |  |  |  |  |  |
| GO:0051834\_evasion\_or\_tolerance\_of\_defenses\_of\_other\_organism\_during\_symbiotic\_interaction | 2 | 0 |  |  |  |  |  |  |  |  |
| GO:0051883\_killing\_of\_cells\_in\_other\_organism\_during\_symbiotic\_interaction | 2 | 0 |  |  |  |  |  |  |  |  |
| GO:0051890\_regulation\_of\_cardioblast\_differentiation | 2 | 0 |  |  |  |  |  |  |  |  |
| GO:0051891\_positive\_regulation\_of\_cardioblast\_differentiation | 2 | 0 |  |  |  |  |  |  |  |  |
| GO:0051900\_regulation\_of\_mitochondrial\_depolarization | 2 | 0 |  |  |  |  |  |  |  |  |
| GO:0051902\_negative\_regulation\_of\_mitochondrial\_depolarization | 2 | 0 |  |  |  |  |  |  |  |  |
| GO:0051927\_negative\_regulation\_of\_calcium\_ion\_transport\_via\_voltage-gated\_calcium\_channel\_activity | 2 | 0 |  |  |  |  |  |  |  |  |
| GO:0051932\_synaptic\_transmission\_\_GABAergic | 2 | 0 |  |  |  |  |  |  |  |  |
| GO:0051934\_catecholamine\_uptake\_during\_transmission\_of\_nerve\_impulse | 2 | 0 |  |  |  |  |  |  |  |  |
| GO:0051940\_regulation\_of\_catecholamine\_uptake\_during\_transmission\_of\_nerve\_impulse | 2 | 0 |  |  |  |  |  |  |  |  |
| GO:0051967\_negative\_regulation\_of\_synaptic\_transmission\_\_glutamatergic | 2 | 0 |  |  |  |  |  |  |  |  |
| GO:0052025\_modification\_by\_symbiont\_of\_host\_cell\_membrane | 2 | 0 |  |  |  |  |  |  |  |  |
| GO:0052043\_modification\_by\_symbiont\_of\_host\_cellular\_component | 2 | 0 |  |  |  |  |  |  |  |  |
| GO:0052111\_modification\_by\_symbiont\_of\_host\_structure | 2 | 0 |  |  |  |  |  |  |  |  |
| GO:0052173\_response\_to\_defenses\_of\_other\_organism\_during\_symbiotic\_interaction | 2 | 0 |  |  |  |  |  |  |  |  |
| GO:0052185\_modification\_of\_structure\_of\_other\_organism\_during\_symbiotic\_interaction | 2 | 0 |  |  |  |  |  |  |  |  |
| GO:0052188\_modification\_of\_cellular\_component\_in\_other\_organism\_during\_symbiotic\_interaction | 2 | 0 |  |  |  |  |  |  |  |  |
| GO:0052200\_response\_to\_host\_defenses | 2 | 0 |  |  |  |  |  |  |  |  |
| GO:0052331\_hemolysis\_by\_organism\_of\_erythrocytes\_in\_other\_organism\_during\_symbiotic\_interaction | 2 | 0 |  |  |  |  |  |  |  |  |
| GO:0052332\_modification\_by\_organism\_of\_cell\_membrane\_in\_other\_organism\_during\_symbiotic\_interaction | 2 | 0 |  |  |  |  |  |  |  |  |
| GO:0055007\_cardiac\_muscle\_cell\_differentiation | 2 | 0 |  |  |  |  |  |  |  |  |
| GO:0055075\_potassium\_ion\_homeostasis | 2 | 0 |  |  |  |  |  |  |  |  |
| GO:0055090\_acylglycerol\_homeostasis | 2 | 0 |  |  |  |  |  |  |  |  |
| GO:0055098\_response\_to\_low\_density\_lipoprotein\_stimulus | 2 | 0 |  |  |  |  |  |  |  |  |
| GO:0060004\_reflex | 2 | 0 |  |  |  |  |  |  |  |  |
| GO:0060026\_convergent\_extension | 2 | 0 |  |  |  |  |  |  |  |  |
| GO:0060027\_convergent\_extension\_involved\_in\_gastrulation | 2 | 0 |  |  |  |  |  |  |  |  |
| GO:0060033\_anatomical\_structure\_regression | 2 | 0 |  |  |  |  |  |  |  |  |
| GO:0060037\_pharyngeal\_system\_development | 2 | 0 |  |  |  |  |  |  |  |  |
| GO:0060044\_negative\_regulation\_of\_cardiac\_muscle\_cell\_proliferation | 2 | 0 |  |  |  |  |  |  |  |  |
| GO:0060045\_positive\_regulation\_of\_cardiac\_muscle\_cell\_proliferation | 2 | 0 |  |  |  |  |  |  |  |  |
| GO:0060052\_neurofilament\_cytoskeleton\_organization | 2 | 0 |  |  |  |  |  |  |  |  |
| GO:0060056\_mammary\_gland\_involution | 2 | 0 |  |  |  |  |  |  |  |  |
| GO:0060079\_regulation\_of\_excitatory\_postsynaptic\_membrane\_potential | 2 | 0 |  |  |  |  |  |  |  |  |
| GO:0060080\_regulation\_of\_inhibitory\_postsynaptic\_membrane\_potential | 2 | 0 |  |  |  |  |  |  |  |  |
| GO:0060081\_membrane\_hyperpolarization | 2 | 0 |  |  |  |  |  |  |  |  |
| GO:0060087\_relaxation\_of\_vascular\_smooth\_muscle | 2 | 0 |  |  |  |  |  |  |  |  |
| GO:0060117\_auditory\_receptor\_cell\_development | 2 | 0 |  |  |  |  |  |  |  |  |
| GO:0060122\_inner\_ear\_receptor\_stereocilium\_organization | 2 | 0 |  |  |  |  |  |  |  |  |
| GO:0060151\_peroxisome\_localization | 2 | 0 |  |  |  |  |  |  |  |  |
| GO:0060152\_microtubule-based\_peroxisome\_localization | 2 | 0 |  |  |  |  |  |  |  |  |
| GO:0060159\_regulation\_of\_dopamine\_receptor\_signaling\_pathway | 2 | 0 |  |  |  |  |  |  |  |  |
| GO:0060261\_positive\_regulation\_of\_transcription\_initiation\_from\_RNA\_polymerase\_II\_promoter | 2 | 0 |  |  |  |  |  |  |  |  |
| GO:0060264\_regulation\_of\_respiratory\_burst\_during\_acute\_inflammatory\_response | 2 | 0 |  |  |  |  |  |  |  |  |
| GO:0060271\_cilium\_morphogenesis | 2 | 0 |  |  |  |  |  |  |  |  |
| GO:0060272\_embryonic\_skeletal\_joint\_morphogenesis | 2 | 0 |  |  |  |  |  |  |  |  |
| GO:0060285\_ciliary\_cell\_motility | 2 | 0 |  |  |  |  |  |  |  |  |
| GO:0060297\_regulation\_of\_sarcomere\_organization | 2 | 0 |  |  |  |  |  |  |  |  |
| GO:0060337\_type\_I\_interferon-mediated\_signaling\_pathway | 2 | 0 |  |  |  |  |  |  |  |  |
| GO:0060338\_regulation\_of\_type\_I\_interferon-mediated\_signaling\_pathway | 2 | 0 |  |  |  |  |  |  |  |  |
| GO:0060370\_susceptibility\_to\_T\_cell\_mediated\_cytotoxicity | 2 | 0 |  |  |  |  |  |  |  |  |
| GO:0060443\_mammary\_gland\_morphogenesis | 2 | 0 |  |  |  |  |  |  |  |  |
| GO:0060457\_negative\_regulation\_of\_digestive\_system\_process | 2 | 0 |  |  |  |  |  |  |  |  |
| GO:0060544\_regulation\_of\_necroptosis | 2 | 0 |  |  |  |  |  |  |  |  |
| GO:0060545\_positive\_regulation\_of\_necroptosis | 2 | 0 |  |  |  |  |  |  |  |  |
| GO:0060553\_induction\_of\_necroptosis | 2 | 0 |  |  |  |  |  |  |  |  |
| GO:0060555\_induction\_of\_necroptosis\_by\_extracellular\_signals | 2 | 0 |  |  |  |  |  |  |  |  |
| GO:0060556\_regulation\_of\_vitamin\_D\_biosynthetic\_process | 2 | 0 |  |  |  |  |  |  |  |  |
| GO:0060557\_positive\_regulation\_of\_vitamin\_D\_biosynthetic\_process | 2 | 0 |  |  |  |  |  |  |  |  |
| GO:0060584\_regulation\_of\_prostaglandin-endoperoxide\_synthase\_activity | 2 | 0 |  |  |  |  |  |  |  |  |
| GO:0060585\_positive\_regulation\_of\_prostaglandin-endoperoxidase\_synthase\_activity | 2 | 0 |  |  |  |  |  |  |  |  |
| GO:0060620\_regulation\_of\_cholesterol\_import | 2 | 0 |  |  |  |  |  |  |  |  |
| GO:0060621\_negative\_regulation\_of\_cholesterol\_import | 2 | 0 |  |  |  |  |  |  |  |  |
| GO:0060696\_regulation\_of\_phospholipid\_catabolic\_process | 2 | 0 |  |  |  |  |  |  |  |  |
| GO:0060752\_intestinal\_phytosterol\_absorption | 2 | 0 |  |  |  |  |  |  |  |  |
| GO:0060759\_regulation\_of\_response\_to\_cytokine\_stimulus | 2 | 0 |  |  |  |  |  |  |  |  |
| GO:0060840\_artery\_development | 2 | 0 |  |  |  |  |  |  |  |  |
| GO:0070059\_apoptosis\_in\_response\_to\_endoplasmic\_reticulum\_stress | 2 | 0 |  |  |  |  |  |  |  |  |
| GO:0070070\_proton-transporting\_V-type\_ATPase\_complex\_assembly | 2 | 0 |  |  |  |  |  |  |  |  |
| GO:0070072\_vacuolar\_proton-transporting\_V-type\_ATPase\_complex\_assembly | 2 | 0 |  |  |  |  |  |  |  |  |
| GO:0070166\_enamel\_mineralization | 2 | 0 |  |  |  |  |  |  |  |  |
| GO:0070170\_regulation\_of\_tooth\_mineralization | 2 | 0 |  |  |  |  |  |  |  |  |
| GO:0070230\_positive\_regulation\_of\_lymphocyte\_apoptosis | 2 | 0 |  |  |  |  |  |  |  |  |
| GO:0070266\_necroptosis | 2 | 0 |  |  |  |  |  |  |  |  |
| GO:0070483\_detection\_of\_hypoxia | 2 | 0 |  |  |  |  |  |  |  |  |
| GO:0070493\_thrombin\_receptor\_signaling\_pathway | 2 | 0 |  |  |  |  |  |  |  |  |
| GO:0070536\_protein\_K63-linked\_deubiquitination | 2 | 0 |  |  |  |  |  |  |  |  |
| GO:0070574\_cadmium\_ion\_transmembrane\_transport | 2 | 0 |  |  |  |  |  |  |  |  |
| GO:0070586\_cell-cell\_adhesion\_involved\_in\_gastrulation | 2 | 0 |  |  |  |  |  |  |  |  |
| GO:0070587\_regulation\_of\_cell-cell\_adhesion\_involved\_in\_gastrulation | 2 | 0 |  |  |  |  |  |  |  |  |
| GO:0070637\_pyridine\_nucleoside\_metabolic\_process | 2 | 0 |  |  |  |  |  |  |  |  |
| GO:0070638\_pyridine\_nucleoside\_catabolic\_process | 2 | 0 |  |  |  |  |  |  |  |  |
| GO:0070874\_negative\_regulation\_of\_glycogen\_metabolic\_process | 2 | 0 |  |  |  |  |  |  |  |  |
| GO:0075136\_response\_to\_host | 2 | 0 |  |  |  |  |  |  |  |  |
| GO:0006171\_cAMP\_biosynthetic\_process | 59 | 0 | 0.000000 | 0.000000 | 540 | 455.561090 | 514.94 | 574.318910 | 0.953593 |
| GO:0006820\_anion\_transport | 59 | 0 | 0.000000 | 0.000000 | 540 | 455.561090 | 514.94 | 574.318910 | 0.953593 |
| GO:0007409\_axonogenesis | 59 | 0 | 0.000000 | 0.000000 | 540 | 455.561090 | 514.94 | 574.318910 | 0.953593 |
| GO:0033673\_negative\_regulation\_of\_kinase\_activity | 59 | 0 | 0.000000 | 0.000000 | 540 | 455.561090 | 514.94 | 574.318910 | 0.953593 |
| GO:0043281\_regulation\_of\_caspase\_activity | 59 | 0 | 0.000000 | 0.000000 | 540 | 455.561090 | 514.94 | 574.318910 | 0.953593 |
| GO:0051604\_protein\_maturation | 59 | 0 | 0.000000 | 0.000000 | 540 | 455.561090 | 514.94 | 574.318910 | 0.953593 |
| GO:0001822\_kidney\_development | 20 | 0 | 0.000000 | 0.000000 | 581 | 498.042400 | 556.56 | 615.077600 | 0.957935 |
| GO:0002703\_regulation\_of\_leukocyte\_mediated\_immunity | 20 | 0 | 0.000000 | 0.000000 | 581 | 498.042400 | 556.56 | 615.077600 | 0.957935 |
| GO:0002706\_regulation\_of\_lymphocyte\_mediated\_immunity | 20 | 0 | 0.000000 | 0.000000 | 581 | 498.042400 | 556.56 | 615.077600 | 0.957935 |
| GO:0005977\_glycogen\_metabolic\_process | 20 | 0 | 0.000000 | 0.000000 | 581 | 498.042400 | 556.56 | 615.077600 | 0.957935 |
| GO:0006096\_glycolysis | 20 | 0 | 0.000000 | 0.000000 | 581 | 498.042400 | 556.56 | 615.077600 | 0.957935 |
| GO:0006305\_DNA\_alkylation | 20 | 0 | 0.000000 | 0.000000 | 581 | 498.042400 | 556.56 | 615.077600 | 0.957935 |
| GO:0006306\_DNA\_methylation | 20 | 0 | 0.000000 | 0.000000 | 581 | 498.042400 | 556.56 | 615.077600 | 0.957935 |
| GO:0006733\_oxidoreduction\_coenzyme\_metabolic\_process | 20 | 0 | 0.000000 | 0.000000 | 581 | 498.042400 | 556.56 | 615.077600 | 0.957935 |
| GO:0006885\_regulation\_of\_pH | 20 | 0 | 0.000000 | 0.000000 | 581 | 498.042400 | 556.56 | 615.077600 | 0.957935 |
| GO:0007159\_leukocyte\_adhesion | 20 | 0 | 0.000000 | 0.000000 | 581 | 498.042400 | 556.56 | 615.077600 | 0.957935 |
| GO:0007215\_glutamate\_signaling\_pathway | 20 | 0 | 0.000000 | 0.000000 | 581 | 498.042400 | 556.56 | 615.077600 | 0.957935 |
| GO:0008633\_activation\_of\_pro-apoptotic\_gene\_products | 20 | 0 | 0.000000 | 0.000000 | 581 | 498.042400 | 556.56 | 615.077600 | 0.957935 |
| GO:0009064\_glutamine\_family\_amino\_acid\_metabolic\_process | 20 | 0 | 0.000000 | 0.000000 | 581 | 498.042400 | 556.56 | 615.077600 | 0.957935 |
| GO:0009144\_purine\_nucleoside\_triphosphate\_metabolic\_process | 20 | 0 | 0.000000 | 0.000000 | 581 | 498.042400 | 556.56 | 615.077600 | 0.957935 |
| GO:0009205\_purine\_ribonucleoside\_triphosphate\_metabolic\_process | 20 | 0 | 0.000000 | 0.000000 | 581 | 498.042400 | 556.56 | 615.077600 | 0.957935 |
| GO:0009583\_detection\_of\_light\_stimulus | 20 | 0 | 0.000000 | 0.000000 | 581 | 498.042400 | 556.56 | 615.077600 | 0.957935 |
| GO:0009584\_detection\_of\_visible\_light | 20 | 0 | 0.000000 | 0.000000 | 581 | 498.042400 | 556.56 | 615.077600 | 0.957935 |
| GO:0010594\_regulation\_of\_endothelial\_cell\_migration | 20 | 0 | 0.000000 | 0.000000 | 581 | 498.042400 | 556.56 | 615.077600 | 0.957935 |
| GO:0016202\_regulation\_of\_striated\_muscle\_tissue\_development | 20 | 0 | 0.000000 | 0.000000 | 581 | 498.042400 | 556.56 | 615.077600 | 0.957935 |
| GO:0021700\_developmental\_maturation | 20 | 0 | 0.000000 | 0.000000 | 581 | 498.042400 | 556.56 | 615.077600 | 0.957935 |
| GO:0031047\_gene\_silencing\_by\_RNA | 20 | 0 | 0.000000 | 0.000000 | 581 | 498.042400 | 556.56 | 615.077600 | 0.957935 |
| GO:0032409\_regulation\_of\_transporter\_activity | 20 | 0 | 0.000000 | 0.000000 | 581 | 498.042400 | 556.56 | 615.077600 | 0.957935 |
| GO:0032535\_regulation\_of\_cellular\_component\_size | 20 | 0 | 0.000000 | 0.000000 | 581 | 498.042400 | 556.56 | 615.077600 | 0.957935 |
| GO:0033692\_cellular\_polysaccharide\_biosynthetic\_process | 20 | 0 | 0.000000 | 0.000000 | 581 | 498.042400 | 556.56 | 615.077600 | 0.957935 |
| GO:0034367\_macromolecular\_complex\_remodeling | 20 | 0 | 0.000000 | 0.000000 | 581 | 498.042400 | 556.56 | 615.077600 | 0.957935 |
| GO:0034368\_protein-lipid\_complex\_remodeling | 20 | 0 | 0.000000 | 0.000000 | 581 | 498.042400 | 556.56 | 615.077600 | 0.957935 |
| GO:0034369\_plasma\_lipoprotein\_particle\_remodeling | 20 | 0 | 0.000000 | 0.000000 | 581 | 498.042400 | 556.56 | 615.077600 | 0.957935 |
| GO:0042594\_response\_to\_starvation | 20 | 0 | 0.000000 | 0.000000 | 581 | 498.042400 | 556.56 | 615.077600 | 0.957935 |
| GO:0043244\_regulation\_of\_protein\_complex\_disassembly | 20 | 0 | 0.000000 | 0.000000 | 581 | 498.042400 | 556.56 | 615.077600 | 0.957935 |
| GO:0043900\_regulation\_of\_multi-organism\_process | 20 | 0 | 0.000000 | 0.000000 | 581 | 498.042400 | 556.56 | 615.077600 | 0.957935 |
| GO:0045165\_cell\_fate\_commitment | 20 | 0 | 0.000000 | 0.000000 | 581 | 498.042400 | 556.56 | 615.077600 | 0.957935 |
| GO:0045840\_positive\_regulation\_of\_mitosis | 20 | 0 | 0.000000 | 0.000000 | 581 | 498.042400 | 556.56 | 615.077600 | 0.957935 |
| GO:0046209\_nitric\_oxide\_metabolic\_process | 20 | 0 | 0.000000 | 0.000000 | 581 | 498.042400 | 556.56 | 615.077600 | 0.957935 |
| GO:0046323\_glucose\_import | 20 | 0 | 0.000000 | 0.000000 | 581 | 498.042400 | 556.56 | 615.077600 | 0.957935 |
| GO:0046519\_sphingoid\_metabolic\_process | 20 | 0 | 0.000000 | 0.000000 | 581 | 498.042400 | 556.56 | 615.077600 | 0.957935 |
| GO:0046661\_male\_sex\_differentiation | 20 | 0 | 0.000000 | 0.000000 | 581 | 498.042400 | 556.56 | 615.077600 | 0.957935 |
| GO:0048634\_regulation\_of\_muscle\_development | 20 | 0 | 0.000000 | 0.000000 | 581 | 498.042400 | 556.56 | 615.077600 | 0.957935 |
| GO:0050804\_regulation\_of\_synaptic\_transmission | 20 | 0 | 0.000000 | 0.000000 | 581 | 498.042400 | 556.56 | 615.077600 | 0.957935 |
| GO:0050866\_negative\_regulation\_of\_cell\_activation | 20 | 0 | 0.000000 | 0.000000 | 581 | 498.042400 | 556.56 | 615.077600 | 0.957935 |
| GO:0051320\_S\_phase | 20 | 0 | 0.000000 | 0.000000 | 581 | 498.042400 | 556.56 | 615.077600 | 0.957935 |
| GO:0051785\_positive\_regulation\_of\_nuclear\_division | 20 | 0 | 0.000000 | 0.000000 | 581 | 498.042400 | 556.56 | 615.077600 | 0.957935 |
| GO:0051172\_negative\_regulation\_of\_nitrogen\_compound\_metabolic\_process | 298 | 0 | 0.000000 | 0.000000 | 582 | 498.680872 | 557.08 | 615.479128 | 0.957182 |
| GO:0006575\_cellular\_amino\_acid\_derivative\_metabolic\_process | 76 | 0 | 0.000000 | 0.000000 | 587 | 505.155865 | 563.01 | 620.864135 | 0.959131 |
| GO:0006979\_response\_to\_oxidative\_stress | 76 | 0 | 0.000000 | 0.000000 | 587 | 505.155865 | 563.01 | 620.864135 | 0.959131 |
| GO:0051051\_negative\_regulation\_of\_transport | 76 | 0 | 0.000000 | 0.000000 | 587 | 505.155865 | 563.01 | 620.864135 | 0.959131 |
| GO:0051101\_regulation\_of\_DNA\_binding | 76 | 0 | 0.000000 | 0.000000 | 587 | 505.155865 | 563.01 | 620.864135 | 0.959131 |
| GO:0070838\_divalent\_metal\_ion\_transport | 76 | 0 | 0.000000 | 0.000000 | 587 | 505.155865 | 563.01 | 620.864135 | 0.959131 |
| GO:0007179\_transforming\_growth\_factor\_beta\_receptor\_signaling\_pathway | 72 | 0 | 0.000000 | 0.000000 | 589 | 508.285516 | 565.87 | 623.454484 | 0.960730 |
| GO:0060249\_anatomical\_structure\_homeostasis | 72 | 0 | 0.000000 | 0.000000 | 589 | 508.285516 | 565.87 | 623.454484 | 0.960730 |
| GO:0009891\_positive\_regulation\_of\_biosynthetic\_process | 359 | 0 | 0.000000 | 0.000000 | 590 | 509.079967 | 566.45 | 623.820033 | 0.960085 |
| GO:0008380\_RNA\_splicing | 192 | 0 | 0.000000 | 0.000000 | 591 | 509.716716 | 567.12 | 624.523284 | 0.959594 |
| GO:0010629\_negative\_regulation\_of\_gene\_expression | 289 | 0 | 0.000000 | 0.000000 | 592 | 510.342975 | 567.7 | 625.057025 | 0.958953 |
| GO:0009100\_glycoprotein\_metabolic\_process | 139 | 0 | 0.000000 | 0.000000 | 593 | 512.057131 | 569.21 | 626.362869 | 0.959882 |
| GO:0031399\_regulation\_of\_protein\_modification\_process | 144 | 0 | 0.000000 | 0.000000 | 594 | 513.843640 | 570.78 | 627.716360 | 0.960909 |
| GO:0031324\_negative\_regulation\_of\_cellular\_metabolic\_process | 404 | 0 | 0.000000 | 0.000000 | 595 | 515.135521 | 571.87 | 628.604479 | 0.961126 |
| GO:0000087\_M\_phase\_of\_mitotic\_cell\_cycle | 118 | 0 | 0.000000 | 0.000000 | 598 | 521.344130 | 577.65 | 633.955870 | 0.965970 |
| GO:0010608\_posttranscriptional\_regulation\_of\_gene\_expression | 118 | 0 | 0.000000 | 0.000000 | 598 | 521.344130 | 577.65 | 633.955870 | 0.965970 |
| GO:0048285\_organelle\_fission | 118 | 0 | 0.000000 | 0.000000 | 598 | 521.344130 | 577.65 | 633.955870 | 0.965970 |
| GO:0008654\_phospholipid\_biosynthetic\_process | 63 | 0 | 0.000000 | 0.000000 | 602 | 525.041131 | 581.23 | 637.418869 | 0.965498 |
| GO:0030522\_intracellular\_receptor-mediated\_signaling\_pathway | 63 | 0 | 0.000000 | 0.000000 | 602 | 525.041131 | 581.23 | 637.418869 | 0.965498 |
| GO:0042445\_hormone\_metabolic\_process | 63 | 0 | 0.000000 | 0.000000 | 602 | 525.041131 | 581.23 | 637.418869 | 0.965498 |
| GO:0051348\_negative\_regulation\_of\_transferase\_activity | 63 | 0 | 0.000000 | 0.000000 | 602 | 525.041131 | 581.23 | 637.418869 | 0.965498 |
| GO:0000956\_nuclear-transcribed\_mRNA\_catabolic\_process | 15 | 0 | 0.000000 | 0.000000 | 664 | 587.182871 | 642.36 | 697.537129 | 0.967410 |
| GO:0001701\_in\_utero\_embryonic\_development | 15 | 0 | 0.000000 | 0.000000 | 664 | 587.182871 | 642.36 | 697.537129 | 0.967410 |
| GO:0001937\_negative\_regulation\_of\_endothelial\_cell\_proliferation | 15 | 0 | 0.000000 | 0.000000 | 664 | 587.182871 | 642.36 | 697.537129 | 0.967410 |
| GO:0006611\_protein\_export\_from\_nucleus | 15 | 0 | 0.000000 | 0.000000 | 664 | 587.182871 | 642.36 | 697.537129 | 0.967410 |
| GO:0006612\_protein\_targeting\_to\_membrane | 15 | 0 | 0.000000 | 0.000000 | 664 | 587.182871 | 642.36 | 697.537129 | 0.967410 |
| GO:0006692\_prostanoid\_metabolic\_process | 15 | 0 | 0.000000 | 0.000000 | 664 | 587.182871 | 642.36 | 697.537129 | 0.967410 |
| GO:0006693\_prostaglandin\_metabolic\_process | 15 | 0 | 0.000000 | 0.000000 | 664 | 587.182871 | 642.36 | 697.537129 | 0.967410 |
| GO:0006942\_regulation\_of\_striated\_muscle\_contraction | 15 | 0 | 0.000000 | 0.000000 | 664 | 587.182871 | 642.36 | 697.537129 | 0.967410 |
| GO:0006956\_complement\_activation | 15 | 0 | 0.000000 | 0.000000 | 664 | 587.182871 | 642.36 | 697.537129 | 0.967410 |
| GO:0007043\_cell-cell\_junction\_assembly | 15 | 0 | 0.000000 | 0.000000 | 664 | 587.182871 | 642.36 | 697.537129 | 0.967410 |
| GO:0007098\_centrosome\_cycle | 15 | 0 | 0.000000 | 0.000000 | 664 | 587.182871 | 642.36 | 697.537129 | 0.967410 |
| GO:0007219\_Notch\_signaling\_pathway | 15 | 0 | 0.000000 | 0.000000 | 664 | 587.182871 | 642.36 | 697.537129 | 0.967410 |
| GO:0007369\_gastrulation | 15 | 0 | 0.000000 | 0.000000 | 664 | 587.182871 | 642.36 | 697.537129 | 0.967410 |
| GO:0007569\_cell\_aging | 15 | 0 | 0.000000 | 0.000000 | 664 | 587.182871 | 642.36 | 697.537129 | 0.967410 |
| GO:0007585\_respiratory\_gaseous\_exchange | 15 | 0 | 0.000000 | 0.000000 | 664 | 587.182871 | 642.36 | 697.537129 | 0.967410 |
| GO:0007589\_body\_fluid\_secretion | 15 | 0 | 0.000000 | 0.000000 | 664 | 587.182871 | 642.36 | 697.537129 | 0.967410 |
| GO:0008584\_male\_gonad\_development | 15 | 0 | 0.000000 | 0.000000 | 664 | 587.182871 | 642.36 | 697.537129 | 0.967410 |
| GO:0008637\_apoptotic\_mitochondrial\_changes | 15 | 0 | 0.000000 | 0.000000 | 664 | 587.182871 | 642.36 | 697.537129 | 0.967410 |
| GO:0009060\_aerobic\_respiration | 15 | 0 | 0.000000 | 0.000000 | 664 | 587.182871 | 642.36 | 697.537129 | 0.967410 |
| GO:0009311\_oligosaccharide\_metabolic\_process | 15 | 0 | 0.000000 | 0.000000 | 664 | 587.182871 | 642.36 | 697.537129 | 0.967410 |
| GO:0009820\_alkaloid\_metabolic\_process | 15 | 0 | 0.000000 | 0.000000 | 664 | 587.182871 | 642.36 | 697.537129 | 0.967410 |
| GO:0009952\_anterior\_posterior\_pattern\_formation | 15 | 0 | 0.000000 | 0.000000 | 664 | 587.182871 | 642.36 | 697.537129 | 0.967410 |
| GO:0010720\_positive\_regulation\_of\_cell\_development | 15 | 0 | 0.000000 | 0.000000 | 664 | 587.182871 | 642.36 | 697.537129 | 0.967410 |
| GO:0010927\_cellular\_component\_assembly\_involved\_in\_morphogenesis | 15 | 0 | 0.000000 | 0.000000 | 664 | 587.182871 | 642.36 | 697.537129 | 0.967410 |
| GO:0016441\_posttranscriptional\_gene\_silencing | 15 | 0 | 0.000000 | 0.000000 | 664 | 587.182871 | 642.36 | 697.537129 | 0.967410 |
| GO:0016571\_histone\_methylation | 15 | 0 | 0.000000 | 0.000000 | 664 | 587.182871 | 642.36 | 697.537129 | 0.967410 |
| GO:0019321\_pentose\_metabolic\_process | 15 | 0 | 0.000000 | 0.000000 | 664 | 587.182871 | 642.36 | 697.537129 | 0.967410 |
| GO:0022407\_regulation\_of\_cell-cell\_adhesion | 15 | 0 | 0.000000 | 0.000000 | 664 | 587.182871 | 642.36 | 697.537129 | 0.967410 |
| GO:0022898\_regulation\_of\_transmembrane\_transporter\_activity | 15 | 0 | 0.000000 | 0.000000 | 664 | 587.182871 | 642.36 | 697.537129 | 0.967410 |
| GO:0030101\_natural\_killer\_cell\_activation | 15 | 0 | 0.000000 | 0.000000 | 664 | 587.182871 | 642.36 | 697.537129 | 0.967410 |
| GO:0030512\_negative\_regulation\_of\_transforming\_growth\_factor\_beta\_receptor\_signaling\_pathway | 15 | 0 | 0.000000 | 0.000000 | 664 | 587.182871 | 642.36 | 697.537129 | 0.967410 |
| GO:0030641\_regulation\_of\_cellular\_pH | 15 | 0 | 0.000000 | 0.000000 | 664 | 587.182871 | 642.36 | 697.537129 | 0.967410 |
| GO:0031341\_regulation\_of\_cell\_killing | 15 | 0 | 0.000000 | 0.000000 | 664 | 587.182871 | 642.36 | 697.537129 | 0.967410 |
| GO:0031346\_positive\_regulation\_of\_cell\_projection\_organization | 15 | 0 | 0.000000 | 0.000000 | 664 | 587.182871 | 642.36 | 697.537129 | 0.967410 |
| GO:0032387\_negative\_regulation\_of\_intracellular\_transport | 15 | 0 | 0.000000 | 0.000000 | 664 | 587.182871 | 642.36 | 697.537129 | 0.967410 |
| GO:0032663\_regulation\_of\_interleukin-2\_production | 15 | 0 | 0.000000 | 0.000000 | 664 | 587.182871 | 642.36 | 697.537129 | 0.967410 |
| GO:0032677\_regulation\_of\_interleukin-8\_production | 15 | 0 | 0.000000 | 0.000000 | 664 | 587.182871 | 642.36 | 697.537129 | 0.967410 |
| GO:0032963\_collagen\_metabolic\_process | 15 | 0 | 0.000000 | 0.000000 | 664 | 587.182871 | 642.36 | 697.537129 | 0.967410 |
| GO:0033261\_regulation\_of\_S\_phase | 15 | 0 | 0.000000 | 0.000000 | 664 | 587.182871 | 642.36 | 697.537129 | 0.967410 |
| GO:0035194\_posttranscriptional\_gene\_silencing\_by\_RNA | 15 | 0 | 0.000000 | 0.000000 | 664 | 587.182871 | 642.36 | 697.537129 | 0.967410 |
| GO:0042100\_B\_cell\_proliferation | 15 | 0 | 0.000000 | 0.000000 | 664 | 587.182871 | 642.36 | 697.537129 | 0.967410 |
| GO:0042116\_macrophage\_activation | 15 | 0 | 0.000000 | 0.000000 | 664 | 587.182871 | 642.36 | 697.537129 | 0.967410 |
| GO:0042168\_heme\_metabolic\_process | 15 | 0 | 0.000000 | 0.000000 | 664 | 587.182871 | 642.36 | 697.537129 | 0.967410 |
| GO:0042503\_tyrosine\_phosphorylation\_of\_Stat3\_protein | 15 | 0 | 0.000000 | 0.000000 | 664 | 587.182871 | 642.36 | 697.537129 | 0.967410 |
| GO:0042516\_regulation\_of\_tyrosine\_phosphorylation\_of\_Stat3\_protein | 15 | 0 | 0.000000 | 0.000000 | 664 | 587.182871 | 642.36 | 697.537129 | 0.967410 |
| GO:0043242\_negative\_regulation\_of\_protein\_complex\_disassembly | 15 | 0 | 0.000000 | 0.000000 | 664 | 587.182871 | 642.36 | 697.537129 | 0.967410 |
| GO:0043484\_regulation\_of\_RNA\_splicing | 15 | 0 | 0.000000 | 0.000000 | 664 | 587.182871 | 642.36 | 697.537129 | 0.967410 |
| GO:0043574\_peroxisomal\_transport | 15 | 0 | 0.000000 | 0.000000 | 664 | 587.182871 | 642.36 | 697.537129 | 0.967410 |
| GO:0044259\_multicellular\_organismal\_macromolecule\_metabolic\_process | 15 | 0 | 0.000000 | 0.000000 | 664 | 587.182871 | 642.36 | 697.537129 | 0.967410 |
| GO:0045739\_positive\_regulation\_of\_DNA\_repair | 15 | 0 | 0.000000 | 0.000000 | 664 | 587.182871 | 642.36 | 697.537129 | 0.967410 |
| GO:0045778\_positive\_regulation\_of\_ossification | 15 | 0 | 0.000000 | 0.000000 | 664 | 587.182871 | 642.36 | 697.537129 | 0.967410 |
| GO:0048041\_focal\_adhesion\_formation | 15 | 0 | 0.000000 | 0.000000 | 664 | 587.182871 | 642.36 | 697.537129 | 0.967410 |
| GO:0048146\_positive\_regulation\_of\_fibroblast\_proliferation | 15 | 0 | 0.000000 | 0.000000 | 664 | 587.182871 | 642.36 | 697.537129 | 0.967410 |
| GO:0048515\_spermatid\_differentiation | 15 | 0 | 0.000000 | 0.000000 | 664 | 587.182871 | 642.36 | 697.537129 | 0.967410 |
| GO:0048592\_eye\_morphogenesis | 15 | 0 | 0.000000 | 0.000000 | 664 | 587.182871 | 642.36 | 697.537129 | 0.967410 |
| GO:0048736\_appendage\_development | 15 | 0 | 0.000000 | 0.000000 | 664 | 587.182871 | 642.36 | 697.537129 | 0.967410 |
| GO:0050796\_regulation\_of\_insulin\_secretion | 15 | 0 | 0.000000 | 0.000000 | 664 | 587.182871 | 642.36 | 697.537129 | 0.967410 |
| GO:0051004\_regulation\_of\_lipoprotein\_lipase\_activity | 15 | 0 | 0.000000 | 0.000000 | 664 | 587.182871 | 642.36 | 697.537129 | 0.967410 |
| GO:0060048\_cardiac\_muscle\_contraction | 15 | 0 | 0.000000 | 0.000000 | 664 | 587.182871 | 642.36 | 697.537129 | 0.967410 |
| GO:0060173\_limb\_development | 15 | 0 | 0.000000 | 0.000000 | 664 | 587.182871 | 642.36 | 697.537129 | 0.967410 |
| GO:0060393\_regulation\_of\_pathway-restricted\_SMAD\_protein\_phosphorylation | 15 | 0 | 0.000000 | 0.000000 | 664 | 587.182871 | 642.36 | 697.537129 | 0.967410 |
| GO:0070169\_positive\_regulation\_of\_biomineral\_formation | 15 | 0 | 0.000000 | 0.000000 | 664 | 587.182871 | 642.36 | 697.537129 | 0.967410 |
| GO:0015849\_organic\_acid\_transport | 80 | 0 | 0.000000 | 0.000000 | 666 | 589.834624 | 644.94 | 700.045376 | 0.968378 |
| GO:0031175\_neuron\_projection\_development | 80 | 0 | 0.000000 | 0.000000 | 666 | 589.834624 | 644.94 | 700.045376 | 0.968378 |
| GO:0008285\_negative\_regulation\_of\_cell\_proliferation | 202 | 0 | 0.000000 | 0.000000 | 667 | 590.582113 | 645.65 | 700.717887 | 0.967991 |
| GO:0006413\_translational\_initiation | 46 | 0 | 0.000000 | 0.000000 | 681 | 605.474034 | 660.11 | 714.745966 | 0.969325 |
| GO:0007202\_activation\_of\_phospholipase\_C\_activity | 46 | 0 | 0.000000 | 0.000000 | 681 | 605.474034 | 660.11 | 714.745966 | 0.969325 |
| GO:0007588\_excretion | 46 | 0 | 0.000000 | 0.000000 | 681 | 605.474034 | 660.11 | 714.745966 | 0.969325 |
| GO:0010038\_response\_to\_metal\_ion | 46 | 0 | 0.000000 | 0.000000 | 681 | 605.474034 | 660.11 | 714.745966 | 0.969325 |
| GO:0010638\_positive\_regulation\_of\_organelle\_organization | 46 | 0 | 0.000000 | 0.000000 | 681 | 605.474034 | 660.11 | 714.745966 | 0.969325 |
| GO:0010639\_negative\_regulation\_of\_organelle\_organization | 46 | 0 | 0.000000 | 0.000000 | 681 | 605.474034 | 660.11 | 714.745966 | 0.969325 |
| GO:0010863\_positive\_regulation\_of\_phospholipase\_C\_activity | 46 | 0 | 0.000000 | 0.000000 | 681 | 605.474034 | 660.11 | 714.745966 | 0.969325 |
| GO:0030335\_positive\_regulation\_of\_cell\_migration | 46 | 0 | 0.000000 | 0.000000 | 681 | 605.474034 | 660.11 | 714.745966 | 0.969325 |
| GO:0030384\_phosphoinositide\_metabolic\_process | 46 | 0 | 0.000000 | 0.000000 | 681 | 605.474034 | 660.11 | 714.745966 | 0.969325 |
| GO:0042157\_lipoprotein\_metabolic\_process | 46 | 0 | 0.000000 | 0.000000 | 681 | 605.474034 | 660.11 | 714.745966 | 0.969325 |
| GO:0042254\_ribosome\_biogenesis | 46 | 0 | 0.000000 | 0.000000 | 681 | 605.474034 | 660.11 | 714.745966 | 0.969325 |
| GO:0042391\_regulation\_of\_membrane\_potential | 46 | 0 | 0.000000 | 0.000000 | 681 | 605.474034 | 660.11 | 714.745966 | 0.969325 |
| GO:0046879\_hormone\_secretion | 46 | 0 | 0.000000 | 0.000000 | 681 | 605.474034 | 660.11 | 714.745966 | 0.969325 |
| GO:0065004\_protein-DNA\_complex\_assembly | 46 | 0 | 0.000000 | 0.000000 | 681 | 605.474034 | 660.11 | 714.745966 | 0.969325 |
| GO:0006260\_DNA\_replication | 153 | 0 | 0.000000 | 0.000000 | 683 | 607.097117 | 661.61 | 716.122883 | 0.968682 |
| GO:0019932\_second-messenger-mediated\_signaling | 153 | 0 | 0.000000 | 0.000000 | 683 | 607.097117 | 661.61 | 716.122883 | 0.968682 |
| GO:0030030\_cell\_projection\_organization | 127 | 0 | 0.000000 | 0.000000 | 684 | 607.925538 | 662.38 | 716.834462 | 0.968392 |
| GO:0006140\_regulation\_of\_nucleotide\_metabolic\_process | 60 | 0 | 0.000000 | 0.000000 | 687 | 611.791169 | 665.88 | 719.968831 | 0.969258 |
| GO:0044087\_regulation\_of\_cellular\_component\_biogenesis | 60 | 0 | 0.000000 | 0.000000 | 687 | 611.791169 | 665.88 | 719.968831 | 0.969258 |
| GO:0046058\_cAMP\_metabolic\_process | 60 | 0 | 0.000000 | 0.000000 | 687 | 611.791169 | 665.88 | 719.968831 | 0.969258 |
| GO:0002526\_acute\_inflammatory\_response | 31 | 0 | 0.000000 | 0.000000 | 700 | 627.088117 | 680.73 | 734.371883 | 0.972471 |
| GO:0006633\_fatty\_acid\_biosynthetic\_process | 31 | 0 | 0.000000 | 0.000000 | 700 | 627.088117 | 680.73 | 734.371883 | 0.972471 |
| GO:0006888\_ER\_to\_Golgi\_vesicle-mediated\_transport | 31 | 0 | 0.000000 | 0.000000 | 700 | 627.088117 | 680.73 | 734.371883 | 0.972471 |
| GO:0007173\_epidermal\_growth\_factor\_receptor\_signaling\_pathway | 31 | 0 | 0.000000 | 0.000000 | 700 | 627.088117 | 680.73 | 734.371883 | 0.972471 |
| GO:0007200\_activation\_of\_phospholipase\_C\_activity\_by\_G-protein\_coupled\_receptor\_protein\_signaling\_pathway\_coupled\_to\_IP3\_second\_messenger | 31 | 0 | 0.000000 | 0.000000 | 700 | 627.088117 | 680.73 | 734.371883 | 0.972471 |
| GO:0007423\_sensory\_organ\_development | 31 | 0 | 0.000000 | 0.000000 | 700 | 627.088117 | 680.73 | 734.371883 | 0.972471 |
| GO:0008629\_induction\_of\_apoptosis\_by\_intracellular\_signals | 31 | 0 | 0.000000 | 0.000000 | 700 | 627.088117 | 680.73 | 734.371883 | 0.972471 |
| GO:0031668\_cellular\_response\_to\_extracellular\_stimulus | 31 | 0 | 0.000000 | 0.000000 | 700 | 627.088117 | 680.73 | 734.371883 | 0.972471 |
| GO:0042742\_defense\_response\_to\_bacterium | 31 | 0 | 0.000000 | 0.000000 | 700 | 627.088117 | 680.73 | 734.371883 | 0.972471 |
| GO:0044403\_symbiosis\_\_encompassing\_mutualism\_through\_parasitism | 31 | 0 | 0.000000 | 0.000000 | 700 | 627.088117 | 680.73 | 734.371883 | 0.972471 |
| GO:0044419\_interspecies\_interaction\_between\_organisms | 31 | 0 | 0.000000 | 0.000000 | 700 | 627.088117 | 680.73 | 734.371883 | 0.972471 |
| GO:0045185\_maintenance\_of\_protein\_location | 31 | 0 | 0.000000 | 0.000000 | 700 | 627.088117 | 680.73 | 734.371883 | 0.972471 |
| GO:0046883\_regulation\_of\_hormone\_secretion | 31 | 0 | 0.000000 | 0.000000 | 700 | 627.088117 | 680.73 | 734.371883 | 0.972471 |
| GO:0001655\_urogenital\_system\_development | 23 | 0 | 0.000000 | 0.000000 | 722 | 650.572356 | 703.59 | 756.607644 | 0.974501 |
| GO:0001906\_cell\_killing | 23 | 0 | 0.000000 | 0.000000 | 722 | 650.572356 | 703.59 | 756.607644 | 0.974501 |
| GO:0006023\_aminoglycan\_biosynthetic\_process | 23 | 0 | 0.000000 | 0.000000 | 722 | 650.572356 | 703.59 | 756.607644 | 0.974501 |
| GO:0006509\_membrane\_protein\_ectodomain\_proteolysis | 23 | 0 | 0.000000 | 0.000000 | 722 | 650.572356 | 703.59 | 756.607644 | 0.974501 |
| GO:0006641\_triglyceride\_metabolic\_process | 23 | 0 | 0.000000 | 0.000000 | 722 | 650.572356 | 703.59 | 756.607644 | 0.974501 |
| GO:0007190\_activation\_of\_adenylate\_cyclase\_activity | 23 | 0 | 0.000000 | 0.000000 | 722 | 650.572356 | 703.59 | 756.607644 | 0.974501 |
| GO:0007218\_neuropeptide\_signaling\_pathway | 23 | 0 | 0.000000 | 0.000000 | 722 | 650.572356 | 703.59 | 756.607644 | 0.974501 |
| GO:0007623\_circadian\_rhythm | 23 | 0 | 0.000000 | 0.000000 | 722 | 650.572356 | 703.59 | 756.607644 | 0.974501 |
| GO:0016579\_protein\_deubiquitination | 23 | 0 | 0.000000 | 0.000000 | 722 | 650.572356 | 703.59 | 756.607644 | 0.974501 |
| GO:0018130\_heterocycle\_biosynthetic\_process | 23 | 0 | 0.000000 | 0.000000 | 722 | 650.572356 | 703.59 | 756.607644 | 0.974501 |
| GO:0030166\_proteoglycan\_biosynthetic\_process | 23 | 0 | 0.000000 | 0.000000 | 722 | 650.572356 | 703.59 | 756.607644 | 0.974501 |
| GO:0033344\_cholesterol\_efflux | 23 | 0 | 0.000000 | 0.000000 | 722 | 650.572356 | 703.59 | 756.607644 | 0.974501 |
| GO:0033619\_membrane\_protein\_proteolysis | 23 | 0 | 0.000000 | 0.000000 | 722 | 650.572356 | 703.59 | 756.607644 | 0.974501 |
| GO:0034637\_cellular\_carbohydrate\_biosynthetic\_process | 23 | 0 | 0.000000 | 0.000000 | 722 | 650.572356 | 703.59 | 756.607644 | 0.974501 |
| GO:0043473\_pigmentation | 23 | 0 | 0.000000 | 0.000000 | 722 | 650.572356 | 703.59 | 756.607644 | 0.974501 |
| GO:0043627\_response\_to\_estrogen\_stimulus | 23 | 0 | 0.000000 | 0.000000 | 722 | 650.572356 | 703.59 | 756.607644 | 0.974501 |
| GO:0045740\_positive\_regulation\_of\_DNA\_replication | 23 | 0 | 0.000000 | 0.000000 | 722 | 650.572356 | 703.59 | 756.607644 | 0.974501 |
| GO:0046467\_membrane\_lipid\_biosynthetic\_process | 23 | 0 | 0.000000 | 0.000000 | 722 | 650.572356 | 703.59 | 756.607644 | 0.974501 |
| GO:0048871\_multicellular\_organismal\_homeostasis | 23 | 0 | 0.000000 | 0.000000 | 722 | 650.572356 | 703.59 | 756.607644 | 0.974501 |
| GO:0050707\_regulation\_of\_cytokine\_secretion | 23 | 0 | 0.000000 | 0.000000 | 722 | 650.572356 | 703.59 | 756.607644 | 0.974501 |
| GO:0051262\_protein\_tetramerization | 23 | 0 | 0.000000 | 0.000000 | 722 | 650.572356 | 703.59 | 756.607644 | 0.974501 |
| GO:0051353\_positive\_regulation\_of\_oxidoreductase\_activity | 23 | 0 | 0.000000 | 0.000000 | 722 | 650.572356 | 703.59 | 756.607644 | 0.974501 |
| GO:0051259\_protein\_oligomerization | 83 | 0 | 0.000000 | 0.000000 | 724 | 653.434284 | 706.19 | 758.945716 | 0.975401 |
| GO:0051329\_interphase\_of\_mitotic\_cell\_cycle | 83 | 0 | 0.000000 | 0.000000 | 724 | 653.434284 | 706.19 | 758.945716 | 0.975401 |
| GO:0009101\_glycoprotein\_biosynthetic\_process | 109 | 0 | 0.000000 | 0.000000 | 727 | 655.970665 | 708.66 | 761.349335 | 0.974773 |
| GO:0009790\_embryonic\_development | 109 | 0 | 0.000000 | 0.000000 | 727 | 655.970665 | 708.66 | 761.349335 | 0.974773 |
| GO:0010817\_regulation\_of\_hormone\_levels | 109 | 0 | 0.000000 | 0.000000 | 727 | 655.970665 | 708.66 | 761.349335 | 0.974773 |
| GO:0007605\_sensory\_perception\_of\_sound | 54 | 0 | 0.000000 | 0.000000 | 729 | 658.513327 | 711.23 | 763.946673 | 0.975624 |
| GO:0050954\_sensory\_perception\_of\_mechanical\_stimulus | 54 | 0 | 0.000000 | 0.000000 | 729 | 658.513327 | 711.23 | 763.946673 | 0.975624 |
| GO:0006066\_alcohol\_metabolic\_process | 206 | 0 | 0.000000 | 0.000000 | 730 | 660.957439 | 713.4 | 765.842561 | 0.977260 |
| GO:0051128\_regulation\_of\_cellular\_component\_organization | 237 | 0 | 0.000000 | 0.000000 | 731 | 661.713277 | 714.03 | 766.346723 | 0.976785 |
| GO:0045859\_regulation\_of\_protein\_kinase\_activity | 213 | 0 | 0.000000 | 0.000000 | 734 | 664.812290 | 716.64 | 768.467710 | 0.976349 |
| GO:0051254\_positive\_regulation\_of\_RNA\_metabolic\_process | 213 | 0 | 0.000000 | 0.000000 | 734 | 664.812290 | 716.64 | 768.467710 | 0.976349 |
| GO:0051726\_regulation\_of\_cell\_cycle | 213 | 0 | 0.000000 | 0.000000 | 734 | 664.812290 | 716.64 | 768.467710 | 0.976349 |
| GO:0009725\_response\_to\_hormone\_stimulus | 129 | 0 | 0.000000 | 0.000000 | 737 | 667.995932 | 719.69 | 771.384068 | 0.976513 |
| GO:0015672\_monovalent\_inorganic\_cation\_transport | 129 | 0 | 0.000000 | 0.000000 | 737 | 667.995932 | 719.69 | 771.384068 | 0.976513 |
| GO:0051347\_positive\_regulation\_of\_transferase\_activity | 129 | 0 | 0.000000 | 0.000000 | 737 | 667.995932 | 719.69 | 771.384068 | 0.976513 |
| GO:0006812\_cation\_transport | 246 | 0 | 0.000000 | 0.000000 | 739 | 670.066570 | 721.61 | 773.153430 | 0.976468 |
| GO:0009887\_organ\_morphogenesis | 246 | 0 | 0.000000 | 0.000000 | 739 | 670.066570 | 721.61 | 773.153430 | 0.976468 |
| GO:0007010\_cytoskeleton\_organization | 275 | 0 | 0.000000 | 0.000000 | 740 | 671.285914 | 722.67 | 774.054086 | 0.976581 |
| GO:0070887\_cellular\_response\_to\_chemical\_stimulus | 157 | 0 | 0.000000 | 0.000000 | 741 | 672.956750 | 724.25 | 775.543250 | 0.977395 |
| GO:0042180\_cellular\_ketone\_metabolic\_process | 291 | 0 | 0.000000 | 0.000000 | 742 | 673.576864 | 724.86 | 776.143136 | 0.976900 |
| GO:0006357\_regulation\_of\_transcription\_from\_RNA\_polymerase\_II\_promoter | 351 | 0 | 0.000000 | 0.000000 | 743 | 674.207158 | 725.4 | 776.592842 | 0.976312 |
| GO:0046486\_glycerolipid\_metabolic\_process | 97 | 0 | 0.000000 | 0.000000 | 745 | 676.052913 | 727.11 | 778.167087 | 0.975987 |
| GO:0051098\_regulation\_of\_binding | 97 | 0 | 0.000000 | 0.000000 | 745 | 676.052913 | 727.11 | 778.167087 | 0.975987 |
| GO:0000082\_G1\_S\_transition\_of\_mitotic\_cell\_cycle | 36 | 0 | 0.000000 | 0.000000 | 753 | 686.071880 | 736.6 | 787.128120 | 0.978220 |
| GO:0000910\_cytokinesis | 36 | 0 | 0.000000 | 0.000000 | 753 | 686.071880 | 736.6 | 787.128120 | 0.978220 |
| GO:0001819\_positive\_regulation\_of\_cytokine\_production | 36 | 0 | 0.000000 | 0.000000 | 753 | 686.071880 | 736.6 | 787.128120 | 0.978220 |
| GO:0006334\_nucleosome\_assembly | 36 | 0 | 0.000000 | 0.000000 | 753 | 686.071880 | 736.6 | 787.128120 | 0.978220 |
| GO:0006814\_sodium\_ion\_transport | 36 | 0 | 0.000000 | 0.000000 | 753 | 686.071880 | 736.6 | 787.128120 | 0.978220 |
| GO:0009566\_fertilization | 36 | 0 | 0.000000 | 0.000000 | 753 | 686.071880 | 736.6 | 787.128120 | 0.978220 |
| GO:0034097\_response\_to\_cytokine\_stimulus | 36 | 0 | 0.000000 | 0.000000 | 753 | 686.071880 | 736.6 | 787.128120 | 0.978220 |
| GO:0034103\_regulation\_of\_tissue\_remodeling | 36 | 0 | 0.000000 | 0.000000 | 753 | 686.071880 | 736.6 | 787.128120 | 0.978220 |
| GO:0000302\_response\_to\_reactive\_oxygen\_species | 21 | 0 | 0.000000 | 0.000000 | 799 | 732.023699 | 781.46 | 830.896301 | 0.978048 |
| GO:0000718\_nucleotide-excision\_repair\_\_DNA\_damage\_removal | 21 | 0 | 0.000000 | 0.000000 | 799 | 732.023699 | 781.46 | 830.896301 | 0.978048 |
| GO:0001654\_eye\_development | 21 | 0 | 0.000000 | 0.000000 | 799 | 732.023699 | 781.46 | 830.896301 | 0.978048 |
| GO:0001936\_regulation\_of\_endothelial\_cell\_proliferation | 21 | 0 | 0.000000 | 0.000000 | 799 | 732.023699 | 781.46 | 830.896301 | 0.978048 |
| GO:0002819\_regulation\_of\_adaptive\_immune\_response | 21 | 0 | 0.000000 | 0.000000 | 799 | 732.023699 | 781.46 | 830.896301 | 0.978048 |
| GO:0002822\_regulation\_of\_adaptive\_immune\_response\_based\_on\_somatic\_recombination\_of\_immune\_receptors\_built\_from\_immunoglobulin\_superfamily\_domains | 21 | 0 | 0.000000 | 0.000000 | 799 | 732.023699 | 781.46 | 830.896301 | 0.978048 |
| GO:0002831\_regulation\_of\_response\_to\_biotic\_stimulus | 21 | 0 | 0.000000 | 0.000000 | 799 | 732.023699 | 781.46 | 830.896301 | 0.978048 |
| GO:0006024\_glycosaminoglycan\_biosynthetic\_process | 21 | 0 | 0.000000 | 0.000000 | 799 | 732.023699 | 781.46 | 830.896301 | 0.978048 |
| GO:0006040\_amino\_sugar\_metabolic\_process | 21 | 0 | 0.000000 | 0.000000 | 799 | 732.023699 | 781.46 | 830.896301 | 0.978048 |
| GO:0006073\_cellular\_glucan\_metabolic\_process | 21 | 0 | 0.000000 | 0.000000 | 799 | 732.023699 | 781.46 | 830.896301 | 0.978048 |
| GO:0006109\_regulation\_of\_carbohydrate\_metabolic\_process | 21 | 0 | 0.000000 | 0.000000 | 799 | 732.023699 | 781.46 | 830.896301 | 0.978048 |
| GO:0006282\_regulation\_of\_DNA\_repair | 21 | 0 | 0.000000 | 0.000000 | 799 | 732.023699 | 781.46 | 830.896301 | 0.978048 |
| GO:0006284\_base-excision\_repair | 21 | 0 | 0.000000 | 0.000000 | 799 | 732.023699 | 781.46 | 830.896301 | 0.978048 |
| GO:0006402\_mRNA\_catabolic\_process | 21 | 0 | 0.000000 | 0.000000 | 799 | 732.023699 | 781.46 | 830.896301 | 0.978048 |
| GO:0006493\_protein\_amino\_acid\_O-linked\_glycosylation | 21 | 0 | 0.000000 | 0.000000 | 799 | 732.023699 | 781.46 | 830.896301 | 0.978048 |
| GO:0006664\_glycolipid\_metabolic\_process | 21 | 0 | 0.000000 | 0.000000 | 799 | 732.023699 | 781.46 | 830.896301 | 0.978048 |
| GO:0006775\_fat-soluble\_vitamin\_metabolic\_process | 21 | 0 | 0.000000 | 0.000000 | 799 | 732.023699 | 781.46 | 830.896301 | 0.978048 |
| GO:0006892\_post-Golgi\_vesicle-mediated\_transport | 21 | 0 | 0.000000 | 0.000000 | 799 | 732.023699 | 781.46 | 830.896301 | 0.978048 |
| GO:0006903\_vesicle\_targeting | 21 | 0 | 0.000000 | 0.000000 | 799 | 732.023699 | 781.46 | 830.896301 | 0.978048 |
| GO:0007292\_female\_gamete\_generation | 21 | 0 | 0.000000 | 0.000000 | 799 | 732.023699 | 781.46 | 830.896301 | 0.978048 |
| GO:0008156\_negative\_regulation\_of\_DNA\_replication | 21 | 0 | 0.000000 | 0.000000 | 799 | 732.023699 | 781.46 | 830.896301 | 0.978048 |
| GO:0008360\_regulation\_of\_cell\_shape | 21 | 0 | 0.000000 | 0.000000 | 799 | 732.023699 | 781.46 | 830.896301 | 0.978048 |
| GO:0009199\_ribonucleoside\_triphosphate\_metabolic\_process | 21 | 0 | 0.000000 | 0.000000 | 799 | 732.023699 | 781.46 | 830.896301 | 0.978048 |
| GO:0010675\_regulation\_of\_cellular\_carbohydrate\_metabolic\_process | 21 | 0 | 0.000000 | 0.000000 | 799 | 732.023699 | 781.46 | 830.896301 | 0.978048 |
| GO:0014031\_mesenchymal\_cell\_development | 21 | 0 | 0.000000 | 0.000000 | 799 | 732.023699 | 781.46 | 830.896301 | 0.978048 |
| GO:0016338\_calcium-independent\_cell-cell\_adhesion | 21 | 0 | 0.000000 | 0.000000 | 799 | 732.023699 | 781.46 | 830.896301 | 0.978048 |
| GO:0030048\_actin\_filament-based\_movement | 21 | 0 | 0.000000 | 0.000000 | 799 | 732.023699 | 781.46 | 830.896301 | 0.978048 |
| GO:0030111\_regulation\_of\_Wnt\_receptor\_signaling\_pathway | 21 | 0 | 0.000000 | 0.000000 | 799 | 732.023699 | 781.46 | 830.896301 | 0.978048 |
| GO:0030148\_sphingolipid\_biosynthetic\_process | 21 | 0 | 0.000000 | 0.000000 | 799 | 732.023699 | 781.46 | 830.896301 | 0.978048 |
| GO:0032768\_regulation\_of\_monooxygenase\_activity | 21 | 0 | 0.000000 | 0.000000 | 799 | 732.023699 | 781.46 | 830.896301 | 0.978048 |
| GO:0032886\_regulation\_of\_microtubule-based\_process | 21 | 0 | 0.000000 | 0.000000 | 799 | 732.023699 | 781.46 | 830.896301 | 0.978048 |
| GO:0035295\_tube\_development | 21 | 0 | 0.000000 | 0.000000 | 799 | 732.023699 | 781.46 | 830.896301 | 0.978048 |
| GO:0042398\_cellular\_amino\_acid\_derivative\_biosynthetic\_process | 21 | 0 | 0.000000 | 0.000000 | 799 | 732.023699 | 781.46 | 830.896301 | 0.978048 |
| GO:0042439\_ethanolamine\_and\_derivative\_metabolic\_process | 21 | 0 | 0.000000 | 0.000000 | 799 | 732.023699 | 781.46 | 830.896301 | 0.978048 |
| GO:0043523\_regulation\_of\_neuron\_apoptosis | 21 | 0 | 0.000000 | 0.000000 | 799 | 732.023699 | 781.46 | 830.896301 | 0.978048 |
| GO:0044042\_glucan\_metabolic\_process | 21 | 0 | 0.000000 | 0.000000 | 799 | 732.023699 | 781.46 | 830.896301 | 0.978048 |
| GO:0045649\_regulation\_of\_macrophage\_differentiation | 21 | 0 | 0.000000 | 0.000000 | 799 | 732.023699 | 781.46 | 830.896301 | 0.978048 |
| GO:0046427\_positive\_regulation\_of\_JAK-STAT\_cascade | 21 | 0 | 0.000000 | 0.000000 | 799 | 732.023699 | 781.46 | 830.896301 | 0.978048 |
| GO:0048520\_positive\_regulation\_of\_behavior | 21 | 0 | 0.000000 | 0.000000 | 799 | 732.023699 | 781.46 | 830.896301 | 0.978048 |
| GO:0048762\_mesenchymal\_cell\_differentiation | 21 | 0 | 0.000000 | 0.000000 | 799 | 732.023699 | 781.46 | 830.896301 | 0.978048 |
| GO:0051238\_sequestering\_of\_metal\_ion | 21 | 0 | 0.000000 | 0.000000 | 799 | 732.023699 | 781.46 | 830.896301 | 0.978048 |
| GO:0051297\_centrosome\_organization | 21 | 0 | 0.000000 | 0.000000 | 799 | 732.023699 | 781.46 | 830.896301 | 0.978048 |
| GO:0055072\_iron\_ion\_homeostasis | 21 | 0 | 0.000000 | 0.000000 | 799 | 732.023699 | 781.46 | 830.896301 | 0.978048 |
| GO:0060485\_mesenchyme\_development | 21 | 0 | 0.000000 | 0.000000 | 799 | 732.023699 | 781.46 | 830.896301 | 0.978048 |
| GO:0070167\_regulation\_of\_biomineral\_formation | 21 | 0 | 0.000000 | 0.000000 | 799 | 732.023699 | 781.46 | 830.896301 | 0.978048 |
| GO:0070507\_regulation\_of\_microtubule\_cytoskeleton\_organization | 21 | 0 | 0.000000 | 0.000000 | 799 | 732.023699 | 781.46 | 830.896301 | 0.978048 |
| GO:0006366\_transcription\_from\_RNA\_polymerase\_II\_promoter | 506 | 0 | 0.000000 | 0.000000 | 800 | 732.990985 | 782.27 | 831.549015 | 0.977838 |
| GO:0019226\_transmission\_of\_nerve\_impulse | 209 | 0 | 0.000000 | 0.000000 | 801 | 736.807596 | 785.61 | 834.412404 | 0.980787 |
| GO:0032940\_secretion\_by\_cell | 155 | 0 | 0.000000 | 0.000000 | 802 | 738.542528 | 787.18 | 835.817472 | 0.981521 |
| GO:0030334\_regulation\_of\_cell\_migration | 89 | 0 | 0.000000 | 0.000000 | 807 | 743.126286 | 791.55 | 839.973714 | 0.980855 |
| GO:0034660\_ncRNA\_metabolic\_process | 89 | 0 | 0.000000 | 0.000000 | 807 | 743.126286 | 791.55 | 839.973714 | 0.980855 |
| GO:0043405\_regulation\_of\_MAP\_kinase\_activity | 89 | 0 | 0.000000 | 0.000000 | 807 | 743.126286 | 791.55 | 839.973714 | 0.980855 |
| GO:0051240\_positive\_regulation\_of\_multicellular\_organismal\_process | 89 | 0 | 0.000000 | 0.000000 | 807 | 743.126286 | 791.55 | 839.973714 | 0.980855 |
| GO:0051325\_interphase | 89 | 0 | 0.000000 | 0.000000 | 807 | 743.126286 | 791.55 | 839.973714 | 0.980855 |
| GO:0006082\_organic\_acid\_metabolic\_process | 290 | 0 | 0.000000 | 0.000000 | 808 | 744.467114 | 792.77 | 841.072886 | 0.981151 |
| GO:0007507\_heart\_development | 68 | 0 | 0.000000 | 0.000000 | 813 | 749.831776 | 798.01 | 846.188224 | 0.981562 |
| GO:0009991\_response\_to\_extracellular\_stimulus | 68 | 0 | 0.000000 | 0.000000 | 813 | 749.831776 | 798.01 | 846.188224 | 0.981562 |
| GO:0043062\_extracellular\_structure\_organization | 68 | 0 | 0.000000 | 0.000000 | 813 | 749.831776 | 798.01 | 846.188224 | 0.981562 |
| GO:0048858\_cell\_projection\_morphogenesis | 68 | 0 | 0.000000 | 0.000000 | 813 | 749.831776 | 798.01 | 846.188224 | 0.981562 |
| GO:0051439\_regulation\_of\_ubiquitin-protein\_ligase\_activity\_during\_mitotic\_cell\_cycle | 68 | 0 | 0.000000 | 0.000000 | 813 | 749.831776 | 798.01 | 846.188224 | 0.981562 |
| GO:0019935\_cyclic-nucleotide-mediated\_signaling | 82 | 0 | 0.000000 | 0.000000 | 816 | 753.492550 | 801.52 | 849.547450 | 0.982255 |
| GO:0048193\_Golgi\_vesicle\_transport | 82 | 0 | 0.000000 | 0.000000 | 816 | 753.492550 | 801.52 | 849.547450 | 0.982255 |
| GO:0048514\_blood\_vessel\_morphogenesis | 82 | 0 | 0.000000 | 0.000000 | 816 | 753.492550 | 801.52 | 849.547450 | 0.982255 |
| GO:0031327\_negative\_regulation\_of\_cellular\_biosynthetic\_process | 332 | 0 | 0.000000 | 0.000000 | 817 | 754.104721 | 801.99 | 849.875279 | 0.981628 |
| GO:0000077\_DNA\_damage\_checkpoint | 37 | 0 | 0.000000 | 0.000000 | 826 | 765.702900 | 813.09 | 860.477100 | 0.984370 |
| GO:0006937\_regulation\_of\_muscle\_contraction | 37 | 0 | 0.000000 | 0.000000 | 826 | 765.702900 | 813.09 | 860.477100 | 0.984370 |
| GO:0007584\_response\_to\_nutrient | 37 | 0 | 0.000000 | 0.000000 | 826 | 765.702900 | 813.09 | 860.477100 | 0.984370 |
| GO:0009913\_epidermal\_cell\_differentiation | 37 | 0 | 0.000000 | 0.000000 | 826 | 765.702900 | 813.09 | 860.477100 | 0.984370 |
| GO:0015918\_sterol\_transport | 37 | 0 | 0.000000 | 0.000000 | 826 | 765.702900 | 813.09 | 860.477100 | 0.984370 |
| GO:0030301\_cholesterol\_transport | 37 | 0 | 0.000000 | 0.000000 | 826 | 765.702900 | 813.09 | 860.477100 | 0.984370 |
| GO:0032956\_regulation\_of\_actin\_cytoskeleton\_organization | 37 | 0 | 0.000000 | 0.000000 | 826 | 765.702900 | 813.09 | 860.477100 | 0.984370 |
| GO:0046489\_phosphoinositide\_biosynthetic\_process | 37 | 0 | 0.000000 | 0.000000 | 826 | 765.702900 | 813.09 | 860.477100 | 0.984370 |
| GO:0046700\_heterocycle\_catabolic\_process | 37 | 0 | 0.000000 | 0.000000 | 826 | 765.702900 | 813.09 | 860.477100 | 0.984370 |
| GO:0003006\_reproductive\_developmental\_process | 51 | 0 | 0.000000 | 0.000000 | 836 | 775.005929 | 822.02 | 869.034071 | 0.983278 |
| GO:0006898\_receptor-mediated\_endocytosis | 51 | 0 | 0.000000 | 0.000000 | 836 | 775.005929 | 822.02 | 869.034071 | 0.983278 |
| GO:0007059\_chromosome\_segregation | 51 | 0 | 0.000000 | 0.000000 | 836 | 775.005929 | 822.02 | 869.034071 | 0.983278 |
| GO:0016052\_carbohydrate\_catabolic\_process | 51 | 0 | 0.000000 | 0.000000 | 836 | 775.005929 | 822.02 | 869.034071 | 0.983278 |
| GO:0018212\_peptidyl-tyrosine\_modification | 51 | 0 | 0.000000 | 0.000000 | 836 | 775.005929 | 822.02 | 869.034071 | 0.983278 |
| GO:0030518\_steroid\_hormone\_receptor\_signaling\_pathway | 51 | 0 | 0.000000 | 0.000000 | 836 | 775.005929 | 822.02 | 869.034071 | 0.983278 |
| GO:0031279\_regulation\_of\_cyclase\_activity | 51 | 0 | 0.000000 | 0.000000 | 836 | 775.005929 | 822.02 | 869.034071 | 0.983278 |
| GO:0032147\_activation\_of\_protein\_kinase\_activity | 51 | 0 | 0.000000 | 0.000000 | 836 | 775.005929 | 822.02 | 869.034071 | 0.983278 |
| GO:0046474\_glycerophospholipid\_biosynthetic\_process | 51 | 0 | 0.000000 | 0.000000 | 836 | 775.005929 | 822.02 | 869.034071 | 0.983278 |
| GO:0070482\_response\_to\_oxygen\_levels | 51 | 0 | 0.000000 | 0.000000 | 836 | 775.005929 | 822.02 | 869.034071 | 0.983278 |
| GO:0006364\_rRNA\_processing | 39 | 0 | 0.000000 | 0.000000 | 847 | 786.318467 | 833.26 | 880.201533 | 0.983778 |
| GO:0006401\_RNA\_catabolic\_process | 39 | 0 | 0.000000 | 0.000000 | 847 | 786.318467 | 833.26 | 880.201533 | 0.983778 |
| GO:0006497\_protein\_amino\_acid\_lipidation | 39 | 0 | 0.000000 | 0.000000 | 847 | 786.318467 | 833.26 | 880.201533 | 0.983778 |
| GO:0009310\_amine\_catabolic\_process | 39 | 0 | 0.000000 | 0.000000 | 847 | 786.318467 | 833.26 | 880.201533 | 0.983778 |
| GO:0009792\_embryonic\_development\_ending\_in\_birth\_or\_egg\_hatching | 39 | 0 | 0.000000 | 0.000000 | 847 | 786.318467 | 833.26 | 880.201533 | 0.983778 |
| GO:0030509\_BMP\_signaling\_pathway | 39 | 0 | 0.000000 | 0.000000 | 847 | 786.318467 | 833.26 | 880.201533 | 0.983778 |
| GO:0032970\_regulation\_of\_actin\_filament-based\_process | 39 | 0 | 0.000000 | 0.000000 | 847 | 786.318467 | 833.26 | 880.201533 | 0.983778 |
| GO:0042158\_lipoprotein\_biosynthetic\_process | 39 | 0 | 0.000000 | 0.000000 | 847 | 786.318467 | 833.26 | 880.201533 | 0.983778 |
| GO:0043009\_chordate\_embryonic\_development | 39 | 0 | 0.000000 | 0.000000 | 847 | 786.318467 | 833.26 | 880.201533 | 0.983778 |
| GO:0046365\_monosaccharide\_catabolic\_process | 39 | 0 | 0.000000 | 0.000000 | 847 | 786.318467 | 833.26 | 880.201533 | 0.983778 |
| GO:0051960\_regulation\_of\_nervous\_system\_development | 39 | 0 | 0.000000 | 0.000000 | 847 | 786.318467 | 833.26 | 880.201533 | 0.983778 |
| GO:0006732\_coenzyme\_metabolic\_process | 65 | 0 | 0.000000 | 0.000000 | 854 | 793.643338 | 840.39 | 887.136662 | 0.984063 |
| GO:0016054\_organic\_acid\_catabolic\_process | 65 | 0 | 0.000000 | 0.000000 | 854 | 793.643338 | 840.39 | 887.136662 | 0.984063 |
| GO:0022415\_viral\_reproductive\_process | 65 | 0 | 0.000000 | 0.000000 | 854 | 793.643338 | 840.39 | 887.136662 | 0.984063 |
| GO:0045596\_negative\_regulation\_of\_cell\_differentiation | 65 | 0 | 0.000000 | 0.000000 | 854 | 793.643338 | 840.39 | 887.136662 | 0.984063 |
| GO:0046395\_carboxylic\_acid\_catabolic\_process | 65 | 0 | 0.000000 | 0.000000 | 854 | 793.643338 | 840.39 | 887.136662 | 0.984063 |
| GO:0051235\_maintenance\_of\_location | 65 | 0 | 0.000000 | 0.000000 | 854 | 793.643338 | 840.39 | 887.136662 | 0.984063 |
| GO:0051437\_positive\_regulation\_of\_ubiquitin-protein\_ligase\_activity\_during\_mitotic\_cell\_cycle | 65 | 0 | 0.000000 | 0.000000 | 854 | 793.643338 | 840.39 | 887.136662 | 0.984063 |
| GO:0006397\_mRNA\_processing | 180 | 0 | 0.000000 | 0.000000 | 857 | 797.031895 | 843.36 | 889.688105 | 0.984084 |
| GO:0051253\_negative\_regulation\_of\_RNA\_metabolic\_process | 180 | 0 | 0.000000 | 0.000000 | 857 | 797.031895 | 843.36 | 889.688105 | 0.984084 |
| GO:0051336\_regulation\_of\_hydrolase\_activity | 180 | 0 | 0.000000 | 0.000000 | 857 | 797.031895 | 843.36 | 889.688105 | 0.984084 |
| GO:0006520\_cellular\_amino\_acid\_metabolic\_process | 108 | 0 | 0.000000 | 0.000000 | 864 | 803.052851 | 849.22 | 895.387149 | 0.982894 |
| GO:0006644\_phospholipid\_metabolic\_process | 108 | 0 | 0.000000 | 0.000000 | 864 | 803.052851 | 849.22 | 895.387149 | 0.982894 |
| GO:0007346\_regulation\_of\_mitotic\_cell\_cycle | 108 | 0 | 0.000000 | 0.000000 | 864 | 803.052851 | 849.22 | 895.387149 | 0.982894 |
| GO:0008202\_steroid\_metabolic\_process | 108 | 0 | 0.000000 | 0.000000 | 864 | 803.052851 | 849.22 | 895.387149 | 0.982894 |
| GO:0019637\_organophosphate\_metabolic\_process | 108 | 0 | 0.000000 | 0.000000 | 864 | 803.052851 | 849.22 | 895.387149 | 0.982894 |
| GO:0032446\_protein\_modification\_by\_small\_protein\_conjugation | 108 | 0 | 0.000000 | 0.000000 | 864 | 803.052851 | 849.22 | 895.387149 | 0.982894 |
| GO:0044106\_cellular\_amine\_metabolic\_process | 108 | 0 | 0.000000 | 0.000000 | 864 | 803.052851 | 849.22 | 895.387149 | 0.982894 |
| GO:0006790\_sulfur\_metabolic\_process | 62 | 0 | 0.000000 | 0.000000 | 876 | 814.015811 | 859.98 | 905.944189 | 0.981712 |
| GO:0007420\_brain\_development | 62 | 0 | 0.000000 | 0.000000 | 876 | 814.015811 | 859.98 | 905.944189 | 0.981712 |
| GO:0007586\_digestion | 62 | 0 | 0.000000 | 0.000000 | 876 | 814.015811 | 859.98 | 905.944189 | 0.981712 |
| GO:0009190\_cyclic\_nucleotide\_biosynthetic\_process | 62 | 0 | 0.000000 | 0.000000 | 876 | 814.015811 | 859.98 | 905.944189 | 0.981712 |
| GO:0019216\_regulation\_of\_lipid\_metabolic\_process | 62 | 0 | 0.000000 | 0.000000 | 876 | 814.015811 | 859.98 | 905.944189 | 0.981712 |
| GO:0031145\_anaphase-promoting\_complex-dependent\_proteasomal\_ubiquitin-dependent\_protein\_catabolic\_process | 62 | 0 | 0.000000 | 0.000000 | 876 | 814.015811 | 859.98 | 905.944189 | 0.981712 |
| GO:0031667\_response\_to\_nutrient\_levels | 62 | 0 | 0.000000 | 0.000000 | 876 | 814.015811 | 859.98 | 905.944189 | 0.981712 |
| GO:0043406\_positive\_regulation\_of\_MAP\_kinase\_activity | 62 | 0 | 0.000000 | 0.000000 | 876 | 814.015811 | 859.98 | 905.944189 | 0.981712 |
| GO:0044242\_cellular\_lipid\_catabolic\_process | 62 | 0 | 0.000000 | 0.000000 | 876 | 814.015811 | 859.98 | 905.944189 | 0.981712 |
| GO:0051436\_negative\_regulation\_of\_ubiquitin-protein\_ligase\_activity\_during\_mitotic\_cell\_cycle | 62 | 0 | 0.000000 | 0.000000 | 876 | 814.015811 | 859.98 | 905.944189 | 0.981712 |
| GO:0052548\_regulation\_of\_endopeptidase\_activity | 62 | 0 | 0.000000 | 0.000000 | 876 | 814.015811 | 859.98 | 905.944189 | 0.981712 |
| GO:0060191\_regulation\_of\_lipase\_activity | 62 | 0 | 0.000000 | 0.000000 | 876 | 814.015811 | 859.98 | 905.944189 | 0.981712 |
| GO:0003012\_muscle\_system\_process | 141 | 0 | 0.000000 | 0.000000 | 879 | 817.365580 | 863.07 | 908.774420 | 0.981877 |
| GO:0016049\_cell\_growth | 141 | 0 | 0.000000 | 0.000000 | 879 | 817.365580 | 863.07 | 908.774420 | 0.981877 |
| GO:0051603\_proteolysis\_involved\_in\_cellular\_protein\_catabolic\_process | 141 | 0 | 0.000000 | 0.000000 | 879 | 817.365580 | 863.07 | 908.774420 | 0.981877 |
| GO:0006091\_generation\_of\_precursor\_metabolites\_and\_energy | 163 | 0 | 0.000000 | 0.000000 | 880 | 818.164087 | 863.81 | 909.455913 | 0.981602 |
| GO:0000122\_negative\_regulation\_of\_transcription\_from\_RNA\_polymerase\_II\_promoter | 113 | 0 | 0.000000 | 0.000000 | 882 | 820.738281 | 866.24 | 911.741719 | 0.982132 |
| GO:0006631\_fatty\_acid\_metabolic\_process | 113 | 0 | 0.000000 | 0.000000 | 882 | 820.738281 | 866.24 | 911.741719 | 0.982132 |
| GO:0034984\_cellular\_response\_to\_DNA\_damage\_stimulus | 215 | 0 | 0.000000 | 0.000000 | 883 | 821.471820 | 866.91 | 912.348180 | 0.981778 |
| GO:0001932\_regulation\_of\_protein\_amino\_acid\_phosphorylation | 101 | 0 | 0.000000 | 0.000000 | 884 | 823.306434 | 868.56 | 913.813566 | 0.982534 |
| GO:0000028\_ribosomal\_small\_subunit\_assembly | 1 | 0 |  |  |  |  |  |  |  |  |
| GO:0000042\_protein\_targeting\_to\_Golgi | 1 | 0 |  |  |  |  |  |  |  |  |
| GO:0000046\_autophagic\_vacuole\_fusion | 1 | 0 |  |  |  |  |  |  |  |  |
| GO:0000052\_citrulline\_metabolic\_process | 1 | 0 |  |  |  |  |  |  |  |  |
| GO:0000054\_ribosome\_export\_from\_nucleus | 1 | 0 |  |  |  |  |  |  |  |  |
| GO:0000056\_ribosomal\_small\_subunit\_export\_from\_nucleus | 1 | 0 |  |  |  |  |  |  |  |  |
| GO:0000072\_M\_phase\_specific\_microtubule\_process | 1 | 0 |  |  |  |  |  |  |  |  |
| GO:0000093\_mitotic\_telophase | 1 | 0 |  |  |  |  |  |  |  |  |
| GO:0000098\_sulfur\_amino\_acid\_catabolic\_process | 1 | 0 |  |  |  |  |  |  |  |  |
| GO:0000114\_regulation\_of\_transcription\_during\_G1\_phase\_of\_mitotic\_cell\_cycle | 1 | 0 |  |  |  |  |  |  |  |  |
| GO:0000115\_regulation\_of\_transcription\_during\_S-phase\_of\_mitotic\_cell\_cycle | 1 | 0 |  |  |  |  |  |  |  |  |
| GO:0000117\_regulation\_of\_transcription\_during\_G2\_M-phase\_of\_mitotic\_cell\_cycle | 1 | 0 |  |  |  |  |  |  |  |  |
| GO:0000132\_establishment\_of\_mitotic\_spindle\_orientation | 1 | 0 |  |  |  |  |  |  |  |  |
| GO:0000154\_rRNA\_modification | 1 | 0 |  |  |  |  |  |  |  |  |
| GO:0000160\_two-component\_signal\_transduction\_system\_(phosphorelay) | 1 | 0 |  |  |  |  |  |  |  |  |
| GO:0000161\_MAPKKK\_cascade\_involved\_in\_osmosensory\_signaling\_pathway | 1 | 0 |  |  |  |  |  |  |  |  |
| GO:0000173\_inactivation\_of\_MAPK\_activity\_involved\_in\_osmosensory\_signaling\_pathway | 1 | 0 |  |  |  |  |  |  |  |  |
| GO:0000212\_meiotic\_spindle\_organization | 1 | 0 |  |  |  |  |  |  |  |  |
| GO:0000255\_allantoin\_metabolic\_process | 1 | 0 |  |  |  |  |  |  |  |  |
| GO:0000270\_peptidoglycan\_metabolic\_process | 1 | 0 |  |  |  |  |  |  |  |  |
| GO:0000296\_spermine\_transport | 1 | 0 |  |  |  |  |  |  |  |  |
| GO:0000301\_retrograde\_transport\_\_vesicle\_recycling\_within\_Golgi | 1 | 0 |  |  |  |  |  |  |  |  |
| GO:0000303\_response\_to\_superoxide | 1 | 0 |  |  |  |  |  |  |  |  |
| GO:0000320\_re-entry\_into\_mitotic\_cell\_cycle | 1 | 0 |  |  |  |  |  |  |  |  |
| GO:0000338\_protein\_deneddylation | 1 | 0 |  |  |  |  |  |  |  |  |
| GO:0000395\_nuclear\_mRNA\_5'-splice\_site\_recognition | 1 | 0 |  |  |  |  |  |  |  |  |
| GO:0000710\_meiotic\_mismatch\_repair | 1 | 0 |  |  |  |  |  |  |  |  |
| GO:0000717\_nucleotide-excision\_repair\_\_DNA\_duplex\_unwinding | 1 | 0 |  |  |  |  |  |  |  |  |
| GO:0000722\_telomere\_maintenance\_via\_recombination | 1 | 0 |  |  |  |  |  |  |  |  |
| GO:0000746\_conjugation | 1 | 0 |  |  |  |  |  |  |  |  |
| GO:0000747\_conjugation\_with\_cellular\_fusion | 1 | 0 |  |  |  |  |  |  |  |  |
| GO:0000912\_formation\_of\_actomyosin\_apparatus\_involved\_in\_cytokinesis | 1 | 0 |  |  |  |  |  |  |  |  |
| GO:0000915\_cytokinesis\_\_contractile\_ring\_formation | 1 | 0 |  |  |  |  |  |  |  |  |
| GO:0000921\_septin\_ring\_assembly | 1 | 0 |  |  |  |  |  |  |  |  |
| GO:0000966\_RNA\_5'-end\_processing | 1 | 0 |  |  |  |  |  |  |  |  |
| GO:0001315\_age-dependent\_response\_to\_reactive\_oxygen\_species | 1 | 0 |  |  |  |  |  |  |  |  |
| GO:0001519\_peptide\_amidation | 1 | 0 |  |  |  |  |  |  |  |  |
| GO:0001560\_regulation\_of\_cell\_growth\_by\_extracellular\_stimulus | 1 | 0 |  |  |  |  |  |  |  |  |
| GO:0001574\_ganglioside\_biosynthetic\_process | 1 | 0 |  |  |  |  |  |  |  |  |
| GO:0001575\_globoside\_metabolic\_process | 1 | 0 |  |  |  |  |  |  |  |  |
| GO:0001658\_branching\_involved\_in\_ureteric\_bud\_morphogenesis | 1 | 0 |  |  |  |  |  |  |  |  |
| GO:0001662\_behavioral\_fear\_response | 1 | 0 |  |  |  |  |  |  |  |  |
| GO:0001675\_acrosome\_assembly | 1 | 0 |  |  |  |  |  |  |  |  |
| GO:0001692\_histamine\_metabolic\_process | 1 | 0 |  |  |  |  |  |  |  |  |
| GO:0001694\_histamine\_biosynthetic\_process | 1 | 0 |  |  |  |  |  |  |  |  |
| GO:0001732\_formation\_of\_translation\_initiation\_complex | 1 | 0 |  |  |  |  |  |  |  |  |
| GO:0001757\_somite\_specification | 1 | 0 |  |  |  |  |  |  |  |  |
| GO:0001774\_microglial\_cell\_activation | 1 | 0 |  |  |  |  |  |  |  |  |
| GO:0001782\_B\_cell\_homeostasis | 1 | 0 |  |  |  |  |  |  |  |  |
| GO:0001787\_natural\_killer\_cell\_proliferation | 1 | 0 |  |  |  |  |  |  |  |  |
| GO:0001823\_mesonephros\_development | 1 | 0 |  |  |  |  |  |  |  |  |
| GO:0001832\_blastocyst\_growth | 1 | 0 |  |  |  |  |  |  |  |  |
| GO:0001833\_inner\_cell\_mass\_cell\_proliferation | 1 | 0 |  |  |  |  |  |  |  |  |
| GO:0001839\_neural\_plate\_morphogenesis | 1 | 0 |  |  |  |  |  |  |  |  |
| GO:0001845\_phagolysosome\_formation | 1 | 0 |  |  |  |  |  |  |  |  |
| GO:0001865\_NK\_T\_cell\_differentiation | 1 | 0 |  |  |  |  |  |  |  |  |
| GO:0001866\_NK\_T\_cell\_proliferation | 1 | 0 |  |  |  |  |  |  |  |  |
| GO:0001887\_selenium\_metabolic\_process | 1 | 0 |  |  |  |  |  |  |  |  |
| GO:0001892\_embryonic\_placenta\_development | 1 | 0 |  |  |  |  |  |  |  |  |
| GO:0001911\_negative\_regulation\_of\_leukocyte\_mediated\_cytotoxicity | 1 | 0 |  |  |  |  |  |  |  |  |
| GO:0001915\_negative\_regulation\_of\_T\_cell\_mediated\_cytotoxicity | 1 | 0 |  |  |  |  |  |  |  |  |
| GO:0001920\_negative\_regulation\_of\_receptor\_recycling | 1 | 0 |  |  |  |  |  |  |  |  |
| GO:0001941\_postsynaptic\_membrane\_organization | 1 | 0 |  |  |  |  |  |  |  |  |
| GO:0001958\_endochondral\_ossification | 1 | 0 |  |  |  |  |  |  |  |  |
| GO:0001973\_adenosine\_receptor\_signaling\_pathway | 1 | 0 |  |  |  |  |  |  |  |  |
| GO:0001977\_renal\_system\_process\_involved\_in\_regulation\_of\_blood\_volume | 1 | 0 |  |  |  |  |  |  |  |  |
| GO:0001980\_regulation\_of\_systemic\_arterial\_blood\_pressure\_by\_ischemic\_conditions | 1 | 0 |  |  |  |  |  |  |  |  |
| GO:0001993\_regulation\_of\_systemic\_arterial\_blood\_pressure\_by\_norepinephrine-epinephrine | 1 | 0 |  |  |  |  |  |  |  |  |
| GO:0001996\_positive\_regulation\_of\_heart\_rate\_by\_epinephrine-norepinephrine | 1 | 0 |  |  |  |  |  |  |  |  |
| GO:0001999\_renal\_response\_to\_blood\_flow\_during\_renin-angiotensin\_regulation\_of\_systemic\_arterial\_blood\_pressure | 1 | 0 |  |  |  |  |  |  |  |  |
| GO:0002001\_renin\_secretion\_into\_blood\_stream | 1 | 0 |  |  |  |  |  |  |  |  |
| GO:0002017\_regulation\_of\_blood\_volume\_by\_renal\_aldosterone | 1 | 0 |  |  |  |  |  |  |  |  |
| GO:0002018\_renin-angiotensin\_regulation\_of\_aldosterone\_production | 1 | 0 |  |  |  |  |  |  |  |  |
| GO:0002031\_G-protein\_coupled\_receptor\_internalization | 1 | 0 |  |  |  |  |  |  |  |  |
| GO:0002035\_brain\_renin-angiotensin\_system | 1 | 0 |  |  |  |  |  |  |  |  |
| GO:0002042\_cell\_migration\_involved\_in\_sprouting\_angiogenesis | 1 | 0 |  |  |  |  |  |  |  |  |
| GO:0002052\_positive\_regulation\_of\_neuroblast\_proliferation | 1 | 0 |  |  |  |  |  |  |  |  |
| GO:0002053\_positive\_regulation\_of\_mesenchymal\_cell\_proliferation | 1 | 0 |  |  |  |  |  |  |  |  |
| GO:0002063\_chondrocyte\_development | 1 | 0 |  |  |  |  |  |  |  |  |
| GO:0002064\_epithelial\_cell\_development | 1 | 0 |  |  |  |  |  |  |  |  |
| GO:0002074\_extraocular\_skeletal\_muscle\_development | 1 | 0 |  |  |  |  |  |  |  |  |
| GO:0002077\_acrosome\_matrix\_dispersal | 1 | 0 |  |  |  |  |  |  |  |  |
| GO:0002082\_regulation\_of\_oxidative\_phosphorylation | 1 | 0 |  |  |  |  |  |  |  |  |
| GO:0002084\_protein\_depalmitoylation | 1 | 0 |  |  |  |  |  |  |  |  |
| GO:0002088\_lens\_development\_in\_camera-type\_eye | 1 | 0 |  |  |  |  |  |  |  |  |
| GO:0002089\_lens\_morphogenesis\_in\_camera-type\_eye | 1 | 0 |  |  |  |  |  |  |  |  |
| GO:0002093\_auditory\_receptor\_cell\_morphogenesis | 1 | 0 |  |  |  |  |  |  |  |  |
| GO:0002209\_behavioral\_defense\_response | 1 | 0 |  |  |  |  |  |  |  |  |
| GO:0002220\_innate\_immune\_response\_activating\_cell\_surface\_receptor\_signaling\_pathway | 1 | 0 |  |  |  |  |  |  |  |  |
| GO:0002223\_stimulatory\_C-type\_lectin\_receptor\_signaling\_pathway | 1 | 0 |  |  |  |  |  |  |  |  |
| GO:0002312\_B\_cell\_activation\_during\_immune\_response | 1 | 0 |  |  |  |  |  |  |  |  |
| GO:0002313\_mature\_B\_cell\_differentiation\_during\_immune\_response | 1 | 0 |  |  |  |  |  |  |  |  |
| GO:0002318\_myeloid\_progenitor\_cell\_differentiation | 1 | 0 |  |  |  |  |  |  |  |  |
| GO:0002320\_lymphoid\_progenitor\_cell\_differentiation | 1 | 0 |  |  |  |  |  |  |  |  |
| GO:0002326\_B\_cell\_lineage\_commitment | 1 | 0 |  |  |  |  |  |  |  |  |
| GO:0002328\_pro-B\_cell\_differentiation | 1 | 0 |  |  |  |  |  |  |  |  |
| GO:0002335\_mature\_B\_cell\_differentiation | 1 | 0 |  |  |  |  |  |  |  |  |
| GO:0002355\_detection\_of\_tumor\_cell | 1 | 0 |  |  |  |  |  |  |  |  |
| GO:0002368\_B\_cell\_cytokine\_production | 1 | 0 |  |  |  |  |  |  |  |  |
| GO:0002424\_T\_cell\_mediated\_immune\_response\_to\_tumor\_cell | 1 | 0 |  |  |  |  |  |  |  |  |
| GO:0002431\_Fc\_receptor\_mediated\_stimulatory\_signaling\_pathway | 1 | 0 |  |  |  |  |  |  |  |  |
| GO:0002437\_inflammatory\_response\_to\_antigenic\_stimulus | 1 | 0 |  |  |  |  |  |  |  |  |
| GO:0002439\_chronic\_inflammatory\_response\_to\_antigenic\_stimulus | 1 | 0 |  |  |  |  |  |  |  |  |
| GO:0002447\_eosinophil\_mediated\_immunity | 1 | 0 |  |  |  |  |  |  |  |  |
| GO:0002455\_humoral\_immune\_response\_mediated\_by\_circulating\_immunoglobulin | 1 | 0 |  |  |  |  |  |  |  |  |
| GO:0002467\_germinal\_center\_formation | 1 | 0 |  |  |  |  |  |  |  |  |
| GO:0002468\_dendritic\_cell\_antigen\_processing\_and\_presentation | 1 | 0 |  |  |  |  |  |  |  |  |
| GO:0002478\_antigen\_processing\_and\_presentation\_of\_exogenous\_peptide\_antigen | 1 | 0 |  |  |  |  |  |  |  |  |
| GO:0002495\_antigen\_processing\_and\_presentation\_of\_peptide\_antigen\_via\_MHC\_class\_II | 1 | 0 |  |  |  |  |  |  |  |  |
| GO:0002513\_tolerance\_induction\_to\_self\_antigen | 1 | 0 |  |  |  |  |  |  |  |  |
| GO:0002514\_B\_cell\_tolerance\_induction | 1 | 0 |  |  |  |  |  |  |  |  |
| GO:0002517\_T\_cell\_tolerance\_induction | 1 | 0 |  |  |  |  |  |  |  |  |
| GO:0002523\_leukocyte\_migration\_during\_inflammatory\_response | 1 | 0 |  |  |  |  |  |  |  |  |
| GO:0002566\_somatic\_diversification\_of\_immune\_receptors\_via\_somatic\_mutation | 1 | 0 |  |  |  |  |  |  |  |  |
| GO:0002568\_somatic\_diversification\_of\_T\_cell\_receptor\_genes | 1 | 0 |  |  |  |  |  |  |  |  |
| GO:0002576\_platelet\_degranulation | 1 | 0 |  |  |  |  |  |  |  |  |
| GO:0002577\_regulation\_of\_antigen\_processing\_and\_presentation | 1 | 0 |  |  |  |  |  |  |  |  |
| GO:0002578\_negative\_regulation\_of\_antigen\_processing\_and\_presentation | 1 | 0 |  |  |  |  |  |  |  |  |
| GO:0002580\_regulation\_of\_antigen\_processing\_and\_presentation\_of\_peptide\_or\_polysaccharide\_antigen\_via\_MHC\_class\_II | 1 | 0 |  |  |  |  |  |  |  |  |
| GO:0002581\_negative\_regulation\_of\_antigen\_processing\_and\_presentation\_of\_peptide\_or\_polysaccharide\_antigen\_via\_MHC\_class\_II | 1 | 0 |  |  |  |  |  |  |  |  |
| GO:0002604\_regulation\_of\_dendritic\_cell\_antigen\_processing\_and\_presentation | 1 | 0 |  |  |  |  |  |  |  |  |
| GO:0002605\_negative\_regulation\_of\_dendritic\_cell\_antigen\_processing\_and\_presentation | 1 | 0 |  |  |  |  |  |  |  |  |
| GO:0002634\_regulation\_of\_germinal\_center\_formation | 1 | 0 |  |  |  |  |  |  |  |  |
| GO:0002649\_regulation\_of\_tolerance\_induction\_to\_self\_antigen | 1 | 0 |  |  |  |  |  |  |  |  |
| GO:0002651\_positive\_regulation\_of\_tolerance\_induction\_to\_self\_antigen | 1 | 0 |  |  |  |  |  |  |  |  |
| GO:0002661\_regulation\_of\_B\_cell\_tolerance\_induction | 1 | 0 |  |  |  |  |  |  |  |  |
| GO:0002663\_positive\_regulation\_of\_B\_cell\_tolerance\_induction | 1 | 0 |  |  |  |  |  |  |  |  |
| GO:0002664\_regulation\_of\_T\_cell\_tolerance\_induction | 1 | 0 |  |  |  |  |  |  |  |  |
| GO:0002666\_positive\_regulation\_of\_T\_cell\_tolerance\_induction | 1 | 0 |  |  |  |  |  |  |  |  |
| GO:0002674\_negative\_regulation\_of\_acute\_inflammatory\_response | 1 | 0 |  |  |  |  |  |  |  |  |
| GO:0002681\_somatic\_recombination\_of\_T\_cell\_receptor\_gene\_segments | 1 | 0 |  |  |  |  |  |  |  |  |
| GO:0002686\_negative\_regulation\_of\_leukocyte\_migration | 1 | 0 |  |  |  |  |  |  |  |  |
| GO:0002691\_regulation\_of\_cellular\_extravasation | 1 | 0 |  |  |  |  |  |  |  |  |
| GO:0002693\_positive\_regulation\_of\_cellular\_extravasation | 1 | 0 |  |  |  |  |  |  |  |  |
| GO:0002701\_negative\_regulation\_of\_production\_of\_molecular\_mediator\_of\_immune\_response | 1 | 0 |  |  |  |  |  |  |  |  |
| GO:0002719\_negative\_regulation\_of\_cytokine\_production\_during\_immune\_response | 1 | 0 |  |  |  |  |  |  |  |  |
| GO:0002725\_negative\_regulation\_of\_T\_cell\_cytokine\_production | 1 | 0 |  |  |  |  |  |  |  |  |
| GO:0002759\_regulation\_of\_antimicrobial\_humoral\_response | 1 | 0 |  |  |  |  |  |  |  |  |
| GO:0002775\_antimicrobial\_peptide\_production | 1 | 0 |  |  |  |  |  |  |  |  |
| GO:0002777\_antimicrobial\_peptide\_biosynthetic\_process | 1 | 0 |  |  |  |  |  |  |  |  |
| GO:0002778\_antibacterial\_peptide\_production | 1 | 0 |  |  |  |  |  |  |  |  |
| GO:0002780\_antibacterial\_peptide\_biosynthetic\_process | 1 | 0 |  |  |  |  |  |  |  |  |
| GO:0002784\_regulation\_of\_antimicrobial\_peptide\_production | 1 | 0 |  |  |  |  |  |  |  |  |
| GO:0002786\_regulation\_of\_antibacterial\_peptide\_production | 1 | 0 |  |  |  |  |  |  |  |  |
| GO:0002805\_regulation\_of\_antimicrobial\_peptide\_biosynthetic\_process | 1 | 0 |  |  |  |  |  |  |  |  |
| GO:0002807\_positive\_regulation\_of\_antimicrobial\_peptide\_biosynthetic\_process | 1 | 0 |  |  |  |  |  |  |  |  |
| GO:0002808\_regulation\_of\_antibacterial\_peptide\_biosynthetic\_process | 1 | 0 |  |  |  |  |  |  |  |  |
| GO:0002815\_biosynthetic\_process\_of\_antibacterial\_peptides\_active\_against\_Gram-positive\_bacteria | 1 | 0 |  |  |  |  |  |  |  |  |
| GO:0002816\_regulation\_of\_biosynthetic\_process\_of\_antibacterial\_peptides\_active\_against\_Gram-positive\_bacteria | 1 | 0 |  |  |  |  |  |  |  |  |
| GO:0002832\_negative\_regulation\_of\_response\_to\_biotic\_stimulus | 1 | 0 |  |  |  |  |  |  |  |  |
| GO:0002840\_regulation\_of\_T\_cell\_mediated\_immune\_response\_to\_tumor\_cell | 1 | 0 |  |  |  |  |  |  |  |  |
| GO:0002842\_positive\_regulation\_of\_T\_cell\_mediated\_immune\_response\_to\_tumor\_cell | 1 | 0 |  |  |  |  |  |  |  |  |
| GO:0002901\_mature\_B\_cell\_apoptosis | 1 | 0 |  |  |  |  |  |  |  |  |
| GO:0002904\_positive\_regulation\_of\_B\_cell\_apoptosis | 1 | 0 |  |  |  |  |  |  |  |  |
| GO:0002905\_regulation\_of\_mature\_B\_cell\_apoptosis | 1 | 0 |  |  |  |  |  |  |  |  |
| GO:0002906\_negative\_regulation\_of\_mature\_B\_cell\_apoptosis | 1 | 0 |  |  |  |  |  |  |  |  |
| GO:0003010\_voluntary\_skeletal\_muscle\_contraction | 1 | 0 |  |  |  |  |  |  |  |  |
| GO:0003051\_angiotensin-mediated\_drinking\_behavior | 1 | 0 |  |  |  |  |  |  |  |  |
| GO:0003058\_hormonal\_regulation\_of\_the\_force\_of\_heart\_contraction | 1 | 0 |  |  |  |  |  |  |  |  |
| GO:0003062\_regulation\_of\_heart\_rate\_by\_chemical\_signal | 1 | 0 |  |  |  |  |  |  |  |  |
| GO:0003065\_positive\_regulation\_of\_heart\_rate\_by\_epinephrine | 1 | 0 |  |  |  |  |  |  |  |  |
| GO:0003071\_renal\_system\_process\_involved\_in\_regulation\_of\_systemic\_arterial\_blood\_pressure | 1 | 0 |  |  |  |  |  |  |  |  |
| GO:0003085\_negative\_regulation\_of\_systemic\_arterial\_blood\_pressure | 1 | 0 |  |  |  |  |  |  |  |  |
| GO:0003099\_positive\_regulation\_of\_the\_force\_of\_heart\_contraction\_by\_chemical\_signal | 1 | 0 |  |  |  |  |  |  |  |  |
| GO:0003108\_negative\_regulation\_of\_the\_force\_of\_heart\_contraction\_by\_chemical\_signal | 1 | 0 |  |  |  |  |  |  |  |  |
| GO:0005981\_regulation\_of\_glycogen\_catabolic\_process | 1 | 0 |  |  |  |  |  |  |  |  |
| GO:0005982\_starch\_metabolic\_process | 1 | 0 |  |  |  |  |  |  |  |  |
| GO:0005983\_starch\_catabolic\_process | 1 | 0 |  |  |  |  |  |  |  |  |
| GO:0005988\_lactose\_metabolic\_process | 1 | 0 |  |  |  |  |  |  |  |  |
| GO:0005989\_lactose\_biosynthetic\_process | 1 | 0 |  |  |  |  |  |  |  |  |
| GO:0005991\_trehalose\_metabolic\_process | 1 | 0 |  |  |  |  |  |  |  |  |
| GO:0005993\_trehalose\_catabolic\_process | 1 | 0 |  |  |  |  |  |  |  |  |
| GO:0006010\_glucose\_6-phosphate\_utilization | 1 | 0 |  |  |  |  |  |  |  |  |
| GO:0006013\_mannose\_metabolic\_process | 1 | 0 |  |  |  |  |  |  |  |  |
| GO:0006021\_inositol\_biosynthetic\_process | 1 | 0 |  |  |  |  |  |  |  |  |
| GO:0006037\_cell\_wall\_chitin\_metabolic\_process | 1 | 0 |  |  |  |  |  |  |  |  |
| GO:0006042\_glucosamine\_biosynthetic\_process | 1 | 0 |  |  |  |  |  |  |  |  |
| GO:0006045\_N-acetylglucosamine\_biosynthetic\_process | 1 | 0 |  |  |  |  |  |  |  |  |
| GO:0006048\_UDP-N-acetylglucosamine\_biosynthetic\_process | 1 | 0 |  |  |  |  |  |  |  |  |
| GO:0006050\_mannosamine\_metabolic\_process | 1 | 0 |  |  |  |  |  |  |  |  |
| GO:0006051\_N-acetylmannosamine\_metabolic\_process | 1 | 0 |  |  |  |  |  |  |  |  |
| GO:0006059\_hexitol\_metabolic\_process | 1 | 0 |  |  |  |  |  |  |  |  |
| GO:0006060\_sorbitol\_metabolic\_process | 1 | 0 |  |  |  |  |  |  |  |  |
| GO:0006062\_sorbitol\_catabolic\_process | 1 | 0 |  |  |  |  |  |  |  |  |
| GO:0006065\_UDP-glucuronate\_biosynthetic\_process | 1 | 0 |  |  |  |  |  |  |  |  |
| GO:0006083\_acetate\_metabolic\_process | 1 | 0 |  |  |  |  |  |  |  |  |
| GO:0006085\_acetyl-CoA\_biosynthetic\_process | 1 | 0 |  |  |  |  |  |  |  |  |
| GO:0006103\_2-oxoglutarate\_metabolic\_process | 1 | 0 |  |  |  |  |  |  |  |  |
| GO:0006106\_fumarate\_metabolic\_process | 1 | 0 |  |  |  |  |  |  |  |  |
| GO:0006107\_oxaloacetate\_metabolic\_process | 1 | 0 |  |  |  |  |  |  |  |  |
| GO:0006116\_NADH\_oxidation | 1 | 0 |  |  |  |  |  |  |  |  |
| GO:0006145\_purine\_base\_catabolic\_process | 1 | 0 |  |  |  |  |  |  |  |  |
| GO:0006148\_inosine\_catabolic\_process | 1 | 0 |  |  |  |  |  |  |  |  |
| GO:0006154\_adenosine\_catabolic\_process | 1 | 0 |  |  |  |  |  |  |  |  |
| GO:0006166\_purine\_ribonucleoside\_salvage | 1 | 0 |  |  |  |  |  |  |  |  |
| GO:0006172\_ADP\_biosynthetic\_process | 1 | 0 |  |  |  |  |  |  |  |  |
| GO:0006173\_dADP\_biosynthetic\_process | 1 | 0 |  |  |  |  |  |  |  |  |
| GO:0006188\_IMP\_biosynthetic\_process | 1 | 0 |  |  |  |  |  |  |  |  |
| GO:0006189\_'de\_novo'\_IMP\_biosynthetic\_process | 1 | 0 |  |  |  |  |  |  |  |  |
| GO:0006196\_AMP\_catabolic\_process | 1 | 0 |  |  |  |  |  |  |  |  |
| GO:0006198\_cAMP\_catabolic\_process | 1 | 0 |  |  |  |  |  |  |  |  |
| GO:0006207\_'de\_novo'\_pyrimidine\_base\_biosynthetic\_process | 1 | 0 |  |  |  |  |  |  |  |  |
| GO:0006214\_thymidine\_catabolic\_process | 1 | 0 |  |  |  |  |  |  |  |  |
| GO:0006216\_cytidine\_catabolic\_process | 1 | 0 |  |  |  |  |  |  |  |  |
| GO:0006222\_UMP\_biosynthetic\_process | 1 | 0 |  |  |  |  |  |  |  |  |
| GO:0006241\_CTP\_biosynthetic\_process | 1 | 0 |  |  |  |  |  |  |  |  |
| GO:0006256\_UDP\_catabolic\_process | 1 | 0 |  |  |  |  |  |  |  |  |
| GO:0006265\_DNA\_topological\_change | 1 | 0 |  |  |  |  |  |  |  |  |
| GO:0006272\_leading\_strand\_elongation | 1 | 0 |  |  |  |  |  |  |  |  |
| GO:0006287\_base-excision\_repair\_\_gap-filling | 1 | 0 |  |  |  |  |  |  |  |  |
| GO:0006313\_transposition\_\_DNA-mediated | 1 | 0 |  |  |  |  |  |  |  |  |
| GO:0006336\_DNA\_replication-independent\_nucleosome\_assembly | 1 | 0 |  |  |  |  |  |  |  |  |
| GO:0006343\_establishment\_of\_chromatin\_silencing | 1 | 0 |  |  |  |  |  |  |  |  |
| GO:0006344\_maintenance\_of\_chromatin\_silencing | 1 | 0 |  |  |  |  |  |  |  |  |
| GO:0006346\_methylation-dependent\_chromatin\_silencing | 1 | 0 |  |  |  |  |  |  |  |  |
| GO:0006348\_chromatin\_silencing\_at\_telomere | 1 | 0 |  |  |  |  |  |  |  |  |
| GO:0006361\_transcription\_initiation\_from\_RNA\_polymerase\_I\_promoter | 1 | 0 |  |  |  |  |  |  |  |  |
| GO:0006369\_termination\_of\_RNA\_polymerase\_II\_transcription | 1 | 0 |  |  |  |  |  |  |  |  |
| GO:0006393\_termination\_of\_mitochondrial\_transcription | 1 | 0 |  |  |  |  |  |  |  |  |
| GO:0006407\_rRNA\_export\_from\_nucleus | 1 | 0 |  |  |  |  |  |  |  |  |
| GO:0006408\_snRNA\_export\_from\_nucleus | 1 | 0 |  |  |  |  |  |  |  |  |
| GO:0006409\_tRNA\_export\_from\_nucleus | 1 | 0 |  |  |  |  |  |  |  |  |
| GO:0006419\_alanyl-tRNA\_aminoacylation | 1 | 0 |  |  |  |  |  |  |  |  |
| GO:0006420\_arginyl-tRNA\_aminoacylation | 1 | 0 |  |  |  |  |  |  |  |  |
| GO:0006423\_cysteinyl-tRNA\_aminoacylation | 1 | 0 |  |  |  |  |  |  |  |  |
| GO:0006431\_methionyl-tRNA\_aminoacylation | 1 | 0 |  |  |  |  |  |  |  |  |
| GO:0006432\_phenylalanyl-tRNA\_aminoacylation | 1 | 0 |  |  |  |  |  |  |  |  |
| GO:0006434\_seryl-tRNA\_aminoacylation | 1 | 0 |  |  |  |  |  |  |  |  |
| GO:0006435\_threonyl-tRNA\_aminoacylation | 1 | 0 |  |  |  |  |  |  |  |  |
| GO:0006436\_tryptophanyl-tRNA\_aminoacylation | 1 | 0 |  |  |  |  |  |  |  |  |
| GO:0006437\_tyrosyl-tRNA\_aminoacylation | 1 | 0 |  |  |  |  |  |  |  |  |
| GO:0006447\_regulation\_of\_translational\_initiation\_by\_iron | 1 | 0 |  |  |  |  |  |  |  |  |
| GO:0006448\_regulation\_of\_translational\_elongation | 1 | 0 |  |  |  |  |  |  |  |  |
| GO:0006450\_regulation\_of\_translational\_fidelity | 1 | 0 |  |  |  |  |  |  |  |  |
| GO:0006494\_protein\_amino\_acid\_terminal\_glycosylation | 1 | 0 |  |  |  |  |  |  |  |  |
| GO:0006496\_protein\_amino\_acid\_terminal\_N-glycosylation | 1 | 0 |  |  |  |  |  |  |  |  |
| GO:0006499\_N-terminal\_protein\_myristoylation | 1 | 0 |  |  |  |  |  |  |  |  |
| GO:0006500\_N-terminal\_protein\_palmitoylation | 1 | 0 |  |  |  |  |  |  |  |  |
| GO:0006526\_arginine\_biosynthetic\_process | 1 | 0 |  |  |  |  |  |  |  |  |
| GO:0006528\_asparagine\_metabolic\_process | 1 | 0 |  |  |  |  |  |  |  |  |
| GO:0006530\_asparagine\_catabolic\_process | 1 | 0 |  |  |  |  |  |  |  |  |
| GO:0006534\_cysteine\_metabolic\_process | 1 | 0 |  |  |  |  |  |  |  |  |
| GO:0006543\_glutamine\_catabolic\_process | 1 | 0 |  |  |  |  |  |  |  |  |
| GO:0006545\_glycine\_biosynthetic\_process | 1 | 0 |  |  |  |  |  |  |  |  |
| GO:0006547\_histidine\_metabolic\_process | 1 | 0 |  |  |  |  |  |  |  |  |
| GO:0006549\_isoleucine\_metabolic\_process | 1 | 0 |  |  |  |  |  |  |  |  |
| GO:0006556\_S-adenosylmethionine\_biosynthetic\_process | 1 | 0 |  |  |  |  |  |  |  |  |
| GO:0006562\_proline\_catabolic\_process | 1 | 0 |  |  |  |  |  |  |  |  |
| GO:0006564\_L-serine\_biosynthetic\_process | 1 | 0 |  |  |  |  |  |  |  |  |
| GO:0006577\_betaine\_metabolic\_process | 1 | 0 |  |  |  |  |  |  |  |  |
| GO:0006580\_ethanolamine\_metabolic\_process | 1 | 0 |  |  |  |  |  |  |  |  |
| GO:0006585\_dopamine\_biosynthetic\_process\_from\_tyrosine | 1 | 0 |  |  |  |  |  |  |  |  |
| GO:0006591\_ornithine\_metabolic\_process | 1 | 0 |  |  |  |  |  |  |  |  |
| GO:0006597\_spermine\_biosynthetic\_process | 1 | 0 |  |  |  |  |  |  |  |  |
| GO:0006598\_polyamine\_catabolic\_process | 1 | 0 |  |  |  |  |  |  |  |  |
| GO:0006610\_ribosomal\_protein\_import\_into\_nucleus | 1 | 0 |  |  |  |  |  |  |  |  |
| GO:0006614\_SRP-dependent\_cotranslational\_protein\_targeting\_to\_membrane | 1 | 0 |  |  |  |  |  |  |  |  |
| GO:0006616\_SRP-dependent\_cotranslational\_protein\_targeting\_to\_membrane\_\_translocation | 1 | 0 |  |  |  |  |  |  |  |  |
| GO:0006617\_SRP-dependent\_cotranslational\_protein\_targeting\_to\_membrane\_\_signal\_sequence\_recognition | 1 | 0 |  |  |  |  |  |  |  |  |
| GO:0006627\_mitochondrial\_protein\_processing\_during\_import | 1 | 0 |  |  |  |  |  |  |  |  |
| GO:0006646\_phosphatidylethanolamine\_biosynthetic\_process | 1 | 0 |  |  |  |  |  |  |  |  |
| GO:0006655\_phosphatidylglycerol\_biosynthetic\_process | 1 | 0 |  |  |  |  |  |  |  |  |
| GO:0006657\_CDP-choline\_pathway | 1 | 0 |  |  |  |  |  |  |  |  |
| GO:0006667\_sphinganine\_metabolic\_process | 1 | 0 |  |  |  |  |  |  |  |  |
| GO:0006668\_sphinganine-1-phosphate\_metabolic\_process | 1 | 0 |  |  |  |  |  |  |  |  |
| GO:0006669\_sphinganine-1-phosphate\_biosynthetic\_process | 1 | 0 |  |  |  |  |  |  |  |  |
| GO:0006670\_sphingosine\_metabolic\_process | 1 | 0 |  |  |  |  |  |  |  |  |
| GO:0006689\_ganglioside\_catabolic\_process | 1 | 0 |  |  |  |  |  |  |  |  |
| GO:0006711\_estrogen\_catabolic\_process | 1 | 0 |  |  |  |  |  |  |  |  |
| GO:0006713\_glucocorticoid\_catabolic\_process | 1 | 0 |  |  |  |  |  |  |  |  |
| GO:0006734\_NADH\_metabolic\_process | 1 | 0 |  |  |  |  |  |  |  |  |
| GO:0006741\_NADP\_biosynthetic\_process | 1 | 0 |  |  |  |  |  |  |  |  |
| GO:0006746\_FADH2\_metabolic\_process | 1 | 0 |  |  |  |  |  |  |  |  |
| GO:0006768\_biotin\_metabolic\_process | 1 | 0 |  |  |  |  |  |  |  |  |
| GO:0006771\_riboflavin\_metabolic\_process | 1 | 0 |  |  |  |  |  |  |  |  |
| GO:0006781\_succinyl-CoA\_pathway | 1 | 0 |  |  |  |  |  |  |  |  |
| GO:0006789\_bilirubin\_conjugation | 1 | 0 |  |  |  |  |  |  |  |  |
| GO:0006797\_polyphosphate\_metabolic\_process | 1 | 0 |  |  |  |  |  |  |  |  |
| GO:0006837\_serotonin\_transport | 1 | 0 |  |  |  |  |  |  |  |  |
| GO:0006842\_tricarboxylic\_acid\_transport | 1 | 0 |  |  |  |  |  |  |  |  |
| GO:0006843\_mitochondrial\_citrate\_transport | 1 | 0 |  |  |  |  |  |  |  |  |
| GO:0006848\_pyruvate\_transport | 1 | 0 |  |  |  |  |  |  |  |  |
| GO:0006862\_nucleotide\_transport | 1 | 0 |  |  |  |  |  |  |  |  |
| GO:0006867\_asparagine\_transport | 1 | 0 |  |  |  |  |  |  |  |  |
| GO:0006868\_glutamine\_transport | 1 | 0 |  |  |  |  |  |  |  |  |
| GO:0006876\_cellular\_cadmium\_ion\_homeostasis | 1 | 0 |  |  |  |  |  |  |  |  |
| GO:0006926\_virus-infected\_cell\_apoptosis | 1 | 0 |  |  |  |  |  |  |  |  |
| GO:0006931\_substrate-bound\_cell\_migration\_\_cell\_attachment\_to\_substrate | 1 | 0 |  |  |  |  |  |  |  |  |
| GO:0006948\_induction\_by\_virus\_of\_host\_cell-cell\_fusion | 1 | 0 |  |  |  |  |  |  |  |  |
| GO:0006958\_complement\_activation\_\_classical\_pathway | 1 | 0 |  |  |  |  |  |  |  |  |
| GO:0006963\_positive\_regulation\_of\_antibacterial\_peptide\_biosynthetic\_process | 1 | 0 |  |  |  |  |  |  |  |  |
| GO:0006965\_positive\_regulation\_of\_biosynthetic\_process\_of\_antibacterial\_peptides\_active\_against\_Gram-positive\_bacteria | 1 | 0 |  |  |  |  |  |  |  |  |
| GO:0006987\_activation\_of\_signaling\_protein\_activity\_involved\_in\_unfolded\_protein\_response | 1 | 0 |  |  |  |  |  |  |  |  |
| GO:0006990\_positive\_regulation\_of\_gene-specific\_transcription\_involved\_in\_unfolded\_protein\_response | 1 | 0 |  |  |  |  |  |  |  |  |
| GO:0006991\_response\_to\_sterol\_depletion | 1 | 0 |  |  |  |  |  |  |  |  |
| GO:0006994\_positive\_regulation\_of\_transcription\_via\_sterol\_regulatory\_element\_binding\_involved\_in\_ER-nuclear\_sterol\_response\_pathway | 1 | 0 |  |  |  |  |  |  |  |  |
| GO:0007039\_vacuolar\_protein\_catabolic\_process | 1 | 0 |  |  |  |  |  |  |  |  |
| GO:0007068\_negative\_regulation\_of\_transcription\_\_mitotic | 1 | 0 |  |  |  |  |  |  |  |  |
| GO:0007097\_nuclear\_migration | 1 | 0 |  |  |  |  |  |  |  |  |
| GO:0007100\_mitotic\_centrosome\_separation | 1 | 0 |  |  |  |  |  |  |  |  |
| GO:0007108\_cytokinesis\_\_initiation\_of\_separation | 1 | 0 |  |  |  |  |  |  |  |  |
| GO:0007109\_cytokinesis\_\_completion\_of\_separation | 1 | 0 |  |  |  |  |  |  |  |  |
| GO:0007132\_meiotic\_metaphase\_I | 1 | 0 |  |  |  |  |  |  |  |  |
| GO:0007135\_meiosis\_II | 1 | 0 |  |  |  |  |  |  |  |  |
| GO:0007136\_meiotic\_prophase\_II | 1 | 0 |  |  |  |  |  |  |  |  |
| GO:0007161\_calcium-independent\_cell-matrix\_adhesion | 1 | 0 |  |  |  |  |  |  |  |  |
| GO:0007196\_inhibition\_of\_adenylate\_cyclase\_activity\_by\_metabotropic\_glutamate\_receptor\_signaling\_pathway | 1 | 0 |  |  |  |  |  |  |  |  |
| GO:0007197\_inhibition\_of\_adenylate\_cyclase\_activity\_by\_muscarinic\_acetylcholine\_receptor\_signaling\_pathway | 1 | 0 |  |  |  |  |  |  |  |  |
| GO:0007258\_JUN\_phosphorylation | 1 | 0 |  |  |  |  |  |  |  |  |
| GO:0007321\_sperm\_displacement | 1 | 0 |  |  |  |  |  |  |  |  |
| GO:0007343\_egg\_activation | 1 | 0 |  |  |  |  |  |  |  |  |
| GO:0007386\_compartment\_specification | 1 | 0 |  |  |  |  |  |  |  |  |
| GO:0007387\_anterior\_compartment\_specification | 1 | 0 |  |  |  |  |  |  |  |  |
| GO:0007388\_posterior\_compartment\_specification | 1 | 0 |  |  |  |  |  |  |  |  |
| GO:0007402\_ganglion\_mother\_cell\_fate\_determination | 1 | 0 |  |  |  |  |  |  |  |  |
| GO:0007406\_negative\_regulation\_of\_neuroblast\_proliferation | 1 | 0 |  |  |  |  |  |  |  |  |
| GO:0007424\_open\_tracheal\_system\_development | 1 | 0 |  |  |  |  |  |  |  |  |
| GO:0007440\_foregut\_morphogenesis | 1 | 0 |  |  |  |  |  |  |  |  |
| GO:0007443\_Malpighian\_tubule\_morphogenesis | 1 | 0 |  |  |  |  |  |  |  |  |
| GO:0007444\_imaginal\_disc\_development | 1 | 0 |  |  |  |  |  |  |  |  |
| GO:0007447\_imaginal\_disc\_pattern\_formation | 1 | 0 |  |  |  |  |  |  |  |  |
| GO:0007494\_midgut\_development | 1 | 0 |  |  |  |  |  |  |  |  |
| GO:0007497\_posterior\_midgut\_development | 1 | 0 |  |  |  |  |  |  |  |  |
| GO:0007499\_ectoderm\_and\_mesoderm\_interaction | 1 | 0 |  |  |  |  |  |  |  |  |
| GO:0007501\_mesodermal\_cell\_fate\_specification | 1 | 0 |  |  |  |  |  |  |  |  |
| GO:0007509\_mesoderm\_migration | 1 | 0 |  |  |  |  |  |  |  |  |
| GO:0007518\_myoblast\_cell\_fate\_determination | 1 | 0 |  |  |  |  |  |  |  |  |
| GO:0007538\_primary\_sex\_determination | 1 | 0 |  |  |  |  |  |  |  |  |
| GO:0007597\_blood\_coagulation\_\_intrinsic\_pathway | 1 | 0 |  |  |  |  |  |  |  |  |
| GO:0007616\_long-term\_memory | 1 | 0 |  |  |  |  |  |  |  |  |
| GO:0007617\_mating\_behavior | 1 | 0 |  |  |  |  |  |  |  |  |
| GO:0007624\_ultradian\_rhythm | 1 | 0 |  |  |  |  |  |  |  |  |
| GO:0007638\_mechanosensory\_behavior | 1 | 0 |  |  |  |  |  |  |  |  |
| GO:0008045\_motor\_axon\_guidance | 1 | 0 |  |  |  |  |  |  |  |  |
| GO:0008057\_eye\_pigment\_granule\_organization | 1 | 0 |  |  |  |  |  |  |  |  |
| GO:0008063\_Toll\_signaling\_pathway | 1 | 0 |  |  |  |  |  |  |  |  |
| GO:0008065\_establishment\_of\_blood-nerve\_barrier | 1 | 0 |  |  |  |  |  |  |  |  |
| GO:0008090\_retrograde\_axon\_cargo\_transport | 1 | 0 |  |  |  |  |  |  |  |  |
| GO:0008215\_spermine\_metabolic\_process | 1 | 0 |  |  |  |  |  |  |  |  |
| GO:0008292\_acetylcholine\_biosynthetic\_process | 1 | 0 |  |  |  |  |  |  |  |  |
| GO:0008295\_spermidine\_biosynthetic\_process | 1 | 0 |  |  |  |  |  |  |  |  |
| GO:0008298\_intracellular\_mRNA\_localization | 1 | 0 |  |  |  |  |  |  |  |  |
| GO:0008356\_asymmetric\_cell\_division | 1 | 0 |  |  |  |  |  |  |  |  |
| GO:0008592\_regulation\_of\_Toll\_signaling\_pathway | 1 | 0 |  |  |  |  |  |  |  |  |
| GO:0008611\_ether\_lipid\_biosynthetic\_process | 1 | 0 |  |  |  |  |  |  |  |  |
| GO:0008614\_pyridoxine\_metabolic\_process | 1 | 0 |  |  |  |  |  |  |  |  |
| GO:0008615\_pyridoxine\_biosynthetic\_process | 1 | 0 |  |  |  |  |  |  |  |  |
| GO:0008627\_induction\_of\_apoptosis\_by\_ionic\_changes | 1 | 0 |  |  |  |  |  |  |  |  |
| GO:0008655\_pyrimidine\_salvage | 1 | 0 |  |  |  |  |  |  |  |  |
| GO:0009052\_pentose-phosphate\_shunt\_\_non-oxidative\_branch | 1 | 0 |  |  |  |  |  |  |  |  |
| GO:0009067\_aspartate\_family\_amino\_acid\_biosynthetic\_process | 1 | 0 |  |  |  |  |  |  |  |  |
| GO:0009075\_histidine\_family\_amino\_acid\_metabolic\_process | 1 | 0 |  |  |  |  |  |  |  |  |
| GO:0009128\_purine\_nucleoside\_monophosphate\_catabolic\_process | 1 | 0 |  |  |  |  |  |  |  |  |
| GO:0009129\_pyrimidine\_nucleoside\_monophosphate\_metabolic\_process | 1 | 0 |  |  |  |  |  |  |  |  |
| GO:0009130\_pyrimidine\_nucleoside\_monophosphate\_biosynthetic\_process | 1 | 0 |  |  |  |  |  |  |  |  |
| GO:0009133\_nucleoside\_diphosphate\_biosynthetic\_process | 1 | 0 |  |  |  |  |  |  |  |  |
| GO:0009135\_purine\_nucleoside\_diphosphate\_metabolic\_process | 1 | 0 |  |  |  |  |  |  |  |  |
| GO:0009136\_purine\_nucleoside\_diphosphate\_biosynthetic\_process | 1 | 0 |  |  |  |  |  |  |  |  |
| GO:0009138\_pyrimidine\_nucleoside\_diphosphate\_metabolic\_process | 1 | 0 |  |  |  |  |  |  |  |  |
| GO:0009140\_pyrimidine\_nucleoside\_diphosphate\_catabolic\_process | 1 | 0 |  |  |  |  |  |  |  |  |
| GO:0009147\_pyrimidine\_nucleoside\_triphosphate\_metabolic\_process | 1 | 0 |  |  |  |  |  |  |  |  |
| GO:0009148\_pyrimidine\_nucleoside\_triphosphate\_biosynthetic\_process | 1 | 0 |  |  |  |  |  |  |  |  |
| GO:0009153\_purine\_deoxyribonucleotide\_biosynthetic\_process | 1 | 0 |  |  |  |  |  |  |  |  |
| GO:0009157\_deoxyribonucleoside\_monophosphate\_biosynthetic\_process | 1 | 0 |  |  |  |  |  |  |  |  |
| GO:0009158\_ribonucleoside\_monophosphate\_catabolic\_process | 1 | 0 |  |  |  |  |  |  |  |  |
| GO:0009159\_deoxyribonucleoside\_monophosphate\_catabolic\_process | 1 | 0 |  |  |  |  |  |  |  |  |
| GO:0009169\_purine\_ribonucleoside\_monophosphate\_catabolic\_process | 1 | 0 |  |  |  |  |  |  |  |  |
| GO:0009173\_pyrimidine\_ribonucleoside\_monophosphate\_metabolic\_process | 1 | 0 |  |  |  |  |  |  |  |  |
| GO:0009174\_pyrimidine\_ribonucleoside\_monophosphate\_biosynthetic\_process | 1 | 0 |  |  |  |  |  |  |  |  |
| GO:0009179\_purine\_ribonucleoside\_diphosphate\_metabolic\_process | 1 | 0 |  |  |  |  |  |  |  |  |
| GO:0009180\_purine\_ribonucleoside\_diphosphate\_biosynthetic\_process | 1 | 0 |  |  |  |  |  |  |  |  |
| GO:0009182\_purine\_deoxyribonucleoside\_diphosphate\_metabolic\_process | 1 | 0 |  |  |  |  |  |  |  |  |
| GO:0009183\_purine\_deoxyribonucleoside\_diphosphate\_biosynthetic\_process | 1 | 0 |  |  |  |  |  |  |  |  |
| GO:0009186\_deoxyribonucleoside\_diphosphate\_metabolic\_process | 1 | 0 |  |  |  |  |  |  |  |  |
| GO:0009188\_ribonucleoside\_diphosphate\_biosynthetic\_process | 1 | 0 |  |  |  |  |  |  |  |  |
| GO:0009189\_deoxyribonucleoside\_diphosphate\_biosynthetic\_process | 1 | 0 |  |  |  |  |  |  |  |  |
| GO:0009193\_pyrimidine\_ribonucleoside\_diphosphate\_metabolic\_process | 1 | 0 |  |  |  |  |  |  |  |  |
| GO:0009195\_pyrimidine\_ribonucleoside\_diphosphate\_catabolic\_process | 1 | 0 |  |  |  |  |  |  |  |  |
| GO:0009208\_pyrimidine\_ribonucleoside\_triphosphate\_metabolic\_process | 1 | 0 |  |  |  |  |  |  |  |  |
| GO:0009209\_pyrimidine\_ribonucleoside\_triphosphate\_biosynthetic\_process | 1 | 0 |  |  |  |  |  |  |  |  |
| GO:0009214\_cyclic\_nucleotide\_catabolic\_process | 1 | 0 |  |  |  |  |  |  |  |  |
| GO:0009222\_pyrimidine\_ribonucleotide\_catabolic\_process | 1 | 0 |  |  |  |  |  |  |  |  |
| GO:0009231\_riboflavin\_biosynthetic\_process | 1 | 0 |  |  |  |  |  |  |  |  |
| GO:0009253\_peptidoglycan\_catabolic\_process | 1 | 0 |  |  |  |  |  |  |  |  |
| GO:0009256\_10-formyltetrahydrofolate\_metabolic\_process | 1 | 0 |  |  |  |  |  |  |  |  |
| GO:0009258\_10-formyltetrahydrofolate\_catabolic\_process | 1 | 0 |  |  |  |  |  |  |  |  |
| GO:0009265\_2'-deoxyribonucleotide\_biosynthetic\_process | 1 | 0 |  |  |  |  |  |  |  |  |
| GO:0009292\_genetic\_transfer | 1 | 0 |  |  |  |  |  |  |  |  |
| GO:0009294\_DNA\_mediated\_transformation | 1 | 0 |  |  |  |  |  |  |  |  |
| GO:0009296\_flagellum\_assembly | 1 | 0 |  |  |  |  |  |  |  |  |
| GO:0009298\_GDP-mannose\_biosynthetic\_process | 1 | 0 |  |  |  |  |  |  |  |  |
| GO:0009304\_tRNA\_transcription | 1 | 0 |  |  |  |  |  |  |  |  |
| GO:0009313\_oligosaccharide\_catabolic\_process | 1 | 0 |  |  |  |  |  |  |  |  |
| GO:0009372\_quorum\_sensing | 1 | 0 |  |  |  |  |  |  |  |  |
| GO:0009386\_translational\_attenuation | 1 | 0 |  |  |  |  |  |  |  |  |
| GO:0009397\_folic\_acid\_and\_derivative\_catabolic\_process | 1 | 0 |  |  |  |  |  |  |  |  |
| GO:0009399\_nitrogen\_fixation | 1 | 0 |  |  |  |  |  |  |  |  |
| GO:0009404\_toxin\_metabolic\_process | 1 | 0 |  |  |  |  |  |  |  |  |
| GO:0009435\_NAD\_biosynthetic\_process | 1 | 0 |  |  |  |  |  |  |  |  |
| GO:0009437\_carnitine\_metabolic\_process | 1 | 0 |  |  |  |  |  |  |  |  |
| GO:0009441\_glycolate\_metabolic\_process | 1 | 0 |  |  |  |  |  |  |  |  |
| GO:0009624\_response\_to\_nematode | 1 | 0 |  |  |  |  |  |  |  |  |
| GO:0009642\_response\_to\_light\_intensity | 1 | 0 |  |  |  |  |  |  |  |  |
| GO:0009648\_photoperiodism | 1 | 0 |  |  |  |  |  |  |  |  |
| GO:0009720\_detection\_of\_hormone\_stimulus | 1 | 0 |  |  |  |  |  |  |  |  |
| GO:0009726\_detection\_of\_endogenous\_stimulus | 1 | 0 |  |  |  |  |  |  |  |  |
| GO:0009730\_detection\_of\_carbohydrate\_stimulus | 1 | 0 |  |  |  |  |  |  |  |  |
| GO:0009732\_detection\_of\_hexose\_stimulus | 1 | 0 |  |  |  |  |  |  |  |  |
| GO:0009826\_unidimensional\_cell\_growth | 1 | 0 |  |  |  |  |  |  |  |  |
| GO:0009912\_auditory\_receptor\_cell\_fate\_commitment | 1 | 0 |  |  |  |  |  |  |  |  |
| GO:0009954\_proximal\_distal\_pattern\_formation | 1 | 0 |  |  |  |  |  |  |  |  |
| GO:0009972\_cytidine\_deamination | 1 | 0 |  |  |  |  |  |  |  |  |
| GO:0010107\_potassium\_ion\_import | 1 | 0 |  |  |  |  |  |  |  |  |
| GO:0010259\_multicellular\_organismal\_aging | 1 | 0 |  |  |  |  |  |  |  |  |
| GO:0010269\_response\_to\_selenium\_ion | 1 | 0 |  |  |  |  |  |  |  |  |
| GO:0010273\_detoxification\_of\_copper\_ion | 1 | 0 |  |  |  |  |  |  |  |  |
| GO:0010383\_cell\_wall\_polysaccharide\_metabolic\_process | 1 | 0 |  |  |  |  |  |  |  |  |
| GO:0010430\_fatty\_acid\_omega-oxidation | 1 | 0 |  |  |  |  |  |  |  |  |
| GO:0010463\_mesenchymal\_cell\_proliferation | 1 | 0 |  |  |  |  |  |  |  |  |
| GO:0010464\_regulation\_of\_mesenchymal\_cell\_proliferation | 1 | 0 |  |  |  |  |  |  |  |  |
| GO:0010507\_negative\_regulation\_of\_autophagy | 1 | 0 |  |  |  |  |  |  |  |  |
| GO:0010509\_polyamine\_homeostasis | 1 | 0 |  |  |  |  |  |  |  |  |
| GO:0010534\_regulation\_of\_activation\_of\_JAK2\_kinase\_activity | 1 | 0 |  |  |  |  |  |  |  |  |
| GO:0010535\_positive\_regulation\_of\_activation\_of\_JAK2\_kinase\_activity | 1 | 0 |  |  |  |  |  |  |  |  |
| GO:0010561\_negative\_regulation\_of\_glycoprotein\_biosynthetic\_process | 1 | 0 |  |  |  |  |  |  |  |  |
| GO:0010569\_regulation\_of\_double-strand\_break\_repair\_via\_homologous\_recombination | 1 | 0 |  |  |  |  |  |  |  |  |
| GO:0010591\_regulation\_of\_lamellipodium\_assembly | 1 | 0 |  |  |  |  |  |  |  |  |
| GO:0010592\_positive\_regulation\_of\_lamellipodium\_assembly | 1 | 0 |  |  |  |  |  |  |  |  |
| GO:0010621\_negative\_regulation\_of\_transcription\_by\_transcription\_factor\_localization | 1 | 0 |  |  |  |  |  |  |  |  |
| GO:0010623\_developmental\_programmed\_cell\_death | 1 | 0 |  |  |  |  |  |  |  |  |
| GO:0010631\_epithelial\_cell\_migration | 1 | 0 |  |  |  |  |  |  |  |  |
| GO:0010632\_regulation\_of\_epithelial\_cell\_migration | 1 | 0 |  |  |  |  |  |  |  |  |
| GO:0010634\_positive\_regulation\_of\_epithelial\_cell\_migration | 1 | 0 |  |  |  |  |  |  |  |  |
| GO:0010658\_striated\_muscle\_cell\_apoptosis | 1 | 0 |  |  |  |  |  |  |  |  |
| GO:0010659\_cardiac\_muscle\_cell\_apoptosis | 1 | 0 |  |  |  |  |  |  |  |  |
| GO:0010662\_regulation\_of\_striated\_muscle\_cell\_apoptosis | 1 | 0 |  |  |  |  |  |  |  |  |
| GO:0010664\_negative\_regulation\_of\_striated\_muscle\_cell\_apoptosis | 1 | 0 |  |  |  |  |  |  |  |  |
| GO:0010665\_regulation\_of\_cardiac\_muscle\_cell\_apoptosis | 1 | 0 |  |  |  |  |  |  |  |  |
| GO:0010667\_negative\_regulation\_of\_cardiac\_muscle\_cell\_apoptosis | 1 | 0 |  |  |  |  |  |  |  |  |
| GO:0010669\_epithelial\_structure\_maintenance | 1 | 0 |  |  |  |  |  |  |  |  |
| GO:0010692\_regulation\_of\_alkaline\_phosphatase\_activity | 1 | 0 |  |  |  |  |  |  |  |  |
| GO:0010693\_negative\_regulation\_of\_alkaline\_phosphatase\_activity | 1 | 0 |  |  |  |  |  |  |  |  |
| GO:0010710\_regulation\_of\_collagen\_catabolic\_process | 1 | 0 |  |  |  |  |  |  |  |  |
| GO:0010711\_negative\_regulation\_of\_collagen\_catabolic\_process | 1 | 0 |  |  |  |  |  |  |  |  |
| GO:0010715\_regulation\_of\_extracellular\_matrix\_disassembly | 1 | 0 |  |  |  |  |  |  |  |  |
| GO:0010716\_negative\_regulation\_of\_extracellular\_matrix\_disassembly | 1 | 0 |  |  |  |  |  |  |  |  |
| GO:0010719\_negative\_regulation\_of\_epithelial\_to\_mesenchymal\_transition | 1 | 0 |  |  |  |  |  |  |  |  |
| GO:0010722\_regulation\_of\_ferrochelatase\_activity | 1 | 0 |  |  |  |  |  |  |  |  |
| GO:0010731\_protein\_amino\_acid\_glutathionylation | 1 | 0 |  |  |  |  |  |  |  |  |
| GO:0010732\_regulation\_of\_protein\_amino\_acid\_glutathionylation | 1 | 0 |  |  |  |  |  |  |  |  |
| GO:0010734\_negative\_regulation\_of\_protein\_amino\_acid\_glutathionylation | 1 | 0 |  |  |  |  |  |  |  |  |
| GO:0010735\_positive\_regulation\_of\_transcription\_via\_serum\_response\_element\_binding | 1 | 0 |  |  |  |  |  |  |  |  |
| GO:0010737\_protein\_kinase\_A\_signaling\_cascade | 1 | 0 |  |  |  |  |  |  |  |  |
| GO:0010738\_regulation\_of\_protein\_kinase\_A\_signaling\_cascade | 1 | 0 |  |  |  |  |  |  |  |  |
| GO:0010739\_positive\_regulation\_of\_protein\_kinase\_A\_signaling\_cascade | 1 | 0 |  |  |  |  |  |  |  |  |
| GO:0010749\_regulation\_of\_nitric\_oxide\_mediated\_signal\_transduction | 1 | 0 |  |  |  |  |  |  |  |  |
| GO:0010751\_negative\_regulation\_of\_nitric\_oxide\_mediated\_signal\_transduction | 1 | 0 |  |  |  |  |  |  |  |  |
| GO:0010752\_regulation\_of\_cGMP-mediated\_signaling | 1 | 0 |  |  |  |  |  |  |  |  |
| GO:0010754\_negative\_regulation\_of\_cGMP-mediated\_signaling | 1 | 0 |  |  |  |  |  |  |  |  |
| GO:0010756\_positive\_regulation\_of\_plasminogen\_activation | 1 | 0 |  |  |  |  |  |  |  |  |
| GO:0010757\_negative\_regulation\_of\_plasminogen\_activation | 1 | 0 |  |  |  |  |  |  |  |  |
| GO:0010758\_regulation\_of\_macrophage\_chemotaxis | 1 | 0 |  |  |  |  |  |  |  |  |
| GO:0010759\_positive\_regulation\_of\_macrophage\_chemotaxis | 1 | 0 |  |  |  |  |  |  |  |  |
| GO:0010766\_negative\_regulation\_of\_sodium\_ion\_transport | 1 | 0 |  |  |  |  |  |  |  |  |
| GO:0010767\_regulation\_of\_transcription\_from\_RNA\_polymerase\_II\_promoter\_in\_response\_to\_UV-induced\_DNA\_damage | 1 | 0 |  |  |  |  |  |  |  |  |
| GO:0010768\_negative\_regulation\_of\_transcription\_from\_RNA\_polymerase\_II\_promoter\_in\_response\_to\_UV-induced\_DNA\_damage | 1 | 0 |  |  |  |  |  |  |  |  |
| GO:0010771\_negative\_regulation\_of\_cell\_morphogenesis\_involved\_in\_differentiation | 1 | 0 |  |  |  |  |  |  |  |  |
| GO:0010793\_regulation\_of\_mRNA\_export\_from\_nucleus | 1 | 0 |  |  |  |  |  |  |  |  |
| GO:0010801\_negative\_regulation\_of\_peptidyl-threonine\_phosphorylation | 1 | 0 |  |  |  |  |  |  |  |  |
| GO:0010803\_regulation\_of\_tumor\_necrosis\_factor-mediated\_signaling\_pathway | 1 | 0 |  |  |  |  |  |  |  |  |
| GO:0010804\_negative\_regulation\_of\_tumor\_necrosis\_factor-mediated\_signaling\_pathway | 1 | 0 |  |  |  |  |  |  |  |  |
| GO:0010813\_neuropeptide\_catabolic\_process | 1 | 0 |  |  |  |  |  |  |  |  |
| GO:0010814\_substance\_P\_catabolic\_process | 1 | 0 |  |  |  |  |  |  |  |  |
| GO:0010816\_calcitonin\_catabolic\_process | 1 | 0 |  |  |  |  |  |  |  |  |
| GO:0010826\_negative\_regulation\_of\_centrosome\_duplication | 1 | 0 |  |  |  |  |  |  |  |  |
| GO:0010830\_regulation\_of\_myotube\_differentiation | 1 | 0 |  |  |  |  |  |  |  |  |
| GO:0010832\_negative\_regulation\_of\_myotube\_differentiation | 1 | 0 |  |  |  |  |  |  |  |  |
| GO:0010835\_regulation\_of\_protein\_amino\_acid\_ADP-ribosylation | 1 | 0 |  |  |  |  |  |  |  |  |
| GO:0010836\_negative\_regulation\_of\_protein\_amino\_acid\_ADP-ribosylation | 1 | 0 |  |  |  |  |  |  |  |  |
| GO:0010837\_regulation\_of\_keratinocyte\_proliferation | 1 | 0 |  |  |  |  |  |  |  |  |
| GO:0010839\_negative\_regulation\_of\_keratinocyte\_proliferation | 1 | 0 |  |  |  |  |  |  |  |  |
| GO:0010840\_regulation\_of\_circadian\_sleep\_wake\_cycle\_\_wakefulness | 1 | 0 |  |  |  |  |  |  |  |  |
| GO:0010841\_positive\_regulation\_of\_circadian\_sleep\_wake\_cycle\_\_wakefulness | 1 | 0 |  |  |  |  |  |  |  |  |
| GO:0010842\_retina\_layer\_formation | 1 | 0 |  |  |  |  |  |  |  |  |
| GO:0010897\_negative\_regulation\_of\_triglyceride\_catabolic\_process | 1 | 0 |  |  |  |  |  |  |  |  |
| GO:0010899\_regulation\_of\_phosphatidylcholine\_catabolic\_process | 1 | 0 |  |  |  |  |  |  |  |  |
| GO:0010900\_negative\_regulation\_of\_phosphatidylcholine\_catabolic\_process | 1 | 0 |  |  |  |  |  |  |  |  |
| GO:0010902\_positive\_regulation\_of\_very-low-density\_lipoprotein\_particle\_remodeling | 1 | 0 |  |  |  |  |  |  |  |  |
| GO:0010919\_regulation\_of\_inositol\_phosphate\_biosynthetic\_process | 1 | 0 |  |  |  |  |  |  |  |  |
| GO:0010920\_negative\_regulation\_of\_inositol\_phosphate\_biosynthetic\_process | 1 | 0 |  |  |  |  |  |  |  |  |
| GO:0010924\_regulation\_of\_inositol-polyphosphate\_5-phosphatase\_activity | 1 | 0 |  |  |  |  |  |  |  |  |
| GO:0010925\_positive\_regulation\_of\_inositol-polyphosphate\_5-phosphatase\_activity | 1 | 0 |  |  |  |  |  |  |  |  |
| GO:0010931\_macrophage\_tolerance\_induction | 1 | 0 |  |  |  |  |  |  |  |  |
| GO:0010932\_regulation\_of\_macrophage\_tolerance\_induction | 1 | 0 |  |  |  |  |  |  |  |  |
| GO:0010933\_positive\_regulation\_of\_macrophage\_tolerance\_induction | 1 | 0 |  |  |  |  |  |  |  |  |
| GO:0010934\_macrophage\_cytokine\_production | 1 | 0 |  |  |  |  |  |  |  |  |
| GO:0010935\_regulation\_of\_macrophage\_cytokine\_production | 1 | 0 |  |  |  |  |  |  |  |  |
| GO:0010936\_negative\_regulation\_of\_macrophage\_cytokine\_production | 1 | 0 |  |  |  |  |  |  |  |  |
| GO:0010944\_negative\_regulation\_of\_transcription\_by\_competitive\_promoter\_binding | 1 | 0 |  |  |  |  |  |  |  |  |
| GO:0010983\_positive\_regulation\_of\_high-density\_lipoprotein\_particle\_clearance | 1 | 0 |  |  |  |  |  |  |  |  |
| GO:0010986\_positive\_regulation\_of\_lipoprotein\_particle\_clearance | 1 | 0 |  |  |  |  |  |  |  |  |
| GO:0010987\_negative\_regulation\_of\_high-density\_lipoprotein\_particle\_clearance | 1 | 0 |  |  |  |  |  |  |  |  |
| GO:0010988\_regulation\_of\_low-density\_lipoprotein\_particle\_clearance | 1 | 0 |  |  |  |  |  |  |  |  |
| GO:0010989\_negative\_regulation\_of\_low-density\_lipoprotein\_particle\_clearance | 1 | 0 |  |  |  |  |  |  |  |  |
| GO:0010990\_regulation\_of\_SMAD\_protein\_complex\_assembly | 1 | 0 |  |  |  |  |  |  |  |  |
| GO:0010991\_negative\_regulation\_of\_SMAD\_protein\_complex\_assembly | 1 | 0 |  |  |  |  |  |  |  |  |
| GO:0014009\_glial\_cell\_proliferation | 1 | 0 |  |  |  |  |  |  |  |  |
| GO:0014010\_Schwann\_cell\_proliferation | 1 | 0 |  |  |  |  |  |  |  |  |
| GO:0014045\_establishment\_of\_endothelial\_blood-brain\_barrier | 1 | 0 |  |  |  |  |  |  |  |  |
| GO:0014055\_acetylcholine\_secretion | 1 | 0 |  |  |  |  |  |  |  |  |
| GO:0014056\_regulation\_of\_acetylcholine\_secretion | 1 | 0 |  |  |  |  |  |  |  |  |
| GO:0014060\_regulation\_of\_epinephrine\_secretion | 1 | 0 |  |  |  |  |  |  |  |  |
| GO:0014067\_negative\_regulation\_of\_phosphoinositide\_3-kinase\_cascade | 1 | 0 |  |  |  |  |  |  |  |  |
| GO:0014071\_response\_to\_cycloalkane | 1 | 0 |  |  |  |  |  |  |  |  |
| GO:0014721\_twitch\_skeletal\_muscle\_contraction | 1 | 0 |  |  |  |  |  |  |  |  |
| GO:0014724\_regulation\_of\_twitch\_skeletal\_muscle\_contraction | 1 | 0 |  |  |  |  |  |  |  |  |
| GO:0014806\_smooth\_muscle\_hyperplasia | 1 | 0 |  |  |  |  |  |  |  |  |
| GO:0014823\_response\_to\_activity | 1 | 0 |  |  |  |  |  |  |  |  |
| GO:0014832\_urinary\_bladder\_smooth\_muscle\_contraction | 1 | 0 |  |  |  |  |  |  |  |  |
| GO:0014834\_satellite\_cell\_maintenance\_involved\_in\_skeletal\_muscle\_regeneration | 1 | 0 |  |  |  |  |  |  |  |  |
| GO:0014848\_urinary\_tract\_smooth\_muscle\_contraction | 1 | 0 |  |  |  |  |  |  |  |  |
| GO:0014850\_response\_to\_muscle\_activity | 1 | 0 |  |  |  |  |  |  |  |  |
| GO:0014873\_response\_to\_muscle\_activity\_involved\_in\_regulation\_of\_muscle\_adaptation | 1 | 0 |  |  |  |  |  |  |  |  |
| GO:0014874\_response\_to\_stimulus\_involved\_in\_regulation\_of\_muscle\_adaptation | 1 | 0 |  |  |  |  |  |  |  |  |
| GO:0014895\_smooth\_muscle\_hypertrophy | 1 | 0 |  |  |  |  |  |  |  |  |
| GO:0014904\_myotube\_cell\_development | 1 | 0 |  |  |  |  |  |  |  |  |
| GO:0014916\_regulation\_of\_lung\_blood\_pressure | 1 | 0 |  |  |  |  |  |  |  |  |
| GO:0015675\_nickel\_ion\_transport | 1 | 0 |  |  |  |  |  |  |  |  |
| GO:0015676\_vanadium\_ion\_transport | 1 | 0 |  |  |  |  |  |  |  |  |
| GO:0015680\_intracellular\_copper\_ion\_transport | 1 | 0 |  |  |  |  |  |  |  |  |
| GO:0015684\_ferrous\_iron\_transport | 1 | 0 |  |  |  |  |  |  |  |  |
| GO:0015692\_lead\_ion\_transport | 1 | 0 |  |  |  |  |  |  |  |  |
| GO:0015693\_magnesium\_ion\_transport | 1 | 0 |  |  |  |  |  |  |  |  |
| GO:0015727\_lactate\_transport | 1 | 0 |  |  |  |  |  |  |  |  |
| GO:0015728\_mevalonate\_transport | 1 | 0 |  |  |  |  |  |  |  |  |
| GO:0015742\_alpha-ketoglutarate\_transport | 1 | 0 |  |  |  |  |  |  |  |  |
| GO:0015746\_citrate\_transport | 1 | 0 |  |  |  |  |  |  |  |  |
| GO:0015747\_urate\_transport | 1 | 0 |  |  |  |  |  |  |  |  |
| GO:0015755\_fructose\_transport | 1 | 0 |  |  |  |  |  |  |  |  |
| GO:0015760\_glucose-6-phosphate\_transport | 1 | 0 |  |  |  |  |  |  |  |  |
| GO:0015782\_CMP-sialic\_acid\_transport | 1 | 0 |  |  |  |  |  |  |  |  |
| GO:0015785\_UDP-galactose\_transport | 1 | 0 |  |  |  |  |  |  |  |  |
| GO:0015789\_UDP-N-acetylgalactosamine\_transport | 1 | 0 |  |  |  |  |  |  |  |  |
| GO:0015790\_UDP-xylose\_transport | 1 | 0 |  |  |  |  |  |  |  |  |
| GO:0015798\_myo-inositol\_transport | 1 | 0 |  |  |  |  |  |  |  |  |
| GO:0015803\_branched-chain\_aliphatic\_amino\_acid\_transport | 1 | 0 |  |  |  |  |  |  |  |  |
| GO:0015805\_S-adenosylmethionine\_transport | 1 | 0 |  |  |  |  |  |  |  |  |
| GO:0015809\_arginine\_transport | 1 | 0 |  |  |  |  |  |  |  |  |
| GO:0015817\_histidine\_transport | 1 | 0 |  |  |  |  |  |  |  |  |
| GO:0015820\_leucine\_transport | 1 | 0 |  |  |  |  |  |  |  |  |
| GO:0015826\_threonine\_transport | 1 | 0 |  |  |  |  |  |  |  |  |
| GO:0015827\_tryptophan\_transport | 1 | 0 |  |  |  |  |  |  |  |  |
| GO:0015846\_polyamine\_transport | 1 | 0 |  |  |  |  |  |  |  |  |
| GO:0015853\_adenine\_transport | 1 | 0 |  |  |  |  |  |  |  |  |
| GO:0015855\_pyrimidine\_transport | 1 | 0 |  |  |  |  |  |  |  |  |
| GO:0015886\_heme\_transport | 1 | 0 |  |  |  |  |  |  |  |  |
| GO:0015888\_thiamin\_transport | 1 | 0 |  |  |  |  |  |  |  |  |
| GO:0015910\_peroxisomal\_long-chain\_fatty\_acid\_import | 1 | 0 |  |  |  |  |  |  |  |  |
| GO:0015919\_peroxisomal\_membrane\_transport | 1 | 0 |  |  |  |  |  |  |  |  |
| GO:0015937\_coenzyme\_A\_biosynthetic\_process | 1 | 0 |  |  |  |  |  |  |  |  |
| GO:0015956\_bis(5'-nucleosidyl)\_oligophosphate\_metabolic\_process | 1 | 0 |  |  |  |  |  |  |  |  |
| GO:0015958\_bis(5'-nucleosidyl)\_oligophosphate\_catabolic\_process | 1 | 0 |  |  |  |  |  |  |  |  |
| GO:0015959\_diadenosine\_polyphosphate\_metabolic\_process | 1 | 0 |  |  |  |  |  |  |  |  |
| GO:0015961\_diadenosine\_polyphosphate\_catabolic\_process | 1 | 0 |  |  |  |  |  |  |  |  |
| GO:0016046\_detection\_of\_fungus | 1 | 0 |  |  |  |  |  |  |  |  |
| GO:0016078\_tRNA\_catabolic\_process | 1 | 0 |  |  |  |  |  |  |  |  |
| GO:0016091\_prenol\_biosynthetic\_process | 1 | 0 |  |  |  |  |  |  |  |  |
| GO:0016094\_polyprenol\_biosynthetic\_process | 1 | 0 |  |  |  |  |  |  |  |  |
| GO:0016108\_tetraterpenoid\_metabolic\_process | 1 | 0 |  |  |  |  |  |  |  |  |
| GO:0016116\_carotenoid\_metabolic\_process | 1 | 0 |  |  |  |  |  |  |  |  |
| GO:0016119\_carotene\_metabolic\_process | 1 | 0 |  |  |  |  |  |  |  |  |
| GO:0016140\_O-glycoside\_metabolic\_process | 1 | 0 |  |  |  |  |  |  |  |  |
| GO:0016142\_O-glycoside\_catabolic\_process | 1 | 0 |  |  |  |  |  |  |  |  |
| GO:0016188\_synaptic\_vesicle\_maturation | 1 | 0 |  |  |  |  |  |  |  |  |
| GO:0016189\_synaptic\_vesicle\_to\_endosome\_fusion | 1 | 0 |  |  |  |  |  |  |  |  |
| GO:0016241\_regulation\_of\_macroautophagy | 1 | 0 |  |  |  |  |  |  |  |  |
| GO:0016242\_negative\_regulation\_of\_macroautophagy | 1 | 0 |  |  |  |  |  |  |  |  |
| GO:0016259\_selenocysteine\_metabolic\_process | 1 | 0 |  |  |  |  |  |  |  |  |
| GO:0016260\_selenocysteine\_biosynthetic\_process | 1 | 0 |  |  |  |  |  |  |  |  |
| GO:0016269\_O-glycan\_processing\_\_core\_3 | 1 | 0 |  |  |  |  |  |  |  |  |
| GO:0016320\_endoplasmic\_reticulum\_membrane\_fusion | 1 | 0 |  |  |  |  |  |  |  |  |
| GO:0016344\_meiotic\_chromosome\_movement\_towards\_spindle\_pole | 1 | 0 |  |  |  |  |  |  |  |  |
| GO:0016446\_somatic\_hypermutation\_of\_immunoglobulin\_genes | 1 | 0 |  |  |  |  |  |  |  |  |
| GO:0016559\_peroxisome\_fission | 1 | 0 |  |  |  |  |  |  |  |  |
| GO:0016560\_protein\_import\_into\_peroxisome\_matrix\_\_docking | 1 | 0 |  |  |  |  |  |  |  |  |
| GO:0016598\_protein\_arginylation | 1 | 0 |  |  |  |  |  |  |  |  |
| GO:0016998\_cell\_wall\_macromolecule\_catabolic\_process | 1 | 0 |  |  |  |  |  |  |  |  |
| GO:0017062\_respiratory\_chain\_complex\_III\_assembly | 1 | 0 |  |  |  |  |  |  |  |  |
| GO:0017185\_peptidyl-lysine\_hydroxylation | 1 | 0 |  |  |  |  |  |  |  |  |
| GO:0018095\_protein\_polyglutamylation | 1 | 0 |  |  |  |  |  |  |  |  |
| GO:0018125\_peptidyl-cysteine\_methylation | 1 | 0 |  |  |  |  |  |  |  |  |
| GO:0018126\_protein\_amino\_acid\_hydroxylation | 1 | 0 |  |  |  |  |  |  |  |  |
| GO:0018146\_keratan\_sulfate\_biosynthetic\_process | 1 | 0 |  |  |  |  |  |  |  |  |
| GO:0018153\_isopeptide\_cross-linking\_via\_N6-(L-isoglutamyl)-L-lysine | 1 | 0 |  |  |  |  |  |  |  |  |
| GO:0018184\_protein\_amino\_acid\_polyamination | 1 | 0 |  |  |  |  |  |  |  |  |
| GO:0018190\_protein\_amino\_acid\_octanoylation | 1 | 0 |  |  |  |  |  |  |  |  |
| GO:0018191\_peptidyl-serine\_octanoylation | 1 | 0 |  |  |  |  |  |  |  |  |
| GO:0018192\_enzyme\_active\_site\_formation\_via\_L-cysteine\_persulfide | 1 | 0 |  |  |  |  |  |  |  |  |
| GO:0018199\_peptidyl-glutamine\_modification | 1 | 0 |  |  |  |  |  |  |  |  |
| GO:0018200\_peptidyl-glutamic\_acid\_modification | 1 | 0 |  |  |  |  |  |  |  |  |
| GO:0018208\_peptidyl-proline\_modification | 1 | 0 |  |  |  |  |  |  |  |  |
| GO:0018262\_isopeptide\_cross-linking | 1 | 0 |  |  |  |  |  |  |  |  |
| GO:0018277\_protein\_amino\_acid\_deamination | 1 | 0 |  |  |  |  |  |  |  |  |
| GO:0018307\_enzyme\_active\_site\_formation | 1 | 0 |  |  |  |  |  |  |  |  |
| GO:0018318\_protein\_amino\_acid\_palmitoylation | 1 | 0 |  |  |  |  |  |  |  |  |
| GO:0018319\_protein\_amino\_acid\_myristoylation | 1 | 0 |  |  |  |  |  |  |  |  |
| GO:0018345\_protein\_palmitoylation | 1 | 0 |  |  |  |  |  |  |  |  |
| GO:0018350\_protein\_amino\_acid\_esterification | 1 | 0 |  |  |  |  |  |  |  |  |
| GO:0018352\_protein-pyridoxal-5-phosphate\_linkage | 1 | 0 |  |  |  |  |  |  |  |  |
| GO:0018377\_protein\_myristoylation | 1 | 0 |  |  |  |  |  |  |  |  |
| GO:0018395\_peptidyl-lysine\_hydroxylation\_to\_5-hydroxy-L-lysine | 1 | 0 |  |  |  |  |  |  |  |  |
| GO:0018401\_peptidyl-proline\_hydroxylation\_to\_4-hydroxy-L-proline | 1 | 0 |  |  |  |  |  |  |  |  |
| GO:0018872\_arsonoacetate\_metabolic\_process | 1 | 0 |  |  |  |  |  |  |  |  |
| GO:0018874\_benzoate\_metabolic\_process | 1 | 0 |  |  |  |  |  |  |  |  |
| GO:0019060\_intracellular\_transport\_of\_viral\_proteins\_in\_host\_cell | 1 | 0 |  |  |  |  |  |  |  |  |
| GO:0019064\_viral\_envelope\_fusion\_with\_host\_membrane | 1 | 0 |  |  |  |  |  |  |  |  |
| GO:0019086\_late\_viral\_mRNA\_transcription | 1 | 0 |  |  |  |  |  |  |  |  |
| GO:0019087\_transformation\_of\_host\_cell\_by\_virus | 1 | 0 |  |  |  |  |  |  |  |  |
| GO:0019089\_transmission\_of\_virus | 1 | 0 |  |  |  |  |  |  |  |  |
| GO:0019098\_reproductive\_behavior | 1 | 0 |  |  |  |  |  |  |  |  |
| GO:0019240\_citrulline\_biosynthetic\_process | 1 | 0 |  |  |  |  |  |  |  |  |
| GO:0019302\_D-ribose\_biosynthetic\_process | 1 | 0 |  |  |  |  |  |  |  |  |
| GO:0019303\_D-ribose\_catabolic\_process | 1 | 0 |  |  |  |  |  |  |  |  |
| GO:0019307\_mannose\_biosynthetic\_process | 1 | 0 |  |  |  |  |  |  |  |  |
| GO:0019310\_inositol\_catabolic\_process | 1 | 0 |  |  |  |  |  |  |  |  |
| GO:0019322\_pentose\_biosynthetic\_process | 1 | 0 |  |  |  |  |  |  |  |  |
| GO:0019323\_pentose\_catabolic\_process | 1 | 0 |  |  |  |  |  |  |  |  |
| GO:0019371\_cyclooxygenase\_pathway | 1 | 0 |  |  |  |  |  |  |  |  |
| GO:0019372\_lipoxygenase\_pathway | 1 | 0 |  |  |  |  |  |  |  |  |
| GO:0019388\_galactose\_catabolic\_process | 1 | 0 |  |  |  |  |  |  |  |  |
| GO:0019405\_alditol\_catabolic\_process | 1 | 0 |  |  |  |  |  |  |  |  |
| GO:0019407\_hexitol\_catabolic\_process | 1 | 0 |  |  |  |  |  |  |  |  |
| GO:0019408\_dolichol\_biosynthetic\_process | 1 | 0 |  |  |  |  |  |  |  |  |
| GO:0019441\_tryptophan\_catabolic\_process\_to\_kynurenine | 1 | 0 |  |  |  |  |  |  |  |  |
| GO:0019471\_4-hydroxyproline\_metabolic\_process | 1 | 0 |  |  |  |  |  |  |  |  |
| GO:0019511\_peptidyl-proline\_hydroxylation | 1 | 0 |  |  |  |  |  |  |  |  |
| GO:0019519\_pentitol\_metabolic\_process | 1 | 0 |  |  |  |  |  |  |  |  |
| GO:0019527\_pentitol\_catabolic\_process | 1 | 0 |  |  |  |  |  |  |  |  |
| GO:0019614\_catechol\_catabolic\_process | 1 | 0 |  |  |  |  |  |  |  |  |
| GO:0019673\_GDP-mannose\_metabolic\_process | 1 | 0 |  |  |  |  |  |  |  |  |
| GO:0019693\_ribose\_phosphate\_metabolic\_process | 1 | 0 |  |  |  |  |  |  |  |  |
| GO:0019695\_choline\_metabolic\_process | 1 | 0 |  |  |  |  |  |  |  |  |
| GO:0019747\_regulation\_of\_isoprenoid\_metabolic\_process | 1 | 0 |  |  |  |  |  |  |  |  |
| GO:0019852\_L-ascorbic\_acid\_metabolic\_process | 1 | 0 |  |  |  |  |  |  |  |  |
| GO:0019856\_pyrimidine\_base\_biosynthetic\_process | 1 | 0 |  |  |  |  |  |  |  |  |
| GO:0019858\_cytosine\_metabolic\_process | 1 | 0 |  |  |  |  |  |  |  |  |
| GO:0019884\_antigen\_processing\_and\_presentation\_of\_exogenous\_antigen | 1 | 0 |  |  |  |  |  |  |  |  |
| GO:0019886\_antigen\_processing\_and\_presentation\_of\_exogenous\_peptide\_antigen\_via\_MHC\_class\_II | 1 | 0 |  |  |  |  |  |  |  |  |
| GO:0021508\_floor\_plate\_formation | 1 | 0 |  |  |  |  |  |  |  |  |
| GO:0021514\_ventral\_spinal\_cord\_interneuron\_differentiation | 1 | 0 |  |  |  |  |  |  |  |  |
| GO:0021521\_ventral\_spinal\_cord\_interneuron\_specification | 1 | 0 |  |  |  |  |  |  |  |  |
| GO:0021522\_spinal\_cord\_motor\_neuron\_differentiation | 1 | 0 |  |  |  |  |  |  |  |  |
| GO:0021527\_spinal\_cord\_association\_neuron\_differentiation | 1 | 0 |  |  |  |  |  |  |  |  |
| GO:0021528\_commissural\_neuron\_differentiation\_in\_the\_spinal\_cord | 1 | 0 |  |  |  |  |  |  |  |  |
| GO:0021533\_cell\_differentiation\_in\_hindbrain | 1 | 0 |  |  |  |  |  |  |  |  |
| GO:0021540\_corpus\_callosum\_morphogenesis | 1 | 0 |  |  |  |  |  |  |  |  |
| GO:0021544\_subpallium\_development | 1 | 0 |  |  |  |  |  |  |  |  |
| GO:0021554\_optic\_nerve\_development | 1 | 0 |  |  |  |  |  |  |  |  |
| GO:0021562\_vestibulocochlear\_nerve\_development | 1 | 0 |  |  |  |  |  |  |  |  |
| GO:0021602\_cranial\_nerve\_morphogenesis | 1 | 0 |  |  |  |  |  |  |  |  |
| GO:0021631\_optic\_nerve\_morphogenesis | 1 | 0 |  |  |  |  |  |  |  |  |
| GO:0021680\_cerebellar\_Purkinje\_cell\_layer\_development | 1 | 0 |  |  |  |  |  |  |  |  |
| GO:0021692\_cerebellar\_Purkinje\_cell\_layer\_morphogenesis | 1 | 0 |  |  |  |  |  |  |  |  |
| GO:0021694\_cerebellar\_Purkinje\_cell\_layer\_formation | 1 | 0 |  |  |  |  |  |  |  |  |
| GO:0021697\_cerebellar\_cortex\_formation | 1 | 0 |  |  |  |  |  |  |  |  |
| GO:0021702\_cerebellar\_Purkinje\_cell\_differentiation | 1 | 0 |  |  |  |  |  |  |  |  |
| GO:0021756\_striatum\_development | 1 | 0 |  |  |  |  |  |  |  |  |
| GO:0021757\_caudate\_nucleus\_development | 1 | 0 |  |  |  |  |  |  |  |  |
| GO:0021758\_putamen\_development | 1 | 0 |  |  |  |  |  |  |  |  |
| GO:0021761\_limbic\_system\_development | 1 | 0 |  |  |  |  |  |  |  |  |
| GO:0021771\_lateral\_geniculate\_nucleus\_development | 1 | 0 |  |  |  |  |  |  |  |  |
| GO:0021775\_smoothened\_signaling\_pathway\_involved\_in\_ventral\_spinal\_cord\_interneuron\_specification | 1 | 0 |  |  |  |  |  |  |  |  |
| GO:0021794\_thalamus\_development | 1 | 0 |  |  |  |  |  |  |  |  |
| GO:0021799\_cerebral\_cortex\_radially\_oriented\_cell\_migration | 1 | 0 |  |  |  |  |  |  |  |  |
| GO:0021800\_cerebral\_cortex\_tangential\_migration | 1 | 0 |  |  |  |  |  |  |  |  |
| GO:0021854\_hypothalamus\_development | 1 | 0 |  |  |  |  |  |  |  |  |
| GO:0021859\_pyramidal\_neuron\_differentiation | 1 | 0 |  |  |  |  |  |  |  |  |
| GO:0021860\_pyramidal\_neuron\_development | 1 | 0 |  |  |  |  |  |  |  |  |
| GO:0021872\_generation\_of\_neurons\_in\_the\_forebrain | 1 | 0 |  |  |  |  |  |  |  |  |
| GO:0021879\_forebrain\_neuron\_differentiation | 1 | 0 |  |  |  |  |  |  |  |  |
| GO:0021884\_forebrain\_neuron\_development | 1 | 0 |  |  |  |  |  |  |  |  |
| GO:0021896\_forebrain\_astrocyte\_differentiation | 1 | 0 |  |  |  |  |  |  |  |  |
| GO:0021897\_forebrain\_astrocyte\_development | 1 | 0 |  |  |  |  |  |  |  |  |
| GO:0021914\_negative\_regulation\_of\_smoothened\_signaling\_pathway\_involved\_in\_ventral\_spinal\_cord\_patterning | 1 | 0 |  |  |  |  |  |  |  |  |
| GO:0021919\_BMP\_signaling\_pathway\_in\_spinal\_cord\_dorsal\_ventral\_patterning | 1 | 0 |  |  |  |  |  |  |  |  |
| GO:0021965\_spinal\_cord\_ventral\_commissure\_morphogenesis | 1 | 0 |  |  |  |  |  |  |  |  |
| GO:0021984\_adenohypophysis\_development | 1 | 0 |  |  |  |  |  |  |  |  |
| GO:0021990\_neural\_plate\_formation | 1 | 0 |  |  |  |  |  |  |  |  |
| GO:0021997\_neural\_plate\_axis\_specification | 1 | 0 |  |  |  |  |  |  |  |  |
| GO:0021999\_neural\_plate\_anterior\_posterior\_pattern\_formation | 1 | 0 |  |  |  |  |  |  |  |  |
| GO:0022009\_central\_nervous\_system\_vasculogenesis | 1 | 0 |  |  |  |  |  |  |  |  |
| GO:0022038\_corpus\_callosum\_development | 1 | 0 |  |  |  |  |  |  |  |  |
| GO:0030007\_cellular\_potassium\_ion\_homeostasis | 1 | 0 |  |  |  |  |  |  |  |  |
| GO:0030011\_maintenance\_of\_cell\_polarity | 1 | 0 |  |  |  |  |  |  |  |  |
| GO:0030026\_cellular\_manganese\_ion\_homeostasis | 1 | 0 |  |  |  |  |  |  |  |  |
| GO:0030033\_microvillus\_assembly | 1 | 0 |  |  |  |  |  |  |  |  |
| GO:0030037\_actin\_filament\_reorganization\_during\_cell\_cycle | 1 | 0 |  |  |  |  |  |  |  |  |
| GO:0030047\_actin\_modification | 1 | 0 |  |  |  |  |  |  |  |  |
| GO:0030070\_insulin\_processing | 1 | 0 |  |  |  |  |  |  |  |  |
| GO:0030103\_vasopressin\_secretion | 1 | 0 |  |  |  |  |  |  |  |  |
| GO:0030186\_melatonin\_metabolic\_process | 1 | 0 |  |  |  |  |  |  |  |  |
| GO:0030187\_melatonin\_biosynthetic\_process | 1 | 0 |  |  |  |  |  |  |  |  |
| GO:0030212\_hyaluronan\_metabolic\_process | 1 | 0 |  |  |  |  |  |  |  |  |
| GO:0030220\_platelet\_formation | 1 | 0 |  |  |  |  |  |  |  |  |
| GO:0030238\_male\_sex\_determination | 1 | 0 |  |  |  |  |  |  |  |  |
| GO:0030259\_lipid\_glycosylation | 1 | 0 |  |  |  |  |  |  |  |  |
| GO:0030302\_deoxynucleotide\_transport | 1 | 0 |  |  |  |  |  |  |  |  |
| GO:0030327\_prenylated\_protein\_catabolic\_process | 1 | 0 |  |  |  |  |  |  |  |  |
| GO:0030389\_fructosamine\_metabolic\_process | 1 | 0 |  |  |  |  |  |  |  |  |
| GO:0030393\_fructoselysine\_metabolic\_process | 1 | 0 |  |  |  |  |  |  |  |  |
| GO:0030432\_peristalsis | 1 | 0 |  |  |  |  |  |  |  |  |
| GO:0030488\_tRNA\_methylation | 1 | 0 |  |  |  |  |  |  |  |  |
| GO:0030517\_negative\_regulation\_of\_axon\_extension | 1 | 0 |  |  |  |  |  |  |  |  |
| GO:0030581\_symbiont\_intracellular\_protein\_transport\_in\_host | 1 | 0 |  |  |  |  |  |  |  |  |
| GO:0030718\_germ-line\_stem\_cell\_maintenance | 1 | 0 |  |  |  |  |  |  |  |  |
| GO:0030728\_ovulation | 1 | 0 |  |  |  |  |  |  |  |  |
| GO:0030824\_negative\_regulation\_of\_cGMP\_metabolic\_process | 1 | 0 |  |  |  |  |  |  |  |  |
| GO:0030825\_positive\_regulation\_of\_cGMP\_metabolic\_process | 1 | 0 |  |  |  |  |  |  |  |  |
| GO:0030827\_negative\_regulation\_of\_cGMP\_biosynthetic\_process | 1 | 0 |  |  |  |  |  |  |  |  |
| GO:0030828\_positive\_regulation\_of\_cGMP\_biosynthetic\_process | 1 | 0 |  |  |  |  |  |  |  |  |
| GO:0030836\_positive\_regulation\_of\_actin\_filament\_depolymerization | 1 | 0 |  |  |  |  |  |  |  |  |
| GO:0030845\_inhibition\_of\_phospholipase\_C\_activity\_involved\_in\_G-protein\_coupled\_receptor\_signaling\_pathway | 1 | 0 |  |  |  |  |  |  |  |  |
| GO:0030854\_positive\_regulation\_of\_granulocyte\_differentiation | 1 | 0 |  |  |  |  |  |  |  |  |
| GO:0030857\_negative\_regulation\_of\_epithelial\_cell\_differentiation | 1 | 0 |  |  |  |  |  |  |  |  |
| GO:0030878\_thyroid\_gland\_development | 1 | 0 |  |  |  |  |  |  |  |  |
| GO:0030885\_regulation\_of\_myeloid\_dendritic\_cell\_activation | 1 | 0 |  |  |  |  |  |  |  |  |
| GO:0030887\_positive\_regulation\_of\_myeloid\_dendritic\_cell\_activation | 1 | 0 |  |  |  |  |  |  |  |  |
| GO:0030903\_notochord\_development | 1 | 0 |  |  |  |  |  |  |  |  |
| GO:0030910\_olfactory\_placode\_formation | 1 | 0 |  |  |  |  |  |  |  |  |
| GO:0030913\_paranodal\_junction\_assembly | 1 | 0 |  |  |  |  |  |  |  |  |
| GO:0030948\_negative\_regulation\_of\_vascular\_endothelial\_growth\_factor\_receptor\_signaling\_pathway | 1 | 0 |  |  |  |  |  |  |  |  |
| GO:0030967\_ER-nuclear\_sterol\_response\_pathway | 1 | 0 |  |  |  |  |  |  |  |  |
| GO:0031017\_exocrine\_pancreas\_development | 1 | 0 |  |  |  |  |  |  |  |  |
| GO:0031063\_regulation\_of\_histone\_deacetylation | 1 | 0 |  |  |  |  |  |  |  |  |
| GO:0031065\_positive\_regulation\_of\_histone\_deacetylation | 1 | 0 |  |  |  |  |  |  |  |  |
| GO:0031076\_embryonic\_camera-type\_eye\_development | 1 | 0 |  |  |  |  |  |  |  |  |
| GO:0031081\_nuclear\_pore\_distribution | 1 | 0 |  |  |  |  |  |  |  |  |
| GO:0031086\_nuclear-transcribed\_mRNA\_catabolic\_process\_\_deadenylation-independent\_decay | 1 | 0 |  |  |  |  |  |  |  |  |
| GO:0031087\_deadenylation-independent\_decapping\_of\_nuclear-transcribed\_mRNA | 1 | 0 |  |  |  |  |  |  |  |  |
| GO:0031106\_septin\_ring\_organization | 1 | 0 |  |  |  |  |  |  |  |  |
| GO:0031115\_negative\_regulation\_of\_microtubule\_polymerization | 1 | 0 |  |  |  |  |  |  |  |  |
| GO:0031117\_positive\_regulation\_of\_microtubule\_depolymerization | 1 | 0 |  |  |  |  |  |  |  |  |
| GO:0031118\_rRNA\_pseudouridine\_synthesis | 1 | 0 |  |  |  |  |  |  |  |  |
| GO:0031125\_rRNA\_3'-end\_processing | 1 | 0 |  |  |  |  |  |  |  |  |
| GO:0031146\_SCF-dependent\_proteasomal\_ubiquitin-dependent\_protein\_catabolic\_process | 1 | 0 |  |  |  |  |  |  |  |  |
| GO:0031179\_peptide\_modification | 1 | 0 |  |  |  |  |  |  |  |  |
| GO:0031282\_regulation\_of\_guanylate\_cyclase\_activity | 1 | 0 |  |  |  |  |  |  |  |  |
| GO:0031284\_positive\_regulation\_of\_guanylate\_cyclase\_activity | 1 | 0 |  |  |  |  |  |  |  |  |
| GO:0031290\_retinal\_ganglion\_cell\_axon\_guidance | 1 | 0 |  |  |  |  |  |  |  |  |
| GO:0031293\_membrane\_protein\_intracellular\_domain\_proteolysis | 1 | 0 |  |  |  |  |  |  |  |  |
| GO:0031335\_regulation\_of\_sulfur\_amino\_acid\_metabolic\_process | 1 | 0 |  |  |  |  |  |  |  |  |
| GO:0031342\_negative\_regulation\_of\_cell\_killing | 1 | 0 |  |  |  |  |  |  |  |  |
| GO:0031424\_keratinization | 1 | 0 |  |  |  |  |  |  |  |  |
| GO:0031441\_negative\_regulation\_of\_mRNA\_3'-end\_processing | 1 | 0 |  |  |  |  |  |  |  |  |
| GO:0031442\_positive\_regulation\_of\_mRNA\_3'-end\_processing | 1 | 0 |  |  |  |  |  |  |  |  |
| GO:0031443\_fast-twitch\_skeletal\_muscle\_fiber\_contraction | 1 | 0 |  |  |  |  |  |  |  |  |
| GO:0031446\_regulation\_of\_fast-twitch\_skeletal\_muscle\_fiber\_contraction | 1 | 0 |  |  |  |  |  |  |  |  |
| GO:0031448\_positive\_regulation\_of\_fast-twitch\_skeletal\_muscle\_fiber\_contraction | 1 | 0 |  |  |  |  |  |  |  |  |
| GO:0031453\_positive\_regulation\_of\_heterochromatin\_formation | 1 | 0 |  |  |  |  |  |  |  |  |
| GO:0031557\_induction\_of\_programmed\_cell\_death\_in\_response\_to\_chemical\_stimulus | 1 | 0 |  |  |  |  |  |  |  |  |
| GO:0031574\_S-M\_checkpoint | 1 | 0 |  |  |  |  |  |  |  |  |
| GO:0031581\_hemidesmosome\_assembly | 1 | 0 |  |  |  |  |  |  |  |  |
| GO:0031627\_telomeric\_loop\_formation | 1 | 0 |  |  |  |  |  |  |  |  |
| GO:0031848\_protection\_from\_non-homologous\_end\_joining\_at\_telomere | 1 | 0 |  |  |  |  |  |  |  |  |
| GO:0031937\_positive\_regulation\_of\_chromatin\_silencing | 1 | 0 |  |  |  |  |  |  |  |  |
| GO:0031943\_regulation\_of\_glucocorticoid\_metabolic\_process | 1 | 0 |  |  |  |  |  |  |  |  |
| GO:0031954\_positive\_regulation\_of\_protein\_amino\_acid\_autophosphorylation | 1 | 0 |  |  |  |  |  |  |  |  |
| GO:0031999\_negative\_regulation\_of\_fatty\_acid\_beta-oxidation | 1 | 0 |  |  |  |  |  |  |  |  |
| GO:0032011\_ARF\_protein\_signal\_transduction | 1 | 0 |  |  |  |  |  |  |  |  |
| GO:0032023\_trypsinogen\_activation | 1 | 0 |  |  |  |  |  |  |  |  |
| GO:0032025\_response\_to\_cobalt\_ion | 1 | 0 |  |  |  |  |  |  |  |  |
| GO:0032048\_cardiolipin\_metabolic\_process | 1 | 0 |  |  |  |  |  |  |  |  |
| GO:0032049\_cardiolipin\_biosynthetic\_process | 1 | 0 |  |  |  |  |  |  |  |  |
| GO:0032060\_bleb\_formation | 1 | 0 |  |  |  |  |  |  |  |  |
| GO:0032066\_nucleolus\_to\_nucleoplasm\_transport | 1 | 0 |  |  |  |  |  |  |  |  |
| GO:0032074\_negative\_regulation\_of\_nuclease\_activity | 1 | 0 |  |  |  |  |  |  |  |  |
| GO:0032075\_positive\_regulation\_of\_nuclease\_activity | 1 | 0 |  |  |  |  |  |  |  |  |
| GO:0032119\_sequestering\_of\_zinc\_ion | 1 | 0 |  |  |  |  |  |  |  |  |
| GO:0032185\_septin\_cytoskeleton\_organization | 1 | 0 |  |  |  |  |  |  |  |  |
| GO:0032196\_transposition | 1 | 0 |  |  |  |  |  |  |  |  |
| GO:0032235\_negative\_regulation\_of\_calcium\_ion\_transport\_via\_store-operated\_calcium\_channel\_activity | 1 | 0 |  |  |  |  |  |  |  |  |
| GO:0032241\_positive\_regulation\_of\_nucleobase\_\_nucleoside\_\_nucleotide\_and\_nucleic\_acid\_transport | 1 | 0 |  |  |  |  |  |  |  |  |
| GO:0032261\_purine\_nucleotide\_salvage | 1 | 0 |  |  |  |  |  |  |  |  |
| GO:0032275\_luteinizing\_hormone\_secretion | 1 | 0 |  |  |  |  |  |  |  |  |
| GO:0032287\_myelin\_maintenance\_in\_the\_peripheral\_nervous\_system | 1 | 0 |  |  |  |  |  |  |  |  |
| GO:0032288\_myelin\_assembly | 1 | 0 |  |  |  |  |  |  |  |  |
| GO:0032314\_regulation\_of\_Rac\_GTPase\_activity | 1 | 0 |  |  |  |  |  |  |  |  |
| GO:0032330\_regulation\_of\_chondrocyte\_differentiation | 1 | 0 |  |  |  |  |  |  |  |  |
| GO:0032331\_negative\_regulation\_of\_chondrocyte\_differentiation | 1 | 0 |  |  |  |  |  |  |  |  |
| GO:0032346\_positive\_regulation\_of\_aldosterone\_metabolic\_process | 1 | 0 |  |  |  |  |  |  |  |  |
| GO:0032347\_regulation\_of\_aldosterone\_biosynthetic\_process | 1 | 0 |  |  |  |  |  |  |  |  |
| GO:0032349\_positive\_regulation\_of\_aldosterone\_biosynthetic\_process | 1 | 0 |  |  |  |  |  |  |  |  |
| GO:0032354\_response\_to\_follicle-stimulating\_hormone\_stimulus | 1 | 0 |  |  |  |  |  |  |  |  |
| GO:0032377\_regulation\_of\_intracellular\_lipid\_transport | 1 | 0 |  |  |  |  |  |  |  |  |
| GO:0032380\_regulation\_of\_intracellular\_sterol\_transport | 1 | 0 |  |  |  |  |  |  |  |  |
| GO:0032383\_regulation\_of\_intracellular\_cholesterol\_transport | 1 | 0 |  |  |  |  |  |  |  |  |
| GO:0032423\_regulation\_of\_mismatch\_repair | 1 | 0 |  |  |  |  |  |  |  |  |
| GO:0032425\_positive\_regulation\_of\_mismatch\_repair | 1 | 0 |  |  |  |  |  |  |  |  |
| GO:0032459\_regulation\_of\_protein\_oligomerization | 1 | 0 |  |  |  |  |  |  |  |  |
| GO:0032460\_negative\_regulation\_of\_protein\_oligomerization | 1 | 0 |  |  |  |  |  |  |  |  |
| GO:0032462\_regulation\_of\_protein\_homooligomerization | 1 | 0 |  |  |  |  |  |  |  |  |
| GO:0032463\_negative\_regulation\_of\_protein\_homooligomerization | 1 | 0 |  |  |  |  |  |  |  |  |
| GO:0032467\_positive\_regulation\_of\_cytokinesis | 1 | 0 |  |  |  |  |  |  |  |  |
| GO:0032468\_Golgi\_calcium\_ion\_homeostasis | 1 | 0 |  |  |  |  |  |  |  |  |
| GO:0032470\_elevation\_of\_endoplasmic\_reticulum\_calcium\_ion\_concentration | 1 | 0 |  |  |  |  |  |  |  |  |
| GO:0032471\_reduction\_of\_endoplasmic\_reticulum\_calcium\_ion\_concentration | 1 | 0 |  |  |  |  |  |  |  |  |
| GO:0032472\_Golgi\_calcium\_ion\_transport | 1 | 0 |  |  |  |  |  |  |  |  |
| GO:0032486\_Rap\_protein\_signal\_transduction | 1 | 0 |  |  |  |  |  |  |  |  |
| GO:0032495\_response\_to\_muramyl\_dipeptide | 1 | 0 |  |  |  |  |  |  |  |  |
| GO:0032498\_detection\_of\_muramyl\_dipeptide | 1 | 0 |  |  |  |  |  |  |  |  |
| GO:0032499\_detection\_of\_peptidoglycan | 1 | 0 |  |  |  |  |  |  |  |  |
| GO:0032528\_microvillus\_organization | 1 | 0 |  |  |  |  |  |  |  |  |
| GO:0032581\_ER-dependent\_peroxisome\_biogenesis | 1 | 0 |  |  |  |  |  |  |  |  |
| GO:0032594\_protein\_transport\_within\_lipid\_bilayer | 1 | 0 |  |  |  |  |  |  |  |  |
| GO:0032595\_B\_cell\_receptor\_transport\_within\_lipid\_bilayer | 1 | 0 |  |  |  |  |  |  |  |  |
| GO:0032596\_protein\_transport\_into\_membrane\_raft | 1 | 0 |  |  |  |  |  |  |  |  |
| GO:0032597\_B\_cell\_receptor\_transport\_into\_membrane\_raft | 1 | 0 |  |  |  |  |  |  |  |  |
| GO:0032599\_protein\_transport\_out\_of\_membrane\_raft | 1 | 0 |  |  |  |  |  |  |  |  |
| GO:0032600\_chemokine\_receptor\_transport\_out\_of\_membrane\_raft | 1 | 0 |  |  |  |  |  |  |  |  |
| GO:0032601\_connective\_tissue\_growth\_factor\_production | 1 | 0 |  |  |  |  |  |  |  |  |
| GO:0032603\_fractalkine\_production | 1 | 0 |  |  |  |  |  |  |  |  |
| GO:0032605\_hepatocyte\_growth\_factor\_production | 1 | 0 |  |  |  |  |  |  |  |  |
| GO:0032610\_interleukin-1\_alpha\_production | 1 | 0 |  |  |  |  |  |  |  |  |
| GO:0032621\_interleukin-18\_production | 1 | 0 |  |  |  |  |  |  |  |  |
| GO:0032639\_TRAIL\_production | 1 | 0 |  |  |  |  |  |  |  |  |
| GO:0032644\_regulation\_of\_fractalkine\_production | 1 | 0 |  |  |  |  |  |  |  |  |
| GO:0032646\_regulation\_of\_hepatocyte\_growth\_factor\_production | 1 | 0 |  |  |  |  |  |  |  |  |
| GO:0032650\_regulation\_of\_interleukin-1\_alpha\_production | 1 | 0 |  |  |  |  |  |  |  |  |
| GO:0032661\_regulation\_of\_interleukin-18\_production | 1 | 0 |  |  |  |  |  |  |  |  |
| GO:0032679\_regulation\_of\_TRAIL\_production | 1 | 0 |  |  |  |  |  |  |  |  |
| GO:0032681\_regulation\_of\_lymphotoxin\_A\_production | 1 | 0 |  |  |  |  |  |  |  |  |
| GO:0032693\_negative\_regulation\_of\_interleukin-10\_production | 1 | 0 |  |  |  |  |  |  |  |  |
| GO:0032703\_negative\_regulation\_of\_interleukin-2\_production | 1 | 0 |  |  |  |  |  |  |  |  |
| GO:0032713\_negative\_regulation\_of\_interleukin-4\_production | 1 | 0 |  |  |  |  |  |  |  |  |
| GO:0032730\_positive\_regulation\_of\_interleukin-1\_alpha\_production | 1 | 0 |  |  |  |  |  |  |  |  |
| GO:0032732\_positive\_regulation\_of\_interleukin-1\_production | 1 | 0 |  |  |  |  |  |  |  |  |
| GO:0032736\_positive\_regulation\_of\_interleukin-13\_production | 1 | 0 |  |  |  |  |  |  |  |  |
| GO:0032753\_positive\_regulation\_of\_interleukin-4\_production | 1 | 0 |  |  |  |  |  |  |  |  |
| GO:0032754\_positive\_regulation\_of\_interleukin-5\_production | 1 | 0 |  |  |  |  |  |  |  |  |
| GO:0032762\_mast\_cell\_cytokine\_production | 1 | 0 |  |  |  |  |  |  |  |  |
| GO:0032763\_regulation\_of\_mast\_cell\_cytokine\_production | 1 | 0 |  |  |  |  |  |  |  |  |
| GO:0032765\_positive\_regulation\_of\_mast\_cell\_cytokine\_production | 1 | 0 |  |  |  |  |  |  |  |  |
| GO:0032784\_regulation\_of\_RNA\_elongation | 1 | 0 |  |  |  |  |  |  |  |  |
| GO:0032786\_positive\_regulation\_of\_RNA\_elongation | 1 | 0 |  |  |  |  |  |  |  |  |
| GO:0032788\_saturated\_monocarboxylic\_acid\_metabolic\_process | 1 | 0 |  |  |  |  |  |  |  |  |
| GO:0032789\_unsaturated\_monocarboxylic\_acid\_metabolic\_process | 1 | 0 |  |  |  |  |  |  |  |  |
| GO:0032790\_ribosome\_disassembly | 1 | 0 |  |  |  |  |  |  |  |  |
| GO:0032792\_negative\_regulation\_of\_CREB\_transcription\_factor\_activity | 1 | 0 |  |  |  |  |  |  |  |  |
| GO:0032793\_positive\_regulation\_of\_CREB\_transcription\_factor\_activity | 1 | 0 |  |  |  |  |  |  |  |  |
| GO:0032804\_negative\_regulation\_of\_low-density\_lipoprotein\_receptor\_catabolic\_process | 1 | 0 |  |  |  |  |  |  |  |  |
| GO:0032805\_positive\_regulation\_of\_low-density\_lipoprotein\_receptor\_catabolic\_process | 1 | 0 |  |  |  |  |  |  |  |  |
| GO:0032812\_positive\_regulation\_of\_epinephrine\_secretion | 1 | 0 |  |  |  |  |  |  |  |  |
| GO:0032835\_glomerulus\_development | 1 | 0 |  |  |  |  |  |  |  |  |
| GO:0032847\_regulation\_of\_cellular\_pH\_reduction | 1 | 0 |  |  |  |  |  |  |  |  |
| GO:0032848\_negative\_regulation\_of\_cellular\_pH\_reduction | 1 | 0 |  |  |  |  |  |  |  |  |
| GO:0032899\_regulation\_of\_neurotrophin\_production | 1 | 0 |  |  |  |  |  |  |  |  |
| GO:0032900\_negative\_regulation\_of\_neurotrophin\_production | 1 | 0 |  |  |  |  |  |  |  |  |
| GO:0032903\_regulation\_of\_nerve\_growth\_factor\_production | 1 | 0 |  |  |  |  |  |  |  |  |
| GO:0032904\_negative\_regulation\_of\_nerve\_growth\_factor\_production | 1 | 0 |  |  |  |  |  |  |  |  |
| GO:0032907\_transforming\_growth\_factor-beta3\_production | 1 | 0 |  |  |  |  |  |  |  |  |
| GO:0032910\_regulation\_of\_transforming\_growth\_factor-beta3\_production | 1 | 0 |  |  |  |  |  |  |  |  |
| GO:0032911\_negative\_regulation\_of\_transforming\_growth\_factor-beta1\_production | 1 | 0 |  |  |  |  |  |  |  |  |
| GO:0032913\_negative\_regulation\_of\_transforming\_growth\_factor-beta3\_production | 1 | 0 |  |  |  |  |  |  |  |  |
| GO:0032926\_negative\_regulation\_of\_activin\_receptor\_signaling\_pathway | 1 | 0 |  |  |  |  |  |  |  |  |
| GO:0032933\_SREBP-mediated\_signaling\_pathway | 1 | 0 |  |  |  |  |  |  |  |  |
| GO:0032938\_negative\_regulation\_of\_translation\_in\_response\_to\_oxidative\_stress | 1 | 0 |  |  |  |  |  |  |  |  |
| GO:0032958\_inositol\_phosphate\_biosynthetic\_process | 1 | 0 |  |  |  |  |  |  |  |  |
| GO:0032976\_release\_of\_matrix\_enzymes\_from\_mitochondria | 1 | 0 |  |  |  |  |  |  |  |  |
| GO:0032980\_keratinocyte\_activation | 1 | 0 |  |  |  |  |  |  |  |  |
| GO:0032988\_ribonucleoprotein\_complex\_disassembly | 1 | 0 |  |  |  |  |  |  |  |  |
| GO:0033029\_regulation\_of\_neutrophil\_apoptosis | 1 | 0 |  |  |  |  |  |  |  |  |
| GO:0033031\_positive\_regulation\_of\_neutrophil\_apoptosis | 1 | 0 |  |  |  |  |  |  |  |  |
| GO:0033079\_immature\_T\_cell\_proliferation | 1 | 0 |  |  |  |  |  |  |  |  |
| GO:0033080\_immature\_T\_cell\_proliferation\_in\_the\_thymus | 1 | 0 |  |  |  |  |  |  |  |  |
| GO:0033083\_regulation\_of\_immature\_T\_cell\_proliferation | 1 | 0 |  |  |  |  |  |  |  |  |
| GO:0033084\_regulation\_of\_immature\_T\_cell\_proliferation\_in\_the\_thymus | 1 | 0 |  |  |  |  |  |  |  |  |
| GO:0033085\_negative\_regulation\_of\_T\_cell\_differentiation\_in\_the\_thymus | 1 | 0 |  |  |  |  |  |  |  |  |
| GO:0033087\_negative\_regulation\_of\_immature\_T\_cell\_proliferation | 1 | 0 |  |  |  |  |  |  |  |  |
| GO:0033088\_negative\_regulation\_of\_immature\_T\_cell\_proliferation\_in\_the\_thymus | 1 | 0 |  |  |  |  |  |  |  |  |
| GO:0033136\_serine\_phosphorylation\_of\_STAT3\_protein | 1 | 0 |  |  |  |  |  |  |  |  |
| GO:0033137\_negative\_regulation\_of\_peptidyl-serine\_phosphorylation | 1 | 0 |  |  |  |  |  |  |  |  |
| GO:0033139\_regulation\_of\_peptidyl-serine\_phosphorylation\_of\_STAT\_protein | 1 | 0 |  |  |  |  |  |  |  |  |
| GO:0033141\_positive\_regulation\_of\_peptidyl-serine\_phosphorylation\_of\_STAT\_protein | 1 | 0 |  |  |  |  |  |  |  |  |
| GO:0033153\_T\_cell\_receptor\_V(D)J\_recombination | 1 | 0 |  |  |  |  |  |  |  |  |
| GO:0033169\_histone\_H3-K9\_demethylation | 1 | 0 |  |  |  |  |  |  |  |  |
| GO:0033173\_calcineurin-NFAT\_signaling\_pathway | 1 | 0 |  |  |  |  |  |  |  |  |
| GO:0033182\_regulation\_of\_histone\_ubiquitination | 1 | 0 |  |  |  |  |  |  |  |  |
| GO:0033206\_cytokinesis\_after\_meiosis | 1 | 0 |  |  |  |  |  |  |  |  |
| GO:0033239\_negative\_regulation\_of\_cellular\_amine\_metabolic\_process | 1 | 0 |  |  |  |  |  |  |  |  |
| GO:0033240\_positive\_regulation\_of\_cellular\_amine\_metabolic\_process | 1 | 0 |  |  |  |  |  |  |  |  |
| GO:0033260\_DNA\_replication\_during\_S\_phase | 1 | 0 |  |  |  |  |  |  |  |  |
| GO:0033262\_regulation\_of\_DNA\_replication\_during\_S\_phase | 1 | 0 |  |  |  |  |  |  |  |  |
| GO:0033292\_T-tubule\_organization | 1 | 0 |  |  |  |  |  |  |  |  |
| GO:0033341\_regulation\_of\_collagen\_binding | 1 | 0 |  |  |  |  |  |  |  |  |
| GO:0033342\_negative\_regulation\_of\_collagen\_binding | 1 | 0 |  |  |  |  |  |  |  |  |
| GO:0033345\_asparagine\_catabolic\_process\_via\_L-aspartate | 1 | 0 |  |  |  |  |  |  |  |  |
| GO:0033366\_protein\_localization\_in\_secretory\_granule | 1 | 0 |  |  |  |  |  |  |  |  |
| GO:0033367\_protein\_localization\_in\_mast\_cell\_secretory\_granule | 1 | 0 |  |  |  |  |  |  |  |  |
| GO:0033368\_protease\_localization\_in\_mast\_cell\_secretory\_granule | 1 | 0 |  |  |  |  |  |  |  |  |
| GO:0033370\_maintenance\_of\_protein\_location\_in\_mast\_cell\_secretory\_granule | 1 | 0 |  |  |  |  |  |  |  |  |
| GO:0033371\_T\_cell\_secretory\_granule\_organization | 1 | 0 |  |  |  |  |  |  |  |  |
| GO:0033373\_maintenance\_of\_protease\_location\_in\_mast\_cell\_secretory\_granule | 1 | 0 |  |  |  |  |  |  |  |  |
| GO:0033374\_protein\_localization\_in\_T\_cell\_secretory\_granule | 1 | 0 |  |  |  |  |  |  |  |  |
| GO:0033375\_protease\_localization\_in\_T\_cell\_secretory\_granule | 1 | 0 |  |  |  |  |  |  |  |  |
| GO:0033377\_maintenance\_of\_protein\_location\_in\_T\_cell\_secretory\_granule | 1 | 0 |  |  |  |  |  |  |  |  |
| GO:0033379\_maintenance\_of\_protease\_location\_in\_T\_cell\_secretory\_granule | 1 | 0 |  |  |  |  |  |  |  |  |
| GO:0033380\_granzyme\_B\_localization\_in\_T\_cell\_secretory\_granule | 1 | 0 |  |  |  |  |  |  |  |  |
| GO:0033382\_maintenance\_of\_granzyme\_B\_location\_in\_T\_cell\_secretory\_granule | 1 | 0 |  |  |  |  |  |  |  |  |
| GO:0033484\_nitric\_oxide\_homeostasis | 1 | 0 |  |  |  |  |  |  |  |  |
| GO:0033504\_floor\_plate\_development | 1 | 0 |  |  |  |  |  |  |  |  |
| GO:0033566\_gamma-tubulin\_complex\_localization | 1 | 0 |  |  |  |  |  |  |  |  |
| GO:0033577\_protein\_amino\_acid\_glycosylation\_in\_endoplasmic\_reticulum | 1 | 0 |  |  |  |  |  |  |  |  |
| GO:0033595\_response\_to\_genistein | 1 | 0 |  |  |  |  |  |  |  |  |
| GO:0033600\_negative\_regulation\_of\_mammary\_gland\_epithelial\_cell\_proliferation | 1 | 0 |  |  |  |  |  |  |  |  |
| GO:0033606\_chemokine\_receptor\_transport\_within\_lipid\_bilayer | 1 | 0 |  |  |  |  |  |  |  |  |
| GO:0033617\_mitochondrial\_respiratory\_chain\_complex\_IV\_assembly | 1 | 0 |  |  |  |  |  |  |  |  |
| GO:0033622\_integrin\_activation | 1 | 0 |  |  |  |  |  |  |  |  |
| GO:0033623\_regulation\_of\_integrin\_activation | 1 | 0 |  |  |  |  |  |  |  |  |
| GO:0033625\_positive\_regulation\_of\_integrin\_activation | 1 | 0 |  |  |  |  |  |  |  |  |
| GO:0033693\_neurofilament\_bundle\_assembly | 1 | 0 |  |  |  |  |  |  |  |  |
| GO:0033750\_ribosome\_localization | 1 | 0 |  |  |  |  |  |  |  |  |
| GO:0033753\_establishment\_of\_ribosome\_localization | 1 | 0 |  |  |  |  |  |  |  |  |
| GO:0033875\_ribonucleoside\_bisphosphate\_metabolic\_process | 1 | 0 |  |  |  |  |  |  |  |  |
| GO:0033962\_cytoplasmic\_mRNA\_processing\_body\_assembly | 1 | 0 |  |  |  |  |  |  |  |  |
| GO:0034032\_purine\_nucleoside\_bisphosphate\_metabolic\_process | 1 | 0 |  |  |  |  |  |  |  |  |
| GO:0034035\_purine\_ribonucleoside\_bisphosphate\_metabolic\_process | 1 | 0 |  |  |  |  |  |  |  |  |
| GO:0034063\_stress\_granule\_assembly | 1 | 0 |  |  |  |  |  |  |  |  |
| GO:0034080\_CenH3-containing\_nucleosome\_assembly\_at\_centromere | 1 | 0 |  |  |  |  |  |  |  |  |
| GO:0034109\_homotypic\_cell-cell\_adhesion | 1 | 0 |  |  |  |  |  |  |  |  |
| GO:0034115\_negative\_regulation\_of\_heterotypic\_cell-cell\_adhesion | 1 | 0 |  |  |  |  |  |  |  |  |
| GO:0034116\_positive\_regulation\_of\_heterotypic\_cell-cell\_adhesion | 1 | 0 |  |  |  |  |  |  |  |  |
| GO:0034122\_negative\_regulation\_of\_toll-like\_receptor\_signaling\_pathway | 1 | 0 |  |  |  |  |  |  |  |  |
| GO:0034123\_positive\_regulation\_of\_toll-like\_receptor\_signaling\_pathway | 1 | 0 |  |  |  |  |  |  |  |  |
| GO:0034142\_toll-like\_receptor\_4\_signaling\_pathway | 1 | 0 |  |  |  |  |  |  |  |  |
| GO:0034143\_regulation\_of\_toll-like\_receptor\_4\_signaling\_pathway | 1 | 0 |  |  |  |  |  |  |  |  |
| GO:0034145\_positive\_regulation\_of\_toll-like\_receptor\_4\_signaling\_pathway | 1 | 0 |  |  |  |  |  |  |  |  |
| GO:0034196\_acylglycerol\_transport | 1 | 0 |  |  |  |  |  |  |  |  |
| GO:0034197\_triglyceride\_transport | 1 | 0 |  |  |  |  |  |  |  |  |
| GO:0034205\_beta-amyloid\_formation | 1 | 0 |  |  |  |  |  |  |  |  |
| GO:0034213\_quinolinate\_catabolic\_process | 1 | 0 |  |  |  |  |  |  |  |  |
| GO:0034231\_islet\_amyloid\_polypeptide\_processing | 1 | 0 |  |  |  |  |  |  |  |  |
| GO:0034238\_macrophage\_fusion | 1 | 0 |  |  |  |  |  |  |  |  |
| GO:0034239\_regulation\_of\_macrophage\_fusion | 1 | 0 |  |  |  |  |  |  |  |  |
| GO:0034241\_positive\_regulation\_of\_macrophage\_fusion | 1 | 0 |  |  |  |  |  |  |  |  |
| GO:0034248\_regulation\_of\_amide\_metabolic\_process | 1 | 0 |  |  |  |  |  |  |  |  |
| GO:0034263\_autophagy\_in\_response\_to\_ER\_overload | 1 | 0 |  |  |  |  |  |  |  |  |
| GO:0034287\_detection\_of\_monosaccharide\_stimulus | 1 | 0 |  |  |  |  |  |  |  |  |
| GO:0034313\_diol\_catabolic\_process | 1 | 0 |  |  |  |  |  |  |  |  |
| GO:0034332\_adherens\_junction\_organization | 1 | 0 |  |  |  |  |  |  |  |  |
| GO:0034333\_adherens\_junction\_assembly | 1 | 0 |  |  |  |  |  |  |  |  |
| GO:0034340\_response\_to\_type\_I\_interferon | 1 | 0 |  |  |  |  |  |  |  |  |
| GO:0034356\_NAD\_biosynthesis\_via\_nicotinamide\_riboside\_salvage\_pathway | 1 | 0 |  |  |  |  |  |  |  |  |
| GO:0034373\_intermediate-density\_lipoprotein\_particle\_remodeling | 1 | 0 |  |  |  |  |  |  |  |  |
| GO:0034378\_chylomicron\_assembly | 1 | 0 |  |  |  |  |  |  |  |  |
| GO:0034436\_glycoprotein\_transport | 1 | 0 |  |  |  |  |  |  |  |  |
| GO:0034439\_lipoprotein\_lipid\_oxidation | 1 | 0 |  |  |  |  |  |  |  |  |
| GO:0034454\_microtubule\_anchoring\_at\_centrosome | 1 | 0 |  |  |  |  |  |  |  |  |
| GO:0034465\_response\_to\_carbon\_monoxide | 1 | 0 |  |  |  |  |  |  |  |  |
| GO:0034509\_centromeric\_core\_chromatin\_formation | 1 | 0 |  |  |  |  |  |  |  |  |
| GO:0034516\_response\_to\_vitamin\_B6 | 1 | 0 |  |  |  |  |  |  |  |  |
| GO:0034551\_mitochondrial\_respiratory\_chain\_complex\_III\_assembly | 1 | 0 |  |  |  |  |  |  |  |  |
| GO:0034552\_respiratory\_chain\_complex\_II\_assembly | 1 | 0 |  |  |  |  |  |  |  |  |
| GO:0034553\_mitochondrial\_respiratory\_chain\_complex\_II\_assembly | 1 | 0 |  |  |  |  |  |  |  |  |
| GO:0034589\_hydroxyproline\_transport | 1 | 0 |  |  |  |  |  |  |  |  |
| GO:0034694\_response\_to\_prostaglandin\_stimulus | 1 | 0 |  |  |  |  |  |  |  |  |
| GO:0034695\_response\_to\_prostaglandin\_E\_stimulus | 1 | 0 |  |  |  |  |  |  |  |  |
| GO:0034698\_response\_to\_gonadotropin\_stimulus | 1 | 0 |  |  |  |  |  |  |  |  |
| GO:0034699\_response\_to\_luteinizing\_hormone\_stimulus | 1 | 0 |  |  |  |  |  |  |  |  |
| GO:0034724\_DNA\_replication-independent\_nucleosome\_organization | 1 | 0 |  |  |  |  |  |  |  |  |
| GO:0034729\_histone\_H3-K79\_methylation | 1 | 0 |  |  |  |  |  |  |  |  |
| GO:0034755\_iron\_ion\_transmembrane\_transport | 1 | 0 |  |  |  |  |  |  |  |  |
| GO:0034764\_positive\_regulation\_of\_transmembrane\_transport | 1 | 0 |  |  |  |  |  |  |  |  |
| GO:0034765\_regulation\_of\_ion\_transmembrane\_transport | 1 | 0 |  |  |  |  |  |  |  |  |
| GO:0034767\_positive\_regulation\_of\_ion\_transmembrane\_transport | 1 | 0 |  |  |  |  |  |  |  |  |
| GO:0034959\_endothelin\_maturation | 1 | 0 |  |  |  |  |  |  |  |  |
| GO:0034982\_mitochondrial\_protein\_processing | 1 | 0 |  |  |  |  |  |  |  |  |
| GO:0034983\_peptidyl-lysine\_deacetylation | 1 | 0 |  |  |  |  |  |  |  |  |
| GO:0035021\_negative\_regulation\_of\_Rac\_protein\_signal\_transduction | 1 | 0 |  |  |  |  |  |  |  |  |
| GO:0035041\_sperm\_chromatin\_decondensation | 1 | 0 |  |  |  |  |  |  |  |  |
| GO:0035042\_fertilization\_\_exchange\_of\_chromosomal\_proteins | 1 | 0 |  |  |  |  |  |  |  |  |
| GO:0035054\_embryonic\_heart\_tube\_anterior\_posterior\_pattern\_formation | 1 | 0 |  |  |  |  |  |  |  |  |
| GO:0035066\_positive\_regulation\_of\_histone\_acetylation | 1 | 0 |  |  |  |  |  |  |  |  |
| GO:0035082\_axoneme\_assembly | 1 | 0 |  |  |  |  |  |  |  |  |
| GO:0035087\_RNA\_interference\_\_siRNA\_loading\_onto\_RISC | 1 | 0 |  |  |  |  |  |  |  |  |
| GO:0035090\_maintenance\_of\_apical\_basal\_cell\_polarity | 1 | 0 |  |  |  |  |  |  |  |  |
| GO:0035093\_spermatogenesis\_\_exchange\_of\_chromosomal\_proteins | 1 | 0 |  |  |  |  |  |  |  |  |
| GO:0035104\_positive\_regulation\_of\_transcription\_via\_sterol\_regulatory\_element\_binding | 1 | 0 |  |  |  |  |  |  |  |  |
| GO:0035110\_leg\_morphogenesis | 1 | 0 |  |  |  |  |  |  |  |  |
| GO:0035112\_genitalia\_morphogenesis | 1 | 0 |  |  |  |  |  |  |  |  |
| GO:0035116\_embryonic\_hindlimb\_morphogenesis | 1 | 0 |  |  |  |  |  |  |  |  |
| GO:0035137\_hindlimb\_morphogenesis | 1 | 0 |  |  |  |  |  |  |  |  |
| GO:0035238\_vitamin\_A\_biosynthetic\_process | 1 | 0 |  |  |  |  |  |  |  |  |
| GO:0035265\_organ\_growth | 1 | 0 |  |  |  |  |  |  |  |  |
| GO:0035280\_gene\_silencing\_by\_miRNA\_\_miRNA\_loading\_onto\_RISC | 1 | 0 |  |  |  |  |  |  |  |  |
| GO:0040009\_regulation\_of\_growth\_rate | 1 | 0 |  |  |  |  |  |  |  |  |
| GO:0040013\_negative\_regulation\_of\_locomotion | 1 | 0 |  |  |  |  |  |  |  |  |
| GO:0040015\_negative\_regulation\_of\_multicellular\_organism\_growth | 1 | 0 |  |  |  |  |  |  |  |  |
| GO:0040020\_regulation\_of\_meiosis | 1 | 0 |  |  |  |  |  |  |  |  |
| GO:0040023\_establishment\_of\_nucleus\_localization | 1 | 0 |  |  |  |  |  |  |  |  |
| GO:0040030\_regulation\_of\_molecular\_function\_\_epigenetic | 1 | 0 |  |  |  |  |  |  |  |  |
| GO:0040037\_negative\_regulation\_of\_fibroblast\_growth\_factor\_receptor\_signaling\_pathway | 1 | 0 |  |  |  |  |  |  |  |  |
| GO:0040038\_polar\_body\_extrusion\_after\_meiotic\_divisions | 1 | 0 |  |  |  |  |  |  |  |  |
| GO:0042074\_cell\_migration\_involved\_in\_gastrulation | 1 | 0 |  |  |  |  |  |  |  |  |
| GO:0042091\_interleukin-10\_biosynthetic\_process | 1 | 0 |  |  |  |  |  |  |  |  |
| GO:0042118\_endothelial\_cell\_activation | 1 | 0 |  |  |  |  |  |  |  |  |
| GO:0042159\_lipoprotein\_catabolic\_process | 1 | 0 |  |  |  |  |  |  |  |  |
| GO:0042214\_terpene\_metabolic\_process | 1 | 0 |  |  |  |  |  |  |  |  |
| GO:0042225\_interleukin-5\_biosynthetic\_process | 1 | 0 |  |  |  |  |  |  |  |  |
| GO:0042241\_interleukin-18\_biosynthetic\_process | 1 | 0 |  |  |  |  |  |  |  |  |
| GO:0042257\_ribosomal\_subunit\_assembly | 1 | 0 |  |  |  |  |  |  |  |  |
| GO:0042262\_DNA\_protection | 1 | 0 |  |  |  |  |  |  |  |  |
| GO:0042276\_error-prone\_postreplication\_DNA\_repair | 1 | 0 |  |  |  |  |  |  |  |  |
| GO:0042313\_protein\_kinase\_C\_deactivation | 1 | 0 |  |  |  |  |  |  |  |  |
| GO:0042369\_vitamin\_D\_catabolic\_process | 1 | 0 |  |  |  |  |  |  |  |  |
| GO:0042412\_taurine\_biosynthetic\_process | 1 | 0 |  |  |  |  |  |  |  |  |
| GO:0042418\_epinephrine\_biosynthetic\_process | 1 | 0 |  |  |  |  |  |  |  |  |
| GO:0042421\_norepinephrine\_biosynthetic\_process | 1 | 0 |  |  |  |  |  |  |  |  |
| GO:0042424\_catecholamine\_catabolic\_process | 1 | 0 |  |  |  |  |  |  |  |  |
| GO:0042428\_serotonin\_metabolic\_process | 1 | 0 |  |  |  |  |  |  |  |  |
| GO:0042435\_indole\_derivative\_biosynthetic\_process | 1 | 0 |  |  |  |  |  |  |  |  |
| GO:0042474\_middle\_ear\_morphogenesis | 1 | 0 |  |  |  |  |  |  |  |  |
| GO:0042504\_tyrosine\_phosphorylation\_of\_Stat4\_protein | 1 | 0 |  |  |  |  |  |  |  |  |
| GO:0042519\_regulation\_of\_tyrosine\_phosphorylation\_of\_Stat4\_protein | 1 | 0 |  |  |  |  |  |  |  |  |
| GO:0042520\_positive\_regulation\_of\_tyrosine\_phosphorylation\_of\_Stat4\_protein | 1 | 0 |  |  |  |  |  |  |  |  |
| GO:0042524\_negative\_regulation\_of\_tyrosine\_phosphorylation\_of\_Stat5\_protein | 1 | 0 |  |  |  |  |  |  |  |  |
| GO:0042537\_benzene\_and\_derivative\_metabolic\_process | 1 | 0 |  |  |  |  |  |  |  |  |
| GO:0042560\_pteridine\_and\_derivative\_catabolic\_process | 1 | 0 |  |  |  |  |  |  |  |  |
| GO:0042596\_fear\_response | 1 | 0 |  |  |  |  |  |  |  |  |
| GO:0042637\_catagen | 1 | 0 |  |  |  |  |  |  |  |  |
| GO:0042640\_anagen | 1 | 0 |  |  |  |  |  |  |  |  |
| GO:0042670\_retinal\_cone\_cell\_differentiation | 1 | 0 |  |  |  |  |  |  |  |  |
| GO:0042700\_luteinizing\_hormone\_signaling\_pathway | 1 | 0 |  |  |  |  |  |  |  |  |
| GO:0042703\_menstruation | 1 | 0 |  |  |  |  |  |  |  |  |
| GO:0042726\_riboflavin\_and\_derivative\_metabolic\_process | 1 | 0 |  |  |  |  |  |  |  |  |
| GO:0042727\_riboflavin\_and\_derivative\_biosynthetic\_process | 1 | 0 |  |  |  |  |  |  |  |  |
| GO:0042746\_circadian\_sleep\_wake\_cycle\_\_wakefulness | 1 | 0 |  |  |  |  |  |  |  |  |
| GO:0042748\_circadian\_sleep\_wake\_cycle\_\_non-REM\_sleep | 1 | 0 |  |  |  |  |  |  |  |  |
| GO:0042755\_eating\_behavior | 1 | 0 |  |  |  |  |  |  |  |  |
| GO:0042756\_drinking\_behavior | 1 | 0 |  |  |  |  |  |  |  |  |
| GO:0042766\_nucleosome\_mobilization | 1 | 0 |  |  |  |  |  |  |  |  |
| GO:0042780\_tRNA\_3'-end\_processing | 1 | 0 |  |  |  |  |  |  |  |  |
| GO:0042789\_mRNA\_transcription\_from\_RNA\_polymerase\_II\_promoter | 1 | 0 |  |  |  |  |  |  |  |  |
| GO:0042795\_snRNA\_transcription\_from\_RNA\_polymerase\_II\_promoter | 1 | 0 |  |  |  |  |  |  |  |  |
| GO:0042796\_snRNA\_transcription\_from\_RNA\_polymerase\_III\_promoter | 1 | 0 |  |  |  |  |  |  |  |  |
| GO:0042822\_pyridoxal\_phosphate\_metabolic\_process | 1 | 0 |  |  |  |  |  |  |  |  |
| GO:0042823\_pyridoxal\_phosphate\_biosynthetic\_process | 1 | 0 |  |  |  |  |  |  |  |  |
| GO:0042866\_pyruvate\_biosynthetic\_process | 1 | 0 |  |  |  |  |  |  |  |  |
| GO:0042904\_9-cis-retinoic\_acid\_biosynthetic\_process | 1 | 0 |  |  |  |  |  |  |  |  |
| GO:0042905\_9-cis-retinoic\_acid\_metabolic\_process | 1 | 0 |  |  |  |  |  |  |  |  |
| GO:0042985\_negative\_regulation\_of\_amyloid\_precursor\_protein\_biosynthetic\_process | 1 | 0 |  |  |  |  |  |  |  |  |
| GO:0042986\_positive\_regulation\_of\_amyloid\_precursor\_protein\_biosynthetic\_process | 1 | 0 |  |  |  |  |  |  |  |  |
| GO:0042989\_sequestering\_of\_actin\_monomers | 1 | 0 |  |  |  |  |  |  |  |  |
| GO:0042996\_regulation\_of\_Golgi\_to\_plasma\_membrane\_protein\_transport | 1 | 0 |  |  |  |  |  |  |  |  |
| GO:0042997\_negative\_regulation\_of\_Golgi\_to\_plasma\_membrane\_protein\_transport | 1 | 0 |  |  |  |  |  |  |  |  |
| GO:0042999\_regulation\_of\_Golgi\_to\_plasma\_membrane\_CFTR\_protein\_transport | 1 | 0 |  |  |  |  |  |  |  |  |
| GO:0043002\_negative\_regulation\_of\_Golgi\_to\_plasma\_membrane\_CFTR\_protein\_transport | 1 | 0 |  |  |  |  |  |  |  |  |
| GO:0043004\_cytoplasmic\_sequestering\_of\_CFTR\_protein | 1 | 0 |  |  |  |  |  |  |  |  |
| GO:0043012\_regulation\_of\_fusion\_of\_sperm\_to\_egg\_plasma\_membrane | 1 | 0 |  |  |  |  |  |  |  |  |
| GO:0043016\_regulation\_of\_lymphotoxin\_A\_biosynthetic\_process | 1 | 0 |  |  |  |  |  |  |  |  |
| GO:0043017\_positive\_regulation\_of\_lymphotoxin\_A\_biosynthetic\_process | 1 | 0 |  |  |  |  |  |  |  |  |
| GO:0043049\_otic\_placode\_formation | 1 | 0 |  |  |  |  |  |  |  |  |
| GO:0043064\_flagellum\_organization | 1 | 0 |  |  |  |  |  |  |  |  |
| GO:0043116\_negative\_regulation\_of\_vascular\_permeability | 1 | 0 |  |  |  |  |  |  |  |  |
| GO:0043126\_regulation\_of\_1-phosphatidylinositol\_4-kinase\_activity | 1 | 0 |  |  |  |  |  |  |  |  |
| GO:0043128\_positive\_regulation\_of\_1-phosphatidylinositol\_4-kinase\_activity | 1 | 0 |  |  |  |  |  |  |  |  |
| GO:0043129\_surfactant\_homeostasis | 1 | 0 |  |  |  |  |  |  |  |  |
| GO:0043146\_spindle\_stabilization | 1 | 0 |  |  |  |  |  |  |  |  |
| GO:0043148\_mitotic\_spindle\_stabilization | 1 | 0 |  |  |  |  |  |  |  |  |
| GO:0043152\_induction\_of\_bacterial\_agglutination | 1 | 0 |  |  |  |  |  |  |  |  |
| GO:0043173\_nucleotide\_salvage | 1 | 0 |  |  |  |  |  |  |  |  |
| GO:0043174\_nucleoside\_salvage | 1 | 0 |  |  |  |  |  |  |  |  |
| GO:0043181\_vacuolar\_sequestering | 1 | 0 |  |  |  |  |  |  |  |  |
| GO:0043200\_response\_to\_amino\_acid\_stimulus | 1 | 0 |  |  |  |  |  |  |  |  |
| GO:0043217\_myelin\_maintenance | 1 | 0 |  |  |  |  |  |  |  |  |
| GO:0043247\_telomere\_maintenance\_in\_response\_to\_DNA\_damage | 1 | 0 |  |  |  |  |  |  |  |  |
| GO:0043249\_erythrocyte\_maturation | 1 | 0 |  |  |  |  |  |  |  |  |
| GO:0043268\_positive\_regulation\_of\_potassium\_ion\_transport | 1 | 0 |  |  |  |  |  |  |  |  |
| GO:0043299\_leukocyte\_degranulation | 1 | 0 |  |  |  |  |  |  |  |  |
| GO:0043307\_eosinophil\_activation | 1 | 0 |  |  |  |  |  |  |  |  |
| GO:0043308\_eosinophil\_degranulation | 1 | 0 |  |  |  |  |  |  |  |  |
| GO:0043312\_neutrophil\_degranulation | 1 | 0 |  |  |  |  |  |  |  |  |
| GO:0043330\_response\_to\_exogenous\_dsRNA | 1 | 0 |  |  |  |  |  |  |  |  |
| GO:0043353\_enucleate\_erythrocyte\_differentiation | 1 | 0 |  |  |  |  |  |  |  |  |
| GO:0043371\_negative\_regulation\_of\_CD4-positive\_\_alpha\_beta\_T\_cell\_differentiation | 1 | 0 |  |  |  |  |  |  |  |  |
| GO:0043383\_negative\_T\_cell\_selection | 1 | 0 |  |  |  |  |  |  |  |  |
| GO:0043403\_skeletal\_muscle\_regeneration | 1 | 0 |  |  |  |  |  |  |  |  |
| GO:0043418\_homocysteine\_catabolic\_process | 1 | 0 |  |  |  |  |  |  |  |  |
| GO:0043420\_anthranilate\_metabolic\_process | 1 | 0 |  |  |  |  |  |  |  |  |
| GO:0043437\_butanoic\_acid\_metabolic\_process | 1 | 0 |  |  |  |  |  |  |  |  |
| GO:0043455\_regulation\_of\_secondary\_metabolic\_process | 1 | 0 |  |  |  |  |  |  |  |  |
| GO:0043456\_regulation\_of\_pentose-phosphate\_shunt | 1 | 0 |  |  |  |  |  |  |  |  |
| GO:0043457\_regulation\_of\_cellular\_respiration | 1 | 0 |  |  |  |  |  |  |  |  |
| GO:0043517\_positive\_regulation\_of\_DNA\_damage\_response\_\_signal\_transduction\_by\_p53\_class\_mediator | 1 | 0 |  |  |  |  |  |  |  |  |
| GO:0043518\_negative\_regulation\_of\_DNA\_damage\_response\_\_signal\_transduction\_by\_p53\_class\_mediator | 1 | 0 |  |  |  |  |  |  |  |  |
| GO:0043551\_regulation\_of\_phosphoinositide\_3-kinase\_activity | 1 | 0 |  |  |  |  |  |  |  |  |
| GO:0043552\_positive\_regulation\_of\_phosphoinositide\_3-kinase\_activity | 1 | 0 |  |  |  |  |  |  |  |  |
| GO:0043556\_regulation\_of\_translation\_in\_response\_to\_oxidative\_stress | 1 | 0 |  |  |  |  |  |  |  |  |
| GO:0043584\_nose\_development | 1 | 0 |  |  |  |  |  |  |  |  |
| GO:0043586\_tongue\_development | 1 | 0 |  |  |  |  |  |  |  |  |
| GO:0043587\_tongue\_morphogenesis | 1 | 0 |  |  |  |  |  |  |  |  |
| GO:0043647\_inositol\_phosphate\_metabolic\_process | 1 | 0 |  |  |  |  |  |  |  |  |
| GO:0043652\_engulfment\_of\_apoptotic\_cell | 1 | 0 |  |  |  |  |  |  |  |  |
| GO:0043654\_recognition\_of\_apoptotic\_cell | 1 | 0 |  |  |  |  |  |  |  |  |
| GO:0043696\_dedifferentiation | 1 | 0 |  |  |  |  |  |  |  |  |
| GO:0043697\_cell\_dedifferentiation | 1 | 0 |  |  |  |  |  |  |  |  |
| GO:0043901\_negative\_regulation\_of\_multi-organism\_process | 1 | 0 |  |  |  |  |  |  |  |  |
| GO:0043921\_modulation\_by\_host\_of\_viral\_transcription | 1 | 0 |  |  |  |  |  |  |  |  |
| GO:0043923\_positive\_regulation\_by\_host\_of\_viral\_transcription | 1 | 0 |  |  |  |  |  |  |  |  |
| GO:0044007\_dissemination\_or\_transmission\_of\_symbiont\_from\_host | 1 | 0 |  |  |  |  |  |  |  |  |
| GO:0044089\_positive\_regulation\_of\_cellular\_component\_biogenesis | 1 | 0 |  |  |  |  |  |  |  |  |
| GO:0044258\_intestinal\_lipid\_catabolic\_process | 1 | 0 |  |  |  |  |  |  |  |  |
| GO:0044273\_sulfur\_compound\_catabolic\_process | 1 | 0 |  |  |  |  |  |  |  |  |
| GO:0045013\_negative\_regulation\_of\_transcription\_by\_carbon\_catabolites | 1 | 0 |  |  |  |  |  |  |  |  |
| GO:0045014\_negative\_regulation\_of\_transcription\_by\_glucose | 1 | 0 |  |  |  |  |  |  |  |  |
| GO:0045020\_error-prone\_DNA\_repair | 1 | 0 |  |  |  |  |  |  |  |  |
| GO:0045023\_G0\_to\_G1\_transition | 1 | 0 |  |  |  |  |  |  |  |  |
| GO:0045047\_protein\_targeting\_to\_ER | 1 | 0 |  |  |  |  |  |  |  |  |
| GO:0045065\_cytotoxic\_T\_cell\_differentiation | 1 | 0 |  |  |  |  |  |  |  |  |
| GO:0045074\_regulation\_of\_interleukin-10\_biosynthetic\_process | 1 | 0 |  |  |  |  |  |  |  |  |
| GO:0045082\_positive\_regulation\_of\_interleukin-10\_biosynthetic\_process | 1 | 0 |  |  |  |  |  |  |  |  |
| GO:0045132\_meiotic\_chromosome\_segregation | 1 | 0 |  |  |  |  |  |  |  |  |
| GO:0045163\_clustering\_of\_voltage-gated\_potassium\_channels | 1 | 0 |  |  |  |  |  |  |  |  |
| GO:0045175\_basal\_protein\_localization | 1 | 0 |  |  |  |  |  |  |  |  |
| GO:0045188\_regulation\_of\_circadian\_sleep\_wake\_cycle\_\_non-REM\_sleep | 1 | 0 |  |  |  |  |  |  |  |  |
| GO:0045189\_connective\_tissue\_growth\_factor\_biosynthetic\_process | 1 | 0 |  |  |  |  |  |  |  |  |
| GO:0045196\_establishment\_or\_maintenance\_of\_neuroblast\_polarity | 1 | 0 |  |  |  |  |  |  |  |  |
| GO:0045199\_maintenance\_of\_epithelial\_cell\_apical\_basal\_polarity | 1 | 0 |  |  |  |  |  |  |  |  |
| GO:0045200\_establishment\_of\_neuroblast\_polarity | 1 | 0 |  |  |  |  |  |  |  |  |
| GO:0045204\_MAPK\_export\_from\_nucleus | 1 | 0 |  |  |  |  |  |  |  |  |
| GO:0045208\_MAPK\_phosphatase\_export\_from\_nucleus | 1 | 0 |  |  |  |  |  |  |  |  |
| GO:0045209\_MAPK\_phosphatase\_export\_from\_nucleus\_\_leptomycin\_B\_sensitive | 1 | 0 |  |  |  |  |  |  |  |  |
| GO:0045292\_nuclear\_mRNA\_cis\_splicing\_\_via\_spliceosome | 1 | 0 |  |  |  |  |  |  |  |  |
| GO:0045324\_late\_endosome\_to\_vacuole\_transport | 1 | 0 |  |  |  |  |  |  |  |  |
| GO:0045329\_carnitine\_biosynthetic\_process | 1 | 0 |  |  |  |  |  |  |  |  |
| GO:0045345\_positive\_regulation\_of\_MHC\_class\_I\_biosynthetic\_process | 1 | 0 |  |  |  |  |  |  |  |  |
| GO:0045355\_negative\_regulation\_of\_interferon-alpha\_biosynthetic\_process | 1 | 0 |  |  |  |  |  |  |  |  |
| GO:0045360\_regulation\_of\_interleukin-1\_biosynthetic\_process | 1 | 0 |  |  |  |  |  |  |  |  |
| GO:0045362\_positive\_regulation\_of\_interleukin-1\_biosynthetic\_process | 1 | 0 |  |  |  |  |  |  |  |  |
| GO:0045366\_regulation\_of\_interleukin-13\_biosynthetic\_process | 1 | 0 |  |  |  |  |  |  |  |  |
| GO:0045368\_positive\_regulation\_of\_interleukin-13\_biosynthetic\_process | 1 | 0 |  |  |  |  |  |  |  |  |
| GO:0045381\_regulation\_of\_interleukin-18\_biosynthetic\_process | 1 | 0 |  |  |  |  |  |  |  |  |
| GO:0045405\_regulation\_of\_interleukin-5\_biosynthetic\_process | 1 | 0 |  |  |  |  |  |  |  |  |
| GO:0045407\_positive\_regulation\_of\_interleukin-5\_biosynthetic\_process | 1 | 0 |  |  |  |  |  |  |  |  |
| GO:0045425\_positive\_regulation\_of\_granulocyte\_macrophage\_colony-stimulating\_factor\_biosynthetic\_process | 1 | 0 |  |  |  |  |  |  |  |  |
| GO:0045475\_locomotor\_rhythm | 1 | 0 |  |  |  |  |  |  |  |  |
| GO:0045553\_TRAIL\_biosynthetic\_process | 1 | 0 |  |  |  |  |  |  |  |  |
| GO:0045554\_regulation\_of\_TRAIL\_biosynthetic\_process | 1 | 0 |  |  |  |  |  |  |  |  |
| GO:0045556\_positive\_regulation\_of\_TRAIL\_biosynthetic\_process | 1 | 0 |  |  |  |  |  |  |  |  |
| GO:0045575\_basophil\_activation | 1 | 0 |  |  |  |  |  |  |  |  |
| GO:0045583\_regulation\_of\_cytotoxic\_T\_cell\_differentiation | 1 | 0 |  |  |  |  |  |  |  |  |
| GO:0045585\_positive\_regulation\_of\_cytotoxic\_T\_cell\_differentiation | 1 | 0 |  |  |  |  |  |  |  |  |
| GO:0045589\_regulation\_of\_regulatory\_T\_cell\_differentiation | 1 | 0 |  |  |  |  |  |  |  |  |
| GO:0045590\_negative\_regulation\_of\_regulatory\_T\_cell\_differentiation | 1 | 0 |  |  |  |  |  |  |  |  |
| GO:0045602\_negative\_regulation\_of\_endothelial\_cell\_differentiation | 1 | 0 |  |  |  |  |  |  |  |  |
| GO:0045603\_positive\_regulation\_of\_endothelial\_cell\_differentiation | 1 | 0 |  |  |  |  |  |  |  |  |
| GO:0045605\_negative\_regulation\_of\_epidermal\_cell\_differentiation | 1 | 0 |  |  |  |  |  |  |  |  |
| GO:0045617\_negative\_regulation\_of\_keratinocyte\_differentiation | 1 | 0 |  |  |  |  |  |  |  |  |
| GO:0045623\_negative\_regulation\_of\_T-helper\_cell\_differentiation | 1 | 0 |  |  |  |  |  |  |  |  |
| GO:0045629\_negative\_regulation\_of\_T-helper\_2\_cell\_differentiation | 1 | 0 |  |  |  |  |  |  |  |  |
| GO:0045654\_positive\_regulation\_of\_megakaryocyte\_differentiation | 1 | 0 |  |  |  |  |  |  |  |  |
| GO:0045672\_positive\_regulation\_of\_osteoclast\_differentiation | 1 | 0 |  |  |  |  |  |  |  |  |
| GO:0045683\_negative\_regulation\_of\_epidermis\_development | 1 | 0 |  |  |  |  |  |  |  |  |
| GO:0045716\_positive\_regulation\_of\_low-density\_lipoprotein\_receptor\_biosynthetic\_process | 1 | 0 |  |  |  |  |  |  |  |  |
| GO:0045719\_negative\_regulation\_of\_glycogen\_biosynthetic\_process | 1 | 0 |  |  |  |  |  |  |  |  |
| GO:0045738\_negative\_regulation\_of\_DNA\_repair | 1 | 0 |  |  |  |  |  |  |  |  |
| GO:0045747\_positive\_regulation\_of\_Notch\_signaling\_pathway | 1 | 0 |  |  |  |  |  |  |  |  |
| GO:0045750\_positive\_regulation\_of\_S\_phase\_of\_mitotic\_cell\_cycle | 1 | 0 |  |  |  |  |  |  |  |  |
| GO:0045751\_negative\_regulation\_of\_Toll\_signaling\_pathway | 1 | 0 |  |  |  |  |  |  |  |  |
| GO:0045759\_negative\_regulation\_of\_action\_potential | 1 | 0 |  |  |  |  |  |  |  |  |
| GO:0045773\_positive\_regulation\_of\_axon\_extension | 1 | 0 |  |  |  |  |  |  |  |  |
| GO:0045794\_negative\_regulation\_of\_cell\_volume | 1 | 0 |  |  |  |  |  |  |  |  |
| GO:0045799\_positive\_regulation\_of\_chromatin\_assembly\_or\_disassembly | 1 | 0 |  |  |  |  |  |  |  |  |
| GO:0045818\_negative\_regulation\_of\_glycogen\_catabolic\_process | 1 | 0 |  |  |  |  |  |  |  |  |
| GO:0045836\_positive\_regulation\_of\_meiosis | 1 | 0 |  |  |  |  |  |  |  |  |
| GO:0045837\_negative\_regulation\_of\_membrane\_potential | 1 | 0 |  |  |  |  |  |  |  |  |
| GO:0045844\_positive\_regulation\_of\_striated\_muscle\_development | 1 | 0 |  |  |  |  |  |  |  |  |
| GO:0045852\_pH\_elevation | 1 | 0 |  |  |  |  |  |  |  |  |
| GO:0045870\_positive\_regulation\_of\_retroviral\_genome\_replication | 1 | 0 |  |  |  |  |  |  |  |  |
| GO:0045875\_negative\_regulation\_of\_sister\_chromatid\_cohesion | 1 | 0 |  |  |  |  |  |  |  |  |
| GO:0045879\_negative\_regulation\_of\_smoothened\_signaling\_pathway | 1 | 0 |  |  |  |  |  |  |  |  |
| GO:0045896\_regulation\_of\_transcription\_\_mitotic | 1 | 0 |  |  |  |  |  |  |  |  |
| GO:0045907\_positive\_regulation\_of\_vasoconstriction | 1 | 0 |  |  |  |  |  |  |  |  |
| GO:0045910\_negative\_regulation\_of\_DNA\_recombination | 1 | 0 |  |  |  |  |  |  |  |  |
| GO:0045915\_positive\_regulation\_of\_catecholamine\_metabolic\_process | 1 | 0 |  |  |  |  |  |  |  |  |
| GO:0045921\_positive\_regulation\_of\_exocytosis | 1 | 0 |  |  |  |  |  |  |  |  |
| GO:0045945\_positive\_regulation\_of\_transcription\_from\_RNA\_polymerase\_III\_promoter | 1 | 0 |  |  |  |  |  |  |  |  |
| GO:0045956\_positive\_regulation\_of\_calcium\_ion-dependent\_exocytosis | 1 | 0 |  |  |  |  |  |  |  |  |
| GO:0045964\_positive\_regulation\_of\_dopamine\_metabolic\_process | 1 | 0 |  |  |  |  |  |  |  |  |
| GO:0045989\_positive\_regulation\_of\_striated\_muscle\_contraction | 1 | 0 |  |  |  |  |  |  |  |  |
| GO:0045993\_negative\_regulation\_of\_translational\_initiation\_by\_iron | 1 | 0 |  |  |  |  |  |  |  |  |
| GO:0046005\_positive\_regulation\_of\_circadian\_sleep\_wake\_cycle\_\_REM\_sleep | 1 | 0 |  |  |  |  |  |  |  |  |
| GO:0046007\_negative\_regulation\_of\_activated\_T\_cell\_proliferation | 1 | 0 |  |  |  |  |  |  |  |  |
| GO:0046010\_positive\_regulation\_of\_circadian\_sleep\_wake\_cycle\_\_non-REM\_sleep | 1 | 0 |  |  |  |  |  |  |  |  |
| GO:0046031\_ADP\_metabolic\_process | 1 | 0 |  |  |  |  |  |  |  |  |
| GO:0046036\_CTP\_metabolic\_process | 1 | 0 |  |  |  |  |  |  |  |  |
| GO:0046040\_IMP\_metabolic\_process | 1 | 0 |  |  |  |  |  |  |  |  |
| GO:0046048\_UDP\_metabolic\_process | 1 | 0 |  |  |  |  |  |  |  |  |
| GO:0046049\_UMP\_metabolic\_process | 1 | 0 |  |  |  |  |  |  |  |  |
| GO:0046056\_dADP\_metabolic\_process | 1 | 0 |  |  |  |  |  |  |  |  |
| GO:0046085\_adenosine\_metabolic\_process | 1 | 0 |  |  |  |  |  |  |  |  |
| GO:0046087\_cytidine\_metabolic\_process | 1 | 0 |  |  |  |  |  |  |  |  |
| GO:0046101\_hypoxanthine\_biosynthetic\_process | 1 | 0 |  |  |  |  |  |  |  |  |
| GO:0046103\_inosine\_biosynthetic\_process | 1 | 0 |  |  |  |  |  |  |  |  |
| GO:0046104\_thymidine\_metabolic\_process | 1 | 0 |  |  |  |  |  |  |  |  |
| GO:0046108\_uridine\_metabolic\_process | 1 | 0 |  |  |  |  |  |  |  |  |
| GO:0046125\_pyrimidine\_deoxyribonucleoside\_metabolic\_process | 1 | 0 |  |  |  |  |  |  |  |  |
| GO:0046127\_pyrimidine\_deoxyribonucleoside\_catabolic\_process | 1 | 0 |  |  |  |  |  |  |  |  |
| GO:0046133\_pyrimidine\_ribonucleoside\_catabolic\_process | 1 | 0 |  |  |  |  |  |  |  |  |
| GO:0046173\_polyol\_biosynthetic\_process | 1 | 0 |  |  |  |  |  |  |  |  |
| GO:0046184\_aldehyde\_biosynthetic\_process | 1 | 0 |  |  |  |  |  |  |  |  |
| GO:0046203\_spermidine\_catabolic\_process | 1 | 0 |  |  |  |  |  |  |  |  |
| GO:0046219\_indolalkylamine\_biosynthetic\_process | 1 | 0 |  |  |  |  |  |  |  |  |
| GO:0046292\_formaldehyde\_metabolic\_process | 1 | 0 |  |  |  |  |  |  |  |  |
| GO:0046293\_formaldehyde\_biosynthetic\_process | 1 | 0 |  |  |  |  |  |  |  |  |
| GO:0046317\_regulation\_of\_glucosylceramide\_biosynthetic\_process | 1 | 0 |  |  |  |  |  |  |  |  |
| GO:0046318\_negative\_regulation\_of\_glucosylceramide\_biosynthetic\_process | 1 | 0 |  |  |  |  |  |  |  |  |
| GO:0046322\_negative\_regulation\_of\_fatty\_acid\_oxidation | 1 | 0 |  |  |  |  |  |  |  |  |
| GO:0046335\_ethanolamine\_biosynthetic\_process | 1 | 0 |  |  |  |  |  |  |  |  |
| GO:0046337\_phosphatidylethanolamine\_metabolic\_process | 1 | 0 |  |  |  |  |  |  |  |  |
| GO:0046340\_diacylglycerol\_catabolic\_process | 1 | 0 |  |  |  |  |  |  |  |  |
| GO:0046351\_disaccharide\_biosynthetic\_process | 1 | 0 |  |  |  |  |  |  |  |  |
| GO:0046352\_disaccharide\_catabolic\_process | 1 | 0 |  |  |  |  |  |  |  |  |
| GO:0046370\_fructose\_biosynthetic\_process | 1 | 0 |  |  |  |  |  |  |  |  |
| GO:0046380\_N-acetylneuraminate\_biosynthetic\_process | 1 | 0 |  |  |  |  |  |  |  |  |
| GO:0046390\_ribose\_phosphate\_biosynthetic\_process | 1 | 0 |  |  |  |  |  |  |  |  |
| GO:0046399\_glucuronate\_biosynthetic\_process | 1 | 0 |  |  |  |  |  |  |  |  |
| GO:0046434\_organophosphate\_catabolic\_process | 1 | 0 |  |  |  |  |  |  |  |  |
| GO:0046448\_tropane\_alkaloid\_metabolic\_process | 1 | 0 |  |  |  |  |  |  |  |  |
| GO:0046449\_creatinine\_metabolic\_process | 1 | 0 |  |  |  |  |  |  |  |  |
| GO:0046471\_phosphatidylglycerol\_metabolic\_process | 1 | 0 |  |  |  |  |  |  |  |  |
| GO:0046477\_glycosylceramide\_catabolic\_process | 1 | 0 |  |  |  |  |  |  |  |  |
| GO:0046485\_ether\_lipid\_metabolic\_process | 1 | 0 |  |  |  |  |  |  |  |  |
| GO:0046487\_glyoxylate\_metabolic\_process | 1 | 0 |  |  |  |  |  |  |  |  |
| GO:0046491\_L-methylmalonyl-CoA\_metabolic\_process | 1 | 0 |  |  |  |  |  |  |  |  |
| GO:0046501\_protoporphyrinogen\_IX\_metabolic\_process | 1 | 0 |  |  |  |  |  |  |  |  |
| GO:0046511\_sphinganine\_biosynthetic\_process | 1 | 0 |  |  |  |  |  |  |  |  |
| GO:0046514\_ceramide\_catabolic\_process | 1 | 0 |  |  |  |  |  |  |  |  |
| GO:0046549\_retinal\_cone\_cell\_development | 1 | 0 |  |  |  |  |  |  |  |  |
| GO:0046586\_regulation\_of\_calcium-dependent\_cell-cell\_adhesion | 1 | 0 |  |  |  |  |  |  |  |  |
| GO:0046588\_negative\_regulation\_of\_calcium-dependent\_cell-cell\_adhesion | 1 | 0 |  |  |  |  |  |  |  |  |
| GO:0046597\_negative\_regulation\_of\_virion\_penetration\_into\_host\_cell | 1 | 0 |  |  |  |  |  |  |  |  |
| GO:0046600\_negative\_regulation\_of\_centriole\_replication | 1 | 0 |  |  |  |  |  |  |  |  |
| GO:0046606\_negative\_regulation\_of\_centrosome\_cycle | 1 | 0 |  |  |  |  |  |  |  |  |
| GO:0046620\_regulation\_of\_organ\_growth | 1 | 0 |  |  |  |  |  |  |  |  |
| GO:0046636\_negative\_regulation\_of\_alpha-beta\_T\_cell\_activation | 1 | 0 |  |  |  |  |  |  |  |  |
| GO:0046639\_negative\_regulation\_of\_alpha-beta\_T\_cell\_differentiation | 1 | 0 |  |  |  |  |  |  |  |  |
| GO:0046640\_regulation\_of\_alpha-beta\_T\_cell\_proliferation | 1 | 0 |  |  |  |  |  |  |  |  |
| GO:0046641\_positive\_regulation\_of\_alpha-beta\_T\_cell\_proliferation | 1 | 0 |  |  |  |  |  |  |  |  |
| GO:0046655\_folic\_acid\_metabolic\_process | 1 | 0 |  |  |  |  |  |  |  |  |
| GO:0046666\_retinal\_cell\_programmed\_cell\_death | 1 | 0 |  |  |  |  |  |  |  |  |
| GO:0046668\_regulation\_of\_retinal\_cell\_programmed\_cell\_death | 1 | 0 |  |  |  |  |  |  |  |  |
| GO:0046670\_positive\_regulation\_of\_retinal\_cell\_programmed\_cell\_death | 1 | 0 |  |  |  |  |  |  |  |  |
| GO:0046674\_induction\_of\_retinal\_programmed\_cell\_death | 1 | 0 |  |  |  |  |  |  |  |  |
| GO:0046685\_response\_to\_arsenic | 1 | 0 |  |  |  |  |  |  |  |  |
| GO:0046686\_response\_to\_cadmium\_ion | 1 | 0 |  |  |  |  |  |  |  |  |
| GO:0046689\_response\_to\_mercury\_ion | 1 | 0 |  |  |  |  |  |  |  |  |
| GO:0046692\_sperm\_competition | 1 | 0 |  |  |  |  |  |  |  |  |
| GO:0046713\_boron\_transport | 1 | 0 |  |  |  |  |  |  |  |  |
| GO:0046719\_regulation\_of\_viral\_protein\_levels\_in\_host\_cell | 1 | 0 |  |  |  |  |  |  |  |  |
| GO:0046814\_virion\_attachment\_\_binding\_of\_host\_cell\_surface\_coreceptor | 1 | 0 |  |  |  |  |  |  |  |  |
| GO:0046826\_negative\_regulation\_of\_protein\_export\_from\_nucleus | 1 | 0 |  |  |  |  |  |  |  |  |
| GO:0046827\_positive\_regulation\_of\_protein\_export\_from\_nucleus | 1 | 0 |  |  |  |  |  |  |  |  |
| GO:0046833\_positive\_regulation\_of\_RNA\_export\_from\_nucleus | 1 | 0 |  |  |  |  |  |  |  |  |
| GO:0046835\_carbohydrate\_phosphorylation | 1 | 0 |  |  |  |  |  |  |  |  |
| GO:0046838\_phosphorylated\_carbohydrate\_dephosphorylation | 1 | 0 |  |  |  |  |  |  |  |  |
| GO:0046853\_inositol\_and\_derivative\_phosphorylation | 1 | 0 |  |  |  |  |  |  |  |  |
| GO:0046855\_inositol\_phosphate\_dephosphorylation | 1 | 0 |  |  |  |  |  |  |  |  |
| GO:0046856\_phosphoinositide\_dephosphorylation | 1 | 0 |  |  |  |  |  |  |  |  |
| GO:0046898\_response\_to\_cycloheximide | 1 | 0 |  |  |  |  |  |  |  |  |
| GO:0046916\_cellular\_transition\_metal\_ion\_homeostasis | 1 | 0 |  |  |  |  |  |  |  |  |
| GO:0046931\_pore\_complex\_biogenesis | 1 | 0 |  |  |  |  |  |  |  |  |
| GO:0046939\_nucleotide\_phosphorylation | 1 | 0 |  |  |  |  |  |  |  |  |
| GO:0046946\_hydroxylysine\_metabolic\_process | 1 | 0 |  |  |  |  |  |  |  |  |
| GO:0046947\_hydroxylysine\_biosynthetic\_process | 1 | 0 |  |  |  |  |  |  |  |  |
| GO:0046963\_3'-phosphoadenosine\_5'-phosphosulfate\_transport | 1 | 0 |  |  |  |  |  |  |  |  |
| GO:0046984\_regulation\_of\_hemoglobin\_biosynthetic\_process | 1 | 0 |  |  |  |  |  |  |  |  |
| GO:0046986\_negative\_regulation\_of\_hemoglobin\_biosynthetic\_process | 1 | 0 |  |  |  |  |  |  |  |  |
| GO:0048013\_ephrin\_receptor\_signaling\_pathway | 1 | 0 |  |  |  |  |  |  |  |  |
| GO:0048070\_regulation\_of\_pigmentation\_during\_development | 1 | 0 |  |  |  |  |  |  |  |  |
| GO:0048073\_regulation\_of\_eye\_pigmentation | 1 | 0 |  |  |  |  |  |  |  |  |
| GO:0048075\_positive\_regulation\_of\_eye\_pigmentation | 1 | 0 |  |  |  |  |  |  |  |  |
| GO:0048087\_positive\_regulation\_of\_pigmentation\_during\_development | 1 | 0 |  |  |  |  |  |  |  |  |
| GO:0048160\_primary\_follicle\_stage\_\_oogenesis | 1 | 0 |  |  |  |  |  |  |  |  |
| GO:0048170\_positive\_regulation\_of\_long-term\_neuronal\_synaptic\_plasticity | 1 | 0 |  |  |  |  |  |  |  |  |
| GO:0048172\_regulation\_of\_short-term\_neuronal\_synaptic\_plasticity | 1 | 0 |  |  |  |  |  |  |  |  |
| GO:0048175\_hepatocyte\_growth\_factor\_biosynthetic\_process | 1 | 0 |  |  |  |  |  |  |  |  |
| GO:0048176\_regulation\_of\_hepatocyte\_growth\_factor\_biosynthetic\_process | 1 | 0 |  |  |  |  |  |  |  |  |
| GO:0048178\_negative\_regulation\_of\_hepatocyte\_growth\_factor\_biosynthetic\_process | 1 | 0 |  |  |  |  |  |  |  |  |
| GO:0048203\_vesicle\_targeting\_\_trans-Golgi\_to\_endosome | 1 | 0 |  |  |  |  |  |  |  |  |
| GO:0048210\_Golgi\_vesicle\_fusion\_to\_target\_membrane | 1 | 0 |  |  |  |  |  |  |  |  |
| GO:0048241\_epinephrine\_transport | 1 | 0 |  |  |  |  |  |  |  |  |
| GO:0048242\_epinephrine\_secretion | 1 | 0 |  |  |  |  |  |  |  |  |
| GO:0048245\_eosinophil\_chemotaxis | 1 | 0 |  |  |  |  |  |  |  |  |
| GO:0048265\_response\_to\_pain | 1 | 0 |  |  |  |  |  |  |  |  |
| GO:0048289\_isotype\_switching\_to\_IgE\_isotypes | 1 | 0 |  |  |  |  |  |  |  |  |
| GO:0048293\_regulation\_of\_isotype\_switching\_to\_IgE\_isotypes | 1 | 0 |  |  |  |  |  |  |  |  |
| GO:0048295\_positive\_regulation\_of\_isotype\_switching\_to\_IgE\_isotypes | 1 | 0 |  |  |  |  |  |  |  |  |
| GO:0048302\_regulation\_of\_isotype\_switching\_to\_IgG\_isotypes | 1 | 0 |  |  |  |  |  |  |  |  |
| GO:0048304\_positive\_regulation\_of\_isotype\_switching\_to\_IgG\_isotypes | 1 | 0 |  |  |  |  |  |  |  |  |
| GO:0048311\_mitochondrion\_distribution | 1 | 0 |  |  |  |  |  |  |  |  |
| GO:0048339\_paraxial\_mesoderm\_development | 1 | 0 |  |  |  |  |  |  |  |  |
| GO:0048340\_paraxial\_mesoderm\_morphogenesis | 1 | 0 |  |  |  |  |  |  |  |  |
| GO:0048388\_endosomal\_lumen\_acidification | 1 | 0 |  |  |  |  |  |  |  |  |
| GO:0048478\_replication\_fork\_protection | 1 | 0 |  |  |  |  |  |  |  |  |
| GO:0048483\_autonomic\_nervous\_system\_development | 1 | 0 |  |  |  |  |  |  |  |  |
| GO:0048485\_sympathetic\_nervous\_system\_development | 1 | 0 |  |  |  |  |  |  |  |  |
| GO:0048499\_synaptic\_vesicle\_membrane\_organization | 1 | 0 |  |  |  |  |  |  |  |  |
| GO:0048535\_lymph\_node\_development | 1 | 0 |  |  |  |  |  |  |  |  |
| GO:0048539\_bone\_marrow\_development | 1 | 0 |  |  |  |  |  |  |  |  |
| GO:0048549\_positive\_regulation\_of\_pinocytosis | 1 | 0 |  |  |  |  |  |  |  |  |
| GO:0048553\_negative\_regulation\_of\_metalloenzyme\_activity | 1 | 0 |  |  |  |  |  |  |  |  |
| GO:0048566\_embryonic\_gut\_development | 1 | 0 |  |  |  |  |  |  |  |  |
| GO:0048596\_embryonic\_camera-type\_eye\_morphogenesis | 1 | 0 |  |  |  |  |  |  |  |  |
| GO:0048617\_embryonic\_foregut\_morphogenesis | 1 | 0 |  |  |  |  |  |  |  |  |
| GO:0048619\_embryonic\_hindgut\_morphogenesis | 1 | 0 |  |  |  |  |  |  |  |  |
| GO:0048636\_positive\_regulation\_of\_muscle\_development | 1 | 0 |  |  |  |  |  |  |  |  |
| GO:0048639\_positive\_regulation\_of\_developmental\_growth | 1 | 0 |  |  |  |  |  |  |  |  |
| GO:0048640\_negative\_regulation\_of\_developmental\_growth | 1 | 0 |  |  |  |  |  |  |  |  |
| GO:0048665\_neuron\_fate\_specification | 1 | 0 |  |  |  |  |  |  |  |  |
| GO:0048679\_regulation\_of\_axon\_regeneration | 1 | 0 |  |  |  |  |  |  |  |  |
| GO:0048681\_negative\_regulation\_of\_axon\_regeneration | 1 | 0 |  |  |  |  |  |  |  |  |
| GO:0048703\_embryonic\_viscerocranium\_morphogenesis | 1 | 0 |  |  |  |  |  |  |  |  |
| GO:0048745\_smooth\_muscle\_tissue\_development | 1 | 0 |  |  |  |  |  |  |  |  |
| GO:0048755\_branching\_morphogenesis\_of\_a\_nerve | 1 | 0 |  |  |  |  |  |  |  |  |
| GO:0048793\_pronephros\_development | 1 | 0 |  |  |  |  |  |  |  |  |
| GO:0048807\_female\_genitalia\_morphogenesis | 1 | 0 |  |  |  |  |  |  |  |  |
| GO:0048818\_positive\_regulation\_of\_hair\_follicle\_maturation | 1 | 0 |  |  |  |  |  |  |  |  |
| GO:0048819\_regulation\_of\_hair\_follicle\_maturation | 1 | 0 |  |  |  |  |  |  |  |  |
| GO:0048821\_erythrocyte\_development | 1 | 0 |  |  |  |  |  |  |  |  |
| GO:0048845\_venous\_blood\_vessel\_morphogenesis | 1 | 0 |  |  |  |  |  |  |  |  |
| GO:0048853\_forebrain\_morphogenesis | 1 | 0 |  |  |  |  |  |  |  |  |
| GO:0048865\_stem\_cell\_fate\_commitment | 1 | 0 |  |  |  |  |  |  |  |  |
| GO:0048867\_stem\_cell\_fate\_determination | 1 | 0 |  |  |  |  |  |  |  |  |
| GO:0048874\_homeostasis\_of\_number\_of\_cells\_in\_a\_free-living\_population | 1 | 0 |  |  |  |  |  |  |  |  |
| GO:0048875\_chemical\_homeostasis\_within\_a\_tissue | 1 | 0 |  |  |  |  |  |  |  |  |
| GO:0050427\_3'-phosphoadenosine\_5'-phosphosulfate\_metabolic\_process | 1 | 0 |  |  |  |  |  |  |  |  |
| GO:0050652\_dermatan\_sulfate\_proteoglycan\_biosynthetic\_process\_\_polysaccharide\_chain\_biosynthetic\_process | 1 | 0 |  |  |  |  |  |  |  |  |
| GO:0050666\_regulation\_of\_homocysteine\_metabolic\_process | 1 | 0 |  |  |  |  |  |  |  |  |
| GO:0050674\_urothelial\_cell\_proliferation | 1 | 0 |  |  |  |  |  |  |  |  |
| GO:0050675\_regulation\_of\_urothelial\_cell\_proliferation | 1 | 0 |  |  |  |  |  |  |  |  |
| GO:0050677\_positive\_regulation\_of\_urothelial\_cell\_proliferation | 1 | 0 |  |  |  |  |  |  |  |  |
| GO:0050685\_positive\_regulation\_of\_mRNA\_processing | 1 | 0 |  |  |  |  |  |  |  |  |
| GO:0050687\_negative\_regulation\_of\_defense\_response\_to\_virus | 1 | 0 |  |  |  |  |  |  |  |  |
| GO:0050689\_negative\_regulation\_of\_defense\_response\_to\_virus\_by\_host | 1 | 0 |  |  |  |  |  |  |  |  |
| GO:0050713\_negative\_regulation\_of\_interleukin-1\_beta\_secretion | 1 | 0 |  |  |  |  |  |  |  |  |
| GO:0050722\_regulation\_of\_interleukin-1\_beta\_biosynthetic\_process | 1 | 0 |  |  |  |  |  |  |  |  |
| GO:0050725\_positive\_regulation\_of\_interleukin-1\_beta\_biosynthetic\_process | 1 | 0 |  |  |  |  |  |  |  |  |
| GO:0050751\_fractalkine\_biosynthetic\_process | 1 | 0 |  |  |  |  |  |  |  |  |
| GO:0050752\_regulation\_of\_fractalkine\_biosynthetic\_process | 1 | 0 |  |  |  |  |  |  |  |  |
| GO:0050754\_positive\_regulation\_of\_fractalkine\_biosynthetic\_process | 1 | 0 |  |  |  |  |  |  |  |  |
| GO:0050756\_fractalkine\_metabolic\_process | 1 | 0 |  |  |  |  |  |  |  |  |
| GO:0050757\_thymidylate\_synthase\_biosynthetic\_process | 1 | 0 |  |  |  |  |  |  |  |  |
| GO:0050758\_regulation\_of\_thymidylate\_synthase\_biosynthetic\_process | 1 | 0 |  |  |  |  |  |  |  |  |
| GO:0050760\_negative\_regulation\_of\_thymidylate\_synthase\_biosynthetic\_process | 1 | 0 |  |  |  |  |  |  |  |  |
| GO:0050765\_negative\_regulation\_of\_phagocytosis | 1 | 0 |  |  |  |  |  |  |  |  |
| GO:0050774\_negative\_regulation\_of\_dendrite\_morphogenesis | 1 | 0 |  |  |  |  |  |  |  |  |
| GO:0050783\_cocaine\_metabolic\_process | 1 | 0 |  |  |  |  |  |  |  |  |
| GO:0050822\_peptide\_stabilization | 1 | 0 |  |  |  |  |  |  |  |  |
| GO:0050823\_peptide\_antigen\_stabilization | 1 | 0 |  |  |  |  |  |  |  |  |
| GO:0050832\_defense\_response\_to\_fungus | 1 | 0 |  |  |  |  |  |  |  |  |
| GO:0050855\_regulation\_of\_B\_cell\_receptor\_signaling\_pathway | 1 | 0 |  |  |  |  |  |  |  |  |
| GO:0050858\_negative\_regulation\_of\_antigen\_receptor-mediated\_signaling\_pathway | 1 | 0 |  |  |  |  |  |  |  |  |
| GO:0050860\_negative\_regulation\_of\_T\_cell\_receptor\_signaling\_pathway | 1 | 0 |  |  |  |  |  |  |  |  |
| GO:0050861\_positive\_regulation\_of\_B\_cell\_receptor\_signaling\_pathway | 1 | 0 |  |  |  |  |  |  |  |  |
| GO:0050883\_musculoskeletal\_movement\_\_spinal\_reflex\_action | 1 | 0 |  |  |  |  |  |  |  |  |
| GO:0050884\_neuromuscular\_process\_controlling\_posture | 1 | 0 |  |  |  |  |  |  |  |  |
| GO:0050893\_sensory\_processing | 1 | 0 |  |  |  |  |  |  |  |  |
| GO:0050902\_leukocyte\_adhesive\_activation | 1 | 0 |  |  |  |  |  |  |  |  |
| GO:0050910\_detection\_of\_mechanical\_stimulus\_involved\_in\_sensory\_perception\_of\_sound | 1 | 0 |  |  |  |  |  |  |  |  |
| GO:0050922\_negative\_regulation\_of\_chemotaxis | 1 | 0 |  |  |  |  |  |  |  |  |
| GO:0050923\_regulation\_of\_negative\_chemotaxis | 1 | 0 |  |  |  |  |  |  |  |  |
| GO:0050924\_positive\_regulation\_of\_negative\_chemotaxis | 1 | 0 |  |  |  |  |  |  |  |  |
| GO:0050929\_induction\_of\_negative\_chemotaxis | 1 | 0 |  |  |  |  |  |  |  |  |
| GO:0050951\_sensory\_perception\_of\_temperature\_stimulus | 1 | 0 |  |  |  |  |  |  |  |  |
| GO:0050955\_thermoception | 1 | 0 |  |  |  |  |  |  |  |  |
| GO:0050974\_detection\_of\_mechanical\_stimulus\_involved\_in\_sensory\_perception | 1 | 0 |  |  |  |  |  |  |  |  |
| GO:0050983\_spermidine\_catabolic\_process\_to\_deoxyhypusine\_\_using\_deoxyhypusine\_synthase | 1 | 0 |  |  |  |  |  |  |  |  |
| GO:0051013\_microtubule\_severing | 1 | 0 |  |  |  |  |  |  |  |  |
| GO:0051029\_rRNA\_transport | 1 | 0 |  |  |  |  |  |  |  |  |
| GO:0051030\_snRNA\_transport | 1 | 0 |  |  |  |  |  |  |  |  |
| GO:0051031\_tRNA\_transport | 1 | 0 |  |  |  |  |  |  |  |  |
| GO:0051036\_regulation\_of\_endosome\_size | 1 | 0 |  |  |  |  |  |  |  |  |
| GO:0051040\_regulation\_of\_calcium-independent\_cell-cell\_adhesion | 1 | 0 |  |  |  |  |  |  |  |  |
| GO:0051041\_positive\_regulation\_of\_calcium-independent\_cell-cell\_adhesion | 1 | 0 |  |  |  |  |  |  |  |  |
| GO:0051066\_dihydrobiopterin\_metabolic\_process | 1 | 0 |  |  |  |  |  |  |  |  |
| GO:0051085\_chaperone\_mediated\_protein\_folding\_requiring\_cofactor | 1 | 0 |  |  |  |  |  |  |  |  |
| GO:0051089\_constitutive\_protein\_ectodomain\_proteolysis | 1 | 0 |  |  |  |  |  |  |  |  |
| GO:0051102\_DNA\_ligation\_during\_DNA\_recombination | 1 | 0 |  |  |  |  |  |  |  |  |
| GO:0051105\_regulation\_of\_DNA\_ligation | 1 | 0 |  |  |  |  |  |  |  |  |
| GO:0051106\_positive\_regulation\_of\_DNA\_ligation | 1 | 0 |  |  |  |  |  |  |  |  |
| GO:0051125\_regulation\_of\_actin\_nucleation | 1 | 0 |  |  |  |  |  |  |  |  |
| GO:0051126\_negative\_regulation\_of\_actin\_nucleation | 1 | 0 |  |  |  |  |  |  |  |  |
| GO:0051136\_regulation\_of\_NK\_T\_cell\_differentiation | 1 | 0 |  |  |  |  |  |  |  |  |
| GO:0051138\_positive\_regulation\_of\_NK\_T\_cell\_differentiation | 1 | 0 |  |  |  |  |  |  |  |  |
| GO:0051155\_positive\_regulation\_of\_striated\_muscle\_cell\_differentiation | 1 | 0 |  |  |  |  |  |  |  |  |
| GO:0051156\_glucose\_6-phosphate\_metabolic\_process | 1 | 0 |  |  |  |  |  |  |  |  |
| GO:0051160\_L-xylitol\_catabolic\_process | 1 | 0 |  |  |  |  |  |  |  |  |
| GO:0051164\_L-xylitol\_metabolic\_process | 1 | 0 |  |  |  |  |  |  |  |  |
| GO:0051193\_regulation\_of\_cofactor\_metabolic\_process | 1 | 0 |  |  |  |  |  |  |  |  |
| GO:0051196\_regulation\_of\_coenzyme\_metabolic\_process | 1 | 0 |  |  |  |  |  |  |  |  |
| GO:0051204\_protein\_insertion\_into\_mitochondrial\_membrane | 1 | 0 |  |  |  |  |  |  |  |  |
| GO:0051290\_protein\_heterotetramerization | 1 | 0 |  |  |  |  |  |  |  |  |
| GO:0051292\_nuclear\_pore\_complex\_assembly | 1 | 0 |  |  |  |  |  |  |  |  |
| GO:0051294\_establishment\_of\_spindle\_orientation | 1 | 0 |  |  |  |  |  |  |  |  |
| GO:0051295\_establishment\_of\_meiotic\_spindle\_localization | 1 | 0 |  |  |  |  |  |  |  |  |
| GO:0051315\_attachment\_of\_spindle\_microtubules\_to\_kinetochore\_during\_mitosis | 1 | 0 |  |  |  |  |  |  |  |  |
| GO:0051326\_telophase | 1 | 0 |  |  |  |  |  |  |  |  |
| GO:0051342\_regulation\_of\_cyclic-nucleotide\_phosphodiesterase\_activity | 1 | 0 |  |  |  |  |  |  |  |  |
| GO:0051344\_negative\_regulation\_of\_cyclic-nucleotide\_phosphodiesterase\_activity | 1 | 0 |  |  |  |  |  |  |  |  |
| GO:0051445\_regulation\_of\_meiotic\_cell\_cycle | 1 | 0 |  |  |  |  |  |  |  |  |
| GO:0051450\_myoblast\_proliferation | 1 | 0 |  |  |  |  |  |  |  |  |
| GO:0051454\_intracellular\_pH\_elevation | 1 | 0 |  |  |  |  |  |  |  |  |
| GO:0051458\_adrenocorticotropin\_secretion | 1 | 0 |  |  |  |  |  |  |  |  |
| GO:0051459\_regulation\_of\_adrenocorticotropin\_secretion | 1 | 0 |  |  |  |  |  |  |  |  |
| GO:0051461\_positive\_regulation\_of\_adrenocorticotropin\_secretion | 1 | 0 |  |  |  |  |  |  |  |  |
| GO:0051531\_NFAT\_protein\_import\_into\_nucleus | 1 | 0 |  |  |  |  |  |  |  |  |
| GO:0051532\_regulation\_of\_NFAT\_protein\_import\_into\_nucleus | 1 | 0 |  |  |  |  |  |  |  |  |
| GO:0051533\_positive\_regulation\_of\_NFAT\_protein\_import\_into\_nucleus | 1 | 0 |  |  |  |  |  |  |  |  |
| GO:0051542\_elastin\_biosynthetic\_process | 1 | 0 |  |  |  |  |  |  |  |  |
| GO:0051560\_mitochondrial\_calcium\_ion\_homeostasis | 1 | 0 |  |  |  |  |  |  |  |  |
| GO:0051561\_elevation\_of\_mitochondrial\_calcium\_ion\_concentration | 1 | 0 |  |  |  |  |  |  |  |  |
| GO:0051582\_positive\_regulation\_of\_neurotransmitter\_uptake | 1 | 0 |  |  |  |  |  |  |  |  |
| GO:0051586\_positive\_regulation\_of\_dopamine\_uptake | 1 | 0 |  |  |  |  |  |  |  |  |
| GO:0051590\_positive\_regulation\_of\_neurotransmitter\_transport | 1 | 0 |  |  |  |  |  |  |  |  |
| GO:0051594\_detection\_of\_glucose | 1 | 0 |  |  |  |  |  |  |  |  |
| GO:0051642\_centrosome\_localization | 1 | 0 |  |  |  |  |  |  |  |  |
| GO:0051645\_Golgi\_localization | 1 | 0 |  |  |  |  |  |  |  |  |
| GO:0051647\_nucleus\_localization | 1 | 0 |  |  |  |  |  |  |  |  |
| GO:0051664\_nuclear\_pore\_localization | 1 | 0 |  |  |  |  |  |  |  |  |
| GO:0051708\_intracellular\_protein\_transport\_in\_other\_organism\_during\_symbiotic\_interaction | 1 | 0 |  |  |  |  |  |  |  |  |
| GO:0051764\_actin\_crosslink\_formation | 1 | 0 |  |  |  |  |  |  |  |  |
| GO:0051767\_nitric-oxide\_synthase\_biosynthetic\_process | 1 | 0 |  |  |  |  |  |  |  |  |
| GO:0051768\_nitric-oxide\_synthase\_2\_biosynthetic\_process | 1 | 0 |  |  |  |  |  |  |  |  |
| GO:0051769\_regulation\_of\_nitric-oxide\_synthase\_biosynthetic\_process | 1 | 0 |  |  |  |  |  |  |  |  |
| GO:0051771\_negative\_regulation\_of\_nitric-oxide\_synthase\_biosynthetic\_process | 1 | 0 |  |  |  |  |  |  |  |  |
| GO:0051772\_regulation\_of\_nitric-oxide\_synthase\_2\_biosynthetic\_process | 1 | 0 |  |  |  |  |  |  |  |  |
| GO:0051773\_positive\_regulation\_of\_nitric-oxide\_synthase\_2\_biosynthetic\_process | 1 | 0 |  |  |  |  |  |  |  |  |
| GO:0051781\_positive\_regulation\_of\_cell\_division | 1 | 0 |  |  |  |  |  |  |  |  |
| GO:0051782\_negative\_regulation\_of\_cell\_division | 1 | 0 |  |  |  |  |  |  |  |  |
| GO:0051788\_response\_to\_misfolded\_protein | 1 | 0 |  |  |  |  |  |  |  |  |
| GO:0051790\_short-chain\_fatty\_acid\_biosynthetic\_process | 1 | 0 |  |  |  |  |  |  |  |  |
| GO:0051791\_medium-chain\_fatty\_acid\_metabolic\_process | 1 | 0 |  |  |  |  |  |  |  |  |
| GO:0051792\_medium-chain\_fatty\_acid\_biosynthetic\_process | 1 | 0 |  |  |  |  |  |  |  |  |
| GO:0051794\_regulation\_of\_catagen | 1 | 0 |  |  |  |  |  |  |  |  |
| GO:0051795\_positive\_regulation\_of\_catagen | 1 | 0 |  |  |  |  |  |  |  |  |
| GO:0051821\_dissemination\_or\_transmission\_of\_organism\_from\_other\_organism\_during\_symbiotic\_interaction | 1 | 0 |  |  |  |  |  |  |  |  |
| GO:0051894\_positive\_regulation\_of\_focal\_adhesion\_formation | 1 | 0 |  |  |  |  |  |  |  |  |
| GO:0051930\_regulation\_of\_sensory\_perception\_of\_pain | 1 | 0 |  |  |  |  |  |  |  |  |
| GO:0051931\_regulation\_of\_sensory\_perception | 1 | 0 |  |  |  |  |  |  |  |  |
| GO:0051944\_positive\_regulation\_of\_catecholamine\_uptake\_during\_transmission\_of\_nerve\_impulse | 1 | 0 |  |  |  |  |  |  |  |  |
| GO:0051962\_positive\_regulation\_of\_nervous\_system\_development | 1 | 0 |  |  |  |  |  |  |  |  |
| GO:0051965\_positive\_regulation\_of\_synaptogenesis | 1 | 0 |  |  |  |  |  |  |  |  |
| GO:0051977\_lysophospholipid\_transport | 1 | 0 |  |  |  |  |  |  |  |  |
| GO:0051988\_regulation\_of\_attachment\_of\_spindle\_microtubules\_to\_kinetochore | 1 | 0 |  |  |  |  |  |  |  |  |
| GO:0052097\_interspecies\_quorum\_sensing | 1 | 0 |  |  |  |  |  |  |  |  |
| GO:0052106\_quorum\_sensing\_during\_interaction\_with\_host | 1 | 0 |  |  |  |  |  |  |  |  |
| GO:0052312\_modulation\_of\_transcription\_in\_other\_organism\_during\_symbiotic\_interaction | 1 | 0 |  |  |  |  |  |  |  |  |
| GO:0052472\_modulation\_by\_host\_of\_symbiont\_transcription | 1 | 0 |  |  |  |  |  |  |  |  |
| GO:0055009\_atrial\_cardiac\_muscle\_morphogenesis | 1 | 0 |  |  |  |  |  |  |  |  |
| GO:0055012\_ventricular\_cardiac\_muscle\_cell\_differentiation | 1 | 0 |  |  |  |  |  |  |  |  |
| GO:0055071\_manganese\_ion\_homeostasis | 1 | 0 |  |  |  |  |  |  |  |  |
| GO:0055073\_cadmium\_ion\_homeostasis | 1 | 0 |  |  |  |  |  |  |  |  |
| GO:0055076\_transition\_metal\_ion\_homeostasis | 1 | 0 |  |  |  |  |  |  |  |  |
| GO:0055089\_fatty\_acid\_homeostasis | 1 | 0 |  |  |  |  |  |  |  |  |
| GO:0055095\_lipoprotein\_mediated\_signaling | 1 | 0 |  |  |  |  |  |  |  |  |
| GO:0055096\_low\_density\_lipoprotein\_mediated\_signaling | 1 | 0 |  |  |  |  |  |  |  |  |
| GO:0055099\_response\_to\_high\_density\_lipoprotein\_stimulus | 1 | 0 |  |  |  |  |  |  |  |  |
| GO:0055118\_negative\_regulation\_of\_cardiac\_muscle\_contraction | 1 | 0 |  |  |  |  |  |  |  |  |
| GO:0055119\_relaxation\_of\_cardiac\_muscle | 1 | 0 |  |  |  |  |  |  |  |  |
| GO:0060003\_copper\_ion\_export | 1 | 0 |  |  |  |  |  |  |  |  |
| GO:0060022\_hard\_palate\_development | 1 | 0 |  |  |  |  |  |  |  |  |
| GO:0060039\_pericardium\_development | 1 | 0 |  |  |  |  |  |  |  |  |
| GO:0060055\_angiogenesis\_involved\_in\_wound\_healing | 1 | 0 |  |  |  |  |  |  |  |  |
| GO:0060059\_embryonic\_retina\_morphogenesis\_in\_camera-type\_eye | 1 | 0 |  |  |  |  |  |  |  |  |
| GO:0060065\_uterus\_development | 1 | 0 |  |  |  |  |  |  |  |  |
| GO:0060068\_vagina\_development | 1 | 0 |  |  |  |  |  |  |  |  |
| GO:0060082\_eye\_blink\_reflex | 1 | 0 |  |  |  |  |  |  |  |  |
| GO:0060083\_smooth\_muscle\_contraction\_involved\_in\_micturition | 1 | 0 |  |  |  |  |  |  |  |  |
| GO:0060088\_auditory\_receptor\_cell\_stereocilium\_organization | 1 | 0 |  |  |  |  |  |  |  |  |
| GO:0060120\_inner\_ear\_receptor\_cell\_fate\_commitment | 1 | 0 |  |  |  |  |  |  |  |  |
| GO:0060135\_maternal\_process\_involved\_in\_female\_pregnancy | 1 | 0 |  |  |  |  |  |  |  |  |
| GO:0060142\_regulation\_of\_syncytium\_formation\_by\_plasma\_membrane\_fusion | 1 | 0 |  |  |  |  |  |  |  |  |
| GO:0060143\_positive\_regulation\_of\_syncytium\_formation\_by\_plasma\_membrane\_fusion | 1 | 0 |  |  |  |  |  |  |  |  |
| GO:0060157\_urinary\_bladder\_development | 1 | 0 |  |  |  |  |  |  |  |  |
| GO:0060160\_negative\_regulation\_of\_dopamine\_receptor\_signaling\_pathway | 1 | 0 |  |  |  |  |  |  |  |  |
| GO:0060161\_positive\_regulation\_of\_dopamine\_receptor\_signaling\_pathway | 1 | 0 |  |  |  |  |  |  |  |  |
| GO:0060167\_regulation\_of\_adenosine\_receptor\_signaling\_pathway | 1 | 0 |  |  |  |  |  |  |  |  |
| GO:0060169\_negative\_regulation\_of\_adenosine\_receptor\_signaling\_pathway | 1 | 0 |  |  |  |  |  |  |  |  |
| GO:0060216\_definitive\_hemopoiesis | 1 | 0 |  |  |  |  |  |  |  |  |
| GO:0060219\_camera-type\_eye\_photoreceptor\_cell\_differentiation | 1 | 0 |  |  |  |  |  |  |  |  |
| GO:0060231\_mesenchymal\_to\_epithelial\_transition | 1 | 0 |  |  |  |  |  |  |  |  |
| GO:0060254\_regulation\_of\_N-terminal\_protein\_palmitoylation | 1 | 0 |  |  |  |  |  |  |  |  |
| GO:0060259\_regulation\_of\_feeding\_behavior | 1 | 0 |  |  |  |  |  |  |  |  |
| GO:0060262\_negative\_regulation\_of\_N-terminal\_protein\_palmitoylation | 1 | 0 |  |  |  |  |  |  |  |  |
| GO:0060265\_positive\_regulation\_of\_respiratory\_burst\_during\_acute\_inflammatory\_response | 1 | 0 |  |  |  |  |  |  |  |  |
| GO:0060266\_negative\_regulation\_of\_respiratory\_burst\_during\_acute\_inflammatory\_response | 1 | 0 |  |  |  |  |  |  |  |  |
| GO:0060268\_negative\_regulation\_of\_respiratory\_burst | 1 | 0 |  |  |  |  |  |  |  |  |
| GO:0060286\_flagellar\_cell\_motility | 1 | 0 |  |  |  |  |  |  |  |  |
| GO:0060298\_positive\_regulation\_of\_sarcomere\_organization | 1 | 0 |  |  |  |  |  |  |  |  |
| GO:0060299\_negative\_regulation\_of\_sarcomere\_organization | 1 | 0 |  |  |  |  |  |  |  |  |
| GO:0060300\_regulation\_of\_cytokine\_activity | 1 | 0 |  |  |  |  |  |  |  |  |
| GO:0060302\_negative\_regulation\_of\_cytokine\_activity | 1 | 0 |  |  |  |  |  |  |  |  |
| GO:0060305\_regulation\_of\_cell\_diameter | 1 | 0 |  |  |  |  |  |  |  |  |
| GO:0060306\_regulation\_of\_membrane\_repolarization | 1 | 0 |  |  |  |  |  |  |  |  |
| GO:0060307\_regulation\_of\_ventricular\_cardiomyocyte\_membrane\_repolarization | 1 | 0 |  |  |  |  |  |  |  |  |
| GO:0060309\_elastin\_catabolic\_process | 1 | 0 |  |  |  |  |  |  |  |  |
| GO:0060310\_regulation\_of\_elastin\_catabolic\_process | 1 | 0 |  |  |  |  |  |  |  |  |
| GO:0060311\_negative\_regulation\_of\_elastin\_catabolic\_process | 1 | 0 |  |  |  |  |  |  |  |  |
| GO:0060312\_regulation\_of\_blood\_vessel\_remodeling | 1 | 0 |  |  |  |  |  |  |  |  |
| GO:0060313\_negative\_regulation\_of\_blood\_vessel\_remodeling | 1 | 0 |  |  |  |  |  |  |  |  |
| GO:0060315\_negative\_regulation\_of\_ryanodine-sensitive\_calcium-release\_channel\_activity | 1 | 0 |  |  |  |  |  |  |  |  |
| GO:0060316\_positive\_regulation\_of\_ryanodine-sensitive\_calcium-release\_channel\_activity | 1 | 0 |  |  |  |  |  |  |  |  |
| GO:0060318\_definitive\_erythrocyte\_differentiation | 1 | 0 |  |  |  |  |  |  |  |  |
| GO:0060322\_head\_development | 1 | 0 |  |  |  |  |  |  |  |  |
| GO:0060324\_face\_development | 1 | 0 |  |  |  |  |  |  |  |  |
| GO:0060336\_negative\_regulation\_of\_interferon-gamma-mediated\_signaling\_pathway | 1 | 0 |  |  |  |  |  |  |  |  |
| GO:0060349\_bone\_morphogenesis | 1 | 0 |  |  |  |  |  |  |  |  |
| GO:0060350\_endochondral\_bone\_morphogenesis | 1 | 0 |  |  |  |  |  |  |  |  |
| GO:0060356\_leucine\_import | 1 | 0 |  |  |  |  |  |  |  |  |
| GO:0060368\_regulation\_of\_Fc\_receptor\_mediated\_stimulatory\_signaling\_pathway | 1 | 0 |  |  |  |  |  |  |  |  |
| GO:0060369\_positive\_regulation\_of\_Fc\_receptor\_mediated\_stimulatory\_signaling\_pathway | 1 | 0 |  |  |  |  |  |  |  |  |
| GO:0060380\_regulation\_of\_single-stranded\_telomeric\_DNA\_binding | 1 | 0 |  |  |  |  |  |  |  |  |
| GO:0060381\_positive\_regulation\_of\_single-stranded\_telomeric\_DNA\_binding | 1 | 0 |  |  |  |  |  |  |  |  |
| GO:0060382\_regulation\_of\_DNA\_strand\_elongation | 1 | 0 |  |  |  |  |  |  |  |  |
| GO:0060383\_positive\_regulation\_of\_DNA\_strand\_elongation | 1 | 0 |  |  |  |  |  |  |  |  |
| GO:0060397\_JAK-STAT\_cascade\_involved\_in\_growth\_hormone\_signaling\_pathway | 1 | 0 |  |  |  |  |  |  |  |  |
| GO:0060398\_regulation\_of\_growth\_hormone\_receptor\_signaling\_pathway | 1 | 0 |  |  |  |  |  |  |  |  |
| GO:0060425\_lung\_morphogenesis | 1 | 0 |  |  |  |  |  |  |  |  |
| GO:0060433\_bronchus\_development | 1 | 0 |  |  |  |  |  |  |  |  |
| GO:0060438\_trachea\_development | 1 | 0 |  |  |  |  |  |  |  |  |
| GO:0060441\_branching\_involved\_in\_lung\_morphogenesis | 1 | 0 |  |  |  |  |  |  |  |  |
| GO:0060445\_branching\_involved\_in\_salivary\_gland\_morphogenesis | 1 | 0 |  |  |  |  |  |  |  |  |
| GO:0060502\_epithelial\_cell\_proliferation\_involved\_in\_lung\_morphogenesis | 1 | 0 |  |  |  |  |  |  |  |  |
| GO:0060503\_bud\_dilation\_involved\_in\_lung\_branching | 1 | 0 |  |  |  |  |  |  |  |  |
| GO:0060560\_developmental\_growth\_involved\_in\_morphogenesis | 1 | 0 |  |  |  |  |  |  |  |  |
| GO:0060579\_ventral\_spinal\_cord\_interneuron\_fate\_commitment | 1 | 0 |  |  |  |  |  |  |  |  |
| GO:0060586\_multicellular\_organismal\_iron\_ion\_homeostasis | 1 | 0 |  |  |  |  |  |  |  |  |
| GO:0060587\_regulation\_of\_lipoprotein\_lipid\_oxidation | 1 | 0 |  |  |  |  |  |  |  |  |
| GO:0060588\_negative\_regulation\_of\_lipoprotein\_lipid\_oxidation | 1 | 0 |  |  |  |  |  |  |  |  |
| GO:0060638\_mesenchymal-epithelial\_cell\_signaling | 1 | 0 |  |  |  |  |  |  |  |  |
| GO:0060665\_regulation\_of\_branching\_involved\_in\_salivary\_gland\_morphogenesis\_by\_mesenchymal-epithelial\_signaling | 1 | 0 |  |  |  |  |  |  |  |  |
| GO:0060675\_ureteric\_bud\_morphogenesis | 1 | 0 |  |  |  |  |  |  |  |  |
| GO:0060688\_regulation\_of\_morphogenesis\_of\_a\_branching\_structure | 1 | 0 |  |  |  |  |  |  |  |  |
| GO:0060693\_regulation\_of\_branching\_involved\_in\_salivary\_gland\_morphogenesis | 1 | 0 |  |  |  |  |  |  |  |  |
| GO:0060694\_regulation\_of\_cholesterol\_transporter\_activity | 1 | 0 |  |  |  |  |  |  |  |  |
| GO:0060695\_negative\_regulation\_of\_cholesterol\_transporter\_activity | 1 | 0 |  |  |  |  |  |  |  |  |
| GO:0060697\_positive\_regulation\_of\_phospholipid\_catabolic\_process | 1 | 0 |  |  |  |  |  |  |  |  |
| GO:0060729\_intestinal\_epithelial\_structure\_maintenance | 1 | 0 |  |  |  |  |  |  |  |  |
| GO:0060730\_regulation\_of\_intestinal\_epithelial\_structure\_maintenance | 1 | 0 |  |  |  |  |  |  |  |  |
| GO:0060731\_positive\_regulation\_of\_intestinal\_epithelial\_structure\_maintenance | 1 | 0 |  |  |  |  |  |  |  |  |
| GO:0060760\_positive\_regulation\_of\_response\_to\_cytokine\_stimulus | 1 | 0 |  |  |  |  |  |  |  |  |
| GO:0060761\_negative\_regulation\_of\_response\_to\_cytokine\_stimulus | 1 | 0 |  |  |  |  |  |  |  |  |
| GO:0060788\_ectodermal\_placode\_formation | 1 | 0 |  |  |  |  |  |  |  |  |
| GO:0060841\_venous\_blood\_vessel\_development | 1 | 0 |  |  |  |  |  |  |  |  |
| GO:0060856\_establishment\_of\_blood-brain\_barrier | 1 | 0 |  |  |  |  |  |  |  |  |
| GO:0060896\_neural\_plate\_pattern\_specification | 1 | 0 |  |  |  |  |  |  |  |  |
| GO:0065001\_specification\_of\_axis\_polarity | 1 | 0 |  |  |  |  |  |  |  |  |
| GO:0070075\_tear\_secretion | 1 | 0 |  |  |  |  |  |  |  |  |
| GO:0070076\_histone\_lysine\_demethylation | 1 | 0 |  |  |  |  |  |  |  |  |
| GO:0070077\_histone\_arginine\_demethylation | 1 | 0 |  |  |  |  |  |  |  |  |
| GO:0070078\_histone\_H3-R2\_demethylation | 1 | 0 |  |  |  |  |  |  |  |  |
| GO:0070079\_histone\_H4-R3\_demethylation | 1 | 0 |  |  |  |  |  |  |  |  |
| GO:0070086\_ubiquitin-dependent\_endocytosis | 1 | 0 |  |  |  |  |  |  |  |  |
| GO:0070091\_glucagon\_secretion | 1 | 0 |  |  |  |  |  |  |  |  |
| GO:0070103\_regulation\_of\_interleukin-6-mediated\_signaling\_pathway | 1 | 0 |  |  |  |  |  |  |  |  |
| GO:0070104\_negative\_regulation\_of\_interleukin-6-mediated\_signaling\_pathway | 1 | 0 |  |  |  |  |  |  |  |  |
| GO:0070106\_interleukin-27-mediated\_signaling\_pathway | 1 | 0 |  |  |  |  |  |  |  |  |
| GO:0070162\_adiponectin\_secretion | 1 | 0 |  |  |  |  |  |  |  |  |
| GO:0070163\_regulation\_of\_adiponectin\_secretion | 1 | 0 |  |  |  |  |  |  |  |  |
| GO:0070165\_positive\_regulation\_of\_adiponectin\_secretion | 1 | 0 |  |  |  |  |  |  |  |  |
| GO:0070172\_positive\_regulation\_of\_tooth\_mineralization | 1 | 0 |  |  |  |  |  |  |  |  |
| GO:0070173\_regulation\_of\_enamel\_mineralization | 1 | 0 |  |  |  |  |  |  |  |  |
| GO:0070189\_kynurenine\_metabolic\_process | 1 | 0 |  |  |  |  |  |  |  |  |
| GO:0070212\_protein\_amino\_acid\_poly-ADP-ribosylation | 1 | 0 |  |  |  |  |  |  |  |  |
| GO:0070213\_protein\_amino\_acid\_auto-ADP-ribosylation | 1 | 0 |  |  |  |  |  |  |  |  |
| GO:0070232\_regulation\_of\_T\_cell\_apoptosis | 1 | 0 |  |  |  |  |  |  |  |  |
| GO:0070234\_positive\_regulation\_of\_T\_cell\_apoptosis | 1 | 0 |  |  |  |  |  |  |  |  |
| GO:0070242\_thymocyte\_apoptosis | 1 | 0 |  |  |  |  |  |  |  |  |
| GO:0070243\_regulation\_of\_thymocyte\_apoptosis | 1 | 0 |  |  |  |  |  |  |  |  |
| GO:0070245\_positive\_regulation\_of\_thymocyte\_apoptosis | 1 | 0 |  |  |  |  |  |  |  |  |
| GO:0070267\_oncosis | 1 | 0 |  |  |  |  |  |  |  |  |
| GO:0070286\_axonemal\_dynein\_complex\_assembly | 1 | 0 |  |  |  |  |  |  |  |  |
| GO:0070314\_G1\_to\_G0\_transition | 1 | 0 |  |  |  |  |  |  |  |  |
| GO:0070327\_thyroid\_hormone\_transport | 1 | 0 |  |  |  |  |  |  |  |  |
| GO:0070407\_oxidation-dependent\_protein\_catabolic\_process | 1 | 0 |  |  |  |  |  |  |  |  |
| GO:0070408\_carbamoyl\_phosphate\_metabolic\_process | 1 | 0 |  |  |  |  |  |  |  |  |
| GO:0070409\_carbamoyl\_phosphate\_biosynthetic\_process | 1 | 0 |  |  |  |  |  |  |  |  |
| GO:0070509\_calcium\_ion\_import | 1 | 0 |  |  |  |  |  |  |  |  |
| GO:0070527\_platelet\_aggregation | 1 | 0 |  |  |  |  |  |  |  |  |
| GO:0070528\_protein\_kinase\_C\_signaling\_cascade | 1 | 0 |  |  |  |  |  |  |  |  |
| GO:0070534\_protein\_K63-linked\_ubiquitination | 1 | 0 |  |  |  |  |  |  |  |  |
| GO:0070535\_histone\_H2A\_K63-linked\_ubiquitination | 1 | 0 |  |  |  |  |  |  |  |  |
| GO:0070537\_histone\_H2A\_K63-linked\_deubiquitination | 1 | 0 |  |  |  |  |  |  |  |  |
| GO:0070560\_protein\_secretion\_by\_platelet | 1 | 0 |  |  |  |  |  |  |  |  |
| GO:0070562\_regulation\_of\_vitamin\_D\_receptor\_signaling\_pathway | 1 | 0 |  |  |  |  |  |  |  |  |
| GO:0070564\_positive\_regulation\_of\_vitamin\_D\_receptor\_signaling\_pathway | 1 | 0 |  |  |  |  |  |  |  |  |
| GO:0070570\_regulation\_of\_neuron\_projection\_regeneration | 1 | 0 |  |  |  |  |  |  |  |  |
| GO:0070571\_negative\_regulation\_of\_neuron\_projection\_regeneration | 1 | 0 |  |  |  |  |  |  |  |  |
| GO:0070601\_centromeric\_sister\_chromatid\_cohesion | 1 | 0 |  |  |  |  |  |  |  |  |
| GO:0070602\_regulation\_of\_centromeric\_sister\_chromatid\_cohesion | 1 | 0 |  |  |  |  |  |  |  |  |
| GO:0070625\_zymogen\_granule\_exocytosis | 1 | 0 |  |  |  |  |  |  |  |  |
| GO:0070684\_seminal\_clot\_liquefaction | 1 | 0 |  |  |  |  |  |  |  |  |
| GO:0070715\_sodium-dependent\_organic\_cation\_transport | 1 | 0 |  |  |  |  |  |  |  |  |
| GO:0070813\_hydrogen\_sulfide\_metabolic\_process | 1 | 0 |  |  |  |  |  |  |  |  |
| GO:0070814\_hydrogen\_sulfide\_biosynthetic\_process | 1 | 0 |  |  |  |  |  |  |  |  |
| GO:0070846\_Hsp90\_deacetylation | 1 | 0 |  |  |  |  |  |  |  |  |
| GO:0090030\_regulation\_of\_steroid\_hormone\_biosynthetic\_process | 1 | 0 |  |  |  |  |  |  |  |  |
| GO:0090031\_positive\_regulation\_of\_steroid\_hormone\_biosynthetic\_process | 1 | 0 |  |  |  |  |  |  |  |  |
| GO:0001944\_vasculature\_development | 88 | 0 | 0.000000 | 0.000000 | 885 | 824.202508 | 869.44 | 914.677492 | 0.982418 |
| GO:0000085\_G2\_phase\_of\_mitotic\_cell\_cycle | 5 | 0 | 0.000000 | 0.000000 | 1165 | 1103.479066 | 1146.85 | 1190.220934 | 0.984421 |
| GO:0000389\_nuclear\_mRNA\_3'-splice\_site\_recognition | 5 | 0 | 0.000000 | 0.000000 | 1165 | 1103.479066 | 1146.85 | 1190.220934 | 0.984421 |
| GO:0000768\_syncytium\_formation\_by\_plasma\_membrane\_fusion | 5 | 0 | 0.000000 | 0.000000 | 1165 | 1103.479066 | 1146.85 | 1190.220934 | 0.984421 |
| GO:0001504\_neurotransmitter\_uptake | 5 | 0 | 0.000000 | 0.000000 | 1165 | 1103.479066 | 1146.85 | 1190.220934 | 0.984421 |
| GO:0001580\_detection\_of\_chemical\_stimulus\_involved\_in\_sensory\_perception\_of\_bitter\_taste | 5 | 0 | 0.000000 | 0.000000 | 1165 | 1103.479066 | 1146.85 | 1190.220934 | 0.984421 |
| GO:0001672\_regulation\_of\_chromatin\_assembly\_or\_disassembly | 5 | 0 | 0.000000 | 0.000000 | 1165 | 1103.479066 | 1146.85 | 1190.220934 | 0.984421 |
| GO:0001754\_eye\_photoreceptor\_cell\_differentiation | 5 | 0 | 0.000000 | 0.000000 | 1165 | 1103.479066 | 1146.85 | 1190.220934 | 0.984421 |
| GO:0001783\_B\_cell\_apoptosis | 5 | 0 | 0.000000 | 0.000000 | 1165 | 1103.479066 | 1146.85 | 1190.220934 | 0.984421 |
| GO:0001954\_positive\_regulation\_of\_cell-matrix\_adhesion | 5 | 0 | 0.000000 | 0.000000 | 1165 | 1103.479066 | 1146.85 | 1190.220934 | 0.984421 |
| GO:0001960\_negative\_regulation\_of\_cytokine-mediated\_signaling\_pathway | 5 | 0 | 0.000000 | 0.000000 | 1165 | 1103.479066 | 1146.85 | 1190.220934 | 0.984421 |
| GO:0001964\_startle\_response | 5 | 0 | 0.000000 | 0.000000 | 1165 | 1103.479066 | 1146.85 | 1190.220934 | 0.984421 |
| GO:0001991\_regulation\_of\_systemic\_arterial\_blood\_pressure\_by\_circulatory\_renin-angiotensin | 5 | 0 | 0.000000 | 0.000000 | 1165 | 1103.479066 | 1146.85 | 1190.220934 | 0.984421 |
| GO:0002369\_T\_cell\_cytokine\_production | 5 | 0 | 0.000000 | 0.000000 | 1165 | 1103.479066 | 1146.85 | 1190.220934 | 0.984421 |
| GO:0002702\_positive\_regulation\_of\_production\_of\_molecular\_mediator\_of\_immune\_response | 5 | 0 | 0.000000 | 0.000000 | 1165 | 1103.479066 | 1146.85 | 1190.220934 | 0.984421 |
| GO:0002720\_positive\_regulation\_of\_cytokine\_production\_during\_immune\_response | 5 | 0 | 0.000000 | 0.000000 | 1165 | 1103.479066 | 1146.85 | 1190.220934 | 0.984421 |
| GO:0002724\_regulation\_of\_T\_cell\_cytokine\_production | 5 | 0 | 0.000000 | 0.000000 | 1165 | 1103.479066 | 1146.85 | 1190.220934 | 0.984421 |
| GO:0003091\_renal\_water\_homeostasis | 5 | 0 | 0.000000 | 0.000000 | 1165 | 1103.479066 | 1146.85 | 1190.220934 | 0.984421 |
| GO:0006012\_galactose\_metabolic\_process | 5 | 0 | 0.000000 | 0.000000 | 1165 | 1103.479066 | 1146.85 | 1190.220934 | 0.984421 |
| GO:0006027\_glycosaminoglycan\_catabolic\_process | 5 | 0 | 0.000000 | 0.000000 | 1165 | 1103.479066 | 1146.85 | 1190.220934 | 0.984421 |
| GO:0006098\_pentose-phosphate\_shunt | 5 | 0 | 0.000000 | 0.000000 | 1165 | 1103.479066 | 1146.85 | 1190.220934 | 0.984421 |
| GO:0006105\_succinate\_metabolic\_process | 5 | 0 | 0.000000 | 0.000000 | 1165 | 1103.479066 | 1146.85 | 1190.220934 | 0.984421 |
| GO:0006206\_pyrimidine\_base\_metabolic\_process | 5 | 0 | 0.000000 | 0.000000 | 1165 | 1103.479066 | 1146.85 | 1190.220934 | 0.984421 |
| GO:0006221\_pyrimidine\_nucleotide\_biosynthetic\_process | 5 | 0 | 0.000000 | 0.000000 | 1165 | 1103.479066 | 1146.85 | 1190.220934 | 0.984421 |
| GO:0006268\_DNA\_unwinding\_during\_replication | 5 | 0 | 0.000000 | 0.000000 | 1165 | 1103.479066 | 1146.85 | 1190.220934 | 0.984421 |
| GO:0006271\_DNA\_strand\_elongation\_during\_DNA\_replication | 5 | 0 | 0.000000 | 0.000000 | 1165 | 1103.479066 | 1146.85 | 1190.220934 | 0.984421 |
| GO:0006544\_glycine\_metabolic\_process | 5 | 0 | 0.000000 | 0.000000 | 1165 | 1103.479066 | 1146.85 | 1190.220934 | 0.984421 |
| GO:0006563\_L-serine\_metabolic\_process | 5 | 0 | 0.000000 | 0.000000 | 1165 | 1103.479066 | 1146.85 | 1190.220934 | 0.984421 |
| GO:0006595\_polyamine\_metabolic\_process | 5 | 0 | 0.000000 | 0.000000 | 1165 | 1103.479066 | 1146.85 | 1190.220934 | 0.984421 |
| GO:0006613\_cotranslational\_protein\_targeting\_to\_membrane | 5 | 0 | 0.000000 | 0.000000 | 1165 | 1103.479066 | 1146.85 | 1190.220934 | 0.984421 |
| GO:0006677\_glycosylceramide\_metabolic\_process | 5 | 0 | 0.000000 | 0.000000 | 1165 | 1103.479066 | 1146.85 | 1190.220934 | 0.984421 |
| GO:0006740\_NADPH\_regeneration | 5 | 0 | 0.000000 | 0.000000 | 1165 | 1103.479066 | 1146.85 | 1190.220934 | 0.984421 |
| GO:0006743\_ubiquinone\_metabolic\_process | 5 | 0 | 0.000000 | 0.000000 | 1165 | 1103.479066 | 1146.85 | 1190.220934 | 0.984421 |
| GO:0006744\_ubiquinone\_biosynthetic\_process | 5 | 0 | 0.000000 | 0.000000 | 1165 | 1103.479066 | 1146.85 | 1190.220934 | 0.984421 |
| GO:0006760\_folic\_acid\_and\_derivative\_metabolic\_process | 5 | 0 | 0.000000 | 0.000000 | 1165 | 1103.479066 | 1146.85 | 1190.220934 | 0.984421 |
| GO:0006817\_phosphate\_transport | 5 | 0 | 0.000000 | 0.000000 | 1165 | 1103.479066 | 1146.85 | 1190.220934 | 0.984421 |
| GO:0006835\_dicarboxylic\_acid\_transport | 5 | 0 | 0.000000 | 0.000000 | 1165 | 1103.479066 | 1146.85 | 1190.220934 | 0.984421 |
| GO:0006884\_cell\_volume\_homeostasis | 5 | 0 | 0.000000 | 0.000000 | 1165 | 1103.479066 | 1146.85 | 1190.220934 | 0.984421 |
| GO:0006977\_DNA\_damage\_response\_\_signal\_transduction\_by\_p53\_class\_mediator\_resulting\_in\_cell\_cycle\_arrest | 5 | 0 | 0.000000 | 0.000000 | 1165 | 1103.479066 | 1146.85 | 1190.220934 | 0.984421 |
| GO:0007016\_cytoskeletal\_anchoring\_at\_plasma\_membrane | 5 | 0 | 0.000000 | 0.000000 | 1165 | 1103.479066 | 1146.85 | 1190.220934 | 0.984421 |
| GO:0007064\_mitotic\_sister\_chromatid\_cohesion | 5 | 0 | 0.000000 | 0.000000 | 1165 | 1103.479066 | 1146.85 | 1190.220934 | 0.984421 |
| GO:0007140\_male\_meiosis | 5 | 0 | 0.000000 | 0.000000 | 1165 | 1103.479066 | 1146.85 | 1190.220934 | 0.984421 |
| GO:0007171\_activation\_of\_transmembrane\_receptor\_protein\_tyrosine\_kinase\_activity | 5 | 0 | 0.000000 | 0.000000 | 1165 | 1103.479066 | 1146.85 | 1190.220934 | 0.984421 |
| GO:0007220\_Notch\_receptor\_processing | 5 | 0 | 0.000000 | 0.000000 | 1165 | 1103.479066 | 1146.85 | 1190.220934 | 0.984421 |
| GO:0007350\_blastoderm\_segmentation | 5 | 0 | 0.000000 | 0.000000 | 1165 | 1103.479066 | 1146.85 | 1190.220934 | 0.984421 |
| GO:0007351\_tripartite\_regional\_subdivision | 5 | 0 | 0.000000 | 0.000000 | 1165 | 1103.479066 | 1146.85 | 1190.220934 | 0.984421 |
| GO:0007431\_salivary\_gland\_development | 5 | 0 | 0.000000 | 0.000000 | 1165 | 1103.479066 | 1146.85 | 1190.220934 | 0.984421 |
| GO:0007435\_salivary\_gland\_morphogenesis | 5 | 0 | 0.000000 | 0.000000 | 1165 | 1103.479066 | 1146.85 | 1190.220934 | 0.984421 |
| GO:0007598\_blood\_coagulation\_\_extrinsic\_pathway | 5 | 0 | 0.000000 | 0.000000 | 1165 | 1103.479066 | 1146.85 | 1190.220934 | 0.984421 |
| GO:0007603\_phototransduction\_\_visible\_light | 5 | 0 | 0.000000 | 0.000000 | 1165 | 1103.479066 | 1146.85 | 1190.220934 | 0.984421 |
| GO:0007635\_chemosensory\_behavior | 5 | 0 | 0.000000 | 0.000000 | 1165 | 1103.479066 | 1146.85 | 1190.220934 | 0.984421 |
| GO:0008089\_anterograde\_axon\_cargo\_transport | 5 | 0 | 0.000000 | 0.000000 | 1165 | 1103.479066 | 1146.85 | 1190.220934 | 0.984421 |
| GO:0008210\_estrogen\_metabolic\_process | 5 | 0 | 0.000000 | 0.000000 | 1165 | 1103.479066 | 1146.85 | 1190.220934 | 0.984421 |
| GO:0008211\_glucocorticoid\_metabolic\_process | 5 | 0 | 0.000000 | 0.000000 | 1165 | 1103.479066 | 1146.85 | 1190.220934 | 0.984421 |
| GO:0008228\_opsonization | 5 | 0 | 0.000000 | 0.000000 | 1165 | 1103.479066 | 1146.85 | 1190.220934 | 0.984421 |
| GO:0008542\_visual\_learning | 5 | 0 | 0.000000 | 0.000000 | 1165 | 1103.479066 | 1146.85 | 1190.220934 | 0.984421 |
| GO:0008589\_regulation\_of\_smoothened\_signaling\_pathway | 5 | 0 | 0.000000 | 0.000000 | 1165 | 1103.479066 | 1146.85 | 1190.220934 | 0.984421 |
| GO:0008595\_determination\_of\_anterior\_posterior\_axis\_\_embryo | 5 | 0 | 0.000000 | 0.000000 | 1165 | 1103.479066 | 1146.85 | 1190.220934 | 0.984421 |
| GO:0008634\_negative\_regulation\_of\_survival\_gene\_product\_expression | 5 | 0 | 0.000000 | 0.000000 | 1165 | 1103.479066 | 1146.85 | 1190.220934 | 0.984421 |
| GO:0009068\_aspartate\_family\_amino\_acid\_catabolic\_process | 5 | 0 | 0.000000 | 0.000000 | 1165 | 1103.479066 | 1146.85 | 1190.220934 | 0.984421 |
| GO:0009083\_branched\_chain\_family\_amino\_acid\_catabolic\_process | 5 | 0 | 0.000000 | 0.000000 | 1165 | 1103.479066 | 1146.85 | 1190.220934 | 0.984421 |
| GO:0009084\_glutamine\_family\_amino\_acid\_biosynthetic\_process | 5 | 0 | 0.000000 | 0.000000 | 1165 | 1103.479066 | 1146.85 | 1190.220934 | 0.984421 |
| GO:0009126\_purine\_nucleoside\_monophosphate\_metabolic\_process | 5 | 0 | 0.000000 | 0.000000 | 1165 | 1103.479066 | 1146.85 | 1190.220934 | 0.984421 |
| GO:0009167\_purine\_ribonucleoside\_monophosphate\_metabolic\_process | 5 | 0 | 0.000000 | 0.000000 | 1165 | 1103.479066 | 1146.85 | 1190.220934 | 0.984421 |
| GO:0009218\_pyrimidine\_ribonucleotide\_metabolic\_process | 5 | 0 | 0.000000 | 0.000000 | 1165 | 1103.479066 | 1146.85 | 1190.220934 | 0.984421 |
| GO:0009988\_cell-cell\_recognition | 5 | 0 | 0.000000 | 0.000000 | 1165 | 1103.479066 | 1146.85 | 1190.220934 | 0.984421 |
| GO:0010043\_response\_to\_zinc\_ion | 5 | 0 | 0.000000 | 0.000000 | 1165 | 1103.479066 | 1146.85 | 1190.220934 | 0.984421 |
| GO:0010165\_response\_to\_X-ray | 5 | 0 | 0.000000 | 0.000000 | 1165 | 1103.479066 | 1146.85 | 1190.220934 | 0.984421 |
| GO:0010559\_regulation\_of\_glycoprotein\_biosynthetic\_process | 5 | 0 | 0.000000 | 0.000000 | 1165 | 1103.479066 | 1146.85 | 1190.220934 | 0.984421 |
| GO:0010575\_positive\_regulation\_vascular\_endothelial\_growth\_factor\_production | 5 | 0 | 0.000000 | 0.000000 | 1165 | 1103.479066 | 1146.85 | 1190.220934 | 0.984421 |
| GO:0010714\_positive\_regulation\_of\_collagen\_metabolic\_process | 5 | 0 | 0.000000 | 0.000000 | 1165 | 1103.479066 | 1146.85 | 1190.220934 | 0.984421 |
| GO:0010811\_positive\_regulation\_of\_cell-substrate\_adhesion | 5 | 0 | 0.000000 | 0.000000 | 1165 | 1103.479066 | 1146.85 | 1190.220934 | 0.984421 |
| GO:0010871\_negative\_regulation\_of\_receptor\_biosynthetic\_process | 5 | 0 | 0.000000 | 0.000000 | 1165 | 1103.479066 | 1146.85 | 1190.220934 | 0.984421 |
| GO:0010893\_positive\_regulation\_of\_steroid\_biosynthetic\_process | 5 | 0 | 0.000000 | 0.000000 | 1165 | 1103.479066 | 1146.85 | 1190.220934 | 0.984421 |
| GO:0010898\_positive\_regulation\_of\_triglyceride\_catabolic\_process | 5 | 0 | 0.000000 | 0.000000 | 1165 | 1103.479066 | 1146.85 | 1190.220934 | 0.984421 |
| GO:0010953\_regulation\_of\_protein\_maturation\_by\_peptide\_bond\_cleavage | 5 | 0 | 0.000000 | 0.000000 | 1165 | 1103.479066 | 1146.85 | 1190.220934 | 0.984421 |
| GO:0010984\_regulation\_of\_lipoprotein\_particle\_clearance | 5 | 0 | 0.000000 | 0.000000 | 1165 | 1103.479066 | 1146.85 | 1190.220934 | 0.984421 |
| GO:0014003\_oligodendrocyte\_development | 5 | 0 | 0.000000 | 0.000000 | 1165 | 1103.479066 | 1146.85 | 1190.220934 | 0.984421 |
| GO:0014073\_response\_to\_tropane | 5 | 0 | 0.000000 | 0.000000 | 1165 | 1103.479066 | 1146.85 | 1190.220934 | 0.984421 |
| GO:0014821\_phasic\_smooth\_muscle\_contraction | 5 | 0 | 0.000000 | 0.000000 | 1165 | 1103.479066 | 1146.85 | 1190.220934 | 0.984421 |
| GO:0014829\_vascular\_smooth\_muscle\_contraction | 5 | 0 | 0.000000 | 0.000000 | 1165 | 1103.479066 | 1146.85 | 1190.220934 | 0.984421 |
| GO:0014866\_skeletal\_myofibril\_assembly | 5 | 0 | 0.000000 | 0.000000 | 1165 | 1103.479066 | 1146.85 | 1190.220934 | 0.984421 |
| GO:0015696\_ammonium\_transport | 5 | 0 | 0.000000 | 0.000000 | 1165 | 1103.479066 | 1146.85 | 1190.220934 | 0.984421 |
| GO:0015780\_nucleotide-sugar\_transport | 5 | 0 | 0.000000 | 0.000000 | 1165 | 1103.479066 | 1146.85 | 1190.220934 | 0.984421 |
| GO:0015781\_pyrimidine\_nucleotide-sugar\_transport | 5 | 0 | 0.000000 | 0.000000 | 1165 | 1103.479066 | 1146.85 | 1190.220934 | 0.984421 |
| GO:0015851\_nucleobase\_transport | 5 | 0 | 0.000000 | 0.000000 | 1165 | 1103.479066 | 1146.85 | 1190.220934 | 0.984421 |
| GO:0015858\_nucleoside\_transport | 5 | 0 | 0.000000 | 0.000000 | 1165 | 1103.479066 | 1146.85 | 1190.220934 | 0.984421 |
| GO:0015872\_dopamine\_transport | 5 | 0 | 0.000000 | 0.000000 | 1165 | 1103.479066 | 1146.85 | 1190.220934 | 0.984421 |
| GO:0015911\_plasma\_membrane\_long-chain\_fatty\_acid\_transport | 5 | 0 | 0.000000 | 0.000000 | 1165 | 1103.479066 | 1146.85 | 1190.220934 | 0.984421 |
| GO:0015985\_energy\_coupled\_proton\_transport\_\_down\_electrochemical\_gradient | 5 | 0 | 0.000000 | 0.000000 | 1165 | 1103.479066 | 1146.85 | 1190.220934 | 0.984421 |
| GO:0015986\_ATP\_synthesis\_coupled\_proton\_transport | 5 | 0 | 0.000000 | 0.000000 | 1165 | 1103.479066 | 1146.85 | 1190.220934 | 0.984421 |
| GO:0016246\_RNA\_interference | 5 | 0 | 0.000000 | 0.000000 | 1165 | 1103.479066 | 1146.85 | 1190.220934 | 0.984421 |
| GO:0018065\_protein-cofactor\_linkage | 5 | 0 | 0.000000 | 0.000000 | 1165 | 1103.479066 | 1146.85 | 1190.220934 | 0.984421 |
| GO:0018196\_peptidyl-asparagine\_modification | 5 | 0 | 0.000000 | 0.000000 | 1165 | 1103.479066 | 1146.85 | 1190.220934 | 0.984421 |
| GO:0018279\_protein\_amino\_acid\_N-linked\_glycosylation\_via\_asparagine | 5 | 0 | 0.000000 | 0.000000 | 1165 | 1103.479066 | 1146.85 | 1190.220934 | 0.984421 |
| GO:0018342\_protein\_prenylation | 5 | 0 | 0.000000 | 0.000000 | 1165 | 1103.479066 | 1146.85 | 1190.220934 | 0.984421 |
| GO:0019369\_arachidonic\_acid\_metabolic\_process | 5 | 0 | 0.000000 | 0.000000 | 1165 | 1103.479066 | 1146.85 | 1190.220934 | 0.984421 |
| GO:0019835\_cytolysis | 5 | 0 | 0.000000 | 0.000000 | 1165 | 1103.479066 | 1146.85 | 1190.220934 | 0.984421 |
| GO:0020027\_hemoglobin\_metabolic\_process | 5 | 0 | 0.000000 | 0.000000 | 1165 | 1103.479066 | 1146.85 | 1190.220934 | 0.984421 |
| GO:0022410\_circadian\_sleep\_wake\_cycle\_process | 5 | 0 | 0.000000 | 0.000000 | 1165 | 1103.479066 | 1146.85 | 1190.220934 | 0.984421 |
| GO:0022614\_membrane\_to\_membrane\_docking | 5 | 0 | 0.000000 | 0.000000 | 1165 | 1103.479066 | 1146.85 | 1190.220934 | 0.984421 |
| GO:0030194\_positive\_regulation\_of\_blood\_coagulation | 5 | 0 | 0.000000 | 0.000000 | 1165 | 1103.479066 | 1146.85 | 1190.220934 | 0.984421 |
| GO:0030219\_megakaryocyte\_differentiation | 5 | 0 | 0.000000 | 0.000000 | 1165 | 1103.479066 | 1146.85 | 1190.220934 | 0.984421 |
| GO:0030816\_positive\_regulation\_of\_cAMP\_metabolic\_process | 5 | 0 | 0.000000 | 0.000000 | 1165 | 1103.479066 | 1146.85 | 1190.220934 | 0.984421 |
| GO:0030819\_positive\_regulation\_of\_cAMP\_biosynthetic\_process | 5 | 0 | 0.000000 | 0.000000 | 1165 | 1103.479066 | 1146.85 | 1190.220934 | 0.984421 |
| GO:0030838\_positive\_regulation\_of\_actin\_filament\_polymerization | 5 | 0 | 0.000000 | 0.000000 | 1165 | 1103.479066 | 1146.85 | 1190.220934 | 0.984421 |
| GO:0031018\_endocrine\_pancreas\_development | 5 | 0 | 0.000000 | 0.000000 | 1165 | 1103.479066 | 1146.85 | 1190.220934 | 0.984421 |
| GO:0031057\_negative\_regulation\_of\_histone\_modification | 5 | 0 | 0.000000 | 0.000000 | 1165 | 1103.479066 | 1146.85 | 1190.220934 | 0.984421 |
| GO:0031058\_positive\_regulation\_of\_histone\_modification | 5 | 0 | 0.000000 | 0.000000 | 1165 | 1103.479066 | 1146.85 | 1190.220934 | 0.984421 |
| GO:0031112\_positive\_regulation\_of\_microtubule\_polymerization\_or\_depolymerization | 5 | 0 | 0.000000 | 0.000000 | 1165 | 1103.479066 | 1146.85 | 1190.220934 | 0.984421 |
| GO:0031113\_regulation\_of\_microtubule\_polymerization | 5 | 0 | 0.000000 | 0.000000 | 1165 | 1103.479066 | 1146.85 | 1190.220934 | 0.984421 |
| GO:0031345\_negative\_regulation\_of\_cell\_projection\_organization | 5 | 0 | 0.000000 | 0.000000 | 1165 | 1103.479066 | 1146.85 | 1190.220934 | 0.984421 |
| GO:0031397\_negative\_regulation\_of\_protein\_ubiquitination | 5 | 0 | 0.000000 | 0.000000 | 1165 | 1103.479066 | 1146.85 | 1190.220934 | 0.984421 |
| GO:0031507\_heterochromatin\_formation | 5 | 0 | 0.000000 | 0.000000 | 1165 | 1103.479066 | 1146.85 | 1190.220934 | 0.984421 |
| GO:0031639\_plasminogen\_activation | 5 | 0 | 0.000000 | 0.000000 | 1165 | 1103.479066 | 1146.85 | 1190.220934 | 0.984421 |
| GO:0031645\_negative\_regulation\_of\_neurological\_system\_process | 5 | 0 | 0.000000 | 0.000000 | 1165 | 1103.479066 | 1146.85 | 1190.220934 | 0.984421 |
| GO:0031998\_regulation\_of\_fatty\_acid\_beta-oxidation | 5 | 0 | 0.000000 | 0.000000 | 1165 | 1103.479066 | 1146.85 | 1190.220934 | 0.984421 |
| GO:0032055\_negative\_regulation\_of\_translation\_in\_response\_to\_stress | 5 | 0 | 0.000000 | 0.000000 | 1165 | 1103.479066 | 1146.85 | 1190.220934 | 0.984421 |
| GO:0032094\_response\_to\_food | 5 | 0 | 0.000000 | 0.000000 | 1165 | 1103.479066 | 1146.85 | 1190.220934 | 0.984421 |
| GO:0032095\_regulation\_of\_response\_to\_food | 5 | 0 | 0.000000 | 0.000000 | 1165 | 1103.479066 | 1146.85 | 1190.220934 | 0.984421 |
| GO:0032098\_regulation\_of\_appetite | 5 | 0 | 0.000000 | 0.000000 | 1165 | 1103.479066 | 1146.85 | 1190.220934 | 0.984421 |
| GO:0032148\_activation\_of\_protein\_kinase\_B\_activity | 5 | 0 | 0.000000 | 0.000000 | 1165 | 1103.479066 | 1146.85 | 1190.220934 | 0.984421 |
| GO:0032202\_telomere\_assembly | 5 | 0 | 0.000000 | 0.000000 | 1165 | 1103.479066 | 1146.85 | 1190.220934 | 0.984421 |
| GO:0032210\_regulation\_of\_telomere\_maintenance\_via\_telomerase | 5 | 0 | 0.000000 | 0.000000 | 1165 | 1103.479066 | 1146.85 | 1190.220934 | 0.984421 |
| GO:0032274\_gonadotropin\_secretion | 5 | 0 | 0.000000 | 0.000000 | 1165 | 1103.479066 | 1146.85 | 1190.220934 | 0.984421 |
| GO:0032364\_oxygen\_homeostasis | 5 | 0 | 0.000000 | 0.000000 | 1165 | 1103.479066 | 1146.85 | 1190.220934 | 0.984421 |
| GO:0032366\_intracellular\_sterol\_transport | 5 | 0 | 0.000000 | 0.000000 | 1165 | 1103.479066 | 1146.85 | 1190.220934 | 0.984421 |
| GO:0032367\_intracellular\_cholesterol\_transport | 5 | 0 | 0.000000 | 0.000000 | 1165 | 1103.479066 | 1146.85 | 1190.220934 | 0.984421 |
| GO:0032414\_positive\_regulation\_of\_ion\_transmembrane\_transporter\_activity | 5 | 0 | 0.000000 | 0.000000 | 1165 | 1103.479066 | 1146.85 | 1190.220934 | 0.984421 |
| GO:0032429\_regulation\_of\_phospholipase\_A2\_activity | 5 | 0 | 0.000000 | 0.000000 | 1165 | 1103.479066 | 1146.85 | 1190.220934 | 0.984421 |
| GO:0032430\_positive\_regulation\_of\_phospholipase\_A2\_activity | 5 | 0 | 0.000000 | 0.000000 | 1165 | 1103.479066 | 1146.85 | 1190.220934 | 0.984421 |
| GO:0032438\_melanosome\_organization | 5 | 0 | 0.000000 | 0.000000 | 1165 | 1103.479066 | 1146.85 | 1190.220934 | 0.984421 |
| GO:0032465\_regulation\_of\_cytokinesis | 5 | 0 | 0.000000 | 0.000000 | 1165 | 1103.479066 | 1146.85 | 1190.220934 | 0.984421 |
| GO:0032479\_regulation\_of\_type\_I\_interferon\_production | 5 | 0 | 0.000000 | 0.000000 | 1165 | 1103.479066 | 1146.85 | 1190.220934 | 0.984421 |
| GO:0032490\_detection\_of\_molecule\_of\_bacterial\_origin | 5 | 0 | 0.000000 | 0.000000 | 1165 | 1103.479066 | 1146.85 | 1190.220934 | 0.984421 |
| GO:0032570\_response\_to\_progesterone\_stimulus | 5 | 0 | 0.000000 | 0.000000 | 1165 | 1103.479066 | 1146.85 | 1190.220934 | 0.984421 |
| GO:0032606\_type\_I\_interferon\_production | 5 | 0 | 0.000000 | 0.000000 | 1165 | 1103.479066 | 1146.85 | 1190.220934 | 0.984421 |
| GO:0032607\_interferon-alpha\_production | 5 | 0 | 0.000000 | 0.000000 | 1165 | 1103.479066 | 1146.85 | 1190.220934 | 0.984421 |
| GO:0032612\_interleukin-1\_production | 5 | 0 | 0.000000 | 0.000000 | 1165 | 1103.479066 | 1146.85 | 1190.220934 | 0.984421 |
| GO:0032647\_regulation\_of\_interferon-alpha\_production | 5 | 0 | 0.000000 | 0.000000 | 1165 | 1103.479066 | 1146.85 | 1190.220934 | 0.984421 |
| GO:0032722\_positive\_regulation\_of\_chemokine\_production | 5 | 0 | 0.000000 | 0.000000 | 1165 | 1103.479066 | 1146.85 | 1190.220934 | 0.984421 |
| GO:0032801\_receptor\_catabolic\_process | 5 | 0 | 0.000000 | 0.000000 | 1165 | 1103.479066 | 1146.85 | 1190.220934 | 0.984421 |
| GO:0032856\_activation\_of\_Ras\_GTPase\_activity | 5 | 0 | 0.000000 | 0.000000 | 1165 | 1103.479066 | 1146.85 | 1190.220934 | 0.984421 |
| GO:0032862\_activation\_of\_Rho\_GTPase\_activity | 5 | 0 | 0.000000 | 0.000000 | 1165 | 1103.479066 | 1146.85 | 1190.220934 | 0.984421 |
| GO:0032967\_positive\_regulation\_of\_collagen\_biosynthetic\_process | 5 | 0 | 0.000000 | 0.000000 | 1165 | 1103.479066 | 1146.85 | 1190.220934 | 0.984421 |
| GO:0033028\_myeloid\_cell\_apoptosis | 5 | 0 | 0.000000 | 0.000000 | 1165 | 1103.479066 | 1146.85 | 1190.220934 | 0.984421 |
| GO:0033160\_positive\_regulation\_of\_protein\_import\_into\_nucleus\_\_translocation | 5 | 0 | 0.000000 | 0.000000 | 1165 | 1103.479066 | 1146.85 | 1190.220934 | 0.984421 |
| GO:0034379\_very-low-density\_lipoprotein\_particle\_assembly | 5 | 0 | 0.000000 | 0.000000 | 1165 | 1103.479066 | 1146.85 | 1190.220934 | 0.984421 |
| GO:0034380\_high-density\_lipoprotein\_particle\_assembly | 5 | 0 | 0.000000 | 0.000000 | 1165 | 1103.479066 | 1146.85 | 1190.220934 | 0.984421 |
| GO:0034382\_chylomicron\_remnant\_clearance | 5 | 0 | 0.000000 | 0.000000 | 1165 | 1103.479066 | 1146.85 | 1190.220934 | 0.984421 |
| GO:0034383\_low-density\_lipoprotein\_particle\_clearance | 5 | 0 | 0.000000 | 0.000000 | 1165 | 1103.479066 | 1146.85 | 1190.220934 | 0.984421 |
| GO:0034390\_smooth\_muscle\_cell\_apoptosis | 5 | 0 | 0.000000 | 0.000000 | 1165 | 1103.479066 | 1146.85 | 1190.220934 | 0.984421 |
| GO:0034391\_regulation\_of\_smooth\_muscle\_cell\_apoptosis | 5 | 0 | 0.000000 | 0.000000 | 1165 | 1103.479066 | 1146.85 | 1190.220934 | 0.984421 |
| GO:0034643\_mitochondrion\_localization\_\_microtubule-mediated | 5 | 0 | 0.000000 | 0.000000 | 1165 | 1103.479066 | 1146.85 | 1190.220934 | 0.984421 |
| GO:0034968\_histone\_lysine\_methylation | 5 | 0 | 0.000000 | 0.000000 | 1165 | 1103.479066 | 1146.85 | 1190.220934 | 0.984421 |
| GO:0034969\_histone\_arginine\_methylation | 5 | 0 | 0.000000 | 0.000000 | 1165 | 1103.479066 | 1146.85 | 1190.220934 | 0.984421 |
| GO:0035095\_behavioral\_response\_to\_nicotine | 5 | 0 | 0.000000 | 0.000000 | 1165 | 1103.479066 | 1146.85 | 1190.220934 | 0.984421 |
| GO:0035176\_social\_behavior | 5 | 0 | 0.000000 | 0.000000 | 1165 | 1103.479066 | 1146.85 | 1190.220934 | 0.984421 |
| GO:0035278\_gene\_silencing\_by\_miRNA\_\_negative\_regulation\_of\_translation | 5 | 0 | 0.000000 | 0.000000 | 1165 | 1103.479066 | 1146.85 | 1190.220934 | 0.984421 |
| GO:0040018\_positive\_regulation\_of\_multicellular\_organism\_growth | 5 | 0 | 0.000000 | 0.000000 | 1165 | 1103.479066 | 1146.85 | 1190.220934 | 0.984421 |
| GO:0040033\_negative\_regulation\_of\_translation\_\_ncRNA-mediated | 5 | 0 | 0.000000 | 0.000000 | 1165 | 1103.479066 | 1146.85 | 1190.220934 | 0.984421 |
| GO:0042026\_protein\_refolding | 5 | 0 | 0.000000 | 0.000000 | 1165 | 1103.479066 | 1146.85 | 1190.220934 | 0.984421 |
| GO:0042104\_positive\_regulation\_of\_activated\_T\_cell\_proliferation | 5 | 0 | 0.000000 | 0.000000 | 1165 | 1103.479066 | 1146.85 | 1190.220934 | 0.984421 |
| GO:0042117\_monocyte\_activation | 5 | 0 | 0.000000 | 0.000000 | 1165 | 1103.479066 | 1146.85 | 1190.220934 | 0.984421 |
| GO:0042130\_negative\_regulation\_of\_T\_cell\_proliferation | 5 | 0 | 0.000000 | 0.000000 | 1165 | 1103.479066 | 1146.85 | 1190.220934 | 0.984421 |
| GO:0042220\_response\_to\_cocaine | 5 | 0 | 0.000000 | 0.000000 | 1165 | 1103.479066 | 1146.85 | 1190.220934 | 0.984421 |
| GO:0042347\_negative\_regulation\_of\_NF-kappaB\_import\_into\_nucleus | 5 | 0 | 0.000000 | 0.000000 | 1165 | 1103.479066 | 1146.85 | 1190.220934 | 0.984421 |
| GO:0042359\_vitamin\_D\_metabolic\_process | 5 | 0 | 0.000000 | 0.000000 | 1165 | 1103.479066 | 1146.85 | 1190.220934 | 0.984421 |
| GO:0042364\_water-soluble\_vitamin\_biosynthetic\_process | 5 | 0 | 0.000000 | 0.000000 | 1165 | 1103.479066 | 1146.85 | 1190.220934 | 0.984421 |
| GO:0042471\_ear\_morphogenesis | 5 | 0 | 0.000000 | 0.000000 | 1165 | 1103.479066 | 1146.85 | 1190.220934 | 0.984421 |
| GO:0042490\_mechanoreceptor\_differentiation | 5 | 0 | 0.000000 | 0.000000 | 1165 | 1103.479066 | 1146.85 | 1190.220934 | 0.984421 |
| GO:0042535\_positive\_regulation\_of\_tumor\_necrosis\_factor\_biosynthetic\_process | 5 | 0 | 0.000000 | 0.000000 | 1165 | 1103.479066 | 1146.85 | 1190.220934 | 0.984421 |
| GO:0042744\_hydrogen\_peroxide\_catabolic\_process | 5 | 0 | 0.000000 | 0.000000 | 1165 | 1103.479066 | 1146.85 | 1190.220934 | 0.984421 |
| GO:0042745\_circadian\_sleep\_wake\_cycle | 5 | 0 | 0.000000 | 0.000000 | 1165 | 1103.479066 | 1146.85 | 1190.220934 | 0.984421 |
| GO:0042749\_regulation\_of\_circadian\_sleep\_wake\_cycle | 5 | 0 | 0.000000 | 0.000000 | 1165 | 1103.479066 | 1146.85 | 1190.220934 | 0.984421 |
| GO:0042769\_DNA\_damage\_response\_\_detection\_of\_DNA\_damage | 5 | 0 | 0.000000 | 0.000000 | 1165 | 1103.479066 | 1146.85 | 1190.220934 | 0.984421 |
| GO:0043029\_T\_cell\_homeostasis | 5 | 0 | 0.000000 | 0.000000 | 1165 | 1103.479066 | 1146.85 | 1190.220934 | 0.984421 |
| GO:0043044\_ATP-dependent\_chromatin\_remodeling | 5 | 0 | 0.000000 | 0.000000 | 1165 | 1103.479066 | 1146.85 | 1190.220934 | 0.984421 |
| GO:0043094\_cellular\_metabolic\_compound\_salvage | 5 | 0 | 0.000000 | 0.000000 | 1165 | 1103.479066 | 1146.85 | 1190.220934 | 0.984421 |
| GO:0043171\_peptide\_catabolic\_process | 5 | 0 | 0.000000 | 0.000000 | 1165 | 1103.479066 | 1146.85 | 1190.220934 | 0.984421 |
| GO:0043487\_regulation\_of\_RNA\_stability | 5 | 0 | 0.000000 | 0.000000 | 1165 | 1103.479066 | 1146.85 | 1190.220934 | 0.984421 |
| GO:0043488\_regulation\_of\_mRNA\_stability | 5 | 0 | 0.000000 | 0.000000 | 1165 | 1103.479066 | 1146.85 | 1190.220934 | 0.984421 |
| GO:0043496\_regulation\_of\_protein\_homodimerization\_activity | 5 | 0 | 0.000000 | 0.000000 | 1165 | 1103.479066 | 1146.85 | 1190.220934 | 0.984421 |
| GO:0043550\_regulation\_of\_lipid\_kinase\_activity | 5 | 0 | 0.000000 | 0.000000 | 1165 | 1103.479066 | 1146.85 | 1190.220934 | 0.984421 |
| GO:0043555\_regulation\_of\_translation\_in\_response\_to\_stress | 5 | 0 | 0.000000 | 0.000000 | 1165 | 1103.479066 | 1146.85 | 1190.220934 | 0.984421 |
| GO:0043616\_keratinocyte\_proliferation | 5 | 0 | 0.000000 | 0.000000 | 1165 | 1103.479066 | 1146.85 | 1190.220934 | 0.984421 |
| GO:0044253\_positive\_regulation\_of\_multicellular\_organismal\_metabolic\_process | 5 | 0 | 0.000000 | 0.000000 | 1165 | 1103.479066 | 1146.85 | 1190.220934 | 0.984421 |
| GO:0045008\_depyrimidination | 5 | 0 | 0.000000 | 0.000000 | 1165 | 1103.479066 | 1146.85 | 1190.220934 | 0.984421 |
| GO:0045071\_negative\_regulation\_of\_viral\_genome\_replication | 5 | 0 | 0.000000 | 0.000000 | 1165 | 1103.479066 | 1146.85 | 1190.220934 | 0.984421 |
| GO:0045109\_intermediate\_filament\_organization | 5 | 0 | 0.000000 | 0.000000 | 1165 | 1103.479066 | 1146.85 | 1190.220934 | 0.984421 |
| GO:0045161\_neuronal\_ion\_channel\_clustering | 5 | 0 | 0.000000 | 0.000000 | 1165 | 1103.479066 | 1146.85 | 1190.220934 | 0.984421 |
| GO:0045176\_apical\_protein\_localization | 5 | 0 | 0.000000 | 0.000000 | 1165 | 1103.479066 | 1146.85 | 1190.220934 | 0.984421 |
| GO:0045187\_regulation\_of\_circadian\_sleep\_wake\_cycle\_\_sleep | 5 | 0 | 0.000000 | 0.000000 | 1165 | 1103.479066 | 1146.85 | 1190.220934 | 0.984421 |
| GO:0045349\_interferon-alpha\_biosynthetic\_process | 5 | 0 | 0.000000 | 0.000000 | 1165 | 1103.479066 | 1146.85 | 1190.220934 | 0.984421 |
| GO:0045351\_type\_I\_interferon\_biosynthetic\_process | 5 | 0 | 0.000000 | 0.000000 | 1165 | 1103.479066 | 1146.85 | 1190.220934 | 0.984421 |
| GO:0045354\_regulation\_of\_interferon-alpha\_biosynthetic\_process | 5 | 0 | 0.000000 | 0.000000 | 1165 | 1103.479066 | 1146.85 | 1190.220934 | 0.984421 |
| GO:0045426\_quinone\_cofactor\_biosynthetic\_process | 5 | 0 | 0.000000 | 0.000000 | 1165 | 1103.479066 | 1146.85 | 1190.220934 | 0.984421 |
| GO:0045620\_negative\_regulation\_of\_lymphocyte\_differentiation | 5 | 0 | 0.000000 | 0.000000 | 1165 | 1103.479066 | 1146.85 | 1190.220934 | 0.984421 |
| GO:0045648\_positive\_regulation\_of\_erythrocyte\_differentiation | 5 | 0 | 0.000000 | 0.000000 | 1165 | 1103.479066 | 1146.85 | 1190.220934 | 0.984421 |
| GO:0045713\_low-density\_lipoprotein\_receptor\_biosynthetic\_process | 5 | 0 | 0.000000 | 0.000000 | 1165 | 1103.479066 | 1146.85 | 1190.220934 | 0.984421 |
| GO:0045741\_positive\_regulation\_of\_epidermal\_growth\_factor\_receptor\_activity | 5 | 0 | 0.000000 | 0.000000 | 1165 | 1103.479066 | 1146.85 | 1190.220934 | 0.984421 |
| GO:0045777\_positive\_regulation\_of\_blood\_pressure | 5 | 0 | 0.000000 | 0.000000 | 1165 | 1103.479066 | 1146.85 | 1190.220934 | 0.984421 |
| GO:0045821\_positive\_regulation\_of\_glycolysis | 5 | 0 | 0.000000 | 0.000000 | 1165 | 1103.479066 | 1146.85 | 1190.220934 | 0.984421 |
| GO:0045843\_negative\_regulation\_of\_striated\_muscle\_development | 5 | 0 | 0.000000 | 0.000000 | 1165 | 1103.479066 | 1146.85 | 1190.220934 | 0.984421 |
| GO:0045922\_negative\_regulation\_of\_fatty\_acid\_metabolic\_process | 5 | 0 | 0.000000 | 0.000000 | 1165 | 1103.479066 | 1146.85 | 1190.220934 | 0.984421 |
| GO:0045933\_positive\_regulation\_of\_muscle\_contraction | 5 | 0 | 0.000000 | 0.000000 | 1165 | 1103.479066 | 1146.85 | 1190.220934 | 0.984421 |
| GO:0045948\_positive\_regulation\_of\_translational\_initiation | 5 | 0 | 0.000000 | 0.000000 | 1165 | 1103.479066 | 1146.85 | 1190.220934 | 0.984421 |
| GO:0045974\_regulation\_of\_translation\_\_ncRNA-mediated | 5 | 0 | 0.000000 | 0.000000 | 1165 | 1103.479066 | 1146.85 | 1190.220934 | 0.984421 |
| GO:0046112\_nucleobase\_biosynthetic\_process | 5 | 0 | 0.000000 | 0.000000 | 1165 | 1103.479066 | 1146.85 | 1190.220934 | 0.984421 |
| GO:0046131\_pyrimidine\_ribonucleoside\_metabolic\_process | 5 | 0 | 0.000000 | 0.000000 | 1165 | 1103.479066 | 1146.85 | 1190.220934 | 0.984421 |
| GO:0046321\_positive\_regulation\_of\_fatty\_acid\_oxidation | 5 | 0 | 0.000000 | 0.000000 | 1165 | 1103.479066 | 1146.85 | 1190.220934 | 0.984421 |
| GO:0046329\_negative\_regulation\_of\_JNK\_cascade | 5 | 0 | 0.000000 | 0.000000 | 1165 | 1103.479066 | 1146.85 | 1190.220934 | 0.984421 |
| GO:0046339\_diacylglycerol\_metabolic\_process | 5 | 0 | 0.000000 | 0.000000 | 1165 | 1103.479066 | 1146.85 | 1190.220934 | 0.984421 |
| GO:0046513\_ceramide\_biosynthetic\_process | 5 | 0 | 0.000000 | 0.000000 | 1165 | 1103.479066 | 1146.85 | 1190.220934 | 0.984421 |
| GO:0046530\_photoreceptor\_cell\_differentiation | 5 | 0 | 0.000000 | 0.000000 | 1165 | 1103.479066 | 1146.85 | 1190.220934 | 0.984421 |
| GO:0046580\_negative\_regulation\_of\_Ras\_protein\_signal\_transduction | 5 | 0 | 0.000000 | 0.000000 | 1165 | 1103.479066 | 1146.85 | 1190.220934 | 0.984421 |
| GO:0046782\_regulation\_of\_viral\_transcription | 5 | 0 | 0.000000 | 0.000000 | 1165 | 1103.479066 | 1146.85 | 1190.220934 | 0.984421 |
| GO:0046884\_follicle-stimulating\_hormone\_secretion | 5 | 0 | 0.000000 | 0.000000 | 1165 | 1103.479066 | 1146.85 | 1190.220934 | 0.984421 |
| GO:0046902\_regulation\_of\_mitochondrial\_membrane\_permeability | 5 | 0 | 0.000000 | 0.000000 | 1165 | 1103.479066 | 1146.85 | 1190.220934 | 0.984421 |
| GO:0047497\_mitochondrion\_transport\_along\_microtubule | 5 | 0 | 0.000000 | 0.000000 | 1165 | 1103.479066 | 1146.85 | 1190.220934 | 0.984421 |
| GO:0048010\_vascular\_endothelial\_growth\_factor\_receptor\_signaling\_pathway | 5 | 0 | 0.000000 | 0.000000 | 1165 | 1103.479066 | 1146.85 | 1190.220934 | 0.984421 |
| GO:0048011\_nerve\_growth\_factor\_receptor\_signaling\_pathway | 5 | 0 | 0.000000 | 0.000000 | 1165 | 1103.479066 | 1146.85 | 1190.220934 | 0.984421 |
| GO:0048069\_eye\_pigmentation | 5 | 0 | 0.000000 | 0.000000 | 1165 | 1103.479066 | 1146.85 | 1190.220934 | 0.984421 |
| GO:0048168\_regulation\_of\_neuronal\_synaptic\_plasticity | 5 | 0 | 0.000000 | 0.000000 | 1165 | 1103.479066 | 1146.85 | 1190.220934 | 0.984421 |
| GO:0048261\_negative\_regulation\_of\_receptor-mediated\_endocytosis | 5 | 0 | 0.000000 | 0.000000 | 1165 | 1103.479066 | 1146.85 | 1190.220934 | 0.984421 |
| GO:0048286\_lung\_alveolus\_development | 5 | 0 | 0.000000 | 0.000000 | 1165 | 1103.479066 | 1146.85 | 1190.220934 | 0.984421 |
| GO:0048488\_synaptic\_vesicle\_endocytosis | 5 | 0 | 0.000000 | 0.000000 | 1165 | 1103.479066 | 1146.85 | 1190.220934 | 0.984421 |
| GO:0048565\_gut\_development | 5 | 0 | 0.000000 | 0.000000 | 1165 | 1103.479066 | 1146.85 | 1190.220934 | 0.984421 |
| GO:0048599\_oocyte\_development | 5 | 0 | 0.000000 | 0.000000 | 1165 | 1103.479066 | 1146.85 | 1190.220934 | 0.984421 |
| GO:0048635\_negative\_regulation\_of\_muscle\_development | 5 | 0 | 0.000000 | 0.000000 | 1165 | 1103.479066 | 1146.85 | 1190.220934 | 0.984421 |
| GO:0048675\_axon\_extension | 5 | 0 | 0.000000 | 0.000000 | 1165 | 1103.479066 | 1146.85 | 1190.220934 | 0.984421 |
| GO:0048753\_pigment\_granule\_organization | 5 | 0 | 0.000000 | 0.000000 | 1165 | 1103.479066 | 1146.85 | 1190.220934 | 0.984421 |
| GO:0048754\_branching\_morphogenesis\_of\_a\_tube | 5 | 0 | 0.000000 | 0.000000 | 1165 | 1103.479066 | 1146.85 | 1190.220934 | 0.984421 |
| GO:0048813\_dendrite\_morphogenesis | 5 | 0 | 0.000000 | 0.000000 | 1165 | 1103.479066 | 1146.85 | 1190.220934 | 0.984421 |
| GO:0050655\_dermatan\_sulfate\_proteoglycan\_metabolic\_process | 5 | 0 | 0.000000 | 0.000000 | 1165 | 1103.479066 | 1146.85 | 1190.220934 | 0.984421 |
| GO:0050691\_regulation\_of\_defense\_response\_to\_virus\_by\_host | 5 | 0 | 0.000000 | 0.000000 | 1165 | 1103.479066 | 1146.85 | 1190.220934 | 0.984421 |
| GO:0050766\_positive\_regulation\_of\_phagocytosis | 5 | 0 | 0.000000 | 0.000000 | 1165 | 1103.479066 | 1146.85 | 1190.220934 | 0.984421 |
| GO:0050772\_positive\_regulation\_of\_axonogenesis | 5 | 0 | 0.000000 | 0.000000 | 1165 | 1103.479066 | 1146.85 | 1190.220934 | 0.984421 |
| GO:0050802\_circadian\_sleep\_wake\_cycle\_\_sleep | 5 | 0 | 0.000000 | 0.000000 | 1165 | 1103.479066 | 1146.85 | 1190.220934 | 0.984421 |
| GO:0050807\_regulation\_of\_synapse\_organization | 5 | 0 | 0.000000 | 0.000000 | 1165 | 1103.479066 | 1146.85 | 1190.220934 | 0.984421 |
| GO:0050820\_positive\_regulation\_of\_coagulation | 5 | 0 | 0.000000 | 0.000000 | 1165 | 1103.479066 | 1146.85 | 1190.220934 | 0.984421 |
| GO:0050829\_defense\_response\_to\_Gram-negative\_bacterium | 5 | 0 | 0.000000 | 0.000000 | 1165 | 1103.479066 | 1146.85 | 1190.220934 | 0.984421 |
| GO:0050853\_B\_cell\_receptor\_signaling\_pathway | 5 | 0 | 0.000000 | 0.000000 | 1165 | 1103.479066 | 1146.85 | 1190.220934 | 0.984421 |
| GO:0050854\_regulation\_of\_antigen\_receptor-mediated\_signaling\_pathway | 5 | 0 | 0.000000 | 0.000000 | 1165 | 1103.479066 | 1146.85 | 1190.220934 | 0.984421 |
| GO:0050869\_negative\_regulation\_of\_B\_cell\_activation | 5 | 0 | 0.000000 | 0.000000 | 1165 | 1103.479066 | 1146.85 | 1190.220934 | 0.984421 |
| GO:0050891\_multicellular\_organismal\_water\_homeostasis | 5 | 0 | 0.000000 | 0.000000 | 1165 | 1103.479066 | 1146.85 | 1190.220934 | 0.984421 |
| GO:0050930\_induction\_of\_positive\_chemotaxis | 5 | 0 | 0.000000 | 0.000000 | 1165 | 1103.479066 | 1146.85 | 1190.220934 | 0.984421 |
| GO:0051005\_negative\_regulation\_of\_lipoprotein\_lipase\_activity | 5 | 0 | 0.000000 | 0.000000 | 1165 | 1103.479066 | 1146.85 | 1190.220934 | 0.984421 |
| GO:0051016\_barbed-end\_actin\_filament\_capping | 5 | 0 | 0.000000 | 0.000000 | 1165 | 1103.479066 | 1146.85 | 1190.220934 | 0.984421 |
| GO:0051058\_negative\_regulation\_of\_small\_GTPase\_mediated\_signal\_transduction | 5 | 0 | 0.000000 | 0.000000 | 1165 | 1103.479066 | 1146.85 | 1190.220934 | 0.984421 |
| GO:0051096\_positive\_regulation\_of\_helicase\_activity | 5 | 0 | 0.000000 | 0.000000 | 1165 | 1103.479066 | 1146.85 | 1190.220934 | 0.984421 |
| GO:0051103\_DNA\_ligation\_during\_DNA\_repair | 5 | 0 | 0.000000 | 0.000000 | 1165 | 1103.479066 | 1146.85 | 1190.220934 | 0.984421 |
| GO:0051123\_transcriptional\_preinitiation\_complex\_assembly | 5 | 0 | 0.000000 | 0.000000 | 1165 | 1103.479066 | 1146.85 | 1190.220934 | 0.984421 |
| GO:0051281\_positive\_regulation\_of\_release\_of\_sequestered\_calcium\_ion\_into\_cytosol | 5 | 0 | 0.000000 | 0.000000 | 1165 | 1103.479066 | 1146.85 | 1190.220934 | 0.984421 |
| GO:0051310\_metaphase\_plate\_congression | 5 | 0 | 0.000000 | 0.000000 | 1165 | 1103.479066 | 1146.85 | 1190.220934 | 0.984421 |
| GO:0051319\_G2\_phase | 5 | 0 | 0.000000 | 0.000000 | 1165 | 1103.479066 | 1146.85 | 1190.220934 | 0.984421 |
| GO:0051403\_stress-activated\_MAPK\_cascade | 5 | 0 | 0.000000 | 0.000000 | 1165 | 1103.479066 | 1146.85 | 1190.220934 | 0.984421 |
| GO:0051496\_positive\_regulation\_of\_stress\_fiber\_formation | 5 | 0 | 0.000000 | 0.000000 | 1165 | 1103.479066 | 1146.85 | 1190.220934 | 0.984421 |
| GO:0051654\_establishment\_of\_mitochondrion\_localization | 5 | 0 | 0.000000 | 0.000000 | 1165 | 1103.479066 | 1146.85 | 1190.220934 | 0.984421 |
| GO:0051693\_actin\_filament\_capping | 5 | 0 | 0.000000 | 0.000000 | 1165 | 1103.479066 | 1146.85 | 1190.220934 | 0.984421 |
| GO:0051893\_regulation\_of\_focal\_adhesion\_formation | 5 | 0 | 0.000000 | 0.000000 | 1165 | 1103.479066 | 1146.85 | 1190.220934 | 0.984421 |
| GO:0051898\_negative\_regulation\_of\_protein\_kinase\_B\_signaling\_cascade | 5 | 0 | 0.000000 | 0.000000 | 1165 | 1103.479066 | 1146.85 | 1190.220934 | 0.984421 |
| GO:0051926\_negative\_regulation\_of\_calcium\_ion\_transport | 5 | 0 | 0.000000 | 0.000000 | 1165 | 1103.479066 | 1146.85 | 1190.220934 | 0.984421 |
| GO:0051954\_positive\_regulation\_of\_amine\_transport | 5 | 0 | 0.000000 | 0.000000 | 1165 | 1103.479066 | 1146.85 | 1190.220934 | 0.984421 |
| GO:0055091\_phospholipid\_homeostasis | 5 | 0 | 0.000000 | 0.000000 | 1165 | 1103.479066 | 1146.85 | 1190.220934 | 0.984421 |
| GO:0055117\_regulation\_of\_cardiac\_muscle\_contraction | 5 | 0 | 0.000000 | 0.000000 | 1165 | 1103.479066 | 1146.85 | 1190.220934 | 0.984421 |
| GO:0060041\_retina\_development\_in\_camera-type\_eye | 5 | 0 | 0.000000 | 0.000000 | 1165 | 1103.479066 | 1146.85 | 1190.220934 | 0.984421 |
| GO:0060113\_inner\_ear\_receptor\_cell\_differentiation | 5 | 0 | 0.000000 | 0.000000 | 1165 | 1103.479066 | 1146.85 | 1190.220934 | 0.984421 |
| GO:0060123\_regulation\_of\_growth\_hormone\_secretion | 5 | 0 | 0.000000 | 0.000000 | 1165 | 1103.479066 | 1146.85 | 1190.220934 | 0.984421 |
| GO:0060260\_regulation\_of\_transcription\_initiation\_from\_RNA\_polymerase\_II\_promoter | 5 | 0 | 0.000000 | 0.000000 | 1165 | 1103.479066 | 1146.85 | 1190.220934 | 0.984421 |
| GO:0060390\_regulation\_of\_SMAD\_protein\_nuclear\_translocation | 5 | 0 | 0.000000 | 0.000000 | 1165 | 1103.479066 | 1146.85 | 1190.220934 | 0.984421 |
| GO:0060391\_positive\_regulation\_of\_SMAD\_protein\_nuclear\_translocation | 5 | 0 | 0.000000 | 0.000000 | 1165 | 1103.479066 | 1146.85 | 1190.220934 | 0.984421 |
| GO:0065002\_intracellular\_protein\_transmembrane\_transport | 5 | 0 | 0.000000 | 0.000000 | 1165 | 1103.479066 | 1146.85 | 1190.220934 | 0.984421 |
| GO:0070071\_proton-transporting\_two-sector\_ATPase\_complex\_assembly | 5 | 0 | 0.000000 | 0.000000 | 1165 | 1103.479066 | 1146.85 | 1190.220934 | 0.984421 |
| GO:0070120\_ciliary\_neurotrophic\_factor-mediated\_signaling\_pathway | 5 | 0 | 0.000000 | 0.000000 | 1165 | 1103.479066 | 1146.85 | 1190.220934 | 0.984421 |
| GO:0070228\_regulation\_of\_lymphocyte\_apoptosis | 5 | 0 | 0.000000 | 0.000000 | 1165 | 1103.479066 | 1146.85 | 1190.220934 | 0.984421 |
| GO:0070301\_cellular\_response\_to\_hydrogen\_peroxide | 5 | 0 | 0.000000 | 0.000000 | 1165 | 1103.479066 | 1146.85 | 1190.220934 | 0.984421 |
| GO:0070303\_negative\_regulation\_of\_stress-activated\_protein\_kinase\_signaling\_pathway | 5 | 0 | 0.000000 | 0.000000 | 1165 | 1103.479066 | 1146.85 | 1190.220934 | 0.984421 |
| GO:0070584\_mitochondrion\_morphogenesis | 5 | 0 | 0.000000 | 0.000000 | 1165 | 1103.479066 | 1146.85 | 1190.220934 | 0.984421 |
| GO:0070667\_negative\_regulation\_of\_mast\_cell\_proliferation | 5 | 0 | 0.000000 | 0.000000 | 1165 | 1103.479066 | 1146.85 | 1190.220934 | 0.984421 |
| GO:0070828\_heterochromatin\_organization | 5 | 0 | 0.000000 | 0.000000 | 1165 | 1103.479066 | 1146.85 | 1190.220934 | 0.984421 |
| GO:0006333\_chromatin\_assembly\_or\_disassembly | 52 | 0 | 0.000000 | 0.000000 | 1171 | 1109.254783 | 1152.42 | 1195.585217 | 0.984133 |
| GO:0006473\_protein\_amino\_acid\_acetylation | 52 | 0 | 0.000000 | 0.000000 | 1171 | 1109.254783 | 1152.42 | 1195.585217 | 0.984133 |
| GO:0022904\_respiratory\_electron\_transport\_chain | 52 | 0 | 0.000000 | 0.000000 | 1171 | 1109.254783 | 1152.42 | 1195.585217 | 0.984133 |
| GO:0042089\_cytokine\_biosynthetic\_process | 52 | 0 | 0.000000 | 0.000000 | 1171 | 1109.254783 | 1152.42 | 1195.585217 | 0.984133 |
| GO:0044270\_nitrogen\_compound\_catabolic\_process | 52 | 0 | 0.000000 | 0.000000 | 1171 | 1109.254783 | 1152.42 | 1195.585217 | 0.984133 |
| GO:0060284\_regulation\_of\_cell\_development | 52 | 0 | 0.000000 | 0.000000 | 1171 | 1109.254783 | 1152.42 | 1195.585217 | 0.984133 |
| GO:0018193\_peptidyl-amino\_acid\_modification | 117 | 0 | 0.000000 | 0.000000 | 1172 | 1110.053198 | 1153.16 | 1196.266802 | 0.983925 |
| GO:0006665\_sphingolipid\_metabolic\_process | 44 | 0 | 0.000000 | 0.000000 | 1179 | 1117.294493 | 1160.08 | 1202.865507 | 0.983953 |
| GO:0006730\_one-carbon\_metabolic\_process | 44 | 0 | 0.000000 | 0.000000 | 1179 | 1117.294493 | 1160.08 | 1202.865507 | 0.983953 |
| GO:0006959\_humoral\_immune\_response | 44 | 0 | 0.000000 | 0.000000 | 1179 | 1117.294493 | 1160.08 | 1202.865507 | 0.983953 |
| GO:0016125\_sterol\_metabolic\_process | 44 | 0 | 0.000000 | 0.000000 | 1179 | 1117.294493 | 1160.08 | 1202.865507 | 0.983953 |
| GO:0050900\_leukocyte\_migration | 44 | 0 | 0.000000 | 0.000000 | 1179 | 1117.294493 | 1160.08 | 1202.865507 | 0.983953 |
| GO:0051301\_cell\_division | 44 | 0 | 0.000000 | 0.000000 | 1179 | 1117.294493 | 1160.08 | 1202.865507 | 0.983953 |
| GO:0051321\_meiotic\_cell\_cycle | 44 | 0 | 0.000000 | 0.000000 | 1179 | 1117.294493 | 1160.08 | 1202.865507 | 0.983953 |
| GO:0003013\_circulatory\_system\_process | 133 | 0 | 0.000000 | 0.000000 | 1182 | 1119.899413 | 1162.56 | 1205.220587 | 0.983553 |
| GO:0008015\_blood\_circulation | 133 | 0 | 0.000000 | 0.000000 | 1182 | 1119.899413 | 1162.56 | 1205.220587 | 0.983553 |
| GO:0044262\_cellular\_carbohydrate\_metabolic\_process | 133 | 0 | 0.000000 | 0.000000 | 1182 | 1119.899413 | 1162.56 | 1205.220587 | 0.983553 |
| GO:0006367\_transcription\_initiation\_from\_RNA\_polymerase\_II\_promoter | 66 | 0 | 0.000000 | 0.000000 | 1194 | 1131.957623 | 1173.91 | 1215.862377 | 0.983174 |
| GO:0006813\_potassium\_ion\_transport | 66 | 0 | 0.000000 | 0.000000 | 1194 | 1131.957623 | 1173.91 | 1215.862377 | 0.983174 |
| GO:0010562\_positive\_regulation\_of\_phosphorus\_metabolic\_process | 66 | 0 | 0.000000 | 0.000000 | 1194 | 1131.957623 | 1173.91 | 1215.862377 | 0.983174 |
| GO:0010564\_regulation\_of\_cell\_cycle\_process | 66 | 0 | 0.000000 | 0.000000 | 1194 | 1131.957623 | 1173.91 | 1215.862377 | 0.983174 |
| GO:0014706\_striated\_muscle\_tissue\_development | 66 | 0 | 0.000000 | 0.000000 | 1194 | 1131.957623 | 1173.91 | 1215.862377 | 0.983174 |
| GO:0031589\_cell-substrate\_adhesion | 66 | 0 | 0.000000 | 0.000000 | 1194 | 1131.957623 | 1173.91 | 1215.862377 | 0.983174 |
| GO:0045937\_positive\_regulation\_of\_phosphate\_metabolic\_process | 66 | 0 | 0.000000 | 0.000000 | 1194 | 1131.957623 | 1173.91 | 1215.862377 | 0.983174 |
| GO:0048667\_cell\_morphogenesis\_involved\_in\_neuron\_differentiation | 66 | 0 | 0.000000 | 0.000000 | 1194 | 1131.957623 | 1173.91 | 1215.862377 | 0.983174 |
| GO:0048812\_neuron\_projection\_morphogenesis | 66 | 0 | 0.000000 | 0.000000 | 1194 | 1131.957623 | 1173.91 | 1215.862377 | 0.983174 |
| GO:0051090\_regulation\_of\_transcription\_factor\_activity | 66 | 0 | 0.000000 | 0.000000 | 1194 | 1131.957623 | 1173.91 | 1215.862377 | 0.983174 |
| GO:0052547\_regulation\_of\_peptidase\_activity | 66 | 0 | 0.000000 | 0.000000 | 1194 | 1131.957623 | 1173.91 | 1215.862377 | 0.983174 |
| GO:0090046\_regulation\_of\_transcription\_regulator\_activity | 66 | 0 | 0.000000 | 0.000000 | 1194 | 1131.957623 | 1173.91 | 1215.862377 | 0.983174 |
| GO:0006006\_glucose\_metabolic\_process | 53 | 0 | 0.000000 | 0.000000 | 1205 | 1143.406184 | 1184.89 | 1226.373816 | 0.983311 |
| GO:0016485\_protein\_processing | 53 | 0 | 0.000000 | 0.000000 | 1205 | 1143.406184 | 1184.89 | 1226.373816 | 0.983311 |
| GO:0022900\_electron\_transport\_chain | 53 | 0 | 0.000000 | 0.000000 | 1205 | 1143.406184 | 1184.89 | 1226.373816 | 0.983311 |
| GO:0030258\_lipid\_modification | 53 | 0 | 0.000000 | 0.000000 | 1205 | 1143.406184 | 1184.89 | 1226.373816 | 0.983311 |
| GO:0042107\_cytokine\_metabolic\_process | 53 | 0 | 0.000000 | 0.000000 | 1205 | 1143.406184 | 1184.89 | 1226.373816 | 0.983311 |
| GO:0043434\_response\_to\_peptide\_hormone\_stimulus | 53 | 0 | 0.000000 | 0.000000 | 1205 | 1143.406184 | 1184.89 | 1226.373816 | 0.983311 |
| GO:0045786\_negative\_regulation\_of\_cell\_cycle | 53 | 0 | 0.000000 | 0.000000 | 1205 | 1143.406184 | 1184.89 | 1226.373816 | 0.983311 |
| GO:0048015\_phosphoinositide-mediated\_signaling | 53 | 0 | 0.000000 | 0.000000 | 1205 | 1143.406184 | 1184.89 | 1226.373816 | 0.983311 |
| GO:0051047\_positive\_regulation\_of\_secretion | 53 | 0 | 0.000000 | 0.000000 | 1205 | 1143.406184 | 1184.89 | 1226.373816 | 0.983311 |
| GO:0051339\_regulation\_of\_lyase\_activity | 53 | 0 | 0.000000 | 0.000000 | 1205 | 1143.406184 | 1184.89 | 1226.373816 | 0.983311 |
| GO:0060193\_positive\_regulation\_of\_lipase\_activity | 53 | 0 | 0.000000 | 0.000000 | 1205 | 1143.406184 | 1184.89 | 1226.373816 | 0.983311 |
| GO:0046903\_secretion | 218 | 0 | 0.000000 | 0.000000 | 1206 | 1144.183395 | 1185.59 | 1226.996605 | 0.983076 |
| GO:0030163\_protein\_catabolic\_process | 330 | 0 | 0.000000 | 0.000000 | 1207 | 1145.349076 | 1186.73 | 1228.110924 | 0.983206 |
| GO:0048646\_anatomical\_structure\_formation\_involved\_in\_morphogenesis | 111 | 0 | 0.000000 | 0.000000 | 1208 | 1146.205091 | 1187.55 | 1228.894909 | 0.983071 |
| GO:0019941\_modification-dependent\_protein\_catabolic\_process | 138 | 0 | 0.000000 | 0.000000 | 1210 | 1147.872685 | 1189.21 | 1230.547315 | 0.982818 |
| GO:0043632\_modification-dependent\_macromolecule\_catabolic\_process | 138 | 0 | 0.000000 | 0.000000 | 1210 | 1147.872685 | 1189.21 | 1230.547315 | 0.982818 |
| GO:0022414\_reproductive\_process | 365 | 0 | 0.000000 | 0.000000 | 1211 | 1148.382133 | 1189.67 | 1230.957867 | 0.982386 |
| GO:0006120\_mitochondrial\_electron\_transport\_\_NADH\_to\_ubiquinone | 42 | 0 | 0.000000 | 0.000000 | 1224 | 1162.764720 | 1203.72 | 1244.675280 | 0.983431 |
| GO:0006865\_amino\_acid\_transport | 42 | 0 | 0.000000 | 0.000000 | 1224 | 1162.764720 | 1203.72 | 1244.675280 | 0.983431 |
| GO:0006887\_exocytosis | 42 | 0 | 0.000000 | 0.000000 | 1224 | 1162.764720 | 1203.72 | 1244.675280 | 0.983431 |
| GO:0006944\_membrane\_fusion | 42 | 0 | 0.000000 | 0.000000 | 1224 | 1162.764720 | 1203.72 | 1244.675280 | 0.983431 |
| GO:0007608\_sensory\_perception\_of\_smell | 42 | 0 | 0.000000 | 0.000000 | 1224 | 1162.764720 | 1203.72 | 1244.675280 | 0.983431 |
| GO:0008203\_cholesterol\_metabolic\_process | 42 | 0 | 0.000000 | 0.000000 | 1224 | 1162.764720 | 1203.72 | 1244.675280 | 0.983431 |
| GO:0016072\_rRNA\_metabolic\_process | 42 | 0 | 0.000000 | 0.000000 | 1224 | 1162.764720 | 1203.72 | 1244.675280 | 0.983431 |
| GO:0032259\_methylation | 42 | 0 | 0.000000 | 0.000000 | 1224 | 1162.764720 | 1203.72 | 1244.675280 | 0.983431 |
| GO:0032868\_response\_to\_insulin\_stimulus | 42 | 0 | 0.000000 | 0.000000 | 1224 | 1162.764720 | 1203.72 | 1244.675280 | 0.983431 |
| GO:0042692\_muscle\_cell\_differentiation | 42 | 0 | 0.000000 | 0.000000 | 1224 | 1162.764720 | 1203.72 | 1244.675280 | 0.983431 |
| GO:0043414\_biopolymer\_methylation | 42 | 0 | 0.000000 | 0.000000 | 1224 | 1162.764720 | 1203.72 | 1244.675280 | 0.983431 |
| GO:0048585\_negative\_regulation\_of\_response\_to\_stimulus | 42 | 0 | 0.000000 | 0.000000 | 1224 | 1162.764720 | 1203.72 | 1244.675280 | 0.983431 |
| GO:0051271\_negative\_regulation\_of\_cell\_motion | 42 | 0 | 0.000000 | 0.000000 | 1224 | 1162.764720 | 1203.72 | 1244.675280 | 0.983431 |
| GO:0000723\_telomere\_maintenance | 26 | 0 | 0.000000 | 0.000000 | 1249 | 1189.917560 | 1230.11 | 1270.302440 | 0.984876 |
| GO:0006720\_isoprenoid\_metabolic\_process | 26 | 0 | 0.000000 | 0.000000 | 1249 | 1189.917560 | 1230.11 | 1270.302440 | 0.984876 |
| GO:0007162\_negative\_regulation\_of\_cell\_adhesion | 26 | 0 | 0.000000 | 0.000000 | 1249 | 1189.917560 | 1230.11 | 1270.302440 | 0.984876 |
| GO:0007194\_negative\_regulation\_of\_adenylate\_cyclase\_activity | 26 | 0 | 0.000000 | 0.000000 | 1249 | 1189.917560 | 1230.11 | 1270.302440 | 0.984876 |
| GO:0007498\_mesoderm\_development | 26 | 0 | 0.000000 | 0.000000 | 1249 | 1189.917560 | 1230.11 | 1270.302440 | 0.984876 |
| GO:0009150\_purine\_ribonucleotide\_metabolic\_process | 26 | 0 | 0.000000 | 0.000000 | 1249 | 1189.917560 | 1230.11 | 1270.302440 | 0.984876 |
| GO:0009166\_nucleotide\_catabolic\_process | 26 | 0 | 0.000000 | 0.000000 | 1249 | 1189.917560 | 1230.11 | 1270.302440 | 0.984876 |
| GO:0009309\_amine\_biosynthetic\_process | 26 | 0 | 0.000000 | 0.000000 | 1249 | 1189.917560 | 1230.11 | 1270.302440 | 0.984876 |
| GO:0009582\_detection\_of\_abiotic\_stimulus | 26 | 0 | 0.000000 | 0.000000 | 1249 | 1189.917560 | 1230.11 | 1270.302440 | 0.984876 |
| GO:0018105\_peptidyl-serine\_phosphorylation | 26 | 0 | 0.000000 | 0.000000 | 1249 | 1189.917560 | 1230.11 | 1270.302440 | 0.984876 |
| GO:0030225\_macrophage\_differentiation | 26 | 0 | 0.000000 | 0.000000 | 1249 | 1189.917560 | 1230.11 | 1270.302440 | 0.984876 |
| GO:0031280\_negative\_regulation\_of\_cyclase\_activity | 26 | 0 | 0.000000 | 0.000000 | 1249 | 1189.917560 | 1230.11 | 1270.302440 | 0.984876 |
| GO:0031281\_positive\_regulation\_of\_cyclase\_activity | 26 | 0 | 0.000000 | 0.000000 | 1249 | 1189.917560 | 1230.11 | 1270.302440 | 0.984876 |
| GO:0031344\_regulation\_of\_cell\_projection\_organization | 26 | 0 | 0.000000 | 0.000000 | 1249 | 1189.917560 | 1230.11 | 1270.302440 | 0.984876 |
| GO:0032504\_multicellular\_organism\_reproduction | 26 | 0 | 0.000000 | 0.000000 | 1249 | 1189.917560 | 1230.11 | 1270.302440 | 0.984876 |
| GO:0042326\_negative\_regulation\_of\_phosphorylation | 26 | 0 | 0.000000 | 0.000000 | 1249 | 1189.917560 | 1230.11 | 1270.302440 | 0.984876 |
| GO:0043241\_protein\_complex\_disassembly | 26 | 0 | 0.000000 | 0.000000 | 1249 | 1189.917560 | 1230.11 | 1270.302440 | 0.984876 |
| GO:0045927\_positive\_regulation\_of\_growth | 26 | 0 | 0.000000 | 0.000000 | 1249 | 1189.917560 | 1230.11 | 1270.302440 | 0.984876 |
| GO:0048609\_reproductive\_process\_in\_a\_multicellular\_organism | 26 | 0 | 0.000000 | 0.000000 | 1249 | 1189.917560 | 1230.11 | 1270.302440 | 0.984876 |
| GO:0050663\_cytokine\_secretion | 26 | 0 | 0.000000 | 0.000000 | 1249 | 1189.917560 | 1230.11 | 1270.302440 | 0.984876 |
| GO:0050673\_epithelial\_cell\_proliferation | 26 | 0 | 0.000000 | 0.000000 | 1249 | 1189.917560 | 1230.11 | 1270.302440 | 0.984876 |
| GO:0051053\_negative\_regulation\_of\_DNA\_metabolic\_process | 26 | 0 | 0.000000 | 0.000000 | 1249 | 1189.917560 | 1230.11 | 1270.302440 | 0.984876 |
| GO:0051258\_protein\_polymerization | 26 | 0 | 0.000000 | 0.000000 | 1249 | 1189.917560 | 1230.11 | 1270.302440 | 0.984876 |
| GO:0051350\_negative\_regulation\_of\_lyase\_activity | 26 | 0 | 0.000000 | 0.000000 | 1249 | 1189.917560 | 1230.11 | 1270.302440 | 0.984876 |
| GO:0051402\_neuron\_apoptosis | 26 | 0 | 0.000000 | 0.000000 | 1249 | 1189.917560 | 1230.11 | 1270.302440 | 0.984876 |
| GO:0001952\_regulation\_of\_cell-matrix\_adhesion | 13 | 0 | 0.000000 | 0.000000 | 1321 | 1262.171399 | 1301.55 | 1340.928601 | 0.985276 |
| GO:0002200\_somatic\_diversification\_of\_immune\_receptors | 13 | 0 | 0.000000 | 0.000000 | 1321 | 1262.171399 | 1301.55 | 1340.928601 | 0.985276 |
| GO:0002263\_cell\_activation\_during\_immune\_response | 13 | 0 | 0.000000 | 0.000000 | 1321 | 1262.171399 | 1301.55 | 1340.928601 | 0.985276 |
| GO:0002366\_leukocyte\_activation\_during\_immune\_response | 13 | 0 | 0.000000 | 0.000000 | 1321 | 1262.171399 | 1301.55 | 1340.928601 | 0.985276 |
| GO:0002456\_T\_cell\_mediated\_immunity | 13 | 0 | 0.000000 | 0.000000 | 1321 | 1262.171399 | 1301.55 | 1340.928601 | 0.985276 |
| GO:0002705\_positive\_regulation\_of\_leukocyte\_mediated\_immunity | 13 | 0 | 0.000000 | 0.000000 | 1321 | 1262.171399 | 1301.55 | 1340.928601 | 0.985276 |
| GO:0002708\_positive\_regulation\_of\_lymphocyte\_mediated\_immunity | 13 | 0 | 0.000000 | 0.000000 | 1321 | 1262.171399 | 1301.55 | 1340.928601 | 0.985276 |
| GO:0003014\_renal\_system\_process | 13 | 0 | 0.000000 | 0.000000 | 1321 | 1262.171399 | 1301.55 | 1340.928601 | 0.985276 |
| GO:0003044\_regulation\_of\_systemic\_arterial\_blood\_pressure\_mediated\_by\_a\_chemical\_signal | 13 | 0 | 0.000000 | 0.000000 | 1321 | 1262.171399 | 1301.55 | 1340.928601 | 0.985276 |
| GO:0006014\_D-ribose\_metabolic\_process | 13 | 0 | 0.000000 | 0.000000 | 1321 | 1262.171399 | 1301.55 | 1340.928601 | 0.985276 |
| GO:0006081\_cellular\_aldehyde\_metabolic\_process | 13 | 0 | 0.000000 | 0.000000 | 1321 | 1262.171399 | 1301.55 | 1340.928601 | 0.985276 |
| GO:0006090\_pyruvate\_metabolic\_process | 13 | 0 | 0.000000 | 0.000000 | 1321 | 1262.171399 | 1301.55 | 1340.928601 | 0.985276 |
| GO:0006278\_RNA-dependent\_DNA\_replication | 13 | 0 | 0.000000 | 0.000000 | 1321 | 1262.171399 | 1301.55 | 1340.928601 | 0.985276 |
| GO:0006376\_mRNA\_splice\_site\_selection | 13 | 0 | 0.000000 | 0.000000 | 1321 | 1262.171399 | 1301.55 | 1340.928601 | 0.985276 |
| GO:0006607\_NLS-bearing\_substrate\_import\_into\_nucleus | 13 | 0 | 0.000000 | 0.000000 | 1321 | 1262.171399 | 1301.55 | 1340.928601 | 0.985276 |
| GO:0006783\_heme\_biosynthetic\_process | 13 | 0 | 0.000000 | 0.000000 | 1321 | 1262.171399 | 1301.55 | 1340.928601 | 0.985276 |
| GO:0006900\_membrane\_budding | 13 | 0 | 0.000000 | 0.000000 | 1321 | 1262.171399 | 1301.55 | 1340.928601 | 0.985276 |
| GO:0006901\_vesicle\_coating | 13 | 0 | 0.000000 | 0.000000 | 1321 | 1262.171399 | 1301.55 | 1340.928601 | 0.985276 |
| GO:0006940\_regulation\_of\_smooth\_muscle\_contraction | 13 | 0 | 0.000000 | 0.000000 | 1321 | 1262.171399 | 1301.55 | 1340.928601 | 0.985276 |
| GO:0006953\_acute-phase\_response | 13 | 0 | 0.000000 | 0.000000 | 1321 | 1262.171399 | 1301.55 | 1340.928601 | 0.985276 |
| GO:0007004\_telomere\_maintenance\_via\_telomerase | 13 | 0 | 0.000000 | 0.000000 | 1321 | 1262.171399 | 1301.55 | 1340.928601 | 0.985276 |
| GO:0007052\_mitotic\_spindle\_organization | 13 | 0 | 0.000000 | 0.000000 | 1321 | 1262.171399 | 1301.55 | 1340.928601 | 0.985276 |
| GO:0007091\_mitotic\_metaphase\_anaphase\_transition | 13 | 0 | 0.000000 | 0.000000 | 1321 | 1262.171399 | 1301.55 | 1340.928601 | 0.985276 |
| GO:0007193\_inhibition\_of\_adenylate\_cyclase\_activity\_by\_G-protein\_signaling | 13 | 0 | 0.000000 | 0.000000 | 1321 | 1262.171399 | 1301.55 | 1340.928601 | 0.985276 |
| GO:0007263\_nitric\_oxide\_mediated\_signal\_transduction | 13 | 0 | 0.000000 | 0.000000 | 1321 | 1262.171399 | 1301.55 | 1340.928601 | 0.985276 |
| GO:0009065\_glutamine\_family\_amino\_acid\_catabolic\_process | 13 | 0 | 0.000000 | 0.000000 | 1321 | 1262.171399 | 1301.55 | 1340.928601 | 0.985276 |
| GO:0009262\_deoxyribonucleotide\_metabolic\_process | 13 | 0 | 0.000000 | 0.000000 | 1321 | 1262.171399 | 1301.55 | 1340.928601 | 0.985276 |
| GO:0009746\_response\_to\_hexose\_stimulus | 13 | 0 | 0.000000 | 0.000000 | 1321 | 1262.171399 | 1301.55 | 1340.928601 | 0.985276 |
| GO:0009749\_response\_to\_glucose\_stimulus | 13 | 0 | 0.000000 | 0.000000 | 1321 | 1262.171399 | 1301.55 | 1340.928601 | 0.985276 |
| GO:0010810\_regulation\_of\_cell-substrate\_adhesion | 13 | 0 | 0.000000 | 0.000000 | 1321 | 1262.171399 | 1301.55 | 1340.928601 | 0.985276 |
| GO:0010833\_telomere\_maintenance\_via\_telomere\_lengthening | 13 | 0 | 0.000000 | 0.000000 | 1321 | 1262.171399 | 1301.55 | 1340.928601 | 0.985276 |
| GO:0014065\_phosphoinositide\_3-kinase\_cascade | 13 | 0 | 0.000000 | 0.000000 | 1321 | 1262.171399 | 1301.55 | 1340.928601 | 0.985276 |
| GO:0015914\_phospholipid\_transport | 13 | 0 | 0.000000 | 0.000000 | 1321 | 1262.171399 | 1301.55 | 1340.928601 | 0.985276 |
| GO:0018149\_peptide\_cross-linking | 13 | 0 | 0.000000 | 0.000000 | 1321 | 1262.171399 | 1301.55 | 1340.928601 | 0.985276 |
| GO:0019439\_aromatic\_compound\_catabolic\_process | 13 | 0 | 0.000000 | 0.000000 | 1321 | 1262.171399 | 1301.55 | 1340.928601 | 0.985276 |
| GO:0030201\_heparan\_sulfate\_proteoglycan\_metabolic\_process | 13 | 0 | 0.000000 | 0.000000 | 1321 | 1262.171399 | 1301.55 | 1340.928601 | 0.985276 |
| GO:0030239\_myofibril\_assembly | 13 | 0 | 0.000000 | 0.000000 | 1321 | 1262.171399 | 1301.55 | 1340.928601 | 0.985276 |
| GO:0030260\_entry\_into\_host\_cell | 13 | 0 | 0.000000 | 0.000000 | 1321 | 1262.171399 | 1301.55 | 1340.928601 | 0.985276 |
| GO:0030833\_regulation\_of\_actin\_filament\_polymerization | 13 | 0 | 0.000000 | 0.000000 | 1321 | 1262.171399 | 1301.55 | 1340.928601 | 0.985276 |
| GO:0031334\_positive\_regulation\_of\_protein\_complex\_assembly | 13 | 0 | 0.000000 | 0.000000 | 1321 | 1262.171399 | 1301.55 | 1340.928601 | 0.985276 |
| GO:0031365\_N-terminal\_protein\_amino\_acid\_modification | 13 | 0 | 0.000000 | 0.000000 | 1321 | 1262.171399 | 1301.55 | 1340.928601 | 0.985276 |
| GO:0031398\_positive\_regulation\_of\_protein\_ubiquitination | 13 | 0 | 0.000000 | 0.000000 | 1321 | 1262.171399 | 1301.55 | 1340.928601 | 0.985276 |
| GO:0031575\_G1\_S\_transition\_checkpoint | 13 | 0 | 0.000000 | 0.000000 | 1321 | 1262.171399 | 1301.55 | 1340.928601 | 0.985276 |
| GO:0032488\_Cdc42\_protein\_signal\_transduction | 13 | 0 | 0.000000 | 0.000000 | 1321 | 1262.171399 | 1301.55 | 1340.928601 | 0.985276 |
| GO:0034284\_response\_to\_monosaccharide\_stimulus | 13 | 0 | 0.000000 | 0.000000 | 1321 | 1262.171399 | 1301.55 | 1340.928601 | 0.985276 |
| GO:0035107\_appendage\_morphogenesis | 13 | 0 | 0.000000 | 0.000000 | 1321 | 1262.171399 | 1301.55 | 1340.928601 | 0.985276 |
| GO:0035108\_limb\_morphogenesis | 13 | 0 | 0.000000 | 0.000000 | 1321 | 1262.171399 | 1301.55 | 1340.928601 | 0.985276 |
| GO:0042095\_interferon-gamma\_biosynthetic\_process | 13 | 0 | 0.000000 | 0.000000 | 1321 | 1262.171399 | 1301.55 | 1340.928601 | 0.985276 |
| GO:0042743\_hydrogen\_peroxide\_metabolic\_process | 13 | 0 | 0.000000 | 0.000000 | 1321 | 1262.171399 | 1301.55 | 1340.928601 | 0.985276 |
| GO:0042982\_amyloid\_precursor\_protein\_metabolic\_process | 13 | 0 | 0.000000 | 0.000000 | 1321 | 1262.171399 | 1301.55 | 1340.928601 | 0.985276 |
| GO:0043462\_regulation\_of\_ATPase\_activity | 13 | 0 | 0.000000 | 0.000000 | 1321 | 1262.171399 | 1301.55 | 1340.928601 | 0.985276 |
| GO:0044409\_entry\_into\_host | 13 | 0 | 0.000000 | 0.000000 | 1321 | 1262.171399 | 1301.55 | 1340.928601 | 0.985276 |
| GO:0045766\_positive\_regulation\_of\_angiogenesis | 13 | 0 | 0.000000 | 0.000000 | 1321 | 1262.171399 | 1301.55 | 1340.928601 | 0.985276 |
| GO:0045861\_negative\_regulation\_of\_proteolysis | 13 | 0 | 0.000000 | 0.000000 | 1321 | 1262.171399 | 1301.55 | 1340.928601 | 0.985276 |
| GO:0046456\_icosanoid\_biosynthetic\_process | 13 | 0 | 0.000000 | 0.000000 | 1321 | 1262.171399 | 1301.55 | 1340.928601 | 0.985276 |
| GO:0046470\_phosphatidylcholine\_metabolic\_process | 13 | 0 | 0.000000 | 0.000000 | 1321 | 1262.171399 | 1301.55 | 1340.928601 | 0.985276 |
| GO:0046503\_glycerolipid\_catabolic\_process | 13 | 0 | 0.000000 | 0.000000 | 1321 | 1262.171399 | 1301.55 | 1340.928601 | 0.985276 |
[truncated: 287,301 more chars]
