# Supplementary material for: Concordance of Gene Expression and Functional Correlation Patterns across the NCI-60 Cell Lines and the Cancer Genome Atlas Glioblastoma Samples
Source: PLoS One. 2012 Jul 26;7(7):e40062. doi: 10.1371/journal.pone.0040062 (PMC3406063; doi:10.1371/journal.pone.0040062)
Supplement: Figure S1 — Full version of Figure 1A. (PDF) [file pone.0040062.s001.pdf]

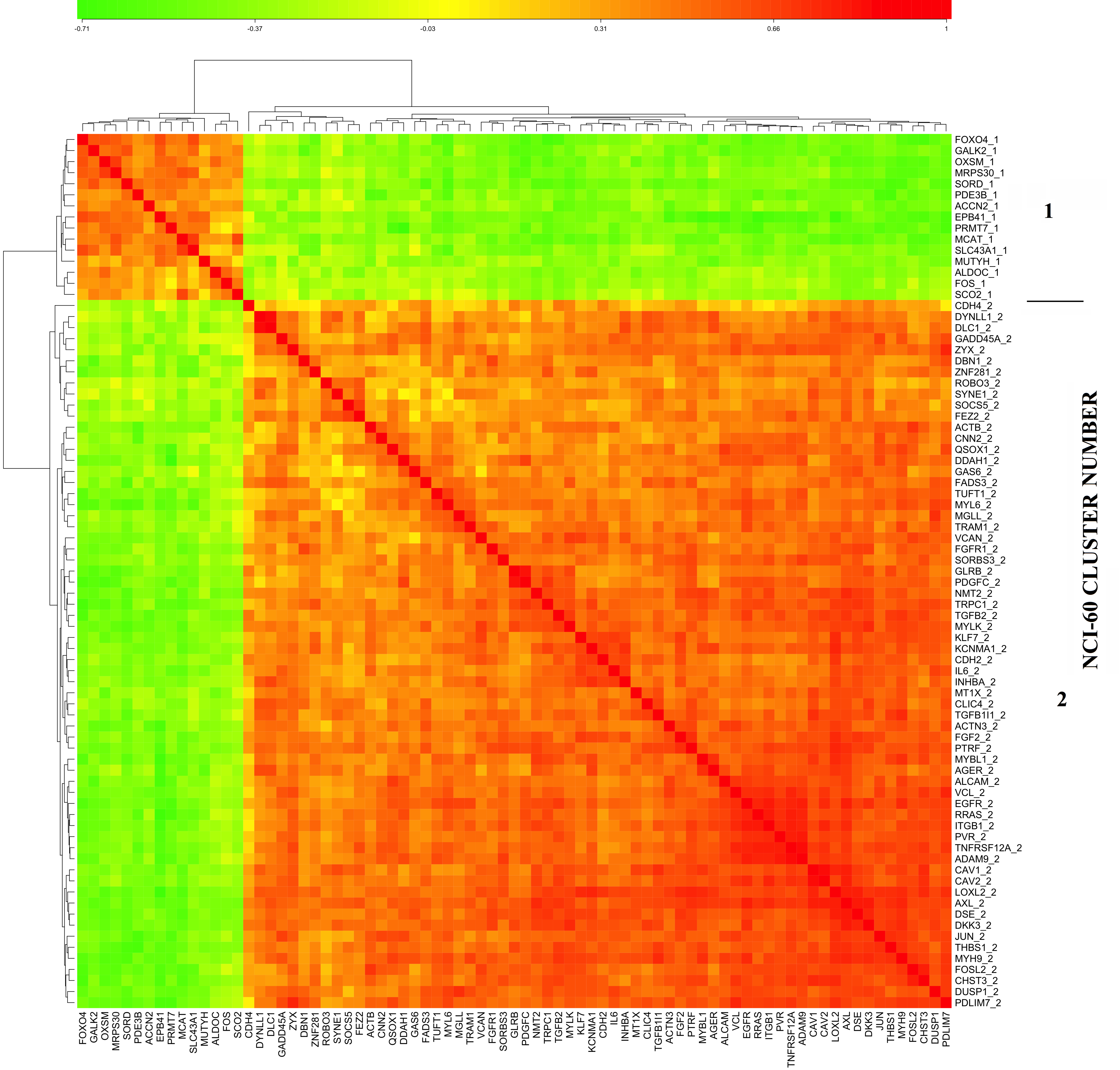

-0.71
-0.37
-0.03
0.31
0.66
1
FOXO4_1
GALK2_1
OXSM_1
MRPS30_1
SORD_1
PDE3B_1
ACCN2_1
EPB41_1
PRMT7_1
MCAT_1
SLC43A1_1
MUTYH_1
ALDOC_1
FOS_1
SCO2_1
CDH4_2
DYNLL1_2
DLC1_2
GADD45A_2
ZYX_2
DBN1_2
ZNF281_2
ROBO3_2
SYNE1_2
SOCS5_2
FEZ2_2
ACTB_2
CNN2_2
QSOX1_2
DDAH1_2
GAS6_2
FADS3_2
TUFT1_2
MYL6_2
MGLL_2
TRAM1_2
VCAN_2
FGFR1_2
SORBS3_2
GLRB_2
PDGFC_2
NMT2_2
TRPC1_2
TGFB2_2
MYLK_2
KLF7_2
KCNMA1_2
CDH2_2
IL6_2
INHBA_2
MT1X_2
CLIC4_2
TGFB1I1_2
ACTN3_2
FGF2_2
PTRF_2
MYBL1_2
AGER_2
ALCAM_2
VCL_2
EGFR_2
RRAS_2
ITGB1_2
PVR_2
TNFRSF12A_2
ADAM9_2
CAV1_2
CAV2_2
LOXL2_2
AXL_2
DSE_2
DKK3_2
JUN_2
THBS1_2
MYH9_2
FOSL2_2
CHST3_2
DUSP1_2
PDLIM7_2
1
2
NCI-60 CLUSTER NUMBER
FOXO4
GALK2
OXSM
MRPS30
SORD
PDE3B
ACCN2
EPB41
PRMT7
MCAT
SLC43A1
MUTYH
ALDOC
FOS
SCO2
CDH4
DYNLL1
DLC1
GADD45A
ZYX
DBN1
ZNF281
ROBO3
SYNE1
SOCS5
FEZ2
ACTB
CNN2
QSOX1
DDAH1
GAS6
FADS3
TUFT1
MYL6
MGLL
TRAM1
VCAN
FGFR1
SORBS3
GLRB
PDGFC
NMT2
TRPC1
TGFB2
MYLK
KLF7
KCNMA1
CDH2
IL6
INHBA
MT1X
CLIC4
TGFB1I1
ACTN3
FGF2
PTRF
MYBL1
AGER
ALCAM
VCL
EGFR
RRAS
ITGB1
PVR
TNFRSF12A
ADAM9
CAV1
CAV2
LOXL2
AXL
DSE
DKK3
JUN
THBS1
MYH9
FOSL2
CHST3
DUSP1
PDLIM7
