# Supplementary material for: Concordance of Gene Expression and Functional Correlation Patterns across the NCI-60 Cell Lines and the Cancer Genome Atlas Glioblastoma Samples
Source: PLoS One. 2012 Jul 26;7(7):e40062. doi: 10.1371/journal.pone.0040062 (PMC3406063; doi:10.1371/journal.pone.0040062)
Supplement: Figure S3 — Full version of Figure 2A. (PDF) [file pone.0040062.s003.pdf]

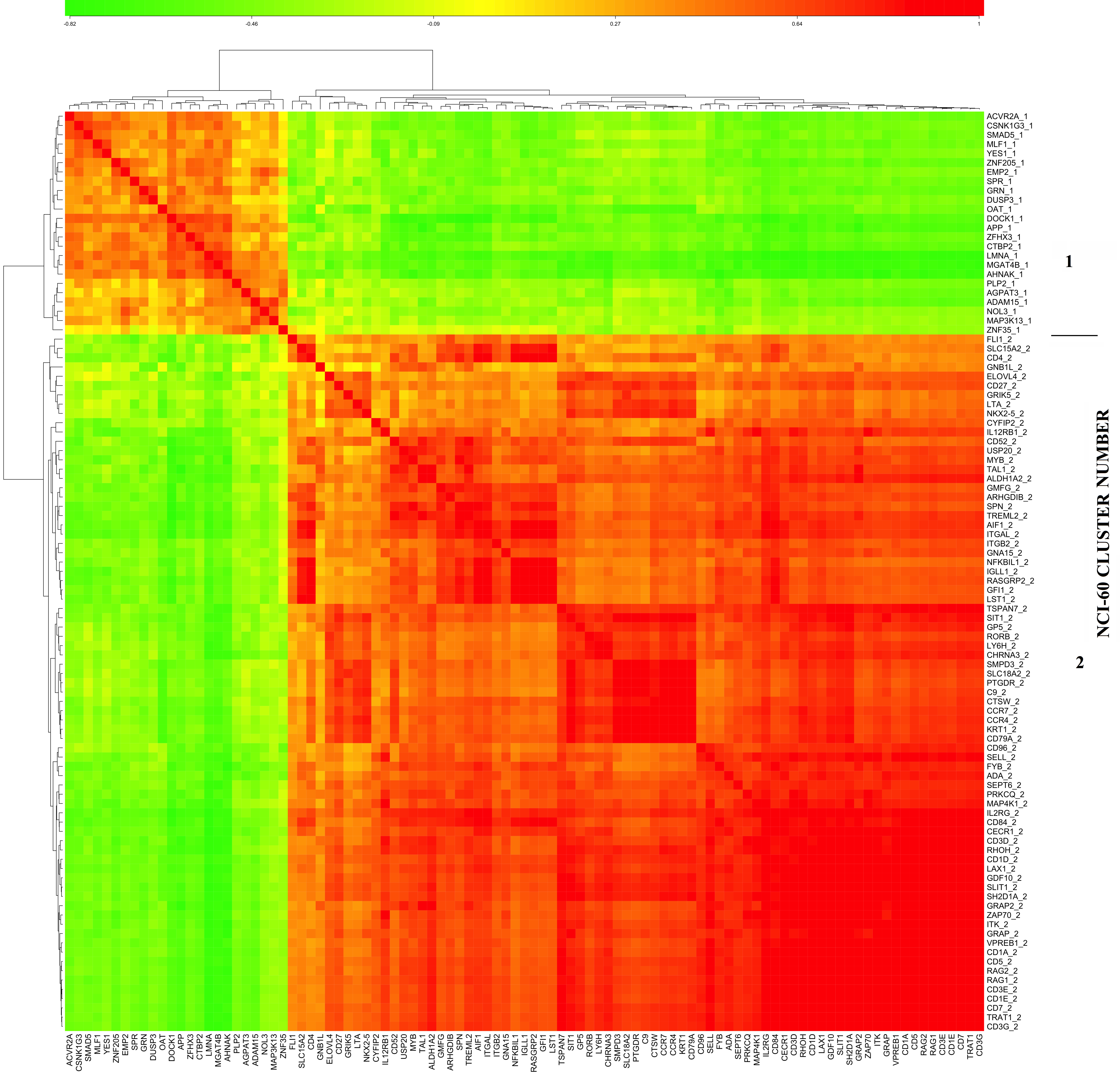
-0.82
-0.46
-0.09
0.27
0.64
1
ACVR2A_1
CSNK1G3_1
SMAD5_1
MLF1_1
YES1_1
ZNF205_1
EMP2_1
SPR_1
GRN_1
DUSP3_1
OAT_1
DOCK1_1
APP_1
ZFHX3_1
CTBP2_1
LMNA_1
MGAT4B_1
AHNAK_1
PLP2_1
AGPAT3_1
ADAM15_1
NOL3_1
MAP3K13_1
ZNF35_1
FLI1_2
SLC15A2_2
CD4_2
GNB1L_2
ELOVL4_2
CD27_2
GRIK5_2
LTA_2
NKX2-5_2
CYFIP2_2
IL12RB1_2
CD52_2
USP20_2
MYB_2
TAL1_2
ALDH1A2_2
GMFG_2
ARHGDIB_2
SPN_2
TREML2_2
AIF1_2
ITGAL_2
ITGB2_2
GNA15_2
NFKBIL1_2
IGLL1_2
RASGRP2_2
GFI1_2
LST1_2
TSPAN7_2
SIT1_2
GP5_2
RORB_2
LY6H_2
CHRNA3_2
SMPD3_2
SLC18A2_2
PTGDR_2
C9_2
CTSW_2
CCR7_2
CCR4_2
KRT1_2
CD79A_2
CD96_2
SELL_2
FYB_2
ADA_2
SEPT6_2
PRKCQ_2
MAP4K1_2
IL2RG_2
CD84_2
CECR1_2
CD3D_2
RHOH_2
CD1D_2
LAX1_2
GDF10_2
SLIT1_2
SH2D1A_2
GRAP2_2
ZAP70_2
ITK_2
GRAP_2
VPREB1_2
CD1A_2
CD5_2
RAG2_2
RAG1_2
CD3E_2
CD1E_2
CD7_2
TRAT1_2
CD3G_2
1
2
NCI-60 CLUSTER NUMBER
ACVR2A
CSNK1G3
SMAD5
MLF1
YES1
ZNF205
EMP2
SPR
GRN
DUSP3
OAT
DOCK1
APP
ZFHX3
CTBP2
LMNA
MGAT4B
AHNAK
PLP2
AGPAT3
ADAM15
NOL3
MAP3K13
ZNF35
FLI1
SLC15A2
CD4
GNB1L
ELOVL4
CD27
GRIK5
LTA
NKX2-5
CYFIP2
IL12RB1
CD52
USP20
MYB
TAL1
ALDH1A2
GMFG
ARHGDIB
SPN
TREML2
AIF1
ITGAL
ITGB2
GNA15
NFKBIL1
IGLL1
RASGRP2
GFI1
LST1
TSPAN7
SIT1
GP5
RORB
LY6H
CHRNA3
SMPD3
SLC18A2
PTGDR
C9
CTSW
CCR7
CCR4
KRT1
CD79A
CD96
SELL
FYB
ADA
SEPT6
PRKCQ
MAP4K1
IL2RG
CD84
CECR1
CD3D
RHOH
CD1D
LAX1
GDF10
SLIT1
SH2D1A
GRAP2
ZAP70
ITK
GRAP
VPREB1
CD1A
CD5
RAG2
RAG1
CD3E
CD1E
CD7
TRAT1
CD3G
