# Supplementary material for: Concordance of Gene Expression and Functional Correlation Patterns across the NCI-60 Cell Lines and the Cancer Genome Atlas Glioblastoma Samples
Source: PLoS One. 2012 Jul 26;7(7):e40062. doi: 10.1371/journal.pone.0040062 (PMC3406063; doi:10.1371/journal.pone.0040062)
Supplement: Figure S4 — Full version of Figure 2B. (PDF) [file pone.0040062.s004.pdf]

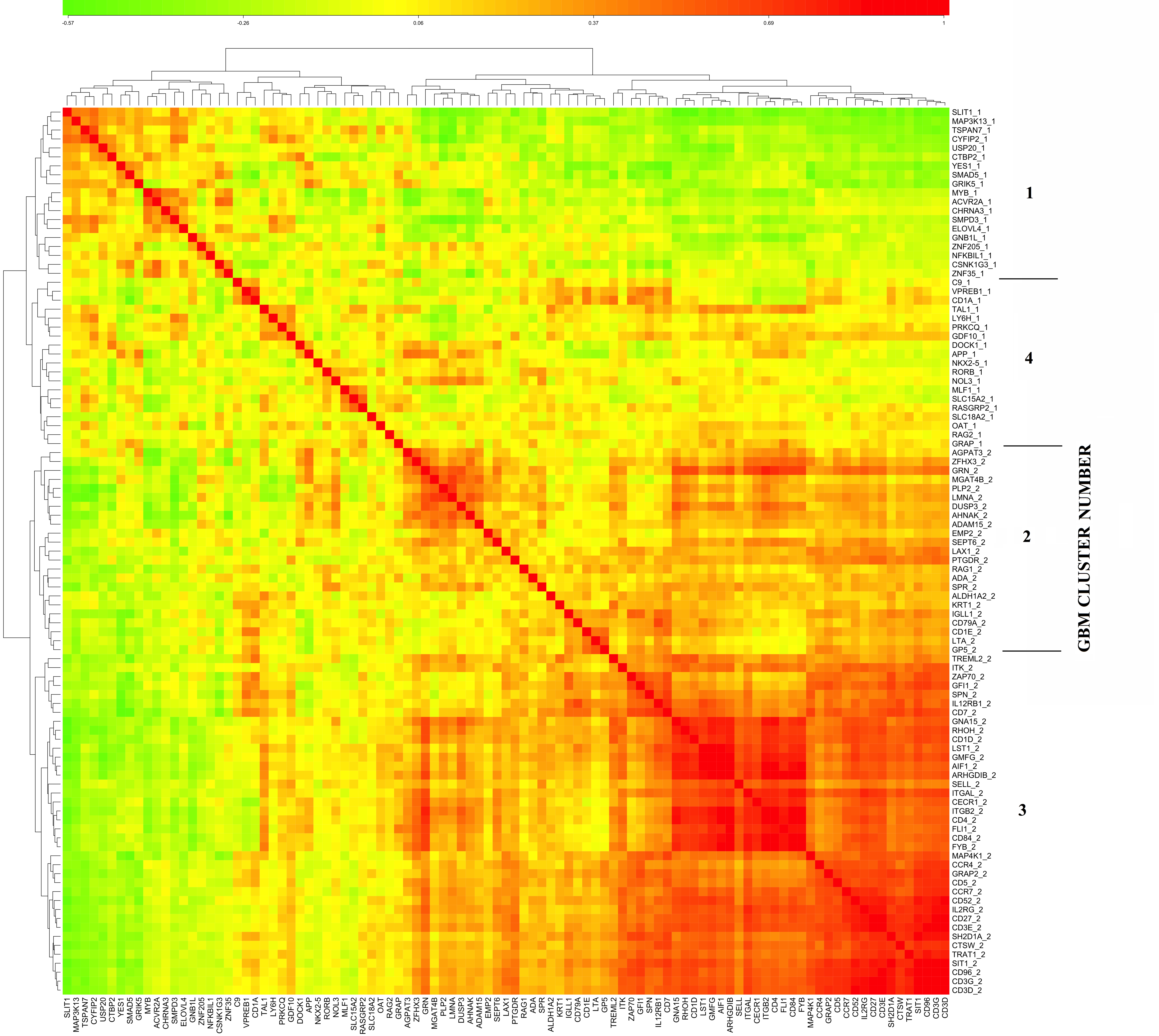
-0.57
-0.26
0.06
0.37
0.69
1
SLIT1_1
MAP3K13_1
TSPAN7_1
CYFIP2_1
USP20_1
CTBP2_1
YES1_1
SMAD5_1
GRIK5_1
MYB_1
ACVR2A_1
CHRNA3_1
SMPD3_1
ELOVL4_1
GNB1L_1
ZNF205_1
NFKBIL1_1
CSNK1G3_1
ZNF35_1
C9_1
VPREB1_1
CD1A_1
TAL1_1
LY6H_1
PRKCQ_1
GDF10_1
DOCK1_1
APP_1
NKX2-5_1
RORB_1
NOL3_1
MLF1_1
SLC15A2_1
RASGRP2_1
SLC18A2_1
OAT_1
RAG2_1
GRAP_1
AGPAT3_2
ZFHX3_2
GRN_2
MGAT4B_2
PLP2_2
LMNA_2
DUSP3_2
AHNAK_2
ADAM15_2
EMP2_2
SEPT6_2
LAX1_2
PTGDR_2
RAG1_2
ADA_2
SPR_2
ALDH1A2_2
KRT1_2
IGLL1_2
CD79A_2
CD1E_2
LTA_2
GP5_2
TREML2_2
ITK_2
ZAP70_2
GFI1_2
SPN_2
IL12RB1_2
CD7_2
GNA15_2
RHOH_2
CD1D_2
LST1_2
GMFG_2
AIF1_2
ARHGDIB_2
SELL_2
ITGAL_2
CECR1_2
ITGB2_2
CD4_2
FLI1_2
CD84_2
FYB_2
MAP4K1_2
CCR4_2
GRAP2_2
CD5_2
CCR7_2
CD52_2
IL2RG_2
CD27_2
CD3E_2
SH2D1A_2
CTSW_2
TRAT1_2
SIT1_2
CD96_2
CD3G_2
CD3D_2
1
4
2
3
GBM CLUSTER NUMBER
